# Identification and mechanistic basis of non-ACE2 blocking neutralizing antibodies from COVID-19 patients with deep RNA sequencing and molecular dynamics simulations

**DOI:** 10.3389/fmolb.2022.1080964

**Published:** 2022-12-16

**Authors:** Alger M. Fredericks, Kyle W. East, Yuanjun Shi, Jinchan Liu, Federica Maschietto, Alfred Ayala, William G. Cioffi, Maya Cohen, William G. Fairbrother, Craig T. Lefort, Gerard J. Nau, Mitchell M. Levy, Jimin Wang, Victor S. Batista, George P. Lisi, Sean F. Monaghan

**Affiliations:** ^1^ Department of Surgery, Division of Surgical Research, The Miriam Hospital, Alpert Medical School of Brown University, Providence, RI, United States; ^2^ Department of Molecular Biology, Cell Biology and Biochemistry, Brown University, Providence, RI, United States; ^3^ Department of Chemistry, Yale University, New Haven, CT, United States; ^4^ Department of Molecular Biophysics and Biochemistry, Yale University, New Haven, CT, United States; ^5^ Department of Surgery, Division of Surgical Research, Alpert Medical School of Brown University and Rhode Island Hospital, Providence, RI, United States; ^6^ Department of Medicine, Division of Pulmonary, Critical Care, and Sleep Medicine, Alpert Medical School of Brown University and Rhode Island Hospital, Providence, RI, United States; ^7^ Department of Medicine, Division of Infectious Disease, Alpert Medical School of Brown University and Rhode Island Hospital, Providence, RI, United States

**Keywords:** RNA sequencing, antibodies, COVID-19, sepsis, intensive care

## Abstract

Variants of severe acute respiratory syndrome coronavirus-2 (SARS-CoV-2) continue to cause disease and impair the effectiveness of treatments. The therapeutic potential of convergent neutralizing antibodies (NAbs) from fully recovered patients has been explored in several early stages of novel drugs. Here, we identified initially elicited NAbs (Ig Heavy, Ig lambda, Ig kappa) in response to COVID-19 infection in patients admitted to the intensive care unit at a single center with deep RNA sequencing (>100 million reads) of peripheral blood as a diagnostic tool for predicting the severity of the disease and as a means to pinpoint specific compensatory NAb treatments. Clinical data were prospectively collected at multiple time points during ICU admission, and amino acid sequences for the NAb CDR3 segments were identified. Patients who survived severe COVID-19 had significantly more of a Class 3 antibody (C135) to SARS-CoV-2 compared to non-survivors (15059.4 vs. 1412.7, *p* = 0.016). In addition to highlighting the utility of RNA sequencing in revealing unique NAb profiles in COVID-19 patients with different outcomes, we provided a physical basis for our findings *via* atomistic modeling combined with molecular dynamics simulations. We established the interactions of the Class 3 NAb C135 with the SARS-CoV-2 spike protein, proposing a mechanistic basis for inhibition *via* multiple conformations that can effectively prevent ACE2 from binding to the spike protein, despite C135 not directly blocking the ACE2 binding motif. Overall, we demonstrate that deep RNA sequencing combined with structural modeling offers the new potential to identify and understand novel therapeutic(s) NAbs in individuals lacking certain immune responses due to their poor endogenous production. Our results suggest a possible window of opportunity for administration of such NAbs when their full sequence becomes available. A method involving rapid deep RNA sequencing of patients infected with SARS-CoV-2 or its variants at the earliest infection time could help to develop personalized treatments using the identified specific NAbs.

## Introduction

Severe acute respiratory syndrome coronavirus 2 (SARS-CoV-2), responsible for coronavirus disease 2019 (COVID-19), continues to cause critical illness requiring intensive care unit (ICU) admission, which is currently driven, in part, by variants. (Krause et al., 2021). Few direct treatments against COVID-19 are available (Willyard, 2021) and with variants comes further potential for escape from current treatments and vaccines. (Acevedo et al., 2021; Starr et al., 2021a). Variants have caused an increase in cases, led to recommendations for additional vaccine doses, and blunted the efficacy of two current antibody treatments. Quickly identifying novel antibodies to target the new variants could aid in care for these patients, reducing viral load and preventing hypoxemia. This is important on two levels; first because the majority of antibodies target the SARS-CoV-2 spike protein receptor-binding domain (RBD) and mutations in this region are present in variants, (Liu et al., 2021; Starr et al., 2021b), and second, because the *de novo* discovery and testing of antibodies is typically a very time-consuming process. (Weinreich et al., 2020).

Long-lived, strongly neutralizing antibodies (Nabs) against the spike protein of SARS-CoV-2 have been developed for therapeutic use, (Brouwer et al., 2020; Cao et al., 2020; Liu et al., 2020; Deshpande et al., 2021), and the number of studies of potential Nabs increases rapidly due to well-developed methodologies for single-cell RNA sequencing and production of monoclonal antibodies with predefined specificity. (Kohler and Milstein, 1975; Tang et al., 2010a; Tang et al., 2010b). The measured binding affinity for some of these NAb-spike protein complexes is as tight as 7.2 *p*mol (*K*_D_), (Deshpande et al., 2021), about three-orders of magnitude tighter than the ACE2-spike complex. Most of these NAbs bind the receptor-binding motif (RBM) site of the spike protein receptor-binding domain (RBD), *i.e.* the ACE2-binding site, and sterically block ACE2 binding. These “ACE2 blocking” NAbs can easily strip bound ACE2 from a spike protein complex to free the virus from host cell attachment.

The binding of multiple ACE2 blocking NAbs to three “up” positioned RBD opens the central pore of the spike trimer, permitting the central stalk of the S2 fragment of the spike protein to extend (*i.e.* the postfusion state) relative to the bent prefusion state. (Tai et al., 2021; Wang et al., 2021; Yang et al., 2022). When this occurs away from a host cell membrane, it permanently disarms the spike trimer and also exposes more epitopes for binding of additional NAbs. However, the ACE2 blocking NAbs cannot bind the spike protein when its three RBDs are in “down” positions. Thus, immunity must also rely on NAbs of other types that bind different regions of the RBD or different domains of the spike protein entirely, which are often referred to as non-ACE2 blocking NAbs, a class of NAbs that have not been well studied.

In the treatment of viruses, NAb therapeutics are often cocktails of two non-competing NAbs that can simultaneously bind, for example, different locations of the SARS-CoV-2 spike protein RBD, one at an ACE2 blocking site and the other at a non-ACE2 blocking site. (Baum et al., 2020; Hansen et al., 2020; Liu et al., 2020; Wu et al., 2020). The rationale for the latter NAb is to reduce the probability of spontaneous mutations that become resistant to ACE2 blocking. Because the ACE2 binding site is often conserved in variants of concern, most known spike mutations are located distal to this site and do not alter the binding affinity of ACE2-blocking NAbs significantly. Thus, it is presently unclear how non-ACE2 blocking NAbs work and whether they play a dominant role in NAb cocktails.

Efforts to identify both types of convalescent NAbs have been carried out using single-cell RNA sequencing (RNAseq) of B- or T-cell samples from recovered patients infected with SARS-CoV-2. At least one study included patients who did not survive. (Garcia-Beltran et al., 2021). However, the systematic time-course evolution of NAbs in patients has not been evaluated previously, which became our motivation for this study. We used RNAseq data from critically ill COVID-19 patients in the ICU to identify novel antibodies elicited during the course of the disease, with particular focus on NAbs generated by patients who survived, to inform the severity of the disease and future antibody or vaccine development. We found that time-course evolution of NAbs differs between surviving and non-surviving COVID-19 patients. COVID-19 survivors expressed a very high level of non-ACE2 blocking NAbs at the time of initial infection that helped them completely fend off additional persistent infections. The level of non-ACE2 blocking NAbs was much higher than non-survivors, suggesting that non-ACE2 blocking NAbs, and not necessarily the convergent ACE2 blocking NAbs, are critical for recovery. We also used the recently proposed structural classification of COVID-19 antibodies (Barnes et al., 2020) to categorize potential efficacious antibodies and provide a possible rationale for this effect. Using NAb C135 as a representative of the non-ACE2 blocking class, (Barnes et al., 2020; Robbiani et al., 2020), we carried out full atomistic modeling and molecular dynamics (MD) simulations of the NAb-spike protein complex, the results of which provide a biophysical basis for how non-ACE2 blocking NAbs neutralize SARS-CoV-2.

## Materials and methods

### Patient study design, population, and setting

ICU participants in April-June 2020 at a single tertiary care hospital were enrolled after themselves or surrogates provided informed consent (IRB Approval # 411616). SARS-CoV-2 infection was based on positive PCR from the nasopharynx. Patients were followed until discharge or death and clinical information was collected prospectively. Blood samples were collected on Day 0 of ICU admission and Day 3 whenever possible.

### RNA extraction, sequencing, data protection and quality assurance

Blood was collected directly from the patient into PAXgene tubes (Qiagen, Germantown, MD) on both Day 0 and Day 3 and was sent to Genewiz (South Plainfield, NJ) for RNA extraction, ribosomal RNA depletion, and sequencing on Illumina HiSeq machines with greater than 100 million reads per sample. In order to ensure security of the genomic data to HIPAA standards, the raw files were returned on password protected external hard drives and all analysis was done on servers within the hospital firewalls. As previously described with some of the data, (Monaghan et al., 2021), quality was assessed by using FastQC. (Andrews, 2014). Sequencing data was aligned using STAR aligner with standard parameters. Unmapped reads were included in the output.

### Identification of antibodies

Alignment files were then parsed for reads mapping onto the V(D)J locus using ImReP (Mandric et al., 2020) to identify novel antibodies produced by the patients with active COVID-19 disease. The resulting CDR3 sequences were then compared across survivors and non-survivors retrospectively. Only sequences that appeared in every patient in each group were considered distinct to that group. Comparison was done using NCBI blast, with a threshold of 66% length match and 70% sequence match. Querying sequences by time point, across time points, further filtered blast output and survivors vs non-survivors by time point and across time points.

### Classification of CDR3 regions and model of human neutralizing antibodies (NAb)

The patient-derived light chain CDR3 sequences were aligned to known CDR3 regions of previously identified neutralizing antibodies (Supplementary Table S1). CDR3 sequences were classified according to primary sequence similarity to the CDR3 classifications of known three-dimensional structures based on their interactions within NAb-spike protein complexes. (Barnes et al., 2020). If the primary sequence did not align to any of those sequences, the CDR3 was left unclassified. It was found that C135 has the most closely related sequences with the fewest sequence differences that we had manually mutated in our computations.

### Statistical analysis

SigmaPlot 14.5 (Sysstat Software) was used for analysis. T-tests were used to compare survivors and non-survivors, but paired t-tests were used to compare across time points. Alpha was set at 0.05.

### Molecular dynamics simulations

**FIGURE 1:**
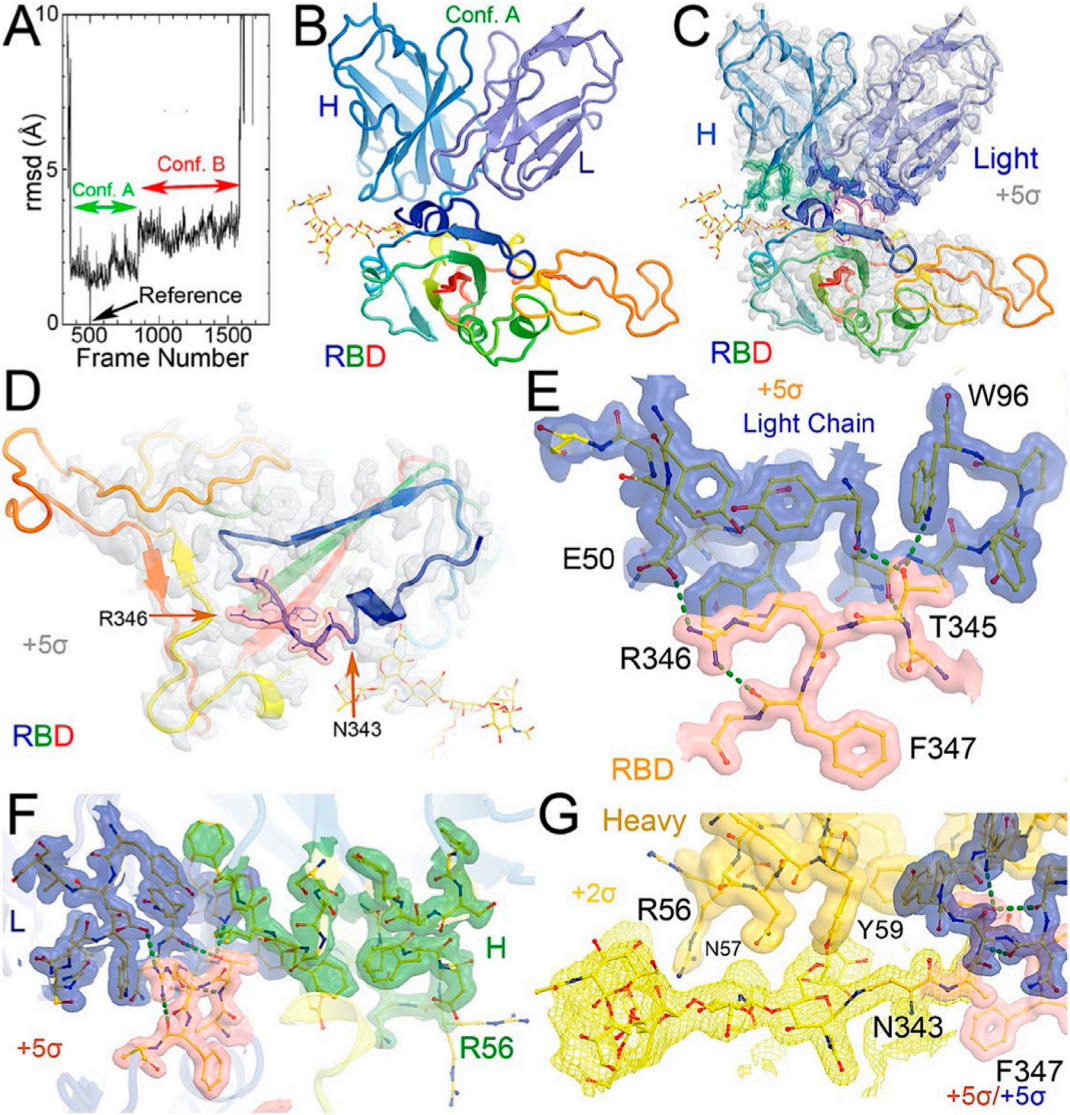
MD-derived electrostatic potential (ESP) maps and the equilibrated structure for NAb conformation. **(A)** Frames corresponding to conformation A (green arrow) and conformation B (red arrow). **(B)** Overall structure of conformation A between the spike RBD (rainbow colors with glycosylated N343 in ball-and-stick) and heavy (slate)/light (light blue) chains of the C135 Fab. **(C)** MD-derived ESP maps contoured at +5σ. Contact interface maps are colored: light chain in blue, heavy chain in green, and RBD in pink. **(D)** Closeup view of the RBD with the map from **(C)**. **(E)** Interactions between the RBD and the NAb light chain. **(F)** Interactions among RBD, heavy/light chains with maps contoured +5σ. **(G)**. Reduced contouring level to +2.5σ for the heavy chain to show the features for N343 glycan.

**FIGURE 2:**
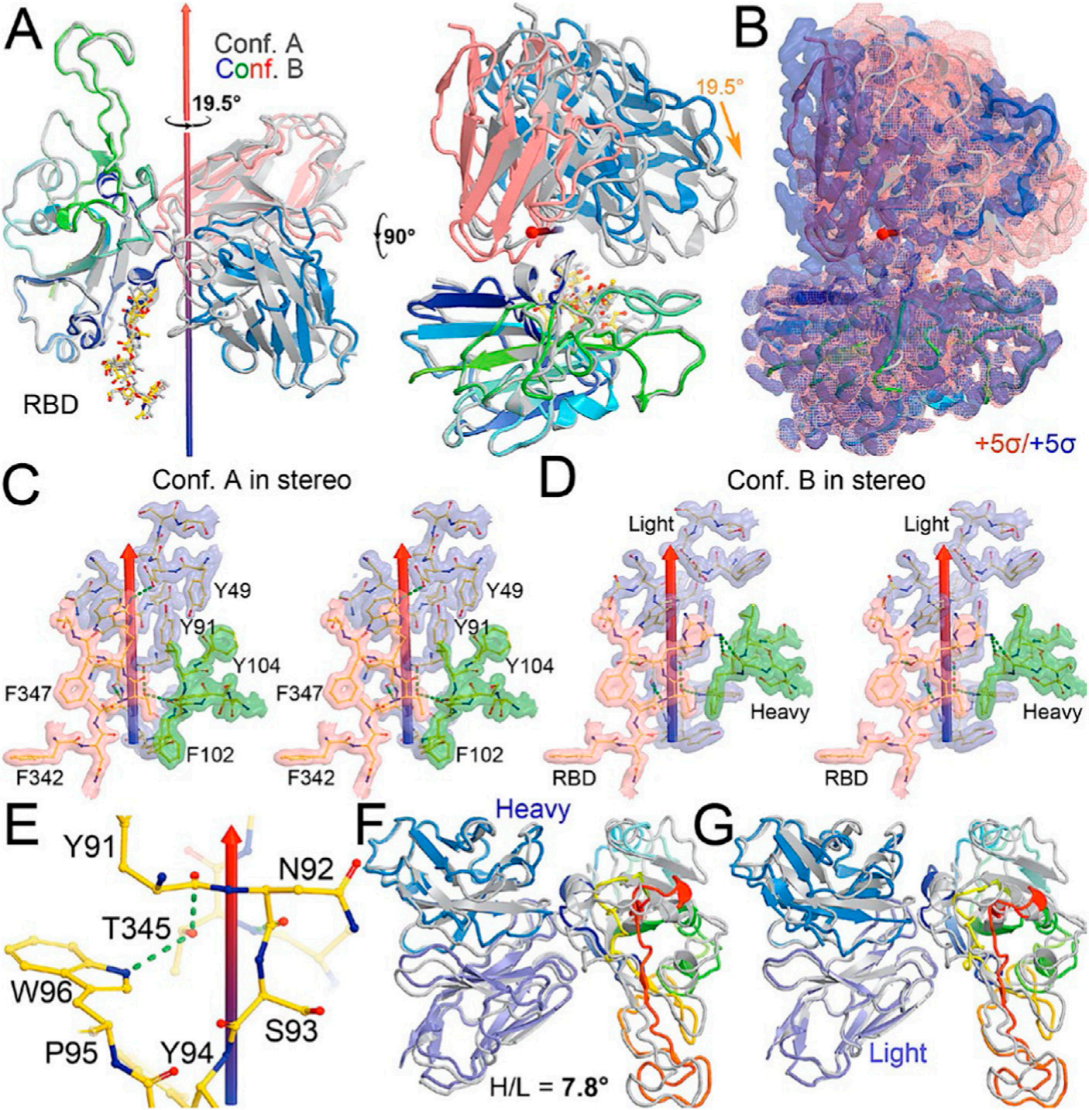
Comparison between conformations A and B of the NAb-spike complex. **(A)** Two orthogonal views of comparison with rotational axis and angle indicated upon alignment of the RBD (rainbow color) between conformation A (grey) and B (multicolor). **(B)** Corresponding MD-ESP maps contoured at +5σ. **(C)** Interactions of the RBD and heavy/light chains near the rotational axis in stereodiagram of conformation (A). **(D)** Conformation B in stereodiagram. **(E)** Conserved interaction surrounding T345 in the two conformations. **(F)** Alignment of heavy chain between the two conformations. **(G)** Alignment of light chain.

**FIGURE 3:**
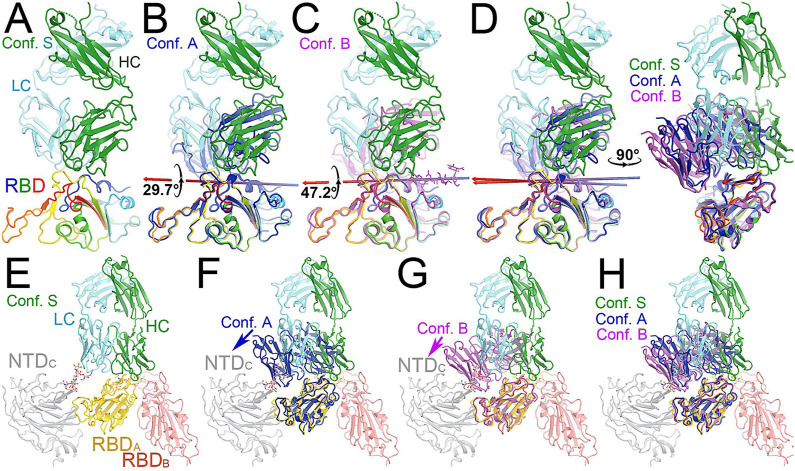
Comparison of MD-derived conformation A (blue) and B (magenta) with the starting conformation S (multicolor). **(A)** Starting conformation S. **(B)** Superposition between conformations S and (A). **(C)** Superposition between conformations S and (B). **(D)** Two orthogonal views of conformations S, A and (B). **(E)** Conformation S in context of the spike trimer with NTD and RBD of neighboring subunits included. **(F)** Superposition of conformations S and A in the context of the spike protein. **(G)** Superposition of conformations S and B in the trimer. **(H)** Compiled view of conformation S and A/B in the trimer.

**FIGURE 4:**
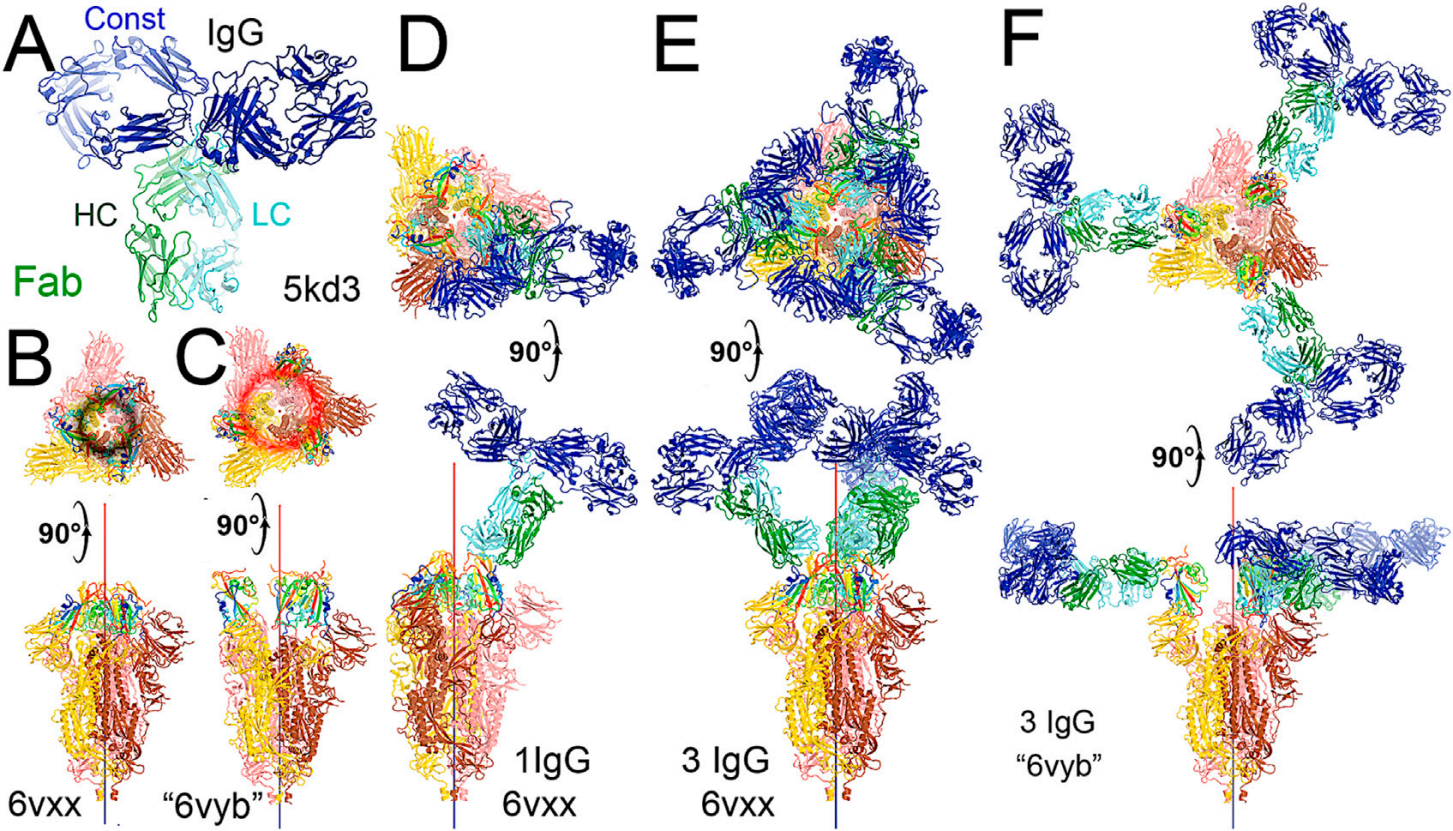
Binding of the full-length NAb C135 to the spike trimer in two conformations. **(A)** Using the full-length anti-PD1 IgG4 to represent the full-length C135, constant domains are in blue, heavy chain in green, and light chain in cyan. **(B)** Two orthogonal views of the closed spike trimer (from PDB: 6vxx coordinates). **(C)** Two orthogonal views of the fully open (open-1) spike trimer (using symmetrized PDB: 6vyb coordinates). **(D)** Binding of one NAb C135 to the closed spike trimer in two orthogonal views. **(E)** Binding of three NAb C135 to the closed spike trimer in two orthogonal views. **(F)** Binding of three NAb C135 to the open spike trimer.

A complete NAb-spike protein model was built for MD simulations primarily based on the cryo-EM map (emd22736) reported at 3.5-Å resolution, starting with the corresponding PDB 7k8z coordinates (Figures 1, 2, 3, 4) Any missing residues, particularly charged residues often located on surface loops, could incorrectly modulate MD trajectories. Rebuilding was therefore extensive due to the highly incomplete nature of the deposited 7k8z coordinates, which likely resulted from a lack of confidence in the experimental map at relatively low local resolution, particularly near the RBD/NAb C135 interface. Our rebuilt model included two missing loops (S443-G447 and E471-F489), one single-residue gap (V502), and many truncated large residues to those containing only a single Cβ sidechain atom (i.e., to alanine) even in the most ordered version of the RBDs, including R346 and N440 (these two sidechains were not present in the 7k8z coordinates although they were included in published figures). The first missing loop and the single-residue gap are located at the RBD/NAb C135 interface and the second missing loop is at the RBD/RBD interface mediated by glycans. Rebuilding also included the entire Fab fragment of NAb C135 for the “up” positioned RBD and half of the Fab fragment for each of the two “down” positioned RBDs (Supplementary Figures S1, S2). This structure contains one “up” and two “down” RBDs. The only variable loop-containing half of the C135 Fab fragment that is in direct contact with the RBD was included in our MD simulations (Supplementary Figure S3). A slightly truncated version of the RBD (N334 to L516, a few residues shorter than the conventional definition after excluding two paired strands at its N- and C-termini) as well as the glycan attached to N343 was included for the RBD in the RBD-NAb complex in our simulations.

The entire C135 Fab fragment bound to each of the three RBDs was clearly visible in the experimental map at +2σ contouring level for confident rebuilding (Supplementary Figures S1, S2). Although a high-resolution crystal structure of NAb C135 was docked into the cryo-EM map of the 7k8z complex, the conformation of this NAb C135 in the complex clearly differs in variable loop three of the heavy chain from that of the isolated NAb C135 structure, suggesting an induced-fit conformational change of this Fab fragment. This induced-fit conformational change was to justify additional MD simulations. Following complete RBD rebuilding, we mapped Omicron variant mutations and found that two of these are located at the RBD/NAb C135 interface and five additional mutations are nearby (Supplementary Figures S4E, F).

After obtaining the more complete coordinates, a glycan moiety was attached to N343 according to the experimental map using the Glycan Reader and Modeler module in CHARM-GUI. (Jo et al., 2008). Topology and parameters were built generated using the PSFGEN tool in VMD. (Humphrey et al., 1996). The system was solvated in the middle of TIP3P water box (∼104 Å × 104 Å X 104 Å). Five cycles of equilibration were run using NAMD. (Kale et al., 1999; Phillips et al., 2005). The MD simulations were run using a 2 fs time step and under 1.013225 bar (NPT ensemble). During the first cycle (500 ps), the entire system was frozen with the exception of waters and ions. For the second cycle (15 ns), the protein sidechains and residues within 8 Å of the C1325/RBD interface were released. For the third cycle (10 ns), all restraints were released. For the fourth cycle (500 ps), the full released system was heated from 1 K to 298 K. The freed system from the equilibration run was taken as the starting point for the production run, which was carried out on GPU and used the AMBER force field parameters. (Cornell et al., 1995). ParmEd was used to convert the parameters and hydrogen mass repartitioning (HMR) was used to enable the MD simulations to employ 14 fs step. (Cornell et al., 1995). The overall production run was carried out for 465 ns. The resulting MD trajectories were analyzed using MD-derived ESP maps as described elsewhere. (Wang et al., 2022a; Wang et al., 2022b; Wang et al., 2022c).

## Results

### A new pattern of time-course evolution of non-ACE2 blocking NAbs

A total of 15 patients had samples collected on Day 0 initially admitted in the ICU, 12 of which also had samples collected on Day 3 (2 patients were discharged from the ICU and one succumbed to COVID-19 death). Clinical and demographic data were previously reported (Monaghan et al., 2021) and are also presented in Table 1. We analyzed antibody (Ig) counts of Ig Heavy, Ig kappa, and Ig lambda chains in COVID-19 survivors and non-survivors (Table 1) in aggregate and compared across Days 0 and three time points. There was no difference in the number of Ig Heavy, Ig kappa, or Ig lambda counts between survivors and non-survivors on any given day, but when comparing *across time points*, we found a significant increase in Ig lambda across all patients (16590 vs. 33610, *p* = 0.009). When analyzing survivors alone, we observed the same trend (11313 vs. 31,087, *p* = 0.0426), which was not present in non-survivors (21868 vs. 36,133, *p* = 0.1504).

**TABLE 1:** Demographic data and antibody types per group (survivor vs non-survivor) at ICU Day 0 and ICU Day 3.

Demographics and antibody types	Day 0		Day 3	
	Survivor (8)	Non-survivor (7)	Survivor (6)	Non-survivor (6)
Median age (SD)	53.9 (22.7)	64.1 (13.8)	53.5 (18.5)	60.9 (11.8)
Female—no. (%)	2 (25)	2 (29)	2 (33)	2 (33)
Race				
White—no. (%)	2 (25)	4 (57)	1 (17)	3 (50)
Black or African American—no. (%)	1 (13)	1 (14)	1 (17)	1 (17)
Other Race—no. (%)	5 (62)	2 (29)	4 (66)	2 (33)
Ethnicity				
Hispanic—no. (%)	4 (50)	5 (71)	3 (50)	4 (66)
Median BMI (SD)	28.1 (4.1)	31.5 (4.9)	26.6 (3.6)	31.7 (5.4)
Antibody reads				
Ig Heavy (SD)	1877.6 (31136.3)	17247 (17780)	45029.8 (48919.6)	39982.1 (33397.3)
Ig Kappa (SD)	70548.6 (136600.2)	43630.7 (44591.6)	65278.3 (83406)	34784.3 (22616.4)
Ig Lambda (SD)	9289.9 (11302.4)	19000.9 (24621.1)	31087.3 (29572.1)	36133 (30801)
Total Reads (SD)	99478.5 (178615)	80942.6 (86484.6)	142306.8 (141072.9)	111939.2 (84022.7)

We analyzed the complementarity-determining region (CDR3) sequence counts of each patient (i.e., a variable amino acid sequence that NAbs use to recognize their antigen) and categorized the antibody types using a recently published classification system (Barnes et al., 2020) on Day 0 and Day 3 (Supplementary Tables S2, S3, respectively). The resulting categorizations of antibody types, antibody classes, and NAb structure are reported for Day 0 and Day 3 in Supplementary Tables S2, S3, respectively. Most NAbs detected in survivors on Day 0 of ICU admission categorize as Class 3 (non-ACE2 blocking), while the most abundant NAbs in non-survivors belong to Class 4 (a different type of non-ACE2 blocking NAb). When assessing the number of counts that align to the non-ACE2 blocking C135 antibody, a SARS-CoV-2-specific NAb, survivors had significantly more (15059.4) than non-survivors (1,412.7, *p* = 0.016) on Day 0 but not on Day 3, suggesting an initial phase of NAb production may be critical for surviving COVID-19 ICU admission. All other distinct SARS-CoV-2 antibodies (as described in Supplementary Table S1) had no difference across survivors vs non-survivors or across time points.

Top NAbs identified as unique to survivors and non-survivors on Day 0 are presented in Table 2 (a full list is presented in Supplementary Table S4 for survivors and Supplementary Table S5 for non-survivors). Among survivors, most antibodies categorize as non-ACE2 blocking Class 3 while many of the top antibodies found in non-survivors were categorized as mainly Class 4 with some other classes of antibodies having fewer reads. In order to understand how non-ACE2 blocking NAbs would interact with the SARS-CoV-2 spike protein, we carried out MD simulations on the complex after modeling the CDR3 sequence most frequently found in survivors into an existing cryo-EM structure and report our findings as follows.

**TABLE 2:** CDR3 alignments exclusive to survivors day 0.

Alignment	ClusterCounts	CDR3 counts	Class	ID
CDR3 Alignments Exclusive to Survivors Day 0				
CQQYNNYWAF	43	75616	C3	C135
CQQYNNWPPLTF	52	60021	C3	REGN10987
CQQYDNHF	42	48510	C3	REGN10987
CQQYNRYWTF	26	44085	C3	C135
CQQYNNWPPLFTF	31	36103	C3	REGN10987
CQQYNNWPPGFTF	35	35532	C3	REGN10987
CQQLNSYPGGTF	26	35395	C1	B38
CQQYDSYPWTF	26	34909	C3	C135
CQQYNSYSRTF	32	34521	C3	C135
CQQYGSFF	25	33616	C1	C102
CQQYDKWPFF	25	30823	C4	S2A4
CMQALQTPLTF	18	26607	C2	C002
CMQALQTPRTF	17	26599	C2	C104
CQQRSNWPPLTF	39	20622	C3	REGN10987
CHQYNNW	30	19583	C3	REGN10987
CQQFNTWPPE	17	19345	C1	B38
CQQYQTWPPLF	28	17810	C1	B38
CQQFNNWPPGFSF	17	16145	C1	B38
CQSVDSSGIYI	27	15458	C4	S2A4
CQSADSSGTYPDVVF	33	15132	C4	S2A4
CDR3 Alignments Exclusive to Non-Survivors Day 0				
CQQSYSTPLFAF	28	140569	C4	EY6A
CQQYYTTPMYAF	23	125715	C4	CR3022
CQQYYSTPPSF	34	110321	C4	CR3022
CQSADSSGTWVF	37	62077	C4	S2A4
CQSADTSGTYGNWVF	22	61809	C3	S309
CQSADTSGPYRDVVF	17	61626	C3	S309
CQSPDSSATYQV	11	61577	C4	S2A4
CQSADNSGSYGIF	22	51193	C4	S2A4
CQQYGSSVTF	27	48316	C1	C102
CQSADSSGTYKMLF	16	30329	C4	S2A4
CQRYNNYPYIF	15	25022	C3	C110
CQRYNTAPYTF	12	22628	C3	C110
CQQVYDTPRTF	12	19935	C2	C002
CMQGTHWPPIIF	19	14254	C1	B38
CIQGTHWPPSAF	14	14235	C1	B38
CMQGTHWPPSTF	16	14,233	C1	B38
CMQGTHWPPAF	20	14141	C1	B38
CMQGTLWPPSF	13	14091	C1	B38
CMQGTHWPPGLTF	13	14077	C1	B38
CMQGTHWPPSFTF	14	14076	C1	B38

Top 20 CDR3 alignments that were exclusive to survivors and non-survivors on ICU Day 0. The alignment is the common sequence to which a number of CDR3 from each patient aligned (ClusterCounts). The CDR3 counts represent the number of times a CDR3 mapped from the survivors or non-survivors. The class and ID are defined (Barnes et al., 2020) and references in Supplementary Table S1.

### Non-ACE2 blocking C135 NAbs can still preclude ACE2 binding

The cryo-EM structure reported for the spike/NAb C135 Fab fragment (PDB: 7k8z) shows that binding sites for the Fab fragment and the ACE2 receptor domain do not overlap. (Barnes et al., 2020). However, the 7k8z model built for C135 represents only about one-half of its Fab fragment and about one-sixth of its entire NAb IgG1. (Barnes et al., 2020). The ACE2 receptor domain also represents only a fraction of the neutral amino acid transporter (N^0^AT1)/full-length ACE2 complex, which is a membrane-bound dimer that contains two ACE2 receptor domains. (Yan et al., 2020). To determine whether binding of the intact NAb C135 IgG1 to the RBD of the spike trimer interferes with the binding of ACE2 receptors attached to the host cell membrane, we considered 1) the size of the intact NAb IgG1, and 2) the geometry and curvature of the host membrane and membrane-bound N^0^AT1/ACE2 dimer, as discussed previously. (Wang et al., 2021).

To visualize the complete NAb C135 IgG1, we used the recently reported monoclonal therapeutic anti-PD1 (Parkinson Disease) full-length IgG4 structure, as it is a good representation of the size of NAb C135 IgG1 for our modeling, (Scapin et al., 2015), although the elbow angle between the constant domain and Fab fragment may differ (Figure 1A). We modeled one, two, or three complete NAbs C135 IgG1 bound to one, two, or three RBDs of the spike trimer in its two different conformations (Figure 1). The first conformation was taken from the PDB: 6vxx coordinates, which represents the fully closed central pore with all three RBDs in the “down” position (Figure 1B). (Walls et al., 2020) The second conformation is the symmetrized version of PDB: 6vyb (i.e., “6vyb”), which represents a partially opened central pore (open-1) with all three RBDs in the “up” position after rotating two “down” positioned RBDs (Figure 1C). (Walls et al., 2020; Wang et al., 2021) Another fully opened conformation (open-2) was observed, which was essential for the productive host-viral membrane fusion reaction. (Kirchdoerfer et al., 2018; Wang et al., 2021).

The maximal dimension of the intact NAb C135 IgG is comparable with that of the ectodomain of the spike protein (Figure 1D). When one NAb C135 IgG binds one RBD of the fully closed spike trimer or to one “down” RBD in the mixed up-down asymmetric spike trimers, the large size of the NAb C135 IgG sterically blocks the virus from approaching the host membrane and thereby prevents the viral spike protein from binding to the N^0^AT1/ACE2 dimer (Figure 1D). Therefore, binding of a single NAb C135 IgG1 to one “down” positioned RBD (as well as multiple NAb C135 molecules) is sufficient to completely block binding of the N^0^AT1/ACE2 dimer and thereby prevents the ACE2-dependent host-viral membrane fusion (Figure 1D).

### Non-ACE2 blocking NAb C135 can open the spike trimer and block membrane fusion

As long as one RBD is in a “down” position, the bound NAb C135 IgG1 will prevent the virus from approaching any host cell membrane from the direction of the occupied RBD. However, NAb C135 binds RBD independent of its conformational state (Figure 1), and when bound to an “up” positioned RBD, it will not prevent the virus from approaching the host cell membrane. If this RBD already has an ACE2 receptor bound, the Nab is still able to bind (Figure 2A). Binding of either a non-ACE2 blocking NAb or the ACE2 receptor can shift the down-to-up equilibrium of the neighboring RBDs and thereby cooperatively open the central pore of the spike trimer (Figure 1). Therefore, this NAb can permanently disarm the triggering mechanism of the spike trimer with or without assistance of the ACE2 receptor. As MD simulations described below, the binding of NAb C135 IgG1 weakens inter-subunit interactions that constrain the RBDs in “down” positions within the spike trimer.

The fusion reaction of two negatively charged membranes is an energetically unfavorable process that is made possible when coupled with the highly exergonic conversion of the prefusion to postfusion states of the S2 fragment of the spike protein. A single spike trimer can fuse two membranes but cannot open a connecting pore between them to transfer genetic material from the virus to the host cell. (Wang et al., 2021). This requires formation of supercomplexes containing minimal three spike trimers and six N^0^AT1/ACE2 dimers, involving binding of two NAbs to each ACE2 dimer. (Wang et al., 2021). Modeling of the full-length NAb C135 IgG1 and the full-length N^0^AT1/ACE2 dimer shows that two IgG1 molecules would generate steric clashes within the ACE2 dimers that could explain the ability of the NAb to block this step of membrane fusion (Figure 2).

### Two stable states of the Nab C135-RBD complex are revealed by MD simulations

After a three-step minimization of the NAb C135-RBD complex, MD simulations were carried out for 465 ns and coordinates were written every 200 ps. During the MD simulations, the complex rapidly evolved away from the starting configuration (conformation S) *via* short-lived intermediates and rapidly settled into two stable configurations (conformation A and B) according to a root-mean-square fluctuation (rmsF) analysis in reference to either frame one or frame 500 (or to frame 1000, data not shown). Conformation A begins at frame 360 and lasts for 98 ns; conformation B begins at frame 849 and lasts for 149 ns (Figure 3). In our initial analysis, we combined the two conformations into a single state that represents 52% of the total trajectory frames. Each of the two conformations in the MD trajectories could have persisted for much longer, but we were unable to reliably estimate their duration. However, during interpretation of the MD-derived electrostatic potential (ESP) maps, we found two clear and closely related conformations (Figures 3, 4, Supplementary Table S5).

When MD-derived ESP maps are visualized in continuous contouring levels of 10σ, 5σ, 2.5σ, and 1.25σ, the NAb C135/RBD interface remains the most ordered part of the complex (Supplementary Table S5) This region of the equilibrated structure has the smallest rmsF and the smallest atomic B-factors after the fitted models were refined. While the interface area buried between the RBD and NAb C135 is 8,132 Å^2^ (heavy chain, 4,536 Å^2^, light chain, 3,596 Å^2^, including 2,387 Å^2^ between the heavy chain and N343 glycans) for conformation A, and 6,476 Å^2^ (heavy chain, 1681 Å^2^, and light chain 4794 Å^2^, including only 432 Å^2^ between the heavy atom and N343 glycans) for conformation B, multiple hydrogen (H)-bonds are observed. Inter-subunit H-bonds include R346 of the RBD and E50 of light chain (numbering of antibody residues in this study is according to their genomic coding sequence, but it does not include gaps and insertions in the aligned antibody structure) and T346 of the RBD with both the W96 sidechain and Y91 backbone carbonyl and N92 backbone carbonyl of the light chain (Figure 2E). There are also extensive interactions between the heavy chain and the glycan units attached to N343 of the RBD when the MD-derived ESP maps are viewed at reduced contouring levels (Figure 3G). In conformation B, the H-bond pattern of T346 of the RBD remains unchanged relative to conformation A, but R346 of the RBD is repositioned to form H-bonds in one of the two sidechain rotamers with the carbonyl groups of both G101 and F102 of the heavy chain (Figures 4C,D).

The two conformations differ by a rotation of 19.5° around an axis passing through the conserved H-bond network of T346 (Figure 2A). This rotation is evident in the two MD-derived maps even before the maps were interpreted and the coordinates were built (Figure 2B). In addition, there is a small rigid rotation of the heavy and light chains within NAb C135 (Figures 2F,G), revealing that dynamics of this complex are largely associated with rigid-body rotations of domains with a small rmsF value of ∼1.8 Å for the entire complex in MD trajectories of each conformational state.

### Functional roles of glycans in binding of NAb C135 to the RBD

Viruses often hijack host glycosylation machinery to hide epitopes of their spike proteins. The majority of NAbs do not recognize glycans because these are components of host cellular proteins and specified by the individual’s blood type. We found, unexpectedly, that R56, N57, and Y59 of the heavy chain of NAb C135 make favorable transient interactions with glycosylated residue N343 for a significant duration of MD trajectories only in conformation A (Figure 3G). Likewise, the light chain of NAb C135 would also interact with glycosylated N165 of the neighboring N-terminal domain (NTD) (Supplementary Figures S3, S6), which was not included in our current MD simulations. In fact, interactions of a given NAb with glycans may help to recruit the NAb before it fully recognizes the underlying epitope, i.e., this binding represents the first step of antibody-epitope recognition. Much of the heavy chain-glycan interactions are lost in the most persistent, and likely more stable, conformation B. Our MD simulations reveal stable antibody-epitope interactions after NAb C135 peels away from the N343 glycans and likely also the N165 glycans according to the observed domain rotations.

**FIGURE 5:**
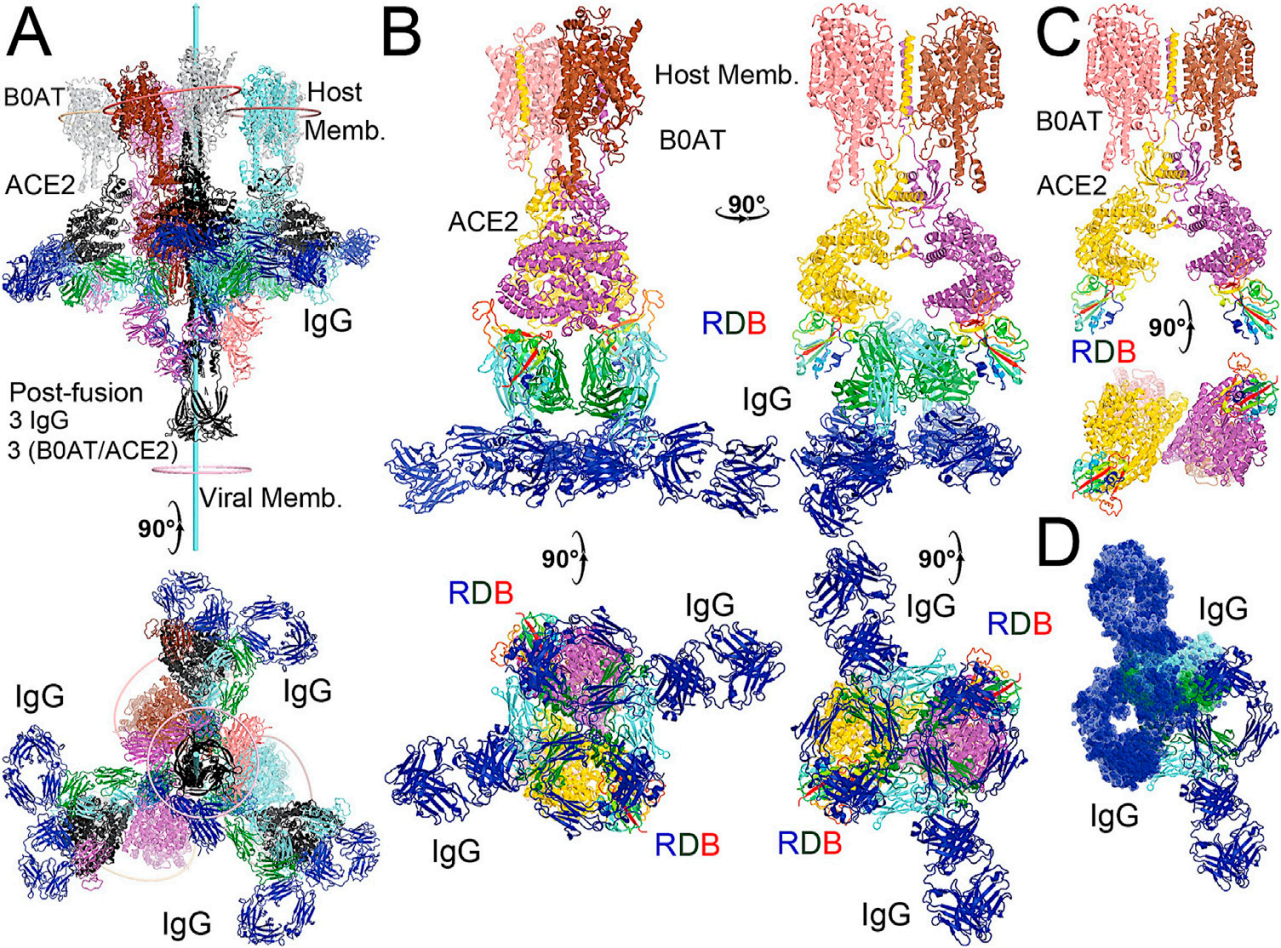
Modeling of the full-length NAb C135 with the full-length N^0^AT1/ACE2/spiker supercomplexes. **(A)** Two orthogonal views of three NAb C135 (blue, forest, and cyan) and three B^0^AT1/ACE2 (magenta, pink, and grey) dimers bound to one open spike trimer (black for the extended S2 central stalk, and multicolor for NTD and rainbow colors for RBDs). **(B)** Two NAb C135 bound to the B^0^AT1/ACE2 dimer in the opened ACE2 cleft in four orthogonal views. **(C)** B^0^AT1/ACE2 dimer with the spike RBD (rainbow color), but without the modeled NAb C135. **(D)** Two simultaneously modeled NAb C135 on one ACE2 dimer showing steric clashes between the constant domains and heavy/light chains of NAb C135.

Comparison with the starting configuration (conformation S) taken from the NAb C135/spike complex structure reveals how NAb C135 fully recognizes the epitope of the RBD. Recognition involves a rotation of NAb C135 in conformation A by 29.7° and then conformation B by 47.2° with two closely related axes that are similar to the rotational axis separating the conformations (Figure 5). The direction of these NAb C135 rotations relative to the RBD would place the light chain of the NAb in a partially overlapping position with both its NTD and N165 glycans within the NAb C135/spike complex. Because the RBD interacts with the N165 glycans of a neighboring NTD within the complex (Supplementary Figures S3, S6), an initial binding of NAb C135 weakens this interaction and gradually pushes the NTD away from the spike RBD to facilitate full NAb C135-epitope recognition. This frees the RBD from constraints of its inter-subunit interactions within its trimer and allows the down-to-up conversion of the RBD.

The transition from initial binding of the NAb C135 to the spike trimer to full recognition revealed in our MD simulations involves the gradual engagement of the NAb light chain *via* specific hydrogen bonds and extensive hydrophobic interactions, as the heavy chain become less engaged. The interface between the heavy chain of the NAb and the spike RBD includes an induced-fit recognition of the NAb in variable loop three of the heavy chain, *i.e.* the conformation of this loop in the complex differs significantly in the unbound NAb (Supplementary Figure S6).

The NAb C135/spike complex obtained through our MD simulations only partially represents the full NAb/epitope recognition mode because formation of the stable RBD-NAb C135 complex in its full recognition mode provides greater rotational freedom of the spike RBD relative to the remaining part of the spike trimer. With this increased rotational freedom, the stable complex gradually becomes invisible in the cryo-EM map, indicating significantly reduced local resolution. In fact, the “up” positioned RBDs always have relatively low local resolution with respect to the more stable “down” positioned RBDs in all cryo-EM structures of the spike trimer and its complexes with NAbs or ACE2.

## Discussion

Deep RNA sequencing is a novel tool to identify antibodies elicited in response to infections, including COVID-19. Typical identification of antibodies is time consuming. (Shrestha et al., 2021). In addition, antibodies identified from plasma do not provide as much detail as is gleaned from the RNA sequencing data among Ig classes and the actual amino acid sequence of the antibody. By identifying antibodies that are unique to COVID-19 survivors, we propose the CDR3 sequences most likely to positively impact infection. Utilizing this amino acid sequence created *in vivo* by a critically ill patient for models of a NAb/spike complex, we are able to establish the molecular details of the binding interaction *via* MD simulations.

The majority of prior work assessing immunoglobulin light chain types of lambda and kappa has been limited to hematologic malignancies and HIV infection. (Müller and Köhler, 1997; Terrier et al., 2014; Andrei and Wang, 2019). The impact of an increase of Ig lambda at ICU Day 3 in survivors is unknown. However, this could also be used as a potential marker of recovery. In addition, the use of an RNAseq-based workflow could enhance the study of class types in antibodies not only during infectious disease but also to bolster previous findings in hematologic malignancies and transplant medicine.

Classes of NAbs (Class 1, 2, 3, 4) against SARS-CoV-2 are catalogued based on their interactions with the spike protein, (Barnes et al., 2020), especially related to the conformation of the spike RBD to which they bind - 1) NAbs encoded by VH3-53 gene with short CDRH3 loops and bind only to “up” RBDs to block interaction with the ACE2 receptor; 2) NAbs that bind both “up” and “down” RBDs to block ACE2 and contact RBDs from adjacent spike subunits; 3) NAbs that bind outside the ACE2 site and recognize both “up” and “down” RBDs; and 4) NAbs that bind only “up” RBDs but do not block ACE2 binding. When assessing top counts of unique NAbs on Day 0 between COVID-19 survivors and non-survivors, the most abundant NAbs among survivors belong to Class 3 (including the non-ACE2 blocking C135-type described above), while the most abundant NAbs among non-survivors belong to both Class 2 and Class 4. Unexpectedly, there is no detectable difference in Class 1 ACE2-blocking NAb production between surviving and non-surviving patients. Class 4 NAbs bind the opposite side of the RBD that does not overlap with either Class 1 or Class 3 NAbs, requiring “up” positioned RBDs to expose the otherwise buried epitopes. According to our atomistic modeling, the binding of this class of NAbs could actually sterically block the partially open central pore of the RBDs and thereby physically obstructing the extension of the S2 central stalk during the prefusion-to-postfusion transition. However, this Class 4 NAbs are not expected to prevent the virus from approaching the host cell membranes, unlike Class 3.

Antibodies that bind at the ACE2 site on the spike RBD are also known to be influenced by viral mutations. (Starr et al., 2021a). With the proliferation of variants (Drews et al., 2021) as the pandemic continues, it is important to ensure that antibodies exist that are not disrupted by mutations at the ACE2-binding motif. Class 3 and 4 antibodies bind to an epitope orthogonal to the ACE2 binding site and are known to attenuate the virus in cells that overexpress ACE2. (Starr et al., 2021a). ACE inhibitors are known to increase ACE2 expression (Vaduganathan et al., 2020) but there has been no definitive correlation with patient outcome. (Reynolds et al., 2020). This lack of impact of ACE inhibitors may be due to a different class of antibodies that a patient produces in response to infection. Unfortunately, this sample size is not sufficient to assess the impact of pre-infection ACE inhibitor use. However, since NAb C135 binds to the spike protein distal to the ACE2 binding site and is produced in substantially higher levels in survivors, this suggests a unique ‘ACE2-independent’ mechanism of antibody mediated protection.

The spike protein forms a trimer where all must be in the “up” position for RBD binding to ACE2. (Lobo and Warwicker, 2021). Simulations of spike trimers showed RBD’s down-to-up transition as well as locked, stabilized “down” states, where acidic-pH confers stability to the locked structure. (Lobo and Warwicker, 2021). Class 1 and 4 NAbs are only able to bind in the “up” conformation, while Class 2 and 3 NAbs are able to bind the spike protein in both the “up” and “down” conformations, which is the most effective for neutralizing the virus. (Brouwer et al., 2020; Liu et al., 2020). It is also proposed that utilizing a spike protein in the “down” position may result more effective vaccines. (Juraszek et al., 2021).

Cellular transmission of both SARS-CoV and SARS-CoV-2 are pH (*i.e.* protonation state) dependent, as is that of many other coronaviruses. (Yang et al., 2004; Jimenez et al., 2021). At low pH, viruses can transmit locally by cell-to-cell spreading through cell fusion and endocytosis. (White et al., 1981; Schonichen et al., 2013). However, different coronaviruses have distinct pH sensors that exhibit unique pH dependencies. For example, helical repeat 1 (HR1) and helical repeat 2 (HR2) of the central stalk of the influenzae spike protein (i.e., hemagglutinin) become extended as pH decreases, (Bullough et al., 1994), which is caused by changes in protonation states of its pH-sensing residues. (Wiley and Skehel, 1987; Chen et al., 1995; Schonichen et al., 2013). However, lower pH values stabilize the spike protein of SARS-CoV-2 in the fully closed conformation with its three RBDs in the “down” position and its HR1 and HR2 of the S2 central stalk in a non-extended conformation. (Zhou et al., 2020).

Patients who are critically ill are typically acidotic. Thus, NAbs such as the Class 3 C135 NAb, which are able to efficiently bind the “down”-positioned spike RBD and to remain bound should a pH-driven conformational change occur, could be ideal therapeutics for ICU patients. The down-to-up pH-dependent equilibrium of the spike protein is also highly sensitive to spontaneous mutations. For example, the spike protein of the Omicron variant is in an open conformation at pH 7.5 (100% with one RBD “up” and two RBDs “down”) whereas the spike protein of the prototype SARS-CoV-2 (Wuhan strain plus the D614G mutation) is 50.3% in the open conformation and 49.7% in the fully closed conformation, (Ye et al., 2022), highlighting different mechanisms of cellular transmission between the two variants. Moreover, mature virions of SARS-CoV-2 are released from acidified Golgi *via* exocytosis during which the spike protein is also stabilized in the closed state. (Sicari et al., 2020a; Sicari et al., 2020b). Interestingly, fatalities from severe infection often result from non-local cell-to-cell transmission that is dependent on ACE2 while initial SARS-CoV-2 infection involves mainly local cell-to-cell transmission that is largely independent of ACE2. (Zeng et al., 2022). Thus, ACE2 blocking NAbs likely have little effect on this initial step of local cellular transmission, where only non-ACE2 blocking NAbs can exhibit dual functions, inhibiting both the local and non-local cellular transmission upon their binding. This highlights the importance of including non-ACE2 blocking NAbs in therapeutic treatments of SARS-CoV-2. Moreover, NAbs elicited in response to an immunogen of the closed state-stabilized spike trimer are distinct from those produced in response to the mRNA-based or protein-based RBD-only proteins used in current vaccine designs. (Carnell et al., 2021). Our new understanding the molecular mechanism(s) of non-ACE2 blocking NAbs could help to design the next-generation of immunogens for more effective vaccines.

Some engineered antibodies appear to already utilize sites that mimic a cocktail of both Class 1 and 4 antibodies. (Rappazzo et al., 2021). However, based on our current study, it appears that focusing on Class 3 antibodies, with further structural work, could reveal the ideal COVID-19 antibody. The widely reported Regeneron Antibody (Weinreich et al., 2020) is one of Class 3, but distinct from C135, (Kohler and Milstein, 1975) which was also elevated in COVID-19 survivors relative to non-survivors on ICU Day 0. In one patient with a low level of C135 compared to other survivors, an increase in another Class 3 antibody similar to REGN10987 was found (Supplementary Tables S2, S3), highlighting the importance of Class 3 NAbs. This suggests that if patients with low levels of Class 3 antibodies, specifically C135, are identified by RNAseq on ICU Day 0, they could be treated by supplementing other commercially available Class 3 NAbs. With further analysis of antibody profiles from patients with new SARS-CoV-2 variants and extensive structural assessments, more versatile monoclonal antibodies could be produced using recombinant techniques for treatment of critically ill patients.

Such a need is illustrated when considering a SARS-CoV-2 variant such as Omicron, with 29 single amino acid mutations in its spike protein, as well as several insertions/deletions. Nearly half of these mutations occur within the spike RBD and several at the RBM, which could alter interactions between the RBD and ACE2 that are essential for viral reentry to the host cells. Since prior biophysical studies have suggested that conformational flexibility of the RBM is essential for the stability of the RBDs in the “down” state, Omicron mutations could destabilize the “down” state, attenuating the ability of NAbs to promote this conformation and facilitating viral entry into cells. RNAseq from patients who survive an infection of future SARS-CoV-2 variants could elucidate antibodies created to solve this problem, particularly if those NAbs are capable of binding to both “up” and “down” spike conformations (i.e., such as C135). This scenario takes on heightened importance as a significant body of work has shown that NAbs specific for “wild-type” SARS-CoV-2 are less effective against variants. Mutant spike protein from SARS-CoV-2 is resistant to sera from convalescent or mRNA vaccine recipients but not to patients infected and then vaccinated. (Schmidt et al., 2021). In addition, SARS-CoV-2 variants have shown some NAbs to be less effective, requiring additional vaccine doses and highlighting the importance of both antibody development and vaccination in the care of COVID-19 patients. RNAseq with analysis for CDR3 sequences could, if optimized, uncover novel antibodies in real-time as patients are hospitalized with critical illness from future variants. RNAseq could also assess response to vaccines, especially in immunosuppressed patients, (Yahav et al., 2021), providing insight into a cocktail of antibodies that may be beneficial in treating COVID-19, in addition to ubiquitous vaccination.

## Data Availability

The coordinates for equilibrated structures derived from the MD-derived ESP maps as well as our starting model are available in the supporting materials.

## References

[B1] Acevedo M. L. Alonso-Palomares L. Bustamante A. Gaggero A. Paredes F. Cortés C. P. (2021). Infectivity and immune escape of the new SARS-CoV-2 variant of interest Lambda. medRxiv. 10.1101/2021.06.28.21259673

[B2] Andrei M. Wang J. C. (2019). Cutaneous light chain amyloidosis with multiple myeloma: A concise review. Hematol. Oncol. Stem Cell Ther. 12 (2), 71–81. 10.1016/j.hemonc.2018.09.003 30261180

[B3] Andrews S. (2014). A quality control tool for high throughput sequence data. FastQC. Available at: http://www.bioinformatics.babraham.ac.uk/projects/fastqc/ (Accessed June 16, 2020).

[B4] Barnes C. O. Jette C. A. Abernathy M. E. Dam K. M. A. Esswein S. R. Gristick H. B. (2020). SARS-CoV-2 neutralizing antibody structures inform therapeutic strategies. Nature 588 (7839), 682–687. 10.1038/s41586-020-2852-1 33045718 PMC8092461

[B5] Baum A. Fulton B. O. Wloga E. Copin R. Pascal K. E. Russo V. (2020). Antibody cocktail to SARS-CoV-2 spike protein prevents rapid mutational escape seen with individual antibodies. Science 369 (6506), 1014–1018. 10.1126/science.abd0831 32540904 PMC7299283

[B6] Brouwer P. J. M. Caniels T. G. van der Straten K. Snitselaar J. L. Aldon Y. Bangaru S. (2020). Potent neutralizing antibodies from COVID-19 patients define multiple targets of vulnerability. Science 369 (6504), 643–650. 10.1126/science.abc5902 32540902 PMC7299281

[B7] Bullough P. A. Hughson F. M. Skehel J. J. Wiley D. C. (1994). Structure of influenza haemagglutinin at the pH of membrane fusion. Nature 371 (6492), 37–43. 10.1038/371037a0 8072525

[B8] Cao Y. Su B. Guo X. Sun W. Deng Y. Bao L. (2020). Potent neutralizing antibodies against SARS-CoV-2 identified by high-throughput single-cell sequencing of convalescent patients' B cells. Cell 182 (1), 73–84. 10.1016/j.cell.2020.05.025 32425270 PMC7231725

[B9] Carnell G. W. Ciazynska K. A. Wells D. A. Xiong X. Aguinam E. T. McLaughlin S. H. (2021). SARS-CoV-2 spike protein stabilized in the closed state induces potent neutralizing responses. J. Virol. 95 (15), e0020321. 10.1128/JVI.00203-21 33963055 PMC8274612

[B10] Chen J. Wharton S. A. Weissenhorn W. Calder L. J. Hughson F. M. Skehel J. J. (1995). A soluble domain of the membrane-anchoring chain of influenza virus hemagglutinin (HA2) folds in *Escherichia coli* into the low-pH-induced conformation. Proc. Natl. Acad. Sci. U. S. A. 92 (26), 12205–12209. 10.1073/pnas.92.26.12205 8618870 PMC40325

[B11] Cornell W. D. Cieplak P. Bayly C. I. Gould I. R. Merz K. M. Ferguson D. M. (1995). A second generation force field for the simulation of proteins, nucleic acids, and organic molecules. J. Am. Chem. Soc. 117 (19), 5179–5197. 10.1021/ja00124a002

[B12] Deshpande A. Harris B. D. Martinez-Sobrido L. Kobie J. J. Walter M. R. (2021). Epitope classification and RBD binding properties of neutralizing antibodies against SARS-CoV-2 variants of concern. Front. Immunol. 12, 691715. 10.3389/fimmu.2021.691715 34149735 PMC8212047

[B13] Drews S. J. Abe K. T. Hu Q. Samson R. Gingras A. C. Colwill K. (2021). Resistance of SARS-CoV-2 Beta and Gamma variants to plasma collected from Canadian blood donors during the Spring of 2020. Transfusion 62, 37–43. 10.1111/trf.16713 34662434 PMC8662190

[B14] Garcia-Beltran W. F. Lam E. C. Astudillo M. G. Yang D. Miller T. E. Feldman J. (2021). COVID-19-neutralizing antibodies predict disease severity and survival. Cell 184 (2), 476–488.e11. 10.1016/j.cell.2020.12.015 33412089 PMC7837114

[B15] Hansen J. Baum A. Pascal K. E. Russo V. Giordano S. Wloga E. (2020). Studies in humanized mice and convalescent humans yield a SARS-CoV-2 antibody cocktail. Science 369 (6506), 1010–1014. 10.1126/science.abd0827 32540901 PMC7299284

[B16] Humphrey W. Dalke A. Schulten K. (1996). Vmd: Visual molecular dynamics. J. Mol. Graph. 14 (133-8), 33–38. 10.1016/0263-7855(96)00018-5 8744570

[B17] Jimenez L. Campos Codo A. Sampaio V. S. Oliveira A. E. R. Ferreira L. K. K. Davanzo G. G. (2021). Acid pH increases SARS-CoV-2 infection and the risk of death by COVID-19. Front. Med. 8, 637885. 10.3389/fmed.2021.637885 PMC8417536 34490283

[B18] Jo S. Kim T. Iyer V. G. Im W. (2008). CHARMM-GUI: A web-based graphical user interface for CHARMM. J. Comput. Chem. 29, 1859–1865. 10.1002/jcc.20945 18351591

[B19] Juraszek J. Rutten L. Blokland S. Bouchier P. Voorzaat R. Ritschel T. (2021). Stabilizing the closed SARS-CoV-2 spike trimer. Nat. Commun. 12 (1), 244. 10.1038/s41467-020-20321-x 33431842 PMC7801441

[B20] Kale L. Skeel R. Bhandarkar M. Brunner R. Gursoy A. Krawetz N. (1999). NAMD2: Greater scalability for parallel molecular dynamics. J. Comput. Phys. 151, 283–312. 10.1006/jcph.1999.6201

[B21] Kirchdoerfer R. N. Wang N. Pallesen J. Wrapp D. Turner H. L. Cottrell C. A. (2018). Stabilized coronavirus spikes are resistant to conformational changes induced by receptor recognition or proteolysis. Sci. Rep. 8 (1), 15701. 10.1038/s41598-018-34171-7 30356097 PMC6200764

[B22] Kohler G. Milstein C. (1975). Continuous cultures of fused cells secreting antibody of predefined specificity. Nature 256 (5517), 495–497. 10.1038/256495a0 1172191

[B23] Krause P. R. Fleming T. R. Longini I. M. Peto R. Briand S. Heymann D. L. (2021). SARS-CoV-2 variants and vaccines. N. Engl. J. Med. 385 (2), 179–186. 10.1056/NEJMsr2105280 34161052 PMC8262623

[B24] Liu L. Wang P. Nair M. S. Yu J. Rapp M. Wang Q. (2020). Potent neutralizing antibodies against multiple epitopes on SARS-CoV-2 spike. Nature 584 (7821), 450–456. 10.1038/s41586-020-2571-7 32698192

[B25] Liu L. Iketani S. Guo Y. Chan J. F. W. Wang M. Liu L. (2021). Striking antibody evasion manifested by the omicron variant of SARS-CoV-2. Nature 602, 676–681. 10.1038/s41586-021-04388-0 35016198

[B26] Lobo V. R. Warwicker J. (2021). Predicted pH-dependent stability of SARS-CoV-2 spike protein trimer from interfacial acidic groups. Comput. Struct. Biotechnol. J. 19, 5140–5148. 10.1016/j.csbj.2021.08.049 34490059 PMC8410215

[B27] Mandric I. Rotman J. Yang H. T. Strauli N. Montoya D. J. Van Der Wey W. (2020). Author Correction: Profiling immunoglobulin repertoires across multiple human tissues using RNA sequencing. Nat. Commun. 11 (1), 4499. 10.1038/s41467-020-18509-2 32887888 PMC7474053

[B28] Monaghan S. F. Fredericks A. M. Jentzsch M. S. Cioffi W. G. Cohen M. Fairbrother W. G. (2021). Deep RNA sequencing of intensive care unit patients with COVID-19. medRxiv. 10.1101/2021.01.11.21249276 PMC9491252 36130991

[B29] Müller S. Köhler H. (1997). B cell superantigens in HIV-1 infection. Int. Rev. Immunol. 14 (4), 339–349. 10.3109/08830189709116524 9186785

[B30] Phillips J. C. Braun R. Wang W. Gumbart J. Tajkhorshid E. Villa E. (2005). Scalable molecular dynamics with NAMD. J. Comput. Chem. 26 (16), 1781–1802. 10.1002/jcc.20289 16222654 PMC2486339

[B31] Rappazzo C. G. Tse L. V. Kaku C. I. Wrapp D. Sakharkar M. Huang D. (2021). Broad and potent activity against SARS-like viruses by an engineered human monoclonal antibody. Sci. (New York, NY) 371 (6531), 823–829. 10.1126/science.abf4830 PMC7963221 33495307

[B32] Reynolds H. R. Adhikari S. Pulgarin C. Troxel A. B. Iturrate E. Johnson S. B. (2020). Renin-angiotensin-aldosterone system inhibitors and risk of covid-19. N. Engl. J. Med. 382 (25), 2441–2448. 10.1056/NEJMoa2008975 32356628 PMC7206932

[B33] Robbiani D. F. Gaebler C. Muecksch F. Lorenzi J. C. C. Wang Z. Cho A. (2020). Convergent antibody responses to SARS-CoV-2 in convalescent individuals. Nature 584 (7821), 437–442. 10.1038/s41586-020-2456-9 32555388 PMC7442695

[B34] Scapin G. Yang X. Prosise W. W. McCoy M. Reichert P. Johnston J. M. (2015). Structure of full-length human anti-PD1 therapeutic IgG4 antibody pembrolizumab. Nat. Struct. Mol. Biol. 22 (12), 953–958. 10.1038/nsmb.3129 26595420

[B35] Schmidt F. Weisblum Y. Rutkowska M. Poston D. DaSilva J. Zhang F. (2021). High genetic barrier to SARS-CoV-2 polyclonal neutralizing antibody escape. Nature 600, 512–516. 10.1038/s41586-021-04005-0 34544114 PMC9241107

[B36] Schonichen A. Webb B. A. Jacobson M. P. Barber D. L. (2013). Considering protonation as a posttranslational modification regulating protein structure and function. Annu. Rev. Biophys. 42, 289–314. 10.1146/annurev-biophys-050511-102349 23451893 PMC4041481

[B37] Shrestha L. B. Tedla N. Bull R. A. (2021). Broadly-neutralizing antibodies against emerging SARS-CoV-2 variants. Front. Immunol. 12, 752003. 10.3389/fimmu.2021.752003 34646276 PMC8502962

[B38] Sicari D. Chatziioannou A. Koutsandreas T. Sitia R. Chevet E. (2020). Correction: Role of the early secretory pathway in SARS-CoV-2 infection. J. Cell Biol. 219 (9), e20200600508132020c. 10.1083/jcb.20200600508132020c PMC7480093 32841314

[B39] Sicari D. Chatziioannou A. Koutsandreas T. Sitia R. Chevet E. (2020). Role of the early secretory pathway in SARS-CoV-2 infection. J. Cell Biol. 219 (9), e202006005. 10.1083/jcb.202006005 32725137 PMC7480111

[B40] Starr T. N. Czudnochowski N. Liu Z. Zatta F. Park Y. J. Addetia A. (2021). SARS-CoV-2 RBD antibodies that maximize breadth and resistance to escape. Nature 597, 97–102. 10.1038/s41586-021-03807-6 34261126 PMC9282883

[B41] Starr T. N. Greaney A. J. Addetia A. Hannon W. W. Choudhary M. C. Dingens A. S. (2021). Prospective mapping of viral mutations that escape antibodies used to treat COVID-19. Sci. (New York, NY) 371 (6531), 850–854. 10.1126/science.abf9302 PMC7963219 33495308

[B42] Tai L. Zhu G. Yang M. Cao L. Xing X. Yin G. (2021). Nanometer-resolution *in situ* structure of the SARS-CoV-2 postfusion spike protein. Proc. Natl. Acad. Sci. U. S. A. 118 (48), e2112703118. 10.1073/pnas.2112703118 34782481 PMC8640741

[B43] Tang F. Barbacioru C. Bao S. Lee C. Nordman E. Wang X. (2010). Tracing the derivation of embryonic stem cells from the inner cell mass by single-cell RNA-Seq analysis. Cell Stem Cell 6 (5), 468–478. 10.1016/j.stem.2010.03.015 20452321 PMC2954317

[B44] Tang F. Barbacioru C. Nordman E. Li B. Xu N. Bashkirov V. I. (2010). RNA-Seq analysis to capture the transcriptome landscape of a single cell. Nat. Protoc. 5 (3), 516–535. 10.1038/nprot.2009.236 20203668 PMC3847604

[B45] Terrier B. Chaara W. Dufat L. Geri G. Rosenzwajg M. Musset L. (2014). Serum biomarker signature identifies patients with B-cell non-Hodgkin lymphoma associated with cryoglobulinemia vasculitis in chronic HCV infection. Autoimmun. Rev. 13 (3), 319–326. 10.1016/j.autrev.2013.11.001 24220267

[B46] Vaduganathan M. Vardeny O. Michel T. McMurray J. J. V. Pfeffer M. A. Solomon S. D. (2020). Renin-angiotensin-aldosterone system inhibitors in patients with covid-19. N. Engl. J. Med. 382 (17), 1653–1659. 10.1056/NEJMsr2005760 32227760 PMC7121452

[B47] Walls A. C. Park Y. J. Tortorici M. A. Wall A. McGuire A. T. Veesler D. (2020). Structure, function, and antigenicity of the SARS-CoV-2 spike glycoprotein. Cell 181 (2), 281–292. 10.1016/j.cell.2020.02.058 32155444 PMC7102599

[B48] Wang J. Maschietto F. Guberman-Pfeffer M. J. Reiss K. Allen B. Xiong Y. (2021). Computational insights into the membrane fusion mechanism of SARS-CoV-2 at the cellular level. Comput. Struct. Biotechnol. J. 19, 5019–5028. 10.1016/j.csbj.2021.08.053 34540146 PMC8442599

[B49] Wang J. Skeens E. Arantes P. R. Maschietto F. Allen B. Kyro G. W. (2022a). Structural basis for reduced dynamics of three engineered HNH endonuclease lys-to-ala mutants for the clustered regularly interspaced short palindromic repeat (CRISPR)-Associated 9 (CRISPR/Cas9) enzyme. Biochemistry 61 (9), 785–794. 10.1021/acs.biochem.2c00127 35420793 PMC9069930

[B50] Wang J. Shi Y. Reiss K. Maschietto F. Lolis E. Konigsberg W. H. (2022b). Structural insights into binding of remdesivir triphosphate within the replication-transcription complex of SARS-COV-2. Biochemistry 61, 1966–1973. 10.1021/acs.biochem.2c00341 36044776 PMC9469760

[B51] Wang J. Shi Y. Reiss K. Allen B. Maschietto F. Lolis E. (2022c). Insights into binding of single-stranded viral RNA template to the replication-transcription complex of SARS-CoV-2 for the priming reaction from molecular dynamics simulations. Biochemistry 61 (6), 424–432. 10.1021/acs.biochem.1c00755 35199520

[B52] Weinreich D. M. Sivapalasingam S. Norton T. Ali S. Gao H. Bhore R. (2020). REGN-COV2, a neutralizing antibody cocktail, in outpatients with covid-19. N. Engl. J. Med. 384 (3), 238–251. 10.1056/NEJMoa2035002 33332778 PMC7781102

[B53] White J. Matlin K. Helenius A. (1981). Cell fusion by Semliki Forest, influenza, and vesicular stomatitis viruses. J. Cell Biol. 89 (3), 674–679. 10.1083/jcb.89.3.674 6265470 PMC2111813

[B54] Wiley D. C. Skehel J. J. (1987). The structure and function of the hemagglutinin membrane glycoprotein of influenza virus. Annu. Rev. Biochem. 56, 365–394. 10.1146/annurev.bi.56.070187.002053 3304138

[B55] Willyard C. (2021). How antiviral pill molnupiravir shot ahead in the COVID drug hunt. Nature. Online ahead of print. 10.1038/d41586-021-02783-1 34625735

[B56] Wu Y. Wang F. Shen C. Peng W. Li D. Zhao C. (2020). A noncompeting pair of human neutralizing antibodies block COVID-19 virus binding to its receptor ACE2. Science 368 (6496), 1274–1278. 10.1126/science.abc2241 32404477 PMC7223722

[B57] Yahav D. Rozen-Zvi B. Mashraki T. Atamna A. Ben-Zvi H. Bar-Haim E. (2021). Immunosuppression reduction when administering a booster dose of the BNT162b2 mRNA SARS-CoV-2 vaccine in kidney transplant recipients without adequate humoral response following two vaccine doses: Protocol for a randomised controlled trial (BECAME study). BMJ open 11 (10), e055611. 10.1136/bmjopen-2021-055611 PMC8506046 34635537

[B58] Yan R. Zhang Y. Li Y. Xia L. Guo Y. Zhou Q. (2020). Structural basis for the recognition of SARS-CoV-2 by full-length human ACE2. Science 367 (6485), 1444–1448. 10.1126/science.abb2762 32132184 PMC7164635

[B59] Yang Z. Y. Huang Y. Ganesh L. Leung K. Kong W. P. Schwartz O. (2004). pH-dependent entry of severe acute respiratory syndrome coronavirus is mediated by the spike glycoprotein and enhanced by dendritic cell transfer through DC-SIGN. J. Virol. 78 (11), 5642–5650. 10.1128/JVI.78.11.5642-5650.2004 15140961 PMC415834

[B60] Yang K. Wang C. White K. I. Pfuetzner R. A. Esquivies L. Brunger A. T. (2022). Structural conservation among variants of the SARS-CoV-2 spike postfusion bundle. Proc. Natl. Acad. Sci. U. S. A. 119 (16), e2119467119. 10.1073/pnas.2119467119 35363556 PMC9169775

[B61] Ye G. Liu B. Li F. (2022). Cryo-EM structure of a SARS-CoV-2 omicron spike protein ectodomain. Nat. Commun. 13 (1), 1214. 10.1038/s41467-022-28882-9 35241675 PMC8894419

[B62] Zeng C. Evans J. P. King T. Zheng Y. M. Oltz E. M. Whelan S. P. J. (2022). SARS-CoV-2 spreads through cell-to-cell transmission. Proc. Natl. Acad. Sci. U. S. A. 119 (1), e2111400119. 10.1073/pnas.2111400119 34937699 PMC8740724

[B63] Zhou T. Tsybovsky Y. Gorman J. Rapp M. Cerutti G. Chuang G. Y. (2020). Cryo-EM structures of SARS-CoV-2 spike without and with ACE2 reveal a pH-dependent switch to mediate endosomal positioning of receptor-binding domains. Cell Host Microbe 28 (6), 867–879. 10.1016/j.chom.2020.11.004 33271067 PMC7670890

