# Supplementary material for: Identification and mechanistic basis of non-ACE2 blocking neutralizing antibodies from COVID-19 patients with deep RNA sequencing and molecular dynamics simulations
Source: Front Mol Biosci. 2022 Dec 16;9:1080964. doi: 10.3389/fmolb.2022.1080964 (PMC9800910; doi:10.3389/fmolb.2022.1080964)
Supplement: Supplementary file 1 [file DataSheet4.pdf]

```
ATOM      1  N   GLN X   1       0.000   0.000   0.000  1.00  0.00
N
ATOM      2  HT1 GLN X   1       0.825  -0.422   0.472  1.00  0.00
H
ATOM      3  HT2 GLN X   1      -0.837  -0.599   0.147  1.00  0.00
H
ATOM      4  HT3 GLN X   1      -0.217   0.945   0.374  1.00  0.00
H
ATOM      5  CA  GLN X   1       0.200   0.097  -1.444  1.00  0.00
C
ATOM      6  HA  GLN X   1      -0.677   0.510  -1.921  1.00  0.00
H
ATOM      7  CB  GLN X   1       1.301   1.230  -1.745  1.00  0.00
C
ATOM      8  HB1 GLN X   1       1.062   2.114  -1.117  1.00  0.00
H
ATOM      9  HB2 GLN X   1       1.102   1.672  -2.745  1.00  0.00
H
ATOM     10  CG  GLN X   1       2.831   0.871  -1.602  1.00  0.00
C
ATOM     11  HG1 GLN X   1       3.550   1.672  -1.878  1.00  0.00
H
ATOM     12  HG2 GLN X   1       2.990  -0.048  -2.206  1.00  0.00
H
ATOM     13  CD  GLN X   1       3.228   0.352  -0.164  1.00  0.00
C
ATOM     14  OE1 GLN X   1       2.757  -0.615   0.309  1.00  0.00
O
ATOM     15  NE2 GLN X   1       4.363   0.925   0.428  1.00  0.00
N
ATOM     16 HE21 GLN X   1       4.663   0.625   1.334  1.00  0.00
H
ATOM     17 HE22 GLN X   1       4.623   1.858   0.178  1.00  0.00
H
ATOM     18  C   GLN X   1       0.511  -1.150  -2.183  1.00  0.00
C
ATOM     19  O   GLN X   1       0.858  -2.197  -1.587  1.00  0.00
O
ATOM     20  N   VAL X   2       0.440  -1.093  -3.522  1.00  0.00
N
ATOM     21  HN  VAL X   2       0.316  -0.242  -4.027  1.00  0.00
H
ATOM     22  CA  VAL X   2       0.489  -2.225  -4.328  1.00  0.00
C
ATOM     23  HA  VAL X   2       0.438  -3.210  -3.888  1.00  0.00
H
ATOM     24  CB  VAL X   2      -0.662  -1.984  -5.338  1.00  0.00
C
ATOM     25  HB  VAL X   2      -0.485  -1.087  -5.969  1.00  0.00
H
```

```
ATOM     26  CG1 VAL X   2      -0.738  -3.209  -6.326  1.00  0.00
C
ATOM     27 HG11 VAL X   2      -0.080  -2.949  -7.182  1.00  0.00
H
ATOM     28 HG12 VAL X   2      -1.763  -3.221  -6.754  1.00  0.00
H
ATOM     29 HG13 VAL X   2      -0.630  -4.179  -5.794  1.00  0.00
H
ATOM     30  CG2 VAL X   2      -1.953  -1.751  -4.579  1.00  0.00
C
ATOM     31 HG21 VAL X   2      -2.064  -2.531  -3.796  1.00  0.00
H
ATOM     32 HG22 VAL X   2      -2.866  -1.812  -5.208  1.00  0.00
H
ATOM     33 HG23 VAL X   2      -2.071  -0.752  -4.107  1.00  0.00
H
ATOM     34  C   VAL X   2       1.852  -2.135  -5.045  1.00  0.00
C
ATOM     35  O   VAL X   2       2.123  -1.166  -5.769  1.00  0.00
O
ATOM     36  N   GLN X   3       2.678  -3.181  -4.888  1.00  0.00
N
ATOM     37  HN  GLN X   3       2.316  -3.886  -4.284  1.00  0.00
H
ATOM     38  CA  GLN X   3       3.995  -3.351  -5.524  1.00  0.00
C
ATOM     39  HA  GLN X   3       4.001  -2.882  -6.497  1.00  0.00
H
ATOM     40  CB  GLN X   3       5.111  -2.702  -4.652  1.00  0.00
C
ATOM     41  HB1 GLN X   3       5.147  -3.292  -3.711  1.00  0.00
H
ATOM     42  HB2 GLN X   3       4.848  -1.647  -4.424  1.00  0.00
H
ATOM     43  CG  GLN X   3       6.441  -2.731  -5.363  1.00  0.00
C
ATOM     44  HG1 GLN X   3       6.784  -3.786  -5.440  1.00  0.00
H
ATOM     45  HG2 GLN X   3       7.173  -2.089  -4.828  1.00  0.00
H
ATOM     46  CD  GLN X   3       6.307  -2.283  -6.757  1.00  0.00
C
ATOM     47  OE1 GLN X   3       6.312  -3.178  -7.643  1.00  0.00
O
ATOM     48  NE2 GLN X   3       6.226  -0.953  -7.043  1.00  0.00
N
ATOM     49 HE21 GLN X   3       6.097  -0.642  -7.984  1.00  0.00
H
ATOM     50 HE22 GLN X   3       6.195  -0.251  -6.331  1.00  0.00
H
```

```
ATOM      51  C   GLN X   3       4.167  −4.873  −5.518  1.00  0.00
C
ATOM      52  O   GLN X   3       3.484  −5.529  −4.710  1.00  0.00
O
ATOM      53  N   LEU X   4       4.947  −5.394  −6.448  1.00  0.00
N
ATOM      54  HN  LEU X   4       5.385  −4.826  −7.141  1.00  0.00
H
ATOM      55  CA  LEU X   4       5.159  −6.856  −6.531  1.00  0.00
C
ATOM      56  HA  LEU X   4       4.762  −7.412  −5.694  1.00  0.00
H
ATOM      57  CB  LEU X   4       4.547  −7.454  −7.859  1.00  0.00
C
ATOM      58  HB1 LEU X   4       4.878  −8.511  −7.932  1.00  0.00
H
ATOM      59  HB2 LEU X   4       5.052  −6.966  −8.720  1.00  0.00
H
ATOM      60  CG  LEU X   4       3.006  −7.453  −7.918  1.00  0.00
C
ATOM      61  HG  LEU X   4       2.647  −7.529  −6.869  1.00  0.00
H
ATOM      62  CD1 LEU X   4       2.429  −6.147  −8.580  1.00  0.00
C
ATOM      63 HD11 LEU X   4       2.808  −6.023  −9.617  1.00  0.00
H
ATOM      64 HD12 LEU X   4       2.771  −5.253  −8.015  1.00  0.00
H
ATOM      65 HD13 LEU X   4       1.320  −6.135  −8.649  1.00  0.00
H
ATOM      66  CD2 LEU X   4       2.493  −8.601  −8.758  1.00  0.00
C
ATOM      67 HD21 LEU X   4       1.382  −8.602  −8.733  1.00  0.00
H
ATOM      68 HD22 LEU X   4       2.779  −9.562  −8.280  1.00  0.00
H
ATOM      69 HD23 LEU X   4       2.771  −8.501  −9.829  1.00  0.00
H
ATOM      70  C   LEU X   4       6.647  −7.152  −6.611  1.00  0.00
C
ATOM      71  O   LEU X   4       7.549  −6.352  −6.800  1.00  0.00
O
ATOM      72  N   VAL X   5       6.924  −8.440  −6.146  1.00  0.00
N
ATOM      73  HN  VAL X   5       6.195  −8.965  −5.713  1.00  0.00
H
ATOM      74  CA  VAL X   5       8.294  −8.981  −6.070  1.00  0.00
C
ATOM      75  HA  VAL X   5       9.018  −8.181  −6.099  1.00  0.00
H
```

```
ATOM     76  CB  VAL X   5       8.479  -9.728  -4.773  1.00  0.00
C
ATOM     77  HB  VAL X   5       7.935 -10.697  -4.782  1.00  0.00
H
ATOM     78  CG1 VAL X   5       9.995 -10.052  -4.464  1.00  0.00
C
ATOM     79 HG11 VAL X   5      10.032 -10.452  -3.428  1.00  0.00
H
ATOM     80 HG12 VAL X   5      10.554  -9.106  -4.303  1.00  0.00
H
ATOM     81 HG13 VAL X   5      10.502 -10.718  -5.195  1.00  0.00
H
ATOM     82  CG2 VAL X   5       8.039  -8.762  -3.649  1.00  0.00
C
ATOM     83 HG21 VAL X   5       6.979  -8.903  -3.347  1.00  0.00
H
ATOM     84 HG22 VAL X   5       8.283  -7.699  -3.864  1.00  0.00
H
ATOM     85 HG23 VAL X   5       8.667  -9.058  -2.781  1.00  0.00
H
ATOM     86  C   VAL X   5       8.551  -9.987  -7.220  1.00  0.00
C
ATOM     87  O   VAL X   5       7.857 -10.913  -7.413  1.00  0.00
O
ATOM     88  N   GLU X   6       9.579  -9.702  -8.079  1.00  0.00
N
ATOM     89  HN  GLU X   6      10.198  -8.998  -7.739  1.00  0.00
H
ATOM     90  CA  GLU X   6       9.840 -10.394  -9.357  1.00  0.00
C
ATOM     91  HA  GLU X   6       8.932 -10.692  -9.860  1.00  0.00
H
ATOM     92  CB  GLU X   6      10.566  -9.448 -10.289  1.00  0.00
C
ATOM     93  HB1 GLU X   6      10.922  -9.993 -11.190  1.00  0.00
H
ATOM     94  HB2 GLU X   6      11.465  -9.012  -9.802  1.00  0.00
H
ATOM     95  CG  GLU X   6       9.598  -8.322 -10.710  1.00  0.00
C
ATOM     96  HG1 GLU X   6       9.168  -7.932  -9.763  1.00  0.00
H
ATOM     97  HG2 GLU X   6       8.782  -8.760 -11.323  1.00  0.00
H
ATOM     98  CD  GLU X   6      10.365  -7.142 -11.319  1.00  0.00
C
ATOM     99  OE1 GLU X   6      10.352  -5.973 -10.814  1.00  0.00
O
ATOM    100  OE2 GLU X   6      10.944  -7.405 -12.400  1.00  0.00
O
```

```
ATOM    101  C   GLU X   6      10.730 -11.652  -9.241  1.00  0.00
C
ATOM    102  O   GLU X   6      11.581 -11.729  -8.414  1.00  0.00
O
ATOM    103  N   SER X   7      10.472 -12.671 -10.121  1.00  0.00
N
ATOM    104  HN  SER X   7       9.635 -12.749 -10.657  1.00  0.00
H
ATOM    105  CA  SER X   7      11.349 -13.850 -10.326  1.00  0.00
C
ATOM    106  HA  SER X   7      12.334 -13.552  -9.997  1.00  0.00
H
ATOM    107  CB  SER X   7      10.754 -15.064  -9.589  1.00  0.00
C
ATOM    108  HB1 SER X   7      10.005 -15.570 -10.234  1.00  0.00
H
ATOM    109  HB2 SER X   7      10.167 -14.761  -8.696  1.00  0.00
H
ATOM    110  OG  SER X   7      11.742 -16.001  -9.275  1.00  0.00
O
ATOM    111  HG1 SER X   7      12.174 -15.821  -8.436  1.00  0.00
H
ATOM    112  C   SER X   7      11.529 -14.316 -11.792  1.00  0.00
C
ATOM    113  O   SER X   7      10.799 -13.800 -12.623  1.00  0.00
O
ATOM    114  N   GLY X   8      12.447 -15.216 -12.069  1.00  0.00
N
ATOM    115  HN  GLY X   8      12.953 -15.561 -11.282  1.00  0.00
H
ATOM    116  CA  GLY X   8      12.745 -15.541 -13.501  1.00  0.00
C
ATOM    117  HA1 GLY X   8      11.881 -15.744 -14.116  1.00  0.00
H
ATOM    118  HA2 GLY X   8      13.308 -16.458 -13.399  1.00  0.00
H
ATOM    119  C   GLY X   8      13.567 -14.458 -14.255  1.00  0.00
C
ATOM    120  O   GLY X   8      13.925 -13.417 -13.683  1.00  0.00
O
ATOM    121  N   GLY X   9      13.937 -14.635 -15.522  1.00  0.00
N
ATOM    122  HN  GLY X   9      13.740 -15.423 -16.101  1.00  0.00
H
ATOM    123  CA  GLY X   9      14.646 -13.676 -16.368  1.00  0.00
C
ATOM    124  HA1 GLY X   9      14.919 -12.818 -15.773  1.00  0.00
H
ATOM    125  HA2 GLY X   9      14.139 -13.388 -17.277  1.00  0.00
H
```

ATOM	126	C	GLY	X	9	15.906	-14.297	-16.837	1.00	0.00	C
ATOM	127	O	GLY	X	9	16.077	-15.505	-16.809	1.00	0.00	O
ATOM	128	N	GLY	X	10	16.862	-13.338	-17.256	1.00	0.00	N
ATOM	129	HN	GLY	X	10	16.397	-12.457	-17.300	1.00	0.00	H
ATOM	130	CA	GLY	X	10	18.241	-13.667	-17.638	1.00	0.00	C
ATOM	131	HA1	GLY	X	10	18.647	-14.214	-16.800	1.00	0.00	H
ATOM	132	HA2	GLY	X	10	18.688	-12.698	-17.804	1.00	0.00	H
ATOM	133	C	GLY	X	10	18.407	-14.237	-19.010	1.00	0.00	C
ATOM	134	O	GLY	X	10	17.502	-14.247	-19.821	1.00	0.00	O
ATOM	135	N	VAL	X	11	19.628	-14.745	-19.357	1.00	0.00	N
ATOM	136	HN	VAL	X	11	20.330	-14.706	-18.651	1.00	0.00	H
ATOM	137	CA	VAL	X	11	20.035	-15.298	-20.614	1.00	0.00	C
ATOM	138	HA	VAL	X	11	19.702	-14.654	-21.415	1.00	0.00	H
ATOM	139	CB	VAL	X	11	21.559	-15.490	-20.488	1.00	0.00	C
ATOM	140	HB	VAL	X	11	21.845	-16.235	-19.715	1.00	0.00	H
ATOM	141	CG1	VAL	X	11	22.119	-16.120	-21.726	1.00	0.00	C
ATOM	142	HG11	VAL	X	11	23.218	-16.252	-21.640	1.00	0.00	H
ATOM	143	HG12	VAL	X	11	21.952	-15.474	-22.615	1.00	0.00	H
ATOM	144	HG13	VAL	X	11	21.620	-17.097	-21.900	1.00	0.00	H
ATOM	145	CG2	VAL	X	11	22.307	-14.222	-20.109	1.00	0.00	C
ATOM	146	HG21	VAL	X	11	22.187	-13.989	-19.029	1.00	0.00	H
ATOM	147	HG22	VAL	X	11	22.061	-13.322	-20.712	1.00	0.00	H
ATOM	148	HG23	VAL	X	11	23.365	-14.403	-20.394	1.00	0.00	H
ATOM	149	C	VAL	X	11	19.345	-16.654	-20.949	1.00	0.00	C
ATOM	150	O	VAL	X	11	19.577	-17.699	-20.338	1.00	0.00	O

```
ATOM    151  N   VAL X  12      18.600 -16.586 -22.056  1.00  0.00
N
ATOM    152  HN  VAL X  12      18.523 -15.717 -22.539  1.00  0.00
H
ATOM    153  CA  VAL X  12      18.000 -17.814 -22.616  1.00  0.00
C
ATOM    154  HA  VAL X  12      18.149 -18.580 -21.870  1.00  0.00
H
ATOM    155  CB  VAL X  12      16.505 -17.584 -22.830  1.00  0.00
C
ATOM    156  HB  VAL X  12      16.291 -16.741 -23.522  1.00  0.00
H
ATOM    157  CG1 VAL X  12      15.941 -18.806 -23.541  1.00  0.00
C
ATOM    158 HG11 VAL X  12      16.119 -18.662 -24.628  1.00  0.00
H
ATOM    159 HG12 VAL X  12      14.836 -18.786 -23.431  1.00  0.00
H
ATOM    160 HG13 VAL X  12      16.386 -19.749 -23.156  1.00  0.00
H
ATOM    161  CG2 VAL X  12      15.825 -17.551 -21.452  1.00  0.00
C
ATOM    162 HG21 VAL X  12      16.161 -16.602 -20.982  1.00  0.00
H
ATOM    163 HG22 VAL X  12      16.157 -18.460 -20.906  1.00  0.00
H
ATOM    164 HG23 VAL X  12      14.723 -17.612 -21.579  1.00  0.00
H
ATOM    165  C   VAL X  12      18.685 -18.337 -23.858  1.00  0.00
C
ATOM    166  O   VAL X  12      18.751 -17.734 -24.913  1.00  0.00
O
ATOM    167  N   GLN X  13      19.145 -19.612 -23.758  1.00  0.00
N
ATOM    168  HN  GLN X  13      19.037 -20.141 -22.919  1.00  0.00
H
ATOM    169  CA  GLN X  13      19.603 -20.258 -24.960  1.00  0.00
C
ATOM    170  HA  GLN X  13      20.473 -19.672 -25.216  1.00  0.00
H
ATOM    171  CB  GLN X  13      20.179 -21.752 -24.747  1.00  0.00
C
ATOM    172  HB1 GLN X  13      20.381 -22.295 -25.695  1.00  0.00
H
ATOM    173  HB2 GLN X  13      19.293 -22.157 -24.213  1.00  0.00
H
ATOM    174  CG  GLN X  13      21.460 -21.809 -23.890  1.00  0.00
C
ATOM    175  HG1 GLN X  13      21.234 -21.378 -22.891  1.00  0.00
H
```

ATOM 176 HG2 GLN X 13 22.227 −21.262 −24.479 1.00 0.00
H
ATOM 177 CD GLN X 13 21.732 −23.226 −23.553 1.00 0.00
C
ATOM 178 OE1 GLN X 13 21.479 −23.647 −22.440 1.00 0.00
O
ATOM 179 NE2 GLN X 13 22.270 −24.026 −24.446 1.00 0.00
N
ATOM 180 HE21 GLN X 13 22.720 −23.629 −25.246 1.00 0.00
H
ATOM 181 HE22 GLN X 13 22.414 −24.965 −24.134 1.00 0.00
H
ATOM 182 C GLN X 13 18.630 −20.285 −26.166 1.00 0.00
C
ATOM 183 O GLN X 13 17.402 −20.537 −25.898 1.00 0.00
O
ATOM 184 N PRO X 14 18.960 −19.895 −27.473 1.00 0.00
N
ATOM 185 CD PRO X 14 20.040 −18.957 −27.772 1.00 0.00
C
ATOM 186 HD1 PRO X 14 20.957 −19.445 −27.380 1.00 0.00
H
ATOM 187 HD2 PRO X 14 19.881 −17.975 −27.277 1.00 0.00
H
ATOM 188 CA PRO X 14 18.073 −19.948 −28.702 1.00 0.00
C
ATOM 189 HA PRO X 14 17.424 −19.097 −28.559 1.00 0.00
H
ATOM 190 CB PRO X 14 18.981 −19.734 −29.897 1.00 0.00
C
ATOM 191 HB1 PRO X 14 18.510 −19.511 −30.878 1.00 0.00
H
ATOM 192 HB2 PRO X 14 19.669 −20.598 −30.015 1.00 0.00
H
ATOM 193 CG PRO X 14 20.111 −18.752 −29.375 1.00 0.00
C
ATOM 194 HG1 PRO X 14 21.091 −18.899 −29.877 1.00 0.00
H
ATOM 195 HG2 PRO X 14 19.770 −17.772 −29.771 1.00 0.00
H
ATOM 196 C PRO X 14 17.197 −21.157 −28.831 1.00 0.00
C
ATOM 197 O PRO X 14 17.665 −22.269 −28.644 1.00 0.00
O
ATOM 198 N GLY X 15 15.877 −20.911 −29.053 1.00 0.00
N
ATOM 199 HN GLY X 15 15.597 −19.970 −28.878 1.00 0.00
H
ATOM 200 CA GLY X 15 14.825 −21.878 −29.173 1.00 0.00
C

```
ATOM    201  HA1 GLY X  15      15.237 -22.729 -29.695  1.00  0.00
H
ATOM    202  HA2 GLY X  15      14.059 -21.276 -29.639  1.00  0.00
H
ATOM    203  C   GLY X  15      14.286 -22.495 -27.894  1.00  0.00
C
ATOM    204  O   GLY X  15      13.723 -23.578 -27.898  1.00  0.00
O
ATOM    205  N   ARG X  16      14.539 -21.873 -26.695  1.00  0.00
N
ATOM    206  HN  ARG X  16      14.990 -20.984 -26.711  1.00  0.00
H
ATOM    207  CA  ARG X  16      14.057 -22.552 -25.451  1.00  0.00
C
ATOM    208  HA  ARG X  16      13.594 -23.488 -25.726  1.00  0.00
H
ATOM    209  CB  ARG X  16      15.260 -22.881 -24.400  1.00  0.00
C
ATOM    210  HB1 ARG X  16      14.856 -23.112 -23.391  1.00  0.00
H
ATOM    211  HB2 ARG X  16      15.936 -22.000 -24.401  1.00  0.00
H
ATOM    212  CG  ARG X  16      16.167 -24.159 -24.797  1.00  0.00
C
ATOM    213  HG1 ARG X  16      15.389 -24.951 -24.827  1.00  0.00
H
ATOM    214  HG2 ARG X  16      16.824 -24.211 -23.902  1.00  0.00
H
ATOM    215  CD  ARG X  16      17.063 -23.943 -25.997  1.00  0.00
C
ATOM    216  HD1 ARG X  16      17.657 -23.016 -25.850  1.00  0.00
H
ATOM    217  HD2 ARG X  16      16.420 -23.904 -26.902  1.00  0.00
H
ATOM    218  NE  ARG X  16      17.920 -25.141 -26.124  1.00  0.00
N
ATOM    219  HE  ARG X  16      17.950 -25.815 -25.387  1.00  0.00
H
ATOM    220  CZ  ARG X  16      18.865 -25.279 -26.994  1.00  0.00
C
ATOM    221  NH1 ARG X  16      19.122 -24.477 -27.918  1.00  0.00
N
ATOM    222 HH11 ARG X  16      18.510 -23.715 -28.129  1.00  0.00
H
ATOM    223 HH12 ARG X  16      19.833 -24.695 -28.586  1.00  0.00
H
ATOM    224  NH2 ARG X  16      19.606 -26.307 -26.804  1.00  0.00
N
ATOM    225 HH21 ARG X  16      19.534 -26.676 -25.877  1.00  0.00
H
```

```
ATOM    226 HH22 ARG X  16      20.442 -26.407 -27.343  1.00  0.00
H
ATOM    227  C   ARG X  16      12.938 -21.889 -24.748  1.00  0.00
C
ATOM    228  O   ARG X  16      12.522 -20.786 -25.125  1.00  0.00
O
ATOM    229  N   SER X  17      12.374 -22.460 -23.669  1.00  0.00
N
ATOM    230  HN  SER X  17      12.768 -23.255 -23.215  1.00  0.00
H
ATOM    231  CA  SER X  17      11.119 -21.980 -23.103  1.00  0.00
C
ATOM    232  HA  SER X  17      10.683 -21.293 -23.813  1.00  0.00
H
ATOM    233  CB  SER X  17      10.158 -23.064 -22.842  1.00  0.00
C
ATOM    234  HB1 SER X  17      10.564 -23.752 -22.070  1.00  0.00
H
ATOM    235  HB2 SER X  17      10.043 -23.707 -23.741  1.00  0.00
H
ATOM    236  OG  SER X  17       8.823 -22.690 -22.317  1.00  0.00
O
ATOM    237  HG1 SER X  17       8.680 -21.775 -22.571  1.00  0.00
H
ATOM    238  C   SER X  17      11.379 -21.325 -21.722  1.00  0.00
C
ATOM    239  O   SER X  17      12.168 -21.837 -20.953  1.00  0.00
O
ATOM    240  N   LEU X  18      10.688 -20.137 -21.510  1.00  0.00
N
ATOM    241  HN  LEU X  18      10.079 -19.691 -22.161  1.00  0.00
H
ATOM    242  CA  LEU X  18      11.038 -19.477 -20.221  1.00  0.00
C
ATOM    243  HA  LEU X  18      11.764 -20.045 -19.659  1.00  0.00
H
ATOM    244  CB  LEU X  18      11.743 -18.136 -20.464  1.00  0.00
C
ATOM    245  HB1 LEU X  18      11.023 -17.416 -20.909  1.00  0.00
H
ATOM    246  HB2 LEU X  18      12.648 -18.219 -21.103  1.00  0.00
H
ATOM    247  CG  LEU X  18      12.376 -17.421 -19.221  1.00  0.00
C
ATOM    248  HG  LEU X  18      11.654 -17.385 -18.378  1.00  0.00
H
ATOM    249  CD1 LEU X  18      13.563 -18.172 -18.711  1.00  0.00
C
ATOM    250 HD11 LEU X  18      14.029 -17.574 -17.899  1.00  0.00
H
```

```
ATOM    251 HD12 LEU X  18      14.416 -18.460 -19.361  1.00  0.00
H
ATOM    252 HD13 LEU X  18      13.300 -19.115 -18.184  1.00  0.00
H
ATOM    253  CD2 LEU X  18      12.771 -15.981 -19.369  1.00  0.00
C
ATOM    254 HD21 LEU X  18      13.278 -15.526 -18.491  1.00  0.00
H
ATOM    255 HD22 LEU X  18      11.968 -15.358 -19.817  1.00  0.00
H
ATOM    256 HD23 LEU X  18      13.457 -15.899 -20.239  1.00  0.00
H
ATOM    257  C   LEU X  18       9.841 -19.254 -19.262  1.00  0.00
C
ATOM    258  O   LEU X  18       8.775 -18.815 -19.672  1.00  0.00
O
ATOM    259  N   ARG X  19      10.098 -19.351 -17.951  1.00  0.00
N
ATOM    260  HN  ARG X  19      10.971 -19.521 -17.501  1.00  0.00
H
ATOM    261  CA  ARG X  19       9.089 -18.991 -16.961  1.00  0.00
C
ATOM    262  HA  ARG X  19       8.104 -18.818 -17.368  1.00  0.00
H
ATOM    263  CB  ARG X  19       9.185 -20.200 -15.931  1.00  0.00
C
ATOM    264  HB1 ARG X  19      10.139 -20.126 -15.366  1.00  0.00
H
ATOM    265  HB2 ARG X  19       9.157 -21.218 -16.375  1.00  0.00
H
ATOM    266  CG  ARG X  19       8.006 -20.147 -14.952  1.00  0.00
C
ATOM    267  HG1 ARG X  19       7.024 -19.889 -15.404  1.00  0.00
H
ATOM    268  HG2 ARG X  19       8.085 -19.325 -14.208  1.00  0.00
H
ATOM    269  CD  ARG X  19       7.715 -21.486 -14.181  1.00  0.00
C
ATOM    270  HD1 ARG X  19       8.441 -21.653 -13.357  1.00  0.00
H
ATOM    271  HD2 ARG X  19       7.721 -22.318 -14.918  1.00  0.00
H
ATOM    272  NE  ARG X  19       6.353 -21.313 -13.461  1.00  0.00
N
ATOM    273  HE  ARG X  19       6.402 -21.394 -12.465  1.00  0.00
H
ATOM    274  CZ  ARG X  19       5.221 -21.394 -14.086  1.00  0.00
C
ATOM    275  NH1 ARG X  19       5.082 -21.394 -15.403  1.00  0.00
N
```

```
ATOM    276 HH11 ARG X  19       4.205 -21.594 -15.840  1.00  0.00
H
ATOM    277 HH12 ARG X  19       5.871 -21.056 -15.916  1.00  0.00
H
ATOM    278  NH2 ARG X  19       4.177 -21.544 -13.288  1.00  0.00
N
ATOM    279 HH21 ARG X  19       4.358 -21.707 -12.318  1.00  0.00
H
ATOM    280 HH22 ARG X  19       3.266 -21.721 -13.658  1.00  0.00
H
ATOM    281  C   ARG X  19       9.471 -17.712 -16.178  1.00  0.00
C
ATOM    282  O   ARG X  19      10.432 -17.595 -15.474  1.00  0.00
O
ATOM    283  N   LEU X  20       8.544 -16.670 -16.293  1.00  0.00
N
ATOM    284  HN  LEU X  20       7.773 -16.725 -16.922  1.00  0.00
H
ATOM    285  CA  LEU X  20       8.703 -15.310 -15.771  1.00  0.00
C
ATOM    286  HA  LEU X  20       9.620 -15.188 -15.214  1.00  0.00
H
ATOM    287  CB  LEU X  20       8.631 -14.240 -16.828  1.00  0.00
C
ATOM    288  HB1 LEU X  20       7.755 -14.359 -17.502  1.00  0.00
H
ATOM    289  HB2 LEU X  20       9.465 -14.302 -17.559  1.00  0.00
H
ATOM    290  CG  LEU X  20       8.660 -12.784 -16.276  1.00  0.00
C
ATOM    291  HG  LEU X  20       7.847 -12.586 -15.545  1.00  0.00
H
ATOM    292  CD1 LEU X  20       9.918 -12.468 -15.562  1.00  0.00
C
ATOM    293 HD11 LEU X  20       9.987 -11.403 -15.255  1.00  0.00
H
ATOM    294 HD12 LEU X  20      10.819 -12.644 -16.188  1.00  0.00
H
ATOM    295 HD13 LEU X  20      10.091 -13.188 -14.734  1.00  0.00
H
ATOM    296  CD2 LEU X  20       8.421 -11.698 -17.320  1.00  0.00
C
ATOM    297 HD21 LEU X  20       9.386 -11.494 -17.830  1.00  0.00
H
ATOM    298 HD22 LEU X  20       8.123 -10.707 -16.915  1.00  0.00
H
ATOM    299 HD23 LEU X  20       7.641 -12.041 -18.034  1.00  0.00
H
ATOM    300  C   LEU X  20       7.551 -15.156 -14.754  1.00  0.00
C
```

ATOM	301	O	LEU	X	20	6.380	−15.505	−14.996	1.00	0.00	O
ATOM	302	N	SER	X	21	7.827	−14.562	−13.561	1.00	0.00	N
ATOM	303	HN	SER	X	21	8.679	−14.165	−13.229	1.00	0.00	H
ATOM	304	CA	SER	X	21	6.771	−14.355	−12.639	1.00	0.00	C
ATOM	305	HA	SER	X	21	5.941	−13.960	−13.206	1.00	0.00	H
ATOM	306	CB	SER	X	21	6.476	−15.570	−11.764	1.00	0.00	C
ATOM	307	HB1	SER	X	21	6.182	−16.291	−12.557	1.00	0.00	H
ATOM	308	HB2	SER	X	21	5.615	−15.419	−11.078	1.00	0.00	H
ATOM	309	OG	SER	X	21	7.557	−15.860	−10.965	1.00	0.00	O
ATOM	310	HG1	SER	X	21	8.199	−16.323	−11.507	1.00	0.00	H
ATOM	311	C	SER	X	21	6.960	−13.097	−11.743	1.00	0.00	C
ATOM	312	O	SER	X	21	8.075	−12.538	−11.630	1.00	0.00	O
ATOM	313	N	CYS	X	22	5.900	−12.750	−11.024	1.00	0.00	N
ATOM	314	HN	CYS	X	22	4.970	−13.088	−11.141	1.00	0.00	H
ATOM	315	CA	CYS	X	22	5.989	−11.743	−9.953	1.00	0.00	C
ATOM	316	HA	CYS	X	22	6.874	−11.986	−9.383	1.00	0.00	H
ATOM	317	CB	CYS	X	22	6.085	−10.243	−10.496	1.00	0.00	C
ATOM	318	HB1	CYS	X	22	7.010	−10.352	−11.103	1.00	0.00	H
ATOM	319	HB2	CYS	X	22	6.475	−9.564	−9.709	1.00	0.00	H
ATOM	320	SG	CYS	X	22	4.633	−9.700	−11.478	1.00	0.00	S
ATOM	321	C	CYS	X	22	4.874	−11.973	−8.980	1.00	0.00	C
ATOM	322	O	CYS	X	22	3.856	−12.563	−9.280	1.00	0.00	O
ATOM	323	N	ALA	X	23	5.082	−11.610	−7.679	1.00	0.00	N
ATOM	324	HN	ALA	X	23	5.953	−11.248	−7.357	1.00	0.00	H
ATOM	325	CA	ALA	X	23	4.203	−12.041	−6.606	1.00	0.00	C

```
ATOM    326  HA  ALA X  23       3.313 -12.534  -6.967  1.00  0.00
H
ATOM    327  CB  ALA X  23       4.979 -12.862  -5.613  1.00  0.00
C
ATOM    328  HB1 ALA X  23       4.544 -12.838  -4.590  1.00  0.00
H
ATOM    329  HB2 ALA X  23       6.048 -12.612  -5.439  1.00  0.00
H
ATOM    330  HB3 ALA X  23       4.962 -13.922  -5.943  1.00  0.00
H
ATOM    331  C   ALA X  23       3.763 -10.784  -5.816  1.00  0.00
C
ATOM    332  O   ALA X  23       4.653  -9.969  -5.451  1.00  0.00
O
ATOM    333  N   ALA X  24       2.496 -10.673  -5.460  1.00  0.00
N
ATOM    334  HN  ALA X  24       1.893 -11.425  -5.715  1.00  0.00
H
ATOM    335  CA  ALA X  24       2.029  -9.626  -4.666  1.00  0.00
C
ATOM    336  HA  ALA X  24       2.198  -8.797  -5.337  1.00  0.00
H
ATOM    337  CB  ALA X  24       0.491  -9.664  -4.391  1.00  0.00
C
ATOM    338  HB1 ALA X  24      -0.122  -9.558  -5.311  1.00  0.00
H
ATOM    339  HB2 ALA X  24       0.110  -9.127  -3.496  1.00  0.00
H
ATOM    340  HB3 ALA X  24       0.293 -10.719  -4.103  1.00  0.00
H
ATOM    341  C   ALA X  24       2.737  -9.406  -3.277  1.00  0.00
C
ATOM    342  O   ALA X  24       2.962 -10.365  -2.533  1.00  0.00
O
ATOM    343  N   SER X  25       3.078  -8.195  -2.833  1.00  0.00
N
ATOM    344  HN  SER X  25       3.075  -7.443  -3.487  1.00  0.00
H
ATOM    345  CA  SER X  25       3.386  -7.843  -1.436  1.00  0.00
C
ATOM    346  HA  SER X  25       3.146  -8.613  -0.718  1.00  0.00
H
ATOM    347  CB  SER X  25       4.891  -7.637  -1.352  1.00  0.00
C
ATOM    348  HB1 SER X  25       5.328  -6.904  -2.064  1.00  0.00
H
ATOM    349  HB2 SER X  25       5.338  -8.590  -1.708  1.00  0.00
H
ATOM    350  OG  SER X  25       5.421  -7.303  -0.055  1.00  0.00
O
```

```
ATOM    351  HG1 SER X  25       5.237  -6.362  -0.009  1.00  0.00
H
ATOM    352  C   SER X  25       2.784  -6.515  -0.962  1.00  0.00
C
ATOM    353  O   SER X  25       3.251  -5.424  -1.263  1.00  0.00
O
ATOM    354  N   GLY X  26       1.699  -6.573  -0.131  1.00  0.00
N
ATOM    355  HN  GLY X  26       1.325  -7.459   0.133  1.00  0.00
H
ATOM    356  CA  GLY X  26       1.034  -5.441   0.584  1.00  0.00
C
ATOM    357  HA1 GLY X  26       1.445  -4.526   0.185  1.00  0.00
H
ATOM    358  HA2 GLY X  26       1.174  -5.532   1.651  1.00  0.00
H
ATOM    359  C   GLY X  26      -0.470  -5.384   0.397  1.00  0.00
C
ATOM    360  O   GLY X  26      -1.062  -4.388   0.739  1.00  0.00
O
ATOM    361  N   PHE X  27      -1.078  -6.359  -0.228  1.00  0.00
N
ATOM    362  HN  PHE X  27      -0.596  -7.210  -0.420  1.00  0.00
H
ATOM    363  CA  PHE X  27      -2.456  -6.255  -0.775  1.00  0.00
C
ATOM    364  HA  PHE X  27      -3.003  -5.656  -0.062  1.00  0.00
H
ATOM    365  CB  PHE X  27      -2.404  -5.386  -2.013  1.00  0.00
C
ATOM    366  HB1 PHE X  27      -1.812  -4.456  -1.874  1.00  0.00
H
ATOM    367  HB2 PHE X  27      -3.402  -5.045  -2.360  1.00  0.00
H
ATOM    368  CG  PHE X  27      -1.787  -6.026  -3.225  1.00  0.00
C
ATOM    369  CD1 PHE X  27      -0.359  -5.985  -3.426  1.00  0.00
C
ATOM    370  HD1 PHE X  27       0.288  -5.690  -2.613  1.00  0.00
H
ATOM    371  CE1 PHE X  27       0.142  -6.392  -4.630  1.00  0.00
C
ATOM    372  HE1 PHE X  27       1.215  -6.293  -4.701  1.00  0.00
H
ATOM    373  CZ  PHE X  27      -0.630  -6.753  -5.746  1.00  0.00
C
ATOM    374  HZ  PHE X  27      -0.213  -7.082  -6.686  1.00  0.00
H
ATOM    375  CD2 PHE X  27      -2.526  -6.665  -4.202  1.00  0.00
C
```

```
ATOM    376  HD2 PHE X  27       -3.567  -6.792  -3.944  1.00  0.00
H
ATOM    377  CE2 PHE X  27       -1.939  -7.094  -5.492  1.00  0.00
C
ATOM    378  HE2 PHE X  27       -2.393  -7.615  -6.322  1.00  0.00
H
ATOM    379  C   PHE X  27       -3.133  -7.610  -0.872  1.00  0.00
C
ATOM    380  O   PHE X  27       -2.529  -8.650  -0.687  1.00  0.00
O
ATOM    381  N   THR X  28       -4.418  -7.595  -1.195  1.00  0.00
N
ATOM    382  HN  THR X  28       -4.976  -6.768  -1.184  1.00  0.00
H
ATOM    383  CA  THR X  28       -5.154  -8.896  -1.556  1.00  0.00
C
ATOM    384  HA  THR X  28       -4.728  -9.653  -0.915  1.00  0.00
H
ATOM    385  CB  THR X  28       -6.584  -8.809  -1.141  1.00  0.00
C
ATOM    386  HB  THR X  28       -7.209  -8.422  -1.975  1.00  0.00
H
ATOM    387  OG1 THR X  28       -6.886  -7.935  -0.011  1.00  0.00
O
ATOM    388  HG1 THR X  28       -7.706  -8.265   0.362  1.00  0.00
H
ATOM    389  CG2 THR X  28       -7.199 -10.134  -0.843  1.00  0.00
C
ATOM    390 HG21 THR X  28       -8.296 -10.043  -0.694  1.00  0.00
H
ATOM    391 HG22 THR X  28       -6.783 -10.673   0.034  1.00  0.00
H
ATOM    392 HG23 THR X  28       -7.021 -10.852  -1.672  1.00  0.00
H
ATOM    393  C   THR X  28       -5.071  -9.214  -3.061  1.00  0.00
C
ATOM    394  O   THR X  28       -5.693  -8.607  -3.944  1.00  0.00
O
ATOM    395  N   PHE X  29       -4.249 -10.235  -3.463  1.00  0.00
N
ATOM    396  HN  PHE X  29       -3.730 -10.816  -2.840  1.00  0.00
H
ATOM    397  CA  PHE X  29       -4.056 -10.421  -4.935  1.00  0.00
C
ATOM    398  HA  PHE X  29       -3.564  -9.490  -5.177  1.00  0.00
H
ATOM    399  CB  PHE X  29       -3.062 -11.619  -5.088  1.00  0.00
C
ATOM    400  HB1 PHE X  29       -3.622 -12.520  -4.758  1.00  0.00
H
```

```
ATOM    401  HB2 PHE X  29      −2.140 −11.514  −4.477  1.00  0.00
H
ATOM    402  CG  PHE X  29      −2.565 −11.799  −6.532  1.00  0.00
C
ATOM    403  CD1 PHE X  29      −2.663 −13.059  −7.144  1.00  0.00
C
ATOM    404  HD1 PHE X  29      −3.179 −13.833  −6.597  1.00  0.00
H
ATOM    405  CE1 PHE X  29      −2.094 −13.383  −8.372  1.00  0.00
C
ATOM    406  HE1 PHE X  29      −2.127 −14.398  −8.739  1.00  0.00
H
ATOM    407  CZ  PHE X  29      −1.364 −12.419  −8.947  1.00  0.00
C
ATOM    408  HZ  PHE X  29      −0.868 −12.636  −9.881  1.00  0.00
H
ATOM    409  CD2 PHE X  29      −1.722 −10.810  −7.096  1.00  0.00
C
ATOM    410  HD2 PHE X  29      −1.491  −9.899  −6.563  1.00  0.00
H
ATOM    411  CE2 PHE X  29      −1.100 −11.166  −8.350  1.00  0.00
C
ATOM    412  HE2 PHE X  29      −0.421 −10.448  −8.784  1.00  0.00
H
ATOM    413  C   PHE X  29      −5.253 −10.600  −5.817  1.00  0.00
C
ATOM    414  O   PHE X  29      −5.381 −10.111  −6.923  1.00  0.00
O
ATOM    415  N   SER X  30      −6.210 −11.294  −5.209  1.00  0.00
N
ATOM    416  HN  SER X  30      −5.933 −11.704  −4.344  1.00  0.00
H
ATOM    417  CA  SER X  30      −7.542 −11.752  −5.721  1.00  0.00
C
ATOM    418  HA  SER X  30      −7.264 −12.298  −6.611  1.00  0.00
H
ATOM    419  CB  SER X  30      −8.356 −12.683  −4.731  1.00  0.00
C
ATOM    420  HB1 SER X  30      −7.669 −13.539  −4.562  1.00  0.00
H
ATOM    421  HB2 SER X  30      −9.245 −13.132  −5.224  1.00  0.00
H
ATOM    422  OG  SER X  30      −8.707 −12.041  −3.550  1.00  0.00
O
ATOM    423  HG1 SER X  30      −9.234 −11.253  −3.701  1.00  0.00
H
ATOM    424  C   SER X  30      −8.311 −10.583  −6.244  1.00  0.00
C
ATOM    425  O   SER X  30      −9.004 −10.721  −7.249  1.00  0.00
O
```

```
ATOM    426  N   SER X  31       -8.230  -9.364  -5.616  1.00  0.00
N
ATOM    427  HN  SER X  31       -7.642  -9.377  -4.811  1.00  0.00
H
ATOM    428  CA  SER X  31       -8.904  -8.075  -6.057  1.00  0.00
C
ATOM    429  HA  SER X  31       -9.966  -8.271  -6.065  1.00  0.00
H
ATOM    430  CB  SER X  31       -8.734  -6.860  -5.053  1.00  0.00
C
ATOM    431  HB1 SER X  31       -9.384  -5.995  -5.303  1.00  0.00
H
ATOM    432  HB2 SER X  31       -7.635  -6.699  -5.069  1.00  0.00
H
ATOM    433  OG  SER X  31       -8.941  -7.440  -3.742  1.00  0.00
O
ATOM    434  HG1 SER X  31       -9.000  -6.711  -3.121  1.00  0.00
H
ATOM    435  C   SER X  31       -8.439  -7.435  -7.316  1.00  0.00
C
ATOM    436  O   SER X  31       -9.203  -6.681  -7.894  1.00  0.00
O
ATOM    437  N   TYR X  32       -7.172  -7.779  -7.711  1.00  0.00
N
ATOM    438  HN  TYR X  32       -6.691  -8.597  -7.404  1.00  0.00
H
ATOM    439  CA  TYR X  32       -6.575  -7.088  -8.854  1.00  0.00
C
ATOM    440  HA  TYR X  32       -7.247  -6.307  -9.180  1.00  0.00
H
ATOM    441  CB  TYR X  32       -5.133  -6.722  -8.548  1.00  0.00
C
ATOM    442  HB1 TYR X  32       -4.650  -6.359  -9.480  1.00  0.00
H
ATOM    443  HB2 TYR X  32       -4.613  -7.596  -8.101  1.00  0.00
H
ATOM    444  CG  TYR X  32       -5.179  -5.550  -7.594  1.00  0.00
C
ATOM    445  CD1 TYR X  32       -5.398  -5.769  -6.204  1.00  0.00
C
ATOM    446  HD1 TYR X  32       -5.375  -6.761  -5.778  1.00  0.00
H
ATOM    447  CE1 TYR X  32       -5.407  -4.751  -5.210  1.00  0.00
C
ATOM    448  HE1 TYR X  32       -5.590  -4.968  -4.168  1.00  0.00
H
ATOM    449  CZ  TYR X  32       -5.308  -3.452  -5.764  1.00  0.00
C
ATOM    450  OH  TYR X  32       -5.574  -2.384  -4.977  1.00  0.00
O
```

```
ATOM    451  HH  TYR X  32      -5.727  -2.669  -4.073  1.00  0.00
H
ATOM    452  CD2 TYR X  32      -5.035  -4.255  -8.020  1.00  0.00
C
ATOM    453  HD2 TYR X  32      -5.020  -4.051  -9.080  1.00  0.00
H
ATOM    454  CE2 TYR X  32      -5.089  -3.162  -7.106  1.00  0.00
C
ATOM    455  HE2 TYR X  32      -5.005  -2.121  -7.381  1.00  0.00
H
ATOM    456  C   TYR X  32      -6.562  -7.949 -10.096  1.00  0.00
C
ATOM    457  O   TYR X  32      -6.285  -9.092 -10.089  1.00  0.00
O
ATOM    458  N   ALA X  33      -6.792  -7.173 -11.182  1.00  0.00
N
ATOM    459  HN  ALA X  33      -7.139  -6.245 -11.066  1.00  0.00
H
ATOM    460  CA  ALA X  33      -6.520  -7.572 -12.526  1.00  0.00
C
ATOM    461  HA  ALA X  33      -6.927  -8.551 -12.731  1.00  0.00
H
ATOM    462  CB  ALA X  33      -7.228  -6.696 -13.532  1.00  0.00
C
ATOM    463  HB1 ALA X  33      -7.026  -7.043 -14.567  1.00  0.00
H
ATOM    464  HB2 ALA X  33      -6.869  -5.646 -13.478  1.00  0.00
H
ATOM    465  HB3 ALA X  33      -8.333  -6.652 -13.426  1.00  0.00
H
ATOM    466  C   ALA X  33      -5.007  -7.500 -12.691  1.00  0.00
C
ATOM    467  O   ALA X  33      -4.362  -6.660 -12.016  1.00  0.00
O
ATOM    468  N   MET X  34      -4.457  -8.408 -13.461  1.00  0.00
N
ATOM    469  HN  MET X  34      -4.935  -9.179 -13.874  1.00  0.00
H
ATOM    470  CA  MET X  34      -2.944  -8.470 -13.535  1.00  0.00
C
ATOM    471  HA  MET X  34      -2.522  -7.607 -13.041  1.00  0.00
H
ATOM    472  CB  MET X  34      -2.525  -9.811 -12.927  1.00  0.00
C
ATOM    473  HB1 MET X  34      -1.484 -10.158 -13.099  1.00  0.00
H
ATOM    474  HB2 MET X  34      -3.109 -10.662 -13.339  1.00  0.00
H
ATOM    475  CG  MET X  34      -2.702  -9.687 -11.323  1.00  0.00
C
```

```
ATOM    476  HG1 MET X  34      -2.280 -10.638 -10.934  1.00  0.00
H
ATOM    477  HG2 MET X  34      -3.800  -9.620 -11.169  1.00  0.00
H
ATOM    478  SD  MET X  34      -1.744  -8.335 -10.501  1.00  0.00
S
ATOM    479  CE  MET X  34      -0.079  -8.582 -11.313  1.00  0.00
C
ATOM    480  HE1 MET X  34      -0.155  -8.081 -12.302  1.00  0.00
H
ATOM    481  HE2 MET X  34       0.064  -9.683 -11.273  1.00  0.00
H
ATOM    482  HE3 MET X  34       0.748  -8.011 -10.840  1.00  0.00
H
ATOM    483  C   MET X  34      -2.551  -8.544 -14.962  1.00  0.00
C
ATOM    484  O   MET X  34      -3.276  -9.151 -15.718  1.00  0.00
O
ATOM    485  N   HSD X  35      -1.416  -7.928 -15.324  1.00  0.00
N
ATOM    486  HN  HSD X  35      -0.796  -7.522 -14.657  1.00  0.00
H
ATOM    487  CA  HSD X  35      -0.942  -7.695 -16.694  1.00  0.00
C
ATOM    488  HA  HSD X  35      -1.355  -8.575 -17.166  1.00  0.00
H
ATOM    489  CB  HSD X  35      -1.651  -6.496 -17.474  1.00  0.00
C
ATOM    490  HB1 HSD X  35      -2.306  -6.876 -18.288  1.00  0.00
H
ATOM    491  HB2 HSD X  35      -0.813  -5.962 -17.972  1.00  0.00
H
ATOM    492  ND1 HSD X  35      -2.155  -4.108 -16.397  1.00  0.00
N
ATOM    493  HD1 HSD X  35      -1.355  -3.607 -16.726  1.00  0.00
H
ATOM    494  CG  HSD X  35      -2.423  -5.490 -16.593  1.00  0.00
C
ATOM    495  CE1 HSD X  35      -3.261  -3.478 -15.875  1.00  0.00
C
ATOM    496  HE1 HSD X  35      -3.453  -2.430 -15.648  1.00  0.00
H
ATOM    497  NE2 HSD X  35      -4.235  -4.421 -15.671  1.00  0.00
N
ATOM    498  CD2 HSD X  35      -3.675  -5.630 -16.123  1.00  0.00
C
ATOM    499  HD2 HSD X  35      -4.212  -6.569 -16.066  1.00  0.00
H
ATOM    500  C   HSD X  35       0.625  -7.600 -16.712  1.00  0.00
C
```

```
ATOM    501  O   HSD X  35       1.297  -7.454 -15.755  1.00  0.00
O
ATOM    502  N   TRP X  36       1.208  -7.682 -17.909  1.00  0.00
N
ATOM    503  HN  TRP X  36       0.647  -7.892 -18.705  1.00  0.00
H
ATOM    504  CA  TRP X  36       2.634  -7.375 -18.159  1.00  0.00
C
ATOM    505  HA  TRP X  36       2.956  -6.825 -17.288  1.00  0.00
H
ATOM    506  CB  TRP X  36       3.376  -8.695 -18.496  1.00  0.00
C
ATOM    507  HB1 TRP X  36       4.312  -8.563 -19.080  1.00  0.00
H
ATOM    508  HB2 TRP X  36       2.771  -9.233 -19.257  1.00  0.00
H
ATOM    509  CG  TRP X  36       3.424  -9.710 -17.417  1.00  0.00
C
ATOM    510  CD1 TRP X  36       2.686 -10.908 -17.386  1.00  0.00
C
ATOM    511  HD1 TRP X  36       1.892 -11.098 -18.092  1.00  0.00
H
ATOM    512  NE1 TRP X  36       3.183 -11.781 -16.381  1.00  0.00
N
ATOM    513  HE1 TRP X  36       2.895 -12.683 -16.146  1.00  0.00
H
ATOM    514  CE2 TRP X  36       4.310 -11.155 -15.815  1.00  0.00
C
ATOM    515  CD2 TRP X  36       4.513  -9.900 -16.458  1.00  0.00
C
ATOM    516  CE3 TRP X  36       5.666  -9.132 -16.123  1.00  0.00
C
ATOM    517  HE3 TRP X  36       5.877  -8.169 -16.563  1.00  0.00
H
ATOM    518  CZ3 TRP X  36       6.548  -9.587 -15.147  1.00  0.00
C
ATOM    519  HZ3 TRP X  36       7.396  -8.958 -14.920  1.00  0.00
H
ATOM    520  CZ2 TRP X  36       5.201 -11.523 -14.767  1.00  0.00
C
ATOM    521  HZ2 TRP X  36       4.832 -12.317 -14.134  1.00  0.00
H
ATOM    522  CH2 TRP X  36       6.292 -10.779 -14.405  1.00  0.00
C
ATOM    523  HH2 TRP X  36       7.030 -10.965 -13.639  1.00  0.00
H
ATOM    524  C   TRP X  36       2.862  -6.353 -19.310  1.00  0.00
C
ATOM    525  O   TRP X  36       2.081  -6.312 -20.262  1.00  0.00
O
```

```
ATOM    526  N   VAL X  37       3.933  -5.599 -19.264  1.00  0.00
N
ATOM    527  HN  VAL X  37       4.547  -5.583 -18.478  1.00  0.00
H
ATOM    528  CA  VAL X  37       4.369  -4.680 -20.258  1.00  0.00
C
ATOM    529  HA  VAL X  37       4.008  -4.885 -21.255  1.00  0.00
H
ATOM    530  CB  VAL X  37       4.071  -3.218 -19.753  1.00  0.00
C
ATOM    531  HB  VAL X  37       4.463  -3.060 -18.726  1.00  0.00
H
ATOM    532  CG1 VAL X  37       4.628  -2.173 -20.666  1.00  0.00
C
ATOM    533 HG11 VAL X  37       4.212  -1.168 -20.436  1.00  0.00
H
ATOM    534 HG12 VAL X  37       4.432  -2.339 -21.747  1.00  0.00
H
ATOM    535 HG13 VAL X  37       5.707  -1.979 -20.487  1.00  0.00
H
ATOM    536  CG2 VAL X  37       2.558  -3.096 -19.695  1.00  0.00
C
ATOM    537 HG21 VAL X  37       2.162  -3.720 -20.525  1.00  0.00
H
ATOM    538 HG22 VAL X  37       2.107  -2.088 -19.816  1.00  0.00
H
ATOM    539 HG23 VAL X  37       2.086  -3.420 -18.742  1.00  0.00
H
ATOM    540  C   VAL X  37       5.798  -4.871 -20.409  1.00  0.00
C
ATOM    541  O   VAL X  37       6.422  -5.599 -19.632  1.00  0.00
O
ATOM    542  N   ARG X  38       6.426  -4.347 -21.445  1.00  0.00
N
ATOM    543  HN  ARG X  38       5.968  -3.826 -22.161  1.00  0.00
H
ATOM    544  CA  ARG X  38       7.872  -4.413 -21.640  1.00  0.00
C
ATOM    545  HA  ARG X  38       8.393  -4.180 -20.723  1.00  0.00
H
ATOM    546  CB  ARG X  38       8.415  -5.765 -22.127  1.00  0.00
C
ATOM    547  HB1 ARG X  38       7.997  -6.532 -21.440  1.00  0.00
H
ATOM    548  HB2 ARG X  38       9.515  -5.900 -22.205  1.00  0.00
H
ATOM    549  CG  ARG X  38       7.895  -6.193 -23.597  1.00  0.00
C
ATOM    550  HG1 ARG X  38       8.280  -5.389 -24.260  1.00  0.00
H
```

```
ATOM    551  HG2 ARG X  38       6.789  -6.102 -23.652  1.00  0.00
H
ATOM    552  CD  ARG X  38       8.444  -7.504 -24.079  1.00  0.00
C
ATOM    553  HD1 ARG X  38       7.840  -8.388 -23.784  1.00  0.00
H
ATOM    554  HD2 ARG X  38       9.531  -7.620 -23.878  1.00  0.00
H
ATOM    555  NE  ARG X  38       8.453  -7.550 -25.601  1.00  0.00
N
ATOM    556  HE  ARG X  38       8.321  -6.700 -26.112  1.00  0.00
H
ATOM    557  CZ  ARG X  38       8.692  -8.657 -26.353  1.00  0.00
C
ATOM    558  NH1 ARG X  38       8.674  -9.909 -26.006  1.00  0.00
N
ATOM    559 HH11 ARG X  38       8.479 -10.168 -25.060  1.00  0.00
H
ATOM    560 HH12 ARG X  38       8.685 -10.533 -26.787  1.00  0.00
H
ATOM    561  NH2 ARG X  38       8.807  -8.477 -27.655  1.00  0.00
N
ATOM    562 HH21 ARG X  38       8.556  -7.585 -28.030  1.00  0.00
H
ATOM    563 HH22 ARG X  38       8.575  -9.230 -28.270  1.00  0.00
H
ATOM    564  C   ARG X  38       8.337  -3.382 -22.681  1.00  0.00
C
ATOM    565  O   ARG X  38       7.477  -2.823 -23.429  1.00  0.00
O
ATOM    566  N   GLN X  39       9.674  -3.271 -22.782  1.00  0.00
N
ATOM    567  HN  GLN X  39      10.262  -3.668 -22.081  1.00  0.00
H
ATOM    568  CA  GLN X  39      10.342  -2.709 -23.990  1.00  0.00
C
ATOM    569  HA  GLN X  39       9.654  -2.853 -24.810  1.00  0.00
H
ATOM    570  CB  GLN X  39      10.450  -1.208 -23.821  1.00  0.00
C
ATOM    571  HB1 GLN X  39       9.436  -0.913 -23.477  1.00  0.00
H
ATOM    572  HB2 GLN X  39      10.648  -0.727 -24.802  1.00  0.00
H
ATOM    573  CG  GLN X  39      11.506  -0.677 -22.785  1.00  0.00
C
ATOM    574  HG1 GLN X  39      12.582  -0.876 -22.978  1.00  0.00
H
ATOM    575  HG2 GLN X  39      11.324  -1.026 -21.746  1.00  0.00
H
```

ATOM	576	CD	GLN	X	39	11.507	0.871	−22.728	1.00	0.00	C
ATOM	577	OE1	GLN	X	39	11.749	1.271	−21.548	1.00	0.00	O
ATOM	578	NE2	GLN	X	39	11.223	1.644	−23.752	1.00	0.00	N
ATOM	579	HE21	GLN	X	39	11.297	2.629	−23.598	1.00	0.00	H
ATOM	580	HE22	GLN	X	39	11.111	1.277	−24.676	1.00	0.00	H
ATOM	581	C	GLN	X	39	11.784	−3.337	−24.278	1.00	0.00	C
ATOM	582	O	GLN	X	39	12.472	−3.820	−23.370	1.00	0.00	O
ATOM	583	N	ALA	X	40	12.108	−3.335	−25.529	1.00	0.00	N
ATOM	584	HN	ALA	X	40	11.627	−2.783	−26.205	1.00	0.00	H
ATOM	585	CA	ALA	X	40	13.424	−3.821	−25.914	1.00	0.00	C
ATOM	586	HA	ALA	X	40	13.745	−4.540	−25.174	1.00	0.00	H
ATOM	587	CB	ALA	X	40	13.388	−4.547	−27.281	1.00	0.00	C
ATOM	588	HB1	ALA	X	40	14.389	−4.945	−27.552	1.00	0.00	H
ATOM	589	HB2	ALA	X	40	12.987	−3.784	−27.981	1.00	0.00	H
ATOM	590	HB3	ALA	X	40	12.653	−5.378	−27.342	1.00	0.00	H
ATOM	591	C	ALA	X	40	14.354	−2.627	−25.974	1.00	0.00	C
ATOM	592	O	ALA	X	40	13.915	−1.502	−26.239	1.00	0.00	O
ATOM	593	N	PRO	X	41	15.712	−2.653	−25.804	1.00	0.00	N
ATOM	594	CD	PRO	X	41	16.336	−3.805	−25.215	1.00	0.00	C
ATOM	595	HD1	PRO	X	41	15.860	−4.761	−25.520	1.00	0.00	H
ATOM	596	HD2	PRO	X	41	16.193	−3.647	−24.125	1.00	0.00	H
ATOM	597	CA	PRO	X	41	16.670	−1.564	−25.997	1.00	0.00	C
ATOM	598	HA	PRO	X	41	16.628	−0.966	−25.099	1.00	0.00	H
ATOM	599	CB	PRO	X	41	18.029	−2.371	−26.145	1.00	0.00	C
ATOM	600	HB1	PRO	X	41	18.762	−1.772	−25.564	1.00	0.00	H

ATOM	601	HB2	PRO	X	41	18.310	−2.264	−27.215	1.00	0.00	H
ATOM	602	CG	PRO	X	41	17.767	−3.758	−25.694	1.00	0.00	C
ATOM	603	HG1	PRO	X	41	17.733	−4.503	−26.518	1.00	0.00	H
ATOM	604	HG2	PRO	X	41	18.478	−4.129	−24.925	1.00	0.00	H
ATOM	605	C	PRO	X	41	16.336	−0.523	−27.107	1.00	0.00	C
ATOM	606	O	PRO	X	41	16.197	−0.942	−28.218	1.00	0.00	O
ATOM	607	N	GLY	X	42	16.223	0.776	−26.774	1.00	0.00	N
ATOM	608	HN	GLY	X	42	16.125	1.033	−25.816	1.00	0.00	H
ATOM	609	CA	GLY	X	42	16.039	1.814	−27.815	1.00	0.00	C
ATOM	610	HA1	GLY	X	42	16.796	1.742	−28.582	1.00	0.00	H
ATOM	611	HA2	GLY	X	42	16.035	2.726	−27.237	1.00	0.00	H
ATOM	612	C	GLY	X	42	14.730	1.908	−28.512	1.00	0.00	C
ATOM	613	O	GLY	X	42	14.728	2.645	−29.487	1.00	0.00	O
ATOM	614	N	LYS	X	43	13.761	1.102	−28.150	1.00	0.00	N
ATOM	615	HN	LYS	X	43	13.983	0.403	−27.474	1.00	0.00	H
ATOM	616	CA	LYS	X	43	12.388	1.275	−28.657	1.00	0.00	C
ATOM	617	HA	LYS	X	43	12.319	2.128	−29.315	1.00	0.00	H
ATOM	618	CB	LYS	X	43	12.010	−0.086	−29.294	1.00	0.00	C
ATOM	619	HB1	LYS	X	43	10.929	−0.154	−29.542	1.00	0.00	H
ATOM	620	HB2	LYS	X	43	12.138	−0.874	−28.521	1.00	0.00	H
ATOM	621	CG	LYS	X	43	12.948	−0.593	−30.406	1.00	0.00	C
ATOM	622	HG1	LYS	X	43	12.675	−1.652	−30.599	1.00	0.00	H
ATOM	623	HG2	LYS	X	43	14.005	−0.719	−30.089	1.00	0.00	H
ATOM	624	CD	LYS	X	43	13.029	0.207	−31.742	1.00	0.00	C
ATOM	625	HD1	LYS	X	43	13.706	−0.394	−32.386	1.00	0.00	H

```
ATOM    626  HD2 LYS X  43      13.632   1.100 -31.470  1.00  0.00
H
ATOM    627  CE  LYS X  43      11.695   0.621 -32.374  1.00  0.00
C
ATOM    628  HE1 LYS X  43      11.891   1.282 -33.245  1.00  0.00
H
ATOM    629  HE2 LYS X  43      11.115   1.240 -31.656  1.00  0.00
H
ATOM    630  NZ  LYS X  43      10.998  -0.577 -32.918  1.00  0.00
N
ATOM    631  HZ1 LYS X  43      11.680  -1.275 -33.277  1.00  0.00
H
ATOM    632  HZ2 LYS X  43      10.258  -0.327 -33.605  1.00  0.00
H
ATOM    633  HZ3 LYS X  43      10.468  -1.037 -32.151  1.00  0.00
H
ATOM    634  C   LYS X  43      11.342   1.461 -27.548  1.00  0.00
C
ATOM    635  O   LYS X  43      11.621   1.090 -26.370  1.00  0.00
O
ATOM    636  N   GLY X  44      10.186   2.039 -27.883  1.00  0.00
N
ATOM    637  HN  GLY X  44      10.096   2.369 -28.820  1.00  0.00
H
ATOM    638  CA  GLY X  44       9.028   2.361 -27.000  1.00  0.00
C
ATOM    639  HA1 GLY X  44       8.271   2.805 -27.630  1.00  0.00
H
ATOM    640  HA2 GLY X  44       9.264   3.029 -26.186  1.00  0.00
H
ATOM    641  C   GLY X  44       8.409   1.127 -26.318  1.00  0.00
C
ATOM    642  O   GLY X  44       8.703  -0.032 -26.527  1.00  0.00
O
ATOM    643  N   LEU X  45       7.514   1.432 -25.358  1.00  0.00
N
ATOM    644  HN  LEU X  45       7.340   2.350 -25.010  1.00  0.00
H
ATOM    645  CA  LEU X  45       6.909   0.313 -24.575  1.00  0.00
C
ATOM    646  HA  LEU X  45       7.653  -0.454 -24.424  1.00  0.00
H
ATOM    647  CB  LEU X  45       6.392   0.797 -23.181  1.00  0.00
C
ATOM    648  HB1 LEU X  45       5.829  -0.005 -22.659  1.00  0.00
H
ATOM    649  HB2 LEU X  45       5.695   1.649 -23.333  1.00  0.00
H
ATOM    650  CG  LEU X  45       7.526   1.257 -22.319  1.00  0.00
C
```

```
ATOM    651  HG  LEU X  45       8.493   0.876 -22.711  1.00  0.00
H
ATOM    652  CD1 LEU X  45       7.445   2.779 -22.213  1.00  0.00
C
ATOM    653 HD11 LEU X  45       6.431   2.966 -21.799  1.00  0.00
H
ATOM    654 HD12 LEU X  45       7.561   3.388 -23.135  1.00  0.00
H
ATOM    655 HD13 LEU X  45       8.247   3.205 -21.573  1.00  0.00
H
ATOM    656  CD2 LEU X  45       7.511   0.622 -20.913  1.00  0.00
C
ATOM    657 HD21 LEU X  45       7.831  -0.442 -20.872  1.00  0.00
H
ATOM    658 HD22 LEU X  45       6.481   0.601 -20.497  1.00  0.00
H
ATOM    659 HD23 LEU X  45       8.147   1.210 -20.217  1.00  0.00
H
ATOM    660  C   LEU X  45       5.764  -0.371 -25.385  1.00  0.00
C
ATOM    661  O   LEU X  45       5.156   0.200 -26.341  1.00  0.00
O
ATOM    662  N   GLU X  46       5.514  -1.660 -25.057  1.00  0.00
N
ATOM    663  HN  GLU X  46       6.050  -2.217 -24.427  1.00  0.00
H
ATOM    664  CA  GLU X  46       4.414  -2.435 -25.699  1.00  0.00
C
ATOM    665  HA  GLU X  46       3.688  -1.798 -26.183  1.00  0.00
H
ATOM    666  CB  GLU X  46       4.923  -3.399 -26.786  1.00  0.00
C
ATOM    667  HB1 GLU X  46       4.053  -4.031 -27.065  1.00  0.00
H
ATOM    668  HB2 GLU X  46       5.666  -4.012 -26.232  1.00  0.00
H
ATOM    669  CG  GLU X  46       5.320  -2.567 -28.050  1.00  0.00
C
ATOM    670  HG1 GLU X  46       6.099  -1.811 -27.816  1.00  0.00
H
ATOM    671  HG2 GLU X  46       4.488  -1.941 -28.437  1.00  0.00
H
ATOM    672  CD  GLU X  46       5.807  -3.500 -29.030  1.00  0.00
C
ATOM    673  OE1 GLU X  46       5.372  -3.496 -30.201  1.00  0.00
O
ATOM    674  OE2 GLU X  46       6.606  -4.348 -28.669  1.00  0.00
O
ATOM    675  C   GLU X  46       3.688  -3.282 -24.677  1.00  0.00
C
```

ATOM 676 O GLU X 46 4.306 −3.916 −23.792 1.00 0.00
O
ATOM 677 N TRP X 47 2.365 −3.263 −24.653 1.00 0.00
N
ATOM 678 HN TRP X 47 1.954 −2.706 −25.370 1.00 0.00
H
ATOM 679 CA TRP X 47 1.541 −4.176 −23.819 1.00 0.00
C
ATOM 680 HA TRP X 47 1.923 −4.255 −22.812 1.00 0.00
H
ATOM 681 CB TRP X 47 0.174 −3.488 −23.720 1.00 0.00
C
ATOM 682 HB1 TRP X 47 −0.426 −3.440 −24.655 1.00 0.00
H
ATOM 683 HB2 TRP X 47 0.270 −2.445 −23.352 1.00 0.00
H
ATOM 684 CG TRP X 47 −0.792 −4.192 −22.793 1.00 0.00
C
ATOM 685 CD1 TRP X 47 −1.143 −3.734 −21.600 1.00 0.00
C
ATOM 686 HD1 TRP X 47 −0.656 −2.917 −21.089 1.00 0.00
H
ATOM 687 NE1 TRP X 47 −2.264 −4.447 −21.112 1.00 0.00
N
ATOM 688 HE1 TRP X 47 −2.815 −4.028 −20.424 1.00 0.00
H
ATOM 689 CE2 TRP X 47 −2.667 −5.319 −22.058 1.00 0.00
C
ATOM 690 CD2 TRP X 47 −1.723 −5.253 −23.129 1.00 0.00
C
ATOM 691 CE3 TRP X 47 −1.897 −6.054 −24.228 1.00 0.00
C
ATOM 692 HE3 TRP X 47 −1.228 −5.942 −25.068 1.00 0.00
H
ATOM 693 CZ3 TRP X 47 −2.973 −6.949 −24.299 1.00 0.00
C
ATOM 694 HZ3 TRP X 47 −3.025 −7.696 −25.078 1.00 0.00
H
ATOM 695 CZ2 TRP X 47 −3.844 −6.029 −22.257 1.00 0.00
C
ATOM 696 HZ2 TRP X 47 −4.585 −5.901 −21.482 1.00 0.00
H
ATOM 697 CH2 TRP X 47 −3.916 −6.928 −23.312 1.00 0.00
C
ATOM 698 HH2 TRP X 47 −4.751 −7.607 −23.400 1.00 0.00
H
ATOM 699 C TRP X 47 1.551 −5.614 −24.282 1.00 0.00
C
ATOM 700 O TRP X 47 1.377 −5.989 −25.472 1.00 0.00
O

```
ATOM    701  N   VAL X  48       1.685  -6.546 -23.342  1.00  0.00
N
ATOM    702  HN  VAL X  48       2.007  -6.306 -22.430  1.00  0.00
H
ATOM    703  CA  VAL X  48       1.898  -7.994 -23.579  1.00  0.00
C
ATOM    704  HA  VAL X  48       2.324  -8.131 -24.562  1.00  0.00
H
ATOM    705  CB  VAL X  48       2.860  -8.680 -22.623  1.00  0.00
C
ATOM    706  HB  VAL X  48       2.643  -8.616 -21.535  1.00  0.00
H
ATOM    707  CG1 VAL X  48       3.051 -10.124 -23.001  1.00  0.00
C
ATOM    708 HG11 VAL X  48       3.781 -10.700 -22.395  1.00  0.00
H
ATOM    709 HG12 VAL X  48       3.270 -10.261 -24.082  1.00  0.00
H
ATOM    710 HG13 VAL X  48       2.100 -10.697 -23.039  1.00  0.00
H
ATOM    711  CG2 VAL X  48       4.193  -7.880 -22.776  1.00  0.00
C
ATOM    712 HG21 VAL X  48       4.842  -8.468 -22.092  1.00  0.00
H
ATOM    713 HG22 VAL X  48       4.274  -6.898 -22.264  1.00  0.00
H
ATOM    714 HG23 VAL X  48       4.657  -7.901 -23.785  1.00  0.00
H
ATOM    715  C   VAL X  48       0.537  -8.725 -23.458  1.00  0.00
C
ATOM    716  O   VAL X  48       0.012  -9.370 -24.379  1.00  0.00
O
ATOM    717  N   ALA X  49       0.040  -8.841 -22.178  1.00  0.00
N
ATOM    718  HN  ALA X  49       0.558  -8.437 -21.427  1.00  0.00
H
ATOM    719  CA  ALA X  49      -1.151  -9.640 -21.924  1.00  0.00
C
ATOM    720  HA  ALA X  49      -1.975  -9.334 -22.551  1.00  0.00
H
ATOM    721  CB  ALA X  49      -0.805 -11.149 -21.922  1.00  0.00
C
ATOM    722  HB1 ALA X  49      -0.248 -11.419 -22.845  1.00  0.00
H
ATOM    723  HB2 ALA X  49      -1.765 -11.700 -21.834  1.00  0.00
H
ATOM    724  HB3 ALA X  49      -0.054 -11.325 -21.122  1.00  0.00
H
ATOM    725  C   ALA X  49      -1.826  -9.247 -20.587  1.00  0.00
C
```

ATOM 726 O ALA X 49 −1.195 −8.767 −19.698 1.00 0.00
O
ATOM 727 N VAL X 50 −3.199 −9.507 −20.358 1.00 0.00
N
ATOM 728 HN VAL X 50 −3.814 −9.798 −21.087 1.00 0.00
H
ATOM 729 CA VAL X 50 −3.959 −9.454 −19.111 1.00 0.00
C
ATOM 730 HA VAL X 50 −3.341 −9.178 −18.269 1.00 0.00
H
ATOM 731 CB VAL X 50 −5.158 −8.509 −19.189 1.00 0.00
C
ATOM 732 HB VAL X 50 −4.752 −7.495 −19.393 1.00 0.00
H
ATOM 733 CG1 VAL X 50 −6.160 −8.692 −20.330 1.00 0.00
C
ATOM 734 HG11 VAL X 50 −5.601 −8.663 −21.290 1.00 0.00
H
ATOM 735 HG12 VAL X 50 −7.008 −8.006 −20.122 1.00 0.00
H
ATOM 736 HG13 VAL X 50 −6.592 −9.715 −20.284 1.00 0.00
H
ATOM 737 CG2 VAL X 50 −5.916 −8.378 −17.857 1.00 0.00
C
ATOM 738 HG21 VAL X 50 −6.405 −7.381 −17.810 1.00 0.00
H
ATOM 739 HG22 VAL X 50 −5.216 −8.295 −16.998 1.00 0.00
H
ATOM 740 HG23 VAL X 50 −6.625 −9.222 −17.720 1.00 0.00
H
ATOM 741 C VAL X 50 −4.406 −10.945 −18.653 1.00 0.00
C
ATOM 742 O VAL X 50 −4.934 −11.765 −19.371 1.00 0.00
O
ATOM 743 N ILE X 51 −4.266 −11.200 −17.335 1.00 0.00
N
ATOM 744 HN ILE X 51 −3.778 −10.613 −16.694 1.00 0.00
H
ATOM 745 CA ILE X 51 −5.049 −12.307 −16.893 1.00 0.00
C
ATOM 746 HA ILE X 51 −5.629 −12.799 −17.660 1.00 0.00
H
ATOM 747 CB ILE X 51 −4.161 −13.522 −16.406 1.00 0.00
C
ATOM 748 HB ILE X 51 −3.685 −13.882 −17.343 1.00 0.00
H
ATOM 749 CG2 ILE X 51 −3.260 −13.096 −15.238 1.00 0.00
C
ATOM 750 HG21 ILE X 51 −3.901 −12.839 −14.368 1.00 0.00
H

```
ATOM    751 HG22 ILE X  51      -2.556 -12.266 -15.463  1.00  0.00
H
ATOM    752 HG23 ILE X  51      -2.559 -13.930 -15.022  1.00  0.00
H
ATOM    753  CG1 ILE X  51      -5.044 -14.680 -15.940  1.00  0.00
C
ATOM    754 HG11 ILE X  51      -5.724 -14.425 -15.099  1.00  0.00
H
ATOM    755 HG12 ILE X  51      -4.328 -15.448 -15.576  1.00  0.00
H
ATOM    756  CD  ILE X  51      -5.730 -15.322 -17.270  1.00  0.00
C
ATOM    757  HD1 ILE X  51      -6.204 -16.311 -17.096  1.00  0.00
H
ATOM    758  HD2 ILE X  51      -4.865 -15.547 -17.929  1.00  0.00
H
ATOM    759  HD3 ILE X  51      -6.409 -14.583 -17.747  1.00  0.00
H
ATOM    760  C   ILE X  51      -5.946 -11.623 -15.851  1.00  0.00
C
ATOM    761  O   ILE X  51      -5.466 -11.100 -14.862  1.00  0.00
O
ATOM    762  N   PRO X  52      -7.287 -11.648 -16.088  1.00  0.00
N
ATOM    763  CD  PRO X  52      -7.845 -11.812 -17.417  1.00  0.00
C
ATOM    764  HD1 PRO X  52      -7.842 -12.899 -17.651  1.00  0.00
H
ATOM    765  HD2 PRO X  52      -7.157 -11.294 -18.119  1.00  0.00
H
ATOM    766  CA  PRO X  52      -8.269 -11.225 -15.171  1.00  0.00
C
ATOM    767  HA  PRO X  52      -8.087 -10.172 -15.015  1.00  0.00
H
ATOM    768  CB  PRO X  52      -9.601 -11.648 -15.950  1.00  0.00
C
ATOM    769  HB1 PRO X  52     -10.516 -11.223 -15.484  1.00  0.00
H
ATOM    770  HB2 PRO X  52      -9.830 -12.726 -15.806  1.00  0.00
H
ATOM    771  CG  PRO X  52      -9.284 -11.247 -17.348  1.00  0.00
C
ATOM    772  HG1 PRO X  52     -10.007 -11.756 -18.021  1.00  0.00
H
ATOM    773  HG2 PRO X  52      -9.302 -10.170 -17.621  1.00  0.00
H
ATOM    774  C   PRO X  52      -8.295 -11.872 -13.763  1.00  0.00
C
ATOM    775  O   PRO X  52      -7.385 -12.554 -13.408  1.00  0.00
O
```

```
ATOM    776  N   PHE X  53      -9.273 -11.437 -12.873  1.00  0.00
N
ATOM    777  HN  PHE X  53      -9.979 -10.795 -13.163  1.00  0.00
H
ATOM    778  CA  PHE X  53      -9.266 -11.781 -11.426  1.00  0.00
C
ATOM    779  HA  PHE X  53      -8.423 -11.263 -10.993  1.00  0.00
H
ATOM    780  CB  PHE X  53     -10.544 -11.181 -10.668  1.00  0.00
C
ATOM    781  HB1 PHE X  53     -10.257 -11.067  -9.601  1.00  0.00
H
ATOM    782  HB2 PHE X  53     -11.443 -11.827 -10.764  1.00  0.00
H
ATOM    783  CG  PHE X  53     -11.002  -9.812 -11.143  1.00  0.00
C
ATOM    784  CD1 PHE X  53     -10.235  -8.705 -10.688  1.00  0.00
C
ATOM    785  HD1 PHE X  53      -9.373  -8.837 -10.052  1.00  0.00
H
ATOM    786  CE1 PHE X  53     -10.515  -7.367 -11.059  1.00  0.00
C
ATOM    787  HE1 PHE X  53      -9.866  -6.617 -10.632  1.00  0.00
H
ATOM    788  CZ  PHE X  53     -11.470  -7.078 -11.971  1.00  0.00
C
ATOM    789  HZ  PHE X  53     -11.706  -6.051 -12.210  1.00  0.00
H
ATOM    790  CD2 PHE X  53     -12.044  -9.526 -12.023  1.00  0.00
C
ATOM    791  HD2 PHE X  53     -12.501 -10.376 -12.507  1.00  0.00
H
ATOM    792  CE2 PHE X  53     -12.326  -8.266 -12.467  1.00  0.00
C
ATOM    793  HE2 PHE X  53     -13.101  -7.965 -13.157  1.00  0.00
H
ATOM    794  C   PHE X  53      -9.187 -13.294 -11.182  1.00  0.00
C
ATOM    795  O   PHE X  53      -8.475 -13.736 -10.301  1.00  0.00
O
ATOM    796  N   ASP X  54      -9.908 -14.097 -11.968  1.00  0.00
N
ATOM    797  HN  ASP X  54     -10.510 -13.705 -12.659  1.00  0.00
H
ATOM    798  CA  ASP X  54      -9.942 -15.549 -11.823  1.00  0.00
C
ATOM    799  HA  ASP X  54      -9.506 -15.844 -10.880  1.00  0.00
H
ATOM    800  CB  ASP X  54     -11.379 -16.126 -11.858  1.00  0.00
C
```

```
ATOM    801  HB1 ASP X  54     -11.381 -17.235 -11.921  1.00  0.00
H
ATOM    802  HB2 ASP X  54     -11.796 -15.587 -12.735  1.00  0.00
H
ATOM    803  CG  ASP X  54     -12.212 -15.860 -10.659  1.00  0.00
C
ATOM    804  OD1 ASP X  54     -11.742 -15.446  -9.552  1.00  0.00
O
ATOM    805  OD2 ASP X  54     -13.479 -16.056 -10.848  1.00  0.00
O
ATOM    806  C   ASP X  54      -9.048 -16.106 -12.903  1.00  0.00
C
ATOM    807  O   ASP X  54      -8.314 -17.044 -12.672  1.00  0.00
O
ATOM    808  N   GLY X  55      -9.250 -15.570 -14.117  1.00  0.00
N
ATOM    809  HN  GLY X  55      -9.941 -14.880 -14.317  1.00  0.00
H
ATOM    810  CA  GLY X  55      -8.595 -15.920 -15.385  1.00  0.00
C
ATOM    811  HA1 GLY X  55      -7.674 -16.430 -15.145  1.00  0.00
H
ATOM    812  HA2 GLY X  55      -8.375 -15.008 -15.919  1.00  0.00
H
ATOM    813  C   GLY X  55      -9.250 -16.824 -16.290  1.00  0.00
C
ATOM    814  O   GLY X  55      -8.634 -17.507 -17.107  1.00  0.00
O
ATOM    815  N   ARG X  56     -10.573 -16.898 -16.300  1.00  0.00
N
ATOM    816  HN  ARG X  56     -11.029 -16.203 -15.749  1.00  0.00
H
ATOM    817  CA  ARG X  56     -11.327 -17.701 -17.303  1.00  0.00
C
ATOM    818  HA  ARG X  56     -10.846 -18.667 -17.355  1.00  0.00
H
ATOM    819  CB  ARG X  56     -12.778 -17.835 -16.903  1.00  0.00
C
ATOM    820  HB1 ARG X  56     -13.294 -18.460 -17.663  1.00  0.00
H
ATOM    821  HB2 ARG X  56     -13.362 -16.893 -16.829  1.00  0.00
H
ATOM    822  CG  ARG X  56     -12.895 -18.409 -15.489  1.00  0.00
C
ATOM    823  HG1 ARG X  56     -13.983 -18.346 -15.270  1.00  0.00
H
ATOM    824  HG2 ARG X  56     -12.379 -17.814 -14.706  1.00  0.00
H
ATOM    825  CD  ARG X  56     -12.624 -19.907 -15.255  1.00  0.00
C
```

```
ATOM    826  HD1 ARG X  56      -11.539 -20.032 -15.462  1.00  0.00
H
ATOM    827  HD2 ARG X  56      -13.146 -20.504 -16.033  1.00  0.00
H
ATOM    828  NE  ARG X  56      -13.144 -20.281 -13.912  1.00  0.00
N
ATOM    829  HE  ARG X  56      -13.231 -19.580 -13.204  1.00  0.00
H
ATOM    830  CZ  ARG X  56      -12.539 -21.376 -13.346  1.00  0.00
C
ATOM    831  NH1 ARG X  56      -12.049 -21.205 -12.114  1.00  0.00
N
ATOM    832 HH11 ARG X  56      -12.320 -20.350 -11.672  1.00  0.00
H
ATOM    833 HH12 ARG X  56      -11.723 -21.990 -11.587  1.00  0.00
H
ATOM    834  NH2 ARG X  56      -12.444 -22.558 -13.964  1.00  0.00
N
ATOM    835 HH21 ARG X  56      -12.697 -22.549 -14.931  1.00  0.00
H
ATOM    836 HH22 ARG X  56      -12.165 -23.325 -13.386  1.00  0.00
H
ATOM    837  C   ARG X  56      -11.161 -17.292 -18.810  1.00  0.00
C
ATOM    838  O   ARG X  56      -11.119 -18.142 -19.678  1.00  0.00
O
ATOM    839  N   ASN X  57      -11.156 -15.968 -19.048  1.00  0.00
N
ATOM    840  HN  ASN X  57      -11.228 -15.395 -18.235  1.00  0.00
H
ATOM    841  CA  ASN X  57      -10.806 -15.326 -20.309  1.00  0.00
C
ATOM    842  HA  ASN X  57      -10.667 -16.132 -21.014  1.00  0.00
H
ATOM    843  CB  ASN X  57      -11.874 -14.342 -20.778  1.00  0.00
C
ATOM    844  HB1 ASN X  57      -11.656 -13.975 -21.804  1.00  0.00
H
ATOM    845  HB2 ASN X  57      -12.041 -13.509 -20.063  1.00  0.00
H
ATOM    846  CG  ASN X  57      -13.250 -15.014 -20.949  1.00  0.00
C
ATOM    847  OD1 ASN X  57      -14.234 -14.441 -20.539  1.00  0.00
O
ATOM    848  ND2 ASN X  57      -13.362 -16.216 -21.521  1.00  0.00
N
ATOM    849 HD21 ASN X  57      -12.521 -16.732 -21.677  1.00  0.00
H
ATOM    850 HD22 ASN X  57      -14.274 -16.318 -21.920  1.00  0.00
H
```

ATOM 851 C ASN X 57 −9.484 −14.679 −20.219 1.00 0.00
C
ATOM 852 O ASN X 57 −9.079 −14.139 −19.192 1.00 0.00
O
ATOM 853 N LYS X 58 −8.650 −14.793 −21.312 1.00 0.00
N
ATOM 854 HN LYS X 58 −9.043 −15.147 −22.157 1.00 0.00
H
ATOM 855 CA LYS X 58 −7.262 −14.348 −21.299 1.00 0.00
C
ATOM 856 HA LYS X 58 −7.018 −13.776 −20.416 1.00 0.00
H
ATOM 857 CB LYS X 58 −6.274 −15.503 −21.329 1.00 0.00
C
ATOM 858 HB1 LYS X 58 −5.244 −15.101 −21.223 1.00 0.00
H
ATOM 859 HB2 LYS X 58 −6.179 −16.077 −22.275 1.00 0.00
H
ATOM 860 CG LYS X 58 −6.452 −16.620 −20.354 1.00 0.00
C
ATOM 861 HG1 LYS X 58 −7.386 −17.181 −20.568 1.00 0.00
H
ATOM 862 HG2 LYS X 58 −6.613 −16.139 −19.365 1.00 0.00
H
ATOM 863 CD LYS X 58 −5.255 −17.636 −20.271 1.00 0.00
C
ATOM 864 HD1 LYS X 58 −4.287 −17.136 −20.056 1.00 0.00
H
ATOM 865 HD2 LYS X 58 −5.062 −18.046 −21.285 1.00 0.00
H
ATOM 866 CE LYS X 58 −5.205 −18.812 −19.256 1.00 0.00
C
ATOM 867 HE1 LYS X 58 −6.118 −19.444 −19.246 1.00 0.00
H
ATOM 868 HE2 LYS X 58 −5.101 −18.483 −18.200 1.00 0.00
H
ATOM 869 NZ LYS X 58 −4.063 −19.695 −19.512 1.00 0.00
N
ATOM 870 HZ1 LYS X 58 −4.044 −20.205 −20.418 1.00 0.00
H
ATOM 871 HZ2 LYS X 58 −3.857 −20.364 −18.743 1.00 0.00
H
ATOM 872 HZ3 LYS X 58 −3.224 −19.082 −19.470 1.00 0.00
H
ATOM 873 C LYS X 58 −6.921 −13.546 −22.569 1.00 0.00
C
ATOM 874 O LYS X 58 −7.008 −13.999 −23.688 1.00 0.00
O
ATOM 875 N TYR X 59 −6.477 −12.305 −22.418 1.00 0.00
N

```
ATOM    876  HN  TYR X  59      -6.447 -11.824 -21.545  1.00  0.00
H
ATOM    877  CA  TYR X  59      -6.238 -11.400 -23.543  1.00  0.00
C
ATOM    878  HA  TYR X  59      -6.490 -11.964 -24.429  1.00  0.00
H
ATOM    879  CB  TYR X  59      -7.151 -10.156 -23.640  1.00  0.00
C
ATOM    880  HB1 TYR X  59      -7.050  -9.696 -24.646  1.00  0.00
H
ATOM    881  HB2 TYR X  59      -6.816  -9.340 -22.965  1.00  0.00
H
ATOM    882  CG  TYR X  59      -8.553 -10.554 -23.449  1.00  0.00
C
ATOM    883  CD1 TYR X  59      -9.182 -10.362 -22.202  1.00  0.00
C
ATOM    884  HD1 TYR X  59      -8.691  -9.884 -21.366  1.00  0.00
H
ATOM    885  CE1 TYR X  59     -10.511 -10.828 -22.058  1.00  0.00
C
ATOM    886  HE1 TYR X  59     -11.012 -10.585 -21.133  1.00  0.00
H
ATOM    887  CZ  TYR X  59     -11.249 -11.356 -23.108  1.00  0.00
C
ATOM    888  OH  TYR X  59     -12.621 -11.581 -22.948  1.00  0.00
O
ATOM    889  HH  TYR X  59     -12.973 -12.365 -23.376  1.00  0.00
H
ATOM    890  CD2 TYR X  59      -9.244 -11.099 -24.485  1.00  0.00
C
ATOM    891  HD2 TYR X  59      -8.651 -11.362 -25.348  1.00  0.00
H
ATOM    892  CE2 TYR X  59     -10.592 -11.487 -24.335  1.00  0.00
C
ATOM    893  HE2 TYR X  59     -11.048 -11.960 -25.192  1.00  0.00
H
ATOM    894  C   TYR X  59      -4.806 -11.148 -23.639  1.00  0.00
C
ATOM    895  O   TYR X  59      -4.177 -10.729 -22.688  1.00  0.00
O
ATOM    896  N   TYR X  60      -4.291 -11.304 -24.881  1.00  0.00
N
ATOM    897  HN  TYR X  60      -4.975 -11.732 -25.467  1.00  0.00
H
ATOM    898  CA  TYR X  60      -2.965 -11.205 -25.352  1.00  0.00
C
ATOM    899  HA  TYR X  60      -2.427 -10.918 -24.460  1.00  0.00
H
ATOM    900  CB  TYR X  60      -2.447 -12.646 -26.011  1.00  0.00
C
```

ATOM 901 HB1 TYR X 60 −1.420 −12.490 −26.406 1.00 0.00
H
ATOM 902 HB2 TYR X 60 −3.058 −13.016 −26.862 1.00 0.00
H
ATOM 903 CG TYR X 60 −2.472 −13.868 −25.107 1.00 0.00
C
ATOM 904 CD1 TYR X 60 −3.622 −14.604 −24.904 1.00 0.00
C
ATOM 905 HD1 TYR X 60 −4.514 −14.363 −25.463 1.00 0.00
H
ATOM 906 CE1 TYR X 60 −3.661 −15.699 −24.074 1.00 0.00
C
ATOM 907 HE1 TYR X 60 −4.621 −16.159 −23.896 1.00 0.00
H
ATOM 908 CZ TYR X 60 −2.513 −16.053 −23.339 1.00 0.00
C
ATOM 909 OH TYR X 60 −2.661 −17.243 −22.569 1.00 0.00
O
ATOM 910 HH TYR X 60 −3.396 −17.694 −22.991 1.00 0.00
H
ATOM 911 CD2 TYR X 60 −1.278 −14.333 −24.495 1.00 0.00
C
ATOM 912 HD2 TYR X 60 −0.317 −13.894 −24.716 1.00 0.00
H
ATOM 913 CE2 TYR X 60 −1.318 −15.440 −23.585 1.00 0.00
C
ATOM 914 HE2 TYR X 60 −0.362 −15.693 −23.150 1.00 0.00
H
ATOM 915 C TYR X 60 −2.836 −10.111 −26.544 1.00 0.00
C
ATOM 916 O TYR X 60 −3.768 −9.904 −27.287 1.00 0.00
O
ATOM 917 N ALA X 61 −1.694 −9.477 −26.691 1.00 0.00
N
ATOM 918 HN ALA X 61 −0.951 −9.671 −26.055 1.00 0.00
H
ATOM 919 CA ALA X 61 −1.334 −8.784 −27.934 1.00 0.00
C
ATOM 920 HA ALA X 61 −2.190 −8.178 −28.191 1.00 0.00
H
ATOM 921 CB ALA X 61 −0.096 −7.811 −27.708 1.00 0.00
C
ATOM 922 HB1 ALA X 61 0.169 −7.380 −28.697 1.00 0.00
H
ATOM 923 HB2 ALA X 61 0.649 −8.398 −27.129 1.00 0.00
H
ATOM 924 HB3 ALA X 61 −0.511 −6.920 −27.189 1.00 0.00
H
ATOM 925 C ALA X 61 −1.086 −9.774 −29.098 1.00 0.00
C

```
ATOM    926  O   ALA X  61      -0.605 -10.908 -28.932  1.00  0.00
O
ATOM    927  N   ASP X  62      -1.449  -9.360 -30.350  1.00  0.00
N
ATOM    928  HN  ASP X  62      -1.871  -8.464 -30.460  1.00  0.00
H
ATOM    929  CA  ASP X  62      -1.425 -10.166 -31.523  1.00  0.00
C
ATOM    930  HA  ASP X  62      -2.036 -11.038 -31.343  1.00  0.00
H
ATOM    931  CB  ASP X  62      -1.887  -9.315 -32.697  1.00  0.00
C
ATOM    932  HB1 ASP X  62      -1.745  -9.896 -33.634  1.00  0.00
H
ATOM    933  HB2 ASP X  62      -1.356  -8.346 -32.820  1.00  0.00
H
ATOM    934  CG  ASP X  62      -3.417  -9.047 -32.672  1.00  0.00
C
ATOM    935  OD1 ASP X  62      -4.218  -9.506 -31.817  1.00  0.00
O
ATOM    936  OD2 ASP X  62      -3.830  -8.448 -33.687  1.00  0.00
O
ATOM    937  C   ASP X  62       0.019 -10.635 -31.835  1.00  0.00
C
ATOM    938  O   ASP X  62       0.287 -11.758 -32.332  1.00  0.00
O
ATOM    939  N   SER X  63       1.041  -9.856 -31.537  1.00  0.00
N
ATOM    940  HN  SER X  63       0.891  -8.910 -31.263  1.00  0.00
H
ATOM    941  CA  SER X  63       2.387 -10.334 -31.792  1.00  0.00
C
ATOM    942  HA  SER X  63       2.359 -10.630 -32.830  1.00  0.00
H
ATOM    943  CB  SER X  63       3.458  -9.167 -31.776  1.00  0.00
C
ATOM    944  HB1 SER X  63       4.435  -9.618 -32.052  1.00  0.00
H
ATOM    945  HB2 SER X  63       3.585  -8.785 -30.741  1.00  0.00
H
ATOM    946  OG  SER X  63       3.150  -8.102 -32.626  1.00  0.00
O
ATOM    947  HG1 SER X  63       2.459  -7.643 -32.144  1.00  0.00
H
ATOM    948  C   SER X  63       2.933 -11.449 -30.893  1.00  0.00
C
ATOM    949  O   SER X  63       3.981 -12.034 -31.216  1.00  0.00
O
ATOM    950  N   VAL X  64       2.262 -11.775 -29.761  1.00  0.00
N
```

```
ATOM    951  HN  VAL X  64       1.306 −11.536 −29.611  1.00  0.00
H
ATOM    952  CA  VAL X  64       2.860 −12.593 −28.744  1.00  0.00
C
ATOM    953  HA  VAL X  64       3.841 −12.949 −29.022  1.00  0.00
H
ATOM    954  CB  VAL X  64       3.100 −11.788 −27.411  1.00  0.00
C
ATOM    955  HB  VAL X  64       3.313 −12.592 −26.675  1.00  0.00
H
ATOM    956  CG1 VAL X  64       4.270 −10.930 −27.407  1.00  0.00
C
ATOM    957 HG11 VAL X  64       4.395 −10.355 −26.464  1.00  0.00
H
ATOM    958 HG12 VAL X  64       4.132 −10.230 −28.259  1.00  0.00
H
ATOM    959 HG13 VAL X  64       5.257 −11.430 −27.514  1.00  0.00
H
ATOM    960  CG2 VAL X  64       1.906 −11.001 −27.127  1.00  0.00
C
ATOM    961 HG21 VAL X  64       1.110 −11.765 −26.999  1.00  0.00
H
ATOM    962 HG22 VAL X  64       1.527 −10.266 −27.869  1.00  0.00
H
ATOM    963 HG23 VAL X  64       2.146 −10.507 −26.161  1.00  0.00
H
ATOM    964  C   VAL X  64       2.024 −13.892 −28.458  1.00  0.00
C
ATOM    965  O   VAL X  64       2.453 −14.826 −27.808  1.00  0.00
O
ATOM    966  N   THR X  65       0.817 −13.986 −29.059  1.00  0.00
N
ATOM    967  HN  THR X  65       0.615 −13.296 −29.749  1.00  0.00
H
ATOM    968  CA  THR X  65      −0.082 −15.093 −29.145  1.00  0.00
C
ATOM    969  HA  THR X  65      −0.448 −15.108 −28.129  1.00  0.00
H
ATOM    970  CB  THR X  65      −1.206 −14.796 −30.129  1.00  0.00
C
ATOM    971  HB  THR X  65      −1.701 −15.698 −30.549  1.00  0.00
H
ATOM    972  OG1 THR X  65      −0.607 −14.234 −31.313  1.00  0.00
O
ATOM    973  HG1 THR X  65      −1.318 −14.250 −31.959  1.00  0.00
H
ATOM    974  CG2 THR X  65      −2.239 −13.873 −29.477  1.00  0.00
C
ATOM    975 HG21 THR X  65      −2.896 −13.497 −30.290  1.00  0.00
H
```

```
ATOM    976 HG22 THR X  65      -1.726 -12.977 -29.067  1.00  0.00
H
ATOM    977 HG23 THR X  65      -2.784 -14.455 -28.703  1.00  0.00
H
ATOM    978  C   THR X  65       0.532 -16.420 -29.459  1.00  0.00
C
ATOM    979  O   THR X  65       1.295 -16.588 -30.352  1.00  0.00
O
ATOM    980  N   GLY X  66       0.198 -17.445 -28.631  1.00  0.00
N
ATOM    981  HN  GLY X  66      -0.559 -17.397 -27.984  1.00  0.00
H
ATOM    982  CA  GLY X  66       0.727 -18.779 -28.881  1.00  0.00
C
ATOM    983  HA1 GLY X  66       0.837 -19.000 -29.933  1.00  0.00
H
ATOM    984  HA2 GLY X  66      -0.014 -19.443 -28.463  1.00  0.00
H
ATOM    985  C   GLY X  66       2.058 -19.074 -28.333  1.00  0.00
C
ATOM    986  O   GLY X  66       2.197 -20.091 -27.623  1.00  0.00
O
ATOM    987  N   ARG X  67       3.048 -18.187 -28.489  1.00  0.00
N
ATOM    988  HN  ARG X  67       2.802 -17.343 -28.959  1.00  0.00
H
ATOM    989  CA  ARG X  67       4.326 -18.317 -27.934  1.00  0.00
C
ATOM    990  HA  ARG X  67       4.832 -19.265 -28.046  1.00  0.00
H
ATOM    991  CB  ARG X  67       5.369 -17.331 -28.442  1.00  0.00
C
ATOM    992  HB1 ARG X  67       6.280 -17.600 -27.866  1.00  0.00
H
ATOM    993  HB2 ARG X  67       5.084 -16.272 -28.261  1.00  0.00
H
ATOM    994  CG  ARG X  67       5.648 -17.596 -29.888  1.00  0.00
C
ATOM    995  HG1 ARG X  67       5.065 -16.832 -30.447  1.00  0.00
H
ATOM    996  HG2 ARG X  67       5.136 -18.510 -30.257  1.00  0.00
H
ATOM    997  CD  ARG X  67       7.130 -17.441 -30.270  1.00  0.00
C
ATOM    998  HD1 ARG X  67       7.236 -18.018 -31.213  1.00  0.00
H
ATOM    999  HD2 ARG X  67       7.718 -17.865 -29.428  1.00  0.00
H
ATOM   1000  NE  ARG X  67       7.364 -15.996 -30.488  1.00  0.00
N
```

```
ATOM   1001  HE  ARG X  67       6.608 -15.510 -30.926  1.00  0.00
H
ATOM   1002  CZ  ARG X  67       8.490 -15.372 -30.260  1.00  0.00
C
ATOM   1003  NH1 ARG X  67       9.587 -15.950 -29.748  1.00  0.00
N
ATOM   1004 HH11 ARG X  67       9.600 -16.947 -29.679  1.00  0.00
H
ATOM   1005 HH12 ARG X  67      10.445 -15.441 -29.683  1.00  0.00
H
ATOM   1006  NH2 ARG X  67       8.560 -14.089 -30.603  1.00  0.00
N
ATOM   1007 HH21 ARG X  67       7.661 -13.661 -30.699  1.00  0.00
H
ATOM   1008 HH22 ARG X  67       9.330 -13.539 -30.281  1.00  0.00
H
ATOM   1009  C   ARG X  67       4.198 -18.233 -26.337  1.00  0.00
C
ATOM   1010  O   ARG X  67       4.910 -18.888 -25.598  1.00  0.00
O
ATOM   1011  N   PHE X  68       3.315 -17.256 -25.863  1.00  0.00
N
ATOM   1012  HN  PHE X  68       2.835 -16.647 -26.490  1.00  0.00
H
ATOM   1013  CA  PHE X  68       2.974 -17.152 -24.505  1.00  0.00
C
ATOM   1014  HA  PHE X  68       3.796 -17.578 -23.949  1.00  0.00
H
ATOM   1015  CB  PHE X  68       2.868 -15.618 -24.108  1.00  0.00
C
ATOM   1016  HB1 PHE X  68       2.733 -15.694 -23.008  1.00  0.00
H
ATOM   1017  HB2 PHE X  68       2.141 -15.019 -24.698  1.00  0.00
H
ATOM   1018  CG  PHE X  68       4.107 -14.712 -24.296  1.00  0.00
C
ATOM   1019  CD1 PHE X  68       5.363 -15.172 -24.815  1.00  0.00
C
ATOM   1020  HD1 PHE X  68       5.498 -16.174 -25.195  1.00  0.00
H
ATOM   1021  CE1 PHE X  68       6.454 -14.290 -25.034  1.00  0.00
C
ATOM   1022  HE1 PHE X  68       7.330 -14.617 -25.574  1.00  0.00
H
ATOM   1023  CZ  PHE X  68       6.235 -12.903 -24.951  1.00  0.00
C
ATOM   1024  HZ  PHE X  68       7.021 -12.227 -25.255  1.00  0.00
H
ATOM   1025  CD2 PHE X  68       4.006 -13.321 -24.078  1.00  0.00
C
```

```
ATOM   1026  HD2 PHE X  68       3.029 -12.986 -23.763  1.00  0.00
H
ATOM   1027  CE2 PHE X  68       5.004 -12.401 -24.403  1.00  0.00
C
ATOM   1028  HE2 PHE X  68       4.995 -11.349 -24.158  1.00  0.00
H
ATOM   1029  C   PHE X  68       1.712 -17.903 -24.095  1.00  0.00
C
ATOM   1030  O   PHE X  68       0.719 -17.926 -24.807  1.00  0.00
O
ATOM   1031  N   THR X  69       1.779 -18.541 -22.936  1.00  0.00
N
ATOM   1032  HN  THR X  69       2.580 -19.002 -22.561  1.00  0.00
H
ATOM   1033  CA  THR X  69       0.677 -18.873 -22.080  1.00  0.00
C
ATOM   1034  HA  THR X  69      -0.170 -18.450 -22.599  1.00  0.00
H
ATOM   1035  CB  THR X  69       0.294 -20.371 -21.925  1.00  0.00
C
ATOM   1036  HB  THR X  69      -0.224 -20.818 -22.801  1.00  0.00
H
ATOM   1037  OG1 THR X  69      -0.706 -20.684 -20.904  1.00  0.00
O
ATOM   1038  HG1 THR X  69      -1.172 -21.447 -21.253  1.00  0.00
H
ATOM   1039  CG2 THR X  69       1.537 -21.142 -21.663  1.00  0.00
C
ATOM   1040 HG21 THR X  69       1.956 -20.681 -20.743  1.00  0.00
H
ATOM   1041 HG22 THR X  69       2.384 -21.039 -22.374  1.00  0.00
H
ATOM   1042 HG23 THR X  69       1.428 -22.218 -21.411  1.00  0.00
H
ATOM   1043  C   THR X  69       0.718 -18.110 -20.705  1.00  0.00
C
ATOM   1044  O   THR X  69       1.723 -18.081 -20.036  1.00  0.00
O
ATOM   1045  N   ILE X  70      -0.378 -17.401 -20.337  1.00  0.00
N
ATOM   1046  HN  ILE X  70      -1.248 -17.522 -20.808  1.00  0.00
H
ATOM   1047  CA  ILE X  70      -0.424 -16.548 -19.150  1.00  0.00
C
ATOM   1048  HA  ILE X  70       0.511 -16.592 -18.611  1.00  0.00
H
ATOM   1049  CB  ILE X  70      -0.740 -15.094 -19.462  1.00  0.00
C
ATOM   1050  HB  ILE X  70      -0.065 -14.805 -20.296  1.00  0.00
H
```

```
ATOM   1051  CG2 ILE X  70      -2.226 -14.845 -19.826  1.00  0.00
C
ATOM   1052 HG21 ILE X  70      -2.501 -15.443 -20.721  1.00  0.00
H
ATOM   1053 HG22 ILE X  70      -2.295 -13.749 -19.992  1.00  0.00
H
ATOM   1054 HG23 ILE X  70      -2.865 -15.179 -18.981  1.00  0.00
H
ATOM   1055  CG1 ILE X  70      -0.299 -14.203 -18.249  1.00  0.00
C
ATOM   1056 HG11 ILE X  70       0.666 -14.594 -17.864  1.00  0.00
H
ATOM   1057 HG12 ILE X  70      -1.165 -14.329 -17.564  1.00  0.00
H
ATOM   1058  CD  ILE X  70      -0.144 -12.722 -18.510  1.00  0.00
C
ATOM   1059  HD1 ILE X  70       0.301 -12.205 -17.633  1.00  0.00
H
ATOM   1060  HD2 ILE X  70      -1.165 -12.294 -18.615  1.00  0.00
H
ATOM   1061  HD3 ILE X  70       0.504 -12.654 -19.410  1.00  0.00
H
ATOM   1062  C   ILE X  70      -1.469 -17.080 -18.191  1.00  0.00
C
ATOM   1063  O   ILE X  70      -2.364 -17.757 -18.634  1.00  0.00
O
ATOM   1064  N   SER X  71      -1.133 -17.019 -16.845  1.00  0.00
N
ATOM   1065  HN  SER X  71      -0.308 -16.522 -16.587  1.00  0.00
H
ATOM   1066  CA  SER X  71      -1.956 -17.512 -15.725  1.00  0.00
C
ATOM   1067  HA  SER X  71      -2.986 -17.290 -15.961  1.00  0.00
H
ATOM   1068  CB  SER X  71      -1.839 -19.057 -15.475  1.00  0.00
C
ATOM   1069  HB1 SER X  71      -2.259 -19.640 -16.323  1.00  0.00
H
ATOM   1070  HB2 SER X  71      -2.404 -19.406 -14.585  1.00  0.00
H
ATOM   1071  OG  SER X  71      -0.535 -19.525 -15.281  1.00  0.00
O
ATOM   1072  HG1 SER X  71       0.052 -19.333 -16.016  1.00  0.00
H
ATOM   1073  C   SER X  71      -1.645 -16.785 -14.421  1.00  0.00
C
ATOM   1074  O   SER X  71      -0.714 -15.963 -14.250  1.00  0.00
O
ATOM   1075  N   ARG X  72      -2.415 -17.115 -13.363  1.00  0.00
N
```

ATOM	1076	HN	ARG	X	72	−3.106	−17.817	−13.515	1.00	0.00	H
ATOM	1077	CA	ARG	X	72	−2.220	−16.565	−12.039	1.00	0.00	C
ATOM	1078	HA	ARG	X	72	−1.153	−16.409	−11.981	1.00	0.00	H
ATOM	1079	CB	ARG	X	72	−3.067	−15.311	−11.944	1.00	0.00	C
ATOM	1080	HB1	ARG	X	72	−2.699	−14.773	−12.844	1.00	0.00	H
ATOM	1081	HB2	ARG	X	72	−2.805	−14.796	−10.995	1.00	0.00	H
ATOM	1082	CG	ARG	X	72	−4.586	−15.567	−11.928	1.00	0.00	C
ATOM	1083	HG1	ARG	X	72	−4.849	−16.098	−10.988	1.00	0.00	H
ATOM	1084	HG2	ARG	X	72	−4.815	−16.265	−12.761	1.00	0.00	H
ATOM	1085	CD	ARG	X	72	−5.505	−14.209	−11.965	1.00	0.00	C
ATOM	1086	HD1	ARG	X	72	−6.580	−14.483	−11.901	1.00	0.00	H
ATOM	1087	HD2	ARG	X	72	−5.329	−13.624	−12.893	1.00	0.00	H
ATOM	1088	NE	ARG	X	72	−5.103	−13.467	−10.720	1.00	0.00	N
ATOM	1089	HE	ARG	X	72	−4.917	−14.069	−9.943	1.00	0.00	H
ATOM	1090	CZ	ARG	X	72	−5.201	−12.167	−10.542	1.00	0.00	C
ATOM	1091	NH1	ARG	X	72	−5.590	−11.298	−11.404	1.00	0.00	N
ATOM	1092	HH11	ARG	X	72	−5.905	−11.439	−12.343	1.00	0.00	H
ATOM	1093	HH12	ARG	X	72	−5.689	−10.341	−11.133	1.00	0.00	H
ATOM	1094	NH2	ARG	X	72	−5.072	−11.928	−9.234	1.00	0.00	N
ATOM	1095	HH21	ARG	X	72	−4.982	−12.709	−8.616	1.00	0.00	H
ATOM	1096	HH22	ARG	X	72	−5.211	−11.011	−8.860	1.00	0.00	H
ATOM	1097	C	ARG	X	72	−2.639	−17.523	−10.952	1.00	0.00	C
ATOM	1098	O	ARG	X	72	−3.530	−18.294	−11.193	1.00	0.00	O
ATOM	1099	N	ASP	X	73	−1.948	−17.416	−9.776	1.00	0.00	N
ATOM	1100	HN	ASP	X	73	−1.308	−16.671	−9.606	1.00	0.00	H

ATOM 1101 CA ASP X 73 −2.275 −18.280 −8.666 1.00 0.00
C
ATOM 1102 HA ASP X 73 −3.154 −18.875 −8.864 1.00 0.00
H
ATOM 1103 CB ASP X 73 −1.119 −19.244 −8.237 1.00 0.00
C
ATOM 1104 HB1 ASP X 73 −0.126 −18.766 −8.091 1.00 0.00
H
ATOM 1105 HB2 ASP X 73 −0.992 −19.942 −9.092 1.00 0.00
H
ATOM 1106 CG ASP X 73 −1.414 −20.063 −6.995 1.00 0.00
C
ATOM 1107 OD1 ASP X 73 −0.548 −20.991 −6.786 1.00 0.00
O
ATOM 1108 OD2 ASP X 73 −2.254 −19.890 −6.119 1.00 0.00
O
ATOM 1109 C ASP X 73 −2.750 −17.307 −7.598 1.00 0.00
C
ATOM 1110 O ASP X 73 −2.096 −16.351 −7.226 1.00 0.00
O
ATOM 1111 N ASN X 74 −4.032 −17.443 −7.242 1.00 0.00
N
ATOM 1112 HN ASN X 74 −4.581 −18.130 −7.712 1.00 0.00
H
ATOM 1113 CA ASN X 74 −4.700 −16.630 −6.305 1.00 0.00
C
ATOM 1114 HA ASN X 74 −4.186 −15.681 −6.311 1.00 0.00
H
ATOM 1115 CB ASN X 74 −6.149 −16.399 −6.701 1.00 0.00
C
ATOM 1116 HB1 ASN X 74 −6.709 −15.853 −5.912 1.00 0.00
H
ATOM 1117 HB2 ASN X 74 −6.575 −17.415 −6.844 1.00 0.00
H
ATOM 1118 CG ASN X 74 −6.264 −15.629 −7.973 1.00 0.00
C
ATOM 1119 OD1 ASN X 74 −5.389 −14.780 −8.406 1.00 0.00
O
ATOM 1120 ND2 ASN X 74 −7.310 −15.883 −8.742 1.00 0.00
N
ATOM 1121 HD21 ASN X 74 −7.293 −15.597 −9.700 1.00 0.00
H
ATOM 1122 HD22 ASN X 74 −7.993 −16.573 −8.502 1.00 0.00
H
ATOM 1123 C ASN X 74 −4.631 −17.217 −4.909 1.00 0.00
C
ATOM 1124 O ASN X 74 −5.178 −16.510 −4.013 1.00 0.00
O
ATOM 1125 N SER X 75 −3.936 −18.430 −4.664 1.00 0.00
N

ATOM 1126 HN SER X 75 −3.488 −18.793 −5.478 1.00 0.00
H
ATOM 1127 CA SER X 75 −3.692 −18.890 −3.322 1.00 0.00
C
ATOM 1128 HA SER X 75 −4.405 −18.460 −2.634 1.00 0.00
H
ATOM 1129 CB SER X 75 −3.753 −20.440 −3.279 1.00 0.00
C
ATOM 1130 HB1 SER X 75 −3.424 −20.742 −2.261 1.00 0.00
H
ATOM 1131 HB2 SER X 75 −3.189 −20.974 −4.072 1.00 0.00
H
ATOM 1132 OG SER X 75 −5.162 −20.835 −3.422 1.00 0.00
O
ATOM 1133 HG1 SER X 75 −5.249 −21.790 −3.372 1.00 0.00
H
ATOM 1134 C SER X 75 −2.300 −18.482 −2.830 1.00 0.00
C
ATOM 1135 O SER X 75 −2.186 −18.013 −1.709 1.00 0.00
O
ATOM 1136 N LYS X 76 −1.268 −18.723 −3.675 1.00 0.00
N
ATOM 1137 HN LYS X 76 −1.488 −19.177 −4.535 1.00 0.00
H
ATOM 1138 CA LYS X 76 0.070 −18.270 −3.374 1.00 0.00
C
ATOM 1139 HA LYS X 76 0.252 −18.250 −2.310 1.00 0.00
H
ATOM 1140 CB LYS X 76 1.183 −19.188 −3.893 1.00 0.00
C
ATOM 1141 HB1 LYS X 76 2.180 −18.699 −3.918 1.00 0.00
H
ATOM 1142 HB2 LYS X 76 1.052 −19.427 −4.970 1.00 0.00
H
ATOM 1143 CG LYS X 76 1.325 −20.458 −3.134 1.00 0.00
C
ATOM 1144 HG1 LYS X 76 1.429 −20.185 −2.062 1.00 0.00
H
ATOM 1145 HG2 LYS X 76 2.214 −21.060 −3.424 1.00 0.00
H
ATOM 1146 CD LYS X 76 0.170 −21.367 −3.263 1.00 0.00
C
ATOM 1147 HD1 LYS X 76 −0.099 −21.372 −4.341 1.00 0.00
H
ATOM 1148 HD2 LYS X 76 −0.592 −20.853 −2.639 1.00 0.00
H
ATOM 1149 CE LYS X 76 0.410 −22.888 −2.888 1.00 0.00
C
ATOM 1150 HE1 LYS X 76 0.628 −22.963 −1.801 1.00 0.00
H

```
ATOM   1151  HE2 LYS X  76       1.250 -23.335  -3.462  1.00  0.00
H
ATOM   1152  NZ  LYS X  76      -0.853 -23.591  -3.203  1.00  0.00
N
ATOM   1153  HZ1 LYS X  76      -1.016 -23.448  -4.220  1.00  0.00
H
ATOM   1154  HZ2 LYS X  76      -1.671 -23.294  -2.634  1.00  0.00
H
ATOM   1155  HZ3 LYS X  76      -0.768 -24.605  -2.990  1.00  0.00
H
ATOM   1156  C   LYS X  76       0.421 -16.770  -3.946  1.00  0.00
C
ATOM   1157  O   LYS X  76       1.463 -16.200  -3.733  1.00  0.00
O
ATOM   1158  N   ASN X  77      -0.489 -16.149  -4.713  1.00  0.00
N
ATOM   1159  HN  ASN X  77      -1.346 -16.620  -4.908  1.00  0.00
H
ATOM   1160  CA  ASN X  77      -0.547 -14.738  -4.838  1.00  0.00
C
ATOM   1161  HA  ASN X  77      -1.472 -14.493  -5.339  1.00  0.00
H
ATOM   1162  CB  ASN X  77      -0.391 -13.887  -3.516  1.00  0.00
C
ATOM   1163  HB1 ASN X  77      -0.297 -12.791  -3.674  1.00  0.00
H
ATOM   1164  HB2 ASN X  77       0.525 -14.179  -2.958  1.00  0.00
H
ATOM   1165  CG  ASN X  77      -1.665 -14.132  -2.683  1.00  0.00
C
ATOM   1166  OD1 ASN X  77      -2.684 -14.610  -3.233  1.00  0.00
O
ATOM   1167  ND2 ASN X  77      -1.613 -13.854  -1.345  1.00  0.00
N
ATOM   1168 HD21 ASN X  77      -2.484 -13.820  -0.854  1.00  0.00
H
ATOM   1169 HD22 ASN X  77      -0.877 -13.614  -0.712  1.00  0.00
H
ATOM   1170  C   ASN X  77       0.436 -14.214  -5.884  1.00  0.00
C
ATOM   1171  O   ASN X  77       1.145 -13.211  -5.756  1.00  0.00
O
ATOM   1172  N   THR X  78       0.558 -14.979  -6.951  1.00  0.00
N
ATOM   1173  HN  THR X  78       0.057 -15.822  -7.130  1.00  0.00
H
ATOM   1174  CA  THR X  78       1.642 -14.841  -7.882  1.00  0.00
C
ATOM   1175  HA  THR X  78       1.857 -13.784  -7.826  1.00  0.00
H
```

```
ATOM   1176  CB  THR X  78       2.619 -15.951  -7.655  1.00  0.00
C
ATOM   1177  HB  THR X  78       2.149 -16.886  -8.030  1.00  0.00
H
ATOM   1178  OG1 THR X  78       3.076 -16.013  -6.339  1.00  0.00
O
ATOM   1179  HG1 THR X  78       2.426 -15.596  -5.769  1.00  0.00
H
ATOM   1180  CG2 THR X  78       3.914 -15.676  -8.464  1.00  0.00
C
ATOM   1181 HG21 THR X  78       4.457 -14.782  -8.089  1.00  0.00
H
ATOM   1182 HG22 THR X  78       3.702 -15.412  -9.522  1.00  0.00
H
ATOM   1183 HG23 THR X  78       4.626 -16.529  -8.462  1.00  0.00
H
ATOM   1184  C   THR X  78       1.039 -14.863  -9.319  1.00  0.00
C
ATOM   1185  O   THR X  78       0.038 -15.490  -9.695  1.00  0.00
O
ATOM   1186  N   LEU X  79       1.628 -14.106 -10.266  1.00  0.00
N
ATOM   1187  HN  LEU X  79       2.330 -13.516  -9.873  1.00  0.00
H
ATOM   1188  CA  LEU X  79       1.273 -14.038 -11.688  1.00  0.00
C
ATOM   1189  HA  LEU X  79       0.353 -14.527 -11.974  1.00  0.00
H
ATOM   1190  CB  LEU X  79       1.293 -12.600 -12.196  1.00  0.00
C
ATOM   1191  HB1 LEU X  79       2.086 -12.012 -11.686  1.00  0.00
H
ATOM   1192  HB2 LEU X  79       0.456 -12.047 -11.718  1.00  0.00
H
ATOM   1193  CG  LEU X  79       1.348 -12.267 -13.706  1.00  0.00
C
ATOM   1194  HG  LEU X  79       2.328 -12.581 -14.125  1.00  0.00
H
ATOM   1195  CD1 LEU X  79       0.248 -12.893 -14.465  1.00  0.00
C
ATOM   1196 HD11 LEU X  79       0.323 -13.981 -14.674  1.00  0.00
H
ATOM   1197 HD12 LEU X  79       0.147 -12.406 -15.459  1.00  0.00
H
ATOM   1198 HD13 LEU X  79      -0.735 -12.805 -13.956  1.00  0.00
H
ATOM   1199  CD2 LEU X  79       1.381 -10.734 -13.895  1.00  0.00
C
ATOM   1200 HD21 LEU X  79       0.467 -10.335 -13.405  1.00  0.00
H
```

```
ATOM   1201 HD22 LEU X  79       1.390 -10.355 -14.939  1.00  0.00
H
ATOM   1202 HD23 LEU X  79       2.241 -10.216 -13.418  1.00  0.00
H
ATOM   1203  C   LEU X  79       2.337 -14.717 -12.491  1.00  0.00
C
ATOM   1204  O   LEU X  79       3.511 -14.472 -12.205  1.00  0.00
O
ATOM   1205  N   TYR X  80       2.048 -15.621 -13.456  1.00  0.00
N
ATOM   1206  HN  TYR X  80       1.088 -15.874 -13.548  1.00  0.00
H
ATOM   1207  CA  TYR X  80       2.941 -16.332 -14.340  1.00  0.00
C
ATOM   1208  HA  TYR X  80       3.896 -15.946 -14.016  1.00  0.00
H
ATOM   1209  CB  TYR X  80       2.817 -17.743 -14.171  1.00  0.00
C
ATOM   1210  HB1 TYR X  80       3.699 -18.206 -14.664  1.00  0.00
H
ATOM   1211  HB2 TYR X  80       1.877 -18.166 -14.587  1.00  0.00
H
ATOM   1212  CG  TYR X  80       3.017 -18.104 -12.715  1.00  0.00
C
ATOM   1213  CD1 TYR X  80       4.259 -18.464 -12.295  1.00  0.00
C
ATOM   1214  HD1 TYR X  80       5.079 -18.344 -12.988  1.00  0.00
H
ATOM   1215  CE1 TYR X  80       4.429 -18.996 -10.961  1.00  0.00
C
ATOM   1216  HE1 TYR X  80       5.374 -19.181 -10.472  1.00  0.00
H
ATOM   1217  CZ  TYR X  80       3.308 -19.210 -10.090  1.00  0.00
C
ATOM   1218  OH  TYR X  80       3.497 -19.817  -8.823  1.00  0.00
O
ATOM   1219  HH  TYR X  80       4.443 -19.980  -8.822  1.00  0.00
H
ATOM   1220  CD2 TYR X  80       1.931 -18.366 -11.852  1.00  0.00
C
ATOM   1221  HD2 TYR X  80       0.987 -18.311 -12.373  1.00  0.00
H
ATOM   1222  CE2 TYR X  80       2.060 -18.835 -10.582  1.00  0.00
C
ATOM   1223  HE2 TYR X  80       1.196 -19.004  -9.957  1.00  0.00
H
ATOM   1224  C   TYR X  80       2.772 -16.034 -15.789  1.00  0.00
C
ATOM   1225  O   TYR X  80       1.620 -16.052 -16.222  1.00  0.00
O
```

ATOM 1226 N LEU X 81 3.896 −15.831 −16.556 1.00 0.00
N
ATOM 1227 HN LEU X 81 4.749 −15.834 −16.041 1.00 0.00
H
ATOM 1228 CA LEU X 81 3.976 −15.742 −17.968 1.00 0.00
C
ATOM 1229 HA LEU X 81 3.008 −15.992 −18.376 1.00 0.00
H
ATOM 1230 CB LEU X 81 4.488 −14.338 −18.461 1.00 0.00
C
ATOM 1231 HB1 LEU X 81 5.422 −13.997 −17.966 1.00 0.00
H
ATOM 1232 HB2 LEU X 81 3.618 −13.710 −18.174 1.00 0.00
H
ATOM 1233 CG LEU X 81 4.576 −14.156 −19.984 1.00 0.00
C
ATOM 1234 HG LEU X 81 5.401 −14.643 −20.548 1.00 0.00
H
ATOM 1235 CD1 LEU X 81 3.285 −14.544 −20.650 1.00 0.00
C
ATOM 1236 HD11 LEU X 81 2.478 −14.181 −19.978 1.00 0.00
H
ATOM 1237 HD12 LEU X 81 3.189 −15.648 −20.735 1.00 0.00
H
ATOM 1238 HD13 LEU X 81 3.153 −14.037 −21.630 1.00 0.00
H
ATOM 1239 CD2 LEU X 81 4.750 −12.632 −20.186 1.00 0.00
C
ATOM 1240 HD21 LEU X 81 3.909 −11.980 −19.866 1.00 0.00
H
ATOM 1241 HD22 LEU X 81 4.726 −12.500 −21.289 1.00 0.00
H
ATOM 1242 HD23 LEU X 81 5.617 −12.149 −19.686 1.00 0.00
H
ATOM 1243 C LEU X 81 4.956 −16.852 −18.389 1.00 0.00
C
ATOM 1244 O LEU X 81 6.050 −16.894 −17.886 1.00 0.00
O
ATOM 1245 N GLN X 82 4.568 −17.867 −19.248 1.00 0.00
N
ATOM 1246 HN GLN X 82 3.618 −17.961 −19.536 1.00 0.00
H
ATOM 1247 CA GLN X 82 5.399 −18.968 −19.686 1.00 0.00
C
ATOM 1248 HA GLN X 82 6.373 −18.831 −19.240 1.00 0.00
H
ATOM 1249 CB GLN X 82 4.787 −20.320 −19.244 1.00 0.00
C
ATOM 1250 HB1 GLN X 82 3.754 −20.422 −19.639 1.00 0.00
H

```
ATOM   1251  HB2 GLN X  82       4.788 -20.315 -18.133  1.00  0.00
H
ATOM   1252  CG  GLN X  82       5.662 -21.607 -19.575  1.00  0.00
C
ATOM   1253  HG1 GLN X  82       6.057 -21.511 -20.609  1.00  0.00
H
ATOM   1254  HG2 GLN X  82       4.985 -22.488 -19.579  1.00  0.00
H
ATOM   1255  CD  GLN X  82       6.840 -21.860 -18.625  1.00  0.00
C
ATOM   1256  OE1 GLN X  82       6.821 -21.352 -17.495  1.00  0.00
O
ATOM   1257  NE2 GLN X  82       7.903 -22.616 -19.053  1.00  0.00
N
ATOM   1258 HE21 GLN X  82       8.766 -22.759 -18.570  1.00  0.00
H
ATOM   1259 HE22 GLN X  82       7.822 -22.867 -20.018  1.00  0.00
H
ATOM   1260  C   GLN X  82       5.490 -18.867 -21.208  1.00  0.00
C
ATOM   1261  O   GLN X  82       4.575 -19.088 -22.056  1.00  0.00
O
ATOM   1262  N   MET X  83       6.789 -18.699 -21.682  1.00  0.00
N
ATOM   1263  HN  MET X  83       7.530 -18.747 -21.017  1.00  0.00
H
ATOM   1264  CA  MET X  83       7.079 -18.362 -22.985  1.00  0.00
C
ATOM   1265  HA  MET X  83       6.291 -18.016 -23.638  1.00  0.00
H
ATOM   1266  CB  MET X  83       8.196 -17.202 -23.013  1.00  0.00
C
ATOM   1267  HB1 MET X  83       8.475 -16.867 -24.035  1.00  0.00
H
ATOM   1268  HB2 MET X  83       9.098 -17.554 -22.468  1.00  0.00
H
ATOM   1269  CG  MET X  83       7.822 -15.974 -22.065  1.00  0.00
C
ATOM   1270  HG1 MET X  83       7.908 -16.407 -21.045  1.00  0.00
H
ATOM   1271  HG2 MET X  83       6.747 -15.735 -22.212  1.00  0.00
H
ATOM   1272  SD  MET X  83       8.846 -14.514 -22.254  1.00  0.00
S
ATOM   1273  CE  MET X  83       8.559 -13.662 -20.631  1.00  0.00
C
ATOM   1274  HE1 MET X  83       8.505 -14.441 -19.840  1.00  0.00
H
ATOM   1275  HE2 MET X  83       7.573 -13.171 -20.486  1.00  0.00
H
```

```
ATOM   1276  HE3 MET X  83       9.436 -13.047 -20.334  1.00  0.00
H
ATOM   1277  C   MET X  83       7.621 -19.622 -23.709  1.00  0.00
C
ATOM   1278  O   MET X  83       8.368 -20.249 -23.037  1.00  0.00
O
ATOM   1279  N   ASN X  84       7.327 -19.740 -24.973  1.00  0.00
N
ATOM   1280  HN  ASN X  84       6.746 -19.059 -25.413  1.00  0.00
H
ATOM   1281  CA  ASN X  84       7.869 -20.845 -25.818  1.00  0.00
C
ATOM   1282  HA  ASN X  84       8.455 -21.492 -25.182  1.00  0.00
H
ATOM   1283  CB  ASN X  84       6.609 -21.586 -26.384  1.00  0.00
C
ATOM   1284  HB1 ASN X  84       6.988 -22.303 -27.142  1.00  0.00
H
ATOM   1285  HB2 ASN X  84       5.859 -20.951 -26.901  1.00  0.00
H
ATOM   1286  CG  ASN X  84       5.880 -22.313 -25.263  1.00  0.00
C
ATOM   1287  OD1 ASN X  84       6.274 -23.433 -24.942  1.00  0.00
O
ATOM   1288  ND2 ASN X  84       4.923 -21.636 -24.511  1.00  0.00
N
ATOM   1289 HD21 ASN X  84       4.812 -20.650 -24.630  1.00  0.00
H
ATOM   1290 HD22 ASN X  84       4.616 -22.024 -23.642  1.00  0.00
H
ATOM   1291  C   ASN X  84       8.694 -20.275 -26.968  1.00  0.00
C
ATOM   1292  O   ASN X  84       8.478 -19.110 -27.369  1.00  0.00
O
ATOM   1293  N   SER X  85       9.701 -21.102 -27.419  1.00  0.00
N
ATOM   1294  HN  SER X  85       9.671 -22.048 -27.104  1.00  0.00
H
ATOM   1295  CA  SER X  85      10.647 -20.773 -28.465  1.00  0.00
C
ATOM   1296  HA  SER X  85      11.523 -21.390 -28.323  1.00  0.00
H
ATOM   1297  CB  SER X  85      10.127 -21.312 -29.798  1.00  0.00
C
ATOM   1298  HB1 SER X  85      10.689 -20.831 -30.627  1.00  0.00
H
ATOM   1299  HB2 SER X  85       9.061 -21.042 -29.957  1.00  0.00
H
ATOM   1300  OG  SER X  85      10.294 -22.715 -29.928  1.00  0.00
O
```

```
ATOM   1301  HG1 SER X  85       9.887 -22.930 -30.770  1.00  0.00
H
ATOM   1302  C   SER X  85      11.227 -19.416 -28.690  1.00  0.00
C
ATOM   1303  O   SER X  85      10.860 -18.688 -29.627  1.00  0.00
O
ATOM   1304  N   LEU X  86      12.166 -18.947 -27.730  1.00  0.00
N
ATOM   1305  HN  LEU X  86      12.431 -19.484 -26.932  1.00  0.00
H
ATOM   1306  CA  LEU X  86      12.650 -17.619 -27.761  1.00  0.00
C
ATOM   1307  HA  LEU X  86      11.891 -17.039 -28.266  1.00  0.00
H
ATOM   1308  CB  LEU X  86      12.975 -16.968 -26.351  1.00  0.00
C
ATOM   1309  HB1 LEU X  86      13.264 -15.915 -26.554  1.00  0.00
H
ATOM   1310  HB2 LEU X  86      13.898 -17.372 -25.884  1.00  0.00
H
ATOM   1311  CG  LEU X  86      11.827 -16.858 -25.430  1.00  0.00
C
ATOM   1312  HG  LEU X  86      11.301 -17.830 -25.316  1.00  0.00
H
ATOM   1313  CD1 LEU X  86      12.407 -16.410 -24.066  1.00  0.00
C
ATOM   1314 HD11 LEU X  86      11.571 -16.310 -23.341  1.00  0.00
H
ATOM   1315 HD12 LEU X  86      12.830 -15.384 -24.025  1.00  0.00
H
ATOM   1316 HD13 LEU X  86      13.157 -17.053 -23.558  1.00  0.00
H
ATOM   1317  CD2 LEU X  86      10.775 -15.989 -25.983  1.00  0.00
C
ATOM   1318 HD21 LEU X  86      10.269 -16.408 -26.879  1.00  0.00
H
ATOM   1319 HD22 LEU X  86      11.083 -14.926 -26.078  1.00  0.00
H
ATOM   1320 HD23 LEU X  86       9.948 -15.952 -25.241  1.00  0.00
H
ATOM   1321  C   LEU X  86      13.870 -17.450 -28.711  1.00  0.00
C
ATOM   1322  O   LEU X  86      14.726 -18.313 -28.907  1.00  0.00
O
ATOM   1323  N   ARG X  87      13.905 -16.339 -29.484  1.00  0.00
N
ATOM   1324  HN  ARG X  87      13.315 -15.570 -29.249  1.00  0.00
H
ATOM   1325  CA  ARG X  87      14.837 -16.057 -30.553  1.00  0.00
C
```

```
ATOM   1326  HA  ARG X  87      15.626 -16.788 -30.647  1.00  0.00
H
ATOM   1327  CB  ARG X  87      14.009 -15.899 -31.929  1.00  0.00
C
ATOM   1328  HB1 ARG X  87      14.631 -15.671 -32.821  1.00  0.00
H
ATOM   1329  HB2 ARG X  87      13.285 -15.058 -31.883  1.00  0.00
H
ATOM   1330  CG  ARG X  87      13.164 -17.193 -32.251  1.00  0.00
C
ATOM   1331  HG1 ARG X  87      12.453 -16.970 -33.074  1.00  0.00
H
ATOM   1332  HG2 ARG X  87      12.625 -17.423 -31.307  1.00  0.00
H
ATOM   1333  CD  ARG X  87      13.892 -18.391 -32.718  1.00  0.00
C
ATOM   1334  HD1 ARG X  87      14.770 -18.577 -32.064  1.00  0.00
H
ATOM   1335  HD2 ARG X  87      14.307 -18.080 -33.701  1.00  0.00
H
ATOM   1336  NE  ARG X  87      12.965 -19.582 -32.797  1.00  0.00
N
ATOM   1337  HE  ARG X  87      11.970 -19.487 -32.824  1.00  0.00
H
ATOM   1338  CZ  ARG X  87      13.400 -20.771 -33.094  1.00  0.00
C
ATOM   1339  NH1 ARG X  87      14.701 -21.134 -33.160  1.00  0.00
N
ATOM   1340 HH11 ARG X  87      15.437 -20.473 -33.305  1.00  0.00
H
ATOM   1341 HH12 ARG X  87      14.891 -22.109 -33.271  1.00  0.00
H
ATOM   1342  NH2 ARG X  87      12.525 -21.714 -33.031  1.00  0.00
N
ATOM   1343 HH21 ARG X  87      11.558 -21.489 -33.155  1.00  0.00
H
ATOM   1344 HH22 ARG X  87      12.836 -22.633 -33.277  1.00  0.00
H
ATOM   1345  C   ARG X  87      15.435 -14.688 -30.400  1.00  0.00
C
ATOM   1346  O   ARG X  87      14.951 -13.951 -29.505  1.00  0.00
O
ATOM   1347  N   ALA X  88      16.506 -14.259 -31.170  1.00  0.00
N
ATOM   1348  HN  ALA X  88      16.765 -14.899 -31.889  1.00  0.00
H
ATOM   1349  CA  ALA X  88      17.493 -13.229 -30.842  1.00  0.00
C
ATOM   1350  HA  ALA X  88      18.096 -13.596 -30.024  1.00  0.00
H
```

```
ATOM   1351  CB  ALA X  88      18.449 -13.021 -32.051  1.00  0.00
C
ATOM   1352  HB1 ALA X  88      19.233 -12.350 -31.637  1.00  0.00
H
ATOM   1353  HB2 ALA X  88      18.041 -12.519 -32.954  1.00  0.00
H
ATOM   1354  HB3 ALA X  88      18.870 -14.027 -32.260  1.00  0.00
H
ATOM   1355  C   ALA X  88      16.841 -11.825 -30.565  1.00  0.00
C
ATOM   1356  O   ALA X  88      17.270 -11.058 -29.730  1.00  0.00
O
ATOM   1357  N   GLU X  89      15.667 -11.487 -31.226  1.00  0.00
N
ATOM   1358  HN  GLU X  89      15.251 -12.167 -31.825  1.00  0.00
H
ATOM   1359  CA  GLU X  89      15.049 -10.142 -31.090  1.00  0.00
C
ATOM   1360  HA  GLU X  89      15.762  -9.444 -30.678  1.00  0.00
H
ATOM   1361  CB  GLU X  89      14.502  -9.714 -32.477  1.00  0.00
C
ATOM   1362  HB1 GLU X  89      14.209  -8.656 -32.305  1.00  0.00
H
ATOM   1363  HB2 GLU X  89      13.586 -10.261 -32.787  1.00  0.00
H
ATOM   1364  CG  GLU X  89      15.546  -9.733 -33.660  1.00  0.00
C
ATOM   1365  HG1 GLU X  89      15.921 -10.776 -33.739  1.00  0.00
H
ATOM   1366  HG2 GLU X  89      16.403  -9.135 -33.282  1.00  0.00
H
ATOM   1367  CD  GLU X  89      15.279  -9.162 -35.043  1.00  0.00
C
ATOM   1368  OE1 GLU X  89      16.122  -9.348 -35.928  1.00  0.00
O
ATOM   1369  OE2 GLU X  89      14.265  -8.459 -35.121  1.00  0.00
O
ATOM   1370  C   GLU X  89      13.880 -10.088 -30.076  1.00  0.00
C
ATOM   1371  O   GLU X  89      13.017  -9.228 -30.081  1.00  0.00
O
ATOM   1372  N   ASP X  90      13.772 -11.209 -29.277  1.00  0.00
N
ATOM   1373  HN  ASP X  90      14.395 -11.981 -29.372  1.00  0.00
H
ATOM   1374  CA  ASP X  90      12.795 -11.327 -28.082  1.00  0.00
C
ATOM   1375  HA  ASP X  90      12.021 -10.598 -28.270  1.00  0.00
H
```

```
ATOM   1376  CB  ASP X  90      12.257 -12.805 -27.940  1.00  0.00
C
ATOM   1377  HB1 ASP X  90      11.483 -12.787 -27.144  1.00  0.00
H
ATOM   1378  HB2 ASP X  90      13.086 -13.460 -27.596  1.00  0.00
H
ATOM   1379  CG  ASP X  90      11.615 -13.315 -29.166  1.00  0.00
C
ATOM   1380  OD1 ASP X  90      10.873 -12.531 -29.757  1.00  0.00
O
ATOM   1381  OD2 ASP X  90      11.742 -14.524 -29.464  1.00  0.00
O
ATOM   1382  C   ASP X  90      13.492 -10.812 -26.838  1.00  0.00
C
ATOM   1383  O   ASP X  90      13.030 -10.921 -25.670  1.00  0.00
O
ATOM   1384  N   THR X  91      14.649 -10.076 -26.901  1.00  0.00
N
ATOM   1385  HN  THR X  91      15.062  -9.897 -27.791  1.00  0.00
H
ATOM   1386  CA  THR X  91      15.408  -9.509 -25.791  1.00  0.00
C
ATOM   1387  HA  THR X  91      15.446 -10.211 -24.971  1.00  0.00
H
ATOM   1388  CB  THR X  91      16.881  -9.178 -26.020  1.00  0.00
C
ATOM   1389  HB  THR X  91      16.989  -8.630 -26.980  1.00  0.00
H
ATOM   1390  OG1 THR X  91      17.596 -10.360 -26.239  1.00  0.00
O
ATOM   1391  HG1 THR X  91      18.215 -10.160 -26.945  1.00  0.00
H
ATOM   1392  CG2 THR X  91      17.480  -8.295 -24.835  1.00  0.00
C
ATOM   1393 HG21 THR X  91      18.569  -8.334 -24.618  1.00  0.00
H
ATOM   1394 HG22 THR X  91      17.227  -8.882 -23.926  1.00  0.00
H
ATOM   1395 HG23 THR X  91      17.194  -7.225 -24.914  1.00  0.00
H
ATOM   1396  C   THR X  91      14.738  -8.251 -25.263  1.00  0.00
C
ATOM   1397  O   THR X  91      14.571  -7.281 -26.030  1.00  0.00
O
ATOM   1398  N   ALA X  92      14.458  -8.144 -24.004  1.00  0.00
N
ATOM   1399  HN  ALA X  92      14.582  -8.943 -23.420  1.00  0.00
H
ATOM   1400  CA  ALA X  92      13.572  -6.998 -23.550  1.00  0.00
C
```

ATOM 1401 HA ALA X 92 13.940 −6.051 −23.917 1.00 0.00
H
ATOM 1402 CB ALA X 92 12.100 −7.174 −23.988 1.00 0.00
C
ATOM 1403 HB1 ALA X 92 11.485 −6.308 −23.662 1.00 0.00
H
ATOM 1404 HB2 ALA X 92 11.639 −8.089 −23.558 1.00 0.00
H
ATOM 1405 HB3 ALA X 92 12.010 −7.213 −25.095 1.00 0.00
H
ATOM 1406 C ALA X 92 13.543 −6.891 −22.025 1.00 0.00
C
ATOM 1407 O ALA X 92 13.692 −7.825 −21.208 1.00 0.00
O
ATOM 1408 N VAL X 93 13.340 −5.673 −21.494 1.00 0.00
N
ATOM 1409 HN VAL X 93 13.193 −4.866 −22.060 1.00 0.00
H
ATOM 1410 CA VAL X 93 12.966 −5.565 −20.057 1.00 0.00
C
ATOM 1411 HA VAL X 93 13.234 −6.409 −19.438 1.00 0.00
H
ATOM 1412 CB VAL X 93 13.535 −4.306 −19.370 1.00 0.00
C
ATOM 1413 HB VAL X 93 14.603 −4.585 −19.246 1.00 0.00
H
ATOM 1414 CG1 VAL X 93 13.270 −3.179 −20.280 1.00 0.00
C
ATOM 1415 HG11 VAL X 93 13.739 −3.201 −21.287 1.00 0.00
H
ATOM 1416 HG12 VAL X 93 13.872 −2.374 −19.807 1.00 0.00
H
ATOM 1417 HG13 VAL X 93 12.183 −3.031 −20.457 1.00 0.00
H
ATOM 1418 CG2 VAL X 93 12.872 −4.096 −17.913 1.00 0.00
C
ATOM 1419 HG21 VAL X 93 12.908 −5.100 −17.440 1.00 0.00
H
ATOM 1420 HG22 VAL X 93 11.829 −3.714 −17.901 1.00 0.00
H
ATOM 1421 HG23 VAL X 93 13.487 −3.420 −17.282 1.00 0.00
H
ATOM 1422 C VAL X 93 11.446 −5.602 −19.919 1.00 0.00
C
ATOM 1423 O VAL X 93 10.679 −4.836 −20.553 1.00 0.00
O
ATOM 1424 N TYR X 94 10.921 −6.421 −18.992 1.00 0.00
N
ATOM 1425 HN TYR X 94 11.549 −6.957 −18.434 1.00 0.00
H

```
ATOM   1426  CA  TYR X  94       9.559  -6.629 -18.637  1.00  0.00
C
ATOM   1427  HA  TYR X  94       8.937  -6.350 -19.474  1.00  0.00
H
ATOM   1428  CB  TYR X  94       9.135  -8.078 -18.534  1.00  0.00
C
ATOM   1429  HB1 TYR X  94       8.159  -8.169 -18.010  1.00  0.00
H
ATOM   1430  HB2 TYR X  94       9.872  -8.601 -17.888  1.00  0.00
H
ATOM   1431  CG  TYR X  94       9.011  -8.786 -19.807  1.00  0.00
C
ATOM   1432  CD1 TYR X  94      10.164  -9.305 -20.437  1.00  0.00
C
ATOM   1433  HD1 TYR X  94      11.120  -9.331 -19.935  1.00  0.00
H
ATOM   1434  CE1 TYR X  94      10.138 -10.122 -21.505  1.00  0.00
C
ATOM   1435  HE1 TYR X  94      11.029 -10.468 -22.006  1.00  0.00
H
ATOM   1436  CZ  TYR X  94       8.920 -10.326 -22.177  1.00  0.00
C
ATOM   1437  OH  TYR X  94       8.700 -11.047 -23.378  1.00  0.00
O
ATOM   1438  HH  TYR X  94       9.550 -11.451 -23.567  1.00  0.00
H
ATOM   1439  CD2 TYR X  94       7.779  -9.051 -20.416  1.00  0.00
C
ATOM   1440  HD2 TYR X  94       6.907  -8.675 -19.901  1.00  0.00
H
ATOM   1441  CE2 TYR X  94       7.717  -9.835 -21.587  1.00  0.00
C
ATOM   1442  HE2 TYR X  94       6.806  -9.960 -22.153  1.00  0.00
H
ATOM   1443  C   TYR X  94       9.201  -5.884 -17.358  1.00  0.00
C
ATOM   1444  O   TYR X  94       9.945  -5.799 -16.412  1.00  0.00
O
ATOM   1445  N   TYR X  95       7.936  -5.389 -17.326  1.00  0.00
N
ATOM   1446  HN  TYR X  95       7.335  -5.463 -18.118  1.00  0.00
H
ATOM   1447  CA  TYR X  95       7.352  -4.722 -16.149  1.00  0.00
C
ATOM   1448  HA  TYR X  95       7.954  -4.832 -15.259  1.00  0.00
H
ATOM   1449  CB  TYR X  95       7.170  -3.163 -16.407  1.00  0.00
C
ATOM   1450  HB1 TYR X  95       6.373  -2.644 -15.832  1.00  0.00
H
```

```
ATOM   1451  HB2 TYR X  95       6.777  -3.094 -17.444  1.00  0.00
H
ATOM   1452  CG  TYR X  95       8.451  -2.340 -16.485  1.00  0.00
C
ATOM   1453  CD1 TYR X  95       9.163  -2.149 -17.664  1.00  0.00
C
ATOM   1454  HD1 TYR X  95       8.651  -2.428 -18.572  1.00  0.00
H
ATOM   1455  CE1 TYR X  95      10.308  -1.334 -17.743  1.00  0.00
C
ATOM   1456  HE1 TYR X  95      10.824  -1.258 -18.689  1.00  0.00
H
ATOM   1457  CZ  TYR X  95      10.825  -0.888 -16.534  1.00  0.00
C
ATOM   1458  OH  TYR X  95      12.029  -0.202 -16.656  1.00  0.00
O
ATOM   1459  HH  TYR X  95      12.305  -0.098 -17.570  1.00  0.00
H
ATOM   1460  CD2 TYR X  95       8.921  -1.776 -15.302  1.00  0.00
C
ATOM   1461  HD2 TYR X  95       8.375  -1.739 -14.371  1.00  0.00
H
ATOM   1462  CE2 TYR X  95      10.145  -1.123 -15.269  1.00  0.00
C
ATOM   1463  HE2 TYR X  95      10.640  -0.779 -14.372  1.00  0.00
H
ATOM   1464  C   TYR X  95       6.007  -5.301 -15.869  1.00  0.00
C
ATOM   1465  O   TYR X  95       5.211  -5.276 -16.788  1.00  0.00
O
ATOM   1466  N   CYS X  96       5.805  -5.801 -14.602  1.00  0.00
N
ATOM   1467  HN  CYS X  96       6.582  -5.835 -13.979  1.00  0.00
H
ATOM   1468  CA  CYS X  96       4.570  -6.295 -14.085  1.00  0.00
C
ATOM   1469  HA  CYS X  96       4.279  -7.037 -14.813  1.00  0.00
H
ATOM   1470  CB  CYS X  96       4.923  -7.062 -12.793  1.00  0.00
C
ATOM   1471  HB1 CYS X  96       5.138  -6.382 -11.941  1.00  0.00
H
ATOM   1472  HB2 CYS X  96       5.846  -7.637 -13.023  1.00  0.00
H
ATOM   1473  SG  CYS X  96       3.579  -8.323 -12.468  1.00  0.00
S
ATOM   1474  C   CYS X  96       3.497  -5.179 -13.897  1.00  0.00
C
ATOM   1475  O   CYS X  96       3.842  -3.995 -13.712  1.00  0.00
O
```

```
ATOM   1476  N   ALA X  97       2.214  -5.508 -14.127  1.00  0.00
N
ATOM   1477  HN  ALA X  97       1.913  -6.373 -14.521  1.00  0.00
H
ATOM   1478  CA  ALA X  97       1.215  -4.514 -14.002  1.00  0.00
C
ATOM   1479  HA  ALA X  97       1.530  -3.757 -13.300  1.00  0.00
H
ATOM   1480  CB  ALA X  97       1.067  -3.747 -15.334  1.00  0.00
C
ATOM   1481  HB1 ALA X  97       2.011  -3.224 -15.600  1.00  0.00
H
ATOM   1482  HB2 ALA X  97       0.369  -2.906 -15.133  1.00  0.00
H
ATOM   1483  HB3 ALA X  97       0.911  -4.343 -16.259  1.00  0.00
H
ATOM   1484  C   ALA X  97      -0.033  -5.000 -13.358  1.00  0.00
C
ATOM   1485  O   ALA X  97      -0.399  -6.131 -13.458  1.00  0.00
O
ATOM   1486  N   SER X  98      -0.683  -4.073 -12.637  1.00  0.00
N
ATOM   1487  HN  SER X  98      -0.352  -3.141 -12.505  1.00  0.00
H
ATOM   1488  CA  SER X  98      -1.845  -4.355 -11.875  1.00  0.00
C
ATOM   1489  HA  SER X  98      -2.322  -5.284 -12.149  1.00  0.00
H
ATOM   1490  CB  SER X  98      -1.638  -4.389 -10.305  1.00  0.00
C
ATOM   1491  HB1 SER X  98      -1.257  -5.417 -10.121  1.00  0.00
H
ATOM   1492  HB2 SER X  98      -2.615  -4.200  -9.810  1.00  0.00
H
ATOM   1493  OG  SER X  98      -0.727  -3.387  -9.832  1.00  0.00
O
ATOM   1494  HG1 SER X  98       0.129  -3.557 -10.233  1.00  0.00
H
ATOM   1495  C   SER X  98      -2.971  -3.314 -12.086  1.00  0.00
C
ATOM   1496  O   SER X  98      -2.821  -2.166 -12.517  1.00  0.00
O
ATOM   1497  N   SER X  99      -4.198  -3.780 -11.893  1.00  0.00
N
ATOM   1498  HN  SER X  99      -4.444  -4.745 -11.929  1.00  0.00
H
ATOM   1499  CA  SER X  99      -5.344  -2.743 -12.104  1.00  0.00
C
ATOM   1500  HA  SER X  99      -4.962  -1.818 -11.697  1.00  0.00
H
```

```
ATOM   1501  CB  SER X  99      -5.841  -2.367 -13.498  1.00  0.00
C
ATOM   1502  HB1 SER X  99      -5.031  -1.995 -14.161  1.00  0.00
H
ATOM   1503  HB2 SER X  99      -6.630  -1.608 -13.314  1.00  0.00
H
ATOM   1504  OG  SER X  99      -6.461  -3.448 -14.253  1.00  0.00
O
ATOM   1505  HG1 SER X  99      -5.716  -3.930 -14.620  1.00  0.00
H
ATOM   1506  C   SER X  99      -6.526  -3.055 -11.182  1.00  0.00
C
ATOM   1507  O   SER X  99      -6.820  -4.224 -11.049  1.00  0.00
O
ATOM   1508  N   SER X 100      -7.304  -2.039 -10.685  1.00  0.00
N
ATOM   1509  HN  SER X 100      -7.070  -1.084 -10.854  1.00  0.00
H
ATOM   1510  CA  SER X 100      -8.544  -2.355  -9.936  1.00  0.00
C
ATOM   1511  HA  SER X 100      -8.563  -3.378  -9.593  1.00  0.00
H
ATOM   1512  CB  SER X 100      -8.619  -1.318  -8.849  1.00  0.00
C
ATOM   1513  HB1 SER X 100      -8.824  -0.285  -9.202  1.00  0.00
H
ATOM   1514  HB2 SER X 100      -7.615  -1.415  -8.384  1.00  0.00
H
ATOM   1515  OG  SER X 100      -9.718  -1.596  -7.959  1.00  0.00
O
ATOM   1516  HG1 SER X 100      -9.407  -2.160  -7.247  1.00  0.00
H
ATOM   1517  C   SER X 100      -9.807  -2.154 -10.850  1.00  0.00
C
ATOM   1518  O   SER X 100      -9.927  -1.174 -11.603  1.00  0.00
O
ATOM   1519  N   GLY X 101     -10.710  -3.097 -10.927  1.00  0.00
N
ATOM   1520  HN  GLY X 101     -10.714  -3.851 -10.275  1.00  0.00
H
ATOM   1521  CA  GLY X 101     -11.838  -3.087 -11.945  1.00  0.00
C
ATOM   1522  HA1 GLY X 101     -12.447  -2.213 -11.766  1.00  0.00
H
ATOM   1523  HA2 GLY X 101     -12.392  -4.014 -11.929  1.00  0.00
H
ATOM   1524  C   GLY X 101     -11.527  -2.911 -13.303  1.00  0.00
C
ATOM   1525  O   GLY X 101     -12.208  -2.248 -14.094  1.00  0.00
O
```

```
ATOM   1526  N   TYR X 102     -10.425  -3.509 -13.707  1.00  0.00
N
ATOM   1527  HN  TYR X 102      -9.824  -3.910 -13.021  1.00  0.00
H
ATOM   1528  CA  TYR X 102      -9.882  -3.413 -15.086  1.00  0.00
C
ATOM   1529  HA  TYR X 102      -8.975  -3.997 -15.032  1.00  0.00
H
ATOM   1530  CB  TYR X 102     -10.996  -3.835 -16.109  1.00  0.00
C
ATOM   1531  HB1 TYR X 102     -10.529  -3.867 -17.117  1.00  0.00
H
ATOM   1532  HB2 TYR X 102     -11.924  -3.260 -16.317  1.00  0.00
H
ATOM   1533  CG  TYR X 102     -11.324  -5.430 -15.886  1.00  0.00
C
ATOM   1534  CD1 TYR X 102     -12.676  -5.768 -15.932  1.00  0.00
C
ATOM   1535  HD1 TYR X 102     -13.454  -5.022 -16.001  1.00  0.00
H
ATOM   1536  CE1 TYR X 102     -13.113  -7.134 -15.905  1.00  0.00
C
ATOM   1537  HE1 TYR X 102     -14.162  -7.368 -16.012  1.00  0.00
H
ATOM   1538  CZ  TYR X 102     -12.070  -8.124 -15.988  1.00  0.00
C
ATOM   1539  OH  TYR X 102     -12.244  -9.519 -16.085  1.00  0.00
O
ATOM   1540  HH  TYR X 102     -13.182  -9.722 -16.124  1.00  0.00
H
ATOM   1541  CD2 TYR X 102     -10.329  -6.389 -15.846  1.00  0.00
C
ATOM   1542  HD2 TYR X 102      -9.295  -6.095 -15.738  1.00  0.00
H
ATOM   1543  CE2 TYR X 102     -10.730  -7.714 -15.968  1.00  0.00
C
ATOM   1544  HE2 TYR X 102      -9.954  -8.462 -16.037  1.00  0.00
H
ATOM   1545  C   TYR X 102      -9.319  -2.077 -15.611  1.00  0.00
C
ATOM   1546  O   TYR X 102      -9.019  -1.898 -16.767  1.00  0.00
O
ATOM   1547  N   LEU X 103      -9.132  -1.089 -14.744  1.00  0.00
N
ATOM   1548  HN  LEU X 103      -9.233  -1.208 -13.760  1.00  0.00
H
ATOM   1549  CA  LEU X 103      -8.956   0.323 -15.125  1.00  0.00
C
ATOM   1550  HA  LEU X 103      -9.095   0.452 -16.189  1.00  0.00
H
```

```
ATOM   1551  CB  LEU X 103     -10.011   1.128 -14.343  1.00  0.00
C
ATOM   1552  HB1 LEU X 103      -9.775   0.981 -13.267  1.00  0.00
H
ATOM   1553  HB2 LEU X 103     -11.039   0.766 -14.558  1.00  0.00
H
ATOM   1554  CG  LEU X 103      -9.960   2.664 -14.503  1.00  0.00
C
ATOM   1555  HG  LEU X 103      -8.918   3.013 -14.335  1.00  0.00
H
ATOM   1556  CD1 LEU X 103     -10.642   3.179 -15.843  1.00  0.00
C
ATOM   1557 HD11 LEU X 103     -11.689   2.809 -15.872  1.00  0.00
H
ATOM   1558 HD12 LEU X 103     -10.083   2.655 -16.648  1.00  0.00
H
ATOM   1559 HD13 LEU X 103     -10.537   4.254 -16.102  1.00  0.00
H
ATOM   1560  CD2 LEU X 103     -10.796   3.321 -13.386  1.00  0.00
C
ATOM   1561 HD21 LEU X 103     -11.077   4.339 -13.732  1.00  0.00
H
ATOM   1562 HD22 LEU X 103     -10.249   3.379 -12.421  1.00  0.00
H
ATOM   1563 HD23 LEU X 103     -11.783   2.843 -13.210  1.00  0.00
H
ATOM   1564  C   LEU X 103      -7.662   0.866 -14.773  1.00  0.00
C
ATOM   1565  O   LEU X 103      -7.262   0.893 -13.614  1.00  0.00
O
ATOM   1566  N   PHE X 104      -6.932   1.319 -15.788  1.00  0.00
N
ATOM   1567  HN  PHE X 104      -7.180   1.260 -16.752  1.00  0.00
H
ATOM   1568  CA  PHE X 104      -5.671   2.052 -15.655  1.00  0.00
C
ATOM   1569  HA  PHE X 104      -4.849   1.510 -15.211  1.00  0.00
H
ATOM   1570  CB  PHE X 104      -5.318   2.346 -17.174  1.00  0.00
C
ATOM   1571  HB1 PHE X 104      -6.134   3.059 -17.421  1.00  0.00
H
ATOM   1572  HB2 PHE X 104      -5.401   1.491 -17.879  1.00  0.00
H
ATOM   1573  CG  PHE X 104      -4.005   3.129 -17.293  1.00  0.00
C
ATOM   1574  CD1 PHE X 104      -3.898   4.551 -17.248  1.00  0.00
C
ATOM   1575  HD1 PHE X 104      -4.833   5.089 -17.295  1.00  0.00
H
```

```
ATOM   1576  CE1 PHE X 104      -2.705   5.226 -17.266  1.00  0.00
C
ATOM   1577  HE1 PHE X 104      -2.837   6.298 -17.252  1.00  0.00
H
ATOM   1578  CZ  PHE X 104      -1.495   4.571 -17.254  1.00  0.00
C
ATOM   1579  HZ  PHE X 104      -0.522   5.038 -17.316  1.00  0.00
H
ATOM   1580  CD2 PHE X 104      -2.763   2.416 -17.291  1.00  0.00
C
ATOM   1581  HD2 PHE X 104      -2.829   1.339 -17.261  1.00  0.00
H
ATOM   1582  CE2 PHE X 104      -1.555   3.076 -17.228  1.00  0.00
C
ATOM   1583  HE2 PHE X 104      -0.634   2.517 -17.164  1.00  0.00
H
ATOM   1584  C   PHE X 104      -5.649   3.259 -14.746  1.00  0.00
C
ATOM   1585  O   PHE X 104      -6.601   4.124 -14.833  1.00  0.00
O
ATOM   1586  N   HSD X 105      -4.578   3.468 -14.010  1.00  0.00
N
ATOM   1587  HN  HSD X 105      -3.952   2.692 -14.020  1.00  0.00
H
ATOM   1588  CA  HSD X 105      -4.153   4.551 -13.224  1.00  0.00
C
ATOM   1589  HA  HSD X 105      -3.552   5.128 -13.911  1.00  0.00
H
ATOM   1590  CB  HSD X 105      -5.198   5.337 -12.343  1.00  0.00
C
ATOM   1591  HB1 HSD X 105      -5.950   5.851 -12.979  1.00  0.00
H
ATOM   1592  HB2 HSD X 105      -4.670   6.122 -11.760  1.00  0.00
H
ATOM   1593  ND1 HSD X 105      -5.736   3.999 -10.145  1.00  0.00
N
ATOM   1594  HD1 HSD X 105      -5.129   4.444  -9.487  1.00  0.00
H
ATOM   1595  CG  HSD X 105      -6.046   4.423 -11.443  1.00  0.00
C
ATOM   1596  CE1 HSD X 105      -6.821   3.415  -9.681  1.00  0.00
C
ATOM   1597  HE1 HSD X 105      -6.932   3.142  -8.632  1.00  0.00
H
ATOM   1598  NE2 HSD X 105      -7.879   3.537 -10.477  1.00  0.00
N
ATOM   1599  CD2 HSD X 105      -7.330   4.146 -11.640  1.00  0.00
C
ATOM   1600  HD2 HSD X 105      -7.910   4.309 -12.539  1.00  0.00
H
```

```
ATOM   1601  C   HSD X 105      -3.165   3.965 -12.297  1.00  0.00
C
ATOM   1602  O   HSD X 105      -3.049   2.777 -12.230  1.00  0.00
O
ATOM   1603  N   SER X 106      -2.375   4.867 -11.671  1.00  0.00
N
ATOM   1604  HN  SER X 106      -2.459   5.852 -11.801  1.00  0.00
H
ATOM   1605  CA  SER X 106      -1.224   4.564 -10.715  1.00  0.00
C
ATOM   1606  HA  SER X 106      -1.089   5.509 -10.210  1.00  0.00
H
ATOM   1607  CB  SER X 106      -1.650   3.744  -9.538  1.00  0.00
C
ATOM   1608  HB1 SER X 106      -1.808   2.672  -9.786  1.00  0.00
H
ATOM   1609  HB2 SER X 106      -2.541   4.142  -9.007  1.00  0.00
H
ATOM   1610  OG  SER X 106      -0.539   3.714  -8.578  1.00  0.00
O
ATOM   1611  HG1 SER X 106       0.004   2.969  -8.843  1.00  0.00
H
ATOM   1612  C   SER X 106       0.034   4.038 -11.402  1.00  0.00
C
ATOM   1613  O   SER X 106       0.084   2.945 -11.934  1.00  0.00
O
ATOM   1614  N   ASP X 107       1.170   4.742 -11.310  1.00  0.00
N
ATOM   1615  HN  ASP X 107       1.184   5.678 -10.968  1.00  0.00
H
ATOM   1616  CA  ASP X 107       2.388   4.240 -11.926  1.00  0.00
C
ATOM   1617  HA  ASP X 107       2.219   3.359 -12.527  1.00  0.00
H
ATOM   1618  CB  ASP X 107       2.967   5.321 -12.893  1.00  0.00
C
ATOM   1619  HB1 ASP X 107       2.240   5.516 -13.710  1.00  0.00
H
ATOM   1620  HB2 ASP X 107       3.907   5.024 -13.405  1.00  0.00
H
ATOM   1621  CG  ASP X 107       3.188   6.700 -12.354  1.00  0.00
C
ATOM   1622  OD1 ASP X 107       2.771   7.044 -11.210  1.00  0.00
O
ATOM   1623  OD2 ASP X 107       4.000   7.462 -12.919  1.00  0.00
O
ATOM   1624  C   ASP X 107       3.576   3.836 -10.975  1.00  0.00
C
ATOM   1625  O   ASP X 107       4.763   4.056 -11.329  1.00  0.00
O
```

ATOM 1626 N TYR X 108 3.275 3.108 −9.842 1.00 0.00
N
ATOM 1627 HN TYR X 108 2.414 2.650 −9.634 1.00 0.00
H
ATOM 1628 CA TYR X 108 4.392 2.640 −8.944 1.00 0.00
C
ATOM 1629 HA TYR X 108 5.135 3.423 −8.997 1.00 0.00
H
ATOM 1630 CB TYR X 108 4.012 2.487 −7.461 1.00 0.00
C
ATOM 1631 HB1 TYR X 108 3.283 1.650 −7.412 1.00 0.00
H
ATOM 1632 HB2 TYR X 108 3.546 3.375 −6.982 1.00 0.00
H
ATOM 1633 CG TYR X 108 5.072 2.099 −6.469 1.00 0.00
C
ATOM 1634 CD1 TYR X 108 4.634 1.334 −5.286 1.00 0.00
C
ATOM 1635 HD1 TYR X 108 3.572 1.192 −5.146 1.00 0.00
H
ATOM 1636 CE1 TYR X 108 5.518 1.053 −4.240 1.00 0.00
C
ATOM 1637 HE1 TYR X 108 5.185 0.480 −3.388 1.00 0.00
H
ATOM 1638 CZ TYR X 108 6.868 1.306 −4.442 1.00 0.00
C
ATOM 1639 OH TYR X 108 7.800 0.812 −3.464 1.00 0.00
O
ATOM 1640 HH TYR X 108 7.351 0.323 −2.770 1.00 0.00
H
ATOM 1641 CD2 TYR X 108 6.442 2.458 −6.544 1.00 0.00
C
ATOM 1642 HD2 TYR X 108 6.850 2.983 −7.395 1.00 0.00
H
ATOM 1643 CE2 TYR X 108 7.299 2.076 −5.521 1.00 0.00
C
ATOM 1644 HE2 TYR X 108 8.368 2.218 −5.593 1.00 0.00
H
ATOM 1645 C TYR X 108 5.076 1.413 −9.540 1.00 0.00
C
ATOM 1646 O TYR X 108 4.778 0.299 −9.062 1.00 0.00
O
ATOM 1647 N TRP X 109 5.899 1.629 −10.541 1.00 0.00
N
ATOM 1648 HN TRP X 109 5.955 2.546 −10.929 1.00 0.00
H
ATOM 1649 CA TRP X 109 6.558 0.584 −11.278 1.00 0.00
C
ATOM 1650 HA TRP X 109 5.776 −0.135 −11.475 1.00 0.00
H

```
ATOM   1651  CB  TRP X 109       7.247   0.878 -12.562  1.00  0.00
C
ATOM   1652  HB1 TRP X 109       7.642  -0.068 -12.990  1.00  0.00
H
ATOM   1653  HB2 TRP X 109       8.173   1.459 -12.364  1.00  0.00
H
ATOM   1654  CG  TRP X 109       6.345   1.638 -13.492  1.00  0.00
C
ATOM   1655  CD1 TRP X 109       6.455   2.901 -13.979  1.00  0.00
C
ATOM   1656  HD1 TRP X 109       7.152   3.673 -13.689  1.00  0.00
H
ATOM   1657  NE1 TRP X 109       5.381   3.122 -14.813  1.00  0.00
N
ATOM   1658  HE1 TRP X 109       5.268   3.997 -15.231  1.00  0.00
H
ATOM   1659  CE2 TRP X 109       4.587   1.982 -14.890  1.00  0.00
C
ATOM   1660  CD2 TRP X 109       5.164   1.030 -14.057  1.00  0.00
C
ATOM   1661  CE3 TRP X 109       4.554  -0.236 -13.947  1.00  0.00
C
ATOM   1662  HE3 TRP X 109       4.992  -1.069 -13.418  1.00  0.00
H
ATOM   1663  CZ3 TRP X 109       3.490  -0.555 -14.811  1.00  0.00
C
ATOM   1664  HZ3 TRP X 109       3.174  -1.582 -14.919  1.00  0.00
H
ATOM   1665  CZ2 TRP X 109       3.454   1.676 -15.672  1.00  0.00
C
ATOM   1666  HZ2 TRP X 109       3.018   2.480 -16.248  1.00  0.00
H
ATOM   1667  CH2 TRP X 109       2.825   0.433 -15.548  1.00  0.00
C
ATOM   1668  HH2 TRP X 109       1.971   0.028 -16.071  1.00  0.00
H
ATOM   1669  C   TRP X 109       7.535  -0.174 -10.409  1.00  0.00
C
ATOM   1670  O   TRP X 109       8.159   0.325  -9.466  1.00  0.00
O
ATOM   1671  N   GLY X 110       7.600  -1.531 -10.581  1.00  0.00
N
ATOM   1672  HN  GLY X 110       6.982  -1.990 -11.215  1.00  0.00
H
ATOM   1673  CA  GLY X 110       8.651  -2.360 -10.044  1.00  0.00
C
ATOM   1674  HA1 GLY X 110       8.439  -3.384 -10.314  1.00  0.00
H
ATOM   1675  HA2 GLY X 110       8.633  -2.199  -8.976  1.00  0.00
H
```

```
ATOM   1676  C   GLY X 110       9.998  -2.039 -10.493  1.00  0.00
C
ATOM   1677  O   GLY X 110      10.242  -1.243 -11.349  1.00  0.00
O
ATOM   1678  N   GLN X 111      10.956  -2.850  -9.942  1.00  0.00
N
ATOM   1679  HN  GLN X 111      10.784  -3.606  -9.316  1.00  0.00
H
ATOM   1680  CA  GLN X 111      12.337  -2.787 -10.350  1.00  0.00
C
ATOM   1681  HA  GLN X 111      12.669  -1.772 -10.189  1.00  0.00
H
ATOM   1682  CB  GLN X 111      13.340  -3.607  -9.457  1.00  0.00
C
ATOM   1683  HB1 GLN X 111      14.353  -3.588  -9.914  1.00  0.00
H
ATOM   1684  HB2 GLN X 111      13.002  -4.665  -9.484  1.00  0.00
H
ATOM   1685  CG  GLN X 111      13.436  -3.216  -7.961  1.00  0.00
C
ATOM   1686  HG1 GLN X 111      14.310  -3.754  -7.534  1.00  0.00
H
ATOM   1687  HG2 GLN X 111      12.519  -3.644  -7.503  1.00  0.00
H
ATOM   1688  CD  GLN X 111      13.629  -1.697  -7.784  1.00  0.00
C
ATOM   1689  OE1 GLN X 111      14.773  -1.221  -7.874  1.00  0.00
O
ATOM   1690  NE2 GLN X 111      12.565  -0.964  -7.470  1.00  0.00
N
ATOM   1691 HE21 GLN X 111      12.530   0.002  -7.725  1.00  0.00
H
ATOM   1692 HE22 GLN X 111      11.731  -1.488  -7.296  1.00  0.00
H
ATOM   1693  C   GLN X 111      12.588  -3.092 -11.846  1.00  0.00
C
ATOM   1694  O   GLN X 111      13.344  -2.373 -12.553  1.00  0.00
O
ATOM   1695  N   GLY X 112      11.930  -4.102 -12.349  1.00  0.00
N
ATOM   1696  HN  GLY X 112      11.257  -4.525 -11.746  1.00  0.00
H
ATOM   1697  CA  GLY X 112      12.082  -4.505 -13.820  1.00  0.00
C
ATOM   1698  HA1 GLY X 112      12.374  -3.665 -14.433  1.00  0.00
H
ATOM   1699  HA2 GLY X 112      11.087  -4.870 -14.029  1.00  0.00
H
ATOM   1700  C   GLY X 112      13.069  -5.593 -14.092  1.00  0.00
C
```

```
ATOM   1701  O   GLY X 112      14.182  -5.688 -13.534  1.00  0.00
O
ATOM   1702  N   THR X 113      12.733  -6.478 -15.017  1.00  0.00
N
ATOM   1703  HN  THR X 113      11.862  -6.481 -15.502  1.00  0.00
H
ATOM   1704  CA  THR X 113      13.655  -7.610 -15.133  1.00  0.00
C
ATOM   1705  HA  THR X 113      14.660  -7.425 -14.784  1.00  0.00
H
ATOM   1706  CB  THR X 113      13.008  -8.892 -14.670  1.00  0.00
C
ATOM   1707  HB  THR X 113      12.031  -9.035 -15.179  1.00  0.00
H
ATOM   1708  OG1 THR X 113      12.931  -9.027 -13.295  1.00  0.00
O
ATOM   1709  HG1 THR X 113      12.157  -8.568 -12.962  1.00  0.00
H
ATOM   1710  CG2 THR X 113      13.815 -10.106 -15.098  1.00  0.00
C
ATOM   1711 HG21 THR X 113      13.267 -11.007 -14.749  1.00  0.00
H
ATOM   1712 HG22 THR X 113      14.836 -10.190 -14.669  1.00  0.00
H
ATOM   1713 HG23 THR X 113      14.031 -10.265 -16.176  1.00  0.00
H
ATOM   1714  C   THR X 113      13.866  -7.781 -16.626  1.00  0.00
C
ATOM   1715  O   THR X 113      12.918  -7.748 -17.427  1.00  0.00
O
ATOM   1716  N   LEU X 114      15.163  -7.813 -17.061  1.00  0.00
N
ATOM   1717  HN  LEU X 114      15.926  -7.805 -16.419  1.00  0.00
H
ATOM   1718  CA  LEU X 114      15.617  -8.025 -18.436  1.00  0.00
C
ATOM   1719  HA  LEU X 114      14.809  -7.635 -19.038  1.00  0.00
H
ATOM   1720  CB  LEU X 114      17.028  -7.424 -18.605  1.00  0.00
C
ATOM   1721  HB1 LEU X 114      17.729  -7.914 -17.897  1.00  0.00
H
ATOM   1722  HB2 LEU X 114      17.042  -6.345 -18.341  1.00  0.00
H
ATOM   1723  CG  LEU X 114      17.748  -7.509 -19.931  1.00  0.00
C
ATOM   1724  HG  LEU X 114      17.932  -8.541 -20.298  1.00  0.00
H
ATOM   1725  CD1 LEU X 114      17.189  -6.657 -21.075  1.00  0.00
C
```

```
ATOM   1726 HD11 LEU X 114      16.848  -5.644 -20.772  1.00  0.00
H
ATOM   1727 HD12 LEU X 114      16.244  -7.139 -21.405  1.00  0.00
H
ATOM   1728 HD13 LEU X 114      17.879  -6.549 -21.939  1.00  0.00
H
ATOM   1729  CD2 LEU X 114      19.190  -6.913 -19.773  1.00  0.00
C
ATOM   1730 HD21 LEU X 114      19.625  -7.059 -20.785  1.00  0.00
H
ATOM   1731 HD22 LEU X 114      19.713  -7.433 -18.942  1.00  0.00
H
ATOM   1732 HD23 LEU X 114      19.127  -5.807 -19.690  1.00  0.00
H
ATOM   1733  C   LEU X 114      15.691  -9.585 -18.709  1.00  0.00
C
ATOM   1734  O   LEU X 114      16.405 -10.355 -18.005  1.00  0.00
O
ATOM   1735  N   VAL X 115      14.980  -9.964 -19.709  1.00  0.00
N
ATOM   1736  HN  VAL X 115      14.490  -9.280 -20.243  1.00  0.00
H
ATOM   1737  CA  VAL X 115      15.069 -11.308 -20.356  1.00  0.00
C
ATOM   1738  HA  VAL X 115      15.595 -12.007 -19.723  1.00  0.00
H
ATOM   1739  CB  VAL X 115      13.749 -11.872 -20.694  1.00  0.00
C
ATOM   1740  HB  VAL X 115      13.369 -11.237 -21.523  1.00  0.00
H
ATOM   1741  CG1 VAL X 115      13.861 -13.336 -21.247  1.00  0.00
C
ATOM   1742 HG11 VAL X 115      14.317 -14.068 -20.548  1.00  0.00
H
ATOM   1743 HG12 VAL X 115      14.274 -13.295 -22.278  1.00  0.00
H
ATOM   1744 HG13 VAL X 115      12.824 -13.681 -21.450  1.00  0.00
H
ATOM   1745  CG2 VAL X 115      12.805 -11.816 -19.542  1.00  0.00
C
ATOM   1746 HG21 VAL X 115      13.280 -12.451 -18.763  1.00  0.00
H
ATOM   1747 HG22 VAL X 115      11.863 -12.403 -19.592  1.00  0.00
H
ATOM   1748 HG23 VAL X 115      12.581 -10.753 -19.309  1.00  0.00
H
ATOM   1749  C   VAL X 115      15.925 -11.077 -21.617  1.00  0.00
C
ATOM   1750  O   VAL X 115      15.727 -10.110 -22.370  1.00  0.00
O
```

```
ATOM   1751  N   THR X 116      16.997 -11.915 -21.816  1.00  0.00
N
ATOM   1752  HN  THR X 116      17.218 -12.696 -21.237  1.00  0.00
H
ATOM   1753  CA  THR X 116      17.876 -11.628 -22.891  1.00  0.00
C
ATOM   1754  HA  THR X 116      17.348 -11.055 -23.639  1.00  0.00
H
ATOM   1755  CB  THR X 116      19.052 -10.808 -22.464  1.00  0.00
C
ATOM   1756  HB  THR X 116      18.678  -9.816 -22.133  1.00  0.00
H
ATOM   1757  OG1 THR X 116      19.946 -10.468 -23.499  1.00  0.00
O
ATOM   1758  HG1 THR X 116      19.421  -9.916 -24.083  1.00  0.00
H
ATOM   1759  CG2 THR X 116      19.921 -11.505 -21.375  1.00  0.00
C
ATOM   1760 HG21 THR X 116      20.675 -10.848 -20.891  1.00  0.00
H
ATOM   1761 HG22 THR X 116      20.461 -12.423 -21.691  1.00  0.00
H
ATOM   1762 HG23 THR X 116      19.343 -11.824 -20.482  1.00  0.00
H
ATOM   1763  C   THR X 116      18.140 -12.895 -23.666  1.00  0.00
C
ATOM   1764  O   THR X 116      18.379 -13.988 -23.151  1.00  0.00
O
ATOM   1765  N   VAL X 117      17.933 -12.891 -25.038  1.00  0.00
N
ATOM   1766  HN  VAL X 117      17.777 -12.061 -25.568  1.00  0.00
H
ATOM   1767  CA  VAL X 117      18.049 -14.126 -25.821  1.00  0.00
C
ATOM   1768  HA  VAL X 117      18.031 -14.990 -25.174  1.00  0.00
H
ATOM   1769  CB  VAL X 117      16.958 -14.279 -26.845  1.00  0.00
C
ATOM   1770  HB  VAL X 117      16.944 -13.476 -27.613  1.00  0.00
H
ATOM   1771  CG1 VAL X 117      17.346 -15.611 -27.586  1.00  0.00
C
ATOM   1772 HG11 VAL X 117      16.615 -15.925 -28.361  1.00  0.00
H
ATOM   1773 HG12 VAL X 117      17.419 -16.520 -26.951  1.00  0.00
H
ATOM   1774 HG13 VAL X 117      18.214 -15.456 -28.261  1.00  0.00
H
ATOM   1775  CG2 VAL X 117      15.566 -14.390 -26.116  1.00  0.00
C
```

```
ATOM   1776 HG21 VAL X 117      15.607 -15.342 -25.545  1.00  0.00
H
ATOM   1777 HG22 VAL X 117      14.763 -14.394 -26.884  1.00  0.00
H
ATOM   1778 HG23 VAL X 117      15.357 -13.482 -25.511  1.00  0.00
H
ATOM   1779  C   VAL X 117      19.464 -14.184 -26.502  1.00  0.00
C
ATOM   1780  O   VAL X 117      19.714 -13.306 -27.297  1.00  0.00
O
ATOM   1781  N   SER X 118      20.240 -15.213 -26.152  1.00  0.00
N
ATOM   1782  HN  SER X 118      20.037 -15.799 -25.372  1.00  0.00
H
ATOM   1783  CA  SER X 118      21.613 -15.291 -26.673  1.00  0.00
C
ATOM   1784  HA  SER X 118      21.696 -16.368 -26.662  1.00  0.00
H
ATOM   1785  CB  SER X 118      22.011 -14.750 -28.112  1.00  0.00
C
ATOM   1786  HB1 SER X 118      21.924 -13.642 -28.129  1.00  0.00
H
ATOM   1787  HB2 SER X 118      21.220 -15.104 -28.807  1.00  0.00
H
ATOM   1788  OG  SER X 118      23.229 -15.342 -28.624  1.00  0.00
O
ATOM   1789  HG1 SER X 118      23.431 -14.793 -29.385  1.00  0.00
H
ATOM   1790  C   SER X 118      22.644 -14.870 -25.590  1.00  0.00
C
ATOM   1791  O   SER X 118      22.323 -14.064 -24.698  1.00  0.00
O
ATOM   1792  N   SER X 119      23.857 -15.433 -25.674  1.00  0.00
N
ATOM   1793  HN  SER X 119      23.956 -16.086 -26.421  1.00  0.00
H
ATOM   1794  CA  SER X 119      24.995 -15.003 -24.836  1.00  0.00
C
ATOM   1795  HA  SER X 119      24.702 -14.996 -23.796  1.00  0.00
H
ATOM   1796  CB  SER X 119      26.247 -15.950 -25.126  1.00  0.00
C
ATOM   1797  HB1 SER X 119      26.105 -17.052 -25.166  1.00  0.00
H
ATOM   1798  HB2 SER X 119      26.995 -15.627 -24.371  1.00  0.00
H
ATOM   1799  OG  SER X 119      26.889 -15.734 -26.364  1.00  0.00
O
ATOM   1800  HG1 SER X 119      27.532 -16.443 -26.447  1.00  0.00
H
```

```
ATOM   1801  C    SER X 119      25.304 −13.560 −25.279  1.00  0.00
C
ATOM   1802  O    SER X 119      24.766 −13.061 −26.307  1.00  0.00
O
ATOM   1803  NT   SER X 119      26.208 −12.879 −24.526  1.00  0.00
N
ATOM   1804  HNT  SER X 119      26.712 −13.343 −23.802  1.00  0.00
H
ATOM   1805  CAT  SER X 119      26.405 −11.419 −24.803  1.00  0.00
C
ATOM   1806  HT1  SER X 119      26.661 −11.410 −25.884  1.00  0.00
H
ATOM   1807  HT2  SER X 119      27.229 −10.936 −24.236  1.00  0.00
H
ATOM   1808  HT3  SER X 119      25.556 −10.768 −24.500  1.00  0.00
H
ATOM   1809  N    ASP X 120      −5.756  −4.217 −33.193  1.00  0.00
N
ATOM   1810  HT1  ASP X 120      −5.245  −4.115 −34.093  1.00  0.00
H
ATOM   1811  HT2  ASP X 120      −6.772  −4.195 −33.414  1.00  0.00
H
ATOM   1812  HT3  ASP X 120      −5.577  −5.137 −32.744  1.00  0.00
H
ATOM   1813  CA   ASP X 120      −5.494  −3.098 −32.140  1.00  0.00
C
ATOM   1814  HA   ASP X 120      −6.125  −3.429 −31.328  1.00  0.00
H
ATOM   1815  CB   ASP X 120      −4.025  −3.172 −31.627  1.00  0.00
C
ATOM   1816  HB1  ASP X 120      −3.938  −4.222 −31.274  1.00  0.00
H
ATOM   1817  HB2  ASP X 120      −3.999  −2.469 −30.768  1.00  0.00
H
ATOM   1818  CG   ASP X 120      −2.912  −2.775 −32.451  1.00  0.00
C
ATOM   1819  OD1  ASP X 120      −1.742  −2.969 −32.052  1.00  0.00
O
ATOM   1820  OD2  ASP X 120      −3.127  −2.327 −33.638  1.00  0.00
O
ATOM   1821  C    ASP X 120      −5.942  −1.744 −32.645  1.00  0.00
C
ATOM   1822  O    ASP X 120      −6.563  −1.658 −33.681  1.00  0.00
O
ATOM   1823  N    ILE X 121      −5.650  −0.657 −31.939  1.00  0.00
N
ATOM   1824  HN   ILE X 121      −5.179  −0.693 −31.060  1.00  0.00
H
ATOM   1825  CA   ILE X 121      −5.725   0.650 −32.511  1.00  0.00
C
```

ATOM 1826 HA ILE X 121 −6.098 0.741 −33.521 1.00 0.00
H
ATOM 1827 CB ILE X 121 −6.381 1.659 −31.535 1.00 0.00
C
ATOM 1828 HB ILE X 121 −5.773 1.695 −30.606 1.00 0.00
H
ATOM 1829 CG2 ILE X 121 −6.310 3.106 −32.114 1.00 0.00
C
ATOM 1830 HG21 ILE X 121 −5.297 3.369 −32.487 1.00 0.00
H
ATOM 1831 HG22 ILE X 121 −6.603 3.787 −31.286 1.00 0.00
H
ATOM 1832 HG23 ILE X 121 −7.102 3.346 −32.856 1.00 0.00
H
ATOM 1833 CG1 ILE X 121 −7.859 1.199 −31.322 1.00 0.00
C
ATOM 1834 HG11 ILE X 121 −7.812 0.213 −30.812 1.00 0.00
H
ATOM 1835 HG12 ILE X 121 −8.321 0.810 −32.254 1.00 0.00
H
ATOM 1836 CD ILE X 121 −8.807 2.166 −30.546 1.00 0.00
C
ATOM 1837 HD1 ILE X 121 −8.640 3.232 −30.813 1.00 0.00
H
ATOM 1838 HD2 ILE X 121 −8.683 2.057 −29.447 1.00 0.00
H
ATOM 1839 HD3 ILE X 121 −9.836 2.050 −30.950 1.00 0.00
H
ATOM 1840 C ILE X 121 −4.261 1.122 −32.638 1.00 0.00
C
ATOM 1841 O ILE X 121 −3.560 1.229 −31.669 1.00 0.00
O
ATOM 1842 N GLN X 122 −3.759 1.430 −33.844 1.00 0.00
N
ATOM 1843 HN GLN X 122 −4.442 1.438 −34.570 1.00 0.00
H
ATOM 1844 CA GLN X 122 −2.518 2.009 −34.103 1.00 0.00
C
ATOM 1845 HA GLN X 122 −1.899 1.676 −33.283 1.00 0.00
H
ATOM 1846 CB GLN X 122 −1.807 1.412 −35.328 1.00 0.00
C
ATOM 1847 HB1 GLN X 122 −1.680 0.310 −35.266 1.00 0.00
H
ATOM 1848 HB2 GLN X 122 −0.747 1.724 −35.439 1.00 0.00
H
ATOM 1849 CG GLN X 122 −2.641 1.601 −36.622 1.00 0.00
C
ATOM 1850 HG1 GLN X 122 −2.560 2.637 −37.014 1.00 0.00
H

```
ATOM   1851  HG2 GLN X 122      -3.725   1.365 -36.566  1.00  0.00
H
ATOM   1852  CD  GLN X 122      -2.214   0.697 -37.857  1.00  0.00
C
ATOM   1853  OE1 GLN X 122      -1.379   0.993 -38.727  1.00  0.00
O
ATOM   1854  NE2 GLN X 122      -2.744  -0.534 -37.766  1.00  0.00
N
ATOM   1855 HE21 GLN X 122      -3.189  -0.703 -36.887  1.00  0.00
H
ATOM   1856 HE22 GLN X 122      -2.343  -1.236 -38.355  1.00  0.00
H
ATOM   1857  C   GLN X 122      -2.490   3.505 -34.058  1.00  0.00
C
ATOM   1858  O   GLN X 122      -3.214   4.228 -34.708  1.00  0.00
O
ATOM   1859  N   MET X 123      -1.555   4.050 -33.310  1.00  0.00
N
ATOM   1860  HN  MET X 123      -0.861   3.505 -32.847  1.00  0.00
H
ATOM   1861  CA  MET X 123      -1.427   5.411 -32.849  1.00  0.00
C
ATOM   1862  HA  MET X 123      -2.188   5.983 -33.358  1.00  0.00
H
ATOM   1863  CB  MET X 123      -1.715   5.645 -31.375  1.00  0.00
C
ATOM   1864  HB1 MET X 123      -1.564   6.740 -31.265  1.00  0.00
H
ATOM   1865  HB2 MET X 123      -1.005   5.061 -30.751  1.00  0.00
H
ATOM   1866  CG  MET X 123      -3.012   5.154 -31.036  1.00  0.00
C
ATOM   1867  HG1 MET X 123      -2.896   4.066 -30.843  1.00  0.00
H
ATOM   1868  HG2 MET X 123      -3.802   5.175 -31.817  1.00  0.00
H
ATOM   1869  SD  MET X 123      -3.737   6.167 -29.738  1.00  0.00
S
ATOM   1870  CE  MET X 123      -2.643   5.870 -28.388  1.00  0.00
C
ATOM   1871  HE1 MET X 123      -2.985   4.850 -28.111  1.00  0.00
H
ATOM   1872  HE2 MET X 123      -2.801   6.520 -27.502  1.00  0.00
H
ATOM   1873  HE3 MET X 123      -1.557   5.840 -28.620  1.00  0.00
H
ATOM   1874  C   MET X 123      -0.169   6.060 -33.283  1.00  0.00
C
ATOM   1875  O   MET X 123       0.928   5.581 -33.166  1.00  0.00
O
```

```
ATOM   1876  N   THR X 124      -0.170   7.284 -33.887  1.00  0.00
N
ATOM   1877  HN  THR X 124      -0.993   7.794 -34.128  1.00  0.00
H
ATOM   1878  CA  THR X 124       1.031   7.950 -34.349  1.00  0.00
C
ATOM   1879  HA  THR X 124       1.850   7.249 -34.408  1.00  0.00
H
ATOM   1880  CB  THR X 124       0.838   8.424 -35.767  1.00  0.00
C
ATOM   1881  HB  THR X 124       0.665   7.491 -36.346  1.00  0.00
H
ATOM   1882  OG1 THR X 124       2.049   9.070 -36.281  1.00  0.00
O
ATOM   1883  HG1 THR X 124       2.781   8.586 -35.893  1.00  0.00
H
ATOM   1884  CG2 THR X 124      -0.335   9.461 -35.790  1.00  0.00
C
ATOM   1885 HG21 THR X 124      -0.342  10.276 -35.035  1.00  0.00
H
ATOM   1886 HG22 THR X 124      -1.324   8.956 -35.808  1.00  0.00
H
ATOM   1887 HG23 THR X 124      -0.339   9.923 -36.800  1.00  0.00
H
ATOM   1888  C   THR X 124       1.349   9.069 -33.381  1.00  0.00
C
ATOM   1889  O   THR X 124       0.528   9.640 -32.676  1.00  0.00
O
ATOM   1890  N   GLN X 125       2.663   9.462 -33.294  1.00  0.00
N
ATOM   1891  HN  GLN X 125       3.428   9.288 -33.910  1.00  0.00
H
ATOM   1892  CA  GLN X 125       2.932  10.732 -32.635  1.00  0.00
C
ATOM   1893  HA  GLN X 125       2.037  11.240 -32.306  1.00  0.00
H
ATOM   1894  CB  GLN X 125       3.767  10.558 -31.362  1.00  0.00
C
ATOM   1895  HB1 GLN X 125       4.060  11.541 -30.936  1.00  0.00
H
ATOM   1896  HB2 GLN X 125       4.637   9.896 -31.563  1.00  0.00
H
ATOM   1897  CG  GLN X 125       2.851   9.866 -30.310  1.00  0.00
C
ATOM   1898  HG1 GLN X 125       2.352   8.913 -30.590  1.00  0.00
H
ATOM   1899  HG2 GLN X 125       2.049  10.615 -30.138  1.00  0.00
H
ATOM   1900  CD  GLN X 125       3.462   9.609 -28.893  1.00  0.00
C
```

```
ATOM   1901  OE1 GLN X 125       3.343   8.489 -28.441  1.00  0.00
O
ATOM   1902  NE2 GLN X 125       4.254  10.646 -28.423  1.00  0.00
N
ATOM   1903 HE21 GLN X 125       4.721  10.508 -27.550  1.00  0.00
H
ATOM   1904 HE22 GLN X 125       4.644  11.278 -29.094  1.00  0.00
H
ATOM   1905  C   GLN X 125       3.677  11.656 -33.498  1.00  0.00
C
ATOM   1906  O   GLN X 125       4.471  11.226 -34.323  1.00  0.00
O
ATOM   1907  N   SER X 126       3.417  13.031 -33.322  1.00  0.00
N
ATOM   1908  HN  SER X 126       2.743  13.326 -32.649  1.00  0.00
H
ATOM   1909  CA  SER X 126       4.014  14.149 -34.107  1.00  0.00
C
ATOM   1910  HA  SER X 126       4.882  13.716 -34.582  1.00  0.00
H
ATOM   1911  CB  SER X 126       2.967  14.610 -35.168  1.00  0.00
C
ATOM   1912  HB1 SER X 126       2.168  15.154 -34.621  1.00  0.00
H
ATOM   1913  HB2 SER X 126       2.709  13.739 -35.808  1.00  0.00
H
ATOM   1914  OG  SER X 126       3.595  15.533 -36.017  1.00  0.00
O
ATOM   1915  HG1 SER X 126       3.074  16.328 -36.148  1.00  0.00
H
ATOM   1916  C   SER X 126       4.341  15.325 -33.226  1.00  0.00
C
ATOM   1917  O   SER X 126       3.624  15.519 -32.232  1.00  0.00
O
ATOM   1918  N   PRO X 127       5.431  16.063 -33.418  1.00  0.00
N
ATOM   1919  CD  PRO X 127       5.648  17.299 -32.673  1.00  0.00
C
ATOM   1920  HD1 PRO X 127       5.303  17.246 -31.618  1.00  0.00
H
ATOM   1921  HD2 PRO X 127       5.227  18.220 -33.130  1.00  0.00
H
ATOM   1922  CA  PRO X 127       6.626  15.599 -34.185  1.00  0.00
C
ATOM   1923  HA  PRO X 127       6.155  15.383 -35.132  1.00  0.00
H
ATOM   1924  CB  PRO X 127       7.605  16.823 -34.070  1.00  0.00
C
ATOM   1925  HB1 PRO X 127       7.365  17.470 -34.941  1.00  0.00
H
```

```
ATOM   1926  HB2 PRO X 127       8.699  16.628 -34.087  1.00  0.00
H
ATOM   1927  CG  PRO X 127       7.173  17.618 -32.769  1.00  0.00
C
ATOM   1928  HG1 PRO X 127       7.626  17.097 -31.899  1.00  0.00
H
ATOM   1929  HG2 PRO X 127       7.315  18.717 -32.696  1.00  0.00
H
ATOM   1930  C   PRO X 127       7.292  14.332 -33.659  1.00  0.00
C
ATOM   1931  O   PRO X 127       7.148  13.841 -32.533  1.00  0.00
O
ATOM   1932  N   SER X 128       8.054  13.719 -34.535  1.00  0.00
N
ATOM   1933  HN  SER X 128       8.270  13.983 -35.472  1.00  0.00
H
ATOM   1934  CA  SER X 128       8.761  12.454 -34.100  1.00  0.00
C
ATOM   1935  HA  SER X 128       8.011  11.861 -33.598  1.00  0.00
H
ATOM   1936  CB  SER X 128       9.282  11.593 -35.288  1.00  0.00
C
ATOM   1937  HB1 SER X 128       9.917  12.336 -35.818  1.00  0.00
H
ATOM   1938  HB2 SER X 128       8.484  11.327 -36.014  1.00  0.00
H
ATOM   1939  OG  SER X 128       9.956  10.396 -34.894  1.00  0.00
O
ATOM   1940  HG1 SER X 128      10.503  10.139 -35.640  1.00  0.00
H
ATOM   1941  C   SER X 128       9.892  12.780 -33.128  1.00  0.00
C
ATOM   1942  O   SER X 128      10.129  12.127 -32.153  1.00  0.00
O
ATOM   1943  N   THR X 129      10.665  13.805 -33.529  1.00  0.00
N
ATOM   1944  HN  THR X 129      10.322  14.327 -34.306  1.00  0.00
H
ATOM   1945  CA  THR X 129      11.796  14.244 -32.745  1.00  0.00
C
ATOM   1946  HA  THR X 129      11.565  14.001 -31.718  1.00  0.00
H
ATOM   1947  CB  THR X 129      13.146  13.556 -32.982  1.00  0.00
C
ATOM   1948  HB  THR X 129      13.040  12.495 -32.671  1.00  0.00
H
ATOM   1949  OG1 THR X 129      14.193  14.079 -32.150  1.00  0.00
O
ATOM   1950  HG1 THR X 129      14.974  13.561 -32.357  1.00  0.00
H
```

```
ATOM   1951  CG2 THR X 129      13.568  13.731 -34.499  1.00  0.00
C
ATOM   1952 HG21 THR X 129      12.746  13.244 -35.066  1.00  0.00
H
ATOM   1953 HG22 THR X 129      14.539  13.207 -34.631  1.00  0.00
H
ATOM   1954 HG23 THR X 129      13.753  14.767 -34.856  1.00  0.00
H
ATOM   1955  C   THR X 129      11.897  15.794 -32.892  1.00  0.00
C
ATOM   1956  O   THR X 129      11.604  16.374 -33.906  1.00  0.00
O
ATOM   1957  N   LEU X 130      12.202  16.443 -31.806  1.00  0.00
N
ATOM   1958  HN  LEU X 130      12.418  15.868 -31.021  1.00  0.00
H
ATOM   1959  CA  LEU X 130      12.306  17.867 -31.685  1.00  0.00
C
ATOM   1960  HA  LEU X 130      12.303  18.347 -32.653  1.00  0.00
H
ATOM   1961  CB  LEU X 130      11.100  18.344 -30.846  1.00  0.00
C
ATOM   1962  HB1 LEU X 130      11.086  18.052 -29.774  1.00  0.00
H
ATOM   1963  HB2 LEU X 130      10.193  17.965 -31.362  1.00  0.00
H
ATOM   1964  CG  LEU X 130      11.041  19.855 -30.757  1.00  0.00
C
ATOM   1965  HG  LEU X 130      11.880  20.237 -30.136  1.00  0.00
H
ATOM   1966  CD1 LEU X 130      10.953  20.522 -32.141  1.00  0.00
C
ATOM   1967 HD11 LEU X 130      10.026  20.219 -32.673  1.00  0.00
H
ATOM   1968 HD12 LEU X 130      11.845  20.272 -32.755  1.00  0.00
H
ATOM   1969 HD13 LEU X 130      10.937  21.629 -32.052  1.00  0.00
H
ATOM   1970  CD2 LEU X 130       9.711  20.129 -29.900  1.00  0.00
C
ATOM   1971 HD21 LEU X 130       9.857  19.582 -28.944  1.00  0.00
H
ATOM   1972 HD22 LEU X 130       8.869  19.728 -30.505  1.00  0.00
H
ATOM   1973 HD23 LEU X 130       9.625  21.224 -29.731  1.00  0.00
H
ATOM   1974  C   LEU X 130      13.640  18.287 -31.035  1.00  0.00
C
ATOM   1975  O   LEU X 130      14.003  17.678 -30.005  1.00  0.00
O
```

ATOM 1976 N SER X 131 14.403 19.215 −31.621 1.00 0.00
N
ATOM 1977 HN SER X 131 14.172 19.624 −32.500 1.00 0.00
H
ATOM 1978 CA SER X 131 15.628 19.647 −30.962 1.00 0.00
C
ATOM 1979 HA SER X 131 15.912 18.868 −30.270 1.00 0.00
H
ATOM 1980 CB SER X 131 16.689 19.782 −31.968 1.00 0.00
C
ATOM 1981 HB1 SER X 131 16.606 20.700 −32.588 1.00 0.00
H
ATOM 1982 HB2 SER X 131 16.684 18.873 −32.606 1.00 0.00
H
ATOM 1983 OG SER X 131 17.951 19.765 −31.373 1.00 0.00
O
ATOM 1984 HG1 SER X 131 18.206 18.852 −31.524 1.00 0.00
H
ATOM 1985 C SER X 131 15.227 20.920 −30.289 1.00 0.00
C
ATOM 1986 O SER X 131 14.891 21.899 −30.903 1.00 0.00
O
ATOM 1987 N ALA X 132 15.204 20.983 −28.928 1.00 0.00
N
ATOM 1988 HN ALA X 132 15.524 20.310 −28.265 1.00 0.00
H
ATOM 1989 CA ALA X 132 14.643 22.141 −28.172 1.00 0.00
C
ATOM 1990 HA ALA X 132 14.801 22.983 −28.829 1.00 0.00
H
ATOM 1991 CB ALA X 132 13.160 21.961 −27.860 1.00 0.00
C
ATOM 1992 HB1 ALA X 132 12.746 22.988 −27.776 1.00 0.00
H
ATOM 1993 HB2 ALA X 132 12.795 21.271 −27.070 1.00 0.00
H
ATOM 1994 HB3 ALA X 132 12.558 21.512 −28.679 1.00 0.00
H
ATOM 1995 C ALA X 132 15.578 22.559 −27.046 1.00 0.00
C
ATOM 1996 O ALA X 132 16.572 21.934 −26.678 1.00 0.00
O
ATOM 1997 N SER X 133 15.194 23.693 −26.468 1.00 0.00
N
ATOM 1998 HN SER X 133 14.403 24.198 −26.804 1.00 0.00
H
ATOM 1999 CA SER X 133 16.076 24.464 −25.589 1.00 0.00
C
ATOM 2000 HA SER X 133 16.842 23.844 −25.148 1.00 0.00
H

```
ATOM   2001  CB  SER X 133      16.659  25.563 -26.512  1.00  0.00
C
ATOM   2002  HB1 SER X 133      15.816  26.139 -26.949  1.00  0.00
H
ATOM   2003  HB2 SER X 133      17.170  25.133 -27.400  1.00  0.00
H
ATOM   2004  OG  SER X 133      17.600  26.420 -25.891  1.00  0.00
O
ATOM   2005  HG1 SER X 133      17.989  26.959 -26.584  1.00  0.00
H
ATOM   2006  C   SER X 133      15.234  24.892 -24.381  1.00  0.00
C
ATOM   2007  O   SER X 133      14.001  24.922 -24.468  1.00  0.00
O
ATOM   2008  N   VAL X 134      16.048  25.305 -23.329  1.00  0.00
N
ATOM   2009  HN  VAL X 134      17.040  25.222 -23.262  1.00  0.00
H
ATOM   2010  CA  VAL X 134      15.481  26.052 -22.122  1.00  0.00
C
ATOM   2011  HA  VAL X 134      14.792  25.429 -21.571  1.00  0.00
H
ATOM   2012  CB  VAL X 134      16.568  26.415 -21.127  1.00  0.00
C
ATOM   2013  HB  VAL X 134      17.143  27.243 -21.592  1.00  0.00
H
ATOM   2014  CG1 VAL X 134      15.924  26.974 -19.793  1.00  0.00
C
ATOM   2015 HG11 VAL X 134      15.385  26.132 -19.308  1.00  0.00
H
ATOM   2016 HG12 VAL X 134      15.144  27.743 -19.984  1.00  0.00
H
ATOM   2017 HG13 VAL X 134      16.701  27.215 -19.037  1.00  0.00
H
ATOM   2018  CG2 VAL X 134      17.393  25.179 -20.826  1.00  0.00
C
ATOM   2019 HG21 VAL X 134      16.652  24.516 -20.330  1.00  0.00
H
ATOM   2020 HG22 VAL X 134      18.180  25.366 -20.064  1.00  0.00
H
ATOM   2021 HG23 VAL X 134      17.954  24.777 -21.696  1.00  0.00
H
ATOM   2022  C   VAL X 134      14.790  27.308 -22.605  1.00  0.00
C
ATOM   2023  O   VAL X 134      15.374  28.213 -23.197  1.00  0.00
O
ATOM   2024  N   GLY X 135      13.460  27.375 -22.353  1.00  0.00
N
ATOM   2025  HN  GLY X 135      13.067  26.604 -21.858  1.00  0.00
H
```

```
ATOM   2026  CA  GLY X 135      12.583  28.470 −22.782  1.00  0.00
C
ATOM   2027  HA1 GLY X 135      13.050  29.342 −23.217  1.00  0.00
H
ATOM   2028  HA2 GLY X 135      12.055  28.900 −21.944  1.00  0.00
H
ATOM   2029  C   GLY X 135      11.481  28.054 −23.845  1.00  0.00
C
ATOM   2030  O   GLY X 135      10.384  28.634 −23.899  1.00  0.00
O
ATOM   2031  N   ASP X 136      11.808  26.922 −24.560  1.00  0.00
N
ATOM   2032  HN  ASP X 136      12.618  26.372 −24.373  1.00  0.00
H
ATOM   2033  CA  ASP X 136      10.962  26.464 −25.577  1.00  0.00
C
ATOM   2034  HA  ASP X 136      10.599  27.321 −26.125  1.00  0.00
H
ATOM   2035  CB  ASP X 136      11.629  25.479 −26.576  1.00  0.00
C
ATOM   2036  HB1 ASP X 136      10.854  25.079 −27.264  1.00  0.00
H
ATOM   2037  HB2 ASP X 136      11.984  24.598 −25.999  1.00  0.00
H
ATOM   2038  CG  ASP X 136      12.814  26.065 −27.328  1.00  0.00
C
ATOM   2039  OD1 ASP X 136      13.112  27.286 −27.086  1.00  0.00
O
ATOM   2040  OD2 ASP X 136      13.480  25.239 −28.000  1.00  0.00
O
ATOM   2041  C   ASP X 136       9.603  25.871 −25.224  1.00  0.00
C
ATOM   2042  O   ASP X 136       9.435  24.999 −24.442  1.00  0.00
O
ATOM   2043  N   ARG X 137       8.553  26.376 −25.947  1.00  0.00
N
ATOM   2044  HN  ARG X 137       8.802  27.219 −26.416  1.00  0.00
H
ATOM   2045  CA  ARG X 137       7.128  25.973 −25.824  1.00  0.00
C
ATOM   2046  HA  ARG X 137       6.893  25.496 −24.884  1.00  0.00
H
ATOM   2047  CB  ARG X 137       6.167  27.167 −26.151  1.00  0.00
C
ATOM   2048  HB1 ARG X 137       5.192  26.845 −26.575  1.00  0.00
H
ATOM   2049  HB2 ARG X 137       6.655  27.735 −26.972  1.00  0.00
H
ATOM   2050  CG  ARG X 137       5.851  28.093 −24.913  1.00  0.00
C
```

ATOM 2051 HG1 ARG X 137 5.538 27.470 −24.048 1.00 0.00
H
ATOM 2052 HG2 ARG X 137 4.936 28.672 −25.160 1.00 0.00
H
ATOM 2053 CD ARG X 137 7.024 28.981 −24.606 1.00 0.00
C
ATOM 2054 HD1 ARG X 137 7.118 29.758 −25.393 1.00 0.00
H
ATOM 2055 HD2 ARG X 137 7.944 28.401 −24.377 1.00 0.00
H
ATOM 2056 NE ARG X 137 6.660 29.761 −23.432 1.00 0.00
N
ATOM 2057 HE ARG X 137 5.750 29.792 −23.018 1.00 0.00
H
ATOM 2058 CZ ARG X 137 7.426 30.724 −22.920 1.00 0.00
C
ATOM 2059 NH1 ARG X 137 8.677 30.815 −23.188 1.00 0.00
N
ATOM 2060 HH11 ARG X 137 9.174 29.954 −23.295 1.00 0.00
H
ATOM 2061 HH12 ARG X 137 9.131 31.429 −22.543 1.00 0.00
H
ATOM 2062 NH2 ARG X 137 6.871 31.615 −22.179 1.00 0.00
N
ATOM 2063 HH21 ARG X 137 5.918 31.423 −21.944 1.00 0.00
H
ATOM 2064 HH22 ARG X 137 7.254 32.492 −21.889 1.00 0.00
H
ATOM 2065 C ARG X 137 6.854 24.863 −26.805 1.00 0.00
C
ATOM 2066 O ARG X 137 6.762 25.000 −28.016 1.00 0.00
O
ATOM 2067 N VAL X 138 6.665 23.739 −26.249 1.00 0.00
N
ATOM 2068 HN VAL X 138 6.603 23.633 −25.259 1.00 0.00
H
ATOM 2069 CA VAL X 138 6.597 22.439 −26.964 1.00 0.00
C
ATOM 2070 HA VAL X 138 6.747 22.501 −28.032 1.00 0.00
H
ATOM 2071 CB VAL X 138 7.489 21.448 −26.345 1.00 0.00
C
ATOM 2072 HB VAL X 138 7.328 21.418 −25.246 1.00 0.00
H
ATOM 2073 CG1 VAL X 138 7.452 20.031 −26.941 1.00 0.00
C
ATOM 2074 HG11 VAL X 138 8.224 19.382 −26.476 1.00 0.00
H
ATOM 2075 HG12 VAL X 138 7.449 20.040 −28.051 1.00 0.00
H

```
ATOM   2076 HG13 VAL X 138       6.545  19.480 -26.612  1.00  0.00
H
ATOM   2077  CG2 VAL X 138       8.971  21.889 -26.563  1.00  0.00
C
ATOM   2078 HG21 VAL X 138       9.190  22.185 -27.611  1.00  0.00
H
ATOM   2079 HG22 VAL X 138       9.657  21.061 -26.286  1.00  0.00
H
ATOM   2080 HG23 VAL X 138       9.303  22.712 -25.894  1.00  0.00
H
ATOM   2081  C   VAL X 138       5.272  21.866 -26.872  1.00  0.00
C
ATOM   2082  O   VAL X 138       4.751  21.705 -25.811  1.00  0.00
O
ATOM   2083  N   THR X 139       4.664  21.434 -28.001  1.00  0.00
N
ATOM   2084  HN  THR X 139       5.045  21.569 -28.912  1.00  0.00
H
ATOM   2085  CA  THR X 139       3.330  20.716 -27.955  1.00  0.00
C
ATOM   2086  HA  THR X 139       3.097  20.472 -26.929  1.00  0.00
H
ATOM   2087  CB  THR X 139       2.080  21.435 -28.458  1.00  0.00
C
ATOM   2088  HB  THR X 139       2.293  21.999 -29.391  1.00  0.00
H
ATOM   2089  OG1 THR X 139       1.730  22.349 -27.471  1.00  0.00
O
ATOM   2090  HG1 THR X 139       2.335  23.078 -27.625  1.00  0.00
H
ATOM   2091  CG2 THR X 139       0.826  20.505 -28.718  1.00  0.00
C
ATOM   2092 HG21 THR X 139      -0.015  21.222 -28.837  1.00  0.00
H
ATOM   2093 HG22 THR X 139       0.625  19.998 -27.750  1.00  0.00
H
ATOM   2094 HG23 THR X 139       0.889  19.915 -29.657  1.00  0.00
H
ATOM   2095  C   THR X 139       3.499  19.438 -28.805  1.00  0.00
C
ATOM   2096  O   THR X 139       4.019  19.505 -29.919  1.00  0.00
O
ATOM   2097  N   ILE X 140       3.148  18.282 -28.167  1.00  0.00
N
ATOM   2098  HN  ILE X 140       2.798  18.304 -27.234  1.00  0.00
H
ATOM   2099  CA  ILE X 140       3.340  17.019 -28.918  1.00  0.00
C
ATOM   2100  HA  ILE X 140       3.682  17.244 -29.917  1.00  0.00
H
```

```
ATOM   2101  CB  ILE X 140       4.111  16.117 -28.114  1.00  0.00
C
ATOM   2102  HB  ILE X 140       3.585  15.917 -27.157  1.00  0.00
H
ATOM   2103  CG2 ILE X 140       4.201  14.676 -28.832  1.00  0.00
C
ATOM   2104 HG21 ILE X 140       4.690  14.053 -28.053  1.00  0.00
H
ATOM   2105 HG22 ILE X 140       4.650  14.705 -29.848  1.00  0.00
H
ATOM   2106 HG23 ILE X 140       3.176  14.251 -28.891  1.00  0.00
H
ATOM   2107  CG1 ILE X 140       5.504  16.650 -27.871  1.00  0.00
C
ATOM   2108 HG11 ILE X 140       5.649  17.704 -27.552  1.00  0.00
H
ATOM   2109 HG12 ILE X 140       6.227  16.509 -28.702  1.00  0.00
H
ATOM   2110  CD  ILE X 140       6.072  15.846 -26.575  1.00  0.00
C
ATOM   2111  HD1 ILE X 140       6.244  14.768 -26.780  1.00  0.00
H
ATOM   2112  HD2 ILE X 140       5.388  16.008 -25.714  1.00  0.00
H
ATOM   2113  HD3 ILE X 140       7.084  16.228 -26.318  1.00  0.00
H
ATOM   2114  C   ILE X 140       1.946  16.422 -29.083  1.00  0.00
C
ATOM   2115  O   ILE X 140       1.097  16.453 -28.226  1.00  0.00
O
ATOM   2116  N   THR X 141       1.666  15.845 -30.276  1.00  0.00
N
ATOM   2117  HN  THR X 141       2.379  15.760 -30.968  1.00  0.00
H
ATOM   2118  CA  THR X 141       0.360  15.569 -30.744  1.00  0.00
C
ATOM   2119  HA  THR X 141      -0.391  15.741 -29.987  1.00  0.00
H
ATOM   2120  CB  THR X 141      -0.054  16.374 -31.973  1.00  0.00
C
ATOM   2121  HB  THR X 141       0.562  16.162 -32.873  1.00  0.00
H
ATOM   2122  OG1 THR X 141       0.001  17.799 -31.809  1.00  0.00
O
ATOM   2123  HG1 THR X 141       0.949  17.944 -31.781  1.00  0.00
H
ATOM   2124  CG2 THR X 141      -1.575  16.117 -32.247  1.00  0.00
C
ATOM   2125 HG21 THR X 141      -1.924  16.736 -33.101  1.00  0.00
H
```

```
ATOM   2126 HG22 THR X 141      -2.147  16.528 -31.387  1.00  0.00
H
ATOM   2127 HG23 THR X 141      -1.700  15.038 -32.479  1.00  0.00
H
ATOM   2128  C   THR X 141       0.377  14.140 -31.111  1.00  0.00
C
ATOM   2129  O   THR X 141       1.245  13.641 -31.828  1.00  0.00
O
ATOM   2130  N   CYS X 142      -0.592  13.348 -30.577  1.00  0.00
N
ATOM   2131  HN  CYS X 142      -1.262  13.692 -29.923  1.00  0.00
H
ATOM   2132  CA  CYS X 142      -0.801  11.912 -30.838  1.00  0.00
C
ATOM   2133  HA  CYS X 142      -0.029  11.482 -31.459  1.00  0.00
H
ATOM   2134  CB  CYS X 142      -0.909  11.230 -29.492  1.00  0.00
C
ATOM   2135  HB1 CYS X 142      -1.792  11.476 -28.863  1.00  0.00
H
ATOM   2136  HB2 CYS X 142       0.019  11.541 -28.966  1.00  0.00
H
ATOM   2137  SG  CYS X 142      -0.948   9.410 -29.680  1.00  0.00
S
ATOM   2138  C   CYS X 142      -2.214  11.849 -31.587  1.00  0.00
C
ATOM   2139  O   CYS X 142      -3.194  12.546 -31.248  1.00  0.00
O
ATOM   2140  N   ARG X 143      -2.274  10.887 -32.578  1.00  0.00
N
ATOM   2141  HN  ARG X 143      -1.580  10.176 -32.664  1.00  0.00
H
ATOM   2142  CA  ARG X 143      -3.563  10.576 -33.273  1.00  0.00
C
ATOM   2143  HA  ARG X 143      -4.423  10.994 -32.771  1.00  0.00
H
ATOM   2144  CB  ARG X 143      -3.690  11.249 -34.684  1.00  0.00
C
ATOM   2145  HB1 ARG X 143      -4.637  10.890 -35.141  1.00  0.00
H
ATOM   2146  HB2 ARG X 143      -2.848  10.843 -35.285  1.00  0.00
H
ATOM   2147  CG  ARG X 143      -3.617  12.748 -34.608  1.00  0.00
C
ATOM   2148  HG1 ARG X 143      -2.538  12.892 -34.386  1.00  0.00
H
ATOM   2149  HG2 ARG X 143      -4.276  13.049 -33.765  1.00  0.00
H
ATOM   2150  CD  ARG X 143      -4.027  13.455 -35.940  1.00  0.00
C
```

```
ATOM   2151  HD1 ARG X 143      -5.134  13.352 -35.958  1.00  0.00
H
ATOM   2152  HD2 ARG X 143      -3.535  13.027 -36.839  1.00  0.00
H
ATOM   2153  NE  ARG X 143      -3.726  14.896 -35.784  1.00  0.00
N
ATOM   2154  HE  ARG X 143      -4.393  15.494 -35.340  1.00  0.00
H
ATOM   2155  CZ  ARG X 143      -2.628  15.514 -36.188  1.00  0.00
C
ATOM   2156  NH1 ARG X 143      -1.581  14.891 -36.583  1.00  0.00
N
ATOM   2157 HH11 ARG X 143      -1.544  13.904 -36.738  1.00  0.00
H
ATOM   2158 HH12 ARG X 143      -0.826  15.394 -37.004  1.00  0.00
H
ATOM   2159  NH2 ARG X 143      -2.697  16.881 -36.151  1.00  0.00
N
ATOM   2160 HH21 ARG X 143      -3.492  17.105 -35.588  1.00  0.00
H
ATOM   2161 HH22 ARG X 143      -1.839  17.393 -36.176  1.00  0.00
H
ATOM   2162  C   ARG X 143      -3.865   9.087 -33.331  1.00  0.00
C
ATOM   2163  O   ARG X 143      -3.034   8.312 -33.080  1.00  0.00
O
ATOM   2164  N   ALA X 144      -5.202   8.818 -33.542  1.00  0.00
N
ATOM   2165  HN  ALA X 144      -5.776   9.607 -33.749  1.00  0.00
H
ATOM   2166  CA  ALA X 144      -5.602   7.418 -33.400  1.00  0.00
C
ATOM   2167  HA  ALA X 144      -4.791   6.704 -33.417  1.00  0.00
H
ATOM   2168  CB  ALA X 144      -6.318   7.304 -32.072  1.00  0.00
C
ATOM   2169  HB1 ALA X 144      -5.614   7.674 -31.296  1.00  0.00
H
ATOM   2170  HB2 ALA X 144      -6.501   6.287 -31.663  1.00  0.00
H
ATOM   2171  HB3 ALA X 144      -7.217   7.957 -32.066  1.00  0.00
H
ATOM   2172  C   ALA X 144      -6.506   6.970 -34.616  1.00  0.00
C
ATOM   2173  O   ALA X 144      -7.405   7.629 -35.095  1.00  0.00
O
ATOM   2174  N   SER X 145      -6.325   5.690 -35.011  1.00  0.00
N
ATOM   2175  HN  SER X 145      -5.613   5.206 -34.507  1.00  0.00
H
```

ATOM	2176	CA	SER	X	145	−7.063	4.995	−36.149	1.00	0.00	C
ATOM	2177	HA	SER	X	145	−6.860	5.601	−37.019	1.00	0.00	H
ATOM	2178	CB	SER	X	145	−6.514	3.553	−36.446	1.00	0.00	C
ATOM	2179	HB1	SER	X	145	−5.432	3.794	−36.523	1.00	0.00	H
ATOM	2180	HB2	SER	X	145	−7.023	3.116	−37.332	1.00	0.00	H
ATOM	2181	OG	SER	X	145	−6.562	2.720	−35.344	1.00	0.00	O
ATOM	2182	HG1	SER	X	145	−7.285	2.091	−35.405	1.00	0.00	H
ATOM	2183	C	SER	X	145	−8.550	4.937	−35.936	1.00	0.00	C
ATOM	2184	O	SER	X	145	−9.344	5.085	−36.836	1.00	0.00	O
ATOM	2185	N	GLN	X	146	−8.956	4.587	−34.699	1.00	0.00	N
ATOM	2186	HN	GLN	X	146	−8.207	4.490	−34.048	1.00	0.00	H
ATOM	2187	CA	GLN	X	146	−10.351	4.624	−34.231	1.00	0.00	C
ATOM	2188	HA	GLN	X	146	−10.894	5.114	−35.026	1.00	0.00	H
ATOM	2189	CB	GLN	X	146	−10.733	3.144	−34.004	1.00	0.00	C
ATOM	2190	HB1	GLN	X	146	−11.698	3.081	−33.458	1.00	0.00	H
ATOM	2191	HB2	GLN	X	146	−9.863	2.751	−33.435	1.00	0.00	H
ATOM	2192	CG	GLN	X	146	−11.007	2.249	−35.258	1.00	0.00	C
ATOM	2193	HG1	GLN	X	146	−11.170	1.202	−34.925	1.00	0.00	H
ATOM	2194	HG2	GLN	X	146	−10.058	2.332	−35.830	1.00	0.00	H
ATOM	2195	CD	GLN	X	146	−12.051	2.677	−36.311	1.00	0.00	C
ATOM	2196	OE1	GLN	X	146	−12.854	3.676	−36.136	1.00	0.00	O
ATOM	2197	NE2	GLN	X	146	−12.072	1.952	−37.436	1.00	0.00	N
ATOM	2198	HE21	GLN	X	146	−11.578	1.087	−37.523	1.00	0.00	H
ATOM	2199	HE22	GLN	X	146	−12.911	2.088	−37.962	1.00	0.00	H
ATOM	2200	C	GLN	X	146	−10.468	5.534	−33.029	1.00	0.00	C

ATOM 2201 O GLN X 146 −9.478 5.648 −32.266 1.00 0.00
O
ATOM 2202 N SER X 147 −11.631 6.229 −32.693 1.00 0.00
N
ATOM 2203 HN SER X 147 −12.437 6.162 −33.276 1.00 0.00
H
ATOM 2204 CA SER X 147 −11.870 7.176 −31.607 1.00 0.00
C
ATOM 2205 HA SER X 147 −11.112 7.937 −31.722 1.00 0.00
H
ATOM 2206 CB SER X 147 −13.229 7.936 −31.684 1.00 0.00
C
ATOM 2207 HB1 SER X 147 −13.179 8.678 −30.858 1.00 0.00
H
ATOM 2208 HB2 SER X 147 −14.015 7.218 −31.366 1.00 0.00
H
ATOM 2209 OG SER X 147 −13.545 8.631 −32.866 1.00 0.00
O
ATOM 2210 HG1 SER X 147 −13.675 7.957 −33.537 1.00 0.00
H
ATOM 2211 C SER X 147 −11.739 6.456 −30.244 1.00 0.00
C
ATOM 2212 O SER X 147 −12.220 5.390 −29.991 1.00 0.00
O
ATOM 2213 N ILE X 148 −11.001 7.069 −29.282 1.00 0.00
N
ATOM 2214 HN ILE X 148 −10.503 7.911 −29.479 1.00 0.00
H
ATOM 2215 CA ILE X 148 −10.705 6.507 −27.973 1.00 0.00
C
ATOM 2216 HA ILE X 148 −11.188 5.559 −27.786 1.00 0.00
H
ATOM 2217 CB ILE X 148 −9.292 6.185 −27.796 1.00 0.00
C
ATOM 2218 HB ILE X 148 −8.964 5.665 −26.871 1.00 0.00
H
ATOM 2219 CG2 ILE X 148 −8.875 5.095 −28.830 1.00 0.00
C
ATOM 2220 HG21 ILE X 148 −7.889 4.662 −28.556 1.00 0.00
H
ATOM 2221 HG22 ILE X 148 −8.695 5.585 −29.811 1.00 0.00
H
ATOM 2222 HG23 ILE X 148 −9.665 4.325 −28.958 1.00 0.00
H
ATOM 2223 CG1 ILE X 148 −8.484 7.471 −28.186 1.00 0.00
C
ATOM 2224 HG11 ILE X 148 −8.915 8.358 −27.672 1.00 0.00
H
ATOM 2225 HG12 ILE X 148 −8.426 7.681 −29.275 1.00 0.00
H

ATOM 2226 CD ILE X 148 -7.030 7.425 -27.591 1.00 0.00
C
ATOM 2227 HD1 ILE X 148 -6.317 6.990 -28.325 1.00 0.00
H
ATOM 2228 HD2 ILE X 148 -6.840 6.927 -26.616 1.00 0.00
H
ATOM 2229 HD3 ILE X 148 -6.701 8.484 -27.534 1.00 0.00
H
ATOM 2230 C ILE X 148 -11.162 7.393 -26.839 1.00 0.00
C
ATOM 2231 O ILE X 148 -10.581 7.231 -25.754 1.00 0.00
O
ATOM 2232 N SER X 149 -12.277 8.211 -26.986 1.00 0.00
N
ATOM 2233 HN SER X 149 -12.624 8.134 -27.917 1.00 0.00
H
ATOM 2234 CA SER X 149 -12.873 9.154 -26.097 1.00 0.00
C
ATOM 2235 HA SER X 149 -13.589 9.828 -26.544 1.00 0.00
H
ATOM 2236 CB SER X 149 -13.798 8.388 -25.051 1.00 0.00
C
ATOM 2237 HB1 SER X 149 -12.995 7.905 -24.454 1.00 0.00
H
ATOM 2238 HB2 SER X 149 -14.504 7.576 -25.328 1.00 0.00
H
ATOM 2239 OG SER X 149 -14.532 9.283 -24.220 1.00 0.00
O
ATOM 2240 HG1 SER X 149 -15.363 9.370 -24.694 1.00 0.00
H
ATOM 2241 C SER X 149 -11.830 9.998 -25.499 1.00 0.00
C
ATOM 2242 O SER X 149 -10.912 10.392 -26.238 1.00 0.00
O
ATOM 2243 N ASN X 150 -11.884 10.308 -24.165 1.00 0.00
N
ATOM 2244 HN ASN X 150 -12.729 9.984 -23.746 1.00 0.00
H
ATOM 2245 CA ASN X 150 -10.972 10.968 -23.379 1.00 0.00
C
ATOM 2246 HA ASN X 150 -10.492 11.697 -24.014 1.00 0.00
H
ATOM 2247 CB ASN X 150 -11.573 11.695 -22.131 1.00 0.00
C
ATOM 2248 HB1 ASN X 150 -10.653 11.960 -21.566 1.00 0.00
H
ATOM 2249 HB2 ASN X 150 -12.368 11.024 -21.741 1.00 0.00
H
ATOM 2250 CG ASN X 150 -12.311 13.020 -22.556 1.00 0.00
C

ATOM 2251 OD1 ASN X 150 -12.793 13.161 -23.648 1.00 0.00
O
ATOM 2252 ND2 ASN X 150 -12.335 14.009 -21.589 1.00 0.00
N
ATOM 2253 HD21 ASN X 150 -12.685 14.858 -21.985 1.00 0.00
H
ATOM 2254 HD22 ASN X 150 -11.685 14.138 -20.840 1.00 0.00
H
ATOM 2255 C ASN X 150 -9.954 9.903 -22.878 1.00 0.00
C
ATOM 2256 O ASN X 150 -9.038 10.276 -22.170 1.00 0.00
O
ATOM 2257 N TRP X 151 -10.137 8.679 -23.307 1.00 0.00
N
ATOM 2258 HN TRP X 151 -10.703 8.452 -24.095 1.00 0.00
H
ATOM 2259 CA TRP X 151 -9.479 7.543 -22.736 1.00 0.00
C
ATOM 2260 HA TRP X 151 -9.376 7.869 -21.712 1.00 0.00
H
ATOM 2261 CB TRP X 151 -10.244 6.229 -22.778 1.00 0.00
C
ATOM 2262 HB1 TRP X 151 -9.807 5.576 -21.993 1.00 0.00
H
ATOM 2263 HB2 TRP X 151 -10.125 5.698 -23.747 1.00 0.00
H
ATOM 2264 CG TRP X 151 -11.734 6.451 -22.501 1.00 0.00
C
ATOM 2265 CD1 TRP X 151 -12.754 5.669 -22.993 1.00 0.00
C
ATOM 2266 HD1 TRP X 151 -12.753 4.923 -23.773 1.00 0.00
H
ATOM 2267 NE1 TRP X 151 -14.026 6.118 -22.550 1.00 0.00
N
ATOM 2268 HE1 TRP X 151 -14.873 5.853 -22.956 1.00 0.00
H
ATOM 2269 CE2 TRP X 151 -13.850 7.106 -21.661 1.00 0.00
C
ATOM 2270 CD2 TRP X 151 -12.426 7.344 -21.583 1.00 0.00
C
ATOM 2271 CE3 TRP X 151 -11.971 8.220 -20.632 1.00 0.00
C
ATOM 2272 HE3 TRP X 151 -10.923 8.213 -20.370 1.00 0.00
H
ATOM 2273 CZ3 TRP X 151 -12.880 8.957 -19.880 1.00 0.00
C
ATOM 2274 HZ3 TRP X 151 -12.613 9.613 -19.064 1.00 0.00
H
ATOM 2275 CZ2 TRP X 151 -14.732 7.939 -21.028 1.00 0.00
C

```
ATOM   2276  HZ2 TRP X 151     -15.804   7.856 -21.130  1.00  0.00
H
ATOM   2277  CH2 TRP X 151     -14.287   8.851 -20.007  1.00  0.00
C
ATOM   2278  HH2 TRP X 151     -15.032   9.385 -19.436  1.00  0.00
H
ATOM   2279  C   TRP X 151      -8.000   7.257 -23.078  1.00  0.00
C
ATOM   2280  O   TRP X 151      -7.678   6.217 -23.620  1.00  0.00
O
ATOM   2281  N   LEU X 152      -7.010   8.188 -22.671  1.00  0.00
N
ATOM   2282  HN  LEU X 152      -7.183   8.975 -22.084  1.00  0.00
H
ATOM   2283  CA  LEU X 152      -5.626   8.022 -22.990  1.00  0.00
C
ATOM   2284  HA  LEU X 152      -5.293   7.003 -23.122  1.00  0.00
H
ATOM   2285  CB  LEU X 152      -5.458   8.800 -24.284  1.00  0.00
C
ATOM   2286  HB1 LEU X 152      -5.994   9.770 -24.200  1.00  0.00
H
ATOM   2287  HB2 LEU X 152      -5.966   8.211 -25.077  1.00  0.00
H
ATOM   2288  CG  LEU X 152      -4.000   8.964 -24.878  1.00  0.00
C
ATOM   2289  HG  LEU X 152      -3.422   8.074 -24.550  1.00  0.00
H
ATOM   2290  CD1 LEU X 152      -4.084   9.113 -26.397  1.00  0.00
C
ATOM   2291 HD11 LEU X 152      -3.124   9.504 -26.796  1.00  0.00
H
ATOM   2292 HD12 LEU X 152      -4.763   9.969 -26.599  1.00  0.00
H
ATOM   2293 HD13 LEU X 152      -4.437   8.154 -26.832  1.00  0.00
H
ATOM   2294  CD2 LEU X 152      -3.338  10.255 -24.304  1.00  0.00
C
ATOM   2295 HD21 LEU X 152      -4.025  11.113 -24.467  1.00  0.00
H
ATOM   2296 HD22 LEU X 152      -2.269  10.363 -24.587  1.00  0.00
H
ATOM   2297 HD23 LEU X 152      -3.290  10.136 -23.200  1.00  0.00
H
ATOM   2298  C   LEU X 152      -4.831   8.581 -21.834  1.00  0.00
C
ATOM   2299  O   LEU X 152      -5.316   9.497 -21.142  1.00  0.00
O
ATOM   2300  N   ALA X 153      -3.576   8.109 -21.579  1.00  0.00
N
```

ATOM 2301 HN ALA X 153 -3.202 7.499 -22.273 1.00 0.00
H
ATOM 2302 CA ALA X 153 -2.699 8.476 -20.566 1.00 0.00
C
ATOM 2303 HA ALA X 153 -2.999 9.385 -20.065 1.00 0.00
H
ATOM 2304 CB ALA X 153 -2.467 7.167 -19.803 1.00 0.00
C
ATOM 2305 HB1 ALA X 153 -2.124 6.360 -20.485 1.00 0.00
H
ATOM 2306 HB2 ALA X 153 -3.393 6.873 -19.264 1.00 0.00
H
ATOM 2307 HB3 ALA X 153 -1.643 7.351 -19.081 1.00 0.00
H
ATOM 2308 C ALA X 153 -1.362 8.962 -21.241 1.00 0.00
C
ATOM 2309 O ALA X 153 -1.098 8.623 -22.434 1.00 0.00
O
ATOM 2310 N TRP X 154 -0.539 9.862 -20.622 1.00 0.00
N
ATOM 2311 HN TRP X 154 -0.961 10.084 -19.747 1.00 0.00
H
ATOM 2312 CA TRP X 154 0.759 10.318 -21.182 1.00 0.00
C
ATOM 2313 HA TRP X 154 0.960 9.764 -22.088 1.00 0.00
H
ATOM 2314 CB TRP X 154 0.811 11.768 -21.691 1.00 0.00
C
ATOM 2315 HB1 TRP X 154 1.868 11.969 -21.969 1.00 0.00
H
ATOM 2316 HB2 TRP X 154 0.504 12.528 -20.941 1.00 0.00
H
ATOM 2317 CG TRP X 154 0.055 12.180 -22.981 1.00 0.00
C
ATOM 2318 CD1 TRP X 154 -1.111 12.859 -23.036 1.00 0.00
C
ATOM 2319 HD1 TRP X 154 -1.816 12.892 -22.219 1.00 0.00
H
ATOM 2320 NE1 TRP X 154 -1.369 13.234 -24.286 1.00 0.00
N
ATOM 2321 HE1 TRP X 154 -2.048 13.895 -24.522 1.00 0.00
H
ATOM 2322 CE2 TRP X 154 -0.270 12.993 -25.072 1.00 0.00
C
ATOM 2323 CD2 TRP X 154 0.648 12.318 -24.246 1.00 0.00
C
ATOM 2324 CE3 TRP X 154 1.922 11.998 -24.758 1.00 0.00
C
ATOM 2325 HE3 TRP X 154 2.654 11.451 -24.181 1.00 0.00
H

ATOM 2326 CZ3 TRP X 154 2.219 12.398 −26.098 1.00 0.00
C
ATOM 2327 HZ3 TRP X 154 3.203 12.133 −26.455 1.00 0.00
H
ATOM 2328 CZ2 TRP X 154 0.039 13.442 −26.338 1.00 0.00
C
ATOM 2329 HZ2 TRP X 154 −0.550 14.129 −26.926 1.00 0.00
H
ATOM 2330 CH2 TRP X 154 1.334 13.225 −26.776 1.00 0.00
C
ATOM 2331 HH2 TRP X 154 1.688 13.451 −27.771 1.00 0.00
H
ATOM 2332 C TRP X 154 1.916 10.206 −20.297 1.00 0.00
C
ATOM 2333 O TRP X 154 1.942 10.631 −19.144 1.00 0.00
O
ATOM 2334 N PHE X 155 3.018 9.573 −20.930 1.00 0.00
N
ATOM 2335 HN PHE X 155 2.988 9.282 −21.883 1.00 0.00
H
ATOM 2336 CA PHE X 155 4.296 9.262 −20.242 1.00 0.00
C
ATOM 2337 HA PHE X 155 4.234 9.462 −19.183 1.00 0.00
H
ATOM 2338 CB PHE X 155 4.594 7.780 −20.508 1.00 0.00
C
ATOM 2339 HB1 PHE X 155 5.569 7.458 −20.083 1.00 0.00
H
ATOM 2340 HB2 PHE X 155 4.691 7.631 −21.605 1.00 0.00
H
ATOM 2341 CG PHE X 155 3.607 6.789 −19.911 1.00 0.00
C
ATOM 2342 CD1 PHE X 155 3.038 5.738 −20.651 1.00 0.00
C
ATOM 2343 HD1 PHE X 155 3.156 5.681 −21.723 1.00 0.00
H
ATOM 2344 CE1 PHE X 155 2.270 4.719 −19.998 1.00 0.00
C
ATOM 2345 HE1 PHE X 155 1.806 3.980 −20.634 1.00 0.00
H
ATOM 2346 CZ PHE X 155 2.257 4.729 −18.578 1.00 0.00
C
ATOM 2347 HZ PHE X 155 1.764 3.933 −18.040 1.00 0.00
H
ATOM 2348 CD2 PHE X 155 3.447 6.781 −18.518 1.00 0.00
C
ATOM 2349 HD2 PHE X 155 3.834 7.593 −17.919 1.00 0.00
H
ATOM 2350 CE2 PHE X 155 2.946 5.695 −17.796 1.00 0.00
C

```
ATOM   2351  HE2 PHE X 155       3.061   5.664 −16.722  1.00  0.00
H
ATOM   2352  C   PHE X 155       5.586   9.953 −20.604  1.00  0.00
C
ATOM   2353  O   PHE X 155       5.894   9.981 −21.828  1.00  0.00
O
ATOM   2354  N   GLN X 156       6.384  10.474 −19.679  1.00  0.00
N
ATOM   2355  HN  GLN X 156       6.051  10.569 −18.744  1.00  0.00
H
ATOM   2356  CA  GLN X 156       7.712  10.990 −19.809  1.00  0.00
C
ATOM   2357  HA  GLN X 156       7.865  11.282 −20.837  1.00  0.00
H
ATOM   2358  CB  GLN X 156       7.954  12.123 −18.780  1.00  0.00
C
ATOM   2359  HB1 GLN X 156       7.759  11.731 −17.759  1.00  0.00
H
ATOM   2360  HB2 GLN X 156       7.191  12.920 −18.912  1.00  0.00
H
ATOM   2361  CG  GLN X 156       9.495  12.544 −18.883  1.00  0.00
C
ATOM   2362  HG1 GLN X 156       9.725  13.043 −19.849  1.00  0.00
H
ATOM   2363  HG2 GLN X 156      10.206  11.696 −18.789  1.00  0.00
H
ATOM   2364  CD  GLN X 156       9.790  13.434 −17.632  1.00  0.00
C
ATOM   2365  OE1 GLN X 156      10.553  13.025 −16.713  1.00  0.00
O
ATOM   2366  NE2 GLN X 156       9.192  14.653 −17.647  1.00  0.00
N
ATOM   2367 HE21 GLN X 156       8.738  14.990 −18.472  1.00  0.00
H
ATOM   2368 HE22 GLN X 156       9.549  15.290 −16.964  1.00  0.00
H
ATOM   2369  C   GLN X 156       8.656   9.820 −19.579  1.00  0.00
C
ATOM   2370  O   GLN X 156       8.641   9.226 −18.477  1.00  0.00
O
ATOM   2371  N   GLN X 157       9.531   9.508 −20.565  1.00  0.00
N
ATOM   2372  HN  GLN X 157       9.426   9.971 −21.442  1.00  0.00
H
ATOM   2373  CA  GLN X 157      10.579   8.556 −20.335  1.00  0.00
C
ATOM   2374  HA  GLN X 157      10.490   8.172 −19.330  1.00  0.00
H
ATOM   2375  CB  GLN X 157      10.339   7.284 −21.240  1.00  0.00
C
```

ATOM 2376 HB1 GLN X 157 10.118 7.710 −22.242 1.00 0.00
H
ATOM 2377 HB2 GLN X 157 9.524 6.634 −20.855 1.00 0.00
H
ATOM 2378 CG GLN X 157 11.558 6.344 −21.170 1.00 0.00
C
ATOM 2379 HG1 GLN X 157 11.921 6.315 −20.120 1.00 0.00
H
ATOM 2380 HG2 GLN X 157 12.401 6.795 −21.736 1.00 0.00
H
ATOM 2381 CD GLN X 157 11.280 5.010 −21.820 1.00 0.00
C
ATOM 2382 OE1 GLN X 157 11.031 4.822 −22.991 1.00 0.00
O
ATOM 2383 NE2 GLN X 157 11.214 4.013 −20.903 1.00 0.00
N
ATOM 2384 HE21 GLN X 157 10.978 3.130 −21.308 1.00 0.00
H
ATOM 2385 HE22 GLN X 157 11.564 4.114 −19.972 1.00 0.00
H
ATOM 2386 C GLN X 157 11.946 9.124 −20.415 1.00 0.00
C
ATOM 2387 O GLN X 157 12.391 9.472 −21.495 1.00 0.00
O
ATOM 2388 N LYS X 158 12.627 9.253 −19.280 1.00 0.00
N
ATOM 2389 HN LYS X 158 12.053 9.253 −18.465 1.00 0.00
H
ATOM 2390 CA LYS X 158 14.069 9.637 −19.253 1.00 0.00
C
ATOM 2391 HA LYS X 158 14.263 10.437 −19.952 1.00 0.00
H
ATOM 2392 CB LYS X 158 14.413 10.107 −17.847 1.00 0.00
C
ATOM 2393 HB1 LYS X 158 15.424 10.569 −17.826 1.00 0.00
H
ATOM 2394 HB2 LYS X 158 14.345 9.206 −17.201 1.00 0.00
H
ATOM 2395 CG LYS X 158 13.521 11.232 −17.225 1.00 0.00
C
ATOM 2396 HG1 LYS X 158 13.771 11.553 −16.191 1.00 0.00
H
ATOM 2397 HG2 LYS X 158 12.457 10.915 −17.211 1.00 0.00
H
ATOM 2398 CD LYS X 158 13.617 12.442 −18.125 1.00 0.00
C
ATOM 2399 HD1 LYS X 158 12.664 12.991 −17.971 1.00 0.00
H
ATOM 2400 HD2 LYS X 158 13.486 12.244 −19.210 1.00 0.00
H

ATOM 2401 CE LYS X 158 14.842 13.354 −17.924 1.00 0.00
C
ATOM 2402 HE1 LYS X 158 15.033 14.216 −18.598 1.00 0.00
H
ATOM 2403 HE2 LYS X 158 15.752 12.738 −18.093 1.00 0.00
H
ATOM 2404 NZ LYS X 158 15.028 13.788 −16.460 1.00 0.00
N
ATOM 2405 HZ1 LYS X 158 15.322 13.016 −15.829 1.00 0.00
H
ATOM 2406 HZ2 LYS X 158 14.098 14.045 −16.071 1.00 0.00
H
ATOM 2407 HZ3 LYS X 158 15.708 14.561 −16.315 1.00 0.00
H
ATOM 2408 C LYS X 158 14.910 8.467 −19.647 1.00 0.00
C
ATOM 2409 O LYS X 158 14.347 7.411 −19.876 1.00 0.00
O
ATOM 2410 N PRO X 159 16.214 8.674 −19.795 1.00 0.00
N
ATOM 2411 CD PRO X 159 16.864 9.955 −20.070 1.00 0.00
C
ATOM 2412 HD1 PRO X 159 17.156 10.407 −19.098 1.00 0.00
H
ATOM 2413 HD2 PRO X 159 16.195 10.633 −20.641 1.00 0.00
H
ATOM 2414 CA PRO X 159 17.154 7.605 −20.110 1.00 0.00
C
ATOM 2415 HA PRO X 159 16.829 7.093 −21.004 1.00 0.00
H
ATOM 2416 CB PRO X 159 18.578 8.302 −20.221 1.00 0.00
C
ATOM 2417 HB1 PRO X 159 19.129 7.755 −21.016 1.00 0.00
H
ATOM 2418 HB2 PRO X 159 19.208 8.144 −19.320 1.00 0.00
H
ATOM 2419 CG PRO X 159 18.178 9.680 −20.730 1.00 0.00
C
ATOM 2420 HG1 PRO X 159 19.053 10.352 −20.599 1.00 0.00
H
ATOM 2421 HG2 PRO X 159 17.957 9.582 −21.814 1.00 0.00
H
ATOM 2422 C PRO X 159 17.115 6.388 −19.146 1.00 0.00
C
ATOM 2423 O PRO X 159 17.347 6.555 −17.921 1.00 0.00
O
ATOM 2424 N GLY X 160 16.773 5.113 −19.572 1.00 0.00
N
ATOM 2425 HN GLY X 160 16.488 5.041 −20.524 1.00 0.00
H

ATOM 2426 CA GLY X 160 16.894 3.880 −18.835 1.00 0.00
C
ATOM 2427 HA1 GLY X 160 17.827 3.769 −18.301 1.00 0.00
H
ATOM 2428 HA2 GLY X 160 16.908 3.216 −19.687 1.00 0.00
H
ATOM 2429 C GLY X 160 15.753 3.565 −17.873 1.00 0.00
C
ATOM 2430 O GLY X 160 15.284 2.409 −17.730 1.00 0.00
O
ATOM 2431 N LYS X 161 15.333 4.480 −17.143 1.00 0.00
N
ATOM 2432 HN LYS X 161 15.684 5.405 −17.266 1.00 0.00
H
ATOM 2433 CA LYS X 161 14.200 4.516 −16.179 1.00 0.00
C
ATOM 2434 HA LYS X 161 14.365 3.950 −15.273 1.00 0.00
H
ATOM 2435 CB LYS X 161 14.027 5.929 −15.680 1.00 0.00
C
ATOM 2436 HB1 LYS X 161 13.130 5.884 −15.026 1.00 0.00
H
ATOM 2437 HB2 LYS X 161 13.688 6.600 −16.498 1.00 0.00
H
ATOM 2438 CG LYS X 161 15.142 6.588 −14.834 1.00 0.00
C
ATOM 2439 HG1 LYS X 161 16.009 6.966 −15.417 1.00 0.00
H
ATOM 2440 HG2 LYS X 161 15.636 5.841 −14.177 1.00 0.00
H
ATOM 2441 CD LYS X 161 14.564 7.678 −13.877 1.00 0.00
C
ATOM 2442 HD1 LYS X 161 13.667 7.220 −13.409 1.00 0.00
H
ATOM 2443 HD2 LYS X 161 14.255 8.580 −14.448 1.00 0.00
H
ATOM 2444 CE LYS X 161 15.599 8.211 −12.874 1.00 0.00
C
ATOM 2445 HE1 LYS X 161 16.315 8.936 −13.317 1.00 0.00
H
ATOM 2446 HE2 LYS X 161 16.111 7.352 −12.389 1.00 0.00
H
ATOM 2447 NZ LYS X 161 14.850 8.917 −11.817 1.00 0.00
N
ATOM 2448 HZ1 LYS X 161 15.236 8.904 −10.851 1.00 0.00
H
ATOM 2449 HZ2 LYS X 161 13.921 8.450 −11.821 1.00 0.00
H
ATOM 2450 HZ3 LYS X 161 14.641 9.923 −11.975 1.00 0.00
H

```
ATOM   2451  C   LYS X 161      12.831   3.901 −16.608  1.00  0.00
C
ATOM   2452  O   LYS X 161      12.522   3.950 −17.815  1.00  0.00
O
ATOM   2453  N   ALA X 162      11.994   3.469 −15.723  1.00  0.00
N
ATOM   2454  HN  ALA X 162      12.352   3.304 −14.807  1.00  0.00
H
ATOM   2455  CA  ALA X 162      10.539   3.250 −15.945  1.00  0.00
C
ATOM   2456  HA  ALA X 162      10.404   2.540 −16.748  1.00  0.00
H
ATOM   2457  CB  ALA X 162       9.938   2.529 −14.780  1.00  0.00
C
ATOM   2458  HB1 ALA X 162       8.837   2.382 −14.794  1.00  0.00
H
ATOM   2459  HB2 ALA X 162      10.198   3.062 −13.840  1.00  0.00
H
ATOM   2460  HB3 ALA X 162      10.291   1.493 −14.589  1.00  0.00
H
ATOM   2461  C   ALA X 162       9.739   4.487 −16.287  1.00  0.00
C
ATOM   2462  O   ALA X 162      10.081   5.612 −15.924  1.00  0.00
O
ATOM   2463  N   PRO X 163       8.763   4.478 −17.189  1.00  0.00
N
ATOM   2464  CD  PRO X 163       8.252   3.240 −17.730  1.00  0.00
C
ATOM   2465  HD1 PRO X 163       8.388   2.452 −16.958  1.00  0.00
H
ATOM   2466  HD2 PRO X 163       8.883   3.219 −18.644  1.00  0.00
H
ATOM   2467  CA  PRO X 163       7.902   5.636 −17.560  1.00  0.00
C
ATOM   2468  HA  PRO X 163       8.466   6.480 −17.930  1.00  0.00
H
ATOM   2469  CB  PRO X 163       7.005   4.968 −18.569  1.00  0.00
C
ATOM   2470  HB1 PRO X 163       7.482   5.082 −19.566  1.00  0.00
H
ATOM   2471  HB2 PRO X 163       6.070   5.545 −18.733  1.00  0.00
H
ATOM   2472  CG  PRO X 163       6.808   3.481 −18.095  1.00  0.00
C
ATOM   2473  HG1 PRO X 163       6.070   3.386 −17.270  1.00  0.00
H
ATOM   2474  HG2 PRO X 163       6.619   2.748 −18.908  1.00  0.00
H
ATOM   2475  C   PRO X 163       7.217   6.434 −16.421  1.00  0.00
C
```

```
ATOM   2476  O   PRO X 163       6.714   5.808 −15.501  1.00  0.00
O
ATOM   2477  N   LYS X 164       7.186   7.747 −16.477  1.00  0.00
N
ATOM   2478  HN  LYS X 164       7.738   8.166 −17.194  1.00  0.00
H
ATOM   2479  CA  LYS X 164       6.611   8.673 −15.519  1.00  0.00
C
ATOM   2480  HA  LYS X 164       6.357   8.216 −14.575  1.00  0.00
H
ATOM   2481  CB  LYS X 164       7.636   9.812 −15.230  1.00  0.00
C
ATOM   2482  HB1 LYS X 164       7.955  10.120 −16.248  1.00  0.00
H
ATOM   2483  HB2 LYS X 164       8.545   9.362 −14.777  1.00  0.00
H
ATOM   2484  CG  LYS X 164       7.007  10.945 −14.391  1.00  0.00
C
ATOM   2485  HG1 LYS X 164       6.207  11.490 −14.937  1.00  0.00
H
ATOM   2486  HG2 LYS X 164       7.765  11.732 −14.190  1.00  0.00
H
ATOM   2487  CD  LYS X 164       6.532  10.563 −13.036  1.00  0.00
C
ATOM   2488  HD1 LYS X 164       5.821   9.711 −12.982  1.00  0.00
H
ATOM   2489  HD2 LYS X 164       5.810  11.357 −12.748  1.00  0.00
H
ATOM   2490  CE  LYS X 164       7.700  10.485 −11.946  1.00  0.00
C
ATOM   2491  HE1 LYS X 164       8.291  11.426 −11.967  1.00  0.00
H
ATOM   2492  HE2 LYS X 164       8.351   9.608 −12.150  1.00  0.00
H
ATOM   2493  NZ  LYS X 164       7.140  10.264 −10.599  1.00  0.00
N
ATOM   2494  HZ1 LYS X 164       7.901   9.983  −9.949  1.00  0.00
H
ATOM   2495  HZ2 LYS X 164       6.477   9.474 −10.735  1.00  0.00
H
ATOM   2496  HZ3 LYS X 164       6.689  11.128 −10.235  1.00  0.00
H
ATOM   2497  C   LYS X 164       5.254   9.192 −16.047  1.00  0.00
C
ATOM   2498  O   LYS X 164       5.145   9.946 −16.995  1.00  0.00
O
ATOM   2499  N   LEU X 165       4.180   8.818 −15.397  1.00  0.00
N
ATOM   2500  HN  LEU X 165       4.267   8.330 −14.532  1.00  0.00
H
```

```
ATOM   2501  CA  LEU X 165       2.885   9.195 -15.924  1.00  0.00
C
ATOM   2502  HA  LEU X 165       2.829   9.109 -16.999  1.00  0.00
H
ATOM   2503  CB  LEU X 165       1.833   8.343 -15.199  1.00  0.00
C
ATOM   2504  HB1 LEU X 165       1.758   8.615 -14.124  1.00  0.00
H
ATOM   2505  HB2 LEU X 165       2.146   7.281 -15.285  1.00  0.00
H
ATOM   2506  CG  LEU X 165       0.362   8.348 -15.669  1.00  0.00
C
ATOM   2507  HG  LEU X 165      -0.124   9.318 -15.433  1.00  0.00
H
ATOM   2508  CD1 LEU X 165       0.172   8.416 -17.235  1.00  0.00
C
ATOM   2509 HD11 LEU X 165       0.523   7.520 -17.790  1.00  0.00
H
ATOM   2510 HD12 LEU X 165       0.565   9.270 -17.827  1.00  0.00
H
ATOM   2511 HD13 LEU X 165      -0.907   8.557 -17.457  1.00  0.00
H
ATOM   2512  CD2 LEU X 165      -0.340   7.180 -15.108  1.00  0.00
C
ATOM   2513 HD21 LEU X 165      -1.313   6.948 -15.591  1.00  0.00
H
ATOM   2514 HD22 LEU X 165      -0.369   7.210 -13.998  1.00  0.00
H
ATOM   2515 HD23 LEU X 165       0.262   6.263 -15.285  1.00  0.00
H
ATOM   2516  C   LEU X 165       2.588  10.700 -15.506  1.00  0.00
C
ATOM   2517  O   LEU X 165       2.608  11.145 -14.374  1.00  0.00
O
ATOM   2518  N   LEU X 166       2.400  11.530 -16.530  1.00  0.00
N
ATOM   2519  HN  LEU X 166       2.460  11.237 -17.482  1.00  0.00
H
ATOM   2520  CA  LEU X 166       2.029  12.959 -16.512  1.00  0.00
C
ATOM   2521  HA  LEU X 166       2.362  13.407 -15.587  1.00  0.00
H
ATOM   2522  CB  LEU X 166       2.825  13.797 -17.692  1.00  0.00
C
ATOM   2523  HB1 LEU X 166       2.647  14.893 -17.729  1.00  0.00
H
ATOM   2524  HB2 LEU X 166       2.400  13.364 -18.622  1.00  0.00
H
ATOM   2525  CG  LEU X 166       4.338  13.625 -17.848  1.00  0.00
C
```

ATOM 2526 HG LEU X 166 4.515 12.532 −17.943 1.00 0.00
H
ATOM 2527 CD1 LEU X 166 4.801 14.221 −19.167 1.00 0.00
C
ATOM 2528 HD11 LEU X 166 5.902 14.152 −19.295 1.00 0.00
H
ATOM 2529 HD12 LEU X 166 4.493 15.259 −18.918 1.00 0.00
H
ATOM 2530 HD13 LEU X 166 4.194 13.901 −20.041 1.00 0.00
H
ATOM 2531 CD2 LEU X 166 5.171 14.282 −16.767 1.00 0.00
C
ATOM 2532 HD21 LEU X 166 4.848 15.337 −16.632 1.00 0.00
H
ATOM 2533 HD22 LEU X 166 6.239 14.208 −17.065 1.00 0.00
H
ATOM 2534 HD23 LEU X 166 5.157 13.796 −15.768 1.00 0.00
H
ATOM 2535 C LEU X 166 0.607 13.244 −16.603 1.00 0.00
C
ATOM 2536 O LEU X 166 0.097 14.134 −15.947 1.00 0.00
O
ATOM 2537 N ILE X 167 −0.052 12.553 −17.550 1.00 0.00
N
ATOM 2538 HN ILE X 167 0.315 11.908 −18.215 1.00 0.00
H
ATOM 2539 CA ILE X 167 −1.435 12.873 −17.873 1.00 0.00
C
ATOM 2540 HA ILE X 167 −1.776 13.676 −17.237 1.00 0.00
H
ATOM 2541 CB ILE X 167 −1.593 13.418 −19.294 1.00 0.00
C
ATOM 2542 HB ILE X 167 −1.376 12.541 −19.940 1.00 0.00
H
ATOM 2543 CG2 ILE X 167 −3.003 13.812 −19.591 1.00 0.00
C
ATOM 2544 HG21 ILE X 167 −3.547 12.930 −19.989 1.00 0.00
H
ATOM 2545 HG22 ILE X 167 −3.060 14.522 −20.443 1.00 0.00
H
ATOM 2546 HG23 ILE X 167 −3.435 14.282 −18.682 1.00 0.00
H
ATOM 2547 CG1 ILE X 167 −0.642 14.638 −19.551 1.00 0.00
C
ATOM 2548 HG11 ILE X 167 −0.851 14.952 −20.597 1.00 0.00
H
ATOM 2549 HG12 ILE X 167 0.417 14.385 −19.332 1.00 0.00
H
ATOM 2550 CD ILE X 167 −0.911 15.885 −18.658 1.00 0.00
C

ATOM 2551 HD1 ILE X 167 −0.677 15.732 −17.583 1.00 0.00
H
ATOM 2552 HD2 ILE X 167 −1.927 16.298 −18.829 1.00 0.00
H
ATOM 2553 HD3 ILE X 167 −0.294 16.757 −18.963 1.00 0.00
H
ATOM 2554 C ILE X 167 −2.364 11.654 −17.721 1.00 0.00
C
ATOM 2555 O ILE X 167 −2.089 10.642 −18.337 1.00 0.00
O
ATOM 2556 N TYR X 168 −3.541 11.716 −17.049 1.00 0.00
N
ATOM 2557 HN TYR X 168 −3.629 12.498 −16.437 1.00 0.00
H
ATOM 2558 CA TYR X 168 −4.549 10.658 −16.929 1.00 0.00
C
ATOM 2559 HA TYR X 168 −4.346 9.797 −17.548 1.00 0.00
H
ATOM 2560 CB TYR X 168 −4.684 10.235 −15.439 1.00 0.00
C
ATOM 2561 HB1 TYR X 168 −4.838 11.174 −14.866 1.00 0.00
H
ATOM 2562 HB2 TYR X 168 −3.659 9.834 −15.286 1.00 0.00
H
ATOM 2563 CG TYR X 168 −5.728 9.183 −15.290 1.00 0.00
C
ATOM 2564 CD1 TYR X 168 −6.838 9.431 −14.408 1.00 0.00
C
ATOM 2565 HD1 TYR X 168 −6.859 10.362 −13.860 1.00 0.00
H
ATOM 2566 CE1 TYR X 168 −7.832 8.514 −14.168 1.00 0.00
C
ATOM 2567 HE1 TYR X 168 −8.609 8.671 −13.435 1.00 0.00
H
ATOM 2568 CZ TYR X 168 −7.615 7.187 −14.728 1.00 0.00
C
ATOM 2569 OH TYR X 168 −8.421 6.144 −14.219 1.00 0.00
O
ATOM 2570 HH TYR X 168 −7.810 5.455 −14.491 1.00 0.00
H
ATOM 2571 CD2 TYR X 168 −5.554 7.906 −15.846 1.00 0.00
C
ATOM 2572 HD2 TYR X 168 −4.682 7.671 −16.440 1.00 0.00
H
ATOM 2573 CE2 TYR X 168 −6.535 6.934 −15.627 1.00 0.00
C
ATOM 2574 HE2 TYR X 168 −6.518 6.021 −16.204 1.00 0.00
H
ATOM 2575 C TYR X 168 −5.836 11.290 −17.473 1.00 0.00
C

ATOM 2576 O TYR X 168 −5.957 12.520 −17.476 1.00 0.00
O
ATOM 2577 N GLU X 169 −6.789 10.555 −17.932 1.00 0.00
N
ATOM 2578 HN GLU X 169 −6.836 9.579 −17.733 1.00 0.00
H
ATOM 2579 CA GLU X 169 −7.933 10.921 −18.707 1.00 0.00
C
ATOM 2580 HA GLU X 169 −8.202 10.008 −19.218 1.00 0.00
H
ATOM 2581 CB GLU X 169 −9.097 11.314 −17.716 1.00 0.00
C
ATOM 2582 HB1 GLU X 169 −9.802 12.062 −18.138 1.00 0.00
H
ATOM 2583 HB2 GLU X 169 −8.691 11.846 −16.830 1.00 0.00
H
ATOM 2584 CG GLU X 169 −9.871 10.095 −17.083 1.00 0.00
C
ATOM 2585 HG1 GLU X 169 −9.220 9.391 −16.522 1.00 0.00
H
ATOM 2586 HG2 GLU X 169 −10.326 9.433 −17.851 1.00 0.00
H
ATOM 2587 CD GLU X 169 −10.915 10.555 −16.115 1.00 0.00
C
ATOM 2588 OE1 GLU X 169 −11.460 9.717 −15.395 1.00 0.00
O
ATOM 2589 OE2 GLU X 169 −11.204 11.795 −15.958 1.00 0.00
O
ATOM 2590 C GLU X 169 −7.751 11.906 −19.808 1.00 0.00
C
ATOM 2591 O GLU X 169 −8.644 12.701 −20.085 1.00 0.00
O
ATOM 2592 N ALA X 170 −6.596 11.852 −20.569 1.00 0.00
N
ATOM 2593 HN ALA X 170 −5.881 11.165 −20.464 1.00 0.00
H
ATOM 2594 CA ALA X 170 −6.295 12.845 −21.570 1.00 0.00
C
ATOM 2595 HA ALA X 170 −5.275 12.632 −21.852 1.00 0.00
H
ATOM 2596 CB ALA X 170 −7.069 12.624 −22.859 1.00 0.00
C
ATOM 2597 HB1 ALA X 170 −6.864 11.574 −23.156 1.00 0.00
H
ATOM 2598 HB2 ALA X 170 −6.704 13.115 −23.786 1.00 0.00
H
ATOM 2599 HB3 ALA X 170 −8.169 12.637 −22.697 1.00 0.00
H
ATOM 2600 C ALA X 170 −6.381 14.337 −21.269 1.00 0.00
C

```
ATOM   2601  O   ALA X 170      -6.450  15.157 -22.195  1.00  0.00
O
ATOM   2602  N   SER X 171      -6.312  14.671 -19.969  1.00  0.00
N
ATOM   2603  HN  SER X 171      -6.505  14.081 -19.189  1.00  0.00
H
ATOM   2604  CA  SER X 171      -6.233  16.027 -19.481  1.00  0.00
C
ATOM   2605  HA  SER X 171      -5.316  16.396 -19.918  1.00  0.00
H
ATOM   2606  CB  SER X 171      -7.444  16.974 -19.886  1.00  0.00
C
ATOM   2607  HB1 SER X 171      -7.556  16.919 -20.990  1.00  0.00
H
ATOM   2608  HB2 SER X 171      -7.219  17.992 -19.502  1.00  0.00
H
ATOM   2609  OG  SER X 171      -8.657  16.458 -19.343  1.00  0.00
O
ATOM   2610  HG1 SER X 171      -8.931  15.636 -19.757  1.00  0.00
H
ATOM   2611  C   SER X 171      -6.057  16.274 -17.930  1.00  0.00
C
ATOM   2612  O   SER X 171      -5.809  17.446 -17.502  1.00  0.00
O
ATOM   2613  N   SER X 172      -6.133  15.227 -17.155  1.00  0.00
N
ATOM   2614  HN  SER X 172      -6.210  14.303 -17.523  1.00  0.00
H
ATOM   2615  CA  SER X 172      -5.878  15.287 -15.738  1.00  0.00
C
ATOM   2616  HA  SER X 172      -6.289  16.168 -15.268  1.00  0.00
H
ATOM   2617  CB  SER X 172      -6.699  14.357 -14.880  1.00  0.00
C
ATOM   2618  HB1 SER X 172      -6.331  13.395 -15.298  1.00  0.00
H
ATOM   2619  HB2 SER X 172      -7.730  14.692 -15.121  1.00  0.00
H
ATOM   2620  OG  SER X 172      -6.440  14.466 -13.496  1.00  0.00
O
ATOM   2621  HG1 SER X 172      -5.612  13.997 -13.371  1.00  0.00
H
ATOM   2622  C   SER X 172      -4.429  15.108 -15.410  1.00  0.00
C
ATOM   2623  O   SER X 172      -3.807  14.115 -15.728  1.00  0.00
O
ATOM   2624  N   LEU X 173      -3.809  16.170 -14.792  1.00  0.00
N
ATOM   2625  HN  LEU X 173      -4.310  16.989 -14.524  1.00  0.00
H
```

```
ATOM   2626  CA  LEU X 173      -2.398  16.123 -14.421  1.00  0.00
C
ATOM   2627  HA  LEU X 173      -1.839  15.557 -15.152  1.00  0.00
H
ATOM   2628  CB  LEU X 173      -1.907  17.647 -14.156  1.00  0.00
C
ATOM   2629  HB1 LEU X 173      -2.510  18.042 -13.311  1.00  0.00
H
ATOM   2630  HB2 LEU X 173      -1.938  18.211 -15.112  1.00  0.00
H
ATOM   2631  CG  LEU X 173      -0.446  17.856 -13.737  1.00  0.00
C
ATOM   2632  HG  LEU X 173      -0.345  17.355 -12.751  1.00  0.00
H
ATOM   2633  CD1 LEU X 173       0.637  17.270 -14.687  1.00  0.00
C
ATOM   2634 HD11 LEU X 173       0.335  17.360 -15.752  1.00  0.00
H
ATOM   2635 HD12 LEU X 173       0.659  16.177 -14.488  1.00  0.00
H
ATOM   2636 HD13 LEU X 173       1.620  17.788 -14.663  1.00  0.00
H
ATOM   2637  CD2 LEU X 173      -0.039  19.374 -13.526  1.00  0.00
C
ATOM   2638 HD21 LEU X 173      -0.369  19.582 -12.485  1.00  0.00
H
ATOM   2639 HD22 LEU X 173      -0.435  20.016 -14.341  1.00  0.00
H
ATOM   2640 HD23 LEU X 173       1.070  19.434 -13.513  1.00  0.00
H
ATOM   2641  C   LEU X 173      -2.189  15.280 -13.107  1.00  0.00
C
ATOM   2642  O   LEU X 173      -2.813  15.589 -12.058  1.00  0.00
O
ATOM   2643  N   GLU X 174      -1.267  14.253 -13.115  1.00  0.00
N
ATOM   2644  HN  GLU X 174      -0.932  13.940 -14.000  1.00  0.00
H
ATOM   2645  CA  GLU X 174      -0.853  13.474 -12.001  1.00  0.00
C
ATOM   2646  HA  GLU X 174      -1.818  13.275 -11.557  1.00  0.00
H
ATOM   2647  CB  GLU X 174      -0.100  12.169 -12.431  1.00  0.00
C
ATOM   2648  HB1 GLU X 174       0.265  11.657 -11.515  1.00  0.00
H
ATOM   2649  HB2 GLU X 174       0.802  12.467 -13.007  1.00  0.00
H
ATOM   2650  CG  GLU X 174      -1.013  11.053 -13.019  1.00  0.00
C
```

```
ATOM   2651  HG1 GLU X 174      -0.433  10.161 -13.337  1.00  0.00
H
ATOM   2652  HG2 GLU X 174      -1.486  11.358 -13.977  1.00  0.00
H
ATOM   2653  CD  GLU X 174      -2.071  10.471 -12.062  1.00  0.00
C
ATOM   2654  OE1 GLU X 174      -3.237  10.312 -12.446  1.00  0.00
O
ATOM   2655  OE2 GLU X 174      -1.701  10.160 -10.878  1.00  0.00
O
ATOM   2656  C   GLU X 174      -0.135  14.329 -10.939  1.00  0.00
C
ATOM   2657  O   GLU X 174       0.357  15.397 -11.107  1.00  0.00
O
ATOM   2658  N   SER X 175      -0.228  13.948  -9.682  1.00  0.00
N
ATOM   2659  HN  SER X 175      -0.458  12.988  -9.545  1.00  0.00
H
ATOM   2660  CA  SER X 175       0.410  14.527  -8.530  1.00  0.00
C
ATOM   2661  HA  SER X 175       0.352  15.605  -8.516  1.00  0.00
H
ATOM   2662  CB  SER X 175      -0.289  14.020  -7.172  1.00  0.00
C
ATOM   2663  HB1 SER X 175      -0.029  12.955  -6.989  1.00  0.00
H
ATOM   2664  HB2 SER X 175      -1.347  14.212  -7.452  1.00  0.00
H
ATOM   2665  OG  SER X 175       0.051  14.795  -6.045  1.00  0.00
O
ATOM   2666  HG1 SER X 175       0.123  14.166  -5.323  1.00  0.00
H
ATOM   2667  C   SER X 175       1.926  14.130  -8.408  1.00  0.00
C
ATOM   2668  O   SER X 175       2.272  13.031  -8.779  1.00  0.00
O
ATOM   2669  N   GLY X 176       2.789  15.074  -8.106  1.00  0.00
N
ATOM   2670  HN  GLY X 176       2.401  15.916  -7.739  1.00  0.00
H
ATOM   2671  CA  GLY X 176       4.280  14.942  -8.224  1.00  0.00
C
ATOM   2672  HA1 GLY X 176       4.574  13.904  -8.170  1.00  0.00
H
ATOM   2673  HA2 GLY X 176       4.736  15.424  -7.371  1.00  0.00
H
ATOM   2674  C   GLY X 176       4.863  15.525  -9.464  1.00  0.00
C
ATOM   2675  O   GLY X 176       5.957  15.259  -9.862  1.00  0.00
O
```

ATOM	2676	N	VAL	X	177	4.027	16.190	−10.326	1.00	0.00	N
ATOM	2677	HN	VAL	X	177	3.038	16.248	−10.212	1.00	0.00	H
ATOM	2678	CA	VAL	X	177	4.334	16.625	−11.659	1.00	0.00	C
ATOM	2679	HA	VAL	X	177	5.330	16.246	−11.831	1.00	0.00	H
ATOM	2680	CB	VAL	X	177	3.478	16.053	−12.818	1.00	0.00	C
ATOM	2681	HB	VAL	X	177	2.413	16.340	−12.689	1.00	0.00	H
ATOM	2682	CG1	VAL	X	177	4.050	16.487	−14.216	1.00	0.00	C
ATOM	2683	HG11	VAL	X	177	4.085	17.596	−14.274	1.00	0.00	H
ATOM	2684	HG12	VAL	X	177	3.454	16.116	−15.077	1.00	0.00	H
ATOM	2685	HG13	VAL	X	177	5.123	16.220	−14.322	1.00	0.00	H
ATOM	2686	CG2	VAL	X	177	3.584	14.505	−12.679	1.00	0.00	C
ATOM	2687	HG21	VAL	X	177	3.150	14.241	−11.691	1.00	0.00	H
ATOM	2688	HG22	VAL	X	177	4.668	14.272	−12.751	1.00	0.00	H
ATOM	2689	HG23	VAL	X	177	3.108	13.974	−13.531	1.00	0.00	H
ATOM	2690	C	VAL	X	177	4.207	18.123	−11.560	1.00	0.00	C
ATOM	2691	O	VAL	X	177	3.184	18.572	−10.977	1.00	0.00	O
ATOM	2692	N	PRO	X	178	5.121	18.971	−12.065	1.00	0.00	N
ATOM	2693	CD	PRO	X	178	6.417	18.511	−12.492	1.00	0.00	C
ATOM	2694	HD1	PRO	X	178	6.363	18.278	−13.577	1.00	0.00	H
ATOM	2695	HD2	PRO	X	178	6.768	17.627	−11.917	1.00	0.00	H
ATOM	2696	CA	PRO	X	178	4.934	20.390	−12.189	1.00	0.00	C
ATOM	2697	HA	PRO	X	178	4.739	20.848	−11.231	1.00	0.00	H
ATOM	2698	CB	PRO	X	178	6.303	20.858	−12.887	1.00	0.00	C
ATOM	2699	HB1	PRO	X	178	6.579	21.890	−12.581	1.00	0.00	H
ATOM	2700	HB2	PRO	X	178	6.250	20.801	−13.995	1.00	0.00	H

ATOM 2701 CG PRO X 178 7.243 19.791 −12.419 1.00 0.00
C
ATOM 2702 HG1 PRO X 178 8.171 19.562 −12.985 1.00 0.00
H
ATOM 2703 HG2 PRO X 178 7.509 19.939 −11.351 1.00 0.00
H
ATOM 2704 C PRO X 178 3.761 20.853 −13.082 1.00 0.00
C
ATOM 2705 O PRO X 178 3.465 20.263 −14.093 1.00 0.00
O
ATOM 2706 N SER X 179 3.175 21.985 −12.749 1.00 0.00
N
ATOM 2707 HN SER X 179 3.612 22.340 −11.926 1.00 0.00
H
ATOM 2708 CA SER X 179 2.015 22.646 −13.326 1.00 0.00
C
ATOM 2709 HA SER X 179 1.262 21.874 −13.380 1.00 0.00
H
ATOM 2710 CB SER X 179 1.465 23.845 −12.471 1.00 0.00
C
ATOM 2711 HB1 SER X 179 1.268 23.250 −11.553 1.00 0.00
H
ATOM 2712 HB2 SER X 179 0.498 24.284 −12.796 1.00 0.00
H
ATOM 2713 OG SER X 179 2.375 24.876 −12.040 1.00 0.00
O
ATOM 2714 HG1 SER X 179 2.739 24.634 −11.186 1.00 0.00
H
ATOM 2715 C SER X 179 2.119 23.202 −14.703 1.00 0.00
C
ATOM 2716 O SER X 179 1.059 23.279 −15.303 1.00 0.00
O
ATOM 2717 N ARG X 180 3.316 23.463 −15.201 1.00 0.00
N
ATOM 2718 HN ARG X 180 4.020 23.495 −14.496 1.00 0.00
H
ATOM 2719 CA ARG X 180 3.533 23.744 −16.579 1.00 0.00
C
ATOM 2720 HA ARG X 180 2.930 24.594 −16.862 1.00 0.00
H
ATOM 2721 CB ARG X 180 5.012 24.129 −16.896 1.00 0.00
C
ATOM 2722 HB1 ARG X 180 5.146 25.087 −16.349 1.00 0.00
H
ATOM 2723 HB2 ARG X 180 5.177 24.390 −17.963 1.00 0.00
H
ATOM 2724 CG ARG X 180 6.046 23.031 −16.489 1.00 0.00
C
ATOM 2725 HG1 ARG X 180 5.990 22.047 −17.001 1.00 0.00
H

```
ATOM   2726  HG2 ARG X 180       5.855  22.768 −15.427  1.00  0.00
H
ATOM   2727  CD  ARG X 180       7.490  23.606 −16.727  1.00  0.00
C
ATOM   2728  HD1 ARG X 180       7.792  24.409 −16.021  1.00  0.00
H
ATOM   2729  HD2 ARG X 180       7.657  24.120 −17.698  1.00  0.00
H
ATOM   2730  NE  ARG X 180       8.547  22.477 −16.561  1.00  0.00
N
ATOM   2731  HE  ARG X 180       8.977  21.991 −17.322  1.00  0.00
H
ATOM   2732  CZ  ARG X 180       9.145  22.197 −15.401  1.00  0.00
C
ATOM   2733  NH1 ARG X 180       8.815  22.870 −14.324  1.00  0.00
N
ATOM   2734 HH11 ARG X 180       8.399  23.772 −14.437  1.00  0.00
H
ATOM   2735 HH12 ARG X 180       9.357  22.598 −13.528  1.00  0.00
H
ATOM   2736  NH2 ARG X 180      10.141  21.369 −15.450  1.00  0.00
N
ATOM   2737 HH21 ARG X 180      10.365  20.850 −16.275  1.00  0.00
H
ATOM   2738 HH22 ARG X 180      10.629  21.189 −14.596  1.00  0.00
H
ATOM   2739  C   ARG X 180       3.219  22.582 −17.520  1.00  0.00
C
ATOM   2740  O   ARG X 180       3.028  22.836 −18.739  1.00  0.00
O
ATOM   2741  N   PHE X 181       3.073  21.315 −17.050  1.00  0.00
N
ATOM   2742  HN  PHE X 181       2.973  21.247 −16.060  1.00  0.00
H
ATOM   2743  CA  PHE X 181       2.705  20.167 −17.857  1.00  0.00
C
ATOM   2744  HA  PHE X 181       2.988  20.379 −18.877  1.00  0.00
H
ATOM   2745  CB  PHE X 181       3.229  18.882 −17.248  1.00  0.00
C
ATOM   2746  HB1 PHE X 181       2.640  17.994 −17.564  1.00  0.00
H
ATOM   2747  HB2 PHE X 181       3.220  19.053 −16.150  1.00  0.00
H
ATOM   2748  CG  PHE X 181       4.752  18.748 −17.542  1.00  0.00
C
ATOM   2749  CD1 PHE X 181       5.164  18.124 −18.729  1.00  0.00
C
ATOM   2750  HD1 PHE X 181       4.454  17.796 −19.475  1.00  0.00
H
```

```
ATOM   2751  CE1 PHE X 181       6.545  17.952 −19.016  1.00  0.00
C
ATOM   2752  HE1 PHE X 181       6.863  17.484 −19.936  1.00  0.00
H
ATOM   2753  CZ  PHE X 181       7.501  18.362 −17.980  1.00  0.00
C
ATOM   2754  HZ  PHE X 181       8.536  18.061 −18.047  1.00  0.00
H
ATOM   2755  CD2 PHE X 181       5.673  19.063 −16.572  1.00  0.00
C
ATOM   2756  HD2 PHE X 181       5.251  19.303 −15.607  1.00  0.00
H
ATOM   2757  CE2 PHE X 181       7.041  18.916 −16.771  1.00  0.00
C
ATOM   2758  HE2 PHE X 181       7.713  19.351 −16.046  1.00  0.00
H
ATOM   2759  C   PHE X 181       1.124  20.044 −17.927  1.00  0.00
C
ATOM   2760  O   PHE X 181       0.436  19.941 −16.964  1.00  0.00
O
ATOM   2761  N   SER X 182       0.560  20.167 −19.183  1.00  0.00
N
ATOM   2762  HN  SER X 182       1.045  20.332 −20.038  1.00  0.00
H
ATOM   2763  CA  SER X 182      −0.853  19.988 −19.407  1.00  0.00
C
ATOM   2764  HA  SER X 182      −1.308  19.594 −18.510  1.00  0.00
H
ATOM   2765  CB  SER X 182      −1.614  21.393 −19.587  1.00  0.00
C
ATOM   2766  HB1 SER X 182      −1.289  22.013 −18.725  1.00  0.00
H
ATOM   2767  HB2 SER X 182      −2.712  21.222 −19.584  1.00  0.00
H
ATOM   2768  OG  SER X 182      −1.149  22.171 −20.722  1.00  0.00
O
ATOM   2769  HG1 SER X 182      −0.409  22.675 −20.374  1.00  0.00
H
ATOM   2770  C   SER X 182      −1.166  19.069 −20.563  1.00  0.00
C
ATOM   2771  O   SER X 182      −0.364  19.005 −21.540  1.00  0.00
O
ATOM   2772  N   GLY X 183      −2.349  18.428 −20.595  1.00  0.00
N
ATOM   2773  HN  GLY X 183      −3.074  18.644 −19.945  1.00  0.00
H
ATOM   2774  CA  GLY X 183      −2.844  17.814 −21.791  1.00  0.00
C
ATOM   2775  HA1 GLY X 183      −2.844  16.766 −21.531  1.00  0.00
H
```

ATOM 2776 HA2 GLY X 183 -2.266 18.106 -22.655 1.00 0.00
H
ATOM 2777 C GLY X 183 -4.295 18.134 -22.172 1.00 0.00
C
ATOM 2778 O GLY X 183 -5.045 18.678 -21.360 1.00 0.00
O
ATOM 2779 N SER X 184 -4.643 17.744 -23.426 1.00 0.00
N
ATOM 2780 HN SER X 184 -3.929 17.317 -23.976 1.00 0.00
H
ATOM 2781 CA SER X 184 -5.916 18.061 -24.066 1.00 0.00
C
ATOM 2782 HA SER X 184 -6.693 17.975 -23.321 1.00 0.00
H
ATOM 2783 CB SER X 184 -5.985 19.615 -24.589 1.00 0.00
C
ATOM 2784 HB1 SER X 184 -5.418 19.782 -25.530 1.00 0.00
H
ATOM 2785 HB2 SER X 184 -5.450 20.228 -23.831 1.00 0.00
H
ATOM 2786 OG SER X 184 -7.354 19.969 -24.808 1.00 0.00
O
ATOM 2787 HG1 SER X 184 -7.367 20.560 -25.564 1.00 0.00
H
ATOM 2788 C SER X 184 -6.355 17.136 -25.187 1.00 0.00
C
ATOM 2789 O SER X 184 -5.512 16.485 -25.735 1.00 0.00
O
ATOM 2790 N GLY X 185 -7.616 17.108 -25.467 1.00 0.00
N
ATOM 2791 HN GLY X 185 -8.253 17.646 -24.921 1.00 0.00
H
ATOM 2792 CA GLY X 185 -8.172 16.513 -26.674 1.00 0.00
C
ATOM 2793 HA1 GLY X 185 -7.415 16.348 -27.426 1.00 0.00
H
ATOM 2794 HA2 GLY X 185 -8.923 17.218 -26.998 1.00 0.00
H
ATOM 2795 C GLY X 185 -8.978 15.275 -26.473 1.00 0.00
C
ATOM 2796 O GLY X 185 -8.807 14.487 -25.508 1.00 0.00
O
ATOM 2797 N SER X 186 -9.971 14.975 -27.375 1.00 0.00
N
ATOM 2798 HN SER X 186 -9.986 15.658 -28.101 1.00 0.00
H
ATOM 2799 CA SER X 186 -10.742 13.800 -27.367 1.00 0.00
C
ATOM 2800 HA SER X 186 -10.340 12.966 -26.812 1.00 0.00
H

```
ATOM   2801  CB  SER X 186     -12.179  14.221 -26.762  1.00  0.00
C
ATOM   2802  HB1 SER X 186     -12.843  14.677 -27.527  1.00  0.00
H
ATOM   2803  HB2 SER X 186     -12.084  14.900 -25.888  1.00  0.00
H
ATOM   2804  OG  SER X 186     -12.922  13.016 -26.373  1.00  0.00
O
ATOM   2805  HG1 SER X 186     -12.966  13.027 -25.414  1.00  0.00
H
ATOM   2806  C   SER X 186     -10.872  13.137 -28.738  1.00  0.00
C
ATOM   2807  O   SER X 186     -10.672  13.799 -29.761  1.00  0.00
O
ATOM   2808  N   GLY X 187     -11.363  11.939 -28.781  1.00  0.00
N
ATOM   2809  HN  GLY X 187     -11.597  11.511 -27.911  1.00  0.00
H
ATOM   2810  CA  GLY X 187     -11.714  11.150 -29.959  1.00  0.00
C
ATOM   2811  HA1 GLY X 187     -12.387  11.734 -30.569  1.00  0.00
H
ATOM   2812  HA2 GLY X 187     -12.310  10.289 -29.695  1.00  0.00
H
ATOM   2813  C   GLY X 187     -10.619  10.600 -30.775  1.00  0.00
C
ATOM   2814  O   GLY X 187     -10.080   9.491 -30.452  1.00  0.00
O
ATOM   2815  N   THR X 188     -10.169  11.421 -31.821  1.00  0.00
N
ATOM   2816  HN  THR X 188     -10.511  12.349 -31.949  1.00  0.00
H
ATOM   2817  CA  THR X 188      -8.978  11.152 -32.592  1.00  0.00
C
ATOM   2818  HA  THR X 188      -8.598  10.189 -32.285  1.00  0.00
H
ATOM   2819  CB  THR X 188      -9.241  10.995 -34.016  1.00  0.00
C
ATOM   2820  HB  THR X 188      -8.331  10.705 -34.583  1.00  0.00
H
ATOM   2821  OG1 THR X 188      -9.777  12.152 -34.615  1.00  0.00
O
ATOM   2822  HG1 THR X 188     -10.171  11.804 -35.418  1.00  0.00
H
ATOM   2823  CG2 THR X 188     -10.390   9.940 -34.261  1.00  0.00
C
ATOM   2824 HG21 THR X 188     -10.652   9.364 -33.348  1.00  0.00
H
ATOM   2825 HG22 THR X 188     -10.057   9.173 -34.992  1.00  0.00
H
```

```
ATOM   2826 HG23 THR X 188     -11.381  10.368 -34.526  1.00  0.00
H
ATOM   2827  C   THR X 188      -7.674  11.956 -32.488  1.00  0.00
C
ATOM   2828  O   THR X 188      -6.571  11.746 -33.091  1.00  0.00
O
ATOM   2829  N   GLU X 189      -7.609  13.002 -31.653  1.00  0.00
N
ATOM   2830  HN  GLU X 189      -8.440  13.318 -31.202  1.00  0.00
H
ATOM   2831  CA  GLU X 189      -6.459  13.925 -31.520  1.00  0.00
C
ATOM   2832  HA  GLU X 189      -5.530  13.414 -31.723  1.00  0.00
H
ATOM   2833  CB  GLU X 189      -6.491  15.082 -32.569  1.00  0.00
C
ATOM   2834  HB1 GLU X 189      -7.524  15.451 -32.746  1.00  0.00
H
ATOM   2835  HB2 GLU X 189      -6.188  14.447 -33.428  1.00  0.00
H
ATOM   2836  CG  GLU X 189      -5.600  16.298 -32.189  1.00  0.00
C
ATOM   2837  HG1 GLU X 189      -4.546  16.006 -31.990  1.00  0.00
H
ATOM   2838  HG2 GLU X 189      -6.078  16.724 -31.281  1.00  0.00
H
ATOM   2839  CD  GLU X 189      -5.552  17.319 -33.297  1.00  0.00
C
ATOM   2840  OE1 GLU X 189      -5.945  18.512 -33.086  1.00  0.00
O
ATOM   2841  OE2 GLU X 189      -5.062  16.993 -34.436  1.00  0.00
O
ATOM   2842  C   GLU X 189      -6.093  14.410 -30.157  1.00  0.00
C
ATOM   2843  O   GLU X 189      -6.835  15.000 -29.414  1.00  0.00
O
ATOM   2844  N   PHE X 190      -4.843  14.075 -29.657  1.00  0.00
N
ATOM   2845  HN  PHE X 190      -4.255  13.493 -30.212  1.00  0.00
H
ATOM   2846  CA  PHE X 190      -4.465  14.157 -28.316  1.00  0.00
C
ATOM   2847  HA  PHE X 190      -5.211  14.688 -27.743  1.00  0.00
H
ATOM   2848  CB  PHE X 190      -4.218  12.809 -27.647  1.00  0.00
C
ATOM   2849  HB1 PHE X 190      -3.969  12.842 -26.565  1.00  0.00
H
ATOM   2850  HB2 PHE X 190      -3.521  12.165 -28.225  1.00  0.00
H
```

```
ATOM   2851  CG  PHE X 190      -5.531  12.127 -27.748  1.00  0.00
C
ATOM   2852  CD1 PHE X 190      -5.789  11.281 -28.867  1.00  0.00
C
ATOM   2853  HD1 PHE X 190      -5.081  11.068 -29.655  1.00  0.00
H
ATOM   2854  CE1 PHE X 190      -7.083  10.730 -28.899  1.00  0.00
C
ATOM   2855  HE1 PHE X 190      -7.377   9.996 -29.635  1.00  0.00
H
ATOM   2856  CZ  PHE X 190      -8.069  11.038 -27.971  1.00  0.00
C
ATOM   2857  HZ  PHE X 190      -9.003  10.499 -27.909  1.00  0.00
H
ATOM   2858  CD2 PHE X 190      -6.518  12.437 -26.799  1.00  0.00
C
ATOM   2859  HD2 PHE X 190      -6.393  13.206 -26.050  1.00  0.00
H
ATOM   2860  CE2 PHE X 190      -7.738  11.857 -26.910  1.00  0.00
C
ATOM   2861  HE2 PHE X 190      -8.471  12.089 -26.152  1.00  0.00
H
ATOM   2862  C   PHE X 190      -3.238  15.075 -28.241  1.00  0.00
C
ATOM   2863  O   PHE X 190      -2.246  14.713 -28.839  1.00  0.00
O
ATOM   2864  N   THR X 191      -3.300  16.236 -27.515  1.00  0.00
N
ATOM   2865  HN  THR X 191      -4.019  16.390 -26.841  1.00  0.00
H
ATOM   2866  CA  THR X 191      -2.141  17.043 -27.379  1.00  0.00
C
ATOM   2867  HA  THR X 191      -1.342  16.712 -28.026  1.00  0.00
H
ATOM   2868  CB  THR X 191      -2.278  18.483 -27.786  1.00  0.00
C
ATOM   2869  HB  THR X 191      -1.429  19.151 -27.529  1.00  0.00
H
ATOM   2870  OG1 THR X 191      -3.426  19.110 -27.163  1.00  0.00
O
ATOM   2871  HG1 THR X 191      -3.066  19.562 -26.397  1.00  0.00
H
ATOM   2872  CG2 THR X 191      -2.505  18.582 -29.313  1.00  0.00
C
ATOM   2873 HG21 THR X 191      -2.962  19.563 -29.562  1.00  0.00
H
ATOM   2874 HG22 THR X 191      -3.105  17.747 -29.735  1.00  0.00
H
ATOM   2875 HG23 THR X 191      -1.521  18.602 -29.827  1.00  0.00
H
```

```
ATOM   2876  C   THR X 191      -1.558  16.880 -25.983  1.00  0.00
C
ATOM   2877  O   THR X 191      -2.313  16.772 -25.023  1.00  0.00
O
ATOM   2878  N   LEU X 192      -0.241  16.801 -25.920  1.00  0.00
N
ATOM   2879  HN  LEU X 192       0.341  16.715 -26.725  1.00  0.00
H
ATOM   2880  CA  LEU X 192       0.427  17.077 -24.616  1.00  0.00
C
ATOM   2881  HA  LEU X 192      -0.331  17.142 -23.849  1.00  0.00
H
ATOM   2882  CB  LEU X 192       1.445  15.947 -24.291  1.00  0.00
C
ATOM   2883  HB1 LEU X 192       1.980  15.608 -25.204  1.00  0.00
H
ATOM   2884  HB2 LEU X 192       0.898  14.996 -24.122  1.00  0.00
H
ATOM   2885  CG  LEU X 192       2.560  16.110 -23.170  1.00  0.00
C
ATOM   2886  HG  LEU X 192       3.230  16.955 -23.437  1.00  0.00
H
ATOM   2887  CD1 LEU X 192       2.015  16.116 -21.748  1.00  0.00
C
ATOM   2888 HD11 LEU X 192       1.453  17.047 -21.521  1.00  0.00
H
ATOM   2889 HD12 LEU X 192       2.754  16.042 -20.922  1.00  0.00
H
ATOM   2890 HD13 LEU X 192       1.244  15.330 -21.599  1.00  0.00
H
ATOM   2891  CD2 LEU X 192       3.439  14.836 -23.197  1.00  0.00
C
ATOM   2892 HD21 LEU X 192       3.003  13.963 -22.666  1.00  0.00
H
ATOM   2893 HD22 LEU X 192       4.325  14.934 -22.534  1.00  0.00
H
ATOM   2894 HD23 LEU X 192       3.804  14.591 -24.217  1.00  0.00
H
ATOM   2895  C   LEU X 192       1.194  18.416 -24.728  1.00  0.00
C
ATOM   2896  O   LEU X 192       1.758  18.769 -25.773  1.00  0.00
O
ATOM   2897  N   THR X 193       1.226  19.185 -23.627  1.00  0.00
N
ATOM   2898  HN  THR X 193       0.597  19.047 -22.865  1.00  0.00
H
ATOM   2899  CA  THR X 193       1.823  20.540 -23.730  1.00  0.00
C
ATOM   2900  HA  THR X 193       2.481  20.689 -24.574  1.00  0.00
H
```

ATOM 2901 CB THR X 193 0.811 21.668 −23.596 1.00 0.00
C
ATOM 2902 HB THR X 193 0.005 21.408 −22.877 1.00 0.00
H
ATOM 2903 OG1 THR X 193 0.109 21.722 −24.814 1.00 0.00
O
ATOM 2904 HG1 THR X 193 0.793 22.011 −25.422 1.00 0.00
H
ATOM 2905 CG2 THR X 193 1.423 23.049 −23.286 1.00 0.00
C
ATOM 2906 HG21 THR X 193 0.539 23.721 −23.312 1.00 0.00
H
ATOM 2907 HG22 THR X 193 2.169 23.384 −24.038 1.00 0.00
H
ATOM 2908 HG23 THR X 193 1.868 23.098 −22.269 1.00 0.00
H
ATOM 2909 C THR X 193 2.787 20.707 −22.623 1.00 0.00
C
ATOM 2910 O THR X 193 2.507 20.605 −21.409 1.00 0.00
O
ATOM 2911 N ILE X 194 4.066 21.051 −22.968 1.00 0.00
N
ATOM 2912 HN ILE X 194 4.440 21.035 −23.892 1.00 0.00
H
ATOM 2913 CA ILE X 194 5.027 21.495 −21.901 1.00 0.00
C
ATOM 2914 HA ILE X 194 4.736 21.267 −20.886 1.00 0.00
H
ATOM 2915 CB ILE X 194 6.495 20.926 −22.120 1.00 0.00
C
ATOM 2916 HB ILE X 194 6.935 21.427 −23.008 1.00 0.00
H
ATOM 2917 CG2 ILE X 194 7.300 21.330 −20.823 1.00 0.00
C
ATOM 2918 HG21 ILE X 194 8.386 21.100 −20.874 1.00 0.00
H
ATOM 2919 HG22 ILE X 194 6.911 20.850 −19.900 1.00 0.00
H
ATOM 2920 HG23 ILE X 194 7.333 22.440 −20.793 1.00 0.00
H
ATOM 2921 CG1 ILE X 194 6.562 19.420 −22.346 1.00 0.00
C
ATOM 2922 HG11 ILE X 194 5.940 19.131 −23.220 1.00 0.00
H
ATOM 2923 HG12 ILE X 194 6.215 18.930 −21.411 1.00 0.00
H
ATOM 2924 CD ILE X 194 7.961 18.831 −22.566 1.00 0.00
C
ATOM 2925 HD1 ILE X 194 8.472 18.837 −21.580 1.00 0.00
H

```
ATOM   2926  HD2 ILE X 194       8.610  19.242 -23.368  1.00  0.00
H
ATOM   2927  HD3 ILE X 194       7.883  17.764 -22.868  1.00  0.00
H
ATOM   2928  C   ILE X 194       5.136  22.983 -21.971  1.00  0.00
C
ATOM   2929  O   ILE X 194       5.715  23.628 -22.911  1.00  0.00
O
ATOM   2930  N   SER X 195       4.622  23.782 -21.006  1.00  0.00
N
ATOM   2931  HN  SER X 195       4.244  23.491 -20.131  1.00  0.00
H
ATOM   2932  CA  SER X 195       4.778  25.240 -20.927  1.00  0.00
C
ATOM   2933  HA  SER X 195       4.746  25.548 -21.962  1.00  0.00
H
ATOM   2934  CB  SER X 195       3.688  26.073 -20.145  1.00  0.00
C
ATOM   2935  HB1 SER X 195       4.091  27.100 -20.018  1.00  0.00
H
ATOM   2936  HB2 SER X 195       3.556  25.621 -19.139  1.00  0.00
H
ATOM   2937  OG  SER X 195       2.430  26.046 -20.761  1.00  0.00
O
ATOM   2938  HG1 SER X 195       2.472  26.562 -21.569  1.00  0.00
H
ATOM   2939  C   SER X 195       6.201  25.712 -20.463  1.00  0.00
C
ATOM   2940  O   SER X 195       6.376  26.096 -19.295  1.00  0.00
O
ATOM   2941  N   SER X 196       7.142  25.802 -21.436  1.00  0.00
N
ATOM   2942  HN  SER X 196       6.860  25.287 -22.242  1.00  0.00
H
ATOM   2943  CA  SER X 196       8.394  26.392 -21.226  1.00  0.00
C
ATOM   2944  HA  SER X 196       8.863  26.476 -22.195  1.00  0.00
H
ATOM   2945  CB  SER X 196       8.461  27.851 -20.566  1.00  0.00
C
ATOM   2946  HB1 SER X 196       8.101  27.973 -19.522  1.00  0.00
H
ATOM   2947  HB2 SER X 196       7.651  28.401 -21.092  1.00  0.00
H
ATOM   2948  OG  SER X 196       9.779  28.517 -20.695  1.00  0.00
O
ATOM   2949  HG1 SER X 196      10.413  28.057 -20.140  1.00  0.00
H
ATOM   2950  C   SER X 196       9.444  25.472 -20.507  1.00  0.00
C
```

```
ATOM   2951  O   SER X 196       9.434  25.308 −19.309  1.00  0.00
O
ATOM   2952  N   LEU X 197      10.225  24.796 −21.289  1.00  0.00
N
ATOM   2953  HN  LEU X 197      10.410  25.048 −22.235  1.00  0.00
H
ATOM   2954  CA  LEU X 197      11.230  23.773 −20.805  1.00  0.00
C
ATOM   2955  HA  LEU X 197      10.698  22.872 −20.538  1.00  0.00
H
ATOM   2956  CB  LEU X 197      12.088  23.100 −21.935  1.00  0.00
C
ATOM   2957  HB1 LEU X 197      12.575  22.171 −21.569  1.00  0.00
H
ATOM   2958  HB2 LEU X 197      12.943  23.757 −22.201  1.00  0.00
H
ATOM   2959  CG  LEU X 197      11.479  22.639 −23.282  1.00  0.00
C
ATOM   2960  HG  LEU X 197      11.185  23.598 −23.758  1.00  0.00
H
ATOM   2961  CD1 LEU X 197      12.451  21.849 −24.124  1.00  0.00
C
ATOM   2962 HD11 LEU X 197      12.736  20.876 −23.669  1.00  0.00
H
ATOM   2963 HD12 LEU X 197      13.460  22.313 −24.164  1.00  0.00
H
ATOM   2964 HD13 LEU X 197      12.077  21.455 −25.093  1.00  0.00
H
ATOM   2965  CD2 LEU X 197      10.172  21.810 −23.152  1.00  0.00
C
ATOM   2966 HD21 LEU X 197       9.277  22.414 −22.888  1.00  0.00
H
ATOM   2967 HD22 LEU X 197      10.253  21.013 −22.382  1.00  0.00
H
ATOM   2968 HD23 LEU X 197       9.714  21.509 −24.118  1.00  0.00
H
ATOM   2969  C   LEU X 197      12.194  24.134 −19.703  1.00  0.00
C
ATOM   2970  O   LEU X 197      12.619  25.248 −19.634  1.00  0.00
O
ATOM   2971  N   GLN X 198      12.540  23.175 −18.870  1.00  0.00
N
ATOM   2972  HN  GLN X 198      11.975  22.353 −18.888  1.00  0.00
H
ATOM   2973  CA  GLN X 198      13.654  23.361 −17.960  1.00  0.00
C
ATOM   2974  HA  GLN X 198      14.096  24.332 −18.127  1.00  0.00
H
ATOM   2975  CB  GLN X 198      13.102  23.047 −16.547  1.00  0.00
C
```

```
ATOM   2976  HB1 GLN X 198      13.965  22.955 -15.854  1.00  0.00
H
ATOM   2977  HB2 GLN X 198      12.543  22.089 -16.606  1.00  0.00
H
ATOM   2978  CG  GLN X 198      12.201  24.220 -16.011  1.00  0.00
C
ATOM   2979  HG1 GLN X 198      11.840  23.894 -15.012  1.00  0.00
H
ATOM   2980  HG2 GLN X 198      11.355  24.282 -16.728  1.00  0.00
H
ATOM   2981  CD  GLN X 198      12.892  25.595 -15.758  1.00  0.00
C
ATOM   2982  OE1 GLN X 198      14.136  25.728 -15.911  1.00  0.00
O
ATOM   2983  NE2 GLN X 198      12.063  26.559 -15.515  1.00  0.00
N
ATOM   2984 HE21 GLN X 198      12.412  27.488 -15.390  1.00  0.00
H
ATOM   2985 HE22 GLN X 198      11.069  26.451 -15.504  1.00  0.00
H
ATOM   2986  C   GLN X 198      14.736  22.349 -18.309  1.00  0.00
C
ATOM   2987  O   GLN X 198      14.462  21.473 -19.133  1.00  0.00
O
ATOM   2988  N   PRO X 199      15.984  22.571 -17.934  1.00  0.00
N
ATOM   2989  CD  PRO X 199      16.463  23.699 -17.140  1.00  0.00
C
ATOM   2990  HD1 PRO X 199      15.964  23.701 -16.147  1.00  0.00
H
ATOM   2991  HD2 PRO X 199      16.157  24.706 -17.497  1.00  0.00
H
ATOM   2992  CA  PRO X 199      17.093  21.804 -18.528  1.00  0.00
C
ATOM   2993  HA  PRO X 199      17.022  21.907 -19.601  1.00  0.00
H
ATOM   2994  CB  PRO X 199      18.383  22.426 -18.087  1.00  0.00
C
ATOM   2995  HB1 PRO X 199      18.712  23.025 -18.963  1.00  0.00
H
ATOM   2996  HB2 PRO X 199      19.252  21.769 -17.864  1.00  0.00
H
ATOM   2997  CG  PRO X 199      17.915  23.507 -16.957  1.00  0.00
C
ATOM   2998  HG1 PRO X 199      18.285  23.110 -15.988  1.00  0.00
H
ATOM   2999  HG2 PRO X 199      18.415  24.493 -17.064  1.00  0.00
H
ATOM   3000  C   PRO X 199      17.033  20.294 -18.344  1.00  0.00
C
```

ATOM 3001 O PRO X 199 17.804 19.605 −19.042 1.00 0.00
O
ATOM 3002 N ASP X 200 16.266 19.804 −17.342 1.00 0.00
N
ATOM 3003 HN ASP X 200 15.745 20.486 −16.835 1.00 0.00
H
ATOM 3004 CA ASP X 200 16.018 18.341 −17.048 1.00 0.00
C
ATOM 3005 HA ASP X 200 16.819 17.815 −17.545 1.00 0.00
H
ATOM 3006 CB ASP X 200 15.984 18.304 −15.509 1.00 0.00
C
ATOM 3007 HB1 ASP X 200 15.068 18.834 −15.170 1.00 0.00
H
ATOM 3008 HB2 ASP X 200 16.881 18.863 −15.167 1.00 0.00
H
ATOM 3009 CG ASP X 200 15.982 16.863 −14.984 1.00 0.00
C
ATOM 3010 OD1 ASP X 200 16.465 15.967 −15.735 1.00 0.00
O
ATOM 3011 OD2 ASP X 200 15.616 16.652 −13.836 1.00 0.00
O
ATOM 3012 C ASP X 200 14.689 17.850 −17.612 1.00 0.00
C
ATOM 3013 O ASP X 200 14.226 16.740 −17.395 1.00 0.00
O
ATOM 3014 N ASP X 201 13.967 18.657 −18.441 1.00 0.00
N
ATOM 3015 HN ASP X 201 14.352 19.536 −18.710 1.00 0.00
H
ATOM 3016 CA ASP X 201 12.753 18.268 −19.205 1.00 0.00
C
ATOM 3017 HA ASP X 201 12.132 17.662 −18.561 1.00 0.00
H
ATOM 3018 CB ASP X 201 11.807 19.399 −19.600 1.00 0.00
C
ATOM 3019 HB1 ASP X 201 10.926 19.036 −20.172 1.00 0.00
H
ATOM 3020 HB2 ASP X 201 12.345 20.215 −20.128 1.00 0.00
H
ATOM 3021 CG ASP X 201 11.159 19.969 −18.329 1.00 0.00
C
ATOM 3022 OD1 ASP X 201 11.148 19.334 −17.216 1.00 0.00
O
ATOM 3023 OD2 ASP X 201 10.696 21.157 −18.420 1.00 0.00
O
ATOM 3024 C ASP X 201 13.014 17.465 −20.410 1.00 0.00
C
ATOM 3025 O ASP X 201 12.079 16.850 −20.856 1.00 0.00
O

ATOM 3026 N PHE X 202 14.201 17.366 −20.944 1.00 0.00
N
ATOM 3027 HN PHE X 202 15.049 17.710 −20.548 1.00 0.00
H
ATOM 3028 CA PHE X 202 14.456 16.562 −22.138 1.00 0.00
C
ATOM 3029 HA PHE X 202 13.702 16.842 −22.859 1.00 0.00
H
ATOM 3030 CB PHE X 202 15.887 16.872 −22.670 1.00 0.00
C
ATOM 3031 HB1 PHE X 202 15.964 16.304 −23.621 1.00 0.00
H
ATOM 3032 HB2 PHE X 202 16.718 16.620 −21.976 1.00 0.00
H
ATOM 3033 CG PHE X 202 16.037 18.361 −22.953 1.00 0.00
C
ATOM 3034 CD1 PHE X 202 17.110 19.164 −22.423 1.00 0.00
C
ATOM 3035 HD1 PHE X 202 17.866 18.720 −21.793 1.00 0.00
H
ATOM 3036 CE1 PHE X 202 17.132 20.472 −22.711 1.00 0.00
C
ATOM 3037 HE1 PHE X 202 17.972 21.042 −22.342 1.00 0.00
H
ATOM 3038 CZ PHE X 202 16.288 21.058 −23.612 1.00 0.00
C
ATOM 3039 HZ PHE X 202 16.358 22.078 −23.960 1.00 0.00
H
ATOM 3040 CD2 PHE X 202 15.151 18.940 −23.873 1.00 0.00
C
ATOM 3041 HD2 PHE X 202 14.473 18.416 −24.530 1.00 0.00
H
ATOM 3042 CE2 PHE X 202 15.325 20.287 −24.168 1.00 0.00
C
ATOM 3043 HE2 PHE X 202 14.725 20.679 −24.976 1.00 0.00
H
ATOM 3044 C PHE X 202 14.363 15.085 −21.926 1.00 0.00
C
ATOM 3045 O PHE X 202 15.060 14.396 −21.156 1.00 0.00
O
ATOM 3046 N ALA X 203 13.466 14.546 −22.703 1.00 0.00
N
ATOM 3047 HN ALA X 203 12.969 15.191 −23.278 1.00 0.00
H
ATOM 3048 CA ALA X 203 12.972 13.181 −22.616 1.00 0.00
C
ATOM 3049 HA ALA X 203 13.759 12.518 −22.289 1.00 0.00
H
ATOM 3050 CB ALA X 203 11.844 13.040 −21.491 1.00 0.00
C

```
ATOM   3051  HB1 ALA X 203      12.292  13.317 −20.513  1.00  0.00
H
ATOM   3052  HB2 ALA X 203      11.431  12.011 −21.417  1.00  0.00
H
ATOM   3053  HB3 ALA X 203      11.046  13.759 −21.774  1.00  0.00
H
ATOM   3054  C   ALA X 203      12.337  12.620 −23.907  1.00  0.00
C
ATOM   3055  O   ALA X 203      12.153  13.270 −24.935  1.00  0.00
O
ATOM   3056  N   THR X 204      11.945  11.357 −23.820  1.00  0.00
N
ATOM   3057  HN  THR X 204      12.202  10.781 −23.048  1.00  0.00
H
ATOM   3058  CA  THR X 204      10.993  10.780 −24.813  1.00  0.00
C
ATOM   3059  HA  THR X 204      11.011  11.349 −25.731  1.00  0.00
H
ATOM   3060  CB  THR X 204      11.414   9.446 −25.369  1.00  0.00
C
ATOM   3061  HB  THR X 204      11.398   8.586 −24.665  1.00  0.00
H
ATOM   3062  OG1 THR X 204      12.830   9.376 −25.520  1.00  0.00
O
ATOM   3063  HG1 THR X 204      13.112  10.269 −25.734  1.00  0.00
H
ATOM   3064  CG2 THR X 204      10.851   9.118 −26.691  1.00  0.00
C
ATOM   3065 HG21 THR X 204      11.184   9.909 −27.397  1.00  0.00
H
ATOM   3066 HG22 THR X 204       9.740   9.136 −26.668  1.00  0.00
H
ATOM   3067 HG23 THR X 204      11.232   8.107 −26.951  1.00  0.00
H
ATOM   3068  C   THR X 204       9.591  10.567 −24.277  1.00  0.00
C
ATOM   3069  O   THR X 204       9.307  10.115 −23.153  1.00  0.00
O
ATOM   3070  N   TYR X 205       8.562  10.885 −25.096  1.00  0.00
N
ATOM   3071  HN  TYR X 205       8.681  11.208 −26.032  1.00  0.00
H
ATOM   3072  CA  TYR X 205       7.200  10.918 −24.528  1.00  0.00
C
ATOM   3073  HA  TYR X 205       7.125  10.463 −23.551  1.00  0.00
H
ATOM   3074  CB  TYR X 205       6.660  12.303 −24.532  1.00  0.00
C
ATOM   3075  HB1 TYR X 205       5.596  12.351 −24.218  1.00  0.00
H
```

```
ATOM   3076  HB2 TYR X 205       6.691  12.812 −25.519  1.00  0.00
H
ATOM   3077  CG  TYR X 205       7.490  13.268 −23.583  1.00  0.00
C
ATOM   3078  CD1 TYR X 205       8.582  13.945 −24.023  1.00  0.00
C
ATOM   3079  HD1 TYR X 205       9.001  13.778 −25.005  1.00  0.00
H
ATOM   3080  CE1 TYR X 205       9.287  14.888 −23.213  1.00  0.00
C
ATOM   3081  HE1 TYR X 205      10.193  15.345 −23.582  1.00  0.00
H
ATOM   3082  CZ  TYR X 205       8.920  15.010 −21.869  1.00  0.00
C
ATOM   3083  OH  TYR X 205       9.641  15.811 −20.906  1.00  0.00
O
ATOM   3084  HH  TYR X 205      10.462  16.178 −21.241  1.00  0.00
H
ATOM   3085  CD2 TYR X 205       6.955  13.525 −22.342  1.00  0.00
C
ATOM   3086  HD2 TYR X 205       6.007  13.093 −22.055  1.00  0.00
H
ATOM   3087  CE2 TYR X 205       7.673  14.346 −21.421  1.00  0.00
C
ATOM   3088  HE2 TYR X 205       7.242  14.644 −20.477  1.00  0.00
H
ATOM   3089  C   TYR X 205       6.350  10.014 −25.363  1.00  0.00
C
ATOM   3090  O   TYR X 205       6.239  10.225 −26.576  1.00  0.00
O
ATOM   3091  N   TYR X 206       5.639   9.097 −24.687  1.00  0.00
N
ATOM   3092  HN  TYR X 206       5.692   9.136 −23.692  1.00  0.00
H
ATOM   3093  CA  TYR X 206       4.554   8.204 −25.189  1.00  0.00
C
ATOM   3094  HA  TYR X 206       4.593   8.199 −26.268  1.00  0.00
H
ATOM   3095  CB  TYR X 206       4.719   6.685 −24.761  1.00  0.00
C
ATOM   3096  HB1 TYR X 206       3.943   5.992 −25.148  1.00  0.00
H
ATOM   3097  HB2 TYR X 206       4.819   6.714 −23.655  1.00  0.00
H
ATOM   3098  CG  TYR X 206       6.043   6.021 −25.282  1.00  0.00
C
ATOM   3099  CD1 TYR X 206       6.100   5.400 −26.527  1.00  0.00
C
ATOM   3100  HD1 TYR X 206       5.156   5.238 −27.025  1.00  0.00
H
```

```
ATOM   3101  CE1 TYR X 206       7.326   5.082 −27.097  1.00  0.00
C
ATOM   3102  HE1 TYR X 206       7.389   4.673 −28.094  1.00  0.00
H
ATOM   3103  CZ  TYR X 206       8.482   5.464 −26.442  1.00  0.00
C
ATOM   3104  OH  TYR X 206       9.787   5.216 −27.056  1.00  0.00
O
ATOM   3105  HH  TYR X 206      10.415   5.438 −26.365  1.00  0.00
H
ATOM   3106  CD2 TYR X 206       7.248   6.159 −24.558  1.00  0.00
C
ATOM   3107  HD2 TYR X 206       7.296   6.535 −23.547  1.00  0.00
H
ATOM   3108  CE2 TYR X 206       8.499   5.936 −25.156  1.00  0.00
C
ATOM   3109  HE2 TYR X 206       9.447   6.229 −24.729  1.00  0.00
H
ATOM   3110  C   TYR X 206       3.198   8.560 −24.760  1.00  0.00
C
ATOM   3111  O   TYR X 206       2.883   8.950 −23.654  1.00  0.00
O
ATOM   3112  N   CYS X 207       2.204   8.323 −25.571  1.00  0.00
N
ATOM   3113  HN  CYS X 207       2.494   8.140 −26.507  1.00  0.00
H
ATOM   3114  CA  CYS X 207       0.784   8.245 −25.232  1.00  0.00
C
ATOM   3115  HA  CYS X 207       0.538   8.578 −24.234  1.00  0.00
H
ATOM   3116  CB  CYS X 207      −0.126   9.114 −26.227  1.00  0.00
C
ATOM   3117  HB1 CYS X 207       0.200  10.164 −26.069  1.00  0.00
H
ATOM   3118  HB2 CYS X 207      −1.192   9.021 −25.926  1.00  0.00
H
ATOM   3119  SG  CYS X 207       0.007   8.828 −28.003  1.00  0.00
S
ATOM   3120  C   CYS X 207       0.328   6.754 −25.285  1.00  0.00
C
ATOM   3121  O   CYS X 207       0.667   6.090 −26.266  1.00  0.00
O
ATOM   3122  N   GLN X 208      −0.516   6.350 −24.389  1.00  0.00
N
ATOM   3123  HN  GLN X 208      −0.823   6.979 −23.680  1.00  0.00
H
ATOM   3124  CA  GLN X 208      −1.029   4.950 −24.379  1.00  0.00
C
ATOM   3125  HA  GLN X 208      −0.828   4.505 −25.342  1.00  0.00
H
```

```
ATOM   3126  CB  GLN X 208      -0.458   4.131 -23.185  1.00  0.00
C
ATOM   3127  HB1 GLN X 208      -0.568   4.783 -22.293  1.00  0.00
H
ATOM   3128  HB2 GLN X 208       0.653   4.118 -23.165  1.00  0.00
H
ATOM   3129  CG  GLN X 208      -0.904   2.705 -22.886  1.00  0.00
C
ATOM   3130  HG1 GLN X 208      -0.082   1.990 -23.104  1.00  0.00
H
ATOM   3131  HG2 GLN X 208      -1.838   2.533 -23.461  1.00  0.00
H
ATOM   3132  CD  GLN X 208      -1.323   2.423 -21.394  1.00  0.00
C
ATOM   3133  OE1 GLN X 208      -0.450   2.373 -20.527  1.00  0.00
O
ATOM   3134  NE2 GLN X 208      -2.581   2.264 -21.031  1.00  0.00
N
ATOM   3135 HE21 GLN X 208      -2.694   2.246 -20.037  1.00  0.00
H
ATOM   3136 HE22 GLN X 208      -3.280   2.458 -21.719  1.00  0.00
H
ATOM   3137  C   GLN X 208      -2.518   5.185 -24.204  1.00  0.00
C
ATOM   3138  O   GLN X 208      -3.044   6.023 -23.426  1.00  0.00
O
ATOM   3139  N   GLN X 209      -3.295   4.472 -25.045  1.00  0.00
N
ATOM   3140  HN  GLN X 209      -2.903   3.802 -25.670  1.00  0.00
H
ATOM   3141  CA  GLN X 209      -4.755   4.469 -24.733  1.00  0.00
C
ATOM   3142  HA  GLN X 209      -5.069   5.440 -24.378  1.00  0.00
H
ATOM   3143  CB  GLN X 209      -5.517   4.303 -26.006  1.00  0.00
C
ATOM   3144  HB1 GLN X 209      -5.198   5.144 -26.658  1.00  0.00
H
ATOM   3145  HB2 GLN X 209      -6.611   4.497 -25.993  1.00  0.00
H
ATOM   3146  CG  GLN X 209      -5.065   3.011 -26.792  1.00  0.00
C
ATOM   3147  HG1 GLN X 209      -4.101   2.672 -26.357  1.00  0.00
H
ATOM   3148  HG2 GLN X 209      -5.005   3.254 -27.875  1.00  0.00
H
ATOM   3149  CD  GLN X 209      -6.124   1.915 -26.587  1.00  0.00
C
ATOM   3150  OE1 GLN X 209      -7.090   2.123 -25.904  1.00  0.00
O
```

ATOM 3151 NE2 GLN X 209 −5.883 0.707 −27.238 1.00 0.00
N
ATOM 3152 HE21 GLN X 209 −5.185 0.678 −27.954 1.00 0.00
H
ATOM 3153 HE22 GLN X 209 −6.559 −0.021 −27.128 1.00 0.00
H
ATOM 3154 C GLN X 209 −5.243 3.472 −23.626 1.00 0.00
C
ATOM 3155 O GLN X 209 −4.540 2.515 −23.359 1.00 0.00
O
ATOM 3156 N TYR X 210 −6.384 3.637 −22.969 1.00 0.00
N
ATOM 3157 HN TYR X 210 −6.899 4.484 −23.079 1.00 0.00
H
ATOM 3158 CA TYR X 210 −6.897 2.584 −22.093 1.00 0.00
C
ATOM 3159 HA TYR X 210 −6.637 1.579 −22.391 1.00 0.00
H
ATOM 3160 CB TYR X 210 −6.594 2.906 −20.588 1.00 0.00
C
ATOM 3161 HB1 TYR X 210 −5.503 2.987 −20.394 1.00 0.00
H
ATOM 3162 HB2 TYR X 210 −6.912 2.019 −20.000 1.00 0.00
H
ATOM 3163 CG TYR X 210 −7.189 4.166 −20.052 1.00 0.00
C
ATOM 3164 CD1 TYR X 210 −6.472 5.390 −20.119 1.00 0.00
C
ATOM 3165 HD1 TYR X 210 −5.563 5.280 −20.691 1.00 0.00
H
ATOM 3166 CE1 TYR X 210 −6.988 6.616 −19.664 1.00 0.00
C
ATOM 3167 HE1 TYR X 210 −6.360 7.489 −19.764 1.00 0.00
H
ATOM 3168 CZ TYR X 210 −8.079 6.520 −18.855 1.00 0.00
C
ATOM 3169 OH TYR X 210 −8.393 7.702 −18.140 1.00 0.00
O
ATOM 3170 HH TYR X 210 −9.094 7.555 −17.500 1.00 0.00
H
ATOM 3171 CD2 TYR X 210 −8.263 4.130 −19.190 1.00 0.00
C
ATOM 3172 HD2 TYR X 210 −8.852 3.226 −19.142 1.00 0.00
H
ATOM 3173 CE2 TYR X 210 −8.756 5.328 −18.646 1.00 0.00
C
ATOM 3174 HE2 TYR X 210 −9.687 5.214 −18.111 1.00 0.00
H
ATOM 3175 C TYR X 210 −8.389 2.554 −22.383 1.00 0.00
C

```
ATOM   3176  O   TYR X 210      -9.160   2.181 -21.508  1.00  0.00
O
ATOM   3177  N   ASN X 211      -8.788   2.931 -23.634  1.00  0.00
N
ATOM   3178  HN  ASN X 211      -8.122   3.362 -24.238  1.00  0.00
H
ATOM   3179  CA  ASN X 211     -10.157   2.677 -24.150  1.00  0.00
C
ATOM   3180  HA  ASN X 211     -10.893   3.091 -23.477  1.00  0.00
H
ATOM   3181  CB  ASN X 211     -10.333   3.251 -25.565  1.00  0.00
C
ATOM   3182  HB1 ASN X 211      -9.602   2.859 -26.304  1.00  0.00
H
ATOM   3183  HB2 ASN X 211     -10.235   4.357 -25.596  1.00  0.00
H
ATOM   3184  CG  ASN X 211     -11.729   2.970 -26.176  1.00  0.00
C
ATOM   3185  OD1 ASN X 211     -12.605   3.779 -26.141  1.00  0.00
O
ATOM   3186  ND2 ASN X 211     -11.920   1.809 -26.885  1.00  0.00
N
ATOM   3187 HD21 ASN X 211     -11.140   1.194 -26.992  1.00  0.00
H
ATOM   3188 HD22 ASN X 211     -12.832   1.426 -27.037  1.00  0.00
H
ATOM   3189  C   ASN X 211     -10.364   1.164 -24.184  1.00  0.00
C
ATOM   3190  O   ASN X 211     -11.482   0.751 -23.969  1.00  0.00
O
ATOM   3191  N   SER X 212      -9.313   0.319 -24.462  1.00  0.00
N
ATOM   3192  HN  SER X 212      -8.405   0.623 -24.741  1.00  0.00
H
ATOM   3193  CA  SER X 212      -9.671  -1.083 -24.866  1.00  0.00
C
ATOM   3194  HA  SER X 212     -10.290  -1.445 -24.058  1.00  0.00
H
ATOM   3195  CB  SER X 212     -10.527  -1.214 -26.238  1.00  0.00
C
ATOM   3196  HB1 SER X 212     -11.540  -0.857 -25.956  1.00  0.00
H
ATOM   3197  HB2 SER X 212     -10.612  -2.247 -26.639  1.00  0.00
H
ATOM   3198  OG  SER X 212      -9.854  -0.432 -27.291  1.00  0.00
O
ATOM   3199  HG1 SER X 212      -9.048  -0.941 -27.410  1.00  0.00
H
ATOM   3200  C   SER X 212      -8.491  -1.965 -25.043  1.00  0.00
C
```

ATOM 3201 O SER X 212 -7.400 -1.529 -25.426 1.00 0.00
O
ATOM 3202 N TYR X 213 -8.529 -3.278 -24.639 1.00 0.00
N
ATOM 3203 HN TYR X 213 -9.333 -3.592 -24.139 1.00 0.00
H
ATOM 3204 CA TYR X 213 -7.522 -4.208 -24.923 1.00 0.00
C
ATOM 3205 HA TYR X 213 -6.606 -3.877 -24.457 1.00 0.00
H
ATOM 3206 CB TYR X 213 -8.007 -5.538 -24.352 1.00 0.00
C
ATOM 3207 HB1 TYR X 213 -7.194 -6.255 -24.598 1.00 0.00
H
ATOM 3208 HB2 TYR X 213 -8.986 -5.771 -24.822 1.00 0.00
H
ATOM 3209 CG TYR X 213 -8.207 -5.630 -22.849 1.00 0.00
C
ATOM 3210 CD1 TYR X 213 -7.358 -5.001 -21.918 1.00 0.00
C
ATOM 3211 HD1 TYR X 213 -6.524 -4.394 -22.240 1.00 0.00
H
ATOM 3212 CE1 TYR X 213 -7.598 -5.097 -20.537 1.00 0.00
C
ATOM 3213 HE1 TYR X 213 -6.847 -4.578 -19.961 1.00 0.00
H
ATOM 3214 CZ TYR X 213 -8.702 -5.708 -20.060 1.00 0.00
C
ATOM 3215 OH TYR X 213 -8.814 -5.888 -18.669 1.00 0.00
O
ATOM 3216 HH TYR X 213 -8.119 -5.398 -18.222 1.00 0.00
H
ATOM 3217 CD2 TYR X 213 -9.336 -6.288 -22.292 1.00 0.00
C
ATOM 3218 HD2 TYR X 213 -10.021 -6.845 -22.915 1.00 0.00
H
ATOM 3219 CE2 TYR X 213 -9.521 -6.338 -20.892 1.00 0.00
C
ATOM 3220 HE2 TYR X 213 -10.313 -7.021 -20.623 1.00 0.00
H
ATOM 3221 C TYR X 213 -7.085 -4.484 -26.394 1.00 0.00
C
ATOM 3222 O TYR X 213 -7.980 -4.695 -27.164 1.00 0.00
O
ATOM 3223 N PRO X 214 -5.808 -4.724 -26.837 1.00 0.00
N
ATOM 3224 CD PRO X 214 -5.582 -5.242 -28.166 1.00 0.00
C
ATOM 3225 HD1 PRO X 214 -5.726 -4.358 -28.823 1.00 0.00
H

ATOM 3226 HD2 PRO X 214 −6.254 −6.027 −28.576 1.00 0.00
H
ATOM 3227 CA PRO X 214 −4.553 −4.281 −26.322 1.00 0.00
C
ATOM 3228 HA PRO X 214 −4.265 −4.820 −25.432 1.00 0.00
H
ATOM 3229 CB PRO X 214 −3.421 −4.720 −27.281 1.00 0.00
C
ATOM 3230 HB1 PRO X 214 −2.450 −4.955 −26.795 1.00 0.00
H
ATOM 3231 HB2 PRO X 214 −3.312 −3.868 −27.987 1.00 0.00
H
ATOM 3232 CG PRO X 214 −4.183 −5.821 −28.152 1.00 0.00
C
ATOM 3233 HG1 PRO X 214 −3.855 −6.016 −29.196 1.00 0.00
H
ATOM 3234 HG2 PRO X 214 −4.202 −6.768 −27.571 1.00 0.00
H
ATOM 3235 C PRO X 214 −4.395 −2.858 −25.925 1.00 0.00
C
ATOM 3236 O PRO X 214 −4.752 −2.013 −26.703 1.00 0.00
O
ATOM 3237 N TRP X 215 −3.937 −2.518 −24.737 1.00 0.00
N
ATOM 3238 HN TRP X 215 −3.672 −3.161 −24.023 1.00 0.00
H
ATOM 3239 CA TRP X 215 −3.621 −1.102 −24.286 1.00 0.00
C
ATOM 3240 HA TRP X 215 −4.481 −0.513 −24.567 1.00 0.00
H
ATOM 3241 CB TRP X 215 −3.506 −1.017 −22.721 1.00 0.00
C
ATOM 3242 HB1 TRP X 215 −3.000 −0.051 −22.508 1.00 0.00
H
ATOM 3243 HB2 TRP X 215 −2.754 −1.783 −22.434 1.00 0.00
H
ATOM 3244 CG TRP X 215 −4.677 −1.079 −21.934 1.00 0.00
C
ATOM 3245 CD1 TRP X 215 −5.965 −1.276 −22.312 1.00 0.00
C
ATOM 3246 HD1 TRP X 215 −6.241 −1.388 −23.350 1.00 0.00
H
ATOM 3247 NE1 TRP X 215 −6.791 −1.311 −21.251 1.00 0.00
N
ATOM 3248 HE1 TRP X 215 −7.753 −1.147 −21.226 1.00 0.00
H
ATOM 3249 CE2 TRP X 215 −6.056 −1.352 −20.075 1.00 0.00
C
ATOM 3250 CD2 TRP X 215 −4.693 −1.101 −20.437 1.00 0.00
C

```
ATOM   3251  CE3 TRP X 215      -3.745  -0.837 -19.465  1.00  0.00
C
ATOM   3252  HE3 TRP X 215      -2.709  -0.573 -19.619  1.00  0.00
H
ATOM   3253  CZ3 TRP X 215      -4.192  -0.937 -18.077  1.00  0.00
C
ATOM   3254  HZ3 TRP X 215      -3.438  -0.687 -17.346  1.00  0.00
H
ATOM   3255  CZ2 TRP X 215      -6.410  -1.372 -18.752  1.00  0.00
C
ATOM   3256  HZ2 TRP X 215      -7.445  -1.530 -18.489  1.00  0.00
H
ATOM   3257  CH2 TRP X 215      -5.532  -1.250 -17.734  1.00  0.00
C
ATOM   3258  HH2 TRP X 215      -5.835  -1.283 -16.698  1.00  0.00
H
ATOM   3259  C   TRP X 215      -2.374  -0.564 -25.039  1.00  0.00
C
ATOM   3260  O   TRP X 215      -1.325  -0.412 -24.465  1.00  0.00
O
ATOM   3261  N   THR X 216      -2.611  -0.224 -26.261  1.00  0.00
N
ATOM   3262  HN  THR X 216      -3.523  -0.118 -26.651  1.00  0.00
H
ATOM   3263  CA  THR X 216      -1.607   0.212 -27.214  1.00  0.00
C
ATOM   3264  HA  THR X 216      -0.739  -0.424 -27.120  1.00  0.00
H
ATOM   3265  CB  THR X 216      -1.989   0.276 -28.649  1.00  0.00
C
ATOM   3266  HB  THR X 216      -1.379   0.998 -29.233  1.00  0.00
H
ATOM   3267  OG1 THR X 216      -3.293   0.872 -28.821  1.00  0.00
O
ATOM   3268  HG1 THR X 216      -3.520   0.753 -29.746  1.00  0.00
H
ATOM   3269  CG2 THR X 216      -1.965  -1.134 -29.162  1.00  0.00
C
ATOM   3270 HG21 THR X 216      -0.977  -1.554 -28.877  1.00  0.00
H
ATOM   3271 HG22 THR X 216      -2.237  -1.077 -30.238  1.00  0.00
H
ATOM   3272 HG23 THR X 216      -2.781  -1.695 -28.658  1.00  0.00
H
ATOM   3273  C   THR X 216      -1.008   1.571 -26.877  1.00  0.00
C
ATOM   3274  O   THR X 216      -1.727   2.542 -26.658  1.00  0.00
O
ATOM   3275  N   PHE X 217       0.319   1.620 -27.152  1.00  0.00
N
```

```
ATOM   3276  HN  PHE X 217       0.826   0.794 -27.386  1.00  0.00
H
ATOM   3277  CA  PHE X 217       1.164   2.756 -26.929  1.00  0.00
C
ATOM   3278  HA  PHE X 217       0.753   3.556 -26.332  1.00  0.00
H
ATOM   3279  CB  PHE X 217       2.553   2.447 -26.319  1.00  0.00
C
ATOM   3280  HB1 PHE X 217       3.167   3.373 -26.331  1.00  0.00
H
ATOM   3281  HB2 PHE X 217       3.156   1.652 -26.807  1.00  0.00
H
ATOM   3282  CG  PHE X 217       2.493   1.927 -24.851  1.00  0.00
C
ATOM   3283  CD1 PHE X 217       3.071   2.714 -23.815  1.00  0.00
C
ATOM   3284  HD1 PHE X 217       3.369   3.726 -24.045  1.00  0.00
H
ATOM   3285  CE1 PHE X 217       3.082   2.383 -22.524  1.00  0.00
C
ATOM   3286  HE1 PHE X 217       3.565   2.953 -21.744  1.00  0.00
H
ATOM   3287  CZ  PHE X 217       2.551   1.119 -22.094  1.00  0.00
C
ATOM   3288  HZ  PHE X 217       2.582   0.889 -21.039  1.00  0.00
H
ATOM   3289  CD2 PHE X 217       2.049   0.637 -24.509  1.00  0.00
C
ATOM   3290  HD2 PHE X 217       1.635  -0.145 -25.128  1.00  0.00
H
ATOM   3291  CE2 PHE X 217       1.990   0.301 -23.125  1.00  0.00
C
ATOM   3292  HE2 PHE X 217       1.678  -0.659 -22.741  1.00  0.00
H
ATOM   3293  C   PHE X 217       1.434   3.441 -28.312  1.00  0.00
C
ATOM   3294  O   PHE X 217       1.606   2.779 -29.336  1.00  0.00
O
ATOM   3295  N   GLY X 218       1.428   4.733 -28.428  1.00  0.00
N
ATOM   3296  HN  GLY X 218       1.323   5.347 -27.649  1.00  0.00
H
ATOM   3297  CA  GLY X 218       1.767   5.465 -29.622  1.00  0.00
C
ATOM   3298  HA1 GLY X 218       1.531   6.512 -29.502  1.00  0.00
H
ATOM   3299  HA2 GLY X 218       1.236   5.080 -30.480  1.00  0.00
H
ATOM   3300  C   GLY X 218       3.281   5.413 -29.939  1.00  0.00
C
```

ATOM   3301 O   GLY X 218   4.093   4.713 −29.312 1.00 0.00
O
ATOM   3302 N   GLN X 219   3.743   6.052 −31.072 1.00 0.00
N
ATOM   3303 HN GLN X 219   3.117   6.414 −31.758 1.00 0.00
H
ATOM   3304 CA GLN X 219   5.122   5.951 −31.643 1.00 0.00
C
ATOM   3305 HA GLN X 219   5.161   4.873 −31.692 1.00 0.00
H
ATOM   3306 CB GLN X 219   5.368   6.485 −33.117 1.00 0.00
C
ATOM   3307 HB1 GLN X 219   6.470   6.387 −33.221 1.00 0.00
H
ATOM   3308 HB2 GLN X 219   5.019   7.539 −33.099 1.00 0.00
H
ATOM   3309 CG GLN X 219   4.575   5.637 −34.164 1.00 0.00
C
ATOM   3310 HG1 GLN X 219   3.477   5.572 −34.008 1.00 0.00
H
ATOM   3311 HG2 GLN X 219   5.159   4.708 −34.336 1.00 0.00
H
ATOM   3312 CD GLN X 219   4.555   6.360 −35.482 1.00 0.00
C
ATOM   3313 OE1 GLN X 219   4.075   7.515 −35.703 1.00 0.00
O
ATOM   3314 NE2 GLN X 219   5.004   5.686 −36.613 1.00 0.00
N
ATOM   3315 HE21 GLN X 219   5.195   4.705 −36.592 1.00 0.00
H
ATOM   3316 HE22 GLN X 219   5.072   6.206 −37.465 1.00 0.00
H
ATOM   3317 C   GLN X 219   6.227   6.410 −30.689 1.00 0.00
C
ATOM   3318 O   GLN X 219   7.323   5.870 −30.747 1.00 0.00
O
ATOM   3319 N   GLY X 220   6.062   7.487 −29.944 1.00 0.00
N
ATOM   3320 HN GLY X 220   5.197   7.955 −29.781 1.00 0.00
H
ATOM   3321 CA GLY X 220   7.191   7.955 −29.124 1.00 0.00
C
ATOM   3322 HA1 GLY X 220   8.015   7.271 −28.981 1.00 0.00
H
ATOM   3323 HA2 GLY X 220   6.706   8.213 −28.194 1.00 0.00
H
ATOM   3324 C   GLY X 220   7.587   9.312 −29.691 1.00 0.00
C
ATOM   3325 O   GLY X 220   7.959   9.319 −30.847 1.00 0.00
O

```
ATOM   3326  N   THR X 221       7.667  10.359 -28.884  1.00  0.00
N
ATOM   3327  HN  THR X 221       7.382  10.258 -27.934  1.00  0.00
H
ATOM   3328  CA  THR X 221       8.102  11.710 -29.323  1.00  0.00
C
ATOM   3329  HA  THR X 221       8.488  11.658 -30.330  1.00  0.00
H
ATOM   3330  CB  THR X 221       7.059  12.822 -29.306  1.00  0.00
C
ATOM   3331  HB  THR X 221       6.633  12.761 -28.281  1.00  0.00
H
ATOM   3332  OG1 THR X 221       6.150  12.579 -30.370  1.00  0.00
O
ATOM   3333  HG1 THR X 221       6.590  13.057 -31.078  1.00  0.00
H
ATOM   3334  CG2 THR X 221       7.690  14.202 -29.518  1.00  0.00
C
ATOM   3335 HG21 THR X 221       8.338  14.466 -28.655  1.00  0.00
H
ATOM   3336 HG22 THR X 221       6.920  14.988 -29.672  1.00  0.00
H
ATOM   3337 HG23 THR X 221       8.276  14.250 -30.460  1.00  0.00
H
ATOM   3338  C   THR X 221       9.324  12.043 -28.533  1.00  0.00
C
ATOM   3339  O   THR X 221       9.256  12.257 -27.306  1.00  0.00
O
ATOM   3340  N   LYS X 222      10.454  12.237 -29.182  1.00  0.00
N
ATOM   3341  HN  LYS X 222      10.515  12.095 -30.167  1.00  0.00
H
ATOM   3342  CA  LYS X 222      11.744  12.636 -28.563  1.00  0.00
C
ATOM   3343  HA  LYS X 222      11.882  12.259 -27.560  1.00  0.00
H
ATOM   3344  CB  LYS X 222      12.940  12.253 -29.467  1.00  0.00
C
ATOM   3345  HB1 LYS X 222      13.803  12.858 -29.116  1.00  0.00
H
ATOM   3346  HB2 LYS X 222      12.807  12.436 -30.554  1.00  0.00
H
ATOM   3347  CG  LYS X 222      13.311  10.763 -29.494  1.00  0.00
C
ATOM   3348  HG1 LYS X 222      12.584  10.106 -30.017  1.00  0.00
H
ATOM   3349  HG2 LYS X 222      13.361  10.402 -28.445  1.00  0.00
H
ATOM   3350  CD  LYS X 222      14.666  10.542 -30.193  1.00  0.00
C
```

```
ATOM   3351  HD1 LYS X 222      15.445  11.100 -29.631  1.00  0.00
H
ATOM   3352  HD2 LYS X 222      14.707  10.963 -31.220  1.00  0.00
H
ATOM   3353  CE  LYS X 222      15.032   9.088 -30.329  1.00  0.00
C
ATOM   3354  HE1 LYS X 222      14.962   8.716 -29.285  1.00  0.00
H
ATOM   3355  HE2 LYS X 222      16.036   8.787 -30.698  1.00  0.00
H
ATOM   3356  NZ  LYS X 222      14.139   8.315 -31.117  1.00  0.00
N
ATOM   3357  HZ1 LYS X 222      14.461   7.350 -30.901  1.00  0.00
H
ATOM   3358  HZ2 LYS X 222      14.120   8.544 -32.131  1.00  0.00
H
ATOM   3359  HZ3 LYS X 222      13.152   8.320 -30.790  1.00  0.00
H
ATOM   3360  C   LYS X 222      11.703  14.157 -28.425  1.00  0.00
C
ATOM   3361  O   LYS X 222      11.229  14.866 -29.331  1.00  0.00
O
ATOM   3362  N   VAL X 223      12.256  14.787 -27.422  1.00  0.00
N
ATOM   3363  HN  VAL X 223      12.530  14.228 -26.644  1.00  0.00
H
ATOM   3364  CA  VAL X 223      12.529  16.177 -27.239  1.00  0.00
C
ATOM   3365  HA  VAL X 223      12.478  16.695 -28.186  1.00  0.00
H
ATOM   3366  CB  VAL X 223      11.628  16.758 -26.122  1.00  0.00
C
ATOM   3367  HB  VAL X 223      11.735  16.483 -25.051  1.00  0.00
H
ATOM   3368  CG1 VAL X 223      11.761  18.318 -26.047  1.00  0.00
C
ATOM   3369 HG11 VAL X 223      12.812  18.650 -25.907  1.00  0.00
H
ATOM   3370 HG12 VAL X 223      11.252  18.736 -25.152  1.00  0.00
H
ATOM   3371 HG13 VAL X 223      11.491  18.774 -27.024  1.00  0.00
H
ATOM   3372  CG2 VAL X 223      10.103  16.372 -26.374  1.00  0.00
C
ATOM   3373 HG21 VAL X 223       9.863  15.301 -26.201  1.00  0.00
H
ATOM   3374 HG22 VAL X 223       9.802  16.724 -27.384  1.00  0.00
H
ATOM   3375 HG23 VAL X 223       9.578  16.963 -25.593  1.00  0.00
H
```

```
ATOM   3376  C   VAL X 223      13.966  16.266 −26.805  1.00  0.00
C
ATOM   3377  O   VAL X 223      14.251  15.721 −25.727  1.00  0.00
O
ATOM   3378  N   GLU X 224      14.900  16.710 −27.635  1.00  0.00
N
ATOM   3379  HN  GLU X 224      14.527  17.103 −28.472  1.00  0.00
H
ATOM   3380  CA  GLU X 224      16.370  16.726 −27.502  1.00  0.00
C
ATOM   3381  HA  GLU X 224      16.570  15.920 −26.811  1.00  0.00
H
ATOM   3382  CB  GLU X 224      17.131  16.243 −28.755  1.00  0.00
C
ATOM   3383  HB1 GLU X 224      18.228  16.350 −28.611  1.00  0.00
H
ATOM   3384  HB2 GLU X 224      16.941  16.783 −29.707  1.00  0.00
H
ATOM   3385  CG  GLU X 224      16.829  14.771 −29.109  1.00  0.00
C
ATOM   3386  HG1 GLU X 224      15.934  14.784 −29.766  1.00  0.00
H
ATOM   3387  HG2 GLU X 224      16.625  14.147 −28.212  1.00  0.00
H
ATOM   3388  CD  GLU X 224      17.878  14.212 −30.048  1.00  0.00
C
ATOM   3389  OE1 GLU X 224      18.479  13.102 −29.704  1.00  0.00
O
ATOM   3390  OE2 GLU X 224      18.047  14.706 −31.201  1.00  0.00
O
ATOM   3391  C   GLU X 224      16.756  18.034 −26.981  1.00  0.00
C
ATOM   3392  O   GLU X 224      16.024  19.060 −27.025  1.00  0.00
O
ATOM   3393  N   ILE X 225      17.982  18.110 −26.452  1.00  0.00
N
ATOM   3394  HN  ILE X 225      18.560  17.311 −26.299  1.00  0.00
H
ATOM   3395  CA  ILE X 225      18.664  19.361 −26.236  1.00  0.00
C
ATOM   3396  HA  ILE X 225      17.934  20.089 −25.916  1.00  0.00
H
ATOM   3397  CB  ILE X 225      19.686  19.270 −25.060  1.00  0.00
C
ATOM   3398  HB  ILE X 225      19.122  18.771 −24.243  1.00  0.00
H
ATOM   3399  CG2 ILE X 225      20.912  18.388 −25.462  1.00  0.00
C
ATOM   3400 HG21 ILE X 225      21.563  19.075 −26.044  1.00  0.00
H
```

ATOM 3401 HG22 ILE X 225 20.488 17.498 −25.975 1.00 0.00
H
ATOM 3402 HG23 ILE X 225 21.551 18.064 −24.613 1.00 0.00
H
ATOM 3403 CG1 ILE X 225 20.193 20.625 −24.492 1.00 0.00
C
ATOM 3404 HG11 ILE X 225 19.389 21.338 −24.211 1.00 0.00
H
ATOM 3405 HG12 ILE X 225 20.656 21.201 −25.322 1.00 0.00
H
ATOM 3406 CD ILE X 225 21.171 20.466 −23.356 1.00 0.00
C
ATOM 3407 HD1 ILE X 225 20.576 19.915 −22.598 1.00 0.00
H
ATOM 3408 HD2 ILE X 225 21.402 21.448 −22.890 1.00 0.00
H
ATOM 3409 HD3 ILE X 225 22.120 19.917 −23.537 1.00 0.00
H
ATOM 3410 C ILE X 225 19.281 19.937 −27.555 1.00 0.00
C
ATOM 3411 O ILE X 225 19.857 19.185 −28.337 1.00 0.00
O
ATOM 3412 N LYS X 226 18.967 21.225 −27.904 1.00 0.00
N
ATOM 3413 HN LYS X 226 18.433 21.693 −27.204 1.00 0.00
H
ATOM 3414 CA LYS X 226 19.546 21.847 −29.087 1.00 0.00
C
ATOM 3415 HA LYS X 226 19.622 21.043 −29.803 1.00 0.00
H
ATOM 3416 CB LYS X 226 18.516 22.913 −29.609 1.00 0.00
C
ATOM 3417 HB1 LYS X 226 18.187 23.554 −28.764 1.00 0.00
H
ATOM 3418 HB2 LYS X 226 17.626 22.287 −29.835 1.00 0.00
H
ATOM 3419 CG LYS X 226 18.984 23.731 −30.810 1.00 0.00
C
ATOM 3420 HG1 LYS X 226 19.663 23.154 −31.474 1.00 0.00
H
ATOM 3421 HG2 LYS X 226 19.391 24.693 −30.431 1.00 0.00
H
ATOM 3422 CD LYS X 226 17.720 24.142 −31.513 1.00 0.00
C
ATOM 3423 HD1 LYS X 226 16.940 24.496 −30.805 1.00 0.00
H
ATOM 3424 HD2 LYS X 226 17.198 23.295 −32.008 1.00 0.00
H
ATOM 3425 CE LYS X 226 17.662 25.193 −32.660 1.00 0.00
C

ATOM 3426 HE1 LYS X 226 18.424 24.899 −33.414 1.00 0.00
H
ATOM 3427 HE2 LYS X 226 17.875 26.221 −32.298 1.00 0.00
H
ATOM 3428 NZ LYS X 226 16.305 25.381 −33.240 1.00 0.00
N
ATOM 3429 HZ1 LYS X 226 16.283 25.656 −34.243 1.00 0.00
H
ATOM 3430 HZ2 LYS X 226 15.674 26.048 −32.752 1.00 0.00
H
ATOM 3431 HZ3 LYS X 226 15.733 24.513 −33.232 1.00 0.00
H
ATOM 3432 C LYS X 226 20.939 22.379 −28.948 1.00 0.00
C
ATOM 3433 O LYS X 226 21.344 22.895 −27.902 1.00 0.00
O
ATOM 3434 NT LYS X 226 21.788 22.255 −30.022 1.00 0.00
N
ATOM 3435 HNT LYS X 226 21.566 21.617 −30.756 1.00 0.00
H
ATOM 3436 CAT LYS X 226 23.108 22.876 −30.142 1.00 0.00
C
ATOM 3437 HT1 LYS X 226 23.003 23.891 −30.581 1.00 0.00
H
ATOM 3438 HT2 LYS X 226 23.881 22.363 −30.754 1.00 0.00
H
ATOM 3439 HT3 LYS X 226 23.730 22.940 −29.224 1.00 0.00
H
ATOM 3440 CAY ASN X 227 −21.538 −6.896 −44.154 1.00 0.00
C
ATOM 3441 HY1 ASN X 227 −22.277 −6.686 −43.351 1.00 0.00
H
ATOM 3442 HY2 ASN X 227 −22.034 −7.533 −44.918 1.00 0.00
H
ATOM 3443 HY3 ASN X 227 −21.189 −6.033 −44.761 1.00 0.00
H
ATOM 3444 CY ASN X 227 −20.300 −7.593 −43.663 1.00 0.00
C
ATOM 3445 OY ASN X 227 −19.879 −8.633 −44.178 1.00 0.00
O
ATOM 3446 N ASN X 227 −19.756 −6.978 −42.676 1.00 0.00
N
ATOM 3447 HN ASN X 227 −20.057 −6.148 −42.211 1.00 0.00
H
ATOM 3448 CA ASN X 227 −18.562 −7.460 −41.897 1.00 0.00
C
ATOM 3449 HA ASN X 227 −18.317 −8.464 −42.211 1.00 0.00
H
ATOM 3450 CB ASN X 227 −17.322 −6.579 −42.228 1.00 0.00
C

```
ATOM   3451  HB1 ASN X 227     -16.427  -6.964 -41.693  1.00  0.00
H
ATOM   3452  HB2 ASN X 227     -17.559  -5.637 -41.689  1.00  0.00
H
ATOM   3453  CG  ASN X 227     -17.109  -6.364 -43.710  1.00  0.00
C
ATOM   3454  OD1 ASN X 227     -17.292  -5.242 -44.150  1.00  0.00
O
ATOM   3455  ND2 ASN X 227     -16.852  -7.421 -44.469  1.00  0.00
N
ATOM   3456 HD21 ASN X 227     -16.427  -8.261 -44.131  1.00  0.00
H
ATOM   3457 HD22 ASN X 227     -16.911  -7.245 -45.452  1.00  0.00
H
ATOM   3458  C   ASN X 227     -18.873  -7.575 -40.437  1.00  0.00
C
ATOM   3459  O   ASN X 227     -19.671  -6.805 -39.900  1.00  0.00
O
ATOM   3460  N   LEU X 228     -18.280  -8.558 -39.797  1.00  0.00
N
ATOM   3461  HN  LEU X 228     -17.593  -9.109 -40.266  1.00  0.00
H
ATOM   3462  CA  LEU X 228     -18.555  -8.840 -38.366  1.00  0.00
C
ATOM   3463  HA  LEU X 228     -19.625  -8.985 -38.357  1.00  0.00
H
ATOM   3464  CB  LEU X 228     -17.924 -10.252 -37.965  1.00  0.00
C
ATOM   3465  HB1 LEU X 228     -16.845 -10.089 -38.176  1.00  0.00
H
ATOM   3466  HB2 LEU X 228     -18.411 -10.909 -38.717  1.00  0.00
H
ATOM   3467  CG  LEU X 228     -18.231 -10.661 -36.519  1.00  0.00
C
ATOM   3468  HG  LEU X 228     -17.570 -10.044 -35.874  1.00  0.00
H
ATOM   3469  CD1 LEU X 228     -19.666 -10.293 -36.040  1.00  0.00
C
ATOM   3470 HD11 LEU X 228     -19.860 -10.800 -35.071  1.00  0.00
H
ATOM   3471 HD12 LEU X 228     -20.566 -10.484 -36.662  1.00  0.00
H
ATOM   3472 HD13 LEU X 228     -19.490  -9.215 -35.838  1.00  0.00
H
ATOM   3473  CD2 LEU X 228     -17.726 -12.057 -36.298  1.00  0.00
C
ATOM   3474 HD21 LEU X 228     -16.914 -12.398 -36.975  1.00  0.00
H
ATOM   3475 HD22 LEU X 228     -18.631 -12.659 -36.528  1.00  0.00
H
```

ATOM 3476 HD23 LEU X 228 -17.449 -12.221 -35.235 1.00 0.00
H
ATOM 3477 C LEU X 228 -18.050 -7.705 -37.396 1.00 0.00
C
ATOM 3478 O LEU X 228 -16.853 -7.501 -37.126 1.00 0.00
O
ATOM 3479 N CYS X 229 -19.029 -7.033 -36.780 1.00 0.00
N
ATOM 3480 HN CYS X 229 -19.981 -7.230 -37.002 1.00 0.00
H
ATOM 3481 CA CYS X 229 -18.834 -5.973 -35.787 1.00 0.00
C
ATOM 3482 HA CYS X 229 -18.311 -5.193 -36.320 1.00 0.00
H
ATOM 3483 CB CYS X 229 -20.208 -5.491 -35.456 1.00 0.00
C
ATOM 3484 HB1 CYS X 229 -20.271 -4.805 -34.584 1.00 0.00
H
ATOM 3485 HB2 CYS X 229 -20.896 -6.332 -35.223 1.00 0.00
H
ATOM 3486 SG CYS X 229 -20.965 -4.590 -36.862 1.00 0.00
S
ATOM 3487 C CYS X 229 -18.143 -6.466 -34.485 1.00 0.00
C
ATOM 3488 O CYS X 229 -18.366 -7.513 -33.887 1.00 0.00
O
ATOM 3489 N PRO X 230 -17.240 -5.677 -33.931 1.00 0.00
N
ATOM 3490 CD PRO X 230 -16.607 -4.577 -34.782 1.00 0.00
C
ATOM 3491 HD1 PRO X 230 -17.303 -3.722 -34.925 1.00 0.00
H
ATOM 3492 HD2 PRO X 230 -16.277 -4.982 -35.763 1.00 0.00
H
ATOM 3493 CA PRO X 230 -16.336 -6.070 -32.854 1.00 0.00
C
ATOM 3494 HA PRO X 230 -16.264 -7.138 -32.711 1.00 0.00
H
ATOM 3495 CB PRO X 230 -14.997 -5.551 -33.416 1.00 0.00
C
ATOM 3496 HB1 PRO X 230 -14.528 -6.378 -33.991 1.00 0.00
H
ATOM 3497 HB2 PRO X 230 -14.286 -5.448 -32.568 1.00 0.00
H
ATOM 3498 CG PRO X 230 -15.321 -4.220 -34.008 1.00 0.00
C
ATOM 3499 HG1 PRO X 230 -15.649 -3.581 -33.161 1.00 0.00
H
ATOM 3500 HG2 PRO X 230 -14.432 -3.822 -34.542 1.00 0.00
H

```
ATOM   3501  C   PRO X 230     -16.826  -5.550 -31.495  1.00  0.00
C
ATOM   3502  O   PRO X 230     -16.155  -4.773 -30.741  1.00  0.00
O
ATOM   3503  N   PHE X 231     -18.124  -5.910 -31.073  1.00  0.00
N
ATOM   3504  HN  PHE X 231     -18.612  -6.559 -31.652  1.00  0.00
H
ATOM   3505  CA  PHE X 231     -18.763  -5.589 -29.893  1.00  0.00
C
ATOM   3506  HA  PHE X 231     -18.828  -4.511 -29.875  1.00  0.00
H
ATOM   3507  CB  PHE X 231     -20.226  -6.079 -29.764  1.00  0.00
C
ATOM   3508  HB1 PHE X 231     -20.765  -5.583 -28.929  1.00  0.00
H
ATOM   3509  HB2 PHE X 231     -20.284  -7.160 -29.514  1.00  0.00
H
ATOM   3510  CG  PHE X 231     -21.123  -5.940 -30.920  1.00  0.00
C
ATOM   3511  CD1 PHE X 231     -22.090  -6.899 -31.191  1.00  0.00
C
ATOM   3512  HD1 PHE X 231     -22.023  -7.857 -30.696  1.00  0.00
H
ATOM   3513  CE1 PHE X 231     -23.224  -6.578 -31.908  1.00  0.00
C
ATOM   3514  HE1 PHE X 231     -24.042  -7.282 -31.953  1.00  0.00
H
ATOM   3515  CZ  PHE X 231     -23.391  -5.428 -32.626  1.00  0.00
C
ATOM   3516  HZ  PHE X 231     -24.238  -5.206 -33.258  1.00  0.00
H
ATOM   3517  CD2 PHE X 231     -21.235  -4.703 -31.559  1.00  0.00
C
ATOM   3518  HD2 PHE X 231     -20.669  -3.820 -31.304  1.00  0.00
H
ATOM   3519  CE2 PHE X 231     -22.395  -4.495 -32.500  1.00  0.00
C
ATOM   3520  HE2 PHE X 231     -22.546  -3.498 -32.886  1.00  0.00
H
ATOM   3521  C   PHE X 231     -17.974  -6.042 -28.615  1.00  0.00
C
ATOM   3522  O   PHE X 231     -18.092  -5.351 -27.595  1.00  0.00
O
ATOM   3523  N   GLY X 232     -17.080  -7.032 -28.723  1.00  0.00
N
ATOM   3524  HN  GLY X 232     -17.047  -7.592 -29.547  1.00  0.00
H
ATOM   3525  CA  GLY X 232     -16.077  -7.456 -27.715  1.00  0.00
C
```

ATOM 3526 HA1 GLY X 232 −15.304 −7.953 −28.282 1.00 0.00
H
ATOM 3527 HA2 GLY X 232 −16.534 −8.103 −26.981 1.00 0.00
H
ATOM 3528 C GLY X 232 −15.426 −6.317 −26.962 1.00 0.00
C
ATOM 3529 O GLY X 232 −15.250 −6.290 −25.712 1.00 0.00
O
ATOM 3530 N GLU X 233 −15.005 −5.299 −27.776 1.00 0.00
N
ATOM 3531 HN GLU X 233 −15.262 −5.320 −28.738 1.00 0.00
H
ATOM 3532 CA GLU X 233 −14.126 −4.214 −27.245 1.00 0.00
C
ATOM 3533 HA GLU X 233 −13.512 −4.487 −26.400 1.00 0.00
H
ATOM 3534 CB GLU X 233 −13.255 −3.562 −28.315 1.00 0.00
C
ATOM 3535 HB1 GLU X 233 −12.494 −2.967 −27.767 1.00 0.00
H
ATOM 3536 HB2 GLU X 233 −13.880 −2.879 −28.931 1.00 0.00
H
ATOM 3537 CG GLU X 233 −12.432 −4.475 −29.270 1.00 0.00
C
ATOM 3538 HG1 GLU X 233 −13.067 −5.184 −29.844 1.00 0.00
H
ATOM 3539 HG2 GLU X 233 −11.781 −5.206 −28.746 1.00 0.00
H
ATOM 3540 CD GLU X 233 −11.677 −3.612 −30.333 1.00 0.00
C
ATOM 3541 OE1 GLU X 233 −12.264 −3.337 −31.406 1.00 0.00
O
ATOM 3542 OE2 GLU X 233 −10.482 −3.294 −30.025 1.00 0.00
O
ATOM 3543 C GLU X 233 −15.081 −3.109 −26.734 1.00 0.00
C
ATOM 3544 O GLU X 233 −14.641 −2.327 −25.956 1.00 0.00
O
ATOM 3545 N VAL X 234 −16.374 −3.004 −27.112 1.00 0.00
N
ATOM 3546 HN VAL X 234 −16.787 −3.750 −27.629 1.00 0.00
H
ATOM 3547 CA VAL X 234 −17.324 −1.997 −26.464 1.00 0.00
C
ATOM 3548 HA VAL X 234 −16.903 −1.008 −26.362 1.00 0.00
H
ATOM 3549 CB VAL X 234 −18.476 −1.813 −27.430 1.00 0.00
C
ATOM 3550 HB VAL X 234 −19.077 −2.746 −27.481 1.00 0.00
H

```
ATOM   3551  CG1 VAL X 234     -19.464  -0.807 -26.916  1.00  0.00
C
ATOM   3552 HG11 VAL X 234     -19.899  -1.150 -25.953  1.00  0.00
H
ATOM   3553 HG12 VAL X 234     -20.259  -0.660 -27.677  1.00  0.00
H
ATOM   3554 HG13 VAL X 234     -18.986   0.187 -26.781  1.00  0.00
H
ATOM   3555  CG2 VAL X 234     -17.974  -1.254 -28.842  1.00  0.00
C
ATOM   3556 HG21 VAL X 234     -18.876  -1.052 -29.458  1.00  0.00
H
ATOM   3557 HG22 VAL X 234     -17.201  -1.873 -29.346  1.00  0.00
H
ATOM   3558 HG23 VAL X 234     -17.604  -0.222 -28.661  1.00  0.00
H
ATOM   3559  C   VAL X 234     -17.884  -2.507 -25.123  1.00  0.00
C
ATOM   3560  O   VAL X 234     -17.688  -1.862 -24.175  1.00  0.00
O
ATOM   3561  N   PHE X 235     -18.373  -3.761 -25.104  1.00  0.00
N
ATOM   3562  HN  PHE X 235     -18.500  -4.276 -25.949  1.00  0.00
H
ATOM   3563  CA  PHE X 235     -18.764  -4.503 -23.918  1.00  0.00
C
ATOM   3564  HA  PHE X 235     -19.522  -3.855 -23.504  1.00  0.00
H
ATOM   3565  CB  PHE X 235     -19.366  -5.881 -24.370  1.00  0.00
C
ATOM   3566  HB1 PHE X 235     -19.431  -6.585 -23.513  1.00  0.00
H
ATOM   3567  HB2 PHE X 235     -18.828  -6.413 -25.184  1.00  0.00
H
ATOM   3568  CG  PHE X 235     -20.801  -5.621 -24.804  1.00  0.00
C
ATOM   3569  CD1 PHE X 235     -21.053  -5.197 -26.140  1.00  0.00
C
ATOM   3570  HD1 PHE X 235     -20.233  -5.009 -26.817  1.00  0.00
H
ATOM   3571  CE1 PHE X 235     -22.395  -5.046 -26.583  1.00  0.00
C
ATOM   3572  HE1 PHE X 235     -22.469  -4.769 -27.624  1.00  0.00
H
ATOM   3573  CZ  PHE X 235     -23.423  -5.438 -25.774  1.00  0.00
C
ATOM   3574  HZ  PHE X 235     -24.434  -5.530 -26.143  1.00  0.00
H
ATOM   3575  CD2 PHE X 235     -21.944  -5.906 -23.974  1.00  0.00
C
```

```
ATOM   3576  HD2 PHE X 235     -21.676  -6.178 -22.963  1.00  0.00
H
ATOM   3577  CE2 PHE X 235     -23.228  -5.943 -24.496  1.00  0.00
C
ATOM   3578  HE2 PHE X 235     -24.039  -6.363 -23.920  1.00  0.00
H
ATOM   3579  C   PHE X 235     -17.664  -4.620 -22.794  1.00  0.00
C
ATOM   3580  O   PHE X 235     -17.901  -4.314 -21.648  1.00  0.00
O
ATOM   3581  N   ASN X 236     -16.452  -5.041 -23.151  1.00  0.00
N
ATOM   3582  HN  ASN X 236     -16.296  -5.148 -24.130  1.00  0.00
H
ATOM   3583  CA  ASN X 236     -15.308  -5.245 -22.242  1.00  0.00
C
ATOM   3584  HA  ASN X 236     -15.742  -5.050 -21.272  1.00  0.00
H
ATOM   3585  CB  ASN X 236     -14.634  -6.570 -22.449  1.00  0.00
C
ATOM   3586  HB1 ASN X 236     -13.776  -6.708 -21.756  1.00  0.00
H
ATOM   3587  HB2 ASN X 236     -14.317  -6.719 -23.503  1.00  0.00
H
ATOM   3588  CG  ASN X 236     -15.655  -7.681 -22.025  1.00  0.00
C
ATOM   3589  OD1 ASN X 236     -16.023  -7.695 -20.883  1.00  0.00
O
ATOM   3590  ND2 ASN X 236     -15.940  -8.632 -22.902  1.00  0.00
N
ATOM   3591 HD22 ASN X 236     -15.535  -8.699 -23.810  1.00  0.00
H
ATOM   3592  C   ASN X 236     -14.269  -4.121 -22.317  1.00  0.00
C
ATOM   3593  O   ASN X 236     -13.031  -4.195 -21.993  1.00  0.00
O
ATOM   3594  N   ALA X 237     -14.737  -2.938 -22.799  1.00  0.00
N
ATOM   3595  HN  ALA X 237     -15.704  -2.757 -22.958  1.00  0.00
H
ATOM   3596  CA  ALA X 237     -13.913  -1.764 -22.810  1.00  0.00
C
ATOM   3597  HA  ALA X 237     -13.074  -1.913 -23.474  1.00  0.00
H
ATOM   3598  CB  ALA X 237     -14.627  -0.426 -23.304  1.00  0.00
C
ATOM   3599  HB1 ALA X 237     -15.499  -0.150 -22.673  1.00  0.00
H
ATOM   3600  HB2 ALA X 237     -15.024  -0.666 -24.314  1.00  0.00
H
```

ATOM	3601	HB3	ALA	X	237	-13.989	0.483	-23.294	1.00	0.00	H
ATOM	3602	C	ALA	X	237	-13.425	-1.389	-21.457	1.00	0.00	C
ATOM	3603	O	ALA	X	237	-14.049	-1.605	-20.478	1.00	0.00	O
ATOM	3604	N	THR	X	238	-12.246	-0.825	-21.419	1.00	0.00	N
ATOM	3605	HN	THR	X	238	-11.804	-0.621	-22.289	1.00	0.00	H
ATOM	3606	CA	THR	X	238	-11.461	-0.774	-20.152	1.00	0.00	C
ATOM	3607	HA	THR	X	238	-11.811	-1.568	-19.509	1.00	0.00	H
ATOM	3608	CB	THR	X	238	-9.997	-0.946	-20.276	1.00	0.00	C
ATOM	3609	HB	THR	X	238	-9.408	-0.586	-19.405	1.00	0.00	H
ATOM	3610	OG1	THR	X	238	-9.470	-0.358	-21.487	1.00	0.00	O
ATOM	3611	HG1	THR	X	238	-9.536	0.598	-21.426	1.00	0.00	H
ATOM	3612	CG2	THR	X	238	-9.816	-2.473	-20.528	1.00	0.00	C
ATOM	3613	HG21	THR	X	238	-10.134	-2.765	-21.552	1.00	0.00	H
ATOM	3614	HG22	THR	X	238	-10.337	-3.117	-19.788	1.00	0.00	H
ATOM	3615	HG23	THR	X	238	-8.748	-2.772	-20.462	1.00	0.00	H
ATOM	3616	C	THR	X	238	-11.777	0.536	-19.350	1.00	0.00	C
ATOM	3617	O	THR	X	238	-11.388	0.748	-18.201	1.00	0.00	O
ATOM	3618	N	ARG	X	239	-12.445	1.490	-19.944	1.00	0.00	N
ATOM	3619	HN	ARG	X	239	-12.507	1.434	-20.937	1.00	0.00	H
ATOM	3620	CA	ARG	X	239	-13.109	2.589	-19.288	1.00	0.00	C
ATOM	3621	HA	ARG	X	239	-13.179	2.468	-18.217	1.00	0.00	H
ATOM	3622	CB	ARG	X	239	-12.299	3.869	-19.567	1.00	0.00	C
ATOM	3623	HB1	ARG	X	239	-12.514	4.266	-20.583	1.00	0.00	H
ATOM	3624	HB2	ARG	X	239	-11.241	3.540	-19.648	1.00	0.00	H
ATOM	3625	CG	ARG	X	239	-12.303	5.006	-18.581	1.00	0.00	C

ATOM 3626 HG1 ARG X 239 −11.738 5.891 −18.943 1.00 0.00
H
ATOM 3627 HG2 ARG X 239 −11.768 4.614 −17.689 1.00 0.00
H
ATOM 3628 CD ARG X 239 −13.710 5.519 −18.143 1.00 0.00
C
ATOM 3629 HD1 ARG X 239 −14.390 4.711 −17.798 1.00 0.00
H
ATOM 3630 HD2 ARG X 239 −14.257 6.000 −18.982 1.00 0.00
H
ATOM 3631 NE ARG X 239 −13.603 6.488 −17.018 1.00 0.00
N
ATOM 3632 HE ARG X 239 −12.673 6.609 −16.669 1.00 0.00
H
ATOM 3633 CZ ARG X 239 −14.576 7.340 −16.809 1.00 0.00
C
ATOM 3634 NH1 ARG X 239 −14.227 8.389 −16.013 1.00 0.00
N
ATOM 3635 HH11 ARG X 239 −13.305 8.596 −15.685 1.00 0.00
H
ATOM 3636 HH12 ARG X 239 −14.745 9.227 −16.187 1.00 0.00
H
ATOM 3637 NH2 ARG X 239 −15.730 7.282 −17.284 1.00 0.00
N
ATOM 3638 HH21 ARG X 239 −16.050 6.496 −17.813 1.00 0.00
H
ATOM 3639 HH22 ARG X 239 −16.365 8.054 −17.259 1.00 0.00
H
ATOM 3640 C ARG X 239 −14.516 2.775 −19.899 1.00 0.00
C
ATOM 3641 O ARG X 239 −14.541 3.002 −21.115 1.00 0.00
O
ATOM 3642 N PHE X 240 −15.630 2.755 −19.117 1.00 0.00
N
ATOM 3643 HN PHE X 240 −15.510 2.413 −18.188 1.00 0.00
H
ATOM 3644 CA PHE X 240 −16.961 3.094 −19.502 1.00 0.00
C
ATOM 3645 HA PHE X 240 −17.056 2.838 −20.547 1.00 0.00
H
ATOM 3646 CB PHE X 240 −17.993 2.236 −18.756 1.00 0.00
C
ATOM 3647 HB1 PHE X 240 −18.895 2.798 −18.433 1.00 0.00
H
ATOM 3648 HB2 PHE X 240 −17.510 1.792 −17.859 1.00 0.00
H
ATOM 3649 CG PHE X 240 −18.439 1.127 −19.669 1.00 0.00
C
ATOM 3650 CD1 PHE X 240 −17.526 0.092 −20.125 1.00 0.00
C

```
ATOM   3651  HD1 PHE X 240     -16.528   0.163 -19.720  1.00  0.00
H
ATOM   3652  CE1 PHE X 240     -18.041  -0.947 -20.875  1.00  0.00
C
ATOM   3653  HE1 PHE X 240     -17.421  -1.783 -21.165  1.00  0.00
H
ATOM   3654  CZ  PHE X 240     -19.253  -0.928 -21.490  1.00  0.00
C
ATOM   3655  HZ  PHE X 240     -19.539  -1.663 -22.228  1.00  0.00
H
ATOM   3656  CD2 PHE X 240     -19.777   1.152 -20.248  1.00  0.00
C
ATOM   3657  HD2 PHE X 240     -20.505   1.863 -19.884  1.00  0.00
H
ATOM   3658  CE2 PHE X 240     -20.128   0.147 -21.234  1.00  0.00
C
ATOM   3659  HE2 PHE X 240     -21.047   0.198 -21.799  1.00  0.00
H
ATOM   3660  C   PHE X 240     -17.312   4.641 -19.472  1.00  0.00
C
ATOM   3661  O   PHE X 240     -16.847   5.313 -18.572  1.00  0.00
O
ATOM   3662  N   ALA X 241     -18.111   5.269 -20.325  1.00  0.00
N
ATOM   3663  HN  ALA X 241     -18.431   4.641 -21.030  1.00  0.00
H
ATOM   3664  CA  ALA X 241     -18.451   6.697 -20.356  1.00  0.00
C
ATOM   3665  HA  ALA X 241     -17.591   7.309 -20.129  1.00  0.00
H
ATOM   3666  CB  ALA X 241     -19.016   7.016 -21.763  1.00  0.00
C
ATOM   3667  HB1 ALA X 241     -18.901   8.087 -22.035  1.00  0.00
H
ATOM   3668  HB2 ALA X 241     -20.051   6.706 -22.023  1.00  0.00
H
ATOM   3669  HB3 ALA X 241     -18.413   6.544 -22.569  1.00  0.00
H
ATOM   3670  C   ALA X 241     -19.635   7.036 -19.363  1.00  0.00
C
ATOM   3671  O   ALA X 241     -20.564   6.195 -19.078  1.00  0.00
O
ATOM   3672  N   SER X 242     -19.553   8.210 -18.815  1.00  0.00
N
ATOM   3673  HN  SER X 242     -18.824   8.817 -19.120  1.00  0.00
H
ATOM   3674  CA  SER X 242     -20.636   8.770 -18.085  1.00  0.00
C
ATOM   3675  HA  SER X 242     -20.955   8.079 -17.318  1.00  0.00
H
```

```
ATOM   3676  CB  SER X 242     -20.382  10.098 -17.341  1.00  0.00
C
ATOM   3677  HB1 SER X 242     -21.259  10.488 -16.782  1.00  0.00
H
ATOM   3678  HB2 SER X 242     -20.001  10.940 -17.958  1.00  0.00
H
ATOM   3679  OG  SER X 242     -19.394   9.934 -16.290  1.00  0.00
O
ATOM   3680  HG1 SER X 242     -18.583   9.649 -16.719  1.00  0.00
H
ATOM   3681  C   SER X 242     -21.884   8.994 -18.902  1.00  0.00
C
ATOM   3682  O   SER X 242     -21.841   9.302 -20.036  1.00  0.00
O
ATOM   3683  N   VAL X 243     -23.122   8.760 -18.283  1.00  0.00
N
ATOM   3684  HN  VAL X 243     -23.144   8.578 -17.303  1.00  0.00
H
ATOM   3685  CA  VAL X 243     -24.407   8.797 -19.008  1.00  0.00
C
ATOM   3686  HA  VAL X 243     -24.265   8.036 -19.762  1.00  0.00
H
ATOM   3687  CB  VAL X 243     -25.629   8.382 -18.123  1.00  0.00
C
ATOM   3688  HB  VAL X 243     -25.322   7.353 -17.838  1.00  0.00
H
ATOM   3689  CG1 VAL X 243     -25.771   9.273 -16.876  1.00  0.00
C
ATOM   3690 HG11 VAL X 243     -26.752   8.929 -16.484  1.00  0.00
H
ATOM   3691 HG12 VAL X 243     -25.741  10.364 -17.086  1.00  0.00
H
ATOM   3692 HG13 VAL X 243     -24.975   9.023 -16.143  1.00  0.00
H
ATOM   3693  CG2 VAL X 243     -26.934   8.189 -18.999  1.00  0.00
C
ATOM   3694 HG21 VAL X 243     -27.726   7.660 -18.427  1.00  0.00
H
ATOM   3695 HG22 VAL X 243     -26.574   7.722 -19.941  1.00  0.00
H
ATOM   3696 HG23 VAL X 243     -27.402   9.164 -19.251  1.00  0.00
H
ATOM   3697  C   VAL X 243     -24.661  10.108 -19.750  1.00  0.00
C
ATOM   3698  O   VAL X 243     -25.021  10.082 -20.875  1.00  0.00
O
ATOM   3699  N   TYR X 244     -24.414  11.269 -19.169  1.00  0.00
N
ATOM   3700  HN  TYR X 244     -24.031  11.398 -18.257  1.00  0.00
H
```

ATOM 3701 CA TYR X 244 −24.605 12.578 −19.781 1.00 0.00
C
ATOM 3702 HA TYR X 244 −25.627 12.606 −20.132 1.00 0.00
H
ATOM 3703 CB TYR X 244 −24.443 13.692 −18.723 1.00 0.00
C
ATOM 3704 HB1 TYR X 244 −24.862 13.369 −17.747 1.00 0.00
H
ATOM 3705 HB2 TYR X 244 −25.037 14.613 −18.905 1.00 0.00
H
ATOM 3706 CG TYR X 244 −22.996 14.036 −18.568 1.00 0.00
C
ATOM 3707 CD1 TYR X 244 −22.122 13.344 −17.629 1.00 0.00
C
ATOM 3708 HD1 TYR X 244 −22.406 12.462 −17.074 1.00 0.00
H
ATOM 3709 CE1 TYR X 244 −20.780 13.765 −17.649 1.00 0.00
C
ATOM 3710 HE1 TYR X 244 −20.094 13.086 −17.164 1.00 0.00
H
ATOM 3711 CZ TYR X 244 −20.366 14.963 −18.300 1.00 0.00
C
ATOM 3712 OH TYR X 244 −18.994 15.211 −18.360 1.00 0.00
O
ATOM 3713 HH TYR X 244 −18.785 16.004 −18.860 1.00 0.00
H
ATOM 3714 CD2 TYR X 244 −22.626 15.300 −19.068 1.00 0.00
C
ATOM 3715 HD2 TYR X 244 −23.328 15.933 −19.590 1.00 0.00
H
ATOM 3716 CE2 TYR X 244 −21.352 15.714 −18.975 1.00 0.00
C
ATOM 3717 HE2 TYR X 244 −21.049 16.623 −19.473 1.00 0.00
H
ATOM 3718 C TYR X 244 −23.803 12.792 −21.042 1.00 0.00
C
ATOM 3719 O TYR X 244 −24.311 13.440 −21.975 1.00 0.00
O
ATOM 3720 N ALA X 245 −22.501 12.360 −21.047 1.00 0.00
N
ATOM 3721 HN ALA X 245 −22.213 11.774 −20.293 1.00 0.00
H
ATOM 3722 CA ALA X 245 −21.478 12.459 −22.050 1.00 0.00
C
ATOM 3723 HA ALA X 245 −21.669 13.329 −22.661 1.00 0.00
H
ATOM 3724 CB ALA X 245 −20.101 12.801 −21.300 1.00 0.00
C
ATOM 3725 HB1 ALA X 245 −20.186 13.785 −20.791 1.00 0.00
H

```
ATOM   3726  HB2 ALA X 245     -19.325  12.932 -22.085  1.00  0.00
H
ATOM   3727  HB3 ALA X 245     -19.858  11.925 -20.662  1.00  0.00
H
ATOM   3728  C   ALA X 245     -21.277  11.325 -22.970  1.00  0.00
C
ATOM   3729  O   ALA X 245     -20.432  11.400 -23.830  1.00  0.00
O
ATOM   3730  N   TRP X 246     -22.166  10.294 -22.859  1.00  0.00
N
ATOM   3731  HN  TRP X 246     -22.980  10.524 -22.332  1.00  0.00
H
ATOM   3732  CA  TRP X 246     -22.167   9.009 -23.527  1.00  0.00
C
ATOM   3733  HA  TRP X 246     -21.635   8.281 -22.931  1.00  0.00
H
ATOM   3734  CB  TRP X 246     -23.733   8.667 -23.754  1.00  0.00
C
ATOM   3735  HB1 TRP X 246     -24.119   8.607 -22.714  1.00  0.00
H
ATOM   3736  HB2 TRP X 246     -23.780   7.668 -24.238  1.00  0.00
H
ATOM   3737  CG  TRP X 246     -24.573   9.566 -24.564  1.00  0.00
C
ATOM   3738  CD1 TRP X 246     -25.407  10.624 -24.112  1.00  0.00
C
ATOM   3739  HD1 TRP X 246     -25.536  11.024 -23.118  1.00  0.00
H
ATOM   3740  NE1 TRP X 246     -26.091  11.109 -25.218  1.00  0.00
N
ATOM   3741  HE1 TRP X 246     -26.887  11.650 -25.051  1.00  0.00
H
ATOM   3742  CE2 TRP X 246     -25.671  10.437 -26.368  1.00  0.00
C
ATOM   3743  CD2 TRP X 246     -24.795   9.439 -25.996  1.00  0.00
C
ATOM   3744  CE3 TRP X 246     -24.266   8.536 -26.943  1.00  0.00
C
ATOM   3745  HE3 TRP X 246     -23.648   7.683 -26.705  1.00  0.00
H
ATOM   3746  CZ3 TRP X 246     -24.802   8.626 -28.257  1.00  0.00
C
ATOM   3747  HZ3 TRP X 246     -24.533   7.766 -28.853  1.00  0.00
H
ATOM   3748  CZ2 TRP X 246     -26.242  10.579 -27.630  1.00  0.00
C
ATOM   3749  HZ2 TRP X 246     -26.887  11.393 -27.929  1.00  0.00
H
ATOM   3750  CH2 TRP X 246     -25.730   9.599 -28.585  1.00  0.00
C
```

ATOM 3751 HH2 TRP X 246 -26.191 9.737 -29.552 1.00 0.00
H
ATOM 3752 C TRP X 246 -21.446 8.840 -24.873 1.00 0.00
C
ATOM 3753 O TRP X 246 -21.578 9.574 -25.828 1.00 0.00
O
ATOM 3754 N ASN X 247 -20.554 7.858 -24.956 1.00 0.00
N
ATOM 3755 HN ASN X 247 -20.361 7.393 -24.096 1.00 0.00
H
ATOM 3756 CA ASN X 247 -19.854 7.376 -26.055 1.00 0.00
C
ATOM 3757 HA ASN X 247 -19.483 8.179 -26.675 1.00 0.00
H
ATOM 3758 CB ASN X 247 -18.678 6.455 -25.598 1.00 0.00
C
ATOM 3759 HB1 ASN X 247 -18.963 5.432 -25.270 1.00 0.00
H
ATOM 3760 HB2 ASN X 247 -18.210 6.896 -24.692 1.00 0.00
H
ATOM 3761 CG ASN X 247 -17.518 6.363 -26.517 1.00 0.00
C
ATOM 3762 OD1 ASN X 247 -17.328 7.061 -27.534 1.00 0.00
O
ATOM 3763 ND2 ASN X 247 -16.514 5.628 -26.087 1.00 0.00
N
ATOM 3764 HD21 ASN X 247 -15.726 5.516 -26.692 1.00 0.00
H
ATOM 3765 HD22 ASN X 247 -16.664 5.244 -25.176 1.00 0.00
H
ATOM 3766 C ASN X 247 -20.712 6.607 -27.097 1.00 0.00
C
ATOM 3767 O ASN X 247 -21.669 5.937 -26.734 1.00 0.00
O
ATOM 3768 N ARG X 248 -20.246 6.648 -28.366 1.00 0.00
N
ATOM 3769 HN ARG X 248 -19.449 7.208 -28.578 1.00 0.00
H
ATOM 3770 CA ARG X 248 -20.849 5.745 -29.398 1.00 0.00
C
ATOM 3771 HA ARG X 248 -21.013 4.817 -28.870 1.00 0.00
H
ATOM 3772 CB ARG X 248 -22.167 6.204 -30.086 1.00 0.00
C
ATOM 3773 HB1 ARG X 248 -22.943 6.374 -29.310 1.00 0.00
H
ATOM 3774 HB2 ARG X 248 -22.579 5.426 -30.764 1.00 0.00
H
ATOM 3775 CG ARG X 248 -22.086 7.524 -30.756 1.00 0.00
C

ATOM 3776 HG1 ARG X 248 -21.066 7.907 -30.975 1.00 0.00
H
ATOM 3777 HG2 ARG X 248 -22.356 8.342 -30.054 1.00 0.00
H
ATOM 3778 CD ARG X 248 -23.002 7.863 -31.980 1.00 0.00
C
ATOM 3779 HD1 ARG X 248 -22.697 7.415 -32.950 1.00 0.00
H
ATOM 3780 HD2 ARG X 248 -23.157 8.957 -32.095 1.00 0.00
H
ATOM 3781 NE ARG X 248 -24.361 7.242 -31.714 1.00 0.00
N
ATOM 3782 HE ARG X 248 -24.290 6.516 -31.030 1.00 0.00
H
ATOM 3783 CZ ARG X 248 -25.431 7.606 -32.361 1.00 0.00
C
ATOM 3784 NH1 ARG X 248 -25.559 8.569 -33.324 1.00 0.00
N
ATOM 3785 HH11 ARG X 248 -24.686 8.761 -33.773 1.00 0.00
H
ATOM 3786 HH12 ARG X 248 -26.410 8.521 -33.846 1.00 0.00
H
ATOM 3787 NH2 ARG X 248 -26.560 6.978 -32.189 1.00 0.00
N
ATOM 3788 HH21 ARG X 248 -26.565 6.247 -31.508 1.00 0.00
H
ATOM 3789 HH22 ARG X 248 -27.372 7.149 -32.748 1.00 0.00
H
ATOM 3790 C ARG X 248 -19.896 5.459 -30.444 1.00 0.00
C
ATOM 3791 O ARG X 248 -18.849 5.978 -30.453 1.00 0.00
O
ATOM 3792 N LYS X 249 -20.173 4.598 -31.387 1.00 0.00
N
ATOM 3793 HN LYS X 249 -21.061 4.156 -31.294 1.00 0.00
H
ATOM 3794 CA LYS X 249 -19.497 4.353 -32.566 1.00 0.00
C
ATOM 3795 HA LYS X 249 -19.204 5.365 -32.802 1.00 0.00
H
ATOM 3796 CB LYS X 249 -18.272 3.330 -32.475 1.00 0.00
C
ATOM 3797 HB1 LYS X 249 -17.499 3.747 -31.795 1.00 0.00
H
ATOM 3798 HB2 LYS X 249 -17.706 3.210 -33.424 1.00 0.00
H
ATOM 3799 CG LYS X 249 -18.571 1.852 -32.017 1.00 0.00
C
ATOM 3800 HG1 LYS X 249 -19.035 1.536 -32.976 1.00 0.00
H

ATOM 3801 HG2 LYS X 249 −19.275 1.890 −31.159 1.00 0.00
H
ATOM 3802 CD LYS X 249 −17.367 1.053 −31.704 1.00 0.00
C
ATOM 3803 HD1 LYS X 249 −17.732 0.013 −31.566 1.00 0.00
H
ATOM 3804 HD2 LYS X 249 −16.909 1.421 −30.761 1.00 0.00
H
ATOM 3805 CE LYS X 249 −16.333 1.049 −32.916 1.00 0.00
C
ATOM 3806 HE1 LYS X 249 −15.906 2.070 −33.015 1.00 0.00
H
ATOM 3807 HE2 LYS X 249 −16.718 0.608 −33.860 1.00 0.00
H
ATOM 3808 NZ LYS X 249 −15.002 0.366 −32.489 1.00 0.00
N
ATOM 3809 HZ1 LYS X 249 −14.496 0.220 −33.386 1.00 0.00
H
ATOM 3810 HZ2 LYS X 249 −15.309 −0.556 −32.117 1.00 0.00
H
ATOM 3811 HZ3 LYS X 249 −14.432 0.765 −31.716 1.00 0.00
H
ATOM 3812 C LYS X 249 −20.493 3.984 −33.680 1.00 0.00
C
ATOM 3813 O LYS X 249 −21.514 3.395 −33.359 1.00 0.00
O
ATOM 3814 N ARG X 250 −20.233 4.383 −34.927 1.00 0.00
N
ATOM 3815 HN ARG X 250 −19.343 4.789 −35.119 1.00 0.00
H
ATOM 3816 CA ARG X 250 −21.141 4.116 −35.980 1.00 0.00
C
ATOM 3817 HA ARG X 250 −22.033 3.618 −35.629 1.00 0.00
H
ATOM 3818 CB ARG X 250 −21.594 5.454 −36.616 1.00 0.00
C
ATOM 3819 HB1 ARG X 250 −20.683 6.086 −36.688 1.00 0.00
H
ATOM 3820 HB2 ARG X 250 −22.366 5.918 −35.966 1.00 0.00
H
ATOM 3821 CG ARG X 250 −22.291 5.405 −38.032 1.00 0.00
C
ATOM 3822 HG1 ARG X 250 −22.952 6.296 −37.987 1.00 0.00
H
ATOM 3823 HG2 ARG X 250 −22.944 4.506 −38.067 1.00 0.00
H
ATOM 3824 CD ARG X 250 −21.340 5.241 −39.291 1.00 0.00
C
ATOM 3825 HD1 ARG X 250 −20.857 4.241 −39.325 1.00 0.00
H

ATOM 3826 HD2 ARG X 250 −20.454 5.906 −39.210 1.00 0.00
H
ATOM 3827 NE ARG X 250 −22.121 5.475 −40.529 1.00 0.00
N
ATOM 3828 HE ARG X 250 −23.088 5.731 −40.515 1.00 0.00
H
ATOM 3829 CZ ARG X 250 −21.544 5.584 −41.705 1.00 0.00
C
ATOM 3830 NH1 ARG X 250 −20.328 5.134 −42.057 1.00 0.00
N
ATOM 3831 HH11 ARG X 250 −19.813 4.577 −41.407 1.00 0.00
H
ATOM 3832 HH12 ARG X 250 −19.975 5.060 −42.990 1.00 0.00
H
ATOM 3833 NH2 ARG X 250 −22.289 5.991 −42.722 1.00 0.00
N
ATOM 3834 HH21 ARG X 250 −22.957 6.626 −42.335 1.00 0.00
H
ATOM 3835 HH22 ARG X 250 −21.886 6.287 −43.588 1.00 0.00
H
ATOM 3836 C ARG X 250 −20.441 3.202 −36.972 1.00 0.00
C
ATOM 3837 O ARG X 250 −19.495 3.521 −37.713 1.00 0.00
O
ATOM 3838 N ILE X 251 −20.895 1.911 −36.977 1.00 0.00
N
ATOM 3839 HN ILE X 251 −21.642 1.568 −36.413 1.00 0.00
H
ATOM 3840 CA ILE X 251 −20.235 0.864 −37.657 1.00 0.00
C
ATOM 3841 HA ILE X 251 −19.195 1.095 −37.836 1.00 0.00
H
ATOM 3842 CB ILE X 251 −20.218 −0.426 −36.791 1.00 0.00
C
ATOM 3843 HB ILE X 251 −21.217 −0.910 −36.846 1.00 0.00
H
ATOM 3844 CG2 ILE X 251 −19.175 −1.383 −37.450 1.00 0.00
C
ATOM 3845 HG21 ILE X 251 −19.390 −1.616 −38.515 1.00 0.00
H
ATOM 3846 HG22 ILE X 251 −19.024 −2.384 −36.993 1.00 0.00
H
ATOM 3847 HG23 ILE X 251 −18.242 −0.806 −37.276 1.00 0.00
H
ATOM 3848 CG1 ILE X 251 −19.979 −0.234 −35.174 1.00 0.00
C
ATOM 3849 HG11 ILE X 251 −20.931 0.227 −34.833 1.00 0.00
H
ATOM 3850 HG12 ILE X 251 −19.061 0.352 −34.954 1.00 0.00
H

```
ATOM   3851  CD  ILE X 251     -19.815  -1.621 -34.556  1.00  0.00
C
ATOM   3852  HD1 ILE X 251     -18.837  -2.042 -34.873  1.00  0.00
H
ATOM   3853  HD2 ILE X 251     -20.591  -2.311 -34.952  1.00  0.00
H
ATOM   3854  HD3 ILE X 251     -20.062  -1.536 -33.476  1.00  0.00
H
ATOM   3855  C   ILE X 251     -20.913   0.579 -38.992  1.00  0.00
C
ATOM   3856  O   ILE X 251     -22.054   0.220 -39.018  1.00  0.00
O
ATOM   3857  N   SER X 252     -20.229   0.823 -40.124  1.00  0.00
N
ATOM   3858  HN  SER X 252     -19.346   1.286 -40.124  1.00  0.00
H
ATOM   3859  CA  SER X 252     -20.736   0.612 -41.515  1.00  0.00
C
ATOM   3860  HA  SER X 252     -21.789   0.826 -41.400  1.00  0.00
H
ATOM   3861  CB  SER X 252     -19.948   1.412 -42.562  1.00  0.00
C
ATOM   3862  HB1 SER X 252     -18.865   1.171 -42.629  1.00  0.00
H
ATOM   3863  HB2 SER X 252     -20.003   2.441 -42.147  1.00  0.00
H
ATOM   3864  OG  SER X 252     -20.509   1.381 -43.838  1.00  0.00
O
ATOM   3865  HG1 SER X 252     -21.420   1.668 -43.742  1.00  0.00
H
ATOM   3866  C   SER X 252     -20.703  -0.817 -41.998  1.00  0.00
C
ATOM   3867  O   SER X 252     -19.678  -1.508 -41.922  1.00  0.00
O
ATOM   3868  N   ASN X 253     -21.857  -1.356 -42.570  1.00  0.00
N
ATOM   3869  HN  ASN X 253     -22.587  -0.691 -42.710  1.00  0.00
H
ATOM   3870  CA  ASN X 253     -22.051  -2.637 -43.094  1.00  0.00
C
ATOM   3871  HA  ASN X 253     -23.084  -2.845 -43.332  1.00  0.00
H
ATOM   3872  CB  ASN X 253     -21.319  -2.792 -44.503  1.00  0.00
C
ATOM   3873  HB1 ASN X 253     -21.573  -3.803 -44.887  1.00  0.00
H
ATOM   3874  HB2 ASN X 253     -20.254  -2.578 -44.268  1.00  0.00
H
ATOM   3875  CG  ASN X 253     -21.815  -1.819 -45.573  1.00  0.00
C
```

```
ATOM   3876  OD1 ASN X 253     -22.809  -1.114 -45.408  1.00  0.00
O
ATOM   3877  ND2 ASN X 253     -21.132  -1.969 -46.732  1.00  0.00
N
ATOM   3878 HD21 ASN X 253     -21.223  -1.334 -47.499  1.00  0.00
H
ATOM   3879 HD22 ASN X 253     -20.412  -2.647 -46.591  1.00  0.00
H
ATOM   3880  C   ASN X 253     -21.805  -3.788 -42.039  1.00  0.00
C
ATOM   3881  O   ASN X 253     -20.709  -4.342 -41.923  1.00  0.00
O
ATOM   3882  N   CYS X 254     -22.804  -4.181 -41.226  1.00  0.00
N
ATOM   3883  HN  CYS X 254     -23.657  -3.666 -41.260  1.00  0.00
H
ATOM   3884  CA  CYS X 254     -22.593  -4.830 -39.950  1.00  0.00
C
ATOM   3885  HA  CYS X 254     -21.526  -4.960 -39.847  1.00  0.00
H
ATOM   3886  CB  CYS X 254     -22.983  -3.864 -38.818  1.00  0.00
C
ATOM   3887  HB1 CYS X 254     -24.031  -3.528 -38.974  1.00  0.00
H
ATOM   3888  HB2 CYS X 254     -22.208  -3.072 -38.899  1.00  0.00
H
ATOM   3889  SG  CYS X 254     -22.816  -4.694 -37.157  1.00  0.00
S
ATOM   3890  C   CYS X 254     -23.388  -6.134 -39.951  1.00  0.00
C
ATOM   3891  O   CYS X 254     -24.629  -6.128 -40.136  1.00  0.00
O
ATOM   3892  N   VAL X 255     -22.708  -7.250 -39.633  1.00  0.00
N
ATOM   3893  HN  VAL X 255     -21.722  -7.136 -39.538  1.00  0.00
H
ATOM   3894  CA  VAL X 255     -23.198  -8.594 -39.267  1.00  0.00
C
ATOM   3895  HA  VAL X 255     -24.261  -8.746 -39.387  1.00  0.00
H
ATOM   3896  CB  VAL X 255     -22.530  -9.697 -40.175  1.00  0.00
C
ATOM   3897  HB  VAL X 255     -21.439  -9.495 -40.126  1.00  0.00
H
ATOM   3898  CG1 VAL X 255     -22.962 -11.112 -39.741  1.00  0.00
C
ATOM   3899 HG11 VAL X 255     -22.520 -11.873 -40.419  1.00  0.00
H
ATOM   3900 HG12 VAL X 255     -24.061 -11.135 -39.576  1.00  0.00
H
```

```
ATOM   3901 HG13 VAL X 255     -22.448 -11.321 -38.778  1.00  0.00
H
ATOM   3902  CG2 VAL X 255     -23.071  -9.360 -41.556  1.00  0.00
C
ATOM   3903 HG21 VAL X 255     -22.988 -10.189 -42.291  1.00  0.00
H
ATOM   3904 HG22 VAL X 255     -22.556  -8.470 -41.975  1.00  0.00
H
ATOM   3905 HG23 VAL X 255     -24.151  -9.126 -41.440  1.00  0.00
H
ATOM   3906  C   VAL X 255     -22.691  -8.812 -37.825  1.00  0.00
C
ATOM   3907  O   VAL X 255     -21.715  -8.204 -37.363  1.00  0.00
O
ATOM   3908  N   ALA X 256     -23.485  -9.520 -36.986  1.00  0.00
N
ATOM   3909  HN  ALA X 256     -24.354  -9.890 -37.304  1.00  0.00
H
ATOM   3910  CA  ALA X 256     -23.161  -9.597 -35.613  1.00  0.00
C
ATOM   3911  HA  ALA X 256     -22.111  -9.410 -35.440  1.00  0.00
H
ATOM   3912  CB  ALA X 256     -23.870  -8.454 -34.899  1.00  0.00
C
ATOM   3913  HB1 ALA X 256     -23.912  -8.582 -33.796  1.00  0.00
H
ATOM   3914  HB2 ALA X 256     -24.903  -8.482 -35.307  1.00  0.00
H
ATOM   3915  HB3 ALA X 256     -23.349  -7.509 -35.162  1.00  0.00
H
ATOM   3916  C   ALA X 256     -23.469 -10.866 -34.864  1.00  0.00
C
ATOM   3917  O   ALA X 256     -24.277 -11.787 -35.204  1.00  0.00
O
ATOM   3918  N   ASP X 257     -22.787 -10.936 -33.735  1.00  0.00
N
ATOM   3919  HN  ASP X 257     -22.195 -10.168 -33.504  1.00  0.00
H
ATOM   3920  CA  ASP X 257     -22.776 -12.089 -32.909  1.00  0.00
C
ATOM   3921  HA  ASP X 257     -23.213 -12.931 -33.427  1.00  0.00
H
ATOM   3922  CB  ASP X 257     -21.271 -12.552 -32.705  1.00  0.00
C
ATOM   3923  HB1 ASP X 257     -20.773 -11.769 -32.096  1.00  0.00
H
ATOM   3924  HB2 ASP X 257     -20.744 -12.634 -33.680  1.00  0.00
H
ATOM   3925  CG  ASP X 257     -21.144 -13.793 -31.821  1.00  0.00
C
```

ATOM 3926 OD1 ASP X 257 −19.967 −14.040 −31.422 1.00 0.00
O
ATOM 3927 OD2 ASP X 257 −22.133 −14.569 −31.526 1.00 0.00
O
ATOM 3928 C ASP X 257 −23.464 −11.936 −31.558 1.00 0.00
C
ATOM 3929 O ASP X 257 −23.004 −11.426 −30.552 1.00 0.00
O
ATOM 3930 N TYR X 258 −24.690 −12.450 −31.483 1.00 0.00
N
ATOM 3931 HN TYR X 258 −25.301 −12.844 −32.165 1.00 0.00
H
ATOM 3932 CA TYR X 258 −25.417 −12.412 −30.298 1.00 0.00
C
ATOM 3933 HA TYR X 258 −24.979 −11.787 −29.535 1.00 0.00
H
ATOM 3934 CB TYR X 258 −26.883 −11.962 −30.480 1.00 0.00
C
ATOM 3935 HB1 TYR X 258 −27.422 −11.905 −29.511 1.00 0.00
H
ATOM 3936 HB2 TYR X 258 −27.471 −12.712 −31.051 1.00 0.00
H
ATOM 3937 CG TYR X 258 −26.967 −10.737 −31.220 1.00 0.00
C
ATOM 3938 CD1 TYR X 258 −26.903 −10.680 −32.587 1.00 0.00
C
ATOM 3939 HD1 TYR X 258 −26.784 −11.523 −33.251 1.00 0.00
H
ATOM 3940 CE1 TYR X 258 −27.138 −9.463 −33.350 1.00 0.00
C
ATOM 3941 HE1 TYR X 258 −26.883 −9.315 −34.389 1.00 0.00
H
ATOM 3942 CZ TYR X 258 −27.282 −8.264 −32.579 1.00 0.00
C
ATOM 3943 OH TYR X 258 −27.204 −6.988 −33.271 1.00 0.00
O
ATOM 3944 HH TYR X 258 −27.297 −7.161 −34.211 1.00 0.00
H
ATOM 3945 CD2 TYR X 258 −27.190 −9.566 −30.501 1.00 0.00
C
ATOM 3946 HD2 TYR X 258 −27.276 −9.551 −29.424 1.00 0.00
H
ATOM 3947 CE2 TYR X 258 −27.312 −8.317 −31.206 1.00 0.00
C
ATOM 3948 HE2 TYR X 258 −27.441 −7.420 −30.618 1.00 0.00
H
ATOM 3949 C TYR X 258 −25.467 −13.765 −29.484 1.00 0.00
C
ATOM 3950 O TYR X 258 −26.227 −13.926 −28.553 1.00 0.00
O

```
ATOM   3951  N   SER X 259      -24.581 -14.751 -29.747  1.00  0.00
N
ATOM   3952  HN  SER X 259      -23.929 -14.580 -30.482  1.00  0.00
H
ATOM   3953  CA  SER X 259      -24.477 -15.959 -28.934  1.00  0.00
C
ATOM   3954  HA  SER X 259      -25.416 -16.486 -29.023  1.00  0.00
H
ATOM   3955  CB  SER X 259      -23.303 -16.968 -29.474  1.00  0.00
C
ATOM   3956  HB1 SER X 259      -23.476 -17.068 -30.567  1.00  0.00
H
ATOM   3957  HB2 SER X 259      -23.329 -17.973 -29.002  1.00  0.00
H
ATOM   3958  OG  SER X 259      -21.967 -16.426 -29.414  1.00  0.00
O
ATOM   3959  HG1 SER X 259      -22.117 -15.700 -30.024  1.00  0.00
H
ATOM   3960  C   SER X 259      -24.154 -15.687 -27.377  1.00  0.00
C
ATOM   3961  O   SER X 259      -24.781 -16.197 -26.468  1.00  0.00
O
ATOM   3962  N   VAL X 260      -23.129 -14.894 -27.042  1.00  0.00
N
ATOM   3963  HN  VAL X 260      -22.457 -14.630 -27.730  1.00  0.00
H
ATOM   3964  CA  VAL X 260      -22.984 -14.552 -25.688  1.00  0.00
C
ATOM   3965  HA  VAL X 260      -23.025 -15.436 -25.068  1.00  0.00
H
ATOM   3966  CB  VAL X 260      -21.659 -13.794 -25.443  1.00  0.00
C
ATOM   3967  HB  VAL X 260      -21.646 -12.695 -25.606  1.00  0.00
H
ATOM   3968  CG1 VAL X 260      -21.299 -13.895 -23.957  1.00  0.00
C
ATOM   3969 HG11 VAL X 260      -21.275 -14.978 -23.706  1.00  0.00
H
ATOM   3970 HG12 VAL X 260      -22.158 -13.413 -23.443  1.00  0.00
H
ATOM   3971 HG13 VAL X 260      -20.355 -13.358 -23.724  1.00  0.00
H
ATOM   3972  CG2 VAL X 260      -20.487 -14.311 -26.385  1.00  0.00
C
ATOM   3973 HG21 VAL X 260      -20.571 -15.375 -26.694  1.00  0.00
H
ATOM   3974 HG22 VAL X 260      -19.492 -14.088 -25.945  1.00  0.00
H
ATOM   3975 HG23 VAL X 260      -20.694 -13.764 -27.330  1.00  0.00
H
```

```
ATOM   3976  C   VAL X 260     -24.140 -13.602 -25.240  1.00  0.00
C
ATOM   3977  O   VAL X 260     -24.552 -13.615 -24.060  1.00  0.00
O
ATOM   3978  N   LEU X 261     -24.690 -12.682 -26.006  1.00  0.00
N
ATOM   3979  HN  LEU X 261     -24.250 -12.562 -26.893  1.00  0.00
H
ATOM   3980  CA  LEU X 261     -25.688 -11.666 -25.710  1.00  0.00
C
ATOM   3981  HA  LEU X 261     -25.375 -11.158 -24.810  1.00  0.00
H
ATOM   3982  CB  LEU X 261     -25.771 -10.615 -26.859  1.00  0.00
C
ATOM   3983  HB1 LEU X 261     -26.342  -9.782 -26.397  1.00  0.00
H
ATOM   3984  HB2 LEU X 261     -26.288 -11.105 -27.712  1.00  0.00
H
ATOM   3985  CG  LEU X 261     -24.387 -10.045 -27.173  1.00  0.00
C
ATOM   3986  HG  LEU X 261     -23.727 -10.845 -27.570  1.00  0.00
H
ATOM   3987  CD1 LEU X 261     -24.538  -8.979 -28.334  1.00  0.00
C
ATOM   3988 HD11 LEU X 261     -23.545  -8.499 -28.471  1.00  0.00
H
ATOM   3989 HD12 LEU X 261     -25.221  -8.174 -27.987  1.00  0.00
H
ATOM   3990 HD13 LEU X 261     -24.879  -9.441 -29.285  1.00  0.00
H
ATOM   3991  CD2 LEU X 261     -23.794  -9.296 -25.967  1.00  0.00
C
ATOM   3992 HD21 LEU X 261     -24.545  -8.701 -25.404  1.00  0.00
H
ATOM   3993 HD22 LEU X 261     -22.918  -8.704 -26.308  1.00  0.00
H
ATOM   3994 HD23 LEU X 261     -23.457 -10.085 -25.261  1.00  0.00
H
ATOM   3995  C   LEU X 261     -27.017 -12.121 -25.396  1.00  0.00
C
ATOM   3996  O   LEU X 261     -27.756 -11.568 -24.542  1.00  0.00
O
ATOM   3997  N   TYR X 262     -27.330 -13.251 -25.981  1.00  0.00
N
ATOM   3998  HN  TYR X 262     -26.816 -13.530 -26.788  1.00  0.00
H
ATOM   3999  CA  TYR X 262     -28.465 -14.179 -25.548  1.00  0.00
C
ATOM   4000  HA  TYR X 262     -29.316 -13.525 -25.428  1.00  0.00
H
```

ATOM 4001 CB TYR X 262 -28.857 -15.151 -26.798 1.00 0.00
C
ATOM 4002 HB1 TYR X 262 -29.537 -15.961 -26.458 1.00 0.00
H
ATOM 4003 HB2 TYR X 262 -27.869 -15.630 -26.968 1.00 0.00
H
ATOM 4004 CG TYR X 262 -29.327 -14.525 -28.086 1.00 0.00
C
ATOM 4005 CD1 TYR X 262 -30.249 -13.382 -28.162 1.00 0.00
C
ATOM 4006 HD1 TYR X 262 -30.476 -12.891 -27.227 1.00 0.00
H
ATOM 4007 CE1 TYR X 262 -30.786 -12.925 -29.346 1.00 0.00
C
ATOM 4008 HE1 TYR X 262 -31.292 -11.975 -29.436 1.00 0.00
H
ATOM 4009 CZ TYR X 262 -30.492 -13.674 -30.527 1.00 0.00
C
ATOM 4010 OH TYR X 262 -31.034 -13.220 -31.729 1.00 0.00
O
ATOM 4011 HH TYR X 262 -31.715 -12.584 -31.498 1.00 0.00
H
ATOM 4012 CD2 TYR X 262 -29.017 -15.139 -29.269 1.00 0.00
C
ATOM 4013 HD2 TYR X 262 -28.451 -16.058 -29.225 1.00 0.00
H
ATOM 4014 CE2 TYR X 262 -29.621 -14.787 -30.513 1.00 0.00
C
ATOM 4015 HE2 TYR X 262 -29.319 -15.372 -31.369 1.00 0.00
H
ATOM 4016 C TYR X 262 -28.293 -14.953 -24.255 1.00 0.00
C
ATOM 4017 O TYR X 262 -29.265 -15.500 -23.808 1.00 0.00
O
ATOM 4018 N ASN X 263 -27.156 -14.836 -23.587 1.00 0.00
N
ATOM 4019 HN ASN X 263 -26.304 -14.408 -23.881 1.00 0.00
H
ATOM 4020 CA ASN X 263 -27.002 -15.495 -22.270 1.00 0.00
C
ATOM 4021 HA ASN X 263 -27.591 -16.397 -22.347 1.00 0.00
H
ATOM 4022 CB ASN X 263 -25.564 -16.218 -22.149 1.00 0.00
C
ATOM 4023 HB1 ASN X 263 -24.760 -15.510 -22.446 1.00 0.00
H
ATOM 4024 HB2 ASN X 263 -25.449 -17.055 -22.870 1.00 0.00
H
ATOM 4025 CG ASN X 263 -25.127 -16.675 -20.795 1.00 0.00
C

```
ATOM   4026  OD1 ASN X 263     -25.558 -16.244 -19.686  1.00  0.00
O
ATOM   4027  ND2 ASN X 263     -24.078 -17.523 -20.741  1.00  0.00
N
ATOM   4028 HD21 ASN X 263     -23.842 -18.053 -19.927  1.00  0.00
H
ATOM   4029 HD22 ASN X 263     -23.589 -17.753 -21.581  1.00  0.00
H
ATOM   4030  C   ASN X 263     -27.624 -14.715 -21.093  1.00  0.00
C
ATOM   4031  O   ASN X 263     -27.575 -13.480 -21.035  1.00  0.00
O
ATOM   4032  N   SER X 264     -28.321 -15.285 -20.138  1.00  0.00
N
ATOM   4033  HN  SER X 264     -28.449 -16.274 -20.116  1.00  0.00
H
ATOM   4034  CA  SER X 264     -29.120 -14.504 -19.248  1.00  0.00
C
ATOM   4035  HA  SER X 264     -29.387 -13.495 -19.528  1.00  0.00
H
ATOM   4036  CB  SER X 264     -30.506 -15.107 -19.189  1.00  0.00
C
ATOM   4037  HB1 SER X 264     -30.988 -15.129 -20.190  1.00  0.00
H
ATOM   4038  HB2 SER X 264     -31.259 -14.584 -18.561  1.00  0.00
H
ATOM   4039  OG  SER X 264     -30.403 -16.434 -18.622  1.00  0.00
O
ATOM   4040  HG1 SER X 264     -30.303 -17.111 -19.295  1.00  0.00
H
ATOM   4041  C   SER X 264     -28.531 -14.512 -17.927  1.00  0.00
C
ATOM   4042  O   SER X 264     -29.017 -13.770 -16.945  1.00  0.00
O
ATOM   4043  N   ALA X 265     -27.357 -15.160 -17.749  1.00  0.00
N
ATOM   4044  HN  ALA X 265     -26.874 -15.570 -18.519  1.00  0.00
H
ATOM   4045  CA  ALA X 265     -26.658 -14.984 -16.537  1.00  0.00
C
ATOM   4046  HA  ALA X 265     -27.322 -14.706 -15.732  1.00  0.00
H
ATOM   4047  CB  ALA X 265     -25.921 -16.320 -16.256  1.00  0.00
C
ATOM   4048  HB1 ALA X 265     -26.627 -17.173 -16.160  1.00  0.00
H
ATOM   4049  HB2 ALA X 265     -25.399 -16.183 -15.285  1.00  0.00
H
ATOM   4050  HB3 ALA X 265     -25.176 -16.527 -17.053  1.00  0.00
H
```

```
ATOM   4051  C   ALA X 265     -25.629 -13.828 -16.836  1.00  0.00
C
ATOM   4052  O   ALA X 265     -25.543 -12.874 -16.052  1.00  0.00
O
ATOM   4053  N   SER X 266     -24.882 -13.785 -17.943  1.00  0.00
N
ATOM   4054  HN  SER X 266     -24.972 -14.637 -18.453  1.00  0.00
H
ATOM   4055  CA  SER X 266     -24.070 -12.747 -18.459  1.00  0.00
C
ATOM   4056  HA  SER X 266     -23.358 -12.562 -17.668  1.00  0.00
H
ATOM   4057  CB  SER X 266     -23.299 -13.024 -19.792  1.00  0.00
C
ATOM   4058  HB1 SER X 266     -22.914 -12.060 -20.189  1.00  0.00
H
ATOM   4059  HB2 SER X 266     -24.012 -13.363 -20.573  1.00  0.00
H
ATOM   4060  OG  SER X 266     -22.217 -13.848 -19.589  1.00  0.00
O
ATOM   4061  HG1 SER X 266     -22.386 -14.435 -18.849  1.00  0.00
H
ATOM   4062  C   SER X 266     -24.864 -11.497 -18.604  1.00  0.00
C
ATOM   4063  O   SER X 266     -24.385 -10.458 -18.208  1.00  0.00
O
ATOM   4064  N   PHE X 267     -26.062 -11.515 -19.138  1.00  0.00
N
ATOM   4065  HN  PHE X 267     -26.497 -12.347 -19.473  1.00  0.00
H
ATOM   4066  CA  PHE X 267     -26.855 -10.345 -19.392  1.00  0.00
C
ATOM   4067  HA  PHE X 267     -26.390  -9.433 -19.047  1.00  0.00
H
ATOM   4068  CB  PHE X 267     -27.057 -10.075 -20.895  1.00  0.00
C
ATOM   4069  HB1 PHE X 267     -27.552  -9.085 -20.988  1.00  0.00
H
ATOM   4070  HB2 PHE X 267     -27.575 -10.865 -21.480  1.00  0.00
H
ATOM   4071  CG  PHE X 267     -25.661  -9.814 -21.533  1.00  0.00
C
ATOM   4072  CD1 PHE X 267     -24.944  -8.709 -21.178  1.00  0.00
C
ATOM   4073  HD1 PHE X 267     -25.510  -8.070 -20.516  1.00  0.00
H
ATOM   4074  CE1 PHE X 267     -23.686  -8.486 -21.629  1.00  0.00
C
ATOM   4075  HE1 PHE X 267     -23.073  -7.677 -21.259  1.00  0.00
H
```

```
ATOM   4076  CZ  PHE X 267     -23.069  -9.403 -22.475  1.00  0.00
C
ATOM   4077  HZ  PHE X 267     -22.253  -8.903 -22.976  1.00  0.00
H
ATOM   4078  CD2 PHE X 267     -25.036 -10.906 -22.302  1.00  0.00
C
ATOM   4079  HD2 PHE X 267     -25.543 -11.784 -22.675  1.00  0.00
H
ATOM   4080  CE2 PHE X 267     -23.778 -10.590 -22.849  1.00  0.00
C
ATOM   4081  HE2 PHE X 267     -23.246 -11.397 -23.331  1.00  0.00
H
ATOM   4082  C   PHE X 267     -28.084 -10.333 -18.584  1.00  0.00
C
ATOM   4083  O   PHE X 267     -28.993 -11.148 -18.844  1.00  0.00
O
ATOM   4084  N   SER X 268     -28.104  -9.442 -17.573  1.00  0.00
N
ATOM   4085  HN  SER X 268     -27.339  -8.831 -17.383  1.00  0.00
H
ATOM   4086  CA  SER X 268     -29.249  -9.099 -16.713  1.00  0.00
C
ATOM   4087  HA  SER X 268     -29.324  -9.903 -15.996  1.00  0.00
H
ATOM   4088  CB  SER X 268     -29.112  -7.867 -15.737  1.00  0.00
C
ATOM   4089  HB1 SER X 268     -29.849  -7.727 -14.918  1.00  0.00
H
ATOM   4090  HB2 SER X 268     -29.003  -6.896 -16.266  1.00  0.00
H
ATOM   4091  OG  SER X 268     -27.989  -7.993 -14.924  1.00  0.00
O
ATOM   4092  HG1 SER X 268     -28.008  -8.935 -14.740  1.00  0.00
H
ATOM   4093  C   SER X 268     -30.581  -8.832 -17.396  1.00  0.00
C
ATOM   4094  O   SER X 268     -31.622  -9.311 -17.007  1.00  0.00
O
ATOM   4095  N   THR X 269     -30.590  -8.061 -18.450  1.00  0.00
N
ATOM   4096  HN  THR X 269     -29.733  -7.666 -18.771  1.00  0.00
H
ATOM   4097  CA  THR X 269     -31.780  -7.905 -19.295  1.00  0.00
C
ATOM   4098  HA  THR X 269     -32.127  -8.928 -19.287  1.00  0.00
H
ATOM   4099  CB  THR X 269     -32.948  -7.122 -18.766  1.00  0.00
C
ATOM   4100  HB  THR X 269     -33.074  -7.490 -17.725  1.00  0.00
H
```

ATOM 4101 OG1 THR X 269 -34.176 -7.502 -19.339 1.00 0.00
O
ATOM 4102 HG1 THR X 269 -34.480 -8.202 -18.756 1.00 0.00
H
ATOM 4103 CG2 THR X 269 -32.730 -5.571 -18.761 1.00 0.00
C
ATOM 4104 HG21 THR X 269 -33.521 -5.041 -18.190 1.00 0.00
H
ATOM 4105 HG22 THR X 269 -32.710 -4.990 -19.708 1.00 0.00
H
ATOM 4106 HG23 THR X 269 -31.806 -5.370 -18.177 1.00 0.00
H
ATOM 4107 C THR X 269 -31.440 -7.499 -20.676 1.00 0.00
C
ATOM 4108 O THR X 269 -30.555 -6.656 -20.773 1.00 0.00
O
ATOM 4109 N PHE X 270 -32.166 -7.981 -21.753 1.00 0.00
N
ATOM 4110 HN PHE X 270 -32.944 -8.571 -21.551 1.00 0.00
H
ATOM 4111 CA PHE X 270 -31.932 -7.832 -23.158 1.00 0.00
C
ATOM 4112 HA PHE X 270 -31.337 -6.945 -23.320 1.00 0.00
H
ATOM 4113 CB PHE X 270 -31.006 -9.043 -23.644 1.00 0.00
C
ATOM 4114 HB1 PHE X 270 -31.468 -10.023 -23.397 1.00 0.00
H
ATOM 4115 HB2 PHE X 270 -30.048 -8.970 -23.087 1.00 0.00
H
ATOM 4116 CG PHE X 270 -30.630 -9.120 -25.151 1.00 0.00
C
ATOM 4117 CD1 PHE X 270 -30.664 -7.990 -25.993 1.00 0.00
C
ATOM 4118 HD1 PHE X 270 -31.036 -7.054 -25.605 1.00 0.00
H
ATOM 4119 CE1 PHE X 270 -30.232 -7.923 -27.322 1.00 0.00
C
ATOM 4120 HE1 PHE X 270 -30.291 -6.942 -27.769 1.00 0.00
H
ATOM 4121 CZ PHE X 270 -29.745 -9.132 -27.901 1.00 0.00
C
ATOM 4122 HZ PHE X 270 -29.456 -9.085 -28.941 1.00 0.00
H
ATOM 4123 CD2 PHE X 270 -30.077 -10.224 -25.771 1.00 0.00
C
ATOM 4124 HD2 PHE X 270 -29.982 -11.115 -25.168 1.00 0.00
H
ATOM 4125 CE2 PHE X 270 -29.667 -10.259 -27.115 1.00 0.00
C

ATOM 4126 HE2 PHE X 270 −29.104 −11.096 −27.499 1.00 0.00
H
ATOM 4127 C PHE X 270 −33.185 −7.630 −23.909 1.00 0.00
C
ATOM 4128 O PHE X 270 −34.047 −8.474 −24.028 1.00 0.00
O
ATOM 4129 N LYS X 271 −33.412 −6.450 −24.433 1.00 0.00
N
ATOM 4130 HN LYS X 271 −32.753 −5.701 −24.434 1.00 0.00
H
ATOM 4131 CA LYS X 271 −34.700 −5.949 −24.963 1.00 0.00
C
ATOM 4132 HA LYS X 271 −35.257 −6.801 −25.324 1.00 0.00
H
ATOM 4133 CB LYS X 271 −35.334 −5.025 −23.895 1.00 0.00
C
ATOM 4134 HB1 LYS X 271 −36.305 −4.724 −24.342 1.00 0.00
H
ATOM 4135 HB2 LYS X 271 −34.792 −4.056 −23.839 1.00 0.00
H
ATOM 4136 CG LYS X 271 −35.516 −5.582 −22.526 1.00 0.00
C
ATOM 4137 HG1 LYS X 271 −35.820 −4.743 −21.864 1.00 0.00
H
ATOM 4138 HG2 LYS X 271 −34.544 −5.881 −22.078 1.00 0.00
H
ATOM 4139 CD LYS X 271 −36.542 −6.717 −22.559 1.00 0.00
C
ATOM 4140 HD1 LYS X 271 −36.145 −7.641 −23.033 1.00 0.00
H
ATOM 4141 HD2 LYS X 271 −37.495 −6.381 −23.021 1.00 0.00
H
ATOM 4142 CE LYS X 271 −37.115 −7.246 −21.204 1.00 0.00
C
ATOM 4143 HE1 LYS X 271 −37.772 −6.459 −20.775 1.00 0.00
H
ATOM 4144 HE2 LYS X 271 −36.310 −7.488 −20.478 1.00 0.00
H
ATOM 4145 NZ LYS X 271 −37.936 −8.426 −21.536 1.00 0.00
N
ATOM 4146 HZ1 LYS X 271 −38.660 −8.699 −20.841 1.00 0.00
H
ATOM 4147 HZ2 LYS X 271 −37.247 −9.156 −21.807 1.00 0.00
H
ATOM 4148 HZ3 LYS X 271 −38.555 −8.096 −22.304 1.00 0.00
H
ATOM 4149 C LYS X 271 −34.575 −5.315 −26.354 1.00 0.00
C
ATOM 4150 O LYS X 271 −33.687 −4.548 −26.591 1.00 0.00
O

```
ATOM   4151  N   CYS X 272      -35.494  -5.697 -27.190  1.00  0.00
N
ATOM   4152  HN  CYS X 272      -36.190  -6.304 -26.815  1.00  0.00
H
ATOM   4153  CA  CYS X 272      -35.424  -5.256 -28.600  1.00  0.00
C
ATOM   4154  HA  CYS X 272      -34.704  -4.477 -28.799  1.00  0.00
H
ATOM   4155  CB  CYS X 272      -35.214  -6.281 -29.743  1.00  0.00
C
ATOM   4156  HB1 CYS X 272      -35.373  -5.907 -30.777  1.00  0.00
H
ATOM   4157  HB2 CYS X 272      -35.968  -7.091 -29.636  1.00  0.00
H
ATOM   4158  SG  CYS X 272      -33.429  -6.919 -29.615  1.00  0.00
S
ATOM   4159  C   CYS X 272      -36.771  -4.670 -28.892  1.00  0.00
C
ATOM   4160  O   CYS X 272      -37.800  -4.951 -28.303  1.00  0.00
O
ATOM   4161  N   TYR X 273      -36.689  -3.548 -29.642  1.00  0.00
N
ATOM   4162  HN  TYR X 273      -35.883  -3.165 -30.087  1.00  0.00
H
ATOM   4163  CA  TYR X 273      -37.756  -2.646 -29.913  1.00  0.00
C
ATOM   4164  HA  TYR X 273      -38.658  -3.100 -29.530  1.00  0.00
H
ATOM   4165  CB  TYR X 273      -37.527  -1.275 -29.178  1.00  0.00
C
ATOM   4166  HB1 TYR X 273      -38.400  -0.610 -29.352  1.00  0.00
H
ATOM   4167  HB2 TYR X 273      -36.527  -0.945 -29.531  1.00  0.00
H
ATOM   4168  CG  TYR X 273      -37.412  -1.479 -27.689  1.00  0.00
C
ATOM   4169  CD1 TYR X 273      -36.265  -1.955 -27.093  1.00  0.00
C
ATOM   4170  HD1 TYR X 273      -35.330  -1.820 -27.617  1.00  0.00
H
ATOM   4171  CE1 TYR X 273      -36.206  -2.300 -25.718  1.00  0.00
C
ATOM   4172  HE1 TYR X 273      -35.276  -2.394 -25.178  1.00  0.00
H
ATOM   4173  CZ  TYR X 273      -37.397  -2.112 -25.011  1.00  0.00
C
ATOM   4174  OH  TYR X 273      -37.305  -2.291 -23.627  1.00  0.00
O
ATOM   4175  HH  TYR X 273      -38.214  -2.245 -23.322  1.00  0.00
H
```

ATOM 4176 CD2 TYR X 273 -38.619 -1.317 -26.995 1.00 0.00
C
ATOM 4177 HD2 TYR X 273 -39.505 -0.880 -27.432 1.00 0.00
H
ATOM 4178 CE2 TYR X 273 -38.588 -1.494 -25.630 1.00 0.00
C
ATOM 4179 HE2 TYR X 273 -39.395 -1.188 -24.981 1.00 0.00
H
ATOM 4180 C TYR X 273 -37.898 -2.492 -31.354 1.00 0.00
C
ATOM 4181 O TYR X 273 -36.967 -2.373 -32.080 1.00 0.00
O
ATOM 4182 N GLY X 274 -39.187 -2.495 -31.925 1.00 0.00
N
ATOM 4183 HN GLY X 274 -39.959 -2.574 -31.299 1.00 0.00
H
ATOM 4184 CA GLY X 274 -39.465 -2.562 -33.352 1.00 0.00
C
ATOM 4185 HA1 GLY X 274 -38.833 -1.883 -33.905 1.00 0.00
H
ATOM 4186 HA2 GLY X 274 -40.507 -2.448 -33.616 1.00 0.00
H
ATOM 4187 C GLY X 274 -39.239 -3.951 -33.882 1.00 0.00
C
ATOM 4188 O GLY X 274 -40.231 -4.679 -34.154 1.00 0.00
O
ATOM 4189 N VAL X 275 -37.914 -4.257 -34.094 1.00 0.00
N
ATOM 4190 HN VAL X 275 -37.218 -3.561 -33.937 1.00 0.00
H
ATOM 4191 CA VAL X 275 -37.397 -5.461 -34.513 1.00 0.00
C
ATOM 4192 HA VAL X 275 -37.779 -5.712 -35.491 1.00 0.00
H
ATOM 4193 CB VAL X 275 -35.988 -5.248 -34.933 1.00 0.00
C
ATOM 4194 HB VAL X 275 -35.944 -4.396 -35.644 1.00 0.00
H
ATOM 4195 CG1 VAL X 275 -35.161 -4.947 -33.655 1.00 0.00
C
ATOM 4196 HG11 VAL X 275 -35.038 -5.914 -33.122 1.00 0.00
H
ATOM 4197 HG12 VAL X 275 -35.590 -4.319 -32.845 1.00 0.00
H
ATOM 4198 HG13 VAL X 275 -34.162 -4.588 -33.981 1.00 0.00
H
ATOM 4199 CG2 VAL X 275 -35.249 -6.359 -35.731 1.00 0.00
C
ATOM 4200 HG21 VAL X 275 -34.434 -5.924 -36.348 1.00 0.00
H

```
ATOM   4201 HG22 VAL X 275     -36.048  -6.774 -36.383  1.00  0.00
H
ATOM   4202 HG23 VAL X 275     -34.783  -7.198 -35.171  1.00  0.00
H
ATOM   4203  C   VAL X 275     -37.648  -6.589 -33.568  1.00  0.00
C
ATOM   4204  O   VAL X 275     -37.732  -6.335 -32.421  1.00  0.00
O
ATOM   4205  N   SER X 276     -37.821  -7.806 -34.043  1.00  0.00
N
ATOM   4206  HN  SER X 276     -37.803  -7.929 -35.032  1.00  0.00
H
ATOM   4207  CA  SER X 276     -37.760  -8.984 -33.166  1.00  0.00
C
ATOM   4208  HA  SER X 276     -38.493  -8.882 -32.380  1.00  0.00
H
ATOM   4209  CB  SER X 276     -38.184 -10.340 -33.855  1.00  0.00
C
ATOM   4210  HB1 SER X 276     -37.536 -10.460 -34.749  1.00  0.00
H
ATOM   4211  HB2 SER X 276     -39.220 -10.230 -34.241  1.00  0.00
H
ATOM   4212  OG  SER X 276     -38.033 -11.511 -32.962  1.00  0.00
O
ATOM   4213  HG1 SER X 276     -38.924 -11.647 -32.632  1.00  0.00
H
ATOM   4214  C   SER X 276     -36.372  -9.177 -32.480  1.00  0.00
C
ATOM   4215  O   SER X 276     -35.355  -8.811 -33.132  1.00  0.00
O
ATOM   4216  N   PRO X 277     -36.098  -9.747 -31.251  1.00  0.00
N
ATOM   4217  CD  PRO X 277     -37.094  -9.839 -30.211  1.00  0.00
C
ATOM   4218  HD1 PRO X 277     -38.106 -10.161 -30.539  1.00  0.00
H
ATOM   4219  HD2 PRO X 277     -37.213  -8.875 -29.673  1.00  0.00
H
ATOM   4220  CA  PRO X 277     -34.737 -10.176 -30.821  1.00  0.00
C
ATOM   4221  HA  PRO X 277     -33.991  -9.464 -31.141  1.00  0.00
H
ATOM   4222  CB  PRO X 277     -34.848 -10.443 -29.316  1.00  0.00
C
ATOM   4223  HB1 PRO X 277     -34.490  -9.557 -28.749  1.00  0.00
H
ATOM   4224  HB2 PRO X 277     -34.192 -11.291 -29.026  1.00  0.00
H
ATOM   4225  CG  PRO X 277     -36.358 -10.741 -29.173  1.00  0.00
C
```

ATOM 4226 HG1 PRO X 277 -36.571 -11.762 -29.557 1.00 0.00
H
ATOM 4227 HG2 PRO X 277 -36.758 -10.482 -28.170 1.00 0.00
H
ATOM 4228 C PRO X 277 -34.332 -11.379 -31.705 1.00 0.00
C
ATOM 4229 O PRO X 277 -33.157 -11.711 -31.593 1.00 0.00
O
ATOM 4230 N THR X 278 -35.272 -12.157 -32.382 1.00 0.00
N
ATOM 4231 HN THR X 278 -36.245 -11.941 -32.405 1.00 0.00
H
ATOM 4232 CA THR X 278 -34.919 -13.313 -33.118 1.00 0.00
C
ATOM 4233 HA THR X 278 -34.172 -13.838 -32.541 1.00 0.00
H
ATOM 4234 CB THR X 278 -36.005 -14.331 -33.355 1.00 0.00
C
ATOM 4235 HB THR X 278 -35.624 -15.318 -33.693 1.00 0.00
H
ATOM 4236 OG1 THR X 278 -36.981 -13.982 -34.325 1.00 0.00
O
ATOM 4237 HG1 THR X 278 -37.441 -13.195 -34.024 1.00 0.00
H
ATOM 4238 CG2 THR X 278 -36.777 -14.561 -32.057 1.00 0.00
C
ATOM 4239 HG21 THR X 278 -36.097 -14.963 -31.277 1.00 0.00
H
ATOM 4240 HG22 THR X 278 -37.536 -15.367 -32.155 1.00 0.00
H
ATOM 4241 HG23 THR X 278 -37.201 -13.723 -31.463 1.00 0.00
H
ATOM 4242 C THR X 278 -34.184 -13.043 -34.396 1.00 0.00
C
ATOM 4243 O THR X 278 -34.141 -11.913 -34.915 1.00 0.00
O
ATOM 4244 N LYS X 279 -33.381 -14.020 -34.936 1.00 0.00
N
ATOM 4245 HN LYS X 279 -33.113 -14.915 -34.588 1.00 0.00
H
ATOM 4246 CA LYS X 279 -32.737 -13.887 -36.201 1.00 0.00
C
ATOM 4247 HA LYS X 279 -32.076 -14.741 -36.227 1.00 0.00
H
ATOM 4248 CB LYS X 279 -33.753 -13.964 -37.372 1.00 0.00
C
ATOM 4249 HB1 LYS X 279 -33.187 -13.966 -38.328 1.00 0.00
H
ATOM 4250 HB2 LYS X 279 -34.330 -13.015 -37.344 1.00 0.00
H

ATOM 4251 CG LYS X 279 -34.768 -15.144 -37.332 1.00 0.00
C
ATOM 4252 HG1 LYS X 279 -35.391 -15.084 -36.415 1.00 0.00
H
ATOM 4253 HG2 LYS X 279 -34.140 -16.061 -37.326 1.00 0.00
H
ATOM 4254 CD LYS X 279 -35.748 -15.241 -38.578 1.00 0.00
C
ATOM 4255 HD1 LYS X 279 -36.571 -14.511 -38.426 1.00 0.00
H
ATOM 4256 HD2 LYS X 279 -36.157 -16.274 -38.564 1.00 0.00
H
ATOM 4257 CE LYS X 279 -35.071 -14.949 -39.939 1.00 0.00
C
ATOM 4258 HE1 LYS X 279 -34.703 -13.901 -39.903 1.00 0.00
H
ATOM 4259 HE2 LYS X 279 -35.855 -14.990 -40.725 1.00 0.00
H
ATOM 4260 NZ LYS X 279 -34.081 -15.914 -40.297 1.00 0.00
N
ATOM 4261 HZ1 LYS X 279 -33.212 -15.908 -39.726 1.00 0.00
H
ATOM 4262 HZ2 LYS X 279 -33.845 -15.782 -41.301 1.00 0.00
H
ATOM 4263 HZ3 LYS X 279 -34.402 -16.902 -40.353 1.00 0.00
H
ATOM 4264 C LYS X 279 -31.759 -12.701 -36.516 1.00 0.00
C
ATOM 4265 O LYS X 279 -31.535 -12.373 -37.695 1.00 0.00
O
ATOM 4266 N LEU X 280 -31.154 -12.064 -35.496 1.00 0.00
N
ATOM 4267 HN LEU X 280 -31.384 -12.272 -34.548 1.00 0.00
H
ATOM 4268 CA LEU X 280 -30.252 -10.938 -35.701 1.00 0.00
C
ATOM 4269 HA LEU X 280 -30.683 -10.224 -36.387 1.00 0.00
H
ATOM 4270 CB LEU X 280 -30.105 -10.138 -34.394 1.00 0.00
C
ATOM 4271 HB1 LEU X 280 -29.415 -9.306 -34.649 1.00 0.00
H
ATOM 4272 HB2 LEU X 280 -29.577 -10.806 -33.681 1.00 0.00
H
ATOM 4273 CG LEU X 280 -31.384 -9.600 -33.832 1.00 0.00
C
ATOM 4274 HG LEU X 280 -32.163 -10.382 -33.702 1.00 0.00
H
ATOM 4275 CD1 LEU X 280 -30.972 -8.931 -32.457 1.00 0.00
C

```
ATOM   4276 HD11 LEU X 280     -31.888  -8.708 -31.869  1.00  0.00
H
ATOM   4277 HD12 LEU X 280     -30.255  -8.090 -32.567  1.00  0.00
H
ATOM   4278 HD13 LEU X 280     -30.556  -9.830 -31.954  1.00  0.00
H
ATOM   4279  CD2 LEU X 280     -32.013  -8.531 -34.756  1.00  0.00
C
ATOM   4280 HD21 LEU X 280     -32.458  -9.160 -35.556  1.00  0.00
H
ATOM   4281 HD22 LEU X 280     -31.218  -7.836 -35.102  1.00  0.00
H
ATOM   4282 HD23 LEU X 280     -32.880  -8.015 -34.291  1.00  0.00
H
ATOM   4283  C   LEU X 280     -28.937 -11.246 -36.494  1.00  0.00
C
ATOM   4284  O   LEU X 280     -28.609 -10.549 -37.452  1.00  0.00
O
ATOM   4285  N   ASN X 281     -28.245 -12.302 -36.168  1.00  0.00
N
ATOM   4286  HN  ASN X 281     -28.388 -12.869 -35.361  1.00  0.00
H
ATOM   4287  CA  ASN X 281     -27.041 -12.846 -36.775  1.00  0.00
C
ATOM   4288  HA  ASN X 281     -26.273 -12.120 -36.553  1.00  0.00
H
ATOM   4289  CB  ASN X 281     -26.651 -14.150 -36.055  1.00  0.00
C
ATOM   4290  HB1 ASN X 281     -27.372 -14.995 -36.023  1.00  0.00
H
ATOM   4291  HB2 ASN X 281     -26.478 -13.972 -34.973  1.00  0.00
H
ATOM   4292  CG  ASN X 281     -25.388 -14.820 -36.639  1.00  0.00
C
ATOM   4293  OD1 ASN X 281     -25.483 -15.861 -37.220  1.00  0.00
O
ATOM   4294  ND2 ASN X 281     -24.198 -14.099 -36.708  1.00  0.00
N
ATOM   4295 HD21 ASN X 281     -23.373 -14.622 -36.923  1.00  0.00
H
ATOM   4296 HD22 ASN X 281     -24.307 -13.200 -36.284  1.00  0.00
H
ATOM   4297  C   ASN X 281     -27.195 -13.026 -38.390  1.00  0.00
C
ATOM   4298  O   ASN X 281     -26.297 -12.768 -39.153  1.00  0.00
O
ATOM   4299  N   ASP X 282     -28.409 -13.578 -38.774  1.00  0.00
N
ATOM   4300  HN  ASP X 282     -29.087 -13.605 -38.044  1.00  0.00
H
```

```
ATOM   4301  CA  ASP X 282     -28.791 -13.870 -40.123  1.00  0.00
C
ATOM   4302  HA  ASP X 282     -28.182 -14.599 -40.636  1.00  0.00
H
ATOM   4303  CB  ASP X 282     -30.214 -14.505 -40.245  1.00  0.00
C
ATOM   4304  HB1 ASP X 282     -30.645 -14.615 -41.262  1.00  0.00
H
ATOM   4305  HB2 ASP X 282     -30.875 -13.674 -39.917  1.00  0.00
H
ATOM   4306  CG  ASP X 282     -30.388 -15.791 -39.482  1.00  0.00
C
ATOM   4307  OD1 ASP X 282     -31.580 -16.136 -39.210  1.00  0.00
O
ATOM   4308  OD2 ASP X 282     -29.446 -16.491 -39.133  1.00  0.00
O
ATOM   4309  C   ASP X 282     -28.785 -12.607 -40.966  1.00  0.00
C
ATOM   4310  O   ASP X 282     -28.559 -12.580 -42.153  1.00  0.00
O
ATOM   4311  N   LEU X 283     -29.046 -11.466 -40.277  1.00  0.00
N
ATOM   4312  HN  LEU X 283     -29.208 -11.636 -39.308  1.00  0.00
H
ATOM   4313  CA  LEU X 283     -29.322 -10.249 -41.044  1.00  0.00
C
ATOM   4314  HA  LEU X 283     -29.708 -10.455 -42.031  1.00  0.00
H
ATOM   4315  CB  LEU X 283     -30.502  -9.620 -40.328  1.00  0.00
C
ATOM   4316  HB1 LEU X 283     -30.737  -8.724 -40.941  1.00  0.00
H
ATOM   4317  HB2 LEU X 283     -30.288  -9.413 -39.258  1.00  0.00
H
ATOM   4318  CG  LEU X 283     -31.830 -10.383 -40.362  1.00  0.00
C
ATOM   4319  HG  LEU X 283     -31.777 -11.385 -39.885  1.00  0.00
H
ATOM   4320  CD1 LEU X 283     -32.835  -9.536 -39.540  1.00  0.00
C
ATOM   4321 HD11 LEU X 283     -32.964  -8.503 -39.926  1.00  0.00
H
ATOM   4322 HD12 LEU X 283     -32.391  -9.455 -38.525  1.00  0.00
H
ATOM   4323 HD13 LEU X 283     -33.770 -10.110 -39.362  1.00  0.00
H
ATOM   4324  CD2 LEU X 283     -32.419 -10.637 -41.732  1.00  0.00
C
ATOM   4325 HD21 LEU X 283     -32.592  -9.660 -42.233  1.00  0.00
H
```

ATOM 4326 HD22 LEU X 283 -33.397 -11.160 -41.661 1.00 0.00
H
ATOM 4327 HD23 LEU X 283 -31.732 -11.037 -42.507 1.00 0.00
H
ATOM 4328 C LEU X 283 -28.083 -9.342 -41.260 1.00 0.00
C
ATOM 4329 O LEU X 283 -27.092 -9.398 -40.569 1.00 0.00
O
ATOM 4330 N CYS X 284 -28.166 -8.448 -42.354 1.00 0.00
N
ATOM 4331 HN CYS X 284 -29.040 -8.348 -42.824 1.00 0.00
H
ATOM 4332 CA CYS X 284 -27.170 -7.491 -42.592 1.00 0.00
C
ATOM 4333 HA CYS X 284 -26.411 -7.699 -41.852 1.00 0.00
H
ATOM 4334 CB CYS X 284 -26.636 -7.899 -44.017 1.00 0.00
C
ATOM 4335 HB1 CYS X 284 -27.431 -7.789 -44.785 1.00 0.00
H
ATOM 4336 HB2 CYS X 284 -26.281 -8.948 -43.922 1.00 0.00
H
ATOM 4337 SG CYS X 284 -25.352 -6.787 -44.620 1.00 0.00
S
ATOM 4338 HG1 CYS X 284 -26.198 -6.008 -45.279 1.00 0.00
H
ATOM 4339 C CYS X 284 -27.701 -6.092 -42.569 1.00 0.00
C
ATOM 4340 O CYS X 284 -28.676 -5.777 -43.213 1.00 0.00
O
ATOM 4341 N PHE X 285 -27.028 -5.188 -41.757 1.00 0.00
N
ATOM 4342 HN PHE X 285 -26.197 -5.533 -41.328 1.00 0.00
H
ATOM 4343 CA PHE X 285 -27.561 -3.836 -41.482 1.00 0.00
C
ATOM 4344 HA PHE X 285 -28.527 -3.801 -41.964 1.00 0.00
H
ATOM 4345 CB PHE X 285 -27.658 -3.674 -39.919 1.00 0.00
C
ATOM 4346 HB1 PHE X 285 -27.950 -2.623 -39.709 1.00 0.00
H
ATOM 4347 HB2 PHE X 285 -26.714 -3.959 -39.407 1.00 0.00
H
ATOM 4348 CG PHE X 285 -28.706 -4.580 -39.318 1.00 0.00
C
ATOM 4349 CD1 PHE X 285 -30.029 -4.329 -39.543 1.00 0.00
C
ATOM 4350 HD1 PHE X 285 -30.298 -3.441 -40.096 1.00 0.00
H

ATOM 4351 CE1 PHE X 285 −30.996 −5.251 −39.051 1.00 0.00
C
ATOM 4352 HE1 PHE X 285 −32.036 −4.994 −39.186 1.00 0.00
H
ATOM 4353 CZ PHE X 285 −30.553 −6.451 −38.432 1.00 0.00
C
ATOM 4354 HZ PHE X 285 −31.231 −7.049 −37.841 1.00 0.00
H
ATOM 4355 CD2 PHE X 285 −28.276 −5.811 −38.737 1.00 0.00
C
ATOM 4356 HD2 PHE X 285 −27.239 −6.107 −38.679 1.00 0.00
H
ATOM 4357 CE2 PHE X 285 −29.225 −6.725 −38.276 1.00 0.00
C
ATOM 4358 HE2 PHE X 285 −28.928 −7.673 −37.852 1.00 0.00
H
ATOM 4359 C PHE X 285 −26.630 −2.717 −41.894 1.00 0.00
C
ATOM 4360 O PHE X 285 −25.416 −2.680 −41.657 1.00 0.00
O
ATOM 4361 N THR X 286 −27.129 −1.756 −42.655 1.00 0.00
N
ATOM 4362 HN THR X 286 −28.114 −1.603 −42.636 1.00 0.00
H
ATOM 4363 CA THR X 286 −26.324 −0.825 −43.346 1.00 0.00
C
ATOM 4364 HA THR X 286 −25.690 −1.330 −44.060 1.00 0.00
H
ATOM 4365 CB THR X 286 −27.087 0.164 −44.194 1.00 0.00
C
ATOM 4366 HB THR X 286 −27.804 0.591 −43.461 1.00 0.00
H
ATOM 4367 OG1 THR X 286 −27.950 −0.466 −45.219 1.00 0.00
O
ATOM 4368 HG1 THR X 286 −27.466 −1.109 −45.742 1.00 0.00
H
ATOM 4369 CG2 THR X 286 −26.281 1.141 −44.844 1.00 0.00
C
ATOM 4370 HG21 THR X 286 −26.905 1.750 −45.533 1.00 0.00
H
ATOM 4371 HG22 THR X 286 −25.414 0.626 −45.311 1.00 0.00
H
ATOM 4372 HG23 THR X 286 −25.818 1.773 −44.056 1.00 0.00
H
ATOM 4373 C THR X 286 −25.330 −0.062 −42.468 1.00 0.00
C
ATOM 4374 O THR X 286 −24.174 0.058 −42.778 1.00 0.00
O
ATOM 4375 N ASN X 287 −25.746 0.429 −41.349 1.00 0.00
N

```
ATOM   4376  HN  ASN X 287     -26.732   0.396 -41.205  1.00  0.00
H
ATOM   4377  CA  ASN X 287     -24.860   0.968 -40.329  1.00  0.00
C
ATOM   4378  HA  ASN X 287     -23.869   0.543 -40.271  1.00  0.00
H
ATOM   4379  CB  ASN X 287     -24.609   2.545 -40.454  1.00  0.00
C
ATOM   4380  HB1 ASN X 287     -23.868   2.901 -39.707  1.00  0.00
H
ATOM   4381  HB2 ASN X 287     -25.501   3.164 -40.221  1.00  0.00
H
ATOM   4382  CG  ASN X 287     -23.935   2.997 -41.765  1.00  0.00
C
ATOM   4383  OD1 ASN X 287     -22.807   2.791 -42.199  1.00  0.00
O
ATOM   4384  ND2 ASN X 287     -24.822   3.774 -42.546  1.00  0.00
N
ATOM   4385 HD21 ASN X 287     -24.640   4.119 -43.467  1.00  0.00
H
ATOM   4386 HD22 ASN X 287     -25.644   4.167 -42.134  1.00  0.00
H
ATOM   4387  C   ASN X 287     -25.482   0.606 -39.002  1.00  0.00
C
ATOM   4388  O   ASN X 287     -26.663   0.881 -38.652  1.00  0.00
O
ATOM   4389  N   VAL X 288     -24.607   0.177 -38.069  1.00  0.00
N
ATOM   4390  HN  VAL X 288     -23.647   0.026 -38.292  1.00  0.00
H
ATOM   4391  CA  VAL X 288     -25.108  -0.096 -36.719  1.00  0.00
C
ATOM   4392  HA  VAL X 288     -26.139   0.195 -36.581  1.00  0.00
H
ATOM   4393  CB  VAL X 288     -25.014  -1.621 -36.317  1.00  0.00
C
ATOM   4394  HB  VAL X 288     -24.015  -2.033 -36.575  1.00  0.00
H
ATOM   4395  CG1 VAL X 288     -25.397  -1.877 -34.897  1.00  0.00
C
ATOM   4396 HG11 VAL X 288     -26.397  -1.466 -34.646  1.00  0.00
H
ATOM   4397 HG12 VAL X 288     -24.649  -1.344 -34.271  1.00  0.00
H
ATOM   4398 HG13 VAL X 288     -25.363  -2.928 -34.538  1.00  0.00
H
ATOM   4399  CG2 VAL X 288     -26.032  -2.381 -37.140  1.00  0.00
C
ATOM   4400 HG21 VAL X 288     -27.021  -1.883 -37.046  1.00  0.00
H
```

```
ATOM   4401 HG22 VAL X 288     -26.157  -3.391 -36.696  1.00  0.00
H
ATOM   4402 HG23 VAL X 288     -25.763  -2.442 -38.217  1.00  0.00
H
ATOM   4403  C   VAL X 288     -24.419   0.822 -35.774  1.00  0.00
C
ATOM   4404  O   VAL X 288     -23.175   0.920 -35.835  1.00  0.00
O
ATOM   4405  N   TYR X 289     -25.112   1.545 -34.941  1.00  0.00
N
ATOM   4406  HN  TYR X 289     -26.098   1.402 -34.918  1.00  0.00
H
ATOM   4407  CA  TYR X 289     -24.692   2.482 -33.903  1.00  0.00
C
ATOM   4408  HA  TYR X 289     -23.649   2.695 -34.088  1.00  0.00
H
ATOM   4409  CB  TYR X 289     -25.608   3.754 -33.691  1.00  0.00
C
ATOM   4410  HB1 TYR X 289     -25.227   4.508 -32.969  1.00  0.00
H
ATOM   4411  HB2 TYR X 289     -26.604   3.367 -33.389  1.00  0.00
H
ATOM   4412  CG  TYR X 289     -25.811   4.402 -35.029  1.00  0.00
C
ATOM   4413  CD1 TYR X 289     -26.810   4.130 -35.958  1.00  0.00
C
ATOM   4414  HD1 TYR X 289     -27.417   3.255 -35.779  1.00  0.00
H
ATOM   4415  CE1 TYR X 289     -26.972   4.992 -37.040  1.00  0.00
C
ATOM   4416  HE1 TYR X 289     -27.788   4.634 -37.650  1.00  0.00
H
ATOM   4417  CZ  TYR X 289     -26.136   6.094 -37.254  1.00  0.00
C
ATOM   4418  OH  TYR X 289     -26.333   7.040 -38.419  1.00  0.00
O
ATOM   4419  HH  TYR X 289     -27.203   6.940 -38.812  1.00  0.00
H
ATOM   4420  CD2 TYR X 289     -25.000   5.548 -35.261  1.00  0.00
C
ATOM   4421  HD2 TYR X 289     -24.295   5.789 -34.479  1.00  0.00
H
ATOM   4422  CE2 TYR X 289     -25.096   6.291 -36.389  1.00  0.00
C
ATOM   4423  HE2 TYR X 289     -24.435   7.131 -36.543  1.00  0.00
H
ATOM   4424  C   TYR X 289     -24.735   1.801 -32.599  1.00  0.00
C
ATOM   4425  O   TYR X 289     -25.831   1.351 -32.209  1.00  0.00
O
```

ATOM 4426 N ALA X 290 -23.501 1.702 -31.954 1.00 0.00
N
ATOM 4427 HN ALA X 290 -22.620 2.065 -32.247 1.00 0.00
H
ATOM 4428 CA ALA X 290 -23.446 1.074 -30.663 1.00 0.00
C
ATOM 4429 HA ALA X 290 -24.426 0.766 -30.328 1.00 0.00
H
ATOM 4430 CB ALA X 290 -22.477 -0.089 -30.570 1.00 0.00
C
ATOM 4431 HB1 ALA X 290 -22.713 -0.619 -29.622 1.00 0.00
H
ATOM 4432 HB2 ALA X 290 -21.468 0.376 -30.582 1.00 0.00
H
ATOM 4433 HB3 ALA X 290 -22.605 -0.708 -31.483 1.00 0.00
H
ATOM 4434 C ALA X 290 -23.070 2.199 -29.600 1.00 0.00
C
ATOM 4435 O ALA X 290 -22.106 2.881 -29.780 1.00 0.00
O
ATOM 4436 N ASP X 291 -24.035 2.438 -28.689 1.00 0.00
N
ATOM 4437 HN ASP X 291 -24.794 1.792 -28.696 1.00 0.00
H
ATOM 4438 CA ASP X 291 -24.017 3.520 -27.766 1.00 0.00
C
ATOM 4439 HA ASP X 291 -23.302 4.209 -28.190 1.00 0.00
H
ATOM 4440 CB ASP X 291 -25.245 4.457 -27.902 1.00 0.00
C
ATOM 4441 HB1 ASP X 291 -25.034 5.411 -27.372 1.00 0.00
H
ATOM 4442 HB2 ASP X 291 -26.181 4.090 -27.430 1.00 0.00
H
ATOM 4443 CG ASP X 291 -25.669 4.987 -29.331 1.00 0.00
C
ATOM 4444 OD1 ASP X 291 -25.086 4.680 -30.365 1.00 0.00
O
ATOM 4445 OD2 ASP X 291 -26.742 5.612 -29.507 1.00 0.00
O
ATOM 4446 C ASP X 291 -23.769 3.012 -26.356 1.00 0.00
C
ATOM 4447 O ASP X 291 -24.549 2.228 -25.876 1.00 0.00
O
ATOM 4448 N SER X 292 -22.642 3.339 -25.661 1.00 0.00
N
ATOM 4449 HN SER X 292 -21.979 3.969 -26.057 1.00 0.00
H
ATOM 4450 CA SER X 292 -22.278 2.643 -24.359 1.00 0.00
C

ATOM 4451 HA SER X 292 −23.132 2.068 −24.030 1.00 0.00
H
ATOM 4452 CB SER X 292 −20.974 1.726 −24.447 1.00 0.00
C
ATOM 4453 HB1 SER X 292 −21.185 0.756 −24.946 1.00 0.00
H
ATOM 4454 HB2 SER X 292 −20.876 1.545 −23.355 1.00 0.00
H
ATOM 4455 OG SER X 292 −19.723 2.313 −24.879 1.00 0.00
O
ATOM 4456 HG1 SER X 292 −19.747 2.422 −25.832 1.00 0.00
H
ATOM 4457 C SER X 292 −22.201 3.571 −23.121 1.00 0.00
C
ATOM 4458 O SER X 292 −21.574 4.613 −23.155 1.00 0.00
O
ATOM 4459 N PHE X 293 −22.943 3.170 −22.000 1.00 0.00
N
ATOM 4460 HN PHE X 293 −23.526 2.364 −22.071 1.00 0.00
H
ATOM 4461 CA PHE X 293 −22.975 3.996 −20.774 1.00 0.00
C
ATOM 4462 HA PHE X 293 −21.947 4.314 −20.681 1.00 0.00
H
ATOM 4463 CB PHE X 293 −23.921 5.293 −20.984 1.00 0.00
C
ATOM 4464 HB1 PHE X 293 −23.441 5.917 −21.768 1.00 0.00
H
ATOM 4465 HB2 PHE X 293 −23.971 5.781 −19.988 1.00 0.00
H
ATOM 4466 CG PHE X 293 −25.293 4.963 −21.372 1.00 0.00
C
ATOM 4467 CD1 PHE X 293 −26.330 4.583 −20.395 1.00 0.00
C
ATOM 4468 HD1 PHE X 293 −26.040 4.499 −19.358 1.00 0.00
H
ATOM 4469 CE1 PHE X 293 −27.651 4.460 −20.841 1.00 0.00
C
ATOM 4470 HE1 PHE X 293 −28.492 4.227 −20.205 1.00 0.00
H
ATOM 4471 CZ PHE X 293 −27.893 4.586 −22.193 1.00 0.00
C
ATOM 4472 HZ PHE X 293 −28.910 4.604 −22.554 1.00 0.00
H
ATOM 4473 CD2 PHE X 293 −25.618 4.981 −22.733 1.00 0.00
C
ATOM 4474 HD2 PHE X 293 −24.889 5.111 −23.519 1.00 0.00
H
ATOM 4475 CE2 PHE X 293 −26.946 4.879 −23.130 1.00 0.00
C

ATOM 4476 HE2 PHE X 293 -27.201 4.931 -24.178 1.00 0.00
H
ATOM 4477 C PHE X 293 -23.157 3.178 -19.455 1.00 0.00
C
ATOM 4478 O PHE X 293 -23.195 1.973 -19.458 1.00 0.00
O
ATOM 4479 N VAL X 294 -23.306 3.927 -18.357 1.00 0.00
N
ATOM 4480 HN VAL X 294 -23.204 4.909 -18.495 1.00 0.00
H
ATOM 4481 CA VAL X 294 -23.452 3.481 -17.013 1.00 0.00
C
ATOM 4482 HA VAL X 294 -23.609 2.419 -17.125 1.00 0.00
H
ATOM 4483 CB VAL X 294 -22.234 3.689 -16.093 1.00 0.00
C
ATOM 4484 HB VAL X 294 -21.801 4.674 -16.371 1.00 0.00
H
ATOM 4485 CG1 VAL X 294 -22.339 3.458 -14.613 1.00 0.00
C
ATOM 4486 HG11 VAL X 294 -21.317 3.388 -14.185 1.00 0.00
H
ATOM 4487 HG12 VAL X 294 -22.979 2.580 -14.382 1.00 0.00
H
ATOM 4488 HG13 VAL X 294 -22.818 4.367 -14.190 1.00 0.00
H
ATOM 4489 CG2 VAL X 294 -21.150 2.611 -16.594 1.00 0.00
C
ATOM 4490 HG21 VAL X 294 -21.604 1.603 -16.484 1.00 0.00
H
ATOM 4491 HG22 VAL X 294 -20.212 2.755 -16.016 1.00 0.00
H
ATOM 4492 HG23 VAL X 294 -20.906 2.789 -17.663 1.00 0.00
H
ATOM 4493 C VAL X 294 -24.632 4.219 -16.339 1.00 0.00
C
ATOM 4494 O VAL X 294 -24.830 5.368 -16.493 1.00 0.00
O
ATOM 4495 N ILE X 295 -25.506 3.436 -15.607 1.00 0.00
N
ATOM 4496 HN ILE X 295 -25.485 2.448 -15.475 1.00 0.00
H
ATOM 4497 CA ILE X 295 -26.557 4.084 -14.825 1.00 0.00
C
ATOM 4498 HA ILE X 295 -26.382 5.078 -14.441 1.00 0.00
H
ATOM 4499 CB ILE X 295 -27.915 4.200 -15.520 1.00 0.00
C
ATOM 4500 HB ILE X 295 -28.655 4.533 -14.761 1.00 0.00
H

ATOM 4501 CG2 ILE X 295 -27.838 5.353 -16.562 1.00 0.00
C
ATOM 4502 HG21 ILE X 295 -27.566 5.002 -17.580 1.00 0.00
H
ATOM 4503 HG22 ILE X 295 -27.121 6.162 -16.304 1.00 0.00
H
ATOM 4504 HG23 ILE X 295 -28.829 5.853 -16.622 1.00 0.00
H
ATOM 4505 CG1 ILE X 295 -28.379 2.791 -16.056 1.00 0.00
C
ATOM 4506 HG11 ILE X 295 -28.824 2.191 -15.234 1.00 0.00
H
ATOM 4507 HG12 ILE X 295 -27.565 2.184 -16.508 1.00 0.00
H
ATOM 4508 CD ILE X 295 -29.472 2.893 -17.117 1.00 0.00
C
ATOM 4509 HD1 ILE X 295 -29.135 3.346 -18.074 1.00 0.00
H
ATOM 4510 HD2 ILE X 295 -30.368 3.407 -16.708 1.00 0.00
H
ATOM 4511 HD3 ILE X 295 -29.726 1.838 -17.355 1.00 0.00
H
ATOM 4512 C ILE X 295 -26.690 3.163 -13.596 1.00 0.00
C
ATOM 4513 O ILE X 295 -26.194 2.049 -13.598 1.00 0.00
O
ATOM 4514 N ARG X 296 -27.437 3.629 -12.572 1.00 0.00
N
ATOM 4515 HN ARG X 296 -27.907 4.508 -12.581 1.00 0.00
H
ATOM 4516 CA ARG X 296 -27.810 2.778 -11.414 1.00 0.00
C
ATOM 4517 HA ARG X 296 -27.021 2.069 -11.207 1.00 0.00
H
ATOM 4518 CB ARG X 296 -27.996 3.627 -10.146 1.00 0.00
C
ATOM 4519 HB1 ARG X 296 -27.214 4.395 -9.965 1.00 0.00
H
ATOM 4520 HB2 ARG X 296 -27.889 2.973 -9.254 1.00 0.00
H
ATOM 4521 CG ARG X 296 -29.410 4.366 -10.129 1.00 0.00
C
ATOM 4522 HG1 ARG X 296 -30.213 3.603 -10.037 1.00 0.00
H
ATOM 4523 HG2 ARG X 296 -29.567 4.912 -11.084 1.00 0.00
H
ATOM 4524 CD ARG X 296 -29.475 5.121 -8.776 1.00 0.00
C
ATOM 4525 HD1 ARG X 296 -28.804 6.002 -8.865 1.00 0.00
H

```
ATOM   4526  HD2 ARG X 296     -29.131   4.412  -7.993  1.00  0.00
H
ATOM   4527  NE  ARG X 296     -30.924   5.539  -8.510  1.00  0.00
N
ATOM   4528  HE  ARG X 296     -31.156   6.368  -9.019  1.00  0.00
H
ATOM   4529  CZ  ARG X 296     -31.891   4.734  -8.179  1.00  0.00
C
ATOM   4530  NH1 ARG X 296     -31.729   3.464  -7.869  1.00  0.00
N
ATOM   4531 HH11 ARG X 296     -30.848   3.046  -8.090  1.00  0.00
H
ATOM   4532 HH12 ARG X 296     -32.541   2.941  -7.611  1.00  0.00
H
ATOM   4533  NH2 ARG X 296     -33.158   5.195  -8.227  1.00  0.00
N
ATOM   4534 HH21 ARG X 296     -33.300   6.082  -8.666  1.00  0.00
H
ATOM   4535 HH22 ARG X 296     -33.973   4.624  -8.125  1.00  0.00
H
ATOM   4536  C   ARG X 296     -28.952   1.841 -11.742  1.00  0.00
C
ATOM   4537  O   ARG X 296     -29.823   2.103 -12.621  1.00  0.00
O
ATOM   4538  N   GLY X 297     -29.005   0.696 -11.034  1.00  0.00
N
ATOM   4539  HN  GLY X 297     -28.256   0.430 -10.431  1.00  0.00
H
ATOM   4540  CA  GLY X 297     -29.697  -0.521 -11.487  1.00  0.00
C
ATOM   4541  HA1 GLY X 297     -29.657  -1.252 -10.693  1.00  0.00
H
ATOM   4542  HA2 GLY X 297     -29.195  -0.964 -12.334  1.00  0.00
H
ATOM   4543  C   GLY X 297     -31.191  -0.298 -11.786  1.00  0.00
C
ATOM   4544  O   GLY X 297     -31.753  -0.894 -12.725  1.00  0.00
O
ATOM   4545  N   ASP X 298     -31.805   0.611 -11.011  1.00  0.00
N
ATOM   4546  HN  ASP X 298     -31.322   0.995 -10.229  1.00  0.00
H
ATOM   4547  CA  ASP X 298     -33.169   0.953 -11.098  1.00  0.00
C
ATOM   4548  HA  ASP X 298     -33.770   0.058 -11.166  1.00  0.00
H
ATOM   4549  CB  ASP X 298     -33.719   1.757 -10.015  1.00  0.00
C
ATOM   4550  HB1 ASP X 298     -34.814   1.929 -10.089  1.00  0.00
H
```

```
ATOM   4551  HB2 ASP X 298     -33.152   2.703  -9.890  1.00  0.00
H
ATOM   4552  CG  ASP X 298     -33.907   0.927  -8.695  1.00  0.00
C
ATOM   4553  OD1 ASP X 298     -33.920   1.642  -7.664  1.00  0.00
O
ATOM   4554  OD2 ASP X 298     -34.093  -0.326  -8.673  1.00  0.00
O
ATOM   4555  C   ASP X 298     -33.505   1.683 -12.413  1.00  0.00
C
ATOM   4556  O   ASP X 298     -34.674   1.621 -12.834  1.00  0.00
O
ATOM   4557  N   GLU X 299     -32.509   2.362 -13.047  1.00  0.00
N
ATOM   4558  HN  GLU X 299     -31.563   2.313 -12.735  1.00  0.00
H
ATOM   4559  CA  GLU X 299     -32.801   3.161 -14.230  1.00  0.00
C
ATOM   4560  HA  GLU X 299     -33.828   3.491 -14.194  1.00  0.00
H
ATOM   4561  CB  GLU X 299     -31.871   4.442 -14.146  1.00  0.00
C
ATOM   4562  HB1 GLU X 299     -31.774   4.910 -15.149  1.00  0.00
H
ATOM   4563  HB2 GLU X 299     -30.867   4.005 -13.959  1.00  0.00
H
ATOM   4564  CG  GLU X 299     -32.201   5.363 -12.915  1.00  0.00
C
ATOM   4565  HG1 GLU X 299     -32.132   4.990 -11.871  1.00  0.00
H
ATOM   4566  HG2 GLU X 299     -33.276   5.644 -12.949  1.00  0.00
H
ATOM   4567  CD  GLU X 299     -31.552   6.728 -12.710  1.00  0.00
C
ATOM   4568  OE1 GLU X 299     -30.272   6.758 -12.643  1.00  0.00
O
ATOM   4569  OE2 GLU X 299     -32.268   7.724 -12.789  1.00  0.00
O
ATOM   4570  C   GLU X 299     -32.680   2.374 -15.527  1.00  0.00
C
ATOM   4571  O   GLU X 299     -32.909   2.927 -16.600  1.00  0.00
O
ATOM   4572  N   VAL X 300     -32.449   1.066 -15.410  1.00  0.00
N
ATOM   4573  HN  VAL X 300     -32.192   0.734 -14.505  1.00  0.00
H
ATOM   4574  CA  VAL X 300     -32.403   0.068 -16.502  1.00  0.00
C
ATOM   4575  HA  VAL X 300     -31.657   0.438 -17.190  1.00  0.00
H
```

ATOM 4576 CB VAL X 300 -32.148 -1.300 -15.989 1.00 0.00
C
ATOM 4577 HB VAL X 300 -32.943 -1.354 -15.214 1.00 0.00
H
ATOM 4578 CG1 VAL X 300 -32.370 -2.368 -17.028 1.00 0.00
C
ATOM 4579 HG11 VAL X 300 -33.475 -2.349 -17.148 1.00 0.00
H
ATOM 4580 HG12 VAL X 300 -32.141 -3.417 -16.743 1.00 0.00
H
ATOM 4581 HG13 VAL X 300 -31.890 -2.130 -18.001 1.00 0.00
H
ATOM 4582 CG2 VAL X 300 -30.699 -1.363 -15.463 1.00 0.00
C
ATOM 4583 HG21 VAL X 300 -30.428 -2.355 -15.042 1.00 0.00
H
ATOM 4584 HG22 VAL X 300 -30.494 -0.577 -14.705 1.00 0.00
H
ATOM 4585 HG23 VAL X 300 -29.986 -1.308 -16.312 1.00 0.00
H
ATOM 4586 C VAL X 300 -33.747 -0.004 -17.215 1.00 0.00
C
ATOM 4587 O VAL X 300 -33.830 0.136 -18.416 1.00 0.00
O
ATOM 4588 N ARG X 301 -34.795 -0.120 -16.372 1.00 0.00
N
ATOM 4589 HN ARG X 301 -34.608 -0.061 -15.394 1.00 0.00
H
ATOM 4590 CA ARG X 301 -36.142 -0.125 -16.798 1.00 0.00
C
ATOM 4591 HA ARG X 301 -36.305 -1.035 -17.356 1.00 0.00
H
ATOM 4592 CB ARG X 301 -37.048 -0.334 -15.568 1.00 0.00
C
ATOM 4593 HB1 ARG X 301 -38.120 -0.130 -15.780 1.00 0.00
H
ATOM 4594 HB2 ARG X 301 -36.836 0.308 -14.687 1.00 0.00
H
ATOM 4595 CG ARG X 301 -36.992 -1.674 -14.923 1.00 0.00
C
ATOM 4596 HG1 ARG X 301 -37.803 -1.862 -14.188 1.00 0.00
H
ATOM 4597 HG2 ARG X 301 -36.110 -1.941 -14.303 1.00 0.00
H
ATOM 4598 CD ARG X 301 -37.066 -2.872 -15.831 1.00 0.00
C
ATOM 4599 HD1 ARG X 301 -36.095 -3.095 -16.322 1.00 0.00
H
ATOM 4600 HD2 ARG X 301 -37.683 -2.710 -16.740 1.00 0.00
H

```
ATOM   4601  NE  ARG X 301     -37.531  -4.077 -15.072  1.00  0.00
N
ATOM   4602  HE  ARG X 301     -36.769  -4.616 -14.714  1.00  0.00
H
ATOM   4603  CZ  ARG X 301     -38.776  -4.463 -14.722  1.00  0.00
C
ATOM   4604  NH1 ARG X 301     -39.850  -3.752 -15.005  1.00  0.00
N
ATOM   4605 HH11 ARG X 301     -39.808  -2.756 -15.077  1.00  0.00
H
ATOM   4606 HH12 ARG X 301     -40.708  -4.081 -14.611  1.00  0.00
H
ATOM   4607  NH2 ARG X 301     -38.968  -5.519 -14.025  1.00  0.00
N
ATOM   4608 HH21 ARG X 301     -38.310  -6.250 -13.848  1.00  0.00
H
ATOM   4609 HH22 ARG X 301     -39.903  -5.728 -13.736  1.00  0.00
H
ATOM   4610  C   ARG X 301     -36.695   0.990 -17.621  1.00  0.00
C
ATOM   4611  O   ARG X 301     -37.579   0.751 -18.386  1.00  0.00
O
ATOM   4612  N   GLN X 302     -36.150   2.244 -17.484  1.00  0.00
N
ATOM   4613  HN  GLN X 302     -35.327   2.427 -16.952  1.00  0.00
H
ATOM   4614  CA  GLN X 302     -36.473   3.457 -18.267  1.00  0.00
C
ATOM   4615  HA  GLN X 302     -37.457   3.234 -18.653  1.00  0.00
H
ATOM   4616  CB  GLN X 302     -36.534   4.645 -17.304  1.00  0.00
C
ATOM   4617  HB1 GLN X 302     -37.448   4.638 -16.672  1.00  0.00
H
ATOM   4618  HB2 GLN X 302     -36.623   5.516 -17.988  1.00  0.00
H
ATOM   4619  CG  GLN X 302     -35.408   4.814 -16.356  1.00  0.00
C
ATOM   4620  HG1 GLN X 302     -34.530   4.941 -17.025  1.00  0.00
H
ATOM   4621  HG2 GLN X 302     -35.167   3.911 -15.756  1.00  0.00
H
ATOM   4622  CD  GLN X 302     -35.527   6.027 -15.441  1.00  0.00
C
ATOM   4623  OE1 GLN X 302     -35.209   7.156 -15.740  1.00  0.00
O
ATOM   4624  NE2 GLN X 302     -36.102   5.776 -14.248  1.00  0.00
N
ATOM   4625 HE21 GLN X 302     -36.537   4.876 -14.230  1.00  0.00
H
```

ATOM 4626 HE22 GLN X 302 −36.556 6.547 −13.801 1.00 0.00
H
ATOM 4627 C GLN X 302 −35.638 3.684 −19.576 1.00 0.00
C
ATOM 4628 O GLN X 302 −35.768 4.619 −20.317 1.00 0.00
O
ATOM 4629 N ILE X 303 −34.708 2.728 −19.872 1.00 0.00
N
ATOM 4630 HN ILE X 303 −34.471 2.032 −19.199 1.00 0.00
H
ATOM 4631 CA ILE X 303 −33.985 2.731 −21.110 1.00 0.00
C
ATOM 4632 HA ILE X 303 −33.771 3.750 −21.396 1.00 0.00
H
ATOM 4633 CB ILE X 303 −32.530 2.234 −20.971 1.00 0.00
C
ATOM 4634 HB ILE X 303 −32.508 1.127 −20.888 1.00 0.00
H
ATOM 4635 CG2 ILE X 303 −31.772 2.526 −22.261 1.00 0.00
C
ATOM 4636 HG21 ILE X 303 −31.901 3.613 −22.451 1.00 0.00
H
ATOM 4637 HG22 ILE X 303 −32.083 1.893 −23.118 1.00 0.00
H
ATOM 4638 HG23 ILE X 303 −30.671 2.423 −22.157 1.00 0.00
H
ATOM 4639 CG1 ILE X 303 −31.651 2.727 −19.838 1.00 0.00
C
ATOM 4640 HG11 ILE X 303 −30.646 2.258 −19.908 1.00 0.00
H
ATOM 4641 HG12 ILE X 303 −32.054 2.411 −18.852 1.00 0.00
H
ATOM 4642 CD ILE X 303 −31.575 4.248 −19.769 1.00 0.00
C
ATOM 4643 HD1 ILE X 303 −30.812 4.693 −20.443 1.00 0.00
H
ATOM 4644 HD2 ILE X 303 −31.438 4.576 −18.716 1.00 0.00
H
ATOM 4645 HD3 ILE X 303 −32.535 4.666 −20.140 1.00 0.00
H
ATOM 4646 C ILE X 303 −34.646 2.078 −22.290 1.00 0.00
C
ATOM 4647 O ILE X 303 −34.346 0.903 −22.555 1.00 0.00
O
ATOM 4648 N ALA X 304 −35.584 2.718 −22.997 1.00 0.00
N
ATOM 4649 HN ALA X 304 −35.819 3.662 −22.776 1.00 0.00
H
ATOM 4650 CA ALA X 304 −36.412 2.197 −24.016 1.00 0.00
C

ATOM 4651 HA ALA X 304 −35.812 1.538 −24.627 1.00 0.00
H
ATOM 4652 CB ALA X 304 −37.526 1.267 −23.308 1.00 0.00
C
ATOM 4653 HB1 ALA X 304 −38.262 1.817 −22.684 1.00 0.00
H
ATOM 4654 HB2 ALA X 304 −36.993 0.595 −22.602 1.00 0.00
H
ATOM 4655 HB3 ALA X 304 −38.037 0.694 −24.111 1.00 0.00
H
ATOM 4656 C ALA X 304 −36.990 3.402 −24.781 1.00 0.00
C
ATOM 4657 O ALA X 304 −36.844 4.501 −24.215 1.00 0.00
O
ATOM 4658 N PRO X 305 −37.507 3.316 −25.915 1.00 0.00
N
ATOM 4659 CD PRO X 305 −37.305 2.172 −26.817 1.00 0.00
C
ATOM 4660 HD1 PRO X 305 −37.901 1.288 −26.502 1.00 0.00
H
ATOM 4661 HD2 PRO X 305 −36.273 1.774 −26.925 1.00 0.00
H
ATOM 4662 CA PRO X 305 −38.414 4.283 −26.592 1.00 0.00
C
ATOM 4663 HA PRO X 305 −37.779 5.143 −26.747 1.00 0.00
H
ATOM 4664 CB PRO X 305 −38.892 3.524 −27.840 1.00 0.00
C
ATOM 4665 HB1 PRO X 305 −39.177 4.195 −28.679 1.00 0.00
H
ATOM 4666 HB2 PRO X 305 −39.810 2.944 −27.604 1.00 0.00
H
ATOM 4667 CG PRO X 305 −37.625 2.731 −28.172 1.00 0.00
C
ATOM 4668 HG1 PRO X 305 −37.877 1.925 −28.894 1.00 0.00
H
ATOM 4669 HG2 PRO X 305 −36.834 3.407 −28.561 1.00 0.00
H
ATOM 4670 C PRO X 305 −39.626 4.714 −25.755 1.00 0.00
C
ATOM 4671 O PRO X 305 −40.098 3.900 −24.988 1.00 0.00
O
ATOM 4672 N GLY X 306 −39.989 5.963 −25.823 1.00 0.00
N
ATOM 4673 HN GLY X 306 −39.488 6.543 −26.461 1.00 0.00
H
ATOM 4674 CA GLY X 306 −41.213 6.429 −25.183 1.00 0.00
C
ATOM 4675 HA1 GLY X 306 −42.023 5.741 −25.372 1.00 0.00
H

ATOM	4676	HA2	GLY	X	306	−41.460	7.403	−25.579	1.00	0.00	H
ATOM	4677	C	GLY	X	306	−41.267	6.621	−23.705	1.00	0.00	C
ATOM	4678	O	GLY	X	306	−42.304	6.449	−23.070	1.00	0.00	O
ATOM	4679	N	GLN	X	307	−40.095	6.937	−23.144	1.00	0.00	N
ATOM	4680	HN	GLN	X	307	−39.422	7.353	−23.751	1.00	0.00	H
ATOM	4681	CA	GLN	X	307	−39.909	7.011	−21.749	1.00	0.00	C
ATOM	4682	HA	GLN	X	307	−40.692	6.533	−21.178	1.00	0.00	H
ATOM	4683	CB	GLN	X	307	−38.615	6.118	−21.467	1.00	0.00	C
ATOM	4684	HB1	GLN	X	307	−38.203	6.249	−20.443	1.00	0.00	H
ATOM	4685	HB2	GLN	X	307	−37.857	6.728	−22.002	1.00	0.00	H
ATOM	4686	CG	GLN	X	307	−38.793	4.600	−21.844	1.00	0.00	C
ATOM	4687	HG1	GLN	X	307	−37.886	4.007	−21.599	1.00	0.00	H
ATOM	4688	HG2	GLN	X	307	−38.769	4.434	−22.943	1.00	0.00	H
ATOM	4689	CD	GLN	X	307	−40.104	3.938	−21.344	1.00	0.00	C
ATOM	4690	OE1	GLN	X	307	−40.224	3.791	−20.099	1.00	0.00	O
ATOM	4691	NE2	GLN	X	307	−41.025	3.602	−22.272	1.00	0.00	N
ATOM	4692	HE21	GLN	X	307	−40.818	3.772	−23.236	1.00	0.00	H
ATOM	4693	HE22	GLN	X	307	−41.791	2.998	−22.053	1.00	0.00	H
ATOM	4694	C	GLN	X	307	−39.883	8.466	−21.091	1.00	0.00	C
ATOM	4695	O	GLN	X	307	−39.609	9.403	−21.824	1.00	0.00	O
ATOM	4696	N	THR	X	308	−40.028	8.603	−19.808	1.00	0.00	N
ATOM	4697	HN	THR	X	308	−40.192	7.844	−19.183	1.00	0.00	H
ATOM	4698	CA	THR	X	308	−39.717	9.871	−19.154	1.00	0.00	C
ATOM	4699	HA	THR	X	308	−39.056	10.471	−19.761	1.00	0.00	H
ATOM	4700	CB	THR	X	308	−40.944	10.824	−18.845	1.00	0.00	C

```
ATOM   4701  HB  THR X 308     -41.596  10.935 -19.738  1.00  0.00
H
ATOM   4702  OG1 THR X 308     -40.514  12.140 -18.568  1.00  0.00
O
ATOM   4703  HG1 THR X 308     -40.762  12.629 -19.357  1.00  0.00
H
ATOM   4704  CG2 THR X 308     -41.849  10.295 -17.730  1.00  0.00
C
ATOM   4705 HG21 THR X 308     -42.739  10.932 -17.542  1.00  0.00
H
ATOM   4706 HG22 THR X 308     -41.334  10.166 -16.755  1.00  0.00
H
ATOM   4707 HG23 THR X 308     -42.149   9.276 -18.056  1.00  0.00
H
ATOM   4708  C   THR X 308     -38.781   9.665 -17.931  1.00  0.00
C
ATOM   4709  O   THR X 308     -38.474   8.520 -17.584  1.00  0.00
O
ATOM   4710  N   GLY X 309     -38.401  10.787 -17.335  1.00  0.00
N
ATOM   4711  HN  GLY X 309     -38.651  11.645 -17.777  1.00  0.00
H
ATOM   4712  CA  GLY X 309     -37.426  10.873 -16.180  1.00  0.00
C
ATOM   4713  HA1 GLY X 309     -37.173   9.830 -16.062  1.00  0.00
H
ATOM   4714  HA2 GLY X 309     -37.977  11.359 -15.388  1.00  0.00
H
ATOM   4715  C   GLY X 309     -36.162  11.589 -16.581  1.00  0.00
C
ATOM   4716  O   GLY X 309     -36.085  12.106 -17.690  1.00  0.00
O
ATOM   4717  N   LYS X 310     -35.301  11.666 -15.571  1.00  0.00
N
ATOM   4718  HN  LYS X 310     -35.366  11.142 -14.725  1.00  0.00
H
ATOM   4719  CA  LYS X 310     -34.079  12.487 -15.637  1.00  0.00
C
ATOM   4720  HA  LYS X 310     -34.351  13.511 -15.849  1.00  0.00
H
ATOM   4721  CB  LYS X 310     -33.353  12.542 -14.179  1.00  0.00
C
ATOM   4722  HB1 LYS X 310     -32.427  13.126 -14.365  1.00  0.00
H
ATOM   4723  HB2 LYS X 310     -33.016  11.520 -13.902  1.00  0.00
H
ATOM   4724  CG  LYS X 310     -34.187  13.287 -13.109  1.00  0.00
C
ATOM   4725  HG1 LYS X 310     -33.520  13.191 -12.226  1.00  0.00
H
```

```
ATOM   4726  HG2 LYS X 310     -35.129  12.776 -12.816  1.00  0.00
H
ATOM   4727  CD  LYS X 310     -34.363  14.796 -13.525  1.00  0.00
C
ATOM   4728  HD1 LYS X 310     -35.011  14.803 -14.428  1.00  0.00
H
ATOM   4729  HD2 LYS X 310     -33.349  15.034 -13.911  1.00  0.00
H
ATOM   4730  CE  LYS X 310     -34.883  15.728 -12.465  1.00  0.00
C
ATOM   4731  HE1 LYS X 310     -34.862  16.772 -12.844  1.00  0.00
H
ATOM   4732  HE2 LYS X 310     -34.298  15.696 -11.521  1.00  0.00
H
ATOM   4733  NZ  LYS X 310     -36.301  15.413 -12.103  1.00  0.00
N
ATOM   4734  HZ1 LYS X 310     -36.924  15.457 -12.935  1.00  0.00
H
ATOM   4735  HZ2 LYS X 310     -36.593  16.061 -11.344  1.00  0.00
H
ATOM   4736  HZ3 LYS X 310     -36.420  14.427 -11.794  1.00  0.00
H
ATOM   4737  C   LYS X 310     -33.143  12.034 -16.715  1.00  0.00
C
ATOM   4738  O   LYS X 310     -32.468  12.836 -17.391  1.00  0.00
O
ATOM   4739  N   ILE X 311     -33.007  10.740 -16.969  1.00  0.00
N
ATOM   4740  HN  ILE X 311     -33.413  10.035 -16.393  1.00  0.00
H
ATOM   4741  CA  ILE X 311     -32.159  10.185 -17.971  1.00  0.00
C
ATOM   4742  HA  ILE X 311     -31.163  10.596 -17.917  1.00  0.00
H
ATOM   4743  CB  ILE X 311     -31.894   8.675 -17.759  1.00  0.00
C
ATOM   4744  HB  ILE X 311     -32.795   8.027 -17.814  1.00  0.00
H
ATOM   4745  CG2 ILE X 311     -31.044   8.046 -18.912  1.00  0.00
C
ATOM   4746 HG21 ILE X 311     -31.456   8.273 -19.918  1.00  0.00
H
ATOM   4747 HG22 ILE X 311     -30.902   6.953 -18.770  1.00  0.00
H
ATOM   4748 HG23 ILE X 311     -30.018   8.471 -18.920  1.00  0.00
H
ATOM   4749  CG1 ILE X 311     -31.139   8.413 -16.468  1.00  0.00
C
ATOM   4750 HG11 ILE X 311     -30.997   7.332 -16.256  1.00  0.00
H
```

ATOM 4751 HG12 ILE X 311 -31.691 8.777 -15.575 1.00 0.00
H
ATOM 4752 CD ILE X 311 -29.875 9.312 -16.215 1.00 0.00
C
ATOM 4753 HD1 ILE X 311 -29.123 9.129 -17.012 1.00 0.00
H
ATOM 4754 HD2 ILE X 311 -29.394 9.154 -15.226 1.00 0.00
H
ATOM 4755 HD3 ILE X 311 -30.072 10.405 -16.193 1.00 0.00
H
ATOM 4756 C ILE X 311 -32.763 10.649 -19.368 1.00 0.00
C
ATOM 4757 O ILE X 311 -31.991 11.086 -20.234 1.00 0.00
O
ATOM 4758 N ALA X 312 -34.077 10.523 -19.650 1.00 0.00
N
ATOM 4759 HN ALA X 312 -34.633 10.110 -18.932 1.00 0.00
H
ATOM 4760 CA ALA X 312 -34.793 10.834 -20.894 1.00 0.00
C
ATOM 4761 HA ALA X 312 -34.286 10.319 -21.696 1.00 0.00
H
ATOM 4762 CB ALA X 312 -36.227 10.303 -20.817 1.00 0.00
C
ATOM 4763 HB1 ALA X 312 -36.853 10.571 -21.695 1.00 0.00
H
ATOM 4764 HB2 ALA X 312 -36.805 10.663 -19.939 1.00 0.00
H
ATOM 4765 HB3 ALA X 312 -36.209 9.195 -20.741 1.00 0.00
H
ATOM 4766 C ALA X 312 -34.879 12.299 -21.369 1.00 0.00
C
ATOM 4767 O ALA X 312 -34.647 12.657 -22.537 1.00 0.00
O
ATOM 4768 N ASP X 313 -35.169 13.185 -20.413 1.00 0.00
N
ATOM 4769 HN ASP X 313 -35.527 12.807 -19.563 1.00 0.00
H
ATOM 4770 CA ASP X 313 -35.245 14.643 -20.600 1.00 0.00
C
ATOM 4771 HA ASP X 313 -35.743 14.756 -21.551 1.00 0.00
H
ATOM 4772 CB ASP X 313 -36.192 15.348 -19.610 1.00 0.00
C
ATOM 4773 HB1 ASP X 313 -36.124 16.448 -19.749 1.00 0.00
H
ATOM 4774 HB2 ASP X 313 -35.995 14.994 -18.575 1.00 0.00
H
ATOM 4775 CG ASP X 313 -37.660 14.975 -20.001 1.00 0.00
C

ATOM 4776 OD1 ASP X 313 −38.042 14.255 −20.970 1.00 0.00
O
ATOM 4777 OD2 ASP X 313 −38.416 15.375 −19.100 1.00 0.00
O
ATOM 4778 C ASP X 313 −33.861 15.279 −20.708 1.00 0.00
C
ATOM 4779 O ASP X 313 −33.548 16.187 −21.471 1.00 0.00
O
ATOM 4780 N TYR X 314 −32.869 14.765 −19.906 1.00 0.00
N
ATOM 4781 HN TYR X 314 −33.060 14.109 −19.180 1.00 0.00
H
ATOM 4782 CA TYR X 314 −31.637 15.419 −19.601 1.00 0.00
C
ATOM 4783 HA TYR X 314 −31.569 16.242 −20.297 1.00 0.00
H
ATOM 4784 CB TYR X 314 −31.524 16.014 −18.162 1.00 0.00
C
ATOM 4785 HB1 TYR X 314 −30.695 16.752 −18.143 1.00 0.00
H
ATOM 4786 HB2 TYR X 314 −31.380 15.426 −17.230 1.00 0.00
H
ATOM 4787 CG TYR X 314 −32.654 17.020 −17.928 1.00 0.00
C
ATOM 4788 CD1 TYR X 314 −33.051 17.976 −18.900 1.00 0.00
C
ATOM 4789 HD1 TYR X 314 −32.394 17.988 −19.756 1.00 0.00
H
ATOM 4790 CE1 TYR X 314 −34.138 18.795 −18.769 1.00 0.00
C
ATOM 4791 HE1 TYR X 314 −34.441 19.451 −19.571 1.00 0.00
H
ATOM 4792 CZ TYR X 314 −34.966 18.680 −17.669 1.00 0.00
C
ATOM 4793 OH TYR X 314 −36.196 19.374 −17.443 1.00 0.00
O
ATOM 4794 HH TYR X 314 −36.414 19.759 −18.295 1.00 0.00
H
ATOM 4795 CD2 TYR X 314 −33.423 17.000 −16.759 1.00 0.00
C
ATOM 4796 HD2 TYR X 314 −33.316 16.214 −16.026 1.00 0.00
H
ATOM 4797 CE2 TYR X 314 −34.551 17.837 −16.589 1.00 0.00
C
ATOM 4798 HE2 TYR X 314 −35.224 17.609 −15.776 1.00 0.00
H
ATOM 4799 C TYR X 314 −30.400 14.638 −19.898 1.00 0.00
C
ATOM 4800 O TYR X 314 −29.305 15.182 −19.675 1.00 0.00
O

ATOM 4801 N ASN X 315 -30.420 13.337 -20.320 1.00 0.00
N
ATOM 4802 HN ASN X 315 -31.230 12.758 -20.377 1.00 0.00
H
ATOM 4803 CA ASN X 315 -29.077 12.719 -20.515 1.00 0.00
C
ATOM 4804 HA ASN X 315 -28.357 13.508 -20.679 1.00 0.00
H
ATOM 4805 CB ASN X 315 -28.747 11.744 -19.340 1.00 0.00
C
ATOM 4806 HB1 ASN X 315 -27.769 11.281 -19.589 1.00 0.00
H
ATOM 4807 HB2 ASN X 315 -29.453 10.890 -19.255 1.00 0.00
H
ATOM 4808 CG ASN X 315 -28.595 12.427 -18.020 1.00 0.00
C
ATOM 4809 OD1 ASN X 315 -27.431 12.581 -17.538 1.00 0.00
O
ATOM 4810 ND2 ASN X 315 -29.641 12.862 -17.441 1.00 0.00
N
ATOM 4811 HD21 ASN X 315 -29.575 13.550 -16.718 1.00 0.00
H
ATOM 4812 HD22 ASN X 315 -30.580 12.792 -17.777 1.00 0.00
H
ATOM 4813 C ASN X 315 -28.993 11.819 -21.819 1.00 0.00
C
ATOM 4814 O ASN X 315 -28.068 12.018 -22.615 1.00 0.00
O
ATOM 4815 N TYR X 316 -29.846 10.816 -22.039 1.00 0.00
N
ATOM 4816 HN TYR X 316 -30.555 10.630 -21.364 1.00 0.00
H
ATOM 4817 CA TYR X 316 -29.932 10.129 -23.304 1.00 0.00
C
ATOM 4818 HA TYR X 316 -29.494 10.614 -24.164 1.00 0.00
H
ATOM 4819 CB TYR X 316 -29.239 8.668 -23.157 1.00 0.00
C
ATOM 4820 HB1 TYR X 316 -29.646 8.119 -22.281 1.00 0.00
H
ATOM 4821 HB2 TYR X 316 -28.189 8.883 -22.864 1.00 0.00
H
ATOM 4822 CG TYR X 316 -29.159 7.852 -24.464 1.00 0.00
C
ATOM 4823 CD1 TYR X 316 -28.336 8.283 -25.514 1.00 0.00
C
ATOM 4824 HD1 TYR X 316 -27.747 9.185 -25.437 1.00 0.00
H
ATOM 4825 CE1 TYR X 316 -27.996 7.413 -26.552 1.00 0.00
C

ATOM 4826 HE1 TYR X 316 -27.279 7.552 -27.348 1.00 0.00
H
ATOM 4827 CZ TYR X 316 -28.633 6.191 -26.635 1.00 0.00
C
ATOM 4828 OH TYR X 316 -28.458 5.318 -27.715 1.00 0.00
O
ATOM 4829 HH TYR X 316 -27.858 5.659 -28.383 1.00 0.00
H
ATOM 4830 CD2 TYR X 316 -29.844 6.688 -24.596 1.00 0.00
C
ATOM 4831 HD2 TYR X 316 -30.569 6.533 -23.810 1.00 0.00
H
ATOM 4832 CE2 TYR X 316 -29.590 5.873 -25.650 1.00 0.00
C
ATOM 4833 HE2 TYR X 316 -30.187 5.004 -25.885 1.00 0.00
H
ATOM 4834 C TYR X 316 -31.439 9.974 -23.710 1.00 0.00
C
ATOM 4835 O TYR X 316 -32.250 9.494 -23.040 1.00 0.00
O
ATOM 4836 N LYS X 317 -31.753 10.212 -24.964 1.00 0.00
N
ATOM 4837 HN LYS X 317 -31.138 10.719 -25.563 1.00 0.00
H
ATOM 4838 CA LYS X 317 -33.075 10.014 -25.475 1.00 0.00
C
ATOM 4839 HA LYS X 317 -33.656 9.367 -24.834 1.00 0.00
H
ATOM 4840 CB LYS X 317 -33.789 11.404 -25.521 1.00 0.00
C
ATOM 4841 HB1 LYS X 317 -33.203 12.113 -26.145 1.00 0.00
H
ATOM 4842 HB2 LYS X 317 -33.709 11.899 -24.529 1.00 0.00
H
ATOM 4843 CG LYS X 317 -35.243 11.369 -25.899 1.00 0.00
C
ATOM 4844 HG1 LYS X 317 -35.216 11.091 -26.974 1.00 0.00
H
ATOM 4845 HG2 LYS X 317 -35.624 12.408 -25.803 1.00 0.00
H
ATOM 4846 CD LYS X 317 -36.130 10.515 -24.922 1.00 0.00
C
ATOM 4847 HD1 LYS X 317 -35.932 10.933 -23.912 1.00 0.00
H
ATOM 4848 HD2 LYS X 317 -35.720 9.482 -24.925 1.00 0.00
H
ATOM 4849 CE LYS X 317 -37.699 10.472 -25.083 1.00 0.00
C
ATOM 4850 HE1 LYS X 317 -38.192 9.712 -24.440 1.00 0.00
H

```
ATOM   4851  HE2 LYS X 317     -37.936  10.161 -26.123  1.00  0.00
H
ATOM   4852  NZ  LYS X 317     -38.281  11.789 -24.955  1.00  0.00
N
ATOM   4853  HZ1 LYS X 317     -38.110  12.356 -25.811  1.00  0.00
H
ATOM   4854  HZ2 LYS X 317     -38.013  12.391 -24.151  1.00  0.00
H
ATOM   4855  HZ3 LYS X 317     -39.314  11.679 -24.916  1.00  0.00
H
ATOM   4856  C   LYS X 317     -33.059   9.357 -26.849  1.00  0.00
C
ATOM   4857  O   LYS X 317     -32.344   9.742 -27.748  1.00  0.00
O
ATOM   4858  N   LEU X 318     -33.913   8.319 -27.007  1.00  0.00
N
ATOM   4859  HN  LEU X 318     -34.326   8.018 -26.151  1.00  0.00
H
ATOM   4860  CA  LEU X 318     -34.130   7.603 -28.254  1.00  0.00
C
ATOM   4861  HA  LEU X 318     -33.233   7.729 -28.841  1.00  0.00
H
ATOM   4862  CB  LEU X 318     -34.413   6.046 -28.056  1.00  0.00
C
ATOM   4863  HB1 LEU X 318     -34.441   5.591 -29.070  1.00  0.00
H
ATOM   4864  HB2 LEU X 318     -35.378   5.895 -27.528  1.00  0.00
H
ATOM   4865  CG  LEU X 318     -33.278   5.267 -27.360  1.00  0.00
C
ATOM   4866  HG  LEU X 318     -33.183   5.784 -26.381  1.00  0.00
H
ATOM   4867  CD1 LEU X 318     -33.829   3.855 -27.218  1.00  0.00
C
ATOM   4868 HD11 LEU X 318     -33.728   3.403 -28.227  1.00  0.00
H
ATOM   4869 HD12 LEU X 318     -34.832   3.954 -26.749  1.00  0.00
H
ATOM   4870 HD13 LEU X 318     -33.169   3.323 -26.500  1.00  0.00
H
ATOM   4871  CD2 LEU X 318     -31.983   5.391 -28.210  1.00  0.00
C
ATOM   4872 HD21 LEU X 318     -31.204   4.708 -27.809  1.00  0.00
H
ATOM   4873 HD22 LEU X 318     -31.664   6.455 -28.194  1.00  0.00
H
ATOM   4874 HD23 LEU X 318     -32.180   5.156 -29.278  1.00  0.00
H
ATOM   4875  C   LEU X 318     -35.206   8.307 -29.031  1.00  0.00
C
```

```
ATOM   4876  O   LEU X 318     -36.104   8.873 -28.433  1.00  0.00
O
ATOM   4877  N   PRO X 319     -35.180   8.270 -30.370  1.00  0.00
N
ATOM   4878  CD  PRO X 319     -34.112   7.669 -31.219  1.00  0.00
C
ATOM   4879  HD1 PRO X 319     -33.741   6.709 -30.800  1.00  0.00
H
ATOM   4880  HD2 PRO X 319     -33.265   8.384 -31.308  1.00  0.00
H
ATOM   4881  CA  PRO X 319     -36.401   8.426 -31.113  1.00  0.00
C
ATOM   4882  HA  PRO X 319     -36.885   9.382 -30.976  1.00  0.00
H
ATOM   4883  CB  PRO X 319     -35.938   8.257 -32.535  1.00  0.00
C
ATOM   4884  HB1 PRO X 319     -35.709   9.279 -32.903  1.00  0.00
H
ATOM   4885  HB2 PRO X 319     -36.586   7.861 -33.347  1.00  0.00
H
ATOM   4886  CG  PRO X 319     -34.752   7.319 -32.528  1.00  0.00
C
ATOM   4887  HG1 PRO X 319     -35.105   6.265 -32.542  1.00  0.00
H
ATOM   4888  HG2 PRO X 319     -34.049   7.388 -33.386  1.00  0.00
H
ATOM   4889  C   PRO X 319     -37.482   7.404 -30.680  1.00  0.00
C
ATOM   4890  O   PRO X 319     -37.131   6.338 -30.190  1.00  0.00
O
ATOM   4891  N   ASP X 320     -38.702   7.733 -30.878  1.00  0.00
N
ATOM   4892  HN  ASP X 320     -39.077   8.581 -31.244  1.00  0.00
H
ATOM   4893  CA  ASP X 320     -39.891   6.833 -30.778  1.00  0.00
C
ATOM   4894  HA  ASP X 320     -39.884   6.384 -29.796  1.00  0.00
H
ATOM   4895  CB  ASP X 320     -41.237   7.639 -30.936  1.00  0.00
C
ATOM   4896  HB1 ASP X 320     -41.286   8.183 -29.968  1.00  0.00
H
ATOM   4897  HB2 ASP X 320     -42.170   7.041 -31.021  1.00  0.00
H
ATOM   4898  CG  ASP X 320     -41.290   8.657 -32.031  1.00  0.00
C
ATOM   4899  OD1 ASP X 320     -42.534   9.064 -32.236  1.00  0.00
O
ATOM   4900  OD2 ASP X 320     -40.313   9.204 -32.530  1.00  0.00
O
```

ATOM 4901 C ASP X 320 −39.937 5.712 −31.765 1.00 0.00
C
ATOM 4902 O ASP X 320 −40.430 4.591 −31.453 1.00 0.00
O
ATOM 4903 N ASP X 321 −39.473 5.974 −32.955 1.00 0.00
N
ATOM 4904 HN ASP X 321 −39.416 6.949 −33.155 1.00 0.00
H
ATOM 4905 CA ASP X 321 −39.551 5.051 −34.083 1.00 0.00
C
ATOM 4906 HA ASP X 321 −40.387 4.376 −33.973 1.00 0.00
H
ATOM 4907 CB ASP X 321 −39.644 5.766 −35.380 1.00 0.00
C
ATOM 4908 HB1 ASP X 321 −39.491 4.977 −36.147 1.00 0.00
H
ATOM 4909 HB2 ASP X 321 −38.895 6.577 −35.506 1.00 0.00
H
ATOM 4910 CG ASP X 321 −41.061 6.287 −35.641 1.00 0.00
C
ATOM 4911 OD1 ASP X 321 −42.060 5.462 −35.560 1.00 0.00
O
ATOM 4912 OD2 ASP X 321 −41.240 7.498 −35.931 1.00 0.00
O
ATOM 4913 C ASP X 321 −38.239 4.162 −34.151 1.00 0.00
C
ATOM 4914 O ASP X 321 −37.997 3.372 −35.054 1.00 0.00
O
ATOM 4915 N PHE X 322 −37.391 4.199 −33.132 1.00 0.00
N
ATOM 4916 HN PHE X 322 −37.555 4.923 −32.466 1.00 0.00
H
ATOM 4917 CA PHE X 322 −36.245 3.380 −32.853 1.00 0.00
C
ATOM 4918 HA PHE X 322 −35.423 3.678 −33.488 1.00 0.00
H
ATOM 4919 CB PHE X 322 −35.817 3.617 −31.387 1.00 0.00
C
ATOM 4920 HB1 PHE X 322 −36.725 3.466 −30.766 1.00 0.00
H
ATOM 4921 HB2 PHE X 322 −35.642 4.698 −31.195 1.00 0.00
H
ATOM 4922 CG PHE X 322 −34.658 2.744 −30.962 1.00 0.00
C
ATOM 4923 CD1 PHE X 322 −33.320 3.121 −30.954 1.00 0.00
C
ATOM 4924 HD1 PHE X 322 −33.026 4.060 −31.399 1.00 0.00
H
ATOM 4925 CE1 PHE X 322 −32.341 2.282 −30.363 1.00 0.00
C

```
ATOM   4926  HE1 PHE X 322     -31.312   2.599 -30.278  1.00  0.00
H
ATOM   4927  CZ  PHE X 322     -32.698   1.012 -29.838  1.00  0.00
C
ATOM   4928  HZ  PHE X 322     -31.951   0.324 -29.470  1.00  0.00
H
ATOM   4929  CD2 PHE X 322     -35.066   1.573 -30.308  1.00  0.00
C
ATOM   4930  HD2 PHE X 322     -36.111   1.340 -30.164  1.00  0.00
H
ATOM   4931  CE2 PHE X 322     -34.068   0.666 -29.765  1.00  0.00
C
ATOM   4932  HE2 PHE X 322     -34.199  -0.326 -29.360  1.00  0.00
H
ATOM   4933  C   PHE X 322     -36.366   1.989 -33.319  1.00  0.00
C
ATOM   4934  O   PHE X 322     -37.436   1.327 -33.258  1.00  0.00
O
ATOM   4935  N   THR X 323     -35.327   1.466 -33.997  1.00  0.00
N
ATOM   4936  HN  THR X 323     -34.748   2.062 -34.547  1.00  0.00
H
ATOM   4937  CA  THR X 323     -35.108   0.058 -34.300  1.00  0.00
C
ATOM   4938  HA  THR X 323     -35.937  -0.570 -34.009  1.00  0.00
H
ATOM   4939  CB  THR X 323     -35.035  -0.179 -35.778  1.00  0.00
C
ATOM   4940  HB  THR X 323     -34.152   0.377 -36.157  1.00  0.00
H
ATOM   4941  OG1 THR X 323     -36.304   0.256 -36.324  1.00  0.00
O
ATOM   4942  HG1 THR X 323     -36.298   1.211 -36.228  1.00  0.00
H
ATOM   4943  CG2 THR X 323     -34.833  -1.702 -36.142  1.00  0.00
C
ATOM   4944 HG21 THR X 323     -35.519  -2.196 -35.421  1.00  0.00
H
ATOM   4945 HG22 THR X 323     -33.830  -2.087 -35.857  1.00  0.00
H
ATOM   4946 HG23 THR X 323     -35.217  -2.002 -37.140  1.00  0.00
H
ATOM   4947  C   THR X 323     -33.781  -0.333 -33.702  1.00  0.00
C
ATOM   4948  O   THR X 323     -32.717   0.167 -34.000  1.00  0.00
O
ATOM   4949  N   GLY X 324     -33.843  -1.371 -32.878  1.00  0.00
N
ATOM   4950  HN  GLY X 324     -34.706  -1.704 -32.508  1.00  0.00
H
```

```
ATOM   4951  CA  GLY X 324     -32.635  -1.974 -32.378  1.00  0.00
C
ATOM   4952  HA1 GLY X 324     -31.933  -1.157 -32.290  1.00  0.00
H
ATOM   4953  HA2 GLY X 324     -32.273  -2.748 -33.038  1.00  0.00
H
ATOM   4954  C   GLY X 324     -32.899  -2.486 -31.025  1.00  0.00
C
ATOM   4955  O   GLY X 324     -34.023  -2.617 -30.585  1.00  0.00
O
ATOM   4956  N   CYS X 325     -31.772  -2.757 -30.286  1.00  0.00
N
ATOM   4957  HN  CYS X 325     -30.871  -2.659 -30.700  1.00  0.00
H
ATOM   4958  CA  CYS X 325     -31.727  -3.347 -28.941  1.00  0.00
C
ATOM   4959  HA  CYS X 325     -32.767  -3.386 -28.653  1.00  0.00
H
ATOM   4960  CB  CYS X 325     -31.234  -4.775 -28.906  1.00  0.00
C
ATOM   4961  HB1 CYS X 325     -31.542  -5.227 -27.940  1.00  0.00
H
ATOM   4962  HB2 CYS X 325     -30.131  -4.801 -29.039  1.00  0.00
H
ATOM   4963  SG  CYS X 325     -31.928  -5.700 -30.290  1.00  0.00
S
ATOM   4964  C   CYS X 325     -31.128  -2.480 -27.839  1.00  0.00
C
ATOM   4965  O   CYS X 325     -30.419  -1.518 -28.005  1.00  0.00
O
ATOM   4966  N   VAL X 326     -31.426  -2.884 -26.585  1.00  0.00
N
ATOM   4967  HN  VAL X 326     -31.973  -3.717 -26.548  1.00  0.00
H
ATOM   4968  CA  VAL X 326     -30.881  -2.344 -25.328  1.00  0.00
C
ATOM   4969  HA  VAL X 326     -30.065  -1.687 -25.591  1.00  0.00
H
ATOM   4970  CB  VAL X 326     -32.023  -1.533 -24.623  1.00  0.00
C
ATOM   4971  HB  VAL X 326     -32.885  -2.194 -24.389  1.00  0.00
H
ATOM   4972  CG1 VAL X 326     -31.468  -1.045 -23.200  1.00  0.00
C
ATOM   4973 HG11 VAL X 326     -32.332  -0.535 -22.723  1.00  0.00
H
ATOM   4974 HG12 VAL X 326     -30.620  -0.341 -23.337  1.00  0.00
H
ATOM   4975 HG13 VAL X 326     -31.045  -1.855 -22.568  1.00  0.00
H
```

```
ATOM   4976  CG2 VAL X 326      -32.475  -0.409 -25.568  1.00  0.00
C
ATOM   4977 HG21 VAL X 326      -31.587   0.097 -26.002  1.00  0.00
H
ATOM   4978 HG22 VAL X 326      -33.253   0.302 -25.216  1.00  0.00
H
ATOM   4979 HG23 VAL X 326      -32.996  -0.875 -26.431  1.00  0.00
H
ATOM   4980  C   VAL X 326      -30.434  -3.577 -24.540  1.00  0.00
C
ATOM   4981  O   VAL X 326      -31.304  -4.337 -24.243  1.00  0.00
O
ATOM   4982  N   ILE X 327      -29.113  -3.636 -24.080  1.00  0.00
N
ATOM   4983  HN  ILE X 327      -28.350  -3.020 -24.258  1.00  0.00
H
ATOM   4984  CA  ILE X 327      -28.676  -4.785 -23.223  1.00  0.00
C
ATOM   4985  HA  ILE X 327      -29.477  -5.272 -22.686  1.00  0.00
H
ATOM   4986  CB  ILE X 327      -28.009  -5.766 -24.126  1.00  0.00
C
ATOM   4987  HB  ILE X 327      -28.667  -6.251 -24.878  1.00  0.00
H
ATOM   4988  CG2 ILE X 327      -26.717  -5.118 -24.899  1.00  0.00
C
ATOM   4989 HG21 ILE X 327      -26.369  -5.947 -25.552  1.00  0.00
H
ATOM   4990 HG22 ILE X 327      -25.907  -4.942 -24.159  1.00  0.00
H
ATOM   4991 HG23 ILE X 327      -27.110  -4.215 -25.413  1.00  0.00
H
ATOM   4992  CG1 ILE X 327      -27.390  -6.999 -23.355  1.00  0.00
C
ATOM   4993 HG11 ILE X 327      -28.061  -7.094 -22.474  1.00  0.00
H
ATOM   4994 HG12 ILE X 327      -26.389  -6.766 -22.932  1.00  0.00
H
ATOM   4995  CD  ILE X 327      -27.346  -8.255 -24.204  1.00  0.00
C
ATOM   4996  HD1 ILE X 327      -28.357  -8.600 -24.511  1.00  0.00
H
ATOM   4997  HD2 ILE X 327      -26.777  -9.065 -23.699  1.00  0.00
H
ATOM   4998  HD3 ILE X 327      -26.723  -8.081 -25.107  1.00  0.00
H
ATOM   4999  C   ILE X 327      -27.903  -4.293 -22.101  1.00  0.00
C
ATOM   5000  O   ILE X 327      -26.923  -3.632 -22.277  1.00  0.00
O
```

ATOM 5001 N ALA X 328 −28.461 −4.705 −20.893 1.00 0.00
N
ATOM 5002 HN ALA X 328 −29.226 −5.344 −20.863 1.00 0.00
H
ATOM 5003 CA ALA X 328 −27.944 −4.244 −19.642 1.00 0.00
C
ATOM 5004 HA ALA X 328 −27.078 −3.631 −19.840 1.00 0.00
H
ATOM 5005 CB ALA X 328 −28.839 −3.158 −19.013 1.00 0.00
C
ATOM 5006 HB1 ALA X 328 −28.345 −2.916 −18.048 1.00 0.00
H
ATOM 5007 HB2 ALA X 328 −29.888 −3.449 −18.791 1.00 0.00
H
ATOM 5008 HB3 ALA X 328 −28.977 −2.251 −19.640 1.00 0.00
H
ATOM 5009 C ALA X 328 −27.512 −5.337 −18.705 1.00 0.00
C
ATOM 5010 O ALA X 328 −28.156 −6.372 −18.678 1.00 0.00
O
ATOM 5011 N TRP X 329 −26.390 −5.246 −17.966 1.00 0.00
N
ATOM 5012 HN TRP X 329 −25.911 −4.377 −18.061 1.00 0.00
H
ATOM 5013 CA TRP X 329 −25.932 −6.155 −17.028 1.00 0.00
C
ATOM 5014 HA TRP X 329 −26.768 −6.762 −16.712 1.00 0.00
H
ATOM 5015 CB TRP X 329 −24.796 −7.035 −17.523 1.00 0.00
C
ATOM 5016 HB1 TRP X 329 −25.196 −7.583 −18.402 1.00 0.00
H
ATOM 5017 HB2 TRP X 329 −24.447 −7.753 −16.750 1.00 0.00
H
ATOM 5018 CG TRP X 329 −23.541 −6.311 −17.909 1.00 0.00
C
ATOM 5019 CD1 TRP X 329 −22.303 −6.150 −17.160 1.00 0.00
C
ATOM 5020 HD1 TRP X 329 −22.035 −6.507 −16.177 1.00 0.00
H
ATOM 5021 NE1 TRP X 329 −21.405 −5.565 −17.946 1.00 0.00
N
ATOM 5022 HE1 TRP X 329 −20.537 −5.338 −17.561 1.00 0.00
H
ATOM 5023 CE2 TRP X 329 −21.882 −5.261 −19.237 1.00 0.00
C
ATOM 5024 CD2 TRP X 329 −23.229 −5.575 −19.206 1.00 0.00
C
ATOM 5025 CE3 TRP X 329 −24.075 −5.196 −20.230 1.00 0.00
C

ATOM 5026 HE3 TRP X 329 -25.137 -5.380 -20.166 1.00 0.00
H
ATOM 5027 CZ3 TRP X 329 -23.557 -4.308 -21.223 1.00 0.00
C
ATOM 5028 HZ3 TRP X 329 -24.212 -3.871 -21.961 1.00 0.00
H
ATOM 5029 CZ2 TRP X 329 -21.280 -4.456 -20.176 1.00 0.00
C
ATOM 5030 HZ2 TRP X 329 -20.274 -4.062 -20.147 1.00 0.00
H
ATOM 5031 CH2 TRP X 329 -22.107 -3.990 -21.262 1.00 0.00
C
ATOM 5032 HH2 TRP X 329 -21.650 -3.324 -21.979 1.00 0.00
H
ATOM 5033 C TRP X 329 -25.379 -5.527 -15.674 1.00 0.00
C
ATOM 5034 O TRP X 329 -24.962 -4.357 -15.735 1.00 0.00
O
ATOM 5035 N ASN X 330 -25.294 -6.281 -14.561 1.00 0.00
N
ATOM 5036 HN ASN X 330 -25.489 -7.258 -14.590 1.00 0.00
H
ATOM 5037 CA ASN X 330 -24.685 -5.827 -13.307 1.00 0.00
C
ATOM 5038 HA ASN X 330 -25.222 -4.920 -13.073 1.00 0.00
H
ATOM 5039 CB ASN X 330 -24.985 -6.833 -12.169 1.00 0.00
C
ATOM 5040 HB1 ASN X 330 -24.475 -7.802 -12.357 1.00 0.00
H
ATOM 5041 HB2 ASN X 330 -26.089 -6.957 -12.172 1.00 0.00
H
ATOM 5042 CG ASN X 330 -24.619 -6.497 -10.776 1.00 0.00
C
ATOM 5043 OD1 ASN X 330 -24.092 -5.432 -10.528 1.00 0.00
O
ATOM 5044 ND2 ASN X 330 -25.014 -7.353 -9.811 1.00 0.00
N
ATOM 5045 HD21 ASN X 330 -24.747 -7.096 -8.882 1.00 0.00
H
ATOM 5046 HD22 ASN X 330 -25.470 -8.204 -10.072 1.00 0.00
H
ATOM 5047 C ASN X 330 -23.189 -5.561 -13.251 1.00 0.00
C
ATOM 5048 O ASN X 330 -22.415 -6.463 -13.602 1.00 0.00
O
ATOM 5049 N SER X 331 -22.780 -4.325 -13.008 1.00 0.00
N
ATOM 5050 HN SER X 331 -23.377 -3.529 -12.943 1.00 0.00
H

```
ATOM   5051  CA  SER X 331     -21.343  -3.980 -13.131  1.00  0.00
C
ATOM   5052  HA  SER X 331     -20.803  -4.827 -13.526  1.00  0.00
H
ATOM   5053  CB  SER X 331     -20.945  -2.957 -14.252  1.00  0.00
C
ATOM   5054  HB1 SER X 331     -21.321  -2.022 -13.783  1.00  0.00
H
ATOM   5055  HB2 SER X 331     -21.575  -3.117 -15.153  1.00  0.00
H
ATOM   5056  OG  SER X 331     -19.553  -2.892 -14.535  1.00  0.00
O
ATOM   5057  HG1 SER X 331     -19.322  -1.961 -14.569  1.00  0.00
H
ATOM   5058  C   SER X 331     -20.768  -3.660 -11.779  1.00  0.00
C
ATOM   5059  O   SER X 331     -19.733  -3.059 -11.768  1.00  0.00
O
ATOM   5060  N   ASN X 332     -21.335  -4.039 -10.523  1.00  0.00
N
ATOM   5061  HN  ASN X 332     -22.166  -4.585 -10.592  1.00  0.00
H
ATOM   5062  CA  ASN X 332     -20.811  -3.822  -9.164  1.00  0.00
C
ATOM   5063  HA  ASN X 332     -20.980  -2.790  -8.894  1.00  0.00
H
ATOM   5064  CB  ASN X 332     -21.449  -4.915  -8.299  1.00  0.00
C
ATOM   5065  HB1 ASN X 332     -21.174  -5.942  -8.619  1.00  0.00
H
ATOM   5066  HB2 ASN X 332     -22.535  -5.004  -8.516  1.00  0.00
H
ATOM   5067  CG  ASN X 332     -21.169  -4.810  -6.808  1.00  0.00
C
ATOM   5068  OD1 ASN X 332     -20.720  -3.715  -6.326  1.00  0.00
O
ATOM   5069  ND2 ASN X 332     -21.318  -5.919  -6.012  1.00  0.00
N
ATOM   5070 HD21 ASN X 332     -20.750  -5.940  -5.189  1.00  0.00
H
ATOM   5071 HD22 ASN X 332     -21.610  -6.807  -6.367  1.00  0.00
H
ATOM   5072  C   ASN X 332     -19.359  -4.036  -9.189  1.00  0.00
C
ATOM   5073  O   ASN X 332     -18.588  -3.174  -8.711  1.00  0.00
O
ATOM   5074  N   ASN X 333     -18.859  -5.194  -9.589  1.00  0.00
N
ATOM   5075  HN  ASN X 333     -19.440  -5.947  -9.885  1.00  0.00
H
```

ATOM 5076 CA ASN X 333 -17.444 -5.644 -9.324 1.00 0.00
C
ATOM 5077 HA ASN X 333 -17.462 -5.572 -8.247 1.00 0.00
H
ATOM 5078 CB ASN X 333 -17.352 -7.285 -9.633 1.00 0.00
C
ATOM 5079 HB1 ASN X 333 -18.213 -7.728 -9.088 1.00 0.00
H
ATOM 5080 HB2 ASN X 333 -16.391 -7.494 -9.116 1.00 0.00
H
ATOM 5081 CG ASN X 333 -17.178 -7.552 -11.133 1.00 0.00
C
ATOM 5082 OD1 ASN X 333 -16.075 -7.837 -11.595 1.00 0.00
O
ATOM 5083 ND2 ASN X 333 -18.336 -7.535 -11.850 1.00 0.00
N
ATOM 5084 HD21 ASN X 333 -18.449 -7.699 -12.830 1.00 0.00
H
ATOM 5085 HD22 ASN X 333 -19.137 -7.612 -11.256 1.00 0.00
H
ATOM 5086 C ASN X 333 -16.375 -4.684 -9.902 1.00 0.00
C
ATOM 5087 O ASN X 333 -15.265 -4.549 -9.330 1.00 0.00
O
ATOM 5088 N LEU X 334 -16.688 -3.977 -10.938 1.00 0.00
N
ATOM 5089 HN LEU X 334 -17.622 -4.132 -11.248 1.00 0.00
H
ATOM 5090 CA LEU X 334 -15.835 -3.056 -11.685 1.00 0.00
C
ATOM 5091 HA LEU X 334 -14.807 -3.329 -11.502 1.00 0.00
H
ATOM 5092 CB LEU X 334 -16.250 -3.193 -13.216 1.00 0.00
C
ATOM 5093 HB1 LEU X 334 -15.522 -2.636 -13.844 1.00 0.00
H
ATOM 5094 HB2 LEU X 334 -17.282 -2.794 -13.314 1.00 0.00
H
ATOM 5095 CG LEU X 334 -16.303 -4.575 -13.878 1.00 0.00
C
ATOM 5096 HG LEU X 334 -17.176 -5.183 -13.558 1.00 0.00
H
ATOM 5097 CD1 LEU X 334 -16.483 -4.424 -15.446 1.00 0.00
C
ATOM 5098 HD11 LEU X 334 -15.627 -3.828 -15.829 1.00 0.00
H
ATOM 5099 HD12 LEU X 334 -17.404 -3.840 -15.657 1.00 0.00
H
ATOM 5100 HD13 LEU X 334 -16.570 -5.440 -15.889 1.00 0.00
H

ATOM   5101 CD2 LEU X 334   -15.006 -5.344 -13.537 1.00 0.00
C
ATOM   5102 HD21 LEU X 334   -14.922 -6.215 -14.221 1.00 0.00
H
ATOM   5103 HD22 LEU X 334   -15.107 -5.594 -12.459 1.00 0.00
H
ATOM   5104 HD23 LEU X 334   -14.076 -4.739 -13.590 1.00 0.00
H
ATOM   5105 C   LEU X 334   -16.035 -1.595 -11.349 1.00 0.00
C
ATOM   5106 O   LEU X 334   -15.056 -0.910 -11.000 1.00 0.00
O
ATOM   5107 N   ASP X 335   -17.201 -1.056 -11.370 1.00 0.00
N
ATOM   5108 HN ASP X 335   -17.945 -1.613 -11.730 1.00 0.00
H
ATOM   5109 CA ASP X 335   -17.548   0.317 -11.236 1.00 0.00
C
ATOM   5110 HA ASP X 335   -16.744   1.011 -11.429 1.00 0.00
H
ATOM   5111 CB ASP X 335   -18.718   0.604 -12.201 1.00 0.00
C
ATOM   5112 HB1 ASP X 335   -19.058   1.659 -12.131 1.00 0.00
H
ATOM   5113 HB2 ASP X 335   -19.505 -0.159 -12.019 1.00 0.00
H
ATOM   5114 CG ASP X 335   -18.213   0.388 -13.603 1.00 0.00
C
ATOM   5115 OD1 ASP X 335   -17.258   1.100 -13.945 1.00 0.00
O
ATOM   5116 OD2 ASP X 335   -18.823 -0.296 -14.393 1.00 0.00
O
ATOM   5117 C   ASP X 335   -18.097   0.692 -9.829 1.00 0.00
C
ATOM   5118 O   ASP X 335   -18.343   1.889 -9.538 1.00 0.00
O
ATOM   5119 N   SER X 336   -18.295 -0.221 -8.853 1.00 0.00
N
ATOM   5120 HN SER X 336   -17.953 -1.134 -9.059 1.00 0.00
H
ATOM   5121 CA SER X 336   -18.750   0.086 -7.513 1.00 0.00
C
ATOM   5122 HA SER X 336   -19.192   1.067 -7.424 1.00 0.00
H
ATOM   5123 CB SER X 336   -19.750 -0.912 -6.900 1.00 0.00
C
ATOM   5124 HB1 SER X 336   -19.235 -1.823 -6.527 1.00 0.00
H
ATOM   5125 HB2 SER X 336   -20.369 -1.211 -7.772 1.00 0.00
H

```
ATOM   5126  OG  SER X 336     -20.453  -0.294  -5.818  1.00  0.00
O
ATOM   5127  HG1 SER X 336     -21.341  -0.638  -5.938  1.00  0.00
H
ATOM   5128  C   SER X 336     -17.523   0.021  -6.586  1.00  0.00
C
ATOM   5129  O   SER X 336     -16.713  -0.883  -6.664  1.00  0.00
O
ATOM   5130  N   LYS X 337     -17.513   0.948  -5.634  1.00  0.00
N
ATOM   5131  HN  LYS X 337     -18.203   1.664  -5.701  1.00  0.00
H
ATOM   5132  CA  LYS X 337     -16.342   1.171  -4.805  1.00  0.00
C
ATOM   5133  HA  LYS X 337     -15.801   0.240  -4.720  1.00  0.00
H
ATOM   5134  CB  LYS X 337     -15.380   2.288  -5.314  1.00  0.00
C
ATOM   5135  HB1 LYS X 337     -14.452   2.385  -4.710  1.00  0.00
H
ATOM   5136  HB2 LYS X 337     -15.950   3.241  -5.275  1.00  0.00
H
ATOM   5137  CG  LYS X 337     -14.823   2.108  -6.790  1.00  0.00
C
ATOM   5138  HG1 LYS X 337     -14.384   3.036  -7.214  1.00  0.00
H
ATOM   5139  HG2 LYS X 337     -15.709   1.965  -7.445  1.00  0.00
H
ATOM   5140  CD  LYS X 337     -13.861   0.923  -6.866  1.00  0.00
C
ATOM   5141  HD1 LYS X 337     -13.743   0.690  -7.945  1.00  0.00
H
ATOM   5142  HD2 LYS X 337     -14.289   0.012  -6.394  1.00  0.00
H
ATOM   5143  CE  LYS X 337     -12.417   1.164  -6.202  1.00  0.00
C
ATOM   5144  HE1 LYS X 337     -12.497   1.236  -5.096  1.00  0.00
H
ATOM   5145  HE2 LYS X 337     -11.933   2.079  -6.603  1.00  0.00
H
ATOM   5146  NZ  LYS X 337     -11.535   0.091  -6.439  1.00  0.00
N
ATOM   5147  HZ1 LYS X 337     -10.681   0.044  -5.848  1.00  0.00
H
ATOM   5148  HZ2 LYS X 337     -11.228  -0.001  -7.428  1.00  0.00
H
ATOM   5149  HZ3 LYS X 337     -12.040  -0.808  -6.301  1.00  0.00
H
ATOM   5150  C   LYS X 337     -16.791   1.590  -3.397  1.00  0.00
C
```

```
ATOM   5151  O   LYS X 337     -17.549   2.549  -3.120  1.00  0.00
O
ATOM   5152  N   VAL X 338     -16.288   0.900  -2.333  1.00  0.00
N
ATOM   5153  HN  VAL X 338     -15.601   0.194  -2.484  1.00  0.00
H
ATOM   5154  CA  VAL X 338     -16.371   1.291  -0.957  1.00  0.00
C
ATOM   5155  HA  VAL X 338     -17.374   1.301  -0.557  1.00  0.00
H
ATOM   5156  CB  VAL X 338     -15.625   0.295  -0.074  1.00  0.00
C
ATOM   5157  HB  VAL X 338     -14.563   0.171  -0.376  1.00  0.00
H
ATOM   5158  CG1 VAL X 338     -15.495   0.676   1.379  1.00  0.00
C
ATOM   5159 HG11 VAL X 338     -15.484  -0.265   1.970  1.00  0.00
H
ATOM   5160 HG12 VAL X 338     -16.354   1.321   1.663  1.00  0.00
H
ATOM   5161 HG13 VAL X 338     -14.527   1.188   1.565  1.00  0.00
H
ATOM   5162  CG2 VAL X 338     -16.348  -1.134  -0.143  1.00  0.00
C
ATOM   5163 HG21 VAL X 338     -16.313  -1.369  -1.228  1.00  0.00
H
ATOM   5164 HG22 VAL X 338     -17.385  -0.941   0.205  1.00  0.00
H
ATOM   5165 HG23 VAL X 338     -15.702  -1.813   0.454  1.00  0.00
H
ATOM   5166  C   VAL X 338     -15.998   2.762  -0.720  1.00  0.00
C
ATOM   5167  O   VAL X 338     -15.008   3.214  -1.284  1.00  0.00
O
ATOM   5168  N   GLY X 339     -16.716   3.498   0.016  1.00  0.00
N
ATOM   5169  HN  GLY X 339     -17.519   3.001   0.335  1.00  0.00
H
ATOM   5170  CA  GLY X 339     -16.579   5.016   0.209  1.00  0.00
C
ATOM   5171  HA1 GLY X 339     -15.530   5.269   0.234  1.00  0.00
H
ATOM   5172  HA2 GLY X 339     -16.966   5.285   1.181  1.00  0.00
H
ATOM   5173  C   GLY X 339     -17.235   5.873  -0.803  1.00  0.00
C
ATOM   5174  O   GLY X 339     -17.381   7.086  -0.550  1.00  0.00
O
ATOM   5175  N   GLY X 340     -17.791   5.317  -1.902  1.00  0.00
N
```

```
ATOM   5176  HN  GLY X 340     -17.694   4.326  -1.948  1.00  0.00
H
ATOM   5177  CA  GLY X 340     -18.447   5.804  -3.079  1.00  0.00
C
ATOM   5178  HA1 GLY X 340     -18.853   6.737  -2.716  1.00  0.00
H
ATOM   5179  HA2 GLY X 340     -19.198   5.093  -3.391  1.00  0.00
H
ATOM   5180  C   GLY X 340     -17.502   6.023  -4.248  1.00  0.00
C
ATOM   5181  O   GLY X 340     -16.331   6.411  -3.988  1.00  0.00
O
ATOM   5182  N   ASN X 341     -17.859   5.799  -5.514  1.00  0.00
N
ATOM   5183  HN  ASN X 341     -18.749   5.354  -5.573  1.00  0.00
H
ATOM   5184  CA  ASN X 341     -17.003   6.057  -6.617  1.00  0.00
C
ATOM   5185  HA  ASN X 341     -16.017   5.729  -6.322  1.00  0.00
H
ATOM   5186  CB  ASN X 341     -17.466   5.094  -7.702  1.00  0.00
C
ATOM   5187  HB1 ASN X 341     -18.421   5.484  -8.115  1.00  0.00
H
ATOM   5188  HB2 ASN X 341     -17.535   4.071  -7.275  1.00  0.00
H
ATOM   5189  CG  ASN X 341     -16.437   4.948  -8.791  1.00  0.00
C
ATOM   5190  OD1 ASN X 341     -15.291   5.408  -8.725  1.00  0.00
O
ATOM   5191  ND2 ASN X 341     -16.847   4.265  -9.893  1.00  0.00
N
ATOM   5192 HD21 ASN X 341     -16.116   3.801 -10.394  1.00  0.00
H
ATOM   5193 HD22 ASN X 341     -17.773   3.889  -9.905  1.00  0.00
H
ATOM   5194  C   ASN X 341     -17.070   7.576  -6.989  1.00  0.00
C
ATOM   5195  O   ASN X 341     -18.122   8.103  -7.189  1.00  0.00
O
ATOM   5196  N   TYR X 342     -15.952   8.311  -7.146  1.00  0.00
N
ATOM   5197  HN  TYR X 342     -15.067   7.935  -6.885  1.00  0.00
H
ATOM   5198  CA  TYR X 342     -15.975   9.669  -7.672  1.00  0.00
C
ATOM   5199  HA  TYR X 342     -16.882  10.092  -7.267  1.00  0.00
H
ATOM   5200  CB  TYR X 342     -14.787  10.559  -7.323  1.00  0.00
C
```

```
ATOM   5201  HB1 TYR X 342     -14.850  11.474  -7.950  1.00  0.00
H
ATOM   5202  HB2 TYR X 342     -13.907   9.933  -7.584  1.00  0.00
H
ATOM   5203  CG  TYR X 342     -14.994  10.891  -5.904  1.00  0.00
C
ATOM   5204  CD1 TYR X 342     -14.011  10.588  -4.968  1.00  0.00
C
ATOM   5205  HD1 TYR X 342     -13.265   9.845  -5.212  1.00  0.00
H
ATOM   5206  CE1 TYR X 342     -14.170  10.920  -3.633  1.00  0.00
C
ATOM   5207  HE1 TYR X 342     -13.493  10.489  -2.911  1.00  0.00
H
ATOM   5208  CZ  TYR X 342     -15.252  11.615  -3.155  1.00  0.00
C
ATOM   5209  OH  TYR X 342     -15.565  11.617  -1.812  1.00  0.00
O
ATOM   5210  HH  TYR X 342     -14.796  11.544  -1.242  1.00  0.00
H
ATOM   5211  CD2 TYR X 342     -16.060  11.714  -5.510  1.00  0.00
C
ATOM   5212  HD2 TYR X 342     -16.933  11.844  -6.133  1.00  0.00
H
ATOM   5213  CE2 TYR X 342     -16.175  12.116  -4.177  1.00  0.00
C
ATOM   5214  HE2 TYR X 342     -17.127  12.523  -3.867  1.00  0.00
H
ATOM   5215  C   TYR X 342     -16.249   9.734  -9.245  1.00  0.00
C
ATOM   5216  O   TYR X 342     -16.708  10.684  -9.739  1.00  0.00
O
ATOM   5217  N   ASN X 343     -15.986   8.559  -9.960  1.00  0.00
N
ATOM   5218  HN  ASN X 343     -15.764   7.699  -9.507  1.00  0.00
H
ATOM   5219  CA  ASN X 343     -16.358   8.499 -11.370  1.00  0.00
C
ATOM   5220  HA  ASN X 343     -16.086   9.433 -11.840  1.00  0.00
H
ATOM   5221  CB  ASN X 343     -15.639   7.324 -12.176  1.00  0.00
C
ATOM   5222  HB1 ASN X 343     -16.006   7.208 -13.218  1.00  0.00
H
ATOM   5223  HB2 ASN X 343     -15.821   6.421 -11.554  1.00  0.00
H
ATOM   5224  CG  ASN X 343     -14.190   7.608 -12.124  1.00  0.00
C
ATOM   5225  OD1 ASN X 343     -13.638   8.580 -11.679  1.00  0.00
O
```

ATOM 5226 ND2 ASN X 343 −13.434 6.560 −12.581 1.00 0.00
N
ATOM 5227 HD21 ASN X 343 −12.455 6.759 −12.634 1.00 0.00
H
ATOM 5228 HD22 ASN X 343 −13.876 5.703 −12.844 1.00 0.00
H
ATOM 5229 C ASN X 343 −17.902 8.383 −11.662 1.00 0.00
C
ATOM 5230 O ASN X 343 −18.697 8.023 −10.809 1.00 0.00
O
ATOM 5231 N TYR X 344 −18.382 8.859 −12.841 1.00 0.00
N
ATOM 5232 HN TYR X 344 −17.823 9.497 −13.366 1.00 0.00
H
ATOM 5233 CA TYR X 344 −19.786 8.698 −13.171 1.00 0.00
C
ATOM 5234 HA TYR X 344 −19.671 8.764 −14.243 1.00 0.00
H
ATOM 5235 CB TYR X 344 −20.339 7.243 −13.040 1.00 0.00
C
ATOM 5236 HB1 TYR X 344 −21.324 7.253 −13.552 1.00 0.00
H
ATOM 5237 HB2 TYR X 344 −20.605 6.958 −11.999 1.00 0.00
H
ATOM 5238 CG TYR X 344 −19.361 6.285 −13.680 1.00 0.00
C
ATOM 5239 CD1 TYR X 344 −19.368 6.199 −15.066 1.00 0.00
C
ATOM 5240 HD1 TYR X 344 −20.039 6.867 −15.587 1.00 0.00
H
ATOM 5241 CE1 TYR X 344 −18.542 5.202 −15.702 1.00 0.00
C
ATOM 5242 HE1 TYR X 344 −18.443 5.085 −16.772 1.00 0.00
H
ATOM 5243 CZ TYR X 344 −17.731 4.316 −14.955 1.00 0.00
C
ATOM 5244 OH TYR X 344 −17.067 3.249 −15.508 1.00 0.00
O
ATOM 5245 HH TYR X 344 −16.968 2.553 −14.853 1.00 0.00
H
ATOM 5246 CD2 TYR X 344 −18.538 5.375 −12.918 1.00 0.00
C
ATOM 5247 HD2 TYR X 344 −18.522 5.298 −11.841 1.00 0.00
H
ATOM 5248 CE2 TYR X 344 −17.769 4.401 −13.560 1.00 0.00
C
ATOM 5249 HE2 TYR X 344 −17.220 3.693 −12.958 1.00 0.00
H
ATOM 5250 C TYR X 344 −20.781 9.703 −12.684 1.00 0.00
C

```
ATOM   5251  O   TYR X 344     -21.199   9.703 -11.519  1.00  0.00
O
ATOM   5252  N   LEU X 345     -21.221  10.542 -13.652  1.00  0.00
N
ATOM   5253  HN  LEU X 345     -20.943  10.397 -14.598  1.00  0.00
H
ATOM   5254  CA  LEU X 345     -22.089  11.681 -13.512  1.00  0.00
C
ATOM   5255  HA  LEU X 345     -22.487  11.831 -12.519  1.00  0.00
H
ATOM   5256  CB  LEU X 345     -21.230  12.948 -13.781  1.00  0.00
C
ATOM   5257  HB1 LEU X 345     -21.755  13.919 -13.657  1.00  0.00
H
ATOM   5258  HB2 LEU X 345     -20.877  12.874 -14.832  1.00  0.00
H
ATOM   5259  CG  LEU X 345     -20.001  13.143 -12.756  1.00  0.00
C
ATOM   5260  HG  LEU X 345     -20.043  12.189 -12.187  1.00  0.00
H
ATOM   5261  CD1 LEU X 345     -18.649  13.331 -13.451  1.00  0.00
C
ATOM   5262 HD11 LEU X 345     -17.753  13.323 -12.794  1.00  0.00
H
ATOM   5263 HD12 LEU X 345     -18.603  14.241 -14.087  1.00  0.00
H
ATOM   5264 HD13 LEU X 345     -18.684  12.432 -14.102  1.00  0.00
H
ATOM   5265  CD2 LEU X 345     -20.271  14.256 -11.767  1.00  0.00
C
ATOM   5266 HD21 LEU X 345     -21.095  14.091 -11.040  1.00  0.00
H
ATOM   5267 HD22 LEU X 345     -20.419  15.210 -12.318  1.00  0.00
H
ATOM   5268 HD23 LEU X 345     -19.361  14.465 -11.165  1.00  0.00
H
ATOM   5269  C   LEU X 345     -23.300  11.607 -14.452  1.00  0.00
C
ATOM   5270  O   LEU X 345     -23.399  10.994 -15.459  1.00  0.00
O
ATOM   5271  N   TYR X 346     -24.307  12.376 -14.100  1.00  0.00
N
ATOM   5272  HN  TYR X 346     -24.442  12.650 -13.151  1.00  0.00
H
ATOM   5273  CA  TYR X 346     -25.574  12.386 -14.787  1.00  0.00
C
ATOM   5274  HA  TYR X 346     -25.457  12.168 -15.839  1.00  0.00
H
ATOM   5275  CB  TYR X 346     -26.744  11.411 -14.323  1.00  0.00
C
```

ATOM 5276 HB1 TYR X 346 -26.457 10.346 -14.459 1.00 0.00
H
ATOM 5277 HB2 TYR X 346 -27.711 11.616 -14.830 1.00 0.00
H
ATOM 5278 CG TYR X 346 -27.079 11.594 -12.822 1.00 0.00
C
ATOM 5279 CD1 TYR X 346 -26.499 10.736 -11.801 1.00 0.00
C
ATOM 5280 HD1 TYR X 346 -25.742 10.039 -12.128 1.00 0.00
H
ATOM 5281 CE1 TYR X 346 -26.918 10.837 -10.452 1.00 0.00
C
ATOM 5282 HE1 TYR X 346 -26.526 10.144 -9.723 1.00 0.00
H
ATOM 5283 CZ TYR X 346 -27.829 11.799 -10.120 1.00 0.00
C
ATOM 5284 OH TYR X 346 -28.202 11.908 -8.780 1.00 0.00
O
ATOM 5285 HH TYR X 346 -27.937 11.041 -8.465 1.00 0.00
H
ATOM 5286 CD2 TYR X 346 -28.147 12.533 -12.384 1.00 0.00
C
ATOM 5287 HD2 TYR X 346 -28.544 13.214 -13.122 1.00 0.00
H
ATOM 5288 CE2 TYR X 346 -28.445 12.633 -11.071 1.00 0.00
C
ATOM 5289 HE2 TYR X 346 -29.179 13.321 -10.677 1.00 0.00
H
ATOM 5290 C TYR X 346 -25.975 13.860 -14.591 1.00 0.00
C
ATOM 5291 O TYR X 346 -25.614 14.490 -13.569 1.00 0.00
O
ATOM 5292 N ARG X 347 -26.760 14.353 -15.496 1.00 0.00
N
ATOM 5293 HN ARG X 347 -26.989 13.801 -16.294 1.00 0.00
H
ATOM 5294 CA ARG X 347 -27.273 15.672 -15.527 1.00 0.00
C
ATOM 5295 HA ARG X 347 -26.644 16.175 -14.809 1.00 0.00
H
ATOM 5296 CB ARG X 347 -27.120 16.323 -16.926 1.00 0.00
C
ATOM 5297 HB1 ARG X 347 -27.740 15.793 -17.680 1.00 0.00
H
ATOM 5298 HB2 ARG X 347 -26.060 16.113 -17.184 1.00 0.00
H
ATOM 5299 CG ARG X 347 -27.446 17.807 -16.999 1.00 0.00
C
ATOM 5300 HG1 ARG X 347 -26.627 18.389 -16.525 1.00 0.00
H

```
ATOM   5301  HG2 ARG X 347     -28.416  18.053 -16.518  1.00  0.00
H
ATOM   5302  CD  ARG X 347     -27.650  18.437 -18.397  1.00  0.00
C
ATOM   5303  HD1 ARG X 347     -27.808  19.530 -18.276  1.00  0.00
H
ATOM   5304  HD2 ARG X 347     -28.534  18.017 -18.922  1.00  0.00
H
ATOM   5305  NE  ARG X 347     -26.453  18.266 -19.287  1.00  0.00
N
ATOM   5306  HE  ARG X 347     -26.432  17.554 -19.989  1.00  0.00
H
ATOM   5307  CZ  ARG X 347     -25.361  18.966 -19.156  1.00  0.00
C
ATOM   5308  NH1 ARG X 347     -25.308  20.159 -18.583  1.00  0.00
N
ATOM   5309 HH11 ARG X 347     -26.108  20.750 -18.484  1.00  0.00
H
ATOM   5310 HH12 ARG X 347     -24.450  20.672 -18.543  1.00  0.00
H
ATOM   5311  NH2 ARG X 347     -24.319  18.426 -19.734  1.00  0.00
N
ATOM   5312 HH21 ARG X 347     -23.445  18.905 -19.819  1.00  0.00
H
ATOM   5313 HH22 ARG X 347     -24.453  17.734 -20.443  1.00  0.00
H
ATOM   5314  C   ARG X 347     -28.709  15.789 -14.992  1.00  0.00
C
ATOM   5315  O   ARG X 347     -29.587  15.102 -15.419  1.00  0.00
O
ATOM   5316  N   LEU X 348     -28.941  16.718 -14.037  1.00  0.00
N
ATOM   5317  HN  LEU X 348     -28.152  17.244 -13.728  1.00  0.00
H
ATOM   5318  CA  LEU X 348     -30.081  16.698 -13.159  1.00  0.00
C
ATOM   5319  HA  LEU X 348     -30.747  15.879 -13.386  1.00  0.00
H
ATOM   5320  CB  LEU X 348     -29.597  16.804 -11.658  1.00  0.00
C
ATOM   5321  HB1 LEU X 348     -29.014  17.741 -11.788  1.00  0.00
H
ATOM   5322  HB2 LEU X 348     -28.849  16.021 -11.409  1.00  0.00
H
ATOM   5323  CG  LEU X 348     -30.757  16.680 -10.604  1.00  0.00
C
ATOM   5324  HG  LEU X 348     -31.535  17.447 -10.808  1.00  0.00
H
ATOM   5325  CD1 LEU X 348     -31.441  15.332 -10.613  1.00  0.00
C
```

ATOM 5326 HD11 LEU X 348 -31.971 15.120 -11.566 1.00 0.00
H
ATOM 5327 HD12 LEU X 348 -32.234 15.312 -9.834 1.00 0.00
H
ATOM 5328 HD13 LEU X 348 -30.840 14.403 -10.517 1.00 0.00
H
ATOM 5329 CD2 LEU X 348 -30.307 17.047 -9.124 1.00 0.00
C
ATOM 5330 HD21 LEU X 348 -29.527 16.318 -8.818 1.00 0.00
H
ATOM 5331 HD22 LEU X 348 -31.215 17.208 -8.505 1.00 0.00
H
ATOM 5332 HD23 LEU X 348 -29.660 17.950 -9.157 1.00 0.00
H
ATOM 5333 C LEU X 348 -30.957 17.879 -13.480 1.00 0.00
C
ATOM 5334 O LEU X 348 -32.165 17.840 -13.336 1.00 0.00
O
ATOM 5335 N PHE X 349 -30.319 18.930 -13.909 1.00 0.00
N
ATOM 5336 HN PHE X 349 -29.369 19.105 -13.661 1.00 0.00
H
ATOM 5337 CA PHE X 349 -31.067 20.137 -14.230 1.00 0.00
C
ATOM 5338 HA PHE X 349 -32.144 20.071 -14.170 1.00 0.00
H
ATOM 5339 CB PHE X 349 -30.472 21.291 -13.340 1.00 0.00
C
ATOM 5340 HB1 PHE X 349 -30.769 22.333 -13.588 1.00 0.00
H
ATOM 5341 HB2 PHE X 349 -29.387 21.289 -13.579 1.00 0.00
H
ATOM 5342 CG PHE X 349 -30.709 21.046 -11.810 1.00 0.00
C
ATOM 5343 CD1 PHE X 349 -31.970 21.144 -11.252 1.00 0.00
C
ATOM 5344 HD1 PHE X 349 -32.738 21.288 -11.997 1.00 0.00
H
ATOM 5345 CE1 PHE X 349 -32.148 21.135 -9.885 1.00 0.00
C
ATOM 5346 HE1 PHE X 349 -33.111 21.250 -9.408 1.00 0.00
H
ATOM 5347 CZ PHE X 349 -31.042 20.963 -8.991 1.00 0.00
C
ATOM 5348 HZ PHE X 349 -31.145 20.992 -7.916 1.00 0.00
H
ATOM 5349 CD2 PHE X 349 -29.577 20.969 -10.968 1.00 0.00
C
ATOM 5350 HD2 PHE X 349 -28.566 21.040 -11.342 1.00 0.00
H

ATOM   5351 CE2 PHE X 349   -29.743 20.870 -9.564 1.00 0.00
C
ATOM   5352 HE2 PHE X 349   -28.899 20.580 -8.956 1.00 0.00
H
ATOM   5353 C   PHE X 349   -30.679 20.566 -15.576 1.00 0.00
C
ATOM   5354 O   PHE X 349   -29.473 20.534 -15.972 1.00 0.00
O
ATOM   5355 N   ARG X 350   -31.709 21.005 -16.409 1.00 0.00
N
ATOM   5356 HN ARG X 350   -32.640 20.883 -16.074 1.00 0.00
H
ATOM   5357 CA ARG X 350   -31.399 21.592 -17.710 1.00 0.00
C
ATOM   5358 HA ARG X 350   -30.485 22.168 -17.700 1.00 0.00
H
ATOM   5359 CB ARG X 350   -31.232 20.595 -18.871 1.00 0.00
C
ATOM   5360 HB1 ARG X 350   -32.211 20.298 -19.305 1.00 0.00
H
ATOM   5361 HB2 ARG X 350   -30.806 19.628 -18.530 1.00 0.00
H
ATOM   5362 CG ARG X 350   -30.445 21.144 -20.063 1.00 0.00
C
ATOM   5363 HG1 ARG X 350   -29.516 21.480 -19.555 1.00 0.00
H
ATOM   5364 HG2 ARG X 350   -30.863 22.100 -20.444 1.00 0.00
H
ATOM   5365 CD ARG X 350   -30.252 20.234 -21.231 1.00 0.00
C
ATOM   5366 HD1 ARG X 350   -31.104 19.703 -21.707 1.00 0.00
H
ATOM   5367 HD2 ARG X 350   -29.717 19.471 -20.626 1.00 0.00
H
ATOM   5368 NE ARG X 350   -29.459 20.855 -22.196 1.00 0.00
N
ATOM   5369 HE ARG X 350   -28.520 21.097 -21.950 1.00 0.00
H
ATOM   5370 CZ ARG X 350   -29.790 21.112 -23.469 1.00 0.00
C
ATOM   5371 NH1 ARG X 350   -30.999 20.924 -23.971 1.00 0.00
N
ATOM   5372 HH11 ARG X 350   -31.783 20.676 -23.402 1.00 0.00
H
ATOM   5373 HH12 ARG X 350   -31.069 21.481 -24.799 1.00 0.00
H
ATOM   5374 NH2 ARG X 350   -28.800 21.526 -24.234 1.00 0.00
N
ATOM   5375 HH21 ARG X 350   -27.855 21.531 -23.906 1.00 0.00
H

ATOM 5376 HH22 ARG X 350 -29.082 21.901 -25.117 1.00 0.00
H
ATOM 5377 C ARG X 350 -32.622 22.516 -18.094 1.00 0.00
C
ATOM 5378 O ARG X 350 -33.738 22.442 -17.573 1.00 0.00
O
ATOM 5379 N LYS X 351 -32.493 23.459 -19.072 1.00 0.00
N
ATOM 5380 HN LYS X 351 -31.549 23.663 -19.320 1.00 0.00
H
ATOM 5381 CA LYS X 351 -33.611 24.370 -19.385 1.00 0.00
C
ATOM 5382 HA LYS X 351 -34.353 24.411 -18.602 1.00 0.00
H
ATOM 5383 CB LYS X 351 -33.068 25.738 -19.586 1.00 0.00
C
ATOM 5384 HB1 LYS X 351 -33.849 26.416 -19.992 1.00 0.00
H
ATOM 5385 HB2 LYS X 351 -32.265 25.641 -20.348 1.00 0.00
H
ATOM 5386 CG LYS X 351 -32.472 26.486 -18.326 1.00 0.00
C
ATOM 5387 HG1 LYS X 351 -32.021 27.472 -18.570 1.00 0.00
H
ATOM 5388 HG2 LYS X 351 -31.636 25.894 -17.897 1.00 0.00
H
ATOM 5389 CD LYS X 351 -33.573 26.822 -17.294 1.00 0.00
C
ATOM 5390 HD1 LYS X 351 -33.970 25.880 -16.859 1.00 0.00
H
ATOM 5391 HD2 LYS X 351 -34.427 27.269 -17.846 1.00 0.00
H
ATOM 5392 CE LYS X 351 -33.064 27.727 -16.119 1.00 0.00
C
ATOM 5393 HE1 LYS X 351 -32.044 27.371 -15.860 1.00 0.00
H
ATOM 5394 HE2 LYS X 351 -33.704 27.708 -15.212 1.00 0.00
H
ATOM 5395 NZ LYS X 351 -32.932 29.078 -16.557 1.00 0.00
N
ATOM 5396 HZ1 LYS X 351 -33.432 29.758 -15.950 1.00 0.00
H
ATOM 5397 HZ2 LYS X 351 -33.045 29.207 -17.583 1.00 0.00
H
ATOM 5398 HZ3 LYS X 351 -31.925 29.332 -16.495 1.00 0.00
H
ATOM 5399 C LYS X 351 -34.420 23.843 -20.564 1.00 0.00
C
ATOM 5400 O LYS X 351 -35.503 24.327 -20.888 1.00 0.00
O

ATOM 5401 N SER X 352 -33.889 22.943 -21.338 1.00 0.00
N
ATOM 5402 HN SER X 352 -33.005 22.487 -21.278 1.00 0.00
H
ATOM 5403 CA SER X 352 -34.688 22.350 -22.478 1.00 0.00
C
ATOM 5404 HA SER X 352 -35.718 22.541 -22.217 1.00 0.00
H
ATOM 5405 CB SER X 352 -34.338 22.980 -23.861 1.00 0.00
C
ATOM 5406 HB1 SER X 352 -33.368 22.589 -24.236 1.00 0.00
H
ATOM 5407 HB2 SER X 352 -34.263 24.085 -23.771 1.00 0.00
H
ATOM 5408 OG SER X 352 -35.380 22.735 -24.827 1.00 0.00
O
ATOM 5409 HG1 SER X 352 -36.223 22.869 -24.389 1.00 0.00
H
ATOM 5410 C SER X 352 -34.443 20.841 -22.559 1.00 0.00
C
ATOM 5411 O SER X 352 -33.300 20.402 -22.290 1.00 0.00
O
ATOM 5412 N ASN X 353 -35.376 19.997 -22.851 1.00 0.00
N
ATOM 5413 HN ASN X 353 -36.305 20.320 -23.014 1.00 0.00
H
ATOM 5414 CA ASN X 353 -35.323 18.544 -22.973 1.00 0.00
C
ATOM 5415 HA ASN X 353 -34.838 18.132 -22.100 1.00 0.00
H
ATOM 5416 CB ASN X 353 -36.716 17.811 -23.115 1.00 0.00
C
ATOM 5417 HB1 ASN X 353 -36.547 16.713 -23.152 1.00 0.00
H
ATOM 5418 HB2 ASN X 353 -37.256 18.023 -24.062 1.00 0.00
H
ATOM 5419 CG ASN X 353 -37.622 18.209 -22.025 1.00 0.00
C
ATOM 5420 OD1 ASN X 353 -37.187 18.502 -20.817 1.00 0.00
O
ATOM 5421 ND2 ASN X 353 -38.937 18.279 -22.171 1.00 0.00
N
ATOM 5422 HD21 ASN X 353 -39.453 18.601 -21.377 1.00 0.00
H
ATOM 5423 HD22 ASN X 353 -39.399 18.333 -23.056 1.00 0.00
H
ATOM 5424 C ASN X 353 -34.424 18.135 -24.159 1.00 0.00
C
ATOM 5425 O ASN X 353 -34.355 18.812 -25.137 1.00 0.00
O

ATOM 5426 N LEU X 354 -33.856 16.855 -24.173 1.00 0.00
N
ATOM 5427 HN LEU X 354 -33.703 16.329 -23.340 1.00 0.00
H
ATOM 5428 CA LEU X 354 -33.036 16.417 -25.261 1.00 0.00
C
ATOM 5429 HA LEU X 354 -32.518 17.328 -25.524 1.00 0.00
H
ATOM 5430 CB LEU X 354 -32.012 15.406 -24.917 1.00 0.00
C
ATOM 5431 HB1 LEU X 354 -31.333 15.316 -25.791 1.00 0.00
H
ATOM 5432 HB2 LEU X 354 -32.448 14.416 -24.666 1.00 0.00
H
ATOM 5433 CG LEU X 354 -31.101 15.917 -23.742 1.00 0.00
C
ATOM 5434 HG LEU X 354 -31.663 15.837 -22.787 1.00 0.00
H
ATOM 5435 CD1 LEU X 354 -29.905 14.915 -23.554 1.00 0.00
C
ATOM 5436 HD11 LEU X 354 -29.439 14.938 -22.545 1.00 0.00
H
ATOM 5437 HD12 LEU X 354 -29.166 15.037 -24.374 1.00 0.00
H
ATOM 5438 HD13 LEU X 354 -30.200 13.845 -23.582 1.00 0.00
H
ATOM 5439 CD2 LEU X 354 -30.482 17.293 -23.788 1.00 0.00
C
ATOM 5440 HD21 LEU X 354 -29.748 17.495 -22.978 1.00 0.00
H
ATOM 5441 HD22 LEU X 354 -31.265 18.069 -23.926 1.00 0.00
H
ATOM 5442 HD23 LEU X 354 -29.782 17.348 -24.649 1.00 0.00
H
ATOM 5443 C LEU X 354 -33.816 15.891 -26.443 1.00 0.00
C
ATOM 5444 O LEU X 354 -34.965 15.418 -26.257 1.00 0.00
O
ATOM 5445 N LYS X 355 -33.366 16.095 -27.662 1.00 0.00
N
ATOM 5446 HN LYS X 355 -32.519 16.554 -27.919 1.00 0.00
H
ATOM 5447 CA LYS X 355 -33.967 15.415 -28.851 1.00 0.00
C
ATOM 5448 HA LYS X 355 -35.013 15.284 -28.615 1.00 0.00
H
ATOM 5449 CB LYS X 355 -33.670 16.357 -30.035 1.00 0.00
C
ATOM 5450 HB1 LYS X 355 -34.192 15.898 -30.902 1.00 0.00
H

ATOM 5451 HB2 LYS X 355 −32.581 16.397 −30.256 1.00 0.00
H
ATOM 5452 CG LYS X 355 −34.053 17.871 −29.902 1.00 0.00
C
ATOM 5453 HG1 LYS X 355 −33.530 18.349 −29.047 1.00 0.00
H
ATOM 5454 HG2 LYS X 355 −35.154 17.857 −29.750 1.00 0.00
H
ATOM 5455 CD LYS X 355 −33.654 18.601 −31.193 1.00 0.00
C
ATOM 5456 HD1 LYS X 355 −34.322 18.322 −32.035 1.00 0.00
H
ATOM 5457 HD2 LYS X 355 −32.653 18.382 −31.624 1.00 0.00
H
ATOM 5458 CE LYS X 355 −33.639 20.123 −30.931 1.00 0.00
C
ATOM 5459 HE1 LYS X 355 −34.684 20.397 −30.670 1.00 0.00
H
ATOM 5460 HE2 LYS X 355 −33.333 20.684 −31.840 1.00 0.00
H
ATOM 5461 NZ LYS X 355 −32.587 20.462 −29.931 1.00 0.00
N
ATOM 5462 HZ1 LYS X 355 −32.139 21.367 −30.177 1.00 0.00
H
ATOM 5463 HZ2 LYS X 355 −31.905 19.678 −29.979 1.00 0.00
H
ATOM 5464 HZ3 LYS X 355 −32.841 20.570 −28.928 1.00 0.00
H
ATOM 5465 C LYS X 355 −33.300 14.003 −28.995 1.00 0.00
C
ATOM 5466 O LYS X 355 −32.237 13.713 −28.422 1.00 0.00
O
ATOM 5467 N PRO X 356 −33.953 13.138 −29.728 1.00 0.00
N
ATOM 5468 CD PRO X 356 −35.371 13.280 −30.322 1.00 0.00
C
ATOM 5469 HD1 PRO X 356 −35.423 14.170 −30.984 1.00 0.00
H
ATOM 5470 HD2 PRO X 356 −36.201 13.322 −29.584 1.00 0.00
H
ATOM 5471 CA PRO X 356 −33.266 11.956 −30.358 1.00 0.00
C
ATOM 5472 HA PRO X 356 −33.378 11.046 −29.787 1.00 0.00
H
ATOM 5473 CB PRO X 356 −34.014 11.836 −31.703 1.00 0.00
C
ATOM 5474 HB1 PRO X 356 −33.882 10.825 −32.145 1.00 0.00
H
ATOM 5475 HB2 PRO X 356 −33.758 12.583 −32.485 1.00 0.00
H

```
ATOM   5476  CG  PRO X 356     -35.531  12.128 -31.278  1.00  0.00
C
ATOM   5477  HG1 PRO X 356     -36.227  12.432 -32.089  1.00  0.00
H
ATOM   5478  HG2 PRO X 356     -35.784  11.188 -30.743  1.00  0.00
H
ATOM   5479  C   PRO X 356     -31.766  11.971 -30.618  1.00  0.00
C
ATOM   5480  O   PRO X 356     -31.337  12.597 -31.615  1.00  0.00
O
ATOM   5481  N   PHE X 357     -30.942  11.179 -29.857  1.00  0.00
N
ATOM   5482  HN  PHE X 357     -31.289  10.408 -29.328  1.00  0.00
H
ATOM   5483  CA  PHE X 357     -29.439  11.117 -30.069  1.00  0.00
C
ATOM   5484  HA  PHE X 357     -28.999  10.538 -29.271  1.00  0.00
H
ATOM   5485  CB  PHE X 357     -29.136  10.488 -31.484  1.00  0.00
C
ATOM   5486  HB1 PHE X 357     -28.032  10.389 -31.565  1.00  0.00
H
ATOM   5487  HB2 PHE X 357     -29.417  11.199 -32.289  1.00  0.00
H
ATOM   5488  CG  PHE X 357     -29.747   9.144 -31.716  1.00  0.00
C
ATOM   5489  CD1 PHE X 357     -29.463   8.002 -30.936  1.00  0.00
C
ATOM   5490  HD1 PHE X 357     -28.891   8.034 -30.020  1.00  0.00
H
ATOM   5491  CE1 PHE X 357     -29.999   6.745 -31.241  1.00  0.00
C
ATOM   5492  HE1 PHE X 357     -29.801   6.013 -30.472  1.00  0.00
H
ATOM   5493  CZ  PHE X 357     -30.783   6.608 -32.394  1.00  0.00
C
ATOM   5494  HZ  PHE X 357     -31.259   5.654 -32.566  1.00  0.00
H
ATOM   5495  CD2 PHE X 357     -30.530   8.978 -32.854  1.00  0.00
C
ATOM   5496  HD2 PHE X 357     -30.618   9.819 -33.526  1.00  0.00
H
ATOM   5497  CE2 PHE X 357     -30.999   7.777 -33.246  1.00  0.00
C
ATOM   5498  HE2 PHE X 357     -31.627   7.728 -34.124  1.00  0.00
H
ATOM   5499  C   PHE X 357     -28.727  12.410 -29.871  1.00  0.00
C
ATOM   5500  O   PHE X 357     -27.653  12.591 -30.367  1.00  0.00
O
```

ATOM 5501 N GLU X 358 -29.245 13.334 -29.084 1.00 0.00
N
ATOM 5502 HN GLU X 358 -30.186 13.195 -28.787 1.00 0.00
H
ATOM 5503 CA GLU X 358 -28.661 14.640 -28.776 1.00 0.00
C
ATOM 5504 HA GLU X 358 -27.965 14.925 -29.552 1.00 0.00
H
ATOM 5505 CB GLU X 358 -29.825 15.597 -28.588 1.00 0.00
C
ATOM 5506 HB1 GLU X 358 -30.321 15.383 -27.618 1.00 0.00
H
ATOM 5507 HB2 GLU X 358 -30.463 15.439 -29.484 1.00 0.00
H
ATOM 5508 CG GLU X 358 -29.342 17.026 -28.709 1.00 0.00
C
ATOM 5509 HG1 GLU X 358 -28.751 17.223 -29.630 1.00 0.00
H
ATOM 5510 HG2 GLU X 358 -28.687 17.443 -27.914 1.00 0.00
H
ATOM 5511 CD GLU X 358 -30.484 17.967 -28.817 1.00 0.00
C
ATOM 5512 OE1 GLU X 358 -30.619 18.665 -29.822 1.00 0.00
O
ATOM 5513 OE2 GLU X 358 -31.306 17.873 -27.865 1.00 0.00
O
ATOM 5514 C GLU X 358 -27.859 14.547 -27.439 1.00 0.00
C
ATOM 5515 O GLU X 358 -28.222 13.999 -26.415 1.00 0.00
O
ATOM 5516 N ARG X 359 -26.644 15.160 -27.520 1.00 0.00
N
ATOM 5517 HN ARG X 359 -26.382 15.497 -28.421 1.00 0.00
H
ATOM 5518 CA ARG X 359 -25.670 15.172 -26.446 1.00 0.00
C
ATOM 5519 HA ARG X 359 -26.164 14.760 -25.579 1.00 0.00
H
ATOM 5520 CB ARG X 359 -24.424 14.333 -26.749 1.00 0.00
C
ATOM 5521 HB1 ARG X 359 -23.726 14.879 -27.419 1.00 0.00
H
ATOM 5522 HB2 ARG X 359 -24.664 13.281 -27.016 1.00 0.00
H
ATOM 5523 CG ARG X 359 -23.555 14.108 -25.497 1.00 0.00
C
ATOM 5524 HG1 ARG X 359 -24.131 13.404 -24.858 1.00 0.00
H
ATOM 5525 HG2 ARG X 359 -23.368 15.063 -24.962 1.00 0.00
H

```
ATOM   5526  CD  ARG X 359     -22.166  13.488 -25.610  1.00  0.00
C
ATOM   5527  HD1 ARG X 359     -22.053  12.478 -26.059  1.00  0.00
H
ATOM   5528  HD2 ARG X 359     -21.825  13.365 -24.560  1.00  0.00
H
ATOM   5529  NE  ARG X 359     -21.371  14.584 -26.254  1.00  0.00
N
ATOM   5530  HE  ARG X 359     -21.762  15.376 -26.720  1.00  0.00
H
ATOM   5531  CZ  ARG X 359     -20.018  14.593 -26.306  1.00  0.00
C
ATOM   5532  NH1 ARG X 359     -19.368  15.492 -26.990  1.00  0.00
N
ATOM   5533 HH11 ARG X 359     -19.960  16.110 -27.507  1.00  0.00
H
ATOM   5534 HH12 ARG X 359     -18.374  15.527 -26.894  1.00  0.00
H
ATOM   5535  NH2 ARG X 359     -19.312  13.698 -25.617  1.00  0.00
N
ATOM   5536 HH21 ARG X 359     -19.726  12.870 -25.237  1.00  0.00
H
ATOM   5537 HH22 ARG X 359     -18.327  13.769 -25.770  1.00  0.00
H
ATOM   5538  C   ARG X 359     -25.382  16.624 -26.055  1.00  0.00
C
ATOM   5539  O   ARG X 359     -25.098  17.523 -26.877  1.00  0.00
O
ATOM   5540  N   ASP X 360     -25.464  16.939 -24.727  1.00  0.00
N
ATOM   5541  HN  ASP X 360     -25.909  16.293 -24.112  1.00  0.00
H
ATOM   5542  CA  ASP X 360     -25.065  18.252 -24.185  1.00  0.00
C
ATOM   5543  HA  ASP X 360     -24.767  18.976 -24.930  1.00  0.00
H
ATOM   5544  CB  ASP X 360     -26.251  18.828 -23.348  1.00  0.00
C
ATOM   5545  HB1 ASP X 360     -26.237  18.187 -22.441  1.00  0.00
H
ATOM   5546  HB2 ASP X 360     -27.208  18.820 -23.913  1.00  0.00
H
ATOM   5547  CG  ASP X 360     -25.840  20.269 -22.843  1.00  0.00
C
ATOM   5548  OD1 ASP X 360     -24.658  20.680 -22.984  1.00  0.00
O
ATOM   5549  OD2 ASP X 360     -26.691  20.991 -22.259  1.00  0.00
O
ATOM   5550  C   ASP X 360     -23.809  17.828 -23.350  1.00  0.00
C
```

```
ATOM   5551  O   ASP X 360     -23.863  17.064 -22.450  1.00  0.00
O
ATOM   5552  N   ILE X 361     -22.615  18.314 -23.669  1.00  0.00
N
ATOM   5553  HN  ILE X 361     -22.384  18.795 -24.511  1.00  0.00
H
ATOM   5554  CA  ILE X 361     -21.382  17.944 -22.948  1.00  0.00
C
ATOM   5555  HA  ILE X 361     -21.518  17.021 -22.405  1.00  0.00
H
ATOM   5556  CB  ILE X 361     -20.229  17.711 -23.970  1.00  0.00
C
ATOM   5557  HB  ILE X 361     -20.672  17.053 -24.748  1.00  0.00
H
ATOM   5558  CG2 ILE X 361     -19.769  19.071 -24.737  1.00  0.00
C
ATOM   5559 HG21 ILE X 361     -19.381  18.992 -25.775  1.00  0.00
H
ATOM   5560 HG22 ILE X 361     -19.126  19.620 -24.017  1.00  0.00
H
ATOM   5561 HG23 ILE X 361     -20.688  19.694 -24.788  1.00  0.00
H
ATOM   5562  CG1 ILE X 361     -19.018  16.925 -23.355  1.00  0.00
C
ATOM   5563 HG11 ILE X 361     -18.452  17.556 -22.637  1.00  0.00
H
ATOM   5564 HG12 ILE X 361     -18.314  16.746 -24.195  1.00  0.00
H
ATOM   5565  CD  ILE X 361     -19.541  15.690 -22.626  1.00  0.00
C
ATOM   5566  HD1 ILE X 361     -20.313  15.183 -23.244  1.00  0.00
H
ATOM   5567  HD2 ILE X 361     -19.856  15.862 -21.575  1.00  0.00
H
ATOM   5568  HD3 ILE X 361     -18.700  14.965 -22.669  1.00  0.00
H
ATOM   5569  C   ILE X 361     -20.842  19.110 -22.007  1.00  0.00
C
ATOM   5570  O   ILE X 361     -19.923  19.076 -21.242  1.00  0.00
O
ATOM   5571  N   SER X 362     -21.598  20.243 -22.116  1.00  0.00
N
ATOM   5572  HN  SER X 362     -22.456  20.186 -22.622  1.00  0.00
H
ATOM   5573  CA  SER X 362     -21.409  21.400 -21.234  1.00  0.00
C
ATOM   5574  HA  SER X 362     -20.441  21.790 -21.515  1.00  0.00
H
ATOM   5575  CB  SER X 362     -22.591  22.443 -21.499  1.00  0.00
C
```

ATOM 5576 HB1 SER X 362 -23.514 21.855 -21.306 1.00 0.00
H
ATOM 5577 HB2 SER X 362 -22.536 22.691 -22.581 1.00 0.00
H
ATOM 5578 OG SER X 362 -22.563 23.601 -20.687 1.00 0.00
O
ATOM 5579 HG1 SER X 362 -22.851 24.342 -21.224 1.00 0.00
H
ATOM 5580 C SER X 362 -21.422 21.203 -19.717 1.00 0.00
C
ATOM 5581 O SER X 362 -22.205 20.438 -19.147 1.00 0.00
O
ATOM 5582 N THR X 363 -20.524 21.983 -19.006 1.00 0.00
N
ATOM 5583 HN THR X 363 -19.920 22.609 -19.493 1.00 0.00
H
ATOM 5584 CA THR X 363 -20.433 21.890 -17.519 1.00 0.00
C
ATOM 5585 HA THR X 363 -21.169 21.145 -17.258 1.00 0.00
H
ATOM 5586 CB THR X 363 -19.104 21.456 -17.033 1.00 0.00
C
ATOM 5587 HB THR X 363 -19.005 21.498 -15.927 1.00 0.00
H
ATOM 5588 OG1 THR X 363 -18.052 22.152 -17.705 1.00 0.00
O
ATOM 5589 HG1 THR X 363 -18.096 23.085 -17.487 1.00 0.00
H
ATOM 5590 CG2 THR X 363 -18.896 19.951 -17.588 1.00 0.00
C
ATOM 5591 HG21 THR X 363 -17.879 19.646 -17.260 1.00 0.00
H
ATOM 5592 HG22 THR X 363 -18.984 19.766 -18.680 1.00 0.00
H
ATOM 5593 HG23 THR X 363 -19.504 19.334 -16.893 1.00 0.00
H
ATOM 5594 C THR X 363 -20.868 23.355 -16.943 1.00 0.00
C
ATOM 5595 O THR X 363 -20.746 23.563 -15.780 1.00 0.00
O
ATOM 5596 N GLU X 364 -21.217 24.330 -17.755 1.00 0.00
N
ATOM 5597 HN GLU X 364 -21.325 24.047 -18.704 1.00 0.00
H
ATOM 5598 CA GLU X 364 -21.661 25.615 -17.432 1.00 0.00
C
ATOM 5599 HA GLU X 364 -20.952 26.227 -16.894 1.00 0.00
H
ATOM 5600 CB GLU X 364 -21.946 26.466 -18.769 1.00 0.00
C

ATOM 5601 HB1 GLU X 364 −22.330 27.480 −18.526 1.00 0.00
H
ATOM 5602 HB2 GLU X 364 −22.717 26.114 −19.488 1.00 0.00
H
ATOM 5603 CG GLU X 364 −20.774 26.791 −19.706 1.00 0.00
C
ATOM 5604 HG1 GLU X 364 −20.602 25.845 −20.263 1.00 0.00
H
ATOM 5605 HG2 GLU X 364 −19.895 27.102 −19.101 1.00 0.00
H
ATOM 5606 CD GLU X 364 −21.130 27.905 −20.694 1.00 0.00
C
ATOM 5607 OE1 GLU X 364 −21.216 29.184 −20.389 1.00 0.00
O
ATOM 5608 OE2 GLU X 364 −21.262 27.545 −21.888 1.00 0.00
O
ATOM 5609 C GLU X 364 −22.819 25.680 −16.357 1.00 0.00
C
ATOM 5610 O GLU X 364 −23.693 24.836 −16.297 1.00 0.00
O
ATOM 5611 N ILE X 365 −22.602 26.615 −15.331 1.00 0.00
N
ATOM 5612 HN ILE X 365 −21.763 27.154 −15.347 1.00 0.00
H
ATOM 5613 CA ILE X 365 −23.389 26.549 −14.074 1.00 0.00
C
ATOM 5614 HA ILE X 365 −23.239 25.536 −13.730 1.00 0.00
H
ATOM 5615 CB ILE X 365 −22.788 27.476 −12.890 1.00 0.00
C
ATOM 5616 HB ILE X 365 −21.776 27.719 −13.280 1.00 0.00
H
ATOM 5617 CG2 ILE X 365 −23.413 28.867 −12.893 1.00 0.00
C
ATOM 5618 HG21 ILE X 365 −22.885 29.370 −12.055 1.00 0.00
H
ATOM 5619 HG22 ILE X 365 −24.481 28.909 −12.587 1.00 0.00
H
ATOM 5620 HG23 ILE X 365 −23.158 29.368 −13.851 1.00 0.00
H
ATOM 5621 CG1 ILE X 365 −22.883 26.756 −11.524 1.00 0.00
C
ATOM 5622 HG11 ILE X 365 −22.910 25.650 −11.621 1.00 0.00
H
ATOM 5623 HG12 ILE X 365 −23.876 27.064 −11.131 1.00 0.00
H
ATOM 5624 CD ILE X 365 −21.814 27.084 −10.465 1.00 0.00
C
ATOM 5625 HD1 ILE X 365 −21.931 28.158 −10.206 1.00 0.00
H

```
ATOM   5626  HD2 ILE X 365     -20.818  26.796 -10.866  1.00  0.00
H
ATOM   5627  HD3 ILE X 365     -22.022  26.520  -9.531  1.00  0.00
H
ATOM   5628  C   ILE X 365     -24.875  26.744 -14.369  1.00  0.00
C
ATOM   5629  O   ILE X 365     -25.243  27.654 -15.130  1.00  0.00
O
ATOM   5630  N   TYR X 366     -25.802  26.029 -13.656  1.00  0.00
N
ATOM   5631  HN  TYR X 366     -25.431  25.525 -12.880  1.00  0.00
H
ATOM   5632  CA  TYR X 366     -27.261  26.209 -13.919  1.00  0.00
C
ATOM   5633  HA  TYR X 366     -27.269  26.667 -14.897  1.00  0.00
H
ATOM   5634  CB  TYR X 366     -28.036  24.837 -13.843  1.00  0.00
C
ATOM   5635  HB1 TYR X 366     -27.652  24.520 -12.850  1.00  0.00
H
ATOM   5636  HB2 TYR X 366     -27.553  24.088 -14.506  1.00  0.00
H
ATOM   5637  CG  TYR X 366     -29.550  24.868 -13.859  1.00  0.00
C
ATOM   5638  CD1 TYR X 366     -30.251  24.592 -15.029  1.00  0.00
C
ATOM   5639  HD1 TYR X 366     -29.669  24.435 -15.926  1.00  0.00
H
ATOM   5640  CE1 TYR X 366     -31.671  24.551 -15.129  1.00  0.00
C
ATOM   5641  HE1 TYR X 366     -32.065  24.453 -16.129  1.00  0.00
H
ATOM   5642  CZ  TYR X 366     -32.394  24.731 -13.992  1.00  0.00
C
ATOM   5643  OH  TYR X 366     -33.802  24.657 -14.224  1.00  0.00
O
ATOM   5644  HH  TYR X 366     -34.228  24.980 -13.427  1.00  0.00
H
ATOM   5645  CD2 TYR X 366     -30.343  24.942 -12.683  1.00  0.00
C
ATOM   5646  HD2 TYR X 366     -29.806  25.039 -11.751  1.00  0.00
H
ATOM   5647  CE2 TYR X 366     -31.785  24.909 -12.750  1.00  0.00
C
ATOM   5648  HE2 TYR X 366     -32.423  24.873 -11.880  1.00  0.00
H
ATOM   5649  C   TYR X 366     -27.889  27.252 -12.982  1.00  0.00
C
ATOM   5650  O   TYR X 366     -27.913  27.138 -11.727  1.00  0.00
O
```

ATOM 5651 N GLN X 367 −28.509 28.380 −13.504 1.00 0.00
N
ATOM 5652 HN GLN X 367 −28.455 28.445 −14.498 1.00 0.00
H
ATOM 5653 CA GLN X 367 −29.283 29.367 −12.688 1.00 0.00
C
ATOM 5654 HA GLN X 367 −29.034 29.160 −11.658 1.00 0.00
H
ATOM 5655 CB GLN X 367 −28.980 30.859 −13.141 1.00 0.00
C
ATOM 5656 HB1 GLN X 367 −29.476 31.600 −12.478 1.00 0.00
H
ATOM 5657 HB2 GLN X 367 −29.384 30.885 −14.176 1.00 0.00
H
ATOM 5658 CG GLN X 367 −27.456 31.257 −13.221 1.00 0.00
C
ATOM 5659 HG1 GLN X 367 −26.914 30.412 −13.698 1.00 0.00
H
ATOM 5660 HG2 GLN X 367 −27.239 31.308 −12.133 1.00 0.00
H
ATOM 5661 CD GLN X 367 −27.296 32.601 −13.938 1.00 0.00
C
ATOM 5662 OE1 GLN X 367 −27.009 33.653 −13.340 1.00 0.00
O
ATOM 5663 NE2 GLN X 367 −27.582 32.570 −15.295 1.00 0.00
N
ATOM 5664 HE21 GLN X 367 −27.166 33.296 −15.842 1.00 0.00
H
ATOM 5665 HE22 GLN X 367 −27.922 31.712 −15.681 1.00 0.00
H
ATOM 5666 C GLN X 367 −30.768 29.051 −12.803 1.00 0.00
C
ATOM 5667 O GLN X 367 −31.280 29.097 −13.923 1.00 0.00
O
ATOM 5668 N ALA X 368 −31.468 28.743 −11.687 1.00 0.00
N
ATOM 5669 HN ALA X 368 −31.057 28.826 −10.782 1.00 0.00
H
ATOM 5670 CA ALA X 368 −32.900 28.360 −11.668 1.00 0.00
C
ATOM 5671 HA ALA X 368 −32.907 27.588 −12.424 1.00 0.00
H
ATOM 5672 CB ALA X 368 −33.292 27.457 −10.503 1.00 0.00
C
ATOM 5673 HB1 ALA X 368 −32.489 26.766 −10.169 1.00 0.00
H
ATOM 5674 HB2 ALA X 368 −34.246 26.916 −10.680 1.00 0.00
H
ATOM 5675 HB3 ALA X 368 −33.546 28.207 −9.724 1.00 0.00
H

ATOM 5676 C ALA X 368 -33.892 29.350 -12.104 1.00 0.00
C
ATOM 5677 O ALA X 368 -33.748 30.534 -11.781 1.00 0.00
O
ATOM 5678 N GLY X 369 -34.924 29.009 -12.887 1.00 0.00
N
ATOM 5679 HN GLY X 369 -35.145 28.045 -13.007 1.00 0.00
H
ATOM 5680 CA GLY X 369 -35.917 29.792 -13.581 1.00 0.00
C
ATOM 5681 HA1 GLY X 369 -36.559 30.279 -12.862 1.00 0.00
H
ATOM 5682 HA2 GLY X 369 -36.480 29.109 -14.200 1.00 0.00
H
ATOM 5683 C GLY X 369 -35.361 30.921 -14.426 1.00 0.00
C
ATOM 5684 O GLY X 369 -34.232 31.044 -14.765 1.00 0.00
O
ATOM 5685 N SER X 370 -36.253 31.868 -14.767 1.00 0.00
N
ATOM 5686 HN SER X 370 -37.163 31.747 -14.377 1.00 0.00
H
ATOM 5687 CA SER X 370 -35.957 33.017 -15.614 1.00 0.00
C
ATOM 5688 HA SER X 370 -35.187 32.755 -16.325 1.00 0.00
H
ATOM 5689 CB SER X 370 -37.160 33.402 -16.549 1.00 0.00
C
ATOM 5690 HB1 SER X 370 -37.262 32.568 -17.276 1.00 0.00
H
ATOM 5691 HB2 SER X 370 -36.907 34.285 -17.173 1.00 0.00
H
ATOM 5692 OG SER X 370 -38.338 33.644 -15.797 1.00 0.00
O
ATOM 5693 HG1 SER X 370 -38.633 32.762 -15.562 1.00 0.00
H
ATOM 5694 C SER X 370 -35.681 34.197 -14.647 1.00 0.00
C
ATOM 5695 O SER X 370 -35.364 35.280 -15.116 1.00 0.00
O
ATOM 5696 N THR X 371 -35.636 33.962 -13.359 1.00 0.00
N
ATOM 5697 HN THR X 371 -35.760 33.096 -12.882 1.00 0.00
H
ATOM 5698 CA THR X 371 -35.348 35.050 -12.384 1.00 0.00
C
ATOM 5699 HA THR X 371 -35.734 35.980 -12.773 1.00 0.00
H
ATOM 5700 CB THR X 371 -36.135 34.881 -11.069 1.00 0.00
C

ATOM 5701 HB THR X 371 −36.118 33.842 −10.675 1.00 0.00
H
ATOM 5702 OG1 THR X 371 −37.477 35.196 −11.346 1.00 0.00
O
ATOM 5703 HG1 THR X 371 −37.990 34.663 −10.735 1.00 0.00
H
ATOM 5704 CG2 THR X 371 −35.713 35.961 −9.927 1.00 0.00
C
ATOM 5705 HG21 THR X 371 −35.668 36.982 −10.363 1.00 0.00
H
ATOM 5706 HG22 THR X 371 −34.742 35.616 −9.512 1.00 0.00
H
ATOM 5707 HG23 THR X 371 −36.504 36.013 −9.148 1.00 0.00
H
ATOM 5708 C THR X 371 −33.874 35.160 −12.055 1.00 0.00
C
ATOM 5709 O THR X 371 −33.296 34.119 −11.812 1.00 0.00
O
ATOM 5710 N PRO X 372 −33.156 36.264 −12.026 1.00 0.00
N
ATOM 5711 CD PRO X 372 −33.626 37.563 −12.586 1.00 0.00
C
ATOM 5712 HD1 PRO X 372 −34.313 38.142 −11.932 1.00 0.00
H
ATOM 5713 HD2 PRO X 372 −34.139 37.453 −13.566 1.00 0.00
H
ATOM 5714 CA PRO X 372 −31.804 36.421 −11.531 1.00 0.00
C
ATOM 5715 HA PRO X 372 −31.226 35.898 −12.278 1.00 0.00
H
ATOM 5716 CB PRO X 372 −31.592 37.982 −11.504 1.00 0.00
C
ATOM 5717 HB1 PRO X 372 −30.512 38.241 −11.484 1.00 0.00
H
ATOM 5718 HB2 PRO X 372 −32.006 38.297 −10.522 1.00 0.00
H
ATOM 5719 CG PRO X 372 −32.445 38.394 −12.710 1.00 0.00
C
ATOM 5720 HG1 PRO X 372 −32.617 39.489 −12.646 1.00 0.00
H
ATOM 5721 HG2 PRO X 372 −31.796 38.205 −13.592 1.00 0.00
H
ATOM 5722 C PRO X 372 −31.386 35.740 −10.172 1.00 0.00
C
ATOM 5723 O PRO X 372 −32.298 35.592 −9.304 1.00 0.00
O
ATOM 5724 N CYS X 373 −30.079 35.393 −9.918 1.00 0.00
N
ATOM 5725 HN CYS X 373 −29.446 35.668 −10.638 1.00 0.00
H

```
ATOM   5726  CA  CYS X 373     -29.563  35.020  -8.589  1.00  0.00
C
ATOM   5727  HA  CYS X 373     -29.940  34.049  -8.304  1.00  0.00
H
ATOM   5728  CB  CYS X 373     -27.973  34.799  -8.592  1.00  0.00
C
ATOM   5729  HB1 CYS X 373     -27.581  34.367  -7.646  1.00  0.00
H
ATOM   5730  HB2 CYS X 373     -27.441  35.719  -8.915  1.00  0.00
H
ATOM   5731  SG  CYS X 373     -27.598  33.457  -9.785  1.00  0.00
S
ATOM   5732  C   CYS X 373     -29.767  36.026  -7.491  1.00  0.00
C
ATOM   5733  O   CYS X 373     -29.609  37.242  -7.577  1.00  0.00
O
ATOM   5734  N   ASN X 374     -30.156  35.526  -6.281  1.00  0.00
N
ATOM   5735  HN  ASN X 374     -30.347  34.549  -6.314  1.00  0.00
H
ATOM   5736  CA  ASN X 374     -30.365  36.215  -5.062  1.00  0.00
C
ATOM   5737  HA  ASN X 374     -30.616  37.254  -5.217  1.00  0.00
H
ATOM   5738  CB  ASN X 374     -31.508  35.740  -4.233  1.00  0.00
C
ATOM   5739  HB1 ASN X 374     -31.746  36.416  -3.384  1.00  0.00
H
ATOM   5740  HB2 ASN X 374     -31.356  34.766  -3.722  1.00  0.00
H
ATOM   5741  CG  ASN X 374     -32.839  35.919  -4.880  1.00  0.00
C
ATOM   5742  OD1 ASN X 374     -33.167  36.894  -5.515  1.00  0.00
O
ATOM   5743  ND2 ASN X 374     -33.720  34.916  -4.630  1.00  0.00
N
ATOM   5744 HD21 ASN X 374     -34.699  34.978  -4.826  1.00  0.00
H
ATOM   5745 HD22 ASN X 374     -33.440  34.056  -4.206  1.00  0.00
H
ATOM   5746  C   ASN X 374     -29.054  36.308  -4.128  1.00  0.00
C
ATOM   5747  O   ASN X 374     -28.313  35.348  -3.982  1.00  0.00
O
ATOM   5748  N   GLY X 375     -28.797  37.544  -3.698  1.00  0.00
N
ATOM   5749  HN  GLY X 375     -29.321  38.241  -4.181  1.00  0.00
H
ATOM   5750  CA  GLY X 375     -27.702  38.068  -2.837  1.00  0.00
C
```

```
ATOM   5751  HA1 GLY X 375     -27.878  37.541  -1.911  1.00  0.00
H
ATOM   5752  HA2 GLY X 375     -27.916  39.121  -2.732  1.00  0.00
H
ATOM   5753  C   GLY X 375     -26.296  37.800  -3.295  1.00  0.00
C
ATOM   5754  O   GLY X 375     -26.137  37.165  -4.332  1.00  0.00
O
ATOM   5755  N   VAL X 376     -25.410  38.256  -2.463  1.00  0.00
N
ATOM   5756  HN  VAL X 376     -25.608  38.799  -1.651  1.00  0.00
H
ATOM   5757  CA  VAL X 376     -23.948  38.231  -2.726  1.00  0.00
C
ATOM   5758  HA  VAL X 376     -23.833  38.829  -3.618  1.00  0.00
H
ATOM   5759  CB  VAL X 376     -23.177  38.923  -1.593  1.00  0.00
C
ATOM   5760  HB  VAL X 376     -23.656  39.922  -1.517  1.00  0.00
H
ATOM   5761  CG1 VAL X 376     -23.438  38.471  -0.164  1.00  0.00
C
ATOM   5762 HG11 VAL X 376     -22.861  37.564   0.118  1.00  0.00
H
ATOM   5763 HG12 VAL X 376     -24.476  38.079  -0.101  1.00  0.00
H
ATOM   5764 HG13 VAL X 376     -23.328  39.338   0.522  1.00  0.00
H
ATOM   5765  CG2 VAL X 376     -21.730  39.187  -1.845  1.00  0.00
C
ATOM   5766 HG21 VAL X 376     -21.590  39.569  -2.879  1.00  0.00
H
ATOM   5767 HG22 VAL X 376     -21.299  38.194  -1.594  1.00  0.00
H
ATOM   5768 HG23 VAL X 376     -21.254  39.861  -1.102  1.00  0.00
H
ATOM   5769  C   VAL X 376     -23.451  36.779  -2.832  1.00  0.00
C
ATOM   5770  O   VAL X 376     -22.627  36.377  -3.680  1.00  0.00
O
ATOM   5771  N   GLU X 377     -24.037  35.783  -2.105  1.00  0.00
N
ATOM   5772  HN  GLU X 377     -24.676  36.048  -1.387  1.00  0.00
H
ATOM   5773  CA  GLU X 377     -23.818  34.354  -2.210  1.00  0.00
C
ATOM   5774  HA  GLU X 377     -22.771  34.096  -2.148  1.00  0.00
H
ATOM   5775  CB  GLU X 377     -24.517  33.699  -1.030  1.00  0.00
C
```

ATOM 5776 HB1 GLU X 377 -24.430 32.592 -1.079 1.00 0.00
H
ATOM 5777 HB2 GLU X 377 -25.585 34.000 -1.098 1.00 0.00
H
ATOM 5778 CG GLU X 377 -23.838 34.205 0.351 1.00 0.00
C
ATOM 5779 HG1 GLU X 377 -24.158 35.237 0.608 1.00 0.00
H
ATOM 5780 HG2 GLU X 377 -22.758 34.316 0.118 1.00 0.00
H
ATOM 5781 CD GLU X 377 -24.142 33.245 1.477 1.00 0.00
C
ATOM 5782 OE1 GLU X 377 -24.397 33.706 2.657 1.00 0.00
O
ATOM 5783 OE2 GLU X 377 -23.923 32.067 1.260 1.00 0.00
O
ATOM 5784 C GLU X 377 -24.325 33.608 -3.437 1.00 0.00
C
ATOM 5785 O GLU X 377 -23.878 32.556 -3.841 1.00 0.00
O
ATOM 5786 N GLY X 378 -25.171 34.388 -4.139 1.00 0.00
N
ATOM 5787 HN GLY X 378 -25.239 35.345 -3.868 1.00 0.00
H
ATOM 5788 CA GLY X 378 -25.610 33.960 -5.467 1.00 0.00
C
ATOM 5789 HA1 GLY X 378 -24.702 33.729 -6.004 1.00 0.00
H
ATOM 5790 HA2 GLY X 378 -26.160 34.684 -6.049 1.00 0.00
H
ATOM 5791 C GLY X 378 -26.493 32.795 -5.384 1.00 0.00
C
ATOM 5792 O GLY X 378 -26.136 31.775 -5.906 1.00 0.00
O
ATOM 5793 N PHE X 379 -27.542 32.784 -4.678 1.00 0.00
N
ATOM 5794 HN PHE X 379 -27.693 33.624 -4.163 1.00 0.00
H
ATOM 5795 CA PHE X 379 -28.549 31.751 -4.451 1.00 0.00
C
ATOM 5796 HA PHE X 379 -27.971 30.883 -4.171 1.00 0.00
H
ATOM 5797 CB PHE X 379 -29.565 32.060 -3.317 1.00 0.00
C
ATOM 5798 HB1 PHE X 379 -30.207 31.163 -3.183 1.00 0.00
H
ATOM 5799 HB2 PHE X 379 -30.078 33.020 -3.538 1.00 0.00
H
ATOM 5800 CG PHE X 379 -28.706 32.248 -2.078 1.00 0.00
C

```
ATOM   5801  CD1 PHE X 379     -28.764  33.430  -1.345  1.00  0.00
C
ATOM   5802  HD1 PHE X 379     -29.358  34.222  -1.778  1.00  0.00
H
ATOM   5803  CE1 PHE X 379     -28.230  33.403  -0.015  1.00  0.00
C
ATOM   5804  HE1 PHE X 379     -28.392  34.274   0.603  1.00  0.00
H
ATOM   5805  CZ  PHE X 379     -27.418  32.374   0.363  1.00  0.00
C
ATOM   5806  HZ  PHE X 379     -26.933  32.488   1.322  1.00  0.00
H
ATOM   5807  CD2 PHE X 379     -27.985  31.131  -1.631  1.00  0.00
C
ATOM   5808  HD2 PHE X 379     -28.044  30.146  -2.071  1.00  0.00
H
ATOM   5809  CE2 PHE X 379     -27.282  31.309  -0.449  1.00  0.00
C
ATOM   5810  HE2 PHE X 379     -26.868  30.402  -0.035  1.00  0.00
H
ATOM   5811  C   PHE X 379     -29.443  31.556  -5.661  1.00  0.00
C
ATOM   5812  O   PHE X 379     -29.734  32.436  -6.503  1.00  0.00
O
ATOM   5813  N   ASN X 380     -29.752  30.292  -5.976  1.00  0.00
N
ATOM   5814  HN  ASN X 380     -29.326  29.592  -5.409  1.00  0.00
H
ATOM   5815  CA  ASN X 380     -30.601  29.792  -7.040  1.00  0.00
C
ATOM   5816  HA  ASN X 380     -31.102  28.887  -6.729  1.00  0.00
H
ATOM   5817  CB  ASN X 380     -31.776  30.629  -7.658  1.00  0.00
C
ATOM   5818  HB1 ASN X 380     -32.325  30.015  -8.404  1.00  0.00
H
ATOM   5819  HB2 ASN X 380     -31.419  31.495  -8.256  1.00  0.00
H
ATOM   5820  CG  ASN X 380     -32.588  31.043  -6.500  1.00  0.00
C
ATOM   5821  OD1 ASN X 380     -32.649  30.659  -5.348  1.00  0.00
O
ATOM   5822  ND2 ASN X 380     -33.349  32.160  -6.800  1.00  0.00
N
ATOM   5823 HD21 ASN X 380     -33.147  32.515  -7.713  1.00  0.00
H
ATOM   5824 HD22 ASN X 380     -34.119  32.538  -6.287  1.00  0.00
H
ATOM   5825  C   ASN X 380     -29.661  29.348  -8.201  1.00  0.00
C
```

```
ATOM   5826  O   ASN X 380     -30.039  29.189  -9.287  1.00  0.00
O
ATOM   5827  N   CYS X 381     -28.336  29.124  -7.900  1.00  0.00
N
ATOM   5828  HN  CYS X 381     -28.003  29.523  -7.049  1.00  0.00
H
ATOM   5829  CA  CYS X 381     -27.278  28.785  -8.908  1.00  0.00
C
ATOM   5830  HA  CYS X 381     -27.795  28.724  -9.855  1.00  0.00
H
ATOM   5831  CB  CYS X 381     -26.228  29.865  -9.068  1.00  0.00
C
ATOM   5832  HB1 CYS X 381     -25.479  29.723  -9.876  1.00  0.00
H
ATOM   5833  HB2 CYS X 381     -25.554  30.046  -8.203  1.00  0.00
H
ATOM   5834  SG  CYS X 381     -26.944  31.608  -9.230  1.00  0.00
S
ATOM   5835  C   CYS X 381     -26.681  27.486  -8.501  1.00  0.00
C
ATOM   5836  O   CYS X 381     -26.043  27.490  -7.426  1.00  0.00
O
ATOM   5837  N   TYR X 382     -26.966  26.353  -9.215  1.00  0.00
N
ATOM   5838  HN  TYR X 382     -27.319  26.535 -10.130  1.00  0.00
H
ATOM   5839  CA  TYR X 382     -26.720  24.959  -8.803  1.00  0.00
C
ATOM   5840  HA  TYR X 382     -26.110  25.005  -7.912  1.00  0.00
H
ATOM   5841  CB  TYR X 382     -27.952  24.140  -8.711  1.00  0.00
C
ATOM   5842  HB1 TYR X 382     -27.742  23.067  -8.515  1.00  0.00
H
ATOM   5843  HB2 TYR X 382     -28.464  24.153  -9.697  1.00  0.00
H
ATOM   5844  CG  TYR X 382     -29.011  24.717  -7.770  1.00  0.00
C
ATOM   5845  CD1 TYR X 382     -28.579  25.461  -6.670  1.00  0.00
C
ATOM   5846  HD1 TYR X 382     -27.551  25.557  -6.354  1.00  0.00
H
ATOM   5847  CE1 TYR X 382     -29.560  26.159  -5.895  1.00  0.00
C
ATOM   5848  HE1 TYR X 382     -29.258  26.772  -5.058  1.00  0.00
H
ATOM   5849  CZ  TYR X 382     -30.920  25.994  -6.177  1.00  0.00
C
ATOM   5850  OH  TYR X 382     -31.874  26.693  -5.406  1.00  0.00
O
```

ATOM 5851 HH TYR X 382 -31.335 27.275 -4.865 1.00 0.00
H
ATOM 5852 CD2 TYR X 382 -30.374 24.688 -8.100 1.00 0.00
C
ATOM 5853 HD2 TYR X 382 -30.636 24.167 -9.009 1.00 0.00
H
ATOM 5854 CE2 TYR X 382 -31.300 25.304 -7.333 1.00 0.00
C
ATOM 5855 HE2 TYR X 382 -32.332 25.125 -7.599 1.00 0.00
H
ATOM 5856 C TYR X 382 -25.868 24.331 -9.951 1.00 0.00
C
ATOM 5857 O TYR X 382 -25.871 24.760 -11.090 1.00 0.00
O
ATOM 5858 N PHE X 383 -24.876 23.492 -9.628 1.00 0.00
N
ATOM 5859 HN PHE X 383 -24.522 23.377 -8.703 1.00 0.00
H
ATOM 5860 CA PHE X 383 -24.112 22.757 -10.653 1.00 0.00
C
ATOM 5861 HA PHE X 383 -23.716 23.508 -11.321 1.00 0.00
H
ATOM 5862 CB PHE X 383 -22.834 22.085 -9.978 1.00 0.00
C
ATOM 5863 HB1 PHE X 383 -23.274 21.300 -9.327 1.00 0.00
H
ATOM 5864 HB2 PHE X 383 -22.227 22.832 -9.423 1.00 0.00
H
ATOM 5865 CG PHE X 383 -21.953 21.428 -10.964 1.00 0.00
C
ATOM 5866 CD1 PHE X 383 -21.159 22.095 -11.891 1.00 0.00
C
ATOM 5867 HD1 PHE X 383 -21.130 23.173 -11.960 1.00 0.00
H
ATOM 5868 CE1 PHE X 383 -20.157 21.439 -12.600 1.00 0.00
C
ATOM 5869 HE1 PHE X 383 -19.519 21.928 -13.321 1.00 0.00
H
ATOM 5870 CZ PHE X 383 -20.025 20.076 -12.534 1.00 0.00
C
ATOM 5871 HZ PHE X 383 -19.198 19.575 -13.016 1.00 0.00
H
ATOM 5872 CD2 PHE X 383 -21.855 19.984 -10.948 1.00 0.00
C
ATOM 5873 HD2 PHE X 383 -22.426 19.462 -10.194 1.00 0.00
H
ATOM 5874 CE2 PHE X 383 -20.835 19.380 -11.653 1.00 0.00
C
ATOM 5875 HE2 PHE X 383 -20.604 18.335 -11.510 1.00 0.00
H

ATOM 5876 C PHE X 383 −25.062 21.679 −11.317 1.00 0.00
C
ATOM 5877 O PHE X 383 −25.784 21.010 −10.620 1.00 0.00
O
ATOM 5878 N PRO X 384 −25.097 21.572 −12.650 1.00 0.00
N
ATOM 5879 CD PRO X 384 −24.239 22.365 −13.609 1.00 0.00
C
ATOM 5880 HD1 PRO X 384 −23.202 22.489 −13.230 1.00 0.00
H
ATOM 5881 HD2 PRO X 384 −24.639 23.383 −13.804 1.00 0.00
H
ATOM 5882 CA PRO X 384 −25.963 20.566 −13.377 1.00 0.00
C
ATOM 5883 HA PRO X 384 −26.957 20.727 −12.984 1.00 0.00
H
ATOM 5884 CB PRO X 384 −25.719 21.014 −14.848 1.00 0.00
C
ATOM 5885 HB1 PRO X 384 −26.530 21.771 −14.885 1.00 0.00
H
ATOM 5886 HB2 PRO X 384 −25.971 20.267 −15.631 1.00 0.00
H
ATOM 5887 CG PRO X 384 −24.348 21.569 −14.889 1.00 0.00
C
ATOM 5888 HG1 PRO X 384 −23.594 20.759 −14.991 1.00 0.00
H
ATOM 5889 HG2 PRO X 384 −24.058 22.177 −15.773 1.00 0.00
H
ATOM 5890 C PRO X 384 −25.756 19.080 −13.086 1.00 0.00
C
ATOM 5891 O PRO X 384 −26.695 18.281 −13.103 1.00 0.00
O
ATOM 5892 N LEU X 385 −24.508 18.714 −12.776 1.00 0.00
N
ATOM 5893 HN LEU X 385 −23.780 19.383 −12.902 1.00 0.00
H
ATOM 5894 CA LEU X 385 −24.064 17.305 −12.877 1.00 0.00
C
ATOM 5895 HA LEU X 385 −24.866 16.731 −13.317 1.00 0.00
H
ATOM 5896 CB LEU X 385 −22.791 17.110 −13.843 1.00 0.00
C
ATOM 5897 HB1 LEU X 385 −22.719 16.020 −14.050 1.00 0.00
H
ATOM 5898 HB2 LEU X 385 −21.778 17.355 −13.459 1.00 0.00
H
ATOM 5899 CG LEU X 385 −22.742 17.838 −15.206 1.00 0.00
C
ATOM 5900 HG LEU X 385 −22.689 18.902 −14.891 1.00 0.00
H

```
ATOM   5901  CD1 LEU X 385     -21.475  17.558 -15.875  1.00  0.00
C
ATOM   5902 HD11 LEU X 385     -21.567  18.095 -16.844  1.00  0.00
H
ATOM   5903 HD12 LEU X 385     -21.283  16.486 -16.094  1.00  0.00
H
ATOM   5904 HD13 LEU X 385     -20.625  18.028 -15.335  1.00  0.00
H
ATOM   5905  CD2 LEU X 385     -23.898  17.446 -16.132  1.00  0.00
C
ATOM   5906 HD21 LEU X 385     -23.714  16.491 -16.670  1.00  0.00
H
ATOM   5907 HD22 LEU X 385     -23.989  18.233 -16.912  1.00  0.00
H
ATOM   5908 HD23 LEU X 385     -24.845  17.348 -15.560  1.00  0.00
H
ATOM   5909  C   LEU X 385     -23.830  16.627 -11.587  1.00  0.00
C
ATOM   5910  O   LEU X 385     -23.174  17.142 -10.708  1.00  0.00
O
ATOM   5911  N   GLN X 386     -24.351  15.429 -11.342  1.00  0.00
N
ATOM   5912  HN  GLN X 386     -24.944  14.954 -11.988  1.00  0.00
H
ATOM   5913  CA  GLN X 386     -24.233  14.814 -10.055  1.00  0.00
C
ATOM   5914  HA  GLN X 386     -23.631  15.234  -9.263  1.00  0.00
H
ATOM   5915  CB  GLN X 386     -25.584  14.701  -9.230  1.00  0.00
C
ATOM   5916  HB1 GLN X 386     -25.378  14.247  -8.237  1.00  0.00
H
ATOM   5917  HB2 GLN X 386     -26.292  14.034  -9.766  1.00  0.00
H
ATOM   5918  CG  GLN X 386     -26.342  16.077  -8.969  1.00  0.00
C
ATOM   5919  HG1 GLN X 386     -27.235  15.996  -8.312  1.00  0.00
H
ATOM   5920  HG2 GLN X 386     -26.539  16.419 -10.007  1.00  0.00
H
ATOM   5921  CD  GLN X 386     -25.502  17.273  -8.396  1.00  0.00
C
ATOM   5922  OE1 GLN X 386     -24.882  17.094  -7.361  1.00  0.00
O
ATOM   5923  NE2 GLN X 386     -25.450  18.474  -9.068  1.00  0.00
N
ATOM   5924 HE21 GLN X 386     -24.776  19.096  -8.669  1.00  0.00
H
ATOM   5925 HE22 GLN X 386     -25.867  18.576  -9.971  1.00  0.00
H
```

ATOM 5926 C GLN X 386 −23.712 13.414 −10.190 1.00 0.00
C
ATOM 5927 O GLN X 386 −24.101 12.768 −11.135 1.00 0.00
O
ATOM 5928 N SER X 387 −22.778 13.039 −9.352 1.00 0.00
N
ATOM 5929 HN SER X 387 −22.508 13.531 −8.528 1.00 0.00
H
ATOM 5930 CA SER X 387 −22.118 11.757 −9.458 1.00 0.00
C
ATOM 5931 HA SER X 387 −22.048 11.601 −10.524 1.00 0.00
H
ATOM 5932 CB SER X 387 −20.696 11.425 −9.010 1.00 0.00
C
ATOM 5933 HB1 SER X 387 −19.962 12.079 −9.528 1.00 0.00
H
ATOM 5934 HB2 SER X 387 −20.386 10.361 −9.094 1.00 0.00
H
ATOM 5935 OG SER X 387 −20.581 11.753 −7.578 1.00 0.00
O
ATOM 5936 HG1 SER X 387 −20.320 12.676 −7.539 1.00 0.00
H
ATOM 5937 C SER X 387 −23.003 10.742 −8.785 1.00 0.00
C
ATOM 5938 O SER X 387 −23.647 11.071 −7.796 1.00 0.00
O
ATOM 5939 N TYR X 388 −23.059 9.481 −9.307 1.00 0.00
N
ATOM 5940 HN TYR X 388 −22.561 9.313 −10.154 1.00 0.00
H
ATOM 5941 CA TYR X 388 −23.854 8.390 −8.761 1.00 0.00
C
ATOM 5942 HA TYR X 388 −24.885 8.703 −8.682 1.00 0.00
H
ATOM 5943 CB TYR X 388 −23.798 7.160 −9.611 1.00 0.00
C
ATOM 5944 HB1 TYR X 388 −24.045 6.199 −9.111 1.00 0.00
H
ATOM 5945 HB2 TYR X 388 −22.813 7.058 −10.114 1.00 0.00
H
ATOM 5946 CG TYR X 388 −24.815 7.181 −10.765 1.00 0.00
C
ATOM 5947 CD1 TYR X 388 −26.197 7.181 −10.459 1.00 0.00
C
ATOM 5948 HD1 TYR X 388 −26.481 7.143 −9.417 1.00 0.00
H
ATOM 5949 CE1 TYR X 388 −27.215 7.148 −11.431 1.00 0.00
C
ATOM 5950 HE1 TYR X 388 −28.244 6.970 −11.155 1.00 0.00
H

ATOM 5951 CZ TYR X 388 −26.819 7.278 −12.700 1.00 0.00
C
ATOM 5952 OH TYR X 388 −27.796 7.184 −13.693 1.00 0.00
O
ATOM 5953 HH TYR X 388 −28.634 6.987 −13.269 1.00 0.00
H
ATOM 5954 CD2 TYR X 388 −24.457 7.280 −12.101 1.00 0.00
C
ATOM 5955 HD2 TYR X 388 −23.407 7.410 −12.315 1.00 0.00
H
ATOM 5956 CE2 TYR X 388 −25.471 7.383 −13.049 1.00 0.00
C
ATOM 5957 HE2 TYR X 388 −25.301 7.436 −14.114 1.00 0.00
H
ATOM 5958 C TYR X 388 −23.588 7.929 −7.303 1.00 0.00
C
ATOM 5959 O TYR X 388 −24.440 7.454 −6.545 1.00 0.00
O
ATOM 5960 N GLY X 389 −22.268 7.843 −6.950 1.00 0.00
N
ATOM 5961 HN GLY X 389 −21.501 8.007 −7.565 1.00 0.00
H
ATOM 5962 CA GLY X 389 −21.915 7.496 −5.617 1.00 0.00
C
ATOM 5963 HA1 GLY X 389 −22.660 7.927 −4.965 1.00 0.00
H
ATOM 5964 HA2 GLY X 389 −21.011 8.028 −5.361 1.00 0.00
H
ATOM 5965 C GLY X 389 −21.859 5.969 −5.331 1.00 0.00
C
ATOM 5966 O GLY X 389 −22.011 5.514 −4.215 1.00 0.00
O
ATOM 5967 N PHE X 390 −21.662 5.143 −6.384 1.00 0.00
N
ATOM 5968 HN PHE X 390 −21.403 5.632 −7.214 1.00 0.00
H
ATOM 5969 CA PHE X 390 −21.677 3.743 −6.233 1.00 0.00
C
ATOM 5970 HA PHE X 390 −22.695 3.400 −6.119 1.00 0.00
H
ATOM 5971 CB PHE X 390 −21.168 3.109 −7.638 1.00 0.00
C
ATOM 5972 HB1 PHE X 390 −21.228 2.002 −7.711 1.00 0.00
H
ATOM 5973 HB2 PHE X 390 −20.135 3.350 −7.966 1.00 0.00
H
ATOM 5974 CG PHE X 390 −22.068 3.612 −8.801 1.00 0.00
C
ATOM 5975 CD1 PHE X 390 −23.450 3.387 −8.697 1.00 0.00
C

```
ATOM   5976  HD1 PHE X 390     -23.873   3.091  -7.748  1.00  0.00
H
ATOM   5977  CE1 PHE X 390     -24.248   3.484  -9.871  1.00  0.00
C
ATOM   5978  HE1 PHE X 390     -25.289   3.196  -9.878  1.00  0.00
H
ATOM   5979  CZ  PHE X 390     -23.679   3.930 -10.989  1.00  0.00
C
ATOM   5980  HZ  PHE X 390     -24.205   4.073 -11.921  1.00  0.00
H
ATOM   5981  CD2 PHE X 390     -21.510   4.140  -9.918  1.00  0.00
C
ATOM   5982  HD2 PHE X 390     -20.477   4.445  -9.847  1.00  0.00
H
ATOM   5983  CE2 PHE X 390     -22.351   4.226 -11.099  1.00  0.00
C
ATOM   5984  HE2 PHE X 390     -21.905   4.504 -12.042  1.00  0.00
H
ATOM   5985  C   PHE X 390     -20.774   3.040  -5.205  1.00  0.00
C
ATOM   5986  O   PHE X 390     -19.566   3.217  -5.270  1.00  0.00
O
ATOM   5987  N   GLN X 391     -21.445   2.435  -4.194  1.00  0.00
N
ATOM   5988  HN  GLN X 391     -22.427   2.556  -4.319  1.00  0.00
H
ATOM   5989  CA  GLN X 391     -20.770   1.748  -3.100  1.00  0.00
C
ATOM   5990  HA  GLN X 391     -19.808   1.444  -3.485  1.00  0.00
H
ATOM   5991  CB  GLN X 391     -20.658   2.704  -1.875  1.00  0.00
C
ATOM   5992  HB1 GLN X 391     -20.036   3.571  -2.183  1.00  0.00
H
ATOM   5993  HB2 GLN X 391     -20.342   2.166  -0.956  1.00  0.00
H
ATOM   5994  CG  GLN X 391     -21.998   3.357  -1.437  1.00  0.00
C
ATOM   5995  HG1 GLN X 391     -22.608   2.541  -0.994  1.00  0.00
H
ATOM   5996  HG2 GLN X 391     -22.548   3.761  -2.313  1.00  0.00
H
ATOM   5997  CD  GLN X 391     -21.698   4.417  -0.345  1.00  0.00
C
ATOM   5998  OE1 GLN X 391     -21.458   4.006   0.800  1.00  0.00
O
ATOM   5999  NE2 GLN X 391     -21.905   5.727  -0.597  1.00  0.00
N
ATOM   6000 HE21 GLN X 391     -22.024   5.869  -1.580  1.00  0.00
H
```

ATOM 6001 HE22 GLN X 391 -21.602 6.417 0.060 1.00 0.00
H
ATOM 6002 C GLN X 391 -21.588 0.473 -2.785 1.00 0.00
C
ATOM 6003 O GLN X 391 -22.841 0.539 -2.742 1.00 0.00
O
ATOM 6004 N PRO X 392 -21.084 -0.660 -2.491 1.00 0.00
N
ATOM 6005 CD PRO X 392 -19.575 -0.774 -2.395 1.00 0.00
C
ATOM 6006 HD1 PRO X 392 -19.063 -0.221 -1.578 1.00 0.00
H
ATOM 6007 HD2 PRO X 392 -19.063 -0.552 -3.355 1.00 0.00
H
ATOM 6008 CA PRO X 392 -21.771 -1.943 -2.710 1.00 0.00
C
ATOM 6009 HA PRO X 392 -22.391 -1.833 -3.587 1.00 0.00
H
ATOM 6010 CB PRO X 392 -20.618 -2.939 -2.947 1.00 0.00
C
ATOM 6011 HB1 PRO X 392 -20.191 -2.799 -3.963 1.00 0.00
H
ATOM 6012 HB2 PRO X 392 -20.822 -4.013 -2.746 1.00 0.00
H
ATOM 6013 CG PRO X 392 -19.458 -2.234 -2.075 1.00 0.00
C
ATOM 6014 HG1 PRO X 392 -19.636 -2.443 -0.999 1.00 0.00
H
ATOM 6015 HG2 PRO X 392 -18.481 -2.649 -2.403 1.00 0.00
H
ATOM 6016 C PRO X 392 -22.664 -2.286 -1.555 1.00 0.00
C
ATOM 6017 O PRO X 392 -23.233 -3.337 -1.632 1.00 0.00
O
ATOM 6018 N THR X 393 -22.711 -1.501 -0.417 1.00 0.00
N
ATOM 6019 HN THR X 393 -22.132 -0.705 -0.258 1.00 0.00
H
ATOM 6020 CA THR X 393 -23.595 -1.788 0.704 1.00 0.00
C
ATOM 6021 HA THR X 393 -23.842 -2.824 0.883 1.00 0.00
H
ATOM 6022 CB THR X 393 -22.871 -1.452 2.070 1.00 0.00
C
ATOM 6023 HB THR X 393 -23.613 -1.559 2.890 1.00 0.00
H
ATOM 6024 OG1 THR X 393 -22.345 -0.144 2.000 1.00 0.00
O
ATOM 6025 HG1 THR X 393 -23.054 0.487 2.144 1.00 0.00
H

```
ATOM   6026  CG2 THR X 393     -21.727  -2.435   2.462  1.00  0.00
C
ATOM   6027 HG21 THR X 393     -22.100  -3.443   2.743  1.00  0.00
H
ATOM   6028 HG22 THR X 393     -21.289  -1.948   3.360  1.00  0.00
H
ATOM   6029 HG23 THR X 393     -20.995  -2.577   1.638  1.00  0.00
H
ATOM   6030  C   THR X 393     -24.793  -0.859   0.719  1.00  0.00
C
ATOM   6031  O   THR X 393     -25.615  -0.851   1.593  1.00  0.00
O
ATOM   6032  N   ASN X 394     -25.002  -0.129  -0.348  1.00  0.00
N
ATOM   6033  HN  ASN X 394     -24.344  -0.201  -1.093  1.00  0.00
H
ATOM   6034  CA  ASN X 394     -26.300   0.316  -0.902  1.00  0.00
C
ATOM   6035  HA  ASN X 394     -26.902   0.310  -0.005  1.00  0.00
H
ATOM   6036  CB  ASN X 394     -26.334   1.686  -1.615  1.00  0.00
C
ATOM   6037  HB1 ASN X 394     -27.359   1.909  -1.982  1.00  0.00
H
ATOM   6038  HB2 ASN X 394     -25.543   1.685  -2.395  1.00  0.00
H
ATOM   6039  CG  ASN X 394     -25.928   2.804  -0.627  1.00  0.00
C
ATOM   6040  OD1 ASN X 394     -25.716   2.624   0.628  1.00  0.00
O
ATOM   6041  ND2 ASN X 394     -25.770   4.005  -1.169  1.00  0.00
N
ATOM   6042 HD21 ASN X 394     -25.351   4.781  -0.696  1.00  0.00
H
ATOM   6043 HD22 ASN X 394     -26.196   4.043  -2.073  1.00  0.00
H
ATOM   6044  C   ASN X 394     -26.967  -0.833  -1.686  1.00  0.00
C
ATOM   6045  O   ASN X 394     -26.221  -1.763  -2.094  1.00  0.00
O
ATOM   6046  N   GLY X 395     -28.306  -0.844  -1.884  1.00  0.00
N
ATOM   6047  HN  GLY X 395     -28.787  -0.067  -1.485  1.00  0.00
H
ATOM   6048  CA  GLY X 395     -29.034  -1.878  -2.701  1.00  0.00
C
ATOM   6049  HA1 GLY X 395     -30.084  -1.629  -2.665  1.00  0.00
H
ATOM   6050  HA2 GLY X 395     -28.834  -2.823  -2.218  1.00  0.00
H
```

```
ATOM   6051  C   GLY X 395     -28.551  -2.118  -4.129  1.00  0.00
C
ATOM   6052  O   GLY X 395     -27.838  -1.284  -4.737  1.00  0.00
O
ATOM   6053  N   VAL X 396     -29.106  -3.159  -4.867  1.00  0.00
N
ATOM   6054  HN  VAL X 396     -29.719  -3.819  -4.438  1.00  0.00
H
ATOM   6055  CA  VAL X 396     -28.693  -3.472  -6.235  1.00  0.00
C
ATOM   6056  HA  VAL X 396     -27.634  -3.333  -6.399  1.00  0.00
H
ATOM   6057  CB  VAL X 396     -28.969  -4.954  -6.589  1.00  0.00
C
ATOM   6058  HB  VAL X 396     -28.645  -5.533  -5.699  1.00  0.00
H
ATOM   6059  CG1 VAL X 396     -30.501  -5.282  -6.708  1.00  0.00
C
ATOM   6060 HG11 VAL X 396     -30.491  -6.392  -6.752  1.00  0.00
H
ATOM   6061 HG12 VAL X 396     -31.047  -4.690  -7.474  1.00  0.00
H
ATOM   6062 HG13 VAL X 396     -31.092  -5.153  -5.776  1.00  0.00
H
ATOM   6063  CG2 VAL X 396     -28.134  -5.352  -7.838  1.00  0.00
C
ATOM   6064 HG21 VAL X 396     -28.483  -6.383  -8.062  1.00  0.00
H
ATOM   6065 HG22 VAL X 396     -27.041  -5.157  -7.797  1.00  0.00
H
ATOM   6066 HG23 VAL X 396     -28.565  -4.751  -8.667  1.00  0.00
H
ATOM   6067  C   VAL X 396     -29.293  -2.584  -7.207  1.00  0.00
C
ATOM   6068  O   VAL X 396     -28.738  -2.334  -8.341  1.00  0.00
O
ATOM   6069  N   GLY X 397     -30.397  -1.868  -6.810  1.00  0.00
N
ATOM   6070  HN  GLY X 397     -30.879  -2.195  -6.001  1.00  0.00
H
ATOM   6071  CA  GLY X 397     -30.961  -0.739  -7.565  1.00  0.00
C
ATOM   6072  HA1 GLY X 397     -31.762  -0.406  -6.922  1.00  0.00
H
ATOM   6073  HA2 GLY X 397     -31.179  -1.029  -8.582  1.00  0.00
H
ATOM   6074  C   GLY X 397     -30.093   0.455  -7.577  1.00  0.00
C
ATOM   6075  O   GLY X 397     -30.235   1.280  -8.476  1.00  0.00
O
```

```
ATOM   6076  N   TYR X 398     -29.189   0.531  -6.589  1.00  0.00
N
ATOM   6077  HN  TYR X 398     -29.092  -0.200  -5.918  1.00  0.00
H
ATOM   6078  CA  TYR X 398     -28.316   1.685  -6.467  1.00  0.00
C
ATOM   6079  HA  TYR X 398     -28.519   2.546  -7.086  1.00  0.00
H
ATOM   6080  CB  TYR X 398     -28.226   2.174  -4.967  1.00  0.00
C
ATOM   6081  HB1 TYR X 398     -27.489   3.005  -4.978  1.00  0.00
H
ATOM   6082  HB2 TYR X 398     -27.856   1.353  -4.317  1.00  0.00
H
ATOM   6083  CG  TYR X 398     -29.574   2.637  -4.385  1.00  0.00
C
ATOM   6084  CD1 TYR X 398     -30.055   3.976  -4.370  1.00  0.00
C
ATOM   6085  HD1 TYR X 398     -29.448   4.721  -4.862  1.00  0.00
H
ATOM   6086  CE1 TYR X 398     -31.270   4.308  -3.769  1.00  0.00
C
ATOM   6087  HE1 TYR X 398     -31.614   5.317  -3.938  1.00  0.00
H
ATOM   6088  CZ  TYR X 398     -32.058   3.405  -3.021  1.00  0.00
C
ATOM   6089  OH  TYR X 398     -33.277   3.836  -2.379  1.00  0.00
O
ATOM   6090  HH  TYR X 398     -33.513   3.102  -1.806  1.00  0.00
H
ATOM   6091  CD2 TYR X 398     -30.405   1.694  -3.687  1.00  0.00
C
ATOM   6092  HD2 TYR X 398     -30.117   0.653  -3.666  1.00  0.00
H
ATOM   6093  CE2 TYR X 398     -31.556   2.100  -3.002  1.00  0.00
C
ATOM   6094  HE2 TYR X 398     -32.043   1.227  -2.593  1.00  0.00
H
ATOM   6095  C   TYR X 398     -26.897   1.263  -7.022  1.00  0.00
C
ATOM   6096  O   TYR X 398     -25.948   2.024  -7.020  1.00  0.00
O
ATOM   6097  N   GLN X 399     -26.728   0.047  -7.621  1.00  0.00
N
ATOM   6098  HN  GLN X 399     -27.470  -0.614  -7.708  1.00  0.00
H
ATOM   6099  CA  GLN X 399     -25.541  -0.484  -8.229  1.00  0.00
C
ATOM   6100  HA  GLN X 399     -24.748   0.011  -7.688  1.00  0.00
H
```

ATOM   6101 CB GLN X 399   -25.362 -2.063 -8.000 1.00 0.00
C
ATOM   6102 HB1 GLN X 399   -24.623 -2.503 -8.704 1.00 0.00
H
ATOM   6103 HB2 GLN X 399   -26.333 -2.535 -8.262 1.00 0.00
H
ATOM   6104 CG GLN X 399   -24.919 -2.455 -6.587 1.00 0.00
C
ATOM   6105 HG1 GLN X 399   -24.442 -3.458 -6.610 1.00 0.00
H
ATOM   6106 HG2 GLN X 399   -25.917 -2.528 -6.104 1.00 0.00
H
ATOM   6107 CD GLN X 399   -24.092 -1.429 -5.735 1.00 0.00
C
ATOM   6108 OE1 GLN X 399   -22.871 -1.119 -5.983 1.00 0.00
O
ATOM   6109 NE2 GLN X 399   -24.724 -0.819 -4.680 1.00 0.00
N
ATOM   6110 HE21 GLN X 399   -25.600 -1.181 -4.362 1.00 0.00
H
ATOM   6111 HE22 GLN X 399   -24.107 -0.261 -4.125 1.00 0.00
H
ATOM   6112 C   GLN X 399   -25.321 -0.099 -9.688 1.00 0.00
C
ATOM   6113 O   GLN X 399   -26.226 -0.041 -10.472 1.00 0.00
O
ATOM   6114 N   PRO X 400   -24.053   0.168 -10.125 1.00 0.00
N
ATOM   6115 CD PRO X 400   -22.862 -0.002 -9.343 1.00 0.00
C
ATOM   6116 HD1 PRO X 400   -23.009 -0.587 -8.410 1.00 0.00
H
ATOM   6117 HD2 PRO X 400   -22.752   1.019 -8.919 1.00 0.00
H
ATOM   6118 CA PRO X 400   -23.721   0.356 -11.567 1.00 0.00
C
ATOM   6119 HA PRO X 400   -24.420   1.141 -11.815 1.00 0.00
H
ATOM   6120 CB PRO X 400   -22.190   0.536 -11.555 1.00 0.00
C
ATOM   6121 HB1 PRO X 400   -22.039   1.623 -11.384 1.00 0.00
H
ATOM   6122 HB2 PRO X 400   -21.715   0.280 -12.527 1.00 0.00
H
ATOM   6123 CG PRO X 400   -21.717 -0.165 -10.264 1.00 0.00
C
ATOM   6124 HG1 PRO X 400   -21.595 -1.240 -10.515 1.00 0.00
H
ATOM   6125 HG2 PRO X 400   -20.732   0.245 -9.954 1.00 0.00
H

ATOM 6126 C PRO X 400 -24.140 -0.773 -12.369 1.00 0.00
C
ATOM 6127 O PRO X 400 -23.801 -1.944 -12.196 1.00 0.00
O
ATOM 6128 N TYR X 401 -24.826 -0.484 -13.517 1.00 0.00
N
ATOM 6129 HN TYR X 401 -25.317 0.375 -13.641 1.00 0.00
H
ATOM 6130 CA TYR X 401 -25.128 -1.469 -14.561 1.00 0.00
C
ATOM 6131 HA TYR X 401 -24.685 -2.427 -14.332 1.00 0.00
H
ATOM 6132 CB TYR X 401 -26.712 -1.712 -14.754 1.00 0.00
C
ATOM 6133 HB1 TYR X 401 -26.876 -2.146 -15.764 1.00 0.00
H
ATOM 6134 HB2 TYR X 401 -27.214 -0.770 -14.447 1.00 0.00
H
ATOM 6135 CG TYR X 401 -27.266 -2.748 -13.883 1.00 0.00
C
ATOM 6136 CD1 TYR X 401 -27.110 -2.672 -12.428 1.00 0.00
C
ATOM 6137 HD1 TYR X 401 -26.560 -1.948 -11.846 1.00 0.00
H
ATOM 6138 CE1 TYR X 401 -27.544 -3.743 -11.623 1.00 0.00
C
ATOM 6139 HE1 TYR X 401 -27.425 -3.619 -10.557 1.00 0.00
H
ATOM 6140 CZ TYR X 401 -28.223 -4.793 -12.165 1.00 0.00
C
ATOM 6141 OH TYR X 401 -28.761 -5.867 -11.488 1.00 0.00
O
ATOM 6142 HH TYR X 401 -29.541 -6.078 -12.007 1.00 0.00
H
ATOM 6143 CD2 TYR X 401 -28.034 -3.757 -14.355 1.00 0.00
C
ATOM 6144 HD2 TYR X 401 -28.256 -3.937 -15.397 1.00 0.00
H
ATOM 6145 CE2 TYR X 401 -28.443 -4.830 -13.547 1.00 0.00
C
ATOM 6146 HE2 TYR X 401 -28.907 -5.706 -13.976 1.00 0.00
H
ATOM 6147 C TYR X 401 -24.521 -0.892 -15.797 1.00 0.00
C
ATOM 6148 O TYR X 401 -24.678 0.281 -16.161 1.00 0.00
O
ATOM 6149 N ARG X 402 -23.585 -1.668 -16.413 1.00 0.00
N
ATOM 6150 HN ARG X 402 -23.491 -2.625 -16.153 1.00 0.00
H

```
ATOM   6151  CA  ARG X 402     -23.178  -1.237 -17.751  1.00  0.00
C
ATOM   6152  HA  ARG X 402     -23.008  -0.170 -17.780  1.00  0.00
H
ATOM   6153  CB  ARG X 402     -21.898  -1.977 -18.133  1.00  0.00
C
ATOM   6154  HB1 ARG X 402     -21.721  -1.909 -19.227  1.00  0.00
H
ATOM   6155  HB2 ARG X 402     -22.079  -3.056 -17.940  1.00  0.00
H
ATOM   6156  CG  ARG X 402     -20.665  -1.421 -17.409  1.00  0.00
C
ATOM   6157  HG1 ARG X 402     -20.865  -1.447 -16.317  1.00  0.00
H
ATOM   6158  HG2 ARG X 402     -20.590  -0.329 -17.601  1.00  0.00
H
ATOM   6159  CD  ARG X 402     -19.368  -2.078 -17.585  1.00  0.00
C
ATOM   6160  HD1 ARG X 402     -19.134  -2.288 -18.651  1.00  0.00
H
ATOM   6161  HD2 ARG X 402     -19.365  -3.075 -17.095  1.00  0.00
H
ATOM   6162  NE  ARG X 402     -18.319  -1.278 -16.896  1.00  0.00
N
ATOM   6163  HE  ARG X 402     -18.644  -0.698 -16.149  1.00  0.00
H
ATOM   6164  CZ  ARG X 402     -17.021  -1.297 -17.101  1.00  0.00
C
ATOM   6165  NH1 ARG X 402     -16.466  -2.074 -18.019  1.00  0.00
N
ATOM   6166 HH11 ARG X 402     -16.923  -2.845 -18.462  1.00  0.00
H
ATOM   6167 HH12 ARG X 402     -15.482  -2.207 -17.899  1.00  0.00
H
ATOM   6168  NH2 ARG X 402     -16.246  -0.593 -16.336  1.00  0.00
N
ATOM   6169 HH21 ARG X 402     -16.751  -0.028 -15.683  1.00  0.00
H
ATOM   6170 HH22 ARG X 402     -15.259  -0.590 -16.494  1.00  0.00
H
ATOM   6171  C   ARG X 402     -24.181  -1.601 -18.834  1.00  0.00
C
ATOM   6172  O   ARG X 402     -24.790  -2.665 -18.887  1.00  0.00
O
ATOM   6173  N   VAL X 403     -24.378  -0.592 -19.727  1.00  0.00
N
ATOM   6174  HN  VAL X 403     -23.988   0.287 -19.463  1.00  0.00
H
ATOM   6175  CA  VAL X 403     -25.363  -0.562 -20.741  1.00  0.00
C
```

```
ATOM   6176  HA  VAL X 403     -25.924  -1.482 -20.671  1.00  0.00
H
ATOM   6177  CB  VAL X 403     -26.469   0.526 -20.552  1.00  0.00
C
ATOM   6178  HB  VAL X 403     -26.064   1.553 -20.428  1.00  0.00
H
ATOM   6179  CG1 VAL X 403     -27.614   0.538 -21.612  1.00  0.00
C
ATOM   6180 HG11 VAL X 403     -28.109  -0.455 -21.568  1.00  0.00
H
ATOM   6181 HG12 VAL X 403     -27.304   0.802 -22.645  1.00  0.00
H
ATOM   6182 HG13 VAL X 403     -28.292   1.364 -21.307  1.00  0.00
H
ATOM   6183  CG2 VAL X 403     -27.103   0.238 -19.150  1.00  0.00
C
ATOM   6184 HG21 VAL X 403     -26.443   0.514 -18.299  1.00  0.00
H
ATOM   6185 HG22 VAL X 403     -27.466  -0.809 -19.070  1.00  0.00
H
ATOM   6186 HG23 VAL X 403     -27.948   0.958 -19.191  1.00  0.00
H
ATOM   6187  C   VAL X 403     -24.758  -0.229 -22.135  1.00  0.00
C
ATOM   6188  O   VAL X 403     -24.093   0.791 -22.340  1.00  0.00
O
ATOM   6189  N   VAL X 404     -25.259  -0.985 -23.105  1.00  0.00
N
ATOM   6190  HN  VAL X 404     -25.880  -1.716 -22.833  1.00  0.00
H
ATOM   6191  CA  VAL X 404     -24.958  -0.757 -24.433  1.00  0.00
C
ATOM   6192  HA  VAL X 404     -24.538   0.222 -24.609  1.00  0.00
H
ATOM   6193  CB  VAL X 404     -24.089  -1.879 -25.088  1.00  0.00
C
ATOM   6194  HB  VAL X 404     -24.590  -2.845 -24.864  1.00  0.00
H
ATOM   6195  CG1 VAL X 404     -23.909  -1.686 -26.631  1.00  0.00
C
ATOM   6196 HG11 VAL X 404     -23.548  -0.665 -26.878  1.00  0.00
H
ATOM   6197 HG12 VAL X 404     -24.835  -1.851 -27.223  1.00  0.00
H
ATOM   6198 HG13 VAL X 404     -23.275  -2.505 -27.033  1.00  0.00
H
ATOM   6199  CG2 VAL X 404     -22.737  -1.767 -24.495  1.00  0.00
C
ATOM   6200 HG21 VAL X 404     -21.991  -2.442 -24.967  1.00  0.00
H
```

```
ATOM   6201 HG22 VAL X 404     -22.776  -2.142 -23.450  1.00  0.00
H
ATOM   6202 HG23 VAL X 404     -22.356  -0.731 -24.367  1.00  0.00
H
ATOM   6203  C   VAL X 404     -26.294  -0.865 -25.146  1.00  0.00
C
ATOM   6204  O   VAL X 404     -27.109  -1.796 -25.038  1.00  0.00
O
ATOM   6205  N   VAL X 405     -26.542   0.184 -25.988  1.00  0.00
N
ATOM   6206  HN  VAL X 405     -25.778   0.825 -25.971  1.00  0.00
H
ATOM   6207  CA  VAL X 405     -27.597   0.265 -26.926  1.00  0.00
C
ATOM   6208  HA  VAL X 405     -28.368  -0.408 -26.583  1.00  0.00
H
ATOM   6209  CB  VAL X 405     -28.210   1.698 -27.018  1.00  0.00
C
ATOM   6210  HB  VAL X 405     -27.406   2.464 -27.047  1.00  0.00
H
ATOM   6211  CG1 VAL X 405     -29.345   1.785 -28.033  1.00  0.00
C
ATOM   6212 HG11 VAL X 405     -29.073   1.925 -29.101  1.00  0.00
H
ATOM   6213 HG12 VAL X 405     -30.002   2.638 -27.759  1.00  0.00
H
ATOM   6214 HG13 VAL X 405     -29.960   0.861 -27.977  1.00  0.00
H
ATOM   6215  CG2 VAL X 405     -28.886   1.846 -25.668  1.00  0.00
C
ATOM   6216 HG21 VAL X 405     -28.176   1.635 -24.840  1.00  0.00
H
ATOM   6217 HG22 VAL X 405     -29.723   1.129 -25.525  1.00  0.00
H
ATOM   6218 HG23 VAL X 405     -29.213   2.898 -25.526  1.00  0.00
H
ATOM   6219  C   VAL X 405     -27.041  -0.177 -28.325  1.00  0.00
C
ATOM   6220  O   VAL X 405     -25.914   0.166 -28.685  1.00  0.00
O
ATOM   6221  N   LEU X 406     -27.846  -0.910 -29.079  1.00  0.00
N
ATOM   6222  HN  LEU X 406     -28.693  -1.349 -28.787  1.00  0.00
H
ATOM   6223  CA  LEU X 406     -27.491  -1.177 -30.469  1.00  0.00
C
ATOM   6224  HA  LEU X 406     -26.570  -0.667 -30.710  1.00  0.00
H
ATOM   6225  CB  LEU X 406     -27.375  -2.679 -30.729  1.00  0.00
C
```

```
ATOM   6226  HB1 LEU X 406     -27.132  -2.842 -31.801  1.00  0.00
H
ATOM   6227  HB2 LEU X 406     -28.334  -3.213 -30.559  1.00  0.00
H
ATOM   6228  CG  LEU X 406     -26.221  -3.344 -29.995  1.00  0.00
C
ATOM   6229  HG  LEU X 406     -26.375  -2.957 -28.965  1.00  0.00
H
ATOM   6230  CD1 LEU X 406     -26.380  -4.916 -30.032  1.00  0.00
C
ATOM   6231 HD11 LEU X 406     -27.390  -5.149 -29.633  1.00  0.00
H
ATOM   6232 HD12 LEU X 406     -25.639  -5.351 -29.327  1.00  0.00
H
ATOM   6233 HD13 LEU X 406     -25.976  -5.392 -30.951  1.00  0.00
H
ATOM   6234  CD2 LEU X 406     -24.756  -3.043 -30.415  1.00  0.00
C
ATOM   6235 HD21 LEU X 406     -24.517  -1.970 -30.257  1.00  0.00
H
ATOM   6236 HD22 LEU X 406     -24.655  -3.363 -31.474  1.00  0.00
H
ATOM   6237 HD23 LEU X 406     -24.036  -3.731 -29.920  1.00  0.00
H
ATOM   6238  C   LEU X 406     -28.628  -0.691 -31.450  1.00  0.00
C
ATOM   6239  O   LEU X 406     -29.597  -1.412 -31.573  1.00  0.00
O
ATOM   6240  N   SER X 407     -28.547   0.531 -32.062  1.00  0.00
N
ATOM   6241  HN  SER X 407     -27.833   1.126 -31.702  1.00  0.00
H
ATOM   6242  CA  SER X 407     -29.452   1.005 -33.065  1.00  0.00
C
ATOM   6243  HA  SER X 407     -30.475   0.682 -32.940  1.00  0.00
H
ATOM   6244  CB  SER X 407     -29.603   2.550 -33.006  1.00  0.00
C
ATOM   6245  HB1 SER X 407     -28.655   3.097 -33.198  1.00  0.00
H
ATOM   6246  HB2 SER X 407     -29.822   2.622 -31.919  1.00  0.00
H
ATOM   6247  OG  SER X 407     -30.627   3.066 -33.865  1.00  0.00
O
ATOM   6248  HG1 SER X 407     -30.282   3.370 -34.708  1.00  0.00
H
ATOM   6249  C   SER X 407     -29.088   0.529 -34.468  1.00  0.00
C
ATOM   6250  O   SER X 407     -27.972   0.555 -34.926  1.00  0.00
O
```

```
ATOM   6251  N   PHE X 408      -30.054  -0.004 -35.160  1.00  0.00
N
ATOM   6252  HN  PHE X 408      -31.009  -0.029 -34.876  1.00  0.00
H
ATOM   6253  CA  PHE X 408      -29.908  -0.555 -36.473  1.00  0.00
C
ATOM   6254  HA  PHE X 408      -28.884  -0.623 -36.810  1.00  0.00
H
ATOM   6255  CB  PHE X 408      -30.571  -1.938 -36.655  1.00  0.00
C
ATOM   6256  HB1 PHE X 408      -30.321  -2.399 -37.635  1.00  0.00
H
ATOM   6257  HB2 PHE X 408      -31.674  -1.811 -36.635  1.00  0.00
H
ATOM   6258  CG  PHE X 408      -30.352  -2.916 -35.547  1.00  0.00
C
ATOM   6259  CD1 PHE X 408      -29.169  -3.132 -34.878  1.00  0.00
C
ATOM   6260  HD1 PHE X 408      -28.401  -2.434 -35.178  1.00  0.00
H
ATOM   6261  CE1 PHE X 408      -28.992  -4.123 -33.917  1.00  0.00
C
ATOM   6262  HE1 PHE X 408      -28.085  -4.358 -33.380  1.00  0.00
H
ATOM   6263  CZ  PHE X 408      -30.045  -5.056 -33.788  1.00  0.00
C
ATOM   6264  HZ  PHE X 408      -29.863  -5.836 -33.063  1.00  0.00
H
ATOM   6265  CD2 PHE X 408      -31.394  -3.880 -35.378  1.00  0.00
C
ATOM   6266  HD2 PHE X 408      -32.301  -3.806 -35.960  1.00  0.00
H
ATOM   6267  CE2 PHE X 408      -31.235  -4.961 -34.501  1.00  0.00
C
ATOM   6268  HE2 PHE X 408      -32.043  -5.660 -34.342  1.00  0.00
H
ATOM   6269  C   PHE X 408      -30.414   0.446 -37.460  1.00  0.00
C
ATOM   6270  O   PHE X 408      -31.591   0.931 -37.333  1.00  0.00
O
ATOM   6271  N   GLU X 409      -29.595   0.795 -38.501  1.00  0.00
N
ATOM   6272  HN  GLU X 409      -28.649   0.508 -38.629  1.00  0.00
H
ATOM   6273  CA  GLU X 409      -29.966   1.734 -39.538  1.00  0.00
C
ATOM   6274  HA  GLU X 409      -31.023   1.942 -39.453  1.00  0.00
H
ATOM   6275  CB  GLU X 409      -29.186   3.141 -39.561  1.00  0.00
C
```

```
ATOM   6276  HB1 GLU X 409     -28.113   2.909 -39.388  1.00  0.00
H
ATOM   6277  HB2 GLU X 409     -29.488   3.656 -38.624  1.00  0.00
H
ATOM   6278  CG  GLU X 409     -29.207   4.078 -40.800  1.00  0.00
C
ATOM   6279  HG1 GLU X 409     -30.201   4.563 -40.904  1.00  0.00
H
ATOM   6280  HG2 GLU X 409     -29.219   3.509 -41.754  1.00  0.00
H
ATOM   6281  CD  GLU X 409     -28.140   5.136 -40.688  1.00  0.00
C
ATOM   6282  OE1 GLU X 409     -28.455   6.089 -39.941  1.00  0.00
O
ATOM   6283  OE2 GLU X 409     -27.136   5.110 -41.404  1.00  0.00
O
ATOM   6284  C   GLU X 409     -29.841   1.002 -40.841  1.00  0.00
C
ATOM   6285  O   GLU X 409     -28.773   0.366 -41.164  1.00  0.00
O
ATOM   6286  N   LEU X 410     -30.935   1.068 -41.630  1.00  0.00
N
ATOM   6287  HN  LEU X 410     -31.619   1.747 -41.371  1.00  0.00
H
ATOM   6288  CA  LEU X 410     -30.995   0.409 -42.991  1.00  0.00
C
ATOM   6289  HA  LEU X 410     -29.995   0.065 -43.210  1.00  0.00
H
ATOM   6290  CB  LEU X 410     -32.031  -0.752 -43.118  1.00  0.00
C
ATOM   6291  HB1 LEU X 410     -33.073  -0.464 -42.860  1.00  0.00
H
ATOM   6292  HB2 LEU X 410     -31.826  -1.297 -42.173  1.00  0.00
H
ATOM   6293  CG  LEU X 410     -31.778  -1.642 -44.399  1.00  0.00
C
ATOM   6294  HG  LEU X 410     -31.860  -0.961 -45.273  1.00  0.00
H
ATOM   6295  CD1 LEU X 410     -30.397  -2.407 -44.387  1.00  0.00
C
ATOM   6296 HD11 LEU X 410     -29.488  -1.810 -44.158  1.00  0.00
H
ATOM   6297 HD12 LEU X 410     -30.120  -2.829 -45.377  1.00  0.00
H
ATOM   6298 HD13 LEU X 410     -30.363  -3.259 -43.675  1.00  0.00
H
ATOM   6299  CD2 LEU X 410     -32.906  -2.618 -44.594  1.00  0.00
C
ATOM   6300 HD21 LEU X 410     -33.890  -2.109 -44.677  1.00  0.00
H
```

```
ATOM   6301 HD22 LEU X 410     -32.970  -3.340 -43.752  1.00  0.00
H
ATOM   6302 HD23 LEU X 410     -32.732  -3.171 -45.542  1.00  0.00
H
ATOM   6303  C   LEU X 410     -31.230   1.566 -43.991  1.00  0.00
C
ATOM   6304  O   LEU X 410     -32.296   2.209 -43.952  1.00  0.00
O
ATOM   6305  NT  LEU X 410     -30.232   1.813 -44.811  1.00  0.00
N
ATOM   6306  HNT LEU X 410     -29.472   1.182 -44.672  1.00  0.00
H
ATOM   6307  CAT LEU X 410     -30.400   2.657 -45.925  1.00  0.00
C
ATOM   6308  HT1 LEU X 410     -31.464   2.787 -46.217  1.00  0.00
H
ATOM   6309  HT2 LEU X 410     -29.732   2.460 -46.791  1.00  0.00
H
ATOM   6310  HT3 LEU X 410     -30.090   3.674 -45.603  1.00  0.00
H
ATOM   6311  C1  GLC X 411     -16.826  -9.726 -22.751  1.00  0.00
C
ATOM   6312  H1  GLC X 411     -17.786  -9.263 -22.435  1.00  0.00
H
ATOM   6313  C5  GLC X 411     -17.253 -11.471 -21.200  1.00  0.00
C
ATOM   6314  H5  GLC X 411     -18.205 -10.982 -20.899  1.00  0.00
H
ATOM   6315  O5  GLC X 411     -16.281 -10.585 -21.739  1.00  0.00
O
ATOM   6316  C2  GLC X 411     -17.041 -10.666 -23.998  1.00  0.00
C
ATOM   6317  H2  GLC X 411     -16.027 -10.992 -24.315  1.00  0.00
H
ATOM   6318  N   GLC X 411     -17.663 -10.057 -25.153  1.00  0.00
N
ATOM   6319  HN  GLC X 411     -17.102 -10.102 -25.976  1.00  0.00
H
ATOM   6320  C   GLC X 411     -18.865  -9.338 -25.120  1.00  0.00
C
ATOM   6321  O   GLC X 411     -19.503  -9.077 -24.126  1.00  0.00
O
ATOM   6322  CT  GLC X 411     -19.338  -8.916 -26.427  1.00  0.00
C
ATOM   6323  HT1 GLC X 411     -19.502  -7.822 -26.528  1.00  0.00
H
ATOM   6324  HT2 GLC X 411     -20.342  -9.342 -26.639  1.00  0.00
H
ATOM   6325  HT3 GLC X 411     -18.601  -9.112 -27.235  1.00  0.00
H
```

```
ATOM   6326  C3  GLC X 411     −17.869 −11.970 −23.517  1.00  0.00
C
ATOM   6327  H3  GLC X 411     −18.861 −11.543 −23.255  1.00  0.00
H
ATOM   6328  O3  GLC X 411     −17.828 −12.889 −24.581  1.00  0.00
O
ATOM   6329  HO3 GLC X 411     −17.798 −13.754 −24.166  1.00  0.00
H
ATOM   6330  C4  GLC X 411     −17.408 −12.574 −22.277  1.00  0.00
C
ATOM   6331  H4  GLC X 411     −16.404 −13.044 −22.358  1.00  0.00
H
ATOM   6332  O4  GLC X 411     −18.265 −13.582 −21.795  1.00  0.00
O
ATOM   6333  C6  GLC X 411     −16.669 −12.102 −19.944  1.00  0.00
C
ATOM   6334  H61 GLC X 411     −15.700 −12.609 −20.138  1.00  0.00
H
ATOM   6335  H62 GLC X 411     −17.305 −12.914 −19.530  1.00  0.00
H
ATOM   6336  O6  GLC X 411     −16.511 −11.128 −18.959  1.00  0.00
O
ATOM   6337  C1  FUC X 412     −17.232 −11.394 −17.749  1.00  0.00
C
ATOM   6338  H1  FUC X 412     −16.888 −12.419 −17.495  1.00  0.00
H
ATOM   6339  C5  FUC X 412     −19.097 −10.049 −18.338  1.00  0.00
C
ATOM   6340  H5  FUC X 412     −18.501  −9.598 −19.161  1.00  0.00
H
ATOM   6341  O5  FUC X 412     −18.643 −11.324 −17.952  1.00  0.00
O
ATOM   6342  C2  FUC X 412     −16.771 −10.454 −16.645  1.00  0.00
C
ATOM   6343  H2  FUC X 412     −17.145 −10.750 −15.642  1.00  0.00
H
ATOM   6344  O2  FUC X 412     −15.364 −10.419 −16.471  1.00  0.00
O
ATOM   6345  HO2 FUC X 412     −15.317 −10.726 −15.563  1.00  0.00
H
ATOM   6346  C3  FUC X 412     −17.301  −8.970 −16.798  1.00  0.00
C
ATOM   6347  H3  FUC X 412     −16.754  −8.415 −17.590  1.00  0.00
H
ATOM   6348  O3  FUC X 412     −17.005  −8.254 −15.659  1.00  0.00
O
ATOM   6349  HO3 FUC X 412     −16.084  −8.422 −15.447  1.00  0.00
H
ATOM   6350  C4  FUC X 412     −18.799  −9.001 −17.114  1.00  0.00
C
```

ATOM 6351 H4 FUC X 412 −19.227 −8.063 −17.528 1.00 0.00
H
ATOM 6352 O4 FUC X 412 −19.497 −9.332 −15.920 1.00 0.00
O
ATOM 6353 HO4 FUC X 412 −19.640 −8.617 −15.295 1.00 0.00
H
ATOM 6354 C6 FUC X 412 −20.679 −10.140 −18.600 1.00 0.00
C
ATOM 6355 H61 FUC X 412 −21.191 −9.192 −18.872 1.00 0.00
H
ATOM 6356 H62 FUC X 412 −21.222 −10.606 −17.751 1.00 0.00
H
ATOM 6357 H63 FUC X 412 −20.795 −10.694 −19.556 1.00 0.00
H
ATOM 6358 C1 GLC X 413 −17.800 −14.920 −21.958 1.00 0.00
C
ATOM 6359 H1 GLC X 413 −16.995 −14.976 −21.195 1.00 0.00
H
ATOM 6360 C5 GLC X 413 −16.970 −16.586 −23.276 1.00 0.00
C
ATOM 6361 H5 GLC X 413 −16.213 −16.845 −22.506 1.00 0.00
H
ATOM 6362 O5 GLC X 413 −17.492 −15.286 −23.268 1.00 0.00
O
ATOM 6363 C2 GLC X 413 −18.951 −15.764 −21.446 1.00 0.00
C
ATOM 6364 H2 GLC X 413 −19.750 −15.456 −22.154 1.00 0.00
H
ATOM 6365 N GLC X 413 −19.345 −15.456 −20.111 1.00 0.00
N
ATOM 6366 HN GLC X 413 −20.250 −15.116 −19.865 1.00 0.00
H
ATOM 6367 C GLC X 413 −18.689 −15.668 −18.997 1.00 0.00
C
ATOM 6368 O GLC X 413 −17.505 −15.973 −18.888 1.00 0.00
O
ATOM 6369 CT GLC X 413 −19.461 −15.336 −17.734 1.00 0.00
C
ATOM 6370 HT1 GLC X 413 −18.801 −15.514 −16.858 1.00 0.00
H
ATOM 6371 HT2 GLC X 413 −20.413 −15.894 −17.602 1.00 0.00
H
ATOM 6372 HT3 GLC X 413 −19.750 −14.264 −17.764 1.00 0.00
H
ATOM 6373 C3 GLC X 413 −18.739 −17.261 −21.742 1.00 0.00
C
ATOM 6374 H3 GLC X 413 −18.109 −17.723 −20.951 1.00 0.00
H
ATOM 6375 O3 GLC X 413 −19.970 −17.930 −21.521 1.00 0.00
O

```
ATOM   6376  HO3 GLC X 413     -19.801 -18.863 -21.668  1.00  0.00
H
ATOM   6377  C4  GLC X 413     -18.166 -17.482 -23.064  1.00  0.00
C
ATOM   6378  H4  GLC X 413     -18.924 -17.280 -23.851  1.00  0.00
H
ATOM   6379  O4  GLC X 413     -17.723 -18.860 -23.142  1.00  0.00
O
ATOM   6380  C6  GLC X 413     -16.353 -16.847 -24.628  1.00  0.00
C
ATOM   6381  H61 GLC X 413     -17.185 -16.683 -25.346  1.00  0.00
H
ATOM   6382  H62 GLC X 413     -16.039 -17.909 -24.721  1.00  0.00
H
ATOM   6383  O6  GLC X 413     -15.251 -16.009 -25.018  1.00  0.00
O
ATOM   6384  HO6 GLC X 413     -14.861 -16.506 -25.741  1.00  0.00
H
ATOM   6385  C1  MAN X 414     -18.729 -19.717 -23.711  1.00  0.00
C
ATOM   6386  H1  MAN X 414     -19.227 -19.287 -24.606  1.00  0.00
H
ATOM   6387  C5  MAN X 414     -20.596 -21.155 -23.303  1.00  0.00
C
ATOM   6388  H5  MAN X 414     -21.095 -20.677 -24.173  1.00  0.00
H
ATOM   6389  O5  MAN X 414     -19.668 -20.206 -22.761  1.00  0.00
O
ATOM   6390  C2  MAN X 414     -17.906 -20.954 -24.174  1.00  0.00
C
ATOM   6391  H2  MAN X 414     -17.177 -20.509 -24.884  1.00  0.00
H
ATOM   6392  O2  MAN X 414     -17.046 -21.512 -23.107  1.00  0.00
O
ATOM   6393  HO2 MAN X 414     -17.204 -20.993 -22.316  1.00  0.00
H
ATOM   6394  C3  MAN X 414     -18.788 -22.022 -24.799  1.00  0.00
C
ATOM   6395  H3  MAN X 414     -19.321 -21.619 -25.687  1.00  0.00
H
ATOM   6396  O3  MAN X 414     -18.064 -23.216 -25.293  1.00  0.00
O
ATOM   6397  C4  MAN X 414     -19.912 -22.376 -23.812  1.00  0.00
C
ATOM   6398  H4  MAN X 414     -19.565 -22.985 -22.950  1.00  0.00
H
ATOM   6399  O4  MAN X 414     -20.817 -23.232 -24.503  1.00  0.00
O
ATOM   6400  HO4 MAN X 414     -21.532 -23.464 -23.907  1.00  0.00
H
```

```
ATOM   6401  C6  MAN X 414     -21.727 -21.240 -22.253  1.00  0.00
C
ATOM   6402  H61 MAN X 414     -22.486 -21.948 -22.648  1.00  0.00
H
ATOM   6403  H62 MAN X 414     -22.158 -20.230 -22.088  1.00  0.00
H
ATOM   6404  O6  MAN X 414     -20.988 -21.794 -21.164  1.00  0.00
O
ATOM   6405  C1  MAN X 415     -21.309 -21.285 -19.871  1.00  0.00
C
ATOM   6406  H1  MAN X 415     -21.499 -20.190 -19.884  1.00  0.00
H
ATOM   6407  C5  MAN X 415     -22.373 -23.344 -19.402  1.00  0.00
C
ATOM   6408  H5  MAN X 415     -22.375 -23.630 -20.475  1.00  0.00
H
ATOM   6409  O5  MAN X 415     -22.381 -21.954 -19.180  1.00  0.00
O
ATOM   6410  C2  MAN X 415     -20.033 -21.692 -19.110  1.00  0.00
C
ATOM   6411  H2  MAN X 415     -19.181 -21.154 -19.578  1.00  0.00
H
ATOM   6412  O2  MAN X 415     -20.115 -21.298 -17.735  1.00  0.00
O
ATOM   6413  C3  MAN X 415     -19.811 -23.142 -19.266  1.00  0.00
C
ATOM   6414  H3  MAN X 415     -19.710 -23.424 -20.336  1.00  0.00
H
ATOM   6415  O3  MAN X 415     -18.600 -23.500 -18.638  1.00  0.00
O
ATOM   6416  HO3 MAN X 415     -18.649 -23.576 -17.682  1.00  0.00
H
ATOM   6417  C4  MAN X 415     -21.093 -23.807 -18.715  1.00  0.00
C
ATOM   6418  H4  MAN X 415     -21.198 -23.539 -17.642  1.00  0.00
H
ATOM   6419  O4  MAN X 415     -20.998 -25.232 -18.822  1.00  0.00
O
ATOM   6420  HO4 MAN X 415     -20.238 -25.532 -18.317  1.00  0.00
H
ATOM   6421  C6  MAN X 415     -23.632 -23.847 -18.715  1.00  0.00
C
ATOM   6422  H61 MAN X 415     -23.681 -23.474 -17.669  1.00  0.00
H
ATOM   6423  H62 MAN X 415     -23.612 -24.951 -18.587  1.00  0.00
H
ATOM   6424  O6  MAN X 415     -24.875 -23.506 -19.342  1.00  0.00
O
ATOM   6425  HO6 MAN X 415     -25.021 -22.559 -19.409  1.00  0.00
H
```

```
ATOM   6426  C1  GLC X 416     -19.670 -20.006 -17.458  1.00  0.00
C
ATOM   6427  H1  GLC X 416     -19.962 -19.357 -18.311  1.00  0.00
H
ATOM   6428  C5  GLC X 416     -17.702 -18.875 -16.724  1.00  0.00
C
ATOM   6429  H5  GLC X 416     -17.933 -18.143 -17.527  1.00  0.00
H
ATOM   6430  O5  GLC X 416     -18.266 -20.084 -17.194  1.00  0.00
O
ATOM   6431  C2  GLC X 416     -20.456 -19.695 -16.172  1.00  0.00
C
ATOM   6432  H2  GLC X 416     -20.169 -20.327 -15.305  1.00  0.00
H
ATOM   6433  N   GLC X 416     -21.927 -19.900 -16.195  1.00  0.00
N
ATOM   6434  HN  GLC X 416     -22.403 -20.506 -15.562  1.00  0.00
H
ATOM   6435  C   GLC X 416     -22.688 -19.282 -17.081  1.00  0.00
C
ATOM   6436  O   GLC X 416     -22.162 -18.556 -17.908  1.00  0.00
O
ATOM   6437  CT  GLC X 416     -24.128 -19.509 -17.229  1.00  0.00
C
ATOM   6438  HT1 GLC X 416     -24.329 -20.455 -17.775  1.00  0.00
H
ATOM   6439  HT2 GLC X 416     -24.705 -18.618 -17.557  1.00  0.00
H
ATOM   6440  HT3 GLC X 416     -24.367 -19.744 -16.170  1.00  0.00
H
ATOM   6441  C3  GLC X 416     -19.974 -18.378 -15.643  1.00  0.00
C
ATOM   6442  H3  GLC X 416     -20.271 -17.661 -16.438  1.00  0.00
H
ATOM   6443  O3  GLC X 416     -20.769 -17.981 -14.489  1.00  0.00
O
ATOM   6444  HO3 GLC X 416     -21.193 -17.129 -14.620  1.00  0.00
H
ATOM   6445  C4  GLC X 416     -18.487 -18.334 -15.376  1.00  0.00
C
ATOM   6446  H4  GLC X 416     -18.095 -18.992 -14.571  1.00  0.00
H
ATOM   6447  O4  GLC X 416     -17.982 -17.030 -15.079  1.00  0.00
O
ATOM   6448  HO4 GLC X 416     -17.507 -17.061 -14.245  1.00  0.00
H
ATOM   6449  C6  GLC X 416     -16.169 -19.096 -16.492  1.00  0.00
C
ATOM   6450  H61 GLC X 416     -15.972 -19.737 -15.606  1.00  0.00
H
```

```
ATOM   6451  H62 GLC X 416     −15.778 −18.068 −16.338  1.00  0.00
H
ATOM   6452  O6  GLC X 416     −15.592 −19.659 −17.677  1.00  0.00
O
ATOM   6453  HO6 GLC X 416     −15.801 −20.595 −17.684  1.00  0.00
H
ATOM   6454  C1  MAN X 417     −17.704 −23.283 −26.687  1.00  0.00
C
ATOM   6455  H1  MAN X 417     −18.514 −22.862 −27.320  1.00  0.00
H
ATOM   6456  C5  MAN X 417     −15.443 −23.116 −25.996  1.00  0.00
C
ATOM   6457  H5  MAN X 417     −15.772 −23.107 −24.935  1.00  0.00
H
ATOM   6458  O5  MAN X 417     −16.461 −22.571 −26.840  1.00  0.00
O
ATOM   6459  C2  MAN X 417     −17.511 −24.705 −27.043  1.00  0.00
C
ATOM   6460  H2  MAN X 417     −18.376 −25.279 −26.646  1.00  0.00
H
ATOM   6461  O2  MAN X 417     −17.241 −24.963 −28.464  1.00  0.00
O
ATOM   6462  C3  MAN X 417     −16.360 −25.286 −26.237  1.00  0.00
C
ATOM   6463  H3  MAN X 417     −16.541 −25.157 −25.148  1.00  0.00
H
ATOM   6464  O3  MAN X 417     −16.179 −26.705 −26.264  1.00  0.00
O
ATOM   6465  HO3 MAN X 417     −16.351 −27.006 −27.159  1.00  0.00
H
ATOM   6466  C4  MAN X 417     −15.100 −24.535 −26.462  1.00  0.00
C
ATOM   6467  H4  MAN X 417     −14.855 −24.345 −27.529  1.00  0.00
H
ATOM   6468  O4  MAN X 417     −13.960 −24.849 −25.674  1.00  0.00
O
ATOM   6469  C6  MAN X 417     −14.288 −22.066 −26.134  1.00  0.00
C
ATOM   6470  H61 MAN X 417     −13.810 −22.134 −27.135  1.00  0.00
H
ATOM   6471  H62 MAN X 417     −13.420 −22.168 −25.449  1.00  0.00
H
ATOM   6472  O6  MAN X 417     −14.706 −20.688 −25.932  1.00  0.00
O
ATOM   6473  HO6 MAN X 417     −15.034 −20.406 −26.789  1.00  0.00
H
ATOM   6474  C1  GLC X 418     −12.937 −25.678 −26.129  1.00  0.00
C
ATOM   6475  H1  GLC X 418     −12.716 −25.665 −27.218  1.00  0.00
H
```

```
ATOM   6476  C5  GLC X 418     -12.575 -27.941 -26.524  1.00  0.00
C
ATOM   6477  H5  GLC X 418     -12.583 -27.576 -27.573  1.00  0.00
H
ATOM   6478  O5  GLC X 418     -13.274 -26.964 -25.778  1.00  0.00
O
ATOM   6479  C2  GLC X 418     -11.624 -25.463 -25.388  1.00  0.00
C
ATOM   6480  H2  GLC X 418     -11.775 -25.701 -24.313  1.00  0.00
H
ATOM   6481  N   GLC X 418     -11.215 -24.100 -25.520  1.00  0.00
N
ATOM   6482  HN  GLC X 418     -11.109 -23.587 -24.671  1.00  0.00
H
ATOM   6483  C   GLC X 418     -10.757 -23.488 -26.616  1.00  0.00
C
ATOM   6484  O   GLC X 418     -10.643 -24.042 -27.696  1.00  0.00
O
ATOM   6485  CT  GLC X 418     -10.229 -22.145 -26.468  1.00  0.00
C
ATOM   6486  HT1 GLC X 418      -9.165 -22.280 -26.181  1.00  0.00
H
ATOM   6487  HT2 GLC X 418     -10.678 -21.568 -25.632  1.00  0.00
H
ATOM   6488  HT3 GLC X 418     -10.522 -21.594 -27.387  1.00  0.00
H
ATOM   6489  C3  GLC X 418     -10.592 -26.444 -25.883  1.00  0.00
C
ATOM   6490  H3  GLC X 418     -10.284 -26.225 -26.927  1.00  0.00
H
ATOM   6491  O3  GLC X 418      -9.407 -26.256 -25.159  1.00  0.00
O
ATOM   6492  HO3 GLC X 418      -8.818 -25.984 -25.866  1.00  0.00
H
ATOM   6493  C4  GLC X 418     -11.219 -27.833 -25.949  1.00  0.00
C
ATOM   6494  H4  GLC X 418     -11.311 -28.114 -24.878  1.00  0.00
H
ATOM   6495  O4  GLC X 418     -10.306 -28.772 -26.514  1.00  0.00
O
ATOM   6496  HO4 GLC X 418      -9.816 -29.261 -25.849  1.00  0.00
H
ATOM   6497  C6  GLC X 418     -13.314 -29.202 -26.549  1.00  0.00
C
ATOM   6498  H61 GLC X 418     -12.628 -30.035 -26.815  1.00  0.00
H
ATOM   6499  H62 GLC X 418     -14.092 -29.264 -27.339  1.00  0.00
H
ATOM   6500  O6  GLC X 418     -13.788 -29.596 -25.256  1.00  0.00
O
```

```
ATOM   6501  HO6 GLC X 418     -14.529 -29.018 -25.058  1.00  0.00
H
ATOM   6502  C1  GLC X 419     -18.470 -25.115 -29.181  1.00  0.00
C
ATOM   6503  H1  GLC X 419     -19.206 -24.340 -28.878  1.00  0.00
H
ATOM   6504  C5  GLC X 419     -20.127 -26.567 -29.952  1.00  0.00
C
ATOM   6505  H5  GLC X 419     -20.840 -25.800 -29.583  1.00  0.00
H
ATOM   6506  O5  GLC X 419     -18.934 -26.455 -29.170  1.00  0.00
O
ATOM   6507  C2  GLC X 419     -18.094 -24.843 -30.613  1.00  0.00
C
ATOM   6508  H2  GLC X 419     -17.282 -25.561 -30.856  1.00  0.00
H
ATOM   6509  N   GLC X 419     -17.413 -23.554 -30.804  1.00  0.00
N
ATOM   6510  HN  GLC X 419     -16.473 -23.641 -31.125  1.00  0.00
H
ATOM   6511  C   GLC X 419     -18.030 -22.361 -30.700  1.00  0.00
C
ATOM   6512  O   GLC X 419     -19.209 -22.279 -30.257  1.00  0.00
O
ATOM   6513  CT  GLC X 419     -17.221 -21.212 -31.081  1.00  0.00
C
ATOM   6514  HT1 GLC X 419     -16.851 -20.796 -30.120  1.00  0.00
H
ATOM   6515  HT2 GLC X 419     -17.846 -20.394 -31.499  1.00  0.00
H
ATOM   6516  HT3 GLC X 419     -16.375 -21.387 -31.781  1.00  0.00
H
ATOM   6517  C3  GLC X 419     -19.251 -25.065 -31.531  1.00  0.00
C
ATOM   6518  H3  GLC X 419     -20.069 -24.380 -31.219  1.00  0.00
H
ATOM   6519  O3  GLC X 419     -18.931 -24.815 -32.881  1.00  0.00
O
ATOM   6520  HO3 GLC X 419     -18.973 -23.903 -33.177  1.00  0.00
H
ATOM   6521  C4  GLC X 419     -19.694 -26.513 -31.379  1.00  0.00
C
ATOM   6522  H4  GLC X 419     -18.747 -27.071 -31.540  1.00  0.00
H
ATOM   6523  O4  GLC X 419     -20.743 -26.961 -32.213  1.00  0.00
O
ATOM   6524  HO4 GLC X 419     -20.358 -27.080 -33.084  1.00  0.00
H
ATOM   6525  C6  GLC X 419     -20.593 -28.001 -29.556  1.00  0.00
C
```

```
ATOM   6526  H61 GLC X 419     -21.667 -28.105 -29.823  1.00  0.00
H
ATOM   6527  H62 GLC X 419     -20.553 -28.068 -28.448  1.00  0.00
H
ATOM   6528  O6  GLC X 419     -19.803 -29.085 -30.129  1.00  0.00
O
ATOM   6529  HO6 GLC X 419     -20.045 -29.124 -31.057  1.00  0.00
H
```
