# Supplementary material for: Identification and mechanistic basis of non-ACE2 blocking neutralizing antibodies from COVID-19 patients with deep RNA sequencing and molecular dynamics simulations
Source: Front Mol Biosci. 2022 Dec 16;9:1080964. doi: 10.3389/fmolb.2022.1080964 (PMC9800910; doi:10.3389/fmolb.2022.1080964)
Supplement: Supplementary file 2 [file DataSheet3.pdf]

```
REMARK   3
REMARK   3 REFINEMENT.
REMARK   3   PROGRAM     : PHENIX (1.18.2_3874:
phenix.real_space_refine)
REMARK   3   AUTHORS     :
Adams,Afonine,Bunkoczi,Burnley,Chen,Dar,Davis,
REMARK   3               : Draizen,Echols,Gildea,Gros,Grosse-
Kunstleve,Headd,
REMARK   3               :
Hintze,Hung,Ioerger,Liebschner,McCoy,McKee,Moriarty,
REMARK   3               :
Oeffner,Poon,Read,Richardson,Richardson,Sacchettini,
REMARK   3               :
Sauter,Sobolev,Storoni,Terwilliger,Williams,Zwart
REMARK   3
REMARK   3  SCATTERING TABLE: ELECTRON
REMARK   3
REMARK   3  REFINEMENT TARGET: REAL-SPACE (WEIGHTED MAP SUM AT ATOM
CENTERS)
REMARK   3
REMARK   3  MODEL TO MAP FIT.
REMARK   3    CC_mask  : 0.8735
REMARK   3    CC_volume: 0.8655
REMARK   3    CC_peaks : 0.8341
REMARK   3
REMARK   3 GEOMETRY RESTRAINTS LIBRARY: GEOSTD + MONOMER LIBRARY + CDL
V1.2
REMARK   3 DEVIATIONS FROM IDEAL VALUES.
REMARK   3   BOND      :  0.006   0.067   3383
REMARK   3   ANGLE     :  0.829   7.991   4594
REMARK   3   CHIRALITY :  0.052   0.197    509
REMARK   3   PLANARITY :  0.006   0.077    582
REMARK   3   DIHEDRAL  : 13.560 179.842    505
REMARK   3   MIN NONBONDED DISTANCE : 2.158
REMARK   3
REMARK   3 MOLPROBITY STATISTICS.
REMARK   3   ALL-ATOM CLASHSCORE : 5.60
REMARK   3   RAMACHANDRAN PLOT:
REMARK   3     OUTLIERS :  0.50 %
REMARK   3     ALLOWED  :  5.97 %
REMARK   3     FAVORED  : 93.53 %
REMARK   3   ROTAMER OUTLIERS :  1.14 %
REMARK   3   CBETA DEVIATIONS :  0.00 %
REMARK   3   PEPTIDE PLANE:
REMARK   3     CIS-PROLINE     : 5.56 %
REMARK   3     CIS-GENERAL     : 0.00 %
REMARK   3     TWISTED PROLINE : 0.00 %
REMARK   3     TWISTED GENERAL : 0.52 %
REMARK   3
REMARK   3 RAMA-Z (RAMACHANDRAN PLOT Z-SCORE):
```

```
REMARK   3 INTERPRETATION: BAD |RAMA-Z| > 3; SUSPICIOUS 2 < |RAMA-Z| <
3; GOOD
REMARK   3 SCORES FOR WHOLE/HELIX/SHEET/LOOP ARE SCALED INDEPENDENTLY;
REMARK   3 THEREFORE, THE VALUES ARE NOT RELATED IN A SIMPLE MANNER.
REMARK   3   WHOLE: -0.66 (0.40), RESIDUES: 402
REMARK   3   HELIX: -3.62 (0.68), RESIDUES: 27
REMARK   3   SHEET:  0.68 (0.44), RESIDUES: 140
REMARK   3   LOOP : -0.73 (0.37), RESIDUES: 235
REMARK   3
HELIX    1   1 PRO B  199  ASP B  201  5
3
HELIX    2   2 PHE C  338  PHE C  342  5
5
HELIX    3   3 LEU C  387  ASP C  389  5
3
HELIX    4   4 GLY C  404  GLN C  409  5
6
HELIX    5   5 LYS C  417  TYR C  421  1
5
HELIX    6   6 ASN C  439  ASP C  442  1
4
HELIX    7   7 VAL C  502  TYR C  504  5
3
HELIX    8   8 PHE A   29  SER A   31  5
3
HELIX    9   9 ALA A   88  ASP A   90  5
3
SHEET    1   A 4 MET B 123  GLN B 125  0
SHEET    2   A 4 THR B 139  ALA B 144 -1  N  ARG B 143   O  THR B 124
SHEET    3   A 4 GLU B 189  THR B 193 -1  N  LEU B 192   O  ILE B 140
SHEET    4   A 4 SER B 182  SER B 186 -1  N  SER B 186   O  GLU B 189
SHEET    1   B 2 THR B 129  ALA B 132  0
SHEET    2   B 2 LYS B 222  ILE B 225  1  N  LYS B 222   O  LEU B 130
SHEET    1   C 3 THR B 204  GLN B 209  0
SHEET    2   C 3 LEU B 152  GLN B 157 -1  N  GLN B 157   O  THR B 204
SHEET    3   C 3 LYS B 164  ILE B 167 -1  N  ILE B 167   O  TRP B 154
SHEET    1   D 5 ASN C 354  SER C 359  0
SHEET    2   D 5 ASN C 394  ARG C 403 -1  N  SER C 399   O  ASN C 354
SHEET    3   D 5 PRO C 506  SER C 513 -1  N  SER C 513   O  TYR C 396
SHEET    4   D 5 GLY C 431  ASN C 437 -1  N  TRP C 436   O  ARG C 508
SHEET    5   D 5 THR C 376  TYR C 380 -1  N  TYR C 380   O  GLY C 431
SHEET    1   E 2 LEU C 452  ARG C 454  0
SHEET    2   E 2 LEU C 491  SER C 493 -1  N  GLN C 492   O  TYR C 453
SHEET    1   F 4 GLN A   3  SER A   7  0
SHEET    2   F 4 LEU A  18  SER A  25 -1  N  SER A  25   O  GLN A   3
SHEET    3   F 4 THR A  78  MET A  83 -1  N  MET A  83   O  LEU A  18
SHEET    4   F 4 PHE A  68  ASP A  73 -1  N  ASP A  73   O  THR A  78
SHEET    1   G 5 THR A 113  VAL A 115  0
SHEET    2   G 5 ALA A  92  SER A 100 -1  N  TYR A  94   O  THR A 113
SHEET    3   G 5 TYR A  32  GLN A  39 -1  N  GLN A  39   O  VAL A  93
```

```
SHEET    4   G 5 LEU A  45  ILE A  51 -1  N  ILE A  51   O  MET A  34
SHEET    5   G 5 LYS A  58  TYR A  60 -1  N  TYR A  59   O  VAL A  50
LINK         C1  NAG V   9                 O2  BMA V   8
LINK         C1  BMA V   8                 O3  BMA V   3
LINK         C1  NAG V   2                 O4  NAG V   1
LINK         C1  BMA V   3                 O4  NAG V   2
LINK         C1  FUC V   4                 O6  NAG V   1
LINK         C1  BMA V   5                 O6  BMA V   3
SSBOND   1 CYS B  142    CYS B  207
SSBOND   2 CYS C  336    CYS C  361
SSBOND   3 CYS C  379    CYS C  432
SSBOND   4 CYS C  479    CYS C  487
SSBOND   5 CYS A   22    CYS A   96
CRYST1  100.000  100.000  100.000  90.00  90.00  90.00 P 1
SCALE1      0.010000  0.000000  0.000000        0.00000
SCALE2      0.000000  0.010000  0.000000        0.00000
SCALE3      0.000000  0.000000  0.010000        0.00000
ATOM      1  N   ASP B   1      55.119  43.529  40.482  1.00 27.51
N
ATOM      2  CA  ASP B   1      55.600  44.374  41.565  1.00 27.51
C
ATOM      3  C   ASP B   1      55.829  45.800  41.078  1.00 27.51
C
ATOM      4  O   ASP B   1      56.140  46.019  39.911  1.00 27.51
O
ATOM      5  CB  ASP B   1      56.888  43.805  42.149  1.00 27.51
C
ATOM      6  CG  ASP B   1      57.937  43.561  41.094  1.00 27.51
C
ATOM      7  OD1 ASP B   1      57.556  43.384  39.922  1.00 27.51
O
ATOM      8  OD2 ASP B   1      59.136  43.553  41.434  1.00 27.51
O
ATOM      9  N   ILE B   2      55.674  46.765  41.977  1.00 21.42
N
ATOM     10  CA  ILE B   2      55.865  48.169  41.637  1.00 21.42
C
ATOM     11  C   ILE B   2      57.343  48.508  41.751  1.00 21.42
C
ATOM     12  O   ILE B   2      57.978  48.226  42.771  1.00 21.42
O
ATOM     13  CB  ILE B   2      55.015  49.068  42.546  1.00 21.42
C
ATOM     14  CG1 ILE B   2      53.535  48.717  42.399  1.00 21.42
C
ATOM     15  CG2 ILE B   2      55.263  50.531  42.235  1.00 21.42
C
ATOM     16  CD1 ILE B   2      52.684  49.162  43.548  1.00 21.42
C
ATOM     17  N   GLN B   3      57.896  49.111  40.705  1.00 23.36
```

N
ATOM 18 CA GLN B 3 59.303 49.478 40.686 1.00 23.36
C
ATOM 19 C GLN B 3 59.482 50.877 41.256 1.00 23.36
C
ATOM 20 O GLN B 3 58.771 51.808 40.869 1.00 23.36
O
ATOM 21 CB GLN B 3 59.866 49.411 39.267 1.00 23.36
C
ATOM 22 CG GLN B 3 59.594 48.105 38.557 1.00 23.36
C
ATOM 23 CD GLN B 3 60.007 46.905 39.372 1.00 23.36
C
ATOM 24 OE1 GLN B 3 59.220 45.987 39.583 1.00 23.36
O
ATOM 25 NE2 GLN B 3 61.249 46.902 39.834 1.00 23.36
N
ATOM 26 N MET B 4 60.429 51.016 42.176 1.00 19.78
N
ATOM 27 CA MET B 4 60.734 52.282 42.823 1.00 19.78
C
ATOM 28 C MET B 4 62.144 52.689 42.432 1.00 19.78
C
ATOM 29 O MET B 4 63.060 51.865 42.478 1.00 19.78
O
ATOM 30 CB MET B 4 60.624 52.164 44.347 1.00 19.78
C
ATOM 31 CG MET B 4 59.311 51.592 44.847 1.00 19.78
C
ATOM 32 SD MET B 4 57.901 52.632 44.444 1.00 19.78
S
ATOM 33 CE MET B 4 58.371 54.146 45.255 1.00 19.78
C
ATOM 34 N THR B 5 62.323 53.948 42.045 1.00 21.11
N
ATOM 35 CA THR B 5 63.651 54.417 41.677 1.00 21.11
C
ATOM 36 C THR B 5 63.882 55.811 42.240 1.00 21.11
C
ATOM 37 O THR B 5 62.978 56.642 42.249 1.00 21.11
O
ATOM 38 CB THR B 5 63.849 54.397 40.152 1.00 21.11
C
ATOM 39 OG1 THR B 5 65.210 54.712 39.839 1.00 21.11
O
ATOM 40 CG2 THR B 5 62.929 55.380 39.464 1.00 21.11
C
ATOM 41 N GLN B 6 65.091 56.060 42.725 1.00 20.54
N
ATOM 42 CA GLN B 6 65.445 57.335 43.329 1.00 20.54

C
ATOM 43 C GLN B 6 66.420 58.062 42.414 1.00 20.54
C
ATOM 44 O GLN B 6 67.358 57.456 41.894 1.00 20.54
O
ATOM 45 CB GLN B 6 66.048 57.129 44.719 1.00 20.54
C
ATOM 46 CG GLN B 6 65.073 56.510 45.687 1.00 20.54
C
ATOM 47 CD GLN B 6 65.700 56.116 47.004 1.00 20.54
C
ATOM 48 OE1 GLN B 6 65.498 55.006 47.481 1.00 20.54
O
ATOM 49 NE2 GLN B 6 66.439 57.028 47.611 1.00 20.54
N
ATOM 50 N SER B 7 66.186 59.359 42.211 1.00 28.45
N
ATOM 51 CA SER B 7 66.754 60.036 41.046 1.00 28.45
C
ATOM 52 C SER B 7 68.246 60.308 41.187 1.00 28.45
C
ATOM 53 O SER B 7 69.017 59.866 40.327 1.00 28.45
O
ATOM 54 CB SER B 7 66.001 61.331 40.739 1.00 28.45
C
ATOM 55 OG SER B 7 66.694 62.079 39.757 1.00 28.45
O
ATOM 56 N PRO B 8 68.718 61.030 42.213 1.00 27.25
N
ATOM 57 CA PRO B 8 70.171 61.101 42.408 1.00 27.25
C
ATOM 58 C PRO B 8 70.638 59.847 43.127 1.00 27.25
C
ATOM 59 O PRO B 8 70.325 59.637 44.302 1.00 27.25
O
ATOM 60 CB PRO B 8 70.349 62.363 43.267 1.00 27.25
C
ATOM 61 CG PRO B 8 68.962 62.860 43.587 1.00 27.25
C
ATOM 62 CD PRO B 8 68.013 61.754 43.279 1.00 27.25
C
ATOM 63 N SER B 9 71.350 58.982 42.404 1.00 29.14
N
ATOM 64 CA SER B 9 71.862 57.762 43.019 1.00 29.14
C
ATOM 65 C SER B 9 72.904 58.079 44.079 1.00 29.14
C
ATOM 66 O SER B 9 72.916 57.468 45.151 1.00 29.14
O
ATOM 67 CB SER B 9 72.445 56.838 41.956 1.00 29.14

C
ATOM 68 OG SER B 9 71.421 56.288 41.153 1.00 29.14
O
ATOM 69 N THR B 10 73.791 59.025 43.796 1.00 33.00
N
ATOM 70 CA THR B 10 74.754 59.490 44.777 1.00 33.00
C
ATOM 71 C THR B 10 74.864 61.000 44.659 1.00 33.00
C
ATOM 72 O THR B 10 74.626 61.572 43.595 1.00 33.00
O
ATOM 73 CB THR B 10 76.126 58.831 44.594 1.00 33.00
C
ATOM 74 OG1 THR B 10 77.053 59.389 45.531 1.00 33.00
O
ATOM 75 CG2 THR B 10 76.638 59.051 43.187 1.00 33.00
C
ATOM 76 N LEU B 11 75.203 61.643 45.768 1.00 36.94
N
ATOM 77 CA LEU B 11 75.224 63.096 45.795 1.00 36.94
C
ATOM 78 C LEU B 11 76.231 63.579 46.824 1.00 36.94
C
ATOM 79 O LEU B 11 76.192 63.157 47.982 1.00 36.94
O
ATOM 80 CB LEU B 11 73.831 63.641 46.101 1.00 36.94
C
ATOM 81 CG LEU B 11 73.692 65.126 46.391 1.00 36.94
C
ATOM 82 CD1 LEU B 11 73.955 65.927 45.135 1.00 36.94
C
ATOM 83 CD2 LEU B 11 72.296 65.370 46.876 1.00 36.94
C
ATOM 84 N SER B 12 77.131 64.456 46.396 1.00 41.19
N
ATOM 85 CA SER B 12 78.108 65.065 47.284 1.00 41.19
C
ATOM 86 C SER B 12 77.599 66.426 47.728 1.00 41.19
C
ATOM 87 O SER B 12 77.088 67.202 46.918 1.00 41.19
O
ATOM 88 CB SER B 12 79.465 65.205 46.593 1.00 41.19
C
ATOM 89 OG SER B 12 79.302 65.516 45.223 1.00 41.19
O
ATOM 90 N ALA B 13 77.723 66.704 49.020 1.00 42.09
N
ATOM 91 CA ALA B 13 77.234 67.957 49.571 1.00 42.09
C
ATOM 92 C ALA B 13 78.126 68.372 50.731 1.00 42.09

C
ATOM   93 O   ALA B 13   78.946 67.595 51.223 1.00 42.09
O
ATOM   94 CB ALA B 13   75.777 67.838 50.017 1.00 42.09
C
ATOM   95 N   SER B 14   77.962 69.620 51.158 1.00 42.98
N
ATOM   96 CA SER B 14   78.711 70.175 52.272 1.00 42.98
C
ATOM   97 C   SER B 14   77.755 70.509 53.408 1.00 42.98
C
ATOM   98 O   SER B 14   76.535 70.524 53.237 1.00 42.98
O
ATOM   99 CB SER B 14   79.495 71.425 51.849 1.00 42.98
C
ATOM   100 OG SER B 14   78.634 72.395 51.285 1.00 42.98
O
ATOM   101 N   VAL B 15   78.316 70.758 54.587 1.00 42.15
N
ATOM   102 CA VAL B 15   77.478 71.055 55.738 1.00 42.15
C
ATOM   103 C   VAL B 15   76.749 72.368 55.509 1.00 42.15
C
ATOM   104 O   VAL B 15   77.370 73.416 55.295 1.00 42.15
O
ATOM   105 CB VAL B 15   78.319 71.094 57.019 1.00 42.15
C
ATOM   106 CG1 VAL B 15   77.454 71.480 58.198 1.00 42.15
C
ATOM   107 CG2 VAL B 15   78.969 69.750 57.256 1.00 42.15
C
ATOM   108 N   GLY B 16   75.422 72.315 55.545 1.00 43.40
N
ATOM   109 CA GLY B 16   74.599 73.495 55.439 1.00 43.40
C
ATOM   110 C   GLY B 16   73.805 73.635 54.158 1.00 43.40
C
ATOM   111 O   GLY B 16   72.902 74.478 54.114 1.00 43.40
O
ATOM   112 N   ASP B 17   74.095 72.853 53.120 1.00 43.64
N
ATOM   113 CA ASP B 17   73.324 73.019 51.897 1.00 43.64
C
ATOM   114 C   ASP B 17   71.927 72.422 52.024 1.00 43.64
C
ATOM   115 O   ASP B 17   71.615 71.662 52.943 1.00 43.64
O
ATOM   116 CB ASP B 17   74.014 72.403 50.678 1.00 43.64
C
ATOM   117 CG ASP B 17   75.510 72.553 50.703 1.00 43.64

C
ATOM 118 OD1 ASP B 17 76.208 71.558 50.951 1.00 43.64
O
ATOM 119 OD2 ASP B 17 75.993 73.678 50.475 1.00 43.64
O
ATOM 120 N LYS B 18 71.079 72.811 51.080 1.00 40.48
N
ATOM 121 CA LYS B 18 69.744 72.263 50.929 1.00 40.48
C
ATOM 122 C LYS B 18 69.830 71.068 49.996 1.00 40.48
C
ATOM 123 O LYS B 18 70.401 71.168 48.908 1.00 40.48
O
ATOM 124 CB LYS B 18 68.803 73.315 50.346 1.00 40.48
C
ATOM 125 CG LYS B 18 68.110 74.185 51.368 1.00 40.48
C
ATOM 126 CD LYS B 18 66.871 74.861 50.796 1.00 40.48
C
ATOM 127 CE LYS B 18 66.246 74.044 49.676 1.00 40.48
C
ATOM 128 NZ LYS B 18 64.766 74.185 49.650 1.00 40.48
N
ATOM 129 N ILE B 19 69.271 69.938 50.415 1.00 33.20
N
ATOM 130 CA ILE B 19 69.371 68.704 49.652 1.00 33.20
C
ATOM 131 C ILE B 19 67.962 68.218 49.353 1.00 33.20
C
ATOM 132 O ILE B 19 67.119 68.161 50.254 1.00 33.20
O
ATOM 133 CB ILE B 19 70.175 67.638 50.414 1.00 33.20
C
ATOM 134 CG1 ILE B 19 71.647 68.029 50.432 1.00 33.20
C
ATOM 135 CG2 ILE B 19 70.036 66.287 49.740 1.00 33.20
C
ATOM 136 CD1 ILE B 19 72.280 68.029 49.090 1.00 33.20
C
ATOM 137 N THR B 20 67.705 67.888 48.092 1.00 27.05
N
ATOM 138 CA THR B 20 66.417 67.364 47.662 1.00 27.05
C
ATOM 139 C THR B 20 66.628 66.033 46.959 1.00 27.05
C
ATOM 140 O THR B 20 67.458 65.928 46.053 1.00 27.05
O
ATOM 141 CB THR B 20 65.697 68.349 46.737 1.00 27.05
C
ATOM 142 OG1 THR B 20 65.264 69.480 47.498 1.00 27.05

O
ATOM 143 CG2 THR B 20 64.487 67.698 46.098 1.00 27.05
C
ATOM 144 N ILE B 21 65.879 65.020 47.385 1.00 22.41
N
ATOM 145 CA ILE B 21 65.954 63.672 46.839 1.00 22.41
C
ATOM 146 C ILE B 21 64.586 63.328 46.274 1.00 22.41
C
ATOM 147 O ILE B 21 63.568 63.564 46.929 1.00 22.41
O
ATOM 148 CB ILE B 21 66.377 62.654 47.913 1.00 22.41
C
ATOM 149 CG1 ILE B 21 67.658 63.117 48.607 1.00 22.41
C
ATOM 150 CG2 ILE B 21 66.554 61.281 47.302 1.00 22.41
C
ATOM 151 CD1 ILE B 21 67.982 62.363 49.866 1.00 22.41
C
ATOM 152 N THR B 22 64.557 62.790 45.063 1.00 22.40
N
ATOM 153 CA THR B 22 63.312 62.449 44.393 1.00 22.40
C
ATOM 154 C THR B 22 63.188 60.938 44.269 1.00 22.40
C
ATOM 155 O THR B 22 64.160 60.251 43.951 1.00 22.40
O
ATOM 156 CB THR B 22 63.233 63.090 43.003 1.00 22.40
C
ATOM 157 OG1 THR B 22 63.454 64.498 43.113 1.00 22.40
O
ATOM 158 CG2 THR B 22 61.870 62.852 42.380 1.00 22.40
C
ATOM 159 N CYS B 23 61.991 60.425 44.529 1.00 20.48
N
ATOM 160 CA CYS B 23 61.675 59.018 44.348 1.00 20.48
C
ATOM 161 C CYS B 23 60.459 58.914 43.436 1.00 20.48
C
ATOM 162 O CYS B 23 59.520 59.703 43.548 1.00 20.48
O
ATOM 163 CB CYS B 23 61.427 58.326 45.714 1.00 20.48
C
ATOM 164 SG CYS B 23 60.966 56.566 45.638 1.00 20.48
S
ATOM 165 N ARG B 24 60.491 57.963 42.513 1.00 23.16
N
ATOM 166 CA ARG B 24 59.438 57.792 41.525 1.00 23.16
C
ATOM 167 C ARG B 24 58.980 56.344 41.511 1.00 23.16

```
C
ATOM    168  O   ARG B  24      59.787  55.423  41.667  1.00 23.16
O
ATOM    169  CB  ARG B  24      59.908  58.199  40.117  1.00 23.16
C
ATOM    170  CG  ARG B  24      60.462  59.605  40.028  1.00 23.16
C
ATOM    171  CD  ARG B  24      60.433  60.139  38.606  1.00 23.16
C
ATOM    172  NE  ARG B  24      60.600  61.587  38.585  1.00 23.16
N
ATOM    173  CZ  ARG B  24      61.776  62.197  38.493  1.00 23.16
C
ATOM    174  NH1 ARG B  24      62.888  61.483  38.406  1.00 23.16
N
ATOM    175  NH2 ARG B  24      61.839  63.520  38.486  1.00 23.16
N
ATOM    176  N   ALA B  25      57.681  56.154  41.315  1.00 21.65
N
ATOM    177  CA  ALA B  25      57.058  54.843  41.265  1.00 21.65
C
ATOM    178  C   ALA B  25      56.542  54.569  39.859  1.00 21.65
C
ATOM    179  O   ALA B  25      56.123  55.485  39.148  1.00 21.65
O
ATOM    180  CB  ALA B  25      55.907  54.746  42.268  1.00 21.65
C
ATOM    181  N   SER B  26      56.579  53.299  39.460  1.00 23.50
N
ATOM    182  CA  SER B  26      56.119  52.933  38.124  1.00 23.50
C
ATOM    183  C   SER B  26      54.600  52.955  38.008  1.00 23.50
C
ATOM    184  O   SER B  26      54.072  53.187  36.916  1.00 23.50
O
ATOM    185  CB  SER B  26      56.658  51.556  37.739  1.00 23.50
C
ATOM    186  OG  SER B  26      56.140  50.545  38.578  1.00 23.50
O
ATOM    187  N   GLN B  27      53.889  52.707  39.102  1.00 24.57
N
ATOM    188  CA  GLN B  27      52.440  52.813  39.163  1.00 24.57
C
ATOM    189  C   GLN B  27      52.069  53.724  40.321  1.00 24.57
C
ATOM    190  O   GLN B  27      52.866  53.950  41.231  1.00 24.57
O
ATOM    191  CB  GLN B  27      51.771  51.447  39.348  1.00 24.57
C
ATOM    192  CG  GLN B  27      52.140  50.422  38.305  1.00 24.57
```

C
ATOM 193 CD GLN B 27 51.716 50.840 36.917 1.00 24.57
C
ATOM 194 OE1 GLN B 27 52.483 50.731 35.963 1.00 24.57
O
ATOM 195 NE2 GLN B 27 50.490 51.328 36.797 1.00 24.57
N
ATOM 196 N SER B 28 50.847 54.246 40.287 1.00 21.25
N
ATOM 197 CA SER B 28 50.397 55.157 41.330 1.00 21.25
C
ATOM 198 C SER B 28 50.166 54.407 42.639 1.00 21.25
C
ATOM 199 O SER B 28 49.512 53.364 42.663 1.00 21.25
O
ATOM 200 CB SER B 28 49.121 55.868 40.889 1.00 21.25
C
ATOM 201 OG SER B 28 48.684 56.780 41.874 1.00 21.25
O
ATOM 202 N ILE B 29 50.715 54.945 43.729 1.00 17.66
N
ATOM 203 CA ILE B 29 50.686 54.327 45.055 1.00 17.66
C
ATOM 204 C ILE B 29 50.059 55.272 46.071 1.00 17.66
C
ATOM 205 O ILE B 29 50.452 55.281 47.238 1.00 17.66
O
ATOM 206 CB ILE B 29 52.096 53.909 45.514 1.00 17.66
C
ATOM 207 CG1 ILE B 29 53.069 55.085 45.414 1.00 17.66
C
ATOM 208 CG2 ILE B 29 52.585 52.722 44.714 1.00 17.66
C
ATOM 209 CD1 ILE B 29 54.386 54.839 46.113 1.00 17.66
C
ATOM 210 N SER B 30 49.006 55.981 45.681 1.00 18.78
N
ATOM 211 CA SER B 30 48.857 57.418 45.871 1.00 18.78
C
ATOM 212 C SER B 30 49.615 58.092 47.015 1.00 18.78
C
ATOM 213 O SER B 30 50.496 58.903 46.733 1.00 18.78
O
ATOM 214 CB SER B 30 47.366 57.706 46.051 1.00 18.78
C
ATOM 215 OG SER B 30 46.929 57.324 47.344 1.00 18.78
O
ATOM 216 N ASN B 31 49.348 57.795 48.283 1.00 17.25
N
ATOM 217 CA ASN B 31 50.109 58.442 49.351 1.00 17.25

C
ATOM 218 C ASN B 31 50.935 57.458 50.166 1.00 17.25
C
ATOM 219 O ASN B 31 51.562 57.860 51.149 1.00 17.25
O
ATOM 220 CB ASN B 31 49.195 59.229 50.296 1.00 17.25
C
ATOM 221 CG ASN B 31 48.640 60.491 49.665 1.00 17.25
C
ATOM 222 OD1 ASN B 31 49.220 61.046 48.740 1.00 17.25
O
ATOM 223 ND2 ASN B 31 47.508 60.948 50.170 1.00 17.25
N
ATOM 224 N TRP B 32 50.962 56.188 49.780 1.00 13.87
N
ATOM 225 CA TRP B 32 51.457 55.130 50.659 1.00 13.87
C
ATOM 226 C TRP B 32 52.948 54.877 50.416 1.00 13.87
C
ATOM 227 O TRP B 32 53.368 53.836 49.918 1.00 13.87
O
ATOM 228 CB TRP B 32 50.623 53.870 50.468 1.00 13.87
C
ATOM 229 CG TRP B 32 49.130 54.121 50.329 1.00 13.87
C
ATOM 230 CD1 TRP B 32 48.294 53.542 49.426 1.00 13.87
C
ATOM 231 CD2 TRP B 32 48.315 55.001 51.121 1.00 13.87
C
ATOM 232 NE1 TRP B 32 47.014 54.005 49.597 1.00 13.87
N
ATOM 233 CE2 TRP B 32 47.000 54.902 50.630 1.00 13.87
C
ATOM 234 CE3 TRP B 32 48.570 55.863 52.190 1.00 13.87
C
ATOM 235 CZ2 TRP B 32 45.948 55.631 51.171 1.00 13.87
C
ATOM 236 CZ3 TRP B 32 47.521 56.587 52.722 1.00 13.87
C
ATOM 237 CH2 TRP B 32 46.227 56.465 52.211 1.00 13.87
C
ATOM 238 N LEU B 33 53.752 55.861 50.805 1.00 13.98
N
ATOM 239 CA LEU B 33 55.203 55.795 50.692 1.00 13.98
C
ATOM 240 C LEU B 33 55.836 56.246 51.999 1.00 13.98
C
ATOM 241 O LEU B 33 55.318 57.139 52.670 1.00 13.98
O
ATOM 242 CB LEU B 33 55.703 56.666 49.536 1.00 13.98

C
ATOM 243 CG LEU B 33 57.209 56.700 49.266 1.00 13.98
C
ATOM 244 CD1 LEU B 33 57.488 56.481 47.804 1.00 13.98
C
ATOM 245 CD2 LEU B 33 57.812 58.010 49.730 1.00 13.98
C
ATOM 246 N ALA B 34 56.957 55.626 52.353 1.00 14.00
N
ATOM 247 CA ALA B 34 57.711 55.953 53.551 1.00 14.00
C
ATOM 248 C ALA B 34 59.162 56.217 53.172 1.00 14.00
C
ATOM 249 O ALA B 34 59.666 55.673 52.193 1.00 14.00
O
ATOM 250 CB ALA B 34 57.638 54.824 54.584 1.00 14.00
C
ATOM 251 N TRP B 35 59.818 57.071 53.951 1.00 16.39
N
ATOM 252 CA TRP B 35 61.216 57.440 53.755 1.00 16.39
C
ATOM 253 C TRP B 35 62.030 57.017 54.970 1.00 16.39
C
ATOM 254 O TRP B 35 61.693 57.397 56.099 1.00 16.39
O
ATOM 255 CB TRP B 35 61.387 58.950 53.562 1.00 16.39
C
ATOM 256 CG TRP B 35 60.992 59.519 52.241 1.00 16.39
C
ATOM 257 CD1 TRP B 35 59.815 60.130 51.936 1.00 16.39
C
ATOM 258 CD2 TRP B 35 61.800 59.596 51.064 1.00 16.39
C
ATOM 259 NE1 TRP B 35 59.829 60.559 50.636 1.00 16.39
N
ATOM 260 CE2 TRP B 35 61.038 60.245 50.077 1.00 16.39
C
ATOM 261 CE3 TRP B 35 63.090 59.167 50.744 1.00 16.39
C
ATOM 262 CZ2 TRP B 35 61.522 60.475 48.797 1.00 16.39
C
ATOM 263 CZ3 TRP B 35 63.567 59.397 49.473 1.00 16.39
C
ATOM 264 CH2 TRP B 35 62.786 60.046 48.516 1.00 16.39
C
ATOM 265 N PHE B 36 63.129 56.295 54.711 1.00 17.41
N
ATOM 266 CA PHE B 36 64.059 55.768 55.702 1.00 17.41
C
ATOM 267 C PHE B 36 65.449 56.356 55.499 1.00 17.41

C
ATOM 268 O PHE B 36 65.845 56.677 54.380 1.00 17.41
O
ATOM 269 CB PHE B 36 64.195 54.244 55.605 1.00 17.41
C
ATOM 270 CG PHE B 36 62.944 53.497 55.901 1.00 17.41
C
ATOM 271 CD1 PHE B 36 62.696 53.024 57.172 1.00 17.41
C
ATOM 272 CD2 PHE B 36 62.028 53.234 54.903 1.00 17.41
C
ATOM 273 CE1 PHE B 36 61.553 52.322 57.445 1.00 17.41
C
ATOM 274 CE2 PHE B 36 60.882 52.535 55.175 1.00 17.41
C
ATOM 275 CZ PHE B 36 60.645 52.079 56.443 1.00 17.41
C
ATOM 276 N GLN B 37 66.200 56.441 56.592 1.00 22.43
N
ATOM 277 CA GLN B 37 67.602 56.832 56.584 1.00 22.43
C
ATOM 278 C GLN B 37 68.419 55.695 57.181 1.00 22.43
C
ATOM 279 O GLN B 37 68.020 55.118 58.193 1.00 22.43
O
ATOM 280 CB GLN B 37 67.803 58.120 57.391 1.00 22.43
C
ATOM 281 CG GLN B 37 69.240 58.530 57.635 1.00 22.43
C
ATOM 282 CD GLN B 37 69.346 59.619 58.684 1.00 22.43
C
ATOM 283 OE1 GLN B 37 69.059 59.395 59.854 1.00 22.43
O
ATOM 284 NE2 GLN B 37 69.736 60.812 58.261 1.00 22.43
N
ATOM 285 N GLN B 38 69.545 55.364 56.552 1.00 25.64
N
ATOM 286 CA GLN B 38 70.479 54.372 57.071 1.00 25.64
C
ATOM 287 C GLN B 38 71.868 54.987 57.124 1.00 25.64
C
ATOM 288 O GLN B 38 72.418 55.374 56.088 1.00 25.64
O
ATOM 289 CB GLN B 38 70.500 53.103 56.215 1.00 25.64
C
ATOM 290 CG GLN B 38 71.401 52.010 56.789 1.00 25.64
C
ATOM 291 CD GLN B 38 71.343 50.720 56.005 1.00 25.64
C
ATOM 292 OE1 GLN B 38 71.175 50.726 54.793 1.00 25.64

O
ATOM 293 NE2 GLN B 38 71.489 49.602 56.699 1.00 25.64
N
ATOM 294 N LYS B 39 72.425 55.068 58.322 1.00 33.81
N
ATOM 295 CA LYS B 39 73.792 55.513 58.521 1.00 33.81
C
ATOM 296 C LYS B 39 74.757 54.352 58.310 1.00 33.81
C
ATOM 297 O LYS B 39 74.373 53.188 58.425 1.00 33.81
O
ATOM 298 CB LYS B 39 73.948 56.086 59.921 1.00 33.81
C
ATOM 299 CG LYS B 39 73.227 57.395 60.116 1.00 33.81
C
ATOM 300 CD LYS B 39 73.240 57.808 61.567 1.00 33.81
C
ATOM 301 CE LYS B 39 72.353 59.012 61.804 1.00 33.81
C
ATOM 302 NZ LYS B 39 72.598 59.604 63.143 1.00 33.81
N
ATOM 303 N PRO B 40 76.017 54.640 57.981 1.00 37.75
N
ATOM 304 CA PRO B 40 76.991 53.555 57.774 1.00 37.75
C
ATOM 305 C PRO B 40 77.120 52.687 59.016 1.00 37.75
C
ATOM 306 O PRO B 40 77.411 53.173 60.109 1.00 37.75
O
ATOM 307 CB PRO B 40 78.299 54.292 57.460 1.00 37.75
C
ATOM 308 CG PRO B 40 77.979 55.729 57.361 1.00 37.75
C
ATOM 309 CD PRO B 40 76.525 55.955 57.561 1.00 37.75
C
ATOM 310 N GLY B 41 76.876 51.391 58.841 1.00 38.67
N
ATOM 311 CA GLY B 41 77.014 50.433 59.917 1.00 38.67
C
ATOM 312 C GLY B 41 75.872 50.377 60.906 1.00 38.67
C
ATOM 313 O GLY B 41 76.010 49.719 61.942 1.00 38.67
O
ATOM 314 N LYS B 42 74.750 51.032 60.628 1.00 36.67
N
ATOM 315 CA LYS B 42 73.608 51.048 61.529 1.00 36.67
C
ATOM 316 C LYS B 42 72.380 50.480 60.828 1.00 36.67
C
ATOM 317 O LYS B 42 72.401 50.166 59.637 1.00 36.67

O
ATOM 318 CB LYS B 42 73.319 52.467 62.029 1.00 36.67
C
ATOM 319 CG LYS B 42 74.490 53.149 62.706 1.00 36.67
C
ATOM 320 CD LYS B 42 75.165 52.238 63.712 1.00 36.67
C
ATOM 321 CE LYS B 42 75.625 53.024 64.921 1.00 36.67
C
ATOM 322 NZ LYS B 42 74.587 54.001 65.341 1.00 36.67
N
ATOM 323 N ALA B 43 71.318 50.345 61.580 1.00 30.87
N
ATOM 324 CA ALA B 43 70.055 49.856 61.052 1.00 30.87
C
ATOM 325 C ALA B 43 69.215 51.010 60.510 1.00 30.87
C
ATOM 326 O ALA B 43 69.321 52.139 60.993 1.00 30.87
O
ATOM 327 CB ALA B 43 69.276 49.123 62.136 1.00 30.87
C
ATOM 328 N PRO B 44 68.370 50.750 59.510 1.00 27.80
N
ATOM 329 CA PRO B 44 67.556 51.825 58.928 1.00 27.80
C
ATOM 330 C PRO B 44 66.643 52.479 59.951 1.00 27.80
C
ATOM 331 O PRO B 44 66.123 51.834 60.859 1.00 27.80
O
ATOM 332 CB PRO B 44 66.743 51.108 57.847 1.00 27.80
C
ATOM 333 CG PRO B 44 67.557 49.928 57.496 1.00 27.80
C
ATOM 334 CD PRO B 44 68.223 49.493 58.761 1.00 27.80
C
ATOM 335 N LYS B 45 66.445 53.782 59.780 1.00 24.87
N
ATOM 336 CA LYS B 45 65.607 54.582 60.658 1.00 24.87
C
ATOM 337 C LYS B 45 64.526 55.271 59.837 1.00 24.87
C
ATOM 338 O LYS B 45 64.821 55.854 58.791 1.00 24.87
O
ATOM 339 CB LYS B 45 66.445 55.621 61.403 1.00 24.87
C
ATOM 340 CG LYS B 45 65.664 56.424 62.404 1.00 24.87
C
ATOM 341 CD LYS B 45 66.423 56.509 63.708 1.00 24.87
C
ATOM 342 CE LYS B 45 66.634 55.121 64.283 1.00 24.87

C
ATOM 343 NZ LYS B 45 65.352 54.397 64.480 1.00 24.87
N
ATOM 344 N LEU B 46 63.288 55.219 60.320 1.00 20.28
N
ATOM 345 CA LEU B 46 62.163 55.821 59.614 1.00 20.28
C
ATOM 346 C LEU B 46 62.141 57.330 59.822 1.00 20.28
C
ATOM 347 O LEU B 46 62.229 57.811 60.954 1.00 20.28
O
ATOM 348 CB LEU B 46 60.848 55.206 60.091 1.00 20.28
C
ATOM 349 CG LEU B 46 59.538 55.801 59.565 1.00 20.28
C
ATOM 350 CD1 LEU B 46 59.406 55.613 58.063 1.00 20.28
C
ATOM 351 CD2 LEU B 46 58.348 55.192 60.277 1.00 20.28
C
ATOM 352 N LEU B 47 62.025 58.075 58.725 1.00 20.64
N
ATOM 353 CA LEU B 47 61.926 59.527 58.775 1.00 20.64
C
ATOM 354 C LEU B 47 60.513 60.006 58.483 1.00 20.64
C
ATOM 355 O LEU B 47 59.909 60.696 59.304 1.00 20.64
O
ATOM 356 CB LEU B 47 62.886 60.177 57.774 1.00 20.64
C
ATOM 357 CG LEU B 47 64.384 59.942 57.866 1.00 20.64
C
ATOM 358 CD1 LEU B 47 65.044 60.575 56.662 1.00 20.64
C
ATOM 359 CD2 LEU B 47 64.918 60.526 59.150 1.00 20.64
C
ATOM 360 N ILE B 48 59.970 59.649 57.325 1.00 19.28
N
ATOM 361 CA ILE B 48 58.703 60.208 56.870 1.00 19.28
C
ATOM 362 C ILE B 48 57.730 59.069 56.628 1.00 19.28
C
ATOM 363 O ILE B 48 58.061 58.098 55.947 1.00 19.28
O
ATOM 364 CB ILE B 48 58.864 61.060 55.597 1.00 19.28
C
ATOM 365 CG1 ILE B 48 59.941 62.132 55.788 1.00 19.28
C
ATOM 366 CG2 ILE B 48 57.535 61.691 55.203 1.00 19.28
C
ATOM 367 CD1 ILE B 48 59.493 63.316 56.579 1.00 19.28

C
ATOM 368 N TYR B 49 56.544 59.193 57.182 1.00 21.29
N
ATOM 369 CA TYR B 49 55.462 58.252 56.990 1.00 21.29
C
ATOM 370 C TYR B 49 54.605 58.798 55.851 1.00 21.29
C
ATOM 371 O TYR B 49 55.101 59.604 55.063 1.00 21.29
O
ATOM 372 CB TYR B 49 54.766 58.043 58.328 1.00 21.29
C
ATOM 373 CG TYR B 49 53.857 56.862 58.362 1.00 21.29
C
ATOM 374 CD1 TYR B 49 54.337 55.583 58.127 1.00 21.29
C
ATOM 375 CD2 TYR B 49 52.527 57.016 58.688 1.00 21.29
C
ATOM 376 CE1 TYR B 49 53.495 54.494 58.171 1.00 21.29
C
ATOM 377 CE2 TYR B 49 51.683 55.940 58.742 1.00 21.29
C
ATOM 378 CZ TYR B 49 52.169 54.681 58.484 1.00 21.29
C
ATOM 379 OH TYR B 49 51.317 53.602 58.540 1.00 21.29
O
ATOM 380 N GLU B 50 53.337 58.371 55.747 1.00 21.08
N
ATOM 381 CA GLU B 50 52.485 58.657 54.586 1.00 21.08
C
ATOM 382 C GLU B 50 52.881 59.981 53.964 1.00 21.08
C
ATOM 383 O GLU B 50 52.876 61.011 54.643 1.00 21.08
O
ATOM 384 CB GLU B 50 51.001 58.741 54.969 1.00 21.08
C
ATOM 385 CG GLU B 50 50.437 57.573 55.727 1.00 21.08
C
ATOM 386 CD GLU B 50 49.091 57.885 56.353 1.00 21.08
C
ATOM 387 OE1 GLU B 50 48.601 59.016 56.187 1.00 21.08
O
ATOM 388 OE2 GLU B 50 48.525 56.994 57.000 1.00 21.08
O
ATOM 389 N ALA B 51 53.117 59.993 52.662 1.00 20.61
N
ATOM 390 CA ALA B 51 54.283 60.616 52.037 1.00 20.61
C
ATOM 391 C ALA B 51 54.784 61.940 52.618 1.00 20.61
C
ATOM 392 O ALA B 51 55.837 62.424 52.197 1.00 20.61

```
O
ATOM    393  CB  ALA B  51      53.974  60.827  50.556  1.00 20.61
C
ATOM    394  N   SER B  52      54.057  62.559  53.550  1.00 23.74
N
ATOM    395  CA  SER B  52      54.459  63.862  54.065  1.00 23.74
C
ATOM    396  C   SER B  52      54.428  63.990  55.585  1.00 23.74
C
ATOM    397  O   SER B  52      54.687  65.082  56.098  1.00 23.74
O
ATOM    398  CB  SER B  52      53.577  64.955  53.459  1.00 23.74
C
ATOM    399  OG  SER B  52      53.475  64.795  52.062  1.00 23.74
O
ATOM    400  N   SER B  53      54.129  62.932  56.325  1.00 23.47
N
ATOM    401  CA  SER B  53      53.956  63.036  57.769  1.00 23.47
C
ATOM    402  C   SER B  53      55.263  62.698  58.478  1.00 23.47
C
ATOM    403  O   SER B  53      55.761  61.574  58.368  1.00 23.47
O
ATOM    404  CB  SER B  53      52.830  62.123  58.247  1.00 23.47
C
ATOM    405  OG  SER B  53      52.673  62.218  59.647  1.00 23.47
O
ATOM    406  N   LEU B  54      55.799  63.664  59.219  1.00 27.38
N
ATOM    407  CA  LEU B  54      57.081  63.511  59.893  1.00 27.38
C
ATOM    408  C   LEU B  54      56.929  62.704  61.175  1.00 27.38
C
ATOM    409  O   LEU B  54      55.990  62.908  61.946  1.00 27.38
O
ATOM    410  CB  LEU B  54      57.674  64.888  60.201  1.00 27.38
C
ATOM    411  CG  LEU B  54      59.016  64.965  60.926  1.00 27.38
C
ATOM    412  CD1 LEU B  54      60.134  64.505  60.024  1.00 27.38
C
ATOM    413  CD2 LEU B  54      59.274  66.384  61.408  1.00 27.38
C
ATOM    414  N   GLU B  55      57.862  61.785  61.404  1.00 29.65
N
ATOM    415  CA  GLU B  55      57.807  60.912  62.567  1.00 29.65
C
ATOM    416  C   GLU B  55      58.242  61.641  63.833  1.00 29.65
C
ATOM    417  O   GLU B  55      59.009  62.604  63.790  1.00 29.65
```

O
ATOM 418 CB GLU B 55 58.688 59.683 62.359 1.00 29.65
C
ATOM 419 CG GLU B 55 57.959 58.522 61.752 1.00 29.65
C
ATOM 420 CD GLU B 55 56.856 58.016 62.646 1.00 29.65
C
ATOM 421 OE1 GLU B 55 55.677 58.188 62.280 1.00 29.65
O
ATOM 422 OE2 GLU B 55 57.169 57.454 63.714 1.00 29.65
O
ATOM 423 N SER B 56 57.750 61.157 64.970 1.00 32.55
N
ATOM 424 CA SER B 56 58.068 61.778 66.248 1.00 32.55
C
ATOM 425 C SER B 56 59.507 61.483 66.644 1.00 32.55
C
ATOM 426 O SER B 56 59.999 60.365 66.481 1.00 32.55
O
ATOM 427 CB SER B 56 57.117 61.282 67.332 1.00 32.55
C
ATOM 428 OG SER B 56 57.548 60.040 67.850 1.00 32.55
O
ATOM 429 N GLY B 57 60.180 62.495 67.180 1.00 33.07
N
ATOM 430 CA GLY B 57 61.577 62.370 67.520 1.00 33.07
C
ATOM 431 C GLY B 57 62.533 62.623 66.380 1.00 33.07
C
ATOM 432 O GLY B 57 63.742 62.446 66.560 1.00 33.07
O
ATOM 433 N VAL B 58 62.036 63.023 65.215 1.00 31.11
N
ATOM 434 CA VAL B 58 62.878 63.326 64.062 1.00 31.11
C
ATOM 435 C VAL B 58 63.043 64.839 63.973 1.00 31.11
C
ATOM 436 O VAL B 58 62.062 65.574 64.136 1.00 31.11
O
ATOM 437 CB VAL B 58 62.280 62.744 62.770 1.00 31.11
C
ATOM 438 CG1 VAL B 58 63.161 63.063 61.580 1.00 31.11
C
ATOM 439 CG2 VAL B 58 62.095 61.246 62.906 1.00 31.11
C
ATOM 440 N PRO B 59 64.252 65.341 63.730 1.00 32.83
N
ATOM 441 CA PRO B 59 64.447 66.791 63.611 1.00 32.83
C
ATOM 442 C PRO B 59 63.606 67.401 62.500 1.00 32.83

C
ATOM 443 O PRO B 59 63.350 66.781 61.468 1.00 32.83
O
ATOM 444 CB PRO B 59 65.942 66.915 63.307 1.00 32.83
C
ATOM 445 CG PRO B 59 66.537 65.732 63.963 1.00 32.83
C
ATOM 446 CD PRO B 59 65.533 64.623 63.822 1.00 32.83
C
ATOM 447 N SER B 60 63.188 68.645 62.723 1.00 34.91
N
ATOM 448 CA SER B 60 62.212 69.305 61.867 1.00 34.91
C
ATOM 449 C SER B 60 62.799 69.844 60.571 1.00 34.91
C
ATOM 450 O SER B 60 62.067 70.470 59.802 1.00 34.91
O
ATOM 451 CB SER B 60 61.534 70.446 62.628 1.00 34.91
C
ATOM 452 OG SER B 60 62.463 71.129 63.448 1.00 34.91
O
ATOM 453 N ARG B 61 64.086 69.633 60.303 1.00 33.76
N
ATOM 454 CA ARG B 61 64.614 70.042 59.010 1.00 33.76
C
ATOM 455 C ARG B 61 64.221 69.085 57.891 1.00 33.76
C
ATOM 456 O ARG B 61 64.312 69.457 56.720 1.00 33.76
O
ATOM 457 CB ARG B 61 66.138 70.189 59.065 1.00 33.76
C
ATOM 458 CG ARG B 61 66.894 69.051 59.721 1.00 33.76
C
ATOM 459 CD ARG B 61 68.383 69.362 59.751 1.00 33.76
C
ATOM 460 NE ARG B 61 69.203 68.207 60.094 1.00 33.76
N
ATOM 461 CZ ARG B 61 69.399 67.772 61.333 1.00 33.76
C
ATOM 462 NH1 ARG B 61 68.834 68.395 62.354 1.00 33.76
N
ATOM 463 NH2 ARG B 61 70.162 66.714 61.551 1.00 33.76
N
ATOM 464 N PHE B 62 63.762 67.880 58.220 1.00 29.38
N
ATOM 465 CA PHE B 62 63.317 66.918 57.220 1.00 29.38
C
ATOM 466 C PHE B 62 61.852 67.162 56.883 1.00 29.38
C
ATOM 467 O PHE B 62 61.013 67.255 57.781 1.00 29.38

O
ATOM 468 CB PHE B 62 63.498 65.486 57.725 1.00 29.38
C
ATOM 469 CG PHE B 62 64.921 65.107 57.999 1.00 29.38
C
ATOM 470 CD1 PHE B 62 65.738 64.655 56.984 1.00 29.38
C
ATOM 471 CD2 PHE B 62 65.438 65.191 59.276 1.00 29.38
C
ATOM 472 CE1 PHE B 62 67.043 64.303 57.236 1.00 29.38
C
ATOM 473 CE2 PHE B 62 66.742 64.838 59.531 1.00 29.38
C
ATOM 474 CZ PHE B 62 67.544 64.397 58.509 1.00 29.38
C
ATOM 475 N SER B 63 61.547 67.264 55.594 1.00 26.49
N
ATOM 476 CA SER B 63 60.167 67.383 55.145 1.00 26.49
C
ATOM 477 C SER B 63 59.981 66.552 53.887 1.00 26.49
C
ATOM 478 O SER B 63 60.920 66.367 53.116 1.00 26.49
O
ATOM 479 CB SER B 63 59.781 68.846 54.885 1.00 26.49
C
ATOM 480 OG SER B 63 60.511 69.379 53.800 1.00 26.49
O
ATOM 481 N GLY B 64 58.773 66.030 53.691 1.00 23.79
N
ATOM 482 CA GLY B 64 58.477 65.242 52.518 1.00 23.79
C
ATOM 483 C GLY B 64 57.237 65.761 51.820 1.00 23.79
C
ATOM 484 O GLY B 64 56.408 66.449 52.410 1.00 23.79
O
ATOM 485 N SER B 65 57.122 65.419 50.543 1.00 22.21
N
ATOM 486 CA SER B 65 55.961 65.839 49.770 1.00 22.21
C
ATOM 487 C SER B 65 55.812 64.924 48.567 1.00 22.21
C
ATOM 488 O SER B 65 56.676 64.099 48.282 1.00 22.21
O
ATOM 489 CB SER B 65 56.075 67.305 49.337 1.00 22.21
C
ATOM 490 OG SER B 65 57.230 67.519 48.554 1.00 22.21
O
ATOM 491 N GLY B 66 54.697 65.074 47.870 1.00 21.12
N
ATOM 492 CA GLY B 66 54.432 64.325 46.660 1.00 21.12

C
ATOM 493 C GLY B 66 53.255 63.381 46.818 1.00 21.12
C
ATOM 494 O GLY B 66 52.734 63.157 47.907 1.00 21.12
O
ATOM 495 N SER B 67 52.862 62.812 45.685 1.00 20.97
N
ATOM 496 CA SER B 67 51.684 61.956 45.646 1.00 20.97
C
ATOM 497 C SER B 67 51.631 61.274 44.293 1.00 20.97
C
ATOM 498 O SER B 67 52.193 61.762 43.313 1.00 20.97
O
ATOM 499 CB SER B 67 50.397 62.750 45.898 1.00 20.97
C
ATOM 500 OG SER B 67 49.294 61.885 46.081 1.00 20.97
O
ATOM 501 N GLY B 68 50.972 60.123 44.255 1.00 19.84
N
ATOM 502 CA GLY B 68 50.794 59.448 42.990 1.00 19.84
C
ATOM 503 C GLY B 68 52.002 58.648 42.560 1.00 19.84
C
ATOM 504 O GLY B 68 52.216 57.529 43.028 1.00 19.84
O
ATOM 505 N THR B 69 52.789 59.209 41.644 1.00 21.79
N
ATOM 506 CA THR B 69 53.972 58.547 41.121 1.00 21.79
C
ATOM 507 C THR B 69 55.263 59.304 41.392 1.00 21.79
C
ATOM 508 O THR B 69 56.332 58.812 41.025 1.00 21.79
O
ATOM 509 CB THR B 69 53.839 58.325 39.608 1.00 21.79
C
ATOM 510 OG1 THR B 69 53.462 59.554 38.977 1.00 21.79
O
ATOM 511 CG2 THR B 69 52.794 57.274 39.315 1.00 21.79
C
ATOM 512 N GLU B 70 55.205 60.477 42.014 1.00 23.06
N
ATOM 513 CA GLU B 70 56.387 61.294 42.248 1.00 23.06
C
ATOM 514 C GLU B 70 56.380 61.799 43.681 1.00 23.06
C
ATOM 515 O GLU B 70 55.373 62.346 44.148 1.00 23.06
O
ATOM 516 CB GLU B 70 56.441 62.472 41.271 1.00 23.06
C
ATOM 517 CG GLU B 70 57.774 63.189 41.221 1.00 23.06

C
ATOM 518 CD GLU B 70 58.134 63.644 39.818 1.00 23.06
C
ATOM 519 OE1 GLU B 70 57.215 64.014 39.060 1.00 23.06
O
ATOM 520 OE2 GLU B 70 59.330 63.635 39.473 1.00 23.06
O
ATOM 521 N PHE B 71 57.505 61.619 44.374 1.00 18.78
N
ATOM 522 CA PHE B 71 57.642 61.978 45.773 1.00 18.78
C
ATOM 523 C PHE B 71 59.012 62.604 45.981 1.00 18.78
C
ATOM 524 O PHE B 71 59.954 62.305 45.250 1.00 18.78
O
ATOM 525 CB PHE B 71 57.474 60.746 46.682 1.00 18.78
C
ATOM 526 CG PHE B 71 56.192 59.995 46.458 1.00 18.78
C
ATOM 527 CD1 PHE B 71 55.096 60.221 47.265 1.00 18.78
C
ATOM 528 CD2 PHE B 71 56.085 59.060 45.446 1.00 18.78
C
ATOM 529 CE1 PHE B 71 53.921 59.537 47.062 1.00 18.78
C
ATOM 530 CE2 PHE B 71 54.907 58.377 45.240 1.00 18.78
C
ATOM 531 CZ PHE B 71 53.828 58.617 46.052 1.00 18.78
C
ATOM 532 N THR B 72 59.121 63.472 46.979 1.00 20.60
N
ATOM 533 CA THR B 72 60.372 64.153 47.268 1.00 20.60
C
ATOM 534 C THR B 72 60.599 64.222 48.769 1.00 20.60
C
ATOM 535 O THR B 72 59.654 64.272 49.561 1.00 20.60
O
ATOM 536 CB THR B 72 60.407 65.581 46.697 1.00 20.60
C
ATOM 537 OG1 THR B 72 59.210 66.272 47.064 1.00 20.60
O
ATOM 538 CG2 THR B 72 60.526 65.552 45.192 1.00 20.60
C
ATOM 539 N LEU B 73 61.875 64.233 49.139 1.00 21.84
N
ATOM 540 CA LEU B 73 62.339 64.451 50.499 1.00 21.84
C
ATOM 541 C LEU B 73 63.331 65.606 50.476 1.00 21.84
C
ATOM 542 O LEU B 73 64.261 65.604 49.670 1.00 21.84

```
O
ATOM    543  CB  LEU B  73      62.995  63.181  51.060  1.00 21.84
C
ATOM    544  CG  LEU B  73      63.826  63.285  52.337  1.00 21.84
C
ATOM    545  CD1 LEU B  73      62.944  63.596  53.522  1.00 21.84
C
ATOM    546  CD2 LEU B  73      64.605  62.003  52.578  1.00 21.84
C
ATOM    547  N   THR B  74      63.135  66.589  51.347  1.00 27.83
N
ATOM    548  CA  THR B  74      63.981  67.772  51.413  1.00 27.83
C
ATOM    549  C   THR B  74      64.518  67.928  52.827  1.00 27.83
C
ATOM    550  O   THR B  74      63.781  67.752  53.799  1.00 27.83
O
ATOM    551  CB  THR B  74      63.202  69.029  51.000  1.00 27.83
C
ATOM    552  OG1 THR B  74      62.720  68.874  49.662  1.00 27.83
O
ATOM    553  CG2 THR B  74      64.093  70.254  51.062  1.00 27.83
C
ATOM    554  N   ILE B  75      65.802  68.266  52.941  1.00 33.61
N
ATOM    555  CA  ILE B  75      66.483  68.339  54.225  1.00 33.61
C
ATOM    556  C   ILE B  75      66.746  69.782  54.653  1.00 33.61
C
ATOM    557  O   ILE B  75      66.668  70.103  55.839  1.00 33.61
O
ATOM    558  CB  ILE B  75      67.789  67.520  54.180  1.00 33.61
C
ATOM    559  CG1 ILE B  75      67.486  66.095  53.698  1.00 33.61
C
ATOM    560  CG2 ILE B  75      68.436  67.488  55.543  1.00 33.61
C
ATOM    561  CD1 ILE B  75      68.670  65.175  53.712  1.00 33.61
C
ATOM    562  N   SER B  76      67.105  70.645  53.714  1.00 40.69
N
ATOM    563  CA  SER B  76      67.111  72.101  53.873  1.00 40.69
C
ATOM    564  C   SER B  76      68.231  72.696  54.724  1.00 40.69
C
ATOM    565  O   SER B  76      68.462  73.906  54.653  1.00 40.69
O
ATOM    566  CB  SER B  76      65.776  72.574  54.462  1.00 40.69
C
ATOM    567  OG  SER B  76      64.833  72.847  53.445  1.00 40.69
```

O
ATOM   568 N   SER B 77   68.944 71.890 55.510 1.00 41.56
N
ATOM   569 CA SER B 77   70.230 72.320 56.066 1.00 41.56
C
ATOM   570 C   SER B 77   70.973 71.064 56.514 1.00 41.56
C
ATOM   571 O   SER B 77   70.715 70.556 57.607 1.00 41.56
O
ATOM   572 CB SER B 77   70.049 73.298 57.215 1.00 41.56
C
ATOM   573 OG SER B 77   69.910 74.622 56.732 1.00 41.56
O
ATOM   574 N   LEU B 78   71.896 70.592 55.688 1.00 41.36
N
ATOM   575 CA LEU B 78   72.614 69.372 56.019 1.00 41.36
C
ATOM   576 C   LEU B 78   73.476 69.581 57.256 1.00 41.36
C
ATOM   577 O   LEU B 78   74.199 70.574 57.364 1.00 41.36
O
ATOM   578 CB LEU B 78   73.481 68.934 54.843 1.00 41.36
C
ATOM   579 CG LEU B 78   72.888 67.915 53.878 1.00 41.36
C
ATOM   580 CD1 LEU B 78   73.991 67.284 53.065 1.00 41.36
C
ATOM   581 CD2 LEU B 78   72.111 66.852 54.618 1.00 41.36
C
ATOM   582 N   GLN B 79   73.395 68.646 58.186 1.00 41.11
N
ATOM   583 CA GLN B 79   74.237 68.594 59.365 1.00 41.11
C
ATOM   584 C   GLN B 79   75.222 67.440 59.249 1.00 41.11
C
ATOM   585 O   GLN B 79   75.073 66.565 58.390 1.00 41.11
O
ATOM   586 CB GLN B 79   73.376 68.444 60.623 1.00 41.11
C
ATOM   587 CG GLN B 79   72.425 69.601 60.858 1.00 41.11
C
ATOM   588 CD GLN B 79   73.136 70.939 60.955 1.00 41.11
C
ATOM   589 OE1 GLN B 79   72.912 71.834 60.143 1.00 41.11
O
ATOM   590 NE2 GLN B 79   73.992 71.080 61.955 1.00 41.11
N
ATOM   591 N   PRO B 80   76.266 67.417 60.083 1.00 42.90
N
ATOM   592 CA PRO B 80   77.250 66.328 59.979 1.00 42.90

C
ATOM 593 C PRO B 80 76.664 64.938 60.155 1.00 42.90
C
ATOM 594 O PRO B 80 77.205 63.979 59.597 1.00 42.90
O
ATOM 595 CB PRO B 80 78.248 66.665 61.093 1.00 42.90
C
ATOM 596 CG PRO B 80 78.184 68.136 61.192 1.00 42.90
C
ATOM 597 CD PRO B 80 76.747 68.494 60.968 1.00 42.90
C
ATOM 598 N ASP B 81 75.581 64.795 60.912 1.00 43.30
N
ATOM 599 CA ASP B 81 74.966 63.491 61.120 1.00 43.30
C
ATOM 600 C ASP B 81 74.019 63.084 59.998 1.00 43.30
C
ATOM 601 O ASP B 81 73.502 61.963 60.029 1.00 43.30
O
ATOM 602 CB ASP B 81 74.207 63.477 62.446 1.00 43.30
C
ATOM 603 CG ASP B 81 75.117 63.256 63.632 1.00 43.30
C
ATOM 604 OD1 ASP B 81 76.274 62.837 63.422 1.00 43.30
O
ATOM 605 OD2 ASP B 81 74.676 63.499 64.773 1.00 43.30
O
ATOM 606 N ASP B 82 73.776 63.949 59.020 1.00 37.05
N
ATOM 607 CA ASP B 82 72.786 63.688 57.986 1.00 37.05
C
ATOM 608 C ASP B 82 73.371 63.047 56.739 1.00 37.05
C
ATOM 609 O ASP B 82 72.630 62.801 55.784 1.00 37.05
O
ATOM 610 CB ASP B 82 72.075 64.986 57.604 1.00 37.05
C
ATOM 611 CG ASP B 82 71.368 65.622 58.770 1.00 37.05
C
ATOM 612 OD1 ASP B 82 71.267 64.974 59.826 1.00 37.05
O
ATOM 613 OD2 ASP B 82 70.912 66.772 58.632 1.00 37.05
O
ATOM 614 N PHE B 83 74.667 62.769 56.716 1.00 38.01
N
ATOM 615 CA PHE B 83 75.288 62.151 55.553 1.00 38.01
C
ATOM 616 C PHE B 83 75.107 60.643 55.661 1.00 38.01
C
ATOM 617 O PHE B 83 75.739 59.989 56.494 1.00 38.01

O
ATOM 618 CB PHE B 83 76.754 62.562 55.462 1.00 38.01
C
ATOM 619 CG PHE B 83 76.934 64.020 55.172 1.00 38.01
C
ATOM 620 CD1 PHE B 83 76.995 64.474 53.871 1.00 38.01
C
ATOM 621 CD2 PHE B 83 76.997 64.941 56.200 1.00 38.01
C
ATOM 622 CE1 PHE B 83 77.141 65.815 53.600 1.00 38.01
C
ATOM 623 CE2 PHE B 83 77.145 66.283 55.932 1.00 38.01
C
ATOM 624 CZ PHE B 83 77.216 66.718 54.632 1.00 38.01
C
ATOM 625 N THR B 84 74.228 60.097 54.826 1.00 28.30
N
ATOM 626 CA THR B 84 73.740 58.737 54.997 1.00 28.30
C
ATOM 627 C THR B 84 73.125 58.277 53.683 1.00 28.30
C
ATOM 628 O THR B 84 73.097 59.017 52.698 1.00 28.30
O
ATOM 629 CB THR B 84 72.728 58.666 56.140 1.00 28.30
C
ATOM 630 N THR B 85 72.631 57.042 53.673 1.00 22.95
N
ATOM 631 CA THR B 85 71.894 56.518 52.532 1.00 22.95
C
ATOM 632 C THR B 85 70.404 56.633 52.819 1.00 22.95
C
ATOM 633 O THR B 85 69.940 56.216 53.878 1.00 22.95
O
ATOM 634 CB THR B 85 72.262 55.062 52.246 1.00 22.95
C
ATOM 635 OG1 THR B 85 73.684 54.939 52.137 1.00 22.95
O
ATOM 636 CG2 THR B 85 71.631 54.608 50.950 1.00 22.95
C
ATOM 637 N TYR B 86 69.659 57.204 51.884 1.00 20.45
N
ATOM 638 CA TYR B 86 68.228 57.406 52.056 1.00 20.45
C
ATOM 639 C TYR B 86 67.467 56.465 51.134 1.00 20.45
C
ATOM 640 O TYR B 86 67.796 56.350 49.953 1.00 20.45
O
ATOM 641 CB TYR B 86 67.851 58.862 51.781 1.00 20.45
C
ATOM 642 CG TYR B 86 68.410 59.799 52.822 1.00 20.45

C
ATOM 643 CD1 TYR B 86 67.682 60.122 53.947 1.00 20.45
C
ATOM 644 CD2 TYR B 86 69.682 60.335 52.688 1.00 20.45
C
ATOM 645 CE1 TYR B 86 68.195 60.969 54.907 1.00 20.45
C
ATOM 646 CE2 TYR B 86 70.200 61.178 53.641 1.00 20.45
C
ATOM 647 CZ TYR B 86 69.457 61.489 54.747 1.00 20.45
C
ATOM 648 OH TYR B 86 69.976 62.332 55.698 1.00 20.45
O
ATOM 649 N PHE B 87 66.459 55.787 51.682 1.00 15.92
N
ATOM 650 CA PHE B 87 65.644 54.827 50.949 1.00 15.92
C
ATOM 651 C PHE B 87 64.183 55.263 50.972 1.00 15.92
C
ATOM 652 O PHE B 87 63.713 55.807 51.966 1.00 15.92
O
ATOM 653 CB PHE B 87 65.745 53.420 51.562 1.00 15.92
C
ATOM 654 CG PHE B 87 67.107 52.806 51.476 1.00 15.92
C
ATOM 655 CD1 PHE B 87 67.543 52.230 50.305 1.00 15.92
C
ATOM 656 CD2 PHE B 87 67.938 52.779 52.579 1.00 15.92
C
ATOM 657 CE1 PHE B 87 68.794 51.660 50.226 1.00 15.92
C
ATOM 658 CE2 PHE B 87 69.187 52.198 52.500 1.00 15.92
C
ATOM 659 CZ PHE B 87 69.609 51.648 51.323 1.00 15.92
C
ATOM 660 N CYS B 88 63.467 55.025 49.880 1.00 15.34
N
ATOM 661 CA CYS B 88 62.012 55.128 49.866 1.00 15.34
C
ATOM 662 C CYS B 88 61.435 53.721 49.759 1.00 15.34
C
ATOM 663 O CYS B 88 62.026 52.851 49.121 1.00 15.34
O
ATOM 664 CB CYS B 88 61.502 56.018 48.709 1.00 15.34
C
ATOM 665 SG CYS B 88 62.031 55.521 47.042 1.00 15.34
S
ATOM 666 N GLN B 89 60.305 53.488 50.415 1.00 13.02
N
ATOM 667 CA GLN B 89 59.610 52.211 50.335 1.00 13.02

```
C
ATOM    668  C   GLN B  89      58.124  52.466  50.135  1.00 13.02
C
ATOM    669  O   GLN B  89      57.548  53.320  50.803  1.00 13.02
O
ATOM    670  CB  GLN B  89      59.821  51.363  51.606  1.00 13.02
C
ATOM    671  CG  GLN B  89      59.210  49.969  51.525  1.00 13.02
C
ATOM    672  CD  GLN B  89      59.282  49.191  52.828  1.00 13.02
C
ATOM    673  OE1 GLN B  89      59.306  49.761  53.908  1.00 13.02
O
ATOM    674  NE2 GLN B  89      59.319  47.872  52.719  1.00 13.02
N
ATOM    675  N   GLN B  90      57.499  51.722  49.234  1.00 12.89
N
ATOM    676  CA  GLN B  90      56.051  51.788  49.090  1.00 12.89
C
ATOM    677  C   GLN B  90      55.403  50.651  49.868  1.00 12.89
C
ATOM    678  O   GLN B  90      55.967  49.565  50.000  1.00 12.89
O
ATOM    679  CB  GLN B  90      55.626  51.736  47.617  1.00 12.89
C
ATOM    680  CG  GLN B  90      55.934  50.437  46.882  1.00 12.89
C
ATOM    681  CD  GLN B  90      54.824  49.405  46.987  1.00 12.89
C
ATOM    682  OE1 GLN B  90      53.703  49.719  47.368  1.00 12.89
O
ATOM    683  NE2 GLN B  90      55.141  48.166  46.656  1.00 12.89
N
ATOM    684  N   TYR B  91      54.214  50.916  50.404  1.00 13.84
N
ATOM    685  CA  TYR B  91      53.460  49.888  51.106  1.00 13.84
C
ATOM    686  C   TYR B  91      52.007  49.865  50.646  1.00 13.84
C
ATOM    687  O   TYR B  91      51.107  49.560  51.423  1.00 13.84
O
ATOM    688  CB  TYR B  91      53.559  50.060  52.622  1.00 13.84
C
ATOM    689  CG  TYR B  91      53.193  51.422  53.164  1.00 13.84
C
ATOM    690  CD1 TYR B  91      51.940  51.655  53.707  1.00 13.84
C
ATOM    691  CD2 TYR B  91      54.113  52.461  53.179  1.00 13.84
C
ATOM    692  CE1 TYR B  91      51.604  52.881  54.224  1.00 13.84
```

C
ATOM 693 CE2 TYR B 91 53.784 53.695 53.695 1.00 13.84
C
ATOM 694 CZ TYR B 91 52.525 53.898 54.216 1.00 13.84
C
ATOM 695 OH TYR B 91 52.178 55.119 54.736 1.00 13.84
O
ATOM 696 N ASN B 92 51.783 50.181 49.372 1.00 14.17
N
ATOM 697 CA ASN B 92 50.462 50.056 48.768 1.00 14.17
C
ATOM 698 C ASN B 92 50.034 48.594 48.672 1.00 14.17
C
ATOM 699 O ASN B 92 48.903 48.245 49.022 1.00 14.17
O
ATOM 700 CB ASN B 92 50.487 50.723 47.391 1.00 14.17
C
ATOM 701 CG ASN B 92 49.190 50.575 46.636 1.00 14.17
C
ATOM 702 OD1 ASN B 92 48.278 51.368 46.799 1.00 14.17
O
ATOM 703 ND2 ASN B 92 49.113 49.561 45.791 1.00 14.17
N
ATOM 704 N SER B 93 50.932 47.722 48.220 1.00 14.02
N
ATOM 705 CA SER B 93 50.613 46.311 48.036 1.00 14.02
C
ATOM 706 C SER B 93 51.900 45.498 48.032 1.00 14.02
C
ATOM 707 O SER B 93 52.987 46.029 47.807 1.00 14.02
O
ATOM 708 CB SER B 93 49.829 46.079 46.739 1.00 14.02
C
ATOM 709 OG SER B 93 50.425 46.763 45.657 1.00 14.02
O
ATOM 710 N TYR B 94 51.754 44.196 48.275 1.00 14.41
N
ATOM 711 CA TYR B 94 52.884 43.276 48.283 1.00 14.41
C
ATOM 712 C TYR B 94 53.453 43.090 46.877 1.00 14.41
C
ATOM 713 O TYR B 94 52.714 43.122 45.894 1.00 14.41
O
ATOM 714 CB TYR B 94 52.463 41.908 48.807 1.00 14.41
C
ATOM 715 CG TYR B 94 51.971 41.861 50.228 1.00 14.41
C
ATOM 716 CD1 TYR B 94 52.680 42.458 51.252 1.00 14.41
C
ATOM 717 CD2 TYR B 94 50.803 41.188 50.547 1.00 14.41

C
ATOM 718 CE1 TYR B 94 52.236 42.398 52.548 1.00 14.41
C
ATOM 719 CE2 TYR B 94 50.355 41.125 51.837 1.00 14.41
C
ATOM 720 CZ TYR B 94 51.074 41.726 52.834 1.00 14.41
C
ATOM 721 OH TYR B 94 50.625 41.666 54.128 1.00 14.41
O
ATOM 722 N PRO B 95 54.772 42.886 46.750 1.00 14.04
N
ATOM 723 CA PRO B 95 55.832 43.025 47.754 1.00 14.04
C
ATOM 724 C PRO B 95 56.087 44.492 48.077 1.00 14.04
C
ATOM 725 O PRO B 95 55.923 45.319 47.195 1.00 14.04
O
ATOM 726 CB PRO B 95 57.053 42.399 47.071 1.00 14.04
C
ATOM 727 CG PRO B 95 56.754 42.417 45.638 1.00 14.04
C
ATOM 728 CD PRO B 95 55.276 42.301 45.498 1.00 14.04
C
ATOM 729 N TRP B 96 56.474 44.806 49.310 1.00 13.16
N
ATOM 730 CA TRP B 96 56.734 46.189 49.715 1.00 13.16
C
ATOM 731 C TRP B 96 58.114 46.605 49.216 1.00 13.16
C
ATOM 732 O TRP B 96 59.109 46.491 49.929 1.00 13.16
O
ATOM 733 CB TRP B 96 56.656 46.339 51.227 1.00 13.16
C
ATOM 734 CG TRP B 96 55.309 46.189 51.843 1.00 13.16
C
ATOM 735 CD1 TRP B 96 54.103 46.152 51.210 1.00 13.16
C
ATOM 736 CD2 TRP B 96 55.035 46.081 53.239 1.00 13.16
C
ATOM 737 NE1 TRP B 96 53.094 46.018 52.130 1.00 13.16
N
ATOM 738 CE2 TRP B 96 53.642 45.977 53.384 1.00 13.16
C
ATOM 739 CE3 TRP B 96 55.835 46.065 54.384 1.00 13.16
C
ATOM 740 CZ2 TRP B 96 53.034 45.852 54.624 1.00 13.16
C
ATOM 741 CZ3 TRP B 96 55.230 45.946 55.611 1.00 13.16
C
ATOM 742 CH2 TRP B 96 53.844 45.839 55.725 1.00 13.16

C
ATOM 743 N THR B 97 58.171 47.143 48.002 1.00 13.09
N
ATOM 744 CA THR B 97 59.442 47.365 47.325 1.00 13.09
C
ATOM 745 C THR B 97 60.155 48.623 47.807 1.00 13.09
C
ATOM 746 O THR B 97 59.536 49.659 48.047 1.00 13.09
O
ATOM 747 CB THR B 97 59.232 47.443 45.813 1.00 13.09
C
ATOM 748 OG1 THR B 97 58.003 48.118 45.523 1.00 13.09
O
ATOM 749 CG2 THR B 97 59.186 46.052 45.223 1.00 13.09
C
ATOM 750 N PHE B 98 61.474 48.512 47.941 1.00 13.15
N
ATOM 751 CA PHE B 98 62.358 49.623 48.259 1.00 13.15
C
ATOM 752 C PHE B 98 62.931 50.228 46.983 1.00 13.15
C
ATOM 753 O PHE B 98 63.063 49.554 45.963 1.00 13.15
O
ATOM 754 CB PHE B 98 63.533 49.162 49.126 1.00 13.15
C
ATOM 755 CG PHE B 98 63.168 48.754 50.524 1.00 13.15
C
ATOM 756 CD1 PHE B 98 63.180 49.675 51.550 1.00 13.15
C
ATOM 757 CD2 PHE B 98 62.890 47.435 50.824 1.00 13.15
C
ATOM 758 CE1 PHE B 98 62.887 49.297 52.836 1.00 13.15
C
ATOM 759 CE2 PHE B 98 62.597 47.056 52.110 1.00 13.15
C
ATOM 760 CZ PHE B 98 62.596 47.987 53.114 1.00 13.15
C
ATOM 761 N GLY B 99 63.307 51.498 47.061 1.00 15.83
N
ATOM 762 CA GLY B 99 64.170 52.077 46.052 1.00 15.83
C
ATOM 763 C GLY B 99 65.599 51.581 46.204 1.00 15.83
C
ATOM 764 O GLY B 99 65.947 50.876 47.146 1.00 15.83
O
ATOM 765 N GLN B 100 66.450 51.955 45.252 1.00 19.59
N
ATOM 766 CA GLN B 100 67.829 51.484 45.285 1.00 19.59
C
ATOM 767 C GLN B 100 68.721 52.334 46.179 1.00 19.59

C
ATOM 768 O GLN B 100 69.891 51.991 46.361 1.00 19.59
O
ATOM 769 CB GLN B 100 68.398 51.408 43.862 1.00 19.59
C
ATOM 770 CG GLN B 100 69.067 52.671 43.358 1.00 19.59
C
ATOM 771 CD GLN B 100 68.083 53.768 43.014 1.00 19.59
C
ATOM 772 OE1 GLN B 100 66.874 53.568 43.045 1.00 19.59
O
ATOM 773 NE2 GLN B 100 68.605 54.939 42.680 1.00 19.59
N
ATOM 774 N GLY B 101 68.199 53.414 46.752 1.00 19.60
N
ATOM 775 CA GLY B 101 68.930 54.195 47.728 1.00 19.60
C
ATOM 776 C GLY B 101 69.662 55.391 47.154 1.00 19.60
C
ATOM 777 O GLY B 101 70.191 55.327 46.044 1.00 19.60
O
ATOM 778 N THR B 102 69.690 56.487 47.904 1.00 23.07
N
ATOM 779 CA THR B 102 70.430 57.688 47.534 1.00 23.07
C
ATOM 780 C THR B 102 71.477 57.946 48.603 1.00 23.07
C
ATOM 781 O THR B 102 71.134 58.194 49.760 1.00 23.07
O
ATOM 782 CB THR B 102 69.506 58.900 47.398 1.00 23.07
C
ATOM 783 OG1 THR B 102 68.733 58.779 46.202 1.00 23.07
O
ATOM 784 CG2 THR B 102 70.322 60.197 47.345 1.00 23.07
C
ATOM 785 N LYS B 103 72.747 57.894 48.212 1.00 31.08
N
ATOM 786 CA LYS B 103 73.848 58.171 49.121 1.00 31.08
C
ATOM 787 C LYS B 103 74.134 59.667 49.133 1.00 31.08
C
ATOM 788 O LYS B 103 74.293 60.279 48.075 1.00 31.08
O
ATOM 789 CB LYS B 103 75.103 57.408 48.699 1.00 31.08
C
ATOM 790 CG LYS B 103 74.894 55.924 48.457 1.00 31.08
C
ATOM 791 CD LYS B 103 76.137 55.210 47.901 1.00 31.08
C
ATOM 792 CE LYS B 103 77.483 55.683 48.464 1.00 31.08

C
ATOM 793 NZ LYS B 103 77.495 56.129 49.888 1.00 31.08
N
ATOM 794 N VAL B 104 74.197 60.248 50.324 1.00 33.71
N
ATOM 795 CA VAL B 104 74.598 61.640 50.505 1.00 33.71
C
ATOM 796 C VAL B 104 75.939 61.644 51.224 1.00 33.71
C
ATOM 797 O VAL B 104 76.036 61.193 52.371 1.00 33.71
O
ATOM 798 CB VAL B 104 73.550 62.441 51.287 1.00 33.71
C
ATOM 799 CG1 VAL B 104 74.014 63.877 51.470 1.00 33.71
C
ATOM 800 CG2 VAL B 104 72.215 62.402 50.568 1.00 33.71
C
ATOM 801 N GLU B 105 76.967 62.166 50.562 1.00 39.69
N
ATOM 802 CA GLU B 105 78.334 62.097 51.056 1.00 39.69
C
ATOM 803 C GLU B 105 78.905 63.495 51.250 1.00 39.69
C
ATOM 804 O GLU B 105 78.603 64.418 50.490 1.00 39.69
O
ATOM 805 CB GLU B 105 79.222 61.311 50.098 1.00 39.69
C
ATOM 806 CG GLU B 105 78.546 60.107 49.492 1.00 39.69
C
ATOM 807 CD GLU B 105 79.499 58.967 49.268 1.00 39.69
C
ATOM 808 OE1 GLU B 105 79.824 58.268 50.246 1.00 39.69
O
ATOM 809 OE2 GLU B 105 79.926 58.771 48.114 1.00 39.69
O
ATOM 810 N ILE B 106 79.741 63.633 52.274 1.00 42.73
N
ATOM 811 CA ILE B 106 80.331 64.921 52.621 1.00 42.73
C
ATOM 812 C ILE B 106 81.456 65.244 51.650 1.00 42.73
C
ATOM 813 O ILE B 106 82.260 64.377 51.289 1.00 42.73
O
ATOM 814 CB ILE B 106 80.824 64.912 54.078 1.00 42.73
C
ATOM 815 CG1 ILE B 106 81.488 66.244 54.426 1.00 42.73
C
ATOM 816 CG2 ILE B 106 81.779 63.756 54.320 1.00 42.73
C
ATOM 817 CD1 ILE B 106 81.140 66.745 55.801 1.00 42.73

```
C
ATOM    818  N   LYS B 107      81.503  66.496  51.208  1.00 44.92
N
ATOM    819  CA  LYS B 107      82.525  66.941  50.269  1.00 44.92
C
ATOM    820  C   LYS B 107      83.828  67.271  50.983  1.00 44.92
C
ATOM    821  O   LYS B 107      84.862  66.666  50.708  1.00 44.92
O
ATOM    822  CB  LYS B 107      82.038  68.156  49.483  1.00 44.92
C
ATOM    823  CG  LYS B 107      81.989  67.924  47.990  1.00 44.92
C
ATOM    824  CD  LYS B 107      81.570  69.175  47.250  1.00 44.92
C
ATOM    825  CE  LYS B 107      80.284  69.740  47.810  1.00 44.92
C
ATOM    826  NZ  LYS B 107      79.709  70.769  46.906  1.00 44.92
N
TER     827      LYS B 107
ATOM    828  N   ASN C 334      45.945  38.135  29.827  1.00 45.63
N
ATOM    829  CA  ASN C 334      45.358  39.376  30.311  1.00 45.63
C
ATOM    830  C   ASN C 334      44.438  39.126  31.502  1.00 45.63
C
ATOM    831  O   ASN C 334      43.247  39.410  31.448  1.00 45.63
O
ATOM    832  CB  ASN C 334      44.595  40.078  29.188  1.00 45.63
C
ATOM    833  CG  ASN C 334      43.694  39.135  28.419  1.00 45.63
C
ATOM    834  OD1 ASN C 334      42.800  39.566  27.694  1.00 45.63
O
ATOM    835  ND2 ASN C 334      43.921  37.838  28.579  1.00 45.63
N
ATOM    836  N   LEU C 335      45.001  38.583  32.576  1.00 40.25
N
ATOM    837  CA  LEU C 335      44.265  38.372  33.813  1.00 40.25
C
ATOM    838  C   LEU C 335      44.311  39.643  34.648  1.00 40.25
C
ATOM    839  O   LEU C 335      45.388  40.200  34.879  1.00 40.25
O
ATOM    840  CB  LEU C 335      44.851  37.198  34.596  1.00 40.25
C
ATOM    841  CG  LEU C 335      43.957  36.580  35.672  1.00 40.25
C
ATOM    842  CD1 LEU C 335      42.774  35.873  35.050  1.00 40.25
C
```

ATOM   843 CD2 LEU C 335   44.751 35.620 36.539 1.00 40.25
C
ATOM   844 N   CYS C 336   43.142 40.104 35.086 1.00 34.46
N
ATOM   845 CA CYS C 336   43.074 41.336 35.853 1.00 34.46
C
ATOM   846 C   CYS C 336   43.769 41.109 37.198 1.00 34.46
C
ATOM   847 O   CYS C 336   43.595 40.054 37.810 1.00 34.46
O
ATOM   848 CB CYS C 336   41.613 41.735 36.088 1.00 34.46
C
ATOM   849 SG CYS C 336   40.465 41.763 34.648 1.00 34.46
S
ATOM   850 N   PRO C 337   44.572 42.053 37.675 1.00 27.90
N
ATOM   851 CA PRO C 337   45.272 41.839 38.965 1.00 27.90
C
ATOM   852 C   PRO C 337   44.478 42.260 40.204 1.00 27.90
C
ATOM   853 O   PRO C 337   44.733 43.277 40.842 1.00 27.90
O
ATOM   854 CB PRO C 337   46.531 42.691 38.802 1.00 27.90
C
ATOM   855 CG PRO C 337   46.100 43.807 37.929 1.00 27.90
C
ATOM   856 CD PRO C 337   45.005 43.297 37.020 1.00 27.90
C
ATOM   857 N   PHE C 338   43.490 41.441 40.575 1.00 21.81
N
ATOM   858 CA PHE C 338   42.622 41.782 41.698 1.00 21.81
C
ATOM   859 C   PHE C 338   43.308 41.612 43.045 1.00 21.81
C
ATOM   860 O   PHE C 338   42.921 42.278 44.008 1.00 21.81
O
ATOM   861 CB PHE C 338   41.353 40.930 41.665 1.00 21.81
C
ATOM   862 CG PHE C 338   40.351 41.374 40.643 1.00 21.81
C
ATOM   863 CD1 PHE C 338   40.505 42.564 39.969 1.00 21.81
C
ATOM   864 CD2 PHE C 338   39.246 40.596 40.362 1.00 21.81
C
ATOM   865 CE1 PHE C 338   39.584 42.969 39.034 1.00 21.81
C
ATOM   866 CE2 PHE C 338   38.321 41.002 39.429 1.00 21.81
C
ATOM   867 CZ PHE C 338   38.494 42.185 38.762 1.00 21.81
C

ATOM 868 N GLY C 339 44.316 40.744 43.134 1.00 20.69
N
ATOM 869 CA GLY C 339 44.974 40.513 44.407 1.00 20.69
C
ATOM 870 C GLY C 339 45.614 41.755 44.983 1.00 20.69
C
ATOM 871 O GLY C 339 45.632 41.941 46.199 1.00 20.69
O
ATOM 872 N GLU C 340 46.136 42.623 44.126 1.00 20.09
N
ATOM 873 CA GLU C 340 46.742 43.860 44.585 1.00 20.09
C
ATOM 874 C GLU C 340 45.715 44.949 44.866 1.00 20.09
C
ATOM 875 O GLU C 340 46.090 46.018 45.349 1.00 20.09
O
ATOM 876 CB GLU C 340 47.774 44.335 43.560 1.00 20.09
C
ATOM 877 CG GLU C 340 48.991 43.421 43.486 1.00 20.09
C
ATOM 878 CD GLU C 340 49.932 43.776 42.361 1.00 20.09
C
ATOM 879 OE1 GLU C 340 50.605 44.818 42.451 1.00 20.09
O
ATOM 880 OE2 GLU C 340 49.999 43.008 41.385 1.00 20.09
O
ATOM 881 N VAL C 341 44.441 44.708 44.583 1.00 16.71
N
ATOM 882 CA VAL C 341 43.366 45.603 44.995 1.00 16.71
C
ATOM 883 C VAL C 341 42.706 45.113 46.274 1.00 16.71
C
ATOM 884 O VAL C 341 42.585 45.857 47.243 1.00 16.71
O
ATOM 885 CB VAL C 341 42.331 45.760 43.859 1.00 16.71
C
ATOM 886 CG1 VAL C 341 41.171 46.630 44.315 1.00 16.71
C
ATOM 887 CG2 VAL C 341 42.983 46.344 42.623 1.00 16.71
C
ATOM 888 N PHE C 342 42.297 43.844 46.296 1.00 15.85
N
ATOM 889 CA PHE C 342 41.591 43.298 47.450 1.00 15.85
C
ATOM 890 C PHE C 342 42.514 43.110 48.648 1.00 15.85
C
ATOM 891 O PHE C 342 42.107 43.354 49.787 1.00 15.85
O
ATOM 892 CB PHE C 342 40.926 41.977 47.074 1.00 15.85
C

ATOM	893	CG	PHE	C	342	39.653	42.141	46.301	1.00	15.85	C
ATOM	894	CD1	PHE	C	342	38.432	41.971	46.916	1.00	15.85	C
ATOM	895	CD2	PHE	C	342	39.678	42.482	44.967	1.00	15.85	C
ATOM	896	CE1	PHE	C	342	37.265	42.120	46.205	1.00	15.85	C
ATOM	897	CE2	PHE	C	342	38.510	42.635	44.254	1.00	15.85	C
ATOM	898	CZ	PHE	C	342	37.305	42.455	44.879	1.00	15.85	C
ATOM	899	N	ASN	C	343	43.751	42.666	48.419	1.00	18.79	N
ATOM	900	CA	ASN	C	343	44.714	42.459	49.495	1.00	18.79	C
ATOM	901	C	ASN	C	343	45.747	43.564	49.575	1.00	18.79	C
ATOM	902	O	ASN	C	343	46.859	43.332	50.054	1.00	18.79	O
ATOM	903	CB	ASN	C	343	45.423	41.123	49.341	1.00	18.79	C
ATOM	904	CG	ASN	C	343	44.506	39.987	49.500	1.00	18.79	C
ATOM	905	OD1	ASN	C	343	43.685	39.962	50.411	1.00	18.79	O
ATOM	906	ND2	ASN	C	343	44.634	39.017	48.618	1.00	18.79	N
ATOM	907	N	ALA	C	344	45.408	44.754	49.102	1.00	14.80	N
ATOM	908	CA	ALA	C	344	46.274	45.903	49.288	1.00	14.80	C
ATOM	909	C	ALA	C	344	46.505	46.146	50.773	1.00	14.80	C
ATOM	910	O	ALA	C	344	45.613	45.961	51.601	1.00	14.80	O
ATOM	911	CB	ALA	C	344	45.653	47.135	48.636	1.00	14.80	C
ATOM	912	N	THR	C	345	47.722	46.552	51.117	1.00	13.82	N
ATOM	913	CA	THR	C	345	48.081	46.684	52.521	1.00	13.82	C
ATOM	914	C	THR	C	345	47.774	48.057	53.102	1.00	13.82	C
ATOM	915	O	THR	C	345	47.930	48.245	54.308	1.00	13.82	O
ATOM	916	CB	THR	C	345	49.560	46.346	52.722	1.00	13.82	C
ATOM	917	OG1	THR	C	345	50.345	46.960	51.697	1.00	13.82	O

ATOM 918 CG2 THR C 345 49.738 44.851 52.641 1.00 13.82
C
ATOM 919 N ARG C 346 47.329 49.009 52.287 1.00 14.01
N
ATOM 920 CA ARG C 346 46.786 50.269 52.783 1.00 14.01
C
ATOM 921 C ARG C 346 45.535 50.583 51.979 1.00 14.01
C
ATOM 922 O ARG C 346 45.601 50.656 50.752 1.00 14.01
O
ATOM 923 CB ARG C 346 47.803 51.410 52.653 1.00 14.01
C
ATOM 924 CG ARG C 346 47.887 52.352 53.848 1.00 14.01
C
ATOM 925 CD ARG C 346 46.561 52.959 54.265 1.00 14.01
C
ATOM 926 NE ARG C 346 46.756 54.151 55.088 1.00 14.01
N
ATOM 927 CZ ARG C 346 45.790 54.995 55.426 1.00 14.01
C
ATOM 928 NH1 ARG C 346 44.548 54.784 55.021 1.00 14.01
N
ATOM 929 NH2 ARG C 346 46.060 56.055 56.167 1.00 14.01
N
ATOM 930 N PHE C 347 44.409 50.772 52.660 1.00 12.63
N
ATOM 931 CA PHE C 347 43.157 51.172 52.029 1.00 12.63
C
ATOM 932 C PHE C 347 42.904 52.658 52.260 1.00 12.63
C
ATOM 933 O PHE C 347 43.407 53.254 53.210 1.00 12.63
O
ATOM 934 CB PHE C 347 41.984 50.348 52.571 1.00 12.63
C
ATOM 935 CG PHE C 347 41.639 49.153 51.727 1.00 12.63
C
ATOM 936 CD1 PHE C 347 42.543 48.122 51.562 1.00 12.63
C
ATOM 937 CD2 PHE C 347 40.407 49.060 51.100 1.00 12.63
C
ATOM 938 CE1 PHE C 347 42.230 47.027 50.791 1.00 12.63
C
ATOM 939 CE2 PHE C 347 40.093 47.964 50.328 1.00 12.63
C
ATOM 940 CZ PHE C 347 41.006 46.947 50.172 1.00 12.63
C
ATOM 941 N ALA C 348 42.105 53.251 51.378 1.00 12.03
N
ATOM 942 CA ALA C 348 41.851 54.685 51.390 1.00 12.03
C

ATOM	943	C	ALA	C	348	40.618	55.047	52.218	1.00	12.03	C
ATOM	944	O	ALA	C	348	39.741	54.222	52.466	1.00	12.03	O
ATOM	945	CB	ALA	C	348	41.670	55.202	49.964	1.00	12.03	C
ATOM	946	N	SER	C	349	40.570	56.310	52.643	1.00	13.70	N
ATOM	947	CA	SER	C	349	39.367	56.884	53.235	1.00	13.70	C
ATOM	948	C	SER	C	349	38.301	57.094	52.165	1.00	13.70	C
ATOM	949	O	SER	C	349	38.605	57.281	50.989	1.00	13.70	O
ATOM	950	CB	SER	C	349	39.679	58.222	53.909	1.00	13.70	C
ATOM	951	OG	SER	C	349	40.486	58.052	55.056	1.00	13.70	O
ATOM	952	N	VAL	C	350	37.035	57.089	52.589	1.00	12.50	N
ATOM	953	CA	VAL	C	350	35.945	57.150	51.617	1.00	12.50	C
ATOM	954	C	VAL	C	350	35.905	58.496	50.889	1.00	12.50	C
ATOM	955	O	VAL	C	350	35.525	58.553	49.717	1.00	12.50	O
ATOM	956	CB	VAL	C	350	34.601	56.811	52.292	1.00	12.50	C
ATOM	957	CG1	VAL	C	350	34.210	57.867	53.319	1.00	12.50	C
ATOM	958	CG2	VAL	C	350	33.499	56.619	51.246	1.00	12.50	C
ATOM	959	N	TYR	C	351	36.290	59.596	51.546	1.00	15.24	N
ATOM	960	CA	TYR	C	351	36.325	60.874	50.837	1.00	15.24	C
ATOM	961	C	TYR	C	351	37.436	60.903	49.792	1.00	15.24	C
ATOM	962	O	TYR	C	351	37.281	61.540	48.748	1.00	15.24	O
ATOM	963	CB	TYR	C	351	36.462	62.040	51.826	1.00	15.24	C
ATOM	964	CG	TYR	C	351	37.873	62.387	52.251	1.00	15.24	C
ATOM	965	CD1	TYR	C	351	38.574	63.416	51.636	1.00	15.24	C
ATOM	966	CD2	TYR	C	351	38.492	61.708	53.285	1.00	15.24	C
ATOM	967	CE1	TYR	C	351	39.855	63.738	52.027	1.00	15.24	C

ATOM 968 CE2 TYR C 351 39.771 62.022 53.679 1.00 15.24
C
ATOM 969 CZ TYR C 351 40.446 63.037 53.048 1.00 15.24
C
ATOM 970 OH TYR C 351 41.723 63.351 53.445 1.00 15.24
O
ATOM 971 N ALA C 352 38.532 60.187 50.030 1.00 14.76
N
ATOM 972 CA ALA C 352 39.688 60.176 49.143 1.00 14.76
C
ATOM 973 C ALA C 352 39.827 58.854 48.391 1.00 14.76
C
ATOM 974 O ALA C 352 40.942 58.390 48.155 1.00 14.76
O
ATOM 975 CB ALA C 352 40.955 60.472 49.942 1.00 14.76
C
ATOM 976 N TRP C 353 38.699 58.264 47.989 1.00 13.98
N
ATOM 977 CA TRP C 353 38.642 56.896 47.475 1.00 13.98
C
ATOM 978 C TRP C 353 39.644 56.631 46.353 1.00 13.98
C
ATOM 979 O TRP C 353 39.911 57.500 45.524 1.00 13.98
O
ATOM 980 CB TRP C 353 37.224 56.597 46.982 1.00 13.98
C
ATOM 981 CG TRP C 353 36.614 57.620 46.054 1.00 13.98
C
ATOM 982 CD1 TRP C 353 35.985 58.775 46.407 1.00 13.98
C
ATOM 983 CD2 TRP C 353 36.528 57.538 44.630 1.00 13.98
C
ATOM 984 NE1 TRP C 353 35.531 59.428 45.290 1.00 13.98
N
ATOM 985 CE2 TRP C 353 35.854 58.688 44.185 1.00 13.98
C
ATOM 986 CE3 TRP C 353 36.965 56.607 43.685 1.00 13.98
C
ATOM 987 CZ2 TRP C 353 35.606 58.930 42.840 1.00 13.98
C
ATOM 988 CZ3 TRP C 353 36.719 56.851 42.353 1.00 13.98
C
ATOM 989 CH2 TRP C 353 36.045 58.000 41.943 1.00 13.98
C
ATOM 990 N ASN C 354 40.177 55.407 46.317 1.00 16.15
N
ATOM 991 CA ASN C 354 41.270 55.071 45.404 1.00 16.15
C
ATOM 992 C ASN C 354 40.733 54.313 44.195 1.00 16.15
C

```
ATOM    993  O   ASN C 354      39.927  53.396  44.349  1.00 16.15
O
ATOM    994  CB  ASN C 354      42.325  54.237  46.136  1.00 16.15
C
ATOM    995  CG  ASN C 354      43.643  54.150  45.388  1.00 16.15
C
ATOM    996  OD1 ASN C 354      43.830  54.771  44.349  1.00 16.15
O
ATOM    997  ND2 ASN C 354      44.571  53.380  45.934  1.00 16.15
N
ATOM    998  N   ARG C 355      41.177  54.687  42.995  1.00 17.19
N
ATOM    999  CA  ARG C 355      40.704  54.062  41.763  1.00 17.19
C
ATOM   1000  C   ARG C 355      41.864  53.434  40.999  1.00 17.19
C
ATOM   1001  O   ARG C 355      42.914  54.058  40.833  1.00 17.19
O
ATOM   1002  CB  ARG C 355      39.976  55.069  40.866  1.00 17.19
C
ATOM   1003  CG  ARG C 355      39.820  54.613  39.418  1.00 17.19
C
ATOM   1004  CD  ARG C 355      38.746  55.391  38.678  1.00 17.19
C
ATOM   1005  NE  ARG C 355      37.409  54.966  39.074  1.00 17.19
N
ATOM   1006  CZ  ARG C 355      36.299  55.256  38.411  1.00 17.19
C
ATOM   1007  NH1 ARG C 355      36.352  55.978  37.301  1.00 17.19
N
ATOM   1008  NH2 ARG C 355      35.132  54.819  38.850  1.00 17.19
N
ATOM   1009  N   LYS C 356      41.666  52.201  40.544  1.00 23.19
N
ATOM   1010  CA  LYS C 356      42.578  51.517  39.639  1.00 23.19
C
ATOM   1011  C   LYS C 356      41.839  51.199  38.349  1.00 23.19
C
ATOM   1012  O   LYS C 356      40.653  50.876  38.370  1.00 23.19
O
ATOM   1013  CB  LYS C 356      43.121  50.232  40.258  1.00 23.19
C
ATOM   1014  CG  LYS C 356      43.866  50.444  41.558  1.00 23.19
C
ATOM   1015  CD  LYS C 356      45.315  50.888  41.342  1.00 23.19
C
ATOM   1016  CE  LYS C 356      46.109  50.000  40.384  1.00 23.19
C
ATOM   1017  NZ  LYS C 356      45.660  48.582  40.316  1.00 23.19
N
```

```
ATOM   1018  N   ARG C 357      42.530  51.300  37.222  1.00 33.55
N
ATOM   1019  CA  ARG C 357      41.930  51.006  35.929  1.00 33.55
C
ATOM   1020  C   ARG C 357      42.712  49.886  35.261  1.00 33.55
C
ATOM   1021  O   ARG C 357      43.944  49.882  35.297  1.00 33.55
O
ATOM   1022  CB  ARG C 357      41.906  52.245  35.036  1.00 33.55
C
ATOM   1023  CG  ARG C 357      41.234  52.015  33.700  1.00 33.55
C
ATOM   1024  CD  ARG C 357      41.345  53.233  32.810  1.00 33.55
C
ATOM   1025  NE  ARG C 357      42.734  53.519  32.474  1.00 33.55
N
ATOM   1026  CZ  ARG C 357      43.211  53.544  31.235  1.00 33.55
C
ATOM   1027  NH1 ARG C 357      42.408  53.301  30.209  1.00 33.55
N
ATOM   1028  NH2 ARG C 357      44.491  53.814  31.022  1.00 33.55
N
ATOM   1029  N   ILE C 358      41.998  48.929  34.673  1.00 34.13
N
ATOM   1030  CA  ILE C 358      42.604  47.834  33.925  1.00 34.13
C
ATOM   1031  C   ILE C 358      41.889  47.736  32.588  1.00 34.13
C
ATOM   1032  O   ILE C 358      40.664  47.856  32.529  1.00 34.13
O
ATOM   1033  CB  ILE C 358      42.523  46.497  34.684  1.00 34.13
C
ATOM   1034  CG1 ILE C 358      42.962  46.674  36.134  1.00 34.13
C
ATOM   1035  CG2 ILE C 358      43.412  45.470  34.019  1.00 34.13
C
ATOM   1036  CD1 ILE C 358      41.965  46.170  37.134  1.00 34.13
C
ATOM   1037  N   SER C 359      42.642  47.540  31.514  1.00 39.74
N
ATOM   1038  CA  SER C 359      42.071  47.506  30.176  1.00 39.74
C
ATOM   1039  C   SER C 359      42.231  46.126  29.561  1.00 39.74
C
ATOM   1040  O   SER C 359      43.252  45.464  29.765  1.00 39.74
O
ATOM   1041  CB  SER C 359      42.725  48.548  29.271  1.00 39.74
C
ATOM   1042  OG  SER C 359      42.279  49.849  29.594  1.00 39.74
O
```

ATOM 1043 N ASN C 360 41.210 45.701 28.817 1.00 42.80
N
ATOM 1044 CA ASN C 360 41.240 44.472 28.027 1.00 42.80
C
ATOM 1045 C ASN C 360 41.737 43.290 28.856 1.00 42.80
C
ATOM 1046 O ASN C 360 42.785 42.704 28.587 1.00 42.80
O
ATOM 1047 CB ASN C 360 42.103 44.652 26.775 1.00 42.80
C
ATOM 1048 CG ASN C 360 41.683 45.838 25.944 1.00 42.80
C
ATOM 1049 OD1 ASN C 360 42.469 46.752 25.707 1.00 42.80
O
ATOM 1050 ND2 ASN C 360 40.439 45.831 25.494 1.00 42.80
N
ATOM 1051 N CYS C 361 40.974 42.952 29.889 1.00 41.88
N
ATOM 1052 CA CYS C 361 41.378 41.877 30.779 1.00 41.88
C
ATOM 1053 C CYS C 361 40.192 40.983 31.099 1.00 41.88
C
ATOM 1054 O CYS C 361 39.031 41.358 30.933 1.00 41.88
O
ATOM 1055 CB CYS C 361 42.036 42.419 32.068 1.00 41.88
C
ATOM 1056 SG CYS C 361 40.969 43.215 33.320 1.00 41.88
S
ATOM 1057 N VAL C 362 40.519 39.767 31.524 1.00 39.86
N
ATOM 1058 CA VAL C 362 39.562 38.803 32.036 1.00 39.86
C
ATOM 1059 C VAL C 362 39.908 38.564 33.497 1.00 39.86
C
ATOM 1060 O VAL C 362 41.047 38.749 33.927 1.00 39.86
O
ATOM 1061 CB VAL C 362 39.579 37.483 31.241 1.00 39.86
C
ATOM 1062 CG1 VAL C 362 39.350 37.759 29.770 1.00 39.86
C
ATOM 1063 CG2 VAL C 362 40.898 36.765 31.444 1.00 39.86
C
ATOM 1064 N ALA C 363 38.908 38.159 34.269 1.00 36.27
N
ATOM 1065 CA ALA C 363 39.077 38.098 35.709 1.00 36.27
C
ATOM 1066 C ALA C 363 38.492 36.807 36.254 1.00 36.27
C
ATOM 1067 O ALA C 363 37.677 36.144 35.613 1.00 36.27
O

ATOM 1068 CB ALA C 363 38.433 39.305 36.398 1.00 36.27
C
ATOM 1069 N GLU C 364 38.928 36.464 37.457 1.00 37.01
N
ATOM 1070 CA GLU C 364 38.460 35.290 38.174 1.00 37.01
C
ATOM 1071 C GLU C 364 37.641 35.741 39.377 1.00 37.01
C
ATOM 1072 O GLU C 364 38.159 36.435 40.256 1.00 37.01
O
ATOM 1073 CB GLU C 364 39.643 34.431 38.610 1.00 37.01
C
ATOM 1074 CG GLU C 364 39.376 32.970 38.500 1.00 37.01
C
ATOM 1075 CD GLU C 364 38.321 32.531 39.467 1.00 37.01
C
ATOM 1076 OE1 GLU C 364 38.635 32.428 40.667 1.00 37.01
O
ATOM 1077 OE2 GLU C 364 37.174 32.308 39.036 1.00 37.01
O
ATOM 1078 N TYR C 365 36.374 35.333 39.422 1.00 34.19
N
ATOM 1079 CA TYR C 365 35.457 35.750 40.475 1.00 34.19
C
ATOM 1080 C TYR C 365 35.094 34.625 41.433 1.00 34.19
C
ATOM 1081 O TYR C 365 34.235 34.821 42.296 1.00 34.19
O
ATOM 1082 CB TYR C 365 34.180 36.330 39.867 1.00 34.19
C
ATOM 1083 CG TYR C 365 34.394 37.595 39.077 1.00 34.19
C
ATOM 1084 CD1 TYR C 365 34.830 37.547 37.766 1.00 34.19
C
ATOM 1085 CD2 TYR C 365 34.163 38.838 39.645 1.00 34.19
C
ATOM 1086 CE1 TYR C 365 35.028 38.692 37.041 1.00 34.19
C
ATOM 1087 CE2 TYR C 365 34.358 39.992 38.925 1.00 34.19
C
ATOM 1088 CZ TYR C 365 34.794 39.914 37.625 1.00 34.19
C
ATOM 1089 OH TYR C 365 34.992 41.061 36.898 1.00 34.19
O
ATOM 1090 N SER C 366 35.722 33.455 41.307 1.00 34.39
N
ATOM 1091 CA SER C 366 35.290 32.305 42.094 1.00 34.39
C
ATOM 1092 C SER C 366 35.504 32.533 43.584 1.00 34.39
C

```
ATOM   1093  O   SER C 366      34.664  32.145  44.402  1.00 34.39
O
ATOM   1094  CB  SER C 366      36.015  31.042  41.635  1.00 34.39
C
ATOM   1095  OG  SER C 366      37.404  31.137  41.881  1.00 34.39
O
ATOM   1096  N  AVAL C 367      36.623  33.152  43.962  0.50 31.52
N
ATOM   1097  CA AVAL C 367      36.836  33.437  45.375  0.50 31.52
C
ATOM   1098  C  AVAL C 367      35.881  34.524  45.855  0.50 31.52
C
ATOM   1099  O  AVAL C 367      35.526  34.569  47.037  0.50 31.52
O
ATOM   1100  CB AVAL C 367      38.307  33.806  45.637  0.50 31.52
C
ATOM   1101  CG1AVAL C 367      38.638  35.156  45.038  0.50 31.52
C
ATOM   1102  CG2AVAL C 367      38.596  33.797  47.124  0.50 31.52
C
ATOM   1103  N  BVAL C 367      36.723  33.152  43.962  0.50 31.52
N
ATOM   1104  CA BVAL C 367      36.936  33.437  45.375  0.50 31.52
C
ATOM   1105  C  BVAL C 367      35.981  34.524  45.855  0.50 31.52
C
ATOM   1106  O  BVAL C 367      35.626  34.569  47.037  0.50 31.52
O
ATOM   1107  CB BVAL C 367      38.407  33.806  45.637  0.50 31.52
C
ATOM   1108  CG1BVAL C 367      38.609  34.221  47.078  0.50 31.52
C
ATOM   1109  CG2BVAL C 367      39.313  32.644  45.285  0.50 31.52
C
ATOM   1110  N   LEU C 368      35.442  35.405  44.956  1.00 29.13
N
ATOM   1111  CA  LEU C 368      34.520  36.466  45.342  1.00 29.13
C
ATOM   1112  C   LEU C 368      33.095  35.954  45.521  1.00 29.13
C
ATOM   1113  O   LEU C 368      32.327  36.541  46.287  1.00 29.13
O
ATOM   1114  CB  LEU C 368      34.559  37.596  44.311  1.00 29.13
C
ATOM   1115  CG  LEU C 368      35.940  38.158  43.960  1.00 29.13
C
ATOM   1116  CD1 LEU C 368      35.818  39.305  42.981  1.00 29.13
C
ATOM   1117  CD2 LEU C 368      36.697  38.595  45.197  1.00 29.13
C
```

```
ATOM   1118  N   TYR C 369      32.724  34.869  44.833  1.00 33.58
N
ATOM   1119  CA  TYR C 369      31.417  34.253  45.048  1.00 33.58
C
ATOM   1120  C   TYR C 369      31.272  33.693  46.451  1.00 33.58
C
ATOM   1121  O   TYR C 369      30.148  33.524  46.930  1.00 33.58
O
ATOM   1122  CB  TYR C 369      31.181  33.113  44.057  1.00 33.58
C
ATOM   1123  CG  TYR C 369      31.058  33.527  42.616  1.00 33.58
C
ATOM   1124  CD1 TYR C 369      30.249  34.585  42.244  1.00 33.58
C
ATOM   1125  CD2 TYR C 369      31.741  32.846  41.623  1.00 33.58
C
ATOM   1126  CE1 TYR C 369      30.137  34.963  40.930  1.00 33.58
C
ATOM   1127  CE2 TYR C 369      31.631  33.215  40.304  1.00 33.58
C
ATOM   1128  CZ  TYR C 369      30.826  34.273  39.963  1.00 33.58
C
ATOM   1129  OH  TYR C 369      30.712  34.648  38.647  1.00 33.58
O
ATOM   1130  N   ASN C 370      32.381  33.400  47.114  1.00 33.49
N
ATOM   1131  CA  ASN C 370      32.365  32.622  48.341  1.00 33.49
C
ATOM   1132  C   ASN C 370      31.946  33.483  49.523  1.00 33.49
C
ATOM   1133  O   ASN C 370      32.630  34.452  49.867  1.00 33.49
O
ATOM   1134  CB  ASN C 370      33.745  32.018  48.580  1.00 33.49
C
ATOM   1135  CG  ASN C 370      33.817  31.234  49.859  1.00 33.49
C
ATOM   1136  OD1 ASN C 370      32.985  30.369  50.115  1.00 33.49
O
ATOM   1137  ND2 ASN C 370      34.808  31.537  50.681  1.00 33.49
N
ATOM   1138  N   SER C 371      30.830  33.130  50.149  1.00 34.71
N
ATOM   1139  CA  SER C 371      30.528  33.682  51.456  1.00 34.71
C
ATOM   1140  C   SER C 371      31.497  33.110  52.486  1.00 34.71
C
ATOM   1141  O   SER C 371      32.321  32.244  52.188  1.00 34.71
O
ATOM   1142  CB  SER C 371      29.085  33.386  51.849  1.00 34.71
C
```

```
ATOM   1143  OG  SER C 371      28.241  33.388  50.714  1.00 34.71
O
ATOM   1144  N   ALA C 372      31.421  33.647  53.701  1.00 37.16
N
ATOM   1145  CA  ALA C 372      32.310  33.289  54.804  1.00 37.16
C
ATOM   1146  C   ALA C 372      33.713  33.831  54.562  1.00 37.16
C
ATOM   1147  O   ALA C 372      34.574  33.759  55.443  1.00 37.16
O
ATOM   1148  CB  ALA C 372      32.350  31.773  55.034  1.00 37.16
C
ATOM   1149  N   SER C 373      33.952  34.380  53.380  1.00 32.75
N
ATOM   1150  CA  SER C 373      35.115  35.214  53.134  1.00 32.75
C
ATOM   1151  C   SER C 373      34.744  36.639  52.778  1.00 32.75
C
ATOM   1152  O   SER C 373      35.509  37.552  53.083  1.00 32.75
O
ATOM   1153  CB  SER C 373      35.983  34.633  52.010  1.00 32.75
C
ATOM   1154  OG  SER C 373      35.270  34.579  50.790  1.00 32.75
O
ATOM   1155  N   PHE C 374      33.585  36.850  52.161  1.00 25.92
N
ATOM   1156  CA  PHE C 374      33.109  38.178  51.802  1.00 25.92
C
ATOM   1157  C   PHE C 374      31.707  38.361  52.345  1.00 25.92
C
ATOM   1158  O   PHE C 374      30.807  37.578  52.027  1.00 25.92
O
ATOM   1159  CB  PHE C 374      33.148  38.379  50.290  1.00 25.92
C
ATOM   1160  CG  PHE C 374      34.530  38.398  49.748  1.00 25.92
C
ATOM   1161  CD1 PHE C 374      35.358  39.473  49.991  1.00 25.92
C
ATOM   1162  CD2 PHE C 374      35.025  37.326  49.037  1.00 25.92
C
ATOM   1163  CE1 PHE C 374      36.638  39.492  49.518  1.00 25.92
C
ATOM   1164  CE2 PHE C 374      36.308  37.342  48.559  1.00 25.92
C
ATOM   1165  CZ  PHE C 374      37.117  38.424  48.798  1.00 25.92
C
ATOM   1166  N   SER C 375      31.528  39.398  53.162  1.00 24.99
N
ATOM   1167  CA  SER C 375      30.260  39.595  53.848  1.00 24.99
C
```

ATOM 1168 C SER C 375 29.126 39.836 52.867 1.00 24.99
C
ATOM 1169 O SER C 375 28.012 39.342 53.069 1.00 24.99
O
ATOM 1170 CB SER C 375 30.372 40.751 54.838 1.00 24.99
C
ATOM 1171 OG SER C 375 31.541 40.620 55.623 1.00 24.99
O
ATOM 1172 N THR C 376 29.372 40.595 51.803 1.00 23.46
N
ATOM 1173 CA THR C 376 28.298 40.791 50.843 1.00 23.46
C
ATOM 1174 C THR C 376 28.858 40.978 49.441 1.00 23.46
C
ATOM 1175 O THR C 376 29.957 41.510 49.252 1.00 23.46
O
ATOM 1176 CB THR C 376 27.403 41.976 51.225 1.00 23.46
C
ATOM 1177 OG1 THR C 376 26.332 42.088 50.284 1.00 23.46
O
ATOM 1178 CG2 THR C 376 28.191 43.268 51.241 1.00 23.46
C
ATOM 1179 N PHE C 377 28.071 40.534 48.461 1.00 24.09
N
ATOM 1180 CA PHE C 377 28.451 40.562 47.051 1.00 24.09
C
ATOM 1181 C PHE C 377 27.183 40.793 46.244 1.00 24.09
C
ATOM 1182 O PHE C 377 26.291 39.943 46.247 1.00 24.09
O
ATOM 1183 CB PHE C 377 29.138 39.259 46.647 1.00 24.09
C
ATOM 1184 CG PHE C 377 29.368 39.118 45.169 1.00 24.09
C
ATOM 1185 CD1 PHE C 377 30.385 39.803 44.540 1.00 24.09
C
ATOM 1186 CD2 PHE C 377 28.580 38.276 44.413 1.00 24.09
C
ATOM 1187 CE1 PHE C 377 30.602 39.666 43.186 1.00 24.09
C
ATOM 1188 CE2 PHE C 377 28.795 38.139 43.060 1.00 24.09
C
ATOM 1189 CZ PHE C 377 29.807 38.829 42.450 1.00 24.09
C
ATOM 1190 N LYS C 378 27.094 41.934 45.570 1.00 24.02
N
ATOM 1191 CA LYS C 378 25.927 42.290 44.776 1.00 24.02
C
ATOM 1192 C LYS C 378 26.382 42.748 43.406 1.00 24.02
C

ATOM 1193 O LYS C 378 27.464 43.312 43.271 1.00 24.02
O
ATOM 1194 CB LYS C 378 25.111 43.411 45.432 1.00 24.02
C
ATOM 1195 CG LYS C 378 24.907 43.256 46.916 1.00 24.02
C
ATOM 1196 CD LYS C 378 23.758 44.114 47.392 1.00 24.02
C
ATOM 1197 CE LYS C 378 22.431 43.425 47.148 1.00 24.02
C
ATOM 1198 NZ LYS C 378 22.328 42.161 47.923 1.00 24.02
N
ATOM 1199 N CYS C 379 25.569 42.510 42.388 1.00 24.87
N
ATOM 1200 CA CYS C 379 25.879 42.999 41.056 1.00 24.87
C
ATOM 1201 C CYS C 379 24.669 43.719 40.486 1.00 24.87
C
ATOM 1202 O CYS C 379 23.523 43.368 40.768 1.00 24.87
O
ATOM 1203 CB CYS C 379 26.336 41.866 40.116 1.00 24.87
C
ATOM 1204 SG CYS C 379 27.926 41.106 40.582 1.00 24.87
S
ATOM 1205 N TYR C 380 24.945 44.743 39.690 1.00 24.50
N
ATOM 1206 CA TYR C 380 23.951 45.614 39.087 1.00 24.50
C
ATOM 1207 C TYR C 380 24.205 45.654 37.589 1.00 24.50
C
ATOM 1208 O TYR C 380 25.342 45.866 37.156 1.00 24.50
O
ATOM 1209 CB TYR C 380 24.030 47.030 39.668 1.00 24.50
C
ATOM 1210 CG TYR C 380 23.783 47.106 41.154 1.00 24.50
C
ATOM 1211 CD1 TYR C 380 22.507 47.301 41.654 1.00 24.50
C
ATOM 1212 CD2 TYR C 380 24.828 46.986 42.058 1.00 24.50
C
ATOM 1213 CE1 TYR C 380 22.277 47.367 43.004 1.00 24.50
C
ATOM 1214 CE2 TYR C 380 24.604 47.050 43.412 1.00 24.50
C
ATOM 1215 CZ TYR C 380 23.326 47.242 43.880 1.00 24.50
C
ATOM 1216 OH TYR C 380 23.097 47.307 45.230 1.00 24.50
O
ATOM 1217 N GLY C 381 23.153 45.441 36.802 1.00 29.88
N

ATOM 1218 CA GLY C 381 23.246 45.476 35.362 1.00 29.88
C
ATOM 1219 C GLY C 381 23.723 44.193 34.719 1.00 29.88
C
ATOM 1220 O GLY C 381 23.545 44.021 33.511 1.00 29.88
O
ATOM 1221 N VAL C 382 24.328 43.289 35.488 1.00 31.53
N
ATOM 1222 CA VAL C 382 24.757 41.990 34.992 1.00 31.53
C
ATOM 1223 C VAL C 382 24.382 40.942 36.024 1.00 31.53
C
ATOM 1224 O VAL C 382 24.165 41.241 37.199 1.00 31.53
O
ATOM 1225 CB VAL C 382 26.272 41.922 34.699 1.00 31.53
C
ATOM 1226 CG1 VAL C 382 26.621 42.766 33.493 1.00 31.53
C
ATOM 1227 CG2 VAL C 382 27.071 42.359 35.918 1.00 31.53
C
ATOM 1228 N SER C 383 24.304 39.715 35.575 1.00 35.15
N
ATOM 1229 CA SER C 383 23.993 38.645 36.499 1.00 35.15
C
ATOM 1230 C SER C 383 25.213 38.302 37.349 1.00 35.15
C
ATOM 1231 O SER C 383 26.347 38.355 36.872 1.00 35.15
O
ATOM 1232 CB SER C 383 23.527 37.408 35.740 1.00 35.15
C
ATOM 1233 OG SER C 383 23.168 36.372 36.633 1.00 35.15
O
ATOM 1234 N PRO C 384 25.004 37.942 38.600 1.00 35.16
N
ATOM 1235 CA PRO C 384 26.133 37.598 39.471 1.00 35.16
C
ATOM 1236 C PRO C 384 26.652 36.182 39.276 1.00 35.16
C
ATOM 1237 O PRO C 384 27.310 35.652 40.173 1.00 35.16
O
ATOM 1238 CB PRO C 384 25.550 37.780 40.876 1.00 35.16
C
ATOM 1239 CG PRO C 384 24.094 37.550 40.698 1.00 35.16
C
ATOM 1240 CD PRO C 384 23.752 38.105 39.356 1.00 35.16
C
ATOM 1241 N THR C 385 26.354 35.545 38.141 1.00 39.19
N
ATOM 1242 CA THR C 385 26.592 34.112 37.978 1.00 39.19
C

ATOM 1243 C THR C 385 27.809 33.784 37.118 1.00 39.19
C
ATOM 1244 O THR C 385 28.693 33.042 37.554 1.00 39.19
O
ATOM 1245 CB THR C 385 25.351 33.437 37.379 1.00 39.19
C
ATOM 1246 OG1 THR C 385 24.919 34.168 36.226 1.00 39.19
O
ATOM 1247 CG2 THR C 385 24.231 33.393 38.394 1.00 39.19
C
ATOM 1248 N LYS C 386 27.871 34.304 35.897 1.00 40.42
N
ATOM 1249 CA LYS C 386 28.791 33.739 34.915 1.00 40.42
C
ATOM 1250 C LYS C 386 29.872 34.734 34.518 1.00 40.42
C
ATOM 1251 O LYS C 386 30.167 34.897 33.331 1.00 40.42
O
ATOM 1252 CB LYS C 386 28.010 33.280 33.683 1.00 40.42
C
ATOM 1253 CG LYS C 386 27.162 32.036 33.926 1.00 40.42
C
ATOM 1254 CD LYS C 386 27.953 30.746 33.837 1.00 40.42
C
ATOM 1255 CE LYS C 386 28.355 30.436 32.408 1.00 40.42
C
ATOM 1256 NZ LYS C 386 27.415 31.020 31.414 1.00 40.42
N
ATOM 1257 N LEU C 387 30.464 35.408 35.505 1.00 37.68
N
ATOM 1258 CA LEU C 387 31.346 36.532 35.209 1.00 37.68
C
ATOM 1259 C LEU C 387 32.701 36.087 34.675 1.00 37.68
C
ATOM 1260 O LEU C 387 33.347 36.842 33.943 1.00 37.68
O
ATOM 1261 CB LEU C 387 31.521 37.396 36.458 1.00 37.68
C
ATOM 1262 CG LEU C 387 30.224 37.975 37.024 1.00 37.68
C
ATOM 1263 CD1 LEU C 387 30.449 38.544 38.413 1.00 37.68
C
ATOM 1264 CD2 LEU C 387 29.661 39.035 36.092 1.00 37.68
C
ATOM 1265 N ASN C 388 33.154 34.882 35.031 1.00 39.46
N
ATOM 1266 CA ASN C 388 34.459 34.417 34.567 1.00 39.46
C
ATOM 1267 C ASN C 388 34.509 34.281 33.051 1.00 39.46
C

ATOM 1268 O ASN C 388 35.570 34.472 32.449 1.00 39.46
O
ATOM 1269 CB ASN C 388 34.807 33.082 35.222 1.00 39.46
C
ATOM 1270 CG ASN C 388 35.376 33.247 36.610 1.00 39.46
C
ATOM 1271 OD1 ASN C 388 35.008 34.165 37.338 1.00 39.46
O
ATOM 1272 ND2 ASN C 388 36.282 32.359 36.986 1.00 39.46
N
ATOM 1273 N ASP C 389 33.383 33.956 32.418 1.00 42.00
N
ATOM 1274 CA ASP C 389 33.352 33.758 30.975 1.00 42.00
C
ATOM 1275 C ASP C 389 33.212 35.052 30.188 1.00 42.00
C
ATOM 1276 O ASP C 389 33.328 35.022 28.961 1.00 42.00
O
ATOM 1277 CB ASP C 389 32.213 32.811 30.596 1.00 42.00
C
ATOM 1278 CG ASP C 389 32.319 31.477 31.289 1.00 42.00
C
ATOM 1279 OD1 ASP C 389 31.272 30.868 31.563 1.00 42.00
O
ATOM 1280 OD2 ASP C 389 33.452 31.030 31.553 1.00 42.00
O
ATOM 1281 N LEU C 390 32.967 36.176 30.845 1.00 41.65
N
ATOM 1282 CA LEU C 390 32.867 37.442 30.139 1.00 41.65
C
ATOM 1283 C LEU C 390 34.243 38.079 29.987 1.00 41.65
C
ATOM 1284 O LEU C 390 35.189 37.753 30.704 1.00 41.65
O
ATOM 1285 CB LEU C 390 31.924 38.397 30.871 1.00 41.65
C
ATOM 1286 CG LEU C 390 30.501 37.896 31.115 1.00 41.65
C
ATOM 1287 CD1 LEU C 390 29.744 38.853 32.016 1.00 41.65
C
ATOM 1288 CD2 LEU C 390 29.775 37.718 29.797 1.00 41.65
C
ATOM 1289 N CYS C 391 34.347 38.989 29.025 1.00 42.59
N
ATOM 1290 CA CYS C 391 35.545 39.791 28.831 1.00 42.59
C
ATOM 1291 C CYS C 391 35.138 41.252 28.744 1.00 42.59
C
ATOM 1292 O CYS C 391 34.156 41.590 28.078 1.00 42.59
O

ATOM 1293 CB CYS C 391 36.311 39.378 27.569 1.00 42.59
C
ATOM 1294 SG CYS C 391 37.849 40.288 27.300 1.00 42.59
S
ATOM 1295 N PHE C 392 35.894 42.114 29.415 1.00 37.80
N
ATOM 1296 CA PHE C 392 35.520 43.510 29.582 1.00 37.80
C
ATOM 1297 C PHE C 392 36.563 44.417 28.952 1.00 37.80
C
ATOM 1298 O PHE C 392 37.767 44.199 29.106 1.00 37.80
O
ATOM 1299 CB PHE C 392 35.343 43.854 31.066 1.00 37.80
C
ATOM 1300 CG PHE C 392 34.239 43.090 31.729 1.00 37.80
C
ATOM 1301 CD1 PHE C 392 32.928 43.270 31.339 1.00 37.80
C
ATOM 1302 CD2 PHE C 392 34.513 42.183 32.731 1.00 37.80
C
ATOM 1303 CE1 PHE C 392 31.912 42.563 31.940 1.00 37.80
C
ATOM 1304 CE2 PHE C 392 33.501 41.477 33.336 1.00 37.80
C
ATOM 1305 CZ PHE C 392 32.200 41.666 32.941 1.00 37.80
C
ATOM 1306 N THR C 393 36.085 45.436 28.239 1.00 38.71
N
ATOM 1307 CA THR C 393 36.980 46.396 27.604 1.00 38.71
C
ATOM 1308 C THR C 393 37.788 47.164 28.641 1.00 38.71
C
ATOM 1309 O THR C 393 39.005 47.325 28.508 1.00 38.71
O
ATOM 1310 CB THR C 393 36.169 47.356 26.735 1.00 38.71
C
ATOM 1311 OG1 THR C 393 35.683 46.662 25.580 1.00 38.71
O
ATOM 1312 CG2 THR C 393 37.024 48.524 26.293 1.00 38.71
C
ATOM 1313 N ASN C 394 37.119 47.647 29.685 1.00 35.70
N
ATOM 1314 CA ASN C 394 37.757 48.356 30.783 1.00 35.70
C
ATOM 1315 C ASN C 394 37.101 47.927 32.082 1.00 35.70
C
ATOM 1316 O ASN C 394 35.883 47.806 32.145 1.00 35.70
O
ATOM 1317 CB ASN C 394 37.624 49.875 30.640 1.00 35.70
C

ATOM 1318 CG ASN C 394 38.500 50.438 29.553 1.00 35.70
C
ATOM 1319 OD1 ASN C 394 39.722 50.339 29.612 1.00 35.70
O
ATOM 1320 ND2 ASN C 394 37.880 51.045 28.555 1.00 35.70
N
ATOM 1321 N VAL C 395 37.902 47.713 33.115 1.00 29.17
N
ATOM 1322 CA VAL C 395 37.404 47.474 34.462 1.00 29.17
C
ATOM 1323 C VAL C 395 37.967 48.560 35.363 1.00 29.17
C
ATOM 1324 O VAL C 395 39.183 48.778 35.396 1.00 29.17
O
ATOM 1325 CB VAL C 395 37.790 46.078 34.981 1.00 29.17
C
ATOM 1326 CG1 VAL C 395 37.288 45.886 36.403 1.00 29.17
C
ATOM 1327 CG2 VAL C 395 37.232 45.002 34.072 1.00 29.17
C
ATOM 1328 N TYR C 396 37.088 49.244 36.083 1.00 24.70
N
ATOM 1329 CA TYR C 396 37.488 50.223 37.081 1.00 24.70
C
ATOM 1330 C TYR C 396 37.244 49.626 38.457 1.00 24.70
C
ATOM 1331 O TYR C 396 36.155 49.122 38.727 1.00 24.70
O
ATOM 1332 CB TYR C 396 36.711 51.529 36.925 1.00 24.70
C
ATOM 1333 CG TYR C 396 36.718 52.087 35.524 1.00 24.70
C
ATOM 1334 CD1 TYR C 396 37.711 52.957 35.110 1.00 24.70
C
ATOM 1335 CD2 TYR C 396 35.725 51.749 34.622 1.00 24.70
C
ATOM 1336 CE1 TYR C 396 37.719 53.469 33.830 1.00 24.70
C
ATOM 1337 CE2 TYR C 396 35.725 52.255 33.340 1.00 24.70
C
ATOM 1338 CZ TYR C 396 36.723 53.114 32.953 1.00 24.70
C
ATOM 1339 OH TYR C 396 36.725 53.621 31.675 1.00 24.70
O
ATOM 1340 N ALA C 397 38.254 49.666 39.313 1.00 17.04
N
ATOM 1341 CA ALA C 397 38.168 49.129 40.664 1.00 17.04
C
ATOM 1342 C ALA C 397 38.365 50.276 41.646 1.00 17.04
C

```
ATOM    1343  O   ALA C 397       39.473  50.797  41.779  1.00 17.04
O
ATOM    1344  CB  ALA C 397       39.207  48.031  40.879  1.00 17.04
C
ATOM    1345  N   ASP C 398       37.290  50.675  42.317  1.00 13.76
N
ATOM    1346  CA  ASP C 398       37.322  51.713  43.336  1.00 13.76
C
ATOM    1347  C   ASP C 398       37.308  51.048  44.704  1.00 13.76
C
ATOM    1348  O   ASP C 398       36.585  50.080  44.913  1.00 13.76
O
ATOM    1349  CB  ASP C 398       36.117  52.646  43.199  1.00 13.76
C
ATOM    1350  CG  ASP C 398       35.983  53.231  41.806  1.00 13.76
C
ATOM    1351  OD1 ASP C 398       37.002  53.352  41.108  1.00 13.76
O
ATOM    1352  OD2 ASP C 398       34.857  53.577  41.418  1.00 13.76
O
ATOM    1353  N   SER C 399       38.115  51.544  45.634  1.00 10.98
N
ATOM    1354  CA  SER C 399       38.159  50.902  46.940  1.00 10.98
C
ATOM    1355  C   SER C 399       38.322  51.926  48.055  1.00 10.98
C
ATOM    1356  O   SER C 399       38.963  52.978  47.880  1.00 10.98
O
ATOM    1357  CB  SER C 399       39.272  49.844  47.013  1.00 10.98
C
ATOM    1358  OG  SER C 399       40.530  50.380  46.660  1.00 10.98
O
ATOM    1359  N   PHE C 400       37.713  51.593  49.201  1.00 10.21
N
ATOM    1360  CA  PHE C 400       37.709  52.445  50.393  1.00 10.21
C
ATOM    1361  C   PHE C 400       37.221  51.639  51.594  1.00 10.21
C
ATOM    1362  O   PHE C 400       36.841  50.475  51.469  1.00 10.21
O
ATOM    1363  CB  PHE C 400       36.843  53.701  50.197  1.00 10.21
C
ATOM    1364  CG  PHE C 400       35.505  53.436  49.561  1.00 10.21
C
ATOM    1365  CD1 PHE C 400       34.408  53.087  50.332  1.00 10.21
C
ATOM    1366  CD2 PHE C 400       35.341  53.546  48.192  1.00 10.21
C
ATOM    1367  CE1 PHE C 400       33.176  52.851  49.744  1.00 10.21
C
```

ATOM 1368 CE2 PHE C 400 34.113 53.308 47.601 1.00 10.21
C
ATOM 1369 CZ PHE C 400 33.030 52.962 48.380 1.00 10.21
C
ATOM 1370 N VAL C 401 37.250 52.273 52.766 1.00 12.18
N
ATOM 1371 CA VAL C 401 36.743 51.698 54.010 1.00 12.18
C
ATOM 1372 C VAL C 401 35.562 52.534 54.488 1.00 12.18
C
ATOM 1373 O VAL C 401 35.593 53.765 54.414 1.00 12.18
O
ATOM 1374 CB VAL C 401 37.838 51.629 55.096 1.00 12.18
C
ATOM 1375 CG1 VAL C 401 37.259 51.178 56.432 1.00 12.18
C
ATOM 1376 CG2 VAL C 401 38.948 50.695 54.665 1.00 12.18
C
ATOM 1377 N ILE C 402 34.520 51.864 54.981 1.00 14.54
N
ATOM 1378 CA ILE C 402 33.300 52.532 55.420 1.00 14.54
C
ATOM 1379 C ILE C 402 32.692 51.729 56.565 1.00 14.54
C
ATOM 1380 O ILE C 402 32.978 50.554 56.731 1.00 14.54
O
ATOM 1381 CB ILE C 402 32.324 52.676 54.227 1.00 14.54
C
ATOM 1382 CG1 ILE C 402 31.255 53.717 54.493 1.00 14.54
C
ATOM 1383 CG2 ILE C 402 31.715 51.346 53.853 1.00 14.54
C
ATOM 1384 CD1 ILE C 402 30.453 54.036 53.264 1.00 14.54
C
ATOM 1385 N ARG C 403 31.844 52.367 57.365 1.00 19.81
N
ATOM 1386 CA ARG C 403 31.139 51.642 58.420 1.00 19.81
C
ATOM 1387 C ARG C 403 30.069 50.698 57.869 1.00 19.81
C
ATOM 1388 O ARG C 403 29.504 50.909 56.795 1.00 19.81
O
ATOM 1389 CB ARG C 403 30.542 52.627 59.433 1.00 19.81
C
ATOM 1390 CG ARG C 403 29.188 53.119 59.020 1.00 19.81
C
ATOM 1391 CD ARG C 403 28.446 53.997 60.017 1.00 19.81
C
ATOM 1392 NE ARG C 403 27.749 53.169 60.987 1.00 19.81
N

ATOM 1393 CZ ARG C 403 27.103 53.625 62.050 1.00 19.81
C
ATOM 1394 NH1 ARG C 403 27.060 54.924 62.308 1.00 19.81
N
ATOM 1395 NH2 ARG C 403 26.469 52.769 62.835 1.00 19.81
N
ATOM 1396 N GLY C 404 29.794 49.641 58.641 1.00 18.41
N
ATOM 1397 CA GLY C 404 28.975 48.540 58.156 1.00 18.41
C
ATOM 1398 C GLY C 404 27.553 48.917 57.789 1.00 18.41
C
ATOM 1399 O GLY C 404 26.976 48.334 56.872 1.00 18.41
O
ATOM 1400 N ASP C 405 26.964 49.876 58.501 1.00 20.72
N
ATOM 1401 CA ASP C 405 25.618 50.326 58.158 1.00 20.72
C
ATOM 1402 C ASP C 405 25.585 51.080 56.835 1.00 20.72
C
ATOM 1403 O ASP C 405 24.557 51.086 56.155 1.00 20.72
O
ATOM 1404 CB ASP C 405 25.057 51.212 59.266 1.00 20.72
C
ATOM 1405 CG ASP C 405 24.560 50.415 60.448 1.00 20.72
C
ATOM 1406 OD1 ASP C 405 24.125 49.266 60.247 1.00 20.72
O
ATOM 1407 OD2 ASP C 405 24.611 50.939 61.577 1.00 20.72
O
ATOM 1408 N GLU C 406 26.682 51.721 56.456 1.00 18.81
N
ATOM 1409 CA GLU C 406 26.685 52.571 55.278 1.00 18.81
C
ATOM 1410 C GLU C 406 27.130 51.839 54.018 1.00 18.81
C
ATOM 1411 O GLU C 406 27.186 52.453 52.951 1.00 18.81
O
ATOM 1412 CB GLU C 406 27.542 53.808 55.557 1.00 18.81
C
ATOM 1413 CG GLU C 406 26.949 54.604 56.719 1.00 18.81
C
ATOM 1414 CD GLU C 406 27.511 55.991 56.870 1.00 18.81
C
ATOM 1415 OE1 GLU C 406 28.628 56.121 57.399 1.00 18.81
O
ATOM 1416 OE2 GLU C 406 26.826 56.952 56.475 1.00 18.81
O
ATOM 1417 N VAL C 407 27.399 50.534 54.113 1.00 17.77
N

ATOM 1418 CA VAL C 407 27.621 49.717 52.924 1.00 17.77
C
ATOM 1419 C VAL C 407 26.383 49.722 52.036 1.00 17.77
C
ATOM 1420 O VAL C 407 26.487 49.621 50.810 1.00 17.77
O
ATOM 1421 CB VAL C 407 28.032 48.288 53.342 1.00 17.77
C
ATOM 1422 CG1 VAL C 407 28.134 47.370 52.137 1.00 17.77
C
ATOM 1423 CG2 VAL C 407 29.347 48.323 54.087 1.00 17.77
C
ATOM 1424 N ARG C 408 25.197 49.873 52.631 1.00 24.00
N
ATOM 1425 CA ARG C 408 23.966 49.985 51.856 1.00 24.00
C
ATOM 1426 C ARG C 408 23.858 51.308 51.110 1.00 24.00
C
ATOM 1427 O ARG C 408 23.048 51.414 50.187 1.00 24.00
O
ATOM 1428 CB ARG C 408 22.749 49.819 52.766 1.00 24.00
C
ATOM 1429 CG ARG C 408 22.816 48.629 53.698 1.00 24.00
C
ATOM 1430 CD ARG C 408 21.648 48.645 54.669 1.00 24.00
C
ATOM 1431 NE ARG C 408 21.152 50.000 54.887 1.00 24.00
N
ATOM 1432 CZ ARG C 408 20.919 50.528 56.083 1.00 24.00
C
ATOM 1433 NH1 ARG C 408 21.132 49.812 57.179 1.00 24.00
N
ATOM 1434 NH2 ARG C 408 20.470 51.771 56.186 1.00 24.00
N
ATOM 1435 N GLN C 409 24.638 52.318 51.485 1.00 20.66
N
ATOM 1436 CA GLN C 409 24.630 53.561 50.728 1.00 20.66
C
ATOM 1437 C GLN C 409 25.427 53.458 49.437 1.00 20.66
C
ATOM 1438 O GLN C 409 25.392 54.389 48.631 1.00 20.66
O
ATOM 1439 CB GLN C 409 25.180 54.711 51.578 1.00 20.66
C
ATOM 1440 CG GLN C 409 24.407 54.955 52.860 1.00 20.66
C
ATOM 1441 CD GLN C 409 22.917 55.026 52.623 1.00 20.66
C
ATOM 1442 OE1 GLN C 409 22.420 55.979 52.032 1.00 20.66
O

ATOM 1443 NE2 GLN C 409 22.197 54.008 53.070 1.00 20.66
N
ATOM 1444 N ILE C 410 26.164 52.370 49.241 1.00 15.82
N
ATOM 1445 CA ILE C 410 26.955 52.186 48.019 1.00 15.82
C
ATOM 1446 C ILE C 410 26.065 51.414 47.052 1.00 15.82
C
ATOM 1447 O ILE C 410 26.177 50.202 46.869 1.00 15.82
O
ATOM 1448 CB ILE C 410 28.285 51.485 48.303 1.00 15.82
C
ATOM 1449 CG1 ILE C 410 28.966 52.136 49.514 1.00 15.82
C
ATOM 1450 CG2 ILE C 410 29.174 51.507 47.065 1.00 15.82
C
ATOM 1451 CD1 ILE C 410 29.276 53.605 49.337 1.00 15.82
C
ATOM 1452 N ALA C 411 25.179 52.147 46.393 1.00 18.90
N
ATOM 1453 CA ALA C 411 24.237 51.584 45.438 1.00 18.90
C
ATOM 1454 C ALA C 411 23.683 52.724 44.601 1.00 18.90
C
ATOM 1455 O ALA C 411 23.710 53.878 45.034 1.00 18.90
O
ATOM 1456 CB ALA C 411 23.103 50.833 46.151 1.00 18.90
C
ATOM 1457 N PRO C 412 23.199 52.441 43.393 1.00 21.18
N
ATOM 1458 CA PRO C 412 22.589 53.505 42.587 1.00 21.18
C
ATOM 1459 C PRO C 412 21.345 54.072 43.258 1.00 21.18
C
ATOM 1460 O PRO C 412 20.614 53.371 43.956 1.00 21.18
O
ATOM 1461 CB PRO C 412 22.248 52.808 41.263 1.00 21.18
C
ATOM 1462 CG PRO C 412 22.362 51.357 41.524 1.00 21.18
C
ATOM 1463 CD PRO C 412 23.310 51.174 42.654 1.00 21.18
C
ATOM 1464 N GLY C 413 21.125 55.365 43.050 1.00 21.99
N
ATOM 1465 CA GLY C 413 19.939 56.044 43.548 1.00 21.99
C
ATOM 1466 C GLY C 413 19.825 56.177 45.052 1.00 21.99
C
ATOM 1467 O GLY C 413 18.717 56.104 45.592 1.00 21.99
O

ATOM 1468 N GLN C 414 20.939 56.386 45.745 1.00 21.99
N
ATOM 1469 CA GLN C 414 20.937 56.547 47.189 1.00 21.99
C
ATOM 1470 C GLN C 414 21.212 57.997 47.554 1.00 21.99
C
ATOM 1471 O GLN C 414 21.743 58.772 46.759 1.00 21.99
O
ATOM 1472 CB GLN C 414 21.980 55.640 47.849 1.00 21.99
C
ATOM 1473 CG GLN C 414 21.716 54.165 47.678 1.00 21.99
C
ATOM 1474 CD GLN C 414 20.340 53.764 48.148 1.00 21.99
C
ATOM 1475 OE1 GLN C 414 19.993 53.944 49.310 1.00 21.99
O
ATOM 1476 NE2 GLN C 414 19.546 53.213 47.245 1.00 21.99
N
ATOM 1477 N THR C 415 20.841 58.358 48.778 1.00 22.25
N
ATOM 1478 CA THR C 415 21.054 59.706 49.271 1.00 22.25
C
ATOM 1479 C THR C 415 21.491 59.639 50.727 1.00 22.25
C
ATOM 1480 O THR C 415 21.230 58.665 51.431 1.00 22.25
O
ATOM 1481 CB THR C 415 19.796 60.570 49.107 1.00 22.25
C
ATOM 1482 OG1 THR C 415 20.128 61.943 49.336 1.00 22.25
O
ATOM 1483 CG2 THR C 415 18.716 60.136 50.075 1.00 22.25
C
ATOM 1484 N GLY C 416 22.180 60.683 51.165 1.00 20.12
N
ATOM 1485 CA GLY C 416 22.848 60.702 52.446 1.00 20.12
C
ATOM 1486 C GLY C 416 24.271 61.192 52.306 1.00 20.12
C
ATOM 1487 O GLY C 416 24.756 61.475 51.214 1.00 20.12
O
ATOM 1488 N LYS C 417 24.956 61.267 53.449 1.00 19.00
N
ATOM 1489 CA LYS C 417 26.261 61.922 53.501 1.00 19.00
C
ATOM 1490 C LYS C 417 27.276 61.245 52.582 1.00 19.00
C
ATOM 1491 O LYS C 417 27.982 61.917 51.825 1.00 19.00
O
ATOM 1492 CB LYS C 417 26.769 61.946 54.941 1.00 19.00
C

ATOM 1493 CG LYS C 417 25.897 62.743 55.897 1.00 19.00
C
ATOM 1494 CD LYS C 417 25.736 64.168 55.423 1.00 19.00
C
ATOM 1495 CE LYS C 417 25.141 65.046 56.510 1.00 19.00
C
ATOM 1496 NZ LYS C 417 23.678 64.852 56.623 1.00 19.00
N
ATOM 1497 N ILE C 418 27.359 59.914 52.628 1.00 15.37
N
ATOM 1498 CA ILE C 418 28.328 59.210 51.789 1.00 15.37
C
ATOM 1499 C ILE C 418 27.995 59.388 50.313 1.00 15.37
C
ATOM 1500 O ILE C 418 28.877 59.661 49.494 1.00 15.37
O
ATOM 1501 CB ILE C 418 28.400 57.721 52.173 1.00 15.37
C
ATOM 1502 CG1 ILE C 418 28.887 57.547 53.611 1.00 15.37
C
ATOM 1503 CG2 ILE C 418 29.302 56.969 51.207 1.00 15.37
C
ATOM 1504 CD1 ILE C 418 30.221 58.198 53.899 1.00 15.37
C
ATOM 1505 N ALA C 419 26.719 59.249 49.951 1.00 17.49
N
ATOM 1506 CA ALA C 419 26.327 59.370 48.550 1.00 17.49
C
ATOM 1507 C ALA C 419 26.393 60.813 48.061 1.00 17.49
C
ATOM 1508 O ALA C 419 26.791 61.063 46.920 1.00 17.49
O
ATOM 1509 CB ALA C 419 24.925 58.799 48.349 1.00 17.49
C
ATOM 1510 N GLU C 420 26.004 61.778 48.900 1.00 21.76
N
ATOM 1511 CA GLU C 420 26.007 63.174 48.466 1.00 21.76
C
ATOM 1512 C GLU C 420 27.407 63.759 48.388 1.00 21.76
C
ATOM 1513 O GLU C 420 27.727 64.476 47.436 1.00 21.76
O
ATOM 1514 CB GLU C 420 25.155 64.034 49.396 1.00 21.76
C
ATOM 1515 CG GLU C 420 23.765 64.221 48.905 1.00 21.76
C
ATOM 1516 CD GLU C 420 22.916 63.043 49.190 1.00 21.76
C
ATOM 1517 OE1 GLU C 420 22.453 62.919 50.337 1.00 21.76
O

```
ATOM   1518  OE2 GLU C 420      22.717  62.233  48.271  1.00 21.76
O
ATOM   1519  N   TYR C 421      28.249  63.491  49.383  1.00 20.01
N
ATOM   1520  CA  TYR C 421      29.477  64.245  49.543  1.00 20.01
C
ATOM   1521  C   TYR C 421      30.760  63.437  49.417  1.00 20.01
C
ATOM   1522  O   TYR C 421      31.836  64.035  49.456  1.00 20.01
O
ATOM   1523  CB  TYR C 421      29.492  64.946  50.909  1.00 20.01
C
ATOM   1524  CG  TYR C 421      28.315  65.863  51.170  1.00 20.01
C
ATOM   1525  CD1 TYR C 421      27.759  66.626  50.153  1.00 20.01
C
ATOM   1526  CD2 TYR C 421      27.767  65.962  52.435  1.00 20.01
C
ATOM   1527  CE1 TYR C 421      26.684  67.467  50.398  1.00 20.01
C
ATOM   1528  CE2 TYR C 421      26.696  66.798  52.687  1.00 20.01
C
ATOM   1529  CZ  TYR C 421      26.161  67.543  51.666  1.00 20.01
C
ATOM   1530  OH  TYR C 421      25.094  68.373  51.917  1.00 20.01
O
ATOM   1531  N   ASN C 422      30.701  62.109  49.322  1.00 14.94
N
ATOM   1532  CA  ASN C 422      31.929  61.317  49.305  1.00 14.94
C
ATOM   1533  C   ASN C 422      32.087  60.469  48.051  1.00 14.94
C
ATOM   1534  O   ASN C 422      33.094  60.601  47.355  1.00 14.94
O
ATOM   1535  CB  ASN C 422      31.984  60.436  50.556  1.00 14.94
C
ATOM   1536  CG  ASN C 422      32.105  61.245  51.826  1.00 14.94
C
ATOM   1537  OD1 ASN C 422      33.190  61.403  52.360  1.00 14.94
O
ATOM   1538  ND2 ASN C 422      30.986  61.759  52.313  1.00 14.94
N
ATOM   1539  N   TYR C 423      31.128  59.606  47.737  1.00 12.60
N
ATOM   1540  CA  TYR C 423      31.268  58.683  46.614  1.00 12.60
C
ATOM   1541  C   TYR C 423      29.888  58.412  46.042  1.00 12.60
C
ATOM   1542  O   TYR C 423      29.019  57.890  46.740  1.00 12.60
O
```

```
ATOM   1543  CB  TYR C 423      31.941  57.380  47.059  1.00 12.60
C
ATOM   1544  CG  TYR C 423      32.128  56.351  45.964  1.00 12.60
C
ATOM   1545  CD1 TYR C 423      33.153  56.471  45.039  1.00 12.60
C
ATOM   1546  CD2 TYR C 423      31.289  55.255  45.867  1.00 12.60
C
ATOM   1547  CE1 TYR C 423      33.332  55.528  44.045  1.00 12.60
C
ATOM   1548  CE2 TYR C 423      31.462  54.309  44.873  1.00 12.60
C
ATOM   1549  CZ  TYR C 423      32.485  54.452  43.968  1.00 12.60
C
ATOM   1550  OH  TYR C 423      32.662  53.510  42.982  1.00 12.60
O
ATOM   1551  N   LYS C 424      29.687  58.771  44.778  1.00 16.56
N
ATOM   1552  CA  LYS C 424      28.382  58.691  44.136  1.00 16.56
C
ATOM   1553  C   LYS C 424      28.456  57.777  42.921  1.00 16.56
C
ATOM   1554  O   LYS C 424      29.327  57.948  42.067  1.00 16.56
O
ATOM   1555  CB  LYS C 424      27.898  60.086  43.725  1.00 16.56
C
ATOM   1556  CG  LYS C 424      26.573  60.106  42.976  1.00 16.56
C
ATOM   1557  CD  LYS C 424      25.438  59.649  43.857  1.00 16.56
C
ATOM   1558  CE  LYS C 424      24.088  59.953  43.227  1.00 16.56
C
ATOM   1559  NZ  LYS C 424      22.985  59.400  44.047  1.00 16.56
N
ATOM   1560  N   LEU C 425      27.552  56.820  42.851  1.00 18.45
N
ATOM   1561  CA  LEU C 425      27.407  55.942  41.699  1.00 18.45
C
ATOM   1562  C   LEU C 425      26.375  56.504  40.724  1.00 18.45
C
ATOM   1563  O   LEU C 425      25.422  57.164  41.137  1.00 18.45
O
ATOM   1564  CB  LEU C 425      26.973  54.548  42.140  1.00 18.45
C
ATOM   1565  CG  LEU C 425      27.980  53.697  42.916  1.00 18.45
C
ATOM   1566  CD1 LEU C 425      27.375  52.358  43.274  1.00 18.45
C
ATOM   1567  CD2 LEU C 425      29.243  53.508  42.106  1.00 18.45
C
```

ATOM 1568 N PRO C 426 26.545 56.255 39.427 1.00 23.31
N
ATOM 1569 CA PRO C 426 25.524 56.662 38.455 1.00 23.31
C
ATOM 1570 C PRO C 426 24.224 55.897 38.647 1.00 23.31
C
ATOM 1571 O PRO C 426 24.180 54.817 39.235 1.00 23.31
O
ATOM 1572 CB PRO C 426 26.159 56.324 37.102 1.00 23.31
C
ATOM 1573 CG PRO C 426 27.597 56.218 37.351 1.00 23.31
C
ATOM 1574 CD PRO C 426 27.766 55.765 38.773 1.00 23.31
C
ATOM 1575 N ASP C 427 23.143 56.488 38.131 1.00 28.74
N
ATOM 1576 CA ASP C 427 21.839 55.835 38.196 1.00 28.74
C
ATOM 1577 C ASP C 427 21.789 54.574 37.343 1.00 28.74
C
ATOM 1578 O ASP C 427 21.111 53.610 37.710 1.00 28.74
O
ATOM 1579 CB ASP C 427 20.746 56.807 37.760 1.00 28.74
C
ATOM 1580 CG ASP C 427 20.553 57.941 38.738 1.00 28.74
C
ATOM 1581 OD1 ASP C 427 21.143 57.894 39.833 1.00 28.74
O
ATOM 1582 OD2 ASP C 427 19.811 58.884 38.411 1.00 28.74
O
ATOM 1583 N ASP C 428 22.503 54.555 36.219 1.00 30.12
N
ATOM 1584 CA ASP C 428 22.519 53.423 35.292 1.00 30.12
C
ATOM 1585 C ASP C 428 23.780 52.582 35.445 1.00 30.12
C
ATOM 1586 O ASP C 428 24.356 52.112 34.463 1.00 30.12
O
ATOM 1587 CB ASP C 428 22.370 53.917 33.857 1.00 30.12
C
ATOM 1588 CG ASP C 428 21.080 54.678 33.636 1.00 30.12
C
ATOM 1589 OD1 ASP C 428 20.018 54.182 34.060 1.00 30.12
O
ATOM 1590 OD2 ASP C 428 21.131 55.773 33.043 1.00 30.12
O
ATOM 1591 N PHE C 429 24.214 52.383 36.685 1.00 22.48
N
ATOM 1592 CA PHE C 429 25.493 51.754 36.990 1.00 22.48
C

ATOM 1593 C PHE C 429 25.516 50.281 36.582 1.00 22.48
C
ATOM 1594 O PHE C 429 24.567 49.538 36.841 1.00 22.48
O
ATOM 1595 CB PHE C 429 25.754 51.915 38.492 1.00 22.48
C
ATOM 1596 CG PHE C 429 26.917 51.128 39.020 1.00 22.48
C
ATOM 1597 CD1 PHE C 429 28.212 51.501 38.732 1.00 22.48
C
ATOM 1598 CD2 PHE C 429 26.709 50.046 39.849 1.00 22.48
C
ATOM 1599 CE1 PHE C 429 29.275 50.792 39.239 1.00 22.48
C
ATOM 1600 CE2 PHE C 429 27.772 49.335 40.356 1.00 22.48
C
ATOM 1601 CZ PHE C 429 29.052 49.710 40.047 1.00 22.48
C
ATOM 1602 N THR C 430 26.613 49.859 35.954 1.00 23.67
N
ATOM 1603 CA THR C 430 26.867 48.457 35.637 1.00 23.67
C
ATOM 1604 C THR C 430 28.140 48.024 36.351 1.00 23.67
C
ATOM 1605 O THR C 430 29.208 48.589 36.107 1.00 23.67
O
ATOM 1606 CB THR C 430 27.010 48.243 34.126 1.00 23.67
C
ATOM 1607 OG1 THR C 430 25.818 48.675 33.464 1.00 23.67
O
ATOM 1608 CG2 THR C 430 27.250 46.777 33.814 1.00 23.67
C
ATOM 1609 N GLY C 431 28.032 47.021 37.208 1.00 21.79
N
ATOM 1610 CA GLY C 431 29.184 46.558 37.953 1.00 21.79
C
ATOM 1611 C GLY C 431 28.749 45.818 39.201 1.00 21.79
C
ATOM 1612 O GLY C 431 27.572 45.614 39.439 1.00 21.79
O
ATOM 1613 N CYS C 432 29.732 45.448 40.005 1.00 19.61
N
ATOM 1614 CA CYS C 432 29.482 44.707 41.228 1.00 19.61
C
ATOM 1615 C CYS C 432 30.074 45.445 42.419 1.00 19.61
C
ATOM 1616 O CYS C 432 31.111 46.095 42.320 1.00 19.61
O
ATOM 1617 CB CYS C 432 30.058 43.285 41.157 1.00 19.61
C

ATOM 1618 SG CYS C 432 29.386 42.286 39.791 1.00 19.61
S
ATOM 1619 N VAL C 433 29.392 45.338 43.547 1.00 15.67
N
ATOM 1620 CA VAL C 433 29.855 45.871 44.818 1.00 15.67
C
ATOM 1621 C VAL C 433 30.144 44.692 45.728 1.00 15.67
C
ATOM 1622 O VAL C 433 29.275 43.841 45.942 1.00 15.67
O
ATOM 1623 CB VAL C 433 28.811 46.809 45.448 1.00 15.67
C
ATOM 1624 CG1 VAL C 433 29.296 47.323 46.799 1.00 15.67
C
ATOM 1625 CG2 VAL C 433 28.508 47.950 44.516 1.00 15.67
C
ATOM 1626 N ILE C 434 31.353 44.634 46.263 1.00 15.85
N
ATOM 1627 CA ILE C 434 31.747 43.566 47.167 1.00 15.85
C
ATOM 1628 C ILE C 434 32.347 44.186 48.417 1.00 15.85
C
ATOM 1629 O ILE C 434 33.085 45.173 48.342 1.00 15.85
O
ATOM 1630 CB ILE C 434 32.723 42.576 46.491 1.00 15.85
C
ATOM 1631 CG1 ILE C 434 32.967 41.357 47.386 1.00 15.85
C
ATOM 1632 CG2 ILE C 434 33.983 43.260 46.062 1.00 15.85
C
ATOM 1633 CD1 ILE C 434 32.996 40.052 46.654 1.00 15.85
C
ATOM 1634 N ALA C 435 31.990 43.638 49.573 1.00 15.92
N
ATOM 1635 CA ALA C 435 32.423 44.228 50.829 1.00 15.92
C
ATOM 1636 C ALA C 435 32.631 43.136 51.864 1.00 15.92
C
ATOM 1637 O ALA C 435 31.952 42.104 51.843 1.00 15.92
O
ATOM 1638 CB ALA C 435 31.407 45.252 51.340 1.00 15.92
C
ATOM 1639 N TRP C 436 33.562 43.388 52.782 1.00 15.31
N
ATOM 1640 CA TRP C 436 33.845 42.421 53.836 1.00 15.31
C
ATOM 1641 C TRP C 436 34.234 43.132 55.123 1.00 15.31
C
ATOM 1642 O TRP C 436 34.838 44.204 55.102 1.00 15.31
O

ATOM 1643 CB TRP C 436 34.939 41.425 53.412 1.00 15.31
C
ATOM 1644 CG TRP C 436 36.313 41.997 53.168 1.00 15.31
C
ATOM 1645 CD1 TRP C 436 37.369 41.964 54.021 1.00 15.31
C
ATOM 1646 CD2 TRP C 436 36.783 42.639 51.977 1.00 15.31
C
ATOM 1647 NE1 TRP C 436 38.462 42.558 53.449 1.00 15.31
N
ATOM 1648 CE2 TRP C 436 38.128 42.982 52.192 1.00 15.31
C
ATOM 1649 CE3 TRP C 436 36.192 42.968 50.757 1.00 15.31
C
ATOM 1650 CZ2 TRP C 436 38.891 43.637 51.235 1.00 15.31
C
ATOM 1651 CZ3 TRP C 436 36.953 43.618 49.808 1.00 15.31
C
ATOM 1652 CH2 TRP C 436 38.288 43.942 50.051 1.00 15.31
C
ATOM 1653 N ASN C 437 33.884 42.510 56.249 1.00 18.59
N
ATOM 1654 CA ASN C 437 34.202 43.067 57.556 1.00 18.59
C
ATOM 1655 C ASN C 437 35.706 43.054 57.793 1.00 18.59
C
ATOM 1656 O ASN C 437 36.395 42.088 57.463 1.00 18.59
O
ATOM 1657 CB ASN C 437 33.483 42.280 58.657 1.00 18.59
C
ATOM 1658 CG ASN C 437 33.714 42.862 60.036 1.00 18.59
C
ATOM 1659 OD1 ASN C 437 34.784 42.714 60.612 1.00 18.59
O
ATOM 1660 ND2 ASN C 437 32.710 43.529 60.567 1.00 18.59
N
ATOM 1661 N SER C 438 36.214 44.135 58.376 1.00 20.64
N
ATOM 1662 CA SER C 438 37.645 44.340 58.552 1.00 20.64
C
ATOM 1663 C SER C 438 37.976 44.736 59.987 1.00 20.64
C
ATOM 1664 O SER C 438 38.953 45.442 60.229 1.00 20.64
O
ATOM 1665 CB SER C 438 38.150 45.399 57.572 1.00 20.64
C
ATOM 1666 OG SER C 438 39.553 45.388 57.478 1.00 20.64
O
ATOM 1667 N ASN C 439 37.171 44.272 60.944 1.00 25.50
N

ATOM 1668 CA ASN C 439 37.271 44.753 62.320 1.00 25.50
C
ATOM 1669 C ASN C 439 38.646 44.484 62.922 1.00 25.50
C
ATOM 1670 O ASN C 439 39.212 45.350 63.596 1.00 25.50
O
ATOM 1671 CB ASN C 439 36.182 44.105 63.168 1.00 25.50
C
ATOM 1672 CG ASN C 439 36.081 44.714 64.548 1.00 25.50
C
ATOM 1673 OD1 ASN C 439 36.419 45.877 64.756 1.00 25.50
O
ATOM 1674 ND2 ASN C 439 35.619 43.923 65.504 1.00 25.50
N
ATOM 1675 N ASN C 440 39.199 43.294 62.687 1.00 28.43
N
ATOM 1676 CA ASN C 440 40.505 42.958 63.248 1.00 28.43
C
ATOM 1677 C ASN C 440 41.610 43.858 62.715 1.00 28.43
C
ATOM 1678 O ASN C 440 42.579 44.136 63.427 1.00 28.43
O
ATOM 1679 CB ASN C 440 40.836 41.497 62.954 1.00 28.43
C
ATOM 1680 CG ASN C 440 39.913 40.545 63.667 1.00 28.43
C
ATOM 1681 OD1 ASN C 440 39.586 40.735 64.837 1.00 28.43
O
ATOM 1682 ND2 ASN C 440 39.490 39.502 62.966 1.00 28.43
N
ATOM 1683 N LEU C 441 41.488 44.325 61.476 1.00 23.03
N
ATOM 1684 CA LEU C 441 42.561 45.084 60.851 1.00 23.03
C
ATOM 1685 C LEU C 441 42.439 46.586 61.060 1.00 23.03
C
ATOM 1686 O LEU C 441 43.448 47.262 61.271 1.00 23.03
O
ATOM 1687 CB LEU C 441 42.609 44.788 59.350 1.00 23.03
C
ATOM 1688 CG LEU C 441 42.579 43.325 58.922 1.00 23.03
C
ATOM 1689 CD1 LEU C 441 42.544 43.215 57.417 1.00 23.03
C
ATOM 1690 CD2 LEU C 441 43.788 42.587 59.481 1.00 23.03
C
ATOM 1691 N ASP C 442 41.227 47.133 60.994 1.00 22.63
N
ATOM 1692 CA ASP C 442 41.046 48.571 60.874 1.00 22.63
C

ATOM 1693 C ASP C 442 40.531 49.241 62.139 1.00 22.63
C
ATOM 1694 O ASP C 442 40.315 50.455 62.132 1.00 22.63
O
ATOM 1695 CB ASP C 442 40.105 48.871 59.702 1.00 22.63
C
ATOM 1696 CG ASP C 442 40.761 48.616 58.366 1.00 22.63
C
ATOM 1697 OD1 ASP C 442 41.937 48.986 58.210 1.00 22.63
O
ATOM 1698 OD2 ASP C 442 40.109 48.040 57.478 1.00 22.63
O
ATOM 1699 N SER C 443 40.348 48.502 63.221 1.00 29.81
N
ATOM 1700 CA SER C 443 39.953 49.086 64.493 1.00 29.81
C
ATOM 1701 C SER C 443 41.153 49.161 65.430 1.00 29.81
C
ATOM 1702 O SER C 443 42.036 48.302 65.406 1.00 29.81
O
ATOM 1703 CB SER C 443 38.839 48.270 65.140 1.00 29.81
C
ATOM 1704 OG SER C 443 39.360 47.079 65.692 1.00 29.81
O
ATOM 1705 N LYS C 444 41.179 50.206 66.252 1.00 34.62
N
ATOM 1706 CA LYS C 444 42.234 50.408 67.234 1.00 34.62
C
ATOM 1707 C LYS C 444 41.616 50.664 68.601 1.00 34.62
C
ATOM 1708 O LYS C 444 40.562 51.293 68.714 1.00 34.62
O
ATOM 1709 CB LYS C 444 43.140 51.583 66.858 1.00 34.62
C
ATOM 1710 CG LYS C 444 43.995 51.357 65.629 1.00 34.62
C
ATOM 1711 CD LYS C 444 44.692 50.013 65.675 1.00 34.62
C
ATOM 1712 CE LYS C 444 45.976 50.037 64.866 1.00 34.62
C
ATOM 1713 NZ LYS C 444 46.079 51.263 64.031 1.00 34.62
N
ATOM 1714 N VAL C 445 42.289 50.174 69.643 1.00 38.02
N
ATOM 1715 CA VAL C 445 41.843 50.426 71.008 1.00 38.02
C
ATOM 1716 C VAL C 445 41.987 51.910 71.310 1.00 38.02
C
ATOM 1717 O VAL C 445 43.035 52.514 71.052 1.00 38.02
O

```
ATOM   1718  CB  VAL C 445      42.636  49.567  72.002  1.00 38.02
C
ATOM   1719  CG1 VAL C 445      42.511  50.126  73.407  1.00 38.02
C
ATOM   1720  CG2 VAL C 445      42.149  48.130  71.956  1.00 38.02
C
ATOM   1721  N   GLY C 446      40.929  52.509  71.847  1.00 37.02
N
ATOM   1722  CA  GLY C 446      40.888  53.942  72.017  1.00 37.02
C
ATOM   1723  C   GLY C 446      40.424  54.710  70.804  1.00 37.02
C
ATOM   1724  O   GLY C 446      40.414  55.946  70.840  1.00 37.02
O
ATOM   1725  N   GLY C 447      40.048  54.024  69.728  1.00 34.62
N
ATOM   1726  CA  GLY C 447      39.513  54.682  68.555  1.00 34.62
C
ATOM   1727  C   GLY C 447      40.533  54.925  67.465  1.00 34.62
C
ATOM   1728  O   GLY C 447      41.477  55.696  67.647  1.00 34.62
O
ATOM   1729  N   ASN C 448      40.350  54.271  66.322  1.00 30.72
N
ATOM   1730  CA  ASN C 448      41.210  54.521  65.176  1.00 30.72
C
ATOM   1731  C   ASN C 448      40.945  55.914  64.625  1.00 30.72
C
ATOM   1732  O   ASN C 448      39.805  56.377  64.579  1.00 30.72
O
ATOM   1733  CB  ASN C 448      40.975  53.464  64.099  1.00 30.72
C
ATOM   1734  CG  ASN C 448      42.033  53.484  63.014  1.00 30.72
C
ATOM   1735  OD1 ASN C 448      42.851  54.395  62.942  1.00 30.72
O
ATOM   1736  ND2 ASN C 448      42.013  52.474  62.158  1.00 30.72
N
ATOM   1737  N   TYR C 449      42.009  56.585  64.208  1.00 36.23
N
ATOM   1738  CA  TYR C 449      41.933  57.962  63.748  1.00 36.23
C
ATOM   1739  C   TYR C 449      42.045  58.086  62.230  1.00 36.23
C
ATOM   1740  O   TYR C 449      41.901  59.189  61.697  1.00 36.23
O
ATOM   1741  CB  TYR C 449      43.020  58.775  64.470  1.00 36.23
C
ATOM   1742  CG  TYR C 449      43.527  60.016  63.782  1.00 36.23
C
```

ATOM 1743 CD1 TYR C 449 42.781 61.186 63.776 1.00 36.23
C
ATOM 1744 CD2 TYR C 449 44.766 60.025 63.157 1.00 36.23
C
ATOM 1745 CE1 TYR C 449 43.249 62.326 63.152 1.00 36.23
C
ATOM 1746 CE2 TYR C 449 45.242 61.158 62.533 1.00 36.23
C
ATOM 1747 CZ TYR C 449 44.481 62.306 62.533 1.00 36.23
C
ATOM 1748 OH TYR C 449 44.953 63.438 61.911 1.00 36.23
O
ATOM 1749 N ASN C 450 42.217 56.975 61.514 1.00 28.99
N
ATOM 1750 CA ASN C 450 42.622 57.041 60.112 1.00 28.99
C
ATOM 1751 C ASN C 450 41.483 57.437 59.173 1.00 28.99
C
ATOM 1752 O ASN C 450 41.570 58.451 58.475 1.00 28.99
O
ATOM 1753 CB ASN C 450 43.222 55.702 59.684 1.00 28.99
C
ATOM 1754 CG ASN C 450 44.733 55.725 59.685 1.00 28.99
C
ATOM 1755 OD1 ASN C 450 45.343 56.763 59.446 1.00 28.99
O
ATOM 1756 ND2 ASN C 450 45.344 54.582 59.948 1.00 28.99
N
ATOM 1757 N TYR C 451 40.421 56.647 59.125 1.00 19.56
N
ATOM 1758 CA TYR C 451 39.458 56.721 58.029 1.00 19.56
C
ATOM 1759 C TYR C 451 38.448 57.844 58.259 1.00 19.56
C
ATOM 1760 O TYR C 451 37.757 57.860 59.281 1.00 19.56
O
ATOM 1761 CB TYR C 451 38.760 55.373 57.895 1.00 19.56
C
ATOM 1762 CG TYR C 451 39.721 54.235 57.644 1.00 19.56
C
ATOM 1763 CD1 TYR C 451 40.350 54.095 56.421 1.00 19.56
C
ATOM 1764 CD2 TYR C 451 40.003 53.306 58.633 1.00 19.56
C
ATOM 1765 CE1 TYR C 451 41.226 53.062 56.184 1.00 19.56
C
ATOM 1766 CE2 TYR C 451 40.878 52.268 58.405 1.00 19.56
C
ATOM 1767 CZ TYR C 451 41.486 52.151 57.179 1.00 19.56
C

ATOM 1768 OH TYR C 451 42.354 51.115 56.949 1.00 19.56
O
ATOM 1769 N LEU C 452 38.349 58.771 57.307 1.00 18.47
N
ATOM 1770 CA LEU C 452 37.510 59.956 57.439 1.00 18.47
C
ATOM 1771 C LEU C 452 36.465 60.019 56.329 1.00 18.47
C
ATOM 1772 O LEU C 452 36.662 59.482 55.239 1.00 18.47
O
ATOM 1773 CB LEU C 452 38.348 61.242 57.401 1.00 18.47
C
ATOM 1774 CG LEU C 452 39.435 61.416 58.460 1.00 18.47
C
ATOM 1775 CD1 LEU C 452 40.780 61.413 57.799 1.00 18.47
C
ATOM 1776 CD2 LEU C 452 39.225 62.706 59.218 1.00 18.47
C
ATOM 1777 N TYR C 453 35.353 60.691 56.617 1.00 18.27
N
ATOM 1778 CA TYR C 453 34.311 60.965 55.639 1.00 18.27
C
ATOM 1779 C TYR C 453 33.983 62.452 55.658 1.00 18.27
C
ATOM 1780 O TYR C 453 34.092 63.108 56.693 1.00 18.27
O
ATOM 1781 CB TYR C 453 33.039 60.125 55.904 1.00 18.27
C
ATOM 1782 CG TYR C 453 32.353 60.378 57.232 1.00 18.27
C
ATOM 1783 CD1 TYR C 453 31.233 61.186 57.312 1.00 18.27
C
ATOM 1784 CD2 TYR C 453 32.810 59.783 58.395 1.00 18.27
C
ATOM 1785 CE1 TYR C 453 30.599 61.408 58.518 1.00 18.27
C
ATOM 1786 CE2 TYR C 453 32.182 60.003 59.605 1.00 18.27
C
ATOM 1787 CZ TYR C 453 31.080 60.814 59.656 1.00 18.27
C
ATOM 1788 OH TYR C 453 30.454 61.033 60.861 1.00 18.27
O
ATOM 1789 N ARG C 454 33.594 62.987 54.504 1.00 20.12
N
ATOM 1790 CA ARG C 454 33.233 64.398 54.410 1.00 20.12
C
ATOM 1791 C ARG C 454 31.803 64.586 54.896 1.00 20.12
C
ATOM 1792 O ARG C 454 30.873 63.980 54.360 1.00 20.12
O

ATOM 1793 CB ARG C 454 33.380 64.912 52.980 1.00 20.12
C
ATOM 1794 CG ARG C 454 33.180 66.428 52.848 1.00 20.12
C
ATOM 1795 CD ARG C 454 33.333 66.908 51.417 1.00 20.12
C
ATOM 1796 NE ARG C 454 34.640 66.557 50.877 1.00 20.12
N
ATOM 1797 CZ ARG C 454 35.747 67.267 51.057 1.00 20.12
C
ATOM 1798 NH1 ARG C 454 35.717 68.393 51.754 1.00 20.12
N
ATOM 1799 NH2 ARG C 454 36.885 66.851 50.535 1.00 20.12
N
ATOM 1800 N LEU C 455 31.625 65.438 55.901 1.00 22.80
N
ATOM 1801 CA LEU C 455 30.314 65.684 56.483 1.00 22.80
C
ATOM 1802 C LEU C 455 29.626 66.921 55.921 1.00 22.80
C
ATOM 1803 O LEU C 455 28.396 66.955 55.870 1.00 22.80
O
ATOM 1804 CB LEU C 455 30.441 65.808 58.005 1.00 22.80
C
ATOM 1805 CG LEU C 455 29.183 65.933 58.864 1.00 22.80
C
ATOM 1806 CD1 LEU C 455 28.204 64.816 58.577 1.00 22.80
C
ATOM 1807 CD2 LEU C 455 29.564 65.932 60.328 1.00 22.80
C
ATOM 1808 N PHE C 456 30.381 67.923 55.474 1.00 27.83
N
ATOM 1809 CA PHE C 456 29.817 69.164 54.956 1.00 27.83
C
ATOM 1810 C PHE C 456 30.368 69.458 53.569 1.00 27.83
C
ATOM 1811 O PHE C 456 31.565 69.299 53.325 1.00 27.83
O
ATOM 1812 CB PHE C 456 30.127 70.356 55.878 1.00 27.83
C
ATOM 1813 CG PHE C 456 29.667 70.171 57.291 1.00 27.83
C
ATOM 1814 CD1 PHE C 456 28.336 70.315 57.623 1.00 27.83
C
ATOM 1815 CD2 PHE C 456 30.567 69.856 58.286 1.00 27.83
C
ATOM 1816 CE1 PHE C 456 27.914 70.149 58.922 1.00 27.83
C
ATOM 1817 CE2 PHE C 456 30.149 69.689 59.585 1.00 27.83
C

ATOM 1818 CZ PHE C 456 28.820 69.834 59.903 1.00 27.83
C
ATOM 1819 N ARG C 457 29.491 69.896 52.669 1.00 27.52
N
ATOM 1820 CA ARG C 457 29.904 70.380 51.360 1.00 27.52
C
ATOM 1821 C ARG C 457 28.846 71.340 50.831 1.00 27.52
C
ATOM 1822 O ARG C 457 27.669 71.234 51.181 1.00 27.52
O
ATOM 1823 CB ARG C 457 30.124 69.231 50.371 1.00 27.52
C
ATOM 1824 CG ARG C 457 31.006 69.600 49.202 1.00 27.52
C
ATOM 1825 CD ARG C 457 31.250 68.430 48.273 1.00 27.52
C
ATOM 1826 NE ARG C 457 32.258 68.764 47.276 1.00 27.52
N
ATOM 1827 CZ ARG C 457 31.983 69.098 46.022 1.00 27.52
C
ATOM 1828 NH1 ARG C 457 30.729 69.136 45.603 1.00 27.52
N
ATOM 1829 NH2 ARG C 457 32.964 69.385 45.185 1.00 27.52
N
ATOM 1830 N LYS C 458 29.278 72.267 49.972 1.00 35.49
N
ATOM 1831 CA LYS C 458 28.361 73.270 49.433 1.00 35.49
C
ATOM 1832 C LYS C 458 27.338 72.645 48.492 1.00 35.49
C
ATOM 1833 O LYS C 458 26.137 72.914 48.599 1.00 35.49
O
ATOM 1834 CB LYS C 458 29.143 74.368 48.712 1.00 35.49
C
ATOM 1835 CG LYS C 458 30.029 75.209 49.610 1.00 35.49
C
ATOM 1836 CD LYS C 458 29.355 75.516 50.927 1.00 35.49
C
ATOM 1837 CE LYS C 458 28.335 76.629 50.786 1.00 35.49
C
ATOM 1838 NZ LYS C 458 28.425 77.595 51.913 1.00 35.49
N
ATOM 1839 N SER C 459 27.794 71.820 47.556 1.00 31.43
N
ATOM 1840 CA SER C 459 26.916 71.159 46.605 1.00 31.43
C
ATOM 1841 C SER C 459 27.277 69.683 46.522 1.00 31.43
C
ATOM 1842 O SER C 459 28.361 69.265 46.929 1.00 31.43
O

ATOM 1843 CB SER C 459 27.002 71.797 45.216 1.00 31.43
C
ATOM 1844 OG SER C 459 28.341 71.851 44.768 1.00 31.43
O
ATOM 1845 N ASN C 460 26.349 68.901 45.982 1.00 27.26
N
ATOM 1846 CA ASN C 460 26.527 67.459 45.892 1.00 27.26
C
ATOM 1847 C ASN C 460 27.600 67.091 44.874 1.00 27.26
C
ATOM 1848 O ASN C 460 27.783 67.761 43.857 1.00 27.26
O
ATOM 1849 CB ASN C 460 25.209 66.791 45.516 1.00 27.26
C
ATOM 1850 CG ASN C 460 24.149 66.960 46.579 1.00 27.26
C
ATOM 1851 OD1 ASN C 460 24.389 67.565 47.618 1.00 27.26
O
ATOM 1852 ND2 ASN C 460 22.970 66.418 46.325 1.00 27.26
N
ATOM 1853 N LEU C 461 28.316 66.004 45.163 1.00 23.51
N
ATOM 1854 CA LEU C 461 29.314 65.473 44.247 1.00 23.51
C
ATOM 1855 C LEU C 461 28.642 64.753 43.088 1.00 23.51
C
ATOM 1856 O LEU C 461 27.644 64.055 43.269 1.00 23.51
O
ATOM 1857 CB LEU C 461 30.244 64.498 44.973 1.00 23.51
C
ATOM 1858 CG LEU C 461 31.590 64.973 45.507 1.00 23.51
C
ATOM 1859 CD1 LEU C 461 32.344 63.791 46.098 1.00 23.51
C
ATOM 1860 CD2 LEU C 461 32.417 65.642 44.437 1.00 23.51
C
ATOM 1861 N LYS C 462 29.202 64.912 41.900 1.00 24.28
N
ATOM 1862 CA LYS C 462 28.701 64.211 40.732 1.00 24.28
C
ATOM 1863 C LYS C 462 29.217 62.774 40.699 1.00 24.28
C
ATOM 1864 O LYS C 462 30.212 62.451 41.346 1.00 24.28
O
ATOM 1865 CB LYS C 462 29.113 64.954 39.466 1.00 24.28
C
ATOM 1866 CG LYS C 462 28.495 66.340 39.342 1.00 24.28
C
ATOM 1867 CD LYS C 462 28.570 66.853 37.917 1.00 24.28
C

ATOM 1868 CE LYS C 462 30.008 67.017 37.468 1.00 24.28
C
ATOM 1869 NZ LYS C 462 30.772 67.912 38.374 1.00 24.28
N
ATOM 1870 N PRO C 463 28.543 61.891 39.962 1.00 21.90
N
ATOM 1871 CA PRO C 463 29.006 60.503 39.853 1.00 21.90
C
ATOM 1872 C PRO C 463 30.470 60.393 39.447 1.00 21.90
C
ATOM 1873 O PRO C 463 30.914 60.998 38.473 1.00 21.90
O
ATOM 1874 CB PRO C 463 28.081 59.920 38.783 1.00 21.90
C
ATOM 1875 CG PRO C 463 26.815 60.661 38.975 1.00 21.90
C
ATOM 1876 CD PRO C 463 27.192 62.055 39.396 1.00 21.90
C
ATOM 1877 N PHE C 464 31.217 59.604 40.221 1.00 20.55
N
ATOM 1878 CA PHE C 464 32.633 59.303 40.008 1.00 20.55
C
ATOM 1879 C PHE C 464 33.525 60.538 40.064 1.00 20.55
C
ATOM 1880 O PHE C 464 34.633 60.527 39.530 1.00 20.55
O
ATOM 1881 CB PHE C 464 32.853 58.543 38.697 1.00 20.55
C
ATOM 1882 CG PHE C 464 32.280 57.159 38.703 1.00 20.55
C
ATOM 1883 CD1 PHE C 464 32.399 56.352 39.820 1.00 20.55
C
ATOM 1884 CD2 PHE C 464 31.613 56.665 37.600 1.00 20.55
C
ATOM 1885 CE1 PHE C 464 31.871 55.084 39.831 1.00 20.55
C
ATOM 1886 CE2 PHE C 464 31.086 55.397 37.607 1.00 20.55
C
ATOM 1887 CZ PHE C 464 31.215 54.604 38.725 1.00 20.55
C
ATOM 1888 N GLU C 465 33.079 61.595 40.726 1.00 24.23
N
ATOM 1889 CA GLU C 465 33.907 62.758 40.995 1.00 24.23
C
ATOM 1890 C GLU C 465 34.442 62.677 42.420 1.00 24.23
C
ATOM 1891 O GLU C 465 33.771 62.171 43.320 1.00 24.23
O
ATOM 1892 CB GLU C 465 33.106 64.044 40.789 1.00 24.23
C

```
ATOM   1893  CG  GLU C 465      33.806  65.313  41.211  1.00 24.23
C
ATOM   1894  CD  GLU C 465      33.033  66.555  40.816  1.00 24.23
C
ATOM   1895  OE1 GLU C 465      33.607  67.417  40.122  1.00 24.23
O
ATOM   1896  OE2 GLU C 465      31.848  66.667  41.195  1.00 24.23
O
ATOM   1897  N   ARG C 466      35.664  63.158  42.616  1.00 21.04
N
ATOM   1898  CA  ARG C 466      36.341  63.060  43.900  1.00 21.04
C
ATOM   1899  C   ARG C 466      36.772  64.439  44.373  1.00 21.04
C
ATOM   1900  O   ARG C 466      37.277  65.243  43.588  1.00 21.04
O
ATOM   1901  CB  ARG C 466      37.555  62.137  43.810  1.00 21.04
C
ATOM   1902  CG  ARG C 466      38.207  61.842  45.139  1.00 21.04
C
ATOM   1903  CD  ARG C 466      39.244  60.754  44.999  1.00 21.04
C
ATOM   1904  NE  ARG C 466      40.363  61.193  44.176  1.00 21.04
N
ATOM   1905  CZ  ARG C 466      41.577  60.664  44.227  1.00 21.04
C
ATOM   1906  NH1 ARG C 466      41.836  59.674  45.065  1.00 21.04
N
ATOM   1907  NH2 ARG C 466      42.537  61.129  43.441  1.00 21.04
N
ATOM   1908  N   ASP C 467      36.580  64.705  45.662  1.00 23.47
N
ATOM   1909  CA  ASP C 467      36.950  65.976  46.275  1.00 23.47
C
ATOM   1910  C   ASP C 467      37.811  65.688  47.498  1.00 23.47
C
ATOM   1911  O   ASP C 467      37.322  65.135  48.485  1.00 23.47
O
ATOM   1912  CB  ASP C 467      35.704  66.778  46.653  1.00 23.47
C
ATOM   1913  CG  ASP C 467      36.025  68.183  47.120  1.00 23.47
C
ATOM   1914  OD1 ASP C 467      37.215  68.536  47.205  1.00 23.47
O
ATOM   1915  OD2 ASP C 467      35.075  68.932  47.408  1.00 23.47
O
ATOM   1916  N   ILE C 468      39.087  66.064  47.441  1.00 24.85
N
ATOM   1917  CA  ILE C 468      40.005  65.847  48.551  1.00 24.85
C
```

```
ATOM   1918  C   ILE C 468      40.316  67.139  49.294  1.00 24.85
C
ATOM   1919  O   ILE C 468      41.213  67.158  50.137  1.00 24.85
O
ATOM   1920  CB  ILE C 468      41.304  65.165  48.091  1.00 24.85
C
ATOM   1921  CG1 ILE C 468      41.934  65.931  46.929  1.00 24.85
C
ATOM   1922  CG2 ILE C 468      41.050  63.718  47.715  1.00 24.85
C
ATOM   1923  CD1 ILE C 468      43.381  65.578  46.703  1.00 24.85
C
ATOM   1924  N   SER C 469      39.588  68.215  49.014  1.00 29.02
N
ATOM   1925  CA  SER C 469      39.883  69.502  49.629  1.00 29.02
C
ATOM   1926  C   SER C 469      39.577  69.481  51.121  1.00 29.02
C
ATOM   1927  O   SER C 469      38.613  68.857  51.567  1.00 29.02
O
ATOM   1928  CB  SER C 469      39.083  70.608  48.949  1.00 29.02
C
ATOM   1929  OG  SER C 469      37.695  70.391  49.086  1.00 29.02
O
ATOM   1930  N   THR C 470      40.411  70.173  51.896  1.00 35.44
N
ATOM   1931  CA  THR C 470      40.250  70.264  53.340  1.00 35.44
C
ATOM   1932  C   THR C 470      39.895  71.676  53.794  1.00 35.44
C
ATOM   1933  O   THR C 470      40.018  71.989  54.979  1.00 35.44
O
ATOM   1934  CB  THR C 470      41.516  69.790  54.049  1.00 35.44
C
ATOM   1935  OG1 THR C 470      42.653  70.451  53.484  1.00 35.44
O
ATOM   1936  CG2 THR C 470      41.687  68.297  53.887  1.00 35.44
C
ATOM   1937  N   GLU C 471      39.443  72.524  52.875  1.00 44.82
N
ATOM   1938  CA  GLU C 471      39.149  73.912  53.203  1.00 44.82
C
ATOM   1939  C   GLU C 471      37.991  74.012  54.188  1.00 44.82
C
ATOM   1940  O   GLU C 471      37.049  73.218  54.157  1.00 44.82
O
ATOM   1941  CB  GLU C 471      38.820  74.693  51.935  1.00 44.82
C
ATOM   1942  CG  GLU C 471      39.947  74.729  50.930  1.00 44.82
C
```

ATOM 1943 CD GLU C 471 39.553 75.424 49.648 1.00 44.82
C
ATOM 1944 OE1 GLU C 471 40.201 75.173 48.612 1.00 44.82
O
ATOM 1945 OE2 GLU C 471 38.594 76.223 49.673 1.00 44.82
O
ATOM 1946 N ILE C 472 38.069 75.015 55.066 1.00 50.35
N
ATOM 1947 CA ILE C 472 37.063 75.192 56.104 1.00 50.35
C
ATOM 1948 C ILE C 472 35.698 75.479 55.485 1.00 50.35
C
ATOM 1949 O ILE C 472 35.585 76.114 54.431 1.00 50.35
O
ATOM 1950 CB ILE C 472 37.488 76.321 57.060 1.00 50.35
C
ATOM 1951 CG1 ILE C 472 38.914 76.083 57.545 1.00 50.35
C
ATOM 1952 CG2 ILE C 472 36.553 76.408 58.250 1.00 50.35
C
ATOM 1953 CD1 ILE C 472 39.024 74.979 58.564 1.00 50.35
C
ATOM 1954 N TYR C 473 34.651 74.999 56.148 1.00 48.20
N
ATOM 1955 CA TYR C 473 33.276 75.139 55.693 1.00 48.20
C
ATOM 1956 C TYR C 473 32.570 76.211 56.514 1.00 48.20
C
ATOM 1957 O TYR C 473 32.724 76.269 57.737 1.00 48.20
O
ATOM 1958 CB TYR C 473 32.540 73.799 55.808 1.00 48.20
C
ATOM 1959 CG TYR C 473 31.064 73.842 55.509 1.00 48.20
C
ATOM 1960 CD1 TYR C 473 30.604 73.839 54.207 1.00 48.20
C
ATOM 1961 CD2 TYR C 473 30.130 73.850 56.532 1.00 48.20
C
ATOM 1962 CE1 TYR C 473 29.261 73.862 53.931 1.00 48.20
C
ATOM 1963 CE2 TYR C 473 28.787 73.875 56.265 1.00 48.20
C
ATOM 1964 CZ TYR C 473 28.357 73.882 54.962 1.00 48.20
C
ATOM 1965 OH TYR C 473 27.014 73.904 54.685 1.00 48.20
O
ATOM 1966 N GLN C 474 31.791 77.052 55.838 1.00 63.59
N
ATOM 1967 CA GLN C 474 31.176 78.230 56.445 1.00 63.59
C

ATOM 1968 C GLN C 474 29.667 78.045 56.498 1.00 63.59
C
ATOM 1969 O GLN C 474 29.004 78.032 55.457 1.00 63.59
O
ATOM 1970 CB GLN C 474 31.531 79.489 55.661 1.00 63.59
C
ATOM 1971 CG GLN C 474 32.793 80.170 56.128 1.00 63.59
C
ATOM 1972 CD GLN C 474 33.905 80.066 55.120 1.00 63.59
C
ATOM 1973 OE1 GLN C 474 33.695 80.271 53.927 1.00 63.59
O
ATOM 1974 NE2 GLN C 474 35.101 79.743 55.592 1.00 63.59
N
ATOM 1975 N GLY C 475 29.125 77.921 57.709 1.00 76.33
N
ATOM 1976 CA GLY C 475 27.680 77.870 57.856 1.00 76.33
C
ATOM 1977 C GLY C 475 27.007 79.167 57.445 1.00 76.33
C
ATOM 1978 O GLY C 475 25.903 79.158 56.895 1.00 76.33
O
ATOM 1979 N SER C 476 27.660 80.298 57.707 1.00 91.44
N
ATOM 1980 CA SER C 476 27.158 81.602 57.297 1.00 91.44
C
ATOM 1981 C SER C 476 28.341 82.540 57.089 1.00 91.44
C
ATOM 1982 O SER C 476 29.503 82.153 57.240 1.00 91.44
O
ATOM 1983 CB SER C 476 26.174 82.170 58.325 1.00 91.44
C
ATOM 1984 OG SER C 476 25.633 83.404 57.885 1.00 91.44
O
ATOM 1985 N THR C 477 28.031 83.789 56.739 1.00 98.63
N
ATOM 1986 CA THR C 477 29.076 84.769 56.449 1.00 98.63
C
ATOM 1987 C THR C 477 29.979 85.088 57.638 1.00 98.63
C
ATOM 1988 O THR C 477 31.210 85.076 57.463 1.00 98.63
O
ATOM 1989 CB THR C 477 28.435 86.040 55.880 1.00 98.63
C
ATOM 1990 OG1 THR C 477 27.215 86.321 56.576 1.00 98.63
O
ATOM 1991 CG2 THR C 477 28.136 85.863 54.401 1.00 98.63
C
ATOM 1992 N PRO C 478 29.469 85.379 58.857 1.00 99.32
N

```
ATOM   1993  CA  PRO C 478      30.372  85.846  59.920  1.00 99.32
C
ATOM   1994  C   PRO C 478      31.273  84.747  60.459  1.00 99.32
C
ATOM   1995  O   PRO C 478      31.038  84.216  61.549  1.00 99.32
O
ATOM   1996  CB  PRO C 478      29.405  86.353  60.994  1.00 99.32
C
ATOM   1997  CG  PRO C 478      28.222  85.479  60.835  1.00 99.32
C
ATOM   1998  CD  PRO C 478      28.083  85.265  59.352  1.00 99.32
C
ATOM   1999  N   CYS C 479      32.316  84.405  59.709  1.00 93.30
N
ATOM   2000  CA  CYS C 479      33.175  83.280  60.036  1.00 93.30
C
ATOM   2001  C   CYS C 479      34.614  83.767  59.969  1.00 93.30
C
ATOM   2002  O   CYS C 479      35.078  84.191  58.905  1.00 93.30
O
ATOM   2003  CB  CYS C 479      32.933  82.138  59.046  1.00 93.30
C
ATOM   2004  SG  CYS C 479      33.010  80.428  59.634  1.00 93.30
S
ATOM   2005  N   ASN C 480      35.315  83.718  61.098  1.00 97.56
N
ATOM   2006  CA  ASN C 480      36.671  84.245  61.205  1.00 97.56
C
ATOM   2007  C   ASN C 480      37.641  83.087  61.387  1.00 97.56
C
ATOM   2008  O   ASN C 480      37.532  82.328  62.357  1.00 97.56
O
ATOM   2009  CB  ASN C 480      36.786  85.238  62.361  1.00 97.56
C
ATOM   2010  CG  ASN C 480      37.310  86.587  61.917  1.00 97.56
C
ATOM   2011  OD1 ASN C 480      37.845  86.726  60.817  1.00 97.56
O
ATOM   2012  ND2 ASN C 480      37.166  87.589  62.775  1.00 97.56
N
ATOM   2013  N   GLY C 481      38.591  82.968  60.466  1.00 95.10
N
ATOM   2014  CA  GLY C 481      39.598  81.926  60.564  1.00 95.10
C
ATOM   2015  C   GLY C 481      38.949  80.559  60.603  1.00 95.10
C
ATOM   2016  O   GLY C 481      38.215  80.163  59.690  1.00 95.10
O
ATOM   2017  N   VAL C 482      39.215  79.825  61.678  1.00 93.70
N
```

ATOM 2018 CA VAL C 482 38.601 78.524 61.891 1.00 93.70
C
ATOM 2019 C VAL C 482 37.728 78.486 63.140 1.00 93.70
C
ATOM 2020 O VAL C 482 37.022 77.487 63.348 1.00 93.70
O
ATOM 2021 CB VAL C 482 39.668 77.412 61.949 1.00 93.70
C
ATOM 2022 CG1 VAL C 482 40.551 77.472 60.716 1.00 93.70
C
ATOM 2023 CG2 VAL C 482 40.507 77.557 63.197 1.00 93.70
C
ATOM 2024 N GLU C 483 37.749 79.526 63.971 1.00 98.63
N
ATOM 2025 CA GLU C 483 36.904 79.630 65.157 1.00 98.63
C
ATOM 2026 C GLU C 483 35.847 80.719 64.995 1.00 98.63
C
ATOM 2027 O GLU C 483 35.552 81.464 65.931 1.00 98.63
O
ATOM 2028 CB GLU C 483 37.741 79.899 66.405 1.00 98.63
C
ATOM 2029 CG GLU C 483 39.136 79.298 66.397 1.00 98.63
C
ATOM 2030 CD GLU C 483 39.126 77.793 66.581 1.00 98.63
C
ATOM 2031 OE1 GLU C 483 40.146 77.147 66.264 1.00 98.63
O
ATOM 2032 OE2 GLU C 483 38.099 77.255 67.045 1.00 98.63
O
ATOM 2033 N GLY C 484 35.262 80.821 63.807 1.00 94.01
N
ATOM 2034 CA GLY C 484 34.343 81.902 63.516 1.00 94.01
C
ATOM 2035 C GLY C 484 32.910 81.650 63.932 1.00 94.01
C
ATOM 2036 O GLY C 484 32.001 82.343 63.464 1.00 94.01
O
ATOM 2037 N PHE C 485 32.694 80.624 64.761 1.00 93.62
N
ATOM 2038 CA PHE C 485 31.398 80.325 65.366 1.00 93.62
C
ATOM 2039 C PHE C 485 30.436 79.757 64.325 1.00 93.62
C
ATOM 2040 O PHE C 485 29.371 79.231 64.661 1.00 93.62
O
ATOM 2041 CB PHE C 485 30.837 81.586 66.048 1.00 93.62
C
ATOM 2042 CG PHE C 485 29.348 81.586 66.219 1.00 93.62
C

ATOM	2043	CD1	PHE	C	485	28.745	80.790	67.177	1.00	93.62	C
ATOM	2044	CD2	PHE	C	485	28.550	82.384	65.417	1.00	93.62	C
ATOM	2045	CE1	PHE	C	485	27.373	80.791	67.332	1.00	93.62	C
ATOM	2046	CE2	PHE	C	485	27.178	82.390	65.567	1.00	93.62	C
ATOM	2047	CZ	PHE	C	485	26.588	81.592	66.526	1.00	93.62	C
ATOM	2048	N	ASN	C	486	30.837	79.801	63.056	1.00	84.13	N
ATOM	2049	CA	ASN	C	486	30.105	79.146	61.980	1.00	84.13	C
ATOM	2050	C	ASN	C	486	31.051	78.365	61.078	1.00	84.13	C
ATOM	2051	O	ASN	C	486	30.609	77.766	60.094	1.00	84.13	O
ATOM	2052	CB	ASN	C	486	29.307	80.163	61.160	1.00	84.13	C
ATOM	2053	CG	ASN	C	486	27.884	80.324	61.657	1.00	84.13	C
ATOM	2054	OD1	ASN	C	486	27.334	79.433	62.303	1.00	84.13	O
ATOM	2055	ND2	ASN	C	486	27.280	81.466	61.357	1.00	84.13	N
ATOM	2056	N	CYS	C	487	32.345	78.381	61.387	1.00	75.18	N
ATOM	2057	CA	CYS	C	487	33.355	77.652	60.635	1.00	75.18	C
ATOM	2058	C	CYS	C	487	33.396	76.202	61.100	1.00	75.18	C
ATOM	2059	O	CYS	C	487	33.435	75.925	62.302	1.00	75.18	O
ATOM	2060	CB	CYS	C	487	34.738	78.298	60.793	1.00	75.18	C
ATOM	2061	SG	CYS	C	487	34.793	80.098	60.526	1.00	75.18	S
ATOM	2062	N	TYR	C	488	33.381	75.278	60.140	1.00	52.10	N
ATOM	2063	CA	TYR	C	488	33.433	73.851	60.423	1.00	52.10	C
ATOM	2064	C	TYR	C	488	34.520	73.199	59.582	1.00	52.10	C
ATOM	2065	O	TYR	C	488	34.672	73.513	58.399	1.00	52.10	O
ATOM	2066	CB	TYR	C	488	32.093	73.168	60.133	1.00	52.10	C
ATOM	2067	CG	TYR	C	488	30.967	73.569	61.054	1.00	52.10	C

ATOM 2068 CD1 TYR C 488 31.010 73.271 62.406 1.00 52.10
C
ATOM 2069 CD2 TYR C 488 29.856 74.235 60.566 1.00 52.10
C
ATOM 2070 CE1 TYR C 488 29.980 73.635 63.248 1.00 52.10
C
ATOM 2071 CE2 TYR C 488 28.823 74.602 61.397 1.00 52.10
C
ATOM 2072 CZ TYR C 488 28.889 74.299 62.736 1.00 52.10
C
ATOM 2073 OH TYR C 488 27.857 74.665 63.566 1.00 52.10
O
ATOM 2074 N PHE C 489 35.279 72.309 60.203 1.00 34.83
N
ATOM 2075 CA PHE C 489 36.185 71.446 59.455 1.00 34.83
C
ATOM 2076 C PHE C 489 35.367 70.362 58.757 1.00 34.83
C
ATOM 2077 O PHE C 489 34.584 69.672 59.414 1.00 34.83
O
ATOM 2078 CB PHE C 489 37.214 70.822 60.390 1.00 34.83
C
ATOM 2079 CG PHE C 489 38.315 70.092 59.683 1.00 34.83
C
ATOM 2080 CD1 PHE C 489 39.159 70.755 58.814 1.00 34.83
C
ATOM 2081 CD2 PHE C 489 38.510 68.739 59.894 1.00 34.83
C
ATOM 2082 CE1 PHE C 489 40.173 70.083 58.168 1.00 34.83
C
ATOM 2083 CE2 PHE C 489 39.522 68.065 59.249 1.00 34.83
C
ATOM 2084 CZ PHE C 489 40.352 68.735 58.386 1.00 34.83
C
ATOM 2085 N PRO C 490 35.507 70.187 57.439 1.00 29.56
N
ATOM 2086 CA PRO C 490 34.527 69.371 56.706 1.00 29.56
C
ATOM 2087 C PRO C 490 34.671 67.868 56.883 1.00 29.56
C
ATOM 2088 O PRO C 490 33.725 67.144 56.551 1.00 29.56
O
ATOM 2089 CB PRO C 490 34.764 69.776 55.247 1.00 29.56
C
ATOM 2090 CG PRO C 490 36.189 70.163 55.204 1.00 29.56
C
ATOM 2091 CD PRO C 490 36.515 70.772 56.541 1.00 29.56
C
ATOM 2092 N LEU C 491 35.791 67.371 57.393 1.00 24.34
N

ATOM 2093 CA LEU C 491 36.034 65.938 57.485 1.00 24.34
C
ATOM 2094 C LEU C 491 35.834 65.455 58.914 1.00 24.34
C
ATOM 2095 O LEU C 491 36.202 66.143 59.869 1.00 24.34
O
ATOM 2096 CB LEU C 491 37.446 65.592 57.013 1.00 24.34
C
ATOM 2097 CG LEU C 491 37.817 66.060 55.608 1.00 24.34
C
ATOM 2098 CD1 LEU C 491 39.263 65.731 55.302 1.00 24.34
C
ATOM 2099 CD2 LEU C 491 36.894 65.439 54.577 1.00 24.34
C
ATOM 2100 N GLN C 492 35.251 64.271 59.056 1.00 23.00
N
ATOM 2101 CA GLN C 492 34.951 63.681 60.349 1.00 23.00
C
ATOM 2102 C GLN C 492 35.455 62.248 60.374 1.00 23.00
C
ATOM 2103 O GLN C 492 35.339 61.527 59.382 1.00 23.00
O
ATOM 2104 CB GLN C 492 33.446 63.719 60.625 1.00 23.00
C
ATOM 2105 CG GLN C 492 33.040 63.171 61.969 1.00 23.00
C
ATOM 2106 CD GLN C 492 33.706 63.905 63.111 1.00 23.00
C
ATOM 2107 OE1 GLN C 492 33.700 65.134 63.162 1.00 23.00
O
ATOM 2108 NE2 GLN C 492 34.294 63.154 64.031 1.00 23.00
N
ATOM 2109 N SER C 493 36.002 61.833 61.506 1.00 22.74
N
ATOM 2110 CA SER C 493 36.536 60.488 61.644 1.00 22.74
C
ATOM 2111 C SER C 493 35.482 59.544 62.203 1.00 22.74
C
ATOM 2112 O SER C 493 34.636 59.931 63.010 1.00 22.74
O
ATOM 2113 CB SER C 493 37.767 60.482 62.549 1.00 22.74
C
ATOM 2114 OG SER C 493 38.387 59.211 62.546 1.00 22.74
O
ATOM 2115 N TYR C 494 35.534 58.293 61.747 1.00 20.11
N
ATOM 2116 CA TYR C 494 34.620 57.277 62.251 1.00 20.11
C
ATOM 2117 C TYR C 494 34.953 56.873 63.682 1.00 20.11
C

ATOM 2118 O TYR C 494 34.047 56.578 64.465 1.00 20.11
O
ATOM 2119 CB TYR C 494 34.653 56.048 61.346 1.00 20.11
C
ATOM 2120 CG TYR C 494 33.784 56.129 60.109 1.00 20.11
C
ATOM 2121 CD1 TYR C 494 32.416 56.302 60.209 1.00 20.11
C
ATOM 2122 CD2 TYR C 494 34.333 55.995 58.843 1.00 20.11
C
ATOM 2123 CE1 TYR C 494 31.619 56.358 59.087 1.00 20.11
C
ATOM 2124 CE2 TYR C 494 33.543 56.049 57.716 1.00 20.11
C
ATOM 2125 CZ TYR C 494 32.190 56.226 57.845 1.00 20.11
C
ATOM 2126 OH TYR C 494 31.392 56.279 56.729 1.00 20.11
O
ATOM 2127 N GLY C 495 36.235 56.841 64.035 1.00 24.70
N
ATOM 2128 CA GLY C 495 36.655 56.418 65.358 1.00 24.70
C
ATOM 2129 C GLY C 495 36.442 54.946 65.644 1.00 24.70
C
ATOM 2130 O GLY C 495 35.917 54.587 66.699 1.00 24.70
O
ATOM 2131 N PHE C 496 36.846 54.084 64.713 1.00 24.03
N
ATOM 2132 CA PHE C 496 36.641 52.650 64.865 1.00 24.03
C
ATOM 2133 C PHE C 496 37.393 52.111 66.080 1.00 24.03
C
ATOM 2134 O PHE C 496 38.591 52.352 66.239 1.00 24.03
O
ATOM 2135 CB PHE C 496 37.108 51.917 63.606 1.00 24.03
C
ATOM 2136 CG PHE C 496 36.309 52.240 62.369 1.00 24.03
C
ATOM 2137 CD1 PHE C 496 34.940 52.060 62.341 1.00 24.03
C
ATOM 2138 CD2 PHE C 496 36.939 52.698 61.228 1.00 24.03
C
ATOM 2139 CE1 PHE C 496 34.213 52.341 61.201 1.00 24.03
C
ATOM 2140 CE2 PHE C 496 36.214 52.982 60.088 1.00 24.03
C
ATOM 2141 CZ PHE C 496 34.855 52.802 60.076 1.00 24.03
C
ATOM 2142 N GLN C 497 36.686 51.365 66.921 1.00 32.09
N

```
ATOM   2143  CA  GLN C 497      37.252  50.723  68.099  1.00 32.09
C
ATOM   2144  C   GLN C 497      36.568  49.378  68.299  1.00 32.09
C
ATOM   2145  O   GLN C 497      35.389  49.225  67.963  1.00 32.09
O
ATOM   2146  CB  GLN C 497      37.100  51.603  69.352  1.00 32.09
C
ATOM   2147  CG  GLN C 497      35.678  51.949  69.729  1.00 32.09
C
ATOM   2148  CD  GLN C 497      35.604  53.132  70.675  1.00 32.09
C
ATOM   2149  OE1 GLN C 497      36.623  53.699  71.057  1.00 32.09
O
ATOM   2150  NE2 GLN C 497      34.393  53.504  71.060  1.00 32.09
N
ATOM   2151  N   PRO C 498      37.276  48.392  68.856  1.00 34.19
N
ATOM   2152  CA  PRO C 498      36.821  46.995  68.732  1.00 34.19
C
ATOM   2153  C   PRO C 498      35.502  46.671  69.417  1.00 34.19
C
ATOM   2154  O   PRO C 498      34.931  45.614  69.127  1.00 34.19
O
ATOM   2155  CB  PRO C 498      37.974  46.192  69.351  1.00 34.19
C
ATOM   2156  CG  PRO C 498      38.769  47.172  70.132  1.00 34.19
C
ATOM   2157  CD  PRO C 498      38.627  48.478  69.430  1.00 34.19
C
ATOM   2158  N   THR C 499      34.994  47.517  70.308  1.00 36.05
N
ATOM   2159  CA  THR C 499      33.862  47.120  71.136  1.00 36.05
C
ATOM   2160  C   THR C 499      32.497  47.524  70.591  1.00 36.05
C
ATOM   2161  O   THR C 499      31.485  47.119  71.169  1.00 36.05
O
ATOM   2162  CB  THR C 499      34.009  47.697  72.546  1.00 36.05
C
ATOM   2163  OG1 THR C 499      34.269  49.103  72.466  1.00 36.05
O
ATOM   2164  CG2 THR C 499      35.147  47.013  73.278  1.00 36.05
C
ATOM   2165  N   ASN C 500      32.418  48.299  69.514  1.00 31.99
N
ATOM   2166  CA  ASN C 500      31.099  48.733  69.077  1.00 31.99
C
ATOM   2167  C   ASN C 500      30.440  47.669  68.197  1.00 31.99
C
```

ATOM 2168 O ASN C 500 31.075 46.717 67.742 1.00 31.99
O
ATOM 2169 CB ASN C 500 31.186 50.070 68.349 1.00 31.99
C
ATOM 2170 CG ASN C 500 32.419 50.184 67.497 1.00 31.99
C
ATOM 2171 OD1 ASN C 500 32.772 49.254 66.779 1.00 31.99
O
ATOM 2172 ND2 ASN C 500 33.088 51.324 67.572 1.00 31.99
N
ATOM 2173 N GLY C 501 29.144 47.845 67.965 1.00 27.34
N
ATOM 2174 CA GLY C 501 28.333 46.842 67.306 1.00 27.34
C
ATOM 2175 C GLY C 501 28.655 46.670 65.831 1.00 27.34
C
ATOM 2176 O GLY C 501 29.457 47.389 65.238 1.00 27.34
O
ATOM 2177 N VAL C 502 27.980 45.688 65.228 1.00 24.63
N
ATOM 2178 CA VAL C 502 28.327 45.227 63.886 1.00 24.63
C
ATOM 2179 C VAL C 502 28.097 46.309 62.834 1.00 24.63
C
ATOM 2180 O VAL C 502 28.857 46.406 61.864 1.00 24.63
O
ATOM 2181 CB VAL C 502 27.558 43.933 63.561 1.00 24.63
C
ATOM 2182 CG1 VAL C 502 26.064 44.172 63.602 1.00 24.63
C
ATOM 2183 CG2 VAL C 502 27.980 43.386 62.217 1.00 24.63
C
ATOM 2184 N GLY C 503 27.066 47.138 62.995 1.00 22.27
N
ATOM 2185 CA GLY C 503 26.839 48.220 62.054 1.00 22.27
C
ATOM 2186 C GLY C 503 27.829 49.357 62.177 1.00 22.27
C
ATOM 2187 O GLY C 503 27.939 50.171 61.256 1.00 22.27
O
ATOM 2188 N TYR C 504 28.542 49.437 63.294 1.00 24.99
N
ATOM 2189 CA TYR C 504 29.591 50.419 63.515 1.00 24.99
C
ATOM 2190 C TYR C 504 30.977 49.897 63.167 1.00 24.99
C
ATOM 2191 O TYR C 504 31.943 50.652 63.264 1.00 24.99
O
ATOM 2192 CB TYR C 504 29.574 50.878 64.979 1.00 24.99
C

ATOM	2193	CG	TYR	C	504	28.309	51.578	65.406	1.00	24.99	C
ATOM	2194	CD1	TYR	C	504	28.161	52.945	65.241	1.00	24.99	C
ATOM	2195	CD2	TYR	C	504	27.237	50.861	65.917	1.00	24.99	C
ATOM	2196	CE1	TYR	C	504	26.999	53.590	65.627	1.00	24.99	C
ATOM	2197	CE2	TYR	C	504	26.068	51.492	66.292	1.00	24.99	C
ATOM	2198	CZ	TYR	C	504	25.953	52.855	66.139	1.00	24.99	C
ATOM	2199	OH	TYR	C	504	24.791	53.489	66.512	1.00	24.99	O
ATOM	2200	N	GLN	C	505	31.101	48.631	62.781	1.00	22.64	N
ATOM	2201	CA	GLN	C	505	32.398	48.055	62.457	1.00	22.64	C
ATOM	2202	C	GLN	C	505	32.885	48.539	61.094	1.00	22.64	C
ATOM	2203	O	GLN	C	505	32.080	48.866	60.222	1.00	22.64	O
ATOM	2204	CB	GLN	C	505	32.326	46.533	62.436	1.00	22.64	C
ATOM	2205	CG	GLN	C	505	31.704	45.913	63.646	1.00	22.64	C
ATOM	2206	CD	GLN	C	505	32.685	45.710	64.746	1.00	22.64	C
ATOM	2207	OE1	GLN	C	505	33.612	46.493	64.913	1.00	22.64	O
ATOM	2208	NE2	GLN	C	505	32.482	44.657	65.530	1.00	22.64	N
ATOM	2209	N	PRO	C	506	34.198	48.572	60.883	1.00	19.38	N
ATOM	2210	CA	PRO	C	506	34.712	48.890	59.546	1.00	19.38	C
ATOM	2211	C	PRO	C	506	34.498	47.744	58.571	1.00	19.38	C
ATOM	2212	O	PRO	C	506	34.665	46.572	58.905	1.00	19.38	O
ATOM	2213	CB	PRO	C	506	36.204	49.142	59.787	1.00	19.38	C
ATOM	2214	CG	PRO	C	506	36.521	48.402	61.036	1.00	19.38	C
ATOM	2215	CD	PRO	C	506	35.277	48.400	61.871	1.00	19.38	C
ATOM	2216	N	TYR	C	507	34.125	48.102	57.348	1.00	16.00	N
ATOM	2217	CA	TYR	C	507	34.006	47.193	56.221	1.00	16.00	C

ATOM 2218 C TYR C 507 34.847 47.750 55.083 1.00 16.00
C
ATOM 2219 O TYR C 507 34.748 48.938 54.761 1.00 16.00
O
ATOM 2220 CB TYR C 507 32.551 47.046 55.759 1.00 16.00
C
ATOM 2221 CG TYR C 507 31.731 46.052 56.547 1.00 16.00
C
ATOM 2222 CD1 TYR C 507 31.194 44.935 55.934 1.00 16.00
C
ATOM 2223 CD2 TYR C 507 31.487 46.238 57.895 1.00 16.00
C
ATOM 2224 CE1 TYR C 507 30.441 44.026 56.646 1.00 16.00
C
ATOM 2225 CE2 TYR C 507 30.733 45.331 58.614 1.00 16.00
C
ATOM 2226 CZ TYR C 507 30.219 44.231 57.980 1.00 16.00
C
ATOM 2227 OH TYR C 507 29.467 43.326 58.693 1.00 16.00
O
ATOM 2228 N ARG C 508 35.673 46.899 54.488 1.00 13.44
N
ATOM 2229 CA ARG C 508 36.398 47.264 53.280 1.00 13.44
C
ATOM 2230 C ARG C 508 35.502 47.000 52.079 1.00 13.44
C
ATOM 2231 O ARG C 508 34.870 45.944 51.987 1.00 13.44
O
ATOM 2232 CB ARG C 508 37.711 46.481 53.177 1.00 13.44
C
ATOM 2233 CG ARG C 508 38.766 47.008 54.130 1.00 13.44
C
ATOM 2234 CD ARG C 508 40.045 46.182 54.197 1.00 13.44
C
ATOM 2235 NE ARG C 508 40.934 46.765 55.198 1.00 13.44
N
ATOM 2236 CZ ARG C 508 42.248 46.582 55.254 1.00 13.44
C
ATOM 2237 NH1 ARG C 508 42.861 45.821 54.364 1.00 13.44
N
ATOM 2238 NH2 ARG C 508 42.948 47.174 56.204 1.00 13.44
N
ATOM 2239 N VAL C 509 35.423 47.975 51.176 1.00 11.00
N
ATOM 2240 CA VAL C 509 34.531 47.928 50.026 1.00 11.00
C
ATOM 2241 C VAL C 509 35.361 48.072 48.764 1.00 11.00
C
ATOM 2242 O VAL C 509 36.230 48.952 48.679 1.00 11.00
O

ATOM 2243 CB VAL C 509 33.457 49.033 50.084 1.00 11.00
C
ATOM 2244 CG1 VAL C 509 32.511 48.932 48.886 1.00 11.00
C
ATOM 2245 CG2 VAL C 509 32.677 48.941 51.370 1.00 11.00
C
ATOM 2246 N VAL C 510 35.088 47.202 47.795 1.00 11.84
N
ATOM 2247 CA VAL C 510 35.583 47.328 46.433 1.00 11.84
C
ATOM 2248 C VAL C 510 34.378 47.381 45.502 1.00 11.84
C
ATOM 2249 O VAL C 510 33.496 46.519 45.563 1.00 11.84
O
ATOM 2250 CB VAL C 510 36.519 46.166 46.051 1.00 11.84
C
ATOM 2251 CG1 VAL C 510 36.967 46.307 44.613 1.00 11.84
C
ATOM 2252 CG2 VAL C 510 37.719 46.124 46.979 1.00 11.84
C
ATOM 2253 N VAL C 511 34.343 48.396 44.653 1.00 13.04
N
ATOM 2254 CA VAL C 511 33.323 48.557 43.628 1.00 13.04
C
ATOM 2255 C VAL C 511 33.995 48.350 42.278 1.00 13.04
C
ATOM 2256 O VAL C 511 34.935 49.070 41.931 1.00 13.04
O
ATOM 2257 CB VAL C 511 32.652 49.936 43.710 1.00 13.04
C
ATOM 2258 CG1 VAL C 511 31.577 50.063 42.645 1.00 13.04
C
ATOM 2259 CG2 VAL C 511 32.067 50.163 45.111 1.00 13.04
C
ATOM 2260 N LEU C 512 33.521 47.368 41.522 1.00 18.27
N
ATOM 2261 CA LEU C 512 34.039 47.060 40.196 1.00 18.27
C
ATOM 2262 C LEU C 512 33.020 47.523 39.165 1.00 18.27
C
ATOM 2263 O LEU C 512 31.905 47.010 39.129 1.00 18.27
O
ATOM 2264 CB LEU C 512 34.298 45.561 40.048 1.00 18.27
C
ATOM 2265 CG LEU C 512 35.258 44.878 41.024 1.00 18.27
C
ATOM 2266 CD1 LEU C 512 35.236 43.373 40.833 1.00 18.27
C
ATOM 2267 CD2 LEU C 512 36.663 45.404 40.846 1.00 18.27
C

ATOM 2268 N SER C 513 33.396 48.485 38.336 1.00 23.26
N
ATOM 2269 CA SER C 513 32.563 48.941 37.232 1.00 23.26
C
ATOM 2270 C SER C 513 33.118 48.379 35.927 1.00 23.26
C
ATOM 2271 O SER C 513 34.335 48.347 35.731 1.00 23.26
O
ATOM 2272 CB SER C 513 32.509 50.464 37.181 1.00 23.26
C
ATOM 2273 OG SER C 513 31.453 50.899 36.351 1.00 23.26
O
ATOM 2274 N PHE C 514 32.225 47.957 35.030 1.00 29.30
N
ATOM 2275 CA PHE C 514 32.566 46.967 34.007 1.00 29.30
C
ATOM 2276 C PHE C 514 32.713 47.491 32.587 1.00 29.30
C
ATOM 2277 O PHE C 514 33.596 47.020 31.880 1.00 29.30
O
ATOM 2278 CB PHE C 514 31.533 45.840 34.005 1.00 29.30
C
ATOM 2279 CG PHE C 514 31.727 44.863 35.111 1.00 29.30
C
ATOM 2280 CD1 PHE C 514 32.955 44.743 35.722 1.00 29.30
C
ATOM 2281 CD2 PHE C 514 30.687 44.076 35.547 1.00 29.30
C
ATOM 2282 CE1 PHE C 514 33.143 43.855 36.745 1.00 29.30
C
ATOM 2283 CE2 PHE C 514 30.872 43.187 36.569 1.00 29.30
C
ATOM 2284 CZ PHE C 514 32.102 43.074 37.168 1.00 29.30
C
ATOM 2285 N GLU C 515 31.852 48.379 32.101 1.00 37.37
N
ATOM 2286 CA GLU C 515 32.015 48.955 30.757 1.00 37.37
C
ATOM 2287 C GLU C 515 32.280 47.881 29.695 1.00 37.37
C
ATOM 2288 O GLU C 515 33.368 47.782 29.132 1.00 37.37
O
ATOM 2289 CB GLU C 515 33.138 49.994 30.739 1.00 37.37
C
ATOM 2290 CG GLU C 515 32.880 51.158 29.812 1.00 37.37
C
ATOM 2291 CD GLU C 515 34.004 52.166 29.826 1.00 37.37
C
ATOM 2292 OE1 GLU C 515 35.120 51.821 29.391 1.00 37.37
O

ATOM 2293 OE2 GLU C 515 33.773 53.309 30.263 1.00 37.37
O
ATOM 2294 N LEU C 516 31.270 47.048 29.467 1.00 43.16
N
ATOM 2295 CA LEU C 516 31.363 45.995 28.456 1.00 43.16
C
ATOM 2296 C LEU C 516 31.651 46.567 27.071 1.00 43.16
C
ATOM 2297 O LEU C 516 32.262 45.908 26.231 1.00 43.16
O
ATOM 2298 CB LEU C 516 30.075 45.174 28.411 1.00 43.16
C
ATOM 2299 CG LEU C 516 29.627 44.538 29.724 1.00 43.16
C
ATOM 2300 CD1 LEU C 516 28.425 45.273 30.272 1.00 43.16
C
ATOM 2301 CD2 LEU C 516 29.304 43.076 29.515 1.00 43.16
C
TER 2302 LEU C 516
ATOM 2303 N GLN A 1 59.036 47.697 74.253 1.00 56.72
N
ATOM 2304 CA GLN A 1 59.634 46.556 74.935 1.00 56.72
C
ATOM 2305 C GLN A 1 59.618 45.333 74.018 1.00 56.72
C
ATOM 2306 O GLN A 1 60.017 44.236 74.409 1.00 56.72
O
ATOM 2307 CB GLN A 1 58.897 46.276 76.252 1.00 56.72
C
ATOM 2308 CG GLN A 1 59.506 45.188 77.140 1.00 56.72
C
ATOM 2309 CD GLN A 1 60.985 45.395 77.432 1.00 56.72
C
ATOM 2310 OE1 GLN A 1 61.514 46.501 77.313 1.00 56.72
O
ATOM 2311 NE2 GLN A 1 61.661 44.318 77.812 1.00 56.72
N
ATOM 2312 N VAL A 2 59.169 45.536 72.779 1.00 48.14
N
ATOM 2313 CA VAL A 2 59.157 44.442 71.819 1.00 48.14
C
ATOM 2314 C VAL A 2 60.583 43.968 71.568 1.00 48.14
C
ATOM 2315 O VAL A 2 61.535 44.759 71.550 1.00 48.14
O
ATOM 2316 CB VAL A 2 58.454 44.872 70.520 1.00 48.14
C
ATOM 2317 CG1 VAL A 2 57.194 45.649 70.846 1.00 48.14
C
ATOM 2318 CG2 VAL A 2 59.371 45.704 69.653 1.00 48.14

C
ATOM 2319 N GLN A 3 60.738 42.658 71.416 1.00 45.04
N
ATOM 2320 CA GLN A 3 62.036 42.028 71.232 1.00 45.04
C
ATOM 2321 C GLN A 3 61.928 40.979 70.139 1.00 45.04
C
ATOM 2322 O GLN A 3 61.016 40.148 70.163 1.00 45.04
O
ATOM 2323 CB GLN A 3 62.523 41.374 72.528 1.00 45.04
C
ATOM 2324 CG GLN A 3 63.951 41.699 72.902 1.00 45.04
C
ATOM 2325 CD GLN A 3 64.259 43.179 72.824 1.00 45.04
C
ATOM 2326 OE1 GLN A 3 63.481 44.012 73.286 1.00 45.04
O
ATOM 2327 NE2 GLN A 3 65.396 43.515 72.230 1.00 45.04
N
ATOM 2328 N LEU A 4 62.854 41.017 69.187 1.00 36.15
N
ATOM 2329 CA LEU A 4 62.978 39.991 68.162 1.00 36.15
C
ATOM 2330 C LEU A 4 64.411 39.494 68.164 1.00 36.15
C
ATOM 2331 O LEU A 4 65.343 40.290 68.026 1.00 36.15
O
ATOM 2332 CB LEU A 4 62.603 40.528 66.779 1.00 36.15
C
ATOM 2333 CG LEU A 4 61.179 41.049 66.608 1.00 36.15
C
ATOM 2334 CD1 LEU A 4 61.044 41.806 65.301 1.00 36.15
C
ATOM 2335 CD2 LEU A 4 60.207 39.898 66.650 1.00 36.15
C
ATOM 2336 N VAL A 5 64.589 38.188 68.317 1.00 33.49
N
ATOM 2337 CA VAL A 5 65.906 37.580 68.439 1.00 33.49
C
ATOM 2338 C VAL A 5 66.115 36.674 67.237 1.00 33.49
C
ATOM 2339 O VAL A 5 65.442 35.646 67.105 1.00 33.49
O
ATOM 2340 CB VAL A 5 66.052 36.798 69.749 1.00 33.49
C
ATOM 2341 CG1 VAL A 5 67.421 36.150 69.828 1.00 33.49
C
ATOM 2342 CG2 VAL A 5 65.815 37.714 70.938 1.00 33.49
C
ATOM 2343 N GLU A 6 67.040 37.054 66.362 1.00 31.93

N
ATOM 2344 CA GLU A 6 67.342 36.263 65.181 1.00 31.93
C
ATOM 2345 C GLU A 6 68.316 35.145 65.527 1.00 31.93
C
ATOM 2346 O GLU A 6 69.078 35.234 66.491 1.00 31.93
O
ATOM 2347 CB GLU A 6 67.926 37.149 64.082 1.00 31.93
C
ATOM 2348 CG GLU A 6 66.912 38.049 63.412 1.00 31.93
C
ATOM 2349 CD GLU A 6 67.099 39.506 63.763 1.00 31.93
C
ATOM 2350 OE1 GLU A 6 67.861 39.801 64.703 1.00 31.93
O
ATOM 2351 OE2 GLU A 6 66.480 40.360 63.101 1.00 31.93
O
ATOM 2352 N SER A 7 68.276 34.078 64.734 1.00 33.01
N
ATOM 2353 CA SER A 7 69.133 32.925 64.963 1.00 33.01
C
ATOM 2354 C SER A 7 69.233 32.124 63.678 1.00 33.01
C
ATOM 2355 O SER A 7 68.350 32.174 62.817 1.00 33.01
O
ATOM 2356 CB SER A 7 68.609 32.044 66.098 1.00 33.01
C
ATOM 2357 OG SER A 7 68.604 32.747 67.326 1.00 33.01
O
ATOM 2358 N GLY A 8 70.301 31.347 63.586 1.00 33.45
N
ATOM 2359 CA GLY A 8 70.669 30.718 62.340 1.00 33.45
C
ATOM 2360 C GLY A 8 71.530 31.640 61.512 1.00 33.45
C
ATOM 2361 O GLY A 8 71.757 32.806 61.838 1.00 33.45
O
ATOM 2362 N GLY A 9 72.070 31.088 60.438 1.00 36.48
N
ATOM 2363 CA GLY A 9 72.768 31.902 59.467 1.00 36.48
C
ATOM 2364 C GLY A 9 74.243 32.071 59.757 1.00 36.48
C
ATOM 2365 O GLY A 9 74.624 32.796 60.679 1.00 36.48
O
ATOM 2366 N THR A 10 75.077 31.447 58.934 1.00 42.78
N
ATOM 2367 CA THR A 10 76.534 31.465 59.017 1.00 42.78
C
ATOM 2368 C THR A 10 77.056 31.211 57.610 1.00 42.78

C
ATOM 2369 O THR A 10 76.318 31.360 56.630 1.00 42.78
O
ATOM 2370 CB THR A 10 77.078 30.408 60.000 1.00 42.78
C
ATOM 2371 OG1 THR A 10 76.760 29.094 59.522 1.00 42.78
O
ATOM 2372 CG2 THR A 10 76.564 30.590 61.427 1.00 42.78
C
ATOM 2373 N VAL A 11 78.332 30.842 57.507 1.00 42.87
N
ATOM 2374 CA VAL A 11 78.849 30.383 56.231 1.00 42.87
C
ATOM 2375 C VAL A 11 78.095 29.128 55.816 1.00 42.87
C
ATOM 2376 O VAL A 11 77.649 28.336 56.653 1.00 42.87
O
ATOM 2377 CB VAL A 11 80.361 30.121 56.318 1.00 42.87
C
ATOM 2378 CG1 VAL A 11 80.965 30.071 54.935 1.00 42.87
C
ATOM 2379 CG2 VAL A 11 81.027 31.191 57.157 1.00 42.87
C
ATOM 2380 N LEU A 12 77.935 28.951 54.510 1.00 44.63
N
ATOM 2381 CA LEU A 12 77.215 27.798 53.996 1.00 44.63
C
ATOM 2382 C LEU A 12 77.733 27.467 52.607 1.00 44.63
C
ATOM 2383 O LEU A 12 77.819 28.348 51.749 1.00 44.63
O
ATOM 2384 CB LEU A 12 75.708 28.059 53.953 1.00 44.63
C
ATOM 2385 CG LEU A 12 74.917 26.835 53.509 1.00 44.63
C
ATOM 2386 CD1 LEU A 12 73.954 26.398 54.592 1.00 44.63
C
ATOM 2387 CD2 LEU A 12 74.190 27.112 52.214 1.00 44.63
C
ATOM 2388 N GLN A 13 78.074 26.202 52.395 1.00 48.35
N
ATOM 2389 CA GLN A 13 78.584 25.782 51.105 1.00 48.35
C
ATOM 2390 C GLN A 13 77.481 25.853 50.052 1.00 48.35
C
ATOM 2391 O GLN A 13 76.314 25.595 50.349 1.00 48.35
O
ATOM 2392 CB GLN A 13 79.138 24.364 51.189 1.00 48.35
C
ATOM 2393 CG GLN A 13 79.819 24.052 52.511 1.00 48.35

C
ATOM 2394 CD GLN A 13 80.979 24.983 52.812 1.00 48.35
C
ATOM 2395 OE1 GLN A 13 81.627 25.499 51.904 1.00 48.35
O
ATOM 2396 NE2 GLN A 13 81.245 25.201 54.093 1.00 48.35
N
ATOM 2397 N PRO A 14 77.828 26.203 48.816 1.00 48.21
N
ATOM 2398 CA PRO A 14 76.801 26.377 47.783 1.00 48.21
C
ATOM 2399 C PRO A 14 75.980 25.114 47.577 1.00 48.21
C
ATOM 2400 O PRO A 14 76.490 23.995 47.663 1.00 48.21
O
ATOM 2401 CB PRO A 14 77.615 26.728 46.534 1.00 48.21
C
ATOM 2402 CG PRO A 14 78.869 27.321 47.067 1.00 48.21
C
ATOM 2403 CD PRO A 14 79.165 26.593 48.339 1.00 48.21
C
ATOM 2404 N GLY A 15 74.691 25.308 47.313 1.00 47.60
N
ATOM 2405 CA GLY A 15 73.770 24.224 47.081 1.00 47.60
C
ATOM 2406 C GLY A 15 73.120 23.657 48.325 1.00 47.60
C
ATOM 2407 O GLY A 15 72.116 22.948 48.210 1.00 47.60
O
ATOM 2408 N ARG A 16 73.652 23.954 49.506 1.00 47.46
N
ATOM 2409 CA ARG A 16 73.078 23.444 50.739 1.00 47.46
C
ATOM 2410 C ARG A 16 71.800 24.204 51.093 1.00 47.46
C
ATOM 2411 O ARG A 16 71.491 25.257 50.531 1.00 47.46
O
ATOM 2412 CB ARG A 16 74.087 23.546 51.881 1.00 47.46
C
ATOM 2413 CG ARG A 16 75.351 22.734 51.681 1.00 47.46
C
ATOM 2414 CD ARG A 16 75.060 21.386 51.038 1.00 47.46
C
ATOM 2415 NE ARG A 16 76.192 20.822 50.296 1.00 47.46
N
ATOM 2416 CZ ARG A 16 77.436 20.673 50.754 1.00 47.46
C
ATOM 2417 NH1 ARG A 16 77.763 21.004 51.998 1.00 47.46
N
ATOM 2418 NH2 ARG A 16 78.361 20.152 49.961 1.00 47.46

N
ATOM 2419 N SER A 17 71.049 23.648 52.036 1.00 41.01
N
ATOM 2420 CA SER A 17 69.841 24.270 52.555 1.00 41.01
C
ATOM 2421 C SER A 17 70.122 24.887 53.916 1.00 41.01
C
ATOM 2422 O SER A 17 71.021 24.457 54.641 1.00 41.01
O
ATOM 2423 CB SER A 17 68.703 23.255 52.669 1.00 41.01
C
ATOM 2424 OG SER A 17 68.137 22.980 51.403 1.00 41.01
O
ATOM 2425 N LEU A 18 69.346 25.912 54.257 1.00 35.63
N
ATOM 2426 CA LEU A 18 69.543 26.612 55.515 1.00 35.63
C
ATOM 2427 C LEU A 18 68.187 27.040 56.052 1.00 35.63
C
ATOM 2428 O LEU A 18 67.227 27.192 55.297 1.00 35.63
O
ATOM 2429 CB LEU A 18 70.472 27.821 55.333 1.00 35.63
C
ATOM 2430 CG LEU A 18 71.048 28.490 56.579 1.00 35.63
C
ATOM 2431 CD1 LEU A 18 71.821 27.496 57.414 1.00 35.63
C
ATOM 2432 CD2 LEU A 18 71.928 29.662 56.193 1.00 35.63
C
ATOM 2433 N ARG A 19 68.108 27.203 57.367 1.00 32.71
N
ATOM 2434 CA ARG A 19 66.901 27.693 58.016 1.00 32.71
C
ATOM 2435 C ARG A 19 67.277 28.780 59.008 1.00 32.71
C
ATOM 2436 O ARG A 19 68.120 28.558 59.879 1.00 32.71
O
ATOM 2437 CB ARG A 19 66.143 26.564 58.733 1.00 32.71
C
ATOM 2438 CG ARG A 19 64.641 26.807 58.841 1.00 32.71
C
ATOM 2439 CD ARG A 19 63.896 25.600 59.393 1.00 32.71
C
ATOM 2440 NE ARG A 19 64.024 25.499 60.840 1.00 32.71
N
ATOM 2441 CZ ARG A 19 63.143 24.888 61.623 1.00 32.71
C
ATOM 2442 NH1 ARG A 19 62.066 24.326 61.097 1.00 32.71
N
ATOM 2443 NH2 ARG A 19 63.338 24.841 62.932 1.00 32.71

N
ATOM 2444 N LEU A 20 66.662 29.951 58.874 1.00 26.93
N
ATOM 2445 CA LEU A 20 66.765 30.972 59.904 1.00 26.93
C
ATOM 2446 C LEU A 20 65.492 31.012 60.726 1.00 26.93
C
ATOM 2447 O LEU A 20 64.411 30.651 60.261 1.00 26.93
O
ATOM 2448 CB LEU A 20 67.022 32.371 59.340 1.00 26.93
C
ATOM 2449 CG LEU A 20 67.845 32.633 58.100 1.00 26.93
C
ATOM 2450 CD1 LEU A 20 67.632 34.094 57.700 1.00 26.93
C
ATOM 2451 CD2 LEU A 20 69.297 32.334 58.371 1.00 26.93
C
ATOM 2452 N SER A 21 65.633 31.485 61.953 1.00 26.10
N
ATOM 2453 CA SER A 21 64.503 31.596 62.852 1.00 26.10
C
ATOM 2454 C SER A 21 64.598 32.917 63.591 1.00 26.10
C
ATOM 2455 O SER A 21 65.671 33.503 63.715 1.00 26.10
O
ATOM 2456 CB SER A 21 64.451 30.424 63.838 1.00 26.10
C
ATOM 2457 OG SER A 21 65.405 30.588 64.864 1.00 26.10
O
ATOM 2458 N CYS A 22 63.454 33.391 64.060 1.00 26.66
N
ATOM 2459 CA CYS A 22 63.355 34.663 64.757 1.00 26.66
C
ATOM 2460 C CYS A 22 62.310 34.498 65.848 1.00 26.66
C
ATOM 2461 O CYS A 22 61.151 34.209 65.553 1.00 26.66
O
ATOM 2462 CB CYS A 22 62.988 35.784 63.769 1.00 26.66
C
ATOM 2463 SG CYS A 22 62.337 37.323 64.438 1.00 26.66
S
ATOM 2464 N ALA A 23 62.719 34.651 67.101 1.00 30.10
N
ATOM 2465 CA ALA A 23 61.846 34.427 68.247 1.00 30.10
C
ATOM 2466 C ALA A 23 61.400 35.765 68.814 1.00 30.10
C
ATOM 2467 O ALA A 23 62.216 36.674 68.979 1.00 30.10
O
ATOM 2468 CB ALA A 23 62.553 33.601 69.322 1.00 30.10

C
ATOM 2469 N ALA A 24 60.111 35.885 69.108 1.00 34.23
N
ATOM 2470 CA ALA A 24 59.512 37.155 69.484 1.00 34.23
C
ATOM 2471 C ALA A 24 59.146 37.172 70.962 1.00 34.23
C
ATOM 2472 O ALA A 24 58.812 36.142 71.551 1.00 34.23
O
ATOM 2473 CB ALA A 24 58.266 37.442 68.647 1.00 34.23
C
ATOM 2474 N SER A 25 59.210 38.362 71.550 1.00 39.64
N
ATOM 2475 CA SER A 25 58.851 38.558 72.945 1.00 39.64
C
ATOM 2476 C SER A 25 58.331 39.973 73.122 1.00 39.64
C
ATOM 2477 O SER A 25 58.664 40.872 72.348 1.00 39.64
O
ATOM 2478 CB SER A 25 60.044 38.315 73.873 1.00 39.64
C
ATOM 2479 OG SER A 25 60.520 36.990 73.744 1.00 39.64
O
ATOM 2480 N GLY A 26 57.508 40.164 74.147 1.00 39.48
N
ATOM 2481 CA GLY A 26 57.023 41.487 74.467 1.00 39.48
C
ATOM 2482 C GLY A 26 55.867 41.986 73.634 1.00 39.48
C
ATOM 2483 O GLY A 26 55.541 43.174 73.713 1.00 39.48
O
ATOM 2484 N PHE A 27 55.248 41.132 72.824 1.00 33.26
N
ATOM 2485 CA PHE A 27 54.008 41.490 72.150 1.00 33.26
C
ATOM 2486 C PHE A 27 53.255 40.207 71.835 1.00 33.26
C
ATOM 2487 O PHE A 27 53.799 39.107 71.929 1.00 33.26
O
ATOM 2488 CB PHE A 27 54.254 42.342 70.894 1.00 33.26
C
ATOM 2489 CG PHE A 27 54.996 41.634 69.789 1.00 33.26
C
ATOM 2490 CD1 PHE A 27 54.314 41.109 68.706 1.00 33.26
C
ATOM 2491 CD2 PHE A 27 56.375 41.527 69.817 1.00 33.26
C
ATOM 2492 CE1 PHE A 27 54.992 40.470 67.685 1.00 33.26
C
ATOM 2493 CE2 PHE A 27 57.054 40.889 68.798 1.00 33.26

C
ATOM 2494 CZ PHE A 27 56.360 40.361 67.733 1.00 33.26
C
ATOM 2495 N THR A 28 51.981 40.362 71.496 1.00 30.28
N
ATOM 2496 CA THR A 28 51.126 39.218 71.196 1.00 30.28
C
ATOM 2497 C THR A 28 51.442 38.752 69.781 1.00 30.28
C
ATOM 2498 O THR A 28 50.960 39.322 68.802 1.00 30.28
O
ATOM 2499 CB THR A 28 49.657 39.586 71.357 1.00 30.28
C
ATOM 2500 OG1 THR A 28 49.417 39.992 72.709 1.00 30.28
O
ATOM 2501 CG2 THR A 28 48.782 38.389 71.051 1.00 30.28
C
ATOM 2502 N PHE A 29 52.252 37.696 69.688 1.00 24.75
N
ATOM 2503 CA PHE A 29 52.784 37.228 68.410 1.00 24.75
C
ATOM 2504 C PHE A 29 51.672 36.845 67.442 1.00 24.75
C
ATOM 2505 O PHE A 29 51.776 37.093 66.237 1.00 24.75
O
ATOM 2506 CB PHE A 29 53.718 36.045 68.679 1.00 24.75
C
ATOM 2507 CG PHE A 29 54.420 35.499 67.467 1.00 24.75
C
ATOM 2508 CD1 PHE A 29 55.498 36.160 66.911 1.00 24.75
C
ATOM 2509 CD2 PHE A 29 54.039 34.287 66.926 1.00 24.75
C
ATOM 2510 CE1 PHE A 29 56.157 35.638 65.821 1.00 24.75
C
ATOM 2511 CE2 PHE A 29 54.694 33.762 65.838 1.00 24.75
C
ATOM 2512 CZ PHE A 29 55.754 34.440 65.283 1.00 24.75
C
ATOM 2513 N SER A 30 50.588 36.260 67.955 1.00 25.64
N
ATOM 2514 CA SER A 30 49.535 35.727 67.101 1.00 25.64
C
ATOM 2515 C SER A 30 48.760 36.805 66.359 1.00 25.64
C
ATOM 2516 O SER A 30 48.175 36.513 65.315 1.00 25.64
O
ATOM 2517 CB SER A 30 48.563 34.887 67.929 1.00 25.64
C
ATOM 2518 OG SER A 30 49.255 33.905 68.672 1.00 25.64

O
ATOM 2519 N SER A 31 48.733 38.033 66.865 1.00 25.71
N
ATOM 2520 CA SER A 31 47.966 39.102 66.245 1.00 25.71
C
ATOM 2521 C SER A 31 48.809 39.980 65.328 1.00 25.71
C
ATOM 2522 O SER A 31 48.338 41.032 64.895 1.00 25.71
O
ATOM 2523 CB SER A 31 47.293 39.959 67.318 1.00 25.71
C
ATOM 2524 OG SER A 31 48.253 40.636 68.104 1.00 25.71
O
ATOM 2525 N TYR A 32 50.036 39.572 65.020 1.00 23.10
N
ATOM 2526 CA TYR A 32 50.923 40.328 64.148 1.00 23.10
C
ATOM 2527 C TYR A 32 51.300 39.492 62.933 1.00 23.10
C
ATOM 2528 O TYR A 32 51.536 38.289 63.047 1.00 23.10
O
ATOM 2529 CB TYR A 32 52.192 40.752 64.886 1.00 23.10
C
ATOM 2530 CG TYR A 32 52.033 41.982 65.739 1.00 23.10
C
ATOM 2531 CD1 TYR A 32 52.671 43.161 65.409 1.00 23.10
C
ATOM 2532 CD2 TYR A 32 51.244 41.964 66.877 1.00 23.10
C
ATOM 2533 CE1 TYR A 32 52.534 44.285 66.183 1.00 23.10
C
ATOM 2534 CE2 TYR A 32 51.101 43.087 67.657 1.00 23.10
C
ATOM 2535 CZ TYR A 32 51.746 44.242 67.303 1.00 23.10
C
ATOM 2536 OH TYR A 32 51.607 45.365 68.081 1.00 23.10
O
ATOM 2537 N ALA A 33 51.334 40.133 61.769 1.00 17.88
N
ATOM 2538 CA ALA A 33 51.905 39.527 60.577 1.00 17.88
C
ATOM 2539 C ALA A 33 53.418 39.704 60.609 1.00 17.88
C
ATOM 2540 O ALA A 33 53.919 40.729 61.071 1.00 17.88
O
ATOM 2541 CB ALA A 33 51.317 40.167 59.320 1.00 17.88
C
ATOM 2542 N MET A 34 54.150 38.700 60.139 1.00 17.58
N
ATOM 2543 CA MET A 34 55.601 38.692 60.293 1.00 17.58

```
C
ATOM   2544  C   MET A  34      56.271  38.616  58.929  1.00 17.58
C
ATOM   2545  O   MET A  34      55.790  37.928  58.033  1.00 17.58
O
ATOM   2546  CB  MET A  34      56.057  37.522  61.172  1.00 17.58
C
ATOM   2547  CG  MET A  34      55.427  37.486  62.563  1.00 17.58
C
ATOM   2548  SD  MET A  34      55.813  38.909  63.596  1.00 17.58
S
ATOM   2549  CE  MET A  34      57.580  38.724  63.773  1.00 17.58
C
ATOM   2550  N   HIS A  35      57.390  39.316  58.767  1.00 16.19
N
ATOM   2551  CA  HIS A  35      58.039  39.344  57.464  1.00 16.19
C
ATOM   2552  C   HIS A  35      59.546  39.514  57.599  1.00 16.19
C
ATOM   2553  O   HIS A  35      60.076  39.812  58.669  1.00 16.19
O
ATOM   2554  CB  HIS A  35      57.403  40.404  56.560  1.00 16.19
C
ATOM   2555  CG  HIS A  35      56.946  41.629  57.276  1.00 16.19
C
ATOM   2556  ND1 HIS A  35      57.727  42.752  57.416  1.00 16.19
N
ATOM   2557  CD2 HIS A  35      55.762  41.915  57.867  1.00 16.19
C
ATOM   2558  CE1 HIS A  35      57.051  43.673  58.076  1.00 16.19
C
ATOM   2559  NE2 HIS A  35      55.854  43.193  58.356  1.00 16.19
N
ATOM   2560  N   TRP A  36      60.231  39.260  56.488  1.00 16.35
N
ATOM   2561  CA  TRP A  36      61.687  39.304  56.425  1.00 16.35
C
ATOM   2562  C   TRP A  36      62.127  40.339  55.401  1.00 16.35
C
ATOM   2563  O   TRP A  36      61.522  40.466  54.337  1.00 16.35
O
ATOM   2564  CB  TRP A  36      62.280  37.941  56.042  1.00 16.35
C
ATOM   2565  CG  TRP A  36      62.169  36.875  57.085  1.00 16.35
C
ATOM   2566  CD1 TRP A  36      61.168  35.965  57.215  1.00 16.35
C
ATOM   2567  CD2 TRP A  36      63.111  36.586  58.121  1.00 16.35
C
ATOM   2568  NE1 TRP A  36      61.418  35.134  58.274  1.00 16.35
```

```
N
ATOM   2569  CE2 TRP A  36      62.605  35.496  58.849  1.00 16.35
C
ATOM   2570  CE3 TRP A  36      64.329  37.148  58.509  1.00 16.35
C
ATOM   2571  CZ2 TRP A  36      63.273  34.955  59.939  1.00 16.35
C
ATOM   2572  CZ3 TRP A  36      64.988  36.609  59.592  1.00 16.35
C
ATOM   2573  CH2 TRP A  36      64.459  35.525  60.295  1.00 16.35
C
ATOM   2574  N   VAL A  37      63.184  41.071  55.732  1.00 15.71
N
ATOM   2575  CA  VAL A  37      63.831  42.009  54.824  1.00 15.71
C
ATOM   2576  C   VAL A  37      65.296  41.606  54.773  1.00 15.71
C
ATOM   2577  O   VAL A  37      65.804  40.999  55.711  1.00 15.71
O
ATOM   2578  CB  VAL A  37      63.649  43.470  55.300  1.00 15.71
C
ATOM   2579  CG1 VAL A  37      64.468  44.446  54.466  1.00 15.71
C
ATOM   2580  CG2 VAL A  37      62.185  43.852  55.264  1.00 15.71
C
ATOM   2581  N   ARG A  38      65.969  41.878  53.662  1.00 17.46
N
ATOM   2582  CA  ARG A  38      67.382  41.539  53.573  1.00 17.46
C
ATOM   2583  C   ARG A  38      68.151  42.660  52.894  1.00 17.46
C
ATOM   2584  O   ARG A  38      67.585  43.496  52.191  1.00 17.46
O
ATOM   2585  CB  ARG A  38      67.609  40.219  52.826  1.00 17.46
C
ATOM   2586  CG  ARG A  38      67.445  40.308  51.335  1.00 17.46
C
ATOM   2587  CD  ARG A  38      67.762  38.981  50.674  1.00 17.46
C
ATOM   2588  NE  ARG A  38      67.484  39.027  49.247  1.00 17.46
N
ATOM   2589  CZ  ARG A  38      67.506  37.970  48.445  1.00 17.46
C
ATOM   2590  NH1 ARG A  38      67.800  36.774  48.925  1.00 17.46
N
ATOM   2591  NH2 ARG A  38      67.234  38.114  47.160  1.00 17.46
N
ATOM   2592  N   GLN A  39      69.458  42.660  53.129  1.00 20.75
N
ATOM   2593  CA  GLN A  39      70.374  43.627  52.537  1.00 20.75
```

C
ATOM 2594 C GLN A 39 71.662 42.911 52.169 1.00 20.75
C
ATOM 2595 O GLN A 39 72.343 42.379 53.046 1.00 20.75
O
ATOM 2596 CB GLN A 39 70.661 44.772 53.509 1.00 20.75
C
ATOM 2597 CG GLN A 39 71.638 45.790 52.986 1.00 20.75
C
ATOM 2598 CD GLN A 39 71.596 47.067 53.779 1.00 20.75
C
ATOM 2599 OE1 GLN A 39 71.422 47.048 54.993 1.00 20.75
O
ATOM 2600 NE2 GLN A 39 71.747 48.187 53.097 1.00 20.75
N
ATOM 2601 N LEU A 40 71.978 42.881 50.910 1.00 24.03
N
ATOM 2602 CA LEU A 40 73.227 42.283 50.463 1.00 24.03
C
ATOM 2603 C LEU A 40 74.357 43.308 50.509 1.00 24.03
C
ATOM 2604 O LEU A 40 74.111 44.511 50.411 1.00 24.03
O
ATOM 2605 CB LEU A 40 73.077 41.746 49.045 1.00 24.03
C
ATOM 2606 N PRO A 41 75.605 42.870 50.672 1.00 26.76
N
ATOM 2607 CA PRO A 41 76.723 43.824 50.673 1.00 26.76
C
ATOM 2608 C PRO A 41 76.795 44.589 49.359 1.00 26.76
C
ATOM 2609 O PRO A 41 76.754 44.004 48.276 1.00 26.76
O
ATOM 2610 CB PRO A 41 77.955 42.934 50.877 1.00 26.76
C
ATOM 2611 CG PRO A 41 77.439 41.663 51.430 1.00 26.76
C
ATOM 2612 CD PRO A 41 76.054 41.487 50.898 1.00 26.76
C
ATOM 2613 N GLY A 42 76.894 45.909 49.463 1.00 28.41
N
ATOM 2614 CA GLY A 42 76.944 46.735 48.276 1.00 28.41
C
ATOM 2615 C GLY A 42 75.630 46.909 47.555 1.00 28.41
C
ATOM 2616 O GLY A 42 75.626 47.348 46.403 1.00 28.41
O
ATOM 2617 N LYS A 43 74.511 46.568 48.186 1.00 26.43
N
ATOM 2618 CA LYS A 43 73.200 46.750 47.586 1.00 26.43

C
ATOM 2619 C LYS A 43 72.260 47.342 48.623 1.00 26.43
C
ATOM 2620 O LYS A 43 72.561 47.380 49.816 1.00 26.43
O
ATOM 2621 CB LYS A 43 72.634 45.436 47.042 1.00 26.43
C
ATOM 2622 CG LYS A 43 73.523 44.737 46.041 1.00 26.43
C
ATOM 2623 CD LYS A 43 72.711 43.843 45.130 1.00 26.43
C
ATOM 2624 CE LYS A 43 73.570 43.269 44.017 1.00 26.43
C
ATOM 2625 NZ LYS A 43 74.835 44.034 43.832 1.00 26.43
N
ATOM 2626 N GLY A 44 71.111 47.811 48.152 1.00 20.13
N
ATOM 2627 CA GLY A 44 70.102 48.356 49.031 1.00 20.13
C
ATOM 2628 C GLY A 44 69.233 47.282 49.662 1.00 20.13
C
ATOM 2629 O GLY A 44 69.417 46.084 49.465 1.00 20.13
O
ATOM 2630 N LEU A 45 68.259 47.743 50.438 1.00 16.62
N
ATOM 2631 CA LEU A 45 67.336 46.856 51.135 1.00 16.62
C
ATOM 2632 C LEU A 45 66.403 46.149 50.158 1.00 16.62
C
ATOM 2633 O LEU A 45 65.990 46.709 49.143 1.00 16.62
O
ATOM 2634 CB LEU A 45 66.516 47.651 52.147 1.00 16.62
C
ATOM 2635 CG LEU A 45 67.302 48.358 53.246 1.00 16.62
C
ATOM 2636 CD1 LEU A 45 66.528 49.555 53.761 1.00 16.62
C
ATOM 2637 CD2 LEU A 45 67.597 47.390 54.373 1.00 16.62
C
ATOM 2638 N GLU A 46 66.064 44.902 50.479 1.00 18.11
N
ATOM 2639 CA GLU A 46 65.142 44.116 49.669 1.00 18.11
C
ATOM 2640 C GLU A 46 64.164 43.378 50.571 1.00 18.11
C
ATOM 2641 O GLU A 46 64.566 42.785 51.572 1.00 18.11
O
ATOM 2642 CB GLU A 46 65.893 43.112 48.788 1.00 18.11
C
ATOM 2643 CG GLU A 46 66.669 43.740 47.653 1.00 18.11

C
ATOM 2644 CD GLU A 46 67.177 42.710 46.671 1.00 18.11
C
ATOM 2645 OE1 GLU A 46 67.573 41.614 47.114 1.00 18.11
O
ATOM 2646 OE2 GLU A 46 67.171 42.995 45.460 1.00 18.11
O
ATOM 2647 N TRP A 47 62.888 43.409 50.206 1.00 14.93
N
ATOM 2648 CA TRP A 47 61.829 42.717 50.931 1.00 14.93
C
ATOM 2649 C TRP A 47 61.739 41.272 50.458 1.00 14.93
C
ATOM 2650 O TRP A 47 61.842 40.993 49.264 1.00 14.93
O
ATOM 2651 CB TRP A 47 60.512 43.460 50.700 1.00 14.93
C
ATOM 2652 CG TRP A 47 59.284 43.037 51.458 1.00 14.93
C
ATOM 2653 CD1 TRP A 47 58.810 43.581 52.614 1.00 14.93
C
ATOM 2654 CD2 TRP A 47 58.317 42.067 51.050 1.00 14.93
C
ATOM 2655 NE1 TRP A 47 57.631 42.982 52.970 1.00 14.93
N
ATOM 2656 CE2 TRP A 47 57.304 42.050 52.025 1.00 14.93
C
ATOM 2657 CE3 TRP A 47 58.220 41.195 49.968 1.00 14.93
C
ATOM 2658 CZ2 TRP A 47 56.209 41.198 51.945 1.00 14.93
C
ATOM 2659 CZ3 TRP A 47 57.133 40.350 49.892 1.00 14.93
C
ATOM 2660 CH2 TRP A 47 56.143 40.358 50.875 1.00 14.93
C
ATOM 2661 N VAL A 48 61.548 40.350 51.400 1.00 15.82
N
ATOM 2662 CA VAL A 48 61.666 38.927 51.098 1.00 15.82
C
ATOM 2663 C VAL A 48 60.298 38.249 51.070 1.00 15.82
C
ATOM 2664 O VAL A 48 59.830 37.829 50.009 1.00 15.82
O
ATOM 2665 CB VAL A 48 62.616 38.256 52.111 1.00 15.82
C
ATOM 2666 CG1 VAL A 48 62.717 36.778 51.894 1.00 15.82
C
ATOM 2667 CG2 VAL A 48 64.002 38.869 52.007 1.00 15.82
C
ATOM 2668 N ALA A 49 59.625 38.179 52.214 1.00 15.41

N
ATOM 2669 CA ALA A 49 58.382 37.425 52.299 1.00 15.41
C
ATOM 2670 C ALA A 49 57.612 37.849 53.539 1.00 15.41
C
ATOM 2671 O ALA A 49 58.183 38.387 54.490 1.00 15.41
O
ATOM 2672 CB ALA A 49 58.645 35.919 52.337 1.00 15.41
C
ATOM 2673 N VAL A 50 56.304 37.569 53.519 1.00 16.30
N
ATOM 2674 CA VAL A 50 55.409 37.873 54.630 1.00 16.30
C
ATOM 2675 C VAL A 50 54.476 36.693 54.879 1.00 16.30
C
ATOM 2676 O VAL A 50 54.025 36.013 53.946 1.00 16.30
O
ATOM 2677 CB VAL A 50 54.598 39.167 54.386 1.00 16.30
C
ATOM 2678 CG1 VAL A 50 53.648 39.002 53.208 1.00 16.30
C
ATOM 2679 CG2 VAL A 50 53.838 39.573 55.643 1.00 16.30
C
ATOM 2680 N ILE A 51 54.207 36.450 56.161 1.00 17.81
N
ATOM 2681 CA ILE A 51 53.243 35.453 56.615 1.00 17.81
C
ATOM 2682 C ILE A 51 52.194 36.105 57.517 1.00 17.81
C
ATOM 2683 O ILE A 51 52.533 36.806 58.485 1.00 17.81
O
ATOM 2684 CB ILE A 51 53.947 34.271 57.306 1.00 17.81
C
ATOM 2685 CG1 ILE A 51 52.946 33.157 57.609 1.00 17.81
C
ATOM 2686 CG2 ILE A 51 54.699 34.699 58.561 1.00 17.81
C
ATOM 2687 CD1 ILE A 51 53.584 31.818 57.800 1.00 17.81
C
ATOM 2688 N PRO A 52 50.907 35.931 57.191 1.00 20.70
N
ATOM 2689 CA PRO A 52 49.813 36.430 58.039 1.00 20.70
C
ATOM 2690 C PRO A 52 49.670 35.759 59.400 1.00 20.70
C
ATOM 2691 O PRO A 52 50.491 34.928 59.792 1.00 20.70
O
ATOM 2692 CB PRO A 52 48.580 36.157 57.171 1.00 20.70
C
ATOM 2693 CG PRO A 52 49.073 36.332 55.792 1.00 20.70

```
C
ATOM   2694  CD  PRO A  52      50.476  35.769  55.795  1.00 20.70
C
ATOM   2695  N   PHE A  53      48.605  36.133  60.122  1.00 22.17
N
ATOM   2696  CA  PHE A  53      48.444  35.763  61.529  1.00 22.17
C
ATOM   2697  C   PHE A  53      48.384  34.254  61.724  1.00 22.17
C
ATOM   2698  O   PHE A  53      49.020  33.712  62.632  1.00 22.17
O
ATOM   2699  CB  PHE A  53      47.177  36.406  62.094  1.00 22.17
C
ATOM   2700  CG  PHE A  53      46.994  37.828  61.691  1.00 22.17
C
ATOM   2701  CD1 PHE A  53      47.918  38.782  62.053  1.00 22.17
C
ATOM   2702  CD2 PHE A  53      45.900  38.214  60.948  1.00 22.17
C
ATOM   2703  CE1 PHE A  53      47.760  40.089  61.682  1.00 22.17
C
ATOM   2704  CE2 PHE A  53      45.738  39.524  60.577  1.00 22.17
C
ATOM   2705  CZ  PHE A  53      46.667  40.462  60.945  1.00 22.17
C
ATOM   2706  N   ASP A  54      47.603  33.563  60.903  1.00 30.50
N
ATOM   2707  CA  ASP A  54      47.511  32.116  61.019  1.00 30.50
C
ATOM   2708  C   ASP A  54      48.454  31.395  60.070  1.00 30.50
C
ATOM   2709  O   ASP A  54      48.843  30.257  60.346  1.00 30.50
O
ATOM   2710  CB  ASP A  54      46.071  31.654  60.777  1.00 30.50
C
ATOM   2711  CG  ASP A  54      45.601  31.929  59.370  1.00 30.50
C
ATOM   2712  OD1 ASP A  54      44.773  31.151  58.859  1.00 30.50
O
ATOM   2713  OD2 ASP A  54      46.052  32.926  58.773  1.00 30.50
O
ATOM   2714  N   GLY A  55      48.839  32.030  58.970  1.00 31.94
N
ATOM   2715  CA  GLY A  55      49.796  31.445  58.057  1.00 31.94
C
ATOM   2716  C   GLY A  55      49.211  30.606  56.948  1.00 31.94
C
ATOM   2717  O   GLY A  55      49.948  29.838  56.324  1.00 31.94
O
ATOM   2718  N  AARG A  56      47.910  30.723  56.680  0.50 42.12
```

N
ATOM 2719 CA AARG A 56 47.298 29.923 55.625 0.50 42.12
C
ATOM 2720 C AARG A 56 47.715 30.411 54.243 0.50 42.12
C
ATOM 2721 O AARG A 56 47.947 29.603 53.339 0.50 42.12
O
ATOM 2722 CB AARG A 56 45.779 29.946 55.776 0.50 42.12
C
ATOM 2723 CG AARG A 56 45.056 28.947 54.902 0.50 42.12
C
ATOM 2724 CD AARG A 56 43.561 28.967 55.166 0.50 42.12
C
ATOM 2725 NE AARG A 56 42.803 28.482 54.018 0.50 42.12
N
ATOM 2726 CZ AARG A 56 41.477 28.496 53.938 0.50 42.12
C
ATOM 2727 NH1AARG A 56 40.755 28.971 54.943 0.50 42.12
N
ATOM 2728 NH2AARG A 56 40.874 28.035 52.853 0.50 42.12
N
ATOM 2729 N BARG A 56 48.010 30.723 56.680 0.50 42.12
N
ATOM 2730 CA BARG A 56 47.398 29.923 55.625 0.50 42.12
C
ATOM 2731 C BARG A 56 47.815 30.411 54.243 0.50 42.12
C
ATOM 2732 O BARG A 56 48.047 29.603 53.339 0.50 42.12
O
ATOM 2733 CB BARG A 56 45.879 29.946 55.776 0.50 42.12
C
ATOM 2734 CG BARG A 56 45.376 29.330 57.061 0.50 42.12
C
ATOM 2735 CD BARG A 56 45.660 27.839 57.103 0.50 42.12
C
ATOM 2736 NE BARG A 56 45.159 27.228 58.329 0.50 42.12
N
ATOM 2737 CZ BARG A 56 45.466 25.999 58.732 0.50 42.12
C
ATOM 2738 NH1BARG A 56 46.275 25.243 58.003 0.50 42.12
N
ATOM 2739 NH2BARG A 56 44.963 25.528 59.862 0.50 42.12
N
ATOM 2740 N ASN A 57 47.806 31.722 54.057 1.00 33.59
N
ATOM 2741 CA ASN A 57 48.312 32.322 52.834 1.00 33.59
C
ATOM 2742 C ASN A 57 49.702 32.885 53.106 1.00 33.59
C
ATOM 2743 O ASN A 57 50.013 33.266 54.233 1.00 33.59

```
O
ATOM   2744  CB  ASN A  57      47.379  33.430  52.335  1.00 33.59
C
ATOM   2745  CG  ASN A  57      45.970  32.932  52.073  1.00 33.59
C
ATOM   2746  OD1 ASN A  57      45.694  32.346  51.033  1.00 33.59
O
ATOM   2747  ND2 ASN A  57      45.077  33.161  53.022  1.00 33.59
N
ATOM   2748  N   LYS A  58      50.548  32.901  52.080  1.00 26.66
N
ATOM   2749  CA  LYS A  58      51.920  33.383  52.208  1.00 26.66
C
ATOM   2750  C   LYS A  58      52.271  34.193  50.972  1.00 26.66
C
ATOM   2751  O   LYS A  58      51.860  33.834  49.868  1.00 26.66
O
ATOM   2752  CB  LYS A  58      52.906  32.218  52.363  1.00 26.66
C
ATOM   2753  CG  LYS A  58      52.614  31.320  53.542  1.00 26.66
C
ATOM   2754  CD  LYS A  58      53.387  30.023  53.466  1.00 26.66
C
ATOM   2755  CE  LYS A  58      53.230  29.220  54.741  1.00 26.66
C
ATOM   2756  NZ  LYS A  58      51.976  28.426  54.745  1.00 26.66
N
ATOM   2757  N   TYR A  59      53.035  35.276  51.138  1.00 22.55
N
ATOM   2758  CA  TYR A  59      53.344  36.119  49.987  1.00 22.55
C
ATOM   2759  C   TYR A  59      54.845  36.361  49.878  1.00 22.55
C
ATOM   2760  O   TYR A  59      55.517  36.590  50.885  1.00 22.55
O
ATOM   2761  CB  TYR A  59      52.578  37.443  50.061  1.00 22.55
C
ATOM   2762  CG  TYR A  59      51.077  37.248  50.045  1.00 22.55
C
ATOM   2763  CD1 TYR A  59      50.319  37.443  51.186  1.00 22.55
C
ATOM   2764  CD2 TYR A  59      50.426  36.843  48.889  1.00 22.55
C
ATOM   2765  CE1 TYR A  59      48.951  37.255  51.175  1.00 22.55
C
ATOM   2766  CE2 TYR A  59      49.060  36.651  48.870  1.00 22.55
C
ATOM   2767  CZ  TYR A  59      48.328  36.858  50.014  1.00 22.55
C
ATOM   2768  OH  TYR A  59      46.966  36.668  50.000  1.00 22.55
```

O
ATOM 2769 N TYR A 60 55.362 36.307 48.645 1.00 23.35
N
ATOM 2770 CA TYR A 60 56.793 36.349 48.339 1.00 23.35
C
ATOM 2771 C TYR A 60 57.074 37.419 47.285 1.00 23.35
C
ATOM 2772 O TYR A 60 56.160 38.066 46.770 1.00 23.35
O
ATOM 2773 CB TYR A 60 57.297 34.991 47.841 1.00 23.35
C
ATOM 2774 CG TYR A 60 57.047 33.836 48.776 1.00 23.35
C
ATOM 2775 CD1 TYR A 60 57.978 33.489 49.738 1.00 23.35
C
ATOM 2776 CD2 TYR A 60 55.893 33.074 48.677 1.00 23.35
C
ATOM 2777 CE1 TYR A 60 57.765 32.436 50.584 1.00 23.35
C
ATOM 2778 CE2 TYR A 60 55.671 32.016 49.519 1.00 23.35
C
ATOM 2779 CZ TYR A 60 56.609 31.696 50.469 1.00 23.35
C
ATOM 2780 OH TYR A 60 56.391 30.640 51.314 1.00 23.35
O
ATOM 2781 N ALA A 61 58.355 37.581 46.927 1.00 28.48
N
ATOM 2782 CA ALA A 61 58.798 38.687 46.081 1.00 28.48
C
ATOM 2783 C ALA A 61 59.374 38.264 44.724 1.00 28.48
C
ATOM 2784 O ALA A 61 60.317 38.899 44.244 1.00 28.48
O
ATOM 2785 CB ALA A 61 59.828 39.536 46.822 1.00 28.48
C
ATOM 2786 N ASP A 62 58.856 37.204 44.109 1.00 34.97
N
ATOM 2787 CA ASP A 62 59.034 36.937 42.677 1.00 34.97
C
ATOM 2788 C ASP A 62 60.432 36.503 42.261 1.00 34.97
C
ATOM 2789 O ASP A 62 60.627 36.061 41.127 1.00 34.97
O
ATOM 2790 CB ASP A 62 58.674 38.172 41.848 1.00 34.97
C
ATOM 2791 CG ASP A 62 57.242 38.181 41.414 1.00 34.97
C
ATOM 2792 OD1 ASP A 62 56.522 37.216 41.734 1.00 34.97
O
ATOM 2793 OD2 ASP A 62 56.836 39.155 40.754 1.00 34.97

O
ATOM 2794 N SER A 63 61.405 36.602 43.151 1.00 33.18
N
ATOM 2795 CA SER A 63 62.747 36.132 42.868 1.00 33.18
C
ATOM 2796 C SER A 63 63.236 35.145 43.906 1.00 33.18
C
ATOM 2797 O SER A 63 64.240 34.466 43.670 1.00 33.18
O
ATOM 2798 CB SER A 63 63.728 37.310 42.773 1.00 33.18
C
ATOM 2799 OG SER A 63 64.475 37.450 43.964 1.00 33.18
O
ATOM 2800 N LEU A 64 62.559 35.051 45.043 1.00 31.30
N
ATOM 2801 CA LEU A 64 62.778 33.998 46.014 1.00 31.30
C
ATOM 2802 C LEU A 64 61.642 32.993 46.027 1.00 31.30
C
ATOM 2803 O LEU A 64 61.675 32.063 46.835 1.00 31.30
O
ATOM 2804 CB LEU A 64 62.888 34.553 47.430 1.00 31.30
C
ATOM 2805 CG LEU A 64 61.689 35.433 47.750 1.00 31.30
C
ATOM 2806 CD1 LEU A 64 61.552 35.411 49.197 1.00 31.30
C
ATOM 2807 CD2 LEU A 64 61.941 36.852 47.343 1.00 31.30
C
ATOM 2808 N THR A 65 60.622 33.180 45.184 1.00 33.19
N
ATOM 2809 CA THR A 65 59.404 32.380 45.288 1.00 33.19
C
ATOM 2810 C THR A 65 59.697 30.891 45.175 1.00 33.19
C
ATOM 2811 O THR A 65 59.054 30.073 45.840 1.00 33.19
O
ATOM 2812 CB THR A 65 58.402 32.803 44.215 1.00 33.19
C
ATOM 2813 OG1 THR A 65 58.176 34.212 44.298 1.00 33.19
O
ATOM 2814 CG2 THR A 65 57.083 32.082 44.406 1.00 33.19
C
ATOM 2815 N GLY A 66 60.667 30.519 44.343 1.00 33.63
N
ATOM 2816 CA GLY A 66 60.953 29.107 44.159 1.00 33.63
C
ATOM 2817 C GLY A 66 61.652 28.479 45.350 1.00 33.63
C
ATOM 2818 O GLY A 66 61.260 27.408 45.819 1.00 33.63

O
ATOM 2819 N ARG A 67 62.688 29.137 45.863 1.00 35.90
N
ATOM 2820 CA ARG A 67 63.618 28.495 46.780 1.00 35.90
C
ATOM 2821 C ARG A 67 63.397 28.845 48.245 1.00 35.90
C
ATOM 2822 O ARG A 67 63.998 28.199 49.109 1.00 35.90
O
ATOM 2823 CB ARG A 67 65.057 28.851 46.392 1.00 35.90
C
ATOM 2824 CG ARG A 67 65.468 28.329 45.022 1.00 35.90
C
ATOM 2825 CD ARG A 67 66.928 28.603 44.740 1.00 35.90
C
ATOM 2826 NE ARG A 67 67.209 30.031 44.645 1.00 35.90
N
ATOM 2827 CZ ARG A 67 68.129 30.657 45.368 1.00 35.90
C
ATOM 2828 NH1 ARG A 67 68.858 29.984 46.243 1.00 35.90
N
ATOM 2829 NH2 ARG A 67 68.320 31.958 45.215 1.00 35.90
N
ATOM 2830 N PHE A 68 62.568 29.839 48.551 1.00 30.72
N
ATOM 2831 CA PHE A 68 62.376 30.314 49.915 1.00 30.72
C
ATOM 2832 C PHE A 68 60.942 30.039 50.344 1.00 30.72
C
ATOM 2833 O PHE A 68 60.007 30.307 49.586 1.00 30.72
O
ATOM 2834 CB PHE A 68 62.678 31.815 50.026 1.00 30.72
C
ATOM 2835 CG PHE A 68 64.135 32.169 49.866 1.00 30.72
C
ATOM 2836 CD1 PHE A 68 64.835 32.761 50.898 1.00 30.72
C
ATOM 2837 CD2 PHE A 68 64.801 31.927 48.682 1.00 30.72
C
ATOM 2838 CE1 PHE A 68 66.165 33.101 50.747 1.00 30.72
C
ATOM 2839 CE2 PHE A 68 66.130 32.259 48.535 1.00 30.72
C
ATOM 2840 CZ PHE A 68 66.811 32.843 49.569 1.00 30.72
C
ATOM 2841 N THR A 69 60.770 29.507 51.550 1.00 26.02
N
ATOM 2842 CA THR A 69 59.444 29.300 52.118 1.00 26.02
C
ATOM 2843 C THR A 69 59.412 29.794 53.560 1.00 26.02

C
ATOM 2844 O THR A 69 60.316 29.509 54.346 1.00 26.02
O
ATOM 2845 CB THR A 69 59.021 27.818 52.041 1.00 26.02
C
ATOM 2846 OG1 THR A 69 57.827 27.612 52.804 1.00 26.02
O
ATOM 2847 CG2 THR A 69 60.108 26.916 52.564 1.00 26.02
C
ATOM 2848 N ILE A 70 58.362 30.529 53.907 1.00 20.50
N
ATOM 2849 CA ILE A 70 58.229 31.147 55.221 1.00 20.50
C
ATOM 2850 C ILE A 70 57.194 30.370 56.025 1.00 20.50
C
ATOM 2851 O ILE A 70 56.222 29.857 55.463 1.00 20.50
O
ATOM 2852 CB ILE A 70 57.851 32.637 55.085 1.00 20.50
C
ATOM 2853 CG1 ILE A 70 57.895 33.348 56.438 1.00 20.50
C
ATOM 2854 CG2 ILE A 70 56.499 32.803 54.399 1.00 20.50
C
ATOM 2855 CD1 ILE A 70 57.869 34.858 56.325 1.00 20.50
C
ATOM 2856 N SER A 71 57.412 30.263 57.335 1.00 21.23
N
ATOM 2857 CA SER A 71 56.469 29.562 58.200 1.00 21.23
C
ATOM 2858 C SER A 71 56.519 30.166 59.597 1.00 21.23
C
ATOM 2859 O SER A 71 57.419 30.934 59.924 1.00 21.23
O
ATOM 2860 CB SER A 71 56.761 28.057 58.239 1.00 21.23
C
ATOM 2861 OG SER A 71 57.693 27.745 59.250 1.00 21.23
O
ATOM 2862 N ARG A 72 55.521 29.842 60.418 1.00 21.11
N
ATOM 2863 CA ARG A 72 55.459 30.375 61.772 1.00 21.11
C
ATOM 2864 C ARG A 72 54.854 29.347 62.715 1.00 21.11
C
ATOM 2865 O ARG A 72 54.071 28.491 62.303 1.00 21.11
O
ATOM 2866 CB ARG A 72 54.639 31.680 61.843 1.00 21.11
C
ATOM 2867 CG ARG A 72 53.148 31.521 61.551 1.00 21.11
C
ATOM 2868 CD ARG A 72 52.428 32.863 61.506 1.00 21.11

C
ATOM 2869 NE ARG A 72 52.422 33.521 62.806 1.00 21.11
N
ATOM 2870 CZ ARG A 72 52.230 34.823 62.990 1.00 21.11
C
ATOM 2871 NH1 ARG A 72 52.246 35.324 64.210 1.00 21.11
N
ATOM 2872 NH2 ARG A 72 52.027 35.621 61.956 1.00 21.11
N
ATOM 2873 N ASP A 73 55.229 29.447 63.987 1.00 26.52
N
ATOM 2874 CA ASP A 73 54.645 28.659 65.066 1.00 26.52
C
ATOM 2875 C ASP A 73 54.186 29.630 66.144 1.00 26.52
C
ATOM 2876 O ASP A 73 55.013 30.236 66.834 1.00 26.52
O
ATOM 2877 CB ASP A 73 55.648 27.648 65.623 1.00 26.52
C
ATOM 2878 CG ASP A 73 54.992 26.584 66.484 1.00 26.52
C
ATOM 2879 OD1 ASP A 73 55.559 25.480 66.590 1.00 26.52
O
ATOM 2880 OD2 ASP A 73 53.916 26.846 67.055 1.00 26.52
O
ATOM 2881 N ASN A 74 52.868 29.784 66.279 1.00 26.33
N
ATOM 2882 CA ASN A 74 52.301 30.699 67.259 1.00 26.33
C
ATOM 2883 C ASN A 74 52.351 30.147 68.676 1.00 26.33
C
ATOM 2884 O ASN A 74 52.301 30.927 69.631 1.00 26.33
O
ATOM 2885 CB ASN A 74 50.859 31.040 66.881 1.00 26.33
C
ATOM 2886 CG ASN A 74 50.774 31.895 65.635 1.00 26.33
C
ATOM 2887 OD1 ASN A 74 51.754 32.514 65.236 1.00 26.33
O
ATOM 2888 ND2 ASN A 74 49.608 31.929 65.012 1.00 26.33
N
ATOM 2889 N SER A 75 52.457 28.828 68.834 1.00 28.76
N
ATOM 2890 CA SER A 75 52.576 28.255 70.170 1.00 28.76
C
ATOM 2891 C SER A 75 53.936 28.561 70.780 1.00 28.76
C
ATOM 2892 O SER A 75 54.039 28.813 71.984 1.00 28.76
O
ATOM 2893 CB SER A 75 52.328 26.749 70.120 1.00 28.76

C
ATOM 2894 OG SER A 75 53.412 26.068 69.522 1.00 28.76
O
ATOM 2895 N LYS A 76 54.989 28.552 69.964 1.00 29.03
N
ATOM 2896 CA LYS A 76 56.337 28.865 70.415 1.00 29.03
C
ATOM 2897 C LYS A 76 56.731 30.313 70.161 1.00 29.03
C
ATOM 2898 O LYS A 76 57.823 30.719 70.566 1.00 29.03
O
ATOM 2899 CB LYS A 76 57.346 27.940 69.733 1.00 29.03
C
ATOM 2900 CG LYS A 76 56.870 26.513 69.593 1.00 29.03
C
ATOM 2901 CD LYS A 76 57.989 25.607 69.136 1.00 29.03
C
ATOM 2902 CE LYS A 76 57.607 24.153 69.307 1.00 29.03
C
ATOM 2903 NZ LYS A 76 56.580 23.727 68.319 1.00 29.03
N
ATOM 2904 N ASN A 77 55.868 31.096 69.510 1.00 27.35
N
ATOM 2905 CA ASN A 77 56.158 32.486 69.155 1.00 27.35
C
ATOM 2906 C ASN A 77 57.416 32.597 68.293 1.00 27.35
C
ATOM 2907 O ASN A 77 58.286 33.429 68.539 1.00 27.35
O
ATOM 2908 CB ASN A 77 56.284 33.363 70.403 1.00 27.35
C
ATOM 2909 CG ASN A 77 54.999 33.438 71.196 1.00 27.35
C
ATOM 2910 OD1 ASN A 77 53.939 33.046 70.720 1.00 27.35
O
ATOM 2911 ND2 ASN A 77 55.090 33.948 72.412 1.00 27.35
N
ATOM 2912 N THR A 78 57.507 31.756 67.265 1.00 24.95
N
ATOM 2913 CA THR A 78 58.717 31.698 66.454 1.00 24.95
C
ATOM 2914 C THR A 78 58.391 31.800 64.971 1.00 24.95
C
ATOM 2915 O THR A 78 57.455 31.170 64.483 1.00 24.95
O
ATOM 2916 CB THR A 78 59.501 30.408 66.732 1.00 24.95
C
ATOM 2917 OG1 THR A 78 59.799 30.334 68.128 1.00 24.95
O
ATOM 2918 CG2 THR A 78 60.804 30.396 65.961 1.00 24.95

```
C
ATOM   2919  N   LEU A  79      59.186  32.591  64.258  1.00 21.29
N
ATOM   2920  CA  LEU A  79      59.093  32.761  62.818  1.00 21.29
C
ATOM   2921  C   LEU A  79      60.247  32.016  62.159  1.00 21.29
C
ATOM   2922  O   LEU A  79      61.359  32.017  62.680  1.00 21.29
O
ATOM   2923  CB  LEU A  79      59.144  34.250  62.467  1.00 21.29
C
ATOM   2924  CG  LEU A  79      59.212  34.684  61.009  1.00 21.29
C
ATOM   2925  CD1 LEU A  79      57.894  34.414  60.328  1.00 21.29
C
ATOM   2926  CD2 LEU A  79      59.577  36.157  60.934  1.00 21.29
C
ATOM   2927  N   TYR A  80      59.988  31.379  61.018  1.00 23.26
N
ATOM   2928  CA  TYR A  80      60.979  30.579  60.312  1.00 23.26
C
ATOM   2929  C   TYR A  80      61.058  30.988  58.852  1.00 23.26
C
ATOM   2930  O   TYR A  80      60.035  31.258  58.218  1.00 23.26
O
ATOM   2931  CB  TYR A  80      60.646  29.082  60.359  1.00 23.26
C
ATOM   2932  CG  TYR A  80      60.483  28.499  61.735  1.00 23.26
C
ATOM   2933  CD1 TYR A  80      61.583  28.242  62.532  1.00 23.26
C
ATOM   2934  CD2 TYR A  80      59.227  28.180  62.228  1.00 23.26
C
ATOM   2935  CE1 TYR A  80      61.439  27.697  63.784  1.00 23.26
C
ATOM   2936  CE2 TYR A  80      59.075  27.638  63.481  1.00 23.26
C
ATOM   2937  CZ  TYR A  80      60.184  27.400  64.254  1.00 23.26
C
ATOM   2938  OH  TYR A  80      60.036  26.857  65.507  1.00 23.26
O
ATOM   2939  N   LEU A  81      62.275  30.982  58.312  1.00 24.24
N
ATOM   2940  CA  LEU A  81      62.502  31.085  56.878  1.00 24.24
C
ATOM   2941  C   LEU A  81      63.383  29.918  56.456  1.00 24.24
C
ATOM   2942  O   LEU A  81      64.447  29.702  57.037  1.00 24.24
O
ATOM   2943  CB  LEU A  81      63.157  32.422  56.504  1.00 24.24
```

```
C
ATOM   2944  CG  LEU A  81      63.353  32.721  55.016  1.00 24.24
C
ATOM   2945  CD1 LEU A  81      62.025  32.823  54.291  1.00 24.24
C
ATOM   2946  CD2 LEU A  81      64.162  33.995  54.825  1.00 24.24
C
ATOM   2947  N   GLN A  82      62.933  29.165  55.461  1.00 32.03
N
ATOM   2948  CA  GLN A  82      63.648  28.011  54.933  1.00 32.03
C
ATOM   2949  C   GLN A  82      64.130  28.344  53.531  1.00 32.03
C
ATOM   2950  O   GLN A  82      63.324  28.707  52.669  1.00 32.03
O
ATOM   2951  CB  GLN A  82      62.738  26.780  54.913  1.00 32.03
C
ATOM   2952  CG  GLN A  82      63.352  25.535  54.294  1.00 32.03
C
ATOM   2953  CD  GLN A  82      64.504  24.984  55.095  1.00 32.03
C
ATOM   2954  OE1 GLN A  82      64.454  24.933  56.320  1.00 32.03
O
ATOM   2955  NE2 GLN A  82      65.557  24.570  54.404  1.00 32.03
N
ATOM   2956  N   MET A  83      65.434  28.227  53.301  1.00 35.31
N
ATOM   2957  CA  MET A  83      66.017  28.469  51.992  1.00 35.31
C
ATOM   2958  C   MET A  83      66.651  27.193  51.466  1.00 35.31
C
ATOM   2959  O   MET A  83      67.370  26.505  52.196  1.00 35.31
O
ATOM   2960  CB  MET A  83      67.065  29.578  52.021  1.00 35.31
C
ATOM   2961  CG  MET A  83      66.821  30.705  52.988  1.00 35.31
C
ATOM   2962  SD  MET A  83      68.392  31.477  53.394  1.00 35.31
S
ATOM   2963  CE  MET A  83      67.878  32.558  54.684  1.00 35.31
C
ATOM   2964  N   ASN A  84      66.402  26.906  50.195  1.00 41.01
N
ATOM   2965  CA  ASN A  84      66.873  25.697  49.537  1.00 41.01
C
ATOM   2966  C   ASN A  84      67.929  26.042  48.498  1.00 41.01
C
ATOM   2967  O   ASN A  84      67.713  26.926  47.663  1.00 41.01
O
ATOM   2968  CB  ASN A  84      65.710  24.968  48.869  1.00 41.01
```

C
ATOM 2969 CG ASN A 84 64.682 24.491 49.860 1.00 41.01
C
ATOM 2970 OD1 ASN A 84 65.014 24.112 50.980 1.00 41.01
O
ATOM 2971 ND2 ASN A 84 63.420 24.518 49.459 1.00 41.01
N
ATOM 2972 N SER A 85 69.063 25.338 48.550 1.00 43.61
N
ATOM 2973 CA SER A 85 70.098 25.389 47.516 1.00 43.61
C
ATOM 2974 C SER A 85 70.614 26.818 47.317 1.00 43.61
C
ATOM 2975 O SER A 85 70.392 27.460 46.291 1.00 43.61
O
ATOM 2976 CB SER A 85 69.582 24.798 46.199 1.00 43.61
C
ATOM 2977 OG SER A 85 68.698 25.693 45.553 1.00 43.61
O
ATOM 2978 N LEU A 86 71.294 27.304 48.350 1.00 42.85
N
ATOM 2979 CA LEU A 86 71.771 28.679 48.349 1.00 42.85
C
ATOM 2980 C LEU A 86 72.818 28.905 47.265 1.00 42.85
C
ATOM 2981 O LEU A 86 73.733 28.099 47.081 1.00 42.85
O
ATOM 2982 CB LEU A 86 72.343 29.029 49.721 1.00 42.85
C
ATOM 2983 CG LEU A 86 71.258 29.238 50.780 1.00 42.85
C
ATOM 2984 CD1 LEU A 86 71.842 29.741 52.080 1.00 42.85
C
ATOM 2985 CD2 LEU A 86 70.221 30.208 50.262 1.00 42.85
C
ATOM 2986 N ARG A 87 72.675 30.017 46.545 1.00 45.02
N
ATOM 2987 CA ARG A 87 73.621 30.441 45.526 1.00 45.02
C
ATOM 2988 C ARG A 87 74.525 31.543 46.073 1.00 45.02
C
ATOM 2989 O ARG A 87 74.364 32.017 47.198 1.00 45.02
O
ATOM 2990 CB ARG A 87 72.889 30.930 44.278 1.00 45.02
C
ATOM 2991 CG ARG A 87 71.642 30.159 43.922 1.00 45.02
C
ATOM 2992 CD ARG A 87 70.999 30.753 42.684 1.00 45.02
C
ATOM 2993 NE ARG A 87 69.720 30.131 42.360 1.00 45.02

N
ATOM 2994 CZ ARG A 87 69.591 28.940 41.784 1.00 45.02
C
ATOM 2995 NH1 ARG A 87 70.667 28.232 41.470 1.00 45.02
N
ATOM 2996 NH2 ARG A 87 68.385 28.455 41.523 1.00 45.02
N
ATOM 2997 N ALA A 88 75.478 31.974 45.243 1.00 44.00
N
ATOM 2998 CA ALA A 88 76.434 32.993 45.667 1.00 44.00
C
ATOM 2999 C ALA A 88 75.752 34.331 45.927 1.00 44.00
C
ATOM 3000 O ALA A 88 76.131 35.058 46.851 1.00 44.00
O
ATOM 3001 CB ALA A 88 77.534 33.149 44.619 1.00 44.00
C
ATOM 3002 N GLU A 89 74.749 34.674 45.124 1.00 44.35
N
ATOM 3003 CA GLU A 89 74.059 35.948 45.274 1.00 44.35
C
ATOM 3004 C GLU A 89 73.097 35.975 46.453 1.00 44.35
C
ATOM 3005 O GLU A 89 72.541 37.036 46.748 1.00 44.35
O
ATOM 3006 CB GLU A 89 73.294 36.292 43.995 1.00 44.35
C
ATOM 3007 CG GLU A 89 74.120 36.187 42.729 1.00 44.35
C
ATOM 3008 CD GLU A 89 75.528 36.712 42.908 1.00 44.35
C
ATOM 3009 OE1 GLU A 89 75.683 37.907 43.231 1.00 44.35
O
ATOM 3010 OE2 GLU A 89 76.481 35.927 42.729 1.00 44.35
O
ATOM 3011 N ASP A 90 72.876 34.844 47.120 1.00 38.14
N
ATOM 3012 CA ASP A 90 71.997 34.808 48.278 1.00 38.14
C
ATOM 3013 C ASP A 90 72.659 35.351 49.539 1.00 38.14
C
ATOM 3014 O ASP A 90 71.979 35.492 50.559 1.00 38.14
O
ATOM 3015 CB ASP A 90 71.509 33.375 48.514 1.00 38.14
C
ATOM 3016 CG ASP A 90 70.645 32.862 47.380 1.00 38.14
C
ATOM 3017 OD1 ASP A 90 70.236 33.669 46.528 1.00 38.14
O
ATOM 3018 OD2 ASP A 90 70.369 31.647 47.339 1.00 38.14

```
O
ATOM   3019  N   THR A  91      73.953  35.658  49.490  1.00 32.30
N
ATOM   3020  CA  THR A  91      74.661  36.231  50.630  1.00 32.30
C
ATOM   3021  C   THR A  91      74.067  37.581  51.015  1.00 32.30
C
ATOM   3022  O   THR A  91      74.021  38.497  50.191  1.00 32.30
O
ATOM   3023  CB  THR A  91      76.140  36.396  50.284  1.00 32.30
C
ATOM   3024  OG1 THR A  91      76.726  35.109  50.052  1.00 32.30
O
ATOM   3025  CG2 THR A  91      76.875  37.093  51.405  1.00 32.30
C
ATOM   3026  N   ALA A  92      73.644  37.714  52.271  1.00 23.56
N
ATOM   3027  CA  ALA A  92      72.968  38.932  52.710  1.00 23.56
C
ATOM   3028  C   ALA A  92      72.791  38.910  54.220  1.00 23.56
C
ATOM   3029  O   ALA A  92      72.874  37.859  54.856  1.00 23.56
O
ATOM   3030  CB  ALA A  92      71.604  39.094  52.030  1.00 23.56
C
ATOM   3031  N   VAL A  93      72.521  40.081  54.780  1.00 20.17
N
ATOM   3032  CA  VAL A  93      72.068  40.206  56.158  1.00 20.17
C
ATOM   3033  C   VAL A  93      70.546  40.203  56.153  1.00 20.17
C
ATOM   3034  O   VAL A  93      69.922  40.973  55.420  1.00 20.17
O
ATOM   3035  CB  VAL A  93      72.610  41.491  56.802  1.00 20.17
C
ATOM   3036  CG1 VAL A  93      72.168  41.591  58.242  1.00 20.17
C
ATOM   3037  CG2 VAL A  93      74.122  41.532  56.702  1.00 20.17
C
ATOM   3038  N   TYR A  94      69.948  39.341  56.966  1.00 19.75
N
ATOM   3039  CA  TYR A  94      68.501  39.180  57.028  1.00 19.75
C
ATOM   3040  C   TYR A  94      67.974  39.786  58.321  1.00 19.75
C
ATOM   3041  O   TYR A  94      68.486  39.492  59.403  1.00 19.75
O
ATOM   3042  CB  TYR A  94      68.113  37.702  56.932  1.00 19.75
C
ATOM   3043  CG  TYR A  94      68.269  37.127  55.547  1.00 19.75
```

C
ATOM 3044 CD1 TYR A 94 67.171 36.924 54.727 1.00 19.75
C
ATOM 3045 CD2 TYR A 94 69.519 36.793 55.055 1.00 19.75
C
ATOM 3046 CE1 TYR A 94 67.318 36.411 53.461 1.00 19.75
C
ATOM 3047 CE2 TYR A 94 69.672 36.280 53.795 1.00 19.75
C
ATOM 3048 CZ TYR A 94 68.571 36.088 53.001 1.00 19.75
C
ATOM 3049 OH TYR A 94 68.727 35.578 51.739 1.00 19.75
O
ATOM 3050 N TYR A 95 66.953 40.628 58.202 1.00 21.79
N
ATOM 3051 CA TYR A 95 66.292 41.281 59.318 1.00 21.79
C
ATOM 3052 C TYR A 95 64.868 40.758 59.459 1.00 21.79
C
ATOM 3053 O TYR A 95 64.164 40.566 58.464 1.00 21.79
O
ATOM 3054 CB TYR A 95 66.223 42.794 59.125 1.00 21.79
C
ATOM 3055 CG TYR A 95 67.539 43.497 58.903 1.00 21.79
C
ATOM 3056 CD1 TYR A 95 68.348 43.848 59.969 1.00 21.79
C
ATOM 3057 CD2 TYR A 95 67.940 43.859 57.628 1.00 21.79
C
ATOM 3058 CE1 TYR A 95 69.540 44.514 59.766 1.00 21.79
C
ATOM 3059 CE2 TYR A 95 69.127 44.525 57.417 1.00 21.79
C
ATOM 3060 CZ TYR A 95 69.921 44.847 58.488 1.00 21.79
C
ATOM 3061 OH TYR A 95 71.107 45.511 58.281 1.00 21.79
O
ATOM 3062 N CYS A 96 64.447 40.575 60.703 1.00 21.29
N
ATOM 3063 CA CYS A 96 63.102 40.152 61.067 1.00 21.29
C
ATOM 3064 C CYS A 96 62.245 41.380 61.372 1.00 21.29
C
ATOM 3065 O CYS A 96 62.719 42.325 62.003 1.00 21.29
O
ATOM 3066 CB CYS A 96 63.191 39.230 62.289 1.00 21.29
C
ATOM 3067 SG CYS A 96 61.717 38.366 62.800 1.00 21.29
S
ATOM 3068 N ALA A 97 60.986 41.375 60.928 1.00 17.02

N
ATOM 3069 CA ALA A 97 60.125 42.543 61.085 1.00 17.02
C
ATOM 3070 C ALA A 97 58.677 42.103 61.263 1.00 17.02
C
ATOM 3071 O ALA A 97 58.315 40.973 60.947 1.00 17.02
O
ATOM 3072 CB ALA A 97 60.250 43.487 59.885 1.00 17.02
C
ATOM 3073 N SER A 98 57.841 43.016 61.762 1.00 18.29
N
ATOM 3074 CA SER A 98 56.449 42.698 62.067 1.00 18.29
C
ATOM 3075 C SER A 98 55.534 43.856 61.684 1.00 18.29
C
ATOM 3076 O SER A 98 55.981 44.985 61.493 1.00 18.29
O
ATOM 3077 CB SER A 98 56.268 42.365 63.549 1.00 18.29
C
ATOM 3078 OG SER A 98 56.654 43.450 64.362 1.00 18.29
O
ATOM 3079 N SER A 99 54.240 43.555 61.558 1.00 19.21
N
ATOM 3080 CA SER A 99 53.239 44.576 61.263 1.00 19.21
C
ATOM 3081 C SER A 99 51.890 44.186 61.855 1.00 19.21
C
ATOM 3082 O SER A 99 51.530 43.010 61.905 1.00 19.21
O
ATOM 3083 CB SER A 99 53.091 44.816 59.754 1.00 19.21
C
ATOM 3084 OG SER A 99 52.770 43.626 59.066 1.00 19.21
O
ATOM 3085 N SER A 100 51.133 45.195 62.280 1.00 22.37
N
ATOM 3086 CA SER A 100 49.809 45.001 62.861 1.00 22.37
C
ATOM 3087 C SER A 100 48.737 45.361 61.840 1.00 22.37
C
ATOM 3088 O SER A 100 48.802 46.422 61.218 1.00 22.37
O
ATOM 3089 CB SER A 100 49.639 45.851 64.122 1.00 22.37
C
ATOM 3090 OG SER A 100 48.370 45.635 64.702 1.00 22.37
O
ATOM 3091 N GLY A 101 47.760 44.477 61.668 1.00 22.20
N
ATOM 3092 CA GLY A 101 46.704 44.714 60.697 1.00 22.20
C
ATOM 3093 C GLY A 101 47.177 44.748 59.261 1.00 22.20

C
ATOM   3094 O   GLY A 101   46.541 45.390 58.422 1.00 22.20
O
ATOM   3095 N   PHE A 102   48.284 44.070 58.961 1.00 19.83
N
ATOM   3096 CA PHE A 102   48.964 44.068 57.666 1.00 19.83
C
ATOM   3097 C   PHE A 102   49.500 45.435 57.275 1.00 19.83
C
ATOM   3098 O   PHE A 102   49.954 45.613 56.145 1.00 19.83
O
ATOM   3099 CB PHE A 102   48.067 43.541 56.539 1.00 19.83
C
ATOM   3100 CG PHE A 102   47.617 42.132 56.732 1.00 19.83
C
ATOM   3101 CD1 PHE A 102   48.533 41.119 56.913 1.00 19.83
C
ATOM   3102 CD2 PHE A 102   46.276 41.817 56.714 1.00 19.83
C
ATOM   3103 CE1 PHE A 102   48.119 39.823 57.087 1.00 19.83
C
ATOM   3104 CE2 PHE A 102   45.860 40.520 56.887 1.00 19.83
C
ATOM   3105 CZ PHE A 102   46.782 39.522 57.074 1.00 19.83
C
ATOM   3106 N   LEU A 103   49.470 46.406 58.175 1.00 21.21
N
ATOM   3107 CA LEU A 103   49.796 47.782 57.841 1.00 21.21
C
ATOM   3108 C   LEU A 103   51.161 48.136 58.403 1.00 21.21
C
ATOM   3109 O   LEU A 103   51.436 47.890 59.580 1.00 21.21
O
ATOM   3110 CB LEU A 103   48.736 48.742 58.379 1.00 21.21
C
ATOM   3111 CG LEU A 103   49.050 50.225 58.177 1.00 21.21
C
ATOM   3112 CD1 LEU A 103   49.339 50.516 56.716 1.00 21.21
C
ATOM   3113 CD2 LEU A 103   47.907 51.071 58.676 1.00 21.21
C
ATOM   3114 N   PHE A 104   52.006 48.713 57.560 1.00 24.72
N
ATOM   3115 CA PHE A 104   53.325 49.147 57.992 1.00 24.72
C
ATOM   3116 C   PHE A 104   53.191 50.308 58.969 1.00 24.72
C
ATOM   3117 O   PHE A 104   52.694 51.378 58.606 1.00 24.72
O
ATOM   3118 CB PHE A 104   54.168 49.556 56.786 1.00 24.72

C
ATOM 3119 CG PHE A 104 55.439 50.243 57.158 1.00 24.72
C
ATOM 3120 CD1 PHE A 104 56.396 49.582 57.894 1.00 24.72
C
ATOM 3121 CD2 PHE A 104 55.661 51.558 56.804 1.00 24.72
C
ATOM 3122 CE1 PHE A 104 57.549 50.211 58.258 1.00 24.72
C
ATOM 3123 CE2 PHE A 104 56.812 52.190 57.167 1.00 24.72
C
ATOM 3124 CZ PHE A 104 57.758 51.520 57.894 1.00 24.72
C
ATOM 3125 N HIS A 105 53.572 50.081 60.217 1.00 34.66
N
ATOM 3126 CA HIS A 105 53.597 51.168 61.187 1.00 34.66
C
ATOM 3127 C HIS A 105 54.741 51.083 62.187 1.00 34.66
C
ATOM 3128 O HIS A 105 54.823 51.952 63.058 1.00 34.66
O
ATOM 3129 CB HIS A 105 52.262 51.215 61.942 1.00 34.66
C
ATOM 3130 CG HIS A 105 52.100 50.110 62.937 1.00 34.66
C
ATOM 3131 ND1 HIS A 105 51.854 50.343 64.273 1.00 34.66
N
ATOM 3132 CD2 HIS A 105 52.163 48.765 62.795 1.00 34.66
C
ATOM 3133 CE1 HIS A 105 51.767 49.189 64.909 1.00 34.66
C
ATOM 3134 NE2 HIS A 105 51.950 48.216 64.036 1.00 34.66
N
ATOM 3135 N SER A 106 55.624 50.097 62.099 1.00 41.24
N
ATOM 3136 CA SER A 106 56.598 49.826 63.148 1.00 41.24
C
ATOM 3137 C SER A 106 57.977 50.326 62.744 1.00 41.24
C
ATOM 3138 O SER A 106 58.352 50.257 61.570 1.00 41.24
O
ATOM 3139 CB SER A 106 56.658 48.327 63.446 1.00 41.24
C
ATOM 3140 OG SER A 106 57.305 48.079 64.680 1.00 41.24
O
ATOM 3141 N ASP A 107 58.731 50.846 63.714 1.00 47.45
N
ATOM 3142 CA ASP A 107 60.101 51.211 63.387 1.00 47.45
C
ATOM 3143 C ASP A 107 61.101 50.124 63.763 1.00 47.45

C
ATOM 3144 O ASP A 107 61.552 49.379 62.887 1.00 47.45
O
ATOM 3145 CB ASP A 107 60.458 52.508 64.111 1.00 47.45
C
ATOM 3146 CG ASP A 107 59.889 52.555 65.520 1.00 47.45
C
ATOM 3147 OD1 ASP A 107 60.330 51.747 66.366 1.00 47.45
O
ATOM 3148 OD2 ASP A 107 58.999 53.392 65.779 1.00 47.45
O
ATOM 3149 N PHE A 108 61.451 50.032 65.051 1.00 44.35
N
ATOM 3150 CA PHE A 108 62.131 48.903 65.688 1.00 44.35
C
ATOM 3151 C PHE A 108 62.959 48.039 64.745 1.00 44.35
C
ATOM 3152 O PHE A 108 62.659 46.855 64.578 1.00 44.35
O
ATOM 3153 CB PHE A 108 61.188 47.994 66.470 1.00 44.35
C
ATOM 3154 CG PHE A 108 61.931 46.966 67.279 1.00 44.35
C
ATOM 3155 CD1 PHE A 108 61.793 45.614 67.023 1.00 44.35
C
ATOM 3156 CD2 PHE A 108 62.844 47.365 68.234 1.00 44.35
C
ATOM 3157 CE1 PHE A 108 62.516 44.685 67.741 1.00 44.35
C
ATOM 3158 CE2 PHE A 108 63.562 46.437 68.955 1.00 44.35
C
ATOM 3159 CZ PHE A 108 63.398 45.099 68.707 1.00 44.35
C
ATOM 3160 N TRP A 109 63.959 48.584 64.079 1.00 35.59
N
ATOM 3161 CA TRP A 109 64.770 47.716 63.245 1.00 35.59
C
ATOM 3162 C TRP A 109 65.694 46.884 64.122 1.00 35.59
C
ATOM 3163 O TRP A 109 66.381 47.416 64.997 1.00 35.59
O
ATOM 3164 CB TRP A 109 65.560 48.516 62.222 1.00 35.59
C
ATOM 3165 CG TRP A 109 65.680 47.764 60.963 1.00 35.59
C
ATOM 3166 CD1 TRP A 109 66.509 46.716 60.716 1.00 35.59
C
ATOM 3167 CD2 TRP A 109 64.910 47.958 59.776 1.00 35.59
C
ATOM 3168 NE1 TRP A 109 66.323 46.260 59.438 1.00 35.59

N
ATOM 3169 CE2 TRP A 109 65.345 47.006 58.839 1.00 35.59
C
ATOM 3170 CE3 TRP A 109 63.906 48.854 59.408 1.00 35.59
C
ATOM 3171 CZ2 TRP A 109 64.812 46.923 57.559 1.00 35.59
C
ATOM 3172 CZ3 TRP A 109 63.378 48.771 58.139 1.00 35.59
C
ATOM 3173 CH2 TRP A 109 63.832 47.813 57.228 1.00 35.59
C
ATOM 3174 N GLY A 110 65.701 45.576 63.890 1.00 36.22
N
ATOM 3175 CA GLY A 110 66.450 44.648 64.709 1.00 36.22
C
ATOM 3176 C GLY A 110 67.921 44.608 64.355 1.00 36.22
C
ATOM 3177 O GLY A 110 68.464 45.505 63.706 1.00 36.22
O
ATOM 3178 N GLN A 111 68.576 43.540 64.800 1.00 37.43
N
ATOM 3179 CA GLN A 111 70.011 43.393 64.604 1.00 37.43
C
ATOM 3180 C GLN A 111 70.360 42.739 63.274 1.00 37.43
C
ATOM 3181 O GLN A 111 71.284 43.189 62.591 1.00 37.43
O
ATOM 3182 CB GLN A 111 70.615 42.582 65.753 1.00 37.43
C
ATOM 3183 CG GLN A 111 70.686 43.334 67.073 1.00 37.43
C
ATOM 3184 CD GLN A 111 71.294 44.714 66.930 1.00 37.43
C
ATOM 3185 OE1 GLN A 111 70.772 45.691 67.462 1.00 37.43
O
ATOM 3186 NE2 GLN A 111 72.415 44.798 66.226 1.00 37.43
N
ATOM 3187 N GLY A 112 69.647 41.688 62.896 1.00 30.54
N
ATOM 3188 CA GLY A 112 69.927 40.972 61.672 1.00 30.54
C
ATOM 3189 C GLY A 112 70.912 39.836 61.878 1.00 30.54
C
ATOM 3190 O GLY A 112 71.676 39.797 62.839 1.00 30.54
O
ATOM 3191 N THR A 113 70.878 38.886 60.954 1.00 26.36
N
ATOM 3192 CA THR A 113 71.802 37.764 60.965 1.00 26.36
C
ATOM 3193 C THR A 113 72.374 37.581 59.568 1.00 26.36

C
ATOM 3194 O THR A 113 71.663 37.719 58.573 1.00 26.36
O
ATOM 3195 CB THR A 113 71.122 36.470 61.467 1.00 26.36
C
ATOM 3196 OG1 THR A 113 72.107 35.450 61.658 1.00 26.36
O
ATOM 3197 CG2 THR A 113 70.073 35.968 60.497 1.00 26.36
C
ATOM 3198 N LEU A 114 73.670 37.295 59.497 1.00 27.96
N
ATOM 3199 CA LEU A 114 74.384 37.257 58.230 1.00 27.96
C
ATOM 3200 C LEU A 114 74.391 35.844 57.663 1.00 27.96
C
ATOM 3201 O LEU A 114 74.705 34.887 58.374 1.00 27.96
O
ATOM 3202 CB LEU A 114 75.816 37.763 58.420 1.00 27.96
C
ATOM 3203 CG LEU A 114 76.775 37.641 57.238 1.00 27.96
C
ATOM 3204 CD1 LEU A 114 76.331 38.529 56.103 1.00 27.96
C
ATOM 3205 CD2 LEU A 114 78.192 37.993 57.674 1.00 27.96
C
ATOM 3206 N VAL A 115 74.050 35.717 56.385 1.00 30.11
N
ATOM 3207 CA VAL A 115 74.110 34.457 55.658 1.00 30.11
C
ATOM 3208 C VAL A 115 75.127 34.629 54.540 1.00 30.11
C
ATOM 3209 O VAL A 115 74.984 35.526 53.700 1.00 30.11
O
ATOM 3210 CB VAL A 115 72.737 34.061 55.090 1.00 30.11
C
ATOM 3211 CG1 VAL A 115 72.862 32.866 54.171 1.00 30.11
C
ATOM 3212 CG2 VAL A 115 71.761 33.781 56.211 1.00 30.11
C
ATOM 3213 N THR A 116 76.148 33.774 54.524 1.00 37.66
N
ATOM 3214 CA THR A 116 77.231 33.861 53.552 1.00 37.66
C
ATOM 3215 C THR A 116 77.362 32.545 52.804 1.00 37.66
C
ATOM 3216 O THR A 116 77.530 31.490 53.421 1.00 37.66
O
ATOM 3217 CB THR A 116 78.563 34.199 54.218 1.00 37.66
C
ATOM 3218 OG1 THR A 116 78.357 35.158 55.258 1.00 37.66

```
O
ATOM   3219  CG2 THR A 116      79.513  34.779  53.198  1.00 37.66
C
ATOM   3220  N   ASP A 117      77.298  32.613  51.482  1.00 44.15
N
ATOM   3221  CA  ASP A 117      77.381  31.437  50.624  1.00 44.15
C
ATOM   3222  C   ASP A 117      78.790  31.396  50.041  1.00 44.15
C
ATOM   3223  O   ASP A 117      79.048  31.907  48.952  1.00 44.15
O
ATOM   3224  CB  ASP A 117      76.308  31.496  49.546  1.00 44.15
C
ATOM   3225  CG  ASP A 117      76.391  30.345  48.568  1.00 44.15
C
ATOM   3226  OD1 ASP A 117      77.183  30.429  47.609  1.00 44.15
O
ATOM   3227  OD2 ASP A 117      75.648  29.362  48.748  1.00 44.15
O
ATOM   3228  N   ALA A 118      79.713  30.798  50.788  1.00 47.14
N
ATOM   3229  CA  ALA A 118      81.102  30.691  50.348  1.00 47.14
C
ATOM   3230  C   ALA A 118      81.262  29.576  49.327  1.00 47.14
C
ATOM   3231  O   ALA A 118      81.026  29.777  48.137  1.00 47.14
O
ATOM   3232  CB  ALA A 118      82.017  30.457  51.531  1.00 47.14
C
TER    3233      ALA A 118
ATOM   3234  C1  NAG V   1      42.034  37.874  49.174  1.00 34.73
C
ATOM   3235  C2  NAG V   1      42.236  37.133  47.872  1.00 34.73
C
ATOM   3236  C3  NAG V   1      41.812  35.671  48.022  1.00 34.73
C
ATOM   3237  C4  NAG V   1      42.302  35.060  49.331  1.00 34.73
C
ATOM   3238  C5  NAG V   1      42.141  36.026  50.490  1.00 34.73
C
ATOM   3239  C6  NAG V   1      42.714  35.526  51.794  1.00 34.73
C
ATOM   3240  C7  NAG V   1      41.971  38.037  45.608  1.00 34.73
C
ATOM   3241  C8  NAG V   1      43.378  37.587  45.375  1.00 34.73
C
ATOM   3242  N2  NAG V   1      41.474  37.784  46.818  1.00 34.73
N
ATOM   3243  O3  NAG V   1      42.315  34.916  46.928  1.00 34.73
O
```

ATOM 3244 O4 NAG V 1 41.435 33.990 49.665 1.00 34.73
O
ATOM 3245 O5 NAG V 1 42.770 37.265 50.173 1.00 34.73
O
ATOM 3246 O6 NAG V 1 43.459 36.524 52.473 1.00 34.73
O
ATOM 3247 O7 NAG V 1 41.313 38.595 44.739 1.00 34.73
O
ATOM 3248 C1 NAG V 2 42.076 32.738 49.536 1.00 49.60
C
ATOM 3249 C2 NAG V 2 41.059 31.765 50.114 1.00 49.60
C
ATOM 3250 C3 NAG V 2 41.488 30.321 49.882 1.00 49.60
C
ATOM 3251 C4 NAG V 2 41.924 30.088 48.443 1.00 49.60
C
ATOM 3252 C5 NAG V 2 42.910 31.162 48.014 1.00 49.60
C
ATOM 3253 C6 NAG V 2 43.293 31.070 46.558 1.00 49.60
C
ATOM 3254 C7 NAG V 2 39.880 32.716 52.042 1.00 49.60
C
ATOM 3255 C8 NAG V 2 39.871 32.870 53.531 1.00 49.60
C
ATOM 3256 N2 NAG V 2 40.890 32.010 51.536 1.00 49.60
N
ATOM 3257 O3 NAG V 2 40.386 29.476 50.190 1.00 49.60
O
ATOM 3258 O4 NAG V 2 42.583 28.832 48.361 1.00 49.60
O
ATOM 3259 O5 NAG V 2 42.306 32.444 48.204 1.00 49.60
O
ATOM 3260 O6 NAG V 2 42.144 31.050 45.723 1.00 49.60
O
ATOM 3261 O7 NAG V 2 39.010 33.202 51.332 1.00 49.60
O
ATOM 3262 C1 BMA V 3 41.744 27.896 47.668 1.00 79.42
C
ATOM 3263 C2 BMA V 3 42.688 26.895 46.992 1.00 79.42
C
ATOM 3264 C3 BMA V 3 41.875 25.797 46.333 1.00 79.42
C
ATOM 3265 C4 BMA V 3 40.885 25.177 47.335 1.00 79.42
C
ATOM 3266 C5 BMA V 3 40.007 26.288 47.939 1.00 79.42
C
ATOM 3267 C6 BMA V 3 39.015 25.790 48.974 1.00 79.42
C
ATOM 3268 O2 BMA V 3 43.504 26.268 47.964 1.00 79.42
O

ATOM 3269 O3 BMA V 3 42.718 24.799 45.741 1.00 79.42
O
ATOM 3270 O4 BMA V 3 40.063 24.241 46.675 1.00 79.42
O
ATOM 3271 O5 BMA V 3 40.865 27.247 48.568 1.00 79.42
O
ATOM 3272 O6 BMA V 3 39.736 25.228 50.070 1.00 79.42
O
ATOM 3273 C1 FUC V 4 42.760 37.334 53.466 1.00 36.66
C
ATOM 3274 C2 FUC V 4 41.205 37.071 53.587 1.00 36.66
C
ATOM 3275 C3 FUC V 4 40.591 38.199 54.411 1.00 36.66
C
ATOM 3276 C4 FUC V 4 41.562 38.688 55.516 1.00 36.66
C
ATOM 3277 C5 FUC V 4 42.562 37.572 55.896 1.00 36.66
C
ATOM 3278 C6 FUC V 4 43.535 37.975 56.985 1.00 36.66
C
ATOM 3279 O2 FUC V 4 40.862 35.812 54.129 1.00 36.66
O
ATOM 3280 O3 FUC V 4 40.327 39.303 53.574 1.00 36.66
O
ATOM 3281 O4 FUC V 4 42.260 39.848 55.096 1.00 36.66
O
ATOM 3282 O5 FUC V 4 43.353 37.127 54.766 1.00 36.66
O
ATOM 3283 C1 BMA V 5 39.108 25.572 51.327 1.00 80.56
C
ATOM 3284 C2 BMA V 5 37.595 25.230 51.269 1.00 80.56
C
ATOM 3285 C3 BMA V 5 36.953 25.485 52.630 1.00 80.56
C
ATOM 3286 C4 BMA V 5 37.739 24.807 53.761 1.00 80.56
C
ATOM 3287 C5 BMA V 5 39.219 25.215 53.697 1.00 80.56
C
ATOM 3288 C6 BMA V 5 40.063 24.506 54.738 1.00 80.56
C
ATOM 3289 O2 BMA V 5 37.401 23.855 50.987 1.00 80.56
O
ATOM 3290 O3 BMA V 5 35.600 25.049 52.653 1.00 80.56
O
ATOM 3291 O4 BMA V 5 37.200 25.185 55.019 1.00 80.56
O
ATOM 3292 O5 BMA V 5 39.733 24.874 52.399 1.00 80.56
O
ATOM 3293 O6 BMA V 5 39.428 24.659 56.002 1.00 80.56
O

ATOM 3294 C1 BMA V 8 42.395 24.714 44.336 1.00 99.57
C
ATOM 3295 C2 BMA V 8 43.359 23.721 43.635 1.00 99.57
C
ATOM 3296 C3 BMA V 8 42.528 22.619 42.965 1.00 99.57
C
ATOM 3297 C4 BMA V 8 41.433 23.190 42.062 1.00 99.57
C
ATOM 3298 C5 BMA V 8 40.598 24.235 42.818 1.00 99.57
C
ATOM 3299 C6 BMA V 8 39.119 23.896 42.818 1.00 99.57
C
ATOM 3300 O2 BMA V 8 44.240 23.095 44.571 1.00 99.57
O
ATOM 3301 O3 BMA V 8 41.970 21.728 43.925 1.00 99.57
O
ATOM 3302 O4 BMA V 8 42.021 23.779 40.911 1.00 99.57
O
ATOM 3303 O5 BMA V 8 41.046 24.297 44.183 1.00 99.57
O
ATOM 3304 O6 BMA V 8 38.961 22.602 43.390 1.00 99.57
O
ATOM 3305 C1 NAG V 9 45.518 22.731 43.998 1.00109.04
C
ATOM 3306 C2 NAG V 9 46.167 21.805 45.015 1.00109.04
C
ATOM 3307 C3 NAG V 9 46.501 22.573 46.288 1.00109.04
C
ATOM 3308 C4 NAG V 9 47.371 23.778 45.961 1.00109.04
C
ATOM 3309 C5 NAG V 9 46.689 24.643 44.906 1.00109.04
C
ATOM 3310 C6 NAG V 9 47.566 25.775 44.424 1.00109.04
C
ATOM 3311 C7 NAG V 9 45.229 19.586 44.544 1.00109.04
C
ATOM 3312 C8 NAG V 9 44.280 18.520 45.002 1.00109.04
C
ATOM 3313 N2 NAG V 9 45.300 20.676 45.314 1.00109.04
N
ATOM 3314 O1 NAG V 9 45.413 22.033 42.801 1.00109.04
O
ATOM 3315 O3 NAG V 9 47.179 21.715 47.199 1.00109.04
O
ATOM 3316 O4 NAG V 9 47.597 24.552 47.133 1.00109.04
O
ATOM 3317 O5 NAG V 9 46.375 23.851 43.751 1.00109.04
O
ATOM 3318 O6 NAG V 9 48.746 25.281 43.806 1.00109.04
O

```
ATOM   3319  O7  NAG V   9      45.899  19.466  43.524  1.00109.04
O
END
```
