# Supplementary material for: Identification and mechanistic basis of non-ACE2 blocking neutralizing antibodies from COVID-19 patients with deep RNA sequencing and molecular dynamics simulations
Source: Front Mol Biosci. 2022 Dec 16;9:1080964. doi: 10.3389/fmolb.2022.1080964 (PMC9800910; doi:10.3389/fmolb.2022.1080964)
Supplement: Supplementary file 4 [file DataSheet5.pdf]

```
ATOM  31200  N   GLN H   1      140.726 136.667 218.018  1.00481.07
N
ATOM  31201  CA  GLN H   1      141.321 136.802 216.656  1.00461.16
C
ATOM  31202  CB  GLN H   1      141.311 135.453 215.930  1.00498.67
C
ATOM  31203  CG  GLN H   1      142.081 135.456 214.615  1.00520.94
C
ATOM  31204  CD  GLN H   1      141.934 134.177 213.822  1.00535.06
C
ATOM  31205  OE1 GLN H   1      141.361 133.193 214.285  1.00538.06
O
ATOM  31206  NE2 GLN H   1      142.461 134.185 212.609  1.00556.90
N
ATOM  31207  C   GLN H   1      140.567 137.885 215.885  1.00425.34
C
ATOM  31208  O   GLN H   1      141.013 139.031 215.834  1.00400.10
O
ATOM  31209  N   VAL H   2      139.421 137.502 215.305  1.00391.00
N
ATOM  31210  CA  VAL H   2      138.606 138.399 214.499  1.00361.52
C
ATOM  31211  CB  VAL H   2      138.216 137.794 213.132  1.00313.43
C
ATOM  31212  CG1 VAL H   2      137.335 138.737 212.320  1.00268.87
C
ATOM  31213  CG2 VAL H   2      139.433 137.378 212.313  1.00228.03
C
ATOM  31214  C   VAL H   2      137.384 138.825 215.314  1.00368.19
C
ATOM  31215  O   VAL H   2      136.626 137.996 215.817  1.00314.88
O
ATOM  31216  N   GLN H   3      137.239 140.148 215.448  1.00376.29
N
ATOM  31217  CA  GLN H   3      136.095 140.774 216.088  1.00349.00
C
ATOM  31218  CB  GLN H   3      136.299 140.879 217.605  1.00266.62
C
ATOM  31219  CG  GLN H   3      137.641 141.471 218.026  1.00261.71
C
ATOM  31220  CD  GLN H   3      137.602 142.886 218.557  1.00268.20
C
ATOM  31221  OE1 GLN H   3      136.617 143.615 218.412  1.00260.30
O
ATOM  31222  NE2 GLN H   3      138.697 143.285 219.190  1.00246.43
N
ATOM  31223  C   GLN H   3      135.824 142.116 215.406  1.00379.27
C
ATOM  31224  O   GLN H   3      136.743 142.752 214.894  1.00382.77
O
```

```
ATOM  31225  N   LEU H   4      134.547 142.515 215.395  1.00395.06
N
ATOM  31226  CA  LEU H   4      134.095 143.725 214.722  1.00336.88
C
ATOM  31227  CB  LEU H   4      133.255 143.334 213.498  1.00364.98
C
ATOM  31228  CG  LEU H   4      133.770 142.141 212.685  1.00402.15
C
ATOM  31229  CD1 LEU H   4      132.751 141.683 211.655  1.00430.71
C
ATOM  31230  CD2 LEU H   4      135.105 142.447 212.017  1.00407.89
C
ATOM  31231  C   LEU H   4      133.311 144.585 215.712  1.00312.18
C
ATOM  31232  O   LEU H   4      132.410 144.104 216.401  1.00350.20
O
ATOM  31233  N   VAL H   5      133.692 145.861 215.803  1.00293.41
N
ATOM  31234  CA  VAL H   5      133.085 146.754 216.776  1.00332.83
C
ATOM  31235  CB  VAL H   5      134.137 147.411 217.703  1.00318.41
C
ATOM  31236  CG1 VAL H   5      133.623 148.670 218.397  1.00310.66
C
ATOM  31237  CG2 VAL H   5      134.697 146.429 218.737  1.00199.07
C
ATOM  31238  C   VAL H   5      132.200 147.750 216.028  1.00351.44
C
ATOM  31239  O   VAL H   5      132.609 148.313 215.014  1.00345.03
O
ATOM  31240  N   GLU H   6      130.969 147.906 216.531  1.00367.15
N
ATOM  31241  CA  GLU H   6      129.988 148.829 215.983  1.00362.47
C
ATOM  31242  CB  GLU H   6      128.667 148.095 215.738  1.00424.22
C
ATOM  31243  CG  GLU H   6      128.764 146.987 214.698  1.00498.22
C
ATOM  31244  CD  GLU H   6      129.334 145.659 215.174  1.00527.61
C
ATOM  31245  OE1 GLU H   6      129.420 145.451 216.408  1.00504.12
O
ATOM  31246  OE2 GLU H   6      129.685 144.830 214.314  1.00578.05
O
ATOM  31247  C   GLU H   6      129.840 149.985 216.967  1.00329.64
C
ATOM  31248  O   GLU H   6      129.840 149.762 218.178  1.00257.21
O
ATOM  31249  N   SER H   7      129.764 151.216 216.437  1.00356.84
N
```

```
ATOM  31250  CA  SER H   7      129.656 152.414 217.256  1.00372.34
C
ATOM  31251  CB  SER H   7      130.949 152.722 217.990  1.00359.11
C
ATOM  31252  OG  SER H   7      132.055 152.768 217.103  1.00364.09
O
ATOM  31253  C   SER H   7      129.153 153.608 216.441  1.00364.14
C
ATOM  31254  O   SER H   7      129.700 153.933 215.387  1.00308.49
O
ATOM  31255  N   GLY H   8      128.108 154.262 216.968  1.00373.61
N
ATOM  31256  CA  GLY H   8      127.509 155.431 216.348  1.00379.24
C
ATOM  31257  C   GLY H   8      125.983 155.377 216.357  1.00365.37
C
ATOM  31258  O   GLY H   8      125.329 156.309 215.889  1.00340.80
O
ATOM  31259  N   GLY H   9      125.434 154.280 216.895  1.00347.25
N
ATOM  31260  CA  GLY H   9      123.997 154.084 216.986  1.00356.90
C
ATOM  31261  C   GLY H   9      123.396 154.810 218.189  1.00360.52
C
ATOM  31262  O   GLY H   9      123.880 154.666 219.310  1.00400.39
O
ATOM  31263  N   GLY H  10      122.340 155.596 217.936  1.00336.82
N
ATOM  31264  CA  GLY H  10      121.592 156.301 218.965  1.00270.58
C
ATOM  31265  C   GLY H  10      120.287 156.873 218.412  1.00307.03
C
ATOM  31266  O   GLY H  10      120.137 157.021 217.201  1.00340.45
O
ATOM  31267  N   VAL H  11      119.354 157.193 219.317  1.00334.58
N
ATOM  31268  CA  VAL H  11      118.054 157.764 218.977  1.00363.39
C
ATOM  31269  CB  VAL H  11      117.209 158.030 220.248  1.00333.72
C
ATOM  31270  CG1 VAL H  11      115.809 158.586 219.968  1.00234.71
C
ATOM  31271  CG2 VAL H  11      117.130 156.809 221.154  1.00327.80
C
ATOM  31272  C   VAL H  11      118.271 159.037 218.151  1.00380.43
C
ATOM  31273  O   VAL H  11      119.102 159.873 218.511  1.00342.63
O
ATOM  31274  N   VAL H  12      117.527 159.159 217.039  1.00369.62
N
```

```
ATOM  31275  CA  VAL H  12     117.637 160.300 216.138  1.00333.23
C
ATOM  31276  CB  VAL H  12     118.718 160.061 215.064  1.00265.37
C
ATOM  31277  CG1 VAL H  12     118.240 159.213 213.895  1.00285.13
C
ATOM  31278  CG2 VAL H  12     119.340 161.361 214.588  1.00200.71
C
ATOM  31279  C   VAL H  12     116.250 160.654 215.579  1.00385.76
C
ATOM  31280  O   VAL H  12     115.298 159.901 215.780  1.00434.53
O
ATOM  31281  N   GLN H  13     116.135 161.824 214.920  1.00384.26
N
ATOM  31282  CA  GLN H  13     114.870 162.375 214.431  1.00349.51
C
ATOM  31283  CB  GLN H  13     114.817 163.895 214.638  1.00375.99
C
ATOM  31284  CG  GLN H  13     114.654 164.354 216.086  1.00380.40
C
ATOM  31285  CD  GLN H  13     113.246 164.286 216.635  1.00386.46
C
ATOM  31286  OE1 GLN H  13     112.269 164.153 215.901  1.00406.32
O
ATOM  31287  NE2 GLN H  13     113.134 164.384 217.952  1.00322.89
N
ATOM  31288  C   GLN H  13     114.696 162.080 212.937  1.00292.19
C
ATOM  31289  O   GLN H  13     115.671 162.152 212.188  1.00171.32
O
ATOM  31290  N   PRO H  14     113.458 161.788 212.437  1.00303.10
N
ATOM  31291  CA  PRO H  14     113.248 161.496 211.013  1.00286.75
C
ATOM  31292  CB  PRO H  14     111.718 161.599 210.835  1.00251.76
C
ATOM  31293  CG  PRO H  14     111.154 161.234 212.202  1.00240.16
C
ATOM  31294  CD  PRO H  14     112.192 161.727 213.201  1.00271.27
C
ATOM  31295  C   PRO H  14     114.007 162.472 210.112  1.00249.60
C
ATOM  31296  O   PRO H  14     114.280 163.601 210.505  1.00199.92
O
ATOM  31297  N   GLY H  15     114.389 162.022 208.916  1.00275.96
N
ATOM  31298  CA  GLY H  15     115.090 162.884 207.976  1.00272.27
C
ATOM  31299  C   GLY H  15     116.316 163.545 208.600  1.00261.33
C
```

```
ATOM  31300  O   GLY H  15      116.566 164.730 208.387  1.00185.11
O
ATOM  31301  N   ARG H  16      117.063 162.767 209.392  1.00307.28
N
ATOM  31302  CA  ARG H  16      118.310 163.236 209.975  1.00379.88
C
ATOM  31303  CB  ARG H  16      118.117 163.636 211.446  1.00381.01
C
ATOM  31304  CG  ARG H  16      117.108 164.754 211.697  1.00338.24
C
ATOM  31305  CD  ARG H  16      117.469 166.151 211.216  1.00320.56
C
ATOM  31306  NE  ARG H  16      118.833 166.538 211.549  1.00358.41
N
ATOM  31307  CZ  ARG H  16      119.486 167.561 211.006  1.00398.53
C
ATOM  31308  NH1 ARG H  16      118.892 168.318 210.099  1.00379.69
N
ATOM  31309  NH2 ARG H  16      120.730 167.824 211.369  1.00467.08
N
ATOM  31310  C   ARG H  16      119.383 162.167 209.765  1.00414.96
C
ATOM  31311  O   ARG H  16      119.129 160.982 209.984  1.00379.76
O
ATOM  31312  N   SER H  17      120.570 162.605 209.314  1.00425.11
N
ATOM  31313  CA  SER H  17      121.697 161.724 209.037  1.00366.32
C
ATOM  31314  CB  SER H  17      122.818 162.445 208.310  1.00329.90
C
ATOM  31315  OG  SER H  17      122.832 162.125 206.923  1.00287.32
O
ATOM  31316  C   SER H  17      122.184 161.051 210.321  1.00329.37
C
ATOM  31317  O   SER H  17      121.894 161.504 211.424  1.00325.41
O
ATOM  31318  N   LEU H  18      122.895 159.935 210.157  1.00309.51
N
ATOM  31319  CA  LEU H  18      123.498 159.198 211.255  1.00296.84
C
ATOM  31320  CB  LEU H  18      122.498 158.122 211.698  1.00262.24
C
ATOM  31321  CG  LEU H  18      122.487 157.722 213.171  1.00233.58
C
ATOM  31322  CD1 LEU H  18      121.171 157.004 213.480  1.00172.10
C
ATOM  31323  CD2 LEU H  18      123.698 156.854 213.523  1.00186.91
C
ATOM  31324  C   LEU H  18      124.778 158.572 210.709  1.00317.25
C
```

ATOM 31325 O   LEU H 18   124.906 158.402 209.496 1.00344.95
O
ATOM 31326 N   ARG H 19   125.733 158.251 211.590 1.00288.63
N
ATOM 31327 CA ARG H 19   126.938 157.578 211.131 1.00280.49
C
ATOM 31328 CB ARG H 19   128.141 158.524 211.180 1.00260.10
C
ATOM 31329 CG ARG H 19   129.202 158.214 210.134 1.00259.37
C
ATOM 31330 CD ARG H 19   129.602 159.467 209.370 1.00267.69
C
ATOM 31331 NE ARG H 19   130.747 159.371 208.469 1.00259.02
N
ATOM 31332 CZ ARG H 19   131.843 158.645 208.685 1.00258.31
C
ATOM 31333 NH1 ARG H 19   131.962 157.911 209.787 1.00209.85
N
ATOM 31334 NH2 ARG H 19   132.807 158.657 207.778 1.00233.29
N
ATOM 31335 C   ARG H 19   127.171 156.303 211.938 1.00269.42
C
ATOM 31336 O   ARG H 19   127.110 156.336 213.164 1.00286.21
O
ATOM 31337 N   LEU H 20   127.439 155.195 211.226 1.00256.95
N
ATOM 31338 CA LEU H 20   127.685 153.871 211.793 1.00288.01
C
ATOM 31339 CB LEU H 20   126.608 152.891 211.294 1.00260.98
C
ATOM 31340 CG LEU H 20   125.554 152.364 212.282 1.00230.85
C
ATOM 31341 CD1 LEU H 20   125.747 150.904 212.692 1.00189.01
C
ATOM 31342 CD2 LEU H 20   125.341 153.274 213.482 1.00238.60
C
ATOM 31343 C   LEU H 20   129.079 153.375 211.388 1.00329.48
C
ATOM 31344 O   LEU H 20   129.287 152.970 210.235 1.00255.23
O
ATOM 31345 N   SER H 21   129.994 153.332 212.376 1.00371.98
N
ATOM 31346 CA SER H 21   131.419 153.074 212.201 1.00343.26
C
ATOM 31347 CB SER H 21   132.248 154.077 212.978 1.00360.65
C
ATOM 31348 OG SER H 21   131.582 155.329 213.094 1.00361.50
O
ATOM 31349 C   SER H 21   131.784 151.654 212.635 1.00282.51
C

ATOM 31350 O SER H 21 131.451 151.240 213.742 1.00292.61
O
ATOM 31351 N CYS H 22 132.516 150.940 211.779 1.00247.99
N
ATOM 31352 CA CYS H 22 132.860 149.554 212.057 1.00317.34
C
ATOM 31353 CB CYS H 22 132.336 148.656 210.946 1.00366.28
C
ATOM 31354 SG CYS H 22 132.632 146.898 211.252 1.00439.44
S
ATOM 31355 C CYS H 22 134.375 149.417 212.164 1.00323.16
C
ATOM 31356 O CYS H 22 135.092 150.008 211.362 1.00349.71
O
ATOM 31357 N ALA H 23 134.859 148.650 213.155 1.00307.85
N
ATOM 31358 CA ALA H 23 136.293 148.506 213.386 1.00302.33
C
ATOM 31359 CB ALA H 23 136.680 148.940 214.782 1.00267.28
C
ATOM 31360 C ALA H 23 136.741 147.077 213.115 1.00322.27
C
ATOM 31361 O ALA H 23 136.314 146.150 213.801 1.00344.16
O
ATOM 31362 N ALA H 24 137.636 146.939 212.130 1.00376.16
N
ATOM 31363 CA ALA H 24 138.145 145.655 211.673 1.00419.73
C
ATOM 31364 CB ALA H 24 138.534 145.749 210.216 1.00426.56
C
ATOM 31365 C ALA H 24 139.321 145.218 212.543 1.00397.36
C
ATOM 31366 O ALA H 24 139.942 146.041 213.220 1.00388.17
O
ATOM 31367 N SER H 25 139.592 143.910 212.526 1.00329.94
N
ATOM 31368 CA SER H 25 140.706 143.305 213.236 1.00267.84
C
ATOM 31369 CB SER H 25 140.564 143.515 214.709 1.00252.73
C
ATOM 31370 OG SER H 25 139.209 143.320 215.081 1.00239.96
O
ATOM 31371 C SER H 25 140.732 141.819 212.905 1.00280.32
C
ATOM 31372 O SER H 25 139.674 141.192 212.814 1.00247.76
O
ATOM 31373 N GLY H 26 141.951 141.301 212.709 1.00303.09
N
ATOM 31374 CA GLY H 26 142.207 139.890 212.450 1.00294.73
C

ATOM 31375 C GLY H 26 142.544 139.602 210.987 1.00260.47
C
ATOM 31376 O GLY H 26 143.066 138.539 210.660 1.00230.31
O
ATOM 31377 N PHE H 27 142.236 140.565 210.116 1.00239.61
N
ATOM 31378 CA PHE H 27 142.354 140.381 208.685 1.00222.40
C
ATOM 31379 CB PHE H 27 141.040 139.871 208.091 1.00208.92
C
ATOM 31380 CG PHE H 27 139.894 140.792 208.412 1.00229.30
C
ATOM 31381 CD1 PHE H 27 139.115 140.554 209.534 1.00249.12
C
ATOM 31382 CE1 PHE H 27 138.082 141.413 209.868 1.00236.64
C
ATOM 31383 CZ PHE H 27 137.831 142.520 209.081 1.00249.90
C
ATOM 31384 CD2 PHE H 27 139.643 141.924 207.641 1.00200.85
C
ATOM 31385 CE2 PHE H 27 138.614 142.789 207.976 1.00204.72
C
ATOM 31386 C PHE H 27 142.567 141.737 208.046 1.00233.46
C
ATOM 31387 O PHE H 27 142.210 142.788 208.589 1.00233.31
O
ATOM 31388 N THR H 28 143.109 141.658 206.843 1.00238.59
N
ATOM 31389 CA THR H 28 143.480 142.830 206.094 1.00236.29
C
ATOM 31390 CB THR H 28 144.636 142.429 205.181 1.00249.02
C
ATOM 31391 OG1 THR H 28 144.246 141.136 204.716 1.00237.71
O
ATOM 31392 CG2 THR H 28 145.927 142.286 205.956 1.00254.04
C
ATOM 31393 C THR H 28 142.217 143.377 205.447 1.00228.44
C
ATOM 31394 O THR H 28 141.433 142.627 204.874 1.00196.36
O
ATOM 31395 N PHE H 29 142.029 144.687 205.613 1.00243.36
N
ATOM 31396 CA PHE H 29 140.950 145.437 205.005 1.00217.50
C
ATOM 31397 CB PHE H 29 140.922 146.840 205.604 1.00228.20
C
ATOM 31398 CG PHE H 29 139.552 147.398 205.863 1.00231.45
C
ATOM 31399 CD1 PHE H 29 139.112 148.505 205.150 1.00247.41
C

ATOM 31400 CE1 PHE H 29   137.861 149.048 205.398 1.00324.05
C
ATOM 31401 CZ PHE H 29   137.044 148.475 206.355 1.00338.87
C
ATOM 31402 CD2 PHE H 29   138.724 146.827 206.825 1.00203.34
C
ATOM 31403 CE2 PHE H 29   137.473 147.375 207.073 1.00266.53
C
ATOM 31404 C   PHE H 29   141.101 145.499 203.483 1.00181.55
C
ATOM 31405 O   PHE H 29   140.092 145.553 202.804 1.00189.15
O
ATOM 31406 N   SER H 30   142.331 145.494 202.940 1.00189.48
N
ATOM 31407 CA SER H 30   142.572 145.747 201.515 1.00235.65
C
ATOM 31408 CB SER H 30   144.028 146.074 201.230 1.00283.02
C
ATOM 31409 OG SER H 30   144.624 145.107 200.363 1.00291.96
O
ATOM 31410 C   SER H 30   142.109 144.589 200.625 1.00226.08
C
ATOM 31411 O   SER H 30   141.829 144.767 199.431 1.00170.61
O
ATOM 31412 N   SER H 31   142.108 143.385 201.201 1.00232.25
N
ATOM 31413 CA SER H 31   141.692 142.217 200.448 1.00239.84
C
ATOM 31414 CB SER H 31   142.805 141.180 200.258 1.00263.75
C
ATOM 31415 OG SER H 31   143.731 141.151 201.335 1.00278.42
O
ATOM 31416 C   SER H 31   140.402 141.648 201.035 1.00189.98
C
ATOM 31417 O   SER H 31   140.053 140.504 200.741 1.00201.99
O
ATOM 31418 N   TYR H 32   139.697 142.478 201.828 1.00131.90
N
ATOM 31419 CA TYR H 32   138.386 142.136 202.387 1.00186.26
C
ATOM 31420 CB TYR H 32   138.414 141.991 203.929 1.00181.82
C
ATOM 31421 CG TYR H 32   138.730 140.572 204.314 1.00168.28
C
ATOM 31422 CD1 TYR H 32   137.796 139.730 204.897 1.00157.33
C
ATOM 31423 CE1 TYR H 32   138.099 138.387 205.112 1.00201.02
C
ATOM 31424 CZ TYR H 32   139.333 137.867 204.722 1.00217.45
C

ATOM 31425 OH TYR H 32 139.742 136.565 204.851 1.00188.53
O
ATOM 31426 CE2 TYR H 32 140.257 138.698 204.124 1.00238.70
C
ATOM 31427 CD2 TYR H 32 139.941 140.028 203.918 1.00222.74
C
ATOM 31428 C TYR H 32 137.242 142.903 201.688 1.00191.30
C
ATOM 31429 O TYR H 32 137.439 143.902 201.002 1.00154.66
O
ATOM 31430 N ALA H 33 136.023 142.380 201.788 1.00200.95
N
ATOM 31431 CA ALA H 33 134.880 143.154 201.350 1.00187.24
C
ATOM 31432 CB ALA H 33 134.421 142.702 199.979 1.00196.01
C
ATOM 31433 C ALA H 33 133.815 143.071 202.444 1.00205.88
C
ATOM 31434 O ALA H 33 133.817 142.132 203.265 1.00143.62
O
ATOM 31435 N MET H 34 132.912 144.064 202.420 1.00201.18
N
ATOM 31436 CA MET H 34 132.206 144.519 203.601 1.00151.30
C
ATOM 31437 CB MET H 34 132.900 145.805 204.027 1.00147.34
C
ATOM 31438 CG MET H 34 134.346 145.524 204.399 1.00173.06
C
ATOM 31439 SD MET H 34 134.474 144.086 205.523 1.00251.05
S
ATOM 31440 CE MET H 34 136.017 144.432 206.371 1.00231.93
C
ATOM 31441 C MET H 34 130.717 144.737 203.327 1.00144.75
C
ATOM 31442 O MET H 34 130.371 145.413 202.336 1.00102.00
O
ATOM 31443 N HIS H 35 129.885 144.128 204.219 1.00139.38
N
ATOM 31444 CA HIS H 35 128.417 144.162 204.237 1.00169.18
C
ATOM 31445 CB HIS H 35 127.813 142.759 204.236 1.00125.76
C
ATOM 31446 CG HIS H 35 128.294 141.838 203.181 1.00168.82
C
ATOM 31447 ND1 HIS H 35 129.519 141.201 203.256 1.00238.74
N
ATOM 31448 CE1 HIS H 35 129.660 140.406 202.210 1.00274.82
C
ATOM 31449 NE2 HIS H 35 128.545 140.493 201.465 1.00224.47
N

```
ATOM  31450  CD2 HIS H  35     127.682 141.371 202.074  1.00184.21
C
ATOM  31451  C   HIS H  35     127.812 144.775 205.514  1.00198.96
C
ATOM  31452  O   HIS H  35     128.443 144.751 206.570  1.00186.42
O
ATOM  31453  N   TRP H  36     126.528 145.207 205.421  1.00219.89
N
ATOM  31454  CA  TRP H  36     125.700 145.837 206.457  1.00156.07
C
ATOM  31455  CB  TRP H  36     125.497 147.311 206.112  1.00145.33
C
ATOM  31456  CG  TRP H  36     126.609 148.188 206.566  1.00168.98
C
ATOM  31457  CD1 TRP H  36     127.335 149.056 205.811  1.00202.34
C
ATOM  31458  NE1 TRP H  36     128.262 149.693 206.592  1.00250.84
N
ATOM  31459  CE2 TRP H  36     128.165 149.240 207.882  1.00220.13
C
ATOM  31460  CD2 TRP H  36     127.127 148.280 207.903  1.00191.25
C
ATOM  31461  CE3 TRP H  36     126.827 147.645 209.110  1.00184.32
C
ATOM  31462  CZ3 TRP H  36     127.560 147.971 210.230  1.00183.66
C
ATOM  31463  CH2 TRP H  36     128.584 148.921 210.186  1.00180.95
C
ATOM  31464  CZ2 TRP H  36     128.899 149.584 209.020  1.00186.89
C
ATOM  31465  C   TRP H  36     124.310 145.195 206.544  1.00153.02
C
ATOM  31466  O   TRP H  36     123.485 145.335 205.634  1.00145.42
O
ATOM  31467  N   VAL H  37     123.990 144.519 207.650  1.00136.89
N
ATOM  31468  CA  VAL H  37     122.655 143.924 207.655  1.00201.00
C
ATOM  31469  CB  VAL H  37     122.629 142.379 207.546  1.00224.56
C
ATOM  31470  CG1 VAL H  37     121.214 141.793 207.533  1.00166.10
C
ATOM  31471  CG2 VAL H  37     123.391 141.881 206.327  1.00256.49
C
ATOM  31472  C   VAL H  37     121.916 144.430 208.888  1.00225.37
C
ATOM  31473  O   VAL H  37     122.524 144.435 209.966  1.00161.23
O
ATOM  31474  N   ARG H  38     120.630 144.815 208.681  1.00246.01
N
```

ATOM 31475 CA ARG H 38   119.738 145.472 209.641 1.00204.59
C
ATOM 31476 CB ARG H 38   119.467 146.940 209.272 1.00138.30
C
ATOM 31477 CG ARG H 38   118.136 147.156 208.559 1.00153.60
C
ATOM 31478 CD ARG H 38   118.028 148.453 207.777 1.00211.69
C
ATOM 31479 NE ARG H 38   116.833 148.643 206.947 1.00240.77
N
ATOM 31480 CZ ARG H 38   116.669 149.574 205.995 1.00232.23
C
ATOM 31481 NH1 ARG H 38   117.629 150.435 205.695 1.00201.46
N
ATOM 31482 NH2 ARG H 38   115.531 149.645 205.329 1.00263.02
N
ATOM 31483 C   ARG H 38   118.396 144.736 209.775 1.00206.84
C
ATOM 31484 O   ARG H 38   117.613 144.648 208.825 1.00127.13
O
ATOM 31485 N   GLN H 39   118.093 144.300 211.007 1.00281.66
N
ATOM 31486 CA GLN H 39   116.835 143.642 211.344 1.00318.49
C
ATOM 31487 CB GLN H 39   117.101 142.203 211.802 1.00312.40
C
ATOM 31488 CG GLN H 39   117.883 142.084 213.109 1.00308.83
C
ATOM 31489 CD GLN H 39   117.007 141.984 214.335 1.00344.29
C
ATOM 31490 OE1 GLN H 39   115.868 141.519 214.279 1.00414.41
O
ATOM 31491 NE2 GLN H 39   117.534 142.427 215.463 1.00367.87
N
ATOM 31492 C   GLN H 39   116.047 144.451 212.383 1.00356.39
C
ATOM 31493 O   GLN H 39   116.618 144.934 213.363 1.00429.54
O
ATOM 31494 N   ALA H 40   114.725 144.590 212.170 1.00299.28
N
ATOM 31495 CA ALA H 40   113.828 145.231 213.128 1.00281.83
C
ATOM 31496 CB ALA H 40   112.526 145.597 212.456 1.00224.67
C
ATOM 31497 C   ALA H 40   113.574 144.273 214.288 1.00299.57
C
ATOM 31498 O   ALA H 40   113.355 143.093 214.044 1.00355.72
O
ATOM 31499 N   PRO H 41   113.604 144.712 215.573 1.00295.16
N

ATOM 31500 CA PRO H 41   113.415 143.775 216.693 1.00272.88
C
ATOM 31501 CB PRO H 41   113.554 144.680 217.938 1.00263.44
C
ATOM 31502 CG PRO H 41   114.366 145.883 217.448 1.00233.32
C
ATOM 31503 CD PRO H 41   113.912 146.089 216.016 1.00240.20
C
ATOM 31504 C   PRO H 41   112.215 142.812 216.580 1.00244.69
C
ATOM 31505 O   PRO H 41   111.053 143.226 216.479 1.00158.28
O
ATOM 31506 N   GLY H 42   112.538 141.507 216.517 1.00251.54
N
ATOM 31507 CA GLY H 42   111.588 140.429 216.262 1.00264.62
C
ATOM 31508 C   GLY H 42   111.212 140.224 214.786 1.00327.08
C
ATOM 31509 O   GLY H 42   110.208 139.569 214.510 1.00311.18
O
ATOM 31510 N   LYS H 43   112.023 140.743 213.843 1.00365.80
N
ATOM 31511 CA LYS H 43   111.742 140.705 212.407 1.00333.33
C
ATOM 31512 CB LYS H 43   111.560 142.138 211.888 1.00339.97
C
ATOM 31513 CG LYS H 43   110.446 142.373 210.872 1.00316.99
C
ATOM 31514 CD LYS H 43   109.087 142.609 211.501 1.00317.65
C
ATOM 31515 CE LYS H 43   109.105 143.638 212.613 1.00286.50
C
ATOM 31516 NZ LYS H 43   108.013 143.397 213.583 1.00250.34
N
ATOM 31517 C   LYS H 43   112.914 140.069 211.657 1.00300.12
C
ATOM 31518 O   LYS H 43   114.060 140.213 212.078 1.00323.70
O
ATOM 31519 N   GLY H 44   112.629 139.397 210.529 1.00253.52
N
ATOM 31520 CA GLY H 44   113.669 138.842 209.673 1.00200.94
C
ATOM 31521 C   GLY H 44   114.753 139.872 209.356 1.00223.21
C
ATOM 31522 O   GLY H 44   114.649 141.044 209.717 1.00290.11
O
ATOM 31523 N   LEU H 45   115.794 139.422 208.660 1.00222.40
N
ATOM 31524 CA LEU H 45   116.959 140.245 208.398 1.00197.14
C

ATOM 31525 CB LEU H 45   118.162 139.308 208.401 1.00196.17
C
ATOM 31526 CG LEU H 45   118.814 139.178 209.764 1.00144.79
C
ATOM 31527 CD1 LEU H 45   119.124 137.707 210.114 1.00108.60
C
ATOM 31528 CD2 LEU H 45   120.034 140.077 209.729 1.00127.31
C
ATOM 31529 C   LEU H 45   116.830 140.984 207.069 1.00180.93
C
ATOM 31530 O   LEU H 45   115.881 140.831 206.298 1.00133.68
O
ATOM 31531 N   GLU H 46   117.811 141.825 206.805 1.00175.02
N
ATOM 31532 CA GLU H 46   117.686 142.659 205.636 1.00185.23
C
ATOM 31533 CB GLU H 46   116.731 143.837 205.876 1.00258.38
C
ATOM 31534 CG GLU H 46   116.505 144.736 204.660 1.00301.60
C
ATOM 31535 CD GLU H 46   115.573 145.933 204.853 1.00270.95
C
ATOM 31536 OE1 GLU H 46   114.706 145.866 205.765 1.00194.91
O
ATOM 31537 OE2 GLU H 46   115.710 146.937 204.093 1.00226.49
O
ATOM 31538 C   GLU H 46   119.098 143.078 205.297 1.00161.51
C
ATOM 31539 O   GLU H 46   119.791 143.809 206.005 1.00135.99
O
ATOM 31540 N   TRP H 47   119.559 142.555 204.195 1.00160.23
N
ATOM 31541 CA TRP H 47   120.832 143.127 203.870 1.00154.54
C
ATOM 31542 CB TRP H 47   121.647 142.225 202.958 1.00171.73
C
ATOM 31543 CG TRP H 47   122.574 143.100 202.198 1.00142.17
C
ATOM 31544 CD1 TRP H 47   123.648 143.776 202.708 1.00108.29
C
ATOM 31545 NE1 TRP H 47   124.206 144.553 201.728 1.00105.06
N
ATOM 31546 CE2 TRP H 47   123.474 144.414 200.569 1.00145.40
C
ATOM 31547 CD2 TRP H 47   122.416 143.500 200.826 1.00144.19
C
ATOM 31548 CE3 TRP H 47   121.526 143.129 199.805 1.00107.40
C
ATOM 31549 CZ3 TRP H 47   121.744 143.682 198.565 1.00134.87
C

ATOM 31550 CH2 TRP H 47 122.789 144.589 198.326 1.00164.19
C
ATOM 31551 CZ2 TRP H 47 123.673 144.982 199.308 1.00135.81
C
ATOM 31552 C TRP H 47 120.580 144.493 203.253 1.00146.27
C
ATOM 31553 O TRP H 47 119.697 144.660 202.400 1.00119.07
O
ATOM 31554 N VAL H 48 121.463 145.403 203.656 1.00144.79
N
ATOM 31555 CA VAL H 48 121.401 146.812 203.332 1.00172.53
C
ATOM 31556 CB VAL H 48 121.602 147.527 204.661 1.00159.04
C
ATOM 31557 CG1 VAL H 48 120.764 148.791 204.682 1.00210.40
C
ATOM 31558 CG2 VAL H 48 121.192 146.544 205.756 1.00124.40
C
ATOM 31559 C VAL H 48 122.460 147.238 202.309 1.00195.06
C
ATOM 31560 O VAL H 48 122.143 147.660 201.188 1.00167.45
O
ATOM 31561 N ALA H 49 123.728 147.181 202.734 1.00208.38
N
ATOM 31562 CA ALA H 49 124.842 147.783 202.018 1.00193.43
C
ATOM 31563 CB ALA H 49 125.417 148.892 202.874 1.00133.96
C
ATOM 31564 C ALA H 49 125.894 146.724 201.674 1.00180.34
C
ATOM 31565 O ALA H 49 126.102 145.778 202.429 1.00176.64
O
ATOM 31566 N VAL H 50 126.566 146.883 200.530 1.00173.05
N
ATOM 31567 CA VAL H 50 127.743 146.073 200.285 1.00148.78
C
ATOM 31568 CB VAL H 50 127.412 144.763 199.556 1.00168.78
C
ATOM 31569 CG1 VAL H 50 126.806 145.011 198.168 1.00169.07
C
ATOM 31570 CG2 VAL H 50 128.626 143.828 199.537 1.00138.99
C
ATOM 31571 C VAL H 50 128.844 146.869 199.584 1.00140.82
C
ATOM 31572 O VAL H 50 128.617 147.704 198.670 1.00110.76
O
ATOM 31573 N ILE H 51 130.072 146.542 200.006 1.00103.84
N
ATOM 31574 CA ILE H 51 131.164 147.218 199.343 1.00133.69
C

```
ATOM  31575  CB  ILE H  51     131.572 148.483 200.112  1.00126.43
C
ATOM  31576  CG1 ILE H  51     131.922 149.602 199.126  1.00112.27
C
ATOM  31577  CG2 ILE H  51     132.634 148.177 201.154  1.00117.56
C
ATOM  31578  CD1 ILE H  51     130.853 149.712 198.035  1.00 99.47
C
ATOM  31579  C   ILE H  51     132.312 146.254 199.060  1.00161.97
C
ATOM  31580  O   ILE H  51     132.609 145.400 199.896  1.00179.48
O
ATOM  31581  N   PRO H  52     132.984 146.356 197.876  1.00160.56
N
ATOM  31582  CA  PRO H  52     134.121 145.493 197.579  1.00121.02
C
ATOM  31583  CB  PRO H  52     133.973 145.396 196.051  1.00102.54
C
ATOM  31584  CG  PRO H  52     133.537 146.791 195.608  1.00106.86
C
ATOM  31585  CD  PRO H  52     132.715 147.310 196.772  1.00145.40
C
ATOM  31586  C   PRO H  52     135.368 146.235 198.065  1.00152.76
C
ATOM  31587  O   PRO H  52     135.382 147.474 198.137  1.00144.09
O
ATOM  31588  N   PHE H  53     136.404 145.458 198.418  1.00177.98
N
ATOM  31589  CA  PHE H  53     137.690 145.944 198.926  1.00203.74
C
ATOM  31590  CB  PHE H  53     138.718 144.805 199.040  1.00184.23
C
ATOM  31591  CG  PHE H  53     138.833 143.898 197.846  1.00188.29
C
ATOM  31592  CD1 PHE H  53     138.055 142.744 197.747  1.00220.96
C
ATOM  31593  CE1 PHE H  53     138.148 141.913 196.640  1.00213.44
C
ATOM  31594  CZ  PHE H  53     139.027 142.247 195.626  1.00224.73
C
ATOM  31595  CD2 PHE H  53     139.722 144.213 196.825  1.00146.66
C
ATOM  31596  CE2 PHE H  53     139.807 143.392 195.711  1.00178.97
C
ATOM  31597  C   PHE H  53     138.194 147.223 198.234  1.00171.16
C
ATOM  31598  O   PHE H  53     138.264 148.305 198.819  1.00148.71
O
ATOM  31599  N   ASP H  54     138.571 147.151 196.967  1.00160.74
N
```

ATOM 31600 CA ASP H 54   138.857 148.432 196.350 1.00225.61
C
ATOM 31601 CB ASP H 54   139.220 148.303 194.860 1.00273.90
C
ATOM 31602 CG ASP H 54   138.084 148.135 193.858 1.00247.86
C
ATOM 31603 OD1 ASP H 54   136.912 148.335 194.230 1.00256.25
O
ATOM 31604 OD2 ASP H 54   138.394 147.837 192.686 1.00202.40
O
ATOM 31605 C   ASP H 54   137.857 149.503 196.820 1.00259.11
C
ATOM 31606 O   ASP H 54   138.284 150.532 197.343 1.00277.80
O
ATOM 31607 N   GLY H 55   136.545 149.248 196.677 1.00268.52
N
ATOM 31608 CA GLY H 55   135.501 150.180 197.087 1.00231.33
C
ATOM 31609 C   GLY H 55   134.849 150.861 195.886 1.00217.29
C
ATOM 31610 O   GLY H 55   133.738 151.375 196.001 1.00222.76
O
ATOM 31611 N   ARG H 56   135.565 150.849 194.750 1.00168.77
N
ATOM 31612 CA ARG H 56   135.140 151.458 193.502 1.00190.98
C
ATOM 31613 CB ARG H 56   136.009 150.877 192.367 1.00178.64
C
ATOM 31614 CG ARG H 56   135.489 151.110 190.946 1.00247.06
C
ATOM 31615 CD ARG H 56   136.060 150.321 189.752 1.00242.10
C
ATOM 31616 NE ARG H 56   135.200 149.397 188.991 1.00198.76
N
ATOM 31617 CZ ARG H 56   135.595 148.462 188.102 1.00262.66
C
ATOM 31618 NH1 ARG H 56   134.690 147.703 187.508 1.00319.69
N
ATOM 31619 NH2 ARG H 56   136.871 148.287 187.791 1.00256.72
N
ATOM 31620 C   ARG H 56   133.596 151.447 193.404 1.00257.14
C
ATOM 31621 O   ARG H 56   132.970 152.512 193.521 1.00208.59
O
ATOM 31622 N   ASN H 57   132.970 150.248 193.272 1.00295.40
N
ATOM 31623 CA ASN H 57   131.540 150.048 192.999 1.00225.45
C
ATOM 31624 CB ASN H 57   131.191 148.778 192.205 1.00181.41
C

ATOM 31625 CG ASN H 57 131.596 148.810 190.747 1.00160.15
C
ATOM 31626 OD1 ASN H 57 131.941 147.784 190.162 1.00 92.98
O
ATOM 31627 ND2 ASN H 57 131.598 150.000 190.161 1.00207.01
N
ATOM 31628 C ASN H 57 130.727 149.873 194.274 1.00193.68
C
ATOM 31629 O ASN H 57 130.743 148.838 194.946 1.00137.79
O
ATOM 31630 N LYS H 58 129.915 150.868 194.558 1.00205.02
N
ATOM 31631 CA LYS H 58 129.028 150.652 195.677 1.00215.70
C
ATOM 31632 CB LYS H 58 128.595 152.035 196.183 1.00299.46
C
ATOM 31633 CG LYS H 58 129.684 152.875 196.858 1.00289.06
C
ATOM 31634 CD LYS H 58 130.627 153.668 195.959 1.00243.01
C
ATOM 31635 CE LYS H 58 131.458 154.633 196.778 1.00246.86
C
ATOM 31636 NZ LYS H 58 131.938 153.999 198.032 1.00239.32
N
ATOM 31637 C LYS H 58 127.937 149.687 195.207 1.00165.15
C
ATOM 31638 O LYS H 58 127.689 149.594 193.999 1.00192.28
O
ATOM 31639 N TYR H 59 127.293 148.961 196.135 1.00111.75
N
ATOM 31640 CA TYR H 59 125.920 148.592 195.779 1.00163.98
C
ATOM 31641 CB TYR H 59 125.950 147.474 194.746 1.00177.81
C
ATOM 31642 CG TYR H 59 124.599 146.885 194.462 1.00190.36
C
ATOM 31643 CD1 TYR H 59 123.677 147.542 193.662 1.00154.87
C
ATOM 31644 CE1 TYR H 59 122.435 146.972 193.411 1.00202.22
C
ATOM 31645 CZ TYR H 59 122.094 145.732 193.949 1.00212.13
C
ATOM 31646 OH TYR H 59 120.869 145.135 193.736 1.00145.23
O
ATOM 31647 CE2 TYR H 59 123.010 145.081 194.755 1.00247.08
C
ATOM 31648 CD2 TYR H 59 124.245 145.659 195.004 1.00229.81
C
ATOM 31649 C TYR H 59 124.962 148.245 196.939 1.00195.79
C

ATOM 31650 O TYR H 59 125.122 147.172 197.546 1.00136.19
O
ATOM 31651 N TYR H 60 123.898 149.088 197.128 1.00231.28
N
ATOM 31652 CA TYR H 60 122.901 149.116 198.220 1.00171.99
C
ATOM 31653 CB TYR H 60 122.556 150.545 198.695 1.00129.95
C
ATOM 31654 CG TYR H 60 123.663 151.566 198.508 1.00169.65
C
ATOM 31655 CD1 TYR H 60 124.583 151.839 199.522 1.00146.38
C
ATOM 31656 CE1 TYR H 60 125.622 152.752 199.349 1.00149.46
C
ATOM 31657 CZ TYR H 60 125.784 153.420 198.134 1.00174.10
C
ATOM 31658 OH TYR H 60 126.799 154.343 197.928 1.00126.04
O
ATOM 31659 CE2 TYR H 60 124.884 153.146 197.108 1.00190.56
C
ATOM 31660 CD2 TYR H 60 123.842 152.238 197.297 1.00188.71
C
ATOM 31661 C TYR H 60 121.592 148.465 197.774 1.00156.06
C
ATOM 31662 O TYR H 60 121.363 148.341 196.561 1.00151.91
O
ATOM 31663 N ALA H 61 120.741 148.072 198.751 1.00141.97
N
ATOM 31664 CA ALA H 61 119.427 147.491 198.439 1.00189.66
C
ATOM 31665 CB ALA H 61 118.752 146.732 199.593 1.00 98.28
C
ATOM 31666 C ALA H 61 118.529 148.557 197.804 1.00230.32
C
ATOM 31667 O ALA H 61 118.856 149.742 197.842 1.00299.70
O
ATOM 31668 N ASP H 62 117.432 148.121 197.178 1.00258.08
N
ATOM 31669 CA ASP H 62 116.567 149.028 196.447 1.00243.86
C
ATOM 31670 CB ASP H 62 115.596 148.257 195.557 1.00267.39
C
ATOM 31671 CG ASP H 62 114.502 147.609 196.387 1.00280.52
C
ATOM 31672 OD1 ASP H 62 114.844 146.837 197.312 1.00282.35
O
ATOM 31673 OD2 ASP H 62 113.328 147.927 196.149 1.00285.10
O
ATOM 31674 C ASP H 62 115.791 149.891 197.439 1.00227.37
C

ATOM 31675 O ASP H 62 115.581 151.068 197.163 1.00270.71
O
ATOM 31676 N SER H 63 115.402 149.308 198.590 1.00197.64
N
ATOM 31677 CA SER H 63 114.479 149.948 199.529 1.00209.07
C
ATOM 31678 CB SER H 63 113.489 148.961 200.196 1.00189.20
C
ATOM 31679 OG SER H 63 114.095 148.102 201.158 1.00170.89
O
ATOM 31680 C SER H 63 115.177 150.932 200.484 1.00188.70
C
ATOM 31681 O SER H 63 114.563 151.384 201.461 1.00158.63
O
ATOM 31682 N VAL H 64 116.436 151.291 200.143 1.00165.69
N
ATOM 31683 CA VAL H 64 117.241 152.321 200.804 1.00175.65
C
ATOM 31684 CB VAL H 64 117.962 151.845 202.108 1.00168.87
C
ATOM 31685 CG1 VAL H 64 117.174 150.885 203.001 1.00129.52
C
ATOM 31686 CG2 VAL H 64 119.372 151.314 201.897 1.00187.57
C
ATOM 31687 C VAL H 64 118.185 153.036 199.809 1.00166.47
C
ATOM 31688 O VAL H 64 119.089 153.743 200.243 1.00186.94
O
ATOM 31689 N THR H 65 118.002 152.915 198.477 1.00160.69
N
ATOM 31690 CA THR H 65 119.005 153.458 197.548 1.00215.57
C
ATOM 31691 CB THR H 65 119.218 152.653 196.250 1.00242.00
C
ATOM 31692 OG1 THR H 65 120.324 153.203 195.522 1.00197.88
O
ATOM 31693 CG2 THR H 65 118.009 152.625 195.338 1.00270.86
C
ATOM 31694 C THR H 65 118.824 154.958 197.285 1.00244.96
C
ATOM 31695 O THR H 65 117.701 155.467 197.251 1.00259.01
O
ATOM 31696 N GLY H 66 119.964 155.637 197.061 1.00230.98
N
ATOM 31697 CA GLY H 66 120.089 157.087 197.040 1.00181.47
C
ATOM 31698 C GLY H 66 119.669 157.731 198.364 1.00228.52
C
ATOM 31699 O GLY H 66 119.375 158.920 198.364 1.00265.02
O

ATOM 31700 N ARG H 67 119.616 156.947 199.462 1.00276.02
N
ATOM 31701 CA ARG H 67 119.263 157.406 200.808 1.00275.66
C
ATOM 31702 CB ARG H 67 117.955 156.789 201.332 1.00243.39
C
ATOM 31703 CG ARG H 67 116.672 157.267 200.666 1.00193.50
C
ATOM 31704 CD ARG H 67 115.461 156.803 201.459 1.00225.88
C
ATOM 31705 NE ARG H 67 115.430 155.404 201.891 1.00243.63
N
ATOM 31706 CZ ARG H 67 115.467 154.975 203.156 1.00232.79
C
ATOM 31707 NH1 ARG H 67 115.542 155.842 204.150 1.00185.69
N
ATOM 31708 NH2 ARG H 67 115.421 153.681 203.421 1.00269.20
N
ATOM 31709 C ARG H 67 120.383 157.095 201.808 1.00263.74
C
ATOM 31710 O ARG H 67 120.576 157.829 202.773 1.00297.88
O
ATOM 31711 N PHE H 68 121.085 155.976 201.605 1.00213.14
N
ATOM 31712 CA PHE H 68 122.269 155.674 202.387 1.00161.07
C
ATOM 31713 CB PHE H 68 122.266 154.216 202.834 1.00171.56
C
ATOM 31714 CG PHE H 68 121.347 153.895 203.979 1.00172.19
C
ATOM 31715 CD1 PHE H 68 119.969 154.027 203.859 1.00163.38
C
ATOM 31716 CE1 PHE H 68 119.132 153.712 204.917 1.00194.14
C
ATOM 31717 CZ PHE H 68 119.668 153.257 206.101 1.00245.61
C
ATOM 31718 CD2 PHE H 68 121.872 153.423 205.169 1.00186.77
C
ATOM 31719 CE2 PHE H 68 121.034 153.106 206.227 1.00234.74
C
ATOM 31720 C PHE H 68 123.489 155.937 201.518 1.00172.39
C
ATOM 31721 O PHE H 68 123.322 156.295 200.353 1.00142.08
O
ATOM 31722 N THR H 69 124.678 155.793 202.129 1.00240.21
N
ATOM 31723 CA THR H 69 126.003 155.910 201.523 1.00298.93
C
ATOM 31724 CB THR H 69 126.508 157.369 201.516 1.00302.21
C

ATOM 31725 OG1 THR H 69   125.521 158.274 201.019 1.00349.61
O
ATOM 31726 CG2 THR H 69   127.775 157.596 200.716 1.00267.93
C
ATOM 31727 C   THR H 69   126.945 154.972 202.289 1.00286.84
C
ATOM 31728 O   THR H 69   126.934 154.976 203.522 1.00219.00
O
ATOM 31729 N   ILE H 70   127.732 154.156 201.556 1.00292.66
N
ATOM 31730 CA ILE H 70   128.710 153.232 202.132 1.00238.49
C
ATOM 31731 CB ILE H 70   128.343 151.746 201.886 1.00208.28
C
ATOM 31732 CG1 ILE H 70   129.204 150.800 202.739 1.00199.56
C
ATOM 31733 CG2 ILE H 70   128.400 151.407 200.400 1.00180.92
C
ATOM 31734 CD1 ILE H 70   128.625 149.404 203.002 1.00158.94
C
ATOM 31735 C   ILE H 70   130.130 153.578 201.667 1.00226.88
C
ATOM 31736 O   ILE H 70   130.453 153.487 200.477 1.00197.44
O
ATOM 31737 N   SER H 71   130.963 153.964 202.650 1.00206.83
N
ATOM 31738 CA SER H 71   132.364 154.300 202.457 1.00202.01
C
ATOM 31739 CB SER H 71   132.663 155.730 202.775 1.00187.90
C
ATOM 31740 OG SER H 71   133.550 155.778 203.893 1.00186.04
O
ATOM 31741 C   SER H 71   133.223 153.447 203.377 1.00203.02
C
ATOM 31742 O   SER H 71   132.809 153.062 204.465 1.00185.94
O
ATOM 31743 N   ARG H 72   134.460 153.247 202.934 1.00231.98
N
ATOM 31744 CA ARG H 72   135.473 152.508 203.653 1.00215.96
C
ATOM 31745 CB ARG H 72   135.388 151.019 203.303 1.00243.55
C
ATOM 31746 CG ARG H 72   135.048 150.719 201.843 1.00269.24
C
ATOM 31747 CD ARG H 72   136.028 149.914 200.998 1.00214.95
C
ATOM 31748 NE ARG H 72   136.778 148.977 201.832 1.00215.96
N
ATOM 31749 CZ ARG H 72   136.428 147.720 202.143 1.00209.45
C

```
ATOM  31750  NH1 ARG H  72      135.322 147.163 201.668  1.00190.33
N
ATOM  31751  NH2 ARG H  72      137.218 147.008 202.926  1.00164.40
N
ATOM  31752  C   ARG H  72      136.802 153.051 203.169  1.00222.61
C
ATOM  31753  O   ARG H  72      136.913 153.528 202.042  1.00266.49
O
ATOM  31754  N   ASP H  73      137.787 152.989 204.052  1.00210.31
N
ATOM  31755  CA  ASP H  73      139.148 153.272 203.657  1.00249.94
C
ATOM  31756  CB  ASP H  73      139.564 154.654 204.168  1.00267.42
C
ATOM  31757  CG  ASP H  73      140.889 155.154 203.620  1.00280.58
C
ATOM  31758  OD1 ASP H  73      141.414 154.523 202.681  1.00229.25
O
ATOM  31759  OD2 ASP H  73      141.391 156.169 204.147  1.00347.50
O
ATOM  31760  C   ASP H  73      139.997 152.090 204.118  1.00303.05
C
ATOM  31761  O   ASP H  73      139.763 151.551 205.198  1.00320.17
O
ATOM  31762  N   ASN H  74      140.945 151.677 203.268  1.00373.64
N
ATOM  31763  CA  ASN H  74      141.821 150.539 203.523  1.00391.56
C
ATOM  31764  CB  ASN H  74      142.459 150.004 202.231  1.00402.56
C
ATOM  31765  CG  ASN H  74      141.463 149.636 201.145  1.00361.16
C
ATOM  31766  OD1 ASN H  74      140.288 149.405 201.423  1.00360.64
O
ATOM  31767  ND2 ASN H  74      141.920 149.585 199.900  1.00273.95
N
ATOM  31768  C   ASN H  74      142.868 150.903 204.582  1.00396.62
C
ATOM  31769  O   ASN H  74      143.232 150.066 205.410  1.00357.80
O
ATOM  31770  N   SER H  75      143.340 152.158 204.544  1.00376.68
N
ATOM  31771  CA  SER H  75      144.352 152.669 205.460  1.00297.38
C
ATOM  31772  CB  SER H  75      145.068 153.858 204.866  1.00310.17
C
ATOM  31773  OG  SER H  75      145.786 153.483 203.697  1.00352.70
O
ATOM  31774  C   SER H  75      143.741 153.002 206.822  1.00252.18
C
```

ATOM 31775 O SER H 75 144.456 153.053 207.819 1.00249.31
O
ATOM 31776 N LYS H 76 142.422 153.239 206.857 1.00250.65
N
ATOM 31777 CA LYS H 76 141.689 153.487 208.092 1.00259.55
C
ATOM 31778 CB LYS H 76 140.449 154.357 207.837 1.00313.92
C
ATOM 31779 CG LYS H 76 140.688 155.861 207.697 1.00300.22
C
ATOM 31780 CD LYS H 76 140.320 156.706 208.913 1.00254.52
C
ATOM 31781 CE LYS H 76 141.273 157.860 209.154 1.00218.49
C
ATOM 31782 NZ LYS H 76 142.335 157.518 210.136 1.00181.56
N
ATOM 31783 C LYS H 76 141.305 152.160 208.757 1.00212.97
C
ATOM 31784 O LYS H 76 141.177 152.054 209.977 1.00165.75
O
ATOM 31785 N ASN H 77 141.091 151.119 207.958 1.00219.16
N
ATOM 31786 CA ASN H 77 140.705 149.862 208.576 1.00289.85
C
ATOM 31787 CB ASN H 77 141.853 149.300 209.434 1.00329.13
C
ATOM 31788 CG ASN H 77 141.995 147.789 209.441 1.00357.11
C
ATOM 31789 OD1 ASN H 77 141.176 147.061 208.883 1.00369.65
O
ATOM 31790 ND2 ASN H 77 143.057 147.300 210.066 1.00325.46
N
ATOM 31791 C ASN H 77 139.442 150.127 209.398 1.00286.97
C
ATOM 31792 O ASN H 77 139.300 149.602 210.504 1.00301.13
O
ATOM 31793 N THR H 78 138.567 150.999 208.867 1.00280.46
N
ATOM 31794 CA THR H 78 137.227 151.222 209.400 1.00299.38
C
ATOM 31795 CB THR H 78 137.170 152.333 210.460 1.00342.77
C
ATOM 31796 OG1 THR H 78 137.895 153.472 209.994 1.00391.06
O
ATOM 31797 CG2 THR H 78 137.674 151.909 211.825 1.00373.81
C
ATOM 31798 C THR H 78 136.226 151.483 208.275 1.00242.94
C
ATOM 31799 O THR H 78 136.493 152.269 207.362 1.00172.13
O

ATOM 31800 N   LEU H 79   135.071 150.808 208.382 1.00227.62
N
ATOM 31801 CA LEU H 79   133.965 150.964 207.453 1.00235.67
C
ATOM 31802 CB LEU H 79   133.428 149.592 207.038 1.00166.69
C
ATOM 31803 CG LEU H 79   132.068 149.652 206.340 1.00183.14
C
ATOM 31804 CD1 LEU H 79   132.201 150.121 204.896 1.00146.50
C
ATOM 31805 CD2 LEU H 79   131.326 148.328 206.425 1.00196.12
C
ATOM 31806 C   LEU H 79   132.861 151.822 208.074 1.00289.69
C
ATOM 31807 O   LEU H 79   132.415 151.583 209.202 1.00269.63
O
ATOM 31808 N   TYR H 80   132.408 152.796 207.273 1.00316.51
N
ATOM 31809 CA TYR H 80   131.403 153.775 207.648 1.00268.88
C
ATOM 31810 CB TYR H 80   131.973 155.193 207.500 1.00271.24
C
ATOM 31811 CG TYR H 80   133.331 155.429 208.120 1.00315.02
C
ATOM 31812 CD1 TYR H 80   133.498 155.475 209.498 1.00359.10
C
ATOM 31813 CE1 TYR H 80   134.740 155.699 210.075 1.00374.15
C
ATOM 31814 CZ TYR H 80   135.851 155.883 209.268 1.00345.87
C
ATOM 31815 OH TYR H 80   137.091 156.117 209.793 1.00266.47
O
ATOM 31816 CE2 TYR H 80   135.705 155.844 207.892 1.00339.95
C
ATOM 31817 CD2 TYR H 80   134.457 155.620 207.331 1.00327.57
C
ATOM 31818 C   TYR H 80   130.137 153.553 206.816 1.00217.13
C
ATOM 31819 O   TYR H 80   130.200 153.190 205.641 1.00161.65
O
ATOM 31820 N   LEU H 81   128.980 153.756 207.453 1.00215.55
N
ATOM 31821 CA LEU H 81   127.723 153.884 206.730 1.00255.03
C
ATOM 31822 CB LEU H 81   126.798 152.728 207.141 1.00283.97
C
ATOM 31823 CG LEU H 81   125.390 152.703 206.531 1.00275.24
C
ATOM 31824 CD1 LEU H 81   125.419 152.678 205.006 1.00243.06
C

ATOM 31825 CD2 LEU H 81 124.568 151.538 207.072 1.00229.65
C
ATOM 31826 C LEU H 81 127.108 155.255 207.025 1.00268.53
C
ATOM 31827 O LEU H 81 127.239 155.773 208.136 1.00264.88
O
ATOM 31828 N GLN H 82 126.451 155.843 206.014 1.00280.81
N
ATOM 31829 CA GLN H 82 125.718 157.094 206.163 1.00317.47
C
ATOM 31830 CB GLN H 82 126.328 158.164 205.249 1.00307.40
C
ATOM 31831 CG GLN H 82 125.957 159.598 205.609 1.00298.11
C
ATOM 31832 CD GLN H 82 126.555 160.108 206.900 1.00266.72
C
ATOM 31833 OE1 GLN H 82 127.772 160.087 207.119 1.00200.84
O
ATOM 31834 NE2 GLN H 82 125.689 160.608 207.766 1.00251.82
N
ATOM 31835 C GLN H 82 124.234 156.841 205.882 1.00349.49
C
ATOM 31836 O GLN H 82 123.897 156.205 204.886 1.00387.57
O
ATOM 31837 N MET H 83 123.355 157.339 206.768 1.00308.33
N
ATOM 31838 CA MET H 83 121.916 157.121 206.670 1.00277.35
C
ATOM 31839 CB MET H 83 121.375 156.400 207.912 1.00264.65
C
ATOM 31840 CG MET H 83 122.172 155.178 208.337 1.00283.98
C
ATOM 31841 SD MET H 83 121.333 154.147 209.579 1.00274.22
S
ATOM 31842 CE MET H 83 122.754 153.402 210.378 1.00342.62
C
ATOM 31843 C MET H 83 121.208 158.470 206.554 1.00309.63
C
ATOM 31844 O MET H 83 121.441 159.352 207.376 1.00352.21
O
ATOM 31845 N ASN H 84 120.341 158.626 205.537 1.00319.61
N
ATOM 31846 CA ASN H 84 119.554 159.842 205.349 1.00297.50
C
ATOM 31847 CB ASN H 84 120.008 160.688 204.152 1.00267.65
C
ATOM 31848 CG ASN H 84 121.437 161.183 204.223 1.00244.48
C
ATOM 31849 OD1 ASN H 84 121.770 161.990 205.083 1.00210.86
O

ATOM 31850 ND2 ASN H 84 122.273 160.759 203.289 1.00245.64
N
ATOM 31851 C ASN H 84 118.083 159.498 205.121 1.00291.71
C
ATOM 31852 O ASN H 84 117.768 158.459 204.543 1.00326.35
O
ATOM 31853 N SER H 85 117.198 160.413 205.547 1.00254.96
N
ATOM 31854 CA SER H 85 115.754 160.348 205.328 1.00282.17
C
ATOM 31855 CB SER H 85 115.385 160.180 203.853 1.00282.66
C
ATOM 31856 OG SER H 85 115.954 161.195 203.033 1.00260.79
O
ATOM 31857 C SER H 85 115.101 159.280 206.212 1.00272.48
C
ATOM 31858 O SER H 85 114.049 158.740 205.849 1.00210.61
O
ATOM 31859 N LEU H 86 115.714 159.038 207.388 1.00245.91
N
ATOM 31860 CA LEU H 86 115.411 157.939 208.303 1.00256.81
C
ATOM 31861 CB LEU H 86 116.252 158.108 209.574 1.00256.28
C
ATOM 31862 CG LEU H 86 117.606 157.398 209.598 1.00307.27
C
ATOM 31863 CD1 LEU H 86 118.570 158.108 210.539 1.00277.37
C
ATOM 31864 CD2 LEU H 86 117.458 155.928 209.983 1.00314.83
C
ATOM 31865 C LEU H 86 113.924 157.897 208.653 1.00240.79
C
ATOM 31866 O LEU H 86 113.273 158.935 208.707 1.00254.04
O
ATOM 31867 N ARG H 87 113.403 156.693 208.922 1.00259.14
N
ATOM 31868 CA ARG H 87 111.990 156.507 209.220 1.00271.13
C
ATOM 31869 CB ARG H 87 111.214 156.116 207.952 1.00265.77
C
ATOM 31870 CG ARG H 87 110.666 157.273 207.122 1.00244.25
C
ATOM 31871 CD ARG H 87 111.449 157.720 205.894 1.00215.26
C
ATOM 31872 NE ARG H 87 112.109 156.615 205.208 1.00274.77
N
ATOM 31873 CZ ARG H 87 111.627 155.962 204.151 1.00354.33
C
ATOM 31874 NH1 ARG H 87 110.470 156.318 203.617 1.00390.83
N

ATOM 31875 NH2 ARG H 87 112.312 154.966 203.614 1.00378.72
N
ATOM 31876 C ARG H 87 111.833 155.473 210.337 1.00278.57
C
ATOM 31877 O ARG H 87 112.798 154.777 210.664 1.00210.06
O
ATOM 31878 N ALA H 88 110.606 155.405 210.898 1.00312.83
N
ATOM 31879 CA ALA H 88 110.242 154.585 212.050 1.00311.94
C
ATOM 31880 CB ALA H 88 108.913 155.027 212.646 1.00193.92
C
ATOM 31881 C ALA H 88 110.264 153.095 211.696 1.00348.36
C
ATOM 31882 O ALA H 88 110.485 152.262 212.575 1.00417.02
O
ATOM 31883 N GLU H 89 110.030 152.773 210.415 1.00324.11
N
ATOM 31884 CA GLU H 89 110.200 151.419 209.906 1.00266.32
C
ATOM 31885 CB GLU H 89 109.420 151.208 208.599 1.00256.85
C
ATOM 31886 CG GLU H 89 109.750 152.196 207.484 1.00292.43
C
ATOM 31887 CD GLU H 89 109.144 153.593 207.553 1.00291.27
C
ATOM 31888 OE1 GLU H 89 109.146 154.289 206.512 1.00256.03
O
ATOM 31889 OE2 GLU H 89 108.688 153.999 208.647 1.00309.36
O
ATOM 31890 C GLU H 89 111.694 151.100 209.830 1.00271.07
C
ATOM 31891 O GLU H 89 112.098 149.985 210.174 1.00226.18
O
ATOM 31892 N ASP H 90 112.494 152.124 209.459 1.00281.40
N
ATOM 31893 CA ASP H 90 113.933 152.053 209.215 1.00284.28
C
ATOM 31894 CB ASP H 90 114.475 153.305 208.506 1.00251.12
C
ATOM 31895 CG ASP H 90 113.996 153.582 207.078 1.00261.47
C
ATOM 31896 OD1 ASP H 90 113.754 152.624 206.289 1.00159.72
O
ATOM 31897 OD2 ASP H 90 113.891 154.778 206.740 1.00326.08
O
ATOM 31898 C ASP H 90 114.701 151.866 210.527 1.00284.76
C
ATOM 31899 O ASP H 90 115.925 152.006 210.549 1.00231.00
O

ATOM 31900 N THR H 91 113.960 151.591 211.618 1.00356.07
N
ATOM 31901 CA THR H 91 114.462 151.303 212.959 1.00384.01
C
ATOM 31902 CB THR H 91 113.388 151.585 214.032 1.00347.73
C
ATOM 31903 OG1 THR H 91 113.528 152.916 214.529 1.00374.47
O
ATOM 31904 CG2 THR H 91 113.385 150.643 215.221 1.00256.47
C
ATOM 31905 C THR H 91 114.908 149.845 213.038 1.00401.34
C
ATOM 31906 O THR H 91 114.094 148.934 212.892 1.00413.08
O
ATOM 31907 N ALA H 92 116.199 149.632 213.306 1.00380.10
N
ATOM 31908 CA ALA H 92 116.747 148.290 213.291 1.00350.01
C
ATOM 31909 CB ALA H 92 117.073 147.903 211.869 1.00389.57
C
ATOM 31910 C ALA H 92 117.977 148.230 214.187 1.00309.21
C
ATOM 31911 O ALA H 92 118.267 149.202 214.888 1.00220.73
O
ATOM 31912 N VAL H 93 118.625 147.052 214.187 1.00321.11
N
ATOM 31913 CA VAL H 93 119.985 146.827 214.661 1.00295.36
C
ATOM 31914 CB VAL H 93 120.068 145.626 215.639 1.00266.14
C
ATOM 31915 CG1 VAL H 93 121.313 145.646 216.534 1.00164.14
C
ATOM 31916 CG2 VAL H 93 118.816 145.503 216.502 1.00294.03
C
ATOM 31917 C VAL H 93 120.861 146.653 213.417 1.00274.67
C
ATOM 31918 O VAL H 93 120.489 145.925 212.498 1.00268.17
O
ATOM 31919 N TYR H 94 121.998 147.364 213.379 1.00244.14
N
ATOM 31920 CA TYR H 94 122.902 147.363 212.239 1.00232.83
C
ATOM 31921 CB TYR H 94 123.338 148.785 211.903 1.00161.05
C
ATOM 31922 CG TYR H 94 122.300 149.490 211.077 1.00208.46
C
ATOM 31923 CD1 TYR H 94 121.024 149.666 211.580 1.00236.25
C
ATOM 31924 CE1 TYR H 94 120.042 150.317 210.854 1.00263.45
C

ATOM 31925 CZ TYR H 94 120.330 150.795 209.589 1.00235.64
C
ATOM 31926 OH TYR H 94 119.341 151.441 208.903 1.00247.91
O
ATOM 31927 CE2 TYR H 94 121.599 150.612 209.058 1.00202.87
C
ATOM 31928 CD2 TYR H 94 122.571 149.956 209.798 1.00214.42
C
ATOM 31929 C TYR H 94 124.130 146.501 212.500 1.00296.07
C
ATOM 31930 O TYR H 94 124.927 146.803 213.390 1.00342.41
O
ATOM 31931 N TYR H 95 124.274 145.455 211.676 1.00274.76
N
ATOM 31932 CA TYR H 95 125.390 144.537 211.785 1.00213.36
C
ATOM 31933 CB TYR H 95 124.907 143.090 211.797 1.00169.16
C
ATOM 31934 CG TYR H 95 123.830 142.727 212.783 1.00166.81
C
ATOM 31935 CD1 TYR H 95 124.125 142.126 214.006 1.00197.50
C
ATOM 31936 CE1 TYR H 95 123.122 141.761 214.898 1.00217.23
C
ATOM 31937 CZ TYR H 95 121.790 141.979 214.568 1.00231.49
C
ATOM 31938 OH TYR H 95 120.801 141.598 215.437 1.00197.57
O
ATOM 31939 CE2 TYR H 95 121.479 142.562 213.345 1.00226.09
C
ATOM 31940 CD2 TYR H 95 122.493 142.914 212.461 1.00180.91
C
ATOM 31941 C TYR H 95 126.356 144.769 210.626 1.00232.35
C
ATOM 31942 O TYR H 95 125.929 144.958 209.469 1.00153.32
O
ATOM 31943 N CYS H 96 127.644 144.736 211.021 1.00237.37
N
ATOM 31944 CA CYS H 96 128.838 144.837 210.198 1.00245.86
C
ATOM 31945 CB CYS H 96 129.873 145.694 210.916 1.00278.28
C
ATOM 31946 SG CYS H 96 131.357 146.022 209.935 1.00437.02
S
ATOM 31947 C CYS H 96 129.404 143.443 209.913 1.00260.06
C
ATOM 31948 O CYS H 96 129.562 142.620 210.830 1.00176.51
O
ATOM 31949 N ALA H 97 129.732 143.231 208.622 1.00291.42
N

ATOM 31950 CA ALA H 97 130.002 141.928 208.027 1.00274.05
C
ATOM 31951 CB ALA H 97 128.718 141.336 207.501 1.00321.20
C
ATOM 31952 C ALA H 97 131.062 142.002 206.923 1.00226.40
C
ATOM 31953 O ALA H 97 131.048 142.885 206.049 1.00120.88
O
ATOM 31954 N SER H 98 131.948 140.999 206.961 1.00234.90
N
ATOM 31955 CA SER H 98 133.056 140.886 206.035 1.00239.98
C
ATOM 31956 CB SER H 98 134.365 140.808 206.769 1.00265.85
C
ATOM 31957 OG SER H 98 134.607 142.018 207.470 1.00253.75
O
ATOM 31958 C SER H 98 132.867 139.687 205.110 1.00229.69
C
ATOM 31959 O SER H 98 132.030 138.811 205.356 1.00194.32
O
ATOM 31960 N SER H 99 133.646 139.706 204.020 1.00196.89
N
ATOM 31961 CA SER H 99 133.733 138.592 203.087 1.00205.71
C
ATOM 31962 CB SER H 99 133.278 138.981 201.706 1.00190.70
C
ATOM 31963 OG SER H 99 133.781 138.059 200.734 1.00139.88
O
ATOM 31964 C SER H 99 135.162 138.073 203.011 1.00188.99
C
ATOM 31965 O SER H 99 136.001 138.468 203.815 1.00183.83
O
ATOM 31966 N SER H 100 135.380 137.167 202.040 1.00160.94
N
ATOM 31967 CA SER H 100 136.684 136.734 201.549 1.00225.47
C
ATOM 31968 CB SER H 100 136.723 135.229 201.450 1.00238.41
C
ATOM 31969 OG SER H 100 135.685 134.755 200.599 1.00189.37
O
ATOM 31970 C SER H 100 136.957 137.363 200.178 1.00231.62
C
ATOM 31971 O SER H 100 138.092 137.666 199.792 1.00241.22
O
ATOM 31972 N GLY H 101 135.879 137.484 199.415 1.00172.59
N
ATOM 31973 CA GLY H 101 135.828 138.393 198.294 1.00141.33
C
ATOM 31974 C GLY H 101 134.370 138.789 198.172 1.00155.25
C

```
ATOM  31975  O   GLY H 101     133.534 138.301 198.941  1.00103.41
O
ATOM  31976  N   TYR H 102     134.112 139.637 197.178  1.00179.84
N
ATOM  31977  CA  TYR H 102     132.805 140.190 196.879  1.00162.24
C
ATOM  31978  CB  TYR H 102     132.872 140.899 195.526  1.00177.39
C
ATOM  31979  CG  TYR H 102     131.573 141.529 195.106  1.00188.37
C
ATOM  31980  CD1 TYR H 102     131.058 142.569 195.848  1.00207.20
C
ATOM  31981  CE1 TYR H 102     129.868 143.176 195.498  1.00196.83
C
ATOM  31982  CZ  TYR H 102     129.167 142.755 194.391  1.00129.71
C
ATOM  31983  OH  TYR H 102     128.006 143.424 194.192  1.00 85.53
O
ATOM  31984  CE2 TYR H 102     129.652 141.705 193.633  1.00161.12
C
ATOM  31985  CD2 TYR H 102     130.848 141.098 194.000  1.00185.41
C
ATOM  31986  C   TYR H 102     131.711 139.122 196.819  1.00146.18
C
ATOM  31987  O   TYR H 102     130.596 139.426 197.232  1.00173.71
O
ATOM  31988  N   LEU H 103     132.048 137.910 196.330  1.00100.89
N
ATOM  31989  CA  LEU H 103     131.094 137.026 195.682  1.00 72.91
C
ATOM  31990  CB  LEU H 103     131.488 136.719 194.225  1.00 91.47
C
ATOM  31991  CG  LEU H 103     130.637 137.344 193.112  1.00 88.65
C
ATOM  31992  CD1 LEU H 103     130.437 136.377 191.934  1.00 78.78
C
ATOM  31993  CD2 LEU H 103     129.295 137.743 193.686  1.00 85.79
C
ATOM  31994  C   LEU H 103     130.817 135.756 196.478  1.00 68.66
C
ATOM  31995  O   LEU H 103     129.787 135.185 196.151  1.00 86.95
O
ATOM  31996  N   PHE H 104     131.621 135.319 197.484  1.00 64.72
N
ATOM  31997  CA  PHE H 104     131.088 134.212 198.304  1.00 92.71
C
ATOM  31998  CB  PHE H 104     131.813 132.885 197.976  1.00111.21
C
ATOM  31999  CG  PHE H 104     131.888 131.834 199.078  1.00251.30
C
```

```
ATOM  32000  CD1 PHE H 104     130.758 131.358 199.753  1.00276.97
C
ATOM  32001  CE1 PHE H 104     130.841 130.419 200.787  1.00222.99
C
ATOM  32002  CZ  PHE H 104     132.066 129.906 201.148  1.00234.47
C
ATOM  32003  CD2 PHE H 104     133.112 131.291 199.463  1.00331.90
C
ATOM  32004  CE2 PHE H 104     133.197 130.341 200.481  1.00330.73
C
ATOM  32005  C   PHE H 104     130.728 134.644 199.754  1.00 91.31
C
ATOM  32006  O   PHE H 104     131.028 135.789 200.082  1.00125.00
O
ATOM  32007  N   HIS H 105     130.050 133.798 200.604  1.00 76.70
N
ATOM  32008  CA  HIS H 105     130.456 133.598 202.015  1.00127.95
C
ATOM  32009  CB  HIS H 105     131.972 133.921 202.178  1.00227.51
C
ATOM  32010  CG  HIS H 105     132.606 134.097 203.536  1.00252.52
C
ATOM  32011  ND1 HIS H 105     132.873 135.353 204.091  1.00237.15
N
ATOM  32012  CE1 HIS H 105     133.508 135.207 205.238  1.00284.10
C
ATOM  32013  NE2 HIS H 105     133.711 133.894 205.430  1.00327.37
N
ATOM  32014  CD2 HIS H 105     133.180 133.199 204.370  1.00300.41
C
ATOM  32015  C   HIS H 105     129.802 134.638 202.884  1.00128.20
C
ATOM  32016  O   HIS H 105     129.207 135.546 202.340  1.00189.81
O
ATOM  32017  N   SER H 106     130.047 134.623 204.187  1.00136.56
N
ATOM  32018  CA  SER H 106     129.754 135.856 204.901  1.00153.17
C
ATOM  32019  CB  SER H 106     128.325 135.861 205.288  1.00155.40
C
ATOM  32020  OG  SER H 106     128.016 137.138 205.799  1.00185.53
O
ATOM  32021  C   SER H 106     130.632 136.034 206.132  1.00207.89
C
ATOM  32022  O   SER H 106     131.000 137.173 206.457  1.00137.09
O
ATOM  32023  N   ASP H 107     131.016 134.856 206.663  1.00307.74
N
ATOM  32024  CA  ASP H 107     131.326 134.396 208.015  1.00329.89
C
```

ATOM 32025 CB ASP H 107 132.381 133.286 208.011 1.00392.22
C
ATOM 32026 CG ASP H 107 132.918 132.981 209.395 1.00414.64
C
ATOM 32027 OD1 ASP H 107 132.292 132.163 210.098 1.00519.35
O
ATOM 32028 OD2 ASP H 107 133.939 133.592 209.765 1.00402.63
O
ATOM 32029 C ASP H 107 131.545 135.497 209.047 1.00321.37
C
ATOM 32030 O ASP H 107 130.979 135.433 210.137 1.00411.08
O
ATOM 32031 N TYR H 108 132.382 136.480 208.725 1.00254.31
N
ATOM 32032 CA TYR H 108 132.729 137.476 209.719 1.00244.83
C
ATOM 32033 CB TYR H 108 133.961 138.230 209.253 1.00260.06
C
ATOM 32034 CG TYR H 108 135.203 137.394 209.333 1.00301.84
C
ATOM 32035 CD1 TYR H 108 135.327 136.399 210.288 1.00324.21
C
ATOM 32036 CE1 TYR H 108 136.476 135.633 210.378 1.00392.42
C
ATOM 32037 CZ TYR H 108 137.527 135.875 209.512 1.00419.19
C
ATOM 32038 OH TYR H 108 138.667 135.128 209.597 1.00498.97
O
ATOM 32039 CE2 TYR H 108 137.420 136.876 208.562 1.00402.48
C
ATOM 32040 CD2 TYR H 108 136.261 137.625 208.478 1.00338.78
C
ATOM 32041 C TYR H 108 131.585 138.452 209.960 1.00254.49
C
ATOM 32042 O TYR H 108 131.255 139.243 209.081 1.00239.30
O
ATOM 32043 N TRP H 109 131.013 138.385 211.172 1.00248.55
N
ATOM 32044 CA TRP H 109 129.891 139.211 211.585 1.00198.49
C
ATOM 32045 CB TRP H 109 128.607 138.405 211.754 1.00190.47
C
ATOM 32046 CG TRP H 109 127.748 138.576 210.554 1.00159.01
C
ATOM 32047 CD1 TRP H 109 128.185 138.761 209.278 1.00136.66
C
ATOM 32048 NE1 TRP H 109 127.125 138.872 208.428 1.00136.77
N
ATOM 32049 CE2 TRP H 109 125.967 138.756 209.139 1.00151.39
C

ATOM 32050 CD2 TRP H 109 126.319 138.566 210.497 1.00170.76
C
ATOM 32051 CE3 TRP H 109 125.310 138.438 211.456 1.00180.16
C
ATOM 32052 CZ3 TRP H 109 124.005 138.496 211.021 1.00184.99
C
ATOM 32053 CH2 TRP H 109 123.680 138.693 209.673 1.00188.86
C
ATOM 32054 CZ2 TRP H 109 124.647 138.831 208.703 1.00149.81
C
ATOM 32055 C TRP H 109 130.212 139.829 212.917 1.00199.73
C
ATOM 32056 O TRP H 109 131.211 139.441 213.532 1.00194.43
O
ATOM 32057 N GLY H 110 129.287 140.710 213.326 1.00181.69
N
ATOM 32058 CA GLY H 110 129.454 141.537 214.505 1.00270.05
C
ATOM 32059 C GLY H 110 128.459 141.202 215.610 1.00288.78
C
ATOM 32060 O GLY H 110 127.750 140.194 215.532 1.00277.89
O
ATOM 32061 N GLN H 111 128.485 142.046 216.650 1.00294.14
N
ATOM 32062 CA GLN H 111 127.473 142.031 217.690 1.00320.57
C
ATOM 32063 CB GLN H 111 128.088 142.159 219.086 1.00222.51
C
ATOM 32064 CG GLN H 111 127.118 141.677 220.160 1.00292.72
C
ATOM 32065 CD GLN H 111 126.281 140.480 219.759 1.00316.14
C
ATOM 32066 OE1 GLN H 111 126.784 139.363 219.623 1.00254.55
O
ATOM 32067 NE2 GLN H 111 124.988 140.698 219.572 1.00370.95
N
ATOM 32068 C GLN H 111 126.362 143.029 217.349 1.00373.59
C
ATOM 32069 O GLN H 111 125.187 142.671 217.395 1.00418.15
O
ATOM 32070 N GLY H 112 126.743 144.274 217.017 1.00381.45
N
ATOM 32071 CA GLY H 112 125.878 145.205 216.295 1.00341.85
C
ATOM 32072 C GLY H 112 124.979 146.078 217.177 1.00289.51
C
ATOM 32073 O GLY H 112 124.323 145.573 218.086 1.00230.04
O
ATOM 32074 N THR H 113 124.882 147.368 216.809 1.00278.15
N

ATOM 32075 CA THR H 113   124.289 148.437 217.609 1.00328.41
C
ATOM 32076 CB THR H 113   125.278 149.617 217.697 1.00288.73
C
ATOM 32077 OG1 THR H 113   124.706 150.777 218.305 1.00295.46
O
ATOM 32078 CG2 THR H 113   125.882 150.005 216.363 1.00236.17
C
ATOM 32079 C   THR H 113   122.883 148.789 217.099 1.00382.03
C
ATOM 32080 O   THR H 113   122.712 149.027 215.904 1.00447.58
O
ATOM 32081 N   LEU H 114   121.888 148.817 218.014 1.00356.93
N
ATOM 32082 CA LEU H 114   120.496 149.197 217.753 1.00296.63
C
ATOM 32083 CB LEU H 114   119.640 148.851 218.991 1.00257.72
C
ATOM 32084 CG LEU H 114   118.200 149.386 219.074 1.00260.32
C
ATOM 32085 CD1 LEU H 114   117.205 148.512 218.308 1.00235.82
C
ATOM 32086 CD2 LEU H 114   117.753 149.554 220.531 1.00200.84
C
ATOM 32087 C   LEU H 114   120.444 150.689 217.408 1.00269.72
C
ATOM 32088 O   LEU H 114   121.067 151.478 218.117 1.00161.82
O
ATOM 32089 N   VAL H 115   119.746 151.037 216.302 1.00320.51
N
ATOM 32090 CA VAL H 115   119.481 152.403 215.844 1.00366.14
C
ATOM 32091 CB VAL H 115   119.996 152.656 214.402 1.00317.30
C
ATOM 32092 CG1 VAL H 115   119.390 153.896 213.745 1.00228.66
C
ATOM 32093 CG2 VAL H 115   121.517 152.710 214.333 1.00312.47
C
ATOM 32094 C   VAL H 115   117.978 152.678 215.975 1.00409.67
C
ATOM 32095 O   VAL H 115   117.172 151.988 215.349 1.00423.36
O
ATOM 32096 N   THR H 116   117.610 153.669 216.811 1.00406.72
N
ATOM 32097 CA THR H 116   116.227 154.096 217.033 1.00324.84
C
ATOM 32098 CB THR H 116   115.865 154.193 218.536 1.00250.50
C
ATOM 32099 OG1 THR H 116   116.490 153.254 219.419 1.00185.06
O

ATOM 32100 CG2 THR H 116 114.377 154.151 218.829 1.00186.09
C
ATOM 32101 C THR H 116 115.977 155.425 216.302 1.00345.63
C
ATOM 32102 O THR H 116 116.778 156.355 216.405 1.00284.61
O
ATOM 32103 N VAL H 117 114.860 155.499 215.557 1.00372.06
N
ATOM 32104 CA VAL H 117 114.380 156.718 214.912 1.00330.12
C
ATOM 32105 CB VAL H 117 114.282 156.570 213.374 1.00244.62
C
ATOM 32106 CG1 VAL H 117 113.875 157.859 212.688 1.00216.80
C
ATOM 32107 CG2 VAL H 117 115.568 156.053 212.752 1.00251.97
C
ATOM 32108 C VAL H 117 113.040 157.095 215.552 1.00394.87
C
ATOM 32109 O VAL H 117 112.035 156.407 215.362 1.00478.58
O
ATOM 32110 N SER H 118 113.037 158.184 216.333 1.00364.24
N
ATOM 32111 CA SER H 118 111.864 158.558 217.109 1.00339.19
C
ATOM 32112 CB SER H 118 112.062 158.286 218.588 1.00316.10
C
ATOM 32113 OG SER H 118 111.667 156.956 218.928 1.00228.75
O
ATOM 32114 C SER H 118 111.433 159.994 216.819 1.00330.15
C
ATOM 32115 O SER H 118 112.128 160.950 217.165 1.00314.05
O
ATOM 32116 N SER H 119 110.267 160.097 216.175 1.00328.76
N
ATOM 32117 CA SER H 119 109.526 161.327 215.960 1.00335.80
C
ATOM 32118 CB SER H 119 108.231 160.981 215.293 1.00339.45
C
ATOM 32119 OG SER H 119 107.620 159.891 215.976 1.00307.77
O
ATOM 32120 C SER H 119 109.273 162.023 217.305 1.00340.57
C
ATOM 32121 O SER H 119 108.721 161.353 218.222 1.00288.89
O
ATOM 32122 N ALA H 120 108.942 160.365 218.111 1.00 59.82
N
ATOM 32123 CA ALA H 120 109.126 160.537 219.543 1.00 53.84
C
ATOM 32124 C ALA H 120 110.552 161.009 219.859 1.00 50.77
C

ATOM 32125 O ALA H 120 111.476 160.905 219.050 1.00 54.92
O
ATOM 32126 CB ALA H 120 108.794 159.224 220.257 1.00 55.85
C
ATOM 32127 H ALA H 120 108.887 159.550 217.844 1.00 71.82
H
ATOM 32128 HA ALA H 120 108.529 161.220 219.885 1.00 64.64
H
ATOM 32129 HB1 ALA H 120 108.927 159.340 221.210 1.00 67.05
H
ATOM 32130 HB2 ALA H 120 107.869 158.992 220.077 1.00 67.05
H
ATOM 32131 HB3 ALA H 120 109.381 158.527 219.923 1.00 67.05
H
ATOM 32132 N SER H 121 110.714 161.527 221.068 1.00 50.87
N
ATOM 32133 CA SER H 121 111.928 162.173 221.540 1.00 51.40
C
ATOM 32134 C SER H 121 112.471 161.435 222.742 1.00 57.00
C
ATOM 32135 O SER H 121 111.707 160.855 223.523 1.00 59.47
O
ATOM 32136 CB SER H 121 111.652 163.635 221.975 1.00 55.94
C
ATOM 32137 OG SER H 121 111.070 164.364 220.915 1.00 76.46
O
ATOM 32138 H SER H 121 110.097 161.515 221.667 1.00 61.07
H
ATOM 32139 HA SER H 121 112.586 162.162 220.827 1.00 61.71
H
ATOM 32140 HB2 SER H 121 111.043 163.633 222.730 1.00 67.16
H
ATOM 32141 HB3 SER H 121 112.490 164.055 222.227 1.00 67.16
H
ATOM 32142 HG SER H 121 111.055 165.182 221.102 1.00 91.78
H
ATOM 32143 N THR H 122 113.778 161.533 222.946 1.00 51.07
N
ATOM 32144 CA THR H 122 114.381 160.802 224.045 1.00 53.75
C
ATOM 32145 C THR H 122 113.681 161.130 225.360 1.00 58.64
C
ATOM 32146 O THR H 122 113.385 162.290 225.662 1.00 50.81
O
ATOM 32147 CB THR H 122 115.873 161.118 224.104 1.00 46.46
C
ATOM 32148 OG1 THR H 122 116.443 160.716 222.859 1.00 57.24
O
ATOM 32149 CG2 THR H 122 116.520 160.363 225.187 1.00 50.27
C

ATOM 32150 H THR H 122 114.322 162.004 222.474 1.00 61.31
H
ATOM 32151 HA THR H 122 114.288 159.847 223.904 1.00 64.53
H
ATOM 32152 HB THR H 122 116.016 162.063 224.268 1.00 55.78
H
ATOM 32153 HG1 THR H 122 117.267 160.880 222.855 1.00 68.71
H
ATOM 32154 HG21 THR H 122 117.482 160.469 225.138 1.00 60.35
H
ATOM 32155 HG22 THR H 122 116.211 160.687 226.049 1.00 60.35
H
ATOM 32156 HG23 THR H 122 116.302 159.420 225.110 1.00 60.35
H
ATOM 32157 N LYS H 123 113.381 160.084 226.140 1.00 57.97
N
ATOM 32158 CA LYS H 123 112.633 160.265 227.373 1.00 50.67
C
ATOM 32159 C LYS H 123 112.994 159.152 228.348 1.00 58.25
C
ATOM 32160 O LYS H 123 113.069 157.976 227.966 1.00 52.44
O
ATOM 32161 CB LYS H 123 111.129 160.283 227.081 1.00 60.22
C
ATOM 32162 CG LYS H 123 110.265 160.699 228.276 1.00 59.44
C
ATOM 32163 CD LYS H 123 108.795 160.727 227.887 1.00 72.83
C
ATOM 32164 CE LYS H 123 107.888 161.128 229.056 1.00 83.09
C
ATOM 32165 NZ LYS H 123 108.104 160.305 230.276 1.00 78.83
N
ATOM 32166 H LYS H 123 113.601 159.271 225.971 1.00 69.59
H
ATOM 32167 HA LYS H 123 112.875 161.105 227.794 1.00 60.83
H
ATOM 32168 HB2 LYS H 123 110.959 160.910 226.362 1.00 72.29
H
ATOM 32169 HB3 LYS H 123 110.854 159.390 226.816 1.00 72.29
H
ATOM 32170 HG2 LYS H 123 110.382 160.063 228.999 1.00 71.35
H
ATOM 32171 HG3 LYS H 123 110.523 161.587 228.569 1.00 71.35
H
ATOM 32172 HD2 LYS H 123 108.667 161.370 227.173 1.00 87.42
H
ATOM 32173 HD3 LYS H 123 108.528 159.843 227.588 1.00 87.42
H
ATOM 32174 HE2 LYS H 123 108.064 162.054 229.286 1.00 99.73
H

ATOM 32175 HE3 LYS H 123   106.963 161.022 228.787 1.00 99.73
H
ATOM 32176 HZ1 LYS H 123   107.539 160.555 230.917 1.00 94.62
H
ATOM 32177 HZ2 LYS H 123   107.969 159.444 230.091 1.00 94.62
H
ATOM 32178 HZ3 LYS H 123   108.938 160.409 230.570 1.00 94.62
H
ATOM 32179 N   GLY H 124   113.262 159.535 229.589 1.00 59.78
N
ATOM 32180 CA GLY H 124   113.535 158.600 230.660 1.00 60.58
C
ATOM 32181 C   GLY H 124   112.263 157.967 231.220 1.00 54.15
C
ATOM 32182 O   GLY H 124   111.154 158.520 231.123 1.00 49.37
O
ATOM 32183 H   GLY H 124   113.291 160.359 229.838 1.00 71.76
H
ATOM 32184 HA2 GLY H 124   114.108 157.893 230.328 1.00 72.72
H
ATOM 32185 HA3 GLY H 124   113.987 159.063 231.382 1.00 72.72
H
ATOM 32186 N   PRO H 125   112.400 156.770 231.794 1.00 55.69
N
ATOM 32187 CA PRO H 125   111.217 156.034 232.258 1.00 55.38
C
ATOM 32188 C   PRO H 125   110.696 156.527 233.593 1.00 52.08
C
ATOM 32189 O   PRO H 125   111.450 156.950 234.466 1.00 54.17
O
ATOM 32190 CB PRO H 125   111.737 154.601 232.392 1.00 54.30
C
ATOM 32191 CG PRO H 125   113.159 154.795 232.783 1.00 53.37
C
ATOM 32192 CD PRO H 125   113.636 155.990 231.999 1.00 53.25
C
ATOM 32193 HA PRO H 125   110.511 156.089 231.595 1.00 66.49
H
ATOM 32194 HB2 PRO H 125   111.243 154.125 233.077 1.00 65.19
H
ATOM 32195 HB3 PRO H 125   111.663 154.133 231.544 1.00 65.19
H
ATOM 32196 HG2 PRO H 125   113.217 154.963 233.736 1.00 64.07
H
ATOM 32197 HG3 PRO H 125   113.673 154.005 232.553 1.00 64.07
H
ATOM 32198 HD2 PRO H 125   114.286 156.500 232.506 1.00 63.93
H
ATOM 32199 HD3 PRO H 125   114.017 155.718 231.150 1.00 63.93
H

ATOM 32200 N   SER H 126   109.389 156.406 233.762 1.00 56.19
N
ATOM 32201 CA SER H 126   108.798 156.353 235.092 1.00 57.48
C
ATOM 32202 C   SER H 126   108.773 154.904 235.606 1.00 58.74
C
ATOM 32203 O   SER H 126   108.402 153.995 234.866 1.00 59.19
O
ATOM 32204 CB SER H 126   107.397 156.945 235.033 1.00 63.27
C
ATOM 32205 OG SER H 126   107.471 158.313 234.663 1.00 65.78
O
ATOM 32206 H   SER H 126   108.819 156.354 233.120 1.00 67.46
H
ATOM 32207 HA SER H 126   109.323 156.866 235.727 1.00 69.01
H
ATOM 32208 HB2 SER H 126   106.873 156.463 234.372 1.00 75.95
H
ATOM 32209 HB3 SER H 126   106.980 156.872 235.905 1.00 75.95
H
ATOM 32210 HG SER H 126   106.701 158.611 234.510 1.00 78.96
H
ATOM 32211 N   VAL H 127   109.155 154.694 236.879 1.00 55.85
N
ATOM 32212 CA VAL H 127   109.206 153.364 237.501 1.00 53.05
C
ATOM 32213 C   VAL H 127   108.130 153.263 238.595 1.00 55.51
C
ATOM 32214 O   VAL H 127   108.190 153.946 239.628 1.00 58.00
O
ATOM 32215 CB VAL H 127   110.590 153.054 238.075 1.00 44.16
C
ATOM 32216 CG1 VAL H 127   110.648 151.610 238.582 1.00 50.27
C
ATOM 32217 CG2 VAL H 127   111.715 153.275 237.033 1.00 50.29
C
ATOM 32218 H   VAL H 127   109.397 155.324 237.412 1.00 67.04
H
ATOM 32219 HA VAL H 127   108.985 152.713 236.817 1.00 63.69
H
ATOM 32220 HB VAL H 127   110.740 153.663 238.813 1.00 53.01
H
ATOM 32221 HG11 VAL H 127   111.565 151.394 238.816 1.00 60.35
H
ATOM 32222 HG12 VAL H 127   110.078 151.525 239.360 1.00 60.35
H
ATOM 32223 HG13 VAL H 127   110.340 151.016 237.879 1.00 60.35
H
ATOM 32224 HG21 VAL H 127   112.559 152.982 237.414 1.00 60.37
H

ATOM 32225 HG22 VAL H 127   111.515 152.757 236.238 1.00 60.37
H
ATOM 32226 HG23 VAL H 127   111.761 154.218 236.814 1.00 60.37
H
ATOM 32227 N   PHE H 128   107.158 152.389 238.388 1.00 50.97
N
ATOM 32228 CA PHE H 128   106.054 152.184 239.317 1.00 45.49
C
ATOM 32229 C   PHE H 128   106.110 150.780 239.945 1.00 53.35
C
ATOM 32230 O   PHE H 128   106.448 149.809 239.258 1.00 47.44
O
ATOM 32231 CB PHE H 128   104.710 152.354 238.600 1.00 46.17
C
ATOM 32232 CG PHE H 128   104.535 153.699 237.898 1.00 54.15
C
ATOM 32233 CD1 PHE H 128   104.662 154.899 238.603 1.00 59.03
C
ATOM 32234 CD2 PHE H 128   104.210 153.756 236.544 1.00 54.17
C
ATOM 32235 CE1 PHE H 128   104.486 156.131 237.977 1.00 55.14
C
ATOM 32236 CE2 PHE H 128   104.012 154.997 235.893 1.00 53.69
C
ATOM 32237 CZ PHE H 128   104.165 156.185 236.625 1.00 59.56
C
ATOM 32238 H   PHE H 128   107.110 151.885 237.693 1.00 61.19
H
ATOM 32239 HA PHE H 128   106.112 152.835 240.033 1.00 54.62
H
ATOM 32240 HB2 PHE H 128   104.628 151.661 237.926 1.00 55.43
H
ATOM 32241 HB3 PHE H 128   103.998 152.269 239.252 1.00 55.43
H
ATOM 32242 HD1 PHE H 128   104.870 154.875 239.509 1.00 70.86
H
ATOM 32243 HD2 PHE H 128   104.119 152.967 236.061 1.00 65.03
H
ATOM 32244 HE1 PHE H 128   104.582 156.918 238.465 1.00 66.20
H
ATOM 32245 HE2 PHE H 128   103.784 155.025 234.992 1.00 64.46
H
ATOM 32246 HZ PHE H 128   104.052 157.008 236.205 1.00 71.50
H
ATOM 32247 N   PRO H 129   105.815 150.636 241.245 1.00 50.51
N
ATOM 32248 CA PRO H 129   105.782 149.293 241.842 1.00 54.17
C
ATOM 32249 C   PRO H 129   104.581 148.488 241.370 1.00 45.38
C

ATOM 32250 O PRO H 129 103.499 149.024 241.184 1.00 45.02
O
ATOM 32251 CB PRO H 129 105.671 149.593 243.339 1.00 55.03
C
ATOM 32252 CG PRO H 129 104.942 150.875 243.362 1.00 49.81
C
ATOM 32253 CD PRO H 129 105.558 151.662 242.267 1.00 53.53
C
ATOM 32254 HA PRO H 129 106.598 148.810 241.642 1.00 65.03
H
ATOM 32255 HB2 PRO H 129 105.173 148.894 243.789 1.00 66.06
H
ATOM 32256 HB3 PRO H 129 106.554 149.679 243.733 1.00 66.06
H
ATOM 32257 HG2 PRO H 129 103.998 150.726 243.197 1.00 59.80
H
ATOM 32258 HG3 PRO H 129 105.065 151.314 244.218 1.00 59.80
H
ATOM 32259 HD2 PRO H 129 104.946 152.337 241.936 1.00 64.26
H
ATOM 32260 HD3 PRO H 129 106.385 152.079 242.560 1.00 64.26
H
ATOM 32261 N LEU H 130 104.808 147.210 241.120 1.00 45.88
N
ATOM 32262 CA LEU H 130 103.758 146.193 241.032 1.00 51.06
C
ATOM 32263 C LEU H 130 103.777 145.476 242.400 1.00 45.45
C
ATOM 32264 O LEU H 130 104.609 144.591 242.662 1.00 49.05
O
ATOM 32265 CB LEU H 130 104.001 145.243 239.852 1.00 46.42
C
ATOM 32266 CG LEU H 130 104.184 145.882 238.467 1.00 41.17
C
ATOM 32267 CD1 LEU H 130 104.751 144.874 237.450 1.00 45.33
C
ATOM 32268 CD2 LEU H 130 102.869 146.498 237.992 1.00 47.62
C
ATOM 32269 H LEU H 130 105.595 146.887 240.991 1.00 55.08
H
ATOM 32270 HA LEU H 130 102.885 146.593 240.883 1.00 61.30
H
ATOM 32271 HB2 LEU H 130 104.807 144.737 240.039 1.00 55.74
H
ATOM 32272 HB3 LEU H 130 103.241 144.644 239.788 1.00 55.74
H
ATOM 32273 HG LEU H 130 104.838 146.596 238.531 1.00 49.43
H
ATOM 32274 HD11 LEU H 130 104.839 145.311 236.589 1.00 54.42
H

ATOM 32275 HD12 LEU H 130 105.618 144.570 237.759 1.00 54.42
H
ATOM 32276 HD13 LEU H 130 104.141 144.122 237.380 1.00 54.42
H
ATOM 32277 HD21 LEU H 130 103.007 146.905 237.121 1.00 57.17
H
ATOM 32278 HD22 LEU H 130 102.198 145.800 237.927 1.00 57.17
H
ATOM 32279 HD23 LEU H 130 102.587 147.171 238.629 1.00 57.17
H
ATOM 32280 N ALA H 131 102.926 145.920 243.294 1.00 49.91
N
ATOM 32281 CA ALA H 131 102.992 145.493 244.696 1.00 55.09
C
ATOM 32282 C ALA H 131 102.337 144.119 244.906 1.00 52.53
C
ATOM 32283 O ALA H 131 101.242 143.866 244.402 1.00 54.64
O
ATOM 32284 CB ALA H 131 102.311 146.523 245.595 1.00 48.95
C
ATOM 32285 H ALA H 131 102.289 146.474 243.127 1.00 59.92
H
ATOM 32286 HA ALA H 131 103.924 145.412 244.951 1.00 66.13
H
ATOM 32287 HB1 ALA H 131 102.392 146.239 246.519 1.00 58.77
H
ATOM 32288 HB2 ALA H 131 102.744 147.383 245.475 1.00 58.77
H
ATOM 32289 HB3 ALA H 131 101.374 146.586 245.350 1.00 58.77
H
ATOM 32290 N PRO H 132 102.953 143.240 245.695 1.00 59.50
N
ATOM 32291 CA PRO H 132 102.275 141.988 246.080 1.00 60.19
C
ATOM 32292 C PRO H 132 101.136 142.281 247.038 1.00 56.81
C
ATOM 32293 O PRO H 132 101.086 143.341 247.660 1.00 66.04
O
ATOM 32294 CB PRO H 132 103.383 141.175 246.759 1.00 57.06
C
ATOM 32295 CG PRO H 132 104.301 142.193 247.365 1.00 54.06
C
ATOM 32296 CD PRO H 132 104.232 143.426 246.421 1.00 56.64
C
ATOM 32297 HA PRO H 132 101.951 141.502 245.306 1.00 72.25
H
ATOM 32298 HB2 PRO H 132 102.999 140.605 247.443 1.00 68.50
H
ATOM 32299 HB3 PRO H 132 103.847 140.638 246.100 1.00 68.50
H

ATOM 32300 HG2 PRO H 132   103.996 142.422 248.257 1.00 64.90
H
ATOM 32301 HG3 PRO H 132   105.204 141.839 247.404 1.00 64.90
H
ATOM 32302 HD2 PRO H 132   104.220 144.251 246.932 1.00 68.00
H
ATOM 32303 HD3 PRO H 132   104.980 143.429 245.804 1.00 68.00
H
ATOM 32304 N   SER H 133   100.207 141.333 247.149 1.00 77.36
N
ATOM 32305 CA SER H 133   99.077 141.451 248.101 1.00 88.04
C
ATOM 32306 C   SER H 133   99.469 141.659 249.593 1.00 84.43
C
ATOM 32307 O   SER H 133   100.276 140.913 250.172 1.00 71.71
O
ATOM 32308 CB SER H 133   98.189 140.211 247.983 1.00 91.06
C
ATOM 32309 OG SER H 133   97.372 140.304 246.831 1.00 94.19
O
ATOM 32310 H   SER H 133   100.199 140.607 246.687 1.00 92.86
H
ATOM 32311 HA SER H 133   98.597 142.258 247.855 1.00105.68
H
ATOM 32312 HB2 SER H 133   98.751 139.423 247.912 1.00109.30
H
ATOM 32313 HB3 SER H 133   97.625 140.147 248.769 1.00109.30
H
ATOM 32314 HG SER H 133   96.948 139.587 246.722 1.00113.05
H
ATOM 32315 N   SER H 136   97.508 135.671 251.037 1.00 96.13
N
ATOM 32316 CA SER H 136   97.979 135.099 252.302 1.00115.22
C
ATOM 32317 C   SER H 136   99.499 135.153 252.360 1.00107.69
C
ATOM 32318 O   SER H 136   100.182 134.686 251.449 1.00102.58
O
ATOM 32319 CB SER H 136   97.502 133.648 252.476 1.00104.93
C
ATOM 32320 OG SER H 136   98.155 132.752 251.582 1.00 95.90
O
ATOM 32321 H   SER H 136   96.656 135.625 250.929 1.00115.38
H
ATOM 32322 HA SER H 136   97.607 135.613 253.036 1.00138.29
H
ATOM 32323 HB2 SER H 136   97.690 133.368 253.387 1.00125.94
H
ATOM 32324 HB3 SER H 136   96.548 133.612 252.309 1.00125.94
H

```
ATOM  32325  HG  SER H 136      98.982 132.752 251.730  1.00115.11
H
ATOM  32326  N   THR H 137     100.026 135.698 253.453  1.00101.01
N
ATOM  32327  CA  THR H 137     101.439 136.024 253.546  1.00101.00
C
ATOM  32328  C   THR H 137     102.208 135.078 254.481  1.00 93.10
C
ATOM  32329  O   THR H 137     103.438 135.183 254.581  1.00 75.03
O
ATOM  32330  CB  THR H 137     101.561 137.518 253.944  1.00110.21
C
ATOM  32331  OG1 THR H 137     102.915 137.994 253.783  1.00 82.93
O
ATOM  32332  CG2 THR H 137     101.014 137.799 255.372  1.00 97.98
C
ATOM  32333  H   THR H 137      99.577 135.888 254.161  1.00121.24
H
ATOM  32334  HA  THR H 137     101.882 135.929 252.688  1.00121.23
H
ATOM  32335  HB  THR H 137     101.002 138.034 253.340  1.00132.27
H
ATOM  32336  HG1 THR H 137     103.408 137.658 254.374  1.00 99.54
H
ATOM  32337 HG21 THR H 137     101.151 138.732 255.601  1.00117.60
H
ATOM  32338 HG22 THR H 137     100.065 137.603 255.408  1.00117.60
H
ATOM  32339 HG23 THR H 137     101.476 137.244 256.019  1.00117.60
H
ATOM  32340  N   SER H 138     101.536 134.099 255.099  1.00 95.38
N
ATOM  32341  CA  SER H 138     102.230 133.104 255.923  1.00 90.54
C
ATOM  32342  C   SER H 138     102.903 132.001 255.103  1.00 73.95
C
ATOM  32343  O   SER H 138     103.726 131.248 255.635  1.00 52.22
O
ATOM  32344  CB  SER H 138     101.248 132.455 256.899  1.00 86.85
C
ATOM  32345  OG  SER H 138     100.931 133.330 257.959  1.00 86.26
O
ATOM  32346  H   SER H 138     100.685 133.989 255.058  1.00114.49
H
ATOM  32347  HA  SER H 138     102.917 133.565 256.427  1.00108.67
H
ATOM  32348  HB2 SER H 138     100.434 132.228 256.423  1.00104.25
H
ATOM  32349  HB3 SER H 138     101.653 131.651 257.264  1.00104.25
H
```

ATOM 32350 HG SER H 138 100.351 132.981 258.456 1.00103.54
H
ATOM 32351 N GLY H 139 102.553 131.869 253.839 1.00 69.28
N
ATOM 32352 CA GLY H 139 103.089 130.802 253.024 1.00 63.91
C
ATOM 32353 C GLY H 139 102.622 131.029 251.603 1.00 71.94
C
ATOM 32354 O GLY H 139 101.802 131.910 251.326 1.00 77.08
O
ATOM 32355 H GLY H 139 102.005 132.388 253.428 1.00 83.16
H
ATOM 32356 HA2 GLY H 139 104.060 130.809 253.051 1.00 76.72
H
ATOM 32357 HA3 GLY H 139 102.770 129.942 253.338 1.00 76.72
H
ATOM 32358 N GLY H 140 103.141 130.212 250.707 1.00 67.44
N
ATOM 32359 CA GLY H 140 102.795 130.350 249.314 1.00 61.46
C
ATOM 32360 C GLY H 140 103.788 131.227 248.581 1.00 50.72
C
ATOM 32361 O GLY H 140 104.937 131.427 249.001 1.00 46.02
O
ATOM 32362 H GLY H 140 103.691 129.575 250.881 1.00 80.96
H
ATOM 32363 HA2 GLY H 140 102.784 129.476 248.894 1.00 73.78
H
ATOM 32364 HA3 GLY H 140 101.914 130.749 249.236 1.00 73.78
H
ATOM 32365 N THR H 141 103.342 131.719 247.427 1.00 50.06
N
ATOM 32366 CA THR H 141 104.212 132.404 246.474 1.00 47.54
C
ATOM 32367 C THR H 141 103.620 133.782 246.204 1.00 49.72
C
ATOM 32368 O THR H 141 102.409 133.913 246.008 1.00 49.52
O
ATOM 32369 CB THR H 141 104.364 131.568 245.187 1.00 48.49
C
ATOM 32370 OG1 THR H 141 105.061 130.351 245.505 1.00 54.27
O
ATOM 32371 CG2 THR H 141 105.131 132.336 244.028 1.00 48.45
C
ATOM 32372 H THR H 141 102.524 131.668 247.170 1.00 60.10
H
ATOM 32373 HA THR H 141 105.104 132.533 246.833 1.00 57.07
H
ATOM 32374 HB THR H 141 103.479 131.378 244.840 1.00 58.21
H

ATOM 32375 HG1 THR H 141   105.332 129.983 244.800 1.00 65.15
H
ATOM 32376 HG21 THR H 141   105.275 131.742 243.274 1.00 58.17
H
ATOM 32377 HG22 THR H 141   104.612 133.100 243.735 1.00 58.17
H
ATOM 32378 HG23 THR H 141   105.994 132.647 244.351 1.00 58.17
H
ATOM 32379 N   ALA H 142   104.457 134.814 246.258 1.00 42.79
N
ATOM 32380 CA ALA H 142   104.031 136.176 245.958 1.00 52.32
C
ATOM 32381 C   ALA H 142   104.724 136.626 244.674 1.00 46.22
C
ATOM 32382 O   ALA H 142   105.893 136.318 244.462 1.00 42.83
O
ATOM 32383 CB ALA H 142   104.381 137.120 247.112 1.00 47.27
C
ATOM 32384 H   ALA H 142   105.289 134.749 246.469 1.00 51.38
H
ATOM 32385 HA ALA H 142   103.070 136.215 245.828 1.00 62.81
H
ATOM 32386 HB1 ALA H 142   104.144 138.027 246.862 1.00 56.75
H
ATOM 32387 HB2 ALA H 142   103.885 136.855 247.902 1.00 56.75
H
ATOM 32388 HB3 ALA H 142   105.333 137.066 247.288 1.00 56.75
H
ATOM 32389 N   ALA H 143   104.018 137.344 243.803 1.00 45.92
N
ATOM 32390 CA ALA H 143   104.680 137.984 242.681 1.00 43.45
C
ATOM 32391 C   ALA H 143   104.772 139.486 242.933 1.00 44.97
C
ATOM 32392 O   ALA H 143   103.886 140.087 243.542 1.00 44.10
O
ATOM 32393 CB ALA H 143   103.955 137.665 241.382 1.00 45.95
C
ATOM 32394 H   ALA H 143   103.169 137.469 243.846 1.00 55.13
H
ATOM 32395 HA ALA H 143   105.589 137.661 242.582 1.00 52.16
H
ATOM 32396 HB1 ALA H 143   104.378 138.150 240.657 1.00 55.17
H
ATOM 32397 HB2 ALA H 143   104.009 136.710 241.218 1.00 55.17
H
ATOM 32398 HB3 ALA H 143   103.028 137.934 241.464 1.00 55.17
H
ATOM 32399 N   LEU H 144   105.899 140.064 242.558 1.00 46.03
N

ATOM 32400 CA LEU H 144   106.060 141.500 242.650 1.00 53.60
C
ATOM 32401 C   LEU H 144   106.951 141.960 241.500 1.00 50.94
C
ATOM 32402 O   LEU H 144   107.648 141.160 240.865 1.00 43.91
O
ATOM 32403 CB LEU H 144   106.617 141.919 244.015 1.00 47.16
C
ATOM 32404 CG LEU H 144   107.825 141.212 244.552 1.00 52.52
C
ATOM 32405 CD1 LEU H 144   109.059 141.938 244.157 1.00 57.63
C
ATOM 32406 CD2 LEU H 144   107.741 141.085 246.067 1.00 54.49
C
ATOM 32407 H   LEU H 144   106.583 139.646 242.248 1.00 55.26
H
ATOM 32408 HA LEU H 144   105.201 141.940 242.549 1.00 64.35
H
ATOM 32409 HB2 LEU H 144   106.852 142.859 243.957 1.00 56.62
H
ATOM 32410 HB3 LEU H 144   105.912 141.792 244.669 1.00 56.62
H
ATOM 32411 HG LEU H 144   107.863 140.317 244.180 1.00 63.05
H
ATOM 32412 HD11 LEU H 144   109.826 141.493 244.551 1.00 69.19
H
ATOM 32413 HD12 LEU H 144   109.134 141.933 243.190 1.00 69.19
H
ATOM 32414 HD13 LEU H 144   109.006 142.851 244.481 1.00 69.19
H
ATOM 32415 HD21 LEU H 144   108.541 140.640 246.391 1.00 65.41
H
ATOM 32416 HD22 LEU H 144   107.676 141.973 246.454 1.00 65.41
H
ATOM 32417 HD23 LEU H 144   106.957 140.565 246.297 1.00 65.41
H
ATOM 32418 N   GLY H 145   106.894 143.258 241.213 1.00 51.60
N
ATOM 32419 CA GLY H 145   107.733 143.755 240.146 1.00 51.84
C
ATOM 32420 C   GLY H 145   107.686 145.258 239.999 1.00 47.92
C
ATOM 32421 O   GLY H 145   107.209 145.971 240.875 1.00 45.33
O
ATOM 32422 H   GLY H 145   106.398 143.841 241.609 1.00 61.95
H
ATOM 32423 HA2 GLY H 145   108.653 143.497 240.320 1.00 62.23
H
ATOM 32424 HA3 GLY H 145   107.447 143.360 239.307 1.00 62.23
H

```
ATOM  32425  N   CYS H 146     108.198 145.713 238.850  1.00 44.88
N
ATOM  32426  CA  CYS H 146     108.313 147.120 238.482  1.00 53.54
C
ATOM  32427  C   CYS H 146     107.781 147.308 237.074  1.00 49.54
C
ATOM  32428  O   CYS H 146     108.112 146.534 236.166  1.00 48.49
O
ATOM  32429  CB  CYS H 146     109.773 147.594 238.578  1.00 51.93
C
ATOM  32430  SG  CYS H 146     110.353 147.834 240.329  1.00 72.96
S
ATOM  32431  H   CYS H 146     108.501 145.193 238.235  1.00 53.89
H
ATOM  32432  HA  CYS H 146     107.777 147.663 239.082  1.00 64.27
H
ATOM  32433  HB2 CYS H 146     110.346 146.932 238.161  1.00 62.34
H
ATOM  32434  HB3 CYS H 146     109.859 148.444 238.117  1.00 62.34
H
ATOM  32435  N   LEU H 147     106.923 148.303 236.913  1.00 48.59
N
ATOM  32436  CA  LEU H 147     106.457 148.759 235.609  1.00 44.72
C
ATOM  32437  C   LEU H 147     107.342 149.925 235.184  1.00 51.18
C
ATOM  32438  O   LEU H 147     107.371 150.949 235.860  1.00 48.14
O
ATOM  32439  CB  LEU H 147     105.001 149.173 235.705  1.00 46.32
C
ATOM  32440  CG  LEU H 147     104.328 149.656 234.447  1.00 47.72
C
ATOM  32441  CD1 LEU H 147     104.277 148.524 233.493  1.00 54.16
C
ATOM  32442  CD2 LEU H 147     102.913 150.231 234.750  1.00 47.19
C
ATOM  32443  H   LEU H 147     106.582 148.747 237.567  1.00 58.33
H
ATOM  32444  HA  LEU H 147     106.535 148.052 234.948  1.00 53.69
H
ATOM  32445  HB2 LEU H 147     104.494 148.407 236.017  1.00 55.62
H
ATOM  32446  HB3 LEU H 147     104.940 149.896 236.349  1.00 55.62
H
ATOM  32447  HG  LEU H 147     104.826 150.385 234.047  1.00 57.29
H
ATOM  32448 HD11 LEU H 147     103.688 148.758 232.758  1.00 65.02
H
ATOM  32449 HD12 LEU H 147     105.171 148.351 233.161  1.00 65.02
H
```

ATOM 32450 HD13 LEU H 147   103.936 147.740 233.952 1.00 65.02
H
ATOM 32451 HD21 LEU H 147   102.538 150.595 233.933 1.00 56.65
H
ATOM 32452 HD22 LEU H 147   102.346 149.516 235.082 1.00 56.65
H
ATOM 32453 HD23 LEU H 147   102.992 150.927 235.419 1.00 56.65
H
ATOM 32454 N   VAL H 148   108.110 149.741 234.108 1.00 48.34
N
ATOM 32455 CA VAL H 148   109.049 150.731 233.606 1.00 48.84
C
ATOM 32456 C   VAL H 148   108.409 151.345 232.360 1.00 51.77
C
ATOM 32457 O   VAL H 148   108.430 150.754 231.273 1.00 50.25
O
ATOM 32458 CB VAL H 148   110.415 150.116 233.319 1.00 53.30
C
ATOM 32459 CG1 VAL H 148   111.358 151.170 232.690 1.00 58.30
C
ATOM 32460 CG2 VAL H 148   111.020 149.486 234.592 1.00 43.12
C
ATOM 32461 H   VAL H 148   108.101 149.022 233.638 1.00 58.04
H
ATOM 32462 HA VAL H 148   109.172 151.436 234.261 1.00 58.63
H
ATOM 32463 HB VAL H 148   110.308 149.397 232.677 1.00 63.99
H
ATOM 32464 HG11 VAL H 148   112.240 150.780 232.582 1.00 69.99
H
ATOM 32465 HG12 VAL H 148   111.004 151.435 231.827 1.00 69.99
H
ATOM 32466 HG13 VAL H 148   111.407 151.941 233.278 1.00 69.99
H
ATOM 32467 HG21 VAL H 148   111.923 149.188 234.399 1.00 51.77
H
ATOM 32468 HG22 VAL H 148   111.035 150.151 235.298 1.00 51.77
H
ATOM 32469 HG23 VAL H 148   110.474 148.730 234.862 1.00 51.77
H
ATOM 32470 N   LYS H 149   107.818 152.538 232.516 1.00 50.76
N
ATOM 32471 CA LYS H 149   106.857 153.053 231.559 1.00 53.24
C
ATOM 32472 C   LYS H 149   107.331 154.329 230.848 1.00 56.04
C
ATOM 32473 O   LYS H 149   108.040 155.163 231.408 1.00 50.87
O
ATOM 32474 CB LYS H 149   105.530 153.312 232.257 1.00 53.91
C

ATOM 32475 CG LYS H 149 104.347 153.229 231.304 1.00 61.28
C
ATOM 32476 CD LYS H 149 103.051 153.630 231.915 1.00 57.05
C
ATOM 32477 CE LYS H 149 101.902 153.581 230.865 1.00 61.51
C
ATOM 32478 NZ LYS H 149 101.898 154.769 229.976 1.00 74.07
N
ATOM 32479 H LYS H 149 107.967 153.065 233.178 1.00 60.94
H
ATOM 32480 HA LYS H 149 106.747 152.396 230.854 1.00 63.91
H
ATOM 32481 HB2 LYS H 149 105.403 152.651 232.954 1.00 64.72
H
ATOM 32482 HB3 LYS H 149 105.542 154.202 232.644 1.00 64.72
H
ATOM 32483 HG2 LYS H 149 104.513 153.817 230.550 1.00 73.57
H
ATOM 32484 HG3 LYS H 149 104.257 152.314 230.997 1.00 73.57
H
ATOM 32485 HD2 LYS H 149 102.832 153.023 232.640 1.00 68.48
H
ATOM 32486 HD3 LYS H 149 103.120 154.537 232.253 1.00 68.48
H
ATOM 32487 HE2 LYS H 149 102.011 152.791 230.312 1.00 73.83
H
ATOM 32488 HE3 LYS H 149 101.050 153.549 231.328 1.00 73.83
H
ATOM 32489 HZ1 LYS H 149 102.623 154.766 229.460 1.00 88.91
H
ATOM 32490 HZ2 LYS H 149 101.174 154.760 229.457 1.00 88.91
H
ATOM 32491 HZ3 LYS H 149 101.893 155.514 230.463 1.00 88.91
H
ATOM 32492 N ASP H 150 106.896 154.479 229.597 1.00 55.51
N
ATOM 32493 CA ASP H 150 107.019 155.726 228.828 1.00 56.59
C
ATOM 32494 C ASP H 150 108.464 156.200 228.720 1.00 56.36
C
ATOM 32495 O ASP H 150 108.805 157.322 229.104 1.00 57.82
O
ATOM 32496 CB ASP H 150 106.169 156.848 229.435 1.00 52.53
C
ATOM 32497 CG ASP H 150 104.682 156.580 229.357 1.00 58.88
C
ATOM 32498 OD1 ASP H 150 104.259 155.755 228.520 1.00 63.68
O
ATOM 32499 OD2 ASP H 150 103.927 157.234 230.129 1.00 62.32
O1-

ATOM 32500 H ASP H 150 106.510 153.852 229.152 1.00 66.64
H
ATOM 32501 HA ASP H 150 106.698 155.522 227.935 1.00 67.93
H
ATOM 32502 HB2 ASP H 150 106.406 156.950 230.370 1.00 63.06
H
ATOM 32503 HB3 ASP H 150 106.349 157.672 228.954 1.00 63.06
H
ATOM 32504 N TYR H 151 109.307 155.340 228.170 1.00 54.83
N
ATOM 32505 CA TYR H 151 110.676 155.704 227.862 1.00 54.37
C
ATOM 32506 C TYR H 151 110.931 155.560 226.362 1.00 48.70
C
ATOM 32507 O TYR H 151 110.108 155.025 225.594 1.00 48.40
O
ATOM 32508 CB TYR H 151 111.671 154.886 228.677 1.00 50.08
C
ATOM 32509 CG TYR H 151 111.688 153.403 228.329 1.00 55.77
C
ATOM 32510 CD1 TYR H 151 110.836 152.523 228.969 1.00 50.72
C
ATOM 32511 CD2 TYR H 151 112.535 152.894 227.341 1.00 51.87
C
ATOM 32512 CE1 TYR H 151 110.833 151.174 228.668 1.00 43.43
C
ATOM 32513 CE2 TYR H 151 112.538 151.540 227.023 1.00 51.74
C
ATOM 32514 CZ TYR H 151 111.657 150.672 227.706 1.00 50.86
C
ATOM 32515 OH TYR H 151 111.574 149.317 227.430 1.00 51.35
O
ATOM 32516 H TYR H 151 109.104 154.530 227.964 1.00 65.83
H
ATOM 32517 HA TYR H 151 110.815 156.637 228.093 1.00 65.27
H
ATOM 32518 HB2 TYR H 151 112.563 155.235 228.520 1.00 60.12
H
ATOM 32519 HB3 TYR H 151 111.445 154.966 229.617 1.00 60.12
H
ATOM 32520 HD1 TYR H 151 110.252 152.846 229.618 1.00 60.89
H
ATOM 32521 HD2 TYR H 151 113.107 153.471 226.890 1.00 62.28
H
ATOM 32522 HE1 TYR H 151 110.264 150.601 229.129 1.00 52.15
H
ATOM 32523 HE2 TYR H 151 113.112 151.211 226.369 1.00 62.12
H
ATOM 32524 HH TYR H 151 112.215 149.085 226.937 1.00 61.65
H

ATOM 32525 N PHE H 152 112.070 156.096 225.954 1.00 43.60
N
ATOM 32526 CA PHE H 152 112.449 156.191 224.536 1.00 47.66
C
ATOM 32527 C PHE H 152 113.885 156.669 224.421 1.00 44.26
C
ATOM 32528 O PHE H 152 114.279 157.568 225.168 1.00 47.97
O
ATOM 32529 CB PHE H 152 111.583 157.137 223.727 1.00 49.69
C
ATOM 32530 CG PHE H 152 111.928 157.128 222.239 1.00 57.20
C
ATOM 32531 CD1 PHE H 152 111.211 156.345 221.362 1.00 51.68
C
ATOM 32532 CD2 PHE H 152 112.996 157.879 221.751 1.00 57.63
C
ATOM 32533 CE1 PHE H 152 111.525 156.317 220.024 1.00 51.84
C
ATOM 32534 CE2 PHE H 152 113.344 157.851 220.411 1.00 54.07
C
ATOM 32535 CZ PHE H 152 112.605 157.082 219.543 1.00 61.50
C
ATOM 32536 H PHE H 152 112.660 156.423 226.486 1.00 52.34
H
ATOM 32537 HA PHE H 152 112.339 155.300 224.167 1.00 57.22
H
ATOM 32538 HB2 PHE H 152 110.655 156.873 223.820 1.00 59.66
H
ATOM 32539 HB3 PHE H 152 111.707 158.040 224.059 1.00 59.66
H
ATOM 32540 HD1 PHE H 152 110.504 155.830 221.678 1.00 62.04
H
ATOM 32541 HD2 PHE H 152 113.486 158.410 222.337 1.00 69.19
H
ATOM 32542 HE1 PHE H 152 111.028 155.793 219.437 1.00 62.23
H
ATOM 32543 HE2 PHE H 152 114.067 158.349 220.102 1.00 64.92
H
ATOM 32544 HZ PHE H 152 112.817 157.067 218.639 1.00 73.83
H
ATOM 32545 N PRO H 153 114.737 156.035 223.574 1.00 49.28
N
ATOM 32546 CA PRO H 153 114.445 154.804 222.852 1.00 51.79
C
ATOM 32547 C PRO H 153 114.684 153.568 223.767 1.00 55.82
C
ATOM 32548 O PRO H 153 114.916 153.727 224.982 1.00 51.13
O
ATOM 32549 CB PRO H 153 115.457 154.861 221.697 1.00 50.18
C

ATOM 32550 CG PRO H 153 116.694 155.426 222.380 1.00 43.11
C
ATOM 32551 CD PRO H 153 116.178 156.361 223.458 1.00 49.04
C
ATOM 32552 HA PRO H 153 113.547 154.774 222.486 1.00 62.18
H
ATOM 32553 HB2 PRO H 153 115.619 153.974 221.341 1.00 60.24
H
ATOM 32554 HB3 PRO H 153 115.138 155.447 220.992 1.00 60.24
H
ATOM 32555 HG2 PRO H 153 117.212 154.704 222.772 1.00 51.76
H
ATOM 32556 HG3 PRO H 153 117.234 155.909 221.736 1.00 51.76
H
ATOM 32557 HD2 PRO H 153 116.633 156.196 224.299 1.00 58.88
H
ATOM 32558 HD3 PRO H 153 116.298 157.286 223.191 1.00 58.88
H
ATOM 32559 N GLU H 154 114.636 152.354 223.190 1.00 56.45
N
ATOM 32560 CA GLU H 154 115.234 151.181 223.826 1.00 55.67
C
ATOM 32561 C GLU H 154 116.723 151.410 223.994 1.00 47.10
C
ATOM 32562 O GLU H 154 117.280 152.306 223.353 1.00 57.37
O
ATOM 32563 CB GLU H 154 115.037 149.912 222.984 1.00 48.73
C
ATOM 32564 CG GLU H 154 113.632 149.435 222.847 1.00 50.52
C
ATOM 32565 CD GLU H 154 113.173 148.574 224.037 1.00 69.19
C
ATOM 32566 OE1 GLU H 154 112.466 147.568 223.769 1.00 67.29
O
ATOM 32567 OE2 GLU H 154 113.508 148.900 225.220 1.00 58.43
O1-
ATOM 32568 H GLU H 154 114.264 152.189 222.433 1.00 67.77
H
ATOM 32569 HA GLU H 154 114.807 151.045 224.686 1.00 66.83
H
ATOM 32570 HB2 GLU H 154 115.369 150.086 222.089 1.00 58.50
H
ATOM 32571 HB3 GLU H 154 115.545 149.193 223.392 1.00 58.50
H
ATOM 32572 HG2 GLU H 154 113.044 150.204 222.790 1.00 60.65
H
ATOM 32573 HG3 GLU H 154 113.557 148.899 222.042 1.00 60.65
H
ATOM 32574 N PRO H 155 117.408 150.621 224.874 1.00 54.70
N

ATOM 32575 CA PRO H 155 116.907 149.695 225.898 1.00 51.37
C
ATOM 32576 C PRO H 155 116.934 150.232 227.357 1.00 49.93
C
ATOM 32577 O PRO H 155 117.568 151.227 227.676 1.00 50.37
O
ATOM 32578 CB PRO H 155 117.877 148.539 225.774 1.00 48.82
C
ATOM 32579 CG PRO H 155 119.235 149.297 225.654 1.00 50.84
C
ATOM 32580 CD PRO H 155 118.888 150.559 224.819 1.00 48.76
C
ATOM 32581 HA PRO H 155 115.996 149.443 225.684 1.00 61.68
H
ATOM 32582 HB2 PRO H 155 117.845 147.976 226.563 1.00 58.61
H
ATOM 32583 HB3 PRO H 155 117.687 148.012 224.982 1.00 58.61
H
ATOM 32584 HG2 PRO H 155 119.562 149.539 226.535 1.00 61.03
H
ATOM 32585 HG3 PRO H 155 119.889 148.744 225.199 1.00 61.03
H
ATOM 32586 HD2 PRO H 155 119.280 151.351 225.217 1.00 58.53
H
ATOM 32587 HD3 PRO H 155 119.193 150.459 223.905 1.00 58.53
H
ATOM 32588 N VAL H 156 116.236 149.546 228.253 1.00 55.37
N
ATOM 32589 CA VAL H 156 116.483 149.662 229.688 1.00 59.74
C
ATOM 32590 C VAL H 156 117.086 148.355 230.167 1.00 51.84
C
ATOM 32591 O VAL H 156 116.893 147.298 229.574 1.00 53.35
O
ATOM 32592 CB VAL H 156 115.213 149.967 230.520 1.00 63.54
C
ATOM 32593 CG1 VAL H 156 114.712 151.353 230.265 1.00 53.10
C
ATOM 32594 CG2 VAL H 156 114.112 148.954 230.228 1.00 58.11
C
ATOM 32595 H VAL H 156 115.604 148.997 228.054 1.00 66.47
H
ATOM 32596 HA VAL H 156 117.105 150.394 229.819 1.00 71.72
H
ATOM 32597 HB VAL H 156 115.453 149.901 231.458 1.00 76.27
H
ATOM 32598 HG11 VAL H 156 113.933 151.515 230.821 1.00 63.75
H
ATOM 32599 HG12 VAL H 156 115.412 151.987 230.483 1.00 63.75
H

ATOM 32600 HG13 VAL H 156   114.472 151.433 229.328 1.00 63.75
H
ATOM 32601 HG21 VAL H 156   113.315 149.201 230.722 1.00 69.75
H
ATOM 32602 HG22 VAL H 156   113.925 148.958 229.276 1.00 69.75
H
ATOM 32603 HG23 VAL H 156   114.412 148.074 230.503 1.00 69.75
H
ATOM 32604 N   THR H 157   117.793 148.424 231.280 1.00 57.28
N
ATOM 32605 CA THR H 157   118.198 147.217 231.979 1.00 56.75
C
ATOM 32606 C   THR H 157   117.504 147.216 233.332 1.00 51.76
C
ATOM 32607 O   THR H 157   117.192 148.268 233.889 1.00 52.99
O
ATOM 32608 CB THR H 157   119.734 147.103 232.120 1.00 60.37
C
ATOM 32609 OG1 THR H 157   120.219 148.092 233.022 1.00 62.25
O
ATOM 32610 CG2 THR H 157   120.408 147.283 230.771 1.00 57.39
C
ATOM 32611 H   THR H 157   118.049 149.156 231.650 1.00 68.77
H
ATOM 32612 HA THR H 157   117.930 146.425 231.489 1.00 68.13
H
ATOM 32613 HB THR H 157   119.958 146.222 232.462 1.00 72.47
H
ATOM 32614 HG1 THR H 157   121.049 148.013 233.117 1.00 74.73
H
ATOM 32615 HG21 THR H 157   121.355 147.085 230.843 1.00 68.89
H
ATOM 32616 HG22 THR H 157   120.012 146.685 230.118 1.00 68.89
H
ATOM 32617 HG23 THR H 157   120.300 148.198 230.465 1.00 68.89
H
ATOM 32618 N   VAL H 158   117.156 146.028 233.798 1.00 54.15
N
ATOM 32619 CA VAL H 158   116.534 145.850 235.105 1.00 50.23
C
ATOM 32620 C   VAL H 158   117.289 144.746 235.828 1.00 52.46
C
ATOM 32621 O   VAL H 158   117.475 143.646 235.286 1.00 50.77
O
ATOM 32622 CB VAL H 158   115.040 145.491 235.013 1.00 48.63
C
ATOM 32623 CG1 VAL H 158   114.454 145.493 236.406 1.00 58.59
C
ATOM 32624 CG2 VAL H 158   114.289 146.447 234.154 1.00 56.23
C

ATOM 32625 H   VAL H 158   117.271 145.292 233.367 1.00 65.01
H
ATOM 32626 HA VAL H 158   116.609 146.679 235.603 1.00 60.30
H
ATOM 32627 HB VAL H 158   114.956 144.613 234.609 1.00 58.38
H
ATOM 32628 HG11 VAL H 158   113.488 145.422 236.342 1.00 70.33
H
ATOM 32629 HG12 VAL H 158   114.808 144.737 236.899 1.00 70.33
H
ATOM 32630 HG13 VAL H 158   114.696 146.321 236.849 1.00 70.33
H
ATOM 32631 HG21 VAL H 158   113.348 146.206 234.163 1.00 67.50
H
ATOM 32632 HG22 VAL H 158   114.404 147.344 234.504 1.00 67.50
H
ATOM 32633 HG23 VAL H 158   114.634 146.398 233.249 1.00 67.50
H
ATOM 32634 N   SER H 159   117.769 145.050 237.016 1.00 53.58
N
ATOM 32635 CA SER H 159   118.240 144.039 237.935 1.00 49.62
C
ATOM 32636 C   SER H 159   117.419 144.170 239.208 1.00 51.54
C
ATOM 32637 O   SER H 159   116.612 145.098 239.359 1.00 51.35
O
ATOM 32638 CB SER H 159   119.712 144.206 238.219 1.00 49.73
C
ATOM 32639 OG SER H 159   119.900 145.342 239.020 1.00 55.03
O
ATOM 32640 H   SER H 159   117.833 145.854 237.319 1.00 64.32
H
ATOM 32641 HA SER H 159   118.124 143.149 237.567 1.00 59.57
H
ATOM 32642 HB2 SER H 159   120.042 143.423 238.689 1.00 59.70
H
ATOM 32643 HB3 SER H 159   120.192 144.317 237.383 1.00 59.70
H
ATOM 32644 HG SER H 159   119.669 145.178 239.810 1.00 66.07
H
ATOM 32645 N   TRP H 160   117.624 143.212 240.109 1.00 50.76
N
ATOM 32646 CA TRP H 160   116.898 143.117 241.373 1.00 52.98
C
ATOM 32647 C   TRP H 160   117.887 142.951 242.506 1.00 43.86
C
ATOM 32648 O   TRP H 160   118.809 142.136 242.408 1.00 48.78
O
ATOM 32649 CB TRP H 160   115.928 141.936 241.392 1.00 49.32
C

ATOM 32650 CG TRP H 160 114.734 142.209 240.646 1.00 40.81
C
ATOM 32651 CD1 TRP H 160 114.520 141.912 239.358 1.00 47.74
C
ATOM 32652 CD2 TRP H 160 113.568 142.879 241.112 1.00 42.69
C
ATOM 32653 NE1 TRP H 160 113.280 142.356 238.966 1.00 46.33
N
ATOM 32654 CE2 TRP H 160 112.669 142.940 240.032 1.00 43.74
C
ATOM 32655 CE3 TRP H 160 113.182 143.415 242.347 1.00 51.84
C
ATOM 32656 CZ2 TRP H 160 111.410 143.499 240.138 1.00 43.87
C
ATOM 32657 CZ3 TRP H 160 111.921 143.980 242.456 1.00 50.51
C
ATOM 32658 CH2 TRP H 160 111.052 144.033 241.346 1.00 48.74
C
ATOM 32659 H TRP H 160 118.202 142.584 240.008 1.00 60.95
H
ATOM 32660 HA TRP H 160 116.394 143.936 241.496 1.00 63.61
H
ATOM 32661 HB2 TRP H 160 116.362 141.162 241.000 1.00 59.22
H
ATOM 32662 HB3 TRP H 160 115.676 141.745 242.309 1.00 59.22
H
ATOM 32663 HD1 TRP H 160 115.124 141.467 238.806 1.00 57.32
H
ATOM 32664 HE1 TRP H 160 112.945 142.278 238.178 1.00 55.62
H
ATOM 32665 HE3 TRP H 160 113.759 143.391 243.076 1.00 62.23
H
ATOM 32666 HZ2 TRP H 160 110.827 143.510 239.414 1.00 52.67
H
ATOM 32667 HZ3 TRP H 160 111.645 144.329 243.272 1.00 60.64
H
ATOM 32668 HH2 TRP H 160 110.220 144.440 241.436 1.00 58.52
H
ATOM 32669 N ASN H 161 117.709 143.731 243.573 1.00 47.30
N
ATOM 32670 CA ASN H 161 118.647 143.727 244.703 1.00 48.86
C
ATOM 32671 C ASN H 161 120.101 143.778 244.227 1.00 52.93
C
ATOM 32672 O ASN H 161 120.981 143.086 244.749 1.00 49.63
O
ATOM 32673 CB ASN H 161 118.396 142.512 245.606 1.00 50.46
C
ATOM 32674 CG ASN H 161 117.063 142.579 246.274 1.00 51.70
C

ATOM 32675 OD1 ASN H 161 116.357 143.594 246.169 1.00 59.37
O
ATOM 32676 ND2 ASN H 161 116.693 141.519 246.986 1.00 51.53
N
ATOM 32677 H ASN H 161 117.049 144.275 243.670 1.00 56.79
H
ATOM 32678 HA ASN H 161 118.499 144.530 245.228 1.00 58.66
H
ATOM 32679 HB2 ASN H 161 118.425 141.704 245.070 1.00 60.57
H
ATOM 32680 HB3 ASN H 161 119.078 142.477 246.294 1.00 60.57
H
ATOM 32681 HD21 ASN H 161 115.935 141.515 247.387 1.00 61.86
H
ATOM 32682 HD22 ASN H 161 117.215 140.838 247.043 1.00 61.86
H
ATOM 32683 N SER H 162 120.358 144.607 243.204 1.00 58.50
N
ATOM 32684 CA SER H 162 121.715 144.856 242.730 1.00 60.93
C
ATOM 32685 C SER H 162 122.392 143.559 242.278 1.00 59.08
C
ATOM 32686 O SER H 162 123.560 143.331 242.558 1.00 58.77
O
ATOM 32687 CB SER H 162 122.548 145.553 243.818 1.00 57.09
C
ATOM 32688 OG SER H 162 122.037 146.855 244.099 1.00 72.73
O
ATOM 32689 H SER H 162 119.753 145.037 242.769 1.00 70.23
H
ATOM 32690 HA SER H 162 121.667 145.441 241.957 1.00 73.15
H
ATOM 32691 HB2 SER H 162 122.518 145.020 244.629 1.00 68.54
H
ATOM 32692 HB3 SER H 162 123.465 145.634 243.510 1.00 68.54
H
ATOM 32693 HG SER H 162 122.589 147.286 244.562 1.00 87.30
H
ATOM 32694 N GLY H 163 121.659 142.684 241.593 1.00 55.68
N
ATOM 32695 CA GLY H 163 122.240 141.437 241.139 1.00 53.28
C
ATOM 32696 C GLY H 163 122.094 140.251 242.072 1.00 49.54
C
ATOM 32697 O GLY H 163 122.215 139.121 241.612 1.00 62.59
O
ATOM 32698 H GLY H 163 120.832 142.794 241.383 1.00 66.84
H
ATOM 32699 HA2 GLY H 163 121.825 141.196 240.297 1.00 63.97
H

ATOM 32700 HA3 GLY H 163   123.190 141.577 240.996 1.00 63.97
H
ATOM 32701 N   ALA H 164   121.794 140.459 243.357 1.00 56.25
N
ATOM 32702 CA ALA H 164   121.759 139.345 244.290 1.00 54.25
C
ATOM 32703 C   ALA H 164   120.488 138.509 244.187 1.00 63.06
C
ATOM 32704 O   ALA H 164   120.382 137.485 244.882 1.00 62.91
O
ATOM 32705 CB ALA H 164   121.895 139.856 245.731 1.00 57.33
C
ATOM 32706 H   ALA H 164   121.611 141.225 243.701 1.00 67.53
H
ATOM 32707 HA ALA H 164   122.514 138.768 244.096 1.00 65.13
H
ATOM 32708 HB1 ALA H 164   121.815 139.106 246.341 1.00 68.82
H
ATOM 32709 HB2 ALA H 164   122.764 140.275 245.836 1.00 68.82
H
ATOM 32710 HB3 ALA H 164   121.193 140.501 245.904 1.00 68.82
H
ATOM 32711 N   LEU H 165   119.511 138.948 243.390 1.00 57.44
N
ATOM 32712 CA LEU H 165   118.261 138.225 243.177 1.00 45.85
C
ATOM 32713 C   LEU H 165   118.109 138.052 241.671 1.00 49.44
C
ATOM 32714 O   LEU H 165   117.827 139.013 240.946 1.00 53.51
O
ATOM 32715 CB LEU H 165   117.074 138.951 243.816 1.00 45.10
C
ATOM 32716 CG LEU H 165   115.658 138.386 243.578 1.00 45.68
C
ATOM 32717 CD1 LEU H 165   115.541 136.917 244.015 1.00 45.35
C
ATOM 32718 CD2 LEU H 165   114.620 139.290 244.260 1.00 48.11
C
ATOM 32719 H   LEU H 165   119.554 139.686 242.949 1.00 68.96
H
ATOM 32720 HA LEU H 165   118.294 137.346 243.586 1.00 55.04
H
ATOM 32721 HB2 LEU H 165   117.214 138.953 244.776 1.00 54.14
H
ATOM 32722 HB3 LEU H 165   117.070 139.860 243.479 1.00 54.14
H
ATOM 32723 HG LEU H 165   115.470 138.383 242.626 1.00 54.84
H
ATOM 32724 HD11 LEU H 165   114.613 136.641 243.953 1.00 54.45
H

ATOM 32725 HD12 LEU H 165   116.091 136.369 243.433 1.00 54.45
H
ATOM 32726 HD13 LEU H 165   115.848 136.835 244.932 1.00 54.45
H
ATOM 32727 HD21 LEU H 165   113.730 138.971 244.040 1.00 57.76
H
ATOM 32728 HD22 LEU H 165   114.756 139.259 245.220 1.00 57.76
H
ATOM 32729 HD23 LEU H 165   114.734 140.199 243.939 1.00 57.76
H
ATOM 32730 N   THR H 166   118.347 136.832 241.210 1.00 53.90
N
ATOM 32731 CA THR H 166   118.199 136.462 239.821 1.00 55.85
C
ATOM 32732 C   THR H 166   117.224 135.319 239.603 1.00 48.03
C
ATOM 32733 O   THR H 166   116.580 135.274 238.553 1.00 51.48
O
ATOM 32734 CB THR H 166   119.573 136.088 239.235 1.00 54.09
C
ATOM 32735 OG1 THR H 166   120.185 135.089 240.046 1.00 59.59
O
ATOM 32736 CG2 THR H 166   120.496 137.336 239.185 1.00 59.02
C
ATOM 32737 H   THR H 166   118.606 136.179 241.706 1.00 64.71
H
ATOM 32738 HA THR H 166   117.863 137.220 239.317 1.00 67.05
H
ATOM 32739 HB THR H 166   119.457 135.747 238.334 1.00 64.93
H
ATOM 32740 HG1 THR H 166   119.755 134.370 239.990 1.00 71.53
H
ATOM 32741 HG21 THR H 166   121.377 137.084 238.867 1.00 70.85
H
ATOM 32742 HG22 THR H 166   120.123 138.000 238.583 1.00 70.85
H
ATOM 32743 HG23 THR H 166   120.579 137.723 240.070 1.00 70.85
H
ATOM 32744 N   SER H 167   117.074 134.421 240.570 1.00 54.42
N
ATOM 32745 CA SER H 167   116.170 133.288 240.423 1.00 52.69
C
ATOM 32746 C   SER H 167   114.731 133.760 240.434 1.00 46.80
C
ATOM 32747 O   SER H 167   114.337 134.531 241.314 1.00 52.02
O
ATOM 32748 CB SER H 167   116.383 132.282 241.556 1.00 67.72
C
ATOM 32749 OG SER H 167   115.633 131.105 241.301 1.00 70.61
O

ATOM 32750 H   SER H 167   117.484 134.446 241.325 1.00 65.33
H
ATOM 32751 HA SER H 167   116.355 132.847 239.579 1.00 63.25
H
ATOM 32752 HB2 SER H 167   117.324 132.056 241.610 1.00 81.29
H
ATOM 32753 HB3 SER H 167   116.086 132.674 242.392 1.00 81.29
H
ATOM 32754 HG SER H 167   115.664 130.595 241.967 1.00 84.75
H
ATOM 32755 N   GLY H 168   113.951 133.324 239.437 1.00 52.29
N
ATOM 32756 CA GLY H 168   112.546 133.679 239.357 1.00 49.34
C
ATOM 32757 C   GLY H 168   112.257 135.033 238.741 1.00 51.25
C
ATOM 32758 O   GLY H 168   111.089 135.451 238.702 1.00 48.94
O
ATOM 32759 H   GLY H 168   114.222 132.820 238.796 1.00 62.78
H
ATOM 32760 HA2 GLY H 168   112.087 133.009 238.825 1.00 59.24
H
ATOM 32761 HA3 GLY H 168   112.175 133.679 240.253 1.00 59.24
H
ATOM 32762 N   VAL H 169   113.270 135.710 238.226 1.00 49.38
N
ATOM 32763 CA VAL H 169   113.101 137.021 237.614 1.00 45.79
C
ATOM 32764 C   VAL H 169   112.707 136.826 236.153 1.00 45.43
C
ATOM 32765 O   VAL H 169   113.383 136.112 235.408 1.00 47.79
O
ATOM 32766 CB VAL H 169   114.400 137.844 237.745 1.00 48.51
C
ATOM 32767 CG1 VAL H 169   114.344 139.169 236.949 1.00 46.82
C
ATOM 32768 CG2 VAL H 169   114.666 138.148 239.177 1.00 45.36
C
ATOM 32769 H   VAL H 169   114.082 135.427 238.219 1.00 59.28
H
ATOM 32770 HA VAL H 169   112.387 137.506 238.056 1.00 54.97
H
ATOM 32771 HB VAL H 169   115.119 137.308 237.375 1.00 58.25
H
ATOM 32772 HG11 VAL H 169   115.112 139.712 237.187 1.00 56.21
H
ATOM 32773 HG12 VAL H 169   114.361 138.967 236.001 1.00 56.21
H
ATOM 32774 HG13 VAL H 169   113.526 139.638 237.174 1.00 56.21
H

ATOM 32775 HG21 VAL H 169 115.545 138.553 239.255 1.00 54.46
H
ATOM 32776 HG22 VAL H 169 113.989 138.762 239.500 1.00 54.46
H
ATOM 32777 HG23 VAL H 169 114.635 137.323 239.686 1.00 54.46
H
ATOM 32778 N HIS H 170 111.599 137.442 235.749 1.00 49.39
N
ATOM 32779 CA HIS H 170 111.255 137.584 234.340 1.00 45.11
C
ATOM 32780 C HIS H 170 111.157 139.063 233.989 1.00 51.91
C
ATOM 32781 O HIS H 170 110.289 139.774 234.503 1.00 47.92
O
ATOM 32782 CB HIS H 170 109.926 136.895 234.025 1.00 48.94
C
ATOM 32783 CG HIS H 170 109.943 135.417 234.275 1.00 49.36
C
ATOM 32784 ND1 HIS H 170 110.878 134.578 233.711 1.00 56.74
N
ATOM 32785 CD2 HIS H 170 109.141 134.633 235.038 1.00 56.18
C
ATOM 32786 CE1 HIS H 170 110.655 133.339 234.112 1.00 63.82
C
ATOM 32787 NE2 HIS H 170 109.602 133.342 234.915 1.00 52.76
N
ATOM 32788 H HIS H 170 111.024 137.792 236.282 1.00 59.29
H
ATOM 32789 HA HIS H 170 111.958 137.181 233.807 1.00 54.16
H
ATOM 32790 HB2 HIS H 170 109.233 137.284 234.581 1.00 58.75
H
ATOM 32791 HB3 HIS H 170 109.717 137.036 233.088 1.00 58.75
H
ATOM 32792 HD2 HIS H 170 108.416 134.914 235.549 1.00 67.45
H
ATOM 32793 HE1 HIS H 170 111.153 132.592 233.871 1.00 76.61
H
ATOM 32794 HE2 HIS H 170 109.262 132.651 235.296 1.00 63.34
H
ATOM 32795 N THR H 171 111.990 139.508 233.064 1.00 51.33
N
ATOM 32796 CA THR H 171 111.907 140.863 232.535 1.00 44.91
C
ATOM 32797 C THR H 171 111.426 140.766 231.098 1.00 47.80
C
ATOM 32798 O THR H 171 112.044 140.107 230.268 1.00 46.19
O
ATOM 32799 CB THR H 171 113.248 141.579 232.653 1.00 44.55
C

ATOM 32800 OG1 THR H 171   113.604 141.643 234.047 1.00 45.39
O
ATOM 32801 CG2 THR H 171   113.157 142.990 232.074 1.00 52.68
C
ATOM 32802 H   THR H 171   112.622 139.038 232.721 1.00 61.62
H
ATOM 32803 HA THR H 171   111.264 141.390 233.035 1.00 53.92
H
ATOM 32804 HB THR H 171   113.931 141.102 232.155 1.00 53.49
H
ATOM 32805 HG1 THR H 171   114.429 141.518 234.139 1.00 54.49
H
ATOM 32806 HG21 THR H 171   113.996 143.458 232.208 1.00 63.24
H
ATOM 32807 HG22 THR H 171   112.969 142.948 231.123 1.00 63.24
H
ATOM 32808 HG23 THR H 171   112.447 143.484 232.513 1.00 63.24
H
ATOM 32809 N   PHE H 172   110.349 141.344 230.849 1.00 48.81
N
ATOM 32810 CA PHE H 172   109.636 141.146 229.612 1.00 48.85
C
ATOM 32811 C   PHE H 172   110.144 142.080 228.517 1.00 59.44
C
ATOM 32812 O   PHE H 172   110.707 143.151 228.794 1.00 47.97
O
ATOM 32813 CB PHE H 172   108.156 141.393 229.843 1.00 47.49
C
ATOM 32814 CG PHE H 172   107.500 140.318 230.648 1.00 53.11
C
ATOM 32815 CD1 PHE H 172   107.459 140.397 232.045 1.00 52.08
C
ATOM 32816 CD2 PHE H 172   106.980 139.215 230.027 1.00 51.78
C
ATOM 32817 CE1 PHE H 172   106.870 139.407 232.789 1.00 54.94
C
ATOM 32818 CE2 PHE H 172   106.379 138.203 230.773 1.00 50.29
C
ATOM 32819 CZ PHE H 172   106.338 138.304 232.154 1.00 45.98
C
ATOM 32820 H   PHE H 172   109.962 141.892 231.386 1.00 58.60
H
ATOM 32821 HA PHE H 172   109.768 140.235 229.307 1.00 58.65
H
ATOM 32822 HB2 PHE H 172   108.047 142.232 230.319 1.00 57.01
H
ATOM 32823 HB3 PHE H 172   107.708 141.439 228.984 1.00 57.01
H
ATOM 32824 HD1 PHE H 172   107.837 141.131 232.474 1.00 62.52
H

ATOM 32825 HD2 PHE H 172 107.028 139.139 229.102 1.00 62.17
H
ATOM 32826 HE1 PHE H 172 106.832 139.477 233.717 1.00 65.96
H
ATOM 32827 HE2 PHE H 172 106.009 137.464 230.346 1.00 60.37
H
ATOM 32828 HZ PHE H 172 105.947 137.624 232.654 1.00 55.20
H
ATOM 32829 N PRO H 173 109.941 141.695 227.258 1.00 59.71
N
ATOM 32830 CA PRO H 173 110.306 142.583 226.139 1.00 51.41
C
ATOM 32831 C PRO H 173 109.386 143.779 226.125 1.00 53.20
C
ATOM 32832 O PRO H 173 108.187 143.658 226.389 1.00 47.77
O
ATOM 32833 CB PRO H 173 110.095 141.714 224.893 1.00 54.05
C
ATOM 32834 CG PRO H 173 110.000 140.294 225.384 1.00 61.36
C
ATOM 32835 CD PRO H 173 109.475 140.366 226.801 1.00 55.42
C
ATOM 32836 HA PRO H 173 111.236 142.855 226.187 1.00 61.72
H
ATOM 32837 HB2 PRO H 173 109.275 141.979 224.446 1.00 64.88
H
ATOM 32838 HB3 PRO H 173 110.849 141.820 224.291 1.00 64.88
H
ATOM 32839 HG2 PRO H 173 109.390 139.794 224.818 1.00 73.66
H
ATOM 32840 HG3 PRO H 173 110.880 139.884 225.366 1.00 73.66
H
ATOM 32841 HD2 PRO H 173 108.505 140.316 226.813 1.00 66.53
H
ATOM 32842 HD3 PRO H 173 109.851 139.659 227.346 1.00 66.53
H
ATOM 32843 N ALA H 174 109.955 144.941 225.817 1.00 55.62
N
ATOM 32844 CA ALA H 174 109.154 146.158 225.755 1.00 46.98
C
ATOM 32845 C ALA H 174 108.102 146.035 224.681 1.00 46.31
C
ATOM 32846 O ALA H 174 108.276 145.307 223.704 1.00 57.53
O
ATOM 32847 CB ALA H 174 110.042 147.357 225.468 1.00 47.33
C
ATOM 32848 H ALA H 174 110.789 145.049 225.640 1.00 66.77
H
ATOM 32849 HA ALA H 174 108.715 146.300 226.607 1.00 56.40
H

ATOM 32850 HB1 ALA H 174 109.516 148.167 225.557 1.00 56.82
H
ATOM 32851 HB2 ALA H 174 110.775 147.365 226.104 1.00 56.82
H
ATOM 32852 HB3 ALA H 174 110.388 147.285 224.565 1.00 56.82
H
ATOM 32853 N VAL H 175 106.989 146.751 224.871 1.00 45.48
N
ATOM 32854 CA VAL H 175 106.030 146.971 223.798 1.00 49.68
C
ATOM 32855 C VAL H 175 106.044 148.457 223.470 1.00 60.67
C
ATOM 32856 O VAL H 175 106.337 149.294 224.327 1.00 48.99
O
ATOM 32857 CB VAL H 175 104.579 146.531 224.099 1.00 50.38
C
ATOM 32858 CG1 VAL H 175 104.534 145.093 224.680 1.00 60.74
C
ATOM 32859 CG2 VAL H 175 103.851 147.549 224.986 1.00 64.36
C
ATOM 32860 H VAL H 175 106.773 147.119 225.617 1.00 54.60
H
ATOM 32861 HA VAL H 175 106.326 146.434 223.047 1.00 59.64
H
ATOM 32862 HB VAL H 175 104.090 146.505 223.262 1.00 60.49
H
ATOM 32863 HG11 VAL H 175 103.611 144.852 224.857 1.00 72.91
H
ATOM 32864 HG12 VAL H 175 104.919 144.480 224.036 1.00 72.91
H
ATOM 32865 HG13 VAL H 175 105.046 145.070 225.505 1.00 72.91
H
ATOM 32866 HG21 VAL H 175 103.076 147.123 225.386 1.00 77.26
H
ATOM 32867 HG22 VAL H 175 104.456 147.854 225.681 1.00 77.26
H
ATOM 32868 HG23 VAL H 175 103.570 148.300 224.440 1.00 77.26
H
ATOM 32869 N LEU H 176 105.699 148.771 222.213 1.00 66.69
N
ATOM 32870 CA LEU H 176 105.656 150.136 221.699 1.00 50.95
C
ATOM 32871 C LEU H 176 104.237 150.665 221.863 1.00 58.05
C
ATOM 32872 O LEU H 176 103.291 150.152 221.250 1.00 57.17
O
ATOM 32873 CB LEU H 176 106.111 150.150 220.246 1.00 56.48
C
ATOM 32874 CG LEU H 176 106.415 151.489 219.572 1.00 63.68
C

ATOM 32875 CD1 LEU H 176   107.142 152.438 220.491 1.00 57.22
C
ATOM 32876 CD2 LEU H 176   107.289 151.254 218.349 1.00 61.61
C
ATOM 32877 H   LEU H 176   105.478 148.187 221.623 1.00 80.06
H
ATOM 32878 HA LEU H 176   106.245 150.714 222.209 1.00 61.17
H
ATOM 32879 HB2 LEU H 176   106.928 149.626 220.192 1.00 67.80
H
ATOM 32880 HB3 LEU H 176   105.414 149.730 219.719 1.00 67.80
H
ATOM 32881 HG LEU H 176   105.570 151.895 219.323 1.00 76.44
H
ATOM 32882 HD11 LEU H 176   107.427 153.215 219.984 1.00 68.69
H
ATOM 32883 HD12 LEU H 176   106.542 152.709 221.204 1.00 68.69
H
ATOM 32884 HD13 LEU H 176   107.915 151.986 220.864 1.00 68.69
H
ATOM 32885 HD21 LEU H 176   107.443 152.101 217.903 1.00 73.96
H
ATOM 32886 HD22 LEU H 176   108.134 150.872 218.635 1.00 73.96
H
ATOM 32887 HD23 LEU H 176   106.834 150.642 217.751 1.00 73.96
H
ATOM 32888 N   GLN H 177   104.066 151.674 222.702 1.00 55.07
N
ATOM 32889 CA GLN H 177   102.717 152.197 222.850 1.00 56.55
C
ATOM 32890 C   GLN H 177   102.387 153.131 221.682 1.00 57.12
C
ATOM 32891 O   GLN H 177   103.268 153.560 220.924 1.00 54.39
O
ATOM 32892 CB GLN H 177   102.578 152.907 224.194 1.00 55.00
C
ATOM 32893 CG GLN H 177   103.000 152.017 225.443 1.00 58.74
C
ATOM 32894 CD GLN H 177   103.204 152.841 226.709 1.00 70.50
C
ATOM 32895 OE1 GLN H 177   102.297 152.970 227.543 1.00 74.28
O
ATOM 32896 NE2 GLN H 177   104.396 153.418 226.852 1.00 61.77
N
ATOM 32897 H   GLN H 177   104.679 152.054 223.170 1.00 66.11
H
ATOM 32898 HA GLN H 177   102.071 151.473 222.836 1.00 67.89
H
ATOM 32899 HB2 GLN H 177   103.144 153.695 224.189 1.00 66.03
H

ATOM 32900 HB3 GLN H 177 101.651 153.165 224.315 1.00 66.03
H
ATOM 32901 HG2 GLN H 177 102.302 151.365 225.617 1.00 70.51
H
ATOM 32902 HG3 GLN H 177 103.834 151.564 225.242 1.00 70.51
H
ATOM 32903 HE21 GLN H 177 104.999 153.314 226.248 1.00 74.16
H
ATOM 32904 HE22 GLN H 177 104.563 153.894 227.549 1.00 74.16
H
ATOM 32905 N SER H 178 101.099 153.468 221.540 1.00 56.69
N
ATOM 32906 CA SER H 178 100.681 154.315 220.423 1.00 69.11
C
ATOM 32907 C SER H 178 101.240 155.739 220.516 1.00 69.62
C
ATOM 32908 O SER H 178 101.191 156.469 219.519 1.00 63.11
O
ATOM 32909 CB SER H 178 99.154 154.354 220.339 1.00 60.22
C
ATOM 32910 OG SER H 178 98.605 155.115 221.404 1.00 68.90
O
ATOM 32911 H SER H 178 100.465 153.223 222.067 1.00 68.06
H
ATOM 32912 HA SER H 178 101.013 153.926 219.598 1.00 82.96
H
ATOM 32913 HB2 SER H 178 98.895 154.759 219.497 1.00 72.29
H
ATOM 32914 HB3 SER H 178 98.812 153.448 220.391 1.00 72.29
H
ATOM 32915 HG SER H 178 97.766 155.091 221.372 1.00 82.71
H
ATOM 32916 N SER H 179 101.770 156.139 221.676 1.00 57.37
N
ATOM 32917 CA SER H 179 102.409 157.432 221.867 1.00 62.55
C
ATOM 32918 C SER H 179 103.821 157.497 221.299 1.00 61.19
C
ATOM 32919 O SER H 179 104.445 158.562 221.383 1.00 51.43
O
ATOM 32920 CB SER H 179 102.512 157.724 223.355 1.00 52.43
C
ATOM 32921 OG SER H 179 103.345 156.733 223.965 1.00 57.19
O
ATOM 32922 H SER H 179 101.770 155.658 222.389 1.00 68.87
H
ATOM 32923 HA SER H 179 101.864 158.103 221.428 1.00 75.09
H
ATOM 32924 HB2 SER H 179 102.906 158.600 223.484 1.00 62.95
H

ATOM 32925 HB3 SER H 179 101.629 157.694 223.752 1.00 62.95
H
ATOM 32926 HG SER H 179 103.340 156.827 224.800 1.00 68.65
H
ATOM 32927 N GLY H 180 104.361 156.366 220.828 1.00 53.09
N
ATOM 32928 CA GLY H 180 105.751 156.218 220.432 1.00 46.69
C
ATOM 32929 C GLY H 180 106.696 155.887 221.560 1.00 54.74
C
ATOM 32930 O GLY H 180 107.923 155.818 221.351 1.00 46.05
O
ATOM 32931 H GLY H 180 103.915 155.638 220.726 1.00 63.73
H
ATOM 32932 HA2 GLY H 180 105.813 155.509 219.773 1.00 56.05
H
ATOM 32933 HA3 GLY H 180 106.050 157.049 220.029 1.00 56.05
H
ATOM 32934 N LEU H 181 106.186 155.671 222.750 1.00 55.51
N
ATOM 32935 CA LEU H 181 107.050 155.431 223.892 1.00 51.72
C
ATOM 32936 C LEU H 181 106.978 153.960 224.271 1.00 50.31
C
ATOM 32937 O LEU H 181 105.960 153.306 224.045 1.00 52.37
O
ATOM 32938 CB LEU H 181 106.630 156.333 225.035 1.00 52.73
C
ATOM 32939 CG LEU H 181 106.673 157.831 224.745 1.00 56.52
C
ATOM 32940 CD1 LEU H 181 106.032 158.580 225.910 1.00 56.52
C
ATOM 32941 CD2 LEU H 181 108.115 158.284 224.528 1.00 51.76
C
ATOM 32942 H LEU H 181 105.345 155.658 222.928 1.00 66.64
H
ATOM 32943 HA LEU H 181 107.980 155.623 223.691 1.00 62.09
H
ATOM 32944 HB2 LEU H 181 105.717 156.113 225.277 1.00 63.31
H
ATOM 32945 HB3 LEU H 181 107.220 156.167 225.788 1.00 63.31
H
ATOM 32946 HG LEU H 181 106.181 158.035 223.936 1.00 67.85
H
ATOM 32947 HD11 LEU H 181 106.021 159.529 225.707 1.00 67.85
H
ATOM 32948 HD12 LEU H 181 105.125 158.258 226.031 1.00 67.85
H
ATOM 32949 HD13 LEU H 181 106.551 158.420 226.713 1.00 67.85
H

```
ATOM  32950 HD21 LEU H 181     108.128 159.248 224.411  1.00 62.14
H
ATOM  32951 HD22 LEU H 181     108.643 158.038 225.304  1.00 62.14
H
ATOM  32952 HD23 LEU H 181     108.465 157.849 223.735  1.00 62.14
H
ATOM  32953  N   TYR H 182     108.079 153.445 224.820  1.00 50.58
N
ATOM  32954  CA  TYR H 182     108.163 152.050 225.239  1.00 52.70
C
ATOM  32955  C   TYR H 182     107.717 151.876 226.687  1.00 54.21
C
ATOM  32956  O   TYR H 182     107.899 152.768 227.514  1.00 54.92
O
ATOM  32957  CB  TYR H 182     109.594 151.526 225.092  1.00 50.12
C
ATOM  32958  CG  TYR H 182     110.035 151.446 223.644  1.00 51.84
C
ATOM  32959  CD1 TYR H 182     109.687 150.373 222.846  1.00 49.35
C
ATOM  32960  CD2 TYR H 182     110.753 152.478 223.070  1.00 61.15
C
ATOM  32961  CE1 TYR H 182     110.084 150.298 221.516  1.00 54.61
C
ATOM  32962  CE2 TYR H 182     111.148 152.435 221.740  1.00 61.10
C
ATOM  32963  CZ  TYR H 182     110.815 151.352 220.969  1.00 59.09
C
ATOM  32964  OH  TYR H 182     111.246 151.326 219.676  1.00 55.19
O
ATOM  32965  H   TYR H 182     108.801 153.890 224.960  1.00 60.72
H
ATOM  32966  HA  TYR H 182     107.574 151.524 224.676  1.00 63.27
H
ATOM  32967  HB2 TYR H 182     110.199 152.122 225.559  1.00 60.18
H
ATOM  32968  HB3 TYR H 182     109.645 150.635 225.472  1.00 60.18
H
ATOM  32969  HD1 TYR H 182     109.177 149.684 223.207  1.00 59.25
H
ATOM  32970  HD2 TYR H 182     110.979 153.218 223.587  1.00 73.41
H
ATOM  32971  HE1 TYR H 182     109.865 149.557 220.999  1.00 65.56
H
ATOM  32972  HE2 TYR H 182     111.634 153.137 221.373  1.00 73.35
H
ATOM  32973  HH  TYR H 182     111.905 151.839 219.583  1.00 66.26
H
ATOM  32974  N   SER H 183     107.142 150.707 226.982  1.00 47.23
N
```

ATOM 32975 CA SER H 183 106.862 150.247 228.337 1.00 44.39
C
ATOM 32976 C SER H 183 107.257 148.781 228.467 1.00 51.32
C
ATOM 32977 O SER H 183 107.018 147.988 227.550 1.00 49.86
O
ATOM 32978 CB SER H 183 105.383 150.419 228.662 1.00 58.81
C
ATOM 32979 OG SER H 183 105.062 149.688 229.828 1.00 66.21
O
ATOM 32980 H SER H 183 106.895 150.140 226.384 1.00 56.70
H
ATOM 32981 HA SER H 183 107.389 150.754 228.976 1.00 53.29
H
ATOM 32982 HB2 SER H 183 105.196 151.359 228.812 1.00 70.60
H
ATOM 32983 HB3 SER H 183 104.852 150.088 227.920 1.00 70.60
H
ATOM 32984 HG SER H 183 104.256 149.810 230.030 1.00 79.47
H
ATOM 32985 N LEU H 184 107.869 148.411 229.597 1.00 50.22
N
ATOM 32986 CA LEU H 184 108.055 147.001 229.927 1.00 53.46
C
ATOM 32987 C LEU H 184 107.792 146.744 231.405 1.00 58.23
C
ATOM 32988 O LEU H 184 107.715 147.664 232.226 1.00 48.73
O
ATOM 32989 CB LEU H 184 109.463 146.486 229.596 1.00 47.95
C
ATOM 32990 CG LEU H 184 110.690 146.923 230.382 1.00 53.77
C
ATOM 32991 CD1 LEU H 184 110.797 146.285 231.735 1.00 45.26
C
ATOM 32992 CD2 LEU H 184 111.911 146.587 229.563 1.00 53.22
C
ATOM 32993 H LEU H 184 108.183 148.959 230.182 1.00 60.29
H
ATOM 32994 HA LEU H 184 107.401 146.518 229.398 1.00 64.18
H
ATOM 32995 HB2 LEU H 184 109.432 145.519 229.679 1.00 57.57
H
ATOM 32996 HB3 LEU H 184 109.641 146.734 228.675 1.00 57.57
H
ATOM 32997 HG LEU H 184 110.624 147.877 230.550 1.00 64.55
H
ATOM 32998 HD11 LEU H 184 111.726 146.294 232.017 1.00 54.34
H
ATOM 32999 HD12 LEU H 184 110.256 146.787 232.364 1.00 54.34
H

ATOM 33000 HD13 LEU H 184 110.477 145.372 231.679 1.00 54.34
H
ATOM 33001 HD21 LEU H 184 112.695 146.632 230.131 1.00 63.90
H
ATOM 33002 HD22 LEU H 184 111.812 145.694 229.202 1.00 63.90
H
ATOM 33003 HD23 LEU H 184 111.990 147.230 228.840 1.00 63.90
H
ATOM 33004 N SER H 185 107.738 145.448 231.749 1.00 50.45
N
ATOM 33005 CA SER H 185 107.605 145.018 233.132 1.00 48.59
C
ATOM 33006 C SER H 185 108.691 144.023 233.491 1.00 48.22
C
ATOM 33007 O SER H 185 109.150 143.233 232.661 1.00 49.30
O
ATOM 33008 CB SER H 185 106.204 144.402 233.401 1.00 58.04
C
ATOM 33009 OG SER H 185 105.965 143.358 232.471 1.00 65.48
O
ATOM 33010 H SER H 185 107.777 144.798 231.187 1.00 60.57
H
ATOM 33011 HA SER H 185 107.721 145.786 233.712 1.00 58.33
H
ATOM 33012 HB2 SER H 185 106.179 144.044 234.302 1.00 69.68
H
ATOM 33013 HB3 SER H 185 105.527 145.088 233.297 1.00 69.68
H
ATOM 33014 HG SER H 185 106.624 142.838 232.447 1.00 78.60
H
ATOM 33015 N SER H 186 109.091 144.077 234.759 1.00 46.50
N
ATOM 33016 CA SER H 186 110.001 143.126 235.369 1.00 46.05
C
ATOM 33017 C SER H 186 109.349 142.581 236.641 1.00 53.80
C
ATOM 33018 O SER H 186 108.871 143.361 237.484 1.00 43.80
O
ATOM 33019 CB SER H 186 111.323 143.780 235.737 1.00 46.75
C
ATOM 33020 OG SER H 186 112.220 142.785 236.213 1.00 49.12
O
ATOM 33021 H SER H 186 108.832 144.686 235.308 1.00 55.82
H
ATOM 33022 HA SER H 186 110.172 142.402 234.749 1.00 55.29
H
ATOM 33023 HB2 SER H 186 111.701 144.206 234.954 1.00 56.13
H
ATOM 33024 HB3 SER H 186 111.175 144.438 236.435 1.00 56.13
H

```
ATOM  33025  HG  SER H 186     112.551 142.365 235.566  1.00 58.98
H
ATOM  33026  N   VAL H 187     109.348 141.255 236.787  1.00 44.56
N
ATOM  33027  CA  VAL H 187     108.680 140.586 237.893  1.00 44.79
C
ATOM  33028  C   VAL H 187     109.573 139.517 238.482  1.00 44.10
C
ATOM  33029  O   VAL H 187     110.456 138.972 237.818  1.00 46.68
O
ATOM  33030  CB  VAL H 187     107.350 139.900 237.505  1.00 47.64
C
ATOM  33031  CG1 VAL H 187     106.399 140.883 237.040  1.00 45.73
C
ATOM  33032  CG2 VAL H 187     107.626 138.749 236.458  1.00 45.97
C
ATOM  33033  H   VAL H 187     109.736 140.714 236.243  1.00 53.50
H
ATOM  33034  HA  VAL H 187     108.514 141.279 238.552  1.00 53.77
H
ATOM  33035  HB  VAL H 187     106.945 139.479 238.281  1.00 57.19
H
ATOM  33036 HG11 VAL H 187     105.553 140.444 236.862  1.00 54.90
H
ATOM  33037 HG12 VAL H 187     106.284 141.561 237.724  1.00 54.90
H
ATOM  33038 HG13 VAL H 187     106.739 141.289 236.226  1.00 54.90
H
ATOM  33039 HG21 VAL H 187     106.779 138.409 236.130  1.00 55.19
H
ATOM  33040 HG22 VAL H 187     108.145 139.109 235.722  1.00 55.19
H
ATOM  33041 HG23 VAL H 187     108.122 138.037 236.893  1.00 55.19
H
ATOM  33042  N   VAL H 188     109.263 139.159 239.738  1.00 47.16
N
ATOM  33043  CA  VAL H 188     109.948 138.069 240.433  1.00 46.15
C
ATOM  33044  C   VAL H 188     108.929 137.419 241.346  1.00 44.80
C
ATOM  33045  O   VAL H 188     108.065 138.093 241.927  1.00 44.74
O
ATOM  33046  CB  VAL H 188     111.193 138.565 241.203  1.00 42.75
C
ATOM  33047  CG1 VAL H 188     110.795 139.586 242.208  1.00 56.89
C
ATOM  33048  CG2 VAL H 188     111.915 137.412 241.888  1.00 47.85
C
ATOM  33049  H   VAL H 188     108.653 139.540 240.209  1.00 56.61
H
```

ATOM 33050 HA VAL H 188 110.258 137.410 239.793 1.00 55.41
H
ATOM 33051 HB VAL H 188 111.807 138.965 240.567 1.00 51.33
H
ATOM 33052 HG11 VAL H 188 111.594 139.940 242.631 1.00 68.30
H
ATOM 33053 HG12 VAL H 188 110.315 140.300 241.760 1.00 68.30
H
ATOM 33054 HG13 VAL H 188 110.225 139.171 242.873 1.00 68.30
H
ATOM 33055 HG21 VAL H 188 112.745 137.740 242.266 1.00 57.44
H
ATOM 33056 HG22 VAL H 188 111.349 137.058 242.591 1.00 57.44
H
ATOM 33057 HG23 VAL H 188 112.101 136.722 241.231 1.00 57.44
H
ATOM 33058 N THR H 189 109.000 136.107 241.447 1.00 43.93
N
ATOM 33059 CA THR H 189 108.214 135.388 242.434 1.00 41.39
C
ATOM 33060 C THR H 189 109.114 135.004 243.600 1.00 43.28
C
ATOM 33061 O THR H 189 110.256 134.599 243.404 1.00 46.44
O
ATOM 33062 CB THR H 189 107.567 134.143 241.844 1.00 47.53
C
ATOM 33063 OG1 THR H 189 108.532 133.387 241.142 1.00 50.59
O
ATOM 33064 CG2 THR H 189 106.454 134.502 240.909 1.00 44.33
C
ATOM 33065 H THR H 189 109.499 135.607 240.955 1.00 52.75
H
ATOM 33066 HA THR H 189 107.498 135.959 242.756 1.00 49.70
H
ATOM 33067 HB THR H 189 107.196 133.611 242.566 1.00 57.06
H
ATOM 33068 HG1 THR H 189 108.167 132.726 240.774 1.00 60.74
H
ATOM 33069 HG21 THR H 189 106.073 133.697 240.523 1.00 53.22
H
ATOM 33070 HG22 THR H 189 105.759 134.979 241.389 1.00 53.22
H
ATOM 33071 HG23 THR H 189 106.788 135.068 240.196 1.00 53.22
H
ATOM 33072 N VAL H 190 108.601 135.187 244.813 1.00 42.12
N
ATOM 33073 CA VAL H 190 109.369 134.997 246.039 1.00 41.06
C
ATOM 33074 C VAL H 190 108.465 134.278 247.027 1.00 45.23
C

ATOM 33075 O VAL H 190 107.248 134.232 246.833 1.00 44.03
O
ATOM 33076 CB VAL H 190 109.842 136.338 246.636 1.00 43.28
C
ATOM 33077 CG1 VAL H 190 110.879 137.045 245.677 1.00 43.92
C
ATOM 33078 CG2 VAL H 190 108.617 137.233 246.899 1.00 41.80
C
ATOM 33079 H VAL H 190 107.789 135.429 244.956 1.00 50.58
H
ATOM 33080 HA VAL H 190 110.158 134.458 245.867 1.00 49.30
H
ATOM 33081 HB VAL H 190 110.297 136.181 247.478 1.00 51.97
H
ATOM 33082 HG11 VAL H 190 110.932 137.985 245.907 1.00 52.73
H
ATOM 33083 HG12 VAL H 190 111.748 136.628 245.788 1.00 52.73
H
ATOM 33084 HG13 VAL H 190 110.580 136.945 244.759 1.00 52.73
H
ATOM 33085 HG21 VAL H 190 108.920 138.129 247.113 1.00 50.18
H
ATOM 33086 HG22 VAL H 190 108.063 137.253 246.102 1.00 50.18
H
ATOM 33087 HG23 VAL H 190 108.113 136.868 247.643 1.00 50.18
H
ATOM 33088 N PRO H 191 109.015 133.695 248.088 1.00 42.39
N
ATOM 33089 CA PRO H 191 108.106 133.150 249.104 1.00 46.52
C
ATOM 33090 C PRO H 191 107.341 134.286 249.751 1.00 42.79
C
ATOM 33091 O PRO H 191 107.919 135.282 250.168 1.00 42.92
O
ATOM 33092 CB PRO H 191 109.030 132.436 250.097 1.00 45.77
C
ATOM 33093 CG PRO H 191 110.311 132.242 249.352 1.00 46.07
C
ATOM 33094 CD PRO H 191 110.423 133.361 248.367 1.00 46.71
C
ATOM 33095 HA PRO H 191 107.498 132.494 248.727 1.00 55.86
H
ATOM 33096 HB2 PRO H 191 109.165 132.989 250.881 1.00 54.95
H
ATOM 33097 HB3 PRO H 191 108.645 131.582 250.354 1.00 54.95
H
ATOM 33098 HG2 PRO H 191 111.053 132.265 249.976 1.00 55.31
H
ATOM 33099 HG3 PRO H 191 110.290 131.388 248.893 1.00 55.31
H

ATOM 33100 HD2 PRO H 191 110.889 134.119 248.754 1.00 56.07
H
ATOM 33101 HD3 PRO H 191 110.876 133.069 247.560 1.00 56.07
H
ATOM 33102 N SER H 192 106.030 134.133 249.855 1.00 42.43
N
ATOM 33103 CA SER H 192 105.262 135.257 250.365 1.00 44.64
C
ATOM 33104 C SER H 192 105.665 135.608 251.788 1.00 49.22
C
ATOM 33105 O SER H 192 105.570 136.778 252.174 1.00 49.77
O
ATOM 33106 CB SER H 192 103.762 134.982 250.272 1.00 50.96
C
ATOM 33107 OG SER H 192 103.505 133.634 250.061 1.00 71.68
O
ATOM 33108 H SER H 192 105.583 133.428 249.648 1.00 50.95
H
ATOM 33109 HA SER H 192 105.441 136.029 249.807 1.00 53.59
H
ATOM 33110 HB2 SER H 192 103.342 135.256 251.101 1.00 61.18
H
ATOM 33111 HB3 SER H 192 103.397 135.491 249.530 1.00 61.18
H
ATOM 33112 HG SER H 192 102.698 133.467 250.228 1.00 86.05
H
ATOM 33113 N SER H 193 106.133 134.623 252.583 1.00 51.30
N
ATOM 33114 CA SER H 193 106.572 134.915 253.946 1.00 50.02
C
ATOM 33115 C SER H 193 107.842 135.745 253.971 1.00 47.73
C
ATOM 33116 O SER H 193 108.184 136.310 255.019 1.00 47.20
O
ATOM 33117 CB SER H 193 106.806 133.626 254.726 1.00 49.37
C
ATOM 33118 OG SER H 193 107.825 132.842 254.145 1.00 44.73
O
ATOM 33119 H SER H 193 106.202 133.796 252.354 1.00 61.59
H
ATOM 33120 HA SER H 193 105.866 135.411 254.389 1.00 60.05
H
ATOM 33121 HB2 SER H 193 107.065 133.853 255.633 1.00 59.28
H
ATOM 33122 HB3 SER H 193 105.983 133.114 254.734 1.00 59.28
H
ATOM 33123 HG SER H 193 107.900 132.115 254.560 1.00 53.70
H
ATOM 33124 N SER H 194 108.553 135.828 252.847 1.00 45.14
N

ATOM 33125 CA SER H 194   109.780 136.612 252.818 1.00 42.87
C
ATOM 33126 C   SER H 194   109.530 138.113 252.834 1.00 46.05
C
ATOM 33127 O   SER H 194   110.478 138.866 253.053 1.00 45.08
O
ATOM 33128 CB SER H 194   110.619 136.257 251.586 1.00 49.30
C
ATOM 33129 OG SER H 194   110.094 136.779 250.384 1.00 43.55
O
ATOM 33130 H   SER H 194   108.347 135.445 252.104 1.00 54.20
H
ATOM 33131 HA SER H 194   110.294 136.381 253.607 1.00 51.47
H
ATOM 33132 HB2 SER H 194   111.512 136.616 251.711 1.00 59.19
H
ATOM 33133 HB3 SER H 194   110.658 135.292 251.510 1.00 59.19
H
ATOM 33134 HG SER H 194   109.258 136.698 250.377 1.00 52.28
H
ATOM 33135 N   LEU H 195   108.295 138.565 252.606 1.00 44.51
N
ATOM 33136 CA LEU H 195   108.041 140.000 252.580 1.00 44.16
C
ATOM 33137 C   LEU H 195   108.211 140.632 253.950 1.00 53.74
C
ATOM 33138 O   LEU H 195   108.550 141.824 254.031 1.00 49.68
O
ATOM 33139 CB LEU H 195   106.653 140.262 252.043 1.00 43.18
C
ATOM 33140 CG LEU H 195   106.420 139.702 250.615 1.00 49.53
C
ATOM 33141 CD1 LEU H 195   104.861 139.627 250.279 1.00 55.20
C
ATOM 33142 CD2 LEU H 195   107.202 140.546 249.632 1.00 53.14
C
ATOM 33143 H   LEU H 195   107.604 138.071 252.467 1.00 53.44
H
ATOM 33144 HA LEU H 195   108.674 140.428 251.981 1.00 53.01
H
ATOM 33145 HB2 LEU H 195   106.008 139.845 252.635 1.00 51.85
H
ATOM 33146 HB3 LEU H 195   106.507 141.220 252.014 1.00 51.85
H
ATOM 33147 HG LEU H 195   106.738 138.787 250.543 1.00 59.46
H
ATOM 33148 HD11 LEU H 195   104.748 139.267 249.387 1.00 66.27
H
ATOM 33149 HD12 LEU H 195   104.426 139.051 250.926 1.00 66.27
H

```
ATOM  33150 HD13 LEU H 195     104.485 140.520 250.327  1.00 66.27
H
ATOM  33151 HD21 LEU H 195     106.911 140.332 248.732  1.00 63.80
H
ATOM  33152 HD22 LEU H 195     107.037 141.483 249.818  1.00 63.80
H
ATOM  33153 HD23 LEU H 195     108.146 140.350 249.731  1.00 63.80
H
ATOM  33154  N   GLY H 196     108.077 139.827 255.015  1.00 53.70
N
ATOM  33155  CA  GLY H 196     108.285 140.284 256.375  1.00 48.83
C
ATOM  33156  C   GLY H 196     109.728 140.435 256.793  1.00 50.01
C
ATOM  33157  O   GLY H 196     109.986 141.091 257.806  1.00 52.76
O
ATOM  33158  H   GLY H 196     107.861 138.995 254.967  1.00 64.47
H
ATOM  33159  HA2 GLY H 196     107.858 141.149 256.478  1.00 58.63
H
ATOM  33160  HA3 GLY H 196     107.868 139.652 256.980  1.00 58.63
H
ATOM  33161  N   THR H 197     110.678 139.844 256.054  1.00 51.02
N
ATOM  33162  CA  THR H 197     112.085 139.887 256.438  1.00 49.30
C
ATOM  33163  C   THR H 197     112.990 140.450 255.387  1.00 43.73
C
ATOM  33164  O   THR H 197     114.133 140.786 255.718  1.00 47.38
O
ATOM  33165  CB  THR H 197     112.680 138.493 256.784  1.00 50.70
C
ATOM  33166  OG1 THR H 197     112.385 137.553 255.756  1.00 53.68
O
ATOM  33167  CG2 THR H 197     112.168 137.964 258.074  1.00 52.75
C
ATOM  33168  H   THR H 197     110.527 139.411 255.326  1.00 61.25
H
ATOM  33169  HA  THR H 197     112.108 140.429 257.242  1.00 59.19
H
ATOM  33170  HB  THR H 197     113.641 138.601 256.867  1.00 60.86
H
ATOM  33171  HG1 THR H 197     112.951 136.932 255.762  1.00 64.44
H
ATOM  33172 HG21 THR H 197     112.565 137.098 258.259  1.00 63.33
H
ATOM  33173 HG22 THR H 197     112.392 138.573 258.796  1.00 63.33
H
ATOM  33174 HG23 THR H 197     111.204 137.867 258.033  1.00 63.33
H
```

```
ATOM  33175  N   GLN H 198     112.554 140.528 254.136  1.00 46.05
N
ATOM  33176  CA  GLN H 198     113.452 140.775 253.025  1.00 43.51
C
ATOM  33177  C   GLN H 198     113.056 142.042 252.279  1.00 46.07
C
ATOM  33178  O   GLN H 198     111.872 142.359 252.123  1.00 48.40
O
ATOM  33179  CB  GLN H 198     113.459 139.559 252.071  1.00 47.85
C
ATOM  33180  CG  GLN H 198     114.473 139.583 250.940  1.00 51.79
C
ATOM  33181  CD  GLN H 198     115.914 139.581 251.417  1.00 59.36
C
ATOM  33182  OE1 GLN H 198     116.256 138.893 252.360  1.00 56.53
O
ATOM  33183  NE2 GLN H 198     116.748 140.395 250.789  1.00 71.87
N
ATOM  33184  H   GLN H 198     111.729 140.437 253.907  1.00 55.29
H
ATOM  33185  HA  GLN H 198     114.351 140.923 253.356  1.00 52.25
H
ATOM  33186  HB2 GLN H 198     113.641 138.766 252.599  1.00 57.45
H
ATOM  33187  HB3 GLN H 198     112.581 139.493 251.664  1.00 57.45
H
ATOM  33188  HG2 GLN H 198     114.343 138.797 250.386  1.00 62.18
H
ATOM  33189  HG3 GLN H 198     114.336 140.386 250.414  1.00 62.18
H
ATOM  33190 HE21 GLN H 198     116.464 140.891 250.146  1.00 86.28
H
ATOM  33191 HE22 GLN H 198     117.575 140.429 251.025  1.00 86.28
H
ATOM  33192  N   THR H 199     114.067 142.743 251.807  1.00 49.80
N
ATOM  33193  CA  THR H 199     113.903 143.965 251.031  1.00 56.46
C
ATOM  33194  C   THR H 199     114.020 143.659 249.533  1.00 49.64
C
ATOM  33195  O   THR H 199     114.950 142.977 249.103  1.00 46.83
O
ATOM  33196  CB  THR H 199     114.971 144.989 251.447  1.00 57.83
C
ATOM  33197  OG1 THR H 199     114.830 145.283 252.839  1.00 58.34
O
ATOM  33198  CG2 THR H 199     114.838 146.263 250.658  1.00 50.45
C
ATOM  33199  H   THR H 199     114.891 142.529 251.924  1.00 59.78
H
```

```
ATOM  33200  HA  THR H 199     113.021 144.338 251.187  1.00 67.78
H
ATOM  33201  HB  THR H 199     115.850 144.618 251.273  1.00 69.42
H
ATOM  33202  HG1 THR H 199     115.517 145.674 253.121  1.00 70.04
H
ATOM  33203 HG21 THR H 199     115.394 146.956 251.050  1.00 60.57
H
ATOM  33204 HG22 THR H 199     115.116 146.117 249.741  1.00 60.57
H
ATOM  33205 HG23 THR H 199     113.915 146.562 250.662  1.00 60.57
H
ATOM  33206  N   TYR H 200     113.062 144.134 248.749  1.00 43.28
N
ATOM  33207  CA  TYR H 200     113.086 143.967 247.302  1.00 43.89
C
ATOM  33208  C   TYR H 200     113.155 145.329 246.623  1.00 47.93
C
ATOM  33209  O   TYR H 200     112.236 146.147 246.764  1.00 46.36
O
ATOM  33210  CB  TYR H 200     111.856 143.191 246.855  1.00 44.19
C
ATOM  33211  CG  TYR H 200     111.863 141.771 247.421  1.00 46.76
C
ATOM  33212  CD1 TYR H 200     112.646 140.792 246.864  1.00 45.34
C
ATOM  33213  CD2 TYR H 200     111.099 141.436 248.509  1.00 48.50
C
ATOM  33214  CE1 TYR H 200     112.664 139.488 247.365  1.00 50.15
C
ATOM  33215  CE2 TYR H 200     111.089 140.136 249.017  1.00 44.02
C
ATOM  33216  CZ  TYR H 200     111.890 139.177 248.435  1.00 46.38
C
ATOM  33217  OH  TYR H 200     111.913 137.900 248.907  1.00 47.54
O
ATOM  33218  H   TYR H 200     112.374 144.563 249.037  1.00 51.96
H
ATOM  33219  HA  TYR H 200     113.884 143.482 247.038  1.00 52.70
H
ATOM  33220  HB2 TYR H 200     111.058 143.643 247.171  1.00 53.05
H
ATOM  33221  HB3 TYR H 200     111.844 143.135 245.886  1.00 53.05
H
ATOM  33222  HD1 TYR H 200     113.178 141.002 246.131  1.00 54.44
H
ATOM  33223  HD2 TYR H 200     110.577 142.088 248.917  1.00 58.23
H
ATOM  33224  HE1 TYR H 200     113.201 138.840 246.969  1.00 60.21
H
```

ATOM 33225 HE2 TYR H 200   110.547 139.919 249.740 1.00 52.85
H
ATOM 33226 HH TYR H 200   111.454 137.844 249.607 1.00 57.08
H
ATOM 33227 N   ILE H 201   114.231 145.549 245.878 1.00 48.66
N
ATOM 33228 CA ILE H 201   114.467 146.777 245.128 1.00 51.32
C
ATOM 33229 C   ILE H 201   114.793 146.415 243.685 1.00 48.96
C
ATOM 33230 O   ILE H 201   115.724 145.635 243.430 1.00 49.99
O
ATOM 33231 CB ILE H 201   115.628 147.606 245.715 1.00 53.78
C
ATOM 33232 CG1 ILE H 201   115.364 147.993 247.183 1.00 53.43
C
ATOM 33233 CG2 ILE H 201   115.834 148.876 244.864 1.00 51.68
C
ATOM 33234 CD1 ILE H 201   116.616 148.455 247.887 1.00 51.20
C
ATOM 33235 H   ILE H 201   114.866 144.978 245.788 1.00 58.41
H
ATOM 33236 HA ILE H 201   113.656 147.306 245.149 1.00 61.61
H
ATOM 33237 HB ILE H 201   116.429 147.060 245.694 1.00 64.56
H
ATOM 33238 HG12 ILE H 201   114.717 148.715 247.210 1.00 64.15
H
ATOM 33239 HG13 ILE H 201   115.018 147.220 247.657 1.00 64.15
H
ATOM 33240 HG21 ILE H 201   116.356 149.515 245.374 1.00 62.05
H
ATOM 33241 HG22 ILE H 201   116.304 148.637 244.051 1.00 62.05
H
ATOM 33242 HG23 ILE H 201   114.968 149.254 244.646 1.00 62.05
H
ATOM 33243 HD11 ILE H 201   116.443 148.505 248.838 1.00 61.47
H
ATOM 33244 HD12 ILE H 201   117.328 147.819 247.712 1.00 61.47
H
ATOM 33245 HD13 ILE H 201   116.863 149.330 247.549 1.00 61.47
H
ATOM 33246 N   CYS H 202   114.079 147.026 242.735 1.00 49.98
N
ATOM 33247 CA CYS H 202   114.464 146.891 241.338 1.00 55.44
C
ATOM 33248 C   CYS H 202   115.346 148.065 240.926 1.00 52.15
C
ATOM 33249 O   CYS H 202   115.109 149.201 241.340 1.00 56.41
O

ATOM 33250 CB CYS H 202   113.248 146.779 240.417 1.00 64.33
C
ATOM 33251 SG CYS H 202   112.377 148.269 240.202 1.00 62.60
S
ATOM 33252 H   CYS H 202   113.385 147.512 242.878 1.00 60.00
H
ATOM 33253 HA CYS H 202   114.970 146.072 241.226 1.00 66.55
H
ATOM 33254 HB2 CYS H 202   113.547 146.485 239.543 1.00 77.22
H
ATOM 33255 HB3 CYS H 202   112.634 146.131 240.792 1.00 77.22
H
ATOM 33256 N   ASN H 203   116.379 147.761 240.120 1.00 47.56
N
ATOM 33257 CA ASN H 203   117.371 148.716 239.627 1.00 46.66
C
ATOM 33258 C   ASN H 203   117.148 148.889 238.134 1.00 50.22
C
ATOM 33259 O   ASN H 203   117.293 147.935 237.362 1.00 50.72
O
ATOM 33260 CB ASN H 203   118.776 148.220 239.918 1.00 44.30
C
ATOM 33261 CG ASN H 203   118.858 147.553 241.273 1.00 54.99
C
ATOM 33262 OD1 ASN H 203   119.092 146.341 241.370 1.00 59.93
O
ATOM 33263 ND2 ASN H 203   118.637 148.331 242.330 1.00 54.51
N
ATOM 33264 H   ASN H 203   116.527 146.965 239.834 1.00 57.09
H
ATOM 33265 HA ASN H 203   117.261 149.578 240.059 1.00 56.01
H
ATOM 33266 HB2 ASN H 203   119.035 147.574 239.243 1.00 53.19
H
ATOM 33267 HB3 ASN H 203   119.390 148.971 239.911 1.00 53.19
H
ATOM 33268 HD21 ASN H 203   118.671 148.000 243.122 1.00 65.44
H
ATOM 33269 HD22 ASN H 203   118.459 149.166 242.220 1.00 65.44
H
ATOM 33270 N   VAL H 204   116.768 150.097 237.742 1.00 48.11
N
ATOM 33271 CA VAL H 204   116.443 150.418 236.367 1.00 53.59
C
ATOM 33272 C   VAL H 204   117.469 151.395 235.837 1.00 58.48
C
ATOM 33273 O   VAL H 204   117.842 152.354 236.520 1.00 55.88
O
ATOM 33274 CB VAL H 204   115.019 150.995 236.284 1.00 51.01
C

```
ATOM  33275  CG1 VAL H 204     114.616 151.276 234.825  1.00 53.55
C
ATOM  33276  CG2 VAL H 204     114.046 150.028 236.981  1.00 45.72
C
ATOM  33277  H   VAL H 204     116.692 150.767 238.275  1.00 57.76
H
ATOM  33278  HA  VAL H 204     116.490 149.621 235.815  1.00 64.34
H
ATOM  33279  HB  VAL H 204     114.980 151.850 236.742  1.00 61.24
H
ATOM  33280 HG11 VAL H 204     113.704 151.606 234.806  1.00 64.29
H
ATOM  33281 HG12 VAL H 204     115.216 151.942 234.453  1.00 64.29
H
ATOM  33282 HG13 VAL H 204     114.680 150.453 234.315  1.00 64.29
H
ATOM  33283 HG21 VAL H 204     113.136 150.313 236.802  1.00 54.89
H
ATOM  33284 HG22 VAL H 204     114.187 149.133 236.635  1.00 54.89
H
ATOM  33285 HG23 VAL H 204     114.214 150.043 237.936  1.00 54.89
H
ATOM  33286  N   ASN H 205     117.912 151.155 234.608  1.00 62.52
N
ATOM  33287  CA  ASN H 205     118.870 152.015 233.936  1.00 61.41
C
ATOM  33288  C   ASN H 205     118.411 152.295 232.507  1.00 62.79
C
ATOM  33289  O   ASN H 205     118.108 151.369 231.737  1.00 48.95
O
ATOM  33290  CB  ASN H 205     120.264 151.382 233.937  1.00 67.42
C
ATOM  33291  CG  ASN H 205     121.360 152.359 233.504  1.00 83.73
C
ATOM  33292  OD1 ASN H 205     121.109 153.550 233.258  1.00 88.17
O
ATOM  33293  ND2 ASN H 205     122.586 151.858 233.425  1.00 88.86
N
ATOM  33294  H   ASN H 205     117.663 150.483 234.134  1.00 75.06
H
ATOM  33295  HA  ASN H 205     118.914 152.869 234.398  1.00 73.72
H
ATOM  33296  HB2 ASN H 205     120.471 151.077 234.834  1.00 80.93
H
ATOM  33297  HB3 ASN H 205     120.270 150.633 233.321  1.00 80.93
H
ATOM  33298 HD21 ASN H 205     123.242 152.361 233.187  1.00106.66
H
ATOM  33299 HD22 ASN H 205     122.724 151.031 233.612  1.00106.66
H
```

ATOM 33300 N   HIS H 206   118.390 153.577 232.152 1.00 54.13
N
ATOM 33301 CA HIS H 206   118.123 154.042 230.791 1.00 57.88
C
ATOM 33302 C   HIS H 206   119.296 154.925 230.386 1.00 59.98
C
ATOM 33303 O   HIS H 206   119.227 156.133 230.589 1.00 61.39
O
ATOM 33304 CB HIS H 206   116.809 154.792 230.734 1.00 57.77
C
ATOM 33305 CG HIS H 206   116.335 155.038 229.341 1.00 65.02
C
ATOM 33306 ND1 HIS H 206   115.896 156.269 228.905 1.00 49.03
N
ATOM 33307 CD2 HIS H 206   116.256 154.202 228.276 1.00 58.49
C
ATOM 33308 CE1 HIS H 206   115.528 156.168 227.642 1.00 54.91
C
ATOM 33309 NE2 HIS H 206   115.727 154.923 227.240 1.00 49.94
N
ATOM 33310 H   HIS H 206   118.533 154.222 232.702 1.00 64.99
H
ATOM 33311 HA HIS H 206   118.075 153.286 230.185 1.00 69.49
H
ATOM 33312 HB2 HIS H 206   116.130 154.274 231.194 1.00 69.36
H
ATOM 33313 HB3 HIS H 206   116.920 155.653 231.168 1.00 69.36
H
ATOM 33314 HD2 HIS H 206   116.513 153.309 228.253 1.00 70.22
H
ATOM 33315 HE1 HIS H 206   115.186 156.859 227.121 1.00 65.92
H
ATOM 33316 HE2 HIS H 206   115.552 154.617 226.457 1.00 59.95
H
ATOM 33317 N   LYS H 207   120.376 154.331 229.874 1.00 60.95
N
ATOM 33318 CA LYS H 207   121.537 155.124 229.450 1.00 68.23
C
ATOM 33319 C   LYS H 207   121.178 156.284 228.517 1.00 63.82
C
ATOM 33320 O   LYS H 207   121.623 157.414 228.778 1.00 68.93
O
ATOM 33321 CB LYS H 207   122.577 154.209 228.789 1.00 67.73
C
ATOM 33322 CG LYS H 207   123.129 153.108 229.715 1.00 71.94
C
ATOM 33323 CD LYS H 207   124.181 152.186 229.021 1.00 94.35
C
ATOM 33324 CE LYS H 207   123.631 151.334 227.839 1.00 95.10
C

ATOM 33325 NZ LYS H 207   122.812 150.120 228.241 1.00 92.69
N
ATOM 33326 H   LYS H 207   120.463 153.483 229.763 1.00 73.17
H
ATOM 33327 HA LYS H 207   121.928 155.501 230.253 1.00 81.90
H
ATOM 33328 HB2 LYS H 207   122.168 153.773 228.025 1.00 81.30
H
ATOM 33329 HB3 LYS H 207   123.327 154.751 228.498 1.00 81.30
H
ATOM 33330 HG2 LYS H 207   123.557 153.525 230.479 1.00 86.35
H
ATOM 33331 HG3 LYS H 207   122.393 152.549 230.011 1.00 86.35
H
ATOM 33332 HD2 LYS H 207   124.893 152.743 228.670 1.00113.25
H
ATOM 33333 HD3 LYS H 207   124.535 151.571 229.683 1.00113.25
H
ATOM 33334 HE2 LYS H 207   123.065 151.898 227.292 1.00114.15
H
ATOM 33335 HE3 LYS H 207   124.384 151.013 227.318 1.00114.15
H
ATOM 33336 HZ1 LYS H 207   122.492 149.716 227.516 1.00111.25
H
ATOM 33337 HZ2 LYS H 207   123.320 149.547 228.693 1.00111.25
H
ATOM 33338 HZ3 LYS H 207   122.130 150.371 228.755 1.00111.25
H
ATOM 33339 N   PRO H 208   120.356 156.104 227.476 1.00 60.76
N
ATOM 33340 CA PRO H 208   120.023 157.237 226.598 1.00 63.13
C
ATOM 33341 C   PRO H 208   119.521 158.491 227.313 1.00 71.86
C
ATOM 33342 O   PRO H 208   119.643 159.581 226.734 1.00 68.42
O
ATOM 33343 CB PRO H 208   118.929 156.662 225.679 1.00 59.08
C
ATOM 33344 CG PRO H 208   119.164 155.187 225.638 1.00 53.32
C
ATOM 33345 CD PRO H 208   119.763 154.834 226.995 1.00 56.93
C
ATOM 33346 HA PRO H 208   120.814 157.466 226.085 1.00 75.79
H
ATOM 33347 HB2 PRO H 208   118.055 156.859 226.046 1.00 70.93
H
ATOM 33348 HB3 PRO H 208   119.011 157.046 224.791 1.00 70.93
H
ATOM 33349 HG2 PRO H 208   118.322 154.723 225.502 1.00 64.01
H

ATOM 33350 HG3 PRO H 208 119.781 154.970 224.923 1.00 64.01
H
ATOM 33351 HD2 PRO H 208 119.073 154.527 227.604 1.00 68.35
H
ATOM 33352 HD3 PRO H 208 120.447 154.153 226.900 1.00 68.35
H
ATOM 33353 N SER H 209 118.928 158.385 228.510 1.00 61.23
N
ATOM 33354 CA SER H 209 118.447 159.561 229.241 1.00 56.78
C
ATOM 33355 C SER H 209 119.230 159.819 230.521 1.00 65.21
C
ATOM 33356 O SER H 209 118.833 160.693 231.292 1.00 64.38
O
ATOM 33357 CB SER H 209 116.954 159.455 229.576 1.00 62.85
C
ATOM 33358 OG SER H 209 116.671 158.518 230.609 1.00 60.34
O
ATOM 33359 H SER H 209 118.794 157.640 228.920 1.00 73.51
H
ATOM 33360 HA SER H 209 118.556 160.322 228.648 1.00 68.16
H
ATOM 33361 HB2 SER H 209 116.642 160.328 229.861 1.00 75.45
H
ATOM 33362 HB3 SER H 209 116.480 159.179 228.775 1.00 75.45
H
ATOM 33363 HG SER H 209 117.020 157.777 230.428 1.00 72.44
H
ATOM 33364 N ASN H 210 120.350 159.111 230.734 1.00 58.35
N
ATOM 33365 CA ASN H 210 121.091 159.102 232.007 1.00 70.77
C
ATOM 33366 C ASN H 210 120.185 158.859 233.229 1.00 64.65
C
ATOM 33367 O ASN H 210 120.454 159.357 234.319 1.00 68.40
O
ATOM 33368 CB ASN H 210 121.893 160.401 232.186 1.00 79.60
C
ATOM 33369 CG ASN H 210 122.891 160.637 231.060 1.00 90.91
C
ATOM 33370 OD1 ASN H 210 123.534 159.696 230.592 1.00 95.25
O
ATOM 33371 ND2 ASN H 210 123.011 161.899 230.606 1.00 89.85
N
ATOM 33372 H ASN H 210 120.714 158.610 230.137 1.00 70.05
H
ATOM 33373 HA ASN H 210 121.709 158.354 231.969 1.00 84.95
H
ATOM 33374 HB2 ASN H 210 121.280 161.151 232.205 1.00 95.55
H

```
ATOM  33375  HB3 ASN H 210     122.388 160.355 233.019  1.00 95.55
H
ATOM  33376 HD21 ASN H 210     123.565 162.076 229.970  1.00107.85
H
ATOM  33377 HD22 ASN H 210     122.537 162.527 230.950  1.00107.85
H
ATOM  33378  N   THR H 211     119.117 158.075 233.088  1.00 60.51
N
ATOM  33379  CA  THR H 211     118.221 157.804 234.213  1.00 60.44
C
ATOM  33380  C   THR H 211     118.688 156.550 234.958  1.00 67.27
C
ATOM  33381  O   THR H 211     119.034 155.530 234.348  1.00 60.74
O
ATOM  33382  CB  THR H 211     116.770 157.650 233.736  1.00 66.84
C
ATOM  33383  OG1 THR H 211     116.474 158.662 232.769  1.00 67.74
O
ATOM  33384  CG2 THR H 211     115.695 157.765 234.896  1.00 57.27
C
ATOM  33385  H   THR H 211     118.890 157.689 232.354  1.00 72.64
H
ATOM  33386  HA  THR H 211     118.249 158.549 234.834  1.00 72.55
H
ATOM  33387  HB  THR H 211     116.694 156.759 233.362  1.00 80.24
H
ATOM  33388  HG1 THR H 211     117.050 158.652 232.159  1.00 81.32
H
ATOM  33389 HG21 THR H 211     114.802 157.671 234.531  1.00 68.76
H
ATOM  33390 HG22 THR H 211     115.841 157.067 235.553  1.00 68.76
H
ATOM  33391 HG23 THR H 211     115.768 158.629 235.331  1.00 68.76
H
ATOM  33392  N   LYS H 212     118.741 156.649 236.282  1.00 65.02
N
ATOM  33393  CA  LYS H 212     119.014 155.515 237.155  1.00 63.95
C
ATOM  33394  C   LYS H 212     118.046 155.609 238.318  1.00 66.89
C
ATOM  33395  O   LYS H 212     118.071 156.591 239.064  1.00 63.40
O
ATOM  33396  CB  LYS H 212     120.453 155.499 237.670  1.00 61.01
C
ATOM  33397  CG  LYS H 212     121.366 154.522 236.952  1.00 80.87
C
ATOM  33398  CD  LYS H 212     122.756 154.521 237.560  1.00 84.82
C
ATOM  33399  CE  LYS H 212     123.724 153.666 236.762  1.00 90.68
C
```

ATOM 33400 NZ LYS H 212 124.895 153.293 237.607 1.00 94.48
N
ATOM 33401 H LYS H 212 118.620 157.385 236.709 1.00 78.05
H
ATOM 33402 HA LYS H 212 118.875 154.691 236.663 1.00 76.77
H
ATOM 33403 HB2 LYS H 212 120.830 156.386 237.562 1.00 73.23
H
ATOM 33404 HB3 LYS H 212 120.443 155.254 238.608 1.00 73.23
H
ATOM 33405 HG2 LYS H 212 121.001 153.627 237.022 1.00 97.07
H
ATOM 33406 HG3 LYS H 212 121.442 154.777 236.020 1.00 97.07
H
ATOM 33407 HD2 LYS H 212 123.097 155.430 237.579 1.00101.82
H
ATOM 33408 HD3 LYS H 212 122.710 154.166 238.461 1.00101.82
H
ATOM 33409 HE2 LYS H 212 123.279 152.854 236.470 1.00108.84
H
ATOM 33410 HE3 LYS H 212 124.042 154.164 235.992 1.00108.84
H
ATOM 33411 HZ1 LYS H 212 125.409 152.711 237.174 1.00113.41
H
ATOM 33412 HZ2 LYS H 212 125.374 154.017 237.799 1.00113.41
H
ATOM 33413 HZ3 LYS H 212 124.616 152.922 238.367 1.00113.41
H
ATOM 33414 N VAL H 213 117.187 154.610 238.448 1.00 59.41
N
ATOM 33415 CA VAL H 213 116.184 154.551 239.498 1.00 56.76
C
ATOM 33416 C VAL H 213 116.376 153.253 240.273 1.00 58.80
C
ATOM 33417 O VAL H 213 116.607 152.188 239.682 1.00 52.61
O
ATOM 33418 CB VAL H 213 114.756 154.629 238.917 1.00 57.85
C
ATOM 33419 CG1 VAL H 213 113.701 154.528 240.052 1.00 57.95
C
ATOM 33420 CG2 VAL H 213 114.586 155.888 238.161 1.00 60.78
C
ATOM 33421 H VAL H 213 117.166 153.930 237.921 1.00 71.32
H
ATOM 33422 HA VAL H 213 116.309 155.297 240.106 1.00 68.13
H
ATOM 33423 HB VAL H 213 114.618 153.884 238.312 1.00 69.44
H
ATOM 33424 HG11 VAL H 213 112.820 154.693 239.680 1.00 69.57
H

ATOM 33425 HG12 VAL H 213   113.735 153.639 240.437 1.00 69.57
H
ATOM 33426 HG13 VAL H 213   113.902 155.192 240.731 1.00 69.57
H
ATOM 33427 HG21 VAL H 213   113.669 155.947 237.846 1.00 72.96
H
ATOM 33428 HG22 VAL H 213   114.780 156.637 238.746 1.00 72.96
H
ATOM 33429 HG23 VAL H 213   115.194 155.890 237.406 1.00 72.96
H
ATOM 33430 N   ASP H 214   116.304 153.350 241.594 1.00 57.14
N
ATOM 33431 CA ASP H 214   116.149 152.199 242.481 1.00 56.69
C
ATOM 33432 C   ASP H 214   114.802 152.368 243.173 1.00 61.42
C
ATOM 33433 O   ASP H 214   114.591 153.359 243.876 1.00 56.11
O
ATOM 33434 CB ASP H 214   117.269 152.128 243.513 1.00 49.79
C
ATOM 33435 CG ASP H 214   118.664 152.019 242.890 1.00 72.25
C
ATOM 33436 OD1 ASP H 214   118.827 151.464 241.774 1.00 74.14
O
ATOM 33437 OD2 ASP H 214   119.622 152.472 243.553 1.00 77.35
O1-
ATOM 33438 H   ASP H 214   116.345 154.097 242.018 1.00 68.59
H
ATOM 33439 HA ASP H 214   116.171 151.372 241.974 1.00 68.05
H
ATOM 33440 HB2 ASP H 214   117.249 152.932 244.056 1.00 59.78
H
ATOM 33441 HB3 ASP H 214   117.132 151.348 244.072 1.00 59.78
H
ATOM 33442 N   LYS H 215   113.874 151.440 242.938 1.00 51.25
N
ATOM 33443 CA LYS H 215   112.539 151.512 243.510 1.00 52.65
C
ATOM 33444 C   LYS H 215   112.295 150.294 244.401 1.00 55.15
C
ATOM 33445 O   LYS H 215   112.300 149.144 243.926 1.00 47.73
O
ATOM 33446 CB LYS H 215   111.457 151.610 242.425 1.00 59.36
C
ATOM 33447 CG LYS H 215   110.111 152.236 242.951 1.00 64.61
C
ATOM 33448 CD LYS H 215   110.176 153.801 242.886 1.00 67.46
C
ATOM 33449 CE LYS H 215   108.819 154.497 243.038 1.00 65.20
C

ATOM 33450 NZ LYS H 215 108.145 153.969 244.251 1.00 72.12
N
ATOM 33451 H LYS H 215 114.001 150.748 242.443 1.00 61.53
H
ATOM 33452 HA LYS H 215 112.486 152.308 244.063 1.00 63.21
H
ATOM 33453 HB2 LYS H 215 111.786 152.167 241.703 1.00 71.26
H
ATOM 33454 HB3 LYS H 215 111.264 150.719 242.093 1.00 71.26
H
ATOM 33455 HG2 LYS H 215 109.373 151.933 242.398 1.00 77.56
H
ATOM 33456 HG3 LYS H 215 109.965 151.970 243.872 1.00 77.56
H
ATOM 33457 HD2 LYS H 215 110.750 154.117 243.601 1.00 80.98
H
ATOM 33458 HD3 LYS H 215 110.543 154.060 242.027 1.00 80.98
H
ATOM 33459 HE2 LYS H 215 108.947 155.453 243.138 1.00 78.27
H
ATOM 33460 HE3 LYS H 215 108.265 154.314 242.264 1.00 78.27
H
ATOM 33461 HZ1 LYS H 215 107.353 154.363 244.353 1.00 86.58
H
ATOM 33462 HZ2 LYS H 215 108.024 153.091 244.175 1.00 86.58
H
ATOM 33463 HZ3 LYS H 215 108.642 154.132 244.971 1.00 86.58
H
ATOM 33464 N ARG H 216 112.103 150.547 245.691 1.00 55.04
N
ATOM 33465 CA ARG H 216 111.686 149.489 246.604 1.00 47.34
C
ATOM 33466 C ARG H 216 110.219 149.150 246.359 1.00 49.80
C
ATOM 33467 O ARG H 216 109.378 150.020 246.092 1.00 46.96
O
ATOM 33468 CB ARG H 216 111.920 149.900 248.054 1.00 54.14
C
ATOM 33469 CG ARG H 216 111.672 148.719 248.991 1.00 61.00
C
ATOM 33470 CD ARG H 216 112.005 148.936 250.458 1.00 58.37
C
ATOM 33471 NE ARG H 216 111.467 147.794 251.205 1.00 61.35
N
ATOM 33472 CZ ARG H 216 111.410 147.687 252.525 1.00 56.46
C
ATOM 33473 NH1 ARG H 216 110.866 146.598 253.065 1.00 56.11
N
ATOM 33474 NH2 ARG H 216 111.912 148.641 253.297 1.00 63.79
N

ATOM 33475 H   ARG H 216   112.207 151.316 246.061 1.00 66.07
H
ATOM 33476 HA ARG H 216   112.215 148.692 246.451 1.00 56.84
H
ATOM 33477 HB2 ARG H 216   112.837 150.195 248.163 1.00 64.99
H
ATOM 33478 HB3 ARG H 216   111.311 150.616 248.290 1.00 64.99
H
ATOM 33479 HG2 ARG H 216   110.731 148.489 248.945 1.00 73.23
H
ATOM 33480 HG3 ARG H 216   112.212 147.972 248.685 1.00 73.23
H
ATOM 33481 HD2 ARG H 216   112.967 148.977 250.583 1.00 70.07
H
ATOM 33482 HD3 ARG H 216   111.595 149.754 250.779 1.00 70.07
H
ATOM 33483 HE ARG H 216   111.162 147.134 250.744 1.00 73.64
H
ATOM 33484 HH11 ARG H 216   110.557 145.977 252.557 1.00 67.36
H
ATOM 33485 HH12 ARG H 216   110.823 146.518 253.919 1.00 67.36
H
ATOM 33486 HH21 ARG H 216   112.280 149.332 252.940 1.00 76.58
H
ATOM 33487 HH22 ARG H 216   111.874 148.568 254.152 1.00 76.58
H
ATOM 33488 N   VAL H 217   109.937 147.859 246.341 1.00 49.07
N
ATOM 33489 CA VAL H 217   108.610 147.340 246.039 1.00 50.34
C
ATOM 33490 C   VAL H 217   108.095 146.642 247.281 1.00 47.15
C
ATOM 33491 O   VAL H 217   108.691 145.655 247.728 1.00 49.77
O
ATOM 33492 CB VAL H 217   108.640 146.393 244.823 1.00 47.42
C
ATOM 33493 CG1 VAL H 217   107.255 145.869 244.566 1.00 52.55
C
ATOM 33494 CG2 VAL H 217   109.192 147.122 243.600 1.00 52.49
C
ATOM 33495 H   VAL H 217   110.514 147.243 246.507 1.00 58.92
H
ATOM 33496 HA VAL H 217   108.006 148.071 245.835 1.00 60.43
H
ATOM 33497 HB VAL H 217   109.225 145.639 245.003 1.00 56.94
H
ATOM 33498 HG11 VAL H 217   107.238 145.435 243.699 1.00 63.09
H
ATOM 33499 HG12 VAL H 217   107.025 145.231 245.260 1.00 63.09
H

ATOM 33500 HG13 VAL H 217   106.629 146.611 244.578 1.00 63.09
H
ATOM 33501 HG21 VAL H 217   109.070 146.562 242.817 1.00 63.02
H
ATOM 33502 HG22 VAL H 217   108.712 147.956 243.487 1.00 63.02
H
ATOM 33503 HG23 VAL H 217   110.136 147.297 243.736 1.00 63.02
H
ATOM 33504 N   GLU H 218   107.004 147.156 247.844 1.00 48.80
N
ATOM 33505 CA GLU H 218   106.419 146.678 249.087 1.00 51.58
C
ATOM 33506 C   GLU H 218   104.940 146.408 248.917 1.00 57.87
C
ATOM 33507 O   GLU H 218   104.301 146.983 248.038 1.00 52.51
O
ATOM 33508 CB GLU H 218   106.565 147.688 250.233 1.00 57.03
C
ATOM 33509 CG GLU H 218   107.977 148.020 250.601 1.00 69.57
C
ATOM 33510 CD GLU H 218   108.060 148.697 251.961 1.00 79.31
C
ATOM 33511 OE1 GLU H 218   107.541 148.125 252.961 1.00 80.01
O
ATOM 33512 OE2 GLU H 218   108.638 149.800 252.019 1.00 67.54
O1-
ATOM 33513 H   GLU H 218   106.566 147.813 247.505 1.00 58.58
H
ATOM 33514 HA GLU H 218   106.881 145.857 249.319 1.00 61.92
H
ATOM 33515 HB2 GLU H 218   106.130 148.515 249.971 1.00 68.46
H
ATOM 33516 HB3 GLU H 218   106.136 147.322 251.022 1.00 68.46
H
ATOM 33517 HG2 GLU H 218   108.499 147.203 250.635 1.00 83.51
H
ATOM 33518 HG3 GLU H 218   108.347 148.624 249.938 1.00 83.51
H
ATOM 33519 N   PRO H 219   104.354 145.556 249.764 1.00 58.61
N
ATOM 33520 CA PRO H 219   102.889 145.445 249.777 1.00 63.20
C
ATOM 33521 C   PRO H 219   102.307 146.776 250.215 1.00 65.28
C
ATOM 33522 O   PRO H 219   102.963 147.552 250.905 1.00 69.85
O
ATOM 33523 CB PRO H 219   102.605 144.328 250.792 1.00 58.82
C
ATOM 33524 CG PRO H 219   103.980 143.752 251.192 1.00 63.59
C

ATOM 33525 CD PRO H 219   105.005 144.780 250.841 1.00 57.23
C
ATOM 33526 HA PRO H 219   102.530 145.170 248.918 1.00 75.87
H
ATOM 33527 HB2 PRO H 219   102.150 144.698 251.564 1.00 70.61
H
ATOM 33528 HB3 PRO H 219   102.054 143.645 250.378 1.00 70.61
H
ATOM 33529 HG2 PRO H 219   103.990 143.575 252.147 1.00 76.33
H
ATOM 33530 HG3 PRO H 219   104.140 142.930 250.701 1.00 76.33
H
ATOM 33531 HD2 PRO H 219   105.202 145.347 251.603 1.00 68.70
H
ATOM 33532 HD3 PRO H 219   105.819 144.362 250.520 1.00 68.70
H
ATOM 33533 N   LYS H 220   101.089 147.070 249.765 1.00 85.37
N
ATOM 33534 CA LYS H 220   100.442 148.322 250.146 1.00104.72
C
ATOM 33535 C   LYS H 220   99.708 148.166 251.483 1.00109.79
C
ATOM 33536 O   LYS H 220   99.191 147.094 251.809 1.00101.49
O
ATOM 33537 CB LYS H 220   99.475 148.789 249.053 1.00103.62
C
ATOM 33538 CG LYS H 220   99.123 150.278 249.121 1.00109.98
C
ATOM 33539 CD LYS H 220   97.663 150.539 248.786 1.00110.03
C
ATOM 33540 CE LYS H 220   97.363 150.289 247.315 1.00110.75
C
ATOM 33541 NZ LYS H 220   97.935 151.337 246.406 1.00102.24
N
ATOM 33542 H   LYS H 220   100.623 146.568 249.245 1.00102.47
H
ATOM 33543 HA LYS H 220   101.116 149.011 250.259 1.00125.70
H
ATOM 33544 HB2 LYS H 220   99.881 148.622 248.188 1.00124.37
H
ATOM 33545 HB3 LYS H 220   98.649 148.287 249.135 1.00124.37
H
ATOM 33546 HG2 LYS H 220   99.288 150.604 250.020 1.00132.00
H
ATOM 33547 HG3 LYS H 220   99.671 150.766 248.487 1.00132.00
H
ATOM 33548 HD2 LYS H 220   97.103 149.949 249.313 1.00132.06
H
ATOM 33549 HD3 LYS H 220   97.451 151.465 248.984 1.00132.06
H

ATOM 33550 HE2 LYS H 220   97.741 149.432 247.058 1.00132.93
H
ATOM 33551 HE3 LYS H 220   96.402 150.277 247.189 1.00132.93
H
ATOM 33552 HZ1 LYS H 220   97.724 151.156 245.561 1.00122.71
H
ATOM 33553 HZ2 LYS H 220   97.610 152.137 246.620 1.00122.71
H
ATOM 33554 HZ3 LYS H 220   98.823 151.357 246.485 1.00122.71
H
ATOM 33555 N   SER H 221   99.680 149.266 252.256 1.00109.73
N
ATOM 33556 CA SER H 221   99.052 149.293 253.580 1.00105.19
C
ATOM 33557 C   SER H 221   97.556 149.026 253.498 1.00104.03
C
ATOM 33558 O   SER H 221   96.811 149.823 252.920 1.00111.75
O
ATOM 33559 CB SER H 221   99.279 150.657 254.222 1.00111.13
C
ATOM 33560 OG SER H 221   98.364 151.598 253.674 1.00117.06
O
ATOM 33561 H   SER H 221   100.025 150.021 252.029 1.00131.70
H
ATOM 33562 HA SER H 221   99.458 148.598 254.121 1.00126.25
H
ATOM 33563 HB2 SER H 221   99.135 150.588 255.178 1.00133.38
H
ATOM 33564 HB3 SER H 221   100.186 150.948 254.043 1.00133.38
H
ATOM 33565 HG SER H 221   98.379 151.560 252.834 1.00140.50
H
ATOM 33566 N   CYS H 222   97.108 147.935 254.120 1.00105.85
N
ATOM 33567 CA CYS H 222   95.694 147.538 254.077 1.00106.34
C
ATOM 33568 C   CYS H 222   94.832 148.593 254.766 1.00 92.76
C
ATOM 33569 O   CYS H 222   93.659 148.754 254.440 1.00 96.59
O
ATOM 33570 CB CYS H 222   95.488 146.150 254.734 1.00 95.00
C
ATOM 33571 H   CYS H 222   97.604 147.402 254.579 1.00127.05
H
ATOM 33572 HA CYS H 222   95.412 147.462 253.151 1.00127.64
H
ATOM 33573 HB2 CYS H 222   94.566 145.875 254.611 1.00114.02
H
ATOM 33574 HB3 CYS H 222   96.083 145.509 254.313 1.00114.02
H

ATOM 33575 N ASP L 1 111.917 138.893 191.671 1.00416.90
N
ATOM 33576 CA ASP L 1 113.365 138.825 192.004 1.00324.05
C
ATOM 33577 CB ASP L 1 113.775 140.004 192.889 1.00249.34
C
ATOM 33578 CG ASP L 1 115.189 140.480 192.632 1.00278.94
C
ATOM 33579 OD1 ASP L 1 115.926 140.655 193.624 1.00339.29
O
ATOM 33580 OD2 ASP L 1 115.530 140.705 191.443 1.00256.99
O
ATOM 33581 C ASP L 1 113.657 137.484 192.670 1.00319.83
C
ATOM 33582 O ASP L 1 113.573 136.435 192.028 1.00424.63
O
ATOM 33583 N ILE L 2 113.983 137.525 193.968 1.00198.35
N
ATOM 33584 CA ILE L 2 114.353 136.276 194.605 1.00169.59
C
ATOM 33585 CB ILE L 2 115.844 135.933 194.365 1.00144.80
C
ATOM 33586 CG1 ILE L 2 116.212 134.505 194.787 1.00151.49
C
ATOM 33587 CG2 ILE L 2 116.768 137.012 194.913 1.00116.33
C
ATOM 33588 CD1 ILE L 2 115.476 133.410 194.027 1.00144.77
C
ATOM 33589 C ILE L 2 113.781 136.162 196.024 1.00138.55
C
ATOM 33590 O ILE L 2 114.064 136.916 196.948 1.00111.27
O
ATOM 33591 N GLN L 3 112.893 135.194 196.158 1.00138.34
N
ATOM 33592 CA GLN L 3 112.117 135.034 197.361 1.00161.41
C
ATOM 33593 CB GLN L 3 110.700 134.713 196.874 1.00217.07
C
ATOM 33594 CG GLN L 3 109.677 134.438 197.964 1.00276.12
C
ATOM 33595 CD GLN L 3 109.250 135.704 198.659 1.00305.73
C
ATOM 33596 OE1 GLN L 3 109.255 136.781 198.061 1.00259.48
O
ATOM 33597 NE2 GLN L 3 108.855 135.573 199.917 1.00376.81
N
ATOM 33598 C GLN L 3 112.801 133.921 198.146 1.00161.41
C
ATOM 33599 O GLN L 3 113.485 133.095 197.552 1.00170.58
O

ATOM 33600 N MET L 4 112.628 133.929 199.477 1.00210.32
N
ATOM 33601 CA MET L 4 113.056 132.857 200.375 1.00256.47
C
ATOM 33602 CB MET L 4 114.286 133.267 201.196 1.00255.73
C
ATOM 33603 CG MET L 4 115.349 133.993 200.420 1.00200.38
C
ATOM 33604 SD MET L 4 116.277 132.812 199.460 1.00224.34
S
ATOM 33605 CE MET L 4 117.715 132.690 200.512 1.00175.21
C
ATOM 33606 C MET L 4 111.937 132.499 201.364 1.00264.65
C
ATOM 33607 O MET L 4 111.671 133.233 202.316 1.00266.12
O
ATOM 33608 N THR L 5 111.286 131.346 201.176 1.00237.86
N
ATOM 33609 CA THR L 5 110.364 130.867 202.191 1.00174.91
C
ATOM 33610 CB THR L 5 109.080 130.283 201.596 1.00212.77
C
ATOM 33611 OG1 THR L 5 108.500 131.210 200.677 1.00282.47
O
ATOM 33612 CG2 THR L 5 108.098 129.894 202.682 1.00159.22
C
ATOM 33613 C THR L 5 110.949 129.633 202.852 1.00169.82
C
ATOM 33614 O THR L 5 111.521 128.775 202.186 1.00222.71
O
ATOM 33615 N GLN L 6 110.741 129.515 204.158 1.00186.49
N
ATOM 33616 CA GLN L 6 111.136 128.294 204.840 1.00214.18
C
ATOM 33617 CB GLN L 6 112.105 128.592 205.984 1.00219.14
C
ATOM 33618 CG GLN L 6 113.077 129.713 205.653 1.00223.72
C
ATOM 33619 CD GLN L 6 114.042 130.028 206.768 1.00255.41
C
ATOM 33620 OE1 GLN L 6 114.385 131.188 206.994 1.00233.65
O
ATOM 33621 NE2 GLN L 6 114.496 128.994 207.466 1.00274.76
N
ATOM 33622 C GLN L 6 109.897 127.490 205.242 1.00231.88
C
ATOM 33623 O GLN L 6 108.795 128.029 205.385 1.00213.78
O
ATOM 33624 N SER L 7 110.124 126.188 205.436 1.00237.39
N

```
ATOM  33625  CA  SER L   7      109.113 125.145 205.399  1.00252.29
C
ATOM  33626  CB  SER L   7      109.117 124.426 204.024  1.00251.80
C
ATOM  33627  OG  SER L   7      110.074 124.733 202.979  1.00268.47
O
ATOM  33628  C   SER L   7      109.630 124.061 206.327  1.00288.56
C
ATOM  33629  O   SER L   7      110.556 123.349 205.950  1.00346.43
O
ATOM  33630  N   PRO L   8      109.090 123.864 207.543  1.00306.38
N
ATOM  33631  CA  PRO L   8      107.961 124.645 208.029  1.00300.85
C
ATOM  33632  CB  PRO L   8      107.455 123.799 209.203  1.00321.39
C
ATOM  33633  CG  PRO L   8      108.697 123.114 209.733  1.00307.31
C
ATOM  33634  CD  PRO L   8      109.573 122.877 208.521  1.00320.52
C
ATOM  33635  C   PRO L   8      108.453 125.983 208.557  1.00291.81
C
ATOM  33636  O   PRO L   8      109.620 126.123 208.921  1.00277.80
O
ATOM  33637  N   SER L   9      107.534 126.947 208.579  1.00281.19
N
ATOM  33638  CA  SER L   9      107.769 128.267 209.136  1.00303.50
C
ATOM  33639  CB  SER L   9      106.573 129.111 208.854  1.00290.80
C
ATOM  33640  OG  SER L   9      105.432 128.274 208.729  1.00271.99
O
ATOM  33641  C   SER L   9      108.028 128.164 210.637  1.00319.61
C
ATOM  33642  O   SER L   9      108.892 128.856 211.172  1.00306.31
O
ATOM  33643  N   THR L  10      107.251 127.303 211.301  1.00343.36
N
ATOM  33644  CA  THR L  10      107.434 126.991 212.709  1.00343.42
C
ATOM  33645  CB  THR L  10      106.276 127.551 213.554  1.00348.24
C
ATOM  33646  OG1 THR L  10      106.109 128.951 213.320  1.00322.00
O
ATOM  33647  CG2 THR L  10      106.454 127.328 215.041  1.00326.14
C
ATOM  33648  C   THR L  10      107.647 125.481 212.828  1.00333.55
C
ATOM  33649  O   THR L  10      106.904 124.700 212.230  1.00282.81
O
```

ATOM 33650 N LEU L 11 108.687 125.084 213.575 1.00348.74
N
ATOM 33651 CA LEU L 11 108.959 123.673 213.804 1.00369.44
C
ATOM 33652 CB LEU L 11 110.039 123.183 212.837 1.00325.97
C
ATOM 33653 CG LEU L 11 110.255 121.672 212.855 1.00311.50
C
ATOM 33654 CD1 LEU L 11 108.926 120.939 212.678 1.00227.91
C
ATOM 33655 CD2 LEU L 11 111.252 121.255 211.787 1.00339.78
C
ATOM 33656 C LEU L 11 109.369 123.407 215.253 1.00388.12
C
ATOM 33657 O LEU L 11 110.433 123.836 215.697 1.00336.87
O
ATOM 33658 N SER L 12 108.516 122.661 215.965 1.00396.99
N
ATOM 33659 CA SER L 12 108.805 122.184 217.309 1.00340.59
C
ATOM 33660 CB SER L 12 107.580 122.274 218.189 1.00340.39
C
ATOM 33661 OG SER L 12 107.200 123.623 218.417 1.00317.84
O
ATOM 33662 C SER L 12 109.300 120.745 217.223 1.00303.44
C
ATOM 33663 O SER L 12 108.878 120.002 216.338 1.00266.56
O
ATOM 33664 N ALA L 13 110.212 120.384 218.133 1.00288.70
N
ATOM 33665 CA ALA L 13 110.695 119.020 218.319 1.00306.15
C
ATOM 33666 CB ALA L 13 111.679 118.656 217.225 1.00227.42
C
ATOM 33667 C ALA L 13 111.323 118.900 219.711 1.00341.59
C
ATOM 33668 O ALA L 13 111.618 119.921 220.325 1.00435.22
O
ATOM 33669 N SER L 14 111.524 117.663 220.205 1.00337.28
N
ATOM 33670 CA SER L 14 112.102 117.412 221.524 1.00346.07
C
ATOM 33671 CB SER L 14 111.515 116.168 222.163 1.00305.52
C
ATOM 33672 OG SER L 14 110.155 116.360 222.533 1.00320.88
O
ATOM 33673 C SER L 14 113.633 117.342 221.456 1.00381.87
C
ATOM 33674 O SER L 14 114.189 117.191 220.371 1.00457.87
O

ATOM 33675 N   VAL L 15   114.302 117.443 222.624 1.00407.22
N
ATOM 33676 CA VAL L 15   115.758 117.495 222.762 1.00406.37
C
ATOM 33677 CB VAL L 15   116.195 117.926 224.184 1.00413.70
C
ATOM 33678 CG1 VAL L 15   117.684 117.711 224.449 1.00368.39
C
ATOM 33679 CG2 VAL L 15   115.786 119.352 224.529 1.00412.35
C
ATOM 33680 C   VAL L 15   116.371 116.142 222.387 1.00378.65
C
ATOM 33681 O   VAL L 15   116.109 115.134 223.038 1.00367.78
O
ATOM 33682 N   GLY L 16   117.220 116.137 221.351 1.00377.17
N
ATOM 33683 CA GLY L 16   117.907 114.934 220.900 1.00356.23
C
ATOM 33684 C   GLY L 16   117.432 114.438 219.531 1.00390.67
C
ATOM 33685 O   GLY L 16   118.010 113.497 218.988 1.00343.99
O
ATOM 33686 N   ASP L 17   116.401 115.095 218.971 1.00433.68
N
ATOM 33687 CA ASP L 17   115.720 114.666 217.753 1.00391.56
C
ATOM 33688 CB ASP L 17   114.263 115.138 217.701 1.00371.00
C
ATOM 33689 CG ASP L 17   113.335 114.526 218.734 1.00352.92
C
ATOM 33690 OD1 ASP L 17   113.695 113.485 219.318 1.00433.45
O
ATOM 33691 OD2 ASP L 17   112.245 115.108 218.929 1.00244.23
O
ATOM 33692 C   ASP L 17   116.409 115.222 216.506 1.00361.67
C
ATOM 33693 O   ASP L 17   117.019 116.288 216.539 1.00346.48
O
ATOM 33694 N   ARG L 18   116.250 114.496 215.395 1.00343.22
N
ATOM 33695 CA ARG L 18   116.762 114.862 214.082 1.00343.06
C
ATOM 33696 CB ARG L 18   117.077 113.560 213.327 1.00307.54
C
ATOM 33697 CG ARG L 18   117.886 113.701 212.046 1.00296.80
C
ATOM 33698 CD ARG L 18   117.651 112.550 211.081 1.00270.23
C
ATOM 33699 NE ARG L 18   117.789 112.985 209.691 1.00321.56
N

ATOM 33700 CZ ARG L 18   116.851 113.612 208.973 1.00342.78
C
ATOM 33701 NH1 ARG L 18   115.674 113.894 209.507 1.00353.29
N
ATOM 33702 NH2 ARG L 18   117.101 113.956 207.722 1.00362.42
N
ATOM 33703 C   ARG L 18   115.754 115.765 213.355 1.00350.09
C
ATOM 33704 O   ARG L 18   114.726 115.294 212.855 1.00272.75
O
ATOM 33705 N   VAL L 19   116.081 117.063 213.254 1.00362.36
N
ATOM 33706 CA VAL L 19   115.166 118.063 212.721 1.00310.61
C
ATOM 33707 CB VAL L 19   115.148 119.332 213.596 1.00349.50
C
ATOM 33708 CG1 VAL L 19   114.317 120.440 212.965 1.00355.03
C
ATOM 33709 CG2 VAL L 19   114.641 119.027 214.998 1.00318.95
C
ATOM 33710 C   VAL L 19   115.502 118.368 211.259 1.00271.21
C
ATOM 33711 O   VAL L 19   116.648 118.227 210.825 1.00236.40
O
ATOM 33712 N   THR L 20   114.479 118.786 210.502 1.00236.38
N
ATOM 33713 CA THR L 20   114.653 119.081 209.087 1.00265.64
C
ATOM 33714 CB THR L 20   114.163 117.918 208.206 1.00274.01
C
ATOM 33715 OG1 THR L 20   114.819 116.725 208.631 1.00283.36
O
ATOM 33716 CG2 THR L 20   114.425 118.107 206.727 1.00237.73
C
ATOM 33717 C   THR L 20   114.025 120.431 208.736 1.00241.46
C
ATOM 33718 O   THR L 20   112.813 120.610 208.824 1.00219.80
O
ATOM 33719 N   ILE L 21   114.868 121.378 208.312 1.00223.17
N
ATOM 33720 CA ILE L 21   114.391 122.634 207.757 1.00228.13
C
ATOM 33721 CB ILE L 21   114.956 123.854 208.534 1.00274.30
C
ATOM 33722 CG1 ILE L 21   114.909 123.695 210.057 1.00287.73
C
ATOM 33723 CG2 ILE L 21   114.286 125.154 208.111 1.00286.11
C
ATOM 33724 CD1 ILE L 21   115.400 124.929 210.797 1.00269.65
C

```
ATOM  33725  C   ILE L  21     114.738 122.655 206.260  1.00211.60
C
ATOM  33726  O   ILE L  21     115.832 122.249 205.859  1.00157.48
O
ATOM  33727  N   THR L  22     113.790 123.144 205.442  1.00218.23
N
ATOM  33728  CA  THR L  22     113.894 123.226 203.989  1.00194.30
C
ATOM  33729  CB  THR L  22     112.939 122.201 203.351  1.00222.79
C
ATOM  33730  OG1 THR L  22     112.633 121.120 204.240  1.00209.22
O
ATOM  33731  CG2 THR L  22     113.421 121.675 202.016  1.00238.47
C
ATOM  33732  C   THR L  22     113.546 124.641 203.492  1.00202.49
C
ATOM  33733  O   THR L  22     112.476 125.164 203.797  1.00218.06
O
ATOM  33734  N   CYS L  23     114.442 125.266 202.708  1.00192.83
N
ATOM  33735  CA  CYS L  23     114.192 126.578 202.117  1.00192.96
C
ATOM  33736  CB  CYS L  23     115.475 127.397 202.061  1.00203.15
C
ATOM  33737  SG  CYS L  23     115.201 129.167 201.793  1.00236.25
S
ATOM  33738  C   CYS L  23     113.631 126.428 200.700  1.00207.63
C
ATOM  33739  O   CYS L  23     113.729 125.362 200.101  1.00219.09
O
ATOM  33740  N   ARG L  24     113.046 127.502 200.151  1.00231.26
N
ATOM  33741  CA  ARG L  24     112.388 127.428 198.849  1.00228.94
C
ATOM  33742  CB  ARG L  24     110.910 127.024 198.972  1.00236.46
C
ATOM  33743  CG  ARG L  24     110.213 126.667 197.657  1.00251.35
C
ATOM  33744  CD  ARG L  24     110.156 125.175 197.339  1.00234.31
C
ATOM  33745  NE  ARG L  24     110.714 124.627 198.586  1.00280.17
N
ATOM  33746  CZ  ARG L  24     111.190 123.399 198.747  1.00332.30
C
ATOM  33747  NH1 ARG L  24     111.154 122.559 197.715  1.00369.84
N
ATOM  33748  NH2 ARG L  24     111.676 122.987 199.935  1.00374.39
N
ATOM  33749  C   ARG L  24     112.481 128.762 198.111  1.00201.14
C
```

ATOM 33750 O ARG L 24 111.517 129.546 198.083 1.00139.31
O
ATOM 33751 N ALA L 25 113.624 128.962 197.447 1.00162.99
N
ATOM 33752 CA ALA L 25 113.796 130.149 196.630 1.00173.70
C
ATOM 33753 CB ALA L 25 115.249 130.519 196.592 1.00170.59
C
ATOM 33754 C ALA L 25 113.196 129.974 195.233 1.00182.81
C
ATOM 33755 O ALA L 25 113.120 128.856 194.717 1.00189.18
O
ATOM 33756 N SER L 26 112.847 131.118 194.622 1.00182.11
N
ATOM 33757 CA SER L 26 111.937 131.229 193.488 1.00219.29
C
ATOM 33758 CB SER L 26 111.294 132.588 193.481 1.00187.24
C
ATOM 33759 OG SER L 26 112.258 133.569 193.151 1.00188.89
O
ATOM 33760 C SER L 26 112.585 130.893 192.135 1.00251.49
C
ATOM 33761 O SER L 26 111.900 130.416 191.229 1.00272.02
O
ATOM 33762 N GLN L 27 113.887 131.161 191.965 1.00231.94
N
ATOM 33763 CA GLN L 27 114.568 130.762 190.735 1.00205.14
C
ATOM 33764 CB GLN L 27 114.881 131.957 189.829 1.00184.76
C
ATOM 33765 CG GLN L 27 115.052 133.289 190.552 1.00170.24
C
ATOM 33766 CD GLN L 27 115.987 134.220 189.808 1.00205.81
C
ATOM 33767 OE1 GLN L 27 116.093 134.185 188.574 1.00187.16
O
ATOM 33768 NE2 GLN L 27 116.676 135.070 190.558 1.00189.08
N
ATOM 33769 C GLN L 27 115.720 129.797 191.042 1.00201.11
C
ATOM 33770 O GLN L 27 115.663 129.103 192.055 1.00217.33
O
ATOM 33771 N SER L 28 116.743 129.737 190.163 1.00169.01
N
ATOM 33772 CA SER L 28 117.836 128.761 190.250 1.00187.92
C
ATOM 33773 CB SER L 28 118.163 128.228 188.810 1.00171.51
C
ATOM 33774 OG SER L 28 118.568 126.834 188.717 1.00128.25
O

ATOM 33775 C SER L 28 119.084 129.364 190.938 1.00151.82
C
ATOM 33776 O SER L 28 119.854 129.996 190.210 1.00133.44
O
ATOM 33777 N ILE L 29 119.349 129.122 192.265 1.00114.05
N
ATOM 33778 CA ILE L 29 120.536 129.647 192.993 1.00166.49
C
ATOM 33779 CB ILE L 29 120.220 130.385 194.333 1.00164.21
C
ATOM 33780 CG1 ILE L 29 120.139 129.477 195.575 1.00158.40
C
ATOM 33781 CG2 ILE L 29 119.024 131.321 194.209 1.00185.44
C
ATOM 33782 CD1 ILE L 29 119.750 130.175 196.886 1.00 88.16
C
ATOM 33783 C ILE L 29 121.759 128.704 193.165 1.00196.21
C
ATOM 33784 O ILE L 29 122.356 128.681 194.258 1.00168.17
O
ATOM 33785 N SER L 30 122.246 128.065 192.070 1.00196.85
N
ATOM 33786 CA SER L 30 123.176 126.929 192.085 1.00136.60
C
ATOM 33787 CB SER L 30 124.575 127.215 191.565 1.00109.29
C
ATOM 33788 OG SER L 30 124.596 128.018 190.401 1.00156.24
O
ATOM 33789 C SER L 30 123.360 126.462 193.513 1.00147.09
C
ATOM 33790 O SER L 30 122.430 126.046 194.205 1.00167.04
O
ATOM 33791 N ASN L 31 124.591 126.667 193.953 1.00146.49
N
ATOM 33792 CA ASN L 31 125.145 125.975 195.094 1.00161.71
C
ATOM 33793 CB ASN L 31 126.373 125.192 194.621 1.00173.45
C
ATOM 33794 CG ASN L 31 126.965 124.233 195.629 1.00149.22
C
ATOM 33795 OD1 ASN L 31 126.279 123.752 196.554 1.00118.73
O
ATOM 33796 ND2 ASN L 31 128.252 123.972 195.430 1.00113.83
N
ATOM 33797 C ASN L 31 125.418 127.017 196.172 1.00141.95
C
ATOM 33798 O ASN L 31 126.069 126.734 197.180 1.00134.80
O
ATOM 33799 N TRP L 32 124.926 128.237 195.925 1.00128.08
N

ATOM 33800 CA TRP L 32   125.193 129.331 196.844 1.00128.88
C
ATOM 33801 CB TRP L 32   125.486 130.661 196.117 1.00122.95
C
ATOM 33802 CG TRP L 32   126.512 130.425 195.055 1.00127.94
C
ATOM 33803 CD1 TRP L 32   126.280 130.141 193.741 1.00148.22
C
ATOM 33804 NE1 TRP L 32   127.451 129.869 193.080 1.00106.72
N
ATOM 33805 CE2 TRP L 32   128.468 129.925 193.981 1.00101.42
C
ATOM 33806 CD2 TRP L 32   127.925 130.244 195.234 1.00114.80
C
ATOM 33807 CE3 TRP L 32   128.794 130.325 196.325 1.00150.96
C
ATOM 33808 CZ3 TRP L 32   130.149 130.127 196.154 1.00128.64
C
ATOM 33809 CH2 TRP L 32   130.657 129.820 194.901 1.00142.64
C
ATOM 33810 CZ2 TRP L 32   129.828 129.723 193.804 1.00142.42
C
ATOM 33811 C   TRP L 32   123.987 129.386 197.749 1.00122.48
C
ATOM 33812 O   TRP L 32   122.890 129.162 197.212 1.00103.03
O
ATOM 33813 N   LEU L 33   124.263 129.562 199.068 1.00113.34
N
ATOM 33814 CA LEU L 33   123.329 129.582 200.196 1.00120.69
C
ATOM 33815 CB LEU L 33   122.609 128.235 200.302 1.00 96.68
C
ATOM 33816 CG LEU L 33   121.195 128.294 200.890 1.00107.02
C
ATOM 33817 CD1 LEU L 33   120.133 128.331 199.775 1.00 85.41
C
ATOM 33818 CD2 LEU L 33   120.948 127.136 201.856 1.00122.23
C
ATOM 33819 C   LEU L 33   124.081 129.741 201.509 1.00111.09
C
ATOM 33820 O   LEU L 33   125.126 129.134 201.652 1.00138.34
O
ATOM 33821 N   ALA L 34   123.486 130.389 202.515 1.00117.68
N
ATOM 33822 CA ALA L 34   124.012 130.217 203.865 1.00120.31
C
ATOM 33823 CB ALA L 34   124.744 131.468 204.299 1.00 97.55
C
ATOM 33824 C   ALA L 34   122.924 129.772 204.858 1.00156.51
C

ATOM 33825 O   ALA L 34   121.719 129.934 204.597 1.00160.62
O
ATOM 33826 N   TRP L 35   123.379 129.157 205.977 1.00149.84
N
ATOM 33827 CA TRP L 35   122.574 128.927 207.177 1.00163.92
C
ATOM 33828 CB TRP L 35   122.413 127.431 207.510 1.00126.07
C
ATOM 33829 CG TRP L 35   121.428 126.763 206.595 1.00183.57
C
ATOM 33830 CD1 TRP L 35   121.722 125.856 205.621 1.00207.63
C
ATOM 33831 NE1 TRP L 35   120.588 125.463 204.960 1.00208.58
N
ATOM 33832 CE2 TRP L 35   119.516 126.132 205.478 1.00195.06
C
ATOM 33833 CD2 TRP L 35   120.000 126.974 206.510 1.00218.57
C
ATOM 33834 CE3 TRP L 35   119.077 127.767 207.205 1.00263.85
C
ATOM 33835 CZ3 TRP L 35   117.742 127.696 206.866 1.00247.33
C
ATOM 33836 CH2 TRP L 35   117.303 126.859 205.837 1.00232.28
C
ATOM 33837 CZ2 TRP L 35   118.172 126.063 205.124 1.00173.11
C
ATOM 33838 C   TRP L 35   123.095 129.746 208.362 1.00179.48
C
ATOM 33839 O   TRP L 35   124.286 129.749 208.649 1.00188.66
O
ATOM 33840 N   PHE L 36   122.196 130.462 209.043 1.00207.77
N
ATOM 33841 CA PHE L 36   122.592 131.170 210.246 1.00175.00
C
ATOM 33842 CB PHE L 36   122.561 132.686 210.067 1.00166.24
C
ATOM 33843 CG PHE L 36   123.500 133.232 209.025 1.00182.83
C
ATOM 33844 CD1 PHE L 36   124.764 133.656 209.390 1.00180.14
C
ATOM 33845 CE1 PHE L 36   125.629 134.184 208.443 1.00232.65
C
ATOM 33846 CZ PHE L 36   125.236 134.313 207.125 1.00234.20
C
ATOM 33847 CD2 PHE L 36   123.105 133.388 207.698 1.00214.62
C
ATOM 33848 CE2 PHE L 36   123.969 133.921 206.749 1.00209.63
C
ATOM 33849 C   PHE L 36   121.710 130.748 211.413 1.00204.60
C

ATOM 33850 O PHE L 36 120.637 130.156 211.268 1.00184.23
O
ATOM 33851 N GLN L 37 122.217 131.076 212.589 1.00217.13
N
ATOM 33852 CA GLN L 37 121.532 130.747 213.811 1.00225.61
C
ATOM 33853 CB GLN L 37 122.255 129.565 214.447 1.00237.90
C
ATOM 33854 CG GLN L 37 121.681 129.108 215.773 1.00238.08
C
ATOM 33855 CD GLN L 37 122.676 128.197 216.448 1.00259.46
C
ATOM 33856 OE1 GLN L 37 123.886 128.302 216.248 1.00244.25
O
ATOM 33857 NE2 GLN L 37 122.171 127.297 217.274 1.00284.78
N
ATOM 33858 C GLN L 37 121.526 132.022 214.633 1.00224.74
C
ATOM 33859 O GLN L 37 122.546 132.691 214.794 1.00192.09
O
ATOM 33860 N GLN L 38 120.332 132.403 215.061 1.00237.88
N
ATOM 33861 CA GLN L 38 120.244 133.603 215.853 1.00252.31
C
ATOM 33862 CB GLN L 38 119.519 134.691 215.072 1.00209.71
C
ATOM 33863 CG GLN L 38 119.592 136.031 215.773 1.00218.04
C
ATOM 33864 CD GLN L 38 119.103 137.127 214.868 1.00223.07
C
ATOM 33865 OE1 GLN L 38 118.793 136.875 213.699 1.00194.91
O
ATOM 33866 NE2 GLN L 38 119.067 138.342 215.410 1.00202.41
N
ATOM 33867 C GLN L 38 119.563 133.250 217.164 1.00302.80
C
ATOM 33868 O GLN L 38 118.451 132.724 217.170 1.00332.72
O
ATOM 33869 N LYS L 39 120.271 133.514 218.263 1.00303.27
N
ATOM 33870 CA LYS L 39 119.686 133.380 219.583 1.00289.78
C
ATOM 33871 CB LYS L 39 120.598 132.548 220.481 1.00221.71
C
ATOM 33872 CG LYS L 39 120.699 131.086 220.085 1.00199.64
C
ATOM 33873 CD LYS L 39 121.684 130.358 220.967 1.00272.83
C
ATOM 33874 CE LYS L 39 122.796 129.668 220.200 1.00317.34
C

ATOM 33875 NZ LYS L 39 123.810 129.059 221.099 1.00282.97
N
ATOM 33876 C LYS L 39 119.434 134.764 220.167 1.00318.01
C
ATOM 33877 O LYS L 39 120.179 135.697 219.878 1.00373.42
O
ATOM 33878 N PRO L 40 118.363 134.934 220.973 1.00311.89
N
ATOM 33879 CA PRO L 40 118.097 136.208 221.653 1.00331.74
C
ATOM 33880 CB PRO L 40 117.018 135.777 222.662 1.00338.50
C
ATOM 33881 CG PRO L 40 116.241 134.694 221.925 1.00262.79
C
ATOM 33882 CD PRO L 40 117.323 133.914 221.217 1.00238.93
C
ATOM 33883 C PRO L 40 119.332 136.863 222.288 1.00353.56
C
ATOM 33884 O PRO L 40 119.987 136.265 223.141 1.00441.42
O
ATOM 33885 N GLY L 41 119.655 138.088 221.833 1.00288.23
N
ATOM 33886 CA GLY L 41 120.807 138.847 222.304 1.00312.74
C
ATOM 33887 C GLY L 41 122.120 138.394 221.663 1.00418.21
C
ATOM 33888 O GLY L 41 123.192 138.868 222.042 1.00477.28
O
ATOM 33889 N LYS L 42 122.022 137.461 220.703 1.00423.16
N
ATOM 33890 CA LYS L 42 123.144 137.037 219.879 1.00340.56
C
ATOM 33891 CB LYS L 42 123.354 135.514 219.943 1.00311.06
C
ATOM 33892 CG LYS L 42 123.522 134.914 221.337 1.00301.59
C
ATOM 33893 CD LYS L 42 124.854 135.214 222.004 1.00313.74
C
ATOM 33894 CE LYS L 42 124.790 135.098 223.513 1.00311.53
C
ATOM 33895 NZ LYS L 42 124.044 133.891 223.941 1.00284.28
N
ATOM 33896 C LYS L 42 122.945 137.575 218.458 1.00272.94
C
ATOM 33897 O LYS L 42 121.820 137.697 217.951 1.00189.63
O
ATOM 33898 N ALA L 43 124.058 137.982 217.842 1.00208.21
N
ATOM 33899 CA ALA L 43 124.045 138.289 216.423 1.00220.64
C

ATOM 33900 CB ALA L 43 125.027 139.388 216.141 1.00138.74
C
ATOM 33901 C ALA L 43 124.348 137.006 215.639 1.00278.21
C
ATOM 33902 O ALA L 43 125.153 136.191 216.096 1.00372.02
O
ATOM 33903 N PRO L 44 123.742 136.786 214.439 1.00243.50
N
ATOM 33904 CA PRO L 44 123.689 135.467 213.805 1.00205.71
C
ATOM 33905 CB PRO L 44 122.549 135.548 212.783 1.00167.11
C
ATOM 33906 CG PRO L 44 122.072 136.982 212.828 1.00147.21
C
ATOM 33907 CD PRO L 44 123.102 137.793 213.589 1.00179.11
C
ATOM 33908 C PRO L 44 124.962 135.078 213.061 1.00226.14
C
ATOM 33909 O PRO L 44 125.460 135.828 212.215 1.00173.66
O
ATOM 33910 N LYS L 45 125.448 133.874 213.402 1.00255.37
N
ATOM 33911 CA LYS L 45 126.633 133.262 212.820 1.00229.44
C
ATOM 33912 CB LYS L 45 127.435 132.409 213.815 1.00172.16
C
ATOM 33913 CG LYS L 45 127.714 133.024 215.177 1.00185.75
C
ATOM 33914 CD LYS L 45 126.510 133.010 216.114 1.00216.98
C
ATOM 33915 CE LYS L 45 125.790 131.678 216.203 1.00255.77
C
ATOM 33916 NZ LYS L 45 124.568 131.794 217.032 1.00260.34
N
ATOM 33917 C LYS L 45 126.229 132.343 211.668 1.00241.82
C
ATOM 33918 O LYS L 45 125.079 131.886 211.577 1.00158.89
O
ATOM 33919 N LEU L 46 127.228 132.121 210.797 1.00277.56
N
ATOM 33920 CA LEU L 46 127.240 131.111 209.748 1.00236.56
C
ATOM 33921 CB LEU L 46 128.422 131.323 208.778 1.00194.69
C
ATOM 33922 CG LEU L 46 128.467 130.445 207.516 1.00180.66
C
ATOM 33923 CD1 LEU L 46 127.192 130.584 206.711 1.00242.21
C
ATOM 33924 CD2 LEU L 46 129.645 130.787 206.612 1.00198.75
C

ATOM 33925 C LEU L 46 127.266 129.730 210.402 1.00199.02
C
ATOM 33926 O LEU L 46 127.815 129.527 211.493 1.00126.05
O
ATOM 33927 N LEU L 47 126.581 128.809 209.728 1.00229.28
N
ATOM 33928 CA LEU L 47 126.516 127.418 210.122 1.00226.13
C
ATOM 33929 CB LEU L 47 125.113 127.105 210.653 1.00225.61
C
ATOM 33930 CG LEU L 47 124.943 127.116 212.175 1.00218.72
C
ATOM 33931 CD1 LEU L 47 125.558 128.357 212.850 1.00149.51
C
ATOM 33932 CD2 LEU L 47 123.473 126.935 212.548 1.00222.29
C
ATOM 33933 C LEU L 47 126.890 126.536 208.930 1.00244.95
C
ATOM 33934 O LEU L 47 127.295 125.395 209.146 1.00264.80
O
ATOM 33935 N ILE L 48 126.762 127.061 207.689 1.00249.25
N
ATOM 33936 CA ILE L 48 126.991 126.315 206.448 1.00210.85
C
ATOM 33937 CB ILE L 48 125.751 125.470 206.037 1.00199.30
C
ATOM 33938 CG1 ILE L 48 124.622 125.390 207.085 1.00189.07
C
ATOM 33939 CG2 ILE L 48 126.160 124.125 205.438 1.00167.85
C
ATOM 33940 CD1 ILE L 48 124.748 124.320 208.162 1.00207.74
C
ATOM 33941 C ILE L 48 127.354 127.259 205.290 1.00169.95
C
ATOM 33942 O ILE L 48 126.509 128.053 204.895 1.00132.20
O
ATOM 33943 N TYR L 49 128.557 127.112 204.686 1.00194.62
N
ATOM 33944 CA TYR L 49 128.968 127.774 203.436 1.00183.38
C
ATOM 33945 CB TYR L 49 130.406 128.333 203.497 1.00188.64
C
ATOM 33946 CG TYR L 49 131.529 127.334 203.275 1.00267.83
C
ATOM 33947 CD1 TYR L 49 131.852 126.361 204.218 1.00297.95
C
ATOM 33948 CE1 TYR L 49 132.882 125.447 204.017 1.00299.50
C
ATOM 33949 CZ TYR L 49 133.623 125.485 202.846 1.00317.37
C

ATOM 33950 OH TYR L 49   134.645 124.610 202.598 1.00345.28
O
ATOM 33951 CE2 TYR L 49   133.323 126.443 201.891 1.00290.28
C
ATOM 33952 CD2 TYR L 49   132.292 127.350 202.113 1.00267.19
C
ATOM 33953 C   TYR L 49   128.675 126.879 202.211 1.00132.14
C
ATOM 33954 O   TYR L 49   129.067 125.721 202.142 1.00136.09
O
ATOM 33955 N   GLU L 50   127.953 127.419 201.233 1.00103.79
N
ATOM 33956 CA GLU L 50   127.553 126.726 200.021 1.00 97.86
C
ATOM 33957 CB GLU L 50   128.785 126.189 199.315 1.00 99.60
C
ATOM 33958 CG GLU L 50   129.662 127.272 198.737 1.00124.09
C
ATOM 33959 CD GLU L 50   130.436 126.848 197.493 1.00181.68
C
ATOM 33960 OE1 GLU L 50   130.798 125.640 197.382 1.00211.89
O
ATOM 33961 OE2 GLU L 50   130.683 127.720 196.629 1.00167.81
O
ATOM 33962 C   GLU L 50   126.531 125.623 200.305 1.00122.77
C
ATOM 33963 O   GLU L 50   126.248 124.801 199.424 1.00102.44
O
ATOM 33964 N   ALA L 51   125.983 125.641 201.535 1.00181.21
N
ATOM 33965 CA ALA L 51   124.923 124.772 202.042 1.00241.49
C
ATOM 33966 CB ALA L 51   123.573 125.417 201.900 1.00280.82
C
ATOM 33967 C   ALA L 51   124.983 123.372 201.430 1.00275.75
C
ATOM 33968 O   ALA L 51   123.975 122.764 201.055 1.00285.22
O
ATOM 33969 N   SER L 52   126.215 122.874 201.339 1.00261.27
N
ATOM 33970 CA SER L 52   126.476 121.471 201.099 1.00240.38
C
ATOM 33971 CB SER L 52   126.786 121.221 199.650 1.00232.02
C
ATOM 33972 OG SER L 52   127.744 122.161 199.191 1.00297.55
O
ATOM 33973 C   SER L 52   127.625 121.045 202.004 1.00223.78
C
ATOM 33974 O   SER L 52   127.892 119.851 202.122 1.00264.43
O

ATOM 33975 N SER L 53 128.243 122.031 202.678 1.00207.18
N
ATOM 33976 CA SER L 53 129.557 121.878 203.288 1.00204.49
C
ATOM 33977 CB SER L 53 130.645 122.480 202.388 1.00212.23
C
ATOM 33978 OG SER L 53 130.362 122.369 200.983 1.00138.87
O
ATOM 33979 C SER L 53 129.608 122.511 204.681 1.00168.81
C
ATOM 33980 O SER L 53 129.624 123.731 204.808 1.00119.09
O
ATOM 33981 N LEU L 54 129.720 121.699 205.731 1.00176.22
N
ATOM 33982 CA LEU L 54 130.018 122.313 207.013 1.00218.91
C
ATOM 33983 CB LEU L 54 130.671 121.288 207.951 1.00228.11
C
ATOM 33984 CG LEU L 54 130.334 121.407 209.439 1.00236.29
C
ATOM 33985 CD1 LEU L 54 128.915 121.917 209.643 1.00273.71
C
ATOM 33986 CD2 LEU L 54 130.502 120.045 210.100 1.00196.41
C
ATOM 33987 C LEU L 54 130.906 123.548 206.803 1.00203.94
C
ATOM 33988 O LEU L 54 131.649 123.688 205.816 1.00123.49
O
ATOM 33989 N GLU L 55 130.737 124.487 207.732 1.00227.76
N
ATOM 33990 CA GLU L 55 131.665 125.580 207.930 1.00234.49
C
ATOM 33991 CB GLU L 55 130.877 126.895 208.007 1.00285.23
C
ATOM 33992 CG GLU L 55 131.677 128.157 207.712 1.00304.97
C
ATOM 33993 CD GLU L 55 132.852 128.042 206.758 1.00260.24
C
ATOM 33994 OE1 GLU L 55 133.803 127.297 207.115 1.00151.03
O
ATOM 33995 OE2 GLU L 55 132.816 128.703 205.671 1.00261.77
O
ATOM 33996 C GLU L 55 132.521 125.217 209.147 1.00230.78
C
ATOM 33997 O GLU L 55 132.183 124.296 209.908 1.00188.30
O
ATOM 33998 N SER L 56 133.642 125.932 209.299 1.00215.61
N
ATOM 33999 CA SER L 56 134.644 125.585 210.291 1.00242.53
C

ATOM 34000 CB   SER L 56   136.023 125.925 209.798 1.00240.15
C
ATOM 34001 OG   SER L 56   136.996 125.227 210.559 1.00320.39
O
ATOM 34002 C   SER L 56   134.338 126.251 211.631 1.00234.55
C
ATOM 34003 O   SER L 56   133.937 127.412 211.683 1.00211.58
O
ATOM 34004 N   GLY L 57   134.551 125.498 212.718 1.00237.25
N
ATOM 34005 CA   GLY L 57   134.281 125.986 214.060 1.00242.09
C
ATOM 34006 C   GLY L 57   132.840 125.687 214.463 1.00259.43
C
ATOM 34007 O   GLY L 57   132.466 125.900 215.619 1.00224.99
O
ATOM 34008 N   VAL L 58   132.070 125.187 213.479 1.00271.85
N
ATOM 34009 CA   VAL L 58   130.670 124.800 213.599 1.00271.51
C
ATOM 34010 CB   VAL L 58   129.900 124.999 212.276 1.00239.60
C
ATOM 34011 CG1 VAL L 58   128.775 123.989 212.098 1.00156.13
C
ATOM 34012 CG2 VAL L 58   129.364 126.415 212.136 1.00312.91
C
ATOM 34013 C   VAL L 58   130.614 123.336 214.023 1.00271.31
C
ATOM 34014 O   VAL L 58   131.251 122.500 213.381 1.00222.59
O
ATOM 34015 N   PRO L 59   129.884 122.987 215.113 1.00286.01
N
ATOM 34016 CA   PRO L 59   129.690 121.585 215.492 1.00296.48
C
ATOM 34017 CB   PRO L 59   128.789 121.680 216.727 1.00281.27
C
ATOM 34018 CG   PRO L 59   129.194 123.011 217.320 1.00251.80
C
ATOM 34019 CD   PRO L 59   129.308 123.910 216.106 1.00276.31
C
ATOM 34020 C   PRO L 59   129.107 120.734 214.366 1.00327.10
C
ATOM 34021 O   PRO L 59   128.387 121.232 213.501 1.00360.22
O
ATOM 34022 N   SER L 60   129.441 119.439 214.398 1.00314.82
N
ATOM 34023 CA   SER L 60   129.319 118.570 213.237 1.00280.12
C
ATOM 34024 CB   SER L 60   130.351 117.470 213.255 1.00232.99
C

ATOM 34025 OG SER L 60 130.192 116.646 214.399 1.00255.31
O
ATOM 34026 C SER L 60 127.904 118.016 213.082 1.00294.95
C
ATOM 34027 O SER L 60 127.577 117.487 212.017 1.00293.48
O
ATOM 34028 N ARG L 61 127.083 118.154 214.137 1.00274.19
N
ATOM 34029 CA ARG L 61 125.702 117.695 214.139 1.00236.87
C
ATOM 34030 CB ARG L 61 125.082 117.833 215.531 1.00228.25
C
ATOM 34031 CG ARG L 61 125.350 119.177 216.193 1.00275.74
C
ATOM 34032 CD ARG L 61 124.701 119.291 217.557 1.00320.48
C
ATOM 34033 NE ARG L 61 125.147 120.484 218.264 1.00325.34
N
ATOM 34034 CZ ARG L 61 125.836 120.485 219.405 1.00304.29
C
ATOM 34035 NH1 ARG L 61 126.152 119.341 219.995 1.00207.11
N
ATOM 34036 NH2 ARG L 61 126.196 121.629 219.963 1.00331.07
N
ATOM 34037 C ARG L 61 124.903 118.394 213.034 1.00268.65
C
ATOM 34038 O ARG L 61 124.116 117.733 212.357 1.00227.60
O
ATOM 34039 N PHE L 62 125.133 119.713 212.855 1.00327.88
N
ATOM 34040 CA PHE L 62 124.552 120.521 211.783 1.00327.37
C
ATOM 34041 CB PHE L 62 124.787 122.024 211.995 1.00335.51
C
ATOM 34042 CG PHE L 62 124.184 122.605 213.252 1.00319.68
C
ATOM 34043 CD1 PHE L 62 122.885 123.100 213.263 1.00313.07
C
ATOM 34044 CE1 PHE L 62 122.331 123.620 214.425 1.00282.45
C
ATOM 34045 CZ PHE L 62 123.070 123.649 215.584 1.00257.58
C
ATOM 34046 CD2 PHE L 62 124.916 122.643 214.427 1.00253.04
C
ATOM 34047 CE2 PHE L 62 124.358 123.161 215.586 1.00254.42
C
ATOM 34048 C PHE L 62 125.150 120.108 210.440 1.00277.69
C
ATOM 34049 O PHE L 62 126.364 119.969 210.307 1.00296.25
O

```
ATOM  34050  N   SER L  63     124.286 119.911 209.442  1.00224.39
N
ATOM  34051  CA  SER L  63     124.748 119.423 208.157  1.00241.05
C
ATOM  34052  CB  SER L  63     124.892 117.909 208.115  1.00232.86
C
ATOM  34053  OG  SER L  63     123.702 117.246 208.514  1.00255.89
O
ATOM  34054  C   SER L  63     123.854 119.950 207.045  1.00260.46
C
ATOM  34055  O   SER L  63     122.666 120.193 207.245  1.00271.35
O
ATOM  34056  N   GLY L  64     124.462 120.115 205.870  1.00311.67
N
ATOM  34057  CA  GLY L  64     123.807 120.754 204.744  1.00333.28
C
ATOM  34058  C   GLY L  64     123.569 119.782 203.597  1.00288.10
C
ATOM  34059  O   GLY L  64     124.228 118.748 203.487  1.00305.48
O
ATOM  34060  N   SER L  65     122.611 120.128 202.744  1.00207.48
N
ATOM  34061  CA  SER L  65     122.229 119.226 201.683  1.00208.85
C
ATOM  34062  CB  SER L  65     121.551 118.030 202.273  1.00260.21
C
ATOM  34063  OG  SER L  65     120.909 118.391 203.492  1.00297.65
O
ATOM  34064  C   SER L  65     121.294 119.986 200.760  1.00232.58
C
ATOM  34065  O   SER L  65     120.922 121.122 201.058  1.00193.10
O
ATOM  34066  N   GLY L  66     120.928 119.338 199.649  1.00257.19
N
ATOM  34067  CA  GLY L  66     120.113 119.965 198.623  1.00260.32
C
ATOM  34068  C   GLY L  66     120.916 120.935 197.753  1.00275.69
C
ATOM  34069  O   GLY L  66     122.014 121.356 198.118  1.00297.09
O
ATOM  34070  N   SER L  67     120.328 121.258 196.590  1.00237.08
N
ATOM  34071  CA  SER L  67     120.821 122.158 195.556  1.00243.44
C
ATOM  34072  CB  SER L  67     121.368 121.327 194.385  1.00222.32
C
ATOM  34073  OG  SER L  67     122.790 121.330 194.256  1.00179.22
O
ATOM  34074  C   SER L  67     119.629 123.003 195.084  1.00276.56
C
```

ATOM 34075 O   SER L 67   118.500 122.751 195.502 1.00334.44
O
ATOM 34076 N   GLY L 68   119.867 123.988 194.205 1.00267.21
N
ATOM 34077 CA GLY L 68   118.837 124.463 193.285 1.00217.07
C
ATOM 34078 C   GLY L 68   117.793 125.337 193.968 1.00194.58
C
ATOM 34079 O   GLY L 68   118.163 126.331 194.579 1.00186.02
O
ATOM 34080 N   THR L 69   116.514 124.937 193.859 1.00184.65
N
ATOM 34081 CA THR L 69   115.415 125.652 194.495 1.00204.81
C
ATOM 34082 CB THR L 69   114.153 125.640 193.631 1.00206.42
C
ATOM 34083 OG1 THR L 69   113.696 124.292 193.724 1.00228.08
O
ATOM 34084 CG2 THR L 69   114.406 126.025 192.191 1.00243.31
C
ATOM 34085 C   THR L 69   114.986 124.962 195.788 1.00229.35
C
ATOM 34086 O   THR L 69   113.900 125.250 196.299 1.00214.07
O
ATOM 34087 N   GLU L 70   115.840 124.058 196.298 1.00258.45
N
ATOM 34088 CA GLU L 70   115.510 123.260 197.470 1.00242.74
C
ATOM 34089 CB GLU L 70   114.819 121.974 197.034 1.00225.67
C
ATOM 34090 CG GLU L 70   114.633 121.014 198.185 1.00275.16
C
ATOM 34091 CD GLU L 70   113.717 119.858 197.839 1.00289.50
C
ATOM 34092 OE1 GLU L 70   113.471 119.634 196.614 1.00179.94
O
ATOM 34093 OE2 GLU L 70   113.234 119.193 198.785 1.00352.08
O
ATOM 34094 C   GLU L 70   116.727 122.951 198.351 1.00225.41
C
ATOM 34095 O   GLU L 70   117.522 122.057 198.073 1.00190.67
O
ATOM 34096 N   PHE L 71   116.807 123.637 199.490 1.00259.47
N
ATOM 34097 CA PHE L 71   117.935 123.465 200.389 1.00267.74
C
ATOM 34098 CB PHE L 71   118.721 124.774 200.481 1.00304.54
C
ATOM 34099 CG PHE L 71   119.668 124.985 199.325 1.00356.45
C

ATOM 34100 CD1 PHE L 71 119.306 125.749 198.222 1.00307.51
C
ATOM 34101 CE1 PHE L 71 120.188 125.938 197.165 1.00296.94
C
ATOM 34102 CZ PHE L 71 121.435 125.353 197.196 1.00379.49
C
ATOM 34103 CD2 PHE L 71 120.926 124.397 199.334 1.00437.93
C
ATOM 34104 CE2 PHE L 71 121.806 124.584 198.279 1.00452.19
C
ATOM 34105 C PHE L 71 117.471 122.868 201.716 1.00249.57
C
ATOM 34106 O PHE L 71 116.332 123.066 202.135 1.00205.69
O
ATOM 34107 N THR L 72 118.367 122.117 202.368 1.00270.84
N
ATOM 34108 CA THR L 72 117.992 121.317 203.526 1.00265.75
C
ATOM 34109 CB THR L 72 117.613 119.891 203.102 1.00257.96
C
ATOM 34110 OG1 THR L 72 116.619 120.069 202.086 1.00197.52
O
ATOM 34111 CG2 THR L 72 117.177 118.997 204.251 1.00201.07
C
ATOM 34112 C THR L 72 119.041 121.377 204.641 1.00246.72
C
ATOM 34113 O THR L 72 120.068 120.681 204.617 1.00176.12
O
ATOM 34114 N LEU L 73 118.728 122.186 205.657 1.00208.22
N
ATOM 34115 CA LEU L 73 119.464 122.142 206.904 1.00219.59
C
ATOM 34116 CB LEU L 73 119.211 123.456 207.649 1.00184.77
C
ATOM 34117 CG LEU L 73 119.995 123.647 208.949 1.00222.26
C
ATOM 34118 CD1 LEU L 73 121.307 124.389 208.726 1.00204.02
C
ATOM 34119 CD2 LEU L 73 119.152 124.389 209.970 1.00281.44
C
ATOM 34120 C LEU L 73 118.987 120.920 207.691 1.00270.93
C
ATOM 34121 O LEU L 73 117.788 120.648 207.733 1.00285.06
O
ATOM 34122 N THR L 74 119.940 120.172 208.274 1.00318.79
N
ATOM 34123 CA THR L 74 119.670 118.930 208.994 1.00289.43
C
ATOM 34124 CB THR L 74 119.825 117.707 208.073 1.00298.93
C

ATOM 34125 OG1 THR L 74   118.862 117.771 207.017 1.00272.84
O
ATOM 34126 CG2 THR L 74   119.710 116.386 208.805 1.00270.30
C
ATOM 34127 C   THR L 74   120.562 118.816 210.232 1.00239.85
C
ATOM 34128 O   THR L 74   121.786 118.767 210.123 1.00256.67
O
ATOM 34129 N   ILE L 75   119.925 118.755 211.406 1.00203.35
N
ATOM 34130 CA ILE L 75   120.598 118.548 212.683 1.00278.38
C
ATOM 34131 CB ILE L 75   119.933 119.406 213.796 1.00262.53
C
ATOM 34132 CG1 ILE L 75   119.945 120.915 213.507 1.00246.03
C
ATOM 34133 CG2 ILE L 75   120.497 119.100 215.177 1.00222.91
C
ATOM 34134 CD1 ILE L 75   119.387 121.765 214.642 1.00197.36
C
ATOM 34135 C   ILE L 75   120.520 117.049 212.982 1.00323.24
C
ATOM 34136 O   ILE L 75   119.449 116.460 212.852 1.00385.19
O
ATOM 34137 N   SER L 76   121.642 116.413 213.348 1.00334.27
N
ATOM 34138 CA SER L 76   121.628 114.981 213.629 1.00315.27
C
ATOM 34139 CB SER L 76   122.990 114.359 213.472 1.00292.75
C
ATOM 34140 OG SER L 76   123.935 115.019 214.302 1.00275.67
O
ATOM 34141 C   SER L 76   121.031 114.705 215.008 1.00332.04
C
ATOM 34142 O   SER L 76   120.251 113.757 215.149 1.00253.10
O
ATOM 34143 N   SER L 77   121.406 115.556 215.985 1.00378.35
N
ATOM 34144 CA SER L 77   120.939 115.567 217.367 1.00389.18
C
ATOM 34145 CB SER L 77   121.907 114.837 218.272 1.00356.16
C
ATOM 34146 OG SER L 77   121.934 115.416 219.572 1.00289.64
O
ATOM 34147 C   SER L 77   120.729 117.002 217.854 1.00419.21
C
ATOM 34148 O   SER L 77   121.644 117.821 217.792 1.00420.87
O
ATOM 34149 N   LEU L 78   119.525 117.275 218.378 1.00478.95
N

ATOM 34150 CA LEU L 78 119.140 118.581 218.891 1.00518.65
C
ATOM 34151 CB LEU L 78 117.608 118.666 218.882 1.00633.78
C
ATOM 34152 CG LEU L 78 116.993 120.061 218.742 1.00699.07
C
ATOM 34153 CD1 LEU L 78 117.584 120.813 217.559 1.00691.56
C
ATOM 34154 CD2 LEU L 78 115.481 119.968 218.602 1.00762.65
C
ATOM 34155 C LEU L 78 119.709 118.769 220.298 1.00472.63
C
ATOM 34156 O LEU L 78 119.926 117.794 221.022 1.00469.46
O
ATOM 34157 N GLN L 79 119.976 120.032 220.661 1.00400.62
N
ATOM 34158 CA GLN L 79 120.478 120.388 221.983 1.00363.19
C
ATOM 34159 CB GLN L 79 121.994 120.600 221.960 1.00328.65
C
ATOM 34160 CG GLN L 79 122.774 119.499 221.249 1.00348.51
C
ATOM 34161 CD GLN L 79 123.162 118.316 222.106 1.00338.31
C
ATOM 34162 OE1 GLN L 79 123.183 117.175 221.647 1.00306.44
O
ATOM 34163 NE2 GLN L 79 123.513 118.575 223.357 1.00311.61
N
ATOM 34164 C GLN L 79 119.765 121.646 222.484 1.00422.28
C
ATOM 34165 O GLN L 79 119.194 122.393 221.693 1.00480.01
O
ATOM 34166 N PRO L 80 119.770 121.908 223.814 1.00461.29
N
ATOM 34167 CA PRO L 80 119.125 123.099 224.377 1.00519.28
C
ATOM 34168 CB PRO L 80 119.374 122.961 225.890 1.00516.05
C
ATOM 34169 CG PRO L 80 119.612 121.479 226.098 1.00462.78
C
ATOM 34170 CD PRO L 80 120.356 121.045 224.854 1.00427.01
C
ATOM 34171 C PRO L 80 119.673 124.425 223.844 1.00571.69
C
ATOM 34172 O PRO L 80 118.956 125.426 223.834 1.00622.28
O
ATOM 34173 N ASP L 81 120.938 124.416 223.399 1.00553.45
N
ATOM 34174 CA ASP L 81 121.573 125.587 222.808 1.00507.25
C

ATOM 34175 CB ASP L 81 123.081 125.672 223.102 1.00402.75
C
ATOM 34176 CG ASP L 81 123.857 124.360 223.118 1.00354.79
C
ATOM 34177 OD1 ASP L 81 123.266 123.320 223.489 1.00348.46
O
ATOM 34178 OD2 ASP L 81 125.066 124.392 222.789 1.00272.79
O
ATOM 34179 C ASP L 81 121.206 125.707 221.327 1.00544.76
C
ATOM 34180 O ASP L 81 121.296 126.793 220.756 1.00566.33
O
ATOM 34181 N ASP L 82 120.763 124.592 220.726 1.00573.37
N
ATOM 34182 CA ASP L 82 120.475 124.503 219.301 1.00556.86
C
ATOM 34183 CB ASP L 82 120.352 123.052 218.827 1.00639.41
C
ATOM 34184 CG ASP L 82 121.672 122.368 218.506 1.00721.31
C
ATOM 34185 OD1 ASP L 82 122.716 122.828 219.013 1.00817.92
O
ATOM 34186 OD2 ASP L 82 121.645 121.374 217.750 1.00737.60
O
ATOM 34187 C ASP L 82 119.215 125.291 218.950 1.00486.46
C
ATOM 34188 O ASP L 82 119.155 125.913 217.889 1.00515.86
O
ATOM 34189 N PHE L 83 118.203 125.233 219.825 1.00407.05
N
ATOM 34190 CA PHE L 83 116.964 125.962 219.613 1.00403.52
C
ATOM 34191 CB PHE L 83 116.052 125.878 220.838 1.00511.93
C
ATOM 34192 CG PHE L 83 115.609 124.490 221.222 1.00632.81
C
ATOM 34193 CD1 PHE L 83 114.610 123.839 220.513 1.00719.05
C
ATOM 34194 CE1 PHE L 83 114.199 122.566 220.874 1.00829.63
C
ATOM 34195 CZ PHE L 83 114.779 121.932 221.951 1.00848.11
C
ATOM 34196 CD2 PHE L 83 116.177 123.843 222.309 1.00699.23
C
ATOM 34197 CE2 PHE L 83 115.768 122.568 222.669 1.00784.80
C
ATOM 34198 C PHE L 83 117.282 127.424 219.317 1.00376.49
C
ATOM 34199 O PHE L 83 117.763 128.152 220.188 1.00319.45
O

ATOM 34200 N   ALA L 84   117.034 127.824 218.064 1.00365.08
N
ATOM 34201 CA ALA L 84   117.219 129.198 217.625 1.00303.52
C
ATOM 34202 CB ALA L 84   118.690 129.559 217.591 1.00253.24
C
ATOM 34203 C   ALA L 84   116.547 129.413 216.270 1.00255.23
C
ATOM 34204 O   ALA L 84   115.969 128.497 215.678 1.00203.03
O
ATOM 34205 N   THR L 85   116.614 130.659 215.802 1.00234.91
N
ATOM 34206 CA THR L 85   116.111 130.989 214.482 1.00264.70
C
ATOM 34207 CB THR L 85   115.578 132.425 214.423 1.00288.83
C
ATOM 34208 OG1 THR L 85   115.495 132.770 213.039 1.00268.98
O
ATOM 34209 CG2 THR L 85   116.435 133.411 215.194 1.00215.98
C
ATOM 34210 C   THR L 85   117.180 130.639 213.449 1.00254.21
C
ATOM 34211 O   THR L 85   118.376 130.671 213.744 1.00219.87
O
ATOM 34212 N   TYR L 86   116.720 130.288 212.241 1.00253.65
N
ATOM 34213 CA TYR L 86   117.584 129.847 211.159 1.00210.95
C
ATOM 34214 CB TYR L 86   117.462 128.338 210.972 1.00183.10
C
ATOM 34215 CG TYR L 86   118.073 127.597 212.123 1.00193.40
C
ATOM 34216 CD1 TYR L 86   117.295 126.766 212.915 1.00190.99
C
ATOM 34217 CE1 TYR L 86   117.848 126.088 213.985 1.00223.13
C
ATOM 34218 CZ TYR L 86   119.193 126.268 214.282 1.00267.35
C
ATOM 34219 OH TYR L 86   119.738 125.602 215.338 1.00351.94
O
ATOM 34220 CE2 TYR L 86   119.981 127.116 213.516 1.00198.28
C
ATOM 34221 CD2 TYR L 86   119.411 127.787 212.451 1.00168.99
C
ATOM 34222 C   TYR L 86   117.166 130.491 209.848 1.00215.71
C
ATOM 34223 O   TYR L 86   116.102 130.169 209.324 1.00267.98
O
ATOM 34224 N   TYR L 87   118.047 131.346 209.309 1.00188.42
N

ATOM 34225 CA TYR L 87   117.777 132.104 208.092 1.00202.83
C
ATOM 34226 CB TYR L 87   118.018 133.609 208.305 1.00215.55
C
ATOM 34227 CG TYR L 87   117.180 134.229 209.399 1.00266.65
C
ATOM 34228 CD1 TYR L 87   115.956 134.818 209.116 1.00284.79
C
ATOM 34229 CE1 TYR L 87   115.172 135.374 210.114 1.00270.26
C
ATOM 34230 CZ TYR L 87   115.608 135.340 211.424 1.00242.54
C
ATOM 34231 OH TYR L 87   114.841 135.890 212.399 1.00305.23
O
ATOM 34232 CE2 TYR L 87   116.826 134.762 211.733 1.00258.33
C
ATOM 34233 CD2 TYR L 87   117.595 134.207 210.724 1.00280.64
C
ATOM 34234 C   TYR L 87   118.536 131.492 206.906 1.00169.43
C
ATOM 34235 O   TYR L 87   119.748 131.358 206.934 1.00115.60
O
ATOM 34236 N   CYS L 88   117.805 131.072 205.869 1.00180.76
N
ATOM 34237 CA CYS L 88   118.415 130.707 204.602 1.00154.89
C
ATOM 34238 CB CYS L 88   117.583 129.671 203.837 1.00211.22
C
ATOM 34239 SG CYS L 88   115.830 130.075 203.552 1.00314.23
S
ATOM 34240 C   CYS L 88   118.628 131.981 203.788 1.00184.17
C
ATOM 34241 O   CYS L 88   117.676 132.647 203.372 1.00213.63
O
ATOM 34242 N   GLN L 89   119.902 132.324 203.606 1.00167.09
N
ATOM 34243 CA GLN L 89   120.299 133.498 202.844 1.00170.79
C
ATOM 34244 CB GLN L 89   121.271 134.311 203.715 1.00160.70
C
ATOM 34245 CG GLN L 89   121.549 135.734 203.232 1.00157.17
C
ATOM 34246 CD GLN L 89   123.001 136.020 202.920 1.00133.74
C
ATOM 34247 OE1 GLN L 89   123.882 135.889 203.777 1.00117.47
O
ATOM 34248 NE2 GLN L 89   123.261 136.437 201.689 1.00100.35
N
ATOM 34249 C   GLN L 89   120.912 132.975 201.548 1.00137.53
C

```
ATOM  34250  O   GLN L  89     121.573 131.935 201.598  1.00100.91
O
ATOM  34251  N   GLN L  90     120.669 133.652 200.414  1.00104.73
N
ATOM  34252  CA  GLN L  90     121.321 133.214 199.194  1.00103.90
C
ATOM  34253  CB  GLN L  90     120.236 132.829 198.185  1.00 99.49
C
ATOM  34254  CG  GLN L  90     119.366 133.978 197.750  1.00105.79
C
ATOM  34255  CD  GLN L  90     120.187 135.018 197.026  1.00145.26
C
ATOM  34256  OE1 GLN L  90     121.097 134.716 196.236  1.00133.46
O
ATOM  34257  NE2 GLN L  90     119.870 136.273 197.312  1.00169.50
N
ATOM  34258  C   GLN L  90     122.341 134.264 198.745  1.00 90.78
C
ATOM  34259  O   GLN L  90     121.987 135.423 198.732  1.00103.80
O
ATOM  34260  N   TYR L  91     123.572 133.890 198.360  1.00 98.53
N
ATOM  34261  CA  TYR L  91     124.453 134.858 197.700  1.00137.80
C
ATOM  34262  CB  TYR L  91     125.853 134.858 198.313  1.00186.28
C
ATOM  34263  CG  TYR L  91     126.300 133.630 199.068  1.00171.41
C
ATOM  34264  CD1 TYR L  91     127.261 133.770 200.064  1.00194.17
C
ATOM  34265  CE1 TYR L  91     127.730 132.701 200.802  1.00181.19
C
ATOM  34266  CZ  TYR L  91     127.226 131.446 200.533  1.00218.33
C
ATOM  34267  OH  TYR L  91     127.734 130.425 201.291  1.00298.82
O
ATOM  34268  CE2 TYR L  91     126.264 131.282 199.535  1.00151.59
C
ATOM  34269  CD2 TYR L  91     125.802 132.361 198.803  1.00109.68
C
ATOM  34270  C   TYR L  91     124.552 134.660 196.184  1.00119.92
C
ATOM  34271  O   TYR L  91     125.617 134.781 195.561  1.00 99.48
O
ATOM  34272  N   ASN L  92     123.420 134.350 195.570  1.00 98.14
N
ATOM  34273  CA  ASN L  92     123.598 133.935 194.210  1.00102.66
C
ATOM  34274  CB  ASN L  92     122.516 133.000 193.695  1.00113.00
C
```

```
ATOM  34275  CG  ASN L  92     122.862 132.547 192.292  1.00189.18
C
ATOM  34276  OD1 ASN L  92     122.064 131.910 191.615  1.00255.87
O
ATOM  34277  ND2 ASN L  92     124.049 132.894 191.820  1.00260.22
N
ATOM  34278  C   ASN L  92     123.754 135.207 193.409  1.00112.93
C
ATOM  34279  O   ASN L  92     124.811 135.442 192.844  1.00140.71
O
ATOM  34280  N   SER L  93     122.701 136.021 193.402  1.00138.80
N
ATOM  34281  CA  SER L  93     122.729 137.314 192.724  1.00161.10
C
ATOM  34282  CB  SER L  93     121.870 137.355 191.466  1.00143.85
C
ATOM  34283  OG  SER L  93     121.787 136.087 190.833  1.00152.47
O
ATOM  34284  C   SER L  93     122.250 138.391 193.685  1.00160.96
C
ATOM  34285  O   SER L  93     121.063 138.441 193.998  1.00190.80
O
ATOM  34286  N   TYR L  94     123.150 139.275 194.123  1.00134.97
N
ATOM  34287  CA  TYR L  94     122.669 140.423 194.870  1.00143.80
C
ATOM  34288  CB  TYR L  94     123.687 141.554 194.893  1.00118.12
C
ATOM  34289  CG  TYR L  94     124.706 141.335 195.954  1.00 90.68
C
ATOM  34290  CD1 TYR L  94     124.317 140.950 197.222  1.00 73.28
C
ATOM  34291  CE1 TYR L  94     125.291 140.690 198.175  1.00 94.26
C
ATOM  34292  CZ  TYR L  94     126.656 140.843 197.903  1.00111.28
C
ATOM  34293  OH  TYR L  94     127.652 140.590 198.846  1.00 74.75
O
ATOM  34294  CE2 TYR L  94     127.023 141.271 196.630  1.00112.44
C
ATOM  34295  CD2 TYR L  94     126.049 141.502 195.667  1.00 90.26
C
ATOM  34296  C   TYR L  94     121.440 141.022 194.198  1.00169.55
C
ATOM  34297  O   TYR L  94     121.270 140.880 192.982  1.00173.42
O
ATOM  34298  N   PRO L  95     120.583 141.750 194.961  1.00170.92
N
ATOM  34299  CA  PRO L  95     120.658 141.798 196.432  1.00148.57
C
```

```
ATOM  34300  CB  PRO L  95     119.565 142.826 196.797  1.00198.80
C
ATOM  34301  CG  PRO L  95     118.649 142.930 195.583  1.00168.13
C
ATOM  34302  CD  PRO L  95     119.588 142.700 194.420  1.00154.06
C
ATOM  34303  C   PRO L  95     120.470 140.556 197.314  1.00131.41
C
ATOM  34304  O   PRO L  95     119.770 139.579 196.955  1.00 96.44
O
ATOM  34305  N   TRP L  96     121.035 140.644 198.530  1.00 87.87
N
ATOM  34306  CA  TRP L  96     121.116 139.432 199.333  1.00114.16
C
ATOM  34307  CB  TRP L  96     122.297 139.593 200.277  1.00110.56
C
ATOM  34308  CG  TRP L  96     123.609 138.911 199.976  1.00134.89
C
ATOM  34309  CD1 TRP L  96     124.215 138.558 198.787  1.00105.61
C
ATOM  34310  NE1 TRP L  96     125.466 138.023 199.008  1.00 70.15
N
ATOM  34311  CE2 TRP L  96     125.706 138.062 200.357  1.00100.90
C
ATOM  34312  CD2 TRP L  96     124.560 138.598 201.008  1.00133.22
C
ATOM  34313  CE3 TRP L  96     124.524 138.764 202.413  1.00115.67
C
ATOM  34314  CZ3 TRP L  96     125.612 138.374 203.150  1.00 96.11
C
ATOM  34315  CH2 TRP L  96     126.734 137.853 202.475  1.00155.99
C
ATOM  34316  CZ2 TRP L  96     126.828 137.709 201.094  1.00112.58
C
ATOM  34317  C   TRP L  96     119.867 139.063 200.156  1.00113.13
C
ATOM  34318  O   TRP L  96     119.884 139.311 201.356  1.00124.46
O
ATOM  34319  N   THR L  97     118.865 138.335 199.618  1.00103.56
N
ATOM  34320  CA  THR L  97     117.672 138.054 200.423  1.00116.73
C
ATOM  34321  CB  THR L  97     116.519 137.564 199.549  1.00143.01
C
ATOM  34322  OG1 THR L  97     116.684 136.146 199.543  1.00190.03
O
ATOM  34323  CG2 THR L  97     116.591 138.150 198.156  1.00135.70
C
ATOM  34324  C   THR L  97     118.010 137.155 201.622  1.00114.34
C
```

ATOM 34325 O THR L 97 119.179 137.046 201.965 1.00 94.60
O
ATOM 34326 N PHE L 98 116.980 136.557 202.263 1.00143.88
N
ATOM 34327 CA PHE L 98 117.009 135.795 203.516 1.00155.24
C
ATOM 34328 CB PHE L 98 117.151 136.684 204.754 1.00147.63
C
ATOM 34329 CG PHE L 98 118.540 137.053 205.186 1.00151.96
C
ATOM 34330 CD1 PHE L 98 119.265 136.201 206.010 1.00140.68
C
ATOM 34331 CE1 PHE L 98 120.536 136.571 206.417 1.00189.09
C
ATOM 34332 CZ PHE L 98 121.063 137.792 206.037 1.00228.97
C
ATOM 34333 CD2 PHE L 98 119.069 138.285 204.826 1.00159.28
C
ATOM 34334 CE2 PHE L 98 120.335 138.655 205.246 1.00203.78
C
ATOM 34335 C PHE L 98 115.603 135.318 203.881 1.00159.96
C
ATOM 34336 O PHE L 98 114.629 136.018 203.617 1.00231.57
O
ATOM 34337 N GLY L 99 115.500 134.189 204.589 1.00147.44
N
ATOM 34338 CA GLY L 99 114.207 133.583 204.890 1.00169.10
C
ATOM 34339 C GLY L 99 113.447 134.358 205.962 1.00213.12
C
ATOM 34340 O GLY L 99 114.030 135.214 206.624 1.00237.42
O
ATOM 34341 N GLN L 100 112.136 134.098 206.095 1.00236.88
N
ATOM 34342 CA GLN L 100 111.384 134.633 207.220 1.00209.95
C
ATOM 34343 CB GLN L 100 109.880 134.387 207.129 1.00180.95
C
ATOM 34344 CG GLN L 100 109.510 132.910 207.039 1.00281.82
C
ATOM 34345 CD GLN L 100 109.460 132.354 205.631 1.00363.71
C
ATOM 34346 OE1 GLN L 100 110.055 132.892 204.698 1.00369.17
O
ATOM 34347 NE2 GLN L 100 108.741 131.253 205.467 1.00409.88
N
ATOM 34348 C GLN L 100 111.934 133.991 208.487 1.00227.70
C
ATOM 34349 O GLN L 100 112.104 134.680 209.482 1.00286.81
O

ATOM 34350 N GLY L 101 112.235 132.687 208.429 1.00215.46
N
ATOM 34351 CA GLY L 101 112.973 132.040 209.504 1.00241.89
C
ATOM 34352 C GLY L 101 112.166 130.941 210.190 1.00260.97
C
ATOM 34353 O GLY L 101 110.935 130.992 210.201 1.00270.50
O
ATOM 34354 N THR L 102 112.881 129.941 210.726 1.00258.92
N
ATOM 34355 CA THR L 102 112.290 128.870 211.514 1.00243.23
C
ATOM 34356 CB THR L 102 112.442 127.484 210.860 1.00277.46
C
ATOM 34357 OG1 THR L 102 112.262 127.514 209.443 1.00302.89
O
ATOM 34358 CG2 THR L 102 111.528 126.447 211.477 1.00261.83
C
ATOM 34359 C THR L 102 112.964 128.843 212.880 1.00208.13
C
ATOM 34360 O THR L 102 114.186 128.784 212.974 1.00182.59
O
ATOM 34361 N LYS L 103 112.148 128.891 213.935 1.00262.91
N
ATOM 34362 CA LYS L 103 112.611 128.656 215.293 1.00309.35
C
ATOM 34363 CB LYS L 103 112.072 129.740 216.239 1.00313.15
C
ATOM 34364 CG LYS L 103 112.740 129.843 217.607 1.00285.66
C
ATOM 34365 CD LYS L 103 112.309 131.045 218.437 1.00271.36
C
ATOM 34366 CE LYS L 103 113.008 131.103 219.781 1.00280.27
C
ATOM 34367 NZ LYS L 103 112.832 132.410 220.462 1.00260.48
N
ATOM 34368 C LYS L 103 112.163 127.251 215.693 1.00330.83
C
ATOM 34369 O LYS L 103 111.100 126.791 215.270 1.00331.80
O
ATOM 34370 N VAL L 104 112.995 126.564 216.478 1.00307.69
N
ATOM 34371 CA VAL L 104 112.621 125.260 216.991 1.00277.00
C
ATOM 34372 CB VAL L 104 113.733 124.223 216.773 1.00245.72
C
ATOM 34373 CG1 VAL L 104 113.152 122.995 216.093 1.00265.87
C
ATOM 34374 CG2 VAL L 104 114.891 124.795 215.958 1.00220.35
C

```
ATOM  34375  C   VAL L 104     112.255 125.404 218.465  1.00335.70
C
ATOM  34376  O   VAL L 104     112.929 126.127 219.198  1.00385.37
O
ATOM  34377  N   GLU L 105     111.175 124.724 218.881  1.00368.20
N
ATOM  34378  CA  GLU L 105     110.609 124.906 220.214  1.00421.76
C
ATOM  34379  CB  GLU L 105     109.191 125.477 220.131  1.00492.98
C
ATOM  34380  CG  GLU L 105     109.132 126.965 219.819  1.00535.38
C
ATOM  34381  CD  GLU L 105     108.896 127.340 218.364  1.00568.05
C
ATOM  34382  OE1 GLU L 105     108.814 126.427 217.515  1.00590.18
O
ATOM  34383  OE2 GLU L 105     108.789 128.552 218.083  1.00574.23
O
ATOM  34384  C   GLU L 105     110.606 123.591 220.995  1.00401.53
C
ATOM  34385  O   GLU L 105     110.312 122.543 220.416  1.00277.81
O
ATOM  34386  N   ILE L 106     110.874 123.700 222.317  1.00421.41
N
ATOM  34387  CA  ILE L 106     111.079 122.620 223.285  1.00395.31
C
ATOM  34388  CB  ILE L 106     111.719 123.149 224.607  1.00340.99
C
ATOM  34389  CG1 ILE L 106     112.927 124.078 224.392  1.00307.40
C
ATOM  34390  CG2 ILE L 106     112.031 122.018 225.590  1.00283.14
C
ATOM  34391  CD1 ILE L 106     113.769 124.395 225.632  1.00218.75
C
ATOM  34392  C   ILE L 106     109.756 121.885 223.550  1.00386.19
C
ATOM  34393  O   ILE L 106     108.844 122.459 224.152  1.00271.75
O
ATOM  34394  N   LYS L 107     109.657 120.611 223.120  1.00412.00
N
ATOM  34395  CA  LYS L 107     108.497 119.765 223.396  1.00353.30
C
ATOM  34396  CB  LYS L 107     108.027 118.964 222.170  1.00306.78
C
ATOM  34397  CG  LYS L 107     106.973 119.617 221.284  1.00265.54
C
ATOM  34398  CD  LYS L 107     106.063 120.591 222.006  1.00247.47
C
ATOM  34399  CE  LYS L 107     105.755 121.797 221.138  1.00259.82
C
```

```
ATOM  34400  NZ  LYS L 107     105.124 122.915 221.877  1.00210.25
N
ATOM  34401  C   LYS L 107     108.814 118.795 224.541  1.00345.33
C
ATOM  34402  O   LYS L 107     107.962 118.695 225.448  1.00279.09
O
ATOM  34403  N   ARG L 108     107.767 120.179 225.710  1.00 66.50
N
ATOM  34404  CA  ARG L 108     107.697 119.698 227.083  1.00 64.85
C
ATOM  34405  C   ARG L 108     106.230 119.554 227.448  1.00 57.88
C
ATOM  34406  O   ARG L 108     105.338 119.830 226.636  1.00 64.30
O
ATOM  34407  CB  ARG L 108     108.443 120.631 228.032  1.00 56.65
C
ATOM  34408  CG  ARG L 108     107.834 122.026 228.150  1.00 65.58
C
ATOM  34409  CD  ARG L 108     107.946 122.594 229.570  1.00 58.24
C
ATOM  34410  NE  ARG L 108     106.932 122.071 230.494  1.00 65.12
N
ATOM  34411  CZ  ARG L 108     107.084 122.012 231.819  1.00 63.60
C
ATOM  34412  NH1 ARG L 108     108.217 122.418 232.374  1.00 63.29
N
ATOM  34413  NH2 ARG L 108     106.110 121.528 232.596  1.00 58.04
N
ATOM  34414  H   ARG L 108     107.045 120.544 225.418  1.00 79.83
H
ATOM  34415  HA  ARG L 108     108.112 118.824 227.146  1.00 77.85
H
ATOM  34416  HB2 ARG L 108     108.445 120.235 228.918  1.00 68.00
H
ATOM  34417  HB3 ARG L 108     109.354 120.734 227.714  1.00 68.00
H
ATOM  34418  HG2 ARG L 108     108.299 122.629 227.548  1.00 78.72
H
ATOM  34419  HG3 ARG L 108     106.894 121.984 227.915  1.00 78.72
H
ATOM  34420  HD2 ARG L 108     108.818 122.369 229.930  1.00 69.92
H
ATOM  34421  HD3 ARG L 108     107.841 123.557 229.532  1.00 69.92
H
ATOM  34422  HE  ARG L 108     106.194 121.783 230.161  1.00 78.18
H
ATOM  34423 HH11 ARG L 108     108.853 122.719 231.882  1.00 75.98
H
ATOM  34424 HH12 ARG L 108     108.314 122.381 233.228  1.00 75.98
H
```

```
ATOM  34425 HH21 ARG L 108     105.376 121.251 232.243  1.00 69.68
H
ATOM  34426 HH22 ARG L 108     106.217 121.496 233.449  1.00 69.68
H
ATOM  34427  N   THR L 109     105.982 119.122 228.685  1.00 58.98
N
ATOM  34428  CA  THR L 109     104.618 118.817 229.100  1.00 66.78
C
ATOM  34429  C   THR L 109     103.828 120.085 229.386  1.00 60.95
C
ATOM  34430  O   THR L 109     104.381 121.138 229.712  1.00 62.07
O
ATOM  34431  CB  THR L 109     104.618 117.920 230.337  1.00 68.30
C
ATOM  34432  OG1 THR L 109     105.528 118.442 231.312  1.00 61.90
O
ATOM  34433  CG2 THR L 109     105.053 116.523 229.968  1.00 63.88
C
ATOM  34434  H   THR L 109     106.579 119.000 229.292  1.00 70.81
H
ATOM  34435  HA  THR L 109     104.175 118.345 228.377  1.00 80.17
H
ATOM  34436  HB  THR L 109     103.723 117.888 230.710  1.00 81.99
H
ATOM  34437  HG1 THR L 109     105.609 117.899 231.949  1.00 74.31
H
ATOM  34438 HG21 THR L 109     105.052 115.956 230.755  1.00 76.69
H
ATOM  34439 HG22 THR L 109     104.445 116.150 229.310  1.00 76.69
H
ATOM  34440 HG23 THR L 109     105.949 116.541 229.594  1.00 76.69
H
ATOM  34441  N   VAL L 110     102.509 119.967 229.260  1.00 59.59
N
ATOM  34442  CA  VAL L 110     101.643 121.075 229.620  1.00 59.50
C
ATOM  34443  C   VAL L 110     101.941 121.534 231.039  1.00 66.75
C
ATOM  34444  O   VAL L 110     102.215 120.733 231.940  1.00 55.47
O
ATOM  34445  CB  VAL L 110     100.161 120.702 229.492  1.00 59.58
C
ATOM  34446  CG1 VAL L 110      99.302 121.836 230.041  1.00 60.25
C
ATOM  34447  CG2 VAL L 110      99.826 120.396 228.041  1.00 65.27
C
ATOM  34448  H   VAL L 110     102.100 119.268 228.974  1.00 71.53
H
ATOM  34449  HA  VAL L 110     101.834 121.797 229.001  1.00 71.43
H
```

ATOM 34450 HB VAL L 110   99.969 119.904 230.010 1.00 71.52
H
ATOM 34451 HG11 VAL L 110   98.401 121.745 229.693 1.00 72.32
H
ATOM 34452 HG12 VAL L 110   99.290 121.783 231.009 1.00 72.32
H
ATOM 34453 HG13 VAL L 110   99.681 122.683 229.759 1.00 72.32
H
ATOM 34454 HG21 VAL L 110   98.883 120.174 227.974 1.00 78.35
H
ATOM 34455 HG22 VAL L 110   100.021 121.177 227.500 1.00 78.35
H
ATOM 34456 HG23 VAL L 110   100.364 119.645 227.744 1.00 78.35
H
ATOM 34457 N   ALA L 111   101.871 122.852 231.236 1.00 67.03
N
ATOM 34458 CA ALA L 111   102.093 123.472 232.536 1.00 55.93
C
ATOM 34459 C   ALA L 111   101.220 124.710 232.548 1.00 57.10
C
ATOM 34460 O   ALA L 111   101.420 125.604 231.714 1.00 51.75
O
ATOM 34461 CB ALA L 111   103.562 123.815 232.746 1.00 55.55
C
ATOM 34462 H   ALA L 111   101.694 123.420 230.615 1.00 80.46
H
ATOM 34463 HA ALA L 111   101.839 122.880 233.262 1.00 67.14
H
ATOM 34464 HB1 ALA L 111   103.679 124.163 233.644 1.00 66.69
H
ATOM 34465 HB2 ALA L 111   104.093 123.012 232.629 1.00 66.69
H
ATOM 34466 HB3 ALA L 111   103.826 124.483 232.095 1.00 66.69
H
ATOM 34467 N   ALA L 112   100.240 124.742 233.463 1.00 51.93
N
ATOM 34468 CA ALA L 112   99.282 125.836 233.541 1.00 59.60
C
ATOM 34469 C   ALA L 112   99.925 127.057 234.204 1.00 59.30
C
ATOM 34470 O   ALA L 112   100.841 126.924 235.020 1.00 51.71
O
ATOM 34471 CB ALA L 112   98.041 125.416 234.320 1.00 54.49
C
ATOM 34472 H   ALA L 112   100.111 124.130 234.052 1.00 62.35
H
ATOM 34473 HA ALA L 112   98.999 126.086 232.648 1.00 71.55
H
ATOM 34474 HB1 ALA L 112   97.380 126.122 234.265 1.00 65.42
H

ATOM 34475 HB2 ALA L 112 97.689 124.599 233.934 1.00 65.42
H
ATOM 34476 HB3 ALA L 112 98.287 125.265 235.246 1.00 65.42
H
ATOM 34477 N PRO L 113 99.492 128.259 233.840 1.00 57.69
N
ATOM 34478 CA PRO L 113 100.103 129.456 234.436 1.00 59.04
C
ATOM 34479 C PRO L 113 99.650 129.665 235.874 1.00 53.13
C
ATOM 34480 O PRO L 113 98.518 129.349 236.248 1.00 51.84
O
ATOM 34481 CB PRO L 113 99.607 130.601 233.541 1.00 57.07
C
ATOM 34482 CG PRO L 113 98.283 130.079 232.981 1.00 63.65
C
ATOM 34483 CD PRO L 113 98.502 128.589 232.793 1.00 50.32
C
ATOM 34484 HA PRO L 113 101.071 129.399 234.399 1.00 70.87
H
ATOM 34485 HB2 PRO L 113 99.475 131.404 234.067 1.00 68.52
H
ATOM 34486 HB3 PRO L 113 100.246 130.770 232.830 1.00 68.52
H
ATOM 34487 HG2 PRO L 113 97.566 130.248 233.611 1.00 76.41
H
ATOM 34488 HG3 PRO L 113 98.087 130.512 232.134 1.00 76.41
H
ATOM 34489 HD2 PRO L 113 97.678 128.098 232.931 1.00 60.41
H
ATOM 34490 HD3 PRO L 113 98.858 128.402 231.911 1.00 60.41
H
ATOM 34491 N SER L 114 100.557 130.212 236.675 1.00 51.27
N
ATOM 34492 CA SER L 114 100.170 130.921 237.880 1.00 54.79
C
ATOM 34493 C SER L 114 99.838 132.344 237.479 1.00 48.16
C
ATOM 34494 O SER L 114 100.650 133.010 236.834 1.00 52.40
O
ATOM 34495 CB SER L 114 101.291 130.937 238.921 1.00 55.36
C
ATOM 34496 OG SER L 114 101.805 129.652 239.153 1.00 68.66
O
ATOM 34497 H SER L 114 101.406 130.184 236.539 1.00 61.56
H
ATOM 34498 HA SER L 114 99.410 130.480 238.293 1.00 65.78
H
ATOM 34499 HB2 SER L 114 102.007 131.507 238.600 1.00 66.46
H

```
ATOM  34500  HB3 SER L 114     100.937 131.288 239.755  1.00 66.46
H
ATOM  34501  HG  SER L 114     102.582 129.706 239.469  1.00 82.42
H
ATOM  34502  N   VAL L 115      98.680 132.819 237.914  1.00 51.75
N
ATOM  34503  CA  VAL L 115      98.126 134.089 237.503  1.00 46.88
C
ATOM  34504  C   VAL L 115      98.150 135.053 238.676  1.00 57.97
C
ATOM  34505  O   VAL L 115      97.844 134.681 239.814  1.00 48.62
O
ATOM  34506  CB  VAL L 115      96.696 133.909 236.956  1.00 43.49
C
ATOM  34507  CG1 VAL L 115      96.161 135.207 236.463  1.00 47.53
C
ATOM  34508  CG2 VAL L 115      96.712 132.893 235.825  1.00 47.52
C
ATOM  34509  H   VAL L 115      98.179 132.402 238.474  1.00 62.13
H
ATOM  34510  HA  VAL L 115      98.680 134.470 236.804  1.00 56.29
H
ATOM  34511  HB  VAL L 115      96.119 133.588 237.668  1.00 52.21
H
ATOM  34512 HG11 VAL L 115      95.278 135.062 236.090  1.00 57.06
H
ATOM  34513 HG12 VAL L 115      96.110 135.829 237.205  1.00 57.06
H
ATOM  34514 HG13 VAL L 115      96.756 135.554 235.781  1.00 57.06
H
ATOM  34515 HG21 VAL L 115      95.827 132.848 235.429  1.00 57.05
H
ATOM  34516 HG22 VAL L 115      97.358 133.173 235.158  1.00 57.05
H
ATOM  34517 HG23 VAL L 115      96.958 132.025 236.182  1.00 57.05
H
ATOM  34518  N   PHE L 116      98.511 136.306 238.382  1.00 52.37
N
ATOM  34519  CA  PHE L 116      98.626 137.369 239.371  1.00 51.49
C
ATOM  34520  C   PHE L 116      98.170 138.680 238.740  1.00 52.58
C
ATOM  34521  O   PHE L 116      98.466 138.949 237.575  1.00 48.26
O
ATOM  34522  CB  PHE L 116     100.071 137.566 239.838  1.00 47.10
C
ATOM  34523  CG  PHE L 116     100.728 136.359 240.398  1.00 54.35
C
ATOM  34524  CD1 PHE L 116     101.408 135.459 239.580  1.00 49.99
C
```

ATOM 34525 CD2 PHE L 116   100.756 136.167 241.782 1.00 51.46
C
ATOM 34526 CE1 PHE L 116   102.067 134.334 240.141 1.00 48.17
C
ATOM 34527 CE2 PHE L 116   101.404 135.070 242.342 1.00 47.54
C
ATOM 34528 CZ PHE L 116   102.057 134.152 241.532 1.00 45.14
C
ATOM 34529 H   PHE L 116   98.701 136.568 237.586 1.00 62.87
H
ATOM 34530 HA PHE L 116   98.067 137.147 240.133 1.00 61.81
H
ATOM 34531 HB2 PHE L 116   100.600 137.856 239.079 1.00 56.55
H
ATOM 34532 HB3 PHE L 116   100.079 138.246 240.529 1.00 56.55
H
ATOM 34533 HD1 PHE L 116   101.430 135.597 238.660 1.00 60.01
H
ATOM 34534 HD2 PHE L 116   100.334 136.782 242.337 1.00 61.78
H
ATOM 34535 HE1 PHE L 116   102.501 133.723 239.592 1.00 57.83
H
ATOM 34536 HE2 PHE L 116   101.400 134.953 243.264 1.00 57.07
H
ATOM 34537 HZ PHE L 116   102.488 133.419 241.910 1.00 54.19
H
ATOM 34538 N   ILE L 117   97.512 139.517 239.512 1.00 52.90
N
ATOM 34539 CA ILE L 117   96.983 140.786 239.023 1.00 52.39
C
ATOM 34540 C   ILE L 117   97.512 141.893 239.927 1.00 52.56
C
ATOM 34541 O   ILE L 117   97.655 141.703 241.143 1.00 42.01
O
ATOM 34542 CB ILE L 117   95.439 140.820 238.976 1.00 49.34
C
ATOM 34543 CG1 ILE L 117   94.969 142.050 238.194 1.00 41.72
C
ATOM 34544 CG2 ILE L 117   94.863 140.845 240.382 1.00 49.94
C
ATOM 34545 CD1 ILE L 117   93.525 142.085 237.825 1.00 49.22
C
ATOM 34546 H   ILE L 117   97.351 139.374 240.344 1.00 63.51
H
ATOM 34547 HA ILE L 117   97.301 140.930 238.118 1.00 62.89
H
ATOM 34548 HB ILE L 117   95.126 140.017 238.527 1.00 59.24
H
ATOM 34549 HG12 ILE L 117   95.144 142.837 238.733 1.00 50.10
H

```
ATOM  34550 HG13 ILE L 117      95.476 142.095 237.368  1.00 50.10
H
ATOM  34551 HG21 ILE L 117      93.909 140.678 240.335  1.00 59.95
H
ATOM  34552 HG22 ILE L 117      95.294 140.156 240.912  1.00 59.95
H
ATOM  34553 HG23 ILE L 117      95.028 141.717 240.774  1.00 59.95
H
ATOM  34554 HD11 ILE L 117      93.348 142.892 237.317  1.00 59.10
H
ATOM  34555 HD12 ILE L 117      93.318 141.304 237.288  1.00 59.10
H
ATOM  34556 HD13 ILE L 117      92.992 142.080 238.636  1.00 59.10
H
ATOM  34557  N   PHE L 118      97.827 143.038 239.321  1.00 48.28
N
ATOM  34558  CA  PHE L 118      98.455 144.142 240.036  1.00 55.48
C
ATOM  34559  C   PHE L 118      97.647 145.398 239.751  1.00 47.58
C
ATOM  34560  O   PHE L 118      97.485 145.761 238.571  1.00 47.96
O
ATOM  34561  CB  PHE L 118      99.905 144.356 239.595  1.00 47.29
C
ATOM  34562  CG  PHE L 118     100.818 143.203 239.863  1.00 46.69
C
ATOM  34563  CD1 PHE L 118     101.434 143.059 241.099  1.00 45.92
C
ATOM  34564  CD2 PHE L 118     101.107 142.292 238.865  1.00 52.54
C
ATOM  34565  CE1 PHE L 118     102.312 142.018 241.336  1.00 50.24
C
ATOM  34566  CE2 PHE L 118     101.964 141.232 239.097  1.00 51.03
C
ATOM  34567  CZ  PHE L 118     102.584 141.106 240.342  1.00 50.99
C
ATOM  34568  H   PHE L 118      97.683 143.199 238.488  1.00 57.96
H
ATOM  34569  HA  PHE L 118      98.446 143.943 240.985  1.00 66.60
H
ATOM  34570  HB2 PHE L 118      99.914 144.520 238.639  1.00 56.77
H
ATOM  34571  HB3 PHE L 118     100.258 145.126 240.067  1.00 56.77
H
ATOM  34572  HD1 PHE L 118     101.253 143.670 241.776  1.00 55.13
H
ATOM  34573  HD2 PHE L 118     100.718 142.393 238.025  1.00 63.08
H
ATOM  34574  HE1 PHE L 118     102.720 141.934 242.168  1.00 60.31
H
```

ATOM 34575 HE2 PHE L 118   102.127 140.608 238.428 1.00 61.26
H
ATOM 34576 HZ PHE L 118   103.179 140.409 240.499 1.00 61.21
H
ATOM 34577 N   PRO L 119   97.132 146.085 240.770 1.00 50.22
N
ATOM 34578 CA PRO L 119   96.437 147.349 240.543 1.00 55.97
C
ATOM 34579 C   PRO L 119   97.435 148.429 240.185 1.00 51.00
C
ATOM 34580 O   PRO L 119   98.645 148.235 240.366 1.00 48.61
O
ATOM 34581 CB PRO L 119   95.761 147.660 241.898 1.00 56.76
C
ATOM 34582 CG PRO L 119   95.978 146.433 242.756 1.00 55.21
C
ATOM 34583 CD PRO L 119   97.152 145.724 242.198 1.00 55.96
C
ATOM 34584 HA PRO L 119   95.759 147.255 239.855 1.00 67.20
H
ATOM 34585 HB2 PRO L 119   96.176 148.439 242.301 1.00 68.14
H
ATOM 34586 HB3 PRO L 119   94.815 147.822 241.763 1.00 68.14
H
ATOM 34587 HG2 PRO L 119   96.146 146.704 243.671 1.00 66.28
H
ATOM 34588 HG3 PRO L 119   95.190 145.868 242.716 1.00 66.28
H
ATOM 34589 HD2 PRO L 119   97.970 146.032 242.620 1.00 67.18
H
ATOM 34590 HD3 PRO L 119   97.061 144.766 242.312 1.00 67.18
H
ATOM 34591 N   PRO L 120   96.964 149.583 239.731 1.00 46.07
N
ATOM 34592 CA PRO L 120   97.874 150.712 239.516 1.00 57.30
C
ATOM 34593 C   PRO L 120   98.310 151.342 240.832 1.00 57.65
C
ATOM 34594 O   PRO L 120   97.559 151.398 241.809 1.00 52.94
O
ATOM 34595 CB PRO L 120   97.043 151.688 238.677 1.00 57.17
C
ATOM 34596 CG PRO L 120   95.632 151.350 238.959 1.00 55.96
C
ATOM 34597 CD PRO L 120   95.596 149.871 239.289 1.00 52.43
C
ATOM 34598 HA PRO L 120   98.654 150.431 239.016 1.00 68.79
H
ATOM 34599 HB2 PRO L 120   97.240 152.598 238.943 1.00 68.64
H

ATOM 34600 HB3 PRO L 120 97.245 151.564 237.736 1.00 68.64
H
ATOM 34601 HG2 PRO L 120 95.317 151.874 239.713 1.00 67.18
H
ATOM 34602 HG3 PRO L 120 95.091 151.537 238.177 1.00 67.18
H
ATOM 34603 HD2 PRO L 120 94.962 149.691 240.001 1.00 62.95
H
ATOM 34604 HD3 PRO L 120 95.370 149.348 238.504 1.00 62.95
H
ATOM 34605 N SER L 121 99.568 151.758 240.848 1.00 56.13
N
ATOM 34606 CA SER L 121 100.138 152.428 241.996 1.00 56.23
C
ATOM 34607 C SER L 121 99.539 153.816 242.143 1.00 60.28
C
ATOM 34608 O SER L 121 99.145 154.467 241.170 1.00 57.57
O
ATOM 34609 CB SER L 121 101.659 152.543 241.854 1.00 55.31
C
ATOM 34610 OG SER L 121 102.007 153.475 240.840 1.00 55.50
O
ATOM 34611 H SER L 121 100.119 151.661 240.196 1.00 67.39
H
ATOM 34612 HA SER L 121 99.945 151.916 242.796 1.00 67.51
H
ATOM 34613 HB2 SER L 121 102.034 152.843 242.698 1.00 66.40
H
ATOM 34614 HB3 SER L 121 102.021 151.674 241.621 1.00 66.40
H
ATOM 34615 HG SER L 121 102.843 153.550 240.793 1.00 66.62
H
ATOM 34616 N ASP L 122 99.509 154.283 243.385 1.00 58.15
N
ATOM 34617 CA ASP L 122 99.087 155.648 243.644 1.00 57.71
C
ATOM 34618 C ASP L 122 99.930 156.653 242.868 1.00 55.67
C
ATOM 34619 O ASP L 122 99.399 157.656 242.358 1.00 49.11
O
ATOM 34620 CB ASP L 122 99.152 155.933 245.139 1.00 62.41
C
ATOM 34621 CG ASP L 122 98.184 155.084 245.923 1.00 79.00
C
ATOM 34622 OD1 ASP L 122 98.565 154.677 247.044 1.00 95.20
O
ATOM 34623 OD2 ASP L 122 97.066 154.794 245.404 1.00 71.99
O1-
ATOM 34624 H ASP L 122 99.723 153.834 244.086 1.00 69.81
H

ATOM 34625 HA ASP L 122 98.168 155.750 243.353 1.00 69.28
H
ATOM 34626 HB2 ASP L 122 100.046 155.744 245.460 1.00 74.92
H
ATOM 34627 HB3 ASP L 122 98.931 156.864 245.295 1.00 74.92
H
ATOM 34628 N GLU L 123 101.240 156.403 242.765 1.00 58.85
N
ATOM 34629 CA GLU L 123 102.132 157.353 242.115 1.00 65.97
C
ATOM 34630 C GLU L 123 101.702 157.574 240.668 1.00 65.21
C
ATOM 34631 O GLU L 123 101.538 158.717 240.224 1.00 63.23
O
ATOM 34632 CB GLU L 123 103.574 156.852 242.212 1.00 65.49
C
ATOM 34633 CG GLU L 123 104.652 157.768 241.600 1.00 83.91
C
ATOM 34634 CD GLU L 123 106.050 157.098 241.524 1.00 99.44
C
ATOM 34635 OE1 GLU L 123 106.322 156.169 242.336 1.00 88.71
O
ATOM 34636 OE2 GLU L 123 106.867 157.501 240.649 1.00 89.26
O1−
ATOM 34637 H GLU L 123 101.629 155.695 243.061 1.00 70.65
H
ATOM 34638 HA GLU L 123 102.094 158.210 242.566 1.00 79.19
H
ATOM 34639 HB2 GLU L 123 103.792 156.741 243.151 1.00 78.61
H
ATOM 34640 HB3 GLU L 123 103.630 155.998 241.756 1.00 78.61
H
ATOM 34641 HG2 GLU L 123 104.386 158.006 240.698 1.00100.72
H
ATOM 34642 HG3 GLU L 123 104.733 158.567 242.144 1.00100.72
H
ATOM 34643 N GLN L 124 101.436 156.481 239.942 1.00 60.17
N
ATOM 34644 CA GLN L 124 100.986 156.608 238.560 1.00 62.64
C
ATOM 34645 C GLN L 124 99.611 157.250 238.472 1.00 51.98
C
ATOM 34646 O GLN L 124 99.315 157.935 237.494 1.00 57.06
O
ATOM 34647 CB GLN L 124 100.964 155.238 237.864 1.00 51.24
C
ATOM 34648 CG GLN L 124 100.677 155.365 236.406 1.00 55.25
C
ATOM 34649 CD GLN L 124 100.309 154.070 235.703 1.00 58.65
C

```
ATOM  34650  OE1 GLN L 124      99.917 153.094 236.321  1.00 51.27
O
ATOM  34651  NE2 GLN L 124     100.376 154.092 234.386  1.00 55.31
N
ATOM  34652  H   GLN L 124     101.509 155.673 240.226  1.00 72.24
H
ATOM  34653  HA  GLN L 124     101.620 157.169 238.089  1.00 75.20
H
ATOM  34654  HB2 GLN L 124     101.828 154.813 237.968  1.00 61.51
H
ATOM  34655  HB3 GLN L 124     100.272 154.688 238.265  1.00 61.51
H
ATOM  34656  HG2 GLN L 124      99.932 155.977 236.292  1.00 66.33
H
ATOM  34657  HG3 GLN L 124     101.466 155.719 235.968  1.00 66.33
H
ATOM  34658 HE21 GLN L 124     100.617 154.811 233.979  1.00 66.40
H
ATOM  34659 HE22 GLN L 124     100.178 153.388 233.933  1.00 66.40
H
ATOM  34660  N   LEU L 125      98.741 157.023 239.452  1.00 56.85
N
ATOM  34661  CA  LEU L 125      97.436 157.680 239.400  1.00 58.71
C
ATOM  34662  C   LEU L 125      97.551 159.200 239.571  1.00 60.63
C
ATOM  34663  O   LEU L 125      96.745 159.951 239.012  1.00 68.10
O
ATOM  34664  CB  LEU L 125      96.526 157.097 240.461  1.00 60.38
C
ATOM  34665  CG  LEU L 125      95.980 155.704 240.141  1.00 56.64
C
ATOM  34666  CD1 LEU L 125      95.457 155.073 241.427  1.00 63.51
C
ATOM  34667  CD2 LEU L 125      94.872 155.751 239.093  1.00 66.76
C
ATOM  34668  H   LEU L 125      98.875 156.514 240.133  1.00 68.25
H
ATOM  34669  HA  LEU L 125      97.037 157.512 238.531  1.00 70.48
H
ATOM  34670  HB2 LEU L 125      97.023 157.034 241.291  1.00 72.49
H
ATOM  34671  HB3 LEU L 125      95.766 157.690 240.572  1.00 72.49
H
ATOM  34672  HG  LEU L 125      96.696 155.163 239.771  1.00 68.00
H
ATOM  34673 HD11 LEU L 125      95.046 154.220 241.217  1.00 76.24
H
ATOM  34674 HD12 LEU L 125      96.199 154.941 242.037  1.00 76.24
H
```

ATOM 34675 HD13 LEU L 125   94.802 155.667 241.825 1.00 76.24
H
ATOM 34676 HD21 LEU L 125   94.495 154.862 238.992 1.00 80.14
H
ATOM 34677 HD22 LEU L 125   94.186 156.369 239.386 1.00 80.14
H
ATOM 34678 HD23 LEU L 125   95.248 156.047 238.249 1.00 80.14
H
ATOM 34679 N   LYS L 126   98.538 159.671 240.327 1.00 57.53
N
ATOM 34680 CA LYS L 126   98.727 161.107 240.458 1.00 74.89
C
ATOM 34681 C   LYS L 126   99.050 161.736 239.118 1.00 66.45
C
ATOM 34682 O   LYS L 126   98.712 162.898 238.883 1.00 75.38
O
ATOM 34683 CB LYS L 126   99.845 161.417 241.459 1.00 73.74
C
ATOM 34684 CG LYS L 126   99.376 161.574 242.894 1.00 81.22
C
ATOM 34685 CD LYS L 126   100.357 162.397 243.747 1.00 91.18
C
ATOM 34686 CE LYS L 126   101.761 161.774 243.830 1.00108.96
C
ATOM 34687 NZ LYS L 126   101.874 160.592 244.757 1.00 91.69
N
ATOM 34688 H   LYS L 126   99.100 159.188 240.766 1.00 69.06
H
ATOM 34689 HA LYS L 126   97.908 161.497 240.801 1.00 89.89
H
ATOM 34690 HB2 LYS L 126   100.488 160.691 241.438 1.00 88.51
H
ATOM 34691 HB3 LYS L 126   100.273 162.248 241.198 1.00 88.51
H
ATOM 34692 HG2 LYS L 126   98.518 162.027 242.900 1.00 97.49
H
ATOM 34693 HG3 LYS L 126   99.289 160.696 243.298 1.00 97.49
H
ATOM 34694 HD2 LYS L 126   100.446 163.280 243.359 1.00109.45
H
ATOM 34695 HD3 LYS L 126   100.008 162.464 244.650 1.00109.45
H
ATOM 34696 HE2 LYS L 126   102.017 161.474 242.944 1.00130.79
H
ATOM 34697 HE3 LYS L 126   102.381 162.451 244.145 1.00130.79
H
ATOM 34698 HZ1 LYS L 126   102.692 160.245 244.711 1.00110.05
H
ATOM 34699 HZ2 LYS L 126   101.714 160.847 245.595 1.00110.05
H

```
ATOM  34700  HZ3 LYS L 126     101.283 159.968 244.528  1.00110.05
H
ATOM  34701  N   SER L 127      99.695 160.976 238.234  1.00 63.30
N
ATOM  34702  CA  SER L 127     100.129 161.404 236.909  1.00 59.45
C
ATOM  34703  C   SER L 127      99.043 161.306 235.849  1.00 55.36
C
ATOM  34704  O   SER L 127      99.308 161.665 234.697  1.00 59.06
O
ATOM  34705  CB  SER L 127     101.305 160.542 236.445  1.00 57.64
C
ATOM  34706  OG  SER L 127     102.300 160.530 237.443  1.00 70.15
O
ATOM  34707  H   SER L 127      99.905 160.157 238.389  1.00 75.99
H
ATOM  34708  HA  SER L 127     100.407 162.332 236.978  1.00 71.36
H
ATOM  34709  HB2 SER L 127     100.996 159.637 236.289  1.00 69.19
H
ATOM  34710  HB3 SER L 127     101.673 160.913 235.628  1.00 69.19
H
ATOM  34711  HG  SER L 127     102.931 160.026 237.212  1.00 84.21
H
ATOM  34712  N   GLY L 128      97.869 160.777 236.187  1.00 53.71
N
ATOM  34713  CA  GLY L 128      96.711 160.812 235.318  1.00 56.27
C
ATOM  34714  C   GLY L 128      96.477 159.585 234.464  1.00 64.46
C
ATOM  34715  O   GLY L 128      95.596 159.613 233.595  1.00 60.02
O
ATOM  34716  H   GLY L 128      97.719 160.382 236.936  1.00 64.47
H
ATOM  34717  HA2 GLY L 128      95.922 160.938 235.867  1.00 67.56
H
ATOM  34718  HA3 GLY L 128      96.804 161.569 234.718  1.00 67.56
H
ATOM  34719  N   THR L 129      97.204 158.505 234.696  1.00 61.53
N
ATOM  34720  CA  THR L 129      97.042 157.268 233.949  1.00 56.52
C
ATOM  34721  C   THR L 129      96.909 156.138 234.967  1.00 61.16
C
ATOM  34722  O   THR L 129      97.305 156.285 236.128  1.00 60.88
O
ATOM  34723  CB  THR L 129      98.238 157.066 232.985  1.00 59.94
C
ATOM  34724  OG1 THR L 129      98.173 158.032 231.918  1.00 69.77
O
```

ATOM 34725 CG2 THR L 129 98.267 155.679 232.373 1.00 66.42
C
ATOM 34726 H THR L 129 97.817 158.463 235.298 1.00 73.86
H
ATOM 34727 HA THR L 129 96.240 157.263 233.402 1.00 67.86
H
ATOM 34728 HB THR L 129 99.052 157.178 233.500 1.00 71.96
H
ATOM 34729 HG1 THR L 129 98.854 157.969 231.433 1.00 83.76
H
ATOM 34730 HG21 THR L 129 98.946 155.635 231.683 1.00 79.73
H
ATOM 34731 HG22 THR L 129 98.467 155.020 233.055 1.00 79.73
H
ATOM 34732 HG23 THR L 129 97.404 155.473 231.980 1.00 79.73
H
ATOM 34733 N ALA L 130 96.320 155.012 234.542 1.00 56.38
N
ATOM 34734 CA ALA L 130 96.260 153.792 235.352 1.00 51.58
C
ATOM 34735 C ALA L 130 96.653 152.620 234.469 1.00 54.53
C
ATOM 34736 O ALA L 130 96.027 152.371 233.438 1.00 47.77
O
ATOM 34737 CB ALA L 130 94.875 153.548 235.952 1.00 50.75
C
ATOM 34738 H ALA L 130 95.943 154.931 233.773 1.00 67.68
H
ATOM 34739 HA ALA L 130 96.887 153.877 236.087 1.00 61.92
H
ATOM 34740 HB1 ALA L 130 94.884 152.707 236.435 1.00 60.93
H
ATOM 34741 HB2 ALA L 130 94.662 154.274 236.559 1.00 60.93
H
ATOM 34742 HB3 ALA L 130 94.222 153.513 235.237 1.00 60.93
H
ATOM 34743 N SER L 131 97.726 151.949 234.835 1.00 49.72
N
ATOM 34744 CA SER L 131 98.099 150.701 234.209 1.00 49.99
C
ATOM 34745 C SER L 131 97.740 149.570 235.158 1.00 47.72
C
ATOM 34746 O SER L 131 98.109 149.607 236.339 1.00 51.43
O
ATOM 34747 CB SER L 131 99.584 150.684 233.877 1.00 53.09
C
ATOM 34748 OG SER L 131 99.884 151.674 232.911 1.00 54.65
O
ATOM 34749 H SER L 131 98.264 152.202 235.455 1.00 59.69
H

ATOM 34750 HA SER L 131 97.615 150.577 233.377 1.00 60.01
H
ATOM 34751 HB2 SER L 131 100.093 150.865 234.684 1.00 63.74
H
ATOM 34752 HB3 SER L 131 99.822 149.813 233.524 1.00 63.74
H
ATOM 34753 HG SER L 131 99.183 152.084 232.695 1.00 65.61
H
ATOM 34754 N VAL L 132 96.963 148.615 234.656 1.00 46.82
N
ATOM 34755 CA VAL L 132 96.641 147.380 235.362 1.00 48.65
C
ATOM 34756 C VAL L 132 97.409 146.259 234.695 1.00 46.43
C
ATOM 34757 O VAL L 132 97.395 146.152 233.467 1.00 48.57
O
ATOM 34758 CB VAL L 132 95.132 147.074 235.334 1.00 45.95
C
ATOM 34759 CG1 VAL L 132 94.786 146.073 236.416 1.00 44.50
C
ATOM 34760 CG2 VAL L 132 94.339 148.317 235.476 1.00 46.26
C
ATOM 34761 H VAL L 132 96.598 148.662 233.878 1.00 56.21
H
ATOM 34762 HA VAL L 132 96.918 147.463 236.287 1.00 58.40
H
ATOM 34763 HB VAL L 132 94.902 146.683 234.477 1.00 55.17
H
ATOM 34764 HG11 VAL L 132 93.825 145.948 236.434 1.00 53.43
H
ATOM 34765 HG12 VAL L 132 95.226 145.230 236.220 1.00 53.43
H
ATOM 34766 HG13 VAL L 132 95.091 146.415 237.271 1.00 53.43
H
ATOM 34767 HG21 VAL L 132 93.417 148.084 235.673 1.00 55.54
H
ATOM 34768 HG22 VAL L 132 94.708 148.846 236.200 1.00 55.54
H
ATOM 34769 HG23 VAL L 132 94.383 148.817 234.646 1.00 55.54
H
ATOM 34770 N VAL L 133 98.058 145.417 235.492 1.00 43.73
N
ATOM 34771 CA VAL L 133 99.015 144.441 234.986 1.00 41.24
C
ATOM 34772 C VAL L 133 98.569 143.036 235.394 1.00 49.29
C
ATOM 34773 O VAL L 133 98.145 142.803 236.535 1.00 47.17
O
ATOM 34774 CB VAL L 133 100.448 144.764 235.445 1.00 46.92
C

```
ATOM  34775  CG1 VAL L 133     101.453 143.762 234.919  1.00 42.65
C
ATOM  34776  CG2 VAL L 133     100.877 146.148 234.909  1.00 47.79
C
ATOM  34777  H   VAL L 133      97.958 145.391 236.346  1.00 52.50
H
ATOM  34778  HA  VAL L 133      99.009 144.459 234.016  1.00 49.52
H
ATOM  34779  HB  VAL L 133     100.445 144.742 236.415  1.00 56.33
H
ATOM  34780 HG11 VAL L 133     102.341 144.022 235.209  1.00 51.21
H
ATOM  34781 HG12 VAL L 133     101.235 142.884 235.266  1.00 51.21
H
ATOM  34782 HG13 VAL L 133     101.412 143.754 233.949  1.00 51.21
H
ATOM  34783 HG21 VAL L 133     101.750 146.371 235.268  1.00 57.38
H
ATOM  34784 HG22 VAL L 133     100.918 146.112 233.939  1.00 57.38
H
ATOM  34785 HG23 VAL L 133     100.226 146.812 235.187  1.00 57.38
H
ATOM  34786  N   CYS L 134      98.627 142.111 234.445  1.00 51.75
N
ATOM  34787  CA  CYS L 134      98.269 140.712 234.667  1.00 49.10
C
ATOM  34788  C   CYS L 134      99.472 139.876 234.265  1.00 55.02
C
ATOM  34789  O   CYS L 134     100.030 140.077 233.187  1.00 47.29
O
ATOM  34790  CB  CYS L 134      97.034 140.321 233.861  1.00 59.57
C
ATOM  34791  SG  CYS L 134      96.307 138.672 234.235  1.00 59.94
S
ATOM  34792  H   CYS L 134      98.878 142.273 233.639  1.00 62.13
H
ATOM  34793  HA  CYS L 134      98.060 140.547 235.599  1.00 58.95
H
ATOM  34794  HB2 CYS L 134      96.343 140.981 234.029  1.00 71.52
H
ATOM  34795  HB3 CYS L 134      97.273 140.322 232.923  1.00 71.52
H
ATOM  34796  N   LEU L 135      99.925 139.014 235.172  1.00 48.66
N
ATOM  34797  CA  LEU L 135     101.083 138.168 234.959  1.00 48.54
C
ATOM  34798  C   LEU L 135     100.647 136.716 234.881  1.00 52.28
C
ATOM  34799  O   LEU L 135      99.932 136.239 235.761  1.00 49.55
O
```

ATOM 34800 CB LEU L 135   102.078 138.332 236.090 1.00 51.81
C
ATOM 34801 CG LEU L 135   103.153 137.262 236.145 1.00 57.81
C
ATOM 34802 CD1 LEU L 135   104.045 137.279 234.898 1.00 53.91
C
ATOM 34803 CD2 LEU L 135   103.911 137.490 237.419 1.00 54.88
C
ATOM 34804 H   LEU L 135   99.564 138.902 235.944 1.00 58.42
H
ATOM 34805 HA LEU L 135   101.507 138.410 234.122 1.00 58.28
H
ATOM 34806 HB2 LEU L 135   102.523 139.189 235.988 1.00 62.21
H
ATOM 34807 HB3 LEU L 135   101.597 138.307 236.931 1.00 62.21
H
ATOM 34808 HG LEU L 135   102.773 136.369 236.146 1.00 69.39
H
ATOM 34809 HD11 LEU L 135   104.699 136.566 234.968 1.00 64.72
H
ATOM 34810 HD12 LEU L 135   103.493 137.145 234.112 1.00 64.72
H
ATOM 34811 HD13 LEU L 135   104.496 138.136 234.844 1.00 64.72
H
ATOM 34812 HD21 LEU L 135   104.630 136.842 237.480 1.00 65.88
H
ATOM 34813 HD22 LEU L 135   104.279 138.389 237.411 1.00 65.88
H
ATOM 34814 HD23 LEU L 135   103.307 137.387 238.170 1.00 65.88
H
ATOM 34815 N   LEU L 136   101.082 136.032 233.830 1.00 55.08
N
ATOM 34816 CA LEU L 136   100.934 134.590 233.661 1.00 50.56
C
ATOM 34817 C   LEU L 136   102.306 133.987 233.759 1.00 46.41
C
ATOM 34818 O   LEU L 136   103.129 134.181 232.862 1.00 48.62
O
ATOM 34819 CB LEU L 136   100.336 134.238 232.313 1.00 49.91
C
ATOM 34820 CG LEU L 136   98.843 134.416 232.156 1.00 54.88
C
ATOM 34821 CD1 LEU L 136   98.493 135.865 232.409 1.00 52.27
C
ATOM 34822 CD2 LEU L 136   98.453 133.953 230.762 1.00 55.48
C
ATOM 34823 H   LEU L 136   101.486 136.401 233.166 1.00 66.12
H
ATOM 34824 HA LEU L 136   100.355 134.252 234.361 1.00 60.70
H

```
ATOM  34825  HB2 LEU L 136     100.759 134.798 231.644  1.00 59.93
H
ATOM  34826  HB3 LEU L 136     100.530 133.305 232.135  1.00 59.93
H
ATOM  34827  HG  LEU L 136      98.340 133.887 232.795  1.00 65.88
H
ATOM  34828 HD11 LEU L 136      97.656 136.070 231.964  1.00 62.75
H
ATOM  34829 HD12 LEU L 136      98.403 136.006 233.364  1.00 62.75
H
ATOM  34830 HD13 LEU L 136      99.201 136.428 232.057  1.00 62.75
H
ATOM  34831 HD21 LEU L 136      97.499 134.084 230.640  1.00 66.60
H
ATOM  34832 HD22 LEU L 136      98.944 134.475 230.107  1.00 66.60
H
ATOM  34833 HD23 LEU L 136      98.674 133.013 230.668  1.00 66.60
H
ATOM  34834  N   ASN L 137     102.571 133.270 234.848  1.00 45.37
N
ATOM  34835  CA  ASN L 137     103.915 132.789 235.110  1.00 44.93
C
ATOM  34836  C   ASN L 137     104.069 131.313 234.771  1.00 53.88
C
ATOM  34837  O   ASN L 137     103.228 130.484 235.153  1.00 50.66
O
ATOM  34838  CB  ASN L 137     104.305 133.013 236.563  1.00 51.74
C
ATOM  34839  CG  ASN L 137     105.686 133.495 236.685  1.00 53.33
C
ATOM  34840  OD1 ASN L 137     106.152 134.282 235.852  1.00 62.70
O
ATOM  34841  ND2 ASN L 137     106.379 133.037 237.694  1.00 60.13
N
ATOM  34842  H   ASN L 137     101.991 133.053 235.443  1.00 54.47
H
ATOM  34843  HA  ASN L 137     104.517 133.284 234.532  1.00 53.94
H
ATOM  34844  HB2 ASN L 137     103.715 133.679 236.953  1.00 62.11
H
ATOM  34845  HB3 ASN L 137     104.229 132.179 237.049  1.00 62.11
H
ATOM  34846 HD21 ASN L 137     107.193 133.290 237.808  1.00 72.19
H
ATOM  34847 HD22 ASN L 137     106.021 132.483 238.247  1.00 72.19
H
ATOM  34848  N   ASN L 138     105.157 131.005 234.055  1.00 48.61
N
ATOM  34849  CA  ASN L 138     105.716 129.659 233.893  1.00 51.13
C
```

ATOM 34850 C ASN L 138 104.690 128.654 233.353 1.00 54.09
C
ATOM 34851 O ASN L 138 104.302 127.704 234.026 1.00 55.84
O
ATOM 34852 CB ASN L 138 106.291 129.207 235.237 1.00 54.33
C
ATOM 34853 CG ASN L 138 107.564 129.919 235.570 1.00 62.05
C
ATOM 34854 OD1 ASN L 138 107.748 131.076 235.210 1.00 59.36
O
ATOM 34855 ND2 ASN L 138 108.481 129.219 236.200 1.00 79.17
N
ATOM 34856 H ASN L 138 105.615 131.595 233.630 1.00 58.35
H
ATOM 34857 HA ASN L 138 106.416 129.695 233.223 1.00 61.39
H
ATOM 34858 HB2 ASN L 138 105.648 129.393 235.938 1.00 65.23
H
ATOM 34859 HB3 ASN L 138 106.477 128.255 235.201 1.00 65.23
H
ATOM 34860 HD21 ASN L 138 109.230 129.585 236.412 1.00 95.03
H
ATOM 34861 HD22 ASN L 138 108.333 128.395 236.399 1.00 95.03
H
ATOM 34862 N PHE L 139 104.317 128.836 232.085 1.00 51.44
N
ATOM 34863 CA PHE L 139 103.308 127.992 231.458 1.00 50.15
C
ATOM 34864 C PHE L 139 103.815 127.448 230.130 1.00 55.87
C
ATOM 34865 O PHE L 139 104.787 127.940 229.553 1.00 56.07
O
ATOM 34866 CB PHE L 139 101.988 128.725 231.254 1.00 47.87
C
ATOM 34867 CG PHE L 139 102.068 129.910 230.344 1.00 57.02
C
ATOM 34868 CD1 PHE L 139 101.849 129.767 228.985 1.00 56.39
C
ATOM 34869 CD2 PHE L 139 102.332 131.179 230.851 1.00 52.69
C
ATOM 34870 CE1 PHE L 139 101.922 130.855 228.128 1.00 60.83
C
ATOM 34871 CE2 PHE L 139 102.403 132.277 230.023 1.00 50.00
C
ATOM 34872 CZ PHE L 139 102.202 132.117 228.632 1.00 59.86
C
ATOM 34873 H PHE L 139 104.636 129.446 231.568 1.00 61.75
H
ATOM 34874 HA PHE L 139 103.145 127.233 232.042 1.00 60.21
H

ATOM 34875 HB2 PHE L 139 101.347 128.106 230.872 1.00 57.48
H
ATOM 34876 HB3 PHE L 139 101.673 129.040 232.116 1.00 57.48
H
ATOM 34877 HD1 PHE L 139 101.651 128.926 228.641 1.00 67.69
H
ATOM 34878 HD2 PHE L 139 102.464 131.286 231.766 1.00 63.26
H
ATOM 34879 HE1 PHE L 139 101.784 130.740 227.217 1.00 73.03
H
ATOM 34880 HE2 PHE L 139 102.583 133.119 230.376 1.00 60.03
H
ATOM 34881 HZ PHE L 139 102.256 132.849 228.061 1.00 71.86
H
ATOM 34882 N TYR L 140 103.139 126.397 229.667 1.00 53.27
N
ATOM 34883 CA TYR L 140 103.445 125.719 228.416 1.00 53.11
C
ATOM 34884 C TYR L 140 102.188 125.019 227.930 1.00 54.49
C
ATOM 34885 O TYR L 140 101.511 124.358 228.725 1.00 60.52
O
ATOM 34886 CB TYR L 140 104.571 124.703 228.559 1.00 55.03
C
ATOM 34887 CG TYR L 140 105.038 124.174 227.202 1.00 66.31
C
ATOM 34888 CD1 TYR L 140 104.302 123.197 226.521 1.00 63.00
C
ATOM 34889 CD2 TYR L 140 106.192 124.669 226.588 1.00 59.28
C
ATOM 34890 CE1 TYR L 140 104.698 122.724 225.274 1.00 68.45
C
ATOM 34891 CE2 TYR L 140 106.606 124.186 225.337 1.00 68.90
C
ATOM 34892 CZ TYR L 140 105.850 123.217 224.682 1.00 74.01
C
ATOM 34893 OH TYR L 140 106.244 122.731 223.440 1.00 76.42
O
ATOM 34894 H TYR L 140 102.470 126.046 230.077 1.00 63.95
H
ATOM 34895 HA TYR L 140 103.719 126.384 227.766 1.00 63.76
H
ATOM 34896 HB2 TYR L 140 105.326 125.123 228.998 1.00 66.07
H
ATOM 34897 HB3 TYR L 140 104.257 123.950 229.086 1.00 66.07
H
ATOM 34898 HD1 TYR L 140 103.529 122.858 226.911 1.00 75.63
H
ATOM 34899 HD2 TYR L 140 106.692 125.328 227.014 1.00 71.16
H

```
ATOM  34900  HE1 TYR L 140     104.192 122.076 224.839  1.00 82.17
H
ATOM  34901  HE2 TYR L 140     107.386 124.511 224.948  1.00 82.71
H
ATOM  34902  HH  TYR L 140     107.037 122.960 223.282  1.00 91.73
H
ATOM  34903  N   PRO L 141     101.862 125.105 226.645  1.00 62.35
N
ATOM  34904  CA  PRO L 141     102.619 125.775 225.573  1.00 64.80
C
ATOM  34905  C   PRO L 141     102.381 127.295 225.545  1.00 60.82
C
ATOM  34906  O   PRO L 141     101.640 127.795 226.423  1.00 51.47
O
ATOM  34907  CB  PRO L 141     102.094 125.085 224.306  1.00 63.96
C
ATOM  34908  CG  PRO L 141     100.690 124.734 224.628  1.00 59.37
C
ATOM  34909  CD  PRO L 141     100.631 124.473 226.134  1.00 59.32
C
ATOM  34910  HA  PRO L 141     103.572 125.614 225.650  1.00 77.78
H
ATOM  34911  HB2 PRO L 141     102.138 125.695 223.553  1.00 76.78
H
ATOM  34912  HB3 PRO L 141     102.618 124.289 224.120  1.00 76.78
H
ATOM  34913  HG2 PRO L 141     100.109 125.474 224.388  1.00 71.27
H
ATOM  34914  HG3 PRO L 141     100.434 123.939 224.134  1.00 71.27
H
ATOM  34915  HD2 PRO L 141      99.846 124.886 226.525  1.00 71.21
H
ATOM  34916  HD3 PRO L 141     100.632 123.521 226.319  1.00 71.21
H
ATOM  34917  N   ARG L 142     102.986 127.976 224.546  1.00 67.10
N
ATOM  34918  CA AARG L 142     102.958 129.436 224.525  0.50 63.74
C
ATOM  34919  CA BARG L 142     102.963 129.437 224.457  0.50 63.84
C
ATOM  34920  C   ARG L 142     101.547 129.984 224.368  1.00 60.54
C
ATOM  34921  O   ARG L 142     101.276 131.088 224.832  1.00 57.33
O
ATOM  34922  CB AARG L 142     103.849 129.995 223.403  0.50 65.57
C
ATOM  34923  CB BARG L 142     103.759 129.903 223.221  0.50 65.58
C
ATOM  34924  CG AARG L 142     103.817 131.558 223.266  0.50 60.92
C
```

ATOM 34925 CG BARG L 142   104.890 130.918 223.479 0.50 64.03
C
ATOM 34926 CD AARG L 142   104.583 132.052 222.019 0.50 69.14
C
ATOM 34927 CD BARG L 142   105.300 131.770 222.239 0.50 67.41
C
ATOM 34928 NE AARG L 142   104.114 133.353 221.517 0.50 66.01
N
ATOM 34929 NE BARG L 142   104.855 131.210 220.958 0.50 63.87
N
ATOM 34930 CZ AARG L 142   104.846 134.465 221.467 0.50 57.27
C
ATOM 34931 CZ BARG L 142   105.545 130.334 220.229 0.50 67.38
C
ATOM 34932 NH1AARG L 142   104.308 135.572 220.982 0.50 59.51
N
ATOM 34933 NH1BARG L 142   105.045 129.884 219.077 0.50 69.30
N
ATOM 34934 NH2AARG L 142   106.106 134.484 221.885 0.50 61.37
N
ATOM 34935 NH2BARG L 142   106.727 129.909 220.641 0.50 61.82
N
ATOM 34936 H   AARG L 142   103.406 127.616 223.889 0.50 80.55
H
ATOM 34937 H   BARG L 142   103.419 127.604 223.904 0.50 80.55
H
ATOM 34938 HA AARG L 142   103.315 129.739 225.374 0.50 76.52
H
ATOM 34939 HA BARG L 142   103.382 129.790 225.257 0.50 76.63
H
ATOM 34940 HB2AARG L 142   104.766 129.735 223.580 0.50 78.71
H
ATOM 34941 HB2BARG L 142   104.163 129.122 222.811 0.50 78.72
H
ATOM 34942 HB3AARG L 142   103.553 129.620 222.559 0.50 78.71
H
ATOM 34943 HB3BARG L 142   103.139 130.318 222.601 0.50 78.72
H
ATOM 34944 HG2AARG L 142   102.896 131.853 223.191 0.50 73.13
H
ATOM 34945 HG2BARG L 142   104.601 131.532 224.172 0.50 76.87
H
ATOM 34946 HG3AARG L 142   104.230 131.955 224.050 0.50 73.13
H
ATOM 34947 HG3BARG L 142   105.678 130.434 223.773 0.50 76.87
H
ATOM 34948 HD2AARG L 142   105.522 132.141 222.245 0.50 82.99
H
ATOM 34949 HD2BARG L 142   104.909 132.654 222.325 0.50 80.92
H

ATOM 34950 HD3AARG L 142 104.475 131.402 221.308 0.50 82.99
H
ATOM 34951 HD3BARG L 142 106.268 131.836 222.213 0.50 80.92
H
ATOM 34952 HE AARG L 142 103.305 133.398 221.232 0.50 79.24
H
ATOM 34953 HE BARG L 142 104.092 131.466 220.654 0.50 76.67
H
ATOM 34954 HH11AARG L 142 103.496 135.568 220.702 0.50 71.44
H
ATOM 34955 HH11BARG L 142 104.277 130.159 218.803 0.50 83.19
H
ATOM 34956 HH12AARG L 142 104.773 136.295 220.946 0.50 71.44
H
ATOM 34957 HH12BARG L 142 105.490 129.318 218.607 0.50 83.19
H
ATOM 34958 HH21AARG L 142 106.466 133.766 222.196 0.50 73.68
H
ATOM 34959 HH21BARG L 142 107.054 130.198 221.382 0.50 74.22
H
ATOM 34960 HH22AARG L 142 106.561 135.211 221.844 0.50 73.68
H
ATOM 34961 HH22BARG L 142 107.169 129.343 220.168 0.50 74.22
H
ATOM 34962 N GLU L 143 100.642 129.261 223.735 1.00 63.67
N
ATOM 34963 CA GLU L 143 99.346 129.867 223.440 1.00 59.73
C
ATOM 34964 C GLU L 143 98.581 130.127 224.729 1.00 62.92
C
ATOM 34965 O GLU L 143 98.356 129.215 225.520 1.00 55.20
O
ATOM 34966 CB GLU L 143 98.520 128.978 222.508 1.00 78.12
C
ATOM 34967 CG GLU L 143 99.050 128.846 221.069 1.00109.43
C
ATOM 34968 CD GLU L 143 100.478 128.300 220.979 1.00 92.72
C
ATOM 34969 OE1 GLU L 143 101.343 129.017 220.423 1.00 82.30
O
ATOM 34970 OE2 GLU L 143 100.733 127.163 221.458 1.00 80.09
O1-
ATOM 34971 H GLU L 143 100.742 128.449 223.474 1.00 76.43
H
ATOM 34972 HA GLU L 143 99.493 130.712 222.987 1.00 71.71
H
ATOM 34973 HB2 GLU L 143 98.490 128.084 222.886 1.00 93.77
H
ATOM 34974 HB3 GLU L 143 97.624 129.345 222.452 1.00 93.77
H

ATOM 34975 HG2 GLU L 143 98.471 128.242 220.578 1.00131.35
H
ATOM 34976 HG3 GLU L 143 99.044 129.723 220.654 1.00131.35
H
ATOM 34977 N ALA L 144 98.162 131.369 224.929 1.00 69.04
N
ATOM 34978 CA ALA L 144 97.309 131.724 226.056 1.00 62.58
C
ATOM 34979 C ALA L 144 96.373 132.833 225.620 1.00 56.77
C
ATOM 34980 O ALA L 144 96.677 133.585 224.690 1.00 62.69
O
ATOM 34981 CB ALA L 144 98.141 132.168 227.265 1.00 50.28
C
ATOM 34982 H ALA L 144 98.361 132.034 224.420 1.00 82.87
H
ATOM 34983 HA ALA L 144 96.775 130.960 226.322 1.00 75.12
H
ATOM 34984 HB1 ALA L 144 97.547 132.336 228.013 1.00 60.37
H
ATOM 34985 HB2 ALA L 144 98.768 131.464 227.494 1.00 60.37
H
ATOM 34986 HB3 ALA L 144 98.623 132.979 227.037 1.00 60.37
H
ATOM 34987 N LYS L 145 95.231 132.930 226.288 1.00 60.00
N
ATOM 34988 CA LYS L 145 94.264 133.987 226.038 1.00 60.15
C
ATOM 34989 C LYS L 145 94.041 134.771 227.330 1.00 71.02
C
ATOM 34990 O LYS L 145 93.719 134.188 228.380 1.00 62.97
O
ATOM 34991 CB LYS L 145 92.932 133.431 225.514 1.00 61.44
C
ATOM 34992 CG LYS L 145 92.133 134.436 224.683 1.00 75.48
C
ATOM 34993 CD LYS L 145 90.647 134.562 225.054 1.00 74.94
C
ATOM 34994 CE LYS L 145 89.810 133.456 224.447 1.00 85.58
C
ATOM 34995 NZ LYS L 145 88.330 133.659 224.734 1.00 88.40
N
ATOM 34996 H LYS L 145 94.989 132.380 226.905 1.00 72.03
H
ATOM 34997 HA LYS L 145 94.618 134.588 225.364 1.00 72.20
H
ATOM 34998 HB2 LYS L 145 93.113 132.660 224.953 1.00 73.75
H
ATOM 34999 HB3 LYS L 145 92.384 133.169 226.271 1.00 73.75
H

```
ATOM  35000  HG2 LYS L 145      92.533 135.313 224.795  1.00 90.60
H
ATOM  35001  HG3 LYS L 145      92.178 134.168 223.753  1.00 90.60
H
ATOM  35002  HD2 LYS L 145      90.555 134.515 226.019  1.00 89.95
H
ATOM  35003  HD3 LYS L 145      90.308 135.410 224.729  1.00 89.95
H
ATOM  35004  HE2 LYS L 145      89.936 133.448 223.486  1.00102.72
H
ATOM  35005  HE3 LYS L 145      90.080 132.603 224.824  1.00102.72
H
ATOM  35006  HZ1 LYS L 145      87.864 132.959 224.444  1.00106.10
H
ATOM  35007  HZ2 LYS L 145      88.199 133.750 225.609  1.00106.10
H
ATOM  35008  HZ3 LYS L 145      88.038 134.391 224.321  1.00106.10
H
ATOM  35009  N   VAL L 146      94.200 136.093 227.242  1.00 66.02
N
ATOM  35010  CA  VAL L 146      93.975 137.018 228.350  1.00 62.82
C
ATOM  35011  C   VAL L 146      92.858 137.971 227.955  1.00 59.48
C
ATOM  35012  O   VAL L 146      92.948 138.654 226.933  1.00 71.55
O
ATOM  35013  CB  VAL L 146      95.256 137.795 228.707  1.00 64.13
C
ATOM  35014  CG1 VAL L 146      95.029 138.735 229.883  1.00 66.07
C
ATOM  35015  CG2 VAL L 146      96.346 136.826 229.030  1.00 59.15
C
ATOM  35016  H   VAL L 146      94.445 136.490 226.520  1.00 79.25
H
ATOM  35017  HA  VAL L 146      93.684 136.522 229.131  1.00 75.41
H
ATOM  35018  HB  VAL L 146      95.515 138.336 227.946  1.00 76.99
H
ATOM  35019 HG11 VAL L 146      95.871 139.158 230.115  1.00 79.31
H
ATOM  35020 HG12 VAL L 146      94.379 139.407 229.628  1.00 79.31
H
ATOM  35021 HG13 VAL L 146      94.697 138.223 230.636  1.00 79.31
H
ATOM  35022 HG21 VAL L 146      97.128 137.318 229.327  1.00 71.01
H
ATOM  35023 HG22 VAL L 146      96.043 136.232 229.734  1.00 71.01
H
ATOM  35024 HG23 VAL L 146      96.560 136.315 228.234  1.00 71.01
H
```

ATOM 35025 N   GLN L 147   91.810 138.020 228.763 1.00 54.01
N
ATOM 35026 CA GLN L 147   90.671 138.893 228.548 1.00 58.31
C
ATOM 35027 C   GLN L 147   90.507 139.804 229.761 1.00 58.74
C
ATOM 35028 O   GLN L 147   90.360 139.330 230.891 1.00 54.75
O
ATOM 35029 CB GLN L 147   89.389 138.080 228.309 1.00 65.74
C
ATOM 35030 CG GLN L 147   89.073 137.881 226.817 1.00 79.60
C
ATOM 35031 CD GLN L 147   87.833 137.033 226.559 1.00 80.51
C
ATOM 35032 OE1 GLN L 147   87.135 136.609 227.483 1.00 81.46
O
ATOM 35033 NE2 GLN L 147   87.563 136.781 225.293 1.00 85.72
N
ATOM 35034 H   GLN L 147   91.734 137.538 229.472 1.00 64.84
H
ATOM 35035 HA GLN L 147   90.833 139.447 227.768 1.00 69.99
H
ATOM 35036 HB2 GLN L 147   89.492 137.203 228.713 1.00 78.91
H
ATOM 35037 HB3 GLN L 147   88.640 138.545 228.713 1.00 78.91
H
ATOM 35038 HG2 GLN L 147   88.929 138.748 226.411 1.00 95.55
H
ATOM 35039 HG3 GLN L 147   89.825 137.437 226.396 1.00 95.55
H
ATOM 35040 HE21 GLN L 147   88.074 137.090 224.674 1.00102.89
H
ATOM 35041 HE22 GLN L 147   86.875 136.309 225.086 1.00102.89
H
ATOM 35042 N   TRP L 148   90.518 141.105 229.526 1.00 56.17
N
ATOM 35043 CA TRP L 148   90.298 142.067 230.586 1.00 57.85
C
ATOM 35044 C   TRP L 148   88.819 142.365 230.729 1.00 57.57
C
ATOM 35045 O   TRP L 148   88.104 142.494 229.736 1.00 54.91
O
ATOM 35046 CB TRP L 148   91.068 143.355 230.298 1.00 54.17
C
ATOM 35047 CG TRP L 148   92.545 143.261 230.541 1.00 53.79
C
ATOM 35048 CD1 TRP L 148   93.522 143.079 229.608 1.00 51.83
C
ATOM 35049 CD2 TRP L 148   93.213 143.349 231.814 1.00 44.88
C

ATOM 35050 NE1 TRP L 148   94.768 143.059 230.222 1.00 54.57
N
ATOM 35051 CE2 TRP L 148   94.594 143.215 231.575 1.00 49.06
C
ATOM 35052 CE3 TRP L 148   92.776 143.537 233.123 1.00 53.77
C
ATOM 35053 CZ2 TRP L 148   95.537 143.274 232.599 1.00 51.48
C
ATOM 35054 CZ3 TRP L 148   93.723 143.597 234.146 1.00 47.41
C
ATOM 35055 CH2 TRP L 148   95.073 143.470 233.877 1.00 46.46
C
ATOM 35056 H   TRP L 148   90.652 141.457 228.752 1.00 67.44
H
ATOM 35057 HA TRP L 148   90.614 141.702 231.426 1.00 69.45
H
ATOM 35058 HB2 TRP L 148   90.939 143.590 229.365 1.00 65.04
H
ATOM 35059 HB3 TRP L 148   90.720 144.057 230.869 1.00 65.04
H
ATOM 35060 HD1 TRP L 148   93.376 142.982 228.694 1.00 62.22
H
ATOM 35061 HE1 TRP L 148   95.523 142.965 229.821 1.00 65.52
H
ATOM 35062 HE3 TRP L 148   91.869 143.622 233.311 1.00 64.55
H
ATOM 35063 HZ2 TRP L 148   96.445 143.184 232.425 1.00 61.81
H
ATOM 35064 HZ3 TRP L 148   93.442 143.725 235.023 1.00 56.92
H
ATOM 35065 HH2 TRP L 148   95.681 143.519 234.578 1.00 55.78
H
ATOM 35066 N   LYS L 149   88.359 142.470 231.977 1.00 54.66
N
ATOM 35067 CA LYS L 149   86.978 142.829 232.259 1.00 55.02
C
ATOM 35068 C   LYS L 149   86.921 143.924 233.312 1.00 55.17
C
ATOM 35069 O   LYS L 149   87.593 143.845 234.343 1.00 62.27
O
ATOM 35070 CB LYS L 149   86.195 141.594 232.687 1.00 62.83
C
ATOM 35071 CG LYS L 149   86.046 140.609 231.535 1.00 67.53
C
ATOM 35072 CD LYS L 149   85.167 139.407 231.897 1.00 76.10
C
ATOM 35073 CE LYS L 149   85.631 138.161 231.136 1.00 75.41
C
ATOM 35074 NZ LYS L 149   85.279 136.885 231.855 1.00 85.17
N

ATOM 35075 H   LYS L 149   88.836 142.337 232.680 1.00 65.62
H
ATOM 35076 HA LYS L 149   86.563 143.199 231.465 1.00 66.05
H
ATOM 35077 HB2 LYS L 149   86.665 141.151 233.412 1.00 75.42
H
ATOM 35078 HB3 LYS L 149   85.310 141.860 232.980 1.00 75.42
H
ATOM 35079 HG2 LYS L 149   85.641 141.062 230.779 1.00 81.07
H
ATOM 35080 HG3 LYS L 149   86.924 140.274 231.289 1.00 81.07
H
ATOM 35081 HD2 LYS L 149   85.231 139.232 232.849 1.00 91.35
H
ATOM 35082 HD3 LYS L 149   84.247 139.594 231.656 1.00 91.35
H
ATOM 35083 HE2 LYS L 149   85.206 138.144 230.264 1.00 90.52
H
ATOM 35084 HE3 LYS L 149   86.595 138.192 231.033 1.00 90.52
H
ATOM 35085 HZ1 LYS L 149   85.903 136.693 232.459 1.00102.23
H
ATOM 35086 HZ2 LYS L 149   84.494 136.975 232.268 1.00102.23
H
ATOM 35087 HZ3 LYS L 149   85.223 136.213 231.274 1.00102.23
H
ATOM 35088 N   VAL L 150   86.132 144.959 233.034 1.00 63.78
N
ATOM 35089 CA VAL L 150   85.917 146.076 233.944 1.00 58.84
C
ATOM 35090 C   VAL L 150   84.434 146.107 234.287 1.00 67.06
C
ATOM 35091 O   VAL L 150   83.593 146.285 233.399 1.00 68.32
O
ATOM 35092 CB VAL L 150   86.369 147.406 233.325 1.00 61.27
C
ATOM 35093 CG1 VAL L 150   86.134 148.519 234.305 1.00 68.18
C
ATOM 35094 CG2 VAL L 150   87.858 147.336 232.860 1.00 57.67
C
ATOM 35095 H   VAL L 150   85.696 145.037 232.298 1.00 76.56
H
ATOM 35096 HA VAL L 150   86.420 145.936 234.761 1.00 70.63
H
ATOM 35097 HB VAL L 150   85.844 147.590 232.530 1.00 73.55
H
ATOM 35098 HG11 VAL L 150   86.602 149.312 234.000 1.00 81.84
H
ATOM 35099 HG12 VAL L 150   85.182 148.698 234.359 1.00 81.84
H

ATOM 35100 HG13 VAL L 150   86.471 148.251 235.175 1.00 81.84
H
ATOM 35101 HG21 VAL L 150   88.107 148.188 232.471 1.00 69.23
H
ATOM 35102 HG22 VAL L 150   88.419 147.143 233.628 1.00 69.23
H
ATOM 35103 HG23 VAL L 150   87.949 146.631 232.199 1.00 69.23
H
ATOM 35104 N   ASP L 151   84.116 145.925 235.574 1.00 74.55
N
ATOM 35105 CA ASP L 151   82.735 145.756 236.033 1.00 72.64
C
ATOM 35106 C   ASP L 151   81.992 144.817 235.093 1.00 77.84
C
ATOM 35107 O   ASP L 151   80.851 145.089 234.713 1.00 73.61
O
ATOM 35108 CB ASP L 151   81.999 147.091 236.123 1.00 65.01
C
ATOM 35109 CG ASP L 151   82.440 147.939 237.317 1.00 63.41
C
ATOM 35110 OD1 ASP L 151   82.878 147.394 238.353 1.00 64.63
O
ATOM 35111 OD2 ASP L 151   82.335 149.172 237.217 1.00 74.31
O1-
ATOM 35112 H   ASP L 151   84.692 145.897 236.212 1.00 89.49
H
ATOM 35113 HA ASP L 151   82.751 145.377 236.925 1.00 87.20
H
ATOM 35114 HB2 ASP L 151   82.170 147.599 235.315 1.00 78.04
H
ATOM 35115 HB3 ASP L 151   81.049 146.920 236.214 1.00 78.04
H
ATOM 35116 N   ASN L 152   82.656 143.733 234.694 1.00 80.33
N
ATOM 35117 CA ASN L 152   82.157 142.629 233.866 1.00 85.93
C
ATOM 35118 C   ASN L 152   82.102 142.939 232.376 1.00 81.45
C
ATOM 35119 O   ASN L 152   81.706 142.052 231.607 1.00 74.50
O
ATOM 35120 CB ASN L 152   80.764 142.148 234.295 1.00 91.33
C
ATOM 35121 CG ASN L 152   80.816 140.844 235.037 1.00105.52
C
ATOM 35122 OD1 ASN L 152   80.987 139.782 234.435 1.00 85.42
O
ATOM 35123 ND2 ASN L 152   80.687 140.912 236.357 1.00121.37
N
ATOM 35124 H   ASN L 152   83.477 143.603 234.913 1.00 96.42
H

```
ATOM  35125  HA  ASN L 152      82.793 141.904 233.972  1.00103.14
H
ATOM  35126  HB2 ASN L 152      80.364 142.812 234.879  1.00109.62
H
ATOM  35127  HB3 ASN L 152      80.213 142.024 233.506  1.00109.62
H
ATOM  35128 HD21 ASN L 152      80.711 140.194 236.828  1.00145.67
H
ATOM  35129 HD22 ASN L 152      80.580 141.674 236.739  1.00145.67
H
ATOM  35130  N   ALA L 153      82.499 144.136 231.936  1.00 81.48
N
ATOM  35131  CA  ALA L 153      82.471 144.501 230.523  1.00 75.44
C
ATOM  35132  C   ALA L 153      83.784 144.107 229.865  1.00 66.77
C
ATOM  35133  O   ALA L 153      84.851 144.571 230.285  1.00 61.82
O
ATOM  35134  CB  ALA L 153      82.244 146.003 230.363  1.00 84.00
C
ATOM  35135  H   ALA L 153      82.794 144.761 232.447  1.00 97.81
H
ATOM  35136  HA  ALA L 153      81.748 144.021 230.088  1.00 90.56
H
ATOM  35137  HB1 ALA L 153      82.224 146.220 229.418  1.00100.83
H
ATOM  35138  HB2 ALA L 153      81.399 146.241 230.776  1.00100.83
H
ATOM  35139  HB3 ALA L 153      82.969 146.480 230.797  1.00100.83
H
ATOM  35140  N   LEU L 154      83.712 143.278 228.820  1.00 73.45
N
ATOM  35141  CA  LEU L 154      84.918 142.966 228.061  1.00 65.69
C
ATOM  35142  C   LEU L 154      85.549 144.248 227.546  1.00 59.56
C
ATOM  35143  O   LEU L 154      84.869 145.232 227.256  1.00 71.93
O
ATOM  35144  CB  LEU L 154      84.629 142.023 226.895  1.00 68.78
C
ATOM  35145  CG  LEU L 154      84.858 140.539 227.198  1.00 75.37
C
ATOM  35146  CD1 LEU L 154      83.946 140.092 228.330  1.00 89.93
C
ATOM  35147  CD2 LEU L 154      84.637 139.687 225.953  1.00 75.83
C
ATOM  35148  H   LEU L 154      82.995 142.895 228.539  1.00 88.16
H
ATOM  35149  HA  LEU L 154      85.543 142.509 228.645  1.00 78.86
H
```

ATOM 35150 HB2 LEU L 154   83.700 142.130 226.636 1.00 82.56
H
ATOM 35151 HB3 LEU L 154   85.209 142.263 226.155 1.00 82.56
H
ATOM 35152 HG LEU L 154   85.777 140.410 227.479 1.00 90.47
H
ATOM 35153 HD11 LEU L 154   84.031 139.132 228.443 1.00107.94
H
ATOM 35154 HD12 LEU L 154   84.209 140.546 229.145 1.00107.94
H
ATOM 35155 HD13 LEU L 154   83.031 140.318 228.105 1.00107.94
H
ATOM 35156 HD21 LEU L 154   84.744 138.751 226.185 1.00 91.03
H
ATOM 35157 HD22 LEU L 154   83.740 139.843 225.618 1.00 91.03
H
ATOM 35158 HD23 LEU L 154   85.289 139.937 225.278 1.00 91.03
H
ATOM 35159 N   GLN L 155   86.874 144.247 227.491 1.00 65.32
N
ATOM 35160 CA GLN L 155   87.640 145.381 227.013 1.00 64.40
C
ATOM 35161 C   GLN L 155   88.352 144.937 225.752 1.00 62.08
C
ATOM 35162 O   GLN L 155   88.654 143.754 225.594 1.00 65.42
O
ATOM 35163 CB GLN L 155   88.656 145.865 228.052 1.00 59.61
C
ATOM 35164 CG GLN L 155   88.060 146.224 229.383 1.00 56.62
C
ATOM 35165 CD GLN L 155   87.448 147.635 229.437 1.00 59.35
C
ATOM 35166 OE1 GLN L 155   88.137 148.631 229.245 1.00 59.13
O
ATOM 35167 NE2 GLN L 155   86.149 147.710 229.728 1.00 60.46
N
ATOM 35168 H   GLN L 155   87.362 143.581 227.733 1.00 78.41
H
ATOM 35169 HA GLN L 155   87.062 146.135 226.820 1.00 77.31
H
ATOM 35170 HB2 GLN L 155   89.305 145.159 228.202 1.00 71.56
H
ATOM 35171 HB3 GLN L 155   89.100 146.655 227.707 1.00 71.56
H
ATOM 35172 HG2 GLN L 155   87.355 145.591 229.590 1.00 67.97
H
ATOM 35173 HG3 GLN L 155   88.754 146.179 230.059 1.00 67.97
H
ATOM 35174 HE21 GLN L 155   85.697 146.992 229.874 1.00 72.58
H

ATOM 35175 HE22 GLN L 155 85.761 148.478 229.770 1.00 72.58
H
ATOM 35176 N SER L 156 88.620 145.883 224.851 1.00 63.50
N
ATOM 35177 CA SER L 156 89.396 145.577 223.655 1.00 61.85
C
ATOM 35178 C SER L 156 90.134 146.834 223.214 1.00 60.92
C
ATOM 35179 O SER L 156 89.664 147.953 223.435 1.00 62.95
O
ATOM 35180 CB SER L 156 88.501 145.043 222.507 1.00 62.06
C
ATOM 35181 OG SER L 156 87.500 145.986 222.190 1.00 72.55
O
ATOM 35182 H SER L 156 88.366 146.702 224.912 1.00 76.23
H
ATOM 35183 HA SER L 156 90.053 144.893 223.861 1.00 74.25
H
ATOM 35184 HB2 SER L 156 89.050 144.885 221.724 1.00 74.50
H
ATOM 35185 HB3 SER L 156 88.081 144.215 222.790 1.00 74.50
H
ATOM 35186 HG SER L 156 86.996 145.679 221.591 1.00 87.08
H
ATOM 35187 N GLY L 157 91.307 146.642 222.599 1.00 63.44
N
ATOM 35188 CA GLY L 157 92.072 147.748 222.056 1.00 60.39
C
ATOM 35189 C GLY L 157 92.858 148.558 223.064 1.00 65.23
C
ATOM 35190 O GLY L 157 93.661 149.410 222.657 1.00 64.27
O
ATOM 35191 H GLY L 157 91.676 145.873 222.490 1.00 76.15
H
ATOM 35192 HA2 GLY L 157 92.701 147.397 221.407 1.00 72.49
H
ATOM 35193 HA3 GLY L 157 91.460 148.353 221.606 1.00 72.49
H
ATOM 35194 N ASN L 158 92.669 148.332 224.365 1.00 60.04
N
ATOM 35195 CA ASN L 158 93.356 149.118 225.377 1.00 60.55
C
ATOM 35196 C ASN L 158 94.355 148.291 226.181 1.00 55.77
C
ATOM 35197 O ASN L 158 94.729 148.693 227.291 1.00 54.18
O
ATOM 35198 CB ASN L 158 92.331 149.812 226.294 1.00 53.00
C
ATOM 35199 CG ASN L 158 91.360 148.850 226.962 1.00 61.03
C

```
ATOM  35200  OD1 ASN L 158      91.246 147.665 226.605  1.00 54.60
O
ATOM  35201  ND2 ASN L 158      90.635 149.374 227.952  1.00 59.02
N
ATOM  35202  H   ASN L 158      92.145 147.727 224.680  1.00 72.07
H
ATOM  35203  HA  ASN L 158      93.866 149.815 224.937  1.00 72.69
H
ATOM  35204  HB2 ASN L 158      92.808 150.286 226.994  1.00 63.62
H
ATOM  35205  HB3 ASN L 158      91.811 150.438 225.766  1.00 63.62
H
ATOM  35206 HD21 ASN L 158      90.067 148.886 228.373  1.00 70.86
H
ATOM  35207 HD22 ASN L 158      90.736 150.201 228.168  1.00 70.86
H
ATOM  35208  N   SER L 159      94.822 147.163 225.639  1.00 47.27
N
ATOM  35209  CA  SER L 159      95.893 146.418 226.291  1.00 56.91
C
ATOM  35210  C   SER L 159      96.944 145.929 225.295  1.00 52.42
C
ATOM  35211  O   SER L 159      96.709 145.823 224.092  1.00 56.84
O
ATOM  35212  CB  SER L 159      95.348 145.212 227.087  1.00 62.43
C
ATOM  35213  OG  SER L 159      94.732 144.257 226.232  1.00 60.85
O
ATOM  35214  H   SER L 159      94.537 146.817 224.904  1.00 56.75
H
ATOM  35215  HA  SER L 159      96.341 147.035 226.891  1.00 68.32
H
ATOM  35216  HB2 SER L 159      96.083 144.786 227.553  1.00 74.95
H
ATOM  35217  HB3 SER L 159      94.689 145.529 227.725  1.00 74.95
H
ATOM  35218  HG  SER L 159      94.410 143.628 226.688  1.00 73.05
H
ATOM  35219  N   GLN L 160      98.122 145.625 225.836  1.00 52.86
N
ATOM  35220  CA  GLN L 160      99.250 145.099 225.093  1.00 56.04
C
ATOM  35221  C   GLN L 160      99.877 143.973 225.891  1.00 55.63
C
ATOM  35222  O   GLN L 160      99.876 144.005 227.124  1.00 53.51
O
ATOM  35223  CB  GLN L 160     100.312 146.162 224.822  1.00 53.05
C
ATOM  35224  CG  GLN L 160      99.762 147.348 224.035  1.00 59.03
C
```

```
ATOM  35225  CD  GLN L 160     100.856 148.334 223.745  1.00 63.69
C
ATOM  35226  OE1 GLN L 160     101.134 149.251 224.533  1.00 65.54
O
ATOM  35227  NE2 GLN L 160     101.523 148.127 222.629  1.00 62.83
N
ATOM  35228  H   GLN L 160      98.296 145.723 226.673  1.00 63.47
H
ATOM  35229  HA  GLN L 160      98.936 144.757 224.242  1.00 67.28
H
ATOM  35230  HB2 GLN L 160     100.653 146.493 225.668  1.00 63.69
H
ATOM  35231  HB3 GLN L 160     101.032 145.766 224.306  1.00 63.69
H
ATOM  35232  HG2 GLN L 160      99.393 147.037 223.194  1.00 70.86
H
ATOM  35233  HG3 GLN L 160      99.075 147.793 224.556  1.00 70.86
H
ATOM  35234 HE21 GLN L 160     101.319 147.462 222.123  1.00 75.42
H
ATOM  35235 HE22 GLN L 160     102.163 148.657 222.405  1.00 75.42
H
ATOM  35236  N   GLU L 161     100.446 142.998 225.169  1.00 61.15
N
ATOM  35237  CA  GLU L 161     100.994 141.773 225.741  1.00 59.67
C
ATOM  35238  C   GLU L 161     102.441 141.622 225.330  1.00 48.12
C
ATOM  35239  O   GLU L 161     102.839 142.060 224.260  1.00 54.64
O
ATOM  35240  CB  GLU L 161     100.237 140.531 225.269  1.00 58.45
C
ATOM  35241  CG  GLU L 161      98.784 140.456 225.729  1.00 70.58
C
ATOM  35242  CD  GLU L 161      98.107 139.117 225.376  1.00 79.24
C
ATOM  35243  OE1 GLU L 161      98.791 138.210 224.839  1.00 74.55
O
ATOM  35244  OE2 GLU L 161      96.882 138.983 225.625  1.00 78.41
O1-
ATOM  35245  H   GLU L 161     100.527 143.031 224.313  1.00 73.40
H
ATOM  35246  HA  GLU L 161     100.930 141.829 226.707  1.00 71.63
H
ATOM  35247  HB2 GLU L 161     100.237 140.522 224.298  1.00 70.17
H
ATOM  35248  HB3 GLU L 161     100.692 139.746 225.608  1.00 70.17
H
ATOM  35249  HG2 GLU L 161      98.752 140.560 226.693  1.00 84.72
H
```

ATOM 35250 HG3 GLU L 161   98.280 141.166 225.302 1.00 84.72
H
ATOM 35251 N   SER L 162   103.215 140.980 226.177 1.00 49.14
N
ATOM 35252 CA SER L 162   104.589 140.615 225.881 1.00 53.33
C
ATOM 35253 C   SER L 162   104.862 139.243 226.484 1.00 53.79
C
ATOM 35254 O   SER L 162   104.310 138.900 227.534 1.00 52.78
O
ATOM 35255 CB SER L 162   105.552 141.664 226.452 1.00 49.35
C
ATOM 35256 OG SER L 162   106.828 141.514 225.923 1.00 66.02
O
ATOM 35257 H   SER L 162   102.961 140.733 226.962 1.00 59.00
H
ATOM 35258 HA SER L 162   104.738 140.555 224.923 1.00 64.03
H
ATOM 35259 HB2 SER L 162   105.222 142.548 226.231 1.00 59.25
H
ATOM 35260 HB3 SER L 162   105.597 141.557 227.415 1.00 59.25
H
ATOM 35261 HG SER L 162   107.359 142.051 226.293 1.00 79.25
H
ATOM 35262 N   VAL L 163   105.728 138.469 225.832 1.00 53.29
N
ATOM 35263 CA VAL L 163   106.027 137.106 226.244 1.00 50.75
C
ATOM 35264 C   VAL L 163   107.529 136.909 226.278 1.00 49.48
C
ATOM 35265 O   VAL L 163   108.253 137.373 225.393 1.00 58.30
O
ATOM 35266 CB VAL L 163   105.382 136.054 225.312 1.00 57.11
C
ATOM 35267 CG1 VAL L 163   105.606 134.676 225.861 1.00 59.18
C
ATOM 35268 CG2 VAL L 163   103.923 136.281 225.189 1.00 56.07
C
ATOM 35269 H   VAL L 163   106.162 138.720 225.133 1.00 63.98
H
ATOM 35270 HA VAL L 163   105.680 136.981 227.141 1.00 60.92
H
ATOM 35271 HB VAL L 163   105.790 136.135 224.435 1.00 68.55
H
ATOM 35272 HG11 VAL L 163   105.139 134.035 225.304 1.00 71.04
H
ATOM 35273 HG12 VAL L 163   106.556 134.487 225.859 1.00 71.04
H
ATOM 35274 HG13 VAL L 163   105.261 134.639 226.767 1.00 71.04
H

ATOM 35275 HG21 VAL L 163   103.525 135.530 224.720 1.00 67.31
H
ATOM 35276 HG22 VAL L 163   103.539 136.355 226.076 1.00 67.31
H
ATOM 35277 HG23 VAL L 163   103.771 137.099 224.691 1.00 67.31
H
ATOM 35278 N   THR L 164   107.995 136.202 227.295 1.00 48.84
N
ATOM 35279 CA THR L 164   109.392 135.834 227.409 1.00 49.81
C
ATOM 35280 C   THR L 164   109.744 134.746 226.395 1.00 54.59
C
ATOM 35281 O   THR L 164   108.882 134.118 225.774 1.00 58.51
O
ATOM 35282 CB THR L 164   109.725 135.325 228.825 1.00 51.90
C
ATOM 35283 OG1 THR L 164   108.871 134.224 229.161 1.00 53.46
O
ATOM 35284 CG2 THR L 164   109.536 136.413 229.879 1.00 48.47
C
ATOM 35285 H   THR L 164   107.511 135.920 227.949 1.00 58.64
H
ATOM 35286 HA THR L 164   109.927 136.623 227.232 1.00 59.80
H
ATOM 35287 HB THR L 164   110.654 135.047 228.835 1.00 62.31
H
ATOM 35288 HG1 THR L 164   109.201 133.507 228.878 1.00 64.18
H
ATOM 35289 HG21 THR L 164   109.807 136.082 230.749 1.00 58.19
H
ATOM 35290 HG22 THR L 164   110.074 137.188 229.654 1.00 58.19
H
ATOM 35291 HG23 THR L 164   108.603 136.676 229.918 1.00 58.19
H
ATOM 35292 N   GLU L 165   111.046 134.557 226.223 1.00 52.00
N
ATOM 35293 CA GLU L 165   111.585 133.366 225.608 1.00 60.52
C
ATOM 35294 C   GLU L 165   111.483 132.194 226.583 1.00 59.95
C
ATOM 35295 O   GLU L 165   111.201 132.365 227.766 1.00 57.17
O
ATOM 35296 CB GLU L 165   113.042 133.593 225.215 1.00 65.67
C
ATOM 35297 CG GLU L 165   113.243 134.594 224.059 1.00 72.00
C
ATOM 35298 CD GLU L 165   112.439 134.236 222.809 1.00 81.50
C
ATOM 35299 OE1 GLU L 165   111.423 134.914 222.533 1.00 87.09
O

ATOM 35300 OE2 GLU L 165   112.812 133.264 222.112 1.00 77.65
O1-
ATOM 35301 H   GLU L 165   111.650 135.121 226.463 1.00 62.42
H
ATOM 35302 HA GLU L 165   111.083 133.151 224.806 1.00 72.65
H
ATOM 35303 HB2 GLU L 165   113.520 133.936 225.986 1.00 78.84
H
ATOM 35304 HB3 GLU L 165   113.424 132.745 224.938 1.00 78.84
H
ATOM 35305 HG2 GLU L 165   112.960 135.474 224.352 1.00 86.43
H
ATOM 35306 HG3 GLU L 165   114.181 134.611 223.815 1.00 86.43
H
ATOM 35307 N   GLN L 166   111.731 130.990 226.073 1.00 55.97
N
ATOM 35308 CA GLN L 166   111.683 129.793 226.899 1.00 58.84
C
ATOM 35309 C   GLN L 166   112.711 129.866 228.009 1.00 56.24
C
ATOM 35310 O   GLN L 166   113.892 130.131 227.770 1.00 59.76
O
ATOM 35311 CB GLN L 166   111.934 128.539 226.060 1.00 54.44
C
ATOM 35312 CG GLN L 166   110.700 128.025 225.385 1.00 57.80
C
ATOM 35313 CD GLN L 166   110.910 126.613 224.864 1.00 64.52
C
ATOM 35314 OE1 GLN L 166   111.914 126.345 224.231 1.00 59.09
O
ATOM 35315 NE2 GLN L 166   109.987 125.699 225.170 1.00 52.62
N
ATOM 35316 H   GLN L 166   111.930 130.840 225.250 1.00 67.19
H
ATOM 35317 HA GLN L 166   110.797 129.730 227.290 1.00 70.64
H
ATOM 35318 HB2 GLN L 166   112.586 128.748 225.372 1.00 65.35
H
ATOM 35319 HB3 GLN L 166   112.273 127.838 226.638 1.00 65.35
H
ATOM 35320 HG2 GLN L 166   109.967 128.011 226.020 1.00 69.38
H
ATOM 35321 HG3 GLN L 166   110.479 128.600 224.635 1.00 69.38
H
ATOM 35322 HE21 GLN L 166   109.306 125.919 225.647 1.00 63.17
H
ATOM 35323 HE22 GLN L 166   110.074 124.890 224.891 1.00 63.17
H
ATOM 35324 N   ASP L 167   112.261 129.593 229.227 1.00 58.02
N

ATOM 35325 CA ASP L 167 113.175 129.596 230.359 1.00 63.39
C
ATOM 35326 C ASP L 167 114.229 128.504 230.183 1.00 67.12
C
ATOM 35327 O ASP L 167 113.950 127.415 229.669 1.00 57.84
O
ATOM 35328 CB ASP L 167 112.392 129.404 231.649 1.00 59.93
C
ATOM 35329 CG ASP L 167 113.264 129.510 232.890 1.00 80.31
C
ATOM 35330 OD1 ASP L 167 113.647 130.647 233.253 1.00 92.40
O
ATOM 35331 OD2 ASP L 167 113.547 128.459 233.512 1.00 68.67
O1-
ATOM 35332 H ASP L 167 111.445 129.406 229.420 1.00 69.65
H
ATOM 35333 HA ASP L 167 113.637 130.446 230.420 1.00 76.10
H
ATOM 35334 HB2 ASP L 167 111.705 130.085 231.707 1.00 71.94
H
ATOM 35335 HB3 ASP L 167 111.987 128.522 231.644 1.00 71.94
H
ATOM 35336 N SER L 168 115.452 128.812 230.607 1.00 62.94
N
ATOM 35337 CA SER L 168 116.565 127.891 230.443 1.00 72.32
C
ATOM 35338 C SER L 168 116.454 126.688 231.370 1.00 81.69
C
ATOM 35339 O SER L 168 117.022 125.625 231.079 1.00 83.37
O
ATOM 35340 CB SER L 168 117.873 128.619 230.735 1.00 73.24
C
ATOM 35341 OG SER L 168 118.028 128.714 232.146 1.00 78.44
O
ATOM 35342 H SER L 168 115.664 129.551 230.994 1.00 75.56
H
ATOM 35343 HA SER L 168 116.566 127.573 229.528 1.00 86.81
H
ATOM 35344 HB2 SER L 168 118.615 128.120 230.359 1.00 87.92
H
ATOM 35345 HB3 SER L 168 117.843 129.509 230.351 1.00 87.92
H
ATOM 35346 HG SER L 168 118.777 129.046 232.331 1.00 94.15
H
ATOM 35347 N LYS L 169 115.790 126.847 232.513 1.00 75.97
N
ATOM 35348 CA LYS L 169 115.751 125.779 233.499 1.00 74.25
C
ATOM 35349 C LYS L 169 114.585 124.820 233.284 1.00 75.07
C

ATOM 35350 O   LYS L 169   114.755 123.615 233.496 1.00 70.14
O
ATOM 35351 CB LYS L 169   115.704 126.366 234.924 1.00 78.31
C
ATOM 35352 CG LYS L 169   117.096 126.851 235.442 1.00 89.29
C
ATOM 35353 CD LYS L 169   117.308 126.657 236.957 1.00 89.69
C
ATOM 35354 CE LYS L 169   118.810 126.545 237.343 1.00 85.59
C
ATOM 35355 NZ LYS L 169   119.611 127.715 236.893 1.00 94.80
N
ATOM 35356 H   LYS L 169   115.359 127.557 232.735 1.00 91.19
H
ATOM 35357 HA LYS L 169   116.568 125.261 233.424 1.00 89.12
H
ATOM 35358 HB2 LYS L 169   115.104 127.127 234.928 1.00 94.00
H
ATOM 35359 HB3 LYS L 169   115.382 125.684 235.534 1.00 94.00
H
ATOM 35360 HG2 LYS L 169   117.790 126.354 234.981 1.00107.18
H
ATOM 35361 HG3 LYS L 169   117.186 127.798 235.253 1.00107.18
H
ATOM 35362 HD2 LYS L 169   116.933 127.417 237.430 1.00107.65
H
ATOM 35363 HD3 LYS L 169   116.866 125.840 237.236 1.00107.65
H
ATOM 35364 HE2 LYS L 169   118.885 126.485 238.309 1.00102.73
H
ATOM 35365 HE3 LYS L 169   119.184 125.749 236.933 1.00102.73
H
ATOM 35366 HZ1 LYS L 169   120.446 127.646 237.194 1.00113.79
H
ATOM 35367 HZ2 LYS L 169   119.627 127.747 236.003 1.00113.79
H
ATOM 35368 HZ3 LYS L 169   119.253 128.467 237.202 1.00113.79
H
ATOM 35369 N   ASP L 170   113.410 125.323 232.837 1.00 71.22
N
ATOM 35370 CA ASP L 170   112.196 124.511 232.782 1.00 63.76
C
ATOM 35371 C   ASP L 170   111.394 124.643 231.490 1.00 53.16
C
ATOM 35372 O   ASP L 170   110.313 124.047 231.384 1.00 53.03
O
ATOM 35373 CB ASP L 170   111.291 124.817 234.004 1.00 68.71
C
ATOM 35374 CG ASP L 170   110.625 126.201 233.969 1.00 69.93
C

ATOM 35375 OD1 ASP L 170 110.548 126.865 232.913 1.00 71.93
O
ATOM 35376 OD2 ASP L 170 110.143 126.638 235.045 1.00 84.57
O1−
ATOM 35377 H ASP L 170 113.302 126.131 232.563 1.00 85.49
H
ATOM 35378 HA ASP L 170 112.459 123.579 232.836 1.00 76.54
H
ATOM 35379 HB2 ASP L 170 110.584 124.154 234.040 1.00 82.48
H
ATOM 35380 HB3 ASP L 170 111.830 124.772 234.808 1.00 82.48
H
ATOM 35381 N SER L 171 111.872 125.392 230.507 1.00 56.68
N
ATOM 35382 CA SER L 171 111.294 125.409 229.161 1.00 53.88
C
ATOM 35383 C SER L 171 109.892 125.993 229.116 1.00 51.65
C
ATOM 35384 O SER L 171 109.154 125.765 228.150 1.00 58.10
O
ATOM 35385 CB SER L 171 111.275 124.001 228.533 1.00 52.15
C
ATOM 35386 OG SER L 171 112.574 123.485 228.452 1.00 58.50
O
ATOM 35387 H SER L 171 112.549 125.915 230.594 1.00 68.04
H
ATOM 35388 HA SER L 171 111.863 125.979 228.620 1.00 64.69
H
ATOM 35389 HB2 SER L 171 110.734 123.413 229.085 1.00 62.60
H
ATOM 35390 HB3 SER L 171 110.899 124.056 227.641 1.00 62.60
H
ATOM 35391 HG SER L 171 112.572 122.767 228.015 1.00 70.23
H
ATOM 35392 N THR L 172 109.495 126.770 230.108 1.00 45.19
N
ATOM 35393 CA THR L 172 108.204 127.444 230.049 1.00 49.99
C
ATOM 35394 C THR L 172 108.340 128.891 229.593 1.00 52.46
C
ATOM 35395 O THR L 172 109.439 129.445 229.447 1.00 49.61
O
ATOM 35396 CB THR L 172 107.521 127.456 231.414 1.00 58.26
C
ATOM 35397 OG1 THR L 172 108.296 128.275 232.307 1.00 47.60
O
ATOM 35398 CG2 THR L 172 107.379 126.059 231.931 1.00 46.76
C
ATOM 35399 H THR L 172 109.951 126.923 230.822 1.00 54.25
H

ATOM 35400 HA THR L 172 107.665 126.953 229.410 1.00 60.01
H
ATOM 35401 HB THR L 172 106.626 127.825 231.357 1.00 69.94
H
ATOM 35402 HG1 THR L 172 109.088 127.998 232.336 1.00 57.15
H
ATOM 35403 HG21 THR L 172 106.869 126.061 232.757 1.00 56.14
H
ATOM 35404 HG22 THR L 172 106.918 125.509 231.278 1.00 56.14
H
ATOM 35405 HG23 THR L 172 108.253 125.678 232.103 1.00 56.14
H
ATOM 35406 N TYR L 173 107.172 129.504 229.428 1.00 51.40
N
ATOM 35407 CA TYR L 173 107.007 130.895 229.062 1.00 51.95
C
ATOM 35408 C TYR L 173 106.343 131.662 230.188 1.00 56.58
C
ATOM 35409 O TYR L 173 105.634 131.100 231.020 1.00 51.52
O
ATOM 35410 CB TYR L 173 106.129 131.037 227.826 1.00 51.98
C
ATOM 35411 CG TYR L 173 106.632 130.290 226.626 1.00 57.22
C
ATOM 35412 CD1 TYR L 173 106.133 129.016 226.314 1.00 59.78
C
ATOM 35413 CD2 TYR L 173 107.607 130.843 225.803 1.00 55.68
C
ATOM 35414 CE1 TYR L 173 106.595 128.315 225.209 1.00 65.55
C
ATOM 35415 CE2 TYR L 173 108.083 130.159 224.706 1.00 59.22
C
ATOM 35416 CZ TYR L 173 107.574 128.896 224.408 1.00 71.64
C
ATOM 35417 OH TYR L 173 108.028 128.230 223.301 1.00 64.48
O
ATOM 35418 H TYR L 173 106.418 129.105 229.530 1.00 61.71
H
ATOM 35419 HA TYR L 173 107.884 131.272 228.891 1.00 62.37
H
ATOM 35420 HB2 TYR L 173 105.245 130.698 228.034 1.00 62.41
H
ATOM 35421 HB3 TYR L 173 106.077 131.976 227.587 1.00 62.41
H
ATOM 35422 HD1 TYR L 173 105.480 128.635 226.856 1.00 71.76
H
ATOM 35423 HD2 TYR L 173 107.943 131.689 225.995 1.00 66.84
H
ATOM 35424 HE1 TYR L 173 106.256 127.473 225.006 1.00 78.69
H

ATOM 35425 HE2 TYR L 173   108.739 130.538 224.166 1.00 71.09
H
ATOM 35426 HH TYR L 173   108.622 128.683 222.918 1.00 77.41
H
ATOM 35427 N   SER L 174   106.518 132.971 230.152 1.00 53.46
N
ATOM 35428 CA SER L 174   105.699 133.861 230.953 1.00 50.35
C
ATOM 35429 C   SER L 174   105.200 135.008 230.097 1.00 49.94
C
ATOM 35430 O   SER L 174   105.806 135.389 229.092 1.00 50.35
O
ATOM 35431 CB SER L 174   106.483 134.367 232.175 1.00 59.05
C
ATOM 35432 OG SER L 174   107.093 133.254 232.800 1.00 49.74
O
ATOM 35433 H   SER L 174   107.107 133.369 229.670 1.00 64.18
H
ATOM 35434 HA SER L 174   104.910 133.398 231.281 1.00 60.45
H
ATOM 35435 HB2 SER L 174   107.165 134.995 231.888 1.00 70.89
H
ATOM 35436 HB3 SER L 174   105.876 134.798 232.798 1.00 70.89
H
ATOM 35437 HG SER L 174   107.450 133.490 233.523 1.00 59.72
H
ATOM 35438 N   LEU L 175   104.053 135.510 230.472 1.00 44.82
N
ATOM 35439 CA LEU L 175   103.381 136.528 229.705 1.00 49.35
C
ATOM 35440 C   LEU L 175   102.894 137.616 230.645 1.00 56.29
C
ATOM 35441 O   LEU L 175   102.375 137.341 231.738 1.00 48.56
O
ATOM 35442 CB LEU L 175   102.224 135.934 228.941 1.00 46.74
C
ATOM 35443 CG LEU L 175   101.296 136.860 228.178 1.00 52.43
C
ATOM 35444 CD1 LEU L 175   100.646 136.044 227.079 1.00 63.21
C
ATOM 35445 CD2 LEU L 175   100.241 137.513 229.023 1.00 54.18
C
ATOM 35446 H   LEU L 175   103.633 135.274 231.185 1.00 53.81
H
ATOM 35447 HA LEU L 175   103.989 136.933 229.069 1.00 59.25
H
ATOM 35448 HB2 LEU L 175   102.593 135.317 228.288 1.00 56.12
H
ATOM 35449 HB3 LEU L 175   101.670 135.454 229.575 1.00 56.12
H

```
ATOM  35450  HG  LEU L 175     101.822 137.596 227.826  1.00 62.95
H
ATOM  35451 HD11 LEU L 175     100.042 136.615 226.576  1.00 75.88
H
ATOM  35452 HD12 LEU L 175     101.337 135.697 226.492  1.00 75.88
H
ATOM  35453 HD13 LEU L 175     100.154 135.311 227.479  1.00 75.88
H
ATOM  35454 HD21 LEU L 175      99.523 137.822 228.448  1.00 65.04
H
ATOM  35455 HD22 LEU L 175      99.901 136.865 229.659  1.00 65.04
H
ATOM  35456 HD23 LEU L 175     100.634 138.265 229.494  1.00 65.04
H
ATOM  35457  N   SER L 176     103.036 138.853 230.184  1.00 46.56
N
ATOM  35458  CA  SER L 176     102.592 140.038 230.894  1.00 50.82
C
ATOM  35459  C   SER L 176     101.596 140.769 230.020  1.00 46.32
C
ATOM  35460  O   SER L 176     101.859 140.993 228.836  1.00 61.71
O
ATOM  35461  CB  SER L 176     103.787 140.939 231.233  1.00 50.52
C
ATOM  35462  OG  SER L 176     103.349 142.108 231.885  1.00 56.50
O
ATOM  35463  H   SER L 176     103.404 139.036 229.429  1.00 55.90
H
ATOM  35464  HA  SER L 176     102.154 139.798 231.724  1.00 61.01
H
ATOM  35465  HB2 SER L 176     104.392 140.457 231.820  1.00 60.65
H
ATOM  35466  HB3 SER L 176     104.243 141.185 230.414  1.00 60.65
H
ATOM  35467  HG  SER L 176     103.961 142.684 231.883  1.00 67.82
H
ATOM  35468  N   SER L 177     100.449 141.117 230.585  1.00 51.22
N
ATOM  35469  CA  SER L 177      99.460 141.921 229.891  1.00 51.85
C
ATOM  35470  C   SER L 177      99.163 143.188 230.680  1.00 55.16
C
ATOM  35471  O   SER L 177      98.931 143.130 231.889  1.00 52.07
O
ATOM  35472  CB  SER L 177      98.163 141.161 229.686  1.00 46.88
C
ATOM  35473  OG  SER L 177      97.165 142.088 229.313  1.00 53.81
O
ATOM  35474  H   SER L 177     100.221 140.899 231.384  1.00 61.49
H
```

```
ATOM  35475  HA  SER L 177      99.825 142.166 229.027  1.00 62.24
H
ATOM  35476  HB2 SER L 177      98.278 140.504 228.981  1.00 56.29
H
ATOM  35477  HB3 SER L 177      97.909 140.721 230.511  1.00 56.29
H
ATOM  35478  HG  SER L 177      96.541 141.691 228.914  1.00 64.60
H
ATOM  35479  N   THR L 178      99.123 144.318 229.986  1.00 45.33
N
ATOM  35480  CA  THR L 178      98.866 145.612 230.602  1.00 50.36
C
ATOM  35481  C   THR L 178      97.619 146.241 230.010  1.00 54.28
C
ATOM  35482  O   THR L 178      97.513 146.428 228.792  1.00 53.45
O
ATOM  35483  CB  THR L 178     100.058 146.543 230.417  1.00 47.16
C
ATOM  35484  OG1 THR L 178     101.236 145.830 230.776  1.00 57.82
O
ATOM  35485  CG2 THR L 178      99.931 147.743 231.288  1.00 49.42
C
ATOM  35486  H   THR L 178      99.246 144.362 229.136  1.00 54.42
H
ATOM  35487  HA  THR L 178      98.704 145.474 231.549  1.00 60.46
H
ATOM  35488  HB  THR L 178     100.107 146.846 229.498  1.00 56.62
H
ATOM  35489  HG1 THR L 178     101.129 145.454 231.520  1.00 69.41
H
ATOM  35490 HG21 THR L 178     100.735 148.283 231.229  1.00 59.33
H
ATOM  35491 HG22 THR L 178      99.172 148.278 231.007  1.00 59.33
H
ATOM  35492 HG23 THR L 178      99.800 147.473 232.210  1.00 59.33
H
ATOM  35493  N   LEU L 179      96.683 146.572 230.870  1.00 49.79
N
ATOM  35494  CA  LEU L 179      95.539 147.384 230.503  1.00 49.56
C
ATOM  35495  C   LEU L 179      95.796 148.837 230.888  1.00 54.38
C
ATOM  35496  O   LEU L 179      96.095 149.128 232.047  1.00 56.98
O
ATOM  35497  CB  LEU L 179      94.311 146.842 231.210  1.00 47.15
C
ATOM  35498  CG  LEU L 179      93.003 147.600 231.197  1.00 57.86
C
ATOM  35499  CD1 LEU L 179      92.247 147.288 229.925  1.00 52.89
C
```

ATOM 35500 CD2 LEU L 179   92.198 147.210 232.438 1.00 53.93
C
ATOM 35501 H   LEU L 179   96.684 146.332 231.696 1.00 59.77
H
ATOM 35502 HA LEU L 179   95.392 147.368 229.544 1.00 59.50
H
ATOM 35503 HB2 LEU L 179   94.118 145.977 230.814 1.00 56.60
H
ATOM 35504 HB3 LEU L 179   94.546 146.734 232.145 1.00 56.60
H
ATOM 35505 HG LEU L 179   93.153 148.558 231.221 1.00 69.46
H
ATOM 35506 HD11 LEU L 179   91.398 147.759 229.937 1.00 63.49
H
ATOM 35507 HD12 LEU L 179   92.775 147.580 229.164 1.00 63.49
H
ATOM 35508 HD13 LEU L 179   92.095 146.332 229.874 1.00 63.49
H
ATOM 35509 HD21 LEU L 179   91.303 147.576 232.364 1.00 64.74
H
ATOM 35510 HD22 LEU L 179   92.156 146.242 232.497 1.00 64.74
H
ATOM 35511 HD23 LEU L 179   92.637 147.569 233.226 1.00 64.74
H
ATOM 35512 N   THR L 180   95.661 149.757 229.938 1.00 49.89
N
ATOM 35513 CA THR L 180   95.897 151.170 230.214 1.00 56.21
C
ATOM 35514 C   THR L 180   94.613 151.984 230.120 1.00 50.25
C
ATOM 35515 O   THR L 180   93.900 151.916 229.126 1.00 54.98
O
ATOM 35516 CB THR L 180   96.944 151.734 229.255 1.00 58.71
C
ATOM 35517 OG1 THR L 180   98.174 151.043 229.460 1.00 65.25
O
ATOM 35518 CG2 THR L 180   97.164 153.217 229.515 1.00 67.82
C
ATOM 35519 H   THR L 180   95.433 149.586 229.128 1.00 59.89
H
ATOM 35520 HA THR L 180   96.215 151.253 231.126 1.00 67.48
H
ATOM 35521 HB THR L 180   96.642 151.624 228.340 1.00 70.48
H
ATOM 35522 HG1 THR L 180   98.797 151.441 229.061 1.00 78.33
H
ATOM 35523 HG21 THR L 180   98.022 153.492 229.156 1.00 81.41
H
ATOM 35524 HG22 THR L 180   96.464 153.736 229.088 1.00 81.41
H

```
ATOM  35525 HG23 THR L 180      97.149 153.393 230.469  1.00 81.41
H
ATOM  35526  N   LEU L 181      94.344 152.777 231.148  1.00 46.27
N
ATOM  35527  CA  LEU L 181      93.186 153.640 231.195  1.00 50.15
C
ATOM  35528  C   LEU L 181      93.637 155.004 231.673  1.00 54.37
C
ATOM  35529  O   LEU L 181      94.693 155.157 232.293  1.00 51.03
O
ATOM  35530  CB  LEU L 181      92.070 153.136 232.123  1.00 56.98
C
ATOM  35531  CG  LEU L 181      91.420 151.793 231.834  1.00 66.61
C
ATOM  35532  CD1 LEU L 181      92.251 150.702 232.447  1.00 72.46
C
ATOM  35533  CD2 LEU L 181      90.030 151.729 232.439  1.00 70.12
C
ATOM  35534  H   LEU L 181      94.838 152.832 231.850  1.00 55.55
H
ATOM  35535  HA  LEU L 181      92.809 153.691 230.303  1.00 60.21
H
ATOM  35536  HB2 LEU L 181      92.442 153.074 233.017  1.00 68.40
H
ATOM  35537  HB3 LEU L 181      91.359 153.793 232.105  1.00 68.40
H
ATOM  35538  HG  LEU L 181      91.354 151.673 230.875  1.00 79.96
H
ATOM  35539 HD11 LEU L 181      91.783 149.857 232.351  1.00 86.98
H
ATOM  35540 HD12 LEU L 181      93.106 150.660 231.990  1.00 86.98
H
ATOM  35541 HD13 LEU L 181      92.387 150.899 233.388  1.00 86.98
H
ATOM  35542 HD21 LEU L 181      89.644 150.857 232.251  1.00 84.17
H
ATOM  35543 HD22 LEU L 181      90.096 151.861 233.398  1.00 84.17
H
ATOM  35544 HD23 LEU L 181      89.482 152.425 232.044  1.00 84.17
H
ATOM  35545  N   SER L 182      92.827 155.998 231.350  1.00 54.13
N
ATOM  35546  CA  SER L 182      93.009 157.301 231.962  1.00 58.07
C
ATOM  35547  C   SER L 182      92.559 157.221 233.406  1.00 59.66
C
ATOM  35548  O   SER L 182      91.706 156.403 233.763  1.00 58.43
O
ATOM  35549  CB  SER L 182      92.202 158.361 231.224  1.00 56.66
C
```

ATOM 35550 OG SER L 182   90.830 158.068 231.376 1.00 60.82
O
ATOM 35551 H   SER L 182   92.175 155.945 230.792 1.00 64.98
H
ATOM 35552 HA SER L 182   93.940 157.571 231.920 1.00 69.71
H
ATOM 35553 HB2 SER L 182   92.392 159.235 231.601 1.00 68.01
H
ATOM 35554 HB3 SER L 182   92.435 158.349 230.283 1.00 68.01
H
ATOM 35555 HG SER L 182   90.366 158.668 231.013 1.00 73.01
H
ATOM 35556 N   LYS L 183   93.129 158.082 234.241 1.00 58.74
N
ATOM 35557 CA LYS L 183   92.725 158.087 235.643 1.00 60.93
C
ATOM 35558 C   LYS L 183   91.227 158.259 235.738 1.00 65.12
C
ATOM 35559 O   LYS L 183   90.571 157.654 236.597 1.00 65.00
O
ATOM 35560 CB LYS L 183   93.435 159.204 236.400 1.00 59.30
C
ATOM 35561 CG LYS L 183   93.052 159.375 237.874 1.00 63.64
C
ATOM 35562 CD LYS L 183   93.827 160.577 238.417 1.00 73.30
C
ATOM 35563 CE LYS L 183   93.844 160.646 239.929 1.00 83.55
C
ATOM 35564 NZ LYS L 183   92.542 161.124 240.473 1.00 88.57
N
ATOM 35565 H   LYS L 183   93.733 158.656 234.030 1.00 70.52
H
ATOM 35566 HA LYS L 183   92.982 157.248 236.056 1.00 73.15
H
ATOM 35567 HB2 LYS L 183   94.388 159.029 236.371 1.00 71.19
H
ATOM 35568 HB3 LYS L 183   93.237 160.043 235.954 1.00 71.19
H
ATOM 35569 HG2 LYS L 183   92.101 159.543 237.958 1.00 76.40
H
ATOM 35570 HG3 LYS L 183   93.294 158.583 238.378 1.00 76.40
H
ATOM 35571 HD2 LYS L 183   94.747 160.523 238.111 1.00 87.99
H
ATOM 35572 HD3 LYS L 183   93.417 161.392 238.087 1.00 87.99
H
ATOM 35573 HE2 LYS L 183   94.017 159.761 240.288 1.00100.28
H
ATOM 35574 HE3 LYS L 183   94.538 161.260 240.214 1.00100.28
H

ATOM 35575 HZ1 LYS L 183   92.582 161.168 241.362 1.00106.31
H
ATOM 35576 HZ2 LYS L 183   92.356 161.933 240.152 1.00106.31
H
ATOM 35577 HZ3 LYS L 183   91.891 160.565 240.242 1.00106.31
H
ATOM 35578 N   ALA L 184   90.668 159.066 234.836 1.00 57.98
N
ATOM 35579 CA ALA L 184   89.246 159.377 234.893 1.00 67.12
C
ATOM 35580 C   ALA L 184   88.413 158.134 234.617 1.00 64.42
C
ATOM 35581 O   ALA L 184   87.500 157.802 235.381 1.00 70.02
O
ATOM 35582 CB ALA L 184   88.925 160.488 233.899 1.00 64.74
C
ATOM 35583 H   ALA L 184   91.086 159.442 234.186 1.00 69.60
H
ATOM 35584 HA ALA L 184   89.020 159.698 235.780 1.00 80.57
H
ATOM 35585 HB1 ALA L 184   87.976 160.683 233.939 1.00 77.71
H
ATOM 35586 HB2 ALA L 184   89.436 161.279 234.134 1.00 77.71
H
ATOM 35587 HB3 ALA L 184   89.165 160.192 233.007 1.00 77.71
H
ATOM 35588 N   ASP L 185   88.739 157.420 233.543 1.00 62.86
N
ATOM 35589 CA ASP L 185   88.037 156.178 233.238 1.00 72.35
C
ATOM 35590 C   ASP L 185   88.261 155.117 234.305 1.00 69.65
C
ATOM 35591 O   ASP L 185   87.387 154.276 234.527 1.00 72.03
O
ATOM 35592 CB ASP L 185   88.483 155.649 231.880 1.00 78.07
C
ATOM 35593 CG ASP L 185   87.932 156.462 230.731 1.00 80.51
C
ATOM 35594 OD1 ASP L 185   87.158 157.410 230.993 1.00 89.15
O
ATOM 35595 OD2 ASP L 185   88.271 156.151 229.568 1.00 84.50
O1-
ATOM 35596 H   ASP L 185   89.356 157.629 232.982 1.00 75.46
H
ATOM 35597 HA ASP L 185   87.087 156.366 233.201 1.00 86.85
H
ATOM 35598 HB2 ASP L 185   89.452 155.678 231.831 1.00 93.71
H
ATOM 35599 HB3 ASP L 185   88.174 154.735 231.777 1.00 93.71
H

ATOM 35600 N TYR L 186 89.421 155.134 234.970 1.00 67.68
N
ATOM 35601 CA TYR L 186 89.690 154.144 236.008 1.00 62.62
C
ATOM 35602 C TYR L 186 88.789 154.339 237.212 1.00 68.87
C
ATOM 35603 O TYR L 186 88.271 153.363 237.758 1.00 62.79
O
ATOM 35604 CB TYR L 186 91.152 154.216 236.435 1.00 65.28
C
ATOM 35605 CG TYR L 186 91.527 153.265 237.544 1.00 56.64
C
ATOM 35606 CD1 TYR L 186 91.611 151.906 237.320 1.00 56.56
C
ATOM 35607 CD2 TYR L 186 91.855 153.737 238.796 1.00 60.78
C
ATOM 35608 CE1 TYR L 186 91.989 151.027 238.334 1.00 56.44
C
ATOM 35609 CE2 TYR L 186 92.231 152.873 239.822 1.00 60.84
C
ATOM 35610 CZ TYR L 186 92.282 151.520 239.584 1.00 59.14
C
ATOM 35611 OH TYR L 186 92.657 150.674 240.599 1.00 58.77
O
ATOM 35612 H TYR L 186 90.055 155.699 234.840 1.00 81.25
H
ATOM 35613 HA TYR L 186 89.518 153.264 235.637 1.00 75.17
H
ATOM 35614 HB2 TYR L 186 91.709 154.006 235.670 1.00 78.36
H
ATOM 35615 HB3 TYR L 186 91.339 155.116 236.746 1.00 78.36
H
ATOM 35616 HD1 TYR L 186 91.412 151.571 236.478 1.00 67.90
H
ATOM 35617 HD2 TYR L 186 91.824 154.652 238.960 1.00 72.97
H
ATOM 35618 HE1 TYR L 186 92.042 150.114 238.166 1.00 67.76
H
ATOM 35619 HE2 TYR L 186 92.444 153.209 240.663 1.00 73.03
H
ATOM 35620 HH TYR L 186 92.947 151.118 241.251 1.00 70.55
H
ATOM 35621 N GLU L 187 88.599 155.586 237.653 1.00 73.82
N
ATOM 35622 CA GLU L 187 87.820 155.865 238.859 1.00 69.60
C
ATOM 35623 C GLU L 187 86.317 155.796 238.629 1.00 65.60
C
ATOM 35624 O GLU L 187 85.556 155.858 239.603 1.00 71.40
O

ATOM 35625 CB GLU L 187   88.198 157.233 239.418 1.00 68.27
C
ATOM 35626 CG GLU L 187   89.579 157.234 240.031 1.00 70.47
C
ATOM 35627 CD GLU L 187   90.124 158.615 240.297 1.00 86.94
C
ATOM 35628 OE1 GLU L 187   89.608 159.592 239.702 1.00 90.02
O
ATOM 35629 OE2 GLU L 187   91.075 158.721 241.108 1.00 92.13
O1-
ATOM 35630 H   GLU L 187   88.913 156.290 237.269 1.00 88.61
H
ATOM 35631 HA GLU L 187   88.040 155.200 239.529 1.00 83.55
H
ATOM 35632 HB2 GLU L 187   88.185 157.886 238.701 1.00 81.95
H
ATOM 35633 HB3 GLU L 187   87.561 157.483 240.107 1.00 81.95
H
ATOM 35634 HG2 GLU L 187   89.545 156.761 240.878 1.00 84.60
H
ATOM 35635 HG3 GLU L 187   90.189 156.786 239.425 1.00 84.60
H
ATOM 35636 N   LYS L 188   85.883 155.646 237.376 1.00 74.87
N
ATOM 35637 CA LYS L 188   84.491 155.396 237.016 1.00 71.47
C
ATOM 35638 C   LYS L 188   84.061 153.943 237.203 1.00 72.44
C
ATOM 35639 O   LYS L 188   82.927 153.599 236.857 1.00 72.08
O
ATOM 35640 CB LYS L 188   84.267 155.796 235.552 1.00 63.13
C
ATOM 35641 CG LYS L 188   84.059 157.310 235.356 1.00 73.57
C
ATOM 35642 CD LYS L 188   84.376 157.843 233.951 1.00 80.62
C
ATOM 35643 CE LYS L 188   83.766 157.023 232.828 1.00 92.87
C
ATOM 35644 NZ LYS L 188   84.002 157.667 231.484 1.00 95.92
N
ATOM 35645 H   LYS L 188   86.400 155.690 236.689 1.00 89.88
H
ATOM 35646 HA LYS L 188   83.927 155.941 237.585 1.00 85.80
H
ATOM 35647 HB2 LYS L 188   85.042 155.534 235.032 1.00 75.78
H
ATOM 35648 HB3 LYS L 188   83.476 155.343 235.221 1.00 75.78
H
ATOM 35649 HG2 LYS L 188   83.129 157.518 235.540 1.00 88.31
H

ATOM 35650 HG3 LYS L 188 84.634 157.780 235.981 1.00 88.31
H
ATOM 35651 HD2 LYS L 188 84.034 158.747 233.875 1.00 96.77
H
ATOM 35652 HD3 LYS L 188 85.339 157.842 233.826 1.00 96.77
H
ATOM 35653 HE2 LYS L 188 84.168 156.140 232.819 1.00111.47
H
ATOM 35654 HE3 LYS L 188 82.808 156.950 232.969 1.00111.47
H
ATOM 35655 HZ1 LYS L 188 83.639 158.478 231.468 1.00115.14
H
ATOM 35656 HZ2 LYS L 188 84.875 157.738 231.331 1.00115.14
H
ATOM 35657 HZ3 LYS L 188 83.635 157.172 230.842 1.00115.14
H
ATOM 35658 N HIS L 189 84.926 153.069 237.702 1.00 71.35
N
ATOM 35659 CA HIS L 189 84.601 151.652 237.749 1.00 66.75
C
ATOM 35660 C HIS L 189 85.229 151.034 238.978 1.00 66.52
C
ATOM 35661 O HIS L 189 86.204 151.552 239.539 1.00 65.86
O
ATOM 35662 CB HIS L 189 85.079 150.886 236.519 1.00 64.05
C
ATOM 35663 CG HIS L 189 84.511 151.390 235.232 1.00 77.97
C
ATOM 35664 ND1 HIS L 189 85.136 152.357 234.471 1.00 79.09
N
ATOM 35665 CD2 HIS L 189 83.374 151.063 234.572 1.00 74.70
C
ATOM 35666 CE1 HIS L 189 84.408 152.601 233.395 1.00 74.75
C
ATOM 35667 NE2 HIS L 189 83.341 151.822 233.428 1.00 69.79
N
ATOM 35668 H HIS L 189 85.701 153.273 238.015 1.00 85.64
H
ATOM 35669 HA HIS L 189 83.635 151.576 237.803 1.00 80.13
H
ATOM 35670 HB2 HIS L 189 86.044 150.959 236.463 1.00 76.88
H
ATOM 35671 HB3 HIS L 189 84.821 149.956 236.611 1.00 76.88
H
ATOM 35672 HD1 HIS L 189 85.880 152.740 234.665 1.00 94.94
H
ATOM 35673 HD2 HIS L 189 82.736 150.443 234.843 1.00 89.66
H
ATOM 35674 HE1 HIS L 189 84.613 153.215 232.727 1.00 89.73
H

ATOM 35675 N LYS L 190 84.672 149.888 239.363 1.00 66.16
N
ATOM 35676 CA LYS L 190 84.994 149.247 240.626 1.00 68.94
C
ATOM 35677 C LYS L 190 85.797 147.971 240.423 1.00 59.34
C
ATOM 35678 O LYS L 190 86.895 147.854 240.953 1.00 58.80
O
ATOM 35679 CB LYS L 190 83.679 148.984 241.393 1.00 75.98
C
ATOM 35680 CG LYS L 190 83.829 148.322 242.765 1.00 91.91
C
ATOM 35681 CD LYS L 190 82.484 148.314 243.519 1.00116.02
C
ATOM 35682 CE LYS L 190 82.230 147.011 244.279 1.00111.54
C
ATOM 35683 NZ LYS L 190 80.775 146.815 244.562 1.00140.03
N
ATOM 35684 H LYS L 190 84.093 149.457 238.897 1.00 79.42
H
ATOM 35685 HA LYS L 190 85.543 149.833 241.170 1.00 82.75
H
ATOM 35686 HB2 LYS L 190 83.233 149.834 241.532 1.00 91.21
H
ATOM 35687 HB3 LYS L 190 83.123 148.402 240.852 1.00 91.21
H
ATOM 35688 HG2 LYS L 190 84.124 147.404 242.651 1.00110.32
H
ATOM 35689 HG3 LYS L 190 84.477 148.813 243.293 1.00110.32
H
ATOM 35690 HD2 LYS L 190 82.479 149.041 244.161 1.00139.25
H
ATOM 35691 HD3 LYS L 190 81.764 148.432 242.880 1.00139.25
H
ATOM 35692 HE2 LYS L 190 82.540 146.263 243.746 1.00133.87
H
ATOM 35693 HE3 LYS L 190 82.703 147.035 245.125 1.00133.87
H
ATOM 35694 HZ1 LYS L 190 80.643 146.024 244.946 1.00168.07
H
ATOM 35695 HZ2 LYS L 190 80.482 147.454 245.107 1.00168.07
H
ATOM 35696 HZ3 LYS L 190 80.311 146.848 243.803 1.00168.07
H
ATOM 35697 N VAL L 191 85.297 147.012 239.652 1.00 62.60
N
ATOM 35698 CA VAL L 191 85.881 145.675 239.594 1.00 52.77
C
ATOM 35699 C VAL L 191 86.758 145.545 238.365 1.00 56.77
C

ATOM 35700 O VAL L 191 86.292 145.753 237.234 1.00 55.86
O
ATOM 35701 CB VAL L 191 84.795 144.589 239.586 1.00 59.52
C
ATOM 35702 CG1 VAL L 191 85.455 143.213 239.385 1.00 46.80
C
ATOM 35703 CG2 VAL L 191 83.995 144.650 240.892 1.00 62.73
C
ATOM 35704 H VAL L 191 84.609 147.112 239.146 1.00 75.14
H
ATOM 35705 HA VAL L 191 86.443 145.561 240.378 1.00 63.35
H
ATOM 35706 HB VAL L 191 84.172 144.733 238.857 1.00 71.45
H
ATOM 35707 HG11 VAL L 191 84.835 142.521 239.661 1.00 56.19
H
ATOM 35708 HG12 VAL L 191 85.676 143.103 238.446 1.00 56.19
H
ATOM 35709 HG13 VAL L 191 86.262 143.168 239.921 1.00 56.19
H
ATOM 35710 HG21 VAL L 191 83.322 143.952 240.881 1.00 75.31
H
ATOM 35711 HG22 VAL L 191 84.599 144.514 241.638 1.00 75.31
H
ATOM 35712 HG23 VAL L 191 83.570 145.519 240.962 1.00 75.31
H
ATOM 35713 N TYR L 192 88.016 145.160 238.588 1.00 47.97
N
ATOM 35714 CA TYR L 192 88.992 144.941 237.536 1.00 47.39
C
ATOM 35715 C TYR L 192 89.448 143.492 237.589 1.00 50.81
C
ATOM 35716 O TYR L 192 89.826 143.002 238.656 1.00 56.49
O
ATOM 35717 CB TYR L 192 90.160 145.930 237.706 1.00 52.25
C
ATOM 35718 CG TYR L 192 89.709 147.360 237.417 1.00 55.63
C
ATOM 35719 CD1 TYR L 192 89.122 148.139 238.390 1.00 54.07
C
ATOM 35720 CD2 TYR L 192 89.864 147.911 236.161 1.00 51.99
C
ATOM 35721 CE1 TYR L 192 88.693 149.436 238.111 1.00 59.39
C
ATOM 35722 CE2 TYR L 192 89.430 149.184 235.874 1.00 49.29
C
ATOM 35723 CZ TYR L 192 88.859 149.942 236.836 1.00 54.80
C
ATOM 35724 OH TYR L 192 88.461 151.206 236.480 1.00 63.29
O

ATOM 35725 H   TYR L 192   88.333 145.012 239.373 1.00 57.60
H
ATOM 35726 HA TYR L 192   88.612 145.083 236.656 1.00 56.89
H
ATOM 35727 HB2 TYR L 192   90.488 145.888 238.618 1.00 62.72
H
ATOM 35728 HB3 TYR L 192   90.870 145.700 237.088 1.00 62.72
H
ATOM 35729 HD1 TYR L 192   89.011 147.795 239.247 1.00 64.91
H
ATOM 35730 HD2 TYR L 192   90.273 147.409 235.493 1.00 62.41
H
ATOM 35731 HE1 TYR L 192   88.302 149.955 238.776 1.00 71.29
H
ATOM 35732 HE2 TYR L 192   89.527 149.525 235.014 1.00 59.18
H
ATOM 35733 HH TYR L 192   88.138 151.603 237.146 1.00 75.97
H
ATOM 35734 N   ALA L 193   89.423 142.808 236.446 1.00 49.34
N
ATOM 35735 CA ALA L 193   89.787 141.396 236.414 1.00 54.95
C
ATOM 35736 C   ALA L 193   90.431 140.989 235.087 1.00 51.18
C
ATOM 35737 O   ALA L 193   90.055 141.506 234.027 1.00 57.91
O
ATOM 35738 CB ALA L 193   88.520 140.545 236.687 1.00 54.14
C
ATOM 35739 H   ALA L 193   89.201 143.137 235.683 1.00 59.24
H
ATOM 35740 HA ALA L 193   90.470 141.228 237.082 1.00 65.97
H
ATOM 35741 HB1 ALA L 193   88.778 139.615 236.775 1.00 64.99
H
ATOM 35742 HB2 ALA L 193   88.104 140.853 237.508 1.00 64.99
H
ATOM 35743 HB3 ALA L 193   87.905 140.648 235.946 1.00 64.99
H
ATOM 35744 N   CYS L 194   91.430 140.092 235.141 1.00 50.16
N
ATOM 35745 CA ACYS L 194   91.920 139.405 233.953 0.01 58.37
C
ATOM 35746 CA BCYS L 194   91.918 139.402 233.954 0.99 58.66
C
ATOM 35747 C   CYS L 194   91.513 137.940 234.032 1.00 58.95
C
ATOM 35748 O   CYS L 194   91.639 137.306 235.088 1.00 56.46
O
ATOM 35749 CB ACYS L 194   93.439 139.543 233.772 0.01 60.10
C

ATOM 35750 CB BCYS L 194 93.434 139.507 233.743 0.99 60.49
C
ATOM 35751 SG ACYS L 194 94.528 139.083 235.148 0.01 67.47
S
ATOM 35752 SG BCYS L 194 94.455 139.029 235.073 0.99 70.75
S
ATOM 35753 H ACYS L 194 91.837 139.869 235.865 0.01 60.22
H
ATOM 35754 H BCYS L 194 91.839 139.871 235.865 0.99 60.22
H
ATOM 35755 HA ACYS L 194 91.514 139.804 233.168 0.01 70.07
H
ATOM 35756 HA BCYS L 194 91.507 139.825 233.184 0.99 70.43
H
ATOM 35757 HB2ACYS L 194 93.696 138.986 233.021 0.01 72.15
H
ATOM 35758 HB2BCYS L 194 93.670 138.942 232.990 0.99 72.62
H
ATOM 35759 HB3ACYS L 194 93.625 140.475 233.575 0.01 72.15
H
ATOM 35760 HB3BCYS L 194 93.644 140.431 233.543 0.99 72.62
H
ATOM 35761 N GLU L 195 90.987 137.440 232.923 1.00 58.14
N
ATOM 35762 CA GLU L 195 90.532 136.076 232.757 1.00 60.64
C
ATOM 35763 C GLU L 195 91.503 135.408 231.809 1.00 57.40
C
ATOM 35764 O GLU L 195 91.857 135.965 230.760 1.00 56.09
O
ATOM 35765 CB GLU L 195 89.116 136.044 232.196 1.00 65.96
C
ATOM 35766 CG GLU L 195 88.367 134.704 232.254 1.00 78.25
C
ATOM 35767 CD GLU L 195 87.145 134.728 231.316 1.00 86.12
C
ATOM 35768 OE1 GLU L 195 87.326 135.037 230.109 1.00 82.51
O
ATOM 35769 OE2 GLU L 195 86.006 134.498 231.783 1.00 92.32
O1-
ATOM 35770 H GLU L 195 90.875 137.906 232.210 1.00 69.79
H
ATOM 35771 HA GLU L 195 90.518 135.597 233.601 1.00 72.80
H
ATOM 35772 HB2 GLU L 195 88.584 136.684 232.693 1.00 79.18
H
ATOM 35773 HB3 GLU L 195 89.160 136.300 231.262 1.00 79.18
H
ATOM 35774 HG2 GLU L 195 88.959 133.989 231.973 1.00 93.92
H

ATOM 35775 HG3 GLU L 195   88.058 134.544 233.159 1.00 93.92
H
ATOM 35776 N   VAL L 196   91.963 134.243 232.198 1.00 50.56
N
ATOM 35777 CA VAL L 196   93.025 133.550 231.510 1.00 59.68
C
ATOM 35778 C   VAL L 196   92.495 132.205 231.061 1.00 64.74
C
ATOM 35779 O   VAL L 196   91.937 131.447 231.864 1.00 60.75
O
ATOM 35780 CB VAL L 196   94.248 133.411 232.425 1.00 54.81
C
ATOM 35781 CG1 VAL L 196   95.301 132.537 231.776 1.00 53.16
C
ATOM 35782 CG2 VAL L 196   94.769 134.832 232.783 1.00 56.70
C
ATOM 35783 H   VAL L 196   91.664 133.817 232.883 1.00 60.70
H
ATOM 35784 HA VAL L 196   93.293 134.033 230.713 1.00 71.65
H
ATOM 35785 HB VAL L 196   94.012 132.966 233.253 1.00 65.80
H
ATOM 35786 HG11 VAL L 196   96.146 132.671 232.234 1.00 63.82
H
ATOM 35787 HG12 VAL L 196   95.029 131.611 231.847 1.00 63.82
H
ATOM 35788 HG13 VAL L 196   95.387 132.787 230.843 1.00 63.82
H
ATOM 35789 HG21 VAL L 196   95.605 134.749 233.269 1.00 68.07
H
ATOM 35790 HG22 VAL L 196   94.912 135.332 231.964 1.00 68.07
H
ATOM 35791 HG23 VAL L 196   94.108 135.281 233.333 1.00 68.07
H
ATOM 35792 N   THR L 197   92.645 131.937 229.777 1.00 61.18
N
ATOM 35793 CA THR L 197   92.311 130.660 229.184 1.00 57.37
C
ATOM 35794 C   THR L 197   93.591 129.997 228.696 1.00 61.72
C
ATOM 35795 O   THR L 197   94.443 130.641 228.072 1.00 62.72
O
ATOM 35796 CB THR L 197   91.297 130.859 228.060 1.00 65.38
C
ATOM 35797 OG1 THR L 197   90.004 131.009 228.654 1.00 59.70
O
ATOM 35798 CG2 THR L 197   91.300 129.682 227.046 1.00 59.97
C
ATOM 35799 H   THR L 197   92.951 132.503 229.206 1.00 73.44
H

ATOM 35800 HA THR L 197   91.913 130.060 229.833 1.00 68.88
H
ATOM 35801 HB THR L 197   91.525 131.650 227.546 1.00 78.48
H
ATOM 35802 HG1 THR L 197   89.432 131.172 228.061 1.00 71.66
H
ATOM 35803 HG21 THR L 197   90.503 129.717 226.495 1.00 71.99
H
ATOM 35804 HG22 THR L 197   92.082 129.740 226.473 1.00 71.99
H
ATOM 35805 HG23 THR L 197   91.320 128.837 227.520 1.00 71.99
H
ATOM 35806 N   HIS L 198   93.737 128.721 229.023 1.00 60.66
N
ATOM 35807 CA HIS L 198   94.936 127.980 228.690 1.00 54.74
C
ATOM 35808 C   HIS L 198   94.607 126.504 228.747 1.00 55.03
C
ATOM 35809 O   HIS L 198   93.677 126.081 229.438 1.00 69.07
O
ATOM 35810 CB HIS L 198   96.073 128.301 229.645 1.00 53.24
C
ATOM 35811 CG HIS L 198   97.391 127.757 229.215 1.00 54.81
C
ATOM 35812 ND1 HIS L 198   97.930 126.599 229.738 1.00 58.53
N
ATOM 35813 CD2 HIS L 198   98.301 128.231 228.333 1.00 55.05
C
ATOM 35814 CE1 HIS L 198   99.122 126.391 229.203 1.00 55.98
C
ATOM 35815 NE2 HIS L 198   99.365 127.361 228.338 1.00 51.61
N
ATOM 35816 H   HIS L 198   93.146 128.259 229.444 1.00 72.82
H
ATOM 35817 HA HIS L 198   95.220 128.206 227.791 1.00 65.72
H
ATOM 35818 HB2 HIS L 198   96.160 129.266 229.711 1.00 63.92
H
ATOM 35819 HB3 HIS L 198   95.868 127.925 230.514 1.00 63.92
H
ATOM 35820 HD2 HIS L 198   98.221 129.000 227.818 1.00 66.09
H
ATOM 35821 HE1 HIS L 198   99.691 125.682 229.399 1.00 67.20
H
ATOM 35822 HE2 HIS L 198   100.075 127.437 227.857 1.00 61.95
H
ATOM 35823 N   GLN L 199   95.420 125.731 228.044 1.00 62.13
N
ATOM 35824 CA GLN L 199   95.206 124.293 227.932 1.00 67.14
C

ATOM 35825 C   GLN L 199   95.305 123.596 229.294 1.00 67.93
C
ATOM 35826 O   GLN L 199   94.504 122.706 229.601 1.00 67.76
O
ATOM 35827 CB GLN L 199   96.228 123.739 226.931 1.00 63.21
C
ATOM 35828 CG GLN L 199   96.390 122.241 226.915 1.00 70.25
C
ATOM 35829 CD GLN L 199   97.222 121.750 225.728 1.00 73.17
C
ATOM 35830 OE1 GLN L 199   97.564 122.509 224.807 1.00 71.21
O
ATOM 35831 NE2 GLN L 199   97.556 120.468 225.753 1.00 79.76
N
ATOM 35832 H   GLN L 199   96.111 126.013 227.615 1.00 74.58
H
ATOM 35833 HA GLN L 199   94.313 124.108 227.602 1.00 80.59
H
ATOM 35834 HB2 GLN L 199   95.954 124.006 226.040 1.00 75.88
H
ATOM 35835 HB3 GLN L 199   97.094 124.120 227.140 1.00 75.88
H
ATOM 35836 HG2 GLN L 199   96.838 121.963 227.730 1.00 84.33
H
ATOM 35837 HG3 GLN L 199   95.516 121.827 226.860 1.00 84.33
H
ATOM 35838 HE21 GLN L 199   97.305 119.971 226.410 1.00 95.74
H
ATOM 35839 HE22 GLN L 199   98.022 120.132 225.114 1.00 95.74
H
ATOM 35840 N   GLY L 200   96.268 123.996 230.131 1.00 61.55
N
ATOM 35841 CA GLY L 200   96.504 123.371 231.416 1.00 65.33
C
ATOM 35842 C   GLY L 200   95.498 123.734 232.488 1.00 65.84
C
ATOM 35843 O   GLY L 200   95.647 123.334 233.649 1.00 62.57
O
ATOM 35844 H   GLY L 200   96.805 124.647 229.965 1.00 73.88
H
ATOM 35845 HA2 GLY L 200   96.483 122.408 231.303 1.00 78.43
H
ATOM 35846 HA3 GLY L 200   97.383 123.632 231.734 1.00 78.43
H
ATOM 35847 N   LEU L 201   94.469 124.488 232.104 1.00 64.56
N
ATOM 35848 CA LEU L 201   93.412 124.923 232.997 1.00 61.11
C
ATOM 35849 C   LEU L 201   92.091 124.331 232.524 1.00 70.48
C

ATOM 35850 O   LEU L 201   91.648 124.614 231.404 1.00 68.88
O
ATOM 35851 CB LEU L 201   93.312 126.447 233.029 1.00 66.51
C
ATOM 35852 CG LEU L 201   94.500 127.225 233.573 1.00 65.94
C
ATOM 35853 CD1 LEU L 201   94.300 128.679 233.177 1.00 58.26
C
ATOM 35854 CD2 LEU L 201   94.648 127.062 235.093 1.00 55.37
C
ATOM 35855 H   LEU L 201   94.363 124.768 231.298 1.00 77.49
H
ATOM 35856 HA LEU L 201   93.602 124.601 233.892 1.00 73.35
H
ATOM 35857 HB2 LEU L 201   93.171 126.753 232.119 1.00 79.83
H
ATOM 35858 HB3 LEU L 201   92.548 126.681 233.579 1.00 79.83
H
ATOM 35859 HG LEU L 201   95.333 126.888 233.204 1.00 79.15
H
ATOM 35860 HD11 LEU L 201   95.039 129.203 233.520 1.00 69.93
H
ATOM 35861 HD12 LEU L 201   94.269 128.740 232.209 1.00 69.93
H
ATOM 35862 HD13 LEU L 201   93.466 128.996 233.555 1.00 69.93
H
ATOM 35863 HD21 LEU L 201   95.303 127.700 235.418 1.00 66.47
H
ATOM 35864 HD22 LEU L 201   93.789 127.227 235.514 1.00 66.47
H
ATOM 35865 HD23 LEU L 201   94.942 126.159 235.288 1.00 66.47
H
ATOM 35866 N   SER L 202   91.445 123.548 233.392 1.00 72.88
N
ATOM 35867 CA SER L 202   90.171 122.926 233.026 1.00 75.35
C
ATOM 35868 C   SER L 202   89.116 123.968 232.707 1.00 73.07
C
ATOM 35869 O   SER L 202   88.259 123.745 231.848 1.00 77.28
O
ATOM 35870 CB SER L 202   89.686 122.009 234.156 1.00 69.47
C
ATOM 35871 OG SER L 202   89.741 122.698 235.390 1.00 73.84
O
ATOM 35872 H   SER L 202   91.717 123.364 234.188 1.00 87.48
H
ATOM 35873 HA SER L 202   90.307 122.381 232.236 1.00 90.45
H
ATOM 35874 HB2 SER L 202   88.770 121.742 233.979 1.00 83.40
H

ATOM 35875 HB3 SER L 202 90.258 121.227 234.201 1.00 83.40
H
ATOM 35876 HG SER L 202 89.784 122.149 236.024 1.00 88.64
H
ATOM 35877 N SER L 203 89.153 125.107 233.379 1.00 80.13
N
ATOM 35878 CA SER L 203 88.299 126.214 232.964 1.00 78.19
C
ATOM 35879 C SER L 203 89.042 127.532 233.179 1.00 77.88
C
ATOM 35880 O SER L 203 90.119 127.541 233.794 1.00 75.80
O
ATOM 35881 CB SER L 203 86.967 126.182 233.719 1.00 74.33
C
ATOM 35882 OG SER L 203 87.149 126.135 235.120 1.00 63.86
O
ATOM 35883 H SER L 203 89.652 125.262 234.062 1.00 96.18
H
ATOM 35884 HA SER L 203 88.090 126.141 232.021 1.00 93.86
H
ATOM 35885 HB2 SER L 203 86.465 126.983 233.499 1.00 89.23
H
ATOM 35886 HB3 SER L 203 86.472 125.393 233.444 1.00 89.23
H
ATOM 35887 HG SER L 203 86.441 126.366 235.510 1.00 76.65
H
ATOM 35888 N PRO L 204 88.523 128.650 232.667 1.00 68.10
N
ATOM 35889 CA PRO L 204 89.253 129.915 232.791 1.00 66.28
C
ATOM 35890 C PRO L 204 89.457 130.314 234.249 1.00 68.07
C
ATOM 35891 O PRO L 204 88.568 130.178 235.090 1.00 64.68
O
ATOM 35892 CB PRO L 204 88.365 130.921 232.039 1.00 67.93
C
ATOM 35893 CG PRO L 204 87.597 130.098 231.061 1.00 70.22
C
ATOM 35894 CD PRO L 204 87.354 128.774 231.769 1.00 73.57
C
ATOM 35895 HA PRO L 204 90.116 129.851 232.354 1.00 79.56
H
ATOM 35896 HB2 PRO L 204 87.769 131.367 232.663 1.00 81.55
H
ATOM 35897 HB3 PRO L 204 88.918 131.576 231.584 1.00 81.55
H
ATOM 35898 HG2 PRO L 204 86.757 130.535 230.846 1.00 84.30
H
ATOM 35899 HG3 PRO L 204 88.119 129.970 230.253 1.00 84.30
H

ATOM 35900 HD2 PRO L 204 86.529 128.801 232.278 1.00 88.31
H
ATOM 35901 HD3 PRO L 204 87.329 128.042 231.134 1.00 88.31
H
ATOM 35902 N VAL L 205 90.666 130.782 234.541 1.00 57.04
N
ATOM 35903 CA VAL L 205 91.001 131.350 235.837 1.00 54.45
C
ATOM 35904 C VAL L 205 90.809 132.863 235.787 1.00 60.22
C
ATOM 35905 O VAL L 205 91.323 133.528 234.882 1.00 58.52
O
ATOM 35906 CB VAL L 205 92.444 131.012 236.215 1.00 57.10
C
ATOM 35907 CG1 VAL L 205 92.829 131.828 237.423 1.00 66.54
C
ATOM 35908 CG2 VAL L 205 92.628 129.476 236.437 1.00 54.17
C
ATOM 35909 H VAL L 205 91.325 130.781 233.989 1.00 68.48
H
ATOM 35910 HA VAL L 205 90.402 130.985 236.507 1.00 65.37
H
ATOM 35911 HB VAL L 205 93.046 131.243 235.490 1.00 68.54
H
ATOM 35912 HG11 VAL L 205 93.577 131.399 237.869 1.00 79.88
H
ATOM 35913 HG12 VAL L 205 93.084 132.719 237.134 1.00 79.88
H
ATOM 35914 HG13 VAL L 205 92.071 131.880 238.025 1.00 79.88
H
ATOM 35915 HG21 VAL L 205 93.567 129.288 236.589 1.00 65.03
H
ATOM 35916 HG22 VAL L 205 92.108 129.203 237.209 1.00 65.03
H
ATOM 35917 HG23 VAL L 205 92.320 129.003 235.647 1.00 65.03
H
ATOM 35918 N THR L 206 90.099 133.418 236.765 1.00 56.84
N
ATOM 35919 CA THR L 206 89.947 134.863 236.909 1.00 52.37
C
ATOM 35920 C THR L 206 90.673 135.353 238.166 1.00 56.22
C
ATOM 35921 O THR L 206 90.534 134.765 239.245 1.00 59.07
O
ATOM 35922 CB THR L 206 88.462 135.264 236.966 1.00 69.53
C
ATOM 35923 OG1 THR L 206 87.787 134.852 235.765 1.00 67.93
O
ATOM 35924 CG2 THR L 206 88.316 136.798 237.110 1.00 58.48
C

ATOM 35925 H   THR L 206   89.686 132.967 237.369 1.00 68.24
H
ATOM 35926 HA THR L 206   90.355 135.295 236.143 1.00 62.87
H
ATOM 35927 HB THR L 206   88.056 134.832 237.734 1.00 83.46
H
ATOM 35928 HG1 THR L 206   86.976 135.071 235.802 1.00 81.54
H
ATOM 35929 HG21 THR L 206   87.388 137.056 236.981 1.00 70.20
H
ATOM 35930 HG22 THR L 206   88.598 137.078 237.995 1.00 70.20
H
ATOM 35931 HG23 THR L 206   88.861 137.248 236.448 1.00 70.20
H
ATOM 35932 N   LYS L 207   91.435 136.442 238.037 1.00 55.44
N
ATOM 35933 CA LYS L 207   91.957 137.181 239.190 1.00 54.50
C
ATOM 35934 C   LYS L 207   91.427 138.609 239.103 1.00 52.68
C
ATOM 35935 O   LYS L 207   91.396 139.198 238.020 1.00 52.80
O
ATOM 35936 CB LYS L 207   93.505 137.188 239.254 1.00 57.29
C
ATOM 35937 CG LYS L 207   94.201 135.817 239.342 1.00 52.32
C
ATOM 35938 CD LYS L 207   93.965 135.107 240.708 1.00 56.67
C
ATOM 35939 CE LYS L 207   94.720 133.737 240.792 1.00 54.75
C
ATOM 35940 NZ LYS L 207   94.569 133.098 242.171 1.00 71.88
N
ATOM 35941 H   LYS L 207   91.669 136.775 237.279 1.00 66.56
H
ATOM 35942 HA LYS L 207   91.654 136.761 240.011 1.00 65.43
H
ATOM 35943 HB2 LYS L 207   93.836 137.623 238.452 1.00 68.78
H
ATOM 35944 HB3 LYS L 207   93.771 137.692 240.038 1.00 68.78
H
ATOM 35945 HG2 LYS L 207   93.856 135.240 238.642 1.00 62.81
H
ATOM 35946 HG3 LYS L 207   95.157 135.939 239.230 1.00 62.81
H
ATOM 35947 HD2 LYS L 207   94.289 135.677 241.423 1.00 68.04
H
ATOM 35948 HD3 LYS L 207   93.017 134.939 240.822 1.00 68.04
H
ATOM 35949 HE2 LYS L 207   94.355 133.127 240.132 1.00 65.73
H

ATOM 35950 HE3 LYS L 207 95.664 133.880 240.625 1.00 65.73
H
ATOM 35951 HZ1 LYS L 207 94.973 132.306 242.186 1.00 86.28
H
ATOM 35952 HZ2 LYS L 207 94.934 133.622 242.790 1.00 86.28
H
ATOM 35953 HZ3 LYS L 207 93.706 132.986 242.361 1.00 86.28
H
ATOM 35954 N SER L 208 91.036 139.179 240.239 1.00 45.24
N
ATOM 35955 CA SER L 208 90.411 140.492 240.250 1.00 49.72
C
ATOM 35956 C SER L 208 90.807 141.266 241.497 1.00 52.79
C
ATOM 35957 O SER L 208 91.421 140.735 242.417 1.00 59.49
O
ATOM 35958 CB SER L 208 88.900 140.376 240.191 1.00 53.99
C
ATOM 35959 OG SER L 208 88.468 139.869 241.428 1.00 62.82
O
ATOM 35960 H SER L 208 91.121 138.823 241.017 1.00 54.32
H
ATOM 35961 HA SER L 208 90.743 140.978 239.478 1.00 59.70
H
ATOM 35962 HB2 SER L 208 88.510 141.251 240.039 1.00 64.82
H
ATOM 35963 HB3 SER L 208 88.644 139.771 239.479 1.00 64.82
H
ATOM 35964 HG SER L 208 87.635 139.763 241.421 1.00 75.41
H
ATOM 35965 N PHE L 209 90.474 142.551 241.487 1.00 53.09
N
ATOM 35966 CA PHE L 209 90.508 143.379 242.678 1.00 56.61
C
ATOM 35967 C PHE L 209 89.391 144.398 242.549 1.00 58.18
C
ATOM 35968 O PHE L 209 88.810 144.582 241.475 1.00 59.21
O
ATOM 35969 CB PHE L 209 91.874 144.065 242.879 1.00 56.07
C
ATOM 35970 CG PHE L 209 92.244 145.045 241.784 1.00 57.23
C
ATOM 35971 CD1 PHE L 209 91.844 146.363 241.854 1.00 53.36
C
ATOM 35972 CD2 PHE L 209 93.016 144.633 240.699 1.00 53.84
C
ATOM 35973 CE1 PHE L 209 92.173 147.266 240.866 1.00 52.83
C
ATOM 35974 CE2 PHE L 209 93.370 145.517 239.700 1.00 51.02
C

ATOM 35975 CZ PHE L 209   92.956 146.852 239.779 1.00 55.99
C
ATOM 35976 H   PHE L 209   90.220 142.975 240.784 1.00 63.74
H
ATOM 35977 HA PHE L 209   90.353 142.831 243.462 1.00 67.96
H
ATOM 35978 HB2 PHE L 209   91.854 144.554 243.717 1.00 67.31
H
ATOM 35979 HB3 PHE L 209   92.562 143.383 242.908 1.00 67.31
H
ATOM 35980 HD1 PHE L 209   91.340 146.648 242.582 1.00 64.05
H
ATOM 35981 HD2 PHE L 209   93.297 143.748 240.647 1.00 64.63
H
ATOM 35982 HE1 PHE L 209   91.878 148.145 240.920 1.00 63.43
H
ATOM 35983 HE2 PHE L 209   93.880 145.227 238.979 1.00 61.25
H
ATOM 35984 HZ PHE L 209   93.197 147.458 239.116 1.00 67.21
H
ATOM 35985 N   ASN L 210   89.102 145.066 243.658 1.00 56.44
N
ATOM 35986 CA ASN L 210   88.136 146.156 243.709 1.00 67.21
C
ATOM 35987 C   ASN L 210   88.858 147.472 243.978 1.00 62.90
C
ATOM 35988 O   ASN L 210   89.648 147.570 244.918 1.00 68.29
O
ATOM 35989 CB ASN L 210   87.089 145.879 244.787 1.00 75.68
C
ATOM 35990 CG ASN L 210   86.440 144.532 244.608 1.00 72.30
C
ATOM 35991 OD1 ASN L 210   85.622 144.345 243.717 1.00 74.05
O
ATOM 35992 ND2 ASN L 210   86.834 143.573 245.423 1.00 87.77
N
ATOM 35993 H   ASN L 210   89.466 144.903 244.420 1.00 67.75
H
ATOM 35994 HA ASN L 210   87.686 146.244 242.854 1.00 80.68
H
ATOM 35995 HB2 ASN L 210   87.517 145.896 245.657 1.00 90.84
H
ATOM 35996 HB3 ASN L 210   86.399 146.558 244.743 1.00 90.84
H
ATOM 35997 HD21 ASN L 210   86.492 142.787 245.359 1.00105.35
H
ATOM 35998 HD22 ASN L 210   87.434 143.735 246.018 1.00105.35
H
ATOM 35999 N   ARG L 211   88.614 148.474 243.140 1.00 67.50
N

ATOM 36000 CA ARG L 211   89.325 149.739 243.292 1.00 70.25
C
ATOM 36001 C   ARG L 211   89.112 150.307 244.693 1.00 75.40
C
ATOM 36002 O   ARG L 211   87.984 150.363 245.190 1.00 77.93
O
ATOM 36003 CB ARG L 211   88.857 150.737 242.243 1.00 70.81
C
ATOM 36004 CG ARG L 211   89.701 152.002 242.192 1.00 67.97
C
ATOM 36005 CD ARG L 211   89.241 152.885 241.075 1.00 67.07
C
ATOM 36006 NE ARG L 211   87.792 153.010 241.105 1.00 74.17
N
ATOM 36007 CZ ARG L 211   87.142 153.766 241.978 1.00 73.98
C
ATOM 36008 NH1 ARG L 211   85.817 153.835 241.944 1.00 73.40
N
ATOM 36009 NH2 ARG L 211   87.830 154.451 242.880 1.00 72.22
N
ATOM 36010 H   ARG L 211   88.054 148.448 242.487 1.00 81.03
H
ATOM 36011 HA ARG L 211   90.274 149.589 243.165 1.00 84.32
H
ATOM 36012 HB2 ARG L 211   88.898 150.316 241.370 1.00 85.00
H
ATOM 36013 HB3 ARG L 211   87.945 150.998 242.443 1.00 85.00
H
ATOM 36014 HG2 ARG L 211   89.615 152.486 243.026 1.00 81.59
H
ATOM 36015 HG3 ARG L 211   90.630 151.767 242.039 1.00 81.59
H
ATOM 36016 HD2 ARG L 211   89.633 153.767 241.174 1.00 80.51
H
ATOM 36017 HD3 ARG L 211   89.503 152.500 240.223 1.00 80.51
H
ATOM 36018 HE ARG L 211   87.333 152.571 240.525 1.00 89.03
H
ATOM 36019 HH11 ARG L 211   85.378 153.389 241.353 1.00 88.11
H
ATOM 36020 HH12 ARG L 211   85.398 154.326 242.511 1.00 88.11
H
ATOM 36021 HH21 ARG L 211   88.688 154.402 242.894 1.00 86.69
H
ATOM 36022 HH22 ARG L 211   87.416 154.944 243.451 1.00 86.69
H
ATOM 36023 N   GLY L 212   90.202 150.700 245.342 1.00 83.50
N
ATOM 36024 CA GLY L 212   90.118 151.178 246.713 1.00 86.98
C

ATOM 36025 C   GLY L 212   90.127 150.075 247.747 1.00 99.59
C
ATOM 36026 O   GLY L 212   89.322 150.121 248.682 1.00105.53
O
ATOM 36027 H   GLY L 212   90.996 150.698 245.012 1.00100.22
H
ATOM 36028 HA2 GLY L 212   90.874 151.761 246.889 1.00104.41
H
ATOM 36029 HA3 GLY L 212   89.298 151.685 246.820 1.00104.41
H
ATOM 36030 N   GLU L 213   91.024 149.083 247.596 1.00108.00
N
ATOM 36031 CA GLU L 213   91.158 147.929 248.493 1.00107.66
C
ATOM 36032 C   GLU L 213   89.820 147.526 249.102 1.00118.21
C
ATOM 36033 O   GLU L 213   89.629 147.633 250.317 1.00131.64
O
ATOM 36034 CB GLU L 213   92.164 148.218 249.615 1.00115.92
C
ATOM 36035 CG GLU L 213   92.476 147.027 250.544 1.00111.50
C
ATOM 36036 CD GLU L 213   93.475 146.040 249.939 1.00113.86
C
ATOM 36037 OE1 GLU L 213   94.689 146.344 249.962 1.00 98.38
O
ATOM 36038 OE2 GLU L 213   93.049 144.973 249.436 1.00109.69
O1-
ATOM 36039 H   GLU L 213   91.593 149.057 246.952 1.00129.63
H
ATOM 36040 HA GLU L 213   91.484 147.184 247.965 1.00129.22
H
ATOM 36041 HB2 GLU L 213   93.001 148.497 249.210 1.00139.13
H
ATOM 36042 HB3 GLU L 213   91.810 148.933 250.168 1.00139.13
H
ATOM 36043 HG2 GLU L 213   92.853 147.364 251.374 1.00133.83
H
ATOM 36044 HG3 GLU L 213   91.653 146.545 250.726 1.00133.83
H
ATOM 36045 N   CYS L 214   88.896 147.070 248.266 1.00114.23
N
ATOM 36046 CA CYS L 214   87.564 146.663 248.714 1.00146.10
C
ATOM 36047 C   CYS L 214   87.323 145.212 248.306 1.00125.35
C
ATOM 36048 O   CYS L 214   86.383 144.571 248.765 1.00128.94
O
ATOM 36049 CB CYS L 214   86.491 147.591 248.121 1.00142.25
C

```
ATOM  36050  SG  CYS L 214      84.758 147.103 248.395  1.00137.27
S
ATOM  36051  H   CYS L 214      89.015 146.982 247.419  1.00137.11
H
ATOM  36052  HA  CYS L 214      87.490 146.733 249.678  1.00175.35
H
ATOM  36053  HB2 CYS L 214      86.604 148.471 248.512  1.00170.72
H
ATOM  36054  HB3 CYS L 214      86.627 147.635 247.162  1.00170.72
H
ATOM  36055  HG  CYS L 214      84.571 146.029 247.895  1.00164.75
H
```
