# Supplementary material for: Identification and mechanistic basis of non-ACE2 blocking neutralizing antibodies from COVID-19 patients with deep RNA sequencing and molecular dynamics simulations
Source: Front Mol Biosci. 2022 Dec 16;9:1080964. doi: 10.3389/fmolb.2022.1080964 (PMC9800910; doi:10.3389/fmolb.2022.1080964)
Supplement: Supplementary file 5 [file DataSheet2.docx]

**
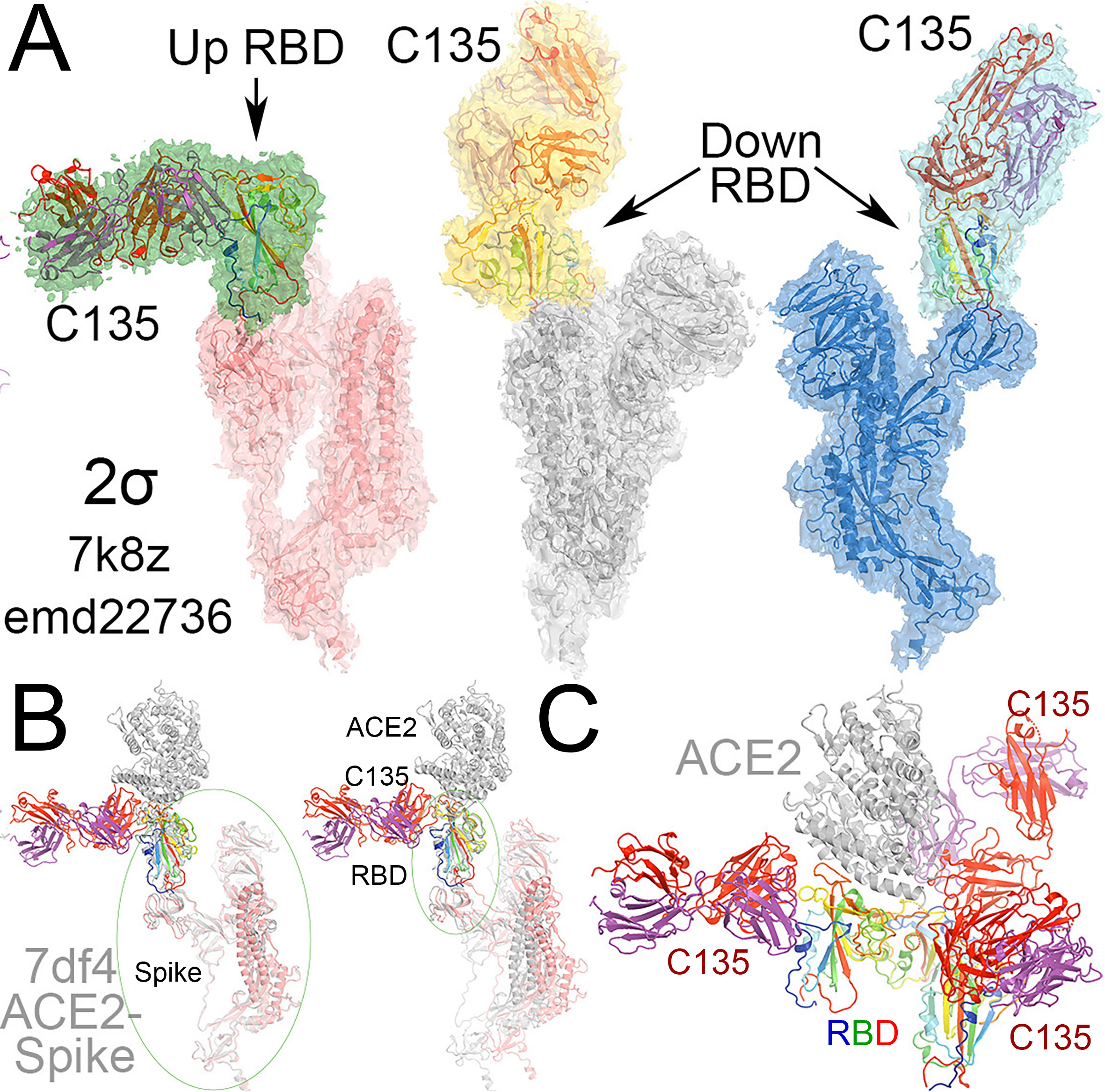
**

**Figure S1.** Refitting atomic coordinates into the experimental cryo-EM map (emd22736) of SARS-CoV-2. (A) Three spike subunits (RBD in rainbow color) with one RBD up (left panel) and two RBDs down (middle and right panels) fitted into the cryo-EM map contoured at +2σ. (B) Comparison of the spike-ACE2 receptor complex in the up conformation (PDB: 7df4) upon alignment of the entire subunit (left) or the RBD-only (right). (C) Modeled ACE2 receptors do not sterically overlap with other RBDs. See Figure S2 for more information.


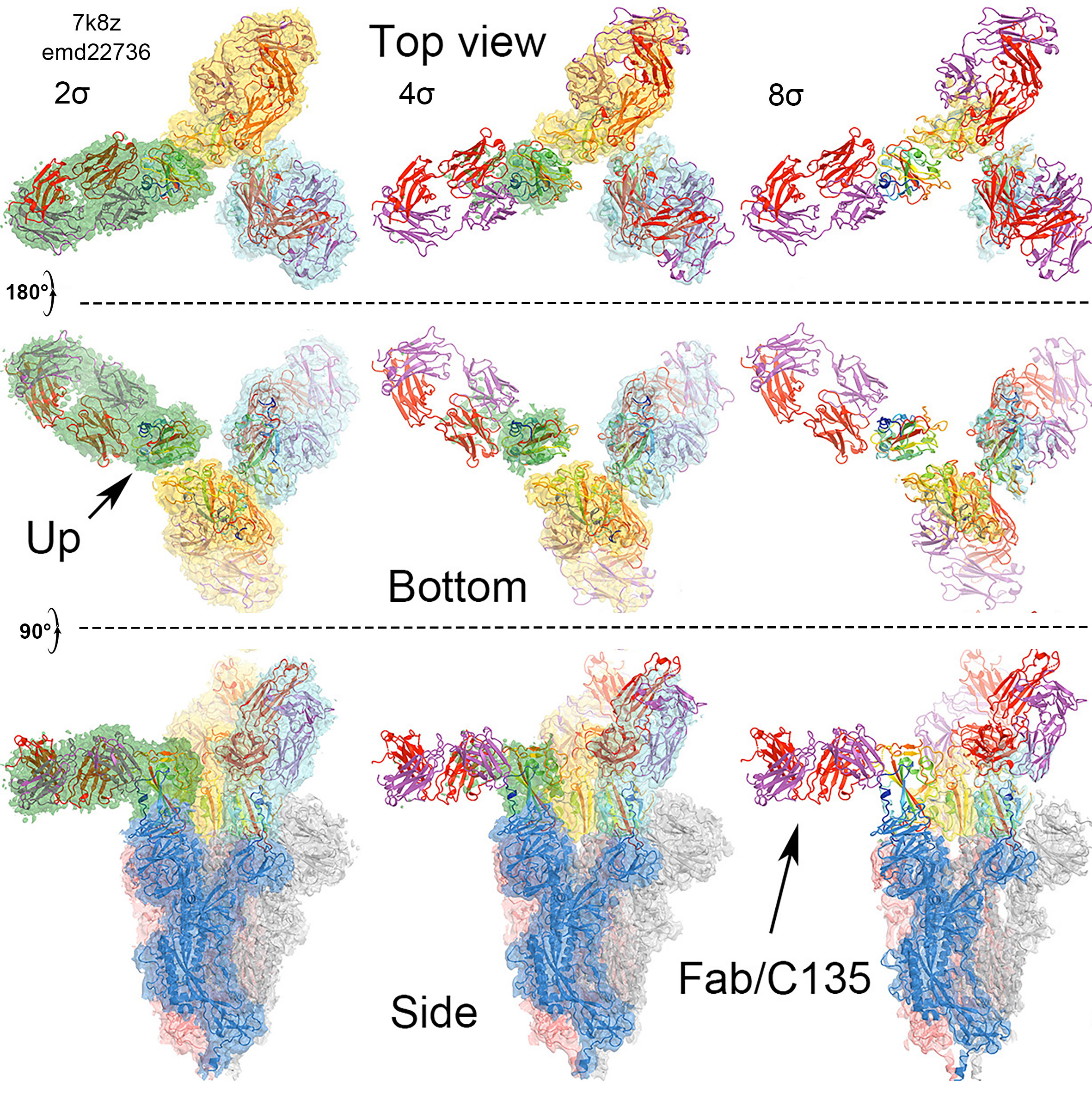


**Figure S2.** Three orthogonal views (top, bottom and side views) of the spike RBD-C135 Fab complex with the map contoured at +2σ (left panel), +4σ (middle), and +8σ (right).

**
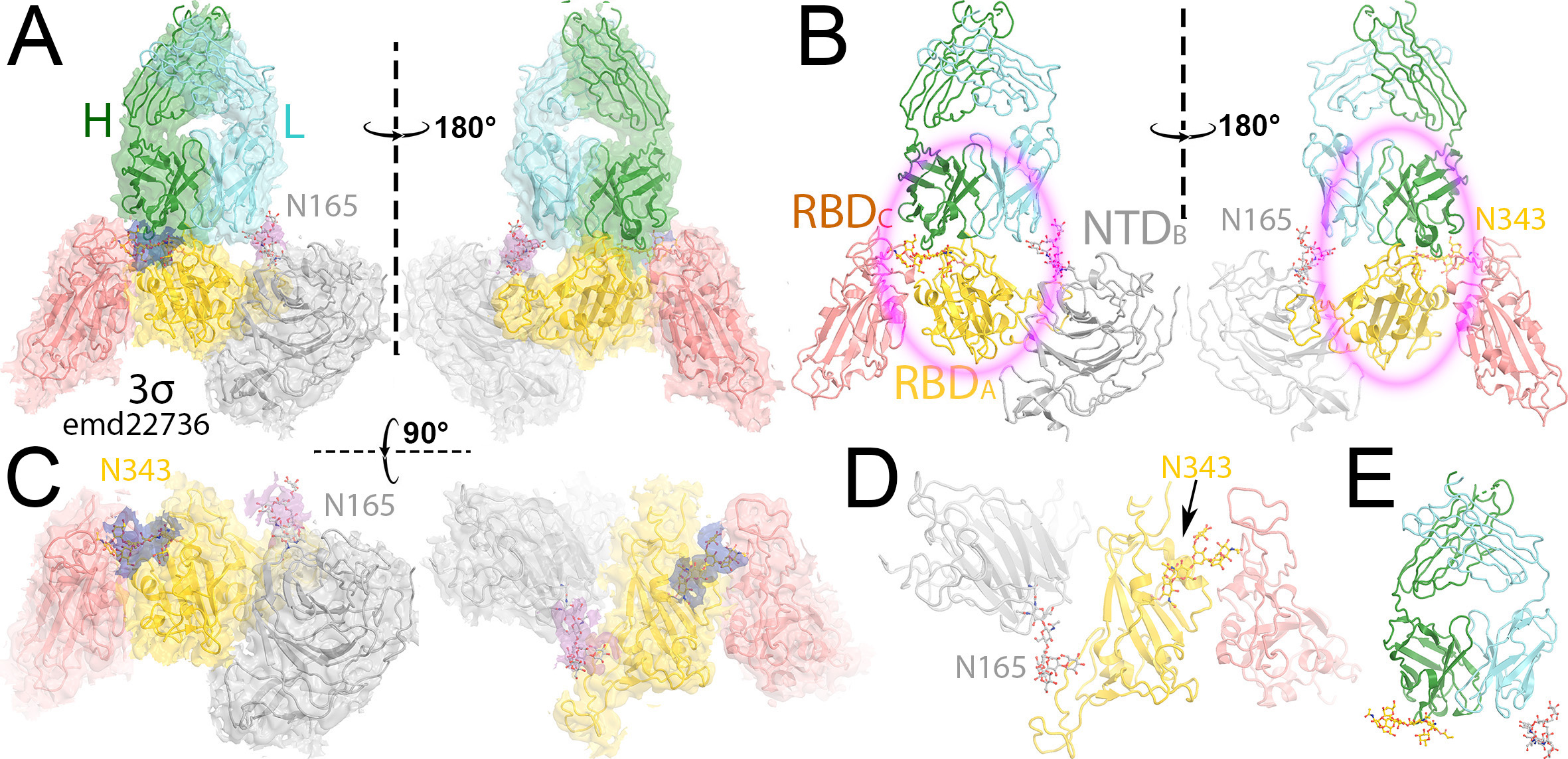
**

**Figure S3.** The RBD-C135 subcomplex within the context of the spike trimer. (A, B) Two front and back views of the subcomplex (middle), including N165 (silver sticks) and one RBD (salmon) with (A) and without (B) experimental maps. (C) Comparison to the experimental map showing the NAb C135 Fab in two orthogonal views. (D) Same view without the experimental map. (E) The relationship between the C135 Fab and the glycan moieties.

**
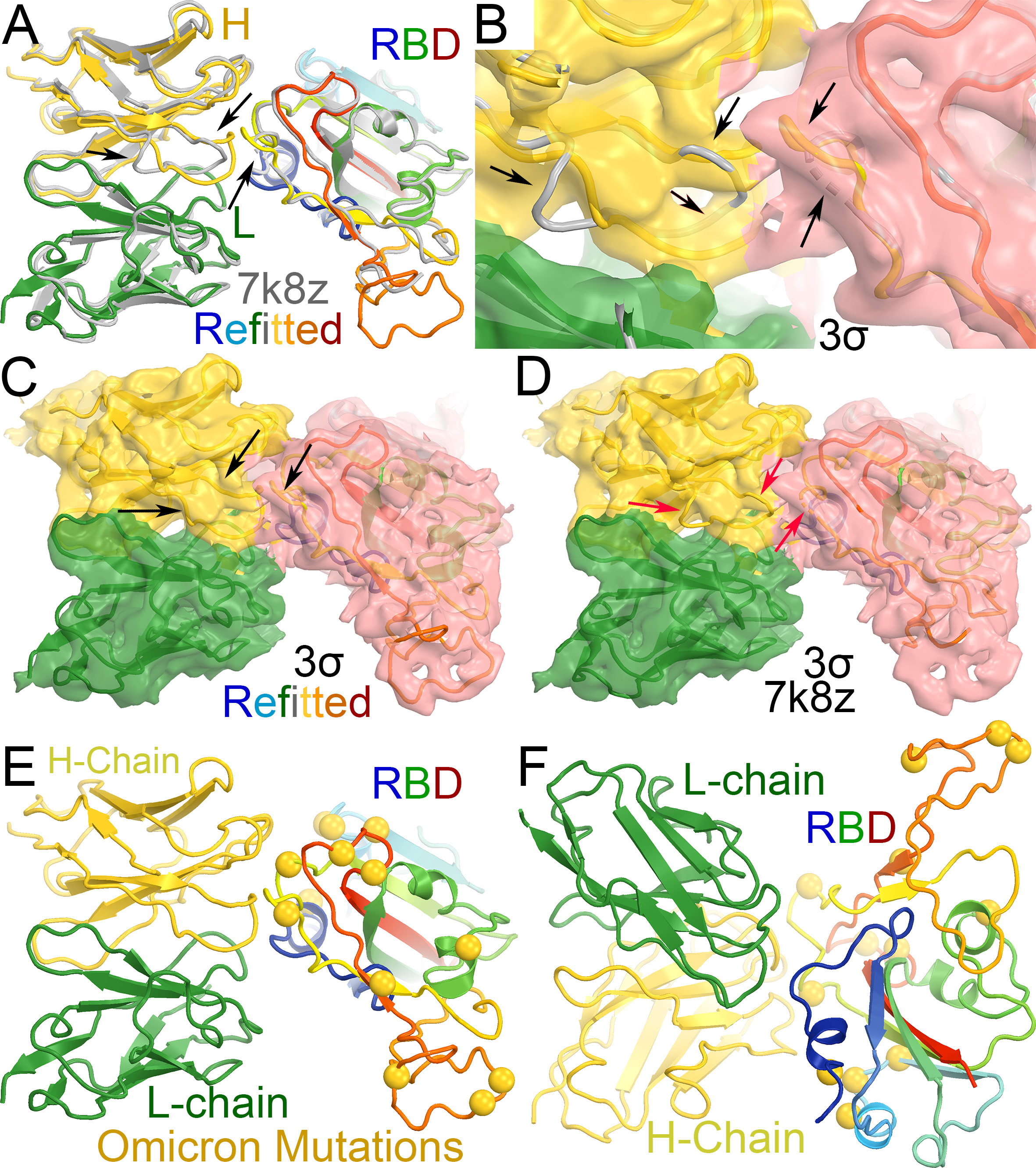
**

**Figure S4.** Comparison of the refitted and 7k8z models. (A) Alignment of the two models (multiple colors for refitted model, silver for the 7k8z model). Arrows indicate major differences. (B) Alignment of the two models with the experimental map contoured at +3σ. (C) Superposition of the refitted model with the experimental map. (D) Superposition of the original 7k8z model with the experimental map. (E, F) Two views of mapped Omicron variant mutations on the complete models derived from the MD simulations.

**
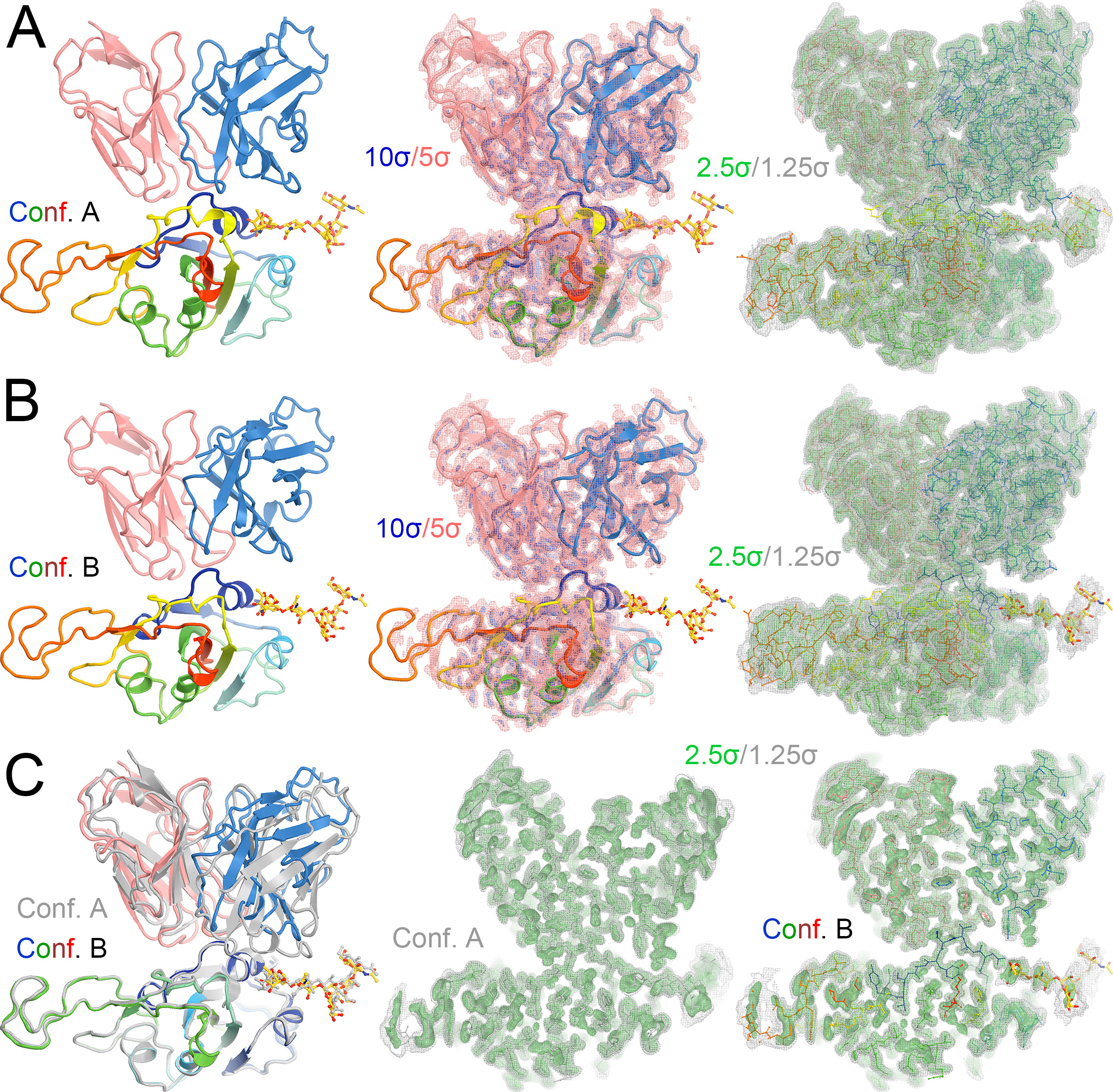
**

**Figure S5.** Two MD conformations of the NAb (A and B). (A) Conformation A, without map, and with MD-derived ESP maps contoured at +10σ (blue), +5σ (salmon), +2.5σ (green), and 1.25σ (silver). (B) Conformation B. (C) Superimposition of the two conformations with slices of MD-derived ESP maps.


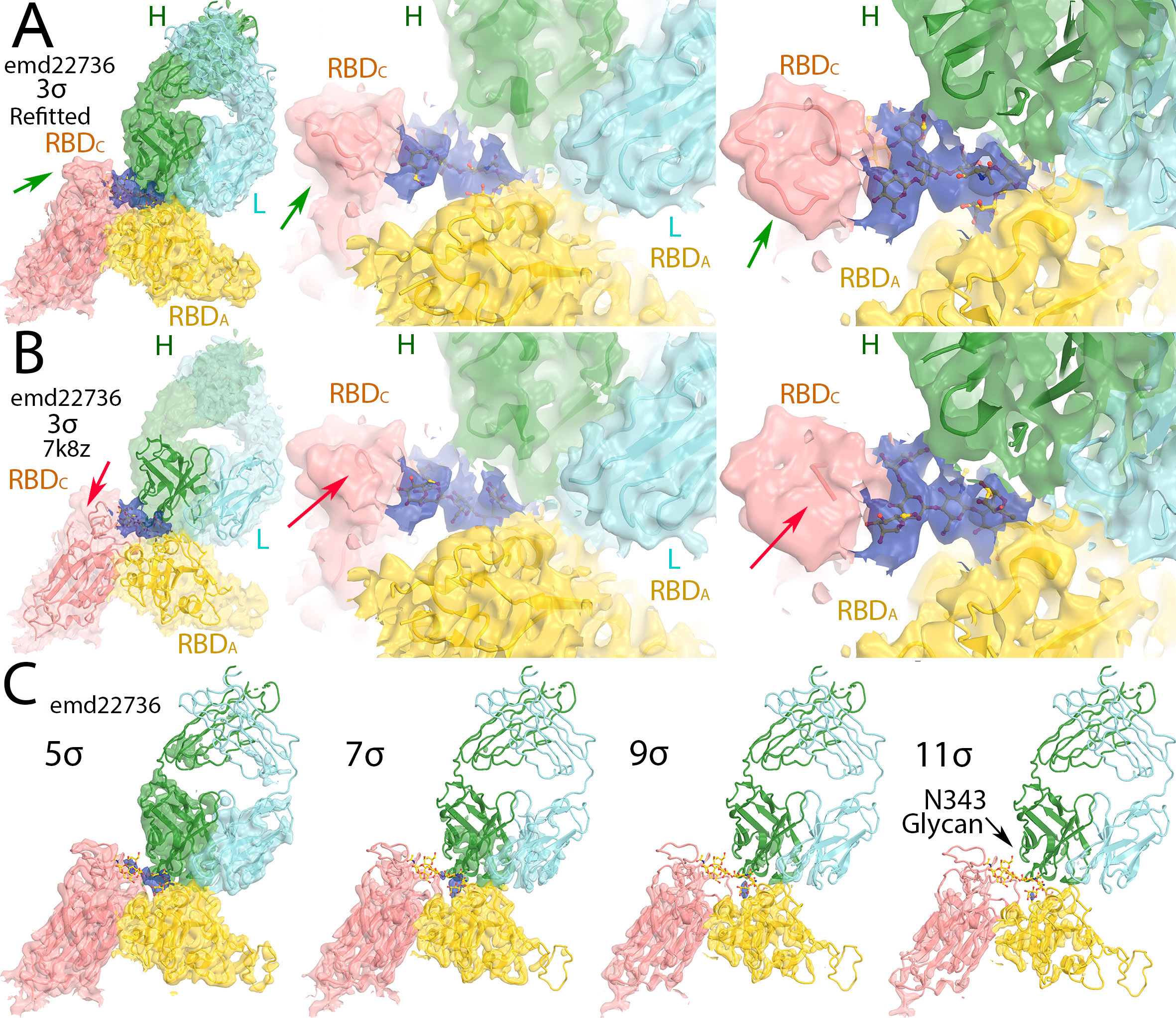


**Figure S6.** Comparison of the MD-derived equilibrated structure with the experimental cryo-EM map. (A) MD-derived structure for neighboring RBD and glycan fitting to the experimental map (successive zoomed views) contoured at +3σ. (B) The original 7k8z incomplete model for the neighboring RBD. (C). Experimental maps are contoured at +5σ, +7σ, +9σ, and +11σ (from left to right panels).
