# Supplementary material for: Identification and mechanistic basis of non-ACE2 blocking neutralizing antibodies from COVID-19 patients with deep RNA sequencing and molecular dynamics simulations
Source: Front Mol Biosci. 2022 Dec 16;9:1080964. doi: 10.3389/fmolb.2022.1080964 (PMC9800910; doi:10.3389/fmolb.2022.1080964)
Supplement: Supplementary file 3 [file DataSheet1.PDF]

Supplemental Table 1<br>Antibodies to COVID-19		
**Antibody**	**PDB**	**CDRL3**
**Class 1**		
C102	7K8N	CQQYGSSPR
C105	6XCM	CSSYEGSNNFV
B38	7BZ5	CQQLNSYPPY
CC12.3	6XC7	CQQYYSTPY
**Class 2**		
C002	7K8O	CQQSYSTPR
C104	7K8U	CQQYGTTPR
C119	7K8W	CSSYTSSSTSV
C121	7K8X	CCSYAGSSTL
C144	7K90	CSSYTSSSTR
COVA2-39	7JMP	CCSYAGSSTW
P2B-2F6	7BWJ	CSSYAGSNNL
Ab2-4	6XEY	CSSYAGSNNL
**Class 3**		
BD23	7BYR	CQQYNSYPY
C135	7K8R	CQQYNSYPW
S309	6WPS	CQQHDTSL
C110	7K8P	CQQYNSYPY
REGN10987	6XDG	CQQYDNLPL
**Class 4**		
CR3022	6W41	CQQYYSTPY
EY6A	6ZDH	CQQSYSTLAL
S304	7JW0	CQQSYVSPTY
S2A4	7JVC	CQSYDSSNHVV

Supplemental Table 2 All Antibodies Identified on ICU Day 0						
Patient	Sequence Identifier	CDR3	Type	Count	COVID Antibody Type	COVID Structure
1	1	CARDRDSGSSVSVYFDLW	IGH	2	None	
1	2	CARGWKVATIFNYYYGMDVW	IGH	2	None	
1	3	CSVSYWNDVFDYW	IGH	2	None	
1	4	CTTSLQYRYSGSYPLDYW	IGH	2	None	
1	6	CARDHSMVRGVRYYYYYGMDVW	IGH	3	None	
1	7	CARLPPFLDQPYYYDSMFFDYW	IGH	3	None	
1	8	CASAGSGSYQDYW	IGH	2	None	
1	9	CARSRDGYIASWEAYYFDYW	IGH	3	None	
1	10	CMHGTHWSYRF	IGK	2	None	
1	13	CASYHGGNSGDFDYW	IGH	2	None	
1	14	CARHSGLTMIVVVGPFWFDPW	IGH	2	None	
1	15	CARPRWDRFDPFDIW	IGH	2	None	
1	16	CAKDLEPHDYHGSGSYLDHW	IGH	24	None	
1	18	CVRLGDYGAYLNFFDHW	IGH	8	None	
1	19	CARSRDGYNRREFDPW	IGH	6	None	
1	20	CAHRRPFVDFDYW	IGH	3	None	
1	21	CAREVSYSNSYDVFDTW	IGH	2	None	
1	22	CARLPDYRNYQGDWFDPW	IGH	2	None	
1	23	CASTRAYIGDDDQSLDYW	IGH	4	None	
1	24	CARVRPGHSYGPLHNFDSW	IGH	2	None	
1	25	CARASSQWLVKYVWFDPW	IGH	11	None	
1	26	CARAKGGYPGWIDPW	IGH	2	None	
1	27	CARAAHCSGGNCYAFRFDPW	IGH	2	None	
1	28	CARHVLPDYYDNSGYYYFHYW	IGH	2	None	
1	29	CARAPWGGYSGNVPRVRSFDDW	IGH	19	None	
1	30	CARGQGGYSSGWTFDYW	IGH	4	None	
1	31	CARDEYNHPQNYYYYGMDVW	IGH	2	None	
1	32	CARDTGDPDRRDGYIPDYW	IGH	34	None	
1	33	CVTRRSASYSDYW	IGH	8	None	
1	34	CARDPLLRDFDWSYFYYMDVW	IGH	2	None	
1	35	CATEAVRFLEWSAYYYYGMDVW	IGH	2	None	
1	36	CARGGYHSSGYYYVGVPQEAFDIW	IGH	6	None	
1	37	CVKERGSSGWLPAPDYW	IGH	4	None	
1	38	CARHEQQGGAFDYW	IGH	4	None	
1	39	CARHRGPIHGGKSMPPDYW	IGH	2	None	
1	40	CAKDWNYEEYYFDYW	IGH	15	None	
1	41	CARELAAAGPPVDYW	IGH	2	None	
1	42	CAHRPFWSGYLAWFDPW	IGH	4	None	
1	43	CARTLYPGTDFDYW	IGH	8	None	
1	44	CARLRARKVFGVVIAGAFDIW	IGH	2	None	
1	45	CSLRSEDTAAYYCARLLGYYDRSDYYSNYFDYW	IGH	2	None	
1	46	CARGDILTGYYKGYYYGMDVW	IGH	2	None	
1	47	CARARRAMTTWAWWFDPW	IGH	2	None	
1	48	CALPILRAEDTAVYYCARLYSGSSAWFDPW	IGH	2	None	
1	49	CARDAVDFYDFWSGYYTRGKNWFDPW	IGH	10	None	
1	50	CARTTVDIVVVTAIIPDYW	IGH	6	None	
1	51	CARDPGLVRVADYYFDYW	IGH	2	None	
1	52	CARHSYYDSSGYPLFYFDYW	IGH	2	None	
1	53	CARDSYSYGSGSYRFDYW	IGH	4	None	
1	54	CTTYSADYW	IGH	32	None	
1	55	CASEREHIVVVTAILDYW	IGH	2	None	
1	56	CAREAGYSSTWYEYLDYW	IGH	553	None	
1	57	CAMDTYYYDSRYFGYFDLW	IGH	2	None	
1	58	CARVYGSEYSSGWYYYYYYMDVW	IGH	3	None	
1	59	CANLGYGDYPEFRYYYYMDVW	IGH	2	None	
1	60	CAREGGIEYTTSSGWDYYYYYMEVW	IGH	2	None	
1	61	CARLTYYDFWSGYLGYFDYW	IGH	2	None	
1	62	CARETEIRGRPNAFDIW	IGH	4	None	
1	63	CARDSRYNWNDEAWYFDLW	IGH	3	None	
1	64	CARVRHNYIPTIFGVVTDYYYYGMDVW	IGH	4	None	
1	65	CARDDADSSADYYYGMDVW	IGH	7	None	
1	66	CAKDGAVAGSSHEMGFDYW	IGH	2	None	
1	67	CVRRGYSVYDPSKGYMDVW	IGH	4	None	
1	68	CAKDPSGLYGDYVGFDYW	IGH	2	None	
1	69	CTTGAVAGPGRRFVHYYYMDVW	IGH	3	None	
1	70	CAHRSVVPAGWFDPW	IGH	3	None	

1	71	CARGGRKVRGVNMLNQYAMDVW	IGH	3	None	
1	72	CAKDRLEYSGGYYDFHYW	IGH	2	None	
1	74	CAKEPQAIAARLHRYYYYYMDVW	IGH	2	None	
1	75	CARANWTKEEMATKQYYYYYYGMDVW	IGH	7	None	
1	76	CATDLAGAKRKVGPYYYYYMDVW	IGH	2	None	
1	77	CARALLNPGLWFHPTSSAGQFDYW	IGH	5	None	
1	78	CQQTYSTLFSF	IGK	553	C4	6ZDH
1	79	CAREYYGSGSYDGYFDYW	IGH	4	None	
1	80	CAREFAFTLTTTYFDSW	IGH	2	None	
1	81	CARGSQFVDFWSGYYTGTDYW	IGH	8	None	
1	82	CARDCVAGFDPW	IGH	2	None	
1	83	CARSGWYHDDFDIW	IGH	10	None	
1	85	CAKDFYGSASPNAFAIW	IGH	2	None	
1	86	CARHEGVVPNYFDFW	IGH	3	None	
1	87	CAREQDYGSTSGYFQHW	IGH	2	None	
1	89	CARDLSSTWPHTFDYW	IGH	3	None	
1	90	CAKDMGIPIPKYSFDYW	IGH	2	None	
1	91	CARESLAVADAFDIW	IGH	2	None	
1	92	CARGLWFFDNW	IGH	4	None	
1	93	CARDDPATGNDYW	IGH	2	None	
1	94	CARPYLMTTVTTNFDYW	IGH	4	None	
1	95	CTTGTDIVLMVYAIGPAHVHAFDIW	IGH	2	None	
1	96	CAKDIAYSSGQFDYW	IGH	5	None	
1	97	CALPILQLWPRHYWYFDLW	IGH	2	None	
1	98	CVTSGWYPTDFKYW	IGH	2	None	
1	99	CTLFPYTTLFRSFTYYYDSAGFRYLDYW	IGH	2	None	
1	100	CARHVDDDRWFDPW	IGH	3	None	
1	101	CMIWPSSAVVF	IGL	32	None	
1	102	CARDFGVFDYAWSYFDFW	IGH	4	None	
1	103	CARDALYDILTGYYTYW	IGH	2	None	
1	105	CAKDREPDGRWDIDYW	IGH	6	None	
1	106	CAKPPSSYDSSGYDNYFDYW	IGH	9	None	
1	107	CARQVTTVGKPGAVAFDIW	IGH	2	None	
1	108	CATSPYSSGWYRPPYYFDYW	IGH	3	None	
1	110	CVVNGEADGYRRFDYW	IGH	2	None	
1	111	CVTGGGTSSRGFDYW	IGH	6	None	
1	112	CARVLDYYDSSGYNWYFDLW	IGH	3	None	
1	114	CSRGRMYYDFWSGYNNWFDPW	IGH	3	None	
1	115	CARHPFDYDTPNFDYW	IGH	2	None	
1	116	CARAGYYDFWSGYPNPDRFFDYW	IGH	2	None	
1	117	CNSRDSNGNHPSYVF	IGL	3	None	
1	118	CARGPRWLLYFYW	IGH	2	None	
1	119	CQSADISGTYRYVF	IGL	26	None	
1	120	CARDYSDWYLDYW	IGH	8	None	
1	122	CARGRAVGASEYFQHW	IGH	2	None	
1	123	CNSRDPSYNHILF	IGL	5	None	
1	124	CAKIAARLEAFDVW	IGH	7	None	
1	125	CLSADSSGIPVVF	IGL	6	None	
1	126	CAREYYYDSSGYYDSLFDYW	IGH	2	None	
1	127	CAKDKIPGVLVNHYYYGMDVW	IGH	2	None	
1	128	CATGTPHYYYYYGMDVW	IGH	7	None	
1	129	CATPLQITAAGIRGLDAFGIW	IGH	3	None	
1	130	CVREGYSSGRAPAFDLW	IGH	9	None	
1	131	CARVGSGWWQWYFDYW	IGH	6	None	
1	132	CARRRWTPYYYYGMDVW	IGH	5	None	
1	133	CVRDRIVLVPAAIRFYFDYW	IGH	2	None	
1	134	CAIGLDDNRGYFYDYW	IGH	2	None	
1	135	CATSSVLRFSNWFDPW	IGH	8	None	
1	136	CARLRSITIFGVVTQGTYMDVW	IGH	2	None	
1	137	CARVGQLRHFDWPYFDYW	IGH	2	None	
1	139	CTRGHSYYDSGGYHSYFDYW	IGH	4	None	
1	140	CQHLNNYPALTF	IGK	4	C1	7BZ5
1	141	CAKGDIVLMVYDPHAFDIW	IGH	2	None	
1	142	CATTSVIVGPRGWFDPW	IGH	8	None	
1	143	CAREVRGVIIKGFNWFDPW	IGH	2	None	
1	144	CAGSEDSSAYSEFDSW	IGH	2	None	
1	145	CARGGATLDTIFGVVIIDEKSIVFDYW	IGH	4	None	
1	147	CATAPPIDSSGSNPIYYYYGMDVW	IGH	2	None	

1	148	CTLFPYTTLFRSYDFWSGYSLAANWFDPW	IGH	2	None	
1	149	CAKGNVFIRLTDYW	IGH	101	None	
1	150	CARDGNDFWNDRYYYYMDVW	IGH	7	None	
1	151	CTRAAVAGTWFYSKNYYYYYMDVW	IGH	2	None	
1	152	CSSYTTSNTLNVLF	IGL	4	C2	7K90
1	153	CARLMVYAMGYYYYYGMDVW	IGH	2	None	
1	154	CARNRPGEMEFYYYHGMDVW	IGH	10	None	
1	155	CAKDGDYDILTGFPGEAYYFDYW	IGH	2	None	
1	156	CAILPDYGDLLDYW	IGH	2	None	
1	157	CATPDGYNSDYYYYMDVW	IGH	2	None	
1	158	CARGIPQWLASNYYYYMDVW	IGH	2	None	
1	159	CARGGDSRYSYGIYYFDYW	IGH	4	None	
1	160	CATASAYWYFDLW	IGH	16	None	
1	161	CARRYYDSSGYYYSYYYYYMDVW	IGH	11	None	
1	162	CTTYGSSSGWYSSFDYW	IGH	2	None	
1	163	CARDTFRDPTPEYSGSYQGLGAFDIW	IGH	2	None	
1	164	CQVWASSGACVF	IGL	9	None	
1	165	CASIAEDYFDYW	IGH	3	None	
1	166	CARVFAVADSCFDYW	IGH	2	None	
1	167	CARVYSSGWSDAFDIW	IGH	15	None	
1	169	CARGEYFYDGSGYYSYWYFDLW	IGH	3	None	
1	170	CATTTPLIYGSSSFDHW	IGH	3	None	
1	171	CARPSLSDSYGPVDAFNVW	IGH	2	None	
1	172	CAKSPGGDNWFDPW	IGH	2	None	
1	173	CARHSLLSEYYYAMDVW	IGH	18	None	
1	174	CQVWDSSSLQELF	IGL	5	None	
1	175	CARSLSGVTMIVTNWFDPW	IGH	2	None	
1	177	CAKLVDKHIFDFW	IGH	2	None	
1	178	CARGLIDSSGYYPDYW	IGH	47	None	
1	179	CGTWDSSLSAGVF	IGL	162	None	
1	180	CARFNFFDSGGNNWFDSW	IGH	3	None	
1	181	CARTSITIFGATYWYFDLW	IGH	2	None	
1	184	CARMVRFFEWPPSPRDNYYMDVW	IGH	2	None	
1	185	CAKDSPYYDFWSGSQYFQHW	IGH	2	None	
1	186	CAKTRSGDFWIIGDFDYW	IGH	2	None	
1	187	CANLAAAGRTDYW	IGH	2	None	
1	188	CARAPNYYDSSGYPLDAFDIW	IGH	8	None	
1	189	CASSKRITMIVVVKSDAFDIW	IGH	74	None	
1	190	CARDPGWYYYDSSGLDYW	IGH	5	None	
1	191	CAKDFQTTVTTWFDYW	IGH	2	None	
1	192	CARTEMATIWGFDFW	IGH	7	None	
1	193	CARHKTGFLEWDNWFDPW	IGH	6	None	
1	194	CARGYLNWFGHWFDPW	IGH	5	None	
1	195	CARASGRITIFGVVIMGAFDIW	IGH	21	None	
1	196	CARPLRDGYYPFDCW	IGH	2	None	
1	197	CASPRGYSYGPLDYW	IGH	13	None	
1	199	CARDGINSHFHGYYYMDVW	IGH	3	None	
1	200	CARDDGLGDLEWFDPW	IGH	2	None	
1	201	CARGGGHYDSGAYYHFFDYW	IGH	3	None	
1	202	CAREDCSGGSCYSNYYYGMDVW	IGH	2	None	
1	203	CASQVAQYLDLLLPFDHW	IGH	4	None	
1	204	CARGGFTGYTRSRFDYW	IGH	2	None	
1	205	CAHRQHGFWSGYWFDPW	IGH	7	None	
1	206	CARAALGMWSYFDYW	IGH	6	None	
1	207	CATNELRYFDWFSPFDHW	IGH	4	None	
1	208	CAKGGYCSGGSCDNWFDPW	IGH	3	None	
1	209	CARHGPLWEPIFYFDYW	IGH	3	None	
1	210	CARLLGVSSSPDQPDYW	IGH	4	None	
1	211	CARDWGLVRAVPDDAFDYW	IGH	15	None	
1	212	CARIGDDFWSGYPYYFDYW	IGH	3	None	
1	213	CAKLGELAGPFDYW	IGH	2	None	
1	214	CAKGARITMIVVVITTFGYFDYW	IGH	4	None	
1	215	CAKKGTDFWSGFDYW	IGH	2	None	
1	216	CTTDYDSSGYYYLYYYYGMDVW	IGH	2	None	
1	217	CARRGYCGTTNCYAWFDPW	IGH	6	None	
1	218	CAGKDLGVITYHSYYYTYGLDVW	IGH	2	None	
1	219	CARGLDIVVVTATLGWFDPW	IGH	2	None	
1	220	CARLKDYRNYYFDYW	IGH	2	None	

1	222	CARDRIATGDRGLGGDYFDSW	IGH	2	None	
1	223	CARVLRGYSGYDLYYYYMDVW	IGH	2	None	
1	224	CAKADGVWMRSGRSCLPDYW	IGH	2	None	
1	225	CARDSYPNPNPFDYW	IGH	2	None	
1	226	CARDIFNYVAPLYYYYGMDVW	IGH	3	None	
1	227	CARVYRYSPRPYQYYLDYW	IGH	10	None	
1	228	CARHHGGVVPAAMGRVYYYYGMDVW	IGH	2	None	
1	229	CARGIPRVRGVLNWFDPW	IGH	2	None	
1	230	CAKSATLLTTVQGWYFDYW	IGH	39	None	
1	231	CAKDMNSGYDPGPNWFDPW	IGH	2	None	
1	232	CAKDGRGGTDYW	IGH	6	None	
1	233	CARDTHSPMRADYW	IGH	6	None	
1	234	CARDSGPGYSYALDYW	IGH	2	None	
1	235	CARPRVSTRGAIYDYW	IGH	22	None	
1	236	CAKDLYCSSTSCYSDGMDVW	IGH	4	None	
1	237	CQQCDSVSTWTF	IGK	3	None	
1	238	CAKDKVGYSYGWVYYYYGMDVW	IGH	34	None	
1	239	CTTTVPLEWYYDFWSGYYIVGYFDYW	IGH	2	None	
1	240	CARQFAYVWGSSMYYYGMDVW	IGH	2	None	
1	241	CARLGKVTIFGVATGGFWFDPW	IGH	7	None	
1	242	CATSPGGYSSGWYYFDYW	IGH	3	None	
1	243	CAKSRSFDTRGWFDPW	IGH	2	None	
1	244	CAKDRGSSGWFFAFDVW	IGH	12	None	
1	245	CQQYTFSPPGEFTF	IGK	32	None	
1	246	CAKLWAVARVDYW	IGH	4	None	
1	247	CARLLVVVVAAALAPDTFDIW	IGH	4	None	
1	248	CAKTEITIFGVAMRGYWYFDLW	IGH	4	None	
1	249	CAEDPSTYHYDSSGFHDYW	IGH	9	None	
1	250	CARGRRDGYRLHYFDSW	IGH	4	None	
1	252	CARGSGALWSGYGWFDPW	IGH	4	None	
1	253	CVKDRGYCSGASCLLTYALDVW	IGH	15	None	
1	254	CAKVGDGYNYGDYW	IGH	9	None	
1	255	CAKVVSRSRSSYSFNYTDVW	IGH	104	None	
1	256	CAKDPIAGSSGYYYNYW	IGH	6	None	
1	257	CTKDMERWLQLARGLFDLW	IGH	2	None	
1	258	CARDVPDDYGETLVDGDYW	IGH	2	None	
1	259	CASAGRQPLGIHLWDDAFDIW	IGH	11	None	
1	261	CARYSLENVAGTPYFDYW	IGH	4	None	
1	262	CARHVRRWLQLIPYWFDPW	IGH	2	None	
1	263	CTRAPSSTWPRDWFDPW	IGH	11	None	
1	264	CAKDILRFSWDGMDVW	IGH	2	None	
1	266	CARDGWNYYDSSGYSTRLTGFDYW	IGH	2	None	
1	267	CARGSTFCPGGVCSWGLREHWFDPW	IGH	4	None	
1	268	CARSAHDFWSGLAFEIW	IGH	2	None	
1	269	CARYGTYYYESIGYYYFDYW	IGH	2	None	
1	270	CARDSPAGNNQQWLVPTPEYYFDYW	IGH	2	None	
1	271	CAHRNWGYAFDIW	IGH	4	None	
1	272	CAKPAGGGSGSFGYYYFDLW	IGH	3	None	
1	273	CARDGLGLLWFGETVDAFDIW	IGH	2	None	
1	274	CARTAGKYNYGPSGRYFDLW	IGH	2	None	
1	275	CARAWDWGSLIDYW	IGH	2	None	
1	276	CARANLVPRDGYNSPFDYW	IGH	2	None	
1	277	CAHRPSGWIDAFDIW	IGH	2	None	
1	278	CARVEARNFDYW	IGH	16	None	
1	279	CAKANFQLERLGYYYYYYMDVW	IGH	2	None	
1	280	CARVVEDIVVLPAAMAHGEDYSYYYAMDVW	IGH	2	None	
1	282	CAKAPTEYSGYDSSTYSYYGMDVW	IGH	4	None	
1	283	CARERVWRDGFDIW	IGH	2	None	
1	284	CAKSVTWMLYNWFDPW	IGH	5	None	
1	285	CARGVCVIATTCEGHFDYW	IGH	4	None	
1	288	CSRTNCPVNFDYW	IGH	2	None	
1	289	CVRDPDTGSFYADYW	IGH	3	None	
1	290	CTTYYYHSDGFSDYW	IGH	12	None	
1	293	CARDRLQLLSSAFDYW	IGH	3	None	
1	294	CQSYDTSLNGDGVF	IGL	10	C4	7JVC
1	295	CARDVDDILTGYSRPYYYYYMDVW	IGH	2	None	
1	296	CARGAPGQQLWLVYFDYW	IGH	2	None	
1	297	CVKHLNYGVRTSNFDYW	IGH	3	None	

1	298	CAKGEVGARADAFDIW	IGH	2	None	
1	299	CARLYDSSGYYGRDDYW	IGH	2	None	
1	300	CARRGDNYGEFDYW	IGH	4	None	
1	301	CARPTHYDFSSFDIW	IGH	4	None	
1	302	CARGMGIAVAGTLIFYYYYYMDVW	IGH	8	None	
1	303	CARDSIAARQWVFVYW	IGH	4	None	
1	305	CARELAKTGYTGFDYW	IGH	2	None	
1	306	CATLGGDYVWGSYPGYW	IGH	6	None	
1	307	CAKGLNWNDANHFDYW	IGH	2	None	
1	308	CAKDAGDYYDSSASFDYW	IGH	3	None	
1	310	CARLPLMVYALDYW	IGH	3	None	
1	311	CTLFPYTTLFRSSDSYFVWFDPW	IGH	3	None	
1	312	CARGPRIAAAGFNWFDPW	IGH	2	None	
1	313	CAKESGVATITDDAFDIW	IGH	2	None	
1	314	CARRDTWPHYFDYW	IGH	2	None	
1	316	CAKGVVLDYHYYYTMDVW	IGH	3	None	
1	317	CARGSLVRLRGAPNFFDPW	IGH	2	None	
1	318	CANHKAALGGYYDFWSGYTVPNYYYYYMDVW	IGH	2	None	
1	320	CARGRRTITIFGVVIPWPTWFDPW	IGH	2	None	
1	321	CANHLRSSFFDYW	IGH	2	None	
1	322	CARSPIFVGWSGNYFIDYW	IGH	2	None	
1	323	CAKDYLRFLEIYYWYFDLW	IGH	3	None	
1	324	CATVFLESPYVGAIYRAYFDYW	IGH	2	None	
1	326	CARARPQYCSGGSCYSGFDYW	IGH	4	None	
1	327	CARDTFAVTTGFYYYYGMDVW	IGH	3	None	
1	329	CAGPYITSILYYFYMDIW	IGH	2	None	
1	330	CARHSWTYSSGWYPSLGFDYW	IGH	2	None	
1	331	CASSGLYYYDSSGYSDYW	IGH	2	None	
1	332	CVRGPTWIYDYW	IGH	8	None	
1	333	CARVWSNTWEGYDYW	IGH	2	None	
1	334	CARHVSPRGSRGYPVPLGIAAAGTVDYW	IGH	3	None	
1	335	CTTVPYYYYYGMDVW	IGH	35	None	
1	337	CARETLGSSWKSGYSSGWYDYW	IGH	2	None	
1	338	CARDPIIGVGNTYYYYGMDVW	IGH	2	None	
1	339	CARGGGSYFLYLGDYFDYW	IGH	2	None	
1	340	CQQSYTTPTYISPWTF	IGK	3	C2	7K8O
1	341	CAKDKPKRFLPLTTFGGHYGFDPW	IGH	2	None	
1	342	CARSMYSSGYQYDYW	IGH	4	None	
1	345	CAREAMNPWRFDYW	IGH	3	None	
1	346	CAGLTSGWLDYW	IGH	3	None	
1	347	CARHSNMITFGGVIVLFDYW	IGH	6	None	
1	348	CARVVRELELPEGFDYW	IGH	4	None	
1	349	CARLGRDGYNRSAFDIW	IGH	4	None	
1	350	CARGSEVWEEQDSVLVWRGFDIW	IGH	5	None	
1	351	CSRLDDQYLVPGDYW	IGH	6	None	
1	352	CARVVGALFTIFGVVIIEDGYFDYW	IGH	3	None	
1	353	CVKDYGDHYNWFDSW	IGH	4	None	
1	354	CAKDKGLDDAFDIW	IGH	14	None	
1	355	CAQGGHLAGRISWFDPW	IGH	2	None	
1	356	CARDPRTTVTTYYYYYYGMDVW	IGH	2	None	
1	357	CARENYYDSSGYFRSW	IGH	5	None	
1	358	CATKSTSYWHFDIW	IGH	4	None	
1	359	CARMRAYYYDSSGYNDYW	IGH	2	None	
1	360	CARVGNGERGYSYGFSAKKYTYYYYYYMDVW	IGH	5	None	
1	361	CARGGYMHGYEVWYFDLW	IGH	6	None	
1	362	CAREGGHNLASGAYSTSFDYW	IGH	2	None	
1	363	CATDVKYYGDSHFDYW	IGH	5	None	
1	364	CARHVYEAAPRDPIIGLIIGSDSGDGGWFDPW	IGH	2	None	
1	366	CARNGSPDYYDSSGYYPATLYFDLW	IGH	3	None	
1	367	CASSRGSRAFDIW	IGH	2	None	
1	368	CARSFRPLVDGMDVW	IGH	4	None	
1	369	CVKDRTGTFSFDFW	IGH	2	None	
1	370	CAKGPIAVAGIFWGDYW	IGH	2	None	
1	371	CARGSAYDVLTHGGFDPW	IGH	2	None	
1	372	CARHPRDDSSEGLWLFDLW	IGH	4	None	
1	373	CAKEGSLAGYMDVW	IGH	4	None	
1	374	CAKDIALPRHDASGYLDSW	IGH	2	None	
1	375	CARDGRYRYYDSSGYSAYFDYW	IGH	2	None	

1	376	CAKDSETLRFLDGRYYYYYYMDVW	IGH	2	None	
1	377	CARAQLRGTEFDSW	IGH	2	None	
1	378	CARDPATYYYDSSGTYFDYW	IGH	3	None	
1	379	CARDTRRRVDAFDIW	IGH	6	None	
1	380	CARVSHPSYYYDSSGYAIDYW	IGH	3	None	
1	382	CARGGGDSGSFSADRSSFAFDIW	IGH	2	None	
1	384	CARGAVVVSTIGPDHFDYW	IGH	2	None	
1	385	CAQSVEGNDWGDAFDMW	IGH	5	None	
1	387	CARDLGDGYNGEIDYW	IGH	5	None	
1	388	CAISDSTISPHPDYW	IGH	5	None	
1	389	CAKDQYVSGNALESWDYW	IGH	2	None	
1	390	CARELSLYNSRIYSLDYW	IGH	2	None	
1	391	CARDRWDSYGLPNYFDYW	IGH	4	None	
1	392	CTLFPYTTLFRSAVYYCARDDCSSTSCPAAFGYW	IGH	2	None	
1	394	CARSYFSDDSSAFRAFDIW	IGH	2	None	
1	395	CARDHQNDGYWYFDLW	IGH	5	None	
1	396	CARNTLYGTYEGAFDIW	IGH	4	None	
1	397	CAKAPGSGWYAENAFDIW	IGH	2	None	
1	398	CQERSNWPPVSITF	IGK	4	None	
1	399	CARAVSWFSPGFDFW	IGH	8	None	
1	400	CARLPYVGYYYSGGFLTGIDYW	IGH	4	None	
1	401	CAKAGGSYAFVFSWFDPW	IGH	3	None	
1	402	CAREIRRGWLDPW	IGH	6	None	
1	403	CARHSYSGELSLYPDYW	IGH	4	None	
1	404	CARSRHDSSSWYGYYFDYW	IGH	5	None	
1	405	CARQGATGDYDSSGYYPDRSFDYW	IGH	2	None	
1	406	CAKDSSYGADYW	IGH	16	None	
1	407	CARHPGSSLYLIDYW	IGH	3	None	
1	408	CAHSLLDIVAAITEWFDPW	IGH	2	None	
1	409	CARDHGLQLVFDYW	IGH	2	None	
1	410	CAREVGGLATKKLYYYYYMDVW	IGH	2	None	
1	411	CARHPSRSGYYYVGDYYFDYW	IGH	4	None	
1	412	CQQYHDWPLLTF	IGK	42	None	
1	413	CAKAWDTMVRGVITLFDSW	IGH	3	None	
1	415	CVRDTSIAARHYYYMDVW	IGH	2	None	
1	416	CARDLYGDYAFDYW	IGH	80	None	
1	417	CVRNSEHNWFDPW	IGH	2	None	
1	419	CARNMEMNLGWFDPW	IGH	4	None	
1	420	CMRIFGYW	IGH	6	None	
1	421	CARDPSSRWLLGNSHLFDYW	IGH	15	None	
1	422	CARLAGVAPAGPLDYW	IGH	5	None	
1	423	CARVVEEAGTPSFDYW	IGH	8	None	
1	425	CASYPGSSGPSGGFDYW	IGH	2	None	
1	426	CTRGGGGYYDSSGYYFRFDNWFDPW	IGH	2	None	
1	427	CARALGYYDSSGYYLSENWFDPW	IGH	2	None	
1	428	CARDLRSYSRSSSVDYW	IGH	11	None	
1	429	CARISFYSSGYYDWYFDLW	IGH	2	None	
1	430	CTLFPYTTLFRSAAANFDYW	IGH	2	None	
1	431	CAHGKKDRSGRSGAYYFDYW	IGH	4	None	
1	433	CAREYYDSSGYPTIDYW	IGH	14	None	
1	434	CARLGSGWYREEYYFDYW	IGH	2	None	
1	435	CARDYDYWSGFDYYYYGMDVW	IGH	2	None	
1	436	CASMPPGSGSHYSFYYYYYMDVW	IGH	2	None	
1	437	CARHWDVVVPAAQYWFDPW	IGH	2	None	
1	438	CARGGVVRRLTQRWRVDRAEYFQHW	IGH	4	None	
1	440	CARGPITIFGVVIELNYYYGMDVW	IGH	2	None	
1	441	CAKGYGAGIMYFDHW	IGH	8	None	
1	442	CYSTDSSGNHRVF	IGL	1435	None	
1	443	CARLETAPYYYMDVW	IGH	16	None	
1	444	CARRKLPLPFDYW	IGH	10	None	
1	445	CARVNYYMWGSYRHPHSAFVIW	IGH	2	None	
1	446	CARGNGPTWQWRYSYFDYW	IGH	2	None	
1	447	CAKASGDYYDSSGPHDYW	IGH	2	None	
1	448	CARQQTHITMIVVVQIHFDYW	IGH	3	None	
1	449	CAHNTIFGFDYW	IGH	4	None	
1	450	CSKDGGTYESPFDYW	IGH	4	None	
1	451	CVKGPDSNYDYVWGTYRYFDYW	IGH	4	None	
1	452	CAKDDYYGPVSSYYYYYGMAVW	IGH	2	None	

1	453	CARVTFSGSYLYMDVW	IGH	2	None	
1	455	CQSTDFSGPYHVVF	IGL	8	None	
1	456	CARETAMATNWFDPW	IGH	6	None	
1	458	CARSYYHGSETYYLLDYW	IGH	4	None	
1	459	CARPSVLGTPVRFDYW	IGH	25	None	
1	460	CAKASLGGYDRGFDYW	IGH	2	None	
1	461	CARGQNYDLLTGELNNWFDPW	IGH	4	None	
1	462	CATTPVAEGWFDPW	IGH	2	None	
1	463	CAKDSDPHFWMMATIYFDYW	IGH	5	None	
1	464	CANGYSYGSHDAFDIW	IGH	4	None	
1	466	CTTGDYSNLGGWVPDAFDIW	IGH	2	None	
1	467	CARLVDTFYYYYGMDVW	IGH	8	None	
1	468	CARLSSGWYGTYYYYYMDVW	IGH	2	None	
1	469	CARLYKEVAILYYYYYIDVW	IGH	2	None	
1	470	CARKMDTSMVKGFDYW	IGH	2	None	
1	471	CTKPSTYDAFHTW	IGH	2	None	
1	472	CARDQGEGLQFMGAFDLW	IGH	2	None	
1	473	CARVMYNYVSGYYFDLW	IGH	2	None	
1	474	CARRGGYRTAATTLALNWFDPW	IGH	3	None	
1	475	CARAVPAAIGVNWFDPW	IGH	4	None	
1	476	CARDRGVAVAGTLYYFDYW	IGH	2	None	
1	477	CAINQRDWNYKWGPYFDYW	IGH	2	None	
1	478	CARHWQAGSLAWVGYYMDVW	IGH	2	None	
1	480	CAKALRGRTTGTRGVYYYYYMDVW	IGH	2	None	
1	481	CARHEIGYSYGRLVYYFDYW	IGH	2	None	
1	482	CAKDQGWLRPFDYW	IGH	20	None	
1	483	CAKDLSPVLGEFDYW	IGH	2	None	
1	484	CARGNHNFVEAAGPSFDHW	IGH	3	None	
1	486	CARDPRDYVSFDYW	IGH	2	None	
1	487	CASLIGDWNPYYFDYW	IGH	2	None	
1	488	CARDGPFSAAGDYW	IGH	4	None	
1	490	CARGLAITIFGVVTMEGWFDPW	IGH	49	None	
1	491	CARVDGASSGYYLADYW	IGH	3	None	
1	492	CATENWYHFDNW	IGH	4	None	
1	493	CARFPRSCSSTSCYRPGLPAAGRIDYW	IGH	2	None	
1	494	CARHNWNSEFDYW	IGH	12	None	
1	495	CARDRRVRIQTVTRDYYYYYMDVW	IGH	2	None	
1	496	CARGHMITFGGVIVINGLDYW	IGH	6	None	
1	497	CQQANSFPGGPLRFTF	IGK	4	C3	7K8R
1	498	CSSNGIRDYW	IGH	4	None	
1	499	CARDGYSYAPDYYYGMDVW	IGH	3	None	
1	500	CARINYDFWSGYYMRAFDIW	IGH	3	None	
1	501	CARHYETQPDYYYGMDVW	IGH	2	None	
1	502	CAAGGGSPIYYYYYYMDVW	IGH	4	None	
1	503	CARDGVFGVPPFYYYYYYYMDVW	IGH	4	None	
1	504	CHQYYSTPQIF	IGK	4	C4	6W41
1	505	CARDKDYYDTIGQQGFDYW	IGH	2	None	
1	506	CARDIGPLYYYDSSVFDYW	IGH	42	None	
1	507	CATWGYSSGWYNNWFDPW	IGH	3	None	
1	508	CTTDKIRGGGILLPYYYFDYW	IGH	113	None	
1	509	CARDSYGDHANTADYW	IGH	2	None	
1	510	CAKGGWGSVAAYFDYW	IGH	2	None	
1	511	CAKDIRSYDFWSGYLDYW	IGH	2	None	
1	512	CARFTMIVGAFDHW	IGH	4	None	
1	513	CARVDYESSGGVWLDPW	IGH	2	None	
1	514	CARDRWEWDDILTGYTAPGWFDPW	IGH	2	None	
1	515	CAKEGWELLTGLYYKYYLDVW	IGH	2	None	
1	516	CARQEVQLGIPADYW	IGH	3	None	
1	517	CAGELTYCGGDCNSRDAFDIW	IGH	3	None	
1	519	CARGGYSGKWAYYIDYW	IGH	6	None	
1	521	CARAPVDTTMFSAFDIW	IGH	6	None	
1	522	CAKDFGPGYTYGLFDYW	IGH	13	None	
1	523	CAKDRLYGIAVAYLHLDYW	IGH	2	None	
1	525	CARDQWPITVVTSLGYYYMDVW	IGH	3	None	
1	526	CARASVTMIVVDANFDYW	IGH	4	None	
1	527	CAKPQGFLVVVAATLGGFDYW	IGH	3	None	
1	528	CASPLNSGRIYKDLDYW	IGH	2	None	
1	529	CARQGRDQLLANWFDPW	IGH	2	None	

1	530	CARIAVGPLPRFDYW	IGH	2	None	
1	531	CAKIDGPTGTCFDYW	IGH	9	None	
1	532	CASFYYRAAAAGPFDSW	IGH	2	None	
1	533	CASPPEYCSSTRCTSAFDIW	IGH	2	None	
1	534	CATGSPVGATSWFDPW	IGH	19	None	
1	535	CARSRRTQQQLPMGFDIW	IGH	2	None	
1	537	CARDSEIFGVVINPFYDYW	IGH	4	None	
1	538	CVREQGNYFDYW	IGH	4	None	
1	539	CASLSWDGNYYYMDVW	IGH	2	None	
1	540	CATGFPPWTSKSPFDYW	IGH	2	None	
1	541	CARGSWTLTGPFDFW	IGH	4	None	
1	542	CARELLSAAGMTTNFDYW	IGH	2	None	
1	543	CAKEYDYIWGSYRRYFDYW	IGH	4	None	
1	544	CARQSAVGSTNFDYW	IGH	10	None	
1	545	CARLPSPDIVLMVYAIRWYFDYW	IGH	2	None	
1	546	CAHQEFGYYEYYFDYW	IGH	6	None	
1	547	CTLFPYTTLFRSNYYGAFDSW	IGH	2	None	
1	548	CARDQRVSIAAAGTVYYYGMDVW	IGH	4	None	
1	549	CARQETGTNWYFDLW	IGH	9	None	
1	550	CARAGRSITIFGVVRDAFDIW	IGH	2	None	
1	551	CARDPSVPAANHDAFDIW	IGH	2	None	
1	553	CARRHRDSWGWFDPW	IGH	9	None	
1	554	CARRASITIFGVVITGTYFDLW	IGH	2	None	
1	555	CARVSTTLEHNWNYGWFDPW	IGH	6	None	
1	556	CARDNKRLRGWPLDYW	IGH	6	None	
1	558	CARAGVVPAPILSEEIPDFDYW	IGH	3	None	
1	559	CARGVVGYSYGYDNWFDPW	IGH	2	None	
1	560	CARLKMTAIPGSFDYW	IGH	2	None	
1	561	CAKDWVPPLYDYAWGNYQSSAFDIW	IGH	7	None	
1	562	CARAVRLRITFGGVIGESDYW	IGH	2	None	
1	563	CARGCSIAVAGIFLPDYYYYMDVW	IGH	10	None	
1	564	CARVNNLNDPEYYYYYMDVW	IGH	2	None	
1	565	CARDDCSSTSCPAAFGYW	IGH	5	None	
1	566	CARDITGTTSYYYYGMDVW	IGH	6	None	
1	567	CARHLEYLGHEGSGYHYWYFDLW	IGH	3	None	
1	568	CARSGFLACLECLIDYW	IGH	2	None	
1	569	CTREASYASSWPEYFDYW	IGH	4	None	
1	570	CARGSSAAWGSRRTNYFDYW	IGH	2	None	
1	571	CARLKRRTEQWLVLSYYYMDVW	IGH	2	None	
1	572	CATVGVWGSYRVFDYW	IGH	2	None	
1	573	CARYHEAPIAAAASYYYYGMDVW	IGH	3	None	
1	574	CARHRQTGTTHHWFDPW	IGH	3	None	
1	575	CTRVSITGGRSLPFDIW	IGH	2	None	
1	576	CSSYRSSNIWVF	IGL	2	None	
1	577	CARDVSNWNIEVDYYYYYGMDVW	IGH	6	None	
1	578	CHSRDTSGNHIYVF	IGL	7	None	
1	579	CARDVGKGITATGTFDYW	IGH	7	None	
1	581	CARAREDIVVVPAATHYYYYGMDVW	IGH	35	None	
1	582	CMQGTHWPPTWTF	IGK	180	None	
1	583	CARNPMVRGEAKFDFW	IGH	4	None	
1	584	CQEYGSSSYTF	IGK	156	C1	7K8N
1	585	CVKGGGSVRLYFDLW	IGH	2	None	
1	586	CARHPITMIEYYFDYW	IGH	2	None	
1	587	CAREAGTSYSAGYFDPW	IGH	16	None	
1	590	CARLMHDFWSGSLVPPHFDYW	IGH	2	None	
1	591	CVRPLSRYLEWLSSAFDLW	IGH	3	None	
1	592	CAHRGSGWLIGYFDYW	IGH	18	None	
1	593	CARGLRANVLRFLEWLFWFDPW	IGH	99	None	
1	594	CARTVLRYFDWLPRDYFDYW	IGH	2	None	
1	595	CARVALRLGEYPDAFDIW	IGH	10	None	
1	596	CARDVPPRYGYVSASPAPELFDYW	IGH	4	None	
1	597	CARSVVVPAAIWSLRYYYYGMDVW	IGH	4	None	
1	599	CAKWNGIAAADNWFDPW	IGH	2	None	
1	600	CARLQGGYSFHYDYW	IGH	2	None	
1	601	CARHFGWPIQDAYYDFWSGSLTRGYYYGMDVW	IGH	2	None	
1	602	CARGGHYSYDNSGYWPDYW	IGH	3	None	
1	603	CVKASGSTYYSAFDHW	IGH	2	None	
1	604	CARDYRGSSDYYYYYGMDVW	IGH	2	None	

1	605	CAKDLLLDYFDYW	IGH	9	None	
1	606	CARDRDNMLTLDYW	IGH	2	None	
1	607	CARSRVTVTTYFSDYW	IGH	2	None	
1	608	CASLGKYGDYVSSSHEYYFDYW	IGH	2	None	
1	610	CARDLHRSRYSSGWYNYYYYMDVW	IGH	2	None	
1	611	CHSEETEGAVADIFDYW	IGH	2	None	
1	612	CARLGPGIAVAGPDLDYW	IGH	2	None	
1	613	CTTVTGPTGYLDYW	IGH	2	None	
1	614	CSRHAGPDYSDSTDLNDYW	IGH	2	None	
1	615	CAREGEYDILPLGFDYW	IGH	2	None	
1	616	CARGSRESSGWLVKWRWFDPW	IGH	2	None	
1	618	CAKFGGLGSGWYEREEWYYYGMDVW	IGH	2	None	
1	619	CARDPPTVLEWFDSW	IGH	2	None	
1	620	CAKDSANYYDSSGHLGWFDPW	IGH	12	None	
1	621	CAKDPLYYDFWSGYLSEQAPYYYYGMDVW	IGH	2	None	
1	622	CASGKRGEPLGDYW	IGH	2	None	
1	624	CARVSHPTFGDVIDYDAFDIW	IGH	3	None	
1	625	CARASIAARPFDYW	IGH	17	None	
1	626	CLQHNSYPLTF	IGK	914	C3	7K8P
1	627	CVSTGLRNAFNIW	IGH	2	None	
1	628	CARSYDFWSGYYIGWFDPW	IGH	4	None	
1	629	CAKVFSWGVGRDGYNSFDFW	IGH	3	None	
1	630	CARGGRAIFGVVITHFDYW	IGH	7	None	
1	631	CARGLMEFFAVVSYWFDPW	IGH	3	None	
1	632	CATTVSPKRGRLDYW	IGH	4	None	
1	633	CARTLVEMATVYYFDYW	IGH	2	None	
1	634	CARDGPSGGGMYSSGWYSFDYW	IGH	4	None	
1	635	CARDSPGHWFDPW	IGH	17	None	
1	636	CAKDMTDYGDYDDAFDIW	IGH	4	None	
1	637	CAKTTILPLAYFDLW	IGH	4	None	
1	638	CARGVVIPYLFDYW	IGH	13	None	
1	639	CAHRQADRVDYYDSSGYYYRSFDYW	IGH	2	None	
1	640	CASLCSGGSCPLFDYW	IGH	4	None	
1	641	CAADPIAAAGVYYYYGMDVW	IGH	2	None	
1	643	CARDWGTMIGDYW	IGH	2	None	
1	644	CAKSSEVSSGWTGPDYW	IGH	2	None	
1	645	CATIGGAGYDQRTYYFESW	IGH	7	None	
1	646	CVRGAPYFDWLYDFYYYGMDVW	IGH	2	None	
1	647	CARAPTTIFGVQWFDPW	IGH	4	None	
1	648	CARDAWIQLWFPDYW	IGH	4	None	
1	650	CTSVQTSTIFGVVIIGGDYW	IGH	4	None	
1	651	CARSPGITIFGVVIIPSWFDPW	IGH	3	None	
1	652	CARDFLRSDMSPVTTIDYW	IGH	6	None	
1	653	CARDLSGWWSFDYW	IGH	38	None	
1	654	CAREGLRDQLLKYYYGMDVW	IGH	7	None	
1	655	CTKGPSGLVIMGHYYFDLW	IGH	2	None	
1	656	CARDRPIRFLEWLSSEPEYYFDYW	IGH	2	None	
1	657	CQQYHSYSPADPF	IGK	2	C3	7K8R
1	659	CARGGVTMTTYLTYYYYYMDVW	IGH	2	None	
1	660	CMQRIEFPPCTF	IGK	17	None	
1	661	CAQQLVLYGWFDPW	IGH	2	None	
1	662	CAHLLIAAGGTLDYW	IGH	2	None	
1	663	CSSYTSSRTLDVF	IGL	1278	C2	7K90
1	664	CANYLQIYSDNSAYYPEYFQHW	IGH	3	None	
1	665	CARIQLWLSYYYYYGMDVW	IGH	2	None	
1	666	CAKDPDYGEPLFDYW	IGH	3	None	
1	667	CARAHYYHTNDAFDFW	IGH	8	None	
1	668	CVRADSQDFDLW	IGH	76	None	
1	669	CAKPSRPYYYHNSGHFDYW	IGH	64	None	
1	670	CARQVEIVVVSDAFDIW	IGH	2	None	
1	671	CARHDMVLMDYDSSGYYYYWFDPW	IGH	5	None	
1	672	CLLYYGGAQLVF	IGL	84	None	
1	673	CATTPAWWGITGTTSFDYW	IGH	2	None	
1	674	CAREGSRLAMIVVVRSYYYYYMDVW	IGH	2	None	
1	675	CARDGEMATIFGAFEIW	IGH	2	None	
1	676	CARDRENGGYDAFDIW	IGH	2	None	
1	678	CARDLVGPVLPTVELSDYW	IGH	2	None	
1	679	CATPGGGHSYHWENWYFDLW	IGH	53	None	

1	680	CARAHNDYYDTSGFYYLDQW	IGH	5	None	
1	681	CARDKAGISVLAPAATLFDFW	IGH	2	None	
1	684	CAKRSAIVGALYWYFDLW	IGH	2	None	
1	685	CARTDYYDSSGFGTLDYW	IGH	2	None	
1	686	CALPICLRAEDTAVYYCARVDYGDFYFDYW	IGH	2	None	
1	687	CARSSGSLTMIVVVLDAFDIW	IGH	13	None	
1	688	CTRPAPWTLSWFDPW	IGH	7	None	
1	689	CARPLGQYYYDSSGYGGDAFDIW	IGH	2	None	
1	690	CARDPRSITIFGVDPGYFDYW	IGH	13	None	
1	691	CAKGDGYSDFDWYFSLW	IGH	6	None	
1	692	CARQGDPGYAGSYHDSW	IGH	92	None	
1	693	CAKVYGGGYDSDYW	IGH	2	None	
1	694	CARASGLGTMIVVVITHFDYW	IGH	3	None	
1	695	CARDALVNYDILTGTRGYYYGMDVW	IGH	3	None	
1	696	CAKAPSPSDSESYFDYW	IGH	2	None	
1	697	CAREVYALYYDILTGQYYFDYW	IGH	2	None	
1	698	CAKDGGDYDSSGYAFDYW	IGH	2	None	
1	699	CARVPREIGSGYYLRYNYYYGLDVW	IGH	2	None	
1	700	CARVAEWQWLSHFDYW	IGH	58	None	
1	701	CARDRHYTGIYGFSFDFW	IGH	142	None	
1	702	CARERTTSYGDYEIPLFDYW	IGH	2	None	
1	703	CARNSRGTNWDYDAFDIW	IGH	3	None	
1	704	CARHGFSWELLVVGVGQYYFDYW	IGH	2	None	
1	705	CARDSDGIAAAGTGYFDYW	IGH	2	None	
1	706	CARHTANAPLGKQLEVLWFDPW	IGH	2	None	
1	707	CARAVGITMIVVVTVGWFDPW	IGH	12	None	
1	708	CARHPYSSPDLFDVW	IGH	3	None	
1	709	CVKGAKYSSGWYGLLVDYW	IGH	44	None	
1	711	CATSPAVAGFFPPDYW	IGH	3	None	
1	712	CARDLKGVAATDHDYW	IGH	2	None	
1	713	CARLGMQPDGRFDPTITTLYYFDYW	IGH	2	None	
1	714	CASLDSSWLPFDYW	IGH	2	None	
1	715	CAKGRRLGDTFYSAFNYW	IGH	7	None	
1	716	CAQVEPDYYDSSGYYSVYW	IGH	10	None	
1	717	CATAGPILGSKLNWFDPW	IGH	6	None	
1	718	CARDATWGSYYYSYMDVW	IGH	2	None	
1	719	CAKGNPYSSGWYAFDYW	IGH	2	None	
1	720	CAKLPYGSGSYVEATDYW	IGH	2	None	
1	721	CTTRLDYYDSSGYYKPFDYW	IGH	3	None	
1	722	CARTPRFITIFGVVPGQDYW	IGH	9	None	
1	723	CARQRREYDTSSSWGFKYYYYMDVW	IGH	19	None	
1	724	CAKRGPGTPMVPGYFDYW	IGH	2	None	
1	725	CAKDNDFWRSSGPYYYYGMDVW	IGH	8	None	
1	726	CARDTTYHYDSSGYYGLDYW	IGH	10	None	
1	727	CARTQTGSNPFYNYYYYMDVW	IGH	2	None	
1	728	CASLSHFITTIIYWYFDLW	IGH	78	None	
1	729	CARLLYYYGFFDYW	IGH	16	None	
1	730	CTRSGLFQAAVTTGFDLW	IGH	2	None	
1	731	CARVRFTAITMIVVVDAFDIW	IGH	71	None	
1	732	CARDSILGVTSTLTFDYW	IGH	29	None	
1	733	CARDRPSYDFWSGSGAFDIW	IGH	2	None	
1	734	CAKGLTLIVVDAIDYW	IGH	2	None	
1	735	CVTLYYSGTYFFDNW	IGH	5	None	
1	736	CAKCFGYSYGYEDLFDYW	IGH	2	None	
1	737	CARGEWGDYADYW	IGH	8	None	
1	738	CARDEGAVAGWGDFDYW	IGH	2	None	
1	739	CTTDGYSYQKGRDYW	IGH	2	None	
1	740	CARYYDFRTGYYYMDVW	IGH	2	None	
1	741	CAAEIEVVEARFDPW	IGH	2	None	
1	743	CARGGGVLRFLEWPSPLDYW	IGH	2	None	
1	744	CADHYGRRSYGLAYFDYW	IGH	4	None	
1	745	CARSSRYYEIVTAYTAGNGFDIW	IGH	2	None	
1	746	CARAPKPTPSEGKFSLIDYW	IGH	2	None	
1	747	CARDQAYCSSTSCYTRLHDYW	IGH	6	None	
1	748	CARGVCGSSSCYEDAFDIW	IGH	7	None	
1	749	CARLGPRNTYGRDWFDPW	IGH	5	None	
1	750	CQQLNSYPGGTF	IGK	183	C1	7BZ5
1	751	CARALIKRFDPW	IGH	9	None	

1	752	CARDSSGWYGEKNWFDPW	IGH	2	None	
1	753	CAREGDHEDPNFDYW	IGH	2	None	
1	755	CARDFPYYFDTSGYYYMDVW	IGH	8	None	
1	756	CARHGRVRDGNNRGYDYW	IGH	2	None	
1	757	CTTDRDNWNPYFDYW	IGH	4	None	
1	758	CARRGPYYYDSSAHWYFDLW	IGH	4	None	
1	759	CATSSISGSNFDYW	IGH	3	None	
1	760	CARHTPLWADSSSSEDFDYW	IGH	2	None	
1	761	CVKDLHSYDTYYLDYW	IGH	9	None	
1	762	CARGDWWEPTWYFDLW	IGH	2	None	
1	764	CARGADHYDFWSGYYRGWFDPW	IGH	2	None	
1	765	CARGNYGDYVPRSYHYYGMDVW	IGH	2	None	
1	766	CASSYSYDSSGYYDYFDYW	IGH	6	None	
1	767	CATAPGIAVAGTRWFDPW	IGH	4	None	
1	769	CVKDALLSGKYYGNYFDYW	IGH	13	None	
1	770	CARDHAEYDNYWFDPW	IGH	3	None	
1	771	CAGEPLVVPAAPAYGSSFIYYYYYMDVW	IGH	4	None	
1	772	CAKAPVAGNPWFDYW	IGH	8	None	
1	773	CARAGYSGYDPAVTGLIDYW	IGH	6	None	
1	774	CTTDLLWQWLVKNYFDYW	IGH	2	None	
1	775	CTTHLYRSTWDRSIYSYYYYMDVW	IGH	31	None	
1	776	CAQRFGEEGFDYW	IGH	2	None	
1	777	CATTHNTGPHYYFDYW	IGH	2	None	
1	778	CARVDLGSPDYYFDYW	IGH	2	None	
1	780	CARDSFYGGNTRVDNPFDYW	IGH	4	None	
1	781	CARAIAAAQHIAAVQHNYHYYYYMDVW	IGH	2	None	
1	782	CAKDPGPTTEMTFDYW	IGH	4	None	
1	783	CAKDHPYDYVWGSLDYW	IGH	6	None	
1	784	CAKLGGSEMGATDYW	IGH	2	None	
1	785	CARDSSGYYYHFDNW	IGH	5	None	
1	786	CARNYGQTTIFGVVISSYYFDYW	IGH	4	None	
1	787	CAKGKQWLVLPPIDYW	IGH	4	None	
1	788	CTTDSDKFDYW	IGH	2	None	
1	789	CNTEVDDGSGYYLVDYW	IGH	2	None	
1	790	CARLAKNGDFWGGPIFDHW	IGH	2	None	
1	791	CAHLVSSSWYEDFLDYW	IGH	4	None	
1	792	CASSLRREYDFWSGYLLFDYW	IGH	2	None	
1	793	CARRGLGYCTNGVCSHFDYW	IGH	2	None	
1	794	CARGRSSGWFEGDYW	IGH	12	None	
1	795	CATGTVGNTNHYTNYAMDVW	IGH	2	None	
1	797	CARGRYYFGSETHLFYYW	IGH	2	None	
1	798	CARRHTLMFGRKSFDPW	IGH	2	None	
1	799	CARASRGYWGPDAFDFW	IGH	3	None	
1	800	CARAPGDVLLWFGAPGGWFDPW	IGH	4	None	
1	801	CARAPRGIFGVVTHFDYW	IGH	88	None	
1	802	CARLRRESHYPGIAVVNYFDYW	IGH	2	None	
1	803	CARGSLLPLEWLFKPTNYFDYW	IGH	2	None	
1	804	CAKAPDGDYDGYW	IGH	2	None	
1	805	CARICTVPIPSGYYFAMDVW	IGH	2	None	
1	806	CARQQQAHHWVSNAYYYYTDVW	IGH	3	None	
1	807	CTTAPSGSSTPPYFDYW	IGH	4	None	
1	808	CAKIMRDYFYNLYYFDYW	IGH	2	None	
1	809	CARLGGDFWSGYYLGGNWFDPW	IGH	6	None	
1	810	CTVRLVPVGQFDYW	IGH	24	None	
1	811	CASPSSSYVDYSINGFDVW	IGH	2	None	
1	812	CAKDSSGSYSDNWFDPW	IGH	2	None	
1	813	CQQYKSYSSTSF	IGK	53	C3	7K8R
1	814	CASLRITRYNWNDVMDYW	IGH	2	None	
1	816	CAVRLYDFWNGNYRTYYYGMDVW	IGH	2	None	
1	817	CQQYNNRPLMFNF	IGK	2	C3	6XDG
1	818	CATSPWNMVADYW	IGH	8	None	
1	819	CARTPVVPGAIIFDYW	IGH	4	None	
1	820	CARGSVPYYDFWSGSSYYFDYW	IGH	4	None	
1	821	CARGNKQWLVLPRNYYYYMDVW	IGH	11	None	
1	822	CARDARRTTVTASFDCW	IGH	2	None	
1	823	CAREGAQTMIVVVVFFDYW	IGH	2	None	
1	824	CTRGYCTRNNCYRDDYW	IGH	6	None	
1	825	CASSRAYSSSWSPFDYW	IGH	3	None	

1	826	CAKDRDWGGAAYFDYW	IGH	2	None	
1	828	CARDADIVVVPARAFDYW	IGH	5	None	
1	829	CARGKRLVAPAAINPNYYYGMDVW	IGH	3	None	
1	830	CARDRYYYDSSGYLINWFDPW	IGH	6	None	
1	831	CARRGIAESGAAHFDYW	IGH	7	None	
1	832	CAKLPSGWDGFDYW	IGH	4	None	
1	833	CMQALHTPFTF	IGK	2385	None	
1	834	CAKMGVPDYGDYFDYW	IGH	14	None	
1	835	CARSLAMIVVGGYAFDIW	IGH	2	None	
1	837	CARGPLYYYRTSGYYGVHYFDYW	IGH	3	None	
1	838	CARTGLRYFDWLTHNWFDPW	IGH	2	None	
1	839	CRVVPAAPFYYYYGMDVW	IGH	4	None	
1	842	CARVTTGTTFFGWFDPW	IGH	2	None	
1	843	CARDQDTALGKVYYFDYW	IGH	2	None	
1	844	CAKDIERITIFGVGPFDYW	IGH	11	None	
1	846	CAREFVLSGEWGHPGGNDYW	IGH	2	None	
1	847	CARGFRDYGDFDYW	IGH	3	None	
1	848	CFARVRFLEWLIDYW	IGH	5	None	
1	850	CAHRPDFSSSMIFDYW	IGH	2	None	
1	851	CATYYYDSSGTYWYFDLW	IGH	6	None	
1	852	CARLYTSGNPFDAW	IGH	2	None	
1	853	CARDGIQLWTTIDYW	IGH	2	None	
1	855	CATEYAYCGGDCPRSFDYW	IGH	3	None	
1	856	CANGPRAGTTLYFDLW	IGH	2	None	
1	857	CATAGAYGSGNYYNSDYW	IGH	3	None	
1	858	CARQEVGSYFDLW	IGH	5	None	
1	859	CYSSGYYYYYYMDVW	IGH	3	None	
1	860	CAKQTGYYDSGGYWGSVEYFQDW	IGH	2	None	
1	861	CARVRLWFGEFFSDSDYW	IGH	5	None	
1	862	CARSGFLEWLLHVHLDYW	IGH	2	None	
1	863	CAKFTGTGEGDYW	IGH	7	None	
1	865	CARGDSYGSVAYFDYW	IGH	2	None	
1	866	CAREIWDTGMTRFYYYGMDVW	IGH	3	None	
1	867	CQVWDSTYDHTIF	IGL	5	None	
1	868	CKNGVCYPESYYHYGMDVW	IGH	2	None	
1	869	CARAYSSGWTSGFDYW	IGH	6	None	
1	870	CATGAAAYFDFW	IGH	6	None	
1	871	CAKAAKWETTVVDYW	IGH	5	None	
1	872	CARQPSLAVPYFDYW	IGH	5	None	
1	873	CAREVRSGSYYYYYSMDVW	IGH	52	None	
1	874	CARLRAQQLAYGPMYYFDYW	IGH	2	None	
1	877	CARVGDSSSSWGFDPW	IGH	2	None	
1	878	CARGRPHNYYDSGSYYNRRMNWFDPW	IGH	5	None	
1	879	CARGYLENSFDLW	IGH	11	None	
1	880	CATLLSGLWFGENDAFDIW	IGH	2	None	
1	882	CTTDRVRVGQWLVVDYYYMDVW	IGH	2	None	
1	883	CATGYYDSSGYYYLTRPFDYW	IGH	2	None	
1	884	CAREAEYYDILTGYYKYAFDMW	IGH	14	None	
1	885	CARHRLSFSFGVVIIDAFDIW	IGH	6	None	
1	886	CAKGFTYYYDSAGFRYLDYW	IGH	847	None	
1	887	CQSSDSSLSAPHVVF	IGL	2	None	
1	888	CATGWLQLREGFQHW	IGH	2	None	
1	889	CARAGKARFLEWLLRPDAFDIW	IGH	4	None	
1	890	CVKEIGAWMGAFDVW	IGH	22	None	
1	891	CAMGGSGYDIILLIDYW	IGH	2	None	
1	892	CAKGEWYYDFWSGYFPFDYW	IGH	6	None	
1	893	CTTWEPDVGVVVTATLDYW	IGH	6	None	
1	894	CARDAGVSVMGWYEVVPAASGDYW	IGH	2	None	
1	896	CARHKSRLQQWLVLDSLYFDYW	IGH	5	None	
1	897	CARAPRQHGAPMVRELEFDYW	IGH	2	None	
1	898	CAHRTLAVAGFLLDYW	IGH	3	None	
1	899	CASDGATAGRSGEYYFDYW	IGH	2	None	
1	900	CARHRGGWEQLPYFDYW	IGH	2	None	
1	901	CTRWATAVDYFDHW	IGH	7	None	
1	902	CARQVDIPMVATFDYW	IGH	2	None	
1	903	CAITYALTTVVSPEKPFFLYW	IGH	3	None	
1	905	CAKDYGTTMEGFDCW	IGH	2	None	
1	906	CARESAYDFWSGYTDGFDPW	IGH	2	None	

1	907	CARRGFLDIYYYYGMDVW	IGH	4	None	
1	908	CARNVDLGLVPALGFDYW	IGH	2	None	
1	909	CARLSRYEPRDFDYW	IGH	3	None	
1	911	CAREVAGGYDRQYYFDYW	IGH	2	None	
1	912	CARVAVAASPQSYYSYGMDVW	IGH	3	None	
1	913	CARVRIEGRYDYW	IGH	18	None	
1	914	CARLLNPYSYIDYW	IGH	18	None	
1	915	CARDTYSSSWYGTKYYFDYW	IGH	2	None	
1	916	CARHHRRWLQLRPDAFDIW	IGH	2	None	
1	917	CTTHTGYSYGYYDPW	IGH	2	None	
1	918	CARLDGNSYVPNYSYYYMDVW	IGH	26	None	
1	919	CARAHSFYNIRSERLENTPYFDYW	IGH	2	None	
1	920	CARDTSGERPLPHFEYW	IGH	2	None	
1	921	CVLYMGSGIWVF	IGL	10	None	
1	922	CQSEDGSGLIFF	IGL	2	None	
1	923	CARAYYDFWSGYSLAANWFDPW	IGH	3	None	
1	924	CARGTVGPTYNYFDNW	IGH	2	None	
1	925	CATAAPVGPVHWFDPW	IGH	2	None	
1	927	CARDHNMSYDFWSGYYPSFCDYW	IGH	2	None	
1	928	CNSRDNGGNHPVIF	IGL	73	None	
1	929	CARLFHFDWLVRHFDYW	IGH	4	None	
1	930	CATSPAMVTISSHWFDPW	IGH	2	None	
1	931	CAHIGTASEDYW	IGH	2	None	
1	932	CAASQPDFSGISGLDYW	IGH	4	None	
1	934	CARRCCAITMISPTNYYYMDVW	IGH	10	None	
1	935	CARLPDTNMVIPYYSDHW	IGH	86	None	
1	936	CARGSVYSRGGDYYYGMDVW	IGH	2	None	
1	937	CAKDSSMIKTKYYFDYW	IGH	2	None	
1	938	CVRDSSGRGGFGDWFDPW	IGH	3	None	
1	939	CARAAYPLYSSSSDWFDPW	IGH	3	None	
1	940	CARDNDNSGSYYSYAMDVW	IGH	8	None	
1	942	CTKIATPGVGTTGTFDDW	IGH	121	None	
1	943	CARAVDYDFLTGNSYYYGMDVW	IGH	3	None	
1	944	CARDRLGSSWQEGFDYW	IGH	4	None	
1	945	CARAPYPYGDYDYW	IGH	6	None	
1	946	CARIGRIHLWSPGHFDSW	IGH	16	None	
1	947	CARLFSTGMAGRGWIDSW	IGH	2	None	
1	948	CARVAEWQQLPTHYSYYGMDVW	IGH	2	None	
1	949	CATRHISITIFGVVTDYFDYW	IGH	7	None	
1	950	CARDDGDYRAGDAFDIW	IGH	4	None	
1	951	CAKAFSADYKMGGQWLVNKAFDYW	IGH	4	None	
1	952	CARDGRYSYGYGRLDNYYYYGMDVW	IGH	12	None	
1	953	CTRGGTSVKRFDLW	IGH	3	None	
1	954	CARSLPIGHFYYYMDVW	IGH	2	None	
1	955	CASPNYYGLRTGFDYW	IGH	4	None	
1	956	CAKGAGFVVAAGIDYW	IGH	2	None	
1	957	CARDLVAITGDNFLDSW	IGH	2	None	
1	958	CANPGYCSSTSCYVFMYYYGMDVW	IGH	4	None	
1	959	CAKDYRSGWSWYYFDYW	IGH	4	None	
1	960	CATALPIYSGYDSNFYYYYGMDVW	IGH	7	None	
1	961	CARDRSMGELWSRRLFLPSYYFDYW	IGH	2	None	
1	962	CARDFGYSSGWYNGYFDYW	IGH	5	None	
1	963	CATWAYCGRSSCYSLDYW	IGH	6	None	
1	964	CARDIAVAGQYYFDYW	IGH	9	None	
1	965	CARDPLSSGWYSAFDIW	IGH	4	None	
1	966	CATHDFWSGYTFDYW	IGH	8	None	
1	968	CASSGMAAADTDTDFDYW	IGH	6	None	
1	969	CARGGISGTTHFNYW	IGH	2	None	
1	970	CARLLGYYDRSDYYSNYFDYW	IGH	5	None	
1	971	CARVSPSGNSWLLDYW	IGH	4	None	
1	972	CASLSAKVVPAAIFAVDSSYYYYMDDW	IGH	2	None	
1	973	CAGDDSSGYYYYNWFDPW	IGH	10	None	
1	974	CAHSQEFDFAWGTYRFHFDYW	IGH	2	None	
1	975	CARVRIRSSYISGWYEWKTDYFDYW	IGH	2	None	
1	976	CAKGPPYSSSWYHPGDYW	IGH	2	None	
1	977	CAKSPFTGTVGPGTFDYW	IGH	2	None	
1	979	CARGTGEWLPLDYW	IGH	38	None	
1	980	CARTRSRTIFGVVSWFDPW	IGH	2	None	

1	981	CAKFPLYYDSSRVDYW	IGH	4	None	
1	982	CAREKGGTWNFDYW	IGH	7	None	
1	983	CARDRTCGGDCQEYYYYGMDVW	IGH	2	None	
1	984	CAMTPHSSGYYGDAFDIW	IGH	3	None	
1	985	CAREGDYYDSSGYYYTGFDYW	IGH	6	None	
1	986	CARSEDYGDYLNPNGAFDIW	IGH	2	None	
1	988	CAREGIASAYDFWSGYQTHYYYYYYMDVW	IGH	4	None	
1	989	CARHATLRMIVVVIPWGPIDYW	IGH	2	None	
1	991	CARKPDYDFWSGYYIANAFDIW	IGH	2	None	
1	992	CARATSVAATDWFFDLW	IGH	2	None	
1	993	CARDGGLGASFDLW	IGH	39	None	
1	994	CAKGQDRSKMATTVQREYYFDYW	IGH	4	None	
1	995	CSSYAGSYNVAF	IGL	10	C2	7BWJ
1	996	CTTEWSFSSGWDYW	IGH	2	None	
1	997	CARGDLRTIFGVVNELDYW	IGH	3	None	
1	998	CAKGYSSGWWWFSDSW	IGH	2	None	
1	999	CQSYDSTLRGLWVF	IGL	6	C4	7JVC
1	1001	CTLFPYTTLFRSRRIAFDIW	IGH	3	None	
1	1002	CARGVENYDFWSGYPGYGFDPW	IGH	2	None	
1	1003	CARTVYSSSPGEFDYW	IGH	3	None	
1	1004	CAHSRIGETSDAFNIW	IGH	2	None	
1	1005	CVKDSLPNVYYYYMDVW	IGH	4	None	
1	1006	CARRAVAYDILWYFDFW	IGH	27	None	
1	1007	CVKGLVGAIHFDYW	IGH	8	None	
1	1008	CARIIWWGNYYFDYW	IGH	4	None	
1	1009	CARILYGEYSSSSEINFDYW	IGH	2	None	
1	1010	CQSAHNSGDYKVF	IGL	8	None	
1	1011	CATFFAVAGTRTQFDYW	IGH	2	None	
1	1012	CQQYDRSPRYNF	IGK	7	C1	7K8N
1	1013	CARLDYGDYWGYFDYW	IGH	24	None	
1	1014	CARDRSGSYTLLFDYW	IGH	2	None	
1	1015	CTTVLYCSGGSCRNIDYW	IGH	2	None	
1	1016	CARAYYYDSSGYYYPNYYYYGMDVW	IGH	10	None	
1	1017	CAKGKYEPGYQDYW	IGH	2	None	
1	1020	CARGPDSGGFFVGLMDSDNWFDSW	IGH	4	None	
1	1021	CARHRTVYDYGGNAIDYYFDYW	IGH	5	None	
1	1022	CVKGGIAARPPRLFDYW	IGH	2	None	
1	1023	CASGIAARRGSFDYW	IGH	4	None	
1	1024	CARDSRAMLGYWYFDLW	IGH	2	None	
1	1025	CARDSHEYYDLLGRALYYFDYW	IGH	14	None	
1	1026	CQSYDSPLSGSAIF	IGL	8	C4	7JVC
1	1028	CATLYCSGGSCYTGSSYFFDQW	IGH	4	None	
1	1029	CAREGHSGGWYGFDYW	IGH	4	None	
1	1030	CAKDIGFGANAPAFDYW	IGH	2	None	
1	1031	CAKDLGLAVGTSPWDYW	IGH	10	None	
1	1032	CARDGISEGDGFDYW	IGH	4	None	
1	1033	CANSRVAHILGVFDYW	IGH	4	None	
1	1034	CARRVSWEYYYYYYMDVW	IGH	4	None	
1	1035	CAAVGVDGDYVRSDYW	IGH	2	None	
1	1036	CARETTYLPTRLFDYW	IGH	8	None	
1	1037	CARLQYSITRFGALMNSYYYMDVW	IGH	6	None	
1	1039	CARVRTIFGVVTAPHHYYYYYMDVW	IGH	2	None	
1	1040	CARDGAFVAARLNYFDPW	IGH	3	None	
1	1041	CANSGNHFDYW	IGH	9	None	
1	1042	CATDPVDTAMVGGDYW	IGH	2	None	
1	1043	CARRIPTNWLRIAVAGTWFDPW	IGH	4	None	
1	1044	CAKRALYYYDSNSAFDIW	IGH	2	None	
1	1046	CARSRTVGDFWSGYYTENYYYGMDVW	IGH	2	None	
1	1047	CETWDSKAFYVF	IGL	12	None	
1	1048	CARGLILTGPLDYYYYYGMDVW	IGH	23	None	
1	1049	CARFSQSRYYYDSSGQLLGYFDYW	IGH	2	None	
1	1050	CASIDYDSSGYHDYW	IGH	3	None	
1	1051	CARDLTYDFWSGYYKGFDYW	IGH	17	None	
1	1052	CARDSSILAFDIW	IGH	10	None	
1	1053	CARLDNNWNNGWGSYFDNW	IGH	2	None	
1	1055	CARDLDTSDWYSWFDPW	IGH	4	None	
1	1056	CAKGTIRFVEWSASGGWDYW	IGH	3	None	
1	1057	CARELQGPREYYFDYW	IGH	2	None	

1	1058	CARDLVVVPATDAFDIW	IGH	10	None	
1	1059	CARLSDIVVVPAAMVSGWYDYW	IGH	6	None	
1	1060	CASRTTGLLHTFTNYHYMDVW	IGH	2	None	
1	1061	CARLQDDGGYQLDYW	IGH	2	None	
1	1062	CAKDEDCSGGSCYSGAFDIW	IGH	8	None	
1	1063	CARANGPWFGESNFDYW	IGH	3	None	
1	1064	CVRDSFDEELVRFDFW	IGH	2	None	
1	1065	CARHPPYGGRWPYYFDFW	IGH	4	None	
1	1066	CAKDTAPCYVPQCYYDSSGFDYW	IGH	2	None	
1	1067	CARAGDYGSGIDFDYW	IGH	8	None	
1	1068	CARGPLRFLEWLFTADYYYGMDVW	IGH	2	None	
1	1069	CAKDSMSSGNCSGGSCYGDFDYW	IGH	7	None	
1	1070	CAKNRGATTTKYYFDYW	IGH	2	None	
1	1072	CQQYHNWPPGHPF	IGK	2	C3	6XDG
1	1073	CAKDLHFSYGDLYNWFDPW	IGH	2	None	
1	1074	CASVSARVGGFYFDHW	IGH	4	None	
1	1075	CAREGRYFDWLLAFDIW	IGH	6	None	
1	1076	CQQYSTYSRSITF	IGK	13	C2	7K8U
1	1077	CARRRTWIQLWLGGYYYYYGMDVW	IGH	2	None	
1	1078	CARELTYYYGSGSCAFDIW	IGH	2	None	
1	1079	CARGTPDLSFDSW	IGH	2	None	
1	1080	CARQIYDSNGIPHFDYW	IGH	2	None	
1	1081	CARGLSPKGQTIVATIRQHNWFDPW	IGH	2	None	
1	1082	CARISGGYMYGSKKPNYYYGMDVW	IGH	4	None	
1	1085	CARGRYCSSPSCYKANWFDPW	IGH	4	None	
1	1086	CARKGGSSGYYYQFDPW	IGH	2	None	
1	1087	CTTDAVSSRRGVDYW	IGH	2	None	
1	1088	CARHRGGEGSSRYDTFEIW	IGH	2	None	
1	1089	CARVVAATRGWFDPW	IGH	7	None	
1	1090	CATSAAAGTIDWFDPW	IGH	2	None	
1	1091	CARVSADDYTAYFFDYW	IGH	6	None	
1	1092	CSSYTTTSTFF	IGL	4	C2	7K90
1	1093	CAPSLRAFVYSSSWYFDYW	IGH	2	None	
1	1094	CVKYGGKYYPGESRFDAW	IGH	2	None	
1	1095	CAKRGYYDSSGYYYEGEYFQHW	IGH	4	None	
1	1096	CAKDLNLVDGSSGWNDYW	IGH	2	None	
1	1097	CARDPGALYSSSSGYYYFDYW	IGH	2	None	
1	1098	CQSYDAYNHRAVF	IGL	11	C4	7JVC
1	1099	CLNGDLQFDYW	IGH	67	None	
1	1100	CAKDLGGYYYYW	IGH	7	None	
1	1101	CARDKRLYGTSWDPTWLDPW	IGH	2	None	
1	1102	CARPHPNSSGYSGAFDIW	IGH	2	None	
1	1103	CARLQYSTTIFGARWNSYYYVDFW	IGH	7	None	
1	1104	CAKDIYYDSSGYPNYW	IGH	4	None	
1	1105	CARVKKTTYYDILTGYLKQYYFDYW	IGH	4	None	
1	1106	CMQDAQDPWITF	IGK	2	None	
1	1107	CAHRRGSSYPNCFDYW	IGH	2	None	
1	1108	CARDHYVAVAGFPVWPPSDYMDVW	IGH	2	None	
1	1109	CARDFQMTDNGYFDYW	IGH	2	None	
1	1110	CAKDQCFRCSSGWYYYYYGMDVW	IGH	42	None	
1	1111	CAADYGDYVRGSYYYGMDVW	IGH	2	None	
1	1112	CARPLTTKDYYYGMDVW	IGH	4	None	
1	1113	CARDRIPYGEIDYW	IGH	6	None	
1	1114	CVRDLSTSWYWGRFDCW	IGH	2	None	
1	1115	CASLRWDYYDSSGFSYYYMDVW	IGH	2	None	
1	1116	CARDLVGYCSSTSCYAGWYFDLW	IGH	2	None	
1	1117	CARGYCTNGVCYLDAFDIW	IGH	2	None	
1	1118	CAGLSGDFYPGSFDVW	IGH	4	None	
1	1119	CASRKMDAAMVADYW	IGH	2	None	
1	1120	CAREFRDITIFGVVIHTFDYW	IGH	9	None	
1	1121	CASFVRYCSSTSCYHFDYW	IGH	2	None	
1	1122	CARSDSYLVQGFDFW	IGH	7	None	
1	1123	CAKGGPLGEQWLIDYW	IGH	2	None	
1	1124	CTTEMWDYGDYFHYW	IGH	2	None	
1	1125	CARDRWSSSGWFDSW	IGH	2	None	
1	1126	CAREARRYNWNLLFDYW	IGH	2	None	
1	1127	CARDLYDSLTGPNNWFDPW	IGH	15	None	
1	1128	CATQPTVTRGGWFDPW	IGH	2	None	

1	1129	CARGYGDNWLADYW	IGH	2	None	
1	1130	CARGGASSHYEYFDYW	IGH	2	None	
1	1132	CAKVGGYAPGGFDYW	IGH	2	None	
1	1133	CSSPSCYKANWFDPW	IGH	2	None	
1	1134	CARDAAQGIAGSLLWYYYYGMDVW	IGH	5	None	
1	1135	CATLRDYGDYW	IGH	13	None	
1	1136	CAREVSVNTAVGSIPYAMDVW	IGH	3	None	
1	1138	CARATRGLIAVQNFDYW	IGH	4	None	
1	1139	CAKDLRTTWLYYFDYW	IGH	6	None	
1	1140	CARFFGQPQWVGRYYFDYW	IGH	2	None	
1	1141	CAHRLTLKAFDYW	IGH	2	None	
1	1142	CARRNYDSSGYYPNAFDIW	IGH	2	None	
1	1143	CARVAPSALRWLLFQGSFDYW	IGH	2	None	
1	1144	CARAPSVGQLWLRWFDPW	IGH	2	None	
1	1145	CAKDQADDGIQLWPYVYYYYGMDVW	IGH	6	None	
1	1146	CARDPYQYSWSYKGLDSKEGFDYW	IGH	4	None	
1	1147	CAREYCGGDCYPGPRPYYYGMDVW	IGH	2	None	
1	1148	CAKDHSSSSYYYYMDVW	IGH	8	None	
1	1149	CQQYVGSPQTWTF	IGK	5	C1	7K8N
1	1150	CARTKLGYCSGGSCYISIVWFDPW	IGH	11	None	
1	1151	CARVLYYYDSSGYQHRGFDYW	IGH	5	None	
1	1152	CARQTAVFGGIADDYW	IGH	2	None	
1	1153	CARLSGTSWGENWFDPW	IGH	2	None	
1	1154	CAGSPGPIRQWLVGPYYYYGMDVW	IGH	2	None	
1	1155	CTLFPYTTLFRSARCSLWRRLEAQVGMATRDTLCGDCWPGWFGPW	IGH	2	None	
1	1156	CVIAARPYYFDYW	IGH	2	None	
1	1157	CAKVYGVQHGFFDYW	IGH	2	None	
1	1158	CARGPLKITVLSGSSWFDPW	IGH	2	None	
1	1159	CARDSSRAVADYW	IGH	6	None	
1	1160	CARQDDFWRDYYAGYFFDYW	IGH	5	None	
1	1161	CAPTDIAALDYW	IGH	4	None	
1	1162	CARGRTGLERLLGFMSSLFYFDYW	IGH	2	None	
1	1163	CARDPYSGSYHFDYW	IGH	5	None	
1	1164	CARDLVDSSGWEYYFDYW	IGH	3	None	
1	1165	CMQATQFPPSLTF	IGK	2	None	
1	1166	CARGHPRFPALVGATDFDYW	IGH	2	None	
1	1167	CARERHCSNGLCHDWHFDLW	IGH	2	None	
1	1168	CARAVMITFGGVIAQAYYYYMDVW	IGH	2	None	
1	1169	CTTSDVVPADPNLDTDYW	IGH	2	None	
1	1170	CARDVRNAVAGTLLFYYYYYMDVW	IGH	2	None	
1	1171	CASPGYSSSLADYW	IGH	3	None	
1	1172	CARVLLEWLLRPQYLYHYYMDVW	IGH	4	None	
1	1173	CARERLSSTVDYW	IGH	3	None	
1	1174	CVTGLPSSNWDDAFGFW	IGH	2	None	
1	1175	CARDPTTVTTIDYYFDYW	IGH	3	None	
1	1176	CAKNLHHFYESSSFDNRFDPW	IGH	2	None	
1	1177	CARGYCSGGSCHEIDYW	IGH	4	None	
1	1178	CARHQSLPNGAFDIW	IGH	3	None	
1	1179	CARSHLTEIQLWTKGYYYYYMDVW	IGH	2	None	
1	1180	CARHPDDSSGYGKFYFDYW	IGH	2	None	
1	1181	CAKDYKYYDFWSGYTRTRSYYYYYDMDVW	IGH	2	None	
1	1182	CARDTTYSSGWYVNTPFDYW	IGH	2	None	
1	1183	CARVDLTTRRNWNYLFDYW	IGH	2	None	
1	1184	CAKGERFLEWLLYGGDYFDYW	IGH	2	None	
1	1185	CARSPYSYDSSGYYFDYW	IGH	29	None	
1	1186	CARDKLAVYDFWSAYHGTDAFDIW	IGH	2	None	
1	1187	CAKEHSYYDFWSGYYGAGWFDPW	IGH	6	None	
1	1188	CARVLSSGWLLWFDPW	IGH	2	None	
1	1189	CAKGMATITLNFDYW	IGH	2	None	
1	1190	CAREAGLAVAGNLFRASIFDYW	IGH	2	None	
1	1191	CAREMIQGFYYFYMDVW	IGH	2	None	
1	1192	CAKDHYYDSSGYPSPFDPW	IGH	2	None	
1	1193	CARRGGSPKYDAFDIW	IGH	26	None	
1	1194	CASPMETDYYDSSGYPGFDYW	IGH	2	None	
1	1195	CARESNSGFTTYFDSW	IGH	2	None	
1	1196	CATLHHPTYYYDSSGSHDYW	IGH	2	None	
1	1197	CVRDLRCSGGSCLYYDSW	IGH	2	None	
1	1198	CAKDGSVGYYDSSGYYYWFDYYYGMDVW	IGH	2	None	

1	1199	CARDLVTVTTHPYYYYGMDVW	IGH	9	None	
1	1200	CTRMFVDIEVSLFINYYYYMDVW	IGH	3	None	
1	1201	CARASSIAARPIGDYFDYW	IGH	2	None	
1	1202	CARSRLAAFRHSSSWYSCNFDYW	IGH	2	None	
1	1203	CAKDSPGGSFLEWLQPEYYYYGMDVW	IGH	2	None	
1	1204	CARAPPIQVLVVVTAIDAFDIW	IGH	8	None	
1	1205	CARDERHCRGGSCMGFDYW	IGH	10	None	
1	1206	CARENLAIRDGYNWPLDYW	IGH	3	None	
1	1207	CAKREKVSGWAPRDYYFDHW	IGH	2	None	
1	1208	CARQYPTRITMIVVAPGAFDIW	IGH	4	None	
1	1209	CVRVRGAYLNYYYMDVW	IGH	2	None	
1	1210	CANPPRGGYDYTW	IGH	2	None	
1	1211	CAKPPDGLRWLPGSDYW	IGH	2	None	
1	1212	CASAYDSSGYYGLSDAFDIW	IGH	3	None	
1	1213	CARLSYSRSSGEWYFDLW	IGH	6	None	
1	1214	CGKDKSDWGSVDYW	IGH	2	None	
1	1215	CAKGGYFCTNGVCLISDYW	IGH	4	None	
1	1216	CARHPQYYSDAFDIW	IGH	4	None	
1	1217	CTRDQFRRSYYDNNGVTYYMDVW	IGH	4	None	
1	1218	CAKGSITIFGVVIGPSDAFDIW	IGH	11	None	
1	1219	CARGIQLWSSLNFDYW	IGH	2	None	
1	1220	CAKVLPYYDFWSGYWDYYYYGMDVW	IGH	2	None	
1	1221	CARHIGGQYSYGGNWLDPW	IGH	4	None	
1	1222	CARLGLRKSIAARRLSNWFDPW	IGH	2	None	
1	1223	CARDLRGHCINGVCYRGLDFDYW	IGH	4	None	
1	1224	CANDQGTYGGFYFDFW	IGH	2	None	
1	1225	CESLRFLEWLPYLIGAFDIW	IGH	2	None	
1	1226	CQQYNPYSPPLTF	IGK	3	C3	7K8R
1	1227	CARDIVGATTGYFDYW	IGH	9	None	
1	1228	CARGPNLWNWFDPW	IGH	9	None	
1	1229	CAKDDPWYSSPFGVGGAFDIW	IGH	2	None	
1	1230	CARTGYMGSGWYYFDYW	IGH	2	None	
1	1231	CTLFPYTTLFRSWYEYLDYW	IGH	6	None	
1	1232	CARGRSSAWFEDAFDVW	IGH	2	None	
1	1233	CARLGYCSGRTCHDYFYHYALDVW	IGH	3	None	
1	1234	CAREQITSSSLLPNWFDPW	IGH	4	None	
1	1235	CARRDGGNYYYYHMDVW	IGH	27	None	
1	1236	CAKDGNVYGSGSYWDNWFDPW	IGH	2	None	
1	1237	CARSLMVLGHYSYAMDVW	IGH	2	None	
1	1238	CAKDNFFWYSSSSKPIDKNWFDPW	IGH	8	None	
1	1239	CASNRYFDWLSSLDYW	IGH	2	None	
1	1240	CARHGGHSITMIVVVIHPYYFDYW	IGH	11	None	
1	1241	CQQYNSISSGYTF	IGK	11	C3	7K8R
1	1243	CAREGYSSAWLDYW	IGH	2	None	
1	1244	CQMWESSSDHILF	IGL	2	None	
1	1245	CARDEGISWPLDYW	IGH	2	None	
1	1246	CARTGYCVNGVCPIGAFDPW	IGH	2	None	
1	1247	CARDSLDYTDSSWYFDTW	IGH	2	None	
1	1248	CVRHAVVAARPRGGYFDSW	IGH	4	None	
1	1249	CARAVIVVDLPAPYFDYW	IGH	2	None	
1	1250	CARERVDDSSGYYRLFDYW	IGH	2	None	
1	1251	CARDPDRYSSGWPNWFDPW	IGH	2	None	
1	1252	CAKGECSSSWCFDHW	IGH	12	None	
1	1253	CAKLPSKGDYPAFDIW	IGH	4	None	
1	1254	CATAPHYYDSSGYYYDWYFDLW	IGH	2	None	
1	1255	CTTAPYYYDSSAYPQTYFDYW	IGH	3	None	
1	1256	CARSYGGDNWNLVRKNWFDPW	IGH	2	None	
1	1257	CARAIMITFGGVPGFDYW	IGH	2	None	
1	1258	CAKGGWYSSGWYGSHFDYW	IGH	2	None	
1	1259	CARDWQGTYYDILTGRYYYYYYMDVW	IGH	2	None	
1	1260	CARVAPDYGDYGNWIDPW	IGH	2	None	
1	1261	CARDLKDILTGERYFDYW	IGH	2	None	
1	1262	CARRVEKLMLSYARLYRTEKYYYYMDVW	IGH	3	None	
1	1263	CVKDARPQGKHYYYFYGMDVW	IGH	2	None	
1	1264	CAKEPRAYCSSTSCYNNWFDPW	IGH	2	None	
1	1265	CAKALFDYYDSSDYW	IGH	2	None	
1	1266	CAQMASSPYYCSGGSCYSEAYFDYW	IGH	2	None	
1	1267	CVRDLTTVTAWMYFDWW	IGH	3	None	

1	1268	CAKELLWFGQLFSYYAMDVW	IGH	2	None	
1	1269	CAKDSRLYASPYYYYYGMDVW	IGH	2	None	
1	1270	CQQKGGSPPYSF	IGK	3	C1	7BZ5
1	1273	CARDPPYDSSGYYGYYFDYW	IGH	2	None	
1	1274	CARGPQFNWVRYYYYYMDVW	IGH	3	None	
1	1275	CATDLPPWELPRYFDYW	IGH	4	None	
1	1276	CAKDPSYYIPPNWFDPW	IGH	17	None	
1	1277	CQVWDTSSGHRVIF	IGL	5	None	
1	1278	CAKDRAYDNWALASYYFDYW	IGH	6	None	
1	1280	CARGKPIGGFTMVRGVIGWFDPW	IGH	4	None	
1	1281	CARHRRQWLARRGLTYFDYW	IGH	2	None	
1	1282	CAREGNIVNYWFDPW	IGH	13	None	
1	1283	CARFHSSGWLDYW	IGH	13	None	
1	1285	CARTGGLQGIGTRGFDFW	IGH	2	None	
1	1286	CAHSPVLRYFDWLYSTGWFDPW	IGH	2	None	
1	1287	CAKFYYYDSSGYYHDDAFDIW	IGH	2	None	
1	1288	CQSYDSSLSGVVF	IGL	3853	C4	7JVC
1	1289	CAREASSSWVDGYFDLW	IGH	2	None	
1	1290	CARESDADVDMIYYFDYW	IGH	2	None	
1	1291	CASSADYDTTGYFDFW	IGH	11	None	
1	1292	CARGLGRIGWGYYFDYW	IGH	2	None	
1	1293	CQQRSNWPPLFTF	IGK	792	C1	7BZ5
1	1294	CARAERKWELLRYFDYW	IGH	2	None	
1	1295	CATTAPHCSSTSCYSNWFDPW	IGH	6	None	
1	1296	CARAPGYSSGYEHYFYYMDVW	IGH	3	None	
1	1297	CTTTGDGSYSDFW	IGH	4	None	
1	1298	CARDRAPHYYDTSDMDENDAFDIW	IGH	2	None	
1	1299	CARYSSGWYDLASRYYFDYW	IGH	2	None	
1	1300	CAKVSSVSSSSDYW	IGH	2	None	
1	1301	CARQVYYTVGSLRGWFDPW	IGH	3	None	
1	1303	CVRGDTGQVPFDSW	IGH	2	None	
1	1304	CARFLRIAAAGTREERYNWFDPW	IGH	2	None	
1	1305	CAKVLYYYDSSGSLDYW	IGH	32	None	
1	1306	CAREGRGNYDFWSGHFEAAFDIW	IGH	2	None	
1	1307	CARRIQAGYSSGWYYYYYGMDVW	IGH	3	None	
1	1308	CARARGFASVYASSYDAFDFW	IGH	2	None	
1	1310	CAKDLARKSISWYLLGGAFDIW	IGH	6	None	
1	1311	CARHGRAFHSKGWFDPW	IGH	4	None	
1	1313	CQSADDSETYPGWVF	IGL	2	None	
1	1314	CAKSVIVVPTYYYGMDVW	IGH	2	None	
1	1316	CATFSTYYYDSSGYYYPFYFDYW	IGH	10	None	
1	1317	CAVCYSNSHYFDYW	IGH	2	None	
1	1318	CARAWWQQLVFNYYYYMDVW	IGH	15	None	
1	1320	CQQFGTSPPMFTF	IGK	13	C2	7K8U
1	1322	CARFDYVGVWFDPW	IGH	4	None	
1	1323	CAREIHGYRFGYSGRDPYYFDYW	IGH	4	None	
1	1324	CQQSYSNPGPWTPGVTF	IGK	7	C2	7K8O
1	1325	CARDLHNYDSRGYYYDFSGFDYW	IGH	6	None	
1	1327	CAKERGSGWSPLPFDYW	IGH	5	None	
1	1328	CATAQIFGVVTWFDPW	IGH	3	None	
1	1329	CQLANSFPPHSTF	IGK	4	C1	7BZ5
1	1330	CATQYSTASDYLYYYLDVW	IGH	3	None	
1	1331	CAAGTNKGRWLQFDAFDIW	IGH	2	None	
1	1332	CAKAWVDFWSGYSPLEDYW	IGH	3	None	
1	1333	CARALLSRSSSLGYFDYW	IGH	2	None	
1	1334	CARDDLLQTGGYIWGSYRFRSSFFDYW	IGH	51	None	
1	1335	CARDRITMVRGVTLTGSYYYYGMDVW	IGH	2	None	
1	1337	CTTRETDCASSTCSMYTAFAYW	IGH	2	None	
1	1338	CARHRVPIWYYYDSSGPLIRGFDPW	IGH	7	None	
1	1339	CAKDLLSGIVGATTSDYW	IGH	2	None	
1	1340	CARHILANDDYVWGSFDYW	IGH	2	None	
1	1341	CATVALTDQSFDHW	IGH	6	None	
1	1342	CAHSLLPRYYYDMCFDYW	IGH	2	None	
1	1343	CARYAVEGWQIHDSNLDAFDMW	IGH	2	None	
1	1344	CTRGHPYDFWSGYPPPYFDYW	IGH	4	None	
1	1345	CATGPTMVRGVIEGTYYYYMDVW	IGH	2	None	
1	1346	CARSRGGPWEYYFDHW	IGH	2	None	
1	1347	CARDSTYRYDTNGYHSDYW	IGH	2	None	

1	1348	CAKQDVFISAFSSYFDYW	IGH	2	None	
1	1349	CARAPLPLRGAGRYFDYW	IGH	2	None	
1	1350	CARRMYSGTNWFVPW	IGH	2	None	
1	1351	CARDPLTYYYDSSGPPYWYFDLW	IGH	7	None	
1	1352	CASSPPNYYDSNNYYYGGIPFDFW	IGH	5	None	
1	1353	CARGPYSGYDDVSWFDPW	IGH	5	None	
1	1354	CARAMLLWFGDLSTPFDYW	IGH	2	None	
1	1355	CASDPFYYNDASGWAYHFDHW	IGH	2	None	
1	1356	CARDRLDQQVATGLDAFDIW	IGH	2	None	
1	1357	CQEYNKWPPWTF	IGK	4	C3	7K8R
1	1359	CARDLYYYDSSGYYYFVRIYYYMDVW	IGH	4	None	
1	1360	CARDYPDGIGVAARLFDYW	IGH	2	None	
1	1361	CARDELQHGWYFDYW	IGH	6	None	
1	1362	CAKGGDWDSYYYYGMDVW	IGH	3	None	
1	1364	CARDLTVTTGTENYYYGMDVW	IGH	3	None	
1	1365	CARHPWVAATPGYFDYW	IGH	4	None	
1	1366	CATAPYGDYFHFDYW	IGH	17	None	
1	1368	CARTVVTLYYYYYYYMDVW	IGH	2	None	
1	1369	CAGHGDTYDSSGYDDYW	IGH	2	None	
1	1370	CAKDITDGYCSGGSCSPGFDYW	IGH	2	None	
1	1371	CVRIFAVAGSRGWFDPW	IGH	11	None	
1	1372	CARGDEVILGSTIFGRGWYLDLW	IGH	7	None	
1	1373	CTLFPYTTLFRSGGYSSSSSYFDYW	IGH	2	None	
1	1374	CATFPHYYDSSGYYPFDYW	IGH	2	None	
1	1375	CQSYDSTTPAVF	IGL	2	C4	7JVC
1	1376	CAVLPKGGSYLIDYW	IGH	2	None	
1	1377	CATEVAIFGVVIYDYW	IGH	5	None	
1	1379	CTTDEAYYDSSGYYYYYYGMDVW	IGH	2	None	
1	1380	CVIVVVTAIDYW	IGH	2	None	
1	1381	CAKQGGVYCSGGSCYSGYFDYW	IGH	26	None	
1	1382	CARILPSPQGLDSFDYW	IGH	2	None	
1	1383	CARDASSNWGTTYYFDYW	IGH	14	None	
1	1385	CAKDIQWELFFDIW	IGH	6	None	
1	1386	CAKTAATGYFDYW	IGH	13	None	
1	1387	CTRDLLIAAAGFLNYYYYYYMDVW	IGH	2	None	
1	1388	CARHPGELLSLDYYYYYIDVW	IGH	2	None	
1	1389	CVRIQDGPGWLQFYDYW	IGH	4	None	
1	1390	CVKSRSSSAYYSSFDYW	IGH	2	None	
1	1391	CARSYSSGWADLDYW	IGH	2	None	
1	1393	CATSKTVAARPSYYYYGMDVW	IGH	2	None	
1	1394	CAKGRYYYDSSGKNWFDPW	IGH	2	None	
1	1395	CAGGGRTLGSIDYPDWFDPW	IGH	8	None	
1	1396	CTRDSSQWLVAYFDYW	IGH	2	None	
1	1397	CARRVSDSSGSSFDYW	IGH	2	None	
1	1398	CTKDKYGSGWYSGWFDPW	IGH	2	None	
1	1399	CVGTFDPW	IGH	18	None	
1	1400	CASEGIYSSSSLNYFDYW	IGH	16	None	
1	1401	CARTPFEGTTKYYDFWSGLNWMAYYFDYW	IGH	6	None	
1	1402	CAAFYYDSSGSYYGDVDYW	IGH	2	None	
1	1403	CARVRLKYFDWTPSSPLIYYFDYW	IGH	3	None	
1	1404	CARGSSRRAQWLVLSYYYMDVW	IGH	2	None	
1	1405	CAKGPFFGVVISSLNWFDPW	IGH	2	None	
1	1406	CARVSLGAMANFDYW	IGH	2	None	
1	1407	CAHSSGYYHTPDYW	IGH	4	None	
1	1408	CTLFPYTTLFRSGVLALLSQCDNWFDPW	IGH	3	None	
1	1409	CARGGAPHYYDSSGYMSYWSSDWFDPW	IGH	4	None	
1	1410	CARRLFGYSSNWYPDYW	IGH	2	None	
1	1411	CARLPDRGGMLFDYW	IGH	2	None	
1	1412	CAKDISRGELYYFDYW	IGH	7	None	
1	1413	CASPLYIVGGTDGMDVW	IGH	3	None	
1	1414	CARGIIDSYVWGPKVEYYFDYW	IGH	3	None	
1	1415	CAHRGYDSSGVFDYW	IGH	2	None	
1	1416	CARGVTAANSWWFDPW	IGH	4	None	
1	1417	CQQDYNLPKLTF	IGK	14	None	
1	1418	CARSPLSYGVVITHIDYW	IGH	2	None	
1	1419	CVRDTQSSSYGGAFDIW	IGH	7	None	
1	1420	CARSGYSSSPTPFDYW	IGH	13	None	
1	1422	CARLEEMSTIWAYFDYW	IGH	2	None	

1	1423	CARGPVWELPYYYYYMDVW	IGH	2	None	
1	1424	CAKKGACGGDCWFDYW	IGH	4	None	
1	1425	CARDREQLVTSYYFDSW	IGH	2	None	
1	1426	CARIFPITMVGKVVWFDPW	IGH	2	None	
1	1427	CAKDRYTGVEVVTYFDYW	IGH	2	None	
1	1428	CTAGNCYPGHFDYW	IGH	2	None	
1	1429	CARHDGSPVFDYW	IGH	9	None	
1	1430	CAGDDYGDSDYWFDPW	IGH	9	None	
1	1431	CARGEEHCSGGRCYYYYGMDVW	IGH	2	None	
1	1432	CQHRSSWPLRITF	IGK	8	None	
1	1433	CAWQECRTTTCHEDVFDYW	IGH	2	None	
1	1434	CASLITMIAYFDYW	IGH	3	None	
1	1435	CVKDAYVLVGGTALDYW	IGH	2	None	
1	1436	CARLYTRPRTMIVVGLYYFDYW	IGH	2	None	
1	1437	CAREPRYRYDASGYQPEDDYW	IGH	3	None	
1	1438	CATSIAVAGTIAYYYYYYGMDVW	IGH	3	None	
1	1439	CARVGDYGSGSYYIFYYYGMDVW	IGH	6	None	
1	1440	CARGGYSSSWYGIKGWFDPW	IGH	4	None	
1	1441	CARLEVAVAGTVLWWFDPW	IGH	4	None	
1	1442	CARDSTYGDYEVGWFDPW	IGH	3	None	
1	1443	CAKDVTMIVVVITCLDYW	IGH	2	None	
1	1445	CARDQGLDGMDVW	IGH	10	None	
1	1446	CARGNSITIIKGAFDVW	IGH	7	None	
1	1447	CVKASRRELLVFLNYYYMDVW	IGH	2	None	
1	1448	CARLDVANWNLDYW	IGH	6	None	
1	1449	CAKDDTPNLTWDPFDSW	IGH	4	None	
1	1450	CAHRQDAAWDYFDPW	IGH	2	None	
1	1452	CARLRGGYSYGPKPQYYFDYW	IGH	4	None	
1	1453	CARTIQYYDYPDAYDIW	IGH	3	None	
1	1454	CASTGGYDPVTGRRHYFYYYMDVW	IGH	2	None	
1	1455	CARDLLYYDFWSGYYPPYYYYGMDVW	IGH	2	None	
1	1456	CARGHYIAARQYYFDYW	IGH	6	None	
1	1457	CASHHGDYPTSGWFDSW	IGH	29	None	
1	1458	CASLNDYGPEQGWFDPW	IGH	2	None	
1	1459	CAKISGGGYYEEGDAFDIW	IGH	6	None	
1	1460	CWSYGDYDDAFDIW	IGH	3	None	
1	1461	CARQGAKNWDRWFDPW	IGH	2	None	
1	1462	CAKETEAAVFDFDYW	IGH	8	None	
1	1463	CAKDPFYDFWSGYYFDYW	IGH	345	None	
1	1464	CARGVLTEKYEINWFGPW	IGH	4	None	
1	1466	CAKSDSNPMYYYDSSGYYDYW	IGH	2	None	
1	1467	CAKVNYYDIYTGTPDAFDIW	IGH	4	None	
1	1468	CAHRSSGWTPGWFDPW	IGH	5	None	
1	1469	CARDHSSSWVENWFDPW	IGH	8	None	
1	1471	CIAVAGTFLGVGGFDYW	IGH	2	None	
1	1472	CVKDRVAVSLYYFHYW	IGH	163	None	
1	1473	CARRHFSSSWYSGDDFDLW	IGH	3	None	
1	1474	CARGGITIFSPYYYYGMDVW	IGH	2	None	
1	1475	CTLEAAVDAFDIW	IGH	2	None	
1	1476	CLQDYTCPSVYTF	IGK	2	None	
1	1477	CARHLTGDDDYW	IGH	97	None	
1	1479	CARALPVYYYVKYNWFDPW	IGH	5	None	
1	1480	CTLFPYTTLFRSYYVPPSWFDPW	IGH	2	None	
1	1481	CVHTLYSVSALDYW	IGH	3	None	
1	1483	CARGLVGATTGYYYYYYMDVW	IGH	2	None	
1	1484	CAKSDLSGWYIDYW	IGH	3	None	
1	1485	CARDGWSSGWFAGDAFDIW	IGH	2	None	
1	1486	CAKDGYDSSGYYLSLINVAHAEYFQHW	IGH	4	None	
1	1487	CAREYYYGSGSYYNYYMDVW	IGH	6	None	
1	1488	CAKRGDGMSMRTLDYW	IGH	12	None	
1	1489	CVHRGRWQDFDFW	IGH	11	None	
1	1490	CAKTPQPSKYSYGYVFDYW	IGH	2	None	
1	1492	CAKDENPYSSTGYFDYW	IGH	2	None	
1	1493	CAKAASRCSSNRCSLPPDYW	IGH	2	None	
1	1494	CARHHRGVYYYDSSPSPLFDYW	IGH	2	None	
1	1495	CARDDSAVIPFDYW	IGH	9	None	
1	1496	CAKAGGSRLGYYYGMDVW	IGH	4	None	
1	1497	CAKDQGDRYYYGMDVW	IGH	7	None	

1	1498	CAKDRYYYDSSGYTYYFDYW	IGH	7	None	
1	1499	CARDRGLFTIFGVVINDAFDIW	IGH	2	None	
1	1500	CARGVSVTTGTLGSFDYW	IGH	2	None	
1	1501	CARSKGITMIVVVIPSAFDIW	IGH	2	None	
1	1502	CTRPRPEAADDYSEYDYW	IGH	2	None	
1	1503	CTRGIYCSRATCHRTGKLFDYW	IGH	4	None	
1	1504	CLANNHYFDFW	IGH	2	None	
1	1505	CAKDDRVFGLVPPYYFDYW	IGH	6	None	
1	1506	CARYSPRLHCSGGSCYSGYW	IGH	4	None	
1	1507	CARDFLNWNDVFGAFDIW	IGH	2	None	
1	1508	CAKDVKYYDFWSGYLTHSNYYYYYGMDVW	IGH	4	None	
1	1510	CARETIVVVGGYGLRYFDYW	IGH	2	None	
1	1512	CAKEEYDSSGYYPPSYYYYGMDVW	IGH	2	None	
1	1513	CAKGGTAWNDLDRNWFDPW	IGH	4	None	
1	1514	CARQQLEYSSSSQHYYMDVW	IGH	2	None	
1	1515	CAIYGGGSSWTLDYW	IGH	3	None	
1	1516	CARQRLNYYDSSGYYYFDYW	IGH	2	None	
1	1517	CARVRPCGGSCYRYYFYYYGMDVW	IGH	34	None	
1	1518	CQTWGTGIRVF	IGL	35	None	
1	1520	CAKPYDGITMIVVVPFDYW	IGH	2	None	
1	1521	CARYNLLHYGDYVVDYW	IGH	2	None	
1	1522	CAKDGHCFDVW	IGH	14	None	
1	1523	CAKPIGPKGLDITIFGVVIIAPFGYW	IGH	3	None	
1	1524	CARIRRVQEFDYW	IGH	2	None	
1	1525	CVRDSSSLGNDYW	IGH	3	None	
1	1526	CAKGHGPSSSYHYMDVW	IGH	2	None	
1	1528	CAKDPGRITMIVVVPTGWYFDLW	IGH	2	None	
1	1529	CAKTLSLGIDPLVYYYYYMDVW	IGH	2	None	
1	1530	CARGGDLQYFDWLVTGFDYW	IGH	14	None	
1	1531	CARDLWDRSIAVGRYYFDYW	IGH	6	None	
1	1532	CARDQSSVTTMLDYW	IGH	2	None	
1	1533	CARETIEEGNGWYFDVW	IGH	2	None	
1	1534	CARSDLWFGERTMSHDYW	IGH	2	None	
1	1535	CTTYQLILWAVASIDYW	IGH	2	None	
1	1536	CARDRLGSHVPSVYYFYYMDVW	IGH	4	None	
1	1537	CAKDGSLMKSAPHFDYW	IGH	4	None	
1	1538	CTTTVDPYDFWSGWAYFDYW	IGH	2	None	
1	1540	CARDPAYYYDSSGSDAFDIW	IGH	4	None	
1	1541	CQSVDHSGTFMF	IGL	17	None	
1	1542	CVTWIVHHFDYW	IGH	20	None	
1	1543	CASQDGYSSNWGHIDYW	IGH	3	None	
1	1544	CARDYSRQWLKKNSKNYYYYYGMDVW	IGH	3	None	
1	1545	CAKNIYCSGGSCYEGIRWFDPW	IGH	2	None	
1	1546	CAREGGITGRGDAFDIW	IGH	2	None	
1	1547	CARGQAEGYYFTRVKENHWFDPW	IGH	6	None	
1	1548	CAKDAVAGNSLFDWFDSW	IGH	4	None	
1	1549	CATGHHYYYDSSGYYYEFDYW	IGH	11	None	
1	1550	CARDSRTYYYDSSGYRHWYFDLW	IGH	2	None	
1	1551	CARAPSESLRFLEWLPYLIGAFDIW	IGH	2	None	
1	1552	CARQRDVYFYDSSGSQRYNNWFDPW	IGH	5	None	
1	1553	CAKDWQWGVRGIFDYW	IGH	2	None	
1	1554	CAKDPSSGYPPSEYFDYW	IGH	2	None	
1	1555	CARQIQRSPTMIVVGYYYMDVW	IGH	2	None	
1	1556	CANLREGGMGYFDYW	IGH	2	None	
1	1557	CARDGYNHVIDYW	IGH	22	None	
1	1558	CARGPVAEDYYDSSGYFHYYYYYGMDVW	IGH	2	None	
1	1559	CAKGFINQYLYFDHW	IGH	2	None	
1	1560	CAKDIHNWSHDYW	IGH	22	None	
1	1562	CAHYEYFSMSGYPVAAFDIW	IGH	5	None	
1	1563	CAKEGLDRWLVPGIFDLW	IGH	7	None	
1	1564	CAKDLGWSSGFLFDYW	IGH	6	None	
1	1565	CARDWGYSSSSNYYYYYMDVW	IGH	2	None	
1	1566	CTTGELLHAFDIW	IGH	2	None	
1	1567	CAKDPHDYGDYVGWVDPW	IGH	2	None	
1	1568	CATYSNSFHFDFW	IGH	8	None	
1	1569	CAKDYEYCSGGRCYIDYW	IGH	2	None	
1	1570	CARGRYFDFEGYYFDLW	IGH	2	None	
1	1571	CARVTPAFDYW	IGH	69	None	

1	1572	CARVRGDTPMALGDYW	IGH	9	None	
1	1573	CAGDRHYYDSSGYYRNAFDIW	IGH	2	None	
1	1574	CAREDWKASGYFDHW	IGH	4	None	
1	1575	CTTAVATSRYYFDFW	IGH	6	None	
1	1576	CARRSYYFDNW	IGH	27	None	
1	1577	CARLRRITIFGVGIRDWFDPW	IGH	2	None	
1	1578	CAKDLPRPDLFYDDTSGYYFDCW	IGH	2	None	
1	1579	CARDPPRSDSSGYDWYFDLW	IGH	2	None	
1	1580	CALPIFSLLDSDLYYFDYW	IGH	2	None	
1	1581	CARRANSSGWYDRYYYYYYYMDVW	IGH	3	None	
1	1582	CAREPLAAQTNNWGFDYW	IGH	3	None	
1	1583	CARDKPSVELSSSWGEYYFDYW	IGH	4	None	
1	1584	CARDLAFSYQFESLWYHYGFDVW	IGH	4	None	
1	1585	CALPIFTMIVVEAFDIW	IGH	2	None	
1	1588	CARQNGLTIFGVVTYGTLFDPW	IGH	13	None	
1	1589	CARDIKASDSGSGSYVDYYYYGMDVW	IGH	3	None	
1	1590	CARSLRDQYCSGGSCSLDAFDIW	IGH	2	None	
1	1592	CAKLGSYYDFWSGYYSQFDYW	IGH	2	None	
1	1593	CARESNRAPAGNDFDSW	IGH	3	None	
1	1594	CASDYYDSSGYFENDAFDIW	IGH	2	None	
1	1596	CARQGFRKPIDYW	IGH	2	None	
1	1597	CARRTVNGPFDFW	IGH	2	None	
1	1598	CVRDRHFEDTTVTTLEFWFDPW	IGH	73	None	
1	1600	CANLPVYYDSSGSHAFDIW	IGH	4	None	
1	1601	CARNSYDFWSGHRRNWYFDLW	IGH	2	None	
1	1602	CARWVAAAVPSKWAEYYYYGMDVW	IGH	5	None	
1	1603	CAKAPYSSSWYLDYW	IGH	2	None	
1	1604	CVRDHGGVGYYFDYW	IGH	4	None	
1	1605	CAKDPGYSYGQYYFDYW	IGH	4	None	
1	1606	CAKETYYYDSSGYRLLDYW	IGH	2	None	
1	1607	CASARASRLSRSPFDYW	IGH	4	None	
1	1608	CARGGDVLLWFGEPIDYW	IGH	10	None	
1	1609	CARRQPIVGGATEGGWFDPW	IGH	3	None	
1	1610	CARTSYYDRSGAFDCW	IGH	2	None	
1	1611	CQQLDSYPESPTF	IGK	7	C1	7BZ5
1	1612	CARPTSSSWYLLGVDFDYW	IGH	2	None	
1	1613	CARVISSPQISYYYYGMDVW	IGH	4	None	
1	1614	CARVEWDLQVPSFHYYMDVW	IGH	2	None	
1	1615	CAPDRGMGGYSYGTFDYW	IGH	2	None	
1	1616	CARHPYYYDSSGYPVGDYYFDYW	IGH	3	None	
1	1618	CARAGGYEHGYVLFDYW	IGH	2	None	
1	1619	CARDAIESSGWLHAFDYW	IGH	6	None	
1	1620	CARVSAVPQVLGFVVAGQFDYW	IGH	5	None	
1	1622	CARRGIADDLDYW	IGH	2	None	
1	1623	CARHYYYDSSGYSGAVDYW	IGH	2	None	
1	1625	CAKDYDILTGYYDHYYYYMDVW	IGH	2	None	
1	1626	CARAGLIFGVVINAFDIW	IGH	3	None	
1	1628	CASLAGSYSSSWLDYW	IGH	2	None	
1	1630	CAKGMYTAMMQNDYW	IGH	2	None	
1	1631	CARLYHTNSYNGAWFDPW	IGH	10	None	
1	1632	CARDRGYVAGLQISDYFDYW	IGH	2	None	
1	1633	CARIELGQWLVPYYFDYW	IGH	2	None	
1	1635	CARDIGYYDSNYNWFDPW	IGH	2	None	
1	1636	CARGRVTMVRGVIIRLHYYYYGMDVW	IGH	4	None	
1	1637	CARATDSNYCKNGVCYPESYYHYGMDVW	IGH	4	None	
1	1638	CAREGPAVTTASFDYW	IGH	2	None	
1	1639	CAREGRRDSALVTFWDEHRNYYYYYMEVW	IGH	2	None	
1	1640	CARDNPYYDSSGYDVGPFDIW	IGH	2	None	
1	1641	CAKETLFKIVVVITREFDYW	IGH	144	None	
1	1642	CARWSLEQQLAFDYW	IGH	2	None	
1	1643	CASYYYDRVDWFDPW	IGH	2	None	
1	1644	CARDLDTHGPDCSDYW	IGH	2	None	
1	1645	CARPLDYGGWSYYFDYW	IGH	4	None	
1	1646	CARLWTFPLFDSDQYYFDSW	IGH	2	None	
1	1647	CYSADISGTGVF	IGL	4	None	
1	1648	CASIKGDYGDYDYYGMDVW	IGH	2	None	
1	1649	CARVLAPTDRAYYNFWSGYYGYYFDRW	IGH	2	None	
1	1650	CARDISSGWDWYFDLW	IGH	2	None	

1	1651	CARDLRRGYSYGAIDYW	IGH	3	None	
1	1652	CTTDPGGTYYGTFDFW	IGH	2	None	
1	1654	CASLWDTAMVNADYW	IGH	4	None	
1	1655	CAKDLLLPDRSMVKYYYNGMDVW	IGH	8	None	
1	1656	CARGPYCSDGRCYYGPFDSW	IGH	2	None	
1	1657	CAKDSHTVEYSSSSSWFDPW	IGH	3	None	
1	1658	CARLWVEYCSSTSGGCFDYW	IGH	4	None	
1	1659	CTRDTYNYLLDYYYGMDVW	IGH	3	None	
1	1660	CAKDSSADYGDYGSSPDYW	IGH	2	None	
1	1661	CARDVAEAGPFIVPTRYFDYW	IGH	2	None	
1	1663	CAKDNDRSGWRKYFQHW	IGH	2	None	
1	1664	CAHRLLVVTGPYFDYW	IGH	2	None	
1	1665	CAKHEYRRRGLLRGPKYTSSSGSPDYW	IGH	5	None	
1	1666	CARDLGRTDYYDSSGYYYDFDYW	IGH	3	None	
1	1667	CARHTPRRMIVVVITLLSAFDIW	IGH	2	None	
1	1668	CARSGRPWLIGSMGVYFDNW	IGH	2	None	
1	1669	CARDETLMDFDYW	IGH	2	None	
1	1671	CARRTVVMSGGLWDYYMDVW	IGH	2	None	
1	1672	CARDREYSLDYW	IGH	9	None	
1	1674	CAKLEYTVTTALPDYW	IGH	4	None	
1	1676	CARVGQTGLGDSLVYFAYW	IGH	6	None	
1	1677	CTQGLHYDFWSGYYFEYW	IGH	2	None	
1	1678	CARPTLPSYFYDSSGYTKGGFDYW	IGH	3	None	
1	1679	CAKDGEAVISRPYYFQYW	IGH	2	None	
1	1680	CARHSFQYSSSWGFDPW	IGH	19	None	
1	1681	CALRTGYDFHWFDPW	IGH	5	None	
1	1682	CVKNADTAMDYW	IGH	2	None	
1	1683	CQVWDSSTVGVLF	IGL	2878	None	
1	1684	CARHRGLLEDGYHGAADYW	IGH	2	None	
1	1685	CAHRGPWFGPYFDYW	IGH	2	None	
1	1686	CVPETGSSYYDSSGYYLFDYW	IGH	2	None	
1	1688	CARQEDDNNYSVYWFDPW	IGH	2	None	
1	1689	CARHVAAAGPPDYFDYW	IGH	4	None	
1	1690	CYSAADNNRVF	IGL	46	None	
1	1691	CAKTGSIAVAGFDYW	IGH	15	None	
1	1692	CAKLTYGDYSGPDYW	IGH	2	None	
1	1693	CARFSSMRTCRGGSCYTSDWYFDLW	IGH	11	None	
1	1694	CARDRLYYGSGNYVYYHYGMDVW	IGH	2	None	
1	1695	CAGWGLATAGTDYW	IGH	2	None	
1	1696	CARDARGLLRSESDYW	IGH	3	None	
1	1697	CARVVVVAATPGVYYYYGMDVW	IGH	3	None	
1	1698	CASGITMIVVVGPPYDYW	IGH	2	None	
1	1699	CARAHPGAKYSSGWYYYYYMDVW	IGH	2	None	
1	1700	CARRTTSSFAFDIW	IGH	36	None	
1	1701	CVKGTHWAGHSHFDYW	IGH	3	None	
1	1702	CARGSATYYDFWRGYFFDCW	IGH	28	None	
1	1703	CARSTKLVTRITMIVVAQLGYFDLW	IGH	20	None	
1	1704	CAKDMGDWRNFDSW	IGH	4	None	
1	1705	CATTSPGSTDAFDIW	IGH	2	None	
1	1706	CTGRCSGGSCYSVDYW	IGH	2	None	
1	1707	CAKNRWIQLWLALLDYW	IGH	5	None	
1	1708	CTRGKEGYYYGSGAFDHW	IGH	2	None	
1	1709	CTRQYYGSFDYW	IGH	2	None	
1	1710	CARASPRGVTNGGRWFDPW	IGH	4	None	
1	1711	CARSRSSRGLLKQYYFDYW	IGH	8	None	
1	1712	CLRAEDTAVYYCARDQGLITIFGVVIKGEMDYW	IGH	2	None	
1	1716	CARLRNGYHNYFYYGMDVW	IGH	4	None	
1	1717	CTRDQKERWLHLPGFLRNWFDPW	IGH	2	None	
1	1718	CARLSYLTDSWDVFDYW	IGH	4	None	
1	1719	CARAQFNWGNFDSW	IGH	2	None	
1	1720	CAHNSGFIYGDYFDYW	IGH	2	None	
1	1721	CAGPQDLQALLMHRGVGIYYYGMDVW	IGH	2	None	
1	1722	CAREVAARSDFEYW	IGH	6	None	
1	1723	CAHMSIGIAAAGSDYW	IGH	2	None	
1	1724	CAKDVESYGDYHYYYGMDVW	IGH	10	None	
1	1725	CARVTVDSTAHDENWFDPW	IGH	2	None	
1	1727	CARSPKTDFWSGLFDHW	IGH	6	None	
1	1729	CARDLEASSGWFNWFDPW	IGH	2	None	

1	1730	CAKNPRGPFYFDYW	IGH	2	None	
1	1731	CARHLSSSWFVDAFDIW	IGH	2	None	
1	1732	CAREKNDILTGYFDYW	IGH	13	None	
1	1734	CARISLWFGELFSLGFDPW	IGH	3	None	
1	1735	CARDISSSWYDYYGMDVW	IGH	3	None	
1	1736	CARHVYSSPGTDAFDIW	IGH	4	None	
1	1737	CAHRKQWLARYDTFDMW	IGH	5	None	
1	1738	CAREEVEMATKDYYYGMDVW	IGH	2	None	
1	1739	CAHSRPDWNTIFGVVIIGGDYFDYW	IGH	2	None	
1	1740	CASLPITMVRGVTYPFQVVLGFDPW	IGH	4	None	
1	1741	CQQYNTYIQYTF	IGK	2	C3	7K8R
1	1742	CTKTGPRMASVLIDAFDIW	IGH	5	None	
1	1743	CARRIGSSSKTVWFDSW	IGH	2	None	
1	1744	CTTDRIYDYVWGSYRYEGYW	IGH	2	None	
1	1745	CARIDFKEGSGWYLVFDYW	IGH	4	None	
1	1746	CARGYYDFWSGLDYW	IGH	6	None	
1	1747	CARHTFRDYVGYYYGMDVW	IGH	2	None	
1	1748	CAKSTPPEWLLLTPMGFDPW	IGH	4	None	
1	1749	CARNDYRYSSGWYYYYGMDVW	IGH	2	None	
1	1750	CARQGSDYYDSSGDYW	IGH	4	None	
1	1751	CARQGHQRGLIAVAAAEGFDYW	IGH	2	None	
1	1752	CAKDIVPAITYFDYW	IGH	2	None	
1	1753	CARDYGDHGEGLHDYW	IGH	2	None	
1	1754	CGGGYSSSSSYFDYW	IGH	14	None	
1	1755	CAREPSRIAAPTHHYYYYGMDVW	IGH	3	None	
1	1756	CAREDLTTATEAFDIW	IGH	6	None	
1	1757	CARDHFYYDILTGYFGAARYYYYYYMDVW	IGH	2	None	
1	1758	CARVYTGYSAWFDPW	IGH	2	None	
1	1759	CARDPSIAAPSFTNWFDPW	IGH	6	None	
1	1760	CARAKSITMIVVVTLDAFDIW	IGH	40	None	
1	1761	CARALKRGRDYFDYW	IGH	2	None	
1	1762	CAKLPDAFGVVSSDYW	IGH	2	None	
1	1763	CARVARRRYFDWFDFDYW	IGH	2	None	
1	1764	CVRPYFGSGHYWHFDLW	IGH	5	None	
1	1765	CVKDMAIAAATFTFDFW	IGH	3	None	
1	1766	CTRDRIVVVRWDAFDIW	IGH	2	None	
1	1768	CAREYSSSWYIWFDPW	IGH	2	None	
1	1769	CARYHSGYDSDNSFDYW	IGH	21	None	
1	1770	CARGEQWLVNFGYW	IGH	2	None	
1	1771	CARDGRYSYGYPYLDYW	IGH	14	None	
1	1772	CAIAARYNWNDFDYW	IGH	2	None	
1	1773	CARAPGAFMDVW	IGH	5	None	
1	1774	CARGDYGDWDWYFDLW	IGH	2	None	
1	1775	CARGAGEQKEDWFDPW	IGH	2	None	
1	1776	CASELHQGYSPFDYW	IGH	2	None	
1	1777	CARCLDGDYGILIGDSPDDAFDIW	IGH	3	None	
1	1778	CTF	IGK	3	None	
1	1779	CARAGAVVRGHYSFDFW	IGH	2	None	
1	1782	CARHRSPVPLVYTWFDPW	IGH	4	None	
1	1783	CARISRGVSWPYYYYYYGMDVW	IGH	2	None	
1	1784	CARLATAMVVFDYW	IGH	11	None	
1	1785	CARADDPYNWNYDGRIDYW	IGH	4	None	
1	1786	CATGVPIAYCSSTSCYVYFDYW	IGH	2	None	
1	1787	CARAGMRLFGAIGEFDYW	IGH	5	None	
1	1789	CAKTLNVLRYFDWLLPRWFDPW	IGH	2	None	
1	1790	CQQFNIWPPEGITF	IGK	43	C1	7BZ5
1	1791	CANTLSMYSSSPGFDFW	IGH	2	None	
1	1792	CAKIYGDSSFDYW	IGH	5	None	
1	1793	CAKDVLKVADLMYYYDSSGPGGDYW	IGH	2	None	
1	1794	CANYLRETYYYDSSGYFRVGAFDIW	IGH	2	None	
1	1795	CLQSKNSLDTF	IGK	2	None	
1	1796	CARRAYYDTLTGYYIDYW	IGH	2	None	
1	1797	CARAPLITGTTGLLGRDDAFDIW	IGH	2	None	
1	1798	CTRDAGAQHLGNFDYW	IGH	2	None	
1	1799	CAKDMGKAEHPYYYYYMDVW	IGH	6	None	
1	1800	CARQPGIAVTPTRFDYW	IGH	7	None	
1	1801	CAKDVPTDYTSGWTLDYW	IGH	6	None	
1	1802	CARVGMGVAVAGNPPPHACDIW	IGH	2	None	

1	1803	CAKDPGYYDISFDYW	IGH	2	None	
1	1804	CARVRRDAYRWGYYFDYW	IGH	2	None	
1	1805	CQQCFSTPPDF	IGK	11	C4	6W41
1	1806	CASGSYSSNSDFDYW	IGH	17	None	
1	1807	CQQYGSFF	IGK	19	C1	7K8N
1	1809	CARGYCSNGECYGAPDYW	IGH	17	None	
1	1810	CASVGSIPTDYW	IGH	2	None	
1	1811	CARYITMVRGVWFDPW	IGH	2	None	
1	1813	CATSPHRGYSSGWTDDYW	IGH	2	None	
1	1815	CTDVVVDSSGYYDDENWFDPW	IGH	2	None	
1	1816	CATLGGDSSGYVGYW	IGH	8	None	
1	1817	CARDELYDFWSGYYPTYGMDVW	IGH	4	None	
1	1818	CARESRYGGIDDYW	IGH	2	None	
1	1819	CARLRWLRLGEIVRAFDIW	IGH	2	None	
1	1821	CARGAYGDSQVGLFDPW	IGH	2	None	
1	1822	CAAWDDSLNGPVF	IGL	872	None	
1	1823	CVKDVTELLKAGYYFDSW	IGH	2	None	
1	1824	CVAMLGPELDAFDMW	IGH	2	None	
1	1826	CAKDIGVAGIGYFDLW	IGH	2	None	
1	1827	CARASRGITMIVVVTPYYYYYMDVW	IGH	5	None	
1	1828	CARDRGILDCSGDRCYPANWFDAW	IGH	2	None	
1	1829	CAKDIGGSIAVAGPYFDYW	IGH	2	None	
1	1830	CTLFPYTTLFRSARFHSSGWLDSW	IGH	2	None	
1	1831	CARSFGSSWYSHFDYW	IGH	2	None	
1	1832	CAKGVVGATSYFDYW	IGH	4	None	
1	1833	CATAPIAAAATGGWFDPW	IGH	8	None	
1	1834	CAREPRELIRGYYFDYW	IGH	19	None	
1	1835	CARHLYSGYDYSYYYMDVW	IGH	2	None	
1	1836	CASPPSFEYDEYFHHW	IGH	6	None	
1	1837	CARVDSSGYYPQDAFDIW	IGH	2	None	
1	1839	CASRGELLPFSYYFDYW	IGH	4	None	
1	1840	CARGTWELPDYFDNW	IGH	2	None	
1	1841	CARGLPPYGANSRFDFW	IGH	4	None	
1	1842	CAKVESDSSGWYGEIDYW	IGH	2	None	
1	1843	CLQAFSFPYSF	IGK	16	None	
1	1844	CAKDDWLVQAGMTHFEYW	IGH	2	None	
1	1845	CQQYYNTLSVAF	IGK	7	C4	6W41
1	1846	CARAPAASGSYSDYW	IGH	4	None	
1	1847	CASRTSSVVVVPATRPAAAGPFEYW	IGH	2	None	
1	1848	CAKGLWNGNSPEDYW	IGH	2	None	
1	1849	CAKDIQGGEYSSGGTDYW	IGH	31	None	
1	1850	CAKDRAYYDILTGYFPTNYYYYYGMDVW	IGH	6	None	
1	1851	CAKRGVSGSWFYFDYW	IGH	2	None	
1	1852	CAKAYAENSWYYFDLW	IGH	2	None	
1	1853	CAASHSSDRFTYFDYW	IGH	6	None	
1	1854	CARAPTWRGDYVHFDYW	IGH	10	None	
1	1855	CTLFPYTTLFRSQMNSLRAEDTAVYYCAKDDGWLRPFHYW	IGH	2	None	
1	1856	CAKDRGQLWSNFDYW	IGH	9	None	
1	1857	CARRILNRITIFGVVIPNWFDPW	IGH	2	None	
1	1858	CARDSHHSSSSLLDYW	IGH	4	None	
1	1859	CAREVGNYGGTLFDYW	IGH	2	None	
1	1860	CAKETFPKYSAYLFDSW	IGH	4	None	
1	1861	CARSHIVTMNWFDPW	IGH	25	None	
1	1862	CARLYCSGGLCYRGYLFDPW	IGH	2	None	
1	1863	CARAPRTTIFGVVLWFDPW	IGH	41	None	
1	1864	CLQDYNYSWVF	IGK	2	None	
1	1865	CARETGYYDSSGYSNWFDPW	IGH	4	None	
1	1866	CASGPRDSNHFDLW	IGH	37	None	
1	1867	CTIYYYDSSGYYWNYW	IGH	4	None	
1	1868	CARVGGVMTSEIHWYFDLW	IGH	2	None	
1	1869	CASADCSSLSCKAGLTPTLEYYLDYW	IGH	2	None	
1	1870	CAKDIGSSSWDVLVDYW	IGH	2	None	
1	1871	CATATGYSSVHWFDPW	IGH	2	None	
1	1872	CARHGDASGSYYGYW	IGH	2	None	
1	1873	CAREDVLRFLLNGAFDIW	IGH	2	None	
1	1874	CAKGPLREDNFYMDVW	IGH	5	None	
1	1875	CARDLPTKRWLQFHFDYW	IGH	2	None	
1	1876	CARGYYDSSGYSSRIRTKNWFDPW	IGH	4	None	

1	1877	CARIQGRKGLASKITTYYFDYW	IGH	2	None	
1	1878	CARDGCSSTSCTDWFDPW	IGH	2	None	
1	1879	CATQHLDFWSGYYNYW	IGH	11	None	
1	1880	CARTHSSSSLHYFDYW	IGH	2	None	
1	1881	CARESSSGVADGMDVW	IGH	4	None	
1	1883	CARDRLDSSSWTMGYFDYW	IGH	5	None	
1	1885	CTTVGIQLWHTFFDYW	IGH	2	None	
1	1886	CAKDRGGAPYCSSTSCYWVDAFDIW	IGH	2	None	
1	1887	CASEGIAVAGTYPAFDYW	IGH	9	None	
1	1889	CARDAFAITMIVGLTDSCFDCW	IGH	8	None	
1	1890	CITDRVIVVPVAMLIFLGAYSDYW	IGH	76	None	
1	1891	CARQGMETDGRFDPTVTTLYYFDFW	IGH	2	None	
1	1892	CARDLVLGPWGSYDRSGYRLDYW	IGH	11	None	
1	1893	CARMCIAALTPLRYFDYW	IGH	3	None	
1	1894	CARHPPASLVWTYEVFHNWYFDLW	IGH	2	None	
1	1895	CATNLRIAGRRDGFDIW	IGH	3	None	
1	1896	CARQMEVEMATIPAEVDYW	IGH	4	None	
1	1899	CARASYSSGWYEEGPDYW	IGH	5	None	
1	1900	CARGRKERALVILYYFDYW	IGH	2	None	
1	1901	CARAGVLRAGGTYNWFDPW	IGH	4	None	
1	1903	CARHGEVGATDYYMDVW	IGH	3	None	
1	1904	CARRVDGSSSWSYYFDYW	IGH	2	None	
1	1905	CARDFKYYDYVWGSYRDDYW	IGH	2	None	
1	1906	CARASGPPPFVDYW	IGH	7	None	
1	1907	CAKDHPSIVLMVYAIGTPDYW	IGH	2	None	
1	1910	CARRYSQYSSGWYYPGVDYW	IGH	4	None	
1	1911	CARQYTIWTGYPYNWFDPW	IGH	2	None	
1	1912	CARDRVGADGSYYFDYW	IGH	4	None	
1	1913	CARVDTTIFYYMDVW	IGH	15	None	
1	1914	CARLPAPQHDYGDYVDYW	IGH	3	None	
1	1915	CARELYNNDCYWFDPW	IGH	2	None	
1	1916	CAKVVGQWRFYFDYW	IGH	2	None	
1	1917	CAKARIQEQLVTNYYFDYW	IGH	2	None	
1	1918	CARGSVTTGADDYYYYYGMDVW	IGH	2	None	
1	1919	CVSWGRTNWYYFDYW	IGH	2	None	
1	1920	CVRDNDGGGYLEYW	IGH	2	None	
1	1921	CAKDSVATSFDYW	IGH	10	None	
1	1922	CARDRVLDIVAHRGHWFDPW	IGH	4	None	
1	1923	CAREKGDGYNSHAFDIW	IGH	2	None	
1	1924	CASLTMVRGTTPYYFDYW	IGH	2	None	
1	1925	CMEALQTPPMFTF	IGK	2	None	
1	1926	CARDYRKGGPFDVW	IGH	3	None	
1	1927	CARDTQWQLTDYW	IGH	2	None	
1	1928	CASDRNYYDILTGSLDYW	IGH	5	None	
1	1929	CQSYDSSLNDIRVVF	IGL	4	C4	7JVC
1	1930	CARDGGFRPWFDPW	IGH	19	None	
1	1931	CATAPAVVAANWFDPW	IGH	7	None	
1	1932	CQHYDNLPPYTF	IGK	502	C3	6XDG
1	1933	CARVTMVTGFDNW	IGH	5	None	
1	1935	CAKVRYYYGHFDYW	IGH	2	None	
1	1936	CARGSYYFDYW	IGH	458	None	
1	1937	CATLSVGSSGWYTPDYW	IGH	2	None	
1	1938	CASYTYYYGSGSYYFDYW	IGH	3	None	
1	1939	CVRVRTYDILTGDYSTADYW	IGH	4	None	
1	1940	CARHYDTSGYYPKGYFQHW	IGH	8	None	
1	1941	CAKLPDSDGLADYW	IGH	6	None	
1	1942	CARVGSDDILTGYSTYW	IGH	3	None	
1	1943	CAKRGGGYYYDSSGYLDYW	IGH	2	None	
1	1945	CARGYYMATMTYYYYYGMDVW	IGH	2	None	
1	1946	CAREGRYFYDSRGYPISLFDYW	IGH	4	None	
1	1947	CAKDIGWSGYYKFTFDYW	IGH	4	None	
1	1948	CARGTLRLPAHPGYFDFW	IGH	2	None	
1	1949	CARDFPVCTGGLCYPNGFDYW	IGH	4	None	
1	1950	CASYDVWSGSHLTGGENWFDPW	IGH	2	None	
1	1951	CAVIIAVAGTDFDYW	IGH	21	None	
1	1953	CARLTYGSGSYIDVW	IGH	140	None	
1	1954	CQQSYLAPRLSF	IGK	6	C2	7K8O
1	1955	CAKVNIGSGSYNGDYW	IGH	2	None	

1	1957	CATMTTVTTYLQYYYYMDVW	IGH	8	None	
1	1958	CARDPTSIAVAEEYYFDYW	IGH	2	None	
1	1959	CAKLSSWYTPFDYW	IGH	8	None	
1	1960	CAKAVMLSYYYYYGLDVW	IGH	2	None	
1	1961	CTRASHYYDMSGYYQPVVTDDSDIW	IGH	65	None	
1	1962	CARARDDFWSGYHNWFAPW	IGH	71	None	
1	1963	CVRGPQFDGVDYW	IGH	3	None	
1	1964	CTLFPYTTLFRSAIYYCAREAGYSSTWYEYLDYW	IGH	7	None	
1	1965	CARQERRITIFGVVISLYMDVW	IGH	5	None	
1	1966	CARTFGLRINSFGYDFDYW	IGH	2	None	
1	1967	CARVGDYVWGSFRDYW	IGH	27	None	
1	1968	CASTTAAWYRYDAFDIW	IGH	2	None	
1	1969	CARENLGDWGNYYFDYW	IGH	4	None	
1	1970	CARVGPRTRVGARFFDSW	IGH	2	None	
1	1971	CAKFQVYDLYYFDYW	IGH	2	None	
1	1972	CARLSRITIFGVAISDLMDVW	IGH	2	None	
1	1974	CAKEAWWGNSVEIDNWFDPW	IGH	2	None	
1	1975	CARGSDCTNGVCYTHYYYYMDVW	IGH	2	None	
1	1976	CQKYNIAPLTF	IGK	190	None	
1	1977	CVRQPGPHMVRGVMWNNWFDPW	IGH	2	None	
1	1979	CATFIVATIWYFDYW	IGH	4	None	
1	1980	CAKADVVVTPRGGFDYW	IGH	2	None	
1	1981	CARHISGWQNPFDSW	IGH	2	None	
1	1982	CASHSSSWWGLYYFDYW	IGH	4	None	
1	1983	CASDYSRSSEGLGYFGFW	IGH	6	None	
1	1985	CAIKMTDFWSGYYNSYYYGMDVW	IGH	2	None	
1	1986	CARDEVEDAMVPELIIPIDYW	IGH	2	None	
1	1987	CARESNCGGDCYFDYW	IGH	4	None	
1	1988	CAKDRGAAAVSDSFDVW	IGH	8	None	
1	1989	CTRPLLQAMAAVGYFDYW	IGH	4	None	
1	1990	CARGDYAGYYYYMDVW	IGH	23	None	
1	1991	CAREEGNLSRRVFDYW	IGH	5	None	
1	1992	CARELRYFDWLLHSDYYYYYGMDVW	IGH	3	None	
1	1993	CARDRRYCGGDCYWAYYYGMDVW	IGH	8	None	
1	1995	CAKAELNYYDSSGYYHFGYW	IGH	2	None	
1	1996	CARGPTIYYDSSGSSFDHW	IGH	2	None	
1	1997	CVHKILRPARLPVSFDSW	IGH	2	None	
1	1999	CARSILESRYSSGWFYYYYYMDVW	IGH	2	None	
1	2000	CARDNRYSGSNEPFDYW	IGH	2	None	
1	2001	CARGGPITIFGVSWYYFDYW	IGH	4	None	
1	2002	CAREAGFSSVYFPGLVVVMTSSFDYW	IGH	4	None	
1	2003	CARFFGIVVVPAARWGFDYW	IGH	2	None	
1	2005	CAHRHQDYDILTGYYSLNWFDPW	IGH	5	None	
1	2006	CASLTYYYDSSGYSVDYFDYW	IGH	2	None	
1	2007	CARIYTRIYNYGDVRDYFFDFW	IGH	2	None	
1	2008	CARFVVVPAAMHYYYGMDVW	IGH	3	None	
1	2009	CARDTIRLGELSLWEDYFDYW	IGH	2	None	
1	2010	CARHFGTYTEENWFDSW	IGH	4	None	
1	2011	CAGQLPGSSWFDPW	IGH	8	None	
1	2012	CARGGLLGIGMYYFDYW	IGH	3	None	
1	2013	CASGPGAISAAVYFDYW	IGH	2	None	
1	2015	CAMVYAILGEYYFDYW	IGH	4	None	
1	2016	CTTVHRDPLDRIMITFGGVIAVYFDYW	IGH	2	None	
1	2017	CARDTYSSGWYFGWFDPW	IGH	6	None	
1	2018	CARARGDIVLMVYAIAYFDYW	IGH	2	None	
1	2019	CQQNYDTPQSTF	IGK	10	C4	6W41
1	2020	CARVYLSGFGTDYW	IGH	2	None	
1	2021	CARLLLNSIRGFDYW	IGH	2	None	
1	2022	CARGYDSFRITVTTPFDPW	IGH	2	None	
1	2023	CAKASGFGMATIGFDYW	IGH	4	None	
1	2025	CARSRREITRIFDFW	IGH	2	None	
1	2026	CAREGLLYYDSSGYYEVQEGIFDYW	IGH	2	None	
1	2027	CARRDHDADGYGWFDPW	IGH	2	None	
1	2028	CAKDKNPIGYVFDYW	IGH	2	None	
1	2029	CARGIYGDYGALDGDWFDPW	IGH	2	None	
1	2030	CARVSRGASYLFDFW	IGH	3	None	
1	2031	CARQGRMIVVLNWFDPW	IGH	4	None	
1	2032	CATSPFSSGWNNYYYYMDVW	IGH	848	None	

1	2033	CAKNLRYYYDTTAWDAFDLW	IGH	2	None	
1	2034	CARDQIRAGGYDTFDYW	IGH	4	None	
1	2035	CSSSYYDSWSPYPSDWYFDLW	IGH	2	None	
1	2036	CARHLSHYDFWSGSGYYYYGMDVW	IGH	2	None	
1	2037	CARGHYDSIGGYFFDYW	IGH	5	None	
1	2038	CARGRAGVFGVVIKAFDIW	IGH	4	None	
1	2039	CARVSSSHYDYFDYW	IGH	4	None	
1	2040	CTTEGSGSLYGVWYSYMDVW	IGH	3	None	
1	2041	CARVLPWDYYDSGTFSSGYYFDYW	IGH	3	None	
1	2042	CAKDISSGIAAAGPLGYFDLW	IGH	2	None	
1	2043	CARERITTLTPSIFYYYYGMDVW	IGH	2	None	
1	2044	CARETTVGYNHFDYW	IGH	9	None	
1	2045	CAHRGPPIAAAAHGFQHW	IGH	5	None	
1	2046	CSLGGLYSYGRQFDYW	IGH	2	None	
1	2047	CARHRGRGSGLGDYYYYMDVW	IGH	2	None	
1	2048	CAREERVYNWNSGAFDIW	IGH	2	None	
1	2049	CQRSNSNPPYTF	IGK	2	C1	7BZ5
1	2050	CVIPSVATFTTTRNMLDYW	IGH	3	None	
1	2051	CAHYYDILTGYPLFDYW	IGH	2	None	
1	2052	CAKKVGPYGDYLYYFDYW	IGH	4	None	
1	2053	CARGRVAAAGTGEDWFDPW	IGH	2	None	
1	2054	CARSSTSDYFDYW	IGH	53	None	
1	2055	CAREFDPWSGYYPDDPFFFDYW	IGH	2	None	
1	2056	CARAPMTAYSGSYSVGALDIW	IGH	2	None	
1	2057	CARLPNYYVGSGYAFGAFDIW	IGH	2	None	
1	2058	CATQSILNVNTPMIYRDYW	IGH	4	None	
1	2060	CAKVPSCCSDDAFDIW	IGH	2	None	
1	2061	CAKVGATTLSPFDYW	IGH	4	None	
1	2062	CARVRLAAAEPGYAFDIW	IGH	4	None	
1	2063	CARVDMGRKPYWYFDLW	IGH	3	None	
1	2064	CAGAFDQFFDSW	IGH	2	None	
1	2065	CARVGTDITMIVVVITAFDIW	IGH	11	None	
1	2066	CAKMITVPVNVGPEKESHWYFDFW	IGH	3	None	
1	2067	CARIPFYDYIWGSFSEAYYFDYW	IGH	2	None	
1	2068	CTRDPFPLTNDYW	IGH	4	None	
1	2069	CARGSTMVRGVIIPPPKIYYYYYYGMDVW	IGH	3	None	
1	2070	CARPGYYDSSAEAFDIW	IGH	2	None	
1	2071	CARDLRIELELTAVGGSISYYYGMDVW	IGH	2	None	
1	2072	CARDRNWNYGWFDPW	IGH	3	None	
1	2073	CACTEWELNSWFDPW	IGH	2	None	
1	2074	CARAREGYGLGFDYW	IGH	2	None	
1	2075	CAGLPAPFITTAGNNYYYAMDVW	IGH	2	None	
1	2076	CSSPHDTDAFDIW	IGH	2	None	
1	2078	CTTDRGYMNWLDPW	IGH	4	None	
1	2079	CARGPLELLEKYFDHW	IGH	2	None	
1	2080	CARDRIEARPGSWDYYYYGMDVW	IGH	3	None	
1	2081	CASGYCAGDCYFDYW	IGH	2	None	
1	2082	CARSVGYCSGTTCYMNWYDAW	IGH	12	None	
1	2083	CARTPIYDHVWGNYRQPNFHFDYW	IGH	3	None	
1	2084	CARLVGDYVWGTYVVGAFDIW	IGH	2	None	
1	2085	CTSPFAPYCSGGSCSLDYW	IGH	5	None	
1	2086	CAKDVHGDYVSDYNWFDPW	IGH	3	None	
1	2087	CARDSGPCSGGTCLFDSW	IGH	2	None	
1	2088	CARQTSWWLTHSNWFDPW	IGH	3	None	
1	2090	CAKNGITMIVVVISRFDYW	IGH	3	None	
1	2091	CAKAARGYCSGGSCYFGYW	IGH	2	None	
1	2092	CAHSPRAAISPFDYW	IGH	2	None	
1	2093	CAKGSRIAVAETSFDYW	IGH	5	None	
1	2095	CARDVGFLEYFEPW	IGH	14	None	
1	2096	CAKDGWDGYNPYYFDYW	IGH	6	None	
1	2097	CARDSYGDLYYYYYGMDVW	IGH	7	None	
1	2098	CARGRAAAGTWDFDYW	IGH	2	None	
1	2099	CAKDQDPNHFDWLLGRSSFDYW	IGH	2	None	
1	2100	CQQDYTTFAF	IGK	2	C4	6ZDH
1	2101	CARDGTVTRSFDYW	IGH	5	None	
1	2102	CAKPHCSSTSCYPNYYYYYGMDVW	IGH	2	None	
1	2103	CARGFSITDSAFDIW	IGH	4	None	
1	2104	CQSADSTATDPLVVF	IGL	6	None	

1	2105	CAKDISGWSVGEVPSGFDVW	IGH	2	None	
1	2106	CARDNLRYNWNYGGNDAFDIW	IGH	2	None	
1	2107	CARVVTVWGSYRPKYWFDPW	IGH	2	None	
1	2108	CTRQQELSIYTGGQYFFDYW	IGH	2	None	
1	2109	CARDPWLIGYNYGFDHW	IGH	10	None	
1	2110	CAKDSQYSSSWNPYWYFDLW	IGH	2	None	
1	2111	CAKTYSGYDDYGDYW	IGH	4	None	
1	2112	CTTGLTFSPFDYW	IGH	13	None	
1	2113	CATAFGYCSSTSCPYPYYW	IGH	7	None	
1	2114	CARESRVVDTAMVVTYYYGMDVW	IGH	2	None	
1	2115	CVKDRNWNDIPSVDYW	IGH	2	None	
1	2116	CQSYDSTYQVLF	IGL	68	C4	7JVC
1	2117	CARSTYDRSDWGGLELKRYRYYYMDVW	IGH	4	None	
1	2118	CARDEYSGYDDHFDYW	IGH	7	None	
1	2119	CARVDGHYDSSGFYQYLDNW	IGH	3	None	
1	2120	CARERGTMIGLVFDYW	IGH	2	None	
1	2121	CATGDRWELTYDIAFDIW	IGH	4	None	
1	2122	CQVWDRSSDHPEVVF	IGL	10	None	
1	2123	CARVDEGITGTTCPDYW	IGH	7	None	
1	2124	CAREAGGSGNAADWFDPW	IGH	2	None	
1	2125	CAKKMAPYCGGDCYTPPFDYW	IGH	3	None	
1	2126	CARYYWAITIFGVVTQAFDYW	IGH	15	None	
1	2127	CARDDRIAVAPLGAFDIW	IGH	2	None	
1	2128	CARELVMGRSQTDAFDLW	IGH	7	None	
1	2129	CATGANTIFGVVIIDAFDIW	IGH	3	None	
1	2130	CARDRVIPAPIDYYYYMDVW	IGH	3	None	
1	2131	CARPRYSGNYGDAFDIW	IGH	4	None	
1	2132	CQQYGNSPFTF	IGK	23446	C1	7K8N
1	2133	CAHSSNHYYYYGMDVW	IGH	3	None	
1	2134	CARDPLHIYDSSGYYYDYW	IGH	4	None	
1	2135	CHQSSRLPFTF	IGK	50	None	
1	2136	CANFRLAEQWLRAFDIW	IGH	2	None	
1	2137	CARDHSYCTNGVCYTEHFDYW	IGH	8	None	
1	2138	CAHTEYNLNYLDLLDYW	IGH	4	None	
1	2139	CASGGSVVVPAAHFDYW	IGH	2	None	
1	2140	CARVPGGLRYYHFFYMDFW	IGH	2	None	
1	2141	CARHNFIAVAGTGFDYW	IGH	3	None	
1	2142	CARDRGYSYGYRRNWFDPW	IGH	18	None	
1	2143	CARHDKKWLARGPPNYYYYMDVW	IGH	5	None	
1	2144	CARSRTPGTIFFYYYGMDVW	IGH	6	None	
1	2145	CARVKGTKITIFGVAVSFFDYW	IGH	6	None	
1	2146	CQSADNSAPNSWVF	IGL	2	None	
1	2148	CAKDIHNYGSGTGPLDYW	IGH	8	None	
1	2149	CARDSGSPIGHFDSW	IGH	4	None	
1	2150	CAHSDWNYVFHAFHVW	IGH	5	None	
1	2154	CARLLEYSSSPRWFDPW	IGH	4	None	
1	2155	CAHLGVHPPIYPRPFDCW	IGH	2	None	
1	2156	CARGYDVLTGYPDYW	IGH	203	None	
1	2157	CTKVSRSWYDEDAFDIW	IGH	10	None	
1	2159	CARAASVTTYYYYGMDVW	IGH	2	None	
1	2160	CAKELEPYYDFWSGYSTLKYFYYGMDVW	IGH	4	None	
1	2163	CAKAISSEQWLVQSDYW	IGH	2	None	
1	2164	CARGSGAAAGDFDYW	IGH	176	None	
1	2165	CARCLPPDFYCSGGSCAPFDYW	IGH	4	None	
1	2166	CAKVAPEGSITTRPEEYFQHW	IGH	2	None	
1	2167	CQQSFTTPPLTF	IGK	119	C2	7K8O
1	2169	CAHRHPYYYPSGYNYGYFDFW	IGH	2	None	
1	2170	CARHRSRILYFAPEFDPW	IGH	4	None	
1	2171	CARGTNCSGGYCSPIAFDMW	IGH	4	None	
1	2172	CARHGVVVVPAPLGIQLWSSDWFDPW	IGH	3	None	
1	2173	CARSHTSGWAGYFQHW	IGH	6	None	
1	2174	CARATFVTTVTNFDYW	IGH	4	None	
1	2176	CAKDGVGELVAAIDYW	IGH	2	None	
1	2177	CARALGGTTIDFDSW	IGH	2	None	
1	2178	CARPPGGSSSSDYW	IGH	3	None	
1	2179	CTKDRDRWTPHIAVAGTAGDYW	IGH	4	None	
1	2180	CARGLRPTTMVRVYYFDYW	IGH	2	None	
1	2181	CARATFNDFWSATLDYW	IGH	2	None	

1	2182	CAAHVNTDFWSGQSGGWFDPW	IGH	2	None	
1	2183	CARDPRYILTVKPGGGGNWFDPW	IGH	2	None	
1	2184	CANEKGGSRDYW	IGH	4	None	
1	2186	CATRWAVAGRGPFDYW	IGH	5	None	
1	2187	CAREHLWIGEVLAPAIDYW	IGH	2	None	
1	2189	CAKEAVAGTNWFDAW	IGH	17	None	
1	2190	CAKHSGTYGVSDYW	IGH	2	None	
1	2191	CAKDSSDYGDYALKGGFDPW	IGH	4	None	
1	2192	CARLVHLSLHYWYFDLW	IGH	2	None	
1	2193	CQQYNNWPRKKLTF	IGK	2	C3	7K8R
1	2194	CARDPRGSNGYHYFDYW	IGH	2	None	
1	2195	CARRRITYITIFGVVDPNRGNWFDPW	IGH	3	None	
1	2196	CARLTEPSITPLTYYYFYMDVW	IGH	7	None	
1	2197	CARHHEVPAAITNWFDPW	IGH	2	None	
1	2198	CAHTQGDIVVVIAATPAFFDYW	IGH	3	None	
1	2200	CARASYSSRDYYYYMDVW	IGH	8	None	
1	2201	CARRLWLRGYFDYW	IGH	26	None	
1	2202	CARVVPNDFWSGYPPHQGHFDYW	IGH	2	None	
1	2203	CARTTWIRGRFDPW	IGH	4	None	
1	2204	CARQVGDSYGFIYYFDYW	IGH	5	None	
1	2205	CVREDSGGYAPIAKFDSW	IGH	2	None	
1	2206	CARLPSGRYNWFDPW	IGH	29	None	
1	2207	CAKLPEWELPHDYW	IGH	2	None	
1	2208	CANLMGYSGYDQHLWFDPW	IGH	3	None	
1	2209	CAKSSEGDVIVVEYAFDIW	IGH	8	None	
1	2210	CARDHVSLMTGSYAGYW	IGH	2	None	
1	2211	CTSSLAAADPPDYW	IGH	6	None	
1	2212	CARDLGYSGYDYRDDYYYYYGMDVW	IGH	6	None	
1	2213	CQHYYNLPPVF	IGK	4	C3	6XDG
1	2214	CAREWIVVVPAAMRFYYYGMDVW	IGH	6	None	
1	2215	CASMSRYGPLDYW	IGH	4	None	
1	2216	CARSVFGDIPTGYCTFDYW	IGH	5	None	
1	2218	CARGPPPGIFFPFDIW	IGH	2	None	
1	2219	CTRSMGTVVTPGYYYYYMDVW	IGH	2	None	
1	2221	CVKDWSPHFDYW	IGH	2	None	
1	2222	CAKEGDSSGYRLPPNDYYMDVW	IGH	4	None	
1	2223	CARHAFLDGIMGSDYW	IGH	8	None	
1	2224	CAKDLLRYNWNLEHYFDFW	IGH	2	None	
1	2225	CARDSLGYCSGGFCPPEINAFFDYW	IGH	3	None	
1	2226	CAKVGRGVLIFVAAGEPYYFDYW	IGH	2	None	
1	2227	CAREGSQDYDILTGTYYYYYYMDVW	IGH	2	None	
1	2228	CLQTYNTPRISF	IGK	3	C2	7K8O
1	2229	CAKSGGAARPEEYYYYYYYMDVW	IGH	2	None	
1	2230	CAKDRDELITIFGGVNWFDPW	IGH	4	None	
1	2231	CARVRNRGITMIVVAYIFYDYW	IGH	3	None	
1	2232	CASGLYDSGGYYYYLAYW	IGH	14	None	
1	2233	CARDNDVTSHYSVFDYW	IGH	3	None	
1	2234	CARDYDSSGYYRFFAFDIW	IGH	3	None	
1	2235	CARVNYGEAPYFDYW	IGH	8	None	
1	2236	CVKVMEWPDDAFDIW	IGH	6	None	
1	2237	CQQRSIEPIFTF	IGK	40	None	
1	2238	CARDFDDYDSSGYYGAFDIW	IGH	13	None	
1	2239	CAKPPGDSEDFDAFDIW	IGH	4	None	
1	2240	CARESSPGATVIPGYDALPIW	IGH	2	None	
1	2241	CARVSRITIFGVVIIGPLDYW	IGH	30	None	
1	2242	CAGSVAIFDGEDAFDIW	IGH	68	None	
1	2243	CARGFRLRAYSLGTYYLDYW	IGH	2	None	
1	2244	CARDLSCGGDCYPWGFDYW	IGH	5	None	
1	2245	CATIAAAGPDYYYYYGMDVW	IGH	7	None	
1	2246	CARGVAFPYYYDSSGYLYADYW	IGH	3	None	
1	2248	CATGPIFGVSNWFDPW	IGH	11	None	
1	2249	CARDHYDSSGYMAGWFDPW	IGH	2	None	
1	2250	CAKQRGLGNSWFDPW	IGH	2	None	
1	2251	CQSADSSRSYHVIF	IGL	6	None	
1	2252	CARDQGLITIFGVVIKGEMDYW	IGH	2	None	
1	2253	CARLFFRRLTSSIAARLEWYFDLW	IGH	4	None	
1	2254	CAHRGSGDYDYW	IGH	6	None	
1	2255	CARGQIAAAVYHFDYW	IGH	2	None	

1	2256	CARHSSGYPSSEFDYW	IGH	5	None	
1	2257	CARDLGVMIVVAKYYFDYW	IGH	6	None	
1	2258	CASGLIYCTTADCYVLNFDSW	IGH	5	None	
1	2259	CVTWFYDESSDDSDVFDVW	IGH	12	None	
1	2260	CATSREGGSYVAVFDYW	IGH	4	None	
1	2261	CQQYVSSTGLTF	IGK	2	None	
1	2262	CARHEGRVVTAIPWGFDPW	IGH	5	None	
1	2263	CASRVATAGMEDYW	IGH	2	None	
1	2264	CARESPFGGVIVPDYW	IGH	2	None	
1	2265	CARAPQYCSSTSCQSGAFDIW	IGH	2	None	
1	2266	CVKDRSSGWHRYFDRW	IGH	27	None	
1	2267	CAKIGRGYSYGLVSVIDYW	IGH	2	None	
1	2268	CARVSGSGWPSDLDYW	IGH	6	None	
1	2269	CAKGLTPTVTTEFDYW	IGH	2	None	
1	2270	CAREKTHNVDSYYYYYMDVW	IGH	2	None	
1	2271	CVKDRAYYDFWSPFDYW	IGH	2	None	
1	2272	CARDWLIAVAGYYYYYGMDVW	IGH	11	None	
1	2273	CAEDTAVYSCVKDRGYCSGASCLLTYALDVW	IGH	3	None	
1	2274	CLSYTSGSTLLF	IGL	2	None	
1	2275	CARDRSYCSSTSCYTEYFDYW	IGH	17	None	
1	2276	CAKGSLSLSGSKGNWFDPW	IGH	2	None	
1	2277	CAKEAPIGEFQGGNYFDSW	IGH	2	None	
1	2278	CARDGLYYYDSSAYFNDAFDIW	IGH	2	None	
1	2279	CAREGGSITGTTSWFDPW	IGH	4	None	
1	2280	CQQLSTFPPHTF	IGK	2	C1	7BZ5
1	2281	CARGHYDFWKSYYDHYMDVW	IGH	2	None	
1	2282	CAKDSYPQLWSEVPGFDLW	IGH	11	None	
1	2283	CARDRDSSSWFYNYYMDVW	IGH	4	None	
1	2284	CARVDTAMVRNWFDPW	IGH	4	None	
1	2285	CARAPWGTWSLLSPGWFDLW	IGH	2	None	
1	2286	CARATPHDGPITIFGVVIPYFDYW	IGH	2	None	
1	2287	CATIEMYSGPDFTSYYFYGMDVW	IGH	26	None	
1	2288	CARGLPRYYDFWSGYSAYW	IGH	2	None	
1	2289	CARGAADSAYDFVNWWFDFW	IGH	2	None	
1	2290	CARLVRHIVVVTAIPVIDYW	IGH	10	None	
1	2291	CARILRSFDWVSSISLGYYAMDVW	IGH	2	None	
1	2292	CARGKRIRGLDPLLYYYDSSDPKREFDYW	IGH	2	None	
1	2293	CARLRRDFWSGPHFDYW	IGH	4	None	
1	2295	CVRELYWQGIGTTPYFDSW	IGH	2	None	
1	2296	CAKDGASGGWDDAFDIW	IGH	2	None	
1	2297	CAKDTYSWIAARPVGPDFDYW	IGH	10	None	
1	2298	CARDSRPRIFSSSWYWFDPW	IGH	6	None	
1	2299	CARDSLFTIFGVVIEYYFDYW	IGH	4	None	
1	2300	CAHRQRYYDETSGLENAFHVW	IGH	2	None	
1	2302	CAKAGLGYDILTGYYSSWYFDLW	IGH	5	None	
1	2303	CAKTSYYDFWSGYLNWFDPW	IGH	2	None	
1	2304	CARGGVGYPDDYW	IGH	5	None	
1	2306	CTTDPTGPKPMVRGVIFDYW	IGH	4	None	
1	2308	CYSTDSTGDHSGVF	IGL	9	None	
1	2309	CAKNDHSITMIVVVTPYYFDYW	IGH	51	None	
1	2310	CVRDDVLRDLEWPNEPLNYYYGMDVW	IGH	9	None	
1	2311	CARHENGWYSLYYFDYW	IGH	2	None	
1	2312	CTADLGDGYSDDAFDIW	IGH	10	None	
1	2313	CMQSIQLPPIFTF	IGK	12	None	
1	2314	CAREQGYGHDRGWFDPW	IGH	76	None	
1	2315	CARIEWRPTDYW	IGH	49	None	
1	2316	CARAGFSYGYDEPSDYW	IGH	6	None	
1	2317	CASDRSVYYYDSSGYDYW	IGH	7	None	
1	2318	CARWLQNTHAFDYW	IGH	4	None	
1	2319	CAKDLGDFEFYFDYW	IGH	3	None	
1	2320	CARTYSNYVYYFDYW	IGH	2	None	
1	2321	CGLFGSYNYPPEIDYW	IGH	2	None	
1	2322	CARDGGQDYDFWSGSNWFDPW	IGH	5	None	
1	2323	CAKDRGMVYAIRLFDYW	IGH	2	None	
1	2324	CASLYSNYGGYW	IGH	2	None	
1	2325	CARDGGVVTPSDWYFDLW	IGH	2	None	
1	2326	CARQEFGYCSGNSCYGDWYFDLW	IGH	2	None	
1	2327	CASSVGYSSSIVRFAFDIW	IGH	4	None	

1	2328	CARRIALPTQAFDYW	IGH	2	None	
1	2329	CARGDNWNVRHYFDYW	IGH	2	None	
1	2330	CANGGMEWELTGAFDIW	IGH	2	None	
1	2331	CTRGPSSVSPANYYYYYYMDVW	IGH	2	None	
1	2332	CARESFWSGYYAVLDYW	IGH	2	None	
1	2333	CARRRLAAADKYYFDYW	IGH	2	None	
1	2334	CASYPAAAGYFDYW	IGH	2	None	
1	2335	CTTSITMIVVAKGDYW	IGH	2	None	
1	2336	CARDPGPKGAFDIW	IGH	14	None	
1	2338	CARGSVVLMVYAIGGWFDPW	IGH	5	None	
1	2339	CAKDPIFRDSTMIVVANYYFDYW	IGH	2	None	
1	2341	CARSDPTSGSYWMPPPNWFDPW	IGH	2	None	
1	2342	CARHQWGYSSSWHETVYYFDYW	IGH	5	None	
1	2343	CARGQVTWSITMIVVVRQNDAFDIW	IGH	8	None	
1	2344	CVREGRRYYDTSGWDYLDSW	IGH	2	None	
1	2345	CARGHNIPTRPQYYNAVDVW	IGH	4	None	
1	2346	CARQEYSSSALLFDFW	IGH	7	None	
1	2347	CARSFNGPSGTNDYW	IGH	2	None	
1	2349	CARDESQSYVVVTATDAFDIW	IGH	2	None	
1	2350	CARDPFYGDNELYYYMDVW	IGH	3	None	
1	2351	CARLTAVAGARWFDPW	IGH	6	None	
1	2352	CATTSSSWPQDYFDYW	IGH	12	None	
1	2353	CAREYDFWSGYYTGMDYW	IGH	4	None	
1	2354	CAKDYRDGYNFGYFDLW	IGH	5	None	
1	2355	CARQIYSSGPGNAFDIW	IGH	2	None	
1	2356	CVKDGLFTIFGVGPPGGHRRIDYW	IGH	15	None	
1	2357	CSGGFGYNTATDYW	IGH	4	None	
1	2358	CARRHYQWLTPHAAFDIW	IGH	2	None	
1	2359	CARSITMVRGYIYLARNYFDYW	IGH	14	None	
1	2361	CARFRYGSSSGLRRGYYYYGMDVW	IGH	3	None	
1	2362	CARALGGAARLLYYFDYW	IGH	5	None	
1	2363	CARDDGYYDQTSGYYYYYGMDVW	IGH	2	None	
1	2364	CARALWFRSYYFDYW	IGH	4	None	
1	2365	CARITFYYYGSGSYNYYGMDVW	IGH	6	None	
1	2366	CASSTIMLPFDYW	IGH	2	None	
1	2367	CARVGQRWYRGLYYFDYW	IGH	7	None	
1	2368	CARGFAGWRGQNYYYYMDVW	IGH	3	None	
1	2369	CAKDPSGSLRYYYYYMDVW	IGH	11	None	
1	2370	CARDDTSLVRFDHW	IGH	7	None	
1	2371	CARHGAYSSSSEVQFDYW	IGH	2	None	
1	2372	CTRGGAVADYSDYW	IGH	5	None	
1	2373	CAREAYYYETSGYYGSRYFDYW	IGH	51	None	
1	2375	CARDHSPVAGLGEDGENWFDPW	IGH	4	None	
1	2376	CASLSYDSSGYGFYYYYGMDVW	IGH	3	None	
1	2377	CATDSLGGSYFPYFDYW	IGH	2	None	
1	2378	CARDEGYSSSSWFWFDPW	IGH	3	None	
1	2379	CARATSITMIVVVITGGFDYW	IGH	2	None	
1	2380	CARENLYTARFDYW	IGH	2	None	
1	2381	CARVAPSYSSRYDYW	IGH	2	None	
1	2382	CARIIAVAGTYRYYGMDVW	IGH	2	None	
1	2383	CARQGSGYLLSFDNW	IGH	4	None	
1	2384	CARSCSRTSCYLQRFDPW	IGH	94	None	
1	2385	CARHQTSYSGYDLYYFDYW	IGH	6	None	
1	2386	CARDRNDCTNGVCYTRLFDYW	IGH	3	None	
1	2387	CARDHYDILTGPTESFDYW	IGH	2	None	
1	2388	CAKYSASWGVLGWLDPW	IGH	2	None	
1	2389	CARDPRVSRVGYYYYYYGMDVW	IGH	2	None	
1	2390	CAREGTGIAVAGSFDYW	IGH	5	None	
1	2391	CAKDRTLGSSRGTYYFDYW	IGH	2	None	
1	2392	CAREGWGHWYAGGYHYYMDVW	IGH	6	None	
1	2393	CAKDRANTYDFWSGYLPRPPEDYW	IGH	2	None	
1	2394	CARDSGIGYYDSSGQDNIDYW	IGH	4	None	
1	2395	CASNYYDSSGLYWGYW	IGH	4	None	
1	2396	CARDVIYKSAGPDYW	IGH	4	None	
1	2397	CEKGWNFWSSYYNDRDWFDPW	IGH	2	None	
1	2398	CAKDKLARYSSSWGYCDYW	IGH	2	None	
1	2399	CARLDRRIAVAGTTRAFDIW	IGH	2	None	
1	2400	CARIVWYNWNVLDYW	IGH	2	None	

1	2401	CARRETYSSGWYFDFW	IGH	2	None	
1	2402	CARRKWERTPGTSDYW	IGH	4	None	
1	2403	CARDPRPVSGYADYW	IGH	11	None	
1	2405	CARDPPHYGDYGVWYFDLW	IGH	6	None	
1	2406	CARKQGDGRGWWTPYYFDYW	IGH	21	None	
1	2407	CTRDPPRTIFGVVIAYYYGMDVW	IGH	2	None	
1	2408	CARDGRLYCTNGVCYIDYW	IGH	2	None	
1	2409	CACGSSWYRESDYW	IGH	5	None	
1	2410	CTWPSGTYTDPAFDIW	IGH	3	None	
1	2411	CARSLTITIFGVVIMGYTFDIW	IGH	10	None	
1	2412	CARGERDFDLW	IGH	47	None	
1	2413	CARVARYSYDSYYFDYW	IGH	8	None	
1	2414	CARDADTGMLTDYYMDVW	IGH	31	None	
1	2415	CARGRRQQLVRLSWFDPW	IGH	2	None	
1	2416	CAREGGHLVAVYDAFDTW	IGH	2	None	
1	2417	CASPHTGSNPYYFDYW	IGH	6	None	
2	1	CARHGILYWWPSDYYYGMDVW	IGH	2	None	
2	2	CARENSSSSDYYYGMDVW	IGH	2	None	
2	5	CTKWGPRDQFEYW	IGH	2	None	
2	6	CARLGIAVAGTFAFDIW	IGH	4	None	
2	7	CTGSRSSDYW	IGH	18	None	
2	10	CARVMAGGLTGYGLYW	IGH	4	None	
2	12	CQQRTNWPRLAWTF	IGK	2	None	
2	14	CARRRGPRVVVPAAPLDYW	IGH	2	None	
2	16	CTRDFFEGITMVQGVIIEDYW	IGH	2	None	
2	17	CARGGYYYGSRGLSNFDSW	IGH	2	None	
2	20	CARLLNLTGYYSWFDPW	IGH	2	None	
2	23	CARLPDTNMVIPYYSDHW	IGH	2	None	
2	24	CATVGFDYGVNLGYYFDNW	IGH	2	None	
2	26	CARVFRSRVTIPYCSGGSCRYYFDYW	IGH	2	None	
2	27	CAKAGYRCSGGSCYSYYFDYW	IGH	5	None	
2	29	CARSLNWNYDSFDYW	IGH	2	None	
2	31	CARDPCHGALDYW	IGH	9	None	
2	37	CARKKYYYDTSSAGWFDLW	IGH	2	None	
2	38	CARDFLSAHAAAAAKEWFDSW	IGH	4	None	
2	39	CAKSQWLALDIFDIW	IGH	4	None	
2	40	CARVTAARLKSHYYYYYGMDVW	IGH	2	None	
2	41	CARGSYGGMVFDYW	IGH	2	None	
2	42	CARSFLHYSGSSGYYSGSFDYW	IGH	3	None	
2	43	CARLRDAVMFDYW	IGH	2	None	
2	44	CASVYGDDGDYW	IGH	2	None	
2	45	CQQLNRYPPWTF	IGK	11	C1	7BZ5
2	46	CAKTLRWLHRVVPKNPSGYYYGMDVW	IGH	2	None	
2	48	CGESHTIDGQVGYVF	IGL	3	None	
2	49	CATLQDDFWSGYQTVDYW	IGH	2	None	
2	51	CTVYNRGYFKYW	IGH	2	None	
2	53	CTRDPAEWELPNDYW	IGH	2	None	
2	55	CARGRANYPHYGSGSFYYYYGMDVW	IGH	2	None	
2	58	CARHGTTVTNPQFDYW	IGH	2	None	
2	60	CATEVTSPYAMDVW	IGH	4	None	
2	61	CARRPPIYYDYIWGSYRPKTSGAFDIW	IGH	2	None	
2	66	CARDEGWYSSSCDYW	IGH	2	None	
2	67	CARGPSYYDSSGYPADYW	IGH	2	None	
2	73	CQVYNTYSAYFF	IGK	7	None	
2	74	CQQYNNWPPGFTF	IGK	81	C3	7K8R
2	75	CARDPGPPGAYFDYW	IGH	2	None	
2	78	CAKHPTYYDILTGYISTYYFDYW	IGH	2	None	
2	82	CARGKFHDSGHYQYPIDGFDVW	IGH	2	None	
2	83	CASRPQYSVYFGVFDYW	IGH	19	None	
2	85	CQVWDSSSDHVVF	IGL	20	C4	7JVC
2	86	CARANWIQLADYW	IGH	2	None	
2	87	CAKLRECTNGVCDANWFDAW	IGH	4	None	
2	88	CARYMAVRGVTGYFDYW	IGH	4	None	
2	89	CATVYSGSYLDAFDIW	IGH	2	None	
2	92	CQQSYRTPRAF	IGK	37	C2	7K8O
2	94	CASLTLTKTLYTDFW	IGH	5	None	
2	95	CQQFYGTPTWTF	IGK	4	C4	6W41
2	96	CARTRPPGIPTGGRHWFDSW	IGH	3	None	

2	97	CARHGYYLDSSGPDLFDSW	IGH	3	None	
2	104	CARLLPIYRGGSFDPW	IGH	2	None	
2	106	CAKALYGGHDYW	IGH	2	None	
2	109	CAHSGITGTRIEFFDYW	IGH	2	None	
2	110	CQHYYSTPTSF	IGK	25	C4	6W41
2	113	CSMESDYYDNNNDYW	IGH	2	None	
2	115	CARDAAFYYFDYW	IGH	2	None	
2	116	CARGYSDFWSDYPHYLDYW	IGH	2	None	
2	121	CQQYDKWPFFTF	IGK	13	C3	6XDG
2	122	CARHFDLLSRQLPHFDYW	IGH	2	None	
2	123	CARDGGGGNYRWYFDYW	IGH	14	None	
2	125	CAKGIRDGYNFDYW	IGH	2	None	
2	127	CARDFGFGSGSYWVYW	IGH	170	None	
2	128	CAKDWGASNGEIFDYW	IGH	2	None	
2	131	CARRIKKGDYFDSW	IGH	3	None	
2	132	CARKHGSNYVWYNWFDPW	IGH	2	None	
2	133	CARGGRVSSGIDYW	IGH	8	None	
2	134	CTRDRIDGFTAQFDYW	IGH	13	None	
2	135	CARGGEWELLIGRYFDLW	IGH	2	None	
2	136	CARFRPVLQLFDYW	IGH	2	None	
2	137	CARAFGSSPLEGYFNYW	IGH	4	None	
2	138	CLQHDNFPLYTF	IGK	56	C3	6XDG
2	140	CARGYDVLTGYPDYW	IGH	15	None	
2	141	CARHEGYYDSSGYLALGMSGFDMW	IGH	2	None	
2	145	CLRAEDTAVYYCAKDALIWSAISQDAFDIW	IGH	2	None	
2	146	CVRATGTAAGTVTDYW	IGH	2	None	
2	147	CARDRGRLRSRWWFDPW	IGH	2	None	
2	148	CARIPSSSWEFDYW	IGH	3	None	
2	150	CARRLGYCGGNKCYIWFDPW	IGH	2	None	
2	151	CQQYDNLPPDITF	IGK	2	C3	6XDG
2	152	CARGSSGYDLWGSLLGYNDAFDIW	IGH	3	None	
2	153	CAKDARIYSSGWSPYYFDYW	IGH	2	None	
2	154	CARHRFGIAVAGFDYW	IGH	2	None	
2	157	CAKDLISSVGATPHDAFDIW	IGH	2	None	
2	160	CAKGFRGPMAGTDYFDYW	IGH	3	None	
2	161	CARGPDTSGYYYFYW	IGH	2	None	
2	163	CAIAGIVVVPGAVEHARRQVQYYFDSW	IGH	3	None	
2	168	CARGYQGFDAFDIW	IGH	2	None	
2	169	CAREGGDIVLMVYAPDYW	IGH	4	None	
2	170	CARTTNYYYDSRGFDYW	IGH	2	None	
2	171	CARGARVGDAGVEYLQHW	IGH	3	None	
2	172	CSARDRSGSFSYW	IGH	2	None	
2	173	CQQAKFFPFTF	IGK	62	None	
2	177	CVREDRKGGAGDYW	IGH	2	None	
2	178	CAREIDTMSYRAFDYW	IGH	2	None	
2	179	CARDPPYGSGSYYNPFDYW	IGH	2	None	
2	180	CARPKRYCSSGSGRCQTYQYYYGMDVW	IGH	4	None	
2	181	CARDDNTHLNLFDLW	IGH	3	None	
2	182	CAKGFHPSLGTSFDYW	IGH	2	None	
2	184	CTIQNFDYW	IGH	2	None	
2	185	CARSDSSWPHYFDFW	IGH	2	None	
2	187	CARGEWLVLWEQENAFDIW	IGH	2	None	
2	188	CTRVMPGSSRPWYFNYW	IGH	2	None	
2	190	CAAWDNILSGAIF	IGL	2	None	
2	191	CQHFDNYPREFTF	IGK	2	C3	6XDG
2	192	CARSPYYDFWSGYYPLQYYYYYYGMDVW	IGH	4	None	
2	195	CAKDISKVRGVYYFDYW	IGH	2	None	
2	196	CARDYDYVWGSYRYGSYYYGMDVW	IGH	4	None	
2	197	CARGKYSGNFYSFDPW	IGH	4	None	
2	199	CQQSYSFRMYTF	IGK	5	C4	6ZDH
2	201	CTRDYYDSSGYYYGFDYW	IGH	2	None	
2	203	CARNFLSAHAAASSKEWFDPW	IGH	2	None	
2	205	CARRDYYYDSSGYYYGLGAFDIW	IGH	3	None	
2	206	CARYGSGSYWSWYFDLW	IGH	2	None	
2	207	CARGSITIYGVLILLPARGWFDRW	IGH	2	None	
2	209	CQEYNDWPPRTF	IGK	8	C3	7K8R
2	211	CTTDLWFTIYGYFQHW	IGH	5	None	
2	212	CARDGGLGWTPLYYFDYW	IGH	4	None	

2	215	CASRDSDEHAIWGNFDFW	IGH	2	None	
2	217	CAKGRKVSSTWFSDYW	IGH	11	None	
2	218	CAQDGAGLPEAVVAAPYNWFDPW	IGH	2	None	
2	219	CAKSYSGYDGNDYW	IGH	2	None	
2	222	CARIDRTRFDPFDIW	IGH	3	None	
2	226	CARVPPGYDSAGYYSLYW	IGH	2	None	
2	227	CAKPLKGGAHYSNYGSPFDYW	IGH	2	None	
2	230	CARGRSIGRGGYDFDYW	IGH	2	None	
2	232	CARLREIGSSSDWFDPW	IGH	2	None	
2	234	CARYCSGGSCYRPWAFDIW	IGH	2	None	
2	236	CQQYGTSPYTF	IGK	3198	C2	7K8U
2	240	CAKIGGTNDYGDYVSFFDYW	IGH	2	None	
2	241	CARNLLSAQAAATTKDWFDPW	IGH	12	None	
2	242	CARDKTFAYYDFWSGSYFDYW	IGH	2	None	
2	243	CARCRSPAYGYFSPNWFDPW	IGH	2	None	
2	245	CARDRGSDDPIDYW	IGH	2	None	
2	247	CARAGGIAAAGLYYYYGMDVW	IGH	3	None	
2	248	CARDWSVAGTSGKNCFDPW	IGH	2	None	
2	250	CAKEYYDYIWGSYRSDAFDIW	IGH	2	None	
2	251	CASGFGLTYGYGHTTHFDSW	IGH	4	None	
2	253	CARTSGSYYSDYIDYW	IGH	2	None	
2	256	CARDPQSPGYSSGWYHDYW	IGH	4	None	
2	258	CAKEENPNDYW	IGH	5	None	
2	259	CARSMKWLDSW	IGH	2	None	
2	260	CARGKGIAAAATPIDYW	IGH	2	None	
2	261	CARPLERDENDAFDIW	IGH	3	None	
2	262	CARATYYTSGWYIFDYW	IGH	2	None	
2	263	CARDLGTVTTGFDYW	IGH	2	None	
2	264	CAAWDDNLSGPVF	IGL	110	None	
2	267	CARLGQWLEADYW	IGH	3	None	
2	273	CATSKGLLQPFDYW	IGH	2	None	
2	274	CAKVLPEQWLVNYFDYW	IGH	3	None	
2	275	CTRDYSSSSAEDYW	IGH	2	None	
2	279	CVRWGRVSCSVKRCFLDFDLW	IGH	4	None	
2	280	CARDRDYYGSGSSEFDYW	IGH	2	None	
2	283	CARDRGTYYYDSSGLDYW	IGH	3	None	
2	286	CARDNLRQQLEEYYYYYGMDVW	IGH	2	None	
2	287	CARPGCGSPSCYNSWFDPW	IGH	2	None	
2	288	CARDEKSYDSRGYRDNYYFDYW	IGH	8	None	
2	289	CARRFLSSLAVYYYGMDVW	IGH	2	None	
2	290	CARVVEMATTLYYFDYW	IGH	6	None	
2	296	CARGDNMYSSSWYFYW	IGH	2	None	
2	299	CHQYNNWLLTF	IGK	2	None	
2	300	CARDYGDCRVQPPCFDYW	IGH	2	None	
2	301	CARDAPNCGGDCYTESFFDYW	IGH	2	None	
2	302	CAKDQTAFNRVYDSFDMW	IGH	2	None	
2	303	CARGPRVVPGVVIKFYFDYW	IGH	2	None	
2	304	CARGHGGNSGNAFDIW	IGH	4	None	
2	305	CAVSTIFGVVTWFDPW	IGH	2	None	
2	306	CMEGAHWPYTF	IGK	4	None	
2	307	CAKAINLAVPGSSFDQW	IGH	2	None	
2	308	CAKVSYLSTASSLDYW	IGH	19	None	
2	310	CAPAEDYGDHLGFDYW	IGH	2	None	
2	313	CARDSKFLTWSPQTGRYYYGMDVW	IGH	2	None	
2	314	CARDDTYYYDSSGYFFDYW	IGH	12	None	
2	316	CARHSSASVVFDLW	IGH	4	None	
2	320	CARAIERYSSSSGFDYW	IGH	3	None	
2	321	CAHTHSEFSSSSGRGYFFDSW	IGH	2	None	
2	322	CATDRKDYGVFDYW	IGH	2	None	
2	323	CCAHSTGYCSGGICYRLFDHW	IGH	2	None	
2	325	CARKLNVVGTPLDYW	IGH	2	None	
2	326	CHQSSRLPFTF	IGK	41	None	
2	327	CAKGTIGHCTGARCYPFDVW	IGH	2	None	
2	329	CAREREDDFWSGSYSPGSEGFDPW	IGH	2	None	
2	330	CARWKQYGDYYFDYW	IGH	2	None	
2	331	CARGRQCVVDTCYFETW	IGH	16	None	
2	334	CARDISMVQVVIRLLYQYYYYGVDVW	IGH	2	None	
2	335	CAKDIIAAAGKGGFDYW	IGH	2	None	

2	337	CSRANTVTMPPSIDYW	IGH	2	None	
2	338	CANLCMTTVTCLDYW	IGH	2	None	
2	339	CARDRSVIAIPVGLYFDYW	IGH	2	None	
2	343	CARVLRVAYATHDAFDIW	IGH	2	None	
2	344	CAAGYSSSWSYYYYGMDVW	IGH	3	None	
2	345	CARTLGSGFLYSFDSW	IGH	2	None	
2	346	CAKDDPSNGWNNFDYW	IGH	3	None	
2	347	CARASGSNHAHFAYW	IGH	2	None	
2	350	CIKDSQDTGGYCRGATCYGTGPFDHW	IGH	2	None	
2	351	CQSADSSGSYVVF	IGL	87	None	
2	352	CASFPRYDFWSGSSSDYW	IGH	2	None	
2	353	CARGTAGSGRDTSGYYSLDYW	IGH	2	None	
2	356	CARAGRGSHSWEYYFDYW	IGH	4	None	
2	359	CAVTPHDSSGYSDYW	IGH	2	None	
2	360	CARQNGLTIFGVVTYGTLFDPW	IGH	2	None	
2	361	CVRDSPGAHFDYW	IGH	2	None	
2	362	CARDNRYDSSGLDYW	IGH	2	None	
2	363	CAKDSERRITIFGVVNDAFDIW	IGH	2	None	
2	365	CARVVCRSSCYDAFDIW	IGH	7	None	
2	366	CAKSIIWGGDYGDYEWYFDLW	IGH	2	None	
2	367	CATYLGYCSCTSCLGTDNWFDPW	IGH	2	None	
2	370	CKVPFRRYDYIRDYW	IGH	26	None	
2	371	CARGSRSGPDDFMITFGGVPDYW	IGH	2	None	
2	373	CARGHHSLSLWYFDLW	IGH	2	None	
2	376	CARDRMPPWFDPW	IGH	6	None	
2	377	CARLKMEGVYGSGTYTHYW	IGH	4	None	
2	379	CARGGKGAAYSSGWYYFDYW	IGH	2	None	
2	381	CARHGTKSNGFDYW	IGH	5	None	
2	382	CARSTEMITPLGGSTALPYFDYW	IGH	2	None	
2	383	CASTSHLYSYGTYYFDYW	IGH	4	None	
2	385	CAKGGTYGDSWWFDPW	IGH	2	None	
2	386	CAKDRTVVVPAAIRAHYYYYGMDVW	IGH	2	None	
2	387	CARDESSPFDAFDVW	IGH	6	None	
2	388	CANGRFGRLFTGFDSW	IGH	2	None	
2	389	CARYSGYDGYWFDPW	IGH	2	None	
2	390	CARYSSGYYSTSFDYW	IGH	2	None	
2	392	CARGLGGDRWFDPW	IGH	11	None	
2	394	CTRDWGEMGSTNAFDIW	IGH	2	None	
2	395	CAKQSIPVSGDWVHFDNW	IGH	3	None	
2	397	CARGNYVWDYYYGMDVW	IGH	2	None	
2	398	CARGYCRSTTCYFDYW	IGH	2	None	
2	400	CVRDRGVERWLHYSSLDYW	IGH	2	None	
2	402	CARGLRSFTIFREYAFDIW	IGH	2	None	
2	404	CAPLPLYYDFPNWFDPW	IGH	2	None	
2	405	CMIWPSNAWVF	IGL	7	None	
2	408	CARVHSYGSGSYFSYYGMDVW	IGH	3	None	
2	410	CQKYDSAHRTF	IGK	612	None	
2	411	CARDRYDSSGDYLAYDIW	IGH	3	None	
2	416	CARGCCSSPRLSSNWFDPW	IGH	2	None	
2	418	CTRDRVQPDYW	IGH	5	None	
2	420	CQKYGGSPPPSITF	IGK	8	C1	7K8N
2	421	CTRFSATNGWLFDYW	IGH	4	None	
2	423	CYSARDNDLRVF	IGL	13	None	
2	425	CARGEFDPW	IGH	21	None	
2	426	CARTGVSGYDLDYFDYW	IGH	2	None	
2	427	CATTGFQGSYRDNDCGGDCYVRRGFDCW	IGH	10	None	
2	428	CARPIASRRIDAFDIW	IGH	2	None	
2	432	CQTWGTGTQVF	IGL	13	None	
2	437	CARAHGIAVAAQEAFDIW	IGH	2	None	
2	438	CARDSGKSVWPTNHLDYW	IGH	2	None	
2	439	CVKGLLGGVLFAMDVW	IGH	2	None	
2	441	CARVVHDYGIGLGGAYDVW	IGH	2	None	
2	442	CARQVATTVVSNFDYW	IGH	2	None	
2	443	CARQVRFLEWPFDYW	IGH	3	None	
2	445	CARPITATPNSPVDYW	IGH	3	None	
2	446	CAKDIGYISPLSTLKNGRGDAFDIW	IGH	2	None	
2	448	CARRGQSDPVITPENWFDPW	IGH	3	None	
2	449	CQQYHSNPPALTF	IGK	4	C4	6W41

2	452	CASGFYDSSGYYYGYW	IGH	2	None	
2	456	CARGEYPYWFSYW	IGH	2	None	
2	457	CQQSFSTLGITF	IGK	6	C2	7K8O
2	459	CARDRQRERRSPRYYYYGMDVW	IGH	3	None	
2	461	CGTWDSSLLVVMF	IGL	2	None	
2	462	CASEGTGRVFDYW	IGH	2	None	
2	464	CARCLGDRYYTGGGYYCRGRFDYW	IGH	4	None	
2	465	CVRDGSYYDTSGYYYLYW	IGH	274	None	
2	466	CARTPKIAVAGTKPPYYYYGMDVW	IGH	2	None	
2	467	CQQRTTWPPNPF	IGK	11	C1	7BZ5
2	469	CAKKGAMAPAPKHFDYW	IGH	3	None	
2	470	CAHRRVSPWSISGPARNSGSGRKRAYYFDYW	IGH	2	None	
2	472	CARHEKIAAPFDYW	IGH	2	None	
2	473	CARKTGKYPFDYW	IGH	25	None	
2	474	CARGHLGLGIAAAGNDYW	IGH	2	None	
2	475	CMQALQAPRTF	IGK	604	None	
2	476	CAKGLYGSYYESDAFDIW	IGH	2	None	
2	477	CAKDPTEGVRDYW	IGH	2	None	
2	479	CARGKAYYDFWSAQTGFDPW	IGH	4	None	
2	480	CAKCMTTVPVIAFDIW	IGH	2	None	
2	482	CCSYAGSYTRNWVF	IGL	3	C2	7K8X
2	484	CQQYDASPPMYTF	IGK	43	C3	6XDG
2	485	CARRVIVVVITGDYFDYW	IGH	2	None	
2	489	CQQYDTLHISF	IGK	2	C3	6XDG
2	491	CARDVYCSGGSCYSVPFDYW	IGH	6	None	
2	492	CARELVRGKYYYYYGMDVW	IGH	9	None	
2	493	CARDLLIEDPSGGSCFDYW	IGH	4	None	
2	494	CARFSRDDYINYWYLGLW	IGH	3	None	
2	495	CARLLGYRYDYVDYFDYW	IGH	2	None	
2	496	CAKDFQGHYFGIGSHRAFDYW	IGH	2	None	
2	497	CAKAVYYYDRSGYPLYYYHGMDVW	IGH	2	None	
2	499	CARDDYEYWSGVYASGHFDYW	IGH	2	None	
2	500	CAKDIFVYSSGWYFDYW	IGH	2	None	
2	501	CARMGTYYYDSGGYSEAFDIW	IGH	3	None	
2	502	CARLGIYGDYPDYW	IGH	4	None	
2	504	CVKNKVPFSLMVFAAADAFDIW	IGH	2	None	
2	505	CARRTRFIAARPQWYFDLW	IGH	2	None	
2	506	CARDSGASTDDSSLGYYFDYW	IGH	2	None	
2	509	CVRYFQSKWPYYFDYW	IGH	19	None	
2	510	CARGESRDYGGPRGFDYW	IGH	4	None	
2	512	CARGPYYDFWSDLGTRYYYYYGMDVW	IGH	2	None	
2	513	CAKGPSSGYGFDYFDSW	IGH	4	None	
2	515	CAGPAVAGTKRIDYW	IGH	12	None	
2	516	CARHYIWGSYEEAWFDPW	IGH	2	None	
2	517	CAKANCGGDCRVLDYW	IGH	2	None	
2	518	CAKDALIWSAISQDAFDIW	IGH	2	None	
2	519	CAKDQFAGNGLYDAFDIW	IGH	4	None	
2	521	CARGYYDSYGDYVVFDFDYW	IGH	2	None	
2	522	CGSYDILTGTDFDYW	IGH	2	None	
2	524	CARDVLTMVQGALDYW	IGH	4	None	
2	527	CARPEGGYSTSWVDYW	IGH	6	None	
2	528	CARHYGSGSSDYW	IGH	2	None	
2	529	CARVHTVTSHDAFDIW	IGH	2	None	
2	530	CCSYAGSSTYVF	IGL	203	C2	7K8X
2	531	CQQYNSHSSF	IGK	8	C3	7K8R
2	533	CAREAKTSPPNFDYW	IGH	3	None	
2	535	CASMEHPEGILGELSEDYW	IGH	4	None	
2	537	CAMSTARWGRYNWFDPW	IGH	4	None	
2	538	CAKCFDLWSGYPVGCKWFDPW	IGH	4	None	
2	539	CNSRDSSGNLPF	IGL	49	None	
2	540	CARDSPDGYYDFWSGYYSHYFDYW	IGH	2	None	
2	541	CARTKLSVIAEDYW	IGH	11	None	
2	542	CARHVSEYSGYDPYYYFDYW	IGH	2	None	
2	543	CAKGSPCGVDCVSFDQW	IGH	2	None	
2	544	CARDDNSGRGGSEITPFDYW	IGH	2	None	
2	545	CSLRSEDTAVYYCAPAEDYGDHLGFDYW	IGH	2	None	
2	546	CAKWNGPHYNNWHVDYW	IGH	3	None	
2	548	CARGTNSSPNWFDPW	IGH	2	None	

2	552	CQQYKSYSRGFTF	IGK	9	C3	7K8R
2	554	CLLSYSGARVF	IGL	32	None	
2	555	CARDISSSWYPNWFDPW	IGH	6	None	
2	556	CAAERLGYCSGDSCRNWLDPW	IGH	2	None	
2	557	CAKAGGGVPQNYFHSW	IGH	10	None	
2	558	CVRGCSGTRCYKNFDYW	IGH	6	None	
2	559	CAKDLAYYGDYASDAFDIW	IGH	2	None	
2	560	CAKDPLEIPAATGGRYNWFDPW	IGH	2	None	
2	561	CAKVFIAVAGYFDYW	IGH	2	None	
2	562	CARAAYCGGDCYSGHDYW	IGH	2	None	
2	563	CTRQAVAVPIDYW	IGH	2	None	
2	564	CAKFQLWPHTKNNWFDRW	IGH	2	None	
2	567	CAREYYYDSSGTFDYW	IGH	3	None	
2	569	CARAESSGPYETYNWFDPW	IGH	2	None	
2	572	CVKGVLPYCTGALCYEFDSW	IGH	6	None	
2	573	CARLDGELEQWSGSYYYYGMDVW	IGH	2	None	
2	574	CARVKDDYSNYVPFDYW	IGH	2	None	
2	576	CASPHYGDYRSPFDYW	IGH	10	None	
2	577	CTRHPHNSGDTFDVW	IGH	2	None	
2	579	CANSRGYSYGLRFDYW	IGH	2	None	
2	582	CARVIIAGQFLEWTHFDYW	IGH	2	None	
2	583	CVKNVPNRHISSSAKDYFDNW	IGH	14	None	
2	584	CAREGRPGKSYDDYVGLDYW	IGH	2	None	
2	585	CARVPLFSDRTTVLFDYW	IGH	2	None	
2	588	CSTWDSSLRAWVF	IGL	2	None	
2	594	CTRLNLQDYGDYAFDYW	IGH	2	None	
2	596	CANARGSSAITKIDYW	IGH	2	None	
2	599	CARGQYYDILTGYYHNKNNWFDPW	IGH	2	None	
2	600	CARLSYYDFWSGTGDYW	IGH	4	None	
2	601	CARDYDRSGYYPDFW	IGH	3	None	
2	604	CARVWRWYIDYW	IGH	2	None	
2	609	CARGPHYGDRSDYFDYW	IGH	3	None	
2	610	CAKNQGNGGPFYYAMDVW	IGH	4	None	
2	611	CQQYNHWPIIF	IGK	3	C3	7K8R
2	613	CAKDCGYGSGVRAFDIW	IGH	3	None	
2	614	CARVPRGFYYYGMDVW	IGH	12	None	
2	615	CAKCTGRSGGSCSPFDPW	IGH	2	None	
2	616	CARLTMAASPGFRFDSW	IGH	3	None	
2	617	CAHKMDYVRRAFDIW	IGH	2	None	
2	619	CMQRVEFPSITF	IGK	7	None	
2	620	CAKDGDGSGRYTHNWFDSW	IGH	4	None	
2	622	CASRRTPYDSAFSGVVFDIW	IGH	3	None	
2	625	CASQYCSGGSCYLSQYYYYGMDVW	IGH	2	None	
2	626	CASGYCSGGSCYPPIDYW	IGH	2	None	
3	1	CARGFQMGISDTYQYYYGLDVW	IGH	2	None	
3	2	CAKEDQWERRAFDIW	IGH	4	None	
3	3	CARGGDYYDSSGYYYPHYYYGMDVW	IGH	2	None	
3	4	CARDSWPTIGRYFDLW	IGH	6	None	
3	7	CATAPVDYYGSGSYWYFDLW	IGH	2	None	
3	8	CAKITQPVEPPFDIW	IGH	8	None	
3	9	CAKDHNWGSSGWSTFDYW	IGH	2	None	
3	10	CARVYGDYRDWYGFDVW	IGH	2	None	
3	11	CAKIFSSSWYTDYW	IGH	36	None	
3	12	CTPYNSDGFHIW	IGH	4	None	
3	13	CARRAYTYGFNYAMDVW	IGH	2	None	
3	14	CARLFHDGTYSWLDPW	IGH	4	None	
3	17	CARSLALGYIYGYLNYW	IGH	4	None	
3	19	CARDYHYDTRGYVGYW	IGH	11	None	
3	20	CARDMDYYDSSGYYQDYW	IGH	26	None	
3	21	CARDRTDSGWPYYFDYW	IGH	13	None	
3	22	CARDGGYYDSSGHEWFDPW	IGH	2	None	
3	23	CGTWHSNSKTQVF	IGL	16	None	
3	24	CAKDIYPIAAAGTSYFDYW	IGH	2	None	
3	25	CAFCSGGSCYNWFDPW	IGH	2	None	
3	26	CASVRRGGGTSYYFDYW	IGH	2	None	
3	27	CARSIAVSGYYFDYW	IGH	6	None	
3	28	CAAKAAGPYNWFDPW	IGH	11	None	
3	29	CARDYSSGWLGEHFDYW	IGH	3	None	

3	30	CLQGTDWPLSF	IGK	59	None	
3	31	CARGPREVRGVIVGYYFDYW	IGH	3	None	
3	32	CARDAGSDRWTNFDHW	IGH	2	None	
3	33	CARLGTPVLRYFDWLTTYYYGMDVW	IGH	4	None	
3	34	CARLPRLHSFDIW	IGH	2	None	
3	35	CAKSVDYYGSGSYYWYW	IGH	6	None	
3	36	CAKDMPYDILTGYYYYYGMDVW	IGH	3	None	
3	37	CARVLPQIGNFFEGRESTWFDPW	IGH	2	None	
3	38	CLRASDSALYYCAKYARESYYYYYAMDVW	IGH	2	None	
3	39	CARESWDRGYEAFDIW	IGH	2	None	
3	40	CARHRNSGWFFPAFDFW	IGH	3	None	
3	42	CAKLAVTPRDAFDIW	IGH	4	None	
3	43	CARSPESPDGYFDYW	IGH	4	None	
3	44	CARWSSTSRYGHFDYW	IGH	2	None	
3	45	CAHRGPNSIGWYTFYFDYW	IGH	2	None	
3	46	CARELVTQAKAGVYYYSGMDVW	IGH	2	None	
3	47	CAKDKGLVAVVTFDYW	IGH	3	None	
3	49	CALMALSPTDTYFPSW	IGH	3	None	
3	50	CARSPTVTVNSLFDNW	IGH	4	None	
3	51	CARETEDYTSSYFDYW	IGH	2	None	
3	52	CAKLGVGATHPEVDYW	IGH	4	None	
3	53	CAKEPAADQRDYW	IGH	6	None	
3	54	CAKVGGYLWFGEFNDAFDIW	IGH	2	None	
3	55	CAKEAGWSEYFDYW	IGH	2	None	
3	56	CARAYGDYRGGFDYW	IGH	15	None	
3	57	CAREVDYGSPFDPW	IGH	4	None	
3	58	CVRSTSLWIELYATGHFDFW	IGH	4	None	
3	59	CARGSNSSAWVNWFDPW	IGH	4	None	
3	60	CAKEMYIGFGEFPPNYFDYW	IGH	10	None	
3	61	CAKAEGYTSGWYFDYW	IGH	2	None	
3	62	CAKAYSTVTTDYYYYGMDVW	IGH	3	None	
3	63	CQQYVNLPPGSF	IGK	306	C3	6XDG
3	64	CTTDEVVVGLFDYW	IGH	4	None	
3	65	CARRLSTSYYFYGMDVW	IGH	10	None	
3	66	CAKTIAVAGTNYFDYW	IGH	4	None	
3	67	CARGGIMRWLEGDGMDVW	IGH	3	None	
3	68	CAKEAPPWGLFDYW	IGH	2	None	
3	69	CARDTPPTYYYDSSGYPDYW	IGH	9	None	
3	70	CARSLYDYVWGSYRRALYYYYGMDVW	IGH	2	None	
3	71	CARWFHTFDIW	IGH	2	None	
3	72	CASQPTDSSGNDYW	IGH	2	None	
3	73	CARGPIKYSGYDYANYFDYW	IGH	2	None	
3	74	CARVRGFLDGIFTGDWLDPW	IGH	7	None	
3	75	CARALLLDGGRLKFDSW	IGH	2	None	
3	76	CQQSDTAPSVF	IGK	9	None	
3	77	CQSYDGSLSAFMIF	IGL	7	C4	7JVC
3	78	CAKDKLGGTTRGWFDPW	IGH	4	None	
3	79	CTRDPYGYTPNDSFDIW	IGH	2	None	
3	80	CQQANSFPLDLFTF	IGK	12	C3	7K8P
3	81	CASFMLKFPGDDYW	IGH	3	None	
3	82	CARLKSDSSSSGRRGYFDSW	IGH	2	None	
3	84	CARALLWFGEPPWFDPW	IGH	6	None	
3	85	CAKLRGYSGYDYPGDYW	IGH	12	None	
3	87	CVRRTSTDNLSFGGPEGFDHW	IGH	2	None	
3	88	CARALLPLYSSSWLDYW	IGH	2	None	
3	89	CARLVVSHWPPDYW	IGH	3	None	
3	90	CASWVNSHWFDYW	IGH	13	None	
3	91	CATEDDSENHNLSIFDYW	IGH	4	None	
3	92	CARGDPTALVFDYW	IGH	2	None	
3	94	CAKEAIGYSSGWYPPFEFVWFDPW	IGH	2	None	
3	95	CARRGANWSHARFDLW	IGH	2	None	
3	96	CARAYCGGECYNGIFDSW	IGH	2	None	
3	97	CASSLLMVYASFDYW	IGH	4	None	
3	99	CTKGRIDQWLAPDYW	IGH	2	None	
3	100	CARGGAGPAAIYDYW	IGH	6	None	
3	101	CSRHILNHRSIEDYFDSW	IGH	2	None	
3	102	CAKDSSDGYGYVPYYYGMDVW	IGH	2	None	
3	104	CAREEQPLIMRFDPW	IGH	4	None	

3	105	CAKTKWVQGLTFNWFDPW	IGH	2	None	
3	106	CATLYDSSGYPHSSTVDYW	IGH	4	None	
3	109	CMQVVQFPPSFIF	IGK	2	None	
3	110	CAREHGDGKQFGYFDYW	IGH	37	None	
3	111	CVREPFRGIIGWYFDLW	IGH	5	None	
3	112	CARDRYYDILTGYQAPYNWFDPW	IGH	3	None	
3	113	CAKLPGGNTYYYDSSGYSFDYW	IGH	2	None	
3	114	CAKDVPYYDSSGPTGYFDYW	IGH	2	None	
3	115	CARTYYVSGYYFDNW	IGH	2	None	
3	116	CQQANSFVFTF	IGK	486	C3	7K8P
3	117	CARQWQWLGIDYW	IGH	2	None	
3	118	CAKGSEYGGIFDYW	IGH	8	None	
3	119	CASESRPDHRFDYW	IGH	3	None	
3	120	CARVHNYYDSSGLLYYYYGMDVW	IGH	5	None	
3	121	CAKDPLIGYDILTGGTFDYW	IGH	4	None	
3	122	CARDPDTYYYDSSGYYYENYFDYW	IGH	2	None	
3	124	CATPRRSGYDEGFDNW	IGH	9	None	
3	125	CAKDTLVGEGRFDYW	IGH	15	None	
3	126	CAREDMNLPHFDYW	IGH	22	None	
3	127	CARGGTYYYNNDGPHYFDYW	IGH	4	None	
3	128	CATERAGCPGGSCYHYFDYW	IGH	6	None	
3	129	CQQYDNLPPGKLTF	IGK	10	C3	6XDG
3	130	CARSEFITGSNRELDYW	IGH	2	None	
3	131	CATDREYCSGGAKCYSAFDYW	IGH	3	None	
3	132	CARTPDYTNYGWFDPW	IGH	2	None	
3	133	CATSLFWGQPFDYW	IGH	8	None	
3	134	CARVGVPVTGSLFRAWLDPW	IGH	2	None	
3	135	CARQARGPDIVGASYFDYW	IGH	3	None	
3	136	CARGTVGASGVARDPAGRAIQGFLYYFDYW	IGH	2	None	
3	137	CARHSLVVPAAVRYNWFDPW	IGH	2	None	
3	139	CQKYNSALRAPALTF	IGK	4	None	
3	140	CHQYNSYRTWTF	IGK	5	C3	7K8R
3	142	CRQYNNWPRGAF	IGK	3	C3	7K8R
3	143	CARDRQVPAENYYAMDVW	IGH	6	None	
3	144	CATSYFGLGNYYYGMDVW	IGH	3	None	
3	145	CAKDIIAVAGPFDYW	IGH	33	None	
3	146	CAKHTSGYDLDGMDVW	IGH	4	None	
3	147	CARDQYYDGNPYSHFDYW	IGH	2	None	
3	148	CQSADSSGTYPDVVF	IGL	21	None	
3	149	CARDLRSSFYDETAFNVW	IGH	4	None	
3	150	CAKFPGGEGSSWTVHPFDYW	IGH	5	None	
3	151	CAHRPTLGYCSGGSCYRLAWFDPW	IGH	9	None	
3	152	CATFHFYDYVWGSYRPTYAFDVW	IGH	5	None	
3	153	CAKVDSSGYYQGLADYW	IGH	2	None	
3	154	CAKDSYYDSWDWFDPW	IGH	10	None	
3	155	CATQGGVTDYYYGMDVW	IGH	2	None	
3	157	CAKSRQVGRTAAGNFDYW	IGH	4	None	
3	158	CANSNCSSTSCYAGEFDYW	IGH	7	None	
3	160	CARMNPYYEDSSGFYYDHW	IGH	7	None	
3	161	CARDPDYYDSSGWAFDIW	IGH	13	None	
3	162	CARVAFYGSGSYAMDVW	IGH	3	None	
3	163	CAKVAPIDYDTYKNGFDFW	IGH	2	None	
3	164	CAKVGVYYDSSGLVDYW	IGH	2	None	
3	165	CATGTLYEVTTDYYYGMDVW	IGH	2	None	
3	166	CARDAGAGYYPYYGMDVW	IGH	2	None	
3	167	CARDSYQLPRRGNWFDPW	IGH	2	None	
3	168	CAKLLWFGELNDYW	IGH	2	None	
3	169	CASHIADYYYYGMDVW	IGH	15	None	
3	170	CARGHSTSGPWFDPW	IGH	2	None	
3	171	CAAIAAFSQTVDYW	IGH	4	None	
3	172	CVKASHDSSPYYYFDSW	IGH	2	None	
3	173	CARLLDANNAVPFDNW	IGH	4	None	
3	174	CARAHSTVYYYGSATGWFDPW	IGH	2	None	
3	175	CASEAPLLGAFDIW	IGH	6	None	
3	176	CAHKVYVWGRERYFDYW	IGH	2	None	
3	177	CAKDLLPTGASFDYW	IGH	4	None	
3	178	CARTPTGPGETYFDYW	IGH	6	None	
3	179	CARHDRDTAMVPLVTFDYW	IGH	3	None	

3	180	CAKLDTAMGYDYW	IGH	4	None	
3	181	CAKGEMATIWAYFDYW	IGH	8	None	
3	182	CTRMYGLGWPDYW	IGH	2	None	
3	183	CARHKSGGTYPLDYW	IGH	2	None	
3	184	CARPLWRGYFFPFDIW	IGH	2	None	
3	185	CQQFNGWPPYSF	IGK	55	C1	7BZ5
3	186	CAKDLFSGSYSKPLFDYW	IGH	3	None	
3	188	CARDKTMFGGTDFDYW	IGH	2	None	
3	189	CARTYAWNSLIDYW	IGH	4	None	
3	190	CTRDRPPGIAAAGPGIAAAGPDYYYYYGMDVW	IGH	4	None	
3	191	CAKLDPWFGGPHDAFDIW	IGH	4	None	
3	192	CARRRTPIVGATDYW	IGH	2	None	
3	193	CALPISGNSGYYSGSFDSW	IGH	6	None	
3	194	CAKDLGPSIEAHGSWFDPW	IGH	6	None	
3	195	CAHSSYYDSSSDYW	IGH	8	None	
3	197	CARDFWPPYYGSGSYLPLDYW	IGH	2	None	
3	198	CARGPNNGYKVIDYW	IGH	3	None	
3	199	CARPLITLTGAFEIW	IGH	4	None	
3	200	CARDVSSTSCYDYW	IGH	2	None	
3	201	CARDWTTVTTDWFDPW	IGH	2	None	
3	204	CQRYNSNSYAF	IGK	51	None	
3	206	CAKPSLYGAPDYW	IGH	2	None	
3	207	CAKDPNPMVWGYFDYW	IGH	2	None	
3	209	CASPMSGHSDYW	IGH	5	None	
3	211	CASPGRAASFDYW	IGH	4	None	
3	212	CASNRITKSFDMW	IGH	2	None	
3	213	CVKDGFGGVIVPGGWFDPW	IGH	2	None	
3	216	CLLYYDGGQPHWVF	IGL	7	None	
3	218	CARDPPTVTTYFDYW	IGH	4	None	
3	219	CARVEDFTIFGVVIEEVWFDPW	IGH	4	None	
3	220	CAKGELGSSGWEGAFDIW	IGH	2	None	
3	221	CARRDTWPHYFDYW	IGH	3	None	
3	222	CARGGYVVHSGSYPTPVDYW	IGH	2	None	
3	223	CARHGRDTMVRGPQGFDPW	IGH	2	None	
3	224	CAKLIDILTGEPDAFDIW	IGH	4	None	
3	225	CAKDIGTSLLGWFDPW	IGH	2	None	
3	227	CAHRHRTITVATYFDYW	IGH	2	None	
3	228	CHKGILAGYDAFDIW	IGH	2	None	
3	229	CATISTTGRRGYYYYYGMDVW	IGH	2	None	
3	231	CARGGEDDILTGYYFGYW	IGH	6	None	
3	232	CAISDDDSGYYYPKYW	IGH	4	None	
3	233	CVREQYYHRSGNTYNEENWFDPW	IGH	4	None	
3	234	CAKDISPNYGVLTGPGGWLDSW	IGH	3	None	
3	235	CARANHELELPWFDPW	IGH	2	None	
3	236	CARVRGAGAMGWQLYFYYGMDVW	IGH	4	None	
3	237	CMQSVQLLLFGF	IGK	4	None	
3	238	CAKAQYFDWLDTAFDYW	IGH	2	None	
3	239	CARDTFDSHTGWFDPW	IGH	4	None	
3	240	CARSYYGGTFYWYFDLW	IGH	2	None	
3	241	CASSEQQLAYWYFDLW	IGH	2	None	
3	242	CAKDYFAGSGTYSYWFDPW	IGH	13	None	
3	243	CARVPHYGDHLGYFDYW	IGH	3	None	
3	245	CAARYCSGHNCHFFFDYW	IGH	5	None	
3	246	CARDETYCSSASCYYYYGMDVW	IGH	2	None	
3	247	CSGVPHYNLVDYW	IGH	2	None	
3	248	CARHPPGSSAPTGSYFFDYW	IGH	2	None	
3	249	CARRSYGQYYFDYW	IGH	2	None	
3	251	CANSARSYYYDSSGPFDYW	IGH	2	None	
3	252	CAHSVDNWNFYYYYGIDVW	IGH	2	None	
3	253	CAKVWGTSGGDYNDYW	IGH	2	None	
3	254	CSRESHCSRGRCSPPTDYW	IGH	4	None	
3	255	CACLIRGSFSIDYW	IGH	4	None	
3	256	CARPGHGYNDFYYW	IGH	11	None	
3	257	CVTYYYDSSGNLGKDYW	IGH	4	None	
3	258	CARDRYYGVNPGELDYW	IGH	3	None	
3	260	CARLHCGDDSCPDSSFFDLW	IGH	6	None	
3	261	CASLTHYDILTGYPYYYYYGMDVW	IGH	5	None	
3	262	CAKVVSTPGHWYFDIW	IGH	3	None	

3	263	CGKDILSTDMVTIDYW	IGH	4	None	
3	264	CARLKKADTGNFFTFDPW	IGH	8	None	
3	265	CARSVDDSDGYWNRYYFDYW	IGH	2	None	
3	266	CALPISAVYYCARSFLHYSGSSGYYSGSFDYW	IGH	10	None	
3	267	CARSGYSSSWYDFVSWFDPW	IGH	10	None	
3	268	CATLGATLGLHYYYYYGMDVW	IGH	2	None	
3	269	CAREEYDYSLGALDIW	IGH	2	None	
3	271	CAKESGITTPEGFDYW	IGH	2	None	
3	272	CQSTDSSGSWLF	IGL	27	None	
3	273	CAKDTYYDSSGVDYW	IGH	28	None	
3	274	CVREGLSSKWNGYFHHW	IGH	17	None	
3	275	CVREGSGSSASGTGFDYW	IGH	2	None	
3	276	CQSYDWGLSGWAF	IGL	3	None	
3	277	CARDVLSPSSWQYYFDYW	IGH	3	None	
3	278	CARAFPRRLEREAWRGGWFDPW	IGH	4	None	
3	279	CQSADTIGAEMFF	IGL	3	None	
3	280	CARDGYSYGSDGMDVW	IGH	5	None	
3	281	CAKCQVPAAMSNLDDFYYYHGMDVW	IGH	4	None	
3	282	CAKDSYGSGSYYRASLDYW	IGH	4	None	
3	284	CAKDLYGSGSYYPSDYW	IGH	43	None	
3	285	CAKMPGDPYSSGWPALYYYGMDVW	IGH	2	None	
3	286	CASRFRSSGTTGNYFGPW	IGH	2	None	
3	287	CARDQANYYDSSGYLNWFDPW	IGH	2	None	
3	288	CAKAPLEYCSGGTCYPFDYW	IGH	4	None	
3	289	CARHPNWALDLDYW	IGH	2	None	
3	290	CAKDREVLMVYAEAFGIYYYYGMDVW	IGH	2	None	
3	291	CTTLMWCSGDCYRSYFDYW	IGH	2	None	
3	292	CARAPLDNPGGNDYW	IGH	5	None	
3	293	CARLYFHETSGYVDYW	IGH	4	None	
3	294	CATEKFCPEEESCYSFDPW	IGH	2	None	
3	295	CAKDLSPSYGPLDYW	IGH	2	None	
3	296	CAKLVSSYSGANDYW	IGH	2	None	
3	297	CAREIRSSGWAGSPNSDNWFDPW	IGH	4	None	
3	298	CARILTFYGPGSEYYSGTIDYW	IGH	2	None	
3	299	CAKPNRITMIVSPDAFDIW	IGH	2	None	
3	300	CARARFCGGVTCYSDFW	IGH	2	None	
3	301	CAKDPFRYCSGGSCSYFDYW	IGH	2	None	
3	302	CARHAAPYYYDSSGYRSWFDPW	IGH	8	None	
3	303	CVKRLFIAGASHGFDIW	IGH	2	None	
3	304	CARGRLGRDNWNYVFDYW	IGH	4	None	
3	306	CAKDALPYGSGLYDYW	IGH	2	None	
3	307	CARIRLSDSSGYYVVDFDYW	IGH	2	None	
3	308	CAVWDDSLSVDWLF	IGL	4	None	
3	309	CARLYSGYDLGDYW	IGH	7	None	
3	311	CARDSDYGDYGGTDYYYYYGMDVW	IGH	6	None	
3	312	CARQKPNSYWAFDYW	IGH	18	None	
3	313	CAKDILPGIAVAGDYW	IGH	15	None	
3	314	CAKERWDTRTSYYLDSW	IGH	2	None	
3	315	CVHSDILGGYSISLFDYW	IGH	2	None	
3	317	CARTHGYSYGTPYFDYW	IGH	4	None	
3	318	CAKLPVWDSSGYNDYW	IGH	2	None	
3	319	CARVHYYDSSGYPRIDYW	IGH	2	None	
3	320	CAKVGGSGYYAYYVDSW	IGH	9	None	
3	322	CARSDVLTGKDAFDIW	IGH	14	None	
3	323	CAKETPSYYYDSSGYHGAFDYW	IGH	2	None	
3	324	CAKDIDVEMATILDYW	IGH	2	None	
3	326	CAKDFFLRDYVWGSQGSLDAFDIW	IGH	2	None	
3	327	CARGYYDSSGYIYYYYGMDVW	IGH	6	None	
3	328	CARRGLITSAFHVW	IGH	2	None	
3	329	CVKDKPDASAFDAFDFW	IGH	4	None	
3	330	CAKDVEEDYGDSQRFEYW	IGH	2	None	
3	331	CARDVRSCSGSICYSRTYYYYGVDVW	IGH	6	None	
3	332	CARDERGQTRITGTTTYFDYW	IGH	4	None	
3	333	CQQYNSYSLTWTF	IGK	147	C3	7K8R
3	334	CARDLLRYFEWLGAFDIW	IGH	4	None	
3	335	CVRDRLRFDSW	IGH	3	None	
3	336	CTTDRRPYHYDTSGYFSAW	IGH	6	None	
3	337	CAKADRPSITMIVVVTSDYW	IGH	12	None	

3	338	CAKEVITMVRGGPDYW	IGH	6	None	
3	339	CAGDILTGYYSYGMDVW	IGH	2	None	
3	340	CAVFDFEIEAGGRKWFDPW	IGH	2	None	
3	341	CAKMSRQAASTYYFDSW	IGH	3	None	
3	342	CAKVDIVATITDYW	IGH	6	None	
3	343	CARERRELLRSGFDIW	IGH	4	None	
3	344	CAKDILGIVGATTLFDYW	IGH	15	None	
3	345	CAKDITIWDGMDVW	IGH	2	None	
3	346	CAKDGSGWYSGAFDIW	IGH	6	None	
3	347	CARHEASARLFNPFYFDSW	IGH	4	None	
3	349	CARGSVGASGRIDYW	IGH	5	None	
3	350	CAKDSSNYDILTGYSSFDYW	IGH	18	None	
3	351	CARERVTVTTLGWFDIW	IGH	12	None	
3	352	CATLGYCSGGSCPGSDGAHNFDYW	IGH	9	None	
3	353	CAGYCTGGVCPGGSFDFW	IGH	2	None	
3	354	CARDRRIQLWLGPGWFDPW	IGH	2	None	
3	355	CARYSSDDARGYSYGYESYW	IGH	7	None	
3	356	CQSADIDGTRYIF	IGL	19	None	
3	358	CARGGTESFYGDLGFFDYW	IGH	9	None	
3	359	CASTFSYYYYGVDVW	IGH	4	None	
3	360	CAKDTRYYDILTGYYNWDYYYGMDVW	IGH	4	None	
3	361	CAKSFDYGSESNYVTFDQW	IGH	2	None	
3	362	CAKNLRNQMPYYEYW	IGH	6	None	
3	363	CARVGSYGYPYYYGLDVW	IGH	48	None	
3	364	CARDDGSFYDTFNGPFQWLDPW	IGH	3	None	
3	365	CTRDCLTHSCARFDYW	IGH	2	None	
3	367	CMQGTHWPPLLAF	IGK	12	None	
3	368	CALRRVPVGGTGAFPEFDPW	IGH	6	None	
3	370	CVADMITFGAGGYYFDYW	IGH	4	None	
3	371	CAKGLYDSSGYSLEAFDIW	IGH	2	None	
3	372	CATYARPGGSNWFDSW	IGH	2	None	
3	373	CAKGAGPVGAMDGIDYW	IGH	2	None	
3	374	CARSYYSNYFHYW	IGH	2	None	
3	375	CKIGAFENGAHSDYW	IGH	2	None	
3	377	CARDPSGHVTGWSPTYFDYW	IGH	2	None	
3	378	CARDRRGYCSGGSCYSGFIDYW	IGH	2	None	
3	379	CAKDSQYYYDSSGYYGADYW	IGH	10	None	
3	380	CARGSRNYRDSLDIW	IGH	2	None	
3	381	CARDDFGVHDSFDIW	IGH	2	None	
3	382	CALPILNSLRVEDTGLYFCVKDFRPLMSPTFDHW	IGH	2	None	
3	383	CSGGSCHSYYYGMDVW	IGH	6	None	
3	384	CTTDKIRGGGILLPYYYFDYW	IGH	2	None	
3	385	CARQWWLHMYYFDYW	IGH	2	None	
3	386	CGTWDSSLSAWVF	IGL	216	None	
3	387	CARDSYNGFDVW	IGH	15	None	
3	388	CARLGYSAGWYTPIGDYW	IGH	7	None	
3	389	CAKLPVASGWNDAFDIW	IGH	2	None	
3	390	CARDDSARGYWFFDFW	IGH	9	None	
3	391	CAKTQQEAVAGPPFDYW	IGH	15	None	
3	392	CAKDRVPAAGPLDYW	IGH	39	None	
3	393	CARDGAYYLGSSGFYYYFDYW	IGH	4	None	
3	395	CAREYDSSGPWFDYW	IGH	10	None	
3	396	CGRRLSRPASFDYW	IGH	5	None	
3	398	CARVRFEDYYYYGMDVW	IGH	3	None	
3	400	CAKDSQHLYYDSSAGWFDPW	IGH	4	None	
3	402	CARQKYSSSWNLGEDWFDPW	IGH	2	None	
3	403	CHQRGNWPF	IGK	134	None	
3	404	CLRAEDTALYYCAKDSYYDSWDWFDPW	IGH	2	None	
3	405	CARGYYPGDGYNLFDYW	IGH	4	None	
3	406	CAKDSGDYYGSGHHGPLDYW	IGH	4	None	
3	407	CTTGNSFSDYGSSYYDFW	IGH	54	None	
3	408	CASGSSDNWFDPW	IGH	97	None	
3	409	CAKFGSSYYDSSGPDYW	IGH	5	None	
3	410	CARRLVGANHAFDLW	IGH	2	None	
3	411	CARDDLDNRGYPNWFDPW	IGH	2	None	
3	412	CATDFYDGTDYADYW	IGH	5	None	
3	414	CAHLPFHLGNLSPFDYW	IGH	3	None	
3	415	CAKVAYYDSNFDYW	IGH	4	None	

3	416	CVKDMGAPGDYSGYFDYW	IGH	2	None	
3	417	CAREFMTLVGATPEFDYW	IGH	4	None	
3	418	CAKVDGGYGGFDYW	IGH	16	None	
3	419	CAKVDSYCSGGSCYSEGFDYW	IGH	4	None	
3	420	CARDNGGVFLTGYSPTYYYYYGMDVW	IGH	2	None	
3	421	CARDGIVYGDYQDSFFDLW	IGH	8	None	
3	422	CARDGDSYHRYTWNYVDYFAYW	IGH	3	None	
3	423	CASCRICSSTSCYGLDNWFDPW	IGH	2	None	
3	424	CAKGYGSGSYAIYYYGMDVW	IGH	6	None	
3	425	CARDGEVRGIISSFDYW	IGH	2	None	
3	426	CAKVGGILTGSDAFDIW	IGH	4	None	
3	427	CQEYGSSSFVF	IGK	6	C1	7K8N
3	429	CARHGSGILWFGELPFCDYW	IGH	4	None	
3	430	CARLQDETRSRIMEGDYW	IGH	2	None	
3	431	CARDRHGSAYSNNWFDPW	IGH	4	None	
3	432	CHVWVSSTAWGVF	IGL	4	None	
3	433	CAKADDYAQQRGAFDIW	IGH	7	None	
3	434	CARRSYDVLSGSYYFDYW	IGH	4	None	
3	435	CARPDYYGSGRRTFDIW	IGH	2	None	
3	436	CAKDRKVVIQLELDYW	IGH	2	None	
3	437	CGRGQSGYTYIDHW	IGH	2	None	
3	438	CARLGRGTTHYYYYGMDVW	IGH	3	None	
3	439	CAREFYSEKTGWYFDLW	IGH	6	None	
3	440	CAKEEGPPAVAGGFDYW	IGH	4	None	
3	441	CARRPPHNGDYRRGAFDIW	IGH	4	None	
3	442	CTTSRGQWFGESWIFDYW	IGH	6	None	
3	443	CARVEGGDYHFDYW	IGH	2	None	
3	444	CATLTGYSSSWYYYYYGMDVW	IGH	4	None	
3	445	CTTDPVWYNWNDDDYW	IGH	4	None	
3	446	CASGCTMTSCHTYFDPW	IGH	2	None	
3	447	CARLTAEGSSPSWGWFDPW	IGH	4	None	
3	448	CTRSRQYTGGWYNYLDYW	IGH	7	None	
3	449	CARQRGFITMVRGNYFDYW	IGH	2	None	
3	450	CAHSPGPESSSWKRAFDIW	IGH	2	None	
3	451	CAKDHDSNGYYCYFDYW	IGH	2	None	
3	452	CAKEMFPSLSGVDYW	IGH	3	None	
3	453	CAFVHNSTTWYGGFDSW	IGH	6	None	
3	454	CAKGSSVVTAITDYW	IGH	11	None	
3	455	CNSRDSGGHHLVIF	IGL	6	None	
3	456	CARHDYGSVRGVIISWFDPW	IGH	11	None	
3	457	CAKDIVPAITYFDYW	IGH	24	None	
3	458	CQHYSSYPLSF	IGK	196	C3	7K8R
3	459	CARSPGIAARGTNYFDYW	IGH	2	None	
3	460	CARALYDSSGYYWGYW	IGH	3	None	
3	461	CAKDIGIAVAGDYYYYGMDVW	IGH	5	None	
3	462	CAKDQIAAAGTPPYYYGMDVW	IGH	2	None	
3	463	CAKDLPVVPAAPFDYW	IGH	2	None	
3	464	CARSRSGSGTHFDFW	IGH	2	None	
3	465	CAGGRPSYYDMLTGHNWFDPW	IGH	9	None	
3	466	CLQYYFTPQSF	IGK	9	None	
3	467	CARDFGDNDSSGYYFPHWFDPW	IGH	2	None	
3	468	CATYSSTYYYAFDVW	IGH	2	None	
3	469	CAKYARESYYYYYAMDVW	IGH	66	None	
3	470	CARVEDIYCSGGTCYPYNWFDPW	IGH	2	None	
3	471	CAKEMDGAGTVFDYW	IGH	4	None	
3	472	CARYSSPGRYYYGMDVW	IGH	3	None	
3	473	CAKDAFGSGSPSWFDPW	IGH	4	None	
3	474	CSSRDSSGDLVLF	IGL	7	None	
3	475	CAGPHDIHALLMHWGVGIYYYGMDVW	IGH	2	None	
3	476	CARSVPYYYDSSPHDWYFDLW	IGH	2	None	
3	477	CARDAGLIAAALDYW	IGH	14	None	
3	479	CARGGDSSGYLYNWFDPW	IGH	4	None	
3	480	CARASPGTYCRSSNCRNWFDSW	IGH	2	None	
3	482	CARVTSGYSSGLSYNWFDPW	IGH	4	None	
3	483	CAKAGILPGRFDYW	IGH	9	None	
3	484	CARVGNSFYYYFDEW	IGH	2	None	
3	485	CAKGILGIAAAGTGFFDYW	IGH	4	None	
3	487	CARYSYDSRGYYFFDSW	IGH	9	None	

3	488	CTREPSATDYYFDYW	IGH	4	None	
3	489	CATVDTAMDTYYYYGMDVW	IGH	7	None	
3	490	CASHRILYYDSSVFDYW	IGH	2	None	
3	491	CAKGVYDSSGYYSSAFGYW	IGH	2	None	
3	492	CARGGCSGGSCYWGGTWLDPW	IGH	2	None	
3	493	CTTAPRDYVWGSYRQWDYW	IGH	9	None	
3	494	CAREPGQWRGGWFDPW	IGH	2	None	
3	495	CAKLPFGELLNWFDPW	IGH	7	None	
3	496	CTRGTPRLNFYDTSGENDAFDIW	IGH	3	None	
3	497	CARDIVHYDSSGYYNYYYGMDVW	IGH	2	None	
3	498	CAKGKFESEGYCSSSSCSFFFDYW	IGH	6	None	
3	499	CARLYYYDSSGYSPPSAFDIW	IGH	4	None	
3	500	CVKGSSRLYDSSGYYFENW	IGH	3	None	
3	501	CARDPLLVPGAFDIW	IGH	9	None	
3	502	CAKDIWPLMGIAAAFDYW	IGH	6	None	
3	503	CARDPNCSSARCYGPKSRIYYYYGFDVW	IGH	4	None	
3	504	CARSSGGASFHYW	IGH	4	None	
3	505	CAKASYPILNWGGAFDIW	IGH	3	None	
3	507	CARDASRGSYYDSTGYSDYW	IGH	3	None	
3	508	CQAWDNSFAFF	IGL	6	None	
3	510	CQQHDNEPRNSF	IGK	2	C3	6XDG
3	511	CARQNEDGSVSYYNDYYYYYGMDVW	IGH	4	None	
3	512	CAKLTSIAASDDSSDYW	IGH	10	None	
3	513	CAKSPHSYGSSLYDYW	IGH	2	None	
3	514	CAKGRDPSTTSLFDYW	IGH	3	None	
3	515	CARDRGGRGTTDGMDVW	IGH	3	None	
3	516	CATASLFYDRSDYYFDYW	IGH	3	None	
3	517	CAKDSLQRWLQFENPNWFDPW	IGH	4	None	
3	518	CARDAGIVGAHNWFDPW	IGH	2	None	
3	519	CAREVSTTAQSGFDIW	IGH	2	None	
3	520	CAKDLGYSSSRGPFDYW	IGH	3	None	
3	521	CAKGQTGSTGSASDYW	IGH	2	None	
3	522	CAKMGEYYYDSSGYSDYW	IGH	4	None	
3	524	CATDRDSCNGGRNCYSAFEYW	IGH	2	None	
3	525	CFGGNPDAFDIW	IGH	2	None	
3	526	CARAQYGSGFLGWVDPW	IGH	4	None	
3	527	CAREWCSSSSCDSNWFDPW	IGH	4	None	
3	528	CVRHELTGDFDFW	IGH	2	None	
3	530	CAKASPADPYFDYW	IGH	19	None	
3	531	CAKSTHTYYDILTGGFDYW	IGH	6	None	
3	532	CAKGSYYDYVWGSYRQNWFDPW	IGH	2	None	
3	533	CGRVDDSRAYRIDYW	IGH	4	None	
3	534	CAKDTESDSTPLGAFNIW	IGH	2	None	
3	535	CARNLLRYFDWLFRGNWFDPW	IGH	2	None	
3	536	CAREGYRGWSTRSNGAFDMW	IGH	10	None	
3	537	CAKVLRYFDWPYAFDIW	IGH	2	None	
3	538	CAKDDFAFGEPAGYFVDW	IGH	5	None	
3	539	CARRSSSWYGTPGDWFDPW	IGH	8	None	
3	540	CARASRYFDWWAFDYW	IGH	5	None	
3	542	CARESGYYYDSSGYQYYFDYW	IGH	5	None	
3	543	CAKDAGYCRNSRCEGIDYW	IGH	3	None	
3	544	CVKDVVLRYYDKLTYFGSW	IGH	2	None	
3	545	CAKLLRASSGWHGDYFDYW	IGH	3	None	
3	546	CAKDSPSYSSSWYAWFDPW	IGH	4	None	
3	547	CARTTMSPTSYDFWSGYPNWFDPW	IGH	2	None	
3	548	CTKGRHKYDYLTRYYEYW	IGH	2	None	
3	549	CANMYMIRGVSPFDYW	IGH	4	None	
3	550	CAKDLDIVVVVAATRYYYYGMDVW	IGH	8	None	
3	551	CQQVNNNPVF	IGK	4	None	
3	554	CARTDHPEENYYESSGYYYYFDYW	IGH	10	None	
3	555	CARDGESSGQQLAYLKFDYW	IGH	7	None	
3	556	CARGGSSSWYDPYYYYGMDVW	IGH	2	None	
3	557	CARLWSGYSYGSYDAFDIW	IGH	4	None	
3	558	CARSSTDSPGAFDVW	IGH	2	None	
3	561	CAKVGYSYGDDFYDYW	IGH	4	None	
3	562	CVKDMAPMHIAGKGFDIW	IGH	2	None	
3	563	CAREPYIGGPYFDYW	IGH	8	None	
3	564	CARGWTTLLTDRRFDAFDIW	IGH	2	None	

3	565	CARDTTDKNYYDSSGEDEGFDPW	IGH	5	None	
3	566	CARDGDSYGYLPLDYW	IGH	2	None	
3	567	CARGHTPYRGVTLFDSW	IGH	2	None	
3	568	CAKEIDYYDSSGYRNWFDPW	IGH	4	None	
3	569	CAKATAVTTLFDCW	IGH	3	None	
3	570	CARDLLLGYCSSTSCSRGWFDPW	IGH	2	None	
3	571	CARDVHDYGSWDAFDIW	IGH	2	None	
3	572	CVKDLDDDDSSEGGFDHW	IGH	2	None	
3	573	CARGDSRGFYFESW	IGH	3	None	
3	574	CARRSYGDSNLDYW	IGH	15	None	
3	575	CARPVTTVTYWYFDLW	IGH	3	None	
3	576	CARDNYTNDHSSGWFGLFAVQPISAIDYW	IGH	2	None	
3	577	CATLESDYYDSSGPNWFDPW	IGH	2	None	
3	578	CARTINYYGSGSPDYW	IGH	4	None	
3	579	CQQFGTSPPMYSF	IGK	20	C2	7K8U
3	581	CAKDQTLDAFDIW	IGH	41	None	
3	582	CAKATGDYNTEFDYW	IGH	4	None	
3	583	CARSAHQWLIQRGGYFDLW	IGH	4	None	
3	585	CARFYFVVVSREYYFDYW	IGH	2	None	
3	586	CAREWKNSYGVTFFDYW	IGH	2	None	
3	587	CARDESSGYGYYW	IGH	6	None	
3	589	CAKGFTYYYDSAGFRYLDYW	IGH	2	None	
3	590	CARGPDFDRSGYYYVGYW	IGH	2	None	
3	591	CQQYDPLFPFNF	IGK	9	C3	6XDG
3	592	CAKDISRSGSFNWFDPW	IGH	6	None	
3	593	CVVSGGAIWYWYYGMDVW	IGH	2	None	
3	594	CARGGTHIVGAIPSFDYW	IGH	2	None	
3	595	CARGLGHCSGGTCYVHPFDIW	IGH	3	None	
3	597	CARSWNDYGGNGLDYW	IGH	2	None	
3	598	CAKDASPVATGPDYW	IGH	2	None	
3	599	CAKVPYGSGVAGDYW	IGH	2	None	
3	600	CTTVYSSSWYWYFDLW	IGH	3	None	
3	601	CANLGSGPGDYW	IGH	2	None	
3	602	CARLRTQLDTAMKPYYYYYGMDVW	IGH	2	None	
3	603	CARVPPQNPWGHLYYYYGLDVW	IGH	2	None	
3	604	CAKASFDYGDLGDYW	IGH	2	None	
3	605	CARTFSQNYYGSGSYLDFW	IGH	2	None	
3	606	CARDAQASADYW	IGH	11	None	
3	607	CQSYDSSLGGLHVIF	IGL	2	C4	7JVC
3	608	CARMICDDYRCPPFDSW	IGH	3	None	
3	609	CALPICLRAEDTALYYCANLVMATRGGDYW	IGH	2	None	
3	612	CARNKGSTGIRDPMNRWFDPW	IGH	4	None	
3	613	CAKDLSSWEPHSLFDYW	IGH	2	None	
3	614	CAHIDLLRGVLKGFYFDYW	IGH	4	None	
3	615	CAAWASGSYSPWFDPW	IGH	2	None	
3	616	CARYNWNDVSSPRFDYW	IGH	2	None	
3	617	CVRVDHCDRGICYYYGMDVW	IGH	2	None	
3	619	CAKDLVATGYSYGYLFDYW	IGH	2	None	
3	620	CARGTYYDFWSGFPYYFDYW	IGH	12	None	
3	621	CARLPRYYDSSGWYFDYW	IGH	2	None	
3	622	CQQYNDFSGGYSGAF	IGK	2	C3	7K8R
3	623	CATDIPQDYYYGMDVW	IGH	2	None	
3	624	CARASYPSPGLWCSSTSCPHWFDPW	IGH	2	None	
3	625	CSAWDSGLSVWVF	IGL	109	None	
3	626	CARQYSSSQGYYYLDVW	IGH	6	None	
3	627	CQTYDRSLSGGSWVF	IGL	2	None	
3	628	CQQSYSIGQLSTF	IGK	2	C4	6ZDH
3	629	CARASYYSSGWYAFDYW	IGH	4	None	
3	630	CARDVLPGTAARGDAFDLW	IGH	3	None	
3	631	CAKDSKYSNSFGWFDPW	IGH	2	None	
3	632	CARDKVGGWLGLDYW	IGH	7	None	
3	633	CARGSRLLTGYLGTYFDYW	IGH	2	None	
3	634	CVSTFTIIVVDEAFDIW	IGH	5	None	
3	635	CAKDTDYDMLYWFDPW	IGH	7	None	
3	636	CAKIGYNGWEFDFW	IGH	4	None	
3	638	CAKLGYDSSGYYSRDYW	IGH	8	None	
3	639	CARMRIRGSGVYRDDAFDFW	IGH	2	None	
3	640	CARNKNYYHTSGYFDSW	IGH	2	None	

3	643	CAALSYGDSAPDYW	IGH	6	None	
3	644	CAKFSSWYSTGDYW	IGH	9	None	
3	645	CAKLAQLAVDYW	IGH	21	None	
3	646	CARAFGDTYYYYYYGMDVW	IGH	5	None	
3	647	CAKEQWLVPNWFDPW	IGH	2	None	
3	648	CARDLAVHKQQLGGYWFDPW	IGH	2	None	
3	650	CAKDDAAGSYGGWIDSW	IGH	2	None	
3	651	CAKDMAPVAGTIAFDYW	IGH	4	None	
3	652	CAKDLKTEMKIFGMLISNHNAFDVW	IGH	10	None	
3	654	CVKSRPQYNWKTAAFDIW	IGH	6	None	
3	655	CAKGSSDYGGNSGAFDVW	IGH	4	None	
3	656	CASSVAPDSSGYYYYLFDYW	IGH	2	None	
3	657	CAKAAGYCSSTSCYSWDYW	IGH	2	None	
3	658	CARGTLITMIADYW	IGH	4	None	
3	659	CARVERDRSRPIYYYYGMDVW	IGH	2	None	
3	660	CMLALETPPLYTF	IGK	8	None	
3	661	CATHRDGMDVW	IGH	3	None	
3	662	CATAPGIAVTGGWFDPW	IGH	5	None	
3	664	CARGGTYNRYW	IGH	4	None	
3	665	CAKGGAVAGTWFDPW	IGH	4	None	
3	666	CARSYDFWSGFYQNYYYGMDVW	IGH	2	None	
3	667	CNSRDRSGNSYVF	IGL	6	None	
3	668	CARERSWGSFSADYW	IGH	5	None	
3	669	CARVDLRCRGFDYW	IGH	2	None	
3	670	CARQGYGDLTFDHW	IGH	3	None	
3	671	CATFEEDGGRWYAFDYW	IGH	4	None	
3	672	CAKDRVWFSYYDAFDIW	IGH	3	None	
3	673	CAGGILWFDIDYW	IGH	2	None	
3	674	CATEFTVTASPFDYW	IGH	2	None	
3	675	CARVEKYYYGSGSYDYW	IGH	2	None	
3	676	CAKDLVGGLMIAFDSW	IGH	4	None	
3	677	CALPICLRSEDTAIYYCAREIRAPRGSFDPW	IGH	3	None	
3	679	CAKSASVGLRLGEYPFDYW	IGH	2	None	
3	680	CSSVATIGVEDYW	IGH	4	None	
3	681	CARPAATWGLGNQYSYYGMDVW	IGH	2	None	
3	682	CASVESAGDYW	IGH	10	None	
3	683	CARDAPLYGDCRDYFEYW	IGH	2	None	
3	684	CAKSPYLDVLTGTTFFDYW	IGH	13	None	
3	686	CVKKSFSDWYFDLW	IGH	4	None	
3	687	CAREPAGSDFDYYFYGMDVW	IGH	2	None	
3	688	CARGLTGRGHYYYYGLDVW	IGH	2	None	
3	689	CARRQRFCSGGSRTCWFDPW	IGH	2	None	
3	690	CAKAGTDGYDSSGYYYRTYFDYW	IGH	11	None	
3	691	CARVSRYGGYGALLSWLDYW	IGH	4	None	
3	692	CARDRAPYCSNGVCYVDAMDVW	IGH	2	None	
3	693	CARERDDYDSSGYYSLTSASWFDPW	IGH	6	None	
3	694	CAKDQLGYCSSTSCYASYNWFDPW	IGH	2	None	
3	695	CASGFGVVTPFDYW	IGH	2	None	
3	696	CARDPLRFLEWLPKLQNWFDPW	IGH	2	None	
3	697	CALGKGYSNMDFDYW	IGH	7	None	
3	698	CARGPNDILTGYLEYYFDSW	IGH	2	None	
3	699	CTKEAQDISMSGLFDNW	IGH	3	None	
3	701	CQSADNSGVPNWFF	IGL	4	None	
3	702	CQYYHNWPPSITF	IGK	4	None	
3	703	CAKVMAEVTTVTTTWYFDLW	IGH	2	None	
3	705	CARPISITLQYYFYFDYW	IGH	2	None	
3	706	CARGFYGDYYDDW	IGH	6	None	
3	707	CTKIRRARNYYDYAMDVW	IGH	6	None	
3	708	CARDGGRKTYYYGSGIDYW	IGH	2	None	
3	709	CARVFGWRGDYW	IGH	26	None	
3	710	CAKDMTLPSSSWYFDYW	IGH	3	None	
3	711	CLRAEDTAVYFCVRDGYGVVAAARSGFDVW	IGH	4	None	
3	712	CARDLETMVRGYMDVW	IGH	4	None	
3	713	CTRDPADFVPRHYYYYGMDVW	IGH	2	None	
3	714	CASYWNYNFDYW	IGH	8	None	
3	715	CARSRQSDEYPDFDYW	IGH	4	None	
3	716	CARLVPYGSSGYFNWFDPW	IGH	3	None	
3	718	CAKLVDSYGPSDYW	IGH	11	None	

3	719	CAKDSILGDSSGYLLYW	IGH	4	None	
3	720	CASPLDGDFLYW	IGH	4	None	
3	721	CARGGVNYYDPGRNGAFDIW	IGH	4	None	
3	722	CVKDAWRYYDILTGYNRDYYYGMDVW	IGH	7	None	
3	724	CAKLGDGYNYFDYW	IGH	62	None	
3	725	CAKDPTYYDILTGYSQTPDAFDIW	IGH	4	None	
3	726	CAKDHPYYDSSGYYLRGNAFDIW	IGH	5	None	
3	727	CMKGTHGPFSF	IGK	6	None	
3	728	CVLYMGSGIWVF	IGL	48	None	
3	729	CARGDYQARAVDNWFDPW	IGH	2	None	
3	730	CARDLRDLLYSSGWEYYFDYW	IGH	8	None	
3	731	CQQGNTFPAFTF	IGK	19	C3	7K8R
3	732	CARVFVSVRVRHLDYW	IGH	2	None	
3	734	CAKDPSSGMGYVGSDYW	IGH	6	None	
3	735	CARRDVVVDAMDVW	IGH	2	None	
3	736	CQQTYGLPRSF	IGK	10	C2	7K8O
3	737	CAKESYSSSGYYYGMDVW	IGH	2	None	
3	738	CAKGAYYDYVWGSYRARYPDYFDYW	IGH	2	None	
3	739	CGADHGSGSDFVRVF	IGL	25	None	
3	740	CARGSRERRHFDRGGYRDAFDIW	IGH	2	None	
3	741	CARARSITMIVVGDSGGFDPW	IGH	7	None	
3	742	CVKETFSMVRQGGSFDKW	IGH	39	None	
3	743	CARRPYYDSSGYYPYYFDYW	IGH	4	None	
3	745	CASMVGEGEDDYW	IGH	4	None	
3	746	CVKDRWEVLSLDYW	IGH	3	None	
3	747	CARSLKNYGGNPYFYFYGMDVW	IGH	5	None	
3	748	CQKYDNAPWTF	IGK	734	C3	6XDG
3	749	CAKILGYCSGGSCYSGDYW	IGH	11	None	
3	750	CMQALQRWAF	IGK	9	None	
3	751	CARDRTPYRNTAAPDYFDYW	IGH	2	None	
3	752	CALPILRVEDTAVYYCARGSRLLTGYLGTYFDYW	IGH	2	None	
3	753	CAKSIVVVIPYYFDYW	IGH	12	None	
3	754	CAKDSGTYGSSFDYW	IGH	9	None	
3	755	CARAGYGIGGDPFDYW	IGH	6	None	
3	756	CAHSITYNWNDLAYSFDPW	IGH	2	None	
3	757	CARQFSAGRHWFDPW	IGH	6	None	
3	760	CAKLGSSGWYYYGSGSTDYW	IGH	2	None	
3	761	CVRDQGTGWPFNWFGPW	IGH	2	None	
3	762	CARELTSKFELDYW	IGH	6	None	
3	763	CAAMDYYDWLPDDAFDIW	IGH	4	None	
3	764	CAKVQNDILTGYYSYYYYAMDVW	IGH	2	None	
3	765	CATGRPVDMATISFGFDYW	IGH	2	None	
3	766	CARLPSRDGYNPSDYW	IGH	4	None	
3	767	CARHGCRGNVCDTDDYYAMDVW	IGH	2	None	
3	768	CAKDQSPVYYGSGSYFKQW	IGH	2	None	
3	769	CAKDAKFYSDSRGYWGLFDYW	IGH	2	None	
3	770	CSSYAGSNNLGVLF	IGL	129	C2	7BWJ
3	771	CQTWGSGVVDVVF	IGL	4	None	
3	772	CTTVDGGTELRLDYYYGMDVW	IGH	2	None	
3	773	CARDSGGGTTFDIW	IGH	2	None	
3	774	CVRTGRDGYKYDYW	IGH	14	None	
3	775	CARHASRGLYYYDSSGYYFDYW	IGH	2	None	
3	776	CAREAGYCSSTSCSRRGFDYW	IGH	2	None	
3	777	CATALPGNYYDSSGYSEFDYW	IGH	3	None	
3	778	CARDKYDNSGSPLDYW	IGH	193	None	
3	779	CAKGVKNFDWYYFDYW	IGH	4	None	
3	780	CAKFGDHSSSIVPDYW	IGH	3	None	
3	781	CAREVYGSARNPGYSYARYFDLW	IGH	2	None	
3	782	CAWGRNAVFYNWFDPW	IGH	6	None	
3	783	CAKTDGTQLWLQGRHVLYNYYAMDVW	IGH	6	None	
3	784	CARDTPGFYYYFDNW	IGH	2	None	
3	785	CAKSPGRQKPYYFDYW	IGH	2	None	
3	786	CANGIPVGPPTHYYYYFYGMDVW	IGH	2	None	
3	787	CAKPRGAVAGGDYW	IGH	13	None	
3	788	CAARSPPAVVVITGWYFDLW	IGH	2	None	
3	790	CMQDAQDPPITF	IGK	3	None	
3	791	CARGYCSSTSCRPWYYYYGMDVW	IGH	4	None	
3	792	CTTGRVGGYFDTW	IGH	2	None	

3	793	CARGDWLLRDQFDYW	IGH	5	None	
3	794	CAGQLSPYYYGMDVW	IGH	75	None	
3	796	CAKIWGYDYGDHEVDAFDIW	IGH	7	None	
3	797	CAYIGGTAGTTNNYFDYW	IGH	2	None	
3	798	CAREYSGTYSQVYFDSW	IGH	2	None	
3	799	CAKLPSSHNIEGGDAFDIW	IGH	2	None	
3	800	CAKDSFIAAAANGGFDYW	IGH	3	None	
3	801	CAKEHHSSGWSYYYYGMDVW	IGH	13	None	
3	802	CAHSEYYGSGSYDGGFWFDPW	IGH	4	None	
3	803	CARQVSNWNSRRFDYW	IGH	2	None	
3	804	CAKDISSYGLFDYW	IGH	47	None	
3	805	CGTWDSSVSSAHLIF	IGL	6	None	
3	806	CYCAADSNRVF	IGL	89	None	
3	808	CVRDRGVTRSMDWFDPW	IGH	2	None	
3	811	CAKLVASEYYYDSSGRDYW	IGH	2	None	
3	813	CARGIKYDSRGRNYYFDYW	IGH	3	None	
3	814	CMQALQTPQTPPMYTF	IGK	3	None	
3	815	CAKDLVSLTTVTGYFQHW	IGH	6	None	
3	816	CQQSAYSPVSHTF	IGK	5	None	
3	817	CTSRARIVGGRISDFDTW	IGH	3	None	
3	818	CARDRCSSTSCSGGDYW	IGH	2	None	
3	819	CAREVYLYYDSSGPIDYW	IGH	8	None	
3	820	CTTEPSGWYDDYW	IGH	2	None	
3	821	CARVFGHRIIFRSTPHNWFDPW	IGH	2	None	
3	822	CAKLADYESEYFQHW	IGH	4	None	
3	823	CVKLIHLWTQDYFDYW	IGH	2	None	
3	824	CARADSYCDSKSCNYFGPFDSW	IGH	3	None	
3	825	CAKVSIAAAGPLDYW	IGH	2	None	
3	827	CAKFSYGSGSGTSIDYW	IGH	6	None	
3	828	CAKDLYHDSGGAYFDYW	IGH	6	None	
3	829	CARSLLYYSSSSGYYTGSFDYW	IGH	7	None	
3	830	CAAWDDSRNGPHVVF	IGL	8	None	
3	831	CATNYGLWSASSYFFDVW	IGH	3	None	
3	832	CASDLGDRPFDYW	IGH	16	None	
3	833	CARDRPDASLAEQPMFDYW	IGH	4	None	
3	834	CAKLEDIVVVPAVSDYYFDYW	IGH	5	None	
3	835	CARGHPGAENYGSGTYTYYYYYYGFDVW	IGH	2	None	
3	836	CATIRTDYYESEDYW	IGH	10	None	
3	838	CATTDYGDYASYW	IGH	4	None	
3	839	CQSYDSSLSVWVF	IGL	1338	C4	7JVC
3	840	CAAVPGPGVYYYYYGMDVW	IGH	2	None	
3	841	CAKDRGAYCGGDCYSWWYFDYW	IGH	5	None	
3	842	CARQRLSDITIFGVVIHNWFDPW	IGH	10	None	
3	843	CARDEVEMATPSGYYYGMDVW	IGH	2	None	
3	844	CQSYDSSLSASGSRVF	IGL	17	C4	7JVC
3	845	CARVNAEYYYDSSGYLDYW	IGH	2	None	
3	846	CQQSSIVPRIF	IGK	2	None	
3	847	CARSLSGYGYAFDLW	IGH	642	None	
3	848	CARVSDDYADYRQRPFDYW	IGH	7	None	
3	849	CASAPLVWFGVPINFDYW	IGH	2	None	
3	850	CAKHRLRLVVVVPFDYW	IGH	4	None	
3	851	CARHLRGTFLYYFDSW	IGH	2	None	
3	852	CAHRRSDHYFDYW	IGH	54	None	
3	853	CAREGIPYCSGDNCHSSHFDYW	IGH	2	None	
3	854	CTTELLWFGDPLGFDYW	IGH	2	None	
3	855	CAHDDDGRHYYYVSWFDHW	IGH	2	None	
3	856	CSRTLSMTRGYGVFNAFDVW	IGH	2	None	
3	857	CARQTQRRGIAVARDVFDLW	IGH	2	None	
3	858	CTTGLFYDWAFDIW	IGH	2	None	
3	859	CAREGWELLAYFDYW	IGH	3	None	
3	860	CAKLRYFDWFTDGAAFDIW	IGH	2	None	
3	861	CAKEINRGWPDAFDIW	IGH	2	None	
3	863	CARSPGYCVGGICTYYYGMDVW	IGH	2	None	
3	864	CARGDILTGYYPFDYW	IGH	11	None	
3	865	CARENGDPSSWFDPW	IGH	7	None	
3	866	CAKGHILTTPDWFDPW	IGH	4	None	
3	867	CAKDAAAGNYGDYVWDYW	IGH	12	None	
3	868	CAKEARWFGGYYFDSW	IGH	2	None	

3	869	CVGDPPQSGYAFDCW	IGH	6	None	
3	870	CAKEKGYNYGYYYYGMDVW	IGH	2	None	
3	872	CARMMYDYDLSGLDYW	IGH	5	None	
3	873	CALPISKPVGATDFDYW	IGH	2	None	
3	874	CIGYGSGSYRDYW	IGH	15	None	
3	875	CAGSPRYGDHFESFDYW	IGH	2	None	
3	876	CARSVKWYFDVW	IGH	2	None	
3	877	CARSRRTDGYNFAYW	IGH	4	None	
3	879	CAKDIPYYDSSGYPFAGAFDIW	IGH	3	None	
3	880	CARGSTSYGDFKYW	IGH	3	None	
3	881	CAKDSGDGYNFYYFDYW	IGH	18	None	
3	882	CTSPRNPDAYYYYGMDVW	IGH	4	None	
3	883	CARHPKDYDFWSGYSYNWFDPW	IGH	4	None	
3	884	CARDPSSKWPYSYYYYGMDVW	IGH	2	None	
3	885	CARDRWERIARGDGWFDPW	IGH	2	None	
3	886	CAREHPDYYDSSGCNDYW	IGH	7	None	
3	888	CAKLSSYDSSGYYYRGWFDPW	IGH	5	None	
3	889	CQSYDFSVSGPSYVF	IGL	2	None	
3	890	CTTDRLYDYVWGSYRYPDYW	IGH	10	None	
3	891	CARDQLLLEGGFDYW	IGH	6	None	
3	892	CAKDGFYGSGTYYNVYYDYW	IGH	2	None	
3	893	CAATSSSWYNWYFDLW	IGH	4	None	
3	894	CARRSSSSWSYFDFW	IGH	10	None	
3	895	CARQQRYYFDAFNMW	IGH	3	None	
3	896	CARDSKMVVDKYYYGMDVW	IGH	2	None	
3	897	CAKGTSDISTGYYHFYYGMDVW	IGH	3	None	
3	898	CARSWYSSGWYLRKFDFW	IGH	6	None	
3	899	CARIRVDTPSDYLDYW	IGH	2	None	
3	901	CARYRYGDYKSNFDYW	IGH	3	None	
3	902	CAKGGVLLWFEHFDYW	IGH	5	None	
3	903	CAQLPGGIVGATRDYW	IGH	6	None	
3	904	CARGGGYRGPFDLW	IGH	11	None	
3	905	CARSKYDSSTSAGDYW	IGH	2	None	
3	907	CVHSTYYYESPWAIDIW	IGH	2	None	
3	908	CATDIVGDHSYGFDYW	IGH	2	None	
3	910	CARDGGWYLQHDNEFDYW	IGH	2	None	
3	911	CARDAASTTDWYFDLW	IGH	2	None	
3	912	CAKVWHSGWHFDYW	IGH	2	None	
3	913	CQQYNSCSPRGMYTF	IGK	2	C3	7K8R
3	914	CARGGLIDYFPNNWRDPFDVW	IGH	2	None	
3	915	CAREDPLLDGMDVW	IGH	7	None	
3	917	CAKDRYMRGGSYGHDAFDIW	IGH	2	None	
3	918	CAKESAKYSGYHWSDYW	IGH	2	None	
3	919	CARTRGVASLFWYFDLW	IGH	2	None	
3	920	CAKASEEGQTDYW	IGH	2	None	
3	921	CARRSAGNMGRFFDPW	IGH	2	None	
3	922	CARDLVDTGFDYW	IGH	47	None	
3	923	CARGDSRYPRSNFDIW	IGH	4	None	
3	924	CASPGIGFDIW	IGH	8	None	
3	925	CARDSSIWHWYFDLW	IGH	6	None	
3	926	CQSADNGGGFWVF	IGL	4	None	
3	927	CAKWEAGKGVFDMW	IGH	4	None	
3	928	CARSIGISGWSPFDSW	IGH	4	None	
3	929	CAKGRVPVADLVPIYYYYYGLDVW	IGH	2	None	
3	930	CARTHDLSGNTWFDPW	IGH	4	None	
3	931	CTRSGKFFDYFDPW	IGH	2	None	
3	932	CAKDPSEFRWLRRDYYYGMDVW	IGH	3	None	
3	933	CARVAAVAGNGAFDIW	IGH	4	None	
3	934	CTRVVHYGSGSYQILGELYFDYW	IGH	4	None	
3	935	CAKDISSYGYSMTNDYW	IGH	6	None	
3	936	CAKDGGLRFLEWSVGPYYYYGMDVW	IGH	11	None	
3	937	CVKDRSATYDERYYFDYW	IGH	4	None	
3	938	CAKIGAGYQPLFDYW	IGH	2	None	
3	939	CARQSQVGAEASDYFDYW	IGH	2	None	
3	940	CQQYYSIQGYTF	IGK	12	None	
3	941	CARALYYFETSGVMFDFW	IGH	2	None	
3	942	CARSPGYSTNWYLPNFDSW	IGH	3	None	
3	944	CATSPYFNRPEGAFDPW	IGH	2	None	

3	945	CASHRYSYWGLFDYW	IGH	2	None	
3	946	CARLDGDVVAARRGWFDPW	IGH	33	None	
3	947	CYSAESSGNYWVF	IGL	756	None	
3	948	CAHSHVLRYFDWLLHTDQHSFFDYW	IGH	2	None	
3	949	CARDCGGNCYSFQYW	IGH	2	None	
3	950	CTSTNGGYYDFWSGPQRNFDYW	IGH	2	None	
3	951	CVRQDADTAFVPLVTFDSW	IGH	19	None	
3	952	CARGQIKWLQYYSDTW	IGH	2	None	
3	953	CYSYTSSSTPYVFGPYVF	IGL	4	C2	7K90
3	954	CARGRTVTTFYYYYGMDVW	IGH	13	None	
3	955	CARLGGTGTTLFDYW	IGH	15	None	
3	956	CARFLYSGFHKYYFDYW	IGH	7	None	
3	957	CLQHNSYPALTWTF	IGK	17	C3	7K8R
3	958	CARAVWGSGSYYKDYYYYGMDVW	IGH	4	None	
3	959	CAKLGAEAAWSDYW	IGH	3	None	
3	960	CAKLGTVTTSHGLYYFDYW	IGH	4	None	
3	961	CAREVSPTDFDIGYFDYW	IGH	2	None	
3	963	CARLAPPMVRGVIASPIYYFDYW	IGH	10	None	
3	964	CAKASSTDTSYFYYYDMDVW	IGH	8	None	
3	965	CQQYQTWPPLF	IGK	12	C1	7BZ5
3	966	CARDSGEHDFWTFDSW	IGH	2	None	
3	967	CQVWDTSSDPPVIF	IGL	24	None	
3	968	CARDPYSTNDFLTGYFPDYW	IGH	3	None	
3	969	CARVVVVPAEAGSFDYW	IGH	2	None	
3	970	CARGYSSSPYRTDFFDYW	IGH	2	None	
3	972	CAKDRATIFGVVNPDYW	IGH	4	None	
3	973	CAKGANWNRCDYW	IGH	19	None	
3	975	CTRPVHYYYDSSNSPDYW	IGH	2	None	
3	977	CAKLGGYSYGSGPFDYW	IGH	8	None	
3	978	CVRPVGKTDFLDYW	IGH	40	None	
3	979	CAKGAVDLFDYW	IGH	78	None	
3	980	CARPAGFCSTTSCLDWFDPW	IGH	2	None	
3	981	CARDKEEEGFGSGNYFEFW	IGH	5	None	
3	982	CARGTVYNSLTCPAGWFDPW	IGH	2	None	
3	983	CQAWDTTTASGVF	IGL	9	None	
3	984	CAKQAAAGTYYYYGMDVW	IGH	11	None	
3	985	CAHKLDYRHYFDYW	IGH	3	None	
3	986	CARRTRADDADYMAKRYFDWW	IGH	4	None	
3	987	CARINGFCTGGRCYALWFDTW	IGH	2	None	
3	988	CAKVILRAYSYDHRGFDYW	IGH	2	None	
3	989	CHHRSNWPPRGTF	IGK	2	None	
3	990	CARDTYSSGWLGGNWFDPW	IGH	4	None	
3	991	CTKDRTTGTPADYFHYAMDVW	IGH	6	None	
3	992	CARDFATHYYESSGYSNFDYW	IGH	2	None	
3	993	CAHIKRVFDYW	IGH	6	None	
3	994	CARYGDAKSGWRSFDFW	IGH	2	None	
3	995	CARLSHFGYDKGRIDYW	IGH	8	None	
3	996	CTTEAVYCSGSRCPGYFDFW	IGH	3	None	
3	997	CARERDYGDYEGWFDPW	IGH	7	None	
3	998	CASSYYYDSSGQPDYW	IGH	6	None	
3	999	CAKLLDSRWGDAFDIW	IGH	5	None	
3	1000	CARNLYSNWIYFDYW	IGH	6	None	
3	1001	CARDQRSERWLQPFDYW	IGH	4	None	
3	1002	CASKTAMAYYFDYW	IGH	8	None	
3	1003	CAKDTIPVAAAGYLDYW	IGH	4	None	
3	1004	CAKDLYFDWLSRWGPGANYQYAVDVW	IGH	42	None	
3	1005	CAKDLRDSGGHWGYIDSW	IGH	14	None	
3	1006	CAKETPRYDILTGLDYW	IGH	2	None	
3	1007	CAREEVGAGPNNDFDVW	IGH	2	None	
3	1008	CQQGHRLPVTF	IGK	14	None	
3	1009	CAKSNEFFWFGKFKRDAFDLW	IGH	4	None	
3	1010	CGKDEGYLRWQTVAMDVW	IGH	2	None	
3	1011	CHQSDNLPWTF	IGK	196	C3	6XDG
3	1012	CARDRPGIAADDYYYYGMDVW	IGH	2	None	
3	1013	CATLSSAATYYYYYGMDVW	IGH	4	None	
3	1014	CQSSESSGAYGVF	IGL	3	None	
3	1015	CARDFSPDGSDIYYDALDIW	IGH	3	None	
3	1016	CAKDHGVDENWFDPW	IGH	2	None	

3	1017	CTHRRGYGSTARFDYW	IGH	5	None	
3	1018	CAKDLLSDDSSNFDIW	IGH	2	None	
3	1019	CGESHTIDGQVGYVF	IGL	3	None	
3	1021	CARWGSYVPTSYEMSIDYW	IGH	4	None	
3	1022	CAAFFGGARSSEADAFDIW	IGH	3	None	
3	1023	CAKVFDSGYVLYYFDYW	IGH	4	None	
3	1024	CARDDNVGGGSGYLTYYYYGMDVW	IGH	2	None	
3	1025	CATLYSVTARQYYYYYYGMDVW	IGH	4	None	
3	1026	CAKDLRQWLDPYGEFDLW	IGH	3	None	
3	1027	CARVPYYILTGSHNYFEHW	IGH	4	None	
3	1028	CARELGGSYIFDYW	IGH	37	None	
3	1029	CARQPVGSPFDCW	IGH	2	None	
3	1030	CQQFYSYQWAF	IGK	3	C3	7K8R
3	1031	CAREGGDGYNFFFDYW	IGH	5	None	
3	1032	CARNPLTVGAPYRFDLW	IGH	2	None	
3	1033	CAKDSPIVVVPAAIWGEVDDAFDIW	IGH	2	None	
3	1035	CQHFENYPHSFPF	IGK	4	None	
3	1036	CQRYGSSAYMYTF	IGK	19	C1	7K8N
3	1037	CAAELAGEIDYW	IGH	15	None	
3	1039	CARADCRSMSCYDFYYYYGMDVW	IGH	2	None	
3	1040	CAKDGELYYDSSGYFNFDPW	IGH	3	None	
3	1041	CARDMVGTTVRTVHNWFDPW	IGH	2	None	
3	1042	CARDPGSPNCSGGSCYSGWYFDLW	IGH	2	None	
3	1043	CARVVYDYYGVNFDYW	IGH	6	None	
3	1044	CAHRIYDSGGYYDYYFDYW	IGH	4	None	
3	1045	CARRDTRVKFDPW	IGH	5	None	
3	1046	CARDDPYGGNSRWFDPW	IGH	4	None	
3	1047	CARDPFTLTVLRGFDYW	IGH	2	None	
3	1048	CAKGLFYYDAQAFDIW	IGH	5	None	
3	1049	CAKDPYASYGGNSIDYW	IGH	2	None	
3	1050	CARDLEPGPSLRLGELSSYYYYGMDVW	IGH	3	None	
3	1051	CARQAFGSTWHLNWFDPW	IGH	5	None	
3	1052	CARVAVAGIPHWFDPW	IGH	2	None	
3	1054	CAKDLGPVAGLFDYW	IGH	2	None	
3	1055	CARAVVVTALGYFDPW	IGH	22	None	
3	1056	CASGYYYDSSGYGIFDYW	IGH	12	None	
3	1057	CAKGKTGTRMAPFDYW	IGH	2	None	
3	1058	CASQGYSSFWYW	IGH	4	None	
3	1060	CAKVLFRFGDYYYGMDVW	IGH	3	None	
3	1061	CAKASDFNGYSYGPDFDYW	IGH	3	None	
3	1062	CAHCAYGSGSFNYW	IGH	2	None	
3	1063	CARGWMHDFWSGYLWFDPW	IGH	4	None	
3	1064	CVRERQDYTDYYLDYW	IGH	281	None	
3	1065	CGKDLEDGYTNWGWFDPW	IGH	3	None	
3	1066	CAKDDVEYSSGWQREDAFDFW	IGH	2	None	
3	1067	CARGLLMYSSGWDDAFDIW	IGH	2	None	
3	1068	CARALAGASFDSW	IGH	3	None	
3	1070	CTKMDLHLPVYSSGRYWPDYW	IGH	2	None	
3	1072	CAKGLYDFWSGIDYW	IGH	16	None	
3	1073	CVRGKYQVLGNDVFEIW	IGH	3	None	
3	1074	CVTAGDSSGSSPYYFDNW	IGH	4	None	
3	1075	CGKDGQPMVRGHYFYYFAMDVW	IGH	2	None	
3	1076	CARLDFDWSSYLGYNRLDPW	IGH	6	None	
3	1077	CARDRRYSYGHDQYYYYGMDVW	IGH	5	None	
3	1078	CARKYDYVWGSYRLVGGSFDYW	IGH	2	None	
3	1080	CARDLAVAESSSELDYW	IGH	2	None	
3	1081	CAHSLGCGPTNCYWTFDYW	IGH	6	None	
3	1082	CARERGSGLGDHFDYW	IGH	6	None	
3	1083	CARYYYGPGSYDNGPIFDSW	IGH	4	None	
3	1084	CAKESGPVPTYVDHW	IGH	6	None	
3	1085	CARGDKVLAAPPDYYYGMDVW	IGH	2	None	
3	1086	CARPSSGYYLGYFDYW	IGH	4	None	
3	1087	CANQEGTTLFYYYYGMDVW	IGH	2	None	
3	1089	CARAPYYDILTGNTPNWFDPW	IGH	3	None	
3	1091	CAKAGQYLSWFDPW	IGH	14	None	
3	1093	CARLVAAASGWFDPW	IGH	5	None	
3	1094	CAVRLHVNKQRPNWFDSW	IGH	4	None	
3	1095	CARVTYYYASGTYYFNWFDPW	IGH	3	None	

3	1096	CASLSGWYVDYW	IGH	4	None	
3	1097	CAKVHSSGWSNWFDPW	IGH	7	None	
3	1098	CASSVLLWFGDFDYW	IGH	49	None	
3	1100	CAKARDVAGTYYFYYYDMDVW	IGH	2	None	
3	1101	CAKDGGDTTGTALFDYW	IGH	6	None	
3	1102	CAPFMASDGMLQGYFHHW	IGH	3	None	
3	1103	CARDLYGSGSYQQFDYW	IGH	2	None	
3	1104	CAREIRAPRGSFDPW	IGH	974	None	
3	1105	CSRDAAGFSTGLFRDDAFDVW	IGH	2	None	
3	1106	CAKDLGGNRLTHYYALDVW	IGH	2	None	
3	1107	CARSVLSFSGKSGYYSGSFDSW	IGH	2	None	
3	1108	CAKDNEYCGGDCYSTWFDPW	IGH	7	None	
3	1109	CARLVGYYDGRSGYIFDYW	IGH	2	None	
3	1110	CAKLAVAGTSRVDYW	IGH	10	None	
3	1111	CAKPLVLHSGCFDYW	IGH	4	None	
3	1112	CARDPETWLQLHYFDYW	IGH	4	None	
3	1113	CAKFGDDYTPLDYW	IGH	8	None	
3	1114	CARDFIVCSTSCYGIGWFDPW	IGH	6	None	
3	1115	CARVWWVHREFDYW	IGH	4	None	
3	1116	CARMGRDHYKEFDYW	IGH	2	None	
3	1117	CARHVSYQDSTGFYKYYFDSW	IGH	3	None	
3	1118	CAKEHYYGSGSYFEYW	IGH	2	None	
3	1119	CAKEVLVAGTLPWFDPW	IGH	42	None	
3	1120	CARVNNFHVWSGYYSAMDPSFFDYW	IGH	2	None	
3	1121	CAKDQSPGVATMDAFDYW	IGH	2	None	
3	1122	CQQYNDHSCIF	IGK	40	None	
3	1123	CVKPVGATKPVGATDFDYW	IGH	8	None	
3	1124	CVRENVLRFSEWLDYW	IGH	2	None	
3	1125	CARDRSAGNGDYVDYFDSW	IGH	3	None	
3	1126	CVRGPSYYILTGYLNSDAFDIW	IGH	24	None	
3	1127	CMQRVEFPPYTF	IGK	82	None	
3	1128	CARDIVVVPAAMGHSTGFDYW	IGH	3	None	
3	1129	CARGTYYYGSGIYYTGVPWFDPW	IGH	3	None	
3	1130	CARDSCSSTSCYGYWYFDLW	IGH	2	None	
3	1131	CAKDSVTDYGDYSWFDPW	IGH	2	None	
3	1132	CVRGYYGDLGLPYYLDSW	IGH	2	None	
3	1133	CARGLSSSWYDYW	IGH	18	None	
3	1134	CTTPILYYGSGSYYNAWRPLDYW	IGH	8	None	
3	1135	CRKAGGMVRGVDAFDIW	IGH	10	None	
3	1136	CAKRGPYYDFWTLDYW	IGH	2	None	
3	1137	CAKLPLRNYYDSSGSEFDYW	IGH	2	None	
3	1138	CAHSELVRGNFDYW	IGH	2	None	
3	1139	CARDSSNGGTRNWYFDLW	IGH	2	None	
3	1140	CARDPTLGRIESYYYALDVW	IGH	2	None	
3	1141	CARQARYNWNLDYW	IGH	2	None	
3	1142	CASLSTLTAAGTDYW	IGH	3	None	
3	1143	CVGGGPYQYYYYGMDVW	IGH	2	None	
3	1145	CARGGYKWKDRLLRYYFDLW	IGH	2	None	
3	1146	CARLVDDYAEEYYFDYW	IGH	3	None	
3	1147	CAKEKSGNNYGDFQYW	IGH	33	None	
3	1148	CAKDSYGITMVRGPFDYW	IGH	2	None	
3	1150	CQHLTSYPQVAF	IGK	50	C1	7BZ5
3	1151	CTTGYDSTATAFDYW	IGH	3	None	
3	1152	CARSQNIAAAPIYYYYYGMDVW	IGH	4	None	
3	1153	CTTDGLVVYAAFDFW	IGH	2	None	
3	1154	CARERTSGSYYNSDHFYYYGMDVW	IGH	3	None	
3	1155	CVRLIKGGAFDFW	IGH	8	None	
3	1156	CAKGNGYHSNDAFDIW	IGH	13	None	
3	1157	CTTDLYCDTTPCPSRWFDPW	IGH	4	None	
3	1158	CQSYDNSLGDNYVF	IGL	4	C4	7JVC
3	1159	CVKHLTGPTRWSSYDYW	IGH	6	None	
3	1160	CAAWDDGLSVSYVF	IGL	2	None	
3	1161	CAHRLRSTMFTGNWFDPGNWFDPW	IGH	2	None	
3	1162	CARILYSSGLGFDPW	IGH	2	None	
3	1163	CMQSIQLPLSF	IGK	44	None	
3	1164	CARIPLPYCTSTTCYWFDTW	IGH	3	None	
3	1166	CARQRDTMIVVVEYYFDYW	IGH	2	None	
3	1167	CARVSWPMEAFDYW	IGH	34	None	

3	1168	CAKDPGDHDQGWFDPW	IGH	5	None	
3	1170	CARMIGPQYTTSWWFDPW	IGH	10	None	
3	1171	CAKDMLPIAAAGHGLDYW	IGH	5	None	
3	1172	CAKEAYYDILTGPFDYW	IGH	2	None	
3	1173	CLQGIALPYAF	IGK	2	None	
3	1174	CARDKMVRTIIITGPLDYW	IGH	2	None	
3	1175	CTTEGYDYGSAIDYW	IGH	5	None	
3	1176	CATVTDFRWDGERYFDNW	IGH	2	None	
3	1177	CSKDRDGYREYW	IGH	2	None	
3	1178	CASAKGDSTMITYFDYW	IGH	2	None	
3	1179	CAKEPWPQLVHWTYYFDYW	IGH	13	None	
3	1180	CARVERFGELRWFDPW	IGH	6	None	
3	1181	CAKFDDYDDRGVDYW	IGH	3	None	
3	1182	CASYDILTGSLDYW	IGH	2	None	
3	1183	CARHGRITGSSWFDPW	IGH	2	None	
3	1184	CAKGIFGVVTIDGMDVW	IGH	4	None	
3	1185	CARVEQWPVGFDYW	IGH	3	None	
3	1187	CATALGGFDPW	IGH	2	None	
3	1188	CARLGDYGTSSYNWYDPW	IGH	2	None	
3	1189	CAKDIYGDYDGHVDYW	IGH	6	None	
3	1190	CARRPSYSGGGGLDYW	IGH	3	None	
3	1191	CARRPNTSSGKFDQW	IGH	4	None	
3	1192	CAKADSDSYFPPSYFDSW	IGH	5	None	
3	1193	CAKDFTIFGVVIPLGLERRYYYYGMDVW	IGH	3	None	
3	1194	CARHLRENFEGYYFDYW	IGH	6	None	
3	1195	CARVGAGVETTIDYW	IGH	2	None	
3	1196	CAKDIQPYSGYDFDYW	IGH	2	None	
3	1198	CARDRYSGYEYRDFDQW	IGH	2	None	
3	1199	CTRYYDYVWGTFGYW	IGH	2	None	
3	1200	CARDREAYSNHGGFDQW	IGH	4	None	
3	1201	CAKAVNDYGDYLPLLRPDNYYYAMDVW	IGH	2	None	
3	1202	CQTWGTGIRVF	IGL	82	None	
3	1203	CARFHRDGYNLFSDYW	IGH	14	None	
3	1204	CQHYDNLRMSSF	IGK	8	C3	6XDG
3	1205	CAKGRGYSGYGDAFDIW	IGH	2	None	
3	1206	CARDLNNVFQYW	IGH	2	None	
3	1207	CAKNFNNCGGDCWYYYGMDVW	IGH	2	None	
3	1208	CAKDNGGNSGWYFDVW	IGH	4	None	
3	1209	CAKGPIRWLQLVNYYFDYW	IGH	2	None	
3	1210	CARGRSVFEPSYRLLLLGYFDYW	IGH	3	None	
3	1213	CARRYCPGGSCYDAFDIW	IGH	4	None	
3	1214	CARESRAAAGPPDAFDIW	IGH	6	None	
3	1215	CAKWVTGDSGDYW	IGH	7	None	
3	1216	CARELRYKAYYYGMDVW	IGH	7	None	
3	1217	CVRGKTPQWATAGVFEYW	IGH	2	None	
3	1218	CARHPRPELYSSYHFDYW	IGH	2	None	
3	1219	CAREGSGTTFRPYNWFDPW	IGH	2	None	
3	1220	CAKDGGPVAGTHGGAFDYW	IGH	3	None	
3	1221	CTRHSTTTFGGQIVEPGGFDFW	IGH	2	None	
3	1222	CARGKTIGAAGLYYFDYW	IGH	2	None	
3	1223	CARVSQFRFLDYW	IGH	2	None	
3	1224	CANMGVTIWGRYFDYW	IGH	4	None	
3	1225	CATGPQVVAATYYYYYGMDVW	IGH	3	None	
3	1226	CTSQFNTWYVFDYW	IGH	3	None	
3	1227	CARDPYNWRAPGDWYFDLW	IGH	2	None	
3	1228	CARGATYYHDNDGPHFFDPW	IGH	10	None	
3	1229	CVRGVGATGYFANW	IGH	4	None	
3	1230	CAKDPDDGYSSSWSFDYW	IGH	8	None	
3	1232	CQQGSNWLRFTF	IGK	2	None	
3	1233	CAKVGPCTTCYYYFYYPMDVW	IGH	5	None	
3	1234	CARVSFAYYYDSSGYYKGYFDYW	IGH	2	None	
3	1237	CARGSGATANDAFDIW	IGH	2	None	
3	1238	CVRSVVCYVTSYCYFDYW	IGH	3	None	
3	1239	CARQRGALGWFDPW	IGH	9	None	
3	1241	CAKEHYGDYYYYYGMDVW	IGH	5	None	
3	1242	CARAGDITLSGGFDYW	IGH	5	None	
3	1243	CARGPTWDGGGYYFDYW	IGH	4	None	
3	1244	CAAWNDSLSGPNWVF	IGL	2	None	

3	1246	CARSGTYSGYDDFDPW	IGH	4	None	
3	1247	CTRHPGYSGPYYYGMDVW	IGH	2	None	
3	1248	CARDMGSGGTCSDYW	IGH	3	None	
3	1249	CAHSPKLLWFGEKRGWFDPW	IGH	8	None	
3	1250	CARVLNYVPNWFDPW	IGH	15	None	
3	1251	CAGHSSFKDYW	IGH	2	None	
3	1252	CARFTRGYSAAPGGWVRYYFDNW	IGH	2	None	
3	1253	CARTTARPQWLYYYYGMDVW	IGH	2	None	
3	1254	CAKVSRYEPSEFSFYFDDW	IGH	4	None	
3	1255	CARGGVDATTSFDMW	IGH	4	None	
3	1256	CARSVISERVRGVAFDFW	IGH	7	None	
3	1257	CARGDIVVVPAALDYW	IGH	4	None	
3	1258	CARLSIVSESVAVWYYYGMDVW	IGH	12	None	
3	1259	CARSPDEQWVAAYYFDYW	IGH	2	None	
3	1260	CARGYSGSHWYNYFDYW	IGH	28	None	
3	1261	CQQHDNLPQKGITF	IGK	16	C3	6XDG
3	1262	CARHRENSGWYTTPFDYW	IGH	6	None	
3	1263	CARASFQYGPGSYLDHW	IGH	2	None	
3	1264	CARSISDCPISLCLAKGYFDRW	IGH	2	None	
3	1266	CARDASYYDILTGYYNSGLFDYW	IGH	3	None	
3	1267	CARDRDHIYSDAHYDAYDIW	IGH	4	None	
3	1268	CARDPGYCSGGSCYTPLYYFDYW	IGH	2	None	
3	1269	CTTVRAWYYFDYW	IGH	3	None	
3	1270	CQSDDGSNWVF	IGL	19	None	
3	1271	CAKARLGGYEPNWFDPW	IGH	8	None	
3	1273	CARDRGVLRPPVAFDIW	IGH	2	None	
3	1274	CAKGSWPALVGYFDYW	IGH	2	None	
3	1275	CTTDRDPYSSSSGGDYW	IGH	6	None	
3	1276	CARSPPGGNAVLYYFDAW	IGH	2	None	
3	1277	CQQSYSTEIPAFTF	IGK	15	C4	6ZDH
3	1278	CARVRYTYYYAMDVW	IGH	3	None	
3	1279	CARVMRYFDWLLEYYYYGMDVW	IGH	2	None	
3	1280	CAKTDSVDLTFDYW	IGH	4	None	
3	1282	CAKDRQGYDYSDYVEYFFDSW	IGH	2	None	
3	1283	CTSYTSSSTFVVF	IGL	2317	C2	7K90
3	1284	CATLFGASGSLLPYYYYGMDVW	IGH	2	None	
3	1285	CSSMVRGVDAFDIW	IGH	2	None	
3	1286	CLQHNSPHWAF	IGK	5	None	
3	1287	CAAWDESLNGFCLF	IGL	5	None	
3	1288	CQQRTSWPRRLTF	IGK	7	C3	7K8P
3	1289	CATLRDGYNPDLDYW	IGH	7	None	
3	1290	CARAMAGKNFFDYW	IGH	24	None	
3	1291	CAKDSPEEYSSGWYWGFDYW	IGH	4	None	
3	1293	CARISRDGFNLYYFDYW	IGH	2	None	
3	1294	CAKIPSTAMVYDAFDIW	IGH	10	None	
3	1295	CATTRIYLMEPVIYYFDSW	IGH	3	None	
3	1297	CSSADSSRNRLF	IGL	2	None	
3	1298	CAKLGTTGSPTGDYW	IGH	14	None	
3	1299	CATLGRLGELSLPTPPEDYW	IGH	2	None	
3	1300	CARDAPLQPLPAARYYYYYYGMDVW	IGH	4	None	
3	1301	CTAAPNYYYFNYW	IGH	3	None	
3	1302	CARAPGGYCSSTSCYGSYYYYGMDVW	IGH	2	None	
3	1303	CAKEKGTNWYYFDSW	IGH	4	None	
3	1304	CAKAPTLLWFGDLLSFFDSW	IGH	2	None	
3	1305	CARLRFVGGTAFGFDYW	IGH	2	None	
3	1306	CARGQYCSGGKCNLPPLNWFDPW	IGH	3	None	
3	1307	CTRLPHNYCSRGTCGPDYYYYGMDVW	IGH	20	None	
3	1308	CAKVQVEYTGGFYLDYW	IGH	4	None	
3	1309	CTTTVRGDFWSGYYSTTYYYYGIDVW	IGH	2	None	
3	1310	CARVDILMAYNWLDPW	IGH	2	None	
3	1311	CALYYCAKYARESYYYYYAMDVW	IGH	3	None	
3	1312	CAGVRGSNNDAFDIW	IGH	6	None	
3	1313	CARGGYSYGCPDYW	IGH	18	None	
3	1314	CSKEIYPLKAIGPGWFDPW	IGH	3	None	
3	1315	CAKDLMVRGYGYGCFDYW	IGH	2	None	
3	1316	CASVVQHLEGHYFDYW	IGH	2	None	
3	1317	CATLSSAVAPYFDYW	IGH	6	None	
3	1318	CAKGDQSTPYSSSWYQNWFDPW	IGH	4	None	

3	1322	CLRGMYQYESAGYRGWLDPW	IGH	13	None	
3	1323	CARCPRGTQYNIFDVW	IGH	13	None	
3	1324	CATPSSGSPHFAMDVW	IGH	2	None	
3	1325	CARLHYDFWSGEDSQLDYW	IGH	2	None	
3	1326	CLRAEDTAVYFCARSLSGYGYAFDLW	IGH	2	None	
3	1327	CAKDSGTSCLEGPCYYYGMDVW	IGH	2	None	
3	1328	CAKELYSDGSGDYYVPYAAFDSW	IGH	2	None	
3	1329	CAHSYYDILTGIAIYFDYW	IGH	6	None	
3	1330	CASFRYNWNDGIDYW	IGH	6	None	
3	1331	CARHYQGSSTGWYYYYYGMDVW	IGH	2	None	
3	1332	CAKVAGYLKSPYYFDYW	IGH	2	None	
3	1333	CARDILTGYYYNWFDPW	IGH	7	None	
3	1334	CQQYNRYWTF	IGK	31863	C3	7K8R
3	1335	CARQADYNLLTGYSSGHYLDFW	IGH	3	None	
3	1336	CVKDLSPAAIRPLFDDW	IGH	11	None	
3	1338	CAKDQSSDWSLNWYLDVW	IGH	2	None	
3	1339	CARHVRAVEMAERSYFQHW	IGH	2	None	
3	1340	CARDRGWELPGFDYW	IGH	2	None	
3	1341	CQQTNNFPPYIF	IGK	19	C1	7BZ5
3	1342	CAKLASGDGYTRGDAFDIW	IGH	2	None	
3	1343	CQDYANWPLHTF	IGK	22	C3	6XDG
3	1344	CARLNLYFDLW	IGH	31	None	
3	1345	CARVPTYPFCSSVSCYGFDPW	IGH	12	None	
3	1346	CARGRHHYDRSGYSDYW	IGH	2	None	
3	1347	CAKDIWPTSSGGYPDYW	IGH	6	None	
3	1348	CARDRYYYDTIGYLAYW	IGH	3	None	
3	1349	CATVRDMFHLGESSPKFDSW	IGH	38	None	
3	1350	CANRGDSSTAFDIW	IGH	9	None	
3	1352	CQQYITYSTMLTF	IGK	2	None	
3	1353	CARGKGSIDWDPRIPPTFYFDYW	IGH	2	None	
3	1354	CATIKGPNYYDSSGHVDYW	IGH	15	None	
3	1355	CARDREYYGSGSSDGMDVW	IGH	2	None	
3	1356	CARLEYDYVWGSYRRTTNYFDYW	IGH	4	None	
3	1357	CWSTSCYGMEACFDYW	IGH	4	None	
3	1358	CAIRGNSRTTCRSGRCTYDFKNWFDPW	IGH	6	None	
3	1359	CARISNFGYFDWLLRTDYYYYGMDVW	IGH	2	None	
3	1360	CAKLAGFSYYDSSEDYW	IGH	3	None	
3	1361	CARAPSGSGSYYFYYW	IGH	13	None	
3	1362	CANLPAVAGTPSAFDIW	IGH	2	None	
3	1363	CAAQLIASLDYW	IGH	3	None	
3	1364	CARSLHVVRYFDAFDVW	IGH	4	None	
3	1365	CARVDWFGDILNPFFWYFDPW	IGH	5	None	
3	1366	CAKGFGGFSDILTGYYDYW	IGH	2	None	
3	1367	CAGALELFFDFW	IGH	2	None	
3	1368	CAKASNYYDSSGYYWYFDLW	IGH	37	None	
3	1369	CAKGQIAAAGTTFDYW	IGH	10	None	
3	1370	CAKDLRPADFSGYQLEYYFDTW	IGH	4	None	
3	1371	CARGGAWIFDFW	IGH	4	None	
3	1372	CALPISAVYYCARAKQYYYDSSGYYFGYW	IGH	2	None	
3	1373	CASPGFIAAAGTFAFDIW	IGH	4	None	
3	1374	CAKDGYPGLEYFQHW	IGH	3	None	
3	1375	CASEKMDYDSSGYYSPFVYW	IGH	2	None	
3	1376	CARVVGSTNDPNFDHW	IGH	3	None	
3	1377	CARAKYDILTGYSLGYFFDYW	IGH	8	None	
3	1378	CAKELGDGYNWGPFDYW	IGH	2	None	
3	1379	CAKYIGSCNGLGCSRYFDPW	IGH	3	None	
3	1380	CAKDILRSEVWRSSSSGSDYW	IGH	4	None	
3	1381	CAKLSGSYHNFDYW	IGH	7	None	
3	1382	CAKGGYGDGYHKGFDYW	IGH	6	None	
3	1383	CAKDRDDIFWSGYAMDVW	IGH	4	None	
3	1384	CQSADYIGSYRVF	IGL	4	None	
3	1385	CARFTKFSLVRAGFDPW	IGH	4	None	
3	1386	CARSVIQRYYYFQYW	IGH	3	None	
3	1387	CAKGHVLLWFGELSARENWFDPW	IGH	2	None	
3	1388	CARDSYFDHIWGPYRFDFW	IGH	2	None	
3	1389	CAREKGGHFDSTGNYFQSRGFDLW	IGH	6	None	
3	1391	CSVDFHALTGYYSLDYW	IGH	3	None	
3	1392	CARLGASGDNYYAMDVW	IGH	2	None	

3	1393	CARDITMIVVVPYFDYW	IGH	4	None	
3	1394	CASPNYDILTGYPINVGYYYGMDVW	IGH	2	None	
3	1395	CTKAYIIGTPSGFDYW	IGH	8	None	
3	1396	CAKDLPGSYYLAYYFDYW	IGH	4	None	
3	1397	CARSGPRGYNYGYRAFDLW	IGH	3	None	
3	1398	CARMGEYCSRSSCYFDFW	IGH	4	None	
3	1399	CAKDMQFGELRDWFDPW	IGH	8	None	
3	1400	CARVRGGIPAAIDYW	IGH	6	None	
3	1401	CAKGNDPLLVGATPYYFDYW	IGH	3	None	
3	1402	CQAWDSSTVVF	IGL	590	C4	7JVC
3	1403	CQEYGDSPAF	IGK	23	C1	7K8N
3	1404	CARGSIVVVPAAKPVRYYYGMDVW	IGH	2	None	
3	1405	CARQISQRGYPIGDYFDYW	IGH	6	None	
3	1406	CTKVARNSGWYSFFDYW	IGH	4	None	
3	1407	CARDDDYYDSSGYILPFDYW	IGH	2	None	
3	1408	CAHSRVLRYFDWLFGDNWFDPW	IGH	2	None	
3	1409	CARQEGNCIGVRCYAIWFDPW	IGH	7	None	
3	1412	CAIYDSSGYYLIEPFDYW	IGH	6	None	
3	1413	CARGYDVLTGYPDYW	IGH	143	None	
3	1414	CANLYYDSSGYSPGYYYYGMDVW	IGH	2	None	
3	1415	CARARFLSRGRETVTAFDHW	IGH	4	None	
3	1416	CARVDYGDYIWLTPGDYW	IGH	8	None	
3	1417	CAKERGRSSGWDFDYW	IGH	2	None	
3	1419	CARNSRSDTPWCDSW	IGH	2	None	
3	1421	CTRFTWSSSGHTDFDFW	IGH	3	None	
3	1422	CAKVDFWSGYSPDYW	IGH	8	None	
3	1423	CARGRDLLAAAGNDAFDIW	IGH	2	None	
3	1424	CAKDFVFNWNQFKFDKW	IGH	3	None	
3	1425	CAKDISSGWYVHYFDYW	IGH	3	None	
3	1426	CQVWESSSDHRWVF	IGL	15	None	
3	1427	CAKVSSGGAYDKHQKNWFDPW	IGH	4	None	
3	1428	CATLGDYGDYYFDYW	IGH	5	None	
3	1429	CAKESTVTHSYYYYGLDVW	IGH	5	None	
3	1430	CAKEPYYDNREGCFDAW	IGH	2	None	
3	1431	CGIMPGWTAYAFDIW	IGH	2	None	
3	1432	CARIPPPYYFNNSDYYSRRSGPAAFDIW	IGH	7	None	
3	1433	CANVGGGWAPDYW	IGH	3	None	
3	1434	CAKGFDGYGVPSLDYW	IGH	3	None	
3	1435	CARRPGRTSGYSAADYW	IGH	3	None	
3	1436	CARDSPYYDFFTGSIFDLW	IGH	4	None	
3	1437	CARAKQYYYDSSGYYFGYW	IGH	23	None	
3	1438	CAKVEPTGLTTVTTHGAFDIW	IGH	2	None	
3	1439	CATSIAVAGTLGSYYYYGMDVW	IGH	2	None	
3	1440	CAKSSDYESLFLGVYQYQFYFAYW	IGH	10	None	
3	1441	CAIERERGSFWFDPW	IGH	3	None	
3	1442	CANPPGDYW	IGH	145	None	
3	1443	CAHESGYDFWSGYYGFDYW	IGH	6	None	
3	1444	CARLYDNSGYSPQYYFDYW	IGH	2	None	
3	1445	CARDPQEVVAALSYFDYW	IGH	2	None	
3	1446	CAKDPTRFDFAREGWFDPW	IGH	2	None	
3	1447	CCSYAGSYTFRVVF	IGL	36	C2	7K8X
3	1448	CSSTSCRPWYYYYGMDVW	IGH	3	None	
3	1449	CTRDPPGYCSGGSCSLFDLW	IGH	2	None	
3	1450	CAREDSSTSRLALDYW	IGH	4	None	
3	1451	CASLGTTVVTSYYYGMDVW	IGH	2	None	
3	1452	CARVNTMVRGVIIDNWFDPW	IGH	2	None	
3	1453	CMESIQLPAF	IGK	2	None	
3	1454	CARRVWRVAASRDRKSGPNYFDLW	IGH	3	None	
3	1456	CARFLDYHFSESGGQYFYYHYGMDVW	IGH	4	None	
3	1457	CAKDRVGVVTASYVDYW	IGH	4	None	
3	1458	CAATTGYYLGAFDIW	IGH	2	None	
3	1459	CAKVPGKYYDGGPFDYW	IGH	2	None	
3	1460	CLLYYGDAQFWVF	IGL	10	None	
3	1461	CARTNQRGVGVAGKSYYFYGMDVW	IGH	2	None	
3	1462	CARCGSNWQPFDLW	IGH	2	None	
3	1463	CARVPDTWYDYW	IGH	90	None	
3	1465	CARDRGKVVPLGAATDAFDLW	IGH	5	None	
3	1466	CARIKGGVYDVPGSFDFW	IGH	2	None	

3	1467	CARDDRSRTRCCGWWPDPW	IGH	3	None	
3	1468	CMQITHWSPAFTF	IGK	9	None	
3	1469	CARPQDYGEYISYYYGMDVW	IGH	4	None	
3	1470	CAIWYSNTYVF	IGL	49	None	
3	1471	CATGPNYYDSSGYPNWFDPW	IGH	8	None	
3	1472	CTTDPAPDGFFDYW	IGH	4	None	
3	1473	CASSSYIYGTFGTRYYAIDVW	IGH	9	None	
3	1474	CQSVDTSGTSLVF	IGL	813	None	
3	1475	CARFYRRDGFFDYW	IGH	3	None	
3	1476	CTRASFYNYGYWWFDPW	IGH	16	None	
3	1477	CAKARSEFGSMRSSFDIW	IGH	13	None	
3	1479	CTKGPVSSDRGWYFDLW	IGH	6	None	
3	1480	CQQYNNLREFTF	IGK	21	C3	6XDG
3	1481	CMDALNTPSIF	IGK	10	None	
3	1482	CARRGGFTIFDPIYFDYW	IGH	2	None	
3	1483	CAKIQVLRYFEAYFDLW	IGH	6	None	
3	1484	CAKDTRPWGYYYDSSGYLDAFDIW	IGH	2	None	
3	1485	CARDRYGYYYDSSSNYFDYW	IGH	4	None	
3	1486	CAREDYWIGETSGSYLGALDIW	IGH	2	None	
3	1487	CAKESQLCSTTSCYFPFDSW	IGH	2	None	
3	1488	CAKVATNWPGPSFDYW	IGH	28	None	
3	1489	CAKDISPYNSLFDYW	IGH	20	None	
3	1490	CARTNSGFWSGYYEDYW	IGH	5	None	
3	1491	CMQVLQTPVMYTF	IGK	45	None	
3	1493	CVKERDASGTWSGLDYW	IGH	4	None	
3	1494	CGPRNYYYGMDVW	IGH	2	None	
3	1496	CAKDQMTGNRGLGVFDVW	IGH	2	None	
3	1497	CAKGHSSSWFYLDSW	IGH	2	None	
3	1499	CASSLYPGAPFDYW	IGH	13	None	
3	1502	CARDLNSIWVAAIESYFDYW	IGH	3	None	
3	1503	CAKDMRLEGRYFDLSQYYYAMDVW	IGH	2	None	
3	1504	CGRDHRYDSRAFVDYW	IGH	4	None	
3	1505	CARTAYSSSKRGGWFDPW	IGH	2	None	
3	1506	CARSFLHYSGSSGYYSGSFDYW	IGH	3647	None	
3	1507	CQSRSDWPVAF	IGK	11	None	
3	1509	CGRVTSHYGVGVLGTFDYW	IGH	2	None	
3	1510	CARDGGYTYGSARFDYW	IGH	2	None	
3	1511	CARGRKLGGNFDYW	IGH	2	None	
3	1513	CARENRITMVRGVIDYW	IGH	2	None	
3	1514	CQHYNNGPPVMYTF	IGK	5	C3	6XDG
3	1516	CARDGRFGEYWLIDYW	IGH	2	None	
3	1517	CAKDRRGSSGWWYFDVW	IGH	5	None	
3	1518	CARDLLGITGPDYW	IGH	11	None	
3	1519	CGRPLYSPNEDGDYW	IGH	2	None	
3	1521	CVKKAQYYDYVRKHYRYTAFDQW	IGH	6	None	
3	1522	CAKAAWIQLWLRSNYFDYW	IGH	3	None	
3	1523	CAKSGNSVFDQW	IGH	4	None	
3	1524	CAKDMWAAPAYYFDSW	IGH	9	None	
3	1525	CAKDGIPANGRWDSFDLW	IGH	4	None	
3	1526	CARHPRAAAGRLDYW	IGH	2	None	
3	1527	CARDDSSGYLDAFDIW	IGH	16	None	
3	1528	CAHSIVGPVRATDSAAGTFDYW	IGH	2	None	
3	1529	CAKDLGPLWFGNGYYYYGMDVW	IGH	4	None	
3	1530	CVRDFGNGTPYYFDFW	IGH	10	None	
3	1532	CAKPSLDGYTPGYFDYW	IGH	17	None	
3	1533	CAHRPDISLYYFDYW	IGH	3	None	
3	1534	CARGGINLWDTHPFDHW	IGH	2	None	
3	1535	CQVWDSATEHVLF	IGL	3	None	
3	1537	CATLQRQQPYDYW	IGH	12	None	
3	1538	CTRDSSRAFGYW	IGH	24	None	
3	1539	CATARFHTSGTYQIGDYW	IGH	2	None	
3	1540	CATPRASGWVYYFAYW	IGH	2	None	
3	1541	CAKQDSGPVHDNWFDPW	IGH	6	None	
3	1542	CQQYKNWPTNSWPTF	IGK	2	C3	6XDG
3	1543	CATMTTPSYYYALDVW	IGH	2	None	
3	1544	CAKLGAEGLPGDYYYGMDVW	IGH	3	None	
3	1545	CAKDPTGYYYDSSGYYYDYW	IGH	6	None	
3	1546	CAKDLDSGYNLGETHDSFDMW	IGH	13	None	

3	1547	CARDIRESSSWYYYYGMDVW	IGH	10	None	
3	1548	CARVHDSSGPVHDTFDFW	IGH	2	None	
3	1549	CAKDQSWFGEYRYKMDAFDMW	IGH	3	None	
3	1550	CAKQARTTDEFDYW	IGH	2	None	
3	1551	CAHRGLVRVGHLTGETNSFDAW	IGH	2	None	
3	1552	CAKDLTSNTWPSLYFDSW	IGH	5	None	
3	1553	CSKDQNVNYPYNWFDPW	IGH	2	None	
3	1554	CARGIGDWYDYYFDSW	IGH	2	None	
3	1555	CARDQGLVLNYYYGMDVW	IGH	2	None	
3	1556	CETWDSDSRVF	IGL	22	None	
3	1557	CATVDRRTDPNSFDYW	IGH	2	None	
3	1558	CAKDIYPVQGSADYW	IGH	4	None	
3	1559	CAHRGSSSSWYTEFDYW	IGH	2	None	
3	1560	CATLAAAGGDYW	IGH	32	None	
3	1561	CARDYLWGTYPYFFDSW	IGH	4	None	
3	1563	CARLTTLHDYVWGSYRFNWFDPW	IGH	4	None	
3	1564	CAKVRYTFGGVIANFDYW	IGH	3	None	
3	1565	CAKDLGDGYCSSTSCYGGLDYW	IGH	4	None	
3	1566	CAADLRNYYDSSGYADYW	IGH	2	None	
3	1567	CVSSSWYGYYFDYW	IGH	4	None	
3	1569	CGAWGSSLSAMIF	IGL	6	None	
3	1570	CAKGYSYGDETAYYFDYW	IGH	12	None	
3	1571	CQQFASSPRLIF	IGK	4	C1	7K8N
3	1572	CQQADSHSTF	IGK	29	None	
3	1573	CASGFAIPRFGYSIDYW	IGH	2	None	
3	1574	CARDRKTVGFSYYYYGMDVW	IGH	4	None	
3	1575	CQLRSKWPTLTF	IGK	4	None	
3	1576	CARDLAMAPRYFDYW	IGH	3	None	
3	1578	CQSYDITLSGGEVF	IGL	5	None	
3	1579	CARHSPYCGGGSCPFDLW	IGH	2	None	
3	1580	CAILGHDYGDYYYGMDVW	IGH	3	None	
3	1581	CALAQHRTYTFDYW	IGH	2	None	
3	1582	CAKATLLLTGYLYYFDYW	IGH	6	None	
3	1583	CAKDIGSDSGSYSGETDYW	IGH	4	None	
3	1585	CSSF	IGL	5	None	
3	1587	CATDRGSSGWPFDYW	IGH	20	None	
3	1588	CARAPITMIVMYYFDYW	IGH	2	None	
3	1589	CTTTPFYYDYW	IGH	28	None	
3	1590	CAKEGMIIANSYDPW	IGH	3	None	
3	1592	CARPAVAGTNDAFDVW	IGH	2	None	
3	1593	CAHRRVGATRGDYFDYW	IGH	6	None	
3	1594	CARDRLNYDILTGPQYYYYGMDVW	IGH	5	None	
3	1595	CARVRSESGYSLDYW	IGH	8	None	
3	1596	CATGGGNSDVIWDYW	IGH	4	None	
3	1597	CATSLLLTTFDYW	IGH	4	None	
3	1598	CVKDHEQWLLEYFHRW	IGH	5	None	
3	1599	CASTHVEVTTIFAYYYYGLDVW	IGH	2	None	
3	1600	CTTDGGTLRYSDIDGYYRSGGFDIW	IGH	2	None	
3	1601	CAKGGDIVLMVYAYYFDYW	IGH	8	None	
3	1602	CARENSWNSAMVAYYFDYW	IGH	4	None	
3	1603	CYSTDRSQNHTGVF	IGL	9	None	
3	1604	CARTFDSGTFYMDFFDFW	IGH	2	None	
3	1605	CAKTLPWVTGNNWFDPW	IGH	7	None	
3	1606	CSRSTTSADFDFW	IGH	8	None	
3	1607	CARDFDNGEWLTLWDYW	IGH	2	None	
3	1608	CARAPGAGSYYDYW	IGH	4	None	
3	1609	CATTYYDFWSGYPYYYYYGMDVW	IGH	2	None	
3	1610	CARDYGESWGYYYYGMDVW	IGH	6	None	
3	1611	CASSPPGGSYVNAFDIW	IGH	5	None	
3	1612	CARDESNVWRWFDPW	IGH	14	None	
3	1613	CARDLEGATTTFFDYW	IGH	4	None	
3	1614	CARLSYRYNLPRAYFFDHW	IGH	4	None	
3	1615	CARDRDCSSTSCYRGWFDPW	IGH	6	None	
3	1616	CAKIAGSYYLHDFDYW	IGH	6	None	
3	1617	CAKRQRWSFDYW	IGH	4	None	
3	1618	CALLVGATRDPGIDYW	IGH	3	None	
3	1619	CTRDVYSKIRGDDDWLDPW	IGH	4	None	
3	1620	CQQYDSLPPGSAF	IGK	32	C3	6XDG

3	1621	CAKDDPPDRAFDYW	IGH	2	None	
3	1622	CARDVLGNGNAFDIW	IGH	2	None	
3	1623	CAKTANYGDYVYFDYW	IGH	5	None	
3	1624	CARVRYVSPWLVPLCRGGCSNWFDPW	IGH	3	None	
3	1625	CAHTHHEYYFDYW	IGH	4	None	
3	1626	CAKRRGSSVSTIDYW	IGH	3	None	
3	1627	CAKDATPLMVRGVTLDYW	IGH	6	None	
3	1628	CARDRSGYVRFDNW	IGH	5	None	
3	1629	CATQQVLLEWSSFDYW	IGH	2	None	
3	1630	CARHSRSFDTFDIW	IGH	2	None	
3	1631	CAKDRFYDSSVQDYW	IGH	2	None	
3	1632	CAKSAGLAATKGVYYYYGMDVW	IGH	2	None	
3	1633	CARQVREGSGYDYDYFDYW	IGH	2	None	
3	1634	CARDDKETISFDFW	IGH	7	None	
3	1635	CTAMAPSPGRRGWTDFDYW	IGH	3	None	
3	1636	CQQYYSIRSVAF	IGK	2	C4	6W41
3	1637	CARDSRITMSRGDGLDVW	IGH	2	None	
3	1638	CARDPYYYDGDAYTGTLDYW	IGH	2	None	
3	1639	CARVAWAVAAPGWFDPW	IGH	2	None	
3	1640	CARHVHSAIDLGIGYFKHW	IGH	4	None	
3	1641	CAREYLAGLEAAAGFWFDPW	IGH	6	None	
3	1642	CAKVTSEGDFDFDYW	IGH	4	None	
3	1643	CARDLRYCSGGSCYSAYFDYW	IGH	11	None	
3	1644	CAKDGDPVIIASQNIPYLDYW	IGH	2	None	
3	1645	CTTGMRYFDSTGYTRIDYW	IGH	4	None	
3	1646	CARSILPAAKDAFDIW	IGH	14	None	
3	1648	CARRHFYDSNGYYFFDFW	IGH	6	None	
3	1649	CQQYGSSQPPTLVTF	IGK	2	C1	7K8N
3	1650	CAKAMTAAGTDAFDIW	IGH	2	None	
3	1652	CARERWFGDLEGGATDIW	IGH	6	None	
3	1653	CLRAEYGYTLGRFDPW	IGH	4	None	
3	1654	CAKESPEYWAFDIW	IGH	2	None	
3	1655	CQQSYTVPSF	IGK	383	C2	7K8O
3	1656	CAKGVGYYDSSGYFQYFDYW	IGH	4	None	
3	1657	CARGLPDSSGYWVPYYYGMDVW	IGH	3	None	
3	1658	CARVDRRYYDSSGPGAFDIW	IGH	2	None	
3	1659	CGAPYIFGYFDAW	IGH	2	None	
3	1660	CARGVTVTSDYYEYYGMDVW	IGH	2	None	
3	1661	CASGLPGYTSRPHFDYW	IGH	18	None	
3	1662	CARHRFLADPFDYW	IGH	14	None	
3	1663	CITEWFDPW	IGH	5	None	
3	1664	CARVSFGRNWFDSW	IGH	3	None	
3	1665	CQQYDSFSRWTF	IGK	47	C3	7K8R
3	1666	CAKMGNEYGDYDDYW	IGH	4	None	
3	1667	CAKEGTWEYSYGFDYW	IGH	4	None	
3	1668	CARDKSQRSGYTYWFDPW	IGH	5	None	
3	1669	CARPPDSGSYYGGFHYW	IGH	2	None	
3	1671	CARDRDYGDLDALDIW	IGH	2	None	
3	1672	CARYNGGGYSSDSRFDYW	IGH	3	None	
3	1673	CATAGIAVAGTSSYYYGMDVW	IGH	3	None	
3	1675	CAKGPIQWFGELSDYW	IGH	2	None	
3	1676	CARGYSSSWEDGMDVW	IGH	5	None	
3	1677	CAIALLWFGNPFDYW	IGH	3	None	
3	1678	CAKDGLEFDGSGWQTGVDYW	IGH	4	None	
3	1679	CARNSQIYDTSGYYYSHLDYW	IGH	3	None	
3	1680	CMQALQTPRTF	IGK	3901	None	
3	1681	CASLYSSNWYSWFDPW	IGH	4	None	
3	1682	CARDYRGGGYYVGDDYW	IGH	8	None	
3	1683	CAKLTGDPLDYYYGMDVW	IGH	4	None	
3	1684	CAMIAVAGTIHASRNDYW	IGH	2	None	
3	1685	CAKASWDGTYYDSSGYGLDYW	IGH	2	None	
3	1688	CAKSALHVPLGPGNWFDPW	IGH	4	None	
3	1689	CARGAMLGGDLFDFW	IGH	2	None	
3	1690	CAHNYILTDYWDAFDLW	IGH	3	None	
3	1691	CARDREDYCSSTSCFHFDYW	IGH	12	None	
3	1692	CASLYSDIVATGLLFDYW	IGH	4	None	
3	1693	CARIRYTVTGYYFDYW	IGH	3	None	
3	1695	CVTQRNDYIDYW	IGH	8	None	

3	1696	CTTDPLYYYDSRTFFDYW	IGH	3	None	
3	1697	CVTTVETLMVTRFDYW	IGH	2	None	
3	1698	CAKGPWDLRRAPDYW	IGH	2	None	
3	1699	CVRVGGVLWFAEFVDGMDVW	IGH	4	None	
3	1700	CAKDPSPITTLIDYW	IGH	5	None	
3	1701	CAKDSHYYGSGSYVTYYYGMDVW	IGH	2	None	
3	1702	CARQKSTSRSFDYW	IGH	3	None	
3	1703	CARPYGSGSTNWFDPW	IGH	8	None	
3	1704	CASNKYDILTEFDYW	IGH	5	None	
3	1705	CAKDDYPYSSGYHDYW	IGH	2	None	
3	1707	CAKLSDRGVYVDSW	IGH	2	None	
3	1708	CARDSYYYDSSGYFDYW	IGH	54	None	
3	1709	CLRSEDTAIYYCAREIRAPRGSFDPW	IGH	4	None	
3	1710	CARSQRVRYYYDSSGYYADVW	IGH	3	None	
3	1711	CAKDNLGDGYNEMDYW	IGH	9	None	
3	1712	CTRDEYAVRGVIITEPFDYW	IGH	8	None	
3	1713	CARDRLGDYDWFDPW	IGH	2	None	
3	1714	CAKGHRVIDYW	IGH	3	None	
3	1715	CARTQRSTMIVVAGDAFDIW	IGH	2	None	
3	1716	CAKSYSSGGALLRYFDSW	IGH	3	None	
3	1717	CTRLISGYGWFDPW	IGH	2	None	
3	1718	CAKGYWAVAGSFIDYW	IGH	7	None	
3	1720	CAKDIGCSSTSCYPDYW	IGH	13	None	
3	1722	CARTRDSSNYYYNPFDNW	IGH	4	None	
3	1724	CARRHYYDSSGYSWDPNFDYW	IGH	2	None	
3	1725	CATWDDSLNGYAVF	IGL	1732	None	
3	1726	CQQYNNWPPTWTF	IGK	2393	C3	7K8R
3	1728	CAKDIFVSHFSDSSGDYESAGDVFDFW	IGH	4	None	
3	1729	CAHTGPRRTWYRGAYYFDSW	IGH	2	None	
3	1730	CASSSRKRYCSSTSCPGYFDYW	IGH	2	None	
3	1731	CARDRDVRSDIIVEIAARAFDYW	IGH	2	None	
3	1732	CAISWRGVLVTDLPYWFFDFW	IGH	2	None	
3	1733	CAKDMRNWKWEPFSAFDIW	IGH	4	None	
3	1736	CAKVGIAAGDFSLLFDYW	IGH	2	None	
3	1737	CAHRHGYYGSGSYYNGDYFDYW	IGH	3	None	
3	1738	CARVHSGSYTGPFDYW	IGH	2	None	
3	1739	CAKDKRVYSSSCVDYW	IGH	6	None	
3	1740	CARDKIFGDGYWVFDYW	IGH	6	None	
3	1742	CVKEISSHGYGDYW	IGH	2	None	
3	1743	CARDVVRFGHRTQFDYW	IGH	10	None	
3	1745	CVREGSAAVPEFDPW	IGH	4	None	
3	1746	CARTPPATNNGRQYFYAYGMDVW	IGH	2	None	
3	1747	CARDDPSDLSSSWPYYFDYW	IGH	14	None	
3	1748	CAKLKVYGDSVFDYW	IGH	2	None	
3	1749	CARDIGNSGYHYYYYGLDVW	IGH	2	None	
3	1750	CAGPQDLQALLMHRGVGIYYYGMDVW	IGH	156	None	
3	1751	CAKDFNGDGYPLRAFDIW	IGH	2	None	
3	1752	CQQYGGSPPTFTF	IGK	353	C1	7K8N
3	1753	CFSTDSNGNHRGFF	IGL	13	None	
3	1754	CAKVAGYSYEGDYW	IGH	12	None	
3	1755	CANFGDEWSPRDYW	IGH	14	None	
3	1756	CARDAFYYSSGTYSGAPNWFDPW	IGH	2	None	
3	1757	CAKTSSLSRSWNDYWYFDLW	IGH	4	None	
3	1758	CARGLARDGSFPWFDPW	IGH	2	None	
3	1759	CTSQSMSFFDYW	IGH	4	None	
3	1760	CAKAASITFVRGVILSDAFDIW	IGH	6	None	
3	1761	CALPICLVSSYYYGMDVW	IGH	3	None	
3	1762	CTTTYSADTFDLW	IGH	2	None	
3	1763	CTTDGEIRPKNRYFYHYGMDVW	IGH	2	None	
3	1764	CARETNTYSFWDAFDIW	IGH	4	None	
3	1765	CQQYSSTSSCTF	IGK	20	None	
3	1766	CQQRGHWPPNSF	IGK	11	C1	7BZ5
3	1767	CARIPDWFHVYYYGMDVW	IGH	2	None	
3	1768	CATLNLDSSGYYFDYW	IGH	7	None	
3	1769	CARDTHDSGWFGYW	IGH	3	None	
3	1770	CNSRDASGKHPF	IGL	2	None	
3	1771	CARDQHTGYSSGWTYYYYGMDVW	IGH	4	None	
3	1772	CARKRAQGHDGSGSTLGPFDYW	IGH	4	None	

3	1773	CASPGPDSSGYYTYW	IGH	2	None	
3	1775	CAKRPAAYPWYYFDYW	IGH	2	None	
3	1776	CARDWKKGPYSGSYYHADYW	IGH	2	None	
3	1777	CSVDLYCIVGRCEFFDHW	IGH	11	None	
3	1778	CAHRRFGLYDTSDFFGFFDSW	IGH	4	None	
3	1779	CAKGWHSSGGGFYFDYW	IGH	5	None	
3	1780	CATHPKDCSGGSCYYYYYGMDVW	IGH	2	None	
3	1781	CAKGYSGSYYGAVDYW	IGH	3	None	
3	1783	CANCLYSSAWYPTPFDYW	IGH	4	None	
3	1784	CAREGLVGGADDAADIW	IGH	6	None	
3	1785	CARHSSTYYDILTGSPPSRFDYW	IGH	6	None	
3	1786	CARRQYQTYYYDSSGYYYVPNFDYW	IGH	2	None	
3	1787	CARVPDTNGHYDAYYYFGMDVW	IGH	4	None	
3	1788	CAKSSAAWLRHFDNW	IGH	44	None	
3	1789	CANSIGDYYGSGSSDYW	IGH	2	None	
3	1790	CQVFDTSSDHLVF	IGL	2	None	
3	1791	CAKDNRGPVGWWYLDPW	IGH	4	None	
3	1792	CAKDSYDSSGYYWFDPW	IGH	4	None	
3	1793	CARDRGAAGSWYFDLW	IGH	2	None	
3	1794	CARDPNWNYEGTWFDPW	IGH	2	None	
3	1795	CAHNLAYYDFW	IGH	8	None	
3	1796	CARESSPGATVIPGYDALPIW	IGH	2	None	
3	1797	CVKQSHGLDYFDYW	IGH	7	None	
3	1798	CARGIAAAATYFDYW	IGH	2	None	
3	1800	CARALTFGELFAFDYW	IGH	6	None	
3	1801	CAKLVVSSLTYYYYGMDVW	IGH	2	None	
3	1802	CARDIDGSGSYYNEYFGWW	IGH	4	None	
3	1803	CARALVVVVVEDYFDYW	IGH	3	None	
3	1804	CVHRQGVQLERRAPRFDFW	IGH	4	None	
3	1805	CAKDWGPETGLSIFGVVTPDFDHW	IGH	2	None	
3	1806	CAKAEEDSSGYYWYFDYW	IGH	7	None	
3	1807	CARDRDDYVWGSYRIFDYW	IGH	3	None	
3	1808	CAKDTGLAVPRGYFDLW	IGH	9	None	
3	1809	CVRPPPRFDYDISGFFLYYFDSW	IGH	2	None	
3	1810	CAKATEEDSSGWYYYYYGMDVW	IGH	2	None	
3	1812	CGRVASTGDIFDYW	IGH	4	None	
3	1813	CARDATDPTSPGYYDYW	IGH	11	None	
3	1815	CAKGRYDRSGYSVGGFDYW	IGH	2	None	
3	1816	CATWVTTDAFDIW	IGH	8	None	
3	1817	CAKDSDAYSSSYKFDPW	IGH	4	None	
3	1818	CARQSRLGDTRNAFDIW	IGH	13	None	
3	1819	CARRKFRVAVGGWFDPW	IGH	2	None	
3	1820	CARDGGETMKDGEAPDIW	IGH	6	None	
3	1821	CATLFPGGSGYYHYW	IGH	2	None	
3	1822	CAKSPSILWPSDWFDPW	IGH	6	None	
3	1823	CTILSEHVGTTGFDYW	IGH	2	None	
3	1824	CAKDLWNYYVLDAFDIW	IGH	2	None	
3	1825	CARTGISEYFYSWQFDPW	IGH	28	None	
3	1826	CQSYDFRLSGFVIF	IGL	4	None	
3	1827	CARRYISPGGGDWFDPW	IGH	12	None	
3	1828	CAKAKFGSSLAQDWFDSW	IGH	4	None	
3	1829	CTTYKRRDGYNWDYW	IGH	3	None	
3	1830	CQQYDTVPRSITF	IGK	7	C3	6WPS
3	1831	CATLYHYYDSSGYYSPLDYW	IGH	6	None	
3	1832	CAGGMATISDYW	IGH	4	None	
3	1833	CATNILTGYYNFFDYW	IGH	6	None	
3	1834	CARDLQGWGPGAGWFDPW	IGH	2	None	
3	1836	CAKGQTSTSSFYFGDW	IGH	5	None	
3	1837	CSRGGSSHFDYW	IGH	4	None	
3	1838	CVSSSWSDYW	IGH	5	None	
3	1840	CAKADRIAVAIAAGGVDYW	IGH	2	None	
3	1841	CAKEDSSGFGTSFPLVDYW	IGH	6	None	
3	1842	CLLSYSGVWVF	IGL	297	None	
3	1843	CATNFRYFDRW	IGH	10	None	
3	1844	CTAYYDILTGQGDTYYFDYW	IGH	10	None	
3	1845	CARDRGDGSGRLTWFDPW	IGH	2	None	
3	1846	CAKDTYPIISAPFDYW	IGH	4	None	
3	1847	CAKERQTDYYDSSGFDYW	IGH	2	None	

3	1849	CARHGPTAAYYYAMDVW	IGH	4	None	
3	1850	CAREAPGTYYFDFW	IGH	12	None	
3	1851	CARASRDGYINAAFDYW	IGH	2	None	
3	1852	CARDFQYYDSTQLPWYFDLW	IGH	4	None	
3	1853	CAREVRGNYMASVPGDWFDPW	IGH	2	None	
3	1855	CARSRGYSGYDSSPNYYFDYW	IGH	2	None	
3	1856	CAKGHGDFEDNYFDYW	IGH	4	None	
3	1857	CARGDCGGDCYYHYYYYGMDVW	IGH	2	None	
3	1858	CAKDTRVVPAAMGYYYGMDVW	IGH	2	None	
3	1859	CTKSVYPYSNYAGGTVDYW	IGH	4	None	
3	1860	CAKIEDDYGDSTDYW	IGH	8	None	
3	1861	CVRGGIPVAGLDGFDIW	IGH	13	None	
3	1862	CAKPHPYYYDSSGSGGYFDYW	IGH	4	None	
3	1863	CARCDAGGDPRNDYGDYINLDYW	IGH	2	None	
3	1864	CAKDIYGSGSYPDVW	IGH	4	None	
3	1865	CAKEGGDWNDLWYYYYYGMDVW	IGH	2	None	
3	1866	CARVSGDYVWGSYRPYFDYW	IGH	2	None	
3	1867	CATNRIGPFDCW	IGH	4	None	
3	1868	CAHAATRPDSVFDFW	IGH	3	None	
3	1869	CARDRAHTAMVMNYFDYW	IGH	2	None	
3	1870	CSRTQWLSTYWYFDFW	IGH	2	None	
3	1872	CAKDRRDGYNSGDYYYYGMDVW	IGH	6	None	
3	1873	CAHTVYHDPKFDPW	IGH	2	None	
3	1874	CAKYGDYAQGNTNYFDYW	IGH	2	None	
3	1876	CARHDYGSEVFDYW	IGH	25	None	
3	1877	CQQLANYPPTTF	IGK	2	C1	7BZ5
3	1878	CAKDNTGYSGPFDYW	IGH	2	None	
3	1879	CAKVSSGRGGMADYW	IGH	4	None	
3	1880	CAKLACSGGSCYDPFDYW	IGH	9	None	
3	1881	CAKARIYYHFDCW	IGH	6	None	
3	1882	CALPISAVYFCARSLSGYGYAFDLW	IGH	3	None	
3	1884	CAKDTTVTFRGLFFDYW	IGH	2	None	
3	1885	CARDARAAAGYSGYDSPRGYYYGMDVW	IGH	3	None	
3	1886	CVSYSDDYGDADYW	IGH	7	None	
3	1887	CAKGMVPTAMDSFDYW	IGH	4	None	
3	1888	CAKAHWDSVGATNAFDIW	IGH	3	None	
3	1889	CARDASYGDFSFDYW	IGH	9	None	
3	1890	CAKEKITMVRGVILYYYGMDVW	IGH	3	None	
3	1891	CAKDRTRGWERLTVDYW	IGH	2	None	
3	1892	CAKDPSSPGDYW	IGH	51	None	
3	1893	CAHRLASSSWYEDYFDYW	IGH	2	None	
3	1894	CARSSPLYCIGRECTYWNFDLW	IGH	3	None	
3	1895	CARDAWVQSAAAGYHFDYW	IGH	2	None	
3	1897	CTKPRSTVFGDGLDVW	IGH	3	None	
3	1898	CWGILTGYYTYFDYW	IGH	2	None	
3	1899	CAKLSGGSSYHPSTHDYW	IGH	8	None	
3	1900	CAKLAGYSSGWAYSYMDVW	IGH	6	None	
3	1901	CARHSLFVVAAAAQPGDFDYW	IGH	2	None	
3	1903	CARSHLTLGPSSQLDGMDVW	IGH	61	None	
3	1904	CARAHVLRFFSYW	IGH	3	None	
3	1905	CALPISKISYLTGSTDLPDYW	IGH	2	None	
3	1906	CATTRSGNERDFDYW	IGH	18	None	
3	1907	CARDSYYDEDYFDYW	IGH	5	None	
3	1908	CAKDMGSYSGSNGLDYW	IGH	9	None	
3	1909	CARDPYYYDSRGYSGDYW	IGH	2	None	
3	1910	CLQDYNYPYTF	IGK	1907	None	
3	1911	CAKVEGSGYYYDFDYW	IGH	2	None	
3	1912	CARISVGGYDWAFDYW	IGH	12	None	
3	1913	CARELTISDPFDYW	IGH	3	None	
3	1914	CAPSPGVYGSGSYSPIDYW	IGH	2	None	
3	1916	CARAPTGTSNPYNFDYW	IGH	2	None	
3	1917	CAKGYGTYRYNYYAMDVW	IGH	2	None	
3	1918	CAKVIDPYSSSDYW	IGH	10	None	
3	1919	CAKDGSTSYDILTGYYFFDYW	IGH	2	None	
3	1920	CHQYFNWPPLFPF	IGK	25	None	
3	1921	CTKGYCSSISCGGDYW	IGH	3	None	
3	1922	CAKDNHATADYYWFDPW	IGH	3	None	
3	1923	CARDYFGSAHAFDPW	IGH	4	None	

3	1924	CANGNYYLSGYYYGMDVW	IGH	4	None	
3	1925	CAHSPMVRGVIRGWFDPW	IGH	6	None	
3	1926	CQTWGTGGTGIPLFVF	IGL	2	None	
3	1927	CARFAAPGFRYFDYW	IGH	11	None	
3	1928	CAKDIQAITIHEGWFDPW	IGH	2	None	
3	1929	CARDSTGYYGSGSPLDYW	IGH	6	None	
3	1930	CAKGVYGYSTSDFDYW	IGH	4	None	
3	1931	CVRDGYGVVAAARSGFDVW	IGH	20	None	
3	1932	CARQFHESVAGNDYW	IGH	4	None	
3	1933	CAREQPNTCYYDNW	IGH	4	None	
3	1934	CARTLGYCSSTSCPTPLYYYYGKDVW	IGH	2	None	
3	1935	CATAPAAIWVWWFDPW	IGH	2	None	
3	1936	CAKGVATGYYIIPFDYW	IGH	4	None	
3	1937	CARGFAAAGTPPNYFDYW	IGH	2	None	
3	1938	CARNLRGSDVFDIW	IGH	2	None	
3	1939	CARHPGIGAAAGPFDPW	IGH	3	None	
4	1	CARDLGRITMVRGGYYFDYW	IGH	2	None	
4	2	CAKGVGAPPLNWFDPW	IGH	2	None	
4	3	CARLVRSGWYLGAKSYYYMDVW	IGH	4	None	
4	4	CARGPPYCTNGVCYDYW	IGH	2	None	
4	6	CAKDITPNVYGGNSVFDYW	IGH	2	None	
4	7	CARANSVAGYYDSSGSYGYFFDFW	IGH	4	None	
4	8	CARAHVDYVSNPHYFDYW	IGH	2	None	
4	9	CARLRSGYFYPDLDYW	IGH	3	None	
4	10	CASYSSSWYDWYYYYYMDVW	IGH	2	None	
4	11	CARDFEGRWESTFDNW	IGH	2	None	
4	12	CARRSKLELTFPYW	IGH	4	None	
4	13	CARHRPEYSSSSDFDYW	IGH	2	None	
4	14	CAVLPPSHQDYW	IGH	2	None	
4	15	CAKYCTTKSCYRGFDYW	IGH	2	None	
4	16	CAHRQGSVGDMSFDYW	IGH	6	None	
4	18	CARRGYCSGGSCYVPFEYW	IGH	2	None	
4	19	CQHLNRYPFTF	IGK	876	C1	7BZ5
4	20	CARPMAQYKYDAYDIW	IGH	4	None	
4	22	CARGAALYGDYPTRDFYYMDVW	IGH	2	None	
4	23	CQSADSGGAYRNVVF	IGL	2	None	
4	24	CAKVCSSSTGDFDYW	IGH	2	None	
4	28	CARGVPSTHWYGSPFDYW	IGH	2	None	
4	30	CARDRTIFGVVRSSLDYW	IGH	2	None	
4	31	CAREFFDYYGSGSPLYYFDYW	IGH	2	None	
4	32	CARDGSSWYGDHYNYLDVW	IGH	4	None	
4	34	CQSADRSGTVIF	IGL	64	None	
4	35	CARGGSSGYYLYWYFDLW	IGH	2	None	
4	36	CAKAVGSGSGWNFDLW	IGH	2	None	
4	38	CTDYDFWSGYSHDDAFDIW	IGH	2	None	
4	39	CARRGGTTAPFEHFDYW	IGH	4	None	
4	40	CATSPWNLGVFFVYW	IGH	2	None	
4	41	CQQRSIWPLAF	IGK	44	None	
4	42	CASTWKRTPSFDIW	IGH	3	None	
4	44	CAKDGTMLGDYW	IGH	2	None	
4	45	CARDLQGDEIGDIDYW	IGH	2	None	
4	46	CAKVAGRVAARHWYFDLW	IGH	2	None	
4	47	CARHSRRWLQSSNDVFDIW	IGH	2	None	
4	48	CARFGPYCSGGGCYYFDYW	IGH	2	None	
4	49	CARDQYLKKDIVVVLAARPAGRVDYW	IGH	2	None	
4	50	CARGTYGSGSYYVVYW	IGH	2	None	
4	51	CVRRLENYVFDIW	IGH	2	None	
4	52	CAKDRRIAVAGPFDYW	IGH	2	None	
4	53	CVPLPVWFGHLLSFDSW	IGH	4	None	
4	55	CAKYYDFWSGYPWFDPW	IGH	3	None	
4	59	CQSTDSTGTIRVF	IGL	3	None	
4	60	CARDVPEGYCSGDSCLDYHQYYMDVW	IGH	3	None	
4	61	CQRSYSVPFTF	IGK	7	C2	7K8O
4	62	CSRLSTGVTGVLGMLSPWYFDLW	IGH	11	None	
4	63	CASDSSGYFQHW	IGH	2	None	
4	65	CARDVSFGQSDYW	IGH	2	None	
4	66	CARAVRGSSSPPFDYW	IGH	2	None	
4	67	CAKLGGIVDPDIVTPYYFDHW	IGH	2	None	

4	68	CVRGGPGTGLDYW	IGH	2	None	
4	70	CQQYYSIFVSF	IGK	42	C4	6W41
4	71	CITSGLYDTSDYFYVGLDYW	IGH	2	None	
4	72	CATSVRGVIPDYW	IGH	2	None	
4	74	CARGREYSGHLPDYW	IGH	2	None	
4	76	CAHRPLSYRGSSHYFDFW	IGH	2	None	
4	77	CARHGIAVALPDWFDPW	IGH	2	None	
4	78	CATVGTRYNWVPGYMDVW	IGH	3	None	
4	79	CAKDSLLYYDSSGYFDYW	IGH	2	None	
4	80	CATFVKGNWYFDIW	IGH	4	None	
4	82	CVRDLYYSDSSGYSYYLDYW	IGH	2	None	
4	83	CARRPLGYCDGGTCYHFDYW	IGH	6	None	
4	84	CQQHSSYSAHTF	IGK	6	None	
4	85	CARKSTDPYYYYMDVW	IGH	2	None	
4	86	CARDSEGNNWFDPW	IGH	16	None	
4	90	CARHWWYLVPGSSDFFDSW	IGH	2	None	
4	91	CARAQKLPDYSSSSRSAFDIW	IGH	8	None	
4	94	CQQFGSSRITF	IGK	44	C1	7K8N
4	96	CARDSSHLWFFDYW	IGH	6	None	
4	98	CTSYTSSSLLWLF	IGL	4	C2	7K90
4	99	CARGLRFGVDDAFDVW	IGH	3	None	
4	101	CAKGLSVGGSWSLDAFHIW	IGH	3	None	
4	103	CETWDSSGAKVF	IGL	10	None	
4	104	CVKAGGPGPYYFEYW	IGH	4	None	
4	105	CARLTLLYNSWYYFDHW	IGH	2	None	
4	107	CATFDRGSAFDYW	IGH	3	None	
4	109	CQSASSSGIVTF	IGL	6	None	
4	110	CARHTSATVVTPFDYW	IGH	10	None	
4	111	CARRWYDGSAYYAFDIW	IGH	2	None	
4	112	CATSRDGPFDYW	IGH	7	None	
4	113	CAIHIVDLDYW	IGH	2	None	
4	114	CARDITSYYYAKSDYFEDAFDIW	IGH	2	None	
4	115	CAHYGITLHYFDYW	IGH	4	None	
4	116	CARHTQMVFSGFDVW	IGH	4	None	
4	117	CQKYSSGPQPF	IGK	4	None	
4	119	CAKVSADNGKYSYYYYYMDVW	IGH	2	None	
4	120	CANSGDPVTSVYYYYYMDVW	IGH	2	None	
4	121	CARLYVDTAPDFDYW	IGH	2	None	
4	122	CQAWDSSGEHMLF	IGL	2	C4	7JVC
4	123	CARDSSSFEGWFDPW	IGH	4	None	
4	125	CAKGHRYYYDSSGYYGYFDYW	IGH	4	None	
4	126	CARPGGSCSGRGCYMGGWFDHW	IGH	2	None	
4	127	CLQYNVWPRYTF	IGK	20	C3	7K8P
4	129	CTTPLVLWFGERDYW	IGH	2	None	
4	130	CASSDWNDGWFDPW	IGH	4	None	
4	131	CARERGAPRYAFDIW	IGH	4	None	
4	132	CAKRRAVFMLYSGNEAFDIW	IGH	30	None	
4	133	CVKDLLFETQEDYAFDVW	IGH	9	None	
4	134	CARDPYYDFWSGLYYYYYYMDVW	IGH	2	None	
4	135	CAREYYYYYMDVW	IGH	28	None	
4	136	CARLPYSGIPIPYYYYYMDVW	IGH	2	None	
4	137	CQRYGSSPLSPRFTF	IGK	3	C1	7K8N
4	138	CARGDCRYCSRYLFYYMDVW	IGH	2	None	
4	139	CARARRGVGFGAPYSYYLDVW	IGH	2	None	
4	140	CAREVWSGGYPWYFDLW	IGH	2	None	
4	141	CAREVGNGDYPHNHYFDPW	IGH	13	None	
4	142	CTRGGNWNSLVDSFDIW	IGH	2	None	
4	143	CASHGSGSYYNPVDYW	IGH	2	None	
4	144	CQQYNSDSSEYSF	IGK	5	C3	7K8R
4	145	CTRPSYYYDSSGYQPDYW	IGH	4	None	
4	146	CQSAESGGPYQVF	IGL	3	None	
4	147	CARLWPSYDFWSGFGPLANDPLDIW	IGH	2	None	
4	148	CGRDEDYAADYW	IGH	2	None	
4	149	CAKDIECGGDCSDWYFDLW	IGH	3	None	
4	152	CTAYMELSSLRSEDTAVYYCARTSSSWYIDYW	IGH	2	None	
4	153	CATLTLNRILFNAFDIW	IGH	3	None	
4	154	CARVNPFFYERGGFDYW	IGH	2	None	
4	155	CVKDLYSEVEMATFDYW	IGH	4	None	

4	156	CARPGLDSSDLYDPFDIW	IGH	2	None	
4	157	CARRSSSSYGHDYVPFDSW	IGH	5	None	
4	158	CAKDLCGSYFRALLCYFDYW	IGH	2	None	
4	159	CAASSREQPDFDYW	IGH	2	None	
4	160	CTRGSSGWSSAAFDYW	IGH	3	None	
4	161	CARLPFLRLSTYSYYQYMDVW	IGH	2	None	
4	163	CAKLPDYYDSSGYPGDGFDIW	IGH	2	None	
4	164	CVLYLGSGIWVF	IGL	33	None	
4	165	CARHHTITMVRGVIIRGYYDYW	IGH	2	None	
4	166	CARSLVRRWLQFGAFDIW	IGH	2	None	
4	167	CAHRHRSSNGWYSLDYW	IGH	2	None	
4	168	CARGQRYGSGSSDRFDYW	IGH	2	None	
4	169	CAKDPYASYGGNSIDYW	IGH	2	None	
4	171	CAKGLGGNSGSLDYW	IGH	4	None	
4	172	CASLATYHDASGFHDAFDVW	IGH	3	None	
4	174	CAKRKAADIW	IGH	2	None	
4	175	CVRRGVGASTRFDYW	IGH	2	None	
4	176	CARDAAVSGSYSTDWFDPW	IGH	2	None	
4	177	CAKDLIRGWSNFDYW	IGH	2	None	
4	178	CAKRGRVFMLYENSHYMDVW	IGH	30	None	
4	179	CAKDKRRLFGVVIWDYW	IGH	2	None	
4	180	CARVNTVSLAATDTWWFDPW	IGH	2	None	
4	181	CARVRKESYNDYW	IGH	4	None	
4	182	CARGADSSGWSRGYMDVW	IGH	2	None	
4	183	CARTSKGYSYGRIDYW	IGH	2	None	
4	184	CAVGASVDFDYW	IGH	2	None	
4	185	CAKDVGPKRSGWYPVVFDYW	IGH	2	None	
4	186	CARDSNMLVYSDEYFQHW	IGH	4	None	
4	187	CAKAQGYSYGRDAFDIW	IGH	2	None	
4	188	CARFTIAAASNAFDYW	IGH	2	None	
4	189	CARTQWLELWLSGWYFDLW	IGH	3	None	
4	191	CARDLRQYSSSYYFDYW	IGH	2	None	
4	192	CATERILAAAGYYYYYMDVW	IGH	2	None	
4	197	CVRTPYHNAFDYW	IGH	2	None	
4	198	CARIIAAAGTSDYW	IGH	3	None	
4	200	CASDGIAAAVRPLDWFDPW	IGH	2	None	
4	201	CHSFDSSNQVVF	IGL	2	C4	7JVC
4	202	CTTDPRRDGYSDFDYW	IGH	2	None	
4	204	CQVRGNWPRIFTF	IGK	2	None	
4	205	CQVWDRSSDQRSSVHWVF	IGL	4	None	
4	207	CARDLVGAKATWFDPW	IGH	2	None	
4	208	CARIAAAGLFDYW	IGH	19	None	
4	209	CARVTGYYDSRADYW	IGH	3	None	
4	212	CTTALREYGDYRAPFDYW	IGH	2	None	
4	213	CARDLDWHNFDLW	IGH	3	None	
4	214	CAKDGGYSSSWSPAEYYFDYW	IGH	2	None	
4	218	CARATIVGATDYFDYW	IGH	5	None	
4	219	CVRVSPEYCIVTSCSAPDYW	IGH	2	None	
4	220	CQHYGGSPLYTF	IGK	283	C1	7K8N
4	221	CARHFLTDYIDFW	IGH	3	None	
4	222	CASLNDDAFDIW	IGH	9	None	
4	223	CAKDLSTDFWSPSTPSTFDYW	IGH	2	None	
4	226	CTRESLYNWDDRYYYMDVW	IGH	2	None	
4	227	CASLYYYDSSRLPFDYW	IGH	2	None	
4	228	CTTAPQIVLIPAGNSYDSTDVW	IGH	2	None	
4	229	CARGAVRLELRLYYYYYMDVW	IGH	2	None	
4	230	CARDGGAQNDYGIDVDAFDIW	IGH	2	None	
4	231	CARSLAYCGTFDYW	IGH	2	None	
4	232	CLQDHNYVLSF	IGK	9	None	
4	233	CAKDRAVAGTPLYYNWFDSW	IGH	7	None	
4	234	CARQKTGTPTGSDYW	IGH	2	None	
4	235	CARWDGKDGWSNFDYW	IGH	4	None	
4	236	CASVGEGYHDILTGYQGSYYFDYW	IGH	3	None	
4	237	CTTYSSGYYYGDYW	IGH	5	None	
4	238	CARDPTSLLWSGYSPPDYW	IGH	2	None	
4	239	CASGRDSSSWYDYW	IGH	2	None	
4	240	CASPISGSSYSDTSNYYPRAFDIW	IGH	2	None	
4	241	CATYPHPSPPVEPWLAPFDFW	IGH	8	None	

4	242	CARANGGGYYVYW	IGH	2	None	
4	243	CQQAKNFPTYTF	IGK	6	C1	7BZ5
4	245	CAADLWRDGIWHVFDIW	IGH	2	None	
4	246	CARGGTWIQLWSNYFDYW	IGH	2	None	
4	247	CILSFDYW	IGH	2	None	
4	248	CARQHGDYGDYW	IGH	6	None	
4	250	CARRYCSTTSCLIDYW	IGH	4	None	
4	252	CARVFMRAPGTPENWFDPW	IGH	2	None	
4	253	CAHRHELYDYVWGSYRYEAYVGYYFDYW	IGH	2	None	
4	255	CAIRLSTASWYGMFAFDLW	IGH	3	None	
4	257	CAKDIRLGIGVSDYW	IGH	2	None	
4	258	CVKDTRSGSSWSSIIFDYW	IGH	2	None	
4	259	CARENNSDYSFNFDNW	IGH	2	None	
4	260	CARMAYDSSGNISPFDYW	IGH	2	None	
4	261	CARILDQKYYDYDWGRYRYTGLFDYW	IGH	4	None	
4	262	CARDQRRGYYGSGSLDYW	IGH	4	None	
4	263	CARDCLMVYAEDHYYYYYMDVW	IGH	2	None	
4	264	CARDLGSTGPQLCDYW	IGH	2	None	
4	265	CVRNYNTYSAYYYYMDVW	IGH	2	None	
4	266	CAGGVVVAATPSMGWFDPW	IGH	2	None	
4	267	CAREGYSSSSPFFDYW	IGH	6	None	
4	268	CARHAVVRGVGYYFYYMDVW	IGH	4	None	
4	269	CARGVPTGVDYFDYW	IGH	4	None	
4	271	CAVMNHYDSSAYHSAKAFDFW	IGH	2	None	
4	274	CARGEEIVVAPDTIPVEMLTGATHHWFDLW	IGH	2	None	
4	275	CATNFPIVYASGASGDYW	IGH	2	None	
4	276	CAKARTTNWGSARPYWYFDLW	IGH	4	None	
4	278	CAREFRYSSGWYGGDAFDIW	IGH	4	None	
4	279	CATDHNRYFEYW	IGH	2	None	
4	280	CARMVYYDYVWGSYPLYYFDYW	IGH	4	None	
4	282	CVRGEYDLWRDYYNDYW	IGH	2	None	
4	283	CATSRDDSNSFDSW	IGH	2	None	
4	284	CSSYSSGSTLILF	IGL	4	None	
4	285	CAKDRYSSSWYDNYYFDYW	IGH	2	None	
4	286	CVKIAVSGLWYFDYW	IGH	2	None	
4	288	CARAESHMVRGVITYYFDYW	IGH	2	None	
4	289	CARLSREMATGSDWFFDLW	IGH	2	None	
4	290	CARATPYMDVW	IGH	10	None	
4	291	CTRDRVALGRHLVEFDYW	IGH	2	None	
4	292	CARASFSHYYYSGNYYPHYYFDYW	IGH	9	None	
4	293	CQSAGTSGGHVYLVF	IGL	2	None	
4	294	CARNHTSSIFDSW	IGH	2	None	
4	295	CVRLDGDNWFDPW	IGH	4	None	
4	296	CARDAYRSSYYFESW	IGH	3	None	
4	298	CQQFNTYPQGGTF	IGK	90	C3	7K8R
4	299	CARPLGVVTAYDAFDIW	IGH	2	None	
4	300	CARGLLLWTGEPPYYIDIW	IGH	2	None	
4	303	CAREMSTVPTRRGIDYW	IGH	2	None	
4	304	CATVRWFVDEMILDYW	IGH	2	None	
4	306	CVQASIAAAGIFDYW	IGH	4	None	
4	308	CARSIVVFSDAFDIW	IGH	2	None	
4	310	CAKSPGRTVTTYRYYFEYW	IGH	2	None	
4	311	CARERRLVTTTKYYFDYW	IGH	2	None	
4	312	CARGGLSHLGENPDYYYYYMDVW	IGH	4	None	
4	315	CARSYSGSDRGDYW	IGH	4	None	
4	317	CQQRANWPPSTPITF	IGK	4	None	
4	318	CMQALHTPLFSF	IGK	7	None	
4	319	CASGYCSDGVCYSALDYW	IGH	2	None	
4	320	CAREELLGRYYFDSW	IGH	2	None	
4	321	CARSFSGSYSYYFDYW	IGH	2	None	
4	322	CAKDPRRYGDYVGFPFDYW	IGH	2	None	
4	323	CARQQRLGASHSPFDYW	IGH	2	None	
4	324	CARFAAKWFDSW	IGH	2	None	
4	326	CARDLGYTYGSNFFDPW	IGH	4	None	
4	327	CARESYCGGDCSYYFDYW	IGH	4	None	
4	328	CTRDEDCSGDGCSQYHFDYW	IGH	3	None	
4	329	CAKAYYYDSSYYYYYAFDIW	IGH	2	None	
4	330	CARGGSGVWQSFDSW	IGH	3	None	

4	331	CQQLNNYLAPTF	IGK	13	C1	7BZ5
4	333	CARDADVSGFLRSSHFDYW	IGH	2	None	
4	334	CLQINGYPALSF	IGK	6	C1	7BZ5
4	336	CAKATYYYDSGKLKRNWFDPW	IGH	3	None	
4	337	CTRLLLPRTFDYW	IGH	6	None	
4	338	CAKNGNTYGWEYYYYMDVW	IGH	2	None	
4	339	CAKGAGQQWLIDYW	IGH	4	None	
4	341	CAHRRGGIFYDFDYW	IGH	3	None	
4	342	CARRRRRLFDYW	IGH	3	None	
4	343	CQQYGTPPPYIF	IGK	18	C2	7K8U
4	344	CAKDTSSGWLSGDFDYW	IGH	4	None	
4	345	CAGSYSYYDSSGYELPFDYW	IGH	2	None	
4	347	CARGYFGKLLSDAFEMW	IGH	4	None	
4	348	CLQHDNGGTF	IGK	2	None	
4	349	CARESLSGSPPYMDVW	IGH	2	None	
4	350	CAKDPSIAAPSGDYW	IGH	2	None	
4	351	CAKDGGDLYAFDIW	IGH	7	None	
4	352	CLTHNIYPYSF	IGK	2	None	
4	353	CARVYYGGNPYADAFDIW	IGH	2	None	
4	356	CAKGPLLGHYYFDYW	IGH	2	None	
4	357	CARSPAPSGSYFKRGDNWFDPW	IGH	2	None	
4	358	CAKSSGQWLANWFDPW	IGH	2	None	
4	359	CARAYSGGRHHYYYYLDVW	IGH	4	None	
4	360	CAKGQGSSSLNFDYW	IGH	2	None	
4	363	CARETPYETGFDPW	IGH	2	None	
4	364	CASPGYSYGTALFDYW	IGH	3	None	
4	365	CARSPMTTVVTPGDYYYYFDVW	IGH	2	None	
4	366	CARDYVQWAVAGTRYYMDVW	IGH	2	None	
4	367	CTTAPGIVVVPVAPGAFHYMDAW	IGH	2	None	
4	368	CTRRYYHDSSGNFQNDYW	IGH	10	None	
4	369	CARDDRYSGSYSRNFDYW	IGH	2	None	
4	370	CAAWWELQDAFDIW	IGH	2	None	
4	371	CAKSESQLWFPLVVGAFDIW	IGH	2	None	
4	374	CARDGSAIDDFWSRTRSFAWMPDYW	IGH	2	None	
4	375	CARDQMVRGGAFDYW	IGH	2	None	
4	376	CARNIGGYSFWFDPW	IGH	2	None	
4	377	CASGLQLVRLDYW	IGH	5	None	
4	378	CARVVLGVGATPAVRTYWYFDLW	IGH	2	None	
4	379	CQHYDDLPRFTF	IGK	70	C3	6XDG
4	380	CASLPHIVVVPDDAFDIW	IGH	2	None	
4	382	CAGEPLVVELSATGEGAFDIW	IGH	2	None	
4	384	CARARNGHPPDAFDIW	IGH	2	None	
4	386	CQAWDSSSDHRYVLF	IGL	2	None	
4	387	CCQQYNNWPQDAF	IGK	3	C3	7K8R
4	389	CARTPKLGKCFVGDCFAGGWFDPW	IGH	2	None	
4	390	CQQCGSPPKF	IGK	4	None	
4	392	CARDAYCSSTSCDRSWRHGWFDPW	IGH	2	None	
4	393	CGADHGSGSNFDWVF	IGL	7	None	
4	394	CQSYGSSLNGLVLF	IGL	4	C1	7K8N
4	396	CVRDPDFGSGTYLGYSDYW	IGH	4	None	
4	397	CARDEPFISGSHYYFHYW	IGH	3	None	
4	399	CARHEIVVVPAAIPSSRYDWFDPW	IGH	2	None	
4	400	CTRVDVSNYWFDSW	IGH	8	None	
4	401	CARDAGDTGWSRDCFDHW	IGH	2	None	
4	402	CARVVVVVPEQPAAIVSYYYYMDVW	IGH	2	None	
4	403	CARVHGNDPKTLFDYW	IGH	2	None	
4	404	CARENFRPTHWFDPW	IGH	4	None	
4	405	CTQVGASDYW	IGH	4	None	
4	406	CQSYHSTNPLWVF	IGL	6	C4	7JVC
4	407	CGTWHSNSKTQVWVF	IGL	2	None	
4	408	CARGYSPWSKYSSSSGPFDYW	IGH	2	None	
4	409	CAKDTTTVASDAFDIW	IGH	2	None	
4	410	CATSLYCSGGSCYSEYFQHW	IGH	2	None	
4	411	CTTISGPDYGGNQQTEDRDFDYW	IGH	2	None	
4	412	CGAGYYARFDYW	IGH	2	None	
4	413	CARDRGRPDSFDLW	IGH	6	None	
4	415	CTTDRILTGYVRYFDYW	IGH	6	None	
4	416	CARGQGNHLDYW	IGH	2	None	

4	418	CAKDIGYSYARYYFDYW	IGH	6	None	
4	419	CAAYYYHTSGYDYW	IGH	2	None	
4	420	CARDIGWELLRESDYW	IGH	2	None	
4	421	CARSPGNFWTANYDYW	IGH	2	None	
4	422	CAREPRWCVSGRCYVEYW	IGH	2	None	
4	423	CQSADSSGSFPWMF	IGL	6	None	
4	424	CARWLQFTSIDYW	IGH	11	None	
4	425	CARETGYTYGHPYFDFW	IGH	10	None	
4	428	CARMGSYGSGSYFDYW	IGH	19	None	
4	431	CARASLYGDFFDSW	IGH	10	None	
4	432	CASSPGYRSGTTSFDYW	IGH	2	None	
4	434	CARESGTGTHDYW	IGH	3	None	
4	435	CQQYNNWPPLFTF	IGK	610	C3	7K8R
4	436	CARLPTLYYYDSSYWYFDLW	IGH	2	None	
4	439	CTTRTLNRIMITFGPFDYW	IGH	2	None	
4	441	CARSRIAAVDIVATYYFDYW	IGH	2	None	
4	442	CQTWGTGLQVF	IGL	114	None	
4	443	CARRLHRDDGDTFDIW	IGH	6	None	
4	444	CARLEYSSSFDYW	IGH	10	None	
4	445	CAKSGILTGYRFDPW	IGH	2	None	
4	446	CAKDASRGSGHYVYAFDIW	IGH	2	None	
4	447	CAKETPNWNYVLGFDPW	IGH	4	None	
4	448	CGTWDSSLSVYVF	IGL	838	None	
4	449	CARQKVYYDGSGFFFDPW	IGH	8	None	
4	450	CARPRPDSSGYDPPFDYW	IGH	2	None	
4	451	CARHSYITMIVVVIPHAFDIW	IGH	2	None	
4	452	CARHSAQQLTQPFDIW	IGH	2	None	
4	453	CARSPDYSDYYFDYW	IGH	4	None	
4	454	CARDVIHSSSWTYYFDYW	IGH	2	None	
4	455	CAKSPGAYYFDYW	IGH	9	None	
4	456	CARDQSITMIVVVPIGFDAFDIW	IGH	2	None	
4	457	CARELESAFDIW	IGH	54	None	
4	458	CARDQSSLYGSAPLDYW	IGH	2	None	
4	459	CVRDQSSFCGSDCYHFDFW	IGH	2	None	
4	460	CARRVVPAAIGYYFDYW	IGH	2	None	
4	461	CARDRAARLLFGLDYW	IGH	2	None	
4	462	CARAGGIAAAGLYYYYGMDVW	IGH	5	None	
4	464	CASLNYYGSGEYFLHW	IGH	2	None	
4	465	CSSYVGSNNHYVF	IGL	11	C2	7BWJ
4	466	CARGPYCGGDCYSRLGTFDIW	IGH	2	None	
4	467	CARSPRYYYSSAYYPLAFDIW	IGH	3	None	
4	468	CARVVTMVRGVIITGYFDYW	IGH	4	None	
4	469	CARFYYGSGSYPSDDYW	IGH	2	None	
4	470	CARAPRGYCSSTSCARNAFDIW	IGH	2	None	
4	471	CARGDDYMTGFSYYMDVW	IGH	2	None	
4	472	CASLATIEEDYW	IGH	2	None	
4	473	CAKAYYGSGSYYKEFFDNW	IGH	6	None	
4	475	CVRDSLSYHESSGYYGSDPFAYDIW	IGH	2	None	
4	476	CARHSVAAAGANWFDPW	IGH	4	None	
4	477	CARDGDETYDVFDIW	IGH	2	None	
4	479	CARDPDYGGNSPPPSDFDYW	IGH	2	None	
4	480	CAREMDPHYNFDYW	IGH	2	None	
4	481	CARLLRSSGCYDYW	IGH	2	None	
4	482	CVKDRPAPSISDYW	IGH	2	None	
4	483	CARNYCSGGSCYTHAFDLW	IGH	2	None	
4	484	CQQLNNYPSGGFTF	IGK	4	C1	7BZ5
4	485	CARDNGGPGDAFDVW	IGH	4	None	
4	487	CARDRRDGWVPKAGFDPW	IGH	2	None	
4	488	CEKQNPPVLAAAGYFDYW	IGH	2	None	
4	492	CARGGDCSSTSCYPTIYYYYYMDVW	IGH	2	None	
4	493	CARTGLFWSGYYRWFDPW	IGH	4	None	
4	494	CARQRGGAIPYWYFDLW	IGH	2	None	
4	497	CTRERAGTSWFDPW	IGH	2	None	
4	499	CATCPGYYDGCPFDYW	IGH	2	None	
4	500	CARHYFDFWSGHFYFDNW	IGH	2	None	
4	501	CARAREQLRGPFRGYPLDYW	IGH	2	None	
4	502	CARFPDRQKPDYYYMDVW	IGH	4	None	
4	503	CTTVITIFGLYYFDYW	IGH	2	None	

4	504	CAAWDDSVNAFWVF	IGL	2	None	
4	505	CAKGRDFGVIIVDLDYW	IGH	2	None	
4	506	CARPVSRGPAYFDYW	IGH	9	None	
4	507	CVRDPSATCTTTHCYGPHHAFDVW	IGH	2	None	
4	508	CARGAPNWGSNYFDYW	IGH	9	None	
4	509	CARAATRNYYDSRGYFYYFDYW	IGH	2	None	
4	511	CARSFLHYSGSSGYYSGSFDYW	IGH	5	None	
4	512	CQSYDTSLSGPKYVF	IGL	13	C4	7JVC
4	513	CGRSSGWFTWYYFDYW	IGH	4	None	
4	514	CASKYGSYYDYW	IGH	50	None	
4	515	CAKEGLRFLEWLPHYMDVW	IGH	2	None	
4	519	CARGSRYSSSWYVGAYWFDPW	IGH	2	None	
4	520	CARHTSIAVAYYYYMDVW	IGH	2	None	
4	521	CTTPPGYYGTSPFDFW	IGH	4	None	
4	525	CAREVPGRYCNGGSCYGSVDYW	IGH	2	None	
4	526	CARQLLGGDSSGSGCFDYW	IGH	6	None	
4	527	CVKDDTYGSSSPFDYW	IGH	2	None	
4	528	CARGLYYYDTTGHSPNFDHW	IGH	2	None	
4	530	CARRVAAGPTRYFDHW	IGH	4	None	
4	531	CARDRGITGAEPYYFDYW	IGH	2	None	
4	532	CARESRAHWVDPW	IGH	2	None	
4	533	CARGRQGGYYRNYYYYYMDVW	IGH	2	None	
4	534	CQSADASGPDIHWVF	IGL	2	None	
4	536	CARGYTSGWYDFDYW	IGH	15	None	
4	537	CARDKADTAMVSYNSFDSW	IGH	4	None	
4	538	CTRDSGSSPFDFWSGYFVPFDPW	IGH	2	None	
4	540	CARAAGYGSNWAPPDTFDYW	IGH	6	None	
4	541	CAKTATGRHGYFDLW	IGH	2	None	
4	542	CAKVGSGYYYGSRELLPPYYYMDVW	IGH	2	None	
4	544	CGRHLDRYNAFDLW	IGH	9	None	
4	545	CAKMVPARFWGYFDYW	IGH	2	None	
4	546	CASFPDDFWSGYYEPGDWFESW	IGH	2	None	
4	547	CARGGGGSYSTLRDAFDIW	IGH	2	None	
4	548	CARGGYSSSSGNWFDPW	IGH	4	None	
4	549	CARDGRCSGGNCYSGLLDYW	IGH	2	None	
4	550	CAKALGHCTNGVCYWDRYHFDYW	IGH	50	None	
4	551	CAREYCTSTTCVVDYW	IGH	8	None	
4	552	CASQTSYSFDIW	IGH	2	None	
4	553	CVQGLQLPLALSF	IGK	2	None	
4	555	CASLSSKCWYLCGGVDYW	IGH	2	None	
4	557	CMQGTHWPPYTF	IGK	1381	None	
4	558	CARDRGTGTSSSWLDPW	IGH	10	None	
4	559	CARVESNGWSDYYFDYW	IGH	6	None	
4	560	CARPFTDSRHYYYYYLDVW	IGH	4	None	
4	561	CANENYYGSGSYIDHW	IGH	2	None	
4	563	CARSRCSGGSCYPTGSFDYW	IGH	2	None	
4	564	CAREWAEAVPWDTFDMW	IGH	2	None	
4	565	CTTDGVTIFGVVIIQNFDYW	IGH	2	None	
4	566	CSRDRIDLAYCSNPRCSNFHYW	IGH	4	None	
4	567	CARIPAVGPSYAVDIW	IGH	2	None	
4	568	CSQTLPRFDLW	IGH	4	None	
4	570	CARVGAARGPLYYYYMDVW	IGH	4	None	
4	572	CARHESSNIAAAAPDAFDIW	IGH	2	None	
4	573	CAKVVMVRGLSHYFDKW	IGH	2	None	
4	574	CATASPNRSLEFSYEHSGYMNFYYYMDVW	IGH	3	None	
4	575	CARSIAVAAKGYFQHW	IGH	2	None	
4	577	CASWDDSRNGVLF	IGL	5	None	
4	579	CASTDYYGSGTPYMDDW	IGH	2	None	
4	580	CARDGYDSSGYYNWFDPW	IGH	2	None	
4	582	CYSTDISDNHTGVF	IGL	2	None	
4	585	CAKNKDADKYSSPFDYW	IGH	2	None	
4	587	CARMVPHSEVILKDDAFDIW	IGH	2	None	
4	589	CARGPSNGDLWFGEAHAFDIW	IGH	2	None	
4	591	CVNDRATSGWSYFGSW	IGH	2	None	
4	592	CARETFFGSPFDFW	IGH	11	None	
4	593	CARHGIAEEYYFDYW	IGH	4	None	
4	594	CARVINYDILTGNRPFDYW	IGH	2	None	
4	595	CARLPPSWAVAGTLDYW	IGH	2	None	

4	596	CVRLDTSGWYRPYYDHW	IGH	3	None	
4	597	CARQLREPHDAFDIW	IGH	2	None	
4	598	CARLIAARPAFDIW	IGH	4	None	
4	599	CARGEFDEQFAFDPW	IGH	4	None	
4	601	CLLRGWSPSYYYMDVW	IGH	4	None	
4	602	CQQYDTIPPRITF	IGK	9	C3	6WPS
4	603	CARDGANRDFDYW	IGH	15	None	
4	604	CARSPTYSTSWSDYYYYMDVW	IGH	2	None	
4	605	CARERGHRPSSYADSW	IGH	2	None	
4	606	CARGSSASGDYGDNW	IGH	11	None	
4	607	CARDTPPGGEFYLDHW	IGH	2	None	
4	608	CTKDIPLYSSSSHPPVDYW	IGH	2	None	
4	609	CARERYCSSTSCFRTISYFDYW	IGH	2	None	
4	610	CAKVSGDTSDWYGSPDVFDIW	IGH	2	None	
4	613	CARDSGDTSPFDPW	IGH	2	None	
4	614	CAREWYSSGWYGPFDIW	IGH	6	None	
4	615	CVKDRGNTWFFDSW	IGH	3	None	
4	616	CARRLNWSYEQYFDTW	IGH	2	None	
4	617	CARDNRYYDSSGYSDAFDIW	IGH	2	None	
4	619	CARHFFRGARDWFDPW	IGH	2	None	
4	621	CARSARHNSAHFDYW	IGH	2	None	
4	622	CAARWYFFEYW	IGH	2	None	
4	623	CARNYCDGGSCYRKYYHNYYMDVW	IGH	2	None	
4	624	CAKGLIGHYDSSAYYLELFDYW	IGH	2	None	
4	625	CARPPRLPSSRHGSGHHYAALYFDLW	IGH	2	None	
4	626	CARDPYDNSGYGVRNFDYW	IGH	2	None	
4	627	CMIWHSSAWVF	IGL	24	None	
4	629	CARDILSSWYDHFDYW	IGH	7	None	
4	630	CQVWDSDSDHAIF	IGL	35	None	
4	632	CARGSDVGNGFDYW	IGH	8	None	
4	633	CARAGVTGLKFDPW	IGH	2	None	
4	634	CQVSDSSSDVVF	IGL	259	None	
4	635	CTGELADSYYFDNW	IGH	11	None	
4	636	CARDSLGTYFYMDVW	IGH	2	None	
4	637	CARDYCGTMACDIEGAFDVW	IGH	6	None	
4	638	CARIRRAIEVPYYFDHW	IGH	2	None	
4	639	CARGRPGRQYYYDSSGWYYFDYW	IGH	3	None	
4	640	CARVAVAGKKIDYW	IGH	2	None	
4	642	CARHIADSSSSKFDFW	IGH	2	None	
4	643	CARHWGGGVDYW	IGH	2	None	
4	644	CTSSPLIYQFDYW	IGH	12	None	
4	646	CASRYSSSWYRGSYFDYW	IGH	3	None	
4	647	CATLSRYSVYFDYW	IGH	2	None	
4	648	CARQPRSSSGWWPHDAFDIW	IGH	2	None	
4	649	CAREPIPILGINFDYW	IGH	2	None	
4	650	CARVWDYYYDFAVDVW	IGH	2	None	
4	651	CARTYYGSGKWFDPW	IGH	2	None	
4	652	CAKDKFSGRSWFPFDHW	IGH	2	None	
4	654	CVKDPDYGSGAYLGYFDSW	IGH	3	None	
4	655	CARVGDYGGKTPSDWFYWYFDLW	IGH	2	None	
4	656	CAKDLDWNAGGFDYW	IGH	2	None	
4	657	CYSRDYSDNRIF	IGL	4	None	
4	658	CEQLSSYPMYTF	IGK	6	C1	7BZ5
4	660	CIRVRDGGAFDLW	IGH	2	None	
4	661	CARAGRTNYYDTSGSDFDFW	IGH	4	None	
4	662	CARDQGWFGQLGALDYW	IGH	2	None	
4	663	CARDSYSSGPTFDYW	IGH	2	None	
4	664	CARDGGIAAIDQSEGDYFDYW	IGH	2	None	
4	666	CARSGVITGPARGGYYYYMDVW	IGH	2	None	
4	667	CARDPGNWSPWYFDLW	IGH	2	None	
4	668	CAKLHGSGSYSSDDYFDPW	IGH	4	None	
4	669	CAALYSSSSRVDYW	IGH	2	None	
4	670	CQQLNAHPTTF	IGK	2	C1	7BZ5
4	671	CAKELGSATWIFDYW	IGH	2	None	
4	673	CQQYNTNSPYNF	IGK	10	C1	7BZ5
4	675	CARDFWGTFRYNDHW	IGH	7	None	
4	677	CARHDARPPDYGSGGWFDPW	IGH	2	None	
4	679	CAREKGIYGYNLHAFDIW	IGH	6	None	

4	680	CARVRRDSSSGRYMDVW	IGH	2	None	
4	681	CHHSSSLPAF	IGK	6	None	
4	682	CARVWAWRYYDSSGYYDYW	IGH	6	None	
4	683	CARDPPGTTSRWFDPW	IGH	2	None	
4	684	CARFLGVVFNWFDPW	IGH	2	None	
4	685	CAREGIKVRGRPIYDYW	IGH	2	None	
4	686	CAKHGGTYAIDYW	IGH	2	None	
4	687	CAKDFQAYSSHLHETYYDYW	IGH	2	None	
4	688	CARLHTMAIYLDYW	IGH	2	None	
4	689	CTGDREGTYYFEHW	IGH	5	None	
4	691	CQQTVSFPPWTF	IGK	16	C1	7BZ5
4	692	CAKIMVRGPSDYW	IGH	4	None	
4	693	CARHFGTYTEENWFDSW	IGH	2	None	
4	694	CARENWASFDFW	IGH	4	None	
4	695	CARPYNSGWYTHYYFDLW	IGH	4	None	
4	696	CASYLTRPGYPTTNFFDYW	IGH	2	None	
4	697	CAREDWDNEGYYFDWW	IGH	6	None	
4	698	CGRVNIVFPDYW	IGH	3	None	
4	699	CSKEPSSWARGDEATDVW	IGH	3	None	
4	700	CARVPTMREVYVDYW	IGH	2	None	
4	701	CASDLSLTYPDIW	IGH	3	None	
4	702	CARPRSAARHPGLYDAFDIW	IGH	6	None	
4	703	CARHRIGIQDFDYW	IGH	3	None	
4	705	CARDSGYCSIGVCLSNWFDPW	IGH	3	None	
4	708	CARLIVVVTAAYSDAFDIW	IGH	2	None	
4	710	CVKDFRFLEWARTGYFDYW	IGH	3	None	
4	711	CARGIRTGVTAFDSW	IGH	4	None	
4	712	CARLRSTNSKWFDPW	IGH	4	None	
4	713	CARQAVGRDDWPPFDYW	IGH	9	None	
4	715	CARQGYADPRIDYW	IGH	2	None	
4	716	CARTFAVYYYDSSGIDYW	IGH	2	None	
4	717	CARDLMSIAVAVTPDYW	IGH	2	None	
4	719	CAHRRSGSYFDYW	IGH	7	None	
4	720	CARQGTTVASIFFDYW	IGH	3	None	
4	722	CARPHRGGPLSAFDPW	IGH	2	None	
4	723	CARGRLITYSSSWYYRPRFDPW	IGH	4	None	
4	725	CARQVVVLGRWFDPW	IGH	2	None	
4	726	CATQRYTGSYYEVWYFDLW	IGH	8	None	
4	728	CARRHGLWKHPFDSW	IGH	2	None	
4	729	CARSPPLSAVTTPHYFDYW	IGH	2	None	
4	730	CAKDKIVHSYGNAFDMW	IGH	3	None	
4	731	CAKDRWSGYCSSTSCQDPIDNWFDPW	IGH	2	None	
4	732	CTREEVEGGYAEYFQHW	IGH	5	None	
4	733	CARVTALDYVWGTHDYW	IGH	2	None	
4	735	CQSANSSDSYWVF	IGL	49	None	
4	737	CAKLIAAAFRWFDPW	IGH	2	None	
4	738	CARDRSGTALTSHWYFDLW	IGH	2	None	
4	739	CNSRDSSGNHSPVF	IGL	146	None	
4	740	CARILHYYDSSGYYYWGFDYW	IGH	2	None	
4	741	CARGPTKGNAFDVW	IGH	3	None	
4	742	CARYSIANYFDYW	IGH	7	None	
4	743	CARDKKRITIFGVVTTGFYYYMDVW	IGH	8	None	
4	744	CARRFNDLLTGSLGRYFYMDVW	IGH	2	None	
4	745	CAGEQKAFDLW	IGH	2	None	
4	746	CARVGCTSPSCYDGWFDPW	IGH	4	None	
4	747	CAKDFWVSGLVVTEDGYFDYW	IGH	2	None	
4	748	CARQVSPPGYHFDYW	IGH	4	None	
4	750	CAGGRSRVDYW	IGH	2	None	
4	751	CARTGSSSGWGFWFDPW	IGH	2	None	
4	752	CARRTLYNTGPYFDYW	IGH	3	None	
4	753	CAREAYDSTGYRLGYFEKW	IGH	5	None	
4	754	CARAPLQGWRHTLNWFDPW	IGH	6	None	
4	755	CARHRYCSSTSCRRLAFDYW	IGH	4	None	
4	756	CARDPILRPMIVVANPADYFDYW	IGH	2	None	
4	757	CARHVYYDFWSGPINWFDPW	IGH	4	None	
4	758	CQQANSFPRITF	IGK	233	C3	7K8R
4	760	CAKMARSATCNPCRWFGPW	IGH	2	None	
4	763	CVRHTAFWYSSSYGWFDPW	IGH	3	None	

4	765	CQSTDSSATRDWVF	IGL	4	None	
4	766	CARGYDVLTGYPDYW	IGH	21	None	
4	767	CAKDINWSYVRYFDLW	IGH	3	None	
4	768	CARGYTYYYDSSGSYPWYFDYW	IGH	2	None	
4	769	CAKDRVAVAYFIDYW	IGH	4	None	
4	772	CARAGVSWLLRALNYFDYW	IGH	2	None	
4	773	CFCAADNNQGGF	IGL	5	None	
4	774	CARTLSEHQPLYYFDYW	IGH	2	None	
4	776	CAREIWYARSLFDIW	IGH	2	None	
4	777	CARDGVRATPSYFDYW	IGH	2	None	
4	779	CMEALQTRQWTF	IGK	3	None	
4	780	CAKGDYGSVLMVPFDYW	IGH	4	None	
4	783	CAKDVSWELPYYFDYW	IGH	2	None	
4	784	CAKGIAVAGTCDYW	IGH	2	None	
4	786	CGSYTSTSTLGLIF	IGL	2	C2	7K90
4	787	CQVWDSSSDQVGLF	IGL	8	None	
4	788	CARDSGDDCSGGSCYSGDYW	IGH	2	None	
4	789	CQQANTF	IGK	2	None	
4	790	CAKETSSGWQGDAFDIW	IGH	2	None	
4	791	CARFNRGVKAAGLFANWFDPW	IGH	2	None	
4	792	CTRDWEHDYW	IGH	2	None	
4	795	CAREIDRGAPKYFDPW	IGH	2	None	
4	796	CAKALREQKPLDYW	IGH	2	None	
4	797	CARGKYQEPPGWLDSW	IGH	2	None	
4	799	CARAGIPYHGAFDIW	IGH	2	None	
4	800	CAKEYCSSADCYADHFDFW	IGH	2	None	
4	803	CARLLDDYGDYRYFDYW	IGH	2	None	
4	804	CARKSGYFSGQYYLDVW	IGH	2	None	
4	805	CARMTITGTTDWFDPW	IGH	2	None	
4	806	CVKEALEYSSSYDDYW	IGH	2	None	
4	807	CARRDRNTNYFDYW	IGH	7	None	
4	809	CARDQIPVLRYFDWLLPFDYW	IGH	4	None	
4	810	CALPISAVYYCAKDLDGGESAFDIW	IGH	4	None	
4	811	CARHPDLAKPFDVW	IGH	4	None	
4	812	CARDRQAAAGRGGLDYW	IGH	2	None	
4	813	CARGVRAGFGVVLPRSNWFDPW	IGH	2	None	
4	814	CARQSFLVAPGAFDIW	IGH	3	None	
4	815	CARHRRRSGYYFDAFDMW	IGH	2	None	
4	816	CARDPGIAIYGVVYYNYLDVW	IGH	3	None	
4	817	CARWRRDGYNLDAFDIW	IGH	2	None	
4	818	CARDQIGRDWGSSTAFDVW	IGH	2	None	
4	819	CAREIIVVVPAARNWFDPW	IGH	2	None	
4	820	CGTWHSNSMF	IGL	2	None	
4	821	CASRDFWSGNAYSYMDVW	IGH	4	None	
4	822	CAKDQGYDFWSGYYSEFDYW	IGH	2	None	
4	823	CARLPGIAAPVGWFDPW	IGH	2	None	
4	824	CARDTYDNTGYYYKDFDYW	IGH	4	None	
4	828	CARQRTAAVGTDFDFW	IGH	2	None	
4	829	CARVFVTTDGYDYW	IGH	2	None	
4	831	CARVIAAAASGWFDPW	IGH	2	None	
4	832	CAHRRLELLFDPW	IGH	2	None	
4	833	CAKVGLWFGWYYYYMDVW	IGH	2	None	
4	835	CARAVPTLTTPLDAFDIW	IGH	4	None	
4	837	CARGIVAASGALFEYW	IGH	2	None	
4	838	CQQYNTYSQHTF	IGK	100	C3	7K8R
4	839	CAKVYDFWSGYYTGYYYYMDVW	IGH	2	None	
4	841	CATSAGTYYYGSALPFDIW	IGH	4	None	
4	842	CARPDGRSSTGYDPFDVW	IGH	2	None	
4	843	CAGSKKYTDGGGFGYYYYSMDVW	IGH	4	None	
4	844	CAREPPAVVHDAFDIW	IGH	4	None	
4	847	CAKDRRRSSSWSMPHDAFDIW	IGH	2	None	
4	848	CARQIWSGYSAKFDYW	IGH	3	None	
4	849	CARDESGYYDSSGYYYSAFDIW	IGH	4	None	
4	850	CVRDTEVIPTYYFDYW	IGH	2	None	
4	853	CTTLDPVNQDFDVW	IGH	3	None	
4	854	CARVFIGGSYGPFDYW	IGH	2	None	
4	855	CAKDRVGIDSYGLRYFDYW	IGH	2	None	
4	856	CARGSSTVTHFDYW	IGH	4	None	

4	858	CAKGGDYGGPRGPFDHW	IGH	2	None	
4	861	CTRLDHVGATRAFDVW	IGH	3	None	
4	862	CVKEAGSSSWRNYFDYW	IGH	2	None	
4	863	CAKVGEGWPPSYYKYYMDVW	IGH	4	None	
4	865	CQPYNSLPIIF	IGK	4	C3	7K8R
4	866	CARLSGTYEWYFDFW	IGH	2	None	
4	867	CVKSNGPMETFGGSNPNTHFDSW	IGH	2	None	
4	868	CARLARPYYYDSSGYRNWFDPW	IGH	2	None	
4	869	CARDPRIELWKRGYYMDVW	IGH	2	None	
4	871	CARDLGMGSYFDYW	IGH	7	None	
4	873	CARDRFLGRGWLQFNAFDIW	IGH	2	None	
4	874	CGTGGAEGLDYW	IGH	5	None	
4	875	CARSSLGSYGDYVGYW	IGH	2	None	
4	876	CARDRRGSDYYYYYMDVW	IGH	4	None	
4	877	CQRYNSDPPFSF	IGK	35	C3	7K8R
4	878	CARQDYESSGPSPYFYYYMDVW	IGH	2	None	
4	879	CARYYYDILTGYLHFDYW	IGH	2	None	
4	880	CARLASRITIFGVASRGWFDPW	IGH	4	None	
4	881	CARDLSGSYYPSFDYW	IGH	4	None	
4	882	CARDAAPLAGPDYW	IGH	8	None	
4	884	CARGRLGYCNDGSCHGHAFDIW	IGH	5	None	
4	885	CARKVRGVIIPNDAFDIW	IGH	2	None	
4	886	CASGPRITRTNDYW	IGH	4	None	
4	888	CAKDLTGRSLIDAFDIW	IGH	3	None	
4	889	CATYGGNEWGPFDYW	IGH	4	None	
4	890	CAKGPMEWKTRQLAPLDYW	IGH	6	None	
4	891	CTTSLGWWAFNYW	IGH	3	None	
4	892	CARDRKSIAARRGADYW	IGH	2	None	
4	894	CGTRLSGKTIDYW	IGH	2	None	
4	895	CARDRGPPAANDYW	IGH	24	None	
4	896	CATTSADFYQTSGYEAGPLDPFDLW	IGH	2	None	
4	897	CQHFDSYPLAF	IGK	11	C3	7K8R
4	898	CARVSQGYYDSSGYHYFDYW	IGH	16	None	
4	899	CARLPHNTAMVPFDYW	IGH	2	None	
4	900	CARSSDPTYGAKRGAFDLW	IGH	4	None	
4	901	CATYGYGSGSYSSYYFDYW	IGH	2	None	
4	902	CARVPTPRDVYFEFW	IGH	2	None	
4	903	CAREDRILTRFDPW	IGH	3	None	
4	904	CAAIPLNYYGSSGSWSYYFDYW	IGH	6	None	
4	905	CARDVGWEWLGLDYMDVW	IGH	2	None	
4	906	CAKGSTHGFPPNWYFDLW	IGH	4	None	
4	907	CAKDWSSSWYTPTDYW	IGH	2	None	
4	908	CGTWDSILNAGVF	IGL	20	None	
4	909	CARLSGSWYLDFW	IGH	13	None	
4	910	CARESYYDSSGHYSLGALDIW	IGH	2	None	
4	911	CHQYYSTPLVTF	IGK	29	C4	6W41
4	913	CASWRSSSWSKGIYAFDIW	IGH	2	None	
4	914	CARVTSGSDYSFEYW	IGH	4	None	
4	915	CARRLSRRSSGWYRSVDAFDIW	IGH	3	None	
4	916	CARQGGLDMKVVGAFDIW	IGH	2	None	
4	917	CAKGFSPTYMTVNTGPDYW	IGH	2	None	
4	918	CARDVQYTTSYGDYW	IGH	2	None	
4	919	CVRGYFLTPYRRPYFDHW	IGH	2	None	
4	920	CARTPLQWLVFDYW	IGH	20	None	
4	921	CASENFGESYYYYYMDVW	IGH	2	None	
4	922	CARSRYDWNDRYFDAW	IGH	2	None	
4	923	CARRAGWDIVATTFDYW	IGH	2	None	
4	924	CQERSDWPRMYAF	IGK	8	None	
4	925	CARERSRGAEDDAFDIW	IGH	2	None	
4	929	CAKDRLVYGSGDGLFDHW	IGH	2	None	
4	931	CAKPLTGSSSNYLDYW	IGH	4	None	
4	932	CANGDSIEGILFDYW	IGH	8	None	
4	935	CQHYNDWPPGHLRTF	IGK	6	C3	7K8R
4	936	CAKGLEGGSWTFDIW	IGH	2	None	
4	937	CQRFKSYPLSYTF	IGK	4	C3	7K8R
4	938	CAKETAARGWGYDAFDIW	IGH	2	None	
4	940	CARQANCGGNCPFDYW	IGH	2	None	
4	943	CANYYGDYAIFDYW	IGH	3	None	

4	945	CARSYSSGWYVGYYFDYW	IGH	2	None	
4	946	CASTRWPKYYFDYW	IGH	2	None	
4	947	CARTLYSYGLHYYYYMDVW	IGH	2	None	
4	950	CARLGGRQQWFDYW	IGH	2	None	
4	951	CQQSSDTPPLTF	IGK	19	None	
4	952	CAKDQTPGLGDLYFFDFW	IGH	9	None	
4	953	CARDRKWELLSNWFDPW	IGH	2	None	
4	954	CTQKIHLPLTF	IGK	25	None	
4	955	CAKATTPGSVWNFFDSW	IGH	4	None	
4	956	CARGSGGSALVGNNWFDPW	IGH	2	None	
4	957	CARVVFGIAAAPPYYYYYMDVW	IGH	2	None	
4	958	CARGVTGGWLIDYW	IGH	2	None	
4	959	CASWDDSPNGRNWVF	IGL	4	None	
4	960	CATDKDSAYVREAFDIW	IGH	4	None	
4	962	CQSVDSTGAHVIF	IGL	5	None	
4	966	CARHMVNYTPAYFLDW	IGH	2	None	
4	967	CARLSTPAAGVLEMLSPWYFDLW	IGH	4	None	
4	968	CARDRVVVTAHYYMDVW	IGH	2	None	
4	969	CSQGYCTRDSCYDSDYW	IGH	2	None	
4	970	CAKATADGPGSYIVDYW	IGH	2	None	
4	971	CSTWDYSLSAPNWVF	IGL	6	None	
4	972	CARAQGNTIVVTLGTYYYYYMDVW	IGH	3	None	
4	973	CARDRAGDGSGYYDCW	IGH	3	None	
4	974	CAGQEWTPGEFDSW	IGH	2	None	
4	975	CARDQNGAVAGIDYW	IGH	2	None	
4	976	CARPVVAGTHGFDYW	IGH	2	None	
4	977	CVRETYYYDSSGYYIDPW	IGH	2	None	
4	978	CARFYLGTTTPNLDYW	IGH	2	None	
4	979	CASLPRDGDPYFDSW	IGH	2	None	
4	980	CARAGGYGPAFDYW	IGH	2	None	
4	981	CAISGGSSSENYYYYYMDVW	IGH	2	None	
4	982	CAHSYYDFWSGYYDYW	IGH	2	None	
4	984	CAKDLDGGESAFDIW	IGH	7	None	
4	985	CARGSEMSTLTNWFDPW	IGH	2	None	
4	986	CARVRLAVDYCPNAVCLGGFDIW	IGH	3	None	
4	987	CCSYAGSYTFPWVF	IGL	12	C2	7K8X
4	988	CQTADTSGDDWVF	IGL	2	None	
4	989	CARVDDSSGYYLDAFDIW	IGH	2	None	
4	991	CARDLWQQLAQGFDYW	IGH	2	None	
4	993	CARDWGRDSVVTSYNWFDPW	IGH	6	None	
4	994	CAKAANSWYLYYFDYW	IGH	2	None	
4	996	CANLGYCLNTTCQGEDYW	IGH	2	None	
4	997	CAKGYSGPNVGLDWFDPW	IGH	2	None	
4	999	CARDGDDFWSGYYPPFDYW	IGH	4	None	
4	1001	CMQATHFPHPVTF	IGK	2	None	
4	1002	CARDSDGWYLNDAFDIW	IGH	2	None	
4	1003	CARESIAARPIDYW	IGH	2	None	
4	1005	CARKVGYRSSNGYYYMDVW	IGH	4	None	
4	1007	CARAAEQLGTPSGHDYW	IGH	4	None	
4	1008	CARFSWQMDAFDIW	IGH	4	None	
4	1009	CQPYNSFSFSYTF	IGK	6	None	
4	1010	CATSSGWSYYYMDVW	IGH	2	None	
4	1011	CARLGCTNGVCYRDDLYTYYMDVW	IGH	2	None	
4	1012	CVRDDSFAFDYW	IGH	3	None	
4	1013	CAKDWEFSINWFQGGFDHW	IGH	2	None	
4	1014	CASLYSTSSPYFDYW	IGH	2	None	
4	1015	CVKLGLDCSDGDCRDYGDDVDYW	IGH	2	None	
4	1016	CARLDDSSGYYSYFDYW	IGH	4	None	
4	1017	CARGRPAAGGPYYMDVW	IGH	2	None	
4	1018	CAKDRLKHSYGSDFDYW	IGH	2	None	
4	1019	CTTDLLMVVPAAMMLNYFDYW	IGH	2	None	
4	1020	CAKSEDYDSSGYYGGYW	IGH	2	None	
4	1021	CARVAAGELYYAFDIW	IGH	2	None	
4	1023	CLQGSHWPPFTF	IGK	8	None	
4	1024	CARKPRYSSGWGWFDPW	IGH	2	None	
4	1027	CARDRNWYGSGSLYFDHW	IGH	2	None	
4	1028	CMQGIDGPPYLF	IGK	23	None	
4	1029	CARLSMVAFDPW	IGH	5	None	

4	1030	CARARIYAWSGSDYDYW	IGH	2	None	
4	1031	CARLVVVVVADGWFDPW	IGH	2	None	
4	1032	CARMMTFGGVIVSDAFDIW	IGH	2	None	
4	1035	CARVSMDIVATKTFDYW	IGH	2	None	
4	1036	CLQDYNHLGTF	IGK	11	None	
4	1038	CAKDIGGGRDSHFYYMDVW	IGH	4	None	
4	1040	CARVGRFGELRGQYYFDYW	IGH	4	None	
4	1041	CARHVMFFGVVIINYFDYW	IGH	2	None	
4	1045	CARGLLGYCSGGSCYPAVFDYW	IGH	2	None	
4	1046	CARDAGGGDWHYFDYW	IGH	2	None	
4	1048	CQSYDYNNWVF	IGL	21	C4	7JVC
4	1049	CARGRQQPRFDPW	IGH	2	None	
4	1050	CAKVSFPEAKTRYGLDYW	IGH	2	None	
4	1051	CLLSYSGAWVF	IGL	63	None	
4	1052	CARGGDWNRYFDCW	IGH	2	None	
4	1054	CQQFNNWPPGFSF	IGK	2	C1	7BZ5
4	1055	CARDAKGESSGYNYFFDHW	IGH	4	None	
4	1056	CARDHRGDENAFDIW	IGH	2	None	
4	1058	CARHVKSTAVSSLDVFDIW	IGH	4	None	
4	1060	CAGRNWNVKNWFDPW	IGH	2	None	
4	1061	CALPIWDLAGRQFLPGVLALLSQCDNWFDPW	IGH	2	None	
4	1062	CARGRGSSWYMVGATGGVYFDYW	IGH	2	None	
4	1063	CARAYGVTMVRGYYNWFDPW	IGH	2	None	
4	1065	CARAAHSGTYRIFDYW	IGH	2	None	
4	1066	CARGSAGSLGYDDILTGYFPRW	IGH	4	None	
4	1067	CARGRAYGDRRMGFDPW	IGH	2	None	
4	1068	CQQYYSTLIIF	IGK	127	C4	6W41
4	1069	CATVAYFYDSSAYVHPGAFDFW	IGH	2	None	
4	1070	CARVVIRVGFDYW	IGH	2	None	
4	1072	CARIIAVAGAWAFDIW	IGH	4	None	
4	1074	CARIGYCSSTSCFRGNDYW	IGH	2	None	
4	1076	CARVTVKTASISQFDHW	IGH	3	None	
4	1078	CYTAADNHPYVVF	IGL	3	None	
4	1079	CARGRGYSPYEGYYSDYW	IGH	5	None	
4	1081	CNSRDSSTGNLWMF	IGL	2	None	
4	1082	CAVGQVAGSPNLRFAYW	IGH	2	None	
4	1083	CAAWDDSRTGPCVTF	IGL	8	None	
4	1084	CARDVRFGGPDYVANWFDPW	IGH	2	None	
4	1085	CAMPLLWFGEWAPFNYW	IGH	2	None	
4	1086	CAKVPGVWIQPYTRTFFDYW	IGH	2	None	
4	1087	CAISVWTYRKADDPDYW	IGH	2	None	
4	1088	CARDGGALGEIYPGYYDYW	IGH	2	None	
4	1089	CAKVPPLSRSWYWEGAFDIW	IGH	2	None	
4	1090	CARTRAYYDFFYFDYW	IGH	2	None	
4	1091	CALGGRWSDHDAFDIW	IGH	2	None	
4	1092	CASNYGYTLTNPFDIW	IGH	7	None	
4	1094	CARPSVGARAFDVW	IGH	4	None	
4	1095	CARVPPTEYYFDYW	IGH	15	None	
4	1096	CARRSVPVATSYSFDYW	IGH	2	None	
4	1097	CARFFPGGNLDHYFDYW	IGH	4	None	
4	1099	CARGYAYYFLYMDVW	IGH	2	None	
4	1102	CVASDKYYDFWSGYPSDAFDIW	IGH	4	None	
4	1103	CASWDDSVSGAIF	IGL	2	None	
4	1105	CARGFSSSWYWAYW	IGH	25	None	
4	1106	CARCNWNWGDNWFDPW	IGH	4	None	
4	1107	CARDRAYLDRELHNYYYMDVW	IGH	2	None	
4	1108	CTTVGYDFWSNYYNYFDHW	IGH	2	None	
4	1109	CARVVQDYYDSSGYEDYW	IGH	2	None	
4	1110	CVRQSSSGYDYW	IGH	2	None	
4	1111	CARGLDYYDSSGLGQIFDYW	IGH	2	None	
4	1112	CATEGRGFAYW	IGH	2	None	
4	1113	CARTPSESNWGFDYW	IGH	2	None	
4	1115	CARGSSASVWGNGFDMW	IGH	9	None	
4	1116	CAKDGHETQWSYYMDVW	IGH	4	None	
4	1118	CARDGVEWLVSFDYW	IGH	2	None	
4	1119	CARAKPRSPPEKFFDYW	IGH	2	None	
4	1120	CARGFNLGSSFDYW	IGH	9	None	
4	1121	CVRSCYGSGNFYKICDFDYW	IGH	2	None	

4	1123	CARDLLVCSSTSCYRGDSSSSYYFDYW	IGH	2	None	
4	1124	CAREPVGDGVCCSFDYW	IGH	3	None	
4	1125	CARGRLVTFFDYW	IGH	17	None	
4	1126	CAGSSDYW	IGH	102	None	
4	1128	CAKDLAADNAYRYYMDVW	IGH	2	None	
4	1130	CYSLDSSGQPIF	IGL	5	None	
4	1132	CAKAIGDYQKNLWFNYYMDVW	IGH	4	None	
4	1133	CARGRLKGWLAGPKSKFDYW	IGH	2	None	
4	1134	CAGSKMIFPMVLDYW	IGH	2	None	
4	1135	CARGYSPLFYDDTGYYSYFDNW	IGH	11	None	
4	1137	CARYYFVPGNAPYMDVW	IGH	4	None	
4	1139	CAKDREWELLGLLDYW	IGH	3	None	
4	1141	CARDYACISTSCSTDTKFDYW	IGH	7	None	
4	1143	CARGRRPGYFQHW	IGH	2	None	
4	1144	CARRAPYYYDSSGPYAFDIW	IGH	2	None	
4	1146	CATERWELLWFDFW	IGH	2	None	
4	1147	CARFKESATSEGYYFDYFDYW	IGH	2	None	
4	1149	CAHVSTPLPDSSKYYFDYW	IGH	2	None	
4	1150	CAREWRSSYCSGGSCFNNWFDPW	IGH	3	None	
4	1151	CARVRSFNILTGYYFDAFDIW	IGH	2	None	
4	1152	CARHFGSSSEFAYW	IGH	2	None	
4	1153	CARGKTHPLGCFDYW	IGH	2	None	
4	1155	CAREGPVTGDFVDYW	IGH	2	None	
4	1157	CAREQLGVLSAAGTDYW	IGH	9	None	
4	1158	CARDFCSGGSCYPDYW	IGH	3	None	
4	1159	CARQDTYGLFYFDYW	IGH	2	None	
4	1160	CARVVAARGTFGAFDIW	IGH	6	None	
4	1161	CARLTAGVVVAAIDYW	IGH	9	None	
4	1162	CATSQHREWLLDAFDIW	IGH	2	None	
4	1163	CARGLAAAGTLNWFDPW	IGH	3	None	
4	1164	CAVRGDRNTIFGVVPDVFDIW	IGH	10	None	
4	1165	CGRYDTIRHASDYW	IGH	11	None	
4	1167	CLLSYSGPNGIPRVVF	IGL	2	None	
4	1168	CARHDSTGYFLGHLDYW	IGH	2	None	
4	1169	CMQGTHLYSF	IGK	68	None	
4	1171	CAHRLGGYNWDDCYFDYW	IGH	4	None	
4	1172	CARDDHYYDSSGYDYW	IGH	3	None	
4	1174	CASVAAAGPLHYYYFYMDVW	IGH	2	None	
4	1175	CARAGRVRGVMKNAFDIW	IGH	2	None	
4	1176	CARGYHYDSSGYYLYYYYYMDVW	IGH	3	None	
4	1178	CAKDDRPDGRFEIDYW	IGH	3	None	
4	1180	CARLNDYDFWSASLDGPDFDYW	IGH	6	None	
4	1181	CARNCNGGSCYPAGLDYW	IGH	2	None	
4	1182	CARVAITMIVVVKGDAFDIW	IGH	2	None	
4	1186	CARHGVAVAGTLVDYW	IGH	2	None	
4	1187	CASLEKGRGDYW	IGH	2	None	
4	1189	CARTPRGYGFYFRHW	IGH	2	None	
4	1190	CATAHHPYPCPFDPW	IGH	2	None	
4	1191	CSRRSPRNCSGGTCYPRTFDKW	IGH	2	None	
4	1192	CARDSHDSAASGWYFDLW	IGH	4	None	
4	1194	CANEKAVTGRFDNW	IGH	4	None	
4	1195	CARDGAAAAKGSCDYW	IGH	6	None	
4	1196	CARDTHYYDNSGYYYPRFGYW	IGH	2	None	
4	1197	CTSEWFDVFAIW	IGH	4	None	
4	1198	CARDLRTRPVTWGQSANDAFDLW	IGH	2	None	
4	1200	CARPAMYGGDSNGFDPW	IGH	2	None	
4	1201	CARGRDDSGTYSTPFDFW	IGH	6	None	
4	1203	CVKDRMIRSFTIALRPWYFEDW	IGH	4	None	
4	1204	CQKYNNAPPLTF	IGK	252	C3	7K8R
4	1205	CARRLRVALDYRNYYFDYW	IGH	3	None	
4	1206	CAREAVGIQLWSPLDYW	IGH	4	None	
4	1207	CARASYSVFGVAQANFDYW	IGH	2	None	
4	1209	CGSYTTSSTFVF	IGL	446	C2	7K90
4	1210	CARQAGAALSRTYYFDYW	IGH	2	None	
4	1211	CQEYHNWPFWTF	IGK	4	C3	6XDG
4	1212	CVRAQLMRHAWERLFDYW	IGH	3	None	
4	1213	CARDGGCSGGSCYPFFWFDPW	IGH	3	None	
4	1214	CARQINVPNDALDIW	IGH	2	None	

4	1215	CARDAINSDIVVVPNWFDPW	IGH	2	None	
4	1217	CARDARAVAGPSYWYFDLW	IGH	3	None	
4	1218	CQRYNSVPFTF	IGK	10769	C3	7K8R
4	1219	CARVYCSSDCSRPSHFHYYMDVW	IGH	2	None	
4	1221	CVRDAYSILDYW	IGH	3	None	
4	1222	CQQYHNWPGDF	IGK	108	C3	6XDG
4	1223	CARAITGSSGWYPDYW	IGH	3	None	
4	1224	CQAYDSSLSAAF	IGL	3	C4	7JVC
4	1225	CAKDSRGDFWSDDYFDYW	IGH	6	None	
4	1226	CVSDDYITLSHFNYW	IGH	17	None	
4	1227	CASSSNYDFWSGYYSRNAFDIW	IGH	4	None	
4	1228	CTRGPQLPQYFQHW	IGH	6	None	
4	1229	CVKGGRILDFDWLITFDYW	IGH	3	None	
4	1230	CQELNSFPRYTF	IGK	9	C1	7BZ5
4	1231	CARQLELRPRAPYYFDYW	IGH	2	None	
4	1232	CARVGGTYGGTYLSLVYYYYMDVW	IGH	2	None	
4	1233	CARGQSISWPDAFDIW	IGH	2	None	
4	1234	CARENPAAINWFDPW	IGH	2	None	
4	1235	CARDPPYYYDSSGYYGWFDPW	IGH	2	None	
4	1236	CAKSVLFVRYNYDVNWFDPW	IGH	2	None	
4	1237	CARVGEEDDSSGYYSPQFDYW	IGH	2	None	
4	1238	CARGERHYEFSTGYYDTLDYW	IGH	6	None	
4	1239	CVQQMATIGEYIDYW	IGH	2	None	
4	1240	CATSEYSTPLFDFW	IGH	3	None	
4	1242	CAKGQLAVLGRLGFDSW	IGH	2	None	
4	1244	CAREYWAPDYW	IGH	6	None	
4	1245	CAHRETYGVLRYFRNSYYFDYW	IGH	2	None	
4	1246	CARVLELGATPIFGYW	IGH	3	None	
4	1247	CARFLVELREFDPW	IGH	2	None	
4	1248	CARSQPWYSSNCDYW	IGH	2	None	
4	1249	CVNFIAAAADYW	IGH	2	None	
4	1250	CARVSMVRGIILFDYW	IGH	2	None	
4	1252	CARDRRYFGSGRPDAFDIW	IGH	2	None	
4	1253	CARGTYSSSDDAFDIW	IGH	6	None	
4	1254	CAKLYNNSPYYFDSW	IGH	2	None	
4	1256	CARHTSNLVRPYYFDYW	IGH	4	None	
4	1257	CARVTPYNWNRGWYFDLW	IGH	4	None	
4	1258	CARFAPPSSSWYAQRGAFDIW	IGH	4	None	
4	1259	CAYRKIAGGSEGFDYW	IGH	2	None	
4	1260	CVMDTSMYFDHW	IGH	2	None	
4	1263	CARIQEDIVVVPAAPDYYYYMDVW	IGH	2	None	
4	1264	CGAWDSSLSGGHYVF	IGL	4	None	
4	1265	CARDRGAARIYYFDYW	IGH	2	None	
4	1266	CAKEGRVGSSWYSLGDYW	IGH	6	None	
4	1267	CAKDHFGSVTYFSLDAYDMW	IGH	4	None	
4	1268	CAKKLAMGTFDYWYFDLW	IGH	6	None	
4	1270	CQHLHSYPRSF	IGK	37	C1	7BZ5
4	1271	CVRDNMGSFDHW	IGH	2	None	
4	1272	CARAPYEVVVAATGYFDYW	IGH	4	None	
4	1273	CARSTGGGGAFDIW	IGH	2	None	
4	1274	CAREGSGWYRYYFDYW	IGH	13	None	
4	1275	CARDSGSGRGWFDPW	IGH	7	None	
4	1276	CQQRSSWRSISF	IGK	2	None	
4	1277	CARAPYYYDSSGYYPSSWYFDLW	IGH	2	None	
4	1278	CARPRRVNSWSDFDYW	IGH	2	None	
4	1279	CARAPWNSKTDAFDIW	IGH	2	None	
4	1280	CAKDDYLPAWGCSGGNCLSDFFDYW	IGH	2	None	
4	1281	CAKNSSPYYYGSGSYYHYFDYW	IGH	2	None	
4	1282	CTTKGMSVDHAFDFW	IGH	2	None	
4	1284	CARDFAWLLAADWFDPW	IGH	4	None	
4	1286	CAAESSIFGVVTPRRYAFDVW	IGH	5	None	
4	1287	CARSSIKLELPIAGDYW	IGH	2	None	
4	1288	CAIPNGGDSSSWSDAFDIW	IGH	2	None	
4	1289	CARFRLHSSSSPSDWYFDLW	IGH	2	None	
4	1290	CERDPRGTPPTDYW	IGH	2	None	
4	1292	CQSYDSVQSASVVF	IGL	10	C4	7JVC
4	1293	CTKSRALAGNEGFDHW	IGH	2	None	
4	1294	CAGQVYYDFWSGRHYYMDVW	IGH	2	None	

4	1297	CVKDRGSYHYYYYMDVW	IGH	2	None	
4	1298	CQRANTFPLTF	IGK	9	C3	7K8P
4	1300	CVRGRPRYYMDVW	IGH	2	None	
4	1301	CQLYGNSPMTTF	IGK	5	C1	7K8N
4	1302	CARDPELGTYDYW	IGH	2	None	
4	1304	CARDGDTGVRRMDYW	IGH	2	None	
4	1305	CARQYQLLLPLDYW	IGH	4	None	
4	1306	CQTSDSSGFYPELF	IGL	4	None	
4	1307	CARVHDILTGYPIFGFDYW	IGH	2	None	
4	1308	CARCDEYSRGLATGFDPW	IGH	4	None	
4	1309	CARGYISASYIITYHDYW	IGH	2	None	
4	1310	CARLGRNLSYFDLW	IGH	2	None	
4	1313	CSSYTSTNTLAVVF	IGL	6	C2	7K90
4	1314	CARDFPYYYDSSGYYFDAFDIW	IGH	2	None	
4	1315	CAREVPLDDAFDIW	IGH	6	None	
4	1316	CARTGDSPGYYAFDIW	IGH	20	None	
4	1317	CQSYDSSLSGWVF	IGL	619	C4	7JVC
4	1318	CASIVTAMVRGRNFDYW	IGH	6	None	
4	1320	CAKSYHGEQHLVWGNWFDPW	IGH	2	None	
4	1321	CYSAADNNLRVF	IGL	106	None	
4	1322	CAKAFYFDFWSGYYGPSDYW	IGH	6	None	
4	1323	CARAPYYTFGPYYYYYMDVW	IGH	2	None	
4	1324	CTTIAAAGTTPFDYW	IGH	2	None	
4	1325	CAKAGSGWFTDYW	IGH	7	None	
4	1326	CARRAAGRHYYYYYYMDVW	IGH	2	None	
4	1328	CARDSSSRRGDYW	IGH	14	None	
4	1329	CAREIGGGYGFDYW	IGH	7	None	
4	1331	CQQYYSTPEALTF	IGK	2	C4	6W41
4	1332	CARSHYDILTHKGDAFDIW	IGH	2	None	
5	1	CARRFDYDSNDYWGYW	IGH	2	None	
5	3	CARGPVFALVRQMTMFDFW	IGH	2	None	
5	4	CAKGAWSDYNLARSGTPFDYW	IGH	3	None	
5	8	CHQYYSTPSF	IGK	37	C4	6W41
5	11	CAKGYSHGHKDGMDVW	IGH	2	None	
5	12	CAREGRRTSSGWVYYYGMDVW	IGH	4	None	
5	15	CYSTDSSGNHRVF	IGL	113	None	
5	18	CARPTYDYDTRGYFPFDLW	IGH	2	None	
5	21	CATGPDYYDSSGFYYW	IGH	2	None	
5	24	CARGLGSSSWFRGWFDPW	IGH	2	None	
5	25	CQSADTSVSYWMF	IGL	4	None	
5	26	CARVQQWLDFGAFDIW	IGH	2	None	
5	29	CARVIAQWPGRDYWYFDLW	IGH	2	None	
5	30	CARWVVITATGAFDIW	IGH	3	None	
5	31	CAKYSRIFGVVNNYALDVW	IGH	3	None	
5	33	CARHRISYSSQSDFDYW	IGH	2	None	
5	35	CARKISVIGRSGFDHW	IGH	2	None	
5	38	CTKGPYCRSASCYAYYHYGMDVW	IGH	2	None	
5	41	CARDLAVAASDAFDIW	IGH	2	None	
5	42	CARERWYYGSGNYHPADYW	IGH	3	None	
5	44	CARGHISFFGVVTGAFDLW	IGH	2	None	
5	45	CARVGSIAAAGPYYYGMDVW	IGH	4	None	
5	46	CARPYVGGKAFDMW	IGH	3	None	
5	49	CARTNFFGSGSYGQLDYW	IGH	5	None	
5	50	CARQPSDWAAFDVW	IGH	4	None	
5	51	CAHRQARYGDSRINLFDPW	IGH	2	None	
5	55	CARTPDSSGYYDHQIDYW	IGH	4	None	
5	56	CARDWGYGDYGLHYFDYW	IGH	2	None	
5	58	CARHVGTAMLDYW	IGH	9	None	
5	61	CAKVPSPYTWYFDYW	IGH	2	None	
5	63	CARDRYYYDRSGSTAGDYW	IGH	2	None	
5	65	CAKDAPPGTGYNNDAWYFDLW	IGH	11	None	
5	67	CARSGYSLFDYW	IGH	14	None	
5	68	CASAIFGVVDDAFDVW	IGH	6	None	
5	70	CAREKTLDISAFDLW	IGH	19	None	
5	71	CARDRRTTGTYIDDYW	IGH	4	None	
5	72	CARLGGTTAGYNWFDPW	IGH	4	None	
5	74	CHQYNNWLRAF	IGK	15	None	
5	78	CARRDGYSMSSEGYSYYYPMDVW	IGH	2	None	

5	79	CQQTNSPPYSF	IGK	37	C3	7K8P
5	82	CVRDDSSLPGFIYW	IGH	4	None	
5	83	CAKANMFFWFGQFKKDAFDLW	IGH	17	None	
5	87	CAKETEDYTSSYFDYW	IGH	552	None	
5	89	CARDDSGWFDPW	IGH	6	None	
5	91	CATYFRGAWYGHDYW	IGH	2	None	
5	93	CQQYDNWLRGLTF	IGK	28	C3	6XDG
5	96	CARGLNRFGGNPFGYW	IGH	3	None	
5	98	CARSSSRGGASCYAACNWFDPW	IGH	2	None	
5	99	CLQTYSTPHCTF	IGK	7	C2	7K8O
5	100	CQQFNSYLF	IGK	137	C1	7BZ5
5	102	CQQRSNWPPFTF	IGK	98	C1	7BZ5
5	108	CQSGDSRGPSVVF	IGL	2	None	
5	111	CARASDWGYDYW	IGH	3	None	
5	112	CNSRDSRSNHQVF	IGL	8	None	
5	113	CARCAIAAAATTGGCFDPW	IGH	2	None	
5	114	CQSATTSGPYRIF	IGL	3	None	
5	115	CATLIDSSVVVFPTYFDYW	IGH	4	None	
5	117	CAKDAIAANSVFDWFDSW	IGH	2	None	
5	119	CARDRRCGSCGDYEGYW	IGH	4	None	
5	120	CASWGGNSFDYW	IGH	2	None	
5	121	CAAQDDGYTGYDLDYW	IGH	2	None	
5	122	CARPADGGTGIYYYYGMDVW	IGH	4	None	
5	123	CAKFRGVFDFWSSRAMDVW	IGH	14	None	
5	125	CQQSYTNPRTF	IGK	3444	C2	7K8O
5	128	CAKDINNWNFDNW	IGH	6	None	
5	132	CARDLLGEYTGYDLQGGFDYW	IGH	2	None	
5	133	CARSIAVAGITPKKSGWFDPW	IGH	2	None	
5	135	CARDVSSPDYVAYW	IGH	4	None	
5	142	CARGPGLVTSNWFDPW	IGH	3	None	
5	144	CAREIIFYYAMDVW	IGH	2	None	
5	146	CVRHFSSIWTLSGYYYLDYW	IGH	5	None	
5	148	CQPYNVYSGTF	IGK	8	None	
5	153	CAKDTAMVPDYW	IGH	2	None	
5	154	CGTWDSSLSAWVF	IGL	48	None	
5	156	CARSRDGYNYGDYW	IGH	9	None	
5	157	CARDLHSADYFYYW	IGH	3	None	
5	158	CSRGLIPETDLASRLFRYFDSW	IGH	4	None	
5	159	CASGSYPYYFDYW	IGH	9	None	
5	160	CARIIAAAGPHNLDYW	IGH	2	None	
5	162	CQQYSSSAAFTF	IGK	12	None	
5	164	CGTSVHGSGYAFDHW	IGH	3	None	
5	165	CMQGTRWPNSF	IGK	3	None	
5	167	CARGRPVTTFYYYYGMDVW	IGH	2	None	
5	170	CARLDTDSNKWFWFDPW	IGH	14	None	
5	172	CARGLVLTGHYFDNW	IGH	2	None	
5	174	CQQYGSLFSITF	IGK	6	C1	7K8N
5	175	CMQGTHWPPAF	IGK	764	None	
5	176	CAKDLHTSGWFPSASFDFW	IGH	7	None	
5	177	CARDMIRGDGEFDYW	IGH	12	None	
5	179	CAAWDDSLNGWVF	IGL	124	None	
5	182	CARAAHSRPDYYYYDMDVW	IGH	3	None	
5	183	CARQRLLRFLGGMTNDAFDVW	IGH	4	None	
5	184	CARDTSVAGRFHYW	IGH	2	None	
5	186	CTSASSTLHYGGNSGDYW	IGH	53	None	
5	188	CAKDKISGDGYWVFDSW	IGH	2	None	
5	189	CARRRGAVFDPW	IGH	9	None	
5	190	CARYTSHCTPTTCWMWFDPW	IGH	2	None	
5	192	CARELPYGGHYGSDYW	IGH	2	None	
5	193	CARLRYYDGTTYFPDYW	IGH	2	None	
5	194	CAKDFQGDYYYYGMDVW	IGH	2	None	
5	196	CQSYDRSLNIWVF	IGL	57	C4	7JVC
5	197	CASNRPGTTQDYW	IGH	4	None	
5	201	CARDKVYDDFTGYSYYFDHW	IGH	2	None	
5	205	CARRPPWSTVYQSGFDPW	IGH	3	None	
5	206	CHSRDSSGNHRGFVF	IGL	4	None	
5	208	CARDSSYYDILTGSYPSYW	IGH	5	None	
5	209	CAKEVRGYSYGLGLFDYW	IGH	2	None	

5	211	CARMGFRSFSYW	IGH	2	None	
5	214	CAIEGFVVADYW	IGH	2	None	
5	217	CSSYTSSSTLYVF	IGL	314	C2	7K90
5	218	CARSLSLGLRPGDYW	IGH	6	None	
5	219	CQLYGGSPLWTF	IGK	457	C1	7K8N
5	221	CASSYYYDSSGYFAPFDYW	IGH	2	None	
5	222	CARDGDYTGNSDFDYW	IGH	6	None	
5	223	CSSSASSSTLDVVF	IGL	2	None	
5	224	CAREGPHQYSSEFDYW	IGH	19	None	
5	230	CAKDQSFFALSGKRSLDYW	IGH	35	None	
5	231	CARRTIGSSNKFDPW	IGH	2	None	
5	233	CARTYCTGGSCYFRAFDYW	IGH	10	None	
5	234	CARENPLNKNAFDFW	IGH	3	None	
5	235	CAKGRPETYYDILTGYQLTSNFLGSPGDYW	IGH	2	None	
5	237	CARVFYDSNGVDYNFFDNW	IGH	2	None	
5	238	CARSIGSSTSCVFDYW	IGH	5	None	
5	239	CATTTGEYYDFWSGYAYYFDYW	IGH	5	None	
5	240	CARDHDFWSALDYW	IGH	4	None	
5	241	CTTGGYMTTVTTAYYYYGMDVW	IGH	2	None	
5	242	CARDPGDTGNEGDGMDVW	IGH	4	None	
5	244	CATDAGEYGDYLRYW	IGH	7	None	
5	246	CARGNPKQWLVTSAFDIW	IGH	2	None	
5	247	CARRGNYEFWSGYVAGDDYW	IGH	2	None	
5	248	CTSRCSGGNCYSDTYFPFDYW	IGH	2	None	
5	249	CAKFGLPGFGFPWFDPW	IGH	5	None	
5	250	CARELAVSATVGDYW	IGH	2	None	
5	251	CARKAPYYYGSGGHSNYCDYW	IGH	2	None	
5	254	CSSYADNNNVVF	IGL	17	C2	7BWJ
5	255	CARDVFYYDGGNWNWDFDLW	IGH	5	None	
5	256	CAKDTYDSSGYDWDVFDIW	IGH	4	None	
5	257	CARHKSGGTYPLDYW	IGH	8	None	
5	258	CARDVGSTTSESGYFDYW	IGH	4	None	
5	259	CGSQRYFALW	IGH	6	None	
5	260	CARNDILTGYYTGLRDNWFDPW	IGH	2	None	
5	261	CARIMAVAGTIDYW	IGH	2	None	
5	263	CWPTAPNDAFDMW	IGH	3	None	
5	267	CQVWDRSSDHSDSDHPVF	IGL	8	None	
5	268	CARINTDRGGFSRRPIDYW	IGH	3	None	
5	269	CARGYYYDILSGSKYYHYAMDVW	IGH	2	None	
5	270	CAGFEYSSSSGAFSYYFDYW	IGH	2	None	
5	271	CQQSRSFPPITF	IGK	3	C1	7BZ5
5	273	CAREVWSGYYAVFDYW	IGH	5	None	
5	274	CARFVGSGTYDSTGSDWYFDLW	IGH	6	None	
5	276	CARIVSSPGGYFRFDQW	IGH	6	None	
5	277	CSRDLNWGSRGASHEFQYYAMDVW	IGH	3	None	
5	278	CARHYGTGNYHYYYGLDVW	IGH	4	None	
5	279	CARDNYYYDSSGYYYGYYYYYGMDVW	IGH	3	None	
5	280	CMQRIEFPYTF	IGK	7	None	
5	283	CAKEGVGPTGKIFDYW	IGH	4	None	
5	284	CAKDLRQAEIVVVPAASYRFDPW	IGH	2	None	
5	287	CARENTLSSTGFDIW	IGH	3	None	
5	289	CARDPNEPHYYGSENWFDPW	IGH	2	None	
5	290	CVRWPYCSTGTCYSAMLDYW	IGH	2	None	
5	293	CAKDSSRRPIGFDLW	IGH	6	None	
5	294	CTHDSSGFYSFHYW	IGH	2	None	
5	295	CARVTKLTSVEGVDWFDTW	IGH	2	None	
5	296	CANSVTNDDFWGGYYRYFYYGMDVW	IGH	2	None	
5	297	CHQSDALPYTF	IGK	31	None	
5	298	CAKLPWELLRGLGGYFDYW	IGH	4	None	
5	302	CAKESYWKPTDYFDSW	IGH	2	None	
5	304	CHVWDSRTAVF	IGL	4	None	
5	306	CAKDHLPGTFFDFW	IGH	2	None	
5	307	CVREGIDCSDGMCPFDYW	IGH	6	None	
5	308	CAARPGDHYGPLDYW	IGH	4	None	
5	309	CAKEATDSKTRIDWFDPW	IGH	11	None	
5	313	CARSFLHYSGSSGYYSGSFDYW	IGH	2	None	
5	314	CASNFRKYCSGGSCYSPAFDIW	IGH	3	None	
5	318	CQSADTSGTYGNWVF	IGL	329	None	

5	320	CARGRVFSGHFFDPW	IGH	2	None	
5	321	CARDKLLYHAMDVW	IGH	2	None	
5	323	CARQNLVSSTTEDWFDPW	IGH	3	None	
5	324	CTTDRSHYYYDSSGYYPRDAFDIW	IGH	2	None	
5	325	CQQYGSTRNF	IGK	2	C2	7K8U
5	328	CARVHSYDRVWGNYRHFDYW	IGH	5	None	
5	329	CAREWVRVAVPGAISNNWFDPW	IGH	3	None	
5	330	CARDRNYYGSGERFDPW	IGH	4	None	
5	331	CAKDWLSMTTVTYFDNW	IGH	6	None	
5	332	CARQGIAARRWWFDPW	IGH	2	None	
5	334	CARDISRGDAFDIW	IGH	4	None	
5	336	CAHSHRSGWIGHFDYW	IGH	2	None	
5	338	CARESGNSGTLDYW	IGH	2	None	
5	340	CVRGPRKSCMTPNCYPWAFDVW	IGH	6	None	
5	341	CARGHVTMIPSLFDYW	IGH	2	None	
5	342	CQKYDSALVTF	IGK	35	None	
5	346	CARLFGVDDYW	IGH	2	None	
5	348	CARDFSSRWYWGYSDYW	IGH	46	None	
5	349	CAKVSGCSGRNCFFRWWFDPW	IGH	2	None	
5	352	CAKIKTGGASFDYW	IGH	6	None	
5	354	CARSLEWLYPYYGMDVW	IGH	4	None	
5	355	CARDRRPIRGGIIDYW	IGH	6	None	
5	359	CARISYDYVRGSFRYFDYW	IGH	2	None	
5	361	CCSYAGSSTRPWVF	IGL	3	C2	7K8X
5	363	CQSYDNSLGGWKLF	IGL	9	C4	7JVC
5	364	CARGPPSGARGGDYW	IGH	7	None	
5	365	CQQSYNTRRYTF	IGK	2	C4	6ZDH
5	367	CAKVRREDYNYDYFDYW	IGH	2	None	
5	371	CVRDLGHCPGGNCYPYFGYW	IGH	2	None	
5	372	CAREDSGNIDKHGIDYW	IGH	4	None	
5	373	CARFFNGGGCDYW	IGH	42	None	
5	374	CAKVRFSSGWPDIDYW	IGH	2	None	
5	376	CARLGWELPFDYW	IGH	2	None	
5	377	CFTDDSGLPDYW	IGH	3	None	
5	382	CARDSFRVAVAGTTGWFDSW	IGH	2	None	
5	383	CARDQHQQIEYFQHW	IGH	2	None	
5	388	CGLYMGSGIYVF	IGL	8	None	
5	389	CALVSTVTTTFYFASW	IGH	4	None	
5	391	CARASNSLLAKDDWFDPW	IGH	2	None	
5	396	CARGYDCSGGSCYPDYW	IGH	2	None	
5	398	CARGGDSGVLWGDYW	IGH	3	None	
5	399	CMIWPSNAWVF	IGL	27	None	
5	400	CARVARSSLELLRFDPW	IGH	2	None	
5	402	CLQHNTYPFTF	IGK	142	C3	7K8R
5	403	CQQYDDVPQYVF	IGK	21	C3	6XDG
5	406	CARGRDIVVEPSDRDAFDIW	IGH	73	None	
5	407	CAREEYDSSGSSFDYW	IGH	8	None	
5	408	CARGKAPDGMDVW	IGH	2	None	
5	412	CAKDRPDYYDSSGHYSPADSFDIW	IGH	2	None	
5	415	CARDRGIDYGDQFDYW	IGH	2	None	
5	416	CAKETLFKIVVVITREFDYW	IGH	2	None	
5	418	CAKEGPFAVTTLDYW	IGH	5	None	
5	420	CARGYCSGGRCPVSIDYYYGMDVW	IGH	2	None	
5	421	CARVNAATDLDYW	IGH	4	None	
5	424	CAKKFSGWSKEFDYW	IGH	3	None	
5	425	CVRDGGLTGDYW	IGH	3	None	
5	427	CQQYRSAPDRF	IGK	3	C4	6W41
5	428	CAKGAQLNIETGDYYYYGMDVW	IGH	4	None	
5	429	CAHRLPVAGSYYFDYW	IGH	2	None	
5	431	CARGYCGGDCYWGSDAFDWW	IGH	2	None	
5	433	CARHNMAYRLGNDYW	IGH	2	None	
5	434	CARVNPTMVMMSPTDFKRSNYFDSW	IGH	3	None	
5	436	CARMMIPPIAMAGTPATMWLDPW	IGH	4	None	
5	438	CAKLSRMTTVDYW	IGH	10	None	
5	439	CVRGNSRYYDSTGYFPFDYW	IGH	4	None	
5	440	CNSRDSSDDHLEVF	IGL	3	None	
5	441	CARDRVEYDLWSGPWRNYYYHGMDVW	IGH	3	None	
5	443	CQQYNNYSPVYSPGTF	IGK	2	C3	7K8R

5	445	CARHAVLRRPYYYFAMDVW	IGH	2	None	
5	446	CARHSGSGWYSPFDYW	IGH	2	None	
5	448	CETWDNNIQVVF	IGL	4	None	
5	449	CARGGYVWGSYRFDDW	IGH	3	None	
5	450	CAKDLRYSSRWTGARYYYGMDVW	IGH	2	None	
5	451	CARLRRVVINNWFDLW	IGH	12	None	
5	454	CARASSSGGYYYAMDVW	IGH	2	None	
5	455	CARRVVVRPYYFDYW	IGH	2	None	
5	456	CARGRRPLWDHSSSWYRYNYLDSW	IGH	8	None	
5	457	CARAVLAKYYDYVWGSYRHSERDFDYW	IGH	2	None	
5	459	CQVWDSSSDHWVF	IGL	43	None	
5	460	CAKDRGPYSSGYPDAFDIW	IGH	2	None	
5	462	CARDRWSTSCLFDYW	IGH	2	None	
5	465	CARRRLVYFGEAGPDYW	IGH	2	None	
5	466	CHSRDSSGHHLLF	IGL	37	None	
5	468	CAKDIIPSKPQRGIQLWFNNFDYW	IGH	2	None	
5	470	CAKSPPQTALRAAFDIW	IGH	2	None	
5	474	CARDAVALALDYW	IGH	6	None	
5	477	CATAPIAGYYGSGSYSGFDNW	IGH	2	None	
5	479	CARDKPDSITIFGVVTSHYALDVW	IGH	3	None	
5	480	CARMPATTIFGVIIPGKRNWFDSW	IGH	11	None	
5	481	CARYCSGANCYGTLFGYW	IGH	2	None	
5	483	CAKGRRGIPNGGNGIDSW	IGH	2	None	
5	484	CVGQDTYGTFDYW	IGH	6	None	
5	485	CAKDVCSGGGCYYQYGMDVW	IGH	2	None	
5	486	CQHFNTYPFNF	IGK	4	C1	7BZ5
5	487	CARGKSIMITFGGVMETYYFDYW	IGH	2	None	
5	488	CAKDYRQWPGSFDYW	IGH	2	None	
5	489	CARDLSMIRGIFPYYYGMDVW	IGH	134	None	
5	491	CSKLEGGHNYGDRGSYWGDNWFDPW	IGH	2	None	
5	495	CARVHHIVVLTAILGWLDPW	IGH	4	None	
5	496	CARSRRSKRRITTMATSDWFDPW	IGH	2	None	
5	500	CAREAYSTGYNWFDPW	IGH	4	None	
5	501	CARDPMPGSSWPRFDPW	IGH	2	None	
5	502	CATSGYSPDGYFDPW	IGH	4	None	
5	504	CAKDHFSGNWYFDLW	IGH	2	None	
5	506	CQPYNNWPQYTF	IGK	15	None	
5	508	CARDAGYSSSWYGGFDPW	IGH	2	None	
5	509	CAHVIAERAFEYW	IGH	2	None	
5	510	CAREIYGGNSRPFDYW	IGH	4	None	
5	512	CARDPFRYYDSSGYQFDYW	IGH	2	None	
5	513	CAKGRFPAAAIWIDSW	IGH	2	None	
5	514	CARQRVDTTLAPYYFYAMDVW	IGH	2	None	
5	515	CARDTSRRDGYNFFDYW	IGH	2	None	
5	516	CARGAKLDHGSGDYYYYGMDVW	IGH	2	None	
5	517	CYSAIDNSQVF	IGL	4	None	
5	518	CARSDFESLTGTLDYW	IGH	2	None	
5	519	CQQYYNAPQLSF	IGK	6	C4	6W41
5	521	CARAPIGHCDGGSCYFSYW	IGH	4	None	
5	522	CARRCMGRDGYNSEDFDYW	IGH	4	None	
5	524	CQTWGTGIQVF	IGL	621	None	
5	526	CAKTVGGASDAFDVW	IGH	4	None	
5	528	CARSPRPLARVPSWYFDYW	IGH	3	None	
5	530	CTTDSTLRYFDAW	IGH	4	None	
5	531	CAREGLQIRGIVTHYYALDVW	IGH	7	None	
5	532	CARRLTTLDRAKQFDYW	IGH	2	None	
5	534	CARTYGSNPYFAMDVW	IGH	2	None	
5	536	CARVNNPFYYYYAMDVW	IGH	2	None	
5	537	CARGTGDGLTGYLYYFDYW	IGH	2	None	
5	538	CARYVPNSDDYGFDYW	IGH	2	None	
5	539	CAHRKVTVTVGTYFDNW	IGH	3	None	
5	540	CARVGSYDSAGYYFTHW	IGH	2	None	
5	542	CARVNYDFWSGYDGMDVW	IGH	2	None	
5	543	CTTDGGESRPEFDHW	IGH	8	None	
5	547	CAKGTIWSGLLRGSFDSW	IGH	4	None	
5	550	CARGLGYDDDGFDFW	IGH	2	None	
5	551	CARYKTPDINSFDIW	IGH	4	None	
5	553	CARQKDSSGYYRFDPW	IGH	4	None	

5	554	CARDRSDYDNIWGTYRHTPDYW	IGH	2	None	
5	559	CARRQTSTIRSDAFEAW	IGH	2	None	
5	561	CAREREFGINYHFDFW	IGH	2	None	
5	566	CARMVGQYCSSTSCYNYFDYW	IGH	4	None	
5	567	CLQHSCFPPMYTF	IGK	12	None	
5	569	CARGLGYYSKWGPDYW	IGH	3	None	
5	570	CAREVYSSHCYFDYW	IGH	21	None	
5	571	CAREGYSSDWYGPDYW	IGH	2	None	
5	572	CAKKKQRFSSSWTFDYW	IGH	2	None	
5	573	CARIPSLTTDLYIDYW	IGH	4	None	
5	575	CARDPAQYGDYGYNNAFDIW	IGH	2	None	
5	577	CATELDASGWYDLFDYW	IGH	4	None	
5	578	CARLSGYYDAELDYW	IGH	4	None	
5	579	CARRLSRGWYDWFDPW	IGH	2	None	
5	580	CAKGVWVTFGGVIPRPYYYGMDVW	IGH	2	None	
5	582	CARGIQWSYDHGDYYLDYW	IGH	2	None	
5	584	CAKVHGSYWGGAFDIW	IGH	6	None	
5	586	CARHGPRYCSSTSCYGWGRYFDLW	IGH	2	None	
5	588	CAREEGAPRHFDTW	IGH	2	None	
5	591	CTIGTNWFDPW	IGH	4	None	
6	1	CASHGPRYYDILTGLYYYYGMDVW	IGH	2	None	
6	2	CARDVDYYGSGGIDYW	IGH	2	None	
6	3	CQQTDINPGTF	IGK	25	None	
6	4	CAKNAAIEEFPITIFGVVTLNYFDYW	IGH	2	None	
6	5	CTTDTLPTYYYDSSGYRPFDYW	IGH	2	None	
6	7	CATAPPIAVAGTGNWFDPW	IGH	4	None	
6	8	CARLRADSSGPTALDYW	IGH	4	None	
6	9	CARDQDTAMEPYYYGMDVW	IGH	2	None	
6	10	CARGREIVVVVAATPAGFDYW	IGH	2	None	
6	11	CARDRIQLWSKPYYFDYW	IGH	4	None	
6	12	CARDTTSGSSSWYSLTGYYYYGMDVW	IGH	2	None	
6	13	CARLRYYDSTGYLDSW	IGH	5	None	
6	14	CATGGYGSGSQSRFNYW	IGH	4	None	
6	18	CARQFLSCSGGSCYSGFDYW	IGH	5	None	
6	19	CARDSGQLVRGVGGWYFDFW	IGH	9	None	
6	20	CARTRQYSGSSLAFDLW	IGH	2	None	
6	21	CARRERGSYYFDYW	IGH	6	None	
6	22	CTTLDLILVIEANLGGWFCPW	IGH	3	None	
6	23	CARHFSARASSLAADWFDPW	IGH	2	None	
6	24	CAREQVPIRDPTPPYYYYGLDVW	IGH	2	None	
6	25	CARHSAWRWLPQPNEYYFDYW	IGH	2	None	
6	26	CARDSSLDGGNYDYVWGSYRYFDWFDPW	IGH	3	None	
6	27	CATSVLRAGTTGGGWFDLLRTRPTGGGWFDPW	IGH	3	None	
6	29	CARHAWHGGNHLDYW	IGH	2	None	
6	30	CYSAADNNLLF	IGL	3	None	
6	31	CTGKNEGFWSGFLGDNWFDPW	IGH	4	None	
6	32	CARHERPYYYDSSGYYNYW	IGH	6	None	
6	33	CASNSGYSYGHYYGMDVW	IGH	4	None	
6	34	CTTPVEGSGSYYNPFDYW	IGH	2	None	
6	35	CARHHIDGYNFGFGYW	IGH	2	None	
6	36	CVRQWLDLRGYYYYGMDVW	IGH	2	None	
6	40	CASPKGHYYDSGDFDYW	IGH	2	None	
6	41	CQVWDSSGDSSSDHLWVF	IGL	2	None	
6	45	CARGSPLYDFWSGYWPSVDYYYGMDVW	IGH	2	None	
6	46	CARDRLYFDSVDYW	IGH	27	None	
6	47	CATAPPYCGGDCYSWFDPW	IGH	3	None	
6	48	CTTDSPFAGFDWLFGSDYYYGMDVW	IGH	2	None	
6	49	CARSYDFWSGFYANYYYGMDVW	IGH	16	None	
6	50	CARDYDPLEDLITTLVVVVSTRSASDIW	IGH	2	None	
6	51	CARHGRSYYHPYVDFW	IGH	2	None	
6	52	CAKFTQALDFDYW	IGH	5	None	
6	53	CARDRSYNWIEYYFDYW	IGH	2	None	
6	54	CATLHSWQQVVGGDYW	IGH	2	None	
6	55	CASQLRPTGWFDPW	IGH	2	None	
6	56	CARGFWSGSKINYYYYGMDVW	IGH	4	None	
6	57	CASLLWFGEENDALDVW	IGH	2	None	
6	60	CAKGPLMSYDILTGYYYYYYGMDVW	IGH	13	None	
6	62	CARIPRDKVVNGPLAYNYYYYGMDVW	IGH	3	None	

6	65	CALPISGSQSRFNYW	IGH	2	None	
6	66	CQQFGTSPRFTF	IGK	11	C2	7K8U
6	67	CGTWHSNSKTRVF	IGL	3	None	
6	68	CARDVRPSSSWQLFDYW	IGH	8	None	
6	70	CAKDEDSSGYFTPFDYW	IGH	2	None	
6	71	CAIIGYCSGGSCYSLPYYYYGMNVW	IGH	4	None	
6	74	CQQYNSYSPGMSTF	IGK	2	C3	7K8R
6	75	CARLKYYDILTRYYFDYW	IGH	2	None	
6	76	CARDTLESRAYDTHYFELW	IGH	4	None	
6	77	CTTGPYSSSWEPRFLGFGLRILDYW	IGH	4	None	
6	78	CMQGTHFYSF	IGK	6	None	
6	80	CVNPSPKGLFDYW	IGH	17	None	
6	82	CARGPRITMIVVNPFRAFDIW	IGH	3	None	
6	83	CVRDSEAPESDIDYW	IGH	6	None	
6	86	CVRGGPGTGLDYW	IGH	2	None	
6	87	CAKDLTYYDFWSGYLTTEIYYYYYGMDVW	IGH	2	None	
6	88	CARDPPRIAAAGTEQWLVPAPSYYFDYW	IGH	4	None	
6	90	CAKESSEITGYYVFGDPYDIW	IGH	12	None	
6	91	CARVSSYCDDGKCYSSNWFDPW	IGH	2	None	
6	92	CARLGIAAAALDFDYW	IGH	10	None	
6	93	CARGGSPELLWFGELFLDAFDIW	IGH	7	None	
6	94	CARDLRFLEWLSFDYW	IGH	6	None	
6	96	CARGHSSFNFEHHLDYW	IGH	4	None	
6	97	CATVLPPSYYDSSGYYYWFDYW	IGH	5	None	
6	99	CIQGTHWPPSAF	IGK	136	None	
6	101	CVRVGRMGYCSSASCSKNWFDPW	IGH	2	None	
6	103	CARQGPAFKEGATQFPFDYW	IGH	2	None	
6	105	CARENYYDSGGYMDVW	IGH	5	None	
6	107	CQQYSTSPPWRF	IGK	2	C2	7K8U
6	109	CASYSSGWYSPFDYW	IGH	2	None	
6	110	CARGDPQSLTLAPRTFFDYW	IGH	2	None	
6	111	CAKDGDDSGFDYW	IGH	3	None	
6	112	CQQSYGTPRKIAF	IGK	2	C2	7K8O
6	113	CARDGGDRDWTLFDHW	IGH	2	None	
6	114	CARVPRSANRSRTLDYW	IGH	2	None	
6	115	CVRDNQRRQGRFDSW	IGH	2	None	
6	116	CARFLVVVITTPPDYW	IGH	2	None	
6	118	CVSQDNWKISYYFDSW	IGH	2	None	
6	119	CTTQNRITMIVVVITEPDYW	IGH	2	None	
6	121	CARSIEYAYLFDAW	IGH	2	None	
6	122	CAGLVRYFDWLTATYYYYGMDVW	IGH	3	None	
6	123	CVRERQDYTDYYLDYW	IGH	2	None	
6	124	CARLAITFGESDNWFDPW	IGH	2	None	
6	126	CQQSSNWPPKVTF	IGK	238	C1	7BZ5
6	129	CATSSLYSSSADPYWYFDLW	IGH	2	None	
6	131	CATITGLTYFDHW	IGH	2	None	
6	132	CATNSAMVRGVTPPGWFDPW	IGH	3	None	
6	134	CQHYDNWPLAF	IGK	15	C3	6XDG
6	135	CARDPMTTVISDYYYYGMDVW	IGH	2	None	
6	136	CAAWDDILNGWVF	IGL	88	None	
6	137	CARDLDHLYYDFWSGYYLPGVYYYYGMDVW	IGH	2	None	
6	138	CARDPESGVLGPDYWYFDLW	IGH	4	None	
6	139	CTRRGQNGAFDIW	IGH	4	None	
6	141	CAKARDEAARRSVDYW	IGH	6	None	
6	142	CARTRGYTYGNGFDFW	IGH	2	None	
6	143	CARHRSSWSALSPFDDW	IGH	2	None	
6	144	CVRVRLGPPSLKYFDHW	IGH	2	None	
6	145	CARDLTVVTAILYDYYYGMDVW	IGH	9	None	
6	147	CATTVTTHSYFDPW	IGH	2	None	
6	149	CAKARVMGTLDAFDIW	IGH	2	None	
6	151	CARLLLRYFDWLLTSDDAFDIW	IGH	6	None	
6	152	CAALPPYYDFWSGFPDDAFDIW	IGH	6	None	
6	153	CAKYITKWMVTAPGFDYW	IGH	2	None	
6	154	CVKGVAAMVTYYFDYW	IGH	3	None	
6	155	CQQFNNYLGFTF	IGK	3	C3	7K8R
6	157	CQQYNSYNSYSLFTF	IGK	13	C3	7K8R
6	158	CQSADSFDTYRNVLF	IGL	2	None	
6	160	CATSSAMVRLGWFDPW	IGH	2	None	

6	161	CVKDNSRGLGGMDFQYW	IGH	2	None	
6	162	CASQMATITEPGSDYW	IGH	3	None	
6	164	CSSYAGSNNPLYVF	IGL	2	C2	7BWJ
6	166	CTRRRAAAGKYHFDFW	IGH	3	None	
6	167	CASPYSSSWYLVYW	IGH	2	None	
6	168	CARGFCDGGTCYLEYW	IGH	2	None	
6	169	CTTDRWTIDYGDYEDQDSYSYYGMDVW	IGH	2	None	
6	171	CQQVYDTPRAF	IGK	40	C2	7K8O
6	172	CARFDYFDSSGFWIDAFDIW	IGH	4	None	
6	174	CATPRSFGYYDSSGAFDYW	IGH	2	None	
6	175	CQVWDSISDRLFF	IGL	2	None	
6	176	CYSTDSSGNHRVF	IGL	88	None	
6	179	CARLQLFDYYYYYGMDVW	IGH	6	None	
6	180	CAKDIGYYDSSGYYYVEASYYYYGMDVW	IGH	6	None	
6	182	CARLGRGYSGYDRIAAALGSPYYYYYYGMDVW	IGH	2	None	
6	185	CARVRRSSSWYDYW	IGH	2	None	
6	186	CARDRDGSSWNKYYFDYW	IGH	10	None	
6	188	CARHRTYGTYAMDVW	IGH	3	None	
6	189	CARENGGSDGDYHYYYYYGMDVW	IGH	2	None	
6	190	CARVIPGNDYGDYPDYW	IGH	4	None	
6	191	CARTPGYSSSLLSSYIDYW	IGH	2	None	
6	195	CARDLSRVDYGVLSGYYQGGILDYW	IGH	2	None	
6	196	CVKHGKELELRSYFDLW	IGH	2	None	
6	197	CARVLDTAIVKYYFDYW	IGH	6	None	
6	198	CGTWDSSLSAGVF	IGL	94	None	
6	199	CQHFHSYPYTF	IGK	89	C3	7K8P
6	201	CATYAASVALYFDYW	IGH	2	None	
6	202	CATAPSYGGTGWFDPW	IGH	9	None	
6	205	CARSPGIAARLYYFDYW	IGH	2	None	
6	208	CAKSYSGYDSVDIW	IGH	2	None	
6	210	CAREGLGPMGYYFDYW	IGH	2	None	
6	212	CQSADNSGVYGVLF	IGL	5	None	
6	213	CATVPYYYASSAQGGFDSW	IGH	2	None	
6	214	CARDVENYFDPW	IGH	6	None	
6	215	CAKDPEPSGSYSDYFDYW	IGH	3	None	
6	216	CARGRPTEYYYDSTPLFDYW	IGH	3	None	
6	217	CARPPYDSSSDGAFDIW	IGH	2	None	
6	218	CAKETYYDIWSGYYRDYYYYYGMDVW	IGH	2	None	
6	219	CARTHGGSYSSPLDYW	IGH	2	None	
6	220	CATAVAYYYYGMDVW	IGH	6	None	
6	222	CARNPTVEWSPYYYYYGMDVW	IGH	7	None	
6	223	CARVGVTIFDQW	IGH	6	None	
6	224	CARQSRGDIVLMVYENWFDPW	IGH	2	None	
6	226	CAKIKNSGWPHDGLDVW	IGH	2	None	
6	227	CARCGSPTGRKTDYW	IGH	2	None	
6	228	CARGYCRSTTCYFDYW	IGH	8	None	
6	229	CARGPYDSSGYYHYGMDVW	IGH	2	None	
6	230	CQQSHSTVMWTF	IGK	11	C4	6ZDH
6	231	CASAHEFSPYNWFDPW	IGH	35	None	
6	232	CARHESYCGGDCYSRMFWFDPW	IGH	2	None	
6	233	CARHRAFQAFSDPLDVW	IGH	2	None	
6	234	CTHSSQNYSSGLTDSRPFDIW	IGH	2	None	
6	236	CVRTLRTHTVTTTGVDYW	IGH	3	None	
6	237	CARALWSGDQQLRARKQAYYYYGMDVW	IGH	3	None	
6	238	CVRNRPSIMDLIMFDYW	IGH	19	None	
6	241	CYSTDSSDDHYRVF	IGL	3	None	
6	242	CHGDYVLSSHTEYYYGMDVW	IGH	6	None	
6	243	CARMPRDTEVRIAVAVDIW	IGH	2	None	
6	245	CATGVYADGRGYYYGRIDYW	IGH	3	None	
6	247	CARLGYSSPQLYYYYYGMDVW	IGH	3	None	
6	248	CAKAIMITFGGVIAPLYYFDYW	IGH	4	None	
6	249	CARVGKQPTLYYFYYAMDVW	IGH	13	None	
6	250	CARDYYGSGSYEGFDYW	IGH	2	None	
6	251	CQQYGSSPMGGMYTF	IGK	2	C1	7K8N
6	252	CMHGTQFPSLTF	IGK	33	None	
6	253	CSSYTSSRTIVF	IGL	144	C2	7K90
6	256	CTGGSSHWAYRFDYW	IGH	3	None	
6	260	CARGRTYWGSDYW	IGH	3	None	

6	261	CARSPFSTVILVVHLEAYYFDYW	IGH	3	None	
6	263	CAKDRGNDYGDQLDYW	IGH	51	None	
6	264	CAKGEGMTYDFWNGHYTGFDDW	IGH	3	None	
6	266	CARDSTIAIDQGDNDFDYW	IGH	5	None	
6	269	CATGSPSKVTGWFDPW	IGH	2	None	
6	271	CARNAANYYDSSGYYYPPVDYYYYYGMDVW	IGH	2	None	
6	273	CARLEYSSSWYGLDYW	IGH	6	None	
6	274	CMEAAQFPRLSF	IGK	17	None	
6	275	CAHRPRYFDWLFESDLGHGWFDPW	IGH	4	None	
6	276	CARVTVTTGGDYW	IGH	2	None	
6	277	CATSPAYCSSTSCYRGWFDPW	IGH	8	None	
6	278	CARDGGNWGNYWYFDLW	IGH	2	None	
6	280	CVRDLYYDSSGYFLSW	IGH	2	None	
6	282	CAREKYSYGFALDYW	IGH	6	None	
6	284	CARHDYGDYFGAFDIW	IGH	4	None	
6	285	CAKDLFFHPNQYYFDYW	IGH	2	None	
6	287	CGREARRISTYDPPFFDSW	IGH	2	None	
6	290	CARDFGAKVASRGRYSYYGMDVW	IGH	2	None	
6	291	CASVGRDGGSWGYWLDPW	IGH	2	None	
6	292	CARGYSGYDDLLRSYYYYYGMDVW	IGH	2	None	
6	293	CAREAHCSGGSCYSGWFDPW	IGH	2	None	
6	294	CTTDSGRNCGGDCSDDYW	IGH	3	None	
6	296	CARDRHGFDFDMSLEYNDAFDLW	IGH	2	None	
6	300	CAKDPSYYDFWSGYLPRVGPYYYYYGMDVW	IGH	2	None	
6	302	CARMGGYFEILTGLKGLDYW	IGH	12	None	
6	303	CARERAIFCEGDCYEEDFDIW	IGH	198	None	
6	305	CAKVLFDKSNFDPWYFDVW	IGH	2	None	
6	307	CARHWSYYSFDYW	IGH	2	None	
6	308	CMQGLQTPSMYAF	IGK	2	None	
6	309	CQQYDNLLLLTF	IGK	38	C3	6XDG
6	312	CARPSTGSSTSEFDYW	IGH	6	None	
6	313	CARKEGILHLVPYHIDYW	IGH	156	None	
6	314	CVKETACSGTHCYPDFW	IGH	2	None	
6	315	CVKDDYYASGNFASNFDCW	IGH	7	None	
6	317	CANQANRGPTFYWYFVLW	IGH	5	None	
6	318	CMEGAHWPAITF	IGK	2	None	
6	319	CARPQAEWDLLLPFDMW	IGH	8	None	
6	321	CARERTIFGVVIHFDYW	IGH	2	None	
6	322	CARGYYYDSSGYYRNWYFDLW	IGH	2	None	
6	324	CARHEVDPFMITFGGVIPRGFDPW	IGH	2	None	
6	325	CARDPRPLYDILTGYHPPDYW	IGH	2	None	
6	327	CARSLDFWTANYGRAPGDYFDSW	IGH	2	None	
6	328	CARLPGFYSYGPYYYYSGMDVW	IGH	2	None	
6	329	CARAHGDYVINWFDPW	IGH	4	None	
6	330	CARHRPYGGRSYYFDVW	IGH	5	None	
6	331	CARAPYYYDMLGKQPFDYW	IGH	4	None	
6	333	CMQGTHWGARSNFITF	IGK	2	None	
6	334	CAREGGAYCGGDCLSAFDIW	IGH	2	None	
6	335	CAKRAGVSPLSSGWYHPIDYW	IGH	2	None	
6	341	CATVFAIFGVVTRFDYW	IGH	20	None	
6	342	CARGDGSGNYFLDYNYYAMDVW	IGH	2	None	
6	343	CAREGIAARRPWFDPW	IGH	2	None	
6	344	CAHRPYRGDYYDSSGYPGGDFDYW	IGH	2	None	
6	346	CATDLRYRISRLGYNSYIDYW	IGH	3	None	
6	347	CAKSMATYSSGWYEEYW	IGH	2	None	
6	348	CARHELYSSGSILFDYW	IGH	2	None	
6	350	CARHRLGGDPFDFW	IGH	4	None	
6	357	CARDNHWSIDYW	IGH	4	None	
6	362	CARATYDRSADPFDFW	IGH	7	None	
6	363	CARDSQSFFYGTVTYYGTMDYW	IGH	2	None	
6	364	CARDPPEPPFQYSSGFYYYYGMDVW	IGH	2	None	
6	365	CAHRVGYCTTSSCLWGVGFDYW	IGH	6	None	
6	366	CARDYSQLGYDFWSGYSSYYYGMDVW	IGH	2	None	
6	367	CARAGWASSSDDDVFDIW	IGH	33	None	
6	368	CATGPGYSSSWYPNWFDPW	IGH	3	None	
6	369	CARSFNYGTYYNAFDSW	IGH	3	None	
6	371	CARDSTVHYYYGMDVW	IGH	6	None	
6	372	CAREDRYCSGGNCYYDYW	IGH	2	None	

6	374	CAHPLTTRLHDAFDIW	IGH	2	None	
6	375	CARLYCSGGSCYTTDNWFDPW	IGH	2	None	
6	376	CQQYYTAPHMYTF	IGK	7	C4	6W41
6	377	CARDSSDTAMVSFDYW	IGH	2	None	
6	378	CARAGYCSTSSCYYLVYW	IGH	4	None	
6	379	CQSHDRSNHDWVF	IGL	7	C4	7JVC
6	380	CASAPAAIRTINDYW	IGH	4	None	
6	381	CAIQLETLMAPTDGMDVW	IGH	2	None	
6	383	CAKLVVNSGNSGADYW	IGH	2	None	
6	384	CAKARGLMVYARKDYYFDYW	IGH	2	None	
6	385	CQQSYSTHREYTF	IGK	7	C4	6ZDH
6	386	CASRREDYDSSGYFSLPALYFDYW	IGH	2	None	
6	387	CQQSYSFDHGTF	IGK	2	None	
6	388	CAKEPHVLRYFDWYEKDYW	IGH	2	None	
6	390	CASLPLRMVGMLDYYYGMDVW	IGH	4	None	
6	391	CARDSDYYDSSGYLAPGDYW	IGH	5	None	
6	392	CARDLLGVLEPHRGYFDYW	IGH	2	None	
6	393	CARDRVVGAPPNYYYGMDVW	IGH	3	None	
6	394	CARGSLIALAGTGFDYW	IGH	3	None	
6	395	CASSRLPYGDSRGVFDYW	IGH	2	None	
6	396	CATTGPGPFDIW	IGH	2	None	
6	397	CARDGSGSSDDAFDIW	IGH	4	None	
6	398	CASIAAAGSSSYYYYGMDVW	IGH	6	None	
6	400	CQQAHALPFTF	IGK	2	None	
6	402	CASSYDSSGYYWYYFDYW	IGH	2	None	
6	403	CQQVYDTPRTF	IGK	14408	C2	7K8O
6	404	CARSFLHYSGSSGYYSGSFDYW	IGH	2	None	
6	405	CARGTGIRYYDSSGYHLGEYYFDYW	IGH	3	None	
6	406	CAAWDDTLSGLLYVF	IGL	2	None	
6	407	CTTDRYYDYVWGSYRLVDYW	IGH	2	None	
6	410	CAKGDQLLFPCWFDPW	IGH	2	None	
6	412	CAVLWRVEMATIYGEVDYW	IGH	2	None	
6	413	CLQHDNFPLLYTF	IGK	15	C3	6XDG
6	418	CQQYHDYPRSF	IGK	52	C3	7K8R
6	420	CASNRGVATIHSPIDYW	IGH	2	None	
6	421	CAKMAGDGYTLNYYFDLW	IGH	125	None	
6	422	CARDQIVAGEFEDYYYGMDVW	IGH	2	None	
6	424	CTTEYSSSSPYFDYW	IGH	2	None	
6	426	CQQRSNWLGAF	IGK	8	None	
6	427	CARHPGLLSGGWYNGSPGSDYW	IGH	2	None	
6	428	CAIQRSSSWYSGFDYW	IGH	2	None	
6	430	CAREVCGGDCYPDSW	IGH	2	None	
6	431	CASRLENLSLSAFDFW	IGH	2	None	
6	433	CARQIRVAGLLYYFDYW	IGH	6	None	
6	434	CARGDDFWSGYYDYW	IGH	2	None	
6	435	CAKVSPRVVTILMYYFDYW	IGH	4	None	
6	436	CARRSYDSSYYFDYW	IGH	13	None	
6	437	CATPRGYSGYGLFDYW	IGH	15	None	
6	439	CARSAAVRGHCTNGVCPDYYGSGNWFDPW	IGH	2	None	
6	440	CATGSAVAGTSLYYYYYYGMDVW	IGH	6	None	
6	442	CAIPRHSSGWYGIWYYFDYW	IGH	2	None	
6	445	CARLPYDFWSPSDYW	IGH	2	None	
6	448	CARDYDPIDDLITVLAVVVNTRSAIDIW	IGH	2	None	
6	449	CARIRALYYSDTSGYPMSSWFDPW	IGH	3	None	
6	452	CATEKMVYAGIAAAGTIFDYW	IGH	2	None	
6	453	CATTAAYSSGSSILRTLDFDYW	IGH	2	None	
6	454	CASSGWYVVYW	IGH	6	None	
6	455	CAKILGGFDYW	IGH	2	None	
6	456	CARDSNSWYAPFDYW	IGH	6	None	
6	458	CARLLPPYSSGWYYYYGMDVW	IGH	4	None	
6	459	CARGKAYGGYDSFDYW	IGH	5	None	
6	460	CVRDHFSEDAFDIW	IGH	3	None	
6	462	CARGLYSGYEYYFDYW	IGH	4	None	
6	465	CARDGHANYDILTGYPFDPW	IGH	2	None	
6	467	CARHTMEAVAGTMTFLFDYW	IGH	3	None	
6	468	CARDGFPYTSSAPFDHW	IGH	2	None	
6	469	CARARRPLWEQQLDFAFDIW	IGH	2	None	
6	470	CARHAYYDFWSGYYFSGKQYYYGMDVW	IGH	5	None	

6	471	CARGIIEYDYVWGSYVDAFDIW	IGH	2	None	
6	472	CAQDRWNVDTPGYSYYYGMDVW	IGH	4	None	
6	473	CAKDVSWFGELLSPLFDYW	IGH	3	None	
6	474	CARSHIVVVVAASYYYGMDVW	IGH	6	None	
6	478	CARQYGYYYDSSGYPDYW	IGH	2	None	
6	483	CARHHSYSGYRFFDYW	IGH	2	None	
6	484	CARHASHWIGAAALNWFDPW	IGH	6	None	
6	486	CARRGFNSADHWFDPW	IGH	5	None	
6	488	CARDVYSYGPPHDYW	IGH	3	None	
6	490	CARDLRRTTGTTLNWFDPW	IGH	2	None	
6	492	CARHEGSYYDILTGYYPGDYW	IGH	4	None	
6	493	CARHAPLAVAKTWYNPHFDYW	IGH	2	None	
6	495	CQHRANWPKITF	IGK	7	None	
6	496	CTTDSGIDYGDYLNYFDYW	IGH	2	None	
6	497	CAASAVLYCSSTSCYATNYYYYGMDVW	IGH	7	None	
6	498	CAKDWGQGRWLQLEDYFDYW	IGH	2	None	
6	499	CARGLGILDYW	IGH	17	None	
6	501	CARLLKPQYQRPLYYFDYW	IGH	2	None	
6	502	CVREGELDGDYGADYW	IGH	2	None	
6	503	CARCSPDYYDTSGYPRRPFDYW	IGH	3	None	
6	504	CARDYPDTAMVTLPIDYW	IGH	2	None	
6	506	CARVYYYDSSGLDYFDYW	IGH	2	None	
6	507	CARVPRLITISNWFDPW	IGH	2	None	
6	508	CARYRGGNNYDKYLDFW	IGH	4	C1	6XCM
6	509	CARVPYYYDSSGYYYPPNYYYYYGMDVW	IGH	3	None	
6	510	CQEYGSSTQVTF	IGK	52	C1	7K8N
6	512	CARVLGRCSNGVCYWDYNRFDPW	IGH	35	None	
6	513	CARSLGFVASNSVGDFFDFW	IGH	6	None	
6	515	CARHPIVVVPAAVGSYAFDIW	IGH	2	None	
6	518	CARGQFYYSGVYSDREYYQHW	IGH	2	None	
6	519	CVKDSQVYDYLGGSAFDVW	IGH	2	None	
6	520	CQQYGPSVIYTF	IGK	2	C1	7K8N
6	521	CARDRYSSSSYYYYGMDVW	IGH	5	None	
6	522	CATQFVVTAKKPVDYW	IGH	2	None	
6	523	CAKGGRVAGMDYW	IGH	2	None	
6	524	CAKETEWEPLTRWFDSW	IGH	2	None	
6	526	CARSSGYDPEYYLDYW	IGH	5	None	
6	527	CARAVQDIVLMVYVYDWFDPW	IGH	4	None	
6	528	CAKIGDDYGDYGDSYYFDYW	IGH	4	None	
6	529	CARAGLSGCNHVGCFTSFYYYGMDVW	IGH	2	None	
6	530	CQQSYIAPDFPF	IGK	6	C4	7JW0
6	532	CAVLETRSYSFRYW	IGH	2	None	
6	534	CQSADDSETYPGWVF	IGL	246	None	
6	535	CARLHDSSGYYFGFDYW	IGH	12	None	
6	536	CVRRLNDVFTGYYSPGWFDPW	IGH	6	None	
6	537	CARFYGLDGFDIW	IGH	6	None	
6	538	CARHGLYCSGGSCYNWFDPW	IGH	3	None	
6	539	CQSYDTSLRAWVF	IGL	449	C4	7JVC
6	540	CARLKREYSSGWSYYFDYW	IGH	5	None	
6	543	CARGDSLRYDYSNGYYYYAMDVW	IGH	5	None	
6	544	CTAYLQWSSLKASDTAMYYCARLSVAVAGSDYW	IGH	2	None	
6	545	CVRTSHYYDSSGNPPFDFW	IGH	2	None	
6	546	CAREYTSISQWFGELTRGRYFDYW	IGH	4	None	
6	547	CYSYAGSYTPF	IGL	2	None	
6	548	CARDQSSGWYSNWFDPW	IGH	4	None	
6	549	CASSRYGDFDSW	IGH	2	None	
6	550	CARHRFSFPSITGTTGSPYYYYGMDVW	IGH	2	None	
6	551	CARRTDEVAGFDYW	IGH	2	None	
6	552	CAKDSRVFLLGTDYYFDSW	IGH	7	None	
6	553	CARGPLYLGYSDDAFDIW	IGH	2	None	
6	556	CVKVGIQLWLMYYFDYW	IGH	4	None	
6	557	CATVFCIAAAGCNFDYW	IGH	2	None	
6	558	CAKMRWELLRVGHNWFDPW	IGH	4	None	
6	559	CARGGQSGSYYTYYYGMDVW	IGH	4	None	
6	561	CARSPGYYGGWSFDYW	IGH	2	None	
6	562	CVKNKVPFSLMVFAAADAFDIW	IGH	2	None	
6	563	CATVVGATGAFDIW	IGH	3	None	
6	565	CAHRRGHDILTGYFNAFDIW	IGH	7	None	

6	566	CARDVVGQDYGDYVDYW	IGH	6	None	
6	567	CARLRRSITMVRGVPDYW	IGH	2	None	
6	568	CARIYKQQLVYFDYYYGMDVW	IGH	2	None	
6	569	CAKDGHYYGSYYFDYW	IGH	4	None	
6	570	CSLRSEDTAMYYCARERAIFCEGDCYEEDFDIW	IGH	5	None	
6	571	CARDPIAAIHYYYYGMDVW	IGH	2	None	
6	572	CARHASPAWYCSGGSCRGFDYW	IGH	2	None	
6	575	CASRREYNYDFDHW	IGH	7	None	
6	576	CARGSFMITFGGVIVSPTGAFDIW	IGH	2	None	
6	578	CANFNPMTMGASDIW	IGH	3	None	
6	580	CARRNFDFWRQYSNYLDPW	IGH	2	None	
6	581	CARDRDSNWNDPRSYYYGMDVW	IGH	4	None	
6	582	CARSPPNTIFGVVIFPSAFDIW	IGH	8	None	
6	583	CARDQLDSSGWQEYYFDYW	IGH	4	None	
6	584	CAKFCSSSNCYADSW	IGH	2	None	
6	585	CARGSLSISMMVVVFIGGSFYLDSW	IGH	2	None	
6	586	CARRYSSSWGRYRPKHRYYFDYW	IGH	2	None	
6	587	CTTEGYSSGWNHGSKFDPW	IGH	2	None	
6	590	CQQYGNSPPKSSF	IGK	3	C1	7K8N
6	591	CARDAAYCGGDCWDALNYYYYYGMDVW	IGH	3	None	
6	594	CVKVSLLGADDYW	IGH	8	None	
6	595	CARIRSDYTILTGFQYYFDYW	IGH	3	None	
6	596	CTTVAHYYDSSGYYLDYW	IGH	2	None	
6	597	CMLSYNGARVF	IGL	45	None	
6	598	CLQYNSWPPQCSF	IGK	2	C3	7K8R
6	599	CVKENRMGVAASGTFLQYDIFDIW	IGH	2	None	
6	600	CARTDYGGKLNAFDIW	IGH	3	None	
6	602	CARASITIFGVGERVDYW	IGH	2	None	
6	603	CARVGTIFGVAHNWFDPW	IGH	2	None	
6	605	CTPQRVYDYVWGSYRYLDYW	IGH	2	None	
6	606	CARQRIVPAKTYYYGSGSYYTLDYFDYW	IGH	2	None	
6	607	CARPKLPYYGSGRGAEWFDPW	IGH	2	None	
6	608	CARMRGGYGFYFDYW	IGH	6	None	
6	609	CARPGYSYYYFGMDVW	IGH	2	None	
6	610	CARASYSTAYDYW	IGH	7	None	
6	611	CARDSVVGAFDIW	IGH	13	None	
6	612	CATSYYYDSSGYLDYW	IGH	3	None	
6	613	CATSTAAGTVSWFDPW	IGH	3	None	
6	614	CAKHEGELLYYYYYYGMDVW	IGH	2	None	
6	615	CQHYNEYSSPF	IGK	2	None	
6	616	CMQGTHWPPSTF	IGK	1580	None	
6	617	CAADTYGDYKSPLFDYW	IGH	4	None	
6	618	CARDEDYGDYGLRLYYFDYW	IGH	2	None	
6	619	CVRGYSSPDYW	IGH	4	None	
6	620	CARDTEQPMLRGFSFDSW	IGH	2	None	
6	621	CQTWGIDILAVF	IGL	2	None	
6	622	CARERVNDYYDSSGYHIDYW	IGH	2	None	
6	624	CATPRGYYFHDAFDIW	IGH	4	None	
6	625	CARDPDRGYCSNPNCPGSYYYGMDVW	IGH	2	None	
6	626	CARDSVRVSEHNYYYYYGMDVW	IGH	2	None	
6	627	CARESGVVATIIVLADYYYGMDVW	IGH	4	None	
6	628	CMQSIQLPIAF	IGK	272	None	
6	631	CAREVPTPWYDILTGYSVPTNAFDIW	IGH	5	None	
6	632	CAHYTSSYYYDSSGYPFFDYW	IGH	2	None	
6	634	CAKVPPHWSYHWYFDHW	IGH	2	None	
6	635	CARIDDGSSWTGDAFDIW	IGH	4	None	
6	636	CSRAGSVFHFRSAYSHSDYW	IGH	56	None	
6	637	CATAPGYSSGWSGWFDPW	IGH	2	None	
6	638	CARVRFLDFYAFDFW	IGH	9	None	
6	639	CVRSPPNLSGQSWFDPW	IGH	2	None	
6	640	CASSGDQYDSSASDYW	IGH	2	None	
6	641	CARTGSGSYYGPPPFDYW	IGH	6	None	
6	642	CARAAGGSYLANFDYW	IGH	4	None	
6	643	CARVIVVSSGWRPVYYYYGMDVW	IGH	6	None	
6	644	CARDDVSTLAFGSVFDYW	IGH	2	None	
6	645	CARDTMTGYSSSWPRDYNWFDPW	IGH	2	None	
6	646	CQEYNNWPPMYIF	IGK	23	C3	7K8R
6	647	CARDSPNNWFDPW	IGH	7	None	

6	648	CARVLWFGYLLEPWLGYFDYW	IGH	24	None	
6	650	CARVWFGEFNPNPLDYW	IGH	2	None	
6	653	CARQPPLFCSSTSCLGQYYYYYYGMDVW	IGH	2	None	
6	654	CQQYDDFPPSPTF	IGK	17	C3	6XDG
6	655	CMQRIEFPSYTF	IGK	2	None	
6	656	CAWEGPLGTHYFDYW	IGH	2	None	
6	657	CARLSVAVAGSDYW	IGH	2	None	
6	658	CQYRTNWPQAWTF	IGK	2	None	
6	661	CQYFGSSPPTTF	IGK	64	C1	7K8N
6	662	CATAPPVAGFFWFDPW	IGH	4	None	
6	663	CARRLDILTGFDGMDVW	IGH	3	None	
6	664	CARGDQKPAFSLDYW	IGH	3	None	
6	665	CAKVSYLSTASSLDYW	IGH	59	None	
6	666	CARDPYYDFWSGYYHSFDYW	IGH	2	None	
6	668	CARVAGIVATNYFDYW	IGH	2	None	
6	669	CARDWGLVRTVPDDALDYW	IGH	2	None	
6	671	CAREDGTYLHFDYW	IGH	2	None	
6	672	CARDVPEDNWNDSHFDYW	IGH	4	None	
6	674	CLQHNTYPWTF	IGK	231	C3	7K8R
6	677	CCSYAGSYTFDWVF	IGL	2	C2	7K8X
6	678	CARDPSVPGGATIAFDFW	IGH	2	None	
6	681	CAKESTNYYDSSAYNYGHYFDHW	IGH	2	None	
6	683	CARDTPSLSRNFDWLAPELFDYFDFW	IGH	2	None	
6	684	CARALPYYDILTGYHPYYFDYW	IGH	4	None	
6	685	CARSEMATISFDYW	IGH	2	None	
6	686	CARVQSTSSDYW	IGH	2	None	
6	689	CARARASYDSSGYWPYYFDYW	IGH	2	None	
6	692	CARGPDVLRYFDWLFNPFDYW	IGH	5	None	
6	693	CQHYGASPYPF	IGK	6	C1	7K8N
6	695	CARDLNRGPDVFDVW	IGH	4	None	
6	696	CARASSMVRGVIPFRWFDPW	IGH	2	None	
6	697	CSLRSEDTAVYYCARGPRITMIVVNPFRAFDIW	IGH	3	None	
6	699	CATGLAVAEPAYYYYGMDVW	IGH	3	None	
6	700	CAREGKGYYDSSGYYRVVDYYFDYW	IGH	2	None	
6	701	CATSGTVEHGTREWLDPW	IGH	2	None	
6	705	CAKHSKYYLGSGLDYW	IGH	4	None	
6	707	CARLVDYYDSSGYRDLGWPAPDYW	IGH	3	None	
6	709	CAKIVVGGALDIW	IGH	2	None	
6	710	CAHSLLWFGELLPYYFDYW	IGH	4	None	
6	711	CARGVAKESSNSWNAYYYADYW	IGH	3	None	
6	715	CTRGSGTSYGDYGLFPYW	IGH	3	None	
6	717	CARDPSGYDHLEKDPDWFDPW	IGH	2	None	
6	718	CARGISGANVAAAGSLEYYFDYW	IGH	2	None	
6	719	CARTTVTPDTFDYW	IGH	10	None	
6	720	CTTRTYYYGTNGYSADYW	IGH	2	None	
6	721	CAKDISGYYRPFDCW	IGH	6	None	
6	722	CHQSSLLPYTF	IGK	16	None	
6	723	CARPHFAMGTNWFDPW	IGH	2	None	
6	725	CARIDYYESGGRYPTAPKRYFFDYW	IGH	2	None	
6	726	CAKIGGGESMGWYFDYW	IGH	2	None	
6	727	CQRYNRAPYTF	IGK	247	C3	7K8P
6	729	CASINTAMVTGVGYNWFDPW	IGH	4	None	
6	730	CHHYNNWPPLFTF	IGK	8	None	
6	732	CAKDSPFYGDYEYYYYYGMDVW	IGH	2	None	
6	733	CARAPQYYASGIGFDYW	IGH	2	None	
6	734	CARDYYDSSGYYNNWFDPW	IGH	17	None	
6	735	CQTWGTVCVF	IGL	14	None	
6	737	CARGGDYGQFEYW	IGH	4	None	
6	738	CAKHYDPIDDLITMMAVVISSRSAFDIW	IGH	12	None	
6	739	CARGDMYYYDTNGYPDYW	IGH	22	None	
6	741	CQVWDSSNDHPVF	IGL	125	C4	7JVC
6	742	CARATWPRTVYYYYGMDVW	IGH	2	None	
6	743	CAKDIAPFGAVAGHFDHW	IGH	2	None	
6	744	CVLYMGSGIWVF	IGL	16	None	
6	745	CARRGSGNSWSLFDYW	IGH	2	None	
6	746	CARDRYYYDSSGYYFPTYYYYGMDVW	IGH	2	None	
6	747	CAKEELPMFLHIASSGWSGFDQW	IGH	4	None	
6	748	CATGARRGYYLYYMDVW	IGH	5	None	

6	749	CAKDYDPIEDLISMMVVVASSRSAFDVW	IGH	6	None	
6	751	CAAMGATELDYW	IGH	2	None	
6	752	CQKYNDYSTWTF	IGK	4	None	
6	753	CARDHPDNDYGDYAGYW	IGH	7	None	
6	754	CTRGNFRQMLLEYHYYGMDVW	IGH	28	None	
6	755	CASDMGATDPDAFDIW	IGH	2	None	
6	756	CASGIVVLPAANSEMGTGYYYYGMDVW	IGH	3	None	
6	757	CARVVRELRTCSGESCQYRRSEEYYFDYW	IGH	3	None	
6	758	CAKFSYDYESGTYVFDSW	IGH	2	None	
6	760	CARWENYYDSSGYTDYW	IGH	11	None	
6	761	CAKDFLAVAGRTSLYFDYW	IGH	2	None	
7	1	CASHKGGSKYYYGSGNWFDPW	IGH	4	None	
7	2	CARDLGTADSSGWYDDYYYYYGMDVW	IGH	4	None	
7	4	CHQRTNWPMGSF	IGK	5	None	
7	5	CATSLTLVRGVIRNDAFDIW	IGH	2	None	
7	6	CARDSTFRLHLFRVCCGYFDYW	IGH	39	None	
7	7	CAKDPKKIMITFGGVIVIPAFDYW	IGH	3	None	
7	8	CARGRGTWFGELLDYW	IGH	3	None	
7	9	CAKLAGYCSGGNCYTRGYFDYW	IGH	3	None	
7	10	CAKAGRSAMVTLDYW	IGH	8	None	
7	11	CARDRFCSGGSCPHYFDYW	IGH	7	None	
7	15	CARSFGTYFDFWSGFYYYYAMDVW	IGH	3	None	
7	17	CARDGSGTWHLDYW	IGH	4	None	
7	18	CAKDAISVIVVVTTFDYW	IGH	11	None	
7	20	CARSTFTVAANPFDYW	IGH	9	None	
7	21	CATSRATRLQRHPFDYW	IGH	26	None	
7	23	CARRVGALPVDAFDIW	IGH	2	None	
7	24	CARVWPQGYCSGGSCYSYYYYGMDVW	IGH	2	None	
7	25	CARDPGGYSYGPHFDYW	IGH	2	None	
7	26	CARDRPWFLYSYVTDSGAFDIW	IGH	3	None	
7	28	CAKLITMIGGDDYW	IGH	3	None	
7	29	CARDKSGYSSGWSLFGSYYFDYW	IGH	6	None	
7	30	CARFRLVDIVATPAFDIW	IGH	2	None	
7	31	CARGGWYIIQKSAFDIW	IGH	3	None	
7	32	CARRGSGYRQYYFDYW	IGH	2	None	
7	33	CAREGVGQIAVAVYYYYGMDVW	IGH	3	None	
7	35	CAKGLGSGIDLLTGDEAFDIW	IGH	2	None	
7	36	CARHPLAVAGGSFDYW	IGH	8	None	
7	38	CAKGDQWLTPSIDYW	IGH	4	None	
7	39	CARYGSGTYHEPFDYW	IGH	3	None	
7	40	CVRAEWDLTFDAFDIW	IGH	2	None	
7	42	CCSYAGSYTF	IGL	26	C2	7K8X
7	43	CARDSQVVPAAGGADYYYYYGIDVW	IGH	2	None	
7	44	CAHSPLVWFGELFAAFDIW	IGH	10	None	
7	45	CAKSGSGNVGIDYW	IGH	7	None	
7	46	CAHTPITNYGDYSFDYW	IGH	2	None	
7	47	CARSRKLRLGELSRIVDHNYYFDFW	IGH	2	None	
7	49	CARDLDGSGSYWGWTYYYYGMDVW	IGH	2	None	
7	50	CAAWDDSLSGWVF	IGL	672	None	
7	52	CVGMVRGVTRPPELNWFDPW	IGH	5	None	
7	53	CARSLAGERFFDYW	IGH	5	None	
7	54	CARGMTTTVFDYW	IGH	11	None	
7	55	CARDSEWFGKLKSHFDYW	IGH	8	None	
7	56	CARDWDDSSGIDAFDIW	IGH	2	None	
7	57	CAKDMWYSSSWPLDYW	IGH	15	None	
7	58	CARDTYYDILTGYYEPLMEDYW	IGH	3	None	
7	59	CARDLDYYGSGTQYDRGYFDYW	IGH	2	None	
7	60	CAAPHCSSTSCYDAFDIW	IGH	2	None	
7	62	CASGLSAVRGIMRDYW	IGH	3	None	
7	63	CARGPRVITFGGVISNFDYW	IGH	4	None	
7	64	CARDRKGGSTYGSALDYW	IGH	6	None	
7	66	CAREPTWRAVGSGWLDYFDYW	IGH	2	None	
7	67	CQQFNNFLGTF	IGK	93	None	
7	68	CACNNHGSGIYGWFDPW	IGH	3	None	
7	71	CARGYGDLHWDYW	IGH	8	None	
7	72	CARALTATGNGDFDFW	IGH	2	None	
7	73	CARTRRGYRHGFDGDRTGPFDSW	IGH	5	None	
7	74	CARDGGDNSGWFRYW	IGH	2	None	

7	75	CASTASMAGLDYW	IGH	2	None	
7	77	CAKDKLSLRYCSGGSCWGVFDYW	IGH	2	None	
7	78	CARGLALLYDILTGYWWFDPW	IGH	2	None	
7	79	CARIRSSSWYRWAEGNWYFDLW	IGH	102	None	
7	81	CASEHHSSGWYLFPPSFDYW	IGH	4	None	
7	82	CARRNPAYSSSWSGPDAFDIW	IGH	2	None	
7	83	CARVFRGGFDPFDIW	IGH	2	None	
7	84	CARDNGYYGSGTYAVDYW	IGH	2	None	
7	85	CQQYKSYSF	IGK	435	C3	7K8R
7	86	CARGGLGYSYGGWYFDYW	IGH	3	None	
7	87	CAKDYSRRILGFIAVAGTPAHFDYW	IGH	9	None	
7	88	CATTNRIRYFDWLLGDDAFDIW	IGH	2	None	
7	91	CAREDYGDYVNYW	IGH	3	None	
7	92	CQQYDDWPLMCTF	IGK	24	C3	6XDG
7	94	CAKSFSLAVAGMRAMEDWFDPW	IGH	2	None	
7	95	CARLLLSTVTTNWYFDLW	IGH	4	None	
7	96	CLHDYNYPLAF	IGK	8	None	
7	97	CGTWHSNSKTWVF	IGL	3	None	
7	98	CASRYDYVWGSYRHRQFDYW	IGH	4	None	
7	99	CARGVIVVVTATPFVYFDYW	IGH	5	None	
7	100	CARLPLYNYGPNFDYW	IGH	2	None	
7	101	CARVPVGPIDYW	IGH	4	None	
7	102	CAHASLYSGYDLTNWFDPW	IGH	2	None	
7	103	CAKDVWPHVDRPPFDYW	IGH	6	None	
7	104	CAKVKYSYGYGLFDYW	IGH	4	None	
7	105	CARAGPTYVSAYFDAW	IGH	2	None	
7	107	CRLVYGSGSTYDYW	IGH	2	None	
7	108	CARDWGSDSSGYPQPFDYW	IGH	4	None	
7	109	CARDNGRVDITMIVVVIGAFDIW	IGH	2	None	
7	111	CARDGRGGYSYGENYFDYW	IGH	2	None	
7	112	CARDYYDSSGYLGTFDSW	IGH	3	None	
7	113	CAREKFCNNGSCSWGYYYGMDVW	IGH	2	None	
7	114	CTKDVVDRIEVAGLEGFDVW	IGH	2	None	
7	115	CAKVEGPEYSYGNYYFDYW	IGH	2	None	
7	116	CARYYYDSSGYYYPSYGMDVW	IGH	2	None	
7	117	CQQYNSLLTFLTF	IGK	3	C3	7K8R
7	118	CAKDRVIIRGRHYMDVW	IGH	2	None	
7	119	CARDWSRTRLTIFGVAGRDYW	IGH	2	None	
7	120	CARDGVVVVAAPNYYYYMDVW	IGH	2	None	
7	121	CAKDLYTVTPEYFQHW	IGH	2	None	
7	122	CARGQRVLRYFDWLLFFDYW	IGH	3	None	
7	123	CARTLIGITFGGVIVKTPFDYW	IGH	7	None	
7	124	CGTWDSSLSAGVF	IGL	150	None	
7	125	CAGLRSFEILTGYYSRLDDAFEIW	IGH	4	None	
7	126	CARESRYYNTNWKFFHDAFDLW	IGH	10	None	
7	127	CFHSRVRGADVFDIW	IGH	2	None	
7	128	CAKRSRYYDVLTGYYLEGGFDYW	IGH	4	None	
7	130	CARGYYYDISGNGGGFDIW	IGH	5	None	
7	131	CVLYMGSGIWVF	IGL	81	None	
7	133	CARGSLSFDPW	IGH	5	None	
7	134	CARAVRLDGQYYFDYW	IGH	2	None	
7	135	CARVRIAAIDYFEYW	IGH	2	None	
7	136	CARDLDDILTGYLSPHAFDIW	IGH	4	None	
7	137	CARDGLLRYFDWSPRGRYYYYMDVW	IGH	3	None	
7	138	CAKGQFVYTVTTLDYW	IGH	4	None	
7	139	CARDPLNYDILTGYYSYYYMDVW	IGH	3	None	
7	140	CARDPKPSYSSGLLGETYFDYW	IGH	2	None	
7	141	CAKDAQTYYYDSSGYYYGSDYFDYW	IGH	3	None	
7	143	CASGRKYYDSSGIAKPNPESTAPFDYW	IGH	4	None	
7	144	CARGYLKTGFDIW	IGH	3	None	
7	146	CAKFSSGSEVGLPRGLHFDFW	IGH	5	None	
7	149	CARADGSITIFGVVIRNPSDYW	IGH	3	None	
7	150	CARHPPGYCTGGTCYWYFDVW	IGH	6	None	
7	151	CARDGEALKWFGDYHPPDYW	IGH	2	None	
7	152	CMSAHSSGSFWVF	IGL	5	None	
7	153	CARVFTPSGVRYFDWLFQPYYFDYW	IGH	3	None	
7	154	CARERRVGYNGPFDYW	IGH	6	None	
7	156	CARDRDPVRFLYMAARVGFDYW	IGH	3	None	

7	157	CARIPKQWLVLHREYYFDYW	IGH	17	None	
7	158	CAKLPPGSSGWYFDLW	IGH	6	None	
7	159	CLQHDNFLGRF	IGK	10	None	
7	160	CARDNRGGSSWSHRNWFDPW	IGH	4	None	
7	161	CARNGETNAFDIW	IGH	47	None	
7	162	CAKDRGNDYGDQLDYW	IGH	10	None	
7	163	CARAPSSRPRSIAVAGLFDYW	IGH	3	None	
7	164	CAKDIGIVVGQAEFDYW	IGH	2	None	
7	165	CAKDIIEGYSRFGFDYW	IGH	5	None	
7	166	CARDRSLRYFDWLSQIDAFDIW	IGH	8	None	
7	167	CASHTLLRYFDWLSPDAFDIW	IGH	2	None	
7	168	CAHRQFRGWNYYPADRNNWFDPW	IGH	2	None	
7	169	CTTFSRPDYW	IGH	6	None	
7	170	CARDSRPRFGDFLPFDLW	IGH	33	None	
7	171	CARDDIVVVPANPNNFDYW	IGH	6	None	
7	172	CAKLGGTRPRFDYW	IGH	2	None	
7	173	CARDPRVVPDAFDIW	IGH	2	None	
7	174	CTTLLGSWQPLDYW	IGH	5	None	
7	175	CQQYNNWFRGGTF	IGK	8	C3	7K8P
7	176	CAKGDESSGYYADFW	IGH	4	None	
7	177	CARAGGQWLPYFDYW	IGH	7	None	
7	178	CARHSYITMIVVVIPHAFDIW	IGH	221	None	
7	179	CAKDRSVIQLWSEVDFDYW	IGH	4	None	
7	180	CARDGIAVAGFSFDYW	IGH	8	None	
7	181	CARLNWNYESFDYW	IGH	3	None	
7	182	CAKTGTYGDGAFDIW	IGH	5	None	
7	183	CARDREDCSSTSCRWGWYYYYYGMDVW	IGH	54	None	
7	185	CARQFPLFMIVVVPTYFDYW	IGH	10	None	
7	186	CARDAHSRKYYYDSSGYYGDAFDIW	IGH	2	None	
7	187	CARSYYYGSGNIVFDYW	IGH	6	None	
7	188	CASVGVWFGEFLDYW	IGH	2	None	
7	190	CAGHKRMAIFDIW	IGH	3	None	
7	191	CARHKIYCSGGSCYSFYFDYW	IGH	3	None	
7	192	CAKIPRWGITMESIHYFDYW	IGH	21	None	
7	193	CARVKSSYCSRGKCNRYAFDIW	IGH	2	None	
7	194	CTRPSGYNYGLGGFDLW	IGH	2	None	
7	195	CARVAPVLRYFDWLVTTTGVVPVFDYW	IGH	10	None	
7	196	CARKCLGYSSGWYGVSDYYQNWFDPW	IGH	2	None	
7	197	CARDFGSGRIFDYW	IGH	2	None	
7	198	CARTRLIVVAGNDAVHFDYW	IGH	5	None	
7	200	CARDNRPYSGYDYYYYYYMDVW	IGH	6	None	
7	201	CARLGYGDYFLKNAFDIW	IGH	3	None	
7	202	CARDEDDYGDYRSFDYW	IGH	38	None	
7	203	CAKTDSSGYYLLDYW	IGH	2	None	
7	204	CARDQPSPQSLYQPLLYAQPFDYW	IGH	2	None	
7	206	CAHSKTILVRGVIIVYQGVVTFDIW	IGH	4	None	
7	207	CARTLAVTRGLPYDYW	IGH	2	None	
7	208	CAKGPFTISLYYFDYW	IGH	3	None	
7	209	CARGVGAVTTIKYYFDYW	IGH	4	None	
7	210	CARLVPGAMGRNWFDPW	IGH	2	None	
7	211	CARDPDDYFTVGDAFDIW	IGH	2	None	
7	212	CAKALIGTYYFDYW	IGH	3	None	
7	213	CARLMGHDYGYYFDHW	IGH	2	None	
7	214	CARDYSSSWYVEGIDYW	IGH	4	None	
7	215	CAKRRNYDFWSDPYYYYGMDVW	IGH	5	None	
7	216	CARHKAGRWLRSRGDWFDPW	IGH	2	None	
7	217	CARGYKRTFWFFDLW	IGH	2	None	
7	218	CARWADSGSYYDGFDIW	IGH	2	None	
7	219	CARSNETFEWYYDTW	IGH	2	None	
7	220	CARVDPTIVADRGFDIW	IGH	2	None	
7	221	CARDRGEELRYFDWLSPVHNWFDPW	IGH	2	None	
7	223	CASLPDYGDYPFDYW	IGH	2	None	
7	224	CGRDRPGGTSGNSWFDPW	IGH	2	None	
7	225	CAIGWRRWYVARDNHDAFDIW	IGH	2	None	
7	226	CARAGITIFGVVRQFDYW	IGH	10	None	
7	227	CARNYGGSTFDYW	IGH	21	None	
7	228	CARLWFGELLDAFDIW	IGH	3	None	
7	229	CARDSTTTVPYYYYYYMDVW	IGH	4	None	

7	230	CARSSDGWYVWFDSW	IGH	17	None	
7	231	CARSRVVPAADYMDVW	IGH	2	None	
7	233	CARARSGWWLTTPDYYFDYW	IGH	2	None	
7	234	CARMIAVGGLAYYFDFW	IGH	341	None	
7	235	CARTVMDAFGGVILPFDYW	IGH	22	None	
7	237	CAKDIGRYCNSVSCYPYYFDFW	IGH	4	None	
7	238	CVKVGFGDYGGGFDDW	IGH	2	None	
7	239	CARRNWNYVLYYFDYW	IGH	2	None	
7	240	CARGGDFWSGYYVISGGNDYW	IGH	2	None	
7	241	CARKADSSGWQPANYFDYW	IGH	2	None	
7	242	CTKSEQMAGEFDSW	IGH	4	None	
7	243	CARFWVLEGFDYW	IGH	6	None	
7	244	CSRGSFDTSPEYNWFDPW	IGH	4	None	
7	245	CARGDGVLLWFGEVWFDPW	IGH	2	None	
7	246	CARGAPVGFWSGSPINYYYYYGMDVW	IGH	6	None	
7	247	CARFRAVSSWPHSAAFDYW	IGH	5	None	
7	248	CARHSGSTYYDSSGYHIDYW	IGH	2	None	
7	249	CARHSGYSYGPPWSAFDIW	IGH	3	None	
7	250	CARAPRITMGGLNWFDPW	IGH	2	None	
7	251	CARVAPHYYGAFDLW	IGH	2	None	
7	252	CARGPRIVVVPAAMPDDAFDIW	IGH	3	None	
7	255	CARDLIAVAGTTPDYW	IGH	23	None	
7	256	CARDSVGESDLFDIW	IGH	2	None	
7	257	CARASGERITIFGVVIQSFDYW	IGH	10	None	
7	258	CAKDRYSSSWNDAFDIW	IGH	15	None	
7	259	CARVPQGIAAEGSFDYW	IGH	2	None	
7	260	CVRNGDIAAAGTADYW	IGH	4	None	
7	261	CAREPEEDYFDYW	IGH	6	None	
7	262	CAKDVRGVIINPGAFDIW	IGH	2	None	
7	263	CARGSMVRGRSPSLDYW	IGH	2	None	
7	264	CARGLYYYGSGQRGFDPW	IGH	5	None	
7	265	CARDFFGVSQQLGGYFDLW	IGH	4	None	
7	267	CQVYGSSLLAF	IGK	2	C1	7K8N
7	268	CARMATNPHGYYWYFDLW	IGH	2	None	
7	269	CARGGNIVVVTAIPSDFDYW	IGH	4	None	
7	270	CARDDYVWGSYRYGFFNWFDPW	IGH	146	None	
7	271	CAKGRWMYSSSWYDYW	IGH	21	None	
7	272	CAREGSGGTELPYYFDYW	IGH	3	None	
7	273	CARGSSILWWWPPDYW	IGH	5	None	
7	275	CAKDIISSSWNKGSGVFDYW	IGH	4	None	
7	276	CAGLHDYADYW	IGH	9	None	
7	278	CARANRDYYDSSVYYFDYW	IGH	2	None	
7	279	CARHRIPSYDFWSGSGFDSW	IGH	6	None	
7	280	CQTMYSTSQWKDAFDIW	IGH	3	None	
7	281	CARYIRIFGVVSGRSFDYW	IGH	2	None	
7	282	CATIRLERRAYFEYW	IGH	3	None	
7	283	CAKDRNTVTIYYFDYW	IGH	3	None	
7	285	CAHRLSVAGPYFDYW	IGH	2	None	
7	286	CARPLGGDPRLRYAFDIW	IGH	2	None	
7	289	CARMRRDFALFYTSSWHYLDYW	IGH	2	None	
7	290	CVRDLGQWLVQADYW	IGH	6	None	
7	292	CATWDSRTWVF	IGL	4	None	
7	293	CQQYTSSSGHSF	IGK	2	None	
7	295	CAGDHRGLVWFGTDGFDIW	IGH	2	None	
7	296	CARDQRGYYDSSGYFDYW	IGH	2	None	
7	297	CAMTMNYDSSGYGFVYW	IGH	2	None	
7	298	CAHRRRRQWFGESPAFDIW	IGH	4	None	
7	299	CARDRIDSSWWGMDAFDIW	IGH	2	None	
7	301	CARSEGMLRSVDYW	IGH	2	None	
7	302	CTIWYGDYFYYFHSW	IGH	2	None	
7	303	CARGGDSSSWHNNWFDPW	IGH	2	None	
7	304	CVKEIGQQVGGLWASAFDVW	IGH	6	None	
7	305	CARPLTRWLRHAFDVW	IGH	11	None	
7	307	CARGRSRLEDIVVVVAALSYYFDYW	IGH	4	None	
7	308	CAAARRITIFGVVGEFDYW	IGH	10	None	
7	310	CAATYYDILTGYLDYW	IGH	2	None	
7	311	CAKDIGYSSGFPDYW	IGH	55	None	
7	312	CARWGDYYDSSGQHTGFDYW	IGH	2	None	

7	313	CARASVGAWLVIEHFDLW	IGH	4	None	
7	314	CARGIKVRGVIIRGGWFDPW	IGH	2	None	
7	315	CAKLSWDYVDGMDVW	IGH	15	None	
7	316	CARSSGYSYGSYRDHFDYW	IGH	2	None	
7	317	CAKDWDYSFDAFDIW	IGH	2	None	
7	318	CASSLYYDSSGPEDTGPFDYW	IGH	2	None	
7	319	CAKAGAVANPFEAVDVW	IGH	6	None	
7	320	CARGPVISSSWYGLDYW	IGH	2	None	
7	321	CAKAPPEVLGYFDSW	IGH	2	None	
7	322	CARDIIYQGVAPFGPDAFDIW	IGH	7	None	
7	323	CARHVLGSGSYRLDYYYGMDVW	IGH	11	None	
7	324	CARDRSGYDFRNGDYW	IGH	2	None	
7	325	CARDGNRYSYAFDYW	IGH	2	None	
7	326	CARDLPIYCSGGSCYVDYW	IGH	2	None	
7	327	CAKARVIISPHFDYW	IGH	3	None	
7	328	CAKDYNSYGYWTGFWDYW	IGH	2	None	
7	329	CARDAASLTGSLDYW	IGH	26	None	
7	330	CARNYGSGNFYYAFDIW	IGH	7	None	
7	331	CARDRTGSYYDYYYYYMDVW	IGH	9	None	
7	332	CVRGSVHRPLTDSHLNWYFDLW	IGH	3	None	
7	333	CAKGGYCSGGSCDNWFDPW	IGH	43	None	
7	334	CARAGYGPAEYFQHW	IGH	8	None	
7	335	CARDRLSTFDWLLSTPDAFDIW	IGH	6	None	
7	336	CSSYTSSSTWVF	IGL	1359	C2	7K90
7	337	CARLSGALGTMIYDYW	IGH	4	None	
7	338	CARNRFTSGWYPDYFDYW	IGH	4	None	
7	339	CARVSFGSGSYPYYYGMDVW	IGH	2	None	
7	340	CARDATSCSGGGCYTRSRAEYFQHW	IGH	4	None	
7	341	CARDSRGVFSGSRDYW	IGH	2	None	
7	342	CATGITILRGTPYFDYW	IGH	2	None	
7	343	CAREINVHVLRSDIGNFDYW	IGH	2	None	
7	345	CAKMRATTVTTRDDYW	IGH	2	None	
7	346	CASLWFGELWDNWFDPW	IGH	2	None	
7	347	CTRDRFGAYDSSGFDLYYFDYW	IGH	2	None	
7	348	CARADLVCSGGSCDWFDPW	IGH	3	None	
7	349	CARDEGLYSSSQRAFDIW	IGH	2	None	
7	351	CARVRSQFRSSGWDYYFDYW	IGH	2	None	
7	353	CARGTSIQQWLVRHFDYW	IGH	2	None	
7	354	CAYDYGGNLFDYW	IGH	2	None	
7	355	CASIQLSPYVDYW	IGH	2	None	
7	356	CARILTPQSGSWWFDPW	IGH	2	None	
7	358	CLQYNSWPPQCSF	IGK	51	C3	7K8R
7	359	CAREGYSSSWYSKLELNDAFDIW	IGH	4	None	
7	360	CAKGAYSYGEHRSVDYW	IGH	23	None	
7	361	CARESQLLFLDYW	IGH	2	None	
7	362	CAKHARLYTSSWPYFDYW	IGH	2	None	
7	363	CARLGYIVATIPSGFDYW	IGH	2	None	
7	364	CARATTRSWSNFDYW	IGH	2	None	
7	365	CAKTPESALSWYFDYW	IGH	2	None	
7	366	CTREQQLLPNSIGFDPW	IGH	2	None	
7	367	CARDPDEVGWELSPFDYW	IGH	5	None	
7	368	CARDGDGSTPNGYCSGGSCWKPADAFDIW	IGH	4	None	
7	369	CARIFSSWTPYYYYYMDVW	IGH	3	None	
7	370	CARHPRRLDPFDYW	IGH	2	None	
7	371	CAKVENYYDSSGYYSNDAYDMW	IGH	5	None	
7	372	CQQYESYSPTFGQGTNLEPTF	IGK	9	C3	7K8R
7	373	CAHSNIVATVNFDYW	IGH	5	None	
7	374	CARPQRVIIISGTERDAFDMW	IGH	5	None	
7	376	CAVWDDSLNDLNWLF	IGL	2	None	
7	377	CARDSGSSSSFAFDIW	IGH	3	None	
7	378	CARHSIRLFYGSGSYPTRGIDYW	IGH	2	None	
7	380	CQVFDSSPAMIF	IGL	3	None	
7	381	CQQDYNLPPEFTF	IGK	9	None	
7	382	CARSEIDIVPILDYW	IGH	3	None	
7	384	CARGHKYSSWYISYW	IGH	2	None	
7	385	CARLPYILTGRFDYW	IGH	15	None	
7	386	CATSTSYNTGHYYFDYW	IGH	3	None	
7	387	CQKYTTAPRGITF	IGK	6	C2	7K8U

7	388	CARDGRTPAGVRFDYW	IGH	2	None	
7	389	CARVPYYSGSGSPYYFDYW	IGH	3	None	
7	390	CAKDPPEADYDYVWGSYRSYYFDYW	IGH	2	None	
7	391	CANVWIVEDHDAFDIW	IGH	2	None	
7	393	CARTHYDILTGYLTIFDYW	IGH	8	None	
7	395	CAKWFMYSSGSYVDNW	IGH	3	None	
7	396	CARDVDPRAGIAQWLVPIVNYWFDPW	IGH	2	None	
7	397	CAKAAQFSMIVVVITYFDYW	IGH	2	None	
7	398	CARPMSLNWDDAFDIW	IGH	2	None	
7	400	CLQHNSYPPADTF	IGK	1536	C1	7BZ5
7	401	CAGDIAAAGFFDHW	IGH	3	None	
7	402	CARDVRWLQMADYW	IGH	5	None	
7	403	CARGMMGSPANYYYSYMDVW	IGH	2	None	
7	404	CARGHAFNFWRSPDLKNYALDVW	IGH	2	None	
7	405	CARGHRGSNYYYYYMDVW	IGH	2	None	
7	406	CARSDVRGYSYGRDYYYYYMDVW	IGH	2	None	
7	407	CASDSVATKEFDYW	IGH	2	None	
7	408	CARVLLWFGDAFDIW	IGH	5	None	
7	409	CARFGLDFTSPRAFDYW	IGH	4	None	
7	410	CARVAVYCSSTSCYGWWFDPW	IGH	4	None	
7	411	CTKALYYDILTAYLDSW	IGH	2	None	
7	412	CARVRDQYYYDSSGPFDIW	IGH	2	None	
7	413	CARDPWHYYDSSGYYPDARFDYW	IGH	3	None	
7	414	CARVLSLYSSTWDAFDIW	IGH	6	None	
7	415	CARHVAHCSSTSCSYYYYYMDVW	IGH	2	None	
7	416	CANFRAPSSWYVYYYYGMDVW	IGH	3	None	
7	417	CASSVAGITADYW	IGH	2	None	
7	418	CARVPRQSYYNYGMDVW	IGH	24	None	
7	420	CARHHPATVAGKKYYFDQW	IGH	5	None	
7	422	CASQVDTAMVFDYW	IGH	2	None	
7	423	CARDREHSSGWYYERYGMDVW	IGH	3	None	
7	424	CASRRGEVLLLRVLQYFDYW	IGH	12	None	
7	425	CIQGTQWPLIF	IGK	2	None	
7	427	CARFGRYSSGWYLDPTDYW	IGH	2	None	
7	428	CARHLNDYGDFRDDYW	IGH	4	None	
7	429	CATYYYGSGRNDYFDYW	IGH	25	None	
7	430	CTRLVPSSGCDYW	IGH	5	None	
7	432	CARILMGYCSGGSCYGIDAFDIW	IGH	2	None	
7	433	CAGQARYDYVWGSYGPAQKRVVSNWFDPW	IGH	2	None	
7	434	CFVLPSEGDVGDAFDIW	IGH	2	None	
7	435	CARDISSWYSHWYFDLW	IGH	3	None	
7	436	CARHLTDTAMVNNWFDPW	IGH	2	None	
7	437	CTIITMVRGAAWETDYW	IGH	3	None	
7	438	CARHAVGTIFGVVIIQAFDIW	IGH	2	None	
7	439	CANFPAAGFDYW	IGH	5	None	
7	440	CARDPTYYYGSGSNDYW	IGH	9	None	
7	441	CARVRTVVAAVDDAFDIW	IGH	23	None	
7	442	CARDRGYSGYDWLRAYYMDVW	IGH	3	None	
7	443	CAVLIWFGDLCYFDYW	IGH	3	None	
7	444	CARHSEGDTGCDYW	IGH	2	None	
7	445	CAKGLSGWYDYYYYGMDVW	IGH	2	None	
7	446	CARGRRGAISLDMARELKRGPKIFDYW	IGH	13	None	
7	447	CAKGRPYSGSYSGDAFDIW	IGH	4	None	
7	448	CATAIYESDVWYWFDHW	IGH	2	None	
7	449	CAKEATDTMVRGVLDAFDIW	IGH	2	None	
7	450	CAAVPLGSFRPNYMDVW	IGH	2	None	
7	451	CATPVFVAGSYYFDHW	IGH	3	None	
7	452	CARESESGSSYGYTAVFEYW	IGH	2	None	
7	453	CASFGFVDSSGALFDYW	IGH	3	None	
7	454	CTRDFNSFGSRWGFDYW	IGH	10	None	
7	455	CQTWGTGAHWVF	IGL	148	None	
7	456	CAREYYDILTGLTPYDYW	IGH	2	None	
7	458	CARHPRIPYLPYGDYRWAGFYMDVW	IGH	2	None	
7	459	CAKSPAGQWLVPRSFDYW	IGH	2	None	
7	460	CAHHNSGVFDVW	IGH	4	None	
7	461	CAHRHGYYDAFDIW	IGH	9	None	
7	462	CARQETVSLGGRKRTTWFDPW	IGH	6	None	
7	463	CLAWDTSPRAFWVF	IGL	6	None	

7	464	CAKDVTMIVVVITAFDYW	IGH	4	None	
7	465	CARGPITFGGVRVFAGLKDYW	IGH	2	None	
7	466	CAKRYSSGWYGVDYW	IGH	4	None	
7	467	CARDGSLGLRYFDWRAPGPNWFDPW	IGH	2	None	
7	468	CARHLHMGYDDSSGYHISLFDYW	IGH	3	None	
7	469	CAKAVLRAYDWEDAFDFW	IGH	2	None	
7	470	CAKLNYQFSSGWSDYW	IGH	7	None	
7	471	CARSHVVVTAIPFDYW	IGH	2	None	
7	472	CATVAAYYGSGRLPDYW	IGH	4	None	
7	473	CARQRGYCSSTSCYDAFDIW	IGH	5	None	
7	474	CARKVPVTGPLVVSTFDIW	IGH	3	None	
7	476	CARSRFYGPGSFDSW	IGH	2	None	
7	477	CARDGGEQWLVLSYFDYW	IGH	3	None	
7	478	CARPDFDGSGDYW	IGH	2	None	
7	479	CAIDLDGYGKEGFDIW	IGH	4	None	
7	480	CATTQTPVTASPGGGDYW	IGH	2	None	
7	481	CARTSDSANTNFDYW	IGH	5	None	
7	482	CARVYCGGDCYSGGNWFDPW	IGH	2	None	
7	483	CARDRQLWDYYYGMDVW	IGH	4	None	
7	484	CARVSSPANYVWGSYRFGYFDYW	IGH	2	None	
7	485	CQQSYSALKITF	IGK	112	C4	6ZDH
7	488	CARAQTNYYYYYYMDVW	IGH	2	None	
7	489	CARKVRGDFYMDVW	IGH	11	None	
7	490	CAKDPPADYGGDYW	IGH	2	None	
7	491	CARASQFLPRERKYYYDSSGYQAFDYW	IGH	2	None	
7	492	CTRRVWFGELARDYW	IGH	7	None	
7	493	CVRSRRYFDWTLPPSDYW	IGH	9	None	
7	494	CAREDTVRGKFDYW	IGH	2	None	
7	495	CGYSPPYTF	IGK	2	None	
7	496	CALGGFGTAMIKGGRWFDPW	IGH	2	None	
7	497	CAKDPVLRYFDWFRDFDYW	IGH	11	None	
7	499	CARASTLGYYSDSGTHFDTFDLW	IGH	2	None	
7	500	CARDLWGHGDPKVGSDYW	IGH	2	None	
7	501	CATSSPHCSSTSCFTNWFDPW	IGH	8	None	
7	502	CAKAPAPNIAAAGIPFRYAKIYYFDYW	IGH	2	None	
7	505	CAKAWDYYGSGSYYNPFDPW	IGH	4	None	
7	506	CQQLNSYPPVYTF	IGK	158	C1	7BZ5
7	507	CARIWIYDSSGYFFYATPYYFDYW	IGH	2	None	
7	508	CARGYKITMIVVVIPITTHWFDPW	IGH	4	None	
7	509	CVSTTPSGSNWFDPW	IGH	2	None	
7	510	CATVWYTAMVGYYFDLW	IGH	2	None	
7	512	CARGPPSQLWFGELLPFDYW	IGH	2	None	
7	513	CARDIESGSSGWYYYYYGMDVW	IGH	6	None	
7	515	CARGQRKELIVVVISYYYYGMDVW	IGH	4	None	
7	516	CARGLQSVAMVKTYYFDYW	IGH	2	None	
7	517	CARDGRSDSYNWIDPW	IGH	2	None	
7	518	CATGRPPVLLWFGDDWFDPW	IGH	6	None	
7	519	CASNNNWIYAFDIW	IGH	2	None	
7	522	CARHKRPTYSLQSYYYMDVW	IGH	11	None	
7	523	CARDPRGVAADYW	IGH	52	None	
7	524	CAKDRYSSGWYYFDYW	IGH	46	None	
7	525	CARVTRQLWSLVGLPRYYYMDVW	IGH	3	None	
7	526	CAKVLTPVYYENDYW	IGH	2	None	
7	527	CARALVMVAATGFFDYW	IGH	2	None	
7	528	CARINQKITMIVYNNWFDPW	IGH	4	None	
7	529	CAKDRRTSSLRIAVAGDFDYW	IGH	2	None	
7	530	CAKGKQWLSEDAFDIW	IGH	13	None	
7	531	CAKVDRNSSGWDYW	IGH	5	None	
7	532	CARDPAGYGFGYW	IGH	2	None	
7	533	CARLNGFGEFWYFDLW	IGH	2	None	
7	534	CARAGWGLVPFDNW	IGH	2	None	
7	535	CAHRRTAMVTVYDYW	IGH	2	None	
7	536	CARLHLDWDTVTTPYFDLW	IGH	3	None	
7	537	CAPAPYDILTGRDDYW	IGH	2	None	
7	538	CARYHDDYGDNDWFDPW	IGH	6	None	
7	539	CAKDLGVLRYFDWLSSAFDIW	IGH	2	None	
7	540	CARRRRLTVATKDYAFDYW	IGH	3	None	
7	541	CARTYPYNSSPGCFDHW	IGH	6	None	

7	542	CAKPELGQWLVHYYFDYW	IGH	2	None	
7	543	CAKMIVVADTFDIW	IGH	2	None	
7	544	CAKEGYSGYYFDYW	IGH	12	None	
7	545	CAILDPQGLVAFDIW	IGH	6	None	
7	546	CARASFVYSSGWYNDYW	IGH	3	None	
7	547	CARVWDYADYFDSW	IGH	10	None	
7	548	CARGRLRQWLVRQYYFDYW	IGH	3	None	
7	549	CVRDLPGLSSGGDYW	IGH	2	None	
7	550	CQSYDSSPLF	IGL	19	C4	7JVC
7	551	CARLLTAKYSGSYYVTLYYFDYW	IGH	2	None	
7	552	CARPYSSGWYRRGLFPAEYFDYW	IGH	2	None	
7	553	CSALDSSLSAPHVVF	IGL	11	None	
7	554	CTREGSFYDERGYYRDAFDIW	IGH	5	None	
7	555	CARMGDGAYDSYFDSW	IGH	2	None	
7	556	CARAAVTMVRGVAWRYYYYMDVW	IGH	4	None	
7	557	CARVTAGHDATLDYW	IGH	2	None	
7	558	CARDRQEELSSSWPVYWFDPW	IGH	2	None	
7	559	CVRAQINYFGSGSYSLLDYW	IGH	8	None	
7	560	CAHVPIRTAFDIW	IGH	2	None	
7	561	CARAGGVTTLAFDIW	IGH	2	None	
7	562	CARGSFIFRLVIQYYFDYW	IGH	5	None	
7	563	CARFTPGLLRFDYW	IGH	4	None	
7	565	CARPNYYGSGSYGFDPW	IGH	4	None	
7	566	CARALLVTMIVEAHWFDPW	IGH	4	None	
7	569	CARLGYYGSGTPGDYW	IGH	4	None	
7	570	CAKDRYRTPKTFDYW	IGH	2	None	
7	571	CARARGLTFVLMVYAMEYYFDYW	IGH	14	None	
7	572	CARIVRDSSSWYRDDYW	IGH	2	None	
7	573	CARDVEMATKHAFDIW	IGH	2	None	
7	575	CARHGGELLWFGEPSTIYFDYW	IGH	2	None	
7	576	CARHEGSSWEPIRFDPW	IGH	3	None	
7	577	CARDAPSYCSGGSCYLPGYFDYW	IGH	3	None	
7	579	CAREKRGGNWYFDLW	IGH	3	None	
7	580	CARTRLAQWAFDPW	IGH	2	None	
7	581	CARDPSTYRFGELLSFDYW	IGH	2	None	
7	582	CAKDKLYYYYDSSGYYDYW	IGH	7	None	
7	583	CARDRLVVVPAAMSIAHFDYW	IGH	2	None	
7	584	CAKTTQYNWNYGSPFDYW	IGH	3	None	
7	585	CARHVSGVTYSPFDPW	IGH	2	None	
7	586	CAREIYIAVAGSFDYW	IGH	8	None	
7	587	CAREGYYYDSSGYSNAFDIW	IGH	2	None	
7	588	CARDGSFRFLEWLPLDYW	IGH	2	None	
7	590	CARGAGIGNLPFDYW	IGH	3	None	
7	591	CARNMPGYYASGRHILMDAFDIW	IGH	2	None	
7	592	CARVDKYYDILTGYYALGHFDYW	IGH	2	None	
7	593	CARGRDTMVRARGGGMFDLW	IGH	2	None	
7	594	CARGYCSGGSCYLGYYYYYYMDVW	IGH	14	None	
7	595	CARAGIVPAARSEYDYW	IGH	3	None	
7	596	CARGSVAGTSPYYYYYMDVW	IGH	6	None	
7	597	CAHKAKVQPTIGAFDIW	IGH	2	None	
7	598	CARSNLFELLIDYW	IGH	2	None	
7	599	CATDLTLFGVVRFDYW	IGH	2	None	
7	600	CARHNTKLRPWPNFDYW	IGH	4	None	
7	603	CQQSDNVPPITF	IGK	133	C3	6XDG
7	604	CARAGEMTTVKRFDYW	IGH	7	None	
7	605	CATDVYSGLGSYFFYW	IGH	2	None	
7	606	CAKALWYCSSTSCQDDAFDIW	IGH	3	None	
7	609	CAHSVRGYSGYDSSFDYW	IGH	16	None	
7	610	CAKDMLHQMAGANFDYW	IGH	2	None	
7	611	CAGGEATFGSDGYLTKGAFDRW	IGH	2	None	
7	612	CARDPSYYYGSGSYLLRFGPKDWFDPW	IGH	2	None	
7	614	CARESIMITFGGVIVANWFDPW	IGH	2	None	
7	615	CARFSTAVGGFDFW	IGH	2	None	
7	616	CARRRGYKYGFDFW	IGH	3	None	
7	617	CARDYYDSSGKNYYYYYGMDVW	IGH	2	None	
7	619	CTTGYYYDSSGYYYADGAFDIW	IGH	2	None	
7	620	CARDFDDYGDYGLERFDYW	IGH	2	None	
7	621	CQQFGDSPMYSF	IGK	4	C1	7K8N

7	622	CAAWDDSLNGNGRGVVF	IGL	2	None	
7	623	CARDLPSAMVTGVAFDIW	IGH	3	None	
7	624	CARDSNVLRYFDWSTDDAFDIW	IGH	2	None	
7	625	CARGSRYGTGWYYYDYW	IGH	23	None	
7	626	CAKDMLGVVAATPLDYW	IGH	2	None	
7	627	CMQALQTPRRCSF	IGK	2	None	
7	628	CARAPAKWQWLVQWFDPW	IGH	16	None	
7	629	CATYPDTGYESDFYYYYLDVW	IGH	2	None	
7	630	CVKAYSSSWSGFFDYW	IGH	2	None	
7	631	CARGSSPPLFITMVRGVFDYW	IGH	11	None	
7	632	CARQISFTMIVVVIPNYFDYW	IGH	2	None	
7	633	CAREKYYDSRRYYFDYW	IGH	3	None	
7	636	CARLSGIVATIEHFDYW	IGH	4	None	
7	640	CARSYCSGGNCVLSEDYW	IGH	15	None	
7	641	CAREREWEPVKFDSW	IGH	2	None	
7	642	CAHRSGYSSGWYNWFDPW	IGH	5	None	
7	643	CARDPTWYSSSWYSGPSGGPLDYW	IGH	2	None	
7	644	CQQSYSTPRVGTGTF	IGK	7	C4	6ZDH
7	645	CAHRGRIAHFDYW	IGH	2	None	
7	646	CARHARFGLDYW	IGH	13	None	
7	647	CARGGGDIIITVVTKAPQSSFDYW	IGH	2	None	
7	648	CARGDVGPDNWYFDLW	IGH	6	None	
7	649	CARISPTMVRGVRFHYYYYYMDVW	IGH	4	None	
7	650	CARDRGLLRTNYYFDYW	IGH	4	None	
7	651	CARHPYDFWSDDAFDIW	IGH	2	None	
7	652	CARGNVGAAAWCLYYYGMDVW	IGH	2	None	
7	653	CARARGSNYGFFDSW	IGH	4	None	
7	654	CVKDVHADWFQYFDRW	IGH	2	None	
7	655	CSREGNYDTLNDFDYW	IGH	2	None	
7	656	CTTAKGHSPYYDILTGYSREDAFDIW	IGH	2	None	
7	657	CARDPVKIFGVVIFYYFDYW	IGH	10	None	
7	658	CARGTLRGIYFDSW	IGH	2	None	
7	659	CARDVTVLDWSFKYW	IGH	17	None	
7	660	CARLPYSSNWYGAFDIW	IGH	3	None	
7	662	CAKESLTAAGGFDYW	IGH	2	None	
7	663	CATDYTESGTFYNPWNFFDNW	IGH	2	None	
7	664	CARAFAYQVLPFDSW	IGH	3	None	
7	665	CAKEQYSYGYIKYYFDYW	IGH	8	None	
7	666	CARDYLNSGWYEVGYYFDYW	IGH	2	None	
7	667	CATRGIREDFRYAFDIW	IGH	3	None	
7	668	CARDFDDYVWGSYRYGEHYFDYW	IGH	2	None	
7	669	CATGAATSYRGLDTFDFW	IGH	5	None	
7	672	CARHHRTVYTGYSSGWYGPPFDYW	IGH	2	None	
7	673	CAAYGDEGGYFDYW	IGH	2	None	
7	674	CAKDRNFGIVGATALDYW	IGH	2	None	
7	676	CARDGQISIGYYRPSDSFDIW	IGH	4	None	
7	677	CVRDGYYDTSGYINGIFHYW	IGH	2	None	
7	678	CARQVKRHGYFDWLEIPYFDYW	IGH	2	None	
7	679	CARGDKGSGSYFVDYW	IGH	5	None	
7	680	CAKDYGWGSSWHYFDYW	IGH	2	None	
7	681	CASTSQLPDYFDYW	IGH	4	None	
7	682	CARSCSRTSCYLQRFDPW	IGH	2	None	
7	683	CARARELYDSSGYYYSRPSYFDYW	IGH	5	None	
7	684	CAHRPWFNDAFDIW	IGH	2	None	
7	685	CARGTDRWTYYFDYW	IGH	8	None	
7	686	CATLLLTLKLGDAFDVW	IGH	11	None	
7	687	CARLAGGREYGDTPRFDPW	IGH	2	C2	7K8U
7	688	CARDEFLGSGTGGWFDPW	IGH	4	None	
7	689	CARGSGYSSSGWFDPW	IGH	3	None	
7	690	CAAGIAVAGRDDYYYYYGMDVW	IGH	5	None	
7	692	CARDRDPTSGWFEAPCDYW	IGH	2	None	
7	693	CAKDVNYDSSGYFNLDYW	IGH	2	None	
7	694	CARFRLVLLWFGESLGFDYW	IGH	2	None	
7	695	CARDTYCTNGVCYTTWFDPW	IGH	6	None	
7	696	CAHSQHTGWYNFDYW	IGH	2	None	
7	697	CARHQSGYSYGRFDYW	IGH	10	None	
7	698	CALPIYSKNTLYLQMNSLRAEDTAVYYCARDTGSGCDYW	IGH	2	None	
7	699	CKNSLYLQMNSLRTEDTALYYCAKGAYSYGEHRSVDYW	IGH	2	None	

7	700	CARAEYYYDSSGYPLYWYFDLW	IGH	2	None	
7	701	CARDLSGPIGTGWFDSW	IGH	2	None	
7	702	CARQRIAAAGGFDCW	IGH	2	None	
7	703	CAKQPLLGVGGSGLAPDYW	IGH	4	None	
7	705	CARAPQRHVIVVVVAATLKYYYGMDVW	IGH	8	None	
7	706	CAKDIFYGSGIDAFDIW	IGH	2	None	
7	707	CATFRGQEWLQTLRAFDVW	IGH	2	None	
7	708	CAKSSSTSCYVGWFDPW	IGH	2	None	
7	709	CVREEIDFDTW	IGH	2	None	
7	710	CARTRVEVEKTSLDYW	IGH	2	None	
7	712	CARGRFLEWLSPEDDYW	IGH	3	None	
7	713	CARVRFGDRKTFDSW	IGH	9	None	
7	714	CARISLYYGSGSHQFDYW	IGH	2	None	
7	715	CARSLAAAGRGDYW	IGH	7	None	
7	716	CACLIVAVGDFDSW	IGH	2	None	
7	717	CMQGTHWPPMYTF	IGK	28	None	
7	718	CAKLSWGYSSGYYDYW	IGH	4	None	
7	719	CARETAAADANWFDPW	IGH	2	None	
7	721	CARDPRGGAQLWLFAENVYFDYW	IGH	2	None	
7	722	CARDRTGTTFHDYW	IGH	6	None	
7	723	CAKDGSGWYHDAFDIW	IGH	5	None	
7	724	CARRGYYDSSGYPQYYFDYW	IGH	2	None	
7	725	CARLRIVVVPAAMLDAGDGIDYW	IGH	2	None	
7	726	CAGSGESAGWYVFFDQW	IGH	2	None	
7	728	CAKQMIAAASALDYW	IGH	4	None	
7	729	CARGEPGGDCCSFDYW	IGH	2	None	
7	730	CARSSEGSYYNVPFDSW	IGH	4	None	
7	731	CQHYYDYPRTF	IGK	71	None	
7	733	CARDLSAVAVAGLGIDYW	IGH	3	None	
7	735	CAKRGQTVGTGDYW	IGH	2	None	
7	736	CARDMRQWLPEDAFDIW	IGH	2	None	
7	738	CARDELVLRYFDWYPMGHDYW	IGH	4	None	
7	739	CARGVRYFTIFGVRQYYFDYW	IGH	2	None	
7	740	CAKVRPLYYDFWSGYSQRPYYFDYW	IGH	8	None	
7	742	CARMSLYYYDSSGQKGAFDIW	IGH	2	None	
7	744	CARALRQQWLVFKYYFDYW	IGH	2	None	
7	745	CAKDLDPSYYGSGSPFDYW	IGH	4	None	
7	746	CAKDQYPLYYDPCFDIW	IGH	4	None	
7	747	CARELLWFGESIPPFDYW	IGH	3	None	
7	748	CARPRELAGWGVFDIW	IGH	5	None	
7	750	CARDSAPYYYDTSGYLDYW	IGH	4	None	
7	752	CARASIQLWLRGRFFDYW	IGH	7	None	
7	753	CARDSITYGSGSYLQDAFDIW	IGH	6	None	
7	754	CASPRGYSYASPWDYW	IGH	2	None	
7	755	CARAISPDGQYAFDVW	IGH	2	None	
7	756	CARPSWGYSYGGPILEPPDYW	IGH	2	None	
7	757	CARAGERSGSYYDDYYFDYW	IGH	5	None	
7	758	CARTNSDYSLVFDYW	IGH	2	None	
7	759	CTSLYYDSRGYGFDPW	IGH	2	None	
7	760	CARVYWSGNGRGFPDYW	IGH	3	None	
7	761	CARSYGDYGVGWFDPW	IGH	4	None	
7	762	CARLMTHSTSDYW	IGH	2	None	
7	763	CARYGVVRGVINAFDIW	IGH	5	None	
7	764	CARDSPFSRGPLGELSFGAFDIW	IGH	6	None	
7	765	CARVQFDWLLQYSPGYFDYW	IGH	2	None	
7	766	CARSLRAFSGYFDYW	IGH	2	None	
7	767	CAAQRITMIVRDYW	IGH	2	None	
7	768	CARATRGWLQLFFGAFDIW	IGH	3	None	
7	770	CQQANSFPPETWTF	IGK	2	C1	7BZ5
7	771	CARRRYDFWSGNPGAFDIW	IGH	2	None	
7	772	CARDSWFGELFGRFDPW	IGH	2	None	
7	773	CLQYFSTPSFTF	IGK	2	C4	6W41
7	774	CAKAGPGWYFDSW	IGH	2	None	
7	775	CARDPGLADPYYDFWSLGKRPGRSDAAFDIW	IGH	2	None	
7	776	CARIIASGGVTVDRQYYFDSW	IGH	2	None	
7	777	CARDHAGESSSWSDLFDYW	IGH	2	None	
7	778	CARGGRITMIVVVIKGAYFDYW	IGH	3	None	
7	779	CAKLRIAVAVVDYW	IGH	2	None	

7	780	CVKDMISRDYYPSLFDLW	IGH	29	None	
7	782	CAMSTGYYWTFDYW	IGH	4	None	
7	783	CASWSDGEDAFDIW	IGH	12	None	
7	784	CAKDQDYGSGSPNDYW	IGH	15	None	
7	785	CTTAASGSWPRAWGYFDYW	IGH	2	None	
7	788	CVRDRAAAGGDWFDPW	IGH	2	None	
7	789	CAKDLWPRDIVVVPAASFDYW	IGH	2	None	
7	790	CARGRYDMYAFDIW	IGH	3	None	
7	791	CARPLYTSGRYGGAFDIW	IGH	5	None	
7	792	CARDTNDYDDQTYYYFGMDVW	IGH	4	None	
7	793	CAKDSHVGLWELNRFDYW	IGH	5	None	
7	794	CARHLRGYSYDDAFDIW	IGH	4	None	
7	795	CVKYSSGLRTFDYW	IGH	3	None	
7	796	CAREMGYEHDYGDFNWFDPW	IGH	2	None	
7	798	CAKSGSGWTQNQFDYW	IGH	3	None	
7	799	CQHYNTYSPMYTF	IGK	6	C3	7K8R
7	800	CQQHSNWPPITFITF	IGK	5	C3	6XDG
7	801	CVKGTGWYPDFDYW	IGH	2	None	
7	802	CAKKRLRYFDWSPLPFDYW	IGH	4	None	
7	803	CAMRLATLIDGPVDYW	IGH	2	None	
7	804	CQQRSSWPPESSF	IGK	2	C1	7BZ5
7	805	CVRAKTTFGGIIVQYYFDYW	IGH	2	None	
7	806	CAHSVHYDILTGYYNPAYFDYW	IGH	15	None	
7	807	CAKDSSGSYGESWFDPW	IGH	3	None	
7	808	CTRDLPVGYSNGWFSDSW	IGH	4	None	
7	809	CARDPANEWLLSWFDPW	IGH	3	None	
7	810	CARMPIYGSGEEYFQHW	IGH	2	None	
7	811	CARLNYQWLIRVDYFDYW	IGH	2	None	
7	812	CARGVSTTQSYYYYYYYMDVW	IGH	2	None	
7	813	CARGWKYDFWSGVRQVPEYYFDYW	IGH	2	None	
7	814	CARARLQDYYYFYGMDVW	IGH	2	None	
7	815	CAKDRLVLSSSQIDYW	IGH	4	None	
7	816	CARERLSRYCSGGSCYWGFDVW	IGH	2	None	
7	817	CARAVDTGNDAFDIW	IGH	6	None	
7	819	CARHITMVRGAIRPLYYMDVW	IGH	2	None	
7	820	CARDPLDIWFDDFDYW	IGH	10	None	
7	821	CARETGTTATLLDALDIW	IGH	7	None	
7	822	CARFIVAPGGGSGFPDYW	IGH	2	None	
7	823	CAHMTTVTTSFDYW	IGH	18	None	
7	824	CATRAGSSTVTDYWYFDLW	IGH	2	None	
7	825	CAKVKSRDTAMADDYW	IGH	8	None	
7	827	CTTAAELWFGRYDAFNIW	IGH	2	None	
7	831	CVRDLGRGYTSARTFDIW	IGH	2	None	
7	833	CAIYPGYSSSWPIDYW	IGH	6	None	
7	834	CATVYAYNWNTRGWFDPW	IGH	4	None	
7	839	CATHPGYSSSWYLSYW	IGH	2	None	
7	840	CARGGDSSGYYSWYFDLW	IGH	2	None	
7	842	CAKDSYGLYWYFDLW	IGH	4	None	
7	843	CAAGYDSNWLQYFDYW	IGH	3	None	
7	844	CAREFRGASEGNYLDPW	IGH	2	None	
7	845	CAREPDYYGSGSYPNDAFDIW	IGH	10	None	
7	846	CARDGSAAGYWYAFDYW	IGH	3	None	
7	847	CARSRPRYYMDVW	IGH	2	None	
7	848	CARDQSTTYYDVLTGYYIPEDAFDIW	IGH	4	None	
7	849	CAKGGADDWAYFDYW	IGH	28	None	
7	850	CAHRQIIGYSGFMSFDYW	IGH	2	None	
7	851	CARVGRSVRGDPFDYW	IGH	2	None	
7	852	CARGFGDYVWGSYRALAEYFQHW	IGH	2	None	
7	853	CARAVGFGVSSGSARVDYYFDYW	IGH	2	None	
7	854	CARRYYDVGPFDNW	IGH	3	None	
7	855	CQHCNSYSMCSF	IGK	24	None	
7	856	CATDIRGSSYDMFDIW	IGH	4	None	
7	857	CARVKSSSPKYTPPPYYYYGMDVW	IGH	2	None	
7	859	CAKDPSSATYYYYYYMDVW	IGH	2	None	
7	860	CARVGLTYYYGSGSYYSDYFDYW	IGH	10	None	
7	862	CARDGYCSSTSCSGAYYYYYMDVW	IGH	2	None	
7	863	CAGSYGSSIEYFQHW	IGH	2	None	
7	864	CARGDLGFDWLFHYW	IGH	16	None	

7	865	CAGGSRSHYNSGIGAFDIW	IGH	10	None	
7	867	CARDESATKSFGYW	IGH	2	None	
7	868	CASVAAIFGEFGASEWFQHW	IGH	2	None	
7	869	CAKDLRVRGGYSYGHDAFDIW	IGH	2	None	
7	870	CARDAGKFLTFIDYW	IGH	4	None	
7	871	CQQRSNWPPLTF	IGK	656	C1	7BZ5
7	872	CAKIVSVIVDVDAFDIW	IGH	2	None	
7	873	CARIARSGWRGDWFDPW	IGH	10	None	
7	874	CASSASGGVIGYDAFDIW	IGH	2	None	
7	875	CARPRAFPSTGAIYWYFDLW	IGH	4	None	
7	876	CAKDFGSGSLGYNWLDPW	IGH	4	None	
7	877	CAREVSSGWYDGDGFDYW	IGH	4	None	
7	878	CARQTLRITIFGVVTTVFDYW	IGH	10	None	
7	879	CARRLLTLRFLEWLRAFDIW	IGH	4	None	
7	880	CARRSAGYKRYYMDVW	IGH	12	None	
7	881	CAKSRLDHLAMGYNWFDPW	IGH	2	None	
7	882	CAKASIAAAGPDAFDIW	IGH	19	None	
7	883	CAHRNYIAAAFDIW	IGH	2	None	
7	884	CQQYDSYPWTF	IGK	27040	C3	7K8R
7	885	CARMNTAMGLDYW	IGH	2	None	
7	886	CAKDLRTGYYRGDAFDIW	IGH	2	None	
7	887	CAHRMTTVRDYYMDVW	IGH	2	None	
7	888	CARVNYYDYVWGSYRYTNFDYW	IGH	14	None	
7	889	CAHTPGDCTNTSCYLSYFDKW	IGH	2	None	
7	891	CARDRTPATDTSWWYFDLW	IGH	6	None	
7	892	CASPIIAWFGEFGDDYW	IGH	2	None	
7	894	CARDRGSGWDGEVDAFDIW	IGH	4	None	
7	895	CAKDASLLRYFVHFDYW	IGH	2	None	
7	896	CAGEPTTKCTSCYPFNDAFDIW	IGH	2	None	
7	897	CAREVRSGSYYYYYSMDVW	IGH	125	None	
7	898	CTCRPSSFWSHYPAMSFFDYW	IGH	4	None	
7	899	CYSTDSNENHEVF	IGL	3	None	
7	900	CARGRLRYFDWFASRDAFDIW	IGH	3	None	
7	901	CAKDPHYDFWSDYFFDYW	IGH	8	None	
7	902	CAKVYHGPGNYYKGYFDYW	IGH	2	None	
7	903	CTRGGGRGWYGLYW	IGH	7	None	
7	904	CARRKLPGFGAFDIW	IGH	2	None	
7	905	CARGWASGWSYYFDYW	IGH	3	None	
7	906	CARRALGYCSGGSCYSAFDYW	IGH	17	None	
7	907	CAHQDRAPSKAVAFDYW	IGH	3	None	
7	908	CARGRVPWQYYDFWSGYHNVPFDYW	IGH	6	None	
7	909	CTTDRVRAVATIFFLATYYFDYW	IGH	6	None	
7	910	CVRDGSGGFSYYFDNW	IGH	2	None	
7	911	CARGSWGVLVRSAFDVW	IGH	3	None	
7	912	CARLSPGGYVPPNGFDVW	IGH	3	None	
7	913	CARDPLWFGERYDAFDIW	IGH	3	None	
7	914	CATVEATDFGDYEDGYFDSW	IGH	6	None	
7	915	CARGGIAAAGIPYYYGMDVW	IGH	4	None	
7	916	CQQYNDYRALTF	IGK	4	C3	7K8R
7	917	CVKDMKSWISYYFNYW	IGH	13	None	
7	918	CARELYTFGPDYW	IGH	2	None	
7	919	CASQGVDSSGYSEFDYW	IGH	2	None	
7	920	CHQSSSLPFTF	IGK	283	None	
7	922	CAHSPVASAVTWFDPW	IGH	3	None	
7	923	CAKGTYCSGGSCYIFDYW	IGH	4	None	
7	925	CAKHKGDFGPTEGYYSDYW	IGH	3	None	
7	926	CARGIRDHCSSISCYTWDYW	IGH	2	None	
7	927	CASDYGDYARYIDYW	IGH	6	None	
7	928	CARGVYGVLAFDYW	IGH	10	None	
7	929	CARWAHTAMDSSVYYYYGMDVW	IGH	2	None	
7	930	CAKTGTIAVAGFDYW	IGH	2	None	
7	931	CARDSRWVNGWFGELRVLGYYFDYW	IGH	5	None	
7	932	CAKAMYSSGWYLRNNAFDIW	IGH	7	None	
7	933	CARHRIAVAGIDYW	IGH	3	None	
7	934	CARVSRRYFDWFGPGAFDIW	IGH	6	None	
7	935	CARGGIQLWLWYFDYW	IGH	4	None	
7	936	CASRSESVTLRFFEWLSIYWYFDLW	IGH	2	None	
7	937	CNSRDSSGNHWVF	IGL	396	None	

7	939	CARHRWLRMIDAFDIW	IGH	2	None	
7	940	CARESVTYGDFWSGYYTPFDYW	IGH	2	None	
7	941	CASGGQYYYEGFDIW	IGH	2	None	
7	942	CAKDILGSGSLAPDAFDIW	IGH	2	None	
7	943	CARVGGRGYYYGSEDYW	IGH	2	None	
7	944	CESHKSDAFDIW	IGH	2	None	
7	945	CQSYDESLKTVLF	IGL	2	C4	7JVC
7	947	CARVFSFWWLVRSGYFDYW	IGH	2	None	
7	948	CARDPTIGYYYGSGSYYFDYW	IGH	2	None	
7	949	CARDRDLTDTAMDTGWYFDLW	IGH	2	None	
7	951	CARGRRRKEGAADFDYW	IGH	2	None	
7	952	CASETGVDYW	IGH	2	None	
7	953	CAKFIGSGYFFYYMDVW	IGH	5	None	
7	955	CAKDVNTMTLRYFDLW	IGH	2	None	
7	956	CARVAYGWGSFPYFFDFW	IGH	5	None	
7	957	CARVRVFTTLRGVSYGFDPW	IGH	3	None	
7	958	CARDVHDSSGWPNGFDYW	IGH	2	None	
7	959	CARHPSEYYYGSGSYYNVMFDYW	IGH	6	None	
7	961	CARDQDTMIVYDAFDIW	IGH	3	None	
7	962	CQHYYSYSWTF	IGK	205	C3	7K8R
7	963	CARFRIQLWLLVYYYYNMDVW	IGH	2	None	
7	965	CARDFNDFWSGYYDYW	IGH	4	None	
7	966	CANLWFGELFDYW	IGH	15	None	
7	967	CARGPSSSWYGDNWFDPW	IGH	2	None	
7	969	CAREVAAAGSRLHFDYW	IGH	2	None	
7	970	CAKYELIAAAGPNFDYW	IGH	12	None	
7	972	CARELAAAGMVNDYW	IGH	2	None	
7	973	CQAWDSSTAFRVVF	IGL	4	C4	7JVC
7	974	CARDEWDYYYYYGMDVW	IGH	2	None	
7	975	CAKLIRWDSDFDYW	IGH	2	None	
7	976	CARDLGGYFDYW	IGH	127	None	
7	977	CARALVSFAELTDSAFDYW	IGH	48	None	
7	978	CAKGTWVTTISWFDPW	IGH	2	None	
7	979	CAVVPAYCSGGSCLDTSDAFGMW	IGH	4	None	
7	980	CAHRPKGWNDIWFDPW	IGH	6	None	
7	981	CVKDVAPLYSGSQTWYFDLW	IGH	4	None	
7	982	CARHRKEQWLVRTGGEYFQHW	IGH	3	None	
7	983	CMQDAHWPPAF	IGK	8	None	
7	984	CHQYNTWPVTF	IGK	2	C3	7K8R
7	985	CVRGDGFHSTYYFDYW	IGH	2	None	
7	986	CARNTGYSSSWCDAFDIW	IGH	3	None	
7	987	CARLEYPDTSGFYYW	IGH	4	None	
7	988	CQQFSNYLFTF	IGK	2	None	
7	989	CAKDGFGGLDYW	IGH	4	None	
7	990	CAKDSSSWPPWYFQHW	IGH	3	None	
7	991	CARAGTAAVDAFDIW	IGH	14	None	
7	992	CLSADSTGSYPVF	IGL	9	None	
7	994	CQHFDSYPRVFTF	IGK	2	C3	7K8R
7	995	CARHGMKTLGGVNFPHYAFDIW	IGH	4	None	
7	996	CARVRRQWLENGPSFFDYW	IGH	2	None	
7	997	CAKASVVVITCLDYW	IGH	2	None	
7	998	CARVGLGSPLFGLVYDAFDIW	IGH	6	None	
7	999	CAKDGVYGDSHFYYFDYW	IGH	2	None	
7	1000	CVKLKGSPASAFDIW	IGH	11	None	
7	1001	CASLNFIRTWMFDYW	IGH	2	None	
7	1002	CQQYHEWPPRNTF	IGK	5	C1	7BZ5
7	1003	CARHLYDYVWGSYRYTDAFDIW	IGH	3	None	
7	1004	CAHAVIAALFDYW	IGH	23	None	
7	1005	CATILTSGMGYFDYW	IGH	7	None	
7	1006	CMQGIEFQTF	IGK	24	None	
7	1008	CAKDARKFGHFKFFDYW	IGH	2	None	
7	1009	CARRVQSGRYNSDNSVYSRFDFW	IGH	3	None	
7	1010	CARIQAFSWIQLPDAFDIW	IGH	5	None	
7	1011	CARGSTIFGVVIKHIDYW	IGH	3	None	
7	1012	CAREAGYTAYDSAALDYW	IGH	4	None	
7	1013	CAKDLRAVLDPNYFDYW	IGH	3	None	
7	1015	CLLSYSDTRPLF	IGL	2	None	
7	1016	CARVQGDPNWFDPW	IGH	11	None	

7	1017	CARTMVRGVPRLDYW	IGH	2	None	
7	1018	CARGRQWEPLAFDIW	IGH	2	None	
7	1019	CARPGHVGKYCGGDCNFDYW	IGH	2	None	
7	1020	CAKDMGSGWLIHFDYW	IGH	4	None	
7	1021	CARAEASLLRYFDWLQKGVWNYFDYW	IGH	8	None	
7	1022	CARHAGASFDTW	IGH	2	None	
7	1023	CARGGGGNYLFLDYW	IGH	2	None	
7	1024	CTRHVEYFDWFYFDYW	IGH	2	None	
7	1025	CARDEPPHGSSWYYVQSRGFDYW	IGH	2	None	
7	1026	CARDGDMGGGTYYFDYW	IGH	4	None	
7	1027	CARDRKYYYDSSGYQLIDAFDIW	IGH	9	None	
7	1028	CAKRSWGYSVAFDIW	IGH	2	None	
7	1029	CARGEVTAMVTDAFDIW	IGH	4	None	
7	1030	CTRDGNFDWLLYLDYW	IGH	2	None	
7	1031	CAREGDAQDYGDYLSYFDYW	IGH	2	None	
7	1032	CARDPTNCSGGSCYPGDYW	IGH	2	None	
7	1033	CARRYYGSGLGGWFDPW	IGH	35	None	
7	1034	CARELYIVVVVAAYFDYW	IGH	2	None	
7	1035	CARHRRDYQRDSYYSDYW	IGH	2	None	
7	1037	CARDHRVAGNQSPYYYYGMDVW	IGH	4	None	
7	1038	CVRGPGSLGAAGPPYDYW	IGH	4	None	
7	1039	CARDLARYNWNDVPFDYW	IGH	2	None	
7	1040	CARDLEISGGSSGSYYRSGFLDYW	IGH	3	None	
7	1043	CAKDHKGYYGSGNYFDSW	IGH	12	None	
7	1044	CAKQADYYDSSGYSWSQDYW	IGH	3	None	
7	1045	CASSIAVAPTRDAFDIW	IGH	2	None	
7	1046	CAREGCSSTSCYQTPDYW	IGH	2	None	
7	1048	CASFAGAAATLYYFDYW	IGH	2	None	
7	1049	CARDPDGYNWMNDAFDIW	IGH	2	None	
7	1051	CAREYSSSWPNDYW	IGH	2	None	
7	1052	CAKADYDSSGYYYYFDYW	IGH	2	None	
7	1054	CARLSFSGWSPYYYYGMDVW	IGH	2	None	
7	1055	CARLQACSGYDCPIDYW	IGH	2	None	
7	1056	CARGGKRIVVVTAIRAFDIW	IGH	2	None	
7	1057	CARYNIEMTSNLFDYW	IGH	4	None	
7	1058	CARNLYFDSGSYYNSRVWFDPW	IGH	2	None	
7	1059	CARTGVTYYYGSGSYFPDAFDIW	IGH	2	None	
7	1060	CTTDLGLLPGTYFDYW	IGH	8	None	
7	1061	CARAGWVDGDTRSWFFDYW	IGH	4	None	
7	1063	CARPGAAGTGYYYYYMDVW	IGH	2	None	
7	1064	CARVKGIGYSGYEWELDYW	IGH	2	None	
7	1065	CARANVGGWLRGLGWYFDLW	IGH	2	None	
7	1066	CARHLQYYYVSELAFDIW	IGH	6	None	
7	1067	CATDGYSITWPYFDFW	IGH	38	None	
7	1068	CARDFDVLSGDYNAYW	IGH	2	None	
7	1069	CARNRLERLALGYNFFDPW	IGH	5	None	
7	1070	CAKDRGGQGLVLSYFDNW	IGH	2	None	
7	1071	CAKDEGPNALSSGWTYFDYW	IGH	2	None	
7	1073	CARSVDTAMVFRPRLFYYMDVW	IGH	3	None	
7	1074	CARGGLRLRVWYFDLW	IGH	2	None	
7	1076	CARHRYDYVWGIHEGEIDYW	IGH	4	None	
7	1077	CAKDMITFGGVGQSFAFDIW	IGH	6	None	
7	1079	CARDPVYGSGSYLGPPQPLYAIDIW	IGH	3	None	
7	1080	CASLGSGSYHLDYW	IGH	10	None	
7	1081	CAKVPPPDSGSYVSYFDYW	IGH	4	None	
7	1082	CAKDIGAAAGKGDDAFDIW	IGH	2	None	
7	1083	CARVGDYGPGNYFSDRISLLADASDIW	IGH	5	None	
7	1084	CTRGGGGRITYFSRFDSW	IGH	2	None	
7	1085	CARTLIRGDPYYYYGMDVW	IGH	4	None	
7	1086	CARVLGSRGTIFGVAIGSDYFDYW	IGH	9	None	
7	1088	CATAPPIAAAGNWFDPW	IGH	8	None	
7	1089	CQRYNKWPLLFTF	IGK	4	C3	7K8R
7	1090	CAISNNYYRFNWFDPW	IGH	2	None	
7	1091	CARIAAAGTGVPFFDYW	IGH	2	None	
7	1092	CARDLTMTTGWFDPW	IGH	3	None	
7	1093	CARGTATKLGPFDYW	IGH	2	None	
7	1094	CAHTVWGAYVDYW	IGH	4	None	
7	1095	CARDWQLGNDYW	IGH	3	None	

7	1096	CARVLPSYDILTGYYMAYFDYW	IGH	2	None	
7	1097	CAKDHTWLNSRTYGVFFDHW	IGH	3	None	
7	1098	CARDDPRIEAAGCDYW	IGH	18	None	
7	1099	CARDVHYDILTGYYNYW	IGH	18	None	
7	1100	CVKDLRIGHGSGNFILHPFDSW	IGH	2	None	
7	1101	CARPTSGQMATISPFDYW	IGH	4	None	
7	1102	CAREVGVAGPWNYYYYYMDVW	IGH	2	None	
7	1104	CARGVTIAAAGILLPYYYYYMDVW	IGH	3	None	
7	1105	CAKVDSSSWHFDYW	IGH	15	None	
7	1106	CARGYDYVWGSYRYTDYFDYW	IGH	6	None	
7	1107	CARDRWERYTTVTQIINAFDIW	IGH	2	None	
7	1108	CARGWRYCSGGSCFVGQYYYYMDVW	IGH	4	None	
7	1109	CASFYYGSGSYAAFDIW	IGH	4	None	
7	1110	CAKDDFGTTASFDYW	IGH	2	None	
7	1111	CARGEETYYDFWSGYLIDYW	IGH	4	None	
7	1112	CQQYNTESTWTF	IGK	84	None	
7	1113	CASFGSWYTNYW	IGH	2	None	
7	1114	CAKDIDGSGSYSNEFWYDAFDIW	IGH	2	None	
7	1115	CARAGTTFPDYW	IGH	19	None	
7	1116	CAKVGGDYGHLIYDYW	IGH	2	None	
7	1117	CAGVYDSSGYYLAEYFQHW	IGH	2	None	
7	1121	CARVVRSSWREIDYW	IGH	14	None	
7	1122	CARLSPRLLWFGELHLSGGFDPW	IGH	2	None	
7	1123	CQETSIFPPGTF	IGK	6	None	
7	1124	CARQIRYFDRVQYNYYMDVW	IGH	2	None	
7	1125	CARERRDSREFVPDYW	IGH	2	None	
7	1126	CARTLYYYDSGGYFYPNYFDYW	IGH	2	None	
7	1127	CARLRGSSWMYYFDSW	IGH	3	None	
7	1129	CARGRGAVLRFLEWSGDYW	IGH	2	None	
7	1130	CARGISNVLLWFGELLGHYYYYMDVW	IGH	2	None	
7	1131	CAHRRDTSVWYTVFDPW	IGH	3	None	
7	1132	CARVNFPGYDSSGYGFDYW	IGH	3	None	
7	1133	CAKDRYLGVATTYYFDYW	IGH	3	None	
7	1134	CARGRHKRVIVVVVAANIGHHFDYW	IGH	3	None	
7	1135	CARTADDSSGFFDYW	IGH	2	None	
7	1136	CARDDVQETLDYW	IGH	2	None	
7	1137	CARDLGVQVVTPQTDDYYYYYGMDVW	IGH	4	None	
7	1138	CARIRYSSNALDYW	IGH	2	None	
7	1140	CAHIFIRLEDYGPQTYYFDYW	IGH	3	None	
7	1141	CAKDLGSWSPNGDYW	IGH	2	None	
7	1142	CARQGGGYGSGHKDWFDPW	IGH	4	None	
7	1143	CMEGTHWPFFAF	IGK	2	None	
7	1144	CARDTFGWLQNRGAFDIW	IGH	8	None	
7	1145	CARVDTAMASAPSFDYW	IGH	2	None	
7	1146	CARHAYGSDRGDFDYW	IGH	2	None	
7	1147	CARTRGYSGYDYFDYW	IGH	4	None	
7	1148	CARDSDGSGSYLETEDAFDIW	IGH	2	None	
7	1149	CAREGNYYGSGSSILKVDAFDIW	IGH	2	None	
7	1150	CYCYGSDYGDYGGVDYW	IGH	2	None	
7	1151	CAKHFVVVTATGTYSGSPDFDLW	IGH	6	None	
7	1152	CARAPRGYDYVWGSYRSWFFDYW	IGH	2	None	
7	1154	CMQALQTPPLTF	IGK	2220	None	
7	1155	CARAQSITMIVVVIITAFDIW	IGH	34	None	
7	1156	CARGDYDILTDSVPRYYSFYYMDFW	IGH	2	None	
7	1157	CVRESLAPDSNSWPDYW	IGH	12	None	
7	1158	CAKVPEYSSSWYYFDYW	IGH	9	None	
7	1159	CARVYRGYKFGFPDSYYYYYMDVW	IGH	4	None	
7	1160	CARERDSSSLLGAFDIW	IGH	2	None	
7	1161	CAKDSVAGHKNYYYYGMDVW	IGH	4	None	
7	1162	CAREPQGGYFDWLLWAYFDYW	IGH	4	None	
7	1163	CACGVGYGGNFVWYFQHW	IGH	2	None	
7	1164	CASTWYTSDHAFDFW	IGH	13	None	
7	1165	CARDFSSLNLRVLAYSGYDYWDYFDYW	IGH	3	None	
7	1166	CARDPCKIQLWLQCYSYFDYW	IGH	2	None	
7	1167	CARDRGIAVAGTLLLRNWYFDLW	IGH	3	None	
7	1168	CARLPWGVGAASNYYFDYW	IGH	2	None	
7	1169	CAKVPGGPRGVGHSFDPW	IGH	2	None	
7	1170	CQQYDNHF	IGK	18	C3	6XDG

7	1171	CATTQVATPGAFDIW	IGH	3	None	
7	1172	CAREILNTDGMDVW	IGH	5	None	
7	1173	CARAHCSSSSCYEIDSFDIW	IGH	4	None	
7	1175	CAKANYYDSSGYIDYW	IGH	14	None	
7	1176	CAKDVSWGIAVAGTQDYW	IGH	2	None	
7	1177	CVRQLYSSSWYQFDYW	IGH	4	None	
7	1178	CGGSPLYNGDWYYFDHW	IGH	2	None	
7	1180	CTRDSRGYYDILTGYYVLPAPFDYW	IGH	2	None	
7	1181	CARHGGFGEFDAFDIW	IGH	4	None	
7	1182	CARHNGRGGIAVANDAFDIW	IGH	4	None	
7	1183	CARIYLRGYSDYW	IGH	3	None	
7	1184	CAKVRSIATATPFFFDSW	IGH	3	None	
7	1185	CARVQVLGISPYYFDFW	IGH	2	None	
7	1187	CARGFSGVVVAALGLDYYYYYGMDVW	IGH	2	None	
7	1188	CARVYSAYYYGSGSYYDDAFDIW	IGH	10	None	
7	1189	CLQSDSTPLAF	IGK	6	None	
7	1191	CATAGPEYSSSRPMYNWFDPW	IGH	3	None	
7	1192	CAVLETRSYSFRYW	IGH	2	None	
7	1193	CQKCDNLPPSCSF	IGK	2	C3	6XDG
7	1194	CASPTYCSGGYCFDYW	IGH	2	None	
7	1195	CAILHPDGYKLFDYW	IGH	6	None	
7	1197	CARVPNLLWFGESNYAFDIW	IGH	2	None	
7	1198	CAKIMTTVTTKDYW	IGH	2	None	
7	1199	CARSVLVGSGYSSHSGSGFDYW	IGH	4	None	
7	1200	CAKGGVWFGELRSGKYYFDYW	IGH	2	None	
7	1201	CQRYNDWPPTWTF	IGK	8	C3	7K8R
7	1202	CARVLGDRGGLFDYW	IGH	11	None	
7	1203	CAKDQAGYSSGWYDGYYFDYW	IGH	22	None	
7	1204	CARDQHQSISGKLKYNYFDCW	IGH	3	None	
7	1205	CARALISYSILTGYYPQSFDIW	IGH	2	None	
7	1206	CARSPLVAAADAGYYYYYYYMDVW	IGH	18	None	
7	1207	CASHRRIGMIVPVDSYFDYW	IGH	22	None	
7	1208	CASPTPWYSSTWHDAFHIW	IGH	2	None	
7	1210	CARGLTQIREQPNRSDAFDIW	IGH	2	None	
7	1211	CARVRSTIIMILRSDAFDVW	IGH	5	None	
7	1212	CAKETRITAADSFDHW	IGH	2	None	
7	1213	CAAYYDILTGYRPPNQRFDYW	IGH	2	None	
7	1214	CARGEEGPRWFGESSFDYW	IGH	4	None	
7	1215	CARLGRGYSSSWYSLRFSAFDIW	IGH	2	None	
7	1217	CQQYYNAPPALSF	IGK	2	C4	6W41
7	1218	CVREVFRVVTTYFDYW	IGH	2	None	
7	1219	CAREDYVWGILISRGAFDIW	IGH	4	None	
7	1220	CARGGGSGLIYYYYMDVW	IGH	2	None	
7	1221	CSRAGYYRFDYW	IGH	9	None	
7	1223	CATGGGISYYFYMDVW	IGH	3	None	
7	1224	CARAILIGSPPQVWSWGPKQKVRRNCYFDFW	IGH	2	None	
7	1225	CARILGYSNYHVDYW	IGH	2	None	
7	1226	CARLLKANWGLHYMAFDIW	IGH	2	None	
7	1227	CAKDISLGYGSGTHFDSW	IGH	3	None	
7	1228	CGRADKGYCAGGSCYFEYW	IGH	2	None	
7	1229	CQQYMHWPSYTF	IGK	6	None	
7	1230	CARWLRGDTNYYMDVW	IGH	5	None	
7	1231	CARDRIDRIAAAGTNDYW	IGH	2	None	
7	1232	CARNEARRITIFGVVILPDAFDIW	IGH	2	None	
7	1234	CAKVQHGFVWFGDSYFDYW	IGH	2	None	
7	1235	CARMPTRLWFGNFDYW	IGH	2	None	
7	1236	CAREPDSSGWYKYFQHW	IGH	2	None	
7	1237	CARGDISTTAGFWYFALW	IGH	5	None	
7	1238	CARSYCSGGSCYNDPRDFDYW	IGH	2	None	
7	1239	CAKDLHPSQGAEYYYGSGSYCQADYW	IGH	2	None	
7	1240	CARAGSSSMWLAATDYFDYW	IGH	5	None	
7	1241	CLKASDTAMYYCARHDRIGLPAAHNWFDPW	IGH	2	None	
7	1242	CAKAAMEVITTFDYW	IGH	2	None	
7	1243	CARDRYGDYGVDYW	IGH	20	None	
7	1244	CARHKLSFYDISTGYSDGFDIW	IGH	2	None	
7	1245	CATGGAKADFWSGYYYLQAFDYW	IGH	2	None	
7	1247	CAHRSSGFYAAYFDYW	IGH	4	None	
7	1248	CTTVGAGYNAFDIW	IGH	6	None	

7	1249	CAREVHYDILTGYYYSFDYW	IGH	18	None	
7	1250	CARLLPSWSDFRNSRNWFDPW	IGH	4	None	
7	1251	CASLSRDYDILTGYYFDYW	IGH	4	None	
7	1252	CARVPSDIVATFPGFIYYYYYMDVW	IGH	6	None	
7	1253	CAKVTVHTAMVLGAFDIW	IGH	2	None	
7	1254	CARDPVVPAARTRQGHFDYW	IGH	2	None	
7	1256	CARQPGYTFDIW	IGH	2	None	
7	1257	CAKDPVEGYSSGWYYYYYGMDVW	IGH	6	None	
7	1258	CQAWDSSTVVF	IGL	2247	C4	7JVC
7	1259	CARGNRQWLARSYWYFDLW	IGH	14	None	
7	1260	CARHIRYFDFKRAFDIW	IGH	2	None	
7	1261	CARDVKDTTMSWYFDLW	IGH	2	None	
7	1262	CAKALSGYEGYAPGPFDIW	IGH	2	None	
7	1263	CARVMGWFGKNYYYYMDVW	IGH	3	None	
7	1264	CARIHAEPYGRGWFIEYYHYLYMDVW	IGH	2	None	
7	1265	CARHPGYSSGWYFSNWFDPW	IGH	4	None	
7	1266	CARAIVPFMITFGGGFDYW	IGH	2	None	
7	1267	CARANWMYWYFDLW	IGH	47	None	
7	1268	CAKVATYSSSYFDYW	IGH	11	None	
7	1269	CVKDNDGDFDYW	IGH	2	None	
7	1270	CVKDKGSGWPQPHWNFDLW	IGH	2	None	
7	1271	CSRGAWVQLTSYFDLW	IGH	3	None	
7	1272	CAHIDLLWFGEFLVAFDIW	IGH	7	None	
7	1273	CAKRRSFCSGENCYETSFDQW	IGH	2	None	
7	1274	CAKGSIFGVVIIGGGWFDPW	IGH	2	None	
7	1275	CARAPLWFGELLPDNWFDPW	IGH	3	None	
7	1276	CANAGFAEDAFDIW	IGH	2	None	
7	1277	CARHLSEGGVPFFFDYW	IGH	240	None	
7	1279	CARGHDILTGYNWFDPW	IGH	8	None	
7	1280	CARFYGVARDAFDLW	IGH	3	None	
7	1281	CVTEVSYFLTTYYYVYW	IGH	12	None	
7	1282	CARQSNSGWYYFDHW	IGH	2	None	
7	1283	CARARCSGGSCYPNYFDSW	IGH	2	None	
7	1285	CAKNMGGSGSYYNLDYW	IGH	2	None	
7	1286	CARRGSYPIGGFDHW	IGH	2	None	
7	1287	CARASYGDYIRPYDYW	IGH	3	None	
7	1288	CARHEDFVEYLWTNSYYYMDVW	IGH	7	None	
7	1289	CAHRRDWGWFGDAFDIW	IGH	13	None	
7	1290	CATVGATGTDFDYW	IGH	15	None	
7	1291	CARDQYGPATLYYYYYMDVW	IGH	6	None	
7	1292	CARTTMIRGVPGYYFDYW	IGH	4	None	
7	1293	CAKGGEWLRYGIDYW	IGH	2	None	
7	1294	CARFSSWPYTYYFDYW	IGH	2	None	
7	1295	CARDPPRHPGNDAFDIW	IGH	2	None	
7	1296	CARHDRIGLPAAHNWFDPW	IGH	2	None	
7	1298	CARGGDGYNLGSWYFDLW	IGH	2	None	
7	1299	CAKENGDYVSFDYW	IGH	7	None	
7	1300	CASERSGYSYGYKGTFDYW	IGH	6	None	
7	1301	CVKDQNSVWYWYFDLW	IGH	2	None	
7	1302	CSSYAGSNNF	IGL	28	C2	7BWJ
7	1303	CARHGNEYCSGGSCYPNYYYYYGMDVW	IGH	5	None	
7	1304	CARTPARFYFDSSGPYWYFDVW	IGH	3	None	
7	1305	CAHLVSSGWYGDAFDIW	IGH	15	None	
7	1306	CAKDDARLTGYYQAFDYW	IGH	2	None	
7	1307	CATYYNYDAFDIW	IGH	4	None	
7	1308	CARHVGYVLRYFDWLDAFDIW	IGH	4	None	
7	1309	CAKSPPRVVAIFDYW	IGH	27	None	
7	1310	CARMFYGSGTPKADYW	IGH	2	None	
7	1311	CARQLQLYYFDFW	IGH	12	None	
7	1312	CTTLAGRYFDWLLYPGYW	IGH	4	None	
7	1313	CAKVRYDFWSGYYPPSNWFDPW	IGH	2	None	
7	1315	CARDNQYYYGSGSYYRSYWYFDLW	IGH	4	None	
7	1316	CAKDIGVTTVITSDYW	IGH	10	None	
7	1317	CARHWVKVQIPFYYYYGMDVW	IGH	3	None	
7	1318	CTRADFGSGSFSNEPYYFRYW	IGH	2	None	
7	1319	CRAANEGGLDYW	IGH	3	None	
7	1320	CAKQFRGVITFDAFDIW	IGH	2	None	
7	1322	CAHTGPQDWAGYYFDYW	IGH	4	None	

7	1324	CARKGGDYVSAFDLW	IGH	2	None	
7	1325	CARGQRVYQPKAGGNSPPGDYW	IGH	2	None	
7	1326	CTTHSWGYGSGSSSSFDYW	IGH	2	None	
7	1327	CAKVPPTVTSVGALDYW	IGH	2	None	
7	1328	CTTDLGIYSSSWYYYYYGMDVW	IGH	4	None	
7	1331	CATAGRFRFDIW	IGH	43	None	
7	1334	CARSPFSSAYEIPSGAFDLW	IGH	3	None	
7	1335	CARDSSNTYYYDSSGYYYVWPTNAFDIW	IGH	5	None	
7	1336	CARDLSYYTSGWNWYFDLW	IGH	2	None	
7	1337	CARPATQWLALHPFDYW	IGH	2	None	
7	1338	CARHSSSDSYTGGLLDYW	IGH	3	None	
7	1339	CAKDTYSSGWYKFYAFDIW	IGH	2	None	
7	1340	CARVPGAVRGVRTNAFDIW	IGH	2	None	
7	1341	CVRYDQEVADGFDVW	IGH	2	None	
7	1342	CARPLGPSTAMGIFDYW	IGH	2	None	
7	1343	CARSRRVVDLRAFDIW	IGH	7	None	
7	1344	CAKDVSPYDYVSYMDVW	IGH	2	None	
7	1345	CARGASYEITFGGVIVIRQGVDYW	IGH	2	None	
7	1346	CARDNAAGHLQYFDWLSRSDYYMDVW	IGH	2	None	
7	1347	CQQYYFPPFNF	IGK	7	C4	6W41
7	1348	CARVRDSSGWSYYYYYYYMDVW	IGH	6	None	
7	1349	CAREGEAAAGTRRYYFDYW	IGH	2	None	
7	1350	CARRGFGELLMVSAFDIW	IGH	3	None	
7	1351	CARDREAYGDYPDAFDIW	IGH	2	None	
7	1353	CARSFLHYSGSSGYYSGSFDYW	IGH	3	None	
7	1355	CAKIGDFYYYYMDVW	IGH	5	None	
7	1357	CARELRFLEWLLALQPYYYGMDVW	IGH	21	None	
7	1358	CARTTTYYDILTGYSAEEPSDYW	IGH	2	None	
7	1359	CAKDSNSISIAAAGTWFDPW	IGH	2	None	
7	1360	CRSADSGGIWVF	IGL	2	None	
7	1361	CATSIAAAGVLGYFQHW	IGH	2	None	
7	1362	CARDYYDSSGYSDGMDVW	IGH	2	None	
7	1363	CLRYCSNPSCSETAFDPW	IGH	2	None	
7	1364	CARDVIVASLTVFDYW	IGH	4	None	
7	1365	CARIHTVVAGHLDYW	IGH	4	None	
7	1366	CARHDFEGEAVAGANAFDIW	IGH	2	None	
7	1367	CARVGYCGWCFDYW	IGH	3	None	
7	1368	CVRDLFRQYSAYSDSW	IGH	2	None	
7	1369	CATGFSWHVAATWGGFDYW	IGH	2	None	
7	1370	CAKVPFTITMDYMDVW	IGH	3	None	
7	1371	CAKISGYGSWSYYNYYW	IGH	2	None	
7	1372	CARDYGGGSVRGFDYW	IGH	7	None	
7	1373	CARLLGTGYRGYDFGDWYFDLW	IGH	2	None	
7	1375	CARDQSVDYYGSGSYYKVLILGAFDIW	IGH	3	None	
7	1376	CAHQASSGWYGYYFDYW	IGH	3	None	
7	1377	CARILFQPGLRVDWYVDLW	IGH	2	None	
7	1378	CARMYGARNQFDYW	IGH	7	None	
7	1379	CARSASGSYAKGHALYYYYYFMDFW	IGH	8	None	
7	1380	CVRGGPTTTDPLDIW	IGH	2	None	
7	1381	CARQSSLGAVITWDDAFDTW	IGH	2	None	
7	1382	CAKDPRAGQLWLGYYFDYW	IGH	3	None	
7	1383	CAITSAAAAGKRWDYFDYW	IGH	2	None	
7	1385	CARGGYCGGRSCYSGISYYFDFW	IGH	2	None	
7	1386	CAKVLAVAGTGDYW	IGH	26	None	
7	1387	CAKDGGYSYGFGKEYFQHW	IGH	4	None	
7	1388	CARDPMEGGADYW	IGH	2	None	
7	1389	CARPNSGSYFGAFDIW	IGH	5	None	
7	1390	CQESYITPIF	IGK	7	C4	7JW0
7	1391	CARDRRLAVAGYDAFDIW	IGH	3	None	
7	1392	CARTSRPYYPYYVDYW	IGH	5	None	
7	1394	CAGRYCTNGVCYNPYYFDYW	IGH	4	None	
7	1395	CAIQTTADGYFDYW	IGH	19	None	
7	1396	CARGIGTARGDYW	IGH	4	None	
7	1397	CARGPEYDYVWGTLGGVYAFDIW	IGH	2	None	
7	1398	CARMRAVLNAFDIW	IGH	6	None	
7	1399	CARARMTAMVPTPCDYW	IGH	2	None	
7	1400	CARGVLMVYNDAFDIW	IGH	2	None	
7	1401	CARDREYDYVWGSSVAFDIW	IGH	2	None	

7	1402	CGADHGSGSNFVWVF	IGL	42	None	
7	1403	CAKDARVGAQVFDYW	IGH	2	None	
7	1406	CARDLSTVVTSHFDYW	IGH	4	None	
7	1408	CARDLPTTDYYGSGSYVDYW	IGH	2	None	
7	1409	CAKMLSSSTDFDYW	IGH	10	None	
7	1410	CARHNPYYDILTGYYYDAFDIW	IGH	5	None	
7	1411	CQQFGSSPVMYTF	IGK	86	C1	7K8N
7	1412	CAKDTSSSLYIAALFDYW	IGH	4	None	
7	1413	CARPGYSSSWYGGNAFDIW	IGH	2	None	
7	1414	CTRDLDSSGWYKDAFDIW	IGH	3	None	
7	1416	CARCNEITMIVGNWFDPW	IGH	2	None	
7	1417	CARAQWLPQSHFDYW	IGH	6	None	
7	1418	CQQLNSYPRYANDF	IGK	8	C1	7BZ5
7	1419	CAREVPGYFDWLLYLDYW	IGH	3	None	
7	1420	CASSILGYCSGGTCYSFGYW	IGH	2	None	
7	1421	CARQDCRSARCYFNNAFDIW	IGH	2	None	
7	1422	CTRVYETTVTTAGWFDPW	IGH	4	None	
7	1423	CARALSRSKQLVTKYYFDYW	IGH	6	None	
7	1424	CARDNVVVAATQSHYYYGMDVW	IGH	2	None	
7	1425	CAKDRWVMTPGYFQHW	IGH	2	None	
7	1426	CVTWGSGSYYNPQDYFDYW	IGH	2	None	
7	1427	CARDGRYCSSTSCSNHFDYW	IGH	5	None	
7	1428	CATTVYSRGWYSEYW	IGH	2	None	
7	1429	CATMGAYGDYVVDYW	IGH	2	None	
7	1430	CARDGSIDAIDRAFDVW	IGH	2	None	
7	1431	CVRDKGSSGLFDLW	IGH	2	None	
7	1432	CTTVYSYYYDSSGYYYAILSLGAFKDYYYYGMDVW	IGH	2	None	
7	1433	CAKGRGPWYSGDWFDPW	IGH	3	None	
7	1435	CQQSYSTLFGYTF	IGK	33	C4	6ZDH
7	1436	CAHQSIAAAVNFDYW	IGH	6	None	
7	1438	CVHRHGYTYDFYW	IGH	2	None	
7	1440	CARTKDGYNPSSGSWHFDLW	IGH	2	None	
7	1441	CARVRLSGWDPRRFDYW	IGH	7	None	
7	1442	CARQSCTGGSCYWSFDYW	IGH	6	None	
7	1443	CAKPLVGAVTGEYFQHW	IGH	2	None	
7	1444	CARALWFGIKNEYYFDYW	IGH	3	None	
7	1445	CAKEGFGQRHLYYYYYYMDVW	IGH	4	None	
7	1446	CQAWDSNTAIF	IGL	16	None	
7	1447	CTRDHGDPEAYWYFDLW	IGH	8	None	
7	1448	CAKDYGDSGALAFPTEYFHPW	IGH	2	None	
7	1449	CARGRWLQLPNYYYYYYGMDVW	IGH	4	None	
7	1450	CARGGGNYYDGRDFDCW	IGH	2	None	
7	1452	CARDAPLSFSTGSDPLPIALLDAFDIW	IGH	2	None	
7	1453	CARDGAGSRNFDLW	IGH	4	None	
7	1454	CARESYDILTGYLYAFDIW	IGH	11	None	
7	1455	CVVWFGELLSFDYW	IGH	2	None	
7	1456	CAHVDYYGAGSYPDLFDYW	IGH	2	None	
7	1457	CARHSPVFIWFGPGGFDYW	IGH	7	None	
7	1458	CARNSRGIGWQYFDYW	IGH	6	None	
7	1459	CAKRRAVSSWYKTAFDTW	IGH	2	None	
7	1460	CAKNIIGQWLVPTSVDYW	IGH	2	None	
7	1462	CARQETSRSGWDLFYFDYW	IGH	8	None	
7	1464	CARESRITIFGVVIYEYFQHW	IGH	2	None	
7	1465	CAREGREQWLVQFDYW	IGH	4	None	
7	1466	CAKDMKWLGRGEVDYW	IGH	2	None	
7	1467	CARVRHSILWWAHWYFDLW	IGH	2	None	
7	1468	CARESGWFGELFDYYYYYMDVW	IGH	2	None	
7	1469	CARARYPELHDYW	IGH	5	None	
7	1470	CARAGAPGIYDYW	IGH	3	None	
7	1471	CSRVRSDMITFGGPSDYW	IGH	2	None	
7	1472	CARGTGFGELSVRWFDPW	IGH	2	None	
7	1473	CARGGGIAAAGSGDYW	IGH	2	None	
7	1474	CASDSGRYDYGDFYYFDYW	IGH	2	None	
7	1475	CASHVPFTIFGVVIVVGWFDPW	IGH	2	None	
7	1476	CARHVYCSSPTCYSPPYFDYW	IGH	2	None	
7	1477	CARGRRGYETYNWFDPW	IGH	2	None	
7	1478	CANQGWVYGDYDGYFDYW	IGH	2	None	
7	1479	CAKEPTLYTSRWYFDYW	IGH	9	None	

7	1480	CAKTGYSYGYPIPYFDYW	IGH	5	None	
7	1481	CAKQSFTLSSSWYDYW	IGH	9	None	
7	1482	CAREVGTSVDYW	IGH	26	None	
7	1483	CARDLGATAQEPFDYW	IGH	5	None	
7	1484	CVRAVGTATPSSDAFDIW	IGH	7	None	
7	1486	CARGSPRSGGSCRRSWCRYFDLW	IGH	2	None	
7	1487	CARHRYSYGHDYW	IGH	13	None	
7	1489	CAKGPPGQQLDAEYFQHW	IGH	2	None	
7	1490	CARALKTSAWLFEYW	IGH	4	None	
7	1491	CARTATRAVAGILRPLYYYYMDVW	IGH	42	None	
7	1492	CARSRYSSGWFVYW	IGH	8	None	
7	1494	CAATGYSSGWYNAFDIW	IGH	15	None	
7	1495	CARLNCGGDCYGFDIW	IGH	2	None	
7	1496	CARTLSSGWYYKNAFDIW	IGH	4	None	
7	1497	CAKSLSYSSSWYSDYW	IGH	4	None	
7	1499	CARQRIPGLVVIKLLPFDYW	IGH	21	None	
7	1500	CARLRGVTGDFDIW	IGH	8	None	
7	1501	CARIVRQWLENSYYFDYW	IGH	7	None	
7	1502	CARAGGRITIFGVVIIHAFDIW	IGH	2	None	
7	1503	CARGRVGLGWQLGSYYYYGMDVW	IGH	3	None	
7	1504	CQQRSNLISF	IGK	10	None	
7	1506	CARRSGYYESSGYYLFGYW	IGH	4	None	
7	1507	CARGDSSGWYGNPYYFDYW	IGH	5	None	
7	1508	CARGRGITIFGVLLAPYYFEYW	IGH	3	None	
7	1509	CARDPPYSGSAPRDAFDIW	IGH	2	None	
7	1510	CAHRGGVGGSIDYW	IGH	6	None	
7	1511	CARDMRLDRYKFDTTAYLDNW	IGH	4	None	
7	1512	CARDFDDSSGYYYFDYW	IGH	26	None	
7	1513	CARIRLDSSSADAFDIW	IGH	2	None	
7	1514	CAKVGTAMGGTIDYW	IGH	2	None	
7	1515	CLRLFEYNGSFFFDYW	IGH	2	None	
7	1516	CAKVASSANAFDIW	IGH	6	None	
7	1517	CAKELSDQWLNDAFDIW	IGH	6	None	
7	1518	CARHGLSSITIFGVVTQNKYFDYW	IGH	4	None	
7	1519	CSREDYW	IGH	23	None	
7	1520	CARVYYDILTGYYIFFDYW	IGH	93	None	
7	1521	CARMLQVWFGRSPAGWFFDLW	IGH	2	None	
7	1522	CAHKIPGWYSFDYW	IGH	3	None	
7	1523	CAREVDDFVWGNARQAYYFDFW	IGH	4	None	
7	1524	CARRIAAAGPQKYDAFDIW	IGH	2	None	
7	1525	CQVWDSSSDHWVF	IGL	460	None	
7	1526	CARVRPFHPYYFDFW	IGH	7	None	
7	1527	CARRHYGSGSYWYEDYFDYW	IGH	4	None	
7	1528	CASHLPEDGFDIW	IGH	4	None	
7	1529	CTKVLFGSGLGSGYYDSW	IGH	4	None	
7	1530	CAREEGILTGYYNGPFDYW	IGH	2	None	
7	1531	CARHLRMGVVVAAPYYFDYW	IGH	11	None	
7	1532	CAAFVDTAVNPRGAFDVW	IGH	2	None	
7	1533	CAKVGRFFAIYTFVTGVAEYLDHW	IGH	2	None	
7	1534	CAKDSGFGIVVVTVDYW	IGH	4	None	
7	1535	CAHSGGDWYRDFEYW	IGH	2	None	
7	1536	CARARIMITFGGVYPNWFDPW	IGH	2	None	
7	1537	CARSGEAHSSAGLQTNAFDIW	IGH	4	None	
7	1538	CARHIPPTIHIVVVTRQGWFDPW	IGH	4	None	
7	1539	CAHRHSHLFAWVVGFDYW	IGH	2	None	
7	1540	CARGLTTVTIFDQLKNWFDPW	IGH	4	None	
7	1541	CAKVPRWSGWFFNAFDIW	IGH	2	None	
7	1542	CARGNIAAAEAGNDYYYYYGMDVW	IGH	2	None	
7	1543	CARDGGNVATTDDWYFDLW	IGH	2	None	
7	1544	CAKANFITIFVMLHDYW	IGH	2	None	
7	1545	CAKDLGGYDLDWFDPW	IGH	4	None	
7	1546	CARRSVRYSSGWYESYYFDYW	IGH	3	None	
7	1547	CARQPLYGSGSHYNWFDPW	IGH	2	None	
7	1549	CAKDIRWRSQAAAGIDYW	IGH	2	None	
7	1550	CARGRNDYYDDSGYWEESYDFDIW	IGH	4	None	
7	1551	CARDSSGWKIDYW	IGH	13	None	
7	1552	CARERAPYDYAFDIW	IGH	2	None	
7	1553	CARHMPVYGGFEYW	IGH	3	None	

7	1554	CARGGTASGFDYW	IGH	25	None	
7	1555	CAKTSGWSAPYFDYW	IGH	2	None	
7	1557	CARGYSSGWYTIHFDYW	IGH	13	None	
7	1558	CAKVNSWYGSYFDYW	IGH	3	None	
7	1559	CTRDGPGYCTNGVCYTADEPSDYW	IGH	2	None	
7	1560	CARDVGFSWYYYDSSGYYQYW	IGH	2	None	
7	1561	CARDRVLLWFGELSRFRIDYW	IGH	2	None	
7	1562	CARSVPEWTRHCGGDCYSDYW	IGH	2	None	
7	1563	CATRSVEGDLWFGEILYPLTNWFDPW	IGH	3	None	
7	1565	CARPRGYDLLTGQPIDDYW	IGH	2	None	
7	1566	CTRLPWGVGATSKYYLDNW	IGH	2	None	
7	1567	CARHSEAYSGSYSTAYFDYW	IGH	2	None	
7	1568	CARGGGIGWGGYYFDYW	IGH	2	None	
7	1569	CAHGTGTWYFWSGESTYYFDYW	IGH	5	None	
7	1570	CAKVSSSGWYRVSAFDIW	IGH	2	None	
7	1572	CARDRVTMVRGVEWGSYYYYYYGMDVW	IGH	2	None	
7	1573	CARASLRRSGWPTFGYYYYGMDVW	IGH	2	None	
7	1575	CARHAKLLWFGEGYFDYW	IGH	5	None	
7	1576	CATSPEHHDNSGSYYRYYYYYMDVW	IGH	2	None	
7	1578	CVSGSGNYRRSTFDFW	IGH	2	None	
7	1579	CARGSDSPEELSYYFDSW	IGH	26	None	
7	1580	CARFLSITAAENHYYYYYSLDVW	IGH	2	None	
7	1581	CARRPNNFDWLFPDAIDIW	IGH	2	None	
7	1583	CAKVTFIAVANDAFDIW	IGH	4	None	
7	1584	CATGRLRYFDWFGYW	IGH	2	None	
7	1585	CAKGSSWYWEFDTW	IGH	4	None	
7	1586	CARAWFGEQNYYYYYYMDVW	IGH	2	None	
7	1587	CARDNVDIVATTMGDAFDIW	IGH	2	None	
7	1588	CAKDQPRGVAGSFDYW	IGH	2	None	
7	1589	CTRRNWDVFDYW	IGH	2	None	
7	1590	CAKAGNYGSFYFDYW	IGH	3	None	
7	1591	CARVDYPGIAVAGSLDYW	IGH	4	None	
7	1593	CARHPLAKNYYYYYYMDVW	IGH	2	None	
7	1595	CATSDSSGWYGLIGAFDIW	IGH	2	None	
7	1596	CAKDRYGDYFFADPYFDYW	IGH	2	None	
7	1597	CARGGWIPPTTTKFDYW	IGH	2	None	
7	1598	CARHSGWVPSGWYQPDWYFDLW	IGH	6	None	
7	1599	CARVISDYTSGPFIYYFDYW	IGH	4	None	
7	1600	CAREGPAMVGFDPW	IGH	6	None	
7	1601	CARDRHYGSGSYALIDAFDIW	IGH	2	None	
7	1602	CARETSRPNTMVRGFWDYW	IGH	7	None	
7	1603	CQQYNNYWAF	IGK	32	C3	7K8R
7	1604	CARDVAAAGPWAFDIW	IGH	10	None	
7	1605	CVREGELDGDYGADYW	IGH	2	None	
7	1606	CARDTSGWYLFYYYYYMDVW	IGH	2	None	
7	1607	CARDLFSGIVVVVAATPINGAFDIW	IGH	3	None	
7	1608	CARGPDRRFLTGYYDYW	IGH	2	None	
7	1609	CARHPPVDYYGSGRRYYFDYW	IGH	2	None	
7	1610	CAKDTEGVLKLAFDIW	IGH	2	None	
7	1611	CAKAEAAYSSSWFVWYFDLW	IGH	4	None	
7	1613	CVKDRGSSRVRGSFDIW	IGH	8	None	
7	1614	CARAPWDPVRLDYFDYW	IGH	2	None	
7	1616	CARVPIVVVPAALGMGIYYYYYYYMDVW	IGH	2	None	
7	1617	CARVYETYYDILTGYYSGYFDYW	IGH	2	None	
7	1618	CAKDVYSNTRDAFNIW	IGH	3	None	
7	1619	CASLTTYYYGSGSYFSRHQTADWFDPW	IGH	3	None	
7	1620	CARESHDYGDFLAEYFQDW	IGH	12	None	
7	1621	CARDGGDDYGDYGWEVEYYFDYW	IGH	3	None	
7	1622	CASLAQHIAAAGPPSDYW	IGH	2	None	
7	1623	CARLVATTVTSRPGQGLAYYYYMDVW	IGH	3	None	
7	1624	CARHEQYYYGSGSYGAFDIW	IGH	4	None	
7	1626	CQQSYSLSGQWTF	IGK	7	None	
7	1628	CARQLPMGDYFDYW	IGH	2	None	
7	1629	CVRDLQLTVEYFDSW	IGH	2	None	
7	1630	CAQGPLYGPFDYW	IGH	9	None	
7	1631	CQQTYSSPRLCTF	IGK	2	C2	7K8O
7	1632	CARPDANDYGDYTADYW	IGH	2	None	
7	1633	CAKDRIYFVDTAMVGDYW	IGH	2	None	

7	1634	CVRTELRGSFDFW	IGH	2	None	
7	1635	CARGGFDSRSPFDLW	IGH	10	None	
7	1636	CARGRRQWLTRDYYYYMDVW	IGH	6	None	
7	1637	CARHSAVHSSSHYYYYYGLDVW	IGH	2	None	
7	1638	CARDVITFGGVIVIPGAFDIW	IGH	2	None	
7	1640	CAGPQDLQALLMHRGVGIYYYGMDVW	IGH	2	None	
7	1642	CARGMTTLTTQDFNYYMDVW	IGH	3	None	
7	1643	CARVEYSSGLRYWYFDLW	IGH	8	None	
7	1644	CAKASGIAAAGQNWDYW	IGH	2	None	
7	1645	CATGPPDYGDYVGYWFDPW	IGH	4	None	
7	1646	CATYRGDYLGDVFDIW	IGH	2	None	
7	1647	CARGWAYSSSWYAYTYFDYW	IGH	7	None	
7	1648	CARDPLTTVTPVLFDYW	IGH	3	None	
7	1649	CARGGGYSYCLGALDAFDIW	IGH	2	None	
7	1650	CAKDRRGFVAVAGNNWFDPW	IGH	2	None	
7	1651	CARLPPIGVAGGFDFW	IGH	2	None	
7	1652	CARGGGSSWYAAYAFDIW	IGH	2	None	
7	1653	CVHEGSAHRDALDVW	IGH	2	None	
7	1654	CAKDGRHLADPLLYYYSAMDVW	IGH	3	None	
7	1655	CVREGGSGFEGGFDIW	IGH	2	None	
7	1656	CAKVGSWYPDYW	IGH	17	None	
7	1657	CAQHTVAGIFDYW	IGH	13	None	
7	1658	CARDRRERTYDSQRYFEGW	IGH	3	None	
7	1659	CAREGAHILTGYYKTWYFDLW	IGH	2	None	
7	1660	CAKESGDRDMTKGFDYW	IGH	5	None	
7	1661	CARDIRDLMEAYPATGYFDLW	IGH	4	None	
7	1662	CARAPGCSSTSCYHYDYW	IGH	2	None	
7	1663	CAKDTGGYSSGWYARFDYW	IGH	3	None	
7	1664	CAKATNDFWSGYYTPTYYFDYW	IGH	2	None	
7	1667	CAKGSGSGIRHLYYFDYW	IGH	2	None	
7	1668	CAREKWGYSGYDLGTMDAFDIW	IGH	2	None	
7	1669	CMIWHSSASVF	IGL	17	None	
7	1670	CAKDPYASGPNWFDPW	IGH	8	None	
7	1671	CARRGDYGWYFDYW	IGH	4	None	
7	1672	CARDQDDYVWGSSYYFDYW	IGH	5	None	
7	1673	CARHDSWGYNWLDYW	IGH	2	None	
7	1674	CQQLKSHPLSF	IGK	2	C1	7BZ5
7	1675	CAKQRIQKQYFDYW	IGH	2	None	
7	1676	CARRGIRYYGSGSYYQVGAFDIW	IGH	2	None	
7	1677	CASSFVGIYCSSTSCPYDYW	IGH	2	None	
7	1678	CARDSTAMAYSFDYW	IGH	3	None	
7	1679	CARLLTTVTTGGLYFDYW	IGH	2	None	
7	1680	CARQPTPDIVLLVYAIRYYFDYW	IGH	4	None	
7	1681	CTTAGHVVRGVIITFDNPYYFDYW	IGH	5	None	
7	1683	CARVTGHTNGLRGFFDFW	IGH	2	None	
7	1684	CARHHYSYGLRGKKNWYFDLW	IGH	2	None	
7	1685	CARDDYGGIRESGFDLW	IGH	2	None	
7	1686	CARDPLAYGDRHFDYW	IGH	6	None	
7	1687	CAKGGGAYSGSYVDYW	IGH	2	None	
7	1688	CATNKEFCSGGSCSPYYFDTW	IGH	2	None	
7	1689	CARVGDRSGWSPEGRNLLNWFDPW	IGH	2	None	
7	1690	CARDPATYSSSWYLSYYFDYW	IGH	3	None	
7	1691	CARVRRGPGNVFEGFDSW	IGH	2	None	
7	1692	CQAFDNSTALYVF	IGL	3	None	
7	1693	CARAVLWFGDPTPPYYFDYW	IGH	6	None	
7	1694	CARDSGVLDYYGSGSYYRLFDYW	IGH	2	None	
7	1695	CAKGPSTFSLEGIDYW	IGH	4	None	
7	1696	CARGLPHDTYYYNSKTYSNTLLDYW	IGH	2	None	
7	1698	CARVTIHSSWYSGSLDYW	IGH	2	None	
7	1699	CAHQMAGNPFLFDYW	IGH	4	None	
7	1700	CAREPGYSSGWSFGYW	IGH	3	None	
7	1702	CTTDSSDFDFLTGYYYYFDYW	IGH	4	None	
7	1703	CARGAPQIVVVVAVLYYYYYMDVW	IGH	16	None	
7	1704	CARPLYITSSGNSVWDAFDIW	IGH	2	None	
7	1705	CASGYSYGYTIDYW	IGH	9	None	
7	1706	CARDSIRYYYGSGSYQPFDYW	IGH	2	None	
7	1707	CQQTNSLPPITF	IGK	5	C1	7BZ5
7	1708	CARHLRFLIWFDPW	IGH	4	None	

7	1709	CAHSRRYGDYDYW	IGH	7	None	
7	1710	CTGRVWFGESSGPYYYGMDVW	IGH	2	None	
7	1711	CARGVLRSVNWFDTW	IGH	4	None	
7	1712	CAKSGKYYYDSSGYYLFDLW	IGH	4	None	
7	1713	CVHRVSYELRGNYYYMDVW	IGH	2	None	
7	1714	CARGTRQWLVLYRLRHYFDYW	IGH	2	None	
7	1716	CARGDPYDFWSGPPDYW	IGH	2	None	
7	1717	CVRVNPDLNSHFDYW	IGH	2	None	
7	1718	CARARRITMIVVVTNYYFDYW	IGH	2	None	
7	1719	CARVEGVAAAAPPAWYYFDYW	IGH	2	None	
7	1720	CAHRQGSWTKSGFDYW	IGH	3	None	
7	1721	CAKTIKGDYARDAFDIW	IGH	4	None	
7	1722	CARDHSPNDHGPTYYYYGMDVW	IGH	2	None	
7	1724	CARQLYSYGQDAFDIW	IGH	9	None	
7	1726	CAKDGNSSSWYVVRWFDPW	IGH	3	None	
7	1727	CAKDMDGSGSYYLSPFDYW	IGH	3	None	
7	1728	CARGRGYCSGGSCYSSSYYYYYMDVW	IGH	2	None	
7	1729	CARGHARPTLITMFRGVIINPLDYW	IGH	6	None	
7	1730	CARDLGGGTLGDYW	IGH	2	None	
7	1731	CARRVDSSWYGDWFDPW	IGH	3	None	
7	1733	CVRQAFPGYTSFWLPGFDVW	IGH	2	None	
7	1734	CARMWGVTTAIDYW	IGH	8	None	
7	1735	CARQRGRSIAARPGFDYW	IGH	2	None	
7	1736	CVHRQDWNYFSFW	IGH	3	None	
7	1737	CVKDILGYYDSRYISVGYFQHW	IGH	2	None	
7	1738	CAKAPFRATNWYFDLW	IGH	3	None	
7	1739	CQSYDRSLRGVVF	IGL	33	C4	7JVC
7	1740	CARVKVDLVTGFWTQDAFDIW	IGH	2	None	
7	1741	CARHVREDWYFDLW	IGH	4	None	
7	1742	CAKDIVYGSGSYFPYYFDYW	IGH	2	None	
7	1743	CARSGRRITMIVVVTDDNWFDPW	IGH	5	None	
7	1744	CARLARRLTIFGPNDYW	IGH	4	None	
7	1745	CAKDIGFDTAMVTGAFDIW	IGH	4	None	
7	1746	CARDGLRWYQGYFDLW	IGH	3	None	
7	1748	CAKESTPAIFIRPDFFDMW	IGH	2	None	
7	1749	CAKEIGLLYGDYPGYW	IGH	2	None	
7	1751	CARDRAGYYGSGTAFDIW	IGH	3	None	
7	1752	CAREEGGGDCSGAACSVVWYFDLW	IGH	3	None	
7	1753	CARGRTLWFGELLSAFGAFDIW	IGH	4	None	
7	1754	CARVKARETCSGGSCYPFFDFW	IGH	4	None	
7	1755	CYSAADNNLRVF	IGL	230	None	
7	1756	CAKWQGLVVALIDYW	IGH	4	None	
7	1757	CAHMDLLWFGELLCGFDYW	IGH	6	None	
7	1758	CAHRRRDYANYW	IGH	2	None	
7	1759	CARDPSYSNYGDYW	IGH	2	None	
8	1	CAKSGVYYDILTGYYFFDYW	IGH	2	None	
8	2	CARGGDGTSSSGWEYYYYLDVW	IGH	2	None	
8	3	CATMSRYDRSGYKSDYW	IGH	2	None	
8	4	CAKDPHPRPKTSVQGFWFAPW	IGH	2	None	
8	5	CASDAYERLSDDAFDIW	IGH	6	None	
8	6	CARFVALSNSGSYHFDYW	IGH	10	None	
8	7	CARAGDIFSGYYPPFDKW	IGH	2	None	
8	8	CAKGAQTMWDALDIW	IGH	2	None	
8	9	CAKYTIETVTIWGTFDSW	IGH	4	None	
8	10	CARPRWDSGGFYSAFDVW	IGH	13	None	
8	11	CAKVYCSGGSCHYHAFDVW	IGH	3	None	
8	12	CARGRIGGWYLHFDYW	IGH	2	None	
8	13	CVSSYGSRRRYYYMDVW	IGH	2	None	
8	14	CARVLSPPVTTSSYFDSW	IGH	6	None	
8	15	CARDQLRYCSSGSCLTLNYFDYW	IGH	2	None	
8	16	CARERGHSAYDFNGHCGADCYLDSW	IGH	2	None	
8	17	CAKDEGEDVSQGHFDFW	IGH	6	None	
8	18	CVRVGPAGATPGYYYYYMDVW	IGH	6	None	
8	19	CARDIDTSSHYGWFDPW	IGH	2	None	
8	21	CAKDISAYFREAFDIW	IGH	4	None	
8	22	CARASRKGSSSGRLEFDYW	IGH	3	None	
8	23	CVYDILTGYVYYYSMDVW	IGH	2	None	
8	24	CARDSRLTPNEYSSGWTYDALDIW	IGH	2	None	

8	26	CAKDELGYCSAGSCYRGVDYW	IGH	4	None	
8	27	CNSRDDSGFHLNVIF	IGL	4	None	
8	28	CAREGGTGNYYDSSGYFLDYFDFW	IGH	3	None	
8	29	CARGPLWVVMPVARMYSFDIW	IGH	2	None	
8	30	CATRLRLYYDSSGYGGLDAFDIW	IGH	2	None	
8	31	CVLVVTASPDAFDIW	IGH	2	None	
8	32	CARAYATDWYVWYFDLW	IGH	8	None	
8	33	CAVEGGIAAAATGWFDPW	IGH	3	None	
8	34	CARLYWTTGWYGAFDAFDIW	IGH	2	None	
8	35	CQQLGSSHWVF	IGK	20	None	
8	36	CARQTYNKIDYW	IGH	6	None	
8	37	CARGSLWSKYGPASYYYDHW	IGH	3	None	
8	39	CLQATEFPWSF	IGK	75	None	
8	40	CARPDSIHTAYAFDVW	IGH	2	None	
8	41	CARGGGDTAHFDHW	IGH	4	None	
8	43	CAHRPRHFDSGGYYHYYFDLW	IGH	2	None	
8	44	CAKDTSLGIEGYFDSW	IGH	2	None	
8	45	CARDRRPTVTTYRSYGLAFDIW	IGH	3	None	
8	46	CARDITLIRGALWWFDPW	IGH	3	None	
8	47	CQSIDSDGSFLVLF	IGL	2	None	
8	48	CVRECPVYDLFTDSRLCFDSW	IGH	3	None	
8	49	CQQTDTLPSVIF	IGK	4	C3	6XDG
8	50	CARQKAVASYKEYYMDVW	IGH	2	None	
8	51	CARLGDDGDYGTLDYW	IGH	3	None	
8	52	CAITRLGKVFGEFTGYFDNW	IGH	2	None	
8	53	CQQYGSSPGTFGQGTKLGTF	IGK	7	C1	7K8N
8	54	CATVPRGGTYQEAFNIW	IGH	2	None	
8	55	CARGYSAYDYAFDYW	IGH	11	None	
8	56	CARRGYYGSGSYYNVFDAFDIW	IGH	2	None	
8	57	CARQPGELEPFDFW	IGH	2	None	
8	58	CARHPASYFDDRNGYRGENFDYW	IGH	4	None	
8	59	CSREWGYFYQSTTYYRSDSFDSW	IGH	2	None	
8	60	CAKGLTVTTSLDAFDIW	IGH	6	None	
8	62	CVKGGEGAAAGDPFDYW	IGH	2	None	
8	63	CARMYNNKWYLDSW	IGH	2	None	
8	64	CAREPPDSSGYFTEHYFDYW	IGH	2	None	
8	65	CVRRGVGYSSAFDIW	IGH	4	None	
8	67	CARVAHVYDSGGFHFDSW	IGH	2	None	
8	68	CATGMNGRTGGLTKFYIDVW	IGH	2	None	
8	69	CATSTNYADKDWYFDLW	IGH	2	None	
8	70	CARVGSAWGAFCFDPW	IGH	2	None	
8	71	CTSSRPMTFIAVATTYYYSYMDVW	IGH	2	None	
8	72	CARDTIIAAGTTFDYW	IGH	23	None	
8	74	CATSSSSTYYLRTFDIW	IGH	9	None	
8	75	CARGYSYYYFYYLDLW	IGH	2	None	
8	76	CARDYFDSSVYWVPQRPYFDYW	IGH	3	None	
8	78	CAKDIGDWGYYFHSW	IGH	2	None	
8	79	CAATYDISGYREYW	IGH	17	None	
8	80	CARDREGLWPFDAFHIW	IGH	4	None	
8	81	CARSVSEQWLGPFDIW	IGH	2	None	
8	82	CATPPRLDYYDSSDGYHAPFHYW	IGH	3	None	
8	83	CARVISAFRELLASGYWFDPW	IGH	5	None	
8	86	CARLNFYDSSPGEAFDIW	IGH	16	None	
8	87	CAILSRPGWNARFDLW	IGH	3	None	
8	89	CTRYSVEMGTIHAFDIW	IGH	3	None	
8	90	CAKDHNTNFGVAGHGFDYW	IGH	6	None	
8	92	CARGRSFCTSNTCYPHPFDYW	IGH	2	None	
8	93	CARHVEMATINSYYYYYYMDVW	IGH	6	None	
8	94	CAKGLGMNYYYMDVW	IGH	2	None	
8	95	CQQYGSSPPTLGGGTTTF	IGK	2	C1	7K8N
8	96	CAKVRTTLTTGWFDPW	IGH	2	None	
8	97	CARGQIGYCSRTTCYAFDDW	IGH	4	None	
8	98	CARGNLRNYQLYYMDVW	IGH	4	None	
8	99	CARLGEIAVVPTGWGYMDVW	IGH	2	None	
8	101	CARGPSNKIVAPGFDSW	IGH	2	None	
8	102	CARGGYCSGGSCSPDDYW	IGH	6	None	
8	103	CAREAEGVGVGHFYYMDVW	IGH	6	None	
8	106	CARDLFYYGSGTYIYYFDHW	IGH	9	None	

8	107	CARDGIVNGAFDIW	IGH	7	None	
8	108	CARDVDTAMVMAFRAFDIW	IGH	2	None	
8	109	CARDGSGYSFHYW	IGH	12	None	
8	110	CTRVGRESESSGITIFGVVTKSHYFDYW	IGH	4	None	
8	111	CARLCTIAVAGLAFDYW	IGH	2	None	
8	112	CTRDLPGVMEHYFDSW	IGH	4	None	
8	113	CATDIPGFSSETFDIW	IGH	2	None	
8	114	CARGLVDTALDYWYYMDVW	IGH	2	None	
8	115	CARPIDMSTITEGFDIW	IGH	2	None	
8	116	CAHRPGGFWSGYYGYW	IGH	2	None	
8	117	CSSAGTVFHYFDYW	IGH	2	None	
8	118	CARDPMHDTSGRDYFDYW	IGH	6	None	
8	120	CARDLDTNSRYSWLDPW	IGH	2	None	
8	121	CARERNDGISWYYFAFW	IGH	2	None	
8	122	CGRDYGVSLGVDYW	IGH	2	None	
8	123	CQQYNNWREGLAF	IGK	3	C3	7K8P
8	124	CARHPLAWFGEFPHYFDYW	IGH	2	None	
8	125	CARENLDTSRPFEYW	IGH	9	None	
8	126	CAKVSYNVLTGYFLPQYYYYYSMDVW	IGH	4	None	
8	127	CLQRTHWPWTF	IGK	148	None	
8	129	CVRHGPREYCSGGCLEYFDIW	IGH	3	None	
8	130	CATDSATVTTWDAFDIW	IGH	13	None	
8	131	CAKDLLIQLWLWDYW	IGH	2	None	
8	132	CTRRLPGNNSGWTFDFW	IGH	2	None	
8	133	CASPNFYDSSGYPGYW	IGH	3	None	
8	134	CARDRFPAPGSWYEKVFDNW	IGH	4	None	
8	136	CARDRNYFDNSASYPHYW	IGH	4	None	
8	137	CARDGDRSARGYCSSTSCYAFDIW	IGH	3	None	
8	138	CAHRIANFWSGYFDYW	IGH	3	None	
8	139	CAKEWKTGEKAFDIW	IGH	7	None	
8	140	CATYCGGDCFNDGFDLW	IGH	2	None	
8	141	CATTLRVYSTNWYSFDYW	IGH	9	None	
8	142	CRQSYSPPYNF	IGK	2	C2	7K8O
8	144	CQQLYTYPPLHF	IGK	6	C1	7BZ5
8	145	CARHAGATLVGLDYW	IGH	2	None	
8	146	CARINAEVTQFDPW	IGH	3	None	
8	147	CAHRRNYDMAFPFDPW	IGH	4	None	
8	148	CVREDTELVFGYYLDLW	IGH	211	None	
8	149	CARYHFYDSSGYWIDAFDIW	IGH	16	None	
8	150	CISILPHTSSHNYMDVW	IGH	6	None	
8	153	CTTDPGGNFYYYNNSGSYYFDYW	IGH	3	None	
8	154	CASGPIKYSSGLTYFDSW	IGH	11	None	
8	155	CARAGLAAVAGTASFDFW	IGH	2	None	
8	156	CARYSSASLISFDYW	IGH	11	None	
8	157	CARFLYSGSGNYYPYYFDYW	IGH	5	None	
8	158	CARVDSSGYSLMDDAFDIW	IGH	2	None	
8	159	CVKDMHEGDGFDIW	IGH	8	None	
8	160	CARGLLLVEPTQAFDYW	IGH	2	None	
8	161	CARTVDYGSWNQFDYW	IGH	14	None	
8	162	CQTWGTGIWVF	IGL	116	None	
8	163	CARTPWNQIDPSYFDYW	IGH	3	None	
8	164	CAKDMRDGYNDDFFAFHIW	IGH	2	None	
8	165	CARALLEWLFSAPVINWFDPW	IGH	2	None	
8	166	CASGLERDFIQPEAFDIW	IGH	8	None	
8	167	CVREAYYTLWTGYRRTGLNNWFDPW	IGH	2	None	
8	168	CAHKAYYGVLTGYSKYNWFAPW	IGH	20	None	
8	169	CVRDDRGSSGWHFDNW	IGH	2	None	
8	170	CARTPSLWFGESLLYYFDYW	IGH	4	None	
8	171	CARGPYYYKRAMPFDSW	IGH	2	None	
8	172	CARESSRDYYYDSSGYYSNPYYFDYW	IGH	2	None	
8	173	CARDFLYCATISCHHDYW	IGH	2	None	
8	174	CQVWNSSIDHSYLF	IGL	2	None	
8	175	CAKHGGLDPRSYMDVW	IGH	4	None	
8	176	CAKERPEYNGFDSHFFDYW	IGH	8	None	
8	177	CARAWFGYARFDYW	IGH	6	None	
8	178	CAKYGAVIRYSYGHFDYW	IGH	6	None	
8	179	CARATSYDYSGSPHDAFDIW	IGH	2	None	
8	180	CARGVIRGVTEIDYW	IGH	3	None	

8	181	CAKIGSYYSGRIPDYW	IGH	5	None	
8	182	CAATIRGKDYGDYRDFDYW	IGH	2	None	
8	183	CARQLKLLDEDYYMDVW	IGH	4	None	
8	184	CARVSVGKHYSGYDLDYW	IGH	7	None	
8	185	CARDENNNYYFDYW	IGH	25	None	
8	187	CVASLSIDWTRYDYW	IGH	2	None	
8	188	CARGPRSYYGGIYYFDYW	IGH	2	None	
8	189	CARGDSIFGVIRRFDPW	IGH	4	None	
8	191	CARGPGRPDVYDSSGYQHYW	IGH	4	None	
8	193	CAAFSEVWEQCEETPSDNFGYW	IGH	3	None	
8	194	CAKLSDFLSDFDFW	IGH	2	None	
8	195	CAKKSTGGLDNGDDAFDIW	IGH	5	None	
8	196	CAAATDYFDSSIHNYFAPW	IGH	5	None	
8	197	CSSFTSSSTRDVVF	IGL	2	C2	7K90
8	198	CVKDLLHWGPDYW	IGH	52	None	
8	199	CARVPVLRRQWLDYFHHYMDVW	IGH	2	None	
8	200	CAKDNEYLVEMPSVSYYYYYMDVW	IGH	2	None	
8	201	CAREAYTYGYVWFDPW	IGH	2	None	
8	202	CARIRPVTGMHYFDYW	IGH	2	None	
8	203	CARLGWYESWYFDLW	IGH	2	None	
8	205	CSRDWLHIGTWQFDFW	IGH	2	None	
8	206	CARQEWELPTTEYYFDYW	IGH	2	None	
8	207	CARDQGGYYPDSSGYYSHTFDYW	IGH	2	None	
8	208	CTTFRVTMVRGVIRGDYW	IGH	2	None	
8	209	CAVVPAAMSDYW	IGH	5	None	
8	211	CARISRYDTRTFDYW	IGH	3	None	
8	212	CARTKGAGYKGFDSW	IGH	2	None	
8	213	CVHYPGSGRFDPW	IGH	2	None	
8	214	CQHYGTSPRSSF	IGK	21	C2	7K8U
8	217	CATGAGYTSSRFDYW	IGH	2	None	
8	219	CARASIQSRYWFFDLW	IGH	2	None	
8	220	CAKDAFTTFGEQGYFDPW	IGH	4	None	
8	222	CARVKYSYDSSGPSNYAFDIW	IGH	2	None	
8	224	CQSYDGRLSGPYFF	IGL	4	None	
8	225	CARYPSMIVMAGLDYW	IGH	2	None	
8	226	CAHSSGYSGYDKKFDYW	IGH	26	None	
8	227	CARDGGPTYYYGTGSYYPFDHW	IGH	2	None	
8	228	CATDAPGFADYASFAYW	IGH	3	None	
8	229	CARTHSSGWYYFDYW	IGH	75	None	
8	230	CARGRASGEQFFDQW	IGH	3	None	
8	231	CAKGGYGSGSYFLTDYW	IGH	2	None	
8	232	CAGWGYSGYDKYFQHW	IGH	4	None	
8	233	CARDVAYLNYYGFTDHYSGCDYW	IGH	3	None	
8	234	CARVNKGHYYDTSAYDYIDYW	IGH	5	None	
8	235	CTSRGSTYSAYDPSFDFW	IGH	2	None	
8	236	CAGSVAAAGRYFEHW	IGH	2	None	
8	237	CAKEAMIVVAYREYFDYW	IGH	2	None	
8	238	CARDGDSFAANYYYYYLDVW	IGH	4	None	
8	239	CARGWSETPDSSSSYMDVW	IGH	2	None	
8	241	CARGDWNDRDAFDIW	IGH	5	None	
8	242	CVKDSANYYDSSRFLFDYW	IGH	3	None	
8	243	CANLGAYYISDYW	IGH	435	None	
8	244	CQQGYNTPPWMF	IGK	19	C4	6W41
8	245	CSSYAGNNNLIF	IGL	38	C2	7BWJ
8	246	CIIYNYESSDVFDIW	IGH	4	None	
8	247	CARWGTLAGNLDFDYW	IGH	3	None	
8	248	CVKDSSGGGLKWFFDLW	IGH	2	None	
8	249	CQQSNSVPSLAF	IGK	4	None	
8	250	CATHGVLRYFDWLLHSGYFDYW	IGH	3	None	
8	251	CAKEHPPFGELFEYYFDYW	IGH	2	None	
8	252	CAREGALNKYYYDSSGYSISYYFDYW	IGH	2	None	
8	253	CQQLDSYLHPLTF	IGK	78	C1	7BZ5
8	254	CARAMVRGVNGWFDPW	IGH	4	None	
8	255	CARQDDGSSWYIFDYW	IGH	4	None	
8	256	CARVGSGKRGYDFYMDVW	IGH	2	None	
8	257	CARDPADSSDYYYYYMDVW	IGH	2	None	
8	258	CARLTKDILTGYYSQYYYYMDVW	IGH	2	None	
8	259	CARELSYYYDTGGYYYGYFQHW	IGH	2	None	

8	260	CVREVWYFGLW	IGH	2	None	
8	261	CARLPLNYFGSGSYNYYFDYW	IGH	2	None	
8	262	CAAPGSGQLLCFGYW	IGH	2	None	
8	263	CQQHDSFPPRF	IGK	23	C3	6WPS
8	264	CARRGGYYYDSGRPAGWFDPW	IGH	2	None	
8	265	CAKGQVEMTTMTFDDW	IGH	8	None	
8	266	CAKNTMILGSKRWLIFDYW	IGH	2	None	
8	268	CARENNFWSVPTSGPSGLSYYYYIDVW	IGH	2	None	
8	269	CARDDAPGGNVFDFW	IGH	2	None	
8	270	CASHQMLATEGIFYYFYYMDVW	IGH	2	None	
8	271	CARSFYSDSETFRKITSNWFDSW	IGH	36	None	
8	272	CAGTTVTTYWYFDLW	IGH	8	None	
8	273	CAKPGWGIAAAGGAFDFW	IGH	2	None	
8	275	CARDSSRSPGSYVFDYW	IGH	3	None	
8	276	CASPDQWGVFIDYW	IGH	2	None	
8	277	CARHLIPGSSFSDIFDFW	IGH	2	None	
8	278	CARNAGPQSWGSYRRYYYMDVW	IGH	8	None	
8	279	CAHRSALYGLDYW	IGH	2	None	
8	280	CARESDLHSGYDSYYIDYW	IGH	4	None	
8	281	CMQHTDWPRTTF	IGK	3	None	
8	282	CAKAHETTSLMLRGAHFDYW	IGH	32	None	
8	284	CARGPEYTGELRDIWTLDYW	IGH	4	None	
8	285	CARHGGATWLSEYYFDYW	IGH	3	None	
8	286	CARDSSSSGWSGGAYDIW	IGH	2	None	
8	288	CARDPSIGLNGAFDIW	IGH	2	None	
8	289	CARGQWGQWLHDGEYYFDYW	IGH	2	None	
8	290	CARDRFTSTWTTVTRFDYW	IGH	2	None	
8	291	CARDSVYESSVCHYYFDYW	IGH	2	None	
8	292	CARAYDTNGFITEYFQYW	IGH	4	None	
8	293	CVRDSETSGSQGDAFDIW	IGH	2	None	
8	294	CAKEKTVYDSPFDYW	IGH	20	None	
8	295	CAKDRSTYKSGWFAFDNW	IGH	6	None	
8	296	CARDRDYESSDYYPRYLDLW	IGH	14	None	
8	297	CARDGHSSGWSPVFDSW	IGH	6	None	
8	298	CAKDINRYSDFDYYFDSW	IGH	3	None	
8	301	CAKDAGYHYDTNRRSQPAHYFDHW	IGH	6	None	
8	302	CARHPNYLWFGESKFDYW	IGH	2	None	
8	303	CARSPSRVTVIVRSSRYYFDYW	IGH	2	None	
8	305	CARISSRITGTASDAFDIW	IGH	6	None	
8	306	CASPWGSSGYYRYFQHW	IGH	2	None	
8	307	CAKETMVAATYDAFDIW	IGH	5	None	
8	308	CARLPHYYGAGSYYPVDYW	IGH	3	None	
8	309	CAICFGGHYYFDYW	IGH	3	None	
8	310	CARTQSHHLIAAAGMLNTFDTW	IGH	3	None	
8	311	CARVQSVYDYDAFDVW	IGH	78	None	
8	312	CARDATGLVYHYYYYMDVW	IGH	3	None	
8	313	CARVNYLDSTGSDYW	IGH	3	None	
8	314	CAREGGIAVAGTDYLDYW	IGH	2	None	
8	316	CATFREYQLLARYWYFDLW	IGH	2	None	
8	317	CVRGREYGDFRGDYW	IGH	4	None	
8	319	CAKDSSRSFGFCSTPDCYLNAFDFW	IGH	6	None	
8	320	CATTLGYRVVDDLLTGYYFDNW	IGH	2	None	
8	321	CAKSVPGTIWYYYYYMDVW	IGH	4	None	
8	322	CTTDGGSSTNDYW	IGH	2	None	
8	323	CAKASWAGYDSSGYQYFEYW	IGH	12	None	
8	324	CARLRYSYADDAFDIW	IGH	34	None	
8	325	CARRFLVGALGYYFDYW	IGH	3	None	
8	327	CSRHEHTDSTGYYILPNWYFDLW	IGH	4	None	
8	328	CARDSGPCSGGTCLFDSW	IGH	4	None	
8	330	CAKAAEATTNMVRGAHFDYW	IGH	16	None	
8	331	CARSKQGKWLQSHWGNWFDPW	IGH	2	None	
8	332	CALPISAVYYCARDRGFCTGGECYSSYMDVW	IGH	2	None	
8	333	CARDGPPSAYDYPRLFDYW	IGH	4	None	
8	335	CAARRFTAMAFFDYW	IGH	2	None	
8	336	CAKGSYGSRRKYYFDYW	IGH	2	None	
8	337	CANRHQQLAMFDYW	IGH	2	None	
8	338	CVKDVYVIRGNDYSPHDDYW	IGH	3	None	
8	339	CARTTVTTFREYYNWFDPW	IGH	4	None	

8	340	CARHAFYCSDGSCLHYLDYW	IGH	7	None	
8	341	CQSYDNGLSGSGSVF	IGL	2	C3	6XDG
8	343	CQSYDTSLSASGIF	IGL	11	C4	7JVC
8	344	CVRQPREGGSYYFRVSDYW	IGH	2	None	
8	347	CARQETRSPQQFDYW	IGH	2	None	
8	348	CVKGALRDIVATIGPQMDYW	IGH	2	None	
8	350	CAKVQQWPRRHFDYW	IGH	2	None	
8	351	CVRDGEGLLSSSNAFDLW	IGH	2	None	
8	352	CVRDDFIGWFDPW	IGH	2	None	
8	353	CARDGVGYVDSIDALDMW	IGH	2	None	
8	354	CASALTTITFESFDYW	IGH	2	None	
8	355	CAKKIRITSYLDNW	IGH	2	None	
8	356	CASDRWYKSDRSGYYYEDYW	IGH	8	None	
8	357	CARDLGDDGLDIW	IGH	4	None	
8	359	CARHRGIGATVTEWYFDLW	IGH	2	None	
8	360	CARHLPSYPHYDESSRSIGSYFDFW	IGH	4	None	
8	361	CQKYGFSPPTYSF	IGK	2	C1	7K8N
8	362	CARDRDNRLFYDSSGGFDYW	IGH	6	None	
8	363	CQTYDSNLSGFWVF	IGL	2	None	
8	364	CVRDHWDQYAGGGQNYWYFDLW	IGH	4	None	
8	365	CAREEVDSGYDFDPW	IGH	3	None	
8	366	CARERRYYDSSGYYYVSYYFDYW	IGH	2	None	
8	367	CVCYYESSGYYLDNW	IGH	2	None	
8	368	CVGSSSSSSGWFDPW	IGH	6	None	
8	370	CARSSGWSSGSFDIW	IGH	5	None	
8	371	CARLDERNDLSFYYYMDVW	IGH	2	None	
8	372	CARRLYSGSWYDWYFDLW	IGH	2	None	
8	373	CARRAYVREVPYYYYYYMDVW	IGH	4	None	
8	374	CAKGSDAIFNWFDPW	IGH	6	None	
8	375	CARGSRWESFDYW	IGH	4	None	
8	376	CATHDQSYDMLTGFDPHYYFDYW	IGH	2	None	
8	378	CARDGLRYFDWSPRDAFDLW	IGH	2	None	
8	379	CARAGWSYDILTGYYKEPFDYW	IGH	4	None	
8	380	CASEDKYCSSTSCYGFDYW	IGH	2	None	
8	381	CVRGFYYDTSGYYEYW	IGH	2	None	
8	382	CARGVQSSSWPPASDYW	IGH	4	None	
8	383	CARLTTVTTSSAFDIW	IGH	3	None	
8	384	CAKDLWWELLLNFDYW	IGH	4	None	
8	385	CVKDQEWRRLFEAFDVW	IGH	4	None	
8	386	CARAVFNWNYWYMDVW	IGH	4	None	
8	388	CGRRPSSGTWYFDYW	IGH	2	None	
8	389	CARERMKNYDPLTGYFLPNWFDPW	IGH	4	None	
8	390	CARAGNSGYPIDYW	IGH	6	None	
8	391	CARTYNIYSAYCGGDCYSGFDYW	IGH	10	None	
8	392	CARLGCSAGSCWEGLDYW	IGH	2	None	
8	393	CVNDRGWLTFDSW	IGH	57	None	
8	394	CARAYCGGDCYSGAFDIW	IGH	2	None	
8	395	CARHSTAMVVGFLLPQYSYYYMDVW	IGH	2	None	
8	396	CARMTGIVVARAFDYW	IGH	2	None	
8	397	CARYGEEVFNPSFWHFDLW	IGH	2	None	
8	398	CAKDFGLDYSGNFFDYW	IGH	2	None	
8	399	CAKVSYLSTASSLDYW	IGH	31	None	
8	400	CATLTSGAGEYYNFWNGKDYW	IGH	3	None	
8	401	CQQRAYWPQVTF	IGK	2	None	
8	402	CARGENFWSGYRPFDYW	IGH	4	None	
8	403	CAKDGSGSDYNVPADFDYW	IGH	2	None	
8	404	CSSRDSSGDHRHVLF	IGL	6	None	
8	405	CARRLRSSALGARDSFDIW	IGH	8	None	
8	408	CARLGYGGPASRDYW	IGH	2	None	
8	409	CARDGSSDYFQIW	IGH	3	None	
8	411	CARSCDTIFGVVISCFDYW	IGH	2	None	
8	412	CAKTDALYQWLNKDYYFDYW	IGH	2	None	
8	413	CAGDTLMLNAFDIW	IGH	4	None	
8	414	CAKGAAAGTRGNADWFDPW	IGH	2	None	
8	415	CARERDTSGYYFFDTW	IGH	2	None	
8	416	CARGGDSTDNDRFYYYYYMDVW	IGH	10	None	
8	417	CATDLRGGYYDYW	IGH	12	None	
8	418	CASPNPTNYYDSSGYYMDYW	IGH	2	None	

8	419	CARHNFCSGGTCNGLRAFDIW	IGH	2	None	
8	420	CATTNRVTSCGGDCPPDYW	IGH	2	None	
8	421	CASRSPPSEQWLVLEGAFDIW	IGH	6	None	
8	422	CVRGSPLHGDYVGYW	IGH	2	None	
8	423	CARDLLSVFANNDIMTGPLDYW	IGH	2	None	
8	424	CARVIEGGSHFEAFDIW	IGH	2	None	
8	425	CAKMIAGAVAGTQSAFDIW	IGH	6	None	
8	426	CARAGTTFWNFDYW	IGH	2	None	
8	427	CAKVARITMIVVVIREYNAFDYW	IGH	2	None	
8	428	CSTDPSIFYYYYYMDVW	IGH	2	None	
8	429	CARGKDIWAIDSSASGQYFQHW	IGH	3	None	
8	430	CAKLRGYCSSSTCLNFDYW	IGH	6	None	
8	431	CVSTLNSESSGYYSSFDFW	IGH	37	None	
8	432	CARDGDYYDSSGSPYYFDYW	IGH	15	None	
8	433	CARDGYFHSSGYPGYW	IGH	2	None	
8	434	CARLRHRYSITSYRSFDIW	IGH	2	None	
8	436	CARHPRLQFLEWEFDYW	IGH	2	None	
8	438	CATRGAGIVPYCSDYW	IGH	2	None	
8	439	CASYYSDRSGHYYGYFGYW	IGH	8	None	
8	440	CVRAPVRGLIINAFDIW	IGH	9	None	
8	441	CARRSGIYYYDSSGYYTYYFDYW	IGH	2	None	
8	442	CARGPYSRGSWFDSW	IGH	2	None	
8	443	CARIIPTRGSNSWYYFDYW	IGH	2	None	
8	444	CARDYFSSWNLYDYW	IGH	2	None	
8	445	CGRTSPPGVQLWKAFIDYW	IGH	3	None	
8	446	CARDFLMGASDNFDYW	IGH	2	None	
8	447	CVKDRELIFWSGFAFDIW	IGH	12	None	
8	448	CAREGDYYGSGTYYRPYYFDYW	IGH	2	None	
8	449	CARLMGRVDAFDIW	IGH	2	None	
8	450	CVKDRQCVSMACHGGFEYW	IGH	2	None	
8	451	CVRRSERYFDGTTYYFDSW	IGH	2	None	
8	453	CAKPDRMIRGVADAFDIW	IGH	2	None	
8	454	CAREVRTGTPGGDFYFYMDVW	IGH	6	None	
8	455	CVKGPDNYYHDDSGYRDDAFDIW	IGH	3	None	
8	456	CARDLSYYDILTGYTGPETTNWFDLW	IGH	8	None	
8	457	CQQLNSYREFTF	IGK	708	C3	7K8R
8	458	CARDMNSACHNPGDYW	IGH	2	None	
8	459	CARGYLELRDAFDVW	IGH	3	None	
8	460	CARWIRPVDTQLLDAFDLW	IGH	6	None	
8	461	CAREYYDSSGYSYDAFAIW	IGH	2	None	
8	462	CARHHRISTMIGVATGGWFDPW	IGH	4	None	
8	463	CAKLRRIGYHYYIDVW	IGH	4	None	
8	464	CARTSRDDYNYDAFDIW	IGH	2	None	
8	465	CHKYGSSPPSLTF	IGK	4	C1	7K8N
8	466	CARHGRFGDLLYYFFDYW	IGH	6	None	
8	467	CASLTYYYDITGSIADYW	IGH	10	None	
8	468	CARDWGTEMGDYW	IGH	2	None	
8	469	CARGNSVVVPAALGFGFYYYYYMDVW	IGH	8	None	
8	470	CARDPYSSSGSYWFDPW	IGH	4	None	
8	471	CARDPEHYNFWSGWFGRQAFDIW	IGH	4	None	
8	472	CARADSHFDWLLSFDSW	IGH	2	None	
8	473	CARVELQSWYFDYW	IGH	5	None	
8	474	CARTGDYGGNSGIVDYW	IGH	3	None	
8	475	CQRYDNIPPEFTF	IGK	11	C3	6XDG
8	477	CARVPRQCGGGPCYYFDDW	IGH	4	None	
8	478	CARLYYFDTTGLYYPPGYFDNW	IGH	4	None	
8	479	CARDGTGAAMDVW	IGH	2	None	
8	480	CQSIDSSFTHHVIF	IGL	2	None	
8	481	CARNYAGPYYDILTGYYLDFW	IGH	6	None	
8	482	CARHSGYFGSGHNWFDPW	IGH	2	None	
8	483	CQHANAFPVF	IGK	5	None	
8	485	CATGYHYNGPKHPSYMDVW	IGH	2	None	
8	486	CARHHPGHTSGPRYFDYW	IGH	2	None	
8	487	CALPISAVYYCARHGNGRPFDYW	IGH	2	None	
8	488	CAKDMSVDGTLEAGAFDMW	IGH	2	None	
8	489	CARASGSYYDVHFDSW	IGH	2	None	
8	490	CARALPDILTGGVIFDYW	IGH	2	None	
8	491	CARELDTANIYYYYMDVW	IGH	2	None	

8	492	CVKGENRVFDIW	IGH	2	None	
8	493	CMQGTHWPPSFTF	IGK	36	None	
8	494	CARLKVYTGYDFDDYW	IGH	3	None	
8	496	CARGGPPLYSSGPSDWFDPW	IGH	2	None	
8	497	CARHRARGSLVDYW	IGH	6	None	
8	498	CAHMDYYDSSGYVRLDAFDIW	IGH	3	None	
8	499	CAKGAVLPFLEWLAEYFNHW	IGH	6	None	
8	500	CAKVSQEDIVLVRVAQYYFDYW	IGH	2	None	
8	501	CARYQVLTIFGVVQTPGYFDYW	IGH	3	None	
8	503	CATFYSGRGVDDAFDIW	IGH	4	None	
8	504	CARGPQYNDRSGYYHPYFDYW	IGH	8	None	
8	505	CAKDSSEYYYYMDVW	IGH	13	None	
8	506	CARHPLRDYTSPYYFDFW	IGH	2	None	
8	507	CARPLDESGNYFGYW	IGH	2	None	
8	508	CCSYAGSSTSTSDWVF	IGL	4	C2	7K8X
8	509	CARVFIMDGGVIRGFDPW	IGH	12	None	
8	510	CARDPGIAATGTRQWFDPW	IGH	2	None	
8	511	CARDLAFDIW	IGH	22	None	
8	512	CQQYISYPLAITF	IGK	4	C3	7K8P
8	513	CLKDFGPYYNRWSGHYFDSW	IGH	2	None	
8	514	CARDEDFVVIPATIGFDHW	IGH	4	None	
8	515	CARSQWFGDYHNWFDPW	IGH	5	None	
8	516	CARGSGWYGGVVDYW	IGH	4	None	
8	517	CARAESYYYDSSGYYYTENDYW	IGH	4	None	
8	518	CARRWGSWYRETGYYFDYW	IGH	10	None	
8	519	CAKDRATYISSCFDYW	IGH	2	None	
8	520	CASQSVDHYFGSGFDYW	IGH	2	None	
8	521	CTTSSAWHGRLDYW	IGH	2	None	
8	523	CARHGRDYSNWDYFDYW	IGH	2	None	
8	524	CAAGRVGTTTAFDYW	IGH	8	None	
8	525	CARESSGWELLRYFDNW	IGH	2	None	
8	526	CATMMGAEYSSGPGNWFDFW	IGH	2	None	
8	527	CAKGGGPQILTGYLRPFDFW	IGH	2	None	
8	528	CTSSHDYSNHDVFDIW	IGH	2	None	
8	529	CARHHSINYADYFHHW	IGH	4	None	
8	530	CARHSYYDFLTGYSPFDPW	IGH	2	None	
8	531	CHQDNNWPPEFTF	IGK	11	C1	7BZ5
8	532	CATTFFDLLTGVSHNYYMDVW	IGH	2	None	
8	533	CSTGPDLDVSGYRESFLFDYW	IGH	9	None	
8	534	CAKGLREMATIPFFDYW	IGH	2	None	
8	535	CARELRDYNFWSGQGPMDYW	IGH	2	None	
8	536	CARGKDFIYYMDVW	IGH	2	None	
8	537	CARTHSTSWPFFAYW	IGH	4	None	
8	539	CAKDHGSGDFQIKDFWYFDLW	IGH	3	None	
8	540	CARDLRSGYSPFYSYYYMDVW	IGH	2	None	
8	541	CARVSVAAAGTSWFDPW	IGH	2	None	
8	542	CAHWGGDRTYYLGYW	IGH	13	None	
8	543	CARGTTVIMGFDFW	IGH	2	None	
8	544	CLQHDNFPPLHTF	IGK	6	C3	6XDG
8	545	CARGRDCSGGSCYSPWFDPW	IGH	2	None	
8	546	CATARATYDNFDYW	IGH	2	None	
8	547	CARVSGFCYSTSCYVDYW	IGH	2	None	
8	548	CARQDYNTLTTGFSPTVFDYW	IGH	4	None	
8	549	CKTEVFEFLEWLEDYW	IGH	4	None	
8	550	CQQDYNYHRTF	IGK	4	None	
8	551	CARGGLFGGHSDSDDFYHYYMDVW	IGH	2	None	
8	552	CQVWDNTSDHYVF	IGL	577	None	
8	553	CAKVPGYDSNGYLYYFDSW	IGH	4	None	
8	554	CARQGDQSLFRRGWLYCFDYW	IGH	3	None	
8	555	CARWAGVVPNFDSW	IGH	3	None	
8	556	CARRPIVLMVYAHFDYW	IGH	3	None	
8	557	CAKDPFPHNILTGFSAHGDNYFDYW	IGH	2	None	
8	558	CAKGGGLRYFDWLQNNWFDPW	IGH	2	None	
8	559	CAKDISDSSGFYSFDSW	IGH	2	None	
8	560	CAREGGYYHDSNTYYYPVFFDYW	IGH	4	None	
8	561	CARDYDTLTGYWFDSRAFNIW	IGH	2	None	
8	562	CARIPTRYNNRWYWYFDSW	IGH	2	None	
8	563	CARARRYFGEYAFDYW	IGH	8	None	

8	564	CARGISAWSYFDYW	IGH	16	None	
8	565	CARGDSSGWYGPDAFDIW	IGH	2	None	
8	566	CAKNLGRTFDIW	IGH	2	None	
8	567	CYSTDGSGNPVF	IGL	13	None	
8	569	CARQQQLVYQGAFDIW	IGH	2	None	
8	570	CARGVYYYDRSGSYYGEFDHW	IGH	2	None	
8	571	CAREGNSGNWYYFDFW	IGH	2	None	
8	572	CAKGACRGGTCYDRTYHYDYYMDVW	IGH	3	None	
8	573	CATHYYDSSGGAYFFDFW	IGH	2	None	
8	574	CQHYNPYSPNSSSANTF	IGK	3	None	
8	575	CAKGRGSMTEGYFDYW	IGH	2	None	
8	576	CARSPSLYYDILTGSIIGGFFDYW	IGH	6	None	
8	577	CARDLYDFSTGWEQDAFDIW	IGH	2	None	
8	579	CAKARGDPDKLISFGDLIFDWW	IGH	2	None	
8	580	CARVPSWLRASKNYYMDVW	IGH	2	None	
8	581	CVRGSLGTLLSYSDSFDIW	IGH	2	None	
8	582	CARHRSAIPGTRSDYYYYIDLW	IGH	12	None	
8	583	CARNMLTYYNFDGSYYYYSMDVW	IGH	4	None	
8	585	CAKRNTMILGYREYNWFDPW	IGH	2	None	
8	586	CARQDFSGITIFGVVTPRGWFDPW	IGH	2	None	
8	587	CAKASTSVVTLDYYYYMDVW	IGH	2	None	
8	588	CATDIQKYYNNSNGYYYNYW	IGH	6	None	
8	589	CVKGSGRYLLGYSFDYW	IGH	2	None	
8	590	CASGRHYYYDSSEPDFDLW	IGH	4	None	
8	591	CARWDYGLGPNRNRFDPW	IGH	2	None	
8	592	CVNGRQRYFDWLLTDDFDIW	IGH	2	None	
8	593	CAREYTYIHNSVGYAIFDEW	IGH	2	None	
8	594	CVRGMGAAVPGLFDFW	IGH	3	None	
8	595	CARRVYCGGDCTRLNWFDPW	IGH	2	None	
8	596	CARRPRGSPGLDYW	IGH	3	None	
8	597	CQSYDSSLVNSPVVF	IGL	17	C4	7JVC
8	598	CAGWSVYFKWSSNYWFDPW	IGH	2	None	
8	600	CARGTWVVAGTPIYYYYMDVW	IGH	2	None	
8	601	CARDGTPRYYDSSGYYYGVLDYW	IGH	4	None	
8	602	CASSAVDMTSIREYYLDYW	IGH	4	None	
8	603	CARVDLYTRVGAFDIW	IGH	7	None	
8	605	CVRRWQVQPRLFDYW	IGH	2	None	
8	606	CARLYCGSDCKYFDLW	IGH	2	None	
8	607	CGSDHYYGSGVTEDFDIW	IGH	2	None	
8	609	CQAWDNSAAVF	IGL	26	None	
8	610	CAKVPYGGNTGGLFFYSYMDVW	IGH	2	None	
8	611	CATLRQSYDILTGYSPRYYFDNW	IGH	7	None	
8	612	CARRKLVATAPHFDSW	IGH	2	None	
8	613	CARDVDAYGRDYW	IGH	3	None	
8	614	CARHVRTYYYDSGTYKPYCYYMDVW	IGH	5	None	
8	615	CAREGGYYDSSGYELDYW	IGH	6	None	
8	616	CARGHNYDISGFVFDYW	IGH	2	None	
8	617	CARSWGTTGSRNYFDTW	IGH	4	None	
8	619	CARTNGDYLRHGFDIW	IGH	5	None	
8	620	CARDGVLRHFDDYYYYIDVW	IGH	3	None	
8	621	CGRAAFFYDYSGPNPPYYYYMDVW	IGH	2	None	
8	623	CSLKPEDTAVYYCATEENLLDYW	IGH	2	None	
8	624	CARRLDAPELNSAFDVW	IGH	2	None	
8	625	CARNRRQWLVNDAFDIW	IGH	2	None	
8	626	CARLFRENSRYFDYW	IGH	2	None	
8	627	CARHYYDSSGDAFDIW	IGH	16	None	
8	628	CVRDNWGIDYW	IGH	8	None	
8	629	CARLRFLLLWFGESRYYFDYW	IGH	2	None	
8	631	CARAAWGMATYYFDYW	IGH	2	None	
8	632	CARQHGDVTEFNWLGPW	IGH	6	None	
8	633	CAKEIRPNDYW	IGH	15	None	
8	634	CARLPRASCGGDCYSELGDAFDLW	IGH	2	None	
8	636	CARGQVERRVSLETGHSYPSTRGWFDPW	IGH	2	None	
8	637	CQQYNSYFISF	IGK	1075	C3	7K8R
8	638	CAKADSMEDAFDIW	IGH	2	None	
8	639	CARGLLSWREYQLLSDWFDPW	IGH	4	None	
8	640	CARMRSEITFGGAVLDYW	IGH	2	None	
8	641	CARIGVPAGSYYQYYHMDVW	IGH	3	None	

8	642	CARTYDSSGYYLSYYFDYW	IGH	2	None	
8	643	CAKDSSGYSVRFDYW	IGH	2	None	
8	644	CAHRADYSSAWYKTFDSW	IGH	2	None	
8	645	CARERDNKGLDYW	IGH	2	None	
8	646	CAKQFDGLDYW	IGH	4	None	
8	647	CARGGYGNWYPYFFDYW	IGH	2	None	
8	648	CARREVASAEYFHHW	IGH	24	None	
8	649	CARDYDPTEHYLFDYW	IGH	2	None	
8	650	CATDGSVTTMGHKYNWFDPW	IGH	2	None	
8	651	CVKDGGLLWFGDPKADAFDIW	IGH	2	None	
8	652	CARSGLGLCLGVTCPDAFDIW	IGH	8	None	
8	653	CARHDYDILTAYVFNWFDPW	IGH	4	None	
8	654	CAKGSARITGTFLFDYW	IGH	2	None	
8	655	CARLNNYDSIGYYCDYW	IGH	2	None	
8	656	CAKDKGDSRGYAFDFW	IGH	2	None	
8	657	CARGYYISGDTEAFDLW	IGH	8	None	
8	659	CARGKYSSGWYSADYYYYMDVW	IGH	4	None	
8	660	CARGGSGGYSYGFDHW	IGH	3	None	
8	662	CARGGYVWGSYRALDYW	IGH	6	None	
8	663	CMQALQTPYTF	IGK	2070	None	
8	664	CARRQSGASSPRPFDIW	IGH	4	None	
8	665	CARDRRMVRGITKTFYFDYW	IGH	4	None	
8	666	CARAHDYDMSGYFEGRMYYFDHW	IGH	6	None	
8	667	CARQAPGYSDSWYGFDPW	IGH	6	None	
8	668	CARDSVIVRGLMESPNWFDPW	IGH	2	None	
8	669	CAKSACGYDCYPDYW	IGH	2	None	
8	671	CVSLYGWDYFFYYMDVW	IGH	2	None	
8	672	CAKEGYSHGRTPNFDFW	IGH	16	None	
8	673	CARSSGEWLSLLPPDYW	IGH	7	None	
8	674	CAREGQEWLETYYYYYMDVW	IGH	2	None	
8	675	CAHSRGARGVSYYFDYW	IGH	2	None	
8	676	CARGWVVVVPAAPFDYW	IGH	2	None	
8	677	CARVLTGIPPFWDYW	IGH	2	None	
8	679	CQSADTSGPYRDVVF	IGL	2	None	
8	680	CHSADSSGSSELF	IGL	3	None	
8	681	CARVMTSVTNGWFDPW	IGH	2	None	
8	682	CAREFTVRGVRIGFDYW	IGH	2	None	
8	683	CAKDRPTVVMFREYAFDIW	IGH	2	None	
8	684	CARGAPHCNGGTCYPHNWFDSW	IGH	6	None	
8	685	CARDGSVGYSIDYYYMDVW	IGH	3	None	
8	686	CAGGISYNIDGTRGGFDIW	IGH	2	None	
8	687	CARGSGRQYYFESW	IGH	2	None	
8	688	CVSSRNPTVINGVFDYW	IGH	2	None	
8	689	CARVRRGNSGWYCWFDLW	IGH	11	None	
8	690	CAKGGHLSFYYFDYW	IGH	4	None	
8	691	CARDQIAVGPGKAFDIW	IGH	2	None	
8	693	CVKGSGGLKLLFYYFESW	IGH	4	None	
8	695	CAKVSRYGDQKWYFDLW	IGH	2	None	
8	696	CQSTDSSSAMGVF	IGL	3	None	
8	697	CTNTPTYSKEFDHW	IGH	2	None	
8	698	CIHKPRGGTDFDQW	IGH	2	None	
8	700	CARSHAHVNDGSTYYLDHW	IGH	2	None	
8	701	CNSRDSSENRWVF	IGL	735	None	
8	702	CARDNPSSGYGDDAFDIW	IGH	2	None	
8	703	CASLFPTENYFDFW	IGH	2	None	
8	704	CARHRIHLWFLMNWFDPW	IGH	3	None	
8	705	CARSMTTVDWDGVYFDYW	IGH	5	None	
8	706	CVTPSLYHSETRQDYW	IGH	2	None	
8	707	CASGSGYASDAFDIW	IGH	12	None	
8	708	CARDDDDGDEAPFDYW	IGH	2	None	
8	709	CARCDRVRRGPDAFDMW	IGH	2	None	
8	710	CARGRRITGRTRNYFEYW	IGH	4	None	
8	711	CAKDRQPHTGWPYYFVNW	IGH	3	None	
8	712	CATEENLLDYW	IGH	4	None	
8	713	CQHFHSYPQEYTF	IGK	5	C3	7K8R
8	714	CARYDILNGAYPFEYW	IGH	53	None	
8	715	CARDEGLHGDSYFDSW	IGH	4	None	
8	716	CQSYDNSLGGHFLF	IGL	2	C4	7JVC

8	717	CARALEVAAAGDYFDYW	IGH	7	None	
8	718	CVREDLSNTYYDYW	IGH	2	None	
8	719	CARGRMSAFYSYYYYMDVW	IGH	2	None	
8	721	CAVTFSHYGSGSHFDYW	IGH	2	None	
8	722	CARHFYGDYVLQGHWFAPW	IGH	2	None	
8	724	CAREALYFSDRGPYYHDAFDMW	IGH	5	None	
8	725	CAHRPPFFSGPYFDSW	IGH	3	None	
8	726	CARGSQDGRRSSGWYLDSW	IGH	2	None	
8	727	CAREWGSSGYYYDDYW	IGH	2	None	
8	728	CARERGRAYNDPDVPDSYDIW	IGH	2	None	
8	729	CAKDWEVYCGRDCYNAFDIW	IGH	6	None	
8	730	CARNARIGFSGTDFWFDPW	IGH	2	None	
8	731	CARSASYGFDFDYW	IGH	2	None	
8	733	CAKGPYITSHYYYYYMDVW	IGH	4	None	
8	734	CARGLHGDYEPYYFDFW	IGH	2	None	
8	736	CARSVAPVLRYFDWLSPNYYYYMDVW	IGH	2	None	
8	737	CQVWDSGTQCYVF	IGL	2	None	
8	738	CAHSRSSGWYSIYYFDVW	IGH	2	None	
8	739	CARETMVAAGRGFDYW	IGH	4	None	
8	740	CTRDGHLGSATFDYW	IGH	4	None	
8	741	CAKSGVQLWSFFDYW	IGH	6	None	
8	742	CTTVGSFYNFWSGYFDHW	IGH	2	None	
8	744	CAYSATGNNHRASWYDPW	IGH	7	None	
8	745	CASWDDSLKGYVF	IGL	532	None	
8	746	CAASWGAFKSVFGYW	IGH	4	None	
8	747	CARHQSSRGWIDVLDIW	IGH	3	None	
8	749	CARISGQFYFDSSGSYFDYW	IGH	8	None	
8	750	CAFLANSIGWYHYFDYW	IGH	2	None	
8	752	CLQYGNSPYAF	IGK	23	C1	7K8N
8	753	CQSTDSSVNYVLF	IGL	27	None	
8	756	CARVHGSGSYYGLDYFDYW	IGH	6	None	
8	757	CARSTRRSHFYYYYMDVW	IGH	2	None	
8	758	CAKDWSGSGSYYQNAFDIW	IGH	6	None	
8	759	CARGHSGWFLDFEYW	IGH	2	None	
8	760	CARQPMPQTYYYMDVW	IGH	2	None	
8	762	CARSLNYAILTGYGTRGFDYW	IGH	2	None	
8	763	CAKSRSAWSIKRYWYFDLW	IGH	4	None	
8	764	CARDNDVSAYSFDYW	IGH	3	None	
8	765	CVRDYWSGYDISDYW	IGH	2	None	
8	766	CARAPLSVVAAAAPFDNW	IGH	4	None	
8	768	CARGAVTAMVYYYYYYMDVW	IGH	2	None	
8	769	CYLTDSSINHRVVF	IGL	4	None	
8	770	CARHVSDSGGRHTDYYHHYLDVW	IGH	2	None	
8	772	CTRGGRTYNFDITKSRTWFVPW	IGH	2	None	
8	774	CASLGSTARHSFYYYIDVW	IGH	3	None	
8	775	CARRGVAPTKDDAFDIW	IGH	2	None	
8	777	CVRDNGDIAVVPTAFEYW	IGH	7	None	
8	779	CARDWNGRVRGAMDYW	IGH	3	None	
8	780	CAKDAFAVDMEATYFDSW	IGH	3	None	
8	781	CTNCRKTYGECAFDVW	IGH	4	None	
8	782	CARQSSNFWSGYLNWFDPW	IGH	2	None	
8	783	CARPALYDSSGYYYYW	IGH	16	None	
8	785	CAIDTGYSSGWPKYYFDHW	IGH	2	None	
8	786	CASLPMVSFRYSDYRNYYHYYYMDVW	IGH	2	None	
8	787	CTSLFKTYSSLTVADYW	IGH	2	None	
8	788	CCARCVDLAKFFDYYSYMDVW	IGH	2	None	
8	789	CAVDASGTATGFHYW	IGH	2	None	
8	790	CARVGRNDYGDDHFDYW	IGH	2	None	
8	791	CAKDTDSSGYYPYFDYW	IGH	10	None	
8	792	CVRGAQRGYSVGWFNWFDSW	IGH	2	None	
8	795	CARTLQTNYYDSSGYYPDAFDIW	IGH	5	None	
8	796	CATTEWDQLPRDTFDIW	IGH	6	None	
8	797	CARRVQDQWLARMGTFYFYMDVW	IGH	2	None	
8	799	CAKDLSSFREFDAFDIW	IGH	2	None	
8	801	CARLGDSSGYVFDYW	IGH	9	None	
8	802	CARRGDYEGSGSYYQDAFGLW	IGH	2	None	
8	803	CARASMVRAPGHYYYYLDVW	IGH	2	None	
8	804	CARSGYYASGSYYNADYYYYMDVW	IGH	3	None	

8	805	CARHIIRYCTSAECYLDGFDIW	IGH	5	None	
8	806	CATSPDDSSGYIPSYFDHW	IGH	2	None	
8	807	CAKSGSNYDYYYYYLDVW	IGH	2	None	
8	808	CYTALNYFDGPDLKQIDYW	IGH	2	None	
8	809	CAKVPPYSTLKRHFDSW	IGH	2	None	
8	811	CARPTGYYSAFYMDVW	IGH	2	None	
8	812	CAKDRGASGSCLDYW	IGH	6	None	
8	813	CASGPFSSGYSHNFDYW	IGH	2	None	
8	814	CAKIGGYDILTGFVDYW	IGH	6	None	
8	815	CAKDLMRAGGNSLDYW	IGH	2	None	
8	816	CQQFNSYPHQLSF	IGK	14	C3	7K8R
8	817	CQQYDNFRLAF	IGK	17	C3	6XDG
8	818	CQSADSIDAPSWVF	IGL	19	None	
8	819	CARDQGFDNWLDPW	IGH	2	None	
8	820	CAKGLNILAINDAFDLW	IGH	2	None	
8	821	CARQWLEDGFDIW	IGH	2	None	
8	822	CAHFLPIYDAFDIW	IGH	2	None	
8	823	CARATRITIFGVVTGGGNFDYW	IGH	10	None	
8	824	CAHRQYYYNSGISSGDFQYW	IGH	2	None	
8	825	CARIRDYLNSGNYWNWFGPW	IGH	4	None	
8	826	CALPICLRADDTALYYCATMSRYDRSGYKSDYW	IGH	2	None	
8	827	CARDLCDSSCPDAFDLW	IGH	2	None	
8	828	CARHIRLVVIPDFDYW	IGH	2	None	
8	829	CAREGLGKAPYFEDW	IGH	2	None	
8	830	CASDRSTYYYDSSGYDYW	IGH	2	None	
8	831	CASQGLFDTLTGVDYW	IGH	3	None	
8	832	CQRYNTAPYTF	IGK	21534	C3	7K8P
8	833	CARSPGGMVFPHYTFDSW	IGH	3	None	
8	834	CAKKGGWRCITGTCFSFDNW	IGH	2	None	
8	835	CARDSLQIWVTSSRTLDYW	IGH	2	None	
8	837	CARVPNAYCTGITCYYFYFDLW	IGH	4	None	
8	839	CARAYCYGDCNFDYW	IGH	3	None	
8	840	CARDLPPGTVGPSKDYW	IGH	2	None	
8	841	CARTPFWDSTGHYDYW	IGH	2	None	
8	843	CAKGTISGYRKYYYFDYW	IGH	2	None	
8	844	CARGLEGYSSSWYIDYW	IGH	3	None	
8	845	CARGDSASGYNWLDPW	IGH	4	None	
8	846	CVKNIGSGWAQDNYYYYYMDVW	IGH	2	None	
8	847	CQVWHSSGDHPQLVF	IGL	2	None	
8	848	CARVKRYYYDSRPDCFDLW	IGH	4	None	
8	849	CARGSAKDSTSTIFGVVIIGRPTVPFDYW	IGH	2	None	
8	850	CARDSSEIAVVAELENWFDPW	IGH	2	None	
8	851	CCSYAGTYNFWVF	IGL	27	C2	7JMP
8	852	CAKYDALTTSPFDHW	IGH	3	None	
8	853	CARLDYGDYWGYFDYW	IGH	6	None	
8	854	CAKEPSGYYYYHMDVW	IGH	2	None	
8	855	CARDRQYQLLSLSLNYYYYMDVW	IGH	6	None	
8	856	CAKYRPLTGGRTSGFDFW	IGH	6	None	
8	858	CVKDLETYYYETSGYYAFDIW	IGH	5	None	
8	860	CAKVSVSAWYLEGAFDIW	IGH	2	None	
8	861	CARGGVNIVTTIYDYW	IGH	2	None	
8	863	CVKSTYGDGYGRPYFDYW	IGH	34	None	
8	866	CIRGEPNGYPRFDDW	IGH	8	None	
8	867	CSSSLGWWAFHYW	IGH	5	None	
8	868	CATGEASGYWYFDYW	IGH	8	None	
8	869	CAKDSESRVEQWLVTPGDYW	IGH	2	None	
8	870	CAKGDSSVDYW	IGH	3	None	
8	871	CARLKTGFAYSSHGSGYFPNWFETW	IGH	10	None	
8	872	CATSDPGIGEFFGYW	IGH	2	None	
8	873	CARDHGDGNWYFDLW	IGH	2	None	
8	874	CVRIAFSYSYGLFYFDHW	IGH	2	None	
8	875	CVGDRRHSYGYSWFDPW	IGH	2	None	
8	877	CVKDLNYDSSGDYFSYGFDIW	IGH	3	None	
8	879	CVRDYAWAFDYW	IGH	9	None	
8	880	CARDGAKSRDDRGLLDYW	IGH	4	None	
8	881	CARGHNYFDSRDTDYW	IGH	2	None	
8	883	CARVDMRIWGDYFDHW	IGH	2	None	
8	885	CAKEGMYGDYGHWDYW	IGH	5	None	

8	886	CARGIVPAGKSGYNWFDPW	IGH	6	None	
8	887	CARHLSATGVLSHPDYW	IGH	4	None	
8	888	CATRPPLVGVAPHYMDVW	IGH	2	None	
8	889	CARVLANHYYYYYYMDVW	IGH	2	None	
8	890	CVLYMGSGIWVF	IGL	55	None	
8	891	CAGRRSGRGASDIW	IGH	4	None	
8	892	CARQAAENLAVMTYYYYMDVW	IGH	2	None	
8	893	CARENIASGDYDYW	IGH	2	None	
8	894	CATDGHCDNTTCYTENGYFYYYMDVW	IGH	2	None	
8	895	CARDFRLGIRHFDYW	IGH	2	None	
8	896	CQHYGSSHGSSRPPITF	IGK	2	C1	7K8N
8	897	CAHRLVSAWHFDSW	IGH	2	None	
8	898	CARGYSGGIKNNWFDPW	IGH	6	None	
8	899	CARVDWGPTTAFDYW	IGH	10	None	
8	900	CATGGYDTSGYYANDYW	IGH	2	None	
8	901	CARQWFTRSSNGNGWFDPW	IGH	2	None	
8	902	CAREGEDVNSDDLVYPFDYW	IGH	2	None	
8	903	CVRHDRADRGWGTQYPGDYW	IGH	6	None	
8	904	CARDWANHGYLMDYW	IGH	12	None	
8	905	CAKDHRDYYDNNFSYGHSYYDYW	IGH	21	None	
8	906	CARETYDYSNYRPGYNYYYYMDVW	IGH	2	None	
8	908	CQQRNSWPVVYSF	IGK	10	C3	7K8R
8	911	CAQEGDSAYSTVFDIW	IGH	5	None	
8	912	CAYGSGYADLNWLDPW	IGH	2	None	
8	914	CQYYNNYSTF	IGK	283	None	
8	915	CARGHIKTYYFDSGTYPFDYW	IGH	2	None	
8	916	CARPPDHYLDGFDIW	IGH	3	None	
8	917	CSAWDSITAHVVF	IGL	5	None	
8	918	CAKCTSTTGTPLDYW	IGH	2	None	
8	919	CARAKYYDITNWFDPW	IGH	2	None	
8	920	CAKDVRSLAVAGSDYW	IGH	4	None	
8	922	CQVWDTSSGHPRVLF	IGL	2	None	
8	923	CARERDGYYYDRSASENWFDPW	IGH	4	None	
8	924	CARDRYFDSSGYYEPYYYYMDVW	IGH	2	None	
8	925	CARSRVYDSSGSAFDYW	IGH	2	None	
8	926	CARQGRDDYGPPFDYW	IGH	4	None	
8	927	CASGKAWCTNGVCYTYHYYYMDVW	IGH	4	None	
8	928	CARDNYCSSHGCYLAAPDYW	IGH	2	None	
8	929	CASTSTTYYYDSRGYYSALDIW	IGH	2	None	
8	930	CMQALQTPRGVFTF	IGK	3	None	
8	931	CQHYGSSRTF	IGK	702	C1	7K8N
8	932	CTTDPTYDSSGYYIGYW	IGH	2	None	
8	933	CASQNSGNAFDLW	IGH	2	None	
8	934	CATLEESTTEITNEAFDIW	IGH	2	None	
8	935	CARAFSGWYGIDYW	IGH	3	None	
8	936	CAKDSVFAGSGWYPAITPYYYNYMDVW	IGH	4	None	
8	937	CARTEVAGTVDAFDIW	IGH	4	None	
8	938	CARVSSRFSEESRIYYYYYHMDVW	IGH	2	None	
8	939	CARGWEQWLVGPGPTDYW	IGH	2	None	
8	940	CARVLSGYTRAYFDFW	IGH	2	None	
8	941	CARNEELLYYFDYW	IGH	2	None	
8	942	CARSLSRADWYFDLW	IGH	2	None	
8	943	CAREAKTGPENHDAFDIW	IGH	23	None	
8	944	CASESGYTGYDPFDYW	IGH	26	None	
8	945	CAREAYDGSGSAPFFDYW	IGH	2	None	
8	946	CVKDLFEGYSFGYVPHFDYW	IGH	2	None	
8	947	CAHRLYYNFWSGYYDAFDIW	IGH	8	None	
8	948	CANSEIGANIAAAAEFNYW	IGH	4	None	
8	949	CARAAVWYDSSGTEPDAFDIW	IGH	6	None	
8	950	CARDPQYYDILAGFDVLAFDSW	IGH	8	None	
8	951	CARDRGYKWNDNVFDIW	IGH	2	None	
8	952	CQSYDTSNTTPWVF	IGL	7	C4	7JVC
8	953	CARGRVPSIAARRHYYYYYMDVW	IGH	4	None	
8	954	CAKDASMLRGVLAVTYFDYW	IGH	2	None	
8	955	CARLDCSSTSCYYDYYYYYMDVW	IGH	3	None	
8	956	CAKDRVPYESSGYNNLLDSW	IGH	2	None	
8	957	CARDRPRSDFWSGREIDNDAFDLW	IGH	3	None	
8	958	CLHHNGYPPAF	IGK	4	None	

8	959	CAKVLAGAGSFDVW	IGH	18	None	
8	960	CGNWASGFEKIDYW	IGH	2	None	
8	961	CARQYSSGWYHALDIW	IGH	7	None	
8	962	CQSYDNGNWVF	IGL	30	C4	7JVC
8	963	CVKEIGDIMVVPIAFDSW	IGH	2	None	
8	964	CMQATHWPALSF	IGK	20	None	
8	965	CAKPPRQKFFDTFGLPADGFEIW	IGH	2	None	
8	966	CARHGNGRPFDYW	IGH	30	None	
8	967	CAKERNNFESSSYLDHW	IGH	3	None	
8	968	CARHKAYDFDAFNIW	IGH	3	None	
8	970	CARQWLVPGLFGYW	IGH	2	None	
8	972	CARLLDYGGNPYAFDFW	IGH	2	None	
8	973	CARDRGYMDVW	IGH	23	None	
8	974	CATKKPITFYSDTSGFEFDYW	IGH	3	None	
8	975	CVRGRDYNDYRFYFDFW	IGH	4	None	
8	976	CASQMATIDSPFDFW	IGH	11	None	
8	977	CASPSRADYYGSGSYSAFDYW	IGH	12	None	
8	978	CARVRLGRYYSDSGSLPYWYFDLW	IGH	6	None	
8	979	CARAVDYFDSRIDYW	IGH	2	None	
8	980	CAKYVNSGWYSNFDHW	IGH	4	None	
8	981	CTTRPSYHDVLTGYYPDYW	IGH	6	None	
8	982	CAKGKSLVYDSTVFDYW	IGH	4	None	
8	983	CQHYNSWPPGLTF	IGK	25	C3	7K8R
8	984	CMARGYNDFDYW	IGH	2	None	
8	985	CGTWDTSLSVGVF	IGL	145	None	
8	986	CARLYHAYYMDVW	IGH	5	None	
8	987	CASSPDRYDNSGTYVDCW	IGH	4	None	
8	988	CQLFNSFPHEVTF	IGK	19	C3	7K8R
8	989	CAKYIAVAGAFDYW	IGH	4	None	
8	990	CARGNRYYYDSSGYSKYYYFYMDVW	IGH	2	None	
8	991	CAGDTITHGDYTTFDYW	IGH	2	None	
8	992	CAKVQDYSEIDYW	IGH	5	None	
8	993	CARDSVGYYDSSVYNDHW	IGH	2	None	
8	994	CTRDNRKSDDSIFGSYRYGDALDLW	IGH	4	None	
8	995	CASSSGRMSIEGPFDIW	IGH	8	None	
8	996	CAKDIRGSSGQTNWVDPW	IGH	2	None	
8	997	CARNPHEIKYSYGSRRRYYYMDVW	IGH	4	None	
8	998	CATSRYYYDSSGTGPLDYW	IGH	2	None	
8	999	CARSRDAYNLAVWFFDYW	IGH	7	None	
8	1000	CARDAIVVAGIDGFDIW	IGH	3	None	
8	1001	CQVVDSSSDLF	IGL	2	None	
8	1002	CAKESVSMIEGNPFDIW	IGH	3	None	
8	1003	CARQSPDTTMVLFDYW	IGH	2	None	
8	1004	CAKDQGALHGDYYFENW	IGH	4	None	
8	1005	CARASAGIYGHWFDPW	IGH	2	None	
8	1006	CARVVPFDKNDYSDAFDIW	IGH	4	None	
8	1007	CVRDWNTQMDSGGDYW	IGH	5	None	
8	1008	CARGSHRGSGRDNWFDPW	IGH	2	None	
8	1009	CARKMRSTWVPDAFDIW	IGH	8	None	
8	1010	CSRVYYDIVTGHWGPDVFDVW	IGH	2	None	
8	1011	CARHHRLWYAPDVW	IGH	2	None	
8	1012	CAKDRLRYDGTYTADYW	IGH	8	None	
8	1013	CVKDRASMVGAFDFW	IGH	2	None	
8	1015	CARAPPTTVTSDFDSW	IGH	2	None	
8	1016	CARQRPAVMIGSDAFDFW	IGH	4	None	
8	1018	CARATFPFSFDPW	IGH	2	None	
8	1019	CARMNYDILTAYYDAFDIW	IGH	4	None	
8	1020	CARDPSIAAAGMGWYFDLW	IGH	3	None	
8	1021	CYSATDNSWVF	IGL	30	None	
8	1022	CARGGYNYASDYW	IGH	22	None	
8	1023	CARRLGSYYDSRGYCFDHW	IGH	2	None	
8	1024	CAKSPHSPAYCGGGDCYTDYW	IGH	4	None	
8	1025	CVRGRIAAANGSSEVPSVYYFDFW	IGH	2	None	
8	1026	CAKDSTRYCSGFRCYDFDYW	IGH	4	None	
8	1028	CARDRDGRSPSFDFW	IGH	4	None	
8	1029	CARDLSYGYYPDDAFDIW	IGH	4	None	
8	1030	CATELLYCSTRYCPFEYW	IGH	3	None	
8	1031	CARDYRPRYSSSWYPDYW	IGH	6	None	

8	1032	CARDKGGRDMDIVATVGDALDIW	IGH	15	None	
8	1033	CATYRSGWYTGPPDYW	IGH	4	None	
8	1034	CAKGEYTYGYSEGFDVW	IGH	3	None	
8	1035	CVRDETVDYNLWNGYYWGNDWFDPW	IGH	6	None	
8	1036	CAKRVVDPLVGPYFDYW	IGH	2	None	
8	1037	CAKDPSYYYDSSGSPQIDYW	IGH	6	None	
8	1039	CARDCSSTSCIPSGAFDIW	IGH	4	None	
8	1040	CARGHGFGKLLYDDAFDVW	IGH	2	None	
8	1041	CAKGHDDAWRAAWYFDSW	IGH	4	None	
8	1042	CARDRAAYCSSTSCYRNWFDPW	IGH	4	None	
8	1043	CVRPTGYFDSSNYLEALNWFDLW	IGH	4	None	
8	1044	CAGLSRATRAYSDYDEIDYW	IGH	2	None	
8	1045	CARRVSGSWYASSFHFDYW	IGH	2	None	
8	1046	CVKGFYGSPTTSLYYFDSW	IGH	5	None	
8	1047	CQHYYSDPGF	IGK	15	C4	6W41
8	1048	CARALAVRPSRGTLYMDVW	IGH	3	None	
8	1049	CARAGSYYDSSAYLDDAFDIW	IGH	2	None	
8	1050	CVRFSGDLDCSTTSCYRWDYYYYMDVW	IGH	4	None	
8	1051	CVRQSERGEYLDLDYW	IGH	2	None	
8	1052	CALPILQMNSLRADDTAVYYCAKEIRPNDYW	IGH	2	None	
8	1053	CARAGRRGYTYGGFDFW	IGH	2	None	
8	1055	CATKGPCGGDCFYYFDFW	IGH	16	None	
8	1057	CATHPETWIAYSSSPGGDYW	IGH	4	None	
8	1058	CARIRKFQLLYYFDSW	IGH	2	None	
8	1060	CSLRSDDTAVYYCARAYDTNGFITEYFQYW	IGH	2	None	
8	1061	CARDSVDRAGPSPVDIVAPDFDYW	IGH	4	None	
8	1062	CAGPGRLYGSQTYYRGYFDYW	IGH	3	None	
8	1063	CAKPNYDISTHYHFDYW	IGH	2	None	
8	1064	CQSADSTGTHWVF	IGL	151	None	
8	1065	CGTWENSLSAGRSHVF	IGL	4	None	
8	1066	CARMMATYRDNAFDIW	IGH	2	None	
8	1067	CARVQRGGSCLDYW	IGH	2	None	
8	1068	CARPANRGWYDAYDIW	IGH	2	None	
8	1069	CAKAPRKWELREVFDYW	IGH	4	None	
8	1070	CATLITYYFDSGTYYNPEDALDVW	IGH	5	None	
8	1071	CARAQSLFSDSSFDAFDIW	IGH	2	None	
8	1072	CARGGYSYGYLLDYW	IGH	6	None	
8	1073	CAVTVIRGVIEPYYFHYW	IGH	2	None	
8	1074	CVRGGPGTGLDYW	IGH	12	None	
8	1075	CANVVSPQKRWELLDYW	IGH	4	None	
8	1076	CARDSFILTGYSDYW	IGH	6	None	
8	1077	CARRGRRGYDTSSTSFDYW	IGH	2	None	
8	1081	CARSGTYPYYFEFW	IGH	4	None	
8	1082	CATSWFGKAAGITTNDGFDIW	IGH	4	None	
8	1083	CTRRRQYSSWPDYW	IGH	2	None	
8	1086	CARHGEAGVQKNFDRW	IGH	2	None	
8	1087	CARGAGTLAAGWFDPW	IGH	2	None	
8	1088	CTTDPIAVVRPFDPW	IGH	3	None	
8	1089	CARQYSSSSWFDPW	IGH	23	None	
8	1090	CARNGGSDWYEGWFDPW	IGH	2	None	
8	1091	CARVWILGSSPSYYFDSW	IGH	2	None	
8	1092	CQHFNNYFFTF	IGK	30	None	
8	1093	CSIVHDTSYHNDDFDIW	IGH	2	None	
8	1094	CARDRAITFGGVIVIGDYW	IGH	2	None	
8	1095	CARDPVPGNHRVLYYFKDW	IGH	2	None	
8	1096	CAREDSTSLGLDYW	IGH	15	None	
8	1097	CMQRLQTPPQRLQTPPYTF	IGK	2	None	
8	1098	CVRTGLKQWLVRHYFDYW	IGH	2	None	
8	1099	CVHSLLLRPFDYW	IGH	12	None	
8	1100	CATARLSTTLDYW	IGH	2	None	
8	1101	CARHRFSWAAALNWFDPW	IGH	3	None	
8	1102	CARDTDSYETSGYDYW	IGH	2	None	
8	1103	CAKDSPYYYDSSGFRIFDYW	IGH	12	None	
8	1104	CVRGRLGWYFNLW	IGH	2	None	
8	1105	CALPIWDILLVPIDVPRGGDTYSYYLDVW	IGH	2	None	
8	1106	CQQYHNLPGSF	IGK	108	C3	6XDG
8	1107	CARLDFGDHGGWFDPW	IGH	2	None	
8	1108	CARLYGSATFFDYW	IGH	7	None	

8	1109	CARASVSYYDSSGYYLDYW	IGH	5	None	
8	1110	CAKEGHGSGHCGSFDPW	IGH	4	None	
8	1111	CTRRLVSARPYWYFDLW	IGH	3	None	
8	1112	CARGFCSGNSCFSGPWDYW	IGH	3	None	
8	1113	CAKQVYGDYYFDYW	IGH	5	None	
8	1114	CAKDRPPSSYPGSGPFDYW	IGH	7	None	
8	1116	CARDYGTSRMRYFDFW	IGH	2	None	
8	1118	CARDKVFYYDRSGYYPIDYW	IGH	2	None	
8	1119	CVHGDVLSGYKNDAFDIW	IGH	4	None	
8	1120	CVMTTGRYNWFDPW	IGH	2	None	
8	1121	CAKTLFPGPLASGSYGVVFDIW	IGH	12	None	
8	1122	CARGMRFSEGTFDIW	IGH	2	None	
8	1123	CARDYGSSGDYTPPFDYW	IGH	2	None	
8	1124	CARLGYDYSSGWTPPKIDYW	IGH	4	None	
8	1126	CATGIGRYSGPDAFDIW	IGH	2	None	
8	1127	CAGDRVIIFGGVIINDDGFDIW	IGH	4	None	
8	1128	CTRWGISGCGGDCSTFDSW	IGH	2	None	
8	1129	CARQFNYFHYMDVW	IGH	6	None	
8	1131	CATVPGAPYYFDYW	IGH	4	None	
8	1132	CAALQTYWYDTSAYCDYW	IGH	3	None	
8	1133	CSSYRSSSTPPIVF	IGL	2	C2	7K8W
8	1134	CARLTYCGGDCYPYIDSW	IGH	2	None	
8	1135	CTRDALSSSWYELHDAFDIW	IGH	2	None	
8	1136	CARHSFDSSGYTFYYYYHMDVW	IGH	2	None	
8	1137	CAKDQTTGTRGDAFDVW	IGH	4	None	
8	1138	CAKQSDPYYFDYW	IGH	2	None	
8	1140	CAKDIDGGHNYYALGFDHW	IGH	2	None	
8	1141	CAREEDYSNHFFDFW	IGH	2	None	
8	1142	CARRVDATFDDW	IGH	2	None	
8	1143	CARSQSTGTYSGAFDYW	IGH	4	None	
8	1144	CARVGFRVSDAFDVW	IGH	4	None	
8	1145	CATERGFYFDSGRRAFEIW	IGH	2	None	
8	1146	CARRNHYIAAAIDYW	IGH	3	None	
8	1147	CAKDGETLRDIVVVVAVFFDNW	IGH	3	None	
8	1149	CARSCGGDCYDHFDYW	IGH	3	None	
8	1150	CATVAVAEPVHWFDPW	IGH	3	None	
8	1151	CAREEGGGTYEDNWLDPW	IGH	2	None	
8	1153	CSRDARTWRLVVDSEFLFWFEYW	IGH	3	None	
8	1154	CASQRAPYFDWTHFDYW	IGH	2	None	
8	1155	CARALRSTQYYYENSAPFYFDDW	IGH	2	None	
8	1156	CAKVGDYDILTGSYYSSYYHYYYMDVW	IGH	8	None	
8	1157	CARDKYRSYGSGTHFDYW	IGH	4	None	
8	1158	CARGFGRFGEVGDYW	IGH	2	None	
8	1159	CARQGGNSVQDYWFDPW	IGH	4	None	
8	1160	CATDPYYKFWTDSYVRLGSYMDVW	IGH	3	None	
8	1162	CARPGPEPQLLSLDYW	IGH	3	None	
8	1163	CASHTYGFRYFDYW	IGH	4	None	
8	1165	CVKGSHYESGFATVNYYPLFDYW	IGH	2	None	
8	1166	CARERGRWLHLFDYW	IGH	4	None	
8	1167	CARDAYYSDGGSSHYYYYYMDVW	IGH	2	None	
8	1168	CARDRAPYFDASGYYSEAFNIW	IGH	2	None	
8	1170	CSKGSALGHCTGTDCYHFDSW	IGH	4	None	
8	1171	CAKDLMVRGDLYYLDQW	IGH	4	None	
8	1172	CARRFYSGSHYPSFFDSW	IGH	3	None	
8	1173	CARDTTVLYTSGWDSLDYW	IGH	4	None	
8	1174	CAKDASRPYDYDDSGYHDAFDVW	IGH	3	None	
8	1175	CAMRNFPATTKAFDIW	IGH	2	None	
8	1176	CARHGWNGSYLDWFDPW	IGH	2	None	
8	1177	CAKDIIPSDGYNWGGFDHW	IGH	24	None	
8	1178	CQEANSLPAITF	IGK	37	None	
8	1179	CARDIAWDCNGGRCHSSAFDIW	IGH	2	None	
8	1182	CATFWYYDVFLTGYRRDFDYW	IGH	8	None	
8	1183	CARSNLRGYSYGVDFDYW	IGH	2	None	
8	1184	CAREMRVGAVRYYFDYW	IGH	2	None	
8	1185	CAKDGPQYSYGCFDYW	IGH	2	None	
8	1187	CARVGQYSGSYNVGDYW	IGH	12	None	
8	1189	CARVWSTATGTRNWFDPW	IGH	6	None	
8	1190	CARHVSLYYDILTGYYNPFDSW	IGH	2	None	

8	1191	CARVGPYNSGDRYYYYMDVW	IGH	2	None	
8	1192	CAHRIEMVREYNWFDPW	IGH	3	None	
8	1193	CTRSYGSGTYTGFDVW	IGH	3	None	
8	1194	CTRGKSYCSGNSCYIDLW	IGH	2	None	
8	1195	CARNSPRGERAFDIW	IGH	2	None	
8	1196	CARYGLENYSDYW	IGH	2	None	
8	1197	CARVPQRFYYPFDYW	IGH	2	None	
8	1198	CAKFFQRYDILTGYYDYW	IGH	4	None	
8	1199	CARDSDHYDSIGFDYW	IGH	4	None	
8	1200	CMQVSHWPIAF	IGK	10	None	
8	1201	CAKDSRGLPRGVLGRFDFW	IGH	2	None	
8	1202	CATLILGKTYCGSTSCYAPFDYW	IGH	2	None	
8	1203	CARVLSASDRHAYYYQYMDVW	IGH	3	None	
8	1205	CASVFYDSGIARDYW	IGH	2	None	
8	1206	CARANGESWYDTFDYW	IGH	6	None	
8	1207	CAKDLYYYDSSGYGVDYW	IGH	6	None	
8	1208	CAKIWGSGSYLDSW	IGH	2	None	
8	1209	CARQYLRHYYDSSGYLDYW	IGH	2	None	
8	1210	CASLSATFYYDSGGYFDYW	IGH	6	None	
8	1212	CARHGSIRGVPRIGFGYW	IGH	2	None	
8	1213	CAKGTNYDLFADSYFFDSW	IGH	2	None	
8	1214	CATWGYSYDRSGARAIFDYW	IGH	6	None	
8	1216	CAHGAYCGGDCYPLLFDYW	IGH	2	None	
8	1217	CARGEAVAGMRDYYYYYMDVW	IGH	3	None	
8	1218	CATEMVRSDAYFYDAGTSAQFDYW	IGH	5	None	
8	1219	CARDRGYLILLTNSFDIW	IGH	4	None	
8	1220	CAKDFGYYGSGSYCLDYW	IGH	105	None	
8	1221	CARSDYGASSGSFDYW	IGH	3	None	
8	1222	CASDRCSYGTCYGRHFVYW	IGH	4	None	
8	1223	CQQRTNRPLSLTF	IGK	9	C3	6XDG
8	1224	CASPKVLRYFDWLKGENPDDAFDIW	IGH	2	None	
8	1225	CARASTHYFLSGTYSPCDYW	IGH	2	None	
8	1227	CAREGVSLDRGTPFDYW	IGH	4	None	
8	1229	CQSPDSDGTWAF	IGL	7	None	
8	1232	CAKGPLTPVDVVSTLKGRYFDSW	IGH	5	None	
8	1233	CARFNRGVTAAGLFANWFDPW	IGH	2	None	
8	1234	CTRERAIAVPGTVWYFDLW	IGH	2	None	
8	1235	CARVANGYNSYYLDYW	IGH	10	None	
8	1236	CARLSVVAANMWFDSW	IGH	5	None	
8	1237	CATLSRDDYNSPFDYW	IGH	5	None	
8	1238	CQLWDSSSDFNYVF	IGL	2	None	
8	1239	CGRDPLFSSYYYDYMDVW	IGH	2	None	
8	1240	CARDLLGYFDWLYAFDIW	IGH	2	None	
8	1241	CARVFPGQLLLHDAFDIW	IGH	4	None	
8	1242	CARAENHYDSSVADYW	IGH	2	None	
8	1243	CASGGEMLTIFSDDYW	IGH	2	None	
8	1244	CARDTALHVFPFYYYYMDVW	IGH	2	None	
8	1245	CARGAFCRAALCFAGWVFDPW	IGH	4	None	
8	1246	CARYSGYRGNAQFDFW	IGH	4	None	
8	1248	CARASYYDSGGSYSKFYFEYW	IGH	6	None	
8	1249	CTKDISGSYSRALDYW	IGH	17	None	
8	1250	CATFREFAVTPSEGLNFDYW	IGH	2	None	
8	1251	CARDRARFDYDGSGYYLGNLDYW	IGH	4	None	
8	1252	CARHPTSTFRRAAAGNWFDPW	IGH	4	None	
8	1253	CARGPYPAPGLYFKHW	IGH	2	None	
8	1254	CARYGDYDIWTQTNANKRHFDYW	IGH	2	None	
8	1255	CAKDISKIGWFEGLDYW	IGH	2	None	
8	1256	CMIWHSSAWVF	IGL	51	None	
8	1257	CTRGTQRYGSWSFDPW	IGH	8	None	
8	1260	CARMYDSGGYYSYYLDSW	IGH	2	None	
8	1261	CVKDRTIEGYEAFEVW	IGH	2	None	
8	1262	CTRKNGISGYDPFDVW	IGH	3	None	
8	1263	CARGLLRSSFDFDYW	IGH	4	None	
8	1264	CARWLTSGSYWHYFDSW	IGH	10	None	
8	1266	CASPPLTRIEITAARGWGAPFDYW	IGH	2	None	
8	1268	CAREGYFCGGDCLDAFDIW	IGH	2	None	
8	1270	CARVSFGYYDSSGYLTYW	IGH	2	None	
8	1271	CARDRRLWRASGTYHYMDVW	IGH	2	None	

8	1272	CAKDMTSITMIVVVITVAFDIW	IGH	5	None	
8	1273	CMSPLQTPPNTF	IGK	8	None	
8	1274	CVRGHYGSGSFQGETAFDIW	IGH	2	None	
8	1275	CAGTQWLLFDSW	IGH	4	None	
8	1276	CASWYYYDSSGYPLRTHDAFDIW	IGH	2	None	
8	1277	CARKLQLVRPFDYW	IGH	3	None	
8	1278	CARELLGEAVFEKVDPPDYW	IGH	4	None	
8	1279	CARELDGRGYHYQAFDIW	IGH	2	None	
8	1280	CARARPPRVSSLRELALFDPW	IGH	2	None	
8	1281	CGNWGPGTYSIDYW	IGH	2	None	
8	1282	CARDSGRGSRDFWSSYFTLW	IGH	4	None	
8	1283	CAHHTSSLLDYW	IGH	2	None	
8	1285	CARHYYGSGSYSGIDYW	IGH	8	None	
8	1286	CARGMYGNFLFDFW	IGH	9	None	
8	1287	CAGGHFGVVPAVPGDPGNYYYSYMDVW	IGH	2	None	
8	1288	CSVTAADTAVYYCARHRRSITMVREYRLYYFDYW	IGH	5	None	
8	1289	CANAYGGHDILTAFDYW	IGH	2	None	
8	1290	CVTDYYNSRGRFSDFFQYW	IGH	4	None	
8	1291	CLQYNTWPLYTF	IGK	17	C3	7K8R
8	1292	CATRNDQLHGDAGLDYW	IGH	2	None	
8	1294	CARHGLRFSSGYLWFDPW	IGH	3	None	
8	1296	CAKKLQRGIVVVVVGAHDAFDIW	IGH	2	None	
8	1297	CARLWPLGSYLLEYYFDYW	IGH	2	None	
8	1298	CAKGSSAYYFNHFYYYYMDVW	IGH	2	None	
8	1299	CGSSRGGSNYFYYYIDVW	IGH	4	None	
8	1300	CARTHYGGNVFPADPIPHYYYMDVW	IGH	2	None	
8	1301	CARLARYSGSALRPFDYW	IGH	2	None	
8	1302	CARDKTASLDAFDIW	IGH	2	None	
8	1303	CLQFNNYLFAF	IGK	7	None	
8	1304	CQYYSSSPRINYATSPRITF	IGK	4	C1	7K8N
8	1305	CARGGTHHGFDIW	IGH	43	None	
8	1306	CARHAYGRWASEFDYW	IGH	2	None	
8	1307	CAKNFAGSGWYKSWFDPW	IGH	10	None	
8	1309	CSAWDDSLIAPTF	IGL	4	None	
8	1310	CARSAYSSSNRIDYW	IGH	2	None	
8	1311	CAKDRYSSAYYFDYW	IGH	7	None	
8	1312	CARGDITMEMGARSDAFDLW	IGH	3	None	
8	1313	CARNTISGYREYYMDVW	IGH	5	None	
8	1314	CARQEELWFGAWYFALW	IGH	6	None	
8	1315	CAKTGFDDNSPFFHSYYYMDVW	IGH	3	None	
8	1316	CAHRRAAVAGPYFDYW	IGH	8	None	
8	1317	CAKERNIVVVTAIDPFNYW	IGH	2	None	
8	1318	CTSFIYYYDSSGYYSDAFDIW	IGH	2	None	
8	1319	CGRHHYYDIFGGYTWFDFW	IGH	59	None	
8	1320	CVRDGASNWYEGYFDPW	IGH	5	None	
8	1322	CARSCVGYCSGGSRASLFDYW	IGH	4	None	
8	1323	CARTPFFSSCDYFDSW	IGH	2	None	
8	1324	CARLEGAYYESSGYYCDYW	IGH	4	None	
8	1325	CARLRAPHTYVYYENSAYHFDYW	IGH	4	None	
8	1326	CARDFKLMVRGFLPFDPW	IGH	4	None	
8	1327	CAIYNSGWYSAAYFDYW	IGH	2	None	
8	1328	CARDVAGPGSIYNAPVGASDIW	IGH	4	None	
8	1329	CAKDMGFMIFGVVVDTFDRW	IGH	8	None	
8	1330	CAREIRGYDHGDYFDYW	IGH	2	None	
8	1331	CARVSHFGSGPDHIRQRYYFDYW	IGH	4	None	
8	1332	CVRQHCSGWYCRGNWFDPW	IGH	6	None	
8	1333	CARGGGLGGHDHFDNW	IGH	4	None	
8	1334	CARDHLAPYSSSWYRWFDPW	IGH	4	None	
8	1335	CQSYDPTNQGEVF	IGL	2	C4	7JVC
8	1336	CARSREGGPTGPTGPLDYW	IGH	2	None	
8	1337	CQHYYPTPPITF	IGK	26	C4	6W41
8	1338	CARAHGSGNYYGLHYFDDW	IGH	2	None	
8	1339	CQQYDDLFTLTF	IGK	261	C3	6XDG
8	1341	CAKSYQLLPYREYFDYW	IGH	2	None	
8	1342	CARDRVNYYDGSTPFDSW	IGH	2	None	
8	1343	CARQPIVVVEPDTPGWFDPW	IGH	8	None	
8	1345	CARINRGYSSGWSHFDYW	IGH	8	None	
8	1346	CARLELTTVTSAATFDVW	IGH	6	None	

8	1347	CARVGSGRSGDYYKSFAFDIW	IGH	5	None	
8	1348	CVRVPLCGGDCPHAFDMW	IGH	2	None	
8	1350	CAKDPELLWFGELSKGGLDYW	IGH	20	None	
8	1352	CARIYQTYSGSYYFDYW	IGH	2	None	
8	1353	CARADYLKGVIDYW	IGH	2	None	
8	1354	CARHKGDRGGGNAFDIW	IGH	4	None	
8	1355	CARHLEEKSYYYESIGYYFDYW	IGH	2	None	
8	1356	CARLSPDSNGIYNGLDYW	IGH	8	None	
8	1357	CVRDRVGVNGAVDYW	IGH	2	None	
8	1358	CARKVQYSGYVYFDYW	IGH	2	None	
8	1359	CARDPASTGTTSGDYFDHW	IGH	6	None	
8	1360	CAKDRGDNYNYVWGTYHFDYW	IGH	2	None	
8	1361	CASAKFGYYMGFRYSYMDVW	IGH	2	None	
8	1362	CARSVAYYSDGSSQYTGGYYYMDVW	IGH	2	None	
8	1363	CARARDDYVWGSYPQQYFESW	IGH	3	None	
8	1364	CAKGESEYTGYGYFDQW	IGH	2	None	
8	1365	CARDGGTGPPWYNWFDPW	IGH	3	None	
8	1366	CQYYSGYPEIVF	IGK	2	None	
8	1367	CAKGRSSVLYYFDSW	IGH	2	None	
8	1368	CAKDKPYYNYMDVW	IGH	2	None	
8	1369	CARHGQSAYYTGFDYW	IGH	2	None	
8	1370	CQQYDSFSPTFTF	IGK	21	C3	7K8R
8	1371	CARDLPMIVAWRAFDIW	IGH	5	None	
8	1372	CATGRPGWYSDLW	IGH	4	None	
8	1373	CARVYGAYDFDALTFYYYMDVW	IGH	6	None	
8	1374	CATSRDYADWGTFDYW	IGH	2	None	
8	1375	CAKDGKGASAPYFFDYW	IGH	2	None	
8	1376	CGRDSPYSAYDYVPRFDPW	IGH	2	None	
8	1377	CARARGLAAIFGVVIRDFDYW	IGH	2	None	
8	1378	CARDPVHHYGSGSYLSINWFDPW	IGH	2	None	
8	1379	CMQGIHMGTF	IGK	14	None	
8	1380	CTSYTSSSTLVVF	IGL	737	C2	7K90
8	1382	CTTVVYYDYVWGSYRSSFDYW	IGH	10	None	
8	1383	CAREKAPPTIVATREFDLW	IGH	4	None	
8	1384	CTHNLRITNDAFDVW	IGH	2	None	
8	1385	CVRDLSTGYCFDQW	IGH	2	None	
8	1386	CARVGYYGSGSFLNGNGYLDYW	IGH	4	None	
8	1387	CAKDFGILPGPNFDSW	IGH	2	None	
8	1388	CARPKVAVMNLDAFDMW	IGH	4	None	
8	1389	CTTDDSGYDSPFDYW	IGH	2	None	
8	1390	CAKDIGDDGTFDYW	IGH	2	None	
8	1391	CARQKSVAMNDAFDIW	IGH	4	None	
8	1392	CARGRARYSTNWYVLDYW	IGH	5	None	
8	1393	CTKERQRDGYIPGDYW	IGH	2	None	
8	1395	CATTYCARGTCYKSFDPW	IGH	7	None	
8	1396	CATDPSTYRYYYDTFGTDYW	IGH	29	None	
8	1398	CASDYYGSGSYYHAFDIW	IGH	2	None	
8	1399	CARDGSGWSRDYW	IGH	94	None	
8	1400	CARLASGSHYFRYPQPYYFYYMDVW	IGH	2	None	
8	1401	CAKDGNRGYDIDYW	IGH	7	None	
8	1402	CASPYYNFLNGYYKGGWFDPW	IGH	3	None	
8	1403	CAIASIAAAAPFDYW	IGH	2	None	
8	1404	CAREGMTLSYYYMDVW	IGH	2	None	
8	1405	CARASHGQLAKKHYSSYLDVW	IGH	2	None	
8	1406	CARLHSGSVLGNYFYLYMDVW	IGH	9	None	
8	1407	CAKGTAYGDFVNDFDPW	IGH	2	None	
8	1408	CAKDSAVATWYFFDFW	IGH	2	None	
8	1409	CARQKWGWYFDLW	IGH	9	None	
8	1410	CARTPAAYDSSGYGFDYW	IGH	3	None	
8	1411	CARTTKVGPGGRYYFDFW	IGH	4	None	
8	1412	CAKGVYGSGSYGFDHW	IGH	4	None	
8	1413	CATRRPPYYVDYW	IGH	4	None	
8	1414	CARDTYTYYSDSSGFEHWFDPW	IGH	22	None	
8	1415	CAKDRGGVGATENFDYW	IGH	2	None	
8	1416	CARDTLGSPPDFDYW	IGH	5	None	
8	1417	CARDGASGITVAGHYDYW	IGH	2	None	
8	1419	CAKDLRPDLTSVFEYW	IGH	4	None	
8	1421	CQQANSFRSITF	IGK	8	None	

8	1422	CARVRSLYYDSPGERAFEIW	IGH	4	None	
8	1423	CARDPPYYESNGSSYFDYW	IGH	4	None	
8	1424	CTTDLLFGEATPAFDNW	IGH	5	None	
8	1425	CARLASYREVVVITHFDYW	IGH	2	None	
8	1426	CVRGTNLVEMIVDYW	IGH	40	None	
8	1427	CARHELSRITLIVVAPSYFDYW	IGH	5	None	
8	1428	CARDIQPAQARPGDSITRYNWFDPW	IGH	2	None	
8	1429	CARDYCAGACNSYYFDYW	IGH	2	None	
8	1431	CARAAVGSGYPDYW	IGH	2	None	
8	1432	CARDNLAVAFDYW	IGH	33	None	
8	1433	CAKCAQLFWLGEGKFSFDHW	IGH	2	None	
8	1434	CVRSWNYAGGFDYW	IGH	2	None	
8	1436	CVRGQWELLLGYYYYYLDVW	IGH	2	None	
8	1437	CAHSRMSGFYAYYYVDVW	IGH	2	None	
8	1438	CATSGWYRGSQFDHW	IGH	2	None	
8	1440	CARTVPLRLGEFSYLDSW	IGH	4	None	
8	1441	CARIWFGVAEARDYFDYW	IGH	2	None	
8	1442	CAKDLCYTSDCSDAHFDSW	IGH	2	None	
8	1443	CARLGSNEYYYMDVW	IGH	12	None	
8	1444	CAKRLYSSGWGDVFDSW	IGH	8	None	
8	1445	CARQGPLDRGVVITNPFDSW	IGH	3	None	
8	1446	CAKDSTAAAGIGAFDYW	IGH	2	None	
8	1448	CARIPRDPSSYYYPHDAFDIW	IGH	3	None	
8	1449	CAKGSAIGSCSGSNCFHFDSW	IGH	2	None	
8	1451	CARYDSGPYGAFDFW	IGH	2	None	
8	1452	CARAQYQVLNPFDYW	IGH	5	None	
8	1453	CARDRRDGFNPDYW	IGH	3	None	
8	1454	CAKNRPGSSGWARNDAFDVW	IGH	2	None	
8	1455	CVKAPDYYDILTGYPYYFDAW	IGH	4	None	
8	1456	CARDRAPQVQFIRKHWYFDLW	IGH	6	None	
8	1457	CASAGYSYGSKRKYYYYYYMDVW	IGH	2	None	
8	1459	CAKVGFAFCGGDCYSPYLDDYW	IGH	2	None	
8	1460	CARLFHGGNAYHYYYMDVW	IGH	2	None	
8	1461	CAREGVRITMIVVVRDAFDIW	IGH	4	None	
8	1463	CARTFHPPRYYETRGYNKWFDPW	IGH	2	None	
8	1464	CAHRSIIGMTASFDYW	IGH	3	None	
8	1465	CATSQYQMGLVFDSW	IGH	11	None	
8	1466	CARRLKYYDSLTGHFDHW	IGH	2	None	
8	1467	CARSGAPTATDWHFDVW	IGH	2	None	
8	1468	CAKDCHYYDSSGAFDYW	IGH	3	None	
8	1469	CAKEDSPHCTTSSCYTGAFDNW	IGH	2	None	
8	1470	CARGPSCTSISCPYAFDIW	IGH	4	None	
8	1471	CACSVAGTWSGSDTFDIW	IGH	4	None	
8	1472	CTTGPSYYYDTSGLRGGFDYW	IGH	2	None	
8	1474	CARAVEYCRGAKCADFDFW	IGH	5	None	
8	1475	CAREFIASSTSGYLLYW	IGH	2	None	
8	1476	CARSTEYGGSWYFDVW	IGH	6	None	
8	1477	CARRLAPAGHFFDYW	IGH	2	None	
8	1478	CQHYGISPLEYIF	IGK	4	C1	7K8N
8	1479	CGREGSGNYWGFFDYW	IGH	8	None	
8	1480	CARDYYDSSGYRMNYFDYW	IGH	2	None	
8	1482	CAAVGGSYYFKFW	IGH	2	None	
8	1483	CSSYTIRSTLFYVF	IGL	4	None	
8	1485	CARDFFTAIDYW	IGH	8	None	
8	1486	CAKKGEQWGGYDSSGYSDYW	IGH	2	None	
8	1487	CTSPIVVVPAATLDGLDYW	IGH	4	None	
8	1488	CARGTLSGYQGVNFDSW	IGH	2	None	
8	1489	CQRYNSAPWAF	IGK	621	C3	7K8R
8	1491	CLRSGSSYTGLYFFDYW	IGH	14	None	
8	1492	CAREAFSVIRGVPDYYHYYMDVW	IGH	3	None	
8	1494	CARGDSFGVW	IGH	2	None	
8	1495	CQQYDYSPLGGTF	IGK	10	C3	6XDG
8	1497	CAITHGSGTQEWFDPW	IGH	2	None	
8	1498	CATYTYTYASASGLDYW	IGH	3	None	
8	1499	CQTYDRSLSGLAF	IGL	2	None	
8	1500	CASPLLLYVSGTHYNDAFDSW	IGH	2	None	
8	1502	CARALTKFWSGSSVWFDPW	IGH	3	None	
8	1503	CVRRDDLAFDYW	IGH	11	None	

8	1504	CARDTYGGHYFDYW	IGH	3	None	
8	1505	CARDFSPLSTVTGFDSW	IGH	2	None	
8	1506	CARRRGPEWDLHEFDNW	IGH	9	None	
8	1507	CARHVVTTVVTPRAPFDYW	IGH	4	None	
8	1508	CARPRGYYASGGLWGFDYW	IGH	4	None	
8	1509	CARDPGSLMVFDYW	IGH	3	None	
8	1510	CARGVEPDGDYYMDVW	IGH	2	None	
8	1511	CQQYNNWPPPWTF	IGK	1250	C3	7K8R
8	1512	CARQLGMSAIDIW	IGH	5	None	
8	1513	CAREMREYDIVTGSFRNSELIDYW	IGH	2	None	
8	1514	CARAPAFAGYLAYW	IGH	6	None	
8	1515	CARDTAAVGSNWYFDLW	IGH	4	None	
8	1516	CAKGNYNFWSGYLDYW	IGH	2	None	
8	1517	CVRLPTGFPNWFGPW	IGH	3	None	
8	1518	CAREVRGVHTFFDFW	IGH	2	None	
8	1519	CAKDRCVYSSTSCYVGDYW	IGH	2	None	
8	1520	CARVCRGGGCPELPFFDYW	IGH	2	None	
8	1521	CLLYYGGAHPVF	IGL	84	None	
8	1522	CAKEKTFPWIAPGPYFDYW	IGH	2	None	
8	1523	CAKISGQFYYESRGAYFDYW	IGH	2	None	
8	1525	CARDGKYSSSSVFDFW	IGH	2	None	
8	1526	CALPISRAAGAEAFDIW	IGH	2	None	
8	1527	CARGAGYFDWSLVPQYFQQW	IGH	4	None	
8	1528	CARIRGSPWGQEYYFYYMDVW	IGH	2	None	
8	1529	CARARHSSGYYDIAYAFDIW	IGH	2	None	
8	1530	CARAQQQWLVLDWYFDLW	IGH	4	None	
8	1531	CAKDKRRGSGSWFYYIDVW	IGH	3	None	
8	1532	CAKDRSGGSGSYWGGFDYW	IGH	3	None	
8	1533	CARGGWLKASNAFDIW	IGH	5	None	
8	1534	CAKDNDSGGFPLTDAFDIW	IGH	2	None	
8	1535	CVRSVTNWLDPW	IGH	3	None	
8	1536	CARHLPSRITIFGVVTPNNWFDPW	IGH	2	None	
8	1539	CARVVIAANWFDPW	IGH	5	None	
8	1540	CASPRYFGESDGDAFDIW	IGH	4	None	
8	1541	CARSGDQGPFDIW	IGH	9	None	
8	1542	CVKDDGEDVSSGYFDYW	IGH	2	None	
8	1543	CARALGDYGRGNYFYYYYMDVW	IGH	3	None	
8	1545	CARLSSQHYISSAHYYGWFDPW	IGH	5	None	
8	1547	CARDRNWNYGAPYFDYW	IGH	2	None	
8	1548	CMQVLQPPMYSF	IGK	8	None	
8	1549	CAKDQGIIDFLEHYMDVW	IGH	2	None	
8	1550	CASCFVGTTGGGYFDSW	IGH	3	None	
8	1552	CAKYSTLRGYTYDPFDYW	IGH	18	None	
8	1553	CATRPGSGKYYPGGSYYPFDPW	IGH	2	None	
8	1554	CAKGSYSGYVTLVDYW	IGH	3	None	
8	1555	CHSRDSSANHLGYVF	IGL	3	None	
8	1556	CARPGISSWRHTFDSW	IGH	8	None	
8	1557	CAKLGAGSAYCGGDCFFDYW	IGH	4	None	
8	1558	CASQPLFYFDNSGYYGYW	IGH	11	None	
8	1559	CARDGYCHDSSGDLCGKINDAFDIW	IGH	2	None	
8	1560	CTSGSGDDTIDYW	IGH	6	None	
8	1561	CQHFNHYPPIIF	IGK	6	C1	7BZ5
8	1564	CARDYSGSRSYGSAYFDNW	IGH	2	None	
8	1565	CARNSGWNYFFYYMDVW	IGH	2	None	
8	1566	CARRGLETYLDFHYMDVW	IGH	2	None	
8	1567	CARDGGYTYGSARFDYW	IGH	5	None	
8	1569	CARQGRLGELSSFDFW	IGH	3	None	
8	1570	CATSTVPVSRGDAFDIW	IGH	2	None	
8	1571	CARLSTYTSGPARKNNWIDPW	IGH	4	None	
8	1572	CARASHFYDSGGSRPNWVDPW	IGH	3	None	
8	1573	CAKDALLNYYDSSSYGFDRW	IGH	2	None	
8	1574	CARLVAAPGNPYYYYYYMDVW	IGH	2	None	
8	1575	CQQYFTTPPSFTF	IGK	11	C2	7K8U
8	1576	CVARRDGYPPAAFDIW	IGH	2	None	
8	1577	CARVAGPDGLADYW	IGH	6	None	
8	1579	CARRRCSSIGCYDAPFDYW	IGH	2	None	
8	1580	CARVMGMRELLVDYW	IGH	2	None	
8	1581	CARDPWNYDVFSGSLEGNFYFDSW	IGH	8	None	

8	1582	CVRDFHDSLWDNYPLGDYFDYW	IGH	2	None	
8	1583	CARVFSYHNFWSGSNFDYW	IGH	4	None	
8	1584	CARDGEIERTVLDAFDLW	IGH	2	None	
8	1585	CARSAKLGTDGRSPFDIW	IGH	2	None	
8	1586	CVREKRERGSDAFAIW	IGH	2	None	
8	1587	CARAGGSRNYDGHDYFDYW	IGH	2	None	
8	1588	CARGGAYPDNWFDSW	IGH	2	None	
8	1589	CARAPEGIAVAGHDYW	IGH	2	None	
8	1590	CARIDDIGGFVHFDYW	IGH	2	None	
8	1591	CASRAQGPYNWFDPW	IGH	2	None	
8	1592	CQQYFSTPLYSF	IGK	96	C4	6W41
8	1593	CVRPYGDNEGYYYYHMDVW	IGH	2	None	
8	1594	CAKDSSDLLLWGGYFDSW	IGH	2	None	
8	1595	CARERTATIFGIVYSYYMDVW	IGH	5	None	
8	1596	CVKDIGLALQHQTYDYW	IGH	3	None	
8	1599	CASPPSPSRGDPWFDPW	IGH	2	None	
8	1600	CASWTFGSGKTDASDIW	IGH	2	None	
8	1601	CAKGTTVTYYNYMDVW	IGH	2	None	
8	1602	CAHSGYGSGTYYTNWFGPW	IGH	6	None	
8	1603	CARDTYNWNPGYFDYW	IGH	2	None	
8	1604	CARDRAYCTNGVCYPDWFDPW	IGH	2	None	
8	1605	CARLGAYSSSPFDYW	IGH	2	None	
8	1606	CVRHSRGLYRSSWGAYFQYW	IGH	2	None	
8	1607	CARVGFMREYWVFDYW	IGH	2	None	
8	1608	CAKDQELLWFGELFSFDYW	IGH	2	None	
8	1609	CATLAVAATDHYFDYW	IGH	6	None	
8	1610	CARGPAPLTMVRQFDYW	IGH	92	None	
8	1611	CAKSPGEGYGSGSYYGTHYFDNW	IGH	22	None	
8	1612	CARDSRNRFFDGDHEHILLDYW	IGH	4	None	
8	1613	CAKGLSIASADNIDYW	IGH	3	None	
8	1614	CGRMLKPYNWFDPW	IGH	2	None	
8	1615	CAKDVLPGPVRGPFDPW	IGH	2	None	
8	1616	CAHSSYDNSGFYYFDYW	IGH	3	None	
8	1617	CARHKFIATRGAWLVGTGGDAFDIW	IGH	2	None	
8	1618	CARIPGYSSAQDYW	IGH	2	None	
8	1619	CLQHQSYPYTF	IGK	4312	C3	7K8P
8	1620	CARVQLVGYYYYMDVW	IGH	2	None	
8	1622	CAGYTVHFDYW	IGH	4	None	
8	1624	CTRDSGYFASGDFVVRPLDYW	IGH	3	None	
8	1626	CTKKDLDYGDHGSGAFDIW	IGH	2	None	
8	1627	CQSLGSSGSVF	IGL	5	None	
8	1629	CVKEAHRSGYYPLDYW	IGH	2	None	
8	1630	CARSTGGNGGPLDYW	IGH	2	None	
8	1631	CARDLTSSPTRGFDPW	IGH	16	None	
8	1632	CARDGRFCHSPTCTEGFDFW	IGH	2	None	
8	1633	CARLEYSSSWPNIYYYYMDVW	IGH	2	None	
8	1634	CGADHGSGSNFVWVF	IGL	3	None	
8	1635	CARAYFDFNGEYPNLFDYW	IGH	52	None	
8	1636	CTREAYVDQRGSKKVLITSAFDYW	IGH	14	None	
8	1638	CTRDPDITGSDGFDMW	IGH	27	None	
8	1639	CARLFYGSGELDYW	IGH	6	None	
8	1641	CARRDGTGWFSFDYW	IGH	4	None	
8	1643	CARDRPHYYDSSGPTPFDYW	IGH	17	None	
8	1644	CASDRGDCSTDGCYALDYW	IGH	5	None	
8	1645	CARDWFGHLDPSNYFDYW	IGH	10	None	
8	1646	CARPRAYYYYDSSRPFYNDYW	IGH	2	None	
8	1647	CAIVEDDYADYRTFDYW	IGH	7	None	
8	1648	CAKDIFRWAFDFW	IGH	9	None	
8	1649	CARDLPPELDTAMVVFGVDANNDYW	IGH	4	None	
8	1650	CANPSLSYCSGNSCYHDDYW	IGH	4	None	
8	1651	CAKVSRGYGYGYLGPFDIW	IGH	2	None	
8	1652	CARAAYFTDYRETSVSGPDKGYGFDIW	IGH	3	None	
8	1653	CVHRPSWYYYDSSGFYPVFAYW	IGH	9	None	
8	1654	CARSATPGWFGQLSDIAKYYYYMDVW	IGH	5	None	
8	1655	CARVGRGALNFDYW	IGH	7	None	
8	1657	CARDWTGVAVVGSFDYW	IGH	2	None	
8	1658	CTRGSERFGCSSPSCYAPFDYW	IGH	4	None	
8	1659	CASSPASSSWYLSWFDPW	IGH	2	None	

8	1660	CARDVVRGITTTTGYYMDVW	IGH	2	None	
8	1661	CTRYCGPDCYFDYW	IGH	3	None	
8	1662	CTTRSSGWVGDYW	IGH	2	None	
8	1663	CGTWDSGLSESYWVF	IGL	8	None	
8	1666	CVRAPDYYDSNGDYQYYLDYW	IGH	2	None	
8	1667	CARDTCTGGTCYSSLDSIDFDYW	IGH	5	None	
8	1668	CARINGELSEAFDVW	IGH	2	None	
8	1669	CAKATVAVAGPDSFDFW	IGH	2	None	
8	1670	CARDTDKSYYGSGSPDQDAFDFW	IGH	6	None	
8	1671	CARVGLDYYQSSGYYYNDVFDFW	IGH	2	None	
8	1672	CARGGKTVAHAYYFHSW	IGH	2	None	
8	1673	CARDDGGYTYTTGFDYW	IGH	2	None	
8	1674	CARHNHELNYDYW	IGH	2	None	
8	1675	CTGTNCYHFDSW	IGH	2	None	
8	1676	CARDSRWLQSRAFDIW	IGH	6	None	
8	1677	CARRMFGSSSRWLDPW	IGH	6	None	
8	1678	CARVGREPPSIDSFYFYMDVW	IGH	2	None	
8	1679	CAKVPGFFVVVVADGRPPSNWFDPW	IGH	5	None	
8	1680	CVTATEGRDYGGNYYDYYFDNW	IGH	3	None	
8	1681	CATPGALGFRDGIGAQDAFDIW	IGH	3	None	
8	1682	CVRDATLRYFDFFDNW	IGH	3	None	
8	1683	CARDTVTRREDWFDPW	IGH	4	None	
8	1685	CAKDKNHYDIMTGFYNHFYYMDVW	IGH	6	None	
8	1687	CARDSDTSSHLSQYDYW	IGH	3	None	
8	1688	CATNDAFDIW	IGH	9	None	
8	1689	CARDARSWRVVVATEFLFWFEYW	IGH	2	None	
8	1690	CARDLHSLSNILIRGGVVFDAFDLW	IGH	2	None	
8	1691	CAKGGDVTWDYNILTAKFDLW	IGH	3	None	
8	1692	CARIRYTYGYLYYFDYW	IGH	14	None	
8	1693	CARAQTSSSWAPYYYYYYYMDVW	IGH	4	None	
8	1694	CARDCTYCNDEGGFDYW	IGH	4	None	
8	1695	CARGPHSLHYYDSSGYQILFDYW	IGH	2	None	
8	1696	CARLVLDDSSPYSLDYW	IGH	4	None	
8	1697	CARLPPECSSTSCYPWYYYYMDVW	IGH	2	None	
8	1699	CARGGTTPTSSFDSW	IGH	4	None	
8	1700	CATDYCSDGCLFAEYFQHW	IGH	2	None	
8	1701	CVKDKGYYYDSIGSWDYFDYW	IGH	2	None	
8	1702	CLRQSYSSGEDYW	IGH	3	None	
8	1703	CARMPSSGYESPVDYW	IGH	2	None	
8	1704	CAIIPLGYFDHW	IGH	2	None	
8	1705	CRGSGTHDYW	IGH	5	None	
8	1706	CAGGGGEGYFDYW	IGH	2	None	
8	1708	CARVMSFYYDSSGFQRWFDPW	IGH	2	None	
8	1709	CARWDYCYTSSCYSFDYW	IGH	2	None	
8	1710	CARLCLHSSGWFPDDYW	IGH	3	None	
8	1712	CAKHSSDYYFGSVTFGGYNFFDPW	IGH	2	None	
8	1713	CAKDQCSTADCSDARFDYW	IGH	4	None	
8	1714	CARSLRDTVVVEDASAFYFDYW	IGH	3	None	
8	1715	CTTVLSLYGELYYYYYYMDVW	IGH	6	None	
8	1716	CARVSIGSAYFYYYYMDVW	IGH	3	None	
8	1718	CARPAGGDCCNFDYW	IGH	2	None	
8	1719	CARVSEHAAFDIW	IGH	5	None	
8	1720	CAKKYYHSPGYPYYFDYW	IGH	6	None	
8	1721	CATLGDMTTVTTVFGFDHW	IGH	2	None	
8	1722	CATQLQTRITIFGVVILHNWFDPW	IGH	9	None	
8	1723	CAAGGPGSGSRTFGFDPW	IGH	4	None	
8	1724	CARPILYYDSTGYFVDYW	IGH	2	None	
8	1725	CAKDHHPDSRTTYYSYYYMDVW	IGH	4	None	
8	1726	CAKVSLKTNSGYYFFDSW	IGH	2	None	
8	1727	CARDPLSSRDDFQPYFDYW	IGH	2	None	
8	1728	CAHLGGSPGSFFDYW	IGH	3	None	
8	1730	CARDTADSVRLDYW	IGH	3	None	
8	1731	CARVRQVNWFDLW	IGH	7	None	
8	1733	CARARFEGYDSSGYTHWYFDLW	IGH	3	None	
8	1734	CQYLNSYPLMYTF	IGK	68	C1	7BZ5
8	1735	CARPIVGAITDDAFDFW	IGH	2	None	
8	1738	CMRDRGGRSGCDYW	IGH	7	None	
8	1739	CARLHDYVWGSYRSHDYW	IGH	2	None	

8	1740	CASLYKKNDLLFQDDSFDVW	IGH	7	None	
8	1741	CARERGGGILTGYRWFDPW	IGH	2	None	
8	1742	CVRPRRPKIIYHFDLW	IGH	2	None	
8	1744	CARDIGSGPYTPQYYFDYW	IGH	4	None	
8	1745	CARDKLLSRVVVIVADKNWFDPW	IGH	2	None	
8	1747	CAKEGPPIFGTTPAFDYW	IGH	2	None	
8	1748	CAKYPRSYWYDSSGSSFFYYYYMDVW	IGH	2	None	
8	1749	CARDGRRNSGWFDYYYYMDVW	IGH	2	None	
8	1750	CQQYNNWPPRKNKCQF	IGK	3	C3	7K8R
8	1751	CAKDIKVGPYNSPGGGDAFDIW	IGH	7	None	
8	1752	CATVLFEVGAMPAFDYW	IGH	2	None	
8	1753	CARATRYCGGDCYPDYW	IGH	4	None	
8	1754	CLRDETANLDYW	IGH	4	None	
8	1755	CATYSADEGLSFDPW	IGH	5	None	
8	1756	CAKGAALGSCTGSNCYHFDCW	IGH	2	None	
8	1758	CARDGHLSSYFYFDLW	IGH	4	None	
8	1759	CARHGGWFLGYEFDNW	IGH	2	None	
8	1760	CARGRWQWISPFDYW	IGH	7	None	
8	1761	CAKGKGPGTVDWFDPW	IGH	3	None	
8	1763	CARDIYLEYSNSRYFYYLDVW	IGH	4	None	
8	1764	CARDEGWGSSTGWYSWYFDLW	IGH	2	None	
8	1765	CARDPTRPYYDILTGYYRDSDAFDIW	IGH	2	None	
8	1766	CARIKILAYYDSADAFDIW	IGH	2	None	
8	1767	CAHRKDLDQQFYIQSGYYYNNFFDFW	IGH	8	None	
8	1768	CARDRAEYCSGADCYSDAFDIW	IGH	7	None	
8	1769	CARKKHRDQTGGDAFDIW	IGH	2	None	
8	1770	CARGTTYCTGGWCSRGYFDSW	IGH	15	None	
8	1771	CARLDGYNLGDYW	IGH	4	None	
8	1772	CAKDYGPSAAADPYYFDYW	IGH	2	None	
8	1773	CARGDAGSTWYTAIDYW	IGH	4	None	
8	1774	CARGGVATTIFGVVIIDPDAFDIW	IGH	5	None	
8	1775	CTRILYGTGPPGDYW	IGH	2	None	
8	1777	CQSADISGSFPVIF	IGL	2	None	
8	1778	CAKDFVYYYDSSKDNHAFDIW	IGH	2	None	
8	1779	CARDLYYYDSRGYPHSLDAFNMW	IGH	2	None	
8	1780	CATVYCSGGSCYSARVQYYFDHW	IGH	2	None	
8	1781	CARTLYYHLDSSGYPFDLW	IGH	3	None	
8	1782	CARHWYGSSSPPDYW	IGH	8	None	
8	1784	CARDLDFYYDSSGYYPYYFDYW	IGH	4	None	
8	1786	CAKATFYYGSGTWGDTFDAW	IGH	2	None	
8	1787	CGRDKFGRGSGYYFFDYW	IGH	2	None	
8	1788	CQSYDSSLSNSLF	IGL	1220	C4	7JVC
8	1789	CARASYNSGWFYWYFDLW	IGH	2	None	
8	1790	CARDIGPYSNYAHRGTFDYW	IGH	4	None	
8	1791	CARGPSSIAAFRAFDIW	IGH	6	None	
8	1792	CQQTNNFPSISF	IGK	5	C3	7K8R
8	1793	CARDPRPPWDSSGYETTYYYYMDVW	IGH	5	None	
8	1794	CARINDIVIGYENFDPW	IGH	2	None	
8	1795	CTREAYVGQRERVITSAFDYW	IGH	3	None	
8	1796	CARGGREGYNFWVGTHSYFDYW	IGH	2	None	
8	1797	CARHEKWEQLKGNWFAPW	IGH	2	None	
8	1798	CAKDQAYQSAFDFW	IGH	3	None	
8	1800	CTRDQAYYDSSGYLDYW	IGH	2	None	
8	1801	CAHMFSLDYFDSW	IGH	5	None	
8	1803	CAKDRDGTGWFEGIDYW	IGH	4	None	
8	1804	CARDYYDSGGNYYPFDFW	IGH	4	None	
8	1805	CVKDWNSSGSYFQHW	IGH	4	None	
8	1806	CARLSYYGSGRYFDYW	IGH	15	None	
8	1807	CARDGHRSPDIVVVPAGYIAGYFDYW	IGH	17	None	
8	1809	CARRLIPGPFDIW	IGH	7	None	
8	1810	CARDRAYQLLPGGWFDPW	IGH	2	None	
8	1811	CAKDRLSASWFDHW	IGH	2	None	
8	1812	CATEEGSIWYKFYFNYW	IGH	4	None	
8	1813	CARVKFNWNDASAFDIW	IGH	2	None	
8	1815	CMQTMQLPWGF	IGK	8	None	
8	1816	CARELGREGNAGYFDLW	IGH	4	None	
8	1817	CVRDAGADSGYDPPLDYW	IGH	3	None	
8	1818	CARHGGTYGAGLFYFDYW	IGH	3	None	

8	1819	CQSYDSSLSDSTVSGPGVF	IGL	12	C4	7JVC
8	1820	CARESGLKQWVQRLYFDYW	IGH	2	None	
8	1821	CARDRYSSGWSSGWYFDLW	IGH	5	None	
8	1822	CARTNWNYVDYYYYMDVW	IGH	4	None	
8	1823	CAKDLYTNLHYMDVW	IGH	8	None	
8	1825	CARHGPVTSYWYFDLW	IGH	2	None	
8	1826	CTRDFPPRAYMVYYMDVW	IGH	2	None	
8	1827	CAKGLFAHFGSGYYRDFFDYW	IGH	70	None	
8	1829	CASGPAAAHYSYYYYMDVW	IGH	4	None	
8	1830	CAITMVRGVLLYYFDYW	IGH	4	None	
8	1831	CATSTKKGDYNFWSGYQSHLDYW	IGH	3	None	
8	1832	CVREEYSGNDGPDYW	IGH	2	None	
8	1833	CTKASYSITWYVWFDPW	IGH	5	None	
8	1834	CARESVLTGKHSYFYYYYIDVW	IGH	3	None	
8	1835	CASYGDYPDFW	IGH	4	None	
8	1836	CVKEQVRGSYLAFDIW	IGH	2	None	
8	1837	CARGREGTRVTTIDYW	IGH	4	None	
8	1838	CARDRLYDLLTGYPRGDASDIW	IGH	7	None	
8	1839	CARGYYDLSAGFDYW	IGH	5	None	
8	1840	CARDNRVYTHGSDFDYW	IGH	4	None	
8	1841	CAKDGQWELNYYYYYMDVW	IGH	2	None	
8	1842	CARPDFFDGVDIW	IGH	2	None	
8	1843	CTRDWWRFFFDFW	IGH	2	None	
8	1845	CAKDTTVAGAKGSFDGW	IGH	2	None	
8	1846	CASPDCRWTLCYGDVGPDYW	IGH	5	None	
8	1847	CARKRMEFDYW	IGH	36	None	
8	1848	CARSVGYCADGSCQPTFDYW	IGH	2	None	
8	1849	CATSRPDYPFDNW	IGH	2	None	
8	1850	CARDYYDSSGPYWYFDLW	IGH	4	None	
8	1851	CARQGLVVVAPGDHRWFDPW	IGH	4	None	
8	1852	CAIGNIHSSHYYGWLDPW	IGH	2	None	
8	1854	CARKYYSEGPFDLW	IGH	9	None	
8	1855	CARDWAGPEGRGWFDPW	IGH	3	None	
8	1856	CASGHENCSAGSCYCSFDDW	IGH	4	None	
8	1857	CVRGSSDWAGTDYW	IGH	2	None	
8	1859	CAKRRLWFGELVPISKGGFDYW	IGH	2	None	
8	1860	CARLGYSSSWHPSSYYYYYMDVW	IGH	2	None	
8	1864	CARTTPSEFGNWPLFDYW	IGH	7	None	
8	1866	CTTDRGYYDSSGYYYTYYFDYW	IGH	3	None	
8	1867	CRQLNHYPHTF	IGK	2	C1	7BZ5
8	1868	CARGTYGSANFYAGFDYW	IGH	42	None	
8	1869	CAAWDNSLSGRGLF	IGL	6	None	
8	1871	CARDRGYFDSSGYTQGYYFDYW	IGH	3	None	
8	1872	CARHPDYDSSGPITGYYYYIDVW	IGH	2	None	
8	1873	CARTNGYTSSWNDAFDIW	IGH	4	None	
8	1874	CQQLDGYPPMLIF	IGK	23	C1	7BZ5
8	1875	CVTLAAAGTSDYW	IGH	4	None	
8	1876	CAHRRHYYDSSGRYYYFDYW	IGH	5	None	
8	1877	CAKDMGTQPFNSGRFDSW	IGH	2	None	
8	1878	CARGDYGSGTYPFKYW	IGH	2	None	
8	1879	CVREGGVYSSDSSGRFDYW	IGH	2	None	
8	1881	CVKEHTGTYNGAFDIW	IGH	2	None	
8	1882	CARYHYDSSGHFDYW	IGH	2	None	
8	1883	CARGGYKYGTGWFDPW	IGH	4	None	
8	1884	CGRSNWDYDFDPW	IGH	2	None	
8	1885	CARDSASSLYRDNYIDYW	IGH	2	None	
8	1886	CARSPPTGWVVTAIPYYFDYW	IGH	3	None	
8	1887	CARDPPYYDILTGYSTTYYFDYW	IGH	2	None	
8	1888	CARTPNSFGSVNFDYW	IGH	2	None	
8	1889	CAATKASLYDLLTGYYSGYFDSW	IGH	2	None	
8	1891	CAKDLRSGTYQMYYFDYW	IGH	2	None	
8	1892	CARGYCRSTTCYFDYW	IGH	19	None	
8	1893	CAKGVDIVVATLDYW	IGH	2	None	
8	1894	CARDSEPPAAAGIYADFNWFDPW	IGH	2	None	
8	1896	CAKDSHSGYDVYFYYMDVW	IGH	4	None	
8	1897	CARGDGDYVYPGVFDIW	IGH	2	None	
8	1898	CARDSARFLEWSRPYYYMDVW	IGH	4	None	
8	1899	CARERGTAVSGTDAITLPLYAFDMW	IGH	2	None	

8	1900	CARQKGALWYFDLW	IGH	9	None	
8	1901	CSSSSCYAPFDFW	IGH	3	None	
8	1902	CVKNVGTGWARDNYYYYYLDLW	IGH	2	None	
8	1903	CARGPHYYYYGGYYHPNFDYW	IGH	2	None	
8	1904	CAKGRAAMVIDDAFDIW	IGH	2	None	
8	1905	CVRVDWPYYYYYYMDVW	IGH	2	None	
8	1906	CVRGSDLFSTDWYFDLW	IGH	2	None	
8	1907	CTTNRYSSAMDHSYYYYMDIW	IGH	8	None	
8	1908	CASDGGSGWDFDYW	IGH	3	None	
8	1909	CAKRDGHGDFDYW	IGH	7	None	
8	1910	CARAGRYGGYYMDVW	IGH	11	None	
8	1911	CARVGKLMIYTTDAFDVW	IGH	12	None	
8	1912	CARRPHYYDTSGTAWYFDFW	IGH	2	None	
8	1914	CARDCDSCYGDSDAFDIW	IGH	2	None	
8	1915	CAKDGKGFLSDYW	IGH	8	None	
8	1916	CARQRLRVSKPDYW	IGH	5	None	
8	1917	CTTVDNFDWLFRVDYW	IGH	2	None	
8	1918	CARGPYNYGTYFDNW	IGH	4	None	
8	1919	CARHQVIYYYYMDVW	IGH	20	None	
8	1921	CARSYYYGSETDYW	IGH	4	None	
8	1922	CARDHRENMVRGLMRWFDPW	IGH	2	None	
8	1923	CAKDIEGGDGYNYYPDAFDIW	IGH	3	None	
8	1924	CARTISSYREHYFDYW	IGH	2	None	
8	1925	CARHYGRRYCGGGRCPNFDYW	IGH	2	None	
8	1926	CARDKFFDGSGYRYNGFDAFDVW	IGH	4	None	
8	1927	CLSADSSGSYPFVF	IGL	19	None	
8	1928	CARGGGIVRYYYDSNGFDYW	IGH	2	None	
8	1929	CTTDGLITGTTAHDYW	IGH	2	None	
8	1930	CVRDQWLAPFDYW	IGH	4	None	
8	1931	CAKSLYFDWAQGGTFDYW	IGH	4	None	
8	1932	CARAGGAEAFNIW	IGH	26	None	
8	1934	CIKDVQGITFDAFEVW	IGH	7	None	
8	1935	CARGGITVVREMNLRYYYLDVW	IGH	2	None	
8	1936	CARDLDGITGTTSGDWYFDLW	IGH	2	None	
8	1937	CARDTGRDPLYYYYYMDVW	IGH	2	None	
8	1938	CQEYGYSPHSVTF	IGK	2	C1	7K8N
8	1940	CAKDNFDILTNIYPTQYYYYMDVW	IGH	5	None	
8	1941	CATEGEDRCSGSSCYSGWLDSW	IGH	2	None	
8	1942	CQQNANLPPAF	IGK	9	None	
8	1943	CARHGTYASSGYYFGYFDYW	IGH	2	None	
8	1944	CARSFGSSGSFYYYMDVW	IGH	4	None	
8	1945	CQSYDSSLNVYMIF	IGL	10	C4	7JVC
8	1946	CAREGRITIFGVVTTYYFDYW	IGH	2	None	
8	1947	CANSIRGKDNGDNEYRDFDYW	IGH	2	None	
8	1948	CARVGHEYESGGSRFSTFYFDSW	IGH	2	None	
8	1949	CSRQECTSTSCAAFNFDYW	IGH	2	None	
8	1950	CARVEMAPIKDYW	IGH	2	None	
8	1951	CARGNLGVADLGDYW	IGH	2	None	
8	1952	CLQYNNWPPSLSTF	IGK	2	C3	7K8P
8	1953	CARLAPQFLGGYYFDYW	IGH	2	None	
8	1954	CASLDYYDSSHHFDGRWFDPW	IGH	2	None	
8	1955	CARITWYGSGERAFDIW	IGH	5	None	
8	1956	CAKTPRENANPYWYFDLW	IGH	2	None	
8	1957	CGTWHSNSKTHWVF	IGL	61	None	
8	1958	CARSGGHLPPTEFDPW	IGH	2	None	
8	1959	CARTAAWGGDSPWYFDFW	IGH	62	None	
8	1960	CAKGNHDTSGYYHWLYFDYW	IGH	2	None	
8	1962	CVRDSLPAVSVSGTYYFDYW	IGH	6	None	
8	1963	CARDRSGYSYGYRFDYW	IGH	4	None	
8	1964	CARIRTTDYSDCFDFW	IGH	2	None	
8	1965	CANSIRGKEDSGDYADFDYW	IGH	2	None	
8	1966	CATDRYYFDILTGYENWFDPW	IGH	4	None	
8	1967	CARLGIITVFGVLTVPNWFDPW	IGH	3	None	
8	1968	CARDSSYYDILGPQSYYFDYW	IGH	2	None	
8	1969	CQQRTTWPRLAF	IGK	28	None	
8	1970	CARGTGDVAVEMSVERFVTIRNWFDPW	IGH	2	None	
8	1971	CARGEYTNTMSQRVNWFDPW	IGH	3	None	
8	1972	CARDLCSGGSCYNIDYW	IGH	2	None	

8	1973	CARALLWDRITAASYLDYW	IGH	3	None	
8	1974	CARLPSDYDILGGYSSFRYGFYFDSW	IGH	2	None	
8	1975	CARYPGTSGIMDYW	IGH	2	None	
8	1976	CARCGDVVVSAIDQPFDFW	IGH	2	None	
8	1977	CARVPACSGPGCYIWYFGLW	IGH	3	None	
8	1978	CAKDRAAYSSREVDYW	IGH	2	None	
8	1979	CATSRAHYYLDSFDVW	IGH	4	None	
8	1980	CAKGVIQGVTGFDIW	IGH	3	None	
8	1982	CHQFKSYPHSF	IGK	67	C3	7K8R
8	1983	CARGTSSSAFYDYW	IGH	5	None	
8	1984	CATDTSGYSEYFLHW	IGH	5	None	
8	1985	CARAGITVAGASVGFIFDYW	IGH	2	None	
8	1986	CARDFPLGAARPGYMDVW	IGH	2	None	
8	1987	CAKSGGGGYFYYFDSW	IGH	2	None	
8	1989	CARGVSRGGVGATTDYW	IGH	2	None	
8	1992	CTTYKYGDIFHDYFDYW	IGH	6	None	
8	1994	CARDGYQVRGLDYW	IGH	8	None	
8	1995	CARPGRAPSRPHYYYMDVW	IGH	3	None	
8	1996	CAREGEQHLVVSDASAYFFDYW	IGH	2	None	
8	1997	CQQYNRDSHSITF	IGK	52	None	
8	1998	CARADNHGYFDAFDIW	IGH	3	None	
8	2000	CAIPPPGGYDFDAFNIW	IGH	2	None	
8	2001	CATLGYYYAYW	IGH	2	None	
8	2002	CARHYGDSVNWFDPW	IGH	18	None	
8	2003	CARDLGRDGYNSGSFDYW	IGH	5	None	
8	2004	CAKDLPYSSGADYW	IGH	4	None	
8	2005	CHQYNSHSYTF	IGK	194	C3	7K8P
8	2006	CAKEGIPYTIATKRGYMDVW	IGH	15	None	
8	2007	CARLAGRRMIVVVITGSYYFDYW	IGH	3	None	
8	2008	CARDHSNSDFKRSWGFDYW	IGH	2	None	
8	2009	CAKDESTYLTGYSYFDYW	IGH	6	None	
8	2010	CAKDQGLSWYFFDFW	IGH	4	None	
8	2011	CTTSYVVGADYFDYW	IGH	6	None	
8	2012	CARDRVPFRDHYESSDIDYW	IGH	2	None	
8	2013	CAKFGTTMTVQHYNSFDPW	IGH	2	None	
8	2014	CARDRSSSWSTFHYW	IGH	8	None	
8	2015	CAREVPHGVGSYEVFDMW	IGH	2	None	
8	2017	CAKAAEVAVDLNWFDRW	IGH	5	None	
8	2018	CARQKNAVVRGLLDYW	IGH	2	None	
8	2019	CARLSGTPFFGVNAVRYWFFDLW	IGH	2	None	
8	2020	CARDLKSYNNGWYTADYMDVW	IGH	3	None	
8	2022	CATGRLGELSYGIYFASW	IGH	2	None	
8	2023	CARHYCSSSACRGNWFDPW	IGH	2	None	
8	2024	CARVQRYKSEEYNWFDPW	IGH	2	None	
8	2025	CARRLEFRSDSSGPYFFDLW	IGH	2	None	
8	2026	CQQFNSYREVVLF	IGK	2	C3	7K8R
8	2027	CVRGGFQYGANSFDLW	IGH	2	None	
8	2028	CASDNSFMAEYYFDYW	IGH	2	None	
8	2029	CARVSAQRSSWYIGSLDYW	IGH	5	None	
8	2031	CARESSGWSNFDYW	IGH	15	None	
8	2032	CVRHRYSATTTYSDPFDSW	IGH	2	None	
8	2033	CARIDSRGEAFDVW	IGH	12	None	
8	2034	CARHRRSITMVREYRLYYFDYW	IGH	9	None	
8	2035	CATDRILLVPVAYDAFDVW	IGH	2	None	
8	2036	CARYKKETRDGSGRTFDYW	IGH	3	None	
8	2037	CARGQDGTILTGPDYW	IGH	2	None	
8	2038	CARESGYNRYFENW	IGH	7	None	
8	2039	CARRRWDLLFDYW	IGH	12	None	
8	2040	CARGVEGLGELSPSYREYYYYMDVW	IGH	4	None	
8	2041	CATLWFGEFPFDFW	IGH	2	None	
8	2042	CVRGTSAYYYYYMDVW	IGH	2	None	
8	2044	CAKATRTTWYGDVFDLW	IGH	2	None	
8	2045	CAKRDTSILGVARGFDPW	IGH	4	None	
8	2046	CARPYAPYSYGYFDSW	IGH	2	None	
8	2047	CARLFPAVIRRGLIVGDDYW	IGH	2	None	
8	2048	CASWSRTPGEYYFDYW	IGH	2	None	
8	2049	CAREPGSYGDHYFDKW	IGH	7	None	
8	2052	CARAGYCTGSSCYDAFDIW	IGH	6	None	

8	2053	CATLGPYFFDGSGINPPDYW	IGH	2	None	
8	2054	CAKDEGYCSSVSCYPLFDYW	IGH	3	None	
8	2055	CAISRYSPPSYHYYMDVW	IGH	3	None	
8	2056	CTRDSSLGVGATDYW	IGH	3	None	
8	2057	CAHTIQWFGEFTYYFFDSW	IGH	19	None	
8	2059	CARAGYTYGYFPDAFHIW	IGH	2	None	
8	2060	CAKDYRIVVTGNYFDYW	IGH	3	None	
8	2062	CARRSLGTQYFDSGAEYFHHW	IGH	2	None	
8	2063	CATGIGVAGGGFYFDYW	IGH	2	None	
8	2064	CARQAGFGELLPYFDFW	IGH	5	None	
8	2065	CAKGMGHWKLLSAFHVW	IGH	3	None	
8	2066	CARDRTDGFSHFDYW	IGH	12	None	
8	2067	CARTEYYDSSVPGGFDPW	IGH	2	None	
8	2069	CARGGVGARRRFDHW	IGH	3	None	
8	2070	CARHTYGFDAFDIW	IGH	18	None	
8	2071	CASWAGRTNNWLWGPLDYW	IGH	2	None	
8	2073	CARHKRLGRVGATGVPDYW	IGH	2	None	
8	2074	CAKDITRYLHFRYFDLW	IGH	2	None	
8	2075	CLQYGSSQGITF	IGK	30	None	
8	2076	CAIAGDYYYMDVW	IGH	3	None	
8	2077	CALPILWFGEFWYFDLW	IGH	2	None	
8	2078	CAKDRVVILATTRGFDFW	IGH	2	None	
8	2079	CAKSLDYDILAGYAHYFDYW	IGH	8	None	
8	2080	CATDLKQQVVQFDFW	IGH	2	None	
8	2081	CTRDHVTTDEFDYW	IGH	2	None	
8	2082	CARHPGPNPTRTAAATNYYYYMDVW	IGH	2	None	
8	2083	CAKGSRPTDTWPRGASHFDYW	IGH	3	None	
9	1	CANEDYGATPRAFDIW	IGH	9	C2	7K8U
9	2	CARLGYCDSSCPRGGGFAYW	IGH	4	None	
9	3	CARSDSADDFDIW	IGH	18	None	
9	4	CVREQTPSNHNWFDLW	IGH	2	None	
9	5	CARDGQQGGPWAFDYW	IGH	8	None	
9	6	CARVGVATQTSPFDYW	IGH	5	None	
9	7	CATAYNYVGSWYFDYW	IGH	6	None	
9	8	CAHSRVWSGYSMLFDPW	IGH	4	None	
9	9	CTTGLDDIVVVPAASRDTHYYYYYMDVW	IGH	2	None	
9	10	CQSYDGSLTEPDVIF	IGL	3	C4	7JVC
9	11	CARDSASASYSSSRFSGVYYYGMDVW	IGH	2	None	
9	12	CSAWDKSLQASVF	IGL	2	None	
9	13	CQQSDRSPHAEGYTF	IGK	13	None	
9	14	CARPLRGYSGYELYWYFDLW	IGH	2	None	
9	15	CARRKGSDTFDFW	IGH	4	None	
9	16	CARIQGEYEGSGSSLDYW	IGH	3	None	
9	17	CARATVPGRLDGFDIW	IGH	3	None	
9	19	CQSYDHTNYGVF	IGL	28	C4	7JVC
9	21	CQQYNTYTHITF	IGK	2	C3	7K8R
9	22	CARDGLLMVYSAGNDFDVW	IGH	2	None	
9	23	CARLVGYCSSTSCYTGAFDIW	IGH	8	None	
9	25	CAREGSYEPLEFDPW	IGH	4	None	
9	26	CARGIESYGDPMDVW	IGH	2	None	
9	27	CVRDRSYLDCSIISCPFDYW	IGH	4	None	
9	28	CVKDPRPGSGWLMNYFDYW	IGH	2	None	
9	29	CAKDGFDCSSTSCYYYYYYYMDVW	IGH	7	None	
9	30	CTTSSGSYFWFPTPEFDHW	IGH	2	None	
9	32	CARKSYDSSGYYTGFDYW	IGH	3	None	
9	33	CQHYGSSVQGSF	IGK	24	C1	7K8N
9	34	CARDPYSNSSGGRDYW	IGH	2	None	
9	35	CGKVVVLPSASPDYW	IGH	14	None	
9	36	CARTPPTTNNHLDYW	IGH	7	None	
9	37	CARDFSYGGNYFDYW	IGH	21	None	
9	38	CARRNYDTRGYSSFDYW	IGH	2	None	
9	39	CARDGGGYSGYDTYYYYYMDVW	IGH	2	None	
9	40	CARAGEDYFGSGNYYPDIW	IGH	2	None	
9	41	CARSSGIAEVPQFDYW	IGH	6	None	
9	42	CARNQMTSGDYVDYFDFW	IGH	2	None	
9	43	CVRHHYGWYSMRAFDIW	IGH	2	None	
9	44	CARVVDTAMVYDAFDIW	IGH	2	None	
9	45	CAKFGGLADRQNDYW	IGH	2	None	

9	46	CQQRANWLLSRYTF	IGK	3	None	
9	47	CARVPAMPYGADVPWYFDYW	IGH	2	None	
9	48	CLKESIQVGGPNYFDLW	IGH	14	None	
9	49	CATHPRFGVDAFDIW	IGH	2	None	
9	50	CARLIAAGGGYQSSFDYW	IGH	2	None	
9	51	CARGSITGTLGEDALDIW	IGH	4	None	
9	52	CARARGSGTNSFDYW	IGH	6	None	
9	53	CVKDKGAWESDYFDYW	IGH	4	None	
9	56	CAGGRFITTPLYFDYW	IGH	2	None	
9	57	CARIGRIHLWSPGHFDSW	IGH	2	None	
9	58	CARSSGYDLIAVTHFDYW	IGH	2	None	
9	59	CARDSSAYSSGWYYFDYW	IGH	6	None	
9	60	CNQGTHRPFTF	IGK	2	None	
9	61	CASSIAAAGLPLDYW	IGH	2	None	
9	62	CARTLAYYYASGSYPYYFDYW	IGH	2	None	
9	63	CSTCGSYCDYW	IGH	4	None	
9	64	CARALNDILTGYYNYW	IGH	2	None	
9	65	CASNSGYDSFDNW	IGH	2	None	
9	66	CVKVTPYCSGGSCSDYW	IGH	6	None	
9	67	CQHYGSSPIFTF	IGK	262	C1	7K8N
9	68	CAKDRTGGGPYHFDYW	IGH	6	None	
9	69	CAHAKDIVVVTGYFDYW	IGH	4	None	
9	70	CAKDRVAVAATYFDYW	IGH	6	None	
9	71	CARGRLRFSGWSYFDHW	IGH	5	None	
9	72	CATEDAWGAFDNW	IGH	2	None	
9	73	CGRDDAEMAAVQNDAIDIW	IGH	4	None	
9	74	CTRETYDCSGSSCYTMPLDYW	IGH	2	None	
9	75	CARGIRGVVDYW	IGH	24	None	
9	76	CARSNLGQWLASNAFDVW	IGH	2	None	
9	77	CATTYSGFDVFDLW	IGH	7	None	
9	78	CARAGYCSGGSCNYYFDYW	IGH	5	None	
9	79	CARLAAYDNSGYIYYW	IGH	2	None	
9	80	CAKKTRSGPAAAGLDYW	IGH	2	None	
9	81	CAMGDLWFDPW	IGH	12	None	
9	84	CTTDKQMSPDYW	IGH	2	None	
9	85	CAKRDGYDWGSAFDIW	IGH	2	None	
9	86	CAKQIAAAGTKAGISTEYYFDYW	IGH	2	None	
9	87	CAKDRDFWSGYHPFDYW	IGH	3	None	
9	88	CVRHARLADYW	IGH	8	None	
9	89	CAKESGPVPPYFDCW	IGH	6	None	
9	90	CARRNIAAADHWYFDLW	IGH	2	None	
9	91	CQHSYGTPLFTF	IGK	54	C2	7K8O
9	92	CAKADRGGYCSGAGCYQYIDYW	IGH	23	None	
9	93	CVRIHGTNRNYVFNWFDTW	IGH	2	None	
9	95	CAKDIGRQSVEGSFDYW	IGH	6	None	
9	96	CARDIPYAGSFLYFDFW	IGH	2	None	
9	99	CARRKAYNYEDAIDSW	IGH	4	None	
9	100	CARLHRGGSSTSVGGNWFDPW	IGH	5	None	
9	101	CQQANSFPLFF	IGK	108	C3	7K8P
9	102	CATRTRWYPAYYFDYW	IGH	2	None	
9	103	CAHRAIFGVVSYYFDYW	IGH	2	None	
9	105	CAKLASHYGSGNYYDTFDIW	IGH	4	None	
9	106	CARRVANTIIDYW	IGH	2	None	
9	107	CANIPMTTVTVVAFDIW	IGH	4	None	
9	108	CARVRYHDAFDYW	IGH	11	None	
9	109	CAKDRVAVAGPYWYFDLW	IGH	2	None	
9	110	CARGDCSGGSCSSRLAFDIW	IGH	2	None	
9	111	CARGPHRGYCTNGVCYLGYW	IGH	2	None	
9	113	CAREVGSVTALNAFDIW	IGH	4	None	
9	114	CATLKPSDGAFEVW	IGH	4	None	
9	115	CARHRCSSPSCYGSSWQNWFDPW	IGH	2	None	
9	118	CAKLAPNGFDLW	IGH	5	None	
9	119	CAKDRAIAVAGTSNYFDYW	IGH	2	None	
9	120	CAKAYYDFWSGYSDHFDYW	IGH	12	None	
9	121	CARDPCSGGGCYSALDYW	IGH	2	None	
9	122	CARFQNYYDSSLVAFDIW	IGH	3	None	
9	124	CQQYYTTPQAYTF	IGK	71	C4	6W41
9	125	CAREVVSDYDDFWSGYFDYW	IGH	2	None	

9	126	CAKGRETVATIDAFDFW	IGH	4	None	
9	127	CARHWLVRGRYDAFDIW	IGH	2	None	
9	128	CAKVSFWGWYFDLW	IGH	2	None	
9	129	CSRVRQLGPIYYFDFW	IGH	2	None	
9	130	CARRPYAGGWFDPW	IGH	6	None	
9	131	CQQRTDWPPLSSF	IGK	3	C1	7BZ5
9	132	CAGSKLRFLESYHYMDVW	IGH	2	None	
9	133	CARSKINSLSPSIYYFDYW	IGH	4	None	
9	134	CASSKFGGSTPFDHW	IGH	2	None	
9	135	CARVRRNFSRVIVVASNWFDPW	IGH	2	None	
9	136	CARRVYSYGYGVNSHYYMDVW	IGH	4	None	
9	137	CAKESVVVVPDSSYYFDYW	IGH	2	None	
9	138	CARDPQLPKDTAPGYYFDYW	IGH	2	None	
9	139	CTRGFPIWWAAPFYHFDFW	IGH	3	None	
9	140	CAKDKYGYSGSALDYW	IGH	2	None	
9	141	CARLSCTTASCSMWDSFDLW	IGH	4	None	
9	142	CARRNGRLVLGGFDYW	IGH	2	None	
9	143	CARTGTTAEGHAFDIW	IGH	2	None	
9	144	CARGMYNYGVGSPYQNWFDPW	IGH	2	None	
9	145	CAKGLKGYDSGSYGTYYYYYMDVW	IGH	2	None	
9	146	CQKYSGAPQPLTF	IGK	5	None	
9	147	CDWSSDVCSSDLYNDYFDQW	IGH	2	None	
9	148	CARATITTFNWLDPW	IGH	2	None	
9	149	CARHVHADYFDSESYSDFDYW	IGH	4	None	
9	150	CASGLPSGVIININAFDIW	IGH	2	None	
9	151	CARRGDQWPHWHFDHW	IGH	2	None	
9	152	CATGRPGAGRYFDLW	IGH	2	None	
9	153	CASTEGAYYYDSSGYYFDYW	IGH	6	None	
9	154	CSSSKLSHPQEDGFDVW	IGH	3	None	
9	155	CARGFSPLWAAPFYYFDYW	IGH	4	None	
9	156	CAREVRSGSYYYYYSMDVW	IGH	51	None	
9	157	CARVGTYSSPPADYW	IGH	2	None	
9	158	CAREIVVVPAAIRELLWEDDYW	IGH	2	None	
9	159	CAKLAYNDYAFDIW	IGH	2	None	
9	160	CVRDGDGHYPIDYW	IGH	3	None	
9	161	CAASNRFRDAFEIW	IGH	2	None	
9	162	CARVKTTWPRGWFDPW	IGH	4	None	
9	163	CAKEVFSNYDILTGYDYW	IGH	2	None	
9	164	CARAGSFPMVRGVIFFYW	IGH	2	None	
9	165	CQQYKFYPIAF	IGK	5	C3	7K8R
9	166	CARSAKELVLLNWFDPW	IGH	2	None	
9	167	CAKDLDTYRYGSAFDHW	IGH	2	None	
9	168	CARANGGPYYYMDVW	IGH	4	None	
9	169	CARVTFSHYYHSRNGPDYW	IGH	2	None	
9	170	CAKVRWNDGRIFDYW	IGH	2	None	
9	171	CAKSCYSSSWCNFDYW	IGH	6	None	
9	172	CQSADNSGVYGVLF	IGL	9	None	
9	173	CVLYMGSGIWVF	IGL	88	None	
9	174	CATDHVVVPSALDYW	IGH	2	None	
9	175	CAKDIRFPGTDYMDVW	IGH	13	None	
9	176	CAGCEVCSSVTYSDYW	IGH	2	None	
9	177	CAKSSRSYLGLLYFDYW	IGH	2	None	
9	178	CAKGGLSGIAARLKVLTYFDYW	IGH	2	None	
9	179	CARHARDHFGVGIKDFDPW	IGH	2	None	
9	180	CATFRSGYFFRYW	IGH	2	None	
9	181	CARHPKDIMKLLKGVNAFDIW	IGH	2	None	
9	182	CARVHYYGSGSHPFDYW	IGH	4	None	
9	183	CARWGPANNPGFDYW	IGH	2	None	
9	184	CAREDFYDVADSYSWYFDLW	IGH	2	None	
9	185	CARLAGSSWLRNWYFDLW	IGH	2	None	
9	186	CARHEDTSSRFFDYW	IGH	2	None	
9	187	CARMAATNTFHSFDTW	IGH	2	None	
9	188	CARHWQIRGHSGQDYVFDIW	IGH	2	None	
9	190	CAREPEDGYNFGLDYW	IGH	2	None	
9	191	CARHPVTIFGVDPSYYYYMDVW	IGH	2	None	
9	192	CARGHSSGWNDDAFEIW	IGH	2	None	
9	193	CAKVPVLSWAALVDYW	IGH	5	None	
9	194	CQQFSSYLFTF	IGK	13	C3	7K8R

9	195	CGILAYCSTTSCSGTDYW	IGH	3	None	
9	196	CARHSSRRTEADWIDPW	IGH	2	None	
9	197	CAKCQQAWELRAYDGFGIW	IGH	2	None	
9	198	CARVARGSPSGDLPAFDSW	IGH	2	None	
9	199	CARHLWRGSDNWYLDLW	IGH	2	None	
9	200	CARENAAYYIDYW	IGH	36	None	
9	201	CQKYRSARSAF	IGK	2	None	
9	202	CARLPYSLFYFDFW	IGH	4	None	
9	203	CVRHTSSQEGYFDPW	IGH	4	None	
9	204	CARIRTLVVIASYDAFTIW	IGH	2	None	
9	205	CVKDGGYYYYYMDVW	IGH	2	None	
9	206	CVKDDYYASGNFASNFDCW	IGH	9	None	
9	207	CARTRKHYYDNSAYHDYW	IGH	2	None	
9	208	CARFRFDHGGDSIGGFDYW	IGH	6	None	
9	209	CAKGSHYCSSTSCHRAVDYW	IGH	2	None	
9	210	CTIVDAVGATVLSEFDFW	IGH	2	None	
9	211	CAKVVVAAKATRNYYFDYW	IGH	6	None	
9	212	CTQATLVPWTF	IGK	2	None	
9	213	CARHDCNSVTCYIDYW	IGH	4	None	
9	214	CAKVGPEYSDFWSGYYDFDYW	IGH	4	None	
9	215	CAREIDWNYYFDYW	IGH	13	None	
9	216	CTRDVVYSRGWYYFDSW	IGH	7	None	
9	217	CARVDRFGESLELVYYIDVW	IGH	2	None	
9	218	CARGDRYCSSTSCYLTDAFDIW	IGH	3	None	
9	219	CQQRRSWPPGLAF	IGK	2	C1	7BZ5
9	220	CARHQGFHYSDSSAYDTFNIW	IGH	5	None	
9	221	CAGGNGSPNWFDPW	IGH	8	None	
9	222	CQHFNNWPPRITF	IGK	52	C1	7BZ5
9	223	CARDKVIVVVPAAISYYYYYMDVW	IGH	2	None	
9	224	CARGRAYSSGWSFDDW	IGH	4	None	
9	225	CAKLRPELGANPHYFDSW	IGH	2	None	
9	226	CARLDYDPWSDNNWFDPW	IGH	2	None	
9	228	CVRDRENFKYYFYMDVW	IGH	4	None	
9	229	CARHRHYYDSRGYNAFDIW	IGH	4	None	
9	230	CARLHFVVLVTHRFDSW	IGH	15	None	
9	232	CASAVTTKKIFDYW	IGH	2	None	
9	233	CARNNYGDFSGGNFDYW	IGH	3	None	
9	234	CARYGGYISIDYW	IGH	2	None	
9	235	CAKTESISSVDPFDIW	IGH	3	None	
9	236	CARGTGYCSSTSCYTALDYW	IGH	2	None	
9	237	CQVWDGSGAQYVF	IGL	4	None	
9	238	CARHYYDTTGFYAGDPMDYW	IGH	4	None	
9	239	CARVYSSGWLEYFQHW	IGH	3	None	
9	240	CANVEYSSSWDYW	IGH	2	None	
9	241	CARYPFLYYDSSGYFDYW	IGH	8	None	
9	243	CARARGYDSSRWYYFDSW	IGH	3	None	
9	244	CARPEYSSGWFLDYW	IGH	2	None	
9	245	CAKDNSGYPSPSFDYW	IGH	2	None	
9	247	CAKDLQIAVAYDAFDIW	IGH	2	None	
9	248	CQVWHSSSDLDVVF	IGL	4	None	
9	249	CARNFNYDSSGLDYW	IGH	3	None	
9	250	CARGVYCSGGSCYSVVDYW	IGH	4	None	
9	251	CARVDFNSFDPW	IGH	6	None	
9	252	CARDASYSGSYGTLDAFDLW	IGH	2	None	
9	253	CMQTIELPLCSF	IGK	15	None	
9	254	CVYCGGDCYSSVDAVDIW	IGH	2	None	
9	255	CARTSYSDISGYHYYFDYW	IGH	2	None	
9	256	CQVWDTNTAHPVVF	IGL	11	None	
9	257	CAHISGTTSSFDYW	IGH	3	None	
9	258	CAKDGSHGHLDYFDYW	IGH	2	None	
9	259	CARSRGDWRGFDYW	IGH	2	None	
9	260	CASLTSSDTSSFDYW	IGH	5	None	
9	262	CARGQETAIVRGDYYYFYMDVW	IGH	4	None	
9	263	CARGTTNPGVDYW	IGH	5	None	
9	264	CAKDVRYGDYRPYYFDYW	IGH	2	None	
9	265	CARHSWRGPDYFFDYW	IGH	2	None	
9	266	CARDSSREEWLLYGSSYFYYYMDVW	IGH	3	None	
9	267	CARIPYYGSGSYEDYW	IGH	2	None	

9	268	CAREELLTGVLGIQFKVFDIW	IGH	2	None	
9	270	CARGGDFWSGYPSDYFDYW	IGH	2	None	
9	271	CAKAEDIVVVPTLEIDYW	IGH	7	None	
9	272	CAREGGAEDGRDYYYYMDVW	IGH	2	None	
9	273	CAKGAGDFWTNYNWGTWFDPW	IGH	2	None	
9	274	CARPYSISQIARFDYW	IGH	2	None	
9	275	CAKSGVVCSGSSCYSYYYYIDVW	IGH	2	None	
9	276	CGTWDSSLSAVVF	IGL	318	None	
9	277	CARDLLPGKRVNWFDPW	IGH	2	None	
9	278	CATDLPRSDRQALDYW	IGH	4	None	
9	279	CARGRLSGTIIDAFDIW	IGH	3	None	
9	280	CARPATWGADWYLDLW	IGH	3	None	
9	281	CARASSQYQLFPGNDAFDIW	IGH	2	None	
9	282	CARDRGAPYGDYAFDLW	IGH	2	None	
9	283	CTLFPYTTLFRSVSYYYYIDVW	IGH	2	None	
9	284	CARIRRGPSYGSLPEAFDSW	IGH	4	None	
9	285	CARIHYYDSRSLIAYYFDNW	IGH	21	None	
9	286	CARDVSRVDYGVLSGYYQGGILDYW	IGH	2	None	
9	287	CAIGYQPLHFDYW	IGH	2	None	
9	288	CAKLMIAYGTSSTAIDYW	IGH	2	None	
9	289	CAHRPLSGSGATSAFDIW	IGH	2	None	
9	290	CARGGGSGIPSDAFDIW	IGH	3	None	
9	292	CARAGGYSGSYHADYW	IGH	2	None	
9	293	CAKEVGPTGSRADYW	IGH	3	None	
9	294	CAKDTGYSYGNDAFDIW	IGH	12	None	
9	295	CATITGSSSSWSIPFDPW	IGH	2	None	
9	296	CTPQMPSSGWTLYYFDYW	IGH	4	None	
9	297	CARQLTGDTLAHFDYW	IGH	2	None	
9	298	CAALRQLERLGNDYW	IGH	4	None	
9	299	CARDGSGPSTIGATMWFDYW	IGH	2	None	
9	300	CARIVAGYYFDYW	IGH	85	None	
9	301	CQVEKGRDGYNLHDAFDIW	IGH	2	None	
9	302	CARIAGCGSGSFNWFDTW	IGH	3	None	
9	303	CARRLYEIWLTYFDNW	IGH	4	None	
9	304	CARAYCSDVSCYRRAYYFDYW	IGH	2	None	
9	305	CARSSSLASYDCWEDSW	IGH	2	None	
9	306	CARGPLDYALRFFDYW	IGH	7	None	
9	308	CARQLRFLEWSRADALNIW	IGH	2	None	
9	309	CARHTHGDPLRAYYFDYW	IGH	2	None	
9	310	CARVSGGATRKGEYYFDYW	IGH	2	None	
9	311	CASPTRWDGSFDYW	IGH	3	None	
9	312	CVRTRDSSENYYGAFDIW	IGH	2	None	
9	313	CQTWGTAIEPHVVF	IGL	3	None	
9	314	CATVSSGPNEFHYW	IGH	197	None	
9	315	CARVIFGVVTPYYFDYW	IGH	3	None	
9	316	CARHPGYCTTTTCYGQVYFDYW	IGH	3	None	
9	317	CAKDTGDYNLWSGLDYW	IGH	2	None	
9	318	CVRVAQYFDSDGYYDGDSYWYFDVW	IGH	3	None	
9	319	CAREVIGSYLDYW	IGH	6	None	
9	320	CAKTGGHDYYHYIDVW	IGH	3	None	
9	321	CAKELDQYSSGWFFDYW	IGH	2	None	
9	322	CASSVPSQLGSWFDPW	IGH	2	None	
9	323	CARDVALRYFDWYKGFDPW	IGH	26	None	
9	324	CARVHQDSSGYHGVNWFDPW	IGH	2	None	
9	325	CAKDKVRGGIVVPYYFDDW	IGH	2	None	
9	326	CYIPGVEHLEAFDLW	IGH	3	None	
9	327	CARGQDVASSLADLGEYYMDVW	IGH	2	None	
9	328	CARGGGAYCGADCKRTFDIW	IGH	2	None	
9	329	CARHCILSTCSFRYFDNW	IGH	3	None	
9	331	CAKDLTTAADTGFLDYW	IGH	2	None	
9	332	CARATGYDSSVPNWFDPW	IGH	2	None	
9	333	CARLDCSGTSCFSRSDAFDIW	IGH	3	None	
9	334	CARGPGIAAAGPRYNWFDPW	IGH	4	None	
9	335	CAKEVYLNSRRGIANAFDVW	IGH	2	None	
9	336	CAHSSYSEFWSGFGAFDIW	IGH	2	None	
9	337	CARDQTVQTYVLYDYW	IGH	4	None	
9	338	CQTPDSSGTRWLF	IGL	5	None	
9	339	CARDLPTHCSSTTCSRWFDPW	IGH	4	None	

9	340	CARREYYYGSGMDVW	IGH	5	None	
9	342	CATDLRFGEFWDYW	IGH	2	None	
9	343	CGRLVTSGSHGSFYAPYSSYYMDVW	IGH	2	None	
9	344	CARGLYSSSWRGFDIW	IGH	9	None	
9	345	CARDHFYYGSETYGGGFDPW	IGH	2	None	
9	346	CASHTFGDYPNWFDPW	IGH	2	None	
9	347	CARDSELLRFGTVSFFDYW	IGH	3	None	
9	348	CARDMAAVGSLWLDYW	IGH	2	None	
9	349	CANDRPEYSGYANFDYW	IGH	8	None	
9	350	CVKSYQMLGEHDAFDIW	IGH	2	None	
9	351	CTRVSKFTTIRGVPVDDAFDVW	IGH	2	None	
9	352	CARDSYYFASGFDLW	IGH	14	None	
9	353	CTKEAVDGTPYFDNW	IGH	2	None	
9	355	CARTLRYFDWLLDYW	IGH	4	None	
9	356	CARRLRGYSYGYLDYW	IGH	3	None	
9	357	CSSFTISSTLNWVF	IGL	3	None	
9	358	CAKDASPGSAVAVYFDYW	IGH	6	None	
9	360	CARDGMGGGSYLKYYFDYW	IGH	2	None	
9	361	CQSSDTSLSGSYFF	IGL	10	C3	6WPS
9	363	CARGAYYHDITYKSPDYW	IGH	2	None	
9	364	CARDVPVLPASNQVAVDIW	IGH	2	None	
9	366	CARDSTYSGSSYWYFDLW	IGH	7	None	
9	367	CARIGFCGLSSGGSCYNDAFDIW	IGH	2	None	
9	368	CAKDWSYKSLSSSWNAADAFDIW	IGH	2	None	
9	369	CARFLEGEGECTRISCQGSFDLW	IGH	11	None	
9	370	CASLGGGSYERGNWFDPW	IGH	2	None	
9	371	CARHYCTDDCFYEDWFDAW	IGH	2	None	
9	372	CAHRKQQFLFDYW	IGH	2	None	
9	374	CTRGQWLAPDFDYW	IGH	2	None	
9	375	CARYIVTRTGIWFDPW	IGH	17	None	
9	377	CARPRVETTRIDAFDIW	IGH	2	None	
9	378	CARHKFPTRITIFGVVNPGFDYW	IGH	2	None	
9	379	CARVNVATAGTLPPFCSGDTCHTSRYSYYWYYMDVW	IGH	2	None	
9	380	CATEHGERGNDYW	IGH	2	None	
9	381	CAKDSHVGHWANYFDYW	IGH	2	None	
9	382	CARDKGEFDFWINFYRGRNAFDLW	IGH	2	None	
9	383	CARGQVATTDYW	IGH	19	None	
9	384	CVKDPYSSSSRYFDYW	IGH	2	None	
9	386	CAKDSLQYCSSTNCYGGFDYW	IGH	2	None	
9	387	CAKVLYPQDNSGFYPRDPFDSW	IGH	2	None	
9	388	CAKGRHYYDSSAYRHFDYW	IGH	2	None	
9	389	CARSPLDRNYQFLPVWFDPW	IGH	2	None	
9	390	CARDAAAGNGFALYYYYYMDVW	IGH	2	None	
9	391	CAKRETDDYSNYDSPPDYW	IGH	2	None	
9	392	CVRGFSKTYYFDNW	IGH	5	None	
9	393	CARYCSRSSCNALDYW	IGH	2	None	
9	394	CAKGGSGSFYKVPWYFDYW	IGH	4	None	
9	395	CAVFLVPQIPGLDYW	IGH	21	None	
9	396	CAKAPSEVGVIYYFDSW	IGH	3	None	
9	397	CARHNTQATESVTRANWFDPW	IGH	2	None	
9	399	CTRDASAAAYNWFDPW	IGH	2	None	
9	401	CARDRLQGYYSGPDYW	IGH	2	None	
9	402	CAYRGTTTLGLAGAFDPW	IGH	2	None	
9	404	CAKDICSGGSCYGFFDYW	IGH	3	None	
9	405	CTRPVVPTAPSKHDAFDIW	IGH	6	None	
9	406	CARDLETASGTMGRWFDPW	IGH	2	None	
9	407	CQQYYSNTMYNTMYTF	IGK	2	C4	6W41
9	409	CARGQYSSSFGFDWFDLW	IGH	2	None	
9	410	CARPGDYDFWSGYYTGSNWFDPW	IGH	4	None	
9	412	CWGTTVVTSGSEDYW	IGH	7	None	
9	413	CAKEVHVVNSGSDGFDIW	IGH	2	None	
9	414	CARQGFRDAWYWFDPW	IGH	2	None	
9	416	CAKDRAPYSSSSGQTFDYW	IGH	2	None	
9	417	CARAGAAYGDLNDAFDIW	IGH	2	None	
9	418	CASPPPGYCSSISCFSFDYW	IGH	2	None	
9	420	CVRDSAYVFDIW	IGH	3	None	
9	421	CARDKSRDWNDVGSFDYW	IGH	2	None	
9	422	CARVDISAHVVFDFW	IGH	2	None	

9	423	CTLFPYTTLFRSEDTAVYYCAKIGEGGSYYGYYFDYW	IGH	2	None	
9	424	CARCPSSSWVLDYMDVW	IGH	14	None	
9	425	CARGTQPVASSHFDYW	IGH	2	None	
9	426	CARTRLGNRSSRADFDDW	IGH	2	None	
9	427	CGKDMSPGGLDYW	IGH	5	None	
9	428	CARDPANQDALDIW	IGH	5	None	
9	429	CARGMELLESLDYW	IGH	2	None	
9	430	CAKFPRAATTAEFDYW	IGH	2	None	
9	431	CARGLLEVITGDAFDIW	IGH	2	None	
9	432	CARDSQMCNITSCPPNWFDPW	IGH	2	None	
9	433	CARGGYYHDSSGSSVITYFDIW	IGH	2	None	
9	434	CARHGDSDIVVVPADNYFDYW	IGH	2	None	
9	435	CAKTSGYCSSTSCYTADAFDIW	IGH	2	None	
9	436	CARDPRQTGYSYGLYFDYW	IGH	2	None	
9	437	CAHSGAYCSRDSKCYDHFDSW	IGH	2	None	
9	438	CGGLDSVRGGYDFDYW	IGH	2	None	
9	439	CARDNVAVAGTGYRDAFDIW	IGH	2	None	
9	440	CARGSSRQITRWAAFDIW	IGH	4	None	
9	441	CVHSLTTFSGAILRIHETFDMW	IGH	2	None	
9	442	CQSVDSSGIYIF	IGL	8	None	
9	443	CVSRYYYGVGSYLSHFDSW	IGH	3	None	
9	444	CVKPPGHGSGNYYSSGAGAFDIW	IGH	2	None	
9	447	CARDCPTSRGYMTCFDYW	IGH	2	None	
9	448	CQHLNSIPPRGTF	IGK	2	C1	7BZ5
9	449	CAKPRYGGNSYYYYYYMDVW	IGH	4	None	
9	450	CARRKMTTEEYDYW	IGH	2	None	
9	451	CARDRSGDSDFWSGYYGDDAFDIW	IGH	4	None	
9	452	CAILSSWYDPIDYW	IGH	4	None	
9	453	CARAWGYYRPEYFQHW	IGH	6	None	
9	454	CARGYSNSRYAFDYW	IGH	5	None	
9	456	CARDIDRYCSGESCTWVFDYW	IGH	2	None	
9	458	CAKDSFLRQYTVKMIDYW	IGH	2	None	
9	459	CAIHTNGYSYVEPFDYW	IGH	2	None	
9	460	CAKDRFGGDDYLEYFDYW	IGH	4	None	
9	461	CARPPGVNLDAFDTW	IGH	2	None	
9	462	CARELKGGITTSWFDSW	IGH	2	None	
9	463	CVRDSPAVAGTPFDYW	IGH	2	None	
9	464	CAKDLSSSGWYALDYW	IGH	2	None	
9	465	CAREVVVVSYYFDYW	IGH	12	None	
9	467	CARDRLQGPYCGGDCGYFDSW	IGH	4	None	
9	468	CAREGKYSGSDSGFDYW	IGH	2	None	
9	469	CARDTASSPRRDPYDAFDIW	IGH	2	None	
9	470	CARGRITIFGVVNYFDYW	IGH	3	None	
9	471	CARARIQHYPKPFDYW	IGH	4	None	
9	472	CARDYTSVSGRGGDYW	IGH	2	None	
9	473	CARVNSVTESYSGDAFDIW	IGH	2	None	
9	474	CTRDFSSDYW	IGH	2	None	
9	475	CARSGSDFGSLDYW	IGH	4	None	
9	476	CARDNSATSLYMFWWFDPW	IGH	5	None	
9	477	CAHRLNYIAFDIW	IGH	9	None	
9	478	CARGHTVDFDFW	IGH	4	None	
9	479	CARTKGGGYDSDFDYW	IGH	2	None	
9	480	CARGTDDFWTGYLRGFYFDSW	IGH	2	None	
9	481	CAHKHLPGYYPVSQINFAFDSW	IGH	4	None	
9	482	CARWVDTTMVISHYFDSW	IGH	2	None	
9	483	CATSRVRVGHLGNFDDW	IGH	2	None	
9	484	CQQRSPWPPMCRF	IGK	14	None	
9	485	CARHREVPGYSSGWTFDYW	IGH	3	None	
9	486	CARAAYDFWSGYYPYRNAFDIW	IGH	2	None	
9	488	CQVWGSSTDHYWVF	IGL	10	None	
9	490	CARTMPSGYYPFGYFDYW	IGH	2	None	
9	491	CARGRWITIFGVAITYNWFDPW	IGH	6	None	
9	492	CARHLSYDILTGYVDENNWFDPW	IGH	2	None	
9	493	CARVTGITGTTFFDYW	IGH	4	None	
9	494	CVRASRTSDAFDVW	IGH	4	None	
9	495	CAAWDDSLSGPVF	IGL	1236	None	
9	496	CARATFYDSSGYWYYFDYW	IGH	4	None	
9	497	CTTDFVVGYSAYW	IGH	4	None	

9	499	CHLRSNWPQIAF	IGK	2	None	
9	500	CARSYYSNYFHYW	IGH	2	None	
9	501	CQQSYSSPHSCSF	IGK	48	C4	6ZDH
9	502	CARDRGSWPDNDAFAFW	IGH	2	None	
9	503	CQQTYNTPMSSF	IGK	4	C4	6W41
9	504	CARVHSGWYSNDYW	IGH	6	None	
9	505	CARERYGSGGHQLLYRPYYFDYW	IGH	2	None	
9	506	CTTDPAYVSFIDYW	IGH	4	None	
9	507	CARLGVLEYLQHW	IGH	2	None	
9	508	CARHKGAVTGRRGWFDPW	IGH	2	None	
9	509	CAKVPFKNHGYDIYFDYW	IGH	2	None	
9	510	CARGGHSSSYNYMDVW	IGH	12	None	
9	511	CARDSTRIAAAGTDYW	IGH	5	None	
9	512	CSSYAGSHNQVIF	IGL	14	C2	7BWJ
9	513	CAKEGGSYSSSWYIDYW	IGH	4	None	
9	514	CVRSLYYYDTDNNGHDISDIW	IGH	2	None	
9	515	CARTIFGVVIDIFDYW	IGH	2	None	
9	516	CSRGIAARQFDYW	IGH	8	None	
9	518	CARDRYYGDSLQGFDYW	IGH	2	None	
9	520	CARDDGSGYDRLDYW	IGH	10	None	
9	521	CARHVSPDYLSDYW	IGH	4	None	
9	522	CARVPGILTTDTTTFDIW	IGH	4	None	
9	523	CARDFSADYW	IGH	213	None	
9	524	CTTDVGYGWFDPW	IGH	2	None	
9	525	CAKGPSGYSPYYFDYW	IGH	6	None	
9	526	CARMRRPGPLGYYSNVDYW	IGH	2	None	
9	527	CARDYYGSQSYYPDDTRFDSW	IGH	2	None	
9	528	CARLCSGDCYINRGSYDYW	IGH	2	None	
9	529	CARLGIGNSGHRDYW	IGH	13	None	
9	530	CAKDGIAVRHYYYMDVW	IGH	2	None	
9	531	CQSYHSDNGVFGGGTNHGVF	IGL	2	None	
9	532	CTRTCSDNCYISQPFDYW	IGH	3	None	
9	533	CARRSITARPGWFDPW	IGH	2	None	
9	534	CAKDLGAVAGYFDYW	IGH	6	None	
9	535	CTRQIDPFWSAFQPDYYFDYW	IGH	3	None	
9	536	CAREAKTSPPNFDYW	IGH	3	None	
9	537	CAKVSYLSTASSLDYW	IGH	6	None	
9	538	CVKDLSEIVLMFYAFDYW	IGH	2	None	
9	539	CTRGDSDYVPLDYW	IGH	2	None	
9	540	CARDPAVAGDLDYW	IGH	7	None	
9	541	CAHLLSSDGLNWFDPW	IGH	2	None	
9	542	CVKGPIAVTAQYFDYW	IGH	7	None	
9	544	CARGAGDCSTGICYRQHFDYW	IGH	2	None	
9	545	CARRDSGYDPFDYW	IGH	9	None	
9	546	CARDRLVGAQAYLDHW	IGH	2	None	
9	547	CARMVQLERRGGPAFDIW	IGH	2	None	
9	548	CQKANNFPLTF	IGK	43	C3	6XDG
9	549	CASLRFLEWLSSDAFDIW	IGH	2	None	
9	550	CATVEGTTLGERHYFDSW	IGH	2	None	
9	551	CARDMAEYSSSSTGVVDYW	IGH	2	None	
9	552	CAAGRSEEYCTGASCFDEYW	IGH	3	None	
9	553	CLLSYGGSRVIF	IGL	110	None	
9	554	CARHYSRNHAFDVW	IGH	9	None	
9	555	CATHMTPRDAFDIW	IGH	4	None	
9	557	CARSPLPYCSSTSCYGVDFDYW	IGH	2	None	
9	559	CAKICNYQLLNWFDPW	IGH	4	None	
9	560	CAKGKYTSGPTADYW	IGH	2	None	
9	561	CAKGHCGGTGCYLWIFDYW	IGH	5	None	
9	562	CARQDWGSFDSW	IGH	2	None	
9	563	CAKVASITMVRGVIDYW	IGH	2	None	
9	564	CVSGPGPTNWFDPW	IGH	11	None	
9	565	CARIRNYYDSGTYMQGPFDYW	IGH	2	None	
9	566	CARALDYSDGRGYYNQDYFECW	IGH	2	None	
9	567	CARSPQGRGVIEAFDIW	IGH	2	None	
9	568	CAKQFSGGSYWEYYLDYW	IGH	9	None	
9	569	CASRSCTSGVCPFDYW	IGH	2	None	
9	570	CARDHPDNDYGDYAGYW	IGH	11	None	
9	571	CAKDQGVSREVPRHFEYW	IGH	4	None	

9	573	CASEKWGSGYIDFW	IGH	8	None	
9	574	CAKDVGIAVAGSGGFDYW	IGH	2	None	
9	575	CARVLSYYYDSSGYDHFDYW	IGH	2	None	
9	576	CVKDECSTTDCHFGFYTYMDVW	IGH	3	None	
9	577	CAKDIYSSSFAHYYYYMDVW	IGH	2	None	
9	578	CAREGGSGREGANAFNIW	IGH	2	None	
9	580	CAKVRCSSTSCYHHDAFDIW	IGH	3	None	
9	582	CAKDQSSVVVVPDDAFDIW	IGH	2	None	
9	583	CAKDPLHDYSGYDYW	IGH	2	None	
9	585	CARDLTPRLRYCTNGVCYSNWFDPW	IGH	2	None	
9	586	CARVGYHNEVECDYW	IGH	2	None	
9	587	CATRSGIEAPGIRWFDPW	IGH	2	None	
9	588	CANLFWSGYYKEDYW	IGH	4	None	
9	589	CAKDFWYSSPPSDAFDVW	IGH	2	None	
9	590	CAKGVVVRGIISRYFDYW	IGH	2	None	
9	591	CARAGTGVPSHWFDPW	IGH	2	None	
9	592	CARGKRIAARLLNKGTTVYYFDYW	IGH	2	None	
9	593	CATQYRSVYSSPFDFW	IGH	4	None	
9	594	CAKDPSYDFWSGHHFDYW	IGH	16	None	
9	595	CAKVPSSSWYGYYYYYMDVW	IGH	8	None	
9	596	CARLDDDDNNYENWFDPW	IGH	6	None	
9	597	CARGHFNNYVLNWFDFW	IGH	2	None	
9	598	CARHSGYCSSTSCYMVDDAFDIW	IGH	2	None	
9	599	CAKDLLVPPTPPQYYYYYYMDVW	IGH	2	None	
9	601	CARSTSLDFWSSPPPYYFDYW	IGH	6	None	
9	602	CAKGSTTFGSYYFDYW	IGH	4	None	
9	603	CLQHASYPMCSF	IGK	2	None	
9	604	CARVHSSTFLNWLDPW	IGH	2	None	
9	605	CARTVDCSGGSCYGALVDYW	IGH	2	None	
9	606	CASDPSYYSGSGGFDYW	IGH	2	None	
9	607	CARDWGGSSIMGTGIFDYW	IGH	2	None	
9	608	CARQQCTSVSCRQGNYFYYYYMDVW	IGH	2	None	
9	609	CARRRWSGYDLPEDDGFDIW	IGH	2	None	
9	610	CTTQYCSGTSCTSFDNW	IGH	2	None	
9	611	CARMYHYEGWFDPW	IGH	4	None	
9	612	CKQYNSYPLIF	IGK	7	C3	7K8P
9	613	CARVGRTAYCSSTSCYGLPFDYW	IGH	2	None	
9	614	CVWSATNWFGPW	IGH	2	None	
9	615	CAKSEPQWLVSLGRSHFDYW	IGH	6	None	
9	616	CARDNHHAANSFDYW	IGH	2	None	
9	617	CARDIGQLVGIVNWFDPW	IGH	2	None	
9	618	CVRDSRPYSYSWFDVW	IGH	2	None	
9	619	CAGLGRVVYPKFRDYNYYMDVW	IGH	3	None	
9	620	CARVWSVGWYFDLW	IGH	9	None	
9	621	CNARDSSGHHVIF	IGL	2	None	
9	622	CARTPYDFWSGYSHYYYYYYMDVW	IGH	2	None	
9	623	CAHRRSGYSYGYHAFDIW	IGH	2	None	
9	624	CARDSNRWSYGLPHDAFDIW	IGH	2	None	
9	626	CLQHNSYPLTF	IGK	818	C3	7K8P
9	627	CLKDSRDKDYFDYW	IGH	2	None	
9	628	CARLFPDTAMVWDYYYYYMDVW	IGH	2	None	
9	629	CAREDIVDRRGDAFDFW	IGH	3	None	
9	630	CARNQGRFLADYW	IGH	2	None	
9	631	CVKDMSPWDTSAPPDYW	IGH	3	None	
9	632	CARHESELAVANAWFDAW	IGH	2	None	
9	633	CAAGPVVGEIIGLDYW	IGH	2	None	
9	635	CANRYSGYDHYPFEYW	IGH	2	None	
9	636	CARVYFGSIGVADGWIDPW	IGH	2	None	
9	637	CARLDSSSSSATVHAFDIW	IGH	2	None	
9	638	CARLTTRWEVSRAYNAIDVW	IGH	2	None	
9	640	CARVPGVQFSHRNWFDPW	IGH	11	None	
9	641	CALSSGTYYPYW	IGH	3	None	
9	642	CARMVDSTSWYNWFHPW	IGH	2	None	
9	643	CARDLVAITGDNFLDSW	IGH	16	None	
9	644	CARDSDYAFWSGYYGWFDPW	IGH	2	None	
9	646	CAKGRCSGGSCYWIDSW	IGH	2	None	
9	647	CAKADTAMVGAFDIW	IGH	6	None	
9	648	CARGGWQWRYYYYYMDVW	IGH	2	None	

9	649	CARGYGSTGFDLW	IGH	16	None	
9	650	CATYNGDYIYDYYYMDVW	IGH	6	None	
9	651	CAKHHRTYSSSSSLDYW	IGH	2	None	
9	652	CAKETCEWLVPGLCYFDYW	IGH	2	None	
9	653	CAKDMQYQLLYYFDYW	IGH	8	None	
9	654	CARDIRPGSSWYMGRDAFDIW	IGH	5	None	
9	655	CARARSRYDFWSGYDASDAFDIW	IGH	2	None	
9	656	CARDTGVKGGAFEIW	IGH	6	None	
9	657	CGRSGYSGYEFDFW	IGH	6	None	
9	658	CARETSTGGLRFIPFGYW	IGH	2	None	
9	659	CSGDSGAFDYW	IGH	7	None	
9	660	CARDPVTGYYYYYMDVW	IGH	14	None	
9	661	CVRGHPYCTNSNCFTAHAFDIW	IGH	12	None	
9	663	CARVGADFSVPFDYW	IGH	2	None	
9	664	CAKEQIPPRYSSITTPFDIW	IGH	2	None	
9	665	CAKDYFYDTSGYFYANFDYW	IGH	2	None	
9	666	CARERPDLAYCRDERCYYEGYLDPW	IGH	3	None	
9	667	CAHRTTLFGVIQHFDSW	IGH	2	None	
9	668	CAREENTAMVSGVFDYW	IGH	2	None	
9	669	CAAGSSRLGGSDYW	IGH	2	None	
9	670	CAKGGYCSSTSCYYLPFDYW	IGH	2	None	
9	671	CARGSADCGYSSNVYSGCAFDLW	IGH	2	None	
9	672	CAKDEMDCSSTSCYWGWTYYYYYMDVW	IGH	3	None	
9	673	CAKDIVATISYPDYW	IGH	7	None	
9	674	CARDCRASGGSCYSGIDYW	IGH	2	None	
9	675	CAREADTSGYYYSFDFW	IGH	2	None	
9	676	CARSPRGYSYFDCW	IGH	7	None	
9	677	CARAPGYCSGGSCSWFDPW	IGH	3	None	
9	678	CAKARGGKLGTYHFDYW	IGH	4	None	
9	679	CAKAYSYGYSGFDYW	IGH	2	None	
9	680	CASHRESYGSFDVW	IGH	4	None	
9	681	CARAGKRGYSYGSGDYFDYW	IGH	2	None	
9	682	CAKERSDQVLSSFDYW	IGH	4	None	
9	683	CARHGDEGRIAARGYFDHW	IGH	2	None	
9	684	CQQANSVLGITF	IGK	42	None	
9	685	CAKGTGYSSGWFDYW	IGH	59	None	
9	686	CQQYASSPGRVTF	IGK	2	C1	7K8N
9	687	CNSRDSSGNHLVF	IGL	399	None	
9	688	CQQRASWPVPF	IGK	4	None	
9	689	CATSSYYAFDNW	IGH	2	None	
9	691	CARDSNVIGPGVAHDVFDVW	IGH	3	None	
9	692	CITLYEPYYYYYIDVW	IGH	3	None	
9	693	CARGSSGPAESFFDSW	IGH	2	None	
9	694	CQQYFTTPPGCSF	IGK	12	C2	7K8U
9	695	CARDQGEGGSYGFSDAFDIW	IGH	2	None	
9	696	CARGVRFFQIASYYHYMDVW	IGH	2	None	
9	697	CQQYNNWPPLTF	IGK	14329	C3	7K8R
9	698	CARDYMTTVTKNYFDYW	IGH	4	None	
9	700	CVKDIWPTVITRNYYFDYW	IGH	2	None	
9	701	CANQRSSGWQWWFDPW	IGH	2	None	
9	702	CQQSSNMPQWTF	IGK	22	C3	6XDG
9	703	CARSTWLRGNFDYW	IGH	3	None	
9	704	CAAADVLLWFGEFDYW	IGH	2	None	
9	705	CQRYNRAPYTF	IGK	300	C3	7K8P
9	706	CMQDAQDPPGTF	IGK	10	None	
9	707	CARDFRGTMIQGRVTWYFDLW	IGH	4	None	
9	708	CVKEGCSSSSCQRSDNWFDPW	IGH	2	None	
9	709	CVRNYVALPEDNWFDPW	IGH	2	None	
9	711	CTTVSYAFWSAYYTGEEDYYYMDVW	IGH	2	None	
9	712	CARAFYRGYFDPW	IGH	11	None	
9	713	CAKVPLYSSGGRDYW	IGH	2	None	
9	714	CARAKYPLDAFDIW	IGH	5	None	
9	715	CTRGINFLFDYW	IGH	6	None	
9	716	CARGHGRQDIVVVPAAKGDGFFIQSAFDIW	IGH	2	None	
9	717	CARHALGVPAANCWFDPW	IGH	5	None	
9	718	CARHGIGTNFDYW	IGH	8	None	
9	719	CARGGLSSDWVPHAFDIW	IGH	2	None	
9	720	CARPSLHGSGRDVFDIW	IGH	3	None	

9	721	CARIPQYSGSWSYYFDYW	IGH	2	None	
9	723	CARGMRCSSATCYKDYW	IGH	15	None	
9	724	CAKRVEYRSSSAYFDYW	IGH	4	None	
9	725	CAKSLKVGGATLTYFDSW	IGH	7	None	
9	726	CARPRLAGGYSYVDAFDMW	IGH	2	None	
9	727	CASTMLTMIVVVIRGFDYW	IGH	2	None	
9	728	CARDEGYSGSGSSYNDPNPRFDYW	IGH	2	None	
9	729	CASTSLNFISASDQYYFDYW	IGH	2	None	
9	730	CARGPLGIQLWSIDYW	IGH	2	None	
9	731	CARDSHERGTGWFDPW	IGH	2	None	
9	732	CARDGLPVGSPLNYFDYW	IGH	3	None	
9	733	CARTIEYCGSDSCYPRFDYW	IGH	2	None	
9	734	CARTTYYDILTGSRVFDYW	IGH	4	None	
9	736	CARDYFDASGYFYFDSW	IGH	2	None	
9	737	CARDPRITFNSGWYYFDKW	IGH	3	None	
9	739	CTSLGFTEPHNFDYW	IGH	6	None	
9	741	CARASIPSASGTFDIW	IGH	2	None	
9	742	CARRSDPLGAFDIW	IGH	6	None	
9	743	CARNIYGSENYYIDYW	IGH	4	None	
9	744	CARGQEQIGSGLVDTFDIW	IGH	3	None	
9	745	CARDNEFGGELVFFDYW	IGH	3	None	
9	746	CARDGPFDSLDYW	IGH	5	None	
9	747	CSREYISSWFDYW	IGH	50	None	
9	748	CARCYDFWSGFPNWFDPW	IGH	2	None	
9	749	CVKTIVETSVVFFDSW	IGH	7	None	
9	750	CARGDSSCASTSCPPDYW	IGH	2	None	
9	751	CARDSGHDWRIDYW	IGH	2	None	
9	752	CVKNYRGYCAIGICYTLDYW	IGH	2	None	
9	753	CARTSYDFFRGYSNFDYW	IGH	2	None	
9	754	CASSTLTDAFDLW	IGH	5	None	
9	755	CAKVTYYSDSNSYLFDYW	IGH	2	None	
9	757	CARGYWSGTSWYRFTFDSW	IGH	6	None	
9	758	CARELVPRNFDYW	IGH	15	None	
9	759	CAIQVAADYFNPVVFDYW	IGH	2	None	
9	761	CAKDVRRPFKGTTGTTSVTFDYW	IGH	2	None	
9	762	CARHYCNSTSCYRGGPEGYYYYMDVW	IGH	2	None	
9	763	CAKDSVEHIPDAFDIW	IGH	2	None	
9	764	CTRGDAGDYNFFDYW	IGH	2	None	
9	765	CARDLVVQGSLFGVVSFFDYW	IGH	2	None	
9	768	CVKDSGLGYCTNGVCTAFDHW	IGH	14	None	
9	769	CARHLHGSSWYRVWFDPW	IGH	2	None	
9	770	CAKVLGQLRITGDWFDPW	IGH	2	None	
9	771	CARTSNDFWTGPPDYW	IGH	5	None	
9	772	CARYIRQGDYFDFW	IGH	2	None	
9	774	CARGHNSPFPYSGSYYDYW	IGH	6	None	
9	775	CARMVGELYLAYFDHW	IGH	2	None	
9	776	CARDEGSTYPHPYFDYW	IGH	2	None	
9	777	CALETSFFDYW	IGH	7	None	
9	779	CARDIGVVPAAFLGYYMDVW	IGH	4	None	
9	780	CARHESGRPSYFDHW	IGH	2	None	
9	781	CSRDDSGWHNDAFDMW	IGH	9	None	
9	782	CARDRLDTPKYDVFDVW	IGH	4	None	
9	783	CARDREQYCSGGSCYDALDIW	IGH	4	None	
9	785	CARVGKNRGSFDIW	IGH	2	None	
9	786	CTSPGDNSDYW	IGH	4	None	
9	787	CAAVHDYGDYGPFFDYW	IGH	3	None	
9	788	CAKETPVTVFDYW	IGH	2	None	
9	789	CAKGGVRSNSGINYYFDYW	IGH	6	None	
9	790	CARPLQFFDYYYMDVW	IGH	2	None	
9	791	CATFYDATAYFLQGYDAFDIW	IGH	2	None	
9	792	CARDRLVYCSTTSCAGFDYW	IGH	6	None	
9	793	CAKDGQVPHDRYYNYYMDVW	IGH	4	None	
9	794	CSSYTTTSTLGVEF	IGL	3	C2	7K90
9	797	CARGSPTPDSSGSRRGFDHW	IGH	2	None	
9	798	CAVWIVVVPSAIEYFQPW	IGH	4	None	
9	799	CATISAFGILSKTRFFFDIW	IGH	2	None	
9	800	CAHFCSSNNCYNFDYW	IGH	2	None	
9	802	CQPYGTSPRWAF	IGK	2	C2	7K8U

9	803	CAREGGVPYCSSTSCLTPYYFDYW	IGH	2	None	
9	804	CSKDGQGNYIRYFDYW	IGH	4	None	
9	805	CAQRETGGDWAFDYW	IGH	4	None	
9	806	CAKGSGVVTTVGYNDYW	IGH	2	None	
9	807	CARLGGFNSNYPDVW	IGH	4	None	
9	808	CARAHDRNGGLDFDYW	IGH	2	None	
9	809	CQQYKRWPLLVTF	IGK	2	None	
9	810	CARGPTSTSWFDPW	IGH	3	None	
9	811	CARRRGGTARPEFDYW	IGH	2	None	
9	812	CAKNHLPGWFNVFDIW	IGH	5	None	
9	813	CARDVTLSYCGGDCYLFDYW	IGH	2	None	
9	814	CAKVPIDYGGSRDAFDIW	IGH	2	None	
9	815	CAILSPSLYCAGDCDVDYW	IGH	4	None	
9	816	CARLGRFNILGLIITPFDYW	IGH	5	None	
9	817	CARHEFRDWDAFDIW	IGH	2	None	
9	818	CARDLGLTGTTGDFDYW	IGH	2	None	
9	819	CIKDLRPVSVAPDVW	IGH	4	None	
9	820	CAKFEYSSSSVDYW	IGH	10	None	
9	821	CARGRKGNDGYNWFDSW	IGH	2	None	
9	822	CASEIWDQGGYRNAFDIW	IGH	2	None	
9	823	CAHRLGGYNWNDGYCDYW	IGH	7	None	
9	824	CAHTVRGYFQFW	IGH	2	None	
9	825	CARGPHCSAGNCFSFDSW	IGH	2	None	
9	827	CARDPLRRFDYW	IGH	46	None	
9	828	CARGQVVMLPAATGGLLGVRDAFDMW	IGH	4	None	
9	830	CARGKEGYGGNLDYW	IGH	2	None	
9	831	CARRDYDILTGDYYDALDIW	IGH	2	None	
9	832	CARSGVLWLETNWFDPW	IGH	3	None	
9	833	CAKGHSFNNWNDVSALDIW	IGH	2	None	
9	834	CARGEVRIIDKGPLFDNW	IGH	5	None	
9	836	CARGAVSFVEKTAFDVW	IGH	2	None	
9	837	CARGCRYCSGAQHGDYW	IGH	2	None	
9	838	CAKMGWDIVMEDYYYMDVW	IGH	4	None	
9	840	CARTIWNVFDVW	IGH	7	None	
9	841	CALKRIKLIRGLITGNYFDYW	IGH	5	None	
9	842	CARTPQGDIVVVPAAIGWFDPW	IGH	2	None	
9	844	CATHYDFWSVDYW	IGH	2	None	
9	845	CARLIDGPATSFDHW	IGH	3	None	
9	846	CVRDHPNSRFLVDHAFDIW	IGH	2	None	
9	847	CAHSPAVALGYYFDFW	IGH	4	None	
9	848	CATVGPPAPLFDPW	IGH	3	None	
9	849	CARDRLRFLNRGYYMDVW	IGH	2	None	
9	850	CARVVEQQWLAQGGWFDPW	IGH	2	None	
9	851	CASGYCSSTSCYLNTFDIW	IGH	2	None	
9	852	CTAYLQISSLKAEDTAVYYCARGREDYGFWGDHMDVW	IGH	2	None	
9	853	CARGRRQLSRRGYYFDYW	IGH	3	None	
9	854	CAKDSRPNWGLDYW	IGH	2	None	
9	858	CAKDYCSSTSCYTGYFDYW	IGH	6	None	
9	859	CARRIDVTGTIPNDNWFDLW	IGH	2	None	
9	860	CARATYYTSGWYIFDYW	IGH	2	None	
9	861	CARGGLWLVRGFDYW	IGH	2	None	
9	862	CGKSRLLLHDAFDIW	IGH	2	None	
9	863	CAKDLSSLSPYYMDVW	IGH	3	None	
9	864	CAKGGGTLDYYVDVW	IGH	4	None	
9	865	CASDGESEGVAGQVYYFDYW	IGH	2	None	
9	866	CAKGFTYYYDSAGFRYLDYW	IGH	2	None	
9	867	CAKDRVRWNDEGAFDIW	IGH	2	None	
9	868	CQSADSSGINPWVF	IGL	12	None	
9	869	CALNSGYYLYFDYW	IGH	4	None	
9	870	CARSEGKTGNWYFDLW	IGH	4	None	
9	871	CARCIYDFWSGCHALDVW	IGH	2	None	
9	872	CARSSSSSRPDPFDYW	IGH	4	None	
9	873	CARGDYSQLFHPNDSLSGFYVRAFEIW	IGH	2	None	
9	874	CARGTYDCGSTSCHTGDAFDIW	IGH	4	None	
9	876	CVRGDFWSGYAHDAFDVW	IGH	5	None	
9	877	CARSGVTYDTSGYTFDYW	IGH	2	None	
9	878	CGYSYGAGAFDIW	IGH	4	None	
9	879	CARHMATIYCSSTSCYAFDIW	IGH	2	None	

9	880	CAKSRTSTWSYFDDW	IGH	2	None	
9	882	CASSGYSLVGAFDIW	IGH	2	None	
9	884	CAKGKITTGTTRSEFDYW	IGH	2	None	
9	885	CAKDRGAQNYYDRSGHYFPDVFDMW	IGH	3	None	
9	886	CARRSLLGLAQSNYRTTAFDIW	IGH	2	None	
9	887	CAKDFHVTMIVVVITTLDYW	IGH	2	None	
9	888	CARHRRYCGSTSCYADDFDYW	IGH	4	None	
9	889	CASSSGWYPRLDYW	IGH	2	None	
9	890	CAKLGGYSSGYSDYW	IGH	3	None	
9	891	CAHRPMGYISGWSNCYFDYW	IGH	12	None	
9	892	CAKAQGLYCSSTSCYVPAEYYFDYW	IGH	2	None	
9	893	CARALLRTWSGGSYYDFW	IGH	6	None	
9	894	CVKGSSWSHWDFDYW	IGH	2	None	
9	895	CARGSCPSSNTRCYNLGSWYFDLW	IGH	10	None	
9	897	CVGEPPSTGKFDYW	IGH	5	None	
9	898	CAKGFNSGWTYASYFHHW	IGH	4	None	
9	899	CAKELGYGDYVVDFDYW	IGH	2	None	
9	900	CARGGFGKPFDYW	IGH	5	None	
9	901	CARDSIFWSDYANEDDAFDLW	IGH	2	None	
9	902	CATAFVLAMTHGTYDPFDIW	IGH	3	None	
9	903	CAKSSRGVVVAATRFDYW	IGH	2	None	
9	905	CAKVAGRLISDYIDYW	IGH	2	None	
9	906	CTRHFSLSGDYLGFDYW	IGH	3	None	
9	907	CAKDTEQWLVHPMASFDYW	IGH	2	None	
9	908	CARANRDDGGTYYFDYW	IGH	4	None	
9	909	CQQYNAWPNSF	IGK	1015	C3	7K8R
9	910	CARCRNEQWLEAPSDYW	IGH	2	None	
9	911	CARIGWVQQVVDYW	IGH	2	None	
9	912	CARIRNGDYGFDSW	IGH	5	None	
9	913	CARDVPGNDFWSGSYYYYMDVW	IGH	3	None	
9	914	CQQRSDWPPLTF	IGK	601	C1	7BZ5
9	915	CARAARGGYFPHYHYYHYMDVW	IGH	2	None	
9	917	CARGPTGYSYGLTDYW	IGH	4	None	
9	920	CARTGYCTTATCFRWFDPW	IGH	5	None	
9	921	CAKRMYSGSYFAAFDIW	IGH	2	None	
9	922	CARDNSIEDINWWFDPW	IGH	2	None	
9	923	CARRAMNVVAPFDGHFEYW	IGH	3	None	
9	925	CARQGRGSDPTAPQFDPW	IGH	236	None	
9	926	CARRVGIAVGVNWFDPW	IGH	2	None	
9	927	CMHSLQTPFAF	IGK	12	None	
9	928	CQNYNRAPGCSF	IGK	2	None	
9	929	CARDFAQQLVPGESGYYFDYW	IGH	2	None	
9	930	CARMNCSGPSCYPWFDYW	IGH	2	None	
9	932	CQQYDNYFMYNF	IGK	22	C3	6XDG
9	933	CGREPTIFGASGSFFNWLDPW	IGH	2	None	
9	934	CARDGDCNRSNCYRYYSYYLDVW	IGH	2	None	
9	935	CARDRAPISSSPYYFDYW	IGH	2	None	
9	936	CAKGRDYDILTGYLDGFDIW	IGH	2	None	
9	937	CARSPSSWYPLRFDYW	IGH	10	None	
9	938	CARVRHYGDYVWFDPW	IGH	5	None	
9	939	CTTDVSGYSYVNFDYW	IGH	2	None	
9	940	CARDNRVLRSFDPW	IGH	5	None	
9	941	CARDPTKFSSGWYYFDHW	IGH	8	None	
9	942	CAHRPSDDILTGYSWFDPW	IGH	4	None	
9	943	CARDRIGYMTTVTTDYW	IGH	2	None	
9	944	CAKGRSGYYAGASDYW	IGH	2	None	
9	945	CGKDFGRFSGSLFYLDSW	IGH	2	None	
9	946	CARHLPTDCSILNCRHNWFDPW	IGH	5	None	
9	947	CARLNGGSSAFDPW	IGH	2	None	
9	948	CTITIISGWPNFDFW	IGH	3	None	
9	949	CAKNVPRSIFGVVLLEGYFDYW	IGH	2	None	
9	950	CARRFSYDSSTYYYYFDYW	IGH	2	None	
9	951	CARVRVIDSTEGAFDLW	IGH	6	None	
9	952	CQQHHNLPFF	IGK	23	C3	6XDG
9	953	CQVWDSTSDHPFFAF	IGL	2	None	
9	954	CARQGRDPAIYGVVTTFDYW	IGH	2	None	
9	955	CARMRGVYNKVFDSW	IGH	2	None	
9	956	CAHRRLHIAAAGFDYW	IGH	3	None	

9	957	CTLFPYTTLFRSLLSTRDFDSW	IGH	2	None	
9	958	CARSIAVAVPYYFDYW	IGH	2	None	
9	959	CARDTYYESSGHYEDWFDSW	IGH	2	None	
9	960	CARVDAVAGSYFDYW	IGH	11	None	
9	961	CARGPHIYSDYWSFDFW	IGH	5	None	
9	962	CARLGYNYDFDHDAFDLW	IGH	2	None	
9	963	CARGTGFGFSAADYFNSW	IGH	2	None	
9	964	CAHRRGYSYGLNWFDPW	IGH	2	None	
9	965	CARAGDYSNYQWHVDYW	IGH	2	None	
9	966	CQSADTSGSYLNWKF	IGL	3	None	
9	967	CAGGGGYSYGHIWFDPW	IGH	12	None	
9	968	CARPVVPAAISAGDRGNWFDPW	IGH	2	None	
9	969	CQVWDSSTDSRSDQVVF	IGL	2	None	
9	970	CAKIQLENVDTAMAPGYYYYMDVW	IGH	4	None	
9	971	CAKDRTQAKYNFGYYFDYW	IGH	2	None	
9	972	CQSYDHTNKNWLF	IGL	2	C4	7JVC
9	973	CARGSSNYYSIDYW	IGH	5	None	
9	974	CARVEVYDSGYRAFDIW	IGH	6	None	
9	975	CARAFWYDSSGFPWVFFDSW	IGH	2	None	
9	976	CARSPSRVDTAYNWYFDLW	IGH	6	None	
9	977	CAKDYSSGWEHCFDIW	IGH	2	None	
9	979	CATVEWLASYFYYTDVW	IGH	2	None	
9	980	CAKVLAGLTYYFDYW	IGH	6	None	
9	981	CASSPGSSSWYRHAFDIW	IGH	2	None	
9	982	CARDHKPADSSAWPPSHWYFDLW	IGH	4	None	
9	983	CARDLRIVGATADFDLW	IGH	2	None	
9	984	CARDLGDIVATTHSRGDYW	IGH	2	None	
9	985	CARRYCSGGSCSLDYW	IGH	4	None	
9	987	CAKDTHYDSSGGDYW	IGH	9	None	
9	988	CARGVLQAAGQSGGWYGFDVW	IGH	2	None	
9	989	CAKDKGYSYGHYYMDVW	IGH	4	None	
9	990	CARVYNYCSSTSCYRGFFDYW	IGH	4	None	
9	991	CARGRYYYGSGSYYAPRYFDYW	IGH	2	None	
9	992	CARVQYGDSVLILNWFDPW	IGH	4	None	
9	993	CARDADTLVVVPAATLFDYW	IGH	2	None	
9	994	CQQSYDAPRIVF	IGK	2	C2	7K8O
9	995	CAKDHGLLTYFDLW	IGH	2	None	
9	996	CNSRDSSDNPSLF	IGL	6	None	
9	997	CARVPVATPGMGIDYW	IGH	6	None	
9	998	CARGVSSSYYHYLDYW	IGH	2	None	
9	999	CGGVAGIEIFDYW	IGH	6	None	
9	1002	CANYNWNYAYSDYW	IGH	2	None	
9	1003	CAKAQGYSYGSYGDYYYMDVW	IGH	2	None	
9	1004	CTTLATGDFWGGSWTIHDYYYYMDVW	IGH	2	None	
9	1005	CQSYDSSMMMF	IGL	7	C4	7JVC
9	1006	CARVLLSGRLDYW	IGH	6	None	
9	1007	CAKGSRSSRPYYFDFW	IGH	10	None	
9	1009	CAKDYMVDFWSGYLGYW	IGH	2	None	
9	1010	CAKDRGKNWDYNWLDPW	IGH	8	None	
9	1011	CVESSGSYFYFDYW	IGH	2	None	
9	1012	CAKISSPGYYDFRSAFPSGWYFDLW	IGH	2	None	
9	1013	CAKDYLYCSGDSCHNKAVDVW	IGH	2	None	
9	1014	CAHSLNGAADYW	IGH	5	None	
9	1016	CARLRYNWTDVENAFDIW	IGH	2	None	
9	1017	CARDRHYTGIYGSSFDYW	IGH	2	None	
9	1018	CARQSFKVGPTKGDDAFDIW	IGH	5	None	
9	1019	CARDRDSSGYYLDAFDIW	IGH	7	None	
9	1020	CASGGQVAGTDAFDIW	IGH	2	None	
9	1021	CAREHLGCTTGTTCVDYW	IGH	4	None	
9	1022	CAKHPNSIAEFDHW	IGH	2	None	
9	1024	CARDGWYSSSSVGFSLDYW	IGH	5	None	
9	1025	CARGCSSANCPIEDAFDIW	IGH	2	None	
9	1026	CARGKGPTSIVDLVFEYFFDSW	IGH	3	None	
9	1027	CAAAKVGATIYWDYW	IGH	2	None	
9	1028	CTRPVVPDEKNDAFDIW	IGH	3	None	
9	1029	CNSYGGSNNFLF	IGL	32	C1	6XCM
9	1031	CSEGTPSGYINYDPFDIW	IGH	2	None	
9	1032	CVRGGSVVEIDGVAWFDPW	IGH	2	None	

9	1033	CARYDYSDRSGDPPGAFDIW	IGH	2	None	
9	1034	CAKDTSYVWGSYRLNYFDYW	IGH	3	None	
9	1035	CARVSIPRSGYDYFDFW	IGH	4	None	
9	1036	CAIPITMVPGAANWFDPW	IGH	2	None	
9	1037	CAKLVTSDREPLYYFDFW	IGH	5	None	
9	1038	CARDHHCSGTSCTQRTDYW	IGH	9	None	
9	1039	CAKERGSSWYLFLLFHFDYW	IGH	2	None	
9	1040	CQSADNDGFYHWVF	IGL	11	None	
9	1041	CANLLYFYNMAGPPDEDFQHW	IGH	2	None	
9	1042	CAKADWGGLYYFDYW	IGH	3	None	
9	1043	CAKSTYSGYDWAEFDSW	IGH	2	None	
9	1044	CTREGRGGNFDNW	IGH	2	None	
9	1046	CVKDFTGVPAAETSYHTYYMDVW	IGH	2	None	
9	1047	CARGDYGRVLDYW	IGH	8	None	
9	1048	CARAAGTALDNAFNIW	IGH	2	None	
9	1049	CAKLSPVKKFFDYW	IGH	6	None	
9	1051	CARLDRLLYRDAFDIW	IGH	2	None	
9	1052	CARHPRSVIIFGYYFDYW	IGH	2	None	
9	1053	CTKDRHHRGIASATTFQYW	IGH	2	None	
9	1054	CARSHCSSTSCYYGGWFDPW	IGH	2	None	
9	1056	CARENGYSTSHDDYFDFW	IGH	2	None	
9	1057	CARITVDTAAFDIW	IGH	7	None	
9	1058	CAKGQSRGYSNTWYQEYFQHW	IGH	2	None	
9	1059	CAKTGSWHIVVTHKGGFDPW	IGH	2	None	
9	1060	CARQGVIRGVDTFDIW	IGH	2	None	
9	1061	CVNIGCTSINCYFGAKDVW	IGH	2	None	
9	1062	CGRLAIVPGATKWFDSW	IGH	6	None	
9	1063	CARHRGFCSSTSCFEWYFDYW	IGH	2	None	
9	1064	CAKDLSSVPGYFDLW	IGH	4	None	
9	1065	CAKDHYFGVVISDSYWDYW	IGH	4	None	
9	1066	CARDSSMGWLDPW	IGH	2	None	
9	1067	CARAYHQDYYDSSGYYLHYFDYW	IGH	2	None	
9	1068	CARDGFCNTVACYGVDYYHYMDVW	IGH	3	None	
9	1069	CARLRGLTAMVPADYW	IGH	4	None	
9	1070	CARIRFRDYDVLTGFVLGGFDTW	IGH	3	None	
9	1071	CARHSGYYTEKYYFDYW	IGH	13	None	
9	1072	CSSYADNNSLLF	IGL	3	C2	7BWJ
9	1073	CARHGSSNWPIDYW	IGH	16	None	
9	1074	CARERGIVWSGRGGIDYW	IGH	5	None	
9	1075	CARVHCSTTTCWVYFDYW	IGH	4	None	
9	1076	CTTDLIAVAGDFDYW	IGH	2	None	
9	1077	CAKGDGDNIPWYFDLW	IGH	2	None	
9	1078	CVLYMGTAISAYVF	IGL	2	None	
9	1079	CAKDIGSGSPLFDHW	IGH	2	None	
9	1080	CARDMAHINIIDYW	IGH	2	None	
9	1081	CAKEIRRHFDFDYW	IGH	2	None	
9	1082	CARVFGGDCQIDYW	IGH	3	None	
9	1084	CAHGLYCSGATCSDYFDYW	IGH	2	None	
9	1085	CARVRSSTSCYTSCVDYYYMDVW	IGH	3	None	
9	1086	CARQRYCSGGSCYSSHWYFDLW	IGH	2	None	
9	1087	CTREFLEWLPTSLYNFYYMDVW	IGH	2	None	
9	1088	CAKEGSITLSPIDYW	IGH	2	None	
9	1089	CARKLDSSSSFDPW	IGH	4	None	
9	1090	CARDITAVAGQTFDYW	IGH	2	None	
9	1091	CVRDAEYCSGGSCFSYWYFDLW	IGH	4	None	
9	1092	CARPQAEYSSGWSGIDYW	IGH	2	None	
9	1093	CARLTGFCAGSGCYSFDFW	IGH	4	None	
9	1094	CARMPLWFGELFPDYW	IGH	2	None	
9	1095	CASEITYYYDSSGSPRRAFDIW	IGH	2	None	
9	1096	CARGSSSTSVAAVGPLAVYDYYYMDVW	IGH	6	None	
9	1097	CSSYADSHNFGVF	IGL	8	C2	7BWJ
9	1098	CARRPYNWNDPYYFDYW	IGH	2	None	
9	1100	CARVVPTCGHSSCPENDYW	IGH	2	None	
9	1101	CARGKCGSTSCYFEYW	IGH	2	None	
9	1102	CARMLYGDYSGNMIDYW	IGH	22	None	
9	1103	CARDRKYYDFWSGYQDNWFDPW	IGH	2	None	
9	1104	CARVSYGSYWDYYYYMDVW	IGH	2	None	
9	1105	CARAIYDFWSGYTDAFDIW	IGH	3	None	

9	1106	CVKGRLASSPEAFDIW	IGH	2	None	
9	1107	CARGDLMGFFDIW	IGH	11	None	
9	1108	CARRSRCDSATCYSGDTTWFDPW	IGH	2	None	
9	1110	CARHYSGYSYGYEMNWFDPW	IGH	4	None	
9	1111	CARIPNDYTNYHYYFDYW	IGH	3	None	
9	1112	CARGDSYDSSGWDAFDIW	IGH	2	None	
9	1113	CAKDTQYSSSWDYFDYW	IGH	6	None	
9	1114	CVKEIGYGSGTYDYFDYW	IGH	4	None	
9	1115	CARGSRTSSWSPSFDYW	IGH	2	None	
9	1117	CARSPIAAAPSLLGNWFDPW	IGH	2	None	
9	1118	CAKVQSREEAFDYW	IGH	2	None	
9	1120	CARDSSSRWYDPDNWFDPW	IGH	2	None	
9	1121	CARENIVIVPGAPDYW	IGH	2	None	
9	1122	CAKDSRGYSYGPNDYW	IGH	2	None	
9	1124	CARDYTTVASMAFDIW	IGH	2	None	
9	1125	CARDTTFDIW	IGH	57	None	
9	1126	CARDFGSIISRVTTGAFHIW	IGH	3	None	
9	1127	CAKDTGGNYYDSSGSSHFDYW	IGH	4	None	
9	1129	CAGGWKPGRGAFDIW	IGH	2	None	
9	1130	CAKAIGYNSIAAAGPFDYW	IGH	2	None	
9	1131	CARHSPRYCSDNGWNFGAAEYFHNW	IGH	2	None	
9	1132	CARVYAENGIVVSWFHPW	IGH	2	None	
9	1133	CAKGGMAVADYFDYW	IGH	2	None	
9	1134	CGKLRTYYYVDVW	IGH	4	None	
9	1135	CARGVVPDYDFWSAYEGNWFEPW	IGH	2	None	
9	1136	CARDRLRGLELTNPSDGYYFDYW	IGH	2	None	
9	1137	CARTLWDYYDLNIYHDYW	IGH	4	None	
9	1138	CVKHTNDGQDYDGYSDYFDYW	IGH	2	None	
9	1139	CARGGQQARGNWFDPW	IGH	3	None	
9	1140	CARRYRRHSTDWYFDLW	IGH	2	None	
9	1141	CARDRQDISYYYMDVW	IGH	2	None	
9	1142	CVRGYDYSADYW	IGH	8	None	
9	1143	CTKDRYCSSTSCPLDYW	IGH	4	None	
9	1144	CARDFGYCSRTTCSYFDYW	IGH	3	None	
9	1145	CASRYSNDPFDYW	IGH	11	None	
9	1146	CAREGVVLPDAIAHFDYW	IGH	2	None	
9	1147	CASASSYGRRYFDLW	IGH	2	None	
9	1148	CARFKQQLGYFDLW	IGH	2	None	
9	1149	CAKDLHGGYVKYFDDW	IGH	2	None	
9	1150	CYSAADNNWVF	IGL	105	None	
9	1151	CAKEFDNWNDGAFDIW	IGH	2	None	
9	1154	CTTDKIRGGGILLPYYYFDYW	IGH	2	None	
9	1155	CARNTVAGTHWYFDLW	IGH	2	None	
9	1156	CARTPGYCSSTSCYTYFDYW	IGH	13	None	
9	1157	CAKGSAPYSVSNWFDPW	IGH	5	None	
9	1158	CAKPVGYCSGGSCAAFDWYFDLW	IGH	2	None	
9	1159	CAVWDDSLPGRWLF	IGL	4	None	
9	1160	CAKDLSVGLRILDAFDIW	IGH	2	None	
9	1161	CARHVSANYCSSTSCNYWYFDLW	IGH	2	None	
9	1162	CATIGSLDSFDIW	IGH	4	None	
9	1163	CARHRTTGSYGGTYGVLPPTNYDYW	IGH	2	None	
9	1164	CARVDQWLEAYHFEYW	IGH	2	None	
9	1165	CAHRHSFYYDSLGAFDMW	IGH	4	None	
9	1166	CATHGSLNRDYFDYW	IGH	2	None	
9	1167	CVISIPSVSWGEDYW	IGH	2	None	
9	1168	CARVDCSSTSCYSPARVYFDYW	IGH	2	None	
9	1169	CNSRDRSGNHYWLF	IGL	11	None	
9	1170	CAKADDVAVSDNFEYW	IGH	2	None	
9	1171	CARHDQYSNVDYW	IGH	2	None	
9	1172	CVKEHCGFTDCYYSEYFKHW	IGH	2	None	
9	1174	CAKGAGVLMVYAVDYW	IGH	2	None	
9	1175	CARDWGGSSSSIYDYW	IGH	2	None	
9	1177	CLSYAGSSNFFVVF	IGL	2	C2	7K8X
9	1178	CARDPSNFQTGTTNHYFDYW	IGH	3	None	
9	1179	CAQGGYSNAFDIW	IGH	4	None	
9	1180	CARGKIGAQSVWRYYFDQW	IGH	2	None	
9	1181	CAREFRYNRNYPGFFFDFW	IGH	2	None	
9	1182	CARLVSESSGWYTQGSSFWFDPW	IGH	6	None	

9	1183	CGRDAVTREGPEDFDYW	IGH	8	None	
9	1184	CAKEPAAGSWGFDYW	IGH	5	None	
9	1185	CARIKYCSFPSCYSGLFDIW	IGH	2	None	
9	1186	CAREGGYSYGSPLYYFDYW	IGH	2	None	
9	1187	CTQCDRYLESPPDYW	IGH	2	None	
9	1188	CVREGDCNGPNCYAFDIW	IGH	4	None	
9	1189	CARQKIFGLVFDFW	IGH	2	None	
9	1190	CAKDGELERQYYFDYW	IGH	2	None	
9	1191	CAKSPSPYCSSPRCRRGYYYMDVW	IGH	2	None	
9	1192	CARVLRYASGRTFGYW	IGH	2	None	
9	1193	CAKDRSDGFWSGSSSVHFDYW	IGH	2	None	
9	1196	CAQYDYSNSPLYFDFW	IGH	2	None	
9	1197	CARQNWEGQLALPYDAFDIW	IGH	2	None	
9	1198	CARAEQFCSGATCYLNFDYW	IGH	2	None	
9	1199	CAKNRGFSTTMLHDAFDVW	IGH	7	None	
9	1200	CARAEQSCTSDTCYQNFDYW	IGH	4	None	
9	1201	CARRRGGLAAAGTGTDYFDYW	IGH	2	None	
9	1202	CARERPMFSYDVFDIW	IGH	2	None	
9	1203	CAKGLPYCSSSSCPMPVYYFDLW	IGH	2	None	
9	1204	CAKDLHSARQGVDAFDFW	IGH	2	None	
9	1205	CARDYSYHNLPSGYKDFW	IGH	4	None	
9	1206	CAHKLVVTGTDIPFDSW	IGH	2	None	
9	1207	CARDGGTTGTWDAFDIW	IGH	2	None	
9	1208	CARLEAAAGTYESDYW	IGH	5	None	
9	1209	CARHVGCTNGVCQMLNAFDIW	IGH	3	None	
9	1210	CAKKRNIYTHGWLQDVFHIW	IGH	2	None	
9	1212	CARFGLERRWYYFDYW	IGH	3	None	
9	1213	CTRELDIWSGYSNDYW	IGH	2	None	
9	1216	CARLYSYCITPDCYRFGYFDYW	IGH	4	None	
9	1217	CAQFPGDSFEIW	IGH	3	None	
9	1218	CARRGPGFGELYYFDYW	IGH	2	None	
9	1219	CVRGFKTENAFEIW	IGH	4	None	
9	1220	CARDRSSSAGFFDYW	IGH	4	None	
9	1221	CARDQVYYDSSGSQGYYYYYMDVW	IGH	2	None	
9	1222	CARHQYYYDRSGYYPDYW	IGH	10	None	
9	1223	CARCPYCKDSSCYNPYYYYYMDVW	IGH	10	None	
9	1225	CARDRGPWFGELSTANYFDYW	IGH	2	None	
9	1226	CARTTVTAPPLFDYW	IGH	2	None	
9	1227	CASPITMVRGYDYW	IGH	2	None	
9	1228	CARGAMIANYGDYGPDYW	IGH	2	None	
9	1229	CARRYSSGCIDYW	IGH	9	None	
9	1230	CVKSNGDWLYYYYYYMDVW	IGH	3	None	
9	1231	CARANGQQPLNWFDPW	IGH	2	None	
9	1232	CVHAGSGTYYNDWFHPW	IGH	4	None	
9	1233	CAKVGLMYSSSSYYFDYW	IGH	2	None	
9	1234	CATDYLSPDAFDLW	IGH	2	None	
9	1235	CARGTYDFWSGYYIGHAFDIW	IGH	2	None	
9	1236	CAKDHYYGSGSYYSPDYW	IGH	2	None	
9	1237	CARHYNSGTYPLDYW	IGH	3	None	
9	1238	CARGSPGDCSSTSCPYGPYYYYYMDVW	IGH	2	None	
9	1239	CAKDRAYYESSGCFGYW	IGH	2	None	
9	1240	CAKDLGYCSSTSCGHDAFDIW	IGH	2	None	
9	1242	CAREMWDYYENSGAPIDYW	IGH	3	None	
9	1243	CVRNRGYDLDYFDYW	IGH	6	None	
9	1244	CHQYYSSPPYSF	IGK	36	C4	6W41
9	1245	CARHEVDGYLPFCSSNSCSFNWFDPW	IGH	2	None	
9	1246	CARSGLSSGYYGFDIW	IGH	5	None	
9	1247	CARVVIETRNYYYYYMDVW	IGH	2	None	
9	1248	CVRGNSGWYHPHYYYYLDVW	IGH	2	None	
9	1249	CARGRRLLDSSGYTVFAGFDYW	IGH	2	None	
9	1250	CARHVSDYDILTLNYYYYMDVW	IGH	2	None	
9	1251	CASSWFGELETFDPW	IGH	2	None	
9	1252	CAKDVLRYFDWLNDAFDIW	IGH	4	None	
9	1253	CLGSTYAFDMW	IGH	9	None	
9	1254	CAKDIDPVLVPPSMGIDYW	IGH	9	None	
9	1255	CARHGYYYESRANWFDPW	IGH	2	None	
9	1256	CTTIRSPSSTDYFDYW	IGH	2	None	
9	1257	CVKGGGRYFDLHPAFDIW	IGH	2	None	

9	1258	CARGGTQLVHFDLW	IGH	2	None	
9	1260	CVKEGRHSSGWSYYFDSW	IGH	2	None	
9	1261	CAKDEKIAAAGTGSFDYW	IGH	4	None	
9	1262	CARDSFRIQSYGTSGTDYW	IGH	3	None	
9	1263	CAKDQGSGFGELDAFDIW	IGH	4	None	
9	1264	CAKDNLRPYGDYDYW	IGH	5	None	
9	1265	CAKDYVGYPRGFDYW	IGH	5	None	
9	1266	CARGPICGSGGSCQRTDLDYW	IGH	4	None	
9	1267	CVKDYYYDSSGYYPYFDYW	IGH	3	None	
9	1268	CARSTGLPNFQKYYLDVW	IGH	2	None	
9	1270	CARQGQLWLYHFDYW	IGH	2	None	
9	1271	CARDGERGYTYGFDYW	IGH	7	None	
9	1272	CASEYSSSWYRQIDYW	IGH	2	None	
9	1273	CAKDQSSSSVHNSFDSW	IGH	2	None	
9	1274	CVKDVDTAMVTIFDYW	IGH	2	None	
9	1275	CARGLYGETYNWFDPW	IGH	6	None	
9	1276	CARQHVRYCISISCPSDDAFDIW	IGH	12	None	
9	1277	CVRDVNIGRRFDFW	IGH	3	None	
9	1278	CARDSPSRQTRIPVAGTFRYYYDYHMDVW	IGH	2	None	
9	1279	CVRELRRDQGGNSDSFDIW	IGH	2	None	
9	1280	CTLFPYTTLFRSEYSYFDYW	IGH	7	None	
9	1282	CAREYDFWSGNYKEGNDAFDVW	IGH	3	None	
9	1283	CARSLGIGSASAFDVW	IGH	3	None	
9	1284	CARASSYCINGVCSPEFDYW	IGH	2	None	
9	1285	CARVEQGHVRNDQNYDAFDVW	IGH	2	None	
9	1286	CARDRVSGERNSDWYPRFDPW	IGH	2	None	
9	1287	CARDLHGGEMEYDFWSRYYPIFDYW	IGH	2	None	
9	1288	CAREYYYDSSGYPNPPSAFDIW	IGH	2	None	
9	1289	CARERVVVMSAAIRYFDLW	IGH	2	None	
9	1290	CARDQYQLPTQKGYHWFDPW	IGH	4	None	
9	1291	CARHKTNLLDDDKYYFDSW	IGH	15	None	
9	1292	CAKDTSGYRLGYFDYW	IGH	2	None	
9	1293	CARQGGYDFWSGYYSFAFDIW	IGH	10	None	
9	1294	CARLSVAVAGKYYYYMDVW	IGH	4	None	
9	1295	CARDDDRSGYTSLAFDLW	IGH	2	None	
9	1296	CARDGRGRSGWDPSGNKYFDPW	IGH	2	None	
9	1297	CRLCCSTSWSLTLFNYMDVW	IGH	2	None	
9	1298	CARGDIVVVPAAIPFGAFDIW	IGH	2	None	
9	1299	CAKDSSYYYDSSGYSTDAFDIW	IGH	2	None	
9	1300	CARGGNYYASGNYYPDYW	IGH	4	None	
9	1301	CVRDSIARTATFDYW	IGH	2	None	
9	1302	CARVPPITVAQIYYMDVW	IGH	2	None	
9	1303	CAKERSITGTFGDAFDIW	IGH	2	None	
9	1304	CARGKYNWDDEYARKIYYYYYMDVW	IGH	6	None	
9	1305	CARSSGYYVPLFDHW	IGH	2	None	
9	1307	CARHLLWGSPDAFDIW	IGH	3	None	
9	1308	CARDPVTVSGLITHAMDVW	IGH	2	None	
9	1309	CASGAATYFYYMDVW	IGH	6	None	
9	1310	CATWPRPKITSFDYW	IGH	2	None	
9	1311	CAKALPPHFLFPFDHW	IGH	4	None	
9	1312	CARASGAIGASTIIFDSW	IGH	2	None	
9	1313	CARDGIEGDYGENNWFDPW	IGH	2	None	
9	1314	CARDDPGIAAAGPFFDYW	IGH	2	None	
9	1315	CARDSAYYEFWSGYYYFDYW	IGH	2	None	
9	1316	CTTTTCRPREYYYYMDVW	IGH	2	None	
9	1317	CARDAGLPNFDFW	IGH	4	None	
9	1318	CARVVSGEVAFDIW	IGH	6	None	
9	1319	CAKIIQWLVPSLDYW	IGH	6	None	
9	1320	CARDPLNRGWFDPW	IGH	9	None	
9	1321	CARSSSTYYYNRTPLTTDYW	IGH	2	None	
9	1322	CVHRVHRTLKEDFWSGLDYW	IGH	3	None	
9	1324	CARQSIVEAGWGLFGFDSW	IGH	2	None	
9	1325	CAHRPPDGGPDYW	IGH	2	None	
9	1326	CCATIYYHESSGLPLFFDSW	IGH	2	None	
9	1327	CAKDSIWLGELAFYMDVW	IGH	2	None	
9	1328	CAHSGSSGRYFDYW	IGH	5	None	
9	1329	CAREHCTNGVCYRYFDYW	IGH	2	None	
9	1331	CARGRAVRPTGAGWPGNFDYW	IGH	5	None	

9	1332	CTTLGTSIIVTGRPVPFNYW	IGH	2	None	
9	1333	CARDRGEYSSSLPFGYW	IGH	2	None	
9	1334	CARRAGEGATIYYYNYMDVW	IGH	2	None	
9	1335	CAKATVYCSSTSCPIDYW	IGH	4	None	
9	1336	CARDIVVVPAAAHDAFDIW	IGH	4	None	
9	1338	CARDPGYCSGPSCYYSHFDYW	IGH	4	None	
9	1339	CARREYYDTLTGHYEHWYFDLW	IGH	8	None	
9	1340	CAHKKGSSTSCYDYW	IGH	3	None	
9	1341	CARERSGYYYNYMDVW	IGH	9	None	
9	1342	CARDPHPGWGDYFDYW	IGH	4	None	
9	1343	CARHSNSINWLNPW	IGH	2	None	
9	1344	CARGPGQTILGVIMPRFDNW	IGH	3	None	
9	1345	CAKQPPSPVYSNIRNPYYCDYW	IGH	4	None	
9	1346	CARTVGYSGYNYFDYW	IGH	2	None	
9	1348	CVTGFTYYYGSQNYSLDYW	IGH	2	None	
9	1349	CAKDAKIAPVSGGLFDYW	IGH	6	None	
9	1350	CARGPESSGYTWFDPW	IGH	5	None	
9	1351	CAKGAVSSGWSQCFDYW	IGH	5	None	
9	1352	CARHRYYYDTSGYVGWFAPW	IGH	2	None	
9	1353	CARQPPSTVASLGFDYW	IGH	4	None	
9	1355	CARGGGLRGYYFDYW	IGH	2	None	
9	1356	CARDGSGSHTTSFYFDYW	IGH	3	None	
9	1357	CTQGADFGVGVAFDIW	IGH	4	None	
9	1358	CALPISALYYCARDLVRANRYSGSWIPFDSW	IGH	2	None	
9	1359	CARAHVSDSYGLDYW	IGH	4	None	
9	1360	CAKDQGGGSCVDYW	IGH	2	None	
9	1362	CARDGIWGYDSDYSFDFW	IGH	2	None	
9	1363	CARTQWLQFDVFNIW	IGH	2	None	
9	1364	CVRESYCRTTACHSSGAFDIW	IGH	2	None	
9	1365	CAKMGTTGTTEFDYW	IGH	5	None	
9	1366	CARDIGDYEVEPAAVDYW	IGH	3	None	
9	1367	CARVHHLSSFDIW	IGH	3	None	
9	1368	CARAWLWQSFDYW	IGH	2	None	
9	1370	CARSRVSGGDCYLDYW	IGH	2	None	
9	1371	CAKDARYFDWDYFDYW	IGH	4	None	
9	1372	CAKDRSVYSSGLETWGYFDYW	IGH	2	None	
9	1373	CASGFSSPYYVNWYFDTW	IGH	2	None	
9	1374	CARVGHRPGYCNSISCYLFWFDSW	IGH	2	None	
9	1375	CARHDFDSLIRPTKPELLLPYYFDFW	IGH	9	None	
9	1376	CCVRGTDYDSSGYDAFDDW	IGH	2	None	
9	1377	CVRDVRQSTPNWYFDLW	IGH	4	None	
9	1378	CRATATTHSDTFDIW	IGH	2	None	
9	1379	CAHMRYSGYQRYFFDYW	IGH	2	None	
9	1381	CSRARIVVLPGSIKANYFDYW	IGH	23	None	
9	1382	CARAWSVNTPSFDNW	IGH	2	None	
9	1383	CAKARYCSYTSCYKGFDSW	IGH	4	None	
9	1384	CARDGGTYDSSGYYYGLDYW	IGH	2	None	
9	1385	CARDDAYCSGASCYDYAAIAAATFDYW	IGH	2	None	
9	1386	CARDLYGGSSLGAFDIW	IGH	3	None	
9	1388	CARDGGEYYGSGSLYDYW	IGH	2	None	
9	1389	CASAHILTGYYIYDYW	IGH	4	None	
9	1390	CARDHGDSTRSGDALDIW	IGH	2	None	
9	1391	CSKGVGSWQVYYFDYW	IGH	2	None	
9	1392	CAKVHRTASCCSDYFDDW	IGH	2	None	
9	1393	CTTVSNAFWNGLYYYFYMDVW	IGH	4	None	
9	1394	CAKAMAAVGLNAFDIW	IGH	3	None	
9	1395	CAKDMVVVPAGDGAFDFW	IGH	4	None	
9	1396	CARPSLLGYCSSTSCYWAAFDIW	IGH	2	None	
9	1397	CARSFDHGGGSYFEHW	IGH	4	None	
9	1398	CARQNDFWSGSYYMDVW	IGH	2	None	
9	1399	CARGRHQLIRNAYFQYW	IGH	5	None	
9	1400	CASLEEWFLPW	IGH	3	None	
9	1401	CAKGGPYGSGSYLEDAFDIW	IGH	2	None	
9	1402	CASRHCTSSSCYEYFHHW	IGH	2	None	
9	1403	CQYYGYSPLVIF	IGK	6	C1	7K8N
9	1405	CQSADQSGTFLVF	IGL	75	None	
9	1406	CARLVDYGSGSSGWFDPW	IGH	2	None	
9	1407	CATIISMAGTFYFDYW	IGH	2	None	

9	1408	CARRIAAAGPDWFDPW	IGH	10	None	
9	1409	CATGPIIIGPDTIGHRYWYFDLW	IGH	2	None	
9	1410	CAKDVSYDFWGGYFISW	IGH	3	None	
9	1411	CARIQAHDFWSGYYRSPNFYFDYW	IGH	2	None	
9	1412	CATPRGFPYYFDYW	IGH	3	None	
9	1413	CARGGSYGSGSGRFDYW	IGH	2	None	
9	1414	CAKNMGGSGWYRGGGFDIW	IGH	4	None	
9	1415	CTTGLVRGAIRPDYW	IGH	4	None	
9	1416	CAKGSSWKVDYW	IGH	3	None	
9	1417	CARGGSRHGYPPPDYW	IGH	2	None	
9	1418	CARPYSSTYYSYFDYW	IGH	2	None	
9	1420	CAKTTSPGYCSSTSCYGAFDIW	IGH	2	None	
9	1421	CAREARSGWTIYFDNW	IGH	2	None	
9	1423	CARSWDGDYVVTNYFDYW	IGH	9	None	
9	1424	CATCSSWYTTEYFQHW	IGH	4	None	
9	1426	CARQDAVAGTFELDYW	IGH	3	None	
9	1428	CVRDRSMWYGDFDYW	IGH	2	None	
9	1429	CARDLCAPSACYLLDYW	IGH	4	None	
9	1430	CARQRNSSHDKWVKDVDYW	IGH	2	None	
9	1431	CARETEYCSGEDCSPFYFDYW	IGH	2	None	
9	1432	CVRAGGRSSPFGYW	IGH	2	None	
9	1433	CAKDLSTYYYDTHANDYW	IGH	2	None	
9	1434	CARVQNIGWFDPW	IGH	14	None	
9	1435	CARDRIADSTSSGLDYW	IGH	31	None	
9	1436	CARHNYASGSPLPYWFDPW	IGH	6	None	
9	1437	CARAVIRGKAFDYW	IGH	3	None	
9	1438	CARHGKKQPANWFDPW	IGH	2	None	
9	1439	CSSYAGNYIFVF	IGL	4	C1	6XCM
9	1440	CAKVPYGGGSPFDPW	IGH	2	None	
9	1441	CARELRIAARLLSLYYMDVW	IGH	2	None	
9	1443	CARDHCSSISCYPRVGAFDIW	IGH	2	None	
9	1444	CARSRLRYFTRFDPW	IGH	2	None	
9	1445	CARALWQQLVPLDYW	IGH	3	None	
9	1446	CAKDGIAAAAILAGEWFDPW	IGH	2	None	
9	1447	CARDRTPGIAAAGFDAFDIW	IGH	2	None	
9	1448	CARDFYDSSGYYYGDLDAFDIW	IGH	2	None	
9	1449	CARDSTYYDSSGYIKAFDIW	IGH	3	None	
9	1450	CTMSLGYCTNGVCPDGFAIW	IGH	7	None	
9	1451	CARGTTSFSPVPFDYW	IGH	2	None	
9	1452	CARGMGPDPIFGVAALDYW	IGH	2	None	
9	1453	CARETRYSYGLFDYW	IGH	15	None	
9	1455	CARPIVRGIQADFVYW	IGH	2	None	
9	1456	CTTDPSEIAAAEAIDYW	IGH	4	None	
9	1457	CQQNYSPPFIF	IGK	24	C2	7K8O
9	1459	CARDSGGYCSSTSCLTKKYMVNWFDPW	IGH	3	None	
9	1460	CARGEYCSSTSCYAFEWFDPW	IGH	4	None	
9	1461	CAKSNPSMITVTPSLFDYW	IGH	4	None	
9	1462	CARPQAEWDLLLPFDMW	IGH	6	None	
9	1463	CVKDQSIAAAGAFDIW	IGH	6	None	
9	1464	CARDGARDLLLFDAFDIW	IGH	2	None	
9	1465	CARDPELVGRLGFDYW	IGH	2	None	
9	1466	CARAPGIAGGDWYFDLW	IGH	2	None	
9	1467	CARDPGPTRYYYFDYW	IGH	7	None	
9	1468	CARVRTAKPGGDYW	IGH	2	None	
9	1469	CARNGSPWTSHCSSTSCPHNWFDPW	IGH	3	None	
9	1470	CARGYGGKSYYYYYMDVW	IGH	2	None	
9	1471	CARGSSRTSWKIFGTDCFDYW	IGH	5	None	
9	1472	CARALYDFWSGNTTEYAFDIW	IGH	2	None	
9	1473	CTRGPTGSGTSYFDHW	IGH	2	None	
9	1474	CARYFAGTTFNDAFDIW	IGH	4	None	
9	1475	CARESGTYDNYFDYW	IGH	2	None	
9	1476	CARHESDSSGSYYFDYW	IGH	4	None	
9	1478	CAQRGSRDRTLDYW	IGH	6	None	
9	1479	CAKDPRVYSSSWSWYFDLW	IGH	2	None	
9	1480	CAKDQRHTGSNPGGFDYW	IGH	2	None	
9	1481	CARQRECSSSRCSTSNWFDPW	IGH	8	None	
9	1482	CVRDKGSAAGTWGYNFDYW	IGH	2	None	
9	1483	CARSSSWYLDDYFDYW	IGH	2	None	

9	1485	CARHLASIVVVPAAISRYYFDYW	IGH	2	None	
9	1486	CAGIFGRWFDPW	IGH	2	None	
9	1487	CARLWDTNSWQHFDYW	IGH	2	None	
9	1488	CARLPARAQVWTPKHAFDIW	IGH	2	None	
9	1489	CARPWGRSHQFVFDYW	IGH	2	None	
9	1491	CAKDHGSAGSFDFDYW	IGH	2	None	
9	1492	CVKDRGQWELLYYFDYW	IGH	2	None	
9	1493	CAKDFGVVPRQPHDYW	IGH	2	None	
9	1494	CAKEALNFYYYMDVW	IGH	3	None	
9	1495	CAHRLVYHYYFDYW	IGH	4	None	
9	1496	CARERSGWSLGPDYW	IGH	4	None	
9	1497	CAKTRYSTAYDAFDMW	IGH	5	None	
9	1498	CARAPEFRVFYYLDVW	IGH	2	None	
9	1500	CSIWPSNAWVF	IGL	21	None	
9	1501	CAKLARLSTSDYW	IGH	2	None	
9	1502	CARSIYLTQDAFDIW	IGH	4	None	
9	1503	CAREDLAVVPPAIWFDPW	IGH	4	None	
9	1505	CARVLGNNWNFGPPFDSW	IGH	2	None	
9	1506	CARDRGGTRFDDW	IGH	8	None	
9	1508	CARGGTPYNYDTGDNPYFDSW	IGH	2	None	
9	1509	CQSYDRSNHLHVVF	IGL	2	C4	7JVC
9	1510	CAKGKDSPLYYGAGNYQSFFDSW	IGH	5	None	
9	1511	CVHSLHYCSSTSCFSPLYFDFW	IGH	2	None	
9	1513	CARGWSRFLEWDYFDYW	IGH	2	None	
9	1514	CARGREDYGFWGDHMDVW	IGH	2	None	
9	1515	CARIIVGATGYFDYW	IGH	8	None	
9	1516	CARTPYATSYYFDDW	IGH	2	None	
9	1517	CARGVDFWSGPPPSNWFDPW	IGH	2	None	
9	1518	CARPDVLTGYRDFDYW	IGH	3	None	
9	1521	CAKASRYYYFDYW	IGH	12	None	
9	1522	CVKYHYDGTSYYWDDYFDYW	IGH	2	None	
9	1523	CARDLDSRAGRYFDYW	IGH	2	None	
9	1524	CATNGATDTPLYYFDYW	IGH	2	None	
9	1525	CARQKVEYCDRSSCYGAIDYW	IGH	2	None	
9	1527	CALPISRGAGRGRGQDYW	IGH	2	None	
9	1529	CARVSLDCSSTSCYIQLYYFDYW	IGH	4	None	
9	1530	CAKEDYGGNGWRGVFDYW	IGH	2	None	
9	1531	CTKDTRVVTSVVVVEGWFDSW	IGH	2	None	
9	1532	CTLFPYTTLFRSAKVRTRWLRLDYW	IGH	2	None	
9	1533	CARETNFYGGSYFDYW	IGH	7	None	
9	1534	CAIGICYTLDYW	IGH	2	None	
9	1535	CTRVGHGWSFGSGFDYW	IGH	2	None	
9	1536	CARKQRGQGPTTDFFDFW	IGH	2	None	
9	1537	CARGGCSGGSCYHHNYYFDYW	IGH	4	None	
9	1538	CARQLPIGSSSVTLGFDYW	IGH	2	None	
9	1539	CAGNDYGDLKFEYW	IGH	2	None	
9	1540	CAISDIVLRTASILKSGYFHQW	IGH	4	None	
9	1541	CARARQGSRYTGYYFDYW	IGH	2	None	
9	1543	CQHFGSSPMFAF	IGK	2	C1	7K8N
9	1544	CARVPLKHINMIEVVNEEGWFDPW	IGH	3	None	
9	1545	CAKTISGSGWHDAFDIW	IGH	2	None	
9	1546	CARDSGPTGTGHSYYFDYW	IGH	2	None	
9	1547	CALPICLTAEDTAVYYCARDPHPGWGDYFDYW	IGH	2	None	
9	1548	CVRYYCNSALCYFDYW	IGH	2	None	
9	1549	CAGELSFTSSSRWFDPW	IGH	3	None	
9	1550	CSRIVVPAGIDYW	IGH	2	None	
9	1551	CARVRGPPAGSWLDPW	IGH	2	None	
9	1552	CARTGLDGNRFDPW	IGH	21	None	
9	1553	CAKVADSSGWYWGVDYW	IGH	2	None	
9	1554	CVRDQNPQRVDWYFDLW	IGH	2	None	
9	1555	CAREIWNDRSFYFDHW	IGH	2	None	
9	1556	CAYSMVTGRFGYFDYW	IGH	2	None	
9	1557	CAKDIIRGSSWYDGWFDPW	IGH	2	None	
9	1558	CAKEQGYTLGDEYFDNW	IGH	2	None	
9	1559	CVRGYRGYDYERVLFDLW	IGH	2	None	
9	1560	CAKGLMVRGLNQKDDYW	IGH	2	None	
9	1561	CAREHTFDRYGLSEDFYYYYIDLW	IGH	4	None	
9	1562	CARRWGYSYAQGDWFDPW	IGH	2	None	

9	1563	CQQFYDTPFPTF	IGK	2	C2	7K8O
9	1564	CANAKAPAAIVFYYFDYW	IGH	4	None	
9	1565	CANSAGAGYGLFPTTYSTDYW	IGH	2	None	
9	1566	CASVVVITPTGQSVYYMDIW	IGH	2	None	
9	1567	CARVSYSYGYSGEHWFDPW	IGH	2	None	
9	1568	CARDPATRSGWYAGLDYW	IGH	2	None	
9	1570	CARGAYSLDPFDIW	IGH	2	None	
9	1571	CARAPPYNWNYRYYYMDVW	IGH	5	None	
9	1572	CAGDPGLPTDYW	IGH	4	None	
9	1573	CVRQSSAVGTTLFDNW	IGH	5	None	
9	1574	CARHEPSGYSSSWYMFGYFDYW	IGH	2	None	
9	1576	CARLPVATGYGDFPFDYW	IGH	2	None	
9	1577	CARGYDVLTGYPDYW	IGH	180	None	
9	1578	CTRPSGSYSPLDYW	IGH	4	None	
9	1579	CARDSLELFRALDYW	IGH	4	None	
9	1580	CFSVDSSGHQRVF	IGL	4	None	
9	1581	CAKSSRQFGMVEFDYW	IGH	11	None	
9	1582	CARDSTITTFAVAANFDYW	IGH	21	None	
9	1583	CQVWDSSSHASADQVIF	IGL	2	C4	7JVC
9	1584	CAHYTEKVPRVPFDYW	IGH	2	None	
9	1586	CARLTRDCDRRNCPPYYYFDSW	IGH	2	None	
9	1587	CARDTGDPDRRDGYIPDYW	IGH	2	None	
9	1588	CARDYYDSSGYYPILRYFDYW	IGH	2	None	
9	1589	CQQYNKRPPASCSF	IGK	8	C3	7K8R
9	1590	CARDARASLYCSSINCYRAFDIW	IGH	2	None	
9	1591	CAKEKLEVRPGYYMDVW	IGH	2	None	
9	1592	CARGTSSGWYFGYW	IGH	6	None	
9	1593	CARDGLLGSAWHGSDWFDMW	IGH	2	None	
9	1594	CQYFGSYPYTF	IGK	7	C3	7K8P
9	1595	CASQRGPTARLDYW	IGH	2	None	
9	1596	CAREPHGYFFDTW	IGH	3	None	
9	1597	CAHRLYSRDFWGGHPFDYW	IGH	2	None	
9	1599	CAKDLRPIQQAPDYW	IGH	2	None	
9	1600	CQQYNVYPYTFGQGTNF	IGK	2	C3	7K8P
9	1601	CVTDDSRGWHSFDNW	IGH	2	None	
9	1604	CAKDKRIAPAGYFDYW	IGH	2	None	
9	1605	CARVVPAAMFDPW	IGH	2	None	
9	1606	CARFWSGHGEFDPW	IGH	2	None	
9	1607	CVKVSPGYYDSSGYYYDYW	IGH	2	None	
9	1608	CASMDSSSWFMSDDYW	IGH	2	None	
9	1609	CASAYGGLEFRNAFDIW	IGH	2	None	
9	1610	CAKQTYYDSWSGPLYYFAYW	IGH	3	None	
9	1611	CARDSGVYSLRFGYW	IGH	8	None	
9	1612	CARDRVVGAARESYPDYW	IGH	2	None	
9	1614	CVREELMDWFDPW	IGH	6	None	
9	1615	CATDREVAYPLGWYFDLW	IGH	2	None	
9	1616	CVREQFCTHTTCYYFDYW	IGH	4	None	
9	1617	CARLRGIVVVPATIQEENYFDYW	IGH	3	None	
9	1618	CARDSCGGDCHSPDAFDIW	IGH	4	None	
9	1619	CARTFYGDFGNYIDVW	IGH	7	None	
9	1620	CARHDLEYSSPNWFDPW	IGH	2	None	
9	1621	CARMAWRIHFSFDYW	IGH	4	None	
9	1623	CVATKDYYPKYFDYW	IGH	3	None	
9	1624	CAASDPRFRWYQIDYW	IGH	2	None	
9	1625	CARVRWTRPTSIDYW	IGH	2	None	
9	1626	CARDFGQLGNHYMDLW	IGH	5	None	
9	1627	CARMRGSGSYDDAFDIW	IGH	6	None	
9	1628	CAKDHTPYCNGGNCFSGFDSW	IGH	2	None	
9	1629	CARDLDTVMDLLGTFDIW	IGH	3	None	
9	1630	CVKTITVFGVVTHFDSW	IGH	5	None	
9	1632	CARAHSGYDWGSFFDYW	IGH	2	None	
9	1633	CQHRSLWPPVYTF	IGK	29	None	
9	1634	CARDAYCTSTSCYPDCW	IGH	2	None	
9	1635	CAKAAGYSGYGTYYFDYW	IGH	6	None	
9	1636	CARDMNPVRGYPHAFDIW	IGH	2	None	
9	1637	CVKDIWPYGDYEDAFDSW	IGH	2	None	
9	1638	CARDSPENSPYYYDSSGSYPPGDFDYW	IGH	2	None	
9	1639	CAKIRRLLFDSFHIW	IGH	4	None	

9	1640	CARVRGYSYNYYMDVW	IGH	2	None	
9	1641	CTRDPSFKSDWGVYADSW	IGH	2	None	
9	1642	CAKAGDSYGYNLSIFYYMDAW	IGH	2	None	
9	1643	CAREPGGTGGYDYW	IGH	5	None	
9	1644	CASRKITFGGVIVYYFHDW	IGH	4	None	
9	1646	CQQYNNLPVMCSF	IGK	36	C3	6XDG
9	1647	CARDWEDEGGTFYPLDYW	IGH	2	None	
9	1649	CVKDILPGYCDNTNCHPGPRFDYW	IGH	4	None	
9	1650	CAKVVDIVPTDALDFW	IGH	9	None	
9	1651	CARHAIDTIYDYW	IGH	3	None	
9	1652	CVLVFRNGVRFLEWLTQFDYW	IGH	2	None	
9	1654	CARARIRLLSVYPNNWFDPW	IGH	6	None	
9	1655	CAKGKEAYCGGDCYLFDSW	IGH	4	None	
9	1656	CARSVVVVATSDLHNWADPW	IGH	4	None	
9	1657	CTLFPYTTLFRSYYCARQKIFGLVFDFW	IGH	2	None	
9	1658	CARINLVPWFDPW	IGH	4	None	
9	1659	CARDYSGSYFGWFDPW	IGH	7	None	
9	1661	CMQALQTPLTF	IGK	1274	None	
9	1662	CARETTGSGSQLFDPW	IGH	2	None	
9	1663	CAKDRGYNWNYVAFDIW	IGH	2	None	
9	1664	CARDPADDPSVSLDYW	IGH	2	None	
9	1665	CARDRFSDIVVLPAALPIRNNWFDPW	IGH	2	None	
9	1667	CAREGKEFCTSISCRTYHFDHW	IGH	2	None	
9	1668	CVKDGEEWEVGEKYFDYW	IGH	2	None	
9	1669	CATDGRFGYCSSTSCSFDYW	IGH	2	None	
9	1670	CTKSRYCSSTSCYIDFDPW	IGH	4	None	
9	1671	CARGGYNGYDYDNYFYYYYMDVW	IGH	2	None	
9	1672	CASAEVGTWGYFDYW	IGH	2	None	
9	1673	CARISYYDFWSGYGDWDLISYFDYW	IGH	2	None	
9	1674	CARVGRDDSGFSWFDPW	IGH	3	None	
9	1675	CAKDGRAWMRINWLFDPW	IGH	2	None	
9	1678	CARGDQKPAFSLDYW	IGH	2	None	
9	1679	CTKLLRRDAFDIW	IGH	2	None	
9	1680	CVRDGRCSTGTCYSPYPYYYMDVW	IGH	2	None	
9	1681	CARNLAGVQEWLRGFDFW	IGH	2	None	
9	1682	CARHLADFRRTDYKDYW	IGH	5	None	
9	1683	CTRVVPVAKVDYW	IGH	2	None	
9	1686	CARSGYYENNGYSRNWFDPW	IGH	2	None	
9	1688	CARLLPRYSSSLPPLYFDYW	IGH	2	None	
9	1689	CARPVEMATISAFDIW	IGH	3	None	
9	1690	CARQVVRGAARRIDAFDIW	IGH	2	None	
9	1691	CAKADTPAALSDWFDPW	IGH	2	None	
9	1692	CAKAPIFYYFDFW	IGH	2	None	
9	1693	CVRGPGPPDYW	IGH	17	None	
9	1694	CARVPGFGVVQNYYFDYW	IGH	2	None	
9	1695	CAKDRTMIVVVITSHFDYW	IGH	2	None	
9	1698	CAKDKGDGYTYDSGFDAFDIW	IGH	2	None	
9	1699	CTKIATPGVGTTGTFDDW	IGH	2	None	
9	1700	CAKNIVVVLGAIDYW	IGH	5	None	
9	1701	CAREVFWSTPGKADWYFDLW	IGH	2	None	
9	1702	CARESKVYCSDYW	IGH	3	None	
9	1703	CARRGAHFYDSSGYYPFDYW	IGH	2	None	
9	1704	CARLADSGGYFDAFDTW	IGH	4	None	
9	1705	CARVPQEYAYGYWYFDLW	IGH	6	None	
9	1706	CARDGSGRYYRADYFDYW	IGH	2	None	
9	1708	CARVLSSSTNCPDYW	IGH	2	None	
9	1709	CARAHYQLQRALFDYW	IGH	4	None	
9	1712	CARHGATGYSITIFGGNWFDPW	IGH	3	None	
9	1714	CARARIYNDILTGQYYFDYW	IGH	3	None	
9	1715	CARETYGSGSEDSFDFW	IGH	3	None	
9	1716	CAREDRYQHLDYW	IGH	2	None	
9	1717	CARILSIVGATTGAFDLW	IGH	2	None	
9	1718	CQSGESSGPSWVF	IGL	8	None	
9	1719	CTRGLHYSYDIRSYYSHYFDSW	IGH	2	None	
9	1720	CATVGYCSSTTCYAIDYW	IGH	3	None	
9	1721	CAKDKIGYYYDSSGYYPDHYFDYW	IGH	4	None	
9	1722	CARAAFDWGSYFDYW	IGH	2	None	
9	1723	CARGPHGYDFWSGYSVLIDYW	IGH	2	None	

9	1724	CARAVYSTSSTRDWLDPW	IGH	2	None	
9	1725	CVKGQQLWRRWFDSW	IGH	4	None	
9	1726	CARERYSRSSWDGIDIW	IGH	2	None	
9	1727	CARLSPGVIPAVTPFNWFDPW	IGH	2	None	
9	1728	CARDPQLTNYRGSGTYYFSWFDPW	IGH	2	None	
9	1730	CVKGSKKSGVYFFDYW	IGH	2	None	
9	1731	CAKSRSYCSSTSCYGLYYFDYW	IGH	2	None	
9	1732	CARHSATGGLYDAFDIW	IGH	3	None	
9	1733	CARGSRRPGIALAYASRGYYYYMDVW	IGH	2	None	
9	1734	CARDAIVVVPAAPRNWYFDLW	IGH	2	None	
9	1735	CQAWDSSTVVF	IGL	999	C4	7JVC
9	1736	CARDRTSGYPDSFDLW	IGH	12	None	
9	1737	CCSYAGSYTYVF	IGL	1019	C2	7K8X
9	1738	CAKVPSSIIVVVYFDYW	IGH	6	None	
9	1739	CAKHLVPAAIQGGFDYW	IGH	2	None	
9	1740	CAKTAEDCSGGICYSGYYYYMDVW	IGH	8	None	
9	1741	CAKDLSLDMGGSFDYW	IGH	2	None	
9	1742	CAKDVTDYGDYEGPFDYW	IGH	2	None	
9	1743	CARDHLYRYSSGWYEDWFDPW	IGH	2	None	
9	1744	CARDREDSNYAYYYYYMDVW	IGH	6	None	
9	1745	CARALYDSSGFGYW	IGH	9	None	
9	1746	CARTPLIAVAGRGVYYYMDVW	IGH	2	None	
9	1747	CVRDSFDGTDYHLRENYYFDYW	IGH	4	None	
9	1748	CARIVSDIGPDYW	IGH	10	None	
9	1749	CAKDHTGQYFDNW	IGH	2	None	
9	1750	CAKMSTTTTTTEDDAFEIW	IGH	4	None	
9	1751	CARLYPNLAVAADYW	IGH	5	None	
9	1752	CAKDVTIFGVDPIAFDVW	IGH	2	None	
9	1754	CARDPCASGVCYKLDYW	IGH	3	None	
9	1755	CAKDDDDWGLKGYFDFW	IGH	8	None	
9	1756	CARGTRDIVATTIDYW	IGH	2	None	
9	1757	CTSGLPDPFDIW	IGH	3	None	
9	1758	CVKDIGGLTYSSASSNHFDYW	IGH	8	None	
9	1759	CARQGANTIFGVVIRFDPW	IGH	4	None	
9	1760	CARATTFSYGTLFDYW	IGH	3	None	
9	1762	CARHLPSYSSSSGFNWFDPW	IGH	2	None	
9	1763	CAKDGYWHSLFRAVEHYYYMDVW	IGH	5	None	
9	1764	CAKDDGITIFGVVYPEAIDYW	IGH	2	None	
9	1766	CARGPGYCSSTSCHRGWFDPW	IGH	2	None	
9	1767	CARGGPITGTSGMDYW	IGH	2	None	
9	1768	CARVEGDHYDTTGRYSRRDIFDIW	IGH	3	None	
9	1769	CAKGGARIVLTTVDYW	IGH	4	None	
9	1770	CAKVHFGADFWSGRGGFDSW	IGH	2	None	
9	1772	CARGLYWGFLEWYDDYW	IGH	4	None	
9	1773	CARDQGDFWSGEERDYW	IGH	2	None	
9	1774	CAHRLPFSVDWNVGWFDPW	IGH	7	None	
9	1776	CAREWGQSYDFWSGYSDYW	IGH	8	None	
9	1777	CAKGTGTDYDSGWFDSW	IGH	2	None	
9	1778	CARVGNYYDSSGYYSGLTLYYFDYW	IGH	6	None	
9	1779	CTTDVESRNRGSGNYFDYW	IGH	2	None	
9	1780	CASSSPYNSSALGRAFDIW	IGH	5	None	
9	1781	CAKERGGLSQYDSSGPDYW	IGH	2	None	
9	1782	CLQSYTPPCSF	IGK	13	None	
9	1783	CAHRLYYDFWSGYYNGGVFDYW	IGH	2	None	
9	1784	CARRRGPGDGFDIW	IGH	4	None	
9	1785	CATGDVDTAMAFDYW	IGH	4	None	
9	1786	CARESIITRGSDAFDIW	IGH	2	None	
9	1787	CARAVRMGDDYFDHW	IGH	6	None	
9	1788	CATEINNHANSWFDPW	IGH	4	None	
9	1789	CAKVSDYDFWSGYSYYYYMDVW	IGH	4	None	
9	1790	CAKSTCGGDCGAFDIW	IGH	2	None	
9	1791	CQQLYSEPPVCSF	IGK	5	C1	7BZ5
9	1792	CAKDSRLFLLGADYYFDYW	IGH	6	None	
9	1793	CASLGYYYDSSGYNQFDYW	IGH	2	None	
9	1794	CAKGYSGYDFSRVLVPNDAFDIW	IGH	3	None	
9	1795	CARGRGGQQLVHPYYFDYW	IGH	2	None	
9	1796	CARGKDYDFWSGYYPIYW	IGH	8	None	
9	1799	CSRGISLSQYGLFDYW	IGH	2	None	

9	1800	CGKDYSDGYSSYLTYYFDYW	IGH	2	None	
9	1801	CARVTHKLWFGENWYFDLW	IGH	2	None	
9	1802	CAREGWVGPTFFDYW	IGH	4	None	
9	1803	CARGERRITIFGVVIKGWFDPW	IGH	2	None	
9	1804	CAHTSGQLYAIDYW	IGH	3	None	
9	1805	CVREAYGSGSGLDYW	IGH	2	None	
9	1806	CARGAVLRFLEWLPADYW	IGH	6	None	
9	1807	CARGGDYDFWSGSFDYW	IGH	3	None	
9	1808	CARQIGLYTAYFDPW	IGH	5	None	
9	1809	CARPYRDPARDGPDAFDIW	IGH	2	None	
9	1810	CANLDTSMTYSYYMDVW	IGH	3	None	
9	1811	CVADVPDFEPQADYW	IGH	2	None	
9	1812	CAKEEGYCNGGSCTGIDYW	IGH	8	None	
9	1813	CAREQHHYDSSGFPVDYFDYW	IGH	4	None	
9	1815	CAKDGYCVTHNCPSPGFDSW	IGH	2	None	
9	1816	CAKDLYPTIFGVVHASFHYW	IGH	2	None	
9	1818	CARGKGYCSGGSCLNWIDPW	IGH	3	None	
9	1819	CARGYHSSSWYGVDYW	IGH	2	None	
9	1820	CAKDPGFSGAVAYFEYW	IGH	5	None	
9	1821	CARGSDTAMAAVADYW	IGH	2	None	
9	1822	CAKDILEWLQGYMDVW	IGH	2	None	
9	1824	CAKGYGYYEHDAFDMW	IGH	3	None	
9	1825	CAKRSPTIFGVVPPPGDSFDIW	IGH	2	None	
9	1826	CARHSSYDFWTFDYW	IGH	3	None	
9	1828	CARVGMVRGVISYYFDYW	IGH	2	None	
9	1829	CATEPYCNNTSCSAAKHLIDYW	IGH	2	None	
9	1830	CARDPIHCSSTSCFLFDYW	IGH	2	None	
9	1831	CARHFGPSDSSGWYYYSYW	IGH	2	None	
9	1832	CVKHRGFSTTMLDDSFDLW	IGH	4	None	
9	1833	CTAELGPDNYNNVDYW	IGH	4	None	
9	1835	CARDRLTLRHFDQLPDEPFDLW	IGH	2	None	
9	1836	CARVEEYGDPEYLQHW	IGH	2	None	
9	1837	CARDGRVLVWFGELDYW	IGH	2	None	
9	1839	CATKPITIFGVVIRARWFDYW	IGH	4	None	
9	1840	CAKCYEQYSYGYLGVDYW	IGH	2	None	
9	1842	CVRDQTLVPWGFDAFDVW	IGH	2	None	
9	1843	CASSKWQYQLDYW	IGH	9	None	
9	1844	CARDRDLGIDYYYYMDVW	IGH	4	None	
9	1845	CARDRCSSTSCYNGPRGWFDPW	IGH	2	None	
9	1847	CARLSPSGWYDGGLGYFDYW	IGH	2	None	
9	1848	CARSSDILYYYYMDVW	IGH	5	None	
9	1849	CARDYPGDCSSTNCPGTFDIW	IGH	13	None	
9	1850	CAKGNMVYATPWYFDLW	IGH	2	None	
9	1852	CARLWDVVLTFRDPGWFDPW	IGH	2	None	
9	1853	CARSIAARPNGDIFNIW	IGH	6	None	
9	1854	CAREDVLRYFVYAFDIW	IGH	2	None	
9	1855	CARHSRSARALDIW	IGH	3	None	
9	1856	CARIGVWGSYRYNDWFDPW	IGH	3	None	
9	1857	CAKDLRYCSSPDCYTEDGFDIW	IGH	4	None	
9	1858	CQQYDNALSLSF	IGK	220	C3	6XDG
9	1859	CQQSYSTLPMYTF	IGK	212	C2	7K8O
9	1860	CSKTYMIGSTYNYFDPW	IGH	2	None	
9	1861	CARRTLLYSSGWYYFDHW	IGH	2	None	
9	1862	CAKVLPGSSGWIFDYW	IGH	2	None	
9	1863	CVKEGDSFGYADNW	IGH	4	None	
9	1864	CQSADSGGSWMF	IGL	3	None	
9	1865	CAKDPHSSGYYYDQRKSRDAFDIW	IGH	4	None	
9	1866	CARRLSPHHDTFDIW	IGH	2	None	
9	1867	CAKNIAGSGWYYGVCFDIW	IGH	3	None	
9	1868	CANAVMGAYLFDNW	IGH	4	None	
9	1869	CTRGGFTIFEENWFDPW	IGH	2	None	
9	1870	CARDRSPYDYPSWFDLW	IGH	2	None	
9	1872	CARRKVVPAAPFSTGFDPW	IGH	2	None	
9	1873	CARDFQGIDDAFDVW	IGH	2	None	
9	1874	CAKKPRLSGAFDIW	IGH	2	None	
9	1875	CAYTPGNGYTHFSYW	IGH	2	None	
9	1876	CVRDPEEYCSSTSYRKYYFDYW	IGH	2	None	
9	1878	CARDYYDSSGYSRPFDYW	IGH	14	None	

9	1880	CARLLGPGGDPIDYW	IGH	6	None	
9	1881	CARSHSASGNLEFDYW	IGH	4	None	
9	1882	CARDGGTSGHDWYFDLW	IGH	4	None	
9	1883	CARGGSCSVTTCYPRSTFDIW	IGH	2	None	
9	1884	CARERTGYYAEYW	IGH	2	None	
9	1885	CARIGNSGGGYYDLDYW	IGH	5	None	
9	1886	CARPIFGADYCGRSCHIFDSW	IGH	3	None	
9	1887	CARVGGSSEWYNFDYW	IGH	5	None	
9	1888	CARTIIVVEVLDAFDIW	IGH	2	None	
9	1889	CARQRRIAQEGSPKYYFDYW	IGH	4	None	
9	1890	CAGERPHSLYFQLW	IGH	6	None	
9	1891	CTTATGSGSRRWYFDLW	IGH	2	None	
9	1894	CARDLMTTVTTGDYW	IGH	4	None	
9	1895	CARCPADCSGGTCYFFYYFDYW	IGH	2	None	
9	1896	CAKRWGDADYSDYW	IGH	2	None	
9	1897	CARDCPIPGFWSGYSMYYQYMDVW	IGH	2	None	
9	1898	CARAPWGGYSGNVPRVRSFDDW	IGH	2	None	
9	1899	CARVDNWNYVDYW	IGH	11	None	
9	1900	CARDRGGYSGYDHVAPLIDYW	IGH	4	None	
9	1901	CCSYAGSSTVRWVF	IGL	4	C2	7JMP
9	1903	CAREPDYSAGGFDSW	IGH	2	None	
9	1904	CAKDVDIAMVRLWGALDYW	IGH	2	None	
9	1905	CARTNVDTSMTTRWFDPW	IGH	5	None	
9	1906	CARTHVTGTGLDYW	IGH	2	None	
9	1907	CARVPRKRYCTNGVCPWYFDLW	IGH	4	None	
9	1910	CARPVRGVEVKSDWYFDLW	IGH	2	None	
9	1912	CQSYDSGLTVSWVF	IGL	4	C4	7JVC
9	1913	CARPVYGSGSYPLDLGAFDIW	IGH	2	None	
9	1914	CARDEAGKYQLLWAWVGIDYW	IGH	2	None	
9	1915	CASIYYDSSDYSGFDYW	IGH	3	None	
9	1916	CAKGGCSSTSCSFDYW	IGH	23	None	
9	1917	CASRVSSIYGVLINDVFDLW	IGH	4	None	
9	1918	CARIVRYCTNGVCYHYFDYW	IGH	2	None	
9	1919	CSSTSCQGAFDIW	IGH	6	None	
9	1921	CATQHVGRPFYFDYW	IGH	2	None	
9	1922	CAKLLKLEWLSTPFDYW	IGH	2	None	
9	1923	CARSYGFYYAFDIW	IGH	18	None	
9	1924	CARDIVVVAAARGFEYW	IGH	3	None	
9	1925	CVRDSFMADLDYW	IGH	3	None	
9	1926	CARSHCSTTSCYIGSIAARLRYHYMDVW	IGH	2	None	
9	1927	CARFAVVPSPYFDFW	IGH	3	None	
9	1929	CARIPLYNYDSSGHYGAFDIW	IGH	10	None	
9	1930	CARDIGLSNTRRFDPW	IGH	4	None	
9	1931	CARGGRCSSTSCYFPNDWFDPW	IGH	6	None	
9	1932	CARHGRYYGSSRLYYYMDVW	IGH	2	None	
9	1933	CARHMQMGTIHDAFDIW	IGH	2	None	
9	1934	CARHRTGGAVYYDFWSGPYYFDYW	IGH	4	None	
9	1935	CARHRDYGDPGSFDYW	IGH	14	None	
9	1936	CAHRRPTRITKIGLHGDYFEYW	IGH	3	None	
9	1937	CARLGYCSSTSCQIASDLDYW	IGH	2	None	
9	1938	CARAYRDSSSWYLYFQHW	IGH	2	None	
9	1939	CTRDPGYDRHDWYFDLW	IGH	2	None	
9	1940	CAREVDYDDYVDDAFDIW	IGH	2	None	
9	1941	CARHPGGGRGKYAFDIW	IGH	2	None	
9	1942	CARDSRYDVYYSYYMDVW	IGH	3	None	
9	1943	CARTRASLSGISGNRDAFDIW	IGH	2	None	
9	1944	CTRDPSDYGDYGFDYW	IGH	2	None	
9	1945	CACYYESSGYRLDYW	IGH	2	None	
9	1946	CAREGYHASGSSGSFDYW	IGH	2	None	
9	1947	CARMKSDFAFDYW	IGH	3	None	
9	1948	CARSAAAGKLSTWFDPW	IGH	2	None	
9	1949	CARRDWYGDYEVDYW	IGH	2	None	
9	1950	CARSNPVYSFDPSSYLGFDYW	IGH	2	None	
9	1951	CAKHRVIYDSGPGPLDYW	IGH	2	None	
9	1952	CASGRRGNPYSFDYW	IGH	2	None	
9	1953	CARPLTTARGWYYFDYW	IGH	2	None	
9	1954	CARNKGYDFWSGGIYYYYYYMDVW	IGH	3	None	
9	1955	CARGAYCSGGTCYRPFDYW	IGH	2	None	

9	1956	CAKDRGPTWYFFDSW	IGH	3	None	
9	1957	CQHYYTTPIAF	IGK	18	C4	6W41
9	1958	CARHQYLGMAPVGSGGEYFQHW	IGH	50	None	
9	1959	CAREFPRRSSSWYSPDAFDIW	IGH	2	None	
9	1960	CARGPDYCDSNGYLFDYW	IGH	2	None	
9	1961	CAKILRLGEVWFTYFDYW	IGH	11	None	
9	1963	CSTSFRSGTFDNW	IGH	2	None	
9	1964	CARSAYSSSSAPFDYW	IGH	6	None	
9	1965	CLSADNSGTYLMF	IGL	6	None	
9	1966	CARDEVIAAAAPSLVDYW	IGH	2	None	
9	1967	CAKSITFGGVIVPTDGFDVW	IGH	2	None	
9	1968	CARDLGDYDFWSGYSDDAFDIW	IGH	4	None	
9	1969	CVRDLELLKEWLANDGFDIW	IGH	2	None	
9	1970	CARHQRGYSSRPFDFW	IGH	3	None	
9	1971	CAREPEVRGDEGHFDYW	IGH	2	None	
9	1972	CASAYFDSNFYYYNWYFDLW	IGH	4	None	
9	1973	CATEGDIVVVPAVTYYMDVW	IGH	4	None	
9	1974	CARVAYCSGGSCWNFDSW	IGH	6	None	
9	1975	CVRDKWLLGDAVTDDAFDIW	IGH	4	None	
9	1976	CVKKDSTVIKGGFDHW	IGH	5	None	
9	1977	CYSTDTSGRHRGVF	IGL	7	None	
9	1978	CAKALRQDYTYDDYMDVW	IGH	86	None	
9	1979	CARKPYYYYYMDVW	IGH	46	None	
9	1980	CARSPNFGGWQVSLYFDYW	IGH	2	None	
9	1981	CAKEITRRSSNGWPLDYW	IGH	4	None	
9	1982	CARDPKWLRLDYW	IGH	9	None	
9	1983	CARDIDYYGSGRSRFDPW	IGH	2	None	
9	1984	CARPNNPTVIIGEYYFNYW	IGH	2	None	
9	1985	CAKDGRSSGWPYYFDYW	IGH	3	None	
9	1986	CARRPDSSGYQNAFDIW	IGH	2	None	
9	1987	CARGMAGIPHDYYIDVW	IGH	2	None	
9	1988	CARVRGWQTYRYAYFDSW	IGH	2	None	
9	1989	CQVWDSKSAHGGVF	IGL	4	None	
9	1990	CARDTASRERSDYW	IGH	3	None	
9	1992	CAKAYGGYHFDYW	IGH	14	None	
9	1993	CASLLRSRYVDYW	IGH	2	None	
9	1994	CARVRVYCSSTSCEGSEFDPW	IGH	2	None	
9	1995	CAKDISPIAAAGNNRFDPW	IGH	20	None	
9	1996	CATAGYYDSSFDDW	IGH	5	None	
9	1997	CMQTTQFPHITF	IGK	11	None	
9	1998	CATETSAWYEIFDHW	IGH	2	None	
9	1999	CQQYNSWPLGFTF	IGK	5	C3	7K8R
9	2000	CARVVGAVAGTLVEAFDIW	IGH	4	None	
9	2001	CASVLPTIDYW	IGH	8	None	
9	2002	CAKGAGPYCDSATCYTFDYW	IGH	2	None	
9	2003	CARGEYSSSMAAFDVW	IGH	2	None	
9	2004	CEIDCYDNRGPQDAFDVW	IGH	2	None	
9	2005	CARDKAYFDSSGYYDAFDIW	IGH	6	None	
9	2006	CQSYDTSLSASHVVF	IGL	9	C3	6WPS
9	2007	CARDCSSTSCPWTFDYW	IGH	6	None	
9	2008	CAKDRTRAQTTRTLDYW	IGH	2	None	
9	2010	CMQSQKLPLYTF	IGK	2	None	
9	2011	CQVWDGSSGHRLF	IGL	14	None	
9	2012	CARGSGLCYGATCYQYFDHW	IGH	2	None	
9	2014	CAKDNWGAADIW	IGH	2	None	
9	2015	CATSPPPTVTWSRYFDSW	IGH	2	None	
9	2016	CAKDKGTPFFGVAIDYW	IGH	2	None	
9	2017	CTTHSVLYSNYPTPFDYW	IGH	2	None	
9	2018	CASLVVVFRTDVDYW	IGH	5	None	
9	2019	CARLVTNGWYFDYW	IGH	2	None	
9	2020	CISSTERYRTNFDIW	IGH	2	None	
9	2022	CASSIFDVEAAFDFW	IGH	4	None	
9	2023	CARPRGVERTVTPDAFNIW	IGH	2	None	
9	2024	CARDFSGTVTRPGYCDYW	IGH	2	None	
9	2025	CVKGGGSIDGRTPYIWFFDFW	IGH	2	None	
9	2026	CARHHGGYDYVWGSYRPYYFDYW	IGH	3	None	
9	2027	CAIVVVPATTGHDAFDIW	IGH	2	None	
9	2028	CVRVSRDFQTNNNWFDLW	IGH	7	None	

9	2029	CVKFRSGSRWFDYLDYW	IGH	2	None	
9	2030	CQSHDSSPGAYNYVF	IGL	3	C3	6WPS
9	2031	CARLYNNSWYYFDYW	IGH	13	None	
9	2032	CARDEGGISGTTGGIDYW	IGH	4	None	
9	2033	CVRDYHFGPTWAFDMW	IGH	4	None	
9	2034	CARVYYYESHGRIDYW	IGH	2	None	
9	2035	CARAGQFGDFQSYIDSW	IGH	2	None	
9	2036	CAKIQRLSDFWSGIDYW	IGH	4	None	
9	2037	CARDDSRGVVVPAAIIDYW	IGH	2	None	
9	2038	CAKDGSEWELVTEPFDYW	IGH	8	None	
9	2040	CARLTPYYYDSSGYYYAGNYFDYW	IGH	2	None	
9	2041	CARRGYDSSGYDAFDIW	IGH	3	None	
9	2043	CAREAVTTVSLDYW	IGH	4	None	
9	2044	CAKGGAGSSSWYPYYFDYW	IGH	2	None	
9	2046	CVRDEGRQSQNNFDIW	IGH	2	None	
9	2047	CARRSTSSMTPQDNWFDPW	IGH	4	None	
9	2048	CARDSSIDELVFRTGDYW	IGH	2	None	
9	2049	CTREVAIGGSYFSDYW	IGH	4	None	
9	2050	CAKDQYDTSGSFDYW	IGH	23	None	
9	2051	CTREYFGTVDGAFDVW	IGH	2	None	
9	2052	CAQDATRPYSSSWYYFDYW	IGH	5	None	
9	2053	CAKDYDYVWGSYSVAFDIW	IGH	2	None	
9	2054	CARRQYQLQPDDYW	IGH	2	None	
9	2055	CAKPYYGGNSYDAFDIW	IGH	5	None	
9	2056	CARSRPAARGAFDIW	IGH	5	None	
9	2057	CAREGHDSFGYFRFDYW	IGH	2	None	
9	2058	CAKEPSELAIADYW	IGH	2	None	
9	2059	CARDQATADCSGLSCQYNWFVPW	IGH	2	None	
9	2060	CDWSSDVCSSDLADTAVYYCALKRIKLIRGLITGNYFDYW	IGH	2	None	
9	2061	CAREGCGADCFHDAFEVW	IGH	2	None	
9	2062	CARLIKETLTWSGLYFDYW	IGH	2	None	
9	2063	CTKVASRLNMVRGAPFDYW	IGH	3	None	
9	2064	CAKDNGIVVLPAGFDNW	IGH	2	None	
9	2065	CARVPVPAAKPTTLVGYYYLDVW	IGH	4	None	
9	2066	CARDGILVVPAAIDYW	IGH	2	None	
9	2067	CARHVNRLPVATNNWFDPW	IGH	2	None	
9	2068	CAREHLPYYYFYAMDVW	IGH	2	None	
9	2069	CARKFGSGTYDNGAWIDPW	IGH	3	None	
9	2070	CARLAIAVAGETDDYW	IGH	2	None	
9	2071	CARDVIDCSSMSCLSYNYYMDVW	IGH	2	None	
9	2072	CARSPNYDFWSGYSTYNFDHW	IGH	3	None	
9	2073	CARDRFLQTGQGMKVAAWFDPW	IGH	5	None	
9	2074	CARHVPFGSGRRGDYYYYMDVW	IGH	2	None	
9	2075	CARSPNDWALDYW	IGH	4	None	
9	2076	CAKGSLDFWSGYRNHYFDHW	IGH	2	None	
9	2077	CARYAYCSGTGCPMRQLGFDPW	IGH	5	None	
9	2078	CARLRVTTTRDFDYW	IGH	2	None	
9	2079	CQEYNTYSLEVLLTF	IGK	8	C3	7K8P
9	2080	CAKGEKISGDFDYW	IGH	7	None	
9	2081	CAGPYYYFDIW	IGH	2	None	
9	2082	CARGVPLGYDGTSYYFFSW	IGH	2	None	
9	2083	CASLGIAASGEQTDYW	IGH	9	None	
9	2084	CAKDYHLPVDGKSGYFDNW	IGH	3	None	
9	2085	CARGGSSSSPFYDYW	IGH	12	None	
9	2086	CAKDLSGLTIYGGYFDYW	IGH	8	None	
9	2087	CAGERAWSASPIDYW	IGH	2	None	
9	2089	CARHSLVGLIWNSYFDPW	IGH	2	None	
9	2090	CARFLAVSTSWPPFDHW	IGH	2	None	
9	2091	CARHGGSGWYDDTFHIW	IGH	4	None	
9	2092	CARGTWDSSGYYGEEPAFDSW	IGH	2	None	
9	2093	CARVGNWNPGGIYYFDYW	IGH	4	None	
9	2094	CARSDPGYSYGYGAFDIW	IGH	2	None	
9	2095	CASGVVPAAKHWFDPW	IGH	2	None	
9	2096	CARPGRDSHYYYYMDVW	IGH	2	None	
9	2097	CAREFRSSGDKIYGAFDIW	IGH	2	None	
9	2098	CARDHEDSTEDFDYW	IGH	2	None	
9	2099	CAREECDIAVAPAACFNDYW	IGH	2	None	
9	2100	CTKGHSYKRDDVSAFDIW	IGH	4	None	

9	2101	CQSYDSVHHWLF	IGL	3	C4	7JVC
9	2102	CARDLEGFSYDYYHPFDVW	IGH	3	None	
9	2103	CARVPAVAGSHLSFFDSW	IGH	2	None	
9	2104	CTRSWIREYYYYMDVW	IGH	2	None	
9	2106	CARDSSCSNIECYGVDYYHYMDVW	IGH	3	None	
9	2107	CAKGSRQWLVQTFDYW	IGH	12	None	
9	2108	CARGEIFEVIISLDYW	IGH	3	None	
9	2110	CARGRGTEWATNTFASW	IGH	4	None	
9	2111	CVRDQGIELWLYVFDVW	IGH	2	None	
9	2112	CANPVYDILTGSDRYAFDIW	IGH	2	None	
9	2114	CARDEHLAAAVPNRNAFDIW	IGH	10	None	
9	2115	CAHKRGDNNYEWFDPW	IGH	2	None	
9	2116	CAGGIYSNLNWFDPW	IGH	5	None	
9	2117	CARSRVPASTYYFDYW	IGH	2	None	
9	2118	CATLPRANFGAPGGYFDNW	IGH	2	None	
9	2119	CAKDIRIAVAGTGGAFDIW	IGH	2	None	
9	2120	CAKDAYNKPYYRYYYYMDVW	IGH	2	None	
9	2121	CAKGGWRMRDKFDPW	IGH	5	None	
9	2122	CARGEMANDAQEKYTSYYYMDIW	IGH	2	None	
9	2123	CQQSFTTPLTSF	IGK	3	C2	7K8O
9	2124	CAKEAEQWLAPKDYW	IGH	3	None	
9	2125	CAREIPHDYYPYYFDLW	IGH	12	None	
9	2126	CARGHSSGRYNWFDPW	IGH	10	None	
9	2127	CARVGICSGGDCYYGAFDIW	IGH	2	None	
9	2128	CAKEGAGQLYLYYFDYW	IGH	2	None	
9	2129	CQTWSTGPDWVF	IGL	106	None	
9	2130	CAKEFILTSRYREEPFDYW	IGH	2	None	
9	2131	CARDWGGDVQSDAFDIW	IGH	2	None	
9	2132	CARWGISAAHDALDVW	IGH	3	None	
9	2133	CVLASLVTFDYW	IGH	2	None	
9	2134	CARANSSSADAFDIW	IGH	2	None	
9	2135	CARDPDYHDNRFDPW	IGH	11	None	
9	2136	CQQYGSLWPF	IGK	38	C1	7K8N
9	2137	CARGKINDGPSPDIFDIW	IGH	2	None	
9	2138	CGVFPDYW	IGH	2	None	
9	2139	CQSADTRGNVIF	IGL	3	None	
9	2140	CAAWDNSLSSPNWEF	IGL	2	None	
9	2141	CARDYSSTNGLDYW	IGH	3	None	
9	2142	CAKDNGKRAPYQEQMVYAPPDYW	IGH	2	None	
9	2143	CARDVCTRSSCYSFDYW	IGH	2	None	
9	2144	CMHGIHWPPKRTF	IGK	64	None	
9	2145	CTAEVSTMVRDNWFDPW	IGH	3	None	
9	2146	CQQFNSFPALGF	IGK	3	C3	7K8R
9	2147	CAKPYRMEVLNYYFDFW	IGH	2	None	
9	2148	CARDSFGYNILTGPDYW	IGH	3	None	
9	2149	CSRQETYDVLPDGAFDIW	IGH	5	None	
9	2150	CARELGILVGTATLDYW	IGH	2	None	
9	2151	CARGRVSYYYDSSGYYWGGFDIW	IGH	4	None	
9	2152	CAHRRGAAVIDYW	IGH	5	None	
9	2154	CARAAKLCADGVCSNYYFHYYMDVW	IGH	2	None	
9	2155	CAKSGPYGLSDAFDIW	IGH	7	None	
9	2156	CARGGYCSTIDCHPGTRDAFNVW	IGH	2	None	
9	2157	CARDLVPAAIHFDYW	IGH	14	None	
9	2158	CARHYYDSSSYLIGYACDVW	IGH	2	None	
9	2161	CARGMYSSGWYPDYFDYW	IGH	9	None	
9	2162	CARDWPIGLFDHW	IGH	4	None	
9	2163	CAKDDDYDPSEAFDIW	IGH	2	None	
9	2164	CARGFQTISAFDLW	IGH	2	None	
9	2165	CAKDSADMNTAIDLDYW	IGH	2	None	
9	2166	CARDEPRSHGALDVW	IGH	2	None	
9	2167	CVKLPHLDFWSGYYAYFDCW	IGH	4	None	
9	2168	CAREARQSGLGAFDIW	IGH	2	None	
9	2170	CARRRAVAERLYYFDYW	IGH	4	None	
9	2172	CATTYCSSTSCYKPNAFDIW	IGH	2	None	
9	2173	CARRSSYGHTVFDYW	IGH	2	None	
9	2174	CARVVDYDFWSGYAYYFDYW	IGH	2	None	
9	2175	CVKDPSCSSTSCYSRAFDIW	IGH	3	None	
9	2176	CAKGLGMGCFDYW	IGH	19	None	

9	2177	CATGARRGYDFYYLDVW	IGH	6	None	
9	2180	CAKEPYRSSWYYFDYW	IGH	2	None	
9	2181	CARDPWAYWYFDLW	IGH	8	None	
9	2182	CARDGDLVQLWALDYW	IGH	2	None	
9	2183	CAKGRDLVVPTANDYW	IGH	2	None	
9	2184	CARRRDNWDYFDYW	IGH	7	None	
9	2185	CARESQLNIAEAGQLDYW	IGH	4	None	
9	2186	CARGGVVPAAIWAFDYW	IGH	2	None	
9	2187	CAHLRFLEYFDYW	IGH	4	None	
9	2189	CARGHSSSWRTWFDPW	IGH	13	None	
9	2190	CARDRGSSSGYYYYMDVW	IGH	8	None	
9	2191	CAHAVGRYSSGWYYAFDIW	IGH	2	None	
9	2192	CARDIGDCNTNHCPHFDFW	IGH	4	None	
9	2193	CARDRLEGPYCEGNCGDFDYW	IGH	2	None	
9	2194	CAKDPNSGWYGGYYFDYW	IGH	4	None	
9	2195	CARDRAFPDDVLDIW	IGH	6	None	
9	2196	CQETYSTPRCGF	IGK	4	C2	7K8O
9	2197	CARRIVTAGIWCFDLW	IGH	2	None	
9	2198	CARVEVCRSTSCTKVFDYW	IGH	2	None	
9	2199	CARHVDSSGWRRPGWYFDLW	IGH	2	None	
9	2200	CARGRTPGVFDAFDFW	IGH	2	None	
9	2201	CARGGLELQVLSAFDIW	IGH	2	None	
9	2203	CQAWDISTASLIF	IGL	9	None	
9	2204	CAGDRGDYPTRAFDVW	IGH	2	None	
9	2205	CAKGSDFWSGYYLFEYW	IGH	2	None	
9	2206	CARGADYGDYEVEWWYFQHW	IGH	2	None	
9	2207	CARAYYVSGGYYLYFEYW	IGH	2	None	
9	2209	CARDVAGGSYYPPFDYW	IGH	3	None	
9	2210	CARGVIPAAFYFDSW	IGH	2	None	
9	2211	CARFYCSSTSCHFDYW	IGH	15	None	
9	2212	CARVIRPSGSGIDYW	IGH	5	None	
9	2213	CARDSCSSSSCSPCFDYW	IGH	4	None	
9	2214	CARAHSLGTFYNPLLYFFDYW	IGH	2	None	
9	2216	CARRVSRGSSWFDPW	IGH	5	None	
9	2217	CLQHDDFPQYTF	IGK	10	None	
9	2218	CARVAATRVWYFDLW	IGH	2	None	
9	2219	CARGVALMVHGRVDYFYMDVW	IGH	5	None	
9	2220	CARSVPLASLFDSW	IGH	6	None	
9	2221	CAKDSSNLRYYDTPGSDDYW	IGH	2	None	
9	2222	CVKDYCINTCYMDYW	IGH	3	None	
9	2223	CVRDEWGRDFSHYCFDYW	IGH	3	None	
9	2224	CTTDRGYDFWSGHELGYFDYW	IGH	2	None	
9	2225	CVGLGRGLCNGECYDSFDPW	IGH	2	None	
9	2226	CARDSARFGDSPVNYFDPW	IGH	6	None	
9	2227	CARYCSSTSCYLVPSDAFDIW	IGH	2	None	
9	2228	CARPEGGYSTSWVDYW	IGH	2	None	
9	2230	CARYTDYVDSW	IGH	7	None	
9	2231	CAKDISNSGWSGVDYW	IGH	2	None	
9	2232	CAKVQDSTTYYYSHFDYW	IGH	4	None	
9	2233	CARDSGTTSLGAFDIW	IGH	10	None	
9	2234	CARHLLFGSLPDWFDPW	IGH	2	None	
9	2235	CARGHDIVVVYHYMDVW	IGH	2	None	
9	2238	CAKSPFYGDYVADYYFDYW	IGH	5	None	
9	2239	CARTDYPKGFDIW	IGH	3	None	
9	2240	CARRPYCNSISCYMDFDYW	IGH	2	None	
9	2241	CARISFGSGYHYDAFDIW	IGH	2	None	
9	2242	CTRGGGNLYHFWFDPW	IGH	3	None	
9	2244	CVKDSSGWQTDGYFDYW	IGH	4	None	
9	2245	CAKEGGRTLLDAFDLW	IGH	2	None	
9	2246	CAKGSGIAAAGNTFGYW	IGH	2	None	
9	2247	CTTGLHYYDSSAYTNDAFDIW	IGH	6	None	
9	2248	CMELKSLTSEDSAVYYYIVLNYGSSYTYFDVW	IGH	2	None	
9	2249	CARVDYSNLRDAFDIW	IGH	2	None	
9	2250	CAKEGGGHSGYDVFDYW	IGH	6	None	
9	2251	CAKDRNTAAAVYYFDYW	IGH	5	None	
9	2252	CAKDIKSGDYYDTYYYGMDVW	IGH	2	None	
9	2254	CARHVAASGQIDYW	IGH	5	None	
9	2255	CARPKRRDVRDAFDIW	IGH	2	None	

9	2256	CAKAAIYLTDSYLYMDVW	IGH	10	None	
9	2257	CAKDWDSGSYYNYW	IGH	2	None	
9	2258	CARLPMKYSSSSRIRNWFDPW	IGH	2	None	
9	2259	CATECNTGTRDCFPTTDGFDMW	IGH	2	None	
9	2260	CAHRLLSVQLERGGWFDPW	IGH	2	None	
9	2261	CTREGDYDLRRFDYW	IGH	3	None	
9	2262	CVTQGKYCSAGSCNSLDYW	IGH	3	None	
9	2263	CAKDTHHGTGTTEPFDFW	IGH	2	None	
9	2264	CARRHIEIVGGGLDYW	IGH	4	None	
9	2265	CARGRMALAGDAFDIW	IGH	2	None	
9	2266	CAALRDFWSGKRFDPW	IGH	10	None	
9	2267	CAKDYCSSTSCYRDAFDIW	IGH	7	None	
9	2268	CAREAYGDYVRFDSW	IGH	4	None	
9	2269	CASSGSRERVVDYFDYW	IGH	2	None	
9	2270	CAKGGLALSFDYW	IGH	19	None	
9	2271	CTTEGAAAGWFDPW	IGH	2	None	
9	2272	CARETVEMATTGYFDYW	IGH	2	None	
9	2274	CAKPYSGYDEYFDYW	IGH	8	None	
9	2275	CTTLTQVTADPFDIW	IGH	3	None	
9	2276	CATFSPTQGDAFDVW	IGH	4	None	
9	2277	CGRNPNDYRVYHYYYMDVW	IGH	6	None	
9	2279	CARDISLKAAAGTFDYW	IGH	4	None	
9	2280	CAKSVTLKSDFSTLGYFDYW	IGH	2	None	
9	2281	CQQYLSWPPPLTF	IGK	31	C3	7K8R
9	2282	CARNPACSSTTNCYVFADYW	IGH	4	None	
9	2283	CARGRWNDDGLDYW	IGH	4	None	
9	2285	CARHYGDSSPFDYW	IGH	12	None	
9	2287	CAHNALGELYSLLDYW	IGH	2	None	
9	2288	CARHYVIVLEEDYYDSSGYYFYVANWFDPW	IGH	2	None	
9	2289	CARRVIQSGSFFDPW	IGH	2	None	
9	2290	CARNGFDQLLSSSVGALDIW	IGH	2	None	
9	2291	CAKAPVSDGGYFDRPYYYMDVW	IGH	2	None	
9	2292	CAREDTLFGVITLDAFDIW	IGH	4	None	
9	2293	CARDTWAAANKVLDYW	IGH	9	None	
9	2294	CARIGATEDYYYMDVW	IGH	2	None	
9	2295	CGTWHSNSKTHWVF	IGL	8	None	
9	2296	CARATRGYSYGYNPSFDYW	IGH	2	None	
9	2297	CAKDRPAYNVVVVAVTVLGYFDYW	IGH	4	None	
9	2298	CAKEGGDGQRPFDYW	IGH	2	None	
9	2299	CTRDSGYNAFDIW	IGH	7	None	
9	2300	CARDNPEIAVAAFDYW	IGH	2	None	
9	2301	CTRNEHYTDSSGYLFDIW	IGH	3	None	
9	2302	CARSDSSAWYSPDAFDMW	IGH	3	None	
9	2303	CARDPEWGAVDYW	IGH	4	None	
9	2304	CTTASPNYSVMFELDYW	IGH	2	None	
9	2305	CASQLDVYRAFDFW	IGH	2	None	
9	2306	CHQYSNWPFSF	IGK	54	C3	6XDG
9	2307	CARLLGYSNRFDYW	IGH	8	None	
9	2308	CARDLSVGLPFCGGHCVLDYW	IGH	4	None	
9	2309	CERRHIIVVSSETVIIEGGFDHW	IGH	2	None	
9	2310	CARDSADLVAVPDVLVGEDDAYDIW	IGH	2	None	
9	2311	CARDQDSSWYLDYW	IGH	2	None	
9	2312	CVGATTPVDYW	IGH	2	None	
9	2313	CAKGTMPVALGGFDIW	IGH	146	None	
9	2314	CARVGPSGNYYYYYMDVW	IGH	2	None	
9	2315	CAKTRSGDCYAFDYW	IGH	2	None	
9	2316	CARGTPGFSYRSPGPDYYYYYMDVW	IGH	2	None	
9	2317	CARLNYYETSGYYGYFDYW	IGH	2	None	
9	2318	CAKQDFTRGSHYGDYDYW	IGH	4	None	
9	2319	CARVDNTLWYGHYKGLDAFDVW	IGH	2	None	
9	2320	CARQEPNYRAPFDYW	IGH	4	None	
9	2321	CARPIGIRLQAFDYW	IGH	2	None	
9	2322	CARETITIFMYFDHW	IGH	2	None	
9	2323	CARSRLAARRFWWFDPW	IGH	2	None	
9	2324	CARASARSGWYGGIDYW	IGH	4	None	
9	2326	CARATAARPGFEDFDYW	IGH	2	None	
9	2329	CARSVSAREFDYW	IGH	6	None	
9	2330	CSRGPYSSGWYGASDIW	IGH	3	None	

9	2331	CAHQYSAYEQYDYW	IGH	2	None	
9	2332	CTRDSYGRQPPDTFDIW	IGH	71	None	
9	2333	CATAGEVYYMDVW	IGH	3	None	
9	2334	CASRFLTAALNHDAFDIW	IGH	2	None	
9	2336	CARECTSTSCPGVRAFDLW	IGH	2	None	
9	2337	CAKEINTYVPYYFDYW	IGH	7	None	
9	2338	CARTLYGDFGDWIDPW	IGH	7	None	
9	2339	CVNSNTGFEGFDVW	IGH	2	None	
9	2340	CAREGDGYSSGWYRFNAFDIW	IGH	3	None	
9	2341	CAIGLSYGLGGDYW	IGH	2	None	
9	2342	CARPRCPSTSPHLYCYMDVW	IGH	6	None	
9	2343	CARSRGKIDGYFDYW	IGH	2	None	
9	2344	CARVDWNDIYAFDIW	IGH	2	None	
9	2345	CARARTRSQLLSHWYFDLW	IGH	2	None	
9	2346	CARKNKAAASTVYYYYYMDVW	IGH	2	None	
9	2347	CVREANHYGSGVTSFDIW	IGH	2	None	
9	2348	CARVPVYQFKYFDYW	IGH	12	None	
9	2349	CARDSEDIWSAYYDYW	IGH	2	None	
9	2351	CATIFCSSTSCYPFDYW	IGH	6	None	
9	2352	CAMARNDFWSGSNWARWFDPW	IGH	2	None	
9	2353	CARAYCSVTDCYYAFDSW	IGH	2	None	
9	2354	CAMGEDYPGRFDYW	IGH	5	None	
9	2355	CQSYASGLSGPYVF	IGL	5	None	
9	2356	CARAYDSSGYYPANFDYW	IGH	2	None	
9	2357	CAHTVWIWNGYSAFDYW	IGH	2	None	
9	2358	CARDLGRTYSYGYRPRDRNWFDPW	IGH	2	None	
9	2359	CARRFGSSWYPAFDYW	IGH	3	None	
9	2360	CARDPYHFWSGYSDSW	IGH	2	None	
9	2361	CARDLTVAGIGAFDIW	IGH	2	None	
9	2362	CAREYCNSRRCSKTAFDLW	IGH	4	None	
9	2363	CQQSHITSLGSPEWTF	IGK	2	None	
9	2364	CARVGNWGTKCFDCW	IGH	6	None	
9	2365	CSSISCPNGYNWFDSW	IGH	3	None	
9	2366	CARYRDSSHTFDIW	IGH	2	None	
9	2368	CARNCLPRDEVVPAAIDEGDNWFDPW	IGH	2	None	
9	2370	CAHSSRLDDCSGATCYYFDFW	IGH	2	None	
9	2371	CATHSSGWNWYFDYW	IGH	7	None	
9	2372	CARGSHYDFWSLQYFHYW	IGH	2	None	
9	2373	CARGIGDAVDMW	IGH	3	None	
9	2375	CASRHYGDYDVHAFDMW	IGH	2	None	
9	2376	CQYYDNVPHF	IGK	42	C3	6XDG
9	2377	CARQPKYSSGSNWFDPW	IGH	6	None	
9	2378	CARTLPLVYCSSTSCYTAFDIW	IGH	2	None	
9	2379	CTRRGKTGTVDYW	IGH	2	None	
9	2380	CARVQTGYSSGWYSSDDTFDMW	IGH	2	None	
9	2381	CARGWGYTRSYYMDVW	IGH	2	None	
9	2382	CVRRSSLGRYYFDFW	IGH	2	None	
9	2383	CARSYSRSVRSPFDIW	IGH	2	None	
9	2384	CAKEVTTFGVLTFNYNYMDVW	IGH	4	None	
9	2385	CARDSGICDTLNCLSAPDYW	IGH	2	None	
9	2386	CTNGVCPDGFAIW	IGH	2	None	
9	2388	CAKDGSSWYNEGYFDYW	IGH	2	None	
9	2389	CAREGDFWSGYQYDYW	IGH	21	None	
9	2390	CAREPYDYLSYLEYW	IGH	2	None	
9	2392	CTRDPPGVVTNDYW	IGH	2	None	
9	2393	CAADGKNYSYFYYYYMAVW	IGH	2	None	
9	2394	CAREVPPYSSGYYVYQRFDFW	IGH	2	None	
9	2395	CARIYCSSTSCYGFYYYMDVW	IGH	2	None	
9	2396	CARGGTWNDVDAFDVW	IGH	13	None	
9	2397	CQSADDTGRFIIF	IGL	2	None	
9	2399	CAKVLVRGVIITAFDYW	IGH	2	None	
9	2400	CARAPGKYSSGWYNLPDAFDIW	IGH	2	None	
9	2403	CAGVVVVPYRGFLFDYW	IGH	3	None	
9	2404	CARDDYSNYGLGGQGDYW	IGH	4	None	
9	2405	CASGLLFGSGSYDYW	IGH	2	None	
9	2407	CALISVGSYSGYFDNW	IGH	2	None	
9	2408	CARRGTAEGFQHRFDPW	IGH	2	None	
9	2410	CASQSSSITIFGVVTRYFDYW	IGH	2	None	

9	2411	CVKDSRLQFVGYPFDHW	IGH	6	None	
9	2412	CAKLVWSDYW	IGH	7	None	
9	2414	CAKDADELELTGPIDYW	IGH	6	None	
9	2415	CARDASIYYNSGSYFDLW	IGH	2	None	
9	2416	CVKDFILPTPVGGSHSWFDPW	IGH	2	None	
9	2417	CARVWLQPSHPDAFDVW	IGH	2	None	
9	2418	CVHSVGHCSTTSCYNMGWFDPW	IGH	2	None	
9	2419	CQQSYNAPRIF	IGK	113	C2	7K8O
9	2420	CVRDRFCSSTSCYSGSPFDYW	IGH	2	None	
9	2421	CARWRRSRSLRFFDYW	IGH	4	None	
9	2422	CARDSRADTVVVPAATGWFDPW	IGH	2	None	
9	2423	CARVHDFWSTYPRDYW	IGH	6	None	
9	2424	CARVVRGRLPDQSWLFDYW	IGH	4	None	
9	2425	CAKVKIFGVYCSSTSCSEYYFDYW	IGH	2	None	
9	2426	CAKAGGSTVTRIFDYW	IGH	5	None	
9	2427	CARQGSSGYYPLQYYFDYW	IGH	2	None	
9	2428	CARDQPLGGWGTDAFDIW	IGH	3	None	
9	2429	CARDNGAEILVIPADYFFPYW	IGH	2	None	
9	2430	CAREVLARNVYFDYW	IGH	2	None	
9	2431	CAKDRADGYSSSRDAFDIW	IGH	2	None	
9	2432	CASGGLGCSSTSCYGLDYW	IGH	2	None	
9	2433	CARGLTRIVVVPAALGWFDPW	IGH	2	None	
9	2434	CARGSGITMIVAVADDAFDIW	IGH	4	None	
9	2436	CARHSLFDSW	IGH	3	None	
9	2437	CARVKDFWPSYASDIW	IGH	2	None	
9	2438	CARLPRIAVAGKTNYFDYW	IGH	6	None	
9	2439	CAGRYYYDSSGYQDAFHIW	IGH	10	None	
9	2440	CARDNSRQVDSQLFWWFDPW	IGH	4	None	
9	2441	CARLGVVVPSDITNWYFDLW	IGH	2	None	
9	2442	CAREYDLWSGYSGLSYFYMDVW	IGH	2	None	
9	2444	CVKDRRYCSSTSCHYYYYYNMDVW	IGH	2	None	
9	2445	CAKDGDIVVVPAAMVYYFDYW	IGH	4	None	
9	2446	CANGDYYDSSGAPDYW	IGH	2	None	
9	2447	CAKLRSPMVRGVVGIWRDGLDIW	IGH	2	None	
9	2448	CARVPAAIFFEGNNWFDPW	IGH	4	None	
9	2449	CHQYNTYSGMF	IGK	3	C3	7K8R
9	2450	CAREIAVTGVGPEYFDYW	IGH	6	None	
9	2451	CARSPSLPASSYFYYYMDVW	IGH	2	None	
9	2452	CAKVPSGGFYYGWFDPW	IGH	2	None	
9	2453	CVKDLLLPVEGTTGYLDVW	IGH	2	None	
9	2454	CQSYDSSLSGWVF	IGL	1037	C4	7JVC
9	2455	CAHSRRRISCSGGNCYHFDYW	IGH	2	None	
9	2456	CVRGYCTDRSCRYTYYFDLW	IGH	3	None	
9	2457	CAKDGVTGGWFPRWFDTW	IGH	2	None	
9	2458	CAGVRDYYDSSGYTNDGFDPW	IGH	6	None	
9	2459	CETWDSDTWVF	IGL	44	None	
9	2460	CASTRHGSGDNWFDPW	IGH	4	None	
9	2461	CQVWDTSNDHVRVVF	IGL	4	C4	7JVC
9	2462	CAKGAGSSWYNWFDPW	IGH	6	None	
9	2463	CARQGALWSVPRGDDPFDTW	IGH	2	None	
10	1	CAKDLYSSGWYTLGAFDIW	IGH	6	None	
10	3	CAREKNWNVDGTDDAFDVW	IGH	2	None	
10	4	CARDSASASYSSSRFSGVYYYGMDVW	IGH	4	None	
10	5	CARRGYGGARHYDYW	IGH	2	None	
10	6	CAKAVEWELLPGAFDIW	IGH	4	None	
10	7	CQQYFDTPMYTF	IGK	57	C4	6W41
10	8	CARGSPYQLLTFAFDIW	IGH	4	None	
10	10	CARHESVLVWSGYYLDAFDIW	IGH	2	None	
10	11	CAPLGYALRQFDYW	IGH	3	None	
10	12	CARSFGPFSMITSFMFDPW	IGH	3	None	
10	13	CARGMARWYYYYYMDVW	IGH	10	None	
10	14	CAKDPGTGGGMDDAFDIW	IGH	2	None	
10	15	CAKPPLSITMIVVVNEYYFDYW	IGH	4	None	
10	17	CARELDTSGSYPDAFLLW	IGH	3	None	
10	18	CAKPYYYDSSGYYGVPNYYFDYW	IGH	4	None	
10	19	CAKGRDIDSSGYYPYWYFDLW	IGH	2	None	
10	20	CARGLGYCSGGSCKRGLDYW	IGH	2	None	
10	21	CARRARHKYTNSAGFDYW	IGH	5	None	

10	22	CLRPEDTAVYYCARETIVVDDSRFDYW	IGH	3	None	
10	24	CAKDGIFWAADRYFDYW	IGH	2	None	
10	25	CASPHSTVVTAEYYFDYW	IGH	10	None	
10	26	CARGSAGRYDFWSGYSGVDWFDSW	IGH	2	None	
10	28	CAKCAHSSPICYNDYW	IGH	2	None	
10	29	CARDIPPGDSIVLVVYAIGGPWFDPW	IGH	2	None	
10	30	CARGHSHGLDYW	IGH	4	None	
10	31	CAREMYYYGSGSTTFDSW	IGH	3	None	
10	32	CARGGARGGARRPYRLDYW	IGH	2	None	
10	33	CARSGNVRSTIFGVVIKAPSGFDYW	IGH	4	None	
10	34	CARGHLRFLEWLPHFFDFW	IGH	3	None	
10	35	CAKDIGAAALWSGYYAFDYW	IGH	58	None	
10	36	CATGYRYGDYWAPDYYYYYMDVW	IGH	7	None	
10	37	CARRVRDNSGYCFDYW	IGH	2	None	
10	38	CARDNAVAPDFWSGYYSGRDDAFDIW	IGH	8	None	
10	39	CARPPATMIDYYYYYYMDVW	IGH	2	None	
10	40	CATEGFYDNTIPDYAFDIW	IGH	3	None	
10	41	CARPSGGYSYGFGPLLGFSLDYW	IGH	2	None	
10	42	CARPAWNQQSPFEYW	IGH	3	None	
10	43	CARVVHKRSDFDYW	IGH	4	None	
10	44	CATNWNYDYW	IGH	2	None	
10	46	CAKVGAATDRGTDYW	IGH	4	None	
10	47	CHTDLGSCSGGSCYSFDYW	IGH	2	None	
10	48	CARGFSYDYYDSSGYYYHYW	IGH	2	None	
10	49	CARDRTDSSSWIDAFDIW	IGH	10	None	
10	50	CAKDIGCGGDCYSNYW	IGH	6	None	
10	51	CAKGLHAYSALIDYW	IGH	2	None	
10	52	CAHGHTVRGNAFDIW	IGH	18	None	
10	53	CALWFGESPFDYW	IGH	10	None	
10	54	CLSGDEDNRVF	IGL	152	None	
10	55	CAQARDIVVVVAPNRDAFDIW	IGH	3	None	
10	56	CAKDRHLNYDILTGPRFDYW	IGH	6	None	
10	57	CARGGDIMTATDYW	IGH	2	None	
10	58	CASPSAHLTGYPFFLRPSENYYYYYGMDVW	IGH	2	None	
10	60	CARDNSRGLEFCDYW	IGH	2	None	
10	61	CANLGTIAVADVGSSDYW	IGH	2	None	
10	62	CAKVDSSSVGVVDALGDYW	IGH	2	None	
10	63	CARGSLLVHIVVVTANPSPFDYW	IGH	12	None	
10	64	CVSYDSSGYHRRFDYW	IGH	6	None	
10	65	CARLHGDYSMQSAPPDYW	IGH	8	None	
10	66	CARSPGDHIGPNWFDPW	IGH	2	None	
10	67	CAKDIGGLYDFGALWFDPW	IGH	16	None	
10	68	CAKLTDSSGYYGDAFDIW	IGH	6	None	
10	69	CARDSGSGWFYYFDYW	IGH	33	None	
10	70	CASNFGYCSSTSCYGLSYYFDYW	IGH	5	None	
10	71	CANLGYVDWYFDLW	IGH	2	None	
10	72	CARAADIVATYHYYYYMDVW	IGH	4	None	
10	73	CAHRVVGATPCDAFDIW	IGH	2	None	
10	74	CARRAMVRGGSWFDPW	IGH	5	None	
10	75	CARGGGDMGWHYYYYYMDVW	IGH	24	None	
10	76	CAKGGDLGRIAMDYW	IGH	3	None	
10	77	CARDHTPIVVVPAANDAFDIW	IGH	4	None	
10	78	CARDWPRNDYGDSYYFDYW	IGH	39	None	
10	79	CAKDLTGTDTNWFDPW	IGH	7	None	
10	80	CARQGETIFGVVIIPGRSDYW	IGH	3	None	
10	81	CARDTASTVDLWPDAFDIW	IGH	4	None	
10	82	CAALIPGIAAAGPNNWFDPW	IGH	4	None	
10	83	CARPGVMVRGVTVGYMDVW	IGH	2	None	
10	84	CAKDIYLGQWLAQWAADIW	IGH	6	None	
10	85	CARADYDILTGYEAHAFDIW	IGH	7	None	
10	86	CARVGFRVSGVAEADYW	IGH	3	None	
10	87	CARVGGSGTYEGDWFDPW	IGH	3	None	
10	88	CAARTWGWELTNFDYW	IGH	24	None	
10	89	CAKDKQWRGTREYYFDYW	IGH	4	None	
10	90	CASSKGYSYGHYYMDVW	IGH	3	None	
10	91	CARGPFSDSSRAVYYYYNRAMDVW	IGH	4	None	
10	92	CASSRGDGYNTLCFDYW	IGH	11	None	
10	93	CARTDGGKAAFDYW	IGH	5	None	

10	94	CQQANSFLGLFTF	IGK	2	None	
10	95	CARYSMSTISAASRYYFDYW	IGH	3	None	
10	96	CAHAYDAVNWFDPW	IGH	2	None	
10	97	CARATSIAAAGTLMWYYYYYMDVW	IGH	22	None	
10	98	CGESHTIDGQVGVVF	IGL	5	None	
10	99	CAKISTARLSVDYW	IGH	18	None	
10	100	CAKGFHPSLGTSFDYW	IGH	2	None	
10	102	CATSFSIAARPRIDYW	IGH	39	None	
10	103	CAREGWGDGYNMYYFDYW	IGH	2	None	
10	104	CAKDIQPTASMFDYW	IGH	5	None	
10	105	CAKLPGDYDSSGDAFDIW	IGH	2	None	
10	106	CATAGSPYGDYVFDYW	IGH	3	None	
10	107	CQQYKDWPLLFTF	IGK	11	None	
10	108	CATRSTYAEDYYHYYYMDVW	IGH	2	None	
10	109	CARYLRNTARFARYNWFDPW	IGH	9	None	
10	110	CAKDSQTYYDFWSGYYDYFDYW	IGH	2	None	
10	111	CLQYLTTPGAF	IGK	9	None	
10	112	CARNHAARYSSAYYLSDYW	IGH	3	None	
10	113	CARGHRITIFGVVIIPVWLDPW	IGH	2	None	
10	114	CARDDTVTSTYGDYFDYW	IGH	4	None	
10	115	CAKDNGRQWLAGNWFDPW	IGH	4	None	
10	116	CARASPYYDFWSGYSHFDYW	IGH	7	None	
10	117	CATDNRMRAVDHFDHW	IGH	6	None	
10	118	CASGYCSSTSCYPYYYYYYGMDVW	IGH	8	None	
10	119	CATHPSAGYSGYGSPRFVYYYMDVW	IGH	3	None	
10	120	CARFSSDPSGARRFDYW	IGH	3	None	
10	121	CARDSVPFYSGTYYDAFDIW	IGH	13	None	
10	122	CAKAAEEWDLPLYFNYW	IGH	3	None	
10	123	CARDRSRIQLWLPERDNWFDPW	IGH	2	None	
10	124	CARASPLLWFGELSNAFDIW	IGH	8	None	
10	125	CARGTAAIVNWFDPW	IGH	4	None	
10	126	CARHPRGTNYLDYW	IGH	2	None	
10	127	CSSTSCYPYYYYYYGMDVW	IGH	16	None	
10	128	CARDLGSITMIVVAANWFDPW	IGH	6	None	
10	129	CARGPLPGDYSNYGIDYW	IGH	2	None	
10	130	CARDNRAMVRGVINPWYFDYW	IGH	4	None	
10	131	CAKDRITLSAFDTW	IGH	11	None	
10	132	CDWSSDVCSSDLDTAVYYCATAGSAYYAPFDDW	IGH	3	None	
10	133	CTRDRDGSYPGPTFDYW	IGH	2	None	
10	134	CARAAYYYDSSGYYQHLSNDAFDIW	IGH	6	None	
10	135	CAKDISPLGLRLGELYWFDPW	IGH	4	None	
10	136	CARDYGDYARWFFDVW	IGH	2	None	
10	137	CARDLTPTRVVIIPNYYYGMDVW	IGH	3	None	
10	138	CAREVGITIFGVVADPFDYW	IGH	2	None	
10	139	CARETNCGGDCSVDYW	IGH	2	None	
10	140	CAKVPPGGYDILTGYYLVYYFDYW	IGH	14	None	
10	142	CGSVSIAGEMAAIFDFFYYMDVW	IGH	3	None	
10	143	CARVRCLYYYYGMDVW	IGH	4	None	
10	144	CATAKAYCSSTSCSPNWFDPW	IGH	25	None	
10	145	CARGDYDFGDYSYYLEYW	IGH	2	None	
10	146	CARPPGGLWLHQSSFWYFDLW	IGH	10	None	
10	147	CAKDDLPGDGMGYYYYYGMDVW	IGH	6	None	
10	148	CARDPSSSWLILSDDAFDIW	IGH	2	None	
10	149	CARQASYYDSSGYYHHYLDYW	IGH	8	None	
10	150	CATAYAYYYDSSGNYRRPPFDYW	IGH	7	None	
10	151	CARLLGSGWSTYYDYYMDVW	IGH	2	None	
10	152	CLQDHNYSRAF	IGK	17	None	
10	153	CATAPSPPDWYFDLW	IGH	2	None	
10	154	CVTLYNSGWAFDHW	IGH	4	None	
10	155	CAKVGGSYGGETDYW	IGH	3	None	
10	156	CAKNRGLRFLEWLPDAFDIW	IGH	2	None	
10	157	CARDSLRGYSYLPQYWYFDLW	IGH	6	None	
10	158	CARAAVAAPKSWFDPW	IGH	3	None	
10	159	CAKDSASGYYDLDAFDIW	IGH	2	None	
10	160	CARVQFAFVTIFGVVTRYYMDVW	IGH	2	None	
10	161	CARDAYYYASSGYRNYYFDLW	IGH	2	None	
10	162	CARSPSITMIIAFITTYFDSW	IGH	5	None	
10	163	CARESNIGPVEAHWFDPW	IGH	4	None	

10	164	CSSYTYSNTLGRGTLDVF	IGL	2	None	
10	165	CARSGYSSSWRKYYYYYMDVW	IGH	2	None	
10	166	CALTNLNGDYEWFDPW	IGH	6	None	
10	167	CAKGGGHDSSGYLDYW	IGH	2	None	
10	168	CAKLPDYGGNDGYFDYW	IGH	3	None	
10	169	CARDGLGYCTGGVCFRAFDIW	IGH	2	None	
10	170	CARSPLDCSGGSCYSRRFDYW	IGH	123	None	
10	171	CAKHPTYYDILTGYISTYYFDYW	IGH	100	None	
10	172	CAREGWRYDTNQGTDYW	IGH	6	None	
10	173	CAKGTPPYSSSWYGGFDPW	IGH	2	None	
10	174	CARAQQWLVGWFDPW	IGH	5	None	
10	175	CARGTYLHGSGNWLRIYYLDSW	IGH	2	None	
10	176	CASHPLEWLLLGGGYYYMDVW	IGH	2	None	
10	177	CARVGKYYDSSGYWGRNDAFDIW	IGH	4	None	
10	178	CVKVRWGVGTTRFDYW	IGH	3	None	
10	179	CARGAPTDDILTLYTVEEDWFDPW	IGH	2	None	
10	180	CTTTPGRPLVDYW	IGH	2	None	
10	181	CARSLENHITIFGVVTRYNWFDPW	IGH	23	None	
10	182	CAKIGWLAHDAFDIW	IGH	5	None	
10	183	CARHGADCSGGSCYPFVYYYYGMDVW	IGH	2	None	
10	184	CAKLPDGSGSYYNLDAFDIW	IGH	2	None	
10	185	CASSPLPNDFWSGYYTLYYYYYMDVW	IGH	5	None	
10	186	CARLGHYYDSSGYYYPPRTHYYYYGMDVW	IGH	8	None	
10	187	CAKLPLWFGDSAAFDIW	IGH	2	None	
10	188	CARGEWELPHNWFHPW	IGH	2	None	
10	189	CATVSSWIAAAGYNWFDPW	IGH	11	None	
10	190	CARGGWFTLSAAAGPLRNYNWFDPW	IGH	3	None	
10	191	CARASAAGISPLLYYYYYMDVW	IGH	6	None	
10	192	CAREHVERRDGYNYFDYW	IGH	3	None	
10	194	CAKDNSGYSGYDLGWFDPW	IGH	18	None	
10	195	CAKCGPGGITIFGVVIIQGDFDYW	IGH	2	None	
10	196	CTRGGARHVYCSSPTCQIGRAFNIW	IGH	2	None	
10	197	CARGGARWEVSYFDYW	IGH	8	None	
10	198	CARDQGIQLWLDYYYYGMDVW	IGH	3	None	
10	199	CARASGYSYEFDAFDIW	IGH	2	None	
10	200	CARRSDYYDTDGDRFDYW	IGH	2	None	
10	201	CARDRSGGYYWFDPW	IGH	20	None	
10	202	CARDSGYSYGLPSYYYYMDVW	IGH	4	None	
10	203	CARLPETTVTTWVGIGPQYDYYYYYMDVW	IGH	2	None	
10	205	CARDLSTVAGNYFDSW	IGH	15	None	
10	206	CARVNILTGYGSIPPFDYW	IGH	3	None	
10	207	CANLAVAGDYYYHGMDVW	IGH	4	None	
10	208	CHQYYTTPQSF	IGK	10272	C4	6W41
10	209	CLQRNTYPRYSF	IGK	2	C1	7BZ5
10	210	CARSVDFGDVFDYW	IGH	3	None	
10	211	CARTANQLLLGYYYYGMDVW	IGH	2	None	
10	212	CAKERIDYGSGSYPFDSW	IGH	2	None	
10	213	CAKDSDGTILLDYW	IGH	4	None	
10	214	CARDIEQQLVGTDFDYW	IGH	3	None	
10	215	CAKANDDYGDYYYYYGMDVW	IGH	12	None	
10	216	CARDRYDFWSGYYTRAMDVW	IGH	7	None	
10	217	CANGGGSWAFDIW	IGH	4	None	
10	218	CVQNFCPGGACRRQFFDYW	IGH	5	None	
10	219	CAKDPPNYDYVWGSYREYYFDYW	IGH	6	None	
10	220	CAKDRTYYDFWSGYLQTPSYYYYGMDVW	IGH	4	None	
10	221	CARRLYYYDINGGFDYW	IGH	5	None	
10	222	CARGSQLWFRGYYYYYMDVW	IGH	2	None	
10	225	CVKGGRRFDPW	IGH	4	None	
10	226	CARDLVEPLLWFGELSPVGAFDIW	IGH	5	None	
10	227	CAKVKFELGGYFDFW	IGH	4	None	
10	228	CARVGLDSNTSLYAMDVW	IGH	4	None	
10	229	CATVRDHDALEVW	IGH	8	None	
10	230	CARGPGGLLWFGELPISSLFDYW	IGH	3	None	
10	231	CAKGTTRYPENYCDYW	IGH	4	None	
10	232	CAKWEFGVVFRPAYYFDYW	IGH	2	None	
10	233	CAGKSCSSTSCYSDFDYW	IGH	2	None	
10	234	CARENYCSSTSCYWGYMDVW	IGH	15	None	
10	235	CARDYGDYYFDYW	IGH	722	None	

10	236	CARDPQVNWNFWVADYW	IGH	4	None	
10	237	CARSITMIVVVTWADAFDIW	IGH	2	None	
10	238	CAKAEYCSGGSCYSGGDYW	IGH	14	None	
10	239	CTTDRIHLRYFDWLLYSGHDYW	IGH	2	None	
10	240	CARPFVPVGATIDYFDYW	IGH	4	None	
10	241	CARDRWFGELSPGDYYYYGMDVW	IGH	2	None	
10	242	CHSSWYASGQDAFDIW	IGH	8	None	
10	243	CTTDTGFCSSVNCFYYW	IGH	2	None	
10	244	CARLGARTSGHVFDIW	IGH	4	None	
10	245	CASNYDILTGYYLGGGIDYW	IGH	3	None	
10	246	CARGSREQWLVIDRTFDYW	IGH	2	None	
10	247	CASFSSGLNHYFDYW	IGH	2	None	
10	248	CAKDLYPYYYGMDVW	IGH	29	None	
10	249	CARDFGYSSGWYLDLW	IGH	2	None	
10	250	CARNGVLLDATGATVYYYFNVW	IGH	15	None	
10	251	CAREVPYDSSGYETKTFDYW	IGH	2	None	
10	252	CARDSQSSNWYLQDYW	IGH	4	None	
10	253	CALVVVISGDAFDIW	IGH	4	None	
10	254	CARMYYYKPTHFDYW	IGH	2	None	
10	255	CAKDLTRPTYYYGSGSPDAFDIW	IGH	4	None	
10	256	CAKLEPSIAVAGTRDYW	IGH	6	None	
10	257	CARARIVLMLYAQPFGFDPW	IGH	2	None	
10	258	CARDRGYTYGYIIDYW	IGH	2	None	
10	259	CYSTDSSGNHRVF	IGL	3387	None	
10	260	CAKDSQYYDFWSGYSGATRYYYYYMDVW	IGH	4	None	
10	261	CARDSAPDFGVVIMAYFDYW	IGH	3	None	
10	262	CARGPYYYDSSGSKKGGGAFDIW	IGH	2	None	
10	263	CATSRDGYNFNWGILDYW	IGH	3	None	
10	264	CARMSSPHILTGPFDYW	IGH	2	None	
10	265	CTRGGQTYYYDSSGYYRDEPFDYW	IGH	7	None	
10	266	CANLNTESFDYW	IGH	2	None	
10	267	CATLSVRFLEWLLGSDAFDIW	IGH	2	None	
10	268	CARFYYYSDSSGTYDYW	IGH	2	None	
10	269	CAIWEYYYDSSGYTDYW	IGH	2	None	
10	270	CARWPSVAATPDGMDVW	IGH	10	None	
10	271	CARGNRAHYDSSTYDRSSFQSGAFDIW	IGH	4	None	
10	273	CVQTCGDCYLVDAFDIW	IGH	13	None	
10	274	CASYRGGHTVRYKWFDPW	IGH	3	None	
10	275	CAYSSGWEIGFPWDYYFDYW	IGH	11	None	
10	276	CHSSGWYGQQSFDDW	IGH	4	None	
10	277	CARGGAIGLRDLSTFDDW	IGH	4	None	
10	278	CASRAWECSGRNCYHWFDPW	IGH	34	None	
10	279	CARGRTLVGITFFGVVTKYNWFDPW	IGH	8	None	
10	280	CARAVTFSYDRSTYLIPGLFDHW	IGH	2	None	
10	281	CARIHYDILTGLPVFDYW	IGH	2	None	
10	282	CTTEDPFPYSADDYW	IGH	3	None	
10	283	CARIYSSTVNNWFDPW	IGH	2	None	
10	284	CAKEGIMVGTTWGTSLYHFDYW	IGH	6	None	
10	285	CAKAPFYSSGWYGVDYW	IGH	19	None	
10	286	CAKTLTYYYDSKGPVFDYW	IGH	3	None	
10	287	CDWSSDVCSSDLYDSSGFGYW	IGH	4	None	
10	288	CATGPVVPAATGTDWFDPW	IGH	3	None	
10	289	CAKALGFYGLPLSFDYW	IGH	10	None	
10	290	CARNKLGYCTGGVCHDFPFDPW	IGH	4	None	
10	291	CAKDGYCGGDCFSPYWYFDLW	IGH	2	None	
10	292	CAKLVLAVTTSDAFDIW	IGH	4	None	
10	293	CARVRGYSYGLGGYYFDYW	IGH	2	None	
10	294	CAVVVPAPDYW	IGH	11	None	
10	295	CARSGDEYYYDSSGYRPFDYW	IGH	61	None	
10	296	CATDPIYDYVWGSYRYNFDYW	IGH	158	None	
10	297	CASSILEYSSSSVVDYW	IGH	2	None	
10	298	CATSRGHIPGNLGLWAFDYW	IGH	3	None	
10	299	CARRLAVVVPAAPLGSYYYYMDVW	IGH	2	None	
10	300	CSCGLSSGSDYYYYGMDVW	IGH	8	None	
10	301	CARGTYGDYKFFFDYW	IGH	2	None	
10	302	CAKGYYDSSGPPPAFDPW	IGH	4	None	
10	303	CATAPPIVGANLWWFDPW	IGH	2	None	
10	304	CAREAGTYGGNSGTTFDIW	IGH	7	None	

10	305	CARTRPWYNWNGGIDYW	IGH	8	None	
10	306	CARDIRGGYSYGYAPWFDPW	IGH	13	None	
10	307	CARSDSYGHPYYFDYW	IGH	6	None	
10	308	CARQPTITMIVVVIPSSFDYW	IGH	37	None	
10	309	CARSPAVTMIIELSTEYFDYW	IGH	2	None	
10	310	CATGAPITIFGVVSRWFDPW	IGH	2	None	
10	311	CAKLPENPAAYSSSNFDYW	IGH	3	None	
10	312	CARHGKGITMIVVVIGPYYFDYW	IGH	24	None	
10	313	CAREGIGITMVRGVITDYYYGMDVW	IGH	3	None	
10	314	CARDLGHSYGHGRGWFDPW	IGH	2	None	
10	315	CARHDPSGWIFDYW	IGH	5	None	
10	316	CARDSVTAPRFDYW	IGH	35	None	
10	317	CTTDFLSTYYDVLTGSYTYDYFDYW	IGH	4	None	
10	318	CVGQGLLVMNAFDIW	IGH	2	None	
10	319	CARENDPSSWATYYFDYW	IGH	3	None	
10	321	CDWSSDVCSSDLYYYGMDVW	IGH	10	None	
10	322	CQSGHSSGSYPGVF	IGL	2	None	
10	323	CAHSTVVRFPPGYFQHW	IGH	2	None	
10	324	CARPAWPLYYYGMDVW	IGH	10	None	
10	325	CARDFSDPIAALDYW	IGH	118	None	
10	326	CAREESIQLYYYYGLDVW	IGH	4	None	
10	327	CARLSTSWPMDYW	IGH	2	None	
10	328	CARTTLRYFDWSDIFADKAGDYDAFDIW	IGH	8	None	
10	330	CARSALSGLVVSWFDPW	IGH	2	None	
10	331	CAPDRVTMIVGTFDYW	IGH	8	None	
10	332	CARGRQSGSYLPDDAFDIW	IGH	2	None	
10	333	CAKGHIGAPGNYFDYW	IGH	2	None	
10	334	CAGFGTTVTYSDYW	IGH	2	None	
10	335	CARHAPYSSSWYEAYYYGMDVW	IGH	7	None	
10	336	CAKGVDSRSYYFDYW	IGH	2	None	
10	337	CTRLVGYCGGDCYSGWFDPW	IGH	2	None	
10	338	CARRPVRPWLQGSSDAFDIW	IGH	4	None	
10	339	CVRLGRDGYSGPPYFDYW	IGH	9	None	
10	340	CARALPTGTYYYDSGYSAFDIW	IGH	6	None	
10	341	CAKDNRRFLEWLLWGAFDIW	IGH	3	None	
10	342	CARHREEGDQNWLDPW	IGH	5	None	
10	343	CARDTYDILTGYLLAFDIW	IGH	26	None	
10	344	CARDEAVPNTKDGFDIW	IGH	2	None	
10	345	CASPTSSGWEGNGAFDIW	IGH	2	None	
10	346	CARGVAYSGWTGFIYYYYMDVW	IGH	24	None	
10	347	CAKDHSPVWGEFDYW	IGH	13	None	
10	348	CAKTPVAGNGVDYW	IGH	16	None	
10	349	CARDAVVTTRGYFDYW	IGH	4	None	
10	350	CSRDATGFSGLDWFDPW	IGH	4	None	
10	352	CARVPVGPDDFWTPVLPYYYGMDVW	IGH	2	None	
10	353	CARPKPNDFWSGYSIDYW	IGH	26	None	
10	354	CATYDVGYCSGGSCRQDAFDIW	IGH	4	None	
10	355	CARVAPNYYDSSGYYSRQLDYW	IGH	5	None	
10	356	CATFFVYSHLDSNYFDYW	IGH	2	None	
10	357	CAFLSSSSAGLDYW	IGH	13	None	
10	358	CTAYMELSRLRSDDTAVYYCAREYYYASYGMDVW	IGH	2	None	
10	359	CARAPVLRFLEWLSHDAFDIW	IGH	2	None	
10	360	CAKDRPEGYYDSSGYLPFDYW	IGH	2	None	
10	362	CARLGAARGHEAFDYW	IGH	3	None	
10	363	CAKDMEIVATIGGYFDYW	IGH	4	None	
10	364	CVKDISEGRSFAWLLTHSQGYFDYW	IGH	4	None	
10	365	CARTTYYDILTGYYWGGFDPW	IGH	2	None	
10	366	CAKVAWGGPVNSWTPLFDSW	IGH	2	None	
10	367	CAREPFYCTNGVCYDYYYYGMDVW	IGH	3	None	
10	368	CQSADTSALVF	IGL	16	None	
10	371	CATTSPLWFGELLSWFDPW	IGH	30	None	
10	372	CAREGIAAAGAHFDYW	IGH	19	None	
10	373	CARHVLAGNTFDYW	IGH	14	None	
10	374	CARDHYGDDTWLLGYFDLW	IGH	2	None	
10	375	CARDDDSSSYFDLYWFFDLW	IGH	2	None	
10	376	CARHKFEIRTNAFDIW	IGH	100	None	
10	377	CARDRHREVVAATGDYW	IGH	2	None	
10	378	CARDRYFDWLYNWFDPW	IGH	2	None	

10	379	CAREAPVVVTATYYYYYGMDVW	IGH	2	None	
10	380	CAREISWSYDSSGSRTIYYFDYW	IGH	8	None	
10	381	CTRRSLGYCSSTSCFNFDYW	IGH	16	None	
10	382	CARQYYYDSRDYLHDAFDIW	IGH	3	None	
10	383	CARGGYCSGGSCYSIYYFDYW	IGH	5	None	
10	385	CASTSETLLDWYFDLW	IGH	2	None	
10	386	CASPRGAFGGGITALDYW	IGH	6	None	
10	387	CARVGGYNYDGYFYFDLW	IGH	2	None	
10	388	CQSADNSGSLNWVF	IGL	18	None	
10	389	CAHAPVDSSGWYLSWYFDLW	IGH	8	None	
10	390	CARYGSGTYSYRNLYYFDYW	IGH	7	None	
10	391	CAKDLESSYDILTGYGRDYW	IGH	2	None	
10	392	CATNYAYPTEGSFDFW	IGH	5	None	
10	393	CARALVYAYDGFGELFDAFDIW	IGH	2	None	
10	394	CTTDVYYDFWSGYYSPPWDYW	IGH	4	None	
10	395	CARTPRVAGFDYW	IGH	51	None	
10	396	CARRGYSGNWYYTFDIW	IGH	2	None	
10	397	CAREPTVWNYVWWFDPW	IGH	2	None	
10	398	CAEANSKVDYW	IGH	2	None	
10	399	CARAAGHDYGRPDYW	IGH	2	None	
10	400	CAKDKRSDIWQTLDYW	IGH	2	None	
10	401	CQSYDFSSHSVVF	IGL	2	C4	7JVC
10	402	CARDFMAIANDDGTTLDFDYW	IGH	2	None	
10	403	CTRDPFHDILTGAPGDAFDIW	IGH	4	None	
10	404	CAKDLLPSDYGGSIDYW	IGH	2	None	
10	405	CTTNKVRGVDYW	IGH	19	None	
10	406	CAKDMITFGGVIVNSFFFDYW	IGH	19	None	
10	407	CARAQPLGYCSGGSCREVPYRYYYYYYMDVW	IGH	2	None	
10	408	CAKQVAESSPYWYLDLW	IGH	2	None	
10	409	CARDLTGTTVTTNYYYGMDVW	IGH	9	None	
10	410	CAKDGTLSEQWLARWFDPW	IGH	4	None	
10	411	CGVVVTSRDNAFDFW	IGH	3	None	
10	412	CNSRDSSGIPSVIF	IGL	119	None	
10	413	CARRGDYDFLSGYPGFDSW	IGH	2	None	
10	414	CANVAVAPPEGNVDYW	IGH	2	None	
10	415	CAREEGILAVGNWFDPW	IGH	4	None	
10	416	CAKDLQVVVILSLDYW	IGH	2	None	
10	417	CAKDRVRCSGGSCYSRPPFDYW	IGH	6	None	
10	418	CAHNKRDLLTYYYMDVW	IGH	2	None	
10	419	CAKELVVVPAAMTDYW	IGH	4	None	
10	420	CARGPTPGIAAYFTDYW	IGH	4	None	
10	421	CARDPYYYGSGSYNSQPAFDYW	IGH	3	None	
10	422	CARMWWLGPPADYW	IGH	2	None	
10	423	CATDQYDFWSGYRIPFDPW	IGH	96	None	
10	424	CAKHPALKLLWFGELSGYYFDYW	IGH	10	None	
10	426	CARDWYDISGYYRKYYNYYGMDVW	IGH	3	None	
10	427	CAKAGYFDWLLLPLGNWFDPW	IGH	8	None	
10	428	CATPRERWLQSGFDYW	IGH	2	None	
10	429	CAKDMAHYYDSQEWFDPW	IGH	4	None	
10	430	CARDLSFSTVRGWFDPW	IGH	4	None	
10	431	CARHHKWFGEFHYFDYW	IGH	6	None	
10	432	CAKGVSLDSYGHYYYYYMDVW	IGH	2	None	
10	433	CARGPYCTGSKCQRYYHALDVW	IGH	2	None	
10	434	CARLPMADWYFDLW	IGH	24	None	
10	435	CAREMSGSVGYTSGLRRLDYW	IGH	2	None	
10	436	CARDSRGDYRDWMDVW	IGH	6	None	
10	437	CARDGGVFVQMVYAASHFNHAMDVW	IGH	2	None	
10	438	CAKCPEGIVLMAPDWFDPW	IGH	6	None	
10	439	CVRLRLYSSGWYLTGDFDYW	IGH	5	None	
10	440	CARDGVVVAATLWSRPDITGNFDYW	IGH	4	None	
10	441	CATYVAYYYDSSGYRAFDYW	IGH	9	None	
10	442	CARNIAGYSSGWYHTLIYYYYMDVW	IGH	3	None	
10	443	CVRMGTVFGVVNGYFYYAMDVW	IGH	6	None	
10	444	CAKLPETSSGWYQADYW	IGH	3	None	
10	445	CTTETTVTAAWSFDYW	IGH	16	None	
10	446	CAKDTRRFGELLYGAHDYW	IGH	2	None	
10	447	CARGSPEETEHDFWAVLGALYYYYGMDVW	IGH	5	None	
10	448	CAASGSSWYDIWFDPW	IGH	4	None	

10	449	CARSTALGDYMRVFDYW	IGH	2	None	
10	450	CAKHQYSYGYWVGGFDYW	IGH	6	None	
10	451	CARDERYYDFWSGYEVPPWSNWFDPW	IGH	55	None	
10	452	CAREKSSISYRAFDIW	IGH	4	None	
10	453	CAKRRSAIDFWSAFDSW	IGH	5	None	
10	454	CARDSIYDYVWGSYRPRPSSAFDIW	IGH	28	None	
10	455	CAIQPPQITIFGFGGTIDYW	IGH	4	None	
10	456	CQQYNTYSGFSF	IGK	348	C3	7K8R
10	457	CATRTQSSSSWYLRVAYYYYGMDVW	IGH	16	None	
10	458	CASSHYDFWSGYLDYW	IGH	32	None	
10	459	CARTYYDILTGYQIAHDYW	IGH	29	None	
10	460	CARLYSSSPHDLLDIW	IGH	3	None	
10	461	CATGSPVWLGYSSGKEFDYW	IGH	13	None	
10	462	CARDLADGYKNYYYYMDVW	IGH	20	None	
10	463	CAKDIGAGCNDASCPIVYYYYMDVW	IGH	2	None	
10	464	CARDAFHYSGLDYW	IGH	2	None	
10	465	CAKDHLSTSSGFYPDAFDFW	IGH	2	None	
10	466	CARGGAYYESSGYCDFW	IGH	7	None	
10	467	CARTPVELEPRDYYYYYMDVW	IGH	2	None	
10	468	CAKDQWELQLGYYHYYMDVW	IGH	2	None	
10	469	CTRGRRYNNGWDKTDNWFDPW	IGH	4	None	
10	470	CAAWDGRLNGFYVF	IGL	2	None	
10	471	CAKLLVGATRDYW	IGH	25	None	
10	472	CARGAKLRFLEWSRELYYYYYMDVW	IGH	2	None	
10	473	CVPTPYDTSGYYSLFDYW	IGH	4	None	
10	474	CASMVRGLGMYYFDYW	IGH	3	None	
10	475	CAREPFPTEIGGVIVYDAFDIW	IGH	4	None	
10	476	CARDPGHDFWSGYRRVYFDYW	IGH	8	None	
10	477	CARDFRRLVGLNPAFDIW	IGH	3	None	
10	478	CAKDLVGLAEDYDILTGYYTGDAFDIW	IGH	2	None	
10	479	CVKELDWGAFHVW	IGH	2	None	
10	480	CARIASSAYMHGLDAGFFDIW	IGH	2	None	
10	481	CAKADAGYYFNYW	IGH	2	None	
10	482	CARTAAHTWGDYGVDYFDYW	IGH	16	None	
10	483	CARGHVYYDFWSGYGYISDYW	IGH	3	None	
10	484	CARKGGSSSTFYYYYYGMDVW	IGH	2	None	
10	485	CASSSETNYYYYMDVW	IGH	7	None	
10	486	CARDSGRPQSDFEYW	IGH	163	None	
10	487	CATYDNSGYFDYW	IGH	14	None	
10	488	CATEPAIRGLYYFDYW	IGH	10	None	
10	489	CAKLPDYGDYGVDYW	IGH	28	None	
10	490	CATAPSITGTTPWDHYYYYYGMDVW	IGH	17	None	
10	491	CARLMFPREYSDYDWGRDYW	IGH	5	None	
10	492	CHQYYSTLGWTF	IGK	8	C4	6W41
10	494	CARDSAAAVGGFDYW	IGH	35	None	
10	496	CARDEAWSGYPSYYFDYW	IGH	15	None	
10	497	CARDRGVIELLGWELFGGSYFASW	IGH	3	None	
10	498	CAREDYYDSSGYYLDYYYYGMDVW	IGH	2	None	
10	499	CARPAGSGYYSSFDYW	IGH	33	None	
10	500	CARGPPHYGDYPLYYFDYW	IGH	2	None	
10	501	CQRYHLLLSAFEIW	IGH	2	None	
10	502	CATVVSSWYYYYMDVW	IGH	2	None	
10	503	CAKDALRGFSGNDYYYFFGMDVW	IGH	2	None	
10	504	CAARFGANPFCFDYW	IGH	4	None	
10	505	CAHRLAAPTSPYFDYW	IGH	8	None	
10	506	CARTNYDSAGYYYYNYMDVW	IGH	4	None	
10	507	CAKDYSGWFRFDSW	IGH	8	None	
10	508	CTTDPFLTYYYDSSGYYFDYW	IGH	3	None	
10	509	CAREIYGDYEEFGYW	IGH	2	None	
10	510	CARGSSGSSSAFDVW	IGH	4	None	
10	511	CAKEGRDFSSSFFDYW	IGH	4	None	
10	513	CARGRPLRRVVEQWFDSW	IGH	2	None	
10	514	CARESAMSAYVWGSYRCFDYW	IGH	2	None	
10	515	CTRSKPGYSSSWHGSYYYYGMDVW	IGH	2	None	
10	516	CARLLRRDEYYYDSSGYYFGYFDLW	IGH	6	None	
10	518	CAKDPAPGHYDSGGTGWGYYFEYW	IGH	2	None	
10	519	CARESRYSSSRQWLGQYYFDYW	IGH	4	None	
10	520	CAKDPRITMIVKDWYFDLW	IGH	2	None	

10	521	CARAAPESSTWLELRVIPWFDPW	IGH	2	None	
10	522	CTTESGGLEMATTPFDYW	IGH	3	None	
10	523	CARGGQWLVQDAFDIW	IGH	7	None	
10	524	CARPLYYYDSSGTATKNKYWNFDLW	IGH	2	None	
10	525	CARLLSSWDEYYFDYW	IGH	21	None	
10	526	CQQYYSTPPSF	IGK	95849	C4	6W41
10	528	CARWLGKRGDCYYCAFDIW	IGH	2	None	
10	529	CARDGGSWLQFRGVDYW	IGH	4	None	
10	530	CAKDADIVVVTARYFQHW	IGH	2	None	
10	531	CARDFQDPSRCNGDCLDYW	IGH	3	None	
10	532	CARDLDGDYFAFYYW	IGH	2	None	
10	533	CASPNRGIPGSTSGDYW	IGH	4	None	
10	534	CARRPVAGYWGRPGINYFDYW	IGH	2	None	
10	535	CATHFAVLRSGYDYW	IGH	8	None	
10	536	CARDPSLSWLVGVSWYFDLW	IGH	6	None	
10	537	CQQTYSAPREITF	IGK	24	C2	7K8O
10	538	CAKERGSGSQGTIDAFDIW	IGH	17	None	
10	539	CASIWNDILTGYSHVPFDPW	IGH	2	None	
10	540	CTRSLCPFGSCSYSAMDVW	IGH	2	None	
10	541	CARVIEKGDSSGYYNSFDYW	IGH	28	None	
10	542	CARVINFKYYDSSGYPKEGYYFDYW	IGH	2	None	
10	543	CARGPTDTYDYDSSGYPKMFYLDSW	IGH	3	None	
10	544	CARESVPSIYMGLYCTGGVCPGGYFDYW	IGH	6	None	
10	545	CARPRNDGGLRGYMDVW	IGH	2	None	
10	546	CTRERRNCFDYW	IGH	5	None	
10	547	CASNPYCGGDCLGAFDIW	IGH	2	None	
10	548	CARQPETPTIDEKGFDLW	IGH	6	None	
10	549	CAREREDDFWSGSYSPGSEGFDPW	IGH	136	None	
10	550	CAKDIPHDYGGNWDAFDIW	IGH	3	None	
10	551	CARVKLPWYGGGQFDYW	IGH	2	None	
10	552	CAKDHGMLMVPPSSGPYCGSDCNAFDIW	IGH	2	None	
10	553	CARGPHFDWLLLTWFDPW	IGH	2	None	
10	554	CARDFPGLGYSNYGDYFDYW	IGH	2	None	
10	555	CARTFVGLGDFAPDYW	IGH	2	None	
10	556	CQQYDSSLRGLTF	IGK	5	C4	7JVC
10	557	CATSFSIVGAHPYYYYYYGMDVW	IGH	13	None	
10	558	CQQYNSLSARWTF	IGK	13	C3	7K8R
10	560	CARDTVSHDYGGNSYAFDIW	IGH	3	None	
10	561	CASFPKGGYYTGEAYFDYW	IGH	12	None	
10	562	CAGGKGGIAAGGRGAFDIW	IGH	15	None	
10	563	CATVYVDTAMVGGGLQGFDPW	IGH	3	None	
10	564	CATKHQGSCRGDGCYSPFDYW	IGH	19	None	
10	565	CARSAPDDDSWYGYRLYFDLW	IGH	2	None	
10	566	CQHRSSWPRMFTF	IGK	5	None	
10	567	CAKSASGSYHSPPDYW	IGH	11	None	
10	568	CARFTGFVEWELFIGGPRFIEHDAFDIW	IGH	4	None	
10	569	CATGGWELLSLDYW	IGH	9	None	
10	570	CTRDVGVSIGYSYGPNWFDPW	IGH	2	None	
10	571	CAHRGSSVWHNDDAFDIW	IGH	4	None	
10	572	CARCGYSYGYFHYYYYMDVW	IGH	17	None	
10	573	CAKEKEDGDYGNYYYYGMDVW	IGH	2	None	
10	574	CAKLPNSGSYPGSDYW	IGH	7	None	
10	575	CARGTSIAARKGWYYYYYMDVW	IGH	2	None	
10	576	CANWNPRLDAFDIW	IGH	7	None	
10	577	CARDEGAYLSGYSWFDPW	IGH	8	None	
10	578	CARAIAARQNYYYYYYMDVW	IGH	21	None	
10	579	CATGGMMQHNRLEINSFDYW	IGH	2	None	
10	580	CAKTNPSITIFGVVTRHYYMDVW	IGH	11	None	
10	581	CAKVSQYTVVVVAADHVFDIW	IGH	2	None	
10	582	CATAGITIWETRNNWFDPW	IGH	3	None	
10	583	CARDRAQYYDFWSGPPVWAVQDEPFDYW	IGH	2	None	
10	584	CARGGSESALLVITDGFDFW	IGH	3	None	
10	585	CALPICAIYYCARPEGGYSTSWVDYW	IGH	9	None	
10	586	CAKSPTIAAAGLSWGVFDYW	IGH	6	None	
10	587	CARDGIAEAGTPFHFDYW	IGH	2	None	
10	588	CARVVPQSQDFDTW	IGH	2	None	
10	589	CQQAYSFPGALSF	IGK	2	C2	7K8O
10	590	CTRAHVFELLDAFDIW	IGH	4	None	

10	591	CARGSGYNEANFDYW	IGH	2	None	
10	592	CARLPGGVAAINSWFDPW	IGH	4	None	
10	593	CAKDMHCTNGVCYDVDYYYYGMDVW	IGH	8	None	
10	594	CAKESVVRYFDWLLSQYFDYW	IGH	2	None	
10	595	CAKDVNSLNSFDYW	IGH	2	None	
10	596	CARVGLGGSYLEYYYYGMDVW	IGH	4	None	
10	597	CARGVSPSLYYYGNADAFDIW	IGH	9	None	
10	598	CAREDRAAVFFDSW	IGH	2	None	
10	600	CAREEAECSSTSCYYYGMDVW	IGH	3	None	
10	601	CAKDQSGDGYNEGYFDYW	IGH	27	None	
10	602	CVREMMVSVPNDYYYYMDVW	IGH	8	None	
10	603	CAKGGFADSVGWYFDLW	IGH	11	None	
10	604	CARSSRVGLSYSDFW	IGH	4	None	
10	605	CARDRTPSASMIVVVMGGAAHDAFDIW	IGH	5	None	
10	606	CAKDIEPELYGSGSYSSYW	IGH	2	None	
10	607	CARGTLYCSSTSCYFDYMDVW	IGH	8	None	
10	608	CAKDYSWELPYYYYYGMDVW	IGH	2	None	
10	609	CARDGSPLYYDILTGYPPLDYW	IGH	3	None	
10	610	CTTVTRAYYDFWSGYHNYFDYW	IGH	2	None	
10	611	CARDLKLAYDFWSGLLPNWFDPW	IGH	3	None	
10	612	CSIEPPYYDFWVGYLDYW	IGH	23	None	
10	613	CARQGGVTMIVVVIPGYFDYW	IGH	2	None	
10	614	CARDKAMAVGYALDIW	IGH	2	None	
10	615	CAKDGANGYSGYDSQWLVPVTDYYFDYW	IGH	5	None	
10	616	CTTHGATGLDSEFDYW	IGH	8	None	
10	617	CARGTQTVQLWSSSYEFDYW	IGH	5	None	
10	618	CARDLTGTGEDYYYYGMDVW	IGH	35	None	
10	619	CARAGCSGGVCYSHFDYW	IGH	2	None	
10	620	CATLAPTVVTTPIDYW	IGH	6	None	
10	621	CAKIGTPGWMTTVTTTDYW	IGH	2	None	
10	622	CARGDSSMWYGGDAFDIW	IGH	11	None	
10	623	CARDPTYDYVWGSYVGWFDPW	IGH	2	None	
10	624	CARVRSSSWYGPYYYYYGMDVW	IGH	3	None	
10	625	CARQRGGWELPFYFDYW	IGH	6	None	
10	626	CASWRGTWLFGQGGFDYW	IGH	4	None	
10	627	CATYYDYSNRPFDYW	IGH	7	None	
10	628	CAQRQGTEEYIDWFDPW	IGH	2	None	
10	629	CGKGYSSFANAGLNWFDPW	IGH	6	None	
10	630	CARNNYDISGYYWDHFDVW	IGH	2	None	
10	631	CATGNVIGPVTWFDPW	IGH	4	None	
10	632	CARQHIVVVTASRWFDPW	IGH	3	None	
10	633	CAGFEYSSPLVGPLGYYYGMDVW	IGH	4	None	
10	634	CATAPAIAVAGKARVWWFDPW	IGH	2	None	
10	635	CAKDLHEFRHGLLTISLGAPDYYYYYGMDVW	IGH	2	None	
10	636	CAKMGHHYDFWEPGAFDIW	IGH	5	None	
10	637	CARLSGSSTSWDWFDPW	IGH	4	None	
10	638	CARGLFRWSPFDIW	IGH	13	None	
10	639	CATATAVRGALYYYYYYGMDVW	IGH	2	None	
10	640	CARAETGERITIFGVVNPYYYYGMDVW	IGH	2	None	
10	641	CARRTGSLSSRWFDPW	IGH	34	None	
10	642	CARDSAPPFWSGYLYYYYGMDVW	IGH	2	None	
10	643	CAGSSWYASASYYYMDVW	IGH	21	None	
10	644	CASNYGGYNWKDNRFFYW	IGH	3	None	
10	645	CASTAPGLRAFDIW	IGH	4	None	
10	646	CAKDMLPAAAAPFDYW	IGH	6	None	
10	647	CAGDGDMAVYDDSGYFFSYFVTW	IGH	5	None	
10	648	CAKESHDLWSGQNYFDYW	IGH	8	None	
10	649	CARALSGYDYIYYYYYMDVW	IGH	2	None	
10	650	CASNSDFWSGYYTYYFDYW	IGH	20	None	
10	651	CASGGRGLVEANDAFDMW	IGH	10	None	
10	652	CAKVLYYDILTGYSTDAFDIW	IGH	9	None	
10	653	CAKASGRTSPDSRYLDSW	IGH	3	None	
10	654	CARDRVEDYDLLGIDYW	IGH	8	None	
10	655	CARLGGYYYEAPYLPDYW	IGH	2	None	
10	656	CAKVSYSGSYVGPQAYYFDYW	IGH	2	None	
10	657	CAKGAALGITIFGVVTPWADYW	IGH	2	None	
10	659	CYSTDTTGNHRGMF	IGL	4	None	
10	660	CAKGRDIVLMVYATHFDYW	IGH	13	None	

10	661	CAREGVGDYDILTGFARHYYYYGMDVW	IGH	55	None	
10	662	CAHRLAHYDILTGYYNTQYFDYW	IGH	2	None	
10	663	CAKGYSSGYWGDYW	IGH	2	None	
10	664	CAKVPDSSGYYTSFDHW	IGH	2	None	
10	666	CAKDYGVGPDRDYFDYW	IGH	2	None	
10	667	CARTKLSVIAEDYW	IGH	2	None	
10	668	CAKDIRPILGAIDYW	IGH	2	None	
10	669	CARDWNTAMEFQYYMDVW	IGH	2	None	
10	670	CAKEVGRGYANYW	IGH	2	None	
10	671	CARVAPANWGFQYYFDYW	IGH	2	None	
10	672	CASGYCSGGSCYYHYYYYMDVW	IGH	2	None	
10	673	CAAERLGYCSGDSCRNWLDPW	IGH	2	None	
10	674	CAKDTSPGIYYFDYW	IGH	12	None	
10	675	CADRLKGIGYDWNTGTFDIW	IGH	2	None	
10	676	CARDPNDYDSSGYYWNYW	IGH	29	None	
10	677	CAREPRIGGQWLEDYFDYW	IGH	13	None	
10	678	CAKDIGVAGTGYNWFDPW	IGH	2	None	
10	679	CAREDCTITSCYGTPTFDSW	IGH	10	None	
10	680	CARDTYDSSGLPSAEDYW	IGH	3	None	
10	681	CARDRLVYGGNSYFDYW	IGH	9	None	
10	682	CARHGGSQTWIQLWLLPSVFDYW	IGH	4	None	
10	683	CTTDWNSYYDILTGYYFLDYW	IGH	31	None	
10	684	CARSRATHYGSNFDLW	IGH	2	None	
10	685	CARGRRGGYSYGLFDYW	IGH	3	None	
10	686	CAKAAPPYDFDSSGYCDDW	IGH	6	None	
10	687	CARDSQYYDILTGYLPHPRRYYYYYYMDVW	IGH	7	None	
10	688	CATVNFQGLLWFGELQVNWFDPW	IGH	8	None	
10	689	CARIPVYYDRRGYYRLDAFDIW	IGH	2	None	
10	690	CARGIREPTIFGVVITPPDYW	IGH	2	None	
10	691	CARDLELTGDRYDYW	IGH	2	None	
10	692	CAKDIASVYSSSWYYALDIW	IGH	2	None	
10	693	CAKGHFGETPSIPNPDYYYYGMDVW	IGH	2	None	
10	694	CAKVRDDSDRFMRYFDYW	IGH	2	None	
10	695	CARDRGSGWPYYFEDW	IGH	2	None	
10	696	CATDLLHVLSGYSHYAFDIW	IGH	4	None	
10	697	CARATGRWLQFLGFDYW	IGH	4	None	
10	698	CARVGALHFLEWLSPAFDIW	IGH	2	None	
10	699	CVRAPRHGPLFFDYW	IGH	5	None	
10	700	CARDGPIVTIFGVVRRPSYYYGMDVW	IGH	4	None	
10	701	CAKDMTTVVIPVSSFDYW	IGH	388	None	
10	702	CAKVVSVSGYYIWDAFDIW	IGH	5	None	
10	703	CARGGRVEWDSTTYDYW	IGH	2	None	
10	704	CARDLEYCSGGSCYPRVTGDFDYW	IGH	13	None	
10	705	CARRYCSSTGCSSYYMDVW	IGH	16	None	
10	706	CASSLTLIAVAGPSLDYW	IGH	4	None	
10	707	CAREPRGYYDSSGYYKRAGYFDYW	IGH	2	None	
10	708	CARLFRITMVRGGAFDYW	IGH	4	None	
10	709	CARSLPPPYDYVWGSYLVGPWFDPW	IGH	7	None	
10	710	CARHPISYHLLRGYVENYFDYW	IGH	3	None	
10	711	CATGRGGTAARLYYFYYMDVW	IGH	15	None	
10	712	CARGRLDILTGYSSYYFDYW	IGH	3	None	
10	713	CAREVDIVLMVYGVGYNWFDPW	IGH	4	None	
10	714	CARLQSGGLSYGGYFDYW	IGH	2	None	
10	715	CARGGCSSTSCYDNYYYYMDVW	IGH	2	None	
10	716	CAKLSGYGDYDREFTDYW	IGH	7	None	
10	717	CQHYNSYSEYAF	IGK	4	C3	7K8R
10	719	CARGAPEYYDFWSGYRYYYYYYGMDVW	IGH	2	None	
10	720	CASSYIVVVPAAIIDHYYYYGMDVW	IGH	3	None	
10	721	CVRDTLARVSVFGVVPQARNYFDAW	IGH	3	None	
10	722	CATGIPTIVVVPAANSVPRVYYYGMDVW	IGH	2	None	
10	723	CARDPRIMITFGGVIVSDYFDYW	IGH	4	None	
10	724	CARDRVLGTDYDFWSGYYDYYYGMDVW	IGH	6	None	
10	725	CATDRIPVWITAAGTGGWFDPW	IGH	2	None	
10	726	CARSKPDFDFWSGSYIDAFDIW	IGH	2	None	
10	727	CAREGWFYYDSSGYYRDAFDIW	IGH	5	None	
10	728	CARGVPAAILYDFWSGYYPPHFDYW	IGH	2	None	
10	729	CARVVTSRDFWSGYYFDYW	IGH	8	None	
10	730	CARDREYYDFWSGRRTPSYYYGMDVW	IGH	2	None	

10	731	CATLLFYDSSGYYIFDYW	IGH	7	None	
10	732	CARLVSGYYGDYFDHW	IGH	12	None	
10	733	CAKDPYHYDSSGYSGLGPPYFQHW	IGH	7	None	
10	734	CAREGDGYDKSFSFDYW	IGH	2	None	
10	735	CAAALASDLMITFGGVIVHYFDYW	IGH	3	None	
10	736	CQKYDSSPPSIAF	IGK	12	C1	7K8N
10	737	CTTVSPYSSGPFDYW	IGH	3	None	
10	738	CARGAPLLRLRLWGSYRNPLDYW	IGH	2	None	
10	739	CARDLVYSYGQRLHDAFDIW	IGH	13	None	
10	740	CAKGTSPLHYGDSNFDYW	IGH	15	None	
10	741	CVHLAWYDGVGYYLHFDYW	IGH	26	None	
10	742	CARAPYLYYYDSSGYYDTSGYFQHW	IGH	7	None	
10	743	CAKDSGPYQLLCDYW	IGH	4	None	
10	744	CARGAYSNSALVWFDPW	IGH	2	None	
10	745	CAHSSLGLPWYFDYW	IGH	10	None	
10	746	CAHRRSGYYDSSGYYLDYW	IGH	5	None	
10	747	CSTMAVAVFSETFDIW	IGH	4	None	
10	748	CAKAFGYSYGYGSDYW	IGH	5	None	
10	749	CARGIEGVTMMVAVVPGNDAFDIW	IGH	2	None	
10	750	CATGPTVLRYFEGYNWFDPW	IGH	4	None	
10	751	CARDRPLIGDFWSGYPPLNPNWFDPW	IGH	2	None	
10	752	CARSPNYDILTGYSVSTILFDYW	IGH	4	None	
10	753	CAKDPGIGDSSGWYDRVFDYW	IGH	2	None	
10	754	CATRSWRRYYDSSGYLDYW	IGH	3	None	
10	755	CARELFFGMAIGDYW	IGH	2	None	
10	756	CARGRVDSSGWYDLPWYFDLW	IGH	2	None	
10	757	CARVPVPHYSSAWYYLDYW	IGH	3	None	
10	758	CARMDYYSYHGMDVW	IGH	4	None	
10	759	CARDPLPLVWFGDSEGPYFDYW	IGH	86	None	
10	760	CTTVATRIAVAVAATLQHLPFDYW	IGH	7	None	
10	761	CARDQGCSGGRCFWRETDYYYGMDVW	IGH	2	None	
10	762	CAHDYGDYRAPAFDIW	IGH	18	None	
10	763	CARGSHSSSWYGQKKYFDYW	IGH	2	None	
10	764	CAKERGSSRTINWFDPW	IGH	4	None	
10	765	CARASFYHSSIGLGFTDYW	IGH	3	None	
10	766	CARAAQVWLPLDYW	IGH	7	None	
10	768	CARVDTAMVLNYFDYW	IGH	15	None	
10	769	CAHRELQYSYGPWYYFDYW	IGH	2	None	
10	770	CSRGGSDFWSGYYRPNWLDPW	IGH	2	None	
10	771	CALPISAVYYCASPRTHYYDSSGYSGFDYW	IGH	2	None	
10	772	CARVRVSEVRFHYYYGMDVW	IGH	3	None	
10	773	CAKDLDYGDYEGDYYYGMDVW	IGH	3	None	
10	774	CARVILWWLYPPNAFDIW	IGH	6	None	
10	775	CANTVVAAQGGSRAFDIW	IGH	6	None	
10	776	CARFAVVLRFLAYYYYGMDVW	IGH	2	None	
10	777	CASSTNQWEPWWFDPW	IGH	2	None	
10	778	CARALILPDDSNDNYYYGMDVW	IGH	2	None	
10	779	CARGVRMTVVMTLSRHEAFDSW	IGH	3	None	
10	780	CAKNAVAAGTVGWYVDVW	IGH	4	None	
10	781	CARGIYGRQYSSARNWFDPW	IGH	4	None	
10	782	CARGPQPYDFWSGYPDYW	IGH	2	None	
10	783	CARDLRVTEASSGAFDIW	IGH	8	None	
10	784	CARDRGYCTNGVCYTTPAYAFDIW	IGH	2	None	
10	785	CARVVPFYDFWSGTKPWFDPW	IGH	6	None	
10	786	CDSTTCREVYFYYYMDVW	IGH	3	None	
10	787	CATDLWPPRELGGNDYW	IGH	2	None	
10	788	CARERGTLWFGELFTNWFDPW	IGH	15	None	
10	789	CTRNREYTFWSGYTKDYYYAMDVW	IGH	7	None	
10	790	CVKGTGTIIGYGDYSAFDIW	IGH	4	None	
10	791	CAKSGLRWFSDRREIDYW	IGH	2	None	
10	792	CTREAQSSSTYYYYYMDVW	IGH	6	None	
10	793	CVRDRQPDYHYSYMDVW	IGH	11	None	
10	794	CAKDHRAVAGTGFDYW	IGH	2	None	
10	795	CAKGSIAVADDAFDIW	IGH	7	None	
10	796	CVREAYSFPYYMDVW	IGH	6	None	
10	797	CARDLSYDSWSGYGNKNWFDPW	IGH	12	None	
10	798	CAKDYYDSLLHAFDIW	IGH	3	None	
10	799	CAKVSLRAEDGMDVW	IGH	2	None	

10	801	CANLGSGSSSWYYFDYW	IGH	5	None	
10	802	CARAMVSYGLAAFDIW	IGH	2	None	
10	803	CARHPPEDFWSGYYHWFDPW	IGH	3	None	
10	804	CLAGDSDYW	IGH	2	None	
10	805	CSSTSCSPNWFDPW	IGH	2	None	
10	806	CAPRWGTEDDAFDIW	IGH	6	None	
10	807	CAREVRDNWNDGGVDYW	IGH	28	None	
10	808	CARAGYHWSPGAAGLFDFW	IGH	4	None	
10	809	CASEEYCSSTSCYQGDYYYGMDVW	IGH	5	None	
10	810	CARPSHTADPAYAFDIW	IGH	2	None	
10	811	CAKGVNSEAPYRAFEIW	IGH	3	None	
10	812	CATGPLLWRQASNWFDPW	IGH	4	None	
10	813	CARLRLVGYCSSTSCYMYYFDYW	IGH	3	None	
10	814	CARTYYYDSSGYFRGGDYYYYGMDVW	IGH	2	None	
10	815	CSRGRGDGYNYGAVDYW	IGH	2	None	
10	816	CARDRYCSSTSCYMGFLYYYYGMDVW	IGH	8	None	
10	817	CAKRGGEDAYNYGAFDYW	IGH	2	None	
10	818	CAREGTRAAGTPFDAW	IGH	5	None	
10	819	CAKDQTAYDYVWGSYRSPGDYW	IGH	4	None	
10	820	CARVGHMIFVHDYW	IGH	4	None	
10	821	CQQSFSDPLLAF	IGK	32	C2	7K8O
10	822	CQSADSTIAPMLF	IGL	4	None	
10	823	CARHPDSSGYFRPVVDYW	IGH	4	None	
10	824	CQSADDNGWFF	IGL	88	None	
10	825	CAKAWYQVLTVDAFDIW	IGH	6	None	
10	826	CARLVAEWELPPMGWFDPW	IGH	17	None	
10	827	CALPISAVYYCARPRSGSYFSYFDYW	IGH	4	None	
10	828	CARGFRSSSGYLFDYW	IGH	4	None	
10	829	CAKLSASKGPVDVVSVVWDAFDMW	IGH	2	None	
10	830	CARTSASYYYDTADLFDYW	IGH	2	None	
10	831	CAREPPLIHHYVDYRTTPNYYYYMDVW	IGH	2	None	
10	832	CQHNHNLPPTF	IGK	3	None	
10	833	CARPPPYYYDSSTYAWFDPW	IGH	14	None	
10	834	CALNSYGSFFDYW	IGH	6	None	
10	835	CARGAYGSGNWGYFDYW	IGH	2	None	
10	836	CVRHSYGFDGYYYMDVW	IGH	2	None	
10	837	CTRPPRNYYDSSGYYYEAEYFQHW	IGH	5	None	
10	839	CARDFTVTTSSDGYYYYGMDVW	IGH	2	None	
10	840	CARDRHREQLWLHDAFDIW	IGH	6	None	
10	841	CARRLNTGMINPLFYGFDLW	IGH	2	None	
10	842	CAKNALPDCSSGNCYSRKSGFDSW	IGH	2	None	
10	843	CARDLSYSSSWYRYFDYW	IGH	2	None	
10	844	CAKGSGDGYPISNWFDPW	IGH	3	None	
10	845	CARTRYNWNDGVFDYW	IGH	3	None	
10	846	CARNQWGGKYKPADYW	IGH	2	None	
10	847	CVKDMKWDLLGAFDVW	IGH	3	None	
10	849	CAKAPKGWDDYDFWSGYSYPNDYW	IGH	8	None	
10	850	CGRFYYYYYMEVW	IGH	6	None	
10	851	CAKDHYYYDSSPPIAFDIW	IGH	2	None	
10	852	CARDPVGEQYFDIW	IGH	4	None	
10	853	CARDRLRPGGLFDLW	IGH	18	None	
10	854	CAREGAVGATTGPDYW	IGH	91	None	
10	855	CAREAPTVTTEDDYYYYGMDVW	IGH	5	None	
10	856	CARDLGIVVGATTIPYVFHYW	IGH	2	None	
10	857	CARLNSSSQYYYYYYGMDVW	IGH	4	None	
10	858	CATTTILGVVRVSWFDPW	IGH	7	None	
10	859	CAKEYFDRVTGFDPW	IGH	4	None	
10	860	CARIATFLGSGSYGWFDPW	IGH	2	None	
10	861	CARDRDVLRYFDWLFQGWFDPW	IGH	4	None	
10	862	CARDLYSSGWEYYMDVW	IGH	2	None	
10	863	CARDLSGWYGHYYYYGMDVW	IGH	2	None	
10	864	CAKDRILLWFGAFDYW	IGH	11	None	
10	865	CATEYVLRYFDWLLDYW	IGH	4	None	
10	866	CAKVGSRDYSGRDYFDYW	IGH	2	None	
10	867	CAKEKYGDYALGVSFDIW	IGH	2	None	
10	868	CATAPARIAAAPINWFDPW	IGH	10	None	
10	869	CARMAVGATNHFDYW	IGH	2	None	
10	870	CARVPLGELLYGPDYW	IGH	3	None	

10	871	CANGSPGYGSGTYFDSW	IGH	2	None	
10	872	CAREAGSSWHNNWFDPW	IGH	6	None	
10	873	CANGAYSYGGKAFDMW	IGH	2	None	
10	874	CARHSFRYSSGWYYYYGMDVW	IGH	6	None	
10	875	CTKGSRGDFALFDYW	IGH	2	None	
10	876	CDWSSDVCSSDLDTAVYYCARDSGRPQSDFDYW	IGH	2	None	
10	877	CAKFGVEQQPMGDYW	IGH	2	None	
10	878	CARESHIVVVTAINAFDIW	IGH	3	None	
10	879	CVRVALGGFLEWVPFDYW	IGH	2	None	
10	880	CATAPGSSGWPDYW	IGH	16	None	
10	881	CARDFFYGSGSYYKNDAFDIW	IGH	3	None	
10	882	CAKDGAFYDILTGYLSHYYYYYGMDVW	IGH	2	None	
10	883	CAKYNYDFWSATFDPW	IGH	4	None	
10	884	CARDGGLHYDILTGYYPYYMDVW	IGH	24	None	
10	885	CARDSGIGGYDIDYW	IGH	3	None	
10	886	CARDRRGRVGWYGPHFDYW	IGH	8	None	
10	887	CASCPHPLYGSPDWYFDLW	IGH	2	None	
10	888	CTKASHYDFWGGSAMDVW	IGH	2	None	
10	889	CTTSREYTVRSPFDYW	IGH	10	None	
10	890	CARESAAPLSYDSSGYYHYFDYW	IGH	21	None	
10	891	CAKMLDGLTGTEDYW	IGH	2	None	
10	892	CARPSSGSYKGVVDYW	IGH	3	None	
10	893	CARVYCSSTSCYKKPIDYW	IGH	2	None	
10	894	CATRRYGDYEVLDYYYYMDVW	IGH	2	None	
10	895	CAHRPMPYGDYNWFDPW	IGH	2	None	
10	896	CAKIAAAGTDYW	IGH	113	None	
10	897	CARDPLGGWFGDYW	IGH	4	None	
10	898	CARVRVPDSNDGFWSGYYKYYYYYYMDVW	IGH	10	None	
10	899	CARVPRDYYDSSGYYFFGGLAGLDYW	IGH	4	None	
10	900	CARVPKDGSYDYVWGSYLDYW	IGH	4	None	
10	901	CAKDARYYDFWSGYFEPHYYYYYGMDVW	IGH	6	None	
10	902	CAKDPGYSYGYGGFDYW	IGH	8	None	
10	903	CAINTKRGYSGYDLGDYW	IGH	4	None	
10	904	CARDWRVMPHMTSVTTEAFDIW	IGH	2	None	
10	905	CARANTKDYDILTGYNIGRDDAFDIW	IGH	2	None	
10	906	CATKPPEYDGTGYYYVSYW	IGH	2	None	
10	907	CAKALGYYYDSSGYSPFDYW	IGH	26	None	
10	908	CATLPIAAAGNGFYYYYYMDVW	IGH	4	None	
10	909	CARGMFVDMTSVFDYW	IGH	2	None	
10	910	CARYYGENAGTGWFDPW	IGH	10	None	
10	911	CARGDAQQVANDYW	IGH	3	None	
10	913	CARGRYSSSWPQEWYFDLW	IGH	3	None	
10	914	CQQYNSYSPVPYTF	IGK	18	C3	7K8R
10	915	CQVWESSTDRNVVF	IGL	8	None	
10	916	CAREMFRRSAFDYW	IGH	5	None	
10	917	CARGINTFDDYVWGSYRYTLTERYTYDYW	IGH	2	None	
10	918	CASSPPRCTGGVCYFGGFDYW	IGH	2	None	
10	919	CATAWENGYNPASDYW	IGH	7	None	
10	921	CAKGAQGYFWSGYWDVW	IGH	6	None	
10	922	CARLPSHTLNYDSSGLGFDYW	IGH	3	None	
10	923	CTTVATRIGVVEVATFQRLPFDYW	IGH	2	None	
10	924	CARLALCGGDCHYFDYW	IGH	31	None	
10	925	CARSDDSSEPFYYYYYMDVW	IGH	2	None	
10	926	CAKVDISRTGGSGSSDYW	IGH	2	None	
10	927	CTRRSYYYDSSGYYVYGHTFDYW	IGH	4	None	
10	928	CARGENDYHTSSFLQYFDYW	IGH	2	None	
10	929	CVRDMFYYDYTGSLSHAFDIW	IGH	20	None	
10	930	CASNRSPYYYDSSGYYFSTFDYW	IGH	8	None	
10	931	CAREGNGGSYSIDYW	IGH	25	None	
10	932	CASNWGGGSYPVEAFDIW	IGH	3	None	
10	933	CAHTLQWLGFDIW	IGH	2	None	
10	934	CARIRLRGYDYVWGSYLSDSFDAFDIW	IGH	7	None	
10	935	CAHRRHLYYYDSSPFDYW	IGH	4	None	
10	936	CARVPVSGGWSPFYHFDYW	IGH	3	None	
10	937	CARHSGYSYFYW	IGH	10	None	
10	938	CARVISGSYYAPDDAFDIW	IGH	4	None	
10	939	CAREVVNEGNDYW	IGH	5	None	
10	940	CASQPVLRYFDWLASHQGFDYW	IGH	3	None	

10	941	CARDGVYYDILTGYYPTGRQYYYYYYMDVW	IGH	12	None	
10	942	CANVPPLTTYYFDYW	IGH	3	None	
10	943	CAYCDGLYGSGSVDYW	IGH	2	None	
10	944	CTTVGEYDSSGYYVLEAFDIW	IGH	4	None	
10	945	CAKDFRNSGYDPRFDYW	IGH	2	None	
10	946	CATLYSDSGSYDSYYYYGMDVW	IGH	2	None	
10	947	CAKDTRGDGDYESAFDIW	IGH	6	None	
10	948	CARSALYYGDYLLGGYYDSSGSFADAFDIW	IGH	19	None	
10	949	CANMMDGGNNDYW	IGH	5	None	
10	951	CARDTMSESLDALDIW	IGH	2	None	
10	953	CAHRLASGFFGVVEYYFDYW	IGH	2	None	
10	954	CARGGWEMATISRSPEPKGYYFDYW	IGH	5	None	
10	955	CAIYGDYDDAFDIW	IGH	16	None	
10	956	CVKDTQYSYGFGTAFDNW	IGH	6	None	
10	957	CARSPLWDYYFDYW	IGH	5	None	
10	958	CATGPALGSGYLYNWFDPW	IGH	2	None	
10	960	CQSPESSGIWVF	IGL	7	None	
10	961	CAKGPNIVVVPTAFDYW	IGH	3	None	
10	962	CARIGFDFSSGYSDHYYYMDVW	IGH	2	None	
10	963	CAKHPGGYGDYLAYYFDYW	IGH	4	None	
10	964	CAKVPDQNDYGDQNAFDIW	IGH	3	None	
10	965	CAREWDSSGWGSSYFDYW	IGH	14	None	
10	966	CAKHGIWESYRFDFW	IGH	13	None	
10	967	CASGPDYYDSSGPFDYW	IGH	31	None	
10	968	CAKDSSGLHKSYYFDYW	IGH	8	None	
10	969	CARDIPVVVITTEDDAFDIW	IGH	2	None	
10	970	CAHSQTLLWFGELPLVEDYFDYW	IGH	3	None	
10	971	CARHGDFWSGMNWFDPW	IGH	2	None	
10	972	CARCATNYDFLTGGPFDHW	IGH	2	None	
10	973	CAHRHRIGCLDYW	IGH	7	None	
10	974	CARGDGYYSPFDYW	IGH	84	None	
10	975	CAMIDGPFDPW	IGH	2	None	
10	976	CVREPEYNYDDSAIAGFDPW	IGH	27	None	
10	977	CATGASLRYFDWLLPYYFDYW	IGH	3	None	
10	978	CARDQGGYSYGYVPSDYW	IGH	4	None	
10	979	CARVNNGGYYFDYW	IGH	12	None	
10	980	CARDKPKDPLAVALGGYYYYGMDVW	IGH	8	None	
10	981	CQQYHNWVFTF	IGK	215	None	
10	982	CAKIQAGTKGDYW	IGH	5	None	
10	983	CASLGSSGDYYDSSGYGLSFDYW	IGH	12	None	
10	984	CAKDPLNDYGDYGAFDIW	IGH	8	None	
10	985	CARGRYSSSWYYLGELEGSDCDYW	IGH	2	None	
10	986	CAKDRSHYYYDSSGFDYW	IGH	25	None	
10	987	CQSCDGSLSGSHVIF	IGL	2	None	
10	988	CARGHPQIADRSNDAFDIW	IGH	8	None	
10	989	CARSPEYYDFWNGYYKGNDAFDIW	IGH	2	None	
10	990	CARDTARGDFDHW	IGH	105	None	
10	991	CATWDSSLSVGHSF	IGL	2	None	
10	992	CAHLEMTTPNYFDFW	IGH	2	None	
10	993	CAKSTSRSGYYKIAAFDYW	IGH	7	None	
10	994	CVTSPEWLRLRYFDYW	IGH	4	None	
10	995	CARDYDTSRSPECDLDYW	IGH	6	None	
10	996	CARDAYSSSSGAGFDPW	IGH	2	None	
10	997	CATDIIMYYDSSGYYLPFRYW	IGH	41	None	
10	998	CARDGSIAVRKTFDYW	IGH	2	None	
10	999	CARFPMQWELIAWYFDLW	IGH	3	None	
10	1000	CATTTPHDYGDYGAGWFDPW	IGH	2	None	
10	1001	CARAFWSGYQYTQFDPW	IGH	2	None	
10	1002	CARVLSKVVGPAFDIW	IGH	2	None	
10	1003	CARQIFGVVTPVYYFDYW	IGH	11	None	
10	1004	CATSLTIFGVVKDYYYYYGMDVW	IGH	39	None	
10	1005	CARGPSGSGYSGYDIDYYYYGMDVW	IGH	2	None	
10	1006	CQSYDTSSLGASRVF	IGL	31	C4	7JVC
10	1007	CARHGFWSGLTSLDAFDIW	IGH	2	None	
10	1008	CARDNVRGQWLFLPGDYW	IGH	4	None	
10	1009	CAREYPYDSSGYPVVWFDPW	IGH	3	None	
10	1010	CARISGDYGDYVFDYW	IGH	53	None	
10	1011	CARHDLYYYDDNGYYSGAFDIW	IGH	6	None	

10	1012	CATYYDFWSGGGHAFDIW	IGH	4	None	
10	1013	CARHPFAPANWFDPW	IGH	6	None	
10	1014	CARERAPDSSGYYAFDIW	IGH	3	None	
10	1016	CARGGWSAAAGTYQLGWFDPW	IGH	2	None	
10	1017	CASAGRGIAMVPAAIGAFDIW	IGH	2	None	
10	1018	CARGIAYCGGDCYSIDYW	IGH	4	None	
10	1019	CSSRRHLDERWLQTAFDFW	IGH	41	None	
10	1020	CAREPHLGYSTPVYYYYYGMDVW	IGH	5	None	
10	1021	CGRHTVGFERDAIDLW	IGH	2	None	
10	1022	CAKDMGPAGYRNPFDYW	IGH	4	None	
10	1023	CALPISAVYYCAKQGGYFALERYSYMDVW	IGH	4	None	
10	1024	CARESPTWGDSTGYSHFDYW	IGH	6	None	
10	1025	CAREARYFDWLFGDLGPSSGAHIDYW	IGH	2	None	
10	1026	CARVARLNSGWSIGAFDIW	IGH	10	None	
10	1027	CARDMSNPVVRYCSGGSCPYYYGMDVW	IGH	10	None	
10	1028	CAKFPGTYYDFWSGSQPVPTLYYMDVW	IGH	2	None	
10	1029	CANGGLEYYDFWSASSAGSPFFDYW	IGH	2	None	
10	1030	CARPGTKDYCGGDCSDYFDYW	IGH	7	None	
10	1031	CAKEGDYNYGYLAFEIW	IGH	2	None	
10	1032	CAREPLGYENSGYPYFDLW	IGH	6	None	
10	1033	CAKGDIAVAGGSFSDYW	IGH	2	None	
10	1034	CAHRRSGWSAEYFQHW	IGH	2	None	
10	1035	CAYGGNVWVAEKGPVDYW	IGH	7	None	
10	1036	CARQRRAGWNPVHIVVVTAITHFDYW	IGH	2	None	
10	1037	CATWDDSLTGCWLF	IGL	2	None	
10	1038	CAKARWYVYYYMDVW	IGH	2	None	
10	1039	CAKLLEREHSSFDYW	IGH	4	None	
10	1040	CAKLDSGYDSPLDYW	IGH	22	None	
10	1041	CARDFVLSRGWYEEAEGYFDYW	IGH	2	None	
10	1042	CQSYDNSFGGHFVF	IGL	8	C4	7JVC
10	1043	CARDLPPGSSWYRDWFDPW	IGH	5	None	
10	1044	CAYDSSGLLLGYAFDIW	IGH	2	None	
10	1045	CAHRLRYDYGSYFDYW	IGH	10	None	
10	1046	CAKDRDAYGLLGFDYW	IGH	4	None	
10	1047	CTIGLLDTSSKVDYW	IGH	2	None	
10	1048	CAVLGEGGYDWIPGYFDYW	IGH	7	None	
10	1049	CAKGSFVTMIVVGDFDYW	IGH	2	None	
10	1050	CAKDMASSWYGGVNLDYW	IGH	4	None	
10	1051	CARGPYSSSWYGVRLHFDYW	IGH	3	None	
10	1052	CAKAPVAGSPADYW	IGH	10	None	
10	1053	CARSSYFDWLSNPFDYW	IGH	25	None	
10	1054	CARHRDSSSLIRGAHDYW	IGH	2	None	
10	1055	CARVGFEQYQLLWDFDYW	IGH	2	None	
10	1056	CAKDTNRGLSTTNSFDYW	IGH	2	None	
10	1057	CARVSLLRYFDWPLRYYYMDVW	IGH	2	None	
10	1058	CARAPGGNYGDDAFDIW	IGH	2	None	
10	1059	CANHYYDNSDYYFHDAFDIW	IGH	2	None	
10	1060	CAAQAILGDYW	IGH	12	None	
10	1061	CVTLGGWMATSHYYYYGMDVW	IGH	2	None	
10	1062	CARGHGDPVLKALDYCDYW	IGH	2	None	
10	1063	CARESVVDTAMVLTHAFDIW	IGH	9	None	
10	1064	CVREADCTRTSCPKHFDKW	IGH	2	None	
10	1065	CARDWGPYDSSGYFWASGAFDIW	IGH	7	None	
10	1066	CARSVVGSSLLVDYW	IGH	2	None	
10	1067	CARLTIFGVPDYW	IGH	20	None	
10	1068	CATLGGYEGVGVDYW	IGH	7	None	
10	1069	CARYEGGYSYDNAFDIW	IGH	4	None	
10	1070	CARDTMVTFLYYYYYGMDVW	IGH	2	None	
10	1071	CARDGKYYYDSSGYYQSVPDYW	IGH	10	None	
10	1072	CARLYDSSGYLADYW	IGH	82	None	
10	1073	CASYGDYEAYFDYW	IGH	8	None	
10	1074	CASAGYSSSRWFDPW	IGH	2	None	
10	1075	CARDWPHDFDWFYDAFDIW	IGH	2	None	
10	1076	CARLPTLRITIFGVVGNEYYFDYW	IGH	2	None	
10	1077	CARDQWPARRTSYYYYYYMDVW	IGH	2	None	
10	1078	CTTKAPHYDISGLDAFDIW	IGH	49	None	
10	1079	CASEVIAVAAPSVDYW	IGH	4	None	
10	1080	CARPRGYYYYSDAFDIW	IGH	2	None	

10	1081	CANSWGVTTASYFQHW	IGH	2	None	
10	1082	CARDTLGTAMVTTQGSYNWFDPW	IGH	4	None	
10	1083	CARESSSWLAENWFDPW	IGH	13	None	
10	1084	CAKDLTLGYCSGGSCPGAFDIW	IGH	2	None	
10	1085	CTRAGQQQLVLTDYW	IGH	2	None	
10	1086	CTTITIFGFDYW	IGH	2	None	
10	1087	CARHRVSVWNDRWFDPW	IGH	2	None	
10	1088	CAKHSTTEVYDVFDIW	IGH	4	None	
10	1089	CARGLGYSSSWLHPQVDYW	IGH	2	None	
10	1090	CARALLVATIEDYYYYTDVW	IGH	10	None	
10	1091	CAKDIGTGYSRYYGMDVW	IGH	24	None	
10	1093	CAKDTDFWSGYYPSWFDPW	IGH	91	None	
10	1095	CVRENEDYYKKGAGNWFDPW	IGH	4	None	
10	1096	CARLTGTSFDYW	IGH	151	None	
10	1097	CARDNPGAMALDAFDIW	IGH	2	None	
10	1098	CAKDPPIWCSGGSCVDYW	IGH	3	None	
10	1099	CARDGEHIVVVTALHDDAFDIW	IGH	5	None	
10	1100	CARGSSMIVVVTKIRDAFDIW	IGH	2	None	
10	1101	CAKVVGVMITFGGVIVPLHFDYW	IGH	2	None	
10	1102	CARYVNYGDFNWFDPW	IGH	2	None	
10	1103	CATDTYCSSTSSYTSHYFDYW	IGH	3	None	
10	1104	CARDSPARYCSSTSCYTNNWFDPW	IGH	2	None	
10	1105	CARGDSSSWYVLGAFDYW	IGH	2	None	
10	1106	CARTRLGGSYYFDSW	IGH	4	None	
10	1107	CATSYYYDSSGYRTGIFDYW	IGH	12	None	
10	1108	CAKDMSPATHGHCDYW	IGH	2	None	
10	1109	CARDRYYDFWSAFGDLYYYYGMDVW	IGH	3	None	
10	1110	CARGPPFARSLFYYYYLDVW	IGH	2	None	
10	1111	CARANVLRYFDWLLEPGSFDPW	IGH	2	None	
10	1112	CARFTKEDYYNPRGYYYDYW	IGH	2	None	
10	1113	CALPILHMDSLRPEDTAMYYCARETIVVDDSRFDYW	IGH	2	None	
10	1114	CARHPYCVSSFCPAVPNNWFDPW	IGH	11	None	
10	1115	CAKDINPASGYADYW	IGH	2	None	
10	1116	CARHVVEVVVVVITKIWFDPW	IGH	3	None	
10	1117	CAREQLWETGGGFDYW	IGH	2	None	
10	1118	CAADWGYSYGRDYYYYMDVW	IGH	26	None	
10	1119	CARTSRDGYNWVKKSLFGHAFDIW	IGH	2	None	
10	1120	CARIVIAVAGSPFDYW	IGH	94	None	
10	1121	CARVVGFSGGWDYW	IGH	4	None	
10	1122	CATLFGRSGWELLGVLDYW	IGH	2	None	
10	1123	CATDSYYLISRGRVDYW	IGH	4	None	
10	1124	CAKAQEGSSWSEIDYW	IGH	2	None	
10	1125	CAKDNGNYYGSGNLHAFDIW	IGH	2	None	
10	1126	CAQGQGDGDYYYGMDVW	IGH	6	None	
10	1127	CATGGEYSSSWYVGYYYYYMDVW	IGH	3	None	
10	1128	CARAMGDDSSYGNWLDPW	IGH	3	None	
10	1129	CASSPPSYGDYVSFDYW	IGH	5	None	
10	1130	CAKALDVVGGKTTGFDYW	IGH	3	None	
10	1131	CARGKGVAAAGTGVYYYGMDVW	IGH	2	None	
10	1132	CAKGLVVVRAKSFLGGNDAFDIW	IGH	2	None	
10	1133	CATEGIAAPGGQLVDKYAFDMW	IGH	2	None	
10	1134	CQQSYTTFGGSF	IGK	12	C2	7K8O
10	1135	CARVPGSPHSGYDSSPFDYW	IGH	4	None	
10	1136	CAVSGSFHTGLFPTEYYYAMDVW	IGH	2	None	
10	1137	CAKLAGAAAEEGSDYW	IGH	9	None	
10	1138	CARRGLHNWNDFDYW	IGH	2	None	
10	1139	CTSSSCRRAPRTPFDSW	IGH	3	None	
10	1140	CARIGHNSGWDTEYFQHW	IGH	7	None	
10	1141	CARAPDSPHNNFWFYFDFW	IGH	2	None	
10	1142	CARTYAAAGPVAVGFDPW	IGH	5	None	
10	1143	CAKVVTPIVLVVYAIPHFDYW	IGH	8	None	
10	1144	CQSADSSGGYHYIF	IGL	20	None	
10	1145	CTRDYYDNSGYYIGYW	IGH	4	None	
10	1146	CARDKVYHNYDLYYYGMDVW	IGH	4	None	
10	1147	CARFWWERSTWRNWYFDLW	IGH	2	None	
10	1148	CARPFDNFGSNDYW	IGH	11	None	
10	1149	CARALYYHSTGYHDAFDVW	IGH	4	None	
10	1150	CAKLSEDHYYDSSGYYARDYW	IGH	2	None	

10	1151	CARDYYDSSGYNDAFDIW	IGH	81	None	
10	1152	CTKGSNSYYDISGPDYW	IGH	9	None	
10	1153	CARHLRYYDNIRYNYIDFW	IGH	3	None	
10	1155	CARAEGRPRYSYGYGYIDYW	IGH	2	None	
10	1156	CAKAPPHYDLPHDAFDIW	IGH	2	None	
10	1157	CARARPPATFYSSGHYYYYYYGMDVW	IGH	2	None	
10	1158	CAHRRGWLTPLFDPW	IGH	3	None	
10	1159	CATDRRHRKYNWNVAVFWFDPW	IGH	2	None	
10	1160	CARAIPPFMIVVVITHPDFDYW	IGH	12	None	
10	1161	CTTDPYSGQDFDYW	IGH	2	None	
10	1162	CARDPTSADSSGWSGYFDYW	IGH	4	None	
10	1163	CASSLGAYGSGSYDWGLDAFDIW	IGH	3	None	
10	1164	CTARLIDHGGNNLRTLDYW	IGH	9	None	
10	1165	CARDGEDSSSWPTYYYYMDVW	IGH	2	None	
10	1166	CARGGDGSGSYYSDYGIITEHYFDYW	IGH	2	None	
10	1167	CAKSTRELDYGDSFDYW	IGH	12	None	
10	1168	CAKTLLLRYFDWLFEAGRSYYFDYW	IGH	2	None	
10	1169	CARDLDTYYSDASVYYPDSFHIW	IGH	8	None	
10	1170	CAHRTLIVGATSGFDYW	IGH	2	None	
10	1171	CARDPTKRRYGGNGYFDYW	IGH	7	None	
10	1172	CARSTRDYYDSSGYYYIDYW	IGH	2	None	
10	1173	CARAGGVIVADAFDIW	IGH	2	None	
10	1174	CAREFMTVVVDMPYYYMDVW	IGH	2	None	
10	1175	CARDSRHIPLFGPADTAMPRDYW	IGH	6	None	
10	1176	CARSPCYYDSRGYKRLDAFDLW	IGH	4	None	
10	1177	CTKGPFDYGDPYYFDFW	IGH	38	None	
10	1178	CATSQLWFRLNWFDPW	IGH	3	None	
10	1179	CALARSDYYDSSGAFDYW	IGH	3	None	
10	1180	CAKGIGYDPWGIDYW	IGH	4	None	
10	1181	CAKSADHIVVVPAATLDYW	IGH	128	None	
10	1182	CARADMAALVPYYYMDVW	IGH	2	None	
10	1183	CAREYVRGYSYGILRYFDYW	IGH	2	None	
10	1184	CARADDYGDYVLPGYFDYW	IGH	23	None	
10	1185	CARQNDYDDYDGAFDIW	IGH	2	None	
10	1186	CARQRTSGVSIFGVVTLEGWFDPW	IGH	2	None	
10	1187	CAKPDLGGWHGDAFDIW	IGH	2	None	
10	1188	CAKDISDDFWSGSFDYW	IGH	17	None	
10	1189	CARGAVARQPFDYW	IGH	19	None	
10	1190	CATGSPIQLWEYYYYYYMDVW	IGH	2	None	
10	1191	CARESWGRDFDPW	IGH	27	None	
10	1192	CAKEAAALYYYDSSGYYGTDYNWFDPW	IGH	2	None	
10	1193	CARGPPWYTTDRGGELRGYFVFW	IGH	12	None	
10	1194	CAKDTEAVAGSDDAFDIW	IGH	6	None	
10	1195	CTRDGRQYSDSSGYLNYAFDIW	IGH	28	None	
10	1196	CTTDQSPYDYVWGSYRPLDAFDIW	IGH	2	None	
10	1197	CARDSHYGSGRYFDSW	IGH	7	None	
10	1198	CATKMGTYYYDSSGYYSLDYW	IGH	7	None	
10	1199	CARGRYSSSWYSPAVNWFDPW	IGH	9	None	
10	1200	CAHITIGYITMVRGGAPTFDYW	IGH	4	None	
10	1201	CARVMYYDFWSGYYSSEDGMDVW	IGH	2	None	
10	1202	CARSPPTALYSSSWFHFDYW	IGH	3	None	
10	1203	CARLPSTISPTDNWFDPW	IGH	3	None	
10	1205	CAREKLTMIFDYW	IGH	8	None	
10	1206	CARAPSPTYFFGNSGYHQFDSW	IGH	2	None	
10	1207	CAKGWTDYGTIFGVVIIGPNYFDYW	IGH	2	None	
10	1208	CARAPIIHDVNSYYSYFDSW	IGH	2	None	
10	1209	CARLTSDNRKYFWFDPW	IGH	2	None	
10	1210	CARLNSSSFLSPNDYW	IGH	2	None	
10	1211	CARGKYSGNFYSFDPW	IGH	858	None	
10	1212	CARDPSYTSGWSPHWYFDLW	IGH	10	None	
10	1213	CTRRGMAYYYDSSGYYGGYYFDYW	IGH	2	None	
10	1214	CAHRRGTVTTPFFDYW	IGH	2	None	
10	1215	CAKDKMEGIAAAWYYFDYW	IGH	2	None	
10	1216	CAKEIESGYDLVPYYYYGMDVW	IGH	88	None	
10	1217	CATEPRGTQLWNKDFNYW	IGH	2	None	
10	1218	CARSRYYDFWSGYPADGYYMDVW	IGH	4	None	
10	1219	CARDPVSRCSDGSCSSTYYFDYW	IGH	3	None	
10	1220	CATTDYGDYEDAFDIW	IGH	7	None	

10	1221	CSRPKAATQVLNAFEIW	IGH	2	None	
10	1222	CARGVLLWFGEEYNWFDPW	IGH	29	None	
10	1224	CARLFGLADYFYFDAFDIW	IGH	8	None	
10	1225	CATTYAVWGSYNFDYW	IGH	9	None	
10	1226	CARGSGTYYDILTGYSYGYYFDYW	IGH	4	None	
10	1227	CARVTWPRIPVFGVARGHFFDYW	IGH	4	None	
10	1228	CARHVTSVSNPLQSDYW	IGH	6	None	
10	1229	CTKDVIRFLRGVDYW	IGH	2	None	
10	1230	CARQRGIAVAGTEDWFDPW	IGH	2	None	
10	1231	CVRHMYGYIAFDYW	IGH	2	None	
10	1232	CTTDEMSDFWSGYPHDYW	IGH	24	None	
10	1233	CAKIRSSSWLLHAFDIW	IGH	2	None	
10	1234	CQAAGSSGSYYVF	IGL	4	None	
10	1235	CARDPWGTYRPPFDLW	IGH	2	None	
10	1236	CASNSHYDSSGKYIDYW	IGH	4	None	
10	1238	CARAPYDILTGYVEYYFDYW	IGH	4	None	
10	1239	CATESPRGGGDRYSYGYRTDYW	IGH	2	None	
10	1240	CARVNPPYDFWSGYLFDYW	IGH	24	None	
10	1242	CSLYLQINTLRADDTAVYYCATRGYSDAYFDYW	IGH	2	None	
10	1243	CAKDGIAAVTDYYFDAW	IGH	12	None	
10	1244	CARDHHDTYYDILTGYYPYPYYYYYMDVW	IGH	3	None	
10	1245	CARDASEYSSSSGDYYYYMDVW	IGH	10	None	
10	1246	CARHERGEMTFIAAAGTVNWFDPW	IGH	2	None	
10	1247	CVHEIDYGDYWTGGWYFDYW	IGH	6	None	
10	1248	CARDSVTDDSSGYYYSWFDPW	IGH	6	None	
10	1249	CAREFLMAGHRTYYYYMDVW	IGH	2	None	
10	1250	CARSYSGSNTRRWFDPW	IGH	2	None	
10	1251	CDWSSDVCSSDLDGTGYYLHFDYW	IGH	3	None	
10	1252	CAKVLVGWLLPNDAFDIW	IGH	3	None	
10	1253	CARYRPISGVVYAMDVW	IGH	2	None	
10	1254	CAKPPGFYGDYATRPWYFDLW	IGH	6	None	
10	1255	CARESGLGGWPYPISAFDLW	IGH	2	None	
10	1256	CANLPAAASGYMDVW	IGH	2	None	
10	1257	CAREGIWYYSSSGNYHNVPDSW	IGH	4	None	
10	1258	CAREGTVTTYPLDYW	IGH	2	None	
10	1259	CARRASPLTYYDFWVANWFDPW	IGH	5	None	
10	1260	CVKDRLAGYSNSLVKFDSW	IGH	2	None	
10	1261	CAKLISRFHDAFDIW	IGH	6	None	
10	1262	CAFEWSADGFDIW	IGH	2	None	
10	1263	CARGGGIAVADYFDYW	IGH	11	None	
10	1264	CARNREPGYNYAHYYFDFW	IGH	2	None	
10	1265	CACSGSYYGYW	IGH	6	None	
10	1266	CARDLSIAAADSEYFQHW	IGH	3	None	
10	1267	CARDFLNTAMAINYYYYGMDVW	IGH	4	None	
10	1268	CASGVGYSYAYSLDFAYW	IGH	3	None	
10	1269	CAKADNKGTLIAPSYYFDFW	IGH	3	None	
10	1270	CARDYESVELLWFGELTKSPPHFDPW	IGH	2	None	
10	1271	CARRMGGSSHFDYW	IGH	4	None	
10	1272	CMQGLQTPPRIYTF	IGK	12	None	
10	1274	CARDSQWELLDYW	IGH	53	None	
10	1275	CARYMTTVSRGSSGYAFDIW	IGH	19	None	
10	1276	CAKGADGSGWSSFDYW	IGH	10	None	
10	1277	CARVHYGSGSYYMYYW	IGH	2	None	
10	1278	CARDAQDIVLVPAALRGNYYYYAMDVW	IGH	2	None	
10	1279	CARGLKGRITMVRGVSAIDYW	IGH	2	None	
10	1280	CAKDYSTGHYFDYW	IGH	8	None	
10	1281	CAKLSGYSGYDFRAGAVAEFDYW	IGH	12	None	
10	1282	CAKTGQQLVRGYAVDYW	IGH	2	None	
10	1283	CAKDYDSSGYELYFDYW	IGH	17	None	
10	1284	CARDSSPEDSPSGPYYDAYDLW	IGH	2	None	
10	1286	CTLYLQMNSLRAEDTAVYYCAKGDGDNIPWYFDLW	IGH	4	None	
10	1287	CEQYGDSPPTF	IGK	621	C1	7K8N
10	1288	CARIILEKYDSSGYFDNW	IGH	2	None	
10	1289	CARAYYDTSAYSTLKDWYFDLW	IGH	29	None	
10	1290	CAKVGISWLGDYW	IGH	8	None	
10	1291	CAKDRLRAAMMGANNHFDYW	IGH	4	None	
10	1292	CAREVDGADDAWFDPW	IGH	3	None	
10	1293	CAKDTTFGGSVYSSNPFDSW	IGH	3	None	

10	1294	CATTRDPPAEYFQHW	IGH	2	None	
10	1295	CTTDLPVAGHYFDYW	IGH	2	None	
10	1296	CARETDRWGKWFDPW	IGH	2	None	
10	1297	CARAMGAAGFGNFDYW	IGH	8	None	
10	1298	CARRVVGASRWGGFDIW	IGH	2	None	
10	1299	CARGSNYDSSGPRPPYYYYYMDVW	IGH	2	None	
10	1301	CQSADRIGTYVEF	IGL	110	None	
10	1302	CARDWDASGWLTFGYW	IGH	2	None	
10	1303	CTRGGFLEWLFYYYYGMDVW	IGH	5	None	
10	1304	CARNGGERYFDWLSPPYYYGMDVW	IGH	2	None	
10	1305	CAKDLVPGVIGYYYGMDVW	IGH	7	None	
10	1306	CAIGYSSAWAEFFQYW	IGH	4	None	
10	1307	CAREKRGIAVYIDYW	IGH	4	None	
10	1308	CARPQRPWFGGTDAFDIW	IGH	7	None	
10	1309	CARGVSKTSMIVVVITAAMYYFDSW	IGH	2	None	
10	1310	CQSYDSSLRSYNYVF	IGL	2	C4	7JVC
10	1311	CARALNDFWSGYLGYYYMDVW	IGH	7	None	
10	1312	CARDPPLPYDYVWGSYRYTGFDYW	IGH	5	None	
10	1313	CARVYSGSDIEAHDAFDIW	IGH	4	None	
10	1314	CARDSYSHYYYDSSGPPESWVDYW	IGH	17	None	
10	1315	CAGDEGIFGVVDHYYYGMDVW	IGH	89	None	
10	1316	CARDLSRVDYGVLSGYYQGGILDYW	IGH	5	None	
10	1317	CATWGGSRNPPYYFVYW	IGH	2	None	
10	1318	CATPMYTSGWYYYGMDVW	IGH	13	None	
10	1319	CARGPGIVGAPDYYYYYMDVW	IGH	5	None	
10	1320	CAKMSKGSGYSDYW	IGH	2	None	
10	1321	CARSRLWFGEMILRRFYMDVW	IGH	2	None	
10	1322	CARGYYYYDSSGYGISDAFDIW	IGH	4	None	
10	1323	CTTDKQLSPDYW	IGH	67	None	
10	1324	CAHRPTLPYYYDSSGYGNWFDPW	IGH	4	None	
10	1325	CATDIVVPAVSRYYYYGLDVW	IGH	2	None	
10	1326	CARAERYHMITFGGVIVTDAFDIW	IGH	2	None	
10	1327	CAHRGLFDGSEYSFYFNYW	IGH	2	None	
10	1328	CVKDYGDYPNFDYW	IGH	44	None	
10	1329	CARRIGARGFDYW	IGH	14	None	
10	1330	CAREYNWNYVTVYFDYW	IGH	4	None	
10	1331	CARAGDYGDFW	IGH	4	None	
10	1332	CARDPTVTTFPPHNYYYYGMDVW	IGH	2	None	
10	1333	CAPEVTTVTNFDYW	IGH	4	None	
10	1334	CARHLNSRFDPW	IGH	2	None	
10	1335	CARDLTRNDSSGYYKPADAFDIW	IGH	2	None	
10	1336	CARGLVEQLVPWFDPW	IGH	3	None	
10	1337	CTREERRSIRPFDYW	IGH	2	None	
10	1338	CASTTEKVWFGELFLQGFDPW	IGH	2	None	
10	1339	CASLGSSTVTTYYYYGMDVW	IGH	5	None	
10	1340	CAKDDRPTTLGGYYYYGMDVW	IGH	2	None	
10	1341	CARETIVPVPASIWNMEDYFYYYGMDVW	IGH	9	None	
10	1342	CARGKILTGRPIDAFDIW	IGH	4	None	
10	1343	CARALTRSFSVDYW	IGH	5	None	
10	1344	CARHGTTIFGVVIPNWFDPW	IGH	3	None	
10	1346	CARAPYYYDSSGYRGGSDYW	IGH	11	None	
10	1347	CARDGVLLWFGESGRGAFDIW	IGH	2	None	
10	1348	CAKDYYRWWFGDHTFDYW	IGH	11	None	
10	1349	CATERRMSIAVAGTADYW	IGH	2	None	
10	1350	CARRDGYNVMYYFDYW	IGH	4	None	
10	1351	CAHAIDGNNNFDYW	IGH	2	None	
10	1352	CARGGQQLVLPPYYDYYYYGMDVW	IGH	8	None	
10	1353	CATPRGLYGDYIFDYW	IGH	7	None	
10	1354	CARAPARYSYGGIDYW	IGH	3	None	
10	1355	CARDRHDFWSGSVGWFDPW	IGH	2	None	
10	1356	CAKIGYYDSSGYPGGVDYW	IGH	3	None	
10	1358	CQPYNTYPRFTF	IGK	2	C3	7K8R
10	1359	CARLYLLLGPSTRVVASWFDPW	IGH	8	None	
10	1360	CARGMVMTNMRTFDFW	IGH	2	None	
10	1361	CASTRSEWELQGHDAFDIW	IGH	5	None	
10	1362	CARSSSLFSSFFDSW	IGH	4	None	
10	1364	CATVAQAGAYYYDSSGYYPKDNWFDPW	IGH	2	None	
10	1365	CAKLASWFGELSGMSGDYW	IGH	2	None	

10	1366	CAKDIDWGSEGFGPFDYW	IGH	4	None	
10	1367	CARLQLELLRDGAFDIW	IGH	2	None	
10	1368	CAKDQAIDCSSTSCYLGFDYW	IGH	4	None	
10	1369	CARHAGHLWKHFDYW	IGH	10	None	
10	1370	CARVFAARPDYYLDYW	IGH	2	None	
10	1371	CAGGQLWFDDAFDIW	IGH	7	None	
10	1372	CAKVKPVGDQWELLGGAAYYYYMDVW	IGH	2	None	
10	1373	CAHHSSGWYGDHFDYW	IGH	14	None	
10	1374	CARGQLWLGLMVSDAFDIW	IGH	2	None	
10	1375	CAAVDNGDSSGYQFDYW	IGH	10	None	
10	1376	CAREAEITIFGVVIMGTLPDYW	IGH	2	None	
10	1377	CAHRRGYDYAIFGYW	IGH	2	None	
10	1378	CARDLQYPSGYDYVWGSYRYIFAAFDIW	IGH	10	None	
10	1379	CARTPDYHFDYW	IGH	110	None	
10	1380	CAREYGVGSGWRFDYYYYGMDVW	IGH	9	None	
10	1381	CAKSGTTMIVVVIDDAFDIW	IGH	13	None	
10	1382	CARDRALESPDIVVVPAAILSCWFDPW	IGH	2	None	
10	1383	CARGLIYSYGLGVYYYYGMDVW	IGH	2	None	
10	1384	CARVSVTMIVVVITPYYYYYMDVW	IGH	2	None	
10	1385	CARDFQAVLRFLEWNDAFDIW	IGH	2	None	
10	1386	CLLSYSGAWVF	IGL	1012	None	
10	1387	CARERLPHQHFDYW	IGH	8	None	
10	1388	CAKAWGGDYDQDPYYYYGMDVW	IGH	18	None	
10	1389	CAGSEGSGYQGYFDPW	IGH	2	None	
10	1390	CTTDPGCSSTSCYYYYYYMDVW	IGH	4	None	
10	1392	CAKGTAIAMVRGVLQPFDPW	IGH	3	None	
10	1393	CARFRIGTQESDQYYYDSSGYYYYGMDVW	IGH	5	None	
10	1394	CQQYNSYYGTWAF	IGK	2	C3	7K8R
10	1395	CARAHKQQLVFFHFDYW	IGH	2	None	
10	1396	CAKSIRPIIGDAFDIW	IGH	11	None	
10	1397	CAKLCSSTSCFIDYW	IGH	4	None	
10	1398	CARLRRLYRAFDIW	IGH	3	None	
10	1399	CVPRTVYAFNIW	IGH	2	None	
10	1401	CAKDSGGGDFWSGYSYYFDYW	IGH	8	None	
10	1402	CARLTDDSSSSEELDYW	IGH	5	None	
10	1403	CLRAEDTAVYYCASIAAAGREYYYYYGMDVW	IGH	2	None	
10	1404	CARDAGSSSWDYYFDYW	IGH	26	None	
10	1405	CDWSSDVCSSDLMDSLRPEDTAMYYCARETIVVDDSRFDYW	IGH	5	None	
10	1406	CARLPQTKRGNWFDPW	IGH	2	None	
10	1407	CASPLLPYYYDSSHAFDIW	IGH	3	None	
10	1408	CARAPVSNLAFDIW	IGH	7	None	
10	1409	CATRFAYGDYRGPIDYW	IGH	5	None	
10	1410	CANGPNWNDPGLDAFDIW	IGH	5	None	
10	1411	CAKLMDIVATDYW	IGH	4	None	
10	1412	CALMNGVDTAMDDYYFDYW	IGH	2	None	
10	1413	CARAVEDSRGFFSYYFDSW	IGH	3	None	
10	1414	CAREYIWGSYGREAFDMW	IGH	2	None	
10	1415	CASSRIQLWLGGVFDYW	IGH	5	None	
10	1416	CAKGPGGGYSSSWYWFDPW	IGH	2	None	
10	1417	CAHRHSWTLSYFDYW	IGH	77	None	
10	1418	CARSSPSYYDFWSGTLFIPAFDIW	IGH	8	None	
10	1419	CATTFPLSSIGGGWELLKGVPDYW	IGH	2	None	
10	1420	CARDYSSKEYYYYYGMDVW	IGH	8	None	
10	1421	CARASRIAVPRGMCAFDIW	IGH	2	None	
10	1422	CARYQEGVVVVPTPDHSGGFDCW	IGH	4	None	
10	1423	CANDGPGYYDYW	IGH	3	None	
10	1424	CARVCMVRGVIITNEICDYYYYGMDVW	IGH	2	None	
10	1425	CAGKEGKTSGYYYFDYW	IGH	3	None	
10	1426	CAREGMPTIYYFDYW	IGH	2	None	
10	1427	CARLVLVVYDKETNWFDPW	IGH	23	None	
10	1428	CATLPYSTSSSPYFDYW	IGH	2	None	
10	1429	CASERSSSSGWTFDYW	IGH	11	None	
10	1430	CAKMGGYLGYCSGGSCYGPDYW	IGH	2	None	
10	1431	CATSRVVPPYSNWFDPW	IGH	3	None	
10	1432	CARSLGGTGEVPGGGDWFDPW	IGH	39	None	
10	1433	CARASTGSYASAFDIW	IGH	9	None	
10	1434	CAKERVSRFPLSGVGPQGENDYW	IGH	3	None	
10	1435	CANLGIAVAGAAFDIW	IGH	12	None	

10	1436	CARDLGEVYDFWSGYETLGVFSLDYW	IGH	2	None	
10	1437	CARHSYYGSGSYYNVDWYFDLW	IGH	3	None	
10	1438	CATLTYSSSWFHYYFDYW	IGH	11	None	
10	1440	CARQGFGSGAHDYW	IGH	2	None	
10	1441	CARETRSEYDFWSGYYTGGYFDYW	IGH	2	None	
10	1442	CQLRDNWPRVYTF	IGK	6	None	
10	1443	CARTKSGAYSSSLDYW	IGH	4	None	
10	1444	CAGGSPIDGHQSDYW	IGH	18	None	
10	1445	CAKEYHYDSSGYFFDYW	IGH	2	None	
10	1447	CATQSLGIAAAGVDYW	IGH	5	None	
10	1448	CARVSGGYYYDSSGADAFDIW	IGH	2	None	
10	1449	CANTGYISGWYIDYW	IGH	2	None	
10	1450	CAKVGDYDSFQGHFDSW	IGH	2	None	
10	1451	CARVDSGLTMVRGVIRKTFDYW	IGH	3	None	
10	1452	CARDVRYYYGSGLFDYW	IGH	2	None	
10	1453	CARQYDSSGYYTDDYW	IGH	8	None	
10	1454	CAREWFSVTVVRGLPDFYQYYLDVW	IGH	2	None	
10	1455	CATFRYSVTTWSWFDPW	IGH	2	None	
10	1456	CARSYSSGWYVGFGLDYYYYMDVW	IGH	2	None	
10	1457	CARAYGDYLYNFDYW	IGH	10	None	
10	1458	CAKDVSIAAASQTDDYW	IGH	2	None	
10	1459	CATGLSDFWSGYYNYW	IGH	10	None	
10	1460	CATYPAYYDILTGPSPLDYW	IGH	4	None	
10	1461	CATSFAIVGANPYFDYW	IGH	4	None	
10	1462	CAKSQKVGATLGRAFDIW	IGH	2	None	
10	1463	CARERITIFGVVTSGTYNWFDPW	IGH	7	None	
10	1464	CAREVSEWFGELLPDYW	IGH	4	None	
10	1465	CAREVRRQWLMIIQADGFDVW	IGH	3	None	
10	1466	CAAESSGSYYKSYYYYGMDVW	IGH	3	None	
10	1467	CARGGLLRYFDWLLHHYFDYW	IGH	2	None	
10	1468	CARDRAGYSSGWYSFDFDYW	IGH	6	None	
10	1469	CARESTPSAVAGTSGFDPW	IGH	2	None	
10	1470	CALPISDTAMYYCARPPHGYSSGWYVRDYW	IGH	2	None	
10	1471	CAKDGMRYNYNDGPYFDYW	IGH	5	None	
10	1472	CARDPQPTDIVVVVAATPLYYYYMDVW	IGH	2	None	
10	1473	CAREGDTFDYGSGNDYW	IGH	17	None	
10	1474	CAKARYCTNGVCFARPFDYW	IGH	2	None	
10	1475	CARPKYCSGGSCYFPSDAFDIW	IGH	4	None	
10	1476	CAREGIAAAGICLDYW	IGH	36	None	
10	1477	CAKDKRPDGIWSFDNW	IGH	2	None	
10	1478	CARNYGTGIYNWFDPW	IGH	2	None	
10	1479	CARDRDSNYIWWFDPW	IGH	10	None	
10	1481	CARDTQQWLVIPAFDIW	IGH	4	None	
10	1482	CATVYAIFLASRPFDYW	IGH	40	None	
10	1483	CAKEKELLYGGYDYW	IGH	5	None	
10	1484	CQQRSNWPPGTSPTF	IGK	7	C1	7BZ5
10	1485	CARSGDSSSWSRQYFQHW	IGH	10	None	
10	1486	CASFVDTGTYYEIYFDYW	IGH	4	None	
10	1487	CARDPPRTVTTFWWYFDLW	IGH	2	None	
10	1488	CARGEYDYVWGSYLTTVDYW	IGH	3	None	
10	1489	CQLYRSSPTWTF	IGK	10	None	
10	1490	CARDSSAGYSSSWSSYYYYYYMDVW	IGH	2	None	
10	1491	CARVGLGYYDFWSGQQAWFDPW	IGH	4	None	
10	1492	CARDGGGYSSSWNPLFNWFDPW	IGH	5	None	
10	1493	CATASGEGSPNWFDPW	IGH	48	None	
10	1494	CARDPGDGYNSANFDYW	IGH	21	None	
10	1495	CARGTPTDMSSYYYYMDVW	IGH	2	None	
10	1496	CARQSPDYDRHFDYW	IGH	2	None	
10	1498	CTSPPTNDYGDYVVWDYYYYYGMDVW	IGH	2	None	
10	1499	CATETSTMTPWFDHW	IGH	3	None	
10	1500	CAKDTTVSPFDIW	IGH	4	None	
10	1501	CARESPTSRHSSSLPQDWFDPW	IGH	2	None	
10	1502	CARHFEDLLSGFDYW	IGH	9	None	
10	1503	CAHRVMMAFDYW	IGH	6	None	
10	1504	CARLEFGEAKYQLLLLTVTGAFDIW	IGH	2	None	
10	1505	CARHYYGSGSYYNLGWFDPW	IGH	2	None	
10	1506	CAKAGRIVVVITGDYFDYW	IGH	7	None	
10	1507	CATDTALPGYYTGTVYNWFDPW	IGH	2	None	

10	1508	CARSRITMIVVVEAFDYW	IGH	24	None	
10	1509	CAKLQGPKWSPLFPDAFDIW	IGH	8	None	
10	1510	CARDPFYDSSIRYYYYYYYGMDVW	IGH	4	None	
10	1511	CAKDIQPLTTMTTYGFDFW	IGH	4	None	
10	1512	CAHRLYFSDGSAQWGYYDYW	IGH	3	None	
10	1513	CTRYSVSYYYYGMDVW	IGH	2	None	
10	1514	CARDAPNCGGDCYTESFFDYW	IGH	17	None	
10	1515	CASEEVYCSGGSCHWFDPW	IGH	8	None	
10	1516	CARWINPQQLLYNWFDPW	IGH	26	None	
10	1517	CARGRPDHPLSMYRGSYLDYW	IGH	2	None	
10	1518	CATSPALIVGAISLDYW	IGH	5	None	
10	1519	CARGAHDYSYGPADYW	IGH	2	None	
10	1520	CAKDLLGEWELRNWFDPW	IGH	2	None	
10	1521	CARHELWFGELSHIDYW	IGH	3	None	
10	1522	CARGAPTWGRGKSFGYW	IGH	2	None	
10	1523	CARDAPELIYDFWSGYYIPYYMDVW	IGH	2	None	
10	1524	CARGSGYCSGGSCWGGWFDSW	IGH	2	None	
10	1525	CAKVPPDWDYFDDW	IGH	2	None	
10	1527	CATVYAVRGVYPHGDYW	IGH	4	None	
10	1528	CARYSRVYYYYGMDVW	IGH	170	None	
10	1529	CAKGGYSDWVPYYFDYW	IGH	3	None	
10	1530	CARGYYDSSGYYYEGWSFDYW	IGH	2	None	
10	1531	CAKGSYDSSGYYSPQVDYW	IGH	2	None	
10	1532	CARPHSGSYYSWFDPW	IGH	40	None	
10	1534	CTTDPSVVVVAASYYYMDVW	IGH	2	None	
10	1535	CAKSGYSGYDPYLYYFDYW	IGH	2	None	
10	1537	CARQENYYDSSGYYYSWFDPW	IGH	3	None	
10	1538	CAREANYGNIGGNNGAFDIW	IGH	6	None	
10	1539	CARDSTSSWLGIPDGGYFDYW	IGH	3	None	
10	1540	CAHRPTYYDFWSGDTNWFDPW	IGH	6	None	
10	1541	CQVWDSYTEIF	IGL	18	None	
10	1542	CAKNAENIVGEVLDYW	IGH	4	None	
10	1543	CARNYVHLWGQFNRFDPW	IGH	2	None	
10	1544	CARPQGTTYYDFWSGIYYYYGMDVW	IGH	2	None	
10	1545	CARDRGTVISPNYYYYMDVW	IGH	2	None	
10	1546	CAHRPGIAAAGTGVFDYW	IGH	2	None	
10	1547	CASMPYSSSFGGYYYYGMDVW	IGH	5	None	
10	1548	CARDFPPLNYYDSSGYDGRTDYW	IGH	15	None	
10	1549	CAHRRRDGFNSFDYW	IGH	6	None	
10	1550	CAKSPGRGSYSPGGDYW	IGH	2	None	
10	1551	CARDGAYSNQITGGYYYYYMDVW	IGH	9	None	
10	1553	CAKERYSVQLWSEPFDYW	IGH	2	None	
10	1554	CARARSKYYDFSNWFDPW	IGH	2	None	
10	1555	CARDPHPDYDFWSGLYYYYMDVW	IGH	7	None	
10	1556	CARDGHYVWGSYRPKWDLDYW	IGH	3	None	
10	1557	CAKAGYSSGWEYSFDMW	IGH	2	None	
10	1558	CATTRSMIVVVGLFDYW	IGH	2	None	
10	1559	CSSTSCYWGYMDVW	IGH	2	None	
10	1560	CARPVNVAVADFDFDYW	IGH	2	None	
10	1561	CARGDEFCHDSGCYERWFDPW	IGH	5	None	
10	1562	CAGATVTDNGEYNWFDPW	IGH	2	None	
10	1563	CATLGGYYYDSSGYYVYW	IGH	2	None	
10	1565	CARIGGDGGTYQFAYW	IGH	2	None	
10	1566	CARDRVSSGYSTYYMDVW	IGH	2	None	
10	1568	CARNVLRFLELNIPHLEDAFDIW	IGH	5	None	
10	1569	CATWTYGDSYYFDYW	IGH	3	None	
10	1570	CARDDVIAAAGPNDYW	IGH	11	None	
10	1571	CTTDAYSHAEVFDIW	IGH	8	None	
10	1572	CARYGWNAADDAFDIW	IGH	15	None	
10	1573	CAMPRMKPHTKNDAFDVW	IGH	2	None	
10	1574	CAKHFWSGYSSQDYW	IGH	4	None	
10	1576	CARPSHWPYFDRW	IGH	5	None	
10	1577	CARERGYSSSWSSYYFDYW	IGH	19	None	
10	1578	CAKDSWYYDFWSGYIAPKVYYYYYMDVW	IGH	4	None	
10	1579	CTTDTVAAILFWRSYAEYFQHW	IGH	2	None	
10	1580	CAREGPPYYYDSSGYPGLDYW	IGH	2	None	
10	1581	CATGPPYDSSGWFDPW	IGH	36	None	
10	1582	CAKEKILTGGSFDYW	IGH	2	None	

10	1583	CASIGRDSSGYYGVSAFDIW	IGH	2	None	
10	1584	CARHEIWFGDLLYFDYW	IGH	10	None	
10	1585	CQEAKSFPPLF	IGK	3	C1	7BZ5
10	1586	CAKADEWGRSDYW	IGH	9	None	
10	1587	CARDLYYYDSSGSYYQHSHSIDYW	IGH	6	None	
10	1588	CASLTLSDYDFWSGYPNYYFDYW	IGH	2	None	
10	1589	CAHKSSKSCTTYSCYVANWFDPW	IGH	5	None	
10	1590	CVKDLIFYHDDTAFDSW	IGH	2	None	
10	1591	CARENYYGSGYHDAFDIW	IGH	2	None	
10	1592	CAREVATIEEAYFFDYW	IGH	6	None	
10	1593	CARDCTSPTCNDAFDIW	IGH	3	None	
10	1594	CARDLGSSDFWSGSSLRRGFDPW	IGH	4	None	
10	1595	CAKDLSLRFLEWLPPNYFDYW	IGH	4	None	
10	1596	CAKLPDSSGYFFDYW	IGH	36	None	
10	1597	CARENGRYSYGAADYW	IGH	3	None	
10	1598	CARGMLKYYDFWIPVGSWFDPW	IGH	5	None	
10	1599	CARDNCSSASCYKFYYYYMDVW	IGH	18	None	
10	1600	CARCIAVAGTWSFSLRYDYYGMDVW	IGH	2	None	
10	1601	CATGPVLEWPHWFDPW	IGH	2	None	
10	1602	CARDGQYYDFWSGYSPQYYYYYYYMDVW	IGH	5	None	
10	1603	CAKDIVGVGRDGYNALDYW	IGH	2	None	
10	1604	CAKDLGSGYYFSPLPYDYW	IGH	2	None	
10	1605	CAKAHYYDSSGYYSIVAFDIW	IGH	3	None	
10	1606	CARGGDSLDIVVVVAATPFDYW	IGH	4	None	
10	1607	CAVHLTSSYGVDYW	IGH	5	None	
10	1608	CAKPKDIVLVVYAANWYFDLW	IGH	4	None	
10	1609	CARPYYYDSSGNPQYYFDYW	IGH	2	None	
10	1610	CAGDVTLAMINRKGFDYW	IGH	2	None	
10	1611	CAVFPFGDYYDSSGYYLHSPDYW	IGH	2	None	
10	1612	CARERAYDSSGYYGYYFDYW	IGH	32	None	
10	1613	CARQGDYVWGSYRFDYW	IGH	15	None	
10	1614	CAKLVVVAPGSDYW	IGH	2	None	
10	1615	CARAPDYYDSSAHGYEYAFDIW	IGH	8	None	
10	1616	CARGSYDGGTQWGLLILGVPTGYFDYW	IGH	4	None	
10	1617	CARGPRGYCSSTSCYRGHYFDYW	IGH	2	None	
10	1618	CAKDTGFPYGDYGGVDYW	IGH	4	None	
10	1620	CARATYSYDSNFYPTFDYW	IGH	2	None	
10	1621	CALPISAVYYCARSVRGDSSGLGDSFDVW	IGH	2	None	
10	1622	CARGLREAILGVVTYPSTFDYW	IGH	8	None	
10	1623	CARVSPLADGYNLDYW	IGH	35	None	
10	1624	CAKEYRSSSTHYYYYYNMDVW	IGH	6	None	
10	1625	CARLVVGVDFWSGFKNPPFAMDVW	IGH	2	None	
10	1626	CAKLGLEQGDAFDIW	IGH	8	None	
10	1627	CAKDPKYYDILTGYYIAPSYYYYYYMDVW	IGH	7	None	
10	1628	CASGSLVTVTTLRPYYYYYMDVW	IGH	4	None	
10	1629	CATGIHSSINLFFNYW	IGH	4	None	
10	1630	CARSPGSPYYYYYYGMDVW	IGH	10	None	
10	1631	CATESGNYSFDNAFDIW	IGH	4	None	
10	1632	CAREVSRYSSSWQNLGGYDYW	IGH	2	None	
10	1633	CTTWHNWNYEGAFDIW	IGH	3	None	
10	1634	CTTDQTLRGGVITTDYYYYGMDVW	IGH	12	None	
10	1635	CARNYDSGRYWPIGAFDIW	IGH	6	None	
10	1636	CAKDKSPQLLRWGWFDPW	IGH	4	None	
10	1637	CAKGRGGYYSYFDYW	IGH	2	None	
10	1640	CAKDSHGGTYDYW	IGH	4	None	
10	1641	CQSADISGTYPPVLF	IGL	53	None	
10	1642	CATALGHYGDYLDAFDIW	IGH	2	None	
10	1643	CARDLSTIFGVVREAHTPSDAFDIW	IGH	10	None	
10	1644	CARDKVSIVAAIGPFDCW	IGH	2	None	
10	1645	CAKAPWIQLGDEDGYFDYW	IGH	3	None	
10	1646	CATLPTTVTTDDAFDIW	IGH	9	None	
10	1647	CARHGMYDILTGSSTRYFDYW	IGH	2	None	
10	1648	CARLNALEEEYYFDYW	IGH	2	None	
10	1649	CARDWGTDYDFWSGYSRYYYYYMDVW	IGH	25	None	
10	1650	CDWSSDVCSSDLHYSWFDTW	IGH	18	None	
10	1651	CLQHYSYHWTF	IGK	6825	None	
10	1652	CAKVGRLAVAGTGTETDYW	IGH	2	None	
10	1653	CARPTMVLTGDDAFDIW	IGH	4	None	

10	1654	CVRDQWWGGALFDPW	IGH	4	None	
10	1655	CAREGELASTAMVTGFDYW	IGH	6	None	
10	1656	CARVSSGWQTDYYYYYMDVW	IGH	7	None	
10	1657	CARDRFVAIPGSPVLAWFDPW	IGH	12	None	
10	1658	CAKDRGSTYSSSWYYFDYW	IGH	18	None	
10	1659	CASLRSPYGGNSDYW	IGH	12	None	
10	1660	CQQRSIWPPYMYSF	IGK	23	C1	7BZ5
10	1662	CAKTGGDSSSIEEDPFDYW	IGH	2	None	
10	1664	CARAQRGSTMIVVGYDYW	IGH	2	None	
10	1665	CARDLLYSSGWYKIDYW	IGH	4	None	
10	1666	CARDLYYDSLLDYW	IGH	9	None	
10	1667	CARERGGGWTAAFDYW	IGH	2	None	
10	1668	CAKDLRYYDFWSGYIGGSEARPYYYYYYGMDVW	IGH	2	None	
10	1669	CAKDISSYDSSGSDYW	IGH	17	None	
10	1670	CASGPPYYDFWSGYYPPIDYW	IGH	4	None	
10	1671	CARRVVSYDYVWGSYRWGWFDPW	IGH	2	None	
10	1672	CARYPYYYDSSGQKGAFDYW	IGH	12	None	
10	1673	CARDRARYYYDSSGYPLPDYW	IGH	8	None	
10	1674	CARALRRTRFFYYYYMDVW	IGH	3	None	
10	1675	CQLYGGSPPTFTF	IGK	6	C1	7K8N
10	1676	CASSELGYSSSSLGSPYYYYYVMDVW	IGH	2	None	
10	1677	CARLYDSRVYYYGMDVW	IGH	14	None	
10	1678	CTRTPSSSHGGYYFDYW	IGH	4	None	
10	1679	CAKDQRGYSYGWSYYYFDYW	IGH	2	None	
10	1680	CAGFGYSGYDFLYYMDVW	IGH	2	None	
10	1681	CATGGYCSSTSCPSDYYYGMDVW	IGH	2	None	
10	1683	CVHLYYDSFGGFDYW	IGH	6	None	
10	1684	CAKTPENGYDSSGYYFDYW	IGH	4	None	
10	1685	CARETFVADIVVVRDAADYW	IGH	5	None	
10	1686	CARDGRANLNYDFWSGYPNGGWFDPW	IGH	2	None	
10	1687	CASPPVYSEYYFDYW	IGH	7	None	
10	1688	CARNRALITIPNFDYW	IGH	16	None	
10	1689	CARRVGLRYFDWFKNNWFDPW	IGH	2	None	
10	1690	CARGTVGATTLGFLVYYYYGMDVW	IGH	5	None	
10	1691	CATIPDIAAADWYFDLW	IGH	2	None	
10	1692	CARNLLWFGELLFPDNAFDIW	IGH	2	None	
10	1693	CARVYDSTAFDVW	IGH	5	None	
10	1694	CAKDLMYAVPILGGIAAAGLFDYW	IGH	22	None	
10	1695	CARLSVPFTVTTLSAFDIW	IGH	7	None	
10	1696	CATNLISSGWYHYYW	IGH	2	None	
10	1697	CEKGREYWSDTCFPNDRDVDYW	IGH	2	None	
10	1698	CAIALPEDITMVRGVAPFDYW	IGH	2	None	
10	1699	CTRVSFSGDTYFQHW	IGH	2	None	
10	1700	CARHNAAVSGTVYYYYYMDVW	IGH	11	None	
10	1701	CARDGTYYDFWSGYLAPSIPHYYYYYGMDVW	IGH	11	None	
10	1702	CARRNFDEINYFDPW	IGH	2	None	
10	1703	CARLDDVDTAMVMDYW	IGH	4	None	
10	1705	CAHRPPGWLRPYFDYW	IGH	30	None	
10	1706	CAKDIRPIAVAGYYFDYW	IGH	39	None	
10	1707	CAKDISGYSYGCLDYW	IGH	10	None	
10	1708	CVRDGGYYDGSGFYSDHGAFDIW	IGH	2	None	
10	1709	CVCGYSYGYDPPSNQNDYW	IGH	2	None	
10	1710	CARVPWYYYDSSGYWPGGYYFDYW	IGH	4	None	
10	1711	CAKGSIAVAGTFSDYW	IGH	8	None	
10	1713	CARGPERELFRYYYYYMDVW	IGH	2	None	
10	1714	CAHRHETWSMDDAFDIW	IGH	2	None	
10	1715	CFQATHWPRAF	IGK	17	None	
10	1716	CARGAGDIYYFDYW	IGH	71	None	
10	1717	CAKESKAVYTNSWSHYDAFDVW	IGH	2	None	
10	1719	CARDRGYYYDSFARIGAFDIW	IGH	4	None	
10	1720	CASTRGYTGYILQSYFDNW	IGH	7	None	
10	1721	CQQGSSYPPVFTF	IGK	23	C1	7BZ5
10	1722	CARDPWPIAARPYYFDYW	IGH	2	None	
10	1723	CARGHSSGAVYYYYYMDVW	IGH	2	None	
10	1724	CATDGLFLRWYFDYW	IGH	10	None	
10	1725	CYSARDNDLRVF	IGL	7376	None	
10	1726	CARESFSPYSSSSGTFDNW	IGH	59	None	
10	1727	CAREDLYNISPYVDAW	IGH	9	None	

10	1728	CAKDLRDDYGGWFDPW	IGH	3	None	
10	1729	CARLSNWNYEGSGIFDYW	IGH	2	None	
10	1730	CARLFRYYYDSSGPDYW	IGH	4	None	
10	1731	CARVELPTHNYYYYYGMDVW	IGH	8	None	
10	1732	CTYTSYGPWRAADYW	IGH	3	None	
10	1733	CAKRGNPTTITVFDSW	IGH	2	None	
10	1734	CAKEGTGSHAIDYW	IGH	15	None	
10	1735	CARVTARPVDYFDYW	IGH	2	None	
10	1736	CASGDTFGGLFSGFTYW	IGH	394	None	
10	1737	CAGYYTIGYYFDYW	IGH	44	None	
10	1738	CDWSSDVCSSDLYDSGRNPQIDYW	IGH	2	None	
10	1739	CARALTYYYDSSGYATGVDYW	IGH	3	None	
10	1740	CARDRRDGYQAYYFDSW	IGH	4	None	
10	1741	CAKAPMGLTVYFDYW	IGH	4	None	
10	1742	CAREVPPLFGTHWFDPW	IGH	4	None	
10	1743	CAKFPVAGNAFDIW	IGH	53	None	
10	1744	CARDLEGSTPNPDDMDVW	IGH	4	None	
10	1745	CAREHQLYYDILTGYYYYYYMDVW	IGH	3	None	
10	1746	CHVWDSSSDRWLF	IGL	4	None	
10	1747	CARHLRHSTFIGSYYMSYYMDVW	IGH	2	None	
10	1748	CARHPPDYVWGSYRYQSWFDPW	IGH	4	None	
10	1749	CQQYKKSPPGAAF	IGK	2	C1	7K8N
10	1750	CARVGRRDNSSSDWNYYFDYW	IGH	2	None	
10	1751	CATGPPLRFWDWFDPW	IGH	65	None	
10	1752	CAKGTNYYDSSGYKDDYYYYGMDVW	IGH	5	None	
10	1753	CARGGFEMITFGGDPYFDYW	IGH	11	None	
10	1754	CAKQGRNSGSYFDYW	IGH	4	None	
10	1755	CTTDSRYDFWSGRVFDYW	IGH	7	None	
10	1756	CTSSGWYLGWFDPW	IGH	6	None	
10	1757	CAKLLSGIAVAGTVFDYW	IGH	3	None	
10	1758	CTTDQVGLYYDILTGNPGFDPW	IGH	2	None	
10	1760	CAHKVLVGATAFDIW	IGH	3	None	
10	1761	CAKFQDPQARYYYDSSGYNDYW	IGH	4	None	
10	1762	CARDHGFDSSGFYADYW	IGH	2	None	
10	1763	CARHAGGGYDFTQSTPPTFNYYYMDVW	IGH	2	None	
10	1764	CARDWTWSGYYLEYFQHW	IGH	2	None	
10	1765	CARDALNYGDFGGIDWYFDLW	IGH	15	None	
10	1766	CARVPGDYVWGSYLVPNYFDYW	IGH	5	None	
10	1768	CARHERRWLQLSVSWFDPW	IGH	42	None	
10	1769	CAKDLKQSWHDYVWGIINLDAFDIW	IGH	2	None	
10	1770	CARDGVSYSSGRSVGFDYW	IGH	2	None	
10	1771	CAKDVRCSDISCYYGFDYW	IGH	8	None	
10	1772	CARVSRYSSGWYDMAVIAPYSYMDVW	IGH	2	None	
10	1773	CAKSRGWELPQLPYFDYW	IGH	2	None	
10	1774	CAKVRGSGYSLVDYW	IGH	12	None	
10	1775	CARDYASSGYYAPDVYWYFDLW	IGH	11	None	
10	1776	CARGTKGNFDWLFKTDYYYYMDVW	IGH	2	None	
10	1777	CARGPRTPMVEIDYW	IGH	2	None	
10	1778	CVKDKACRGGSCGTPFDYW	IGH	4	None	
10	1779	CAKGYRQDVPNTRCFDFW	IGH	4	None	
10	1780	CAKDNIVVVTAILTYNWFDPW	IGH	3	None	
10	1781	CARAGVGATLDINAFDIW	IGH	11	None	
10	1782	CASNDIVATMTQNYYYYYGMDVW	IGH	3	None	
10	1783	CARVPIWSIWSGYLDYW	IGH	15	None	
10	1784	CAKEYYDSSRSFVHVFDIW	IGH	2	None	
10	1785	CAKVSYLSTASSLDYW	IGH	27	None	
10	1786	CAHRLKGVGASWNWGDFDYW	IGH	12	None	
10	1787	CAREPQHYDFWSAHTNRLHAMDVW	IGH	4	None	
10	1788	CATGPPYSSGWPEFSNWFDPW	IGH	2	None	
10	1789	CARGQNYNWNYVGFDYW	IGH	6	None	
10	1790	CARHNTPWLVRTYYFDYW	IGH	2	None	
10	1791	CASPGGFLEWLLGPTDAFDIW	IGH	2	None	
10	1792	CARRKQGDSYPPYFDYW	IGH	3	C1	7BZ5
10	1794	CARDQRATVIYDAFDIW	IGH	9	None	
10	1795	CARGPLADCGVDCHVYDYW	IGH	2	None	
10	1796	CTTDRVWMYSSGWYVYWDYW	IGH	14	None	
10	1797	CAKGDTAMVTTMFDYW	IGH	4	None	
10	1798	CARGQIPIVFHWFDPW	IGH	2	None	

10	1799	CARDRSLKRYCSGGSCLMGYYYYYYMDVW	IGH	2	None	
10	1800	CARVEVIAVAGYYYYYGMDVW	IGH	12	None	
10	1801	CQQSDIMPITF	IGK	14	None	
10	1802	CATTTVITLGGWFDPW	IGH	5	None	
10	1803	CARLFNAHAGYFDYW	IGH	6	None	
10	1804	CAKDIRRFGELLKPPYYYYYYMDVW	IGH	2	None	
10	1806	CAKGEFGSSSWQDWYFDLW	IGH	2	None	
10	1807	CARVPFPYSSSWTRPYYYYGMDVW	IGH	2	None	
10	1808	CARLLVVTRDFDYW	IGH	8	None	
10	1809	CARWGGTYSHWYFDLW	IGH	3	None	
10	1810	CAKSDGTAADFDYW	IGH	8	None	
10	1811	CARKKWLAYAFDIW	IGH	2	None	
10	1812	CALPICPFALERYSYMDVW	IGH	3	None	
10	1813	CAKDQSYSYGETYFDYW	IGH	4	None	
10	1814	CQHYGNLPLRVTF	IGK	23	C3	6XDG
10	1815	CARVEMDFWSDGEAFDIW	IGH	16	None	
10	1816	CARDPPPYSSSWSHFDYW	IGH	4	None	
10	1817	CVKDECSTTDCHFGFYTYMDVW	IGH	2	None	
10	1818	CAKDTYDSSGYVTYW	IGH	4	None	
10	1819	CAKEGGVDVTQGRLNFEYW	IGH	2	None	
10	1820	CAKGPSTGGYADYW	IGH	2	None	
10	1821	CARGLLGYCSSTSCDLDYW	IGH	2	None	
10	1822	CMQGTHWPPTWTF	IGK	712	None	
10	1823	CATSRDYGDHGPFDYW	IGH	47	None	
10	1824	CARENYYDSSVGPYFDYW	IGH	16	None	
10	1825	CARDFRKYAYDLWWYPDYYFDYW	IGH	2	None	
10	1826	CAGNIVVVVAAMSAAFDIW	IGH	2	None	
10	1827	CARTAMDFSEPYYYYYYMDVW	IGH	11	None	
10	1828	CARGVSGTEYWFFDLW	IGH	2	None	
10	1829	CAKDIRSCSGGRCYFLGGEHAFDSW	IGH	9	None	
10	1830	CASTLGEYQLLFYYYGMDVW	IGH	7	None	
10	1831	CAKFGDYDFSPADYW	IGH	8	None	
10	1833	CATIGGYSYGHDAFDIW	IGH	6	None	
10	1834	CARMWVDCSGGSCFYWFDPW	IGH	2	None	
10	1835	CARDNTHWSVDYW	IGH	32	None	
10	1836	CAKINWNDEGSLGDYW	IGH	2	None	
10	1837	CQQYDNLPPTGNLITF	IGK	6	C3	6XDG
10	1838	CARTAIPARPGFAMDVW	IGH	2	None	
10	1839	CTTDGAKLRFLEWLLDFDYW	IGH	6	None	
10	1840	CLSADSRGTHPVF	IGL	3	None	
10	1841	CAKTLTVGSLTGPFDYW	IGH	2	None	
10	1842	CARDNHHAANSFDYW	IGH	2	None	
10	1843	CAKEGYIAARHIVLDYW	IGH	2	None	
10	1844	CAKDLGDDYGDYGPDYW	IGH	12	None	
10	1845	CASLLSYSGSYYGYYYGMDVW	IGH	5	None	
10	1846	CARGQPQQQLGYYFDFW	IGH	4	None	
10	1847	CASPGGHYDILTGYYVGTDNYFDYW	IGH	2	None	
10	1848	CAREGDGYNGIDYW	IGH	23	None	
10	1849	CASTLFGVADYYYYYYMDVW	IGH	2	None	
10	1850	CARELNSSDQKWRDYFDYW	IGH	2	None	
10	1851	CAMYGGGVWSDYYYYYMDVW	IGH	3	None	
10	1852	CVKDTAMVHDGMDIW	IGH	2	None	
10	1853	CARINSGWTEVLDGMDVW	IGH	4	None	
10	1854	CAKLPTKSGYSYGSDAFDIW	IGH	3	None	
10	1856	CARDAANSSGWYLNYW	IGH	2	None	
10	1857	CAKGGNYFFDYW	IGH	33	None	
10	1858	CARGVYYQYYYLDVW	IGH	2	None	
10	1860	CARESGEVYSSRGWGFYYYYYYMDVW	IGH	2	None	
10	1861	CARVHGYISTWYDFDYW	IGH	2	None	
10	1863	CATGRQLWVVGWFDPW	IGH	4	None	
10	1864	CARGLPSRTPYSSAYRGAYDIW	IGH	2	None	
10	1865	CAKDTNYYDTSGTDAFDIW	IGH	2	None	
10	1866	CAAFSHGDYVDYW	IGH	2	None	
10	1867	CATDFGGPKDCSSTSCYSPFGYW	IGH	3	None	
10	1868	CGRLNKDWNYFDFW	IGH	2	None	
10	1869	CATPRSPYYYDSSGPLDYW	IGH	2	None	
10	1870	CTTDTGIGSPYSGSQEGSIAFHVW	IGH	2	None	
10	1871	CARDHDDVDDVFDVW	IGH	2	None	

10	1872	CARDGYCSSTTCYNFYYDGLDVW	IGH	3	None	
10	1873	CATRPLHSSSPFDYW	IGH	4	None	
10	1874	CARDYEDGYGTPFFDHW	IGH	2	None	
10	1875	CARAFTTGMTGVWFDPW	IGH	2	None	
10	1876	CAKSLSGSYTPYFDYW	IGH	3	None	
10	1877	CAKDPQRRYYYDSSGYYYGYFQHW	IGH	8	None	
10	1878	CAKLPTIEGLFDYW	IGH	2	None	
10	1879	CARGVVPPVRGKNWFDPW	IGH	2	None	
10	1880	CARDPKALGPSSDGMDVW	IGH	21	None	
10	1881	CANLGQAGTEYYYYYGMDVW	IGH	4	None	
10	1882	CANSLSSSPPGFDYW	IGH	26	None	
10	1883	CARRGTTVTTYDYYYYMDVW	IGH	4	None	
10	1884	CARDPTDDYGDYVGYFDYW	IGH	13	None	
10	1885	CARDLDEDYDFWSGPFGVRSIRYYFDYW	IGH	4	None	
10	1886	CARHAEDIVLVVYAPASYYYMDVW	IGH	15	None	
10	1887	CARQKDIVVVPATRPDYYYYYGMDVW	IGH	4	None	
10	1888	CARHNGYELWNGYAPYYFDYW	IGH	5	None	
10	1889	CAHDDISSGGYDSDAFDIW	IGH	7	None	
10	1890	CDWSSDVCSSDLDTAVYYCTRGNLGDFWTPYYSGRNNWFDPW	IGH	2	None	
10	1891	CARDWGSSTWIFDYW	IGH	9	None	
10	1892	CAKDSIPFYYDSSGYGTNWFDPW	IGH	6	None	
10	1893	CAHRGYYYDSSLLGYFDYW	IGH	4	None	
10	1894	CARHVGVVVVVVITGPYYFDYW	IGH	5	None	
10	1895	CANGATYDSSDYYTGYW	IGH	7	None	
10	1897	CARGGGYYDSSGYHVGFANLAFDYW	IGH	4	None	
10	1898	CARTSSGWRNYYYYMDVW	IGH	4	None	
10	1899	CAKVLYSYGYSTGCFDYW	IGH	6	None	
10	1900	CAKAPWMLEGVFDYW	IGH	2	None	
10	1901	CTRADYGDPPRGEYFDYW	IGH	3	None	
10	1902	CAHRPTYYYDSSGYRRDAFDIW	IGH	13	None	
10	1903	CAKGSITIFGVVISTDAFDIW	IGH	5	None	
10	1904	CARESGDFWSGGFDYW	IGH	61	None	
10	1905	CARHGPHRLTIPTGPAFAMDVW	IGH	2	None	
10	1906	CARDCSSTSCYAEGAFDIW	IGH	6	None	
10	1907	CATSYSVVVPAAIDYW	IGH	6	None	
10	1908	CARGVEMTTNPPDYYYYMDVW	IGH	2	None	
10	1909	CARGTKTYYDILTGYYPFDYW	IGH	21	None	
10	1910	CAKDQYGGNPWYFDYW	IGH	4	None	
10	1911	CAREAPGYCSGGSCYSVGLTFDYW	IGH	6	None	
10	1912	CARYCVDLVATSNWFDPW	IGH	13	None	
10	1913	CASISGTVEVTFDYW	IGH	3	None	
10	1914	CARDRGRGYTGGYISFFDLW	IGH	2	None	
10	1915	CARILSIREGYYMDVW	IGH	2	None	
10	1916	CATVPPPIPAAIGAYYFDYW	IGH	3	None	
10	1917	CATVLHYYGDPVEHWFDPW	IGH	2	None	
10	1918	CARDRRVRVYCSSTSCPTAAFDIW	IGH	2	None	
10	1919	CARGSDMITFGGSNFFDPW	IGH	19	None	
10	1920	CARGPDYYDRSSLGYFDYW	IGH	2	None	
10	1921	CARDDDYYDSSGPYYYRGMDVW	IGH	7	None	
10	1922	CARDAQANYDFWSGDYYYYGMDVW	IGH	2	None	
10	1924	CAREVENDFWSGYRSVNWFDPW	IGH	8	None	
10	1925	CARDSKYYDFWSGYYIEGLSFPDYYYMDVW	IGH	3	None	
10	1926	CAKCQYYDFWSGYQDWYFDLW	IGH	3	None	
10	1927	CQAWDSGTAWGVVF	IGL	2	None	
10	1929	CTTPWEYTSDFDHW	IGH	6	None	
10	1930	CARGLRYFDWSFLSPEYYYYYYMDVW	IGH	4	None	
10	1931	CARLSGVLPFFEYW	IGH	6	None	
10	1932	CARTAMASKFFDYW	IGH	2	None	
10	1933	CATLVGSYFDSRGHYYVAIFDIW	IGH	2	None	
10	1934	CARDPVVGATRGWFDPW	IGH	2	None	
10	1935	CASSSYYYDSSGYLPDGVGDYW	IGH	82	None	
10	1937	CARGRGVFCSSTSCYNSGWFDPW	IGH	3	None	
10	1938	CARCLGYSQWFDLW	IGH	10	None	
10	1939	CLRLGGDSTGDAFDIW	IGH	2	None	
10	1940	CARQHGTWRRDFDYW	IGH	3	None	
10	1941	CARETGTTSYYYYMDVW	IGH	16	None	
10	1942	CARGQTAGWELLRFDYW	IGH	9	None	
10	1944	CARHRFRSGNHYFDNW	IGH	5	None	

10	1945	CTRAVYYYGSGSYYNHPIDYW	IGH	2	None	
10	1946	CARDYDILTGYYTNYYYYYYMDVW	IGH	2	None	
10	1947	CVRAYYDASGLQYLDFW	IGH	3	None	
10	1948	CAIPLYDYVWGSYPTFDYW	IGH	3	None	
10	1950	CARDFGFVGGTMGRGETIDYW	IGH	4	None	
10	1951	CCSSSVEDFQHW	IGH	31	None	
10	1952	CAKTPFFSGSYFRFHYW	IGH	10	None	
10	1953	CTRDRGGDYGYYYYGMDVW	IGH	4	None	
10	1954	CAREDRLLWFGESNYYFDYW	IGH	2	None	
10	1955	CARERIAVAGTEYFQHW	IGH	75	None	
10	1956	CARVSAPWFGVDWFDPW	IGH	2	None	
10	1957	CARGVSDYSGYDFWSGYYTGIYYYYYMDVW	IGH	2	None	
10	1958	CAKEVSDSSGYRDALDIW	IGH	2	None	
10	1959	CVQRSKWPSLTF	IGK	5	None	
10	1961	CAKSTLRSKRIFGVVKDDFFDVW	IGH	2	None	
10	1962	CERDRLDEVTAIPGPINWYFDLW	IGH	2	None	
10	1963	CAKDGLHPSFIVRGILSYYYDSSGYDYW	IGH	2	None	
10	1964	CARARHLRFLEWSIPLYYYYYMDVW	IGH	8	None	
10	1965	CATSFAVYSGWSHTDYW	IGH	2	None	
10	1966	CAHISITGGTVDYW	IGH	7	None	
10	1967	CARPLFPLAARRPISDYW	IGH	2	None	
10	1968	CTRVAKRGYTFASFDYW	IGH	2	None	
10	1969	CAHRQGVFWLPIDYW	IGH	2	None	
10	1970	CAKESSPLHCGGDCYLGDYW	IGH	2	None	
10	1971	CARDGQYYDILTGYFGPRDYYYYYYMDVW	IGH	5	None	
10	1972	CAHRRPTVTVYDAFEIW	IGH	24	None	
10	1973	CARHSRRGVVVPAATWFDPW	IGH	4	None	
10	1974	CAKESHGGVLAGGSFDHW	IGH	2	None	
10	1975	CQVWHSTSDHSVVF	IGL	77	None	
10	1976	CATTDPGALMVYAISGLFLYAFDIW	IGH	4	None	
10	1977	CARGVPRRDSSGWPPPSPYWYFDLW	IGH	2	None	
10	1978	CARVVMPPDFWSGYYMDVW	IGH	4	None	
10	1979	CAALTVTTFNDAFDIW	IGH	8	None	
10	1980	CARVYSNAWRNFDYW	IGH	2	None	
10	1981	CAKGFDFWSGSQNDYW	IGH	2	None	
10	1982	CAKDNYYYDSSGYSNWYSDLW	IGH	6	None	
10	1983	CATAPVIAARNPGGYYYYYGMDVW	IGH	2	None	
10	1984	CARALTYYYCSGGSCYSAEYFQHW	IGH	2	None	
10	1985	CATAWPSRYYYDSSGREMVDYW	IGH	2	None	
10	1986	CATSVETSVITLADYW	IGH	2	None	
10	1987	CAKDYMPFAVVTTHWFDPW	IGH	4	None	
10	1988	CARIRGYSDFWSGYLGFFDYW	IGH	4	None	
10	1989	CAPGGGVGARDYW	IGH	2	None	
10	1990	CARHPRIAADYDYW	IGH	3	None	
10	1991	CARHFVFTIFGVVITPGWFDPW	IGH	15	None	
10	1992	CATDRDIVVVVAATFDYW	IGH	10	None	
10	1993	CARGRIGVTMIVVVFTGAEYYFDYW	IGH	28	None	
10	1994	CARDYPHAHDYGGSDAFDIW	IGH	2	None	
10	1995	CARDASGGSGWYSDWFDPW	IGH	2	None	
10	1996	CARCFGSAWYPDYW	IGH	2	None	
10	1997	CVAVAGFVDYW	IGH	2	None	
10	1998	CARDLGNNWEIYYFDYW	IGH	34	None	
10	1999	CAKLTSSWNYPDYW	IGH	4	None	
10	2000	CAKTNTPMVPGGLDYW	IGH	4	None	
10	2001	CARDLGYSNIRYFYYYGMDVW	IGH	2	None	
10	2002	CARPIALPGTDAFDVW	IGH	2	None	
10	2003	CAKAPRSMVRGVTLTGTAYYYYGMDVW	IGH	8	None	
10	2004	CLRAEDTAVYYCASNPEDSYDLPDSAFDIW	IGH	2	None	
10	2005	CARDAFTLRYSSGWYIPFDYW	IGH	30	None	
10	2007	CAKDNPINYYGSGSYYNWFDPW	IGH	2	None	
10	2008	CAKDPYPSETMAFDYW	IGH	2	None	
10	2009	CAKQTAEVVPINCYFDLW	IGH	4	None	
10	2010	CAGGCSSTGCYGPNWFDPW	IGH	6	None	
10	2011	CAREGGPHYYDSSGYYGYW	IGH	2	None	
10	2012	CATGGGSYFGHNWFDPW	IGH	4	None	
10	2013	CARDLGEYSYGFYYFDYW	IGH	61	None	
10	2014	CARAHWGDYFGAFDIW	IGH	2	None	
10	2015	CARALSSSWYSPQLGDVTSEPSGLDYW	IGH	4	None	

10	2016	CQQSYTSPPAYSF	IGK	13	C4	7JW0
10	2017	CAADPYYYDSSGSPRAYYYYMDVW	IGH	11	None	
10	2019	CARRSALGFGELLGGFDYW	IGH	2	None	
10	2020	CATLERSSGYQLFDYW	IGH	2	None	
10	2021	CARASRLRFYSPVVTEYYFDYW	IGH	13	None	
10	2022	CTCGPPQESGRFDYW	IGH	2	None	
10	2023	CARARVEAGITMIVVVITTYFDYW	IGH	5	None	
10	2024	CAKVPQSPDVVVPPAIRGWFDPW	IGH	3	None	
10	2025	CARGDPPNARNDAFDIW	IGH	4	None	
10	2026	CVRAPYNITIKDSVDYW	IGH	2	None	
10	2027	CATGAQLWTNNWFDPW	IGH	16	None	
10	2028	CAKESIYGATDYW	IGH	3	None	
10	2029	CAKLSGYAESIKSIAVAGTAGGALDYW	IGH	2	None	
10	2030	CARQPIMITFGGVIVRRFDYW	IGH	7	None	
10	2031	CAKLSTITMIVVVIGGVDYW	IGH	4	None	
10	2032	CARQSSRKWELPLHFDSW	IGH	2	None	
10	2033	CASGGITMVRVRNNWFDPW	IGH	12	None	
10	2034	CARGSGHGALYYYNSPTRFDHW	IGH	2	None	
10	2035	CARSAMDYGDYVLRYNWFDPW	IGH	3	None	
10	2036	CARAAGRFGELWIRFDPW	IGH	2	None	
10	2037	CASIGSAAISTGYYYGMDVW	IGH	3	None	
10	2038	CAKDNSDGYDFVGFDYW	IGH	4	None	
10	2039	CAEEQEDGDRSYYFDYW	IGH	2	None	
10	2040	CAKDPGDDSSGYYYDYW	IGH	21	None	
10	2041	CAKSGNPHYYYYMDVW	IGH	14	None	
10	2042	CAKDSTIVVVPSARLDYW	IGH	2	None	
10	2043	CARDIRENYYDSSGYYGAVTTLENWFDPW	IGH	5	None	
10	2044	CTRPFTTVVTPGDYW	IGH	2	None	
10	2045	CAKLDSERTPKDYW	IGH	2	None	
10	2046	CARTEKRFLEWLLYDDAFDIW	IGH	7	None	
10	2047	CATVTAYYAQGRQVDYW	IGH	2	None	
10	2048	CATVFAYNWNPASDYW	IGH	6	None	
10	2049	CARGLIGPASTYYFDYW	IGH	2	None	
10	2050	CAREEYSSGWNEDWFDPW	IGH	2	None	
10	2051	CARSPYYGDRGKFDYW	IGH	2	None	
10	2052	CARDYYDSSGYYYVYAALGDYW	IGH	3	None	
10	2053	CAREVYRGSDYW	IGH	86	None	
10	2054	CANGILDGMDVW	IGH	13	None	
10	2055	CAKDQFPTVTTPFDYW	IGH	3	None	
10	2056	CAKLGSIGSYSSSWYFDYW	IGH	2	None	
10	2057	CARDVGDLRGDAFDIW	IGH	2	None	
10	2059	CASNYYDSSGYYGIFDYW	IGH	15	None	
10	2060	CARSFSYDSSIFYYWSFDSW	IGH	9	None	
10	2061	CARNLKDGYNYGLYYYYMDVW	IGH	2	None	
10	2062	CARGFTIFGVEPNYFDYW	IGH	2	None	
10	2063	CARANRITMIVVTPFDPW	IGH	2	None	
10	2064	CATSPPYSTVTWFDPW	IGH	2	None	
10	2065	CAKDNWGIAVAGIAFDYW	IGH	3	None	
10	2066	CATVAIPVAAPGDFDYW	IGH	11	None	
10	2067	CARGSPTYYYDSSGYYQMGDYW	IGH	2	None	
10	2068	CARGPLPTYCTGGVCYLEYYYYYYYMDVW	IGH	4	None	
10	2069	CARDEEGGWYENYW	IGH	2	None	
10	2070	CARAPYYYDASGYFFGSSQLRHNWFDSW	IGH	3	None	
10	2071	CTAYMELSSLRSEDTAVYYCARTIFGVVNFDYW	IGH	3	None	
10	2072	CARMDYCDSTTCREVYFYYYMDVW	IGH	3	None	
10	2073	CASYSVAGTFDYW	IGH	4	None	
10	2074	CARMDGITIFGVVISATGYYYMDVW	IGH	4	None	
10	2075	CARGSSGYDSSGYYLGFDPW	IGH	4	None	
10	2076	CARGFYYYDTSAYYYDYW	IGH	2	None	
10	2077	CTTELDYGEYDAFDIW	IGH	2	None	
10	2078	CARDEVYYDFWSGYSGAQRIYYYYYMDVW	IGH	3	None	
10	2079	CAKEGDGYNRDFDYW	IGH	9	None	
10	2080	CARGVGTRVGVNWFDPW	IGH	2	None	
10	2081	CAKGLGYSSSFGLDYW	IGH	34	None	
10	2082	CARDQYHSSGFYSHFDYW	IGH	4	None	
10	2083	CANLRHYYDSSGYSEKNNAFDIW	IGH	2	None	
10	2084	CATKTTMYYYDSSGPSPFDYW	IGH	6	None	
10	2085	CARGTYPWGITMIAPSTDFDYW	IGH	2	None	

10	2086	CARSPTTVTSPVGIWYFDYW	IGH	3	None	
10	2087	CARGQWGSYHDHSGQKAFDFW	IGH	8	None	
10	2088	CVNDEVYDDFWSGYYFGSQDRPKKYYYMDVW	IGH	6	None	
10	2089	CTRWLFPGGSGYDHHGFDSW	IGH	5	None	
10	2090	CAREGVVTAMSGLDYW	IGH	2	None	
10	2091	CAKGSLTVVVVDLFDYW	IGH	3	None	
10	2092	CARAVYDSSGYYYYFDFW	IGH	2	None	
10	2093	CARVAYSGYDSLKFTWKDDWFDPW	IGH	4	None	
10	2094	CAREILGYCSSTSCYTWENYYYYYGMDVW	IGH	6	None	
10	2095	CVRECKSGYGCDAFDFW	IGH	2	None	
10	2096	CAKAPPEGSVLYGDCYFDYW	IGH	2	None	
10	2097	CATSRIGDYYDSSGYSDYW	IGH	9	None	
10	2098	CAKGLRYFDWLLYGPPDAFDIW	IGH	2	None	
10	2099	CAKRHRAGDVGYNAFDIW	IGH	4	None	
10	2100	CDWSSDVCSSDLYYCARDTYYDFWSGYYFDYW	IGH	2	None	
10	2101	CATGEYSSSGAFDIW	IGH	8	None	
10	2102	CARLFSQKNTIFGVVTHTPLGGYFDYW	IGH	4	None	
10	2103	CARDRRYDAFDIW	IGH	195	None	
10	2104	CARERLYCGSDSCFWRNWYFDLW	IGH	7	None	
10	2105	CAKTASGSYSDFDFW	IGH	9	None	
10	2106	CARAQYCSGGSCYSYYYYMDVW	IGH	33	None	
10	2107	CARGYYDFWTWGPFDLW	IGH	4	None	
10	2109	CARTNWGIVGAAWFDPW	IGH	2	None	
10	2110	CARRGSGSYFIYNWFDPW	IGH	7	None	
10	2111	CARAPKADFWSGYPYYYMDVW	IGH	40	None	
10	2112	CARDYYGSGSYTHYDYW	IGH	4	None	
10	2113	CARSYVLRFLEWLNWFDPW	IGH	3	None	
10	2115	CARKADYVWGRYRYLSAFDYW	IGH	2	None	
10	2116	CARAAANTRYWFFDLW	IGH	5	None	
10	2117	CARGRAGTPTQLRADFDYW	IGH	4	None	
10	2118	CARDPERYDFWSDLHFDPW	IGH	5	None	
10	2119	CATSRYSSSQSYYYYMDVW	IGH	4	None	
10	2120	CARQGDFWSAYFAPW	IGH	2	None	
10	2121	CASGYDWYPDYW	IGH	133	None	
10	2122	CARATGDRGLLTSLEYHFDHW	IGH	5	None	
10	2123	CDWSSDVCSSDLYCATDLTYYYDSSGQNWFDPW	IGH	2	None	
10	2124	CAREELHLPESYSSSWYGSDAFDIW	IGH	12	None	
10	2125	CARDPSVLRFLEWYGIEAFDIW	IGH	2	None	
10	2126	CARDPRSYCGGDCRWGWFDPW	IGH	2	None	
10	2127	CARGKYDGSGYYFYFDSW	IGH	2	None	
10	2128	CTRGYCTNGVCYDFDYW	IGH	4	None	
10	2129	CARGTYYYDSSGYLVPDYW	IGH	2	None	
10	2131	CARFIAGNRWYYFDYW	IGH	5	None	
10	2132	CAKGDGDNIPWYFDLW	IGH	608	None	
10	2133	CARDNSQYYDILTGYYNFGSPYYYYYYMDVW	IGH	3	None	
10	2134	CAKVSYYYDSSGYYPTLYWYFDLW	IGH	2	None	
10	2136	CARENYDILTGSFDPW	IGH	2	None	
10	2137	CDWSSDVCSSDLADTAVYYCARNRALITIPNFDYW	IGH	2	None	
10	2138	CARANNGGYSYGYDYW	IGH	6	None	
10	2139	CARRLRTYYYGSGSYGWFDPW	IGH	2	None	
10	2140	CARDPGPSSWSDYSFDYW	IGH	4	None	
10	2141	CQQYYSILTWTF	IGK	40	C4	6W41
10	2142	CAKVQKAREMATMSPDYW	IGH	2	None	
10	2143	CARDENYGGTHAFDIW	IGH	5	None	
10	2144	CARYGLQARDGDYALRGAFDIW	IGH	6	None	
10	2145	CARHFRGDRYCSSISCSPFDPW	IGH	2	None	
10	2146	CAREAGDPPEWFDPW	IGH	3	None	
10	2147	CARERSRMFRGVINPDAIDIW	IGH	3	None	
10	2148	CARAWGVVMTAVSGFDPW	IGH	5	None	
10	2149	CARDNTPLWDGVSPLDYW	IGH	2	None	
10	2150	CARDAHDSSGYYGGKIDYW	IGH	4	None	
10	2151	CAKESRYNWNDAAGIDYW	IGH	3	None	
10	2152	CARVYYGSGNFGYYYYYMDVW	IGH	2	None	
10	2153	CARDVGFGGGTGWYQGVFYYYYGMDVW	IGH	4	None	
10	2156	CTTVSTPPERTYMSMVRGILTERRIPRDDFDIW	IGH	3	None	
10	2157	CARDESPVQAAGDAFDIW	IGH	10	None	
10	2158	CARALTLDPQWIQLWPYYYYYGMDVW	IGH	2	None	
10	2159	CARTIAVARWPPSHHFDYW	IGH	8	None	

10	2160	CAIITPIGEGYSYYMDVW	IGH	2	None	
10	2161	CAHSRYDILTGYSIYFDYW	IGH	16	None	
10	2162	CAKEYRGVDTAMVIMPPDYW	IGH	15	None	
10	2163	CAREFSSGWRPYDYW	IGH	5	None	
10	2164	CATIVPNSGDFYSYNYGMDVW	IGH	3	None	
10	2165	CARRGCSTDVCYLDYW	IGH	2	None	
10	2166	CARDKDPRLATARFDYW	IGH	30	None	
10	2167	CAKVSAGPPSIDYW	IGH	11	None	
10	2168	CARDYLGYCSSTSCLPYYYYYMDVW	IGH	4	None	
10	2169	CARVFIFWGEDVFDIW	IGH	4	None	
10	2170	CARGRSDTTDEYNDFWSGYSPLGNNWFDPW	IGH	54	None	
10	2171	CARAGWTTVTYYYYMDVW	IGH	3	None	
10	2172	CARDPRGYGPIWFDPW	IGH	2	None	
10	2173	CARDLIDILTGYSVHDAFDIW	IGH	6	None	
10	2174	CTRPLYDIMTGYPEPFDYW	IGH	2	None	
10	2175	CARDIAIEALDAARFEYW	IGH	2	None	
10	2176	CTIRDFPGYDAFDMW	IGH	2	None	
10	2177	CARSLRQWLSSYNWFDPW	IGH	21	None	
10	2178	CARGGWSRNLVPAALDYYYYYMDVW	IGH	2	None	
10	2179	CATDLKAAAGTYYYYYGMDVW	IGH	2	None	
10	2180	CATSDIVVVPAAIRVGYYYYGMDVW	IGH	4	None	
10	2181	CVRDESISCYRGWFDPW	IGH	2	None	
10	2182	CARTNGWYRGSFDYW	IGH	6	None	
10	2183	CATDLVPPRDRNYYYYMDVW	IGH	2	None	
10	2184	CARGVTGRNGYFDLW	IGH	4	None	
10	2186	CANLVTNYGGNWYFDLW	IGH	17	None	
10	2187	CARGDQKPAFSLDYW	IGH	2	None	
10	2188	CAKGKSSNYPVNYYYHYMDVW	IGH	14	None	
10	2189	CAKDNPRGGYDSSGYYYPQEGFDYW	IGH	4	None	
10	2190	CTTDSLVITMIVVVEYYFDYW	IGH	8	None	
10	2191	CARDSDDSSGYWEWYFDLW	IGH	7	None	
10	2192	CANFPTTEAFDIW	IGH	18	None	
10	2193	CARATGWELPDAFDIW	IGH	9	None	
10	2194	CARVPLPYCGGDCYPYYYYYGMDVW	IGH	2	None	
10	2195	CARGQAASSGWYGYYFDYW	IGH	31	None	
10	2196	CARGNFGSDSSRASDAFDIW	IGH	4	None	
10	2197	CAKGAYWGRITMIVVVISAFDYW	IGH	5	None	
10	2198	CARDGRDYDYVWGSYRPKGFDPW	IGH	21	None	
10	2199	CARGLGYYSSTSYYPPDYW	IGH	2	None	
10	2200	CAREHKEYYDFWSGMKDGALGDYYMDVW	IGH	2	None	
10	2202	CAHRQIGYYDSSGYFRGW	IGH	2	None	
10	2203	CATVGQTYCGGDCSFDYW	IGH	3	None	
10	2204	CARDKMYYDILTGYYPHYYYYGMDVW	IGH	6	None	
10	2205	CAHRSIITGTGTSLDYW	IGH	3	None	
10	2206	CAKAPTLRGFGGVIVNGPLDYW	IGH	2	None	
10	2207	CARDLEYYDILTGYFGGTNYYYYYGMDVW	IGH	9	None	
10	2208	CTRAQDKSWTLYYFDYW	IGH	8	None	
10	2210	CAKVAAGTYYYDSSDYW	IGH	7	None	
10	2211	CTRGPFSITITDYW	IGH	3	None	
10	2212	CAKDLGITMIVVVIIPSFDYW	IGH	3	None	
10	2213	CARSPPPYFYDSSGFYDAFDIW	IGH	2	None	
10	2214	CVHSGPLYGDYYFDYW	IGH	5	None	
10	2215	CAKAFPRDGNYVRDYW	IGH	3	None	
10	2217	CAKLYYYDIAFDIW	IGH	13	None	
10	2218	CAKDQALVVYALVYFQHW	IGH	2	None	
10	2219	CARVRGSGSSDDAFDIW	IGH	2	None	
10	2220	CAHIPDYNLHRYYFDYW	IGH	8	None	
10	2221	CARARIVYDTSGYFSYFDNW	IGH	5	None	
10	2222	CATEAVAGTGSPAFDIW	IGH	3	None	
10	2223	CATGTAVAWYYFDYW	IGH	4	None	
10	2224	CTRDRPTSGSPYFGSW	IGH	2	None	
10	2225	CAREHSAWWYVELDYW	IGH	3	None	
10	2227	CAKSPYFHCGSGDCYSLFDPW	IGH	2	None	
10	2228	CATGAIVATFVDYW	IGH	4	None	
10	2229	CASIGRYAGPHAFDAW	IGH	2	None	
10	2230	CTTDLGYYDSSGYRFDYW	IGH	17	None	
10	2231	CARETREPIAAAWWEVNYYYYYMDVW	IGH	7	None	
10	2232	CSKDPGRGNYVWGSPYFHYW	IGH	2	None	

10	2233	CVKDGAHDVLRFLERPPNYYYSGMDVW	IGH	8	None	
10	2234	CARGRSSFTMIVLVFTGASYYFDSW	IGH	10	None	
10	2235	CQQYNSNLMYTF	IGK	62	C3	7K8R
10	2236	CAGFSGSSGYYYPYYYYGMDVW	IGH	2	None	
10	2237	CTTELLWFGELLALDYW	IGH	2	None	
10	2238	CAKDTTTGYGNNWFFDYW	IGH	2	None	
10	2239	CARDSHQYYDFWSGYYTGRDAARMNWFDPW	IGH	8	None	
10	2240	CASVTGYSYDRSGYYYVGDWFDPW	IGH	9	None	
10	2241	CAKGQLWLPEDYW	IGH	3	None	
10	2242	CALPISDTAVYYCARGFDYW	IGH	3	None	
10	2243	CQQSYGTLWPF	IGK	4	C4	6ZDH
10	2245	CAKDMSPGYCSSTSCYVGYYYYYGMDVW	IGH	11	None	
10	2246	CAKLPHPGYSSSADYW	IGH	4	None	
10	2247	CARASYCSRTTCYGLDYW	IGH	5	None	
10	2248	CARDGAIRWARPVAEFLPLGDTCFDYW	IGH	3	None	
10	2249	CARKIRWPKYYFDYW	IGH	6	None	
10	2250	CAKAPLASCSSTVCYTFDSW	IGH	4	None	
10	2251	CARPRILGGTRTAFDIW	IGH	4	None	
10	2252	CATGFAYYGSGSYYPFDYW	IGH	2	None	
10	2253	CAKDRGTEGSGSYSRDDYYYYYGMDVW	IGH	3	None	
10	2254	CAREGPSSSSWGGGWFDPW	IGH	8	None	
10	2255	CAKHSKGGYSGYGPAYIDYW	IGH	4	None	
10	2256	CAKDKAPDFWSADWYFDLW	IGH	21	None	
10	2257	CARTATAARLYWYFDLW	IGH	2	None	
10	2259	CAGGNLESWAFDYW	IGH	2	None	
10	2260	CQTWGTGTQVF	IGL	3454	None	
10	2261	CARSEDRRWLSLESRHYYFDYW	IGH	2	None	
10	2262	CARFRYYYDSSGYYYVGLFDYW	IGH	4	None	
10	2264	CTRNPYYYDSSGYYVFFDYW	IGH	2	None	
10	2265	CAKGGYIAVAGTPPPDYW	IGH	8	None	
10	2266	CARDPARPNYDFWSGYGPALDILTGYSSSSIAFDIW	IGH	2	None	
10	2267	CARNSATAMVFAYW	IGH	2	None	
10	2268	CAKDAADYYDSSGYFGYW	IGH	2	None	
10	2269	CAKDPLDYGDYRGGFDPW	IGH	5	None	
10	2270	CGRDYRGYSYGTEVDYW	IGH	6	None	
10	2271	CARDHRWPSHGYNYRDYW	IGH	2	None	
10	2272	CARDFGDYVWGSLVSFGWFDPW	IGH	5	None	
10	2273	CATDTLPYDHGYDYW	IGH	3	None	
10	2274	CARESSSGAFVDYW	IGH	46	None	
10	2275	CASTAESSWYTDYW	IGH	2	None	
10	2276	CATEGRYFDWFHLGRYYYYGMDVW	IGH	39	None	
10	2278	CAIVVGGGRHFDYW	IGH	3	None	
10	2279	CAHATGLYYYDSSGYSVEYYFDYW	IGH	3	None	
10	2280	CARAAGSSWYGDAFDIW	IGH	22	None	
10	2281	CARDLPIGVCSSTSCYYDYW	IGH	3	None	
10	2282	CAKVSGWSPNIDYW	IGH	8	None	
10	2283	CSLRSEDTAVYYCATDFAIAVAGTLDYW	IGH	2	None	
10	2284	CATIITIFKGNVVYYFDSW	IGH	4	None	
10	2285	CETWDSNTWVF	IGL	111	None	
10	2286	CHSRDSSNNHHFLF	IGL	4	None	
10	2287	CASGWLLPEYYFDYW	IGH	87	None	
10	2288	CARGGDFEGDAFDLW	IGH	12	None	
10	2290	CARGTPLTFGGVIVSGFDAFDIW	IGH	14	None	
10	2291	CAKLRHSSSSSYFDYW	IGH	4	None	
10	2292	CAHEDYDILTGYTSFFDYW	IGH	2	None	
10	2293	CARVEYYDILTDYW	IGH	5	None	
10	2294	CAKDLFDYFDSSGSPHDAFDIW	IGH	3	None	
10	2295	CARAYNGYALHFDYW	IGH	5	None	
10	2296	CARHMSDYGDFNPFDFW	IGH	2	None	
10	2297	CARQGGSGTWYYVDYW	IGH	3	None	
10	2298	CATSTPFWSARRNWFDPW	IGH	7	None	
10	2299	CARRYYDYVWGSYFYFDYW	IGH	15	None	
10	2300	CARQGVRRGNRVFGAGGFDSW	IGH	6	None	
10	2301	CARDSSSWHNIDYW	IGH	3	None	
10	2302	CARELIGVDDPPGAPGWFDPW	IGH	6	None	
10	2303	CAGGFCSGGSCYYLLLDYW	IGH	8	None	
10	2304	CARDRATWQWLTPNWFDPW	IGH	2	None	
10	2305	CAREGPNGRSDYW	IGH	2	None	

10	2306	CARAGGIAAAGLYYYYGMDVW	IGH	11	None	
10	2307	CARRVSPVTTQPYFDYW	IGH	12	None	
10	2308	CARLRWGTNARGYMDVW	IGH	2	None	
10	2309	CARDGWSSSWDLDAFDIW	IGH	2	None	
10	2310	CARDHPADDYGDYPDYW	IGH	49	None	
10	2311	CARDRDYYDSSGYYYGGAFDIW	IGH	17	None	
10	2312	CATAGGYSYATGYYFDYW	IGH	2	None	
10	2313	CAKLGIYSGYDSSTDYW	IGH	2	None	
10	2314	CARNNLLGIQYYYYYMDVW	IGH	2	None	
10	2316	CAKMLTGDLMQAFDYW	IGH	2	None	
10	2317	CATGGGWIGYLDWFDPW	IGH	2	None	
10	2318	CARGRGAYSVEVRGHTWFDPW	IGH	2	None	
10	2319	CDWSSDVCSSDLDTAVYYCAREYYYDSSGLDYW	IGH	2	None	
10	2320	CAREPLSGWYVYYW	IGH	7	None	
10	2321	CARGNYKWNSYYFDYW	IGH	14	None	
10	2322	CSLKLASVTAADTSVYYCATVPSSRSWSAFYHFDNW	IGH	2	None	
10	2323	CARQRGSAPLYYMDVW	IGH	2	None	
10	2324	CAKLGNTYDFGLEDYW	IGH	3	None	
10	2325	CAREGPDFWGGYYSGRFRWFDPW	IGH	2	None	
10	2326	CANLWFGELLFNRRGYYYMDVW	IGH	7	None	
10	2327	CARVFYGSGSYTTYLFDYW	IGH	4	None	
10	2328	CARSPAGTTHYFDYW	IGH	2	None	
10	2329	CARAAPLDDITDDAFDIW	IGH	13	None	
10	2330	CVRDKTVGALDYW	IGH	3	None	
10	2333	CARQDPYDSSGYYFLLADYW	IGH	2	None	
10	2334	CAKLTASTYDYGDYVDYW	IGH	2	None	
10	2335	CARGLGVSAIFHYYYYMDVW	IGH	3	None	
10	2336	CARSGRSCSGGTCYSSYW	IGH	9	None	
10	2337	CAKDMGPGEVMVDHFDYW	IGH	4	None	
10	2338	CSLRSEDTAVYYCATDPIYDYVWGSYRYNFDYW	IGH	2	None	
10	2340	CVRPYCIGDVCYAHDIW	IGH	5	None	
10	2341	CARHVRYFDWLLAYHEKVTSYYFDYW	IGH	2	None	
10	2342	CARTLQYYYDSSGYYELDYW	IGH	2	None	
10	2343	CAKDPFYDFWSGYYFDYW	IGH	177	None	
10	2344	CARDQTGDSPTVHYYYYMDVW	IGH	4	None	
10	2345	CAKDTTYYDILTGYLPSAHYYYYGMDVW	IGH	2	None	
10	2346	CAKGSIAARPGYFDYW	IGH	26	None	
10	2347	CTTDGVGATSYYYYGMDVW	IGH	25	None	
10	2348	CATELRRYFDWLLIPFDPW	IGH	2	None	
10	2349	CARLVGATPHTNWYFDLW	IGH	2	None	
10	2350	CARHPANDYGDYVVYFDLW	IGH	2	None	
10	2351	CARAGYFGDYIRPFEYW	IGH	5	None	
10	2352	CAKGVGTMVRGVDYW	IGH	4	None	
10	2353	CARTIRGVYHYDSSGYYYGSENPRRDYFDYW	IGH	2	None	
10	2355	CAKLTVAATYDAFDIW	IGH	2	None	
10	2356	CARDPLYSSSFPYYYYGMDVW	IGH	4	None	
10	2357	CARAADTSGYFQNFDHW	IGH	3	None	
10	2359	CVRAGDATGYYPPYDDSFQYMDVW	IGH	2	None	
10	2360	CARALEGYSSSWYVDYW	IGH	2	None	
10	2361	CARDGVLRFLEWLGYFDYW	IGH	27	None	
10	2362	CARDSDLRLGELSFLDYW	IGH	5	None	
10	2363	CAKITEPGIAAAGTFPPVDYW	IGH	6	None	
10	2364	CAREGVVSLGFDYW	IGH	88	None	
10	2365	CAKAPGVVIIYYFDYW	IGH	2	None	
10	2366	CAKDLYDSTGYYSDDYFYYGMDVW	IGH	4	None	
10	2367	CAKEISGISRGAFDIW	IGH	2	None	
10	2368	CATFFYGDYFGRFAFDIW	IGH	4	None	
10	2369	CAHSLPLGDRRGGHVFDIW	IGH	2	None	
10	2370	CARDSSWNDVGALKGLDYW	IGH	3	None	
10	2371	CARVRDSYGIYYYGMDVW	IGH	9	None	
10	2372	CALENYSSNPTGYDFWSGYYSFGFDYW	IGH	5	None	
10	2373	CVRLDSGPYSFDYW	IGH	4	None	
10	2374	CAKVDDSGYDGFDYW	IGH	4	None	
10	2375	CAKIVDPNERDWPIDYW	IGH	3	None	
10	2376	CTTDFWSYGYLAFKDYW	IGH	2	None	
10	2377	CARVAPLGGISTGYYFDHW	IGH	2	None	
10	2378	CARDRALYGDENYFDYW	IGH	3	None	
10	2379	CTKGRDSTGWQYDYW	IGH	2	None	

10	2380	CARDGEGNSSSWYRIPATILLGQFDYW	IGH	2	None	
10	2381	CAKRAYSHYDRGPLDYW	IGH	5	None	
10	2382	CAITQRWLQSSPFDYW	IGH	3	None	
10	2383	CARDLSGSYYRRVYYFDYW	IGH	6	None	
10	2384	CARDWGTHDSSGYYNWFDPW	IGH	2	None	
10	2385	CASPRNYIVVVSPFDYW	IGH	2	None	
10	2386	CARDLNPHLGIMITFGGVIVTSGLDYW	IGH	2	None	
10	2387	CARDELTVGWTFDYW	IGH	9	None	
10	2388	CATVGGPFGVVMQWDYW	IGH	3	None	
10	2389	CAKDGRDCSGGSCYAWDDWFDPW	IGH	6	None	
10	2390	CARGPRGQYSSSSYYFDYW	IGH	2	None	
10	2391	CQHYNAWPEIYTF	IGK	6	C3	7K8R
10	2392	CARDPASDYDFWSGYYILEWDYW	IGH	9	None	
10	2393	CARDFDDFWSGYGGFDYW	IGH	54	None	
10	2394	CAGSLLFSFDYW	IGH	4	None	
10	2395	CARDRGMTTVTTGDYW	IGH	22	None	
10	2396	CARTIWSGYYTVGYYYGMDVW	IGH	2	None	
10	2397	CAHLPYYDTFTGYYLDYFDPW	IGH	5	None	
10	2398	CARGNQWPQRGGYYYGMDVW	IGH	2	None	
10	2399	CAKQTNVTIVVVVAAVYYFDSW	IGH	2	None	
10	2400	CAKLAASSGLLNDMDRFDYW	IGH	6	None	
10	2401	CAVDTVTTGVGGAFDVW	IGH	218	None	
10	2402	CARRPNRVLRFLEWPFDDYYYYGMDVW	IGH	2	None	
10	2403	CARARGIFGVVRSYAFDLW	IGH	6	None	
10	2404	CASNSAAGPRYYYYGMDVW	IGH	8	None	
10	2405	CARTGIAARPDFDYW	IGH	12	None	
10	2406	CAREGEGGSSRQDDYW	IGH	6	None	
10	2407	CAGASGGDYFWDAFDIW	IGH	7	None	
10	2408	CATGVPHSATGVDYDYVWGSYRYSTLDYW	IGH	10	None	
10	2409	CASFYDSSGYSMMSFDIW	IGH	6	None	
10	2410	CAKDERPVPTGVLVDYW	IGH	4	None	
10	2411	CAKIALVRGYAFDIW	IGH	9	None	
10	2412	CAKADCSSTSCRNWFDPW	IGH	29	None	
10	2413	CARDFTFGGDDYFDHW	IGH	7	None	
10	2414	CAKTGDHTGADYW	IGH	11	None	
10	2415	CARDVCSGGSCYLVGWFDPW	IGH	6	None	
10	2416	CARVVEVHWRLFDYW	IGH	8	None	
10	2417	CTRTKNYYDSSGYYHKGIDYW	IGH	2	None	
10	2419	CAKYSIAVAGNIWGAFDYW	IGH	2	None	
10	2420	CAAWVHSLSGQWVF	IGL	2	None	
10	2421	CARGVRYFDWFIERDQDANILDYW	IGH	10	None	
10	2422	CAKDISSGYFKDAFDIW	IGH	3	None	
10	2423	CSITMIESFDYW	IGH	23	None	
10	2424	CARDWGVWGITPNYYMDVW	IGH	4	None	
10	2425	CARGAYYYDSSGYSYYFDYW	IGH	15	None	
10	2426	CARVIAVAGTQYYFDYW	IGH	2	None	
10	2427	CARGYDVLTGYPDYW	IGH	27	None	
10	2428	CARHVIGRVGVDYHDAFDIW	IGH	9	None	
10	2429	CANLATAMEYAFDIW	IGH	3	None	
10	2430	CAKDQGLFYRTQPFDYW	IGH	3	None	
10	2431	CAKDPTYYDFWSGYLGGTLDYYYYGMDVW	IGH	9	None	
10	2432	CARQSMIVVVITTPWFDPW	IGH	6	None	
10	2433	CARGLHTEMSTIPSNQVFDTW	IGH	3	None	
10	2434	CARDRGAYGDFWSGYSLYYYYMDVW	IGH	2	None	
10	2435	CARTTPLEGDDWQGITIFGVVTINPFDYW	IGH	92	None	
10	2437	CAKDGGWELTYYFDYW	IGH	2	None	
10	2438	CAKQGGYFALERYSYMDVW	IGH	752	None	
10	2439	CATSSGYCSSTSCKTWFDPW	IGH	5	None	
10	2440	CARVADDSTGHYPYFDFW	IGH	12	None	
10	2441	CAKVDSGYDSRGHVDYW	IGH	2	None	
10	2442	CAHSRQWPPNYFDYW	IGH	6	None	
10	2443	CQQYEGSPPYAF	IGK	21	C1	7BZ5
10	2444	CVRDPRDNIVTGHSSHYYLDVW	IGH	5	None	
10	2445	CARDHCSGASCYFDFW	IGH	17	None	
10	2446	CARGEPYDFWSGYSFDPW	IGH	7	None	
10	2447	CARGQEYDYVWGSYADYW	IGH	2	None	
10	2448	CARDPTKQWLVDYYYGMDVW	IGH	7	None	
10	2449	CAKDTEHPSCGDYW	IGH	5	None	

10	2451	CARAGAAAGPLWVHGFFDYW	IGH	17	None	
10	2452	CARGVSSGWTEFDYW	IGH	6	None	
10	2453	CARRPGDTGRGEGDYW	IGH	2	None	
10	2454	CAREPHLSMIVVVTPFGAFDIW	IGH	10	None	
10	2455	CAREYRYYELSEGWFDPW	IGH	2	None	
10	2456	CAKGPGEYYDSSGYQYYFDYW	IGH	2	None	
10	2457	CAKDLVTVTTMGDDAFDIW	IGH	2	None	
10	2458	CATARFLEWLFNFDYW	IGH	15	None	
10	2459	CAKVPDSSGWLQDAFDIW	IGH	9	None	
10	2460	CAREDGSQYYYYGMDVW	IGH	19	None	
10	2461	CARTGWSRGDSSGYYYQVFDYW	IGH	2	None	
10	2462	CARDGHYYYDSSGYRNDAFDIW	IGH	324	None	
10	2463	CASILGYCSSTSCYPDYW	IGH	2	None	
10	2464	CARKYFNSWYDDAFNIW	IGH	4	None	
10	2465	CASPRNSYNSGWYHFDYW	IGH	6	None	
10	2466	CAKVTTVTTEQLTDAFDIW	IGH	2	None	
10	2467	CAKVAAADHSDYW	IGH	9	None	
10	2468	CAKARIVVVTATPDAFDIW	IGH	5	None	
10	2469	CASGYYDSSGYSFYYYYGMDVW	IGH	3	None	
10	2470	CARGHVDTAMVPFDYW	IGH	4	None	
10	2471	CTIRPRRQRQFDFW	IGH	2	None	
10	2472	CAKDIRNIGYPVNIDYW	IGH	4	None	
10	2473	CARRLVGGPLDLDYW	IGH	5	None	
10	2474	CAKGDSSIVVYYYGMDVW	IGH	4	None	
10	2475	CARSMGDSSGYLSYYFDYW	IGH	11	None	
10	2476	CARGAFGYNSYGGNWFDPW	IGH	4	None	
10	2477	CARSRSGSYQSPFDPW	IGH	2	None	
10	2478	CAREPDYYSNPFGYYFYYYMDVW	IGH	8	None	
10	2479	CARDTVLLWFGESPYYMDVW	IGH	22	None	
10	2480	CARVEGITFGGVIASYYFDYW	IGH	3	None	
10	2481	CATLPATIFGVPIWNYMDVW	IGH	2	None	
10	2482	CARAPYSMNSWFRFDPW	IGH	5	None	
10	2483	CTRDTVFGVAPFDYW	IGH	4	None	
10	2484	CARSRWDSSGWFGGYYFDYW	IGH	8	None	
10	2485	CARGQYYFDSTGFPQQYHRPDLPQFQYW	IGH	37	None	
10	2486	CAREGVAARRSPPFFDYW	IGH	63	None	
10	2488	CVHTRQDYHFLTGYLNKWFDSW	IGH	2	None	
10	2489	CARFSSTRLDAFDIW	IGH	12	None	
10	2490	CAHRPHDITMIVVGPYNWFDPW	IGH	3	None	
10	2491	CANFRDHFDYW	IGH	3	None	
10	2492	CARDGRPYSSSPTEEDFDYW	IGH	25	None	
10	2493	CAKIAAESIVSGDYW	IGH	9	None	
10	2494	CARGVGGYSGYDLRFFCFDYW	IGH	3	None	
10	2495	CARDFAPVDRITMIVVVVNAFDIW	IGH	9	None	
10	2496	CARIPTLVLRYFDWLPHHTYYYGMDVW	IGH	7	None	
10	2497	CARETMAAAGRDFDYW	IGH	2	None	
10	2498	CARDSESTNDYGDTENWFDPW	IGH	15	None	
10	2499	CARRRSVRNHYDSSGLYDYW	IGH	2	None	
10	2500	CAKRPEFIGEYFDSW	IGH	3	None	
10	2501	CAHRPGSSWYYAFDIW	IGH	7	None	
10	2502	CAREYGDYVFGNWFDPW	IGH	4	None	
10	2503	CAKDDSSSSSPGGDYW	IGH	3	None	
10	2504	CARGIPSKSGPTNWFDPW	IGH	3	None	
10	2505	CATNSVLRYFDWLLTPYAFDLW	IGH	2	None	
10	2506	CARDPIEYSSSTFDVW	IGH	2	None	
10	2507	CANLAGDILTGPNAFDIW	IGH	2	None	
10	2508	CARSDGELPSWFDPW	IGH	4	None	
10	2509	CARDTAGYYGSGKESVWFDPW	IGH	2	None	
10	2510	CTKSSLRLQRIFGLIRGNYFEDW	IGH	2	None	
10	2511	CAKLPGLRAPNFDYW	IGH	2	None	
10	2512	CAKSYYYGSGSYPDYW	IGH	12	None	
10	2513	CARARGGAKNYGSYYFDYW	IGH	2	None	
10	2514	CARHYDSTSYYNYYFDQW	IGH	3	None	
10	2515	CATDFAIAVAGTLDYW	IGH	159	None	
10	2516	CARVASQWSSYYTAFFDFW	IGH	8	None	
10	2517	CTETHDYYYYGMDVW	IGH	2	None	
10	2518	CAAPHCSGGSCHDAFDIW	IGH	54	None	
10	2519	CARGGYYYDSSGPHIDYW	IGH	3	None	

10	2520	CARTPSYYYDSSGYFSFDYW	IGH	6	None	
10	2522	CARQLYNIDSYESRWFDIW	IGH	2	None	
10	2523	CATDLMVVGATVYDYW	IGH	2	None	
10	2524	CARSPPGEGTMIVVAEYYFDYW	IGH	2	None	
10	2525	CARELYYDFWSGYSVAPDYW	IGH	11	None	
10	2526	CARDHASGRSSVDYW	IGH	4	None	
10	2527	CAKTRGPYDSSGLVDYW	IGH	4	None	
10	2528	CTREAESGLRFYMDVW	IGH	4	None	
10	2529	CARNFPGGTIFGVLSYEPSGWFDPW	IGH	5	None	
10	2530	CATSNSYGLTGDYW	IGH	4	None	
10	2531	CARHERNYDYVWGSYRYEYFDYW	IGH	2	None	
10	2532	CADYSNYVGGDEGYYYYMDVW	IGH	3	None	
10	2533	CARGEWLTDYTLKSFTYW	IGH	2	None	
10	2534	CARPRGGSYAGEFDYW	IGH	14	None	
10	2535	CARDAIALYYYDSSGSVDWYFDLW	IGH	5	None	
10	2536	CTYYCVRRGGDLVLGRGYFDLW	IGH	2	None	
10	2537	CAGSYYDFWSGYYTRSNYFDYW	IGH	2	None	
10	2538	CARPTYYYDSSGYYYVGPSTFDHW	IGH	3	None	
10	2539	CARWKSGYSSGWNDYW	IGH	15	None	
10	2541	CANAPDDSSGYYYVLGGAFDIW	IGH	4	None	
10	2542	CARLRGSITMIVVAMGPWFDPW	IGH	6	None	
10	2543	CAKLAPSSSHCDYW	IGH	5	None	
10	2544	CASDPIAVAGLDAFDIW	IGH	2	None	
10	2545	CARFPSHYYYESSGFSADYW	IGH	2	None	
10	2546	CAREVACLGGSGRPRGDCYPNWFDPW	IGH	2	None	
10	2547	CAKDKYQLLSAGFDYW	IGH	2	None	
10	2548	CARDLGFLEWLFRDYYYYMDVW	IGH	28	None	
10	2549	CAKDISGSSGYQYYFDYW	IGH	8	None	
10	2550	CDWSSDVCSSDLDGQRWLQSTDTYYYYYGMDVW	IGH	2	None	
10	2551	CATVPLWFGVFDPW	IGH	33	None	
10	2552	CSKARIYYHDRSGPDYW	IGH	3	None	
10	2553	CARQIPRDNDFWSGYHTGYFDYW	IGH	4	None	
10	2554	CARTAYIFGSSFDYW	IGH	6	None	
10	2555	CAKDPESEYQLPHWFDPW	IGH	6	None	
10	2556	CASPNDSGDYARFDYW	IGH	2	None	
10	2557	CTTQYSSSSNARGNFDYW	IGH	2	None	
10	2558	CAKDRSPYYYDSSGWGDWTLDYW	IGH	4	None	
10	2559	CAIRSGDLLRFLEWVDDAFDIW	IGH	2	None	
10	2560	CATDFAWFGELFFGYW	IGH	63	None	
10	2561	CARSQTGWPHNYYYYYYMDVW	IGH	5	None	
10	2562	CTSMVPMVYAYYFDYW	IGH	4	None	
10	2563	CARDGFWLYYDILTGYLPPYYFDYW	IGH	2	None	
10	2564	CARQSTNYDRNGYFYDWFDPW	IGH	3	None	
10	2565	CARGPNYYDFWSGYYRNWFDPW	IGH	85	None	
10	2566	CATAPVLRSSNTWGHYYYYYGMDVW	IGH	11	None	
10	2567	CAKDLSLATVPYYYYGMDVW	IGH	2	None	
10	2568	CAREVPLNWASYYFDYW	IGH	4	None	
10	2569	CATGPIFGSGWTNWFDPW	IGH	5	None	
10	2570	CAKVSYYYDSSGYPRNHDAFDIW	IGH	5	None	
10	2572	CAKAQSDSSGYSPDYW	IGH	31	None	
10	2573	CTHPTTGFDTAWIPFDYW	IGH	2	None	
10	2574	CARATSTYYDFWSGTERDAGIFDYW	IGH	3	None	
10	2575	CASSPTDPREYYYYYMDVW	IGH	5	None	
10	2576	CTREAATVTIALDYW	IGH	19	None	
10	2578	CARDLWSGYLAYYYGMDVW	IGH	12	None	
10	2579	CARDTVPYYDFWSGYYFSFDPW	IGH	3	None	
10	2580	CAKDFEYSGSVDYW	IGH	28	None	
10	2581	CARHEIVGAGPDWFDPW	IGH	4	None	
10	2582	CARHRLYGGNSNDAFDIW	IGH	2	None	
10	2583	CASQPINEYSSSYWFDPW	IGH	27	None	
10	2584	CAKHGSGRSNWYFDLW	IGH	2	None	
10	2585	CAKAMGEYCGSGCYSRIIDYW	IGH	2	None	
10	2586	CAHINTLITDWYAPFDYW	IGH	3	None	
10	2587	CTGGVCYLEYYYYYYYMDVW	IGH	7	None	
10	2588	CARDRPQVGATDYW	IGH	6	None	
10	2589	CAKGPWFGEPGLTNYYYYGMDVW	IGH	2	None	
10	2590	CNSVSSSIFGVVTGEDYW	IGH	3	None	
10	2591	CAIYVDIVAFFYW	IGH	2	None	

10	2592	CTTEFYDFWSGDAFDIW	IGH	8	None	
10	2593	CATFKTIYSYGTFDYW	IGH	16	None	
10	2594	CARDLEGRANDYDSSNYGRWFDPW	IGH	2	None	
10	2595	CARETEDDVYKNYFDLW	IGH	5	None	
10	2596	CARDSLFRDFWSGSFDYW	IGH	9	None	
10	2597	CTRDAYSRGSYFFFDYW	IGH	7	None	
10	2598	CARWGVLVGSTWRDAFDIW	IGH	50	None	
10	2599	CARGPPTYDFWSGYSPSHWYFDLW	IGH	4	None	
10	2600	CVRGNYNFDYW	IGH	6	None	
10	2601	CARGQGKAVAGLYFDYW	IGH	2	None	
10	2602	CAKGPPASYGSFYFNYMDVW	IGH	25	None	
10	2603	CATGGETIFGVVTSAYYYMDVW	IGH	2	None	
10	2604	CARGMGGDPLTGYSYGLYYFDYW	IGH	2	None	
10	2605	CAKSFDDDFWSGYYYGMDVW	IGH	3	None	
10	2606	CARDDLGTFTAARLGYYYYGMDVW	IGH	5	None	
10	2607	CARRGGAPTYFDYW	IGH	2	None	
10	2608	CARHPEDIYSNYYYYYYMDVW	IGH	4	None	
10	2611	CAKERVLRYFDWTGPPDAFDIW	IGH	3	None	
10	2612	CARVPQYYDIFSPARGSLDYW	IGH	9	None	
10	2613	CQSTDYSGTSRVF	IGL	6	None	
10	2614	CAKDGDGYNESGDYW	IGH	8	None	
10	2615	CARVPEFTVGEYYFDYW	IGH	2	None	
10	2616	CARTMTNANYDNDYW	IGH	3	None	
10	2617	CAKGRWGIVATSSFDYW	IGH	2	None	
10	2618	CQSVDSYGSSPVF	IGL	2	None	
10	2619	CARGYSSSSWFDYYYYYMDVW	IGH	11	None	
10	2620	CARARRITMVRGVIRGFDPW	IGH	2	None	
10	2621	CAKELADTADFDYFDYW	IGH	2	None	
10	2622	CCSYAGSYTFLNWVF	IGL	15	C2	7K8X
10	2623	CAKDSKYYDFWSGYFSEQYYYYYYGMDVW	IGH	6	None	
10	2624	CATESGSSGWPYPISTFDIW	IGH	4	None	
10	2625	CASHHYDILTGIDYW	IGH	2	None	
10	2626	CASLFDSSGYLSSAFDIW	IGH	3	None	
10	2627	CATSYSTTWYVRGAGYSYYGMDVW	IGH	4	None	
10	2628	CARQPKGWLVRPDYYYMDVW	IGH	2	None	
10	2629	CAKGRVRPEPLMITFGGVIVNDAFDIW	IGH	3	None	
10	2630	CAKDMSDAAAAAKFRFDPW	IGH	2	None	
10	2631	CATTMGRLLLWLAIDYW	IGH	2	None	
10	2632	CAREPAAIKEGFDYW	IGH	2	None	
10	2633	CANLPDLNAFDIW	IGH	26	None	
10	2634	CARDGESPSTAFDIW	IGH	2	None	
10	2635	CACLGDGNYYDSSGYLFDYW	IGH	2	None	
10	2636	CQHLNSYQFPF	IGK	3	C1	7BZ5
10	2637	CATGSPVAGTSSWFDPW	IGH	10	None	
10	2638	CAKAFRIQPITMVRGVPLFYYYGMDVW	IGH	2	None	
10	2639	CARDPSALSFYGLGSYFDDW	IGH	5	None	
10	2640	CARQIAYCGGDCSMRFDPW	IGH	4	None	
10	2641	CAKSRLVVIQYNWFDPW	IGH	4	None	
10	2642	CAHRRPNWNDGGYFDYW	IGH	6	None	
10	2643	CVTWDSTLSADWLF	IGL	387	None	
10	2644	CAHSGYSSGVYYFDYW	IGH	4	None	
10	2645	CAGAAAGIGALDYW	IGH	10	None	
10	2646	CARRRDYLDSSAYYYAEYDAFDIW	IGH	2	None	
10	2647	CATLTWTTGYFDPW	IGH	6	None	
10	2648	CAHFFLGYFVYW	IGH	7	None	
10	2649	CAREGVTTTSWFDPW	IGH	7	None	
10	2650	CARGRRTRRRLQGSGLPEAKPTFDIW	IGH	4	None	
10	2651	CVKESQWAVMGLFEYW	IGH	12	None	
10	2652	CARESIPIYFDPNPAYYYGMDVW	IGH	4	None	
10	2653	CAKADIPNSSSWYPLDYW	IGH	2	None	
10	2654	CAKDARIQLWYLYYFDYW	IGH	7	None	
10	2655	CQQFNNFIFTF	IGK	20	None	
10	2656	CVKEGYCRGASCAHFDYW	IGH	5	None	
10	2657	CASSPDWGSTAFYYMDVW	IGH	3	None	
10	2658	CAKDIRTVTDAFDIW	IGH	12	None	
10	2659	CARARGGSYFDAFDIW	IGH	240	None	
10	2660	CARDPTPLESESPLPYYYYGMDVW	IGH	3	None	
10	2661	CATVGDYVWGSYHHDYW	IGH	4	None	

10	2662	CATSFPALWGSYRTPLDYW	IGH	2	None	
10	2663	CAKDGYFDWLFTFDYW	IGH	4	None	
10	2664	CEFSLVVYAIGPNYYFDYW	IGH	2	None	
10	2665	CARVGVDDRYCDNFRCYYMDVW	IGH	2	None	
10	2666	CASLGGLDYYGSGRDYYYYYGMDVW	IGH	2	None	
10	2667	CAHRLYTKRHFDIW	IGH	2	None	
10	2668	CARALVATMIVVQHFDYW	IGH	2	None	
10	2669	CARTNYDYVWGSYRSPTSFDYW	IGH	26	None	
10	2670	CARRVWGSYRYDPTPFDYW	IGH	4	None	
10	2671	CARAPNIAIVAMYFDYW	IGH	2	None	
10	2672	CARQQLKYGSGWDDAFDIW	IGH	2	None	
10	2673	CARMYQLPPDGYYYYYGMDVW	IGH	15	None	
10	2674	CFSTGYLDYW	IGH	10	None	
10	2675	CARRRGFGVVKRNWFDPW	IGH	2	None	
10	2676	CARGASDFAGFFDYW	IGH	6	None	
10	2677	CARDSGGYDSDNYMDVW	IGH	2	None	
10	2678	CARAILDDYGDYFDYW	IGH	15	None	
10	2679	CATESWGNCSSTSCQPYYYYGMDVW	IGH	2	None	
10	2680	CAHLAEWELSDAFDIW	IGH	2	None	
10	2682	CARDSFAARYFDLW	IGH	5	None	
10	2683	CARHRSGSLKGGDYVDYW	IGH	3	None	
10	2684	CATDSRSQWLRTTYFDYW	IGH	87	None	
10	2685	CALDSGYSSSWYGVFDYW	IGH	2	None	
10	2686	CARRKYYYDSSGYYMDVW	IGH	3	None	
10	2687	CARGLVAAAGLGYYYYYGMDVW	IGH	8	None	
10	2688	CARGGGLYGAGSYFDYW	IGH	2	None	
10	2689	CAKDMGGSGWYSVGGLLYSSALDVW	IGH	4	None	
10	2690	CARVQLWLPPYYYYGMDVW	IGH	2	None	
10	2691	CADLTGTLNFDGYW	IGH	2	None	
10	2692	CARVPFPFFSDDFWSGSYYYYGMDVW	IGH	3	None	
10	2693	CVKDRLPGRAVASSGPSFDHW	IGH	2	None	
10	2694	CARDYVWGRTNSTGDYW	IGH	9	None	
10	2695	CATSGDGYNSPPFDYW	IGH	6	None	
10	2696	CARDLRGDGYESLHFDYW	IGH	2	None	
10	2697	CASEGSIAALDYFDYW	IGH	5	None	
10	2698	CARGSTTLVVTPVAFDIW	IGH	2	None	
10	2699	CARADDSDAAYAFDYW	IGH	3	None	
10	2700	CARESGSWYVPGGWFDPW	IGH	11	None	
10	2702	CYSADSGNNYRVF	IGL	14	None	
10	2703	CARRAGAETWNPFSYW	IGH	2	None	
10	2704	CAIRHQWGGSCYSCYAFDIW	IGH	2	None	
10	2705	CARSQWGSYRSCFDYW	IGH	2	None	
10	2706	CAKLPYGDYVAVDYW	IGH	2	None	
10	2707	CARHSRFLENLLSIPWFDPW	IGH	2	None	
10	2708	CARDIGCTSISCYNYYYYGMDVW	IGH	4	None	
10	2710	CAREGWEQWLEYDAFDIW	IGH	2	None	
10	2711	CGRLERDAFDFW	IGH	57	None	
10	2712	CARDGGSPMVRGVIAPPFDYW	IGH	10	None	
10	2713	CTTGWETVTPQQDTFDYW	IGH	2	None	
10	2714	CARKLGGTAHAFDIW	IGH	5	None	
10	2715	CARDNNYGSGSYWNYYYMDVW	IGH	2	None	
10	2716	CAKDIIPVGASYFDYW	IGH	21	None	
10	2717	CVRDGIGISEAPDGFDLW	IGH	5	None	
10	2718	CARDRRYYDFWSGYYIEPVPPLVHYYYGMDVW	IGH	2	None	
10	2719	CARGYSSSWFKEFFQHW	IGH	4	None	
10	2720	CARILGPLGFCSGGSCPYHFDYW	IGH	4	None	
10	2722	CARNVVVPTPIEPGDWFDPW	IGH	5	None	
10	2723	CARVTKLTSVEGVDWFDTW	IGH	2	None	
10	2724	CAKLPPDGYNPDDAFDIW	IGH	2	None	
10	2725	CTTAEAPDIVVVPAAADYW	IGH	2	None	
10	2726	CAKIGQLGNDYW	IGH	23	None	
10	2728	CAKLQTEITGTNDYW	IGH	2	None	
10	2729	CATTYAIAAPQIPDYW	IGH	2	None	
10	2730	CAKESLDDFWSGYPTTYYYYGMDVW	IGH	4	None	
10	2731	CARQCSGGHDNDGFYYWWFDPW	IGH	4	None	
10	2732	CARWAEVERLPDAFDIW	IGH	3	None	
10	2733	CTSSYSGYYIGPGFDPW	IGH	2	None	
10	2734	CARGVQQWLTPGGWFDPW	IGH	2	None	

10	2735	CAREGDIVVVPAAIGVYNYYGMDVW	IGH	5	None	
10	2736	CASRGGSYGDYTFDYW	IGH	8	None	
10	2737	CARARIAARPVGAFDIW	IGH	4	None	
10	2738	CAKSGAMGDYFDYW	IGH	10	None	
10	2739	CAKDNSPVVVAANNWFDPW	IGH	6	None	
10	2740	CARPDTAMGLDAFDIW	IGH	6	None	
10	2741	CAKGTTSVVPGVTYHDYW	IGH	2	None	
10	2742	CAKPATEYSSGWYPLGYYFDYW	IGH	5	None	
10	2743	CAKDPLDYDSSGYTFDYW	IGH	2	None	
10	2744	CARLNSGFDYYYGMDVW	IGH	6	None	
10	2745	CARGDYYDSGGFFSDAFDIW	IGH	2	None	
10	2746	CTRESGVRLGELAIYYYYGMDVW	IGH	3	None	
10	2747	CAAWYYDILTGFIYW	IGH	2	None	
10	2748	CAKTYYYDSSGVPSLVYFDYW	IGH	2	None	
10	2749	CARDVHYYDSSGYLPRLFDYW	IGH	4	None	
10	2750	CARINYYDSSGRVSAFDIW	IGH	6	None	
10	2751	CARAPLYCSSTSCYTGPRYFDLW	IGH	4	None	
10	2752	CAKAYYSNYVSGTATPYDYW	IGH	2	None	
10	2753	CASLRRGPGILTGREAGREAFEIW	IGH	2	None	
10	2754	CQVWDSYSAPSF	IGL	15	None	
10	2756	CAKDRARRFLEWFPLRSDGMDVW	IGH	7	None	
10	2758	CATAVLLWFGADNWFDPW	IGH	16	None	
10	2759	CARVGGTRSSWYFYYYYMDVW	IGH	2	None	
10	2760	CARVRLEYYDFWSGYPPYYYYYYMDVW	IGH	4	None	
10	2761	CARDEGYCSGGSCYDYYGMDVW	IGH	9	None	
10	2763	CAKDGYAITYYFDSSGYYFDNW	IGH	7	None	
10	2765	CARDHPDYDYVWGSYRPRGYFDYW	IGH	18	None	
10	2766	CARANPITGHFDYW	IGH	2	None	
10	2767	CARPEGGYSTSWVDYW	IGH	2085	None	
10	2768	CARAQQLGILIEYFQHW	IGH	4	None	
10	2769	CARDGEFYSGYDKRHYYYYYMDVW	IGH	2	None	
10	2770	CALCSVHNWFDTW	IGH	2	None	
10	2772	CARALPFFDTRPYFDPW	IGH	3	None	
10	2773	CALGYSYDSYYWYFDLW	IGH	2	None	
10	2774	CTTDPSLYRYYYYYYMDVW	IGH	4	None	
10	2775	CQNYNSGPMYNAPMYNF	IGK	11	None	
10	2776	CARGAYSGSGTSFHDAFDIW	IGH	3	None	
10	2777	CTRVRNAYSHPPYYYYGMDVW	IGH	2	None	
10	2778	CARSGRFLDWLWKDHDALDVW	IGH	8	None	
10	2779	CARGPDMLFGELSDGVQYFEYW	IGH	5	None	
10	2780	CARVEYSSGLKYWYFDLW	IGH	3	None	
10	2781	CARHTLYSYGSSLFDYW	IGH	3	None	
10	2782	CARESDFGYNLPDYW	IGH	6	None	
10	2783	CARLGSSGWYSNYFYYIDVW	IGH	2	None	
10	2784	CAKDPTNPAEGVWFDPW	IGH	2	None	
10	2785	CAKDYGDGYSRNPHNWFDPW	IGH	2	None	
10	2786	CARQGDYYDSNGNWFDPW	IGH	5	None	
10	2787	CAKVIYSYGYLESYFDYW	IGH	2	None	
10	2788	CATRITIFGVVTYMDVW	IGH	26	None	
10	2789	CARASDFYYDGSPYYRDAFDIW	IGH	2	None	
10	2790	CAKPPLDPGLTGNAFDIW	IGH	16	None	
10	2791	CARGFHQRYYYGSGSYYWFDPW	IGH	9	None	
10	2792	CARVYLGYW	IGH	13	None	
10	2793	CAKDPILEWLLIPKITHSFDFDYW	IGH	2	None	
10	2794	CAGYSSVQLYYFDFW	IGH	2	None	
10	2795	CASYCGGDCYEYYFDYW	IGH	66	None	
10	2796	CARDMTHDYGDYPLFDYW	IGH	5	None	
10	2797	CARERDSSGHYFDYFDSW	IGH	8	None	
10	2798	CATLQGGYSYGYLGDYW	IGH	12	None	
10	2799	CARLYIVAAWRLGYYFDYW	IGH	4	None	
10	2800	CAKVASTSWYQAYFDYW	IGH	2	None	
10	2801	CARDDRYYDILTGYFPARGNYYYYYMDVW	IGH	5	None	
10	2802	CAKDSARELLSVFGYW	IGH	2	None	
10	2803	CAKGTPGGTVLSSRYTGEQRDAFDIW	IGH	2	None	
10	2804	CAKAGVVTAIGDYW	IGH	2	None	
10	2805	CARRVVVTGEIDYW	IGH	2	None	
10	2806	CAKDYGVDYGDSYFDYW	IGH	3	None	
10	2807	CARALPPLDYYDSSGHKGYAFDIW	IGH	3	None	

10	2809	CARDHLHYYDSSVSFPGVDYW	IGH	4	None	
10	2810	CAGNAPGTPSAFDIW	IGH	2	None	
10	2811	CARDKPVRARTVVRGIIEFDYW	IGH	7	None	
10	2812	CARDNPGADFWSGYYTGPFDYW	IGH	2	None	
10	2813	CARGRYYDTSGSLYFDYW	IGH	15	None	
10	2814	CARTYVFEIFGVVSWHNWFDPW	IGH	3	None	
10	2815	CARERRRRHHFDGGDYSYAFDIW	IGH	2	None	
10	2816	CARSPRGGFSFDIW	IGH	5	None	
10	2817	CAKDRVTIFGVGGEYYFDYW	IGH	2	None	
10	2818	CATESFKNYDSSGYLGYAFDIW	IGH	2	None	
10	2819	CAKDIASNDYGDSRDYW	IGH	4	None	
10	2820	CASLYNWNADGMDVW	IGH	4	None	
10	2821	CARHVGGLLWFGELFHGVHNWYFDLW	IGH	2	None	
10	2822	CARSPLYHYDTRGYFQLFFDYW	IGH	2	None	
10	2823	CAKEYHYSGSYYDMDLAFDIW	IGH	2	None	
10	2824	CARGSGEQQLVHGDYW	IGH	2	None	
10	2825	CARYGVQLWFLVASQYYFDYW	IGH	7	None	
10	2826	CARDISSGWYQEGDYFDYW	IGH	2	None	
10	2827	CATLPTAYYYDSSGYAVDYW	IGH	5	None	
10	2828	CARVPPVPYYDSSGYYYYYYMDVW	IGH	2	None	
10	2830	CTTEQQLVLGLYYYYYYMDVW	IGH	4	None	
10	2831	CANLYGDYVPTLDYW	IGH	2	None	
10	2832	CARERDTADYTLDYW	IGH	2	None	
10	2833	CATALWTAHDSLAFDIW	IGH	4	None	
10	2835	CARSRGYYYDSSGYPVGAFDIW	IGH	4	None	
10	2836	CAKDRQYYDSSGYYHNWFDPW	IGH	14	None	
10	2837	CAKDIRGWYNWFDPW	IGH	6	None	
10	2838	CARDGHYDSSGYYEVDYW	IGH	2	None	
10	2839	CARGPRTLVVPAAINKGDDYW	IGH	2	None	
10	2840	CTRHTDGQRWLQSTDTYYYYYGMDVW	IGH	7	None	
10	2841	CASSQRYFDFWSGLDAFDLW	IGH	2	None	
10	2842	CARDFYEFDNSGYYEDHFDLW	IGH	3	None	
10	2843	CAKLYDSSGYYYDHHFDYW	IGH	2	None	
10	2844	CAKVDRLLWFGELLKGEENWFDPW	IGH	2	None	
10	2845	CAKDLLYYDSSGYYSGFFDYW	IGH	2	None	
10	2846	CARHVSHIALAGTFVAADAFDIW	IGH	2	None	
10	2847	CARVGYEDGRGYYYYYYGMDVW	IGH	3	None	
10	2848	CAIVYNWNYGVEDYW	IGH	4	None	
10	2849	CAAVFSYDWSPAFDIW	IGH	2	None	
10	2850	CARPVLRFLEWLSPAGVWFDPW	IGH	5	None	
10	2851	CAKEGIAVAGTANWFDPW	IGH	6	None	
10	2852	CATDKGWLLFSPSPTNWFDPW	IGH	9	None	
10	2853	CARERSSTIFGVVIHPGAFDIW	IGH	6	None	
10	2854	CTRGFQYYDFWSGYYDILTGSTVDAFDIW	IGH	5	None	
10	2855	CASLAYYYDSSGYYSSAFDIW	IGH	10	None	
10	2856	CARRGSAYYDILTGYYPYYFDYW	IGH	5	None	
10	2857	CAKDPYYYDSSGYQTPGRLFDYW	IGH	4	None	
10	2858	CAREGGYGGLLHFDYW	IGH	5	None	
10	2859	CARDTTYYDFWSGYLRGPWEHYYYYYGMDVW	IGH	8	None	
10	2860	CARQNYDILTGLYYYGMDVW	IGH	19	None	
10	2861	CARLAYCGGDCYSTWYYFDYW	IGH	6	None	
10	2862	CARAEIEVAAPSDYW	IGH	2	None	
10	2863	CARDTTSINMIMVVPGHGWLDPW	IGH	2	None	
10	2864	CARDWAGTGTVWFDPW	IGH	6	None	
10	2865	CAVSTIFGVVTWFDPW	IGH	184	None	
10	2866	CARDPDCGGDCYYDQDYYYYMDVW	IGH	35	None	
10	2867	CAKVGYSIHLLTYYFDYW	IGH	2	None	
10	2868	CATGSPDYDYVWGSYKRGNWFDPW	IGH	2	None	
10	2869	CTREIRPPGYSSDISGYSDYW	IGH	5	None	
10	2871	CTTERGYGGANDAFDIW	IGH	2	None	
10	2872	CQQRSNWLAPIYTF	IGK	2	None	
10	2873	CAREPFYYDSSGLVPGDAFDIW	IGH	2	None	
10	2874	CAKADQGYYDSSGYYGYGFDYW	IGH	15	None	
10	2875	CARDGDGYCSGGSCYKFDYW	IGH	6	None	
10	2876	CATIAAQHALDYW	IGH	2	None	
10	2877	CAKDLASAYYTAFDYW	IGH	7	None	
10	2878	CARGYTGLSLSFHYYYMDVW	IGH	7	None	
10	2879	CARDSYYGDYEVGWFDPW	IGH	94	None	

10	2880	CAKDPHRGIAGVDAFDIW	IGH	2	None	
10	2881	CARDYPIQSHSNKPVSIFDSW	IGH	2	None	
10	2882	CARGEEYSIYFYALDVW	IGH	2	None	
10	2883	CAKEQYYYDSYFDYW	IGH	33	None	
10	2884	CARDVRYYDILTRYYEPGGLDYW	IGH	69	None	
10	2885	CARHHLRHFDWLLHNYFDDW	IGH	3	None	
10	2886	CAKDHGPYSSSWYSRDDDYW	IGH	3	None	
10	2888	CARATNGRLIMITFVGAFDIW	IGH	11	None	
10	2889	CASDRLWFGAGSDVFDFW	IGH	2	None	
10	2890	CARDRPPWYCSGGSCYSDYYYMDVW	IGH	51	None	
10	2891	CQQYSSYWFRF	IGK	29	C3	7K8R
10	2892	CARGPDYDYVWGSYRHDFDYW	IGH	3	None	
10	2893	CARGLGYIMWADAFDIW	IGH	22	None	
10	2894	CAKDSRLFLLGVDYYFDYW	IGH	9	None	
10	2895	CAHRSIQLWFPSFDYW	IGH	5	None	
10	2896	CARQQDYYDSSGRSLDALDIW	IGH	2	None	
10	2897	CARDPSRITIFGVVIDDAFDIW	IGH	10	None	
10	2898	CARGPYDSSAYYYPFYFDYW	IGH	2	None	
10	2899	CASLGPDGYNTNQFDYW	IGH	2	None	
10	2900	CARSVSAVPAAAPTYYFYHYMDVW	IGH	2	None	
10	2901	CARSGVGSGDYYYRIFDYW	IGH	4	None	
10	2902	CARAPTMIVVVIRIHDAFDIW	IGH	6	None	
10	2903	CAKGQGTDYTQLLWFGELYLGAFDYW	IGH	15	None	
10	2904	CEGRDTAMVPPLYHFYYMDVW	IGH	2	None	
10	2905	CARELRYFDWSSFDYW	IGH	17	None	
10	2906	CARLTMLQGLIIPAGPYFEYW	IGH	13	None	
10	2907	CAKDGESGSYGDWFDPW	IGH	6	None	
10	2908	CAMGLAAAGTSFDYW	IGH	3	None	
10	2909	CAKDISTRIAAAGTFDYW	IGH	10	None	
10	2910	CAREWKYSSGWYDLFWFDSW	IGH	4	None	
10	2911	CATKIGAGTMRWSARPDYVDAFDIW	IGH	2	None	
10	2912	CARGIVVVITTYRFDPW	IGH	2	None	
10	2913	CARGGHSSSWYYYYGMDVW	IGH	29	None	
10	2914	CAKAYRWFGEFYYYYGMDVW	IGH	2	None	
10	2915	CARPVGRDGYKRLDYW	IGH	3	None	
10	2916	CARDPYYEKLTGNYIGNYYYMDVW	IGH	9	None	
10	2917	CAREGTYYDFWSGTPDAFDIW	IGH	21	None	
10	2918	CATGTPFQQLVGWWFDPW	IGH	6	None	
10	2919	CARDRGPHYGDPQRGIPGAHDAFEIW	IGH	2	None	
10	2920	CATVTDFWSGYTFDYW	IGH	4	None	
10	2921	CAKLPGDYYDSSGYYMVDYW	IGH	21	None	
10	2922	CATHRTDGYKEYYFDYW	IGH	4	None	
10	2923	CARDSGSSGWSRFDFW	IGH	2	None	
10	2924	CARVSGYSGYDGAFDYW	IGH	7	None	
10	2925	CAKDYGVSDAFDVW	IGH	2	None	
10	2926	CAHTNANGSSGYYYDYW	IGH	5	None	
10	2927	CARWGKCQWLAHYYYYYGMDVW	IGH	2	None	
10	2929	CARHGVTMVRGVIITGYFDYW	IGH	3	None	
10	2930	CARGIAAAGHFNYYYYMDVW	IGH	12	None	
10	2931	CAKDIPWNDYSQVTVDYW	IGH	5	None	
10	2932	CARIILTEQFGLDYW	IGH	30	None	
10	2933	CARGQHPFSLVVVPTFDPW	IGH	3	None	
10	2934	CARDVNSGYDETYYFDYW	IGH	4	None	
10	2935	CAKDRAITMTRGVIKSFDYW	IGH	2	None	
10	2936	CAREMYSSGCCGAFDIW	IGH	2	None	
10	2937	CARGSGSYYYDSSFDYW	IGH	6	None	
10	2938	CAKDLGDYYDSSGHNWFDPW	IGH	35	None	
10	2939	CARLGIASRPQDWFDPW	IGH	6	None	
10	2940	CAKEGDNYYDSSGYYYLLSDAFDIW	IGH	10	None	
10	2941	CAKDPRNGDYDWYFDLW	IGH	3	None	
10	2942	CAITTTVTTSGWFDPW	IGH	66	None	
10	2943	CVREGTEYPGYYFDYW	IGH	2	None	
10	2944	CARDLDSEIVVDAFDIW	IGH	2	None	
10	2945	CARHEVTPTTRPRIYSYYVDVW	IGH	2	None	
10	2946	CAKDIQGIAADSTADAFDIW	IGH	2	None	
10	2947	CARSFLGRYTPDYW	IGH	4	None	
10	2948	CASHRAPYDSSGYFDYW	IGH	30	None	
10	2949	CARHVYGSGSYYSAYDYW	IGH	4	None	

10	2950	CTTVDWRIVVVITEDYW	IGH	4	None	
10	2952	CARDYRRGITMVRTPNRFDPW	IGH	4	None	
10	2953	CARVPARRPYYDSSGYSDYW	IGH	2	None	
10	2954	CARLDWLVLRQDYWFFDLW	IGH	3	None	
10	2955	CARRSYGSSNYYSYYW	IGH	2	None	
10	2956	CAKDQSGGSSDDAFDIW	IGH	8	None	
10	2957	CASGSFDGGLQSRQQDYW	IGH	2	None	
10	2958	CASMHSSSWYIKGYMDVW	IGH	2	None	
10	2959	CAGGEENYDFWSGSIDYW	IGH	3	None	
10	2960	CARVLWFGESLGYMDVW	IGH	2	None	
10	2961	CARDHPSGASSGYSYYFDYW	IGH	4	None	
10	2962	CARVYQGDYGVDYW	IGH	9	None	
10	2963	CAKSNSADYGDYVDEAFDLW	IGH	2	None	
10	2964	CARSRDVFAMSWFDLW	IGH	2	None	
10	2965	CASSTGAAAGPGTQEQDYW	IGH	2	None	
10	2966	CARFVPIRKWTGPPSWYYYYMDVW	IGH	2	None	
10	2967	CARLRFLEWLSPYWYFDLW	IGH	4	None	
10	2968	CARDQALLRYFDWLPSPHDAFDIW	IGH	4	None	
10	2969	CTTVYGGWYNSVDYW	IGH	9	None	
10	2970	CSTGFCTSRSCYGDALDIW	IGH	2	None	
10	2972	CARDQIGVDSSGCDYW	IGH	7	None	
10	2973	CARVAAYCTNFICSHFDTW	IGH	2	None	
10	2974	CAKVGYYYDSSGYFYWYFDLW	IGH	2	None	
10	2975	CAISSPEDNGDSFAFDYW	IGH	4	None	
10	2976	CARDGVFGSGLPVDYW	IGH	54	None	
10	2977	CAKDPGGGVGYCSSTSCYPENWFDPW	IGH	3	None	
10	2978	CARGLAGTEGGYYFDYW	IGH	2	None	
10	2979	CGADHGSGSNFMGVF	IGL	10	None	
10	2980	CARLTYYYDSSGYPNWYFDLW	IGH	4	None	
10	2981	CARAKRITIFGVVIISGFDYW	IGH	2	None	
10	2982	CARDGYCSGGSCVHYFEYW	IGH	3	None	
10	2983	CAHRPYLWLRFDPW	IGH	6	None	
10	2984	CARHVAAAGTHAFDIW	IGH	7	None	
10	2985	CAKETESNYYRNFDYW	IGH	2	None	
10	2986	CALDSGYDGRFVGPFDYW	IGH	4	None	
10	2987	CTRARFGFWGGFHSGWYFDLW	IGH	2	None	
10	2988	CAKISTDGYNYFDYW	IGH	9	None	
10	2989	CARLAAWFGELSDYW	IGH	7	None	
10	2990	CATGAARGVPNWFDPW	IGH	2	None	
10	2991	CTTDRYYDYVWGSYRYPDYW	IGH	134	None	
10	2992	CQQLNVFPPGFTF	IGK	4	C1	7BZ5
10	2993	CATAPGYSGYDLSNWFDPW	IGH	3	None	
10	2994	CVRDTRDSSGWYAIFDSW	IGH	6	None	
10	2995	CAGFEYSSSPGYYYYGMDVW	IGH	8	None	
10	2996	CARDIPVSDIDYDGPDAFDIW	IGH	3	None	
10	2997	CTAYLQWSSLKASDSAIYYCARPEGGYSTSWVDYW	IGH	3	None	
10	2998	CARDKPYDFWSGYYEAYGMDVW	IGH	14	None	
10	2999	CAKFGTAAVRSDYW	IGH	10	None	
10	3000	CAKDRIPAVPDVVDYYMDVW	IGH	8	None	
10	3001	CATALAVADPYYYYYGMDVW	IGH	4	None	
10	3002	CAHRRLSIAAAGFDYW	IGH	23	None	
10	3003	CAKDRPRKVLVVYAIQFIDYW	IGH	8	None	
10	3004	CARREWPNNRFDPW	IGH	5	None	
10	3005	CARDLPPQYNDFWSGYSLYYYYGMDVW	IGH	3	None	
10	3006	CAKDFEYYDFWSGYSYRARSYYYYYYMDVW	IGH	5	None	
10	3007	CASQRSSGWRSYFDLW	IGH	3	None	
10	3008	CTTAPGGYSSSWYDYW	IGH	5	None	
10	3009	CARDRLAGATRIWRDYFDYW	IGH	29	None	
10	3010	CARVTGTPLKENYLGYW	IGH	5	None	
10	3011	CARYPYGDYDDSYYYMDVW	IGH	5	None	
10	3013	CARDGVDSSGYLPGAFDIW	IGH	15	None	
10	3014	CAKDISSGPVADYW	IGH	12	None	
10	3015	CATGPVIQGTDNWFDPW	IGH	86	None	
10	3016	CARDPVGSGWYQGYYYMDVW	IGH	2	None	
10	3017	CARENYYDSSKDFDYW	IGH	34	None	
10	3018	CATAGSAYYAPFDDW	IGH	38	None	
10	3019	CARSAKNMAGAANWVDYW	IGH	15	None	
10	3020	CARIAEYYDSSGYYGYW	IGH	2	None	

10	3021	CASDYYDSSGYYYLGSIVSGLFDYW	IGH	2	None	
10	3022	CARENQMVAAAYGGLSFFDSW	IGH	2	None	
10	3023	CASGSGSYYNENWFDPW	IGH	53	None	
10	3024	CARGNGLPRSSGWVQTKWTNWFDPW	IGH	2	None	
10	3025	CATGFAVVPAALMDYW	IGH	28	None	
10	3026	CAGCTSTNCRIDYW	IGH	3	None	
10	3027	CARDGRSWYMDVW	IGH	13	None	
10	3028	CAASRGYSYGYALPDAFDIW	IGH	6	None	
10	3029	CARTFPYYYGSGSPLDYW	IGH	64	None	
10	3030	CARDLRGYDILTGYYTPYYFDYW	IGH	12	None	
10	3031	CAKDINCSGGSCYEIYAFDIW	IGH	3	None	
10	3032	CTRTNTNYYVDNDYIDYW	IGH	3	None	
10	3033	CARDPSDDYVDGMDVW	IGH	3	None	
10	3034	CARISRCSFGIHYFDTW	IGH	5	None	
10	3035	CARDSTDYYDSSGYPLGGTFDYW	IGH	31	None	
10	3036	CAQTPSGGTPVAHLDYW	IGH	2	None	
10	3037	CAKGDSSRGYFQHW	IGH	2	None	
10	3038	CTTISYDILTGYYGPDYFYYYMDVW	IGH	2	None	
10	3039	CAKGTLRAYYYDSSGYFDYW	IGH	7	None	
10	3040	CAKSGHNYDSIVLTFDYW	IGH	2	None	
10	3041	CAKDRYYDFWSGYYNHDWFDPW	IGH	19	None	
10	3042	CAKDGVYDSSGYTRYFDLW	IGH	2	None	
10	3043	CARAQSDIFSSYSHYSFDSW	IGH	5	None	
10	3044	CARGLSIAARPDDFRFDPW	IGH	2	None	
10	3045	CARNTNYYDSQGPEFDIW	IGH	2	None	
10	3046	CARDGSFYHDYNGMRQYAFDMW	IGH	3	None	
10	3047	CAREGLELRRFDPW	IGH	2	None	
10	3048	CAAASPVIIGWFDPW	IGH	14	None	
10	3049	CAFTAPSDSTVVIFDVW	IGH	2	None	
10	3050	CAKTPSSSGYHYHLDYW	IGH	2	None	
10	3051	CARTLHYGSGGAYYYYMDVW	IGH	4	None	
10	3052	CARQGWEGDSDIHYFDYW	IGH	6	None	
10	3053	CARGHGIQFLKVWSYFDPW	IGH	4	None	
10	3054	CAKVDGGVVAATADYW	IGH	2	None	
10	3055	CAKDLTRFGEFPIDYW	IGH	2	None	
10	3056	CARGPGLNWNAPHHFDYW	IGH	23	None	
10	3057	CARDRITPPGGWFGELIWALAFDYW	IGH	3	None	
10	3058	CARQADILWSPGGWFDPW	IGH	2	None	
10	3059	CARDSLIWSGHHNWFDPW	IGH	3	None	
10	3060	CAKLCEPARRSPSVDYW	IGH	5	None	
10	3061	CAHRPSPSYDFWSGYYFGTEFNYYYGMDVW	IGH	3	None	
10	3062	CVRGSENWTRRYYYYLMDVW	IGH	3	None	
10	3063	CGRDQGIASVGTVVDYW	IGH	2	None	
10	3064	CARCNREGESNYDFWSAYSDYW	IGH	6	None	
10	3065	CASKLAAAGSDDAFDIW	IGH	3	None	
10	3066	CAHGHTRSITMIVVVTGRSLYFQHW	IGH	2	None	
10	3067	CAKARIGGAEGSWDTIFGVVIFDAFDIW	IGH	2	None	
10	3068	CATSFVVVPAAKRYYYYYGMDVW	IGH	3	None	
10	3069	CARRPPSRTDGMDVW	IGH	2	None	
10	3070	CATVNPLNWGSVGDAFDIW	IGH	2	None	
10	3071	CVHRTRGWGADWFDPW	IGH	304	None	
10	3072	CATDLKVTAYCGGDCYSKSYAFDIW	IGH	2	None	
10	3073	CARSILPEHIVVVTASRDAFDIW	IGH	5	None	
10	3074	CARVVVPAGTHYYYYYYMDVW	IGH	2	None	
10	3075	CAKEGSGITGTGDNWFDPW	IGH	3	None	
10	3076	CQSLDSSSPYPWVF	IGL	3	C1	7BZ5
10	3077	CARFNGPTFGRFQFDYW	IGH	4	None	
10	3078	CAKDFTFGGGYYPYYFDYW	IGH	2	None	
10	3079	CASSGEGISSSWYQGIFDYW	IGH	29	None	
10	3080	CARPYYYGSGSDAHDVW	IGH	2	None	
10	3081	CARAVSSKKMGGDYGDYGDYW	IGH	2	None	
10	3082	CASMELRGYYYYMDVW	IGH	2	None	
10	3083	CARDHFYDSSGYYHYFDSW	IGH	29	None	
10	3084	CARTYYDFWSGSKGSFDYW	IGH	3	None	
10	3086	CARPILTGYFDFGYW	IGH	4	None	
10	3087	CAKDMEWELLIRGGKFDYW	IGH	6	None	
10	3088	CARPDHQYYDFWTNSGRDAFDIW	IGH	2	None	
10	3089	CARDLEGSGWSRPFDYW	IGH	22	None	

10	3090	CARRDEGFTVTQTYFDYW	IGH	2	None	
10	3091	CARQRCSGGSCYSKGRFFDYW	IGH	6	None	
10	3092	CAKVTTPDILTGYHPYDYW	IGH	4	None	
10	3093	CAKAHIVVVTAQPPGFDYW	IGH	2	None	
10	3094	CARPPHLDSSGYYPDYW	IGH	2	None	
10	3095	CARDRYINTWGSLDAFDIW	IGH	2	None	
10	3097	CQQYDNLPRKFIATF	IGK	77	C3	6XDG
10	3098	CARGPSQWLVRVVYFDYW	IGH	18	None	
10	3099	CALPISAVYYCAKSGTTMIVVVIDDAFDIW	IGH	2	None	
10	3100	CVRLSRYSSYWYFFDFW	IGH	2	None	
10	3101	CAHSSPPYDFWSGYYDVLGPFDYW	IGH	2	None	
10	3102	CSAWHDSLSAQWVF	IGL	12	None	
10	3103	CQQYANSPSTFGQGTNSPITF	IGK	2	C3	6XDG
10	3104	CAREAPGDYVWGSYVSQYYFDYW	IGH	4	None	
10	3105	CARDQDSSGWLLYYYYGMDVW	IGH	2	None	
10	3107	CARGGTVVVPGADPHWYSDLW	IGH	2	None	
10	3108	CARELPGSSWFWEEAFDIW	IGH	3	None	
10	3109	CAKGPYDSSGYYLADPLFDYW	IGH	7	None	
10	3110	CARNRGACRGDSCYCDSW	IGH	4	None	
10	3111	CAKAATSGSSSSTDYW	IGH	5	None	
10	3112	CARGVHWNSGVDYW	IGH	3	None	
10	3114	CASQRGYSYAIDAFDIW	IGH	4	None	
10	3115	CANGRWEFDRWYYFDYW	IGH	2	None	
10	3116	CARHREYYDSSGYYSFDYW	IGH	6	None	
10	3118	CATLCPAAAGYYYYGMDVW	IGH	3	None	
10	3119	CARDSHRFYGYGGTYGDNW	IGH	3	None	
10	3120	CASSITIFGVVTMGAFDIW	IGH	4	None	
10	3121	CAKDIEDIVVVVAVNYFDYW	IGH	4	None	
10	3122	CLLYYGSTQHVF	IGL	73	None	
10	3124	CVRSGIALAGNLYYFDYW	IGH	2	None	
10	3125	CARHLPEVPATAIPYWFDPW	IGH	3	None	
10	3126	CARARITKDLDYW	IGH	3	None	
10	3127	CARVGYSSGWYPWLETRINYYYYGMDVW	IGH	2	None	
10	3128	CARDLVQLWPPDAFDIW	IGH	2	None	
10	3130	CARDRNYYDNSGYHIPYFDYW	IGH	4	None	
10	3131	CARKEGGVGATATGGLDYW	IGH	6	None	
10	3132	CTTDFPHPFEVIEWELPFDYW	IGH	6	None	
10	3133	CAKDRGSTIPLYFDLW	IGH	2	None	
10	3134	CARGAMSSTSITNDYW	IGH	6	None	
10	3135	CAKGHWGTGTPHYYHYMDVW	IGH	4	None	
10	3136	CARVFGSDRDGDGWGVGPCDYW	IGH	28	None	
10	3137	CSLKASDTAMYYCARGVPAGTGGALFDYW	IGH	2	None	
10	3138	CAKVLRSAAGSASGDYW	IGH	8	None	
10	3140	CARGRGSPRDHFDYW	IGH	10	None	
10	3141	CAKGIRSGWFDWYVDLW	IGH	3	None	
10	3142	CAKSPAPYYRPHFDYW	IGH	2	None	
10	3143	CARDKSLYCSGGSCYSSQANWYFDLW	IGH	5	None	
10	3144	CARAVLVEGSYFFRLTSWFDPW	IGH	2	None	
10	3145	CARDLTVVAATEYYYGMDVW	IGH	17	None	
10	3146	CATQKEDRKILSAFDIW	IGH	2	None	
10	3147	CATAVAWNRRGLDYW	IGH	2	None	
10	3148	CAKIPPDGDYGYFDLW	IGH	2	None	
10	3149	CARHPQFMYSSSSEYYFDYW	IGH	9	None	
10	3152	CTTGYKDVLRFLEWLGGDYFDYW	IGH	4	None	
10	3153	CAHRQIYDSSPFDYW	IGH	2	None	
10	3154	CARSRMGAYRDAFDIW	IGH	6	None	
10	3155	CARDGALVEGIVVVPAAIRDYW	IGH	6	None	
10	3156	CAREDDDWNYWEGGRWFDPW	IGH	4	None	
10	3157	CARDRDIDYWSDYPPYYYGMDVW	IGH	3	None	
10	3158	CARTYDPYHYFDYW	IGH	2	None	
10	3159	CARDPPLFYDILTGYYNRYYFDYW	IGH	3	None	
10	3160	CAREGDASSGYYQFDYW	IGH	15	None	
10	3161	CAKDFQPDLLWFGDGDAFDIW	IGH	2	None	
10	3162	CAITDTAMTKPDAFDIW	IGH	2	None	
10	3163	CARGGLIDILTGYRYNWFDPW	IGH	2	None	
10	3164	CTSQWEIEEFYYDSW	IGH	2	None	
10	3165	CQSADSSGTYKMLF	IGL	15	None	
10	3166	CASFMNYDSSGYYLVADYW	IGH	3	None	

10	3167	CAKISGSSSQFDYW	IGH	65	None	
10	3168	CAKDQSAARPAYYFDYW	IGH	2	None	
10	3169	CAKDPDPQATIFGVVTYHFFDYW	IGH	2	None	
10	3170	CARSAGRCSDGTCYEDYW	IGH	2	None	
10	3171	CAREPLVYDILTGYYTHFDYW	IGH	12	None	
10	3172	CATFYGDYVGAYYYYMDVW	IGH	3	None	
10	3173	CASAPRDCSSTSCYRYYGMDVW	IGH	7	None	
10	3174	CARPGYMSGWYFGTLDYW	IGH	5	None	
10	3175	CASTTVTYRDDAFDIW	IGH	3	None	
10	3176	CARGSGIVIIGRFDYW	IGH	5	None	
10	3177	CARDGYDYVWGSYRYTLLNFFDYW	IGH	3	None	
10	3178	CARGNGIWGSGYDWGFDYW	IGH	3	None	
10	3179	CARLGGYDPLMITFGGVIVAPGYFDYW	IGH	2	None	
10	3180	CATVDSSTYNWNIFYFDCW	IGH	4	None	
10	3181	CARITMIEAPPPQYYYGMDVW	IGH	9	None	
10	3182	CAKDSSVEDLAVITIFGVVIIGYFDYW	IGH	2	None	
10	3183	CARDWKQWGFDYW	IGH	3	None	
10	3184	CARASTRIVGASRFFNWFDPW	IGH	5	None	
10	3185	CAKDPRYYDFWSGYLQASKLEPRPWYFDLW	IGH	9	None	
10	3186	CARIRDGDYSPWYFDLW	IGH	7	None	
10	3188	CARDSSGYYYGPIDYW	IGH	13	None	
10	3189	CARDPYLRGIAVAGRSEYFQHW	IGH	3	None	
10	3190	CARVPGAIWAYNWFDPW	IGH	3	None	
10	3191	CARGPGRSRAHRGLGYYYYMDVW	IGH	3	None	
10	3192	CARDRERRISYIDYW	IGH	2	None	
10	3193	CARDIFGFDWFSPHRYYYYGMDVW	IGH	13	None	
10	3194	CAVLGNSGYDWGLDYW	IGH	2	None	
10	3195	CATLPPLRSGWYDGSYDAFDIW	IGH	7	None	
10	3196	CATETYSSSRSLDYW	IGH	3	None	
10	3197	CARDAYYYDSSGYYYSPYYYYGMDVW	IGH	35	None	
10	3198	CAKDYDSSGYYHWFDPW	IGH	112	None	
10	3199	CARIPLTLYNWFDPW	IGH	4	None	
10	3200	CTRGVVRGVITFAYW	IGH	6	None	
10	3201	CAKFEEQWPGDAFDIW	IGH	3	None	
10	3202	CARDTLEDIVVVPAGMGWFDPW	IGH	5	None	
10	3204	CARERWTGYSYDVGYFDYW	IGH	6	None	
10	3205	CARASHVPPPIEYYYNYMDVW	IGH	4	None	
10	3206	CAKDYITGTTGFAFDIW	IGH	12	None	
10	3207	CTTSQGHSSGWYSFDYW	IGH	6	None	
10	3208	CALPISALYHCASLEQQSFWGEPRYYMDVW	IGH	2	None	
10	3209	CARGHWSGYFPPPPNFDCW	IGH	5	None	
10	3210	CAKDLAVTRREGDYW	IGH	2	None	
10	3211	CARYRLGYFDWLLRSDYYYYGMDVW	IGH	2	None	
10	3212	CARAQDIVVVPAAMSWFDPW	IGH	2	None	
10	3213	CARHPGQDARWDWAQWLVPVPVYFDFW	IGH	2	None	
10	3214	CTAELGGITMIVVVINGDAFDIW	IGH	5	None	
10	3216	CARHIRQWLVQAKLGWFDPW	IGH	4	None	
10	3217	CARAPGQLWLKYFDYW	IGH	2	None	
10	3218	CAKELGYCSAGRCFAQGYFDYW	IGH	2	None	
10	3219	CRAIAVAATDYFDYW	IGH	5	None	
10	3220	CTSVVLVVAVTKGAFYFDHW	IGH	2	None	
10	3221	CAKAAAITMIDVANDVFDIW	IGH	2	None	
10	3223	CASNREYQLLSPIDYW	IGH	18	None	
10	3224	CARGRLFQGGTYHIDYW	IGH	2	None	
10	3225	CVKDMGSSLWHDGFDMW	IGH	11	None	
10	3226	CARTRGQWLENRQFDYW	IGH	14	None	
10	3227	CARGFLFGGVIAFDYW	IGH	14	None	
10	3229	CARDADNYYYYYYMDVW	IGH	11	None	
10	3230	CHDYGDYVRSGFDYW	IGH	2	None	
10	3231	CARVLDRGYCSSTSCIDYW	IGH	2	None	
10	3232	CARDQGTYSYGYGFDPW	IGH	3	None	
10	3233	CASEEREAYDSSGRYYYGMDVW	IGH	41	None	
10	3234	CARLRYCSDGSCQRNFDYW	IGH	2	None	
10	3235	CAKEVHDYAVLYWYFDLW	IGH	35	None	
10	3236	CAREVLAPHWFDPW	IGH	10	None	
10	3237	CQQFHNWPLMYAF	IGK	16	C3	6XDG
10	3238	CAKGDCSGGSCYDYW	IGH	29	None	
10	3239	CARAGWRSGGSQKLMSVNWFDPW	IGH	2	None	

10	3240	CAKGIHFDWLLQTDYYYYYGMDVW	IGH	2	None	
10	3241	CARGDYSGSGSYFSVGWNRPHDYNAMDVW	IGH	5	None	
10	3242	CANRYYDTSGYRVGDDAFHIW	IGH	10	None	
10	3243	CARLDIVVVTAIPVDWYFDLW	IGH	2	None	
10	3244	CALPICLRAEDTAVYYCAKGDGDNIPWYFDLW	IGH	2	None	
10	3245	CQHYNTYPYSF	IGK	124	C3	7K8P
10	3246	CVKDIIPLSYHGFDIW	IGH	75	None	
10	3247	CAREYCGSRNCFYQVDYW	IGH	9	None	
10	3249	CAKDTVWGSSGWSYFDYW	IGH	2	None	
10	3250	CASPRLDFDWLLTFSDYW	IGH	3	None	
10	3251	CARATLGCSGGSCYSYYFDYW	IGH	4	None	
10	3252	CARDLPYYSPDAFDIW	IGH	6	None	
10	3253	CAREAKTSPPNFDYW	IGH	1067	None	
10	3254	CAAGWANYYDSSGYFYW	IGH	2	None	
10	3255	CARVPPYYYDSSGYSDYW	IGH	167	None	
10	3256	CAKPSGATRNWYFDLW	IGH	2	None	
10	3257	CARILLVPSSMTNAFDLW	IGH	6	None	
10	3258	CWATAQNHPFDYW	IGH	2	None	
10	3259	CARGHIDSANYYLPFDLW	IGH	2	None	
10	3260	CARAALTYYDFWSGYWFDPW	IGH	3	None	
10	3261	CAREEVAAAGTNWFDPW	IGH	14	None	
10	3262	CAKVSPGYPTYYFDYW	IGH	5	None	
10	3263	CAKLHYYDSSDPSGVDYW	IGH	2	None	
10	3264	CARAHSLNYGRQWRPLPFDFW	IGH	2	None	
10	3265	CARDSDYYDSSGYYYYGMDVW	IGH	30	None	
10	3266	CAKGLIAARDYYYYGMDVW	IGH	2	None	
10	3267	CARHVGLTGFRFDPW	IGH	2	None	
10	3268	CAKVSGYHEDAFDIW	IGH	20	None	
10	3269	CARGAPHRSYIKLYYFDYW	IGH	3	None	
10	3270	CTRPLLWFDAFDIW	IGH	6	None	
10	3271	CARILVRTDYYDTNSRKNWFDPW	IGH	3	None	
10	3272	CAKLTTPNDYGDYFDYW	IGH	4	None	
10	3274	CARDDYVWGSYRYNGYNWFDPW	IGH	11	None	
10	3275	CASSFSIAVAGTSDRNDYPWPFYYYGMDVW	IGH	21	None	
10	3276	CAKDSVRYNWNHFDYW	IGH	10	None	
10	3277	CAKVGDDSSSLNFDYW	IGH	2	None	
10	3278	CATEIGNDWSPAVRYFPYW	IGH	2	None	
10	3279	CARGCYDSSAYPWDAFDIW	IGH	2	None	
10	3280	CAKLGGGPYYDSRQDAFDIW	IGH	4	None	
10	3281	CASLWELEWTPDYFDYW	IGH	2	None	
10	3282	CARDSSSSWSLWWFDPW	IGH	2	None	
10	3283	CAKSARGYYDSSGYYHDEYYFDYW	IGH	4	None	
10	3285	CARGGTSRADYYDSSGYEERWYNWFDPW	IGH	2	None	
10	3286	CARARYHYDSTGYHFDNW	IGH	2	None	
10	3287	CARDSGLRRAFDYW	IGH	6	None	
10	3288	CARGVERWQQGGARFDPW	IGH	6	None	
10	3289	CARGTASDSGWYFDYW	IGH	2	None	
10	3290	CATLQRQQLVLGWFDPW	IGH	13	None	
10	3291	CVKDTLVRSGWTWYFDLW	IGH	4	None	
10	3292	CARIRPAGQWLAENDAFDIW	IGH	10	None	
10	3293	CARDQYDSSGYSMGFDPW	IGH	121	None	
10	3294	CASIYSGWSQGDAFDIW	IGH	2	None	
10	3295	CARAQYNNNWLNGDGMDVW	IGH	90	None	
10	3296	CARSFSSSQRDHFDYW	IGH	2	None	
10	3297	CALLAWFGELNGIDYW	IGH	4	None	
10	3298	CATTDRPNFDYW	IGH	2	None	
10	3299	CAKVGYSYGEGAFDIW	IGH	2	None	
10	3300	CASPHLGGYAFAIW	IGH	27	None	
10	3302	CVAWDDGLSGLLF	IGL	9	None	
10	3303	CATETPRCSSTSCFYLGGVDYW	IGH	2	None	
10	3304	CARVGDDYGDYPRDSSGYYNDYW	IGH	2	None	
10	3305	CARDRDYVGFFDFW	IGH	2	None	
10	3306	CARDNSGSFDIW	IGH	34	None	
10	3307	CARDLPPSRKTVAGKDAFDIW	IGH	2	None	
10	3308	CARVRVITMIVVDNWFDPW	IGH	2	None	
10	3309	CQQYYSSPQKNTF	IGK	8	C4	6W41
10	3310	CTRSIMITFGGVIVYYYYGMDVW	IGH	3	None	
10	3311	CGGLPDCSSTSCSDNWFDPW	IGH	12	None	

10	3313	CARSSRGTIFGVVIIPYYFDYW	IGH	2	None	
10	3314	CTRDAEPSEMSTMTNWFDPW	IGH	2	None	
10	3315	CARARSPGIIGTTRKNWFDPW	IGH	3	None	
10	3316	CTRCYCTGNKCSREFDYW	IGH	3	None	
10	3317	CARCVTMIGLEWYFDLW	IGH	7	None	
10	3318	CQQYHSLPPMYTF	IGK	51	C4	6W41
10	3319	CATPRGYSGYDFDYW	IGH	143	None	
10	3320	CAHGDWLFTKYYFDYW	IGH	25	None	
10	3321	CARGLYSSSWYGAHYYFDYW	IGH	37	None	
10	3322	CARDAPAYCGGDCYKFFVNYYYYYGMDVW	IGH	2	None	
10	3323	CAREKEVDFVDFWYFDHW	IGH	2	None	
10	3324	CAKDALLQYFDLW	IGH	6	None	
10	3325	CARTIMITFGGVIAHIGAFDIW	IGH	27	None	
10	3326	CASVVVGYMDVW	IGH	2	None	
10	3327	CAKDTITMIVVVITSGVFDYW	IGH	4	None	
10	3328	CAKVSNVLLWFGELLLPFDYW	IGH	5	None	
10	3329	CAKDTSTGVDVDTAMGHNWFDPW	IGH	2	None	
10	3330	CATIGEWELPFDYW	IGH	6	None	
10	3331	CARGQDIYSGYDWGDYYYYGMDVW	IGH	2	None	
10	3332	CARVRATQLWQFDYW	IGH	7	None	
10	3333	CARLADSEYFYYYMDVW	IGH	8	None	
10	3334	CASDCTGGVCRIYGDYW	IGH	2	None	
10	3335	CSSADSSDNPLF	IGL	5	None	
10	3336	CARGSALHYDFWTSYYYFEYW	IGH	7	None	
10	3337	CAKGDSAVAALFDYW	IGH	8	None	
10	3338	CARDHGTYYYDSSGYQRDYW	IGH	4	None	
10	3339	CTTEAGPYSYGPFFDYW	IGH	4	None	
10	3340	CARAYCSSTSCYKYFDYW	IGH	32	None	
10	3341	CARSYDYVWGSYRYTRGFDYW	IGH	2	None	
10	3342	CATDRLTYSSGWSLDEYFQNW	IGH	2	None	
10	3343	CARGRPESSSWYEDYFDYW	IGH	6	None	
10	3344	CARDPPTYYYDSSGYYRDFDYYYGMDVW	IGH	2	None	
10	3345	CAKDLGGSYGEAAFDIW	IGH	24	None	
10	3346	CATYFAIFGVVMTFDYW	IGH	29	None	
10	3347	CARIQDQGGDTAMVFDYW	IGH	2	None	
10	3348	CARDGAQEMATSFDYW	IGH	2	None	
10	3349	CAKRTAYHYMDVW	IGH	2	None	
10	3350	CTTTTYYYDSSGYSNDAFDIW	IGH	4	None	
10	3351	CAKDRVLGINLYYFDYW	IGH	2	None	
10	3352	CATAPALYIVGATPRRDWFDPW	IGH	14	None	
10	3353	CARAPYNWNYDAFDIW	IGH	12	None	
10	3354	CARACKLCDDAFDIW	IGH	6	None	
10	3355	CARECRGSLWYFDLW	IGH	11	None	
10	3356	CARCYHDTTGHKLYYFDYW	IGH	6	None	
10	3357	CARGVNYDFWSGRQDYFDYW	IGH	5	None	
10	3358	CAKDPSITGTILARGWFDPW	IGH	2	None	
10	3360	CARLPDFWSGYSAYW	IGH	39	None	
10	3361	CARARSLVVVILAFDIW	IGH	7	None	
10	3362	CATETKSIFGVVTTIFDYW	IGH	9	None	
10	3363	CANLHDYGDYGAPLYYYYYGMDVW	IGH	11	None	
10	3364	CAREPPGYDILTGYYTPGYFDLW	IGH	2	None	
10	3366	CATGLVAVAGTTLLQKSALYYYYMDVW	IGH	4	None	
10	3367	CARSPSPSGYPGWFDPW	IGH	2	None	
10	3368	CAKLPLDRGDYGSHAFDIW	IGH	2	None	
10	3369	CARPGREDPDYESWFDPW	IGH	5	None	
10	3370	CARDSPQGMMLDHYFYYGMDVW	IGH	63	None	
10	3371	CARDQQWLDTYYFDYW	IGH	4	None	
10	3372	CARGVGQWLVDVRFDPW	IGH	4	None	
10	3373	CQQYLHWPPGITF	IGK	3	None	
10	3374	CARLEGEGTVYYYYGMDVW	IGH	2	None	
10	3375	CARGEDYNFVWGRTGAFDVW	IGH	2	None	
10	3376	CTTEVGRGFDWLFHAAVVTPGAVDIW	IGH	2	None	
10	3377	CATDRGTIAVAGIFDRW	IGH	2	None	
10	3378	CATPSSYYYDSSGFGYW	IGH	2	None	
10	3379	CARASNVIVFPFTHAMDVW	IGH	4	None	
10	3380	CASRRDGDYSGAFDIW	IGH	4	None	
10	3381	CARALDFWIDYW	IGH	13	None	
10	3382	CARVRDGSGSWDWYFDLW	IGH	5	None	

10	3383	CARDGGYSSGWYAFSPGWFDPW	IGH	2	None	
10	3384	CAREMKMGYDSSGSFDYW	IGH	2	None	
10	3385	CAHSDYGDYEVYFFDYW	IGH	8	None	
10	3387	CAKDMYPSLVIIDYW	IGH	6	None	
10	3388	CTIRTVMTSEPFDHW	IGH	16	None	
10	3389	CARVVPAPAGVSYNWFDPW	IGH	4	None	
10	3390	CARGFGRAMVIFDYW	IGH	2	None	
10	3391	CARDRSEYYYDSSGLGVWFDPW	IGH	7	None	
10	3393	CATAPVVITTGWFDPW	IGH	109	None	
10	3394	CVTHYGEIDYW	IGH	11	None	
10	3395	CSLKASDSAIYYCARPEGGYSTSWVDYW	IGH	10	None	
10	3396	CATQTTVTRRHWFDPW	IGH	4	None	
10	3397	CARESRYYDYVWGSYRPEGVDYW	IGH	12	None	
10	3400	CTTEEYYYDSSGEFDYW	IGH	2	None	
10	3401	CAHWDDEGAFDIW	IGH	8	None	
10	3402	CAHSPVDPYDFWSGYYMGPRWFDPW	IGH	4	None	
10	3403	CARHQWELRQGWFDPW	IGH	4	None	
10	3404	CANIIPPIDYW	IGH	14	None	
10	3405	CARVPGDYDILFGTRDYYFDYW	IGH	4	None	
10	3406	CARDGYSGSYLLDYW	IGH	3	None	
10	3407	CARYSSGWYSDSPYYFDYW	IGH	2	None	
10	3408	CARAPIDYGDYVGSYYYYGMDVW	IGH	2	None	
10	3409	CAKDSRYLGYQTPRSFDYW	IGH	2	None	
10	3410	CAKRRGDFGDDSGAFDVW	IGH	2	None	
10	3411	CASSAGYLISGVVTYAMDIW	IGH	16	None	
10	3412	CVSYTSTSTLDMVF	IGL	5	None	
10	3413	CTRVHNVIFVINVEGAASARNWYVDLW	IGH	4	None	
10	3414	CARARLVRTFDPW	IGH	2	None	
10	3415	CARILLLHGDHSFDYW	IGH	11	None	
10	3416	CAKLPHSSGQSYHQYW	IGH	2	None	
10	3417	CAKAIAVADTVFDYW	IGH	29	None	
10	3418	CAQLQVLRHVKSYYFDYW	IGH	7	None	
10	3420	CARDALESIFGGFDYW	IGH	2	None	
10	3421	CAKARSYDYGGPTDWFDPW	IGH	4	None	
10	3422	CARLRRGAITMIVFDYW	IGH	10	None	
10	3423	CASVASPDDYVWGSYRFDYW	IGH	2	None	
10	3424	CARVSGAPLGSHMFDYW	IGH	9	None	
10	3425	CARVLGESYRFFDYW	IGH	3	None	
10	3426	CAADPFYCTGGVCYTNYYYMDVW	IGH	2	None	
10	3427	CASGPYCRGGSCDIYYFNYW	IGH	2	None	
10	3428	CAKKPKTGYSSGWYIPLLNYYFDYW	IGH	2	None	
10	3429	CTADTVAWIQLWLLAFDIW	IGH	17	None	
10	3431	CARHPVRRRFLEWLALAFDIW	IGH	2	None	
10	3432	CARHVRRWLQSSSGTFPRSIGDFDYW	IGH	2	None	
10	3434	CAKIGGTNDYGDYVSFFDYW	IGH	2	None	
10	3435	CVKGLDFQGSGTLLGTGFEYW	IGH	11	None	
10	3436	CAREAHYYDSSGYPNAYFDYW	IGH	6	None	
10	3437	CAKDNRYYDSSGYYYFDYW	IGH	99	None	
10	3438	CARVNGYYLAPYDYW	IGH	5	None	
10	3439	CARVHSDNDWFDQW	IGH	11	None	
10	3440	CAHRIVVVPGFDPW	IGH	5	None	
10	3441	CARRYYYDRSDYDKAFDIW	IGH	4	None	
10	3442	CALPILRAEDTALYYCAKDYDSSGYYHWFDPW	IGH	2	None	
10	3443	CARGSWILTGYYTILPFDYW	IGH	6	None	
10	3444	CARVPKRTYYYDSSGLYYFDYW	IGH	2	None	
10	3445	CARDHYYDERGFDPW	IGH	4	None	
10	3446	CSSPYYRNAFDIW	IGH	5	None	
10	3447	CARVAARSSSWYYFDYW	IGH	4	None	
10	3448	CARVRLGGYAVDYW	IGH	5	None	
10	3449	CAGPDCSGGSCDEIRGYYFDYW	IGH	23	None	
10	3450	CARALYSSGWPRYYYYMDVW	IGH	3	None	
10	3451	CAKDFVRLTPIFGVVIGAFDYW	IGH	2	None	
10	3452	CARATYYTSGWYIFDYW	IGH	420	None	
10	3453	CAKEERDYLDSSGYPEYFGFW	IGH	3	None	
10	3454	CAKGLLVFGVVIIGSHTFDYW	IGH	2	None	
10	3455	CAKGPGGIAAHSYFDYW	IGH	2	None	
10	3456	CARASSGSWYPVRNNWFDPW	IGH	2	None	
10	3457	CVRAFTGTHFDIW	IGH	3	None	

10	3458	CTSATSVYYFEDDYW	IGH	4	None	
10	3459	CARGPTTVTTALTGAFDIW	IGH	3	None	
10	3460	CARLVQDSSSWYWFDPW	IGH	5	None	
10	3461	CAKYSRGYFDSSGYPPFDYW	IGH	61	None	
10	3462	CATAPGTIVGAARGWFDPW	IGH	2	None	
10	3463	CAKDRQTYASDYYYGMDVW	IGH	4	None	
10	3464	CATAPAAIFGTKKNYYYYGMDVW	IGH	2	None	
10	3465	CATLHYYDSSGYYRPDAFDIW	IGH	2	None	
10	3466	CARENAAYYYDSSGLYFDYW	IGH	4	None	
10	3468	CAKGHLSYYGSGSYDYW	IGH	8	None	
10	3469	CANAGSGSYEAGFFDYW	IGH	2	None	
10	3470	CAKDLVPDFGGVITPLSDYW	IGH	7	None	
10	3471	CLAWDTSPRDVF	IGL	2	None	
10	3472	CAKRHYYGSGSHGWFFDLW	IGH	4	None	
10	3473	CARGLRGVTDADYW	IGH	3	None	
10	3474	CTTESAVYSSTDDAFDIW	IGH	3	None	
10	3475	CAISPNYSDGSGYPDAFDIW	IGH	9	None	
10	3476	CARDRSSGRDGLDYW	IGH	2	None	
10	3477	CASPNRDCSSTSCYFVDAFDIW	IGH	2	None	
10	3478	CARGGYHDTSGNWANWFDLW	IGH	2	None	
10	3479	CARDGKYYDILTGYYRGLVYYYYYYMDVW	IGH	3	None	
10	3480	CAIVRVGAFDWLRVFDYW	IGH	2	None	
10	3481	CATTNINWNNYQFDYW	IGH	9	None	
10	3482	CARDPYYDFWSGYYTIYYYGMDVW	IGH	7	None	
10	3483	CARDSYDILTGHEIWFDPW	IGH	42	None	
10	3484	CAKAGDVLSFYSYGFDVW	IGH	2	None	
10	3485	CAKATVGMVRDYW	IGH	2	None	
10	3486	CARDFTWYSEVSGGSPGDDFDYW	IGH	6	None	
10	3487	CARSYYDTGGPFDFW	IGH	2	None	
10	3488	CARGMRVYGDYDSYYYYYMDVW	IGH	4	None	
10	3489	CARGGRSSWYIDYW	IGH	53	None	
10	3490	CARGGQLERSWFDPW	IGH	6	None	
10	3491	CARSLEIYSSSILPDYW	IGH	3	None	
10	3492	CARSGTGWFGELFYFDYW	IGH	2	None	
10	3493	CATGPIAAAGSGTGGWFDPW	IGH	4	None	
10	3494	CSTGHYYDSSAYYWYYSGMDVW	IGH	2	None	
10	3495	CQQSYSRPQVTFGQGARLEIKQVTF	IGK	3	C2	7K8O
10	3496	CASFTTTYVGDAFDLW	IGH	2	None	
10	3497	CTRDRGGSDSHYYFYYMDVW	IGH	2	None	
10	3498	CAGILVGATRAEYFQHW	IGH	4	None	
10	3499	CAREMGLRYFDWSIYYYYMDVW	IGH	5	None	
10	3500	CWLGAVTGNVAFDIW	IGH	3	None	
10	3501	CAKDSGYSHSWAVDYW	IGH	7	None	
10	3502	CASGLRTGSDYW	IGH	2	None	
10	3503	CARLAGDLMDGFHYW	IGH	9	None	
10	3504	CASANLEWFGRDWGYNWFDPW	IGH	2	None	
10	3505	CARDRTMGNPWSYDILTGYDDYFDYW	IGH	32	None	
10	3506	CATFTMIVVVNYRGAFDLW	IGH	4	None	
10	3507	CSKDPKRDSGGRGYFDYW	IGH	2	None	
10	3508	CTTDLSGNILTGYYPFDYW	IGH	4	None	
10	3509	CAGRKMDIVVVVAATNAFDIW	IGH	3	None	
10	3510	CARSSGWSLPHFDYW	IGH	9	None	
10	3511	CAREGTSWLVPAAYPDYW	IGH	6	None	
10	3512	CAKDHYSGYSTSIDYW	IGH	2	None	
10	3513	CARLTYYYDSSGLKRGYYFDYW	IGH	11	None	
10	3514	CIRFSSYDVWSGYFHTW	IGH	2	None	
10	3515	CTTEETLSDSSSWYGELVNWFDPW	IGH	3	None	
10	3516	CAKGQGHTVTTWGAFDIW	IGH	2	None	
10	3517	CVKGSRGSCSGTICYPFDYW	IGH	2	None	
10	3518	CARVRPGIFMGSDYW	IGH	2	None	
10	3519	CARGYYDILTGTYGGDDYW	IGH	2	None	
10	3520	CARSSNIAPAGIYYEDDGLDVW	IGH	4	None	
10	3521	CAKAPDGIAVAGPFDYW	IGH	2	None	
10	3522	CAKTLGEGRPWIQLWDGALGDAFDIW	IGH	2	None	
10	3523	CAKVSTTVTDRTDYW	IGH	4	None	
10	3524	CARGHDIVVVPAAITHWFDPW	IGH	2	None	
10	3525	CATSNPMTTVTSWFDPW	IGH	9	None	
10	3526	CAHTRITIFGVVIIWGANIRPYYFDYW	IGH	2	None	

10	3527	CASLWWLRGYFDYW	IGH	49	None	
10	3528	CARQTHQYYDILTGYSDWDYYYGMDVW	IGH	2	None	
10	3529	CARHVDFWRVHYFFAMDVW	IGH	2	None	
10	3530	CARVMSNGWPGDYW	IGH	11	None	
10	3531	CAGSLESRDYMLNHRFDYW	IGH	2	None	
10	3532	CAHRRSNWNDVAFDIW	IGH	4	None	
10	3533	CAKVPNSYSSSSDYW	IGH	3	None	
10	3534	CAKDLRPYYDSNSRLLAFDVW	IGH	3	None	
10	3537	CAKDRGPIMTTADYW	IGH	5	None	
10	3538	CAKLPSRIAADPFDYW	IGH	2	None	
10	3539	CATLGPIYDFWSGYDAFDIW	IGH	5	None	
10	3540	CAWGGSLAYCGGDCPRSTAFDIW	IGH	2	None	
10	3541	CAREKDLEWLHDPGGFDIW	IGH	31	None	
10	3542	CATPRTLDYYDPNWYFDLW	IGH	2	None	
10	3543	CATVGVTTFFGWEFDYW	IGH	3	None	
10	3544	CASWYSSGWPGGFDPW	IGH	4	None	
10	3545	CATAIAYYYDSSGYYEFDYW	IGH	12	None	
10	3546	CTTEETYCGGDCYSGFDYW	IGH	2	None	
10	3547	CARRGIYLLDDYW	IGH	35	None	
10	3548	CVRDGYNEGGFDYW	IGH	8	None	
10	3549	CAKDKSAGFGVAIPYMDVW	IGH	12	None	
10	3550	CARLNDGIAVAGTDDFDPW	IGH	3	None	
10	3551	CAKDPAYYDFWSGYAAHPPSYYYYGMDVW	IGH	27	None	
10	3552	CALPIWELPISSLFDYW	IGH	2	None	
10	3553	CVAAAATAAFDIW	IGH	4	None	
10	3554	CARLAWLRGLFDTW	IGH	7	None	
10	3555	CASRGGSGWYRLIDYW	IGH	4	None	
10	3556	CARVSISTGYSSSWFFDYW	IGH	3	None	
10	3557	CATSLVATTHPLFDYW	IGH	3	None	
10	3559	CAKKEVWFGELSSYYYGMDVW	IGH	20	None	
10	3560	CATSPGKTTYWYFDLW	IGH	4	None	
10	3561	CARIHPDLATFPIAVAGQIGDDAFDIW	IGH	4	None	
10	3562	CARVPYDFWSGYYTGRDWYFDLW	IGH	2	None	
10	3563	CASPVLFASGAFDIW	IGH	3	None	
10	3564	CARTFQGPQDIVVVPAAIGYYYGMDVW	IGH	11	None	
10	3565	CAKYNSSSYIDSW	IGH	18	None	
10	3567	CAKFASYAQDAFDIW	IGH	3	None	
10	3568	CARGDGHLVGGVLFTSNFDTW	IGH	2	None	
10	3569	CTREKVSSYGYIGPLDFGYW	IGH	19	None	
10	3570	CARGEETYYDILTGYYNVDYFDYW	IGH	4	None	
10	3571	CAKITSGSYSIDYW	IGH	7	None	
10	3572	CTRTRHDSGDYLNAFDVW	IGH	3	None	
10	3573	CARDHGHSSSWDGNYYYYYYMDVW	IGH	11	None	
10	3574	CARSYYASGSYYNAYFDYW	IGH	5	None	
10	3575	CARGRGWGIAAAGTGDYW	IGH	2	None	
10	3576	CARGQQWLVRPADYW	IGH	5	None	
10	3577	CTTEPEDSSSWQRPYYFDYW	IGH	2	None	
10	3578	CQQYNNWPPRQSRTF	IGK	27	C3	7K8R
10	3579	CTRDGVAGTDYW	IGH	8	None	
10	3580	CAKKDPFDYEGSPFDYW	IGH	2	None	
10	3581	CASLELRSGPINWFDPW	IGH	2	None	
10	3582	CANLRGACSSTSCYRSHDAFDIW	IGH	4	None	
10	3583	CAKCFSPLSPGGLLWFGELYCYYGMDVW	IGH	2	None	
10	3584	CAKDITPVHKGFDYW	IGH	16	None	
10	3585	CATNIVGAPWYMDVW	IGH	4	None	
10	3586	CARQASAYSSSLDLDYW	IGH	2	None	
10	3587	CAKAARSGWYNIYYGMDVW	IGH	2	None	
10	3588	CARVYLVAGIRVFDYW	IGH	2	None	
10	3589	CARSVRGDSSGLGDSFDVW	IGH	6	None	
10	3590	CARETADYTSSWYDYW	IGH	18	None	
10	3591	CAKSSGDYDNWAPDYW	IGH	2	None	
10	3593	CARSVGYNSDWAVNALDIW	IGH	6	None	
10	3594	CATLWFGESTIDYYMDVW	IGH	8	None	
10	3595	CAKVADMTTTPSDYW	IGH	8	None	
10	3596	CAKVNSGHKLDAFDIW	IGH	2	None	
10	3597	CARDRGGFNHFDYW	IGH	38	None	
10	3598	CARGQRWLQISFDPW	IGH	6	None	
10	3599	CARSTSSSWSPDAFDIW	IGH	56	None	

10	3600	CARDPTYYDIHWFDPW	IGH	4	None	
10	3601	CASGPRSSSWHASYFDYW	IGH	14	None	
10	3602	CARQRRWLQLGYYFDYW	IGH	9	None	
10	3603	CATAMSTVTTSPTLFVHYYFDYW	IGH	5	None	
10	3604	CARADPATTMIVVAHSPPPDYW	IGH	3	None	
10	3605	CASPGDRYYDFWSGYYPGDYYYYYGMDVW	IGH	2	None	
10	3606	CAKDILRGAGWRWYYYYGMDVW	IGH	2	None	
10	3607	CAGPPTGYYGSGSFHFDYW	IGH	2	None	
10	3608	CTGDGYSSSWYFYYGMDVW	IGH	7	None	
10	3610	CARGGYNYEEYIDFW	IGH	2	None	
10	3611	CALPISAVYYCATDFAIAVAGTLDYW	IGH	2	None	
10	3612	CAKIDGSTSRTHYFDYW	IGH	6	None	
10	3613	CAKLQSGEIFDYW	IGH	19	None	
10	3614	CLRSEDTAVYYCARVVMPPDFWSGYYMDVW	IGH	2	None	
10	3615	CARVGYCSSTSCYPLVSNYYYYGMDVW	IGH	3	None	
10	3616	CARERSYGSSWYYYYMDVW	IGH	9	None	
10	3617	CAKDQEIAVAGDNDYW	IGH	10	None	
10	3619	CANNIPAYCGGDCRRTYYYYYGMDVW	IGH	2	None	
10	3620	CARHLVHTPDKYYEIFGVVTVYYFDYW	IGH	2	None	
10	3621	CARGNYYGAANDYW	IGH	6	None	
10	3622	CAIGGWRLRDGYAYNWFDPW	IGH	3	None	
10	3623	CARLTYCSSTSCYNILLNPFDYW	IGH	2	None	
10	3624	CARLSVWFGENPQEGDAFDIW	IGH	2	None	
10	3625	CARLSHKKYSSSWYFDYW	IGH	3	None	
10	3627	CARDQEEDTIFGVVISEWPDAFDIW	IGH	2	None	
10	3628	CARPYYGGSRRYFDLW	IGH	4	None	
10	3630	CARPHGYSDYGIMGYDAFDLW	IGH	3	None	
10	3631	CARTDLSGYYFMDYW	IGH	3	None	
10	3632	CARSPGGNDFWSGYYYYYYYMDVW	IGH	2	None	
10	3633	CARGIPEYSSFVRLDYW	IGH	5	None	
10	3634	CARGHHSLSLWYFDLW	IGH	14	None	
10	3635	CASSRGPYSSGWYSGFDYW	IGH	7	None	
10	3636	CARAAYDSTGYHLSSFLYW	IGH	11	None	
10	3637	CARGGDDSSGWIAYYFDYW	IGH	10	None	
10	3638	CARENPSGSWFDPW	IGH	11	None	
10	3639	CAKVDVDTAMPHYYFDYW	IGH	3	None	
10	3640	CAKSTTQTYYDFWSGYSFFDSW	IGH	2	None	
10	3641	CARVGYDFWSGYYHIDYW	IGH	3	None	
10	3642	CARDSRISDYSGNHRNLDYW	IGH	4	None	
10	3643	CAMLGYCSGGTCYSFGRWFDPW	IGH	2	None	
10	3644	CARGYNSGWSINPYYLDYW	IGH	2	None	
10	3645	CASSGVNYYDSSGYSIDYW	IGH	6	None	
10	3646	CASLMTTDTRYYYYMDVW	IGH	6	None	
10	3647	CAREGPYIPLRYFDWLTPRTRGHDAFDIW	IGH	8	None	
10	3648	CARHPITMVRGVTRWDFDYW	IGH	19	None	
10	3649	CAKDFAYYDSSAYYPDYW	IGH	3	None	
10	3650	CARATRWLHFDYW	IGH	6	None	
10	3651	CARDLQGELITIFGVVTWTGWFDPW	IGH	5	None	
10	3653	CARDLRSGNSEAFDIW	IGH	2	None	
10	3654	CARDRVAGLYYYYYYGMDVW	IGH	5	None	
10	3655	CAHRPLITGTGWLVAFDIW	IGH	2	None	
10	3656	CARQPVVYAIHLLVPNYYYYYMDVW	IGH	4	None	
10	3658	CTTEGYSNYYYYYGMDVW	IGH	4	None	
10	3659	CAKLQWDSSVLLRDYW	IGH	7	None	
10	3660	CARDRATVTTRTTGGPFRYYYYYGMDVW	IGH	2	None	
10	3661	CARGTFCENSNCRSDGFDLW	IGH	2	None	
10	3662	CARHRDSSGWYKSEFDYW	IGH	2	None	
10	3663	CAVYGGNSDMNYFDYW	IGH	5	None	
10	3664	CARCPPDRYFDWLSYYFDYW	IGH	2	None	
10	3666	CAALPYLRANPADYW	IGH	2	None	
10	3667	CARETALRYFDWSPPGVAYYFDYW	IGH	6	None	
10	3668	CARRPIAARRAAWYYYYGMDVW	IGH	4	None	
10	3670	CARNRGGTFSFFDSW	IGH	2	None	
10	3671	CARDHVDAPEYKITNEWFDPW	IGH	8	None	
10	3672	CSGGSCYSLFPYYYYYYGMDVW	IGH	2	None	
10	3673	CAKSAAYYDFWSHATWPYYFDYW	IGH	3	None	
10	3674	CARDYGEYYDFWSGYGFAFDIW	IGH	20	None	
10	3675	CATGPVSGYQNWFDPW	IGH	229	None	

10	3676	CAKDLYYDSTGYYEYYFDIW	IGH	2	None	
10	3677	CARDPTGRDLPAPPNINWFDPW	IGH	2	None	
10	3678	CAHRPPLRFSSSGEFDYW	IGH	6	None	
10	3679	CARPLPHYDYVWGTNGGFDYW	IGH	2	None	
10	3680	CTTGVYLWLGSDYW	IGH	6	None	
10	3681	CARHFIPERVSYCTTISCPTDYW	IGH	5	None	
10	3683	CAREGGAHYGDFRLDYW	IGH	2	None	
10	3684	CQSADSSGSYVVF	IGL	31378	None	
10	3685	CARDLSSFHYDSSGYWGVSGSFDFW	IGH	2	None	
10	3686	CARDADSSGWYPRRVADYW	IGH	3	None	
10	3687	CATEAWGAFDVW	IGH	10	None	
10	3688	CNSRDYSGNRLLF	IGL	5	None	
10	3689	CARYLGYCTTGVCYPFDYW	IGH	5	None	
10	3690	CARNRDSKGYYNPFDDW	IGH	5	None	
10	3691	CARERDYYDTSGYYFDFW	IGH	2	None	
10	3692	CAGARGSSLDYW	IGH	2	None	
10	3693	CAKDGPYYGDYVGAFDIW	IGH	4	None	
10	3694	CARARDHYDSSSYYGFDYW	IGH	3	None	
10	3695	CMSVDIEDLDYW	IGH	3	None	
10	3696	CAKLGFADLYDYVWGSYRSFDAFDIW	IGH	2	None	
10	3697	CARADQDSLLWFGELPRSSGYFDYW	IGH	4	None	
10	3698	CAKAFFDWDPLDYW	IGH	2	None	
10	3699	CATRGYSDAYFDYW	IGH	37	None	
10	3700	CGVGSSTTSYYYYGMDVW	IGH	5	None	
10	3701	CARGVPYDSSGYSLDAFDIW	IGH	6	None	
10	3702	CARVHGGTGWFDPW	IGH	7	None	
10	3703	CARTTVPGQGYFDYW	IGH	4	None	
10	3704	CAKDDRPDSSSFDYW	IGH	20	None	
10	3705	CARDPGIFGSFDPW	IGH	2	None	
10	3706	CARLISRIAATDTGAFDIW	IGH	24	None	
10	3708	CARGGTQWPDASDIW	IGH	4	None	
10	3709	CARNPYYYSNSAFDLW	IGH	2	None	
10	3710	CARNRNSFYSFDIW	IGH	4	None	
10	3712	CARVIGYDSSGYADAFDIW	IGH	3	None	
10	3713	CAKDRYSSGWAQIDYW	IGH	2	None	
10	3714	CAKANLYSSPHYYFDYW	IGH	4	None	
10	3715	CARDRGRTSFVGSQYDAFGIW	IGH	6	None	
10	3716	CARELRRPYSSGWYEYFQHW	IGH	2	None	
10	3717	CAHLRGYSYGDAFDIW	IGH	2	None	
10	3718	CARSLNYDFWSGYQGVFDPW	IGH	2	None	
10	3719	CARGKQWPLFDPW	IGH	3	None	
10	3720	CATVLFPSSTNWYYGTFDLW	IGH	7	None	
10	3721	CATSSVLRFLGWFDPW	IGH	4	None	
10	3722	CAREPNSSGWFPAFDYW	IGH	2	None	
10	3723	CSSYTSSSYTSTTTPVVF	IGL	3	None	
10	3724	CARAPAFWSGFTEFDPW	IGH	5	None	
10	3725	CALPISAVYYCARDIRGGYSYGYAPWFDPW	IGH	2	None	
10	3726	CATAYRLYIAVAAYFDYW	IGH	7	None	
10	3727	CVRDSSAGYGYFDIW	IGH	8	None	
10	3728	CATGIAMADTSYYFDYW	IGH	4	None	
10	3729	CARDGTLATWPPLYYMDVW	IGH	2	None	
10	3730	CARVPSNYPDSGAFDIW	IGH	2	None	
10	3731	CAKSKSADYGDYHRTPFDHW	IGH	2	None	
10	3732	CAKGWIEGSYYDLKAFDIW	IGH	2	None	
10	3733	CARDGDYYDSSGYWTILFDYW	IGH	8	None	
10	3734	CARAGITIFGVVTSFDYW	IGH	6	None	
10	3735	CTRVRYYDFWSGSRPYAFDIW	IGH	2	None	
10	3736	CQSYDSRLSRSMIF	IGL	5	C4	7JVC
10	3738	CARGVSSATMTVLVFTSAMEYFDHW	IGH	6	None	
10	3739	CAKDMEQHHYDFWSGYYTDYFDYW	IGH	2	None	
10	3740	CARNIRRDGYNLDYW	IGH	31	None	
10	3741	CARDRGAAAGIDYW	IGH	25	None	
10	3742	CARVRGNFYGEYDFDYW	IGH	2	None	
10	3743	CATTPRGLEQDYYFDYW	IGH	3	None	
10	3744	CARGVSRGVVVPAAIYVSGWFDPW	IGH	2	None	
10	3745	CDWSSDVCSSDLKTEDTAVYYCTSTTVTTHDYW	IGH	2	None	
10	3746	CARAYTNHRFDFW	IGH	2	None	
10	3747	CARALPYYYDSSGWAYFDYW	IGH	3	None	

10	3748	CLGGGNPWEDYW	IGH	2	None	
10	3749	CAMLGGGNSRAGAFDIW	IGH	4	None	
10	3750	CARDLGYTNNWYHDFW	IGH	3	None	
10	3751	CASSRERWLPASFDYW	IGH	52	None	
10	3752	CAKVGTAMDGDYW	IGH	16	None	
10	3753	CARDPVSDYDFWSGYIYYYYYGMDVW	IGH	9	None	
10	3754	CARDGGSTTESYNYYYGMDVW	IGH	3	None	
10	3755	CARGATIFGVVMNGFDPW	IGH	2	None	
10	3756	CARDRTDSGWYLGDYYYYGMDVW	IGH	2	None	
10	3758	CMQRIEFPWTF	IGK	672	None	
10	3759	CYSMPGGLAFDIW	IGH	2	None	
10	3760	CARVSWSPSGWKIFDYW	IGH	11	None	
10	3761	CARVPQHCRDFTCYPPVDGFDIW	IGH	12	None	
10	3762	CARASGNYSPFDHW	IGH	5	None	
10	3763	CASGDYVWGSYRYTAAFDIW	IGH	4	None	
10	3764	CARGFPLGGGLLGYFDYW	IGH	2	None	
10	3765	CTTDLYGVVVAATLYDYVWGSGIRPIDYW	IGH	2	None	
10	3766	CARYSGYERVIFDYW	IGH	4	None	
10	3767	CARQISGYDILTGPVLNDAFDIW	IGH	7	None	
10	3768	CVKDIYSYVKAYYFDYW	IGH	2	None	
10	3769	CATLPGDYGDYGPDYW	IGH	15	None	
10	3770	CARELYYDFWSGFGRWGSSAQLNAFDTW	IGH	2	None	
10	3771	CARVRITMIVVAERGGRYFDYW	IGH	2	None	
10	3772	CATGTPFPDSSGYLNWFDPW	IGH	3	None	
10	3773	CATDSYDFWSGYPRYYGMDVW	IGH	29	None	
10	3774	CAKDISAIPSWVAIDYW	IGH	10	None	
10	3775	CARDVGYGGNLVYYFDYW	IGH	11	None	
10	3776	CASNHQLGWGFLFDYW	IGH	11	None	
10	3777	CARAERYSGSFDYW	IGH	2	None	
10	3778	CATVGITGTYPDYW	IGH	7	None	
10	3779	CAKSSGYYSEYFQHW	IGH	4	None	
10	3780	CVKGYYSSGGNTFDIW	IGH	3	None	
10	3781	CAKDLSRGRGWDFDYW	IGH	4	None	
10	3782	CATDSRYYYKNFDSW	IGH	27	None	
10	3783	CAREGRYYDSSSQGNAFDIW	IGH	3	None	
10	3784	CARDSSLGLVVVAMDGFDYW	IGH	2	None	
10	3785	CATAPALSSPTAYNWFDPW	IGH	4	None	
10	3786	CATVRSQLTLHYYYYYMDVW	IGH	2	None	
10	3787	CARNNKYTSTWYEAPFDYW	IGH	8	None	
10	3788	CARHFPGIRYFDWSATTVAWFDPW	IGH	12	None	
10	3789	CARSYDFWSGYPRYYFDYW	IGH	7	None	
10	3790	CAKDTLYDSSGYYLNAFDIW	IGH	7	None	
10	3791	CATSHQLELHLDYW	IGH	8	None	
10	3792	CARDKGFWSGYYTGYYYYGMDVW	IGH	2	None	
10	3793	CASVSGELVADYW	IGH	3	None	
10	3794	CARLTYYSYGYRWFDPW	IGH	2	None	
10	3795	CARGTFQDYGDSASLDYW	IGH	4	None	
10	3796	CTTDLVLPYYDILTGYYPFDYW	IGH	12	None	
10	3797	CARDRSGWYLGYMDVW	IGH	3	None	
10	3798	CAHNNAIFYFDYW	IGH	10	None	
10	3800	CARNPYYYDSSGYYQPALFDYW	IGH	2	None	
10	3801	CARAFAYSSSWYGVPNQDYW	IGH	3	None	
10	3802	CAKDFNYDFWSGQGNYFDYW	IGH	48	None	
10	3803	CARDQNGSWYAVDYW	IGH	6	None	
10	3804	CTKGFINSGHVVGFDPW	IGH	2	None	
10	3805	CASAVYYDFWSGYYLGWFDPW	IGH	8	None	
10	3806	CARRRSLITMIVVVQYFDYW	IGH	68	None	
10	3807	CARAERARGYYDSIGYYLPFDYW	IGH	2	None	
10	3808	CATGPSRPFGVVTPNWFDPW	IGH	4	None	
10	3809	CAKEGYYGDRRSPLPFDPW	IGH	2	None	
10	3810	CARQVGGYDYGAYYYYYMDVW	IGH	4	None	
10	3811	CARGVGWFGELTWFDPW	IGH	3	None	
10	3812	CARPGYSYGPEGEWFDPW	IGH	5	None	
10	3813	CARGDIVVVVAATPNDYW	IGH	28	None	
10	3814	CARDGPKYYYDSSGYYPPSWDYYYMDVW	IGH	7	None	
10	3815	CAKDGGDWNYEVPFDYW	IGH	2	None	
10	3816	CARAAGDSTADYMDVW	IGH	2	None	
10	3817	CALPISPTQRTATHFDYW	IGH	2	None	

10	3818	CARHFATMLRGVLFYFDYW	IGH	2	None	
10	3819	CARHRPTTIFGVVITPSYFDYW	IGH	3	None	
10	3820	CAKDLVRLNQLDYW	IGH	4	None	
10	3821	CTTDGTIDGSYYRYYYYGMDVW	IGH	6	None	
10	3822	CARAPSGISYPNAFDFW	IGH	4	None	
10	3824	CATDLPYSGYGRFDYW	IGH	11	None	
10	3825	CARHLSGFSGLTYYYYYMDVW	IGH	2	None	
10	3826	CVRERGGDYLDYW	IGH	2	None	
10	3827	CASDLRFLEWLYHDAFDIW	IGH	7	None	
10	3828	CAHRRSPAPTDIDWFDPW	IGH	4	None	
10	3829	CARPNGNYFAYFDYW	IGH	3	None	
10	3830	CARMSFENLGDYDYFDYW	IGH	10	None	
10	3831	CATDSAREYQLLFQIHNTDAFDIW	IGH	3	None	
10	3832	CARDRNTVFGVDRTGFAFDYW	IGH	3	None	
10	3833	CANLRLGELSLSDSYFDYW	IGH	10	None	
10	3834	CARERAGFTMIPFDYW	IGH	6	None	
10	3835	CARVPPGRAAAGNDYW	IGH	4	None	
10	3836	CARDLSIVGPTSDAFDIW	IGH	5	None	
10	3837	CVRDLRRDDVSTGFYKWRWFDSW	IGH	2	None	
10	3838	CQSADASTTSVLF	IGL	8	None	
10	3839	CARDRRDGEGDFWSGYSNYYYYMDVW	IGH	14	None	
10	3840	CASHPGNSNYDILTGYFFIPSYGFDYW	IGH	6	None	
10	3841	CQQYDTYSLAWTF	IGK	2	C3	6WPS
10	3842	CARDHSETTYSDSSDYLPDYW	IGH	2	None	
10	3843	CARGPYYDFWTEGWFDPW	IGH	14	None	
10	3844	CARAYYYDTSGYNDFDLW	IGH	11	None	
10	3845	CARDGQLVEADYYYYGMDVW	IGH	2	None	
10	3846	CARRPGYCTGGVCYAFDIW	IGH	9	None	
10	3847	CATHTVTTPNYWYFDLW	IGH	2	None	
10	3848	CARVGWGGSSSFHYYYYMDVW	IGH	4	None	
10	3849	CAKEPILYYYDSSHERYFDYW	IGH	8	None	
10	3850	CAKLVGDDYGDYVVADYW	IGH	2	None	
10	3851	CARDLNSYSANYYRAYYFDYW	IGH	39	None	
10	3852	CAREGGDLSSSEVDYW	IGH	22	None	
10	3854	CAGSSGWPNWFDPW	IGH	6	None	
10	3855	CPQHDNFLWTF	IGK	9	None	
10	3856	CARDKGSYYDSSGYQGWFDTW	IGH	4	None	
10	3857	CDWSSDVCSSDLYCARPEGGYSTSWVDYW	IGH	8	None	
10	3858	CVAGGGSCYDHSYCRDYW	IGH	5	None	
10	3859	CATGPATAIPDWFDPW	IGH	34	None	
10	3860	CAVLRFLEWPLDYW	IGH	2	None	
10	3861	CAKDPHYDYVGMINWELDYW	IGH	2	None	
10	3862	CAKDFGGGGLSIFGVVIIGTFDYW	IGH	4	None	
10	3863	CTITQDTAMVTYYYYGMDVW	IGH	2	None	
10	3864	CATASVVRALSYGSGSYYNGENYYYYYGMDVW	IGH	2	None	
10	3865	CARGQYSDSSGWYRLYGDYYYYGMDVW	IGH	2	None	
10	3866	CTRGVVVTTSPTSYYYYGMDVW	IGH	2	None	
10	3867	CASCSGGDCYNNYYYMDVW	IGH	2	None	
10	3868	CAKGTYYDSSVPLFSDYW	IGH	2	None	
10	3869	CARATLGRNWNGPLDYW	IGH	4	None	
10	3870	CARRHQASSSWYEGAFDIW	IGH	2	None	
10	3871	CARGRRLQLWLGSFFDSW	IGH	3	None	
10	3872	CAKADVVVVTAIHPHAFDLW	IGH	4	None	
10	3873	CARDIYNWNDGEGNWFDPW	IGH	3	None	
10	3874	CARDPCTGYSCYSYFDDW	IGH	2	None	
10	3875	CTRTPTQRTATHFDYW	IGH	91	None	
10	3876	CVKDTTPIAPAGGFDFW	IGH	8	None	
10	3877	CAGISSGWFGGSPNWFDPW	IGH	6	None	
10	3878	CTTDLPSEHYDFWSGYWRDPAYYYGMDVW	IGH	5	None	
10	3879	CAKGDRAVPPTSALDYW	IGH	4	None	
10	3880	CARDTHYWYFDLW	IGH	34	None	
10	3881	CARTPSYYDFWSGYSPRPSTLGAFDIW	IGH	9	None	
10	3882	CAREGSDFNAVPFDYW	IGH	6	None	
10	3883	CARAPEILGPRGVIPYAFDIW	IGH	5	None	
10	3884	CAHRPAIYSSSWYPTAYWFDPW	IGH	4	None	
10	3886	CARIEVDYDYVWGSYRLHSFDYW	IGH	2	None	
10	3888	CMQALQTPLTF	IGK	8486	None	
10	3889	CVKDDYYASGNFASNFDCW	IGH	4	None	

10	3890	CARHPGIEYYDFWSGYSAENWFDPW	IGH	8	None	
10	3891	CAKGPPIGEGYYYGMDVW	IGH	3	None	
10	3892	CQICDFWSGYYSDYW	IGH	5	None	
10	3893	CAKDALVTRLSYYDTLTGQEGWFDPW	IGH	4	None	
10	3894	CARDMRVYGHYYYYMDVW	IGH	4	None	
10	3896	CAKGLSPASMITFGGVIVYPLDYW	IGH	11	None	
10	3897	CARALPDSSGWFDAFDIW	IGH	4	None	
10	3898	CARSGGDTAMVIAYYFDYW	IGH	3	None	
10	3899	CARDLDPHRAAYVDYW	IGH	4	None	
10	3900	CAKDIRYFDWLPNELDYW	IGH	13	None	
10	3901	CARVGITMVRGVIHNWFDPW	IGH	8	None	
10	3902	CAKDLLGVGGSSDYW	IGH	2	None	
10	3903	CARVPPYYYDSSGRFVSAFDIW	IGH	4	None	
10	3904	CARDGSGYYDSSGYLDYW	IGH	33	None	
10	3905	CARGVNVTWNPETAYFAMDVW	IGH	3	None	
10	3907	CAREGRNYDILTGFQYYYGMDVW	IGH	3	None	
10	3908	CARDPPAGNYWSGYYPTGYFENW	IGH	30	None	
10	3909	CAKAPFPYDYGDAFDIW	IGH	8	None	
10	3910	CARDPGIAVAGHHYFDYW	IGH	7	None	
10	3911	CATWIQLWSSYFDYW	IGH	4	None	
10	3913	CAKDSWRWGKRYFDLW	IGH	4	None	
10	3914	CASGSSGYYPASDYW	IGH	15	None	
10	3915	CAKDIFWGENSYGPYYYGMDVW	IGH	4	None	
10	3916	CATPRHPYDFWSGSFDYW	IGH	5	None	
10	3917	CARDPWLKWEPDYW	IGH	2	None	
10	3919	CARDSYYGSGSYRRVGAFDIW	IGH	2	None	
10	3921	CAREAAYQLISGSYYYYGMDVW	IGH	13	None	
10	3922	CAKDHGIYDSSGYYSDDAFDIW	IGH	2	None	
10	3923	CARVIPTYCSSTSCYYADHYYYYGMDVW	IGH	2	None	
10	3924	CAKDIGRFTYGDYSDYW	IGH	9	None	
10	3925	CATLPEVTALVIIDYW	IGH	2	None	
10	3926	CAHRVPLRGWDYW	IGH	15	None	
10	3927	CARASGKYYYDSSGYYHDDAFDIW	IGH	4	None	
10	3928	CAINPTTGGELVDYW	IGH	4	None	
10	3929	CAKEEGFYDSSGYYYPWGAFDIW	IGH	2	None	
10	3931	CARDGTMVRGELDYW	IGH	2	None	
10	3932	CAKDGKVFGYGDHFDYW	IGH	6	None	
10	3933	CARGRTPRYSYGYDWYFDLW	IGH	2	None	
10	3934	CARGRSYYYDSSGYYPPGFDYW	IGH	2	None	
10	3936	CARDFLKWELLGQDYFDYW	IGH	2	None	
10	3937	CARQPFFCDTNSCWLFDSW	IGH	2	None	
10	3938	CARARGPYSSGFYFDHW	IGH	2	None	
10	3939	CARDSSGYGSGWHYFDLW	IGH	4	None	
10	3940	CARVGPFYGDYGWFDPW	IGH	14	None	
10	3941	CARDSGGVVTSFEYW	IGH	2	None	
10	3942	CARESLDTNYYDYFDPW	IGH	2	None	
10	3943	CMQSVQSPPFTF	IGK	25	None	
10	3944	CARGICSSASCVGVNWFDLW	IGH	6	None	
10	3945	CTRVTPGEGQDDYW	IGH	6	None	
10	3946	CAKDRVWGGTWPTGQFDSW	IGH	5	None	
10	3947	CAKDLAYGGNPFFDNW	IGH	2	None	
10	3948	CSGGSCYSIYYFDYW	IGH	2	None	
10	3950	CARDALDSSGWYDWYFDLW	IGH	15	None	
10	3951	CATTPYSYDNSGHYRESWFDSW	IGH	2	None	
10	3952	CTRVRSGYGDYSVGYYFDYW	IGH	2	None	
10	3953	CAQQHIARPFEYW	IGH	2	None	
10	3954	CAKVQVRDIWSGYYDGW	IGH	2	None	
10	3955	CARDIPFSMSEYYMDVW	IGH	15	None	
10	3956	CARVRRIWSGPHPKNWFDPW	IGH	2	None	
10	3957	CARDVPVAPSTEDAFDIW	IGH	4	None	
10	3958	CARDLELSGAFDIW	IGH	14	None	
10	3959	CTTDIYYDFWSGQPPSLFDYW	IGH	3	None	
10	3960	CANLPSANYGAFDIW	IGH	12	None	
10	3961	CARESTLSIAAAHFDPW	IGH	2	None	
10	3962	CATGIGTGNFLGFDSW	IGH	3	None	
10	3963	CYSTDSSANLGVGVF	IGL	5	None	
10	3964	CANLGSGSYGAQIDYW	IGH	2	None	
10	3965	CAKVTETYYDFWSGYYTGPFDYW	IGH	15	None	

10	3966	CARFQSPAITVTAMLFDSW	IGH	5	None	
10	3967	CARDAKYYDFWSGYARGSPQSNYYYYMDVW	IGH	3	None	
10	3968	CARADYCGSGSFCNNFVYW	IGH	4	None	
10	3969	CARVDGVPYDYYILTVLWFRPGIDYFDYW	IGH	2	None	
10	3971	CARDHNTYYDFWSGYYEYYFDYW	IGH	28	None	
10	3972	CAKSERGWGAFEYW	IGH	2	None	
10	3973	CAKGLLIHYGYFDYW	IGH	4	None	
10	3975	CAKDMVYDSSGYYKNGFDYW	IGH	2	None	
10	3976	CALPISAVYYCARWGVLVGSTWRDAFDIW	IGH	2	None	
10	3977	CARTVKMLLWRTAYYYYIDVW	IGH	2	None	
10	3978	CARGRVLRYFDYYYYYYMDVW	IGH	2	None	
10	3979	CAKEGDYYDSGPFAPHFDHW	IGH	6	None	
10	3980	CTRASGGHSYGDYW	IGH	3	None	
10	3981	CAKAPQWLGVWYFDLW	IGH	2	None	
10	3982	CARDFALEGPTSDAFDLW	IGH	2	None	
10	3983	CACIAVAGMGIDYW	IGH	2	None	
10	3984	CARVLPYYDQYDYW	IGH	2	None	
10	3985	CARDSAIVQAADGMDVW	IGH	3	None	
10	3986	CAKDLDYYESSEYYPIDAFEMW	IGH	4	None	
10	3988	CARVMEQLVLFDYW	IGH	7	None	
10	3989	CARQYYDFWSAYSHYYYYYMDVW	IGH	2	None	
10	3990	CAKDPRTAGFLEWLLSPTNFDYW	IGH	3	None	
10	3991	CAKGRVVIIAHYYMDVW	IGH	10	None	
10	3992	CATSVEVTGPDAFDVW	IGH	7	None	
10	3993	CARHIAARLVWFDPW	IGH	2	None	
10	3994	CAKGQVETPIVDRLDYYYGMDVW	IGH	4	None	
10	3995	CARDIGSQEVVVINTDYW	IGH	4	None	
10	3996	CARKYCSGGSCYFDYW	IGH	27	None	
10	3997	CARYCSGASCSGANWFDPW	IGH	2	None	
10	3998	CTRGPRYDYVWGRYFDYW	IGH	5	None	
10	3999	CALPILRAEDTAVYYCARDSGRPQSDFEYW	IGH	3	None	
10	4000	CARLVATGVVVITTPYYFDYW	IGH	9	None	
10	4001	CARFVFVSSGSTRFDLW	IGH	2	None	
10	4002	CAKDINSNYPKDYW	IGH	9	None	
10	4003	CAHRRRPPNLVEGSLRGIWFDPW	IGH	3	None	
10	4004	CARVRSSWYMGEGFDPW	IGH	11	None	
10	4005	CAKFPEAVAGTAPRDYW	IGH	2	None	
10	4006	CARLSVTKISYYYYMDVW	IGH	4	None	
10	4007	CAKYPSGDYGDLSDYW	IGH	4	None	
10	4009	CARHYTSDFWNDGLDVW	IGH	2	None	
10	4010	CARHGLASIWSGRHPRTLDYW	IGH	4	None	
10	4011	CAALPPPWELAPTFFDYW	IGH	2	None	
10	4012	CARERIQLGFLGPDAFDIW	IGH	4	None	
10	4013	CARHENTDWFDPW	IGH	218	None	
10	4014	CARDPTVSPYDYGGEGAIDYW	IGH	2	None	
10	4015	CAKNQGEGHCPSGTCYPENGFDVW	IGH	5	None	
10	4016	CTTGFNVVGDDAPRDRLYKDVW	IGH	5	None	
10	4017	CATGVGIAVAGTDYFDYW	IGH	2	None	
10	4018	CARSATDGALLYYYGSGSYWFDPW	IGH	4	None	
10	4020	CAKISTSDYYDSSGYHDYW	IGH	11	None	
10	4021	CAHRGTAALIGFDYW	IGH	2	None	
10	4023	CARGSLFPYSSSSGGDLDYW	IGH	2	None	
10	4024	CARGTGSQIVVTLDAFDIW	IGH	22	None	
10	4025	CARIGGDYYDSSGYEEDFDYW	IGH	11	None	
10	4026	CARNGDISWSPLYYFDYW	IGH	4	None	
10	4027	CARGPVYYNILAGYYNSPDYFDYW	IGH	4	None	
10	4028	CASSGSYYDTEADYW	IGH	6	None	
10	4029	CANLPSRDGYNGDYW	IGH	2	None	
10	4030	CARGVVCGGDCYFFDYW	IGH	16	None	
10	4031	CAKAPTEEVPSPWVDYW	IGH	16	None	
10	4032	CAHGFYCGGDCSIVGFDYW	IGH	2	None	
10	4033	CASGYYYDSSAYGSGDYW	IGH	4	None	
10	4034	CAKSSVVTAILRDVYFDYW	IGH	2	None	
10	4035	CARDRRPTPNHYYYGMDVW	IGH	3	None	
10	4036	CAKDANSPGFDWLIPRNLYYFDYW	IGH	4	None	
10	4037	CARVEEGVATDLDYW	IGH	2	None	
10	4038	CARRRGGLAAAGTGTDYFDYW	IGH	27	None	
10	4039	CATHSYNLGTTDYW	IGH	2	None	

10	4040	CARDLRDGYTRTTHHAFDIW	IGH	2	None	
10	4041	CAREPLIYDTQYADTSDYLEAFDVW	IGH	2	None	
10	4043	CVTETAPYCSSTSCYTAFDYW	IGH	2	None	
10	4045	CAKDYFISGSYYSPWYFDYW	IGH	2	None	
10	4046	CARLKRITIFGVSVGAWYMDVW	IGH	2	None	
10	4047	CAKAYYYDSSGYLVLGDAFDIW	IGH	2	None	
10	4048	CTTDYFYDRTTYYAFDYW	IGH	2	None	
10	4049	CAKVGGYSYGYEGLGPSSFDPW	IGH	4	None	
10	4050	CARDTAYCSGGSCYSGDYYYGMDVW	IGH	2	None	
10	4051	CARDADGYCSSNSCYLDYW	IGH	7	None	
10	4052	CARCRFWTGYNWFDPW	IGH	7	None	
10	4053	CARHHARAYYDFWSGYYRFDPW	IGH	2	None	
10	4054	CARLQFGPVAGTEWFDPW	IGH	3	None	
10	4055	CARATLGGNYYYMDVW	IGH	2	None	
10	4056	CARGARLGLFDSVGHFDYW	IGH	2	None	
10	4057	CAKSSSSVWYPTRYYYYMDVW	IGH	9	None	
10	4058	CARDLSRDWNQAFDPW	IGH	2	None	
10	4059	CAKAPTVMDWYFDLW	IGH	7	None	
10	4060	CAREISIVATIFPSYGDYQYYFDYW	IGH	13	None	
10	4061	CAKDGNDYGDYKGWFDPW	IGH	12	None	
10	4062	CAEQRITIFGVEIDYW	IGH	6	None	
10	4063	CAKEKEGSGYYQLYYYYGMDVW	IGH	2	None	
10	4064	CAKDSHFDWLLSLSPSYYFDYW	IGH	2	None	
10	4065	CARGVRIAVTSTQYYYYGMDVW	IGH	6	None	
10	4066	CARRAITLFGVGPYYFDNW	IGH	4	None	
10	4067	CARDRPDDYVWGGFDYW	IGH	72	None	
10	4068	CATAPAKEQWLTPYYYYDMDVW	IGH	3	None	
10	4069	CARGLYYYDSSGYRRFSPFDYW	IGH	2	None	
10	4070	CARRNILTGYSPDVFDYW	IGH	4	None	
10	4071	CARDEQWLVQGDYW	IGH	9	None	
10	4072	CVKDIVPGISNAMDVW	IGH	2	None	
10	4073	CQSYDNSPSGSEFVF	IGL	2	C4	7JVC
10	4074	CARSPTTVLAVAATGWFDPW	IGH	2	None	
10	4075	CANGASVAGKGGDYW	IGH	2	None	
10	4076	CATFSWIQLWLRGGYFDYW	IGH	11	None	
10	4077	CAAWDVSTNGYSVF	IGL	8	None	
10	4078	CAREDDSSGRTDAFDIW	IGH	6	None	
10	4079	CASWNYDLTGDYYYYMDVW	IGH	4	None	
10	4080	CAKVVGGIAVAGWYYFDYW	IGH	4	None	
10	4081	CARESIAADPKWPYFDYW	IGH	4	None	
10	4082	CARRDDFWSGYSYYYGMDVW	IGH	4	None	
10	4083	CAREPFTQWQGETIDYW	IGH	23	None	
10	4084	CQQYYTTPQDSGTF	IGK	6	C4	6W41
10	4085	CAKDMERWLQWREDDAFDIW	IGH	6	None	
10	4086	CARDMVDYYDSSGYYGVHAFDIW	IGH	3	None	
10	4087	CARGANDFWSGYYTMRYYYYMDVW	IGH	4	None	
10	4088	CAMLPYHYSSGWYVQLDYW	IGH	2	None	
10	4089	CARDERGSATSGYYSPYYFDPW	IGH	10	None	
10	4090	CAILEMATIPDAFDIW	IGH	2	None	
10	4091	CARVRAVAGSLLHDAFDIW	IGH	6	None	
10	4092	CARDIDTDRWESIGADYW	IGH	2	None	
10	4093	CARDGCAQPCGQYYWYFHLW	IGH	3	None	
10	4094	CAKFSGYHMPSVDYW	IGH	6	None	
10	4095	CARDFSMGSLATVVTLGVDYW	IGH	6	None	
10	4096	CATAHFRFGELLYTPFDYW	IGH	3	None	
10	4097	CARDGGTTGWPDWYFDLW	IGH	8	None	
10	4098	CANPDPYYDDTNEGFDPW	IGH	2	None	
10	4099	CARETIVVDDSRFDYW	IGH	941	None	
10	4100	CARHDITMIVVVTYPYAFDIW	IGH	2	None	
10	4101	CAHMGITMVRGIDYW	IGH	4	None	
10	4102	CALTPGYSSVPFDYW	IGH	2	None	
10	4103	CARGEGYDVLTGFDYW	IGH	2	None	
10	4104	CARQDYFQRRGSAAFDVW	IGH	3	None	
10	4105	CAKVWGRYDTGDYW	IGH	2	None	
10	4106	CARSSLLRTRKFDHW	IGH	10	None	
10	4107	CARGSLTYYDFWSGQRDGMDVW	IGH	2	None	
10	4108	CARGNGEAYGSGDYW	IGH	3	None	
10	4109	CARSLLGSCSGGRCFGYYFDYW	IGH	2	None	

10	4110	CARSRITIFPYTSLDAFDIW	IGH	3	None	
10	4111	CASRSTYYDFWSGYLEGGNWFDPW	IGH	4	None	
10	4112	CARHLEGIITMIVVVIKEGAFDIW	IGH	6	None	
10	4113	CATARYCSGGSCQGSFDYW	IGH	2	None	
10	4114	CARGDYVGQYNWFDPW	IGH	27	None	
10	4115	CARGGYYDSSGYFIRKLYYYYYMDVW	IGH	2	None	
10	4116	CARRVVDTSSDYFDYW	IGH	2	None	
10	4117	CARQLERAHDAFDIW	IGH	9	None	
10	4118	CAREQYYYDSSGFMGHDAFDIW	IGH	12	None	
10	4119	CARLQAYDPFITIFGVVPLSPFDYW	IGH	8	None	
10	4121	CAKDQAVVALDYW	IGH	2	None	
10	4122	CARDEAALFTIFSVVESEFFDYW	IGH	2	None	
10	4123	CARHCSSPRCPKGGFDIW	IGH	4	None	
10	4124	CARTPSDGRFLEWNGCYFDYW	IGH	2	None	
10	4125	CAKLPEPGGDYAFDYW	IGH	6	None	
10	4126	CATTYYYDSSGVLDYW	IGH	2	None	
10	4127	CAKVSIAVAGPTQDAFDIW	IGH	9	None	
10	4128	CAKDLLPFIAAAGTGGGNWFDPW	IGH	4	None	
10	4129	CARDSQLEPLITMIATPYYYMDVW	IGH	10	None	
10	4130	CARDVRLGWPKVYYNYYYALDVW	IGH	6	None	
10	4131	CAREGRALGGVGAGLVFDYW	IGH	148	None	
10	4132	CARIPSSSWEFDYW	IGH	64	None	
10	4133	CTTGELRYFDWLFNTDIYDAFDIW	IGH	2	None	
10	4134	CAKIGGGTEPRGIDYW	IGH	2	None	
10	4135	CTREGSSGSYSPYW	IGH	2	None	
10	4136	CAKGLDDHSSGWSGYYMDVW	IGH	2	None	
10	4137	CAKSAAMVTGWFDPW	IGH	2	None	
10	4138	CAREITGTTVLRGWFDPW	IGH	2	None	
10	4139	CAKLGVERATMGFDYW	IGH	3	None	
10	4140	CARAGFSSARWEPADYW	IGH	11	None	
10	4141	CATDWALTTVTVGGFGYW	IGH	21	None	
10	4143	CARDQEGRDYGDDPFDMW	IGH	2	None	
10	4144	CAKGSDDYGDYVYYW	IGH	4	None	
10	4145	CAKEGIVHTAMVRQYYFDYW	IGH	4	None	
10	4146	CAKGGGKGEILSVNFDCW	IGH	13	None	
10	4147	CTTLGTVEMATTDFDYW	IGH	5	None	
10	4148	CARDPDTTMVNLDYW	IGH	10	None	
10	4149	CARYSSGWFDAFDIW	IGH	44	None	
10	4150	CVRVRRTSSQVAWFDPW	IGH	2	None	
10	4151	CATAPVVVVAANHYYYYYGMDVW	IGH	10	None	
10	4152	CAKDPSGWYGSYW	IGH	5	None	
10	4153	CARAVTYYYDSSGNDAFDIW	IGH	8	None	
10	4154	CARAGWRRCSSTSCLTDYYYYYMDVW	IGH	2	None	
10	4155	CSRSTGYYGAVDYW	IGH	4	None	
10	4156	CVKDIGGRGYSYGSGFDPW	IGH	2	None	
10	4157	CARGPMIDWLFHFDYW	IGH	12	None	
10	4158	CATSLIYYDILTGKITYYYYYMDVW	IGH	7	None	
10	4159	CARASSGWYETNYFDYW	IGH	28	None	
10	4160	CAREIAAADSNWFDPW	IGH	44	None	
10	4161	CATTLRGHYYATQAFDIW	IGH	2	None	
10	4162	CAKDPLKYDSNWYPNWFGPW	IGH	5	None	
10	4163	CAKDGWSSWYLAEEFDYW	IGH	4	None	
10	4164	CARDLYYYDSSGYQTTHWYFDLW	IGH	31	None	
10	4165	CARDPNRDSSGWAFDIW	IGH	2	None	
10	4166	CAREGNLVNWNFLGGYYYYYMDVW	IGH	3	None	
10	4167	CAKDGGYCTGGVCYREYFDYW	IGH	3	None	
10	4168	CARGDLSSGWKYDYW	IGH	3	None	
10	4169	CAKYSSRQQLPNYYFDYW	IGH	4	None	
10	4170	CARGPIIAAAGTPYNWFDPW	IGH	4	None	
10	4171	CARHIGDETLTEYFDYW	IGH	11	None	
10	4172	CARLYDSSGYYYLYYYYYMDVW	IGH	2	None	
10	4173	CAKDLNKQWLGNSYYYYGMDVW	IGH	2	None	
10	4174	CTAYLQWSSLKASDTAMYYCARLALCGGDCHYFDYW	IGH	3	None	
10	4175	CARDQRYYDILTGYFPHPSYYYYYGMDVW	IGH	12	None	
10	4176	CASEEGRITIFGVVITDAFDIW	IGH	23	None	
10	4177	CAKDPGGHDSSASYGTGFLYW	IGH	8	None	
10	4178	CAKVSGSYFSQHYYIPHWFDLW	IGH	13	None	
10	4179	CAKDHKRGGSYSYVFDYW	IGH	4	None	

10	4180	CAKEGGVDVVQGRISFDHW	IGH	29	None	
10	4181	CATDRVGEIVMVEAVHNWFNPW	IGH	17	None	
10	4182	CARPGGDYYDSSDGRFDPW	IGH	2	None	
10	4183	CARGGMATIFIGNWFDPW	IGH	6	None	
10	4184	CARPLGDVYNWNYLGNAFDIW	IGH	3	None	
10	4185	CARGVVDPGPSYYFDYW	IGH	4	None	
10	4186	CAREAKEQQLFDRLHTNNWFDAW	IGH	6	None	
10	4187	CASNLYYYGSGSYHLDYW	IGH	3	None	
10	4188	CAREYSSSWLDGAVGAFDYW	IGH	2	None	
10	4189	CARDSLTQQLVVAVDNAFDIW	IGH	6	None	
10	4190	CAREIRAVAGSGYYYYYGMDVW	IGH	2	None	
10	4191	CARGPNLTSWPRLYYYYGMDVW	IGH	21	None	
10	4192	CAREDTVSDWFDPW	IGH	2	None	
10	4193	CAPAATEGWFDPW	IGH	4	None	
10	4194	CAKDIYGGRAAAGDYW	IGH	5	None	
10	4195	CAPGRFSAVGGPDRGWFDPW	IGH	2	None	
10	4196	CARDPAPYYYDSSGYYGGWFDPW	IGH	2	None	
10	4197	CAGRITMIVVVIRGIAFDIW	IGH	2	None	
10	4198	CATWTVTASEILDYW	IGH	2	None	
10	4199	CAKGDRGYDYGDHGDYW	IGH	2	None	
10	4200	CAHRQGGQRAAGFDSW	IGH	12	None	
10	4201	CAREYSGYDLRPDYW	IGH	2	None	
10	4202	CAKVPYSSSWYGNDYW	IGH	3	None	
10	4203	CATPAGGPNANFDYW	IGH	2	None	
10	4204	CAKDLPPGDNYYYYYGMDVW	IGH	3	None	
10	4205	CARDGEGAVVVVAAYFDYW	IGH	7	None	
10	4206	CARRSTETEWYFDLW	IGH	2	None	
10	4208	CQSADISAFHVVF	IGL	16	None	
10	4209	CAKGLLWFGEYYDYW	IGH	2	None	
10	4210	CARVAVYDFWSGYYHGYGMDVW	IGH	2	None	
10	4211	CAKAFRIVVVMEYYFDYW	IGH	2	None	
10	4212	CARDSPLRYFDWIRFDYW	IGH	2	None	
10	4214	CAREFIGPYCSGGSCHPYNWFDPW	IGH	4	None	
10	4215	CARDPYPLGIAAADGVYYYGMDVW	IGH	2	None	
10	4216	CTRDTHYHDNRGYRNYAFDIW	IGH	2	None	
10	4217	CARVWHIVVVTATDDAFDIW	IGH	2	None	
10	4218	CARHLDSSGYTPPIFDYW	IGH	7	None	
10	4219	CAHQSGYAANWFDPW	IGH	4	None	
10	4220	CARDREDPNYDYVWGSYRYGAFDIW	IGH	3	None	
10	4221	CARVKRDNYYYYYYYMDVW	IGH	9	None	
10	4222	CAKIGEYYDILTGYYRSLNDYW	IGH	2	None	
10	4223	CARITEGDYDDWFDPW	IGH	22	None	
10	4224	CAREKGRTQRSDFMDVW	IGH	2	None	
10	4225	CARGGQYITIFSWSELVTAGWFDPW	IGH	2	None	
10	4226	CARHTTVVTPRNAFDIW	IGH	2	None	
10	4228	CARGGYITMVRGVSTNFDYW	IGH	2	None	
10	4229	CAKDRGIAVAGTGGHFDYW	IGH	2	None	
10	4230	CAKDLPPGYSSSWSYSFDYW	IGH	6	None	
10	4231	CARGRLYNWNYDYW	IGH	2	None	
10	4232	CAKDMNGMDDYGDYVGAFDIW	IGH	3	None	
10	4234	CQSYDTSFAVF	IGL	172	C4	7JVC
10	4235	CAKVSDYYGSGSYPYFDYW	IGH	2	None	
10	4236	CVKPHYVTHGDFYAGKYFFEYW	IGH	3	None	
10	4237	CAHRHRQHGYYDFWSGYYPTLFDYW	IGH	2	None	
10	4238	CTRDRPDMIVVVTTSDYW	IGH	2	None	
10	4239	CLQDYTPPSLTF	IGK	17	None	
10	4240	CARDPTSTVTTSYYYYYGMDVW	IGH	4	None	
10	4241	CARSGASRDGYNRGDAFDIW	IGH	2	None	
10	4242	CARWCFDWYFDLW	IGH	3	None	
10	4243	CATITGDSSGYYWYGMDVW	IGH	2	None	
10	4244	CAKVGYQPLLSPEPDAFDIW	IGH	2	None	
10	4245	CAKFLGGSYYSSLDYW	IGH	3	None	
10	4246	CTQLVDGYYSGTDAFEIW	IGH	5	None	
10	4247	CTKETYDSRPGAFDVW	IGH	2	None	
10	4248	CATSMYCSGDCYKPNDYW	IGH	11	None	
10	4249	CAREGGSYYPDWFDPW	IGH	8	None	
10	4250	CAYAGGPHYYMDVW	IGH	2	None	
10	4251	CARDLYTYSSSWTIDYW	IGH	2	None	

10	4252	CARPPHGYSSGWYVRDYW	IGH	36	None	
10	4253	CAKKMNPSSPDLYFDYW	IGH	2	None	
10	4254	CARSLAAAGHWYFDLW	IGH	2	None	
10	4255	CARDQEDGYKPFDYW	IGH	2	None	
10	4256	CARDSPLGYSSSWSDYW	IGH	2	None	
10	4257	CARFRGSYYDSSGYYSLRTYFDSW	IGH	5	None	
10	4258	CARIYSSGYYYEPYYFDYW	IGH	3	None	
10	4259	CARRGVGTDGFDIW	IGH	3	None	
10	4260	CARYDGNSDDTFDYW	IGH	2	None	
10	4261	CAKAPSYGDYDDNW	IGH	18	None	
10	4262	CARDPGTIFGGNWYFDLW	IGH	7	None	
10	4263	CAKDQESIFGDFPFDYW	IGH	2	None	
10	4264	CAMGPIVEGTYYFDYW	IGH	2	None	
10	4265	CQSADSTDTSDWVF	IGL	83	None	
10	4266	CARHQPWYSSSSDFDYW	IGH	5	None	
10	4267	CARDLSPTVTYDHNAFDIW	IGH	178	None	
10	4268	CARGFFGIAARPVDYW	IGH	2	None	
10	4269	CTTAMTGTWPADFDHW	IGH	3	None	
10	4270	CARDQDWDIWTGFKNWLDPW	IGH	3	None	
10	4271	CAAAGRGVASPLDYW	IGH	5	None	
10	4272	CARGPGYTTPAMDVW	IGH	6	None	
10	4273	CARPSSGGGVVTVEAFDIW	IGH	4	None	
10	4274	CTRHIGPLMDAYYYGMDVW	IGH	4	None	
10	4275	CARTHYDILTGYSWEYDYW	IGH	21	None	
10	4276	CARGPDQYYDILTGYSLGGRFDPW	IGH	15	None	
10	4277	CATGRQYCSSTSCPDWFDPW	IGH	2	None	
10	4278	CTREIYDYVWGSYRPTDYW	IGH	13	None	
10	4279	CAREDHISGYYYVDCW	IGH	4	None	
10	4280	CAKLPDPLDTPRRAFDIW	IGH	2	None	
10	4281	CAKADINTETTPFDYW	IGH	6	None	
10	4282	CAKDIVAWGGDYVALDVW	IGH	3	None	
10	4283	CARKVFEYGKYFDHW	IGH	2	None	
10	4284	CASALFPKVQMAPFDYW	IGH	10	None	
10	4285	CARDNRLGQWPYYYYYMDVW	IGH	4	None	
10	4287	CASFADPDYYDSSGYSRDAFDIW	IGH	8	None	
10	4288	CARVGGYSGSITFDIW	IGH	4	None	
10	4289	CARGDGYSGYDRLVLQLGPYYYYGMDVW	IGH	2	None	
10	4290	CASNRGSGVAVAGTSPFDYW	IGH	2	None	
10	4291	CARAESPWLAHYWYFDLW	IGH	3	None	
10	4292	CAKVGRGYCSSTSCYTPPSFDYW	IGH	2	None	
10	4293	CARDPFEQRLTHFDYW	IGH	2	None	
10	4294	CTTDGLYCSSTSCYFSGYYYYYGMDVW	IGH	15	None	
10	4295	CAKDGLADYDFWSGYFRPPDYYYGMDVW	IGH	4	None	
10	4296	CARGNPKYYYDSSGSHYDYW	IGH	2	None	
10	4297	CAKISSGGVTTDYW	IGH	2	None	
10	4298	CAKDHIAYDSPGFDYW	IGH	2	None	
10	4299	CARVRHSNILSFDFDYW	IGH	36	None	
10	4300	CATDRLEPLLGPLYYMDVW	IGH	5	None	
10	4301	CTRHVGDLGYYYYGMDVW	IGH	3	None	
10	4302	CARGVVVPAAIIGWFDPW	IGH	18	None	
10	4303	CAREEASYGDLWGVYYYYYYMDVW	IGH	6	None	
10	4304	CAKVGDSSGYHWDFDYW	IGH	2	None	
10	4305	CAALPGIFGVVGIDYW	IGH	2	None	
10	4306	CAHREGWNYYFDYW	IGH	61	None	
10	4307	CARVAQTIFGSHANYYYFYQMDVW	IGH	5	None	
10	4308	CATVAPIAWELDWGENWFDPW	IGH	2	None	
10	4309	CARDEGSYGTRDFDYW	IGH	2	None	
10	4310	CAKDRLYYDILTGRFVKFFFDYW	IGH	3	None	
10	4311	CARDLMGDGYNFVYW	IGH	3	None	
10	4312	CASLNTAMDHEAFDYW	IGH	2	None	
10	4313	CAKLAPQKDYGDYFDWGDYW	IGH	5	None	
10	4314	CAKDGSSWFGWFDPW	IGH	4	None	
10	4315	CAREWINYYDSSGYPTYNWFDPW	IGH	4	None	
10	4316	CTTGVRITILEGFDPW	IGH	3	None	
10	4317	CARDSRYYDILTGYYTDDKYYYYYYMDVW	IGH	2	None	
10	4318	CAKDKTTKSTRWYFDLW	IGH	4	None	
10	4319	CARGGSAYCEGDCARPELFDYW	IGH	6	None	
10	4320	CSSYTSSSTYVF	IGL	7139	C2	7K90

10	4321	CARGFSYYNRWYYFDSW	IGH	5	None	
10	4322	CAKDTGYCSGGSCYGTFDYW	IGH	11	None	
10	4323	CARDGSYGHTGDAFDFW	IGH	2	None	
10	4324	CARQRIVATISDAFDIW	IGH	2	None	
10	4325	CQSADRSDTSLLLF	IGL	2	None	
10	4326	CARARPRSDSSWPRFYYYYYYMDVW	IGH	10	None	
10	4328	CVKDRHGSYFERRGFDYW	IGH	2	None	
10	4329	CATPKRDYSNYRDPLDYW	IGH	2	None	
10	4330	CVRRGQLWLEDDYW	IGH	2	None	
10	4331	CARERDRITMIVVATHAAFDIW	IGH	2	None	
10	4332	CARDYDFWSGSDYYYYGMDVW	IGH	12	None	
10	4333	CAILGGIAAAGTDHYMDVW	IGH	2	None	
10	4334	CASLGDSGYDSGSQYYYYGMDVW	IGH	2	None	
10	4335	CARVRVGDTAMVIGNYYYYYYMDVW	IGH	3	None	
10	4336	CARPPYYGSVYYYVDVW	IGH	2	None	
10	4337	CATVRFLEWLSVNNWFDPW	IGH	4	None	
10	4338	CARDIPLYYYYDSSGYDYW	IGH	9	None	
10	4339	CAKTTRGPGSYGLFDYW	IGH	2	None	
10	4340	CARAADYYYYSGGRTRFDAFDIW	IGH	2	None	
10	4341	CAKERTRSSSYLNDGFNIW	IGH	8	None	
10	4342	CARDWGGMVISEVFDYW	IGH	2	None	
10	4344	CAKVQLNIAYCGGDCRFFDYW	IGH	2	None	
10	4345	CARVESLYLHDNNLQRALDYW	IGH	4	None	
10	4346	CARKYYYDIRPLDYW	IGH	3	None	
10	4347	CARGSVVGAIGVWYYYGMDVW	IGH	2	None	
10	4348	CARAVADDSSGYQGATNPDYW	IGH	2	None	
10	4349	CARDRTYYYDSSGYFGYYGMDVW	IGH	8	None	
10	4350	CARGTHLGELPGGFFDYW	IGH	5	None	
10	4351	CALQSAPVVILFDYW	IGH	5	None	
10	4352	CARDCTNGVCLYYYYGMDVW	IGH	2	None	
10	4353	CAKDAIITIFGVVPNWFDPW	IGH	4	None	
10	4354	CARGALLDYDFWSGYYDRFDPW	IGH	2	None	
10	4355	CAHRRGDDPDIGVITPAVLGIHNWFDSW	IGH	2	None	
10	4356	CARGDLPDFRSNFYFDSW	IGH	7	None	
10	4357	CTRVGGPRDITISGIVNYYYGMDVW	IGH	3	None	
10	4358	CDWSSDVCSSDLARLGALPDYW	IGH	2	None	
10	4359	CAHRAWFHDAFDIW	IGH	20	None	
10	4360	CTTLYEAYYFDYW	IGH	3	None	
10	4361	CARDPVQSVTHYMDVW	IGH	2	None	
10	4362	CATSAPWYSSSWSFDYW	IGH	10	None	
10	4363	CARTYRYCTNGVCYSYYYGMDVW	IGH	4	None	
10	4364	CARGHYDILTGYYMGYW	IGH	2	None	
10	4365	CAPFPDPNIAVAGSAFDIW	IGH	12	None	
10	4366	CAKLPKVRGVIITGDDAFDIW	IGH	2	None	
10	4367	CAKFPGGRGLSDYGDSYFDYW	IGH	2	None	
10	4368	CARAFSLPYAFDIW	IGH	9	None	
10	4369	CARDFSTYGSGSDVDYW	IGH	8	None	
10	4370	CARGATVTTFFRGRNWFDPW	IGH	8	None	
10	4371	CATHVDSDYGRRTFDFW	IGH	10	None	
10	4372	CALPISAVYYCARDSQWELLDYW	IGH	2	None	
10	4373	CATVPSSRGWSAFYHFDNW	IGH	4	None	
10	4374	CARDRMEWANYYYYYMDVW	IGH	24	None	
10	4375	CARETAAPEYSSSLVAPAGNWFDPW	IGH	2	None	
10	4376	CASTNYYDSSGYYDGNRFDYW	IGH	3	None	
10	4377	CQQTYNIPSYTF	IGK	2	C4	7JW0
10	4379	CARYRHVLLWFGETGTKIDFDLW	IGH	5	None	
10	4380	CAREYVVALIAATYWFDPW	IGH	7	None	
10	4381	CARDGADSTIFGVPFDYW	IGH	15	None	
10	4382	CARGQTNRDGYNFYPRGAFDIW	IGH	4	None	
10	4383	CARHIQRFLVVVVPLNWFDPW	IGH	2	None	
10	4384	CATLTMTFGVVIIRGYYFDYW	IGH	4	None	
10	4385	CARDTLRGSSSSWFDPW	IGH	2	None	
10	4386	CARPPIYYDSSGYLKHDAFDIW	IGH	2	None	
10	4387	CARSTILNPYYYYGMDVW	IGH	2	None	
10	4388	CTTDPTLLYSSSWITSEAFDIW	IGH	3	None	
10	4389	CAKAAYYYESSGYYSWRDAFDIW	IGH	3	None	
10	4390	CARDYGGNSDYFDYW	IGH	7	None	
10	4391	CAKDMPSSGWSGGIDYW	IGH	4	None	

10	4392	CARDITYYDFWSGYYPPYYYYGMDVW	IGH	14	None	
10	4393	CAADYGDYGRFYYYYGMDVW	IGH	35	None	
10	4394	CTRAVRSCGVNSCSGNWFDPW	IGH	2	None	
10	4396	CAKGSGSWHGGDYW	IGH	4	None	
10	4397	CAKESAYDFWSGYYGVAFDIW	IGH	4	None	
10	4398	CAKHVVPEIRASPFDYW	IGH	3	None	
10	4401	CASLHSYYYDSSGYYYSDYW	IGH	4	None	
10	4402	CAKAPRGSCRSRLCYHFDHW	IGH	3	None	
10	4403	CARDRPPPGDGYNYPDAFDIW	IGH	2	None	
10	4404	CTTDRREDAIAARLFFGLNYYYGMDVW	IGH	4	None	
10	4405	CARDGDTAMVTWYYYYYMDVW	IGH	16	None	
10	4406	CAKAGGYSYYYYMDVW	IGH	21	None	
10	4407	CAKSLLQNYYYYYGMDVW	IGH	4	None	
10	4408	CATYLGRPGWLPDRFDYW	IGH	2	None	
10	4409	CARGTTDSSSWYGWYFDLW	IGH	4	None	
10	4410	CARPQAEWDLLLPFDIW	IGH	12	None	
10	4411	CTKDVQFLNTVITYGFDFW	IGH	8	None	
10	4412	CATASAHMVRGVIISWFDPW	IGH	5	None	
10	4413	CAKNAGIVGATTLDLYYFDYW	IGH	3	None	
10	4414	CARVLWDSSYYDSSGYYFYGMDVW	IGH	6	None	
10	4415	CAKDSMYYDILTGYYKRATTYYYYYMDVW	IGH	3	None	
10	4416	CAKDIMGKFSSGWLYYYYGMDVW	IGH	2	None	
10	4418	CAKEFEQWLIPRASDVW	IGH	14	None	
10	4419	CARQAEWRRVVVQSKRFFDYW	IGH	3	None	
10	4421	CASASSNGFDPW	IGH	22	None	
10	4422	CAHRGVDTAMEFGYW	IGH	4	None	
10	4423	CAAWDDSLSGWVF	IGL	5403	None	
10	4424	CARGPITMIVVVTPGDYW	IGH	3	None	
10	4425	CATAFAAMVKAPFDYW	IGH	2	None	
10	4426	CAKAVSPSGTYSLGFDVW	IGH	2	None	
10	4427	CAADVWSGYYRPDWDYYYGMDVW	IGH	3	None	
10	4428	CARLLWSPGYFDYW	IGH	23	None	
10	4429	CARESLITMIVVVITTTSYFDLW	IGH	2	None	
10	4430	CARENHIGAPVDFDYW	IGH	7	None	
10	4431	CAKLDYWYSSSSNFDYW	IGH	2	None	
10	4432	CQAWDSSRDVSYVF	IGL	2	C4	7JVC
10	4433	CTKDEAYCGGDCSDPDWFDAW	IGH	3	None	
10	4434	CAKSAALFIDSPGNSLFDYW	IGH	4	None	
10	4435	CAVLIVTSYYYMDVW	IGH	6	None	
10	4436	CARDEITSYYDGDYFDLW	IGH	6	None	
10	4437	CARTSTITPFDQW	IGH	171	None	
10	4438	CLASNDPLTDDSYGGEDYW	IGH	5	None	
10	4439	CARQSFSGSDEKGARYWYFDLW	IGH	3	None	
10	4440	CARHLIVALRYFDWVTGWFDPW	IGH	2	None	
10	4441	CARILWFPAPSVDYW	IGH	2	None	
10	4442	CAHSGPAVAVIGGFDYW	IGH	4	None	
10	4443	CAPLGDVVVPAATGYYYYGMDVW	IGH	3	None	
10	4444	CARDSVAAAGTPWKGYYYYYMDVW	IGH	2	None	
10	4445	CAHRRNLPDTPYFDYW	IGH	4	None	
10	4446	CVRDRGDYVHNWFDPW	IGH	11	None	
10	4447	CHQSSSFPLTF	IGK	615	None	
10	4448	CARDPGYASGWYMGGHDYW	IGH	6	None	
10	4449	CSAQWGELASEWCYYMDVW	IGH	2	None	
10	4450	CARKNTVYSSSLGAFDIW	IGH	2	None	
10	4451	CAKLSGVYYDSSGPFDYW	IGH	2	None	
10	4452	CTTGFPGGDYVPDSYYDYW	IGH	2	None	
10	4453	CARTGYTSGWYVDYYYYYMDVW	IGH	5	None	
10	4454	CAKLGAGMATTRGDYW	IGH	4	None	
10	4455	CTTVEFLPYYDFWSGPPFFFDYW	IGH	2	None	
10	4457	CAKEISSGYYNSPLDAFDIW	IGH	9	None	
10	4458	CASHYSGYDLGGPKFDYW	IGH	3	None	
10	4459	CAKDSGPDSGSDYWYFDLW	IGH	4	None	
10	4460	CAKDTITIFGVVITRGGDAFDIW	IGH	2	None	
10	4461	CQQRSNPLIAF	IGK	6	None	
10	4462	CAKAMVAGATTRFDCW	IGH	2	None	
10	4463	CARARGDYDFWSGYQLSGFCDYW	IGH	2	None	
10	4464	CARAELLWFGDLHKPFYFDYW	IGH	4	None	
10	4465	CASNYDYGGNSGSLLFNYW	IGH	2	None	

10	4466	CARVGELGSLDPEYRFDYW	IGH	2	None	
10	4467	CARVRSPLSSSWPLSEYYYMDVW	IGH	2	None	
10	4468	CARGVAGRLDYW	IGH	25	None	
10	4469	CARDFYSVGDYW	IGH	9	None	
10	4470	CARALPYSGYEDYFDYW	IGH	2	None	
10	4471	CAHRGDSSGYYYDRQYYFDYW	IGH	2	None	
10	4472	CARDGGDTAMVSPHFAFDIW	IGH	2	None	
10	4473	CARDQATEDWGYYMDVW	IGH	2	None	
10	4474	CAKFGFSSSSVNPTPDYW	IGH	5	None	
10	4475	CASSGYYYDSSGYYYYTVFDYW	IGH	2	None	
10	4476	CAVYSSGWYFEENAFDIW	IGH	2	None	
10	4478	CARDRMITFGGVIVPNNWFDPW	IGH	60	None	
10	4479	CARGGMTTVTFFDLW	IGH	2	None	
10	4480	CLRSEDTAVYYCATGPPLRFWDWFDPW	IGH	2	None	
10	4481	CARATSPWDYYDSSGYYYPDYW	IGH	6	None	
10	4482	CAREVTTRVPLYNWFDPW	IGH	2	None	
10	4483	CAKGLDDNLIGYPDGFDIW	IGH	2	None	
10	4484	CARDLSTGTTSDLDYW	IGH	4	None	
10	4485	CAKDRLLYSSGWKEYFQHW	IGH	2	None	
10	4486	CASSYYCSSTSCRILSYYYYYGMDVW	IGH	2	None	
10	4487	CTCQSGYSLNNWFDPW	IGH	4	None	
10	4488	CTRVGHWNDADFDSW	IGH	4	None	
10	4489	CARDSRYCSGGSCYSIWFDPW	IGH	6	None	
10	4490	CARPVTIFGFRSWFDPW	IGH	2	None	
10	4491	CARTEYSSHDGGAFDIW	IGH	6	None	
10	4492	CARAPLLYCSGGSCQMPPYYYYYGMDVW	IGH	3	None	
10	4493	CGTWDSSLSAGVF	IGL	809	None	
10	4494	CAKDINNGRQFDYW	IGH	8	None	
10	4495	CARDPRTYYDFWSGFSYYYGMDVW	IGH	2	None	
10	4496	CARDHDDYGDYGPDNWFDPW	IGH	2	None	
10	4497	CARGWAGILVPDGMDVW	IGH	2	None	
10	4498	CARRVRPTYGDKNEYYFDYW	IGH	2	None	
10	4499	CAKDPLPAYCGGDCYAFDIW	IGH	4	None	
10	4500	CVKDIHPRMGYSGDDFAYW	IGH	2	None	
10	4501	CATWGGYYDSSGYYHDYW	IGH	7	None	
10	4502	CAKDPDSSGFSTTHAFDIW	IGH	2	None	
10	4503	CARGEWLPEWYFDLW	IGH	3	None	
10	4504	CASLPAYNWNDEYINRFPTDYW	IGH	2	None	
10	4505	CARVLPHYGDYVDYW	IGH	41	None	
10	4506	CAKGDGVVVPAALKDYYYYGMDVW	IGH	2	None	
10	4509	CARDLWGDYVVGGLDYW	IGH	4	None	
10	4510	CAKDIYREIGDHAFDFW	IGH	6	None	
10	4511	CARETVAGSKYNWFDPW	IGH	9	None	
10	4512	CAKVALSDSTGYYSSYWYFDLW	IGH	2	None	
10	4513	CAKARRYTSSLDASDIW	IGH	3	None	
10	4514	CAKDTGIQYAFDIW	IGH	8	None	
10	4515	CVHSLSSGWELTFDYW	IGH	6	None	
10	4516	CQQSYSISLNF	IGK	256	None	
10	4518	CAKESLDYYGSGSYNQPFDYW	IGH	6	None	
10	4519	CARGRDFFFDYW	IGH	49	None	
10	4520	CAKVHPIFGEADYW	IGH	8	None	
10	4521	CAATTVTPRRPDYW	IGH	8	None	
10	4522	CTTGYCSDGLCYADALHVW	IGH	3	None	
10	4523	CAKGSRVVINWCYFDYW	IGH	2	None	
10	4524	CARGAEYYDFWSGYYSGSYFDYW	IGH	5	None	
10	4525	CQSYDTSLSALF	IGL	261	C4	7JVC
10	4526	CATGPPIAAAGTWFDPW	IGH	41	None	
10	4527	CANGGDIAVAGRDYW	IGH	2	None	
10	4528	CARGNYDFWSGYHHGAFDIW	IGH	2	None	
10	4529	CARAIEGGYSIPPPVWFDPW	IGH	7	None	
10	4531	CAKEGVYCSSTSCGFDTW	IGH	5	None	
10	4532	CARQTRVDEYSGGYYDRGWFDPW	IGH	2	None	
10	4533	CARAIVDFWSGYFGDYYFDYW	IGH	11	None	
10	4534	CAKVGTSSTSVVPYYFDYW	IGH	7	None	
10	4535	CAKDMGGIAAADTDYYFDYW	IGH	59	None	
10	4536	CARTSIELWSGYPTHENWFDPW	IGH	3	None	
10	4537	CDWSSDVCSSDLGNFYSFDPW	IGH	3	None	
10	4538	CTRSLPSSSWYFYYMDVW	IGH	2	None	

10	4539	CAKDISPNYYYDSSGLDYW	IGH	5	None	
10	4540	CASAETMVVTWYYFDYW	IGH	10	None	
10	4541	CAGGWYSFDYW	IGH	18	None	
10	4542	CAKDISSGGNTIFGVAPNWFDPW	IGH	2	None	
10	4543	CARHGTPNFWSGYLPQFDYW	IGH	4	None	
10	4544	CAKDNSSGFEYFQHW	IGH	3	None	
10	4545	CSSYTRSNTLPWVF	IGL	13	None	
10	4546	CARDSKWVPDYYGSGSYYNRFDYW	IGH	9	None	
10	4547	CARPNGNNQYSNSWCYFDHW	IGH	7	None	
10	4548	CAHRPPRSLYSSGWYDSLVGASPGGWFDPW	IGH	5	None	
10	4549	CARDSVRGWDQDAFDIW	IGH	2	None	
10	4551	CANGEAAADYW	IGH	2	None	
10	4552	CARREPDYYTAFDIW	IGH	2	None	
10	4553	CARHLYDTSGYVVVAFDVW	IGH	2	None	
10	4554	CASGGWQLVKRYYYYYYMDVW	IGH	2	None	
10	4555	CARNHPPDYGDYVNWYFDLW	IGH	6	None	
10	4556	CASSPSGQFSLWNDYW	IGH	6	None	
10	4557	CARDCSGGTCKPGGGYDAFDIW	IGH	2	None	
10	4558	CATDQDSRIAIFPYDAFHIW	IGH	2	None	
10	4559	CARASKSSITIFGVVIIPPGFDYW	IGH	3	None	
10	4560	CAKVPSFVYSYDSGRNPQIDYW	IGH	5	None	
10	4561	CAQTLPVMITFGGVTFDPW	IGH	4	None	
10	4562	CARGRRSYYDFWSGYAEYYGMDVW	IGH	6	None	
10	4563	CARRSPGSGWFDHFDHW	IGH	2	None	
10	4564	CAKDGLAFDHDRFDYW	IGH	16	None	
10	4565	CASDYGGNPDTSGPFDYW	IGH	6	None	
10	4566	CAHTGLKTYYDILTGYSRVAYFDYW	IGH	8	None	
10	4567	CARVENSNYVSYW	IGH	10	None	
10	4568	CAREYQLLYYFDYW	IGH	22	None	
10	4569	CARDLAPTYYDFWSGYPYFDYW	IGH	6	None	
10	4570	CARRLYEIWLTYFDNW	IGH	2	None	
10	4571	CASLPLYYYDSSGYHQAFDIW	IGH	3	None	
10	4572	CARHKQLLFSRRTYYFDYW	IGH	2	None	
10	4573	CAADSIAAGDISHYYYGMDVW	IGH	2	None	
10	4574	CARGITFGGVIGDDAFDIW	IGH	12	None	
10	4575	CASESPEAEVDYW	IGH	16	None	
10	4576	CAKDYYRGDYESNFDYW	IGH	3	None	
10	4577	CARDVVADTWPFGFDIW	IGH	2	None	
10	4578	CARAGYSSSSGYYYYYYGMDVW	IGH	4	None	
10	4579	CARQFGYFDWLLLSGSPSDGDNNWFDPW	IGH	4	None	
10	4580	CARGYSYGPYWYFDLW	IGH	9	None	
10	4581	CAKFPGLWNYDSSGQYNYFDYW	IGH	2	None	
10	4582	CARHPADYDYVWGSYRYMAPDYW	IGH	3	None	
10	4583	CAKAPAISYIVLMVYATTYYFDYW	IGH	3	None	
10	4584	CARMSKYFDWLLQHNWFDPW	IGH	2	None	
10	4585	CARAALIGSGSYWADYW	IGH	2	None	
10	4586	CAKDMSPVDTAMVDYW	IGH	8	None	
10	4587	CARVRDGDNDYW	IGH	24	None	
10	4588	CARGPEYYDFWSGYYGYGMDVW	IGH	8	None	
10	4589	CAREHGKRVAATLIDWFDPW	IGH	2	None	
10	4590	CQSYDYSLGGVIF	IGL	5	None	
10	4591	CAKLSIAAAEDRFDYW	IGH	3	None	
10	4592	CTTDEVGTGWSYGDYQIFRRVSDAFDIW	IGH	11	None	
10	4593	CTTTEPIGYCSSTSCYTGAAFIDYW	IGH	74	None	
10	4594	CASLLGYCTSTNCYNWIDPW	IGH	5	None	
10	4595	CAREWGTAVATYNWFDPW	IGH	2	None	
10	4596	CARHGLSITPDAFDIW	IGH	8	None	
10	4597	CVKDNNYDISGYLINWFGPW	IGH	2	None	
10	4598	CAKSRGVTSNLRWFDPW	IGH	2	None	
10	4599	CARDLDPPGWLVDYW	IGH	4	None	
10	4600	CARERPLDFTYYFDDW	IGH	4	None	
10	4601	CARERVPGYTGYDSDYFDYW	IGH	2	None	
10	4602	CAKLDWEGSLYSSPRGASRRFDYW	IGH	2	None	
10	4603	CAKDFGDFTNPDAFDIW	IGH	41	None	
10	4604	CARTFIAAAGTSIDYW	IGH	10	None	
10	4605	CARKAEGSGYYPDYW	IGH	2	None	
10	4606	CAGSTSEGHLSYYYYYGMDVW	IGH	3	None	
10	4607	CASGYCSSTSCSSPSFDPW	IGH	3	None	

10	4608	CARVFALTVVVIHETNWYFDLW	IGH	2	None	
10	4609	CARNIWGGYYTGFDSW	IGH	10	None	
10	4610	CAALGWNDQVLFDYW	IGH	3	None	
10	4611	CARESPMVRGVIRRDGMDVW	IGH	2	None	
10	4612	CAKAHSRHDYGDNFDYW	IGH	8	None	
10	4613	CARLPRGNYDNSGHYGGESFDYW	IGH	2	None	
10	4614	CAKLPDAPAAISPDYW	IGH	6	None	
10	4615	CARESPQGMVVAGIKGADYW	IGH	2	None	
10	4616	CARVVENHNFWSGSGNYFDYW	IGH	2	None	
10	4617	CAIGTIGSVPVPFDYW	IGH	2	None	
10	4618	CARGSNRQAFDIW	IGH	57	None	
10	4620	CARDTGDSSGWNWFDPW	IGH	72	None	
10	4621	CSKTASAWYRPFDSW	IGH	2	None	
10	4622	CARPATTYALDYW	IGH	5	None	
10	4623	CAKGFYGSGNGHIDYW	IGH	2	None	
10	4624	CAKDLHSGYYYFYFDYW	IGH	23	None	
10	4625	CATLHPENYYDSSGYYPDYW	IGH	3	None	
10	4626	CMKRVDSYDSRGYHTAPDYW	IGH	2	None	
10	4627	CQKYDDLPPGITF	IGK	23	C3	6XDG
10	4628	CALQTTVSSAASSYLDDW	IGH	4	None	
10	4629	CAHNFYSNYLFGWFDPW	IGH	2	None	
10	4630	CARNPGWELPPDYW	IGH	4	None	
10	4631	CARGDYCSGGSCYSLFPYYYYYYGMDVW	IGH	15	None	
10	4632	CARYGVSWGHDSSFDYW	IGH	3	None	
10	4633	CARAGWDSRGNKYNWFDPW	IGH	6	None	
10	4634	CARDGGDYDSSSRWDDYYFDYW	IGH	2	None	
10	4636	CARLWPWGFSSGTRYFDFW	IGH	2	None	
10	4637	CAREALRDIVVVVAAPDAFDIW	IGH	4	None	
10	4638	CARDDNDSSGYPGWFLDYW	IGH	2	None	
10	4639	CARGGTIFGVVITSFDYW	IGH	3	None	
10	4640	CARVETLGYYDSWGVTDWFDPW	IGH	9	None	
10	4641	CAKDAQVALNLGSSPFDYW	IGH	7	None	
10	4643	CARIPGGTHPDYW	IGH	21	None	
10	4644	CGKGHWLDSW	IGH	4	None	
10	4645	CATGTRITMIVVVTPLDYW	IGH	5	None	
10	4646	CASSPLGNYDSSGYGLDYW	IGH	4	None	
10	4647	CARANIALCSGGSCYSVTFDYW	IGH	3	None	
10	4648	CAKDMQHDYYFDYW	IGH	2	None	
10	4649	CLRFESRAAWHSYFDYW	IGH	28	None	
10	4650	CARILSAKWVWGLLTYMDVW	IGH	4	None	
10	4651	CARDRGPYDFWSGYYNYYYYYMDVW	IGH	4	None	
10	4652	CARPAASDRYYFDYW	IGH	5	None	
10	4653	CAHSIVSGDRLELRDFDYW	IGH	2	None	
10	4654	CARESGAYIYGVFDNW	IGH	2	None	
10	4656	CAKDQGYSGYDRVDAFDIW	IGH	2	None	
10	4657	CARGFFWQQLVKPDAFDIW	IGH	2	None	
10	4658	CASVRQWRYSDWYFDLW	IGH	2	None	
10	4659	CAKMDRGSAWFDYW	IGH	2	None	
10	4660	CARHGLRDTIFGVARGWNWFDPW	IGH	3	None	
10	4661	CARDPHMVYSYFDYW	IGH	4	None	
10	4662	CAFRSAFGEVLWHESERSDWFDPW	IGH	8	None	
10	4663	CAKDYGMEPLDLGGGSDYW	IGH	18	None	
10	4664	CARSLSISDPLAVGIWFGELWTGIFDYW	IGH	7	None	
10	4665	CARERLATYYYMDVW	IGH	13	None	
10	4666	CARAIDYYDSSGYYPDAFDIW	IGH	71	None	
10	4667	CTRGPFRITMIVVVMEDAFDIW	IGH	2	None	
10	4668	CTRDGAIVTAMEYFHHW	IGH	20	None	
10	4669	CAKEHTDYGGNSPHYFDYW	IGH	2	None	
10	4670	CAHSSSGITIFGVVIPSFDYW	IGH	5	None	
10	4671	CARGQLESHFPYYYGSGSSFDYW	IGH	5	None	
10	4672	CARGLGSMVRGVSRPPYFFDYW	IGH	11	None	
10	4673	CANGPSPRLGFFDYW	IGH	2	None	
10	4674	CARDGLRCSTSTCYRVFDAW	IGH	2	None	
10	4675	CATARDSGYDWAPHYFDSW	IGH	27	None	
10	4676	CARGRRITIFGVVIMSWFDPW	IGH	4	None	
10	4678	CQSTGSSGTSPVIF	IGL	3	None	
10	4679	CARDPAAIRFLEWYLDYW	IGH	2	None	
10	4681	CAKDYMFGELSPFFDYW	IGH	4	None	

10	4682	CAKAVEMATSGDYW	IGH	4	None	
10	4683	CARDIGYIGYDLYGSERDYW	IGH	2	None	
10	4684	CAKGMCYSYGCMYYFDYW	IGH	7	None	
10	4685	CARDSWYYDFWSGYHDAFDIW	IGH	4	None	
10	4686	CAKDWRIAMIVVVPTHFDYW	IGH	2	None	
10	4689	CAKDINYDSSGYYVGFDFDYW	IGH	5	None	
10	4690	CARDRSTVEMATTRYYYYMDVW	IGH	2	None	
10	4691	CQSPDSDGVYWLF	IGL	4	None	
10	4692	CAKGGLSGSYDDAFDVW	IGH	2	None	
10	4693	CAKNQVLLWFGESIEFDYW	IGH	10	None	
10	4694	CARDRSYSGSPPDAFDIW	IGH	9	None	
10	4695	CARDLPVAGTQGDDAFDIW	IGH	2	None	
10	4696	CATGSAIAVASWFDPW	IGH	12	None	
10	4697	CARGCDSRSGYQSGGFDYW	IGH	2	None	
10	4698	CAKGQFYATIEVYFDYW	IGH	5	None	
10	4699	CATWQWLVRYFDNW	IGH	4	None	
10	4700	CASERGIVVPPGNAFDIW	IGH	7	None	
10	4701	CTRDCDSGNAIRCGGALDVW	IGH	2	None	
10	4702	CVKDSSLYGDYVPGYYMDVW	IGH	15	None	
10	4703	CARDVLDPYDFWSGYYSYYYGMDVW	IGH	2	None	
10	4704	CQSADTNGPSNWVF	IGL	2	None	
10	4705	CTRGLGDGYNWWHDTFDIW	IGH	2	None	
10	4706	CARDACSGGNCLDPW	IGH	5	None	
10	4707	CARHPGPYKHDSGVPDYW	IGH	2	None	
10	4708	CARVHIRSDTAMPKRRGYFDYW	IGH	2	None	
10	4709	CATIYYDYVWGSSNGYYFDYW	IGH	2	None	
10	4710	CARYQYYYDRSGVTYYFDFW	IGH	4	None	
10	4711	CAREFGYSSGWYAYFDYW	IGH	3	None	
10	4712	CAREEDIVVVPAALYYYYYGMDVW	IGH	2	None	
10	4713	CARDVYLYYYDSSGQIVFDYW	IGH	8	None	
10	4714	CARDQMGNYGTTIDYW	IGH	207	None	
10	4715	CAYLSPGDWYFDVW	IGH	38	None	
10	4716	CARDPSLQQWLVLVGRSYYFDYW	IGH	3	None	
10	4717	CARDQLPFNYYDSSGHDYW	IGH	6	None	
10	4718	CARAANLFTWELLGDYYYYGMDVW	IGH	3	None	
10	4719	CARLNGYSSGWTSYYYYYYGMDVW	IGH	4	None	
10	4720	CARDQIVLEWLSQPLFDYW	IGH	2	None	
10	4721	CAKNGAYNWNYGYYYYMDVW	IGH	2	None	
10	4724	CAKLSSMIGDRDYW	IGH	2	None	
10	4725	CARTHYRRDGELLWDFDYW	IGH	2	None	
10	4726	CARSQRIITIFGVVTSGIDYW	IGH	14	None	
10	4727	CARQHRSITIFGVAPTGWFDPW	IGH	4	None	
10	4728	CAGSRSPSPVHDAFDIW	IGH	3	None	
10	4729	CARVHSSTMANYYFAYW	IGH	6	None	
10	4730	CARSVTDPNYVLRYFDWLSQGWFDPW	IGH	2	None	
10	4731	CARDGVNDFWSGHYDYW	IGH	3	None	
10	4733	CAVGYYGSGSKNWFDPW	IGH	4	None	
10	4734	CARGPLYCSSTSCYRDHLDYW	IGH	2	None	
10	4735	CARAVGITMIVVVIGAFDYW	IGH	2	None	
10	4736	CARIQREITIFGVVILLFDYW	IGH	2	None	
10	4738	CARVTGYSSSWLGDYFDYW	IGH	5	None	
10	4739	CAKSRPPDCINMRCAWGYAFDIW	IGH	2	None	
10	4740	CAREGLVGARPSYYYYYMDVW	IGH	4	None	
10	4741	CARDAVDSSSSFRPYWYFDLW	IGH	4	None	
10	4742	CAKDIGFALGQLEFDYW	IGH	8	None	
10	4743	CAKFRDGQEDAFDIW	IGH	10	None	
10	4744	CANTRDYYDSSGLRHAFDIW	IGH	2	None	
10	4745	CARTPQWELSLYYYYYMDVW	IGH	4	None	
10	4747	CAPSHRSKGNFDYW	IGH	2	None	
10	4748	CARDKMGMATIFGWFDPW	IGH	2	None	
10	4749	CTDGGNPNYYYYGMDVW	IGH	43	None	
10	4752	CARDMGTFSYASTDQDYW	IGH	7	None	
10	4753	CATAPPFGRINWFDPW	IGH	2	None	
10	4754	CARDPETSYYDSSGPIDYW	IGH	2	None	
10	4755	CARDRRAITFGGGSEGYFDYW	IGH	26	None	
10	4756	CARASIVANRHDAFDIW	IGH	40	None	
10	4757	CAKDFIPMIVVVIGAFDIW	IGH	4	None	
10	4758	CARDRLFRYNRSSGVFYYYMDVW	IGH	3	None	

10	4759	CTTERGYSYGYDGVFDYW	IGH	9	None	
10	4760	CARETHWRSGSYYRSGDAFDVW	IGH	5	None	
10	4761	CARALYENGGSHYDYW	IGH	7	None	
10	4762	CAREGDYFDSSGYYNWFDPW	IGH	2	None	
10	4763	CIRDEGVASSFNYW	IGH	2	None	
10	4764	CARYRRYSSSPIFGYYYYYMDVW	IGH	8	None	
10	4765	CAKDQTYKGTEWFDPW	IGH	4	None	
10	4766	CGTWHSNSKTQGVF	IGL	11	None	
10	4767	CARRHSFENDGFLWGPTDGFDVW	IGH	4	None	
10	4768	CAKSLRKTFLEWLSSQHAFDIW	IGH	3	None	
10	4769	CDWSSDVCSSDLTAVYYCARDDSSDAGNWFDPW	IGH	2	None	
10	4770	CALPISAVYYCARGSITIYGVLILLPARGWFDRW	IGH	2	None	
10	4771	CARVDSGSYFSWVDYW	IGH	7	None	
10	4772	CARMRKDSSGWYFDYW	IGH	32	None	
10	4773	CASKVPDYDILTGYYWVYGAFDIW	IGH	2	None	
10	4774	CQSSDTSGNLYVF	IGL	18	None	
10	4775	CAKDQAPAVDTAISDDAFDIW	IGH	4	None	
10	4776	CARALQRVSESDYYYYYGMDVW	IGH	7	None	
10	4777	CAHRRPGFFAGSFDYW	IGH	11	None	
10	4778	CASIQPVTTWYYYYGMDVW	IGH	2	None	
10	4779	CALGDCSGGSCYSDAFDIW	IGH	7	None	
10	4780	CARVSIAVTGTDFDYW	IGH	2	None	
10	4781	CARDGFRIMITFGGVTVPDYW	IGH	14	None	
10	4782	CTRVETRYDFWSGYYKGEYYFDYW	IGH	2	None	
10	4783	CARLGGYDFWSGYYQTYYFDYW	IGH	11	None	
10	4784	CARVKIDRMATISYAVNFDYW	IGH	2	None	
10	4786	CVSGDGYFQYW	IGH	9	None	
10	4787	CTRGVDLGTFWGDSNYYAMDVW	IGH	4	None	
10	4788	CARDMQEPLITMIVVVITFAFDIW	IGH	2	None	
10	4789	CANHRLDTAKEYYYYYMDVW	IGH	2	None	
10	4790	CTTAADTYYYGSGSYWVSVDYW	IGH	2	None	
10	4791	CVRDLSPYYDFVSGLTDGMDVW	IGH	2	None	
10	4792	CARGGLLYYDILTGYYAVPAAGFDYW	IGH	4	None	
10	4793	CARERDMGDSPFFYW	IGH	2	None	
10	4794	CSTDDYYDSTAYYYVPFDYW	IGH	5	None	
10	4795	CTTEFYVMTTVTAYHFDYW	IGH	2	None	
10	4796	CARDFRGRGYDSYYYYMDVW	IGH	2	None	
10	4797	CASLEQQSFWGEPRYYMDVW	IGH	2	None	
10	4798	CARVGDEYSSPWGGLDYYYYYMDVW	IGH	2	None	
10	4799	CANFGDYYDSSGTDDYW	IGH	8	None	
10	4800	CAKMGGIAAAGTGPGDYW	IGH	14	None	
10	4801	CAKALSGITGTTSDFDYW	IGH	6	None	
10	4802	CAKDGYYDSSGHSQIYFDYW	IGH	2	None	
10	4803	CARGAPDYGDYGGAFDIW	IGH	2	None	
10	4804	CQQYVDWPPKYGF	IGK	2	None	
10	4805	CAKDKRDSGYDYLDDW	IGH	2	None	
10	4806	CAKVSVVGGLRLFYFDYW	IGH	2	None	
10	4807	CASLGLLDTAMEGWALPFDIW	IGH	2	None	
10	4809	CAKDMAGTGTHDAFDVW	IGH	4	None	
10	4810	CARGGCSGGSCFDYRYYYYGMDVW	IGH	9	None	
10	4811	CARDFPLGDILTGYPFDYW	IGH	6	None	
10	4812	CARDHGFGESLNCFDPW	IGH	2	None	
10	4813	CAHRTENYYDSSGYYFSFVYFDYW	IGH	2	None	
10	4814	CARDLETMTPVYAFDIW	IGH	2	None	
10	4815	CARDMPNSSGYYDDAFDIW	IGH	5	None	
10	4816	CAKDPGSVVGADFDYW	IGH	23	None	
10	4817	CAKSSSSSLYNWFDPW	IGH	4	None	
10	4818	CARDYGSGSYYNGEYYFDYW	IGH	9	None	
10	4819	CARAPSGVSHPFHFDYW	IGH	2	None	
10	4820	CARGAYSSSCPDYW	IGH	10	None	
10	4821	CATDGDYYGSGTDYW	IGH	11	None	
10	4822	CARDLYMRGPRLVYYYGMDVW	IGH	4	None	
10	4823	CANVHSDITVGGWFDPW	IGH	2	None	
10	4824	CTTARPGPHYYDSSGPFDYW	IGH	3	None	
10	4825	CARILYGDHSLKFDYW	IGH	5	None	
10	4826	CAKGRHVDTAMVGYFDYW	IGH	10	None	
10	4827	CARDHLYYDFWSGYIPTDSHRHYYYYMDVW	IGH	2	None	
10	4828	CAKDSRSITIFGVVRGDAFDIW	IGH	3	None	

10	4829	CARENYDILTGTETPFDYW	IGH	25	None	
10	4830	CARVIAFGCSSTSCYIGPPEAYYYYGMDVW	IGH	2	None	
10	4831	CARVGRGYCSSTSCYTAFDPW	IGH	6	None	
10	4832	CARARSGSYRAPDDYW	IGH	10	None	
10	4833	CAKDLNEDTKAFDIW	IGH	2	None	
10	4834	CARDQDDSSGWIDYW	IGH	2	None	
10	4835	CARDLGTYSSSWYVPAVGWFDPW	IGH	4	None	
10	4836	CARGYCRSTTCYFDYW	IGH	15	None	
10	4837	CAMRKVGYCSDANCYRMISFDFW	IGH	4	None	
10	4838	CAHRRSGWTSGWFDPW	IGH	15	None	
10	4839	CARAIGWTPRLFYMDVW	IGH	2	None	
10	4840	CARDSSGYYYEPHDAFDIW	IGH	4	None	
10	4841	CAGAREGYCNIISCYEEYW	IGH	2	None	
10	4842	CARLRRHSGYPGYW	IGH	3	None	
10	4844	CARDAGDYYESSGYYLGYFQDW	IGH	2	None	
10	4845	CARDTWVDILTGYPYYYYMDVW	IGH	7	None	
10	4846	CAKTRRNWVHDFDIW	IGH	4	None	
10	4847	CARVGDDFWSGPPEGWWFDPW	IGH	9	None	
10	4848	CARANEWPELFDYW	IGH	2	None	
10	4849	CATESLVVVPAAKLGDYTSYYYGMDVW	IGH	6	None	
10	4850	CARAPAAGSSWYYYYYGMDVW	IGH	4	None	
10	4851	CATEPAPGTGFDYW	IGH	4	None	
10	4852	CARPYCGGDCTYYWYFDLW	IGH	4	None	
10	4853	CASLLGSGYEYAFDIW	IGH	7	None	
10	4854	CARCKTDHYYDSSGYVPEYYFDYW	IGH	2	None	
10	4855	CVREMDGDYDVLTNDYW	IGH	2	None	
10	4856	CARDLRTTLGFYDSSTYPDDYW	IGH	2	None	
10	4857	CAPLDLYSSSSPMFDPW	IGH	5	None	
10	4858	CAKDIGPEVVVAEPFDYW	IGH	4	None	
10	4859	CATSADTVTTFDYW	IGH	3	None	
10	4860	CARANDEWLELGDYYYLDVW	IGH	2	None	
10	4861	CARDGFNHYDSSGYYVFNYW	IGH	9	None	
10	4862	CAKGHSSPYYYYHMDVW	IGH	16	None	
10	4863	CAREPVCSGGSCRYDAFDIW	IGH	3	None	
10	4864	CAKLPNYYDSSGYFTASDYW	IGH	4	None	
10	4865	CARGGSPGIRDHNWLDPW	IGH	4	None	
10	4866	CARLRPNQKIDYW	IGH	4	None	
10	4867	CARDPFETSGYYYYYW	IGH	11	None	
10	4868	CAKSRVEPTMGFYYYYGMDVW	IGH	2	None	
10	4869	CARDNRVRGVDPTYYYYYYMDVW	IGH	2	None	
10	4870	CAREVHSSGWYVGDAFDIW	IGH	4	None	
10	4871	CARADYDSSGRQTFDYW	IGH	4	None	
10	4872	CARGTYCSGSDCRSDGFDIW	IGH	8	None	
10	4873	CARNTILNYMDVW	IGH	5	None	
10	4874	CARQPYYYDSSGYYYVEAFDIW	IGH	8	None	
10	4875	CDWSSDVCSSDLYYCASGGITMVRVRNNWFDPW	IGH	3	None	
10	4876	CQQYNNWHTAWAF	IGK	5	C3	7K8P
10	4877	CARHVSMGMIRAFDYW	IGH	2	None	
10	4878	CARVGVYDSSGYLLPYYFDYW	IGH	2	None	
10	4880	CARDSSSLEFGAYYFDQW	IGH	6	None	
10	4881	CAKDPAPGYDDSSVTGWGYYFDYW	IGH	3	None	
10	4882	CAREYYYASYGMDVW	IGH	42	None	
10	4883	CARLRFEYISSDLFDYW	IGH	2	None	
10	4884	CAKMTTVTTGVDYYYGMDVW	IGH	16	None	
10	4885	CASDGLAAAGTWYYYMDVW	IGH	2	None	
10	4886	CAHRPGIAARLNGFDIW	IGH	5	None	
10	4887	CAKFSFSGFDILTGPDYW	IGH	2	None	
10	4888	CARDNRYGPRKTFDFW	IGH	2	None	
10	4889	CAKDADMVGATWFDSW	IGH	2	None	
10	4890	CAKLAYFETAYCGGDCYFDYW	IGH	2	None	
10	4891	CARDLIDYDSSVIAFDMW	IGH	2	None	
10	4892	CARVGEEICGYSYGCPFDYW	IGH	10	None	
10	4893	CARARQMGLIVVVPDYW	IGH	2	None	
10	4894	CARDRDIVVVPAAIRDYYYGMDVW	IGH	3	None	
10	4895	CARLVGSIGDNSGYYYLSHFDYW	IGH	7	None	
10	4897	CAKGPWGGGNLIDYW	IGH	2	None	
10	4898	CARGDYYDGSGTYYPSYYYYYYMDVW	IGH	11	None	
10	4899	CARDLALVDDYGDYAQDDAFDIW	IGH	22	None	

10	4901	CQKYNSAPHAF	IGK	1867	C3	7K8R
10	4902	CAKIINYYDSSGYYSGAGDYW	IGH	2	None	
10	4903	CARIPRSYYDSSGYYLQVWHFDYW	IGH	15	None	
10	4904	CARDPDYARRSDAFDIW	IGH	2	None	
10	4905	CARVRGYCSGGSCPYYFDYW	IGH	4	None	
10	4906	CAKDIYPIAAAGTWFDPW	IGH	2	None	
10	4907	CARENTGYYMSYW	IGH	44	None	
10	4908	CAKTVLDDRQRGYFDLW	IGH	2	None	
10	4909	CARGHYYGDSYWYFDLW	IGH	9	None	
10	4910	CAHILQLYSSAWARSWFDPW	IGH	2	None	
10	4911	CAKGAAPIAVAGIDYW	IGH	7	None	
10	4912	CAHRDGNVVRGVIAFDYW	IGH	2	None	
10	4913	CARDITVWSGYYTLDAFDIW	IGH	16	None	
10	4914	CAREVTMIVVVDYW	IGH	4	None	
10	4915	CATFDLYYYDSSGQNDPFDYW	IGH	5	None	
10	4916	CAKGQLGILTGCLDYW	IGH	2	None	
10	4917	CARIQSDTAMVTWFDYW	IGH	10	None	
10	4918	CATDELAVASTAWYFDYW	IGH	4	None	
10	4919	CARVVVDCTNGVCFGGDYEYHFDYW	IGH	2	None	
10	4920	CASLPSYSSSWSTHAFDIW	IGH	6	None	
10	4921	CARYSTYYYDSSGYYSEGLDYW	IGH	11	None	
10	4922	CQQYNSYLYSF	IGK	1535	C3	7K8P
10	4923	CATSPAVWGSYRYCDYW	IGH	8	None	
10	4924	CARARSGHWLVPTGDAFDIW	IGH	4	None	
10	4925	CARPLPKLWFGEFHDAFDIW	IGH	4	None	
10	4926	CMQATQFPKGLTF	IGK	5	None	
10	4927	CARDLAVAVPPASWFDPW	IGH	3	None	
10	4928	CVKDLSGKDDYW	IGH	16	None	
10	4929	CVRLGEFPFFSPPDYW	IGH	2	None	
10	4930	CRVVPAAIPYYYYGMDVW	IGH	2	None	
10	4931	CARIRAVWGLRYCSGGSCYGGFDYW	IGH	4	None	
10	4932	CGVDYYYMDVW	IGH	7	None	
10	4933	CATGPPYYYDSSGYYRDCWFDPW	IGH	4	None	
10	4934	CARDWEYSSSSGEYYFDYW	IGH	18	None	
10	4935	CSRDNSPEEFDSW	IGH	2	None	
10	4936	CAKAVVPAAIYYFDYW	IGH	9	None	
10	4937	CAHSNYDILTGYFLVHDYW	IGH	2	None	
10	4938	CATSFAVFSSGWYFDYW	IGH	2	None	
10	4940	CAKDIDLWLAGDPPRNCFDYW	IGH	2	None	
10	4941	CAKIGGSNYYDSSGYYNGPDYW	IGH	5	None	
10	4942	CASSARARQQLVPFDYW	IGH	6	None	
10	4943	CARIQRGYSYGYRDYW	IGH	5	None	
10	4944	CAKSWPIAARPVQASLFFDYW	IGH	2	None	
10	4945	CARGAGYDILTGYKYYYGMDVW	IGH	4	None	
10	4946	CARVEGGAVAGKGHYFDIW	IGH	9	None	
10	4947	CARFNDYGDLIDAVTLDYW	IGH	14	None	
10	4948	CAKGDSTGHRTVTPGADYW	IGH	2	None	
10	4949	CTRLLYYYDSSGYSHYYYGMDVW	IGH	4	None	
10	4950	CAAQYCTSSSCYGDTFDIW	IGH	17	None	
10	4951	CARDGGGSYLGADAFDIW	IGH	2	None	
10	4952	CTRDAPLESSYRGFVQFDYW	IGH	7	None	
10	4953	CARTMGVESGQSDAFDIW	IGH	10	None	
10	4954	CQSYDSRDHQMVF	IGL	4	C4	7JVC
10	4955	CARETGDYYDSSGYYYGGDFDYW	IGH	2	None	
10	4956	CARDQVPRGYSYGDNWFDPW	IGH	3	None	
10	4957	CARAPVYLWFGELRYYYYGMDVW	IGH	2	None	
10	4958	CARGLWSSSWLPHFYFDYW	IGH	4	None	
10	4959	CAKLPSIADYTYFDYW	IGH	8	None	
10	4960	CARSLLGVLRFLECFDYW	IGH	2	None	
10	4961	CARDVGFGVLRGYFDYW	IGH	15	None	
10	4962	CARVRYYHGSEIPYCFDMW	IGH	5	None	
10	4963	CATYGSGSYSSLYMDVW	IGH	2	None	
10	4965	CARVGYYDSSGYHWFDPW	IGH	3	None	
10	4966	CARGDIVATIPPDYW	IGH	2	None	
10	4967	CARGLRFLEWLLDYYYYYGMDVW	IGH	5	None	
10	4968	CARIWCRDGYNYDGAFDIW	IGH	2	None	
10	4969	CATGPYDFWSGPKMQNWFDPW	IGH	2	None	
10	4970	CARTNDVYYYDSSGPLDYW	IGH	4	None	

10	4971	CARNQWGVEGDAFDIW	IGH	6	None	
10	4972	CQTADTSGTFHAVF	IGL	2	None	
10	4973	CASTYYDSSGYHPLPFDYW	IGH	17	None	
10	4974	CAKDIPHTLYYYDGCCWFDPW	IGH	2	None	
10	4975	CAKGGGDHVPTYYYYYYMDVW	IGH	3	None	
10	4976	CAKDSDYDFWSGSNYMDVW	IGH	10	None	
10	4977	CAREYYDFWSGYRSPDSWFDPW	IGH	3	None	
10	4978	CATGVAFPGLGGWFDPW	IGH	2	None	
10	4979	CARGLSIAAAGTLWIYYYYGMDVW	IGH	3	None	
10	4980	CARGNYDILTGYYNGWFDPW	IGH	14	None	
10	4981	CAKDSNDGYCTGGVCYGGSYNWFDPW	IGH	2	None	
10	4982	CARGLVPPWFGAQGAFDIW	IGH	3	None	
10	4983	CARGMSIRYCSSTSCLAGFYYYYYMDVW	IGH	4	None	
10	4984	CARDFSPYSGYDNCDYW	IGH	3	None	
10	4985	CARDRRYDSSGYYLPNFDYW	IGH	7	None	
10	4986	CAREDRLLFRGWFDPW	IGH	7	None	
10	4987	CAHRRRFLEGTSFSGAFDSW	IGH	5	None	
10	4989	CARVKSDYDYVWGSPGHYYYYMDVW	IGH	5	None	
10	4990	CARHYFRAMPRGFFDYW	IGH	2	None	
10	4991	CARFGRGDFWSGYSDAFDIW	IGH	7	None	
10	4992	CAKDGLVDYYGSGSRHWFDPW	IGH	2	None	
10	4993	CATDRLVVVPAAIGTYYYYGMDVW	IGH	10	None	
10	4994	CLQYGPSPSWTF	IGK	6	C1	7K8N
10	4995	CARVSEEYYDFSYAFDIW	IGH	9	None	
10	4997	CAKLYDFWSGYYSTRDYW	IGH	2	None	
10	4998	CARVWGGYRIDYW	IGH	62	None	
10	4999	CAKVFTVTTGLGNDVDYW	IGH	2	None	
10	5000	CARDLVPLDGSLGAFDIW	IGH	2	None	
10	5001	CARGGWDILTGYPYVFDYW	IGH	3	None	
10	5004	CARDSHYGDYVHFDYW	IGH	7	None	
10	5005	CAKDPMYDYVWGSYRQGPYYFDYW	IGH	2	None	
10	5006	CAKDSERGGVVVAATDYW	IGH	3	None	
10	5007	CASLNSIGNGDFDYW	IGH	4	None	
10	5008	CARASFGDSYGTDYYFDYW	IGH	33	None	
10	5009	CAKDPPYYDILTGYYGYYFDYW	IGH	9	None	
10	5010	CARGRDNDIVTGSFGYW	IGH	2	None	
10	5011	CAKDFTPKLELRFVSWFDPW	IGH	2	None	
10	5012	CAKVAHGSSPQEVDYW	IGH	5	None	
10	5013	CAKGLSGSYYGSFDYW	IGH	22	None	
10	5015	CVAVVTGTTGIDYW	IGH	4	None	
10	5016	CASDRGPPGYCSGGSCYGTFDYW	IGH	4	None	
10	5017	CAKDDFFGGVVVVAADRGFDYW	IGH	4	None	
10	5018	CARESMYDFWSGYYFINQRDYYYYGMDVW	IGH	6	None	
10	5019	CARDGVFPYYEGSGEDYW	IGH	2	None	
10	5020	CAKDFLEWYTALDYW	IGH	6	None	
10	5021	CARDYYDFWSAPGLQGWFDPW	IGH	6	None	
10	5022	CHQYNSFSESF	IGK	4	C3	7K8R
10	5023	CAKDRNYADYGLAWWFDPW	IGH	5	None	
10	5024	CARDGVRGYSSTWRLDYW	IGH	2	None	
10	5025	CAKAFYDSSGLDAFDIW	IGH	4	None	
10	5026	CAKEGYYYDSSGYYYGLRYYFDYW	IGH	5	None	
10	5027	CARQIPPGPEFDYW	IGH	3	None	
10	5028	CAREVMGSGWLNDAFDIW	IGH	7	None	
10	5029	CARTYYGDYDKYVDYW	IGH	2	None	
10	5030	CARTRDYGDYDPEWHFDYW	IGH	3	None	
10	5031	CARDSRPLRSYYQRDAFDIW	IGH	2	None	
10	5032	CARSVTTDYHYYMDVW	IGH	4	None	
10	5033	CAAHTPSLEWDYW	IGH	2	None	
10	5034	CAKDEVLRYFDWDYYYYGMDVW	IGH	5	None	
10	5035	CARRKVTPGAFDIW	IGH	2	None	
10	5036	CARHGTWIQLWVDYW	IGH	2	None	
10	5037	CARENSSGPKPYFDYW	IGH	6	None	
10	5038	CARDQDPYYYDSSGYYDAFDIW	IGH	2	None	
10	5039	CAKAVGPYFPEQFDPW	IGH	3	None	
10	5040	CARHFWLGASSYFDYW	IGH	2	None	
10	5041	CARAVRNQLLPDYW	IGH	3	None	
10	5042	CARDWGFGELSEDYW	IGH	3	None	
10	5043	CARQYSSTSLFDWFDYW	IGH	2	None	

10	5044	CARIPNDSSGWYYIDYW	IGH	3	None	
10	5045	CAKDHPMHIVMVTAFFDSW	IGH	174	None	
10	5046	CAKLANGVATITADYW	IGH	5	None	
10	5047	CATGSSVITLEFDIW	IGH	22	None	
10	5048	CAKDLRSLWFGENYYMDVW	IGH	2	None	
10	5049	CASPRTHYYDSSGYSGFDYW	IGH	98	None	
10	5050	CAKDLVHYDFWSGYLTTTYYYYYGMDVW	IGH	5	None	
10	5051	CARVSDCSSTSCSYYMDVW	IGH	4	None	
10	5052	CARDGAAYDFWSGRPLYYYYYYYMDVW	IGH	2	None	
10	5053	CARDGTLSMGPTVVDWFDPW	IGH	8	None	
10	5054	CARVGDYGSGSHFDYW	IGH	5	None	
10	5055	CARDRVDSSGYYPRYYYGMDVW	IGH	4	None	
10	5056	CARIMGSRVVTPIDYW	IGH	3	None	
10	5057	CARVLMITFGGVIVIPGGFDPW	IGH	5	None	
10	5058	CARDRPSYDSSGYYLDYW	IGH	16	None	
10	5059	CTGGVCHDFPFDPW	IGH	2	None	
10	5060	CAKDIGIVATTEFDYW	IGH	13	None	
10	5061	CAKAITIFGVVINPGGFDYW	IGH	13	None	
10	5062	CARSTSEVYSSSSTFDYW	IGH	2	None	
10	5063	CAKDTYHDFWSGYLAFDYW	IGH	2	None	
10	5064	CAREHYDTSSYLSGLDYW	IGH	2	None	
10	5065	CAKLRDDDSYSDYW	IGH	18	None	
10	5066	CVRGDMKTLVGFDLW	IGH	4	None	
10	5068	CARLSVINGDYFAFDVW	IGH	2	None	
10	5069	CARASSGTTNPYNFDYW	IGH	2	None	
10	5070	CDWSSDVCSSDLEDTAMYYCARETIVVDDSRFDYW	IGH	5	None	
10	5071	CAKDIVPLAQTIDYW	IGH	4	None	
10	5072	CARFGGADIVATTGTSYFDYW	IGH	2	None	
10	5073	CAGILRHTKTTYGPPKNWFDPW	IGH	2	None	
10	5074	CARWDDFWSGYNDAFDIW	IGH	12	None	
10	5075	CTYYCARHRGAEEEIDYW	IGH	2	None	
10	5076	CAKSSQLRFLEWLPSEFDSW	IGH	4	None	
10	5077	CVRYTASSSFGLHWFDYW	IGH	5	None	
10	5078	CARDRHSYYDFWSAHKPDYW	IGH	2	None	
10	5079	CVSWDYSNYRPPDYW	IGH	6	None	
10	5080	CAKDREYYDFWSGYLTPSQNAYYYYGMDVW	IGH	2	None	
10	5081	CAKSYSSSWYRYFEHW	IGH	2	None	
10	5082	CVRGSITVFGILIMLPARGWFDPW	IGH	2	None	
10	5083	CARIRAGIAVAGIFDYW	IGH	2	None	
10	5084	CARHGHSSGWDHVLYYYYMDVW	IGH	5	None	
10	5085	CASSGGDYGMGVDYW	IGH	2	None	
10	5086	CAREAWEDYYGSGSYLSAPYYYGMDVW	IGH	3	None	
10	5087	CARDRPLGFFSPDAFDIW	IGH	10	None	
10	5088	CAKGYIAIPMSAFDYW	IGH	2	None	
10	5089	CAGVPLTSPNYWYFDLW	IGH	2	None	
10	5090	CATGPVGSPSYYYYMDVW	IGH	2	None	
10	5091	CAKGFLWSGELLYFDSW	IGH	2	None	
10	5092	CARDIVNGYDSDYFDHW	IGH	2	None	
10	5093	CDWSSDVCSSDLEDTAVYYCARIPSSSWEFDYW	IGH	2	None	
10	5094	CAGALYYDFWSGYPIHPFDYW	IGH	2	None	
10	5095	CARALYDSRTWYYYMDVW	IGH	17	None	
10	5096	CARDGIMITFGGVIENGLVDYW	IGH	6	None	
10	5097	CAKLPHPYGYGDYGYDYW	IGH	6	None	
10	5099	CAVYIPAPGGAGDAFDIW	IGH	6	None	
10	5100	CANLNTPGYCSSTSCWDWFDPW	IGH	3	None	
10	5101	CANMGTFLWFGELYTDYW	IGH	3	None	
10	5102	CARFRDLDEAAAGIDLGPGRFDYMDVW	IGH	3	None	
10	5103	CARLGMEMATRPAYYFDYW	IGH	9	None	
10	5104	CASGTVWLSHVDYW	IGH	2	None	
10	5105	CARVGGSQWFGTSYFDYW	IGH	8	None	
10	5106	CAKEVTIFGVVTPGFDYW	IGH	8	None	
10	5107	CARDLFSDSSSWYGQWFDPW	IGH	35	None	
10	5108	CARAVYGSGSYSRDWYFDLW	IGH	3	None	
10	5109	CARESGFSTVSYPYYYYYFIDVW	IGH	2	None	
10	5110	CAKLLSRYGDYDDAFDIW	IGH	4	None	
10	5111	CAKPGEVRYNWFDPW	IGH	100	None	
10	5112	CARASPYYDILTGYRVGDYW	IGH	7	None	
10	5113	CVKDTSLVEVRSRGFDYW	IGH	2	None	

10	5114	CAKEPFDNWFDPW	IGH	23	None	
10	5115	CAHRQNNDLSFSFDYW	IGH	8	None	
10	5116	CAREDTWDSYGSNYFDSW	IGH	2	None	
10	5117	CATGGLGPYYYYGMDVW	IGH	7	None	
10	5118	CAREGIAVAVDYW	IGH	57	None	
10	5119	CARFGGDGCTTDDCYDYW	IGH	140	None	
10	5120	CARVSRADFWVDYW	IGH	52	None	
10	5121	CARGGDRQWLVDDYW	IGH	10	None	
10	5122	CAPNPFSYWDGPDYW	IGH	2	None	
10	5123	CAKVLCSGCPTYLDYW	IGH	4	None	
10	5125	CQQYDRTPFNF	IGK	263	C2	7K8U
10	5126	CAKLTPYYYDSSNYHDVFHIW	IGH	2	None	
10	5127	CAKDRTLKWVTMIVVANIYPDAFDIW	IGH	11	None	
10	5128	CARDGDYYDSSGYYYPPRLGYYYGMDVW	IGH	2	None	
10	5129	CARVPPLYNYGYATTPNFDYW	IGH	13	None	
10	5130	CAGGLNYQLLYGLDAFDFW	IGH	2	None	
10	5132	CTRGLDSSTWYHPGFDPW	IGH	4	None	
10	5133	CARTEVGITMIVVVSMGAFDTW	IGH	3	None	
10	5134	CARAKPATGWLIDYW	IGH	4	None	
10	5135	CAHRLYRNTYFDYW	IGH	3	None	
10	5136	CARINPGWRISDSFHYSYMDVW	IGH	2	None	
10	5137	CARTLWLFPGFEDSYYFDYW	IGH	15	None	
10	5138	CATAPLTYCSGGSCYPGGWFDPW	IGH	2	None	
10	5139	CARDAALYYYDSSGYHWFDPW	IGH	3	None	
10	5140	CARGGAYDGSAYFFDYW	IGH	2	None	
10	5141	CSRDSTPIFAVENQGPDVFDIW	IGH	6	None	
10	5142	CAHRRVGNWNYWFDPW	IGH	16	None	
10	5144	CARDFYGDPLWYFDLW	IGH	14	None	
10	5145	CAKVADGYNSHFDYW	IGH	4	None	
10	5146	CARDIVVVPAAPGGTNYYYYYGMDVW	IGH	2	None	
10	5147	CARGKDIVLAVIEGQPDSYYYYMDVW	IGH	8	None	
10	5148	CARDHDSSGSPSDTFGYW	IGH	2	None	
10	5149	CAKDRGQLVLGYFDYW	IGH	16	None	
10	5150	CAKATYDQCFDYW	IGH	2	None	
10	5151	CAKAFRITIFGVVTYYFDYW	IGH	2	None	
10	5152	CARDGGGYCSGSSCYYNWFDPW	IGH	4	None	
10	5154	CARDFRHDIVVVPAAISPYYYYYGMDVW	IGH	2	None	
10	5155	CAKLLDSSGYLNGIQDYW	IGH	15	None	
10	5156	CAKDMFAIGVAGTITYFDYW	IGH	2	None	
10	5157	CARHDIAAAGVNYYYYYMDVW	IGH	2	None	
10	5158	CARNGVRDGYNQWWIDYW	IGH	2	None	
10	5159	CQHSYITPYAF	IGK	86	C4	7JW0
10	5160	CARDMTTVTTWRFDYW	IGH	18	None	
10	5161	CALMSDSLNRLRYNWFDPW	IGH	2	None	
10	5162	CASDFGPHGAFDIW	IGH	7	None	
10	5163	CAKVQKQRGYSYEQSDYW	IGH	2	None	
10	5164	CANTQALVTTHHAEYFQHW	IGH	2	None	
10	5165	CARDELVAATSTLGWFDPW	IGH	4	None	
10	5166	CAKLDDRYGDLTDYW	IGH	4	None	
10	5167	CAKGTSIHFYDSNAYYDHW	IGH	2	None	
10	5168	CARDQLGYYYDSSGYWVSWFDPW	IGH	4	None	
10	5169	CARERYDFEAFDIW	IGH	2	None	
10	5170	CQQDYNLF	IGK	36	None	
10	5171	CARVVRDVLRFLEWPTGYYWYGMDVW	IGH	3	None	
10	5172	CARHVLGVTMIVVVITHPSLFDYW	IGH	3	None	
10	5173	CARAGVVPAARSAFDIW	IGH	6	None	
10	5174	CARSAYYDFWSGYLYYYYYMDVW	IGH	8	None	
10	5175	CATGLSVAVTDVLFDAFDVW	IGH	3	None	
10	5176	CAKATVSTMVRGLWPYYYGMDVW	IGH	4	None	
10	5177	CARAIGDGYNLLDPCFDYW	IGH	5	None	
10	5178	CARENDDFWSGYYRWFDPW	IGH	17	None	
10	5179	CARGPYSSSWYGLKNWFDPW	IGH	46	None	
10	5180	CQEFNSYSPWIF	IGK	8	C3	7K8P
10	5181	CARRPWLSTRESVDYW	IGH	2	None	
10	5182	CARDYVWGNSPADFFEIW	IGH	27	None	
10	5183	CAKSERALGAFDIW	IGH	6	None	
10	5184	CARHEVSCDGDCFLNYFDNW	IGH	4	None	
10	5185	CASYPPVRWFGQDAFDIW	IGH	2	None	

10	5186	CAKDMGTAQDVSFDYW	IGH	5	None	
10	5187	CARVRPHDYGDYVSDAFDIW	IGH	2	None	
10	5188	CARGVEYYDFWSGFGPDPKVDAFDIW	IGH	3	None	
10	5189	CALSRFTLFGVVTRFDYW	IGH	6	None	
10	5190	CAVERNLRGPSYGAFDIW	IGH	4	None	
10	5192	CARGADYGGDVFDVW	IGH	3	None	
10	5193	CARRGIPGHFWSGYYTGDAFDIW	IGH	13	None	
10	5194	CARDNAYDSSGYRTDYW	IGH	13	None	
10	5195	CASDSSGSGRDYW	IGH	31	None	
10	5196	CVHKYAGTTGFDPW	IGH	11	None	
10	5197	CARHWTWTYSYGYYFDYW	IGH	2	None	
10	5198	CAKDDIGLGASPFDNW	IGH	2	None	
10	5200	CARDVYDILTGYSRIFEYW	IGH	10	None	
10	5201	CARAGFLQAGYSGYDPYYYYGMDVW	IGH	3	None	
10	5202	CQQYDNLLALTF	IGK	828	C3	6XDG
10	5203	CAHVYYDFWSGYYFDYFDYW	IGH	3	None	
10	5204	CARSGYSSGWYFHKPEEGSTFDYW	IGH	2	None	
10	5205	CAKTFTPSSWYGWFDPW	IGH	2	None	
10	5206	CARELGYCSSTSCYNVGPLGAYYYYYGMDVW	IGH	4	None	
10	5207	CATDAKVNIVATISVDYW	IGH	4	None	
10	5208	CARDTHDYDSSGYYHDAFDIW	IGH	3	None	
10	5209	CLHSPWSFFWGGSFYMDVW	IGH	2	None	
10	5210	CARQNVLGYLTTWGWFDPW	IGH	4	None	
10	5211	CARESLPYPSISYMDVW	IGH	3	None	
10	5212	CANLLTMIVVSIDAFDIW	IGH	2	None	
10	5213	CAKDNGHGAYGDSHGGFFDYW	IGH	2	None	
10	5214	CARQRWHSTAWTAEYFQHW	IGH	16	None	
10	5215	CARQDIVVLPGAMTLDSMDVW	IGH	2	None	
10	5216	CARSPPGYATTVSYFDYW	IGH	9	None	
10	5217	CATATSLGSILSHSSSVGYYFDYW	IGH	15	None	
10	5218	CAKVTSSREGGFDYW	IGH	8	None	
10	5220	CARDPLRYNWNGPDYW	IGH	2	None	
10	5221	CAKDRYSNYRGWFDPW	IGH	12	None	
10	5222	CARVNIAYYYDSSGSVEPFDYW	IGH	18	None	
10	5223	CARGRYYYDSSGYYYVSQFFWFDPW	IGH	2	None	
10	5224	CLLYYDGSQPYWVF	IGL	4	None	
10	5225	CARLFGYHCRGGICQHSYFNYYMDVW	IGH	2	None	
10	5226	CAKVGGAGDDFWSGYYLWYFDLW	IGH	2	None	
10	5227	CARTRGYSGYDFGTLDYW	IGH	2	None	
10	5228	CASARTTLWVPYWFDPW	IGH	3	None	
10	5229	CARGRYMTTSRYFDLW	IGH	5	None	
10	5230	CAKLPENYGGNSNIDYW	IGH	2	None	
10	5231	CARDLILGYSSSWGDFFDYW	IGH	16	None	
10	5232	CARNHDISVVPGNPWENSWSYDRYNWFDPW	IGH	2	None	
10	5233	CATLGSSMNDFWSGTYYYYMDVW	IGH	2	None	
10	5234	CAKMATGLAAADYW	IGH	2	None	
10	5235	CAFSPSGHYFNIW	IGH	49	None	
10	5236	CARDLTVQLTIFGVVSSLGRASGPFDYW	IGH	7	None	
10	5237	CSSYVGSNNPWVF	IGL	97	C2	7BWJ
10	5238	CARGPVQLQDFDYW	IGH	5	None	
10	5239	CAKDTLLLNYYDSSGIDAFDIW	IGH	2	None	
10	5240	CMIWHSSAWVF	IGL	228	None	
10	5241	CARGSTLFGVIYNWFDPW	IGH	3	None	
10	5242	CNSYRSSNHNFVF	IGL	4	C4	7JVC
10	5243	CTQRTQIPVTF	IGK	32	None	
10	5244	CAKAFDSGSSRDYW	IGH	16	None	
10	5245	CARGGDYYDPREGLDYW	IGH	12	None	
10	5246	CARPTNYSDFNYW	IGH	3	None	
10	5247	CARAEHYDFWSGYHPAGLGYFDYW	IGH	5	None	
10	5248	CTTDPATYYDFWSGYYGMITVGGVIVQTSDYW	IGH	2	None	
10	5249	CARDNRILRFLEWLVIGFDYW	IGH	10	None	
10	5250	CDWSSDVCSSDLSTSWVDYW	IGH	6	None	
10	5251	CARAQMGKSYYDSSKEFDYW	IGH	152	None	
10	5252	CARTGGAAGNYYFDYW	IGH	16	None	
10	5253	CARRVGATVYNWFDPW	IGH	17	None	
10	5254	CAKVGGIYSAYDYAFDYW	IGH	2	None	
10	5255	CTTDSSGYFAFDIW	IGH	2	None	
10	5256	CARGFDYW	IGH	424	None	

10	5257	CARHGDYTGSYYDLDYW	IGH	4	None	
10	5259	CARQVAVADDDYFDYW	IGH	7	None	
10	5260	CVRATIRSGSNYEAADYW	IGH	5	None	
10	5261	CARGVLEGIAAAGHPSDFDYW	IGH	5	None	
10	5262	CARDRDRSLEWWRGGFDPW	IGH	2	None	
10	5263	CARDANSGSYSDSGDWYFDLW	IGH	5	None	
10	5264	CAKVSGGVQDQPDYW	IGH	2	None	
10	5265	CARETGEGDFWSGLYNWFDPW	IGH	4	None	
10	5266	CARHLSPYYDRDAYDIW	IGH	4	None	
10	5267	CALWFGETDAFDIW	IGH	5	None	
10	5268	CARVRGNPDRDRIFLAYCGGDCYPLNAFDIW	IGH	6	None	
10	5269	CARHVEEQQLAINWFDPW	IGH	8	None	
10	5270	CARLGYCSSTSCSHPPSYYYGMDVW	IGH	4	None	
10	5271	CANSPALGYCSGGSCYSHAFDIW	IGH	4	None	
10	5272	CASPHSSGWHLFDYW	IGH	5	None	
10	5273	CATDGVRYFDWLVRRPRDYYYYMDVW	IGH	4	None	
10	5275	CARDLQPFYDSSGYTYYYYYYMDVW	IGH	2	None	
10	5276	CAKRRFNGWDDAFDIW	IGH	2	None	
10	5277	CARESYAMVRGLMYNWFDLW	IGH	2	None	
10	5278	CATDAYCSGGDCYLFDYW	IGH	14	None	
10	5279	CAREAYDDYGSLYFDSW	IGH	6	None	
10	5280	CATVLPYCSSTSCHYKTGFDYW	IGH	8	None	
10	5281	CARRADNIEFNYW	IGH	6	None	
10	5282	CAKDTDPHYYYDSSGYYYPDAFDIW	IGH	2	None	
10	5283	CARYSGWHDRPLDYW	IGH	2	None	
10	5284	CARAAYSSGLFHYW	IGH	2	None	
10	5285	CATVPAWIQLWSYDAFDIW	IGH	2	None	
10	5286	CAKSGGVSLLWFGNAAFDIW	IGH	2	None	
10	5287	CARSFMNPSPVGRFDPW	IGH	6	None	
10	5288	CAKATGWYDFDDW	IGH	4	None	
10	5289	CAKATVVTAILLPNAFDIW	IGH	4	None	
10	5290	CARMLYGDYDRLYYFDYW	IGH	2	None	
10	5291	CARIKRSSWYPYYYYYMDVW	IGH	7	None	
10	5292	CAHRLGGFSIAAAGTTFDYW	IGH	2	None	
10	5293	CAREERSSPGDYW	IGH	4	None	
10	5294	CAKLPRSNYYYDSSGYDAFDIW	IGH	8	None	
10	5295	CAKDRVRYQLLEWFDSW	IGH	7	None	
10	5296	CVNLSLLGGWFDPW	IGH	2	None	
10	5297	CQESVSTSWTF	IGK	7	None	
10	5298	CAREAYSSGWYVVDSNNWFDPW	IGH	4	None	
10	5299	CARDQDKDDSSGYYWLDGYFDYW	IGH	2	None	
10	5300	CARAPPYYYDSSGYVSTADNWFDPW	IGH	15	None	
10	5301	CAITSAIPGTRYYYFDYW	IGH	8	None	
10	5302	CAAEPANYGFVWGSLDYW	IGH	4	None	
10	5303	CAREGVVTSLSAHYFDYW	IGH	3	None	
10	5304	CARQRDGDYDDSGYWGYW	IGH	2	None	
10	5305	CQHARSFPITF	IGK	35	None	
10	5306	CAKDTRPGMQEFDYW	IGH	2	None	
10	5307	CAKLPQGGYSYGLGSFDYW	IGH	3	None	
10	5308	CASNPEDSYDLPDSAFDIW	IGH	124	None	
10	5309	CAKDILWELPHTNAFDIW	IGH	13	None	
10	5310	CSSYTTSNTFYDF	IGL	3	C2	7K90
10	5311	CYGSGSYYTPFDYW	IGH	2	None	
10	5312	CAKDTNWNDGGFGYW	IGH	4	None	
10	5313	CAKDPYSYINPSVFDIW	IGH	2	None	
10	5314	CARGQNYDSSGYYHYYYYYYMDVW	IGH	12	None	
10	5315	CARQGDRTRDFDFW	IGH	11	None	
10	5316	CARGPGPYSSGWQLDYW	IGH	5	None	
10	5317	CANAHSIHDDFGVW	IGH	2	None	
10	5318	CAKLPYCSSTSCYGVDYW	IGH	18	None	
10	5319	CAKDTDYSNYFDYW	IGH	18	None	
10	5320	CARDLDYYDSSGYYYEGFDYW	IGH	100	None	
10	5321	CARAVAGITMIVVVIPEIRYDYW	IGH	3	None	
10	5322	CARLQNGGYYRPQWYFDYW	IGH	5	None	
10	5323	CARHMVGATWGRDYYDYW	IGH	4	None	
10	5324	CAKLVVPAASLVDYW	IGH	2	None	
10	5325	CARGGDYYDFWSGYYGPSHWFDPW	IGH	6	None	
10	5326	CAKDIWSYGDYSFGYW	IGH	4	None	

10	5327	CANLAAAGTSYYYYMDVW	IGH	4	None	
10	5328	CLQHTSYPPMYAF	IGK	2	None	
10	5329	CARRVATGDYYDSSGYWGWFDPW	IGH	7	None	
10	5330	CAKPGYDSSGYYYPYWYFDLW	IGH	7	None	
10	5331	CARDLRTAMDTPYYYYYYYMDVW	IGH	9	None	
10	5332	CGGDCYSDYYYYMDVW	IGH	2	None	
10	5333	CAKVQGSDGYNDYW	IGH	2	None	
10	5334	CARGLLRLYCSSTSCPRGAPYNWFDPW	IGH	2	None	
10	5335	CAKVQYGGYSYGRDYW	IGH	2	None	
10	5336	CARGKDTYHSDTNGLDAFDIW	IGH	10	None	
10	5337	CARHRSDYGDYEPELFDYW	IGH	7	None	
10	5338	CARHGWKNYDSSGVSDAFDIW	IGH	8	None	
10	5339	CAKDDSSGPRAYYVDVW	IGH	2	None	
10	5340	CAKGTTTVTTRSNFDYW	IGH	3	None	
10	5341	CAHRPWGIAVAAFDIW	IGH	2	None	
10	5342	CARDLGYYGSGSSYYFDYW	IGH	8	None	
10	5343	CARDGFGYYDSSGYHPPYYYYGMDVW	IGH	6	None	
10	5344	CSHVRVGYCSGGSCSPDYFDYW	IGH	6	None	
10	5345	CATGPSYDSSGYYYLFDYW	IGH	4	None	
10	5346	CVKDIVSGVEPLQAYYYYYMDVW	IGH	2	None	
10	5347	CARDRFGAAAGMNWFDPW	IGH	6	None	
10	5348	CTTESGWYGVYFDYW	IGH	72	None	
10	5349	CQQSYSTLGTWTF	IGK	1126	C2	7K8O
10	5350	CAKEAAVQLERPTYYFDYW	IGH	2	None	
10	5351	CARDPGIAVAGKVFYYYMDVW	IGH	2	None	
10	5352	CARVYCGGDCYSGLWGAFDIW	IGH	3	None	
10	5353	CARGGNYKGFDNW	IGH	4	None	
10	5354	CHHYNNWPPFTF	IGK	39	None	
10	5355	CAADRWVAGHVTDDYW	IGH	2	None	
10	5356	CARHFGTIFGVVTLPGAFDPW	IGH	2	None	
10	5358	CAKDAPPNYDFWSGYYFAPHYYYYYGMDVW	IGH	2	None	
10	5359	CARDLYSYGSGDSYCYMDVW	IGH	11	None	
10	5360	CARSRRPITMIVVVSQDPIDYW	IGH	2	None	
10	5361	CVRDHDSSGYSSGAFDIW	IGH	3	None	
10	5362	CQAWDSSTVVF	IGL	6744	C4	7JVC
10	5363	CVKDRSWLRFRAFLDVW	IGH	2	None	
10	5364	CARESDSSSWSNEYLDYW	IGH	25	None	
10	5365	CAREDESIVVVPAATHGLNWFDPW	IGH	3	None	
10	5366	CARARHPTTVIIGASDAFDIW	IGH	2	None	
10	5367	CARAAVLRYFDWLLDGNWFDPW	IGH	5	None	
10	5368	CVWEDSSGYPVGDYW	IGH	2	None	
10	5369	CARDSWDLLYRFDHW	IGH	7	None	
10	5370	CAKTSKGFGVIIFDMW	IGH	3	None	
10	5372	CARSPNYSSAWWFDPW	IGH	15	None	
10	5373	CARTLGGGYVALDYW	IGH	4	None	
10	5374	CARQIWNSELLWFGELLYSPGLDYW	IGH	3	None	
10	5375	CARDYYDSSGYYYVPTFDYW	IGH	11	None	
10	5377	CAKDLRTIYTGTTKVGYYFDYW	IGH	2	None	
10	5378	CARADYYFAATRFDYW	IGH	11	None	
10	5379	CAREGKSAYSNPFDYW	IGH	14	None	
10	5380	CAKAPSDGNYYDSSGYAFDIW	IGH	4	None	
10	5381	CAKGTLQLWLQPSQFDYW	IGH	3	None	
10	5382	CARDANPYYDSSGYYHSGLDYW	IGH	5	None	
10	5383	CARGVPAGTGGALFDYW	IGH	8	None	
10	5384	CAKVAAPMSRNDYW	IGH	2	None	
10	5385	CARDSSSGWFADYW	IGH	114	None	
10	5386	CAHSQAVPAAISRKNWFDPW	IGH	2	None	
10	5387	CASGRGGPLRYYFDYW	IGH	6	None	
10	5388	CAKIGGGEVAGTDYW	IGH	2	None	
10	5389	CARALVASEYDYVWGSYPPGDYW	IGH	9	None	
10	5390	CARGTPGYDFWSGYWGDFDYW	IGH	2	None	
10	5391	CARGRGRFGPDDDGFNIW	IGH	3	None	
10	5392	CATAPGYCSGGSCPTNWFDPW	IGH	35	None	
10	5393	CVHSRFSGYDFFDSW	IGH	2	None	
10	5394	CARPLGHYDSSGYRLDAFDIW	IGH	2	None	
10	5395	CAHRKYANYWGNDVFDIW	IGH	2	None	
10	5396	CATHGDYGPARFDYW	IGH	3	None	
10	5398	CANLLKLDYYGSGETDYW	IGH	6	None	

10	5399	CAREAEVVRGFGVVFYYYGMDVW	IGH	2	None	
10	5401	CARRGTIGRTVFDPW	IGH	2	None	
10	5402	CARLDYYDSTGKFDYW	IGH	11	None	
10	5404	CGTWDSSPTAGLAVF	IGL	2	None	
10	5405	CARDRPRGFLEWLGYYYGMDVW	IGH	2	None	
10	5406	CAKDSYDGYYYDSSGYLGAFDIW	IGH	2	None	
10	5407	CAKDKLVHYYDSSGYLDYW	IGH	2	None	
10	5408	CARGPLPYNWNYYYGMDVW	IGH	6	None	
10	5409	CATGPPVAVAGMGNWFDPW	IGH	11	None	
10	5410	CARILSITMIVVVTPWGYFDLW	IGH	8	None	
10	5411	CAGSNAVWSENGGLGYYYYYGMDVW	IGH	2	None	
10	5412	CAKVPGAYYYDSSGYYQPSYYYYGMDVW	IGH	2	None	
10	5413	CARVSSPRSRDFWSGYPPGFHWSPDLYYYYGMDVW	IGH	3	None	
10	5414	CATEYALYGGNAPPDYW	IGH	3	None	
10	5415	CVRLSTTSWFYFDYW	IGH	12	None	
10	5416	CTRGWVTIFGGDTRDAFDIW	IGH	3	None	
10	5417	CAVGGSGGFYGLFDYW	IGH	8	None	
10	5418	CAKSSQTQDTALPYYYYYMDVW	IGH	2	None	
10	5419	CAKMDGSGSYDHLPDYW	IGH	2	None	
10	5420	CARHSDLDYYYDSSGFDYW	IGH	12	None	
10	5421	CVKHLLVLRGDPFDSW	IGH	3	None	
10	5422	CAATDYYGVRSNYEGSWFDPW	IGH	8	None	
10	5423	CARVGRTSGYLNWFDPW	IGH	2	None	
10	5424	CARVKIQGIPRYYYMDVW	IGH	2	None	
10	5425	CYCRFGGVVVITDYW	IGH	3	None	
10	5426	CARGRDYSDSSGYYHVIDYW	IGH	4	None	
10	5427	CAKDIGGYSYGSTSDYW	IGH	4	None	
10	5428	CARDGLGVVVAATHPWGYYYGMDVW	IGH	10	None	
10	5429	CARRDPNCSGATCNYFDYW	IGH	2	None	
10	5430	CQSADSSGADPGWVF	IGL	3	None	
10	5431	CASDRYNAVGYYYYGMDVW	IGH	8	None	
10	5432	CARHGTFTIFGVVIMPDYFDYW	IGH	2	None	
10	5433	CARDTGIFTENGPGWFDSW	IGH	3	None	
10	5434	CARRDPVVVVPAVSAGPTYYIDNW	IGH	2	None	
10	5436	CASEEVNSSGWYFDAFDIW	IGH	4	None	
10	5437	CALTPGYDFWSGYRLDYW	IGH	4	None	
10	5438	CASSNYYDSSGYYKEESFDYW	IGH	2	None	
10	5439	CAREGVREQWLAHPLLNFDYW	IGH	2	None	
10	5440	CARDSRYYDFWSGYYGGPSYYYYYYMDVW	IGH	2	None	
10	5441	CVREISNYVGHWFDPW	IGH	2	None	
10	5442	CARDPRQWLGQPYWYFDLW	IGH	3	None	
10	5443	CAKLGSKVITADYW	IGH	6	None	
10	5444	CAREGRLRYCSGGSCYPYYYYGMDVW	IGH	3	None	
10	5445	CSRGKFAPTYYDFWSVYSADIDYW	IGH	3	None	
10	5446	CAKDSGPFSGQTDYW	IGH	48	None	
10	5447	CATASVLVAGTPNWFDPW	IGH	49	None	
10	5448	CAREAHAGGRSHETSPYYFDYW	IGH	2	None	
10	5449	CARQPIQIIAAAGQPFDYW	IGH	2	None	
10	5450	CTRGRGSCIGATCYSYYW	IGH	2	None	
10	5451	CARDGAYYDFWSGYLGRGPYYYYYMDVW	IGH	5	None	
10	5452	CARGDSSGWYVRNDYYFDYW	IGH	28	None	
10	5453	CARRGSSSTFWYFDFW	IGH	3	None	
10	5454	CAKDFRYYDILTGYLSGARYYYYYYMDVW	IGH	2	None	
10	5455	CARSSAYFHDGSGYYDFDYW	IGH	3	None	
10	5456	CARAVRQWLALYYYYMDVW	IGH	5	None	
10	5457	CAHRLWWALGFEDYSNYGWFDPW	IGH	31	None	
10	5458	CARDGSSLNGDSEYFFDDW	IGH	4	None	
10	5459	CARHFPNTIFGVVTASYYMDVW	IGH	2	None	
10	5460	CAKDRSIQLWPPFAGEFDYW	IGH	7	None	
10	5461	CAKDMAPGGWYMDYW	IGH	4	None	
10	5462	CATLGGYDYRDLYYFHYW	IGH	2	None	
10	5463	CATGFSVGYSYGGSYYYYYGMDVW	IGH	3	None	
10	5464	CAILQQQLRPLNDYW	IGH	8	None	
10	5465	CAGLRITMIVVALDAFDIW	IGH	10	None	
10	5466	CARSHHNHDYGDYLPTDYW	IGH	2	None	
10	5467	CARDPPPNCVGSSCQYYYYGIDVW	IGH	4	None	
10	5468	CARIVESMSLPYFFDYW	IGH	2	None	
10	5469	CAKVGSVQLEDDAFDIW	IGH	2	None	

10	5470	CAKDTSPLSGYSSGWYLGPFDYW	IGH	2	None	
10	5471	CARGSVFRIYDSSGYRETYNWFDPW	IGH	5	None	
10	5472	CAHRGDFWSGYFDYW	IGH	62	None	
10	5473	CARDVVVTATGVHYYYGMDVW	IGH	4	None	
10	5474	CASHSRIAAAGQFDYW	IGH	2	None	
10	5475	CARDPSTVTAYWYFDLW	IGH	3	None	
10	5476	CARVFPGQYNSGCLDYW	IGH	2	None	
10	5477	CAREGPRDYDILTGYWARYYYYGMDVW	IGH	4	None	
10	5478	CATSFYMTTVTPPTDYW	IGH	30	None	
10	5479	CARGGVSRSRIARGPDVFDMW	IGH	2	None	
10	5480	CARDVYCSGGTCFAWYFDLW	IGH	2	None	
10	5481	CARQDTGRIRWFDTW	IGH	4	None	
10	5482	CARDEAVGWLQLLSRTYYYYGMDVW	IGH	5	None	
10	5483	CARAEGALERYDYW	IGH	4	None	
10	5485	CARDLGGVSEHWFDPW	IGH	35	None	
10	5486	CARSNGVASRNDYW	IGH	6	None	
10	5487	CAKAGHYYDYTGHRSYYFDYW	IGH	4	None	
10	5488	CARDIRYSGYDYLSLGVDYW	IGH	3	None	
10	5489	CAKDRARGDYKDYDYYYGMDLW	IGH	15	None	
10	5490	CAGQLVATGRNYYPAMDVW	IGH	2	None	
10	5491	CASEYDSSGYYGGAAFDIW	IGH	2	None	
10	5492	CCSYAGSYSF	IGL	22	C2	7K8X
10	5493	CARDLGVSEGGDYW	IGH	7	None	
10	5494	CARRAARWEYNWFDPW	IGH	3	None	
10	5495	CATFIGGSYWKYW	IGH	2	None	
10	5496	CARVFILEWLLYAPDYYYYGMDVW	IGH	4	None	
10	5497	CARTQNNGALDYW	IGH	12	None	
10	5498	CASLMASNYYDSSGYSGYW	IGH	7	None	
10	5499	CARDHGGRARYYYDSSGYYCDYW	IGH	2	None	
10	5500	CARVRRHPPIMASIYDAFDLW	IGH	2	None	
10	5502	CASPWRTSYASGVFDYW	IGH	5	None	
10	5503	CKTDYYDSSGYPGYFDYW	IGH	2	None	
10	5504	CAKDIQQWLVKGGYFDYW	IGH	2	None	
10	5505	CARGLRYDYVWGSYRYGNWFDPW	IGH	2	None	
10	5506	CARFPKYSYGQDDYW	IGH	6	None	
10	5507	CARHTYYDSSGYPYHYGMDVW	IGH	6	None	
10	5508	CARESYNSRSFDLW	IGH	2	None	
10	5509	CAKDGSYYDFWSGYLKPSDYYYYYYMDVW	IGH	6	None	
10	5510	CARDAAVWGDIVVASNWFDPW	IGH	2	None	
10	5511	CAINSNYYYYYGMDVW	IGH	6	None	
10	5513	CAKDGNYDFWSGSLGGYYYYYYMDVW	IGH	9	None	
10	5515	CARHLPSYYYDSSGYRTGFDYW	IGH	6	None	
10	5516	CARPTFGDYYMDVW	IGH	6	None	
10	5517	CAKDSEYGSGSDVHNWFDPW	IGH	8	None	
10	5518	CARGGTSCYLCSDGTNWFDPW	IGH	4	None	
10	5519	CARGEEKYSYGNGYFQHW	IGH	2	None	
10	5520	CAKTPSGDYYGSGSYGLAAFDIW	IGH	2	None	
10	5521	CARDRAAYDYIWGSNRYEGFDYW	IGH	2	None	
10	5522	CARGSITIYGVLILLPARGWFDRW	IGH	730	None	
10	5523	CARDHPADGYNWGWIDYW	IGH	10	None	
10	5524	CAKIEGPAAGTPSGDYW	IGH	2	None	
10	5525	CARDWGILSNLYDSSAHYYYYGMDVW	IGH	12	None	
10	5526	CARLTTSYSSGWYGFDYW	IGH	2	None	
10	5527	CARSHRGITIFGVVHLWYFDLW	IGH	3	None	
10	5528	CATLPLVVPAAPDYW	IGH	2	None	
10	5529	CATSPPLYGSGSYYRYYYYGMDVW	IGH	2	None	
10	5530	CAKGSFSGLGVHFDYW	IGH	2	None	
10	5531	CANPNSGSYYSFFDYW	IGH	3	None	
10	5532	CAKVGYSSSWYVRAFDIW	IGH	2	None	
10	5533	CAREGSGYRSTWSTYYYYYAMDVW	IGH	6	None	
10	5534	CATDLKVPLAAAGPNWFDPW	IGH	2	None	
10	5535	CARSPPRYDFWSGYFFKNWFDPW	IGH	2	None	
10	5536	CQRYNKYPLIF	IGK	10	C3	7K8R
10	5537	CARDCTFYYDSYDTSPGDDLDYW	IGH	2	None	
10	5538	CARGLSQLLYYYYYMDVW	IGH	6	None	
10	5539	CTTQYYYDSSGYYYGDYW	IGH	2	None	
10	5540	CTREVLVLAASQYYYYGMDVW	IGH	2	None	
10	5541	CAKLRYRARPQEYYMDVW	IGH	3	None	

10	5542	CATAPPTDIADWFDPW	IGH	2	None	
10	5543	CARLPPIWFGELLGIYFDYW	IGH	7	None	
10	5544	CARLPSSNYDYVWGSWRAPYFDYW	IGH	3	None	
10	5545	CARDQRGCTNGVCQSNDYW	IGH	5	None	
10	5546	CARESRNNWFDFW	IGH	65	None	
10	5547	CAKLLGPTPHGDSDTDAFDIW	IGH	9	None	
10	5548	CTSGEWEGQDFDYW	IGH	2	None	
10	5549	CATAYAICGGDCYLGYW	IGH	55	None	
10	5550	CARHGDIVVVPESAKDDYYYYGMDVW	IGH	8	None	
10	5551	CARQYDYDSSGYYYYYW	IGH	33	None	
10	5552	CVRTGGDNTAIDAFEIW	IGH	3	None	
10	5553	CARAISSPFGVVLNYYYMDVW	IGH	2	None	
10	5554	CARVVKYDDVLEGIHDYYFDYW	IGH	2	None	
10	5555	CARTRNDYVGSGSYYTSFDQW	IGH	6	None	
10	5556	CAKGEQWLDEKHFDYW	IGH	2	None	
10	5557	CAKLCAGSLTGYYPDDAFDIW	IGH	2	None	
10	5558	CARGPRSESYDFWSPYYYMDVW	IGH	2	None	
10	5559	CATGRIWFGELRRNYFDYW	IGH	24	None	
10	5560	CARDSNWPPFYFDSW	IGH	7	None	
10	5561	CAREYYYDSSGYYSASFFDYW	IGH	2	None	
10	5562	CAKLALVTMIVVVTQGDDAFDIW	IGH	7	None	
10	5563	CAKSEIGEYYDFWSGYLDGGQPFYFDYW	IGH	2	None	
10	5564	CARCHFGVLMYMDVW	IGH	2	None	
10	5565	CAHREYSSSSVPFDYW	IGH	2	None	
10	5566	CAKGAFVMVTGIPAEYFLHW	IGH	2	None	
10	5567	CARDLYYDSSGFDYW	IGH	526	None	
10	5568	CARHSYYYGSGAHPYYYYMDVW	IGH	2	None	
10	5569	CAKFIRGYSYGYWGFDPW	IGH	2	None	
10	5570	CATARYFDWLTALDYW	IGH	2	None	
10	5571	CALPISGSGWFYYFDYW	IGH	2	None	
10	5572	CARDFGCSSTSCYRPTIGSYTYYGMDVW	IGH	2	None	
10	5573	CATDQKFTNFGVVGFYDYW	IGH	2	None	
10	5574	CARSNYDILTGYYTPWDYW	IGH	288	None	
10	5575	CATGDHFGDYGEFDYW	IGH	3	None	
10	5576	CARGVDDGGSRGYFDYW	IGH	2	None	
10	5577	CVRVKMGVAYAFDLW	IGH	2	None	
10	5578	CTTGPERYCSNGVCPAYFDYW	IGH	30	None	
10	5579	CARDEEVNVRYYDSSGYYAFDYW	IGH	2	None	
10	5580	CARTPRFSTMIVVVITSFDYW	IGH	4	None	
10	5581	CARALPGIAVDYW	IGH	3	None	
10	5582	CASPSTIFGVVNYYYGMDVW	IGH	84	None	
10	5583	CAKMDSSGSGRHFDYW	IGH	6	None	
10	5584	CARGRYRSEVDTAMVFVEVSSSWPLDAFDIW	IGH	2	None	
10	5585	CARPSKFIYGLTSFGFDPW	IGH	5	None	
10	5586	CARDRPFTNLEGGWVDPW	IGH	4	None	
10	5587	CAREVSGGYSGIGFDPW	IGH	2	None	
10	5589	CAKDSASSSWYESGLDYW	IGH	3	None	
10	5590	CATAPSLAARSPGWFDPW	IGH	5	None	
10	5591	CAKIGIAAAGSSYDAFDIW	IGH	6	None	
10	5592	CTTLSWYYYDHSAYFPYW	IGH	5	None	
10	5593	CARVKDFWSAYYYDYW	IGH	4	None	
10	5594	CARARYYYGLGSPYCFDMW	IGH	3	None	
10	5595	CASIAAAGREYYYYYGMDVW	IGH	28	None	
10	5596	CARADAYSSAINWFDPW	IGH	2	None	
10	5597	CAKDHLYYDFWSGYSSNPSYYYYYGMDVW	IGH	5	None	
10	5598	CARGGDYYGLKWWFDPW	IGH	13	None	
10	5599	CARVAAVPAAIFSRGLDYYYGMDVW	IGH	4	None	
10	5600	CAPTLGYCTNGVCSYYFDYW	IGH	5	None	
10	5601	CARTPGTIFGVDINHFYYYYMDVW	IGH	2	None	
10	5602	CARPDYGDQHPNDFRFDYW	IGH	65	None	
10	5603	CARGWRLVTLSPYFDYW	IGH	18	None	
10	5605	CATLSPNGAQNGAFDYW	IGH	2	None	
10	5607	CAADNGLQYLFDYW	IGH	4	None	
10	5608	CARDPPSYYYDSSGYYEEYYFDYW	IGH	22	None	
10	5609	CARARPYGSGSYWLDPW	IGH	4	None	
10	5610	CARTPTMMGDYGDHGDYW	IGH	4	None	
10	5611	CARDYSTSWSNWFAPW	IGH	4	None	
10	5612	CAKDIDGPAGSTSFDYW	IGH	13	None	

10	5613	CARQGTYDSSGYGLAHFDYW	IGH	2	None	
10	5614	CAHRGVSSYPSNWFDPW	IGH	2	None	
10	5615	CAKVLRSSPGYDSSGYYYNPGYYYYGMDVW	IGH	3	None	
10	5616	CARDLVALDPWYFDLW	IGH	2	None	
10	5617	CARDSQGLAGSYSYFHYW	IGH	2	None	
10	5618	CTTDQRGYSSLWTYYYYYGMDVW	IGH	2	None	
10	5619	CATSVHIVVVTYAFDIW	IGH	4	None	
10	5620	CARQSGRGGVAAVNAFDIW	IGH	2	None	
10	5621	CARLGDSSGYYLYYFDYW	IGH	33	None	
10	5622	CASLSSITWRWFDPW	IGH	4	None	
10	5623	CAKDLIVLMVYAMVDGGDVW	IGH	18	None	
10	5624	CAKDRREYYDSSEGYFDYW	IGH	4	None	
10	5625	CAKMEFWSGYTLRDAFDIW	IGH	2	None	
10	5626	CARAGWHSGSITDPFDIW	IGH	9	None	
10	5627	CARHVSSDSGYDVWYFQHW	IGH	2	None	
10	5628	CARLVAASHYSDYW	IGH	5	None	
10	5629	CAKDLRVMTTVTTGTEPFDLW	IGH	2	None	
10	5630	CARDPAVLIAAAGSYYFDYW	IGH	4	None	
10	5631	CARHGFFGVVIIQGNWFDPW	IGH	6	None	
10	5632	CARAWTTVTTDWYFDLW	IGH	16	None	
10	5633	CARHFTRYSHPYYFDYW	IGH	11	None	
10	5634	CAKPPVAGTDYYYYYMDVW	IGH	4	None	
10	5635	CARGGWRVPAAGLPHYYYYMDVW	IGH	2	None	
10	5636	CARGRLAVAGRLAFDIW	IGH	3	None	
10	5637	CAKDQGSPKMATIKGPADYW	IGH	2	None	
10	5638	CARPSMVRTVFYYYYAMDVW	IGH	3	None	
10	5639	CARHRLGDFDWLTGLSYLDAFDIW	IGH	2	None	
10	5640	CARDSRWGSSWYDYYYYGMDVW	IGH	5	None	
10	5642	CSRIYYDVSGYYEASLDYW	IGH	2	None	
10	5643	CARAWDHDYPDAFDIW	IGH	2	None	
10	5644	CARAKSITTTPNWFDPW	IGH	2	None	
10	5645	CQTYSSAPSAPLFTF	IGK	4	None	
10	5646	CARVVPYYDFWSGYYGGFDYW	IGH	11	None	
10	5647	CARDRSSVEVIHNYYYYGMDVW	IGH	2	None	
10	5648	CARAHLTNYYNGHAFNIW	IGH	2	None	
10	5649	CARDPSYGSGASYKSFDYW	IGH	39	None	
10	5650	CARIVVVVAGNGDFDYW	IGH	3	None	
10	5651	CANLPMDTAMADYYYMDVW	IGH	2	None	
10	5652	CARMQYYYDSSGYYYVSGNYYYGMDVW	IGH	3	None	
10	5653	CARAFSSGYAFYW	IGH	4	None	
10	5654	CAKDDGKTLAPRLSMAGYYFDYW	IGH	3	None	
10	5655	CARDNSPEVGYSGYDYGWFDPW	IGH	2	None	
10	5656	CARPYSSSWYWYFDLW	IGH	7	None	
10	5657	CARIPEAYYYDSSGYWFDYW	IGH	7	None	
10	5658	CAREGESSSPPFLDYW	IGH	5	None	
10	5659	CARQHVLLWFGELYNRANFDYW	IGH	5	None	
10	5660	CAKTSSGYGGNSGDYW	IGH	8	None	
10	5661	CVLYMGSGIWVF	IGL	48	None	
10	5662	CARGPYCSGTSCPTDDAFDIW	IGH	2	None	
10	5663	CARGPLKNREHIVVVVAATENYYYYYGMDVW	IGH	12	None	
10	5664	CTTAPPGWHGFDYW	IGH	2	None	
10	5665	CARDYVMSPTYDFWSGYSGYYYYYMDVW	IGH	3	None	
10	5666	CAACYSSSSFVGWDAFDIW	IGH	5	None	
10	5667	CARDSRWSSLGGAFDIW	IGH	3	None	
10	5668	CAKDILQYYYDSSGSGAFDIW	IGH	6	None	
10	5670	CARGGDFLTVDCYMDVW	IGH	3	None	
10	5671	CARDNAPKGTYYDFWSGYYLNWFDPW	IGH	10	None	
10	5672	CATLAGSYGSYYYYGMDVW	IGH	3	None	
10	5673	CAKVQGIAAAGSPLDYYYYMDVW	IGH	2	None	
10	5674	CASSSYGFHWYFDLW	IGH	2	None	
10	5675	CATGAFDFWSGMPRSYWFDPW	IGH	5	None	
10	5676	CAKDREIVANFPFDYW	IGH	2	None	
10	5677	CASDRGTREYSSYLGVLDYW	IGH	7	None	
10	5678	CARHWGRGFLSKVSWFDPW	IGH	2	None	
10	5679	CARDIFGSGSYFMVGYMDVW	IGH	3	None	
10	5680	CARGGWRTPYYYYYMDVW	IGH	25	None	
10	5681	CAKDGDFETPDFYYYGMDVW	IGH	4	None	
10	5682	CATSSGWSRVHWFDPW	IGH	2	None	

10	5683	CARGHSSTFDHW	IGH	3	None	
10	5684	CARGHNYDSSGLPIDYW	IGH	2	None	
10	5685	CAKDLGVDYGGNAGDYW	IGH	2	None	
10	5686	CARRDIMIGGFDQW	IGH	8	None	
10	5687	CARGGWTIGAGYYYYYYMDVW	IGH	5	None	
10	5688	CARDWRNSGYPFDGMDVW	IGH	2	None	
10	5689	CAQELKYCSTKTCPGWFDPW	IGH	2	None	
10	5690	CARQDSYNWDFDYW	IGH	6	None	
10	5691	CATNRADIAVNWFDPW	IGH	7	None	
10	5692	CVKDTLAEGYNFPDYW	IGH	10	None	
10	5693	CAKSPESLRVFQYLSYFAYW	IGH	2	None	
10	5694	CAHTGAAMVSAFDIW	IGH	4	None	
10	5695	CARFSVVVVAARDYYFDYW	IGH	4	None	
10	5696	CASPRVREDYDFWSGYYTRAFDYW	IGH	4	None	
10	5697	CARGLYDSSGYFYSDAFDIW	IGH	3	None	
10	5698	CAKDRNYDILTGSSYCFDYW	IGH	2	None	
10	5699	CARGVATWVRGRSFDSW	IGH	2	None	
10	5700	CTTGGTYYYDSSGYYYDLDYW	IGH	2	None	
10	5701	CARVSSHYDILTGYLGLYLDAFDIW	IGH	14	None	
10	5702	CAAWDDSVNALLF	IGL	76	None	
10	5703	CARSEKYYDYVWGSYRLFPFDYW	IGH	7	None	
10	5704	CARVRPPYHCGGDCYSTDYW	IGH	15	None	
10	5705	CARAWTIPPYAFDIW	IGH	6	None	
10	5706	CATVSFPNYYDSSGYYYFDYW	IGH	9	None	
10	5707	CAKDSIGYCSSTSCQGGFDYW	IGH	16	None	
10	5708	CARGPSQSFSRVEEVGEFGYW	IGH	2	None	
10	5709	CATSSPHCTGGVCPNWFDPW	IGH	134	None	
10	5710	CAMTSGTMTDWFDPW	IGH	2	None	
10	5711	CAKSDIINMIVVVGLFDYW	IGH	2	None	
10	5712	CAKDTALYGDYVGIDYW	IGH	4	None	
10	5713	CQQRSNWPPMYTF	IGK	3358	C1	7BZ5
10	5715	CARDSSHVQLWPTNNNWFDPW	IGH	4	None	
10	5716	CARGTDAITPDYW	IGH	5	None	
10	5717	CATLAYSNNVGTQGGYYYYGMDVW	IGH	2	None	
10	5718	CTTEEYQLLSHLFDYW	IGH	2	None	
10	5719	CARESSSGWTVLGAFDIW	IGH	2	None	
10	5720	CASIMVTATYYFDYW	IGH	6	None	
10	5721	CARDAMAVTTVRSYSYYYYYYMDVW	IGH	4	None	
10	5723	CTREDADELGYTYGIPFDYW	IGH	2	None	
10	5724	CATISMIRKTKVDYW	IGH	2	None	
10	5725	CAKDRDSWSGYYAYYYYAMDVW	IGH	3	None	
10	5726	CAHRRTTFVRSAFDIW	IGH	50	None	
10	5727	CTRDSYSSGWYGRLNYYYGMDVW	IGH	3	None	
10	5728	CARGGGYDSSGYYSPFDYW	IGH	26	None	
10	5729	CAKASAVTGPFDHW	IGH	3	None	
10	5730	CARPFSPLGPELDGFDPW	IGH	2	None	
10	5731	CAKITTGIAVDATTDYW	IGH	10	None	
10	5732	CARDLFEVRNSGYYVTAVDIW	IGH	7	None	
10	5733	CAKLGSGTIFGVVIIPTYFDYW	IGH	11	None	
10	5734	CARDEGDSGSLPSFDYW	IGH	17	None	
10	5735	CARGPVAAPNDAFDIW	IGH	13	None	
11	1	CASLSSSSTTDDYW	IGH	3	None	
11	2	CARDGWLANPHGFDIW	IGH	4	None	
11	3	CARFMEDSAFDYW	IGH	10	None	
11	4	CTRVLGVLRGTDYYYYMDVW	IGH	2	None	
11	5	CARAGGSLSLPATMGWYFDLW	IGH	4	None	
11	6	CARSGGHNWNYDVFDYW	IGH	2	None	
11	7	CARADYGDYVDYYYGMDVW	IGH	6	None	
11	8	CARDYDILTGAPYYYYYYMDVW	IGH	4	None	
11	9	CARAPYSSSFTHCFDYW	IGH	4	None	
11	10	CARDLFGYSSSTPRPDKDDYW	IGH	5	None	
11	11	CAKARRSDGDYALDYW	IGH	6	None	
11	13	CARGDDYGDTSFDYW	IGH	121	None	
11	14	CARDQYTGSYPFDYW	IGH	2	None	
11	15	CMRHNPRANPGDYTSYW	IGH	27	None	
11	16	CARDPQRYCGSTSCYHTWFDYW	IGH	2	None	
11	17	CAKEHGDWSPGNYIDSW	IGH	2	None	
11	18	CARGLSASSYDAFEIW	IGH	2	None	

11	19	CARDSMWELVNDAFDIW	IGH	2	None	
11	20	CARDPYIVVVPAAKGYFDYW	IGH	2	None	
11	21	CAKDLDDGSGWPLDYW	IGH	4	None	
11	22	CAHRGGSDYSLYYFDFW	IGH	2	None	
11	23	CATDNYYGSGNYSRWFDPW	IGH	2	None	
11	24	CARERVDYGDYVDYYYYMDVW	IGH	4	None	
11	25	CAHRASGYSGYAIDYW	IGH	4	None	
11	26	CVRGNSDNYYGYFGPW	IGH	4	None	
11	27	CARVESSSGYLSNFDYW	IGH	2	None	
11	29	CARIRNDYGGITIDYW	IGH	10	None	
11	30	CARFRPSNSDYFDYW	IGH	7	None	
11	31	CARDRSDYDFWNGYYTYW	IGH	2	None	
11	32	CARRPEGGIYETFDYW	IGH	5	None	
11	34	CARVSRYCSRTSCYALWHFDYW	IGH	2	None	
11	35	CARQTTARGSYYVDYW	IGH	2	None	
11	36	CARDWGWPRGAFDIW	IGH	9	None	
11	37	CARDYSPRYYDILTGYYNNWFDPW	IGH	50	None	
11	38	CARDAPPWACSGGSCYPDYW	IGH	2	None	
11	39	CLVQGLISRNYFDYW	IGH	3	None	
11	40	CARDGVGSTYYLDYW	IGH	20	None	
11	41	CASGLTAYGIDYW	IGH	16	None	
11	42	CARVGSWYYDSSGNYVSGGPDYW	IGH	2	None	
11	43	CARETPYGSGKFFWFDPW	IGH	2	None	
11	44	CARSDYGDNLGHDAFAIW	IGH	2	None	
11	45	CARSEYSSSRVYYYMDVW	IGH	5	None	
11	46	CARSTTHSYYYYMDVW	IGH	2	None	
11	48	CARVGHSLSRNGARRGHYDYW	IGH	2	None	
11	49	CARQSKVHDALDIW	IGH	3	None	
11	50	CARQVAPGIAVAPGKWFDPW	IGH	2	None	
11	53	CARQSPVAPYEDYFDYW	IGH	10	None	
11	54	CARDIGYCSSTSCRPDYYFDYW	IGH	3	None	
11	55	CARVFFGEMATITHYYYGMDVW	IGH	4	None	
11	56	CASSGQHSSTWYQGYW	IGH	4	None	
11	57	CAKDRDEYCSSTSCYAFVPLLDTIFDYW	IGH	2	None	
11	59	CATSFAYCSSTSCYVVYW	IGH	2	None	
11	60	CARRDLATTSFDYW	IGH	4	None	
11	61	CARNTLLEWFIDYW	IGH	11	None	
11	62	CAKVGSRFRNEGVLNPLYYFDYW	IGH	3	None	
11	63	CAAWDDSLSGWVF	IGL	1587	None	
11	64	CAKLSVGSSSGSFDYW	IGH	8	None	
11	65	CAREDRYYDFSYW	IGH	16	None	
11	66	CSLTSEDTAVYYCARDHYGSGTYLFDYW	IGH	2	None	
11	67	CARDSCNSIGWCGWFDPW	IGH	2	None	
11	68	CARQGGRLSMVQGINWFDPW	IGH	2	None	
11	69	CARDYYYFWSGLHNWFAPW	IGH	2	None	
11	70	CAGHTPLAVAYNWFDPW	IGH	2	None	
11	71	CATEMADALDIW	IGH	2	None	
11	72	CARGNIAVAAYFDFW	IGH	5	None	
11	73	CASRRGSGSYQIVDAFDMW	IGH	6	None	
11	74	CASDYGDYTNYYYYYGMDVW	IGH	2	None	
11	75	CARDHAAATGWFRELNDYFDYW	IGH	3	None	
11	76	CARDEGVIMITIPFDYW	IGH	2	None	
11	78	CARVAGCSGGSCHKPNWFDPW	IGH	4	None	
11	79	CARHSGYSSSGAFDIW	IGH	6	None	
11	80	CARDVRYCGGDCRDYW	IGH	36	None	
11	81	CAKLGCSSTSCYGPDYW	IGH	2	None	
11	82	CARDEVGSLSTPIDYW	IGH	4	None	
11	84	CATEQATVTPTSPYYYGMDVW	IGH	2	None	
11	85	CASPDPLGYYDSSAHPMDYW	IGH	3	None	
11	86	CTTVRRIPIFGVVDYYFDYW	IGH	3	None	
11	87	CARSMVQGVRWYFQHW	IGH	5	None	
11	88	CAKELIGAGNYIPRYWYFDLW	IGH	2	None	
11	89	CARDLGPTVVKPGYYYYGMDVW	IGH	5	None	
11	90	CARVAVTTWEFDLW	IGH	5	None	
11	91	CATWDSSLTAGSVVFGGGSVVF	IGL	2	None	
11	93	CARNSFYCKGGVCHPMRFSWFDPW	IGH	6	None	
11	94	CARGGGIFGVVIISSNPFDYW	IGH	7	None	
11	95	CARLRDILTGFEAFDIW	IGH	2	None	

11	96	CARAGLTSASSRVFFDSW	IGH	5	None	
11	97	CQQYNNRPPLSSF	IGK	2	C3	7K8R
11	98	CARQGGSETYDDYW	IGH	6	None	
11	99	CARGRVLQRLSDAFDIW	IGH	2	None	
11	100	CTTDRDYDYIWGSYRYIDYW	IGH	70	None	
11	101	CARPLTSPSNFHYYYGMDVW	IGH	2	None	
11	102	CARGEGYYGTGNYYKKYYFDYW	IGH	64	None	
11	103	CARSPGPHCISTRCLDLDYW	IGH	10	None	
11	104	CATPRAPYSSSWSSFDYW	IGH	4	None	
11	105	CARGDCSSASCYSWRWFDPW	IGH	2	None	
11	106	CASTSHMRVYFDYW	IGH	2	None	
11	107	CATAHDCGGGRCYSPWFDPW	IGH	6	None	
11	108	CARDRGSSSYFSNFYYMDDW	IGH	2	None	
11	110	CARDPPAVGVEFDYW	IGH	2	None	
11	111	CARARYADYSAEYFQHW	IGH	3	None	
11	114	CARELAPNWGSRWFDPW	IGH	4	None	
11	116	CARTLLISSHLVDYW	IGH	8	None	
11	118	CASLYYFDFSGFFDYW	IGH	49	None	
11	119	CARDEVLGHYFESW	IGH	13	None	
11	120	CAAVPFETLPPLYYFDYW	IGH	2	None	
11	121	CANYDSKDGDGFDIW	IGH	3	None	
11	122	CAKNGYYDSSGFNLDYW	IGH	6	None	
11	123	CARDYFYDFWSAYAMDVW	IGH	2	None	
11	124	CARDLRYCTSDSCYVGYYYYGMDVW	IGH	3	None	
11	126	CAKEAYDYIWGSYRFPYW	IGH	4	None	
11	127	CARHVEDCSSTSCSPRPYYYGMDVW	IGH	2	None	
11	128	CASSSYYYDSSGPYYFDFW	IGH	3	None	
11	130	CARDPGYYGSGSLDAFDIW	IGH	4	None	
11	131	CARQDKYSSSDDYW	IGH	2	None	
11	132	CARESISGPGAPKNWFDPW	IGH	2	None	
11	133	CATAAGTPPYFDYW	IGH	13	None	
11	134	CVRSPASWGFNYFDYW	IGH	2	None	
11	135	CARMGSCGGGSCYSGYFDPW	IGH	2	None	
11	136	CQSYDGSLSESGVF	IGL	546	C4	7JVC
11	137	CARGLYYDRSFNYW	IGH	4	None	
11	138	CARYGYDYIWGSYRPYYYYYMDVW	IGH	2	None	
11	139	CTKSPSSSSYSGADYW	IGH	2	None	
11	140	CDWSSDVCSSDLRYCGGDCRDYW	IGH	2	None	
11	141	CARDRDSSSWSNSYMDVW	IGH	3	None	
11	142	CARHGGPHWVNWYFDLW	IGH	4	None	
11	143	CARGAGCSGGTCYSGYWYFDLW	IGH	8	None	
11	144	CTRKPVDYW	IGH	15	None	
11	145	CARGKSYSTWYYFDYW	IGH	3	None	
11	146	CVRHGGKMFDSW	IGH	4	None	
11	147	CARDLQQQLVIDYYYGMDVW	IGH	2	None	
11	148	CAKDVDDDDYGVFDYW	IGH	2	None	
11	149	CARSRMISDIGYCSSTSCPFDYW	IGH	2	None	
11	150	CARDLFSFYRGPGNYGWFDPW	IGH	2	None	
11	151	CARGKAGVVGATALDYW	IGH	2	None	
11	152	CARAQIVRYFDWLSSVVFDYW	IGH	2	None	
11	153	CARDGSSRYSTSSGLDYW	IGH	3	None	
11	154	CARDARYCTGGVCYDYYYYGMDVW	IGH	3	None	
11	155	CARDHRVGDTECDYW	IGH	2	None	
11	156	CARQYYYDSSRWDEFDPW	IGH	2	None	
11	157	CAKLSGLGDYDDAFDIW	IGH	4	None	
11	158	CAREDMVQASLKYYYYMDVW	IGH	22	None	
11	159	CASQRADNYGSGIYNEYFQHW	IGH	2	None	
11	160	CASGVVGATISEYYFDYW	IGH	2	None	
11	161	CARQPSWCNGDSCYPDYYGMDVW	IGH	2	None	
11	162	CARNRNFDFWSGYYSGHLDYW	IGH	4	None	
11	163	CARLLTTGYWYFDLW	IGH	9	None	
11	164	CVRGEKRLWPRRNYYLDYW	IGH	4	None	
11	165	CARLILSGSGWYGGDYW	IGH	2	None	
11	167	CARAGYTYNYYFDYW	IGH	6	None	
11	168	CARVGTMVQGVTELFLFDYW	IGH	3	None	
11	169	CARDGRPGAYHFWFDCW	IGH	12	None	
11	170	CYSTDSSGNHRVF	IGL	2295	None	
11	171	CARYYCPGSACFGFDYW	IGH	3	None	

11	172	CAVLQTIAARHFDYW	IGH	2	None	
11	173	CARDYSHYEVDTVAKFYSYYYYMDVW	IGH	2	None	
11	175	CARDKGDGGSSGWYDAFDIW	IGH	2	None	
11	176	CQQSYSTPLFAF	IGK	1189	C4	6ZDH
11	177	CARTNLGELSPRGYYYYGMDVW	IGH	2	None	
11	178	CARARSPDSGLVGDFDSW	IGH	32	None	
11	179	CAKGHYSGSYYYFDYW	IGH	12	None	
11	180	CARGMGLPYSRTSSIAARPMDYW	IGH	2	None	
11	181	CAKLISVYAFDYW	IGH	4	None	
11	182	CHQYNVWPPWAF	IGK	92	C3	7K8P
11	183	CATETIFLGGRFDPW	IGH	2	None	
11	184	CARGAYSSSWYGPKYYFDYW	IGH	3	None	
11	185	CADTAVYYCAKAGVAAPDAYFDYW	IGH	2	None	
11	186	CATGSRITIFGVVTTSSVYAFDIW	IGH	2	None	
11	187	CARHGVRSGWDVDFDLW	IGH	2	None	
11	188	CARDRQSIAARPEIDGFDIW	IGH	2	None	
11	189	CANIPLRVAGILDYW	IGH	2	None	
11	190	CARLGHIVATTIYYFDYW	IGH	2	None	
11	191	CAREPMLGTNAFDIW	IGH	2	None	
11	192	CARDCIGGVCPLDYW	IGH	3	None	
11	193	CARVRHDYGDYIDAFDIW	IGH	2	None	
11	194	CARIDSTGWYGDAFDIW	IGH	3	None	
11	196	CATNAHCSGGKCPLYFDHW	IGH	2	None	
11	197	CARGILAFEGNYYMDVW	IGH	2	None	
11	198	CQSADNSGVYGVLF	IGL	2	None	
11	199	CARGVIGWYYFDFW	IGH	48	None	
11	200	CARDRSPRGYDPNYYYGMDVW	IGH	2	None	
11	201	CASPRGYSGYSFDYW	IGH	25	None	
11	202	CARDLYYYDSRRYYDPSHYFDYW	IGH	4	None	
11	203	CAREPGVTHDFWSDYYTEAFDIW	IGH	8	None	
11	204	CARERSTLERYFDYW	IGH	3	None	
11	205	CARGLYGLTSKIDYW	IGH	2	None	
11	206	CAHSGYYDFWSGYYRGHDAFDIW	IGH	2	None	
11	207	CASTSGYDEYYFDYW	IGH	3	None	
11	209	CARASIVGDSPFFGVARRYYFDYW	IGH	2	None	
11	210	CQHYDSAPALF	IGK	5	C3	6XDG
11	211	CARDSSDVYSGYDSGFYFDHW	IGH	2	None	
11	212	CATPRTYGDYPVGDFDYW	IGH	2	None	
11	213	CQQVSIFPYTF	IGK	10	None	
11	214	CAGLTYYYDSSGHTMGHFDYW	IGH	2	None	
11	215	CARWGDDYGDYVDYW	IGH	14	None	
11	217	CAKDPYYDFWSASYYMDVW	IGH	5	None	
11	218	CSSYGGSHNFLLF	IGL	2	C1	6XCM
11	219	CARGPYTVRIFGVVIDFDYW	IGH	2	None	
11	220	CTRRDDYGDRDWDYW	IGH	4	None	
11	221	CARVLPGASFTWWYFDLW	IGH	199	None	
11	222	CARFTGKVESRSSSRYFQEW	IGH	2	None	
11	224	CARRYCSATSCAFDYW	IGH	21	None	
11	225	CAKDSSWADGDYVFDYW	IGH	12	None	
11	226	CARDGPVTGPKGFDYW	IGH	2	None	
11	227	CVREGESEHNDYDRIFDYW	IGH	2	None	
11	228	CARDSVIPYGSGSYRWFDAW	IGH	3	None	
11	229	CSRIAAALDFGYW	IGH	2	None	
11	230	CAREGDGSGSYWNYYYMDVW	IGH	2	None	
11	231	CARGLDGSGSPPDYW	IGH	2	None	
11	232	CAHSPFDYDFWSGYYSGRTYYFDYW	IGH	4	None	
11	233	CAKDQLGLVVIPAAPDYW	IGH	9	None	
11	234	CARALFARSVPRPHYFYMDVW	IGH	5	None	
11	235	CARVTTERGGTFDDW	IGH	2	None	
11	237	CAHFGYDLRSGYYHGFDYW	IGH	2	None	
11	238	CARDHPDNDYGDYAGYW	IGH	13	None	
11	239	CAGSMAPRRFDYW	IGH	4	None	
11	240	CARDRNYGGRSYFDYW	IGH	2	None	
11	241	CARGNYDSSGYSGFLDWYFDLW	IGH	4	None	
11	242	CVKDDYYASGNFASNFDCW	IGH	3	None	
11	243	CARGGTRGPLWAFGYW	IGH	4	None	
11	244	CAVCPLLEWIFDYW	IGH	4	None	
11	245	CAAPRGYDSSGYDAFDIW	IGH	4	None	

11	246	CARAGYSSSPGLIYYYYYMDVW	IGH	4	None	
11	247	CARSYDFSTHGTFDFW	IGH	3	None	
11	248	CAREPTEFGAVAGTRSGYYYMDVW	IGH	4	None	
11	249	CSRKVAGTAFDIW	IGH	4	None	
11	250	CATRGYCTGGVCPESDASDIW	IGH	14	None	
11	251	CARAPVYYYDSSGYYSHFDYW	IGH	3	None	
11	252	CARENSHDGLHAFDIW	IGH	2	None	
11	253	CARVLQGSSGYYFDYW	IGH	15	None	
11	254	CAKEVYISRITIFGVVTPPYYYGMDVW	IGH	3	None	
11	255	CARGSGWYEYYYGMDVW	IGH	2	None	
11	258	CARDNDYIWGSYRSWFDPW	IGH	4	None	
11	259	CATRLAYCGGDCPYHFDYW	IGH	2	None	
11	260	CARFSWSAVPGTTLTEENWFDPW	IGH	2	None	
11	261	CTTDQRGGDFDYW	IGH	2	None	
11	262	CVRGAFLEWFHGFDIW	IGH	2	None	
11	263	CAHITVGSTHDYW	IGH	7	None	
11	264	CARDCSSTSCYARDAFDIW	IGH	11	None	
11	266	CARTAPCGGDCYYFDYW	IGH	70	None	
11	267	CARASLTHCRYGTCAWFDPW	IGH	2	None	
11	268	CARDVPVDTALADAFDIW	IGH	2	None	
11	269	CARDQHYSNYEYYYYYGMDVW	IGH	2	None	
11	270	CATGPLAAAALNWFDPW	IGH	4	None	
11	271	CQSFDRSNHGVF	IGL	6	C4	7JVC
11	272	CARQSEGITNFGVVIIAPYFDYW	IGH	6	None	
11	273	CAKVLYSSSWSPARGFDYW	IGH	4	None	
11	274	CARLSGRDPDYW	IGH	8	None	
11	275	CQRRSKWPPECSF	IGK	43	None	
11	276	CAHSDDSSGWYSWFDPW	IGH	6	None	
11	277	CARHAVSGSYTPPWDYW	IGH	2	None	
11	278	CATGPAATKGDWFDPW	IGH	6	None	
11	279	CARDILRPDYYDSSGYSHYYFDYW	IGH	2	None	
11	280	CARAGVFYDYIWGSYRYTDENFDYW	IGH	7	None	
11	281	CAQTWNYIWGSFDYW	IGH	2	None	
11	282	CARRPAKNDFWSAYFDYW	IGH	4	None	
11	283	CARGSRDGYNLGRIDYW	IGH	4	None	
11	284	CARDRETSYSSGWSYYFDYW	IGH	8	None	
11	285	CARVTLQLGEGYFDYW	IGH	5	None	
11	286	CARQTGYSGWYGGFDFW	IGH	17	None	
11	287	CARSDSGSGSQHDYW	IGH	5	None	
11	288	CAKSLYCTGGTCYVGYIDLW	IGH	15	None	
11	289	CARFHDYGHDNYFDYW	IGH	2	None	
11	290	CARDPNDYGDYGEAFDYW	IGH	2	None	
11	291	CARLGDEWELLLVDYW	IGH	2	None	
11	292	CTRGTYYYDSSGYPNDAFDIW	IGH	2	None	
11	293	CARTGMDFSEPYYYYYYMDVW	IGH	2	None	
11	294	CARDPGKEVRWGYYDSSPSNWFDPW	IGH	2	None	
11	295	CARRSGWLINAFDIW	IGH	4	None	
11	296	CASSISSASCYDYW	IGH	2	None	
11	297	CASQGANLAALDTFHVW	IGH	2	None	
11	298	CAREHYDFWSGSFSSVNWFDLW	IGH	5	None	
11	299	CARDVGSSGYRYVGRFDPW	IGH	4	None	
11	300	CATYYYDSSGYYLDFDYW	IGH	2	None	
11	301	CARTSSISILGVTYYYYYMDVW	IGH	2	None	
11	302	CARHAAICGGGSGGSCYPYNWFDPW	IGH	6	None	
11	303	CANYSGYDSYWYFDLW	IGH	6	None	
11	305	CLRSEDTAVYYCAADPETYYGDYKRYNWFDPW	IGH	3	None	
11	306	CARDDYGDYGALGDYW	IGH	6	None	
11	307	CARLVIAARPSSRGTLLDYYYYMDVW	IGH	2	None	
11	308	CARDHGVEIVATPPFDYW	IGH	3	None	
11	309	CARPRSKYSSSSGEFDYW	IGH	3	None	
11	311	CARDTSYDFWSGYSGTFDYW	IGH	2	None	
11	312	CVRPHDFWSGSNWFDSW	IGH	2	None	
11	313	CVRTSRDGALDYYDYW	IGH	3	None	
11	314	CAHRQDSTLHFDYW	IGH	10	None	
11	317	CTTTLSVYGDYAQGFDYW	IGH	2	None	
11	318	CAHATYYYGSGSYPQIFDYW	IGH	2	None	
11	319	CATTAAPDDSGFPIGFDNW	IGH	9	None	
11	320	CAREDPREDAGNTFDYW	IGH	2	None	

11	321	CARVMYGSSWDTLDYW	IGH	7	None	
11	323	CAKVAQGSSYFDYW	IGH	2	None	
11	324	CARERMVQGNLHFDYW	IGH	2	None	
11	325	CARGHDYGDYVFHWFDPW	IGH	2	None	
11	326	CAQRFGVVIGPYFDYW	IGH	4	None	
11	327	CARPGITVAGILDGFDIW	IGH	4	None	
11	328	CAREGCGADCYSSSRAFDIW	IGH	4	None	
11	329	CVTPYCSGGRCYAFDYW	IGH	5	None	
11	330	CAANGITMFDYW	IGH	10	None	
11	331	CARASVGGYSSFDYW	IGH	28	None	
11	332	CAKGGPSGYCSGGSCGYFDYW	IGH	2	None	
11	333	CAHSKSSAWYQIDYW	IGH	8	None	
11	334	CSRDIYYFDGSACYYQDVW	IGH	31	None	
11	335	CARDVEKLDIWGSYRSYAFDIW	IGH	2	None	
11	336	CARGYYYDSSGYYRNWYFDLW	IGH	3	None	
11	337	CAGAAARTYNCFDPW	IGH	4	None	
11	338	CARDRGYFDWLLRTDRGTYFDYW	IGH	2	None	
11	339	CTREGSGYDYNWFDPW	IGH	4	None	
11	340	CATDSGSYLGAAYYYMDVW	IGH	2	None	
11	341	CAHRMTSVVARAGFDYW	IGH	3	None	
11	342	CARNRRTIVATIKGYFDYW	IGH	6	None	
11	343	CATDSRSSSWLPFRAAYFDLW	IGH	2	None	
11	344	CARQPLSSSWYCPSDYW	IGH	8	None	
11	345	CARVEGLGYDYYYYMDVW	IGH	9	None	
11	346	CTRDQHRYCSGGSCYSPDYW	IGH	2	None	
11	347	CARVPVHYSNAWYSYRYMDVW	IGH	4	None	
11	348	CARDMYAGIPDYFDYW	IGH	2	None	
11	349	CARSDGYWSWFDPW	IGH	6	None	
11	350	CARVGSDFWALDYW	IGH	5	None	
11	351	CARGLRAYCGGDCYSSDYW	IGH	4	None	
11	353	CARVGLYYYDSSGYRSDAFDIW	IGH	7	None	
11	354	CARSRSFYDSSGSESGSYYYYGMDVW	IGH	2	None	
11	355	CATAFDPYYYDSSGSKRGAFDIW	IGH	2	None	
11	356	CAKSAVQTSRFDCW	IGH	2	None	
11	357	CARGREKYCSSTSCTEYYFDYW	IGH	2	None	
11	358	CARHSISIVVVPAAYNWFDPW	IGH	3	None	
11	359	CASSLSVVACFDYW	IGH	2	None	
11	360	CARATVTTYNWFDPW	IGH	14	None	
11	362	CARGPHSGTSYIDYW	IGH	2	None	
11	363	CARDVITFGGVIDDYW	IGH	2	None	
11	364	CAKDFRVGIPGIIYNWFDPW	IGH	7	None	
11	365	CARGTTYYDILTGYYSLGSFDYW	IGH	2	None	
11	366	CARGDYYSTPDWPQFDPW	IGH	4	None	
11	367	CVRGGPGYCSGLSCYPGDYW	IGH	2	None	
11	368	CARDVVTTRLLYYYYSYMDVW	IGH	6	None	
11	369	CAAEVPQGVIYFNYW	IGH	6	None	
11	370	CATRPQITIFGVPDAFDIW	IGH	5	None	
11	371	CARPQIWGPIDYW	IGH	4	None	
11	372	CANGETPSGYFQHW	IGH	2	None	
11	373	CARMAGYCSGNTCYSHYFDYW	IGH	2	None	
11	374	CAADPDFSEYYYYYYMDVW	IGH	2	None	
11	375	CTRAPAGPPYYYMDIW	IGH	4	None	
11	376	CAKDRAGYDILTGYLRSPDYYYYYGMDVW	IGH	2	None	
11	377	CARLYNDRSGGNDQWFDPW	IGH	5	None	
11	378	CARSRYSASYYGGADYW	IGH	2	None	
11	379	CARQITILDAFDIW	IGH	10	None	
11	380	CALTGVFGLPYNWFDPW	IGH	2	None	
11	381	CARVNSDYLYFDLW	IGH	7	None	
11	382	CTTENYYDSSGYLRDYW	IGH	2	None	
11	383	CAREGGGMGTMIGNWFDPW	IGH	2	None	
11	385	CARGEGYDILTDSYYYYYMDVW	IGH	2	None	
11	386	CAKDLGLEPIYYYYYGMDVW	IGH	4	None	
11	387	CARDSAPHYYDSSGYYPKSFDYW	IGH	3	None	
11	388	CARAYDFWSGSIYYYYGMDVW	IGH	9	None	
11	389	CARDNDQEGVTSFRFNYYYGMDVW	IGH	3	None	
11	390	CARTPDYINYGWFDPW	IGH	58	None	
11	392	CARNLYGSGSRAFDIW	IGH	5	None	
11	393	CAHRTFGGWSAFDYW	IGH	7	None	

11	394	CASSHASTWSFVYW	IGH	79	None	
11	395	CARDRIFWSGYTNPYYYYGMDVW	IGH	3	None	
11	396	CARTKGGGYDSDFDYW	IGH	8	None	
11	398	CAKVRVGGTLHAFDMW	IGH	2	None	
11	399	CARDSKLTTYQDAFDIW	IGH	5	None	
11	400	CARDRLVQGVSDYW	IGH	2	None	
11	401	CARDRGEGYSSGWYFDYW	IGH	10	None	
11	402	CARGTRSIAAYFDYW	IGH	2	None	
11	403	CHQYYTTPQSF	IGK	18	C4	6W41
11	404	CVRDPHYGDYDGDAFDIW	IGH	2	None	
11	405	CARAPYCSSTSCWYYFDYW	IGH	6	None	
11	406	CAREASEQWLILGGSYLYFDYW	IGH	4	None	
11	407	CAREVCGRSSCYFDYW	IGH	2	None	
11	408	CARHGRSVSSSRTYFDYW	IGH	5	None	
11	409	CARDRILEWLPPEFDYW	IGH	2	None	
11	410	CAHGYSGYDFYYYFDYW	IGH	2	None	
11	411	CARSSIAARPSLYFDYW	IGH	10	None	
11	412	CARGDHLGELTKSFFDSW	IGH	5	None	
11	413	CARASWGSGDLAPYYYYGMDVW	IGH	7	None	
11	415	CARGYYDFWSGYLGTGYYYYMDVW	IGH	2	None	
11	418	CARDRFPRGWLDPW	IGH	44	None	
11	419	CARRVRGGLRGWNNWFDPW	IGH	2	None	
11	420	CASRKNGYFDYW	IGH	19	None	
11	421	CARGGVEYDRWFDPW	IGH	16	None	
11	422	CASPRSSMAQGLAIAVFDYW	IGH	2	None	
11	423	CVKGGYSSSSSFDYW	IGH	19	None	
11	424	CQQSWQTPIWTF	IGK	13	C2	7K8O
11	426	CAHRPLGSSTVFDFDYW	IGH	2	None	
11	427	CARESVGRGLQFDCW	IGH	3	None	
11	428	CAILTKTNYYMDVW	IGH	6	None	
11	429	CARPLDRYCSGGSCYSNWGLDYW	IGH	3	None	
11	430	CARRPSVDYGDTYYFDHW	IGH	4	None	
11	431	CARAQFNDNWLNGDGMDVW	IGH	2	None	
11	432	CTRVRPDILTGYWGGSAEFHYYYYGMDVW	IGH	2	None	
11	433	CELRDYSGYPLDYW	IGH	3	None	
11	434	CARDHMNFGVVIIDFYYYGMDVW	IGH	6	None	
11	435	CLRSEDTAVYYCARGFAVHDYIWGSYRWGHYFDYW	IGH	3	None	
11	436	CARGGYSSSSGWFDPW	IGH	27	None	
11	438	CTRGGRSGSFRLLGYYFDSW	IGH	2	None	
11	439	CARGYCSSTSCYRTRIDYW	IGH	6	None	
11	440	CASTHDYGDYEVDYW	IGH	14	None	
11	442	CAHLRVSDDAFGIW	IGH	4	None	
11	443	CARDHDSSGYEGWGWFDPW	IGH	2	None	
11	444	CARDMEASGSYYAPNAFDIW	IGH	4	None	
11	445	CARTWRYCSSTTCYDASDAFDIW	IGH	2	None	
11	446	CTRELPMTTVPTFDYW	IGH	2	None	
11	447	CAKGGIDAFHLW	IGH	8	None	
11	448	CARQFLTGELDYW	IGH	23	None	
11	449	CVSSSGYSGWFDPW	IGH	5	None	
11	450	CARDSYSGYDYDLGYGDYGDFDYW	IGH	2	None	
11	451	CAKGGSGWFDPW	IGH	22	None	
11	452	CARGPDQGRYSSTSSNWFDPW	IGH	3	None	
11	453	CARTYYYDSSGYSAWSDYW	IGH	4	None	
11	454	CARVGGDGYNLGQNFDYW	IGH	2	None	
11	456	CTRDPDDYDYSNYPFFDYW	IGH	2	None	
11	457	CARRALRFNVGGADYW	IGH	5	None	
11	458	CARDQPIYGDLRYWYFDLW	IGH	2	None	
11	459	CARTGGLWGIYWYFDLW	IGH	2	None	
11	460	CARDGGLELGFNYW	IGH	6	None	
11	461	CARDSYGDLGYYYYMDVW	IGH	5	None	
11	462	CVTGPKYYFDSW	IGH	2	None	
11	463	CARVSFNNYNVDWYFDFW	IGH	2	None	
11	464	CALPISDTAMYYCARHVLTIFGVFTYMDVW	IGH	3	None	
11	465	CARERGPLKRRYYYFGMDVW	IGH	2	None	
11	466	CADSSGYYSHIDYW	IGH	10	None	
11	467	CARIEGSYHRSGYIDYW	IGH	10	None	
11	468	CARGMGWRDAFDIW	IGH	33	None	
11	469	CARHKRPNENHYYYYMDVW	IGH	4	None	

11	470	CARDPYDSDYYGAFDIW	IGH	2	None	
11	471	CATHRPTYIVVVVAATLYVDYW	IGH	5	None	
11	472	CARTDLHRYNYKTYFDSW	IGH	2	None	
11	473	CARHRYSGYARGPFDYW	IGH	4	None	
11	474	CARPLGSDTLLRGDFDCW	IGH	2	None	
11	475	CASNLRYCSSTSCVPEYFQHW	IGH	2	None	
11	476	CAHTHYGGNLLYNWFGPW	IGH	2	None	
11	478	CAKLVPPIEEGYFDYW	IGH	3	None	
11	479	CARLNPYGGYYYDSSGYYAPHDYW	IGH	2	None	
11	480	CATSPYAAGPFEYW	IGH	2	None	
11	481	CAHRRVAGYQGFDYW	IGH	3	None	
11	482	CARSIAAAGTYMDVW	IGH	4	None	
11	483	CARHKLGNWHFDLW	IGH	2	None	
11	484	CARDGFGGVIAKAFDYW	IGH	18	None	
11	485	CARARGDLGLYYFDYW	IGH	8	None	
11	487	CAKDIYDYTFLHKDAFDVW	IGH	3	None	
11	488	CARSGYSSGWPRGDFDYW	IGH	2	None	
11	489	CTTDVRYSNYSPLDYW	IGH	3	None	
11	490	CAHRLRYENYYPYFDSW	IGH	2	None	
11	491	CAGQNDYGDYWGYW	IGH	2	None	
11	492	CARMKRVGGGDRHFDYW	IGH	16	None	
11	494	CVRDIGGSGTRYFQHW	IGH	2	None	
11	495	CVRGWRGDVFDPVDVW	IGH	3	None	
11	496	CLSGDEDNWVF	IGL	44	None	
11	498	CARQPPQNDFWSASPTYYFDYW	IGH	2	None	
11	500	CAKDLGAYSSGWYGVDYW	IGH	3	None	
11	501	CMQALHCPPCSF	IGK	6	None	
11	502	CARDSFSSVHLGLYYFDYW	IGH	4	None	
11	503	CTTVVVVGYYFDSW	IGH	4	None	
11	505	CARHFWSMVTSPPFDPW	IGH	8	None	
11	506	CARVGYYGSGRVIAHRYFDYW	IGH	4	None	
11	507	CARTSSAYSPFFDYW	IGH	10	None	
11	508	CARDRSPPSSGYLVSAFDIW	IGH	6	None	
11	509	CATTWAHDYSNYLDYW	IGH	3	None	
11	510	CARHWGAHSSGYSGTDYW	IGH	5	None	
11	511	CARAHFYDSRGTDAFDIW	IGH	4	None	
11	512	CARAIGVATYFDHW	IGH	3	None	
11	514	CVRGDSGNFPGTDYW	IGH	4	None	
11	515	CARGHIVASNREWGSGAFDIW	IGH	12	None	
11	516	CARGPGYYDFWSGYYRGWFDPW	IGH	3	None	
11	517	CARLTGPGYNSGWDYW	IGH	5	None	
11	518	CATAGYCNSPTCPKAFFDYW	IGH	2	None	
11	519	CARSTVTTRSPFDSW	IGH	4	None	
11	520	CTTDDVPNCGGNCYFGHW	IGH	10	None	
11	521	CARDPTPVVVPAAISDGLYYYYYGMDVW	IGH	2	None	
11	522	CARLAYDPLRGDAFEIW	IGH	2	None	
11	523	CARHRLNGNWFDPW	IGH	94	None	
11	524	CARDALSGWLVDYW	IGH	7	None	
11	525	CAKAIPTTSIRSYFDLW	IGH	6	None	
11	526	CAKAGNKHDYGDYMDYW	IGH	2	None	
11	527	CAKASSSSGHHCDYW	IGH	5	None	
11	528	CARYYCTGGVCYHFDYW	IGH	32	None	
11	529	CAILMIQGPLNDYW	IGH	4	None	
11	530	CARFNYDSSGYYFGYW	IGH	13	None	
11	531	CARDQIPDHDFWSGYYTWASAYYYYMDVW	IGH	2	None	
11	532	CASCFSPYGDYVFDYW	IGH	8	None	
11	534	CARGSVTTDFYFDYW	IGH	3	None	
11	535	CARIDNILPDNEFGPW	IGH	7	None	
11	538	CARRGYYEYYFDSW	IGH	4	None	
11	539	CARASPLTLDYYDILTGYYIGEAFDIW	IGH	8	None	
11	540	CALQYQSSVYYYGMDVW	IGH	2	None	
11	541	CASLFGMVQGVKHIRHKNDYYYGMDVW	IGH	6	None	
11	542	CARDLGGGVLSWWFDPW	IGH	2	None	
11	543	CARESGSGDLEWLSHLDYW	IGH	2	None	
11	544	CARIGADFWSGSIFTDYW	IGH	2	None	
11	545	CGTYGDWAFDIW	IGH	4	None	
11	546	CTRLLKSSSGFYFRFDYW	IGH	10	None	
11	547	CARSFNCGGDCPLGWFDPW	IGH	2	None	

11	548	CAKVSYLSTASSLDYW	IGH	11	None	
11	549	CARRVRATMIVVDQFDYW	IGH	3	None	
11	550	CARAYSRGWPDAFDIW	IGH	3	None	
11	551	CARDSITMVQGVILVYYYYYMDVW	IGH	5	None	
11	552	CAREDYGDYEFKSAFDIW	IGH	4	None	
11	554	CAKANDDYYDSSGYFDYW	IGH	4	None	
11	555	CARDPGRGYGPFVYYFDYW	IGH	3	None	
11	557	CARYTSGSHDYYFDYW	IGH	4	None	
11	558	CASYPPLKVGNYDDYW	IGH	6	None	
11	559	CARAYLEYYDSSGYYDYFDHW	IGH	7	None	
11	560	CARGHGGSHCSGGSCYPHNWFDPW	IGH	2	None	
11	561	CASFDSSGYYYYHDYW	IGH	3	None	
11	562	CARELAGMTTVVSPFDYW	IGH	2	None	
11	564	CARDPAFGALDYW	IGH	13	None	
11	565	CARAHIRSFDYLQSDWFDPW	IGH	11	None	
11	566	CARLRSCGGDCYYSDNW	IGH	2	None	
11	568	CARVFDVLTGYSILDYW	IGH	3	None	
11	569	CATTPVGRIMITFGGVIAPHFFDYW	IGH	2	None	
11	571	CSRHRTNNFGSGTPFDYW	IGH	2	None	
11	572	CAKCSFSIDQRSGYFHYW	IGH	2	None	
11	573	CARHSWVDYYGSGSPDYW	IGH	10	None	
11	574	CARIPEVPAAMSHYYYGMDVW	IGH	4	None	
11	575	CARHAHFDWLLYPVGGHNWFDPW	IGH	4	None	
11	576	CARAVGGSYYFGSDYW	IGH	2	None	
11	577	CARPRSGTYYNPADYW	IGH	4	None	
11	578	CGRDGGTPRYFQDW	IGH	2	None	
11	579	CVPFFGSRSGYPTLDYW	IGH	4	None	
11	580	CARHETYYGSGSYGVAFDIW	IGH	42	None	
11	582	CARDYGAHGVDYW	IGH	13	None	
11	583	CARGRLRLPTEIDYW	IGH	2	None	
11	585	CARHSASGGYDFWSGHPSDYW	IGH	3	None	
11	586	CARHWRSGTYSQAIDYW	IGH	6	None	
11	587	CAKDPALITGTTGFDSW	IGH	2	None	
11	588	CARSYYSNYFHYW	IGH	1043	None	
11	589	CDWSSDVCSSDLDFWSGYGYYYYYMDVW	IGH	2	None	
11	590	CARRRIATADQTIDYW	IGH	5	None	
11	591	CAKDLDYYGSGKDYW	IGH	7	None	
11	592	CARTYDDSGYYNFKYW	IGH	4	None	
11	593	CARDRPYGEECFDPW	IGH	2	None	
11	594	CARGVSLPPNWFDPW	IGH	4	None	
11	597	CAPEHLQSYCFDYW	IGH	2	None	
11	598	CARFTPYSGYDSVFDYW	IGH	6	None	
11	599	CANYGETAAWFDYW	IGH	6	None	
11	600	CTRPGTAWDDAFDIW	IGH	2	None	
11	601	CTTEYYYYMDVW	IGH	2	None	
11	602	CAREWWYCSSTSCYQPYHFDYW	IGH	2	None	
11	603	CAVNSGYDSNWFDPW	IGH	5	None	
11	604	CARDLPLLAAAGTKIDYW	IGH	2	None	
11	605	CARQLRGSAYDYADDAFDIW	IGH	2	None	
11	606	CVRDENSGWNDFDYW	IGH	52	None	
11	607	CAKNLYGDYPYYYYYMDVW	IGH	5	None	
11	608	CARDDPTELPMGAFDIW	IGH	4	None	
11	609	CARDQHGVKYGDYVPFDYW	IGH	3	None	
11	610	CARAPETHYYGGSGQRHAWDDAFDMW	IGH	2	None	
11	611	CARRQGAGLYIAAAVFDCW	IGH	2	None	
11	613	CARQRIEYPPGYYFDYW	IGH	5	None	
11	614	CARDGCSSTSCYVALDYW	IGH	2	None	
11	615	CARPRYSGYEEGDYYYYMDVW	IGH	2	None	
11	616	CAKEQWPTDWYFDLW	IGH	2	None	
11	617	CVESHTIDGQVGYVF	IGL	2	None	
11	618	CAGGEYYYDSSGYGALDYYYGMDVW	IGH	2	None	
11	619	CARPEGGYSTSWVDYW	IGH	7	None	
11	620	CGTWDTSLNANWVF	IGL	32	None	
11	621	CARDPLLGYYDILTGYSTEWDYW	IGH	2	None	
11	622	CARGDGHCSGGTCYSLDYW	IGH	2	None	
11	623	CARLWGYCSGGNCYPTYNWFDPW	IGH	2	None	
11	624	CTRGRIPAALSYYYYYMDVW	IGH	2	None	
11	626	CVRDSSQRRCSGGCCYDFDSW	IGH	2	None	

11	627	CARVPGSSRVRYDAFDLW	IGH	2	None	
11	628	CAKGGLYCSGGSCWGFDYW	IGH	4	None	
11	629	CAQMCLLLRRYYDFW	IGH	3	None	
11	630	CARTNYYDSNGYYDYW	IGH	21	None	
11	631	CARDRGGDNEYYFDYW	IGH	2	None	
11	632	CARVPQSVQGPRVYTWFDPW	IGH	2	None	
11	633	CATAPVVYAMWGWFDPW	IGH	5	None	
11	634	CARDHPRIFGVLITSLPFDYW	IGH	2	None	
11	635	CARDLEDYGGNSGLSDAFDIW	IGH	2	None	
11	636	CASTVQLRYFDWLLFGAFDIW	IGH	2	None	
11	637	CARRGGELTIDYW	IGH	8	None	
11	638	CAREIWVDLYGSGSYPFDYW	IGH	3	None	
11	639	CARDSGIAAARRNAFDIW	IGH	4	None	
11	640	CARDGRTTYDFWSGYDYYYGMDVW	IGH	4	None	
11	641	CAKSLTVTTPRNWFDPW	IGH	4	None	
11	642	CARDGGGFRELLYHYFDSW	IGH	6	None	
11	643	CARHFEPRYSGTPFDYW	IGH	2	None	
11	644	CARDFRMGDFWSGYHTGNAMDVW	IGH	17	None	
11	645	CARGCSGGNCYRLRSNWYFDLW	IGH	2	None	
11	647	CAGGDYGNYEFDYW	IGH	2	None	
11	648	CARHGAGTTPVTRGYFDYW	IGH	3	None	
11	649	CASESTSGSYYGTFDYW	IGH	2	None	
11	650	CAREILGYGDYPGPFDYW	IGH	4	None	
11	651	CQEYGSSSFVF	IGK	2044	C1	7K8N
11	652	CAHRLGGSRYPWNFGYFDYW	IGH	4	None	
11	653	CAKGGSGSFYIVPWYFDYW	IGH	2	None	
11	654	CARVVTPVYFDYW	IGH	8	None	
11	656	CASYLDIVVVPAAKVYYYGMDVW	IGH	4	None	
11	657	CARDPPGAAPPGGGWYGSFSFDYW	IGH	2	None	
11	658	CAHRQGFAGYFDYW	IGH	4	None	
11	659	CARHGAHGYCSGDTCYPFDYW	IGH	2	None	
11	660	CARSRGASYYGSGNYEFDPW	IGH	4	None	
11	662	CARDYNVEWYINYW	IGH	8	None	
11	663	CARAPFRNYCSNSPCYFYYYMDVW	IGH	22	None	
11	664	CASKGGHCSSTSCYAFDYW	IGH	4	None	
11	665	CAQIRGSRTYCGGDCSHFDFW	IGH	2	None	
11	666	CARAERWLQSHCFDYW	IGH	2	None	
11	667	CAKVVVPNFNWNFDYW	IGH	14	None	
11	668	CARHVLTIFGVFTYMDVW	IGH	4	None	
11	670	CARSLYSDGTPFDYW	IGH	12	None	
11	671	CAKDQRHGYNYGFDFW	IGH	8	None	
11	672	CVRGYYPVSGQSNFDYW	IGH	10	None	
11	673	CAREVAPGSSWPCDYW	IGH	6	None	
11	674	CQVWHRSSDHPTWVF	IGL	2	None	
11	675	CALRPYHDFWSGYLAFFDYW	IGH	5	None	
11	677	CLHHGNFPLWTF	IGK	2	None	
11	678	CAKVLRDSSSWYWHFDLW	IGH	2	None	
11	680	CARVRSIDDASGYWYYFAYW	IGH	2	None	
11	681	CPRSNTGYYRIDYW	IGH	111	None	
11	682	CARGFDSAWELFRYW	IGH	6	None	
11	683	CAKERGTTYYFDNW	IGH	2	None	
11	684	CANYQHGDNFDYW	IGH	2	None	
11	685	CARGPPVGVPGPLDYW	IGH	15	None	
11	686	CARGESYYGSGSYTLEVDWFDPW	IGH	2	None	
11	687	CARPTRPNDGSGQPREYDAFDIW	IGH	6	None	
11	689	CARSGGYCSSTSCYSDAFDIW	IGH	2	None	
11	690	CARDWNNWLELKKSNDAFDIW	IGH	8	None	
11	691	CARDQRYCSGDCYDAFDIW	IGH	2	None	
11	692	CARYSTIAAGDGMDVW	IGH	4	None	
11	693	CASAALYYYDSSGYYNDAFDIW	IGH	12	None	
11	694	CARAGYCAGGVCQTFDSW	IGH	5	None	
11	695	CARGYYSGGTYYAHYYGMDVW	IGH	6	None	
11	696	CARHAVVVVAATSNWFDPW	IGH	2	None	
11	697	CARGEGQQLVYWYFDLW	IGH	2	None	
11	698	CAKDPYGDSYDTFDIW	IGH	3	None	
11	699	CARDRDSGGASSGYYLDLW	IGH	2	None	
11	700	CARWDDYGEEIDDYW	IGH	7	None	
11	703	CAKGSLVQATIQHWYFDLW	IGH	4	None	

11	704	CARELHLGELSFNYW	IGH	3	None	
11	705	CARGGFVRKFDYW	IGH	13	None	
11	706	CVTVVTPSGLAFDYW	IGH	2	None	
11	707	CEINSWSNRDFDYW	IGH	2	None	
11	708	CARDIGYSSGWYDTLDYW	IGH	4	None	
11	709	CARGITGYSASRYGSTFFDYW	IGH	7	None	
11	710	CARISCTGDVCHPDYW	IGH	2	None	
11	711	CAGGPPRCGGICYLDYW	IGH	2	None	
11	712	CARDAGNYYFYYMDVW	IGH	10	None	
11	713	CARDPEYDYMRESGFDYW	IGH	2	None	
11	714	CHEYNNWPPRHTF	IGK	8	C3	7K8P
11	715	CARESTTYYDYIWGSYRSSPPDAFDIW	IGH	4	None	
11	716	CQHHNNYPRFTF	IGK	4	C3	7K8R
11	717	CARGKGDYDILAGSIHRKNSAFDIW	IGH	9	None	
11	718	CVKDHESWGIQFQDYGDYVSTTADYW	IGH	2	None	
11	719	CARLSPDCSGGSCSIYFDYW	IGH	2	None	
11	720	CARDSGIYDILTGFYDEPKGGDYW	IGH	2	None	
11	721	CARPDDSGSYFDSW	IGH	3	None	
11	722	CARGVSGVNMILVVLGFYKYMDVW	IGH	49	None	
11	723	CVKRILWSGYYRDDAFDMW	IGH	2	None	
11	724	CARGNSGYYYAYFAYW	IGH	5	None	
11	725	CDWSSDVCSSDLEDTAVYYCARDLYYGGNYDYW	IGH	3	None	
11	726	CAHTYGGYPGYW	IGH	10	None	
11	727	CGRSGDSGYDWAYYFDYW	IGH	3	None	
11	728	CAREKRGGYDAPPIYYYYYYGMDVW	IGH	2	None	
11	729	CARVGPDFWSDYYSPYDAFDIW	IGH	2	None	
11	730	CASLLENEIDYGAQPFYYYYGMDVW	IGH	2	None	
11	731	CASGGWLRFAYW	IGH	5	None	
11	732	CARMSDAGTTLGFAYW	IGH	2	None	
11	733	CSRGVCHTYYYYGLDVW	IGH	8	None	
11	734	CARESVTTWYYYYYMDVW	IGH	13	None	
11	735	CVRDRGDSSRDYYALGYMDVW	IGH	2	None	
11	736	CVRDREMATAHFDYW	IGH	2	None	
11	737	CARDAEISGYTYYFDYW	IGH	3	None	
11	738	CVRNQGNRGYYGYDYW	IGH	4	None	
11	739	CARTNCTDGHCHYFDYW	IGH	2	None	
11	741	CATASPHYDILTGYSQDWFDPW	IGH	2	None	
11	742	CARDSGRVATMYFDLW	IGH	2	None	
11	743	CARQSGYSQCYFDYW	IGH	2	None	
11	744	CARDVGNLASFDYW	IGH	26	None	
11	745	CARKVAGVGRRRNPRDRWFDPW	IGH	2	None	
11	746	CARGPVATIRDPFDYW	IGH	2	None	
11	747	CARVKQEYSGYDFAYW	IGH	2	None	
11	748	CARDSRDYYDSSGYYVPYYYYGMDVW	IGH	8	None	
11	749	CARLPGWVEHQRYYYYGMDVW	IGH	6	None	
11	750	CARHGNLRGGNVYFDYW	IGH	9	None	
11	751	CARDPHDVWSSKNWFDPW	IGH	3	None	
11	752	CAKDALSGYCSGTSCFDNWFDHW	IGH	2	None	
11	753	CARDSDWLLHNGIDYW	IGH	2	None	
11	754	CARRPGPLGKNWFDPW	IGH	2	None	
11	756	CMQSIQLPLMCSF	IGK	6	None	
11	757	CAHNEGTTSPFNAFDIW	IGH	3	None	
11	758	CAKDRDDSSVYYPRDAFDVW	IGH	4	None	
11	759	CARDHLSSSWYEYYFDYW	IGH	2	None	
11	760	CARPRTGGDFWSGSFYFDYW	IGH	2	None	
11	761	CAKDWVVAATVSYPLLDYW	IGH	2	None	
11	762	CARESFLTFRAPGPHSDYMDVW	IGH	6	None	
11	763	CVRVDHCDRGICYYYGMDVW	IGH	356	None	
11	764	CARDEIDGPVTIFDYW	IGH	17	None	
11	765	CVHSYHDSSAYYYVFDYW	IGH	8	None	
11	766	CARDYSNYEVGGVAGYYYYYYYMDVW	IGH	7	None	
11	767	CANTFGGVIVISFDYW	IGH	2	None	
11	768	CARALLYSTGWYGYFDYW	IGH	8	None	
11	769	CARLGRGLAIEAYYLHYW	IGH	72	None	
11	770	CAKGFGVQLWEFGYYYGMDVW	IGH	2	None	
11	771	CAKDQYSSGWYWYFDLW	IGH	2	None	
11	772	CVRLSTLVQGTTSFDYW	IGH	3	None	
11	773	CARNGDTAIRQFYYYYYGMDVW	IGH	5	None	

11	774	CATEPAFGGNFNFDYW	IGH	2	None	
11	775	CVKDTCITMDQGCHWFDPW	IGH	2	None	
11	776	CARVGRYCSGGSCHSFDYW	IGH	240	None	
11	777	CARARGPYGDYGGYYFDYW	IGH	3	None	
11	778	CATGNGLLWFRESEYDAFDIW	IGH	2	None	
11	779	CARHRGFSTGWYYFDYW	IGH	11	None	
11	780	CARERIAATDSQGEFDSW	IGH	3	None	
11	781	CARERWLRDGDDAFDIW	IGH	2	None	
11	782	CVSRGRQYDDGDYIFYW	IGH	2	None	
11	783	CARSWDGDYRGYNWFDPW	IGH	2	None	
11	784	CAREMRRESGSYDPAFNIW	IGH	2	None	
11	785	CTRDVLGAFSSDWGPVDYW	IGH	2	None	
11	786	CVHPKTSIFGVVKADYYLDVW	IGH	2	None	
11	787	CTIEYSGRDWVIDYW	IGH	2	None	
11	788	CARDFSSSWTDAFDVW	IGH	4	None	
11	789	CATAGGIVVVPAAMEGDYFDYW	IGH	3	None	
11	790	CARVGTTVTTPYYFDYW	IGH	13	None	
11	791	CARGEWLPEYYFDYW	IGH	4	None	
11	792	CAREGPKSFGSGSFDYW	IGH	4	None	
11	793	CASSGISDLDYW	IGH	12	None	
11	794	CARDQRIAGMTMPFDSW	IGH	3	None	
11	795	CQSYDSAVSGLLF	IGL	6	C4	7JVC
11	796	CASSQPPGSGWYPWFDPW	IGH	6	None	
11	797	CARMTTVTTLPPEYYYYGMDVW	IGH	4	None	
11	798	CAKDRISFYLYYW	IGH	4	None	
11	799	CVKVGSSGWYRSNFDYW	IGH	2	None	
11	800	CVGLRFRENYYYMDVW	IGH	2	None	
11	801	CARPASRYCADDGCHSGNFDYW	IGH	5	None	
11	802	CALGGGYDLTYYDLW	IGH	4	None	
11	803	CARDLSSSWHFDYW	IGH	26	None	
11	805	CARLSPPFFAGTTFSDYW	IGH	2	None	
11	806	CARWPSDIPQFKWFDPW	IGH	9	None	
11	807	CARDCSSTSCPPQPRLYYYYGMDVW	IGH	2	None	
11	809	CARVIRELHFDFW	IGH	13	None	
11	810	CAKGPRNTVTHFDYW	IGH	4	None	
11	811	CAHRSLTYYDFWSGYPYFDYW	IGH	2	None	
11	812	CARGSTIFGVNDYW	IGH	8	None	
11	813	CATTGVVLRVDWFDPW	IGH	4	None	
11	814	CARSPITMIVGNWFDPW	IGH	10	None	
11	815	CARILDGYNPGYFDYW	IGH	12	None	
11	816	CARDLCDGGSCYPNYYYGMDVW	IGH	5	None	
11	817	CARRKSGTYYLGDYW	IGH	2	None	
11	818	CARERSPGSSSWYGPLDYW	IGH	2	None	
11	820	CATSTKGSGYDYRFDYW	IGH	4	None	
11	821	CARGNSYGRIAYHHFDYW	IGH	2	None	
11	822	CAREGLFSTSLKWFDPW	IGH	4	None	
11	823	CARTVVHVSSSGYYASVGYFDSW	IGH	2	None	
11	824	CAKDRDASGSYLALFDYW	IGH	2	None	
11	826	CANSVHNWDYDKRGFDPW	IGH	2	None	
11	827	CAKDPFGGRYTYHLDYW	IGH	2	None	
11	828	CARVALSDSSGIDYW	IGH	8	None	
11	829	CVRDPTAPRPGEYFQHW	IGH	4	None	
11	830	CARGRPGSSWTYYYYYGMDVW	IGH	2	None	
11	831	CTRHEPTYYHFYGMDVW	IGH	2	None	
11	832	CAHRPTSGWYFQHW	IGH	4	None	
11	833	CAHSKPSGYDTSFDYW	IGH	2	None	
11	834	CARVPYPSGTFDYW	IGH	6	None	
11	835	CARALRSYSYYYYMDVW	IGH	8	None	
11	836	CARDPRMTMIAVAALGPFDIW	IGH	3	None	
11	837	CARVFNTIFGEGSDYW	IGH	2	None	
11	838	CAKGQISSGSSLRFDPW	IGH	8	None	
11	839	CARPALTEIHDGFDIW	IGH	2	None	
11	840	CARDKDDFWSGYPGGSDYW	IGH	3	None	
11	841	CARARYDFWSSYYPNWFDSW	IGH	2	None	
11	842	CARKIVGCGGDCYPFDYW	IGH	2	None	
11	843	CMQRIEFPLTF	IGK	48	None	
11	844	CARGNDYGDYVPFKYW	IGH	2	None	
11	845	CARPMGGARFDPW	IGH	8	None	

11	846	CARLRYYHFWTTNPFGFFDSW	IGH	2	None	
11	847	CARATTHPDSSGYYLGGWFDPW	IGH	2	None	
11	848	CAKEGNDHGDWDWYFDLW	IGH	2	None	
11	849	CAHRLKGSGGWDRGAFDIW	IGH	2	None	
11	850	CARGARGGRVPYDFDSW	IGH	2	None	
11	851	CARQGEGYCSGGNCYGRAQFDYW	IGH	5	None	
11	852	CTTDGKYDSSTGAFWFDPW	IGH	4	None	
11	854	CARLIQSPKWEHIHYLDQW	IGH	2	None	
11	855	CARDRELRFLEWFPDYW	IGH	6	None	
11	856	CARGYCSGGSCYSGGSDYW	IGH	6	None	
11	858	CARESGDYGSPDYW	IGH	37	None	
11	859	CASVLDDDAFDIW	IGH	3	None	
11	860	CVHRDEGYCADGTCSTWFDPW	IGH	5	None	
11	861	CARAGYCSGGSCSSVYYYYGMDVW	IGH	6	None	
11	862	CQQGYITPQITF	IGK	4	C2	7K8O
11	863	CARDGSTDFWSGHNWFDPW	IGH	3	None	
11	864	CARDGATVTGLDYW	IGH	56	None	
11	865	CARVRGYYYMDVW	IGH	97	None	
11	866	CAHRRQDSRGDYFDYW	IGH	2	None	
11	867	CATGYSYGLGFYYYYMDVW	IGH	2	None	
11	869	CARPQAEWDLLLPFDIW	IGH	9	None	
11	870	CARAGDSSGCCFFDCW	IGH	2	None	
11	871	CASPPTTDYYDFWSGNNSGAFDIW	IGH	3	None	
11	872	CAKDLESYYDSSGTLDYW	IGH	3	None	
11	873	CARDSGYSSGWLASFDYW	IGH	2	None	
11	874	CAHGARVSTYPDYW	IGH	2	None	
11	875	CASFMAAAGTGYYYYGMDVW	IGH	3	None	
11	876	CARDTEGGDFWSGYWFDPW	IGH	2	None	
11	877	CAQLTSDNRKYFWFDPW	IGH	222	None	
11	878	CQVWVSSFDHPAVF	IGL	3	None	
11	879	CAREAPVSTRPGPGYNWFDSW	IGH	3	None	
11	880	CARVSGLDESWYARSVDYW	IGH	3	None	
11	881	CARPPIGVDVNYFDYW	IGH	2	None	
11	882	CARGGYSSGSGGYYFDYW	IGH	52	None	
11	883	CARSLQPRRAMIVVGDAFDIW	IGH	2	None	
11	884	CARVSGGPGSYSFSEVYYFDYW	IGH	3	None	
11	885	CARHPMGGPDYGGRWYFDLW	IGH	7	None	
11	886	CASCESGSYSCFFDYW	IGH	2	None	
11	887	CARDNADDFWSGYLDPW	IGH	2	None	
11	888	CARDRYDSSSYYGILDYW	IGH	3	None	
11	889	CARELYSRSRDLWNAFDIW	IGH	4	None	
11	890	CARVIGPTLARMAFDIW	IGH	2	None	
11	891	CAREPTRIAAPGRGFDYW	IGH	4	None	
11	893	CARVGYCSAGSCDDLYHFYAMDVW	IGH	2	None	
11	894	CARLQGYCSGGSCREWYFDLW	IGH	2	None	
11	895	CARAQVNYYESSGYQDYW	IGH	2	None	
11	896	CVKGLEDYDFWSDPLDYW	IGH	10	None	
11	897	CARDHAVTMVQEDDYW	IGH	2	None	
11	898	CAKDRQYYDFWSGYYLSGTKGEYYYYYMDVW	IGH	2	None	
11	899	CARDDCSDGSCWYFDLW	IGH	4	None	
11	900	CARHSNGDYFFDYW	IGH	129	None	
11	901	CARDSIYRQDYW	IGH	5	None	
11	903	CAKDGAHCSGGSCYHDNFDYW	IGH	2	None	
11	904	CARHVDSSSWYPNWFDPW	IGH	11	None	
11	905	CAKVGSNAKWLKYNWIDIW	IGH	10	None	
11	906	CARVNDFRSALRIDYW	IGH	4	None	
11	907	CAKGAKGADYW	IGH	20	None	
11	908	CARDVSYCSGGSCYELDYW	IGH	7	None	
11	909	CARDEYYYGSGRDYYYYGMDVW	IGH	4	None	
11	910	CVRDRGGLFCSGESCYHNLFDPW	IGH	3	None	
11	911	CARDPKLEQRWDDYYMDVW	IGH	3	None	
11	912	CARDPLYSSGTRAFDVW	IGH	2	None	
11	913	CAKEWGGYDWVQYLATHTYYYYYGMDVW	IGH	2	None	
11	914	CARHTPAARRYWFDPW	IGH	2	None	
11	915	CAHRVDNYGIFDYW	IGH	5	None	
11	917	CARDRILEWLSDPVVDYYMDVW	IGH	3	None	
11	918	CARDGVLEWLLPRYYFDYW	IGH	8	None	
11	919	CARDRDYRDGDAYYDGLDIW	IGH	2	None	

11	920	CARDRWVTTSVSEFDPW	IGH	2	None	
11	921	CARQGVGVRDDFWSGYYTDYYYGMDVW	IGH	3	None	
11	923	CARDVGVVPPASFGYW	IGH	3	None	
11	924	CAREVSSVAAFRAIWFDPW	IGH	5	None	
11	926	CASDPTFTPSPAYYYYYGMDVW	IGH	2	None	
11	927	CAREDSSGYFSWFDPW	IGH	12	None	
11	928	CARDFPRRDAFDIW	IGH	24	None	
11	929	CAKNLGVATFSFDYW	IGH	2	None	
11	930	CARAGAVGREFEYW	IGH	2	None	
11	931	CDWSSDVCSSDLDTAVYYCAQLTSDNKKYFWFDPW	IGH	2	None	
11	932	CARDGLYMIGTTYYYYYMDVW	IGH	10	None	
11	933	CARINYNDFTWRFDRW	IGH	32	None	
11	934	CARDRGASITIFGADYW	IGH	2	None	
11	935	CARDRGYRGYETYYFDYW	IGH	4	None	
11	936	CARGMASGSSFDTFDIW	IGH	19	None	
11	937	CARDPDFETTVTYYYYYMDVW	IGH	4	None	
11	938	CARDFRGCSGGSCYLAWYFDLW	IGH	3	None	
11	939	CAREVGATLRENYYFDYW	IGH	2	None	
11	940	CARRVRSDPVVHETNWFDPW	IGH	3	None	
11	942	CARDLNDILTAYSLFDYW	IGH	24	None	
11	944	CAKTDSGYGYYYYMDVW	IGH	2	None	
11	945	CVRDDHGSGSNWVRCFDYW	IGH	2	None	
11	946	CARQVICTSCYDGDTDAFDIW	IGH	2	None	
11	947	CTTYFYDYIWGSYDYFDYW	IGH	2	None	
11	948	CARVGGYAYSGYDYAFFDNW	IGH	5	None	
11	949	CGTWDSGLSVVLF	IGL	6	None	
11	950	CAISLAPTAATWFDPW	IGH	11	None	
11	951	CARVGYCSGGSCYSGAFDIW	IGH	3	None	
11	952	CAGYDFWSDLGKNSFDPW	IGH	9	None	
11	953	CTRLGPAQFQFDYW	IGH	3	None	
11	955	CARFRFHSDGYSSAWYWDYW	IGH	13	None	
11	956	CARSYDTNGFGRNAFDIW	IGH	2	None	
11	957	CAREVEWFREYAFDIW	IGH	3	None	
11	958	CAKDLSPYLRYYYYGMDVW	IGH	2	None	
11	959	CLRGNAGYGNFDYW	IGH	6	None	
11	961	CARFPFGGVIDYYYYYMDVW	IGH	2	None	
11	962	CAGQSSLVQGIITEPNWFDPW	IGH	2	None	
11	963	CANVGEVQGVDYW	IGH	6	None	
11	964	CARAGEEGYCTGGPCDVYNWFDRW	IGH	2	None	
11	965	CARDRDYYFDHW	IGH	185	None	
11	966	CAREANYDFWSGYYPHYYYGMDVW	IGH	2	None	
11	967	CQVWDSTNDHPLF	IGL	2	None	
11	970	CASRAEDYYYYFMDVW	IGH	2	None	
11	971	CARCSSQGGYYYYYYGMDVW	IGH	41	None	
11	972	CARVLNPGGSHPGVDFDYW	IGH	2	None	
11	973	CARGVRVADTAMVHYFDNW	IGH	4	None	
11	974	CARDGITINYAMDVW	IGH	2	None	
11	975	CAEGGLVGDYW	IGH	7	None	
11	976	CARGSVVAGLFDYW	IGH	5	None	
11	977	CARGAVTSGWYVGDYW	IGH	36	None	
11	979	CATTPSVWGSYRLDYW	IGH	5	None	
11	980	CAREGSRMKLWFYTWFDMW	IGH	2	None	
11	981	CSLSLITIVGVDNLDYW	IGH	2	None	
11	982	CARGAVLAGVVYFDYW	IGH	2	None	
11	983	CARQNVYYIDYW	IGH	14	None	
11	984	CARLERVVASIDYW	IGH	2	None	
11	985	CAKDGYYSGSGGIYFFDYW	IGH	3	None	
11	986	CARDHGSIQNYDSSGYFGYW	IGH	6	None	
11	987	CARVEGYHDALENYFDYW	IGH	2	None	
11	988	CARRPGWTDAFDVW	IGH	2	None	
11	989	CAKDLRFLEWTPPPLDYW	IGH	4	None	
11	990	CAKDRSGWEQLEGADYW	IGH	3	None	
11	992	CARGRTGWDYYYYMDVW	IGH	2	None	
11	993	CAPDILGLGGVSDYW	IGH	2	None	
11	994	CARHRDQWLVKTLFDYW	IGH	2	None	
11	995	CAKDRAYSSPYLDYW	IGH	9	None	
11	996	CARARNYMDVW	IGH	12	None	
11	998	CARTQNMGAFDYW	IGH	2	None	

11	999	CARENMGSGWYYWFDPW	IGH	7	None	
11	1000	CTRHFDSSAYYHEFDYW	IGH	6	None	
11	1001	CARELMGGGDNFLSAAFDIW	IGH	2	None	
11	1002	CARMKGPGSSSYAKDVW	IGH	6	None	
11	1003	CARAPAMAAAARFDYW	IGH	4	None	
11	1004	CAREADYGDYDGSTDYW	IGH	2	None	
11	1005	CARLGSSSSRYFDLW	IGH	5	None	
11	1007	CAREVLANDFWSGYFFDYW	IGH	2	None	
11	1008	CARQRQAAAGRIRDTLLFDPW	IGH	3	None	
11	1009	CAREDDGYNIYW	IGH	2	None	
11	1010	CARADLVVVGAHQIYYDYGMDVW	IGH	2	None	
11	1011	CAHSLPVTNFDYW	IGH	10	None	
11	1012	CTRDYIAARPSEFFDYW	IGH	4	None	
11	1013	CARWVESYAMDVW	IGH	2	None	
11	1014	CARSDHTGGWHRKFDYW	IGH	2	None	
11	1015	CAKDTEIVVVAATSIDYW	IGH	3	None	
11	1016	CAATVHFAYW	IGH	4	None	
11	1019	CARDSENVDTAMGDWYFDLW	IGH	5	None	
11	1020	CARESLPTYGGFSSGFDYW	IGH	6	None	
11	1021	CARGPKLERPYYYYYYMDVW	IGH	4	None	
11	1022	CTTAPMTVVIIEGHNYFYMDVW	IGH	2	None	
11	1023	CARGGMSLGYCSGGSCSWFDPW	IGH	4	None	
11	1026	CASGQRKARQTGGVDYFDYW	IGH	2	None	
11	1027	CAREIPSYFDSW	IGH	3	None	
11	1028	CAHRRLELRKVGFDPW	IGH	3	None	
11	1029	CAEGNAAAGNNWFDPW	IGH	2	None	
11	1030	CARSGYRASAARNRYNWFDPW	IGH	6	None	
11	1031	CARSYYGSGSYLRYYYYGMDVW	IGH	3	None	
11	1032	CAHYGSSTSLTFDYW	IGH	34	None	
11	1033	CARTQTYYSDASGSLSFDYW	IGH	2	None	
11	1034	CVRHGADDILTGHSTPYFDYW	IGH	2	None	
11	1035	CAKDYYRIGQHLAFDYW	IGH	2	None	
11	1039	CARITGDGYNSGAFDIW	IGH	3	None	
11	1040	CARAVGSGWSGLFDYW	IGH	5	None	
11	1041	CARLTIFGVVGGDYFDSW	IGH	2	None	
11	1043	CARRGDIGMHMYNRFDPW	IGH	2	None	
11	1045	CVRASRRAASGLYPDFW	IGH	2	None	
11	1046	CARYPNIVSTNYYDYW	IGH	2	None	
11	1047	CAKHWYHHYYDMNGYFFNFDYW	IGH	3	None	
11	1048	CATSRTFDHW	IGH	9	None	
11	1049	CARDTTPAVLVVYAIEAYNWFDPW	IGH	2	None	
11	1050	CARRYSNGRREDFDYW	IGH	2	None	
11	1051	CARGIQLWLLRSGWYGGFDYW	IGH	2	None	
11	1052	CARDHCGGGGCYDYW	IGH	2	None	
11	1053	CGTWDSSMTAVVF	IGL	7	None	
11	1054	CAREYTDDYSNYPFDYW	IGH	5	None	
11	1055	CARVPYPIVGASRHYFDYW	IGH	2	None	
11	1057	CARDRVATSEDSFEIW	IGH	2	None	
11	1058	CAKDSLTGMTAGYYYYGMDVW	IGH	2	None	
11	1059	CARDLMSLSDYGGNSRGDYW	IGH	10	None	
11	1060	CVRSVDCDSTSCYTFRPGNWFDSW	IGH	4	None	
11	1061	CASSDYYDSSGYYPRDDYW	IGH	6	None	
11	1062	CARDPIFGVVITEAGWFDPW	IGH	2	None	
11	1063	CAKPGTTMVQGVYYFDYW	IGH	10	None	
11	1064	CARGHVGVNMVVVVFIGGEYYFDHW	IGH	3	None	
11	1065	CARDRWGSGYDKALDYW	IGH	6	None	
11	1066	CARLRSSSLGSDYW	IGH	5	None	
11	1067	CARDPPYYDFWSGYAQIRDYYYYYYMDVW	IGH	3	None	
11	1068	CLRAEDTAVYYCAREYSSGCFDYW	IGH	2	None	
11	1069	CARNLHPYCSGGNCYSDFFDYW	IGH	2	None	
11	1070	CARVRWLLDYMDVW	IGH	6	None	
11	1071	CARAHITMIVVVIRSYYYYGMDVW	IGH	2	None	
11	1072	CASGLYGTYWYFDLW	IGH	4	None	
11	1074	CARDPTMVQGVAYYYGMDVW	IGH	9	None	
11	1075	CTKEAGSSWKYYFDYW	IGH	2	None	
11	1076	CATADWNYGFADYW	IGH	80	None	
11	1077	CARGRRRITGDRRAGGRPFDIW	IGH	23	None	
11	1078	CARAIRESGVYFFDYW	IGH	3	None	

11	1079	CARHPSTIVVVPAAIRRGFSGFDIW	IGH	4	None	
11	1080	CARDLRSGGWIVAFDIW	IGH	3	None	
11	1081	CATYDYSGSSTAFDYW	IGH	2	None	
11	1082	CAKRFAGSTYWAPFAYW	IGH	5	None	
11	1083	CAREGRDNSRSADYW	IGH	2	None	
11	1084	CARGVYYYDSSAAVGGAFDIW	IGH	5	None	
11	1085	CARGGPADSGGYYLYFDYW	IGH	5	None	
11	1086	CAKEAAAGHWYFDLW	IGH	4	None	
11	1087	CARDVSYYDSSGHYVHASDIW	IGH	13	None	
11	1088	CASSTQQLVAEFDYW	IGH	3	None	
11	1089	CARPGVVPTDQFDYW	IGH	2	None	
11	1090	CARDTRTYYDILTGYYPSGYFDYW	IGH	3	None	
11	1091	CARSLGMVAADSFDYW	IGH	2	None	
11	1092	CAREGRNYYGSGILLNWFDPW	IGH	5	None	
11	1093	CARSTQETFGSGSHYNLYW	IGH	17	None	
11	1095	CARLRYYGSGRFNRAFDYW	IGH	6	None	
11	1097	CAKDGYEGSGYNWLDPW	IGH	4	None	
11	1098	CARGGNSPPYYDPMDVW	IGH	4	None	
11	1099	CARAKQYQDDAFDIW	IGH	2	None	
11	1100	CARVYCPGGICTHDSSYYYGMDVW	IGH	11	None	
11	1101	CAKGTRQNYFDYW	IGH	8	None	
11	1102	CARDLSSTNGYYYYYYYMDVW	IGH	2	None	
11	1103	CARVGRATETKNYYYYAMDVW	IGH	2	None	
11	1104	CTTEDSSGLCDYW	IGH	4	None	
11	1106	CASSVPYDFWSGKQFDPW	IGH	6	None	
11	1108	CARALGATSKYHYYYGMDVW	IGH	2	None	
11	1109	CARDLRVSGYDTNFDYW	IGH	2	None	
11	1110	CARPLGRLRYFDSLDAFDIW	IGH	2	None	
11	1112	CARSSLGWSSGWYSYYFDYW	IGH	3	None	
11	1113	CARGTIFGVDPGWFDPW	IGH	2	None	
11	1114	CARDYARDTGFKTPYYFDYW	IGH	3	None	
11	1115	CAILVRYFDWLLSSDAFDIW	IGH	4	None	
11	1116	CARGDYYDSSGYPFYFDCW	IGH	2	None	
11	1118	CDWSSDVCSSDLNSLRAEDTAVYYCARDGYDFWSGYQDYW	IGH	2	None	
11	1119	CARHMPRLYDLHAFDIW	IGH	5	None	
11	1120	CARDRYGDYGNWFDPW	IGH	16	None	
11	1121	CARVGYSTDVYWFDPW	IGH	2	None	
11	1122	CAKGPPYSNSFSRYYYGMDVW	IGH	4	None	
11	1123	CAKDLTGYSMLQTGFDPW	IGH	2	None	
11	1125	CARLVVAGPRVTYYFDFW	IGH	18	None	
11	1126	CNSYAGSNNVIF	IGL	47	C2	7BWJ
11	1127	CARTPHYSDILTGYYVYYFDYW	IGH	4	None	
11	1128	CARHRIPGYSYGFDYW	IGH	16	None	
11	1129	CAHRPSSSWYGDWFDPW	IGH	11	None	
11	1130	CARLLPYGDPGYFDLW	IGH	16	None	
11	1131	CTRDLYQGYYFDYW	IGH	13	None	
11	1132	CTRGQTVSGAKYYFDNW	IGH	9	None	
11	1133	CARDSLGSSSSWYYIDSW	IGH	4	None	
11	1134	CARDDYGSGSSWLRYFDFW	IGH	3	None	
11	1135	CALPICARYVDHLPTHLGELPYDLDAFDIW	IGH	2	None	
11	1136	CATRDSITWSPPDAFDIW	IGH	4	None	
11	1137	CARDRGIGYCSGGTCFPDYW	IGH	2	None	
11	1139	CARIRHDILTGRPLYYFDYW	IGH	3	None	
11	1140	CATNPSGYSYGYWYYW	IGH	2	None	
11	1141	CARDLILGSDSSSWYHVDYW	IGH	7	None	
11	1143	CAHRGLAVAGPHFDYW	IGH	12	None	
11	1144	CARDSQIRKSRWSSPYYYYYYGMDVW	IGH	4	None	
11	1145	CARRGPAIVTTTPDYW	IGH	15	None	
11	1146	CARDPQREYSGYDEGNWFDPW	IGH	3	None	
11	1147	CAKGSGSYNRPFDYW	IGH	7	None	
11	1148	CARDFRYGDNHPDFDYW	IGH	2	None	
11	1149	CARSTHNVLRYFDWLLNDAFDIW	IGH	2	None	
11	1150	CTRSYSGSPRVESYFDYW	IGH	5	None	
11	1151	CAKDLYDFWSNYPYFDHW	IGH	2	None	
11	1152	CARGEYYDFWSGYSVAYMDVW	IGH	2	None	
11	1153	CARPRGATGERAFDIW	IGH	2	None	
11	1155	CAISYGGSAGVISFDYW	IGH	5	None	
11	1156	CQHLNTYPFMYIF	IGK	2	C1	7BZ5

11	1157	CARAGYCSGGSCYYLNHFDYW	IGH	2	None	
11	1158	CARRAVQGGYYMDVW	IGH	8	None	
11	1159	CALPILSRLRSDDTAVYYCARDRDYYFDHW	IGH	3	None	
11	1160	CARDISYCSSTSCQEGWFDPW	IGH	5	None	
11	1161	CARHGFRSSGNYPDDYW	IGH	2	None	
11	1162	CAKDARYYDFWSGYSPKAPQAYYYYGMDVW	IGH	9	None	
11	1163	CVKDRRQIRQFCVGASCYIPIFDDW	IGH	2	None	
11	1164	CARVVVQGVTQAIPTHFDYW	IGH	3	None	
11	1165	CASALTTVTTSSFDYW	IGH	2	None	
11	1166	CARHLRQCSGGICQTYYYYAMDVW	IGH	16	None	
11	1167	CARGIGDYGDYLSLYYMDVW	IGH	3	None	
11	1168	CAHLDYGGAKYAFDIW	IGH	2	None	
11	1169	CARCGGGDYYYYCMDVW	IGH	4	None	
11	1170	CARDRDITVSGVDFDYW	IGH	2	None	
11	1171	CARTLRVAAAGYNWFDPW	IGH	3	None	
11	1172	CARPGGWCSTTSCYSAFDFW	IGH	3	None	
11	1173	CARAYNWKTSPNWFDPW	IGH	8	None	
11	1174	CATEVLAARSDALDIW	IGH	10	None	
11	1175	CARDLGQHLSPHAFDIW	IGH	4	None	
11	1176	CTPFSLYYYYGMDVW	IGH	3	None	
11	1177	CARDWRYYGSGSYSYYYGMDVW	IGH	2	None	
11	1178	CARTSEGYCGADCYSGVPDYW	IGH	2	None	
11	1179	CARSYGGYNLTPFDYW	IGH	2	None	
11	1180	CARDYDILTGYYIGNYYYYGMDVW	IGH	4	None	
11	1181	CARDHGTGIMITFGGVIGYYFDYW	IGH	2	None	
11	1182	CARLSPYGSLHFDYW	IGH	4	None	
11	1183	CARAEASGGFYYLLDYW	IGH	2	None	
11	1184	CAHVGVIVVVPYFDYW	IGH	2	None	
11	1185	CAREWHCSGGSCYSFGYW	IGH	4	None	
11	1186	CARGVVAAAIAMSWFDPW	IGH	3	None	
11	1187	CVRLPAGRRFLEVDYW	IGH	2	None	
11	1188	CARELVASKITMIVVVIRPRGFDPW	IGH	5	None	
11	1189	CVKVEGNTNYNFDYW	IGH	10	None	
11	1190	CARDDVVGASSSWPLQLVGPYYYYYGMDVW	IGH	7	None	
11	1191	CARDRGVCSGGSCYPSVFDYW	IGH	12	None	
11	1192	CARGHWTLAGKYFDDW	IGH	4	None	
11	1193	CARGPTVTTSPLYYFDSW	IGH	2	None	
11	1194	CARAQYYYDSSGSNLDYW	IGH	6	None	
11	1195	CVRDSREYAGYDLFDYW	IGH	6	None	
11	1196	CVREPEYNYDDSAIAGFDPW	IGH	2	None	
11	1198	CAHRWGFREAYFDYW	IGH	10	None	
11	1199	CARMYYYGEKGDWFDPW	IGH	25	None	
11	1200	CARLDTDDFWSGYPSRFDPW	IGH	2	None	
11	1201	CARDTSGTAVVKFDYW	IGH	7	None	
11	1203	CARDPISITIPDMGNWFDPW	IGH	2	None	
11	1204	CARHYDGSDYYPKDYYYYGMDVW	IGH	3	None	
11	1205	CARQGVGATSFGWFDPW	IGH	10	None	
11	1207	CARAYNWNDAFDYW	IGH	4	None	
11	1208	CTTEDVTYYYDSSGFHWYFDLW	IGH	2	None	
11	1210	CAKALLGKSPFDYW	IGH	2	None	
11	1211	CARDTSESGYLSGSFDYW	IGH	4	None	
11	1212	CAKGDVLVVVAASLDYW	IGH	2	None	
11	1213	CATSRDTSSPREPFDYW	IGH	2	None	
11	1214	CVQYNNYPMCSF	IGK	2	C3	7K8P
11	1216	CARFCGVLSGYRKYYFDYW	IGH	4	None	
11	1217	CAKDSSGYDYTNFDYW	IGH	3	None	
11	1218	CARHVPYRDGGHYFDYW	IGH	5	None	
11	1219	CTRGSTSGTSALDFDYW	IGH	2	None	
11	1220	CARQQLDYGDYGSYYYYMDVW	IGH	4	None	
11	1222	CTRGPTVFGVVILGWFDPW	IGH	2	None	
11	1223	CARGITYYDILTGSTYYYYMDVW	IGH	2	None	
11	1224	CARGVYTRTWYSNQFDSW	IGH	5	None	
11	1225	CARAPGYINSPGIDYW	IGH	2	None	
11	1226	CARHGGSGYDPPLFDYW	IGH	5	None	
11	1227	CARHMMIEWEPDDAFDIW	IGH	2	None	
11	1228	CARGRDGYRRPFDNW	IGH	7	None	
11	1229	CARSLVPAASPLDYW	IGH	4	None	
11	1230	CARGVTLGELSPYFDYW	IGH	2	None	

11	1232	CAKDFYTSGWSPNVFDYW	IGH	2	None	
11	1233	CATGPGIAVAGKINWFDPW	IGH	2	None	
11	1234	CARQIPSYDFWSGYYFDYW	IGH	8	None	
11	1235	CARVRRAEIHFWSGYYYFDSW	IGH	2	None	
11	1236	CTRSPSSSWYLGYYYMDVW	IGH	2	None	
11	1237	CAHRRVTMRPGANPAFDYW	IGH	2	None	
11	1238	CAREMYYYGSGSPQVNFDYW	IGH	2	None	
11	1239	CASDRVGVPTVKLFDYW	IGH	7	None	
11	1240	CARGVWSDYYHPYFDSW	IGH	2	None	
11	1241	CARAPQTHYYNNNGQKHVWDDAFDIW	IGH	2	None	
11	1242	CTTGTVTTVTDYW	IGH	8	None	
11	1243	CAISSFRSGYYVFDYW	IGH	21	None	
11	1244	CATNSSYTLPFDYW	IGH	4	None	
11	1245	CARAAEGGYASAFDYW	IGH	2	None	
11	1246	CANDQRSGGVCRSLDYW	IGH	41	None	
11	1247	CARLYYYDSSGYGRLENYYYYMDVW	IGH	2	None	
11	1248	CASSTMPGTVVVAAAIAGELMFDSW	IGH	11	None	
11	1249	CARGRSIAVARVAFDIW	IGH	3	None	
11	1250	CARGPYYDFWIDYNWFDPW	IGH	8	None	
11	1252	CARAPPLTIFGVVIFRRHDAFDIW	IGH	3	None	
11	1253	CARSKPCGDGCYNEFDFW	IGH	4	None	
11	1254	CARGFRDDYDLGYYYYYMDVW	IGH	3	None	
11	1256	CASPYTSTGWYDVYW	IGH	10	None	
11	1257	CARAANPSAYYYGSGDETNWFDPW	IGH	2	None	
11	1258	CARHFKHSGSYTTSPSDYW	IGH	4	None	
11	1259	CQQYNTYSGGGF	IGK	370	C3	7K8R
11	1260	CARNMDHSSNWYESYW	IGH	9	None	
11	1262	CAKVDYRKGYTTNWYPFDYW	IGH	3	None	
11	1263	CAKDRTHIYDILTGYAYDSLDYW	IGH	2	None	
11	1265	CAREFFFKPTDSSGYYSPYNWFDPW	IGH	2	None	
11	1266	CTRHWLRDYYFDYW	IGH	5	None	
11	1267	CARDNVVAANYWFDPW	IGH	6	None	
11	1268	CVKDSGLVINPFDSW	IGH	2	None	
11	1269	CARWIILTGHQAFDPW	IGH	6	None	
11	1271	CQQASSFPRAVTF	IGK	64	C3	7K8R
11	1272	CTRIGVIPTVVISNDAFDIW	IGH	2	None	
11	1273	CARDATTTGSIDYW	IGH	15	None	
11	1274	CTSPLHAGAYYYGMDVW	IGH	14	None	
11	1279	CTSLGGGSDYGGDYW	IGH	2	None	
11	1281	CARGGDIVVVPAAAPENYYYYGMDVW	IGH	2	None	
11	1282	CALRGFCSSSRCFGGAYHFDNW	IGH	2	None	
11	1283	CAHRPTSDFSNDWNGGRFDSW	IGH	2	None	
11	1285	CARRQQWLVPEIFDYYYGMDVW	IGH	2	None	
11	1286	CARETPNVYNWNYGAFDYW	IGH	20	None	
11	1287	CAHSPMTTVTLHFDYW	IGH	16	None	
11	1288	CAGQQWLNAFDIW	IGH	5	None	
11	1289	CARDYESRGTDHDYGDRSVYYYYYMDVW	IGH	7	None	
11	1290	CTSREPNDYGDYELGAFDIW	IGH	3	None	
11	1291	CATAPPSVYKYYYGSGPPDYW	IGH	2	None	
11	1292	CARQHNCSGGRCYGYWFDPW	IGH	2	None	
11	1293	CARGGGNFAYYFDYW	IGH	6	None	
11	1294	CARENDYGDYVTLDYW	IGH	18	None	
11	1295	CAKLVTFGGVIVSRAFDIW	IGH	2	None	
11	1296	CTKSNPGVFDYW	IGH	4	None	
11	1297	CTAYLQWSSLKASDTAMYYCARHFDYGDYYYGMDVW	IGH	2	None	
11	1299	CTLSRYSSSWYYFDYW	IGH	4	None	
11	1300	CAHSFNSAIDYW	IGH	11	None	
11	1302	CARISEYQLLYDWYFDLW	IGH	2	None	
11	1303	CARSINCVGGTCYVGYMDVW	IGH	31	None	
11	1304	CARYGSGSYYKEDNWFDPW	IGH	2	None	
11	1305	CARVRLGRELFDYW	IGH	4	None	
11	1306	CASNPAAGKYYYYYGMDVW	IGH	4	None	
11	1307	CARLPPSGYLLGNAFDIW	IGH	2	None	
11	1308	CAMADTYSSSWYIHDYW	IGH	3	None	
11	1309	CARGPTFGGVFNWFDPW	IGH	2	None	
11	1310	CARLGSYYLTPDYW	IGH	5	None	
11	1311	CARDTLGGWFDALDIW	IGH	2	None	
11	1312	CARISTAMYYFDYW	IGH	11	None	

11	1313	CARVRRDSGYDPDYYYYYMDVW	IGH	6	None	
11	1314	CARIDMTTVIYYVDTW	IGH	2	None	
11	1315	CARVGIPAASYYFDQW	IGH	3	None	
11	1316	CARDGSSGWFGGFDGW	IGH	2	None	
11	1317	CAHRPGIAAAGTSGFYFDYW	IGH	2	None	
11	1318	CARDLSEIVYYFDSSDYSFIYW	IGH	6	None	
11	1319	CARAVPAAPYYYGMDVW	IGH	28	None	
11	1320	CARPTHWYSSSWQQDAFDIW	IGH	2	None	
11	1321	CARKPVRGIAAAGLGYYFDYW	IGH	2	None	
11	1322	CARTRGHIVVVPAETRGDFDYW	IGH	2	None	
11	1323	CARDKDPFDLYYYYGMDVW	IGH	3	None	
11	1324	CARSTASFRIYHNLDYW	IGH	2	None	
11	1325	CARDNTARLVAGSLAGAHVVSYYYYYGMDVW	IGH	3	None	
11	1326	CAYNFTVSADWSGARSDWFGPW	IGH	4	None	
11	1327	CARDADRRSSSSGPIDWYFDLW	IGH	3	None	
11	1328	CAKDRTFYYDAGGYYFFQYW	IGH	2	None	
11	1329	CARDAYSSRSSYWYFDLW	IGH	2	None	
11	1330	CARLDAGIGHFDFDYW	IGH	4	None	
11	1331	CARDPVVPAATPLYYFDYW	IGH	7	None	
11	1332	CQQSFSFPCTF	IGK	24865	C2	7K8O
11	1333	CAKRGQLVISHFDYW	IGH	6	None	
11	1334	CARGRIAVAGTVLDYW	IGH	5	None	
11	1335	CAREVDGHNVYFDYW	IGH	6	None	
11	1336	CARDSRGYNYGYSYGFDLW	IGH	6	None	
11	1337	CAKPGPGYSYGCFDYW	IGH	4	None	
11	1338	CARAPTDYGAHTGAFDIW	IGH	2	None	
11	1339	CARHYGDRYETFSWFAPW	IGH	2	None	
11	1340	CATAFMAYYDILTGYHPVRGPEFDYW	IGH	8	None	
11	1341	CARVLGIAARPLLVQYDAFDIW	IGH	6	None	
11	1342	CAHRSPIVVVGFDPW	IGH	3	None	
11	1343	CAKARISGPLYYMDVW	IGH	5	None	
11	1344	CARDIQSWFPGSALDIW	IGH	7	None	
11	1345	CARLGYCSGALCYRADYYYGMDVW	IGH	33	None	
11	1347	CAHSDIAAAGTPWFDPW	IGH	9	None	
11	1348	CARDRAYGSGASSDAFDIW	IGH	9	None	
11	1349	CARLPYLDSSGYYLPFDYW	IGH	4	None	
11	1350	CARAPPSVVGFWVADSDAFDIW	IGH	2	None	
11	1352	CARYVSDIVVVPAAHYYYYGMDVW	IGH	2	None	
11	1353	CARSFNINYYDFWSGSNWFDPW	IGH	2	None	
11	1354	CARVSRNLGVVIIDYW	IGH	2	None	
11	1355	CAKLATWPGGEFDYW	IGH	2	None	
11	1356	CAKDAVATILYYFDYW	IGH	2	None	
11	1357	CAGAPQTFYYDGRGQKQVWDDAFDIW	IGH	6	None	
11	1358	CVRVHGDLFIFDYW	IGH	19	None	
11	1359	CAREEDYYGSGTPDYW	IGH	3	None	
11	1360	CARTAGNSWAGTFDYW	IGH	2	None	
11	1361	CARGPDGYNSHDAFDIW	IGH	5	None	
11	1363	CTRTKILVVAHYYMDVW	IGH	2	None	
11	1364	CARDLFRITVVQGEQYYFDYW	IGH	2	None	
11	1365	CARGHPSYDFFYYYGMDVW	IGH	2	None	
11	1366	CATGPPYGSGSRNWFDPW	IGH	3	None	
11	1367	CARRPREYCSSISCYADYW	IGH	4	None	
11	1368	CARPLLAAYDAFDIW	IGH	2	None	
11	1369	CARPYGSGTYYVPPFAYW	IGH	3	None	
11	1370	CARVGVTTAKHLDYW	IGH	2	None	
11	1371	CAGFAAVTEVFDYW	IGH	2	None	
11	1372	CARVDCTGGVCQDNWFDPW	IGH	2	None	
11	1373	CASFMVVAAKFDYW	IGH	2	None	
11	1374	CLQDYNYPRTF	IGK	1354	None	
11	1375	CASGGKLQWLSCDYW	IGH	4	None	
11	1376	CARGHYYYDTSGYYNFDYW	IGH	42	None	
11	1377	CATDHSPGGGDYDNWFDPW	IGH	4	None	
11	1378	CARDVGLVPGDYPLLIYYYYGMDVW	IGH	2	None	
11	1379	CQSADGNGFWEF	IGL	3	None	
11	1380	CAGGRGWSYGYFDLW	IGH	2	None	
11	1382	CASGYGDYAIDVLDIW	IGH	3	None	
11	1383	CAREGYSAYDFGAFDIW	IGH	12	None	
11	1384	CARDRGVVGSYYVFDYW	IGH	10	None	

11	1385	CVRGEYDKWRYFHYW	IGH	3	None	
11	1387	CARTGAYYYDSSGHPDYW	IGH	9	None	
11	1388	CARIPPAPIGHNNIGQLGDWYFDLW	IGH	4	None	
11	1389	CASTISGWSGAADYW	IGH	2	None	
11	1390	CVRGQGWSQTTHWYFDLW	IGH	2	None	
11	1391	CARDRLDADYYYYGMDVW	IGH	2	None	
11	1392	CATVLYSSSWYSSFDIW	IGH	2	None	
11	1393	CATVYTHTTYYFDYW	IGH	2	None	
11	1394	CARDYYGSGSYLLGYYFDYW	IGH	2	None	
11	1395	CARHIYGSRSANNWFDPW	IGH	3	None	
11	1396	CARIMGDYSTYWFDPW	IGH	27	None	
11	1397	CARDGGSGSGTYRFHYW	IGH	2	None	
11	1398	CARGQRETGYFDSW	IGH	2	None	
11	1399	CARLRQLGIWGSYRPFDYW	IGH	6	None	
11	1400	CAREGAAGIRYYYGMDVW	IGH	2	None	
11	1401	CARAPGSGYVAFDYW	IGH	4	None	
11	1402	CASQYHGDTLEPQSPGEYYFDYW	IGH	2	None	
11	1403	CARVASTSWREFDYW	IGH	4	None	
11	1404	CARHPRIQLWLNYMDVW	IGH	2	None	
11	1405	CAREKSGSYGRGFDYW	IGH	2	None	
11	1406	CARDLASDYYSSSGDDFDFW	IGH	2	None	
11	1407	CARLVVATTGRRFDYW	IGH	9	None	
11	1408	CAKDPSAKYSYGFQGFWFDTW	IGH	3	None	
11	1409	CARDQGPPDYYGSGSHFDYW	IGH	2	None	
11	1410	CVRARLLPPTISSYAFDIW	IGH	8	None	
11	1411	CALPISRSYYSNYFHYW	IGH	4	None	
11	1412	CARDALGYCDTIRCQGKGDTFDYW	IGH	2	None	
11	1413	CARDSYGNNPQDWYFDLW	IGH	26	None	
11	1414	CASQAPGFRTFDYW	IGH	2	None	
11	1415	CARYTTGDGLAFDYW	IGH	4	None	
11	1416	CARLRTGNSRIFDYW	IGH	795	None	
11	1417	CARDLYRSSWYVHDYW	IGH	6	None	
11	1418	CARDRTIAVAEYYFDYW	IGH	4	None	
11	1419	CARIREWDLLGYSSGWYFDYW	IGH	2	None	
11	1420	CAREGAIAAADTPYYYYYMDVW	IGH	2	None	
11	1421	CARDLRDYGDYGNANYYYYYYMDVW	IGH	2	None	
11	1422	CARDRQRVNYFDYW	IGH	2	None	
11	1423	CAREDPMLQALDYW	IGH	4	None	
11	1424	CARDFIVGYTRKKIIPNWFDPW	IGH	4	None	
11	1425	CTTGGTHDYYYYYMDVW	IGH	4	None	
11	1427	CARDQSSGSNPGLDYW	IGH	8	None	
11	1428	CARDLGSSGHYYYYMDVW	IGH	10	None	
11	1429	CYSAADNIWVF	IGL	125	None	
11	1430	CARALDSHDYGDYLDYW	IGH	5	None	
11	1431	CARDASREGFWSGYHSLDAFDIW	IGH	2	None	
11	1432	CARGHYDFWSGYYSTVSFDPW	IGH	2	None	
11	1433	CASLHSSSWQFDYW	IGH	17	None	
11	1434	CARDDYGFNAFDMW	IGH	5	None	
11	1435	CAKDDGGYDFDGSFDYW	IGH	3	None	
11	1436	CDWSSDVCSSDLSTDDAFDIW	IGH	2	None	
11	1437	CARGNYDYIWGNYFDYW	IGH	3	None	
11	1438	CAKVLCGGDCSLIKYFDYW	IGH	7	None	
11	1439	CARDFGYDSSSWNDYW	IGH	2	None	
11	1440	CARDAVGFGGYSFYYMDVW	IGH	4	None	
11	1441	CAKVSGGYGNSPADYW	IGH	2	None	
11	1443	CARRLDKDFDYW	IGH	18	None	
11	1444	CARAVGNGWFDDW	IGH	32	None	
11	1445	CAKDTATVTPRVFDYW	IGH	2	None	
11	1446	CAKDHSTTVTTKGAFDYW	IGH	3	None	
11	1447	CAKEPLAGAPRGAFDIW	IGH	3	None	
11	1448	CARRNPKYQLLDYVYYYHGMDVW	IGH	2	None	
11	1449	CARIPYDYIWGSYRPYYFDYW	IGH	10	None	
11	1450	CTKEGTPQWHFDVW	IGH	5	None	
11	1451	CARGKAAAGQYYFDYW	IGH	12	None	
11	1453	CARGPGSGPYHYFDFW	IGH	3	None	
11	1454	CAHRLHGGKVGGWFDPW	IGH	2	None	
11	1455	CVRDEISDYDSHLDYW	IGH	6	None	
11	1456	CARADGYSGYDLDYW	IGH	8	None	

11	1457	CARHPDDYSNFRYFDYW	IGH	5	None	
11	1458	CARASYPYQWSSSPFDYW	IGH	2	None	
11	1459	CARDSYPGLGYGDRGVFDYW	IGH	9	None	
11	1460	CAKVHELGDWSGPNDYW	IGH	2	None	
11	1461	CARYYYVSGSSIRPYFNYW	IGH	3	None	
11	1462	CATEGGSSGRAPYFDYW	IGH	8	None	
11	1463	CARVPKSITIFGVVDEDYFDYW	IGH	8	None	
11	1464	CASRFSYYDILTESDAFDIW	IGH	4	None	
11	1465	CARDVDLEGSTREDHFDSW	IGH	2	None	
11	1466	CQQYNTWPLLLTF	IGK	12	C3	7K8R
11	1467	CARDWTTGTTFAFDIW	IGH	5	None	
11	1468	CARDNKLAGYGDWYFDLW	IGH	2	None	
11	1469	CAKDESDDTSFAWYLSYFDYW	IGH	2	None	
11	1471	CARAGIVADPKTGFYYSAMDVW	IGH	4	None	
11	1472	CVHQDTMARQVDYW	IGH	4	None	
11	1473	CARGDWAVADAATDLPFDYW	IGH	2	None	
11	1474	CARDSPRGSGDFDPW	IGH	29	None	
11	1475	CARLIVGTRSGFDYW	IGH	2	None	
11	1476	CTTARWDTYYFDHW	IGH	6	None	
11	1477	CNSRDSSSNYLGVVF	IGL	2	None	
11	1478	CAHRTTVTYAFDYW	IGH	8	None	
11	1479	CHQSSSLPFTF	IGK	147	None	
11	1480	CARPLNYGGNADPYYFDYW	IGH	4	None	
11	1481	CARISSSGWGYMDVW	IGH	3	None	
11	1482	CTTGIRYFDWSLAFGSNDYYYYYYMDVW	IGH	3	None	
11	1483	CARLAVAGTWRWYFDLW	IGH	2	None	
11	1484	CAQAGGYDFWSGPNWFDPW	IGH	2	None	
11	1485	CAREGSDFSSGWTDWFDPW	IGH	2	None	
11	1486	CAHRPYDYMGPYFQHW	IGH	3	None	
11	1487	CARIPFYYDSSGYYAGGVDYW	IGH	12	None	
11	1489	CVHLYRDGYGRKAFFDYW	IGH	3	None	
11	1490	CQAWDSNTVVF	IGL	2212	None	
11	1492	CARHVRLHLGELSSLDYW	IGH	6	None	
11	1493	CARATTRGGAFDIW	IGH	21	None	
11	1494	CARGGYCISGSCSDLYYYYGVDVW	IGH	2	None	
11	1495	CAHYTMTNDAFDIW	IGH	2	None	
11	1496	CASGSWHYPDLW	IGH	4	None	
11	1497	CAKNPTVPTHLSFYYYYMDVW	IGH	8	None	
11	1499	CARDTSNWYHLYYFDYW	IGH	6	None	
11	1500	CARGRGGYCSGGSCYPDAFDIW	IGH	5	None	
11	1501	CARHSSLAAREDYYYYMDVW	IGH	4	None	
11	1502	CARRGPDSSSHFDYW	IGH	4	None	
11	1503	CQHLNSYLLCSF	IGK	4	C1	7BZ5
11	1504	CARDIRLSGFSFDRW	IGH	9	None	
11	1505	CARVEMATIAYFDYW	IGH	5	None	
11	1508	CASQIYMVQGVINGPDYW	IGH	2	None	
11	1509	CARDPTTVTTGDYYYGMDVW	IGH	2	None	
11	1510	CARGGTGYGDYAAFDIW	IGH	2	None	
11	1511	CAHRPPAWNYGYFDYW	IGH	6	None	
11	1512	CAREDCSGGSCYSVGWFDPW	IGH	7	None	
11	1513	CARPPVLMAIVGTRIDYW	IGH	5	None	
11	1514	CARDVTIFGGGGGWWFDPW	IGH	2	None	
11	1515	CTTDPVSSYGDAFDIW	IGH	2	None	
11	1516	CARDRATSGSYSYYFDYW	IGH	5	None	
11	1517	CQAWDDTTAHVVF	IGL	32	None	
11	1518	CARDQRFYDSTNAFDIW	IGH	2	None	
11	1519	CAREGGDGFHPRAFDIW	IGH	6	None	
11	1520	CARPAYAWMEDAFDIW	IGH	2	None	
11	1521	CASVMDYYDSSGYHSFDYW	IGH	7	None	
11	1522	CQQRSKWPPECSF	IGK	2494	C1	7BZ5
11	1523	CARQGSNWLSDYW	IGH	41	None	
11	1524	CARDGRYGSGSFNIDYW	IGH	2	None	
11	1525	CAHSSYYSNSGSFKHAFDVW	IGH	3	None	
11	1526	CARDLVSLSRYSSSSGYYFDYW	IGH	4	None	
11	1527	CARRGSTWENWYFDLW	IGH	2	None	
11	1528	CATGGLVRNPYYFDNW	IGH	3	None	
11	1529	CARLSFRRDGYNYGTPDYYYGMDVW	IGH	3	None	
11	1530	CVKDLELGIRLYQQLVPRPFDYW	IGH	2	None	

11	1531	CARAHDFNFDYW	IGH	35	None	
11	1533	CAREKAPVLEWLSEELDYW	IGH	2	None	
11	1534	CARDREWQRWRLRGGGFDLW	IGH	2	None	
11	1535	CARDRVYYDFWSGYLPRQAPTPYYYYGMDVW	IGH	5	None	
11	1536	CARGTAARRISMQSGDYW	IGH	5	None	
11	1537	CTTVDDYGDYYYMDVW	IGH	3	None	
11	1538	CVRADSQDFDSW	IGH	6	None	
11	1539	CAKGPDEDSDFWSGFYYW	IGH	2	None	
11	1540	CARMSGGDWEFDYW	IGH	14	None	
11	1541	CAHMDRLGYCSGGSCLPTDYW	IGH	2	None	
11	1542	CAREIAVAGIGNAFDIW	IGH	2	None	
11	1543	CAKEIAVTATTTPVDYW	IGH	5	None	
11	1544	CAAEGYMRGTDYW	IGH	2	None	
11	1545	CARYADPNSNYGFLGWFDPW	IGH	2	None	
11	1546	CLRDEDTAVYYCARLADDFWTHPYYYYGMDVW	IGH	2	None	
11	1547	CARVRRDSSGQSIDYW	IGH	19	None	
11	1548	CAKDYSSGWPSFDYW	IGH	10	None	
11	1549	CARESYSSGWQIDYW	IGH	29	None	
11	1550	CAHRRGTTFWGDWFDPW	IGH	4	None	
11	1551	CARARSLNVKWELLQYFFDYW	IGH	15	None	
11	1552	CAKSPLRSPPDYW	IGH	2	None	
11	1553	CARDQGGNNRGIDYW	IGH	3	None	
11	1554	CAAAPISPYFFDYW	IGH	2	None	
11	1557	CARGLRFLEWLNWFDPW	IGH	2	None	
11	1558	CARGAMLGGDLFDCW	IGH	2	None	
11	1559	CARGDCSGGVCLSDYMDGW	IGH	2	None	
11	1560	CATALVSLDWERPLKTNWFDPW	IGH	5	None	
11	1561	CARDYGSGNYDSIDYW	IGH	2	None	
11	1562	CATWDSSLNADHVLF	IGL	2	None	
11	1563	CARKKDDGDYHFDYW	IGH	2	None	
11	1564	CATLHYDFWSGPRPRDAFDIW	IGH	3	None	
11	1565	CARVSRNYGDYDLDYW	IGH	6	None	
11	1566	CATSTTVTTSGWFDPW	IGH	7	None	
11	1567	CTSLLHYYDFRSGFRSQQDSYFDYW	IGH	2	None	
11	1568	CARDFGSGGYLTKDDAFDIW	IGH	6	None	
11	1569	CARPPIFRSSGWYFDYW	IGH	2	None	
11	1570	CSRRYYDFWSGYVGDAFDVW	IGH	2	None	
11	1571	CARTNTERMPVAGTFFSWFDPW	IGH	4	None	
11	1573	CARDLRVGATRPGDYW	IGH	19	None	
11	1574	CARGTLSYGGNFLFDYW	IGH	2	None	
11	1575	CARPLMDSRRDLWSHFHIGFDPW	IGH	2	None	
11	1576	CARDFGTAILEVNQFGPW	IGH	6	None	
11	1578	CARDLEYCSGGSCHTLGYYYYYMDVW	IGH	2	None	
11	1580	CARRPSTNWCLDYW	IGH	2	None	
11	1581	CAKVPFMVQGVGGAYFDYW	IGH	3	None	
11	1582	CETWDSNTRVF	IGL	38	None	
11	1583	CARGLDGDSDYYYYYYMDVW	IGH	2	None	
11	1584	CARDPGYSSSWEEDYFDYW	IGH	2	None	
11	1586	CARRQTWDNWLDPW	IGH	15	None	
11	1587	CARHERGPRVTNFDYW	IGH	7	None	
11	1588	CARQGVGGYGFDIW	IGH	68	None	
11	1589	CAREGVGAADNGDYW	IGH	2	None	
11	1590	CAASHDYVSAFDSW	IGH	24	None	
11	1591	CAREGDYDILTGYLVPLPDYW	IGH	2	None	
11	1592	CARAAADQHFDYW	IGH	4	None	
11	1593	CARDFVGSSGWDEDYYYGMDVW	IGH	2	None	
11	1594	CARIRYDILTGYYFAFDIW	IGH	3	None	
11	1595	CQHLYSFPITF	IGK	308	None	
11	1596	CATTSRGYYYAMDVW	IGH	2	None	
11	1597	CARQGLIAARPFAFDYW	IGH	2	None	
11	1599	CARDLRHLGIVWFDPW	IGH	2	None	
11	1600	CARHLRQCSRGVCHTYYYYGLDVW	IGH	269	None	
11	1601	CARVRDCSGGRCYQNDAFDIW	IGH	2	None	
11	1602	CAHTRVFRFDAW	IGH	26	None	
11	1603	CAHRPGTTVTMYFDYW	IGH	4	None	
11	1604	CATDPLLDYW	IGH	5	None	
11	1605	CARDMSGDWNWYFDLW	IGH	3	None	
11	1606	CARDSLYYDILTGYYPKPYYFDSW	IGH	2	None	

11	1607	CARRYYYASGSAQPHFDYW	IGH	8	None	
11	1608	CARGIDFWSGYSLWEFDYW	IGH	4	None	
11	1609	CATTYAYNWNDGLIDYW	IGH	9	None	
11	1610	CARVPDSDPFYFDYW	IGH	2	None	
11	1611	CTKGYSGYDYAFDIW	IGH	6	None	
11	1612	CAREDYSGYDLEPLDYW	IGH	2	None	
11	1613	CARDYSSSWYLLADYW	IGH	9	None	
11	1614	CARGVDDYVDQYTTIFFDSW	IGH	4	None	
11	1615	CARLSSSGSGWPFDYW	IGH	6	None	
11	1616	CAREGYSGYDYGGYFDYW	IGH	2	None	
11	1618	CQAYDSSLVVLF	IGL	3	C4	7JVC
11	1619	CQQSNRFPRSF	IGK	347	None	
11	1620	CARDLFGAARPLFYFDYW	IGH	2	None	
11	1621	CARRSACGRDCYAADYW	IGH	3	None	
11	1622	CARHYYGSGSYTEVDYW	IGH	4	None	
11	1623	CARDPGITIFGVVMYHYYMDVW	IGH	3	None	
11	1624	CAKDMAPYSGWYDYW	IGH	2	None	
11	1625	CARGLIEYSSSSEGFDYW	IGH	6	None	
11	1626	CVREYCTSSSSCFDTFDLW	IGH	2	None	
11	1627	CARGSFIEESMKIVLNPFDYW	IGH	4	None	
11	1628	CARIKFGGVIVNYYFDYW	IGH	5	None	
11	1630	CARSTNYNGLWSGPDYW	IGH	2	None	
11	1632	CARKGAFWSGPRNAFDIW	IGH	2	None	
11	1634	CATRDPVYSSTWPNWFDSW	IGH	2	None	
11	1635	CARAIWFREPHWYFDLW	IGH	2	None	
11	1636	CAKDRRRKSSCEAFDYW	IGH	2	None	
11	1638	CARDSVAATTNYYYYYYMDVW	IGH	2	None	
11	1639	CAADRGTTGTKLLDYW	IGH	3	None	
11	1640	CQQGNSFTPSF	IGK	2	C1	7BZ5
11	1642	CARENLVGSTCCYFDYW	IGH	2	None	
11	1643	CARVGGYSSSWYMALDYW	IGH	9	None	
11	1644	CARHQGCGGDCPHADYFDYW	IGH	2	None	
11	1645	CARGGIAARPLRNWFDPW	IGH	2	None	
11	1646	CARDRLMATTNQPPDYW	IGH	11	None	
11	1647	CASQYCSSTSWPQWWCMLPFDYW	IGH	7	None	
11	1648	CAKDSQWLVNGWYFDYW	IGH	2	None	
11	1650	CAVSYGEDVGKAFDFW	IGH	22	None	
11	1651	CGLGSYYFDYW	IGH	2	None	
11	1652	CARQVSDSSGWHGGFDPW	IGH	2	None	
11	1653	CARVPIYYDYIWGSYRLDGMDVW	IGH	5	None	
11	1654	CARTSLQGFTTNWFDPW	IGH	5	None	
11	1655	CARDNSSEYSSSSAFDYW	IGH	12	None	
11	1656	CARIASWAFDVW	IGH	2	None	
11	1657	CAREDYGDWIGDYW	IGH	2	None	
11	1658	CQQSYSVPPNVFIF	IGK	17	C2	7K8O
11	1659	CAKDINDSSDYEGWFDSW	IGH	2	None	
11	1661	CATMVEITMVQGVLPTPYFFDFW	IGH	2	None	
11	1662	CAIYTGVVGSTRLSDAFDIW	IGH	3	None	
11	1663	CTATVHTPGFDYW	IGH	5	None	
11	1665	CASQWLVGKGFDYW	IGH	2	None	
11	1666	CASSKGSGSYYNEGWFDPW	IGH	2	None	
11	1667	CMHGTHWPPPGYAF	IGK	2	None	
11	1668	CQHRSDWPWAF	IGK	9	None	
11	1669	CARDINPRNYYDSRAIDYW	IGH	4	None	
11	1670	CVAGGNRFFDYW	IGH	5	None	
11	1671	CARDPDPSRSIIGFDCW	IGH	5	None	
11	1672	CARGAGYSSSRYHLVDYW	IGH	2	None	
11	1673	CATVTSAYYYTYPYYAFDIW	IGH	2	None	
11	1674	CTTDSTYNWNSISGSDYW	IGH	2	None	
11	1675	CARDPGFYSNYFGYW	IGH	4	None	
11	1676	CARDALGGKYFTDYW	IGH	2	None	
11	1677	CARDAVQGSNYFDPW	IGH	2	None	
11	1678	CARDTEYSSSWTPYFDYW	IGH	9	None	
11	1679	CARDQKVDDYGDFLDYW	IGH	4	None	
11	1680	CAKAAASPKGKYHFDHW	IGH	2	None	
11	1682	CARIQGYCTGSSLCQYYFDHW	IGH	2	None	
11	1683	CDWSSDVCSSDLGTYLFDYW	IGH	8	None	
11	1684	CARDSPAPNTLFGVVIRDYYYYGMDVW	IGH	2	None	

11	1686	CARGAGATDAFDIW	IGH	11	None	
11	1687	CARSPCSSPSCYDDYW	IGH	4	None	
11	1688	CARKDPIAVTAPFDPW	IGH	3	None	
11	1689	CARDSPGKVRSIWGSPLPEVGFDYW	IGH	2	None	
11	1690	CARVAGDYYDSSGYWAPHNWFDPW	IGH	3	None	
11	1692	CAKDFSLTTLTYSFDYW	IGH	2	None	
11	1693	CTRGPALRFLEWTLYYFDYW	IGH	2	None	
11	1694	CARPDVYNNHFDYW	IGH	3	None	
11	1695	CTRGKGPQYYFDYW	IGH	3	None	
11	1696	CARAISVHKDHNGYYTMGVDALEIW	IGH	2	None	
11	1697	CARRSAQLFNFDYW	IGH	2	None	
11	1698	CARDLYDFPYYYYYYMDVW	IGH	4	None	
11	1699	CARSSGYDWTHYIWGSYRYPYYFDYW	IGH	2	None	
11	1700	CQQDSNLRTF	IGK	11	None	
11	1701	CALPISNMDPVDTATYYCARSYYSNYFHYW	IGH	7	None	
11	1702	CARHQAEDEPTHIDYW	IGH	2	None	
11	1703	CARPTSSGSYYGFNYW	IGH	7	None	
11	1704	CARELWGDYGRSPFDYW	IGH	2	None	
11	1705	CTRIMITFGGRDYW	IGH	2	None	
11	1706	CARDKGLERHFDYW	IGH	13	None	
11	1707	CATLPPRWPDYYYYMDVW	IGH	2	None	
11	1708	CARARPAWGPDILNWFDPW	IGH	6	None	
11	1709	CARGNNAQWLGLDYW	IGH	3	None	
11	1710	CARGVSDDYRDYDLSGWFDSW	IGH	2	None	
11	1711	CMQDAQDLF	IGK	2	None	
11	1712	CARDWSDFDSGRSLDYW	IGH	4	None	
11	1713	CAVTLRFLEWLLLREYYYGMDVW	IGH	4	None	
11	1714	CARHVDNSYRFDYW	IGH	5	None	
11	1715	CASFNNNFPTNSVPYHYYYYYGMDVW	IGH	2	None	
11	1716	CARQGVVVAVFDFW	IGH	10	None	
11	1717	CSIYGDDLPRYFDYW	IGH	2	None	
11	1718	CARDLESGYGPAGYYYYYMDVW	IGH	11	None	
11	1719	CARGVTPPGRGLVYFDLW	IGH	3	None	
11	1720	CARRSDCSGIKCYLYYYGMDVW	IGH	2	None	
11	1721	CARAWSGSYLSAFDIW	IGH	7	None	
11	1722	CASAPGIPADYNWFDPW	IGH	7	None	
11	1723	CARVQVGDRRRTHSYDYW	IGH	16	None	
11	1724	CARERNDYGDDFDYW	IGH	16	None	
11	1726	CARASRGGYGGYDWDYW	IGH	7	None	
11	1727	CARAGYSSSEDEYFQHW	IGH	2	None	
11	1728	CARMVEGTALPGAGFDYW	IGH	4	None	
11	1730	CASAASYCGGDCYSDAFDIW	IGH	2	None	
11	1731	CARDSARVNFGVVIFDNW	IGH	2	None	
11	1732	CATANSAYCGGECYSGTAPFDAW	IGH	6	None	
11	1733	CQQFNSDPLSITF	IGK	2	C3	7K8P
11	1734	CARQNPKNGDYDILTGRGWHDPW	IGH	2	None	
11	1735	CARDTPDENYYYYMDVW	IGH	2	None	
11	1736	CARENTIFGEEACFDYW	IGH	3	None	
11	1737	CARESTYSGSWYPDYW	IGH	9	None	
11	1738	CARGHIGVNMVAVIFVGGDYYFDHW	IGH	29	None	
11	1739	CARAQQYYYDITAIEAFDYW	IGH	2	None	
11	1740	CARDCRDGYNPPGVDYW	IGH	2	None	
11	1741	CASSSGITYEGDYW	IGH	2	None	
11	1742	CAKDYGSTSSNWFDPW	IGH	13	None	
11	1743	CARAGHGYNSFDSW	IGH	5	None	
11	1744	CAKDRGSIWFYAFDIW	IGH	10	None	
11	1745	CARDLRNYLFFDYW	IGH	11	None	
11	1746	CARGYSSGWLEKWFDPW	IGH	2	None	
11	1750	CARSLSIAAPGLGWFDPW	IGH	2	None	
11	1751	CAGEFALDDTIDYW	IGH	2	None	
11	1752	CARCRYCDSTSYLDYW	IGH	9	None	
11	1753	CARYYGDYKDAFDIW	IGH	6	None	
11	1754	CARDRRVDASGYYYYRYGMDVW	IGH	2	None	
11	1755	CARDKDKGYFDWLPPFDPW	IGH	4	None	
11	1756	CVRGAVTTSYDFDYW	IGH	2	None	
11	1757	CTTDRYWIVEPTRGFDYW	IGH	2	None	
11	1758	CAPGSEYDDYVADYW	IGH	3	None	
11	1759	CAKDLYYYDSSGYPQYYFDYW	IGH	4	None	

11	1760	CATGGTPGRYGSGSYDPTNWFDPW	IGH	2	None	
11	1761	CARGLVSYDFWSGDFDYW	IGH	3	None	
11	1762	CARIQWLRLLGNGIGYYYGMDVW	IGH	2	None	
11	1764	CARTRGGYGDYLNNWIDPW	IGH	2	None	
11	1765	CARVNSGVGGTYYFDYW	IGH	4	None	
11	1766	CARGYCAAGSCYSVYW	IGH	2	None	
11	1767	CAKLLEAGWDGNFDYW	IGH	2	None	
11	1768	CARGPRGVLLGAFDFW	IGH	2	None	
11	1769	CARPMQPGGAYYYYGMDVW	IGH	2	None	
11	1770	CARDARYCSSTSCQGFDPW	IGH	18	None	
11	1771	CTTTGSTVSTRYMDVW	IGH	2	None	
11	1772	CAKSDGTDYYYSGFDYW	IGH	9	None	
11	1773	CARSRDYIWGSYHDPLFDSW	IGH	6	None	
11	1774	CARDRYRYSSSWYFDYW	IGH	159	None	
11	1775	CDWSSDVCSSDLRKYFWFDPW	IGH	3	None	
11	1776	CAKTQTGIAPDYW	IGH	2	None	
11	1777	CGKDVGATTKYYYYGIDVW	IGH	2	None	
11	1778	CARDSGSYYPGDLRFDYW	IGH	2	None	
11	1779	CARIAPDRRGYFPSWFDHW	IGH	4	None	
11	1780	CARESPPRSGNAFDMW	IGH	2	None	
11	1781	CARGMTTVTRPPVRYYYYMDVW	IGH	4	None	
11	1782	CTRAQLYGSSSGGFDYW	IGH	3	None	
11	1783	CARDSPRSLTGDRIAFDIW	IGH	3	None	
11	1784	CVRDRGWQQFDCW	IGH	2	None	
11	1786	CARAFYTRNTSGYFYVDAFDIW	IGH	6	None	
11	1787	CARDRIAAVRYAMDVW	IGH	3	None	
11	1788	CAKGSGAMVQGVGVVLDYW	IGH	2	None	
11	1789	CAREGDREFCSGGSCYLAYW	IGH	2	None	
11	1790	CARGGGPVTPTTKNFDYW	IGH	2	None	
11	1791	CARAGWIFGVVTLTAFDYW	IGH	4	None	
11	1792	CARVKSYYDILTGYYRYYGMDVW	IGH	2	None	
11	1793	CARADYYDSSTPLDYW	IGH	11	None	
11	1794	CARNYYGSGSWGISDYYMDVW	IGH	2	None	
11	1795	CLLYYGGTHMGVF	IGL	36	None	
11	1796	CARSNYDFWSGYYTRGSYFDYW	IGH	2	None	
11	1797	CARDGLTYYDSSGYYTTSIDYW	IGH	4	None	
11	1798	CARYGSGYYYDDAFDIW	IGH	4	None	
11	1799	CAHRVNSNGDWDTGLFDSW	IGH	5	None	
11	1801	CARMMYYYDLSGSYYYYIDVW	IGH	32	None	
11	1802	CARDLVHYYGSGSRGGFFDYW	IGH	4	None	
11	1803	CALPIYQNGPARSLDYW	IGH	2	None	
11	1804	CAKPGALGSTRPGFDYW	IGH	2	None	
11	1805	CAKNTALALFHYW	IGH	2	None	
11	1806	CARDTKVYSDILTDWPPKYYFDYW	IGH	11	None	
11	1807	CTTCSLSDFDIPWYYMDVW	IGH	3	None	
11	1808	CARLGGYCSGTYCYTHTFDYW	IGH	2	None	
11	1809	CARSITTFAVIDYW	IGH	7	None	
11	1810	CARDAEIQLWFGRASYYFDYW	IGH	9	None	
11	1811	CARAYYDYSNYVGFNWFDPW	IGH	2	None	
11	1813	CARELLVVPAAMEYYFDYW	IGH	4	None	
11	1814	CAHRRDHYDYIWGSYRYNWFDPW	IGH	5	None	
11	1815	CAIDRRYYYDSSGYPNPPLDNEYFQHW	IGH	2	None	
11	1817	CARGRYSGYDRSGLGVYYFDYW	IGH	6	None	
11	1818	CARVRYGGRFADAFDIW	IGH	8	None	
11	1819	CATVHDYYASRSLDYFDYW	IGH	15	None	
11	1820	CARGNIAVAGTRAFDYW	IGH	2	None	
11	1821	CARRITIFGVDTDAFDIW	IGH	2	None	
11	1822	CARGPRGVIGSSWSYYFDYW	IGH	3	None	
11	1823	CARKAVTEFWFDPW	IGH	4	None	
11	1824	CARWGIVATNYDAFDIW	IGH	2	None	
11	1825	CAREYCSSTSCYVNWFDPW	IGH	7	None	
11	1826	CARDYGGDQSRPGWVDYW	IGH	18	None	
11	1827	CAKDVLYGDYATFDYW	IGH	7	None	
11	1828	CAGDYGGNFRAFDIW	IGH	2	None	
11	1829	CAKDRGTSGFVPFDIW	IGH	3	None	
11	1830	CARTRQQQLAYNWFDPW	IGH	2	None	
11	1831	CTDIGTSPFDCW	IGH	2	None	
11	1832	CVRRSLYSDRGNWFDPW	IGH	5	None	

11	1833	CARHFWSGYYYGMDVW	IGH	17	None	
11	1834	CARTTYYYDSSGYYHFTAFDIW	IGH	10	None	
11	1835	CAREGIGAEIDYW	IGH	26	None	
11	1836	CASHYGFWSRFDPW	IGH	42	None	
11	1837	CARERGYFYDSSGYDVYDYW	IGH	3	None	
11	1838	CAKAIDYDYFYYYGMDVW	IGH	2	None	
11	1839	CSYAGSYTFVWVF	IGL	30	C2	7K8X
11	1840	CAREGAVADTNYFDYW	IGH	5	None	
11	1841	CARDDPLTTLDYW	IGH	6	None	
11	1842	CARGFRGNSVSGDYW	IGH	16	None	
11	1844	CARVYAGIAAAAVSWFDPW	IGH	2	None	
11	1845	CAREPFSSSWFNAFDLW	IGH	2	None	
11	1846	CVGRYGSGSYMGYW	IGH	2	None	
11	1847	CARAYDSRAYYYFDYW	IGH	16	None	
11	1849	CARLDFGVVIRSYFDYW	IGH	2	None	
11	1850	CVRDDPAIGTGNWFDPW	IGH	2	None	
11	1851	CARAGYGSYGDHNWFDPW	IGH	3	None	
11	1852	CCSRDVSGNHYIF	IGL	2	None	
11	1853	CATTTTPSTQNYDFWSGYYVDYW	IGH	3	None	
11	1854	CARSVGVQGVIMRNNGFDPW	IGH	4	None	
11	1855	CARADRCVGPTCYYEGFDVW	IGH	95	None	
11	1856	CARDYDAFWSGYRIFDYW	IGH	38	None	
11	1857	CAKTYVNIVATGGYYFDYW	IGH	2	None	
11	1858	CQTWGTGIRVF	IGL	243	None	
11	1859	CVRGGPSSGWYYYFDYW	IGH	6	None	
11	1860	CARGVEGSGSYYNSNWFDPW	IGH	4	None	
11	1861	CAHSSGPSYDYIWGSYRYKEDEGYYFDYW	IGH	2	None	
11	1862	CARDRVSLDSGWYNDAFDIW	IGH	2	None	
11	1863	CARGSSNYGFDSW	IGH	2	None	
11	1865	CARAMGYYGSGSYYNGRYFDYW	IGH	2	None	
11	1866	CARILATSYCTGAVCHWWGAFDMW	IGH	13	None	
11	1867	CDWSSDVCSSDLGATTNDYW	IGH	3	None	
11	1868	CARLDYYGSGNEGNYYYYMDVW	IGH	2	None	
11	1869	CARVLMVQGVIIDGFDYW	IGH	20	None	
11	1870	CAKHYIPYYMDVW	IGH	2	None	
11	1871	CARDSDAYYDILNWFDPW	IGH	119	None	
11	1872	CAREPGGLLWFREYEHWYFDLW	IGH	2	None	
11	1874	CTRDDREMNYEDYGDRFDHW	IGH	2	None	
11	1875	CATSGSIAAADYYFHYW	IGH	2	None	
11	1876	CAPNRGYGDYQYMFDYW	IGH	2	None	
11	1877	CASLKATEQWLVGWFDPW	IGH	2	None	
11	1878	CVRIMGDFNSYYFDPW	IGH	2	None	
11	1879	CASGYCGSTICYDSTYYMDVW	IGH	2	None	
11	1881	CAKDSRLFLLGADYYFDYW	IGH	4	None	
11	1882	CQVWDSISDPREVVF	IGL	8	None	
11	1883	CAKDGVHGSAYNYYTDVW	IGH	2	None	
11	1884	CARGWGDADPTAVTMGPHWFDSW	IGH	2	None	
11	1886	CAGMTGPAWIRPGGYNWFDPW	IGH	2	None	
11	1887	CARGLSSSYYDAFDIW	IGH	10	None	
11	1889	CAKGDSSGYFGYW	IGH	11	None	
11	1890	CARDNAIFDRYGASVWFDPW	IGH	6	None	
11	1891	CARVEAGTFSFDPW	IGH	2	None	
11	1894	CASQTSYYDSSGSPSFDYW	IGH	2	None	
11	1895	CARDLYADDYSSYPDYW	IGH	3	None	
11	1896	CARRGLGSSWYLESAFDIW	IGH	3	None	
11	1897	CARVWFRVVTWVFDHW	IGH	4	None	
11	1898	CARSSRGVGDTAMVLGDYW	IGH	4	None	
11	1899	CAHQQWLVRYYFDFW	IGH	2	None	
11	1900	CAAASIDYDYIWGSYRYGAFDIW	IGH	3	None	
11	1901	CARGLTVFGVITPDIGPLFYMDVW	IGH	2	None	
11	1903	CARGGVGATTGAFDIW	IGH	4	None	
11	1904	CARTMHTSSWDYW	IGH	2	None	
11	1905	CARDWTPGDCGGDCLSYFDPW	IGH	2	None	
11	1906	CARSHWAVVPAANDGRGGYYYYGMDVW	IGH	4	None	
11	1907	CAREYDSSFDAFDIW	IGH	17	None	
11	1908	CARAVQGFYDFWSGYYNFDYW	IGH	4	None	
11	1909	CARINYDSNFLSERFDYW	IGH	4	None	
11	1911	CAKAYYYRSGSYWFDYW	IGH	9	None	

11	1913	CARRGGYNFGYDYW	IGH	17	None	
11	1914	CARDLTSMVQGVMAPYYYYMDVW	IGH	10	None	
11	1915	CAKEYYSHTSGYYLAYFDYW	IGH	9	None	
11	1916	CARGPFCSTTTCYDFFDYW	IGH	2	None	
11	1917	CAREGWELWYFDYW	IGH	2	None	
11	1918	CARGFPGELWLAYYFDHW	IGH	12	None	
11	1919	CAHSNHYSTTTIAFDIW	IGH	4	None	
11	1921	CAREADSGYDSAGFDYW	IGH	4	None	
11	1922	CARLGIAAAGTNLPDYW	IGH	7	None	
11	1923	CARQMNGDYFLADNWFDPW	IGH	5	None	
11	1924	CTTDLPNAYYYDSSGYYRNEAFDIW	IGH	2	None	
11	1926	CARDGQQLVEYAFDIW	IGH	13	None	
11	1927	CARTGETTLWQQWLLKAEYFQHW	IGH	2	None	
11	1928	CAKTGEDDSSGWPYYFDYW	IGH	2	None	
11	1929	CARDMGGSSSWPHTNAFDIW	IGH	6	None	
11	1930	CARDQDYYDKLRGGYYYYYMDVW	IGH	2	None	
11	1931	CAKDRSVTMIATDAFDVW	IGH	2	None	
11	1932	CTVAGHPYYYYYYGLDVW	IGH	2	None	
11	1933	CARDINDLIAARPGFDYW	IGH	2	None	
11	1934	CARDPRGYYDSSGYYHPTYYFDYW	IGH	2	None	
11	1936	CARSVAAGYRRSWYYNDYW	IGH	4	None	
11	1937	CARGAGSHFYGSASHDYW	IGH	4	None	
11	1938	CASLYGGNSYYFDYW	IGH	3	None	
11	1939	CAVISGYDSYW	IGH	9	None	
11	1940	CARIRAGYYFEYW	IGH	6	None	
11	1941	CVRDRLVVIPAVPRNFDYFYLDVW	IGH	2	None	
11	1942	CAREGRFLEWLSTYYYYYMDVW	IGH	4	None	
11	1943	CARDGETGYSSSNPTFDYW	IGH	2	None	
11	1944	CARAQSGAYLTDFDYW	IGH	2	None	
11	1946	CANGGSGSYFGYFDYW	IGH	2	None	
11	1947	CAKHTVTTVQYHFDNW	IGH	3	None	
11	1948	CQQYYTTRWAF	IGK	30	C4	6W41
11	1949	CARPSVGATRGYFDYW	IGH	2	None	
11	1950	CARLAISGSYCFDYW	IGH	20	None	
11	1951	CARVGITIFGDERNWFDPW	IGH	3	None	
11	1952	CARDDYDSSGYYYPDALDIW	IGH	2	None	
11	1953	CLIAAPPLADYW	IGH	2	None	
11	1956	CARDQYYDFWSGYSGTKYYFDYW	IGH	2	None	
11	1957	CARYIAVDGRKAFDIW	IGH	12	None	
11	1958	CARDRGVPNAHDYW	IGH	2	None	
11	1959	CARDGSHSGSYHFDYW	IGH	4	None	
11	1960	CARGSAHYDFWSGYYSPLKQNYYYYGMDVW	IGH	3	None	
11	1961	CARHLGDSITSWFDPW	IGH	4	None	
11	1962	CSLDTKRRRAFDYW	IGH	7	None	
11	1963	CARDPGFSSGWSRFDYW	IGH	2	None	
11	1964	CARWRFPELYYFYMDVW	IGH	8	None	
11	1965	CATAGYCSGGSCSEIFYYYAMDVW	IGH	8	None	
11	1966	CAREGMTTVTTSGTNFDYW	IGH	2	None	
11	1967	CARRSYSTVATYDYW	IGH	7	None	
11	1968	CASSPATGYYGNYGMDVW	IGH	2	None	
11	1969	CAREPESPSNFWSGSPGYAFDIW	IGH	4	None	
11	1970	CATDRLGAYCGGDCYKAPLDYW	IGH	4	None	
11	1971	CAHSQGWGPDYW	IGH	7	None	
11	1972	CARHVPLRGLIAARLTFFDSW	IGH	2	None	
11	1973	CARDRVVPAVDPFDYW	IGH	4	None	
11	1974	CAQDPHYDFWSGRLDYW	IGH	2	None	
11	1975	CARLAPGGGSLRFGFRYYYYMDVW	IGH	5	None	
11	1976	CARDQSQGSGINFDYW	IGH	2	None	
11	1977	CARFYNTKQSFDYW	IGH	4	None	
11	1978	CARRRPAASWSRPPSNWFDPW	IGH	2	None	
11	1979	CHQRSYWPPMYTF	IGK	6	None	
11	1980	CAGASGSYWGGLDYW	IGH	6	None	
11	1981	CATGLGISGWFNFAYW	IGH	3	None	
11	1982	CARGPENYDFWSGYYGAFDIW	IGH	2	None	
11	1983	CARGAYLGDLQFPFDFW	IGH	28	None	
11	1984	CARGRTYYYGSESYYNGFDYW	IGH	4	None	
11	1985	CQVWDSSPNLAVIF	IGL	3	None	
11	1986	CVADYGDNQFDYW	IGH	2	None	

11	1987	CARSITIFGVVRYYYYYYMDVW	IGH	2	None	
11	1988	CAHRPPLDIVVVPAAQNWFDPW	IGH	3	None	
11	1989	CARHYFDSGAYYWGYYFDYW	IGH	2	None	
11	1990	CARRYSNYGGFDYW	IGH	12	None	
11	1992	CARDGGYSGYGPFDYW	IGH	8	None	
11	1993	CAAGLIAAHPGLDAFDIW	IGH	2	None	
11	1994	CAVSPPYLIRLRGDYW	IGH	2	None	
11	1996	CARDLGYCTSTSCYSFDYW	IGH	2	None	
11	1998	CARGEIQYNWNDSLDYW	IGH	4	None	
11	1999	CASANYDILTGRPFDFDLW	IGH	2	None	
11	2000	CARDYDFWSGYLNYYYYMDVW	IGH	4	None	
11	2001	CARGSTASGRPLDYW	IGH	11	None	
11	2002	CVRGSISYNFDYW	IGH	35	None	
11	2003	CARKLQVGATESPFDYW	IGH	2	None	
11	2004	CAKDGLGLRYYDILTGYLIPHFDYW	IGH	3	None	
11	2006	CATLPRPVATITDWDYW	IGH	2	None	
11	2007	CARGRGITIFGVVRPTENWFDPW	IGH	2	None	
11	2008	CAAAPQTSYFSSSGQRRVWDDAFHFW	IGH	2	None	
11	2009	CARDFRKGITMVQGVIDGMDVW	IGH	4	None	
11	2010	CAREGPRNWNPDYW	IGH	2	None	
11	2011	CARQRRRDGYNFDFW	IGH	18	None	
11	2012	CGRDAAYRSSFDWYFDLW	IGH	2	None	
11	2013	CASSSGSYYNEGVSYFDYW	IGH	2	None	
11	2014	CAKDGYEGVTAAGTSTLYYMDVW	IGH	5	None	
11	2015	CATLEWLSYHDGFDIW	IGH	3	None	
11	2016	CARSRLAARRFWWFDPW	IGH	5	None	
11	2018	CAHRLVAVAAYWFDPW	IGH	2	None	
11	2019	CATDGPYYYDSSGYSYYMDVW	IGH	2	None	
11	2020	CARDGVAAGLYFDYW	IGH	13	None	
11	2021	CSSAIYYDRGGYYYPPDYW	IGH	5	None	
11	2022	CARLPLVGDDCYYFDFW	IGH	26	None	
11	2023	CARRLDKYSSSAQFDYW	IGH	3	None	
11	2024	CARDLDTIFGVVKQSNWFDPW	IGH	6	None	
11	2025	CARTEDDYNFYYFHYW	IGH	2	None	
11	2027	CAKDLSSMGVGTTATFDYW	IGH	2	None	
11	2028	CATAASRTGVGGDWFDPW	IGH	2	None	
11	2029	CARGVSLGRGGDSWGTFLLDYW	IGH	2	None	
11	2030	CASNPEDSYDLPDSAFDIW	IGH	2	None	
11	2031	CARADYDSSGYALDSFDYW	IGH	18	None	
11	2032	CARGPLLRVFEWLSLNWYFDLW	IGH	2	None	
11	2033	CTRRNYSYWYFDLW	IGH	12	None	
11	2034	CARLGYSGSYFGAFDIW	IGH	15	None	
11	2035	CSRHNCAGNSCYGAFDIW	IGH	18	None	
11	2036	CAPLILDFDFW	IGH	4	None	
11	2037	CARLRYNWNYGYFDYW	IGH	8	None	
11	2038	CARVNRGYCIGDVCAHPWFDPW	IGH	2	None	
11	2039	CAGGRDYGPGSHYRSAFEIW	IGH	2	None	
11	2040	CAKVDTAMGSRVPVAGHFDYW	IGH	63	None	
11	2041	CARDYYYDSSPHDYW	IGH	9	None	
11	2042	CARSTAITIFGVATSGWFDPW	IGH	2	None	
11	2043	CARGSGDYDILTGYPIHFDYW	IGH	2	None	
11	2044	CANCLIAAGYNWFDPW	IGH	2	None	
11	2046	CARSSRGDDYIWGSPTPWDSHSFDYW	IGH	2	None	
11	2047	CASIRYCSSTSCYPFDYW	IGH	8	None	
11	2048	CARVTSAGTFYFDYW	IGH	48	None	
11	2049	CVRNTYDDTNTYNWFDPW	IGH	2	None	
11	2050	CARVGDYSTGWTFDYW	IGH	3	None	
11	2051	CARLEVLRYFDWLFPKHSDYW	IGH	3	None	
11	2052	CARDNAPAVGIDYW	IGH	4	None	
11	2053	CATAAHGLFITGTTIAAFDIW	IGH	2	None	
11	2054	CARDGGASATVPRLGDYW	IGH	2	None	
11	2055	CARDWSAYNSGPDYW	IGH	7	None	
11	2056	CSSYAGSNNWVF	IGL	3340	C2	7BWJ
11	2057	CAKSRLEYCGADCPDSLDVW	IGH	3	None	
11	2058	CARQGSCSSTSCYGLYDYW	IGH	5	None	
11	2059	CAREGVVVTDAFDIW	IGH	5	None	
11	2060	CAGTRQEYSGYDRYFQHW	IGH	2	None	
11	2061	CARDSESNPSRNDAFDIW	IGH	2	None	

11	2062	CVKDVEFFGKHEGSDYW	IGH	3	None	
11	2063	CAKDAKYYDILTGYYPEPSYYYYYGMDVW	IGH	2	None	
11	2064	CARYHAGNYYMDIW	IGH	6	None	
11	2065	CARDKSEWWYEGRVLDAFDIW	IGH	3	None	
11	2066	CAPCSGGVWGQRRDAFDIW	IGH	2	None	
11	2067	CQLYNSYVYTF	IGK	3226	C3	7K8P
11	2068	CARHGGGIYANIDYW	IGH	2	None	
11	2069	CAALGFGDAFELW	IGH	10	None	
11	2070	CSSASCYYYFDYW	IGH	3	None	
11	2071	CARDDPYYDILTGYGVFDPW	IGH	2	None	
11	2072	CARDRGRHCTGGVCSPNTFKFDYW	IGH	2	None	
11	2073	CDWSSDVCSSDLRAEDTAVYYCAREGPAVAGSPFDYW	IGH	2	None	
11	2074	CARWARRTGTTSYYYYYMDVW	IGH	4	None	
11	2075	CARLAFCGGYCYSYGGAFDYW	IGH	8	None	
11	2076	CAHQIEYFRHW	IGH	3	None	
11	2077	CAHSGEFSGHDYVLDYW	IGH	2	None	
11	2078	CQQYYVWPPMITF	IGK	42	C4	6W41
11	2079	CVKGYCSSINCRQNLFEYW	IGH	2	None	
11	2080	CARAGGGDYADWFDPW	IGH	5	None	
11	2081	CTTDELTTVHTDHYQYYYYMDVW	IGH	2	None	
11	2082	CARVLGRYSASEPFDYW	IGH	2	None	
11	2083	CAHSGNYGHDGFDYW	IGH	5	None	
11	2084	CARDLGGASGWDAFDIW	IGH	31	None	
11	2085	CAKLEYCSTTSCYDGEGLYFDYW	IGH	2	None	
11	2086	CASLRRDGSSLSAWYFDYW	IGH	2	None	
11	2088	CARYSSSPYYYYYGMDVW	IGH	11	None	
11	2089	CARDADNYDSHYHFDYW	IGH	2	None	
11	2090	CVRRLTSTSFDFDYW	IGH	3	None	
11	2091	CAKEEDSWYFDLW	IGH	2	None	
11	2092	CARQRVYDSSGYNYYYGMDVW	IGH	2	None	
11	2094	CARDREWNGYYDFWSGYYLPGYYFDYW	IGH	10	None	
11	2095	CAGGPYSGYEDYYYYMDVW	IGH	2	None	
11	2096	CARDNDFWSGYYKNWFDPW	IGH	2	None	
11	2097	CATGDVASGRNNNWFDPW	IGH	2	None	
11	2098	CARLERFLEGIGNWFDPW	IGH	3	None	
11	2099	CARLKQGFEHVHYFDLW	IGH	3	None	
11	2100	CTRDTGYYDSSGFDYW	IGH	2	None	
11	2101	CARVSDLDVLSNYFDYW	IGH	2	None	
11	2102	CARVVDGGNRGGLFDYW	IGH	11	None	
11	2103	CARDSFRNCSGGSCYPDYW	IGH	2	None	
11	2104	CTTYWERLQSREELHPFDYW	IGH	4	None	
11	2105	CASPRSLYSSGWSSPFDYW	IGH	9	None	
11	2106	CARDPGRIQVWVDPFYMDVW	IGH	2	None	
11	2108	CVRDKGQWLWDYW	IGH	2	None	
11	2109	CARSTTVTSWVSDYW	IGH	4	None	
11	2110	CARMDCSSNTCLFYSFDYW	IGH	2	None	
11	2111	CARDTITMVQGVIKLPDAFDIW	IGH	3	None	
11	2112	CAKAPPVNWNYVYYYYMDVW	IGH	2	None	
11	2113	CARSSYVVASSYWYFDFW	IGH	2	None	
11	2114	CARGFGLYGSGSSYYYYMDVW	IGH	4	None	
11	2116	CATWDDTLSGFYVF	IGL	2	None	
11	2117	CAREVSDRRGYFPHYSYFYYMDVW	IGH	2	None	
11	2119	CARRLAGEAIPVAYPFDYW	IGH	2	None	
11	2120	CKTVPNYDSSPDYW	IGH	2	None	
11	2121	CARAPVFGVVISHFDFW	IGH	2	None	
11	2122	CAYQNSSGWFGYYFDYW	IGH	2	None	
11	2123	CARGSGSYYNAYDYW	IGH	36	None	
11	2125	CARDVDTAMVTWYYYYGMDVW	IGH	2	None	
11	2127	CARGNYYDSSGYYFGYYYYGMDVW	IGH	2	None	
11	2128	CARAGPLYDSNGYYLTDAFDIW	IGH	2	None	
11	2129	CAHIIARHYGGQPHYYFDYW	IGH	2	None	
11	2130	CALPISAVYYCVRVDHCDRGICYYYGMDVW	IGH	3	None	
11	2131	CARVLSRPGWFDPW	IGH	15	None	
11	2132	CAREPEYGRSSKCFDSW	IGH	2	None	
11	2133	CARHRPRNGFGYYYYGMDVW	IGH	2	None	
11	2134	CVHRRYDFWSGYYDYW	IGH	5	None	
11	2135	CTRTYFDVWTVNHIFDYW	IGH	29	None	
11	2136	CAGRSYRTDAFDFW	IGH	2	None	

11	2137	CARAPYFDWLSSSFYFDYW	IGH	4	None	
11	2138	CVRSPYSESGAYYFDLW	IGH	2	None	
11	2139	CARSNNYFSNAFDFW	IGH	2	None	
11	2140	CARQSWGVTRNYFDYW	IGH	4	None	
11	2141	CAKPLAYHWNPPFDSW	IGH	10	None	
11	2142	CARQRINILVVPAAIDYW	IGH	2	None	
11	2143	CAKEYNGYDDFDYW	IGH	4	None	
11	2144	CARRAGGSSWSAFDQW	IGH	8	None	
11	2145	CARDLSANPYYDFWSGYPDYW	IGH	4	None	
11	2146	CARIRPTDYAFDSW	IGH	2	None	
11	2147	CARWGASIAARRYYYGMDVW	IGH	8	None	
11	2148	CARGRLWGGGRATTGLGIDYW	IGH	2	None	
11	2149	CARDVTTRYFAADVW	IGH	8	None	
11	2151	CARFRITLGIPDYYYALDVW	IGH	3	None	
11	2152	CARHFENTYYDFWSALGSNWFDPW	IGH	2	None	
11	2153	CTRDYRAFSPTAKFDYW	IGH	6	None	
11	2154	CARARSTVLREGSHAFDIW	IGH	2	None	
11	2155	CARARVWSGYYSFDPW	IGH	2	None	
11	2156	CARHYDSRAILSAFAIW	IGH	2	None	
11	2157	CAKDRSYALFQDAFDIW	IGH	3	None	
11	2158	CASFLREYSSSTYYFDYW	IGH	4	None	
11	2159	CARARLITFGGVIAPEYDAFDIW	IGH	2	None	
11	2160	CARDSNVIPFDYW	IGH	9	None	
11	2161	CARLTGYSSGWYFDYW	IGH	83	None	
11	2162	CARVLAAYNSGWYGYSDYW	IGH	3	None	
11	2163	CAKGVILLGDWYFDLW	IGH	4	None	
11	2165	CARGVPYYDFWSGCWLDPW	IGH	2	None	
11	2166	CARDQPYLWCPDYW	IGH	113	None	
11	2167	CAKDQLAGVVVPDSPFDPW	IGH	2	None	
11	2168	CASHRNEYSSSSPFDYW	IGH	20	None	
11	2169	CARDWGDGGATSGIDYW	IGH	40	None	
11	2170	CVRYGYTYGQYYFDYW	IGH	2	None	
11	2171	CARDCSGGKCYPAYYYYGMDVW	IGH	2	None	
11	2172	CASSMTMVTTYYFDYW	IGH	25	None	
11	2173	CARSPLRFRYNFDYW	IGH	2	None	
11	2174	CARDNFSTGWYVGFDYW	IGH	4	None	
11	2175	CATGPPPSGTIFGVVTNNWFDPW	IGH	2	None	
11	2176	CARDVQPLVDDFWSGFSSKMSAGMMFDYW	IGH	4	None	
11	2177	CARQLSRDYYGSGSYYDGYYGMDVW	IGH	11	None	
11	2178	CAHLPLTGIVGASHFDYW	IGH	2	None	
11	2179	CASCGGDCSLDYW	IGH	6	None	
11	2180	CARWAGSTYQYLDYW	IGH	4	None	
11	2181	CARGLMTTFGGLIALDYW	IGH	3	None	
11	2182	CARHSSSSSGWDAYYFDYW	IGH	8	None	
11	2183	CARDPAGYVGGYDFDYW	IGH	3	None	
11	2185	CQQYKTYPFNF	IGK	2	C3	7K8R
11	2186	CVRDHQNGPARSLDYW	IGH	7	None	
11	2187	CARDWWGTGTSQDYW	IGH	2	None	
11	2188	CARVSLRFFEWSHVRGFFDYW	IGH	2	None	
11	2189	CAKTRGSGTLAFFDLW	IGH	12	None	
11	2190	CASYYGDYSLDYW	IGH	5	None	
11	2191	CARAVSGTSSPLDYW	IGH	15	None	
11	2192	CARESRATARWFDPW	IGH	5	None	
11	2193	CARDKTYCSSGSCYSYDYYYGMDVW	IGH	2	None	
11	2196	CARVSSYYYGSGSYGWFDPW	IGH	2	None	
11	2197	CARPSGTTSLYYYGMDVW	IGH	2	None	
11	2198	CARDHPSDYYGLGSYFDYW	IGH	4	None	
11	2199	CAKSRVPGGHNFDFW	IGH	116	None	
11	2200	CAKSVIGAFRGSDYW	IGH	2	None	
11	2201	CAREGGYSPDDYYYYYGMDVW	IGH	2	None	
11	2202	CASSYDFWSGYPPFDPW	IGH	2	None	
11	2203	CARLPYIGYSSGWYSTGWFDPW	IGH	2	None	
11	2204	CAADSDDYGDYAAYYYYYGMDVW	IGH	2	None	
11	2205	CARDLGGVVNRRAFDIW	IGH	2	None	
11	2206	CQSSDSSGAYPYVF	IGL	8	None	
11	2207	CARAYWGSVDYW	IGH	116	None	
11	2208	CARLQCSGSCNFFDYW	IGH	11	None	
11	2209	CARTDILTGSYMGGVDYW	IGH	6	None	

11	2210	CARVRQWLHYFDSW	IGH	2	None	
11	2211	CARLVDIVSTHFDYW	IGH	4	None	
11	2212	CARELDYNDGTGSNPYFETW	IGH	3	None	
11	2214	CVKDWEEYHYVYAFHVW	IGH	4	None	
11	2215	CARELPRASKLTARLKYYYDYFGMDVW	IGH	2	None	
11	2216	CATGLTRATETDYW	IGH	2	None	
11	2217	CARTHRSYDAFSYYYMDVW	IGH	3	None	
11	2218	CASLDLEDNYYYYGMDVW	IGH	3	None	
11	2219	CAREWREYSGYEDYWYFDLW	IGH	2	None	
11	2220	CARDPATPKYYFDYW	IGH	2	None	
11	2222	CARISVTVGWFDPW	IGH	23	None	
11	2223	CARDITVTDQSEDYYYYYYMDVW	IGH	2	None	
11	2224	CATGRDYYDSSGYRIFDYW	IGH	3	None	
11	2225	CARAAGSTSSFDYW	IGH	28	None	
11	2226	CARDLVAVAGTPTFDYW	IGH	7	None	
11	2227	CAKSVVATIFGARDDDAFDIW	IGH	4	None	
11	2228	CARVGIVAGGYNYFDPW	IGH	2	None	
11	2229	CARDIAARGPFDYW	IGH	6	None	
11	2230	CASWGDVDTANNFDYW	IGH	2	None	
11	2232	CARDQTVSDYGGSVFDYW	IGH	4	None	
11	2233	CQQYGISAGYIF	IGK	2	C1	7K8N
11	2234	CQSADITGTYVLF	IGL	62	None	
11	2235	CARDSSLDILTGYYTLGDAFDIW	IGH	6	None	
11	2236	CARLDTVMVGYAFDIW	IGH	4	None	
11	2237	CAKDTLQWLRLQRGGLDYW	IGH	4	None	
11	2238	CARYPYYYGSGSYYYYMDVW	IGH	5	None	
11	2239	CARDNIVVVPALDYW	IGH	6	None	
11	2240	CAREQYQLPFDYW	IGH	25	None	
11	2241	CAKNIAVTGTWDAFDVW	IGH	3	None	
11	2242	CAREGIVGATTGFDYW	IGH	25	None	
11	2243	CTTDLPSAPDDYW	IGH	2	None	
11	2244	CARHYTSGWNTNFDYW	IGH	4	None	
11	2245	CLKASDTAMYYCARRLTGDSLGSWYFDLW	IGH	2	None	
11	2246	CARGMWKHCSSTSCYTRYYYYGMDVW	IGH	4	None	
11	2247	CAAPQDLQALLMLRGGDYYYYGMDVW	IGH	2	None	
11	2248	CAKVQSRGSGWYWDFDYW	IGH	4	None	
11	2249	CAREGASGGQNPDYW	IGH	4	None	
11	2250	CARVYGGDYGDYW	IGH	34	None	
11	2251	CARRSRAVWGYSSSSKDAFDIW	IGH	2	None	
11	2252	CARVEYCSPDICYTRYYFDYW	IGH	7	None	
11	2253	CAKGFWGGHGPYGPFDYW	IGH	2	None	
11	2255	CARFRERWKAAFDYW	IGH	4	None	
11	2256	CVRGTCYVGYMDVW	IGH	2	None	
11	2257	CAKGGGPRELGYADYW	IGH	2	None	
11	2258	CAAQPFRYASSWSFDYW	IGH	4	None	
11	2259	CARILATSYCSGGVCYWWGASDIW	IGH	9	None	
11	2260	CARDGRVLDYDISWFDPW	IGH	3	None	
11	2261	CAHHRRGYFFDALDFW	IGH	6	None	
11	2263	CARGDYDFWTGSRFNHQSNWFDPW	IGH	125	None	
11	2264	CARSASDWNYVLLVAFDFW	IGH	2	None	
11	2265	CARDQLGVQGVTKGIDYW	IGH	38	None	
11	2266	CARVNGTFSLYYFDYW	IGH	4	None	
11	2267	CAGVVLVAAQGAFDIW	IGH	5	None	
11	2268	CARGPFVVVVAARNWYFDLW	IGH	4	None	
11	2269	CARDQPTGRNGDSDYW	IGH	4	None	
11	2270	CNSRDSDGNTQGLF	IGL	33	None	
11	2271	CARSYSNNYYLLDYW	IGH	4	None	
11	2272	CAHSSNGWYGEYFDYW	IGH	8	None	
11	2273	CARAVVAALTSYWYFDLW	IGH	9	None	
11	2274	CARGYHSSWYWFDRW	IGH	67	None	
11	2275	CARGSITMVQGVIIKGFDPW	IGH	2	None	
11	2276	CAITSGYSSSWWFDPW	IGH	7	None	
11	2277	CASSEIEYSSSSHVRWYYYYGMDVW	IGH	2	None	
11	2278	CATLSPFGVVITHEGDPHYSDVW	IGH	2	None	
11	2279	CARTLNYGDYYHHYYMDVW	IGH	4	None	
11	2280	CARLNMYYDFWSDDFDYW	IGH	8	None	
11	2282	CAKDRTVVLRQAFDIW	IGH	3	None	
11	2283	CARGSPYYYDSSGLYNWFDPW	IGH	11	None	

11	2284	CARENPDYDILTGYYIYVGRRSFDYW	IGH	5	None	
11	2285	CARRSDVYSSSWYVDYW	IGH	3	None	
11	2287	CAREGASDVFDIW	IGH	3	None	
11	2288	CARDLTMEHWYFDYW	IGH	2	None	
11	2289	CARMRFGDNANAFDIW	IGH	11	None	
11	2290	CARASAVRSLTADYW	IGH	36	None	
11	2291	CARDPFARSSSSYFGYW	IGH	6	None	
11	2292	CVKDHLMWQHPNGGDYW	IGH	2	None	
11	2293	CAREPGIFGVVINNWFDPW	IGH	2	None	
11	2294	CAKTDYGDYAVSYMDVW	IGH	12	None	
11	2295	CAREDAWLRNYYYYGMDVW	IGH	4	None	
11	2296	CASLVPSGGYCSGGSCYSDIDYW	IGH	2	None	
11	2297	CATGSMTVTTFTVPPRYYYYMDVW	IGH	2	None	
11	2298	CARDWSIAAHHNDPYFDYW	IGH	4	None	
11	2299	CVREGHSPFFDYW	IGH	2	None	
11	2300	CARDVRSASRYGPFDIW	IGH	3	None	
11	2301	CARSVRSKTYYYYGMDVW	IGH	4	None	
11	2302	CMQGTHWPLTF	IGK	4062	None	
11	2303	CARSEDYGDYTRSNWFDPW	IGH	8	None	
11	2304	CAHRGTSGKLDYW	IGH	52	None	
11	2305	CARDGGITSGGTRFDFW	IGH	2	None	
11	2306	CARVRESRQWLVVPFDYW	IGH	4	None	
11	2307	CAREIEVWGEVIPYNWFDAW	IGH	25	None	
11	2308	CVRDLRRVGCTATSCQVFDNW	IGH	10	None	
11	2309	CARDRYSGSYYHPAYFDYW	IGH	4	None	
11	2310	CATDDPRDWSTRGRFDPW	IGH	4	None	
11	2311	CQSYDTTNQGVVF	IGL	2	C4	7JVC
11	2314	CQSYDSSLASSFF	IGL	4	C4	7JVC
11	2315	CARLGSLDYYDSSGYDAFDIW	IGH	2	None	
11	2316	CARDLRSRKQQITMIVVDGDTIDAFDIW	IGH	3	None	
11	2317	CARRSANVGSGSYYHFDYW	IGH	2	None	
11	2318	CHQANSFPPPIPF	IGK	2	C1	7BZ5
11	2319	CARAVRPKDDFWGGDHNWLDPW	IGH	2	None	
11	2322	CARDWEHYYGSGSYYNFDYW	IGH	2	None	
11	2324	CARYVVKNTVTTGGFDYW	IGH	5	None	
11	2325	CARSPYSGWFGGWFDPW	IGH	2	None	
11	2326	CAKDFIALVLGVISFDPW	IGH	4	None	
11	2327	CANDALPGTGTLLYNWFDPW	IGH	2	None	
11	2328	CARTNYYGSGTYYNFQYYFDYW	IGH	6	None	
11	2330	CARVDYDSSGYFSGFGYW	IGH	2	None	
11	2331	CVRDVSGNSGYYWYYW	IGH	7	None	
11	2332	CARGHFGTVFGMGYLYYYMDVW	IGH	2	None	
11	2333	CARGYCSGGSCYGIYYYYYYMDVW	IGH	2	None	
11	2334	CARGSNSMGPATPFDYW	IGH	7	None	
11	2335	CAKDLGGYGDNW	IGH	2	None	
11	2337	CARMDGYVWSSWRLYNFDYW	IGH	4	None	
11	2338	CAKIGYCSGAVCYPNWFDPW	IGH	2	None	
11	2339	CASSSYYDSSPNPYFDYW	IGH	2	None	
11	2340	CARVRTLQKPPGDGRGYFDYW	IGH	2	None	
11	2341	CARRGGRTSLYYYYYGMDVW	IGH	7	None	
11	2342	CAHRPPPYGDYVWFDPW	IGH	3	None	
11	2343	CAREVAAAESWYFDYW	IGH	8	None	
11	2345	CTHSFGSTYYGGKEFDYW	IGH	2	None	
11	2346	CARSASGAMIVSEVTGDWFDPW	IGH	2	None	
11	2347	CARVIGLRNDYGDHGFDYW	IGH	2	None	
11	2348	CARGSPRVVIIYYFDYW	IGH	4	None	
11	2349	CARDYLTGTTNYFDYW	IGH	3	None	
11	2350	CARPDTTTVTPFDYW	IGH	7	None	
11	2351	CARGIGTPYAFDIW	IGH	2	None	
11	2352	CARGDYGDYGRYFDLW	IGH	2	None	
11	2353	CARAYYYGSGAYSGCDYW	IGH	2	None	
11	2354	CARVGDVLRYFYYMDVW	IGH	2	None	
11	2355	CATAAAIYTVTTGGWFDPW	IGH	4	None	
11	2356	CARDSTYDYIWGSYRGLFDYW	IGH	18	None	
11	2357	CLRAEDTAVYYCARERYYYDSSGYYYFDYW	IGH	2	None	
11	2358	CAKRGGCYGASCYPDYW	IGH	2	None	
11	2359	CATVPPPDYSDSSGDYSSFDYW	IGH	4	None	
11	2360	CARRLTGDSLGSWYFDLW	IGH	191	None	

11	2361	CASTKVCLGGVCYIGYMDVW	IGH	10	None	
11	2362	CASSFGVVSDFDNW	IGH	2	None	
11	2363	CASPRSPMRQGVTLTVFDYW	IGH	10	None	
11	2364	CASISTMTTVTTYFDYW	IGH	14	None	
11	2365	CARDRSGATIGYYYYMDVW	IGH	3	None	
11	2366	CARPMALRGFPYW	IGH	2	None	
11	2367	CARTPFGKTYYSDHW	IGH	2	None	
11	2368	CAREITDSYDSSGYFDYW	IGH	2	None	
11	2369	CAKGPTYGSGSYFDYW	IGH	21	None	
11	2370	CARALEYSSSPGLDYW	IGH	2	None	
11	2371	CTRDFWIDIRGSPPAIDYW	IGH	2	None	
11	2372	CARVRFMAVTTSNWFDPW	IGH	2	None	
11	2374	CARHRLYGGNSYDAFDIW	IGH	2	None	
11	2375	CARTHDYGGNLWFDYW	IGH	3	None	
11	2376	CARDGVESASSGTLREGFKYYYYMDVW	IGH	3	None	
11	2377	CARAKGYNWNDRSAFDIW	IGH	4	None	
11	2378	CARDLSYGDYQLDYW	IGH	3	None	
11	2379	CARDFGPGSSWYFDRW	IGH	2	None	
11	2380	CARHAGQFGGVIVPWFDWFDPW	IGH	7	None	
11	2381	CVRGFCGGATCYPNDAFDIW	IGH	2	None	
11	2382	CARDLWAPPPHGDYDYYYGMDVW	IGH	2	None	
11	2383	CARARSVYVDSSGYYGPNYYYMDVW	IGH	2	None	
11	2384	CAHTTLIAAAPFDYW	IGH	3	None	
11	2385	CARDAVDTAMGGYTLDYW	IGH	2	None	
11	2386	CAKRMVGTGFDYW	IGH	3	None	
11	2388	CARPSGMIRGWRAFDIW	IGH	5	None	
11	2389	CARGGGDYYDSSGYYYGKYYFDYW	IGH	3	None	
11	2391	CARTAVYSGSYYYDYW	IGH	5	None	
11	2392	CAGLQYQNQFDYW	IGH	9	None	
11	2393	CARDSLPYDYIWGSYRPELPTSDYW	IGH	2	None	
11	2394	CAFTGGLRFLEAKYDAFDIW	IGH	4	None	
11	2395	CARGRHSSSWYKSYGWFDPW	IGH	2	None	
11	2396	CARGGSTTITTGFDYW	IGH	2	None	
11	2397	CATASTVASGGIIEEKYLYFDYW	IGH	6	None	
11	2398	CARRYYYDSSGYHPSAYDYW	IGH	3	None	
11	2399	CARDWSPGFDWLFQSDQTKYYFDYW	IGH	10	None	
11	2400	CARSLTYYDSGGNWYFDLW	IGH	10	None	
11	2401	CARVPRDSYGDPKYFFDYW	IGH	2	None	
11	2402	CARVSLDCSSTSCYIQLYYFDYW	IGH	2	None	
11	2403	CARDLSRYDYIWGSYRPNDYW	IGH	2	None	
11	2404	CARTLLDRGSAHFDYW	IGH	2	None	
11	2405	CARGTGYDSSYLTFDIW	IGH	4	None	
11	2406	CARHPRVAGYSDLFDYW	IGH	4	None	
11	2407	CARYYYGSGTSKTLDYW	IGH	26	None	
11	2408	CARGGWGYCTGGVCPRWFDPW	IGH	5	None	
11	2409	CARDIGVHDYVDYW	IGH	7	None	
11	2410	CARATRFRGDYYYYGMDVW	IGH	2	None	
11	2413	CARQFSYYYGSGSLFDAFDIW	IGH	3	None	
11	2415	CAKEESRYCSSTSCYGDYW	IGH	2	None	
11	2416	CARVPIVVVPAAFQARYYYYMDVW	IGH	2	None	
11	2417	CARFSIYSNSWTAPFDYW	IGH	3	None	
11	2418	CARDGRGYSGYESWRFHAFDIW	IGH	2	None	
11	2420	CARGHREDGFAALGIYYYYGMDVW	IGH	3	None	
11	2421	CARGVVYCTGGVCLNYYYYYMDVW	IGH	2	None	
11	2422	CVKHSSGTFDVW	IGH	3	None	
11	2423	CAKKGSSSGYYFDNW	IGH	8	None	
11	2424	CARLTSRAGYNTPDFFDYW	IGH	2	None	
11	2425	CAHRLLLIATPPSFDPW	IGH	2	None	
11	2426	CARVWHYCSGGRCYSGEIDYW	IGH	2	None	
11	2427	CARDYDIAAAGTGAYYYYYMDVW	IGH	2	None	
11	2428	CAKDRVTTVTTFYYW	IGH	2	None	
11	2429	CARDLTYYDGSVYYDRFDYW	IGH	2	None	
11	2430	CGELARSLDAFDIW	IGH	2	None	
11	2431	CAKDPGSGYQARDAFDIW	IGH	6	None	
11	2433	CSLKASDTAMYYCARHREIPAAGTYPDYW	IGH	5	None	
11	2434	CAREVANHYNWFDPW	IGH	2	None	
11	2435	CARGPPYSGLLTGWFDPW	IGH	2	None	
11	2436	CAREDYGGNSGSTIDYW	IGH	7	None	

11	2438	CATLYGGNHYYYYMDVW	IGH	4	None	
11	2439	CAKCRYSSGWDVDYW	IGH	5	None	
11	2440	CARGATGTIFGVVIIPAWFDPW	IGH	2	None	
11	2441	CVRHQAVAGDYFDYW	IGH	4	None	
11	2442	CARDRPDDYVWGGFDYW	IGH	2	None	
11	2443	CARTWSQEWTPDFDYW	IGH	2	None	
11	2444	CVRYNSRTPQLRYYYYMDVW	IGH	4	None	
11	2446	CASGFAIPRFGYSIDYW	IGH	2	None	
11	2447	CTRNDFWSGYWYDYW	IGH	7	None	
11	2448	CAKKGGFSSGWNDYW	IGH	2	None	
11	2449	CATYPAYCGGDCGHYYFDYW	IGH	4	None	
11	2450	CAREELFRIYVTTTYFDYW	IGH	2	None	
11	2451	CANVGVQWLVHFPYW	IGH	2	None	
11	2452	CARASGYCSSTSCRRRYYYGMDVW	IGH	2	None	
11	2453	CARHTKQQLANALDIW	IGH	2	None	
11	2454	CARERAYDGGNLFDPW	IGH	3	None	
11	2455	CARKTGYCSSTSCYTYCSGGSCYFDNW	IGH	11	None	
11	2456	CARDPERSSSCVDYW	IGH	14	None	
11	2457	CASAIAPAAFDYW	IGH	11	None	
11	2458	CARELTIFGVYNWFDPW	IGH	10	None	
11	2460	CVSTSWGDSHPFDFW	IGH	3	None	
11	2461	CAREGGFLEWLGDRGLDYW	IGH	4	None	
11	2463	CAHSPDFWTGYYFDYW	IGH	5	None	
11	2464	CATASPTDYGDYLHREFDYW	IGH	8	None	
11	2466	CARAAGHSNGWYSTLYYFDYW	IGH	2	None	
11	2468	CAKTITMAQGVVINPSYYYGMDVW	IGH	5	None	
11	2469	CARSYSSSWYDPGWFDPW	IGH	2	None	
11	2470	CARDVPDGYGYFYYYYGMDVW	IGH	4	None	
11	2471	CAHRRGPRYFDWSLSYNWFDPW	IGH	2	None	
11	2472	CASWLTRKTTVTSPESYYYYYGMDVW	IGH	2	None	
11	2473	CARVFGSGSYNRGGIDYW	IGH	3	None	
11	2474	CASGTGGDIVVAPPWFDPW	IGH	3	None	
11	2475	CARSLYCRGGSCYSPGIWFDPW	IGH	2	None	
11	2477	CARVPKTVSDWFDPW	IGH	3	None	
11	2478	CARFASSWWQTFDYW	IGH	7	None	
11	2479	CARNSNWNDAWYFDYW	IGH	20	None	
11	2480	CARGSMSTIIFFDYW	IGH	2	None	
11	2481	CARLPDYYDSSGYYYVNFDYW	IGH	3	None	
11	2482	CARKGDRSGQTPDYW	IGH	12	None	
11	2483	CARVEEKKNYYDSSAYYYFDCW	IGH	48	None	
11	2484	CAKDTYGDYGINWYFDLW	IGH	2	None	
11	2485	CARDRHSSSEEFDYW	IGH	17	None	
11	2486	CARHAPNMDDVDYW	IGH	2	None	
11	2487	CAKDRALVVPAAMRYYYYYGMDVW	IGH	2	None	
11	2488	CARVGVLVVSDTYLDYW	IGH	2	None	
11	2490	CARGGYCSGGLCYSFNAFDIW	IGH	8	None	
11	2491	CQQYNSYWETTWTF	IGK	2	C3	7K8R
11	2492	CARKFGESGYDRDWYFDLW	IGH	2	None	
11	2493	CARRQYSGSSHDYW	IGH	5	None	
11	2494	CARLAVAGTSVDYW	IGH	8	None	
11	2495	CARDRNSGDSWWSGGTLKFDYW	IGH	3	None	
11	2496	CAKDSGYYDFWSGYSYFDYW	IGH	8	None	
11	2497	CARSEDGSYRSYFDYW	IGH	45	None	
11	2498	CARSEKYYDYIWGSYRYGPHDYW	IGH	4	None	
11	2499	CARVLASGSSWYPFDYW	IGH	4	None	
11	2500	CARGRIMGATTEYLDYW	IGH	20	None	
11	2501	CARSLSVATSGTKVAFDYW	IGH	8	None	
11	2502	CARLVAYSSSRLSYYFDYW	IGH	123	None	
11	2503	CARTHQRYSSSWRDAFDIW	IGH	2	None	
11	2504	CATERGSSWPPDWYFDLW	IGH	2	None	
11	2505	CAKDSGSGWYKTFDYW	IGH	3	None	
11	2507	CAHSRNIDDIFNIW	IGH	2	None	
11	2508	CARAEIVSTPDAFDIW	IGH	2	None	
11	2509	CARLEGYFDWLLSSYYFDYW	IGH	2	None	
11	2510	CARDLYSSRANWFDPW	IGH	5	None	
11	2511	CARHWERGASTTATHYRAFDYW	IGH	3	None	
11	2512	CARVKGYCSGGSCYYFDPW	IGH	4	None	
11	2513	CAKGDYTNFALFDFW	IGH	2	None	

11	2514	CAREREGDDFWSGYSLYYYYMDVW	IGH	2	None	
11	2515	CARHVISDLGYNWNYGVDYW	IGH	2	None	
11	2516	CAKDRGQWLEGDAFDIW	IGH	2	None	
11	2517	CAKDRWASGSSGGDYW	IGH	4	None	
11	2518	CARHSHSRDSFYYYYMDAW	IGH	2	None	
11	2519	CAKVVGTFFVNEYSGYDANDYW	IGH	2	None	
11	2520	CVRGMGGSYSLDAFEIW	IGH	4	None	
11	2521	CGRWGYSSSPPPDYW	IGH	9	None	
11	2522	CALTEGDGYNYGFDYW	IGH	2	None	
11	2523	CAKFPSSSPSWADYW	IGH	2	None	
11	2525	CARRDYDYIWGSYRHNFDYW	IGH	4	None	
11	2526	CARGTELRYFDWSYYYYMDVW	IGH	4	None	
11	2527	CARDRLWSGYYDFW	IGH	5	None	
11	2528	CARPYSSGYHSAFDIW	IGH	52	None	
11	2529	CARGNYNRGASSYYYYYYMDVW	IGH	2	None	
11	2531	CARVKAVADVWAFDYW	IGH	2	None	
11	2532	CAHLGSSGYYVDFDYW	IGH	4	None	
11	2534	CVRSESCNGPNWFDTW	IGH	2	None	
11	2535	CARDGYDFWSGYQDYW	IGH	44	None	
11	2537	CARDYSDYVSYFDYW	IGH	10	None	
11	2538	CAKERPQPSADALDIW	IGH	16	None	
11	2539	CADLSGSGSYYVGYW	IGH	2	None	
11	2540	CARDIAVTNTKHYYDYW	IGH	23	None	
11	2541	CARRITGAYNWFDRW	IGH	3	None	
11	2542	CARDAYLNSRSYYHFDYW	IGH	4	None	
11	2543	CAAHRIGGCSDRCYMSFYYYGMDVW	IGH	2	None	
11	2544	CARDSRTFGGVIAPGRDAFDIW	IGH	4	None	
11	2545	CAGHRRTPLFWYFDLW	IGH	4	None	
11	2546	CARAILSHDYIWGSYRYKGFDYW	IGH	4	None	
11	2547	CARADDGSGSYFLPFDVW	IGH	30	None	
11	2548	CVKGTTMLSEYYFDYW	IGH	14	None	
11	2549	CQQYNSYSPMYTF	IGK	48	C3	7K8R
11	2550	CARDGVVQGPDSYYYMDVW	IGH	4	None	
11	2551	CARGPYYDFLSGPDAFDIW	IGH	6	None	
11	2552	CARDVLGNGNAFDIW	IGH	54	None	
11	2553	CAWQQLVRGQFDYW	IGH	2	None	
11	2554	CAKDGSGWKPQYYFDYW	IGH	6	None	
11	2555	CATDLIIRGVVDYW	IGH	2	None	
11	2556	CARDLVGTTPAHDAFDVW	IGH	4	None	
11	2557	CARGGDCYSEPFDYW	IGH	2	None	
11	2558	CATWPVPDFVVTLAIGRAFDIW	IGH	2	None	
11	2559	CARDLGAAGGYGADYW	IGH	3	None	
11	2560	CAREAGYLDLTVNNYFDYW	IGH	5	None	
11	2561	CVRESYGETYSGSYYESYSFDFW	IGH	4	None	
11	2562	CAKIPHGGDTFDFW	IGH	6	None	
11	2563	CARGGPHDSLWRWFDPW	IGH	6	None	
11	2564	CARLRDLRYFDYIYYYYGMDVW	IGH	2	None	
11	2565	CARIQRHRWYLDYW	IGH	2	None	
11	2566	CARDRTAVENCSGGSCYGGIDYW	IGH	2	None	
11	2567	CAREADGSPHSDYW	IGH	2	None	
11	2568	CAREGINYSNLLNWFDPW	IGH	2	None	
11	2569	CARAGPLDHLDYW	IGH	7	None	
11	2570	CARGAVFGWWDYYYGMDVW	IGH	2	None	
11	2571	CAKGPMVQGVIITNWFDPW	IGH	2	None	
11	2572	CTRDGTSGYRVTFDVW	IGH	3	None	
11	2573	CAKNFYIGQMGFDGFDIW	IGH	2	None	
11	2574	CARVGFSDGSGYPFDYW	IGH	2	None	
11	2575	CAHSAIVPTTFASDYFDYW	IGH	3	None	
11	2576	CARSDRFVVVPADPSEYYFDYW	IGH	53	None	
11	2577	CARATGQQLGDYW	IGH	2	None	
11	2578	CKRSWNDAVGDYW	IGH	3	None	
11	2579	CASYIGRFLEWLSWFDPW	IGH	6	None	
11	2580	CARGGWARGVVVVAASLTYWYFDLW	IGH	2	None	
11	2581	CAREFRITMVQGVQSYFDYW	IGH	7	None	
11	2582	CAVYSNGWLDYW	IGH	6	None	
11	2583	CAVNRAPYNSGWYYFDYW	IGH	8	None	
11	2584	CVKDLSPAATVVSYNAFDIW	IGH	6	None	
11	2585	CARELSTVTTYFNYW	IGH	11	None	

11	2586	CARDQPLRSFWEPFWFDPW	IGH	2	None	
11	2587	CARGILRGHDYW	IGH	5	None	
11	2588	CAKRDGSGGRFHYMDVW	IGH	2	None	
11	2589	CARVSHYYDSSGYFEDYW	IGH	12	None	
11	2590	CARAAVTMIVVNHFDYW	IGH	2	None	
11	2591	CARLALVEFGYSGYGWSRHFDYW	IGH	9	None	
11	2592	CARELGTGHFDYW	IGH	29	None	
11	2593	CARGAYDYENRAFDIW	IGH	11	None	
11	2594	CARVQRGSSGWFRTEYFQHW	IGH	6	None	
11	2595	CARMSSGYDWNRPDFSFDYW	IGH	2	None	
11	2596	CASIPPESGWDTNWFDPW	IGH	2	None	
11	2597	CAREIRAPRGSFDPW	IGH	2	None	
11	2598	CARTQGGRYYDILTGPSYYYYYMDVW	IGH	96	None	
11	2599	CARGGFYYDSSGIHGDYW	IGH	3	None	
11	2600	CATADFSKSFFDYW	IGH	16	None	
11	2602	CATTASYCTGGVCYPNWFDPW	IGH	11	None	
11	2603	CGIQPYSGANRGDFDYW	IGH	19	None	
11	2604	CARSVGAATEAQNDYW	IGH	4	None	
11	2605	CASWNYHDSSGYKFDYW	IGH	45	None	
11	2606	CAGGLGGSYYLDYYYYMDVW	IGH	2	None	
11	2607	CARETSEWQLEGWYTWFDPW	IGH	2	None	
11	2608	CARDWGGHITSDAFDIW	IGH	5	None	
11	2609	CARDSTEIPQYYYDSSGYSAAFDYW	IGH	4	None	
11	2611	CAKGGMGIPSHFDYW	IGH	18	None	
11	2612	CARYQGGDYDYIWGSYRLGTNYFDYW	IGH	2	None	
11	2613	CAKDKYYDTSDSYSHFDYW	IGH	8	None	
11	2614	CASQGTVATEPYYFDYW	IGH	2	None	
11	2615	CARALQGVYLGIDYW	IGH	2	None	
11	2616	CATDEGEETAMGASLFDSW	IGH	33	None	
11	2617	CARGWGRELLFFYFDYW	IGH	2	None	
11	2618	CARGRSAGSGSQLNFGYW	IGH	3	None	
11	2619	CARDFDILTGYYFDYW	IGH	23	None	
11	2620	CARRGNWNDGSSFDYW	IGH	2	None	
11	2621	CARHSNDILTGYYGGDYYYYYMDVW	IGH	3	None	
11	2622	CARDGPNDYGDYVEIDAFDIW	IGH	9	None	
11	2624	CAKWSSGFSRGLYHYMDVW	IGH	6	None	
11	2625	CAKDYYDSSGYYAFAAFDIW	IGH	8	None	
11	2626	CARYTSSGFFFDFW	IGH	11	None	
11	2628	CAKNGAAAGLGEVDYW	IGH	2	None	
11	2629	CARLMVILATTHFDYW	IGH	7	None	
11	2630	CARDRGHTIFEANAFDIW	IGH	4	None	
11	2631	CARQSGIVTTTDMIDYW	IGH	3	None	
11	2632	CSRSNGYSIGWFLDYW	IGH	13	None	
11	2633	CQQYGSSQGDCSF	IGK	2	C1	7K8N
11	2634	CARHLETGDYYDSSGDNFDYW	IGH	7	None	
11	2635	CAHRRSCTSTSCYRDNWFDPW	IGH	2	None	
11	2636	CARDQGSTATYLDAFDLW	IGH	7	None	
11	2637	CARQSAYCGGYCYFPNHWYFDLW	IGH	2	None	
11	2638	CATAPRYYYDSSGYWGPNDYW	IGH	4	None	
11	2639	CASTILRFLEWSPTIDAFDIW	IGH	2	None	
11	2640	CAQGTILKYYYFDYW	IGH	5	None	
11	2641	CARPRVSDCTETRCREFEYW	IGH	4	None	
11	2642	CAKKENSNYRSFLDYW	IGH	3	None	
11	2643	CARSYDFWSGHFPSDYYYYGMDVW	IGH	3	None	
11	2644	CARDEGIYYDSSGYPRWFDPW	IGH	4	None	
11	2645	CARDPQRGVVINSPNYYYYGMDVW	IGH	3	None	
11	2646	CADRRYCDITRCPGFGYW	IGH	3	None	
11	2647	CAHRPQTTYSNYVVWFDPW	IGH	3	None	
11	2649	CARSFLYYSGTSGYYSGSFDYW	IGH	2	None	
11	2650	CARDPSGNAEYFQHW	IGH	2	None	
11	2651	CARDQHSGGYYVWFDPW	IGH	12	None	
11	2652	CAYQLPGYYYGMDVW	IGH	11	None	
11	2653	CARGTFWSGYRYYFDYW	IGH	5	None	
11	2654	CARFHRRGTVTMYWFDPW	IGH	19	None	
11	2655	CARATRGPRYYYYYYYMDVW	IGH	2	None	
11	2656	CARDFVTTVTYNDAFDIW	IGH	2	None	
11	2657	CAKVAGYDFWRGYSFDQW	IGH	4	None	
11	2658	CARAAAGKIVGATQGCDYW	IGH	2	None	

11	2659	CARARDGYNYLDYW	IGH	6	None	
11	2660	CASNYGDYGSYYYGMDVW	IGH	5	None	
11	2661	CTRVGWSYSRFYYYYMDVW	IGH	11	None	
11	2663	CARAGYVGSWFYYAMDVW	IGH	5	None	
11	2664	CATEDYGDYGSPRFDYW	IGH	4	None	
11	2665	CARVGPYYDFWSGYPIGDYYYYMDVW	IGH	2	None	
11	2666	CARADHSRGPLYYYYGLDVW	IGH	3	None	
11	2667	CTRTTPVTTVPYYYYMDVW	IGH	10	None	
11	2668	CAKVDAAVVRRVSVAGYFDYW	IGH	2	None	
11	2669	CARDGKRRDTLDQRIMITFGGVSFYYFDYW	IGH	2	None	
11	2670	CARYCGGVCYGVNYYYYGMDVW	IGH	3	None	
11	2671	CARLADDFWTHPYYYYGMDVW	IGH	149	None	
11	2672	CAREKYNWNDLDYYYGMDVW	IGH	2	None	
11	2673	CAKGPTLYNDDAFDMW	IGH	4	None	
11	2674	CAHRPSMYSSGHGSWFDPW	IGH	2	None	
11	2675	CARAHPIYSKPRRGYWFDPW	IGH	3	None	
11	2676	CARDKHNGAFDIW	IGH	156	None	
11	2677	CVRQDDYLDYRFDHW	IGH	2	None	
11	2678	CNLGGGSGYEYYYYNMDVW	IGH	2	None	
11	2679	CALTSALYGSGSYYNNWFDPW	IGH	3	None	
11	2680	CAREIILITFGGVITNLQRSQNWFDPW	IGH	5	None	
11	2681	CARDPATMVQGSHFDYW	IGH	2	None	
11	2682	CARADTRGDTDAVDIW	IGH	2	None	
11	2683	CARSLRYCTGGLCYDDDQVYNWFDPW	IGH	2	None	
11	2684	CARDPRYCSGSDCYSLSHYHYMDVW	IGH	2	None	
11	2686	CARDSFSSSGWYGYFQHW	IGH	3	None	
11	2687	CASRYYTKYYYDYW	IGH	3	None	
11	2689	CARDSGSGTYGYGDYRYFDSW	IGH	3	None	
11	2690	CARVEGYSKFFHHHAFDVW	IGH	8	None	
11	2691	CARDGAYAGTWYRYFDLW	IGH	7	None	
11	2692	CAHRGRYSGRYYFDYW	IGH	2	None	
11	2694	CTRGGLRFLEWFIDPDNYYFDYW	IGH	2	None	
11	2695	CARDPAAAGLTLFDYW	IGH	5	None	
11	2696	CTTVKVLRFLETPYYFDYW	IGH	4	None	
11	2697	CARGDDYSGAYYYYLDYW	IGH	2	None	
11	2698	CARTFMVAATRYFLHW	IGH	3	None	
11	2699	CGRLYYYVGSGYYPDYW	IGH	9	None	
11	2700	CARAGRSSGSYSGIDYW	IGH	2	None	
11	2701	CARDKRNPDLEPDAFDIW	IGH	2	None	
11	2702	CAKAPQYSGYEYHIDYW	IGH	7	None	
11	2703	CARGGYSGYHFPFDLW	IGH	2	None	
11	2704	CSGGSCHSFDYW	IGH	37	None	
11	2705	CARDSGTSGYYYEYFQFW	IGH	2	None	
11	2706	CARAAGSGWDSPVDYW	IGH	4	None	
11	2707	CTKEYYGSGSYSDYW	IGH	21	None	
11	2708	CARDLYSSGWYGQPDYW	IGH	3	None	
11	2709	CARLVVLGVYTSFDPW	IGH	19	None	
11	2710	CASSLKYSSSWDFDYW	IGH	8	None	
11	2711	CARVGSGSYSGYDYYYYGMDVW	IGH	2	None	
11	2712	CARDPSSSWDSDSFDTW	IGH	2	None	
11	2713	CARDHGWGGAPNFDYW	IGH	3	None	
11	2714	CARGRCSDAICYGIYFDYW	IGH	4	None	
11	2715	CAKDRAGTRPEDTIFGVVTQYYYGMDVW	IGH	2	None	
11	2716	CARAELLSQDYFDYW	IGH	11	None	
11	2717	CANLAVVDATLGFDLW	IGH	6	None	
11	2718	CARDLAGKQAFDIW	IGH	8	None	
11	2719	CARDGGSSWSMSFDYW	IGH	2	None	
11	2720	CTQQLVRSYDYW	IGH	4	None	
11	2721	CARADHCSSGICYYRALDVW	IGH	8	None	
11	2723	CAREAERAGPYCTGGVCTTAFDIW	IGH	3	None	
11	2724	CARAELNYYDSSGFQYYFDYW	IGH	3	None	
11	2725	CARGRGQFLEWFVFDYW	IGH	8	None	
11	2726	CARDPPLGSNYYYYGMDVW	IGH	2	None	
11	2727	CASSRTSITMVQGVIFDYW	IGH	2	None	
11	2728	CARGLGYCSSTNCRRYYFDYW	IGH	3	None	
11	2729	CATAPLYYYDSSGYFLWFDPW	IGH	5	None	
11	2730	CARVLRITMVQGVHDAFDIW	IGH	2	None	
11	2731	CARGPELGIVVVVRPLSGWFDPW	IGH	2	None	

11	2732	CARVDYSNYYRPPFDPW	IGH	2	None	
11	2733	CASGLLVVYAALDYW	IGH	2	None	
11	2734	CARDRISARDDAFDIW	IGH	15	None	
11	2735	CARDDSEETVERIAIFGVAPLGYAFHIW	IGH	6	None	
11	2736	CARMTRSSWGVFDYW	IGH	3	None	
11	2738	CATDTPVGLYVVYALDYW	IGH	10	None	
11	2739	CQQYDNLLSITF	IGK	316	C3	6XDG
11	2740	CARGYDVLTGYPDYW	IGH	108	None	
11	2741	CARDWGGTTVTPGSLDYW	IGH	5	None	
11	2742	CTRDQEVATPYFFDYW	IGH	2	None	
11	2743	CARSRTHYGSGTYYFDYW	IGH	6	None	
11	2744	CQQFQSYSWTF	IGK	5	C3	7K8R
11	2747	CARVRTTGTRYNYYYMDVW	IGH	2	None	
11	2748	CARVRFPTWAVTEDAFDIW	IGH	5	None	
11	2749	CAKDPRPGYSSSYHYFDYW	IGH	3	None	
11	2750	CAREIYLARPTRASDAFDIW	IGH	2	None	
11	2751	CASLLVPDTAMVKGYYYGMDVW	IGH	6	None	
11	2752	CVQALQTQCSF	IGK	9	None	
11	2753	CARDLLGIAVAGVFDYW	IGH	6	None	
11	2754	CARDPDRDGYSAFDYW	IGH	14	None	
11	2755	CSGGSCHTLGYYYYYMDVW	IGH	2	None	
11	2756	CATGHVGTFDAFDIW	IGH	4	None	
11	2757	CQQANRFLALSF	IGK	4	None	
11	2758	CARVRSGGSCYSSLVCAFDIW	IGH	2	None	
11	2760	CARDQDSIFGLEGFDFW	IGH	3	None	
11	2761	CAREGINDSSGQRGFDYW	IGH	4	None	
11	2762	CASWGATVAFDIW	IGH	5	None	
11	2763	CARQSDSNYDTYLTYW	IGH	15	None	
11	2764	CAHRRIQLWLWDFDYW	IGH	3	None	
11	2765	CARLVGSTSYIQWYLDLW	IGH	3	None	
11	2766	CARYHWNYHADYW	IGH	2	None	
11	2767	CARDLPIVATIEDAFDIW	IGH	2	None	
11	2768	CARYNSGWSWDYW	IGH	13	None	
11	2769	CAREGTSTVLSGPDAFDIW	IGH	3	None	
11	2770	CATPIDDSSGYYSPYAVGFDYW	IGH	39	None	
11	2771	CARGIVVVPAAMAFDPW	IGH	2	None	
11	2772	CVLFMGSGIWMF	IGL	270	None	
11	2773	CTTVNLGGGYYIVYW	IGH	3	None	
11	2774	CARHRAYNWNPTSGFDNW	IGH	2	None	
11	2775	CARLTDGSGTYAFDIW	IGH	2	None	
11	2777	CARRYCSGAHCYDPPYYYYYMDFW	IGH	2	None	
11	2778	CAREAPIPSSHDYW	IGH	2	None	
11	2779	CARDLTYGDPDTDAFDIW	IGH	7	None	
11	2780	CARPYRGVYHSSFDIW	IGH	3	None	
11	2781	CATTPPYCGGYCYENFDFW	IGH	2	None	
11	2782	CAHRNMVQGAYFDYW	IGH	15	None	
11	2783	CARDWESPSPYYYYYMDVW	IGH	4	None	
11	2784	CYHFDYW	IGH	2	None	
11	2785	CARNDYSNYDYMDVW	IGH	2	None	
11	2787	CTRVLSGSGRYGQVNWFDPW	IGH	3	None	
11	2788	CARGGLPGAIGIDAFDTW	IGH	2	None	
11	2789	CARNPDTAMPDYW	IGH	2	None	
11	2790	CAKGDWDSSSSNYFDYW	IGH	9	None	
11	2791	CAKGRVYSNYDKFDYW	IGH	5	None	
11	2792	CARDPGCSSTSCYETWFDPW	IGH	2	None	
11	2793	CARRVGLLHSSRFDPW	IGH	2	None	
11	2794	CAKGYCSGGSCPRGYAFDIW	IGH	4	None	
11	2795	CASYFGRTVTTTTGFDYW	IGH	4	None	
11	2796	CARDGLVGDYW	IGH	528	None	
11	2797	CARGQRGNYDLAAFDIW	IGH	7	None	
11	2798	CAKSRGGPARSGDYW	IGH	6	None	
11	2799	CARDKRQILEYYYGSGSYYFDYW	IGH	2	None	
11	2800	CATNLRPTVYYFDYW	IGH	6	None	
11	2801	CARDLLLLPKMDDYW	IGH	2	None	
11	2802	CARTASNCSGGRCYSSDYW	IGH	2	None	
11	2803	CARVPAPDPLITIFGVPQSGPYDYW	IGH	4	None	
11	2804	CMIWPSNAWVF	IGL	59	None	
11	2805	CALPILVVLGFYKYMDVW	IGH	2	None	

11	2806	CAKAPTVGGTILADYW	IGH	2	None	
11	2807	CARDWRSSRNFDYW	IGH	8	None	
11	2808	CQQSFSAPSYTF	IGK	8	C2	7K8O
11	2810	CARRYTPTHDYSNYPADYW	IGH	3	None	
11	2811	CVKVSQVVVVVNYFDYW	IGH	6	None	
11	2812	CARDVTDYSNYVEYNWFDPW	IGH	2	None	
11	2813	CATRITMVQGVITDNWFDPW	IGH	5	None	
11	2814	CARDDSLSTYDILTGYYPYYFDYW	IGH	2	None	
11	2815	CAKEGDIVATTRYYFDFW	IGH	20	None	
11	2817	CARAGFSSGWYAAFDFW	IGH	2	None	
11	2818	CARERMVQGVASGFDIW	IGH	13	None	
11	2819	CTRHDSLGSYDYAFDIW	IGH	2	None	
11	2820	CARVEGTDYSNYVGYW	IGH	2	None	
11	2821	CARSPSGYDLGYYYFDSW	IGH	5	None	
11	2822	CGVHFNFWSGIFDYW	IGH	2	None	
11	2824	CARVKPLFGGFLEWAFDYW	IGH	6	None	
11	2825	CARHYHGLGIRQDDAFDIW	IGH	9	None	
11	2826	CASRSGRHLYFDLW	IGH	4	None	
11	2828	CARGISNHGEFYFDLW	IGH	2	None	
11	2829	CAHVEDYDFWSGFPSFHYW	IGH	3	None	
11	2831	CARAEGAPEYSLDYW	IGH	2	None	
11	2832	CARDYVVPAAHYYYYYMDVW	IGH	2	None	
11	2833	CARDTVAIIGWGMTHLDYW	IGH	6	None	
11	2834	CARYKWGGTSPSDAFDLW	IGH	2	None	
11	2835	CVRDTNRKIYGYFDFW	IGH	6	None	
11	2836	CARRGGWDKGGGYWHFDLW	IGH	4	None	
11	2837	CQLWDSSSAQVIF	IGL	5	None	
11	2838	CQQYGHWPRWTF	IGK	4	C2	7K8U
11	2839	CARDFSIGSYYPQGYFDYW	IGH	3	None	
11	2840	CARGDYGDPLFFLEFDYW	IGH	12	None	
11	2841	CARVVLTGDHHAFDIW	IGH	8	None	
11	2842	CSTLTTVTVDYW	IGH	2	None	
11	2843	CAKADSSGWSPDFDFW	IGH	2	None	
11	2845	CARDRYCTGGVCHQSWFDRW	IGH	2	None	
11	2847	CARVLVTSRSVRTDVFDIW	IGH	35	None	
11	2848	CARESVLRYFDWLLFGYFDYW	IGH	2	None	
11	2849	CARHRDFGDSNDFDYW	IGH	2	None	
11	2850	CARSNIAARGVWYFDYW	IGH	2	None	
11	2851	CARPAPIFGVYGSPAYFDYW	IGH	2	None	
11	2852	CARVGGNHYGYSRYYFDYW	IGH	8	None	
11	2853	CARSLKTMIQGILYMDVW	IGH	6	None	
11	2855	CATHAVPDYW	IGH	8	None	
11	2856	CARQSDLVVYPYFDYW	IGH	12	None	
11	2857	CAREPPSLGIFGVVLFDYW	IGH	5	None	
11	2858	CATQNGYCTGGVCYEFNWFDPW	IGH	8	None	
11	2859	CARVPGPYYDFWSAQGAFDIW	IGH	2	None	
11	2860	CARDPPGKYDFWSGYSGAYYYYMDVW	IGH	2	None	
11	2861	CVGRWDYFDLW	IGH	3	None	
11	2862	CATGSPYYYGNWFDPW	IGH	2	None	
11	2863	CARDFSITMVQGVIPPEIDYW	IGH	2	None	
11	2864	CARGSGDGGIYNWFDPW	IGH	5	None	
11	2865	CAKSPPTTGPRKYFFDWW	IGH	4	None	
11	2866	CARGLSSLGASSDDYW	IGH	9	None	
11	2867	CARGGWGAARPQYYYYGMDVW	IGH	4	None	
11	2868	CAKVTAVTMWLDYW	IGH	5	None	
11	2869	CARVYGDYEVGRFDPW	IGH	3	None	
11	2870	CVRDLAYHWGSPFDFW	IGH	3	None	
11	2872	CARDWYSLSGGGFDYW	IGH	7	None	
11	2873	CARDRVVGTTTGIDYW	IGH	5	None	
11	2874	CARAGGVVDDAFDFW	IGH	3	None	
11	2875	CARYSGGYSPRYWNFDLW	IGH	3	None	
11	2876	CSALDSSLSARVF	IGL	4	None	
11	2877	CARDPCGSTNCYLDYW	IGH	4	None	
11	2878	CAREESINGYGFDYW	IGH	9	None	
11	2879	CARDREAYSNHGGFDQW	IGH	2	None	
11	2880	CATSRPYSGTYYGAVRIAGFDSW	IGH	2	None	
11	2881	CARLTDEYYYDSSGYLGYW	IGH	4	None	
11	2882	CAKRVDLRDHKYSTSAAPPFDYW	IGH	2	None	

11	2884	CARDRSESTSDDSSWYAYYYYYMDVW	IGH	2	None	
11	2885	CARAQLPSSRYRFDPW	IGH	2	None	
11	2886	CARDFSLMFDLW	IGH	6	None	
11	2887	CARLEYYGDYSYFDYW	IGH	2	None	
11	2888	CARDFRVGGQDDYW	IGH	2	None	
11	2889	CARQNAEMATIRGFDYW	IGH	4	None	
11	2890	CAIQIWFRELLLDYW	IGH	2	None	
11	2891	CARKGDTIFGVVPPDYW	IGH	2	None	
11	2892	CARENPVLRYFDWLPRFDPW	IGH	2	None	
11	2893	CARSGGVADIGVGRWFDPW	IGH	2	None	
11	2894	CTRDDSYRVLSAYFDLW	IGH	2	None	
11	2895	CAHSRGRRLGKDYIGGNYRHNWFDPW	IGH	5	None	
11	2896	CVHIRRSSGWNNWFDPW	IGH	2	None	
11	2897	CARWSYNSGGNIDYW	IGH	2	None	
11	2898	CARVISLAGTQVAAFDYW	IGH	4	None	
11	2899	CARVPPGGHCGSTKCFKMGVFDYW	IGH	2	None	
11	2900	CARHWTTYSGYDLYYFDYW	IGH	18	None	
11	2901	CATTFGSGIAARQITPDYYYYGMDVW	IGH	3	None	
11	2902	CAKDGQLVGTTTYFDYW	IGH	2	None	
11	2903	CARDEYSSSWSDYYFDYW	IGH	12	None	
11	2904	CAHRRRGYDFWSGSPRPFDPW	IGH	6	None	
11	2905	CARGGTEWLPFFDYW	IGH	10	None	
11	2907	CMQGTHWPPIIF	IGK	135	None	
11	2908	CAAIPGIAAAVTNWFDPW	IGH	2	None	
11	2909	CARSPTVTTYAFDIW	IGH	9	None	
11	2910	CARDPRQRHDFWNGYQFDYW	IGH	2	None	
11	2911	CAKERGARYHDYW	IGH	3	None	
11	2912	CARGQHLPEAPDYW	IGH	2	None	
11	2913	CARTYDSTGYYSFDYW	IGH	26	None	
11	2914	CARDRKNYGSGQFDYW	IGH	2	None	
11	2915	CASPIFGVETDAFDIW	IGH	89	None	
11	2916	CARDRMTKTYFDLW	IGH	2	None	
11	2917	CATSFAATLRSSGFDYW	IGH	7	None	
11	2918	CAREYSSGCFDYW	IGH	360	None	
11	2919	CQSADSSGTYWVF	IGL	10631	None	
11	2920	CTTDRIYDYIWGSYRPPFDPW	IGH	6	None	
11	2921	CATDISRQRPEVSGEAVAGSKTDYW	IGH	3	None	
11	2922	CAHVSNKLFDIW	IGH	2	None	
11	2923	CARVDTSGWQSFDYW	IGH	4	None	
11	2924	CARDSETGSGWINPFDYW	IGH	6	None	
11	2925	CAKSGDPGPIHLDYW	IGH	2	None	
11	2926	CARISVHGYGGYDNYFDSW	IGH	10	None	
11	2928	CARGPAAAGNWYFDIW	IGH	4	None	
11	2930	CARALGSSSWYKDYW	IGH	6	None	
11	2932	CARDRNYDSSGSWFDPW	IGH	2	None	
11	2933	CARVVATVTTWANFDYW	IGH	6	None	
11	2934	CARAYGLPVGYSTYFDYW	IGH	2	None	
11	2935	CAREGRQLGSGTWFDPW	IGH	4	None	
11	2936	CARESGELPVLDYW	IGH	2	None	
11	2937	CALVSTVTTTFYFASW	IGH	2	None	
11	2938	CARDSYSGYDDYFDYW	IGH	27	None	
11	2939	CQQYGSSSSWAF	IGK	221	C1	7K8N
11	2941	CAKDVRRSTGTGHLIYYYFGMDVW	IGH	7	None	
11	2942	CARDSYSSSWYGAFDIW	IGH	6	None	
11	2943	CAKQNRDSAYYFDYW	IGH	2	None	
11	2944	CATERRMSIAVAGTADYW	IGH	155	None	
11	2945	CARDSTMVQGVIIFRSLDYW	IGH	2	None	
11	2946	CAREGLSVADISRLDYW	IGH	3	None	
11	2947	CARGEEAFGYYAAAIGFGYW	IGH	2	None	
11	2948	CARAVDDGHPDYYFDYW	IGH	2	None	
11	2949	CAREGPAVAGSPFDYW	IGH	10	None	
11	2950	CARDKRGWYELDYW	IGH	184	None	
11	2951	CSLNLSSVTAADTAVYYCARSINCVGGTCYVGYMDVW	IGH	2	None	
11	2953	CAKDREDSSGSTPPDAFDIW	IGH	2	None	
11	2955	CAKTSSGWYADAFDIW	IGH	2	None	
11	2956	CAKDRTGYYDTHDYW	IGH	2	None	
11	2957	CAKETYHSSGYYFDYW	IGH	2	None	
11	2958	CARDDPFRRTPYSSSWYSFYYYGMDVW	IGH	7	None	

11	2959	CARAGCGGDCFRPLLSHYFDYW	IGH	4	None	
11	2960	CVTGPPDTSGSFDYW	IGH	16	None	
11	2962	CARTIRGYDNFDYW	IGH	8	None	
11	2963	CARDQVDYGPNFDYW	IGH	2	None	
11	2964	CASPSGQYYDYIWGSYRLLPYYFDYW	IGH	4	None	
11	2965	CARLITVFGVVTQRSFDYW	IGH	4	None	
11	2966	CVRDPYYGDYGLSNYFDFW	IGH	2	None	
11	2967	CQESYSTPIFTF	IGK	91	C2	7K8O
11	2968	CARGFAVHDYIWGSYRWGHYFDYW	IGH	5	None	
11	2970	CASLFWSGYYNGYYYGMDVW	IGH	4	None	
11	2971	CAREREVSVVGFKYYYYGIDVW	IGH	2	None	
11	2972	CARGGYGGGLFDYW	IGH	21	None	
11	2973	CARSGWYRSLKTYSSSSNYYYYGMDVW	IGH	2	None	
11	2974	CARHVVEGRPPHYYFDYW	IGH	2	None	
11	2975	CARETNIAVTGGIAFDIW	IGH	2	None	
11	2976	CAKRYYHGSSGYAFDIW	IGH	2	None	
11	2977	CARDTEAGTGRFDPW	IGH	21	None	
11	2978	CAHRREAAYSSNWSWGDFDYW	IGH	2	None	
11	2979	CASGSIIAAAGYYYYYGMDVW	IGH	5	None	
11	2980	CTRDGPVIRDYENFDYW	IGH	2	None	
11	2982	CARDHYGSGTYLFDYW	IGH	572	None	
11	2983	CARGILPAAISPINWFDPW	IGH	8	None	
11	2984	CARDAYGWNHRYFDYW	IGH	15	None	
11	2985	CAKRSCTSTSCYFPLLPVDYW	IGH	6	None	
11	2986	CASLSEGGRAFDYW	IGH	2	None	
11	2987	CGRDRNSYYIDVW	IGH	7	None	
11	2988	CARDETYYYDGSGYYGTGYFEYW	IGH	2	None	
11	2989	CARERIITIFGVAYPQRWYYDLW	IGH	2	None	
11	2990	CATITAYSGYVLYW	IGH	4	None	
11	2991	CATPMGTSYYGSGTWGFDYW	IGH	2	None	
11	2992	CARGWQAANDYW	IGH	2	None	
11	2994	CPQHDNFSMCSF	IGK	4	None	
11	2995	CATGWGGSYGIDYW	IGH	2	None	
11	2996	CATRKNHDFSFDHW	IGH	2	None	
11	2997	CAREGTRQQPVGYFDCW	IGH	4	None	
11	2998	CATLATGEWSHFDQW	IGH	4	None	
11	2999	CARDSYNGGYHPSHYYYYMDVW	IGH	2	None	
11	3000	CVGDHRFYGSGSYNHVGWFDPW	IGH	4	None	
11	3001	CASSRESTVVGYNIARPRLFEYW	IGH	2	None	
11	3002	CARVIGYCTGGVCHTNNWFDPW	IGH	4	None	
11	3003	CARRTSSAHEWDYW	IGH	4	None	
11	3004	CARGDYYGSGSYGHFDYW	IGH	4	None	
11	3005	CTTGIEYSGYDYFDYW	IGH	3	None	
11	3007	CAHRRLGSFWNNYYLTDGFDVW	IGH	2	None	
11	3008	CASWDQEMATGDAFDIW	IGH	2	None	
11	3009	CARHTAAAAGTYFDFW	IGH	6	None	
11	3010	CARDPIRDSSGYYYDYW	IGH	2	None	
11	3011	CAVWDDSLSGRNVVF	IGL	4	None	
11	3012	CARGRDPDYVDSFDIW	IGH	3	None	
11	3013	CAKHLSSGTYRGIDYW	IGH	4	None	
11	3014	CAKDNNWSSDYW	IGH	11	None	
11	3015	CVKEKDTYNLLSHFDYW	IGH	16	None	
11	3016	CAKVPIDYAGLFDYW	IGH	2	None	
11	3017	CARELDPSSIAAAGTGRSDAFDIW	IGH	2	None	
11	3018	CASNIAAAGNWYYYYYMDVW	IGH	2	None	
11	3020	CARDQTSGYSGYGRDYYYYYMDVW	IGH	2	None	
11	3021	CARADYDFWSGYEYYFDYW	IGH	10	None	
11	3022	CARHGGCSGVNCYRPINWFDPW	IGH	2	None	
11	3023	CARDLRYNWNDDWDAFDIW	IGH	4	None	
11	3024	CARDRATHLIDYW	IGH	294	None	
11	3025	CAKDLYQLLSYRMTTVTIAHDYW	IGH	3	None	
11	3026	CARRSSGWPYPFDYW	IGH	3	None	
11	3027	CTRGLPTGTRAFDIW	IGH	2	None	
11	3028	CVTTSCYACAFDIW	IGH	4	None	
11	3029	CAAYSLTTHYWYFGLW	IGH	6	None	
11	3030	CAREYRTYGPKRKNWLDPW	IGH	2	None	
11	3031	CAKDRHCSGGGCYVADYYYHGMDVW	IGH	2	None	
11	3032	CARDRGYCGSGSFYTDYW	IGH	2	None	

11	3033	CAKDTYSSIAAAGSFDYW	IGH	2	None	
11	3034	CARDPGDYGDYLWFDPW	IGH	3	None	
11	3035	CAKAPDIVATIPDYW	IGH	3	None	
11	3036	CAKFSVRWLLPGAFDMW	IGH	3	None	
11	3037	CAKDIELQLVRWGYFDYW	IGH	5	None	
11	3038	CARPHTSGPEGFHAFDIW	IGH	4	None	
11	3039	CTTDPATVTPGGYYYYMDVW	IGH	2	None	
11	3040	CARDRRYYYDTGGYYYAFDYW	IGH	3	None	
11	3041	CARHREIPAAGTYPDYW	IGH	7	None	
11	3042	CARQDDILTGDYYYGMDVW	IGH	2	None	
11	3043	CAREFGYGDYVPQEYYYYYMDVW	IGH	7	None	
11	3044	CTAYLQWSSLKASDTAMYYCASSMTMVTTYYFDYW	IGH	2	None	
11	3045	CARGPMTVLRFDPW	IGH	2	None	
11	3046	CARDGYNYYYYYYMDVW	IGH	20	None	
11	3047	CARQWELLRSFYFDYW	IGH	4	None	
11	3048	CAQRGIVAGNSFDYW	IGH	3	None	
11	3049	CQSADNSASSLYVF	IGL	2	None	
11	3050	CAREGLEMATITAFDIW	IGH	2	None	
11	3052	CARDPRYCSGGSCYLGDNWFDPW	IGH	5	None	
11	3053	CAREGIAVTGIDYFDYW	IGH	2	None	
11	3054	CAADPETYYGDYKRYNWFDPW	IGH	3	None	
11	3055	CARVNRSSRSWYVDYW	IGH	6	None	
11	3056	CARATVENFFGPW	IGH	2	None	
11	3058	CARYSGSYRNFDYW	IGH	102	None	
11	3059	CAREVATVTAWYDYYYKGMDVW	IGH	2	None	
11	3060	CAHKGSSRSLISVEKYYFDYW	IGH	8	None	
11	3061	CARETAGVTWNIGENWFDPW	IGH	2	None	
11	3062	CARSGPKRYYGSVPFDYW	IGH	7	None	
11	3063	CARGPVAALPDAFDIW	IGH	2	None	
12	1	CAKDIWWRGSSAAGTFYYW	IGH	2	None	
12	2	CARARGYSGSYWHYW	IGH	2	None	
12	3	CAKDWYYYGSGSLPQIDYW	IGH	2	None	
12	4	CTTDKVQYYDILTGYYWLDYW	IGH	5	None	
12	5	CTTVILRYFDWLLLRRGRLDDWFDPW	IGH	6	None	
12	7	CQVWDNFSDHYVFF	IGL	37	None	
12	8	CARHGNYDSSGYYPIDWFDPW	IGH	2	None	
12	9	CARLKHSIAVAPLGAFDIW	IGH	2	None	
12	10	CIRHGYSGGWLDYW	IGH	4	None	
12	11	CARDWGVGGAVAGERYYYYGMDVW	IGH	2	None	
12	12	CAKGRVGGYNYWYFDVW	IGH	8	None	
12	13	CARSTTSYCSSTSCYFDYW	IGH	4	None	
12	14	CATDGYDYHYYNGMDVW	IGH	2	None	
12	15	CARDGEYYGSGSYRQFDPW	IGH	7	None	
12	17	CARGIYCSSTSCATYYYYGMDVW	IGH	25	None	
12	18	CARDRDGYNVNYAFDIW	IGH	2	None	
12	19	CAHRRYLGLYPRFDPW	IGH	2	None	
12	20	CARRLIVVVVDDAFDIW	IGH	7	None	
12	21	CAKDRMAAAGSRIYYYCYSMDVW	IGH	2	None	
12	22	CARDPEFGSGSWEFDLW	IGH	2	None	
12	24	CARVGYCSGPTCYPLDYW	IGH	2	None	
12	25	CAKDQYVGYGDPYYYGMDVW	IGH	2	None	
12	26	CARGGSVSDIVATISSFDYW	IGH	2	None	
12	27	CASQLLWFGGGTNYQFDYW	IGH	4	None	
12	28	CATGPVSGYQNWFDPW	IGH	2	None	
12	29	CAREREADSAATGTRYFDFW	IGH	6	None	
12	30	CAKLADYYDSGSYYNAFDYW	IGH	4	None	
12	31	CAREFPYEILTAHKGRGWFDPW	IGH	4	None	
12	32	CARDLADRIAAAGTSNYYYYGMDVW	IGH	21	None	
12	33	CARDTGGSGWYESWFDPW	IGH	12	None	
12	34	CARVRGHAYNYDSFDIW	IGH	4	None	
12	35	CVADVPGGSYPFDYW	IGH	5	None	
12	36	CARHGWVYYDSSHYFDYW	IGH	3	None	
12	37	CTRTSRLWDFDSW	IGH	22	None	
12	38	CAKGGASGFDIW	IGH	7	None	
12	39	CDWSSDVCSSDLDTAVFYCAKGPLGVMVVGASNWYFDLW	IGH	2	None	
12	40	CARGRFSENGGYYPFYFDYW	IGH	8	None	
12	41	CQQYYMYYNF	IGK	2	None	
12	42	CARVSYGSGTPTPGPFDYW	IGH	4	None	

12	43	CARDRVAAAGTDPYYYYGMDVW	IGH	14	None	
12	45	CAHTRGVSGREDYYFDHW	IGH	8	None	
12	46	CARHGKMTTVIPEFDYW	IGH	5	None	
12	47	CHQRSNWRNTF	IGK	2	None	
12	48	CARHSFGEYLFDYW	IGH	5	None	
12	49	CAREPKSHSYPPFDYW	IGH	2	None	
12	50	CAKGSIVGPTTTPSYFDYW	IGH	2	None	
12	51	CAKDFYYYGSGSYMTNWFDPW	IGH	12	None	
12	52	CARQAYCNSDCYSFDFW	IGH	2	None	
12	53	CALQREFDGGYSYYYALDVW	IGH	2	None	
12	54	CARQVGYCSGGSCSYDFDYW	IGH	2	None	
12	55	CARELAWMPVDYW	IGH	2	None	
12	56	CARRGGTFGGAIVETYFDYW	IGH	4	None	
12	57	CAKDWREHFGDYQDAFDMW	IGH	3	None	
12	58	CARVDLRCGGDCYVIDYW	IGH	6	None	
12	59	CARNLEVLDAFDIW	IGH	7	None	
12	60	CARDKYYDSSGYFGHTFDPW	IGH	2	None	
12	61	CTRRWELLYPYYFDYW	IGH	6	None	
12	62	CANTVGYCSGGSCFDYW	IGH	2	None	
12	63	CTTDPGSIITMIGWGYYYYGMDVW	IGH	2	None	
12	64	CTRDDYDSSGYNFDYW	IGH	4	None	
12	65	CAKEALPYYDILTGYLRPYYFDYW	IGH	27	None	
12	66	CARVSDGNSFPFDHW	IGH	26	None	
12	67	CARGTYFGELALDYW	IGH	3	None	
12	68	CARGPGYCNGDKCYAWFDSW	IGH	6	None	
12	69	CMQGSQWPSALTF	IGK	18	None	
12	70	CARVSQQLIKFPDYW	IGH	2	None	
12	71	CAHRRVYTPISAFDIW	IGH	2	None	
12	72	CARESPIGGLFGPDAFDIW	IGH	6	None	
12	73	CARDWARDRFYYFDQW	IGH	8	None	
12	74	CAAWDDVLSGRQVRF	IGL	6	None	
12	76	CAKERSSSTNYYYGMDVW	IGH	16	None	
12	77	CARDYDILTGYDAFDIW	IGH	16	None	
12	78	CAHRRATGPSRYFEYW	IGH	2	None	
12	79	CARDVDGATRVGATFDYW	IGH	3	None	
12	80	CAREGEATVTIYPPRIFDPW	IGH	2	None	
12	81	CARADGWEAAGNNWFDPW	IGH	2	None	
12	82	CARDRAVAGTSSGAFDIW	IGH	4	None	
12	83	CASGIVVVPAATRVTDAFDIW	IGH	2	None	
12	85	CARHGGMDTSGWSYFDFW	IGH	14	None	
12	86	CARDNRLLWFGELSDREVDWFDPW	IGH	5	None	
12	87	CAASENSGDYW	IGH	12	None	
12	88	CAKMLWLLRCFDYW	IGH	12	None	
12	89	CARDGSWAAAAPFDYW	IGH	2	None	
12	90	CARDKYDSSDTKWGVPDYW	IGH	3	None	
12	91	CAKEVVGFAYADYW	IGH	3	None	
12	92	CARVGQQQLVRPYYYYYGMDVW	IGH	3	None	
12	93	CQQYNNLSLSLTF	IGK	7	C3	6XDG
12	94	CTRDMVRDTYYYYYYGMDVW	IGH	2	None	
12	95	CARALPDQYNSSWYDDYFDSW	IGH	2	None	
12	96	CAHTRYSGYDQADYFDYW	IGH	2	None	
12	98	CATVFAAAGSRPPDYW	IGH	2	None	
12	99	CTRSSLRYFDWLFSNLAFDIW	IGH	5	None	
12	100	CARVLPSLSYDILTGYSFDYW	IGH	3	None	
12	101	CAKDLLLGDYGTFDYW	IGH	9	None	
12	102	CARQNSINYYGSGSPRRYWYFDLW	IGH	9	None	
12	103	CASNLYDILTDPSPFDPW	IGH	9	None	
12	104	CATISGYPVDYW	IGH	5	None	
12	105	CATARYSSANNWFDPW	IGH	2	None	
12	106	CAKVGGLPAAIVDYYYYGMDVW	IGH	2	None	
12	107	CAKDLDSALAAGAAFDIW	IGH	3	None	
12	108	CARDRKYSNANFLYW	IGH	2	None	
12	110	CATHSGYSYGEGFDYW	IGH	5	None	
12	111	CARASPGYGSGSYYMDYW	IGH	3	None	
12	112	CTSWDDSLNGLIF	IGL	2	None	
12	113	CARVGAVAFADYYYYYYGMDVW	IGH	4	None	
12	114	CAKQAIPTTRHSGWYDYW	IGH	2	None	
12	115	CLQDYNYPYSF	IGK	891	None	

12	116	CGAPMAGLYYYYYGMDVW	IGH	8	None	
12	117	CARTFSGSYPFVDAFDIW	IGH	3	None	
12	118	CAKSSYYDILRPNDYW	IGH	2	None	
12	119	CAKSPRRDRDIVAAVFDLW	IGH	2	None	
12	120	CQSSDSSNTFSFF	IGL	3	C4	7JVC
12	121	CARQGDGSSWHFDYW	IGH	10	None	
12	123	CARLYGDGYNWVGAAFDYW	IGH	2	None	
12	124	CTGLWFGELFYSPFDHW	IGH	4	None	
12	125	CARVSTSMWTSGWVFDLW	IGH	17	None	
12	126	CARELYGGNSGWFDPW	IGH	4	None	
12	127	CAKDEGGWYGVTYFDYW	IGH	9	None	
12	128	CARYSPDYGEGGNWFDPW	IGH	4	None	
12	129	CAKDYYGSGNNAYYYGMDVW	IGH	5	None	
12	130	CARELEGAINGDYW	IGH	5	None	
12	131	CAREGYCRFGGCYRTHWFDPW	IGH	2	None	
12	132	CAKRYGSGSYSGVPDYW	IGH	2	None	
12	133	CAKRMEGATFFYYYGMDVW	IGH	2	None	
12	134	CAKAVSYDSSAYYMLPQKFDYW	IGH	3	None	
12	135	CAKANLASPFDIW	IGH	2	None	
12	136	CATRIEVAGFDYW	IGH	33	None	
12	137	CARGTYNSSWYFDYW	IGH	2	None	
12	138	CAKDLRVTKFGGGYYFDYW	IGH	6	None	
12	140	CTTWTLDGYYQFDYW	IGH	2	None	
12	141	CSREFYYDSGGLRYDAFKIW	IGH	10	None	
12	142	CARGPVVVPAATTDYYYYYGMDVW	IGH	2	None	
12	143	CFSVADNNLRIF	IGL	6	None	
12	144	CASTNYYDSSGYSGAFDIW	IGH	2	None	
12	145	CARHGYYFDSSGYYEDAFDIW	IGH	4	None	
12	146	CARDSGYYGSGSYFRFDPW	IGH	4	None	
12	147	CAHRLGGTYSGAIFDYW	IGH	2	None	
12	148	CARTSWAVGATIGVGYSYYGMDVW	IGH	2	None	
12	149	CARDARGVDTATYYYYGMDVW	IGH	10	None	
12	150	CQSYATSNMKRVF	IGL	2	C4	7JVC
12	151	CAKDYKYGSSTSLYYYGMDVW	IGH	5	None	
12	152	CAHLVYYYDSSGYYYGPYDHFDYW	IGH	3	None	
12	153	CARDTIAVAGVDAYYYYGMDVW	IGH	3	None	
12	154	CARDLSSGTYDPTRRVWHFDLW	IGH	17	None	
12	156	CARDRFSAPTVVPAVISDYYYGMDVW	IGH	7	None	
12	157	CTATLLYGDNRLHDYW	IGH	8	None	
12	158	CARHSLVTMVRGVINWFDPW	IGH	5	None	
12	160	CATAPVLRSSNTWGHYYYYYGMDVW	IGH	2	None	
12	161	CARQIGLVGPYAFDIW	IGH	2	None	
12	162	CARDIGYCSSTSCQGHYYYYGMDVW	IGH	3	None	
12	164	CAREGGYDYGNAFDIW	IGH	27	None	
12	165	CAKTLSDVFYFDAFDVW	IGH	3	None	
12	166	CVQALQTPPATTF	IGK	24	None	
12	167	CARVSALGTWFDSW	IGH	6	None	
12	168	CVKNGGQWLGYFDFW	IGH	2	None	
12	169	CAREDDFDVSTTIDYW	IGH	10	None	
12	170	CAKDLSSWFGELWGYYYGMDVW	IGH	11	None	
12	171	CAKVGHTWIHLRFLDYW	IGH	2	None	
12	172	CARLLSEVRGAAFDSW	IGH	5	None	
12	173	CARGPLCSSTSCYDGGVYYYGMDVW	IGH	8	None	
12	174	CARGRPYITMVRPYLFNWFDPW	IGH	4	None	
12	175	CAKEDYAYGDYLDYW	IGH	4	None	
12	176	CARHALWFGEPRDYW	IGH	7	None	
12	177	CQQSFSPPPNSF	IGK	21	C2	7K8O
12	178	CAKDYYDSSGYLVFPPWYFDYW	IGH	4	None	
12	179	CAKDLPDFSGSGGYSGFDYW	IGH	4	None	
12	180	CAMTLIVGATFDYW	IGH	3	None	
12	181	CARSTYDILTGYSAPFDYW	IGH	119	None	
12	182	CAKGNYYDSTGYYYGNALDVW	IGH	9	None	
12	183	CAHRGQNGTYPYYFDHW	IGH	5	None	
12	184	CAKGMTTVTTAFDYW	IGH	6	None	
12	185	CARVAGDYWDWYFDLW	IGH	2	None	
12	186	CAKDLVETQLFEWFFDLW	IGH	2	None	
12	187	CARVRAVIGFYYFDLW	IGH	57	None	
12	188	CQQTYTTPHALGGGTTF	IGK	9	C4	6W41

12	189	CARDILDYYDTSGYNYFDCW	IGH	4	None	
12	190	CARVHVAGPDSSPCFDYW	IGH	7	None	
12	191	CAREPATSPDAFDIW	IGH	14	None	
12	192	CATHRGPGHSGGVGFDFW	IGH	4	None	
12	193	CATGLDSSGYYYKNYYGMDVW	IGH	4	None	
12	194	CAREAGVADFDLW	IGH	6	None	
12	195	CAIRLYLTTPYSSGWYWFDPW	IGH	2	None	
12	197	CGRLPKAMEVVLTVDYW	IGH	4	None	
12	198	CAHRRCDCSSTSCYCPFDYW	IGH	3	None	
12	199	CAKADCSSTSCHNQFDPW	IGH	20	None	
12	200	CAKDIAMDPGDDAFDIW	IGH	5	None	
12	201	CARDRIHYYDSSYYYYGMDVW	IGH	9	None	
12	202	CSGGSCYGDYYYYGMDVW	IGH	4	None	
12	203	CARLGGLVISRGYFDYW	IGH	2	None	
12	204	CARDHIPMVRGIVMRLDYW	IGH	8	None	
12	205	CAKDSLSRRGTVTPVDYW	IGH	2	None	
12	206	CTTDAYSHAEVFDIW	IGH	2	None	
12	207	CARDLGSGWYRDFGAFDIW	IGH	2	None	
12	208	CAREGDLDGDYDKNDAFDLW	IGH	3	None	
12	209	CARGIQEGGTGLYYYGMDVW	IGH	5	None	
12	210	CARGGHKYSSAWSLIDYW	IGH	3	None	
12	211	CARGTNSGSTWRIRDNYFDYW	IGH	4	None	
12	213	CASQRNTGEMDYW	IGH	7	None	
12	214	CARDFYDSSGYTSGPGYYYYGMDVW	IGH	16	None	
12	215	CTTDRAVVGATEFDYW	IGH	2	None	
12	216	CAKESDYDSSGYIDYW	IGH	2	None	
12	217	CARDLTSFGELTGGANYYYGMDVW	IGH	3	None	
12	218	CAKVSLRYCSSTSCFTDWSYYFDYW	IGH	4	None	
12	219	CAHRRDLGWFGDYFDYW	IGH	3	None	
12	220	CARISVDYPSGSYWFDPW	IGH	2	None	
12	221	CARDRGYNIWTGYYRGFDNW	IGH	2	None	
12	222	CARDSFSAGGYDSDDYYYYYGMDVW	IGH	2	None	
12	223	CARDGYYDSSGYFDYW	IGH	52	None	
12	224	CATSSGSGWPHHAFDIW	IGH	3	None	
12	225	CARVGYDYVWGSPVNPGYFDYW	IGH	8	None	
12	226	CTWTGYRSGWYYFDYW	IGH	2	None	
12	227	CQQSYSSRNF	IGK	78	C4	6ZDH
12	228	CAKDITVSDSSGYYYYYGMDVW	IGH	4	None	
12	229	CARGSALQNDGAFDIW	IGH	2	None	
12	230	CAFIVVPQTYRRGFDSW	IGH	6	None	
12	232	CARSLESFGYDYW	IGH	2	None	
12	233	CAKDSLIAVAGTSEAINYYYYGMDVW	IGH	28	None	
12	234	CARDSGYDLGTYYYYGMDVW	IGH	10	None	
12	235	CAKGLGYCSGGSCYSDEFDYW	IGH	19	None	
12	236	CARPGCFGASWFCLDAFDIW	IGH	4	None	
12	237	CQQYDSSSSMYSF	IGK	17	C3	6WPS
12	238	CARLVRYCSGGKCSGSGYFDPW	IGH	2	None	
12	239	CARALWRDGTMIPSRGRYYYGMDVW	IGH	2	None	
12	240	CAKASPSLMQRYCSGGSCYSNSFDYW	IGH	2	None	
12	241	CARDKDYGSGKYWFDPW	IGH	9	None	
12	242	CARSWAITFGGVIMPFDSW	IGH	3	None	
12	243	CARLGFYDSGRYVPNVFDTW	IGH	3	None	
12	244	CARHHVLETSSPNWFDPW	IGH	2	None	
12	245	CALPICWPFRERRNSFDIW	IGH	11	None	
12	246	CAREPPSVQQLVYFDYW	IGH	5	None	
12	247	CATGSPHYVVRENWFDPW	IGH	6	None	
12	248	CTPDRGSGWYGGDYW	IGH	2	None	
12	249	CAKVRRELGYHYYYFDYW	IGH	19	None	
12	250	CARTPRYCGGDCLDYW	IGH	2	None	
12	251	CATSPYLLENWFDPW	IGH	3	None	
12	252	CARHLVGAPIDYW	IGH	26	None	
12	253	CARDRLYFDTW	IGH	22	None	
12	254	CATCRGGSSCYADPFEIW	IGH	2	None	
12	255	CAKSAVGSGYLHPYFDSW	IGH	2	None	
12	256	CAKDAFIAAANRYYYYGMDVW	IGH	21	None	
12	258	CAKDYYDSSGSHYYYGMDVW	IGH	111	None	
12	259	CARGYIRITMVRGVIGKEYYFDYW	IGH	2	None	
12	260	CAREGVGGYGERWLDPW	IGH	24	None	

12	261	CAKSNDEYDSTEFDSW	IGH	2	None	
12	262	CARENTSMFYYFDYW	IGH	2	None	
12	263	CATDSPFTTRTTRGFDLW	IGH	2	None	
12	264	CAKDIHGSSSWYGYYYYGMDVW	IGH	2	None	
12	265	CARDRGYYDILTGYYLPSNDAFDIW	IGH	3	None	
12	267	CAKDLTGIVGATMFYYYYGMDVW	IGH	4	None	
12	268	CAKGPQPYYDILTGYYLLYYFDYW	IGH	2	None	
12	269	CARHDTPTLRWAALDYW	IGH	2	None	
12	271	CARTHIVVVTATNYYYYGMDVW	IGH	3	None	
12	273	CARRGSYYESDGFHFDYW	IGH	6	None	
12	274	CARDKWAGGNGGWYLDSW	IGH	2	None	
12	275	CARRGGSQSYYNTYSANYFDFW	IGH	3	None	
12	277	CARVVCSSTSCYRGDFDYW	IGH	2	None	
12	278	CAKDGEGEYGDYVGNWFDPW	IGH	16	None	
12	279	CANQMASYGSGSYYLYYYGMDVW	IGH	2	None	
12	280	CAHRPGLIGWPYFDYW	IGH	3	None	
12	281	CARSHGSDFSSSWYMRSIYAFDIW	IGH	2	None	
12	282	CAAPLGQLWTEGWYFDLW	IGH	5	None	
12	283	CAKLAYSSSWHQASLPDDYW	IGH	5	None	
12	284	CARDQVVVVPAAPGYYYGMDVW	IGH	9	None	
12	285	CSQYSRKTGSAFDIW	IGH	2	None	
12	286	CARFGVVTQTRPPAFDIW	IGH	6	None	
12	287	CAREDYYDSGGHSYYYYGMDVW	IGH	3	None	
12	289	CAREDYYYDSSGYPNYYYYYYGMDVW	IGH	7	None	
12	290	CAREYYYGSGSYRPYW	IGH	2	None	
12	291	CARVVTAIHDEYFDYW	IGH	3	None	
12	292	CARGHYGDSISHFDLW	IGH	2	None	
12	293	CARVFGMIRGVIVPRLDYW	IGH	14	None	
12	294	CARDMSRGYSYGGDYYYYGMDVW	IGH	2	None	
12	295	CARQSAPDWTPPDYW	IGH	2	None	
12	296	CAKGSGDYWYFDLW	IGH	12	None	
12	297	CAKERALINWGLGNAFDIW	IGH	2	None	
12	298	CQQSYGAPSLSFGGGSITF	IGK	2	C2	7K8O
12	299	CARDSMIVVDHNKRYYYYYGMDVW	IGH	2	None	
12	300	CARDIGLLGGGNYYYYGMDVW	IGH	3	None	
12	301	CARENDYPYRFFDYW	IGH	3	None	
12	302	CARDPGYCSGGSCYRDDYW	IGH	11	None	
12	303	CQYFNDYPPPAF	IGK	5	C1	7BZ5
12	304	CARTQSNQKWLDSRKKSGDWFDPW	IGH	2	None	
12	305	CARVIYDSTGSPFDYW	IGH	11	None	
12	306	CAKDRVPGKAQRLYYFDYW	IGH	2	None	
12	307	CAKDTMVRGEGYYYYGMDVW	IGH	6	None	
12	308	CAREDRTTDNYYYGMDVW	IGH	6	None	
12	309	CQQTNSLPPKGFTF	IGK	2	C1	7BZ5
12	310	CAREYSWGDGGFDSW	IGH	4	None	
12	311	CAKDVCSGGSCYEGAFDYW	IGH	2	None	
12	312	CARVFPTYYDILTGYSTDYW	IGH	3	None	
12	313	CARDQVVPAAIALSYYYGMDVW	IGH	5	None	
12	314	CARVMASSSSGWWYYYYGMDVW	IGH	2	None	
12	315	CARDLGRWPQLETYYYYGMDVW	IGH	2	None	
12	316	CARGTSGYDYRGFFDSW	IGH	6	None	
12	317	CASCHGVTYAFDIW	IGH	4	None	
12	318	CAGSGGDYAPSEDFQHW	IGH	2	None	
12	319	CARGGRGYNFANDYW	IGH	3	None	
12	320	CAKDKSGYYGSGSRFDYW	IGH	2	None	
12	321	CARDSTHNWNDRLYYYYGMDVW	IGH	19	None	
12	322	CARDPGSGYGSNSYGAFDIW	IGH	2	None	
12	323	CAREDSSGWFYWFDPW	IGH	31	None	
12	324	CARQSCSRTTCYASFDYW	IGH	5	None	
12	325	CARARILTGYYDWFDPW	IGH	17	None	
12	326	CARSAAAYGDYGDFDDYW	IGH	2	None	
12	327	CAKGGYCSSTSCSKGGPAFDIW	IGH	4	None	
12	328	CASSTHYYDSSGSTLALAFDIW	IGH	4	None	
12	329	CARDRSPSGRYYPDAFDIW	IGH	5	None	
12	330	CARDQGLNAPITMIVVRSGAFDIW	IGH	2	None	
12	331	CANLPLPGYSSGSDDYW	IGH	2	None	
12	332	CAIDNKNDGEPDAFDVW	IGH	4	None	
12	333	CARGGVAVAGSDSPEYFQHW	IGH	4	None	

12	334	CARESGEVPAAIANAFDIW	IGH	2	None	
12	335	CVTLSLDVAALHRDQNAFDVW	IGH	2	None	
12	337	CARDSRVDNGVYAFFDDW	IGH	2	None	
12	338	CARNGPDLRPLDYW	IGH	2	None	
12	340	CARDIYGVAGTRDYYYYGMDVW	IGH	5	None	
12	341	CARDLVARENTAYYYGMDVW	IGH	4	None	
12	342	CSSRDSSPNHAVVF	IGL	4	None	
12	343	CARGWSDGYSYGFDYW	IGH	3	None	
12	344	CASSSGYYYRWFDPW	IGH	24	None	
12	345	CARALWGTMVRGVTYDGPYYYYGMDVW	IGH	3	None	
12	346	CAKDYEAYTSGWYPAFDVW	IGH	5	None	
12	347	CARDTTSSTSPGVYYYYGMDVW	IGH	2	None	
12	348	CTTEEKWELKNWFDPW	IGH	2	None	
12	349	CTRETDCSGSSCYPYYYLGMDVW	IGH	3	None	
12	350	CAKQPQVWFPSRFDYW	IGH	4	None	
12	351	CATQPRGVAGSRFPFEYW	IGH	9	None	
12	353	CARDLGGYDSGYYYGMDVW	IGH	20	None	
12	354	CARVSGYDSLDFDYW	IGH	15	None	
12	355	CARESWYGDYEVGWFDPW	IGH	6	None	
12	356	CARDRLLDYGDYYYTGMDVW	IGH	2	None	
12	357	CVYRQRGDWFDPW	IGH	5	None	
12	358	CARHQGYSGYDSDYNWFDPW	IGH	3	None	
12	359	CARGADVSYSGYDEFDYW	IGH	2	None	
12	360	CARDPNGDYSFNDAFDIW	IGH	2	None	
12	361	CAREGGAGTTRIDAFDIW	IGH	2	None	
12	363	CAKVSSLTPGRYSSSWSDYW	IGH	2	None	
12	364	CTTLDQIVVVITGYYYGMDVW	IGH	5	None	
12	365	CARIFPVTSLYYYYGMDVW	IGH	4	None	
12	366	CARHHYDSSGYYYIFGYYFDYW	IGH	3	None	
12	367	CARDLHASGTYSTVFDYW	IGH	8	None	
12	368	CAREGVVVVITVYYYGMDVW	IGH	6	None	
12	371	CAREPSYSSSWYVGFDYW	IGH	3	None	
12	372	CAKNEGVFDILTGPFDSW	IGH	27	None	
12	374	CARDNYFGAGNHFGWIDSW	IGH	5	None	
12	376	CATVFAWFGESSRFDYW	IGH	3	None	
12	377	CQYYGASPLFAF	IGK	14	C1	7K8N
12	378	CARERWEEYSSGWYVYFDYW	IGH	4	None	
12	379	CARVDGDYVSREDRAYNWFDPW	IGH	2	None	
12	380	CARDSRVLYGDQPPQYYYYYYGMDVW	IGH	2	None	
12	381	CARDYSFGYSSGWDIDYW	IGH	2	None	
12	382	CARRVYYEESYALDIW	IGH	2	None	
12	383	CARIDIRGDWLDPW	IGH	5	None	
12	384	CARHDFTHFYASGGPQTFDIW	IGH	2	None	
12	385	CARVGVTMIVVVIGDAFDIW	IGH	3	None	
12	386	CYQYGNSPPYIF	IGK	19	C1	7K8N
12	387	CARPQSGGDWFDPW	IGH	2	None	
12	388	CAREGEGFGELLSRAFDIW	IGH	2	None	
12	389	CIIGRYSYSYGSYW	IGH	2	None	
12	390	CATDWGSGSSYIVVFDLW	IGH	6	None	
12	391	CAHRSYDILAGTYNVFTGGGFDYW	IGH	2	None	
12	392	CQSADSSGTWVF	IGL	18940	None	
12	393	CARVLTPGFYYDSSSYPLDYW	IGH	2	None	
12	394	CQQRFNWHEYSF	IGK	35	None	
12	395	CARGGWLRFSGHPQFTYFDYW	IGH	10	None	
12	396	CTCISYWDYW	IGH	2	None	
12	397	CARFPIVVITTGFDYW	IGH	6	None	
12	398	CARLSTYYYDSSGQFWRQYYFDYW	IGH	2	None	
12	399	CARTHYYDSSGQDLDYW	IGH	2	None	
12	400	CARDGEDYGGDYYYYGMDVW	IGH	110	None	
12	401	CAKEARPEVEYYFDYW	IGH	2	None	
12	402	CAKDRQWFGEIYDAFNVW	IGH	5	None	
12	403	CARDKYSGSWLEDYYFDYW	IGH	4	None	
12	404	CARRMSVTAPHPFDYW	IGH	13	None	
12	405	CAKDYDILNALSHYYGFDVW	IGH	2	None	
12	406	CAKDRRSGSNEGIGPDYW	IGH	2	None	
12	407	CARDRVTMVRGVIPDYYYYGMDVW	IGH	2	None	
12	409	CAREFSYYFDGDGSDAFDIW	IGH	5	None	
12	410	CARDIPLGVDGEKFDYW	IGH	2	None	

12	411	CTRSFGGNSLGAGQQFDSW	IGH	12	None	
12	412	CARDMYSSGWYDAYYYYGMDVW	IGH	2	None	
12	413	CARDGYCSGPSCYGMGDYW	IGH	6	None	
12	414	CAKEIRVDDIVATNPFDYW	IGH	112	None	
12	415	CARQPPSPSFDFLTGYYDAFPIW	IGH	2	None	
12	416	CAKLWSRGFGELFSDYW	IGH	2	None	
12	417	CASSLAVKQYYFDYW	IGH	2	None	
12	418	CATHARGVLGTTNGAFDMW	IGH	28	None	
12	419	CARRTASISVTYYYGMDVW	IGH	3	None	
12	420	CSTLAAAGNGIDYW	IGH	2	None	
12	421	CVRDPQVVGGSNFDYW	IGH	15	None	
12	422	CARGGYGDYEMDWYFDLW	IGH	2	None	
12	423	CARDYGDYVKGYYYGMDVW	IGH	59	None	
12	424	CAREPSRTTWTLVSSSWNHWYFDLW	IGH	2	None	
12	425	CARDGDGGNSGYYYNGMDVW	IGH	3	None	
12	426	CARGGYGDYLDAFDIW	IGH	7	None	
12	427	CARDFFAVVDIVATMPYYYYYGMDVW	IGH	6	None	
12	428	CARDQVVGATESHYYYYGMDVW	IGH	28	None	
12	429	CAKDGGTHEYSYGLDYW	IGH	2	None	
12	430	CAKDMARYGDYEGLVDYW	IGH	2	None	
12	431	CARDPTIRYSYGLSGTDYFDYW	IGH	2	None	
12	432	CARETWDGDWYFDLW	IGH	3	None	
12	433	CARHNTDSSSWYAYYFDYW	IGH	6	None	
12	434	CARPRAAGPDQPNYFDYW	IGH	2	None	
12	435	CARDSSRVTNYYDSSGYYYDAFDIW	IGH	4	None	
12	436	CARDARYYYDSSGYLVWFDPW	IGH	4	None	
12	437	CAREIVATSVDYW	IGH	9	None	
12	438	CAKDHLSYYDSSGYYFDYW	IGH	4	None	
12	439	CARDIRPDSGYDIDYW	IGH	12	None	
12	440	CARGGYSYGLVTGFDYW	IGH	3	None	
12	441	CARDSMGYCSSTSCHDYYYGMDVW	IGH	3	None	
12	442	CARDVIKYYGSYYGMDVW	IGH	2	None	
12	443	CARDMLILTGYGPDAFDIW	IGH	3	None	
12	444	CARDFKGYYGSGSYYEDGYYYYYGMDVW	IGH	3	None	
12	445	CARDLASYPFDYW	IGH	8	None	
12	446	CARDLIVVVPAAIDSQGYYYGMDVW	IGH	6	None	
12	447	CARSSRLDGVAGTRWFDPW	IGH	4	None	
12	448	CARDINPYCRGDCPTDYW	IGH	2	None	
12	449	CASHRSRIAVAGTFDYW	IGH	33	None	
12	450	CARDGDSSSWQSSLLDYW	IGH	15	None	
12	451	CQQYENFPVAF	IGK	14	C3	6XDG
12	452	CAKDESSDYYGPGRYDYRFDYW	IGH	2	None	
12	453	CAKDDTPILTGYTFDYW	IGH	2	None	
12	455	CTTDRTGSSWYLAGYYFDYW	IGH	2	None	
12	456	CAKSYGGSYYYFDYW	IGH	7	None	
12	457	CASWSLCERSRTPCLGPTFDDYW	IGH	2	None	
12	458	CALPISDTAMYFCARYRPRSGITGYYFDYW	IGH	2	None	
12	460	CARVKYSSSGGSNYFDSW	IGH	2	None	
12	461	CARPQAEWDLLLPFDIW	IGH	5	None	
12	462	CAREGAWYYYDSSGYYSFDYW	IGH	12	None	
12	463	CAKASGGYSSSWWSWWFDPW	IGH	2	None	
12	464	CARVNFPAWNSGWSRDALHIW	IGH	2	None	
12	465	CASSMGNSGYFDAFDIW	IGH	7	None	
12	466	CAKGTDWYYYYGMDVW	IGH	2	None	
12	467	CDWSSDVCSSDLTAVYYCAREGVGGYGERWLDPW	IGH	2	None	
12	468	CARGPPLAATDPLLDYW	IGH	7	None	
12	469	CAKVGGEGDYVWGSYRSYYFDYW	IGH	2	None	
12	471	CAREVYGSSGPLDYW	IGH	14	None	
12	472	CAREATVTTSWFDPW	IGH	6	None	
12	473	CARDGYYYDSSAHKNWYFDLW	IGH	10	None	
12	474	CARSAGSSGYYHFDQW	IGH	8	None	
12	475	CARQVVRGVIKPGPSQNWFDPW	IGH	3	None	
12	476	CARGDGAVTPALLQRVVRGYYFDHW	IGH	2	None	
12	477	CARDHPDPELLWFGEWYFDYW	IGH	3	None	
12	478	CARDPHSSGWYVVGYFDYW	IGH	2	None	
12	479	CASVPAASTTTYYYYGMDVW	IGH	5	None	
12	480	CSKDPHHYYSSPPVDYW	IGH	5	None	
12	481	CARERTGPEAPGDGMDVW	IGH	5	None	

12	483	CAREWTSGWYLFDYW	IGH	5	None	
12	484	CARDVGSGWYQIYFDYW	IGH	2	None	
12	485	CARGKTAIVLYYFDYW	IGH	3	None	
12	486	CARHSHLWFGESEQYYFDYW	IGH	2	None	
12	487	CARGIMTVGPDGYASRGSRTYQDLDYW	IGH	2	None	
12	489	CARVIKGGYRFGPIDYW	IGH	2	None	
12	490	CAKLVLRGVIRDYW	IGH	7	None	
12	491	CARAGDSYDSSSYYPSFDYW	IGH	4	None	
12	492	CARRSEIHNDFDYW	IGH	8	None	
12	493	CARSYYGSGSYTNWFGPW	IGH	5	None	
12	494	CAHRRIILTGPPGFDYW	IGH	2	None	
12	495	CARHIPLVWGGVPAAMPIDGLDVW	IGH	2	None	
12	496	CARDKMIRAGTTDYW	IGH	5	None	
12	497	CARVGAGRKKDGFDIW	IGH	2	None	
12	498	CVPNYCSSTSCYDYW	IGH	11	None	
12	499	CARDMGNEGSSYVYYYYGMDVW	IGH	3	None	
12	500	CARANPLEGIWGGWPFRERRNSFDIW	IGH	1114	None	
12	501	CARGAYIVVVPAATYYYYYGMDVW	IGH	3	None	
12	502	CARDALYCRASRCYSLGAFDIW	IGH	2	None	
12	503	CARRRGYCSGGSCYDYYYYGMDVW	IGH	6	None	
12	504	CATGSQLSGTFDWW	IGH	2	None	
12	506	CAKDPLLNGYFPKYYFDDW	IGH	3	None	
12	508	CLQFNNWPPGTPF	IGK	6	C1	7BZ5
12	510	CARLHCGTTSCFVRDNFFDPW	IGH	2	None	
12	511	CARSTVTTSVPLINYYYGMDVW	IGH	5	None	
12	512	CARRATYYYDSSGYYPTNEWFDPW	IGH	2	None	
12	513	CARGSQEGAVAGSGPPPYYYYGMDVW	IGH	2	None	
12	514	CARDEDPWIKNWFDPW	IGH	4	None	
12	515	CAKDGEAVYSYGYPDYW	IGH	2	None	
12	516	CARDASVAYCGGDCYWFLDYW	IGH	2	None	
12	517	CAKDGLHSTNTYYYYYGMDVW	IGH	2	None	
12	518	CARDDFFDTLTYYYYGMDVW	IGH	3	None	
12	519	CAKATDGYGSGSYYYYW	IGH	4	None	
12	520	CAHGQTKVKLASSWYWFFDLW	IGH	2	None	
12	523	CARDLWYYDSSGYYKSGEYFQHW	IGH	3	None	
12	524	CAKARYGSWSYEGNYFEHW	IGH	5	None	
12	525	CARDLLVGATHPASYYYYGMDVW	IGH	6	None	
12	526	CARRAIEVAGTGLTGFDPW	IGH	4	None	
12	527	CARAPIRYYYDKEARYYFDYW	IGH	4	None	
12	528	CARDSDPIVVVPAAEGYLDYW	IGH	3	None	
12	529	CTKGGTSAYEPEGRYYFDSW	IGH	2	None	
12	530	CAKDSVRGVGWFDPW	IGH	4	None	
12	531	CARVQDSGSYFDYW	IGH	7	None	
12	532	CARPSQTSSFYVGPFDYW	IGH	2	None	
12	533	CATEGPSYSASGSHSSKYLYYGMDVW	IGH	8	None	
12	534	CAREPPNSEGRYFDLW	IGH	2	None	
12	535	CAGHWRGASYVFDIW	IGH	4	None	
12	536	CAKDRYGDYAFDCW	IGH	24	None	
12	537	CAKDGLPDTAMEYYFDYW	IGH	6	None	
12	538	CAKDWSGIVGAPGAAFDIW	IGH	6	None	
12	540	CARTGYFDWHYWFDPW	IGH	2	None	
12	541	CQQTYRTPMYSF	IGK	668	C2	7K8O
12	542	CATESGATNAFDIW	IGH	5	None	
12	543	CARVVELWLGQDGYFDYW	IGH	5	None	
12	544	CARVQTSGWYRDYFDYW	IGH	4	None	
12	545	CAKEQDNYYDTGFTVDYW	IGH	2	None	
12	546	CARLRVEERYNWDDGYDYW	IGH	2	None	
12	549	CAREIPVGSGWYNYYYGMDVW	IGH	4	None	
12	550	CMDGTYWPKPF	IGK	2	None	
12	552	CTTDETEWFGELLGDYFDYW	IGH	2	None	
12	554	CARDRIAVAGTMTDYYYYGMDVW	IGH	13	None	
12	555	CATFLPTNGDAFDNW	IGH	2	None	
12	556	CARAISNSRGYRFDSW	IGH	6	None	
12	557	CAMPVDYYDSGAYRAPFDYW	IGH	2	None	
12	558	CARDNRGYYDSSGYADDAFDIW	IGH	9	None	
12	559	CASLEGGYCSGGSCYFDYW	IGH	14	None	
12	560	CARDRGVYDRSIYYYRGGGFDYW	IGH	2	None	
12	561	CAKDNGNYYGSGNLHAFDIW	IGH	8	None	

12	562	CARDGTTVPNGGYYYYGMDVW	IGH	4	None	
12	563	CQQSYSTLGGITF	IGK	164	C2	7K8O
12	565	CARPRLDYHDSSSYYYGYYYYYGMDVW	IGH	14	None	
12	566	CARGGLLWFGEHFYFDHW	IGH	3	None	
12	567	CARENGYALTATHRYYYGMDVW	IGH	6	None	
12	568	CQSADSSYPFDYVF	IGL	3	None	
12	570	CARVTTDYGGIFDIW	IGH	8	None	
12	571	CARRKVIPGGFGELSPPFDYW	IGH	2	None	
12	572	CANAPGYFYGYNDDYW	IGH	24	None	
12	573	CARHVEQLVHSGFDSW	IGH	2	None	
12	574	CARDQVVPAAMPSYSDGMDVW	IGH	4	None	
12	575	CARDRDYGGNYHLDYW	IGH	9	None	
12	576	CGRRWLQVSEVDYW	IGH	2	None	
12	578	CAIITTAAGERSIDYW	IGH	2	None	
12	579	CASHRQGRGYCTGTSCYMAPDRFDPW	IGH	2	None	
12	580	CARDIHVPLGDSSGYSTETYYYYGMDVW	IGH	52	None	
12	581	CGKSFFAGGRHYIDYW	IGH	8	None	
12	582	CAKDWWGTPAGDYYYYYGMDVW	IGH	6	None	
12	583	CAAWDDSLSGWVF	IGL	1599	None	
12	584	CVRHSYDSSSYFSFDYW	IGH	2	None	
12	585	CMQGINLQTF	IGK	702	None	
12	587	CATELGNGDYGFDAFDIW	IGH	14	None	
12	588	CASRHWGSGGLSFYFRNW	IGH	4	None	
12	589	CARDWTLRGSYYDYW	IGH	6	None	
12	590	CARDQDQWPDYW	IGH	2	None	
12	591	CARTYYDFLWGSYRYFDFW	IGH	2	None	
12	593	CQQSNSTRIYTF	IGK	2	None	
12	594	CAKDLRSAWFGDFVPQAPDYW	IGH	2	None	
12	595	CARSFSNSWFTWFDPW	IGH	7	None	
12	596	CASQYYYDSSGYFRPNWYFDLW	IGH	3	None	
12	597	CAKPGGPYYDSSGYYFGYW	IGH	2	None	
12	598	CVAPRWLQFDYW	IGH	2	None	
12	599	CAKDHLALNGEYYYYGMDVW	IGH	20	None	
12	600	CQSADSNGSSLF	IGL	24	None	
12	602	CARSVLGYGDYSDYFDYW	IGH	2	None	
12	603	CARGSPYNWNDAGGYYYGMDVW	IGH	21	None	
12	604	CYSTDSSGNHRVF	IGL	1468	None	
12	605	CARDIVHYDILTGYYFAVYSDAFDIW	IGH	2	None	
12	606	CARDMVATTAGASYYYYGMDVW	IGH	2	None	
12	607	CARERGSSASDIYYYYGMDVW	IGH	8	None	
12	608	CARDRGTKNWYLDYW	IGH	3	None	
12	609	CARESGGSWSVFDIW	IGH	5	None	
12	610	CARDLATPLTSPDYYYYYGMDVW	IGH	5	None	
12	611	CARDPREWLRISSGGYFDYW	IGH	2	None	
12	612	CARDFAYSYGYYGYW	IGH	2	None	
12	613	CARLSGAWSFDLW	IGH	7	None	
12	614	CARGYPRRPRVDGVSPIKTNHWFDPW	IGH	4	None	
12	615	CQSADGSGAFALF	IGL	61	None	
12	616	CARDSTSNIVVVVAADWFDPW	IGH	7	None	
12	617	CARALSDGTASHWDPNYFFDLW	IGH	2	None	
12	619	CAKVGYDDIWVDAFDIW	IGH	2	None	
12	620	CAKDMGPQPYGSVLNYYYYGMDVW	IGH	2	None	
12	621	CASSQLLWFGEFVYW	IGH	2	None	
12	622	CATEEYQLLSQAVYYYYYGMDVW	IGH	3	None	
12	623	CARDYRIAVADPLDYW	IGH	3	None	
12	624	CAGSTPHHYYYYGMDVW	IGH	8	None	
12	625	CARDPETLTSYSSSWFDPW	IGH	2	None	
12	626	CARTDSSGWFFFDNW	IGH	2	None	
12	627	CAKDAYIAPRDVCSSTSCPGSYYYGMDVW	IGH	5	None	
12	628	CQSTDTSGTNVLF	IGL	2	None	
12	629	CAKLPRLSSGGQHCGRDCFDHW	IGH	2	None	
12	630	CAKGTVTLVPLWFDPW	IGH	4	None	
12	631	CARDGYVGSGSFDPTVDSYYYYGMDVW	IGH	2	None	
12	632	CAKHGYFSDSSGYYYDYYYGMDVW	IGH	4	None	
12	633	CAKVSYLSTASSLDYW	IGH	6	None	
12	634	CAHKGLYWYFDVW	IGH	3	None	
12	635	CQKYNSAPLDCCTF	IGK	2	C3	7K8P
12	636	CARGRGYSYGYRTDAFDIW	IGH	6	None	

12	638	CARFLRGYSGYDPDDYFDYW	IGH	2	None	
12	639	CARGGYSSGWYGHNNWFDPW	IGH	7	None	
12	640	CARDRGSYYRGARDYW	IGH	5	None	
12	641	CARGPPESSGPTDWFDPW	IGH	2	None	
12	642	CARDTTIVVVPAATQGEGYYYGMDVW	IGH	4	None	
12	644	CQHYNKWPSSF	IGK	118	C3	7K8R
12	645	CQQLSSYPPIAF	IGK	2	C1	7BZ5
12	646	CAKDLGSGAYGQYYFDYW	IGH	4	None	
12	647	CAKDVRYGDYEKAYYYYGMDVW	IGH	4	None	
12	648	CARDMKYYDTGSYLGVDTFDIW	IGH	6	None	
12	649	CAFDILTGYYGVYYFDYW	IGH	10	None	
12	650	CAREGYCSGGSCSHYYYYYAMDVW	IGH	3	None	
12	651	CARGVEAPVGYYYFFGMDVW	IGH	2	None	
12	652	CAAERMSLVRGIITPPSFDYW	IGH	3	None	
12	653	CTTDRWFGEFTDYW	IGH	2	None	
12	654	CAKDRSANDYGDDFDYW	IGH	39	None	
12	655	CAKSHSSGYYLIPSWSFDYW	IGH	2	None	
12	656	CGSLPDSRGYYYSYDMDVW	IGH	4	None	
12	657	CAKDVLMVRGVITNNYYYYGMDVW	IGH	2	None	
12	658	CYSYTSRSTLRLF	IGL	5	None	
12	659	CARDALNIAEDGTGVEGFDIW	IGH	4	None	
12	660	CAKAQRPTGTTSGFDYW	IGH	8	None	
12	661	CATLGRITMVRGAHSAIDYW	IGH	4	None	
12	663	CAKDWVEIGSSRFSDGMDVW	IGH	5	None	
12	664	CARGPLPQIWNDPFDYW	IGH	2	None	
12	665	CARGYSSRWSWDFFDYW	IGH	2	None	
12	666	CARDVHYYGSGISTHDYW	IGH	2	None	
12	667	CAKLFGPALTTEAFDIW	IGH	5	None	
12	668	CARGGIAAADPEFDIW	IGH	4	None	
12	669	CARDHPLLLSSFDYW	IGH	4	None	
12	670	CASQGGRGHEPLDYW	IGH	2	None	
12	671	CARDRGDLGELLHYYYYDMDVW	IGH	3	None	
12	672	CQQYYDFKSTF	IGK	4	None	
12	673	CARLTGSGWYAGGNNWFDPW	IGH	2	None	
12	674	CAKDRGDYYDSSGQGGYYFDYW	IGH	6	None	
12	675	CARHLRVYYYDSSGYSISKGYYYYGMDVW	IGH	2	None	
12	676	CARDSITMVRGGEYYYYYGMDVW	IGH	4	None	
12	677	CARDRYNYGYYFDYW	IGH	20	None	
12	678	CARDRSISSVTPEGEAYYYYGMDVW	IGH	4	None	
12	680	CMQSIQLPPCSF	IGK	2	None	
12	681	CQQFDSHSPHSF	IGK	8	None	
12	682	CARDGDSSGSQYYYYYGMDVW	IGH	61	None	
12	683	CARGPPSSSWYGRAGFDYW	IGH	2	None	
12	684	CAKLTVLEPANYDAFDMW	IGH	2	None	
12	685	CVRENSIPGGHYFDYW	IGH	4	None	
12	686	CTRDDRWFGELYSDYW	IGH	2	None	
12	687	CARVSGGNVGNYFDPW	IGH	4	None	
12	688	CARVRGSGWNVIFYFDVW	IGH	3	None	
12	689	CASDPDSSALSNDYW	IGH	2	None	
12	690	CVKDAFDYGDYTYFDYW	IGH	3	None	
12	691	CARDIIWFGESNYYYYGMDVW	IGH	6	None	
12	692	CARGDLLRGGNNWFHPW	IGH	6	None	
12	693	CARWFGELLPPDYW	IGH	39	None	
12	694	CAREDWLRWDRGYYYGMDVW	IGH	3	None	
12	695	CARHVYDILTGYSHAYFDYW	IGH	4	None	
12	696	CALPISAVYYCARDRIDSSGYYQYYGMDVW	IGH	3	None	
12	697	CVRGSHYYASSGPYYFDSW	IGH	2	None	
12	700	CARQSELSHWYFDLW	IGH	4	None	
12	701	CARGGWVTMVRGVIINNWFDPW	IGH	2	None	
12	702	CAKPNAIASPGFFDYW	IGH	2	None	
12	704	CARARESPFRGYSYGLHFDYW	IGH	2	None	
12	706	CARTYTLIAAADHFDYW	IGH	2	None	
12	707	CAKDVMITFGGVRSYYYYYGMDVW	IGH	2	None	
12	708	CAKDHPDYGGNSYYYYGMDVW	IGH	2	None	
12	709	CAKRLMVRGAASSIPNWYFDLW	IGH	2	None	
12	710	CAKDVVATILWGYYFDYW	IGH	4	None	
12	711	CATMFAYSRSHLYFDYW	IGH	3	None	
12	712	CARNYDILTGYYRPDAFDIW	IGH	2	None	

12	713	CARFPYDLDNGPRFDCW	IGH	17	None	
12	714	CARDGPLYSGYEAYYYYYGMDVW	IGH	2	None	
12	715	CAKDLRSLAEYYYYGMDVW	IGH	2	None	
12	716	CARGGWKNYDILTYNWFDPW	IGH	2	None	
12	717	CARGFPHYYIGSGHFYYFDYW	IGH	8	None	
12	719	CARDRENCSSTSCSSYYYYGMDVW	IGH	6	None	
12	720	CARVRHWFDPW	IGH	35	None	
12	721	CARDLAAAGTALSPSYYYYGMDVW	IGH	7	None	
12	722	CARDVLYPNYLTSPTKTVYYYYGMDVW	IGH	2	None	
12	723	CLQRNTYPRYSF	IGK	23	C1	7BZ5
12	725	CARLDPGRLHYGSGSYYNEYYFDYW	IGH	2	None	
12	728	CAKDKTGYSSGWSKDAFDIW	IGH	2	None	
12	729	CARETLVPAANYYYYGMDVW	IGH	3	None	
12	730	CARENDYGDYAGALDIW	IGH	2	None	
12	731	CARHTEIATFFDSW	IGH	6	None	
12	732	CSVRGFTYSHFDYW	IGH	5	None	
12	733	CAKDLMTTKYGWSTLRVFDVW	IGH	4	None	
12	735	CTKTYLSGTVNWFGPW	IGH	2	None	
12	736	CARDTKNRGRFDYW	IGH	7	None	
12	737	CDWSSDVCSSDLYYYGMDVW	IGH	6	None	
12	738	CARQRFVDRSGFDPW	IGH	3	None	
12	740	CARDGSPFDVWFGELLSPYYYYYGMDVW	IGH	4	None	
12	741	CARVTMVRGVMQHYWFDPW	IGH	2	None	
12	742	CARGHYSGSSPYYYYYGMDVW	IGH	2	None	
12	743	CARVFETLGLVDYW	IGH	5	None	
12	744	CARQGDTVAPFDYW	IGH	9	None	
12	745	CAREQQLVPNWFDPW	IGH	12	None	
12	746	CAKDRLRYSSGSGYNWFDPW	IGH	2	None	
12	747	CASDLEVGATHCFGYW	IGH	4	None	
12	748	CARGEGEGDSPYYYGMDVW	IGH	51	None	
12	749	CARDTEQVGDYLYYYGMDVW	IGH	2	None	
12	750	CAKDYVVVTHYYYYGMDVW	IGH	2	None	
12	751	CAKDGDSGGNLAHFDSW	IGH	2	None	
12	752	CAKDDSSSWPTLFDPW	IGH	2	None	
12	753	CARISGWSFLFDYW	IGH	8	None	
12	754	CAKGASIAVAGLVWGVDYW	IGH	4	None	
12	755	CTRAAPSSTRYWYFDLW	IGH	2	None	
12	757	CARGERDCSNGRCSGDWHFDLW	IGH	3	None	
12	758	CNSRDNSGHHLAVF	IGL	7	None	
12	760	CAKGIGRAVAGTQFDSW	IGH	41	None	
12	761	CAKDWVTTGQNYYYYGMDVW	IGH	7	None	
12	763	CQQADNFPPIIF	IGK	20	C3	6XDG
12	764	CAKDIGPYDYVWGSWYYFDYW	IGH	2	None	
12	765	CAREMADTVLDFDYW	IGH	5	None	
12	766	CARDPTPAAGGYFDYW	IGH	2	None	
12	767	CACSYYYDNSGYWDGMDVW	IGH	2	None	
12	768	CAKVRGSFGDLNFDYW	IGH	2	None	
12	769	CARGQYYFDSTGFPQQYHRPDLPQFQYW	IGH	2	None	
12	770	CARDRLFGSYGYFSDFDYW	IGH	2	None	
12	771	CARDELGELARIPFDSW	IGH	2	None	
12	772	CAKDMGCSGGSCPFDYW	IGH	2	None	
12	773	CARVPHYYDSSGPRGGIDYW	IGH	2	None	
12	774	CARAGLGGNLYYYYGMDVW	IGH	8	None	
12	775	CVKDWDTRSDYFDYW	IGH	2	None	
12	776	CAKDGGGGGSYGDAFDIW	IGH	2	None	
12	777	CARHSLRGGNPHDAFDIW	IGH	4	None	
12	778	CARDILYLASVAHYYYYGMDVW	IGH	3	None	
12	779	CARTVTYYYGSGSYFSYFDYW	IGH	3	None	
12	780	CARHKTIVADDAFDIW	IGH	2	None	
12	781	CAKFQRAHDYGDNFDYW	IGH	11	None	
12	782	CLQTFTTPWTF	IGK	14	None	
12	783	CARDSRIQLGLDYW	IGH	2	None	
12	784	CARDLLAYGVNSSYYYYGMDVW	IGH	7	None	
12	785	CMQGLHLPPDMF	IGK	9	None	
12	786	CVRDDDTNSQYSRFDFW	IGH	2	None	
12	787	CARDGQGVSSYYYYYGMDVW	IGH	2	None	
12	788	CARDLAYYDSSGSQTRAYYYYGMDVW	IGH	4	None	
12	789	CAKGLGYNPFYYYFGMDVW	IGH	6	None	

12	790	CARGVVATISADELWSYFDYW	IGH	2	None	
12	791	CATSLAVAPRNSRWYYYYGMDVW	IGH	6	None	
12	792	CARPSSGSYSSWFDPW	IGH	94	None	
12	793	CARDYDIVVVPAAMGFDPW	IGH	12	None	
12	794	CALATPYYDILTGYYQKYYFDYW	IGH	2	None	
12	795	CAKDHYSYGEIYYYYGMDVW	IGH	11	None	
12	796	CARRPLEYSALYYFDFW	IGH	2	None	
12	797	CARGSAVGYSYGYRAFDIW	IGH	7	None	
12	798	CAHRTGSSWYLAFDIW	IGH	2	None	
12	799	CAREYWTQPPKTMVRGVIPPAYYYYGMDVW	IGH	2	None	
12	800	CARSVWGGSGREWFDPW	IGH	2	None	
12	801	CARTRGTGPYGHGDYW	IGH	2	None	
12	802	CARRSVSWYNFDYW	IGH	10	None	
12	803	CARTPRPKLRIQLWLRVDYFDYW	IGH	2	None	
12	804	CARSYYYGSGSPERFDYW	IGH	2	None	
12	805	CARDGGTMVRGVTWFDPW	IGH	2	None	
12	806	CARELAAAGNPTYYYYGMDVW	IGH	2	None	
12	807	CARVFRDAYTLFDYW	IGH	8	None	
12	808	CARGARDTAMDPGSAAPLHYYYYGMDVW	IGH	4	None	
12	809	CARHRYYYGSGSDYKENWFDTW	IGH	3	None	
12	810	CARDGLRYDILTGYYPLYYYYGMDVW	IGH	4	None	
12	811	CARDAGKGVTPYYYYGMDVW	IGH	4	None	
12	812	CARVRSSGWYGPEFDPW	IGH	3	None	
12	813	CAKRPKGYSYDTSGYYYFDYW	IGH	8	None	
12	814	CAKDPPYSRFDYW	IGH	2	None	
12	815	CAKSYSSNWPGYFDSW	IGH	2	None	
12	816	CAKGPWDPREPKFDNW	IGH	4	None	
12	817	CAKEDYVDNYFDSW	IGH	2	None	
12	818	CARRRSSGYSPFDYW	IGH	2	None	
12	819	CAKPPNGYYGPANAFDSW	IGH	2	None	
12	820	CARDHPDNDYGDYAGYW	IGH	4	None	
12	821	CAKDVISSGWYFDYW	IGH	53	None	
12	822	CARSPQGRGVIEAFDIW	IGH	8	None	
12	824	CARDVTPSAAGSYYYYGMDVW	IGH	4	None	
12	825	CARLGSGSYPQLDYW	IGH	8	None	
12	826	CAKESHPESRLLRRRAAAGRGWLDYW	IGH	3	None	
12	827	CALPISATYYCARANPLEGVWGGWPFRERRNSFDIW	IGH	5	None	
12	828	CARHLREKDSNRFDPW	IGH	4	None	
12	829	CARGWSYTADYW	IGH	2	None	
12	830	CARDGDRVRDGSYFDLW	IGH	3	None	
12	831	CARVMVVASSPYYYFGMDVW	IGH	8	None	
12	832	CARERNADGSESYFTDAFDVW	IGH	4	None	
12	833	CARDLRVAGSFDVW	IGH	3	None	
12	834	CAKGLLEVVTALDYW	IGH	3	None	
12	835	CVRSRTFYGSRADYW	IGH	11	None	
12	836	CARTGGLWFGEFRFDYW	IGH	2	None	
12	837	CAREFLDSSSWFNGFDYW	IGH	2	None	
12	838	CQQDYNLPPRSYSF	IGK	2	None	
12	839	CTRALESGGGWLQPTYYYYGMDVW	IGH	2	None	
12	840	CLRAEDTALYYCASSLAVKQYYFDYW	IGH	2	None	
12	841	CQQYNSYLGTWTF	IGK	32	C3	7K8R
12	842	CARSKWLRGHFDYW	IGH	3	None	
12	843	CAKDLTTVVTPDGTYYYYYGMDVW	IGH	5	None	
12	844	CAILFRGTTVAGSRRFGFDPW	IGH	20	None	
12	845	CARILGTGYDSDFDYW	IGH	2	None	
12	846	CARDADPNVDIVATITTWFDPW	IGH	2	None	
12	847	CARILAVAFDASDIW	IGH	6	None	
12	848	CARLSTTSVRNVLMVGGYFDYW	IGH	8	None	
12	849	CAREADSGSSWYGGWFDPW	IGH	4	None	
12	851	CVHRQAEFEDAFDIW	IGH	4	None	
12	852	CARGEGALVRGEIANWLDPW	IGH	6	None	
12	853	CARDSVEMATTNYYYGMDVW	IGH	6	None	
12	854	CARALSRWELSDAFDMW	IGH	2	None	
12	855	CARDSIAAAGGENYYYGMDVW	IGH	8	None	
12	856	CARGWVGTHWVDPW	IGH	2	None	
12	857	CARSYCSSTSCYGYWYFDLW	IGH	10	None	
12	858	CATGPVVGATISWFDPW	IGH	7	None	
12	859	CAIVGVDTTTRGGFDYW	IGH	2	None	

12	860	CARKHISSWHPDYW	IGH	12	None	
12	861	CARGGYGDYSIDYW	IGH	9	None	
12	862	CAREAGYSYVGDYW	IGH	22	None	
12	863	CQTWATGIQVF	IGL	326	None	
12	864	CAKDIVATIDAFDIW	IGH	10	None	
12	865	CARSWNLFSGSKFDYW	IGH	2	None	
12	866	CAREDSSSWSTPVDYW	IGH	10	None	
12	867	CVRDRRGYSDGSYYYYGMDVW	IGH	6	None	
12	868	CARGDIIEDDYGDDPGAFDIW	IGH	2	None	
12	869	CAKGGSSWSFNFDYW	IGH	2	None	
12	870	CARAYYGDYVGTAFDIW	IGH	2	None	
12	871	CHRPRRADSSGDYRDDAFDIW	IGH	7	None	
12	872	CSRLDYGPPYYFDLW	IGH	2	None	
12	873	CAKRAYLTGGGSFDIW	IGH	2	None	
12	874	CARVLGSSGQLIYTDAFDIW	IGH	3	None	
12	875	CTRLDATLHQFDPW	IGH	3	None	
12	876	CAKGIAQGTRGISFDVW	IGH	2	None	
12	877	CMHALQTSITFVTF	IGK	2	None	
12	878	CAKEKGSYNWNDWYYYYYGMDVW	IGH	3	None	
12	879	CASSLIAVADEFDYW	IGH	6	None	
12	880	CARGYYYDSSGPIGGVYYFDYW	IGH	16	None	
12	881	CATDLNGSGSYLRHYYYYYGMDVW	IGH	6	None	
12	882	CAKTYYYDSSGYRHYYYGMDVW	IGH	5	None	
12	883	CAKDPVGATRGPNWFDPW	IGH	2	None	
12	884	CAKVRVEMSTIWDYYFDYW	IGH	2	None	
12	885	CARVFVWDIVVVAPLPGYDYFDYW	IGH	2	None	
12	886	CAHKLYTSSWLLFDYW	IGH	5	None	
12	887	CARDKLQLPLDDAFDIW	IGH	4	None	
12	888	CATASYDYVLLGWGRDFDYW	IGH	2	None	
12	889	CARGITMVREAYYYYGMDVW	IGH	6	None	
12	890	CARHRDYGGNKNWYFDLW	IGH	3	None	
12	891	CAPLLVGTTEDYW	IGH	7	None	
12	892	CAHIVSSAYYSYFHHW	IGH	6	None	
12	893	CAHRLLLWANNWFDPW	IGH	5	None	
12	894	CVREGRNGYYSDYW	IGH	2	None	
12	895	CAKDSLAMTTVTTYFDYW	IGH	3	None	
12	896	CAREGGYGSGGSCYSRWYFDLW	IGH	3	None	
12	897	CAREWGGYSYADDAFDIW	IGH	5	None	
12	898	CARGVGHASGFQGFDSW	IGH	3	None	
12	899	CARWDTAMGNSGFDYW	IGH	7	None	
12	900	CARLGVWGSHRYLVYW	IGH	4	None	
12	901	CARTLSGYAYYYYGMDVW	IGH	4	None	
12	902	CARWGSGAAAGTEYRDYW	IGH	2	None	
12	903	CAHRSSSWSGGGFDYW	IGH	4	None	
12	904	CARDQNTAFDIW	IGH	12	None	
12	905	CARESPGYYCSGGSCHGNWFDPW	IGH	2	None	
12	906	CARLSGPTTSDAFDIW	IGH	20	None	
12	908	CAKDSRIAVAGTITYYYYGMDVW	IGH	4	None	
12	909	CAREFLTATGKGPFDYW	IGH	2	None	
12	910	CAKALRAAGTGIGYDYW	IGH	2	None	
12	911	CARGLDAFDVW	IGH	266	None	
12	912	CARGPCSGWYFPTYYYGMDVW	IGH	2	None	
12	913	CAGLRVTTLEFDPW	IGH	2	None	
12	914	CAKDFLPDIVTTSGVDYW	IGH	8	None	
12	915	CARWWGDDYVWGSYRYFDYW	IGH	3	None	
12	916	CAKSRDIGYDLNYFDYW	IGH	2	None	
12	917	CAKDIGGSGTYYTFDNW	IGH	2	None	
12	918	CALEGGVDAEYFQYW	IGH	2	None	
12	919	CARQPGPLASRYTSGWLDSW	IGH	2	None	
12	920	CQSYDSSTHVIF	IGL	16	C4	7JVC
12	921	CARKPSYSSGWYRFDWYFDLW	IGH	2	None	
12	922	CASGIGMIAKLGFDYW	IGH	123	None	
12	923	CARAGQQLVLSDYW	IGH	4	None	
12	924	CAREFGSGSYYNETFSFYYYYGMDVW	IGH	2	None	
12	926	CAKDFSTYYYDSIDYW	IGH	7	None	
12	927	CARDLPLYYYDDSAYFDYW	IGH	2	None	
12	928	CAKGSRAWYGGPDYW	IGH	4	None	
12	929	CARAAPDYYGSGSYSYPPDYW	IGH	2	None	

12	930	CARRGDATGGFDNW	IGH	5	None	
12	931	CARSGFTSNYDILTGYYAYYFDYW	IGH	2	None	
12	932	CARDQISTWNDEVHYYYGMDVW	IGH	2	None	
12	933	CARDIWAVAVAGTRDDYYYYGMDVW	IGH	3	None	
12	935	CARDSRVVLTGRYYYGMDVW	IGH	5	None	
12	936	CARGPLDGYNFNFDYW	IGH	11	None	
12	937	CAKDWQVVVAATTPGSYYYYGMDVW	IGH	3	None	
12	938	CARTYYDILTGLTSLFDYW	IGH	6	None	
12	939	CARTVWEAAAAPTPYYFDYW	IGH	2	None	
12	940	CVREGSLSSYYYAMDVW	IGH	12	None	
12	943	CARPNSSGFPGIYWYFDLW	IGH	2	None	
12	944	CARDLLSGCCGYGFDYW	IGH	5	None	
12	945	CARDLYEQQLVGAYYYYGMDVW	IGH	8	None	
12	946	CARDDSSGYQDPPYYFDYW	IGH	10	None	
12	949	CARDYGSGGYYVGFDYW	IGH	3	None	
12	950	CAKELWSGTDTPNYYFDYW	IGH	2	None	
12	951	CAKDWGGNWNDGGYYYYGMDVW	IGH	2	None	
12	952	CATGPDISLSFDYW	IGH	2	None	
12	953	CARETCFVGYSSSCPADYW	IGH	2	None	
12	954	CAKEGGLAAGGNHFDYW	IGH	2	None	
12	955	CARPEGGYSTSWVDYW	IGH	3	None	
12	956	CARLDLLIYYYGMDVW	IGH	2	None	
12	957	CAKSGRVAATLYFDYW	IGH	2	None	
12	958	CAKDWYGSGSSQPYYYGMDVW	IGH	65	None	
12	959	CAATTVTTFQYFQHW	IGH	2	None	
12	960	CARQGEYYYGSGAFDYW	IGH	2	None	
12	961	CARETPDTSGSWVDYW	IGH	6	None	
12	962	CATVRYCSRTSCEAGADWFSPW	IGH	9	None	
12	963	CQQSYSGIFWWTF	IGK	8	C4	6ZDH
12	964	CARHAKEMATPLAAFDIW	IGH	2	None	
12	965	CAHRSYGGNGGYYFDYW	IGH	33	None	
12	966	CATGMIAVAGTIVLVPYYFDYW	IGH	3	None	
12	967	CARGPRGGYGHFDYW	IGH	3	None	
12	968	CAKEYSYGPSLIGYYYYGMDVW	IGH	3	None	
12	969	CARGGDIFQAVPATDSFDIW	IGH	8	None	
12	970	CARDNHPWTAYSSSWFDYW	IGH	2	None	
12	971	CARTRDYYGSDIYYFDYW	IGH	2	None	
12	972	CAKADFGYHYDSSGYYFDFW	IGH	4	None	
12	973	CARDSHFSGYDLGYYYYGMDVW	IGH	2	None	
12	974	CTTLSSSSIDYW	IGH	19	None	
12	975	CARDISSGYLDSW	IGH	20	None	
12	976	CARVREISVIRGIIIPSYFDYW	IGH	2	None	
12	977	CARDLHIVVVNTTYYYYYGMDVW	IGH	11	None	
12	978	CARDPVIAAAGSYWYFDLW	IGH	3	None	
12	979	CARVRTGNDYDYW	IGH	2	None	
12	980	CARDDTASAAAGTGYYYYGMDVW	IGH	2	None	
12	981	CARSSMREFPDLFSGEDYFDYW	IGH	2	None	
12	982	CAKSRSAVTTLDPLDYW	IGH	2	None	
12	983	CAKPLIWNEYYFHSW	IGH	8	None	
12	984	CARDYCGGDCYSGTTADAFDIW	IGH	4	None	
12	985	CAKDMYYYDSSGYYPPWFDPW	IGH	2	None	
12	986	CARDQLRYFDWLLQSDYYYYYGMDVW	IGH	2	None	
12	987	CAKDKFITVIGLFEYW	IGH	2	None	
12	988	CARVFGTMVRGVIIGYFDYW	IGH	6	None	
12	989	CSTWDNSLNALAF	IGL	3	None	
12	990	CARDPWGRGDYYYYGMDVW	IGH	13	None	
12	991	CARSAGRPNWFDPW	IGH	7	None	
12	992	CARAYFGGSYYYYYGMDVW	IGH	6	None	
12	993	CARHGPPSIYIGSYSAFDIW	IGH	9	None	
12	994	CARDATVTTSDYYYYYGMDVW	IGH	35	None	
12	995	CARASGVGWLQFEGFAYW	IGH	8	None	
12	996	CTTGDSGYDFKGFDYW	IGH	2	None	
12	998	CARDIFSRADNSAYFHYYGMDVW	IGH	7	None	
12	999	CARLTRQWFLDYW	IGH	2	None	
12	1001	CSSNTSGHAFDIW	IGH	2	None	
12	1003	CAKDRTQLWSPGFDYW	IGH	21	None	
12	1004	CARQGGWYIGIGLFDYW	IGH	2	None	
12	1005	CARQYASNWYSDYFDSW	IGH	7	None	

12	1006	CARHLGYGSGSYYKRIANFDYW	IGH	2	None	
12	1007	CGADHGSGSNFVYWVF	IGL	16	None	
12	1008	CARDIYGDSPNDYYYYYGMDVW	IGH	7	None	
12	1009	CARLNPLILGSGYYYGSVPKYYFDYW	IGH	2	None	
12	1010	CARLLLLAAFDIW	IGH	3	None	
12	1011	CAKDYKVVGANYYYYGMDVW	IGH	12	None	
12	1012	CATSTPIRGVIPWFDPW	IGH	4	None	
12	1014	CAAADVLLWFGEFDYW	IGH	7	None	
12	1015	CAKEDGDYGPDYYYYGMDVW	IGH	4	None	
12	1016	CARAGWGDGYKIHAFDIW	IGH	2	None	
12	1017	CARRLVATNVYYFDYW	IGH	3	None	
12	1018	CAGLYGDYSSPYYYGMDVW	IGH	6	None	
12	1020	CAKDAGYGDYLTPSMRPFDYW	IGH	2	None	
12	1022	CAKGGSYLGYW	IGH	2	None	
12	1023	CARLAWRSRAVGTAEGCFFDYW	IGH	2	None	
12	1024	CSFGSGSYYYYALDVW	IGH	4	None	
12	1025	CGRDRGYSGDNYGFDAFDVW	IGH	12	None	
12	1026	CAHRDWEVSQDAFDMW	IGH	2	None	
12	1027	CAKDFRDYGDTGGYYYYGMDVW	IGH	3	None	
12	1028	CAKDWGSYSGSQDYYYYYGMDVW	IGH	5	None	
12	1029	CYSASDSNESPWVF	IGL	11	None	
12	1030	CAKHRLSDNSGSYYSNFDYW	IGH	4	None	
12	1031	CARDPLAAAGSGGGVYYYTSAMDVW	IGH	2	None	
12	1032	CATVIYYGAEFDYW	IGH	6	None	
12	1033	CTTAATVTFGFDYW	IGH	6	None	
12	1034	CATVKRVATINNYFDYW	IGH	2	None	
12	1035	CAREGIAVGRPPDYW	IGH	2	None	
12	1036	CAREWYGDYGYRYFDLW	IGH	11	None	
12	1037	CAKDLGYYYDSSGYYYEDWFDPW	IGH	14	None	
12	1038	CARDRESFRWRPLDYW	IGH	2	None	
12	1039	CAKESSDYGDYEGSSYYYGMDVW	IGH	3	None	
12	1040	CARTRYSSSYYFDYW	IGH	50	None	
12	1041	CAKAYNSYFDSPFDYW	IGH	3	None	
12	1042	CTRDGHYSSGYSRYYFDYW	IGH	2	None	
12	1044	CARDPSSSWQTYYFDYW	IGH	14	None	
12	1045	CARASWYYDSSGTSEKTLSYYYYGMDVW	IGH	4	None	
12	1046	CAKTPVQRYFDWLFSSGFDYW	IGH	4	None	
12	1047	CAKDLSGWLTTGAFDYW	IGH	7	None	
12	1048	CARGNFGYSSSWYMLDYW	IGH	8	None	
12	1049	CQSADSTNSFPDWVF	IGL	4	None	
12	1050	CARTHLYCSGGSCYLPPFDYW	IGH	4	None	
12	1051	CATSAPIAVAGYGWFDPW	IGH	10	None	
12	1052	CARGYGGNSGVDYW	IGH	29	None	
12	1053	CAKDFKSMVRGITVFDYW	IGH	2	None	
12	1054	CARADYGAPYYYFDFW	IGH	11	None	
12	1055	CAKDSDVLLWFGEPFGDYFDYW	IGH	2	None	
12	1056	CAKEGKAYDYGDYFDYW	IGH	25	None	
12	1057	CATARFVEGTGSYLGYW	IGH	2	None	
12	1058	CAKVQSSGWHLDAFDIW	IGH	3	None	
12	1059	CARVLWQSGGWKLYYYYGMDVW	IGH	2	None	
12	1060	CAKGLYSSSRGGAFDIW	IGH	2	None	
12	1061	CAKDTQHCNGGSCHQNSLNNWFDPW	IGH	2	None	
12	1062	CARTPQYMIVAYVDYW	IGH	2	None	
12	1064	CARDMVAVADTKYYYGMDVW	IGH	13	None	
12	1065	CAREKNSSGWTSAFDIW	IGH	23	None	
12	1066	CLSADSSGTYPYVVF	IGL	4	None	
12	1067	CARLTGSGWSPTTDYW	IGH	5	None	
12	1068	CARVADCSGGSCYHLTNWFDPW	IGH	2	None	
12	1069	CTRARNFAFDYW	IGH	2	None	
12	1070	CAKDMRVTTVTYAGIYYYYGMDVW	IGH	8	None	
12	1071	CARENYGDGMDVW	IGH	13	None	
12	1072	CARDDEEDLGTPGFDYW	IGH	4	None	
12	1073	CARDARTGDGGTYYYYYGMDVW	IGH	2	None	
12	1074	CTRDVYSGYDHFDYW	IGH	2	None	
12	1075	CARQYSSGWYGYYFDYW	IGH	11	None	
12	1076	CARWDDFTAMHDYW	IGH	4	None	
12	1077	CATFPQFCSSTSCYEDYW	IGH	2	None	
12	1078	CARTRVLEYYFDYW	IGH	20	None	

12	1079	CARETHGWSYFDFW	IGH	3	None	
12	1080	CAKDSLAYCGGDCYSDSW	IGH	2	None	
12	1081	CARDSLVVPAASGLESMASYYYYGMDVW	IGH	3	None	
12	1082	CARGYSGYELRGYFDYW	IGH	2	None	
12	1084	CAREVAAAGTVSFDYW	IGH	7	None	
12	1085	CAKEVNYDSSGYLEPYFDYW	IGH	4	None	
12	1086	CYCSSTSCTTDQYYYYGMDVW	IGH	2	None	
12	1088	CAKFGHDYGDYFQGSLDYW	IGH	11	None	
12	1089	CARDRQLERRAFDLW	IGH	2	None	
12	1090	CTTDLTSDIVATIHYYYGMDVW	IGH	5	None	
12	1091	CAREFRGYDGSDYYFVLDYW	IGH	6	None	
12	1092	CARGYSYASGYFQHW	IGH	2	None	
12	1093	CALTDYYDSSGYRNWFDPW	IGH	3	None	
12	1094	CARTRDGDYQLDYW	IGH	4	None	
12	1095	CLQYYSTPPMYTF	IGK	26	C4	6W41
12	1096	CARGYYDSTGYYPYFRDW	IGH	4	None	
12	1097	CVRGSFYGSGSYYKPLDYW	IGH	5	None	
12	1098	CARASTYLDPMTGYYTVIDYW	IGH	4	None	
12	1100	CARDFFAAAGHSESYYYYGMDVW	IGH	4	None	
12	1102	CAKDYYISGDRDYYYYGMDVW	IGH	4	None	
12	1103	CARDRHDYVWGSYRSAFDYW	IGH	2	None	
12	1104	CAKDLSYQPPLNYFDPW	IGH	2	None	
12	1105	CAKAPTPYDFLTGYFDYW	IGH	5	None	
12	1106	CARDSKTAAGTNTYYYYGMDVW	IGH	8	None	
12	1107	CARDHLYALTRNYYYGMDVW	IGH	4	None	
12	1108	CAKDYDSSGSPTLDAFDIW	IGH	2	None	
12	1109	CARDRYCSGGSCYSGNYYYYGMDVW	IGH	10	None	
12	1112	CARDDRLGSGDGIGFDPW	IGH	6	None	
12	1114	CVKIKSHYYYGMDVW	IGH	6	None	
12	1115	CARQQRVSVVTAAWQGPGWHFDLW	IGH	2	None	
12	1116	CARDSYNYHDYW	IGH	20	None	
12	1117	CSRMYDYGSYSDYW	IGH	2	None	
12	1118	CARLGNIAVAGILNPFDIW	IGH	2	None	
12	1119	CARVTDSSGYSDDAFDIW	IGH	11	None	
12	1120	CATFEGPSPAAMLLGYSGYDFHYW	IGH	2	None	
12	1121	CASSGDYSSSWLDYW	IGH	14	None	
12	1123	CAKDALGLPTDRDVYHYYGMDVW	IGH	2	None	
12	1124	CARHTYWKFYYERSGYAIDYW	IGH	6	None	
12	1125	CASWDDSRSGPGVIF	IGL	2	None	
12	1126	CANIIPPIDYW	IGH	2	None	
12	1127	CARETSTQWFGELLTEYYFDYW	IGH	3	None	
12	1128	CARGSTYSSWYGVYWFDPW	IGH	3	None	
12	1129	CARPSSSGWYPDAFDIW	IGH	13	None	
12	1130	CARAFIVGATYAFDIW	IGH	4	None	
12	1131	CAKDKRHCTDAKCSFDSW	IGH	3	None	
12	1133	CARDSGTYYYDSSGPAPPDYW	IGH	17	None	
12	1134	CATIKFSRTCSGGRCYYHYFALDVW	IGH	2	None	
12	1135	CAHRRMGDYDILTGYPLRFDYW	IGH	6	None	
12	1136	CARVSGVEWFGELWWWFDPW	IGH	2	None	
12	1137	CAHRRPNWNGPTNAFDVW	IGH	6	None	
12	1138	CASCGGDCFHDAFDIW	IGH	13	None	
12	1139	CVRGERAGSYVGDYW	IGH	8	None	
12	1140	CARHGPYSSGWYELMYNWFDPW	IGH	2	None	
12	1141	CAKEDSGASYYRLPPFDLW	IGH	2	None	
12	1142	CVRQGTYDILSIGHREFDNW	IGH	31	None	
12	1143	CARVAAAGREINWFDPW	IGH	2	None	
12	1144	CARERYDILTGYYYGMDVW	IGH	17	None	
12	1145	CVRAYSGYDYGDAFEIW	IGH	39	None	
12	1146	CAGDGVGASPGDAFDMW	IGH	2	None	
12	1147	CARGTVVVVAANGTPYYYYYGMDVW	IGH	2	None	
12	1148	CARDILFVDTAYYYGMDVW	IGH	7	None	
12	1149	CAKKQWLAKLFDYW	IGH	3	None	
12	1150	CAKDYGSYSNGWPYYFDYW	IGH	2	None	
12	1151	CAKLGYSGTYTGGYFHSW	IGH	5	None	
12	1152	CAKEVYYGSGSPYYYGMDVW	IGH	2	None	
12	1153	CARDLYCSGGSCYSSGFDYW	IGH	117	None	
12	1154	CQQYDNSPLVYNF	IGK	7	C3	6XDG
12	1155	CARGTVTTPHGDYYYGMDVW	IGH	2	None	

12	1156	CAKDNFGGYSYVFDYW	IGH	2	None	
12	1157	CAKDLAVAGTRGWYFDLW	IGH	4	None	
12	1158	CAKDSGWYAGETYYFDYW	IGH	3	None	
12	1159	CARDGTGIWDYGDYEKNYFDYW	IGH	15	None	
12	1160	CALPILQMNSLRAEDTAVYYCAKDGLEMATILLDYW	IGH	3	None	
12	1162	CAKDRLVYGSGDGLFDHW	IGH	45	None	
12	1163	CARRNSGSGTDAFFDYW	IGH	3	None	
12	1164	CARDWGKGIAAAGTAYYYYGMDVW	IGH	2	None	
12	1165	CARIIAAAGSGWFDPW	IGH	11	None	
12	1166	CARGLGRYDSSGYYSFFDYW	IGH	8	None	
12	1167	CARAPSAPRLRKGYYFDYW	IGH	4	None	
12	1168	CARESYSSYYIGYW	IGH	3	None	
12	1169	CARIKGKDSDGSFDFW	IGH	2	None	
12	1170	CAHRQVAVAGSYFQHW	IGH	5	None	
12	1171	CAKAPRGSTWFGDHFDYW	IGH	8	None	
12	1173	CARGASGLRDFWYFDLW	IGH	2	None	
12	1174	CARLLTRDGVVAATEYFQHW	IGH	8	None	
12	1175	CAVMTIAAAGTALDYW	IGH	2	None	
12	1177	CARDRLTNINYYGSEVYYYYGMDVW	IGH	3	None	
12	1178	CVRGFTTREDYW	IGH	21	None	
12	1179	CARVMWQQLVTLDYW	IGH	3	None	
12	1180	CARVKLPWYGGGQFDYW	IGH	200	None	
12	1181	CARSYEWELPGDYW	IGH	4	None	
12	1182	CAREGRTNLGDSLDYW	IGH	2	None	
12	1183	CAKGSYALFFDYW	IGH	15	None	
12	1184	CARDPKYSSGWYNYYYGMDVW	IGH	2	None	
12	1186	CVRDQPMYYPGSGSSYFDYW	IGH	7	None	
12	1187	CAKGGDIVATIGWFDPW	IGH	6	None	
12	1188	CAHSRTSGWAYFDYW	IGH	2	None	
12	1189	CQQYYTSPGALAF	IGK	3	C4	7JW0
12	1190	CARDLGWYDDYW	IGH	123	None	
12	1191	CAREREARVAGFDSW	IGH	3	None	
12	1192	CARMVITMVRGAPPDYW	IGH	2	None	
12	1193	CARDIADNFAPLYYFDYW	IGH	4	None	
12	1194	CTRIEAHTSDWSRTFDIW	IGH	3	None	
12	1196	CAKIVLVPAEIRDFDYW	IGH	4	None	
12	1197	CARAPYCTGGNCYTYNWFDPW	IGH	4	None	
12	1199	CAITTAVTTVRWFDPW	IGH	8	None	
12	1200	CALDRGTTVTTLDYW	IGH	2	None	
12	1202	CAREATGYSYGLHFGYW	IGH	2	None	
12	1203	CARPRGRSPIAAAGTGFDYW	IGH	2	None	
12	1204	CARHMLTDYDVLTRYSRGTAFDLW	IGH	2	None	
12	1205	CARDEWSAAGINYYYGMDVW	IGH	4	None	
12	1206	CAAGRGSENYYYGMDVW	IGH	2	None	
12	1207	CARSGRGYNTAYYYYYYGMDVW	IGH	7	None	
12	1208	CAKALLLWDDAFDIW	IGH	2	None	
12	1209	CASMPRSTNSKFLVGVYYYYRMDVW	IGH	3	None	
12	1210	CVSTFRIADDRMSFGYW	IGH	3	None	
12	1211	CARGPRMVRGVITPTYFDYW	IGH	2	None	
12	1212	CVREIAAAPGTCSKERSLCNWFDPW	IGH	4	None	
12	1214	CAREFYDYYDSSGPFDYW	IGH	3	None	
12	1215	CAKEGIAVAGDWFDPW	IGH	2	None	
12	1216	CDWSSDVCSSDLTAVYFCAKGNYYDSTGYYYGNALDVW	IGH	2	None	
12	1217	CARAQDYGGNESGFDYW	IGH	2	None	
12	1218	CAKDILGDDYNYGLDYW	IGH	4	None	
12	1219	CARTLWQRHYYDSSGRSNYYYYGMDVW	IGH	20	None	
12	1220	CARHGDSSVWYPLDYW	IGH	2	None	
12	1221	CARDINEETSGYFDYW	IGH	5	None	
12	1222	CARRGRGYSGTVLYYFDYW	IGH	7	None	
12	1223	CARRSGGPFDWLAYYFDYW	IGH	3	None	
12	1224	CAGDLGDGFVYW	IGH	24	None	
12	1225	CARPLDYGDSDAFDIW	IGH	14	None	
12	1226	CARYDSSGYYGSGDYW	IGH	14	None	
12	1227	CAYIPTLHYDYVWGSYRSFDYW	IGH	4	None	
12	1228	CARDREWDSRGSFIPRYYFYGMDVW	IGH	3	None	
12	1229	CAKDSQIFDGTPYYYGMDVW	IGH	2	None	
12	1230	CAGWSYYDPELFDYW	IGH	2	None	
12	1231	CARMSHSGYDRGNFFDHW	IGH	2	None	

12	1233	CARSLLWFGDALDYW	IGH	10	None	
12	1234	CDWSSDVCSSDLANPLEGIWGGWPFRERRNSFDIW	IGH	13	None	
12	1235	CARAGEQQAYHYAMDVW	IGH	8	None	
12	1236	CMEVLQTPYSF	IGK	4	None	
12	1237	CARDYYGSGTYVGTPDDYW	IGH	2	None	
12	1238	CARDSYDSSGYSAKNYWYFDLW	IGH	2	None	
12	1239	CALPISAVYYCARGGWKKSADTAMVNWFDPW	IGH	2	None	
12	1240	CARRAYRYGSSYFDLW	IGH	4	None	
12	1241	CAKAPVLTMIVERYFDLW	IGH	3	None	
12	1242	CARDHFVTGIPEGDYYYYYGMDVW	IGH	2	None	
12	1243	CARDPSGYSYAAYYFDYW	IGH	2	None	
12	1246	CAREHSGSFGLYYYYGMDVW	IGH	6	None	
12	1247	CARVIAVAGNRGFDYW	IGH	3	None	
12	1248	CAKDSMDSGYEVEYYYGMDVW	IGH	2	None	
12	1249	CARGAYSGYDPTMSFDYW	IGH	17	None	
12	1250	CAREGAYGSGSGIDYW	IGH	4	None	
12	1251	CARENHYYDSSGPYSACFDYW	IGH	4	None	
12	1252	CAKGAGDQWLVPFDYW	IGH	2	None	
12	1253	CARDTRTAATTSYYYYGMDVW	IGH	9	None	
12	1254	CAQLHQQLDAEYFQHW	IGH	10	None	
12	1255	CARDRKAAAGIFGPPELDYW	IGH	2	None	
12	1256	CLRELSSSDIGQFDYW	IGH	8	None	
12	1257	CQQYYTSPPSDSF	IGK	480	C4	6W41
12	1258	CARDAMAVAGNLEHYYYYYGMDVW	IGH	2	None	
12	1259	CARERRGSYSGAFDYW	IGH	4	None	
12	1260	CARDRFGYSYGVGLDYW	IGH	2	None	
12	1261	CQQYGSSLEGGITF	IGK	2	C1	7K8N
12	1262	CAKEGLGYCSSTSCYAEKSLFDYW	IGH	9	None	
12	1263	CAKDRSPYSGYDPAGVFDYW	IGH	2	None	
12	1264	CARDHGDLYSSGHHGFDPW	IGH	2	None	
12	1266	CAKDHGVAAADFYYYYGMDVW	IGH	2	None	
12	1267	CARDSRVVEMATSQAYYYGMDVW	IGH	8	None	
12	1268	CARSIVATITGGYYYYGMDVW	IGH	3	None	
12	1269	CASSPLSMALGHGYNWFDPW	IGH	2	None	
12	1270	CARDRTAVVPPLFTYYYYGMDVW	IGH	4	None	
12	1271	CATGNDAFDIW	IGH	54	None	
12	1272	CMGYGGYSDAW	IGH	3	None	
12	1273	CAKAGVPFGSGSHYFDYW	IGH	2	None	
12	1274	CQPSYITPLTF	IGK	3	C4	7JW0
12	1275	CAKDVWGWAFDHW	IGH	53	None	
12	1276	CAKDGLEMATILLDYW	IGH	6	None	
12	1277	CARDAGYSRLTFAYW	IGH	5	None	
12	1278	CARDSPFYDILTGYYQKSYFDYW	IGH	2	None	
12	1279	CARGGWSKYVWGSYPTTWFDPW	IGH	5	None	
12	1280	CARGFSSWPVYLDYW	IGH	2	None	
12	1281	CARQRFGELTIGYYYYGMDVW	IGH	2	None	
12	1282	CARRCGGGCTSCEGVDAFDIW	IGH	4	None	
12	1283	CARGRYSSSWYGAATKFDYW	IGH	3	None	
12	1284	CAHFNKKGAEIPFDYW	IGH	2	None	
12	1285	CARSGTGSGWSVFHYW	IGH	2	None	
12	1286	CAKGWSGSANFWDYW	IGH	6	None	
12	1287	CARGFAGLERLIVDYW	IGH	2	None	
12	1288	CARGGSYCSSTSCYEAYYAMDVW	IGH	5	None	
12	1289	CAGDQGMATIDYYYYGMDVW	IGH	7	None	
12	1291	CARAPHSDLGGYYYYGMDVW	IGH	4	None	
12	1292	CAHTQGRVLAQVIVQLFDFW	IGH	3	None	
12	1294	CAQGGGSNSDYGVYFDNW	IGH	3	None	
12	1295	CATGARRRYDFYYMDVW	IGH	3	None	
12	1296	CVIAVAGTTRIDYW	IGH	4	None	
12	1297	CAKDYGYYGSGSNLYYYYYGMDVW	IGH	2	None	
12	1298	CARDMGIAAAGTDSGFEDYW	IGH	2	None	
12	1299	CARDSPIVGATGGGDYW	IGH	14	None	
12	1300	CARWGDGSGSNIRAFDIW	IGH	3	None	
12	1301	CARSIVVVPAENNPPDYYYGMDVW	IGH	13	None	
12	1302	CASTYCSSTSCYDYYYYYGMDVW	IGH	4	None	
12	1303	CATGQHHYYYYYGMDVW	IGH	13	None	
12	1304	CARDAYSIAAAVYYYGMDVW	IGH	10	None	
12	1305	CARDCTGGICYSDYW	IGH	2	None	

12	1306	CARPTMIVAHAAPADSLDYW	IGH	2	None	
12	1307	CARGHVEKLRYFDWSPRGVDYFDYW	IGH	2	None	
12	1308	CARHPYCSADNCHANRYYYYGMDVW	IGH	3	None	
12	1309	CARDLGRWLQFGSHYYYGMDVW	IGH	7	None	
12	1310	CARHGTPKRITMVRGAQFDYW	IGH	2	None	
12	1312	CAKDHPRGDSSGNYPSASDYW	IGH	2	None	
12	1313	CQQYNHWPSAYTF	IGK	203	C3	7K8R
12	1314	CARGGVSTSPFDSW	IGH	2	None	
12	1315	CARGGYCDGRRCYSGPRDALDMW	IGH	2	None	
12	1316	CAREGALGDAGAFDIW	IGH	35	None	
12	1317	CMQRIEFPLTF	IGK	87	None	
12	1318	CVRDPTGIQTGLLDYW	IGH	3	None	
12	1319	CARDLSRWMSFAGYSSRWGGANYFDYW	IGH	2	None	
12	1321	CARDDYGDNAGYDYW	IGH	3	None	
12	1322	CAHRLMAVADHDAFDIW	IGH	2	None	
12	1323	CARGQPKDIPTLWFFEYW	IGH	6	None	
12	1324	CATGVDTSMVLDYW	IGH	2	None	
12	1325	CTVARYCSRTDCHAGFDWFDPW	IGH	7	None	
12	1326	CARDPDTYYYDSSGYFEDDYW	IGH	2	None	
12	1327	CVKGLYASNWDVPPGDYW	IGH	9	None	
12	1329	CAREWWRRFRGGYDYYYGMDVW	IGH	2	None	
12	1330	CALPISPRHFDWLFLAAYHNWFDPW	IGH	2	None	
12	1331	CALPIYDSTGYYHYYFDYW	IGH	2	None	
12	1332	CASDQLAVDIPPFDYW	IGH	3	None	
12	1333	CATSDYYYDSYDYSDYW	IGH	12	None	
12	1334	CAKDNKWLVPGDAFDIW	IGH	10	None	
12	1335	CSGGDWTDSFDMW	IGH	2	None	
12	1336	CQVWISSSEHHVVF	IGL	39	None	
12	1337	CARGVGGSGSYYTGAWFDPW	IGH	3	None	
12	1338	CTRAEFQLRAFDYW	IGH	4	None	
12	1339	CAHDQVDYDYVWGSYRPYPFDYW	IGH	6	None	
12	1340	CATGRGGSYPNDYW	IGH	5	None	
12	1341	CARDSVETNEVNLYYYGMDVW	IGH	2	None	
12	1342	CARDVRRYQLLNSDAFDIW	IGH	7	None	
12	1343	CAKDPIAVSGTGYFDYW	IGH	13	None	
12	1344	CARRLTASYYFTYSFDFW	IGH	2	None	
12	1345	CARDSVNYGDVDYYYGMDVW	IGH	2	None	
12	1346	CAKVRLVTATLDAFDLW	IGH	2	None	
12	1347	CAKEGPSGEYNPDSFDIW	IGH	33	None	
12	1348	CVTTCSAGACYDKSKDAFDIW	IGH	6	None	
12	1349	CAHRLSGPTGPYFDYW	IGH	15	None	
12	1350	CAREGYDSSGYYYSVWNAFDIW	IGH	2	None	
12	1352	CAGTHFTIFSQAFFTGALDIW	IGH	2	None	
12	1353	CARDQAVYGDYGHYYGMDVW	IGH	5	None	
12	1354	CAKGEVTVHTGVCDYW	IGH	2	None	
12	1355	CARRRLAASPYYFDYW	IGH	11	None	
12	1357	CARHKVATITIDYW	IGH	2	None	
12	1358	CARGGWCSGGSCPNDRSKFYYYYGMDVW	IGH	2	None	
12	1359	CARVVSMVRGVIIFVRKPNWFDPW	IGH	4	None	
12	1360	CASYHYYDSSGYFGPLEYYFDYW	IGH	6	None	
12	1361	CATDGGGSSGPLAPSFFDNW	IGH	4	None	
12	1362	CQSGDSSASSPRVTF	IGL	12	None	
12	1363	CTTGSGSYYKFSYW	IGH	5	None	
12	1365	CARDFMITFGGVIVKGPFDYW	IGH	2	None	
12	1366	CARHGWGYYYYNYALDVW	IGH	7	None	
12	1367	CARDGRYFDWLIIPPTPTTPDYW	IGH	8	None	
12	1368	CAKAMVRGVTLLLDYFDYW	IGH	4	None	
12	1369	CARIRFFVGHDILTGQIVDAFDIW	IGH	9	None	
12	1370	CARDTWSGQFENYYYYYGMDVW	IGH	2	None	
12	1371	CARMRGVNYYDSSGYYHYFDYW	IGH	8	None	
12	1372	CVKSFDNSGWYPDYW	IGH	4	None	
12	1373	CAKGLEVRGIFAYDAFDIW	IGH	6	None	
12	1374	CARVGGTRYCDIW	IGH	2	None	
12	1375	CARAGYSSSWYVPYYFDYW	IGH	10	None	
12	1376	CAKDERGLTYWYLDLW	IGH	4	None	
12	1377	CTRDPCQQQWLRYSDYW	IGH	2	None	
12	1378	CTTAGRGSGSYYNYYAGMDVW	IGH	148	None	
12	1379	CARVSPGYCSGNICYFNTEYFDYW	IGH	2	None	

12	1380	CARHRTGYSSGWDLIYFDYW	IGH	5	None	
12	1381	CARGGTPTTGDNHFDYW	IGH	4	None	
12	1382	CARDLFRSGGSDYYYYYGMDVW	IGH	7	None	
12	1383	CARVLPDYYGSGTHYYYYGMDVW	IGH	3	None	
12	1384	CATASSGWYWDFW	IGH	4	None	
12	1385	CAHIEDRAKHYYYYYGMDVW	IGH	3	None	
12	1386	CAAEPRVSGYVKIYNYYGMDVW	IGH	2	None	
12	1387	CARDDTCSGGDCYYFDYW	IGH	31	None	
12	1388	CAKDALPGENRHFDFW	IGH	4	None	
12	1389	CARDSWGMTHELYYYGMDVW	IGH	4	None	
12	1390	CARAMTYYYGSGSYYRVHDYAFDIW	IGH	4	None	
12	1393	CAKEDGYHDAFDIW	IGH	2	None	
12	1395	CAKVLGYCRGGNCPQETYYYHGMDVW	IGH	40	None	
12	1396	CARSGWGGSGSYYNPNWFDPW	IGH	2	None	
12	1397	CAREYEGSKAYFFDYW	IGH	34	None	
12	1398	CQHYSSYSTWAF	IGK	18	None	
12	1399	CARDFTSIAAAGTLNYYYYGMDVW	IGH	2	None	
12	1400	CASLPLLWFGEWGYFDYW	IGH	3	None	
12	1401	CARGSRSGQGYYYYGMDVW	IGH	7	None	
12	1402	CTRPTPLQTGVTWGGSFDYW	IGH	3	None	
12	1404	CLTSDRKSVRPEADYW	IGH	3	None	
12	1405	CAREFSYGLRLADYYYGMDVW	IGH	4	None	
12	1406	CARDLIVVVPAANATYYYYYGMDVW	IGH	2	None	
12	1408	CARGRGYYDSAGSFFGARESDYNWYFDLW	IGH	4	None	
12	1409	CARHGLGRFTGAGGIDYW	IGH	2	None	
12	1411	CARVYPGCSGGSCYHPGDYW	IGH	6	None	
12	1412	CAKVSSPWLVQNAFDIW	IGH	2	None	
12	1413	CARTDYCPGGICRSHIEFYFDHW	IGH	3	None	
12	1414	CAKDHGSSYGSGSSAQDHYYYYYGMDVW	IGH	3	None	
12	1415	CAEKAAGTRAFDIW	IGH	6	None	
12	1416	CASDTAAELWGGDSGIDYW	IGH	4	None	
12	1417	CQHLNSYLLTF	IGK	50	C1	7BZ5
12	1418	CARDPEVYYGSGTPDYW	IGH	2	None	
12	1419	CARHRERDLPLTWFDPW	IGH	3	None	
12	1420	CGTWDSSLSAGVF	IGL	444	None	
12	1421	CAKTGVRGVIISWFDPW	IGH	2	None	
12	1422	CARRDRHCSGDDCWRWFDPW	IGH	4	None	
12	1423	CARDLYYYDSGGNYQNNWFDPW	IGH	5	None	
12	1424	CARDPRSLSMVRGIFGYW	IGH	2	None	
12	1425	CARDLQQLADRYYYYGMDVW	IGH	4	None	
12	1426	CAHRRTFYGDLDYW	IGH	10	None	
12	1428	CAREMGEKWYYYESSGVDPSDYW	IGH	2	None	
12	1429	CARLRPCSGGSCYSSPPDYW	IGH	3	None	
12	1430	CAKDHGVYGDYYYYYGMDVW	IGH	11	None	
12	1431	CATAYAVAGRTNPDYW	IGH	8	None	
12	1432	CVKDDGCHTSGCPEGYFDYW	IGH	2	None	
12	1433	CAKGRIKFITDLNFDYW	IGH	4	None	
12	1434	CARDASIAAAGSVSWYYYGMDVW	IGH	4	None	
12	1435	CARAHPRGDGYSDDAFDIW	IGH	2	None	
12	1436	CARDTPAYCSSTSCYYYYYGMDVW	IGH	2	None	
12	1437	CVRGGSGYQSYLDYW	IGH	3	None	
12	1438	CAKNPVLTTLTDFDYW	IGH	2	None	
12	1439	CAKDQKFTYHYETSGYYFEYW	IGH	4	None	
12	1440	CARWAPAAGPQDAFDIW	IGH	2	None	
12	1441	CARDRARSEDLAAAMGVYYYYYGMDVW	IGH	2	None	
12	1442	CARDPVRSLWFGEMDYW	IGH	2	None	
12	1443	CARHGGKWDVQPFFDTW	IGH	12	None	
12	1445	CAKAYNYWDYW	IGH	2	None	
12	1446	CARRGDYGGRTHYSFDSW	IGH	7	None	
12	1447	CASGPYGGARFDYW	IGH	11	None	
12	1448	CARGMRLGVVPAAMGVPMGPNWFDPW	IGH	5	None	
12	1449	CATEPTYYYDSSGYYPQYFHHW	IGH	5	None	
12	1450	CARDSRGGIAVAGFDYW	IGH	4	None	
12	1451	CTTEGSSGWYEYFDYW	IGH	2	None	
12	1452	CARDPYCYGDCRQHGYFDHW	IGH	3	None	
12	1453	CARVFSPVKNLPGNVPLRGWFGMEYFSSW	IGH	2	None	
12	1454	CARDLAWTMVRGAADYW	IGH	2	None	
12	1455	CASAPPGGFPYYFDYW	IGH	3	None	

12	1456	CARVPGSGTYGYYYFGMDVW	IGH	2	None	
12	1457	CAKDHGGRWNWWGWFDPW	IGH	4	None	
12	1458	CAHGGIPVLVTKTLFYFDYW	IGH	2	None	
12	1459	CAAIPLNYYGSSGSWSYYFDYW	IGH	2	None	
12	1460	CARERYSGYDYVGLMAFDIW	IGH	2	None	
12	1461	CAKSPPERVDVWGSYRLYYFDYW	IGH	3	None	
12	1462	CATLPDASGGCYYFDYW	IGH	4	None	
12	1463	CARGGPVSTLYYYYYYGMDVW	IGH	3	None	
12	1464	CARVSYNAELLNTHFDYW	IGH	9	None	
12	1466	CARHAQNYHFGSGNDCFDPW	IGH	3	None	
12	1467	CAREDCGGDCYPYRENYFDYW	IGH	2	None	
12	1468	CASLPHYDILTGYSSYNWFDPW	IGH	9	None	
12	1469	CARSTVTTSPFDYW	IGH	26	None	
12	1470	CARDMWGGNSEEYYYYYGMDVW	IGH	2	None	
12	1471	CAHMTYYYGSGSYYTGYFDYW	IGH	2	None	
12	1472	CARGHDYGDLRYYYYYGVDVW	IGH	2	None	
12	1473	CARVEFWGSYYGGWFDFW	IGH	2	None	
12	1474	CASGRWDSTDSRSSSYYFYAMDVW	IGH	40	None	
12	1478	CARTYCGGDCYSYWFDPW	IGH	3	None	
12	1479	CARHLDILTGYYNYYFDYW	IGH	24	None	
12	1480	CARTRGTGYRSVWYFDSW	IGH	3	None	
12	1481	CAKDLGEYCSGGSCQYYYYGMDVW	IGH	2	None	
12	1483	CARDQKGDSSAGLDYW	IGH	2	None	
12	1484	CARDTEIYDSGGFEDFFDSW	IGH	2	None	
12	1485	CAKHGYGSTTYYKRGGFDYW	IGH	2	None	
12	1486	CAKGDCSSTSCYIPDYFDYW	IGH	4	None	
12	1487	CARHLPYGSGSYSPYNFDYW	IGH	4	None	
12	1488	CARQGVAGGHGDHYFDSW	IGH	2	None	
12	1489	CARGVAMIGRDSRFDYW	IGH	3	None	
12	1490	CAKDLSPVGETPYHYYGMDVW	IGH	50	None	
12	1491	CARGFKNIAAILDYW	IGH	4	None	
12	1492	CARRRWVSGSYQNFDCW	IGH	5	None	
12	1493	CARDLGYYDSSGYSQYYYYYGMDVW	IGH	9	None	
12	1494	CARDSVAAAENAYYYYGMDVW	IGH	21	None	
12	1495	CASSQSSYCGGDCPTPFDYW	IGH	2	None	
12	1496	CAKGNPDDSDGPYFSYYYGMDVW	IGH	3	None	
12	1497	CAIHRSDDYGDLNWFDPW	IGH	3	None	
12	1498	CASAKSGWLYFDNW	IGH	4	None	
12	1499	CQSADSDATYIF	IGL	4	None	
12	1500	CALQDNYYDSSGYTMGPFDYW	IGH	2	None	
12	1501	CARDGKSHVDTAMVHYYYYYGMDVW	IGH	21	None	
12	1502	CARDLVGYSSSSQDYYYYYGMDVW	IGH	6	None	
12	1503	CARVAIQLWCDYW	IGH	2	None	
12	1504	CARDRKHDYGDYGSWFDPW	IGH	6	None	
12	1505	CARDREESGFDYW	IGH	58	None	
12	1506	CALVYPVWGSYRPEYFQHW	IGH	2	None	
12	1507	CARDPSRGAENDYW	IGH	2	None	
12	1508	CARDRGGIWFGDANACDIW	IGH	2	None	
12	1509	CAHRPPPTDYYYYGMDVW	IGH	2	None	
12	1510	CAHRYFSGWYGDWFDPW	IGH	62	None	
12	1511	CAKDISALTTVMSPFDHW	IGH	2	None	
12	1512	CARDYYDSSGYYHYYFDYW	IGH	198	None	
12	1513	CARDRAWVDFENEILTGYDYW	IGH	4	None	
12	1514	CARERLPVSLSDALDVW	IGH	6	None	
12	1515	CARSLVAVAGIPFDYW	IGH	5	None	
12	1516	CATALIVGLWEFDYW	IGH	2	None	
12	1517	CARDQKTLAVAGTSGQYYYYGMDVW	IGH	41	None	
12	1518	CAKAGIVGATRFFDYW	IGH	2	None	
12	1519	CARHPRITMIVVVSLYYFDYW	IGH	18	None	
12	1520	CARHGGFVEDYGDYGGQEYFDYW	IGH	2	None	
12	1521	CARDGGSGSYPYYYLDLW	IGH	2	None	
12	1522	CTTDVLSVVVPAAMVPGERYYYYGMDVW	IGH	3	None	
12	1523	CARWTSNLYYFDSW	IGH	5	None	
12	1525	CAWGYGGNSVTLGWYFDYW	IGH	23	None	
12	1526	CARDMVVVPAATQVANNYYYYYGMDVW	IGH	7	None	
12	1527	CARDLHYDSSGWSILHSDYYYYGMDVW	IGH	6	None	
12	1528	CARPLDTAMAFDAFDIW	IGH	2	None	
12	1529	CATRLDYGGNWYFDLW	IGH	4	None	

12	1530	CATGGYYGSDYGSGSGWFDPW	IGH	4	None	
12	1531	CAKDRISLVTSGTFDPW	IGH	2	None	
12	1533	CAKDALIVGATTEPHYYYYGMDVW	IGH	2	None	
12	1534	CTRDIYGSTTLYYFDYW	IGH	3	None	
12	1535	CARTLSGYDSDDAFDIW	IGH	10	None	
12	1536	CASPRGFGYSSPIDYW	IGH	2	None	
12	1537	CAHRRADCNDGDCYRWFDPW	IGH	167	None	
12	1539	CAKDFLVRGYDYGEAAYYYGMDVW	IGH	3	None	
12	1540	CARIRGYDSSGRYFGAFDNW	IGH	4	None	
12	1541	CARDTQRSSGSYYYYGMDVW	IGH	11	None	
12	1542	CARGKWELLGQDAFDVW	IGH	88	None	
12	1543	CARDLRYRDTDGPDYW	IGH	12	None	
12	1544	CARDLTVTTDPSADYW	IGH	27	None	
12	1545	CARSTETYYYYYYGMDVW	IGH	4	None	
12	1546	CARHAGSSAMGWYFALW	IGH	5	None	
12	1547	CARRLRGYASGSYSLDYW	IGH	3	None	
12	1548	CAREAQMPLWFGELLHSYYYYYGMDVW	IGH	2	None	
12	1549	CARLEVGYSSGWFGYW	IGH	4	None	
12	1550	CLQYNYWPPRDGF	IGK	3	None	
12	1551	CARGVLPGTVQSWFDPW	IGH	3	None	
12	1552	CARMGVGATDFDYW	IGH	22	None	
12	1553	CQSYDASLGGSGLF	IGL	7	C4	7JVC
12	1554	CARGYSSGSNYKSAFDIW	IGH	3	None	
12	1555	CARGMAVHGNYMDVW	IGH	9	None	
12	1556	CTTDLLGWFGELFTYW	IGH	4	None	
12	1557	CARAVSCDSISCYEGGWFDPW	IGH	4	None	
12	1558	CAKFRRHDGGYLRSYFDYW	IGH	14	None	
12	1559	CARDPRRTAMVTWYFDYW	IGH	3	None	
12	1560	CARGIVVVPAAIPPQHYYYYGMDVW	IGH	2	None	
12	1562	CQEYENWPRGTF	IGK	34	C3	6XDG
12	1563	CAKDISLETDGGYYYYGMDVW	IGH	20	None	
12	1564	CARDQVEAAAAYYYYGMDVW	IGH	14	None	
12	1565	CARHCIGGNCYAVYYYGMDVW	IGH	2	None	
12	1566	CATSAVAGMVNWFDPW	IGH	5	None	
12	1567	CARGSSGWSPPVPAHFDYW	IGH	3	None	
12	1568	CAQNGDGGNPWGAFDIW	IGH	2	None	
12	1569	CARDSTVTTLGETNYYYYGMDVW	IGH	7	None	
12	1570	CAHGRVLAAGSYYFDYW	IGH	23	None	
12	1571	CARVRGNYADGEPFDIW	IGH	2	None	
12	1572	CARDRLVVVAAMYYYGMDVW	IGH	12	None	
12	1573	CASLYGSGSQDYYYYGMDVW	IGH	2	None	
12	1574	CARDLGSYDNSGFYNYWYFDLW	IGH	8	None	
12	1575	CARSYNYGSGSYFDYW	IGH	28	None	
12	1576	CAKQTPFLTRDLLWFGEPPNYYYGMDVW	IGH	3	None	
12	1577	CVYGSGSYYTPGYYYGMDVW	IGH	2	None	
12	1578	CARSSAAGTKLFDPW	IGH	2	None	
12	1579	CARQVLIAVAGYYYYYGMDVW	IGH	6	None	
12	1580	CVRGGREGVSPFDNW	IGH	2	None	
12	1582	CARLPAGVTPLDYW	IGH	9	None	
12	1583	CARDEGAYCGGNCYPKAFDIW	IGH	2	None	
12	1584	CARSGSFWGVEVTATNYWYFALW	IGH	4	None	
12	1585	CQSGDSTGDANYGVF	IGL	2	None	
12	1586	CAHRNVQKAATGSGGYFDYW	IGH	2	None	
12	1589	CARVSEGYYYDSSGYYFGYW	IGH	6	None	
12	1590	CARGRTTVTTSRYFDLW	IGH	5	None	
12	1591	CARAPRRWLQSPLIYFDYW	IGH	3	None	
12	1592	CAKEGTFGGVIATPFDYW	IGH	4	None	
12	1593	CAREFSANGSGSYYTQYYFDYW	IGH	2	None	
12	1594	CARGYTFYESTGSTSDIFDIW	IGH	2	None	
12	1595	CARRVGSGSYYRSDAFDIW	IGH	5	None	
12	1596	CARAPYYGSAGDAFDMW	IGH	4	None	
12	1597	CAKDYMTTVTTNWFDPW	IGH	10	None	
12	1598	CARHRARMTTVTIYGGRLGYYYGMDVW	IGH	2	None	
12	1599	CAKGGTLVVPAARSYFDYW	IGH	2	None	
12	1600	CARDGYSYGFLFDGMDVW	IGH	2	None	
12	1602	CANTLDYGDSRTFDYW	IGH	4	None	
12	1603	CARGFTMAQTDAFDIW	IGH	2	None	
12	1604	CARRQRPGYYSGWSPFYFDFW	IGH	2	None	

12	1605	CARGGGTYEPFDIW	IGH	2	None	
12	1606	CTTRTYYSENSVYRWFDPW	IGH	2	None	
12	1607	CASDRGTSYYYGSGSLDYW	IGH	9	None	
12	1608	CAGGIAVADAKFDYW	IGH	2	None	
12	1609	CAREDYYYTSSNSNWFDPW	IGH	2	None	
12	1610	CAREKPREYVYGQGLYYYYGMDVW	IGH	3	None	
12	1611	CARGGSGYPDSYYYYGMDVW	IGH	2	None	
12	1612	CARVGLGYYASGTPFDYW	IGH	2	None	
12	1613	CAKDGPPQLLWFGEPPNWFDPW	IGH	2	None	
12	1614	CALPICLRSEDTAVYYCARGGWYSGSGAVSLNWFDPW	IGH	3	None	
12	1615	CARMNGYQVVTAYFDYW	IGH	19	None	
12	1616	CAHSQYSYGSYVDPW	IGH	8	None	
12	1617	CVGTYRDYNYFDYW	IGH	2	None	
12	1619	CARDYIVVVVAATPSYYYYGMDVW	IGH	56	None	
12	1620	CARLYDSSGYYSWYYYGMDVW	IGH	2	None	
12	1621	CARQRGYYDSGGYYYPYYFDYW	IGH	7	None	
12	1622	CARHRQWLIFYYW	IGH	2	None	
12	1623	CARMIYSYQYEDYW	IGH	2	None	
12	1624	CAHRPGIAVAGSLPLYFDYW	IGH	12	None	
12	1625	CAHREDINLLRGIEEPGLTRSSFDYW	IGH	5	None	
12	1626	CARGRRPRLRLGELSPIPWDYW	IGH	3	None	
12	1627	CAKDPRYYGSGSYPAEFDYW	IGH	2	None	
12	1628	CARVSGFGYTRGFDFW	IGH	2	None	
12	1629	CARLKVVAATNWFDPW	IGH	7	None	
12	1630	CATDPKTIVAPSGEWFDPW	IGH	6	None	
12	1631	CAGDQRAGRNYYDSRGYIVDYW	IGH	4	None	
12	1632	CSRAHVWFGELQEADAFDIW	IGH	2	None	
12	1633	CAKDNYDSSGYYLGDYW	IGH	2	None	
12	1634	CARFSGVNLVATTHYWYFDLW	IGH	5	None	
12	1635	CARQRCGGNCYMDYW	IGH	2	None	
12	1636	CARHRYYDSRSSKYYFYYGMDVW	IGH	6	None	
12	1637	CARDKGFSDSSGYYFDNW	IGH	6	None	
12	1638	CAKDVLEGGSGYDHYYYYGMDVW	IGH	8	None	
12	1639	CARDSWGSGSYYDVYYYYGMDVW	IGH	4	None	
12	1640	CARHSTGYSSSWGLFDYW	IGH	11	None	
12	1641	CAKDQGYYGSGSYNFDCW	IGH	3	None	
12	1642	CQLYNSYSESWIF	IGK	10	C3	7K8R
12	1643	CARDSYSSGWNTSYYYYYGMDVW	IGH	6	None	
12	1644	CAKDGVGHIVVVTAPFDYW	IGH	2	None	
12	1645	CARDFLGYDILTGYYYSPSFAYW	IGH	5	None	
12	1646	CSGGSCYSNSSSYYYYYGMDVW	IGH	3	None	
12	1648	CGRDPNTAEDGMDVW	IGH	2	None	
12	1649	CYSAADNNWVF	IGL	208	None	
12	1650	CAREKSSSWAENWFDPW	IGH	3	None	
12	1651	CARMIAAAENDAFDIW	IGH	12	None	
12	1652	CAKDALRGFSGNDYYYFFGMDVW	IGH	25	None	
12	1653	CARAARRVAGSGGYYFDYW	IGH	4	None	
12	1654	CATDFGGDALTSYGGWLGFDPW	IGH	4	None	
12	1655	CARDLVVVTATDAFDIW	IGH	2	None	
12	1656	CTTPAIFCNGSTCYGDYW	IGH	16	None	
12	1657	CARDDSGGIAVAGDYYYYYGMDVW	IGH	2	None	
12	1658	CAREELYLGETTVRDFYYAMDVW	IGH	4	None	
12	1659	CARDLLGYYGDYGPDYW	IGH	6	None	
12	1660	CARVGWEALRRRVWYFDVW	IGH	2	None	
12	1661	CARDYDYVWGSYRYTPPFDYW	IGH	4	None	
12	1662	CAGGYSGYDSRVIDYW	IGH	2	None	
12	1663	CVRVHNTNYGWFAPW	IGH	4	None	
12	1664	CAHCIMGGTWNRRPAHFDDW	IGH	4	None	
12	1665	CTKEPITMIVVFYFEYW	IGH	20	None	
12	1666	CARIWGIKGPFDFW	IGH	3	None	
12	1667	CARDHIAATGFVHYYYYGMDVW	IGH	2	None	
12	1668	CAREGGVIAVAGTRYYYGMDVW	IGH	7	None	
12	1669	CARLRAYCSGGSCPIDYW	IGH	2	None	
12	1670	CARGPTIVVVPAQVGSHTNWFDPW	IGH	2	None	
12	1671	CATWGYDYDRGTPFFDCW	IGH	4	None	
12	1672	CARGDQKPAFSLDYW	IGH	4	None	
12	1673	CARGGWVKSSGRYYYYGMDVW	IGH	4	None	
12	1674	CARNGRGSDSSGYYYYYYYNGMDVW	IGH	2	None	

12	1675	CAKDPRAESSGNFPAWFDPW	IGH	3	None	
12	1676	CATGIAEAREIRGAFGVW	IGH	51	None	
12	1677	CASEFGSTNDYW	IGH	3	None	
12	1678	CARKRQQLVTASFDYW	IGH	4	None	
12	1679	CARGYYYYDSGTYLDKSYYFDYW	IGH	3	None	
12	1680	CAKAQGFSSSWFFDYW	IGH	6	None	
12	1681	CARGGYCSGNNCGGYDYW	IGH	2	None	
12	1682	CAGQSVFVDILPGHQVDYW	IGH	2	None	
12	1683	CAHSGTVSMGYDGIPDYW	IGH	8	None	
12	1684	CAKCQNAHYPRGAFDIW	IGH	2	None	
12	1685	CARASPDWLLSLYFDYW	IGH	6	None	
12	1686	CAYNSGGNYYYYGMDVW	IGH	4	None	
12	1687	CASTRRLSSSWITYNWFDPW	IGH	4	None	
12	1688	CVKDTGTGWSGAHFDYW	IGH	2	None	
12	1689	CARGGDTSGWTYNWFDPW	IGH	68	None	
12	1690	CARAGHCSGGSCFPVPDYW	IGH	8	None	
12	1691	CARQMTWTAPSCFDYW	IGH	2	None	
12	1693	CARGHSDGGDYYDYW	IGH	3	None	
12	1694	CARVDSPYYYDSSGPLPAPQPGAFDIW	IGH	2	None	
12	1695	CAYFDYYGSGSYRSDDYW	IGH	5	None	
12	1696	CVKRNDYGDYYSHYYLYYGLDVW	IGH	2	None	
12	1697	CARDASTPYDTSGYSDHW	IGH	4	None	
12	1698	CAKKQGDYISGCPAPDYW	IGH	20	None	
12	1700	CAKGPLGSGRSNNWFDPW	IGH	2	None	
12	1701	CARISVVRGVSLDYW	IGH	5	None	
12	1702	CASIAETFDYW	IGH	2	None	
12	1703	CAKEPAPTGDYEPYFDYW	IGH	6	None	
12	1704	CARDQYYYDSIFPAFDIW	IGH	5	None	
12	1705	CARDMFAGSSGVNYYYYGMDVW	IGH	9	None	
12	1706	CARVSKGYCSGGSCPAHGYYYYYGMDVW	IGH	3	None	
12	1707	CAKLRNPQAYYDILTGYYKLGYFDYW	IGH	2	None	
12	1708	CAKGLIAYDYVWGSYRLGPFDYW	IGH	5	None	
12	1709	CARGATVDHYYYGMDVW	IGH	4	None	
12	1710	CARRGPIVGPTGGFDFW	IGH	4	None	
12	1711	CAKGEVAAAGTSDYYYYGMDVW	IGH	2	None	
12	1713	CARDHRPDSYGQDYYYGMDVW	IGH	9	None	
12	1714	CARDVPYYYGSGTVFDYW	IGH	2	None	
12	1715	CARGNRRWLQLTSYYFDYW	IGH	2	None	
12	1716	CARGHYYDMYISGYFALW	IGH	2	None	
12	1717	CHQSSSLPFTF	IGK	328	None	
12	1718	CAKDIMGTGTTGELDYW	IGH	2	None	
12	1720	CARGLERSDYVMDQFDPW	IGH	3	None	
12	1721	CEKGGGPTVTTSDDGLDIW	IGH	2	None	
12	1722	CASLYLFGFIAARDDYW	IGH	2	None	
12	1723	CARQPPRNGIDYW	IGH	7	None	
12	1724	CQYRSTWPPTVTF	IGK	11	None	
12	1725	CSRVSMRSSWYPDPEYYFDSW	IGH	5	None	
12	1726	CARDAVAVAGSSNRYYYYGMDVW	IGH	2	None	
12	1727	CARDARYMVTKGLDYW	IGH	2	None	
12	1728	CAKDVGLGNDGSWYYYGMDVW	IGH	7	None	
12	1729	CARGREYSSTYYFDYW	IGH	52	None	
12	1730	CARHGEEAGLDYFDYW	IGH	4	None	
12	1731	CATEDSGYYHYW	IGH	2	None	
12	1732	CTTDPTVAGTKDYW	IGH	2	None	
12	1733	CARDQRARVGCNSTNCFYFDCW	IGH	2	None	
12	1736	CQHYHSSPPYDF	IGK	4	C1	7K8N
12	1737	CARDRRRIAAAGRPWFDPW	IGH	2	None	
12	1738	CLRAEDTAVYYCARDGSPFDVWFGELLSPYYYYYGMDVW	IGH	2	None	
12	1740	CAKEDRGHLDYW	IGH	6	None	
12	1741	CARTNFYGGNSRNAFDIW	IGH	6	None	
12	1742	CTTWGAESRGYYFDHW	IGH	2	None	
12	1743	CARDRTEQTYYDILSGSYHLERFGYW	IGH	2	None	
12	1744	CARRGWLQLDGYFDYW	IGH	9	None	
12	1745	CARAYTVATFWNYNDCDGMDVW	IGH	2	None	
12	1746	CARDGMVVAATLYYYGMDVW	IGH	108	None	
12	1747	CARDRPAYYYASDTYYHYYGMDVW	IGH	5	None	
12	1748	CAKDTRRFRSGIAATFDYW	IGH	2	None	
12	1749	CANAFCSSINCWGGFDYW	IGH	18	None	

12	1751	CTPHPFRYSGGYSPNFGYW	IGH	2	None	
12	1752	CARDRRYYDSSGYFLHAFDIW	IGH	3	None	
12	1753	CARRYRYSGYDYRGAFDFW	IGH	5	None	
12	1754	CARVGDSSGNYYVFDYW	IGH	9	None	
12	1756	CARFGESPEDDYW	IGH	6	None	
12	1757	CARQPPPDGYTQGAFDYW	IGH	2	None	
12	1758	CAKGYNSDSKFSFDIW	IGH	7	None	
12	1759	CATGFQGFPGDLFDTW	IGH	5	None	
12	1760	CAKSSMTTVVTNFDYW	IGH	4	None	
12	1761	CARGNLGSSWYLDYYYYGMDVW	IGH	3	None	
12	1762	CAKVGYYGSGSPNWYFDLW	IGH	4	None	
12	1763	CAREMTTVTIDYFDYW	IGH	2	None	
12	1764	CAHYMLYSYGGFDYW	IGH	6	None	
12	1765	CAKSRLAAMAGNDAFDMW	IGH	4	None	
12	1767	CAKGPHYDSSGYSLHLFDYW	IGH	4	None	
12	1768	CASRYCSGGSCYPVNYYYYGMDVW	IGH	3	None	
12	1770	CARVDNTMVRRIVLDYFDYW	IGH	8	None	
12	1771	CAKDYYGAGSYYFDYW	IGH	7	None	
12	1772	CARTNPPIIYYYGMDVW	IGH	3	None	
12	1773	CVVIEGTFFDYW	IGH	5	None	
12	1774	CARFGEMDYYYYGMDVW	IGH	7	None	
12	1775	CAREAVPVEMATINLIDYW	IGH	16	None	
12	1776	CASVRASYYYDSSGYRSAFDYW	IGH	18	None	
12	1779	CARGRGYGGSEGKIDYW	IGH	4	None	
12	1780	CARGTSMKFGDIFRGYTYGNYYGMDVW	IGH	43	None	
12	1782	CARVSGNEWVRDYYYYGMDVW	IGH	17	None	
12	1783	CARDTTVVPAATRDYYYYGMDVW	IGH	6	None	
12	1784	CARGRWDSSGSSYYYYAMDVW	IGH	12	None	
12	1785	CAKGKRGDCGRQTCHPHFDFW	IGH	5	None	
12	1786	CASNYGDPEAYYYYGMDVW	IGH	4	None	
12	1787	CARVLYFHDSSAFSYYFDSW	IGH	3	None	
12	1788	CAKAKGHYGDYYYGMDVW	IGH	6	None	
12	1789	CARAGIAAAGTSGYYYYGMDVW	IGH	16	None	
12	1790	CARDTPHYYGSPRGYFDRW	IGH	60	None	
12	1791	CARDGAGNPARYGGNFVFDYW	IGH	4	None	
12	1792	CAKDRGTTTGTLFDFW	IGH	2	None	
12	1793	CARHGALGTAEFDYW	IGH	2	None	
12	1794	CQRFYSFPHF	IGK	2	C4	6W41
12	1796	CAAGSYYDILTGYYNLNIPFDYW	IGH	5	None	
12	1797	CARDLPFSDYGDYGDYW	IGH	12	None	
12	1798	CAKDSGYGSGTGGYFDLW	IGH	2	None	
12	1799	CARDNSYLWLGELSGDRPSPCDYW	IGH	2	None	
12	1800	CARINLWGGWFDPW	IGH	5	None	
12	1801	CATVTGVYYGSGSSPYYYYYGMDVW	IGH	2	None	
12	1802	CAREKDGSVDFDYW	IGH	2	None	
12	1803	CARGGVEGFDFW	IGH	8	None	
12	1804	CARVMKVRGYSYGYVDYW	IGH	2	None	
12	1805	CARVVTWNYGAFDIW	IGH	4	None	
12	1806	CARSRTVTTLFFSEPNDYW	IGH	6	None	
12	1808	CARQIPDYDFLTSHYETYYFDYW	IGH	5	None	
12	1810	CARDLGWLQWPNFDYW	IGH	4	None	
12	1811	CANGNILEIFTFGRNFDSW	IGH	7	None	
12	1812	CARLLDFFGSGSYYDFDYW	IGH	5	None	
12	1813	CATDAWIAAAGTRLNNYYFYGMDVW	IGH	10	None	
12	1814	CARDIGEGEDKYYYYGMDVW	IGH	5	None	
12	1815	CARYRPVGYCSGGSCYGPENWFDPW	IGH	8	None	
12	1816	CQQYGSSVTF	IGK	43163	C1	7K8N
12	1817	CARLSEYVPGSFIPFDMW	IGH	36	None	
12	1818	CAKDPSGTYYNYYFDYW	IGH	2	None	
12	1819	CAKDRERYCSGGSCYYYYYGMDVW	IGH	6	None	
12	1820	CANELAGGDYGFDYW	IGH	11	None	
12	1821	CAKGVSSWYEYFQQW	IGH	2	None	
12	1822	CAMARIAVAGTQFYYYYHGMDVW	IGH	3	None	
12	1823	CASFRSVYSSGLGTFDYW	IGH	10	None	
12	1824	CARDGPGSGWLPQFGYW	IGH	4	None	
12	1826	CARDSVRHGDPDAFDFW	IGH	2	None	
12	1827	CVAIAVAGSWFDPW	IGH	3	None	
12	1829	CVHKYYYNSGSYYFDYW	IGH	12	None	

12	1830	CARYRPRSGLTGYYFDYW	IGH	18	None	
12	1831	CAKDLSIHIAVDGDYW	IGH	2	None	
12	1832	CANGLGDTIAVAGSDYW	IGH	2	None	
12	1833	CARLKAPYGSGSYYTGDPYYYYYGMDVW	IGH	2	None	
12	1834	CARDHQIAVLTGYYSYW	IGH	10	None	
12	1835	CARLDNVNGDMGGFGYW	IGH	4	None	
12	1836	CARRIPGYDILTGYYNNWFDPW	IGH	6	None	
12	1838	CARDSSTSSDDYFYYYEMDVW	IGH	3	None	
12	1839	CARASFRDNWALGDYFDYW	IGH	107	None	
12	1840	CTRDETGPYSSESVFDYW	IGH	9	None	
12	1841	CARLGYCSGGSCHWINYYYGMDVW	IGH	4	None	
12	1842	CARETYYYDSSGRTLPYFDYW	IGH	2	None	
12	1844	CAHTASVLNYGDHFDYW	IGH	3	None	
12	1846	CAKHLIIPDAYGDYDPNNWFDPW	IGH	15	None	
12	1848	CARGPVVATRNEGNYYYYYGMDVW	IGH	2	None	
12	1849	CASSLYDYGDYW	IGH	18	None	
12	1850	CVKDRRGHYDSSGYNAVDVW	IGH	3	None	
12	1851	CARVHKDQWLSPAFDVW	IGH	2	None	
12	1852	CARGDYSGYDYYFDYW	IGH	183	None	
12	1853	CARTTVTTSGAYYFDYW	IGH	7	None	
12	1854	CAKAKDPAAVAGQPFDSW	IGH	2	None	
12	1855	CARDLASDYGGKDYYYYGMDVW	IGH	2	None	
12	1856	CARQAAGSSLAYYYYGMDVW	IGH	3	None	
12	1857	CARASYSSSWYAAFDIW	IGH	10	None	
12	1858	CTVNMIVVVTDFDYW	IGH	2	None	
12	1859	CAKDSYCTGGNCFPYDAFDIW	IGH	4	None	
12	1860	CAKDSSGWLKDLGWFDPW	IGH	2	None	
12	1861	CLFWHQDVFDIW	IGH	2	None	
12	1862	CARLRRGDGYKLYYFDCW	IGH	2	None	
12	1863	CARAFHNVWSRDLYYFDYW	IGH	2	None	
12	1864	CARDQAASYNWFDPW	IGH	13	None	
12	1865	CARQIAFCTGGNCYCDHFDHW	IGH	28	None	
12	1866	CARGAPGSGDSFDVW	IGH	2	None	
12	1867	CAKDTGYSYGYSWFDPW	IGH	11	None	
12	1868	CARDSEAAAYSYYYNGMDVW	IGH	2	None	
12	1869	CARGLMSYYDSSGYSPPGFDIW	IGH	5	None	
12	1870	CARLARSYQLAPFDYW	IGH	2	None	
12	1871	CAHRPSTWTKASTWFDPW	IGH	5	None	
12	1872	CAKDGYCSGGSCYYYYGMDVW	IGH	37	None	
12	1873	CAKDQSMNTFGGDDAFDFW	IGH	2	None	
12	1874	CARESYGGYSEGAFDIW	IGH	4	None	
12	1875	CARHGTLLWFGESNQEDAFDIW	IGH	2	None	
12	1877	CTRRVEWVAASAPTNYEGFDIW	IGH	12	None	
12	1878	CAKDDSGDNNDAFDIW	IGH	3	None	
12	1879	CARDFKVVQSGYDLRPSYYYYGMDVW	IGH	8	None	
12	1880	CARDGDYYDSSGYYGWFDPW	IGH	19	None	
12	1882	CARYLFRDYDILTGYYNWFDTW	IGH	2	None	
12	1883	CARDKRGGFYHYYGMDVW	IGH	3	None	
12	1884	CQQSNNYPRLTF	IGK	270	C3	7K8R
12	1885	CVGSDDSHDYW	IGH	2	None	
12	1886	CARGSNHAYSFDFW	IGH	2	None	
12	1887	CARYCRNMACRVPYAFDLW	IGH	2	None	
12	1888	CARDQGWRWLQFGAFDIW	IGH	2	None	
12	1889	CAREPLYNAPWNEPDAFDLW	IGH	2	None	
12	1890	CARETYDSGGYYSPYFDYW	IGH	2	None	
12	1891	CAKQALILSYVTVFDHW	IGH	2	None	
12	1892	CAKAAQSHCSSTSCGYYYYGMDVW	IGH	4	None	
12	1893	CARDGTANCGGDCYYFDYW	IGH	7	None	
12	1894	CCSYAGSSTLVF	IGL	1557	C2	7K8X
12	1895	CASLKGYSSGWYSYYYGMDVW	IGH	6	None	
12	1896	CARGYCSGGSCYPHFDYW	IGH	22	None	
12	1897	CAKDIDVGATNSAVFDVW	IGH	6	None	
12	1898	CARHGNDFQDFDYW	IGH	8	None	
12	1899	CARDYMVAGYMDHW	IGH	3	None	
12	1900	CARDGGRDGMATIIDSLTTFDYW	IGH	10	None	
12	1901	CAKEGDWPAMRFDPW	IGH	87	None	
12	1902	CARDLAAGTTGTSGYYYYYGMDVW	IGH	2	None	
12	1903	CARDSYSSVGTDYYYGMDVW	IGH	16	None	

12	1904	CASEVGSGWTAPLDYW	IGH	6	None	
12	1905	CARDQSYYDSSGRVNWFDPW	IGH	2	None	
12	1906	CVKGRAGYNQGGYFDSW	IGH	4	None	
12	1907	CQQNNAYSSTF	IGK	3	None	
12	1908	CARVVELADLWFGELSGSFDYW	IGH	2	None	
12	1909	CARDRDYGDYVSVGAFDIW	IGH	6	None	
12	1910	CAHTVDDSSGYCFDYW	IGH	5	None	
12	1911	CARPIFGGNAFDFW	IGH	2	None	
12	1913	CAKDSFIAAAGTGYYYYYGMDVW	IGH	6	None	
12	1914	CARGLVSSGGLGYYYYYGMDVW	IGH	2	None	
12	1916	CAKDFTKQQPDLDYW	IGH	2	None	
12	1918	CAMSTLWFGELFPQPNDAFDIW	IGH	2	None	
12	1920	CQQYGSSPLYSF	IGK	1214	C1	7K8N
12	1921	CARRGDYWRFFDFW	IGH	5	None	
12	1922	CAHNQGDGGGNPEEWFDPW	IGH	2	None	
12	1923	CARSLVLLWFGEPEREPYFDYW	IGH	19	None	
12	1924	CARDMGTTPGIAAAGSYYYYGMDVW	IGH	3	None	
12	1925	CARWVRGSGWPPIWYFDLW	IGH	2	None	
12	1927	CAKDGYSGYGYYPDYW	IGH	2	None	
12	1928	CAHRPQGIAAAGYDYW	IGH	2	None	
12	1929	CARDSFQGGATTYYYYGMDVW	IGH	4	None	
12	1930	CATVGGAAAGPFDYW	IGH	19	None	
12	1931	CARDRVDSSSWFLDYYYYGMDVW	IGH	2	None	
12	1932	CARDRLPWRYIAAAGIGYFDLW	IGH	2	None	
12	1933	CAGSLATPYFFDSW	IGH	2	None	
12	1934	CAKAAQLERRQVNYYYGMDVW	IGH	5	None	
12	1935	CARDPPYYDILTGYYWLGLFDYW	IGH	2	None	
12	1936	CASSGPDYGDGDAFDIW	IGH	4	None	
12	1937	CARLRVGEDGYNWDFDYW	IGH	2	None	
12	1938	CVRELLSFEELKHFFDSW	IGH	8	None	
12	1939	CARRTANSGRRYGVSDYW	IGH	2	None	
12	1940	CARDRVPLAVGGAVDAMDVW	IGH	2	None	
12	1941	CAKDPLVAVATSDEKDFDYW	IGH	2	None	
12	1942	CARQPTKRIVVVTASPYYFDYW	IGH	7	None	
12	1943	CARDPYPYGSSHDWYFDLW	IGH	3	None	
12	1944	CARSSQEIWFVGGNYFFDSW	IGH	2	None	
12	1945	CARDRSSEWRDSWSDPW	IGH	2	None	
12	1946	CSRLTGYTISWWGRHDAYDIW	IGH	3	None	
12	1947	CATRVPGDRFFGVFDYW	IGH	2	None	
12	1948	CARYRCSSSSCYIGFDPW	IGH	2	None	
12	1949	CARAYDGGAFDVW	IGH	4	None	
12	1950	CARDQREDYYDSSGYFSEFWFDPW	IGH	2	None	
12	1951	CAKDLQIYDILTGFDYW	IGH	61	None	
12	1952	CARALDGYVYLFDLW	IGH	7	None	
12	1953	CARDGSYAVTSYYYYGMDVW	IGH	10	None	
12	1954	CAKDTKIIAGDPPNYYYYGMDVW	IGH	4	None	
12	1956	CARDRYYDILTGGPYYYYGMDVW	IGH	11	None	
12	1957	CANQGYYYDAGTSFKSRFDYW	IGH	2	None	
12	1958	CMQSKQLRLTF	IGK	211	None	
12	1959	CARGVRGYYDILTGYYKRGEDYW	IGH	3	None	
12	1960	CARRNVVDRRSAFDIW	IGH	3	None	
12	1961	CARQRRGGDYYYDSW	IGH	4	None	
12	1962	CARESTRGGSGDFDYW	IGH	2	None	
12	1963	CARHSHSYSYGWDLAPFDIW	IGH	2	None	
12	1964	CLRSEDTAVYYCARGYSSSWYVYYW	IGH	2	None	
12	1965	CATALAVAGPEGYYYYGMDVW	IGH	5	None	
12	1966	CARSSKLHDMYATLWFDSW	IGH	2	None	
12	1967	CAKDFSVDTAMDGPYYYYYGMDVW	IGH	6	None	
12	1969	CAKDYSDGYNYQFDNW	IGH	4	None	
12	1970	CAKDGPYNWNDNYYYYGMDVW	IGH	4	None	
12	1971	CARLPNFDWLLFDYW	IGH	16	None	
12	1972	CAHSEEYASGHYVPNEHYYFDSW	IGH	11	None	
12	1973	CARVILEPRGYCTSDNCYEYAFDVW	IGH	2	None	
12	1974	CGYGGNSLGGDYW	IGH	2	None	
12	1975	CARAIRGWYGRQPRMLNWFDPW	IGH	2	None	
12	1976	CARNSAAGGPADAFDIW	IGH	6	None	
12	1977	CARSERGGIQLDYW	IGH	31	None	
12	1978	CAHRVGDGAFDYW	IGH	11	None	

12	1979	CARWGDFCSGGSCYAYYFDYW	IGH	2	None	
12	1980	CASASPYYYGSGVDEDNWFDPW	IGH	4	None	
12	1981	CAQYYDILTGYYGSFDYW	IGH	2	None	
12	1982	CARLVDCSGGSCYSGSYYYYYYGMDVW	IGH	2	None	
12	1983	CAKDWAVAGSGQEQYYYYYGMDVW	IGH	3	None	
12	1984	CAKDPDSSGYLTTHFDYW	IGH	10	None	
12	1985	CVRGSGNAYGFGGPFDHW	IGH	2	None	
12	1986	CTTDFLLEGGSYRTFDYW	IGH	2	None	
12	1987	CLKDTVDSGMLEGYFDFW	IGH	4	None	
12	1988	CAKDSAYYGNDWGGYFDIW	IGH	2	None	
12	1989	CDWSSDVCSSDLGWPFRERRNSFDIW	IGH	7	None	
12	1990	CTRPVVEGYNWFDPW	IGH	2	None	
12	1991	CALPICFPQLQTTPHAMQPDNLPKTSSSSCHSSAITKYIIYW	IGH	2	None	
12	1992	CARDGTSNDDFFDYW	IGH	17	None	
12	1993	CATASQLWFSGWFDPW	IGH	3	None	
12	1994	CARHPVDTAMVTGWFDPW	IGH	4	None	
12	1995	CARVGGVRRGGDAFDIW	IGH	3	None	
12	1996	CARDRRYYGSGTPYYYYGMDVW	IGH	5	None	
12	1997	CAAVKGTTFGDYW	IGH	3	None	
12	1998	CAREGLLWFGELLLAGFPVDYW	IGH	3	None	
12	1999	CAKDPSSSWHYWYFDLW	IGH	4	None	
12	2000	CARDGVAATEHMLYYYYGMDVW	IGH	9	None	
12	2001	CAKGKADQQWLVPASYWYFDLW	IGH	2	None	
12	2002	CARAADTSGLGRLPNLSCFDPW	IGH	2	None	
12	2003	CARDRSPERLYYYGMDVW	IGH	2	None	
12	2004	CAREELLHLGELSYLDYW	IGH	2	None	
12	2005	CVKAQTRWPDWFFDLW	IGH	3	None	
12	2006	CAKDLITMVRGNDYW	IGH	2	None	
12	2008	CARDQFNFIGVFGSNPFDPW	IGH	4	None	
12	2009	CARGGYSGYEPSYYYRAMDVW	IGH	2	None	
12	2010	CTTEGAFCGGACHSNQVFDYW	IGH	4	None	
12	2011	CARDKHPTGSTSYYYYGMDVW	IGH	3	None	
12	2012	CVKDITPIAVAATEGFDSW	IGH	3	None	
12	2013	CARGLPYYETSDSPFDYW	IGH	16	None	
12	2014	CAKDTYSSIAAAGSFDYW	IGH	2	None	
12	2015	CQSPDISGDRWVF	IGL	9	None	
12	2016	CAKDRYSGYGDDAFDIW	IGH	5	None	
12	2017	CTTDSNWNDYYYYYGMDVW	IGH	7	None	
12	2018	CAAVDYGSDYW	IGH	2	None	
12	2019	CARVGKYCSGGSCYRTFDYW	IGH	2	None	
12	2021	CARDGNTQWLVRHYFDYW	IGH	5	None	
12	2022	CAIWRRYGSGSLDYW	IGH	3	None	
12	2023	CAGGLGIRSGFEYW	IGH	5	None	
12	2024	CVKVVGAPGGFDQW	IGH	2	None	
12	2025	CAREWPMIRGVINHFDSW	IGH	2	None	
12	2026	CARDKESTVTPYYYYYGMDVW	IGH	18	None	
12	2027	CAKDIGSWFNGAFDMW	IGH	2	None	
12	2028	CASLAGDYEDFDYW	IGH	4	None	
12	2029	CARGQGYDILGTDQLYYYYYGMDVW	IGH	2	None	
12	2030	CARARYYYYDSSGPEGYFDYW	IGH	2	None	
12	2031	CARDEYSYGQMTYYYGMDVW	IGH	2	None	
12	2032	CARDMYTAHDSYYYYYGMDVW	IGH	3	None	
12	2033	CARPRRYSASDSTDAFDLW	IGH	4	None	
12	2034	CAREMGRAPYYDGSGYPDYW	IGH	2	None	
12	2035	CAKTHMGSDLYYYGMDVW	IGH	7	None	
12	2036	CARETTHIAAAGSDSVGYYYYGMDVW	IGH	2	None	
12	2037	CARAKYSSGSRWFDPW	IGH	3	None	
12	2038	CARDLYDSSGYYAGPSVAFDIW	IGH	7	None	
12	2039	CARTDDILTGYYMGIDYW	IGH	3	None	
12	2040	CARAFCRGGAGCYEGFDLW	IGH	2	None	
12	2041	CAGAPLPTWGDYPFFDFW	IGH	4	None	
12	2042	CAVWDDNLSGPGYVF	IGL	2	None	
12	2043	CTSGPRGNFDWLPPLDYW	IGH	2	None	
12	2044	CARVPTLTTDLYFDYW	IGH	7	None	
12	2045	CARHSDYYDSSGYYSPDYYYGMDVW	IGH	2	None	
12	2046	CAHTYDYVGVFDYW	IGH	2	None	
12	2047	CARDSLFYESRYYYGMDVW	IGH	7	None	
12	2048	CARAPRAIAATGYFQYW	IGH	2	None	

12	2049	CAKDQYYYGSGTNGGPNWFDPW	IGH	2	None	
12	2050	CAKDMEASADWALDYW	IGH	4	None	
12	2051	CARDTSQSIAVAGTPYYYYGMDVW	IGH	6	None	
12	2052	CQQSDGMPFAF	IGK	3	None	
12	2053	CARGVVGALYPYYFDSW	IGH	2	None	
12	2054	CARPLRDGYKVEGAFDLW	IGH	3	None	
12	2055	CAKPLYIIYGDSLNPLDYW	IGH	2	None	
12	2056	CAKDLRYFDWDGMDVW	IGH	42	None	
12	2057	CARGLYIWGSGDRSFDVW	IGH	3	None	
12	2058	CARPQDGYSNTYFDYW	IGH	8	None	
12	2059	CQLWDSSSDPPYVF	IGL	3	None	
12	2060	CARVNVGDPNYFDYW	IGH	4	None	
12	2061	CANIQYDILTGYRLKENPFDYW	IGH	2	None	
12	2062	CAREGWRGYSGYDYGGPADYW	IGH	5	None	
12	2063	CARPQGGSYFGQFDYW	IGH	8	None	
12	2065	CAVSRSHYGYFDHW	IGH	4	None	
12	2066	CAHINGRGVVDAFDIW	IGH	2	None	
12	2067	CAHRGWLLPNAFDYW	IGH	3	None	
12	2068	CARTSALRYEPSWYFDLW	IGH	2	None	
12	2069	CAKDGPYSGYDEDYYYYGMDVW	IGH	4	None	
12	2070	CARGPVVPAAATYRSGYYYYGMDVW	IGH	2	None	
12	2071	CARVSFERADSGSYYSPLNFDYW	IGH	2	None	
12	2072	CARDGVMGGTTNYYYYGMDVW	IGH	22	None	
12	2073	CARRAPEHLYASGTYQVLYYLDSW	IGH	2	None	
12	2074	CTREVWGSYGSYFFDYW	IGH	2	None	
12	2075	CARDGWYYYDSSGYYYDGVGIDYW	IGH	2	None	
12	2076	CASSQPGIVVVTLGYLDYW	IGH	2	None	
12	2077	CARVLGSSWYYFDYW	IGH	8	None	
12	2078	CQQYDNLLLFTF	IGK	902	C3	6XDG
12	2079	CARVNVLGAYCGGDCHYYYYGMDVW	IGH	2	None	
12	2080	CQSYDSSVSNSRYVF	IGL	13	C4	7JVC
12	2081	CARHWPNRSYYYDSSGYPNFDYW	IGH	14	None	
12	2082	CAKDLRSGSYDLGVLDYW	IGH	2	None	
12	2083	CARDYIARGTDYYYYGMDVW	IGH	2	None	
12	2084	CAKDGDSSGYIGTYNWFDPW	IGH	4	None	
12	2085	CARGHHDVVGYYWRFFDLW	IGH	2	None	
12	2086	CARDRVRYSDAGDYYSFDYW	IGH	5	None	
12	2087	CARGGTISMTVVVTNDAFHIW	IGH	5	None	
12	2088	CAHSRGRAVGGVIAQFFDFW	IGH	2	None	
12	2089	CAKDAHSWIQLWFFDYW	IGH	15	None	
12	2090	CAVAGTVSGWPIDYW	IGH	2	None	
12	2091	CVTMRAYMSVLYYYGMDVW	IGH	14	None	
12	2092	CARLGSDNRGYKGFDYW	IGH	6	None	
12	2093	CARERRGWLQSDAFDIW	IGH	2	None	
12	2094	CARHPRYCSGGSCYDHDAFDIW	IGH	2	None	
12	2095	CSGAQLGTFDYW	IGH	2	None	
12	2096	CAHGGYTGLAAKTLYYFDSW	IGH	2	None	
12	2097	CARVASYCSSTSCFMDYYYYGMDVW	IGH	2	None	
12	2099	CAREAHYYDSSGYHPYYYYGMDVW	IGH	5	None	
12	2100	CQSADRSGTHLHVIF	IGL	8	None	
12	2101	CTNLEWGGGLDYW	IGH	2	None	
12	2103	CARLIEDDYGDYYLDYW	IGH	6	None	
12	2105	CARDPTYYDILTGYYTQYYYYYYGMDVW	IGH	2	None	
12	2106	CAKGEYCSGGSCYSNSSSYYYYYGMDVW	IGH	2	None	
12	2107	CARGRRAGDYLNKFDSW	IGH	2	None	
12	2108	CLRSEDTAVYYCAREELYLGETTVRDFYYAMDVW	IGH	3	None	
12	2109	CARMRWGYGGNSQYLDYW	IGH	3	None	
12	2110	CARDTTGTTGNWFDPW	IGH	17	None	
12	2111	CARGPYYDILTGYYPNDYW	IGH	2	None	
12	2112	CARDGGPTYGDLDYW	IGH	2	None	
12	2113	CARDRRDGYNPYFGYW	IGH	2	None	
12	2114	CARDSPYRVGRENSYSTPNWFDPW	IGH	2	None	
12	2115	CARDSSTDNWYEIDYW	IGH	3	None	
12	2117	CARDRVPHSTVTIGYYYYYGMDVW	IGH	3	None	
12	2118	CLRAEDTAVYYCARDGVAVAGSYYYYGMDVW	IGH	3	None	
12	2119	CARESGLNVRASGLGYYYYGMDIW	IGH	26	None	
12	2120	CARTVRMQWLAGTEPDYW	IGH	2	None	
12	2121	CARDLRTIVGVFFDYW	IGH	3	None	

12	2122	CAREHISWGAVAGTTFDIW	IGH	2	None	
12	2123	CARMTGDPPNAFDYW	IGH	6	None	
12	2124	CARDFNLGSSWYLFDNW	IGH	6	None	
12	2125	CARHLSGDIVATIGFFDYW	IGH	5	None	
12	2126	CASIVGVTKGDYW	IGH	2	None	
12	2127	CAKDQGRGVVGYYFDYW	IGH	2	None	
12	2129	CVTSLGPLRYALLFDYW	IGH	4	None	
12	2130	CARDPGYCSSTSCYEPFDYW	IGH	2	None	
12	2131	CARDLAGWPPERTYYYYYGMDVW	IGH	99	None	
12	2132	CARDWRVVGADAFDYW	IGH	4	None	
12	2133	CARLLGFNWASGWYFDLW	IGH	3	None	
12	2134	CARGGYYGSGSPKYFQHW	IGH	2	None	
12	2135	CARESYFQGAAYFDYW	IGH	2	None	
12	2136	CAKGTSIAAAGPLFDYW	IGH	3	None	
12	2137	CALPILRSEDTAIYYCTKNYPFCTGGDCYYGFDIW	IGH	2	None	
12	2138	CARRYSSSWPSMGYWFDPW	IGH	4	None	
12	2139	CASSVPDDYGFYYFNYW	IGH	6	None	
12	2140	CARSEVSSGSDLDYW	IGH	6	None	
12	2141	CARDIGAAVAGNEYYYGMDVW	IGH	3	None	
12	2142	CARVPFPYSSSWTRPYYYYGMDVW	IGH	2	None	
12	2143	CAKTYSYGYLPYYGMDVW	IGH	3	None	
12	2144	CMIWHSSAWVF	IGL	36	None	
12	2145	CARESIHRIVGATTFDYW	IGH	12	None	
12	2146	CATRSRISPLVGFDPW	IGH	2	None	
12	2147	CASEMVLGDYFDYW	IGH	14	None	
12	2148	CARTKELPLVHFDSW	IGH	3	None	
12	2149	CATDRNSYGYEGYYYYGMDVW	IGH	3	None	
12	2150	CVKEGGGGWNALDSW	IGH	16	None	
12	2152	CARDLQPMCSGGSCYSEEYYYYGMDVW	IGH	5	None	
12	2155	CARDRHGGSWSPDYW	IGH	2	None	
12	2156	CARGGHNSDWFDPW	IGH	2	None	
12	2157	CARVPQWPRFTFDYW	IGH	4	None	
12	2158	CATSGQWNLHAFDIW	IGH	6	None	
12	2159	CARDGLPHSAYYYHGMDVW	IGH	5	None	
12	2161	CAKDARVGATDDYYYYGMDVW	IGH	3	None	
12	2162	CAKDSDGDTAMVDYW	IGH	2	None	
12	2163	CASKGKSELSDYW	IGH	3	None	
12	2164	CAKDSRLFLLGADYYFDYW	IGH	4	None	
12	2165	CARDIVDTAPREGLYYYGMDVW	IGH	11	None	
12	2166	CARREEQWLAHDYW	IGH	16	None	
12	2167	CAKGAVTNSGSFDSW	IGH	2	None	
12	2168	CARDPLWIQLWAPRFSFDLW	IGH	2	None	
12	2169	CAKPVGSNLKYYFDYW	IGH	9	None	
12	2170	CQSYDSSESASGLF	IGL	4	C4	7JVC
12	2171	CARERERDIVATYDAFDIW	IGH	6	None	
12	2172	CARECSYYDSSGTDAFDIW	IGH	3	None	
12	2174	CARDMGWHSLDYW	IGH	2	None	
12	2175	CATWGDTAMDYYFDYW	IGH	16	None	
12	2176	CARMWYSYGPVFDLW	IGH	33	None	
12	2177	CTRDLCTSTSCVFDHW	IGH	3	None	
12	2179	CARARSLGASYFDYW	IGH	8	None	
12	2180	CASLTGYSSSWYVGDYW	IGH	8	None	
12	2181	CAKAKGPYGSGSLSRTLYYYYGMDVW	IGH	2	None	
12	2182	CATPGYYDILTGPYYFDYW	IGH	3	None	
12	2183	CGAWDDTLNGQYVF	IGL	7	None	
12	2184	CARGFIIAPLSWFDPW	IGH	6	None	
12	2185	CAKDLAPGVGATDYW	IGH	4	None	
12	2186	CNSRDSSHKHLLF	IGL	33	None	
12	2187	CATGTSLYYYDSSGYHNWFDPW	IGH	5	None	
12	2188	CARDMYDYVWGSFVWDYW	IGH	53	None	
12	2190	CARGPPSIVGGFDYW	IGH	2	None	
12	2191	CARDVMVRGDPVFENDYW	IGH	2	None	
12	2192	CAKTQAGYSSGLDYYYGMDVW	IGH	2	None	
12	2193	CVKDIGSGFYSYYGMDVW	IGH	3	None	
12	2194	CARDMAVSGYDHYYYGMDVW	IGH	6	None	
12	2195	CAKDHPPAVLLWFGEPNLHPFDYW	IGH	7	None	
12	2196	CATERITFGGVIGPPFDYW	IGH	5	None	
12	2197	CVSRLDSNFWHTPADFDIW	IGH	3	None	

12	2198	CSLYLQMNSLRPEDTAFYYCAKAMVREVLLNYFDYW	IGH	2	None	
12	2200	CARDWMSRGVYYYFDYW	IGH	4	None	
12	2201	CAKALLDYDSSGYYEGVSDAFDIW	IGH	2	None	
12	2202	CAGYGTVEMATIGDAFDIW	IGH	6	None	
12	2203	CAKEPIFSGDSCCTLGDYW	IGH	3	None	
12	2204	CARHGWELRRTTLFDAW	IGH	3	None	
12	2205	CARDSRHYGSGSLYYYGMDVW	IGH	11	None	
12	2207	CAKGQPGIQLWLHSYFDYW	IGH	6	None	
12	2208	CARERASGERAYFDLW	IGH	2	None	
12	2209	CMEGAHWPRGTF	IGK	7	None	
12	2210	CMQGTHWPPGLTF	IGK	98	None	
12	2211	CAKIPYGSGSSFDYW	IGH	6	None	
12	2212	CAKDEARGPQLLFYYW	IGH	5	None	
12	2213	CARSSTAMEKRGAFDIW	IGH	2	None	
12	2214	CARHGDPYYYDSSGYFDYW	IGH	13	None	
12	2215	CTRGWTHLWLNGFDVW	IGH	2	None	
12	2216	CARVDSTMVPYCFDLW	IGH	4	None	
12	2217	CARAANYGGTAANYGGTSEFDYW	IGH	6	None	
12	2218	CASHLFGTSYDILTGYYNGWFDPW	IGH	3	None	
12	2221	CARDPGDDYGDYGFDYW	IGH	13	None	
12	2222	CAMTTVTRPQYYFDYW	IGH	2	None	
12	2223	CARDRYSSSPTNYYYYGMDVW	IGH	6	None	
12	2224	CAKDGMYSSSWSFDYW	IGH	25	None	
12	2225	CSASYYAITGYVTYGFDMW	IGH	6	None	
12	2226	CARRQSLLLVQAAFDSW	IGH	2	None	
12	2227	CARSRPRMIEAAGPSDYW	IGH	2	None	
12	2228	CQSADISPTYRYVF	IGL	31	C4	7JW0
12	2229	CARGGWTYGSGSYAVNWFDPW	IGH	2	None	
12	2230	CARGLYYGACCAFDIW	IGH	6	None	
12	2231	CARDLYDSSGSQIDYW	IGH	44	None	
12	2232	CAKDAKIAAAGTDGANYYYYGMDVW	IGH	2	None	
12	2233	CTRGPPWYNGYKGGFDYW	IGH	2	None	
12	2234	CARASPQTGYYYGMDVW	IGH	2	None	
12	2235	CAKSDDSSGYYTVIFDYW	IGH	4	None	
12	2236	CAHITRRGYNGYDWDCLDYW	IGH	10	None	
12	2237	CARSSFITFFDFW	IGH	7	None	
12	2238	CARAQAAYFDWQILSGKYYYYYYGMDVW	IGH	15	None	
12	2239	CASINSSGWYDGDAFDIW	IGH	2	None	
12	2240	CAHRSTAMVTFDYW	IGH	10	None	
12	2241	CARDRRARTFYYALDVW	IGH	2	None	
12	2242	CATPGPGSGYTPFDIW	IGH	22	None	
12	2244	CASDQAAAASTDYYYGMDVW	IGH	10	None	
12	2246	CAKEDSSREYGYGAIDYW	IGH	2	None	
12	2247	CTGDGPYRSNSNVLHFYYYYGMDIW	IGH	3	None	
12	2248	CATPQLLGGIAVAGTFDYW	IGH	2	None	
12	2249	CLLSYSGPSGPDVLF	IGL	2	None	
12	2250	CVRGTVNYGYDIELGELYYFDYW	IGH	2	None	
12	2251	CARGSPVGQRTDVFDIW	IGH	62	None	
12	2252	CARGYDVLTGYPDYW	IGH	10	None	
12	2254	CAQRRSRGYYFGPFDPW	IGH	3	None	
12	2255	CSGGSCYRDDYW	IGH	9	None	
12	2256	CAKVLGNYYDSSGISFDYW	IGH	2	None	
12	2257	CARVLTYYYDGRNAFDIW	IGH	2	None	
12	2258	CAKEMDGDIPPFDYW	IGH	6	None	
12	2259	CARDARGVSGWYGSYYYGMDVW	IGH	2	None	
12	2260	CANLLQLWLLGGIEDSYVDYW	IGH	25	None	
12	2261	CQAFDSSLSGWAF	IGL	5	None	
12	2262	CARDMVAVAGESPQNYYYYGMDVW	IGH	21	None	
12	2263	CARGGWDSSWRNNWFDPW	IGH	3	None	
12	2264	CARDLVEELLFDIW	IGH	4	None	
12	2265	CAKERQAVAGTYYYYGMDVW	IGH	3	None	
12	2266	CAKDVRAGALDYW	IGH	3	None	
12	2267	CARDRLPYYESSGSNDAFDIW	IGH	483	None	
12	2268	CARQAWSYTDFPYYFDYW	IGH	2	None	
12	2269	CAKGSYDSSGYYYEGYFHHW	IGH	2	None	
12	2270	CAKDYNVRGWSSMLDYW	IGH	2	None	
12	2271	CARGLVSSTSTNSYYYYGMDVW	IGH	3	None	
12	2272	CARDQSRVNPRRFDYW	IGH	2	None	

12	2274	CARHPIVGAAYPYNWFDPW	IGH	11	None	
12	2275	CAKDHVSGSYLGFDHW	IGH	4	None	
12	2276	CARQGWGDAYSLFDNW	IGH	3	None	
12	2277	CAQGYISGWYGVDYW	IGH	2	None	
12	2278	CARDPLIVADAFDIW	IGH	4	None	
12	2279	CQWYGDSPGSSF	IGK	2	C1	7K8N
12	2280	CARPTSYYDILTGPNGGAFDIW	IGH	2	None	
12	2281	CARDLGDYGSGEGFDPW	IGH	3	None	
12	2282	CARGGPLRDGYNQRGAFDIW	IGH	2	None	
12	2283	CVAERNWNDYW	IGH	4	None	
12	2284	CAREYQLLWTWFDPW	IGH	3	None	
12	2285	CAKDRGYTYGSPDYW	IGH	5	None	
12	2287	CASRFWGSSSHFDYW	IGH	2	None	
12	2288	CALVSTVTTTFYFASW	IGH	2	None	
12	2289	CARGRASYDRLHYYFDYW	IGH	2	None	
12	2290	CARSAVVTPFDPW	IGH	2	None	
12	2292	CATIDYGDYQRGHAFDIW	IGH	2	None	
12	2293	CARSRDSGSYDSDGADYW	IGH	4	None	
12	2294	CAHSHYDILTGYFFGFDYW	IGH	20	None	
12	2295	CAREKRHYEVLTGYYEGYYFDYW	IGH	3	None	
12	2296	CARDCPLPCYDAFDIW	IGH	3	None	
12	2297	CAKDTGYYYGSGTSNFDYW	IGH	2	None	
12	2298	CTAYLQISSLKAEDTAVYYCARERSVIDHPYDAFDIW	IGH	2	None	
12	2299	CAKDLYYEILTGPFLDYW	IGH	4	None	
12	2300	CARDQYGSGSVGTAYYHYGMDVW	IGH	2	None	
12	2301	CAKDFLELKSGYYYYGMDVW	IGH	2	None	
12	2303	CAREKGYSEYFFDAFDIW	IGH	4	None	
12	2304	CARDVRQQLVQGFDPW	IGH	8	None	
12	2305	CARAHQYYDYVWGSPIYYYYGMDVW	IGH	6	None	
12	2306	CARDNQIAAAGTRGFDYW	IGH	20	None	
12	2307	CARDLDGGYGSNYYYYYGMDVW	IGH	3	None	
12	2308	CAMHIPAAIDGVLEYFQHW	IGH	2	None	
12	2309	CGTWDSSLTPGGLF	IGL	4	None	
12	2310	CALLRTHYYYYGMDVW	IGH	4	None	
12	2311	CARDHYGYNYLDAFDIW	IGH	2	None	
12	2313	CETWDSNTRVF	IGL	56	None	
12	2314	CARDEGYCSGGSCLQYYFDYW	IGH	21	None	
12	2315	CARDSGTTAAAAPYYYYGMDVW	IGH	7	None	
12	2316	CARGGTSCYECPFSSWFDPW	IGH	2	None	
12	2317	CARDALGSDFMGPAIDYW	IGH	2	None	
12	2318	CQQANSFQF	IGK	486	None	
12	2319	CAKSNGIAVSNPFDYW	IGH	4	None	
12	2320	CVRVGFGHVYGGYAADYW	IGH	2	None	
12	2321	CARWDRELLGGFDYW	IGH	2	None	
12	2322	CALSTTPHFFDYW	IGH	21	None	
12	2323	CVRGVYSSGWSGYWYFDLW	IGH	2	None	
12	2324	CARGRSAGMAASGFDYW	IGH	13	None	
12	2325	CALPISALYFCARDRLPYYESSGSNDAFDIW	IGH	2	None	
12	2326	CARPLQVGGNYFYW	IGH	6	None	
12	2327	CAKDMVATSSTWYGGIDYW	IGH	14	None	
12	2328	CARDMDWNDIYFDYW	IGH	19	None	
12	2329	CARLGWGSWSYYPFDYW	IGH	3	None	
12	2330	CNSRDSSRNLSYVF	IGL	2	None	
12	2331	CARDLTTVTTVQGTHYYYYGMDVW	IGH	4	None	
12	2332	CARAGYCSSTSCYSMDYW	IGH	49	None	
12	2334	CAKDRVGLRFTFDYW	IGH	6	None	
12	2335	CARESNPNDYW	IGH	22	None	
12	2336	CARSFWGGYNSPFDYW	IGH	4	None	
12	2337	CARESPYYDSYYYGMDVW	IGH	2	None	
12	2338	CAKDHYYGGTSFDCW	IGH	6	None	
12	2339	CARDSTVAGTDSYYYGMDVW	IGH	2	None	
12	2340	CARDGLGITGTTHYYYYGMDVW	IGH	5	None	
12	2342	CARDSRYESGGHLHWYLDLW	IGH	4	None	
12	2343	CARDLPQYYYDSSYHAVFDYW	IGH	2	None	
12	2344	CAKDRSPGVGGDESSFDSW	IGH	3	None	
12	2345	CARDMAVGVAGDLDYW	IGH	8	None	
12	2346	CAKDLQEWLAYAEFFQHW	IGH	9	None	
12	2347	CAKERGSGWYEPQDYW	IGH	2	None	

12	2348	CARDRGAAGPTYYYYGMDVW	IGH	11	None	
12	2349	CTTVARYGDFIDYW	IGH	2	None	
12	2351	CARPEVAVLGRRYAFDIW	IGH	2	None	
12	2352	CVKDITYYYGSGRSYGVAMDVW	IGH	2	None	
12	2353	CQSYDNSLRGRDVLF	IGL	5	C4	7JVC
12	2354	CVKDDYYASGNFASNFDCW	IGH	4	None	
12	2355	CARDWGGRPSMIVVAPDAFDIW	IGH	2	None	
12	2357	CQQSYSTPPALHTF	IGK	88	C4	6ZDH
12	2358	CQQFINHPFTF	IGK	48	None	
12	2359	CAKGLGSTVAHGFDNW	IGH	31	None	
12	2360	CAKDIKMTTQYYYYGMDVW	IGH	3	None	
12	2361	CARLGAYCGGDCYSRGIDYW	IGH	2	None	
12	2362	CARDCIAAECYYYYGMDVW	IGH	5	None	
12	2363	CAKDRGGSYIPFDYW	IGH	34	None	
12	2364	CARGHGGDAYHYFDFW	IGH	15	None	
12	2365	CAKGAGWLVLEVVDYW	IGH	2	None	
12	2366	CAKEAVATWDYYYGMDVW	IGH	2	None	
12	2368	CAKEGDYYGSGKPFDYW	IGH	2	None	
12	2369	CAAELGIGFDYW	IGH	19	None	
12	2370	CAKAGGQGLVGTTHFDYW	IGH	7	None	
12	2371	CARTGNYDDSRDDAFDIW	IGH	2	None	
12	2372	CAKVHSEYSSSWVIYYYYGMDVW	IGH	12	None	
12	2373	CARDIKGAAADDYYYYGMDVW	IGH	6	None	
12	2374	CAHRHANYWGNDLYDVW	IGH	7	None	
12	2375	CARTLVRGAYFVYYYGMDVW	IGH	2	None	
12	2376	CARGRFNGYIHYFDYW	IGH	4	None	
12	2377	CARDFRETVDYW	IGH	5	None	
12	2378	CARDLGSSDPQWGYLSYHQYYALDVW	IGH	2	None	
12	2379	CARRSRRDGCLDYW	IGH	2	None	
12	2380	CARDATAVAGNDNAFDIW	IGH	2	None	
12	2381	CARDRPDYGDYPNFDSW	IGH	10	None	
12	2382	CAGAPDRGYRAFAFRYW	IGH	6	None	
12	2383	CVRQMGYFYDSSGYSSFDYW	IGH	25	None	
12	2384	CAREPDYGDYFDYW	IGH	59	None	
12	2385	CARWDAVVSYFDYW	IGH	6	None	
12	2386	CAKHLKVRSSSFDYW	IGH	2	None	
12	2387	CAKARITMIVVVDYYYYGMDVW	IGH	2	None	
12	2389	CARHFNWGEYWYFDLW	IGH	6	None	
12	2390	CQKYNSALALTF	IGK	413	None	
12	2391	CARGAHYYDSSGYYYLQVSNAFDIW	IGH	2	None	
12	2392	CARGERGTLWFGELNNYFDYW	IGH	2	None	
12	2395	CATTPFGVYYGSGSPWYFDYW	IGH	2	None	
12	2396	CARDGMVRGVTSENYYYYGMDVW	IGH	8	None	
12	2397	CARTYTEHDWPFGYW	IGH	4	None	
12	2398	CARYGEPSSWYLSFSWTLPANNWFDPW	IGH	3	None	
12	2399	CTTMDAPYSDIVVVPADYW	IGH	8	None	
12	2400	CARAPAEYYYGHVDYFGFW	IGH	5	None	
12	2401	CASYYASGNYYSLFDHW	IGH	6	None	
12	2402	CARVGIIDMYFTKWSDYW	IGH	3	None	
12	2403	CAKDIKDTAMAGLDYW	IGH	2	None	
12	2404	CARDGGYRQPHWYFDLW	IGH	4	None	
12	2405	CARGGFGELLYFFDYW	IGH	2	None	
12	2406	CAVGFSSDNPDYW	IGH	4	None	
12	2407	CAKAGKTYTLTDWFDPW	IGH	9	None	
12	2408	CARDHKGGWSPYWYFDLW	IGH	4	None	
12	2410	CAKDALMVRGTPLNYYYYGKDVW	IGH	2	None	
12	2412	CAREASIPRYGSGSYLPYW	IGH	2	None	
12	2413	CARHELWYGSGNNWFDPW	IGH	2	None	
12	2414	CQHYNNWPSYTF	IGK	1476	C3	7K8R
12	2415	CARAEGIYSGSRSIDYW	IGH	4	None	
12	2416	CASIAAADTAFYYYYGMDVW	IGH	2	None	
12	2417	CARDFWFGEILFRDVGHAFDIW	IGH	2	None	
12	2418	CAGVNGDYSSRFDPW	IGH	13	None	
12	2419	CARVDSSSWDGMDVW	IGH	12	None	
12	2420	CATLEYSSGWYSFDYW	IGH	8	None	
12	2421	CATTGSGSYWGYFDYW	IGH	12	None	
12	2422	CARSGIAAADPGRVDYYYHGMDVW	IGH	2	None	
12	2424	CARATVVSRSVLDYW	IGH	2	None	

12	2425	CAQTGLRGWFDPW	IGH	4	None	
12	2426	CAHRGGDYDSGWNEDVFDYW	IGH	4	None	
12	2427	CARGGGIAMDDAFDIW	IGH	2	None	
12	2428	CARGSPVPEYGDYDFDYW	IGH	5	None	
12	2429	CARGFWTSRDGYNWGAFDIW	IGH	2	None	
12	2430	CARDLPYYAILTGYSISPFDYW	IGH	8	None	
12	2431	CAKGPPGSLVRDYFDYW	IGH	4	None	
12	2432	CVRGLGWEHGWFGPW	IGH	2	None	
12	2433	CAGSRQLVWLWFDPW	IGH	7	None	
12	2434	CAREGDGSGSYSWWFDPW	IGH	2	None	
12	2435	CARELSGWPFDYW	IGH	58	None	
12	2436	CARLGSSGSQHVYFDYW	IGH	4	None	
12	2437	CARDNNQWGSGDAFDIW	IGH	6	None	
12	2439	CAKDTDNRLLLWFGELFGSYFDYW	IGH	2	None	
12	2440	CARDLSRRYTYGFHYFDYW	IGH	13	None	
12	2441	CAKARGPSRYFPAFDYW	IGH	6	None	
12	2442	CAKDPGYPIQTLYYYYYGMDVW	IGH	3	None	
12	2443	CAREWLVRDYFDYW	IGH	4	None	
12	2444	CARVVGATKAYYFDYW	IGH	4	None	
12	2445	CARGGYSLRRPLYYYYGMDVW	IGH	3	None	
12	2446	CARDLQDYYDSSGYYDYW	IGH	8	None	
12	2447	CAAAAAGTSYYYGMDVW	IGH	5	None	
12	2448	CARDWAYYYDSSGYYSYDAFDIW	IGH	29	None	
12	2449	CARSPIGAAAKDYW	IGH	6	None	
12	2450	CAREKAPMVPTFDYW	IGH	10	None	
12	2451	CAKVFVAEQWLAPFDYW	IGH	6	None	
12	2452	CARRGIQLWNIFDYW	IGH	10	None	
12	2453	CARWSGWYSSTPSFDLW	IGH	10	None	
12	2454	CQQYTSNSVLTF	IGK	6	None	
12	2455	CASAHRGDCSTGACHNFFDPW	IGH	2	None	
12	2456	CQAWDSSTVVF	IGL	2612	C4	7JVC
12	2457	CAREAEGPLRTDAFDIW	IGH	4	None	
12	2458	CAKDQEPQLLWFGESLYYFDYW	IGH	4	None	
12	2459	CAAAQGLSSGWFDSW	IGH	4	None	
12	2460	CARDIYGRSVGGEPNDYYYYYGMDVW	IGH	2	None	
12	2461	CARDTYSSSSSVYYYGMDVW	IGH	15	None	
12	2462	CARLSSGFYYSVDYW	IGH	2	None	
12	2463	CARGSRFGEFGYW	IGH	2	None	
12	2464	CARGPHYYDSSGFLYFNYW	IGH	2	None	
12	2465	CAREVAGGTCSKERSLCNWFDPW	IGH	4	None	
12	2466	CASQLLPYYYGMDVW	IGH	10	None	
12	2467	CARHGYYYDVSASWYFDYW	IGH	17	None	
12	2468	CARVKLVPAPMGLWYYYGMDVW	IGH	3	None	
12	2469	CASVLRDYSDTLPYYNFDYW	IGH	4	None	
12	2470	CMQGTLWPPSF	IGK	6539	None	
12	2471	CARLTYNYGSGSYGYYFDNW	IGH	3	None	
12	2473	CASLQGRYSSGWYDNWFDPW	IGH	2	None	
12	2474	CAKEVVATEQGGIYYYYGMDVW	IGH	2	None	
12	2475	CTTHPPEWELLFPYYFDYW	IGH	2	None	
12	2476	CASFVAAAVAGGFDYW	IGH	4	None	
12	2477	CVREGYTSGRCGAFDIW	IGH	5	None	
12	2478	CAWSSGYYLYYYGMDVW	IGH	184	None	
12	2479	CARHLVDTAMVRDYWYFDLW	IGH	2	None	
12	2480	CARVTYDRSGSVSGYFDYW	IGH	3	None	
12	2481	CARVGNTAMVIGVYYFENW	IGH	2	None	
12	2483	CARDFKVVPAANGEDETNYYYYGMDVW	IGH	2	None	
12	2484	CARVGEYHFESSGYYDYW	IGH	4	None	
12	2485	CARDRVARGLFITGKYSYYAMDVW	IGH	6	None	
12	2486	CATDATVSHPGYYYGMDVW	IGH	4	None	
12	2487	CAKECGSTCPNLFDYW	IGH	4	None	
12	2488	CARLHDYYDSSGYTVAYAFDIW	IGH	5	None	
12	2489	CARDAGVVSLGEVVTQEYYYYYGMDVW	IGH	2	None	
12	2490	CARVGAARNYYYYGMDVW	IGH	6	None	
12	2491	CAHRGTLYGSSWDDAFDVW	IGH	3	None	
12	2492	CAKEITADIVATKTLGAPEFDYW	IGH	3	None	
12	2493	CVRGGVSGSHDYW	IGH	3	None	
12	2494	CARSVYSSSWAGSRPGWFDPW	IGH	3	None	
12	2495	CARASWRSSGWYERSYYFDYW	IGH	2	None	

12	2496	CARQGYTGPEMGYHFDFW	IGH	4	None	
12	2498	CAKDKASYDYVWGSYRSPLDYW	IGH	4	None	
12	2499	CARDREERQQLALYYYGMDVW	IGH	12	None	
12	2500	CVSPQQYSSGWYDYW	IGH	2	None	
12	2501	CARDLFSGTRGWGLFDYW	IGH	2	None	
12	2502	CARDAPLYGDYPFDYW	IGH	28	None	
12	2503	CARHVWAESYFDYW	IGH	6	None	
12	2504	CAHSSYGSGRYYFDFW	IGH	2	None	
12	2505	CAKALRYSSGWYLPRPSRSPNDWYFDLW	IGH	2	None	
12	2506	CAKHSSSWSAFDHW	IGH	2	None	
12	2507	CAKEYELHIVVVTARWGFDYW	IGH	2	None	
12	2508	CVRHRWELLINAFDLW	IGH	2	None	
12	2509	CASFLQDIVVVTAIARLDAFDIW	IGH	3	None	
12	2510	CATDQGHASDEDYFRYW	IGH	9	None	
12	2511	CAREILSYYDTTSFSNWLDPW	IGH	5	None	
12	2512	CARAVYGGNRHDDAFDIW	IGH	20	None	
12	2513	CARDSLSAAGALDYW	IGH	2	None	
12	2514	CARDPGVVGSTYFDYW	IGH	7	None	
12	2515	CAREGDCSGGSCPLGDAFDIW	IGH	6	None	
12	2516	CARDLYYDSTGYYWGDAFDLW	IGH	6	None	
12	2517	CARAPGSENDGGYYYGMDVW	IGH	2	None	
12	2518	CAKGEVDIVATYYYYYGMDVW	IGH	5	None	
12	2519	CAKDSPWYSSGWGQVDYW	IGH	3	None	
12	2520	CVRGQDRYSGRYRDFDYW	IGH	2	None	
12	2521	CAKDGSGYYYGVYFYFDYW	IGH	2	None	
12	2522	CARDSVIGFGELLSDYW	IGH	2	None	
12	2523	CLLYYGGSQLGVF	IGL	3	None	
12	2524	CARLITYPTYYFDYW	IGH	2	None	
12	2525	CQSYDKSDTDVVF	IGL	2	C4	7JVC
12	2527	CAGPGTAAAMVNDAFDIW	IGH	3	None	
12	2528	CARDMHYGSGSYYNGDYYYYYGMDVW	IGH	4	None	
12	2529	CQSADNSGSYGIF	IGL	106	None	
12	2530	CARGAVAWFGPNWFDPW	IGH	12	None	
12	2531	CARVSWDLVGAFDIW	IGH	3	None	
12	2532	CARHWGPSTGWYFIDYW	IGH	6	None	
12	2533	CALPICSLKASDTAMYYCASGIGMITKLGFDYW	IGH	2	None	
12	2534	CASVGSSYGYWANDYW	IGH	3	None	
12	2535	CARAWPPFGDYGGDAFDFW	IGH	2	None	
12	2536	CARVGSERYEYESFDIW	IGH	2	None	
12	2537	CAHTHRYCSGGSCYSFDYW	IGH	3	None	
12	2538	CARGISIAVAGTLGGWYNWFDPW	IGH	2	None	
12	2539	CAHRVRYTVYEGGGRYYFDHW	IGH	49	None	
12	2540	CARDLGAAAGLNPPTYYYYYGMDVW	IGH	6	None	
12	2542	CARRDLKKNDAFDIW	IGH	2	None	
12	2543	CARDQVGYYDSSGYSSYYYGMDVW	IGH	9	None	
12	2544	CAISPLNKAWYVGYNFDYW	IGH	6	None	
12	2545	CAAAVGADPPDYW	IGH	2	None	
12	2546	CARDGIAVAGTFFGFRYFDYW	IGH	2	None	
12	2547	CARALSYYPIDYW	IGH	18	None	
12	2548	CAHTGGYSSSYFPFAFDIW	IGH	2	None	
12	2550	CARQPYYDSSGYYRGRYYFDYW	IGH	2	None	
12	2551	CAKGQGQLVYEPAFDYW	IGH	4	None	
12	2552	CASISSGWSIYDYW	IGH	8	None	
12	2553	CARTWIGYGDYGDWFDPW	IGH	2	None	
12	2554	CARDLSSNDDMGEGWFDPW	IGH	2	None	
12	2555	CARDGVAGTDDGYYYYGMDVW	IGH	38	None	
12	2556	CQQHDNFSSKTF	IGK	2	C3	6XDG
12	2557	CVRAEWESSGYYPRLDYW	IGH	6	None	
12	2558	CAHRPPYYDILTGPSTYNWFDPW	IGH	2	None	
12	2559	CARETVDTAMVAAFDIW	IGH	2	None	
12	2560	CARYQMPSVDYYYYGMDVW	IGH	5	None	
12	2561	CARDFGLGAPDYFVDW	IGH	2	None	
12	2562	CAREGRIDDVWGSYRTNAFDIW	IGH	6	None	
12	2563	CARDDYVDLDDGFDIW	IGH	3	None	
12	2564	CARLIPGRYSSSWYYYYYGMDVW	IGH	4	None	
12	2565	CARQISFFTGGVYYCDHFDYW	IGH	8	None	
12	2566	CARRYGWVAAGGDGMDVW	IGH	2	None	
12	2567	CVRDKYSSGWYPIDYW	IGH	6	None	

12	2568	CARESLSAGTNYYYYGMDVW	IGH	3	None	
12	2569	CAKVKEAVTGYFDYW	IGH	5	None	
12	2570	CARHSVGFYWFDPW	IGH	12	None	
12	2571	CAKDTYYYDSSGYYPYDYW	IGH	20	None	
12	2572	CARGYYGSGSYYTPYYFDYW	IGH	6	None	
12	2573	CAKDQGTVFDYFDYW	IGH	2	None	
12	2574	CARQHYSDTSDFDYW	IGH	6	None	
12	2575	CARAAKATKVRPNWFDPW	IGH	2	None	
12	2576	CARLSGIGAVAGYFDYW	IGH	2	None	
12	2578	CATSSGSYSQIYFDFW	IGH	11	None	
12	2579	CARTTGRYDRGGLWVEEDYFFDKW	IGH	9	None	
12	2582	CAKDFDGSGYYNGDAFDIW	IGH	4	None	
12	2584	CASWGYSYGSGSFVDYW	IGH	2	None	
12	2585	CAKSVDTTMAPYYYYGMDVW	IGH	2	None	
12	2586	CARSEADYYDSSGYYYAFDIW	IGH	4	None	
12	2587	CARDSRLDYGDYVGGGLDYW	IGH	6	None	
12	2588	CARQIAWCSGDKCYCDYFDFW	IGH	16	None	
12	2589	CARGGSWGDYPLYYFDYW	IGH	45	None	
12	2590	CAHSPCFGTGEGYCSGGSCYCGDWFDPW	IGH	2	None	
12	2591	CARGGRTRFDQW	IGH	4	None	
12	2592	CARDSYSGSPTGPYYYYGMDVW	IGH	3	None	
12	2593	CVGSSEAYYDSSGYVRPDYW	IGH	2	None	
12	2594	CAAWDAGLSGQGAIF	IGL	7	None	
12	2595	CAKEGNSYYFEYW	IGH	148	None	
12	2596	CARGLSGYDDAFDLW	IGH	28	None	
12	2597	CARVGPAAGFYFDDW	IGH	7	None	
12	2598	CVRDGILVSGLAFDVW	IGH	9	None	
12	2599	CARLTDYDSRGHDAFDVW	IGH	2	None	
12	2600	CARWGSQGSGHYYYGMDVW	IGH	4	None	
12	2601	CARENYYDSSGYYYDYW	IGH	6	None	
12	2602	CARHFVVPAAINWFDPW	IGH	3	None	
12	2603	CGLSTGWYTTFDYW	IGH	3	None	
12	2604	CARVPDPYYFDYW	IGH	37	None	
12	2605	CARFARDGYNYGKYYFDYW	IGH	2	None	
12	2607	CAKPADSSGSWGRGWLDPW	IGH	10	None	
12	2608	CAKDFGYYYDSSGYNPQYYYYYGMDVW	IGH	4	None	
12	2610	CVTTPVRGRFDYW	IGH	2	None	
12	2611	CVRDSYSGWTFDYW	IGH	2	None	
12	2612	CARDQSYYYDSGGYYVDYW	IGH	3	None	
12	2613	CARHGLHSYGYDPVDYW	IGH	2	None	
12	2614	CATPRGYSSGPFDYW	IGH	14	None	
12	2615	CARYVFKVGGIFIPDWYFDLW	IGH	2	None	
12	2616	CASLYGSGSYYKEGYYYGMDVW	IGH	2	None	
12	2617	CAREFRIVVVPAAMDSDYYYGMDVW	IGH	2	None	
12	2618	CVLYMGSGFRWVF	IGL	179	None	
12	2619	CARVSGHDVYFDYW	IGH	10	None	
12	2620	CARGSHLQQFDYW	IGH	5	None	
12	2621	CYSTDGSGDHKGLF	IGL	5	None	
12	2622	CARHNGEMVRGVTRHWYFDLW	IGH	3	None	
12	2623	CAHYYFESSGYYPGFDYW	IGH	2	None	
12	2624	CASPNGLRNLIVVVPAAGDDAFDIW	IGH	6	None	
12	2625	CARDRSIAVAGNIGSSDYYYYGMDVW	IGH	4	None	
12	2626	CARHSTQQWLPFDYW	IGH	23	None	
12	2628	CAKFADSSSSSLDYW	IGH	4	None	
12	2629	CARNTWSRRDGYNLDAFDIW	IGH	3	None	
12	2631	CARELEPDCSGGSCYSGYDYW	IGH	2	None	
12	2632	CQSVDSSETFRVWVF	IGL	3	None	
12	2633	CARDRYYYDSSGYYYGFDYW	IGH	33	None	
12	2634	CAKGRAAAGPDDAFNFW	IGH	3	None	
12	2635	CAKDLSQSYYYDSSGYYRPYYYYGMDVW	IGH	5	None	
12	2637	CQSYGSSRSGSNVVF	IGL	8	C1	7K8N
12	2639	CERDTAMVQGDYYYYGMDVW	IGH	3	None	
12	2640	CARTAYDSSGYYLLFYYFDYW	IGH	6	None	
12	2641	CASSHCRGGTCYYFYAMDVW	IGH	2	None	
12	2642	CARVKGYGGFHFDQW	IGH	3	None	
12	2646	CDWSSDVCSSDLWNDIYFDYW	IGH	2	None	
12	2647	CATDIPRPSPFGELLRFVGYYFDYW	IGH	7	None	
12	2648	CASIRRRITVVRGVITLDYW	IGH	2	None	

12	2649	CARGLAVEEDSGYDAGYFDYW	IGH	9	None	
12	2650	CARDSEQWLSNCPDYW	IGH	2	None	
12	2651	CARAIYRGGDYGDYIEAILRGYYYYYGMDVW	IGH	2	None	
12	2652	CARLHYDDNRESFPVGFDYW	IGH	4	None	
12	2655	CARGKHTETTSVTAWHFDLW	IGH	2	None	
12	2656	CANGRLRSPYSFDYW	IGH	2	None	
12	2657	CAKDTTVTTATESYYYGMDVW	IGH	2	None	
12	2658	CARDGVTHEGNYYYYGMDVW	IGH	4	None	
12	2659	CARDNFEGFYGSGNSHFDYW	IGH	4	None	
12	2660	CVRTPTSDWHHYYFDYW	IGH	8	None	
12	2661	CAKYERLLWFGELLSVFDYW	IGH	2	None	
12	2662	CAHRGGQLKGSFDYW	IGH	4	None	
12	2663	CAKDKFYGDYTMVIFDYW	IGH	2	None	
12	2664	CARYSGTYDRSFDYW	IGH	3	None	
12	2665	CAKSPTHIAVAGKGPPFDYW	IGH	3	None	
12	2666	CARLGGTYPDAFDIW	IGH	6	None	
12	2667	CARDKGGSKWYFDLWGRGTLVSVSSGAGTVSLCDAFEMW	IGH	3	None	
12	2668	CARGITRGEWGGPTFYSFDYW	IGH	2	None	
12	2669	CARDFKVTTPQNYYYYGMDVW	IGH	2	None	
12	2670	CALPISKNYPFCTGGDCYYGFDIW	IGH	2	None	
12	2671	CARHRGAAAAPDAFDIW	IGH	2	None	
12	2672	CARDSYCGGDCYYYGMDVW	IGH	18	None	
12	2673	CARDRGGRGPTTTDYW	IGH	6	None	
12	2674	CAKDLETGEVELTAAFDYW	IGH	69	None	
12	2675	CARDGTYSSGWITDFDLW	IGH	7	None	
12	2676	CARYFSDELAVPPSLKDDAFDIW	IGH	2	None	
12	2677	CARDDDYGEGVEYFDYW	IGH	8	None	
12	2678	CAKDRNSITGTTVDYW	IGH	2	None	
12	2679	CARAQRSAWLVDYW	IGH	11	None	
12	2680	CARSTATGWFFDFW	IGH	3	None	
12	2681	CARLDYDSEFVLFDHW	IGH	3	None	
12	2682	CARVWCPNSSSWYAPCSAFDIW	IGH	2	None	
12	2683	CSRKAAGSSRYYYSYGLDVW	IGH	3	None	
12	2684	CAHRRAGYAGSGSYYPGSFDYW	IGH	2	None	
12	2685	CATLWGTKDYW	IGH	22	None	
12	2686	CARDIAVADMRDYYYGMDVW	IGH	8	None	
12	2687	CARDPGPEGGGPFDIW	IGH	2	None	
12	2688	CAHRQDGGWYGNWFDPW	IGH	2	None	
12	2689	CARTYYYDNSGYYGGRSDFDYW	IGH	3	None	
12	2690	CAKDLSAQGYGDAFDIW	IGH	4	None	
12	2691	CARDQMRAAAGGSFYYYGMDVW	IGH	3	None	
12	2692	CGRVLGDSGTEYIDYW	IGH	2	None	
12	2693	CARVEMDNWNDGDAFHFW	IGH	3	None	
12	2694	CARDLVGGRSRSHYYYGMDVW	IGH	2	None	
12	2695	CAKDSRQYGSGSYTSLYYYGMDVW	IGH	3	None	
12	2696	CASGYSYGFDTDYW	IGH	15	None	
12	2697	CARHESMWRIWFDPW	IGH	3	None	
12	2698	CARTNGWYQHFDSW	IGH	9	None	
12	2699	CARWGDTAMVGTGYYFDYW	IGH	2	None	
12	2700	CTGRDGYNYAFDYW	IGH	19	None	
12	2701	CARDCLWCYGGSFDYW	IGH	7	None	
12	2702	CARSGYYYDSSGYPFDYW	IGH	19	None	
12	2703	CARYGAGWEAAAGYYFDYW	IGH	2	None	
12	2704	CVRDLNGYGGYDFDYW	IGH	7	None	
12	2705	CARTLGFSSSPYYFDSW	IGH	11	None	
12	2706	CARGMTTVTTSPLGAFDIW	IGH	2	None	
12	2707	CAREDSSGWTNHFDYW	IGH	21	None	
12	2708	CARDYMATIRDYYYYYGMDVW	IGH	5	None	
12	2709	CARARPDIVATIPWFDPW	IGH	2	None	
12	2710	CARHADPPGYSSSWYGSEIAFDIW	IGH	2	None	
12	2711	CHSAESSGIYRVF	IGL	11	None	
12	2712	CARDVLVRGVIIYYYYGMDVW	IGH	2	None	
12	2713	CARVRITMVRGVVFDYW	IGH	8	None	
12	2714	CAKDIESGSYYGTAFDYW	IGH	2	None	
12	2715	CARDPQNNMIVVVITGDFDYW	IGH	4	None	
12	2716	CARVHIVVVPGATGGWFDPW	IGH	5	None	
12	2717	CARDLGVATIHEQGVYYYYGMDVW	IGH	5	None	
12	2718	CARVYYDYVWGSYRPGYYFDYW	IGH	2	None	

12	2719	CVTHYCSGERCYANWFDPW	IGH	2	None	
12	2720	CARSTLYSSGWYEEGIDYW	IGH	3	None	
12	2721	CARVIYYYDSGGLFRDWFFDLW	IGH	3	None	
12	2722	CARDSMVTATNYYYYYGMDVW	IGH	4	None	
12	2723	CARHRTPYYDSEDAFDVW	IGH	3	None	
12	2724	CATSYYYDATAYYVDQYFFDYW	IGH	4	None	
12	2725	CAKFLRAANWYSSSWYLQPLDYW	IGH	2	None	
12	2726	CARDGSQDGYNPFSFDYW	IGH	2	None	
12	2727	CMQTTHLPHSF	IGK	3	None	
12	2728	CTTKNGFVMIRGVITDYW	IGH	5	None	
12	2729	CARSILTGSVFDYW	IGH	60	None	
12	2730	CAKHPVASLRGVHFFDYW	IGH	2	None	
12	2731	CDWSSDVCSSDLFDIW	IGH	6	None	
12	2732	CAKDWRRYCSSTSCYSIDYW	IGH	5	None	
12	2733	CARVGAPTRFLNKLDWYFDLW	IGH	10	None	
12	2734	CARDRGSSWSRDVHSWYFDLW	IGH	2	None	
12	2736	CARGPLLYYDILTGYYPPNPHYYYGMDVW	IGH	3	None	
12	2737	CASRDCSSTSCSLGNAFDIW	IGH	2	None	
12	2738	CARAVRGVMGFVFYYFDYW	IGH	2	None	
12	2739	CAKGISGYGYASYPEGFDMW	IGH	2	None	
12	2740	CARQPTSVRGFPFDYW	IGH	3	None	
12	2741	CARLLDHYYGSGSPLRENWFDPW	IGH	2	None	
12	2742	CTRVPRGTGIDPLDVW	IGH	2	None	
12	2743	CARDPYSMSGEYFFDYW	IGH	6	None	
12	2744	CAGSGYYWGLIDYW	IGH	10	None	
12	2745	CARDHRRGSPYYYYYYGMDVW	IGH	2	None	
12	2746	CTTENAPYGDYLRLDYW	IGH	2	None	
12	2747	CARDILGIPSYYYYGMDVW	IGH	6	None	
12	2748	CATVAGTPPLYYYYGMDVW	IGH	2	None	
12	2749	CARVGTAMVSFHHYYYGMDVW	IGH	5	None	
12	2750	CAKLTGWGGDCGGDCYPVFDFW	IGH	4	None	
12	2752	CARDSKSFTEGYYYYGMDVW	IGH	10	None	
12	2753	CARDTRESLLYYFEYW	IGH	2	None	
12	2754	CAGGYSSGWYNYYYYGMDVW	IGH	6	None	
12	2755	CAKEGGVYFVSGIYSYFDYW	IGH	3	None	
12	2756	CATVLCSTAICDRDSYYYYALDVW	IGH	3	None	
12	2757	CARGAPGCSGGSCILDYW	IGH	2	None	
12	2758	CAKGEHDYGDYRAYYYGMDVW	IGH	3	None	
12	2759	CATDVRSSGPYYYGMDVW	IGH	10	None	
12	2760	CARVPVGSNWYFINW	IGH	2	None	
12	2761	CARGYSSSWYVYYW	IGH	88	None	
12	2763	CARSRDGYNLWYFDLW	IGH	2	None	
12	2764	CARASQGGGYNPNFDYW	IGH	4	None	
12	2765	CAREEMEAAFDHW	IGH	4	None	
12	2766	CARVGYCSSTSCYGGYNWFDPW	IGH	4	None	
12	2767	CAKDPTYLAAAGIDYW	IGH	2	None	
12	2768	CARWRSVLGATTFDIW	IGH	2	None	
12	2769	CQQYTSYSPWSF	IGK	759	C3	7K8P
12	2770	CARDLGMATIVPPMYYFDYW	IGH	2	None	
12	2772	CARAGDSGIYYDSSGLDYW	IGH	4	None	
12	2773	CARGARYYDSSVYYYGMDVW	IGH	14	None	
12	2774	CASVVVVPATPGYYYYGMDVW	IGH	2	None	
12	2775	CAKDPDYGDYYGILFDYW	IGH	6	None	
12	2776	CARMATITVAFDYW	IGH	22	None	
12	2778	CAHRRGELYYYDSSGYYEYYFDYW	IGH	3	None	
12	2779	CARPAVIRGVVFWFFDLW	IGH	29	None	
12	2780	CARSNYYDKKSWGEAFDIW	IGH	3	None	
12	2781	CAKNRQESAAGVRFDPW	IGH	2	None	
12	2782	CAREVTVTTLGGDWYFDLW	IGH	3	None	
12	2784	CAKESRSGSYEEFDYW	IGH	5	None	
12	2786	CARSSGYSGYDFSRLFCGSCFTFDYW	IGH	2	None	
12	2787	CARDLTGVAAEDYYYGMDVW	IGH	7	None	
12	2790	CAMYDTSGSFDYW	IGH	10	None	
12	2791	CARDRILGRWFGELLHDAFDIW	IGH	2	None	
12	2792	CAKDWGCSGGSCYSSHEGSYYYYYGMDVW	IGH	2	None	
12	2793	CARDNRGYCSGGSCYSEGFDYW	IGH	8	None	
12	2794	CAKDQEYYYDNSGPDYW	IGH	8	None	
12	2795	CAKDLNYGGYIAAAGLCFDYW	IGH	3	None	

12	2796	CARGWQQLKDYYYGMDVW	IGH	2	None	
12	2797	CARGGWKKSADTAMVNWFDPW	IGH	4	None	
12	2798	CARLEDSSSWFNYYYYGMDVW	IGH	2	None	
12	2799	CAKEERVLLWFGELFIDYW	IGH	2	None	
12	2800	CARGRTVTTFYRPRPPPFDYW	IGH	2	None	
12	2801	CARDSVYGDYVYDYW	IGH	2	None	
12	2802	CAREIYYGSGSYPRHFDYW	IGH	2	None	
12	2803	CVRGFYYGSGSYYITSTTIYFDYW	IGH	3	None	
12	2804	CARDGYSGYEGKIYYYYGMDVW	IGH	6	None	
12	2805	CATGHLWFVSW	IGH	2	None	
12	2806	CAKGLIVVVITTPFDYW	IGH	2	None	
12	2807	CARDGTYGGYGVDYYYYGMDVW	IGH	3	None	
12	2809	CARDQVRGVTPQYYYYYGMDVW	IGH	4	None	
12	2810	CARGTIAVAGKEENYYYYYGMDVW	IGH	4	None	
12	2811	CAKSSHGVWGYSYGSCFDYW	IGH	6	None	
12	2812	CAHRRPRYDILTGYTTHNWFDPW	IGH	2	None	
12	2813	CAKTAGTVTTYYYYGMDVW	IGH	14	None	
12	2814	CAKDMGRDSSGWEHYHFYYGMDVW	IGH	4	None	
12	2815	CVTWDSTLSADWLF	IGL	2	None	
12	2816	CARDQGYYDSSGYYIAFDIW	IGH	3	None	
12	2817	CASGTNWGWDYFDYW	IGH	2	None	
12	2818	CAKDSVVRGVYDAFDIW	IGH	17	None	
12	2819	CARGGSSTWSLGIDYW	IGH	2	None	
12	2820	CAKDTRYDDVWGSYRHDAFDIW	IGH	9	None	
12	2821	CARRGSFNWNGIDYW	IGH	5	None	
12	2822	CVRGVPAAVSWGTYYAMDVW	IGH	2	None	
12	2823	CARDLTRTTDPEKYYYYGMDVW	IGH	2	None	
12	2824	CARDPPRSSGGFEEYFQHW	IGH	5	None	
12	2825	CARISWGYWFGELLGALDIW	IGH	2	None	
12	2826	CARHKSMVAIYYFDYW	IGH	2	None	
12	2827	CAKDWGSSSPGYFDNW	IGH	2	None	
12	2828	CAKGEGKRGGYGDYYFDNYFDYW	IGH	2	None	
12	2829	CARDLTGTTGDSYYYGMDVW	IGH	2	None	
12	2830	CARDQYEVRGISSYYYYYGMDVW	IGH	3	None	
12	2831	CARNHGGYVPYYFEYW	IGH	6	None	
12	2832	CARRNVNTTMISAFDIW	IGH	2	None	
12	2834	CAREMSGVPLVLDYW	IGH	4	None	
12	2835	CAKDLGYEISRTFDYW	IGH	2	None	
12	2836	CARAAGFYSSGWYDALDIW	IGH	2	None	
12	2837	CARRDWLAYYFDNW	IGH	2	None	
12	2838	CTREDSGGDWRVDYW	IGH	2	None	
12	2839	CVRDPLSSVWKIGNWYFDLW	IGH	16	None	
12	2840	CVRDGAVRGYSGYDYLPFDYW	IGH	12	None	
12	2841	CARDHYYDSSGYYYGQHNWFDPW	IGH	2	None	
12	2842	CARDSKQWLVSYYYYGMDVW	IGH	2	None	
12	2843	CARALGGSYYSGLDYW	IGH	9	None	
12	2844	CARDLVVVFGDNENHYYYYGMDVW	IGH	4	None	
12	2845	CAKGASFFGSGSPRDYW	IGH	2	None	
12	2846	CARDVYQRASYYYYGMDVW	IGH	2	None	
12	2847	CTSRIEVDGTGFDHW	IGH	3	None	
12	2848	CAKDQSSGYPYDYW	IGH	13	None	
12	2849	CAFGELRPGRFDYW	IGH	2	None	
12	2850	CAKTGYYYDSSGYYADPIDYW	IGH	2	None	
12	2851	CARILGATGFDSW	IGH	2	None	
12	2852	CARGGWYSGSGAVSLNWFDPW	IGH	47	None	
12	2853	CARDLHCSSTSCYASGFDYW	IGH	7	None	
12	2854	CARLRAEIAARIDYW	IGH	2	None	
12	2855	CANDYGDYNGSYYYYGMDVW	IGH	21	None	
12	2856	CAKGTQLWFPLDYW	IGH	5	None	
12	2857	CAKDAAWFGQFHYLDYW	IGH	3	None	
12	2858	CARDKAYAGQYYYYYYGMDVW	IGH	2	None	
12	2859	CAKDMITFGGVIVNVYGYYFDYW	IGH	2	None	
12	2860	CARGNNGDYVEAYYFDSW	IGH	6	None	
12	2861	CSKVAKNGHNLGSSFDHW	IGH	5	None	
12	2862	CAKDDLLTGYHDYW	IGH	6	None	
12	2863	CATGSVYSAYDGFDSW	IGH	2	None	
12	2864	CAHRAVAGHIDYW	IGH	13	None	
12	2865	CARVGNGYSALLFSEGFDIW	IGH	2	None	

12	2866	CARVVRRAAAGTPRFDPW	IGH	11	None	
12	2867	CAGETYKKYSYGTHPNDYW	IGH	2	None	
12	2868	CARAPAAGILGDWFDPW	IGH	2	None	
12	2869	CAKDQGSGSYGYYLSDYW	IGH	3	None	
12	2870	CQSYDTNNHSVF	IGL	93	C4	7JVC
12	2871	CATSRDSSSWIDYW	IGH	4	None	
12	2872	CAKPYYDILTGFDFDYW	IGH	6	None	
12	2873	CARDLFGRVGATLDYW	IGH	4	None	
12	2874	CAKQRRVYDYGDCFDYW	IGH	2	None	
12	2875	CATLISRVSNLDYW	IGH	2	None	
12	2876	CARERRKYYYESSGHFSRYSFDYW	IGH	4	None	
12	2877	CAKGIDRSYGFFYYW	IGH	4	None	
12	2878	CARDENEVVWFGDLAAAGETYYYFGMDVW	IGH	2	None	
12	2879	CALTLPGESGYYSYW	IGH	4	None	
12	2882	CAKDRITHGLGSFYFDYW	IGH	3	None	
12	2883	CAKESSSSVPPYNFDFW	IGH	6	None	
12	2884	CAKDSVWWLPSPGDYW	IGH	2	None	
12	2885	CARCGYSYGWYYYYGMDVW	IGH	19	None	
12	2886	CARDQIAVAGTSDYW	IGH	4	None	
12	2887	CARGGWSTMVRGVLAGWFDPW	IGH	5	None	
12	2888	CARVLNYYGSGSYYNVPSNAFDIW	IGH	2	None	
12	2889	CARAGNYYYSYW	IGH	8	None	
12	2890	CARGIAAAGTKRRSNWFDPW	IGH	2	None	
12	2891	CASRRGIDYGDFFHYYYYGMDVW	IGH	7	None	
12	2893	CARTRRDYDSSGYYPIDYW	IGH	3	None	
12	2895	CARHSGYTYSYVDYW	IGH	3	None	
12	2896	CTTENTEQQLAIDYW	IGH	3	None	
12	2897	CAHRRSHRGWVGHLGFDYW	IGH	2	None	
12	2899	CARDSFSYYGSGSYYQYYYGMDVW	IGH	3	None	
12	2901	CAKAGLFSIHHLDYW	IGH	2	None	
12	2902	CARESWARDTMVRGVIRMDGMDVW	IGH	6	None	
12	2903	CAKEGGPSEYGSGSYFPFGYW	IGH	2	None	
12	2904	CARHLSEMATNDYFDYW	IGH	3	None	
12	2906	CAKDESWHELYYYDSSGGFDYW	IGH	17	None	
12	2907	CAKDGIEAAGTPFYFDSW	IGH	2	None	
12	2909	CARDLSSSTSSTEPHYYYYGMDVW	IGH	9	None	
12	2910	CARLNPNYYGSESYYKILDYW	IGH	3	None	
12	2911	CARLTYYYGSGSYLTFDYW	IGH	2	None	
12	2912	CAKFDRRDDYGDHFDYW	IGH	15	None	
12	2913	CAKAIDITMIVPDYW	IGH	7	None	
12	2916	CARDSADYGDYVLFWGFDYW	IGH	4	None	
12	2917	CTRHLDVQGSGWYFGHYYYGMDVW	IGH	36	None	
12	2918	CARDPGASGSGSYYLDYW	IGH	2	None	
12	2920	CARDRLAVAGTDYYYGMDVW	IGH	514	None	
12	2921	CAKDFDLAIRGVSFDQW	IGH	2	None	
12	2922	CAREGYDYDILTGYKLRADYW	IGH	2	None	
12	2924	CARLDYTSGWYERAIFDYW	IGH	3	None	
12	2925	CAREGVTVTTENDYW	IGH	3	None	
12	2926	CARDSFPYYGSGGFDYW	IGH	4	None	
12	2927	CAKDSHIVATNYYYYGMDVW	IGH	8	None	
12	2928	CAKAAAGETFDYW	IGH	17	None	
12	2929	CARVFVDYDYVWGSYRPDAFDIW	IGH	2	None	
12	2930	CLHYNSYSNNF	IGK	2	None	
12	2931	CARGDQGTAMGHFDYW	IGH	2	None	
12	2932	CEARDGPHDYW	IGH	5	None	
12	2933	CTRGLQRSKYTYGNDAFDIW	IGH	2	None	
12	2934	CAKDSEDYGGNSVGAFDIW	IGH	2	None	
12	2936	CARVRDYGDYLNDAFDIW	IGH	2	None	
12	2937	CTTDAGRTPLTGYPYYFDYW	IGH	7	None	
12	2939	CARTVITPFYYYGMDVW	IGH	5	None	
12	2940	CAKDSAYYEGAFDIW	IGH	18	None	
12	2941	CARELGYCSGGNCPALDYW	IGH	9	None	
12	2943	CAKDSISARLRLPGAFDYW	IGH	2	None	
12	2944	CASHGRGLLGPTNGAFDIW	IGH	6	None	
12	2945	CTTDRVYDYVWGSYRPVDYW	IGH	17	None	
12	2946	CANAILWWVLDYW	IGH	7	None	
12	2947	CATTSPTYYGSGSLYAFDIW	IGH	2	None	
12	2948	CAESKPHCSSTSCYYYGMDVW	IGH	2	None	

12	2949	CAHSIQLWVAGFDYW	IGH	2	None	
12	2951	CCSYAGSSTFVGYVF	IGL	3	C2	7K8X
12	2952	CARLRTGSGSLAIDYW	IGH	4	None	
12	2953	CAKSDVRYPFSDFDYW	IGH	2	None	
12	2954	CARIFPFTYDSSGYYLFDYW	IGH	2	None	
12	2955	CAKDIHYDRSGGFDYW	IGH	2	None	
12	2956	CAKDMGCSSTSCHGWWYYYYGMDVW	IGH	4	None	
12	2957	CARGMFTYYYDSSGYHFDHW	IGH	4	None	
12	2958	CQSADSTVSYGGVF	IGL	2	None	
12	2959	CARDGNTVTTGMGDYW	IGH	2	None	
12	2960	CARDQYSHGRDVHGYYYGMDVW	IGH	6	None	
12	2961	CARDLQSGDCSGGSCYYYGMDVW	IGH	5	None	
12	2962	CARHRVRYFDWLIEAFDIW	IGH	2	None	
12	2963	CAKYAASPYHFDYW	IGH	7	None	
12	2964	CAKDWGPVPGVDYW	IGH	3	None	
12	2965	CAHRRADGDYLRGYFDLW	IGH	2	None	
12	2966	CATGLYIGSYLGAYGPFEYW	IGH	6	None	
12	2967	CAKEGRDGYNFEGCFDYW	IGH	2	None	
12	2968	CAKGDGSGYFGSYIDYW	IGH	2	None	
12	2969	CARDGFRGAATASYETVAGFDSW	IGH	3	None	
12	2970	CARTRAGSGDIGYYYYFGMDVW	IGH	11	None	
12	2971	CARDRPQINYYYGMDVW	IGH	21	None	
12	2972	CARPRDGSYLNAFDIW	IGH	69	None	
12	2973	CAREYYDSSGSQEGYDYW	IGH	2	None	
12	2974	CQSYDSSLSGPVVF	IGL	481	C4	7JVC
12	2975	CAKDRDATVHWDYW	IGH	4	None	
12	2976	CARSGAYYYDSPFPIDAFDIW	IGH	3	None	
12	2977	CTSEGGVGATVGVYYYYYGLDVW	IGH	2	None	
12	2978	CDWSSDVCSSDLGATHPASYYYYGMDVW	IGH	2	None	
12	2979	CAKDAADVVLWFGGFDYW	IGH	7	None	
12	2981	CTRGQAKYYPENSENGDWFDPW	IGH	2	None	
12	2982	CARSSWAAGGRGPSRYYYYGMDVW	IGH	2	None	
12	2983	CARDVSRGFARRDAFDIW	IGH	6	None	
12	2984	CARADYGDSVTRYYYYGMDVW	IGH	2	None	
12	2985	CVRLYAYGTYFYDYW	IGH	2	None	
12	2986	CAAGSSWDYFDYW	IGH	21	None	
12	2987	CTRQDWDYCDHW	IGH	3	None	
12	2988	CTRDIDSSGYYYGMDVW	IGH	4	None	
12	2990	CAKDGYNFSGDYYYYGMDVW	IGH	8	None	
12	2991	CAKVSGSGYNDEGFYFDSW	IGH	25	None	
12	2992	CAKDYRTETTGYYYYAMDVW	IGH	13	None	
12	2995	CASSTDGECFDYW	IGH	6	None	
12	2996	CARHSCSSTSCRGRFDPW	IGH	12	None	
12	2997	CARVGHYYDPFDLW	IGH	2	None	
12	2998	CAHRRRGYPYSFDHW	IGH	2	None	
12	2999	CAREMNDYGVYGWFDPW	IGH	4	None	
12	3000	CARGLRLAAAGTAPFGYW	IGH	3	None	
12	3001	CAKDHLASGSYPDYW	IGH	9	None	
12	3002	CARAGRAVAGDEHYYYYGMDVW	IGH	4	None	
12	3003	CAREVSVAVAGLFYFDNW	IGH	2	None	
12	3004	CARDMLPTVPWGFDYW	IGH	11	None	
12	3005	CARPYNSGWYNAFDIW	IGH	4	None	
12	3006	CARAEWVAAAVAFDYW	IGH	2	None	
12	3007	CAKDCLISGSNLNNLNYYYYGMDVW	IGH	2	None	
12	3008	CAKAAGSGWPSMSWYFDLW	IGH	4	None	
12	3009	CARMRKDYYYHYGLDVW	IGH	4	None	
12	3010	CARAKFVVVTAIDWYFDLW	IGH	2	None	
12	3011	CAKDPWTLEVAGLEWFDPW	IGH	4	None	
12	3012	CARVPQDHNNDAFDIW	IGH	8	None	
12	3013	CLRAEDTALYYCAKDTYSSIAAAGSFDYW	IGH	2	None	
12	3014	CARDLQPATAMSYNWFDPW	IGH	4	None	
12	3015	CAHTRIEYYYDSSGPYYFDYW	IGH	2	None	
12	3016	CARQIGDTGESDYW	IGH	4	None	
12	3017	CARDEIAAAPDGGYYYGMDVW	IGH	7	None	
12	3018	CAKQPVGITMMLVVLFRDEWYFDLW	IGH	214	None	
12	3019	CARVDKHYYDSIAGFWVDYW	IGH	2	None	
12	3020	CARDNVVVVAATPEGDYYYYGMDVW	IGH	3	None	
12	3021	CARGGWRKRDGYNFIAFDIW	IGH	2	None	

12	3022	CARENYYFQNSDYSLTRNAFDIW	IGH	2	None	
12	3023	CASFGLYFYW	IGH	2	None	
12	3024	CAKDRSVTNHYYWYGMDVW	IGH	2	None	
12	3025	CATPARQWLVQPFDYW	IGH	2	None	
12	3027	CARELINYDILTGSPYYYFDYW	IGH	2	None	
12	3028	CARDFPGYSSGWYSITQNSYDAFDIW	IGH	3	None	
12	3029	CSSYAGNNFPVVF	IGL	128	C2	7BWJ
12	3030	CARVTKLTSVEGVDWFDTW	IGH	2	None	
12	3031	CARDPGVLGFVGFDIW	IGH	4	None	
12	3032	CTSPKYYDILTGPFDYW	IGH	37	None	
12	3033	CARDSTEGYGIDYW	IGH	7	None	
12	3034	CARHPYYDSSGYYYKYNWFDPW	IGH	7	None	
12	3036	CAGGLGVVRGVLTPNCFDDW	IGH	13	None	
12	3037	CARELRFCSGGSCYPDDWFDPW	IGH	2	None	
12	3038	CARDLGGGLSRHYYFDYW	IGH	2	None	
12	3039	CARVQGELSSLFYYYYGMDVW	IGH	2	None	
12	3040	CAISWAGDTDGYPPPGDYW	IGH	2	None	
12	3042	CARVQSVDTAMDYYYGMDVW	IGH	3	None	
12	3043	CAKDHNIAVAGLFGDYYFDYW	IGH	2	None	
12	3044	CTSDPDSYGSYW	IGH	5	None	
12	3045	CVKGGTAVADTSTGSDDAFDIW	IGH	4	None	
12	3046	CARNVWDFLGNWFDPW	IGH	2	None	
12	3047	CAKDALYFHGSGSSLPFDVW	IGH	6	None	
12	3048	CASEHTSAYWRAFDGW	IGH	4	None	
12	3049	CQTWDTNTARGVF	IGL	4	None	
12	3050	CARDRVATPSYYFDYW	IGH	16	None	
12	3051	CARERALSAGTLDYW	IGH	2	None	
12	3052	CARDTGFELPGYYYYYGMDVW	IGH	4	None	
12	3053	CARAWGSLHDAFDIW	IGH	2	None	
12	3054	CVKDNFSWDVVVALTATGAFDSW	IGH	4	None	
12	3055	CARDGVVVAANGEGQKYYYYGMDVW	IGH	5	None	
12	3056	CARVADAANIDYW	IGH	9	None	
12	3057	CAKDVRGSSGWYGAFDIW	IGH	2	None	
12	3058	CATLSLRPHVFDFW	IGH	2	None	
12	3059	CALPICDYYFYYGIDVW	IGH	2	None	
12	3060	CARDLTVVNDSYYYYYGMDVW	IGH	11	None	
12	3061	CATWESVWLRSTPFDYW	IGH	19	None	
12	3063	CARLRSYSDAPGYVYLDYW	IGH	2	None	
12	3064	CARQLKRITMVRGVTPFDYW	IGH	2	None	
12	3066	CARGAYTYGIPNFDSW	IGH	3	None	
12	3067	CARSPDYGDYGYYGMDVW	IGH	3	None	
12	3069	CARGEHYGSGKWWWFDPW	IGH	3	None	
12	3070	CARVGWTTVVTKYYYYGMDVW	IGH	5	None	
12	3071	CAKDSLPMRWLQLPRVDYW	IGH	4	None	
12	3072	CAKDSSVGWYSSGWYYFDYW	IGH	8	None	
12	3074	CAHSNVLLWFGERPLLPHQPSERDPYFDYW	IGH	2	None	
12	3075	CARDKALMAAIFPYYYGMDVW	IGH	2	None	
12	3076	CARDTYSDSNYGQVSYYYGMDVW	IGH	4	None	
12	3077	CARDHFGFDVVITPCDYW	IGH	6	None	
12	3078	CARGQTYYDSSGSPNYYYGMDVW	IGH	3	None	
12	3079	CARGGSGYYLEYFQHW	IGH	21	None	
12	3080	CAKDAGPLITMVRGVRYYYYGMDVW	IGH	2	None	
12	3081	CARELLHYYDSSGYELDAFDIW	IGH	6	None	
12	3082	CAKDLGYDWKTHSTFDYW	IGH	2	None	
12	3083	CSKVLAWDMVDYW	IGH	9	None	
12	3085	CQSTDSSSYYRVF	IGL	8	None	
12	3086	CATVMSEAATGVYYFDQW	IGH	5	None	
12	3087	CAKDLASTWYNDPFDYW	IGH	3	None	
12	3088	CARSGLVRGVNIDEFDHW	IGH	7	None	
12	3089	CAKDMGQIYYYYGMDVW	IGH	3	None	
12	3090	CAKGNTAMAYHGDAFDIW	IGH	2	None	
12	3091	CASLGYDSSGYLGAIFDYW	IGH	2	None	
12	3092	CASGTPRYQLRPDGFDIW	IGH	3	None	
12	3093	CAKVSTSEWHFDYW	IGH	2	None	
12	3094	CARDSSIRFGESWYFDLW	IGH	2	None	
12	3095	CARGLVRGSDAFDLW	IGH	8	None	
12	3096	CAKDRGYGDVMEGYFDFW	IGH	6	None	
12	3097	CARRGTSGYYYSFDFW	IGH	17	None	

12	3098	CAKGKQWLVRSYFDYW	IGH	2	None	
12	3099	CARHGGGPRDGFDYW	IGH	5	None	
12	3100	CVRDNWKNAPVFDLW	IGH	2	None	
12	3101	CARSESLFDSAYDSFYFDFW	IGH	2	None	
12	3102	CAKDMWAVTNGSGSYYYYYGMDVW	IGH	5	None	
12	3104	CATTSPYSTGSWFDPW	IGH	3	None	
12	3105	CTTDAVGATWAFDIW	IGH	6	None	
12	3106	CARETSEYYYDSSGYHVYYFDYW	IGH	2	None	
12	3107	CARNGYTYGTTHDAFVIW	IGH	2	None	
12	3108	CARLWYSGYDFPGLFDYW	IGH	16	None	
12	3109	CAHRIYGGNSFPTSDAFDIW	IGH	2	None	
12	3110	CARDSALYCGGDCSSTYAFDIW	IGH	2	None	
12	3111	CARATDSGSYYGYYYYGMDVW	IGH	2	None	
12	3112	CARREVDYYDSSGYGLGAYFDYW	IGH	2	None	
12	3113	CAKGAELGYSYGYYFDYW	IGH	2	None	
12	3114	CARTGYTSAWVLDYW	IGH	13	None	
12	3115	CAKVGSSGYETHEYFDYW	IGH	6	None	
12	3116	CTKNYPFCTGGDCYYGFDIW	IGH	187	None	
12	3117	CQSADRIGAPWVF	IGL	6	None	
12	3118	CAKSSWIDSSGYHYDYW	IGH	5	None	
12	3119	CAKDWHDYGGNSGTGYYYGMDVW	IGH	8	None	
12	3120	CQSADSSTSHPVIF	IGL	8	None	
12	3121	CAREIRFLGPRFDSW	IGH	2	None	
12	3122	CAKRDSSDFLGYFDYW	IGH	7	None	
12	3123	CARGWLTGDDSADYW	IGH	5	None	
12	3124	CAKSFSWWFGEFDYW	IGH	4	None	
12	3125	CAKTSIPYYYYYYGMDVW	IGH	2	None	
12	3126	CARETQTTVPDYYYYYGMDVW	IGH	21	None	
12	3127	CAREGDSSNWYNLRVGWFDPW	IGH	2	None	
12	3129	CARVGWSTGTGSYYYYGMDVW	IGH	15	None	
12	3131	CTTNLGVGTTDFYFDNW	IGH	2	None	
12	3132	CARDVSYYGLRSYEGTDYW	IGH	2	None	
12	3133	CTTTTPSYNFGAGWDYW	IGH	2	None	
12	3134	CAKGPSGSYYDRFDLW	IGH	2	None	
12	3135	CAKDYLAVAGTGGLSQAYYYYYGMDVW	IGH	5	None	
12	3137	CARIQLWLNYYYYYGMDVW	IGH	15	None	
12	3138	CASLSLDTAMPWDYW	IGH	2	None	
12	3139	CARGPGGYFPLDYW	IGH	20	None	
12	3140	CARDTVTGVAGTTYYYGMDVW	IGH	2	None	
12	3141	CARDKRWLPTPESYYYYGMDVW	IGH	5	None	
12	3142	CANSHIAAAGDPFDYW	IGH	23	None	
12	3143	CARDRRQLWLTGEYYYYYYGMDVW	IGH	9	None	
12	3144	CAKAENFGFGIDYW	IGH	9	None	
12	3145	CARERSVIDHPYDAFDIW	IGH	2	None	
12	3146	CAMDTTTWMRADYW	IGH	3	None	
12	3147	CARDSDSIGQSNNYYYYGMDVW	IGH	2	None	
12	3148	CAREGGQGYDSSGYYFESDYW	IGH	4	None	
12	3149	CARDPLPQYDSSSPYYLDSW	IGH	2	None	
12	3150	CAKDQVGYDSAKLDYW	IGH	7	None	
12	3151	CAKDSVRYGHKTIDYW	IGH	2	None	
12	3152	CIREGYRSLKTWRDYW	IGH	3	None	
12	3153	CAKKVYGDYDNWFDPW	IGH	2	None	
12	3154	CAKDNRVVRGVIHPFDYW	IGH	2	None	
12	3155	CVKRSSGSYWDAFHVW	IGH	2	None	
12	3156	CARDMYSGYDGSLYYYGMDVW	IGH	2	None	
12	3157	CARVVGSDSSGYYQVFDFW	IGH	5	None	
12	3158	CARRTYYYDSSGYFAVGGYWYFDLW	IGH	2	None	
12	3159	CARDLEMATIPGVYYYYGMDVW	IGH	7	None	
12	3160	CARDGWGTGGGNLRRDYYYYGMDVW	IGH	2	None	
12	3161	CARGGTPSTPSIADAFDIW	IGH	3	None	
12	3162	CARQSSSSWPIYFDYW	IGH	2	None	
12	3163	CARQKRFFGEPQEFDHW	IGH	4	None	
12	3164	CARADESGGSLLYYYYGMDVW	IGH	4	None	
12	3165	CARGGVSSSSWWAYYFDYW	IGH	4	None	
12	3166	CARVYSSSGVAFDIW	IGH	2	None	
12	3167	CARPMVRGVMSWFDPW	IGH	2	None	
12	3168	CAKDQGGDGSVPGYNGFDYW	IGH	4	None	
12	3169	CARTVDTVMAVWYYFDLW	IGH	2	None	

12	3170	CARDQEGGWYSDYW	IGH	20	None	
12	3171	CARGASNAFDMW	IGH	8	None	
12	3172	CARPTYYYDSSGPSGCFDPW	IGH	2	None	
12	3173	CARGLYSSSWYGSRHWFDPW	IGH	35	None	
12	3174	CARCLLWFGESRNYFDYW	IGH	2	None	
12	3175	CARKYGSGRYYFYYFDNW	IGH	2	None	
12	3177	CAREIYDSSGYYYGLSRAKGTFDYW	IGH	2	None	
12	3178	CARHGEDNYGRVFDYW	IGH	2	None	
12	3179	CARDMITFGGVIADRSDYW	IGH	4	None	
12	3180	CARQDIYSSTPYDYW	IGH	5	None	
12	3181	CARAEDTAMVIGSLDYW	IGH	2	None	
12	3185	CQRYNNWPPMYSF	IGK	37	C3	7K8R
12	3186	CAKDRRRNVDTADYW	IGH	2	None	
12	3187	CARDLGRTTGTNYYYYGMDVW	IGH	9	None	
12	3188	CARDRSPVTTENNWFDPW	IGH	6	None	
12	3190	CARARSLYCGDDSCDAYDIW	IGH	13	None	
12	3191	CASWKLADYGDYGINYYYYGMDVW	IGH	2	None	
12	3192	CARRSLGDYESYFDYW	IGH	3	None	
12	3193	CARIDTGDYYRFDYW	IGH	5	None	
12	3194	CARDGGSYDSSGFHMAGNFDYW	IGH	6	None	
12	3195	CARGKGGIAENWFDPW	IGH	2	None	
12	3196	CQSADSDLAYNWVF	IGL	5	None	
12	3197	CAKDRIAAAGLGFGDYW	IGH	11	None	
12	3198	CARLQYSVYDRGFDSW	IGH	6	None	
12	3199	CVRDFRGLGATGTFYYYGMDVW	IGH	6	None	
12	3201	CARHGPARSWFDLW	IGH	2	None	
12	3204	CSRSYSGSYYYGMDVW	IGH	2	None	
12	3205	CARSGTGYPDYYAMDVW	IGH	4	None	
12	3206	CQQYDSPSPALTF	IGK	29	None	
12	3207	CAKDGYYYDSSGYYSVYYYGMDVW	IGH	2	None	
12	3208	CARVRNVALTGTSGYFDYW	IGH	2	None	
12	3209	CARDRGSTVVTPSFDYW	IGH	2	None	
12	3210	CTTDDPSLYGSGVYYNYYYGMDVW	IGH	3	None	
12	3211	CAREGYYDGDGYYYWYFDLW	IGH	5	None	
12	3212	CARDSYDYVWGSYRYNLDWYFDLW	IGH	6	None	
12	3213	CARDPSSGWVPRFYFDYW	IGH	2	None	
12	3214	CARFNRLGGYSYSPFDYW	IGH	2	None	
12	3215	CAREGHVHGGYSGYDPSYYFDYW	IGH	2	None	
12	3216	CARPVLRYFDWPVGGNNWFDPW	IGH	2	None	
12	3217	CAKDLTYYYDSSGQNDYW	IGH	2	None	
12	3218	CARDFRGWYASGWNPLGYFDLW	IGH	4	None	
12	3219	CTRQGAVAGDYW	IGH	8	None	
12	3220	CARRGDGAFRSSPNYFDYW	IGH	3	None	
12	3221	CARDMLYIAAAGTYYYYYGMDVW	IGH	21	None	
12	3222	CARDQDGFGELLYIYYGMDVW	IGH	6	None	
12	3223	CAKSGRRWELLLYEYYFDYW	IGH	2	None	
12	3224	CARTRPRGPNHPYFFDYW	IGH	72	None	
12	3225	CAKDGDQYSSGWYVSWFDPW	IGH	2	None	
12	3227	CAREDYDSSGYSTGFDYW	IGH	24	None	
12	3228	CARDRSGSYLFWFDPW	IGH	6	None	
12	3229	CAKGDRNIFYFDTGDDYW	IGH	6	None	
12	3231	CAKDALYSGYDWGYFDYW	IGH	6	None	
12	3232	CARDQYGSGWTPDAFDIW	IGH	6	None	
12	3233	CAKEEMTSSGPYSYDYW	IGH	2	None	
12	3234	CARGTDYFEISEDSSAPRRAGYFDLW	IGH	2	None	
12	3235	CESADSSGENVIF	IGL	3	None	
12	3236	CARDHRSDGDYVSYYYGMDVW	IGH	4	None	
12	3237	CARDKGYCSGTSCIFDYW	IGH	2	None	
12	3238	CTVYIELSGLRSEDTAIYYCARVRAVIGFYYFDLW	IGH	2	None	
12	3239	CLQDHNYRSLTF	IGK	4	None	
12	3240	CAKDWTYGSGSYYNHDAFDIW	IGH	2	None	
12	3242	CAKDDLALFGEFTIDYW	IGH	12	None	
12	3243	CARMEGYCSGGSCATPFDYW	IGH	4	None	
12	3245	CAKVDKYYYDISGSFDSW	IGH	2	None	
12	3246	CARRDCSGGSCYGNAFDIW	IGH	5	None	
12	3247	CARWGSGNAGNYYHYGMDVW	IGH	2	None	
12	3248	CARDLGSRGYYYRYYFDYW	IGH	2	None	
12	3249	CAKGVSSGWYLVYFDYW	IGH	4	None	

12	3250	CAKDRHGYIQYYFGYW	IGH	8	None	
12	3252	CARDTAMVSDYYYGMDVW	IGH	23	None	
12	3253	CARDLAVFGNPAKQALYYYYGMDVW	IGH	2	None	
12	3254	CAKAQIPYSSGTYYNFYFDYW	IGH	2	None	
12	3255	CQKRDNWPPGFTF	IGK	5	None	
12	3256	CARDQNRVRFGEPTEDALDIW	IGH	55	None	
12	3257	CAIHSSGWYGGAFEYW	IGH	2	None	
12	3258	CARDPTVTSPSGWDYYYGMDVW	IGH	2	None	
12	3259	CQQSDSLARFTF	IGK	4	None	
12	3261	CAKDQRLEPRSQSGIAVAGWGFDYW	IGH	2	None	
12	3262	CARHSDIVATEEYYFDYW	IGH	2	None	
12	3263	CARGKIGYSGYFVYW	IGH	9	None	
12	3264	CAREGLYYYDSSGYAGVFDYW	IGH	3	None	
12	3265	CAKDSGGIVGAGNFDYW	IGH	2	None	
12	3266	CATERGLLRYFVHSWFDPW	IGH	2	None	
12	3267	CARGYMTTFGFDHW	IGH	5	None	
12	3268	CLLSYSGAWVF	IGL	187	None	
12	3269	CARDTPFHHYYDSSGYFDYW	IGH	2	None	
12	3270	CAHSSSPYDYVWGSYRLRDPLYYFDYW	IGH	2	None	
12	3271	CAREVTTVMGWFDPW	IGH	17	None	
12	3272	CARDDYSSGHAIEGGAFDIW	IGH	4	None	
12	3273	CARDKGGSKWYFDLW	IGH	6	None	
12	3275	CARGRSYYYSSSSVWLDPW	IGH	3	None	
12	3276	CATGVDTAILFDYW	IGH	20	None	
12	3277	CARSGWSTLGYYYYGMDVW	IGH	21	None	
12	3278	CARDGCSGDCYHGYFEHW	IGH	2	None	
12	3279	CARHDLGGVGGIDYW	IGH	2	None	
12	3280	CARVAPSVEPTVPFDYW	IGH	4	None	
12	3281	CAKGLSASGELLSPFDYW	IGH	10	None	
12	3282	CARDLEGGWYSGWFDPW	IGH	3	None	
12	3283	CARGGAWFEYFHHW	IGH	7	None	
12	3284	CARDCSSPTFCYYYYGMDVW	IGH	2	None	
12	3285	CTRDYGNYYWYFDFW	IGH	2	None	
12	3286	CARQPLLYYYDSSGYYPFDYW	IGH	2	None	
12	3288	CAKDTGMATMGDYFDYW	IGH	2	None	
12	3289	CARDFSVRWYSSSPARHQYYYYYGMDVW	IGH	2	None	
12	3290	CAKDGRVVSAGHSWIDPW	IGH	6	None	
12	3291	CAAWDNNLNGRLF	IGL	7	None	
12	3292	CASGFSYGGTADRYFERW	IGH	2	None	
12	3293	CANKGKILTAFPYW	IGH	10	None	
12	3294	CARRFGNYGFFFDTW	IGH	2	None	
12	3295	CAGDNSGWRFGYW	IGH	4	None	
12	3296	CARARHYYDSSGYYLGWYFDLW	IGH	4	None	
12	3297	CASGYSYGYVDYW	IGH	18	None	
12	3298	CARAFWRYGDYYYYYGMDVW	IGH	5	None	
12	3300	CAKSGDSNFLHCSSTSCYAGPFDYW	IGH	2	None	
12	3301	CDWSSDVCSSDLFGEHFYFDHW	IGH	2	None	
12	3302	CAKDRRGYVDFW	IGH	3	None	
12	3303	CARGSSKEEWLANFDYW	IGH	2	None	
12	3304	CDWSSDVCSSDLRAHSFGRDAHGVVREEGDTHLASQISQINPDDYW	IGH	2	None	
12	3305	CSAWDSSLTSWLF	IGL	17	None	
12	3306	CARSVGVSGSYRLFDNW	IGH	2	None	
12	3307	CAKDPLGYCSGGSCPQPDDYW	IGH	5	None	
12	3308	CARVLAAEYYHDGSAYYYPQYFDCW	IGH	7	None	
12	3309	CARDSMGPAPPYYFDYW	IGH	4	None	
12	3310	CARRLYYNSGSFLTGLVFDYW	IGH	2	None	
12	3311	CARVGWFAFRGSVTDALDIW	IGH	4	None	
12	3312	CAKDRKAGDGYSEFDTW	IGH	4	None	
12	3313	CAHSQYYYDSSGYSSYWYFDLW	IGH	2	None	
12	3314	CVRGRDGFNWLDYW	IGH	4	None	
12	3315	CARLRYYDSSGWYYFDYW	IGH	4	None	
12	3316	CARGIIAAAGMVQAVNYYYYGMDVW	IGH	2	None	
12	3317	CARDNVVVTANDGGHYYYYYGMDVW	IGH	2	None	
12	3318	CARGYCRSTTCYFDYW	IGH	3	None	
12	3319	CAKLGMSIAAAGLPEYFQHW	IGH	5	None	
12	3320	CASQSTMVRADDYW	IGH	2	None	
12	3321	CARRPYSSDQFIFDQW	IGH	2	None	
12	3322	CDWSSDVCSSDLLLLLLLHLLLSLLFLLSCLMFLRVHLNAWFLPW	IGH	2	None	

12	3323	CARISIAVAGVDYW	IGH	59	None	
12	3324	CQQLNGSPRF	IGK	4	C1	7K8N
12	3326	CARDPPYYYDSTGDAFDIW	IGH	2	None	
12	3327	CAKEALYYDSSGYLWYFDYW	IGH	2	None	
12	3329	CARHAETPSGYDLYYFDYW	IGH	2	None	
12	3330	CARNPYSSGWRGYYYYSGMDVW	IGH	2	None	
12	3331	CAKDPHGGYDSSGYWYFDYW	IGH	2	None	
12	3332	CAKGLSAWSTISPFDSW	IGH	9	None	
12	3333	CARDPGQPTTFDYW	IGH	4	None	
12	3334	CARDMESRGGMAYYYGMDVW	IGH	2	None	
12	3335	CARQGSQDTSWSWYFDLW	IGH	4	None	
12	3336	CARGNDPPDYGDYFDYW	IGH	2	None	
12	3337	CAKDYLVMDNNYYYYGMDVW	IGH	13	None	
12	3339	CARGIGAYYSVDGVDVW	IGH	4	None	
12	3340	CAKEDSSGGGAYYYGMDVW	IGH	8	None	
12	3342	CAKDASGWYRFDSW	IGH	4	None	
12	3343	CAKDMVRGVLVRGDAFHMW	IGH	2	None	
12	3344	CAKDTFYGGKEGEDYYYYGMDVW	IGH	3	None	
12	3345	CAKDSVDSSGYTAFAGDFGTPNYFDYW	IGH	3	None	
12	3346	CAKERYTGSFGYFFDYW	IGH	2	None	
12	3347	CARPGGQWLVQGSDYFDYW	IGH	4	None	
12	3349	CARDGVVFPPNAFDIW	IGH	2	None	
12	3350	CQQYGSPQPGYRF	IGK	9	C1	7K8N
12	3351	CVRGMSTNRGDWFDPW	IGH	2	None	
12	3352	CARERGIVATPHDAFDIW	IGH	2	None	
12	3353	CAKPGGSQRWLYDYW	IGH	3	None	
12	3355	CAKRRVRGVTITRYYFYAMDVW	IGH	2	None	
12	3356	CTSWLFAHFDYW	IGH	3	None	
12	3357	CARIATYCDGEKCFSGWFDSW	IGH	3	None	
12	3359	CARGRAYGMAWFNYW	IGH	2	None	
12	3360	CAKDKEGYSSSWYWEFDYW	IGH	4	None	
12	3361	CARRFYPDSSGYYFFEYYFDYW	IGH	77	None	
12	3363	CARTKGWLRPDFFDYW	IGH	2	None	
12	3364	CTRDQGMVTDLPNYYYGMDVW	IGH	2	None	
12	3365	CAKDRTYANYDPYFDSW	IGH	13	None	
12	3366	CTRHEDTIFSDDAFDIW	IGH	2	None	
12	3367	CATKLADYDLLTGYSNYYYYGLDVW	IGH	10	None	
12	3368	CARDLFPYYYDSSGYSSGDAFHIW	IGH	2	None	
12	3369	CAREGGYITGLDAFDLW	IGH	4	None	
12	3370	CARSRRVAAAGTPNWFDPW	IGH	4	None	
12	3371	CAKVLDYSYSLYYFDFW	IGH	2	None	
12	3372	CASGSYYYGSTPEYYFDYW	IGH	2	None	
12	3373	CAHAHYDILTGYRWGESFDYW	IGH	2	None	
12	3375	CAKQGGLHWLDYW	IGH	9	None	
12	3376	CARAGRGTLYYFDYW	IGH	22	None	
12	3377	CARGPYFPEEVAGPPPFLGHDYW	IGH	3	None	
12	3378	CAKDFVVVPAGTEGSTNYYYYGMDVW	IGH	2	None	
12	3379	CARDFPTYCSGDSCYPLDAFDLW	IGH	21	None	
12	3380	CAKALYYFDGSSYSYAFDIW	IGH	12	None	
12	3381	CVRGWGLVSGSTWGVGDYW	IGH	2	None	
12	3382	CAKDHQRIANDYYYYGMDVW	IGH	2	None	
12	3383	CARDLRLVDQWIQLWLDVVAYYYYGMDVW	IGH	2	None	
12	3384	CARNYYDSSGYYLQDYYYGMDVW	IGH	2	None	
12	3385	CARLPGSYYNFDYW	IGH	3	None	
12	3386	CGHFHYYSAMDVW	IGH	3	None	
12	3387	CARDGWSPQGGSHYYYYGMDVW	IGH	5	None	
12	3388	CARTYYHDSTGYYSDHW	IGH	13	None	
12	3389	CARDRTLNYYDNPWGDAFDIW	IGH	2	None	
12	3390	CARDDYASGSYSVGDYW	IGH	2	None	
12	3391	CTRRQQVVQGAYYYYGLDVW	IGH	5	None	
12	3392	CATDSSRLYTSAWYYFDYW	IGH	2	None	
12	3393	CASPSVTTYYYYGMDVW	IGH	4	None	
12	3394	CAKRGDRVSVLGAHYFDSW	IGH	4	None	
12	3395	CARVTTDSSGFYHYCDYW	IGH	2	None	
12	3396	CARDLAFSYISGSYDYW	IGH	2	None	
12	3397	CARYLGIQLWLPDYW	IGH	7	None	
12	3398	CASRGVVMMDHFDYW	IGH	2	None	
12	3399	CARDQEERFGESYYYGMDVW	IGH	2	None	

12	3400	CAHNYYYGSGNSNFEYW	IGH	2	None	
12	3401	CAKNSWDYDYVWGSYRYINYFDYW	IGH	5	None	
12	3402	CARDQNICSSTSCRYYYYYGMDVW	IGH	2	None	
12	3403	CARAPTGTYFDYW	IGH	11	None	
12	3404	CGRGAYNYGYDYW	IGH	4	None	
12	3405	CAKATSGSFARFDYW	IGH	2	None	
12	3406	CARDRRWLQFNWFDPW	IGH	6	None	
12	3407	CAKDPGGSSRWEYIFDSW	IGH	7	None	
12	3408	CTRGHVDKVATIQDYYFDYW	IGH	3	None	
12	3409	CAREPLLRSGGRAGWFDPW	IGH	3	None	
12	3410	CAKDGGWPYSSSWYYFDYW	IGH	36	None	
12	3411	CAKASQWGSFGYYYGMDVW	IGH	221	None	
12	3412	CARRSVGATHDAFDIW	IGH	2	None	
12	3413	CVKDHMNGLYNWDEVRDYW	IGH	2	None	
12	3414	CARGETSGYDSFVDYW	IGH	4	None	
12	3415	CAYVIGDLLTGYQNVFSIGSFDYW	IGH	4	None	
12	3416	CAKAMDREALLNYYDYW	IGH	6	None	
12	3417	CARGGRGMLASILDAFDIW	IGH	2	None	
12	3418	CARDLWFGELIFDYW	IGH	9	None	
12	3419	CSRHSEGSGDYFDYW	IGH	4	None	
12	3420	CARVNSPWYYFDNW	IGH	6	None	
12	3421	CGGIVATAGWDGMDVW	IGH	6	None	
12	3422	CAKVRIQLWLLSTGDFDYW	IGH	2	None	
12	3423	CARGGEGSWYRFPFDCW	IGH	2	None	
12	3424	CAKLKQWELLAPFDYW	IGH	13	None	
12	3425	CARDSGSFHFDNW	IGH	9	None	
12	3426	CAKDMPGDNGSDATFDSW	IGH	2	None	
12	3427	CARLRLGQYYYDSSGKNIFDYW	IGH	7	None	
12	3428	CARARRDGYNYYYYYGMDVW	IGH	2	None	
12	3429	CAKAAESGNYALTWFDAW	IGH	2	None	
12	3430	CARWYGDYELGFDDW	IGH	3	None	
12	3431	CLPHNAYPSHF	IGK	2	None	
12	3432	CAKDHFVSGPHSSSYYYYYYGMDVW	IGH	3	None	
12	3434	CAKEWGYGDYDQPTYYYYGMDVW	IGH	2	None	
12	3435	CAREEEQGYSYASDYW	IGH	6	None	
12	3436	CARRRGTDYDTSVYFDHW	IGH	5	None	
12	3437	CATQLLWFGEFTLDAFDIW	IGH	4	None	
12	3438	CARLGVGYYGSGSYLDYW	IGH	2	None	
12	3439	CTTEMYYYGPFDYW	IGH	4	None	
12	3440	CAREGCSGYGLDYW	IGH	11	None	
12	3441	CAKDMGGFSGGIFDYW	IGH	6	None	
12	3442	CAKWDAPAYDSSGYLDSW	IGH	2	None	
12	3443	CARDWGVGSGSYHYYYYGMDVW	IGH	4	None	
12	3444	CARVRTIPAAAFDFW	IGH	3	None	
12	3445	CKSADSSSGYSWVF	IGL	4	None	
12	3446	CARQYGGYVSPTFDYW	IGH	2	None	
12	3447	CARIRPRYCSGGICYSLYYFDYW	IGH	3	None	
12	3448	CAKGRTGDVSNWFDPW	IGH	5	None	
12	3449	CQQYGRSPPSAYGRSPPSLTF	IGK	5	C1	7K8N
12	3450	CASRRGNYYDSSGYYGDAFDIW	IGH	6	None	
12	3451	CVRGGIQVAGIDLFDFW	IGH	2	None	
12	3452	CARIPVPFVTMGMTGFDPW	IGH	2	None	
12	3453	CAKDIGSGEAVAPGFDYW	IGH	2	None	
12	3454	CARDIVFEGEPDDAFDIW	IGH	7	None	
12	3455	CARDGFVVVPAAMIESYYYYGMDVW	IGH	6	None	
13	2	CAREWGYYDSSGYPYYYYGMDVW	IGH	7	None	
13	3	CAREMWEKFGEESAFDVW	IGH	4	None	
13	4	CDWSSDVCSSDLANAAFDIW	IGH	2	None	
13	6	CAKGPDLSDYGSGSYFDYW	IGH	2	None	
13	7	CAKDFRHYYDSSGYPRSYYYYGMDVW	IGH	2	None	
13	9	CQQYGSSSIIF	IGK	145	C1	7K8N
13	10	CARGVRIFGVVEERWFDPW	IGH	5	None	
13	11	CVREGGCSTSNCYESGWFDPW	IGH	4	None	
13	12	CARGFKYDFWSSEYYQDYMDVW	IGH	3	None	
13	15	CARSSCGYGYFDNW	IGH	4	None	
13	16	CARVNLAVTTVTTRRNYHPYYFDYW	IGH	2	None	
13	17	CARGQRLARSQFWFYMDVW	IGH	2	None	
13	19	CANLGDYYDSKIDYW	IGH	2	None	

13	20	CARGFHEFCSGESCYGLGWFDPW	IGH	2	None	
13	22	CAREGRDSYGSTSLCFDYW	IGH	5	None	
13	25	CARDSAVATIGGAYYYYGMDVW	IGH	2	None	
13	26	CARDRSGSYHSYYDYW	IGH	2	None	
13	27	CAHRGPGTYYNWFDPW	IGH	2	None	
13	29	CAKGYYDSSGYYYAGDYYYGMDVW	IGH	2	None	
13	30	CARTTTSTAWSNWFDRW	IGH	6	None	
13	31	CARGIASLSVWFHPW	IGH	2	None	
13	33	CAKLSGPDYYMDVW	IGH	2	None	
13	34	CARDLVGATDYYYYYGMDVW	IGH	8	None	
13	35	CARELDGGSTSCHFDHW	IGH	2	None	
13	36	CFAIPGSLDWFDPW	IGH	4	None	
13	37	CAKDYIVTTTTPIDYYYGMDVW	IGH	2	None	
13	39	CARVAGTAPHLSYYYFDNW	IGH	10	None	
13	42	CAKDKGDGARVYYGGNSGEFDYW	IGH	2	None	
13	43	CARGDGYFEYW	IGH	6	None	
13	45	CARDFCSSINCYGYNYFDSW	IGH	4	None	
13	46	CQQRGSWPPEITF	IGK	265	C1	7BZ5
13	47	CAKDSRVFLLGADYYFDSW	IGH	8	None	
13	48	CQQSYSTPPGALYTF	IGK	2	C4	6ZDH
13	50	CAGEYSSSSWGWYFDLW	IGH	2	None	
13	51	CVRDQRRGYSGYDLDYW	IGH	3	None	
13	52	CARGSADWPRVPDYW	IGH	5	None	
13	53	CARGSNLVMGLDFDYW	IGH	3	None	
13	55	CARQMVVAYFQHW	IGH	2	None	
13	56	CARRSAETYYYDSSGRFDYW	IGH	2	None	
13	57	CSLKASDTAMYYCARTFEGCSSATCQNWFDPW	IGH	3	None	
13	58	CARDIVVVPAARPKPYYYYGMDVW	IGH	3	None	
13	59	CTTLLTISGVVIRAFDVW	IGH	2	None	
13	64	CRAEGELARDYW	IGH	6	None	
13	65	CASGGSSSSSWSGQWVFDSW	IGH	3	None	
13	66	CSSYTSSHSLDVLF	IGL	2	None	
13	67	CASHYIVGPTSLDYW	IGH	2	None	
13	68	CARTGVVLSTSRDDYW	IGH	7	None	
13	69	CARGGGFGEYPYYYGMDVW	IGH	7	None	
13	70	CAKVTVGMYPFTFDIW	IGH	4	None	
13	71	CARINSGYSNWYFDLW	IGH	3	None	
13	72	CARQGYCSGDSCYSWHYYYYMDVW	IGH	2	None	
13	73	CARVASGYYGWFDRW	IGH	2	None	
13	74	CARVPLNNVPYVFDPW	IGH	2	None	
13	75	CASYTSSSSFAVVF	IGL	2	None	
13	77	CARVPLSRDFWSEGYFDYW	IGH	5	None	
13	82	CARDRSGDYYYGMDVW	IGH	12	None	
13	85	CARGRGPSGADYW	IGH	3	None	
13	86	CQLYESYSTWTF	IGK	15	None	
13	88	CARDIIGYTSGCSDYW	IGH	5	None	
13	89	CARVGVYGSGSRDYW	IGH	11	None	
13	90	CARGTTMVRGAPVDYW	IGH	2	None	
13	91	CARIQLYDYVSGSYRYLYYFDSW	IGH	2	None	
13	96	CARLDSINWVDPW	IGH	2	None	
13	98	CARDGTYVSLVFDYW	IGH	2	None	
13	100	CARDGDRIFGVVNEPTDAFDIW	IGH	2	None	
13	102	CARVTINRVRRISEEYYNYAMDIW	IGH	63	None	
13	103	CARDSYIVATTRYYYGMDVW	IGH	4	None	
13	104	CAKADPAMTNGAFDIW	IGH	3	None	
13	105	CTTVAHWHCSGGSCYSGDDAFDIW	IGH	2	None	
13	106	CARDDYGDYGSYDYW	IGH	5	None	
13	109	CARDVIVEGYCSGGTCADYW	IGH	3	None	
13	110	CARRRSLITMIVVVQYFDYW	IGH	2	None	
13	111	CARDYGQCRPGGLCYSDPFDYW	IGH	2	None	
13	112	CARARTDILHYYLDYW	IGH	2	None	
13	116	CAKDGVRGYSYDHFDYW	IGH	2	None	
13	117	CASLYSNYDYYYYGMDVW	IGH	2	None	
13	118	CAKDRGSDYGDQLDYW	IGH	21	None	
13	119	CAKESWILTGYSGLFDYW	IGH	2	None	
13	122	CARDPPSPLTGDGFDIW	IGH	2	None	
13	123	CAKDASDYGDSPSFGYW	IGH	2	None	
13	124	CAKFNDNYDDDGYNYHFDLW	IGH	4	None	

13	126	CAGDWVVAPPANAMDVW	IGH	2	None	
13	127	CAKSESCNGPNWFDAW	IGH	56	None	
13	128	CARHLFIAVASGWYFDLW	IGH	2	None	
13	129	CQQYSSYANRTF	IGK	24	C3	7K8R
13	130	CARSHFNWFLSYYNWFDPW	IGH	2	None	
13	131	CQRYNRAPYTF	IGK	42	C3	7K8P
13	132	CQSYDSGLWGVIF	IGL	117	C4	7JVC
13	134	CARPQAEWDLLLPFDIW	IGH	14	None	
13	135	CAKDVLKGYQLWSADSPFDFW	IGH	2	None	
13	136	CARNGEYQLLFFDYW	IGH	2	None	
13	137	CARGGVVVVPDIFDSSSNWFDPW	IGH	2	None	
13	140	CARVGMTTTRAFDYW	IGH	2	None	
13	142	CAHRHILTGSYFDYW	IGH	9	None	
13	143	CARHGSTTSRWFDPW	IGH	2	None	
13	144	CARIMAVAGTIDYW	IGH	440	None	
13	145	CARGYYYGSGSYYNQYYFDYW	IGH	2	None	
13	146	CARDPGPRGTAYDIW	IGH	2	None	
13	147	CARDERGSWYFGLW	IGH	4	None	
13	148	CTKENYFDSSGYEVGFDYW	IGH	12	None	
13	149	CAIEKWFGYYFADW	IGH	3	None	
13	152	CLLSYSGARLWVF	IGL	12	None	
13	153	CAAYGDNGAHYAFDIW	IGH	2	None	
13	154	CARYYSPYYYYAMDVW	IGH	2	None	
13	155	CARVGEVDDVVYWDLDLW	IGH	4	None	
13	156	CAKGGDYDYYYYYYMDVW	IGH	4	None	
13	158	CAKDYGHCSAGRCYSDPFDYW	IGH	2	None	
13	159	CAKGPVPRYGAFDIW	IGH	2	None	
13	160	CARGGSGYSYGWHYYYGMDVW	IGH	3	None	
13	162	CARDVYSSSSGAYYYGMDVW	IGH	2	None	
13	163	CTRHVHRSNNSYSYYYYLDVW	IGH	6	None	
13	164	CTRGGHDYADVHFDSW	IGH	2	None	
13	168	CVSSYYDSRTHYYLDYW	IGH	3	None	
13	171	CVRDRGWLQFDNW	IGH	6	None	
13	178	CASGKPAGGPPPDYWHFDLW	IGH	14	None	
13	179	CARGIPTHYNYYGMDVW	IGH	3	None	
13	182	CARDLFVASTLYPTTEYYYYGMDVW	IGH	2	None	
13	184	CARDKNYYGSGSRDAFDIW	IGH	2	None	
13	185	CAKDQPVYYYDSSPPGDYW	IGH	4	None	
13	186	CARETNGDFHWFDPW	IGH	2	None	
13	188	CLLYYGGGWAF	IGL	4	None	
13	192	CATLLTGAFDYW	IGH	6	None	
13	193	CQQRTHWPGPF	IGK	3	None	
13	196	CASEPVDVQLERRAYYYYYYGMDVW	IGH	7	None	
13	197	CARGLSRLKVNIVIVPTAIQSNNWFDPW	IGH	4	None	
13	199	CQRLNSYITF	IGK	2	C1	7BZ5
13	202	CARDVVSAGMDYW	IGH	14	None	
13	203	CARDVLKGYQLWSGYSTFDYW	IGH	2	None	
13	204	CDWSSDVCSSDLVTTTFYFASW	IGH	5	None	
13	205	CQQYDNLLLFTF	IGK	32	C3	6XDG
13	206	CVKDTGKMGYYYGGMDVW	IGH	2	None	
13	207	CAKAGFTFQSWYYFDYW	IGH	2	None	
13	208	CAKGITVGGSGWHLRTYYFDYW	IGH	2	None	
13	209	CAHRRSLYRMLSAEDWFDPW	IGH	2	None	
13	210	CVKDLMGAITGAYFDFW	IGH	4	None	
13	213	CAHRPKLDFWSDHYYFDSW	IGH	13	None	
13	214	CATKVATTGTIYFDDW	IGH	4	None	
13	215	CAKVGHYGLSDYFDYW	IGH	2	None	
13	216	CARVGSMVRGVIINFPTHIDYW	IGH	4	None	
13	217	CARASFSSSSRFDPW	IGH	9	None	
13	218	CVAVDPVARTSDYYYMDVW	IGH	2	None	
13	219	CARVGPLLYTNHGVYFFYMDVW	IGH	3	None	
13	220	CARERGRISIFGVVTDPDYFYYYGMDVW	IGH	2	None	
13	222	CARDRGQTGTPGWDAFDIW	IGH	2	None	
13	223	CARVFSSGHYSARTYYFDSW	IGH	2	None	
13	227	CARDRSPELRITGTYEGFDYW	IGH	2	None	
13	229	CQQANNFPFAF	IGK	2	C1	7BZ5
13	230	CARGDSGDCSGESCYSYYGNRFDPW	IGH	4	None	
13	231	CATGGGYTVTTAWGGFDNW	IGH	5	None	

13	232	CQQVDANPFTF	IGK	13	None	
13	233	CVKTSAGGRPYYFDSW	IGH	2	None	
13	234	CQQSYRTLKYSF	IGK	3	C4	6ZDH
13	235	CAKEGDTSIPFDYW	IGH	2	None	
13	236	CHQNSDLPWTF	IGK	94	None	
13	237	CARGYCRSTTCYFDYW	IGH	9	None	
13	240	CARRTVGWATESPFDIW	IGH	2	None	
13	241	CAREYCRAYNCFFDYW	IGH	2	None	
13	242	CARDRDYSVDYW	IGH	6	None	
13	243	CARPDCSSGTCYFDRW	IGH	2	None	
13	244	CARETVTTLDAFDIW	IGH	2	None	
13	245	CARDAYVAGTEGAYYYYYGMDVW	IGH	2	None	
13	246	CARKYYSDGGGFDNW	IGH	3	None	
13	247	CARGTNPKDPLFSLFGVVVTQAYFDYW	IGH	26	None	
13	248	CARVVCATTSCYGMWKAFDLW	IGH	2	None	
13	249	CAKLVSYYGSGSYVDVW	IGH	4	None	
13	252	CARTEDRGDYSNWFDAW	IGH	2	None	
13	253	CAKPGIVEGRAFDIW	IGH	2	None	
13	255	CVKGAYCSSTGCYTTYFDCW	IGH	6	None	
13	256	CARHGLSLSIYGAPNYLDYW	IGH	2	None	
13	258	CARVPTLTTDLYFDYW	IGH	1971	None	
13	260	CGRERTGPLDFDPW	IGH	7	None	
13	261	CALPICALVSTVTTTFYFASW	IGH	15	None	
13	262	CARDFLDYFDSSGYPYFDFW	IGH	2	None	
13	263	CARGLGYCYGATCSGLDYW	IGH	3	None	
13	264	CARERSSDHYVWGSFSPPGYDYYGMDVW	IGH	3	None	
13	266	CARDPVYYYERTNYNNETNPVYYYYFSGMDVW	IGH	3	None	
13	268	CARRPNYDILTGYYPPFDYW	IGH	2	None	
13	269	CARDLYSSSERGDYYYYYGMDVW	IGH	2	None	
13	270	CAKDSVILQRLPYYFDYW	IGH	6	None	
13	272	CARDLKAAAVRRGAFDIW	IGH	2	None	
13	274	CARDHNHMYFDFWSGYRLFDPW	IGH	4	None	
13	275	CARQWRERSLAAFDIW	IGH	2	None	
13	278	CAKDSGDFWSGSLYWYFDLW	IGH	3	None	
13	281	CARDPCTGGNCYGRGDYW	IGH	3	None	
13	282	CMQALETLAITF	IGK	2	None	
13	283	CDWSSDVCSSDLNSYHYYMDIW	IGH	2	None	
13	285	CARDNVYRDYMDVW	IGH	2	None	
13	289	CAHHLSSERTGFDHW	IGH	5	None	
13	290	CARGGNNACDYW	IGH	5	None	
13	291	CARLGPGSAGSDWMFYFDIW	IGH	2	None	
13	294	CARDLIESYSSGWYAPDYW	IGH	4	None	
13	298	CTRVWGSSSWFFGYW	IGH	27	None	
13	300	CARVGPLSCRINGICYSPFDYW	IGH	2	None	
13	302	CQQRSKFPFFTF	IGK	2	None	
13	303	CAKDPYSNYFDYW	IGH	5	None	
13	304	CAKARGIFMLFAAYYGFDVW	IGH	5	None	
13	306	CARVECSGGSCYSIHFDYW	IGH	4	None	
13	308	CARDRFPPGYSFSRIFDIW	IGH	7	None	
13	310	CTRGNSVTPAGYYYYYLDVW	IGH	2	None	
13	311	CARDQEDIVVNGFDYW	IGH	2	None	
13	313	CQQYAKSPPYNF	IGK	36	C1	7K8N
13	314	CVRGAFLEWFHGFDIW	IGH	2	None	
13	315	CQQWSNWPIF	IGK	9	C3	6XDG
13	316	CARVSTRGDYFDYW	IGH	11	None	
13	318	CAKDPGRYCTNGVCRYFDYW	IGH	2	None	
13	320	CQQFNNF	IGK	2	None	
13	321	CAKGSADAFDIW	IGH	10	None	
13	323	CARHGCSGGSCYSFDSW	IGH	2	None	
13	325	CAKEKSGTTSSRFYYALDVW	IGH	2	None	
13	326	CARVGCSTASCHDYW	IGH	2	None	
13	327	CARSYSGSYRDFFDYW	IGH	10	None	
13	328	CAGSPTVTTGFWHFDLW	IGH	3	None	
13	330	CTKGKDGQGDTGNDYW	IGH	4	None	
13	332	CAREAAGQWLVDYW	IGH	2	None	
13	333	CARAARGYTNGPIDYW	IGH	4	None	
13	335	CARDEAAAGTGDYYYYGMDVW	IGH	2	None	
13	337	CARTIYCSGTSCYDIDYW	IGH	2	None	

13	338	CASSTVTMGWYYFGFW	IGH	7	None	
13	340	CAREGYETSGPQDAFDIW	IGH	56	None	
13	341	CARDSAYSNYVDYYYYYGMDVW	IGH	2	None	
13	342	CATGSFIGVVIYAFYIW	IGH	287	None	
13	343	CARKDSYGPGCFDHW	IGH	3	None	
13	345	CVKDFYRCGSYFDQW	IGH	2	None	
13	346	CGSHAGSNNFLF	IGL	124	C2	7BWJ
13	347	CARYAGAKGGLDYW	IGH	3	None	
13	351	CARENSGFDSGFYYYMDVW	IGH	2	None	
13	355	CAKAGTRYYFDYW	IGH	2	None	
13	356	CATEGYSGSYFDSW	IGH	2	None	
13	358	CARDGYNHIAFDIW	IGH	4	None	
13	360	CSSHAGSNNLLF	IGL	1332	C2	7BWJ
13	361	CAKDITAVGYRSFFDYW	IGH	12	None	
13	362	CAKSGGFYHYESSGVTFDIW	IGH	2	None	
13	364	CARVHSGGWSDYW	IGH	2	None	
13	365	CQSADITGTYVIF	IGL	5	None	
13	366	CATTYCGGDCSFDYW	IGH	2	None	
13	367	CARERFEWDKFDYYYIDVW	IGH	2	None	
13	369	CARDIYLASCSGSCYYYYYGMDVW	IGH	3	None	
13	371	CARGDQKPAFSLDYW	IGH	4	None	
13	372	CARRGSGYRDFFDSW	IGH	2	None	
13	373	CARDRATHLIDYW	IGH	2	None	
13	374	CARDSFSYSSSSAYYYYGMDVW	IGH	4	None	
13	376	CARRGDFGSGSLYYYYYMDVW	IGH	6	None	
13	377	CMIWHSSAWVF	IGL	18	None	
13	378	CARGLGEIAVAKTRHWLDPW	IGH	14	None	
13	379	CARSENTLWGVAATWGTYFDYW	IGH	5	None	
13	380	CAKRGYDCFGYYEDAFDIW	IGH	2	None	
13	381	CQVWDCSSDPPGVF	IGL	2	None	
13	382	CQSYDGSIHPVIF	IGL	2	C4	7JVC
13	383	CAKELHTSGWYFDYW	IGH	2	None	
13	384	CQHYKNLPLGF	IGK	20	C3	6XDG
13	385	CVKDRGTGHTYGLFHYW	IGH	2	None	
13	386	CARDSDAYYDILNWFDPW	IGH	2	None	
13	388	CATGDGNWFDPW	IGH	2	None	
13	389	CARTGYSSSSGLEKGYYYYGMDVW	IGH	8	None	
13	390	CARRAADSYGYVGAFDLW	IGH	14	None	
13	392	CARIATLTTSHYFDYW	IGH	18	None	
13	394	CAKDFRSGWYAGWFDPW	IGH	2	None	
13	395	CYSTDSSGNHPQWVF	IGL	4	None	
13	396	CQKYNNWLVTF	IGK	33	None	
13	397	CAKLFYGSVDYW	IGH	4	None	
13	399	CAKGSGIFWIAYQGHSFDYW	IGH	35	None	
13	401	CARAGITIFGVVDYW	IGH	10	None	
13	402	CARDRHSFDNSGYYDYW	IGH	4	None	
13	403	CALPILRAEDTAVYYCAKDLLELLDSDYYYYGMDVW	IGH	2	None	
13	404	CAKDRGGSYYEYYYGMDVW	IGH	4	None	
13	405	CARVTKLTSVEGVDWFDTW	IGH	1019	None	
13	406	CARAQYFDWLLGRGHFDYW	IGH	2	None	
13	407	CARDLYDYDSSGPFDYW	IGH	3	None	
13	408	CARDHPDNDYGDYAGYW	IGH	5	None	
13	409	CATPRGYSYGPFDYW	IGH	17	None	
13	411	CARSPRPNIYDTSSSYPYYFDLW	IGH	5	None	
13	412	CTSYTSSNPPLVF	IGL	2	None	
13	413	CARDSEPLSGDHWFDPW	IGH	40	None	
13	415	CQQSISPPYIF	IGK	128	None	
13	419	CQRYNNYPYIF	IGK	3	C3	7K8P
13	420	CARDLSTTAVKGGAFDIW	IGH	10	None	
13	421	CSSRDSSGNHRLF	IGL	15	None	
13	422	CARGNCSGGSCYSSPFDIW	IGH	3	None	
13	424	CAKDLRAFYDSWGFDYW	IGH	4	None	
13	426	CIKDKIRGLAMGYFDYW	IGH	2	None	
13	427	CQSPDSSATYQVVF	IGL	7	None	
13	428	CAKDLRAYTIIPHFDYW	IGH	2	None	
13	430	CARHGTHTSMALGYAFDIW	IGH	4	None	
13	431	CAHILYVGSARHTFDPW	IGH	6	None	
13	432	CARVSTSFDDW	IGH	32	None	

13	433	CQQSYAAPLDTF	IGK	2	C4	7JW0
13	434	CAKDSWDGYNYDGGYFDYW	IGH	2	None	
13	436	CAKDIGYSSGLPDYW	IGH	3	None	
13	438	CVKDDYYASGNFASNFDCW	IGH	8	None	
13	439	CARTFEGCSSATCQNWFDPW	IGH	54	None	
13	440	CAAYFLTRVFDVW	IGH	2	None	
13	442	CLRYGSSPLFTF	IGK	12	C1	7K8N
13	443	CARDLYTQPDYW	IGH	2	None	
13	444	CAKGTLAAAILNWFDPW	IGH	2	None	
13	446	CAIRDLVFIAGWFFDLW	IGH	5	None	
13	447	CARLPLEGGGAFDIW	IGH	2	None	
13	449	CAGGTSCYTTVTTFDYW	IGH	4	None	
13	450	CARDLYVLGRISAYYYYYGMDVW	IGH	2	None	
13	455	CARDRSSDAYTNYLPDAFDIW	IGH	2	None	
13	456	CARIGETYYDFWSGYLDYW	IGH	2	None	
13	457	CARDAWYGSGHYAFDIW	IGH	2	None	
13	458	CAKGGSRQCSGGSCYSDFW	IGH	2	None	
13	459	CARGPRFLETLSRTYYLDSW	IGH	2	None	
13	460	CAHSTYDILTGYYTLFDYW	IGH	2	None	
13	462	CARQPYGDYVFDSW	IGH	7	None	
13	464	CARVEGSYYYYYMDVW	IGH	3	None	
13	468	CARSSAVTTRYDFDYW	IGH	227	None	
13	473	CASERRWNTVGIIFDYW	IGH	2	None	
13	474	CANLGSFALFPYSSDDHVFDYW	IGH	3	None	
13	476	CAKDISPFRGWFDSW	IGH	4	None	
13	477	CARDQRVATILSYYYGMDVW	IGH	2	None	
13	478	CAKDSSEYTQVIGYFDSW	IGH	2	None	
13	479	CTKRLLGFGNGWYWYFDLW	IGH	4	None	
13	480	CARGGNDYSNPYDSW	IGH	3	None	
13	481	CALPISAADTALFYCARHQRQSNYGDYTPPFDYW	IGH	3	None	
13	485	CARLHNVSPFDSW	IGH	4	None	
13	486	CARDWHVIAVAGFYFDYW	IGH	2	None	
13	487	CARDFCGGDCYNDEYYYGMDVW	IGH	32	None	
13	488	CARFGSGYSKTPTYFFDSW	IGH	8	None	
13	489	CARGHYITTVRGGSYYMDVW	IGH	34	None	
13	490	CARMVGCATTLCHPRFDYW	IGH	7	None	
13	492	CAREDCTGGSCFDNWFDPW	IGH	2	None	
13	493	CARLSYDIWSGSSLYYFDHW	IGH	6	None	
13	495	CARVLKGVGAPGEFDYW	IGH	2	None	
13	498	CAKSYCSSTSCYPPVYTYYYGMDVW	IGH	4	None	
13	500	CARDRLPYYESSGSNDAFDIW	IGH	2	None	
13	501	CARIQRGTSYDISAYPDAW	IGH	2	None	
13	503	CARDRILGSGYYDSW	IGH	23	None	
13	504	CARDGVRGGYAFDMW	IGH	12	None	
13	506	CARLRYDTSAYYHGGYFDFW	IGH	8	None	
13	509	CAKHRGSAYAPGSFDSW	IGH	9	None	
13	510	CARGGVAVVGGGQYFSALDVW	IGH	2	None	
13	511	CAREHRVATIESAMDVW	IGH	2	None	
13	513	CDWSSDVCSSDLTAVYYCAKYREFFHLRWGADAFDIW	IGH	2	None	
13	514	CARARRAFCSGGRCSYYFDYW	IGH	2	None	
13	515	CARELRGTVTTHYYYYGMDVW	IGH	2	None	
13	516	CTRETVDGTPYFDYW	IGH	2	None	
13	518	CGTWDSSRSTGVF	IGL	28	None	
13	520	CARGHIVVVTATQNYYYYYGMDVW	IGH	2	None	
13	521	CQQFNNIPMWTF	IGK	4	C3	6XDG
13	522	CALVSTVTTTFYFASW	IGH	2493	None	
13	523	CARHRYSSSSYYFDYW	IGH	6	None	
13	524	CARTVPPETSGSTGVDYYFDYW	IGH	5	None	
13	525	CVKGYSSGWLASAFDFW	IGH	4	None	
13	528	CAKADYYDSSGDYW	IGH	9	None	
13	531	CAKHGGLLHLWWGKHPFDLW	IGH	8	None	
13	533	CARERRCGGDCDLYYYYYGMDVW	IGH	4	None	
13	534	CAKPPTLMIAWGAFHIW	IGH	2	None	
13	536	CAKYREIFHLRWGADAFDIW	IGH	21	None	
13	538	CAREVVENFFDSW	IGH	31	None	
13	539	CARGGSFHDGSAYHWDFDYW	IGH	4	None	
13	542	CTTDGIVQLRFLEWLSQGHDYW	IGH	4	None	
13	543	CARESGDNAELVTWFDLW	IGH	2	None	

13	545	CAKDYSTTWYIDYW	IGH	10	None	
13	546	CAHRVGSRGDYNWFDPW	IGH	13	None	
13	549	CMKRVDSYDSRGYHTAPDYW	IGH	2	None	
13	550	CAKAMFSSTVTMGNDYW	IGH	2	None	
13	551	CAKGNLGSGYYYGMDVW	IGH	3	None	
13	552	CARHFGSGNYCFDYW	IGH	2	None	
13	553	CARDIMPQFLIRGGSLNSGFDPW	IGH	4	None	
13	555	CQQYDKWPVTF	IGK	8736	C3	6XDG
13	556	CQVWDSTTDHHWVF	IGL	2	None	
13	558	CARASEPVGRTTIGWFGPW	IGH	3	None	
13	559	CAKDQVLWLGELLNIYNYYYGMDVW	IGH	3	None	
13	560	CQSYDSTNYWVF	IGL	18	C4	7JVC
13	562	CATDGPRGFDQW	IGH	4	None	
13	563	CARDLFRYSNYSYYYYYGMDVW	IGH	2	None	
13	566	CARDSLVVPAASGIYYYYGMDVW	IGH	2	None	
13	569	CQHFHSYPLTF	IGK	112	C3	7K8R
13	570	CARGRGIAAAADDAFDIW	IGH	20	None	
13	574	CARDLVGGYSYGTFDYW	IGH	2	None	
13	576	CARDQEGIEGSIDYW	IGH	2	None	
13	577	CARDATPNGFYYDGMDVW	IGH	4	None	
13	578	CSALDSSLSAPVPF	IGL	6	None	
13	580	CMQALQTPLTF	IGK	472	None	
13	582	CAKDRLEDIVVVVAAEWFDPW	IGH	2	None	
13	583	CATRYCSGGSCYGYYFDYW	IGH	3	None	
13	585	CARDFRPNYDFLSTYIFDSW	IGH	2	None	
13	586	CYSAADNNRGVF	IGL	40	None	
13	589	CARATLLGPLTWFDPW	IGH	3	None	
13	590	CARGPLPTLDSSFSYYKDVW	IGH	3	None	
13	594	CTGSSGWYYFDYW	IGH	9	None	
13	595	CALPISSVEGVDWFDTW	IGH	3	None	
13	596	CMQSIQLRGWTF	IGK	11	None	
13	597	CASLEWDNWNYARFVYW	IGH	2	None	
13	598	CARITGTRYYMDVW	IGH	9	None	
13	600	CARDTLVVVPAADYYYYYGMDVW	IGH	2	None	
13	601	CARDYDILAAYPYYW	IGH	6	None	
13	602	CARGHPPGGDFDYW	IGH	66	None	
13	603	CARGGYSSSLYYYYGMDVW	IGH	5	None	
13	605	CARGCGSNICRTKYNDYW	IGH	2	None	
13	606	CHQRRNWPSSTF	IGK	3	None	
13	607	CARRNGYTYAFYFDYW	IGH	26	None	
13	610	CAKDFYSIRIFGVLPPDAFDMW	IGH	2	None	
13	611	CTTLTGYRGIWDYW	IGH	57	None	
13	612	CARPGVYGDRDYW	IGH	180	None	
13	613	CAKDVGLAAAVPHYLDYW	IGH	2	None	
13	614	CTTDPPAILWFGDGLWFDPW	IGH	2	None	
13	615	CAKDFSFIQLYAFDIW	IGH	3	None	
13	616	CARENRGVDDGYSYGYYYGMDVW	IGH	2	None	
13	617	CTKVLSIKATDDYW	IGH	4	None	
13	618	CARQGRGYSYGFFHFTAFDIW	IGH	2	None	
13	619	CARGVVVPAAIIGWFDPW	IGH	2	None	
13	620	CARGSAAAAFDYW	IGH	20	None	
13	623	CQSAGSSGTPPVVF	IGL	15	None	
13	624	CARDGYRRSWYGYYYGMDVW	IGH	4	None	
13	626	CATGARRGYYFYYMDVW	IGH	2	None	
13	627	CARIELWDIVLVPAAPHFDFW	IGH	2	None	
13	630	CTKVSSSSSGRYFFDFW	IGH	13	None	
13	631	CARSSRTFTWFDPW	IGH	2	None	
13	632	CQSTDSSDTYPSWVF	IGL	5	None	
13	634	CAVMRVVHAFYDYW	IGH	2	None	
13	636	CARDLGDFGFYWFDSW	IGH	11	None	
13	637	CARATPRYCSGGSCPDDAFDIW	IGH	2	None	
13	638	CASQDPVSGYCSGGSCYPFDAFDIW	IGH	2	None	
13	639	CARDLRQGSDGQIYYYYGMDVW	IGH	3	None	
13	642	CARVPVATIFGGWFDPW	IGH	4	None	
13	644	CQSTDTSGPHHVIF	IGL	24	None	
13	645	CAKFGHHSSGWYSSDYW	IGH	2	None	
13	646	CMQGIHRYDF	IGK	3	None	
13	648	CAKAAFVYCSSTSCPIDYW	IGH	2	None	

13	650	CARTPDYGDFKYRRYYGMDVW	IGH	4	None	
13	651	CAKDRCSSTSCYYYYGMDVW	IGH	2	None	
13	653	CTRGPKPSHRGFDVW	IGH	2	None	
13	654	CAREEYCSSTSCYYDGMDVW	IGH	6	None	
13	655	CARIPGNSYGYNFFDYW	IGH	2	None	
13	657	CAREHRYLWGSYGGWFDTW	IGH	4	None	
13	658	CATSRPGPCSGGSCYYDFDYW	IGH	3	None	
13	659	CARAWGRSFDSW	IGH	5	None	
13	660	CAKVGGGWHEFDYW	IGH	3	None	
13	661	CNSRDSSGNNWVF	IGL	1433	None	
13	662	CQAWDSSTAVF	IGL	394	C4	7JVC
13	663	CQTWGTGDVVF	IGL	10	None	
13	664	CAKDESSACYWGDYW	IGH	2	None	
13	665	CARSGYSYGYGFDYW	IGH	3	None	
13	666	CARAGVRVRKIVFVTSILNDVFDIW	IGH	3	None	
13	667	CAREGGGLCSGGSCYLSYYYLDVW	IGH	99	None	
13	668	CARGMRDFWSGYSHSYYYYMDVW	IGH	2	None	
13	669	CARDEGYCSGGRCYKIRFDYW	IGH	2	None	
13	671	CTAGTGKSDTDYW	IGH	3	None	
13	673	CARDPSIGNSGSYYKGSSWFDPW	IGH	2	None	
13	674	CAHLSNDRVLDFDYW	IGH	4	None	
13	675	CARVAISQVRGITENSYHYYMDIW	IGH	153	None	
13	676	CAKDWDASSGWSPGDDNYYYYGMDVW	IGH	3	None	
13	677	CSGGSCFLSYYYLDVW	IGH	4	None	
13	679	CARGNTQWLPIYWFDPW	IGH	8	None	
13	680	CARDMKVVPAASVSYYYYYGMDVW	IGH	2	None	
13	681	CARGRGSVTMILVVMTGAYYYFDSW	IGH	3	None	
13	682	CAKSRSTRFGVGDFYYYMDVW	IGH	2	None	
13	683	CMGWLDHHSYIYMDVW	IGH	2	None	
13	684	CARVGINMVRGVIENFYNYAMDVW	IGH	2	None	
13	685	CAHTTNSRFDYW	IGH	10	None	
13	687	CARKGGDLWSGVTFYHLDYW	IGH	6	None	
13	688	CHQYNDWPAITF	IGK	388	C3	7K8R
13	689	CASGYSSGWYVRSEYFQHW	IGH	2	None	
13	693	CAKVSYLSTASSLDYW	IGH	7	None	
13	694	CARQVYYDGSAYYYQHFDYW	IGH	4	None	
13	695	CARAPYASGHSFTRAFDYW	IGH	3	None	
13	697	CARMRRHGYNWASLAADDYW	IGH	4	None	
13	698	CAQIDYYYYMDVW	IGH	2	None	
13	699	CARESSITVFHAFDMW	IGH	10	None	
13	700	CARVWFGYSSSWPYFDYW	IGH	2	None	
13	701	CARGPPLHHFGSGNRHYYFDVW	IGH	2	None	
13	702	CQESYSTPIFTF	IGK	3	C2	7K8O
13	706	CGTWDNSLSGGVF	IGL	1370	None	
13	708	CQQYYTTPMYAF	IGK	16	C4	6W41
13	710	CAKLRGRFRWNDGFDVW	IGH	17	None	
13	712	CARWNSRFAFDIW	IGH	2	None	
13	713	CARQPSVGATYTWYFDLW	IGH	7	None	
13	714	CASGIGIVAKLGFDQW	IGH	2	None	
13	716	CAKSLRDFWSGYSLYYYSYMDVW	IGH	3	None	
13	717	CAKDWEVVTPEGEAFDIW	IGH	2	None	
13	719	CSRGQDYCSLGSCHFHYW	IGH	7	None	
13	720	CVRHNAFEIW	IGH	2	None	
13	723	CAKMSSGGSADFDYW	IGH	2	None	
13	725	CARTQKHPILFYFYYYMDVW	IGH	5	None	
13	729	CARGANTYGYNYMDVW	IGH	3	None	
13	731	CARDKASYNWDALDFW	IGH	2	None	
13	733	CARVGYCSGGSCYSLRHYYYYYGMDVW	IGH	2	None	
13	737	CAKELGLYFGEPYFDSW	IGH	4	None	
13	739	CAGSVDTAMVNPRAFDYW	IGH	2	None	
13	740	CQQYNNFSSIFTF	IGK	6	C3	7K8R
13	741	CATFRYGRWESETLRPDIWFDPW	IGH	2	None	
13	743	CCSYAGSYPFNWVF	IGL	29	C2	7JMP
13	745	CARHGINMIILVFTPDHWFDPW	IGH	135	None	
13	746	CARPVYDSVSRYYIDSW	IGH	2	None	
13	747	CQQSYSILTNMYTF	IGK	350	C4	6ZDH
13	749	CARHQRQSNYGDYTPPFDYW	IGH	190	None	
13	751	CARSSARVALYSSGWNLLCYFDYW	IGH	2	None	

13	753	CAKDQGTCSGDRCYFTSPPFDSW	IGH	2	None	
13	754	CDWSSDVCSSDLFDTW	IGH	3	None	
13	755	CARHGSGTYSSAFYFDYW	IGH	2	None	
13	756	CVRAGVRVRKVFLVPSILNDVFDVW	IGH	33	None	
13	757	CAKGRLWLPVFDSW	IGH	2	None	
13	758	CQSSDSSGSYRIF	IGL	7	None	
13	760	CAREVYSSHCYFDYW	IGH	2	None	
13	761	CARSIVVVPAATENYYYYYGMDVW	IGH	3	None	
13	762	CATDLDYHIDDRWYSAFHIW	IGH	8	None	
13	763	CARERYSGYHTFDYW	IGH	2	None	
13	765	CATDPAIFGVVITGFFEYW	IGH	3	None	
13	767	CARDPDIVVVPAARLGAFDIW	IGH	4	None	
13	768	CAALSSSWLLDYW	IGH	2	None	
13	770	CARDHHHLLGYCSGALCHSLDYW	IGH	4	None	
13	771	CQHYGTSPPKYTF	IGK	41	C2	7K8U
13	772	CARGVAFVPGVMASFDIW	IGH	2	None	
13	773	CMHGTHWPPRYTF	IGK	2	None	
13	775	CTFVPIRVGANDYW	IGH	6	None	
13	777	CARLKPAPMTSLGGWFDPW	IGH	2	None	
13	779	CARGTDPKDPLITLFGVVVIQGYFDYW	IGH	10	None	
13	780	CMQGTRRPPNSF	IGK	4	None	
13	781	CVRQYANWFDPW	IGH	2	None	
13	782	CARSYSHDFWSAFWPSMATPTEHPDEFDIW	IGH	4	None	
13	783	CARDRSRVSGTTGFDYW	IGH	4	None	
14	1	CARDPEWLSLIFDYW	IGH	2	None	
14	2	CAPLGGVLAGLRSGYGDYAVDYW	IGH	2	None	
14	3	CARVWDSCTGGSCFDKDYFDFW	IGH	6	None	
14	4	CVKDGMGELPYGFDFW	IGH	2	None	
14	5	CAKDVRGILGAYFDYW	IGH	9	None	
14	6	CAKVWSSSGELLWFGELLYGPFDYW	IGH	9	None	
14	7	CQQTYSTPPSGF	IGK	378	C4	6W41
14	8	CARGKSYTFDYFDYW	IGH	2	None	
14	9	CARPGSAVFWNGVTFDSW	IGH	2	None	
14	10	CARFRSYPYYFDFW	IGH	5	None	
14	11	CAKDKDIVLMVYAPKGNAFDIW	IGH	2	None	
14	13	CARDPRGWGDWYLDLW	IGH	2	None	
14	15	CARGVLGSGSYAYAFDIW	IGH	2	None	
14	16	CARDTSYYYDSSVGYFDYW	IGH	4	None	
14	17	CAHGSNYYDTSSYSGYFQHW	IGH	2	None	
14	18	CAKGQPGDPNSLFNLPHDYW	IGH	2	None	
14	19	CARDRAYWETSGYYNHASDVW	IGH	3	None	
14	20	CARHAEGGITGIYNLAGFDPW	IGH	2	None	
14	21	CARGFFSAYAPDARRDYW	IGH	8	None	
14	22	CARTVGSLEWRSEVPFDCW	IGH	2	None	
14	24	CQSADSTGMPNWVF	IGL	7	None	
14	25	CARGQRRLGSLVDYW	IGH	7	None	
14	26	CARRPFGSCGTSACYFFDNW	IGH	2	None	
14	27	CSRDVGLSTWAKYMDVW	IGH	2	None	
14	28	CARLMGSIAVPEDYW	IGH	2	None	
14	29	CQQFNTLPSVYTF	IGK	18	C3	6XDG
14	31	CAKDRGYSRADYFDYW	IGH	7	None	
14	32	CARGTESINTMRVVADGFFDYW	IGH	4	None	
14	33	CARDSRVTLGSWFDPW	IGH	2	None	
14	34	CARHQQKTYYDFWSGYVPFDLW	IGH	8	None	
14	35	CARADYYGSGSTGNPYYFDFW	IGH	3	None	
14	36	CAKSGNFYIPTYFDSW	IGH	4	None	
14	37	CARHDFGHAWAFDFW	IGH	85	None	
14	38	CSRLKGLVGWFDPW	IGH	2	None	
14	40	CAKDPDYYDSPNGFDYW	IGH	2	None	
14	41	CARDLGGGRYWAYYFDYW	IGH	4	None	
14	42	CARRKGTNGSFDYW	IGH	2	None	
14	43	CVRSIGWYPLGGYFDYW	IGH	3	None	
14	44	CARVQGGYCSGPSCDSANYYYYYMDVW	IGH	2	None	
14	46	CATAIGAHANVFDYW	IGH	3	None	
14	47	CARVWASIIAVAAGAFDYW	IGH	6	None	
14	49	CATDHHDNYYDRGPSDYW	IGH	3	None	
14	50	CARDVSHSDSLNVFDVW	IGH	3	None	
14	51	CAKENQAWVLGEIDYW	IGH	2	None	

14	52	CAYRIFGLIPYFDSW	IGH	14	None	
14	53	CQQSDRTPMYTF	IGK	102	None	
14	54	CANTVGLRYFDWSSFDCW	IGH	10	None	
14	55	CARPRQRDCSSTNCFDFDDW	IGH	80	None	
14	56	CARDPSIAARPFDYW	IGH	85	None	
14	58	CARDLHYGGNSGFDFW	IGH	4	None	
14	59	CARQYGDFEPRNAIIDYW	IGH	10	None	
14	60	CAKAPQGAVAPDYFDYW	IGH	2	None	
14	62	CARDLVVVAATPFDYW	IGH	2	None	
14	63	CAKDHGDGYTIGRGDYFDYW	IGH	2	None	
14	64	CARGASYGGSYSPIDAFDIW	IGH	2	None	
14	65	CARDRLIFGVIKSPLDYW	IGH	26	None	
14	66	CAKDLDWGGSGWSGIDYW	IGH	2	None	
14	67	CAAFLAVPGTDLFYFEYW	IGH	3	None	
14	68	CATVFRDGYSFPFDNW	IGH	2	None	
14	69	CASDGGYDSSGYYSYFFDNW	IGH	3	None	
14	70	CARLFVSSSSSRPSYYFDYW	IGH	2	None	
14	71	CARTRYNNAHTPHFDHW	IGH	4	None	
14	72	CARDRRDEPSYYYYYYMDVW	IGH	2	None	
14	73	CARSHLVAAGGSYQFDPW	IGH	5	None	
14	74	CARAKGRQWLVQYAFDIW	IGH	2	None	
14	75	CARHSSTTPYSRFDYW	IGH	23	None	
14	76	CARRRISWAQFDYW	IGH	88	None	
14	77	CAHRRLYCSGDSCYSDWDYW	IGH	2	None	
14	78	CGKALLSGGDYDDALDVW	IGH	2	None	
14	79	CAKDGNYYGSGNYYSDNWFDPW	IGH	4	None	
14	80	CDWSSDVCSSDLTGMYYCARDHPDNDYGDYAGYW	IGH	8	None	
14	81	CTRALFYSGSSGYWGYW	IGH	4	None	
14	82	CTAYLQVSSLEADDTAVYFCARTYHYATTGESYFDYW	IGH	14	None	
14	83	CLRGVGATPGSGIDYW	IGH	2	None	
14	84	CALPISRPQAEWDLLLPFDIW	IGH	12	None	
14	85	CARVPIPYCSSTTCYYNWFDPW	IGH	2	None	
14	86	CARGGGSYSVGWYFHYW	IGH	8	None	
14	87	CSPQRGSGSNSRFDFW	IGH	2	None	
14	88	CARDLSYSSSSGGGFDYW	IGH	3	None	
14	89	CARWRSVWSEFDYW	IGH	7	None	
14	90	CARLRETRSSFGVITRFDSW	IGH	6	None	
14	91	CATNRHCSSASCRDDYYYYYMDVW	IGH	2	None	
14	92	CARDSVGGFDPRHWFDPW	IGH	2	None	
14	93	CARDAALYYYDSSGYHWFDPW	IGH	2	None	
14	95	CARDYSNVPLGYYYGMDVW	IGH	2	None	
14	96	CARDTYYDSRGSQYYYYYAMDVW	IGH	4	None	
14	97	CARNIMSADGSVFYHYMDVW	IGH	26	None	
14	98	CAKEKNTATPGPFDYW	IGH	3	None	
14	99	CTAYMELTGLRSEDTAIYYCARKGIKSSWAFDLW	IGH	3	None	
14	100	CASHGENFFDYFDYW	IGH	2	None	
14	101	CARIYGGIYRGLDYW	IGH	67	None	
14	103	CARHPPRGEFLEWLNDAFDIW	IGH	2	None	
14	104	CAKGPTLGYDRGVPFDYW	IGH	2	None	
14	105	CTKQRSGSYSWDYFDFW	IGH	4	None	
14	106	CAKLFDSGYDGSHFDYW	IGH	2	None	
14	107	CTTDYTVGGFDYW	IGH	5	None	
14	108	CAKMTTVSNPGDYW	IGH	2	None	
14	109	CASKYCTGGSCYLGYW	IGH	3	None	
14	110	CQNYNSPSVLTF	IGK	2	None	
14	111	CTRGGDTDGYNHDYW	IGH	2	None	
14	112	CARGHNWNFESWFDPW	IGH	6	None	
14	113	CAISRGYCSGGTCSYYYYMDVW	IGH	2	None	
14	114	CLRAEDTAVYYCARGSAIYYDSSGYFDYW	IGH	4	None	
14	115	CAKDQRGSSLDAFDIW	IGH	2	None	
14	116	CAHKLRENYYGSGSYYPIYDYW	IGH	3	None	
14	117	CARDVMITFEGIIPHAFDTW	IGH	3	None	
14	118	CATSSPWIQLWYIDYW	IGH	7	None	
14	119	CARADTVMGVANYFDYW	IGH	3	None	
14	120	CARDATYYDSSGYYDAFDIW	IGH	8	None	
14	122	CARAEGVGADDAFDIW	IGH	21	None	
14	123	CCRVDDSWSGFYVYRFDYW	IGH	6	None	
14	124	CARPGLYLDDLDYW	IGH	2	None	

14	125	CARMMEWELHKRFDPW	IGH	2	None	
14	126	CTYGGAFYFEYW	IGH	2	None	
14	127	CAKVTYDSSGYYYFDYW	IGH	20	None	
14	128	CARKYSNSHGRCFDPW	IGH	6	None	
14	129	CAREDTSTWYPKKDYW	IGH	2	None	
14	130	CARIPRPREPYSSDMYYFDYW	IGH	2	None	
14	131	CASMYSSGWYEFRFDYW	IGH	2	None	
14	132	CAKERDGVATFGVFEYW	IGH	2	None	
14	133	CARVPFGVATPPDYW	IGH	4	None	
14	134	CASLGERREQPDYW	IGH	2	None	
14	135	CARSLLGYFDFWSGHYIFDYW	IGH	2	None	
14	136	CAKDLDYYDGSNSYYWFFDLW	IGH	7	None	
14	137	CQQANSIHLFTF	IGK	514	None	
14	138	CASASSGYLRADYW	IGH	4	None	
14	140	CARDGYGGPPKYYFDYW	IGH	10	None	
14	141	CARLYWGKGGNSPGVPVYYYYYYMDVW	IGH	2	None	
14	142	CATVGCGGDCYQHYFDYW	IGH	2	None	
14	143	CARPLYPTLEYWYFDLW	IGH	2	None	
14	144	CMQTIQLPPAYTF	IGK	91	None	
14	145	CARGLYYSYAVDVW	IGH	3	None	
14	146	CDWSSDVCSSDLVTVVLRFLEWFPFDYW	IGH	2	None	
14	147	CATRDPQHYGSGSYYHNYYYYMDVW	IGH	7	None	
14	148	CAKLRSGSYLSYFDYW	IGH	3	None	
14	149	CARESSETPPLSSSSGYDYW	IGH	2	None	
14	150	CARHIPPSSYYGTGNYYNTFDSW	IGH	4	None	
14	151	CARVPAWLVSPRYFDLW	IGH	2	None	
14	152	CVRDGGVADHTAFDIW	IGH	5	None	
14	153	CAKAPLSAVDYYDSSGYWAYW	IGH	2	None	
14	154	CARVDTYYMDVW	IGH	9	None	
14	155	CARDNDVYCRGGSCPPDYW	IGH	2	None	
14	156	CAKFSQPGGGHDYW	IGH	2	None	
14	157	CATHLEVTSWFDPW	IGH	20	None	
14	158	CAKESNRGSFTGLDYW	IGH	5	None	
14	159	CAKGASSWYVGYFDSW	IGH	4	None	
14	160	CAKDILGYYGSVTYPDFW	IGH	165	None	
14	161	CARIQRKGIEDYW	IGH	2	None	
14	162	CARSVTAGYSYGDAFDIW	IGH	2	None	
14	163	CARDQGSWFGDRRRGNWFDPW	IGH	2	None	
14	164	CARQLMIRGVINNYFDYW	IGH	26	None	
14	165	CCSYAGSYTFDVVF	IGL	34	C2	7K8X
14	166	CARFYYGSGSNNWFDPW	IGH	3	None	
14	167	CASQMSGYSYGSSHYYYGMDVW	IGH	2	None	
14	168	CDWSSDVCSSDLNNLRAEDTAIYFCAKERGYGAFYFHYW	IGH	2	None	
14	170	CAGGLNYDSSGYYWVNWFDPW	IGH	4	None	
14	171	CARFTRSALYYYYMDVW	IGH	2	None	
14	172	CAHKGPYSSSWFSLDYW	IGH	13	None	
14	173	CAREKGGYSSWGDFEYW	IGH	3	None	
14	174	CARGGYQLPNYYYYGMDVW	IGH	3	None	
14	175	CARDSGMGGTFDWW	IGH	2	None	
14	176	CARCNPNSGYDIFDYW	IGH	16	None	
14	177	CARHKLEYSSSRDYYYYYMDVW	IGH	2	None	
14	178	CAREGTGVNYFDHW	IGH	2	None	
14	179	CAHRTSIAAAGNAFDIW	IGH	3	None	
14	180	CVRGPQSRSSGWYFDYW	IGH	2	None	
14	182	CARQSLGYYDSSGYPPFDYW	IGH	2	None	
14	183	CTTGVYYDSDGYYHAFDIW	IGH	2	None	
14	184	CARMRYSGYNKYYYHYMDVW	IGH	2	None	
14	185	CARAFIPVYGSGSYADYW	IGH	2	None	
14	187	CACLITGWSAPNDYW	IGH	20	None	
14	188	CARRGSVPDTTTWYYFDSW	IGH	9	None	
14	189	CARVYSSGWYDDYW	IGH	19	None	
14	190	CARLGYSHGYYYLDDW	IGH	2	None	
14	191	CAKDKGHTTYWYFDLW	IGH	4	None	
14	192	CAREDSFGLLSGWYFDLW	IGH	2	None	
14	193	CARSPGYCGTTSCYHRPSWFDPW	IGH	2	None	
14	194	CFSLILTSVTAADTAVYYCARGSHTHKIYDILTGHFFDSW	IGH	3	None	
14	195	CAKLDSSAYYDAFDIW	IGH	10	None	
14	196	CVRNPRGYYGSHSPASTDYW	IGH	2	None	

14	197	CARGTFPQLTPPRFDSW	IGH	2	None	
14	198	CARDLGFNSGSYYDPFDMW	IGH	2	None	
14	199	CARVRTSLTIFGVVTPNFDYW	IGH	6	None	
14	200	CAGMRGVAYLDYW	IGH	3	None	
14	201	CAKEYYDILTGYFVPYFDYW	IGH	2	None	
14	202	CARDLRDGHSLNYLDYW	IGH	2	None	
14	203	CARGSHIWYHDSSGRRNHLDYW	IGH	11	None	
14	204	CAKDLGIAARPYLTPNWFDPW	IGH	2	None	
14	205	CARRVFGSTVISGYFDFW	IGH	2	None	
14	207	CAKDRGDGYYYDSSGREYYFDYW	IGH	7	None	
14	208	CARVGVRVRGVINGVFDYW	IGH	7	None	
14	209	CARYVNEDYFDYW	IGH	326	None	
14	210	CARDGGRYYGSGSYLDSW	IGH	2	None	
14	211	CARDSAYHGSGTYYKFDYW	IGH	2	None	
14	212	CLRAEDTAMYYCARDLSRVDYGVLSGYYQGGILDYW	IGH	32	None	
14	213	CAREDYYGSASYYMFNYW	IGH	6	None	
14	214	CARGSRDSYGYLMQFDYW	IGH	4	None	
14	215	CASFGHPRGFDYW	IGH	8	None	
14	216	CAKVRSIAAHVDAFDIW	IGH	2	None	
14	218	CARTHTAMAIFDYYFDYW	IGH	2	None	
14	220	CAKSSSTSFAGGNWFDPW	IGH	3	None	
14	221	CAKDNRDGYNYASFDYW	IGH	14	None	
14	222	CARESGQWLGRSYNYYGMDVW	IGH	2	None	
14	223	CGLISGYDSSGYPHNTGYYFDYW	IGH	6	None	
14	224	CAKVPGPQQPFDYW	IGH	7	None	
14	225	CARGNPVSPTGAYPHIDYW	IGH	2	None	
14	226	CDWSSDVCSSDLGSPPRIDYW	IGH	2	None	
14	227	CTRDIVATIFDYW	IGH	7	None	
14	228	CARITVTGAPWYFDYW	IGH	5	None	
14	229	CARERLQYLPIDYW	IGH	4	None	
14	230	CASQYSRAHWLYAFDIW	IGH	4	None	
14	231	CASYPLGHFASGEYSKRPAKAFDMW	IGH	4	None	
14	232	CARDREGIYDDSSGYYYPDAFDIW	IGH	2	None	
14	233	CAKDGLCDSSGYYCWYFDLW	IGH	2	None	
14	234	CARSGPNTYIGSWYFLYW	IGH	5	None	
14	235	CSSYTSASALGIF	IGL	62	C2	7K90
14	236	CARAVVPGTFAEYFHPW	IGH	7	None	
14	237	CATYIGPHFDYW	IGH	4	None	
14	238	CARDRLRSWGAFDIW	IGH	10	None	
14	239	CARDRAIVSSYYYYYYNMDVW	IGH	2	None	
14	240	CVKDSHSSGYSHSNWFDSW	IGH	7	None	
14	241	CAREFTIFGVAGPYFDDW	IGH	12	None	
14	242	CARGLDNSNYMTYW	IGH	2	None	
14	243	CARAWSSSSWVWDYW	IGH	2	None	
14	244	CAKERGNSGYYGWDAFDIW	IGH	8	None	
14	245	CAREVTMIVVVITNHAFDIW	IGH	3	None	
14	246	CATGLHLWFDFDFW	IGH	2	None	
14	247	CARELVRITIFGVAYYFDYW	IGH	6	None	
14	248	CARSLFMGAFPDSFFYW	IGH	39	None	
14	249	CVRAASVVTGWLTDYW	IGH	2	None	
14	250	CARSDGSGYPFYW	IGH	13	None	
14	251	CARDSVSPPATDIAAATYYYYYGMDVW	IGH	24	None	
14	252	CARLLPWGNWNDAERWFDPW	IGH	5	None	
14	253	CARVVGALSTRNWFDPW	IGH	9	None	
14	254	CARLWFGELLPNYNWFDPW	IGH	3	None	
14	255	CARDSYCGGDCLNDYW	IGH	8	None	
14	256	CARPRAILTGSFDPW	IGH	44	None	
14	257	CALTARPDSYYSDGSGYPFDSW	IGH	2	None	
14	258	CARGGWRQQLQLSYYYMDVW	IGH	3	None	
14	259	CVVLEKFDAFSVW	IGH	8	None	
14	260	CARDFIVHCSGLRCYRFGSDYW	IGH	4	None	
14	261	CARGLWSSSKGTYMDVW	IGH	8	None	
14	262	CQQFSLYPEAITF	IGK	6	C3	7K8R
14	263	CARVQQPLVHGLGSYRGMSDYW	IGH	8	None	
14	264	CAKVAYGEGSADYW	IGH	3	None	
14	265	CVRRAPAAAFDVW	IGH	2	None	
14	266	CASGRTPDYYYDSSGIFDYW	IGH	2	None	
14	267	CARRGIAPGGGWDDFYFDHW	IGH	8	None	

14	268	CARDTEVGSGWSTDYFDSW	IGH	83	None	
14	269	CTTIPATPAKPPAPSKRAFDMW	IGH	4	None	
14	270	CARDQLVVAATDEAAGYYYYGMDVW	IGH	2	None	
14	271	CAKDPSFSTAPGAFDIW	IGH	4	None	
14	272	CAKDQDIVVVPAATAFDYW	IGH	2	None	
14	273	CASRGCSGGYCGIDYW	IGH	5	None	
14	274	CARASVEAVPGRWFDPW	IGH	7	None	
14	275	CARADILMIIGATHFEYW	IGH	3	None	
14	276	CAREFGEWLGLLDYW	IGH	3	None	
14	277	CARVHDYGDYGRFYYAMDVW	IGH	12	None	
14	278	CCSYVSSNTHYVF	IGL	23	None	
14	279	CARHFRRTYCSGGSCRFTHDAFDIW	IGH	2	None	
14	280	CARGEGVINFDLW	IGH	2	None	
14	281	CAAADSTSGWYYFDYW	IGH	3	None	
14	282	CATSRVAAAGEPSHFDYW	IGH	3	None	
14	284	CARDGVCSSTSCYLYYYYGMDVW	IGH	4	None	
14	285	CATPWRGGNYNAFDVW	IGH	2	None	
14	286	CARNRGPVVASYWYFDLW	IGH	4	None	
14	287	CDWSSDVCSSDLDYGDYAGYW	IGH	40	None	
14	289	CARERLGGETGHFDYW	IGH	2	None	
14	290	CARETPHHKGWFDPW	IGH	2	None	
14	291	CAKPGYCTDGVCHYSYYFDYW	IGH	5	None	
14	293	CQQYNTYSGGCTF	IGK	2	C3	7K8R
14	294	CARANPPGRGEVDGMDVW	IGH	6	None	
14	295	CARGIGYCSDGDCYSRHFDLW	IGH	2	None	
14	296	CARQNYGDGVDVW	IGH	4	None	
14	297	CARAPYINYIFGYYFDYW	IGH	6	None	
14	298	CARAIAYYDILAAYHGAEYFDLW	IGH	3	None	
14	299	CARQGVDGVGVDYW	IGH	2	None	
14	300	CARWGEVVPAAGPSYYFDYW	IGH	2	None	
14	301	CARHGFQLYLRGIDYW	IGH	2	None	
14	302	CARRPWDGYNLAFDFW	IGH	2	None	
14	303	CARILDSSGYTADAFDIW	IGH	4	None	
14	304	CARHFGDSSGWYLPWFDPW	IGH	2	None	
14	305	CARFHYVPGGYYSSGSSYFDYW	IGH	2	None	
14	306	CAKLPATYCGDECPDYW	IGH	3	None	
14	307	CAMQGYCTTTSCRGYHEYW	IGH	2	None	
14	308	CARHRRAYCGGGHCHPINDYW	IGH	2	None	
14	309	CARAVIHDHTNPYLYYFYAMDVW	IGH	2	None	
14	310	CARADFWTGGHAFDVW	IGH	7	None	
14	311	CARVRMASGRPWYMDVW	IGH	15	None	
14	312	CALAITVAGKFDYW	IGH	2	None	
14	313	CAVTPYYDFWDGYLFDDW	IGH	5	None	
14	314	CAKDNGSGVSSPWFDYW	IGH	3	None	
14	315	CARGLSYKAVAVYFDSW	IGH	4	None	
14	316	CARDDIVATIVGYMDVW	IGH	55	None	
14	317	CAREEDQLLEAFDYW	IGH	11	None	
14	319	CAREGYGYGYSESPYFDYW	IGH	3	None	
14	320	CVRDPTSGYYDRGFDYW	IGH	2	None	
14	321	CQSADNSGVYGVLF	IGL	10430	None	
14	322	CAGSPWSGYYSAQFDYW	IGH	2	None	
14	323	CARVAYFEWLLNYYYYYMDVW	IGH	2	None	
14	324	CQESYSAPVFSF	IGK	3	C2	7K8O
14	325	CANRLSWSAVVVITPKHYFDYW	IGH	3	None	
14	326	CAKGDCSSTSCSGGFDYW	IGH	4	None	
14	327	CARDFFDFWSGYYTGILDYW	IGH	2	None	
14	328	CARLVVPSHRSYYHSYMDVW	IGH	5	None	
14	329	CARDENVAAAPDYW	IGH	13	None	
14	330	CARSGYLMGYYYYMDVW	IGH	5	None	
14	331	CARRAPTISSAYYFDYW	IGH	10	None	
14	332	CARLRGYYDSSGYYGVAYYFDYW	IGH	28	None	
14	333	CALPISSGNFASNFDCW	IGH	6	None	
14	334	CARVTYYYDSSGRLRANWFDPW	IGH	6	None	
14	335	CAKDRGIVVVPDYFDFW	IGH	4	None	
14	336	CVGWSNNYYYYMDVW	IGH	5	None	
14	337	CARLYNDFWSGESPYNWFDPW	IGH	2	None	
14	338	CAKWDSTDSTGDYW	IGH	2	None	
14	339	CVRGGYTYNYRPDALDIW	IGH	19	None	

14	340	CTTAYDFWSGHFDHRPDYW	IGH	12	None	
14	341	CLSADNSGASRVF	IGL	10	None	
14	342	CARQDFSNSWGSPGYFDFW	IGH	2	None	
14	343	CARNMSAGVRGSVWWFDPW	IGH	2	None	
14	344	CAKDAFKYYYDSGGHYPGSPSDYW	IGH	16	None	
14	345	CARVDCGGDCYYLHYYYYYGMDVW	IGH	3	None	
14	346	CQSADSSGGYSGGYHVFF	IGL	5	None	
14	347	CATDRVGYDTTGHEYYFDYW	IGH	4	None	
14	348	CARAPQLGWYFDLW	IGH	2	None	
14	349	CGRDGDISGWYTGVDYW	IGH	3	None	
14	350	CAKTPYSSSPGSFDYW	IGH	2	None	
14	351	CARDNYCSSSSCSQYYLDSW	IGH	4	None	
14	352	CARDESGYGSLFRFFDLW	IGH	4	None	
14	354	CTTDPAEVYSGYDMDYW	IGH	3	None	
14	355	CARDPGGYCSGGSCYFGWLDSW	IGH	2	None	
14	356	CARGDLMFRYSGYDTFLFHYW	IGH	2	None	
14	357	CAKDMVTAFGVVITSAFDIW	IGH	13	None	
14	358	CAKEFDSTPFRAIYYFDLW	IGH	5	None	
14	359	CNCRDISGNHPF	IGL	2	None	
14	360	CARVSTERVHYYMDVW	IGH	5	None	
14	361	CVKDASRGYNYFDYW	IGH	5	None	
14	362	CAINRGPGHGFDIW	IGH	5	None	
14	363	CAKDEIFFYYDSSASLGDAFDVW	IGH	2	None	
14	364	CATDPVGHFASGDYSKRPARAFDMW	IGH	21	None	
14	365	CAREVLRYFDWLLLVGYYYYYGMDVW	IGH	2	None	
14	366	CAREYCSGAICYFDAFDIW	IGH	8	None	
14	367	CARDADDNSAYYYYFDHW	IGH	2	None	
14	368	CARDAAAGILSGTHGFVDYW	IGH	2	None	
14	369	CAKLAMIVIDYW	IGH	5	None	
14	370	CARMVRGVIINPAFDIW	IGH	6	None	
14	371	CVRGAMTTVYFYYYYMDVW	IGH	4	None	
14	372	CARGDTIFEGLGYYYSYGMDVW	IGH	2	None	
14	373	CARDRDGALPNYFDSW	IGH	5	None	
14	374	CAREILDYYDSSATSGYFDYW	IGH	33	None	
14	375	CAKGGGIQLLWDYW	IGH	5	None	
14	376	CVRGQDGSGYYAALDYW	IGH	19	None	
14	377	CARVFGYCSGGSCYEAKNTKGFYFDYW	IGH	2	None	
14	378	CARDGGTTVTFGGFEYW	IGH	3	None	
14	379	CVTDSTGKALFDYW	IGH	3	None	
14	380	CATAEDYYDSSADYW	IGH	8	None	
14	381	CAKVPGGGSYFDYW	IGH	8	None	
14	382	CARDESSGWSTIYYYYGMDVW	IGH	2	None	
14	383	CARDPNCSGGSCDWALDVW	IGH	7	None	
14	384	CYSAADNNRGVF	IGL	177	None	
14	386	CAKITAISGGGDYW	IGH	7	None	
14	387	CARLNWEGALDIW	IGH	2	None	
14	388	CAKGTHYYVSGNSHNVYFDYW	IGH	4	None	
14	389	CARRPNYDILTGYYPPFDYW	IGH	4	None	
14	390	CAGRVDSGNYYQWYYFDYW	IGH	4	None	
14	391	CAFLLRFLEWSYFDYW	IGH	3	None	
14	392	CAKDAQVVLLGDYW	IGH	2	None	
14	393	CAIMPEGSGYGSIRDYW	IGH	2	None	
14	394	CAADRSGSSDLDYW	IGH	11	None	
14	395	CAKLPYGDYPNFDYW	IGH	13	None	
14	396	CARRFYYDDNHDNNYYYFDYW	IGH	4	None	
14	397	CARVWPDIAVAGTCDYW	IGH	4	None	
14	398	CAREPEALFTNYVFDYW	IGH	2	None	
14	399	CARIPPGPRTPEYLDYW	IGH	2	None	
14	401	CVRDDTLRGYSKGYGWDDYW	IGH	10	None	
14	402	CARGRVAGTKSAYYYYSMDVW	IGH	4	None	
14	403	CAREGDDFWSGYGRGWFDPW	IGH	3	None	
14	404	CASLTYSRSYYFDYW	IGH	9	None	
14	405	CAKSSAGNAFHVW	IGH	29	None	
14	406	CATISQEIAVRPSAVRFDPW	IGH	2	None	
14	407	CARTLSTRPTDRYYYYNMDVW	IGH	6	None	
14	408	CAGSPFVGVPTDYW	IGH	2	None	
14	409	CARLRGTVRQTIFGMAINDYYYYMDAW	IGH	18	None	
14	410	CATGPPIVGATTGWFDPW	IGH	16	None	

14	411	CARDDSAAGKRQYAFDIW	IGH	3	None	
14	412	CARVNYYYGSGSYSYYMDIW	IGH	5	None	
14	413	CARDGDIVVAAQAKVSRSWFDPW	IGH	2	None	
14	414	CAGGVGATLDYW	IGH	19	None	
14	415	CATDLTPGEVAAPFDYW	IGH	2	None	
14	416	CARDFYDSSGYYYRGGDYW	IGH	2	None	
14	418	CASQRIGYCSPSTCQHFDYW	IGH	4	None	
14	419	CAKDVHPDCSVGSCYLDYW	IGH	3	None	
14	420	CVRLPMSDYWNGPRDPFDYW	IGH	5	None	
14	421	CAKGVVPTMKGSPDYW	IGH	12	None	
14	422	CARHPIVVLITDAFDIW	IGH	4	None	
14	423	CARIVYDFWSGYYVREYYFDYW	IGH	2	None	
14	424	CALPISHTVTTTGVDYW	IGH	2	None	
14	425	CARDLRDIVVVPAAHPFDYW	IGH	4	None	
14	426	CARLCPDNSFWSDYYYYMDVW	IGH	17	None	
14	427	CARDGDSNYGIDYW	IGH	4	None	
14	428	CARDLALTQSQVGYYFDYW	IGH	3	None	
14	430	CARDESGSYYRPSEFDYW	IGH	2	None	
14	431	CARDDAPVAGKSKMDYW	IGH	5	None	
14	432	CAKDILAQGGYSYGWYFDLW	IGH	3	None	
14	434	CARLEPIHSSGDYW	IGH	3	None	
14	435	CAREPPWIWSSGGFDPW	IGH	2	None	
14	436	CAKDRGEDIVIVPASITLDYW	IGH	2	None	
14	437	CAKHLGYYDFWSGSYSFDPW	IGH	3	None	
14	438	CARLTSSGYYYPWGRVDYW	IGH	14	None	
14	439	CATEEVPSRYFDFW	IGH	4	None	
14	440	CARGAGYTRGWYDPSPPYYYYMDVW	IGH	2	None	
14	441	CVRDRYCSRTACFTTPPRDFYMDFW	IGH	9	None	
14	442	CAKDIRLYCSSTSCSTFWFDPW	IGH	6	None	
14	443	CARHLTYNDGSGYYYRLYYFDYW	IGH	4	None	
14	444	CAKGAGDYYDSRDDYW	IGH	2	None	
14	445	CAKVQWEVLGWFDPW	IGH	10	None	
14	446	CARDYWSRYCSSLTCSTFDFW	IGH	4	None	
14	447	CQVWHSSSDLDVVF	IGL	20	None	
14	448	CARGYYSTYDSYYFDSW	IGH	4	None	
14	449	CARVVPGYYDTIGYYHFDNW	IGH	3	None	
14	450	CTRESESTDQYYYSYYYMDVW	IGH	3	None	
14	451	CQQSYRTHF	IGK	17	C4	6ZDH
14	452	CARRLMTAVGVPLDYW	IGH	5	None	
14	453	CARHHYSDSRVYVVGYFDYW	IGH	4	None	
14	454	CARGEKYTTGWYSAYW	IGH	3	None	
14	456	CAGKALRVTDAYFDFW	IGH	4	None	
14	457	CTYYYDSSGYYRGGFDYW	IGH	12	None	
14	458	CARHTHPRYSSSPGFDYW	IGH	5	None	
14	459	CARGGGPGRGRFDLW	IGH	3	None	
14	460	CARTAATIAVAFDIW	IGH	10	None	
14	461	CAHTSPFSVVILFDYW	IGH	2	None	
14	462	CATNLRFYYDSSDYYYFEYW	IGH	7	None	
14	463	CARGEPHYDILTGYYPYYFDYW	IGH	4	None	
14	464	CARPFLYHDVFDLW	IGH	2	None	
14	465	CARQEQYDSSGYPLDYW	IGH	11	None	
14	466	CVKMYQVLSSDYDFWSDVKLKGVFDIW	IGH	17	None	
14	467	CAKITMVRGERDYW	IGH	2	None	
14	468	CARMRAGTTTLDYW	IGH	18	None	
14	469	CAKVSDYEAPGDYW	IGH	4	None	
14	470	CAKEAYFDYWNGYYDYW	IGH	3	None	
14	471	CDWSSDVCSSDLAARGHEAFDYW	IGH	2	None	
14	472	CVRVGVPGLRPNYYLDPW	IGH	5	None	
14	473	CASWIVVVPAAISLDAFDIW	IGH	21	None	
14	474	CARVEMLTNFILDDSRFDPW	IGH	31	None	
14	476	CARTYNGWNFGYW	IGH	4	None	
14	478	CAREPENYYDSSGYFCLAGQNPCAIPRTFDYW	IGH	6	None	
14	479	CASLIYDVLTGYQEFDYW	IGH	2	None	
14	480	CARATVDSSGYYHFFDYW	IGH	2	None	
14	481	CKSRDSSGDHPHVVF	IGL	2	None	
14	482	CARDGAGLSSGWYPEYFDYW	IGH	5	None	
14	483	CGAATTLHDYYYMDVW	IGH	2	None	
14	484	CARLMRSCSSTSCFTIDYW	IGH	21	None	

14	485	CSSYTYSNTLGRGTLDVF	IGL	340	None	
14	487	CAKDSVLGATRPVDYW	IGH	7	None	
14	489	CARHLGTVPAAMFDPW	IGH	4	None	
14	490	CASGTYISSSGFDFDIW	IGH	6	None	
14	492	CARDPPGRRSGELSQGHYFDFW	IGH	4	None	
14	493	CAKGQITMIVVVTFDYW	IGH	4	None	
14	494	CAKDFGSSSPLGDYYYYGMDVW	IGH	2	None	
14	495	CARIGGLGDVDYW	IGH	2	None	
14	496	CARDGAVAGTRISGDYW	IGH	4	None	
14	497	CVKDIVVRGALWDAFGLW	IGH	4	None	
14	498	CAIAGTNSVFDSW	IGH	4	None	
14	499	CATYGGITVSGRYLDLW	IGH	2	None	
14	500	CARQWGTWHEPRFDLW	IGH	5	None	
14	501	CARFLKTGTTWDFDYW	IGH	2	None	
14	502	CQHYNNWPPGVTF	IGK	14704	C3	7K8R
14	503	CARAVGWNYGTDAEYFDFW	IGH	2	None	
14	504	CARGYRYDSTGYVYLEYW	IGH	9	None	
14	505	CARQGGRDGSTDYW	IGH	31	None	
14	506	CTSRSDSSGPFFDYW	IGH	2	None	
14	507	CARIIIGSTGTYYYYMDVW	IGH	3	None	
14	508	CACSNTYYHFHYW	IGH	3	None	
14	509	CARISPEVDYYESSVYHFGSDYFDYW	IGH	31	None	
14	510	CAKERTWVAVFDYW	IGH	12	None	
14	511	CQQYQNYPNVAF	IGK	34	C3	7K8R
14	512	CASGGDCGGDCYYFDYW	IGH	8	None	
14	513	CARQRNYYYDSSGYYLDYW	IGH	4	None	
14	514	CAREIDYYGSGRPFDSW	IGH	7	None	
14	515	CAALLRPNSEDWFDPW	IGH	5	None	
14	516	CAKLVPPKGDWFDPW	IGH	118	None	
14	517	CTKEVYSGYDFWGFDYW	IGH	19	None	
14	518	CARNKPLWGYNPYGGNYFDSW	IGH	5	None	
14	519	CTRLAVLPPSINQDAFDIW	IGH	2	None	
14	520	CARDFSGSLPVAGVFDQW	IGH	8	None	
14	521	CATVREKYSIDYSGTFDIW	IGH	4	None	
14	522	CQQHNFYSTWTF	IGK	22	None	
14	523	CARDSLHSDYSNYYYGMDVW	IGH	3	None	
14	524	CARGGGRGYLYFDYW	IGH	5	None	
14	525	CDWSSDVCSSDLDYRDHFDSW	IGH	2	None	
14	527	CARVSVVPAVSNWFDPW	IGH	5	None	
14	528	CTRDPAGYCSGGSCHYLHFDYW	IGH	2	None	
14	529	CARCKNTAMAWGPHDAFDIW	IGH	4	None	
14	530	CARDQSILTISPLYMDVW	IGH	2	None	
14	531	CARESSYCGDDCSDAFDIW	IGH	5	None	
14	532	CAHSPPSRTMFGVVADTLLDYW	IGH	3	None	
14	533	CARDWAYCSGPDCYPAFAYW	IGH	2	None	
14	534	CARDSQSVIAADGTFDYW	IGH	2	None	
14	535	CAHKYCGGDCYFPYW	IGH	2	None	
14	536	CQQYNAYSLTFGPTF	IGK	21	C3	7K8R
14	537	CATLEVKGITMVDINFDYW	IGH	4	None	
14	538	CARRDTWPHYFDYW	IGH	2	None	
14	539	CARFGLWQSSLVDAFDIW	IGH	58	None	
14	540	CVRDRKQQLGISWLDPW	IGH	3	None	
14	541	CATDADYDSNAFDIW	IGH	3	None	
14	542	CAKRYCSGGTCHHHAFDVW	IGH	9	None	
14	543	CAKDSYFFDGDGSYLLDYW	IGH	2	None	
14	544	CAKLVDYGDSSDYW	IGH	37	None	
14	545	CARALYWYDFRSDKINYFDYW	IGH	11	None	
14	546	CARDRAFYYFDSSDLDNFGVDALDIW	IGH	4	None	
14	547	CTTRSYGAISFDYW	IGH	2	None	
14	548	CARDYDAFTGSYYFDYW	IGH	2	None	
14	549	CAREDSYCTGRPCWGFVDYW	IGH	2	None	
14	550	CARERYSTTLGHNWFDPW	IGH	9	None	
14	551	CAREHLVPTASHFDSW	IGH	9	None	
14	552	CARISGSCSRYGPRGVDYW	IGH	2	None	
14	553	CATGFRDYDSSGYSRFDPW	IGH	6	None	
14	554	CTRVAPSGYSEYW	IGH	2	None	
14	555	CVRQYSYGLMGHFPGYFRLW	IGH	4	None	
14	557	CATSRHEEYADYDGVISYYHYAVDVW	IGH	12	None	

14	558	CARDVGYSYGYPYYFDYW	IGH	8	None	
14	559	CATMRPIVMINFDYW	IGH	5	None	
14	560	CAKEGNSAHFDYW	IGH	14	None	
14	561	CATFGGNYFRPNAFDVW	IGH	2	None	
14	562	CARGRGVAVGATVGAFRYW	IGH	2	None	
14	563	CATSAGDYLPFFDFW	IGH	2	None	
14	565	CATRRPKTEVAMTYCYYYMDVW	IGH	2	None	
14	566	CMKALETPLF	IGK	4	None	
14	567	CTRRVGSSVAGPFDYW	IGH	3	None	
14	568	CARLFEGGGAGISSRYLSYYYALDVW	IGH	2	None	
14	569	CARIKSPSSGNYYAFDIW	IGH	10	None	
14	570	CAKASSGWSTPYYYYYMDVW	IGH	2	None	
14	571	CAREIVVVAAASPLTPDSYYYYGLDVW	IGH	3	None	
14	572	CTKAKKTGTADFDGLDVW	IGH	65	None	
14	573	CARDLDIVEEPTTLHYYMDVW	IGH	5	None	
14	574	CARPLGLRRVPDVFNIW	IGH	95	None	
14	575	CARDLVAAGPHYYYYYGMDVW	IGH	11	None	
14	576	CARHLPNYYDSSGYYYSLPPAYYFDYW	IGH	5	None	
14	577	CAKGSWAAAAGYFDYW	IGH	2	None	
14	578	CATLAGIAAAGYYYYYYGMDVW	IGH	4	None	
14	579	CASPAQEGSYRFTQNFDFW	IGH	179	None	
14	580	CAHSFTVVVPAAYAEYFQHW	IGH	3	None	
14	581	CARVPPVNWDHWYFDLW	IGH	2	None	
14	582	CAREGYDLMGDRPYFDYW	IGH	4	None	
14	583	CARLSDMVATMTTHDAFDIW	IGH	2	None	
14	584	CARTLMQYGKSVPVFDYW	IGH	9	None	
14	585	CARLKWFGEVYSDSW	IGH	2	None	
14	586	CYGSGSPDAFDIW	IGH	4	None	
14	588	CAHRPHSGFEPFYFDYW	IGH	2	None	
14	589	CARDDGDYDFWSGYFEPDYW	IGH	4	None	
14	590	CARGGWIQLSIYYMDVW	IGH	3	None	
14	591	CARLLYRSGHYPFDYW	IGH	4	None	
14	592	CARDGQQHRNPYYYDYW	IGH	2	None	
14	593	CATRTSPLRFDAFDIW	IGH	2	None	
14	594	CARDDVRGQWLASTCDYW	IGH	3	None	
14	595	CARGELHGGSGSYNGSDYW	IGH	3	None	
14	596	CASFYPAAGYSSSPGDAFDIW	IGH	3	None	
14	597	CTREAVRGITRTHFDYW	IGH	2	None	
14	598	CASTMVRGTIPDYW	IGH	5	None	
14	600	CATHKIPANNRFFDYW	IGH	2	None	
14	601	CAKSSDWRFGSFDMW	IGH	10	None	
14	602	CAKERIFGVDYYFDYW	IGH	3	None	
14	603	CARQQRWITIFGVDINAFDIW	IGH	31	None	
14	604	CARGSPLLGVNWFDPW	IGH	3	None	
14	605	CAKDLTISSGFCSSTSCLGPFDIW	IGH	6	None	
14	607	CAKDISIVLILGSDAFDIW	IGH	5	None	
14	608	CAREGIYSGTQFEYW	IGH	37	None	
14	609	CAIPYGSSHMNWFDPW	IGH	4	None	
14	610	CATSPGSSRFDPW	IGH	2	None	
14	611	CARGDCSGGSCFDGYYYGMDVW	IGH	4	None	
14	612	CARTPKDGNTAAPFDIW	IGH	5	None	
14	613	CARGITIYGVQFDYW	IGH	4	None	
14	614	CTRDPGGYSSGWSHFDYW	IGH	2	None	
14	615	CARGSPTLGRGYSYDPRGGKAFDIW	IGH	5	None	
14	616	CVQSQSIMPDARAFDIW	IGH	2	None	
14	617	CARDAEAVTMDLDYW	IGH	2	None	
14	618	CARVGYYDNSGSYYWDDAFDIW	IGH	2	None	
14	619	CARWPLNNSDAFDIW	IGH	16	None	
14	620	CARAQYCSSTRCYSLGYYMDVW	IGH	2	None	
14	621	CARDSVTTVAVFDFW	IGH	4	None	
14	622	CARDRGTLELRSHIYYYYGMDVW	IGH	3	None	
14	623	CAREGVSFNYSGSGSAINKYFDSW	IGH	2	None	
14	624	CARETFDSSGYYYSGYW	IGH	7	None	
14	625	CTRAATDSSGYFDYADAFDIW	IGH	16	None	
14	626	CARGDDFWSGYYDYW	IGH	75	None	
14	627	CAREVIVGPTRRLVDYW	IGH	2	None	
14	629	CAREIDSDCYFDPW	IGH	4	None	
14	630	CARGPFSLHVAIFGVVPVDIDYW	IGH	2	None	

14	631	CARQGDTTSWSPIDWFDPW	IGH	5	None	
14	632	CQSYDRTLSGSRWVF	IGL	4	None	
14	633	CVRHGIYRLVGALTPFDYW	IGH	4	None	
14	634	CVRDNSLDFWSGCWLDYW	IGH	2	None	
14	635	CARSPTSISNPYYFDHW	IGH	97	None	
14	636	CARLGWDLPPLYAFDIW	IGH	2	None	
14	637	CASTEGGDPFDVW	IGH	7	None	
14	638	CMEALQLPPNTF	IGK	3	None	
14	639	CARVRQLFFGIIKYFDYW	IGH	14	None	
14	640	CARYQLTTRRYYFDYW	IGH	2	None	
14	641	CARVAPNSRAFPFDYW	IGH	2	None	
14	642	CARLWGTTGTTNFYYW	IGH	3	None	
14	643	CARAVVPYGSSWYYFDYW	IGH	9	None	
14	644	CSKGAGGGQIYYMDVW	IGH	2	None	
14	645	CAKDIFFLGQGIAAAGTGFDYW	IGH	2	None	
14	646	CATSPDVVRGDYW	IGH	2	None	
14	647	CARGLLLGYCSSTSCPGDYW	IGH	12	None	
14	648	CARESGGEGDYYYYYMDVW	IGH	2	None	
14	649	CVRHRSELATKPIDYW	IGH	5	None	
14	650	CVSSTWSINRFDTW	IGH	2	None	
14	651	CASGGYCSSTSCYDNYYYGMDVW	IGH	2	None	
14	652	CARHIITIFGVAILNWFDPW	IGH	2	None	
14	653	CARDFLTLYSYGPDYW	IGH	6	None	
14	654	CVREFSSGWNRAHFDHW	IGH	2	None	
14	655	CVRREAESREYFQHW	IGH	7	None	
14	656	CARVRSGWYLDYW	IGH	54	None	
14	658	CARYWYYHDSSGQYYMDVW	IGH	4	None	
14	659	CALPILKLDSVTAADTAVYYCATVPYYYASSAQGGFDSW	IGH	2	None	
14	660	CATDRTRRRSSSGVFDYW	IGH	2	None	
14	661	CATLYLGLRLFDYW	IGH	2	None	
14	662	CARDRLASDDFHYMDVW	IGH	3	None	
14	663	CAKPLGIVGATVLDYW	IGH	4	None	
14	664	CAKVTGGDWLGYFDLW	IGH	2	None	
14	665	CARGELDYDDYDNVDYYYYYMDVW	IGH	46	None	
14	666	CAKDHSMTFFGVVINPLDSW	IGH	12	None	
14	667	CAKDNSNWYGDYYYGMDVW	IGH	4	None	
14	668	CARDFSVGTSTFDIW	IGH	2	None	
14	669	CARGIRHAITIFGVVIDYW	IGH	2	None	
14	670	CARVRYQGWWDVLIGNYFDSW	IGH	2	None	
14	671	CARDPDLAPTETTYFDYW	IGH	10	None	
14	672	CTRNRIIEMAPIINYYFDYW	IGH	2	None	
14	673	CAKTVVSWYFDLW	IGH	13	None	
14	674	CAKGLYYFETSGYYTRTQTDGFDIW	IGH	3	None	
14	675	CNSRDSSGYHVVF	IGL	3596	None	
14	676	CATSSGGSPIGVIVRGHYFNLW	IGH	2	None	
14	677	CAKGQLYGSSYVDYW	IGH	2	None	
14	679	CARTSVAGNPRVAFDIW	IGH	3	None	
14	680	CARDRLYYYDSSGFFLNDGFDIW	IGH	2	None	
14	681	CARHRGQVVIGANDYW	IGH	6	None	
14	682	CATDGGTIFGVVIINFDYW	IGH	3	None	
14	683	CARDLNTIFGVVPFDYW	IGH	6	None	
14	684	CAKDITRSGTISTIDYW	IGH	2	None	
14	685	CARGDYYDTSGYHDAFDIW	IGH	2	None	
14	686	CAHRDYDRSGPTGDYFDYW	IGH	3	None	
14	687	CQQRSKWPLVMYTF	IGK	17	None	
14	688	CASLVAGSAHLSSNTPNDYW	IGH	2	None	
14	689	CARRGSSGYSINAYWYFDLW	IGH	2	None	
14	690	CARSITMVRGVNNWFDPW	IGH	9	None	
14	691	CARDPNGFSGNFYDSW	IGH	5	None	
14	692	CATKGYYSSSTDYW	IGH	2	None	
14	693	CAHIIGYCSDPSCPEHYFDYW	IGH	5	None	
14	694	CARDANWNRQYFQRW	IGH	3	None	
14	695	CAKSDHADYGGNSGGDYW	IGH	4	None	
14	696	CVLYMSGIYVF	IGL	122	None	
14	697	CAKSVYSSGSYGYSYYYYMDVW	IGH	2	None	
14	698	CQQSNNYSPYSF	IGK	21	C1	7BZ5
14	699	CASGQIGLIAEATGGYYYGMDVW	IGH	2	None	
14	700	CQQYGSETSHTF	IGK	17	C1	7K8N

14	701	CATLGPDYVRYFDYW	IGH	2	None	
14	702	CAHMVWSGPYNWFDPW	IGH	3	None	
14	703	CAREGGWSQADPNYFDPW	IGH	2	None	
14	704	CARALLFSGSSSFDHW	IGH	2	None	
14	705	CARLSPTMIATYYFDYW	IGH	19	None	
14	706	CARLSGVDSGWNRFDYW	IGH	5	None	
14	707	CVRAMVRGEFDSW	IGH	6	None	
14	708	CARCRGWLQEYDAFDVW	IGH	5	None	
14	709	CTVGDKSNYNPRRFDPW	IGH	2	None	
14	710	CARQRYFDWLLSPDYFDSW	IGH	3	None	
14	711	CDWSSDVCSSDLTDRSSWYSRQEDYYFDYW	IGH	2	None	
14	712	CARDKFWSGLRRVTRHFDYW	IGH	3	None	
14	714	CARRAKAGGFDIW	IGH	4	None	
14	715	CARDSHMGYRAFDIW	IGH	12	None	
14	716	CAREILQSGYSGYDKWHFDLW	IGH	2	None	
14	717	CARHSHLTSFGYGTWFDYW	IGH	2	None	
14	718	CARGHRTVNLEMDGMDVW	IGH	2	None	
14	719	CARDPGLTLTVTKGAGGWFDPW	IGH	2	None	
14	720	CARGPIVVVPAARAYYYGMDVW	IGH	2	None	
14	721	CARTDYYESRGADFDYW	IGH	4	None	
14	722	CQQYYGTQYIF	IGK	3	C4	6W41
14	723	CARMKYDILTGYAWGPLDYW	IGH	55	None	
14	724	CAIGARDYSAAVATHEGGGNFDYW	IGH	37	None	
14	725	CARADPYSPGLVVVPVAPDYW	IGH	2	None	
14	726	CARDGRGNYYQDYW	IGH	7	None	
14	727	CLRTLYGSGWNRESHWFDPW	IGH	4	None	
14	728	CARDVKTGTAMVTQHLVWYYYGMDVW	IGH	3	None	
14	729	CVVRVYISSTYYFGHW	IGH	4	None	
14	730	CTVTNDLSAFDVW	IGH	2	None	
14	731	CARDYYYDSSGLLDYW	IGH	4	None	
14	732	CARNSFCTSATCYSWYYYYMDVW	IGH	2	None	
14	733	CARAPPKYYYDSSGYSFDIW	IGH	4	None	
14	735	CARVPPTSVNYAFDIW	IGH	22	None	
14	736	CARLPPFWDYSPQGFDYW	IGH	5	None	
14	737	CATDPLWFGEFRPRGLTDAFDIW	IGH	86	None	
14	739	CARHLRGDSSGYGWFDLW	IGH	2	None	
14	740	CARRTAMVSGNYFDYW	IGH	3	None	
14	741	CARLEGTGYERYW	IGH	2	None	
14	742	CATAPKAVFWSGQIYYYYGMDVW	IGH	6	None	
14	743	CARLGRQVLRFLGSLEGEDGLDIW	IGH	2	None	
14	744	CVIESRLVIDYW	IGH	3	None	
14	746	CAKGAYDSSGYYSVTPEPRFDYW	IGH	6	None	
14	747	CTRAGQKKIFGVVVGDDAFDIW	IGH	2	None	
14	748	CARLIRFTIFGVPSFFDSW	IGH	2	None	
14	749	CSGGGCYSNFYYYFYVDLW	IGH	2	None	
14	750	CTRLSPEADFYESRVYHFGADYFDHW	IGH	17	None	
14	751	CARRYYDHIWGSYVDYW	IGH	5	None	
14	752	CARVVGTKMTRGPPLRYYLDSW	IGH	2	None	
14	753	CAHGTYNSGWYGGYW	IGH	2	None	
14	754	CARDEDELLWFGELFGAFDIW	IGH	3	None	
14	755	CGHISNVWVGEFFDYW	IGH	4	None	
14	756	CARSLPHYSAGGDDYYSLFDPW	IGH	7	None	
14	757	CARDRNDYSGWEYYFDYW	IGH	13	None	
14	758	CARMEISAYNSYYFEYW	IGH	2	None	
14	759	CARGTSIAARPEIDYW	IGH	2	None	
14	760	CARIKDWAQSGLDAFDIW	IGH	14	None	
14	761	CARDLLYYDFGVDYW	IGH	3	None	
14	762	CARTGIAAAATYYYYYMDVW	IGH	4	None	
14	763	CARGLVGYDSSGYYSVPDTFDIW	IGH	2	None	
14	764	CARRPSIWVGEPPGFDFW	IGH	2	None	
14	765	CARDYSSPYFHYYYIDVW	IGH	3	None	
14	766	CASENSTSGQADYW	IGH	2	None	
14	767	CAKDINFVTVAGTLAFDIW	IGH	4	None	
14	768	CVRVPPVMYYYNYYMDVW	IGH	23	None	
14	770	CARVKDGPYGDYQFDLW	IGH	5	None	
14	771	CARQGYCGSDRCYASDYW	IGH	5	None	
14	772	CAKDIGIAARRAFDCW	IGH	2	None	
14	773	CQHFSNYPPLFTF	IGK	79	C1	7BZ5

14	774	CARYGYGERGFDSW	IGH	2	None	
14	775	CARDWGGQLSYLDYW	IGH	32	None	
14	776	CVREERVLQVLDWSSKKFFDYW	IGH	2	None	
14	777	CAFGFSATTVSTSGAPTRNSAYFQHW	IGH	2	None	
14	778	CARVMTNYGGNGYFDSW	IGH	3	None	
14	779	CARDEEVAAGSTPGAFDYW	IGH	6	None	
14	780	CAKCDGDYVRENWYFDLW	IGH	2	None	
14	781	CASPEITTLGAFDIW	IGH	7	None	
14	782	CARGSHLHMIHHFDYW	IGH	2	None	
14	783	CAKDPLRWLSRGYFDYW	IGH	6	None	
14	784	CARLRGDSIVTIFGVSPLGHFDYW	IGH	6	None	
14	785	CARCSISFPWSTFDLW	IGH	3	None	
14	786	CSRVAVVDPGVITGRSKFDYW	IGH	2	None	
14	787	CARGRMSADGSYFYYYMDVW	IGH	2	None	
14	788	CAAEQWLVRRTFEYW	IGH	60	None	
14	789	CARMSHSHPSFHDSVIDAFDIW	IGH	6	None	
14	790	CARDPTLTTVTTVYYYYGMDVW	IGH	2	None	
14	792	CARDRRKVYYYDSSSYLAGSFDIW	IGH	9	None	
14	793	CARVDHWGMYSDHW	IGH	2	None	
14	794	CARGGEMATILIWFDPW	IGH	2	None	
14	795	CTRGLYHDYHHIFDYW	IGH	10	None	
14	796	CARDLGITIFGVVIGLFNYW	IGH	2	None	
14	797	CASEGGGRAVADFDYW	IGH	7	None	
14	799	CQQRSNWPDPVYSF	IGK	2	None	
14	800	CARAVVVTTTTPVRSYYYYGMDVW	IGH	9	None	
14	801	CAKDRGGSYYGNGFDVW	IGH	6	None	
14	802	CATGVAVAAKDYW	IGH	21	None	
14	803	CAREITEGFYDSGSYSWGFDFW	IGH	3	None	
14	804	CARDDGMVYATYYFDCW	IGH	10	None	
14	805	CQVWDASADHPVIF	IGL	4	None	
14	806	CARTLGDQLLNWFDPW	IGH	4	None	
14	807	CARARTVTTAPAHFDYW	IGH	7	None	
14	808	CAKEGGYQDAFDIW	IGH	49	None	
14	809	CARYADTAMALTYYYYYMDVW	IGH	2	None	
14	810	CDWSSDVCSSDLSDTAMYYCARRGMTTEYFDYW	IGH	2	None	
14	811	CARVHRTTISHFDYW	IGH	3	None	
14	812	CVKGWLRIGFDVW	IGH	2	None	
14	813	CAKAQRKALKGIVGATYDYW	IGH	2	None	
14	814	CARRVSILEWLSEDYDAFDIW	IGH	3	None	
14	815	CVRDKGGYGGLFEYW	IGH	8	None	
14	816	CAKRNGLDQWAAFDIW	IGH	4	None	
14	817	CAHRLDGGWYYYFYMDVW	IGH	4	None	
14	818	CVRDLSPTAYNTFDPW	IGH	2	None	
14	819	CASRELPHYNDAFDIW	IGH	2	None	
14	821	CARLGRPGYTSRWYYFDYW	IGH	2	None	
14	822	CATGVELSGTTRIAFDVW	IGH	2	None	
14	823	CAKDSTPSIAVADYW	IGH	10	None	
14	824	CAKDYKGQQWLAHAFDIW	IGH	2	None	
14	825	CAREKAYCGGDCSLGYYYYYGMDVW	IGH	41	None	
14	826	CTREEWELMFNAFDMW	IGH	2	None	
14	827	CAKGAYFGDGRSDYFDYW	IGH	9	None	
14	828	CARDPGSVNIARWFDPW	IGH	9	None	
14	829	CASLEVTMFGSSGSDYW	IGH	3	None	
14	830	CAVAVAGTFGVYYYYGMDVW	IGH	2	None	
14	831	CASSQSAFWNSGPHPLLYSDHW	IGH	2	None	
14	832	CQQYYSYSNQHYSYSSF	IGK	4	None	
14	833	CARDPFDSSAYYANYFDPW	IGH	4	None	
14	834	CARLRRGEPAAISVYYYYYYMDVW	IGH	2	None	
14	835	CAVMALFAFDYW	IGH	5	None	
14	836	CAKVEHGLQLDYW	IGH	2	None	
14	837	CARTGTKANFYYYYYMDVW	IGH	2	None	
14	838	CAHRIVTANDAFDIW	IGH	3	None	
14	839	CAKVSYLSTASSLDYW	IGH	49	None	
14	840	CATASPILLRNWFDPW	IGH	74	None	
14	841	CATARCSGGNCYARFDYW	IGH	2	None	
14	842	CARDRNMAGWGFDYW	IGH	2	None	
14	843	CARFWGSSGRSFDYW	IGH	2	None	
14	844	CARVYSGSYLGWFDPW	IGH	31	None	

14	845	CLQYFTTPYTF	IGK	133	C2	7K8U
14	846	CAALYCSVTTCPLGAYYFYGMDVW	IGH	2	None	
14	847	CARAPYNLDSQGLARRYFDYW	IGH	4	None	
14	848	CAKDRQILTMIVVAHRSRGFDNW	IGH	2	None	
14	849	CARAPGGSYPIDYW	IGH	23	None	
14	850	CARHGNTEIIWNSILNWFDPW	IGH	3	None	
14	851	CAKGFSGFIPGRDYYFDYW	IGH	2	None	
14	852	CAKEGNRHLASAGIGLYFDDW	IGH	2	None	
14	853	CVVPGDDYAAPNPDAFDIW	IGH	2	None	
14	854	CAKAVHSSSSGWYFDLW	IGH	6	None	
14	855	CTTDLEVVAVTGWFDPW	IGH	2	None	
14	856	CARKYYYDGSSFYYIGPDFDYW	IGH	2	None	
14	857	CARLYQPLVTLRYFDYW	IGH	5	None	
14	858	CARALWGSYRPPDYW	IGH	12	None	
14	859	CARETGYRDFNDRTGYYPRAATYYYFAIDVW	IGH	3	None	
14	861	CARGRKNTYDFWSVYYNWFDPW	IGH	2	None	
14	862	CAAHSGYESFEYW	IGH	4	None	
14	863	CARRSHDFWTGYANYFDFW	IGH	2	None	
14	864	CAKESRGGGTTKGYMDVW	IGH	2	None	
14	865	CARGVGSDLATHDAFDIW	IGH	4	None	
14	866	CARIEVGSASRGFDYW	IGH	10	None	
14	867	CTTAPEYSSSWINYFDYW	IGH	2	None	
14	868	CAKEQGQWLVHFDFW	IGH	2	None	
14	869	CAKDISPIQYSATFDYW	IGH	2	None	
14	872	CAKEDPIFGRGFDYW	IGH	2	None	
14	873	CQAWDSSTHVVF	IGL	3807	C4	7JVC
14	874	CARHARIAAGGNYFDSW	IGH	2	None	
14	875	CARHVLCIAARCPFDYW	IGH	19	None	
14	876	CARLLGFWSGYSYFDYW	IGH	8	None	
14	877	CASTWNDHYSQTIDYW	IGH	4	None	
14	878	CARDYYDTSGSHYSHWYFDLW	IGH	4	None	
14	879	CARIPVQQQLAHDYW	IGH	4	None	
14	880	CASSGPSLRQGYRALRKPFDCW	IGH	4	None	
14	881	CASNHDYINLDNWFDPW	IGH	2	None	
14	882	CARFRLPSHIFDYW	IGH	11	None	
14	883	CARTDREMATITFDYW	IGH	5	None	
14	884	CAGSGNDSSGYGFVYW	IGH	5	None	
14	885	CATSDTTGYPMYW	IGH	2	None	
14	887	CATLTPPLAVAQRLLDWLPGDYW	IGH	22	None	
14	888	CARSGWRTTVKLVPFANFDYW	IGH	2	None	
14	889	CSRDLLGFDAFDVW	IGH	6	None	
14	890	CARRIKMFREVLGSFDSW	IGH	33	None	
14	891	CARTVRAVAGREYYFDHW	IGH	4	None	
14	892	CMQDAQDVAF	IGK	12	None	
14	893	CARLIGALNNFYFYMDVW	IGH	3	None	
14	894	CTHSRGCYICPFDIW	IGH	7	None	
14	895	CSLRSEDTAVYYCARVSRNWNLPFDYW	IGH	2	None	
14	897	CARHSMAATVNDAFEIW	IGH	8	None	
14	898	CARVGWVRGYSYGYSYGAFDIW	IGH	3	None	
14	899	CVREEGGSDSYYNWFDPW	IGH	13	None	
14	900	CHARGYIYGRDYW	IGH	2	None	
14	901	CARVSSRNHYCSSSNCYRYMDVW	IGH	8	None	
14	902	CARSRTSYYDSSGYFLHW	IGH	3	None	
14	903	CAKDIVIVAVASPLTAESYYCYGMDVW	IGH	8	None	
14	904	CARVMVRGLMIAFDYW	IGH	24	None	
14	905	CAHSGPETTNWGGWGDDFDYW	IGH	5	None	
14	906	CARHVRAVLYYYGMDVW	IGH	5	None	
14	907	CATELVWYPVHSFDYW	IGH	2	None	
14	908	CVRDWSAGGGYFQHW	IGH	14	None	
14	909	CQQYGRLPPGVSF	IGK	6	C3	6XDG
14	910	CARVFRYSSSSANFDYW	IGH	2	None	
14	911	CQQYDSFPWTFGQGTKLEIKGARTF	IGK	2	C3	7K8R
14	912	CARGRGIVWFGELLTWFDPW	IGH	2	None	
14	914	CVRRFFDSSGHSNALDAFDIW	IGH	5	None	
14	915	CAHRRIMIRFGGMSKWFDPW	IGH	4	None	
14	916	CARGGYDILTGLKTFDYW	IGH	2	None	
14	917	CARGGEDYYGGTNCFDFW	IGH	7	None	
14	918	CARSSPRPKASLNVMVPSALFDYW	IGH	2	None	

14	919	CARQMRQLVGYFDYW	IGH	4	None	
14	920	CASRSGGNALIDYW	IGH	2	None	
14	921	CARVMSSSWYGAGYNWFDPW	IGH	6	None	
14	922	CARDPFSPVVVMPASMQEGDDYW	IGH	4	None	
14	923	CARTIITLWFEGTDAFDIW	IGH	6	None	
14	924	CASGHTAINDYW	IGH	5	None	
14	925	CQSAFSSGPYHVSF	IGL	5	None	
14	926	CARLWQWDGYGYHFYFDIW	IGH	8	None	
14	927	CARGGPTRVLGDTNWFDPW	IGH	3	None	
14	928	CATDMRGFDSW	IGH	2	None	
14	929	CARAGYCSADCCYPRDAPPFDHW	IGH	2	None	
14	930	CARDNWHHTDNYNYYFDYW	IGH	10	None	
14	931	CQQDYNLPPTWTF	IGK	2	None	
14	932	CAKLLDSSGYGNRDYW	IGH	21	None	
14	933	CATSIAARQTPVDYW	IGH	13	None	
14	934	CARDYIPGGDYWSASPTGWFDPW	IGH	3	None	
14	935	CAKDTYCSSTSCPFDYW	IGH	14	None	
14	936	CARARGQPLLYRAYYFDYW	IGH	2	None	
14	937	CTAGPDWAFYFDSW	IGH	2	None	
14	938	CALPISRRGFYFYYMDVW	IGH	6	None	
14	939	CAREVGYSYGYYYYYMDVW	IGH	10	None	
14	940	CATETSSYYLDDAFDIW	IGH	6	None	
14	941	CASNRGEGSGSDFQFDYW	IGH	4	None	
14	942	CAAKPAYCGGDCYFYFDYW	IGH	42	None	
14	943	CASSGWTTPVYFDYW	IGH	7	None	
14	944	CARNTFQYGDPGYFDHW	IGH	10	None	
14	945	CARQYGGGSRAWFDPW	IGH	2	None	
14	946	CQQLITYPVRPTF	IGK	16	C1	7BZ5
14	947	CARTLQLWSNKHDEETFDYW	IGH	21	None	
14	948	CARDRSIWSGLWSIDYW	IGH	11	None	
14	949	CARDPFDSSGYTPPDYW	IGH	4	None	
14	950	CASDNGDYIGYFQHW	IGH	9	None	
14	951	CVRHEEVGATTFDDW	IGH	6	None	
14	952	CVRSERAVYYFYMDVW	IGH	2	None	
14	953	CAKDKDVDTAIGFNYFDYW	IGH	7	None	
14	954	CARLVLGYGSGSYRPEYYFDYW	IGH	3	None	
14	955	CARVICSSSTRCYMPYFDCW	IGH	2	None	
14	956	CAKDNSPILFGGNFDYW	IGH	4	None	
14	957	CARGSHLHMIYDIKTSQYFDYW	IGH	10	None	
14	958	CARPEGGYSTSWVDYW	IGH	2	None	
14	959	CAKIAAADLDAFDIW	IGH	9	None	
14	960	CVRSGAVRVAHFDSW	IGH	2	None	
14	961	CARQRQMGVSYFDSW	IGH	2	None	
14	962	CARGPTDYFYFDSW	IGH	13	None	
14	963	CARHSSSSRPNFDYW	IGH	32	None	
14	964	CAREIPYYDNTGAPQGQDYW	IGH	4	None	
14	965	CARHDRTAKNTQTTRSFLFDYW	IGH	2	None	
14	966	CCSRDSSGDRLLF	IGL	11	None	
14	968	CAKDPATMVRGVIIPSWFDPW	IGH	2	None	
14	969	CAREVDIPVNSDAFDIW	IGH	6	None	
14	970	CEQYDVLPAF	IGK	5	None	
14	971	CARDSVGGFDILDYW	IGH	5	None	
14	972	CARIAPQLAFDYW	IGH	12	None	
14	973	CAKDHYDILTGQPDYW	IGH	2	None	
14	974	CARPNEFWSAYDGFDVW	IGH	6	None	
14	975	CTRHLDDVLGHEYTYGRRVDYW	IGH	4	None	
14	976	CARVPPLLSYCGGDCYYYYMDVW	IGH	2	None	
14	977	CVKDVSGAYDASHFDYW	IGH	3	None	
14	978	CAKAQYRGSYFFDYW	IGH	2	None	
14	979	CARLTRDGSGSRFDYW	IGH	5	None	
14	980	CARDPLRAIVATMSISGIYDQGIPFDYW	IGH	2	None	
14	982	CATLQDITIFGVVTDPLSVRFDLW	IGH	2	None	
14	983	CARTYYYHTSGFPYDAFDIW	IGH	48	None	
14	984	CAKDRTTVVTRGYFDYW	IGH	3	None	
14	985	CARRPFYVEYLSSSLDYW	IGH	3	None	
14	986	CASEIPGEYYFDYW	IGH	27	None	
14	987	CAREEEGGANCAFDIW	IGH	25	None	
14	988	CAKGAFYYDSTGYRHFDQW	IGH	3	None	

14	989	CARCGDIASTGHYFFDSW	IGH	2	None	
14	990	CARGGYCDSSTCYAPYYYMDVW	IGH	4	None	
14	991	CARGLYASSLEPHWFDPW	IGH	4	None	
14	992	CASDYDMVTGYYQAFHIW	IGH	6	None	
14	993	CARHVPEYDSSGYYREYYFDYW	IGH	9	None	
14	994	CAKDGEVGTIFGVVSPCYMDVW	IGH	2	None	
14	995	CARHGTYYYDSSGYYEDYNWFDPW	IGH	2	None	
14	997	CARGRFATVKGYYYYGMDVW	IGH	5	None	
14	998	CAKSQRYQLLSSSPFDIW	IGH	2	None	
14	999	CARDLRVHDILTGVSLYWYLDLW	IGH	10	None	
14	1000	CAMTLLDYYDTAGSFDHW	IGH	2	None	
14	1001	CASLRRCGGIDCSDVYDYW	IGH	3	None	
14	1002	CASLYDSGGYNDFDYW	IGH	2	None	
14	1003	CAKGPEPSSGYYYQNKAFDIW	IGH	2	None	
14	1004	CAREYQLHLVWDYW	IGH	2	None	
14	1005	CAHSRPHKVVVITLDAFDMW	IGH	12	None	
14	1006	CAKDPRHCSNGLCSTGGYYFDYW	IGH	2	None	
14	1008	CAREDCSGGSCYFDYW	IGH	12	None	
14	1009	CAKAPSRYAPSDYW	IGH	3	None	
14	1010	CLLSYSGARVF	IGL	185	None	
14	1011	CAKDLTVFGVVGHALDVW	IGH	2	None	
14	1012	CASTATQYQLPADYW	IGH	5	None	
14	1013	CVRLGTDIGIRAGFDVW	IGH	18	None	
14	1014	CAKTTGTAYRELYIDSW	IGH	13	None	
14	1016	CARLLQSDFWSGYFYTWFDPW	IGH	2	None	
14	1017	CATTERLTVQFDQW	IGH	3	None	
14	1018	CVHTPFYGGGPTYFDYW	IGH	14	None	
14	1019	CAKDRGSGGRYPDYW	IGH	58	None	
14	1020	CARGIVAIPIPPANPFDIW	IGH	6	None	
14	1021	CARHWADIVVVVTANDYLDYW	IGH	2	None	
14	1022	CAIRSGSSLLRFDYW	IGH	2	None	
14	1023	CARHKEGWYYFDDW	IGH	11	None	
14	1024	CARDTPAGGGYQSYYMDVW	IGH	3	None	
14	1025	CARNPDFWRGYDAFDIW	IGH	8	None	
14	1026	CARDWFISKIMLPGPWFDPW	IGH	2	None	
14	1027	CARDQDFGDYVSNFDYW	IGH	8	None	
14	1028	CATDWVDTTTAAGFRLQYRPEYFQQW	IGH	9	None	
14	1029	CAKDLSHGATGDYW	IGH	5	None	
14	1030	CATEKGPYYPRDYW	IGH	3	None	
14	1031	CAKDLYSGYDWVDYW	IGH	2	None	
14	1032	CTHVTGYQSPDFDFW	IGH	3	None	
14	1033	CDWSSDVCSSDLRDLSRVDYGALSGYYQGGILDYW	IGH	39	None	
14	1034	CAKAGEERHPDYW	IGH	6	None	
14	1035	CAREAPRRLGYLNWYIDLW	IGH	3	None	
14	1036	CSLKLNSVTAADTAVYYCARQRYSYGYVLRSFDPW	IGH	4	None	
14	1037	CVRPILGYCSDTSCVDWYFDLW	IGH	10	None	
14	1038	CAKDIRVAATLLGAFDIW	IGH	3	None	
14	1039	CATGYCSITSCPGFDYW	IGH	7	None	
14	1040	CVKDEGSINWYFVTW	IGH	3	None	
14	1041	CAREGTIFGVVIYAFDIW	IGH	20	None	
14	1042	CALGKTLRFSFDYW	IGH	4	None	
14	1043	CASDTIFGVEAPPLYYYMDVW	IGH	7	None	
14	1044	CARDRVNYYVSSGYADFDYW	IGH	12	None	
14	1045	CARFKGDPVGGTEGYYYYLDVW	IGH	2	None	
14	1046	CARDRMVAATGETYYYYYGMDVW	IGH	2	None	
14	1047	CAKDIGAWPYYLDNW	IGH	3	None	
14	1048	CARDKSDFWSGTDAFDIW	IGH	2	None	
14	1049	CARNKNDYGDYGWYFDLW	IGH	33	None	
14	1050	CVTVVLRYFAWLSDLFYYYYNGMDVW	IGH	2	None	
14	1051	CARDHPDNDYGDYAGYW	IGH	9551	None	
14	1052	CAKDRVAFDYYDSSGYLFDYW	IGH	2	None	
14	1053	CARDVEHSDGFSFAFDYW	IGH	35	None	
14	1054	CAKDRYDSSGYILLDAFDIW	IGH	2	None	
14	1055	CAKDRSWVAVAAISAFDIW	IGH	4	None	
14	1057	CARESTHCGGDCYLDYW	IGH	4	None	
14	1058	CARVQYSGYDCHFDYW	IGH	20	None	
14	1059	CARASGSITSGWANFDYW	IGH	4	None	
14	1060	CVHFRVLPTTVWFDPW	IGH	2	None	

14	1061	CAKGHGESVITPLDYW	IGH	2	None	
14	1062	CATDHIFVYGEGLLDYW	IGH	6	None	
14	1063	CARRYDPSGTSSTNWFDPW	IGH	2	None	
14	1064	CANDGVTTGVGFDYW	IGH	3	None	
14	1065	CTRHVHFDFWTTDSAGRNHYYMDVW	IGH	2	None	
14	1066	CAKDREYCSSFGCYSTFYFDSW	IGH	5	None	
14	1067	CARRPWIAAPYYFDYW	IGH	4	None	
14	1068	CARVGGSSWYTYDYW	IGH	16	None	
14	1069	CAKGQEPYYYYTMDVW	IGH	3	None	
14	1070	CARRGGGSGWDFWYDYW	IGH	3	None	
14	1071	CAKDGREQWLVLRFDYW	IGH	2	None	
14	1072	CARERRWLQFQNFDYW	IGH	4	None	
14	1073	CAKEVSQTDDYW	IGH	25	None	
14	1074	CALPISADTAVYYCARFSIYYESSGDYYRDYW	IGH	2	None	
14	1075	CARAISSAYYYDFTYW	IGH	2	None	
14	1076	CARGGFCNGVTCLLQYMDVW	IGH	6	None	
14	1077	CARLLTILASAGWYYFDLW	IGH	6	None	
14	1078	CASIAASSGGVDYW	IGH	2	None	
14	1079	CARSYPTSLNWALAAYDYW	IGH	7	None	
14	1080	CARASVFGTYKSGHYFFDHW	IGH	2	None	
14	1081	CANLASTWLVTDYW	IGH	3	None	
14	1082	CARGYSIDYW	IGH	2019	None	
14	1083	CARDHCSGGFCYIDSW	IGH	3	None	
14	1084	CAKGQLGIRHSNVFDYW	IGH	2	None	
14	1085	CASKPYYDFWSGYYMDGYYYYYMDVW	IGH	5	None	
14	1086	CASSGETVTTWDAFDIW	IGH	3	None	
14	1087	CARQRGSVGAHFDYW	IGH	2	None	
14	1088	CARDGRVAVPNDDFDIW	IGH	6	None	
14	1089	CARVGPGTVGYYYYYYYMDVW	IGH	2	None	
14	1090	CASSYYDSSGYLPVDYW	IGH	5	None	
14	1091	CSVLDCTSTTCQRSGDPQNWFAPW	IGH	2	None	
14	1092	CARNMGYDFWSTFDYW	IGH	6	None	
14	1093	CVRVDISIRSNTGDAFDIW	IGH	3	None	
14	1094	CARLGAARGHEAFDYW	IGH	426	None	
14	1095	CARTRDRAITAAGFFDYW	IGH	5	None	
14	1096	CAREAVGSTWSAYYFDYW	IGH	4	None	
14	1097	CARGRGYSSGEVFDSW	IGH	5	None	
14	1098	CARQNVLFEYSAGPPRLFDVW	IGH	2	None	
14	1100	CARSPGGIVPAGTQNWFDPW	IGH	9	None	
14	1101	CAILKVDSGYHDYW	IGH	4	None	
14	1102	CARTTSLTAPGFDIW	IGH	27	None	
14	1103	CARINYDILTGYIVGLDYW	IGH	48	None	
14	1104	CARDPYDYVFFDIW	IGH	2	None	
14	1105	CARDQAHLRYSFWSGSYFDYW	IGH	2	None	
14	1106	CARDPELDPFYYW	IGH	2	None	
14	1107	CARWRAWEYYFDSW	IGH	2	None	
14	1108	CARDYGDYPSPSFYFDFW	IGH	5	None	
14	1110	CARLGPGSVGSDWMFYFDFW	IGH	206	None	
14	1111	CVRVLGGNYLGYFDYW	IGH	3	None	
14	1112	CARHLRHQEWHLLHSTDFDYW	IGH	2	None	
14	1113	CARVAYSSKYTYYMDVW	IGH	16	None	
14	1114	CARVGLPLWSGYNDYW	IGH	3	None	
14	1115	CAKDHVAGRNVDYW	IGH	25	None	
14	1116	CAKGAIVGLGWVDYW	IGH	2	None	
14	1117	CVSSGWYVSILDYW	IGH	3	None	
14	1118	CARDAMVRGLITRPLNVFDIW	IGH	2	None	
14	1119	CARQRGDGYNDDFEYW	IGH	2	None	
14	1120	CARVLKTNRTFDYW	IGH	4	None	
14	1121	CARELEGHADAFDVW	IGH	5	None	
14	1122	CDWSSDVCSSDLSLRGGDTAVYYCAKDSRLFLMGADYHFDYW	IGH	3	None	
14	1123	CARSEQRGNAFDVW	IGH	6	None	
14	1124	CAKDSHWDSSSWYGRYFDYW	IGH	2	None	
14	1125	CARDQYSSRPTYYYYGMDVW	IGH	10	None	
14	1126	CARHPVVVVAAVVDYW	IGH	8	None	
14	1127	CARDFDHSNSHFDFW	IGH	4	None	
14	1128	CARDKVTLPKPNWFDPW	IGH	2	None	
14	1129	CARERGLNYFDIW	IGH	7	None	
14	1130	CARVGYISSWYHLGNWFDPW	IGH	5	None	

14	1131	CARHAADYSNYPFSLDYYYYMDVW	IGH	2	None	
14	1132	CAKELHQRAVFGVLGFDFW	IGH	3	None	
14	1133	CARSPVYSGALDYYMDVW	IGH	5	None	
14	1134	CATLAVAGQYFFDYW	IGH	4	None	
14	1135	CARQGEAMYYDILTGYYKNEPSSGVVDYW	IGH	2	None	
14	1136	CATSRCSSASCYASGRNKLFDYW	IGH	5	None	
14	1137	CANSRDGYNFDSW	IGH	4	None	
14	1138	CAKGPTLGYGGQLPDYW	IGH	4	None	
14	1139	CSSFAGSNNFPYVF	IGL	4	C2	7BWJ
14	1140	CARAPGGDLTENLDYW	IGH	2	None	
14	1141	CARDGAMVAAAGNYYYYGMDVW	IGH	13	None	
14	1142	CARTIFDYFGSGSSYFDFW	IGH	2	None	
14	1143	CVKDMSYSNLWQTIDYW	IGH	3	None	
14	1144	CASPTWELIREGEFDHW	IGH	5	None	
14	1145	CALNYYDSSGLTTGDYW	IGH	14	None	
14	1146	CARDPSYSSSWSGGADEQDYYYYYMDVW	IGH	2	None	
14	1147	CVKGLSNSPFDSW	IGH	2	None	
14	1148	CSSYTDSPTLPYVF	IGL	10	C2	7K90
14	1149	CARMGSVEKDKYNNYFDTW	IGH	2	None	
14	1150	CARDNGDYQWERLDYW	IGH	2	None	
14	1151	CGRGRYSSGPPGAFDIW	IGH	2	None	
14	1152	CAKLDIASPGDYW	IGH	23	None	
14	1153	CVRGQQELLVNPFDFW	IGH	2	None	
14	1154	CVRAVFNTVWGVSHVGFDSW	IGH	2	None	
14	1155	CARGYGMLNPTVYFDPW	IGH	2	None	
14	1156	CARGVVVVPAAIRDYYYYYMDVW	IGH	3	None	
14	1157	CARRQYYYDSSGSFKGEDWFDPW	IGH	3	None	
14	1158	CARDSPALDYYYGMDVW	IGH	19	None	
14	1159	CARFPGIPSDGLYYMDVW	IGH	2	None	
14	1160	CAKDLPYCSGGSCYSGGHWFDPW	IGH	4	None	
14	1161	CARSLGAYDRFDSW	IGH	2	None	
14	1162	CARVKRTVVITVPVFDYW	IGH	9	None	
14	1163	CARDASSLFADGVCPPYHFDYW	IGH	2	None	
14	1164	CAKETAGSGSFPYDYW	IGH	11	None	
14	1165	CAKPHYYDSSGYSWYFDLW	IGH	10	None	
14	1166	CAKVSPRLAYYDAFDVW	IGH	5	None	
14	1167	CASQAPGFGGNPNTPFDSW	IGH	16	None	
14	1168	CARGFVRNSQDSSAWSLVRPFDSW	IGH	5	None	
14	1169	CAKDTARRAMDVW	IGH	5	None	
14	1170	CVRDQAYYDDERGAFDIW	IGH	4	None	
14	1172	CARGGDSSGWYRQNFFDYW	IGH	6	None	
14	1173	CARATYFSDSSAYYHFDSW	IGH	11	None	
14	1174	CATGRVTVAAATDSPFFEYW	IGH	2	None	
14	1175	CARDSGSSWYFDLW	IGH	29	None	
14	1176	CARQGVRWSWGEEFDSW	IGH	2	None	
14	1177	CARSLGYCTATGCYNLDYW	IGH	2	None	
14	1178	CVRLLGGPSTRAGDYW	IGH	2	None	
14	1179	CARDLYNSKASFDYW	IGH	251	None	
14	1180	CANHDYGGSGGFDYW	IGH	3	None	
14	1181	CTRDSDFWSGYIRYMDVW	IGH	2	None	
14	1182	CARIPYCRSTTCYDYYYMDVW	IGH	12	None	
14	1183	CMQTQQTPPPRYSF	IGK	3	None	
14	1184	CAKLGLRSTVDYW	IGH	2	None	
14	1185	CAKLVATDFVDYW	IGH	2	None	
14	1186	CARDPAQYDSSGLPTDYW	IGH	2	None	
14	1187	CTSWGLDHYYALDVW	IGH	8	None	
14	1188	CASRRYYGSGSYYNYW	IGH	3	None	
14	1189	CARSQIHAKRDAFDIW	IGH	15	None	
14	1190	CARDSSRYCISDVCFDYW	IGH	4	None	
14	1191	CARDRGYYDSSGSHVGWFDPW	IGH	2	None	
14	1192	CARTIVGTKSDAFDIW	IGH	11	None	
14	1193	CARETDDVRISFDHW	IGH	2	None	
14	1194	CTTHTSRDTIGYYLFDHW	IGH	3	None	
14	1195	CARDRDASLSGYYDYW	IGH	3	None	
14	1196	CAREVASGTYWGYFQFW	IGH	3	None	
14	1197	CARYLWFGEFYFFDYW	IGH	6	None	
14	1200	CARTFTSGGFGAPFFDYW	IGH	2	None	
14	1201	CVRDLHDYSRPLFDIW	IGH	2	None	

14	1202	CARYCSGASCHGANWFDPW	IGH	4	None	
14	1203	CTNFDYYYYMDVW	IGH	6	None	
14	1204	CAKDFLYYFDNNFQFDSW	IGH	4	None	
14	1205	CAKPGGLCGGDCFDYW	IGH	2	None	
14	1206	CARDRRVTRLGEGVGQNDYW	IGH	3	None	
14	1207	CARGTRYNRCSSGVCYAHVDAFDIW	IGH	6	None	
14	1208	CARMGVDQDYYFDYW	IGH	2	None	
14	1209	CARDLGSWRRPIAFDIW	IGH	13	None	
14	1210	CARGGEWNQLSIPPDYW	IGH	45	None	
14	1211	CARLTYISGSGLGHWFDPW	IGH	2	None	
14	1212	CARVKGLPIFDNW	IGH	13	None	
14	1213	CVTDAIRGVTGWFDPW	IGH	6	None	
14	1214	CVRMVQGVPSARWFDPW	IGH	2	None	
14	1216	CAKGGQQVTVEPGPYYLDYW	IGH	2	None	
14	1217	CARGGPYSGYDYYGMDVW	IGH	5	None	
14	1218	CASIGGNPPYYYYYMDVW	IGH	4	None	
14	1219	CAKTTVTLSPTPYYFDYW	IGH	6	None	
14	1220	CARRRLGETNRYTFDHW	IGH	2	None	
14	1221	CAKETSGATYYFDYW	IGH	33	None	
14	1222	CARGLTLRFLTERLNWLDPW	IGH	2	None	
14	1223	CAREGGSYRKFDFW	IGH	4	None	
14	1224	CARDGITTRYSSGWYFDYW	IGH	3	None	
14	1225	CARDVRPHSGWYDYW	IGH	3	None	
14	1227	CARLDGCSAGTCYRFDSW	IGH	3	None	
14	1228	CARPVVARGPYAFDIW	IGH	4	None	
14	1229	CAKNFGDYNALWLTYIDYW	IGH	6	None	
14	1230	CAKELPRWSGLGYYYYYMDVW	IGH	2	None	
14	1231	CARSSRGSSGYYYFDNW	IGH	3	None	
14	1232	CAKDEYYYESSGPPDGFDIW	IGH	3	None	
14	1233	CARDNQKRQGRFDYW	IGH	7	None	
14	1234	CAKDSSHYYDSSGTDYW	IGH	22	None	
14	1235	CARGYYDSSTYYPYYYGMDVW	IGH	9	None	
14	1236	CSSYTSSNSLDVIF	IGL	2	C2	7K90
14	1237	CTRAWNMDTAAFDSW	IGH	7	None	
14	1238	CARDRDGDGYYSHSFDYW	IGH	3	None	
14	1239	CARDRSIAARPANWFDPW	IGH	5	None	
14	1240	CARDLWGPTWGSAAASLEDYW	IGH	2	None	
14	1241	CATDQPPYTDYYYYYMDVW	IGH	2	None	
14	1242	CATVPKRFGEWYFDHW	IGH	4	None	
14	1243	CARSSGYSGFDLADAFDIW	IGH	12	None	
14	1244	CAREDSTGWNRASLDYW	IGH	2	None	
14	1245	CARDLPRITMRVVRSRGDVYDVW	IGH	4	None	
14	1247	CAKLYYDSESGDYW	IGH	2	None	
14	1248	CANSPHRSGWFPWGFDSW	IGH	2	None	
14	1249	CARAYSRISLRHFDYLFHAFDVW	IGH	2	None	
14	1250	CYSTDISGNQGVF	IGL	11	None	
14	1251	CAKTTSGSYWSPCDYW	IGH	2	None	
14	1252	CAKDSIYYDFWSGYIGGEVNYYYYGMDVW	IGH	2	None	
14	1253	CFWSGSFYSGDYW	IGH	3	None	
14	1254	CASSYPGGDSWTGYYRYW	IGH	2	None	
14	1255	CVRIGYDGDYEGHYYFDYW	IGH	2	None	
14	1256	CARPSPRTFDLW	IGH	9	None	
14	1257	CAHKGHWNDLGDAFDMW	IGH	3	None	
14	1258	CARGAPSVIADFDSW	IGH	3	None	
14	1259	CVCATDLTVTNYYNDSGFYPRGYFDYW	IGH	21	None	
14	1261	CVRQGCTSTSCSCCGYDPAPFDYW	IGH	4	None	
14	1262	CARDAEFYGSGSSPLDYW	IGH	15	None	
14	1263	CARHLKSIAATWGAFDIW	IGH	9	None	
14	1264	CARGRPSDDFWSGYWGDWFDPW	IGH	4	None	
14	1265	CATGLVGATSLGDYW	IGH	4	None	
14	1266	CAHTPYLRGYSRYDYW	IGH	2	None	
14	1267	CAKNGLYCGGDCNPFDYW	IGH	2	None	
14	1268	CAKYAKVTSDGKYFDLW	IGH	4	None	
14	1269	CAHRSSFRHFDYW	IGH	7	None	
14	1270	CALPILRAEDTAVYYCARGSAIYYDSSGYFDYW	IGH	9	None	
14	1271	CAKALVVVAANYWYFDLW	IGH	2	None	
14	1272	CARDPCSGGQCYGTLDYW	IGH	4	None	
14	1273	CAKDKIVVVPAALSLHFDYW	IGH	2	None	

14	1274	CAREPVGGPTDDYW	IGH	2	None	
14	1275	CLQSSNLPPYTF	IGK	2	None	
14	1276	CARARDFWSGTYYYYYGMDVW	IGH	2	None	
14	1277	CQVWDFSSVHSRGFYFF	IGL	2	None	
14	1278	CARVGGRPLGPFDYW	IGH	4	None	
14	1279	CARHRGSSSRFPIDYW	IGH	11	None	
14	1280	CARIRMGVVIPAAISPFLFDPW	IGH	2	None	
14	1282	CVRGAFLEWFHGFDIW	IGH	501	None	
14	1283	CARESYSPARDYGGNSRAFDYW	IGH	2	None	
14	1284	CAKDEGYCSGDGCYPAYAFDIW	IGH	3	None	
14	1285	CARDYHFLVAGHARPPNWFDPW	IGH	5	None	
14	1286	CTSSSWARFDYW	IGH	2	None	
14	1287	CVRRTTTGDYW	IGH	2	None	
14	1288	CARDYCSITDCYFTFYIDAW	IGH	3	None	
14	1289	CRLEEDCSSTSCYGWYFDYW	IGH	2	None	
14	1290	CAREGILGATRGFDFW	IGH	2	None	
14	1291	CAVSSVVVPAACDYW	IGH	11	None	
14	1292	CARRSVTGTTFVDYW	IGH	2	None	
14	1293	CARGPPLGPYYYYMDVW	IGH	6	None	
14	1294	CVRSPGRTILVPIFDLW	IGH	3	None	
14	1295	CARDGEPGTNVGRFLDYW	IGH	2	None	
14	1296	CARTTPRSSYYSNWYFDLW	IGH	3	None	
14	1297	CAKTPQGTAQDYW	IGH	9	None	
14	1298	CARHPAVNNFDYW	IGH	32	None	
14	1300	CARAISVPAAISSRDYYYYMDVW	IGH	2	None	
14	1301	CARASWYYDSSGYRDYYYYGMDVW	IGH	2	None	
14	1302	CARDIFAGVAAAGTKFYYYGMDVW	IGH	3	None	
14	1304	CARISYYSASSGQGGFDYW	IGH	2	None	
14	1305	CAKAGSGRWPLEYFDYW	IGH	4	None	
14	1306	CMQTLQSPRYIF	IGK	12	None	
14	1308	CTTRDCSGGGCHSDDAFDIW	IGH	2	None	
14	1309	CASPGRPLTVEGFDYW	IGH	2	None	
14	1310	CASRSSRGYYYGMDVW	IGH	3	None	
14	1311	CAKDSGGVAAAVYHFDYW	IGH	2	None	
14	1312	CAKEGSHCTPTNCPRPFDYW	IGH	35	None	
14	1313	CARLWALGRSLDYW	IGH	2	None	
14	1314	CAVVILARSDCPEGACYAYYFDYW	IGH	2	None	
14	1315	CARDQYYESSGYWWGLDYW	IGH	6	None	
14	1316	CAKVSDPSGNTEADYW	IGH	3	None	
14	1317	CAASGFGFNNHGMHWVRQAPGADYYFDSW	IGH	3	None	
14	1318	CAREAYTYGQDPYYLDYW	IGH	2	None	
14	1319	CARESRSERKGAFDIW	IGH	2	None	
14	1320	CARELRVVVVAATQYAFDIW	IGH	2	None	
14	1321	CARDLRDFWSAYDRRFDYW	IGH	4	None	
14	1322	CARDGGSRHYYDSSGLFDYW	IGH	2	None	
14	1323	CARHTPYDFSPYYFDPW	IGH	2	None	
14	1324	CAREDYSPKGTFDQW	IGH	2	None	
14	1325	CARAVGGWYRFDYW	IGH	2	None	
14	1326	CARSRDTALPNDAFDIW	IGH	2	None	
14	1327	CARDDFNWGCFDPW	IGH	3	None	
14	1328	CARLNYAGFFDYW	IGH	2	None	
14	1329	CAKDMLRYSYGCLDYW	IGH	2	None	
14	1330	CARVWEVVDPGAFDMW	IGH	3	None	
14	1331	CARLGYFSDHWERDYW	IGH	5	None	
14	1332	CAREDRRSGWYFRFDYW	IGH	4	None	
14	1333	CARVIIVPAAMVSYYSYYYLDVW	IGH	2	None	
14	1334	CAREVSRYWYFDLW	IGH	4	None	
14	1335	CATDFGGETDWFDPW	IGH	6	None	
14	1336	CATRGRQLEPHAFDIW	IGH	4	None	
14	1337	CARGDFTIFGRGGSVGILDYW	IGH	2	None	
14	1338	CARGRADYYDSRGYYYGAFDIW	IGH	4	None	
14	1339	CTSQFLRGVRGVTVDYW	IGH	2	None	
14	1340	CARSHRSTPAAMEQVDYW	IGH	5	None	
14	1341	CASLIGGNPAGYFDLW	IGH	2	None	
14	1342	CALLYDPFDYW	IGH	34	None	
14	1343	CAKPLLRACCGGDLILDYW	IGH	8	None	
14	1344	CTTAFYYDDTGQVFDIW	IGH	2	None	
14	1345	CARGATGDRWWAFDIW	IGH	8	None	

14	1346	CAGYCSSFDCPSDAFDIW	IGH	37	None	
14	1347	CATALSVRLNGRYNDYW	IGH	29	None	
14	1348	CAVPLKQQLVPPDPYYFDYW	IGH	2	None	
14	1349	CARGVDTAMVQHYYYYYYMDVW	IGH	6	None	
14	1350	CARDQGYDVSTHYSFFFDQW	IGH	8	None	
14	1351	CQSADSGATYRLF	IGL	118	None	
14	1352	CAREDDITGYIDYW	IGH	9	None	
14	1353	CQQRCDWPPRWTF	IGK	15	None	
14	1354	CAKHGSMVRGVRGIYYYYMDVW	IGH	32	None	
14	1355	CATLHGLEVALATIRGTLDYW	IGH	2	None	
14	1356	CAHRPQQAIFDYW	IGH	5	None	
14	1357	CASGREVVPAAMNYYYYYYMDVW	IGH	4	None	
14	1358	CARTNSGTYAGRFDSW	IGH	4	None	
14	1359	CSGIAEENQFDPW	IGH	7	None	
14	1360	CARHRRTVTTADPFDVW	IGH	3	None	
14	1361	CTTDAYNYRSWRGYYSRDAFDIW	IGH	4	None	
14	1362	CAHSSPTYYYAMDVW	IGH	19	None	
14	1363	CARVGLDYGDYPPFDYW	IGH	3	None	
14	1364	CQHYGNSPRTFSRTF	IGK	7	C1	7K8N
14	1365	CAIFHGQQQAFDYW	IGH	2	None	
14	1366	CATDGPTPLGGVVIIPVDYW	IGH	2	None	
14	1368	CARDRGGEYGSSSIWFDPW	IGH	4	None	
14	1369	CAKGGRPSIAAAEIDYW	IGH	5	None	
14	1370	CARILITTDYDSSGYYYDLNAFDIW	IGH	6	None	
14	1371	CARDTGRLGVAGRYFDYW	IGH	2	None	
14	1372	CARRNPGQYDAYDLW	IGH	2	None	
14	1373	CAKYAYYVSEHFDYW	IGH	4	None	
14	1374	CLRAEDTAVYYCAKESTNYYDSSSYNYGHYFDYW	IGH	2	None	
14	1375	CAKDRVGSGWDNDYYYYHMDVW	IGH	2	None	
14	1376	CATTVTTHSYFDPW	IGH	146	None	
14	1377	CARAPVTPYYYYYYMDVW	IGH	6	None	
14	1378	CARFSIYYESSGDYYRDYW	IGH	10	None	
14	1379	CAAVPHFGVVAGTNYYFYYYMDVW	IGH	4	None	
14	1380	CATALWSYYYYYMDVW	IGH	22	None	
14	1381	CAAVPMSYSDKSPFFDYW	IGH	2	None	
14	1382	CTGPRGQPFDYW	IGH	18	None	
14	1383	CARRDHWAESYLDFW	IGH	4	None	
14	1384	CAKDAGHEYYYYYHMDVW	IGH	10	None	
14	1385	CAKVLSGSDMTGDYW	IGH	4	None	
14	1386	CARPSYGAKGLVDYW	IGH	4	None	
14	1387	CTRSLYGTGWNRETYWFDPW	IGH	8	None	
14	1388	CARVGVGAPDWFGPW	IGH	8	None	
14	1389	CATVRDHDALEVW	IGH	2	None	
14	1390	CAGAFNYGFYFDYW	IGH	20	None	
14	1391	CARQRQYCSGGSCYSFWFDPW	IGH	3	None	
14	1392	CARHHYCSGGTCYSAWFDPW	IGH	2	None	
14	1393	CQYYNSYSPPLTF	IGK	1974	None	
14	1394	CAKDPGFLVGPTCYFDYW	IGH	7	None	
14	1395	CARDSSTTTALNHGRFDYW	IGH	3	None	
14	1396	CTRGGYCSSTNCPKVFFDSFDVW	IGH	2	None	
14	1397	CARVRFPPIRIDYW	IGH	2	None	
14	1398	CAKDASPIAVAGTSHFDYW	IGH	2	None	
14	1399	CAKTYQLLSSDYDVWSGVNPEGAFEIW	IGH	55	None	
14	1400	CQQYGCTPPF	IGK	8	C2	7K8U
14	1401	CARDSMAFSTYYMDVW	IGH	2	None	
14	1402	CARDEVTVVRTGMGTFDIW	IGH	2	None	
14	1403	CLRSEDTAIYYCARKGIASSWAFDLW	IGH	2	None	
14	1404	CPQHDTFPYTF	IGK	18	C3	7K8P
14	1405	CARSGYCSSTRCFRVYYYGMDVW	IGH	4	None	
14	1406	CARHHGGYCSSTSCYLWDWFDPW	IGH	2	None	
14	1408	CAGGTAYYYGSRGYSFDYW	IGH	11	None	
14	1409	CARASRLSYGSVWGMSPDAFDVW	IGH	2	None	
14	1411	CARGQGAVVPAAICDYW	IGH	6	None	
14	1412	CARILMPFLFDSW	IGH	6	None	
14	1413	CATEVVVETGSFDIW	IGH	2	None	
14	1414	CAIGASRLIGPNWFDPW	IGH	14	None	
14	1415	CTTVLRSEFDYW	IGH	3	None	
14	1416	CARLGTRPEYFQQW	IGH	4	None	

14	1417	CGRDYYDSSGYYTQYW	IGH	3	None	
14	1418	CARGAPSYSGYDYYAFDMW	IGH	11	None	
14	1419	CARLPQGGYSYMDVW	IGH	2	None	
14	1420	CARNKKDFWFWSASEYFFDYW	IGH	3	None	
14	1421	CARLGGWQQLGFDYW	IGH	2	None	
14	1422	CARGDYGGNSGVGAFDIW	IGH	2	None	
14	1423	CARDASRFGEGPSFDYW	IGH	7	None	
14	1424	CARDDFNDILTGRGGGFDYW	IGH	5	None	
14	1425	CTTDARGVVIRGVIIGFGGHDYW	IGH	2	None	
14	1426	CAKHVHMRAVLPAAPFDIW	IGH	2	None	
14	1427	CARRSSVWGSHRYYLDYW	IGH	2	None	
14	1428	CARAPWEGTRSPFDNW	IGH	4	None	
14	1430	CARDRFWFEERSGQKNYFYAMDVW	IGH	2	None	
14	1431	CARTVTTVVTQDYYFYMDVW	IGH	3	None	
14	1432	CARQPAFHDIVVVVAATFVPWFDPW	IGH	6	None	
14	1433	CARDTTTGFDPW	IGH	61	None	
14	1434	CVTDGGKHWPSNWFDPW	IGH	3	None	
14	1436	CARAEEYGGRQRNDYW	IGH	3	None	
14	1437	CARELAARPPAWFDPW	IGH	4	None	
14	1438	CARDQSGYDFYAFDIW	IGH	2	None	
14	1439	CALPISAVYYCARGDQKPAFSLDYW	IGH	4	None	
14	1440	CARGPDYAFWSGLHAFDIW	IGH	2	None	
14	1441	CGRPGYCSSTSCSLGEEKNYYMDVW	IGH	2	None	
14	1442	CARFSDFTYGSSIVQSAFFDCW	IGH	24	None	
14	1443	CAKDNWGFHYW	IGH	59	None	
14	1444	CARGIVVVVAATTLPPAYYYGMDVW	IGH	2	None	
14	1445	CATLAAHHDFDIW	IGH	10	None	
14	1447	CARHKAQTTSSDYFDYW	IGH	14	None	
14	1448	CARVDGSSALDFDYW	IGH	2	None	
14	1449	CARETYYYDSSGYYYPPYYYYGMDVW	IGH	8	None	
14	1450	CARQSVGNGGMHGGFDIW	IGH	2	None	
14	1451	CVKDPDVFDVW	IGH	3	None	
14	1452	CAGQGTYYDFWSGYYTGINYFDYW	IGH	2	None	
14	1453	CARGGPSEAAAGTDYFDYW	IGH	10	None	
14	1454	CARPRFARYDFAESGTYDYW	IGH	2	None	
14	1455	CAKVYYGSGTNYHFDHW	IGH	4	None	
14	1456	CSSFTSSSTRPYFF	IGL	2	C2	7K90
14	1457	CARIYYGTNAFDIW	IGH	50	None	
14	1458	CASLGDPIAVAGTLDYW	IGH	2	None	
14	1459	CARNSEGNWFDPW	IGH	149	None	
14	1460	CATHNDFWSGQNFDHW	IGH	3	None	
14	1461	CARSVDLHNSAWYYFDYW	IGH	3	None	
14	1462	CASGYSSSWYSAEYFQHW	IGH	2	None	
14	1463	CAREVEAYCGGDCYSAGGSYYYYYMDVW	IGH	2	None	
14	1464	CAKDGDVGGVSEYFHHW	IGH	3	None	
14	1465	CAKDGYSGYEGYYFDYW	IGH	10	None	
14	1466	CAKDLGIAIFGVVVGIFDYW	IGH	4	None	
14	1467	CARGYCRSTTCYFDYW	IGH	11	None	
14	1468	CAKQGPYDSPDYYYDGSDDYW	IGH	2	None	
14	1469	CARGLVHSYASSYYFDYW	IGH	2	None	
14	1470	CVKNGIDPW	IGH	8	None	
14	1471	CARDIVIVSGDSPLTAYSYYFYGMDVW	IGH	2	None	
14	1472	CARVSWTNIRTYQYYYMDVW	IGH	24	None	
14	1473	CARDKGDYFPNWYFDLW	IGH	3	None	
14	1474	CARVPLSYYYYHMDVW	IGH	13	None	
14	1475	CATTNVVPAPFIYW	IGH	4	None	
14	1476	CARDGGGGSGWSDVFDYW	IGH	8	None	
14	1477	CSSYAGNYIFGLFF	IGL	7	None	
14	1478	CARHGIAEAGRFDYW	IGH	42	None	
14	1479	CKNSLYLQMNSLRAEDTAVYYCARGSAIYYDSSGYFDYW	IGH	14	None	
14	1480	CARVKSGTSLIDYW	IGH	2	None	
14	1481	CLRAEDTAVYYCVRTLRTHTVTTTGVDYW	IGH	3	None	
14	1482	CAKEISDYGDYYFDYW	IGH	11	None	
14	1483	CAKDPDTYSSGWSNWFDPW	IGH	2	None	
14	1484	CARSYFDSSGYHYPLGGFDPW	IGH	13	None	
14	1485	CAKAAIVARPDYFDFW	IGH	7	None	
14	1486	CARGPEAAQYYYDSSDPFFRFDPW	IGH	3	None	
14	1487	CAKMIGGNSPGDYW	IGH	4	None	

14	1488	CAKHGRSRFDNRGYHLRDAFDTW	IGH	2	None	
14	1489	CARLSEQLWLPLLDYW	IGH	2	None	
14	1490	CQKLKSYPITF	IGK	50	C1	7BZ5
14	1491	CARESGQWLVRVGYYYYMDVW	IGH	2	None	
14	1492	CASVREWLLRTTHPPTFDYW	IGH	2	None	
14	1493	CARARWELLYFDYW	IGH	3	None	
14	1494	CAKDIKPYSSGWNAFDVW	IGH	4	None	
14	1495	CARERVGATVDSPYYFDQW	IGH	3	None	
14	1496	CNSRDSSSSHLQVLF	IGL	3	None	
14	1497	CAKNLPAGFWSGYPDYW	IGH	2	None	
14	1498	CARDPQFWDGVNFDSW	IGH	2	None	
14	1499	CARHARTAVAGKIFDYW	IGH	2	None	
14	1500	CARGPLYYDGAGYSSLQVPLFDYW	IGH	2	None	
14	1501	CAKDLYVDGYSFGRAFEIW	IGH	4	None	
14	1502	CARDPSPRGWSGYFDYW	IGH	2	None	
14	1503	CARVRITIFGVANDAFDIW	IGH	2	None	
14	1504	CSSTNCPKVFFDSFDVW	IGH	3	None	
14	1505	CATNSLGPYSDYWSGYYKNFDYW	IGH	2	None	
14	1506	CAKDMTVEYSSSWPLDYW	IGH	2	None	
14	1507	CARETQYSFGYGQYDYW	IGH	8	None	
14	1508	CARPSISLARYSLPPIDAFHVW	IGH	3	None	
14	1509	CARVVDSGGMVIYYFDSW	IGH	2	None	
14	1510	CATAPTKSNNHYYYMDVW	IGH	2	None	
14	1511	CARDRGTYDSSGYYYLDYW	IGH	4	None	
14	1512	CARKRITYYYDSSGAVFDYW	IGH	5	None	
14	1513	CARVPTPLLPAQPDFDYW	IGH	2	None	
14	1514	CARHRFYYHNSGLNPDYFDYW	IGH	4	None	
14	1516	CAREGCGGDCYSHGAFDIW	IGH	2	None	
14	1517	CARIRGGGGYFDYW	IGH	68	None	
14	1518	CAKDKLRGVEATPDAFDFW	IGH	4	None	
14	1519	CARVLVPAPTIAGDYYYGMDVW	IGH	2	None	
14	1520	CAKVTYGGYSDIMDVW	IGH	2	None	
14	1521	CARHISSSLNWFDPW	IGH	28	None	
14	1522	CAREVSGVVIEEIQDYYALDVW	IGH	7	None	
14	1523	CARDSQGFLEWLFKAPSVSTGLDYW	IGH	2	None	
14	1524	CARHKRYCASANCFYIDVW	IGH	6	None	
14	1525	CARDFFLAQFEAFDIW	IGH	6	None	
14	1526	CARDLGFLEWLFRDYYYYMDVW	IGH	2	None	
14	1527	CTRDGAGWALPIDYW	IGH	3	None	
14	1528	CARVAWHGDFFRFDPW	IGH	2	None	
14	1529	CQSADTTGSSAVF	IGL	3	None	
14	1530	CSTDITMHTGVDYW	IGH	26	None	
14	1531	CASAGVGFVVRATQDAYDIW	IGH	5	None	
14	1532	CARRRGYSYGGPFDYW	IGH	10	None	
14	1533	CAKGPGSSGSYYNNYYYYYMDVW	IGH	2	None	
14	1534	CAADLEWSYYMDVW	IGH	2	None	
14	1535	CARGDTTYGGYDAFDIW	IGH	4	None	
14	1537	CAKGVYSSVFLVGFDYW	IGH	4	None	
14	1538	CATPRGIQLWSNIFDYW	IGH	2	None	
14	1539	CARQFPRGSTDYFDYW	IGH	3	None	
14	1540	CAREEAYSSSSDYW	IGH	13	None	
14	1541	CQQSFDTPSF	IGK	558	C2	7K8O
14	1542	CAKPSLNSYSDYW	IGH	2	None	
14	1544	CVKGLSPQFLVDAFDVW	IGH	2	None	
14	1545	CARDAADFSSGSYLDYW	IGH	4	None	
14	1546	CAAWDDNLDGSYLF	IGL	6	None	
14	1547	CARDSHLIAGVHYYYYMDVW	IGH	4	None	
14	1548	CARDLGYSSGWPFDYW	IGH	51	None	
14	1549	CARLVRKLELFTGTFDIW	IGH	3	None	
14	1550	CARSRPSSCMYSNGICPYDYW	IGH	2	None	
14	1551	CARRITMVRAIDYW	IGH	11	None	
14	1552	CARGGIVDWYFDLW	IGH	16	None	
14	1553	CAKIDCTNGVCSSDYW	IGH	3	None	
14	1554	CAREITNQNIVNSNWHDPW	IGH	3	None	
14	1555	CAKDPRPLIAAAEAVDYW	IGH	4	None	
14	1556	CARRWAVAGTWPPSYYFDYW	IGH	7	None	
14	1558	CARHVGGTAMVISWFDPW	IGH	12	None	
14	1559	CARHKGRIAASDEIDYW	IGH	4	None	

14	1560	CARDQQYCRSASCFLYYYYYMDVW	IGH	2	None	
14	1561	CARTAGGPSFGFDIW	IGH	3	None	
14	1562	CARGLRYYYGSSGYYYDYW	IGH	2	None	
14	1564	CAKGLGYSSSWFPGQSYFDDW	IGH	2	None	
14	1565	CARGVLRGVITHFDFW	IGH	3	None	
14	1566	CARDRDVVVPAASYYYMDVW	IGH	2	None	
14	1567	CARRCSNSHSCFKWFDPW	IGH	10	None	
14	1568	CARDSLPGIISGYFYPNHFDPW	IGH	2	None	
14	1569	CAKDMGVNWNYDGPSFDYW	IGH	4	None	
14	1570	CARADTASDAFDYW	IGH	4	None	
14	1571	CARHASLSPSIGSSWYMDSW	IGH	26	None	
14	1572	CARRLWYSGSYQGYFDYW	IGH	2	None	
14	1573	CARILYDFYYFDNW	IGH	2	None	
14	1576	CARLHVFTREQFSVRRYYSYFYMDVW	IGH	2	None	
14	1577	CAKIYGYYDFWSGPDTFDYW	IGH	4	None	
14	1578	CARLGNGEKQWLLPEYYFDYW	IGH	2	None	
14	1579	CARALAVARYFDYW	IGH	21	None	
14	1580	CQTYGDSPHSSPRPFGRTF	IGK	2	C1	7K8N
14	1581	CSSYAGSNNLLF	IGL	471	C2	7BWJ
14	1582	CAKDERHQWLRGVVDYW	IGH	2	None	
14	1583	CARRLRPRYCSSTSCYLLFDYW	IGH	2	None	
14	1584	CATDPQSYDTLTGFYSTGFDQW	IGH	2	None	
14	1585	CARVWGFYDSGGYWRNEGFDIW	IGH	2	None	
14	1587	CLVEWLPNFDYW	IGH	4	None	
14	1588	CARDDYGGNYYYYGMDVW	IGH	9	None	
14	1589	CARLGCNYAYYFDHW	IGH	3	None	
14	1590	CTRGRLGDSGSSGYYIHYW	IGH	3	None	
14	1591	CASVPAAPDYYMDVW	IGH	2	None	
14	1592	CGRGYSPIYYYYVDVW	IGH	10	None	
14	1593	CARDLRGPVRLGVWIRAFDPW	IGH	177	None	
14	1594	CARVSRNWNLPFDYW	IGH	4	None	
14	1595	CASALLKDHGDYFTAETFQHW	IGH	8	None	
14	1596	CVRSPDYIGYLEYYMDFW	IGH	2	None	
14	1597	CARGIRYYDSSGYNSSPPDYW	IGH	5	None	
14	1598	CAREDYPGSSWFDPW	IGH	8	None	
14	1599	CARGPRGYNSAWLDSW	IGH	17	None	
14	1601	CAKGLSDVILGHDAFDLW	IGH	3	None	
14	1602	CATRVTGGVDAFDIW	IGH	5	None	
14	1603	CAREKWGDDDTFSYTFDIW	IGH	4	None	
14	1604	CARGRRLYYYDSSGPREKRDAFDIW	IGH	3	None	
14	1605	CQHSYSTLPCSF	IGK	6	C4	6ZDH
14	1606	CAREGTTGDFDYW	IGH	72	None	
14	1607	CVRVKLVGDGRQPEGRYLDYW	IGH	2	None	
14	1608	CASGRTAYSPAFDYW	IGH	2	None	
14	1609	CARVYCVAGVCYRVYDYW	IGH	2	None	
14	1610	CAKDLWEYTTLSPNFDYW	IGH	3	None	
14	1611	CAKSLYSNYRIASDYW	IGH	6	None	
14	1612	CARNIIVAPDAIRYYYGMDVW	IGH	4	None	
14	1614	CARAKHWGGVVMGLYYYYGMDVW	IGH	2	None	
14	1615	CAKEVNWGGVDYW	IGH	16	None	
14	1616	CARARRGYSGYRFDYW	IGH	12	None	
14	1617	CARTHNYNSGSSSNFDYW	IGH	3	None	
14	1618	CVRFALNAYSTSWSHFDSW	IGH	2	None	
14	1619	CAKGRLRFLEWSTPIDYW	IGH	3	None	
14	1620	CDVLWNDETHAFDVW	IGH	3	None	
14	1621	CARQDFRTVRATYLDYW	IGH	20	None	
14	1622	CARQRYSYGYVLRSFDPW	IGH	15	None	
14	1623	CALPISSAAYMELISLRSEDTAVYYCARGDQKPAFSLDYW	IGH	3	None	
14	1624	CAKGKYGSVMIGCFDKW	IGH	3	None	
14	1625	CTRPDGYRPYW	IGH	4	None	
14	1626	CAKGAGAGTGDDYFDSW	IGH	2	None	
14	1627	CARAGGTSERYYFDYW	IGH	11	None	
14	1628	CVRPGSGYYGFSSFDYW	IGH	2	None	
14	1629	CARHGALSVYHIDHTWAYVFDIW	IGH	4	None	
14	1630	CARHVGYTYEVGEEYFDYW	IGH	9	None	
14	1631	CARHSCDSWSDSYWGNWFDPW	IGH	14	None	
14	1632	CATIRGDYSKQMRDYW	IGH	2	None	
14	1633	CVSNGGTRINFQYW	IGH	5	None	

14	1634	CASPADEKKAYFDFLTGYEGPYYFDYW	IGH	2	None	
14	1635	CAKHDVVVIAAGRPRPFDCW	IGH	2	None	
14	1636	CARGGTWIQLWPRFDYW	IGH	4	None	
14	1637	CARDHGEPHGKWFGELLVQYFDYW	IGH	23	None	
14	1638	CARVTDGGYHALDYW	IGH	19	None	
14	1639	CARFFSGFDVW	IGH	17	None	
14	1640	CARRSIAARGPDDAFDIW	IGH	2	None	
14	1642	CAKERSLMGVYSIYEPIDYW	IGH	2	None	
14	1643	CASGYCSSTSCYTRYW	IGH	3	None	
14	1645	CARWAPRGTYVAYW	IGH	2	None	
14	1646	CAKEMEQWLVFYFDSW	IGH	4	None	
14	1647	CAKDRSDTLGYSGYDLPSPYFDYW	IGH	2	None	
14	1648	CARGRSRSSSDGKPYYFDYW	IGH	110	None	
14	1649	CATDLQVVREYFFDYW	IGH	4	None	
14	1650	CARDGGLTDNFRWRYYFDLW	IGH	2	None	
14	1651	CAKIVDTITEFDSW	IGH	18	None	
14	1652	CASNRRIQLWPWAFDIW	IGH	2	None	
14	1653	CASFPLWYYDSSGYNVVYFDYW	IGH	2	None	
14	1654	CGESHTIDGQVGWVF	IGL	13	None	
14	1655	CAREFGGFWSGSHYYFYYMDVW	IGH	8	None	
14	1656	CARDSQSFFYGTVTYYGTMDYW	IGH	4	None	
14	1657	CARCHTSGCPDYW	IGH	14	None	
14	1658	CAKDPGFKTGTPHNWFDPW	IGH	4	None	
14	1659	CAREGTDAALIAWFDPW	IGH	6	None	
14	1660	CALNSYGYDYW	IGH	2	None	
14	1661	CASLAGTDGAYYYYMDVW	IGH	2	None	
14	1662	CARDQGRAGDDDLWNGGSFDYW	IGH	2	None	
14	1663	CASGFGGYGGRYFDLW	IGH	3	None	
14	1664	CARYNRFSYYAIDVW	IGH	29	None	
14	1665	CAHRVTSTLWGVYYFDYW	IGH	2	None	
14	1666	CVRVSVPWVQLQRENDAFDIW	IGH	2	None	
14	1667	CARRVTGTARQFDLW	IGH	2	None	
14	1668	CARPMRGLFLEAMDYW	IGH	2	None	
14	1669	CAKSGALMVRGVSYFDYW	IGH	2	None	
14	1670	CARDNSSPYYYYGMDVW	IGH	4	None	
14	1671	CATDRGRTRYYDSSGPLDYW	IGH	6	None	
14	1672	CARGTWSTSYFFDYW	IGH	3	None	
14	1673	CARQVGGGNYQKPVFWFDPW	IGH	7	None	
14	1674	CARILEDDFWSGYGGYFDYW	IGH	2	None	
14	1675	CAAGGDFWSGLDYW	IGH	15	None	
14	1676	CARVYDSSGYYSGEGDYW	IGH	2	None	
14	1677	CARNILTGYYNYW	IGH	37	None	
14	1678	CARCGPKAGFDYMDVW	IGH	2	None	
14	1679	CARLNDYGVNNWFDPW	IGH	33	None	
14	1680	CARIPSTMIEVVDDAFDIW	IGH	2	None	
14	1681	CQSAASNNIYRVF	IGL	7	None	
14	1682	CAKASGGHFVVVTAEYYFEYW	IGH	2	None	
14	1683	CARSGWLRRDYYYYYYMDVW	IGH	14	None	
14	1684	CARDFYLRSDSSGPPDAFDIW	IGH	2	None	
14	1685	CLQGIHWPRTF	IGK	377	None	
14	1686	CARAGLSIVGATGWFDPW	IGH	8	None	
14	1687	CARELGSSNGQGWFDPW	IGH	6	None	
14	1688	CTAYLHWSGLRASDTAIYYCARLGAARGHEAFDYW	IGH	6	None	
14	1689	CCSYAGSYTWVF	IGL	2332	C2	7JMP
14	1690	CARDDYRFKTVDYW	IGH	2	None	
14	1691	CTTVGDIVLMVYAMIPSQFDYW	IGH	4	None	
14	1692	CAKRGGPGWLASNWFDPW	IGH	2	None	
14	1693	CATALTVFPDYW	IGH	48	None	
14	1694	CARTRFIVPAALALYHDAFDIW	IGH	3	None	
14	1695	CARIPISTTVPATNYYYYALDVW	IGH	3	None	
14	1696	CASLRFLARNAFDWW	IGH	4	None	
14	1697	CARDSAGRYFDWSNYYYYYMDVW	IGH	2	None	
14	1698	CARMPHGWELPIFDYW	IGH	2	None	
14	1699	CARDSHHPWYFDIW	IGH	4	None	
14	1700	CACTVYSSGWTGAFDIW	IGH	2	None	
14	1701	CARESPPGVAPNWFDPW	IGH	4	None	
14	1702	CARLQVPPAMPFDYW	IGH	10	None	
14	1703	CAKLWPSCIVPAGCGYDSSAGAFDIW	IGH	2	None	

14	1704	CARGGRGPAIRGDYFDFW	IGH	3	None	
14	1705	CSSTSCRWLDYW	IGH	4	None	
14	1706	CARHVGFGSTWSLRRGWFDPW	IGH	2	None	
14	1707	CARLRIAVAGSYYFDYW	IGH	4	None	
14	1709	CTMVPLRAGEIFPYW	IGH	2	None	
14	1710	CARGAEMATTFLFDFW	IGH	2	None	
14	1711	CARRNYDVLTSYAIDYW	IGH	2	None	
14	1712	CARDSIVVVTPGGNYYYYYGMDVW	IGH	2	None	
14	1713	CAKGGYSFDAFFDYW	IGH	6	None	
14	1714	CARVMFLRYQLGEYEGWFDPW	IGH	18	None	
14	1715	CARDRTITVFGVITYDAFDIW	IGH	4	None	
14	1716	CQQYGATPQPPTF	IGK	10	C2	7K8U
14	1717	CAIQTYSYDSRGYRTLYFDYW	IGH	6	None	
14	1718	CARPKFGRGIAFDFW	IGH	2	None	
14	1719	CAKDIGDGYNYVFAFDIW	IGH	4	None	
14	1720	CARRYVVVVAAYDAFDIW	IGH	4	None	
14	1721	CARVEGYGSGNYFYYYYGLDVW	IGH	10	None	
14	1722	CARASGQWKALDYW	IGH	3	None	
14	1723	CARDKGATVTTLVNAAYYYSGMDVW	IGH	7	None	
14	1724	CARSKVDILRFLGDVAGENYFDYW	IGH	4	None	
14	1725	CARAVLHSGSSLLDYW	IGH	6	None	
14	1726	CARAFTRGYSHGFFDYW	IGH	27	None	
14	1728	CAKVGYYYDSSGYYYRNTRAEYFQHW	IGH	2	None	
14	1729	CQSTDSSGTFWF	IGL	79	None	
14	1730	CATGRHRAGITDYW	IGH	2	None	
14	1731	CALPISAADTAVYFCARPQAEWDLLLPFDIW	IGH	7	None	
14	1732	CAKDRQFDWIFDFW	IGH	4	None	
14	1733	CARAVVPASESGFDPW	IGH	4	None	
14	1734	CARWKVGSAWFFDYW	IGH	7	None	
14	1735	CARGDLTMAHDYW	IGH	11	None	
14	1736	CDWSSDVCSSDLSRVFLLGTDYYFDSW	IGH	3	None	
14	1737	CSNQPHRDDWFDPW	IGH	2	None	
14	1738	CASWDVSLSGRLF	IGL	9	None	
14	1739	CARDQGGLLVGFDTW	IGH	2	None	
14	1740	CARISGYSSSSAAIHDAFDIW	IGH	3	None	
14	1741	CAKDRRSSSWYYLDHW	IGH	2	None	
14	1742	CASLDITSARLFDYW	IGH	2	None	
14	1743	CARDPSYSSDWFGKDAFDFW	IGH	4	None	
14	1744	CATYRQWLSTVPIFDYW	IGH	2	None	
14	1745	CARSFLSGSYQYYFDYW	IGH	2	None	
14	1746	CQAWHSTTAGVF	IGL	10	None	
14	1747	CASATVGSFHFDYW	IGH	3	None	
14	1748	CARGREYYWNYW	IGH	5	None	
14	1749	CAKGRTDYSNTPDFDCW	IGH	5	None	
14	1750	CARGPIIYDILTSGFDPW	IGH	6	None	
14	1751	CARGPHSGNYYLSDYW	IGH	4	None	
14	1752	CARVYLAAAGTGVNDYW	IGH	11	None	
14	1754	CARDRGSSWSPFDPW	IGH	7	None	
14	1755	CARGACSSTSCYEPYWFDPW	IGH	4	None	
14	1756	CARGVEYTKLHYYYYYMDVW	IGH	3	None	
14	1757	CARDSRHYDFWSGYFPGQSHYYYYGMDVW	IGH	2	None	
14	1760	CARADYYLDVFDVW	IGH	6	None	
14	1761	CARYCTSTSCYTESFDSW	IGH	2	None	
14	1762	CATITQLWLFDYW	IGH	2	None	
14	1763	CARSNSSSWYNDAFDIW	IGH	11	None	
14	1764	CARAVGPQYPAWFDSW	IGH	2	None	
14	1766	CARVLLGGEYSSGLREKGYYYYGMDVW	IGH	3	None	
14	1767	CATGGASSEPDYW	IGH	2	None	
14	1768	CARGPYYSNYPDNWFDPW	IGH	5	None	
14	1769	CATDIPITIFVVVTDDAFDIW	IGH	3	None	
14	1770	CARVPYWGIAAAGTFDYW	IGH	6	None	
14	1771	CARDTVSWLQRRAHDSFDIW	IGH	2	None	
14	1772	CARPLLGVVAALDAFDIW	IGH	4	None	
14	1773	CVKDSGYCSSTRCEEYYYYYYMDVW	IGH	2	None	
14	1774	CAKLGVGRYFDWSDYW	IGH	2	None	
14	1775	CSKGDYGSGRRFDSW	IGH	4	None	
14	1776	CAQITSLEVISSVVRSSAEHFHYW	IGH	10	None	
14	1777	CATGLGSVAVDYFDYW	IGH	2	None	

14	1778	CATTSPYCSSTSCYFHWFDPW	IGH	6	None	
14	1779	CARAHRFEWLIDYW	IGH	4	None	
14	1780	CTALSSHSFFDYW	IGH	5	None	
14	1782	CARRSIAAPSVFDYW	IGH	2	None	
14	1784	CARDPSIVGATTLGDWYFDLW	IGH	5	None	
14	1785	CARQPSLVVVPADIRTNWYFDLW	IGH	3	None	
14	1786	CLQDYNPPYSF	IGK	33	None	
14	1787	CARVGKYFYENESHSYDAFDIW	IGH	3	None	
14	1789	CARVEFLALGSGFFDSW	IGH	5	None	
14	1790	CAKEWGPTATSSQRKTYYDYW	IGH	3	None	
14	1791	CATSPGRTTSVPIFDYW	IGH	140	None	
14	1792	CARDSRDVALAFDLW	IGH	2	None	
14	1793	CARGKLVEANLLLNYYYYMDVW	IGH	2	None	
14	1794	CARDSERYTSTWSSFDYW	IGH	2	None	
14	1795	CARGAYYYDSSVPGDYW	IGH	2	None	
14	1796	CAKDFLKVQLYSSSYFHHFDSW	IGH	3	None	
14	1797	CARDRSYYERSTYYNHAFDIW	IGH	16	None	
14	1798	CARPSSGSRPIYYMDVW	IGH	2	None	
14	1799	CAKDSGEEVRGLSDYW	IGH	2	None	
14	1800	CVRVQAPPAGPRPEPNDYW	IGH	7	None	
14	1801	CMRHVSATNSWFDPW	IGH	3	None	
14	1802	CAKGGLAAWTVSSEYYFDYW	IGH	6	None	
14	1803	CAKDLKFLTGFFDYW	IGH	2	None	
14	1804	CARLDSSSWYDYYFYYMDVW	IGH	17	None	
14	1805	CARSYSNRWPPSGFDPW	IGH	2	None	
14	1806	CAKDMANYYDSSGYPTDAFDIW	IGH	2	None	
14	1807	CAKESGHYCSGGSCYLDYW	IGH	8	None	
14	1808	CAKDPLGGYSSGWYDYW	IGH	4	None	
14	1809	CARGRTLHSSGLDYW	IGH	2	None	
14	1810	CARRAIDFWSGYYKREPGGEFDPW	IGH	3	None	
14	1811	CAKDGSLYSSGWYSDYW	IGH	15	None	
14	1812	CAREAGKTFYYYYGMDVW	IGH	7	None	
14	1813	CVHSGPTARCDDQCWYFHLW	IGH	4	None	
14	1814	CARRLAQLPELYYFDYW	IGH	3	None	
14	1815	CASLRGNGAFDFW	IGH	2	None	
14	1816	CATDRHLYCPNGVCSPGDYW	IGH	16	None	
14	1817	CARRPPRFRGDYFDFW	IGH	2	None	
14	1818	CAISRAGGLFDYW	IGH	5	None	
14	1819	CARGLVYYYDTSAYYYFDYW	IGH	13	None	
14	1820	CARHRGSSSVYASDIW	IGH	4	None	
14	1821	CARVNNYDFWSGYYTYW	IGH	2	None	
14	1822	CVVVPGGTHDAFDVW	IGH	2	None	
14	1823	CAKDSWYQMPTAWYYFDFW	IGH	2	None	
14	1824	CAWPGFCSGGTCPYYLDYW	IGH	33	None	
14	1825	CAKLPQPTISIDYW	IGH	2	None	
14	1826	CATHRRLWGLHTLTYDYW	IGH	2	None	
14	1827	CVGSPSNWYVGWFDPW	IGH	2	None	
14	1828	CTRNPGAYSDTWSPDRFDFW	IGH	2	None	
14	1830	CQQYDNLPRIFTF	IGK	1590	C3	6XDG
14	1831	CARGDWELRGVFFDLW	IGH	8	None	
14	1832	CAKILAAAGTDYW	IGH	15	None	
14	1833	CVRRGQTPIPSFDYW	IGH	3	None	
14	1834	CAKGQSLSILLSGAFDIW	IGH	7	None	
14	1835	CAKDRAEHFPYYYHYMDVW	IGH	6	None	
14	1836	CHQRSTWPRLTF	IGK	5	None	
14	1837	CARANDGSYHYFDYW	IGH	4	None	
14	1838	CAKREIHYDIFTGPIGFDWW	IGH	8	None	
14	1839	CATPGGYTYGPYYFGYW	IGH	2	None	
14	1840	CARDNLEAAAGPIFDYW	IGH	17	None	
14	1841	CARFSRPNYYDKTGYYDNW	IGH	7	None	
14	1842	CGTWDSSLVGVPYVF	IGL	2	None	
14	1843	CARSVRLDRPFDYW	IGH	2	None	
14	1844	CARNSAYVGSTLGFDYW	IGH	4	None	
14	1845	CAKQMSATGYDWYFDNW	IGH	15	None	
14	1846	CAAQLLFYFDYW	IGH	33	None	
14	1847	CASQLVLPGAFDIW	IGH	18	None	
14	1848	CARHNRPLLFGETIDYW	IGH	5	None	
14	1849	CALPIFFLLGADYYFDYW	IGH	14	None	

14	1850	CARHVPISPSIDSSWYFDNW	IGH	2	None	
14	1851	CATVVPAGQPHYYYYGMDVW	IGH	3	None	
14	1852	CARSIVLMDKSDTDYW	IGH	14	None	
14	1853	CAGGNRNMPFDYW	IGH	2	None	
14	1854	CAKVGQVGGSYLVPYLWSFDYW	IGH	2	None	
14	1855	CARDESDYSNYNYYYYMDVW	IGH	2	None	
14	1856	CARLRRGNDAFDIW	IGH	46	None	
14	1857	CAKDSQRSSNWPDAFDIW	IGH	2	None	
14	1858	CARDGGFNAPYTSSSGYNWFDPW	IGH	2	None	
14	1859	CANNDGVYCSSTSCYPYYFDYW	IGH	2	None	
14	1860	CARNLVGATTGDAFDIW	IGH	7	None	
14	1861	CARHPLGYCSGTRCFNWFDSW	IGH	4	None	
14	1862	CAKDITHSLVALDYW	IGH	6	None	
14	1863	CAREVRDDGSGSPSDYW	IGH	2	None	
14	1864	CARIRAVGTVHYFDYW	IGH	4	None	
14	1865	CARRTDVDSSIAAAGYYFDYW	IGH	2	None	
14	1866	CATESFWSGYSPHYYYGMDVW	IGH	31	None	
14	1867	CVRGFFDSRGHYTIDYW	IGH	11	None	
14	1868	CARHNYYVTGSYVFDYW	IGH	5	None	
14	1870	CARGYLYSLGYYDYW	IGH	10	None	
14	1871	CARSWELLWGGGGFFDYW	IGH	2	None	
14	1872	CARQSLRRAFPLLSGIDQKYSEGFDIW	IGH	2	None	
14	1874	CGRLTVDGDLDYW	IGH	2	None	
14	1875	CARVGDSDAFDIW	IGH	223	None	
14	1876	CARGYSSGWLDYYYYGMDVW	IGH	2	None	
14	1878	CARSLRIASHIGYLWTLNNAFDVW	IGH	6	None	
14	1879	CVKDGQVYDYLGGYAFDFW	IGH	5	None	
14	1880	CARGIVVVPAANSNYYYYYGMDVW	IGH	2	None	
14	1881	CAKSGMATIYWYFDLW	IGH	4	None	
14	1882	CATGYSSSWYFYW	IGH	123	None	
14	1883	CARDNGIAARSDAFDIW	IGH	10	None	
14	1884	CARDLPLWEIFGVVIPGRAEYFQHW	IGH	3	None	
14	1885	CATAAEMDFWSGYYTGFDYW	IGH	2	None	
14	1886	CARCLRVASDIGELWSLNNAFDIW	IGH	11	None	
14	1887	CARKADFWSGFSLDYW	IGH	6	None	
14	1888	CARDDYDFWRGIFDYW	IGH	5	None	
14	1889	CARAAQYQLLNDDYYYMDVW	IGH	2	None	
14	1890	CARDNGWLLYPGSFDRW	IGH	4	None	
14	1891	CASDYDTLSSYYQAFDMW	IGH	2	None	
14	1892	CARLKLYCSGGSCYSNYFDYW	IGH	8	None	
14	1893	CQQIYITPVTF	IGK	341958	C4	6W41
14	1894	CARRFYGDNRWFDSW	IGH	3	None	
14	1895	CARANTIFGVVIPAYFDYW	IGH	4	None	
14	1896	CAKDHFVTYLIGTQDKRFDLW	IGH	3	None	
14	1897	CTRDRSGSLPTHFDFW	IGH	60	None	
14	1898	CARERISVTTNFFDYW	IGH	2	None	
14	1900	CARDKAMAVGYALDIW	IGH	509	None	
14	1901	CATDTRDLITIFGVVIRGGPGAFDIW	IGH	19	None	
14	1902	CTRASYYYENSGYTLYDAFDIW	IGH	2	None	
14	1903	CAKDRGPPFYSGTYVFDFW	IGH	22	None	
14	1904	CATAPSVVVPAAIRGWFDPW	IGH	41	None	
14	1905	CAGYGDYIEYYFDYW	IGH	6	None	
14	1906	CETWDSNTLVF	IGL	42	None	
14	1907	CAKARGYSGGPYYFDFW	IGH	2	None	
14	1908	CAISSVPTPLSWWLSPFDSW	IGH	7	None	
14	1909	CAHRRSDYYSLYYFDYW	IGH	3	None	
14	1911	CARSTAVVGTRRDFDHW	IGH	2	None	
14	1912	CARGIWDSSSFEYFQDW	IGH	4	None	
14	1914	CASDSKNGIYGFLLDYW	IGH	8	None	
14	1915	CARRMWLQLGDYYALDVW	IGH	2	None	
14	1917	CARDRALRFLEGSWGSALDIW	IGH	3	None	
14	1918	CAREVGCYGSNCYMVRWFDPW	IGH	2	None	
14	1919	CARTSMYCSSTSCYFGVEENNWFDPW	IGH	2	None	
14	1920	CARVGEGYNPKDNYYYGMDVW	IGH	4	None	
14	1921	CARVPTNYRVAAAGVDDAFHIW	IGH	2	None	
14	1922	CAKDNFWSGYHDAFDVW	IGH	7	None	
14	1923	CAKVPFCSRTSCQYYLDYW	IGH	2	None	
14	1924	CAKGSTWGSYRSLFDYW	IGH	4	None	

14	1925	CLQDAQDPPFTF	IGK	5	None	
14	1927	CAREDGSRYAMHTLKIFDYW	IGH	2	None	
14	1928	CVRQTRSWFDPW	IGH	2	None	
14	1929	CARKVGSGWPNWYFDLW	IGH	2	None	
14	1930	CARHVDNLPRFDYW	IGH	19	None	
14	1931	CAKVSQSSSSDYW	IGH	42	None	
14	1932	CARDLGYYDSSEDLYRGFDYW	IGH	3	None	
14	1933	CARDLKGSIASLYYYYGMDVW	IGH	11	None	
14	1934	CARFSVGSVQRSSGYYYGSGFDYW	IGH	2	None	
14	1935	CARRFGGNSRGYIDVW	IGH	2	None	
14	1936	CAKNSWGGFAEFYYYMDVW	IGH	2	None	
14	1937	CARVGRGFYGFDIW	IGH	2	None	
14	1938	CARLWTNTRWSPFDYW	IGH	8	None	
14	1939	CAKAEESSIWYSLFDYW	IGH	2	None	
14	1940	CARSKIDTLRFLGGVEGEYYFDYW	IGH	19	None	
14	1941	CASLDASDYSSVDYW	IGH	4	None	
14	1942	CTVYMELSSLRSDDTAIYYCARDKAMAVGYALDIW	IGH	2	None	
14	1944	CARDFKVELLDNAFDIW	IGH	14	None	
14	1945	CARISETNGWYYFDYW	IGH	15	None	
14	1946	CQQGNSF	IGK	718	None	
14	1947	CANAIAAVPDYW	IGH	87	None	
14	1948	CTTDHSYYDFWSGYSDYW	IGH	2	None	
14	1949	CARDKIGVGAPDYW	IGH	40	None	
14	1950	CARDSPRLVDALDIW	IGH	2	None	
14	1951	CARILDYGDFWDDYW	IGH	2	None	
14	1952	CARDRRYYNTGYHARDFDLW	IGH	2	None	
14	1953	CAGAEGSPSQLLTTVAPPYWFAPW	IGH	4	None	
14	1954	CARDRVEVGRLDTFHIW	IGH	3	None	
14	1955	CARDSGFSTSYYNIDYW	IGH	3	None	
14	1956	CARSKGYYDILTGYRITYYFDYW	IGH	2	None	
14	1957	CAAGEDIVMIPAVLYFDFW	IGH	2	None	
14	1958	CDWSSDVCSSDLTAVYYCATGARRGYDFYYMDVW	IGH	23	None	
14	1959	CARTSEDCSGGACYYFAFW	IGH	2	None	
14	1960	CATLTDYYDGNGYYSPFDYW	IGH	2	None	
14	1961	CAREARFWGVGNSLDSW	IGH	2	None	
14	1962	CAKGSSSITHYHFDFW	IGH	11	None	
14	1963	CQQRTNWPRGYPF	IGK	124	None	
14	1964	CARTKITIFGVHSSWFDPW	IGH	7	None	
14	1965	CARDRDRSSWKKSFDYW	IGH	2	None	
14	1966	CVRDGSSILVEGGNWFDPW	IGH	19	None	
14	1967	CVRDSYYDGTDNPPYGAEYFYHW	IGH	32	None	
14	1968	CAREEPDTFGGVMWFDPW	IGH	2	None	
14	1969	CARHEAPRWLAPAGSPLDYW	IGH	2	None	
14	1970	CAIAYYDSSGHYRDYW	IGH	30	None	
14	1971	CAKDPYYDFWSGHRGGFDPW	IGH	2	None	
14	1972	CVRAVQYRYYFDNW	IGH	8	None	
14	1973	CAKDLEQWLERWFFDHW	IGH	2	None	
14	1974	CARAFVPDWELEYYFDYW	IGH	2	None	
14	1975	CATSWPYNYLDPW	IGH	30	None	
14	1976	CARALLRAAATATQGHTDYYYYYGMDVW	IGH	3	None	
14	1977	CARGPKGNVYYYYHMDVW	IGH	2	None	
14	1978	CARPPAHGYYGDYVRLFDMW	IGH	2	None	
14	1979	CAREALGVGATGYFASW	IGH	4	None	
14	1980	CARSDLILGYCSGGSCYSCDYW	IGH	6	None	
14	1981	CARTYGAGRHHAFDIW	IGH	2	None	
14	1982	CAREGFCTSGSCFPSEDAFDMW	IGH	3	None	
14	1983	CARGLAAAGTSWGDWLDPW	IGH	2	None	
14	1985	CARPGYSSSSVWFDPW	IGH	2	None	
14	1986	CAKDTTPSLYYFDYW	IGH	10	None	
14	1987	CAKIPGPDITSDAFDIW	IGH	4	None	
14	1988	CARDLARMGYFDIW	IGH	11	None	
14	1989	CTRGYAGVCYDYW	IGH	2	None	
14	1990	CASFLWRYYDSSGKTFYYYYGMDVW	IGH	2	None	
14	1991	CAQTADGSGYWRGSRPFDYW	IGH	4	None	
14	1992	CARDPWSKVGAIQAFDYW	IGH	2	None	
14	1993	CARLTWELDLDYW	IGH	7	None	
14	1994	CAKVAAGTTSDYW	IGH	4	None	
14	1995	CARHNPVAVYYYYYYMDVW	IGH	2	None	

14	1997	CARDQCINGVCFYGSDYW	IGH	2	None	
14	1998	CARDNWNYLPTQAASSDYW	IGH	2	None	
14	1999	CAKDSHYGNPYYFDFW	IGH	2	None	
14	2000	CATPRTYYYGSGSFDYW	IGH	16	None	
14	2001	CTRPLFWYGGTSRDAFDMW	IGH	4	None	
14	2002	CQNYGLSPSNF	IGK	2	C1	7K8N
14	2003	CARHRQYSSSPSFDYW	IGH	2	None	
14	2004	CARALNSGWHSNWFDPW	IGH	4	None	
14	2005	CAKDISSAVAGDGMDVW	IGH	3	None	
14	2006	CAKLPITGTPVDYW	IGH	4	None	
14	2007	CARGLFDVPLYYYYYMDVW	IGH	6	None	
14	2008	CTAYLQWSRLWVSDTAIYYCARSPTSISNPYYFDHW	IGH	3	None	
14	2009	CDWSSDVCSSDLDTAVYFCARPQAEWDLLLPFDIW	IGH	14	None	
14	2010	CARDVREHSYVQDWYFDLW	IGH	8	None	
14	2011	CQPYHSSPPLFTF	IGK	4	None	
14	2012	CTRLYRPTAAPENYYQSYYVDVW	IGH	8	None	
14	2013	CARTKGDLSYSGLDYW	IGH	7	None	
14	2014	CARKFGANIGGGVYYYGMDVW	IGH	2	None	
14	2015	CAKDPLATGTLPGYFDYW	IGH	2	None	
14	2016	CASWWNDFNQYFDNW	IGH	6	None	
14	2017	CARGLAPGYCGSSTCHDDYW	IGH	5	None	
14	2018	CALQICATGARRGYYFYYMDVW	IGH	2	None	
14	2019	CAHKISTTYDNFWSGYFDYW	IGH	2	None	
14	2020	CAKGSHPDFYDTSGRYYYGMDVW	IGH	4	None	
14	2021	CARDQWSDSSGSHDYW	IGH	2	None	
14	2022	CARRNWGADEGGHFDLW	IGH	2	None	
14	2023	CARGPLGGSAWHSPDYFDYW	IGH	2	None	
14	2024	CARITIFGVVIIRGYYFDYW	IGH	2	None	
14	2025	CARGGYCSTTSCYKFRSPDYW	IGH	2	None	
14	2026	CQQYHNWPPKISF	IGK	4	C3	6XDG
14	2027	CAKVVTGTFRGSDYW	IGH	2	None	
14	2028	CAREGGGWDSDPYYFDYW	IGH	6	None	
14	2029	CARGADPQFPIYAMDVW	IGH	3	None	
14	2030	CASPSSHDYYDTSGHPVDYW	IGH	2	None	
14	2031	CARHQYDIFDYFTYW	IGH	12	None	
14	2032	CARQVPHGFDIW	IGH	24	None	
14	2033	CAHRPRATEVIGDYFDYW	IGH	3	None	
14	2034	CARMTMVGASTIGFDIW	IGH	2	None	
14	2035	CARDRMMTNGWNDAFDLW	IGH	4	None	
14	2036	CVRQGGLTAAFDYW	IGH	3	None	
14	2037	CAKNYYDSSGPASYFDYW	IGH	2	None	
14	2038	CARDGGIAARLRGYFDYW	IGH	6	None	
14	2039	CAKDMSVWIAAAGNYFFDNW	IGH	6	None	
14	2040	CATHRDGYNQIFDYW	IGH	10	None	
14	2041	CARESGPRPIVGATMAWYFDLW	IGH	7	None	
14	2043	CARTRMWPGTYDYW	IGH	3	None	
14	2044	CARDIRPGSRGSGDFDYW	IGH	2	None	
14	2045	CARSPYYYDSSGPPIAYFDYW	IGH	2	None	
14	2046	CARINNDYEYDWYFDLW	IGH	8	None	
14	2047	CANGVGYSYGWFFDYW	IGH	2	None	
14	2048	CAKDRPSYSTPELGFPEYYFDYW	IGH	3	None	
14	2050	CARDDRYCSRTNCYMDYW	IGH	4	None	
14	2051	CAKNYGIEADGWDAFDIW	IGH	2	None	
14	2053	CARHYSSSPFYWYFDLW	IGH	3	None	
14	2054	CARHDRKKPVYFDYW	IGH	3	None	
14	2055	CARLIVGAVGGHYWYYMDVW	IGH	5	None	
14	2056	CARSPPQTTAAPPAAYDIW	IGH	2	None	
14	2057	CAHRPPPSSSYFYFDFW	IGH	2	None	
14	2058	CAKAPTYGANTDYW	IGH	2	None	
14	2059	CARLGDYGDTSYFDYW	IGH	2	None	
14	2060	CARMILRSGWSFDLW	IGH	2	None	
14	2061	CARIEMTTGYFDFW	IGH	2	None	
14	2062	CARGLPVAAAGFDSW	IGH	15	None	
14	2063	CASAKLGYSGYDRGGDHAYYMDVW	IGH	2	None	
14	2064	CAREGIVYDWGSDAFDIW	IGH	2	None	
14	2065	CAKRQYGHENYSYYYYIDVW	IGH	2	None	
14	2066	CAKTFQTLSSDYDFWSGANIAGFFDIW	IGH	2	None	
14	2067	CVREGTDSGRPHTTHLNWFDPW	IGH	7	None	

14	2068	CARAPEWLLLGGALDIW	IGH	499	None	
14	2069	CAREPKSLVAPDSFDIW	IGH	3	None	
14	2070	CAKSKADLGSLGNFDYW	IGH	2	None	
14	2071	CATSEAWELTTFDYW	IGH	4	None	
14	2072	CNSRDNGGNHQIF	IGL	4	None	
14	2073	CARASFTIFGDLNWFDPW	IGH	2	None	
14	2074	CAYSGWLDYFQYW	IGH	15	None	
14	2075	CAKSWEPAAIPTYFDYW	IGH	5	None	
14	2076	CARGPSSIRGYSGYVDYW	IGH	4	None	
14	2077	CANLEGAFDLW	IGH	7	None	
14	2078	CARDERSSGYYLDYW	IGH	16	None	
14	2079	CARDRHDYGDYFFDFW	IGH	17	None	
14	2080	CAKGPDPDDCTNGVCSDYW	IGH	2	None	
14	2081	CAKDALQARLLYNWFDPW	IGH	18	None	
14	2082	CARDHPCSGGSCYSVRRFDPW	IGH	2	None	
14	2083	CANWYSTSPKGFDYW	IGH	2	None	
14	2084	CARVGIVVDDAEYFQHW	IGH	8	None	
14	2085	CARDAADGYPAFAYW	IGH	2	None	
14	2086	CAKKRRDSFVFDIW	IGH	8	None	
14	2088	CARGPRALQYNLWSGYFVYW	IGH	20	None	
14	2089	CARHFASSLYIDFW	IGH	2	None	
14	2090	CARQFRMTYTSSSFGYW	IGH	8	None	
14	2091	CAKLSSSSAREIDYW	IGH	4	None	
14	2092	CARGNGGLFVRLYYFDYW	IGH	2	None	
14	2093	CARDLGAPGTKYYYYGLDVW	IGH	2	None	
14	2094	CARDGPHNFGTSGYDYYFDYW	IGH	2	None	
14	2095	CARDLSRVDYGVLSGYYQGGILDYW	IGH	4023	None	
14	2096	CARDPGTVTTHYYYYYMDVW	IGH	4	None	
14	2097	CARDQSYGTSGWHYFDHW	IGH	4	None	
14	2098	CAIGVVGEPGAKFGAFNIW	IGH	2	None	
14	2099	CAREVLAWIGSPPDDALDIW	IGH	3	None	
14	2100	CATFGQQLPTRPYYFDYW	IGH	10	None	
14	2101	CAKLAVAGTPADYW	IGH	13	None	
14	2102	CAARVAVAGTERGYFLDYW	IGH	2	None	
14	2103	CASAGPGFMVRGTQDAFNIW	IGH	2	None	
14	2104	CAKGFYYGSGSTPFDYW	IGH	3	None	
14	2105	CARDWNSGYDSQYYYGMDVW	IGH	2	None	
14	2106	CARDTRYLSSTTYYYYGMDVW	IGH	2	None	
14	2107	CAKNEGLGVWSGYTDAFDVW	IGH	3	None	
14	2108	CVSQVAFLPGSFDYW	IGH	2	None	
14	2109	CATFPVHYYDSSGYYPFDYW	IGH	10	None	
14	2110	CARDVMADEYDFWSGYYLPTPYNWFDPW	IGH	2	None	
14	2111	CAKLPDYGGNSVGWDAFDIW	IGH	2	None	
14	2112	CAHRLDYGSGSPLFDYW	IGH	7	None	
14	2113	CAKDLTPIYINGGLDYW	IGH	2	None	
14	2114	CAKAYMASLRSIYYMDVW	IGH	2	None	
14	2115	CAKGKSGSYWDEIDYW	IGH	4	None	
14	2116	CATVKVGDYYDNGGLRFSNAFDLW	IGH	5	None	
14	2117	CANLPDSSGYRDNW	IGH	20	None	
14	2118	CAGYGDYSDYW	IGH	7	None	
14	2119	CASIAAAGAPTYYYYGMDVW	IGH	57	None	
14	2120	CTRDIAPYGGFWSSYQIRFDPW	IGH	2	None	
14	2121	CAHRRTIFGVVDGFDPW	IGH	8	None	
14	2122	CVRGPYCTYGVCYKYFDPW	IGH	2	None	
14	2123	CARGDQKPAFSLDYW	IGH	6577	None	
14	2124	CARIRREYYDSSGHSPNDAFDIW	IGH	12	None	
14	2125	CTTDESLPGAAAQAFDYW	IGH	2	None	
14	2126	CVRDQTGSSWFKYW	IGH	2	None	
14	2127	CAHRLADSYDFWNLFGYW	IGH	8	None	
14	2128	CVIEAGYCGTNSCIFKNGFDPW	IGH	2	None	
14	2129	CARDEAGGYSYAYHFDYW	IGH	6	None	
14	2130	CALPISLSGYYQGGILDYW	IGH	4	None	
14	2131	CAKAGQLYREDGFDIW	IGH	5	None	
14	2132	CAKMPARFLEWLYLTHFDHW	IGH	2	None	
14	2133	CARATYYDFWSGRTDFDYW	IGH	2	None	
14	2134	CAGATFGVVTKFDYW	IGH	55	None	
14	2135	CARVDDYTNSFDSW	IGH	1012	None	
14	2136	CATPGPGPGLRWDFDYW	IGH	7	None	

14	2137	CARIGDYAIPEDAFDIW	IGH	2	None	
14	2138	CARNPDNISWFIPFDYW	IGH	3	None	
14	2139	CQSADSSDMEFF	IGL	50	None	
14	2140	CTTECYFDSSGAQIDYW	IGH	2	None	
14	2141	CDWSSDVCSSDLFDIW	IGH	7	None	
14	2142	CARHSLYCSSTSCWEGWFDPW	IGH	3	None	
14	2144	CATYTLRKTISTGRAFDIW	IGH	6	None	
14	2145	CAKHSKYGSATEDYW	IGH	2	None	
14	2146	CARDPPRIAGASTSPFTYW	IGH	8	None	
14	2147	CATGAGDWGRPAFDIW	IGH	2	None	
14	2148	CARDPSFWSGYYAYYYGMDVW	IGH	2	None	
14	2149	CARMELLLWLGDHYGGPFDYW	IGH	2	None	
14	2150	CARVSDMRFLEWLLLDYW	IGH	2	None	
14	2151	CARGEWFRSVGLDAFHIW	IGH	2	None	
14	2152	CARDPSTHYCSADGCHPSPRQQYFDYW	IGH	3	None	
14	2153	CARQSVPIFGDDHSDYW	IGH	2	None	
14	2154	CAKGKNVHYYSNSDYDNWYFDLW	IGH	3	None	
14	2155	CATGFSASYFDFW	IGH	3	None	
14	2156	CARGSDYVTFPHYLDYW	IGH	8	None	
14	2157	CLRSEDTAVYYCARGDQKPAFSLDYW	IGH	20	None	
14	2158	CAKDQGPIRGRESSFDYW	IGH	2	None	
14	2159	CARWITDTDPSYYYYGMDVW	IGH	6	None	
14	2160	CARVGHHGTVIVVASPDAFVVW	IGH	2	None	
14	2161	CAREEFYDYIWGNYHATIDYW	IGH	4	None	
14	2162	CASAIMLREDAFDFW	IGH	5	None	
14	2163	CAKDKELGGRIVVVTAFDYW	IGH	2	None	
14	2164	CAREYCRGGYCFYEGFDVW	IGH	28	None	
14	2165	CAKVSSGDFWSVYDAFDIW	IGH	6	None	
14	2166	CASVVIGYCSSTMINCQNNWFDPW	IGH	7	None	
14	2167	CAKLGASTNNWFDPW	IGH	3	None	
14	2168	CAKDGYPIAAVVHGGSYFDYW	IGH	2	None	
14	2169	CLLYYDDAQLGVF	IGL	4	None	
14	2170	CARVVPYYDFWSGSPFDYW	IGH	7	None	
14	2171	CATSKDSFAYW	IGH	2	None	
14	2172	CAKGGYGDYTLLVSYFDLW	IGH	7	None	
14	2173	CARLPTAGTGTWDYW	IGH	4	None	
14	2174	CAKDHQYQLLLSPGAFDIW	IGH	4	None	
14	2175	CARAGGNLGHNWLDPW	IGH	2	None	
14	2176	CARAPVWGGTYNWFDFW	IGH	6	None	
14	2177	CATDRIVVVPAAQNAFDIW	IGH	2	None	
14	2178	CARSYDNTGYRYYYYYMDVW	IGH	4	None	
14	2179	CARSGWKKGLVYYYYGMDVW	IGH	2	None	
14	2180	CARDAATLSGVVIYSYYYMDVW	IGH	2	None	
14	2181	CATVGVGSGYVAYW	IGH	4	None	
14	2182	CARLNYGYSLGSFDIW	IGH	3	None	
14	2183	CARERYCSSTRCSNYYYYMDVW	IGH	2	None	
14	2184	CATLSRYGPYYYYYYMDVW	IGH	2	None	
14	2185	CAVFTYYYDSSGYYFDYW	IGH	19	None	
14	2186	CATVPYYYASSAQGGFDSW	IGH	600	None	
14	2188	CASGDAIRIVGANPFDYW	IGH	4	None	
14	2189	CAVIEGKYWNNYYYRDVW	IGH	6	None	
14	2190	CAKDLSVPWFDPW	IGH	2	None	
14	2191	CTTDGLLWFGELLLGIDYW	IGH	3	None	
14	2192	CAKSTTIFGVLIMGYFDYW	IGH	6	None	
14	2193	CQQHDNWPLSLTF	IGK	2	C3	6XDG
14	2194	CMQGLQTPFTF	IGK	16169	None	
14	2195	CSSSGCNNPWFDPW	IGH	7	None	
14	2196	CARAGRSSSAYFDYW	IGH	4	None	
14	2197	CVKDDMYGGNGWFDPW	IGH	4	None	
14	2198	CVKRAGLIPDRFDFW	IGH	4	None	
14	2199	CARDVGYCSSTSCIKFDYW	IGH	43	None	
14	2200	CARRGGRIDMDTAMVPDFDYW	IGH	24	None	
14	2202	CAKDMAPTQYYDSGGYYYFDYW	IGH	10	None	
14	2203	CATLLVGATNWFDPW	IGH	4	None	
14	2204	CARDRAVHHIDVRGYYANAFDIW	IGH	2	None	
14	2205	CARILGNSGSYYRFDYW	IGH	2	None	
14	2206	CARAPQYLNIVLMVYAPDYW	IGH	25	None	
14	2207	CARPPPSGGWSYAFDIW	IGH	4	None	

14	2208	CAHSKLGISDFDYFDYW	IGH	7	None	
14	2209	CARHDQRDYEYMWETYRYSGVDYW	IGH	4	None	
14	2210	CTRDPWLPRSLKTLAATSFDYW	IGH	8	None	
14	2211	CENDSRVFLLGTDYYFDSW	IGH	3	None	
14	2212	CAGAVAASGDYYYYYGMDVW	IGH	2	None	
14	2213	CAKWADIKSPIDYW	IGH	4	None	
14	2214	CAKDRAWGTYTAPTDAFDLW	IGH	2	None	
14	2215	CAKDITKWMVTAPGFDYW	IGH	201	None	
14	2216	CARVKTNTMIVVVIQFLDYW	IGH	8	None	
14	2217	CAKTPDFWSGFFDYW	IGH	4	None	
14	2218	CNSRDNSGIHLALF	IGL	3	None	
14	2219	CARVCGDCYSGLGDYDAFDIW	IGH	7	None	
14	2220	CARRGMTTEYFDYW	IGH	35	None	
14	2221	CARHVWEYYYDSSGYYDAFDIW	IGH	2	None	
14	2222	CATTPSSGYNVYWYFDLW	IGH	2	None	
14	2223	CHQYNTWPQTF	IGK	183	C3	7K8R
14	2224	CARQLRFDFWSGSYAMDVW	IGH	6	None	
14	2225	CAREPWAGCSGGRCHRDAFDIW	IGH	5	None	
14	2226	CASHRPSDLIWFGEFFDYW	IGH	4	None	
14	2227	CAKFSGDSSGHATAFDIW	IGH	2	None	
14	2228	CSLATPYYFDYW	IGH	6	None	
14	2229	CATTDYGGNLRYFDYW	IGH	2	None	
14	2230	CARHNADSRWFDPW	IGH	39	None	
14	2231	CARLMEMTPFTRPPVYSFDYW	IGH	24	None	
14	2232	CARDLSVCNGTRCYPPADYW	IGH	2	None	
14	2233	CARARKSYLSITARGYFDNW	IGH	4	None	
14	2234	CAKDRGRLQWELLGPFDYW	IGH	2	None	
14	2235	CAKGHSSSWYVCTFDYW	IGH	2	None	
14	2236	CARGIAVANVFDIW	IGH	4	None	
14	2237	CATIPLYYYDSSGYSRRFDYW	IGH	3	None	
14	2238	CARKEKRRIVTYFFDSW	IGH	4	None	
14	2239	CAKGGEGYHHLNFDYW	IGH	3	None	
14	2240	CARHVWDLITTFNFDSW	IGH	7	None	
14	2241	CAKDPVGGYSYGYIDHW	IGH	3	None	
14	2242	CARGLRGGGYFDNW	IGH	13	None	
14	2243	CARDMTMTTVNTVDYW	IGH	2	None	
14	2244	CAKDRIGGNSVFEYW	IGH	3	None	
14	2245	CAKFSDCSGGSCYSRLDYW	IGH	8	None	
14	2246	CATSFAVWGAARPFDYW	IGH	16	None	
14	2247	CGRADRNYWHFDLW	IGH	2	None	
14	2248	CARVPGGGYDLASFDSW	IGH	2	None	
14	2249	CARDKIDYGDYSDYFDYW	IGH	10	None	
14	2250	CARPGCTTTGCHNAFDVW	IGH	5	None	
14	2251	CAKTFQLLSSGYDFRSDIKTEGAFDVW	IGH	3	None	
14	2253	CARDPSTAWVNGVALDYW	IGH	4	None	
14	2254	CARDLSTMTHYYFDYW	IGH	2	None	
14	2255	CARERITMVVGAPSPDYW	IGH	3	None	
14	2256	CASQSELLEPFHYW	IGH	2	None	
14	2257	CARVSSFSSSWYYFDFW	IGH	7	None	
14	2258	CAASSGYYPAYQFDYW	IGH	36	None	
14	2259	CARAPVVTGIKPDFDYW	IGH	2	None	
14	2260	CATRPPEGSFDYW	IGH	28	None	
14	2261	CARDFYCSSTRCYRRQYNHYMDVW	IGH	3	None	
14	2262	CARGRGSNWGRDYFDYW	IGH	49	None	
14	2263	CARDPYGADNWYFDLW	IGH	4	None	
14	2264	CATGTGSSYYFDHW	IGH	36	None	
14	2265	CARVRSGSDPIFDYW	IGH	3	None	
14	2266	CAKDRSRFGELANWFGPW	IGH	2	None	
14	2267	CAKDTAIAVAGGGGIDYW	IGH	7	None	
14	2268	CDWSSDVCSSDLDTAVYYCAKGRSANYLNDFDYW	IGH	2	None	
14	2269	CQQCDDLPQCTF	IGK	4	C3	6XDG
14	2270	CARDRSILGWFGELFGSYHFDYW	IGH	2	None	
14	2272	CTNGVCPFDSW	IGH	2	None	
14	2273	CARRCSSTTCYRAFDNW	IGH	4	None	
14	2274	CAKDKDPVGARYYFDYW	IGH	15	None	
14	2275	CVRDDSDYQYYYGMDVW	IGH	2	None	
14	2276	CARADMTNPYYYAMDVW	IGH	2	None	
14	2277	CASAVVIFDFDSW	IGH	2	None	

14	2278	CSSYFYYAVDVW	IGH	2	None	
14	2279	CAKSESYYYDDSGKYASHFDSW	IGH	5	None	
14	2280	CARIAGSLRGNVFYYYYGMDVW	IGH	4	None	
14	2281	CARDRNKYDLFDALDIW	IGH	4	None	
14	2282	CQQYGSLPRALTF	IGK	2	C1	7K8N
14	2283	CAHRRSGWLGGYFDYW	IGH	4	None	
14	2284	CARGGHCSGTRCNPDNYYFYGMDVW	IGH	58	None	
14	2285	CQERANWPQYSF	IGK	24	None	
14	2286	CVHRSFFPSSGFNAPYFDYW	IGH	23	None	
14	2287	CARGGWGTSCSLYCYYYMDVW	IGH	2	None	
14	2288	CTKDISSGYPRLFDFW	IGH	24	None	
14	2289	CARQNIEVPGTGYSDYW	IGH	2	None	
14	2290	CVRGEGYYDSSGYYWFYFDYW	IGH	4	None	
14	2291	CARGSYDPADYW	IGH	38	None	
14	2292	CVRALAAAGGGVDDYW	IGH	2	None	
14	2293	CAKVGYSYGTDSDYW	IGH	8	None	
14	2294	CQQYDYYSKLTF	IGK	134	None	
14	2295	CAAYMELISLRSEDTAVYYCARGDQKPAFSLDYW	IGH	27	None	
14	2296	CQQSYNVPGWSRTWTF	IGK	10	C2	7K8O
14	2297	CAKAQDVGLVAAPDAFDIW	IGH	2	None	
14	2298	CATERFLEWLLTHAFDIW	IGH	5	None	
14	2299	CARHMGATPHDPFDLW	IGH	2	None	
14	2300	CAAGSFYVIDYW	IGH	3	None	
14	2301	CATSPGGYYYDSSGYPIDYFDYW	IGH	2	None	
14	2302	CACWSLWELTSDYW	IGH	4	None	
14	2303	CARGQLGDEWFFAPFSWFDPW	IGH	2	None	
14	2304	CAREGALSGYDVFDYW	IGH	2	None	
14	2305	CASLWRWELLPDYW	IGH	17	None	
14	2306	CAKDPSTVVTPNFDYW	IGH	6	None	
14	2307	CMQALPTFMYTF	IGK	5	None	
14	2308	CAKGVAMLRDSGSVDYW	IGH	2	None	
14	2309	CAKIESISAPTFDYW	IGH	2	None	
14	2310	CVRQAETVTLFGAALFYDYW	IGH	2	None	
14	2311	CAKDMPLGGCDYW	IGH	2	None	
14	2312	CARAWGPRTQIDYW	IGH	4	None	
14	2313	CASGDSSGSFDYW	IGH	22	None	
14	2314	CARVRNIDYDYDPLDYW	IGH	2	None	
14	2315	CARHHRYVGHPNWFDPW	IGH	2	None	
14	2316	CARSKEESGYWEGLFDNW	IGH	2	None	
14	2317	CARDTDLYSYVPNPLDYW	IGH	2	None	
14	2318	CARPLWFGELEVIDYW	IGH	2	None	
14	2319	CARFQLYNTGLPGAIWFDPW	IGH	2	None	
14	2320	CARDLALNYYETSGYYDDYW	IGH	3	None	
14	2322	CTRLSWRYDLLTDSFIYDVFDIW	IGH	63	None	
14	2323	CARTAAETSQLERFDYW	IGH	9	None	
14	2324	CARVAPDVGETPQRPFDYW	IGH	2	None	
14	2325	CARGHRTLFYSFPTSYYYYYMDVW	IGH	9	None	
14	2327	CALISRDGVPFDYW	IGH	2	None	
14	2328	CARGGWQQPDRFYYYMDVW	IGH	3	None	
14	2329	CAKDSLITIFGVVPTYFDYW	IGH	10	None	
14	2330	CSTADPLSPRALRTDYW	IGH	2	None	
14	2331	CASHLEASKFDHW	IGH	2	None	
14	2332	CARSTSAWFGHAFDIW	IGH	4	None	
14	2333	CTKWTITSAGRAFDIW	IGH	4	None	
14	2334	CAHRTQWLAPWYFDYW	IGH	2	None	
14	2335	CASGTGIAAWDYYYYMDVW	IGH	2	None	
14	2337	CACEDFAVFMWFDPW	IGH	3	None	
14	2338	CAKESSPGYSSGWYEFDYW	IGH	2	None	
14	2339	CARHYCTNGVCHPYFDRW	IGH	5	None	
14	2340	CAREGVTEWFSPRYYYYYMDVW	IGH	4	None	
14	2341	CARYRETTYYWYFDLW	IGH	2	None	
14	2342	CARGHDILTAYHSYYFDYW	IGH	4	None	
14	2343	CARDLPYCSSTSCSPDHYYYGMDVW	IGH	2	None	
14	2344	CARRRGHGAYYMDVW	IGH	2	None	
14	2345	CARLITVFAGQSYFDYW	IGH	2	None	
14	2346	CARHFGVVTTVYYYYGVDVW	IGH	6	None	
14	2347	CARVWNWNYVLDYW	IGH	15	None	
14	2348	CSSYTSYSSTSALF	IGL	5	None	

14	2349	CARGPLGSSGWYGNYFDYW	IGH	2	None	
14	2350	CARLRGSPKGSYYLDYW	IGH	2	None	
14	2351	CATEGIEGSYRRRDFDYW	IGH	2	None	
14	2352	CQEFNSYNTF	IGK	10	C3	7K8R
14	2353	CAKGRGNRLRGVTFDQW	IGH	2	None	
14	2354	CARTLFATKTVDYW	IGH	3	None	
14	2355	CKNSLYLQMSSLRTEDTALYYCAKSSVIAATLSYFDYW	IGH	5	None	
14	2356	CARATARFAAGTVWIAFDYW	IGH	2	None	
14	2357	CARDGRYGNGYYSAFDIW	IGH	2	None	
14	2358	CATSPSSGWLPFDYW	IGH	12	None	
14	2359	CGGIAVAGPSPPYYYYYYMDVW	IGH	2	None	
14	2360	CARVGIAGRPIDYW	IGH	2	None	
14	2361	CVVFGMLTNQGDYMDVW	IGH	28	None	
14	2363	CARDYPLISGWYLDSW	IGH	12	None	
14	2364	CARNTDIVLMVYALRAFDIW	IGH	2	None	
14	2365	CARLQGMSGYFGTLWMYFDLW	IGH	5	None	
14	2366	CAKGISRSTWYYFDYW	IGH	15	None	
14	2367	CARWSGNWDPYNWFDPW	IGH	2	None	
14	2368	CASFLEWSDYYYYGMDVW	IGH	5	None	
14	2369	CARVPGWNYLWFDPW	IGH	2	None	
14	2370	CAKHYYDSASSYPFDYW	IGH	2	None	
14	2371	CARGGDVVVVVAAVRSGFDIW	IGH	2	None	
14	2372	CALPICLRSEDTAVYYCARGDQKPAFSLDYW	IGH	6	None	
14	2373	CAKCWDIVVVPAPFDPW	IGH	10	None	
14	2374	CARIFTRFYFDYW	IGH	9	None	
14	2375	CARKHFQYGDTYGLVDYW	IGH	10	None	
14	2376	CASGSRSAHINAFDIW	IGH	7	None	
14	2378	CATILIAEAGSDPMKVRNYFDYW	IGH	2	None	
14	2379	CARTYCGGDCSSNWFDPW	IGH	2	None	
14	2380	CAKDASDYGDSPSFGYW	IGH	8	None	
14	2381	CAREWSQLAMSGDAFDIW	IGH	3	None	
14	2382	CGAVIHNGYNYGFDLW	IGH	3	None	
14	2383	CATSCSSTSCYTRGFDYYMDVW	IGH	2	None	
14	2384	CQFSLRLTSVTAADAAIYYCVRGAFLEWFHGFDIW	IGH	2	None	
14	2385	CVRDGGVGGTDFFDFW	IGH	2	None	
14	2386	CANWFPLLDAFDIW	IGH	3	None	
14	2387	CAKEKCDDFAPCGYAMDVW	IGH	2	None	
14	2388	CARGGPWGGDYHYYMDVW	IGH	2	None	
14	2389	CAKDIAPFGAVAGHFDHW	IGH	34	None	
14	2390	CARLGEDFYYYYMDLW	IGH	13	None	
14	2391	CARDGRDCNGGICYEGRFDNW	IGH	5	None	
14	2393	CARVAPIPAASPPSVYYYMDVW	IGH	2	None	
14	2395	CAHIEEWGDYFDYW	IGH	5	None	
14	2396	CARFGGDGCTTDDCYDYW	IGH	2	None	
14	2397	CQSYDDKNNMMF	IGL	4	C4	7JVC
14	2398	CARGGRHYGGSDYYYDYW	IGH	2	None	
14	2399	CVKGKYLIDYW	IGH	22	None	
14	2400	CARDSTYYTSALYRNFDFW	IGH	2	None	
14	2401	CARRIRVPAADYYYYYYMDVW	IGH	2	None	
14	2402	CARDRDGYCSSTSCKPRGYLDCW	IGH	2	None	
14	2403	CTTSLIRGSYVFDHW	IGH	2	None	
14	2404	CATDGGAGTIFRYYYYYMDVW	IGH	15	None	
14	2406	CTRVMFGFWSGYNPYYFDYW	IGH	2	None	
14	2407	CARATQDYDTLTGYYNGAFDYW	IGH	2	None	
14	2408	CAKAPDSSVYYYDYWYFDLW	IGH	2	None	
14	2409	CARGGYIYTNFAYW	IGH	2	None	
14	2410	CAKGGLPMVYALAPDYW	IGH	2	None	
14	2411	CAKDIGYDSSGYYVGGYYFDYW	IGH	13	None	
14	2412	CAKDRPYGDEGDGNLDYW	IGH	2	None	
14	2413	CARDQSGSSSPADYW	IGH	2	None	
14	2414	CATNSPVRGIAAAQDYW	IGH	3	None	
14	2415	CARLGDLSGSQSGFNWFDPW	IGH	2	None	
14	2416	CAKDRTRRLRVAIDYW	IGH	4	None	
14	2417	CAKGSTVTTLGHFDQW	IGH	3	None	
14	2418	CARQDFTTIHTTWFDPW	IGH	2	None	
14	2419	CARPTTHWAVADAFDIW	IGH	5	None	
14	2420	CARQMTSIGFDYW	IGH	14	None	
14	2421	CARDRVWLLKSAFDIW	IGH	2	None	

14	2422	CAHGGSYSNYYFDYW	IGH	2	None	
14	2423	CASDGALSGYYGYW	IGH	6	None	
14	2424	CASHFPNYCSSTSCSYYYGMDVW	IGH	2	None	
14	2425	CAKDIRGYCSSTSCPGGFDYW	IGH	2	None	
14	2426	CAKTYQLLSSGYDVWSDITTEGAFHIW	IGH	2	None	
14	2427	CARDPSLSSVMVYAIYTSDAFDVW	IGH	4	None	
14	2428	CAKDATSDYGYYIDYW	IGH	4	None	
14	2429	CATEPKTYYDFWSGPANFDNW	IGH	35	None	
14	2430	CEQWLITDSGSYGFDIW	IGH	2	None	
14	2431	CAKDRIVVVTYPYYYGMDVW	IGH	2	None	
14	2432	CAKEGGNDYSFQPPFDYW	IGH	14	None	
14	2433	CARTLYFGSGTYFDYW	IGH	2	None	
14	2434	CAHRRPGIAARPYDYW	IGH	4	None	
14	2435	CARFGPHDFWSGYHDAFDIW	IGH	2	None	
14	2436	CAKDGWVVPAAIVFSAIGYFDSW	IGH	15	None	
14	2437	CARHGIRFLAWSPLDYW	IGH	2	None	
14	2438	CAKDNWAVAGAFPLDYW	IGH	2	None	
14	2439	CATDCCHPNWFDPW	IGH	2	None	
14	2440	CARHDIYSDSSVYSVYESFDPW	IGH	6	None	
14	2441	CARDQLWVGELLPLDYW	IGH	2	None	
14	2442	CAVPGISVPGSGWYFEIW	IGH	3	None	
14	2443	CARDSSGTYPHYFDYW	IGH	8	None	
14	2444	CGREVDGYVGYFYYGMDVW	IGH	2	None	
14	2445	CATEGRNWGVDAFDIW	IGH	9	None	
14	2446	CARESTFFIPDYYYYYYMDVW	IGH	15	None	
14	2447	CARDYKATGWGVDYW	IGH	3	None	
14	2448	CAAGMYSSSLGYYYYGMDVW	IGH	2	None	
14	2449	CARGYSVGSGYFFDYW	IGH	2	None	
14	2450	CAHQFYYDYWSGYTAPFDYW	IGH	6	None	
14	2451	CARRGSFCSGDCYYWFDPW	IGH	2	None	
14	2452	CARDLYAYYYDDSVYFDVFAYW	IGH	4	None	
14	2453	CARDLRYCSGGGCYSNFYYYFYVDLW	IGH	16	None	
14	2454	CAKGLRGIAAPPVEYFQHW	IGH	2	None	
14	2456	CARISSSHDGLQWYFDLW	IGH	2	None	
14	2457	CARVSPEVDLYESSVYHFGADYCDYW	IGH	2	None	
14	2458	CARDLRGDHGYSIDYW	IGH	2	None	
14	2459	CARLDVVGLLNFDQW	IGH	3	None	
14	2460	CARNVRYYYDSSGYYYYYYMDVW	IGH	6	None	
14	2461	CAKGQFADYEIDYW	IGH	4	None	
14	2462	CARVVGQWELLRYDYW	IGH	6	None	
14	2463	CARVYSTNAYRAAFDSW	IGH	7	None	
14	2464	CARLIVVAPAPRYQGEKKYYFDYW	IGH	7	None	
14	2465	CARLARQVLRFVESLKGEDALDMW	IGH	2	None	
14	2466	CALNDYSNYFGRVHYYYSYVMDVW	IGH	3	None	
14	2467	CAKDLQVLKKTTGGTPPAMITFDVW	IGH	8	None	
14	2468	CAKDTYGDSGHFDYW	IGH	7	None	
14	2469	CAAGPMDATLVAFDIW	IGH	3	None	
14	2472	CARNLAPGVWGSYRPPDYW	IGH	2	None	
14	2473	CAKDRKLVAAISIGDLDYW	IGH	2	None	
14	2474	CTRMSRTYYTSFDNW	IGH	3	None	
14	2475	CARHYISDQMPESWFDPW	IGH	2	None	
14	2476	CDWSSDVCSSDLYYQGGILDYW	IGH	11	None	
14	2477	CAREIAVAGPCIDYW	IGH	48	None	
14	2478	CAPIYVWGTTVDYW	IGH	2	None	
14	2479	CARGGCSSTSCYTPAGYYYYMDVW	IGH	4	None	
14	2480	CARQTMIRGLIRSDYW	IGH	6	None	
14	2481	CTAYMEMSRLRSDDTAVYYCARGIWDSSSFEYFQDW	IGH	2	None	
14	2482	CLQFHSYPLIAF	IGK	2	C3	7K8P
14	2483	CAREPPYSSSWYFDLW	IGH	2	None	
14	2484	CARGPPSSGYYGFFFKDYW	IGH	2	None	
14	2485	CATPMGPYYYDSSGYGADAFDIW	IGH	22	None	
14	2486	CAKEDYTSGFSWFGPW	IGH	14	None	
14	2487	CASEVGYCTNGVCSDYW	IGH	10	None	
14	2488	CVGGDDYDSSGYYYLDYW	IGH	15	None	
14	2489	CAASRLPSALDIW	IGH	2	None	
14	2490	CQQYGSSPVTFGQGSPVTF	IGK	4	C1	7K8N
14	2491	CLRSEDTAVYYCARTHTAMAIFDYYFDYW	IGH	2	None	
14	2492	CQVWDSSGDSSSDHLWVF	IGL	2	None	

14	2493	CARTQGYFYGFDSW	IGH	4	None	
14	2494	CAHLMITYGGVTGDDAFDFW	IGH	2	None	
14	2496	CAKGLNNYDGSGYYYYHYGMDVW	IGH	4	None	
14	2497	CARKGYSGWSIDYW	IGH	2	None	
14	2498	CEQSYSPPRFTF	IGK	10	C2	7K8O
14	2499	CAREKDGGFWGSTHWLDPW	IGH	2	None	
14	2500	CALPISAMYYCARDLSRVDYGALSGYYQGGILDYW	IGH	17	None	
14	2501	CARALWFGDLYYGFDIW	IGH	8	None	
14	2502	CAHSQYYDSSGYYVWFDPW	IGH	2	None	
14	2503	CARDRLSGSYYDTSGGCDYW	IGH	9	None	
14	2504	CQQYDTVPPVFTF	IGK	403	C3	6WPS
14	2505	CARENYGGNPARYTLGYLDYW	IGH	10	None	
14	2506	CARHRVAARDYFSPNKYYFDYW	IGH	2	None	
14	2507	CARAVIFGEIFRPNWFDPW	IGH	6	None	
14	2508	CARLYNWNYADYYYYMDVW	IGH	10	None	
14	2509	CARHFVLMGYSHDYW	IGH	2	None	
14	2510	CQRYDALPRPSPLTF	IGK	7	C3	6XDG
14	2511	CATYDLLIGFHYW	IGH	2	None	
14	2512	CARAQGPLGAYAFDIW	IGH	2	None	
14	2513	CVRDIPRNRYFYDSSDSDFDYW	IGH	2	None	
14	2514	CVSTVGRHSMKIIGGAFDIW	IGH	4	None	
14	2515	CARRDSRSIFDLW	IGH	9	None	
14	2517	CAKNRVDDSDNDYYYVPDAEYLQYW	IGH	2	None	
14	2518	CASRPLDFSIDWYFDFW	IGH	2	None	
14	2519	CAKDNSYDHAFDIW	IGH	14	None	
14	2520	CARDWGGYLGFYYFDYW	IGH	3	None	
14	2521	CASFPVVPAAPGDYYYMDVW	IGH	2	None	
14	2522	CAKVNVVVATINAFDIW	IGH	14	None	
14	2523	CDWSSDVCSSDLNPYYFDHW	IGH	5	None	
14	2524	CAREGVYSGCDLDYW	IGH	14	None	
14	2525	CATGYCSGGSCYHAKTPDYW	IGH	5	None	
14	2526	CGREASFVGTHYESNAFDIW	IGH	2	None	
14	2527	CARGSSAPTTRWFDPW	IGH	4	None	
14	2528	CARDGGPSVGPLHFDYW	IGH	4	None	
14	2529	CARAPTGDGTTWSNAFDIW	IGH	17	None	
14	2530	CAADPHCTGGNCYSYYFAYW	IGH	4	None	
14	2531	CARSRRAVVPAAMAFDYW	IGH	2	None	
14	2532	CARAGSYSSSLVAGAEYYFDYW	IGH	4	None	
14	2533	CARHTDSLSYPGYFDLW	IGH	8	None	
14	2534	CATGENSGWKRTFDYW	IGH	6	None	
14	2535	CAKASEYYFKSRVQWAPDYW	IGH	3	None	
14	2536	CARPTRTYSYFDLW	IGH	5	None	
14	2537	CAKVREVAGPLLLLGYFDYW	IGH	2	None	
14	2538	CAKAPYGSGSPAYFDYW	IGH	2	None	
14	2539	CARGSDPRIVLHFFDYW	IGH	8	None	
14	2540	CTRDVVIAAAGTLIYYYYYYMDVW	IGH	5	None	
14	2541	CARGSYDSRDLFDYW	IGH	2	None	
14	2542	CARGGLYSSGPYGTLDYW	IGH	2	None	
14	2543	CGRGRVVTTVDYW	IGH	24	None	
14	2544	CAREEVGFPRGWFDSW	IGH	2	None	
14	2545	CATSHNSGYPFDW	IGH	4	None	
14	2546	CATKADSGSYSADYW	IGH	2	None	
14	2547	CARGVLAVAAHWGDYW	IGH	2	None	
14	2548	CARGEIGTTYWEIKYFDFW	IGH	4	None	
14	2549	CVTISARYKDATDVW	IGH	8	None	
14	2550	CAKNLLVPSNSELYYDVFDMW	IGH	2	None	
14	2551	CAHDYYDVLSGYYLLDYW	IGH	2	None	
14	2552	CVTESGFSSFWYNFDNW	IGH	3	None	
14	2553	CVNPWLFSGKYYFAYW	IGH	4	None	
14	2554	CVRTLRTHTVTTTGVDYW	IGH	2049	None	
14	2555	CAKDSRLFLMGADYHFDYW	IGH	6526	None	
14	2556	CASERFWSGHYPPRYFDFW	IGH	10	None	
14	2557	CHSYDTSNQVF	IGL	496	C4	7JVC
14	2558	CARAGVIYDVFTGDRDAVDIW	IGH	4	None	
14	2559	CARAGGYDLVDAFDIW	IGH	13	None	
14	2560	CQSPDSSGFYPMIF	IGL	10	None	
14	2561	CARDLQPGITMIVVVITGAYDYW	IGH	2	None	
14	2562	CARHESPRRAAAGSLGAFDIW	IGH	2	None	

14	2563	CATRSPKEEQQLLGPSFDIW	IGH	2	None	
14	2564	CARDPLRDSKPNFDYW	IGH	2	None	
14	2565	CAAISYTSTPGAYFYYGMDVW	IGH	2	None	
14	2566	CARVGRFFGIHAFDIW	IGH	2	None	
14	2567	CAHSPFPYNSGFDPW	IGH	8	None	
14	2568	CATGPQNVWSGYYSFDPW	IGH	4	None	
14	2569	CATQEMATQRGDYW	IGH	7	None	
14	2570	CAKASMIVVVKGWYFDLW	IGH	4	None	
14	2571	CAKAPLSGNWHFDIW	IGH	3	None	
14	2572	CATDLFYLGVGATELFDYW	IGH	4	None	
14	2573	CVKEEYSSSPGGDYW	IGH	3	None	
14	2574	CAKGFGVVVAATDAFDIW	IGH	2	None	
14	2575	CARVANGVWNYYFDYW	IGH	15	None	
14	2576	CQQYGSSLFTFGPGTNLFTF	IGK	8	C1	7K8N
14	2577	CAKVEGEYSSSSGDYW	IGH	11	None	
14	2579	CARHGSLSTAFGFDYW	IGH	3	None	
14	2580	CAKGANPGIAAGLDYW	IGH	2	None	
14	2581	CASDYGGHSFDYW	IGH	15	None	
14	2582	CAKDIRPVDDAFDIW	IGH	8	None	
14	2583	CARHPGYYDFWSGYQYWFDPW	IGH	2	None	
14	2584	CARGLFVINGDVFEIW	IGH	2	None	
14	2585	CARLGGYGDRTDYSYYMDVW	IGH	6	None	
14	2586	CARLLEVFDIW	IGH	2	None	
14	2587	CARGGGGYSYYDWFDPW	IGH	3	None	
14	2588	CAHLQFSPYYFDYW	IGH	6	None	
14	2589	CAKGSRGYSNFYFDYW	IGH	6	None	
14	2590	CARSSYDSSGHYCGGLACPFDYW	IGH	12	None	
14	2591	CARSITMVRDDAFDIW	IGH	12	None	
14	2592	CASGANYYDSSGYYRLLDYW	IGH	6	None	
14	2593	CARDFGKDIVVVPAASDAFDIW	IGH	2	None	
14	2594	CARTRADSTGYSRGYSDYW	IGH	11	None	
14	2596	CVRYHGAERYFDLW	IGH	2	None	
14	2597	CARLVIAAAPRRNYFDYW	IGH	59	None	
14	2598	CARDPPDYSGHGDYYYYMDVW	IGH	2	None	
14	2599	CAKDLASSGWDFDSW	IGH	4	None	
14	2600	CARVEVVVPTLITGRSEFMYW	IGH	6	None	
14	2601	CVRGTPFTAKYSFDYW	IGH	82	None	
14	2602	CAKGVVIARVHYYYMDVW	IGH	2	None	
14	2603	CATKSPGGGYGFDFW	IGH	2	None	
14	2605	CARSPTTHHALNYYFDYW	IGH	5	None	
14	2606	CASLRGTYSYGSQLDYW	IGH	6	None	
14	2607	CARLAYDEANIDMYFDYW	IGH	12	None	
14	2608	CARDGSKYYDFWSGYSHFDYW	IGH	43	None	
14	2609	CARDPSGLGWFGPW	IGH	5	None	
14	2610	CVSRYGGTSDGFDVW	IGH	2	None	
14	2611	CARTPHYYDNVYFDYW	IGH	2	None	
14	2612	CARDLGIAVAGDRQVSWFDPW	IGH	2	None	
14	2613	CARDRCSSTSCYTHNWFDPW	IGH	6	None	
14	2615	CARYPSLTAAGVGGFDSW	IGH	8	None	
14	2616	CAGGNYYYDSSGERPIDYW	IGH	6	None	
14	2617	CARARPDYVGDCFDIW	IGH	2	None	
14	2618	CVRRDYSNSNTYSPLFQYW	IGH	2	None	
14	2619	CARGGYSSSWPHQEHNWFDPW	IGH	13	None	
14	2620	CAKDASYSSSRLPYYFHYW	IGH	5	None	
14	2621	CAKDPRGGSGSYYNMFDYW	IGH	8	None	
14	2622	CVRVNVWFHGSGYPDAFDIW	IGH	2	None	
14	2623	CVSTIVTSYYFDYW	IGH	2	None	
14	2624	CARDRWGSGWFRPLDYW	IGH	5	None	
14	2625	CAKLGMTTVTGRFDPW	IGH	4	None	
14	2626	CASQDWGGSSRAGAFDIW	IGH	2	None	
14	2627	CARIRQDGVFDFW	IGH	2	None	
14	2628	CASGAARRAYWTAPNYYYMDVW	IGH	2	None	
14	2629	CAAATKGWLQWSDAFDIW	IGH	10	None	
14	2630	CQQSVTTPFAF	IGK	98	C2	7K8O
14	2631	CATLGYFYDSSGYLALDYW	IGH	7	None	
14	2632	CARSQYGSSSLKFDYW	IGH	3	None	
14	2633	CASSSRAAILTDVFDVW	IGH	6	None	
14	2634	CARGQLYYDILTGWTHFDYW	IGH	3	None	

14	2635	CGRSTYYYQDTVFDYW	IGH	3	None	
14	2636	CARCKVTGSDAFDLW	IGH	4	None	
14	2637	CTTSHPLRIAAAGTALDYW	IGH	5	None	
14	2638	CARKATISYYFDYW	IGH	3	None	
14	2639	CARLGLVRGVMRGYFDYW	IGH	2	None	
14	2640	CAKGHTAMAQVDYW	IGH	9	None	
14	2642	CARRGSCASTSCNLGGFDIW	IGH	2	None	
14	2643	CAGGGYSYGYSYYW	IGH	16	None	
14	2644	CALPILRTEDTAFYYCVKDDYYASGNFASNFDCW	IGH	7	None	
14	2645	CARDRGTYYDILTGSVDYW	IGH	2	None	
14	2646	CAREPEGGPGPYYYDHW	IGH	3	None	
14	2647	CTRHGPDGKAAPNTDYYYYMDVW	IGH	2	None	
14	2648	CTIDAKEVLVGSMMFITNYFDYW	IGH	2	None	
14	2649	CATRPGAIPSLGQNDYW	IGH	3	None	
14	2650	CARSAVTTLDYW	IGH	70	None	
14	2651	CAKSMVRGVIITFLFDIW	IGH	2	None	
14	2653	CARGKYPYSTPSDWFDPW	IGH	2	None	
14	2654	CLRAEDTAVYYCARGYSIDYW	IGH	5	None	
14	2655	CVISETRDYFDYW	IGH	4	None	
14	2656	CALPISAADTAVYYCATSRVAAAGEPSHFDYW	IGH	2	None	
14	2657	CARTPRSSRESSYFDYW	IGH	2	None	
14	2658	CARIRQYRGHDYW	IGH	4	None	
14	2659	CARRAPDHPEWEGFDPW	IGH	21	None	
14	2660	CARNIQGPFFWHNYYMDLW	IGH	8	None	
14	2661	CARRVRGDNSRNWYFDLW	IGH	2	None	
14	2662	CARDDNNGSSPGRYFDYW	IGH	2	None	
14	2663	CAKLPLGRDYYDSSGSDYW	IGH	2	None	
14	2664	CARHRKGSGAFFSFDRW	IGH	2	None	
14	2665	CASRGNCGGDCNVYYFDYW	IGH	5	None	
14	2666	CVTSPIRGDYHVLYFEQW	IGH	2	None	
14	2667	CVHRPDWEGWFAPW	IGH	3	None	
14	2668	CARGDYYDTRTYSNNAFDVW	IGH	2	None	
14	2669	CARHTSPDFWSGFYIDNW	IGH	4	None	
14	2670	CAAALGCSSTSCYPYYYYYYMDVW	IGH	2	None	
14	2671	CATSRGWITGTPVFDYW	IGH	4	None	
14	2672	CARHAHIGYSMHLDYW	IGH	2	None	
14	2674	CVSRGYGDSAAAFDVW	IGH	3	None	
14	2675	CARAPQGYSFVWLDSW	IGH	2	None	
14	2676	CVHRFPAMLRGREAFDYW	IGH	12	None	
14	2678	CARDGVYYGSGSPNMYFDYW	IGH	5	None	
14	2679	CARQSLFSTSPWEDYW	IGH	6	None	
14	2680	CARLGGSSGYYQYYFDYW	IGH	43	None	
14	2681	CARLLPFYSNPYYYNYYMDVW	IGH	2	None	
14	2682	CQQARSSPYNF	IGK	2	C4	6W41
14	2684	CARMGIAVPTLGYFDSW	IGH	8	None	
14	2685	CAKCKKDYYDGSTYYEYYFDYW	IGH	4	None	
14	2686	CARLTLFGEGTFDHW	IGH	3	None	
14	2687	CMQARYFPLSF	IGK	7	None	
14	2688	CASRYPPTTGYPPKFYFHYW	IGH	2	None	
14	2689	CAIVGTSDFDFW	IGH	49	None	
14	2690	CAADGYEDYGDFLDAFDIW	IGH	3	None	
14	2691	CARRKSRGIVGATSGAFDIW	IGH	2	None	
14	2692	CARGPLYCTGAICHLNWFDPW	IGH	2	None	
14	2693	CARIDYSTQMGGFDYW	IGH	3	None	
14	2694	CARRGVTSGAVPYFDNW	IGH	2	None	
14	2695	CATGEITTVTTWPFDYW	IGH	5	None	
14	2696	CATDTVAGHRPFEYW	IGH	5	None	
14	2697	CARGGWIRTGTTNKPGYYYYYMDVW	IGH	18	None	
14	2699	CVRDNDDVWGSYRSFDHW	IGH	34	None	
14	2700	CATAPGQEYYFDYW	IGH	4	None	
14	2702	CARIRDGYNLYDAFDVW	IGH	6	None	
14	2703	CAKDRIAHGGGVDYW	IGH	5	None	
14	2704	CAKESSDSSAFDSW	IGH	2	None	
14	2705	CTRTGAGSDISYEYFYGMDVW	IGH	2	None	
14	2706	CARLCGSDCSWGNDYW	IGH	2	None	
14	2707	CTTVNHGWSFRAERLFDYW	IGH	23	None	
14	2708	CARVGLKYYYDSTINYKYEGGYYFDQW	IGH	3	None	
14	2709	CARGYDTSYHYPPKKSPPDYW	IGH	7	None	

14	2710	CTRPTYDYDSSGYSYAEYFQHW	IGH	2	None	
14	2711	CAREYYDFTTFSYW	IGH	2	None	
14	2712	CARGYSGFESPFDFW	IGH	6	None	
14	2713	CARGQVYGSANSYPPDYW	IGH	2	None	
14	2714	CATGARRGYDFYYMDVW	IGH	6351	None	
14	2715	CAKGAPRGRQQLGPYNWFDPW	IGH	2	None	
14	2716	CARNTGSYLGDWFDPW	IGH	3	None	
14	2718	CARDKLGYDSSGVAFDIW	IGH	6	None	
14	2719	CATDPVGHFDPGRYHRRPTRAFDIW	IGH	2	None	
14	2720	CVRHVGVLVPAATFAYW	IGH	6	None	
14	2721	CARLDDYDSSGYFVDYW	IGH	14	None	
14	2722	CTTDEGGDTSGDDAFDIW	IGH	2	None	
14	2723	CARHDGRDSSSWSSNPEYYYSGMDVW	IGH	4	None	
14	2724	CAKYAIIFGQFVGDLDYW	IGH	2	None	
14	2725	CAKRAYSHYDRGPLDYW	IGH	2	None	
14	2726	CARGSAIYYDSSGYFDYW	IGH	1506	None	
14	2727	CARDQKVATASALDYW	IGH	2	None	
14	2728	CARSVHHDYLYWYLDLW	IGH	2	None	
14	2729	CARNAVSYCTGAHCNSGHFPYW	IGH	6	None	
14	2730	CNRGQRGAFDIW	IGH	4	None	
14	2731	CARQSWGSGHFDYW	IGH	29	None	
14	2732	CARRDDPRIAAFNTRNWYFDLW	IGH	3	None	
14	2733	CQSFDSSFSGNGVF	IGL	2	C4	7JVC
14	2734	CARGKVAVTIFGVVIARGNWFDPW	IGH	7	None	
14	2736	CARMMAAAGMAADYW	IGH	7	None	
14	2737	CARWRNDSWGPKGFDPW	IGH	4	None	
14	2738	CMYYCARDHPDNDYGDYAGYW	IGH	29	None	
14	2739	CARLGGHYYDSTGYYFDSW	IGH	5	None	
14	2740	CAKIEAMPRAFDIW	IGH	2	None	
14	2741	CAHSVGSRFGSYYFDSW	IGH	9	None	
14	2742	CGTRDSSLSPWVF	IGL	3268	None	
14	2743	CARGNVLRYLEWLSTDPDYFEDW	IGH	2	None	
14	2744	CARRKDTDFWSSHPAYYFDFW	IGH	2	None	
14	2745	CARDKEDFDFWRGTRKYYMDVW	IGH	9	None	
14	2746	CARGSHWDSSSLDYW	IGH	3	None	
14	2747	CAKDGKYCSSTSDYCYFDYW	IGH	2	None	
14	2748	CASYYYDSSGYYFDGFDIW	IGH	5	None	
14	2749	CARVQDYYDSSGYFTNGFDYW	IGH	10	None	
14	2750	CAKDLMYDSWSNYYTGDAFDMW	IGH	13	None	
14	2751	CMRQRGKGSWSFDHW	IGH	3	None	
14	2752	CARDLHQKYCTNGLCSPYYFDYW	IGH	11	None	
14	2753	CARDRAMVVVYDAFDLW	IGH	5	None	
14	2754	CARRRPDSGYATRDDAFDVW	IGH	24	None	
14	2755	CAKSAYVGVYLWAFDVW	IGH	6	None	
14	2757	CARHEMFVIAAAGTSNWFDPW	IGH	2	None	
14	2758	CARWRFSEWLLPYFFDSW	IGH	5	None	
14	2759	CAKARGPVVGLDAFNVW	IGH	6	None	
14	2760	CANFGSVGYNYHAFDIW	IGH	6	None	
14	2761	CARDLVGAYSAYDPFDYW	IGH	5	None	
14	2762	CARPPPFSSGWYLGDAFDIW	IGH	2	None	
14	2763	CAREGHIWEACSGGSCYRSQYFDYW	IGH	5	None	
14	2764	CQSYDIALSGTVIF	IGL	37	None	
14	2765	CVSWGPRDAFDVW	IGH	9	None	
14	2766	CARLTGGRPSDAFDIW	IGH	25	None	
14	2767	CAKDFNQWDLMPFVFDIW	IGH	2	None	
14	2768	CAKGRSENYLNDFDYW	IGH	4	None	
14	2769	CAKDGALVIVTTYYFDYW	IGH	2	None	
14	2770	CARLVSRYYYDSSGYQYYFDYW	IGH	11	None	
14	2771	CAKDSQQLDDRYYYGMDVW	IGH	2	None	
14	2772	CARAGDVLGALNDYW	IGH	18	None	
14	2773	CARTYYYDSSGYYQTPDYFDYW	IGH	2	None	
14	2774	CARDGYYGSGRIQYSDYW	IGH	5	None	
14	2775	CAIRGNCSGGNCYTFDHW	IGH	3	None	
14	2776	CAKDQFCTDSNCFAFDPW	IGH	2	None	
14	2777	CARDERYYYDSSGYSHVPFDIW	IGH	100	None	
14	2778	CARIKHDFDSSGYSDTFDIW	IGH	6	None	
14	2779	CATSEYNYAYAFDMW	IGH	3	None	
14	2780	CARDPLPTTHDGILTSLFDYW	IGH	17	None	

14	2781	CARDRPALGYSYACDYW	IGH	2	None	
14	2782	CASNLPLGYCSSTSCYGGYFDYW	IGH	6	None	
14	2783	CAREAQESTWDFRDDVFDMW	IGH	4	None	
14	2784	CARAGADGQWLPSPHMDYW	IGH	14	None	
14	2785	CARETPCSGTYLCHYFDYW	IGH	2	None	
14	2787	CARRRGYYDSSGYYVSSAFFEDWYFDLW	IGH	2	None	
14	2788	CMQSIQLF	IGK	5	None	
14	2789	CARDCSSTSFYDAFDIW	IGH	31	None	
14	2790	CAKTKHYYDSGALLATEQSFDTW	IGH	2	None	
14	2791	CATDQGSPRWFGELWAFDIW	IGH	2	None	
14	2792	CARASPEMGIHYFDFW	IGH	11	None	
14	2793	CAKGLGYSSASGQDYW	IGH	3	None	
14	2794	CARDSFYDYGGDVDYW	IGH	2	None	
14	2795	CARVFRGDFSAVDHWFDPW	IGH	3	None	
14	2796	CAREPYCTTISCYPRWYFDLW	IGH	49	None	
14	2797	CAKFADYYDSSGSDYW	IGH	24	None	
14	2798	CAKYSSSWLHGFDYW	IGH	2	None	
14	2799	CAIYTIFGVVVAEAFDIW	IGH	2	None	
14	2800	CARVNYALWRGYFTPTNWYFDLW	IGH	2	None	
14	2801	CARDDYYSNYHFDYW	IGH	5	None	
14	2802	CARDVALAGTVTYEYISYAMDVW	IGH	2	None	
14	2803	CARLDYTTSSLFDYW	IGH	16	None	
14	2804	CARHQLAIAPRSAFDIW	IGH	4	None	
14	2805	CARDARIMGNPCDSW	IGH	5	None	
14	2806	CARGGWTSKSYYYYGMDVW	IGH	4	None	
14	2807	CAKSSVIAATLSYFDYW	IGH	79	None	
14	2809	CARDPHSILMVSAGYFDYW	IGH	2	None	
14	2810	CARTSYYYDSTGGSSYNDAFDMW	IGH	2	None	
14	2811	CALPISAVYYCARAKGRISLRYFDWLFHAFDMW	IGH	2	None	
14	2812	CDWSSDVCSSDLAFYYCVKDDYYASGNFASNFDCW	IGH	4	None	
14	2813	CAHSPPSRSWEFDFW	IGH	6	None	
14	2814	CARESETSTSRRLDYW	IGH	2	None	
14	2815	CARRAECGSTSCPNDYW	IGH	2	None	
14	2816	CARTWYSGSYYKLDYW	IGH	2	None	
14	2817	CARSRLGYCSSTSSTCYSPYFQDW	IGH	2	None	
14	2818	CATDRSSWYSRQEDYYFDYW	IGH	6	None	
14	2819	CAKDILGVALGGYPDYW	IGH	8	None	
14	2820	CARGGRYNNGPGVNDYW	IGH	2	None	
14	2821	CVRGSLQGGGYYLDYW	IGH	6	None	
14	2822	CAKGTGDYDFWGGYPLFYFASW	IGH	8	None	
14	2823	CAKDLRILEWLPRAFHIW	IGH	2	None	
14	2824	CAKDVWGAFDIW	IGH	23	None	
14	2825	CTSLTGMVHDYW	IGH	3	None	
14	2826	CATDYYDSSGYYGPTQIFDYW	IGH	7	None	
14	2827	CTSAPVGVMVRGRIVTLDYW	IGH	2	None	
14	2828	CISGRQQWFGAMQIDYW	IGH	2	None	
14	2829	CAKDDLAGTTPRGYLDSW	IGH	11	None	
14	2830	CARHSISYYDFWSGSHFDYW	IGH	6	None	
14	2831	CTTEEVVVAAANDYW	IGH	81	None	
14	2832	CAKDLHSSGWSPNVFDYW	IGH	398	None	
14	2833	CARGGDGGYSDSNGYYYFDYW	IGH	50	None	
14	2834	CATWDDTLSGLNWVF	IGL	14	None	
14	2835	CARSRGGSYPDAFDIW	IGH	13	None	
14	2836	CAKVKDNSGYKFFDIW	IGH	7	None	
14	2838	CARLGPVIPDDILGLRAFDIW	IGH	2	None	
14	2839	CTKRYSYDLGDYW	IGH	2	None	
14	2840	CARGNSGYYSPFPYW	IGH	2	None	
14	2841	CATPLHCSSGNCQLNYYMDVW	IGH	5	None	
14	2842	CARSGQYGGYRSFDYW	IGH	10	None	
14	2843	CARDPDITISGVFIHAFDYW	IGH	10	None	
14	2844	CARSTGLPNFHNYYLDVW	IGH	27	None	
14	2845	CARGLSTLSCGYCPYFDYW	IGH	41	None	
14	2846	CARDLLNRVLYHPDYYYGMDVW	IGH	3	None	
14	2847	CAHRNRRRGRYDFWSGYYTAYFDYW	IGH	3	None	
14	2848	CERDPDSGPTKDAFDLW	IGH	3	None	
14	2849	CAQSAFFYDSSDYSTATTEYFPHW	IGH	2	None	
14	2850	CAKKTRPYGLVDTFDIW	IGH	4	None	
14	2851	CARSGGRPHFVVVIAIGVDYYMDVW	IGH	51	None	

14	2853	CARGTPTTIKPHDYW	IGH	3	None	
14	2854	CAKDRSYYYYDSSGYYHFFDYW	IGH	3	None	
14	2855	CAREIPGIAVAGTSHDYW	IGH	3	None	
14	2856	CAKIEYGYDSSDSLWYFDLW	IGH	3	None	
14	2857	CAHRGILSPFDYW	IGH	4	None	
14	2858	CARDMSYTGNPFYFDHW	IGH	2	None	
14	2859	CAPLSHSSGYYLTDYW	IGH	6	None	
14	2860	CARLITVTTAGYYYYYGMDVW	IGH	2	None	
14	2861	CARGGSRSGSYYDYGMDVW	IGH	7	None	
14	2862	CAPDGGSLMTNLDYW	IGH	3	None	
14	2863	CARRHSTTISGSGDAFDIW	IGH	4	None	
14	2864	CARDGKYLPSLAYYFDNW	IGH	2	None	
14	2865	CARAGSNWNPKGGDYW	IGH	10	None	
14	2866	CARQTNDVLYLDYW	IGH	7	None	
14	2867	CARSGSYYYSDSFDMW	IGH	128	None	
14	2868	CTKSDGGLGISDHFDPW	IGH	8	None	
14	2869	CAKDKVGAPTYVFDYW	IGH	6	None	
14	2870	CARAPTWYSGSEGAFDIW	IGH	9	None	
14	2871	CARRVDGAKYYDFLSGPLFDHW	IGH	14	None	
14	2872	CAVLGGGPPTPRLNWFDPW	IGH	2	None	
14	2873	CARGVVTPTTAEKFYFDFW	IGH	2	None	
14	2874	CAKAAKVALQLSHNYMDVW	IGH	7	None	
14	2875	CNSRDNSANHLGVVF	IGL	17	None	
14	2876	CARHRIAATNSRTDAFDIW	IGH	2	None	
14	2877	CATHYSYSSSWNPDYW	IGH	4	None	
14	2878	CARGGLRFLEWLLHPFDYW	IGH	2	None	
14	2879	CARVRFSQWYGGSSRWFDPW	IGH	2	None	
14	2881	CAKSRVPGGHNFDIW	IGH	2	None	
14	2882	CARYNGRVYRYDYW	IGH	2	None	
14	2884	CGRRGLYDNTRDWYFDLW	IGH	4	None	
14	2885	CARAYLDTTVILGCAAYDIW	IGH	8	None	
14	2887	CARMSYSSSSSGVGWFDPW	IGH	9	None	
14	2889	CARDPYPAALGWETNAFDIW	IGH	2	None	
14	2890	CARDSSMAAAGSAYYFDYW	IGH	2	None	
14	2891	CAREMGRSLGAVAGDDSCDIW	IGH	41	None	
14	2893	CVRPRRSSAFYSLDYW	IGH	2	None	
14	2894	CARMPRRDGYNFDYW	IGH	3	None	
14	2895	CAAFGVLHPLGELLYVGDYW	IGH	2	None	
14	2896	CATSTVTLWGDAFDIW	IGH	5	None	
14	2897	CARESRNFAEYFQHW	IGH	2	None	
14	2898	CAKDMLGEKNAFDIW	IGH	7	None	
14	2899	CACTAMAQGGLDYYYYYGMDVW	IGH	4	None	
14	2900	CARSPEYVAYLEYYMDVW	IGH	2	None	
14	2901	CARHSPKDFDYW	IGH	40	None	
14	2902	CARGHSSDGTAYYFDYW	IGH	4	None	
14	2903	CARKGIASSWAFDLW	IGH	209	None	
14	2904	CVREGDSSRDYAMDVW	IGH	3	None	
14	2905	CAKDSGTSVAGAFDIW	IGH	2	None	
14	2906	CARRYGSDRPRDAFDVW	IGH	4	None	
14	2907	CARREISGNTAGLFDYW	IGH	3	None	
14	2908	CVHISKDYDNSGYQYAAEYFNYW	IGH	2	None	
14	2909	CVKDMSYGGNCDALDIW	IGH	4	None	
14	2910	CATFINYDSSGYGGGFDPW	IGH	2	None	
14	2911	CARARIAAAGTTPLFDYW	IGH	4	None	
14	2912	CASDCSSTTCLAYDAFDIW	IGH	17	None	
14	2913	CARGIDSSGWFVVDYW	IGH	7	None	
14	2914	CARRPEGGNSRDWFFDFW	IGH	2	None	
14	2916	CARDSEYDHDKIGYDLDYW	IGH	14	None	
14	2917	CARDTKPLWATTPFRPYYFDYW	IGH	3	None	
14	2918	CAREGTIFGVVTGNWFDPW	IGH	3	None	
14	2919	CARVGRSSSWYTPGDYW	IGH	2	None	
14	2921	CARGDRYFGSGKGYNWFDPW	IGH	2	None	
14	2922	CAAGPGDLYDFWSGYDYYYYGMDVW	IGH	2	None	
14	2923	CARLSGSGWIDYW	IGH	60	None	
14	2924	CARIPYYDFWSGYYGFDYW	IGH	37	None	
14	2925	CARERVGYGGIPYNFDNW	IGH	2	None	
14	2926	CARGLILGRNYFDAFDLW	IGH	2	None	
14	2927	CARAFGENQLYNWFDPW	IGH	2	None	

14	2928	CARDSIAVAGTNYYYGMDVW	IGH	5	None	
14	2929	CARDFFLQSDTSGPPDGFDLW	IGH	3	None	
14	2931	CARGWFRSGGAGGTYYYYGMDVW	IGH	2	None	
14	2932	CARSDYSNYPYDYW	IGH	66	None	
14	2933	CARSRYCGNDCYSNFDYW	IGH	3	None	
14	2934	CATLSVMTTPIRDYW	IGH	19	None	
14	2935	CAREGSDFWSGYNAFDLW	IGH	2	None	
14	2936	CARDRLSIAAAGIDYYYGMDVW	IGH	2	None	
14	2937	CARGLSEDIVVVPAATPICSFDYW	IGH	2	None	
14	2938	CARDPSVPGAMDVW	IGH	2	None	
14	2939	CARGGGHILTGYYRDYFDYW	IGH	4	None	
14	2940	CARDLLASYGADYYYYYGMDVW	IGH	6	None	
14	2941	CARSLYGITMVRGAPIDYW	IGH	67	None	
14	2942	CARTIHPGIAAAATDYW	IGH	2	None	
14	2943	CARGPHYYDSSATYFDYW	IGH	2	None	
14	2944	CASLPYSLTVASPYYFDYW	IGH	68	None	
14	2945	CARGRGEVVTIDYW	IGH	2	None	
14	2946	CQHYSSYPYIF	IGK	92	C3	7K8P
14	2947	CARDRRIGDTAVVFDYW	IGH	3	None	
14	2948	CAREVVGLGGLDYW	IGH	6	None	
14	2949	CALPIFSLNLYSVTAADTGMYYCARDHPDNDYGDYAGYW	IGH	2	None	
14	2950	CAREGYDSSGYYLTNGGDYW	IGH	2	None	
14	2951	CQQYNNWVALSF	IGK	31	C3	6XDG
14	2952	CARHVEALPSAVRLLEDYYNYGMDVW	IGH	2	None	
14	2953	CARFRELRHENNALRAFDIW	IGH	5	None	
14	2954	CARGIVVVPALIGLFDPW	IGH	2	None	
14	2955	CARDAVLAAVTFFDYW	IGH	45	None	
14	2956	CASILLWFGNYYFDYW	IGH	3	None	
14	2957	CTRTAFVPAAPMGPIYYFDSW	IGH	2	None	
14	2958	CARHHNWDHLYYFDSW	IGH	3	None	
14	2959	CARTVGGYTYGPSLYYFDSW	IGH	8	None	
14	2960	CARGGVIDRNVDLVTTALDYW	IGH	7	None	
14	2961	CARDRGFGEFQPFDYW	IGH	2	None	
14	2962	CMQRIETSYTF	IGK	20	None	
14	2963	CARDRRGIYCSGGPCAFDIW	IGH	2	None	
14	2965	CAMARNSWFNRFDPW	IGH	3	None	
14	2966	CAGGSWGFWSQAKAFDIW	IGH	2	None	
14	2967	CAKVDPNAADYW	IGH	2	None	
14	2968	CARWGIVEADAFDVW	IGH	7	None	
14	2969	CAKDFWSGYPLPSFDPW	IGH	4	None	
14	2970	CAREYKYGGNAAFDAW	IGH	2	None	
14	2971	CARCGYCYSSTCYFFFDFW	IGH	4	None	
14	2972	CQVWDDSRAHYVF	IGL	4	None	
14	2974	CATDCSGGSCYSNYW	IGH	2	None	
14	2976	CARGYRYDSSGYDYFDHW	IGH	9	None	
14	2977	CARGGSYDILTGPFDYW	IGH	4	None	
14	2978	CAKVYYGSGSSFDPFDIW	IGH	11	None	
14	2979	CATTNWNSGNDYW	IGH	3	None	
14	2980	CVKDRTYNYDSGGYSFDFW	IGH	5	None	
14	2981	CARDLYTIFGEKRNWFDPW	IGH	2	None	
14	2982	CARHLGGDSPDAFDIW	IGH	2	None	
14	2983	CARGRRNSGWSQVFDHW	IGH	2	None	
14	2985	CATDYCIGDSCHDVDVFDIW	IGH	4	None	
14	2986	CVRDLTGLLEKFPDAFDIW	IGH	2	None	
14	2987	CAKDNKDIVVIPATYYYYSYMDVW	IGH	3	None	
14	2988	CARDKIKSEMATEDGLYYFDYW	IGH	3	None	
14	2989	CARHCSGRANEACYERDFFEYW	IGH	4	None	
14	2990	CARDHLDHDLTGYYFDYW	IGH	3	None	
14	2991	CARGRGGNYLSYRATGPDYW	IGH	7	None	
14	2992	CARGGSVYGDYGGVMAEENVFDIW	IGH	2	None	
14	2993	CVKDEAGGYSSWYFDYW	IGH	2	None	
14	2994	CAKDGVDTAMVRNYYYYYMDVW	IGH	2	None	
14	2995	CARDLFLLTGYLNGFDYW	IGH	8	None	
14	2996	CAKDIGAINHGSCPDYW	IGH	4	None	
14	2997	CARIRPGWGSTSRRYYFDYW	IGH	4	None	
14	2998	CAKGRYYDNSGYCFDYW	IGH	3	None	
14	2999	CAATRGSNAFDIW	IGH	8	None	
14	3000	CTTEARALRPNAFDIW	IGH	5	None	

14	3001	CARHISRKTTLFGAAITDAFDIW	IGH	3	None	
14	3002	CAKDPSNGWVNAVAFDYW	IGH	29	None	
14	3003	CARARSAIVATKYFDYW	IGH	2	None	
14	3004	CARGQPAKYTGLGSFDYW	IGH	2	None	
14	3005	CAKDAYVRRNTVFGPFDFW	IGH	5	None	
14	3006	CARGYTVGTFDSW	IGH	4	None	
14	3007	CAREATVVHLLPEVWFDSW	IGH	2	None	
14	3008	CARDPGDSSYYYYARGYFNYW	IGH	22	None	
14	3009	CARHGAGGGYYRYYYYAMDVW	IGH	8	None	
14	3010	CVKDDYYASGNFASNFDCW	IGH	8605	None	
14	3011	CARDYLPYYFFCAMDVW	IGH	13	None	
14	3012	CARIQGTESSDFRTDYRIYYFDYW	IGH	2	None	
14	3013	CAQVGAHGSDYW	IGH	2	None	
14	3014	CARVRIAAMDVW	IGH	8	None	
14	3015	CARVSPPYYEYWRGYYFFDQW	IGH	2	None	
14	3016	CAREAYYDSSGYYYFNAFDIW	IGH	2	None	
14	3017	CVRARDIKIAVINDGFDVW	IGH	20	None	
14	3018	CARDGGRWLTTHRCDYW	IGH	3	None	
14	3019	CARGILVEPSANWFDPW	IGH	2	None	
14	3020	CALPIYISSLSWSDYW	IGH	2	None	
14	3021	CARYQDPENWGFRTPNIIDAFDIW	IGH	4	None	
14	3022	CARNFHGSGYYHSDYW	IGH	2	None	
14	3023	CARGSDGWYEYFRHW	IGH	2	None	
14	3024	CARDYSGHYDKSGYCEYW	IGH	3	None	
14	3025	CAKVDSYYDFWSGPDYW	IGH	4	None	
14	3026	CARKDYSPGGLDYW	IGH	9	None	
14	3027	CARATQTRQQLADPLDYW	IGH	4	None	
14	3028	CAREWSDSYDGISYYYGMDVW	IGH	3	None	
14	3029	CARPRSSYCGGNCYYYYFDYW	IGH	25	None	
14	3030	CVKDQNRLTFDSW	IGH	2	None	
14	3031	CAKGNTRFGVVPHYNWFDPW	IGH	8	None	
14	3032	CAREVTTVTTLYFDYW	IGH	7	None	
14	3033	CARSRIQPPYFYYMDVW	IGH	2	None	
14	3034	CAHQIGVGYYGLDYW	IGH	4	None	
14	3035	CARVEFDVSTGRINREFDPW	IGH	4	None	
14	3036	CARGREGAISFVAFDTW	IGH	2	None	
14	3037	CAKDSRLRFLEWPWHDAFDIW	IGH	4	None	
14	3038	CAKDVARGCSTNVCSLSDYYYGMDVW	IGH	2	None	
14	3039	CARDTQLGSWDFGAFDIW	IGH	2	None	
14	3040	CARGGWSSGWATYYMDVW	IGH	2	None	
14	3041	CVRHSGDDVGYYYVYW	IGH	2	None	
14	3042	CARSYYDILTGYYSSFDYW	IGH	44	None	
14	3043	CARDLVIGSSSEYGAFDIW	IGH	2	None	
14	3044	CARGQTYYDKRWAFDIW	IGH	2	None	
14	3045	CVRERCAESCYPGYW	IGH	2	None	
14	3046	CAKGGDTMIVGEYYYYMDVW	IGH	2	None	
14	3047	CAKVDCYSTTCFLKTFDYW	IGH	58	None	
14	3048	CARHSPSYDSSGYYYVDFDYW	IGH	2	None	
14	3049	CARVPLAVMIVVFDYW	IGH	2	None	
14	3050	CATFTFWSGYSVYW	IGH	8	None	
14	3051	CARVCAGDCYVDAFHFW	IGH	9	None	
14	3052	CAKDLGSGNSVRLSDYW	IGH	2	None	
14	3053	CTRMADCGGDSRFDPW	IGH	6	None	
14	3054	CARDADCGGNCYFPLHYAMDVW	IGH	2	None	
14	3055	CARGLPLDWYYDSSGSFILRDAFDMW	IGH	2	None	
14	3056	CARFLVWFGELFTSDYAAFDIW	IGH	6	None	
14	3057	CARCTVRGVASDYW	IGH	4	None	
14	3058	CARDLYSSGWYTSSDAFDIW	IGH	3	None	
14	3059	CAKYWAPGALDYW	IGH	6	None	
14	3060	CAKGPLALGTFFDIW	IGH	4	None	
14	3061	CARVATGTYAFDIW	IGH	5	None	
14	3062	CAKDAQQYQLLPHNWFDPW	IGH	4	None	
14	3063	CARVSVWVPADDGMDVW	IGH	4	None	
14	3064	CARGGSGSFYNGNDYW	IGH	2	None	
14	3065	CARQPLSIAARPDYFDYW	IGH	4	None	
14	3066	CASRPWFGVPSDFDYW	IGH	2	None	
14	3067	CARALPLEEWPLDYW	IGH	6	None	
14	3068	CARDIQYSSPNDYW	IGH	2	None	

14	3070	CASLYCSSTCLSYMDVW	IGH	4	None	
14	3072	CAQGGYYDSSFYYSLFKYW	IGH	4	None	
14	3073	CAKEDRTTVVSYYYYYGMDVW	IGH	2	None	
14	3074	CAKVGYYYDSSGYSRYFDYW	IGH	10	None	
14	3075	CAKGSNFLSGYPVKDAFDFW	IGH	22	None	
14	3076	CASCADTAMVTVFDYW	IGH	8	None	
14	3077	CAKGGGSGYREDNWFDPW	IGH	2	None	
14	3078	CARSKETAGVLWFDPW	IGH	3	None	
14	3079	CVSYNIAVATDYW	IGH	4	None	
14	3080	CANFGNDTPDYW	IGH	4	None	
14	3081	CATTPLKYYDILAGYSPDAFDIW	IGH	4	None	
14	3082	CARLTGSYSWYFDVW	IGH	7	None	
14	3083	CATLGAYYYYYMDVW	IGH	22	None	
14	3084	CARERGDYDFWSGYYFGGGDVVGPPNYYYYGMDVW	IGH	2	None	
14	3085	CARQNDILGVAFDYW	IGH	5	None	
14	3086	CTRDDWYSDNNGYYYVDHW	IGH	2	None	
14	3087	CAHRSLAAAGTENNWFDPW	IGH	2	None	
14	3088	CARNDFWSGSPLRNYMDVW	IGH	2	None	
14	3089	CANLLQGVDYW	IGH	8	None	
14	3090	CARTLCAGDCYSNDAFDVW	IGH	3	None	
14	3091	CARKSGLFCSGGSCYWGAFDIW	IGH	2	None	
14	3092	CARGLLVLATKSLIDYW	IGH	2	None	
14	3093	CARVRPLIAAAGRGFSDYW	IGH	11	None	
14	3094	CARVFDGSYKNYFDYW	IGH	7	None	
14	3095	CARCLGNSPHFDVW	IGH	10	None	
14	3096	CNTFYYHSGGFEDYW	IGH	2	None	
14	3097	CARFGPYCASTTCFTGFDYW	IGH	3	None	
14	3098	CARDVKPHYCSSTSCSLPYYFDYW	IGH	6	None	
14	3099	CANWVYDYGDPGAFDIW	IGH	2	None	
14	3100	CAKEAFFRRSGGSYYLPHFDYW	IGH	2	None	
14	3101	CARDGLTIFGVEYYYYGMDVW	IGH	2	None	
14	3102	CATTRAYSGIDYW	IGH	6	None	
14	3103	CARFYCSGNTCYKAFDYW	IGH	9	None	
14	3104	CARRSSIRIDYWSGNHTPFDHW	IGH	11	None	
14	3105	CARHPLDNKEDYFDYW	IGH	5	None	
14	3106	CARTWRYYYFAMDVW	IGH	5	None	
14	3107	CTTAAGHWVNSNGVWYLFDYW	IGH	2	None	
14	3108	CARDSVTYSSSWYYFDYW	IGH	733	None	
14	3109	CAKIGGGESVGWYFDYW	IGH	117	None	
14	3110	CARLGLWGSSWYYFDNW	IGH	3	None	
14	3111	CARDRQQWLVQGLDYW	IGH	6	None	
14	3112	CQLYGSSSYIF	IGK	14	None	
14	3113	CDWSSDVCSSDLSGNFASNFDCW	IGH	3	None	
14	3115	CARDRGYTYGSIWFDYW	IGH	8	None	
14	3116	CAKDIQAAAGMGNYFDYW	IGH	2	None	
14	3117	CARGRGGGFLEWFPNYYMDVW	IGH	2	None	
14	3118	CAKDHPTVRYSAFDIW	IGH	2	None	
14	3119	CARHRRPRVVPAARTLNWFDPW	IGH	4	None	
14	3120	CARPLKIWHCGGNCYGFDSW	IGH	31	None	
14	3121	CTRRITVITPSHFDSW	IGH	2	None	
14	3122	CMQATQFPPSWTF	IGK	15	None	
14	3123	CAMRGNCSGGICNRFDYW	IGH	29	None	
14	3124	CAREKEGPSMETALYYYYAMDVW	IGH	5	None	
14	3125	CARASIEGSRIEFDYW	IGH	2	None	
14	3126	CQQTYGSPLGAF	IGK	85	None	
14	3127	CARTMRSASEGFDIW	IGH	3	None	
14	3128	CAREEGYTTTTTPLDYW	IGH	11	None	
14	3129	CARPAFNYDFWSNNYYYMDVW	IGH	2	None	
14	3130	CARHVRSGSGSYPDYW	IGH	2	None	
14	3131	CASMTTRFYYYYMDVW	IGH	3	None	
14	3132	CARDQSSTAYHFDYW	IGH	2	None	
14	3133	CTREIIYSSTRNPKDAFDIW	IGH	2	None	
14	3134	CATNLRFSPESSDYYYFDHW	IGH	2	None	
14	3136	CVREERNIMEGRFDYW	IGH	2	None	
14	3137	CATSHTEGGFRAYYYYGMDVW	IGH	6	None	
14	3138	CATPPGYSTTRGAFEIW	IGH	2	None	
14	3139	CARDQDSRDEDWFDPW	IGH	2	None	
14	3140	CAKDFVRYCTTTSCYPYYFDYW	IGH	2	None	

14	3141	CASQEADFWSGYHHPFDHW	IGH	6	None	
14	3142	CARDQFPAVAGIDYW	IGH	4	None	
14	3144	CATVPREYYYDSSGYYKPFDYW	IGH	4	None	
14	3145	CARAQFKAANAGVDAFDIW	IGH	4	None	
14	3146	CAHTNDRHYEGSFDPW	IGH	2	None	
14	3147	CDWSSDVCSSDLTLHLQMNSLRAEDTAVYYCARGYSIDYW	IGH	3	None	
14	3148	CARDRLERRLPYYYYGMDVW	IGH	4	None	
14	3149	CARFALDSYSTSWSNFDFW	IGH	2	None	
14	3150	CAKDNPDCSSTSCYAPDMIFDYW	IGH	2	None	
14	3151	CASLSGTTIIGVVTRGGYFDYW	IGH	4	None	
14	3152	CARDKGGDREIDYW	IGH	3	None	
14	3153	CARSGGSYYIILDYW	IGH	2	None	
14	3154	CARGGRGGDYDWMTFDVW	IGH	2	None	
14	3155	CARNGDPGGRRDYSSYMDVW	IGH	2	None	
14	3156	CAKLGPAVDYYYGMDVW	IGH	2	None	
14	3157	CTKLRSNPRDYLDYW	IGH	15	None	
14	3158	CARDPFCPATSSCVLGADYW	IGH	2	None	
14	3159	CAYWPYSTRDAFDVW	IGH	3	None	
14	3160	CARGPSSWEILNAFDIW	IGH	2	None	
14	3161	CALPICLRAEDTAVYYCARSVSYYYGMDVW	IGH	2	None	
14	3162	CARGGWKTSGGTTRYYYMDVW	IGH	5	None	
14	3164	CVGGSGTYRLSPFDYW	IGH	2	None	
14	3165	CATLESLGYSSGWLDYW	IGH	2	None	
14	3166	CARASSKYYDSSGYHPDYW	IGH	2	None	
14	3167	CAKAEMATTQAPFDYW	IGH	6	None	
14	3168	CARDNRYQLNLRLDYW	IGH	12	None	
14	3169	CATSWTHYYDPSAYYYYFALDIW	IGH	6	None	
14	3170	CARTPTPNVVAMVGAPYYFDPW	IGH	2	None	
14	3171	CARDGGSEKKGGIVLLPAAPDYW	IGH	2	None	
14	3172	CVRSQGLALTAYPLDYW	IGH	8	None	
14	3173	CARLGFINYMDVW	IGH	4	None	
14	3174	CARSPKYHMLYDGFDIW	IGH	3	None	
14	3175	CARDMGTVAAANLGMDYW	IGH	2	None	
14	3176	CATEEVSWTEEGGVTRPLDYW	IGH	2	None	
14	3177	CAKGPVRSAGQYYYYGMDVW	IGH	4	None	
14	3178	CAKDLGQDGGSYHTSSDYW	IGH	4	None	
14	3179	CAREPYMRSSRKFDYW	IGH	11	None	
14	3180	CARGKEDYGSGSYYTNNWFDPW	IGH	73	None	
14	3181	CATTPFGGVIVKDYW	IGH	9	None	
14	3182	CAKVIVVVPAAELGWYFDLW	IGH	3	None	
14	3183	CATGSEPIVVVTATRRAWYFDLW	IGH	2	None	
14	3184	CARGHSGVTRNFDSW	IGH	4	None	
14	3185	CARAHYGDYYYYMDVW	IGH	22	None	
14	3186	CARLPSPIVVVVAAFDYW	IGH	4	None	
14	3187	CAKDFFRDGYIPRLYGAFNIW	IGH	45	None	
14	3189	CASSSDYGDSMQTFDYW	IGH	7	None	
14	3191	CAKGGAPSSSWFFDYW	IGH	8	None	
14	3192	CARVKDSSWYPPFGWYFDLW	IGH	16	None	
14	3193	CARGTRGYQFQYFFDYW	IGH	10	None	
14	3194	CARGQKWAYYFDYW	IGH	15	None	
14	3195	CARISNLVLPHFDSW	IGH	3	None	
14	3196	CASFRFLGGSDYW	IGH	4	None	
14	3197	CARDSNGDYLMSAFDIW	IGH	24	None	
14	3198	CATESPPADFWSGYPFQNYYYYGMDVW	IGH	2	None	
14	3199	CARDKIDGYPSMGWLDPW	IGH	2	None	
14	3200	CARALVNYDFWSGYWDLGAFDIW	IGH	3	None	
14	3201	CAKPGGDDFWSGYYNYW	IGH	4	None	
14	3202	CARGFDFWNNYSPLYLDYW	IGH	10	None	
14	3203	CAHRRSGYSGPFDPW	IGH	4	None	
14	3204	CARGYCTNGVCYAGWDYW	IGH	9	None	
14	3205	CARASTIAVAGRSYFDYW	IGH	4	None	
14	3206	CAGDSQGWGFDIW	IGH	8	None	
14	3207	CVREASTPRDSWSRYQHYYLDVW	IGH	3	None	
14	3208	CATDRSATIGIREDVFDIW	IGH	2	None	
14	3209	CARVLTGYYTDGFDIW	IGH	25	None	
14	3210	CARFRYCGGGTDCWYDFDYW	IGH	3	None	
14	3211	CARDPDTITNPWGGDYW	IGH	6	None	
14	3212	CQSADSSDSYYVIF	IGL	19	None	

14	3213	CARVHILYYDSSGYFAYW	IGH	2	None	
14	3214	CARVTGYYYDSSAYLDYW	IGH	2	None	
14	3215	CAHRRMICSNNRCYSDWDYW	IGH	2	None	
14	3216	CARGDRQPPSSSWSTFYYYYYMDVW	IGH	9	None	
14	3217	CARDAAALGSYFDYW	IGH	5	None	
14	3218	CARGLYYDTAGYYYANAFDTW	IGH	2	None	
14	3219	CASMQEGFGLPDYW	IGH	3	None	
14	3220	CAGSTDMITFGGVIVWGDYW	IGH	2	None	
14	3221	CARHVGFGDYFTSFDFW	IGH	4	None	
14	3222	CRVDYGVLSGYYQGGILDYW	IGH	6	None	
14	3223	CANQHYNFNYYFDLW	IGH	2	None	
14	3224	CARHHDSSSWSPPHFFDYW	IGH	2	None	
14	3225	CAHRPGVDFYELGGYISEDFFDSW	IGH	2	None	
14	3226	CAKDGKSWIQLWSRYLDGYFDYW	IGH	2	None	
14	3227	CARGPHPLNWFDPW	IGH	8	None	
14	3228	CARHFYSNYPYGIDYW	IGH	3	None	
14	3229	CAKVESSWYNYYYAMDVW	IGH	7	None	
14	3230	CATDSLNRGYSSSSWGFDYW	IGH	3	None	
14	3231	CAKGGYDSYFHYW	IGH	2	None	
14	3232	CARDFLAVGEFYYYYGMDVW	IGH	3	None	
14	3234	CAKDGSSYNYPRSIDYW	IGH	4	None	
14	3235	CASTVADKFDYW	IGH	7	None	
14	3236	CMRRRAYHDFWSGYRPLDYW	IGH	2	None	
14	3237	CATVFPPSYYYDSSGYYSYFDYW	IGH	2	None	
14	3238	CTINDALDIW	IGH	2	None	
14	3239	CAIKFYCDYW	IGH	3	None	
14	3240	CATGWTYYYDSSGYFQHW	IGH	2	None	
14	3241	CAKTGGGYWFDSW	IGH	4	None	
14	3242	CLRAEDTAIYYCAKDHVAGRNVDYW	IGH	2	None	
14	3243	CAKDPMVAEDYYYGMDVW	IGH	2	None	
14	3244	CAKEVLMWELTDYW	IGH	4	None	
14	3245	CAKGGRYYDSSGYWGDYYFDYW	IGH	2	None	
14	3246	CARDLAKGGNSGYDWTLDYW	IGH	22	None	
14	3247	CARSPLTYYYDSSGHWSGRATAFDIW	IGH	2	None	
14	3248	CAHSHDFWSGYYVYW	IGH	2	None	
14	3249	CARHANCTSTSCYKAYFQRW	IGH	2	None	
14	3250	CALPICLRASDTAIYYCARLGAARGHEAFDYW	IGH	2	None	
14	3251	CTRMVITPYYFDYW	IGH	8	None	
14	3252	CARDTTRNQGPNYYYYGMDVW	IGH	3	None	
14	3253	CAREEYALGAFDIW	IGH	2	None	
14	3254	CARGDILTDLFDYW	IGH	11	None	
14	3255	CARDTAVASKHYFYLNYIDVW	IGH	2	None	
14	3256	CAGRVSVTEEIGWIDPW	IGH	7	None	
14	3257	CSRPQTGSRPSAFDIW	IGH	2	None	
14	3258	CVRALGSTVLDYW	IGH	10	None	
14	3259	CARLSSLSGFLQWLFDHW	IGH	40	None	
14	3260	CARRGLQNLEWLFPFDPW	IGH	2	None	
14	3261	CARGETALVAPFDSW	IGH	25	None	
14	3262	CARDTYYYDSSGAAYYYGMDVW	IGH	48	None	
14	3265	CALRIAARPLSASYYYYYGMDVW	IGH	2	None	
14	3266	CQTWGTGIHVVF	IGL	442	C4	7JVC
14	3267	CATGSARPAPRGYYYYYYGMDVW	IGH	9	None	
14	3268	CARASTGDYRDAFDIW	IGH	7	None	
14	3269	CTKDINGDYNYWYFDLW	IGH	2	None	
14	3270	CARGGDSSGYSNWRNFDYW	IGH	2	None	
14	3271	CAKDWLATVVTAFDYW	IGH	6	None	
14	3272	CARENYDDYSFDFW	IGH	4	None	
14	3273	CARSQARRIFGVVTYFDYW	IGH	3	None	
14	3274	CARVTFGYSYGINFDYW	IGH	11	None	
14	3275	CQQLNTYLLTF	IGK	619	C1	7BZ5
14	3276	CARGTGGWFPHVFDFW	IGH	7	None	
14	3277	CAVWQYQPLRSFDYW	IGH	2	None	
14	3278	CARGDGWVAPNWFDPW	IGH	2	None	
14	3279	CARAPGDFLDPPFDYW	IGH	2	None	
14	3280	CASVTRYDSSGYYPDYW	IGH	7	None	
14	3281	CAKEPGDFVGYFDKW	IGH	3	None	
14	3283	CATSYCGSTRCSLSSPSRPFDYW	IGH	8	None	
14	3284	CAKLAGEADYYDDHW	IGH	2	None	

14	3285	CARWSGYCSGDNCPPYGFDIW	IGH	2	None	
14	3286	CARVASIAALAGADAFDIW	IGH	2	None	
14	3287	CASSFQSGTFDYW	IGH	3	None	
14	3288	CARGYSGYDDGLNWFDPW	IGH	2	None	
14	3289	CARPQAEWDLLLPFDIW	IGH	11189	None	
14	3290	CARLNYDILTGYSQYYFDYW	IGH	10	None	
14	3291	CVREDALNRARYFSDYMDVW	IGH	3	None	
14	3292	CARARPYSGSWSPFDYW	IGH	7	None	
14	3293	CAKEGEAVTTIRGYFDYW	IGH	2	None	
14	3296	CTTDLARLWGFAFDIW	IGH	8	None	
14	3297	CARDSGSYSSSVWYFDLW	IGH	5	None	
14	3298	CARFSPLGLNDFWSGYYTGAFDIW	IGH	5	None	
14	3299	CARSEGGVHYYDSSGYWFDYW	IGH	2	None	
14	3300	CAKEGYTYGSLDYFQHW	IGH	2	None	
14	3302	CMQTLPTPPDMYTF	IGK	3	None	
14	3303	CVREPTYCGGDCSSDYW	IGH	5	None	
14	3304	CARDPAYCSSTSCHLGTEPFDVW	IGH	2	None	
14	3305	CARQGPTRYCSSTSCSSSFVAPFDYW	IGH	2	None	
14	3306	CAKNFLGPDHDYW	IGH	2	None	
14	3307	CARDSIVLFVAAHFDSW	IGH	13	None	
14	3308	CAKLSLAAAGINDYW	IGH	4	None	
14	3309	CASGLISSSSWLDYW	IGH	2	None	
14	3310	CARGTYYYDSSALAFDIW	IGH	8	None	
14	3311	CQTWGTGIHGGLWVF	IGL	2	None	
14	3312	CARTLSSNYPSVRAYYYYMDVW	IGH	2	None	
14	3313	CARRKYGDFFQGFFDYW	IGH	6	None	
14	3314	CAKNSLVLMVYPDYW	IGH	2	None	
14	3315	CSHTGYDSSLLTPIYFDYW	IGH	2	None	
14	3316	CARTYHYATTGESYFDYW	IGH	484	None	
14	3317	CIRPTLPGGFDYW	IGH	2	None	
14	3318	CQEYYSTASQF	IGK	9	C4	6W41
14	3319	CARGRAEVGLYKRPYYFASW	IGH	2	None	
14	3320	CARVEGACRGDSCYVDPFDIW	IGH	5	None	
14	3321	CARVKSIFGVIVGSDFDYW	IGH	6	None	
14	3322	CARDQLGDSGNGYGYYYGMDVW	IGH	2	None	
14	3323	CAAVGQSSGWYTGYW	IGH	4	None	
14	3324	CARSFHNSAPDYW	IGH	8	None	
14	3325	CAREPPEMATILSYYYYYGMDVW	IGH	5	None	
14	3326	CAHRQPSTSRGWDEGVFDYW	IGH	2	None	
14	3327	CARFPAAGSLYYYYYMDVW	IGH	2	None	
14	3328	CARLDSGGYTFLSSGRKNYPLDYW	IGH	4	None	
14	3329	CATTPSQYSSSDYW	IGH	5	None	
14	3330	CARDEVTLFSVISGYFDYW	IGH	3	None	
14	3331	CARQRGVGFLEWSMPDAFDIW	IGH	6	None	
14	3332	CARTQQQLSLDYW	IGH	7	None	
14	3333	CARGYGTRQEWFDPW	IGH	2	None	
14	3334	CRVVSADFDSW	IGH	10	None	
14	3335	CAGNYYGSGSYNDYW	IGH	6	None	
14	3336	CAKRGSSSSGRVYDAFDVW	IGH	2	None	
14	3337	CTSLTYYDSGRYSDYW	IGH	39	None	
14	3338	CARGSIFRSFHYW	IGH	15	None	
14	3339	CARGEEWIFGVVYPDSW	IGH	130	None	
14	3340	CARVRSTSAPTSYYYYYMDVW	IGH	2	None	
14	3341	CASWFEGGGNSPFGYW	IGH	2	None	
14	3342	CARDAGAWFKWHFDFW	IGH	2	None	
14	3343	CATTTTHYYDPDDFLFDYW	IGH	2	None	
14	3344	CARLPGIAAAGTTFYFDYW	IGH	6	None	
14	3345	CASREYYDSLDVW	IGH	2	None	
14	3346	CARGGYCRGRSCYSFLLDIDYW	IGH	2	None	
14	3347	CAREGSGSGSGNYFHIW	IGH	2	None	
14	3348	CASGPRPHTAFDIW	IGH	2	None	
14	3349	CACGSSHIPVAKPNNNAFDIW	IGH	4	None	
14	3350	CATSIATARRGDYW	IGH	3	None	
14	3351	CMIWPSNALGVF	IGL	185	None	
14	3352	CAKAMYQDFKALGIRRTDFFDIW	IGH	2	None	
14	3354	CARGVFSTIAARSVFDFW	IGH	4	None	
14	3355	CAKVGAAAGKRGYYFDYW	IGH	7	None	
14	3356	CARETIVVDDSRFDYW	IGH	3	None	

14	3357	CARVYRDSSGWFLIDYW	IGH	4	None	
14	3358	CATQKSLVTVNFDSW	IGH	2	None	
14	3359	CAKVGMIGGTMAAFDIW	IGH	2	None	
14	3360	CARGAVAHLGVYYYYGMDVW	IGH	2	None	
14	3361	CATGYGSGSFYDAFDMW	IGH	19	None	
14	3362	CANHPYGYGYVYYYYMDVW	IGH	4	None	
14	3363	CARGYDSSAYYYVAEDFQPW	IGH	2	None	
14	3364	CAREVEMATLDYYYYYMDVW	IGH	2	None	
14	3365	CARTSCTNGVCPFDPW	IGH	4	None	
14	3366	CARDAGIVVVPAAIAGVNAFDIW	IGH	4	None	
14	3367	CARGLAMFGTVIDVFDYW	IGH	2	None	
14	3368	CARVSMWGCSNGVCHTYDYW	IGH	6	None	
14	3369	CQQLTDYPSFTF	IGK	8	C1	7BZ5
14	3370	CARAVGYCSSTSCYPDAFDIW	IGH	2	None	
14	3371	CQHLNSLPPFTF	IGK	85	C1	7BZ5
14	3372	CARDSWDISGRFDYW	IGH	7	None	
14	3373	CARESIDIVVVPAATFDYW	IGH	11	None	
14	3374	CAREATTGTNFFDYW	IGH	2	None	
14	3375	CARANIAVATGLDYW	IGH	2	None	
14	3376	CARHRDYDFSLFDYW	IGH	16	None	
14	3377	CAKDYGPGYSSGWTSFDYW	IGH	13	None	
14	3378	CAISEDFRGAPDYW	IGH	6	None	
14	3379	CARHAKEQQVSAWFDPW	IGH	4	None	
14	3380	CATISRRGVEAYNEWYFDSW	IGH	4	None	
14	3382	CARDFPAPDFGSGYSDGNWFDPW	IGH	7	None	
14	3383	CARDKVHSLNWNYGPDAFDIW	IGH	4	None	
14	3385	CATDRENSGNYFDFW	IGH	4	None	
14	3386	CARHVGGEFKLLDGSYWFDPW	IGH	3	None	
14	3387	CAKDVSGYYFYGMDVW	IGH	2	None	
14	3388	CAKDPPYCTNGVCYPYFFDSW	IGH	4	None	
14	3390	CARNPFYYDSSSYYPIRKNWFDRW	IGH	2	None	
14	3391	CARDLAERWLQFLPFDIW	IGH	4	None	
14	3393	CATLGGSKRQGDTPFDYW	IGH	2	None	
14	3395	CTTGFTWIRKDGYFYGFDVW	IGH	5	None	
14	3396	CARGEEDFWSYTPNPAVVDYW	IGH	2	None	
14	3397	CAREWDVSQGFLDYW	IGH	3	None	
14	3398	CARAGILLWFGEFRHTTFDYW	IGH	4	None	
14	3399	CARRVPSDGTFDMW	IGH	29	None	
14	3400	CARFRMVGRNCFDSW	IGH	2	None	
14	3401	CARYLVVVVDPTEPETVDYW	IGH	2	None	
14	3402	CTTDSLPGSSGYYWGFDYW	IGH	11	None	
14	3403	CARAYGDYPWGVFRDYW	IGH	2	None	
14	3404	CAKGVRPTVSEAIDYW	IGH	2	None	
14	3405	CARTSPIPPDYDILTGYLDYFDYW	IGH	4	None	
14	3406	CVRDYDILTGFDSW	IGH	2	None	
14	3407	CARMRAMVTAFDIW	IGH	5	None	
14	3408	CARSGWTSIDYYYYYMDVW	IGH	9	None	
14	3409	CATLGGQWLAELDYYYYGMDVW	IGH	4	None	
14	3410	CAKEAAVCTNGVCYRHFDYW	IGH	2	None	
14	3411	CTTNRIAEGWFDPW	IGH	2	None	
14	3412	CARHGRYGGDPFLDYW	IGH	2	None	
14	3413	CAKLVVVAATWGHFSDYW	IGH	42	None	
14	3414	CARLSKPWLTSYFYYLDVW	IGH	2	None	
14	3415	CAIRAKYYYDSSGKHVIDYW	IGH	2	None	
14	3416	CALPILRVEDTAIYYCAKIGGGESMGWYFDYW	IGH	3	None	
14	3417	CAREAYDDSTGYYYADCW	IGH	2	None	
14	3418	CTRDDDSYGDFGAYDYW	IGH	2	None	
14	3420	CQSFDSSNSWLF	IGL	3	C4	7JVC
14	3421	CVRDLPLQYDGSGQGPVFNYW	IGH	5	None	
14	3422	CARPHYYGSGNYFMDWYFDLW	IGH	12	None	
14	3423	CAKDLTGWGTGDDPFDVW	IGH	2	None	
14	3424	CARGLPWDYDLLTGFYTPHFDCW	IGH	5	None	
14	3425	CARDYSSGWYLAFDIW	IGH	19	None	
14	3426	CAKDSSLYYYGSGSLYDACDIW	IGH	16	None	
14	3427	CYSRDSSGSLSWVF	IGL	2	None	
14	3428	CARDHFFSPPEEFWSGYFAYW	IGH	2	None	
14	3429	CARDLQYYDFWNGAGGYFDYW	IGH	6	None	
14	3430	CARDWESSSWYGVGYFDYW	IGH	2	None	

14	3431	CASQGGGAIFDWFDPW	IGH	2	None	
14	3432	CARIDGDSSGNGYYYYGMDVW	IGH	3	None	
14	3433	CQQYDHLPPSLSF	IGK	3	C3	6XDG
14	3434	CALPILTAADTGMYYCARDHPDNDYGDYAGYW	IGH	6	None	
14	3435	CASSPGDTAMDLDYW	IGH	2	None	
14	3436	CARSSAFSSSWSYPDYW	IGH	5	None	
14	3437	CQHYNNYSVMYVF	IGK	2	None	
14	3438	CAKDTGWGYYDSSGYYHDAFDIW	IGH	2	None	
14	3439	CARSRERDGHFGTFDYW	IGH	4	None	
14	3440	CARDLLISLIVLMGDYYYGMDVW	IGH	2	None	
14	3441	CATSLMVPRGYFDYW	IGH	2	None	
14	3442	CARGITTTNQLLWFGELGDSAFDIW	IGH	2	None	
14	3443	CATAPLLGLASDEKWSDYYYYGMDVW	IGH	3	None	
14	3444	CAKEGQLVPENYFDSW	IGH	2	None	
14	3445	CARDRLGSSWVHDAFDIW	IGH	2	None	
14	3446	CARDWFGAKIRYYYGMDVW	IGH	3	None	
14	3447	CARGAIETAGNNWLDPW	IGH	7	None	
14	3448	CAGLITPVATRDYW	IGH	16	None	
14	3449	CARDLTTSYFWSGYYDPGLDYW	IGH	5	None	
14	3450	CARTVPGAREGYFANW	IGH	4	None	
14	3451	CAIDRYSYDSNGYPHISSLDYW	IGH	3	None	
14	3453	CAKDDYDSWSGYRTDSGYPSYFFDHW	IGH	2	None	
14	3454	CRVSTGYSSGWYLDYMDVW	IGH	2	None	
14	3455	CANSRIYSSSSGWTFDYW	IGH	4	None	
14	3456	CAKDPYLYSSSWENDYW	IGH	2	None	
14	3457	CAREDSGSDPVGYYYYMDVW	IGH	4	None	
14	3458	CAASGDFYADKSLFDYW	IGH	2	None	
14	3459	CQVWDSFTNYWVF	IGL	8	None	
14	3460	CARSTIFGVVYFDYW	IGH	5	None	
14	3461	CARDLGYDTSGRPFDMW	IGH	12	None	
14	3462	CALPIWTDIGIRAGFDIW	IGH	2	None	
14	3463	CTTDPSHHCRSRDSACFTRFDYW	IGH	6	None	
14	3464	CARHQSTGSIFGVVIIGLFDYW	IGH	6	None	
14	3465	CDWSSDVCSSDLGDQKPAFSLDYW	IGH	5	None	
14	3466	CARDYRDAAHDAFDIW	IGH	2	None	
14	3467	CAHRFYSSGWYDGDWFDPW	IGH	16	None	
14	3468	CARFPQYNDGWQLFDYW	IGH	11	None	
14	3469	CAKALLATTFTIFGVDYSDDAFDIW	IGH	3	None	
14	3470	CARHIRWFGELSPRYYFDYW	IGH	10	None	
14	3471	CARQRAYSDFDFPPDGFDVW	IGH	6	None	
14	3472	CARDRFNAFWNGYKNDAFDIW	IGH	38	None	
14	3473	CARGCSSTNCYRHYFYMDVW	IGH	2	None	
14	3474	CASAIVVVVAATHHAFDIW	IGH	20	None	
14	3475	CVRDAGRGLRGDYW	IGH	5	None	
14	3476	CAKAVDSGYELVAFDFW	IGH	2	None	
14	3477	CVRVFRLLGRPYYFDRW	IGH	9	None	
14	3478	CARLSAATTGWLDPW	IGH	5	None	
14	3479	CQQSYSTPQRLTF	IGK	1654	C4	6ZDH
14	3480	CASFNRDYGRFSTTLLDYW	IGH	2	None	
14	3481	CARVGELYSHDYW	IGH	6	None	
14	3482	CARDSQIYSRGYFDYW	IGH	4	None	
14	3483	CARQAPGYYDSSGYYYVYW	IGH	4	None	
14	3484	CAKDDNWNYKAFDIW	IGH	2	None	
14	3485	CARNPSSAWVTAVGLDYW	IGH	5	None	
14	3487	CARGGWRRITIFGVVINNWFDPW	IGH	2	None	
14	3488	CARDQDLGYSSGWYSDGAFDIW	IGH	4	None	
14	3489	CAHRRALPYFDSRDSLDFDFW	IGH	2	None	
14	3490	CARTTNGFWNGGHIDYW	IGH	5	None	
14	3491	CAKDTSYHYHSLGCSDYW	IGH	5	None	
14	3492	CSTDLPPGWKFDFW	IGH	3	None	
14	3493	CAKPLEYYHFWGTYPFDYW	IGH	2	None	
14	3494	CAHTGWQAAGTYWRYYFDFW	IGH	39	None	
14	3495	CAKEYSTSYITDAFDIW	IGH	2	None	
14	3496	CGADHGSGSNFVWVF	IGL	59	None	
14	3497	CAHHLGYCSSTSCYMNFAYW	IGH	7	None	
14	3498	CARHRPGDSSGINWFDPW	IGH	6	None	
14	3499	CAKDQTVAGTFDDW	IGH	11	None	
14	3500	CAIAEGGHAEYFQHW	IGH	27	None	

14	3501	CARGGYYYDSSGYHLNYFDYW	IGH	5	None	
14	3502	CARRGRIVVVIAMGAFDIW	IGH	2	None	
14	3503	CVRDRAVAGTFWFDPW	IGH	3	None	
14	3504	CAKTSGTTWVWLDPW	IGH	2	None	
14	3505	CARLPVLISASANAFDIW	IGH	3	None	
14	3506	CSSDLPTIPVGANPVPFDYW	IGH	3	None	
14	3507	CARIGMATNDYWFDPW	IGH	2	None	
14	3509	CARDYDFWSAYRGGHAFDVW	IGH	2	None	
14	3510	CARDYHDFWSGYHTDYW	IGH	7	None	
14	3511	CQQYESYLGWSF	IGK	2	C3	7K8R
14	3512	CARVQFSGAVPDAFDIW	IGH	4	None	
14	3513	CAREVFYGGNSLPFDCW	IGH	2	None	
14	3514	CATMGPNWNDDYYYGMDVW	IGH	2	None	
14	3515	CARAAFIGSGGDAFDIW	IGH	19	None	
14	3516	CARGIVEASRLDYW	IGH	2	None	
14	3517	CASSSGHDPYQFDFW	IGH	2	None	
14	3518	CARNGYYETSGYYFSPHYHYMDVW	IGH	4	None	
14	3519	CVRQETAETHLWLQGGNYYFDLW	IGH	4	None	
14	3521	CARIDYLDSSGYYLGPFDWW	IGH	2	None	
14	3522	CAKDLDIVLTISGAFDIW	IGH	3	None	
14	3523	CAKGDRELWLGDLLPYFDHW	IGH	2	None	
14	3524	CAKDSDQGLTGFHFDSW	IGH	5	None	
14	3525	CAILDIAVEPPAREPSPDAFDIW	IGH	2	None	
14	3526	CARSPLDSSNEIYFDSW	IGH	11	None	
14	3527	CAKVPGDSGNYFDYW	IGH	4	None	
14	3528	CAKGSTNLWSGHYDDYYFNYW	IGH	5	None	
14	3529	CARDSLTGRDYYDKSGYYHYW	IGH	2	None	
14	3530	CGRASGTYFGDYW	IGH	21	None	
14	3531	CAKDLSPYSGSPYAFDIW	IGH	6	None	
14	3532	CAKDSNTAMVNFDYW	IGH	12	None	
14	3534	CTRDLDFWSGYALWDYW	IGH	2	None	
14	3535	CAAGFRDYYDSSGDAFDIW	IGH	2	None	
14	3536	CAQTRKGLRFLEWLPFYWFDPW	IGH	2	None	
14	3537	CARHFYGSGSYYSAFDHW	IGH	2	None	
14	3538	CVRFDVW	IGH	10	None	
14	3540	CAKDDEWALLLPDDYW	IGH	2	None	
14	3541	CASLAPVFNYDSSGYYYVDYW	IGH	7	None	
14	3542	CAKDRIRHCSSTSSCHYYYYGMDVW	IGH	5	None	
14	3543	CARIRGTIFGVVLDYW	IGH	4	None	
14	3544	CTKDRYCVGVNCPLDYW	IGH	18	None	
14	3545	CAHRPTVITGFDYW	IGH	6	None	
14	3546	CASPRRFPNYDFWSGYWPSADYYYGMDVW	IGH	3	None	
14	3547	CAKDIGWELGGAFDIW	IGH	2	None	
14	3548	CAAAIWGDPSDSFDMW	IGH	2	None	
14	3549	CARVEVPAAISYFDYW	IGH	4	None	
14	3550	CAKYSAEQWLLTDFLDYW	IGH	2	None	
14	3551	CARDRDGFFCFDYW	IGH	4	None	
14	3553	CTTDPNYSNYEKDYYYGMDVW	IGH	4	None	
14	3554	CARYAPVVHWLRAYFDYW	IGH	5	None	
14	3555	CAAWDDSLNGHVVF	IGL	6295	None	
14	3556	CARGQLLWFGELLANDYW	IGH	2	None	
14	3557	CARLGITTILGYCSSTSCSPGDAFDIW	IGH	2	None	
14	3558	CTTVALLYDFWSGYYFDYW	IGH	2	None	
14	3560	CVKRPDWDPDYW	IGH	3	None	
14	3561	CAKGPEFSYDSSGSIDYW	IGH	2	None	
14	3562	CAKEVERGYSTSDYW	IGH	3	None	
14	3563	CVRDLQGVNSGWYGHDAFDIW	IGH	2	None	
14	3564	CAKAGVGYTSSFAYW	IGH	8	None	
14	3565	CATHRGYSSSWDAFDYW	IGH	3	None	
14	3566	CALPICRLRSEDTAVYYCATGARRGYYFYYMDVW	IGH	11	None	
14	3567	CARHTGSLGGLYFDHW	IGH	4	None	
14	3568	CVRLDYDSSRVDYW	IGH	2	None	
14	3569	CAAGEWLQNLDYYYYGMDVW	IGH	4	None	
14	3570	CAHPATAYDSGFDYW	IGH	2	None	
14	3571	CAKAAVKLWLLRNDAFDIW	IGH	4	None	
14	3572	CARGGNLEWSSLQRNWFDSW	IGH	2	None	
14	3573	CATGVTLVTCFDSW	IGH	11	None	
14	3574	CARAKGVWESYRHYLDYW	IGH	4	None	

14	3575	CARDQGYDFWSGSLDYW	IGH	9	None	
14	3576	CVKDTVGYCSRTINKCGGDASEIW	IGH	3	None	
14	3577	CARADSRLNNRRPFDYW	IGH	3	None	
14	3578	CARGGWARGLVPAASGFWFDPW	IGH	5	None	
14	3579	CAKDGGEWDLVSFDSW	IGH	2	None	
14	3580	CARDSMVRGGFDYW	IGH	11	None	
14	3581	CAKDSSPGEDLSAYDAFDIW	IGH	2	None	
14	3582	CASLLRFLEWASPSHGAFDIW	IGH	5	None	
14	3583	CAKAISQLNYFYYGMDVW	IGH	4	None	
14	3584	CALPISAVYYCARTLRTHTVTTTGVDYW	IGH	2	None	
14	3585	CTTDEIGLRGGNWFDPW	IGH	8	None	
14	3586	CARGRSYGRHFDYW	IGH	154	None	
14	3587	CAKSDSEEQWLVPFDYW	IGH	2	None	
14	3589	CAKGNWDSSGYYTSYYFDYW	IGH	2	None	
14	3590	CARQEYYYDSSGYYREGFFDYW	IGH	3	None	
14	3591	CARDLAVRGVIVRRRFDCW	IGH	2	None	
14	3592	CAKDVWPVAGTGFDYW	IGH	9	None	
14	3593	CARGLYVVAGIGLDYW	IGH	2	None	
14	3594	CARQMGLLGGGITGTIYPYAFDIW	IGH	2	None	
14	3595	CARHLALAGDEYFQPW	IGH	3	None	
14	3596	CAATIEVGARFDAFDIW	IGH	2	None	
14	3597	CARAGGYCSSTSCFDYW	IGH	8	None	
14	3598	CTGSSRGYNYGFQPYWFDPW	IGH	5	None	
14	3599	CARGEGDQLELRWALSTYVGNYYYYYYMDVW	IGH	2	None	
14	3600	CQQSYFTKNFGTF	IGK	9	None	
14	3601	CATEASVDYYDSRYGDYW	IGH	15	None	
14	3602	CARSGSAGYDVWSGHFDLW	IGH	2	None	
14	3603	CAKDLGYEISRTFDYW	IGH	2	None	
14	3605	CARDCPDYGSGSCYFDYW	IGH	2	None	
14	3606	CARDWGGFWSPYFYW	IGH	2	None	
14	3607	CARGGYSSRRRIYYYYGMDVW	IGH	6	None	
14	3608	CAKGKSGQWLVGGPFDVW	IGH	4	None	
14	3609	CAKNGGGQYSTHGFDYW	IGH	9	None	
14	3610	CARIRVSHKGAFDIW	IGH	2	None	
14	3611	CVKDLQTFDETSGYLDDW	IGH	2	None	
14	3612	CASYDSSGYYLDFDYW	IGH	11	C4	7JVC
14	3613	CAKLNGGPGYYDSSGYYNAGSDYW	IGH	6	None	
14	3614	CATLMGPRPFYYYGMDVW	IGH	4	None	
14	3615	CARPGGRSGWSAFWYFDLW	IGH	2	None	
14	3616	CAKDHSEAVAMMGWHFDLW	IGH	2	None	
14	3617	CATDSDSILGSSGYPIRRHFDYW	IGH	21	None	
14	3618	CARLPETATNLESLFDDW	IGH	124	None	
14	3620	CVRSYFDLRSDFYSAFDIW	IGH	2	None	
14	3622	CARGGWVRGVISPYYYYYMDVW	IGH	2	None	
14	3623	CARIPTHDLRAFDIW	IGH	7	None	
14	3624	CAKHSGFGELLNCFDCW	IGH	11	None	
14	3625	CARLVLLWFGEPYDAFDIW	IGH	2	None	
14	3626	CKVVIERFDIW	IGH	2	None	
14	3627	CASGSASIALDAYDIW	IGH	2	None	
14	3628	CAAADVQTWLYAFDFW	IGH	4	None	
14	3629	CATTYYDPSGRGGDYW	IGH	6	None	
14	3630	CAHTRTYQSDLASYNDAFDIW	IGH	4	None	
14	3631	CASSIVGATRYTFRYW	IGH	2	None	
14	3632	CVRVSRGRWLGLDYW	IGH	3	None	
14	3633	CARGEPSSLDLWNGPTPFDDW	IGH	3	None	
14	3634	CAKFASTYSSSSVDYW	IGH	7	None	
14	3635	CASSRYYDSGRAPSPRYFDLW	IGH	2	None	
14	3637	CARQPGVYDSSGYDAFDIW	IGH	3	None	
14	3638	CALPISRVDDYTNSFDSW	IGH	2	None	
14	3639	CARDLVPFGDGYNYDAFDIW	IGH	2	None	
14	3640	CARESQTNYGDYVLYGWFDPW	IGH	4	None	
14	3641	CATRYGDSDPIDYW	IGH	2	None	
14	3643	CANHHLGYGVLYYFDYW	IGH	2	None	
14	3644	CVRARVRGLIVIDAFDFW	IGH	6	None	
14	3645	CAKDITPNYYDSTEAWAFDIW	IGH	2	None	
14	3646	CARIMSETVQMAPYFDNW	IGH	2	None	
14	3648	CARSYNWNYALDFDHW	IGH	11	None	
14	3649	CAREGEATMIGRGFDYW	IGH	5	None	

14	3650	CAKEEYDIVTGSPVLFDHW	IGH	3	None	
14	3651	CARDGSCGGDAYIDYW	IGH	2	None	
14	3652	CARGLTMIVVVQISGFDYW	IGH	9	None	
14	3653	CAKGPYGSGSYYMNYW	IGH	2	None	
14	3654	CARCPGQWLVQSGGIFDYW	IGH	2	None	
14	3655	CAGSGNPGDYW	IGH	11	None	
14	3656	CAKVHSSSWGVFDYW	IGH	3	None	
14	3657	CVRDASAGAYYYMDVW	IGH	4	None	
14	3658	CARDDYDDSESYTYNYYGMDVW	IGH	4	None	
14	3659	CARRRTASTMVRGVTPSWYFDLW	IGH	7	None	
14	3660	CARGRLERDHHYYIDVW	IGH	2	None	
14	3661	CAYYKSNYFFDAFDMW	IGH	2	None	
14	3662	CATARSRGGNYYGSSGFDYW	IGH	9	None	
14	3664	CARRSRTGYLDYW	IGH	6	None	
14	3665	CARVRILTGYYLGWFDPW	IGH	2	None	
14	3666	CAKDIEVMKTVTSYAPYQAFDPW	IGH	6	None	
14	3667	CAKAGGRLASYFDSW	IGH	2	None	
14	3668	CTTDLLVMELWRRGVDYW	IGH	12	None	
14	3669	CAKGIAARSGPFDYW	IGH	3	None	
14	3671	CARDFMATQGYYNYYMDVW	IGH	58	None	
14	3672	CTTQVPGAILSDYW	IGH	2	None	
14	3673	CARHSHSGTYMGGLWYFDYW	IGH	3	None	
14	3674	CASGGGSGWYFEFGYW	IGH	4	None	
14	3675	CARDGGIYEETDYW	IGH	3	None	
14	3676	CATARLRRFDPW	IGH	4	None	
14	3677	CARGRSPLMAVYMDVW	IGH	2	None	
14	3678	CARQVTGVMAADPLDYW	IGH	4	None	
14	3680	CARATPSGGWLSFDYW	IGH	2	None	
14	3681	CALPIWFGELFGAFDIW	IGH	2	None	
14	3682	CARPGGSGTQGGWFDPW	IGH	3	None	
14	3683	CARGPDYSTSKTSFDYW	IGH	5	None	
14	3684	CAKDWGPLYSSCWLDYW	IGH	2	None	
14	3685	CTRPDHLKLNAFDIW	IGH	7	None	
14	3686	CARNVGSSWYEIQWYFDLW	IGH	6	None	
14	3687	CTTGPNDYW	IGH	81	None	
14	3688	CAREGEFTYYYGSGSSYYFDYW	IGH	2	None	
14	3689	CARDRRSAVAGTVSDYW	IGH	9	None	
14	3690	CMRWFRQYQPASQFDPW	IGH	6	None	
14	3691	CTRDVRGGEYYFDYW	IGH	11	None	
14	3692	CTTSHLVAADYW	IGH	5	None	
14	3693	CAKSRGGSGNGDPFRALISLSSGRPWYFDRW	IGH	7	None	
14	3694	CQVFNLYPQGPVTF	IGK	4	None	
14	3695	CAKSLHIVVVTAIFDYW	IGH	2	None	
14	3696	CARELRNSDTSGPPDAFDIW	IGH	5	None	
14	3697	CATHSSSWFEWDYW	IGH	9	None	
14	3698	CAYSKPDYDYVWGSYRPSAIYYYYYGMDVW	IGH	2	None	
14	3699	CASLKRATPSYYYYMDVW	IGH	11	None	
14	3700	CAHRPGAAGGGWFDPW	IGH	2	None	
14	3701	CASPLNPQYDYVWGTYRQLGGPIYYYALDVW	IGH	4	None	
14	3702	CTRDPFLEWFSSFDLW	IGH	4	None	
14	3704	CAAELQTQDYDTSGSYYLDDYW	IGH	4	None	
14	3705	CARAYSYYYDSSGYYAQDWYFDLW	IGH	2	None	
14	3706	CAKDGAYMGRRTYAFDFW	IGH	167	None	
14	3707	CARDPSDGSGWSQADWFDPW	IGH	10	None	
14	3708	CARSPRSTYDFWSGLLPVDYW	IGH	5	None	
14	3709	CMQRIHLSRFTF	IGK	30	None	
14	3710	CARGGGNYLIKNDYW	IGH	4	None	
14	3711	CAKDSAYCSGGSCYGFDYW	IGH	3	None	
14	3712	CQHSDSFPWTF	IGK	34	C3	7K8R
14	3713	CAKLATCSGGSCYSADNDYW	IGH	4	None	
14	3714	CVKDKSPGGADYW	IGH	2	None	
14	3715	CARHWDFNPFEYW	IGH	4	None	
14	3716	CANAEEMYWYDGGAFPNWFDTW	IGH	3	None	
14	3717	CAKDLGGATVGDLDYW	IGH	3	None	
14	3718	CARSSSPIAVAGSDYW	IGH	2	None	
14	3719	CDWSSDVCSSDLAAIYYCVRGAFLEWFHGFDIW	IGH	4	None	
14	3720	CASLAGRSFNNYWYFDLW	IGH	2	None	
14	3721	CARAEVRGYRARGHSYGQKPNYFDYW	IGH	2	None	

14	3722	CAKVRVGDLRRGFDYW	IGH	2	None	
14	3723	CAMFTSGNYFIFDYW	IGH	4	None	
14	3724	CRAVADDYWYFDLW	IGH	9	None	
14	3726	CAPYDYDSSGYIPDYW	IGH	2	None	
14	3727	CARVDMPSKYILDDSWFDLW	IGH	2	None	
14	3728	CASIIVVVPAASWGDDAFEIW	IGH	2	None	
14	3730	CTTDSSRKYYDILTGYYWIDYW	IGH	20	None	
14	3731	CARDVVVVPAAMGVDYW	IGH	2	None	
14	3732	CTKLLVGIELATNFHYW	IGH	3	None	
14	3733	CARMRGFCTKGFDPW	IGH	4	None	
14	3734	CAKSVLGSPIFDAFHIW	IGH	2	None	
14	3735	CAKVSDSGYGDTRDYW	IGH	4	None	
14	3736	CARDRPPGFTIVAYYYYYGMDVW	IGH	2	None	
14	3737	CARHPLRFLEWFFAFDIW	IGH	10	None	
14	3738	CAKAPTSMDWYFDLW	IGH	4	None	
14	3739	CASLYSSGGRLDYW	IGH	4	None	
14	3740	CARDPLAVSSSSDYFYYMDVW	IGH	2	None	
14	3741	CARVGWRTGSSRQYYYYGMDVW	IGH	2	None	
14	3742	CASHYPGLNDFRNGFYGYGWSFDNW	IGH	8	None	
14	3743	CARDWTPVSRPYYYYYYYMDVW	IGH	2	None	
14	3744	CARAPTYCGGDCSPYWYFDLW	IGH	4	None	
14	3745	CAKDGGGAEGAPPRTYFFDSW	IGH	8	None	
14	3746	CVKDQIENYYDNRGYSLQKNYFDWW	IGH	2	None	
14	3747	CARRGPGGDYVWHFDSW	IGH	2	None	
14	3748	CARHPLTGTHRFPHYYYYYMDVW	IGH	2	None	
14	3749	CARVPRIGYCSSTSCYVVDYW	IGH	4	None	
14	3750	CQYRSNWPPGVLTF	IGK	2	None	
14	3752	CARASYYYDSSGFLRDYYYYYYMDVW	IGH	7	None	
14	3753	CLQHDNFPLGGFTF	IGK	31	C3	6XDG
14	3754	CARLKCYSATCHLREHWFDPW	IGH	2	None	
14	3755	CMQSIKTPFFTF	IGK	2	None	
14	3756	CQQYDDFPWSF	IGK	13	C3	7K8R
14	3757	CHQTFSLPFTF	IGK	2	None	
14	3758	CARGTTIFGVVRRWFDPW	IGH	3	None	
14	3759	CAGGLSGMEVPFDYW	IGH	3	None	
14	3760	CAGSKSAVYDSTYFDYW	IGH	6	None	
14	3761	CARHHVVGASDYW	IGH	9	None	
14	3762	CATINYYDSSGYYFPFDYW	IGH	4	None	
14	3763	CASSLISGVDAFDIW	IGH	2	None	
14	3764	CATYYINDPNYFDFW	IGH	4	None	
14	3765	CARVNKGDSSGHYNYFYYGMDVW	IGH	4	None	
14	3766	CARDLGYDPNGRPYDAFAMW	IGH	20	None	
14	3767	CAKGIIAVAANGPDYW	IGH	8	None	
14	3768	CANSLMELFDYW	IGH	2	None	
14	3769	CTTGNRGYDFVGGTYRHFDYW	IGH	2	None	
14	3770	CARAPYYYDSSGFPLPYYFDYW	IGH	6	None	
14	3772	CAKDAYYYDSRRDCYFDLW	IGH	3	None	
14	3773	CAKGSLDSSGYVTGADAFDIW	IGH	2	None	
14	3774	CAKDMSPVAGISPVDYW	IGH	2	None	
14	3775	CARDYVAAMGLLDYW	IGH	2	None	
14	3776	CARVAITYSSSWYLDYW	IGH	3	None	
14	3777	CWCLIFRPFTGDYW	IGH	22	None	
14	3778	CARLTQGLEWELSIDYW	IGH	24	None	
14	3780	CARQSYHVLTGYFNFDYW	IGH	8	None	
14	3781	CAKERITGDFAVLLDYW	IGH	2	None	
14	3782	CARDGRYCGGDCYDDYYYGMDVW	IGH	2	None	
14	3783	CARPPPHSNYYSVFDLW	IGH	6	None	
14	3784	CGRVLKVAGTRWFFDLW	IGH	3	None	
14	3785	CARDCSSTSCYTMSDYW	IGH	5	None	
14	3786	CARDEEGWFGELRGGFDPW	IGH	6	None	
14	3787	CARELPDIAPRSIGRSDDGFDIW	IGH	4	None	
14	3788	CARPRHDSSGYYAIFDYW	IGH	30	None	
14	3789	CARVYYYGSGYPFDHW	IGH	5	None	
14	3790	CASSSITIFGVVIMAAFDIW	IGH	4	None	
14	3791	CARVSRADFWVDYW	IGH	3	None	
14	3792	CVKDVGTSGPHGFDRW	IGH	6	None	
14	3793	CATQISGYRPRDYW	IGH	2	None	
14	3794	CAKGEDSSSWYSTPFLDYW	IGH	4	None	

14	3795	CARPFGLLSMDWYFDLW	IGH	2	None	
14	3796	CAHRLKWQLLYGPWFDPW	IGH	3	None	
14	3797	CSCPPTYYYDSGGFDAFDIW	IGH	2	None	
14	3798	CARDQYAGNNYHPNYFDSW	IGH	2	None	
14	3799	CARHEALVVETSRGTFDIW	IGH	2	None	
14	3800	CAKLVTEIAAAGTADYW	IGH	5	None	
14	3801	CVKGDYNNYVGLLDYW	IGH	3	None	
14	3802	CAKARSPNFRFDAPQVW	IGH	2	None	
14	3803	CAKVARAAAGPFDYW	IGH	2	None	
14	3804	CARDGSGIGQGAFDIW	IGH	2	None	
14	3805	CVLLRQWLAPFDYW	IGH	2	None	
14	3806	CARHIRQFYGDSLVSNPARPPYWYIDVW	IGH	2	None	
14	3807	CARFSMNAYSTSWSNFDYW	IGH	85	None	
14	3809	CAREWPITTACLDYW	IGH	2	None	
14	3810	CASGYGSGSYPSPFGYW	IGH	4	None	
14	3811	CASYIYYYGGTDYGYFDNW	IGH	13	None	
14	3812	CARNPEVVITEFYYFDSW	IGH	3	None	
14	3814	CVRDVACTGVTCLDYW	IGH	11	None	
14	3815	CAAYHDFSPYHFDYW	IGH	12	None	
14	3816	CARDGGRYPLLYWFDQW	IGH	2	None	
14	3817	CDWSSDVCSSDLDTAVYYCARGDQKPAFSLDYW	IGH	3	None	
14	3818	CAKNLHVGTDFVPLDYW	IGH	24	None	
14	3819	CARVRYSSSWYRSYYFDYW	IGH	6	None	
14	3820	CTRHPRDRRVVPRANNRFDPW	IGH	4	None	
14	3821	CAAYHYYPYGMDVW	IGH	2	None	
14	3822	CARDPITMVRGVTPAFDIW	IGH	4	None	
14	3823	CAAWHDRLNGPIF	IGL	2	None	
14	3824	CARELLSSGWYGFDYW	IGH	23	None	
14	3825	CALPISTPKCYKRFYMDVW	IGH	2	None	
14	3826	CASAARYHDDTDGSGLYFFDYW	IGH	8	None	
14	3827	CQVWDSDSDTAIF	IGL	24	None	
14	3828	CATLLQTRGFDIW	IGH	2	None	
14	3829	CQQFHNFPSSITF	IGK	8	C3	6XDG
14	3830	CARAPPMITFGGVIAYYFDYW	IGH	14	None	
14	3831	CARDLMTTVTTGWFDPW	IGH	7	None	
14	3832	CVRGSHRVDYW	IGH	15	None	
14	3833	CARLIRKIAPDLSGAFDIW	IGH	4	None	
14	3834	CARHSDYYGSGTHYPIDYW	IGH	3	None	
14	3835	CALPICARGPRGYNSAWLDSW	IGH	2	None	
14	3836	CARAYHFSGGDYW	IGH	3	None	
14	3837	CARGYCSGYRCYFTFYFDLW	IGH	8	None	
14	3838	CASGTYGDYPSAFDIW	IGH	4	None	
14	3839	CARVLAARTIIDYW	IGH	35	None	
14	3840	CAKASVDDYFDYW	IGH	11	None	
14	3841	CARGTRAVAGLFDYW	IGH	2	None	
14	3842	CTSDTAMAAYYYMDVW	IGH	4	None	
14	3843	CARVIWGGATHFDYW	IGH	3	None	
14	3844	CVRDFNWFDSW	IGH	9	None	
14	3845	CAKVGIMTGTLAPFDIW	IGH	8	None	
14	3846	CLQHNSWPPDAF	IGK	21	C1	7BZ5
14	3847	CAKITGYSSSWYGSAFDIW	IGH	2	None	
14	3848	CAREKGVDLRRVPYDYW	IGH	11	None	
14	3849	CARLLPYLGYSSGWTNDDYW	IGH	2	None	
14	3850	CARHGPEDGVWSGYIDFW	IGH	3	None	
14	3851	CATDFSSSWRNNWFDPW	IGH	4	None	
14	3852	CARVDCSSIISCPLFDYW	IGH	7	None	
14	3854	CASRLYSSSWYVSYW	IGH	2	None	
14	3855	CASQSHYYYDSNWFDPW	IGH	4	None	
14	3856	CARDRNLDIEYFFDFW	IGH	5	None	
14	3857	CAGGLGYTFGYGSFYAMDVW	IGH	2	None	
14	3858	CARGVRYEDEGYWYLDYYYYYMDVW	IGH	2	None	
14	3859	CQQRSGWPVF	IGK	427	None	
14	3860	CARERRSIAAAGYFDYW	IGH	3	None	
14	3861	CQVWDSSSDHPYYVF	IGL	62	None	
14	3862	CAREIRFLEWLKAFDIW	IGH	2	None	
14	3863	CVRGGMAGPANNYGFGNDAFDIW	IGH	2	None	
14	3864	CVRYPHCSSGRCSSYFDYW	IGH	3	None	
14	3866	CAAGGNHQDAFEIW	IGH	2	None	

14	3867	CAKDYYDSSGYSDYAFDIW	IGH	6	None	
14	3868	CHYYGSSPGAF	IGK	13	C1	7K8N
14	3869	CARDRREQQLKQNFYFYYGMDVW	IGH	3	None	
14	3870	CAKDQPERTNPYDILTGYLGAYYYYGMDVW	IGH	43	None	
14	3871	CARAPRPRDYYDSSGPFDYW	IGH	3	None	
14	3872	CARDRLLLPTTRDFWFDPW	IGH	17	None	
14	3873	CARDKLIGRQLPPVGVWFDPW	IGH	2	None	
14	3874	CATLISAGGPSDAVDIW	IGH	16	None	
14	3875	CARDLTYYDLWQVPSYYYYGMDVW	IGH	3	None	
14	3876	CARRIGDYPFDFW	IGH	17	None	
14	3877	CARRRNYYDSSGYPLDAFDIW	IGH	2	None	
14	3878	CARQTNADLAAKYFDYW	IGH	3	None	
14	3879	CARIMMLVLKGWVDPW	IGH	2	None	
14	3880	CDWSSDVCSSDLRRGYDFYYMDVW	IGH	7	None	
14	3881	CAKEDHRDGPNWFDPW	IGH	2	None	
14	3882	CATKGFGPVQGWFDPW	IGH	3	None	
14	3883	CARDKDYYGSGSYSLFDYW	IGH	3	None	
14	3884	CARDLTYQLTKFYFDSW	IGH	2	None	
14	3885	CASWGYSSSWYPGNASYYFDYW	IGH	2	None	
14	3886	CANVFSSGGGDYW	IGH	7	None	
14	3887	CARILRDDSTGYYYYYFDYW	IGH	10	None	
14	3889	CAHRPGVDNYALGSYISEDYFDFW	IGH	2	None	
14	3890	CARHGYSTSWYYFDYW	IGH	8	None	
14	3891	CAREHSQRSNYDYW	IGH	2	None	
14	3892	CARAGYSSSSRKQRYYYYGMDVW	IGH	10	None	
14	3893	CARDVRSVDIVAAAFDIW	IGH	2	None	
14	3894	CASFVGNYGAEDYYYYGMDVW	IGH	4	None	
14	3895	CARALLYNWNYGGGNWFDPW	IGH	7	None	
14	3896	CATQEYSGVHKFDPW	IGH	2	None	
14	3897	CARDKSGSYNENYYYYYMDVW	IGH	4	None	
14	3898	CARVNLDIPYYFDYW	IGH	22	None	
14	3899	CARAKYQLLYDDPFNYFDYW	IGH	2	None	
14	3900	CASLRSMATILSAFDYW	IGH	14	None	
14	3901	CARAKLPYYYDGSGFRADAPDIW	IGH	3	None	
14	3902	CATNQDYDSSGYWGDAFDIW	IGH	2	None	
14	3903	CARDEKFDDSGYYYSYYLDYW	IGH	19	None	
14	3904	CARDYFDSSGNYYVDYW	IGH	7	None	
14	3905	CAKSRTFGLGTYHNGPDAFDVW	IGH	2	None	
14	3906	CARDRTYCSTTTCYNYFDSW	IGH	2	None	
14	3907	CARAKDMIVVVTPSAFDIW	IGH	2	None	
14	3908	CARVQNFDWLPTGYFDYW	IGH	2	None	
14	3909	CAKDLGGHCTNGVCNYYYYGMDVW	IGH	2	None	
14	3910	CVKDMGIAVDTVNLDYW	IGH	6	None	
14	3911	CARGGWKGSSSRYYYYYMDVW	IGH	23	None	
14	3912	CARSTASFRIYHNLDYW	IGH	438	None	
14	3913	CARRNGHDYGSGSRARGLDDAFDIW	IGH	2	None	
14	3914	CAKIPGSGSPNDYW	IGH	4	None	
14	3915	CAKLAEVHAYDFWSAYYNYFDSW	IGH	18	None	
14	3916	CARDMDQQLAPPMAFDVW	IGH	4	None	
14	3917	CARDTSSSSYYYYYYGMDVW	IGH	2	None	
14	3918	CVKMRGATYQWYFDLW	IGH	5	None	
14	3920	CARDSSSEEFYDISGYLDAFDVW	IGH	2	None	
14	3921	CARQHNSGLYFFDYW	IGH	8	None	
14	3922	CAKVWRYSSGWHYFENW	IGH	38	None	
14	3923	CARSSISSVAWTMGAYDAFDLW	IGH	3	None	
14	3924	CARDQQRSYESTAYIDVFDIW	IGH	2	None	
14	3925	CAKMELGTYDSRKNDYW	IGH	5	None	
14	3926	CTTDLHDYGDSDYW	IGH	13	None	
14	3927	CARDGPASYTNGCLDYW	IGH	2	None	
14	3928	CARPFGRLITMIVNTPDAFDIW	IGH	2	None	
14	3929	CAKLDSSSWPSDYW	IGH	2	None	
14	3930	CAKAPPVNWNYVYYYYMDVW	IGH	2	None	
14	3931	CARTPYCDTTSCFFDYW	IGH	3	None	
14	3932	CLSAGSSGAYRIF	IGL	7	None	
14	3933	CARMSGWATQHSGELLYDTYYFDYW	IGH	14	None	
14	3934	CVPKDSSGYFLFDYW	IGH	2	None	
14	3935	CARVVPPLGGITTILYAFDIW	IGH	8	None	
14	3937	CVRHLETEFDYW	IGH	3	None	

14	3938	CARDLRTYCGVDCLFDYW	IGH	2	None	
14	3939	CARFLHGILGFDYW	IGH	2	None	
14	3940	CARDSLYVKPPRGSSGYLDYW	IGH	2	None	
14	3941	CTKLSAGKGDWFDPW	IGH	4	None	
14	3942	CARTERGWLPPTVGDYW	IGH	2	None	
14	3943	CASGSSGWYETIDYW	IGH	4	None	
14	3944	CAHRPSYNDFWSGYYDRYYFDYW	IGH	2	None	
14	3946	CARANGGGGTVYYFYMDVW	IGH	3	None	
14	3947	CARPSRYCSGGSCYPAFDYW	IGH	4	None	
14	3948	CVRPRPFSAARFYIDFW	IGH	2	None	
14	3949	CARGPLYDGRGYYHRPFDIW	IGH	3	None	
14	3950	CARVSTERDFVLGVYGMVAFDAW	IGH	2	None	
14	3951	CAKGKLGIVSAPFDYW	IGH	7	None	
14	3952	CARGPDYYDPEWYFDLW	IGH	3	None	
14	3953	CANGGTYYYDSPPFDIW	IGH	2	None	
14	3954	CARMMCSTSSERFFDYW	IGH	4	None	
14	3955	CAKDTTSGWPYYYYGLDVW	IGH	3	None	
14	3956	CASTILSDWNAYAFHIW	IGH	2	None	
14	3957	CARGGVAARPEAFDYFYMDVW	IGH	2	None	
14	3958	CAKDIGGEYIYQYMDVW	IGH	2	None	
14	3959	CAGHDSLFDIW	IGH	13	None	
14	3960	CAATYYSESSALWFAFDFW	IGH	2	None	
14	3961	CARAGGYCSGGSCSYPWFDPW	IGH	2	None	
14	3962	CTSELFPRYYYLGYW	IGH	2	None	
14	3964	CAKAPDSSGSQHDYW	IGH	2	None	
14	3965	CARHLGYSSGWPNWFDPW	IGH	4	None	
14	3966	CARTGFDNSGFYYFCDYW	IGH	2	None	
14	3967	CARDLESSPLGVLAAMEGYFYYMDVW	IGH	23	None	
14	3968	CASSESSGWDYFNYW	IGH	16	None	
14	3969	CAKLSGRDYYDSNNDYW	IGH	5	None	
14	3970	CARLIRVTYDFWSGSTPPSYYMDVW	IGH	23	None	
14	3971	CARVSGYYDSSGYWRNDAFDIW	IGH	2	None	
14	3972	CAKDSSWGYSYGFFDYW	IGH	8	None	
14	3973	CTTYTPPPGYCRSTSCSSVDYW	IGH	3	None	
14	3974	CAQGYTGYW	IGH	12	None	
14	3975	CARGNADFWSAYPIDYW	IGH	8	None	
14	3977	CARHSTYYDLSSGSENWFDPW	IGH	2	None	
14	3978	CARVRSSKYGAAVESPSYYYYMDVW	IGH	2	None	
14	3979	CARGSKSSFRYCTNGICLQEPFDSW	IGH	10	None	
14	3980	CARSPSIGASQNDYW	IGH	2	None	
14	3981	CARPIGSGNYDAFDIW	IGH	3	None	
14	3982	CATGSPLYYDFWSGHNWFDPW	IGH	2	None	
14	3983	CAKVWEIVLEPGAFLDYW	IGH	47	None	
14	3984	CARLIGYYDSSGYYWFDYW	IGH	2	None	
14	3985	CASTVYYDFWSGYPSPPYFDYW	IGH	17	None	
14	3986	CATGRVRIPESYHQYMDVW	IGH	2	None	
14	3987	CVRDYYDSSGYSGNSYFYYMDVW	IGH	2	None	
14	3988	CARDLTTESKDGGAYDYFDHW	IGH	2	None	
14	3989	CAAQRRPYDAFDIW	IGH	12	None	
14	3990	CARDLNHLDYW	IGH	72	None	
14	3991	CARVDRRAVPAAIIFQHW	IGH	3	None	
14	3992	CVILENMYAAFEIW	IGH	7	None	
14	3993	CARGETYYSFDIW	IGH	3	None	
14	3994	CARDPYYSSYW	IGH	6	None	
14	3995	CARETFYYASGTTTGGFYYYAMDVW	IGH	2	None	
14	3996	CSTYTPLRVGAVFNYHYYLDVW	IGH	2	None	
14	3997	CARSRVPFQLWSFVSGDFDIW	IGH	2	None	
14	3998	CASGFWSGYTDAFDIW	IGH	3	None	
14	3999	CARYRDIVLMVYDSSGIDYW	IGH	2	None	
14	4000	CARGPAPGYSYDYGFEYW	IGH	2	None	
14	4001	CARCGWRYCSGGSCYRRYEAFDIW	IGH	3	None	
14	4002	CAKDGGPYYYDSSGYLLPYYFDYW	IGH	2	None	
14	4003	CAKFIRGLGHSFDYW	IGH	2	None	
14	4004	CAKLATSNGITGTTGDYW	IGH	7	None	
14	4005	CARHQEGHYFDTSNYYYVDSW	IGH	2	None	
14	4006	CAKDQTPIWVPPSFDYW	IGH	2	None	
14	4007	CARTHVVRSSEWFFADRHDAFDVW	IGH	2	None	
14	4008	CAKDIGAVAAYFDYW	IGH	15	None	

14	4009	CAKELNYDFWSGYSPYYLDWW	IGH	5	None	
14	4010	CAKDPHRHYCSGSDCFDNW	IGH	9	None	
14	4011	CARLRCTSTTCYLDYYRNYYMDVW	IGH	3	None	
14	4012	CATDRSPTYWYFDLW	IGH	2	None	
14	4013	CARPSSAYDYFFDHW	IGH	2	None	
14	4014	CARERFSRYTNPFIVLAKKGATSSYFAMDVW	IGH	2	None	
14	4015	CAKVLSGYGEITDYW	IGH	3	None	
14	4016	CARSLSKYTWTTLYFDYW	IGH	7	None	
14	4017	CAREMSEGVLLWFGELGFDYW	IGH	2	None	
14	4018	CAKDAGGGIRVVAIYDYW	IGH	2	None	
14	4019	CARVEELEYCTSRSCSDYYYYYYMDVW	IGH	3	None	
14	4020	CARRNGFSSGPFDHW	IGH	2	None	
14	4021	CDARWNDEIQAFDFW	IGH	2	None	
14	4022	CAKDAVLLPALRLNNHPYYMDVW	IGH	6	None	
14	4023	CATLRAGYGDIPFDYW	IGH	5	None	
14	4024	CARVVYNYPYTEAFDMW	IGH	2	None	
14	4025	CVKDIEKRDGNNYFYYGMDVW	IGH	2	None	
14	4026	CARDDFYYDGQDAFDIW	IGH	12	None	
14	4027	CARAKGRISLRYFDWLFHAFDMW	IGH	280	None	
14	4028	CAKDTAYHFHTGGIDYW	IGH	2	None	
14	4029	CAKRPMVRGVYGEFDYW	IGH	2	None	
14	4030	CAKDVRYCNSATCYDGMDVW	IGH	2	None	
14	4031	CARLFDLGTAAVRWFDPW	IGH	5	None	
14	4032	CARPYSGSYGSYFDYW	IGH	63	None	
14	4033	CATGGGQQYDPNIFGYW	IGH	19	None	
14	4034	CASGAYDSSSGSSGFAFDIW	IGH	2	None	
14	4036	CARDKMSTVDYFQYW	IGH	9	None	
14	4037	CAKHLTGTTSSYDAFDSW	IGH	2	None	
14	4038	CARAHGWLYSFDPW	IGH	5	None	
14	4039	CVKESVAGTMPKTFDVW	IGH	4	None	
14	4040	CATYSRSSYHDSFDIW	IGH	11	None	
14	4041	CQSADTSLTRGVF	IGL	4	C3	6WPS
14	4042	CARDYRYYGSSGYYHDHW	IGH	3	None	
14	4043	CAKGSGYDYRDHFDSW	IGH	2	None	
14	4044	CTTVELSFGEFTFDPW	IGH	8	None	
14	4045	CAATLVGATEDDYW	IGH	4	None	
14	4046	CQQFNTYPSF	IGK	4060	C3	7K8R
14	4047	CANWGSRDCSSTSCPLYYYYMDVW	IGH	9	None	
14	4048	CAKESTNYYDSSGYNYGHYFDYW	IGH	193	None	
14	4049	CARSGSGWSLVTPFDYW	IGH	4	None	
14	4050	CARGVPTVAGLNPFDYW	IGH	2	None	
14	4051	CARHIFAGSSWSRWYCFDYW	IGH	4	None	
14	4052	CAKPYRERVQIPDAFDIW	IGH	4	None	
14	4053	CARDVVSVGAANYYYGMDVW	IGH	2	None	
14	4054	CTRRLDPCSDFWSGYCLVRPFDYW	IGH	5	None	
14	4055	CAKGSGILYSSAFNWFDPW	IGH	3	None	
14	4056	CARGVATITGGTKPDYW	IGH	12	None	
14	4057	CARRRLLWFGDPFDIW	IGH	6	None	
14	4058	CATEGRYFDWLPFDYW	IGH	2	None	
14	4059	CARDAFRAVTPELLIDYW	IGH	2	None	
14	4060	CAREYCSGTTCYSGFAYW	IGH	10	None	
14	4061	CAKDTAPSIYDAFDIW	IGH	12	None	
14	4062	CQHYNSYPFAF	IGK	4896	C3	7K8R
14	4063	CARQREGMVTDGFDIW	IGH	8	None	
14	4064	CARLYGGPAVVGDYYFDFW	IGH	21	None	
14	4065	CAGGLVRASSFDYW	IGH	2	None	
14	4067	CDWSSDVCSSDLAEWDLLLPFDIW	IGH	18	None	
14	4068	CALPIYLQMDSLRAGDTAIYYCAKDSRLFLLGADYYFDYW	IGH	4	None	
14	4069	CARGAYTSSSYFDRW	IGH	4	None	
14	4070	CARDRTYDFWSGYYTGGYFDSW	IGH	3	None	
14	4071	CVKDRVFRADAFDVW	IGH	2	None	
14	4072	CARKDPFGVDDYW	IGH	2	None	
14	4073	CVSVDIGSALVLDYW	IGH	9	None	
14	4074	CVRHGLTGDHYFDLW	IGH	5	None	
14	4076	CHHYNNWPLLTF	IGK	3	None	
14	4077	CATWTPTVKVVDYW	IGH	2	None	
14	4078	CTKDISGDGAAFDIW	IGH	4	None	
14	4079	CVRACSSLDCYIDYW	IGH	10	None	

14	4080	CARPKRRDVRDAFDIW	IGH	14	None	
14	4081	CAKLSGYSYANDYW	IGH	32	None	
14	4082	CTTEGDDARPIDYW	IGH	6	None	
14	4083	CARVYTAGASTPMATSWWYFDIW	IGH	4	None	
14	4084	CAKANVGSYIGDFFDIW	IGH	10	None	
14	4085	CARIRADSSSWYVLGGEKKSHYYFDYW	IGH	50	None	
14	4086	CAKDITPTVTTADYW	IGH	11	None	
14	4087	CARLLPRGDSSGYYYKTYYWYFDLW	IGH	2	None	
14	4088	CARDMAAAWVPGGMSDYW	IGH	6	None	
14	4089	CARADLNPQLRWFDPW	IGH	2	None	
14	4090	CASSSRPGVAAAIDYW	IGH	17	None	
14	4091	CTTEERFLEWLMSSFDYW	IGH	2	None	
14	4093	CARVGTYYYDRDAFDLW	IGH	9	None	
14	4094	CARDKMGLLQWSTRKREFDYW	IGH	2	None	
14	4095	CARDFTGYYDYGSNPAYWFFDLW	IGH	4	None	
14	4096	CTRGPPIAVTATDAFDIW	IGH	7	None	
14	4097	CARDALILLWFGDGNWFDPW	IGH	3	None	
14	4098	CAHSDGGAAATATFDYW	IGH	3	None	
14	4099	CMQALQTPPTFGPGTKVDIKRTF	IGK	5	None	
14	4100	CAKVRELGSSYWYFDLW	IGH	4	None	
14	4101	CARARLRDPYPDYW	IGH	2	None	
14	4102	CAKKQLTPYSTSSIVTPRKQIKRVDAFDVW	IGH	3	None	
14	4103	CARLRGDDFYSGYFDFW	IGH	38	None	
14	4104	CAREIRTTVRGVMINWFDPW	IGH	2	None	
14	4105	CAKGDYDFWNVFSIESRRSFDYW	IGH	4	None	
14	4106	CARHARLGAVGTRFFDYW	IGH	8	None	
14	4107	CIRDRQLASHTYNFDYW	IGH	9	None	
14	4108	CGKDGTSFYEGSGYYLDLW	IGH	5	None	
14	4109	CARGRQYCSGGSCYDAFDIW	IGH	2	None	
14	4110	CARGSSAIGAALGWWGRQFKGNWFDPW	IGH	3	None	
14	4114	CAKDISAVAAQRFDYW	IGH	2	None	
14	4115	CAKDISPYDSSDFYPQDYYYGMDVW	IGH	2	None	
14	4116	CARGSHTHKIYDILTGHFFDSW	IGH	45	None	
14	4117	CQQYNFSPRMYTF	IGK	10	C1	7K8N
14	4118	CARAQDWSYGYYFYMDVW	IGH	4	None	
14	4119	CARELTSLTVFHFDHW	IGH	2	None	
14	4120	CAKYSSSWYGVYFGYW	IGH	2	None	
14	4121	CAHTYDSRAVDVW	IGH	2	None	
14	4122	CARDGATVLPFDFW	IGH	2	None	
14	4123	CARHSDVKQIDYW	IGH	2	None	
14	4124	CARVGLEYWGSVYHFDYW	IGH	2	None	
14	4125	CQQYNSYFRTWTF	IGK	12	C3	7K8R
14	4126	CARCQWEPLLFDFW	IGH	2	None	
14	4127	CARGSVVRGGDYWFDPW	IGH	2	None	
14	4128	CAKDSSPINHYMDVW	IGH	18	None	
14	4129	CAKLIDSSGYEGPFDIW	IGH	2	None	
14	4130	CAKVYRRDIVVVPAAHDYW	IGH	2	None	
14	4131	CDWSSDVCSSDLSDTAIYYCARLGAARGHEAFDYW	IGH	6	None	
14	4132	CARHDTSRESGWGTWFDPW	IGH	5	None	
14	4133	CAKKTSAWYDYW	IGH	2	None	
14	4134	CARHPRPSRNYYYYYMDVW	IGH	2	None	
14	4135	CAKDKEWELLRGPNFDYW	IGH	2	None	
14	4136	CTRAAGITETTRPGYYYGMDVW	IGH	2	None	
14	4137	CATCARNTYYFDSSGLDAFDIW	IGH	2	None	
14	4138	CVRDRAVSNKGGDYW	IGH	4	None	
14	4139	CVRLAPRITIFGEFAYW	IGH	3	None	
14	4140	CATDILPLYCSSTSCYGRRYFDYW	IGH	2	None	
14	4141	CALPICVYYCARRLIAFDIW	IGH	2	None	
14	4142	CARGCGSNICRTKYNDYW	IGH	3	None	
14	4143	CARDPQLAGGEYSYGYLDYYYGMDVW	IGH	2	None	
14	4144	CVSGETTTLPGVFAYW	IGH	6	None	
14	4145	CARDLEVASIRDPFPVYYFDSW	IGH	2	None	
14	4146	CARGRGYYDSSDYNPDYYYYGLDVW	IGH	3	None	
14	4147	CAKGGVVTAIRLPYFDYW	IGH	2	None	
14	4148	CARDSGYNDYRVDYW	IGH	2	None	
14	4149	CARGPLAIAPWYFEYW	IGH	2	None	
14	4150	CAKDILGAVTNAFDYW	IGH	2	None	
14	4151	CQQYYSNALFNF	IGK	5	C4	6W41

14	4152	CTRVPPQGRGPPFDYW	IGH	2	None	
14	4153	CARTINSGWTFDFW	IGH	2	None	
14	4154	CASDYGDYVRRYYYGMDVW	IGH	5	None	
14	4155	CARINFRYSSSSWKYYFDYW	IGH	2	None	
14	4156	CALPILSLKLSSVTAADTAVYFCARPQAEWDLLLPFDIW	IGH	2	None	
14	4157	CARWAGQHSDYIPLFDYW	IGH	2	None	
14	4158	CASFYSGYDYNYYYYMDVW	IGH	2	None	
14	4159	CATWDDSLNSPLAF	IGL	4	None	
14	4160	CAKDGARYYGGNGAFDIW	IGH	13	None	
14	4161	CAHRQITGTTACTFDYW	IGH	15	None	
14	4162	CARHSSQLLWLGGSGGFDIW	IGH	63	None	
14	4163	CARLHSSSFLGLRGAFDIW	IGH	4	None	
14	4164	CARECLGGGDCFDYW	IGH	15	None	
14	4165	CARVSVSGVVAATHYFDYW	IGH	2	None	
14	4166	CSDTSCIDWYFDLW	IGH	3	None	
14	4167	CARWGSGPISYFDYW	IGH	4	None	
14	4168	CAKGLYYDSSGYPTFFDYW	IGH	4	None	
14	4169	CAKDTTGYRSSNYRIEVYYFDSW	IGH	4	None	
14	4170	CALPISRLRRGNDAFDIW	IGH	3	None	
14	4171	CARTFLSTTWYEPFDYW	IGH	2	None	
14	4172	CVTHYDFWSGHHYFDYW	IGH	13	None	
14	4173	CAKSLMVGRPGDDLFDYW	IGH	2	None	
14	4174	CARHHANQYYHSDSPFAYW	IGH	7	None	
14	4175	CAKATPRYYYYGMDVW	IGH	6	None	
14	4176	CASLCGSDCYGIWFDPW	IGH	2	None	
14	4177	CATVSPIYGDYVGWIFDYW	IGH	5	None	
14	4178	CARDRRIILYSSSLNDAFDMW	IGH	2	None	
14	4179	CAKDEGDCTTTSCYFYW	IGH	39	None	
14	4180	CAKDRCSSTTCSSDYW	IGH	3	None	
14	4181	CARDGMRQLDLDAFDFW	IGH	3	None	
14	4182	CAKDYDSSGYSHFDYW	IGH	9	None	
14	4183	CARDKRTTYYYDIDEGPAADYIQHW	IGH	2	None	
14	4184	CALSDYLWGSSPRIDYW	IGH	7	None	
14	4185	CARGALDYTDYVDYGDVDHYYYYMDVW	IGH	3	None	
14	4186	CATSLGLWNDYYLLGTYFDYW	IGH	13	None	
14	4187	CAREAGWTMGFDQW	IGH	20	None	
14	4188	CARHSHNYDSSTYYGEVDYYYYYRMDVW	IGH	11	None	
14	4190	CARDFGPGTYSSGWYWFDPW	IGH	3	None	
14	4191	CAKDGTNIFGVTSLGNFDYW	IGH	3	None	
14	4192	CARVITASGTIDYW	IGH	6	None	
14	4193	CAKDMFPGHIPAAGASHAFDIW	IGH	2	None	
14	4194	CAKASYNDIQFRAFDFW	IGH	4	None	
14	4195	CARDSSWEGVDYW	IGH	8	None	
14	4196	CARDGDIRSSTSCDYW	IGH	2	None	
14	4197	CARHGYISQQLNWFDPW	IGH	10	None	
14	4198	CAKDVSYSNYGFRFDPW	IGH	2	None	
14	4199	CARDHIGEYYDILTGYPLYYYYYGMDVW	IGH	2	None	
14	4200	CARDGEDSPFFDLW	IGH	2	None	
14	4201	CQSADSSGTYWVF	IGL	2948	None	
14	4202	CAHRRLDGYNWADIFDYW	IGH	2	None	
14	4203	CSGEESKGVDYW	IGH	2	None	
14	4204	CARRRTYSRRWLHAFDMW	IGH	4	None	
14	4205	CATGPFCSGIDCYFDYW	IGH	2	None	
14	4206	CAKGSYCSGGSCYSYTVKGLYYFDYW	IGH	2	None	
14	4207	CAKDVDGYNDVGFDYW	IGH	2	None	
14	4208	CARDLGYCSGGNCYSPDYW	IGH	4	None	
14	4209	CARGARITMQVVDHFDYW	IGH	2	None	
14	4210	CALPISAVYYCAKDQLLAPYPYYYYHGPDVW	IGH	3	None	
14	4211	CARNGDFGVIISGSFDYW	IGH	2	None	
14	4212	CVRSSDRFFDFW	IGH	5	None	
14	4213	CARVVEGSGTGSTFDYW	IGH	2	None	
14	4214	CAKHPFTGESLEGYFESW	IGH	5	None	
14	4215	CARESGLITAETTIPFDYW	IGH	3	None	
14	4216	CARDIRYCSSSGCNNPWFDPW	IGH	264	None	
14	4218	CARDTSSTPVAKPNNDAFDIW	IGH	7	None	
14	4219	CAKDPHGLFCSGSSCFDDW	IGH	2	None	
14	4220	CQQSFSISWTF	IGK	173	None	
14	4221	CARHFGDFILTTYYPFDYW	IGH	4	None	

14	4222	CARTQHNPLWWPSHFDYW	IGH	2	None	
14	4223	CARDGGQSTAWPRHSFFDYW	IGH	2	None	
14	4224	CTTGLDYYSDTSDFSGKYCFDHW	IGH	5	None	
14	4225	CASSYSGSYPTDYW	IGH	2	None	
14	4226	CATVEMATINAFDIW	IGH	2	None	
14	4227	CAKSPGYCSSTSCPSEFDPW	IGH	2	None	
14	4229	CAREASGLAAYYYYGLDVW	IGH	8	None	
14	4230	CARVRGELWFIYYYYMDVW	IGH	2	None	
14	4231	CACLHTIPDTFDIW	IGH	15	None	
14	4232	CAKGTYFYDTNGYFGYIDFW	IGH	5	None	
14	4233	CAREPTSYYDTSGYSLPHTYFDSW	IGH	2	None	
14	4234	CARSYYDTVGYYPFDNW	IGH	2	None	
14	4235	CASQEGGSSSWTPPWNYYYGMDVW	IGH	3	None	
14	4236	CARDYCGGDCYSRHYYNYGMDVW	IGH	2	None	
14	4237	CAGELSSSWDYW	IGH	40	None	
14	4238	CARGRVDEYYDAFDIW	IGH	2	None	
14	4239	CARDLQGSLGSYYEFYGMDVW	IGH	9	None	
14	4240	CARDYYDSSGYYNWFDPW	IGH	46	None	
14	4241	CAKDMGLDGSSFDYW	IGH	6	None	
14	4242	CARLDLGDCSSTSCSIDAFDIW	IGH	3	None	
14	4243	CASERGYDFWSGSPFDYW	IGH	40	None	
14	4245	CARVKEFSYGLGDYW	IGH	4	None	
14	4246	CARDDFRDSNFDAFDIW	IGH	14	None	
14	4247	CARSFFRGYCNSASCYVGAFDIW	IGH	2	None	
14	4248	CARRYNIGLGRTFDIW	IGH	2	None	
14	4249	CARLVTTSSYYYYMDVW	IGH	2	None	
14	4250	CHFDRPVDLLDYW	IGH	2	None	
14	4251	CAREGSGYPPTNDAFDIW	IGH	2	None	
14	4253	CARNRGGSRRGYFDYW	IGH	11	None	
14	4254	CAKDPQFIVARPYYFDYW	IGH	2	None	
14	4255	CAKAGGYIVVVPDYW	IGH	4	None	
14	4256	CAKEDYDSTVFDYW	IGH	57	None	
14	4257	CAKAFRRPGSEGDAFDIW	IGH	4	None	
14	4258	CAKGGYDYVWGSYAYDYW	IGH	2	None	
14	4259	CARDKGPHYYGSGRTNYYYYMDVW	IGH	2	None	
14	4260	CARKATGYCGGGSCFDDAFDIW	IGH	4	None	
14	4261	CQYFGSSPPTTF	IGK	13789	C1	7K8N
14	4262	CARDPRVSGITLYLDFW	IGH	3	None	
14	4263	CARTLMIFRISLPVFDHW	IGH	7	None	
14	4264	CAREGGGIAVAPYYFDHW	IGH	6	None	
14	4265	CARHMTTVTKEWFDPW	IGH	3	None	
14	4266	CASRRDYDFWSGSSGAFDIW	IGH	4	None	
14	4267	CAREAYDFSKRYYFDYW	IGH	3	None	
14	4268	CAKEDKLGIFHPLDYW	IGH	6	None	
14	4269	CSRGEELDSGSYMWFDPW	IGH	8	None	
14	4270	CARLSYDSDGSSLGAFYFDSW	IGH	30	None	
14	4271	CATDSMGFSSSFYRFDIW	IGH	2	None	
14	4272	CASLIGYLPDYW	IGH	7	None	
14	4273	CAKPRYPNTYYYANRLDAFDIW	IGH	12	None	
14	4274	CAKDIFGDTNGFDIW	IGH	4	None	
14	4275	CTRDYRPGLNYFDFW	IGH	7	None	
14	4276	CARDRVENDYVWGSYRPYWYFDLW	IGH	2	None	
14	4277	CARVQYDGSGYYETPDYW	IGH	20	None	
14	4278	CATVPRYCSSTSCYSGAFDIW	IGH	4	None	
14	4279	CAKDINPVAGAIDYW	IGH	12	None	
14	4280	CARHVDSDSWSGMLYYFDYW	IGH	2	None	
14	4281	CARVKGSADGSYYVDYW	IGH	2	None	
14	4282	CAREGDSSREYILDYW	IGH	2	None	
14	4283	CARTPDSVSWFDYW	IGH	3	None	
14	4284	CARSLSGLLELLSICFEYW	IGH	15	None	
14	4285	CATVQSWGLHTLVENWFDPW	IGH	8	None	
14	4287	CVRDKAQQRSWSDGIDVW	IGH	6	None	
14	4288	CAIGIVEVPTAKFGAFDFW	IGH	32	None	
14	4289	CAREPPYDFWSGYYPYYYYMDVW	IGH	5	None	
14	4290	CARGSAIFGVVSTETFWYFDLW	IGH	8	None	
14	4291	CGRRPIAFDIW	IGH	10	None	
14	4292	CASSDYSSAWVDYW	IGH	3	None	
14	4293	CAREGLGAVTTEIAFDIW	IGH	4	None	

14	4294	CARDLWAAAGTPHYYYMDVW	IGH	4	None	
14	4297	CAKVFMVRGVITGSTDYW	IGH	2	None	
14	4298	CAKDKLAVAEYYFDYW	IGH	12	None	
14	4299	CATGKDWGTRWFFDLW	IGH	8	None	
14	4300	CATRSPGVMNWFFDLW	IGH	2	None	
14	4301	CALPILRAGDTAVYYCAKDSRLFLLGVEYYFDYW	IGH	5	None	
14	4302	CATHTLERYFDYW	IGH	19	None	
14	4303	CDWSSDVCSSDLTAVYYCARGSAIYYDSSGYFDYW	IGH	4	None	
14	4304	CARERGDRVGPPGNWFDPW	IGH	2	None	
14	4305	CARDTYMSSWYWFDPW	IGH	115	None	
14	4306	CARDLGSGFLEWLSPFDYW	IGH	2	None	
14	4307	CARYSVAGYYYYYMDVW	IGH	8	None	
14	4308	CARPGKRNLPTPGTGIYYFDYW	IGH	5	None	
14	4310	CAKDMGSIGWYYFDYW	IGH	18	None	
14	4311	CARDPGKESVFGVYYYYYMDVW	IGH	5	None	
14	4314	CARASPVAGTRGLLRIFDHW	IGH	4	None	
14	4315	CARAMRYGGNSDAFDIW	IGH	2	None	
14	4316	CAAVSTLDYNSAAFDYW	IGH	2	None	
14	4317	CARRRAGLRTWYLGSFNWFDPW	IGH	5	None	
14	4318	CARDSDPVKDEARAFDYW	IGH	2	None	
14	4319	CTRDAASFCGNGVCPPYHFDYW	IGH	2	None	
14	4320	CQHYDNLLSINF	IGK	24	C3	6XDG
14	4321	CVVGERSSAAPSLDYW	IGH	2	None	
14	4322	CARSLWGPHRTPPGYLDYW	IGH	5	None	
14	4323	CAKDIFPIPRSYFDYW	IGH	5	None	
14	4324	CAKAFVDSHDYW	IGH	21	None	
14	4325	CQAYNMWPRGAF	IGK	5	C3	7K8P
14	4326	CARIHVAAALAFDYW	IGH	191	None	
14	4327	CAKVYYYDTGVDWYFDLW	IGH	4	None	
14	4328	CAGSYSSGPYYYYYYMDVW	IGH	2	None	
14	4329	CARSNHGAIVVDEDAFDIW	IGH	4	None	
14	4330	CVRYRDNWKGLVRWYFDLW	IGH	4	None	
14	4331	CARVDRDYGFDYW	IGH	15	None	
14	4332	CAHRPSPPRGMGTLFDYW	IGH	31	None	
14	4333	CTRAWVFFDFWSGFTSGAGTYLYMDVW	IGH	5	None	
14	4334	CALLLRRIVVVAAPAFDIW	IGH	4	None	
14	4335	CARDFDRSGYKPYYYYMDVW	IGH	2	None	
14	4336	CARLPHENYYYYYYMDVW	IGH	6	None	
14	4337	CARISLGYCSSTSCYSGFDPW	IGH	6	None	
14	4338	CARIRVAEDMSDAFDIW	IGH	2	None	
14	4339	CAKMDTSNPDYFEAW	IGH	19	None	
14	4340	CAKGFPWELTDLHYYYGMDVW	IGH	27	None	
14	4341	CTRDSTIFGVATPDYW	IGH	3	None	
14	4342	CARDSLRCSSTSCYWWFDPW	IGH	4	None	
14	4343	CATHSSSWDSEYFQHW	IGH	2	None	
14	4344	CASVGDYSNGGVDYW	IGH	3	None	
14	4345	CARIAETTGYHHDAFDIW	IGH	5	None	
14	4346	CQKLHSYPPYPF	IGK	4	C1	7BZ5
14	4347	CAHFSIFAVFGDDAFDVW	IGH	4	None	
14	4348	CARASWFGELIFDRW	IGH	4	None	
14	4349	CARDPLSGVYYYYYGMDVW	IGH	2	None	
14	4350	CATDPSSSLLGWFDPW	IGH	9	None	
14	4351	CASTYTAMVPFDYW	IGH	3	None	
14	4352	CAKARGRYCNSISCYSGLDYW	IGH	2	None	
14	4353	CARESMITFGGPSYYFDYW	IGH	2	None	
14	4354	CARESAGGTESYYYNGMDVW	IGH	16	None	
14	4355	CARDQSIVGAMGGTTYYFDYW	IGH	2	None	
14	4356	CARDGTSLTMIVVVITGAIDYW	IGH	4	None	
14	4357	CARARLDSSGYPSLYFDYW	IGH	2	None	
14	4358	CATVRSLYYDSSVSYPSRVFDFW	IGH	2	None	
14	4359	CARVRYCGAECDTLDWYFDLW	IGH	4	None	
14	4360	CARVQYSYYYAMDVW	IGH	152	None	
14	4361	CARAQSPSYISLGYYYGMDVW	IGH	6	None	
14	4362	CARQLFSSSSGTFDLW	IGH	3	None	
14	4363	CAKGEMATILSVFDYW	IGH	3	None	
14	4364	CASLGVGSERGYFDYW	IGH	6	None	
14	4365	CAKHLSALHDYNENLRAFDIW	IGH	3	None	
14	4366	CAKNREWLVRWGGYFDYW	IGH	2	None	

14	4367	CARVPTYRFDSSGYYWGYYFDFW	IGH	8	None	
14	4368	CVKDLHKKFGTEAPDVFDIW	IGH	4	None	
14	4369	CSCIAAAGIGFDPW	IGH	2	None	
14	4370	CARDPIWGFGYYYYMDVW	IGH	13	None	
14	4371	CAHTLIQLRFLQWRSPEYYFDYW	IGH	2	None	
14	4372	CARDYTINWNEYYFDYW	IGH	6	None	
14	4373	CARDSKAGGFFLGPIFDPW	IGH	2	None	
14	4374	CASGRGYCSSTTCLANYFDYW	IGH	5	None	
14	4375	CARHEVYDSSGYYFLDYW	IGH	4	None	
14	4376	CVRDGVYGINNGYFDYW	IGH	2	None	
14	4377	CARQGGYCSDTICYGYFDYW	IGH	6	None	
14	4378	CAKDYSPGIAASAIFEYW	IGH	2	None	
14	4379	CARDRALSGSYYGLWDYW	IGH	4	None	
14	4381	CAKDQIRSSSGYYAFDIW	IGH	2	None	
14	4382	CARHNYETSSPDYW	IGH	2	None	
14	4383	CARYSSSWDFFEYW	IGH	2	None	
14	4384	CARDWNYAGYPDVFDIW	IGH	3	None	
14	4385	CARGDFDWLFPPFDYW	IGH	3	None	
14	4386	CARGKGMILRAISNAFDIW	IGH	6	None	
14	4387	CARQWAGMEPLSDYW	IGH	2	None	
14	4388	CAKDSRRDGYTFWQFDYW	IGH	3	None	
14	4389	CARTLAAAGSSNDYW	IGH	3	None	
14	4390	CAKDRGLAALGPDYW	IGH	4	None	
14	4391	CARATPAENPDYW	IGH	8	None	
14	4392	CQQLNNYSQVTF	IGK	40	C1	7BZ5
14	4393	CGRLHRRKGDYMDVW	IGH	2	None	
14	4394	CARAQYYDLLTGYPTEYFYYMDVW	IGH	2	None	
14	4395	CARTLIKSGYSSSWYGFDYW	IGH	4	None	
14	4396	CARIVRNYYDSSGYYDLDYFDYW	IGH	2	None	
14	4397	CAKDRDSGSGSYTWGYFDYW	IGH	5	None	
14	4398	CARVPYDYGNYAYPDFW	IGH	4	None	
14	4399	CARADYSNTYIDTW	IGH	2	None	
14	4400	CARLNHCTHGVCYTEYYYYYMDVW	IGH	5	None	
14	4401	CAKDGATVFGVRDYYYSYMDVW	IGH	2	None	
14	4402	CARDPGTRVFDYW	IGH	27	None	
14	4403	CARVGEERRSRDYYMDVW	IGH	3	None	
15	1	CAKDLQRLRSYYYGMDVW	IGH	2	None	
15	2	CARGIIRAWFDPW	IGH	6	None	
15	3	CARGKRRVIVLMVYEYYYGMDVW	IGH	5	None	
15	4	CTRAMDTAMIAWDLSDGYVFDIW	IGH	8	None	
15	5	CAKDSSSRAARAWFDPW	IGH	2	None	
15	6	CSSHAGSNNLLF	IGL	5	C2	7BWJ
15	9	CARDTEVGSGWSTDYFDSW	IGH	2	None	
15	10	CARDPVPHYYDSSGYTLGAFDIW	IGH	2	None	
15	11	CARGSGDYAFWSGLGYFFDPW	IGH	2	None	
15	13	CARLGGWSEYFQHW	IGH	7	None	
15	14	CIRRAQDGSHWYTDLW	IGH	48	None	
15	15	CAREGIPPSGARHFFDYW	IGH	2	None	
15	16	CAHASIAVVVDYW	IGH	2	None	
15	17	CAKDRSRYSGYVGEYYFDYW	IGH	2	None	
15	18	CTRDSVLYYFDYW	IGH	4	None	
15	19	CQHYNSYSSWAF	IGK	27	C3	7K8R
15	20	CARPRRPDSSGYLPMDYW	IGH	2	None	
15	21	CARDRGKFPDTFDIW	IGH	6	None	
15	22	CARDTYCSGGSCYSLIRHNWFDPW	IGH	2	None	
15	23	CAKDVTSGESMGLDYW	IGH	2	None	
15	24	CQQRSKWPPECSF	IGK	3	C1	7BZ5
15	25	CAKDRPDPWARITMIVVRSDGMDVW	IGH	2	None	
15	26	CAKDLFTMVRGKPPGLYDYW	IGH	2	None	
15	27	CASSARILPVPFDYW	IGH	2	None	
15	28	CARRSSSGGTWYCDYW	IGH	4	None	
15	29	CALVSTVTTTFYFASW	IGH	3	None	
15	30	CTFGRLWLDSW	IGH	2	None	
15	31	CAAGTFPTGRNAFDIW	IGH	6	None	
15	32	CAKDNRYSSGWYHIFDYW	IGH	6	None	
15	33	CESRDSSGTQLVF	IGL	239	None	
15	34	CARETLYFYDTTEHYYYGMDVW	IGH	2	None	
15	35	CARSLRGSFYGVVAGPYYFDYW	IGH	52	None	

15	36	CVRETAGSGYPHDYW	IGH	2	None	
15	37	CARDRQLWFRSQIDYW	IGH	2	None	
15	38	CAREAYCSRNSCPFDPW	IGH	3	None	
15	40	CARVGYSYGYGMDVW	IGH	4	None	
15	41	CQQYGGSSYSF	IGK	37	C1	7K8N
15	43	CLQFNSFPQITF	IGK	3	C3	7K8R
15	44	CTKDRYCPSIYCPTDYW	IGH	2	None	
15	47	CARDYREEALTGRLKWFDPW	IGH	7	None	
15	49	CARLPEPLYYYYFDLW	IGH	2	None	
15	50	CASQDSSGYFDW	IGH	4	None	
15	51	CAVWDDSLSGRVF	IGL	236	None	
15	52	CARHGIAALGYYYYGMDVW	IGH	2	None	
15	53	CARGGVAAKKGQFDYW	IGH	2	None	
15	54	CAKDVVVVTAIRSYFDYW	IGH	2	None	
15	56	CARDSEPYYYDSSGYHDYW	IGH	9	None	
15	58	CARGDFGMIVPDYW	IGH	4	None	
15	59	CQYNNWPRYTF	IGK	153	None	
15	60	CARYVLRFLESSAFFDYW	IGH	3	None	
15	61	CARGERVALPNSHYNDFW	IGH	2	None	
15	63	CARDFLGSSGWTPMDYW	IGH	4	None	
15	65	CARVRESWFGADDAFDIW	IGH	2	None	
15	66	CAKVDDQDSYGFRSYYYYGMDVW	IGH	2	None	
15	67	CARQKGSGSPLVDYYYYGMDVW	IGH	2	None	
15	68	CARDQFSSGFPPWVDYW	IGH	2	None	
15	69	CASGIAARRLDYW	IGH	4	None	
15	70	CARDTGIAAAGTFDYW	IGH	9	None	
15	71	CATSLGWWALFDPW	IGH	20	None	
15	72	CARHRINYYDSSGPGGYFDYW	IGH	4	None	
15	73	CATRGSRDFWSGYSFDYW	IGH	2	None	
15	74	CARRGYDTPYYNFWFDPW	IGH	2	None	
15	76	CATSPAVVKDNWFDPW	IGH	2	None	
15	77	CATLGYCSSTSCYMAYYYGMDVW	IGH	2	None	
15	78	CNVFAPYSSSWSPFDYW	IGH	2	None	
15	80	CARRFELERVFDYW	IGH	4	None	
15	81	CTTDPALKQVVVIGAFDIW	IGH	2	None	
15	82	CTRYQQRPRLYYYYYYGMDVW	IGH	2	None	
15	83	CATVVTPTDSYFDNW	IGH	2	None	
15	84	CQAWDISTASYVF	IGL	8	None	
15	85	CARDKGDSGYDFYYGMDVW	IGH	2	None	
15	87	CARDHYGSSWSDEYFQHW	IGH	2	None	
15	88	CARGVSGSRYFDKW	IGH	2	None	
15	89	CVKGMAVAGSIYLAMDVW	IGH	2	None	
15	91	CARDWVVISGDYYYGMDVW	IGH	2	None	
15	93	CARDSLSGDTSMEVYYYYGMDVW	IGH	2	None	
15	94	CAKDLNARVLIDPFDIW	IGH	4	None	
15	95	CASTGDLFDVFDYW	IGH	2	None	
15	96	CAKDGAPSPYYYGKTDYFDYW	IGH	2	None	
15	98	CARGGHSSGYYFDYW	IGH	5	None	
15	99	CARDPHDSSGYYLPWYFDLW	IGH	2	None	
15	103	CAKDFGYSSPAAPWFDPW	IGH	2	None	
15	104	CARQRAYCINDCYPGEADYW	IGH	2	None	
15	107	CQVWDIISDHQGVF	IGL	4	None	
15	109	CARGDRELPLDWIDPW	IGH	7	None	
15	110	CAKPGEVRYNWFDPW	IGH	4	None	
15	111	CARDAQHRGFDYW	IGH	2	None	
15	114	CARSAFYGSSGYYYDYW	IGH	6	None	
15	115	CAKGQTAIDPYSYYYGMDVW	IGH	2	None	
15	116	CARGPPNSWSSPGDYW	IGH	2	None	
15	117	CARDLESSGWYDFDPW	IGH	2	None	
15	122	CARSLLLAAAGQRDDAFDIW	IGH	2	None	
15	124	CAKDSRLFLLGADYYFDYW	IGH	7	None	
15	125	CARDRGPFNWYFDLW	IGH	2	None	
15	126	CQRYNRAPYTF	IGK	71	C3	7K8P
15	127	CARGLAGYSSSFDYW	IGH	2	None	
15	128	CDWSSDVCSSDLFDIW	IGH	3	None	
15	129	CAKVPSETLKRITIFGVVISEYYFDYW	IGH	2	None	
15	130	CARDSNYFGSNYYDVFDIW	IGH	4	None	
15	131	CAKLREDSSGYYYYYW	IGH	4	None	

15	132	CGRRQQQSSYWYFDLW	IGH	3	None	
15	134	CARDPSSSAFDIW	IGH	474	None	
15	136	CLQYDNWPQMF	IGK	2	C3	6XDG
15	137	CLQDDNYPLSF	IGK	265	None	
15	138	CARVGVGATHGYYFDYW	IGH	2	None	
15	141	CAKVIVVPAAADYW	IGH	2	None	
15	142	CARVPTAMFNPPQGYYYGMDVW	IGH	2	None	
15	143	CHQYNNWRTF	IGK	7	None	
15	144	CQSYDNNNYVVF	IGL	3	C4	7JVC
15	145	CAHSDSSGYSYYYYYGMDVW	IGH	3	None	
15	149	CARDLRWGDRDDYGDYADYYYYGMDVW	IGH	2	None	
15	150	CATGPAVAGTSNWFDPW	IGH	4	None	
15	151	CYRNPDGDYW	IGH	28	None	
15	152	CQQYDSRSLFTF	IGK	26	C3	6XDG
15	154	CARGPSGSNFFVGDYW	IGH	14	None	
15	156	CARGVPAASDYW	IGH	11	None	
15	159	CARGEMYSSSSHCFDYW	IGH	2	None	
15	160	CARTYILTGYFFDYW	IGH	2	None	
15	161	CETWDSDTQLF	IGL	6	None	
15	163	CAKAPYCSGGSCFPRLFDYW	IGH	6	None	
15	165	CVKTGPNMYYLDTARFSSFDFW	IGH	2	None	
15	166	CAKDSIHNYYYGMDVW	IGH	7	None	
15	167	CTRDHYDYVWGSYRYEDYW	IGH	2	None	
15	169	CARDPTVPAAIVRYYYGMDVW	IGH	2	None	
15	170	CARDLYDSTGYRYLDLW	IGH	6	None	
15	172	CQQRANWPSVIF	IGK	2	None	
15	174	CAKGGSSWFVDYW	IGH	2	None	
15	175	CARNGFLEWHVRFNYW	IGH	2	None	
15	176	CAHSSHIAGWFDPW	IGH	2	None	
15	177	CARAMITFGGVIVGFFDYW	IGH	4	None	
15	178	CGRRTYDSNGHYYFDYW	IGH	38	None	
15	179	CATAGSYRFDSW	IGH	2	None	
15	180	CARDPDDYDSSGYYGSLDYW	IGH	2	None	
15	181	CARHRRWAYYGSGSYFMPSYYYGMDVW	IGH	2	None	
15	182	CAKDESSSWYFYW	IGH	2	None	
15	183	CARAGGGGVEFDYW	IGH	5	None	
15	184	CARHLGPFITPLGAFDIW	IGH	2	None	
15	185	CQQYNSYSRTF	IGK	6099	C3	7K8R
15	187	CVRVFDTAMVSWDLANGNVFDIW	IGH	2	None	
15	188	CARDYDYVWGSQRFDSW	IGH	2	None	
15	189	CARADYGDDHFDYW	IGH	4	None	
15	192	CARDIWSQSVFDPW	IGH	2	None	
15	193	CARVVWLSSGYRGHFDLW	IGH	2	None	
15	196	CAREVRSGSYYYYYSMDVW	IGH	83	None	
15	197	CAKVRTLYRGYAGDLMDVW	IGH	3	None	
15	198	CARVTHCSGGSCYSGAGWFDPW	IGH	2	None	
15	200	CARARGTMIVVAYSVNAFDIW	IGH	4	None	
15	201	CAKDTSPLSGYSSGWYLGPFDYW	IGH	2	None	
15	203	CGTWDSSLSAWVF	IGL	49	None	
15	204	CASMRIYWYFDLW	IGH	2	None	
15	205	CASLKGGHTPWWFDPW	IGH	2	None	
15	206	CTRGQTPGYSYRIAQAPETYYYYYGMDVW	IGH	2	None	
15	208	CARVSSSWSKAYSFDYW	IGH	2	None	
15	209	CASQRGGCSGGSCYGRPFDYW	IGH	2	None	
15	210	CARVPSHHDGSPTRPYFDFW	IGH	2	None	
15	211	CARDGDDLTGHFDYW	IGH	2	None	
15	212	CAVQLVYGSFFDYW	IGH	2	None	
15	213	CASLVGFSSRYAFDIW	IGH	2	None	
15	214	CARLTLRSLDYW	IGH	3	None	
15	215	CAKGGVATQLTYFDYW	IGH	2	None	
15	216	CARAKGDYRSNWFDPW	IGH	4	None	
15	217	CQHYENWPWTF	IGK	5	C3	7K8R
15	218	CQSADNSGVYGVLF	IGL	10	None	
15	219	CATVPYYYASSAQGGFDSW	IGH	2	None	
15	221	CARGPQTGGFWSGDYW	IGH	16	None	
15	223	CARAIRITIFGVVKEFDYW	IGH	7	None	
15	224	CASNEAVVMDSWFDPW	IGH	2	None	
15	225	CARAPRNSWHTFDYW	IGH	2	None	

15	226	CLRVNDPAWDFDLW	IGH	4	None	
15	227	CARRPDSSGYQNAFDIW	IGH	2	None	
15	228	CAISLWGPYYYDSSGYFDYW	IGH	4	None	
15	229	CVKETRGQSFGDRDLYYFESW	IGH	2	None	
15	232	CARAGRHWYFDLW	IGH	2	None	
15	234	CVKADDEYTGYGGFDFW	IGH	2	None	
15	236	CARGYCRSTTCYFDYW	IGH	6	None	
15	237	CAHIDSSGYYEFDYW	IGH	2	None	
15	240	CARIVPVGGNDYW	IGH	3	None	
15	242	CARGRLPIDLMAYAIGDGMDVW	IGH	3	None	
15	244	CAREKPDTYYLDFW	IGH	2	None	
15	245	CARVRGSFNSNYFDYW	IGH	9	None	
15	246	CCSYAGSYTFEGVF	IGL	6	C2	7K8X
15	247	CAREVVYSSSRPNNYYYYGMDVW	IGH	2	None	
15	248	CAKDSGDYGLDYW	IGH	6	None	
15	249	CARDGSAYYAPNWFDPW	IGH	2	None	
15	250	CARVPTLTTDLYFDYW	IGH	2	None	
15	251	CARDSDDSSGYGFDYW	IGH	2	None	
15	252	CARHRSPDRAVISPAHYFDYW	IGH	2	None	
15	253	CAHRLSGFSYGWFDPW	IGH	2	None	
15	254	CAKVPVAATFRYYFDFW	IGH	2	None	
15	255	CAKRMSDCSGGSCYPDNW	IGH	5	None	
15	257	CARDIERITIFGVVDYW	IGH	10	None	
15	258	CQQLNSYSSLYTF	IGK	6	C1	7BZ5
15	260	CARDSSGGRADYYDSSGYFDYW	IGH	2	None	
15	262	CAKRGYSSSWYEGAFDIW	IGH	4	None	
15	263	CARETHGNYDFWSGYYGNPLFDPW	IGH	2	None	
15	265	CLLTYSDARVF	IGL	9	None	
15	266	CARARTRVTIFGVVIQYFDYW	IGH	12	None	
15	267	CARSQSGGGESWFDPW	IGH	2	None	
15	268	CAKGSSDFWSGFDYW	IGH	2	None	
15	269	CARHSERFLQRSTSFEYW	IGH	2	None	
15	270	CATALGAGQWLASPSLNAFDIW	IGH	2	None	
15	271	CARFGYYYYYYGMDVW	IGH	15	None	
15	272	CARGVVRGVINRYNWFDPW	IGH	2	None	
15	273	CATGPFRESYYFDYW	IGH	2	None	
15	274	CARYFRYQLYNWFDPW	IGH	8	None	
15	275	CGRRGNWGGQWYFDLW	IGH	5	None	
15	277	CTTDWVAASGTYYYPDYW	IGH	2	None	
15	279	CAHRRDGHSGPTWDAGTFDPW	IGH	2	None	
15	281	CMQGIHWPPTF	IGK	1971	None	
15	282	CARGSGNYYFDYW	IGH	572	None	
15	283	CARHPTFYYSRGYIVATIGYNWFDPW	IGH	2	None	
15	284	CARGGSKDSSSWYNIDYW	IGH	2	None	
15	285	CTRGPKPSHRGFDVW	IGH	17	None	
15	286	CAKDLSMIVVVRGYFDYW	IGH	2	None	
15	287	CQQFGSSRYPF	IGK	11	C1	7K8N
15	288	CARLPITIFGVVIIPQLSEVGWFDPW	IGH	2	None	
15	290	CARDKRRITIFGVVPRGGYNWFDPW	IGH	2	None	
15	292	CARETIVVDDSRFDFW	IGH	2	None	
15	294	CVVPGRYCSRPGCSYYYALDVW	IGH	3	None	
15	295	CQSYDSDKLWVF	IGL	4	C4	7JVC
15	296	CARERGTLWFGELFTNWFDPW	IGH	2	None	
15	298	CARDGRYCSSTSCSNHFDYW	IGH	2	None	
15	300	CARDQGSGYYLGNFDYW	IGH	2	None	
15	301	CARQADIVLMVYADQGAFDIW	IGH	2	None	
15	302	CARGGVYTSSWYEFDYW	IGH	2	None	
15	304	CARLHVWGSSHYYYYGMDVW	IGH	2	None	
15	306	CARGPVQGSYEYYYYYGMDVW	IGH	2	None	
15	307	CVKRLGGNYSPCYFDYW	IGH	10	None	
15	311	CAKSGGYSYGLYYYGMDVW	IGH	4	None	
15	312	CAREDQYNTYDYW	IGH	2	None	
15	313	CAKDQGIAAVFDYW	IGH	2	None	
15	315	CARQRRQGIVVAHPRYFDYW	IGH	4	None	
15	317	CQHINSFPRF	IGK	4	C3	7K8R
15	318	CAKDQAGYCSSASCYTGSDYW	IGH	2	None	
15	319	CARGGVIRGFFHDWFDPW	IGH	2	None	
15	321	CFSTDSSGYPHVVF	IGL	2	None	

15	322	CARAPERGYSGYWYAFDIW	IGH	4	None	
15	323	CASTLLKAGWFDPW	IGH	2	None	
15	325	CAHRHSSSWYPTDAFDIW	IGH	2	None	
15	327	CAKEVHDYAVLYWYFDLW	IGH	2	None	
15	328	CAKGKGGWYVFDDW	IGH	2	None	
15	329	CTRGQLVSPGPDYYYGMDVW	IGH	4	None	
15	330	CARAQGNIVLMVYVISAFDIW	IGH	3	None	
15	331	CARDRGGASGIGYFDYW	IGH	6	None	
15	332	CARDLAAMVLDYW	IGH	7	None	
15	333	CARDQSGSTVGDYW	IGH	2	None	
15	334	CARGAYGGDRFYWFDPW	IGH	2	None	
15	335	CQQYYGRPLAF	IGK	49	C4	6W41
15	336	CAREAKRERGYFDYW	IGH	2	None	
15	338	CARHGPLSGWYWYFDLW	IGH	2	None	
15	339	CAKENPLYCSSTSCYASGAFDIW	IGH	2	None	
15	340	CAKDLALNYYDSSGPADLFDYW	IGH	2	None	
15	341	CAKDVGSARVFPYYFYGMDVW	IGH	2	None	
15	344	CAATVVITSVGGFDYW	IGH	2	None	
15	349	CAKDVYYYDSSGYYKLHGALRTPRSDAFDIW	IGH	2	None	
15	350	CATESHYDSGAYYYGLFDYW	IGH	2	None	
15	353	CARYDDYDNCFDPW	IGH	2	None	
15	354	CAKVVDCSGGSCYSDFW	IGH	6	None	
15	356	CANLAAADPYFDYW	IGH	2	None	
15	358	CATAAPFSGYYPTKYFQHW	IGH	2	None	
15	359	CARDQIAARPNYYYYGMDVW	IGH	2	None	
15	360	CARDRPGSYWSSDAFDIW	IGH	4	None	
15	361	CVRSHYYQSSGYYLDYW	IGH	54	None	
15	362	CARTLTYYDFWSGYYGRGFDPW	IGH	2	None	
15	363	CAKDSWEVLNWFAPW	IGH	4	None	
15	364	CVLYMGSGIWVF	IGL	90	None	
15	365	CARAHYYDTSSYHFDLW	IGH	2	None	
15	366	CHVWDSSTFF	IGL	7	None	
15	367	CARAAGLVVPAAIFDYW	IGH	2	None	
15	369	CARGRPYSSGCVDYW	IGH	2	None	
15	370	CATLTTANETSGGIFDIW	IGH	2	None	
15	371	CARDKPLPPHYDFWSGYYYGVDYW	IGH	2	None	
15	372	CARQMFDSGSADALDIW	IGH	4	None	
15	374	CARAGLRYFDWSMITFGGDLTYFDYW	IGH	4	None	
15	375	CTRDDFWSGYLPYW	IGH	2	None	
15	376	CARHSGDEDYGDLIDYW	IGH	2	None	
15	377	CARDWHTAMDYW	IGH	2	None	
15	380	CARVGITLIRGVLLSPNWFTPW	IGH	2	None	
15	381	CSSYTDLSTLVF	IGL	861	C2	7K8W
15	382	CARAGYLWGSYRYLSYFDYW	IGH	3	None	
15	384	CQVWDSRSDHSAWVF	IGL	4	None	
15	385	CARDPRSITIFGVAPTKNAFDIW	IGH	3	None	
15	387	CATDRGHDFGNRRLGDYW	IGH	9	None	
15	388	CARELPLDYYGSGRESSTWGFDPW	IGH	2	None	
15	389	CLRAEDTAVYYCVPGGLRYFDSW	IGH	2	None	
15	392	CASSVGYNTNFDYW	IGH	4	None	
15	393	CASTTGAYYYYGMDVW	IGH	2	None	
15	394	CASNVGDHGDAFDLW	IGH	4	None	
15	396	CQQYNNWHHKLTF	IGK	137	C3	7K8P
15	397	CARGYSSGLDWYFDLW	IGH	2	None	
15	399	CARHPPIVVDPYYYYYGMDVW	IGH	2	None	
15	400	CTKELFIMIRGIAIRQYFDAW	IGH	2	None	
15	401	CVREAGHCSSTSCYVGGGWFDSW	IGH	8	None	
15	402	CARDEPTDGLDVW	IGH	3	None	
15	403	CAKDFNVWNDFYYYYGMDVW	IGH	2	None	
15	404	CAKDKSDWTYLDWW	IGH	7	None	
15	405	CARGHQTFWSGYTWGDYYYYYGMDVW	IGH	2	None	
15	406	CARDHPDNDYGDYAGYW	IGH	5	None	
15	408	CARVVGSYYALDYW	IGH	2	None	
15	409	CAKSLDYGDYIVPPDYW	IGH	2	None	
15	410	CAHRGYYDSSGHREWDYW	IGH	2	None	
15	411	CASEVPYSNLGYYYGMDVW	IGH	7	None	
15	412	CARAKGRISLRYIDWLFHAFDMW	IGH	2	None	
15	416	CARSSSSSGIAPYYFDYW	IGH	2	None	

15	418	CARTPYGSGSYYNSYAFDLW	IGH	5	None	
15	419	CQQFESYMWTF	IGK	29	C3	7K8R
15	420	CVREGLVVVTAQDWFFDLW	IGH	2	None	
15	421	CCLSSNNWAYDYW	IGH	24	None	
15	422	CARASKLRYFDWLPFDYW	IGH	2	None	
15	424	CQRYNAYPCIF	IGK	4	C3	7K8R
15	425	CATGARRGYDFYYLDVW	IGH	3	None	
15	427	CAREGGVVQIPPYYYGMDVW	IGH	3	None	
15	428	CARSPYYDILTGYYYIDYW	IGH	2	None	
15	429	CARRPGYCTGGVCYAFDIW	IGH	2	None	
15	431	CAKDSLTYYYDSSGYPQTNYFDYW	IGH	2	None	
15	433	CAKFPFFDGFGEARPHYYFDYW	IGH	4	None	
15	434	CASWLSYCSSTSCDHYYYYYGMDVW	IGH	2	None	
15	435	CYSTDSSANQRGVF	IGL	3	None	
15	436	CLLYYGGAQPWVF	IGL	673	None	
15	438	CARVVRGYSGQYGYFDYW	IGH	4	None	
15	442	CALRYSPTWYGGGNHDAFDIW	IGH	2	None	
15	443	CTLYLEMNSLRPDDTATYYCAKDPRIFLIGKATPPDYW	IGH	2	None	
15	444	CARLGYYYGSGRGDSYWFDPW	IGH	2	None	
15	446	CARGPPFSLWVYFAYW	IGH	2	None	
15	447	CAKATAAGIFYYYGMDVW	IGH	4	None	
15	448	CARLSGDDWYFVLW	IGH	4	None	
15	450	CVRHIPSTVPFDYW	IGH	2	None	
15	451	CARRQGIAAAPTPVLSFDYW	IGH	2	None	
15	452	CATESYYHCDYW	IGH	13	None	
15	453	CALPISKDDIAAAGTLFGYW	IGH	2	None	
15	454	CARDQFPYYGPLIPLLQLDYW	IGH	6	None	
15	455	CARTRLEPTIFGVVFRYPPDYW	IGH	2	None	
15	456	CARHDSGPVGSAQDYW	IGH	2	None	
15	457	CAKLPQGRFDSW	IGH	4	None	
15	458	CARGSFDWLLTRQYYFDYW	IGH	2	None	
15	460	CARDNRFLEWSNSRDTFDIW	IGH	2	None	
15	461	CQSGDSNGARVF	IGL	2	None	
15	462	CAKVSYLSTASSLDYW	IGH	6	None	
15	463	CARDHFYDSSGYYHYFDSW	IGH	2	None	
15	464	CAKGRSQSSSWYWTQYYYYGMDVW	IGH	2	None	
15	465	CARVLGYSRAGWFDPW	IGH	2	None	
15	466	CARDRKEVQLVRIYYYGMDVW	IGH	3	None	
15	467	CTSEGGQVEMATRNWYFDLW	IGH	2	None	
15	469	CARDSWDLVLEPADQFDAFDIW	IGH	2	None	
15	470	CARDFVVPAATDYYYYGMDVW	IGH	2	None	
15	471	CARLDYYDIGRSPFDYW	IGH	6	None	
15	472	CAFYCSSTSCPSFDYW	IGH	2	None	
15	473	CATQIGYGPPIYYYYGMDVW	IGH	2	None	
15	474	CARDNLAAAYFAFDIW	IGH	3	None	
15	475	CATNAITFGGVIDYYYYGMDVW	IGH	2	None	
15	476	CGKRANRGSSWYFDLW	IGH	4	None	
15	477	CVREGSIVGDAFDFW	IGH	2	None	
15	478	CAREVAGYYDSSGSYWFDPW	IGH	2	None	
15	484	CARAQDLAFYYYGMDVW	IGH	2	None	
15	485	CQQFNTWPPEF	IGK	10	C1	7BZ5
15	486	CAREYFGITGTTSAFDIW	IGH	2	None	
15	488	CARAVRQWLALYYYYMDVW	IGH	2	None	
15	489	CYAVWFGEISSGHFDYW	IGH	2	None	
15	490	CARDGIYYYDSSGYYSDAFDIW	IGH	2	None	
15	491	CARAGSIMIEDYFNYW	IGH	2	None	
15	494	CARDRLEWLSYYYYGMDVW	IGH	3	None	
15	496	CTRGKARYDFWSAYLYWYFDLW	IGH	2	None	
15	497	CASYGSYYSSSVGGDWFDPW	IGH	2	None	
15	498	CSSSSYYYYGMDVW	IGH	2	None	
15	501	CSSYTSANTLDVIF	IGL	13	C2	7K90
15	502	CSSGSYLSAFDIW	IGH	2	None	
15	503	CQTWGNGFRVF	IGL	39	None	
15	505	CARPEGGYSTSWVDYW	IGH	4	None	
15	506	CARDSSGWSPFDHW	IGH	11	None	
15	507	CAKVGSGSGWYADYW	IGH	2	None	
15	508	CAKDLTAAAAGYYYGMDVW	IGH	2	None	
15	509	CVREDWAVGGTNNFDHW	IGH	2	None	

15	510	CAKRESSGWYWAFDIW	IGH	3	None	
15	511	CYSARDNDLRVF	IGL	5	None	
15	512	CARDPSATMKVVAPAQAFDIW	IGH	3	None	
15	513	CMQGINLQTF	IGK	4	None	
15	514	CARGSNSNGFDIW	IGH	4	None	
15	516	CARGEVDYYDSSGYPHFDYW	IGH	2	None	
15	517	CARYPPSLAGTSIVDYW	IGH	3	None	
15	518	CATQQRYGDHDYW	IGH	2	None	
15	521	CARDMATGYYYYYGVDVW	IGH	5	None	
15	526	CAREIPELYHYYGMDVW	IGH	3	None	
15	529	CTTDWFAHDYGADYW	IGH	2	None	
15	532	CARARMGAILGGEYYYYGMDVW	IGH	2	None	
15	533	CAKDGVHGGSWHYFPSW	IGH	2	None	
15	537	CAKDISVVVVPIYYFDYW	IGH	2	None	
15	539	CARRGTSGYYYAVDYW	IGH	2	None	
15	540	CQESYSTSSRSTF	IGK	3	C4	6ZDH
15	544	CAKDYRLAVAGRDYFDYW	IGH	4	None	
15	545	CAKDLSWGWFGETRGFDSW	IGH	2	None	
15	546	CQSADSSGTYVVF	IGL	422	None	
15	547	CTTDYQSGTFHFDYW	IGH	4	None	
15	548	CARGALQSSSWYGWYFDLW	IGH	6	None	
15	550	CARHSGYGGNSDFDYW	IGH	2	None	
15	551	CARATGTYAFDIW	IGH	3	None	
15	552	CARDQGHGDYEDYW	IGH	4	None	
15	554	CARVHLGSSWLWAGWFDPW	IGH	2	None	
15	555	CTTGPRITGTTDRDYW	IGH	2	None	
15	556	CAKDRGFGVVMFLFDDW	IGH	7	None	
15	558	CASGYDFWSGYYIKDGMDVW	IGH	2	None	
15	559	CARESRGSYYRDFDYW	IGH	2	None	
15	562	CARVLASGYFDYW	IGH	13	None	
15	563	CARNVLTVDIVATTPRREGWFDPW	IGH	2	None	
15	564	CAREHILTGYYDFWFDPW	IGH	9	None	
15	566	CATRGSFYDSYFNYW	IGH	2	None	
15	567	CATFILVARDYW	IGH	2	None	
15	569	CAKDGVSRWGSIANVVVPAALMRDLWSIDYW	IGH	2	None	
15	570	CARGDGSSWYRNWFRWFDPW	IGH	2	None	
15	571	CQQYESDSGRTF	IGK	4	None	
15	572	CASLAEGGYYPAAFDIW	IGH	2	None	
15	573	CARFVRANINSGSKYYFDNW	IGH	2	None	
15	575	CTTYGITGTPDYW	IGH	2	None	
15	577	CAKVAGPPYFDYW	IGH	3	None	
15	578	CARLGYYDSSGYYKSDGFDIW	IGH	2	None	
15	580	CARFNNWSYRRFDYW	IGH	74	None	
15	582	CARHVGSGWYVPYYFDYW	IGH	6	None	
15	583	CARIRGDDYGDPDAFDIW	IGH	3	None	
15	585	CARSGVRGVNNWFDPW	IGH	2	None	
15	586	CARERMITFGGVIASNFDYW	IGH	2	None	
15	587	CARTSGWSFGQGSFDYW	IGH	2	None	
15	588	CTTDIAQEYYFDYW	IGH	2	None	
15	589	CARDRSPYYYGSGSYYDHW	IGH	2	None	
15	590	CARVKGTIFGTFDPW	IGH	2	None	
15	591	CARDLGYSYGAFDYW	IGH	6	None	
15	592	CAKEHGYYDSSGYYHGFDYW	IGH	2	None	
15	593	CARHPPRWQWLVNFDYW	IGH	2	None	
15	594	CARQNDILGVAFDYW	IGH	2	None	
15	595	CARDSPMVRGVIITAHYYYYGMDVW	IGH	2	None	
15	596	CTRGPSTSGSYYPSDYW	IGH	2	None	
15	597	CARAAGSITMIVVVINAFDIW	IGH	6	None	
15	598	CTTDLRPEELAPYYYYGMDVW	IGH	2	None	
15	600	CARDGISMFRGVELDYW	IGH	2	None	
15	601	CATGNIYWFDIW	IGH	2	None	
15	603	CAKERNDYGDYFDYW	IGH	6	None	
15	606	CARDIGDLYSGSYSVFDYW	IGH	2	None	
15	608	CARLGTPHSSSSDLGWFDPW	IGH	2	None	
15	609	CARGPPSYYYDSSLADYW	IGH	2	None	
15	610	CAKDTGLVPAAIDGAFDIW	IGH	2	None	
15	611	CARVRGRITIFGVVITGNFDYW	IGH	6	None	
15	612	CAKGDGYLSYYFDYW	IGH	2	None	

15	614	CAKDIWGSNDAFDIW	IGH	2	None	
15	615	CARHSLNMGETVFGVVLQSATNYFDPW	IGH	5	None	
15	616	CAHTLTYYYDSSGYYSFDYW	IGH	2	None	
15	617	CAHSSLGIKGERGIWFDPW	IGH	3	None	
15	618	CAHRMGSGYFHYW	IGH	2	None	
15	619	CATVRIAGVPHAFDIW	IGH	2	None	
15	620	CARDLLSGTMGGDAFDIW	IGH	2	None	
15	621	CARLMRSCSSTSCFTIDYW	IGH	2	None	
15	622	CARALGATTRDYYYYGMDVW	IGH	2	None	
15	623	CTPDFDGLREGWFDPW	IGH	6	None	
15	624	CQAWDSSTVVF	IGL	125	C4	7JVC
15	626	CARGRGMALLFDPW	IGH	2	None	
15	627	CARDSDILTGYYYYGMDVW	IGH	2	None	
15	628	CASWSQLWLEVGGHEDAFDIW	IGH	2	None	
15	629	CAHRLLVAGPYFDYW	IGH	2	None	
15	630	CARARITMIVVVREFDYW	IGH	3	None	
15	631	CMQALQTPLGGLFTF	IGK	2	None	
15	632	CAKGIFWPNFWSAYRRAAYYFDYW	IGH	2	None	
15	634	CQSYDNILSGPVF	IGL	14	C3	6XDG
15	635	CARIPDTGMDPIDYW	IGH	2	None	
15	636	CARVWGDDSSGYYYLTGFRPESARFDYW	IGH	4	None	
15	637	CARGPPADYSDISERPRGNWFDPW	IGH	2	None	
15	638	CAKDDYVCFEYW	IGH	2	None	
15	639	CQYFGSSPPTTF	IGK	8	C1	7K8N
15	643	CAREGGTSGWYSFDYW	IGH	4	None	
15	644	CARHRSPWLQLMVAFDIW	IGH	6	None	
15	645	CQCDYSTPPYTF	IGK	12	None	
15	646	CTTDKQMSPDYW	IGH	2	None	
15	647	CAKDQKVLESDYW	IGH	2	None	
15	648	CVRAGYGNSYGLFDYW	IGH	37	None	
15	649	CARDPDDGSGSYYGAFDIW	IGH	3	None	
15	650	CQSTDNSGHRVVF	IGL	2	C4	7JVC
15	651	CARERQQIWFDPW	IGH	6	None	
15	652	CVKDDLDGGHRYAAFDYW	IGH	2	None	
15	653	CARGKEDYGSGSYYTNNWFDPW	IGH	2	None	
15	654	CLRAEDTAVYYCARERGTLWFGELFTNWFDPW	IGH	2	None	
15	655	CAHRRSGVVTPYFDYW	IGH	2	None	
15	656	CQQANSVPRKFTF	IGK	12	C2	7K8O
15	657	CAKDMVPYWDYSSTPFGAFDIW	IGH	2	None	
15	658	CQQSFSTLALTF	IGK	2	C4	6ZDH
15	660	CAKDGGGITIFGVVPDYYYYGMDVW	IGH	4	None	
15	661	CARDLFRNYFHYYGMDVW	IGH	2	None	
15	664	CARHTTGYNWFDPW	IGH	4	None	
15	665	CARDPLLGLGPWYFDLW	IGH	2	None	
15	666	CASLQAPDYGDYVRWFDPW	IGH	2	None	
15	667	CQSFDSSLSGSQVLF	IGL	3	C4	7JVC
15	668	CAREAWDFWSGYYRPIYFDYW	IGH	2	None	
15	669	CARVHCPAGVCFSFGAFDVW	IGH	2	None	
15	670	CARALSVLNYFFALDVW	IGH	2	None	
15	672	CASGLTAYGIDYW	IGH	3	None	
15	674	CAKEKATDSSAYYLFYYYYVMDVW	IGH	2	None	
15	675	CARVQVASGFCSGMHCPNWFDPW	IGH	2	None	
15	677	CAKDARPSRYCSGGSCTRKKYNWFDPW	IGH	2	None	
15	679	CVKDLFGAEYYYDSSAPTGGDYW	IGH	4	None	
15	680	CARHGLAGITIFGVVTMKGWFDPW	IGH	47	None	
15	681	CARALHYYDSSGYSRHDAFDIW	IGH	5	None	
15	682	CATGQGYSSGWYAATHSLDYW	IGH	2	None	
15	683	CARSYIIVGVPPYFDYW	IGH	2	None	
15	685	CARLYDSSGYLDYW	IGH	2	None	
15	686	CLRGTGNYGDWDYW	IGH	9	None	
15	687	CARGSPQGEYYFDYW	IGH	3	None	
15	688	CARDPQVAGGGYFDYW	IGH	3	None	
15	690	CARLGSGFQDAFDIW	IGH	10	None	
15	691	CARGDQKPAFSLDYW	IGH	3	None	
15	693	CARVSPVEDAYYDAYDIW	IGH	2	None	
15	694	CAGLLTSYYDSSDDHYSYYGMDVW	IGH	2	None	
15	695	CQHYGSSTGTF	IGK	5	C1	7K8N
15	696	CARGPIEMATIRPNYFDYW	IGH	2	None	

15	697	CARGNLGIGTRYFDLW	IGH	2	None	
15	698	CARSSSSWSGYYYYGMDVW	IGH	2	None	
15	700	CARVVRRVDRSGNNWFDPW	IGH	9	None	
15	702	CARDNDGGYSYGFSTAPEPYNWFDPW	IGH	2	None	
15	703	CARDPIIAAAGYYYYYGMDVW	IGH	2	None	
15	704	CAKDRWTYSSSWSDYW	IGH	2	None	
15	705	CGRIMAVAGTIDYW	IGH	5	None	
15	706	CARVSQHSGYDTFDYW	IGH	2	None	
15	709	CAGSGNPGDYW	IGH	7	None	
15	710	CAREVRSWTRSYDYW	IGH	2	None	
15	712	CMQGTHWLTWTF	IGK	2	None	
15	713	CAHREVGGWEWFDYW	IGH	2	None	
15	715	CAKESSYGGYPGRNGAFDIW	IGH	4	None	
15	717	CAREIWDEGYSSGWFDYW	IGH	2	None	
15	718	CAKDGATVTMGGLDYW	IGH	2	None	
15	719	CHQYNAYSTWTF	IGK	101	C3	7K8R
15	720	CAREGLPHTLFRLVPAARSDYYYGMDVW	IGH	2	None	
15	721	CARDRQLRYFDWPDAFDIW	IGH	2	None	
15	722	CARDHWGSFEYW	IGH	2	None	
15	725	CAHSQLLTYYYDSSGPHDAFDIW	IGH	2	None	
15	730	CAKDGYDFWSGYYNYW	IGH	4	None	
15	731	CARANVLLRFGELSPFDYW	IGH	8	None	
15	732	CARGDYSGYSSDYYNYLRLPFDYW	IGH	5	None	
15	733	CDWSSDVCSSDLCTRAMDTAMIAWDLSDGYVFDIW	IGH	2	None	
15	734	CARGGGHDFGERRVDYW	IGH	4	None	
15	735	CASLPLRERFDPW	IGH	2	None	
15	736	CAKDGIVQQLDGSPYYFDYW	IGH	2	None	
15	739	CARESGNGSPAFDSW	IGH	7	None	
15	740	CTKDTFGQEDYW	IGH	2	None	
15	741	CATAPRGDYVNDDAFDIW	IGH	2	None	
15	743	CARELTRSSWDPAPFDYW	IGH	4	None	

Supplemental Table 3 All Antibodies Identified on ICU Day 3						
Patient	Sequence Identifier	CDR3	Type	Count	COVID Antibody Type	COVID Structure
1	1	CATSRPNQFLPHEQNFFDRW	IGH	7	None	
1	2	CVRDHKDGDNYHWFFDLW	IGH	2	None	
1	3	CANSFYYDRSDYNDHFDYW	IGH	3	None	
1	4	CARDRRQWLVMPLDYW	IGH	2	None	
1	5	CAKGLIAVAATGWFDPW	IGH	2	None	
1	6	CARDLYDPQYPIDAFDVW	IGH	6	None	
1	7	CARRWLRDNFFDYW	IGH	2	None	
1	9	CARDPVSSSSWPYYYYYGMDVW	IGH	3	None	
1	10	CARKTLGGYSYGSFDFW	IGH	2	None	
1	11	CARDRLGKADHAFDIW	IGH	3	None	
1	12	CAKTFDSSGYYYTCSFDIW	IGH	2	None	
1	13	CASVDPVIPTQGGFGYW	IGH	3	None	
1	14	CQVWESSSDQSWVF	IGL	4	None	
1	15	CARVYGAYSFGHIEFDSW	IGH	2	None	
1	16	CARDAYAGNSEPDPFDIW	IGH	2	None	
1	17	CARDSSHYYDSSGYLDYW	IGH	13	None	
1	18	CARVVAAKNFDYW	IGH	6	None	
1	19	CARQYRRVDTAMVTDYNNYYMDVW	IGH	3	None	
1	20	CARVSGHIVPSWNDAFDIW	IGH	2	None	
1	21	CARGRRQQLVRLSWFDPW	IGH	6	None	
1	22	CARELTVGDYYYGMDVW	IGH	12	None	
1	23	CARASGGITMIVVVTPYYYYYMDVW	IGH	7	None	
1	24	CARDRADLATAFFQYHTDYYYYYMDVW	IGH	4	None	
1	25	CAQDKGLYTSTNRFDPW	IGH	4	None	
1	26	CHQFNSYVEGYSF	IGK	2	C3	7K8R
1	27	CAKAFMIVVVITDFDYW	IGH	2	None	
1	28	CARDGGNYDNSPFDSW	IGH	3	None	
1	29	CARERDADVDMIYYFDYW	IGH	5	None	
1	31	CASWAYVGSRLRVSWDYW	IGH	4	None	
1	33	CVRGISSDGAWFPFRYW	IGH	8	None	
1	34	CARDGGDDYNTWYDYW	IGH	9	None	
1	35	CAIVRGGYRGNYNWFDPW	IGH	4	None	
1	36	CALPICGGSPKYDAFDIW	IGH	2	None	
1	37	CARDTVVVVAANYYYYGMDVW	IGH	6	None	
1	38	CARRGPWERRGPWEMATIFNWFDPW	IGH	2	None	
1	39	CTRVSFRHSSSSLWFDPW	IGH	2	None	
1	40	CASSDYYDRWAFDYW	IGH	17	None	
1	41	CARASSQWLVKYVWFDPW	IGH	6	None	
1	42	CAREIFGYHSSGYLGYLDFW	IGH	26	None	
1	43	CARAGSSAAGLGQLGYLDLW	IGH	6	None	
1	45	CARAIEFIVGLWSDAFDIW	IGH	3	None	
1	46	CAKGRKSWYFDFW	IGH	166	None	
1	47	CARLNYDSSGYSNYYYYYYMDVW	IGH	2	None	
1	48	CARAPWGGYSGNVPRVRSFDDW	IGH	22	None	
1	49	CAGAFDQFFDSW	IGH	3	None	
1	50	CASVVEYCSSTSCEGWFDPW	IGH	2	None	
1	51	CARDRCIGGDCYEIDYW	IGH	15	None	
1	53	CARDRRWAMLAFDFW	IGH	4	None	
1	54	CARVGSSGWYSGGSDYW	IGH	2	None	
1	55	CVKDRTSASSLPHDYW	IGH	2	None	
1	56	CARETNSGFTTYFDYW	IGH	4	None	
1	57	CQQYDNVIISF	IGK	92	C3	6XDG
1	58	CARGRTMIVHFNWFDPW	IGH	6	None	
1	59	CASGIRYCTNGVCPEGPFFDYW	IGH	5	None	
1	61	CARVWELLSDAFDIW	IGH	4	None	
1	62	CARVARRSDENSGYYFDYW	IGH	2	None	
1	63	CASTRGDYNDIGYAFDIW	IGH	2	None	
1	64	CARGSSEDRWFRILFDSW	IGH	3	None	
1	65	CAKDTYSWIAARPVGPDFDYW	IGH	8	None	
1	66	CARSYDFWSGYRGLGYYYYYYGMDVW	IGH	3	None	
1	67	CSRGYNGYPFDAFDIW	IGH	16	None	
1	68	CARERVIYGDSYFVDYW	IGH	2	None	
1	69	CARESQWLIPQYYFDFW	IGH	4	None	
1	70	CARHLGSCSGGSCTPFDYW	IGH	3	None	

1	71	CARDGYEYNWNYGILDYW	IGH	2	None	
1	72	CARDERHCRGGSCMGFDYW	IGH	13	None	
1	73	CARINYYSGYGMDVW	IGH	10	None	
1	75	CARIQGDYYDSSGSVHGWFDPW	IGH	4	None	
1	76	CATAGHHDFWSGYRNYYHYGMDVW	IGH	5	None	
1	77	CASASPYSYGSRYDYVWGSYRSIDYW	IGH	2	None	
1	78	CQQYGSSQGYIF	IGK	14	C1	7K8N
1	79	CVRDVIFSTRFVWFDPW	IGH	7	None	
1	80	CARERLREWELQQDAFDIW	IGH	2	None	
1	81	CSSPGCYEGSGPDYYDGMDVW	IGH	3	None	
1	82	CAKGRGDRLLGIPGYFDLW	IGH	4	None	
1	83	CARIGEVWDYW	IGH	2	None	
1	84	CARQDYSGTYGGYHYKLYMDVW	IGH	4	None	
1	85	CTRDRGSGTIRYNWFDLW	IGH	6	None	
1	86	CAKDIVGLRSIFGVGPFDYW	IGH	4	None	
1	87	CAKGSTHGFPPNWYFDLW	IGH	2	None	
1	89	CARSGGYYDSSGYYVVDAFDIW	IGH	5	None	
1	90	CARFEYGRGAYYTRPSEPFDIW	IGH	2	None	
1	91	CARLINTGTAGLSWVDSW	IGH	3	None	
1	92	CARRKLPLPFDYW	IGH	3	None	
1	93	CAHRDKRIQLWLLGVYAFDIW	IGH	2	None	
1	94	CARVEYSSGWYNKNYYYYYMDVW	IGH	4	None	
1	96	CAREAGYSSTWYEYLDYW	IGH	570	None	
1	97	CARIGRIHLWSPGHFDSW	IGH	14	None	
1	99	CARVGYYYDSSGTNWFDPW	IGH	2	None	
1	101	CARLFVGGHWGYYYYYMDVW	IGH	2	None	
1	102	CAKDGRYCSGGSCYLPFDYW	IGH	2	None	
1	103	CARSKAPGFSYSLYYLDYW	IGH	5	None	
1	104	CARDYSGSSTSPLDYW	IGH	2	None	
1	106	CARDIPDDYGDSPLDGDYW	IGH	5	None	
1	107	CARVRLPGNPVAGYHDYW	IGH	2	None	
1	108	CARDPYDYGDLGWFDPW	IGH	4	None	
1	109	CARDKVYCSGGSCYRYRYYYYYGMDVW	IGH	6	None	
1	110	CARGEIFRGRILEWLSHFDYW	IGH	2	None	
1	111	CARVTTVTNYYYYYMDVW	IGH	4	None	
1	112	CTKIATPGVGTTGSFDDW	IGH	175	None	
1	113	CARPSHGYNSLSAFDIW	IGH	2	None	
1	114	CAPSRYGDRPYYYYGMDVW	IGH	2	None	
1	115	CARHGSSWYIGYYYMDVW	IGH	5	None	
1	116	CARVYQLPPVFDYW	IGH	2	None	
1	117	CARDIDGFSVGATAVFGYW	IGH	2	None	
1	119	CAREDFWSGYYSGYFDYW	IGH	2	None	
1	120	CARDFHDLWSGYYPYFKYW	IGH	4	None	
1	121	CAGDLNLAYGDYPPFDSW	IGH	4	None	
1	122	CARIRGWSPTYSYYYYMDVW	IGH	2	None	
1	123	CARSPPEISGWSHFDYW	IGH	5	None	
1	124	CARVRVQGRHTYFDYW	IGH	6	None	
1	125	CTIDLLPPHIVVVPSAIRHYYYYMDVW	IGH	3	None	
1	126	CARDSRFGDLDDGMDVW	IGH	9	None	
1	127	CARDNDFWSGYPPMDYW	IGH	2	None	
1	128	CATREGYDILTGPFDYW	IGH	15	None	
1	129	CARSTSRYYYDTGYWYFDLW	IGH	3	None	
1	130	CARADTIAVSGSGGLDYW	IGH	11	None	
1	131	CAIDLSIAARPQLDYYYYGMDVW	IGH	6	None	
1	133	CSRTAVSGTTFDYW	IGH	19	None	
1	134	CARGHFGEGWFDPW	IGH	4	None	
1	135	CAKDKGVDIVATTSYYYYGMDVW	IGH	2	None	
1	136	CAKDFKMVGTLDPNDVFDIW	IGH	2	None	
1	137	CARSGSSWQDFDYW	IGH	3	None	
1	138	CARVPAGCSGGSCYSDYW	IGH	6	None	
1	139	CAKDEDCSGGSCYSGAFDIW	IGH	9	None	
1	140	CASRAGGKSDFDTW	IGH	2	None	
1	141	CAMIESSGWYQSIFDYW	IGH	2	None	
1	142	CASPFDGYNKGGAFDIW	IGH	2	None	
1	143	CARGIAPRVLARRDPFDIW	IGH	4	None	
1	144	CARLGPRYSYGRDWFDPW	IGH	7	None	

1	145	CARRGGDKYIFLDWYFDLW	IGH	4	None	
1	147	CARHFGGYYDDAFDIW	IGH	19	None	
1	148	CARSPQTYYYDSSGYSPLYYFDYW	IGH	4	None	
1	149	CARDGAAGLKQWLVPAPSHYFDYW	IGH	3	None	
1	150	CASPRYDFWSGYSRGQFDYW	IGH	5	None	
1	151	CARESLYDYVWGSYRYTYLDYW	IGH	2	None	
1	152	CARSFLYYDFWSGPVYYYYYMDVW	IGH	2	None	
1	153	CQQSYIGPRNSYSTPRTF	IGK	2	C4	7JW0
1	154	CAKWRRAVTPRGDYW	IGH	4	None	
1	155	CARDYTVTTAHIDYYYYGMDVW	IGH	7	None	
1	156	CATIGGGGYDQRTYYFENW	IGH	49	None	
1	157	CAKDREPDGRWDIDYW	IGH	11	None	
1	158	CARVDCSSTSCSAPDYFDYW	IGH	2	None	
1	159	CARGVDRTQYSSGWYDPPPAHPDYFDYW	IGH	8	None	
1	160	CAKDLEPHDYHGSGSYLDHW	IGH	19	None	
1	161	CARKLRGGDTNMDNYNWFDPW	IGH	2	None	
1	162	CARSYCTSTNCYSEGLDWFDPW	IGH	2	None	
1	163	CARGPYDFWTGYYIDYW	IGH	4	None	
1	164	CTTDPPPTRAIVVAISWGDAFDIW	IGH	2	None	
1	166	CAKDSGGSYFYALDYW	IGH	2	None	
1	167	CARDLSSVAGPFDYW	IGH	5	None	
1	168	CASGLFDSGGYYYYFQYW	IGH	12	None	
1	170	CARSRHDSSSWYGYYFDYW	IGH	7	None	
1	171	CARHYYYGSGSYDYW	IGH	6	None	
1	172	CVVNGEADGYRRFDYW	IGH	6	None	
1	174	CARDFSRDGYNSYSFEYW	IGH	3	None	
1	175	CAWGYYYYRYMDAW	IGH	3	None	
1	176	CARDRHTDNCTNGVCYPDWYFDLW	IGH	4	None	
1	178	CARKHIVVVTAILWVDYYYYMDVW	IGH	2	None	
1	179	CAKTAYGTYGYYGMDVW	IGH	2	None	
1	180	CSSYTGSSSFAIF	IGL	9	C1	6XCM
1	181	CAKSIFPSVFGFYAMDVW	IGH	3	None	
1	183	CARPAAGTPYNWFDPW	IGH	30	None	
1	187	CARGSEDNHFWRIDYW	IGH	2	None	
1	188	CARHRTVAGLTHDFDYW	IGH	3	None	
1	189	CARFWGARYSSGWYYYYYYMDVW	IGH	6	None	
1	190	CHQYYSTFYSF	IGK	7	C4	6W41
1	191	CAKDLGAYAHFYIDSW	IGH	2	None	
1	192	CARVYRYSPRPYQYYLDYW	IGH	98	None	
1	193	CTTDRPTRPLDYW	IGH	2	None	
1	194	CARAPSLRWKSNPLYWYFDLW	IGH	2	None	
1	196	CAKDLIGAIPNGYDYYYGMDVW	IGH	4	None	
1	197	CARTGYDSSGYYSYYFDYW	IGH	2	None	
1	198	CQSYDNSLRGPGCIF	IGL	235	C4	7JVC
1	199	CARSMEVVMTPSDAFDFW	IGH	11	None	
1	200	CARAGAALITGVRYFDWTDSDPIDYW	IGH	10	None	
1	201	CARSQEWLVSTLDYW	IGH	6	None	
1	202	CAKGSDSSGWYSLFDYW	IGH	5	None	
1	203	CVRDGGGGLRITIFGVVIVPYFDFW	IGH	5	None	
1	204	CARGQGIGNYNGLDSW	IGH	4	None	
1	205	CARDAGGSYDSRGYYYPNYFDSW	IGH	2	None	
1	206	CGSSPPWTF	IGK	2	None	
1	207	CARSPRIVGAPDYW	IGH	2	None	
1	208	CAKDLRFHINNVHSGAAEFDSW	IGH	6	None	
1	209	CARARDGWYTVRPFDIW	IGH	4	None	
1	210	CMQGTHRF	IGK	12	None	
1	211	CNRARQVSGTYSDFFDFW	IGH	2	None	
1	212	CASRQGYSSSLYYMDVW	IGH	4	None	
1	213	CAGSEDSSAYSEFDSW	IGH	35	None	
1	214	CVTDALTGDHCGGDCADFDYW	IGH	3	None	
1	215	CATGVPPPDHYIWGSHRYNHFYYYMDVW	IGH	2	None	
1	216	CAGRGTKIQDYYDSSGSLRDYW	IGH	2	None	
1	217	CARGVDSSGWYGGYW	IGH	2	None	
1	218	CARDHGGSNYYYYGMDVW	IGH	62	None	
1	219	CARRGDTAMDDAFDIW	IGH	4	None	
1	220	CARDRLGYSYAVFDNW	IGH	2	None	

1	221	CARLYDSTGYYYHYYNHYMDVW	IGH	5	None	
1	222	CAREAPGHAFDIW	IGH	5	None	
1	223	CARDGRNIYSSGFYYYVDVW	IGH	9	None	
1	225	CARDESGGCSHCPYYFVYW	IGH	10	None	
1	226	CAKVSRSWYDEDAFDIW	IGH	55	None	
1	228	CTTFDSSGYYHEYYFDYW	IGH	6	None	
1	230	CLRPVLRGYYDSSVQKADWFDPW	IGH	3	None	
1	231	CARGVCGSSSCYEDAFDIW	IGH	5	None	
1	232	CAKDFGYCSGGWCYPIDSYYFDYW	IGH	5	None	
1	234	CTTARHLVSGDRFDPW	IGH	14	None	
1	235	CAKGRPMIVVVTPGPFDYW	IGH	6	None	
1	236	CARDLYSSSAPRIFDYW	IGH	2	None	
1	238	CARILESQYGLYDFWSGPGQYYYYGMDVW	IGH	2	None	
1	239	CAKRDSQYYCDYW	IGH	11	None	
1	240	CAREGTYYYDSSAEPGWFDPW	IGH	2	None	
1	241	CAKDIWDGYNLVGYYYYGMDVW	IGH	6	None	
1	242	CQQSYSNPGPWTPGVTF	IGK	16	C2	7K8O
1	243	CQTYDSNNRRVIF	IGL	4	C4	7JVC
1	244	CARDVPVEEAFDIW	IGH	5	None	
1	245	CARLPQPAVVLPAPYYFDYW	IGH	2	None	
1	247	CAAWDNGLSGLAF	IGL	2	None	
1	248	CQQFDSVPLHTF	IGK	12	C3	6XDG
1	249	CARDVSDWGWGHWFDPW	IGH	2	None	
1	250	CARAAMYYYDSSGYYTYSFDPW	IGH	2	None	
1	251	CARDNFSHHFLTAYYFDDAFDFW	IGH	2	None	
1	252	CFSTDSSGHHRRGLF	IGL	2	None	
1	253	CARGYHYYDSSGYYPIYYYYYGMDVW	IGH	2	None	
1	254	CARHSLLSEYYYAMDVW	IGH	4	None	
1	255	CASGSPAIRDGFHIW	IGH	2	None	
1	256	CARDVYRGDYVWGSYRFSGSWFDPW	IGH	3	None	
1	257	CARQDLVLEELSSSLNWFGPW	IGH	9	None	
1	258	CANHDCSGGSCFPLDYW	IGH	4	None	
1	259	CARGNSFGRSFDFW	IGH	2	None	
1	260	CARGRYTYGYYFDYW	IGH	56	None	
1	261	CAGGYCSGGSCYRGANWFDHW	IGH	2	None	
1	262	CAKDLWELPPAVYYYYGMDVW	IGH	2	None	
1	263	CARLSAGVVVAAIDYW	IGH	4	None	
1	264	CAKLPDMYYYDSSGYYNNFDYW	IGH	3	None	
1	265	CARGGHTVVGSYFDYW	IGH	2	None	
1	266	CQNYNSAPPDGYTF	IGK	6	C3	7K8R
1	267	CARDGRYSYGYPYLDYW	IGH	2	None	
1	268	CATFAPDITGTTFDYW	IGH	2	None	
1	269	CAKAGYSSGWKQYYFDYW	IGH	3	None	
1	270	CAKGQPGRYSGSYYPFDYW	IGH	2	None	
1	271	CARKGYDSSGYYPFYWYFDLW	IGH	6	None	
1	272	CARDQNLEHGDHPPFDYW	IGH	2	None	
1	273	CAKHEYRRRGLLRGPKYTSSSGSPDYW	IGH	2	None	
1	275	CASSKRITMIVVVKSDAFDIW	IGH	229	None	
1	276	CTTDQFSMTKMTFPFDYW	IGH	3	None	
1	277	CARTEMATIWGFDFW	IGH	12	None	
1	278	CARAQSTYYYDGSGYSYVVDAFDVW	IGH	24	None	
1	279	CVKGITLFPVVTDYW	IGH	4	None	
1	280	CMQATQFPKNQVTF	IGK	5	None	
1	281	CARGYLNWFGHWFDPW	IGH	2	None	
1	282	CARVDYGDHPNSPGHIDYW	IGH	3	None	
1	283	CSSGSYSPFDYW	IGH	7	None	
1	284	CARARGLITASWFDPW	IGH	2	None	
1	285	CTRRGRGDSHIFAFDPW	IGH	2	None	
1	286	CTRVRGSYLHSDYW	IGH	45	None	
1	287	CARGPLLFTIFGVVIDWYFDYW	IGH	3	None	
1	289	CARLRRTSLLAYFDYW	IGH	10	None	
1	290	CARQGGSAAAGIHYYYYMDVW	IGH	2	None	
1	291	CSLRASDTAMYFCARRGGSPKYDAFDIW	IGH	6	None	
1	292	CARVSGGTGWCDYW	IGH	4	None	
1	293	CAKSRGAIFGVVMDAYYYYGMDVW	IGH	3	None	
1	294	CAREVSYSNSYDVFDTW	IGH	17	None	

1	295	CAKPHLARHAFDVW	IGH	4	None	
1	296	CAGASSFNYNYRGFDFW	IGH	3	None	
1	297	CAKDGDYGGNSRLSGNFDFW	IGH	8	None	
1	298	CARKQGDGRGWWTPYYFDYW	IGH	51	None	
1	300	CARDNRYYDSSGYSDAFDIW	IGH	6	None	
1	301	CAKDMGSGSYYADFEYW	IGH	9	None	
1	302	CARDLRVRFLEWLSGNWFDPW	IGH	4	None	
1	303	CQSYDRSLSGSKDVVF	IGL	11	C4	7JVC
1	304	CARDSFSSGFAFDYW	IGH	18	None	
1	305	CASIGWLRGSENNWFDPW	IGH	3	None	
1	306	CASLKYYYDSSGYSTNDAFDIW	IGH	4	None	
1	307	CARHQEDSSSWYQSHARTILDYW	IGH	2	None	
1	308	CATVLGYFFDDW	IGH	2	None	
1	309	CARLFCNSTSCYVYFDHW	IGH	2	None	
1	310	CARDTYTMVRDAPLGFDPW	IGH	4	None	
1	311	CARFHSVRGPSVTYSEIHYWYFDLW	IGH	23	None	
1	312	CARHDGRGNDYNEYW	IGH	5	None	
1	314	CRYYRHTSDSPLSGESFDYW	IGH	2	None	
1	315	CTKSALQLKLQPDVYYYYYMDVW	IGH	17	None	
1	316	CARHQPDPVSAAYSYGHYYYYMDVW	IGH	5	None	
1	317	CTTRLRRASSGEIFDYW	IGH	2	None	
1	318	CAKGGYCSGGSCDNWFDPW	IGH	4	None	
1	319	CQAWHTSTALF	IGL	5	None	
1	320	CAREGLWESSSYVDYW	IGH	6	None	
1	321	CASDSSSAKKPPIYYFDYW	IGH	2	None	
1	322	CALRTGYDFHWFDPW	IGH	2	None	
1	323	CAKDLLLPDRSMVKYYYNGMDVW	IGH	31	None	
1	324	CARQRRDGYSYYYMDVW	IGH	2	None	
1	325	CARDRIVPHGGSYLYYFDYW	IGH	2	None	
1	326	CAKDGRPISGDPYYYYGMDIW	IGH	2	None	
1	327	CVKMEWVLLKKNYFDHW	IGH	2	None	
1	328	CAKEGPTYCGGDCYTSNYLDYW	IGH	2	None	
1	329	CAKPMYSSSWSPAEYFQHW	IGH	2	None	
1	330	CARLGLNYYVRSGYFYW	IGH	2	None	
1	331	CARVDRTYDGFWGGRGNDFDYW	IGH	20	None	
1	333	CARYEGEEMATITYYFDYW	IGH	2	None	
1	334	CAKDWGGGTYSDYW	IGH	6	None	
1	335	CARKGPNSTSWPIDYW	IGH	3	None	
1	336	CVQGALRFLETKDVNWLASW	IGH	2	None	
1	337	CARDRGGDYSNLFDCW	IGH	2	None	
1	338	CARGMGGWYASDGFDIW	IGH	6	None	
1	339	CASFTTGTPRDYW	IGH	2	None	
1	340	CAKDITTWWLPRYYYGMDVW	IGH	2	None	
1	341	CARDQGWSTVEIKESYYYYYMDVW	IGH	3	None	
1	342	CARAYGAPLTFDYW	IGH	7	None	
1	344	CAKDQGGGAAAGTRRMGSYYYYGMDVW	IGH	10	None	
1	345	CARDRQWRVTNYDYW	IGH	2	None	
1	346	CARVEFSYGYVGETNLDYYYGMDVW	IGH	5	None	
1	347	CARFRRPVDAATIAVGWFDPW	IGH	5	None	
1	348	CARENYDSSGTDYW	IGH	2	None	
1	349	CARKTTVTTGPAAKGLDYW	IGH	2	None	
1	350	CAHSLLPRYYYDMCFDYW	IGH	3	None	
1	351	CARDTPVYSSLPYNWFDPW	IGH	4	None	
1	352	CARELTDGGSNWYPGWGWFDPW	IGH	34	None	
1	353	CAKGNDYSNFFLLLGQQKNHYFDYW	IGH	2	None	
1	354	CARDNMDTHYYDSRFDYW	IGH	8	None	
1	355	CANLGYGDYPEFRYSYYMDVW	IGH	4	None	
1	356	CAKLPISGIEGDVTDYW	IGH	5	None	
1	357	CARTGRGTYDYYDSSAYYGDYW	IGH	4	None	
1	358	CARTAGGYFRIQGKRIDYW	IGH	2	None	
1	359	CALNLGYRFNLRYGTIEDYW	IGH	9	None	
1	361	CARSGVGQGYASGNFYYYYGMDVW	IGH	2	None	
1	363	CAKDKVGYSYGWVYYYYGMDVW	IGH	55	None	
1	364	CAKDPVGLVVVTAKDSAHAFDIW	IGH	2	None	
1	365	CAKLAQGQWLGAGANFDYW	IGH	2	None	
1	366	CARGDQRWLQSLVDYW	IGH	2	None	

1	367	CARTLHPIYDSSGYYYFGYW	IGH	2	None	
1	368	CARGTGDFWSGYPHFFDYW	IGH	2	None	
1	369	CARYPFARGDHFDFW	IGH	9	None	
1	370	CATSPGRTYSWRDIYYFNYW	IGH	2	None	
1	371	CVRYGYCSSPSCYQYYYYGMDVW	IGH	2	None	
1	372	CAKALKQWLAPYYFDYW	IGH	3	None	
1	373	CARHGASSGYFASDAFDIW	IGH	2	None	
1	374	CARRIGKWLRIQGSRYYFDYW	IGH	11	None	
1	375	CVHKADWDPVAFDYW	IGH	5	None	
1	376	CQQYTFSPPGEFTF	IGK	63	None	
1	377	CAREWWELMRAYYDYGMDVW	IGH	2	None	
1	378	CARESPLTPHRPHYYGSEYYYYYGMDVW	IGH	3	None	
1	380	CARVAIEYSSPYFDYW	IGH	2	None	
1	381	CAKDTSITMVRGVGGYFDYW	IGH	3	None	
1	382	CAKDGMITIFGVVIMGYFDYW	IGH	5	None	
1	383	CARGSRKGEWFGELLPYYYFDYW	IGH	4	None	
1	385	CARDEVRPNTDFWSGYSSYFDYW	IGH	2	None	
1	386	CARERIYYDVSGYPTQFDFW	IGH	4	None	
1	387	CARGPPGGRFLDWLIRWFDPW	IGH	6	None	
1	388	CASDSGSSAYDSTGLFDYW	IGH	6	None	
1	390	CARHVPPVFLWLGEPGPVRDYYMDVW	IGH	2	None	
1	391	CARGIMFRGVSGLFDYW	IGH	2	None	
1	392	CASLGDSSWNFDYW	IGH	17	None	
1	393	CVSNLVGATQPSPDYW	IGH	2	None	
1	394	CAKDGEDRIQLGALSYYYGMDVW	IGH	2	None	
1	395	CARAGFFGEFILDYW	IGH	8	None	
1	396	CARPLTSGWYFGGGTAFDIW	IGH	4	None	
1	397	CARGRAGKNAQPYYYYMDVW	IGH	4	None	
1	398	CVKGVRGVLSANHYFDYW	IGH	2	None	
1	399	CASPTKRGYYYDSSGYLKDDAFDIW	IGH	2	None	
1	400	CARKPWELLGTHPVRYFDFW	IGH	2	None	
1	401	CAKDSYDFWSGEDYW	IGH	4	None	
1	402	CAKDREAGAMSLELDYW	IGH	2	None	
1	403	CAREMRYSGYDDYYYGMDVW	IGH	2	None	
1	404	CARGDGYSDYGDYW	IGH	2	None	
1	405	CADSHSGGPPYYFDYW	IGH	4	None	
1	406	CAKRGDGMSMRTLDYW	IGH	9	None	
1	407	CARDPRSSSMGLDTFDIW	IGH	5	None	
1	408	CARDRAESSGNWGNYYFDPW	IGH	3	None	
1	409	CAKHGNLGDQIYNYYGMDVW	IGH	17	None	
1	410	CARDRDVYYDSSGYPIFGDAFDIW	IGH	2	None	
1	411	CARGLSYYDYIWGNFRPGDFDHW	IGH	12	None	
1	412	CAKDHSSSSYYYYMDVW	IGH	17	None	
1	413	CARDSPAGNNQQWLVPTPEYYFDYW	IGH	6	None	
1	414	CAKDRGGDKAALDYW	IGH	4	None	
1	415	CSGGSCYSPDYYYGMDVW	IGH	4	None	
1	416	CATRGYSSGWYRAEGDYW	IGH	2	None	
1	417	CGRENIDWIDPW	IGH	2	None	
1	418	CARENYYDSSGYYYDHFDYW	IGH	2	None	
1	419	CVKDQDYDFWTGYYLYW	IGH	4	None	
1	420	CARDGYYDRTALPDYW	IGH	4	None	
1	421	CVKDRDGVWIGGWRHFDHW	IGH	4	None	
1	422	CAKDPSYYGPGSRTNDDYYYSAMDVW	IGH	7	None	
1	423	CARSGPVGATELGDYFDYW	IGH	2	None	
1	424	CAKESQWLVQWYNWFDPW	IGH	4	None	
1	425	CARLMRDASGSSDYW	IGH	3	None	
1	426	CVRIQDGPGWLQFYDYW	IGH	14	None	
1	427	CARDSPVRFTVTTGYFDYW	IGH	3	None	
1	428	CAKDRGSTIFGVDNYYYYGMDVW	IGH	2	None	
1	429	CTTDGSLTNIDHIVSPHYYYYGMDVW	IGH	2	None	
1	430	CMQYTHIPYSF	IGK	3	None	
1	431	CARLGPHDFWSGYYISYFDFW	IGH	7	None	
1	434	CARDRGFYYDSSLHFDYW	IGH	2	None	
1	435	CAKELNTFFLTGSTSDAFDIW	IGH	4	None	
1	436	CAKTTVISLNTYDYW	IGH	26	None	
1	437	CQQYVGSPQTWTF	IGK	8	C1	7K8N

1	438	CQQRNHWPRGF	IGK	20	None	
1	439	CATTTIFGAVNFYLYYYFMDVW	IGH	3	None	
1	440	CAKDLLSHYFDSSGYYSPDYW	IGH	51	None	
1	441	CAKIAAAGTFDYW	IGH	17	None	
1	442	CARTLGYCTNGVCPQPRGWYFDLW	IGH	3	None	
1	443	CARRYCRGGSCHYHPNFDASDIW	IGH	2	None	
1	444	CVRDRIVLVPAAIRFYFDYW	IGH	4	None	
1	445	CARDCPWDTAMEYYYYYYMDVW	IGH	6	None	
1	446	CARWSLEQQLAFDYW	IGH	13	None	
1	447	CASTRWLQRLGYYYYYMDVW	IGH	2	None	
1	448	CARGLVVVTANPFDYW	IGH	2	None	
1	449	CARAKKWVGSFWSGSAEGDVFDIW	IGH	2	None	
1	450	CARRDSGSYNGWFDTW	IGH	10	None	
1	451	CGTWDSSLSAVVF	IGL	586	None	
1	452	CAKDRVEGGSGWYWFDPW	IGH	15	None	
1	453	CAKDVSSVARTYYMDVW	IGH	2	None	
1	454	CARGGNYDLLIGSPGFDFW	IGH	6	None	
1	455	CARDRLLTKYYDRSDGRNWFDPW	IGH	12	None	
1	456	CATSAAILRGYWFDPW	IGH	19	None	
1	457	CARVRFRDGYNYYYYYDMDVW	IGH	7	None	
1	458	CGTWHSNSKTHVVF	IGL	30	None	
1	459	CSRDRSEDDYSNYSPDYYFDYW	IGH	2	None	
1	460	CARSPLPFSSYNYHHYMDVW	IGH	3	None	
1	461	CARYPTFGVGATKDYW	IGH	2	None	
1	462	CARDARIQLWFEGYWFDPW	IGH	4	None	
1	463	CVKGSLASGGRGYYFEKW	IGH	3	None	
1	464	CARDKGSEDESSGHYANDAFDIW	IGH	2	None	
1	465	CASLRSRFLEWFYFDYW	IGH	2	None	
1	466	CARRIALPTQAFDYW	IGH	5	None	
1	467	CAKAHNYGNTFDYW	IGH	4	None	
1	468	CAREVGAIGGDAFDIW	IGH	2	None	
1	469	CATGANTIFGVVIIDAFDIW	IGH	3	None	
1	470	CARDAAPDHGGNFLPFDYW	IGH	2	None	
1	471	CARDGRLGYDFWSGYYGDSW	IGH	5	None	
1	472	CARHPPRSTAGYAFDIW	IGH	2	None	
1	473	CQSLDSSGSYLRLVF	IGL	2	None	
1	474	CAKAHILGATGGYFDHW	IGH	2	None	
1	475	CAKDIGVAGPYYFDSW	IGH	3	None	
1	476	CAAWDDNLNGLSYVF	IGL	2	None	
1	477	CARRGWGALGYYFDYW	IGH	6	None	
1	478	CASTLLYYYDSSGYPLDYW	IGH	2	None	
1	479	CRIIEGGSGAFDIW	IGH	2	None	
1	480	CARDRHPGIVAPGDALAVW	IGH	2	None	
1	481	CARLNWEPYYYMDVW	IGH	7	None	
1	483	CAKDRGSSWYGGLSGYYYYGMDVW	IGH	3	None	
1	484	CARAHYSGSGSYYSRAFDIW	IGH	2	None	
1	485	CARANSWWVVVAAFDYW	IGH	3	None	
1	486	CARAGGYCSSTSCLLPYYYYGLDVW	IGH	2	None	
1	487	CARDQSGRYYSNYFDYW	IGH	12	None	
1	488	CAITAGDNFDSW	IGH	3	None	
1	489	CARLRIAVAHPNYFDYW	IGH	9	None	
1	490	CARDMTTVTTYSDYYYGMDVW	IGH	6	None	
1	492	CSSYAGRNNVLF	IGL	87	C2	7BWJ
1	493	CAREFGQGEDYW	IGH	7	None	
1	494	CAKGETSNSLGGYFDFW	IGH	6	None	
1	495	CARDFDYTNSQIVGDYW	IGH	7	None	
1	496	CARAPWGYGDYFDYW	IGH	9	None	
1	497	CASHRWSEPDRSSSLDYW	IGH	8	None	
1	498	CAKDILTGDYYVGFFDSW	IGH	3	None	
1	499	CARHRLYNWNYVHYW	IGH	2	None	
1	500	CARHGPLWEPIFYFDDW	IGH	8	None	
1	501	CTTDSASGSYNDAFDIW	IGH	2	None	
1	502	CVHSVPYYEYIWGSYYFDYW	IGH	2	None	
1	503	CARVTGIEGVTGWLDPW	IGH	5	None	
1	504	CTRRLHYYYDTGDAFDIW	IGH	7	None	
1	505	CARDRLNYDFWSGYLLDYW	IGH	4	None	

1	506	CAKPVGAVLGFYYYYDMDVW	IGH	3	None	
1	508	CASSRFLEWLLFGAIDYW	IGH	3	None	
1	509	CAIFTVHYYDSSGYYKEAYYYGMDVW	IGH	6	None	
1	510	CARVLGTWTQFDFW	IGH	2	None	
1	511	CARGRATMIIVINDFDSW	IGH	8	None	
1	512	CARADYGVHWFASW	IGH	10	None	
1	513	CVRGPTWIYDYW	IGH	2	None	
1	514	CAREKGGYSSGATCYEDRGSFDTW	IGH	2	None	
1	515	CAKGLQGVPGKAHDFWTGPSGLLDYW	IGH	2	None	
1	516	CARDPTPKWELLLFDYW	IGH	3	None	
1	517	CARGGHYSYDNSGYWPDYW	IGH	3	None	
1	518	CARGDTMVRGVIPLLYFDYW	IGH	2	None	
1	519	CAKGATVTTPMPFDYW	IGH	6	None	
1	520	CARDSDSSGWTPFGDYYYYMDVW	IGH	6	None	
1	521	CNSRDSSGDYLGVVF	IGL	2	None	
1	522	CARAPIFGVVITNFDYW	IGH	19	None	
1	523	CARGGGVLRFLEWPSPLDYW	IGH	8	None	
1	524	CATSSLWLVPGDYW	IGH	2	None	
1	525	CAHILLAAQHLFYYYYYMDVW	IGH	2	None	
1	526	CARPRFSSTWYPFDYW	IGH	2	None	
1	527	CAKDDSEWELLLYFDYW	IGH	2	None	
1	528	CAKEERSSGWSYYYYGMDVW	IGH	2	None	
1	530	CAREPWGIASNWFDPW	IGH	4	None	
1	531	CVRVQGRRVGFDYW	IGH	2	None	
1	532	CQQYNRYCQGF	IGK	12	C3	7K8R
1	533	CAQRAYYGFWRGYSDAFDPW	IGH	11	None	
1	535	CVHRRVTVVVYDAFDVW	IGH	2	None	
1	536	CARVGGGDFENWFDPW	IGH	4	None	
1	537	CTRDRRLAYYKSGWYGQDVFDIW	IGH	5	None	
1	538	CTTEGHPPGLDYW	IGH	3	None	
1	539	CARDPTDYDDYERGLKWFDPW	IGH	23	None	
1	540	CARDRPTRAYSYGDSGPYYFDYW	IGH	5	None	
1	541	CAKESTRYFDWLLGTFDYW	IGH	2	None	
1	542	CASTDCKDGSCYRFDYW	IGH	7	None	
1	543	CAKDAPPHCSSTSCPWTSLWSNWFDPW	IGH	2	None	
1	544	CVRDWGLVRTTPDDAFDSW	IGH	8	None	
1	545	CAAASKSSGSYPFDYW	IGH	4	None	
1	546	CARGSEVWEEQDSVLVWRGFDIW	IGH	16	None	
1	547	CARSTKLVTRITMIVVAQLGYFDLW	IGH	24	None	
1	548	CARDKEASYYDYVWGSYRYNWFDPW	IGH	2	None	
1	549	CTRDVTTPWELLPGNYFYYYMDVW	IGH	17	None	
1	550	CTTIRYFDWLLFGHDYW	IGH	2	None	
1	551	CARHVLHWDYPLYFDFW	IGH	4	None	
1	552	CARYSYYDFNFDYW	IGH	28	None	
1	553	CARDDLIGGGGRNWFDPW	IGH	5	None	
1	554	CARTPTDGGYFSGAFDYW	IGH	3	None	
1	555	CARLGGESYWFDPW	IGH	4	None	
1	556	CARAWIRGSAFDIW	IGH	13	None	
1	557	CAREGVMSLERLPNWFDPW	IGH	6	None	
1	558	CAREIGTLFDLW	IGH	2	None	
1	559	CARTRAGSGDIGYYYYFGMDVW	IGH	5	None	
1	560	CARWFTGGLSSYWYFDLW	IGH	7	None	
1	561	CARGWVRWGARYFDLW	IGH	2	None	
1	563	CATDVKYYGDSHFDYW	IGH	21	None	
1	564	CASLSVGRITMVRGDLYYYYGMDVW	IGH	4	None	
1	565	CAKVGKSGAYSVGYYYYGMDAW	IGH	4	None	
1	566	CARHLDSYVARGHFDPW	IGH	3	None	
1	567	CASNRYLWGPRAFDIW	IGH	2	None	
1	568	CARTRPRWGDLQWTFGDHYFDYW	IGH	3	None	
1	569	CARRGSSWYSPFEYW	IGH	3	None	
1	570	CAKGRLALYCSSTSCYADLFDYW	IGH	2	None	
1	571	CALPIYFCARRGGSPKYDAFDIW	IGH	2	None	
1	572	CAKDGHSSSWYGDNNWFDPW	IGH	4	None	
1	573	CARGQTPMVLDYW	IGH	4	None	
1	574	CAKGQNYYDSSGYYDKFMGIRGNFDYW	IGH	2	None	
1	575	CARGSRKQLWSRYYYGMDVW	IGH	3	None	

1	576	CARDFPYYFDTSGYYYMDVW	IGH	14	None	
1	577	CARPMATIIAVTGTFYFDFW	IGH	4	None	
1	578	CVKDLDSYDTYNFDYW	IGH	11	None	
1	579	CARGGWYIEYW	IGH	8	None	
1	580	CAKDLDYGNMEGTYFDYW	IGH	5	None	
1	584	CAGGGRTLGSIDYPDWFDPW	IGH	4	None	
1	585	CAKDFSIYVYRYFDYW	IGH	3	None	
1	586	CARTRDLEQWLILPSRREPIYYYYMDVW	IGH	3	None	
1	587	CLLHCSGARIF	IGL	8	None	
1	588	CAKADNARGFYFHYW	IGH	6	None	
1	589	CAKDGAKGALYYYGSGSYWVPRDYYYYGMDVW	IGH	2	None	
1	590	CATLHIVGATKGPFDYW	IGH	2	None	
1	591	CARDAGYNRSSGDYYYGMDVW	IGH	3	None	
1	592	CARQTLRYFDWLSAARSPDAFDMW	IGH	2	None	
1	593	CTRYCPTTDCYGGFDFW	IGH	2	None	
1	594	CVIEIRRSWFDPW	IGH	3	None	
1	595	CARDGGYSGIYRGSEYFLTW	IGH	4	None	
1	596	CARDEKTSFSSALYYFDYW	IGH	11	None	
1	597	CAKTRGWLADFDYW	IGH	19	None	
1	598	CAKDIWDGYNADAFDIW	IGH	21	None	
1	599	CACPSLYWNYAFDYW	IGH	4	None	
1	600	CARDMKYYDFWSGYLGPNYYYYYYMDVW	IGH	5	None	
1	601	CARTPPGITVDFDFW	IGH	3	None	
1	602	CARSREVVSYYYMDVW	IGH	4	None	
1	604	CAREGGIRWLQNRVYFDYW	IGH	2	None	
1	605	CARGGVDIVVVPAATYYYGMDVW	IGH	4	None	
1	606	CARGVLTENYEINWFGPW	IGH	4	None	
1	607	CLSADNSGPWVF	IGL	155	None	
1	608	CAAPGQLNYYYYYMDVW	IGH	4	None	
1	609	CARDRWDSYGLPNYFDYW	IGH	2	None	
1	610	CARGVLWYSGQRFDPW	IGH	2	None	
1	611	CASQDDYSSSSFDYW	IGH	6	None	
1	612	CARCPLSGSYFVNW	IGH	2	None	
1	614	CATAYYYDSSGTASFDYW	IGH	2	None	
1	615	CARRSRSCSGGDCSYFFDHW	IGH	3	None	
1	616	CARDHQNDGYWYFDLW	IGH	10	None	
1	617	CATEAAATSTWYMDVW	IGH	7	None	
1	618	CAKDRGAGFLTGYYLEYW	IGH	15	None	
1	619	CARFGRGDRVGFDPW	IGH	4	None	
1	620	CARMPLGYCGTTRCYEVFDYW	IGH	3	None	
1	621	CARRPANYNFWSAYYFDYW	IGH	4	None	
1	623	CATVRPPYYSWNDKDAFQIW	IGH	9	None	
1	624	CTTDRVRVGQWLVVDYYYMDVW	IGH	4	None	
1	625	CAKGMSYYDSSGRLSGTYWYFDLW	IGH	14	None	
1	626	CARDPYDGAFDIW	IGH	14	None	
1	627	CTSRVPSYDSSGYYYHFDYW	IGH	2	None	
1	628	CARLRSGWFEHHFDYW	IGH	6	None	
1	629	CARQESCGGYNSSWCNYYYYYYMDVW	IGH	2	None	
1	630	CARGFGGAVAGILVNYYYYYYMDVW	IGH	16	None	
1	632	CAREGNGDYSYYFDSW	IGH	4	None	
1	633	CARGGRKVRGVNMLNQYAMDVW	IGH	6	None	
1	634	CAREFDPWSGYYPDDPFFFDYW	IGH	36	None	
1	635	CAHTPRYYYDSSGYYYLDYYYMDVW	IGH	3	None	
1	636	CASDGIYRSSSLNYFDYW	IGH	14	None	
1	637	CVRVDNMNVDDYW	IGH	2	None	
1	638	CARVPAFTYNSSWDWRGKKTRFVDYW	IGH	2	None	
1	639	CARHRANYYDSSGYDAFDIW	IGH	2	None	
1	640	CAREHILTGYDIDYW	IGH	2	None	
1	642	CARRRGVDPRSRIAATGSYYYFDYW	IGH	15	None	
1	643	CAREVTRNDYFDYW	IGH	42	None	
1	644	CAKVRIAVAHPGYYYYGMDVW	IGH	7	None	
1	645	CARDRLLVGVGWYLDLW	IGH	2	None	
1	646	CAKDRVFGIAAPEYFQHW	IGH	2	None	
1	647	CAKDTQSYGDYDYYYYSMDVW	IGH	2	None	
1	648	CARHPSRSGYYYVGDYYFDYW	IGH	3	None	
1	649	CARDPGNWSPDYYFDYW	IGH	4	None	

1	650	CARRRGATFHFDSW	IGH	5	None	
1	651	CARDQVGEMDYYGSGFDPW	IGH	2	None	
1	653	CAKGGGAFLDYWFDSW	IGH	2	None	
1	654	CARVVSRLSTSSYYYMDVW	IGH	5	None	
1	655	CARETGPRAPAIAAAGTVGQEYYFDYW	IGH	4	None	
1	656	CHQSSSLPWTF	IGK	579	C3	7K8R
1	657	CAKAPGGSDNWGFFDSW	IGH	2	None	
1	658	CTKDLTTNRIHFFDCW	IGH	2	None	
1	659	CARGEYFYDGSGYYSYWYFDLW	IGH	3	None	
1	661	CARGGELERPDEADYW	IGH	4	None	
1	664	CARLPGPPNRLYWYFDLW	IGH	6	None	
1	665	CMRIFGYW	IGH	6	None	
1	666	CAHRPDYSSSMIFDYW	IGH	2	None	
1	667	CARDQGGPVDAFDFW	IGH	3	None	
1	668	CARDRLGSHVPSVYYFYYIDVW	IGH	2	None	
1	669	CARDRAPTVYGEYVIIGSALDVW	IGH	4	None	
1	670	CARVVEEAGTPSFDYW	IGH	31	None	
1	671	CARVSQGYYDSSGYHYFDYW	IGH	3	None	
1	672	CAKDFGPGYTYGLFDYW	IGH	8	None	
1	673	CAKCKYTYGLSAFDYW	IGH	2	None	
1	674	CASATETYYMDVW	IGH	3	None	
1	675	CASIIASPDGWIDPW	IGH	4	None	
1	676	CARASTYSGSYSAEYFQHW	IGH	2	None	
1	677	CARITWQWLVRHYFDYW	IGH	2	None	
1	678	CARVSKYTMIVVVTPFDIW	IGH	4	None	
1	679	CFARVRFLEWLIDYW	IGH	5	None	
1	680	CLSGDEDNWVF	IGL	30	None	
1	681	CAKDMRAVAGGGRHDAFDIW	IGH	4	None	
1	682	CARETATTITTKWYFDLW	IGH	4	None	
1	683	CTWERPEGDFDSW	IGH	4	None	
1	684	CARGGGKNVGGYGIFDYW	IGH	2	None	
1	685	CARDGGDREEIGWFDPW	IGH	5	None	
1	686	CARHRGTTILPDYW	IGH	3	None	
1	687	CAKGPYGEEWLGGNYFDYW	IGH	3	None	
1	688	CQQTYPTPVF	IGK	412	C4	6W41
1	689	CATPLQKTSAGIRGLDAFGMW	IGH	7	None	
1	690	CAREGDYGTDSPVDYW	IGH	2	None	
1	691	CARAVGEELWGGRQWLRLPPHVGYFDYW	IGH	5	None	
1	692	CATLPHTTGFYTDYYFDYW	IGH	7	None	
1	693	CLHDYYTPWTF	IGK	4	None	
1	694	CASARASRLSRSPFDYW	IGH	46	None	
1	695	CARDGAEEWLQALASHRYYYYGMDVW	IGH	4	None	
1	696	CARSPFRAVAHFDYW	IGH	2	None	
1	697	CARDYDYWSGFDYYYYGMDVW	IGH	4	None	
1	698	CARSEVIYVLSPHLGYFDLW	IGH	2	None	
1	699	CAKAYTRTSNWFDPW	IGH	2	None	
1	700	CTRGNDRWDTKHLFYMDVW	IGH	2	None	
1	701	CAKASNYYLPSRFDYW	IGH	12	None	
1	702	CARDRTVSHGELPSFYYYGMDVW	IGH	50	None	
1	703	CALPIWHNLRASDTAMYFCARRGGSPKYDAFDIW	IGH	2	None	
1	704	CARNQNMPLSGGALRPAFDPW	IGH	2	None	
1	705	CAHSLLYYYESSGSPYFDYW	IGH	6	None	
1	706	CARQGATVVNPYDYW	IGH	2	None	
1	707	CAREKTTGSYRSLDYW	IGH	4	None	
1	708	CATQDVTSLFWRPGRFWYFDLW	IGH	2	None	
1	709	CARTIFGVVINPPDYW	IGH	4	None	
1	710	CARRPLKFTGSDFDYW	IGH	2	None	
1	711	CYSTDSSGNHRVF	IGL	2789	None	
1	712	CARDGPLELRGYFDYW	IGH	2	None	
1	713	CAKGPHTVTQVGSWFDPW	IGH	11	None	
1	714	CARQDDYGANFYFFDYW	IGH	2	None	
1	715	CARAYCGGDCYSPYYYGMDVW	IGH	2	None	
1	716	CATSKWGWEVYFDYW	IGH	3	None	
1	717	CAKDHGAYYYLIVVDGPFDIW	IGH	21	None	
1	718	CASPIRYCTNGGCYSDHYYFDDW	IGH	2	None	
1	719	CARDRIRIAAAGTSVGAFDIW	IGH	2	None	

1	720	CAKGTYWDIVTGYSNFDSW	IGH	4	None	
1	721	CARVALVGALGFSDYW	IGH	2	None	
1	722	CARVPNYYDSSGYPYYFDYW	IGH	20	None	
1	723	CARWGGGYKYKYGMDVW	IGH	2	None	
1	724	CVKLRDLSTWSAFDIW	IGH	2	None	
1	725	CARGYMGMTGSLGYFDYW	IGH	3	None	
1	727	CARDPVSWQQGFDYW	IGH	2	None	
1	728	CARDLGFVMVGDYFDYW	IGH	3	None	
1	729	CAKKIGPYTWNAGDYW	IGH	2	None	
1	730	CARFNAPYSSFIDYW	IGH	2	None	
1	731	CARSTLPSYFYDSGGYTKGGFDYW	IGH	2	None	
1	733	CARRTEYMSGWIPGFDYW	IGH	6	None	
1	734	CQSTDFSGPYHVVF	IGL	50	None	
1	735	CAKDSRRIVGATRSFYYYYGMDVW	IGH	2	None	
1	737	CVREFKSIAAGGMRFDLW	IGH	3	None	
1	738	CAKPYRDGYNPFDYW	IGH	12	None	
1	740	CARSYYHGSETYYLLDYW	IGH	2	None	
1	741	CTRGYCRRDNCYRDDYW	IGH	4	None	
1	742	CARLPITSLELSQGPWFFDSW	IGH	2	None	
1	743	CARSDSYLVQGFDFW	IGH	31	None	
1	744	CARYGTYYYESIGYYYFDYW	IGH	5	None	
1	745	CARDIRDRSSISCYFGYHYYGMDVW	IGH	2	None	
1	747	CARYFRDKMKDYNQYSRYYYFYYMDVW	IGH	2	None	
1	748	CAKDKGVQLERRESYYYYGMDVW	IGH	2	None	
1	749	CTNGRIDFGGFDPW	IGH	4	None	
1	750	CATLTGTTFGYYYGMDVW	IGH	21	None	
1	752	CAHSGIAAAGTVSFDYW	IGH	3	None	
1	753	CAKRLLAYCGGDCYVSDAFDIW	IGH	3	None	
1	754	CARDLTPYGALDYW	IGH	2	None	
1	755	CANIHYGSGNNIQYNWFDPW	IGH	5	None	
1	756	CARLGLELYHYYFDYW	IGH	6	None	
1	757	CARVDLEATLTFDSW	IGH	2	None	
1	758	CAKGLQAMWIKDAFDIW	IGH	2	None	
1	759	CARPPTAGKQLWLLEAFNIW	IGH	6	None	
1	760	CARGRGSGWEEGNWYFDLW	IGH	4	None	
1	761	CYSTDSSDNPEGVF	IGL	99	None	
1	762	CARGRPDSGWELLGALDYW	IGH	6	None	
1	764	CQQRRDWPSVFTF	IGK	66	None	
1	765	CARGGPFTGSYGTFDYW	IGH	2	None	
1	766	CVRDLGSSGYLTYNWFDPW	IGH	2	None	
1	767	CASRYCSIDICFSRSYHYFDLW	IGH	4	None	
1	768	CARERDGYDGFFGYW	IGH	2	None	
1	769	CAGLPAPFITTAGNNYYYAMDVW	IGH	2	None	
1	770	CARDDRIVSKYCVSDCLPGGDAFDFW	IGH	5	None	
1	772	CARDAIESSGWLHAFDYW	IGH	2	None	
1	773	CARATKAWSDSHGQYHYYYGMDVW	IGH	2	None	
1	774	CQKYTSDPPGFTF	IGK	5	None	
1	776	CAKPLIRFLDAFDIW	IGH	2	None	
1	777	CAIHAYRIYTPDSHYYYYGMDVW	IGH	2	None	
1	778	CARGNVEPRRSHALDIW	IGH	4	None	
1	779	CARPPHSSSCHGCDYW	IGH	2	None	
1	780	CASGGGSDYSGYFDYW	IGH	10	None	
1	781	CARSQPYHYYHSGSHRGFDYW	IGH	6	None	
1	782	CARDPRSFRGAFDIW	IGH	9	None	
1	784	CARAPPFVSGTYYIDFW	IGH	2	None	
1	785	CVRDLTTVTAWSYFDFW	IGH	15	None	
1	786	CTSPFAPYCSGGSCSLDYW	IGH	3	None	
1	787	CARVVVLTGYDFDDW	IGH	2	None	
1	788	CAKHGTSSPSPFPLFDPW	IGH	11	None	
1	789	CARDRKLGIGGLDYW	IGH	12	None	
1	790	CARDKLAVYDFWSAYHGTDAFDIW	IGH	3	None	
1	791	CTPLLLWFGEGSDGMDVW	IGH	2	None	
1	792	CARDGPFSAAGDYW	IGH	38	None	
1	793	CARWGRGIAAAGTGLNWFDPW	IGH	3	None	
1	794	CARVVSGGRRWFDSW	IGH	3	None	
1	795	CVKMGDRLLSFVYNWFDSW	IGH	3	None	

1	796	CARHLIAEDYYYYMDVW	IGH	9	None	
1	797	CATIEMYSGPDFTSYYYYGMDVW	IGH	72	None	
1	798	CALYGSSGNDIFDVW	IGH	4	None	
1	799	CVKIEQMFLSALSSFDLW	IGH	6	None	
1	801	CARGPRYYYDSSGYYYVLYYFDYW	IGH	2	None	
1	802	CARDQYSAVTIPSSSPFDYW	IGH	2	None	
1	803	CAKDPLLFLDSSGPNWFDPW	IGH	11	None	
1	804	CATSGTFGGVIAPMDYW	IGH	2	None	
1	805	CARAYYSGWTPRGYFDYW	IGH	2	None	
1	806	CARALAGVTTYFDYW	IGH	2	None	
1	807	CARRPGTTGFGVDYW	IGH	2	None	
1	808	CARDRPDILLWLGQLSKDTYYFYYMDVW	IGH	3	None	
1	809	CAKGWYKSWPQLGGYFDYW	IGH	2	None	
1	810	CEANYYDTSGYHNGPANFDYW	IGH	2	None	
1	811	CARDPGVIAARLTPGLYYYYGMDVW	IGH	2	None	
1	812	CAGELTYCGGDCNSRDAFDIW	IGH	23	None	
1	813	CARLVDTAMRAYFDLW	IGH	3	None	
1	814	CARGGMYSSGWYYFDYW	IGH	16	None	
1	815	CSRSFNDAFDIW	IGH	15	None	
1	816	CARDDKRSAADHFDLW	IGH	7	None	
1	817	CTTDKIRGGGILLPYYYFDYW	IGH	13	None	
1	818	CARGSRVWGTRIVVVPAALSGGDYW	IGH	2	None	
1	819	CAREKHYYDSSGVRWYFDLW	IGH	2	None	
1	820	CARHRFYGDPPDYW	IGH	18	None	
1	821	CARGGYSGNWAYYIDYW	IGH	3	None	
1	822	CARDGGYNDGAPLDYW	IGH	3	None	
1	823	CARDRDYGSGSDYYYYYYGMDVW	IGH	3	None	
1	824	CAKAENYDILSGSHALDVW	IGH	10	None	
1	826	CARKMDTSMVKGFDYW	IGH	3	None	
1	827	CARFFYSNPFDYW	IGH	17	None	
1	828	CARNLYSSGNYFYYYGMDVW	IGH	4	None	
1	829	CARISGDYSYGSYVYWFDSW	IGH	2	None	
1	830	CAKDRGAAAVSDSFDVW	IGH	41	None	
1	831	CAKIMGATTLFDYW	IGH	8	None	
1	832	CARDPSLSGTDHEAFDYW	IGH	4	None	
1	833	CAREYVSAAYDYW	IGH	10	None	
1	834	CARSPPGLLRYFDW	IGH	16	None	
1	835	CAKPSRPYYYHNSGHFDYW	IGH	30	None	
1	836	CASRGYYYDSSGSTLPLDYW	IGH	2	None	
1	837	CARAPVDTTMFSAFDIW	IGH	17	None	
1	838	CAKDPRYGLVQPEYFQHW	IGH	4	None	
1	839	CAKDLDGGKPAGAFDIW	IGH	2	None	
1	840	CARAMKQWPKYNFDHW	IGH	6	None	
1	841	CARRAYCGDDCFPFDYW	IGH	2	None	
1	842	CAKASVVVVAATKLGYYFDFW	IGH	3	None	
1	843	CAKDRAYTSSYYIPKAMDVW	IGH	7	None	
1	844	CARVPMGIHLWYWFDPW	IGH	13	None	
1	845	CAKGLNWKTMYDFQYW	IGH	8	None	
1	847	CAKDEGAWYSASAYYYYGMDVW	IGH	2	None	
1	848	CAKDEEEIAGAGYYFDSW	IGH	2	None	
1	849	CARDALYDILTGYYTYW	IGH	4	None	
1	850	CARLYSGSYYGPYDAFDIW	IGH	2	None	
1	851	CARGGPITILGVAWYYFDYW	IGH	6	None	
1	852	CARALEFLSVEYMWKTYRAGAMDVW	IGH	4	None	
1	853	CARDRPNEYGGNSGSFDPW	IGH	7	None	
1	854	CARHIVYVDGGWYSGPMSWFDPW	IGH	4	None	
1	855	CARGGSRGLMPFDVW	IGH	2	None	
1	856	CTTSTIYDSSGYYDWSIDYW	IGH	6	None	
1	857	CAKSPTYYFESSGYIDFW	IGH	4	None	
1	858	CARSRSSRGLLKQYYFDYW	IGH	40	None	
1	859	CARVSRDGILSYYFDYW	IGH	2	None	
1	860	CQAWDGSTAAYVF	IGL	19	None	
1	861	CASPPEYCSSTRCTSAFDIW	IGH	4	None	
1	862	CAREPPFTIFGVVVQYYYYYMDVW	IGH	2	None	
1	863	CARPRTTWFGESAFDIW	IGH	2	None	
1	864	CARVRRVQLRIQGHFDYW	IGH	3	None	

1	865	CQQYDRSPRYNF	IGK	23	C1	7K8N
1	866	CARDSVPAPRRGQWFDPW	IGH	14	None	
1	867	CARLRLGDLFFAAFDIW	IGH	2	None	
1	868	CAKQPVEITMILVALFRDEWYFDLW	IGH	2	None	
1	869	CARGSRRGDGYNLALDYW	IGH	2	None	
1	870	CARHPSSMVRGVIYYYNYMDVW	IGH	2	None	
1	871	CARGPVAGYDSSGYSFDYW	IGH	3	None	
1	872	CARGRPDEDYYDSSGPQMGYFDYW	IGH	2	None	
1	873	CARAQRGVMWYMDVW	IGH	6	None	
1	874	CARHSPHGGSSGWYDAPTYFDYW	IGH	11	None	
1	875	CTVYSKYYYYYGMDVW	IGH	2	None	
1	876	CARGVNSATSGYSFDPW	IGH	26	None	
1	877	CARDLYVEQWLRSRGWIYYYYGMDVW	IGH	4	None	
1	878	CAKDRQDDYSQTNWFDPW	IGH	10	None	
1	879	CTRGSSGFSEIDYW	IGH	18	None	
1	880	CAREMYGDYAVDVW	IGH	4	None	
1	881	CALGLYCSGGSCYSPDYYYGMDVW	IGH	67	None	
1	883	CARDLYSSSSPTSNYYYYYGMDVW	IGH	2	None	
1	884	CARIRGAKYTSGWYEYYYYMDVW	IGH	2	None	
1	885	CARAYYDYSGWWGFDSW	IGH	2	None	
1	886	CVKDRVVYGDYGDSFDVW	IGH	2	None	
1	887	CARDLFHPVVLWYFDLW	IGH	2	None	
1	888	CARQQYYGVGSLRGWFDPW	IGH	23	None	
1	889	CARAYGPWSNWFGPW	IGH	2	None	
1	890	CARGGGLYFYDGRGYGNWFDPW	IGH	4	None	
1	891	CAHPSQRYKWAIFDYW	IGH	6	None	
1	892	CARAGWEWIQLWFLDYW	IGH	7	None	
1	893	CARILGDSSSGYEGLDYW	IGH	2	None	
1	894	CATDSVVGTAAPLDAFDVW	IGH	18	None	
1	895	CATLNTLPKDWYLDLW	IGH	5	None	
1	896	CSVLIAVAGDFDYW	IGH	5	None	
1	897	CARAPDGGSYYPYYYYYMDVW	IGH	2	None	
1	898	CASAYCSSLDCRAGLTPTLEYYLDYW	IGH	2	None	
1	899	CARGEMTTPYDAFDIW	IGH	2	None	
1	900	CATEWAYYDSSGYLGYW	IGH	3	None	
1	901	CAGGSYYYYYMDVW	IGH	81	None	
1	902	CTRSSVVTFIPPRGYFDYW	IGH	13	None	
1	903	CARDLDYGDYRHRAFDYW	IGH	3	None	
1	904	CARQYNYDETGYYEPASFDYW	IGH	2	None	
1	905	CTRTYDFWSGYSNHDAFDIW	IGH	2	None	
1	906	CARDWARDLLLWSGDLGWFDPW	IGH	2	None	
1	907	CARDASVETAIIHDVFDIW	IGH	12	None	
1	908	CAKEELYYYGSGYFDYW	IGH	2	None	
1	909	CASPPLYSSGWYYFDYW	IGH	2	None	
1	910	CARDRHSSSSGIYYYYYMDVW	IGH	6	None	
1	911	CARGYCSSSSCPEVFAFDIW	IGH	2	None	
1	912	CAKAFYFDFWSDYYGPSDYW	IGH	2	None	
1	913	CARGRISETTDEFDNW	IGH	3	None	
1	914	CARDFSRSYYDSSGYSADYW	IGH	4	None	
1	915	CTVDPYYDTSDGSFSWFDPW	IGH	31	None	
1	916	CAHRRKSGYDSTPFDYW	IGH	2	None	
1	917	CTREATYRTWPEYFDYW	IGH	8	None	
1	918	CARLCTGNTCYHDQW	IGH	5	None	
1	921	CARRVAVRPLYYFDYW	IGH	4	None	
1	922	CARWDSPTTVTTALTPDAFDIW	IGH	2	None	
1	923	CARDIKLELLHHAFDIW	IGH	2	None	
1	924	CARDNVRGDSPFYYYYGLDVW	IGH	6	None	
1	926	CARAPKTSPEDYYYYYAIDVW	IGH	2	None	
1	927	CARASSSGSYASNWFDPW	IGH	2	None	
1	928	CTKEVYCDGDCYSQDGYYYYMDVW	IGH	2	None	
1	929	CCSYAGSYTWMF	IGL	78	C2	7JMP
1	930	CARAIYSSSWHGVVYYYGMDVW	IGH	2	None	
1	931	CARLEYSSSSDVYSYGIDYW	IGH	2	None	
1	932	CVRDSQGFVNTAMPHYFDSW	IGH	5	None	
1	934	CARGYCDSIHCYTDYW	IGH	2	None	
1	935	CTTEEHDYYDSSGYLTFDYW	IGH	2	None	

1	936	CAKAAHFYYDASGYIPYW	IGH	4	None	
1	937	CARDGSPVSPMGAPKSWHFDLW	IGH	3	None	
1	938	CAKEAYDTSGYPFDLW	IGH	7	None	
1	939	CAKGDRAYDYGDSFDYW	IGH	2	None	
1	940	CARDRVAPAPIDYYYYMDVW	IGH	5	None	
1	941	CAKEGGYCSSTSCSRFWGGNWFDPW	IGH	3	None	
1	942	CARGRVGSTTSYDYW	IGH	3	None	
1	943	CARLAHSGIHLWLRRVDYW	IGH	5	None	
1	944	CAKEFTGGRWFFDLW	IGH	2	None	
1	945	CARDRGMPAGNWFDPW	IGH	4	None	
1	946	CARFGVGGSWSAPIGDYW	IGH	4	None	
1	947	CASQPGPYSSSWFDNW	IGH	2	None	
1	948	CARGGSSHYYLNAFDIW	IGH	2	None	
1	949	CARDCRVNYCSSTSCSIPYNWFDPW	IGH	2	None	
1	950	CVRDRTNYESTNSYYFDYW	IGH	9	None	
1	951	CAILDNWNYERFGFDYW	IGH	2	None	
1	952	CAKARPRSFYSYFDPW	IGH	2	None	
1	953	CARSMVRGYYYYGMDVW	IGH	13	None	
1	954	CALPISGYSSTWYEYLDYW	IGH	5	None	
1	955	CAREAGTSYSAGYFDPW	IGH	54	None	
1	957	CARHTRPGSGTYYGTGGWFDLW	IGH	10	None	
1	958	CARHVYYDVSAYGNAFDIW	IGH	2	None	
1	959	CTSSWVVVAATGSGGFDFW	IGH	2	None	
1	961	CVRHVGLSWFDSW	IGH	2	None	
1	962	CARVALRLGEYPDAFDIW	IGH	8	None	
1	963	CARAGIAARRPLDYW	IGH	2	None	
1	964	CARVGRQWELPWGDYFDYW	IGH	2	None	
1	965	CAKGYCSSGSCYGYYGMDVW	IGH	8	None	
1	966	CVRRSQADGRYCSSTSCYPNAFDVW	IGH	2	None	
1	967	CARDGGGLLGATNPWGYYYAMDVW	IGH	5	None	
1	968	CARDLLSHTEGLYYYYGMDVW	IGH	2	None	
1	969	CARGGYCSGGNCYWGEIDYW	IGH	3	None	
1	970	CARRGYNWNTNFDLW	IGH	8	None	
1	971	CARDPNHSSRWGFDYW	IGH	2	None	
1	972	CARALLYCTNGVCYKGGAYFDYW	IGH	6	None	
1	973	CARDKAAHFDLW	IGH	11	None	
1	974	CARLGDYTGGWWKVEFTWFDPW	IGH	2	None	
1	975	CVREGYSSGRAPAFDFW	IGH	8	None	
1	976	CARFIHIISSAVVFDSW	IGH	3	None	
1	977	CVRDSYSYGVIGYFDSW	IGH	2	None	
1	978	CARALSLYYYDSSGYYFDYW	IGH	11	None	
1	979	CAHRLLPSGSQHSYFDYW	IGH	4	None	
1	980	CARDLWFGESLFDYW	IGH	27	None	
1	981	CARQNFEPRARYFDPW	IGH	2	None	
1	982	CTRDNRGYSYGYYAFDIW	IGH	2	None	
1	983	CARGNHNFVEAAGPSFDHW	IGH	3	None	
1	984	CATWGVVSAFDYW	IGH	3	None	
1	985	CARSLYSGPSWFDYW	IGH	2	None	
1	986	CARGAAAGTGAVDYW	IGH	2	None	
1	987	CARGNSSPLYYYYMDVW	IGH	4	None	
1	988	CVSAHSSGYYGFYW	IGH	2	None	
1	989	CARDSPLGSGWYPFDSW	IGH	3	None	
1	990	CAREGGDMVVAAYNYFDYW	IGH	2	None	
1	992	CAKDKGGDSSGYYPFYYYYYGMDVW	IGH	2	None	
1	993	CARIYTRIYNYGDVRDYFFDYW	IGH	20	None	
1	994	CARDLVGPVLPTVELSDYW	IGH	2	None	
1	995	CALPICRGWWTPYYFDYW	IGH	2	None	
1	996	CARLKGSSSFHPFDYW	IGH	4	None	
1	997	CAREREDCTNGVCYTHFDYW	IGH	4	None	
1	998	CARSLLRLGELALLEGAVSPTIFDYW	IGH	6	None	
1	999	CARAKRAARPIDWFDPW	IGH	2	None	
1	1000	CVKEGGYTGGWFYFDYW	IGH	6	None	
1	1001	CAAEPPNCSGDYCYMSWFDPW	IGH	3	None	
1	1003	CARETTRGTGAIDYW	IGH	3	None	
1	1004	CATIYIGGAFDIW	IGH	2	None	
1	1005	CAKEGGVQAVTWYDERVRYFDYW	IGH	2	None	

1	1006	CAKCYDYVWGRYRSDGDFDYW	IGH	2	None	
1	1007	CARRIVVVVAATREVVWFDPW	IGH	3	None	
1	1008	CAKDSRLYASPYYYYYGMDVW	IGH	4	None	
1	1009	CTKVVTNVGHQTVTPVYFFDYW	IGH	3	None	
1	1010	CATIGWPVDNGRDYFEDW	IGH	4	None	
1	1011	CTKAGLPTLAAGVNNWFEPW	IGH	4	None	
1	1012	CASVAYSSSSFYIGLFDYW	IGH	2	None	
1	1013	CAKVFSWGVGRDGYNSFDFW	IGH	9	None	
1	1014	CARQSLSSGATFDYW	IGH	3	None	
1	1015	CATGGVVPAAMLSGYDGLTGFSNFDHW	IGH	3	None	
1	1017	CARGGTGDFDGSWYFDLW	IGH	3	None	
1	1018	CAKGNSQLWSLFDIW	IGH	2	None	
1	1019	CAKARNNWNHGWGVFDYW	IGH	2	None	
1	1020	CASSSYHDSTWFDPW	IGH	2	None	
1	1021	CARHGTSDAARGFDTW	IGH	2	None	
1	1022	CARDDRFLEWLFNYYYYHGLDVW	IGH	3	None	
1	1023	CAREPYYGFWSGYSHFDYW	IGH	2	None	
1	1024	CARGGDFWSGYFPFDYW	IGH	5	None	
1	1025	CARGRENQSSGWTDYW	IGH	2	None	
1	1026	CARGRRGSSGWGTMLSWFDPW	IGH	2	None	
1	1027	CAKTTILPLAYFDLW	IGH	13	None	
1	1028	CARVTLELEAFDYW	IGH	2	None	
1	1029	CARGLGIQYGGPLNWFDPW	IGH	3	None	
1	1030	CARVIDVRSRYYYYYMDVW	IGH	7	None	
1	1031	CARDGSIDYYDSSGYLGAFDIW	IGH	2	None	
1	1032	CTTDLLWQWLVKNYFDYW	IGH	31	None	
1	1033	CARDVPYYYDSSAYYFFDYW	IGH	5	None	
1	1034	CARDGPSVPAAAGGIGWFDPW	IGH	4	None	
1	1035	CARDPPDVPPVGIGSPSDYW	IGH	5	None	
1	1036	CARDMVWLYFDYW	IGH	2	None	
1	1037	CARVGGNPSWFDVW	IGH	2	None	
1	1038	CQSADSTSVYYVF	IGL	4	None	
1	1039	CAARDDCLSGGVLF	IGL	2	None	
1	1040	CAKEGGSGYPYYFDYW	IGH	7	None	
1	1041	CARGPYSGYDDVSWFDPW	IGH	16	None	
1	1042	CAKAPESYGDYYYYMDVW	IGH	4	None	
1	1043	CARSNSVLTGGGFDPW	IGH	2	None	
1	1044	CARDPLIGILTGSDDYW	IGH	2	None	
1	1045	CARHVSRTYYDFWSGSYEKRGAEYFQPW	IGH	3	None	
1	1046	CARYSRPLERNWFDPW	IGH	3	None	
1	1047	CARPYEIGNDAFDIW	IGH	2	None	
1	1048	CARGQRHSYGYYYYYIDVW	IGH	2	None	
1	1049	CTRESSEMTTFVYW	IGH	2	None	
1	1050	CCARAGWNYYRPHDPFDIW	IGH	2	None	
1	1051	CARDFPETGYDFDYW	IGH	7	None	
1	1052	CARERVSCNGGSCTSYLDYW	IGH	4	None	
1	1053	CARGGVSGSSGLHWFDSW	IGH	2	None	
1	1054	CARGPSINRIAIFGVGLHYFDYW	IGH	2	None	
1	1055	CAKDIYDSSGYYGAYFDYW	IGH	7	None	
1	1056	CARRSLYVWGSIRQYYFDTW	IGH	5	None	
1	1057	CAREDVTGPFGYW	IGH	2	None	
1	1058	CARAHDSSGYYYPDLTRLFTGFDYW	IGH	6	None	
1	1059	CATTLGDYSNYEGVFDYW	IGH	2	None	
1	1060	CARVGIVVKPAVSETEYYMDVW	IGH	3	None	
1	1061	CARLVVITSTHYFDYW	IGH	10	None	
1	1062	CVRCMPRRGWFGFAAFDIW	IGH	18	None	
1	1063	CARHLDDSSGYYWGYFDYW	IGH	2	None	
1	1064	CARNYYESSGDAFDIW	IGH	5	None	
1	1065	CARHRGAWGDTSGFYYW	IGH	2	None	
1	1066	CARARSGTSIDFDYW	IGH	14	None	
1	1067	CARKSGSPYHFWSGAAPSWFFDPW	IGH	4	None	
1	1068	CAKDVGTTVISNGFHYW	IGH	5	None	
1	1070	CAREAGRDTAMVPTIDYW	IGH	3	None	
1	1071	CARGVGDGEQYDASGSSHFDYW	IGH	20	None	
1	1072	CSSYTSSRTLDVF	IGL	3101	C2	7K90
1	1073	CAKSATLLTSVQGWYFDYW	IGH	27	None	

1	1074	CARARRDGYNYLATVFDIW	IGH	11	None	
1	1075	CVRADSQDFDLW	IGH	101	None	
1	1076	CARDGSYDFWSGYTYYYYYMDVW	IGH	2	None	
1	1077	CARVGSTISRIHSYSYMDVW	IGH	6	None	
1	1078	CARAANNIESPPGPPVLDYW	IGH	42	None	
1	1079	CARRSLSITIFGVVIRDAFDIW	IGH	6	None	
1	1080	CARSRLLWFGESAEYYFDYW	IGH	2	None	
1	1081	CASPRSPMRQGVILTIFDYW	IGH	2	None	
1	1082	CAKDSGVAGTVVPGAFDIW	IGH	2	None	
1	1083	CASIKRGYDILTGSSSFDYW	IGH	4	None	
1	1084	CARAEDLDTRGGFDAW	IGH	3	None	
1	1085	CASSVAVGATVDYYYFGMDVW	IGH	2	None	
1	1086	CTTDLRITGTEYYFDYW	IGH	4	None	
1	1087	CARDGIGYYGDWVPDYW	IGH	4	None	
1	1088	CQQYNNWPRSSLTF	IGK	10	C3	7K8R
1	1089	CQSSDSSLSAPHVVF	IGL	25	None	
1	1091	CAKDGRSGSFGFYYYYMDVW	IGH	2	None	
1	1092	CARFWTGNWYFDLW	IGH	4	None	
1	1093	CATPGGGHSYHWENWYFDLW	IGH	11	None	
1	1094	CQQYDTVPVPITF	IGK	9	C3	6WPS
1	1095	CARENRGYTDDAFDIW	IGH	2	None	
1	1096	CANQEMAYPGDYW	IGH	6	None	
1	1097	CARIRSWYGGNSGWFDPW	IGH	16	None	
1	1098	CTRSPIFSGYYYYYYMDVW	IGH	4	None	
1	1099	CARVDGHYDSSGFYQYLDNW	IGH	13	None	
1	1100	CQQAHTFPCTF	IGK	8	None	
1	1101	CQSADISGTYRYVF	IGL	269	None	
1	1102	CARDPSEYCTTANCYYFDNW	IGH	3	None	
1	1103	CAKYGYSGSYYHFDFW	IGH	6	None	
1	1104	CARHRRLSNKHIVVVTAHLGYFDYW	IGH	2	None	
1	1105	CARVRHILVVPGNWFDTW	IGH	4	None	
1	1106	CARGHQWEILLRSVETGWFDPW	IGH	2	None	
1	1107	CAKDAVRQILTGPYYFDYW	IGH	7	None	
1	1108	CARLSAESFGSRWIYWFFGLW	IGH	3	None	
1	1109	CAREPNWEQIDYW	IGH	6	None	
1	1110	CAKESSSHYYFAMDVW	IGH	2	None	
1	1111	CARGLRANVLRFLEWLFWFDPW	IGH	28	None	
1	1112	CAKVGGLLWFGEFDDAFDIW	IGH	3	None	
1	1113	CARHERKQLRKNFYFDSW	IGH	2	None	
1	1114	CARYEVVVVADATLYNWFETW	IGH	37	None	
1	1115	CAVTLRPGLPPFDFW	IGH	2	None	
1	1116	CARDSARWRQQKASTGFDYW	IGH	4	None	
1	1117	CASGLIYCTTADCYTRNFDSW	IGH	8	None	
1	1118	CANHKAALGGYYDFWSGYTVPNYYYYYMDVW	IGH	2	None	
1	1119	CAKGGDKIVVVTAFDYW	IGH	2	None	
1	1120	CARIMGVRSGWFTGDAFDIW	IGH	2	None	
1	1121	CARDASPSYCRSSTCSEVDWFDPW	IGH	4	None	
1	1122	CARELRHSGYEFFDYW	IGH	2	None	
1	1123	CAREGWGHWYAGGYHYYMDVW	IGH	9	None	
1	1124	CAGQLPGSSWFDPW	IGH	4	None	
1	1125	CAKVGFRAPSESGYFDLW	IGH	5	None	
1	1126	CARDYSSGWYITFDIW	IGH	6	None	
1	1127	CQSYDGDLSGSKVF	IGL	10	None	
1	1128	CAKEDVWNAPGSEYYYYGMDVW	IGH	9	None	
1	1129	CARYGQATTIRHWFDSW	IGH	2	None	
1	1130	CARIGHMDILYYYYYMDVW	IGH	2	None	
1	1131	CAKGYDILTDVSWEDWFDPW	IGH	2	None	
1	1132	CARALGYCSSTSCPAPDYW	IGH	2	None	
1	1133	CATGKYGDYW	IGH	61	None	
1	1134	CASFYWGLGLDYW	IGH	17	None	
1	1135	CARHLTVGASRPYFQHW	IGH	3	None	
1	1136	CARGLWQWLVLDAFDLW	IGH	2	None	
1	1137	CARLAQNTETTLYYYYFYLDVW	IGH	2	None	
1	1138	CAKDIERITIFGVGPFDYW	IGH	2	None	
1	1139	CSRDSKDSDYYANYPFDYW	IGH	2	None	
1	1140	CARDEGPDSGWFVYW	IGH	2	None	

1	1141	CARDLTVVAATSYYYYGMDVW	IGH	2	None	
1	1142	CATSPAVAGFFPPDYW	IGH	2	None	
1	1143	CTRHPIESDITYFTDYFFDRW	IGH	2	None	
1	1144	CARGEMDDSRFDIW	IGH	3	None	
1	1145	CAKDPSYYIPPNWFDPW	IGH	4	None	
1	1146	CAKEAVAGTNWFDSW	IGH	9	None	
1	1147	CAKGAGMATIYRFDYW	IGH	8	None	
1	1148	CARDGTLRFLEWSPYYYYGMDVW	IGH	2	None	
1	1149	CARDPSYSSTLYWYLDLW	IGH	4	None	
1	1150	CAKDLLIVPAARPPLSDFDHW	IGH	2	None	
1	1151	CSRHIEVGYDSSTFDSW	IGH	2	None	
1	1152	CARHIGGQYSYGGNWLDPW	IGH	17	None	
1	1153	CARDGISGYYYYYMAVW	IGH	2	None	
1	1155	CAASHSSDRFTYFDYW	IGH	4	None	
1	1156	CARSAERYCSGGRCSSDENFQYW	IGH	3	None	
1	1157	CVKDMSNAFNLAPDAFDIW	IGH	2	None	
1	1158	CAKDMVAVTGAMDVW	IGH	2	None	
1	1159	CARVVVGATWWFDPW	IGH	10	None	
1	1160	CAREILDDFSLSLNFDLW	IGH	2	None	
1	1161	CARGYCSGESCRWTVYWYFDLW	IGH	4	None	
1	1162	CAKRIYNYDSSGYWALDYW	IGH	2	None	
1	1163	CTRASHYYDMSGYYQPVVTDDSDIW	IGH	59	None	
1	1164	CARDTTYHYDSSGYYGLDYW	IGH	13	None	
1	1165	CARGWLDDFWNNFSFDQW	IGH	2	None	
1	1166	CATTIFGLVPPRRDAFDIW	IGH	2	None	
1	1167	CLLFDGGAQVWVF	IGL	2	None	
1	1168	CASLSHFITTIIYWYFDLW	IGH	2	None	
1	1169	CARDSSRLGGGWFDPW	IGH	3	None	
1	1170	CARSLGPWLHFDYW	IGH	7	None	
1	1171	CARDQGEQLGAFDYW	IGH	6	None	
1	1173	CAKERVSAMVEYKFDSW	IGH	8	None	
1	1174	CATTVVTPDSNAFDIW	IGH	2	None	
1	1175	CAKDLHFWSANDYW	IGH	22	None	
1	1176	CARAGDGTLGNWFDPW	IGH	2	None	
1	1177	CARAGGNLEWRGWFDPW	IGH	7	None	
1	1178	CARASCFSTRCSAGRPYSYYYAMDVW	IGH	2	None	
1	1179	CAKDVDDGYSSSPGLDYW	IGH	2	None	
1	1180	CATETTVTTMYYYYAMDVW	IGH	2	None	
1	1181	CARGNCGGDCSLFYYYGMDVW	IGH	6	None	
1	1182	CARHIFYESTGYAFDYW	IGH	2	None	
1	1183	CAKPPYYYDSSGYFDYW	IGH	42	None	
1	1184	CTRETYYGSLTAWFDPW	IGH	9	None	
1	1186	CARLTYYDIYDYIYMDVW	IGH	2	None	
1	1187	CARGGDDWGSLDYW	IGH	7	None	
1	1188	CARHSNALDSTGWYYYMDVW	IGH	3	None	
1	1189	CARDLYYDFWSGYYGYYYYGMDVW	IGH	6	None	
1	1190	CAKDVSRIGYSSGDFNYW	IGH	25	None	
1	1191	CARVSYDFWSGYYKHQNYDYYYMDVW	IGH	2	None	
1	1192	CARAAYYQSTDYSYWYFDVW	IGH	2	None	
1	1193	CARVGASPLPPQLHLYYMDVW	IGH	10	None	
1	1194	CAKGDGYSYGSGFVLLDYW	IGH	5	None	
1	1195	CARTASNDYGSLFWFDPW	IGH	2	None	
1	1196	CARTMGRYNNGHPEIHFDYW	IGH	4	None	
1	1197	CARDQWELLQFPRYFFDYW	IGH	2	None	
1	1199	CAAHPVGHSSGWYPLFDYW	IGH	6	None	
1	1200	CARWLGHNSGYFDGW	IGH	6	None	
1	1201	CAHLTYSSSYYYFDYW	IGH	2	None	
1	1202	CARYYYYDSSGYLSAFDYW	IGH	5	None	
1	1203	CAKDPNSITGPFDYW	IGH	7	None	
1	1204	CANARSGSYYYFEFW	IGH	2	None	
1	1205	CAVRLYDFWNGPYRTYYYGMDVW	IGH	3	None	
1	1206	CARVSLAAGDAFDIW	IGH	17	None	
1	1207	CANLLWPDYFDYW	IGH	4	None	
1	1208	CAWDSRNWDSPSYFYYYGMDVW	IGH	2	None	
1	1209	CARALGCGGDCYSSWFDPW	IGH	2	None	
1	1210	CARDQFLYNSGWYHPAYYFDYW	IGH	6	None	

1	1211	CARDNRVEPWFFDYW	IGH	2	None	
1	1212	CAREISFNMMTGGYDRWFDPW	IGH	3	None	
1	1213	CMIWPSNAVVF	IGL	132	None	
1	1214	CARDCCSGWYGSVYYYYYMDVW	IGH	5	None	
1	1217	CARGTGSSFGFAYW	IGH	10	None	
1	1218	CANLGSGPGDYW	IGH	4	None	
1	1221	CVSPPGDCGSASCSFDFW	IGH	2	None	
1	1222	CAKSKGYNHYFDNW	IGH	15	None	
1	1223	CARGFCSSIGCSPDWFDPW	IGH	16	None	
1	1224	CASTLPYSSSSRFDYW	IGH	4	None	
1	1225	CAKETRREWEILQGHFDSW	IGH	2	None	
1	1226	CARLRVQGKRLNWYFDLW	IGH	8	None	
1	1227	CARQQPILQDYFDSSGLDYW	IGH	3	None	
1	1228	CARATFVTTVTNFDYW	IGH	3	None	
1	1229	CVRDRRSYGYPTSFDYW	IGH	2	None	
1	1230	CTRAQRDYW	IGH	3	None	
1	1231	CARGRWRGRPNSVDYFDYW	IGH	3	None	
1	1232	CARPDILTGGGFDYW	IGH	2	None	
1	1233	CASDTTGWYFDFFDSW	IGH	9	None	
1	1234	CARDLTLGAIAANYFDPW	IGH	20	None	
1	1235	CAREPIEEGSGWYFDDW	IGH	31	None	
1	1236	CARDREYGDITPDAFDIW	IGH	2	None	
1	1237	CARVRFTAITMIVVVDAFDIW	IGH	18	None	
1	1238	CARDRSNYYDRSVYYYLRDYYYYMDVW	IGH	4	None	
1	1239	CATRPLLWPWLEEDASAGMAMDPFDYW	IGH	3	None	
1	1240	CARAHNDYYDTSGFYYLDNW	IGH	2	None	
1	1241	CARDPLFYSSSEFYYYYGLDVW	IGH	2	None	
1	1242	CAHRRQLAASSYFDSW	IGH	2	None	
1	1243	CAADRRAVAGTFDYW	IGH	2	None	
1	1244	CASRMPSVTLNRGFPPFDYW	IGH	2	None	
1	1246	CVRSRDYRRSFDTW	IGH	2	None	
1	1247	CAKGLTTVTTRGAFDIW	IGH	2	None	
1	1249	CARVETVPLMDYYYGMDVW	IGH	2	None	
1	1250	CAKERPPRRDRYLSYYFDYW	IGH	3	None	
1	1251	CARHLVGATRAFDIW	IGH	3	None	
1	1252	CARLGITVTVRLDYW	IGH	7	None	
1	1253	CARTETVTSLGPFDYW	IGH	2	None	
1	1254	CANHYYDVGNFDSW	IGH	5	None	
1	1255	CVRAVAGNYFDWW	IGH	3	None	
1	1256	CAKGKQWLVLPPIDYW	IGH	17	None	
1	1257	CARESGVYEHKIDYW	IGH	7	None	
1	1258	CARGWRETWLLRINGHFDYW	IGH	3	None	
1	1259	CARHKEYSSFSNPDDPW	IGH	2	None	
1	1260	CAFIYSSGWSKFDPW	IGH	3	None	
1	1261	CARDRGYCSGGSCYVGWLDYW	IGH	2	None	
1	1262	CARLGGPGWYVVYW	IGH	2	None	
1	1263	CARRVSGSYFGFYYYYLDVW	IGH	5	None	
1	1265	CAKDIAHGYTYEFDPW	IGH	3	None	
1	1266	CARDASRGIAVAGTPFDYW	IGH	9	None	
1	1267	CTTVGWRQLVGAAFDIW	IGH	3	None	
1	1268	CGRRRLASYIDYW	IGH	9	None	
1	1269	CARRKVGRPEERNWFDPW	IGH	2	None	
1	1271	CARLPYVGYYYSGGFLTGIDYW	IGH	2	None	
1	1272	CARVPRYGSGTFAFDIW	IGH	2	None	
1	1273	CAKDTRLGDGSTYGYFDYW	IGH	2	None	
1	1275	CARARGPYDSDGLDYW	IGH	2	None	
1	1276	CIRMGGNSGGFDYW	IGH	2	None	
1	1277	CTKSPYGWELLPLDYW	IGH	2	None	
1	1278	CARAPRGIFGVVTHFDYW	IGH	28	None	
1	1279	CARLRRESHYPGIAVVNYFDYW	IGH	67	None	
1	1280	CARHTGGTTYDYW	IGH	44	None	
1	1281	CARDPLRIQGHHYYYYMDVW	IGH	3	None	
1	1282	CAKSRLGEYPPFDSW	IGH	3	None	
1	1283	CAKATYYYNSGVYQDTYYFDYW	IGH	9	None	
1	1285	CARYLGYPLDYDGVDVW	IGH	4	None	
1	1286	CARAYYYGSGIEGGRFDYW	IGH	2	None	

1	1287	CAKDPGPVIPDAFDIW	IGH	2	None	
1	1288	CARDRVPSSSWTGDFDYW	IGH	2	None	
1	1290	CAREVRPVYNWNDGGFDYW	IGH	3	None	
1	1292	CAKHPRGLLPPFYFDYW	IGH	3	None	
1	1293	CASPSSSYVDYSINGFDVW	IGH	12	None	
1	1294	CARGPVNRYGYAPPIDYW	IGH	4	None	
1	1295	CARARMTTVTYFGYW	IGH	2	None	
1	1296	CAHRLRFTDWAPHDVFDVW	IGH	8	None	
1	1297	CARPGVTTRPVYWFDPW	IGH	4	None	
1	1298	CARRGDYYDSSGPDYW	IGH	4	None	
1	1299	CARDKDDCSGGSCFSTLLEPFDYW	IGH	4	None	
1	1300	CAGSVAIFDGEDAFDIW	IGH	86	None	
1	1302	CARRGVVYDILTGYLIGHDAFDIW	IGH	4	None	
1	1303	CARFLRDGYNARYYFDYW	IGH	2	None	
1	1304	CARVRIQGRRYSGSYWGDYW	IGH	4	None	
1	1305	CAKRERVSGFAPRDYYFEHW	IGH	5	None	
1	1306	CAKGRGYISGYSSNCFDPW	IGH	2	None	
1	1307	CARSGTLWKYWYFDLW	IGH	7	None	
1	1308	CAREYYYNSNSYFITDYW	IGH	2	None	
1	1309	CARHVGIATALSWIDPW	IGH	2	None	
1	1310	CVRSPDSSGWWDFDYW	IGH	5	None	
1	1312	CATAGAFGSGNYYNSDYW	IGH	2	None	
1	1313	CARDDILRWPDYW	IGH	2	None	
1	1314	CSAWDSDLRAWVF	IGL	23	None	
1	1315	CAKKGGSTSPEMYYYYGMDVW	IGH	2	None	
1	1316	CARHISMNRVDWFDPW	IGH	4	None	
1	1317	CLLSYSGVVF	IGL	226	None	
1	1318	CAGYYDSSVYDYGGGSLPFDLW	IGH	2	None	
1	1319	CAKGNVFIRLTDYW	IGH	355	None	
1	1320	CAREDGAGNFPTPDYW	IGH	2	None	
1	1321	CARTMTTVSFYYYYMDVW	IGH	5	None	
1	1322	CARAGVVPAPILSEEIPDFDYW	IGH	2	None	
1	1323	CAREKVDDNWHYVGYW	IGH	7	None	
1	1324	CAKGDTALVPYNWFDPW	IGH	6	None	
1	1325	CAKEATWSFLYFFDSW	IGH	9	None	
1	1326	CVRDLRVYSNLPRTPRYYYMDVW	IGH	2	None	
1	1327	CARVGDYVWGSFRDYW	IGH	8	None	
1	1328	CARFPPYYSDISGQRAFDIW	IGH	2	None	
1	1329	CAREGRERAGTVNDYW	IGH	2	None	
1	1330	CAKDRGEYYYDSSGYYHYW	IGH	3	None	
1	1331	CARSYGYTFGQIDYW	IGH	5	None	
1	1332	CAQGGGYSGSYLFDYW	IGH	2	None	
1	1333	CTTYFDFWSAYYHFDYW	IGH	2	None	
1	1334	CASAYSYGNIDYW	IGH	2	None	
1	1335	CARPNYGDYYFHFW	IGH	3	None	
1	1336	CAKDLRHWDGHDAFDIW	IGH	2	None	
1	1337	CAKDYSGNDISDSAFEYW	IGH	2	None	
1	1338	CARGYSYGYGGMDYFDYW	IGH	2	None	
1	1339	CARMIVAGYWYFDIW	IGH	2	None	
1	1340	CARGVAAGGTGFGYYYYYMDVW	IGH	3	None	
1	1341	CSVLAAAGGVGPPEFDYW	IGH	2	None	
1	1342	CVRDDVLRDLEWPNEPLNYYYGMDVW	IGH	14	None	
1	1343	CAKERVDGMDVW	IGH	4	None	
1	1344	CAKAEYNWNDRQIYYYYGMDVW	IGH	2	None	
1	1345	CAKDSVPRNGDHSATFFDYW	IGH	12	None	
1	1346	CARPLYSSSFFVYW	IGH	2	None	
1	1347	CARASLGSLERPADYW	IGH	2	None	
1	1348	CARDQRTANGWPCASDVW	IGH	2	None	
1	1349	CAKTLNVLRYFDWLLPRWFDPW	IGH	2	None	
1	1350	CAKDVGNHYYYYYGMDVW	IGH	8	None	
1	1352	CARHDISGTHFDYW	IGH	8	None	
1	1353	CARTGLRYFDWLTHNWFDPW	IGH	2	None	
1	1354	CARSGGYCSGGSCYSTREDLNWFDPW	IGH	4	None	
1	1355	CARWSGYRQGAFDIW	IGH	5	None	
1	1356	CAHRQGIVGFTDAFDFW	IGH	2	None	
1	1357	CAREREGAIRFDYW	IGH	6	None	

1	1358	CARGGLPNGDTSTWYFDYW	IGH	9	None	
1	1359	CAGAPHGYCRGGDCSVERMDYW	IGH	2	None	
1	1361	CARREKDGGEVDYW	IGH	3	None	
1	1363	CAKAPPGGWLWRDKYYFDYW	IGH	2	None	
1	1364	CARNRPGEMEFYYYHGMDVW	IGH	130	None	
1	1365	CARGRPHNYYDSGSYYNRRMNWFDPW	IGH	12	None	
1	1366	CARTEAGLIINPDYW	IGH	7	None	
1	1367	CASKGGMPYDSSGYYYYYYGMDVW	IGH	2	None	
1	1368	CFTSSWSYLDYW	IGH	5	None	
1	1369	CARDLTPYDSSGYYYVHDAFDIW	IGH	9	None	
1	1370	CVRHTLPPFVWGSGTQLPYFDLW	IGH	2	None	
1	1371	CARSDYGSGTDNWFDHW	IGH	5	None	
1	1372	CARENGIVVPPSSISKTGLPIGIDYW	IGH	2	None	
1	1374	CARLSHSRFDPW	IGH	49	None	
1	1375	CASGIAAAGKGLGDYW	IGH	3	None	
1	1376	CARGGYCSRTSCYASLKNWLDPW	IGH	2	None	
1	1377	CARGSETDNPGDYTETDALDLW	IGH	5	None	
1	1379	CARDHQPLLYLYYYGMDVW	IGH	2	None	
1	1380	CASDTSMVRDAFDIW	IGH	3	None	
1	1381	CARTRISLEGWLREFDPLDVW	IGH	4	None	
1	1382	CARGSHNYYDSSGYRYAFDIW	IGH	7	None	
1	1383	CAKVGFITVVVAAPNYFDYW	IGH	2	None	
1	1385	CARLGVHYDYVWGSYRSNWFDPW	IGH	4	None	
1	1386	CARGAVVVSSIGPDHFDYW	IGH	34	None	
1	1387	CARDLSRDWDDYGDDDAFDIW	IGH	6	None	
1	1388	CAKVPIYGENDIDYW	IGH	2	None	
1	1389	CAKDFWRRGIAAACPDYW	IGH	2	None	
1	1390	CAKVVSRSRSSYSFNYTDVW	IGH	50	None	
1	1391	CARGGDVLLWFGEPIDYW	IGH	9	None	
1	1392	CVKDRYYIYYHGMDVW	IGH	2	None	
1	1393	CVSLVETADLAFDVW	IGH	2	None	
1	1394	CAKDGDRAVRPSALTYNWFDTW	IGH	4	None	
1	1395	CARAEWLRGDFDYW	IGH	11	None	
1	1396	CATTTPHCSSTSCYSNWFDPW	IGH	3	None	
1	1397	CARLGDWDISGYRDYW	IGH	2	None	
1	1398	CARFAVVGIPLLYNWFDPW	IGH	6	None	
1	1399	CATARQEYMTTVTVLDYW	IGH	6	None	
1	1400	CAAFDIMTGYGAFDVW	IGH	2	None	
1	1401	CTRQSAMAIHGGSWFDPW	IGH	2	None	
1	1402	CARDWGSSSWYLDYW	IGH	2	None	
1	1404	CARDAPFTGGGYYFDYW	IGH	3	None	
1	1405	CARHGGPSYYDFWNGHYIGGSQDAYYYMDVW	IGH	2	None	
1	1406	CARAGNYGSASYNWFDPW	IGH	3	None	
1	1407	CAKGHYYDSNYYSMYYFDYW	IGH	4	None	
1	1408	CARDHADFDGGGWFDPW	IGH	27	None	
1	1411	CARIPPCSDDCFSPYYFDSW	IGH	2	None	
1	1412	CARAKYTFGGHPIVFDYW	IGH	11	None	
1	1413	CARHETYGDTSFDYW	IGH	7	None	
1	1414	CARHPYSGTYWVFDYW	IGH	5	None	
1	1415	CARRSRWGYYFDGW	IGH	6	None	
1	1416	CAREGIVVPWFDPW	IGH	2	None	
1	1417	CARGRRDMIVVVITTRYYYGMDVW	IGH	3	None	
1	1418	CVKANVTAVAGTGPSPKIYYYYYGMDVW	IGH	5	None	
1	1419	CAHTEALEMGEYYFDYW	IGH	2	None	
1	1420	CMKDPSTSYHGQGWFDPW	IGH	2	None	
1	1422	CARDRPRWNFDYW	IGH	3	None	
1	1423	CVKEHYYYDSSGYYRYFDLW	IGH	3	None	
1	1424	CVREHSGGYAPIAKFDYW	IGH	2	None	
1	1426	CARLGSLTSGYYSHFDYW	IGH	5	None	
1	1428	CVKTRGSSSTLYAMDVW	IGH	3	None	
1	1429	CQHYYNWPPRFTF	IGK	23	None	
1	1430	CARRRSGSGAVDYW	IGH	2	None	
1	1431	CAKGFTYYYDSAGFRYLDYW	IGH	221	None	
1	1432	CARDASSNWGTTYYFDYW	IGH	14	None	
1	1433	CARHKAPRPGPYSSGWYGDYW	IGH	2	None	
1	1434	CARDSYYDGSGKERFDYW	IGH	2	None	

1	1435	CARGKPVVTQGFYYYYYMDVW	IGH	2	None	
1	1436	CTKDYGDHYNWFDSW	IGH	2	None	
1	1437	CVKEIGAWMGAFDVW	IGH	174	None	
1	1438	CVRHIPPRPDYGDYFDYW	IGH	2	None	
1	1439	CSRLSYSDILTVSGTFDFW	IGH	3	None	
1	1440	CTKDVLRGVGAQFDHW	IGH	2	None	
1	1441	CAKGIAARYGYYYGMDVW	IGH	2	None	
1	1442	CLAWDTSPRAWGVF	IGL	14	None	
1	1443	CAGGDWEGLQGYFDYW	IGH	2	None	
1	1444	CARYGIAPTGAFTYYIDYW	IGH	2	None	
1	1445	CARAHIPRHYQDINGFRNSDSFDVW	IGH	3	None	
1	1446	CARDRGGDYMGHWFDPW	IGH	12	None	
1	1447	CARETLHDFWRGHYESLDFYYYGMDVW	IGH	2	None	
1	1448	CALGATRKDDAFDIW	IGH	6	None	
1	1449	CVTGGGGCGGDCYWYYFDYW	IGH	2	None	
1	1450	CARRNFNILTGYYNVGGGDDYW	IGH	2	None	
1	1452	CARDSGSGWSTDAFHIW	IGH	5	None	
1	1453	CARQLPSQWLFDSW	IGH	2	None	
1	1454	CARDRGYSGWYSRFLDYW	IGH	2	None	
1	1456	CARESPHHCSSTSCYERHYYYYGMDVW	IGH	3	None	
1	1457	CAADPLYYYDSSGFRFDYW	IGH	3	None	
1	1458	CARAPYFYDSRAFYYSYW	IGH	13	None	
1	1459	CAKEKHRSSSGNDAFDIW	IGH	5	None	
1	1460	CARQGDYYDSSGYYDYW	IGH	19	None	
1	1461	CARQWSLFGKLTYFDSW	IGH	11	None	
1	1462	CAKDKSQQPDYYFDYW	IGH	12	None	
1	1463	CARLSDIAAHPDYW	IGH	6	None	
1	1464	CAKDGPLFCSGACYSAGGYFDPW	IGH	2	None	
1	1465	CARDLGMGYDYVWGSYRNYYYGMDVW	IGH	2	None	
1	1466	CARETYELVSHSRSYYFDYW	IGH	2	None	
1	1467	CARGSHSGSEGDAFDIW	IGH	4	None	
1	1469	CVRDPDTGSFYADYW	IGH	6	None	
1	1470	CAKGITGTTSKYYEYYYGMDVW	IGH	2	None	
1	1471	CARNVDLGLVPALGFDYW	IGH	10	None	
1	1473	CARLSRYEPRDFDYW	IGH	6	None	
1	1474	CARDRHESSGFHFYVGAFDIW	IGH	5	None	
1	1475	CARERGKQFRIDWFDPW	IGH	2	None	
1	1476	CARDSNYDVLTEYYIHYCDYW	IGH	3	None	
1	1478	CARDLWITMIVVVTIPGAFDIW	IGH	8	None	
1	1479	CAKDERGVTPPHFDYW	IGH	2	None	
1	1480	CAKAPRDWKQLDYW	IGH	29	None	
1	1481	CARLRWLQFRSFDYW	IGH	2	None	
1	1482	CATATVGATTVPRNYYYHYLDVW	IGH	28	None	
1	1483	CARGGVVPVDYYFYYMDVW	IGH	4	None	
1	1484	CARLDGNSYVPNYSYYYMDVW	IGH	5	None	
1	1487	CAKLYLSTWSRGNWFDPW	IGH	17	None	
1	1488	CATIPTYSSSWYKWIDYW	IGH	2	None	
1	1489	CVLYMGSGIWVF	IGL	30	None	
1	1490	CVKDRVAAAGTNWFDPW	IGH	2	None	
1	1491	CARVSYSAWARTPTHWYFDLW	IGH	2	None	
1	1492	CTTATIRYCSGGSCLYFDIW	IGH	2	None	
1	1493	CASAEEVVYFDYW	IGH	2	None	
1	1494	CAKDPRYYDFWSGYAGGQPSGGYGYYGMDVW	IGH	3	None	
1	1495	CARQSPYQEIWSDHFRWFDPW	IGH	6	None	
1	1496	CAGGRRRGSGYPVFDYW	IGH	2	None	
1	1497	CAKGSAESGYSSGWYIYYYYMDVW	IGH	2	None	
1	1498	CARTYCSSTSCYLDAFDIW	IGH	4	None	
1	1499	CARFPNDWNWFDPW	IGH	17	None	
1	1500	CARTVREYLSFDYW	IGH	3	None	
1	1501	CARGHDWGTIFGVVILGNNWFDPW	IGH	3	None	
1	1502	CARRESHSYDSSGYFVDPYYFDFW	IGH	67	None	
1	1505	CARGGYGDSYYYYALDVW	IGH	2	None	
1	1506	CAHTGTDYDDHEEGFDYW	IGH	4	None	
1	1508	CAADRSSSSTSCSSTCFWFDPW	IGH	2	None	
1	1510	CARDSGGGYDLYCYYMDVW	IGH	4	None	
1	1511	CATSLPYTNWDRGPLNYYYYMDVW	IGH	4	None	

1	1512	CARRCCAITMISPTNYYYMDVW	IGH	5	None	
1	1513	CARRGSSTANSYNRYFDPW	IGH	2	None	
1	1514	CARVNYYDSSGYLPPDYW	IGH	5	None	
1	1515	CAKGGGAYDFAYYYYGMDVW	IGH	2	None	
1	1516	CAKFSSWLGVDYW	IGH	2	None	
1	1517	CVRDSSGRGGFGDWFDPW	IGH	28	None	
1	1518	CARSVLMNNYAMDVW	IGH	4	None	
1	1519	CARQGDPGYAGSYHDSW	IGH	23	None	
1	1520	CVRHAVVAARPRGDYFDSW	IGH	35	None	
1	1521	CARVSTSFDDW	IGH	29	None	
1	1522	CARGDHAVGATTGWFDPW	IGH	4	None	
1	1523	CARIYNYFYYIDAW	IGH	2	None	
1	1524	CARDQGRDGYNSDWFDPW	IGH	2	None	
1	1525	CVTNKGLRYCSGGRCYRTGSWFDPW	IGH	2	None	
1	1526	CGGPTGTTTITRLKAFDIW	IGH	6	None	
1	1527	CAREVGYSYAYHQTNWFDPW	IGH	4	None	
1	1528	CARVDSRVVVAAQFDPW	IGH	6	None	
1	1529	CARARQVGMTPEYFQNW	IGH	27	None	
1	1530	CARDRPGYGDSSSPHAFDIW	IGH	2	None	
1	1531	CAADPVAGIGDYYYYGMDVW	IGH	2	None	
1	1532	CSWSMPGRSNDYW	IGH	2	None	
1	1533	CATDGYNWRREYYFNSW	IGH	8	None	
1	1535	CARGLRYCSGGSCHGDWFDPW	IGH	2	None	
1	1537	CAKGYPYSGSSDYW	IGH	6	None	
1	1538	CATGGGNAIYYYYYYMDVW	IGH	3	None	
1	1539	CAKSMVPADFPNDYYYYGMDVW	IGH	2	None	
1	1540	CYQYFNWPTF	IGK	5	None	
1	1541	CAKEVYGGYSYGYPYYFDYW	IGH	4	None	
1	1542	CAKDLLGRRAPIAGAIRRGWQKYNFDYW	IGH	2	None	
1	1543	CARLKAPPYWYFDLW	IGH	24	None	
1	1545	CAKANSGSRLDAFDIW	IGH	12	None	
1	1546	CASLRYSSSWYGDDAFDIW	IGH	4	None	
1	1547	CARLPYWNDDGYYYYGLDVW	IGH	3	None	
1	1548	CSSRDSSVNHLEVVF	IGL	4	None	
1	1549	CAKQGGVYCSGGSCYSGYFDYW	IGH	18	None	
1	1550	CARDRIAAAGKAFDIW	IGH	4	None	
1	1552	CQQYGGSSAVTF	IGK	13	C1	7K8N
1	1553	CARVIRDYYGSGSYSYYFDYW	IGH	4	None	
1	1555	CARAYRGYSYTSTMFNWFDTW	IGH	3	None	
1	1556	CARAEWKDHNSFDLW	IGH	2	None	
1	1557	CARLTRSGSPPYYYMDVW	IGH	17	None	
1	1558	CAATRAIGWELQFDYW	IGH	7	None	
1	1559	CTTHLYRSTWDRSIYSYYYYMDVW	IGH	3	None	
1	1560	CARISTGYYKEFDYW	IGH	9	None	
1	1561	CARESIVVVPADENWFDPW	IGH	4	None	
1	1562	CVKGDTIGYYLSRYYFDYW	IGH	2	None	
1	1563	CARDGPAVTPMAYYYYYMDVW	IGH	2	None	
1	1564	CARGAGGFNYWYFHLW	IGH	8	None	
1	1565	CAIATRRYGDYTFDYW	IGH	2	None	
1	1566	CAIHRLTIFGVVTLFDPW	IGH	3	None	
1	1567	CATHDFWSGYTFDYW	IGH	17	None	
1	1568	CARQRRYYHDSGGYYWFFDLW	IGH	6	None	
1	1569	CARAYCSGYNCFSGDDAFDMW	IGH	4	None	
1	1570	CAAGVDTFDYW	IGH	7	None	
1	1571	CARAPYGGDFVVEQYYFDFW	IGH	2	None	
1	1573	CAKVAYTPTYFDDW	IGH	4	None	
1	1574	CTRDPSYSSTWPEYFDYW	IGH	3	None	
1	1575	CAAEPQGEYYYYYGMDVW	IGH	3	None	
1	1576	CVHRPSTPYFDYW	IGH	2	None	
1	1578	CAREKRADRWFGPW	IGH	2	None	
1	1579	CATAMYDFWGSYAADYW	IGH	3	None	
1	1580	CARMHSSGYGGWDYW	IGH	4	None	
1	1581	CARDRRGLGYCSGGSCYPSYNWFDPW	IGH	4	None	
1	1582	CAGDDSSGYYYYNWFDPW	IGH	17	None	
1	1584	CARGHDYGDYENWFDPW	IGH	13	None	
1	1586	CARDLGYSSSVEDYW	IGH	6	None	

1	1587	CARAPKYITMVRGTYYYYYYMDVW	IGH	2	None	
1	1588	CARDRRYDYGDFDWYFDLW	IGH	4	None	
1	1589	CVKGSDLEWLLLPQYFDYW	IGH	2	None	
1	1591	CARTIIDRERSPFDYW	IGH	2	None	
1	1592	CASWENTIFGVVIRKQDPYYFDYW	IGH	2	None	
1	1593	CARHDFSTVAASNWFAPW	IGH	2	None	
1	1594	CATLDYYDYSGRPSRAFGYW	IGH	2	None	
1	1595	CARGTGEWLPLDYW	IGH	74	None	
1	1596	CAKATYMYESSGYQDTNYFDDW	IGH	13	None	
1	1597	CARGRRTVIVVVNYYYYYMDVW	IGH	2	None	
1	1598	CARRAVVGAPHFDSW	IGH	10	None	
1	1599	CAKEIRRGAFDYW	IGH	20	None	
1	1600	CAREWVEYCSGGSCYYSYYYGMDVW	IGH	2	None	
1	1601	CARVWRLGYPAFWYYMDIW	IGH	2	None	
1	1602	CTRAPSSTWPRDWFDPW	IGH	8	None	
1	1603	CARWGLAAAKNYHHAAMDVW	IGH	5	None	
1	1604	CAHSGYYYDSSGYYVWYYFDYW	IGH	3	None	
1	1605	CARDLRDGYNYAFDIW	IGH	2	None	
1	1606	CARDRPRDRYSSGYYYYYYYGMDVW	IGH	2	None	
1	1607	CQHYNSYSQFTF	IGK	115	C3	7K8R
1	1608	CARELDKIRVVVAATVGFDAW	IGH	2	None	
1	1609	CTRDTEYYDNSGYYYPRFGYW	IGH	3	None	
1	1611	CARDPRGYSYGLVDYW	IGH	4	None	
1	1612	CAAYHSYIDVW	IGH	3	None	
1	1613	CASTQLTRNSDAFDIW	IGH	3	None	
1	1614	CARATSVAATDWFFDLW	IGH	5	None	
1	1615	CAKHADPGVDITNFDNW	IGH	2	None	
1	1616	CAKQIGSELRGEAVYYYGMDVW	IGH	4	None	
1	1617	CQPYDILPMYSF	IGK	7	C3	6XDG
1	1618	CATSGLSGYLEYW	IGH	3	None	
1	1619	CARSGFYASNLGWFDPW	IGH	2	None	
1	1620	CVRGGGVHWFDPW	IGH	8	None	
1	1621	CASGRSYGEPRSPFDYW	IGH	7	None	
1	1622	CVKDPSPITPTYYFDYW	IGH	2	None	
1	1623	CAVPRVGVTTPYYFDYW	IGH	2	None	
1	1624	CAKLELGRYSGHDYLGYW	IGH	7	None	
1	1625	CARGPHIVVVTAIFQNWFDPW	IGH	6	None	
1	1626	CARDVWFGELSTSDDYW	IGH	2	None	
1	1627	CARDPGKQQLPYYYYYGMDVW	IGH	2	None	
1	1629	CQVGDSSSDPYYVF	IGL	3	None	
1	1630	CARDRPKQHLGPDHYDHHYYYMDVW	IGH	3	None	
1	1632	CVRGVMTGSYYSGFDQW	IGH	3	None	
1	1634	CVRDRSIAVADPLDYW	IGH	4	None	
1	1638	CAKKSQYLQMSSLRAEDTALYYCAKGRKSWYFDFW	IGH	2	None	
1	1639	CARRAVAYDILWYFDFW	IGH	7	None	
1	1640	CARVSHRPRITMIVGGRLGYFDYW	IGH	2	None	
1	1641	CARERLHDYGFEYW	IGH	2	None	
1	1642	CARGSVVLMVYAIGGWFDPW	IGH	2	None	
1	1643	CAKEWQQLDYW	IGH	8	None	
1	1644	CARNGQYYYDSSGYSVYYYYGMDVW	IGH	2	None	
1	1645	CARGAPPFGESPPFYYYYYGMDVW	IGH	3	None	
1	1646	CARHDLRQLRWNGFDSW	IGH	4	None	
1	1648	CSIIDELELRHWFDPW	IGH	4	None	
1	1649	CARGQGGYPYGTYNSYYYYMDVW	IGH	2	None	
1	1650	CARDRGAWSARPQYFDYW	IGH	2	None	
1	1651	CARGDILTGYYNYRYSYWFDPW	IGH	3	None	
1	1652	CARDGYGSGSYRIYYFDYW	IGH	19	None	
1	1653	CARGHIFVLPAPILGLGPFYTSYYMDVW	IGH	4	None	
1	1654	CTKDVLVGGGTVTTSSSFDYW	IGH	5	None	
1	1655	CAGVSPTMIVVLVGDAFDIW	IGH	2	None	
1	1656	CTKDRDRWTPHIAVAGTAGDYW	IGH	3	None	
1	1657	CMQALQGPDMYTF	IGK	40	None	
1	1658	CARPGPISLGFDPW	IGH	7	None	
1	1660	CAREVSVNTAVGSIPYAMDVW	IGH	37	None	
1	1661	CARDLVGGRPLDYW	IGH	31	None	
1	1662	CASQDGYNSNVGYIDYW	IGH	2	None	

1	1663	CARDYKNSSGNWFDPW	IGH	5	None	
1	1665	CARDSHEYYDLLGRALYYFDYW	IGH	2	None	
1	1666	CLRAEDTAVYYCAKENHEGYYDSSGYYFFFPDLDYW	IGH	2	None	
1	1667	CARGWDFCTSTSCYRFNAFDVW	IGH	2	None	
1	1668	CAKGAGYSDFDWYFDLW	IGH	4	None	
1	1669	CARHETSGGDYDGYYYYYYMDVW	IGH	2	None	
1	1670	CARHARITIFGVVIMGAFDIW	IGH	2	None	
1	1671	CARMVSGSYYDYW	IGH	17	None	
1	1672	CARHSRHLSRFYYFDYW	IGH	3	None	
1	1673	CAKGLWIQLWYPHGFDYW	IGH	2	None	
1	1674	CAKEYYSETYAHYGTPLFDYW	IGH	3	None	
1	1675	CQSYDSSLSRLRVIF	IGL	2	C4	7JVC
1	1676	CARGIAAAQGPGRLYYYYYGLDVW	IGH	3	None	
1	1677	CAHSLFPSCSTTKCYDGGENRGKNWFDSW	IGH	2	None	
1	1679	CARELGTAMVSGSYYYYYGMDVW	IGH	4	None	
1	1680	CAHRPALIYDYVWGSYRYGYHFDYW	IGH	2	None	
1	1681	CAKDISGREMATISGYHYYGMDVW	IGH	20	None	
1	1682	CARDRPYTIFGVVVASESVDYW	IGH	2	None	
1	1683	CAIASDRGIMTTSTERDYW	IGH	2	None	
1	1684	CARQSGYSYGYPDYW	IGH	126	None	
1	1685	CATLYTSSLSYDNNYMDVW	IGH	2	None	
1	1686	CARDGAFVAARLNYFDPW	IGH	6	None	
1	1687	CSRGGWVPGETRSFDIW	IGH	2	None	
1	1688	CARGPRYSTLNTFWFDPW	IGH	4	None	
1	1689	CARNADGSTLVTTKPIRYAYFYYMDVW	IGH	5	None	
1	1690	CARLGGAHSSNYYKESEGYFDYW	IGH	4	None	
1	1691	CTTGVGYLYSYYYGMDVW	IGH	2	None	
1	1692	CQSYDSSLSGVVF	IGL	7353	C4	7JVC
1	1693	CARDFRVGVYDSSGVVFDPW	IGH	3	None	
1	1695	CAKGGLVVDLRAEYFQHW	IGH	2	None	
1	1696	CAHMGDLVAAHSFDYW	IGH	2	None	
1	1697	CARPRWDRFDPFDIW	IGH	11	None	
1	1699	CAKTSCPDGHCHRFNWFDPW	IGH	3	None	
1	1700	CARSDPLNDYTFWHFDLW	IGH	3	None	
1	1701	CAHELGYCTTGVCSLGTYFDYW	IGH	2	None	
1	1703	CARDRRQLGPYNWFDPW	IGH	2	None	
1	1704	CNSRDSSSNHPSYVF	IGL	2	None	
1	1705	CAKDVYYDSSGYYVGGAFDIW	IGH	2	None	
1	1706	CARDLTYDFWSGYYKGFDYW	IGH	45	None	
1	1707	CATGSAYSSSYWFDPW	IGH	5	None	
1	1708	CGRGGGGSTPFDYW	IGH	7	None	
1	1709	CAKDRFTVTIRGWFDPW	IGH	7	None	
1	1710	CARSPMITFGGAIFDSW	IGH	2	None	
1	1711	CARDPLIVTSRIGFYSFDYW	IGH	4	None	
1	1712	CARPSSGWPYWYFDLW	IGH	53	None	
1	1713	CARMYSSLSNFDHW	IGH	2	None	
1	1714	CTTEWSFSSGWDYW	IGH	12	None	
1	1715	CVRRLYGDYGLPGAFDIW	IGH	2	None	
1	1716	CARDTPRAATHPHWYFDLW	IGH	4	None	
1	1717	CAKGHGIAFWSGNYNWFDPW	IGH	3	None	
1	1718	CAKVFTFWSGYMTGQDAFDIW	IGH	4	None	
1	1719	CARGKLVGASRTQYDYYYYMDVW	IGH	4	None	
1	1720	CVRASPSSGYHFFDFW	IGH	5	None	
1	1721	CATDHYDRSDYSGGAFDFW	IGH	3	None	
1	1723	CAKDLELGRLPGGWFDPW	IGH	5	None	
1	1724	CARDLSRRYSSKPKGDYW	IGH	2	None	
1	1725	CTRDRHSRITIFGGAKKGGLYYYGMDVW	IGH	10	None	
1	1726	CAKDGPRFLEWLYQWGNYYYYYGMDVW	IGH	2	None	
1	1727	CVRHEPTYYNYYGMDVW	IGH	4	None	
1	1728	CARLPHYYDSSGYSPSSDYW	IGH	2	None	
1	1729	CARDPPDYGDHVPKPMYYFNYW	IGH	3	None	
1	1730	CAKDKGDVLRFLEWLDAFDIW	IGH	8	None	
1	1731	CARDLGYYYDSSGRSWNWFDPW	IGH	5	None	
1	1732	CARNNYDFWSGYYHGQYYFDYW	IGH	2	None	
1	1733	CARRVKNSWLAAGSRTVSFDYW	IGH	4	None	
1	1734	CARVDSGFSYHYYSYMDVW	IGH	23	None	

1	1735	CQQYYSASGLTF	IGK	136	C4	6W41
1	1736	CARQLQLHSFDYW	IGH	21	None	
1	1737	CARDVENYYDSSAYSSKRPHFYYMDVW	IGH	7	None	
1	1738	CAREGTIAGLSDAFDIW	IGH	3	None	
1	1739	CQQYSTYSRSITF	IGK	3	C2	7K8U
1	1740	CARGRSKISMIVVVITFQSYYFDYW	IGH	2	None	
1	1741	CARDNGIRGQLAHFDYW	IGH	2	None	
1	1742	CTTGDSDSWSGHYSFDYW	IGH	4	None	
1	1743	CARDGVGKQLVPRYYYYYMDVW	IGH	6	None	
1	1744	CARETIVVVGGYGLRYFDYW	IGH	2	None	
1	1745	CAKDTPLIRANCNGDCYSSFDCW	IGH	6	None	
1	1746	CARGVVAVYFDHW	IGH	5	None	
1	1747	CAREYDSGGSHDPHYYYMDVW	IGH	2	None	
1	1748	CARERVRTRGWFDPW	IGH	12	None	
1	1749	CTTDIRLVYGDYVGFDFW	IGH	3	None	
1	1750	CTTVEIFTYYYGSGSYGFDPW	IGH	3	None	
1	1751	CARGVAAAGRFDPW	IGH	6	None	
1	1752	CAMSRPNEMLMHEQNWFDPW	IGH	3	None	
1	1753	CTRGNGYDDTFDYW	IGH	10	None	
1	1754	CARDLMVRGVNLFDPW	IGH	3	None	
1	1755	CASGFDGPNWYFDLW	IGH	3	None	
1	1756	CAKDSRGLEIALFDYW	IGH	2	None	
1	1757	CASSKDYGDYSHYFDYW	IGH	2	None	
1	1758	CARGGLIAVAGRTKINWLDPW	IGH	8	None	
1	1759	CAKYEDFFEDHYYFDFW	IGH	2	None	
1	1760	CASLAVSGTIVDYW	IGH	6	None	
1	1761	CARGDILTGYYKGYYYGMDVW	IGH	3	None	
1	1762	CARDPSLYYHSGTYYFDYW	IGH	2	None	
1	1763	CAKAPKRHDYGDYFDYW	IGH	5	None	
1	1764	CARDRGWELLGAIDVW	IGH	2	None	
1	1765	CVKEAYSSRWSYFDSW	IGH	2	None	
1	1766	CAGRRKTTAAWFDLW	IGH	2	None	
1	1767	CTTYGSSSGWYSSFDYW	IGH	13	None	
1	1768	CAKDLLEWLSLGGYFEYW	IGH	2	None	
1	1769	CARLHSRNYSPYFDSW	IGH	2	None	
1	1770	CARDPSDSSGYHSTPPDYW	IGH	3	None	
1	1771	CARVGSTSGAFDIW	IGH	12	None	
1	1772	CARFRGAGSPAYGGYWYFDLW	IGH	2	None	
1	1773	CARVLRDYSYYYYYMDVW	IGH	6	None	
1	1774	CQSYDAYNHRAVF	IGL	14	C4	7JVC
1	1775	CARYSYCGSDCYSVIDYW	IGH	2	None	
1	1776	CAQVEPDYYDSSGYYSVYW	IGH	44	None	
1	1777	CARDNSHSTPQRVYYTYYYMDAW	IGH	7	None	
1	1778	CAKDWIGRELPHLYYLDYW	IGH	2	None	
1	1779	CASLPYYYDSSGSAFDIW	IGH	4	None	
1	1780	CARDYCINGDCYYFDNW	IGH	3	None	
1	1781	CARGLIDSSGYYPDYW	IGH	15	None	
1	1782	CARLNGDYGFTFYYYYYYMDVW	IGH	3	None	
1	1783	CARAPRMDSSVWYGTGYFDYW	IGH	2	None	
1	1784	CARDAPNSAGYDVYYFDYW	IGH	2	None	
1	1785	CARDPAGYSYGLYYYYYMDVW	IGH	3	None	
1	1786	CATNLAADYFDSSYFVHW	IGH	3	None	
1	1787	CARDLGGRFLEYLFQPDAFDIW	IGH	11	None	
1	1788	CARRDAYCGGDCFGYW	IGH	3	None	
1	1789	CAKDQCFRCSSGWYYYYYGMDVW	IGH	162	None	
1	1790	CARQSCSSTSCYNFDYW	IGH	3	None	
1	1791	CASNYYSSGSYYEYYGMDVW	IGH	2	None	
1	1792	CARDPSIAAPSFTNWFDPW	IGH	4	None	
1	1793	CAKEEGDRYYYDSSVYHPSPFDYW	IGH	5	None	
1	1794	CVGWYYDESGDYSDGFDVW	IGH	8	None	
1	1795	CARLPDYANYQGDWLDPW	IGH	3	None	
1	1796	CAHSEETTVTSWEKPHFFDSW	IGH	10	None	
1	1797	CTRHVDVSEEDYYDSTGYYLFDYW	IGH	4	None	
1	1798	CAKEMNSNYGNTDYW	IGH	6	None	
1	1799	CARDTGLGYCSGGSCYSGYW	IGH	2	None	
1	1800	CARQCDYDNGDWGFDPW	IGH	6	None	

1	1801	CARHPRGWLQLNYYYYYMDVW	IGH	7	None	
1	1802	CARHQAGVPLDYW	IGH	10	None	
1	1803	CAHRPFGIAVAGFDYW	IGH	4	None	
1	1804	CARDLYDSLTGPNNWFDPW	IGH	14	None	
1	1805	CARHLAYYDFWSGSDFYYYYMDVW	IGH	12	None	
1	1806	CARDRLDQQVATGLDAFDIW	IGH	4	None	
1	1808	CARVTHCSGWYRCLDYW	IGH	2	None	
1	1810	CATPLYPGIAAAGIFDYW	IGH	2	None	
1	1811	CVRPYFGSGHYWHFDLW	IGH	8	None	
1	1812	CARFASSPRGGYYREVFDYW	IGH	18	None	
1	1813	CARSARPNYYYYYGMDVW	IGH	3	None	
1	1814	CALEATGTVIIVSLDAFDIW	IGH	4	None	
1	1815	CARYFMGMAVTTTSDYFDSW	IGH	7	None	
1	1817	CARVDQMELSALSSFDLW	IGH	12	None	
1	1818	CARDGDAGNWLDYW	IGH	6	None	
1	1819	CARQKAPAAAGSFYYYYYYMDVW	IGH	2	None	
1	1820	CAKYSASWGVLGWLDPW	IGH	2	None	
1	1821	CAHRRGPEYSGSVVGNAAFDIW	IGH	2	None	
1	1822	CARDYYDSSGYYYYYYGMDVW	IGH	13	None	
1	1823	CARGTPDLSFDHW	IGH	3	None	
1	1824	CQSADISDPYDDVVF	IGL	5	None	
1	1825	CARRASRYGWEEYYFDYW	IGH	7	None	
1	1826	CARQSAVGSTNFDYW	IGH	102	None	
1	1827	CARTAQLRFVKYFFFQNDYW	IGH	2	None	
1	1829	CARDTHSPMRADYW	IGH	9	None	
1	1830	CAHSLLSGYSKSEGFDYW	IGH	5	None	
1	1831	CAKDIGKSGSTQRLSFDSW	IGH	2	None	
1	1832	CAKKVVRINGHFDYW	IGH	6	None	
1	1833	CARDGVRDYDSSGYWGFWFDPW	IGH	3	None	
1	1834	CARGVASGGYCTNDICYPFHYW	IGH	2	None	
1	1835	CARGTWGQLVNFDYW	IGH	10	None	
1	1836	CARAKEGSGYSPEWFDPW	IGH	2	None	
1	1837	CAKLPPLWQLGFDYW	IGH	2	None	
1	1838	CARDPYCGTTTCYSWFDPW	IGH	10	None	
1	1839	CATVYAVVAATPPDYW	IGH	2	None	
1	1840	CARHGLGDYDFWSGYYSRVASLLEGNYYYYMDVW	IGH	2	None	
1	1841	CAKGSGFVLAAGIDYW	IGH	2	None	
1	1842	CARAIWGRRQQVVRYNYYCYMDVW	IGH	5	None	
1	1843	CAKHITIFDWFPAFDYW	IGH	4	None	
1	1844	CTRVSPRWLPDYW	IGH	3	None	
1	1845	CARDLGIAAAGTSTFDIW	IGH	7	None	
1	1846	CAHMKFEAAILSGNYVYYAMDVW	IGH	2	None	
1	1847	CVRTSNYGSSPFDYW	IGH	2	None	
1	1848	CAISQGYGYFDWLLHSLDYW	IGH	2	None	
1	1849	CARDGVGESFYSDYW	IGH	2	None	
1	1850	CAKVGYYDSSGYHLGDYFDYW	IGH	2	None	
1	1851	CATRLSIFDYW	IGH	23	None	
1	1852	CASIVRRSTGRLRYFDWSQGGYFDYW	IGH	2	None	
1	1853	CARDHTTMTFAGYYFYGMDVW	IGH	10	None	
1	1854	CARDFSSESTAYYSLVRAFDLW	IGH	2	None	
1	1855	CARWEQRVPHDYW	IGH	2	None	
1	1856	CAKGQPWQQLDPFDYYYGMDVW	IGH	5	None	
1	1858	CAKDFEWELPGYYFDYW	IGH	4	None	
1	1859	CARVPNFDWLLSENYFYYGMDVW	IGH	2	None	
1	1860	CKQRGNWPPITF	IGK	1085	None	
1	1861	CARGFAGWRGQNYYYYMDVW	IGH	5	None	
1	1862	CQQSYGNPALAF	IGK	4	C2	7K8O
1	1863	CAHIGSLPVGATFDYW	IGH	7	None	
1	1864	CARDRGGLYSSGWYRLGRVDYW	IGH	6	None	
1	1867	CARHYDSSGYIGGASDFDYW	IGH	6	None	
1	1869	CAKDMGMYCSGGSCYSGIGDYW	IGH	8	None	
1	1870	CARAPVWGELLWDGEDGMDVW	IGH	6	None	
1	1871	CARERATSAPGLAQDSWFFDYW	IGH	3	None	
1	1872	CAAVYSLSLNWYFDLW	IGH	2	None	
1	1874	CARDEGDSGYSSRWYISPYYYGMDVW	IGH	4	None	
1	1875	CARDGRTTYYYDSSGSYTYWYFDLW	IGH	2	None	

1	1877	CARETDALECFDNW	IGH	2	None	
1	1878	CARFGGYSGGWYKVDFNWFDPW	IGH	3	None	
1	1879	CARWNGLEMNDWFDPW	IGH	2	None	
1	1880	CARVFQSAPEHNVDVFDYYMDVW	IGH	11	None	
1	1881	CATNSRPGLLHESFDVW	IGH	3	None	
1	1882	CARTGLLVRGLIDFYFDYW	IGH	4	None	
1	1883	CTRTPVLDYGDYKYSYYYGMDVW	IGH	2	None	
1	1884	CAKGAGHIVLMVYLDYW	IGH	4	None	
1	1885	CARLTQLLRYWYLDLW	IGH	2	None	
1	1886	CARDGFRITIFGVVTHDAFDIW	IGH	3	None	
1	1887	CAKVLCGFFGSTSCYLGYYYYGMDVW	IGH	3	None	
1	1888	CARDSFPGASSGPRAFDIW	IGH	3	None	
1	1889	CATLGYCSGDTCLSYFDYW	IGH	6	None	
1	1890	CAKTFGGVIVPYYFDYW	IGH	2	None	
1	1891	CARVVRGYSGYDLYYYYMDVW	IGH	14	None	
1	1892	CALTVANYDFWSGYYPDYW	IGH	4	None	
1	1893	CWGSLWESDYW	IGH	73	None	
1	1894	CARGLYDYADFDHW	IGH	2	None	
1	1895	CARDTAFTEAKGAFDSW	IGH	3	None	
1	1896	CAKDRLWFGESSTIFDYW	IGH	2	None	
1	1897	CARDSDGSGWSFDSW	IGH	10	None	
1	1898	CARVSNYDFWSDLYDHYYMDVW	IGH	2	None	
1	1899	CAKGHIYSSSSYFDSW	IGH	4	None	
1	1900	CARDLSDIVVVEGAFDIW	IGH	3	None	
1	1901	CMQALQTPRGGWTF	IGK	2	None	
1	1902	CAKDVDSSSFWYSDLW	IGH	4	None	
1	1904	CARDPKNVLLWFGESRGWFDPW	IGH	3	None	
1	1905	CAKDLIVIAEYYYDSSGSLDYW	IGH	2	None	
1	1906	CAKDIATKYQLLFLYYYGMDVW	IGH	3	None	
1	1907	CARRHIRVVSATDGFDIW	IGH	3	None	
1	1908	CARGGSERSGWDFDYW	IGH	4	None	
1	1909	CAKQNTVTSTYYYYYMDVW	IGH	4	None	
1	1910	CARGPDYGDYVQHDYW	IGH	2	None	
1	1911	CARRGGSPKYDAFDIW	IGH	1177	None	
1	1912	CARVPRIITMIVVVIPKKADAFDIW	IGH	3	None	
1	1913	CARGQGPVAAIRRAWFDPW	IGH	3	None	
1	1914	CARSTFIAVAGTGFDYW	IGH	10	None	
1	1915	CARMGDGYNFGIDAFDIW	IGH	8	None	
1	1916	CARDENNPHTDFDYW	IGH	5	None	
1	1917	CARMSLGSGNDYW	IGH	10	None	
1	1918	CARPTGTTVPRAFDIW	IGH	4	None	
1	1919	CARLAHDYDGRRSPSTCFYMDVW	IGH	3	None	
1	1920	CAKDRDELITIFGGVNWFDPW	IGH	51	None	
1	1921	CARGRYHPGSGSWFESW	IGH	3	None	
1	1922	CARERSSGWYFHYYYGMDVW	IGH	2	None	
1	1923	CARQTYSDYEIWFDPW	IGH	5	None	
1	1924	CARERGGIYGTDWYFDLW	IGH	2	None	
1	1925	CASSSDYHNSSNYYITPFEYW	IGH	2	None	
1	1926	CASSYGHYYFDYW	IGH	15	None	
1	1927	CARAPPTFRAVGNTVSWYFDLW	IGH	2	None	
1	1928	CVRGEIGYCSGGSCIQDYW	IGH	5	None	
1	1929	CHQSVILPFTF	IGK	19	None	
1	1930	CARGPRPGRYFDHW	IGH	2	None	
1	1932	CATNIVVEAADTPFGNYFDYW	IGH	15	None	
1	1933	CARFRVRDFWSGYPTGWFDSW	IGH	4	None	
1	1934	CTAGNCYPGHFDYW	IGH	4	None	
1	1935	CARRYSQYSSGWYYPGVDYW	IGH	5	None	
1	1936	CAKDTHYYDTSVPSLGFDYW	IGH	6	None	
1	1937	CARHWGIAAADTSLDYFDSW	IGH	3	None	
1	1938	CAKEFFPGYSPGYSHPYLFDYW	IGH	6	None	
1	1939	CAKDFSHGGNGVDYW	IGH	4	None	
1	1940	CATDLGAQDAFNFW	IGH	4	None	
1	1941	CARATYYYDSSPFDYW	IGH	47	None	
1	1942	CASPNTYIAAAGPDYYYGMDVW	IGH	2	None	
1	1943	CSSYTRSSSLPCVF	IGL	7	None	
1	1944	CATSGNWGWPYYLDNW	IGH	10	None	

1	1945	CAKERGGLKPVAATGDFDYW	IGH	3	None	
1	1946	CAREPFVVVPASLTSGEDYYFYYGMDVW	IGH	2	None	
1	1947	CAKEITIFGVVISPVYFDYW	IGH	11	None	
1	1948	CALNVPPMSPIEESGRGWLDPW	IGH	3	None	
1	1949	CARDRLLRFLDCYYYYGMDVW	IGH	2	None	
1	1950	CARDLSGYDPFDPW	IGH	4	None	
1	1952	CARDDYGDYYYYMDVW	IGH	30	None	
1	1953	CARYNGENSLDYW	IGH	6	None	
1	1954	CAKESSSSVYYYYYYMDVW	IGH	3	None	
1	1955	CARDTGDPDRRDGYIPDYW	IGH	5	None	
1	1956	CSKDIGDFWSGYPTGYPDYW	IGH	3	None	
1	1957	CVKDRVAAAAVVTWGGHYFDYW	IGH	3	None	
1	1958	CVKDRVAVSLYYFHYW	IGH	155	None	
1	1959	CARLHYSVTRFGALMNSYYYMDVW	IGH	3	None	
1	1960	CARKYSILDHVGGIPGHYYFYMDVW	IGH	2	None	
1	1961	CTAYMELRSLRSEDTAVYYCAREAYYYETSGYYGSRYFDYW	IGH	3	None	
1	1962	CARGLHRIAYCSRTNCPVNFDYW	IGH	2	None	
1	1963	CARIVGTTPSEAFDIW	IGH	2	None	
1	1964	CARDVENYCDRSACSSQRPSYYCFDVW	IGH	2	None	
1	1965	CARAGGHCTNGVCYTFWDYYYYMDVW	IGH	2	None	
1	1966	CAKIPLPIAAIYFFDDW	IGH	39	None	
1	1967	CTRNAIAVRPQVFYTYYYYYMDVW	IGH	5	None	
1	1968	CARGSGNYYFDHW	IGH	9	None	
1	1969	CAREEWLANGNFDYW	IGH	3	None	
1	1970	CARARLFSAYIAAAGFDYW	IGH	2	None	
1	1972	CAWQECRTTTCHEDVFDYW	IGH	31	None	
1	1973	CARHPGYSYAYIDYW	IGH	6	None	
1	1974	CVRLTVAYHFDLW	IGH	2	None	
1	1975	CATDLNLAHGDKSPFDYW	IGH	11	None	
1	1976	CARASQSGTVVSSNFDYW	IGH	23	None	
1	1977	CARDVVTNHDTAFDIW	IGH	3	None	
1	1978	CARDLARNCGFYFDYW	IGH	2	None	
1	1979	CARDPAGGSDVVIPVADYW	IGH	3	None	
1	1981	CAKEGLDRWLVPGIFDYW	IGH	3	None	
1	1982	CAKRSLSGTFYFDSW	IGH	2	None	
1	1983	CARSLGVAATPGYFQHW	IGH	4	None	
1	1984	CAAQPITVMALGKYYFNYW	IGH	4	None	
1	1985	CAKGYFPDYGGNYRIGHFFDNW	IGH	3	None	
1	1986	CTREYNSGWSYFYFYYYMDVW	IGH	4	None	
1	1987	CARETAGGYSTSGFDYW	IGH	2	None	
1	1988	CARDATTLELLWFGGDYYYGMDVW	IGH	3	None	
1	1989	CARAAPLGYCSGGSCYAFDYW	IGH	4	None	
1	1990	CARGAAMITFGPYNWFAPW	IGH	2	None	
1	1991	CARQQRVAAAGKVYFDYW	IGH	6	None	
1	1992	CARDLYGDYPFDYW	IGH	78	None	
1	1993	CARDKNMAIDYW	IGH	25	None	
1	1994	CASTLGSYGYPFDYW	IGH	8	None	
1	1995	CARLGVSTSVKYSIDYW	IGH	10	None	
1	1996	CATGSPFTLPNWFDPW	IGH	8	None	
1	1997	CLLYYGPAHMFF	IGL	5	None	
1	1998	CAKDLEVRGVIIEGEFDSW	IGH	2	None	
1	1999	CARSTSSWAQEGYWFDFW	IGH	2	None	
1	2000	CARDSLHPNSGTYFYW	IGH	8	None	
1	2001	CARLGQDFGVVISASYYFDYW	IGH	2	None	
1	2002	CATVPPGTIFGVVIIPYYFDYW	IGH	2	None	
1	2003	CARGIAVAGIKLNWYFDLW	IGH	2	None	
1	2004	CARDSDTSGHYDQLDYW	IGH	2	None	
1	2005	CARTTYYDFWSGYYSADYYYYYMDVW	IGH	3	None	
1	2006	CQRLKGNAGDFFGEGYFDSW	IGH	13	None	
1	2007	CAPIWDYPPAFDYW	IGH	4	None	
1	2009	CASDGGYCSSTSCQTNRGIDYW	IGH	3	None	
1	2010	CARDGESDGAGGYYYYGMDVW	IGH	2	None	
1	2011	CARGVGATRKHRVPPFRSYYYYYMDVW	IGH	3	None	
1	2012	CARARVLLAFGGVQCFDYW	IGH	2	None	
1	2013	CARVSGQVIWYNYMDVW	IGH	2	None	
1	2014	CARVRPCGGRCYRYYFYYYGMDVW	IGH	2	None	

1	2015	CARAGVAFKHYFDYW	IGH	23	None	
1	2016	CAKNWNSRRYFYMDVW	IGH	3	None	
1	2017	CQSYDSSVSGPHWVF	IGL	5	C4	7JVC
1	2018	CALLPRLQLGASSSLRDYW	IGH	3	None	
1	2019	CARHGYDFSFWGHFDNW	IGH	3	None	
1	2020	CAKRTESLASRLDLYWFDPW	IGH	2	None	
1	2021	CARDPTTVVTVAYFDYW	IGH	4	None	
1	2022	CVKKRLDNYDSSGYSNDAFDIW	IGH	2	None	
1	2023	CTTGPYYDSSGYWAYYFDYW	IGH	2	None	
1	2024	CAREVRRGTTRVYYYYYYMDVW	IGH	2	None	
1	2025	CSSYSSVSTLAVVF	IGL	15	None	
1	2026	CAREHLWFGELLAPAIDYW	IGH	4	None	
1	2027	CARDDGVGGTLGVPFFDLW	IGH	2	None	
1	2029	CARGHYIAARQYYFDYW	IGH	31	None	
1	2030	CARLGLRKSIAARRLSNWFDPW	IGH	6	None	
1	2031	CARPHPSTIYPYHNYYYYGMDVW	IGH	5	None	
1	2032	CARHSGAILWTNWFDPW	IGH	22	None	
1	2033	CAKGLPYSSGWYDSDAFDIW	IGH	2	None	
1	2034	CVRERVYSSTIWFDPW	IGH	6	None	
1	2035	CARLSTYYEVLSAPDYW	IGH	2	None	
1	2036	CAKTYLGYCTGDSCYSTPNFDVW	IGH	2	None	
1	2038	CATERGLLRYFVHSWFDPW	IGH	2	None	
1	2039	CARDLPITMVRGVLDYW	IGH	2	None	
1	2040	CAKANTLRLLLGYYYYGMDVW	IGH	2	None	
1	2041	CSSYTRRSTLLF	IGL	20	None	
1	2042	CQQKGGSPPYSF	IGK	4	C1	7BZ5
1	2043	CARYSSGWLIVPFDYW	IGH	11	None	
1	2044	CARLKDYRNYYFDYW	IGH	6	None	
1	2045	CARWSQFGGVIEEIDYW	IGH	2	None	
1	2046	CAHRLVGAITDPFDYW	IGH	3	None	
1	2047	CARDKAYYDILTGAKNWFDPW	IGH	2	None	
1	2048	CAKDRIRDSSGYYYVSYYYYGMDVW	IGH	2	None	
1	2050	CSTHMYRSNWDRSIFSYYYYFDVW	IGH	3	None	
1	2051	CAILSADTAMVDYYFFYMDVW	IGH	2	None	
1	2052	CATLEYISSSFAFFDYW	IGH	4	None	
1	2053	CARAEMSKVVNVFGRENYFDPW	IGH	3	None	
1	2055	CAKPPNFYDRSGYSIPDPFDVW	IGH	5	None	
1	2056	CLNGDLQFDYW	IGH	181	None	
1	2057	CQVWDTSSGHRVIF	IGL	39	None	
1	2058	CARPSGSYATYYYYYYMDVW	IGH	6	None	
1	2059	CARGDITAYVWGSYRYAFDVW	IGH	4	None	
1	2060	CTTAAWIVVVIYAFDIW	IGH	2	None	
1	2061	CARGPFDSSGSYFFDYW	IGH	7	None	
1	2062	CARGISSGGYCTNGECFPFRYW	IGH	3	None	
1	2063	CARDFLSDSMAPLSPLDYW	IGH	2	None	
1	2064	CAKVLGLVREFISRNPFDYW	IGH	2	None	
1	2065	CAKGDDAEEYFYGSGKFDFW	IGH	2	None	
1	2066	CATGAAAYFDFW	IGH	173	None	
1	2067	CARWGVEGNTHTQYFDSW	IGH	2	None	
1	2068	CVREVTTGYCFDNW	IGH	8	None	
1	2069	CARKAPDSSGWFSLEFDYW	IGH	7	None	
1	2070	CQSVDSSETYVLF	IGL	19	None	
1	2072	CARGGPSGAYGLDYW	IGH	6	None	
1	2074	CAKDGLMGRSSGFYQRLLYYYMDVW	IGH	2	None	
1	2075	CAREYSSGWDYNWFDPW	IGH	14	None	
1	2076	CSQHVGTEFWYFDLW	IGH	4	None	
1	2077	CAKGKTTMRGVRPYYFDYW	IGH	2	None	
1	2078	CARDLPDKTSPNFHYYYGMDVW	IGH	4	None	
1	2079	CARGQSSIAAAGTEWELPTFDYW	IGH	2	None	
1	2080	CARGIGGSCSGGSCYGSVNFDDW	IGH	8	None	
1	2081	CAREASSSWVDGYFDLW	IGH	4	None	
1	2083	CGQDHDYSTVLPWFDPW	IGH	2	None	
1	2084	CAHLNLASTAVPWRTFDYW	IGH	6	None	
1	2085	CARGNVWGPYIAWFDPW	IGH	2	None	
1	2087	CARCGYLPYYYGMDVW	IGH	7	None	
1	2088	CVKERGATYRMFDSW	IGH	2	None	

1	2089	CARDPDSGRNDPFDIW	IGH	2	None	
1	2090	CAKDPAPAATNWFDPW	IGH	2	None	
1	2091	CAASGWNHYDSSFPFDIW	IGH	3	None	
1	2092	CARVVGDSNSRGGYYSYDGMDVW	IGH	6	None	
1	2093	CARGTYHDLWSAYDMALDPFDIW	IGH	2	None	
1	2094	CAKDVDYSDISDYENWFDPW	IGH	2	None	
1	2095	CARNEQWLGRWFDPW	IGH	8	None	
1	2096	CAKAPSDTAMVTRYYYYGMDVW	IGH	2	None	
1	2098	CARAPGYSSGYEHYFYYMDVW	IGH	12	None	
1	2099	CARHFRYSSPEIDYW	IGH	2	None	
1	2100	CASTTAAWYRYDAFDIW	IGH	2	None	
1	2101	CARSVDRTWNLYYFDYW	IGH	117	None	
1	2103	CARKDYDRGSNYYYYYDMDVW	IGH	2	None	
1	2104	CVKLYPWFGDLLGHYDYW	IGH	5	None	
1	2105	CAKYPLLTYYYDSSGFYFDYW	IGH	2	None	
1	2106	CARRPPGITMIVVVIENAFDIW	IGH	2	None	
1	2107	CARDGGYYGDYVFLYFDLW	IGH	2	None	
1	2108	CTRKGRTWGTAPNAFDVW	IGH	3	None	
1	2109	CVRTGDSPNYYAFDIW	IGH	2	None	
1	2110	CARGQRGVNMILVVIGMSSYFDSW	IGH	4	None	
1	2111	CATGNPSRANYDFWSGSTPSNAFDIW	IGH	2	None	
1	2113	CARDRWSYNGRTGDVFDIW	IGH	22	None	
1	2114	CAKDITIAALFHSHFDYW	IGH	2	None	
1	2115	CARGVDTAMVTWYFDLW	IGH	6	None	
1	2116	CARAVITMIVVEAFDIW	IGH	47	None	
1	2117	CARLTYGSGSYIDVW	IGH	22	None	
1	2118	CAKPLYYYDSRGAFDIW	IGH	5	None	
1	2119	CAKPGDMIVVVITPPHFDYW	IGH	2	None	
1	2120	CARDPRLADGWFYLDYW	IGH	2	None	
1	2122	CAKLDGASAGSFDSW	IGH	4	None	
1	2123	CARYHYGYDSDNSFDYW	IGH	6	None	
1	2124	CARALHGIKLGYCSSTSCPDLSFDIW	IGH	3	None	
1	2125	CAREPNEYEQLDLFDYW	IGH	4	None	
1	2126	CARGEWIQLWSVGNWFDPW	IGH	5	None	
1	2127	CAREMDDSSGYFDAFDMW	IGH	4	None	
1	2128	CARGHYSHYLAAPLEWLDPW	IGH	5	None	
1	2130	CARDLTRDSNDYTPFDYW	IGH	11	None	
1	2131	CARDGRHDIVTGYFLLDYW	IGH	10	None	
1	2132	CARDSGGYKYGYPYWFLDLW	IGH	4	None	
1	2133	CARQQRSHHWNTNAFYYYIDVW	IGH	2	None	
1	2134	CAKDIQGGEYSSGGTDYW	IGH	16	None	
1	2135	CARPSVLGTPVRFDYW	IGH	2	None	
1	2136	CARGGRQWLDSPGLSWFDPW	IGH	4	None	
1	2137	CANIGLQVRIAAAHPNYFDYW	IGH	3	None	
1	2138	CARFSDYDILTGYQQYFDYW	IGH	12	None	
1	2139	CARDLHNYDSRGYYYDFSGFDYW	IGH	6	None	
1	2140	CARISYSSTFYIGSIDYW	IGH	3	None	
1	2141	CAITAGGTIVYYSYYYMDVW	IGH	2	None	
1	2142	CARVVPTGDVDYW	IGH	11	None	
1	2144	CALLFAKDRDYW	IGH	4	None	
1	2145	CAKALTLYSDAFDIW	IGH	13	None	
1	2146	CAGPATAVGITAFDIW	IGH	2	None	
1	2147	CQSFDSDNQGVF	IGL	193	C4	7JVC
1	2148	CARLVEMATIDDAFDIW	IGH	2	None	
1	2149	CARKQSVYTVREGFSAPQNWYFDLW	IGH	4	None	
1	2150	CAKAHKVAGTYSYYFDYW	IGH	2	None	
1	2151	CAKTPLYSSALNWFDPW	IGH	4	None	
1	2152	CARGGSLIGVLNFDFW	IGH	2	None	
1	2153	CAHRRSGWYGPIFDYW	IGH	13	None	
1	2154	CAIGGAVALAKPFDNW	IGH	5	None	
1	2155	CARDLRRRITTPSGSYYYMDVW	IGH	7	None	
1	2156	CARGRQFNVDYVPFDYW	IGH	4	None	
1	2157	CARLGYCSSTSCYGSPNFDYW	IGH	2	None	
1	2158	CASSYCSSTSCYPLYYYYYGMDVW	IGH	2	None	
1	2159	CAKDESTMVGSYFDFW	IGH	4	None	
1	2160	CARSTYGTSFDFW	IGH	2	None	

1	2161	CARPRVSTRGAIYDYW	IGH	6	None	
1	2163	CAKDRGPTIADRATQSYYYYGMDVW	IGH	2	None	
1	2164	CAKGRGRGTYFEYW	IGH	31	None	
1	2165	CVTEDPPPQLGPCRGGRPFDDVFHIW	IGH	23	None	
1	2166	CAREGCPWGNCYYMDVW	IGH	2	None	
1	2167	CASSDILTGYPWYFDYW	IGH	2	None	
1	2168	CARMELLDWSNWFDPW	IGH	2	None	
1	2169	CARLTYYYDRSAYYYDYW	IGH	4	None	
1	2170	CARGINWGPSYFDYW	IGH	5	None	
1	2171	CVTEGSAEPLNGDYAPGFDNW	IGH	6	None	
1	2173	CAKDKEAWNYGLDYSYYMDVW	IGH	14	None	
1	2175	CALVIAVAHLGYFQHW	IGH	2	None	
1	2176	CARSGAVAGGWYYPDYW	IGH	2	None	
1	2177	CARDGSGYNNGYYYYGMDVW	IGH	8	None	
1	2178	CARHVVVTASRQLNYYFYYMDVW	IGH	3	None	
1	2179	CATVGEWELPEPYFFGYW	IGH	2	None	
1	2180	CTRRVPVTSMVTPYFDYW	IGH	2	None	
1	2181	CAREGTIHGFDMW	IGH	2	None	
1	2183	CASHRAVAKVLDYYYMDVW	IGH	2	None	
1	2184	CARARTRPGSWYGGWFDPW	IGH	4	None	
1	2185	CAKVVVVLFDPW	IGH	2	None	
1	2186	CAISRRNDLGINFPLDYW	IGH	3	None	
1	2187	CARVLSSSPGPDAFDFW	IGH	2	None	
1	2189	CARSGREYQLLSWFDPW	IGH	5	None	
1	2190	CTRDRPHYGSGSYYPYFDYW	IGH	8	None	
1	2192	CARAGALEIFGVGLPTYYYYYMDVW	IGH	3	None	
1	2193	CARDLTTVTPLVDYW	IGH	3	None	
1	2194	CQVWDSSGTHPEVVF	IGL	30	None	
1	2195	CARETPDTSGSWVDYW	IGH	4	None	
1	2196	CAKDTYRYGSFGYW	IGH	2	None	
1	2197	CAKDGLTGDFFSWMSIVSFDSW	IGH	2	None	
1	2198	CAREWQLVRGGVDAFDIW	IGH	3	None	
1	2199	CARGDIVVVPGAGKAFDIW	IGH	2	None	
1	2200	CARDRDGELPNHYAFDIW	IGH	2	None	
1	2201	CVRWDLRLFYHYYYMDVW	IGH	3	None	
1	2203	CITDRVIVVPVAMLIFLGAYSDYW	IGH	93	None	
1	2204	CAKDVFHGDYGGYYYGMDVW	IGH	4	None	
1	2205	CATWYSGSYREGYFDYW	IGH	9	None	
1	2206	CARDVRGLGYNLKYYYYMDVW	IGH	2	None	
1	2207	CATVSSMRTCRGGTCYTSYWYSDHW	IGH	5	None	
1	2208	CARHIELVTNYYYYYYMDVW	IGH	4	None	
1	2209	CVKDMGLREWYFDYW	IGH	2	None	
1	2210	CGRGDWFNVW	IGH	2	None	
1	2211	CTRYRPAISGTFYSDAFDIW	IGH	3	None	
1	2213	CAHRRFWSGSIDYW	IGH	3	None	
1	2214	CARHSGYSSSLYYFDHW	IGH	4	None	
1	2215	CASSPGYNYVHFNYW	IGH	5	None	
1	2216	CAREYSNVYGRRFDAW	IGH	3	None	
1	2217	CQESYSRPLSF	IGK	2	C2	7K8O
1	2218	CARLDFGSSGIRIARWYFDLW	IGH	4	None	
1	2220	CARRRTWIQLWLGGYYYYYGMDVW	IGH	8	None	
1	2221	CATYNWIAHRGFDYW	IGH	2	None	
1	2222	CASTRGYSGYDDQSLDYW	IGH	8	None	
1	2223	CARAPRYSSGWYLFEPLFDYW	IGH	4	None	
1	2224	CAREVHCTGGNCQLGLDYW	IGH	4	None	
1	2225	CARQERRITIFGVVISLYMDVW	IGH	9	None	
1	2226	CARRSYGDYVGWYYYYMDVW	IGH	11	None	
1	2227	CARDFDDYDSSGYYGAFDIW	IGH	5	None	
1	2228	CARFRYCSSTSCYRNYYMDVW	IGH	2	None	
1	2229	CARAGVTVFGVRRFDYW	IGH	2	None	
1	2230	CAREPYSSSASFFDYW	IGH	12	None	
1	2232	CMQRIEFPF	IGK	32	None	
1	2233	CVKDQRGNYYMADYW	IGH	4	None	
1	2234	CARESNCGGDCYFDYW	IGH	24	None	
1	2235	CARDRARYNWNYGGMGFDYW	IGH	2	None	
1	2236	CATTYAFGGVISWFDPW	IGH	4	None	

1	2237	CARLAPATTKEAVFQYFFDYW	IGH	5	None	
1	2239	CAKASYGDAYGADYW	IGH	4	None	
1	2240	CATPSPYFWSGYTKYYYYYYGMDVW	IGH	2	None	
1	2241	CAKETNRRITIFGVVLPPRYFDYW	IGH	3	None	
1	2242	CAVLPKGGSYLIDYW	IGH	4	None	
1	2243	CAKSYYHLSSGYYTYFDSW	IGH	12	None	
1	2244	CQTWDSSTPWLVF	IGL	6	C4	7JVC
1	2245	CEQYNSHSRTF	IGK	6	C3	7K8R
1	2246	CTKGWRISPLNDGFDVW	IGH	8	None	
1	2247	CGTTNGWYHFDHW	IGH	4	None	
1	2248	CARVSSGHYDAFDIW	IGH	5	None	
1	2249	CARDRMPQFGDYYYYYGMDVW	IGH	3	None	
1	2250	CARDVSNWNIEVDYYYYYGMDVW	IGH	16	None	
1	2252	CAKVTYDETGYFYFDNW	IGH	4	None	
1	2253	CARGGIKHVYYYYYYMDVW	IGH	3	None	
1	2254	CVRQPGPHMARGVMRNNRFDPW	IGH	4	None	
1	2255	CGESHTIDGQVGWTF	IGL	2	None	
1	2256	CARVKDRSSVVFDIW	IGH	2	None	
1	2257	CARDMEDYGSGTYFADYW	IGH	2	None	
1	2258	CARGKHISWNLYPNDYW	IGH	2	None	
1	2259	CAKVAGLLWFGDERRREHYFDYW	IGH	2	None	
1	2260	CARSVQLWDRGIYYYYYMDVW	IGH	2	None	
1	2261	CAKDLIASPDNLFHYW	IGH	3	None	
1	2262	CATWGMVAAWYFDFW	IGH	2	None	
1	2263	CARDYYGSGSYLPPYFDHW	IGH	4	None	
1	2264	CARDLTDQPLGPYYYYGMDVW	IGH	2	None	
1	2265	CARESKGPYYYDTSGYSTTDAFDIW	IGH	3	None	
1	2266	CVRGPGGADTSASDHPDFDHW	IGH	2	None	
1	2267	CAKVNRAFRYGLDYYYMDVW	IGH	2	None	
1	2268	CTRVFIRYSSAYVHDAFDVW	IGH	5	None	
1	2269	CAIHPGPSDWTPYRWFDTW	IGH	3	None	
1	2271	CMQGSHWPLFTF	IGK	216	None	
1	2272	CAKAGLVCGGPRCYEEDYFDNW	IGH	5	None	
1	2273	CAKDSSVVVVAATGYNWFDPW	IGH	4	None	
1	2274	CARHFDTSGYYYLHSFDPW	IGH	2	None	
1	2275	CARGHVFDSSGFYAANWFDPW	IGH	11	None	
1	2276	CARDRTYCSSTTCYPHAFDIW	IGH	2	None	
1	2277	CAKDRWDSAMVQYVIDYW	IGH	2	None	
1	2278	CTTALRQWLVRDYW	IGH	4	None	
1	2279	CARGIAADVSISWFDPW	IGH	4	None	
1	2280	CARGLGANAIGALDYW	IGH	2	None	
1	2281	CARDPLPRLFGLPPSDAFDIW	IGH	4	None	
1	2282	CAGFKPLRGPHNMDKVEPDYW	IGH	3	None	
1	2283	CARDRYGDYGDAFDIW	IGH	6	None	
1	2284	CVGTFDPW	IGH	73	None	
1	2285	CARDPGAPLRAAAGEYYFDYW	IGH	8	None	
1	2287	CARGGGYQIFLYYYYGMDVW	IGH	6	None	
1	2288	CARPYCSGGDCYPYFFDYW	IGH	13	None	
1	2289	CAREYSYTSGYHYWFDPW	IGH	7	None	
1	2290	CTTVQGQRRSNVLRFLEPGYVRGGYYDYW	IGH	2	None	
1	2291	CATVPAAISYYYYGMDVW	IGH	12	None	
1	2292	CAKTVEVPDRWDPYYLHYYIDVW	IGH	2	None	
1	2293	CASVAGNYDLWSASPGEYYMDVW	IGH	2	None	
1	2295	CARDPPRDYGGEGVYYFDYW	IGH	2	None	
1	2296	CAKDLAMQLWFPTPNFDSW	IGH	4	None	
1	2297	CARDIAVAGTSYYYYYYMDVW	IGH	2	None	
1	2298	CVRCLYNSAPVSPRFEKYFSYYYMDVW	IGH	2	None	
1	2299	CARGPPDSSSWPSYYYSYYMDVW	IGH	2	None	
1	2300	CTRQTFPYFDHW	IGH	2	None	
1	2301	CARLRVVIHLIDYW	IGH	4	None	
1	2302	CCLLPASDYYYGMDVW	IGH	10	None	
1	2303	CASGVSEDKWNYNLDYW	IGH	4	None	
1	2308	CVKDRNIQLWLSYFDYW	IGH	2	None	
1	2309	CAREKAVAGSPLDALDIW	IGH	4	None	
1	2310	CARASAQVDTAMVLYYYYYMDVW	IGH	4	None	
1	2311	CAREPLAAQTNNWGFDYW	IGH	17	None	

1	2312	CARDEGTFTIFGVVIIPSNPIFDYW	IGH	2	None	
1	2313	CTKESEAAVFDFDYW	IGH	2	None	
1	2314	CARKEGLHYYFDYW	IGH	2	None	
1	2315	CTRVEIGGSFYYW	IGH	2	None	
1	2316	CARDQRGGYFSSWPRGNWFDPW	IGH	15	None	
1	2317	CARDGYGRDYTGMVYAFDIW	IGH	4	None	
1	2318	CARRFKGGGYYYYYYMDVW	IGH	4	None	
1	2319	CARDFFSAVPASMIPGRSFDYW	IGH	5	None	
1	2320	CARHPGDYGDYKSAMDVW	IGH	2	None	
1	2321	CQQYSSYLSLFTF	IGK	25	C3	7K8R
1	2322	CVRQRLFRGLLDYW	IGH	2	None	
1	2323	CARGGQLLYGGDFDYW	IGH	6	None	
1	2324	CVRDQYNRVWFESDYW	IGH	4	None	
1	2325	CARDPPDYDEGSGYYHAFDIW	IGH	2	None	
1	2327	CAKDYTTAPGNVNWFDPW	IGH	4	None	
1	2328	CYSATDNSRAFF	IGL	4	None	
1	2329	CARHRVQMDGTYTILTGPKLNWLDPW	IGH	2	None	
1	2330	CARDSVDTAVVKGYYYYGMDVW	IGH	8	None	
1	2331	CTRDHSSWFDFYAFDIW	IGH	2	None	
1	2332	CARHFRMVSWFDPW	IGH	2	None	
1	2333	CAGDDYGDSDYWFDPW	IGH	13	None	
1	2334	CAKAPIFGVVIIPHFDYW	IGH	3	None	
1	2335	CSTLNAVALNDYW	IGH	10	None	
1	2336	CARDPEIVAVPAAAFYYYGMDVW	IGH	10	None	
1	2339	CAVGSTGQFYFDFW	IGH	8	None	
1	2340	CARQPAKYYYDEDRPRIDYYYYYIDVW	IGH	2	None	
1	2341	CARRKPGITMIVVDRDAFDIW	IGH	5	None	
1	2342	CARVLGHEGSSSPFDYW	IGH	2	None	
1	2343	CARQTGGSGWYTAAFDIW	IGH	2	None	
1	2344	CASTTAAAGSDYDYW	IGH	2	None	
1	2345	CATSGYSSHDPFVYW	IGH	2	None	
1	2346	CAREVSYYDSSGYPKDYYYYMDVW	IGH	2	None	
1	2347	CAKASYTYSSSWLDYW	IGH	4	None	
1	2348	CARSVAPGMRVVVLGDYYFDYW	IGH	16	None	
1	2349	CARRRGDDYGSSGYLVAFDIW	IGH	2	None	
1	2350	CARLGYYYDSSGPWRPPFDYW	IGH	4	None	
1	2351	CAHEPHGSLAIFDSW	IGH	5	None	
1	2352	CATGLPTSNLDDAFDIW	IGH	6	None	
1	2353	CARDMAGSLYYFEFW	IGH	2	None	
1	2354	CAKRDYRGNSVAFDIW	IGH	2	None	
1	2355	CARSLYSGYSGYDLPMLTYYFDYW	IGH	2	None	
1	2356	CAKDFGTSYGWYAFDIW	IGH	2	None	
1	2357	CARAYGSSANSYNYYMDVW	IGH	11	None	
1	2358	CVKDRGYCSGASCLLTYALDVW	IGH	31	None	
1	2359	CARAGYSYDSSGYPLDDYW	IGH	6	None	
1	2360	CVRAKDILTGYSFDYW	IGH	2	None	
1	2361	CARLIVGYLNGRRYFDLW	IGH	6	None	
1	2362	CAKDYGDYADEGAFDIW	IGH	2	None	
1	2364	CARGTGGSAYYTGWFDPW	IGH	5	None	
1	2365	CARAQGGTGYYYYYGTDLW	IGH	3	None	
1	2366	CLQHSTYPFMFTF	IGK	2	C3	7K8R
1	2367	CAKPEYRSGWYDFDSW	IGH	2	None	
1	2368	CAKDYVRIAAAHPGAFDIW	IGH	2	None	
1	2369	CARQRREYDTSSSWGFKYYYYMDVW	IGH	26	None	
1	2370	CARRRDWADFTSDYW	IGH	18	None	
1	2371	CATDCGGDCIDYW	IGH	2	None	
1	2372	CARVKDWFYFDFW	IGH	2	None	
1	2373	CARDTAPKPDYYDSSGYPDYW	IGH	2	None	
1	2374	CARVNPKWDLLSSTFDVW	IGH	2	None	
1	2375	CARVRGDTPMALGDYW	IGH	2	None	
1	2376	CACDSSGWYEYYFDYW	IGH	21	None	
1	2377	CARDDRAFDIW	IGH	970	None	
1	2378	CAKDVEYYDILTREKGWLDSW	IGH	4	None	
1	2379	CTRMIDNGGYFFDYW	IGH	4	None	
1	2380	CARAWWQQLVFNYYYYMDVW	IGH	48	None	
1	2381	CARSSGYYSPQFDHW	IGH	3	None	

1	2382	CAREGLTTDGTVTDYW	IGH	6	None	
1	2383	CASGSYSSNSDFDYW	IGH	2	None	
1	2384	CARHVRVGDPRGHFDFW	IGH	30	None	
1	2385	CARGRGIMAGGALQDVHFDYW	IGH	2	None	
1	2387	CAKDYGVLEYSSSSEAFDIW	IGH	4	None	
1	2388	CAKEYTITVTYFYHYGMDVW	IGH	2	None	
1	2389	CTTVRWYYDSSAYPNDAFDIW	IGH	13	None	
1	2390	CAKVAGRVRTRFPFDYW	IGH	2	None	
1	2391	CAKRAVRDGYNSYYFDYW	IGH	2	None	
1	2392	CATDPLSYYDGSAYPRLKDYW	IGH	2	None	
1	2393	CARDLSTTGSDYYYYMDVW	IGH	7	None	
1	2394	CAKDPFYDFWSGYYFDYW	IGH	570	None	
1	2396	CARGRLAARPVAGFDYW	IGH	2	None	
1	2397	CASHHGDYPTSGWFDSW	IGH	2	None	
1	2398	CAKDSNDYGGICDYW	IGH	13	None	
1	2399	CARERSSYGLTYFDYW	IGH	4	None	
1	2400	CAKDMSGYYPDLDYW	IGH	2	None	
1	2401	CAKADTATSLSTDYW	IGH	2	None	
1	2402	CARLPYYDFWRGSSRGYYFDSW	IGH	2	None	
1	2403	CAKDVLIAGSTKSSTFDLW	IGH	3	None	
1	2404	CARDGNCGGDCYSPGSIYYYGMDVW	IGH	2	None	
1	2406	CVRTSNRSAYRKFDHW	IGH	5	None	
1	2407	CAKDKIPFGLIAAATKSKFDYW	IGH	2	None	
1	2409	CAREQDELVKEYYYGMDVW	IGH	2	None	
1	2410	CAKDHIRISGRWVARHPTDYW	IGH	2	None	
1	2411	CTTTPIFISIFGVAPDYW	IGH	2	None	
1	2412	CARDRSYDLWSGYSYDAFDIW	IGH	10	None	
1	2413	CARGDSQPYAEYMWVNYRPRSYFNYW	IGH	2	None	
1	2414	CARHLTGDDDYW	IGH	50	None	
1	2416	CARPSGNPRSPYLDSTAFDYW	IGH	2	None	
1	2417	CARSIMITFGGVIDAACFDLW	IGH	2	None	
1	2418	CARGQSDGSWYEDYW	IGH	4	None	
1	2419	CARDNDSSGYDAFDIW	IGH	10	None	
1	2420	CARRAISTDFHTYYFDYW	IGH	5	None	
1	2421	CAKDRYDILTGPTESFDYW	IGH	31	None	
1	2422	CAKDQRDWYYKWGPHFDSW	IGH	3	None	
1	2423	CQSYDGGLSGEAF	IGL	13	None	
1	2424	CASDDGISASRTKIFDSW	IGH	4	None	
1	2425	CVKDEGSSTWLHNWFDPW	IGH	11	None	
1	2426	CARDRRTSYFIFDFW	IGH	2	None	
1	2427	CAREPRSQYYFDYW	IGH	6	None	
1	2428	CARDWGHDFWSGSLYYFDYW	IGH	2	None	
1	2430	CTTPQQLVGYYFDYW	IGH	4	None	
1	2431	CARAVSSLYYDSSGYNDFW	IGH	2	None	
1	2432	CAKMIYNYGSGYYFDSW	IGH	2	None	
1	2433	CARALSSGWSLAFDYW	IGH	2	None	
1	2434	CARDPSVEDYGGNFYYW	IGH	9	None	
1	2435	CAREAGISFEYW	IGH	5	None	
1	2436	CARDREIAADPFAFDYW	IGH	2	None	
1	2437	CATSSVVVVAWWFDPW	IGH	8	None	
1	2438	CAKDGVGWFGEFIYAMDVW	IGH	4	None	
1	2439	CARHTGGSAAFDYW	IGH	8	None	
1	2440	CARVGDYDYVWGSYRETYYFDYW	IGH	2	None	
1	2441	CARHPPYGGSWPYYFDVW	IGH	24	None	
1	2442	CATVGLHDSGDYYFDSW	IGH	2	None	
1	2443	CAKNIRYYYDSNGYDAFDLW	IGH	8	None	
1	2445	CARAAPGAVGASTEYFQHW	IGH	2	None	
1	2446	CARHFTPSPSSDYMDVW	IGH	3	None	
1	2447	CAKDQWGSSGWYEGDYW	IGH	8	None	
1	2448	CVKGECSSSWCFDYW	IGH	4	None	
1	2449	CANRQEDPRTSAYYSDYW	IGH	5	None	
1	2450	CQQSYSNVMYSF	IGK	3	C2	7K8O
1	2451	CARDALVNYGILTGTRGYYYGMDVW	IGH	8	None	
1	2452	CARDRGLRVMTTFGGLDYW	IGH	4	None	
1	2453	CAREPITMILAVAFDIW	IGH	2	None	
1	2454	CARHFDWGVVVLTAFEYW	IGH	2	None	

1	2455	CAREMLRDAQSRHNYNYYGMDVW	IGH	7	None	
1	2456	CARAVEFSSSNFDYW	IGH	20	None	
1	2457	CAKFGSLSDSTRDYW	IGH	4	None	
1	2462	CAKDSSGSYPHNWFDPW	IGH	8	None	
1	2463	CAKNTIFGVVISSYWYFDLW	IGH	4	None	
1	2464	CARDDPNSSSWYAFDIW	IGH	10	None	
1	2465	CARLGYGCSGDTCYSEIESRDGMDVW	IGH	2	None	
1	2466	CARESHDHWFDFW	IGH	12	None	
1	2467	CARTGLEYYYDSTPEGFDIW	IGH	2	None	
1	2468	CAHSRIGETSDAFNIW	IGH	3	None	
1	2469	CARGRGGPETYYYYGMDVW	IGH	2	None	
1	2470	CARYNLLHYGDYVVDYW	IGH	8	None	
1	2471	CARAPISYCIGVGCNSNFFGPW	IGH	7	None	
1	2472	CQQTLSTPFFTF	IGK	3	C2	7K8O
1	2473	CARHVYSSPGTDAFDIW	IGH	6	None	
1	2474	CAHSRPDWNTIFGVVIIGGDYFDYW	IGH	4	None	
1	2475	CAKDGLRYFDWLSPIHYYYYGMDVW	IGH	3	None	
1	2477	CARVYSSNYYYYYMDVW	IGH	9	None	
1	2479	CAKDKRCNSASCWGGDYW	IGH	6	None	
1	2480	CARDSRYNWNDESWYFDLW	IGH	3	None	
1	2482	CAKMEAQYSGSYPFDYW	IGH	2	None	
1	2484	CVRDSFDEEVVRFDFW	IGH	2	None	
1	2485	CARLIPSSSSLPEYFQHW	IGH	2	None	
1	2486	CARKEAYQLLGSYYYYGMDVW	IGH	4	None	
1	2487	CTRERRATAFDMW	IGH	2	None	
1	2489	CARHLYSGSYFDLW	IGH	6	None	
1	2490	CARDRILGYGASWPDYW	IGH	2	None	
1	2491	CARVRSKWLVNDAFDVW	IGH	2	None	
1	2492	CARQFVGRAGPREDWFDPW	IGH	2	None	
1	2493	CARLQYSTTIFGARWNSYYYVDFW	IGH	5	None	
1	2494	CARGSDKSGPYYFAYW	IGH	2	None	
1	2495	CAKAFKWDRNYYYMDVW	IGH	2	None	
1	2496	CARLGSISWRKFQNYYYYMDVW	IGH	6	None	
1	2497	CVKDGVWNNFDYW	IGH	2	None	
1	2498	CARDLLHDFWRGFYSYFDYW	IGH	5	None	
1	2499	CVKDRSSGWHRYFDRW	IGH	2	None	
1	2500	CAKGSPTIRDNWNYFFRW	IGH	6	None	
1	2501	CQSVDHSGTFMF	IGL	34	None	
1	2502	CAKDNDFWRSGGPYYYYGMDVW	IGH	7	None	
1	2503	CARVRYYYDRSDPSKEIDYW	IGH	2	None	
1	2504	CARAYSSGWLTDIHFDLW	IGH	2	None	
1	2505	CAMGLLHPFFDYW	IGH	2	None	
1	2506	CAREKGWDDYGDQEYYYYYMDVW	IGH	2	None	
1	2507	CQQDYNFWF	IGK	145	None	
1	2508	CARGLDVSDFWTTTHVKKWLDPW	IGH	2	None	
1	2509	CAKPAGGGSGSFGYYYFDLW	IGH	6	None	
1	2510	CAHRVSTAYYYGPFDSW	IGH	2	None	
1	2511	CADGHYDSSGYWGQLDYW	IGH	3	None	
1	2512	CARLRFLEWLFVPVVAWFDPW	IGH	2	None	
1	2514	CARDIGITENAFDIW	IGH	2	None	
1	2515	CAIGFYSGIYFKGVFDIW	IGH	9	None	
1	2516	CAMGGSPSLRVGPYYYYYMDVW	IGH	2	None	
1	2517	CARAPSESLRFLEWLPYLIGAFDIW	IGH	5	None	
1	2518	CARHRYSGYTYGSTYPDWYFDLW	IGH	2	None	
1	2519	CARTLRIPPAATPYFRGWFDYW	IGH	6	None	
1	2520	CVKDKSLLGYCTAGNCYPGHFDYW	IGH	6	None	
1	2521	CVTWFYDESSDDSDAFDIW	IGH	122	None	
1	2522	CAREKNRRDYYYYYGMDVW	IGH	2	None	
1	2523	CARGVGYYYDSSGYYSELFLFDYW	IGH	2	None	
1	2524	CAKVSCPGYYGSGTCYYYGMDVW	IGH	2	None	
1	2525	CATSPWDLLPDYW	IGH	11	None	
1	2526	CAKDEEQYLVLYFYYGMDVW	IGH	3	None	
1	2527	CARHGTMAGWFDPW	IGH	19	None	
1	2528	CSGARGIIKPILHFAYW	IGH	2	None	
1	2529	CARDVTTVTTTRLDYW	IGH	11	None	
1	2530	CAKGGPRSGGRRNFWYFDLW	IGH	2	None	

1	2531	CARERMAYYDFWSGHWYFDLW	IGH	2	None	
1	2532	CARVGEQLVVGGFDIW	IGH	2	None	
1	2533	CARDGLGSSSSCYFDYW	IGH	12	None	
1	2534	CASGAIIARNFDSW	IGH	14	None	
1	2535	CAKASRWLHYYYYGMDVW	IGH	15	None	
1	2536	CARGRYCSSPSCYKANWFDPW	IGH	58	None	
1	2537	CGRVAGTSSPYGYFYIDVW	IGH	3	None	
1	2538	CARRTQHYLDVW	IGH	2	None	
1	2539	CAKDLGWSSGFLFDYW	IGH	33	None	
1	2540	CAKAWDTMVRGVITLFDSW	IGH	8	None	
1	2541	CARGGDYFESAYSFDSW	IGH	2	None	
1	2542	CARGTPSDRWLQFAPFDNW	IGH	4	None	
1	2543	CARATEWLVPNKPDYW	IGH	2	None	
1	2544	CARDHSYCTNGVCYTEHFDYW	IGH	7	None	
1	2545	CARLGYCSASSCPRYYYFYDGMDVW	IGH	3	None	
1	2546	CGRMAASSSSRSYYYMDVW	IGH	43	None	
1	2548	CAKDYEYCSGGRCYIDYW	IGH	2	None	
1	2549	CARQPVGDSVFDYW	IGH	4	None	
1	2550	CARGRDPALASPIDYW	IGH	4	None	
1	2551	CARGKSYDFSISINWFDPW	IGH	3	None	
1	2552	CARHPLRRITMIVVVITPAFDIW	IGH	10	None	
1	2553	CARGPAGVAAPYYFDLW	IGH	3	None	
1	2554	CASQIVVVPAAFRWFDPW	IGH	5	None	
1	2555	CARQGGSPPSYYFDYW	IGH	13	None	
1	2556	CARGSADFWSAGGWFDPW	IGH	6	None	
1	2557	CARALAVYHYDEDIPRGPISFDMW	IGH	2	None	
1	2558	CARVPTAMAAEYFQHW	IGH	2	None	
1	2559	CAKDSQEVRGYSYGYLDYW	IGH	3	None	
1	2560	CATYCGGDCYSGFGYW	IGH	2	None	
1	2561	CVRDRRDSGSDYW	IGH	7	None	
1	2562	CVRGWSAPDYW	IGH	11	None	
1	2564	CARDGGVRGVMFYYYGMDVW	IGH	3	None	
1	2565	CARDRGSSYFLYYFGYW	IGH	13	None	
1	2566	CARDTRLRYCSGGSCYSIYYFDYW	IGH	2	None	
1	2567	CARHSFQYSSSWGFDPW	IGH	4	None	
1	2568	CARERYPQVWWLPGPFDYW	IGH	4	None	
1	2569	CARALWRYCSSTSCFVDYW	IGH	2	None	
1	2571	CARVQNSSGWREGAWDDAFDIW	IGH	2	None	
1	2572	CARAPGYYHYGMDVW	IGH	8	None	
1	2573	CARELSNIAGRENGDPFYYYYMDVW	IGH	3	None	
1	2575	CARAVAALWFDPW	IGH	10	None	
1	2576	CARGGYDFWSGYEFDSW	IGH	2	None	
1	2577	CARQDWATDFDIW	IGH	6	None	
1	2578	CARRANSSGWYDRYYYYYYYMDVW	IGH	10	None	
1	2579	CAKAVSTSHYYYDSW	IGH	10	None	
1	2580	CGRDLVGGHRGRYVGYW	IGH	2	None	
1	2581	CARDMNGQGAGRRYYHYGMDVW	IGH	2	None	
1	2582	CASSQFTYYDSSGFEYW	IGH	10	None	
1	2583	CARGQGNDFWSGLRTNYYYAMDVW	IGH	4	None	
1	2584	CARLGARDGYSPLDYW	IGH	2	None	
1	2585	CSHATFYYGSGGSTWFDPW	IGH	2	None	
1	2587	CARRVAAAGIGVYYYYMDVW	IGH	4	None	
1	2588	CASGPVVVTDGRNYYYYGMDVW	IGH	4	None	
1	2589	CARVYGDSNYFDYW	IGH	12	None	
1	2590	CAREAVALTHYYYYMDVW	IGH	8	None	
1	2591	CARQNGLTIFGVVTYGTLFDPW	IGH	5	None	
1	2592	CAKDAEFMVRGIVKISTVGGFDYW	IGH	5	None	
1	2593	CAKARHALEIDYW	IGH	5	None	
1	2594	CAKDLRGVSPYDYDILTGYNKPARVRYDGLDVW	IGH	3	None	
1	2595	CAKDLRTTWLYYFDYW	IGH	150	None	
1	2596	CAREPLSGGIMKYFFDNW	IGH	2	None	
1	2597	CARHGSFLGVISYFDYW	IGH	7	None	
1	2598	CAKDISGYVRIFDFW	IGH	4	None	
1	2599	CASGSGYMDVW	IGH	9	None	
1	2600	CARGPFPGYKTWEDDYW	IGH	2	None	
1	2601	CARHILGYSSGWARLGRFDYW	IGH	2	None	

1	2603	CARRTVNGPFDFW	IGH	6	None	
1	2604	CVRDRHFEDTTVTTLEFWFDPW	IGH	77	None	
1	2605	CARHVSFFYSGSYHNWFDPW	IGH	4	None	
1	2606	CARGRTERFLGWFDPW	IGH	7	None	
1	2607	CARAEEERWLQFDYW	IGH	4	None	
1	2608	CALWYNLNDRNWFDPW	IGH	7	None	
1	2609	CARVGGFQASFSFDHW	IGH	2	None	
1	2610	CARTSFGVARYYYYYMDVW	IGH	9	None	
1	2611	CARGQFEYCSSTSCPGYYYYYGMDVW	IGH	2	None	
1	2612	CARQSFWMAARPNWFHPW	IGH	4	None	
1	2613	CYSIDDSGNPIF	IGL	7	None	
1	2614	CATATYYYDGSGYSPFDYW	IGH	10	None	
1	2615	CARHAEDRKLRFLDYLSNFDSW	IGH	3	None	
1	2616	CARHFSEPTDYYDSSGYLSGFDYW	IGH	2	None	
1	2619	CARGLFYGMGWYFDLW	IGH	2	None	
1	2620	CARGFITMVRGVIGWFDPW	IGH	4	None	
1	2621	CAGSEGEADYYYYYGMDVW	IGH	4	None	
1	2622	CARDQVHLKDILTGYTPLVYFDYW	IGH	2	None	
1	2623	CMQGINLQTF	IGK	3	None	
1	2624	CARRHYDNSEYYFDSW	IGH	5	None	
1	2625	CALTVGAAHVDYYYYYMHVW	IGH	4	None	
1	2626	CAKEMDILTGYSAFDFW	IGH	2	None	
1	2627	CQQLDSYPESPTF	IGK	2	C1	7BZ5
1	2628	CARDRHYTGIYGFSFDFW	IGH	142	None	
1	2630	CAKDTQWLVHPSFGYW	IGH	2	None	
1	2631	CAKDAGNRYSNYQGNWFDPW	IGH	7	None	
1	2632	CVRAAARTNSYYYMDVW	IGH	2	None	
1	2634	CTVRLVVTPDGFDYW	IGH	6	None	
1	2635	CARGGVFGGSWYIPENWFDPW	IGH	11	None	
1	2636	CARWVGVGATSGLIDYW	IGH	2	None	
1	2637	CAKGKVSADMGHWFDPW	IGH	3	None	
1	2638	CATNLRIAGRRDAFDIW	IGH	4	None	
1	2639	CARLNRLQWLGESSYYYYAMDVW	IGH	4	None	
1	2640	CAIFRQLVISQYFDYW	IGH	2	None	
1	2641	CARSITMIEGGAFDIW	IGH	2	None	
1	2642	CAHFSIIARNAFDYW	IGH	11	None	
1	2643	CARVRASGNYRRYYFDSW	IGH	2	None	
1	2644	CARDLEQWLVRRAFDIW	IGH	14	None	
1	2645	CARREAARPGDYYYYMDVW	IGH	4	None	
1	2646	CARDRGELLLYWYFDLW	IGH	114	None	
1	2647	CARDSRLPWNGYSVSHYYMDVW	IGH	2	None	
1	2648	CAHRPYFDFLMGAFDVW	IGH	4	None	
1	2649	CARGWRQNSFDIW	IGH	6	None	
1	2650	CVTEAEDCPPLLYCRNDSPSFDHW	IGH	5	None	
1	2652	CARDLRYSSPTPHPYFDYW	IGH	2	None	
1	2653	CARAGSGSPSHYYYGMDVW	IGH	4	None	
1	2654	CARDRVITFGGVIVPGWFDPW	IGH	14	None	
1	2657	CAKDLAVEPYYYGMDVW	IGH	5	None	
1	2658	CARDLITWPYNYYYDSSGYQIGWFDPW	IGH	2	None	
1	2659	CARAPRGLVVVLDSFDIW	IGH	6	None	
1	2660	CARDHGNYDSTGFLLPGEVNYFDYW	IGH	2	None	
1	2661	CAKELGYSSSWYMGDAFDIW	IGH	2	None	
1	2662	CARAGLIFGVVINAFDIW	IGH	2	None	
1	2663	CTTDGGNSFGSSAVRYDLDYW	IGH	2	None	
1	2664	CARHAYGDSQSAPVDYW	IGH	3	None	
1	2665	CARRGSERSGYYYMDVW	IGH	2	None	
1	2667	CARDRVIRGVDFDYW	IGH	9	None	
1	2668	CARRRPFYTMVRGVDLSFDYW	IGH	2	None	
1	2669	CARDRAHCSGGTCYLYDYYGMDVW	IGH	5	None	
1	2670	CARGRIGVQHYFYYYYVDVW	IGH	2	None	
1	2671	CVRDQDDDARPNWFDPW	IGH	2	None	
1	2672	CARSLERSGYFGWFDPW	IGH	2	None	
1	2673	CASSRWTHWYFDLW	IGH	4	None	
1	2674	CAREPEYSNNWSSAFDYW	IGH	2	None	
1	2675	CAHRLSLYGDFDYW	IGH	4	None	
1	2676	CARNLGSSSGHGAYFDLW	IGH	2	None	

1	2677	CAKLRIAGAQQWLVEGPMIDAFHIW	IGH	5	None	
1	2678	CARLWRRSWDNWFDPW	IGH	6	None	
1	2679	CVRGRGDYDSGNYYHSYGMDVW	IGH	2	None	
1	2680	CAREGLVGSTFDSW	IGH	2	None	
1	2682	CTRGILTTSNWFDLW	IGH	2	None	
1	2683	CARDSPGYWFDPW	IGH	194	None	
1	2684	CARQSRRGYSYGYMYYW	IGH	2	None	
1	2685	CASYRFVRWLVHPLALEYFQHW	IGH	4	None	
1	2686	CTTQWGQTFYYDTSGYYPLDYW	IGH	5	None	
1	2687	CARRGKITIFGVVKNWFDPW	IGH	2	None	
1	2688	CAKDWDIVVEPDAVDYYYGMDVW	IGH	2	None	
1	2689	CARAAVVDLYEFFYMDVW	IGH	2	None	
1	2690	CAKDHAFGVVIIDYFDYW	IGH	2	None	
1	2691	CACSMIVVVKRAEFDYW	IGH	4	None	
1	2692	CVRESEYYDNTGYPYWYFDLW	IGH	10	None	
1	2693	CARGDFYDVSGYPYYFDYW	IGH	4	None	
1	2694	CATSSRSYFDAFDIW	IGH	2	None	
1	2695	CARGNKTTMIVVAGLVDYW	IGH	2	None	
1	2696	CARVAGDRRRHIVVVTAHPGYFDYW	IGH	6	None	
1	2697	CAKETLFKIVVVITREFDYW	IGH	117	None	
1	2698	CVRGLSHRLTIFGVEDYW	IGH	10	None	
1	2699	CVKSNGAGDFDSW	IGH	2	None	
1	2701	CASLPAVHKRPHSILTNGQYYYYMDVW	IGH	6	None	
1	2702	CAHSLMFQFAFDFW	IGH	5	None	
1	2703	CARGGIVPATTVPYHYYGMDVW	IGH	3	None	
1	2704	CARDGLPYYDFWSGYYFGGGREYYYMDVW	IGH	2	None	
1	2705	CARDSPVVTHLLSPHFDLW	IGH	2	None	
1	2706	CAKAPYSSSWYLHYW	IGH	3	None	
1	2707	CARLPHDSTGYYVPFDYW	IGH	119	None	
1	2708	CTACDDSLNGLWVF	IGL	6	None	
1	2709	CATVTKDYWYFDLW	IGH	6	None	
1	2710	CVRGWDSSENYAIYW	IGH	2	None	
1	2712	CAREKDMEHISAAGNINYFDSW	IGH	14	None	
1	2713	CARESVHPNGPDYW	IGH	2	None	
1	2714	CARHKFCSGAACDFKGFYFDFW	IGH	2	None	
1	2715	CARDWSGSYPHFYYYYMDVW	IGH	4	None	
1	2716	CARATYDYGDYGEGGLWFDPW	IGH	4	None	
1	2718	CAKDLDYGGHSYFDCW	IGH	3	None	
1	2719	CARESETRGYNYGYDYW	IGH	4	None	
1	2720	CTREAAFFGELSTSRWFDPW	IGH	2	None	
1	2721	CQQAFSLPPWSF	IGK	2	C1	7BZ5
1	2722	CARDAHCSSTSCERGDYYYGMDVW	IGH	2	None	
1	2723	CARERPSSWASNFDYW	IGH	2	None	
1	2724	CVKDLVDRDMVSVAFDYW	IGH	2	None	
1	2725	CAREMFSDVLTGYYPSDYW	IGH	4	None	
1	2726	CVRDRRTLGLSYDFEYW	IGH	2	None	
1	2727	CQSYDKSLRGSAF	IGL	14	C4	7JVC
1	2728	CARFGFSDGMDVW	IGH	4	None	
1	2729	CAKGSGYSGYDWLDYW	IGH	2	None	
1	2730	CAKSPYNRATGFDYW	IGH	2	None	
1	2732	CVKGGGSVRLYFDLW	IGH	2	None	
1	2733	CACGYSYGYGGYFEHW	IGH	2	None	
1	2734	CARIRFSGEFDYW	IGH	3	None	
1	2735	CTREEGLFISGWPFDYW	IGH	3	None	
1	2736	CARRVVEVSGQGNWFGPW	IGH	3	None	
1	2737	CARLLKRFGFSNPYYFDCW	IGH	4	None	
1	2738	CARDLTAVAGMDYYYGMDVW	IGH	29	None	
1	2739	CARLDEARASSCDYW	IGH	5	None	
1	2741	CARTSFNVQETGFDSW	IGH	2	None	
1	2742	CARLLIYTSGWYGHLFDYW	IGH	3	None	
1	2743	CARGLMEFFAVVSYWFDPW	IGH	276	None	
1	2744	CHQYNYYSTWTF	IGK	33	None	
1	2745	CQVWDSSTVGVLF	IGL	4102	None	
1	2746	CARSVSEMATIMYFQHW	IGH	2	None	
1	2747	CARAVRDDFWSGYYKNYYYGMDVW	IGH	3	None	
1	2748	CARVRARVQEITIFGVVRAIDAFDIW	IGH	2	None	

1	2749	CATGTFYDIVTGYRGREDFFDSW	IGH	2	None	
1	2750	CAREGYCSGGSCYPDYW	IGH	43	None	
1	2751	CARPFRHYYDSSAFDIW	IGH	8	None	
1	2752	CATSSWALDTSGWYFDYW	IGH	6	None	
1	2753	CAKVVSDDGTHYNLLTGLHVSIDGMDVW	IGH	4	None	
1	2754	CARDRGDVAGLYEFDPW	IGH	4	None	
1	2755	CARWVVVAATGDYYYGMDVW	IGH	9	None	
1	2756	CTRDSQDSSYWFRFWDYW	IGH	3	None	
1	2757	CARVGVDSSSWPYFDYW	IGH	4	None	
1	2758	CARGIYYYDNSGYQYNWFDPW	IGH	2	None	
1	2759	CAKDAHHMVRGVIITFDYW	IGH	2	None	
1	2760	CVTWLFHHFDYW	IGH	75	None	
1	2761	CARGRAYGEDSQAFDIW	IGH	4	None	
1	2762	CARDGWADPGYFQHW	IGH	25	None	
1	2763	CMQGTHWVYTF	IGK	5091	None	
1	2764	CAKGPSSTPWGVFDYW	IGH	2	None	
1	2765	CARDSTYYDFWSGYIDYW	IGH	2	None	
1	2766	CARDLSYCSSTSCYTALFDYW	IGH	9	None	
1	2767	CAKWELQFYFDFW	IGH	2	None	
1	2768	CARRTTSSFAFDIW	IGH	17	None	
1	2769	CATAYKYDGSGPSVEDALDIW	IGH	4	None	
1	2770	CARHRGGEGSSRYDTFEIW	IGH	5	None	
1	2771	CARENFSPDYW	IGH	5	None	
1	2772	CARDRGYCTNGVCPTWYPRYW	IGH	2	None	
1	2773	CAREGDYDSSGSYFQHW	IGH	2	None	
1	2774	CQQYYTTPRAITF	IGK	13	C2	7K8U
1	2775	CARTRGYSYGLIKNWFDPW	IGH	11	None	
1	2776	CARDPMITFGGVIVMDYYYYGMDVW	IGH	7	None	
1	2777	CQQSYNAPPGQAF	IGK	4	C2	7K8O
1	2778	CARLREDYDSSGYSLYFFDYW	IGH	7	None	
1	2779	CASQPQNRSMNRGIKPRYDNFHYYMDVW	IGH	5	None	
1	2780	CARSDMVRGVILSWFDPW	IGH	2	None	
1	2782	CATVRSPYFDSSGYYFDYW	IGH	8	None	
1	2783	CARDARGLLRSESDYW	IGH	2	None	
1	2784	CARLSVVVGATPALWLDYW	IGH	2	None	
1	2785	CARDSSRYCIYLVCYTDFDHW	IGH	2	None	
1	2786	CARSRDGYNRREFDPW	IGH	16	None	
1	2788	CARDPSSRWLLGNSHLFDYW	IGH	34	None	
1	2789	CAIDTSRHGYNFIFDFW	IGH	2	None	
1	2790	CAREGPVRGATGTNPYYFDYW	IGH	2	None	
1	2791	CQHRSDWPPIFTF	IGK	15	None	
1	2792	CARHYFDISGYHSNDAFDVW	IGH	46	None	
1	2793	CARATEGSSYYLDYW	IGH	5	None	
1	2795	CAHSLAYCGADCYDAFHFW	IGH	2	None	
1	2796	CAREVAARSDFEYW	IGH	46	None	
1	2797	CARDWPDAEQWLVTWFDPW	IGH	4	None	
1	2798	CLQHDNLFLWTF	IGK	11	None	
1	2799	CARHLEYSSSFDYW	IGH	112	None	
1	2800	CARQLGQVLSGAIWAPFDIW	IGH	6	None	
1	2801	CASDRRSRKERTRFDYW	IGH	2	None	
1	2802	CAKAPSYSSSSYYFDYW	IGH	11	None	
1	2803	CARPKDNGEWTFDAFDIW	IGH	5	None	
1	2804	CARGSSQWLGLWYFDLW	IGH	3	None	
1	2805	CARGTSCSGGYCSPIAFDIW	IGH	55	None	
1	2806	CVKVPYGGGALKRFYFDYW	IGH	25	None	
1	2807	CARDQGAISITGTTKPDYW	IGH	2	None	
1	2808	CARQLDFWSGYYREYYFDYW	IGH	2	None	
1	2809	CARSSSGRYYRNWYFDLW	IGH	2	None	
1	2810	CARVSSMKTCRGGSCKTSFWYFDLW	IGH	6	None	
1	2811	CQQSDDLPFTF	IGK	41202	C3	6XDG
1	2812	CQSHDISLSGLGVF	IGL	3	C3	6WPS
1	2813	CARGSGTWYPLRFDYW	IGH	9	None	
1	2814	CTRDRGATQRHYADYW	IGH	2	None	
1	2815	CARTIYNFASGYYFDYW	IGH	2	None	
1	2816	CAKDLYCSGTSCFSWSDYW	IGH	2	None	
1	2817	CARRMYSGTNWFVPW	IGH	7	None	

1	2818	CAKDYFIWGRGTYSFDYW	IGH	2	None	
1	2819	CARETLPAAGDVFDVW	IGH	2	None	
1	2820	CARHKTGFLQWDNWFDPW	IGH	4	None	
1	2821	CSRGIDWGGLPFDYW	IGH	4	None	
1	2822	CAKVGRPYGSGTYYNFDFW	IGH	4	None	
1	2823	CARGYSSGWYERWYFDLW	IGH	3	None	
1	2824	CAMHYFDVSGSYGPSHFVYW	IGH	2	None	
1	2825	CARRGPKTRFHFYYYMDVW	IGH	3	None	
1	2826	CARQSAFSSSWYALNWFEFW	IGH	2	None	
1	2827	CARIPAVHFTVFGVGPTHYYYYGMDVW	IGH	2	None	
1	2828	CAKSEMTTVSFGNHFDFW	IGH	4	None	
1	2829	CARIDFKEGSGWYLVFDYW	IGH	5	None	
1	2830	CAKGYCSSTSCYKSDWFDPW	IGH	13	None	
1	2831	CNSRDSTGNREIF	IGL	2	None	
1	2832	CAKPGMLYWLYYFDYW	IGH	2	None	
1	2833	CARHMGAPTGWFDPW	IGH	33	None	
1	2835	CARRNTRAFYDRGHLWFDPW	IGH	2	None	
1	2836	CARLAKFRGVKSPAFDYW	IGH	6	None	
1	2837	CLSADSSGTYQGYVF	IGL	2	None	
1	2838	CARVPNWYGKVDYYFDYW	IGH	2	None	
1	2839	CTTEWEVPTFDYW	IGH	7	None	
1	2840	CARRNILWGWFDPW	IGH	2	None	
1	2841	CAAPVIIAASVDYW	IGH	4	None	
1	2842	CARAIEMQIQYLLLYSPRGYYYMDVW	IGH	2	None	
1	2843	CARQYPSLTMRVVVITLDDAFDSW	IGH	6	None	
1	2844	CAREYYDSSGYYPPVAFDIW	IGH	2	None	
1	2845	CARWCSGYDPEKSRFDYW	IGH	4	None	
1	2846	CVKDKSSSIEVIRDFLYW	IGH	4	None	
1	2847	CARRSCGSISCWLDPW	IGH	3	None	
1	2849	CARGVTSVAARFRNWYFDLW	IGH	2	None	
1	2850	CAKEPYPTAKTYYYYYYMDVW	IGH	2	None	
1	2851	CAGGVYPTPPFDYW	IGH	3	None	
1	2852	CAGVYCSSGSCYIDSW	IGH	3	None	
1	2853	CTTFETVFGVVIDYW	IGH	55	None	
1	2854	CASSAAYQLLFHYLDYW	IGH	2	None	
1	2855	CATHDFWSGLAIDVW	IGH	11	None	
1	2856	CARDGGSGWSGYFDYW	IGH	11	None	
1	2857	CVKEGGAGGWNLVKGRGYFDIW	IGH	3	None	
1	2858	CTTDTGSSWYGYYFDYW	IGH	3	None	
1	2859	CATVALTDQSFDHW	IGH	5	None	
1	2860	CARAVSYYPVYGDYVAYIDYW	IGH	2	None	
1	2861	CARDTHPHYDNWFDPW	IGH	2	None	
1	2862	CARVFRGDTGMVTPLQFDYW	IGH	8	None	
1	2863	CARLTVGGATELDYW	IGH	6	None	
1	2864	CARFHEPDISIGGIYWYFDLW	IGH	5	None	
1	2865	CARDVSECRGTRCIWWSDPW	IGH	4	None	
1	2866	CTTVRAVTTGYFDYW	IGH	5	None	
1	2867	CARAVGITMIVVVTVGWFDPW	IGH	9	None	
1	2868	CAKDLSPLLWFGESRSFDYW	IGH	2	None	
1	2869	CAKDDTPNLTWDPFDYW	IGH	3	None	
1	2870	CARVRPGSSYGPLHYFDNW	IGH	60	None	
1	2871	CARQRGIAAAVDYW	IGH	6	None	
1	2872	CTF	IGK	4	None	
1	2874	CARHVYSSPYNWNQGPYWYFDLW	IGH	2	None	
1	2875	CARLKGDYDPFWYFDLW	IGH	14	None	
1	2876	CVKDKGGRAWPYQNNWFDPW	IGH	2	None	
1	2877	CARTDTSSFGYFDFW	IGH	5	None	
1	2878	CARADDPYNWNYDGRIDYW	IGH	5	None	
1	2879	CTTVTVVVVPDAYAQRPTHYYYYYMDVW	IGH	4	None	
1	2880	CQVWDISSDLYFF	IGL	12	None	
1	2881	CTTVVQYDPWEPAYYFDHW	IGH	2	None	
1	2882	CAREGVDTAMGFDYW	IGH	15	None	
1	2883	CARIRAYCSGGNCYPSSFYFDYW	IGH	6	None	
1	2884	CASGREYRTGTTGFDYW	IGH	2	None	
1	2885	CAKEESAYSSGWQAEDIW	IGH	3	None	
1	2886	CARQLIFGVAFDPW	IGH	4	None	

1	2888	CARQDEAGGYQLLFYFDYW	IGH	4	None	
1	2889	CQQSYTTPTYIPPWTF	IGK	10	C2	7K8O
1	2890	CARGDLVVLTDAFDIW	IGH	3	None	
1	2891	CARAPRTTIFGVVLWFDPW	IGH	19	None	
1	2892	CARGGGYLRFLEWLTYWYFDLW	IGH	3	None	
1	2893	CARDPDGYYYDSSGYYSDAFDIW	IGH	2	None	
1	2894	CARDSGVPAEYDYVWGSYRYTGYYYGMDVW	IGH	3	None	
1	2895	CVRGRREMAAISTFDYW	IGH	2	None	
1	2896	CAKDVLKVADLMYYYDSSGPGGDYW	IGH	16	None	
1	2897	CACQWEEDAFDIW	IGH	29	None	
1	2898	CARHLQGGGHYYQFYMDVW	IGH	4	None	
1	2899	CYSAADNIGLF	IGL	110	None	
1	2900	CAREPFYGDYADAFDIW	IGH	4	None	
1	2901	CARDQRSEQWLTTYFDYW	IGH	2	None	
1	2902	CSRVLSSSSSWGYYFDSW	IGH	3	None	
1	2903	CVRGPVVSLDYGFWSGSAFYFDLW	IGH	3	None	
1	2904	CARHEIAARRYWYFDLW	IGH	8	None	
1	2905	CARPGYSYGMGREWFDPW	IGH	2	None	
1	2907	CARSIALLDLYFDYW	IGH	3	None	
1	2908	CARVMYNYVSGYYFDLW	IGH	4	None	
1	2909	CARQGMETDGRFDPTVTTLYYFDFW	IGH	49	None	
1	2910	CAKAPLPLYGDYGHDAFDIW	IGH	2	None	
1	2911	CAKDITGTTHAGFDYW	IGH	2	None	
1	2913	CARDGNWNDWWFDPW	IGH	2	None	
1	2914	CAGIRLDRLDYW	IGH	4	None	
1	2915	CVKDSEGSGSYGDAFDIW	IGH	2	None	
1	2916	CTRAIPTHYYYYMDVW	IGH	2	None	
1	2917	CQHLTSYPPALTF	IGK	160	C1	7BZ5
1	2919	CARARPHYGDYAPFDYW	IGH	2	None	
1	2920	CARGNYGEKIDYW	IGH	4	None	
1	2921	CATLAPFYDYYYYGMDVW	IGH	5	None	
1	2922	CARLLTDLKRDYPRGYFDLW	IGH	2	None	
1	2923	CARHVVEYSNFPPSYYFDYW	IGH	2	None	
1	2924	CAREVKGSGSYWSAKIGPYFDYW	IGH	2	None	
1	2925	CAKADGDFDYFDYW	IGH	58	None	
1	2926	CARSEEYSDSPEFDYW	IGH	4	None	
1	2927	CSRRDYASFGDWFDPW	IGH	2	None	
1	2928	CAKDRGNIDWFDPW	IGH	12	None	
1	2929	CAHRGQVLVAASFDPW	IGH	2	None	
1	2930	CARDAYFYDGNAYYYIFDVW	IGH	7	None	
1	2931	CARDPGKELPSRYYFDYW	IGH	2	None	
1	2932	CARALQYQLHTDGFDLW	IGH	2	None	
1	2933	CARGYCSNGECYGAPDYW	IGH	9	None	
1	2935	CARRYYDPSGFYFYAFDIW	IGH	13	None	
1	2936	CARAHERQNYYDSSGYYFDYW	IGH	4	None	
1	2937	CAKGVVTISVEPNYMDVW	IGH	6	None	
1	2938	CTRHESTLNGTHIDYW	IGH	2	None	
1	2939	CARDQGAGIRLQEFDYW	IGH	4	None	
1	2940	CARVGSNYADYW	IGH	10	None	
1	2941	CQSTDSSNTYHDVVF	IGL	2	C4	7JVC
1	2942	CAVQAYDYGDYDPYYFDSW	IGH	2	None	
1	2943	CATSGLVWGTKDYYYMDVW	IGH	2	None	
1	2944	CSGGSCETSVYYYYYYMDVW	IGH	3	None	
1	2945	CARGKYNWNYVYFHYW	IGH	2	None	
1	2946	CARFPTSWVTWYSALHAFDIW	IGH	2	None	
1	2947	CAREDRDGYNFDYW	IGH	14	None	
1	2948	CARLSTPAAGVLEMLSPWYFDLW	IGH	2	None	
1	2949	CTRVYSSSWTAQSYFDYW	IGH	4	None	
1	2950	CATPFPTTTITSGYFGQW	IGH	2	None	
1	2951	CAKDMGKAEHPYYYYYMDVW	IGH	4	None	
1	2952	CARDGSDGDYVQYFDYW	IGH	2	None	
1	2953	CAKDWATGMAYDYFDYW	IGH	2	None	
1	2954	CARGALAARPRWFDPW	IGH	2	None	
1	2955	CARGTGPIGNFDYW	IGH	2	None	
1	2957	CVRRLENYVFDIW	IGH	3	None	
1	2958	CARDERGYDVMTDYW	IGH	4	None	

1	2959	CAREEKDHGGYYYGMDVW	IGH	4	None	
1	2961	CANARHSSGWSSFDYW	IGH	2	None	
1	2963	CAILRNWGFDSW	IGH	5	None	
1	2964	CGRRVTPLDYW	IGH	2	None	
1	2965	CARVESRRRQWLVMDYYYMDVW	IGH	13	None	
1	2966	CARDLTPSYGDSYYYYYYMDVW	IGH	2	None	
1	2967	CAREPRELIRGYYFDYW	IGH	3	None	
1	2968	CARDGSYYDSSGYYPNFDYW	IGH	3	None	
1	2969	CAKEPGGYYYSSFYFDSW	IGH	2	None	
1	2970	CAKDGGSAMPTLGWIDPW	IGH	2	None	
1	2971	CSRGSVRRQKGGLFRSGSYSDYYYGMDVW	IGH	5	None	
1	2972	CATPRKLRSGSYSLDYW	IGH	3	None	
1	2973	CSSFAGSNNIGLLF	IGL	3	C2	7BWJ
1	2974	CARGTWELPDYFDNW	IGH	5	None	
1	2975	CTTRMSVTNDYW	IGH	2	None	
1	2976	CAKGRHSSSFFCDYW	IGH	10	None	
1	2978	CAKDSGDIVVIPAAPPGTYYYNYMDVW	IGH	2	None	
1	2979	CASLCGYSNRYYMDVW	IGH	9	None	
1	2980	CARDRRDFGMVIIDYW	IGH	10	None	
1	2981	CQSSDSSGRYSVIF	IGL	27	None	
1	2982	CARESRDWDSSGYYDAW	IGH	2	None	
1	2983	CLQHNSVPLTF	IGK	979	None	
1	2984	CVRVSGYCSPTTCYGGFDYW	IGH	9	None	
1	2985	CARVGSDDILTGYSTYW	IGH	22	None	
1	2986	CARAPFPYYYDSSGYQDAFDIW	IGH	2	None	
1	2987	CARMECIGGICSMYFFDHW	IGH	4	None	
1	2988	CAKDGAKWGFREAFDIW	IGH	2	None	
1	2989	CARPLNQWQAGGYDYW	IGH	2	None	
1	2990	CARDERRYDILTGSDYW	IGH	2	None	
1	2991	CANLYPNYQFDIW	IGH	2	None	
1	2992	CAREQYYYDSSGYYYVGGFDYW	IGH	2	None	
1	2993	CAREPGYCSSTSCPIRAFDIW	IGH	2	None	
1	2994	CAKDRGGYYDSSGYQGYYYYGMDVW	IGH	5	None	
1	2995	CARDSILGVTSTLTFDYW	IGH	66	None	
1	2996	CASSGYSPLSAFDIW	IGH	8	None	
1	2997	CARAWSYNWNYVGFYYYMDVW	IGH	2	None	
1	2998	CARNPLRYSSGWQRQQDDAFDIW	IGH	4	None	
1	2999	CAKDLDPISGYDPFDYW	IGH	4	None	
1	3000	CARRQDYYDSSGYYYDWYFDLW	IGH	5	None	
1	3001	CARVGELLSLDQSHYYYYMDVW	IGH	2	None	
1	3002	CTRSGGIGTYGYQRGFDLW	IGH	5	None	
1	3003	CAKEMASRFWSGYFHYYYGMDVW	IGH	8	None	
1	3004	CARAHYYHTNDAFDIW	IGH	2	None	
1	3005	CAKAALGTCRSTRCYAFDYW	IGH	5	None	
1	3006	CTRHGPAEIPSPPYTGNWFDPW	IGH	4	None	
1	3008	CALPIWGLPQCNYFDFW	IGH	2	None	
1	3009	CAKDIGGGYSYGYHWFDPW	IGH	2	None	
1	3011	CASLPPNYYDSRHYYYGGIPFDSW	IGH	4	None	
1	3012	CARSISSSHSRVAGVHFDYW	IGH	2	None	
1	3013	CARDMGLGAVAGKNRERDLDYW	IGH	4	None	
1	3014	CARDRGSYGYTGVYYYYGMDVW	IGH	2	None	
1	3015	CAKHSRIGYNLDYSDYW	IGH	4	None	
1	3016	CAHRQVFYDEDRSGFYFDYW	IGH	2	None	
1	3017	CARGRGYCSGGSCEHLNWWFDPW	IGH	2	None	
1	3018	CAKGTPSTWIQLWSTGNWFDPW	IGH	4	None	
1	3019	CASSYYGDPKYYYYYGMDVW	IGH	2	None	
1	3020	CAKDIEDVATISGLDYW	IGH	2	None	
1	3021	CARAPVLVTSDAFDIW	IGH	2	None	
1	3022	CARQTYSSGWYPDSW	IGH	17	None	
1	3023	CARDFGSSADRFDYW	IGH	8	None	
1	3026	CARQGRDGYNPSRDDAFDIW	IGH	2	None	
1	3027	CARISGGYMYGSKKPNYYYGMDVW	IGH	2	None	
1	3029	CARDVRTYYDGSDYRFHYYYMDVW	IGH	2	None	
1	3030	CAKGARGYNYGAFDYYYYMDVW	IGH	3	None	
1	3032	CARDRPALAVAEYFQHW	IGH	2	None	
1	3033	CARHVSDRVVPAAITFDYW	IGH	2	None	

1	3034	CATARLAAMEFDYW	IGH	2	None	
1	3035	CAKVPTYYDFWSGEDDAFDIW	IGH	3	None	
1	3036	CASMAGGYNFVRGQYYYHYYMDFW	IGH	3	None	
1	3037	CARGVVTAILSAFDLW	IGH	7	None	
1	3038	CARDHIADKNPDGFDIW	IGH	2	None	
1	3039	CAKDRGPQYCGGDCYSYYYYGMDVW	IGH	4	None	
1	3040	CAKTLPDPFDGFDVW	IGH	4	None	
1	3041	CARDLVLGPWGSYDRSGYRLDYW	IGH	61	None	
1	3042	CVKDPNWTSNHRFDCW	IGH	4	None	
1	3043	CARQRDVYFYDSSGSQRYNNWFDPW	IGH	8	None	
1	3044	CARGRYFDFEGYYFDLW	IGH	7	None	
1	3045	CAGTTTSRPFRGKGTILHAFDIW	IGH	21	None	
1	3046	CASAGRQPLGIHLWDDAFDIW	IGH	41	None	
1	3047	CARKRRHGVVVAATLFDYW	IGH	3	None	
1	3048	CASVSARVGGFYFDHW	IGH	3	None	
1	3049	CQQYHYWPPMYTF	IGK	94	None	
1	3050	CSTDDQSDGTSRYDWDLRRGIFDYW	IGH	5	None	
1	3051	CARASYSSGWYEEGPDYW	IGH	38	None	
1	3052	CSKDTSLELYAFDIW	IGH	2	None	
1	3053	CATQVRGTTRLYYYYFMDVW	IGH	10	None	
1	3054	CASDRRYCSGGSCYGTAFDYW	IGH	2	None	
1	3055	CTTGLTFSPFDYW	IGH	26	None	
1	3056	CATALAIEWPDDYYYGMDVW	IGH	2	None	
1	3057	CARDRVTGTAIFDLW	IGH	17	None	
1	3058	CARVGLGGVGTGTTPYFDYW	IGH	3	None	
1	3059	CARWGLRVAARFDYW	IGH	6	None	
1	3061	CARFIWFGEPHDAFDIW	IGH	12	None	
1	3062	CQSAVSSTTPWVF	IGL	39	None	
1	3063	CAGHWGTVVRGVIDPGYVDYW	IGH	4	None	
1	3064	CARHPQNWNYATDYW	IGH	2	None	
1	3067	CARDFKYYDYVWGSYRDDYW	IGH	25	None	
1	3068	CASHSEWELPTSIFDYW	IGH	10	None	
1	3069	CARDRPLTINTGVYYYAVDVW	IGH	2	None	
1	3070	CARASGPPPFVDYW	IGH	6	None	
1	3072	CAKEAADYSSGWYLFDYW	IGH	3	None	
1	3073	CAKDGIEGLRLGELSFEADYW	IGH	2	None	
1	3074	CVRSESGGYLFYFDFW	IGH	6	None	
1	3076	CARRWFGELGGGWFDSW	IGH	3	None	
1	3077	CGGERVSTKDYFYYGMDVW	IGH	2	None	
1	3078	CALYHTTEGAFDVW	IGH	2	None	
1	3079	CARRGPSMKTFGGVVGHFDHW	IGH	5	None	
1	3080	CARDPNPGEVLNLIWFDPW	IGH	2	None	
1	3081	CARLGVEWDLLSTFDCW	IGH	3	None	
1	3082	CARGRPGGGWLVPLDYW	IGH	3	None	
1	3083	CAKDLETGEVELTAAFDYW	IGH	2	None	
1	3084	CARLSHSSSPKKYYFDFW	IGH	3	None	
1	3085	CARVGGEMAVPRGLDYW	IGH	2	None	
1	3086	CARPRYSYGDYVNNWFDPW	IGH	4	None	
1	3087	CARHKGSDRSGWFSDWYFDLW	IGH	5	None	
1	3088	CQKYGRSPTWTF	IGK	46	C1	7K8N
1	3089	CARHLRSFTLAARQMGSPIPFWYFDLW	IGH	8	None	
1	3091	CTTDDSSSQTSYYYYYYMDVW	IGH	4	None	
1	3092	CARHLAGATYPAYW	IGH	3	None	
1	3093	CARVGQTGLGDSLVYFAYW	IGH	14	None	
1	3094	CVRGLQWDLTRIRFDPW	IGH	3	None	
1	3095	CAHKSGIAAASWFDPW	IGH	2	None	
1	3096	CAKDLFCSGGSCHPQVDSSGYYDDYW	IGH	2	None	
1	3097	CARQYTIWTGYPYNWFDPW	IGH	24	None	
1	3098	CARDRVGADGSYYFDYW	IGH	10	None	
1	3099	CARARATYSDGPYYFDFW	IGH	2	None	
1	3100	CARVLEPVQYRRLAFDIW	IGH	2	None	
1	3101	CARGSPYEILSHGGFDPW	IGH	5	None	
1	3102	CARDGGDYGSSGNDDFDIW	IGH	3	None	
1	3103	CARFEYSSSSDGFDYW	IGH	6	None	
1	3104	CARDAPYYHYDSTGYYKRNNWFDPW	IGH	2	None	
1	3105	CARVYYYDSVGYWGPFDYW	IGH	2	None	

1	3106	CARSRHSKDWFDPW	IGH	2	None	
1	3107	CSGGSCYSIWFDPW	IGH	2	None	
1	3108	CAKDQYSYGLWYFDYW	IGH	9	None	
1	3109	CALPIWELPGLDYW	IGH	2	None	
1	3110	CARVLITMVRGVPHHAFDIW	IGH	2	None	
1	3111	CARILVEYSSSSVTVGFDYW	IGH	2	None	
1	3112	CATSGEEYSSSSVFDYW	IGH	2	None	
1	3113	CARDFWNEAMDVW	IGH	6	None	
1	3114	CARDPMTTVKTFYYYYGMDVW	IGH	5	None	
1	3115	CARDPLGHCSGGRCWKFDYW	IGH	2	None	
1	3116	CARVSAVPQVLGFVVAGQFDYW	IGH	2	None	
1	3118	CAHRTYYYDSSGPAFDYW	IGH	2	None	
1	3119	CVKSMGYYESRGYGWGGALDIW	IGH	2	None	
1	3120	CVAWFHDENSDDSEPFDVW	IGH	35	None	
1	3121	CARELVEGLYYYDTSGYYGEDYW	IGH	2	None	
1	3122	CARVNPSYYDSSGDSNWFDPW	IGH	2	None	
1	3123	CARGAVAIYFYAMDVW	IGH	4	None	
1	3124	CAIAAAGTGDWFDPW	IGH	4	None	
1	3125	CARHSSSSGWFDPW	IGH	23	None	
1	3127	CTTDSIVGPSSPIDVFDVW	IGH	3	None	
1	3128	CTTDLGYCSGTICYTPDFDYW	IGH	2	None	
1	3129	CARGLGDYGTLYYYYYMDVW	IGH	2	None	
1	3130	CARVPTILPGGHTWFDPW	IGH	2	None	
1	3132	CASIKGDYGDYDYYGMDVW	IGH	9	None	
1	3133	CVKDMAIAAATFTFDFW	IGH	34	None	
1	3134	CARHKDYGGNAGFDYW	IGH	12	None	
1	3135	CARVRIVVVTLTWRDYFYYYMDIW	IGH	2	None	
1	3136	CARDYLHCSLTSCFNWFDPW	IGH	4	None	
1	3137	CQHYDNLPPYTF	IGK	2235	C3	6XDG
1	3138	CARLGTYYSGVDYW	IGH	2	None	
1	3139	CARVTMVTGFDNW	IGH	62	None	
1	3140	CAKAGSYYGSGSYPFYYAIDVW	IGH	10	None	
1	3141	CVKDALLSGKYYGNYFDYW	IGH	2	None	
1	3142	CAKGPGGGDYDILTGYYYYGMDVW	IGH	2	None	
1	3143	CAPPRRLRWPSPIDYW	IGH	2	None	
1	3144	CSRGGISGIPLSYFDFW	IGH	4	None	
1	3145	CARDVPVKMSLVQGDYW	IGH	4	None	
1	3146	CARVCGSYYEYYFDYW	IGH	2	None	
1	3147	CARHYDTSGYYPKGYFQHW	IGH	8	None	
1	3148	CARDGPGGGAVTSYYYYGMDVW	IGH	4	None	
1	3149	CYSAVSSGIFVF	IGL	2	None	
1	3150	CARDQKPIVGATTGWFDPW	IGH	2	None	
1	3151	CARAARGEWFLDYW	IGH	2	None	
1	3152	CATAPYGDYFHFDYW	IGH	6	None	
1	3153	CTKDAFVPGTFDLW	IGH	3	None	
1	3154	CAHSSEVGHNWWFDFW	IGH	3	None	
1	3155	CASGGWNSSGRNWYFDLW	IGH	2	None	
1	3156	CLRIVSSGYSVYW	IGH	2	None	
1	3157	CTTDPPYSTSSDDAFDIW	IGH	10	None	
1	3159	CARGDHKSTFFPSYYYYGLDVW	IGH	4	None	
1	3160	CGTWDSSLTTGFVLF	IGL	4	None	
1	3161	CARNPHCGRDCYRYWYFDLW	IGH	7	None	
1	3162	CAKDRSFYIDAFDLW	IGH	3	None	
1	3163	CARARDDFWSGYHNWFAPW	IGH	134	None	
1	3164	CARGGRDGYKIGIDYW	IGH	16	None	
1	3165	CAKDNPPFELAGSSYYYYYYAMDVW	IGH	3	None	
1	3166	CARGGIRAGRLGYFDWW	IGH	3	None	
1	3167	CARHGTWEQRLVPNDAFDIW	IGH	2	None	
1	3169	CAKDQSMEWLSSMWFDPW	IGH	2	None	
1	3170	CVRDSIQVGHTQGYSLPSVNFFDFW	IGH	2	None	
1	3171	CASSGVSANVHLFQYW	IGH	2	None	
1	3172	CSSYTSSSTHVPYVVF	IGL	3	C2	7K8W
1	3173	CARRGIWNGHYRGYFDYW	IGH	2	None	
1	3174	CAREGDSMVRGRYMYYYYGMDVW	IGH	2	None	
1	3175	CAKRPTAFAAGGDFDYW	IGH	2	None	
1	3176	CAKGRGVGAIGFHSFDIW	IGH	3	None	

1	3178	CARRTPLENWNEQAFDIW	IGH	3	None	
1	3179	CASPASLYGSESYYPYW	IGH	4	None	
1	3180	CARPYPRNSGYHHNYYFYGMDVW	IGH	4	None	
1	3181	CVKHAAPGTFITNFDFW	IGH	5	None	
1	3182	CAKDLVAVAHAFDIW	IGH	2	None	
1	3183	CARESSESQVLWGYYYYYGMDVW	IGH	2	None	
1	3184	CAIEHEDSYIDFW	IGH	3	None	
1	3185	CATIGFCLTSVCSAEDYW	IGH	6	None	
1	3186	CAKGMDDSSGFHYIQYW	IGH	4	None	
1	3187	CNSRDNGGNHPVIF	IGL	40	None	
1	3188	CARLDRYSYGHLPNPETFDIW	IGH	2	None	
1	3189	CAKDGGFYSWYFYMDVW	IGH	3	None	
1	3190	CARDYYGFWSARDIGRFDYW	IGH	4	None	
1	3191	CARMKFNDILTGRDPFDIW	IGH	2	None	
1	3192	CRAEEDGGEANYFDYW	IGH	2	None	
1	3193	CATRYCTSTNCYYAYW	IGH	3	None	
1	3194	CVRDGWQYSYGVMGYYYFDYW	IGH	3	None	
1	3197	CVTDALTTLGVVISPDSYYFDHW	IGH	4	None	
1	3198	CAKDTSIAVAGPKDYW	IGH	11	None	
1	3200	CARAVPDIAVLPASGFDLW	IGH	9	None	
1	3201	CGAGYSSSWPPRIDYW	IGH	3	None	
1	3202	CARDWGYSSGWYWGTGFDPW	IGH	2	None	
1	3203	CAKDLAEISSDTWYWGFDYW	IGH	2	None	
1	3204	CAKNSESGITIFGVVTHDYW	IGH	4	None	
1	3205	CARDTPNWTYFDSW	IGH	16	None	
1	3206	CARRSIPLHSSGWYNGPIFDYW	IGH	2	None	
1	3207	CARDTRGYRGLRYYYGMDVW	IGH	2	None	
1	3208	CARHPSLTRGYQRFDPW	IGH	3	None	
1	3210	CAKDRYMFVYTNYGGFDYW	IGH	3	None	
1	3211	CARVFGDYVDYW	IGH	30	None	
1	3212	CARHGKSSSSVYYYYYMDVW	IGH	2	None	
1	3214	CAREGYGSGNYPYDYW	IGH	2	None	
1	3215	CARDPTTSSDWYFDLW	IGH	2	None	
1	3216	CARLPRRLYGAGNYFYNAMDVW	IGH	2	None	
1	3217	CAHRGLPLTTSHKNAFDVW	IGH	3	None	
1	3219	CASFRLWFGELFGFDYW	IGH	2	None	
1	3220	CARGIVSAPGTSRPGFWFDSW	IGH	14	None	
1	3221	CARHRPIYDFWSGRLTYYYYGIDVW	IGH	4	None	
1	3222	CARGSATYYDFWRGYFFDWW	IGH	4	None	
1	3223	CARNRRKDGNLYYFDYW	IGH	2	None	
1	3224	CARLGISGMPGGAFDIW	IGH	4	None	
1	3225	CAKLGYCSGGTCYDYYYHYGLDVW	IGH	4	None	
1	3226	CAKGHGYLQNHFDYW	IGH	4	None	
1	3227	CARDSGLWSRHFDSW	IGH	11	None	
1	3228	CARVWGVAYMDVW	IGH	3	None	
1	3230	CARQDYDFWSAGSRYWFDPW	IGH	2	None	
1	3231	CAKDDSISARQFDYW	IGH	2	None	
1	3232	CARVSHPTFGDVIDYDAFDIW	IGH	2	None	
1	3234	CARSRGSSGWYGFLTYYFDYW	IGH	2	None	
1	3235	CARGGVLVPPLIAPTFDCW	IGH	2	None	
1	3237	CAREVPLKWEPHHWNFDLW	IGH	2	None	
1	3238	CAREDRDGSYAQVYMDVW	IGH	2	None	
1	3239	CVRGGWIQLYDKFDPW	IGH	4	None	
1	3240	CARDWTTGGGCSDPSCYLGSNWFDPW	IGH	2	None	
1	3241	CAKDRDAAGTMGRYFEYW	IGH	2	None	
1	3242	CARARTYTIFGVVIATLPFDYW	IGH	2	None	
1	3243	CASRMVADNLWYFDLW	IGH	2	None	
1	3244	CARRGEMATIEAYDYW	IGH	3	None	
1	3245	CAKEGGSVIAPLSLFNYYSYGMDVW	IGH	2	None	
1	3246	CARLTHCTGTSCRDYW	IGH	2	None	
1	3247	CARWQQWYNWFDRW	IGH	2	None	
1	3248	CARLNYYDSSGYSDANDYFDYW	IGH	5	None	
1	3249	CARLDVANWNLDYW	IGH	5	None	
1	3250	CAREEGNLSRRVFDYW	IGH	9	None	
1	3252	CANSKWLKAEYQYYGMDVW	IGH	2	None	
1	3254	CARHGGITMIVVVIPSWFDPW	IGH	3	None	

1	3255	CARDPRYYHDGAPADYW	IGH	2	None	
1	3256	CARGGIVVVPAAIGRQTEYYYYNGMDVW	IGH	2	None	
1	3257	CARDFYASGWCHFDYW	IGH	2	None	
1	3258	CASTGLRNAFDNW	IGH	4	None	
1	3259	CQSPDSSATYPVVLF	IGL	5	None	
1	3260	CATYPLSAYCTNGVCYIDYW	IGH	3	None	
1	3261	CAKPTSLWFGTPYFHSW	IGH	3	None	
1	3262	CASSTTIFGVVTTAFDYW	IGH	2	None	
1	3263	CARGSGAVAGNFDYW	IGH	1922	None	
1	3264	CARVSGDGPNIYYYYYYMDVW	IGH	2	None	
1	3265	CARDLSRVDYGVLSGYYQGGILDYW	IGH	2	None	
1	3266	CAKDPFSGSYYLGAFDIW	IGH	5	None	
1	3267	CARLSLPEWWFDPW	IGH	4	None	
1	3268	CARRAGYDYGGNEFDPW	IGH	5	None	
1	3269	CARGSNYFESNSYYFSEYFQFW	IGH	2	None	
1	3270	CARDLGYYYGSGSYEDYW	IGH	2	None	
1	3271	CTTDTVYPFDYW	IGH	22	None	
1	3272	CARDRSGYSSGWYVSGLDYW	IGH	4	None	
1	3273	CARDHGVRWWDWYFDLW	IGH	2	None	
1	3274	CSGGFGYNTATDYW	IGH	15	None	
1	3275	CASPARIAVAGLFDYW	IGH	2	None	
1	3276	CGRDVRGMGVTGSRQVRWFDPW	IGH	7	None	
1	3277	CTTDTAMTPYYYDYW	IGH	4	None	
1	3278	CARAPYYDFWSGYYTGYYGMDVW	IGH	2	None	
1	3279	CARHMIGKVDTAMVGDYW	IGH	2	None	
1	3280	CARLRWQPQGSYYYYYGMDVW	IGH	3	None	
1	3281	CARSYSSTTFYYYYMDVW	IGH	2	None	
1	3282	CARSFDVLTVSAFDIW	IGH	3	None	
1	3283	CARHFGTYTEENWFDSW	IGH	6	None	
1	3285	CAKGVSHGAHSSGWYWYFDLW	IGH	4	None	
1	3286	CASHSLMISYGGLIRYFESW	IGH	3	None	
1	3287	CASRRPDNYDSTNHFDYW	IGH	2	None	
1	3288	CARLWTFPLFDSDQYYFDSW	IGH	2	None	
1	3289	CARDSAHNEYVVYW	IGH	3	None	
1	3290	CARDGTSSGWFYYYYYMDVW	IGH	6	None	
1	3291	CASSADYDTTGYFDFW	IGH	110	None	
1	3292	CARDRGAGVALDAFHIW	IGH	2	None	
1	3293	CATFGTNWGHTDLDYW	IGH	4	None	
1	3294	CAKGPDDHHYFDSW	IGH	4	None	
1	3295	CVRQYRSGWCPADYW	IGH	3	None	
1	3296	CARQENWGWGPRDFDLW	IGH	2	None	
1	3297	CARGGVGYPEDYW	IGH	62	None	
1	3298	CQSADTNGIVVF	IGL	16	None	
1	3299	CAKWSSSSGVHNDYW	IGH	2	None	
1	3301	CAKDKVDTAMVTLDYW	IGH	2	None	
1	3302	CARHNPHIITARPPTLKHFDYW	IGH	2	None	
1	3303	CATHSGSRNNDAFDVW	IGH	2	None	
1	3304	CALPILNSLKTEDTAVYYCTTDRGSSWYGYYFDYW	IGH	2	None	
1	3305	CAKDIALPRHDTSGYLDYW	IGH	6	None	
1	3306	CARDTEDGYNLEYYFDYW	IGH	2	None	
1	3307	CQSYDTSHHGIF	IGL	12	C4	7JVC
1	3308	CAKDRFVAGASAFDYW	IGH	2	None	
1	3309	CQKYNIAPLTF	IGK	652	None	
1	3310	CARDLGEPPRKAREGWFDPW	IGH	4	None	
1	3311	CARSMYGSGSQPLDYW	IGH	2	None	
1	3312	CARGERILITFGGVYPTNAMDVW	IGH	13	None	
1	3313	CTRDTRETGTTYHYYNYMDVW	IGH	2	None	
1	3314	CARPDYGGNSHHFDYW	IGH	2	None	
1	3315	CARYTIFGVASHFDYW	IGH	2	None	
1	3316	CARWGGSGYAYYYYYYGMDVW	IGH	4	None	
1	3317	CARLPELYDSSGSNGSFDYW	IGH	6	None	
1	3318	CARGGYRHGYEVWYFDLW	IGH	12	None	
1	3319	CQSAVTSGPSVVF	IGL	6	None	
1	3320	CAKDGGGAMAPRSYYYYYGMDVW	IGH	2	None	
1	3321	CTRGGYCSSPGCYEGSGPDYYDGMDVW	IGH	11	None	
1	3322	CARAREREMDYYMDVW	IGH	3	None	

1	3323	CVRDVRWIQLWAFDYW	IGH	2	None	
1	3324	CAKGPMNGDQWYFDYW	IGH	2	None	
1	3325	CSRGAWERRGRDYYFYYMDVW	IGH	5	None	
1	3326	CARVRSVDTAFDRW	IGH	18	None	
1	3327	CAKVFSASSRPFWYFDYW	IGH	3	None	
1	3329	CARGLRIQGRRFDPW	IGH	6	None	
1	3330	CAKPIHFGVPPCANWFDSW	IGH	2	None	
1	3331	CAGVRHVELFSYSYFDPW	IGH	3	None	
1	3332	CARSSLVHYFETW	IGH	35	None	
1	3333	CATSPFSSGWNNYYYYMDVW	IGH	479	None	
1	3334	CAKDKVPGAALDYW	IGH	6	None	
1	3335	CAKDLPRPDLFYDDTSGYYFDCW	IGH	2	None	
1	3336	CVKDKWAHYDSSGYFDHW	IGH	6	None	
1	3337	CAAPMTTVVALDYW	IGH	3	None	
1	3338	CAREPYGGHDLDYW	IGH	7	None	
1	3339	CAKPPSSYDSSGYDNYFDYW	IGH	13	None	
1	3340	CARGTHMFQPRHLVTFDYW	IGH	2	None	
1	3341	CARAPYYDFWSGYSTVYFDYW	IGH	3	None	
1	3342	CARGGDLRSFDWLITVFDYW	IGH	61	None	
1	3343	CARTHLRGYSYLHVGYYYMDVW	IGH	2	None	
1	3344	CARGDGYSSSWKGECAFDIW	IGH	2	None	
1	3345	CARDDWDCSSGKCLYKWIDPW	IGH	4	None	
1	3346	CARLGPVYYSYYMDVW	IGH	8	None	
1	3347	CARLARVARTSSSCYSDLW	IGH	5	None	
1	3348	CARRAVTPMTLEYYFDYW	IGH	2	None	
1	3349	CARIPRSITIFGVVKGNWFDPW	IGH	5	None	
1	3350	CARPGLGKDAFDIW	IGH	62	None	
1	3352	CQQTYSTLFSF	IGK	516	C4	6ZDH
1	3353	CARAERGTIFGVVTMGLAFDIW	IGH	4	None	
1	3354	CAAWDSSLNAGQF	IGL	3	None	
1	3355	CAKDRGQLVGSGRSGYYYYGMDVW	IGH	2	None	
1	3356	CAKDSYSSSWYHYYYYGMDVW	IGH	5	None	
1	3357	CARETTVGYNHFDYW	IGH	26	None	
1	3359	CTRDMGLELLGYNWFDPW	IGH	8	None	
1	3360	CARDGRYSSSAQFDQW	IGH	4	None	
1	3361	CARGGEVILGSTIFGRGWYFDLW	IGH	10	None	
1	3362	CARGKEYYYGSGSHAIRINGHFDYW	IGH	4	None	
1	3363	CARDPDQLWDDPFDYW	IGH	35	None	
1	3364	CARDTWPNGGKFDCW	IGH	4	None	
1	3365	CASYPITTGYYYYYMDVW	IGH	4	None	
1	3366	CARDVVVRPASGFDPW	IGH	2	None	
1	3367	CARRPNRDLGGGFDRW	IGH	10	None	
1	3369	CASRYYNVLTGYYLNAFDIW	IGH	2	None	
1	3370	CAHHGILTRLEHIIAAAGNPLTYFDYW	IGH	2	None	
1	3371	CAKVLGVRGVTILDYW	IGH	8	None	
1	3372	CAKGHSSSWTEEWLEYYYYYGMDVW	IGH	2	None	
1	3373	CARNLGTDYNYVWGNSPPFYFDNW	IGH	3	None	
1	3374	CARDARRGCIYGSCRPFDYW	IGH	2	None	
1	3375	CAREGRLGNSWTYFDYW	IGH	2	None	
1	3376	CARYYWAITIFGVLTQAFDYW	IGH	29	None	
1	3378	CTTYNTYYDFWSGYYEGWFDPW	IGH	2	None	
1	3379	CANQGSSSSVGRWTYNLLDYW	IGH	7	None	
1	3381	CVRNYYDSSGYSDYW	IGH	2	None	
1	3382	CARWGNPLAFDSW	IGH	2	None	
1	3383	CATQSILNVNTPMIYRDYW	IGH	4	None	
1	3384	CAKLTIAARLTDFDYW	IGH	2	None	
1	3385	CVHRLGYNAGWYVFDYW	IGH	5	None	
1	3386	CARHRQLAGYMDVW	IGH	6	None	
1	3387	CARLRPGLTSGYYVDHW	IGH	3	None	
1	3388	CVKGEGWWPHFYFDSW	IGH	3	None	
1	3389	CARRGPYYYDSSAHWYFDLW	IGH	4	None	
1	3390	CARDSELGYCSGGSCYSTFYYYYGMDVW	IGH	3	None	
1	3391	CARLVEGTPGPNLCFDLW	IGH	5	None	
1	3392	CARLSIAARFETDYW	IGH	2	None	
1	3393	CARDVACSGGSCYYYGMDVW	IGH	8	None	
1	3394	CANWGSGPSNYYYYMDVW	IGH	2	None	

1	3396	CARGPPKMVVKSWFDPW	IGH	2	None	
1	3397	CAKDIGVGGGRTGYYYYGMDVW	IGH	2	None	
1	3398	CARVDMGRKPYWYFDLW	IGH	2	None	
1	3399	CARHESTYSSSWYAQLWDFDYW	IGH	2	None	
1	3400	CAREGVGWLQLRYFDYW	IGH	2	None	
1	3401	CVRNSYCTRTSCYPDYW	IGH	2	None	
1	3402	CARVRIQGTRNYYYYMDVW	IGH	3	None	
1	3403	CQSYHSNNHGWVF	IGL	18	C4	7JVC
1	3404	CATAAPEWFGGVFYYFDYW	IGH	5	None	
1	3405	CARSPKDANYCDYW	IGH	2	None	
1	3406	CASQRGYREATGDFDYW	IGH	2	None	
1	3407	CANYVGATRIFDYW	IGH	2	None	
1	3408	CARQPQRAVAGTGGWVIDYW	IGH	2	None	
1	3409	CAKGVPSHGSTNLSPEGDYW	IGH	13	None	
1	3410	CARGSTMVRGVIIPPPKIYYYYYYGMDVW	IGH	9	None	
1	3411	CARGNFRRADRPWFDPW	IGH	3	None	
1	3412	CAKETDSSFWGFDYW	IGH	4	None	
1	3413	CATETQFDPW	IGH	2	None	
1	3414	CTKDDCSRLNCYFQWNNWFGPW	IGH	2	None	
1	3415	CAKDSGTTTVVVRISYFDYW	IGH	2	None	
1	3416	CASRYCGRGSCYPDYW	IGH	2	None	
1	3417	CARFPGSITIFEVAHDAFNIW	IGH	8	None	
1	3418	CARAIRRVYYDSSGYYSKDWYFDLW	IGH	2	None	
1	3419	CAKENHEGYYDSSGYYFFFPDLDYW	IGH	2	None	
1	3420	CTGSGAARFLGSSRNDFDYW	IGH	3	None	
1	3421	CARNRYEAVAGLLVSYYYSYYMDVW	IGH	2	None	
1	3422	CARDLPDCRSGLCLPSGFDSW	IGH	12	None	
1	3423	CARVGSGWLPDWFDPW	IGH	2	None	
1	3424	CARDSYYYDSNGYGYYYFDYW	IGH	3	None	
1	3425	CARHPPLEYNWNYSMGPIAFDIW	IGH	4	None	
1	3426	CASWGEDRRDGYNYYYYMDVW	IGH	2	None	
1	3427	CQVWDSSPDQRGFF	IGL	538	None	
1	3428	CAHRWTDYGDYAFDVW	IGH	2	None	
1	3429	CSTGAPTFWSGYFAYW	IGH	2	None	
1	3430	CAKDWGYYGSGSSIYYYYGMDVW	IGH	2	None	
1	3431	CQVWASTSDHPDF	IGL	65	None	
1	3432	CARLTTAMVTGLWYFDLW	IGH	4	None	
1	3433	CGTYRYGLGIDYW	IGH	2	None	
1	3434	CARRQPIVGGATEGGWFDPW	IGH	2	None	
1	3435	CARGKDYNSGKFQLNLYFDLW	IGH	10	None	
1	3436	CAREGDSSSWATGIDYW	IGH	2	None	
1	3437	CARMSLCTNNVCYDLDYW	IGH	2	None	
1	3438	CQSVDISGPYPHWVF	IGL	5	None	
1	3439	CARSPERGSYVLDYW	IGH	28	None	
1	3440	CAKDLQRYYYDSSGYYYLAYYFDYW	IGH	2	None	
1	3441	CARFSLMVRGVIRPSGAFDIW	IGH	2	None	
1	3442	CAREDYGEGPDDAFDIW	IGH	9	None	
1	3443	CARDPPAQMMATSPSYFDCW	IGH	2	None	
1	3444	CARDPGYNPYDALDIW	IGH	2	None	
1	3445	CARDYCTGSSCYVRGFDFW	IGH	3	None	
1	3447	CASSSRTLRYGFYYYYMDVW	IGH	12	None	
1	3448	CAKDNGITMVRGVTPLYFDYW	IGH	3	None	
1	3449	CARRITIFGVVITAGVDAFDIW	IGH	2	None	
1	3450	CARHRGRGSGLGDYYYYMDVW	IGH	3	None	
1	3451	CAKVLHQQLVPLDYW	IGH	2	None	
1	3452	CAKGASTYYDSSGYIDYW	IGH	4	None	
1	3453	CAKDIRYFDWDGMDVW	IGH	8	None	
1	3454	CVCYGDYGEDYW	IGH	4	None	
1	3455	CARLLSGHYQAYFDYW	IGH	4	None	
1	3456	CARFSYDFWSGRRIDYW	IGH	2	None	
1	3458	CASLPLEPTGGYWFDPW	IGH	2	None	
1	3459	CARGQRWLQLIRGSWFDPW	IGH	2	None	
1	3460	CARVTRTEWYYYGSGSYPFSYFDYW	IGH	2	None	
1	3461	CARPSSRAVRAFEIW	IGH	4	None	
1	3462	CAKGYSYDDYYYGMDVW	IGH	12	None	
1	3463	CARQGDTDHDFDYW	IGH	12	None	

1	3465	CATPRGPVEGGFDYW	IGH	2	None	
1	3466	CARHGVVVVPAPLGIQLWSSDWFDPW	IGH	3	None	
1	3467	CATSSHCSGGSSCYSYYW	IGH	2	None	
1	3468	CAREGVMATILGHYTWFDPW	IGH	12	None	
1	3469	CARQTRTKYSSLPLDYW	IGH	2	None	
1	3470	CARDRAYYDFWSGYYWVHYYYYMDVW	IGH	3	None	
1	3471	CASGPRDSNHFDLW	IGH	13	None	
1	3472	CAKLPFTFTVVTPDYW	IGH	2	None	
1	3473	CAKDHSSSWYSLDFDYW	IGH	2	None	
1	3474	CARDRLNLVRGYYYYYGMDVW	IGH	2	None	
1	3475	CASLTFWSESPQFDYW	IGH	4	None	
1	3476	CASDAGYSSREPKYYFDYW	IGH	3	None	
1	3477	CTRHYVSAGVWSRSYQTTQPHRFPDYW	IGH	2	None	
1	3479	CYSPDSSGGHRRVF	IGL	3	None	
1	3480	CARDQWPITVVTSLGYYYMDVW	IGH	3	None	
1	3481	CANCYGSGSYFGIAEDFDYW	IGH	4	None	
1	3482	CAKQGGYFALQRSSYMDVW	IGH	2	None	
1	3483	CARRGATPGTGPYYYYYMDVW	IGH	2	None	
1	3484	CANLMGATLLLVRRTYDAFDIW	IGH	57	None	
1	3485	CARGGATLDTIFGVVIIDEKSIVFDYW	IGH	2	None	
1	3486	CASPAEPYYYDSSGYSFDIW	IGH	2	None	
1	3487	CVREGGAFMRWFDPW	IGH	14	None	
1	3488	CAAGRGGYYDTSGHYYDYW	IGH	2	None	
1	3489	CARHDQYSSSFYWFFDLW	IGH	3	None	
1	3490	CARHKGFLQQWLSYFDYW	IGH	6	None	
1	3491	CAKVSRAYSSSWFAFDYW	IGH	2	None	
1	3492	CMHSIRLPRGTF	IGK	17	None	
1	3493	CAKDRGFLDWIAHPDYW	IGH	2	None	
1	3494	CARQDEFWSGSSVGYFFDWW	IGH	7	None	
1	3496	CYSSGYYYYYYMDVW	IGH	2	None	
1	3497	CARDRNTVEMATINWYFDLW	IGH	6	None	
1	3498	CARAVGEQWLYYYYGMDVW	IGH	2	None	
1	3499	CARDKDLGFCSGFTCQGGFDPW	IGH	3	None	
1	3500	CARDPRGYCSGGLCYKDYYYGMDVW	IGH	11	None	
1	3501	CARQAGFTGSYGYMDVW	IGH	2	None	
1	3502	CARLPDTSMVIPYYSDHW	IGH	4	None	
1	3503	CARDQGGDFWSGRLNSDFYYMDVW	IGH	5	None	
1	3504	CAKDIGDPYGDYFVGLDYW	IGH	2	None	
1	3505	CAREALGWLFYDAFDIW	IGH	2	None	
1	3506	CARGWVIHFDYW	IGH	96	None	
1	3507	CAKGSTCIAARPCSYWYFDLW	IGH	2	None	
1	3509	CQQYHDWPLYSF	IGK	2	None	
1	3510	CARHVAAAGPPDYFDYW	IGH	8	None	
1	3511	CQYLNIYPRF	IGK	4	C1	7BZ5
1	3512	CAGQGDDILTHDPDHAFDIW	IGH	2	None	
1	3513	CAKDSMSSGNCSGGSCYGDFDYW	IGH	5	None	
1	3514	CARDLERIRVDVDYW	IGH	3	None	
1	3515	CVREIYDSDLLRNWFDAW	IGH	5	None	
1	3516	CARADGRGSGPYHYYGMDVW	IGH	2	None	
1	3517	CARDLAAAAPDYW	IGH	16	None	
1	3518	CAKDLVDGGYYYDSSGYYEGSVVDYW	IGH	2	None	
1	3519	CNLKASDTAMYYCARQRREYDTSSSWGFKYYYYMDVW	IGH	2	None	
1	3520	CTKEYYGSGSYSDYW	IGH	2	None	
1	3521	CTRGRPRWQDTRHWFDPW	IGH	2	None	
1	3522	CAKERNTVVVTAIDPFNYW	IGH	6	None	
1	3523	CARVRDYYGRVLAFEYW	IGH	3	None	
1	3524	CARVVVVVAATSFSTMFDPW	IGH	8	None	
1	3525	CARGLRVGRQRLPTRSTDYYYYMDVW	IGH	5	None	
1	3526	CATGGDFVGFFDSW	IGH	23	None	
1	3527	CTTEKSSGNYIWFDYW	IGH	2	None	
1	3528	CVREARGFSYSYRRDAFDIW	IGH	13	None	
1	3529	CVRDNDGGGYLEYW	IGH	3	None	
1	3530	CARAPSFRVMGPLYYFDYW	IGH	3	None	
1	3531	CQVWASSGACVF	IGL	201	None	
1	3532	CARDPGYYDNLSFNRLDPW	IGH	2	None	
1	3533	CAKLATVMFGAFDIW	IGH	2	None	

1	3534	CARAIGLSKWEPRPLYDYW	IGH	3	None	
1	3535	CARGSDYGDYNPFYYYYYGMDVW	IGH	3	None	
1	3536	CAKGAPGAAADYYYYGMDVW	IGH	2	None	
1	3537	CARQLPTAIYGVNYFDPW	IGH	4	None	
1	3538	CAKVHSSGYLFDYW	IGH	46	None	
1	3539	CASFNRFDSSGYYYVEDYW	IGH	2	None	
1	3540	CARDSPWLEVYVYYYGMDVW	IGH	2	None	
1	3541	CARDRLNIGGVRADYW	IGH	3	None	
1	3542	CQSGDSGGNSVAF	IGL	9	None	
1	3543	CARDVFGVRSGWYWSYFDYW	IGH	3	None	
1	3544	CAGYGARRGGFDYW	IGH	4	None	
1	3545	CARGPPYVAIPFDYW	IGH	4	None	
1	3546	CVRTKLGYCSGENCYISIVWFDPW	IGH	8	None	
1	3547	CAKDRYTAYPDLGWFDPW	IGH	5	None	
1	3550	CQQSHSAPSFTF	IGK	38	C2	7K8O
1	3551	CARREGPYSSSSKYWYFDLW	IGH	5	None	
1	3552	CASQPVAGTSTGEYYYYYGMDVW	IGH	3	None	
1	3553	CAHTEYNLNFLNLLDYW	IGH	3	None	
1	3554	CAGGLNTAVVTYFDYW	IGH	2	None	
1	3555	CQQYDNWSLTWTF	IGK	3	C3	6XDG
1	3556	CAKKADPHGDHALYYFDYW	IGH	3	None	
1	3557	CARDEGVVVITAGGGLFDVW	IGH	4	None	
1	3558	CAHSDWNYVFHAFHVW	IGH	10	None	
1	3559	CAREAYDSTGYRLGYFEKW	IGH	5	None	
1	3560	CARRRKKGQQPAFFYYYYMDVW	IGH	2	None	
1	3561	CARVWSNTWEGYDYW	IGH	6	None	
1	3562	CARQKRPIGRWFDPW	IGH	2	None	
1	3563	CAKYDVEAPDEDPPTYKWFDPW	IGH	2	None	
1	3564	CARGDWWEPTWYFDLW	IGH	6	None	
1	3565	CAKTFADNGYDFVNWWFDPW	IGH	3	None	
1	3567	CYSTDRSGNQTWVF	IGL	2	None	
1	3568	CAHRCGGDCYNAFDIW	IGH	4	None	
1	3569	CARAGGYDSGYYMDVW	IGH	2	None	
1	3570	CARGKKGIAAAGTVPAFDIW	IGH	2	None	
1	3571	CARVGFYDSSGYYTPDFDYW	IGH	4	None	
1	3572	CQQCNGYPPRFTF	IGK	4	C1	7BZ5
1	3573	CTRDRDTNSQYSWLDPW	IGH	6	None	
1	3574	CARGDTYTSSAFFYW	IGH	6	None	
1	3575	CARHSARREANYFDYW	IGH	3	None	
1	3576	CAKDPVFGDYW	IGH	9	None	
1	3577	CARGTTRVPSYFDSW	IGH	2	None	
1	3578	CAKASGWYVGYYFDYW	IGH	4	None	
1	3579	CARAVGTRVLWSGSWFDPW	IGH	4	None	
1	3580	CARLDYYDGRGHNYRRDYW	IGH	2	None	
1	3581	CVRDRQGVGTSTSFDYW	IGH	8	None	
1	3583	CQQYHSCSPMCTF	IGK	10	None	
1	3584	CAKDRHEIVTGYFDYW	IGH	3	None	
1	3585	CAKDRTLTMIDAFDIW	IGH	6	None	
1	3587	CAREEVVVVVAAPGTKYYYYGMDVW	IGH	4	None	
1	3588	CARDLTQWIQLFGYYYYYGMDVW	IGH	2	None	
1	3589	CARAWTVTKPWGYYYYGMDVW	IGH	3	None	
1	3590	CARHRSRILYFAPEFDPW	IGH	12	None	
1	3591	CAKDGSGYCSGGSCVPHVDYW	IGH	2	None	
1	3592	CAKDRTRFPDYFDHW	IGH	7	None	
1	3593	CARDPYTSSPDNWFDPW	IGH	2	None	
1	3594	CARHAPSIVEVFDYW	IGH	5	None	
1	3595	CARGKVTGTTPVYYFYYW	IGH	3	None	
1	3596	CGREVGATSGDYW	IGH	2	None	
1	3597	CARDGGLGASFDLW	IGH	991	None	
1	3598	CARVFTPPYYDYKGYSDYW	IGH	2	None	
1	3599	CARHEGRVVTAIPWGFDPW	IGH	11	None	
1	3600	CARVIPAVAGPYYYYYGMDVW	IGH	2	None	
1	3601	CARDWGVGNYFDPW	IGH	107	None	
1	3602	CVKDSASAYYSGDYFDSW	IGH	4	None	
1	3604	CAATIAARRLESGGWRIFDYW	IGH	5	None	
1	3605	CAKQEVESYFEYW	IGH	2	None	

1	3606	CARGLDFWSGYYSSRHWSDPW	IGH	2	None	
1	3607	CAKDTGPTPLAHRNAYCGGDCPRGFDYW	IGH	2	None	
1	3608	CARDLPFHCSTTSCTPLFDPW	IGH	13	None	
1	3609	CAAHVNTDFWSGQSGGWFDPW	IGH	11	None	
1	3610	CARDIAPGYCGGDCYSTPLFAFDIW	IGH	2	None	
1	3611	CARSPRLDSPMEGALDIW	IGH	9	None	
1	3612	CAKSSALAVAGEYFDYW	IGH	3	None	
1	3613	CARVQFKVPMDAFDIW	IGH	6	None	
1	3614	CLQDYNFPHIF	IGK	9	None	
1	3615	CKSRDSSGDQVLF	IGL	9	None	
1	3616	CARSLSTSAAPPNDYW	IGH	8	None	
1	3617	CTKDDYYYYMDVW	IGH	20	None	
1	3618	CASSRTNGVSLHYYYYDMDVW	IGH	3	None	
1	3619	CATWGNRAWFFDYW	IGH	8	None	
1	3620	CASPQYTTGWYADVFDIW	IGH	2	None	
1	3621	CARDRDDYYYGSGTFACDIW	IGH	2	None	
1	3622	CARLPSYGDNFKDAFDIW	IGH	2	None	
1	3624	CVRQKRWIDPW	IGH	2	None	
1	3625	CARAASLGELSFYFDYW	IGH	2	None	
1	3626	CARGAITMIVALAAFDVW	IGH	2	None	
1	3627	CARGRGCITIFGVVICPYYFDYW	IGH	3	None	
1	3628	CARNHPLLVPAGSVDYW	IGH	4	None	
1	3629	CARTLWTYGVVLPVGKWFDSW	IGH	9	None	
1	3631	CARLTEPSITPLTYYYFYMDVW	IGH	16	None	
1	3632	CARTGLFTPFDPW	IGH	8	None	
1	3633	CARGGIGVPPYCSGGSCLNYYYYGMDVW	IGH	3	None	
1	3634	CARVPGYCSGGSCREYYFDYW	IGH	2	None	
1	3635	CAKKRGQPVVHGIDFDYW	IGH	2	None	
1	3636	CARGEAVAGFQDFDYW	IGH	2	None	
1	3637	CAKDTRLDYGGNSGFDPW	IGH	4	None	
1	3638	CASPEYYYSTLDAFDIW	IGH	3	None	
1	3639	CMQSIQRLRYSF	IGK	6	None	
1	3640	CAREGYSSSWYSFFYFDYW	IGH	3	None	
1	3642	CARLDDSSDDYYAAFDYW	IGH	6	None	
1	3643	CARGPLGYSDSSNYYPAGINSGYFYYYYMDVW	IGH	3	None	
1	3644	CARQNSHTTIFGVVIKEIDYW	IGH	2	None	
1	3645	CARDSRDSSGYYYGGYYFDYW	IGH	5	None	
1	3646	CARDVGGYRSSKYLPQRSPSYYSYMDVW	IGH	4	None	
1	3647	CAREGGRYCSGGGCLPDYW	IGH	2	None	
1	3648	CARDMGSNGLPFDYW	IGH	2	None	
1	3649	CTTDRGITVRPIFDRW	IGH	4	None	
1	3651	CATRHISITIFGVVTDYFDYW	IGH	66	None	
1	3652	CARAFRGFTPDYW	IGH	4	None	
1	3653	CARASGLGTMIVVVITHFDYW	IGH	9	None	
1	3655	CARAASGVLHGFDYW	IGH	6	None	
1	3656	CAREAYAMDVW	IGH	5	None	
1	3657	CAKAHDWNYFDYFDYW	IGH	8	None	
1	3658	CARDSPYDFWSGYYYANYYYYGMDVW	IGH	2	None	
1	3659	CAKDRVGVSHYYLDSW	IGH	9	None	
1	3660	CARSSTSLAENDAFDIW	IGH	2	None	
1	3661	CATVWGWGSGRFDAW	IGH	3	None	
1	3663	CARDRPAYCGGDCYFDAFDIW	IGH	2	None	
1	3664	CAKDLGSYYENYW	IGH	15	None	
1	3665	CTRGGAVADYSDYW	IGH	9	None	
1	3666	CAARDDSLSGGVVF	IGL	5047	None	
1	3667	CARTFCVNAICESYFDNW	IGH	3	None	
1	3668	CARRNYYDSSDYYWPAYYFDYW	IGH	8	None	
1	3670	CARFVRAIKIVGATEYYFDYW	IGH	6	None	
1	3671	CARGSSAGWGSRRRNYFDHW	IGH	4	None	
1	3672	CARHRERGIRNRTHSYHYYYMDVW	IGH	4	None	
1	3673	CAKGGLRLLFGIDAAFDIW	IGH	2	None	
1	3674	CAREKPDPIAVAGPIDYW	IGH	4	None	
1	3675	CAHSSLYQLEFDYW	IGH	2	None	
1	3676	CARFNFFDSGGNNWFDSW	IGH	3	None	
1	3678	CAKEDEYYYYGMDVW	IGH	18	None	
1	3679	CARMKVVADVWDYW	IGH	2	None	

1	3680	CAGLPGTWYSDFDFW	IGH	4	None	
1	3681	CAKGNIVVVPAAVRRYYYYGMDVW	IGH	4	None	
1	3682	CARDMGGWYNRGPSAFDLW	IGH	2	None	
1	3683	CATDYPDGISVAARLFDYW	IGH	10	None	
1	3684	CARVKHYYGSGGYYFDYW	IGH	2	None	
1	3685	CAGRPPPYNYGLYYFYAMDVW	IGH	2	None	
1	3686	CATLRDSVLTGYSFDYW	IGH	3	None	
1	3687	CAKFGRIAAAGEAGYFDYW	IGH	6	None	
1	3688	CQQFGTSPPMFTF	IGK	16	C2	7K8U
1	3689	CARGVVGHLPGSYFDYW	IGH	4	None	
1	3690	CARTRLSINWFDPW	IGH	16	None	
1	3691	CARSPPIAVAGWGDFDIW	IGH	2	None	
1	3692	CARDQEYYYGSGNIDYW	IGH	4	None	
1	3694	CARDGQWLLLEPFDIW	IGH	22	None	
1	3695	CAGKDLGVITYHSYYYSYGMDVW	IGH	4	None	
1	3697	CARDPEEQMAAAGIYYYYYYMDVW	IGH	2	None	
1	3698	CAREFAFTLTTTYFDSW	IGH	2	None	
1	3699	CARLGRPTLYYYDGMDVW	IGH	2	None	
1	3700	CARDLRFTYYYGSGRRWFDPW	IGH	2	None	
1	3701	CAKDQVDFYYYYGLDVW	IGH	4	None	
1	3702	CARHLNRLHPFDTW	IGH	14	None	
1	3703	CARDVENYYDSSSYSSQRPYYYGMDVW	IGH	2	None	
1	3704	CARAPLMDALDSW	IGH	2	None	
1	3705	CARGPPYYDFWSGPNWFDPW	IGH	9	None	
1	3706	CARLRLFPYFDYW	IGH	8	None	
1	3707	CARSPFRGITIFGVVTTGRYFDLW	IGH	2	None	
1	3708	CAKAITASTLGYFQHW	IGH	6	None	
1	3709	CARRTYYYDSSGYYTWFDPW	IGH	5	None	
1	3711	CVLCRGGWYYYYMDVW	IGH	8	None	
1	3712	CAREYCDGATHCRGGWFFDLW	IGH	2	None	
1	3713	CVSSASYYLEYMDVW	IGH	4	None	
1	3714	CAREAGDSSPWRFDHW	IGH	25	None	
1	3715	CVRRPKDGIVGSQGNFDFW	IGH	2	None	
1	3716	CARRGGYTYAGDYFDYW	IGH	2	None	
1	3717	CARADCTNAACPSDYW	IGH	3	None	
1	3718	CARDSSTWNYYYYHGMDVW	IGH	6	None	
1	3719	CAREGIWFGESPWDYW	IGH	2	None	
1	3720	CARDPFDSSGYYYNWYFDLW	IGH	2	None	
1	3721	CARLSGYYYVDAFDFW	IGH	3	None	
1	3722	CARLEEMSTIWAYFDYW	IGH	7	None	
1	3723	CARVSRITIFGVVIIGPLDYW	IGH	22	None	
1	3724	CATQYAIAARQTMDYW	IGH	2	None	
1	3725	CARHRDVLRYSLFDPW	IGH	4	None	
1	3726	CARDSYDDFWSGYYTVWSLGQKNYYYYAMDVW	IGH	8	None	
1	3727	CARAPQTTDIAAFDPW	IGH	2	None	
1	3728	CAGDLFYYYDSSGPASPIDYW	IGH	2	None	
1	3729	CATIAAAGPDYYYYYGMDVW	IGH	16	None	
1	3730	CARLGEEYSGYDKGPFDYW	IGH	2	None	
1	3731	CAGDLGVAARNLGLYYMDVW	IGH	13	None	
1	3733	CARDFNVYTSSPVDAFDLW	IGH	2	None	
1	3734	CARDYRDRYYHSSGKTFEYW	IGH	28	None	
1	3735	CASMSRYGPFDFW	IGH	4	None	
1	3736	CARELNWNYSPFDYW	IGH	7	None	
1	3737	CAKGVLPTSNYYDSSGYPDYW	IGH	3	None	
1	3739	CARHSSGYPSSEFDYW	IGH	12	None	
1	3741	CARGSYFVYNDYYFDYW	IGH	2	None	
1	3742	CARHTYYYDSSGYYYKHYFDYW	IGH	5	None	
1	3743	CARGADYGAAFDFW	IGH	2	None	
1	3744	CVDAGSGNYYFEYW	IGH	2	None	
1	3745	CARAGVRGTNVYFSDYW	IGH	2	None	
1	3746	CARGVRELSYYYYYYMDVW	IGH	2	None	
1	3748	CAKVLPPGSGSPTEFDFW	IGH	2	None	
1	3749	CAKEEDSDPEWELLRQAHSTFFDYW	IGH	2	None	
1	3750	CASPWGWNRGSYYEYDAFDIW	IGH	2	None	
1	3751	CAGLWSLYCSGGSCSHYVDCW	IGH	4	None	
1	3752	CARGTRGSKAPYYYGMDVW	IGH	6	None	

1	3753	CARQNFIAPIDWFDPW	IGH	9	None	
1	3754	CARGRTTVTTRFGRDGFDIW	IGH	3	None	
1	3755	CARGLRRGYGYGDWFDTW	IGH	4	None	
1	3756	CAREGWEGPYFDLW	IGH	2	None	
1	3757	CATDGSSSLAYYFDYW	IGH	2	None	
1	3758	CARDRYYYGSPGARYYMDVW	IGH	3	None	
1	3759	CARLLEYSSSPRWFDPW	IGH	4	None	
1	3760	CASLTSDWYSDYYGMDVW	IGH	2	None	
1	3761	CTSPQASRDDNSADYW	IGH	3	None	
1	3762	CARHVYEAAPRDPIIGMIIGSDSGDGGWFDPW	IGH	2	None	
1	3763	CARGGYSSGWYGTHNWFDPW	IGH	2	None	
1	3764	CARAGRSIGYSSGWNYFDYW	IGH	10	None	
1	3765	CASDLWAKVEDSSSSDWFDPW	IGH	2	None	
1	3766	CARHKEMAIQKFDPW	IGH	2	None	
1	3767	CARHSLWVPPAGSSSTSFFDSW	IGH	10	None	
1	3768	CAKGVIYDYDPPSAFDIW	IGH	3	None	
1	3769	CARGGMMLLAAGSGGNYYYGMDVW	IGH	3	None	
1	3770	CARVHYDFWSGKEWYFDLW	IGH	2	None	
1	3771	CQQLSTFPPHTF	IGK	32	C1	7BZ5
1	3772	CTRVKGYSSSPFDYW	IGH	55	None	
1	3773	CAKDSYPQLWSEVPGFDLW	IGH	10	None	
1	3774	CARDRGAVTIPNFLFDYW	IGH	4	None	
1	3775	CAREMVFGVVIRWFDPW	IGH	8	None	
1	3776	CARASDYYDETNFDYW	IGH	2	None	
1	3778	CGADHGSGSNFVSPWVF	IGL	5	None	
1	3779	CARRTWSDGGFDYW	IGH	4	None	
1	3780	CARGGGYYDFWSGRGGDYW	IGH	3	None	
1	3781	CAKGNYESYYYYMDVW	IGH	2	None	
1	3782	CARVSPATEIIVPHRGWFDPW	IGH	4	None	
1	3783	CAHSRNPEIGYWFDPW	IGH	8	None	
1	3784	CAKDRLTVVPAAIVIVAPRNYYYYYGMDVW	IGH	2	None	
1	3785	CARDLTVTFGVYYYYGMDVW	IGH	2	None	
1	3787	CATSPNICSGGSCYPYDKYYYYGMDVW	IGH	2	None	
1	3788	CASIGNWFAPW	IGH	3	None	
1	3789	CTKIEGSTLYYNYYYMDVW	IGH	2	None	
1	3790	CARLRLEWYDAKTYYYYGMDVW	IGH	2	None	
1	3791	CARNQWFLYSGSFSYYFDYW	IGH	4	None	
1	3792	CAIGFNDFWSGYGYFDYW	IGH	2	None	
1	3793	CAKETRIYRAFDIW	IGH	2	None	
1	3794	CARAVAGSTFAVVKNWYFDLW	IGH	16	None	
1	3795	CATSPESSGSLDAFDIW	IGH	2	None	
1	3796	CTRDRPYDDFWSGFYYYYYMDVW	IGH	8	None	
1	3797	CARDRDLAGLVCDYW	IGH	2	None	
1	3798	CARDDAIVGTSKGGFDHW	IGH	2	None	
1	3799	CAHSRSSSSGWYVHHFDYW	IGH	2	None	
1	3800	CARLSYSRSSGEWYFDLW	IGH	41	None	
1	3801	CAKDIGGGSTDDAFDIW	IGH	6	None	
1	3802	CAHSVSQLPKLIRIAGSRDYFDYW	IGH	2	None	
1	3803	CQSADNIGTYLLF	IGL	9	None	
1	3804	CARVHCITIFGVVILGGWFDPW	IGH	3	None	
1	3805	CARDLAHIVVVTTHWGYYYGMDVW	IGH	2	None	
1	3806	CARDSLFTIFGVVIEYYFDYW	IGH	7	None	
1	3807	CARHMRIAVAHLDYW	IGH	2	None	
1	3809	CARHSGYSSGWYPGNIDYW	IGH	2	None	
1	3810	CARDGRGSYYSPMTLFDYW	IGH	3	None	
1	3811	CSSYTISSGLGILF	IGL	3	None	
1	3812	CAKGSTRLSDYW	IGH	4	None	
1	3813	CARDRGITGTSGFSLFDIW	IGH	7	None	
1	3814	CATAGHTSYSPYYFDYW	IGH	4	None	
1	3815	CAKTQQYYFDYW	IGH	8	None	
1	3817	CQSADGSAISPWVF	IGL	3	None	
1	3818	CARGVGQLAGYHYYYMDVW	IGH	4	None	
1	3819	CAKDRDIQLWFNYYYGMDVW	IGH	4	None	
1	3820	CARENDQEVRAAPGIIYYYYAVDVW	IGH	9	None	
1	3822	CARGALNWDVQGSDFFDYW	IGH	2	None	
1	3823	CARHELGVTTGFEYW	IGH	2	None	

1	3825	CATRIVSSGWYEFDYW	IGH	11	None	
1	3826	CAREQGYGHDRGWFDPW	IGH	1128	None	
1	3827	CAKPRAYDSSGYYRHWYFDLW	IGH	4	None	
1	3828	CARIEWRPTDYW	IGH	36	None	
1	3829	CARDVHVLLWFGERYYFDYW	IGH	4	None	
1	3831	CARVSFSAWTFDDW	IGH	2	None	
1	3832	CAKEPLFIAVSAAVRFDSW	IGH	2	None	
1	3833	CDVVPSAYNRYFYPDVW	IGH	12	None	
1	3834	CARGNWKLSNDYW	IGH	8	None	
1	3835	CARETYYYDSSGSKPYDYW	IGH	2	None	
1	3836	CARGRINFGGVIDTFFDYW	IGH	5	None	
1	3837	CARLLAAAGQEIDYW	IGH	2	None	
1	3838	CARERVELVATYYWAWFDPW	IGH	2	None	
1	3839	CARETGGSSWYRGVVVGAFDIW	IGH	2	None	
1	3840	CARGDDYYDSSIYRAFDIW	IGH	2	None	
1	3841	CARAFILWWSVGNWFDPW	IGH	2	None	
1	3842	CAALSRVLDHAYSGYEYWFDPW	IGH	4	None	
1	3843	CARAPRAHEIYYYYYGMDVW	IGH	2	None	
1	3844	CARDPYPITMIVVNDYW	IGH	2	None	
1	3845	CARDERSGAMVDYYFDYW	IGH	2	None	
1	3846	CARGHYDSISGYFFDYW	IGH	32	None	
1	3847	CARDVWMPAEWENENFDYW	IGH	2	None	
1	3848	CTSHVDSRYNWFDPW	IGH	2	None	
1	3850	CARGEIEPRSGYYFYHAMDVW	IGH	4	None	
1	3851	CVQALQTLALTF	IGK	69	None	
1	3852	CHVDNSRSHQTIYYMDVW	IGH	2	None	
1	3853	CATDRCSSTSCNPYYYYGMDVW	IGH	2	None	
1	3854	CARDRYYYDSSGYLINWFDPW	IGH	4	None	
1	3855	CARGNDSSNVYYWGFFDKW	IGH	2	None	
1	3856	CARDRSGTTGVFDYW	IGH	17	None	
1	3857	CAKDSVNYYGSGSFDYW	IGH	5	None	
1	3858	CTRGTPTRAKDFYIDVW	IGH	3	None	
1	3859	CTTETAGVGYYYDSSDLKDLYDNAFDIW	IGH	8	None	
1	3860	CARDISKWLTDGMDVW	IGH	2	None	
1	3861	CARHRITWGISAANTHWFDPW	IGH	2	None	
1	3862	CARDRRPPSGSESNYYFDFW	IGH	2	None	
1	3863	CAREVAGGNSLGLDYYYGMDVW	IGH	2	None	
1	3864	CAREIRAPRGSFDPW	IGH	2	None	
1	3865	CAKGSRPRITYYYDNRVDYW	IGH	3	None	
1	3866	CAKDSEIAAAGSTFDYW	IGH	6	None	
1	3867	CARDAFSSPDGRYFDLW	IGH	3	None	
1	3868	CARHGRPLSGSYPTYFDYW	IGH	5	None	
1	3869	CAKNSGSSPIYYFDYW	IGH	2	None	
1	3870	CARGGYKDWYFDLW	IGH	13	None	
1	3871	CATARAVIGYSSSWYWFDPW	IGH	2	None	
1	3872	CAKGQPIYGDYYDYW	IGH	4	None	
1	3873	CARTGGGTHPYMHFDNW	IGH	8	None	
1	3874	CARDNPPSYSSSWYWFDPW	IGH	2	None	
1	3876	CLLDIQLLYRGYW	IGH	2	None	
1	3877	CQTWGTGIQVF	IGL	340	None	
1	3878	CARHLNDPALITGGGLDYW	IGH	2	None	
1	3879	CAAGYYDFWSGHYYVFDYW	IGH	2	None	
1	3880	CTTVFKWELLRDMDYYYYYMDVW	IGH	2	None	
1	3881	CAKDKWSGIAAAGPLDYW	IGH	3	None	
1	3882	CARLDLFSGPIPAANLHGWFDPW	IGH	2	None	
1	3884	CATLQDPWEVQGSYYIDYW	IGH	6	None	
1	3885	CAREIHGYRFGYSGRDPYYFDYW	IGH	3	None	
1	3886	CARATGSRTYYNQPFDYW	IGH	10	None	
1	3887	CVRESTLTWSWFDPW	IGH	5	None	
1	3888	CARGPPLQMATMSRAYWLDYW	IGH	2	None	
1	3889	CTRANMMFWGPFDYW	IGH	5	None	
1	3890	CARDVTYYYGSGSLEDYYYYGMDVW	IGH	2	None	
1	3891	CARGEHGNGYHVVYYYYYMDVW	IGH	3	None	
1	3892	CARGTLRLPAHPGYFDLW	IGH	42	None	
1	3893	CARARGIAVAATIFYYYYGMDVW	IGH	2	None	
1	3894	CATGAEWQLPFDYW	IGH	36	None	

1	3895	CARHGGFRDEYNWGSNTPPFYFEHW	IGH	8	None	
1	3896	CLLYDGGVQIF	IGL	4	None	
1	3897	CARGDPRYISGWPDWFDTW	IGH	4	None	
1	3898	CARFHYYDSSDSSDDAFDIW	IGH	2	None	
1	3899	CARGSVGGWELPAFEIW	IGH	6	None	
1	3900	CARDRLVRVQGTRDRYYYYYYGMDVW	IGH	3	None	
1	3901	CAKDHRRIKIFGDVIKNQYYYAMDVW	IGH	14	None	
1	3902	CAKLHPDIVVAPAPNGGLEAAGAYDYW	IGH	2	None	
1	3903	CAREGSLVAAADNFDYW	IGH	19	None	
1	3905	CVRQQNDYIWGSPWFDPW	IGH	2	None	
1	3908	CARDGGRFLEWTPFDYW	IGH	4	None	
1	3909	CARALWFRSYYFDYW	IGH	3	None	
1	3910	CARSYFSDDSSAFRAFDIW	IGH	3	None	
1	3912	CVKSEEDFSNYFRFW	IGH	4	None	
1	3913	CAKDRWYYDILTGYFPLSHYYGYYGMDVW	IGH	10	None	
1	3915	CAHRPSSGYGYWGGFDYW	IGH	2	None	
1	3916	CALINEMATIFGVENWFDPW	IGH	2	None	
1	3917	CAKDLEAIVGATGLDYW	IGH	2	None	
1	3918	CAKDARLRYSNYLDYW	IGH	2	None	
1	3919	CAKEKLDSVDYYYGMDVW	IGH	3	None	
1	3920	CARDDLLQTGGYIWGSYRFRSSFFDYW	IGH	49	None	
1	3921	CARGITIFGLDFIYWYFDLW	IGH	4	None	
1	3922	CAAWDDRLTGPHVVF	IGL	2	None	
1	3923	CTTWEPDVGVVVTATLDYW	IGH	5	None	
1	3924	CAKDSTNWNYGPNDYW	IGH	27	None	
1	3925	CAKDNTSGDYGISWFDPW	IGH	2	None	
1	3926	CAREAYYYETSGYYGSRYFDYW	IGH	335	None	
1	3929	CAKDRSITMTFGAFDIW	IGH	4	None	
1	3930	CARLSSLVRPARYLDNW	IGH	4	None	
1	3931	CAREKTDPPYDSSGYPRIDYFDYW	IGH	2	None	
1	3932	CATDPYDNTGYYGVQDTFNIW	IGH	5	None	
1	3933	CARHNLRWVYYMDVW	IGH	2	None	
1	3934	CAKSQGHYDFWSGYDYW	IGH	2	None	
1	3935	CAKVGDGYKPPYFDYW	IGH	8	None	
1	3936	CARSGHGDYGGTLDYW	IGH	6	None	
1	3937	CSVWPRYELLTASRYYYYYMDVW	IGH	2	None	
1	3938	CARDLGYSYGLDYW	IGH	10	None	
1	3939	CAKDLSAFYSGSGSSYYYDGMDVW	IGH	2	None	
1	3940	CARFHSSGWLDYW	IGH	123	None	
1	3941	CARHAFLDGIMGSDYW	IGH	8	None	
1	3942	CAKAALIALYYHYYMDVW	IGH	2	None	
1	3943	CAKDRLQAYYHYGMDVW	IGH	5	None	
1	3944	CAASPIYFYDDNGYFSPW	IGH	6	None	
1	3945	CARDLEVGANHWFDSW	IGH	4	None	
1	3946	CAKGDTKGYCSSTSCLTNKYYYYYGMDVW	IGH	2	None	
1	3947	CARSCSRTSCYLQRFDPW	IGH	18	None	
1	3948	CARHQTSYSGYDLYYFDYW	IGH	10	None	
1	3949	CAREVATYYYGSGTYSYYFDYW	IGH	2	None	
1	3950	CARDLFADSGSISVPDYYYMDVW	IGH	3	None	
1	3951	CTRGHSYYDSGGYHSYFDYW	IGH	15	None	
1	3952	CARDGDYYDSSGYYLLATYGAAFDIW	IGH	2	None	
1	3953	CAKVGIFLNSVGHCDYW	IGH	2	None	
1	3954	CARARGEYSSSPKTLDYW	IGH	2	None	
1	3955	CAHRGRWNYAYFDLW	IGH	3	None	
1	3956	CARVSLRFLEWSHYYFDYW	IGH	4	None	
1	3957	CAHQPPCSGANCYYHDTFDIW	IGH	2	None	
1	3958	CMKDRNRNIWGPRQWFDPW	IGH	4	None	
1	3959	CARDLANSEYSSGWYYYYYYMDVW	IGH	4	None	
1	3960	CARHDVGLRWGNFDYW	IGH	7	None	
1	3961	CARRSQYSGAARGYYYYYYMDVW	IGH	4	None	
1	3962	CARDIVVGSETALDYW	IGH	2	None	
1	3963	CAREIGVAVTGTWWTGYFDYW	IGH	2	None	
1	3964	CATGPPLIVGATNWFDPW	IGH	11	None	
1	3965	CARFDYSNSKYAPFFDYW	IGH	2	None	
1	3966	CATFPTSVTNPDYW	IGH	15	None	
1	3967	CASGPSYNWNYVDHW	IGH	12	None	

1	3968	CARSKGANCYSYGGYVGSCRYHFDFW	IGH	6	None	
1	3969	CAGLPTRWTTVIKGYFDYW	IGH	5	None	
1	3970	CARDPRPVSGYADYW	IGH	42	None	
1	3972	CAKNRWIQLWLALLDYW	IGH	2	None	
1	3973	CAKDPRPALLWFGESPSFDPW	IGH	2	None	
1	3974	CVGSFGLERIDYW	IGH	2	None	
1	3975	CAREARYYDFWSGFHYYYYVMDVW	IGH	8	None	
1	3976	CETWDSNPVVF	IGL	104	None	
1	3977	CAKSGDNEGVVGDYW	IGH	2	None	
1	3978	CARGEGEGDQAYYYGMDVW	IGH	3	None	
1	3980	CASSPPNYYDSQNYYFGGIPFDFW	IGH	4	None	
1	3981	CTRAVRGYPPYYFDYW	IGH	2	None	
1	3983	CARGSSYHDSSSAFDIW	IGH	10	None	
1	3984	CARLDIAVTGSFDYW	IGH	2	None	
1	3985	CARRFGELFGTEYYFDYW	IGH	3	None	
1	3986	CARHGGKYSSSSIFDYW	IGH	4	None	
1	3988	CTSGLRWEDSFDFW	IGH	4	None	
1	3989	CARFVDTAMVKENWFDPW	IGH	2	None	
1	3990	CAKDINLAGTYVFDYW	IGH	13	None	
1	3991	CARDVSIAVAGTLLPDYYYYMDVW	IGH	25	None	
1	3992	CARDLGPQNWNYARVINYFDYW	IGH	6	None	
1	3993	CASPHTGSNPYYFDYW	IGH	6	None	
1	3994	CAKDTGSYYYDSSGHTALDYW	IGH	2	None	
3	1	CARTGISEYFYSWQFDPW	IGH	5	None	
3	2	CASSGLGELSFDYW	IGH	4	None	
3	3	CARRSMASGGAINWFDFW	IGH	2	None	
3	4	CARSRSSSWYLIGSWYAFDIW	IGH	3	None	
3	5	CARDSSRPDLAVDYHLFDSW	IGH	3	None	
3	6	CARDGDCSGGSCYSSVNYYYGMDVW	IGH	6	None	
3	8	CAMGGDYVDYW	IGH	16	None	
3	9	CARAVNLWDYLDTW	IGH	2	None	
3	10	CAKGVVPAAIPWFDPW	IGH	22	None	
3	11	CASYPADTRPEKKNYYYYGMDVW	IGH	3	None	
3	12	CAKVFPSDDSRDYW	IGH	3	None	
3	13	CARDGAAHGFDMW	IGH	148	None	
3	14	CATAPGQSGYNWFDPW	IGH	4	None	
3	15	CAKDGSGSYYGALDPFDYW	IGH	4	None	
3	16	CAKLPYQLPSNAFDIW	IGH	4	None	
3	17	CALPISAVYYCARDVGSNYNDYYYYYGMDVW	IGH	2	None	
3	18	CARRNYNIFIGYYGNGYFDNW	IGH	14	None	
3	19	CAKLSGWDGTYWFDPW	IGH	6	None	
3	20	CAKFTVTRGEDYFDYW	IGH	6	None	
3	21	CARHGVPLLPEGPGCFDPW	IGH	5	None	
3	22	CAKSLAPMIVVVIPHAFDIW	IGH	6	None	
3	23	CAREVVPGAIYYYGMDVW	IGH	17	None	
3	24	CTKGYCSSSSCGGDYW	IGH	7	None	
3	25	CARGRLLWFGELLLYNWFDPW	IGH	2	None	
3	26	CAKDYYGSGSYSDAFDIW	IGH	9	None	
3	27	CAKDHKSVQSLLWFGELSIEPWDFDYW	IGH	2	None	
3	28	CMQGIHLRF	IGK	30	None	
3	29	CAKPHPYYYDSSGSGGYFDYW	IGH	2	None	
3	30	CAKDIVPAAMGYFQHW	IGH	2	None	
3	32	CAKDRRYYDFWSGYLSDSTNYYYYGMDVW	IGH	2	None	
3	33	CARDHYYYESSGYYVIHSYSDYW	IGH	14	None	
3	34	CAKGTPRQNYYDSSGYSDYW	IGH	3	None	
3	35	CARGHFGKYDTSGYSELDYW	IGH	14	None	
3	36	CTRLPHNYCSRGTCGPDYYYYGMDVW	IGH	3	None	
3	37	CARDQWPGGGYYYYGMDVW	IGH	9	None	
3	38	CAKETPDYGEGYFDYW	IGH	12	None	
3	39	CAREVVGNYYGLDYW	IGH	2	None	
3	40	CAGEDTATYYYYYGMDVW	IGH	4	None	
3	41	CARDRSAGNGDYVDYFDSW	IGH	5	None	
3	42	CTTNFNYYGSGSYYFGRW	IGH	12	None	
3	43	CAKTRLSTDYYDSSGSFDYW	IGH	2	None	
3	44	CAKDADTEITVIDYW	IGH	2	None	
3	45	CARDPEGSGSYLYYFDYW	IGH	4	None	

3	46	CAKGNGYHSNDAFDIW	IGH	3	None	
3	47	CQSWDSALSGYVF	IGL	7	None	
3	48	CARAISGYDNRGYSRPWFFDLW	IGH	2	None	
3	49	CVRGLYRSSWYYFDYW	IGH	2	None	
3	50	CATFGLKYYDSSGYLGAFDIW	IGH	2	None	
3	51	CAKDHCSGGSCDFIPPAFDPW	IGH	6	None	
3	52	CAKDIMTTVTYYFDYW	IGH	3	None	
3	53	CARYSPCSGDNCYSRDFDYW	IGH	4	None	
3	54	CAREYQLLLYGYYYGMDVW	IGH	30	None	
3	55	CAGGRPSYYDMLTGHNWFDPW	IGH	8	None	
3	56	CMDAFSYW	IGH	2	None	
3	57	CAKDYRIVVTGNYFDYW	IGH	2	None	
3	58	CARGESSGWYESPDYW	IGH	2	None	
3	59	CARMITFGGMGFDPW	IGH	7	None	
3	60	CAKDVWAAGGRSFDHW	IGH	2	None	
3	61	CARSWDVVVPAAMAYFDYW	IGH	2	None	
3	62	CAKDMSVAGNNWFDPW	IGH	2	None	
3	63	CARDRQGYYDSSGYYGWLDYW	IGH	2	None	
3	64	CAREKVEDQLPYYYYYGMDVW	IGH	2	None	
3	65	CVRHRSNRAADYW	IGH	4	None	
3	66	CARAALTSFTFGGVIVRGYFDYW	IGH	2	None	
3	67	CAKDYYDSSGYYFVPVPDYW	IGH	6	None	
3	68	CQQYYNWPPGGYTF	IGK	3	C4	6W41
3	69	CATAFARSGSYSWGYYYYYGMDVW	IGH	6	None	
3	70	CAKDMMGWNSHGYYGYW	IGH	5	None	
3	71	CARDRGGYGDYAVYYYGMDVW	IGH	5	None	
3	72	CARDRAYRGSLSDFDGMDVW	IGH	2	None	
3	73	CARDSLPESGLSSGYYFDYW	IGH	2	None	
3	74	CARDVGIAAAGRDYW	IGH	3	None	
3	75	CAKDNLLLWFGESGYYFDYW	IGH	2	None	
3	76	CATSPIYDSSGYYYGPDYW	IGH	2	None	
3	77	CAKGDVVVPEPWWFDPW	IGH	8	None	
3	78	CASVSPTGRTWYYYGMDVW	IGH	2	None	
3	79	CARTYSSSWPFDYW	IGH	10	None	
3	80	CARMYSLVGAFDYW	IGH	2	None	
3	81	CARMRIYCSGGSCYEPYYYGMDVW	IGH	4	None	
3	82	CARYIGYRQNQYFDSW	IGH	2	None	
3	84	CAHSLGEQWLAARGVYFDYW	IGH	2	None	
3	85	CAKGSSGAGDYYYYGMDVW	IGH	5	None	
3	86	CTMGEWFGGRDFTYYYYAMDVW	IGH	3	None	
3	87	CATTLKLLITYWYFDLW	IGH	39	None	
3	88	CAKDIEVGAAAGVVFEYW	IGH	2	None	
3	89	CARDSINWEGMLPGTPNHIDSW	IGH	2	None	
3	90	CAKAPRLGFIGGHFDYW	IGH	2	None	
3	91	CARYVGLYHAMDVW	IGH	4	None	
3	92	CAKDLSYGDYGASYYYGMDVW	IGH	4	None	
3	93	CARNYGSSAFFDYW	IGH	3	None	
3	94	CAKEMYIGFGEFPPNYFDYW	IGH	71	None	
3	95	CARYGAYTKYYFDYW	IGH	2	None	
3	96	CARDRYYCNDNNCTRGWFDPW	IGH	2	None	
3	97	CAKDLEKYIVATGGRYYYYGMDVW	IGH	11	None	
3	98	CCRADWEFYDSKPFDFW	IGH	6	None	
3	99	CARPSTYYYESSGGAFDIW	IGH	32	None	
3	100	CARSDGRGYPEYFQYW	IGH	4	None	
3	101	CAREGGYRTAHYYYGMDVW	IGH	4	None	
3	102	CAREGITMVRGVPWGYYYGMDVW	IGH	6	None	
3	103	CAKAPTYYYDSSGYSDYYYGMDVW	IGH	2	None	
3	105	CAKGAHYYDSSGYPEFGYW	IGH	3	None	
3	106	CAKERWFGELLNGYYYYGMDVW	IGH	4	None	
3	107	CASDRSQQLVGRTFDYW	IGH	2	None	
3	108	CAKDPYSSGWTDYFDLW	IGH	7	None	
3	109	CAKGWTHYDILTGYLHQWELLKADWFDPW	IGH	2	None	
3	110	CAKDEGIYLYYYDSSGYLDAFDIW	IGH	2	None	
3	111	CASHYSGYYLYW	IGH	12	None	
3	112	CAHGSCSGGSCYWGYYFDSW	IGH	3	None	
3	113	CAKDRYYDILTGPMDAFDIW	IGH	7	None	

3	114	CARALGGGFCSGGSCHFDYW	IGH	2	None	
3	115	CARGGIMRWLEGDGMDVW	IGH	4	None	
3	116	CARDYAQPGYFQQW	IGH	7	None	
3	117	CARYSCSGGNCYSRLFYYYGMDVW	IGH	10	None	
3	118	CARKGYVWGSYRQGNWFDPW	IGH	2	None	
3	119	CAKDGISTRVRGIIDYYAMDVW	IGH	31	None	
3	120	CAKEDRLRYFDWSLAFNYYYYGMDVW	IGH	30	None	
3	121	CARQAICGGDCYPLDYW	IGH	6	None	
3	122	CAKDQGYYYGSGSYGVGDYW	IGH	2	None	
3	123	CAARKVAAVTGYFDYW	IGH	6	None	
3	124	CATGVVAGKTVDYW	IGH	4	None	
3	125	CARVYSGYALAFDIW	IGH	6	None	
3	126	CAREVGNTWANWFDPW	IGH	4	None	
3	127	CAKAGVVVITLGDAYFDYW	IGH	2	None	
3	128	CVKHMAPWSTGPGRGAIISPYYYGMDVW	IGH	4	None	
3	130	CARLNRLLDYYYGMDVW	IGH	2	None	
3	131	CVAWDDSLNGFLLF	IGL	6	None	
3	132	CAHSVDTAMFASDAFNIW	IGH	17	None	
3	133	CQVWDSSSSHRAVIF	IGL	2	None	
3	134	CAIVPLLVPDRYFDYW	IGH	2	None	
3	135	CAKPGGYDDGMDVW	IGH	2	None	
3	137	CAKRGEIFWLGVGRYAMDVW	IGH	115	None	
3	139	CARLVVSHWPPDYW	IGH	2	None	
3	140	CARAPITMIVVTHFDYW	IGH	2	None	
3	141	CARVMTQYSSSWYPPANWFDPW	IGH	6	None	
3	142	CARDYYYDNSAYYDYW	IGH	2	None	
3	143	CAKDKGWTYYYDSSVVPPDAFDIW	IGH	2	None	
3	145	CAKLPEGYNWVDYW	IGH	3	None	
3	146	CQQFNNYALPF	IGK	2	C3	7K8R
3	147	CARDGGGNQDYFDYW	IGH	4	None	
3	148	CARDRIGGRWLQPFDYW	IGH	7	None	
3	149	CAKDLWVVDSSSSGDGMDVW	IGH	4	None	
3	150	CAREIVLRYFDWLLYPPAYPDVW	IGH	2	None	
3	151	CARAYYLDTTRYYFDYW	IGH	2	None	
3	152	CARRGANWSHARFDLW	IGH	11	None	
3	153	CAKVSEPDYFDCW	IGH	6	None	
3	154	CAKEREGQLVLGAFDIW	IGH	4	None	
3	155	CGTWHSNSKTWVF	IGL	30	None	
3	156	CAKDQSRFEQWGLDYFDYW	IGH	5	None	
3	157	CVKGEVDGVAHYLDYW	IGH	6	None	
3	158	CQVWDISSDPYVLF	IGL	2	None	
3	159	CARHLRYGLEYFDYW	IGH	11	None	
3	160	CAKDPRPMATTPGLDYW	IGH	2	None	
3	161	CARAQAMITFGGVIVRYFDYW	IGH	2	None	
3	162	CQSTDSSGSYAIF	IGL	42	None	
3	163	CACLYSSSPYYYGMDVW	IGH	10	None	
3	164	CAYIGGTAGTTNNYFDYW	IGH	6	None	
3	165	CAKGPYLDVLTGTTFFDYW	IGH	48	None	
3	166	CSLKASDSAIYYCAILPQGQHVTLDYW	IGH	2	None	
3	167	CANTLPGARYYFDFW	IGH	2	None	
3	168	CASGEYVAGYDSYYYYGMDVW	IGH	2	None	
3	169	CAAWDDTLTGSMAF	IGL	3	None	
3	170	CVKVISDCPIAACLAKGYFDYW	IGH	3	None	
3	171	CAKTKWVQGLTFNWFDPW	IGH	2	None	
3	172	CATQHQVYYYDRTGYYPEYW	IGH	2	None	
3	173	CQQYNNWPPTWTF	IGK	2727	C3	7K8R
3	175	CAREHGDGKQFGYFDYW	IGH	14	None	
3	176	CARETGVIDLVVSPSGTFDWFDPW	IGH	5	None	
3	178	CATGPVIAAAVINWFDPW	IGH	7	None	
3	179	CARGDSFRTSFYAMDVW	IGH	4	None	
3	180	CARDKEEEAYGSGNYFDSW	IGH	4	None	
3	181	CAKLSSGREPKSYFDYW	IGH	4	None	
3	182	CAKLQGDDYGDTKDYW	IGH	9	None	
3	184	CARDTRYYCSSTSCSYYYYGMDVW	IGH	15	None	
3	185	CARDYYASGEAAMDVW	IGH	4	None	
3	186	CSKDQNVNYPYNWFDPW	IGH	32	None	

3	188	CSSF	IGL	3	None	
3	189	CAKEPRVAAADPYYYYGMDVW	IGH	4	None	
3	190	CARVQTLRLFTMIVVGTFDYW	IGH	2	None	
3	191	CARHCGSGTDGLDYW	IGH	2	None	
3	193	CAHSPLRVFTRFDPW	IGH	4	None	
3	195	CAKEGGPGDYFFDYW	IGH	77	None	
3	196	CAKDPLIGYDILTGGTFDYW	IGH	2	None	
3	197	CVRGLGSRSFSDWLDPW	IGH	2	None	
3	198	CARGRRQWLVQGGNWFDPW	IGH	2	None	
3	199	CARVFDGGFCSSGTCFQNWFDPW	IGH	11	None	
3	201	CARDPGRYDSSGYSDYW	IGH	2	None	
3	202	CTRGGDHFGLFYFDVW	IGH	13	None	
3	203	CARDRGYCDYSAYCSYYFYGMDVW	IGH	2	None	
3	204	CAREDMNLPHFDYW	IGH	111	None	
3	205	CAKGGNCSGGNCYVTQLDYW	IGH	2	None	
3	206	CVRQHDGRRSYQYNYFGPW	IGH	3	None	
3	207	CAKEMTAAHYYFYGMDVW	IGH	2	None	
3	208	CARDSFVTFGGVIDPIPYYFDYW	IGH	2	None	
3	209	CARPSYGSGSKWFDPW	IGH	6	None	
3	210	CAKDQRYMTTGPIGYYYYGMDVW	IGH	2	None	
3	211	CAKDIHPIVGATLGWFDPW	IGH	3	None	
3	212	CGTWDSSLSAWVF	IGL	780	None	
3	213	CAKDEGVWFGELFPDYW	IGH	2	None	
3	214	CARAGIHCGGDCYHFDYW	IGH	2	None	
3	215	CARDEKPDYYYDSSGYSRSYYYYGMDVW	IGH	8	None	
3	216	CARDTTMGYYFDYW	IGH	22	None	
3	217	CARDGGYFDWFDYYYYYGMDVW	IGH	7	None	
3	218	CAKDRFVYGSGTSTYYFDYW	IGH	2	None	
3	219	CARDWDKTWSSGWALDVW	IGH	10	None	
3	220	CARDRLWGEEHDDYW	IGH	7	None	
3	221	CAREFVEMATIVNYYYGMDVW	IGH	5	None	
3	222	CAREADPLSGVNWFDPW	IGH	10	None	
3	223	CARGFWSGPPGHDYYYYYGMDVW	IGH	4	None	
3	224	CAKQLGLYYYGSGTGYFDYW	IGH	6	None	
3	225	CTTSAHILTGRGWYFDYW	IGH	2	None	
3	226	CTREYYYDSSGYYYGDYW	IGH	3	None	
3	227	CARDSPLVRGVHDYW	IGH	2	None	
3	229	CARDPDNGDPTDFDYW	IGH	2	None	
3	230	CSRSSCYFDFW	IGH	8	None	
3	231	CARVGVTTVTLFYYYYGMDVW	IGH	2	None	
3	232	CARGDGFTMIVVVTPTFDYW	IGH	3	None	
3	234	CARGRPDFDAFDIW	IGH	28	None	
3	235	CAKFGSLDSSPVDYW	IGH	3	None	
3	236	CAKDTDRSSPDILTGYYNPYYYYYGMDVW	IGH	2	None	
3	237	CARDGYYFGSGSLGALDVW	IGH	2	None	
3	238	CAKMRSEWLLLPFDYW	IGH	4	None	
3	239	CARERSITIFGVVIEDYYYGMDVW	IGH	3	None	
3	240	CAKIPNPRYCSSPSCYAADYW	IGH	4	None	
3	241	CASNIMVPLGGYYYYGMDVW	IGH	2	None	
3	242	CARAHCSGGSCADWYFDLW	IGH	2	None	
3	243	CQHYNRYSAF	IGK	54	None	
3	244	CAKDRIMILTGYPYYFDYW	IGH	2	None	
3	245	CRQYNNWPRGAF	IGK	4	C3	7K8R
3	246	CSFGGPSSFDYW	IGH	2	None	
3	247	CTRYYDYVWGTFGYW	IGH	2	None	
3	248	CARRQLWPSLFDYW	IGH	2	None	
3	249	CARDLRNNWNPYYYGMDVW	IGH	2	None	
3	250	CARGDSSGYYTHYFDKW	IGH	9	None	
3	251	CAHRLTGLLSFGETNDAFDIW	IGH	4	None	
3	252	CAKFPDMFGGAAFDIW	IGH	2	None	
3	253	CMQSRQVPLFSF	IGK	4	None	
3	254	CATSPGNWLDTDYFDHW	IGH	2	None	
3	255	CARASYPDWLSYLWTYYFDYW	IGH	6	None	
3	256	CARHSDTSGFHDDAFDFW	IGH	3	None	
3	257	CASWLGQQLGWFDPW	IGH	2	None	
3	258	CARDRRSLPPRSSSWYLYDYW	IGH	4	None	

3	259	CARGGTYYYDSSITNVGYYYYGMDVW	IGH	2	None	
3	260	CARVSNWNYPGDYYYGMDVW	IGH	5	None	
3	261	CARVNASRGYGWSGTYYTYYGMDVW	IGH	2	None	
3	262	CTNSRYYYDSSGRGGYFDYW	IGH	4	None	
3	263	CAKDLGFCSGGTCYSAYFDSW	IGH	5	None	
3	264	CARDYGGELWFGEFPFDYW	IGH	7	None	
3	265	CATVFAVRGVHPPDYW	IGH	17	None	
3	266	CARVIRRERQLNWFDHW	IGH	3	None	
3	267	CACLGSAVIIDWFDPW	IGH	2	None	
3	268	CAKDLCSGGSCPPGLDYW	IGH	4	None	
3	269	CARDLWFGELSGWAFDYW	IGH	6	None	
3	270	CARVAGPPSAILTGYTYYYYGMDVW	IGH	2	None	
3	271	CAKGEECGGDCYLSAFDIW	IGH	10	None	
3	272	CAKDVPPYSELLWFGEGRYSLFDYW	IGH	2	None	
3	273	CARGSTYQLLGERGLDYW	IGH	2	None	
3	274	CASARYFDWLGDYW	IGH	10	None	
3	275	CATAGIAVAGTSSYYYGMDVW	IGH	5	None	
3	276	CATGGWELRGIDYW	IGH	6	None	
3	277	CAKVRSGGSSWYVDFDYW	IGH	2	None	
3	278	CARAKPETNCFDYW	IGH	3	None	
3	279	CAKDMSYDSSGYYLDYW	IGH	5	None	
3	280	CAKSFLSYSGTSAYYSGSFDSW	IGH	2	None	
3	281	CARGQEQWLDRPFDYW	IGH	8	None	
3	282	CARGTAGFQWAMIVVADYYYGMDVW	IGH	8	None	
3	283	CAKDIGAATIFDYYYGMDVW	IGH	2	None	
3	284	CATTKSECIGGRCYRIAFDIW	IGH	4	None	
3	285	CARAGHAVIAAAGTGWFDPW	IGH	2	None	
3	286	CARDPFDNVYEPPYFDQW	IGH	6	None	
3	287	CVRGGWAHFDFW	IGH	16	None	
3	288	CTTWLPNPYYYYGVDVW	IGH	2	None	
3	290	CQSYDTSLSALF	IGL	182	C4	7JVC
3	291	CQQRINWSLSF	IGK	2	None	
3	292	CARTSMVVTAIPFDYW	IGH	6	None	
3	293	CASNKYDILTEFDYW	IGH	13	None	
3	294	CAKGESGTGLYYAIFFDHW	IGH	7	None	
3	295	CANLAGGSGWSNWYFDLW	IGH	2	None	
3	296	CAKDSGVRGVIITPYYYYGMDVW	IGH	2	None	
3	297	CARERQWLQYYYHYYGMDVW	IGH	2	None	
3	298	CARDLEYYYILTGYYNPPGSYYYYGMDVW	IGH	2	None	
3	299	CAKDFYHADSDGGAFDIW	IGH	4	None	
3	300	CATPELEWLVFDYW	IGH	4	None	
3	301	CTTPGPDIVVVVAGFDPW	IGH	2	None	
3	304	CARTFGVYTSSPYFDSW	IGH	3	None	
3	305	CAKVLWDTAMVTDAFDIW	IGH	2	None	
3	306	CAAIAAFSQTVDYW	IGH	6	None	
3	307	CAKVPASTDLGDYW	IGH	2	None	
3	308	CARSPANAWWQLVRWRSYFDYW	IGH	2	None	
3	309	CVRDRDLYGDPYYFDYW	IGH	2	None	
3	310	CARDNYRRDGYSTMGRHNWFDPW	IGH	2	None	
3	311	CAVESGFGELPTDYW	IGH	2	None	
3	312	CQQYDNLPPGAAF	IGK	16	C3	6XDG
3	313	CAREDYYDSSGSGYYYYGMDVW	IGH	4	None	
3	314	CAREDPLLDGMDVW	IGH	2	None	
3	315	CATTRSGNERDFDYW	IGH	3	None	
3	316	CARHDRDTAMVPLVTFDYW	IGH	26	None	
3	317	CAKDLSPYLGELSLYGDYW	IGH	6	None	
3	318	CAARSPPAGVVITGWYFDLW	IGH	3	None	
3	319	CARYATPTDALDIW	IGH	2	None	
3	320	CTKQVAESSPYWYFGLW	IGH	2	None	
3	321	CATHRGRFPRGYFEDW	IGH	2	None	
3	322	CATDVEYQDSTGFYKYYFDNW	IGH	2	None	
3	323	CATDRPSCVGSKCELGFDYW	IGH	3	None	
3	324	CASHIKFYAFVFDYW	IGH	31	None	
3	325	CTTLLTTWYFFDHW	IGH	11	None	
3	326	CAKKEALPHYDLLTGYYDYFYYGMDVW	IGH	2	None	
3	327	CARNVAGDFGDHWFDPW	IGH	2	None	

3	328	CAKDLAGRANQRPFIAAALGGYYYYGMDVW	IGH	2	None	
3	329	CTRNIFWSDGYFDYW	IGH	2	None	
3	330	CARESSSAAADLSWFDPW	IGH	18	None	
3	331	CQQFNGWPPYSF	IGK	19	C1	7BZ5
3	332	CATDRIHGDYVGYYYYYGMDVW	IGH	16	None	
3	333	CATKLTYYDILTGYHPFDYW	IGH	4	None	
3	334	CVRGGIPVAGLDGFDIW	IGH	8	None	
3	335	CASSRHHYGIGSSSMDYW	IGH	7	None	
3	336	CAKDNPLGDDFWSGYFWFDPW	IGH	3	None	
3	337	CAATPRSVAGPRWFDSW	IGH	3	None	
3	338	CATGPAYPYYYDSSGNTGGNWFDPW	IGH	7	None	
3	339	CAREGGDYGDWFDPW	IGH	7	None	
3	340	CGRHDDSGSYLSDVFDMW	IGH	17	None	
3	341	CAKFDGDGYNSPVRGNAFDIW	IGH	2	None	
3	342	CARARSGNIGAPPLFDYW	IGH	4	None	
3	343	CATGGYYYDRVGYYFDYW	IGH	2	None	
3	344	CKREGIDYVDHW	IGH	2	None	
3	346	CARHLGKEGYARVALFGVVMFDSW	IGH	2	None	
3	347	CAKDIYSSSWSAYFDFW	IGH	5	None	
3	348	CARTYYGGNSFDYW	IGH	11	None	
3	350	CAKDREVLMVYAEAFGIYYYYGMDVW	IGH	2	None	
3	351	CAALGGITMIPNYYYYGMDVW	IGH	3	None	
3	352	CARGICTGGTCPNDYW	IGH	2	None	
3	353	CARMVVPAAIDYW	IGH	3	None	
3	355	CAKDRRGNWYYIDSW	IGH	17	None	
3	356	CAKDRFYDSSGYLGYW	IGH	3	None	
3	357	CATLFYPYSSSTYYYYYGMDVW	IGH	2	None	
3	358	CAREGYCSSTSCPEDYYYGMDVW	IGH	2	None	
3	359	CARIEYYDSRGWFDPW	IGH	4	None	
3	360	CVRVFTTVTDYW	IGH	6	None	
3	361	CAKTDGTQLWLQGRHVLYNYYAMDVW	IGH	17	None	
3	362	CQRYNSNSYAF	IGK	22	None	
3	363	CATELERWAFDIW	IGH	3	None	
3	365	CARDPRGNDILTGWTYYYYGMDVW	IGH	2	None	
3	366	CARDRGRDGYSSFHPGFFDLW	IGH	2	None	
3	367	CVRAEEMYYYDSSAYFKHW	IGH	4	None	
3	368	CARETKVAATHFDYW	IGH	4	None	
3	369	CAREGGAEDGNLDYW	IGH	4	None	
3	370	CARTPRRAFYLPPPSYTSGSPAGYNWVDSW	IGH	2	None	
3	371	CARVVHGLGHVLLWFGELPPDAFDIW	IGH	4	None	
3	372	CARDGSGSYAHSYYDYYYGMDVW	IGH	5	None	
3	373	CARAEAISRIVTFFDSW	IGH	2	None	
3	374	CANGDRDIVATSRNIDYW	IGH	2	None	
3	375	CAREALPPYYDFWSGYSGELLGGYYYYGMDVW	IGH	4	None	
3	376	CAKLPSYYGDYWFDYW	IGH	6	None	
3	378	CTTGGTYYYDSSGYYSDFDYW	IGH	5	None	
3	379	CARSVAHTIFGVVKAYYYGMDVW	IGH	2	None	
3	380	CARHPYYYDSSGYYTTGESDYW	IGH	6	None	
3	381	CARDYTSIYYYDSSGHFDYW	IGH	3	None	
3	383	CARVWASGIGSEKYYRKIDYW	IGH	3	None	
3	384	CARQEHNGHVFDIW	IGH	9	None	
3	385	CAKDSFSSSLLWFGELYSYGPDYW	IGH	6	None	
3	386	CAKTYSRDYFAHFDSW	IGH	6	None	
3	387	CAKVLHYYDSSGYPGVAAFDIW	IGH	6	None	
3	388	CARDWRLGGRGNYFDYW	IGH	2	None	
3	389	CAKDLSLFRIVVVPAGYYYGMDVW	IGH	4	None	
3	390	CTTLMWCSGDCYRSYFDFW	IGH	2	None	
3	391	CAKDPVAATHADYYYYYGMDVW	IGH	2	None	
3	392	CARGGEDDILTGYYFGYW	IGH	27	None	
3	394	CAIDPMVRGVIKYYYYYYGMDVW	IGH	37	None	
3	395	CAKGGSHYYDRTGRKYDYW	IGH	12	None	
3	396	CAQVAYDILTGHSVWFDPW	IGH	2	None	
3	397	CAKLPMVVAEDAFDIW	IGH	4	None	
3	399	CATSFIVATRPFPYYYYYGMDVW	IGH	4	None	
3	400	CARTLSIAVALDYW	IGH	11	None	
3	402	CARATRGGIIVAHYGHFDYW	IGH	2	None	

3	403	CATLGGYYYDSSGYYLDYFDYW	IGH	4	None	
3	404	CARARDYYDSSGHIGHYYYYGMDVW	IGH	9	None	
3	405	CASLFRSSSWSEYYYYGMDVW	IGH	4	None	
3	406	CTRLEGTVRDYYYGMDVW	IGH	2	None	
3	407	CAHRGDYDILTGYYPGPPTNNWFDPW	IGH	10	None	
3	408	CAKSGYCGGGTCYSDYSDYW	IGH	2	None	
3	409	CARGRLDYDILTGYYQIWFDPW	IGH	4	None	
3	410	CARDLTSTSHIYYYYGMDVW	IGH	2	None	
3	411	CARDFHCSSTSCYANYFDYW	IGH	3	None	
3	412	CARDYGYYYDSSGYYDYGMDVW	IGH	2	None	
3	413	CARDRLDSSRMPPTHYAMDVW	IGH	3	None	
3	416	CARINRLDGYNSRFDPW	IGH	3	None	
3	417	CAKDLWGIAVAGTSFDYW	IGH	2	None	
3	418	CARVVHYGSGSYYDYW	IGH	20	None	
3	419	CAHKIVTGAIFDIW	IGH	2	None	
3	420	CARGDYQARAVDNWFDPW	IGH	18	None	
3	421	CQSADSSVTYRRLF	IGL	13	None	
3	422	CARRIESRDYSFDNW	IGH	2	None	
3	423	CASQPTDSSGNDYW	IGH	6	None	
3	424	CATSPVLDIKDWFDPW	IGH	2	None	
3	425	CAKETPDDYGAAFDYW	IGH	2	None	
3	426	CARVLFPYYDILTGWARDYYYYGMDVW	IGH	5	None	
3	427	CAREIGSSWYIDYW	IGH	3	None	
3	428	CAHQAYTSGWFSDYW	IGH	2	None	
3	429	CAKAGFIGFGASYFDW	IGH	5	None	
3	430	CANAYSFDPGSYYDYW	IGH	3	None	
3	431	CARYYYGPENYHNGAIFDSW	IGH	7	None	
3	432	CANTRRYSGYDLKGYYYNGMDVW	IGH	3	None	
3	433	CAKENLDYGGYYFDNW	IGH	4	None	
3	435	CAKLPTAPSDYYYYGMDVW	IGH	13	None	
3	436	CAKDLDIVVVVAATRYYYYGMDVW	IGH	8	None	
3	437	CARTELAAAVAFDIW	IGH	19	None	
3	438	CATGLAVAGTVHHYYYYGMDVW	IGH	2	None	
3	439	CAKGFFVSVAASTLDYW	IGH	2	None	
3	440	CARGPDGYSSGWLDYW	IGH	2	None	
3	441	CARGNDIVVQPTDTDPFNVW	IGH	5	None	
3	442	CAKDPNDSSSMAADYYYAMDVW	IGH	4	None	
3	443	CAKDIADTAMVNLDYW	IGH	2	None	
3	444	CVHRVRDRPAGQTSGWFGGYFDFW	IGH	2	None	
3	445	CARDLSHYDILTGYYWMYGMDVW	IGH	2	None	
3	446	CARHNWSGGPDYYYGMDVW	IGH	9	None	
3	447	CVFSGSYSWSYFDYW	IGH	2	None	
3	449	CARGVPGYYDSSGSPFDYW	IGH	2	None	
3	450	CALPISAVYYCARSFLHYSGSSGYYSGSFDYW	IGH	2	None	
3	451	CAKLAGGSGWYSGSGYFDLW	IGH	2	None	
3	452	CAKDVRYYYDSSGYAEYFQHW	IGH	2	None	
3	453	CASTGGEVGVAGTSPFDYW	IGH	4	None	
3	455	CARGFSFDHGDYYYYAMDVW	IGH	2	None	
3	456	CARDLDYYDSSGFDYW	IGH	31	None	
3	457	CARDPRYYDILTGYYIPSNYYYYYGMDVW	IGH	2	None	
3	458	CAKDRVGLGITGTTYDYW	IGH	3	None	
3	459	CVRGVVVPASTDYHYYGMDVW	IGH	3	None	
3	460	CARTSSFFGAVATFDYW	IGH	5	None	
3	461	CAKDRGDGYSDTGAFDYW	IGH	2	None	
3	462	CARDHYHYETSGYHVIYSYVDHW	IGH	2	None	
3	463	CAIDGNGSGSYYSPIFDDW	IGH	2	None	
3	465	CARMQAILTGYYRDTWLDPW	IGH	4	None	
3	466	CARATPYYDILTGWNAPVSGGWFDPW	IGH	2	None	
3	467	CARSQYYYDSSGYYYAFYYYYYGMDVW	IGH	2	None	
3	468	CAKEVGYDILTARRRYYYGMDVW	IGH	2	None	
3	469	CARGIFRSGELRFDPW	IGH	2	None	
3	470	CTTGNSFSDYGSSYYDFW	IGH	203	None	
3	471	CAKGAEISSTLGYSFDYW	IGH	4	None	
3	472	CARDLQIVYSSGWYVPNYYYYYGMDVW	IGH	2	None	
3	473	CARSRGFWSGQGWFDPW	IGH	2	None	
3	474	CARDLSSGFTYGHHWFFDLW	IGH	3	None	

3	475	CARDPTYRAILTANTFDIW	IGH	2	None	
3	476	CARDFDWPTYFDYW	IGH	6	None	
3	477	CTTDRYYDYVWGSYRYVDYW	IGH	32	None	
3	478	CARVIGDEYTFDYW	IGH	3	None	
3	480	CARGANDYGDYGDWFDPW	IGH	10	None	
3	482	CAKDRWVDDSSRWFYFDYW	IGH	2	None	
3	483	CAGGERVATIVYYYYGMDVW	IGH	22	None	
3	484	CVKTPGGPFDYW	IGH	2	None	
3	485	CAKGERTYYDILTGYYYYGMDVW	IGH	7	None	
3	486	CAKDSQPNYDILTGYPFDYW	IGH	2	None	
3	487	CARQRFSGYDHLINLRVKYYYYGMDVW	IGH	2	None	
3	488	CARDGGDLWFGESHLDYW	IGH	4	None	
3	489	CARVRRSGWIVEYFDYW	IGH	4	None	
3	490	CARGSHYYDSSGYYYVPPNYYYYGMDVW	IGH	7	None	
3	491	CQTAGSSGTWVF	IGL	3	None	
3	492	CTRVAETTVTRGWFDPW	IGH	2	None	
3	493	CAKEKWNSRNAMDVW	IGH	4	None	
3	494	CTRASRVVVATRAFDIW	IGH	4	None	
3	495	CARDWKKGPYSGSYYHADYW	IGH	12	None	
3	497	CAKDLGVSEDYDILTGYYTRSDYW	IGH	3	None	
3	499	CARSVDTAMVGDNWFDPW	IGH	2	None	
3	500	CAKDVHDFGEYNIKGDALDIW	IGH	2	None	
3	501	CARDLGSITMIVVVHYYYGMDVW	IGH	4	None	
3	502	CARDRAYLFGVVISDVFDFW	IGH	15	None	
3	503	CARSHNDFLTGYNLRGLCAFDIW	IGH	2	None	
3	504	CTTLGHCIAGRCNDYW	IGH	2	None	
3	505	CARQRFPHYNWLAPW	IGH	2	None	
3	506	CARHLPRADGHGLVRMFYYYYALDVW	IGH	3	None	
3	507	CAKDRVPYSSWFIFDLW	IGH	4	None	
3	508	CVRGLTRFGEFDPW	IGH	7	None	
3	509	CARGAKWEGQLELDAFDIW	IGH	14	None	
3	510	CARSMPDYFDNW	IGH	24	None	
3	511	CALPICAKRGEIFWLGVGRYAMDVW	IGH	2	None	
3	512	CARELGAWVDPW	IGH	2	None	
3	513	CARAGSWWVGWFDPW	IGH	7	None	
3	514	CARPQYGGKTYWYFDVW	IGH	2	None	
3	515	CASGICSSTSCYPVLYYYYGMDVW	IGH	2	None	
3	516	CARSSGPMALYWFDPW	IGH	2	None	
3	517	CASWGDDYGDYLDYW	IGH	8	None	
3	519	CMQSIQLPPPMYTF	IGK	6	None	
3	520	CARSFDRVFDPW	IGH	4	None	
3	521	CVRGVGATGYFANW	IGH	3	None	
3	522	CAREGWELLAYFDYW	IGH	3	None	
3	523	CTTFGKAAAGRGYWYYFDYW	IGH	2	None	
3	524	CAKGPSPLYSSGLGWFDPW	IGH	2	None	
3	525	CAHSAGGGGTTRYNWFDPW	IGH	4	None	
3	527	CAKETYGYDSSALFNDGFDMW	IGH	2	None	
3	528	CARMVCDDYRCPPFDSW	IGH	46	None	
3	529	CARKDWHWYFDVW	IGH	3	None	
3	530	CAKIPTNGDYPIYW	IGH	4	None	
3	531	CAKDGIEILLWFGESNGEDYFDYW	IGH	2	None	
3	532	CAREGLGYCSSTSCQRAFDYW	IGH	4	None	
3	533	CARGVDSSGYYRLDDWFDPW	IGH	2	None	
3	534	CARGGEYHLLVRHFDFW	IGH	3	None	
3	535	CAKVGWGYYDTNDAFDIW	IGH	3	None	
3	536	CQQTFSTPPHTF	IGK	18	C4	6W41
3	537	CAKVGVGYCSSTSCPYNWFDPW	IGH	4	None	
3	538	CASHSSSWSGPFDYW	IGH	8	None	
3	539	CTAYLQISSLKAEDTAVYYCASFYYDSRYYFDYW	IGH	3	None	
3	540	CARHLYSYGFWRSNPFDLW	IGH	3	None	
3	541	CAKDQFVGSPLGRDAFDIW	IGH	6	None	
3	542	CARVGSPMVRGVIIAYYYGMDVW	IGH	3	None	
3	543	CATSPAIAARRGGGNWFDPW	IGH	3	None	
3	544	CARHGAPKKDDHYILTGFSLFYYYYGMDVW	IGH	4	None	
3	545	CASFTVTTQYYYYYYGMDVW	IGH	2	None	
3	546	CARLGFDSSGYYPTYYYYGMDVW	IGH	2	None	

3	547	CYSTDSSGNHRVF	IGL	2268	None	
3	549	CAKDVIRNDILTGYFDW	IGH	4	None	
3	550	CVREGNGFDPW	IGH	10	None	
3	551	CATVDSGFDSSGGFDIW	IGH	12	None	
3	552	CYSRDTSGNQIVGVF	IGL	8	None	
3	553	CATGTAYCSSTSCQTNWFDPW	IGH	2	None	
3	554	CARDSYYDYVWGSYPSPGPLDYW	IGH	2	None	
3	555	CAGPPDLQALLMHRGVGIYYYGMDVW	IGH	162	None	
3	556	CARLKKADTGNFFTFDPW	IGH	21	None	
3	557	CAKLLVYSGSYYGDYW	IGH	3	None	
3	558	CAKDRAYYDILTGYLQNTDYYYYGMDVW	IGH	6	None	
3	559	CARVLSSGYYYPSYYFDYW	IGH	2	None	
3	560	CQSADSSGSSPWIF	IGL	2	None	
3	561	CARVRGGLISNWFDSW	IGH	5	None	
3	562	CARDNWSSPGYYYYGMDVW	IGH	8	None	
3	563	CAREIYCSGGSCYKAGAFDIW	IGH	2	None	
3	564	CARDRDYDDYPPDYW	IGH	2	None	
3	565	CARDSSRAFGYW	IGH	116	None	
3	566	CARDFGVSNPENNWFDPW	IGH	2	None	
3	567	CARDGGVNYDILTGYYPYYFDYW	IGH	6	None	
3	568	CARGPVGSNSGGTRFIRYYFDYW	IGH	5	None	
3	569	CVHRWLTPIYFDYW	IGH	23	None	
3	570	CARGRSASDYSDLW	IGH	2	None	
3	572	CARAPTIFGVAPFDYW	IGH	2	None	
3	573	CAKGLGDQNQDYW	IGH	3	None	
3	574	CARLSIVSESVAVWYYYGMDVW	IGH	3	None	
3	575	CARGDYDILTGSRWFDPW	IGH	6	None	
3	576	CAKDSGYDSLPTDYW	IGH	9	None	
3	577	CARVNGGGYNSYFDYW	IGH	4	None	
3	578	CTREARYGRAFDYW	IGH	3	None	
3	579	CARASASGQIDYW	IGH	4	None	
3	580	CAKATGYSSSWYDWYFDYW	IGH	6	None	
3	581	CAKSVRAGGGQDYW	IGH	3	None	
3	582	CAKDGVGSYYDFWNGYHHTWIDPW	IGH	2	None	
3	583	CARERRELLRSGFDIW	IGH	4	None	
3	584	CTAQKPETYYDILTGYYYYYGMDVW	IGH	3	None	
3	585	CAKDNDDGYMVGGIYYFDYW	IGH	4	None	
3	586	CAKDLRPSDFSGYLLEYYFDTW	IGH	8	None	
3	587	CATGPPAADLNWFDPW	IGH	10	None	
3	588	CARNYYDRSGYYPSFDHW	IGH	2	None	
3	589	CARGMDDGFGELFQYYFDYW	IGH	4	None	
3	590	CTTEDTAMVTGTGQIDYW	IGH	14	None	
3	591	CARDPGYSSSWGDWYFDLW	IGH	2	None	
3	592	CARDYPYFADSNKYDLLNWFDPW	IGH	4	None	
3	593	CARVGLHVATIKGVYYYYGMDVW	IGH	2	None	
3	595	CARLNDAHVDYW	IGH	2	None	
3	596	CARDWRYCSGGSCYFWFDPW	IGH	4	None	
3	597	CAYSTPAYGDSLTFDYW	IGH	2	None	
3	598	CARHGDYSDRSGHYYWGYYFDYW	IGH	2	None	
3	599	CARLAPPMVRGVIASPIYYFDYW	IGH	48	None	
3	600	CRRAFVPSATYLDLIPFDYW	IGH	2	None	
3	601	CARDGFYYDFEAGDYLSYNFDFW	IGH	2	None	
3	602	CARDSSNDYYYYYGMDVW	IGH	4	None	
3	603	CAKDADIAVAGTSRHFDYW	IGH	2	None	
3	604	CAKEARIAVAGTHLEEFDYW	IGH	2	None	
3	605	CAKWRLRGDYYYLYGLDVW	IGH	4	None	
3	607	CARGGTHIVGAIPSFDYW	IGH	4	None	
3	609	CQSADIDGTRYIF	IGL	18	None	
3	611	CATAQATPGYFGSKSGFDFW	IGH	2	None	
3	612	CAHSPSVTHEEYGFYYDYW	IGH	3	None	
3	613	CVRDQSLGSDLKSYVDYW	IGH	6	None	
3	614	CARAQRRFPEHTWYFDLW	IGH	2	None	
3	615	CAKSENVLGSFDYW	IGH	2	None	
3	616	CARDERGQTRITGTTTYFDYW	IGH	3	None	
3	617	CEGNYYDSSGYYLSYW	IGH	6	None	
3	618	CAAYLIGTLDYW	IGH	17	None	

3	619	CARDHLEPPRGRESDAFDLW	IGH	2	None	
3	620	CARDRGVLRFLEWTFDYW	IGH	9	None	
3	621	CARLTAQGYFDYW	IGH	17	None	
3	622	CARDFDFWSGYPDYW	IGH	159	None	
3	623	CAKDVYGDYYFDSW	IGH	2	None	
3	624	CARGCSETKCPSDYW	IGH	6	None	
3	625	CAKTYSGWSYYYDGMDVW	IGH	4	None	
3	626	CSRTFGHRYAFDVW	IGH	2	None	
3	627	CAKDGMITVTRGDYFDYW	IGH	4	None	
3	628	CARGDSSSSRGLDYW	IGH	6	None	
3	629	CAKEVRSVIVVVPAARGFDYW	IGH	3	None	
3	630	CNGDTCFSESFYFDHW	IGH	2	None	
3	631	CTTVWGAWYSSSGSDFDYW	IGH	4	None	
3	632	CAKVDSSSWYRSVDYW	IGH	5	None	
3	633	CARDSLLYYYDSSGCFDYW	IGH	8	None	
3	634	CAKLPPLYCSGGSCYLDYW	IGH	6	None	
3	636	CASGGSYYGYYGMDVW	IGH	18	None	
3	637	CAKDENLIGGSMIREMAMDVW	IGH	2	None	
3	638	CASGLFDTSSWPTLFDFW	IGH	2	None	
3	639	CAHLPFHLGNLSPFDYW	IGH	8	None	
3	640	CASAVHEHSYGLPVDYW	IGH	3	None	
3	641	CAKDILAVLLYSYFDHW	IGH	2	None	
3	642	CARVRWPRGGPIYYYAMDVW	IGH	3	None	
3	643	CASYEGAINLTYYYYGMDVW	IGH	2	C2	7BWJ
3	644	CARPNSVVLLWFGESGDDGMDVW	IGH	2	None	
3	645	CARGRFCSTSSCYHHGDWFDYW	IGH	11	None	
3	646	CARDCRYYDILTGYLEGSGYFDYW	IGH	6	None	
3	647	CARLYHREEDIVVVPAAMWAGVVAVAPFDPW	IGH	4	None	
3	648	CAKRHHCPNGICYSPPEDHYYALDVW	IGH	4	None	
3	649	CATIVGAQGVDYW	IGH	2	None	
3	650	CHQRSNWPSGGTF	IGK	4	None	
3	651	CQSTDSSDTWLF	IGL	69	None	
3	652	CARDRYSGYELRDFDFW	IGH	3	None	
3	653	CARDSEPNLYYGWSWFDPW	IGH	5	None	
3	654	CASSGAADSLYYYYGMDVW	IGH	5	None	
3	655	CAKFPDGDYVSYW	IGH	2	None	
3	656	CATNRWLGYQLLALDVW	IGH	2	None	
3	658	CARDWIIYGDYQDAYFDLW	IGH	5	None	
3	659	CAPASDGNSFVDYW	IGH	5	None	
3	661	CAKLDAGPQDYW	IGH	13	None	
3	662	CARVVSSGNYYNRYADYRYYYSMDVW	IGH	5	None	
3	663	CALPILNSLTVEDTAVYFCAAGYGWLVDYW	IGH	3	None	
3	664	CARVAFDWLLYSWFDPW	IGH	8	None	
3	665	CARQVRNGSGYEYLYYYYGMDVW	IGH	3	None	
3	666	CHQRGNWPF	IGK	18	None	
3	667	CATNFRYFDRW	IGH	33	None	
3	668	CARDPANCSGGSCYPGDYW	IGH	7	None	
3	669	CAKDLLLRYFDWLLLGENYYYGMDVW	IGH	4	None	
3	670	CARERAAAGTGGWFDPW	IGH	2	None	
3	671	CARGARYGDSRLLDYW	IGH	8	None	
3	672	CARVPTEARPYDFDYW	IGH	2	None	
3	673	CASGIKYDYGDYNEYYFDYW	IGH	2	None	
3	674	CAREYQGYCSGGSCYPSYYYGMDVW	IGH	2	None	
3	675	CASRPYDFDGMDVW	IGH	2	None	
3	676	CARSGQDCSGGSCYSSDWFDPW	IGH	7	None	
3	677	CAKIMDYGDHLADYW	IGH	6	None	
3	678	CAKGAGPYTYGRYWYSDLW	IGH	2	None	
3	679	CARSSGWYDPDTFDIW	IGH	2	None	
3	680	CASAWGWDNYGGNDWFHPW	IGH	4	None	
3	681	CVRHGYCTSSSCVSFDQW	IGH	3	None	
3	682	CARSRGGSYRDAFDIW	IGH	30	None	
3	685	CARGIGDWYDYYFDSW	IGH	3	None	
3	686	CARRRLWFGELFSILYYYYGMDVW	IGH	3	None	
3	687	CAKVAYYDSNFDYW	IGH	15	None	
3	688	CAKDKTYYDFWSGPLYYYGMDVW	IGH	7	None	
3	689	CARDGSSSWYAYYYGMDVW	IGH	4	None	

3	690	CARESSYDSSGYYLGYYFDYW	IGH	4	None	
3	691	CARGDYYDRYFDLW	IGH	3	None	
3	692	CAAPQDLQALLMLRGGDYYYYGMDVW	IGH	2024	None	
3	693	CARLPDILTLEYYYGMDVW	IGH	2	None	
3	694	CARDGDSYHRYTWNYVDYFAYW	IGH	7	None	
3	695	CARDQGGEQLWGGGYYYYGMDVW	IGH	2	None	
3	696	CARLTHKIPVVVNWFDPW	IGH	2	None	
3	697	CARALYGGNPYYYYGMDVW	IGH	8	None	
3	698	CAKDNGPAAMRGYYYYGMDVW	IGH	3	None	
3	699	CARESAYCGGDCWTYYFDYW	IGH	4	None	
3	701	CARASARRITMIVGSFDYW	IGH	2	None	
3	702	CVRTISGTTFDYW	IGH	13	None	
3	703	CAKLIFSGGASLRFFDYW	IGH	2	None	
3	704	CASVVVPAAAWFDYW	IGH	11	None	
3	705	CAKFLPAAIRDYYYYGMDVW	IGH	5	None	
3	706	CHVWVSSTAWGVF	IGL	2	None	
3	707	CASFYYDSRYYFDYW	IGH	5	None	
3	708	CARVPHYNDSGSLQMGGWFDPW	IGH	2	None	
3	709	CAKDQEFIVVAGIDYW	IGH	4	None	
3	710	CVRDVCSTSTCPPSWFEPW	IGH	3	None	
3	711	CARDRLYYDSSGYHPDYW	IGH	12	None	
3	712	CARDTFDWLLPGDYFDYW	IGH	2	None	
3	713	CAHRPFGYNWNALFDYW	IGH	6	None	
3	714	CARAFLGSLDWLLLNYYYGMDVW	IGH	4	None	
3	715	CAKDSVIGRITVTVYFDYW	IGH	3	None	
3	716	CARQEGNCIGVRCYAIWFDPW	IGH	9	None	
3	717	CARMKNGGTYYGWVFDYW	IGH	3	None	
3	718	CAKGTVAGTRGSAFDIW	IGH	2	None	
3	719	CARYSEISSSWPYYYYYGMDVW	IGH	2	None	
3	720	CARSTWNLGGAFDTW	IGH	2	None	
3	721	CARDPDDSSWSNYYFDYW	IGH	10	None	
3	722	CAKDSSSAWFDYW	IGH	26	None	
3	723	CAKDSWDIVVVPAATGFDYW	IGH	2	None	
3	724	CARPRLRYFDWLLSTYYYGMDVW	IGH	5	None	
3	725	CARTLPGVPSLFEYW	IGH	3	None	
3	726	CVKRPAPLSGGLDGDALDVW	IGH	3	None	
3	727	CATFSGSYWRDYYYYGMDVW	IGH	2	None	
3	728	CARAPNSYGFNDAFDVW	IGH	10	None	
3	729	CAKHDRDYGDSRCDYW	IGH	3	None	
3	730	CARGDEYSGYDDPAGHYYYYGMDVW	IGH	2	None	
3	731	CARFELEMATMDYYYGMDVW	IGH	2	None	
3	732	CAKLLGATPEDYYFDYW	IGH	6	None	
3	733	CARQDFTMIHVGAFDIW	IGH	2	None	
3	735	CARGFSTNYYDSSGYPGDFDYW	IGH	4	None	
3	736	CARVVVAATRGYYYYYYGMDVW	IGH	4	None	
3	737	CARSPHYCGGDCSNWFDPW	IGH	2	None	
3	738	CASNYDILTGYHPQGAFDIW	IGH	2	None	
3	739	CARISSITMIVVVIPGYFDYW	IGH	2	None	
3	740	CARGVGGSYYTPFDYW	IGH	45	None	
3	741	CAFVHNSTTWYGGFDSW	IGH	15	None	
3	742	CMQGTHWPPTWTF	IGK	1023	None	
3	743	CARFGLADYDSSGYYPDYW	IGH	4	None	
3	744	CAKDGGAVGAPFEYW	IGH	6	None	
3	745	CARSSYYEGDAFDIW	IGH	2	None	
3	746	CAKDFTIFGVVIPLGLERRYYYYGMDVW	IGH	6	None	
3	747	CARHRPDEYSNIRFDFW	IGH	6	None	
3	748	CAKDRVWFSYYDAFDIW	IGH	2	None	
3	749	CTTELGYSGYDYFDYW	IGH	2	None	
3	751	CVRDAVPGPARDYFHYW	IGH	10	None	
3	752	CARDLKRITIFGVVMGPNYYYYGMDVW	IGH	3	None	
3	753	CQHYSSYPLSF	IGK	195	C3	7K8R
3	754	CARKRAERWLHDWYFDLW	IGH	2	None	
3	755	CAIERERGSFWFDPW	IGH	3	None	
3	756	CARVVGSTNDPNFDHW	IGH	36	None	
3	757	CARDRRVLGAFDLW	IGH	22	None	
3	759	CARLGSGRSDDYW	IGH	2	None	

3	760	CATYGDFWSGVYWFDPW	IGH	5	None	
3	761	CATASLQLWSYAFDIW	IGH	3	None	
3	762	CAKDPNTKTYYYYFGMDVW	IGH	2	None	
3	763	CARQERTRERQYFDYW	IGH	2	None	
3	764	CAKVSTFDADYGGNGGDFDYW	IGH	2	None	
3	765	CARIGNCYYDSRGPRAFDYW	IGH	2	None	
3	766	CVSVRGPRGAYYYYGMDVW	IGH	2	None	
3	767	CVKPASRGHCRSGICRDYW	IGH	2	None	
3	768	CAKDPVYYETSGYYGVPDYW	IGH	2	None	
3	769	CARALTPRDGMDVW	IGH	3	None	
3	770	CTTDPYYDFWSGYYTEYW	IGH	3	None	
3	771	CAKYARESYYYYYAMDVW	IGH	42	None	
3	772	CARQPAPIFGVIFSSFDSW	IGH	2	None	
3	773	CARSSSPYYYDSSGYLHYYGMDVW	IGH	8	None	
3	774	CARQTRDDTSGYYYFDYW	IGH	2	None	
3	776	CARRVGVGAGGRYFDFW	IGH	3	None	
3	778	CATLQGIFGVVIRAGYYYGMDVW	IGH	2	None	
3	779	CQSAHSSSPYVMF	IGL	8	None	
3	780	CAKLPTYDSSGYQDYW	IGH	13	None	
3	781	CAIEYGDYAPGVVRPATSGAFDIW	IGH	3	None	
3	782	CARGNHLPPWFDPW	IGH	26	None	
3	783	CARNGPYQPFDCW	IGH	3	None	
3	784	CGRGPISLKGSWYYVDYW	IGH	2	None	
3	785	CARTITGDWGGYFDYW	IGH	4	None	
3	786	CQQSYSTPLTF	IGK	18311	C4	6ZDH
3	787	CARQGAGPINYGSGTYSTVYFDPW	IGH	2	None	
3	788	CARRAGPYYYDSSGYYYNYW	IGH	2	None	
3	789	CAKDISPLGIVVVPADHNWFDPW	IGH	3	None	
3	790	CARGRVSWSHYDILTPYYEGFDYW	IGH	9	None	
3	791	CAHVYYDILTGYYLAFDIW	IGH	3	None	
3	792	CAKDVDYGSGTYYHDAFDIW	IGH	2	None	
3	793	CVKAKIDPNYYSSNEFFDAFDVW	IGH	7	None	
3	794	CAKDHLGITMVRGSGYFDYW	IGH	10	None	
3	795	CARDYDIVTGDGAFDVW	IGH	3	None	
3	796	CARLYYFDSSAYKDWYFDLW	IGH	4	None	
3	797	CAKDMAHSSGYSFDYW	IGH	3	None	
3	798	CATPRGVYSSSWYDFDYW	IGH	5	None	
3	799	CASAFLTPVTTPHDALDIW	IGH	5	None	
3	800	CARACFTMVRGEHRGGNYYYYYGMDVW	IGH	2	None	
3	801	CFQTTQFPLTF	IGK	11	None	
3	802	CAHTYLGQSGSYLGFSAFDIW	IGH	2	None	
3	803	CAREFYYDSSGWDNWFDPW	IGH	2	None	
3	804	CAKGASGSYRSAFDYW	IGH	3	None	
3	805	CARDPIGDSVVVTAISDYW	IGH	2	None	
3	806	CTTDRPDYDILTGYSPGAFDIW	IGH	5	None	
3	807	CARDGHSSSWKEDYFDYW	IGH	2	None	
3	808	CAKDPRLVADVGYFDYW	IGH	18	None	
3	809	CARNFHKETVTGYEDYLDIW	IGH	2	None	
3	810	CVKPVGGTDFDYW	IGH	13	None	
3	811	CARHNRRWLQLRPSLFDYW	IGH	2	None	
3	813	CVSSGWFRTSFDYW	IGH	2	None	
3	814	CARMVVATLRIGQYYYYGMDVW	IGH	4	None	
3	815	CARARAGGYYGPFDYW	IGH	10	None	
3	816	CAKDAAIWFGERPDFDYW	IGH	3	None	
3	817	CQQYVNNWGTF	IGK	7	None	
3	818	CTRIGEVVALNWFDPW	IGH	3	None	
3	819	CAALGPTVPVEYLGYYYGMDVW	IGH	2	None	
3	820	CAKLPWGYYYDSSGPTTDAFDIW	IGH	2	None	
3	821	CAREHVSGSPGAMDVW	IGH	4	None	
3	822	CAKLLWLSARGDAFDIW	IGH	14	None	
3	823	CARRRSAYDYDTSGYFPFDYW	IGH	3	None	
3	824	CAKDPLYDSSGYHRAEYFQHW	IGH	4	None	
3	825	CARDASIAVAATYFDSW	IGH	2	None	
3	826	CAKDLSRYSSSWYGAFDIW	IGH	2	None	
3	828	CARLSQNSNYTNGWYYFDYW	IGH	2	None	
3	829	CAHTGMVRYFDWPSSWTYFDYW	IGH	2	None	

3	830	CAKDSIIVEPYYYFDNW	IGH	3	None	
3	831	CARVLSWGNSVPHYFEYW	IGH	5	None	
3	832	CARDGYNWNDGIDYW	IGH	7	None	
3	833	CARVLYDYGDFFDYW	IGH	2	None	
3	834	CARVGRYCSGGSCHSFDYW	IGH	2	None	
3	835	CAKDSDRSGSFYRLFNYW	IGH	2	None	
3	836	CVSGKWQPGYYYGMDVW	IGH	3	None	
3	837	CAKVGDDGSGYYVHFDYW	IGH	2	None	
3	838	CARVTFPDYFDSRGHNWFDPW	IGH	2	None	
3	839	CAKLDYDSVWGSLNYFDNW	IGH	4	None	
3	840	CAKDDDVVVVIAATVIDYW	IGH	2	None	
3	841	CAAILGSPRNWFDPW	IGH	10	None	
3	842	CVKHEWWTSDFYSGYSDAW	IGH	6	None	
3	843	CARDTRWQYYDILTGYHEGYYFDYW	IGH	2	None	
3	844	CASEMITFGGVIVSDYW	IGH	2	None	
3	845	CALYTRGSSWPYFDYW	IGH	6	None	
3	846	CARLSGESIYMGFFDYW	IGH	2	None	
3	847	CVIFTIFGVANPQPLGLHFDCW	IGH	2	None	
3	848	CARGIHSSSWYVWLDPW	IGH	6	None	
3	849	CETWDSNTHEF	IGL	41	C4	7JVC
3	850	CATALAVVVAANLRGDYYYGMDVW	IGH	2	None	
3	851	CSSYAGSNHLEMIF	IGL	10	C2	7BWJ
3	852	CARLTFFAWSGYFDYW	IGH	4	None	
3	853	CQQYGASPVTFGGGTKVEIKLWITF	IGK	2	C1	7K8N
3	854	CAKSAIAAALFYFDYW	IGH	21	None	
3	855	CATGIAVAGTEMNYYYYYGMDVW	IGH	20	None	
3	856	CTVYMELSSLRSEDTAIYYCAREIRAPRGSFDPW	IGH	4	None	
3	857	CARRGLWLLSDYYYYDMDVW	IGH	2	None	
3	858	CARDVGYSYGYNFDPW	IGH	3	None	
3	859	CAKDIAPVVAAYFDYW	IGH	4	None	
3	860	CARDLLGGLEDGMDVW	IGH	2	None	
3	861	CARSSGGASFHYW	IGH	7	None	
3	862	CAREDYGDDESYYFDYW	IGH	5	None	
3	863	CARDGAADYDFWSGTYYFDYW	IGH	3	None	
3	865	CAKDIASCSGGSCYFYYYYYGMDVW	IGH	2	None	
3	866	CAREDIVVVPANDYW	IGH	11	None	
3	867	CARVVLRDTRAYYSHYFDYW	IGH	2	None	
3	869	CARQKPNSYWAFDYW	IGH	3	None	
3	870	CAKDRGPNFGGDSVGGWYYYALDVW	IGH	22	None	
3	871	CAKDYAGVPGYFDYW	IGH	6	None	
3	872	CAIELKTGFDYW	IGH	4	None	
3	873	CVKARGPTSSSAGDYW	IGH	2	None	
3	874	CAREDLWYGDESLDYW	IGH	3	None	
3	875	CARDIHDSSGYPYYYYYGMDVW	IGH	4	None	
3	876	CARVWYYDSSGPRFDPW	IGH	2	None	
3	878	CARDNVAYDFWSGHSQYYKTHWNWFDPW	IGH	5	None	
3	879	CAKGKEGLASSFEYW	IGH	4	None	
3	880	CARTYGSGSYSGWYFDLW	IGH	8	None	
3	881	CAKDLYFDWLSRWGPGANYQYAVDVW	IGH	121	None	
3	882	CQQSYLAPYNF	IGK	12	C4	7JW0
3	883	CAKDMGDGYNPDYIDYW	IGH	37	None	
3	884	CARDPGYYDILTGYYNGAFDIW	IGH	6	None	
3	886	CTTSSSPVDYW	IGH	6	None	
3	887	CASGTFEWLLLSGYFDYW	IGH	4	None	
3	888	CAHTRFYSNAFDYW	IGH	4	None	
3	889	CLKVGYSGYDSNYYYGMDVW	IGH	2	None	
3	890	CVRERQDYTDYYLDYW	IGH	353	None	
3	891	CAHRLRYSGSYYYAFDIW	IGH	5	None	
3	892	CARDSGENDFWSGHTPNFDYW	IGH	4	None	
3	893	CAAGKDTVEVATGTPTPSSSWYGDAFEIW	IGH	5	None	
3	894	CAKVEGGIAVAGTNNFDYW	IGH	2	None	
3	896	CATGGSSSWYTWWFDPW	IGH	5	None	
3	897	CAKSTHTYYDILTGGFDYW	IGH	2	None	
3	898	CARPKITFSCDCSGLFFRRDFDLW	IGH	4	None	
3	899	CAKDNDFWSGYYRGAFDIW	IGH	4	None	
3	900	CMRDRSHKDDSSFYFEGDYW	IGH	3	None	

3	901	CARAYHSNRFLEWLSPHYYYYGMDVW	IGH	3	None	
3	902	CAREDYMITFGGVIEYYYYYGMDVW	IGH	3	None	
3	903	CAREGYRGWSTRSNGAFDMW	IGH	12	None	
3	905	CATRWAVAGKVYYFDYW	IGH	2	None	
3	906	CARVNAYTGLDYW	IGH	8	None	
3	907	CARAKQYYYDSSGYYFGYW	IGH	36	None	
3	908	CAKEATHVYSSGYYYPPYYYYGMDVW	IGH	2	None	
3	909	CARDGQNYYDSSQFDYW	IGH	2	None	
3	910	CQQANTFLTF	IGK	251	None	
3	911	CARDFFWSGTTCGYYYYYGMDVW	IGH	2	None	
3	912	CARPYARGGVVTAIYAFDIW	IGH	2	None	
3	913	CAKVASYQLDLGVDYW	IGH	5	None	
3	914	CATVNFYDTPGDNDYFDYW	IGH	5	None	
3	915	CARDFFDSGGYFSYDYW	IGH	2	None	
3	916	CAKDMTDGYSYGYGGVRGVMFDYW	IGH	2	None	
3	917	CARRITMVRDPIGWGFDYW	IGH	6	None	
3	918	CATPHVVPAAQGDYHYYYYGMDVW	IGH	3	None	
3	919	CATAYDILTGYYEVYGMDVW	IGH	2	None	
3	920	CARDLQWLADEPINDYW	IGH	2	None	
3	921	CATIPDGEEGYYYGMDVW	IGH	2	None	
3	922	CARLYDYSNRYYYGMDVW	IGH	12	None	
3	923	CMQSIRLPQITF	IGK	7	None	
3	924	CARDMGVDMPIANWDVDYW	IGH	9	None	
3	925	CARGLRGNVAFDIW	IGH	3	None	
3	927	CQSYDNSRSTPFVIF	IGL	2	C4	7JVC
3	928	CAKSPPYCSGGSCYYFDYW	IGH	4	None	
3	929	CARSTVDYSSSWHDLWPHSIRPERILSPIFDYW	IGH	4	None	
3	930	CAKTAAILTGYPRHFDYW	IGH	3	None	
3	931	CARDSLLLWFGDLYAPFDYW	IGH	3	None	
3	933	CARATYDIVVVPAAPVDFDYW	IGH	12	None	
3	934	CAQVTKERYSSIDNGDYFDYW	IGH	9	None	
3	935	CAKDTQRYDILTGFDYW	IGH	24	None	
3	936	CARGCQRGIQLWLRIDRYYFDYW	IGH	2	None	
3	937	CARDPTLRLGELSLFGYYYYGMDVW	IGH	2	None	
3	938	CARDPWGENWFDPW	IGH	18	None	
3	939	CARHLGKNWFDTW	IGH	6	None	
3	940	CARAGDYDILTGYYPYYYYGMDVW	IGH	13	None	
3	941	CARLNYYGSDLGLYAMDVW	IGH	5	None	
3	942	CARDPLTDIGGYEPYYFDYW	IGH	6	None	
3	943	CATCSVFATRPPYYYGMDVW	IGH	2	None	
3	944	CAREGASSGGYWEVDAFSGFDPW	IGH	2	None	
3	945	CARVDGYSGYDWPFDYW	IGH	3	None	
3	946	CARDNGGIAVADNWFDPW	IGH	37	None	
3	947	CQSADSTNTQGVF	IGL	5	C4	7JVC
3	948	CAKDGKLPSLLWFGELPIPDGMDVW	IGH	11	None	
3	949	CVRDPVWGMFDYW	IGH	2	None	
3	950	CARHFVVIRGLFDYW	IGH	4	None	
3	951	CARPYCTGTGCSGYYFYYGMDVW	IGH	2	None	
3	952	CARENKYEKSGYYRNAFDIW	IGH	2	None	
3	953	CARAGARSGGYPFDIW	IGH	6	None	
3	954	CASPDSSCGADCSPHAPFDIW	IGH	3	None	
3	955	CAKDGIPLSPWGDTEDYFDYW	IGH	4	None	
3	956	CAKTRGDSYSMNYALDVW	IGH	2	None	
3	957	CARGPWFGDPHIYYYGMDVW	IGH	13	None	
3	958	CAKGRAITIFGAVSDAYDIW	IGH	2	None	
3	959	CARVGVLLDEYYFDYW	IGH	2	None	
3	960	CARDVQGYYGSGSEFDPW	IGH	4	None	
3	961	CARLRGVVTAISTSPNWFDPW	IGH	2	None	
3	962	CATAAPIVVVPAAWREDYYYGMDVW	IGH	2	None	
3	963	CAEGRGGYNYALDYW	IGH	2	None	
3	964	CAREGDDISLGYYYGMDVW	IGH	3	None	
3	965	CEAYYDILTGYLFMLLFDYW	IGH	10	None	
3	966	CARDPPSHDYGGNSRVPWFDPW	IGH	2	None	
3	967	CAREGITIFFNAFDIW	IGH	6	None	
3	968	CAGQQWLVQGYYYYGMDVW	IGH	6	None	
3	969	CAHRRRDGYSDYW	IGH	4	None	

3	970	CARLRTMIVVVTPYYFDYW	IGH	5	None	
3	971	CAKDRFPERAYCGGDCYPFFDYW	IGH	2	None	
3	972	CAREAPDSTWSNWFDPW	IGH	3	None	
3	973	CAKDSSGWYLDPW	IGH	14	None	
3	974	CAKGAGQVTKQWLDYYYGMDVW	IGH	4	None	
3	975	CAKTANILTGYYAQNFDYW	IGH	10	None	
3	976	CTRDTSYAVGTRTNWFDPW	IGH	2	None	
3	977	CAKGMSRITMIVVVITDGMDVW	IGH	3	None	
3	978	CARDRGSGWFPDSW	IGH	7	None	
3	979	CASSNCDGDCFLDYW	IGH	17	None	
3	980	CLRSEDTAIYYCAREIRAPRGSFDPW	IGH	4	None	
3	981	CARGYSGSHWYNYFDYW	IGH	90	None	
3	982	CAPLPSGEDGYNPYFDYW	IGH	2	None	
3	983	CARSPQARYCSSTSCLNYWYFDLW	IGH	2	None	
3	984	CATAPGAPQFSNWFDPW	IGH	2	None	
3	985	CAKVDRYYFKSAGYYSLFDYW	IGH	5	None	
3	986	CAKQSSSWPYYYFDYW	IGH	14	None	
3	987	CVVSGGAIWYWYYGMDVW	IGH	5	None	
3	988	CVKDRNVEICSGGNCLGFFDLW	IGH	5	None	
3	989	CARQTYYYDSSDDAGFDYW	IGH	4	None	
3	990	CARGGDYDSSEDLFDYW	IGH	2	None	
3	991	CAISAGIDCGGDCYSAPDIW	IGH	3	None	
3	992	CARGLGHCSGGTCYVHPFDIW	IGH	6	None	
3	993	CARDLALYCSSTSCFDGMDVW	IGH	2	None	
3	994	CARKLGGRYHLGYFDFW	IGH	8	None	
3	996	CATFHFYDYVWGSYRPTYAFDMW	IGH	4	None	
3	997	CARTRADYAHDAFDLW	IGH	4	None	
3	998	CAAFRYRDAFDIW	IGH	5	None	
3	999	CARHVSSSWYLDAFDIW	IGH	19	None	
3	1000	CAKSRNYVDYFDYW	IGH	2	None	
3	1001	CAHRPAGWELRFDYW	IGH	2	None	
3	1002	CARTSKGIVGGDYW	IGH	4	None	
3	1003	CARLSTYYDFWSGYTGFDYW	IGH	2	None	
3	1004	CAKDLVLQQYAMITFGGVILSGGFDPW	IGH	5	None	
3	1005	CARDRAYCSGGSCYSGYYYYYGMDVW	IGH	5	None	
3	1006	CARTVFGSGYFLGDYYYYGMDVW	IGH	3	None	
3	1007	CCSLAVTGHYYYYYGMDVW	IGH	11	None	
3	1008	CARTKYGDRIAAAGHFDYW	IGH	4	None	
3	1009	CARDRDCVSCSKGDGFDIW	IGH	11	None	
3	1010	CAKDPFPTPCQGCIPYFQNW	IGH	2	None	
3	1011	CARGFRPDYDFWSGYYWDYW	IGH	9	None	
3	1013	CAKDFTMIVPRGAFDIW	IGH	8	None	
3	1014	CARGGGSASYYYHFDDW	IGH	6	None	
3	1015	CAKIAHYYGSGRLGAEYYYYYGMDVW	IGH	2	None	
3	1016	CATGGAAADYNYFDPW	IGH	9	None	
3	1017	CQTYGDSPRSF	IGK	9	C1	7K8N
3	1020	CARQKRSGTYYGAGYNACDPW	IGH	2	None	
3	1021	CARYYDILTAYPPFYYYYGMDVW	IGH	2	None	
3	1022	CARALSPPLWFGEPPAPYWFDPW	IGH	2	None	
3	1023	CARAGLLRLGELSLYRSWDYFDYW	IGH	7	None	
3	1024	CAHRREYTTSTTFDYW	IGH	8	None	
3	1025	CARVTGMFYGDYEDYFDYW	IGH	2	None	
3	1026	CATAAAGTTLGDYYYGMDVW	IGH	3	None	
3	1027	CARDSLRLGELEAFDIW	IGH	2	None	
3	1028	CARAGFTSSRFDTWLDPW	IGH	5	None	
3	1029	CVREGSAAVPEFDPW	IGH	23	None	
3	1030	CAGARYGSGFLGWTDPW	IGH	2	None	
3	1031	CARENRSRPGFDHW	IGH	2	None	
3	1032	CARALSSRYSSPDYW	IGH	10	None	
3	1034	CARDAVGATGAIDYW	IGH	2	None	
3	1035	CARERGYSGYEPVPFDYW	IGH	3	None	
3	1036	CTTDPSDFGDYVWGSYRAVDYW	IGH	2	None	
3	1037	CARHSGIIGYYYGMDVW	IGH	3	None	
3	1038	CASGDGYNAHDYW	IGH	2	None	
3	1039	CAREVPPSYDFWSGYLDYW	IGH	8	None	
3	1040	CARIYDSSGYYYQESDYW	IGH	3	None	

3	1041	CAKLTDMGDSSLLDYW	IGH	2	None	
3	1043	CARDDYSNYDEYYYYGMDVW	IGH	5	None	
3	1045	CARADPITMMDKGDYW	IGH	2	None	
3	1046	CAKVGFMATMDYW	IGH	2	None	
3	1047	CARPLYYYDSSGYYSAFDIW	IGH	24	None	
3	1048	CARGMRYCSSTSCYPPYYYYGMDVW	IGH	2	None	
3	1049	CAMIKGHCSGGTCYDLDYW	IGH	3	None	
3	1050	CVRLYYMDYW	IGH	2	None	
3	1051	CARDGEMLRWNSPMDSFDYW	IGH	2	None	
3	1052	CARDIQTAPDHAFDIW	IGH	3	None	
3	1053	CARNPNYYASPEGWFDSW	IGH	2	None	
3	1054	CSAWDSGLSVWVF	IGL	91	None	
3	1055	CASLFSDHYGDMGVYYFDYW	IGH	9	None	
3	1056	CARESRVDYYGSGHDYW	IGH	4	None	
3	1057	CARATRYDILTGYPMYYGMDVW	IGH	41	None	
3	1058	CARVGDSSGYWNWFDPW	IGH	15	None	
3	1060	CARDKGQLESYYYGMDVW	IGH	2	None	
3	1061	CAKDPTIVVVVAARELDYW	IGH	14	None	
3	1062	CAKEGGLVPTTDSYCFDYW	IGH	2	None	
3	1063	CAKSGTVAGVLGEIDYW	IGH	3	None	
3	1064	CLRYNSAPLFSF	IGK	2	None	
3	1065	CARDSGEDTMVPYNWFDHW	IGH	2	None	
3	1066	CATSSVITFGGVIVSWWFDPW	IGH	4	None	
3	1067	CARGDFDWLLAYQYYFDYW	IGH	2	None	
3	1068	CAKLPTERGSGSYYTDYW	IGH	2	None	
3	1069	CARGGITMVRGVIIETPIYYYYGMDVW	IGH	3	None	
3	1070	CAKDLGYGYFDYW	IGH	55	None	
3	1071	CAKDQATMVRGVIITFDAFDIW	IGH	2	None	
3	1073	CVREVGTGGTWYFDSW	IGH	2	None	
3	1074	CAKAGDYLWGTSRHFDSW	IGH	3	None	
3	1075	CARRLWYGDPRYWSFDLW	IGH	5	None	
3	1076	CAKVDPGGRDSSGSAVDYW	IGH	5	None	
3	1077	CARGKGYCSSTSCQSGYYFDYW	IGH	16	None	
3	1079	CARPYGSVLTGGNWFDPW	IGH	2	None	
3	1080	CARDPYSTNDFLTGYFPDYW	IGH	13	None	
3	1081	CARLSPESPGKLLSQFDPW	IGH	2	None	
3	1082	CARIPLPYCTSTTCYWFDTW	IGH	15	None	
3	1083	CATAPVDYYGSGSYWYFDLW	IGH	5	None	
3	1084	CARRPLASGPYYYGMDVW	IGH	3	None	
3	1085	CARPSRYGGSTFDYW	IGH	4	None	
3	1087	CRDSYNWVDPW	IGH	4	None	
3	1088	CARSLIWFGGSDEADAFDIW	IGH	2	None	
3	1089	CARFHCSGGRCLGFDPW	IGH	18	None	
3	1090	CAREGDTAMVTYVDPW	IGH	4	None	
3	1092	CARLRRLKWFDPW	IGH	3	None	
3	1093	CARCQSSYALYYFDSW	IGH	2	None	
3	1094	CAKVTGDGYSSGLGYFQHW	IGH	7	None	
3	1095	CAKDIPPKYCSGGSCYPEYYYYGMDVW	IGH	5	None	
3	1096	CAKGEDVGVAGPDYW	IGH	4	None	
3	1097	CTTDLLGNAYSSGWPYFDYW	IGH	3	None	
3	1098	CARDLNSYYDLLTGLNHRAFDIW	IGH	4	None	
3	1099	CAHRGGSGWSGASEYFQHW	IGH	6	None	
3	1100	CAKDRERITMARILDYW	IGH	11	None	
3	1101	CVKSRPQYNWKTAAFDIW	IGH	14	None	
3	1102	CAKDTPLDSSGLDYW	IGH	5	None	
3	1103	CASSVAPDSSGYYYYLFDYW	IGH	4	None	
3	1104	CALTPGCTNGVCYTTYYYYYGMDVW	IGH	7	None	
3	1105	CAKVDGGYDYVFDYW	IGH	4	None	
3	1106	CARIEQWPVGFDYW	IGH	2	None	
3	1107	CAKVLPTRRSFYAMDVW	IGH	2	None	
3	1108	CAKVAGSSWSNDAFDIW	IGH	6	None	
3	1109	CAASPGGDLWSGYFNYW	IGH	2	None	
3	1110	CARLSRSSYDDILAGYPELNWFDPW	IGH	4	None	
3	1111	CARGGGGELLDAMAFDYW	IGH	2	None	
3	1112	CAKDPTSFSWYFDYW	IGH	4	None	
3	1113	CAKDEAGIVVVPAGAFDIW	IGH	6	None	

3	1114	CTRGDILTGYRFDPW	IGH	4	None	
3	1115	CARFQWLVNPLSPLAAFDIW	IGH	2	None	
3	1116	CARSYDFWSGFYQNYYYGMDVW	IGH	11	None	
3	1117	CTRDFGSGLSHFFDHW	IGH	3	None	
3	1120	CARRNGGQKAFDLW	IGH	2	None	
3	1121	CTRVSYFSQIHITAAGNDYW	IGH	3	None	
3	1122	CAKSSDYESLFLGVYQYQFNFAYW	IGH	2	None	
3	1123	CAKRGPIYTSGWFFDSW	IGH	3	None	
3	1124	CSKESGPVPTYFDNW	IGH	16	None	
3	1126	CANYSPYYDYVWGSYRPAYDAFDIW	IGH	2	None	
3	1127	CASINSGSYRGAFDIW	IGH	2	None	
3	1128	CATPLDGNFLYW	IGH	6	None	
3	1129	CAKDKLPLTGSPEDYFDYW	IGH	2	None	
3	1131	CARVGVISSPFDFW	IGH	2	None	
3	1132	CVKKSFSDWYFDLW	IGH	10	None	
3	1133	CAKDYFYYDILTGYFPAHKYYYYYGMDVW	IGH	3	None	
3	1134	CAKDRLPLDDSYRSGPAPEDGMDVW	IGH	2	None	
3	1135	CAKEYSSGRWFFDLW	IGH	2	None	
3	1136	CARGTYYYDSSGLGLFDYW	IGH	12	None	
3	1137	CAREGDPGSGWYYYYGMDVW	IGH	2	None	
3	1138	CTKKKAFRGLSPSEFDYW	IGH	2	None	
3	1139	CARDPGLRYFDWLFQASYYYYGMDVW	IGH	4	None	
3	1140	CTLLGIVGALGDYW	IGH	6	None	
3	1141	CASISWGYSSSSGFDYW	IGH	8	None	
3	1142	CARLDEDGDQPFDYW	IGH	4	None	
3	1143	CAKDRAHFRYLRGVPDYW	IGH	2	None	
3	1144	CASGWPYLFDYW	IGH	5	None	
3	1145	CASGFGVVTPFDYW	IGH	11	None	
3	1149	CAKPVGGYCSGGSCYSEDYFDYW	IGH	2	None	
3	1150	CANLESGGNFYMAFDYW	IGH	2	None	
3	1151	CASWQGGGDYYFDYW	IGH	11	None	
3	1152	CARSYGGFCVGGSCLSLDDFDYW	IGH	2	None	
3	1153	CVFRIAARPDYYYGMDVW	IGH	2	None	
3	1154	CAKDFQYSGNYQWGYYFDYW	IGH	8	None	
3	1155	CARRSRMMGLGYNALDVW	IGH	4	None	
3	1156	CANLAGRGGDYW	IGH	7	None	
3	1157	CVRGNYDSSGYYYWFDPW	IGH	28	None	
3	1158	CAREGGDTARKYFDYW	IGH	4	None	
3	1160	CATLELERTFYYYYYGMDVW	IGH	2	None	
3	1161	CARRPEMGTSTEVGGDVFDSW	IGH	2	None	
3	1162	CARDQHTGYSSGWTYYYYGMDVW	IGH	30	None	
3	1163	CARDRNWGDYYYYGMDVW	IGH	12	None	
3	1164	CASPRNPYYYDSSGYWGFDYW	IGH	2	None	
3	1166	CAKLYLSESNYYYDSSGYSDYW	IGH	3	None	
3	1167	CATRSPLVVVADNFDYW	IGH	3	None	
3	1168	CAKRSSLGPHYFEDW	IGH	2	None	
3	1169	CALLYEYVWGSYPNFDYW	IGH	7	None	
3	1170	CATVGWFGEYFDNW	IGH	2	None	
3	1171	CARVAENYDFWSGQNWFDPW	IGH	2	None	
3	1172	CASPWIAFDIW	IGH	8	None	
3	1174	CAREGTGTTGEWFDPW	IGH	2	None	
3	1175	CARLPTSQTNTLDYW	IGH	2	None	
3	1177	CARVVRLFVNHDSTVHFDFW	IGH	3	None	
3	1178	CARGHICSGGSCYSEYYYGMDVW	IGH	3	None	
3	1179	CARHKDIYDLVSGYSGNYAMDVW	IGH	2	None	
3	1180	CARTYYYDSSPLGWDYYYGMDVW	IGH	5	None	
3	1181	CAKDMGILEWVFDYW	IGH	4	None	
3	1182	CAREEKAARNPPPAFDFW	IGH	2	None	
3	1184	CARGGLRGAVIAHFDYW	IGH	2	None	
3	1185	CAKDKFRDYESPSRLDYW	IGH	7	None	
3	1186	CAKDNYDILTGYYTFNWFDPW	IGH	3	None	
3	1187	CARGWYDTTSVWFDSW	IGH	4	None	
3	1188	CARSLWFGELFPYYFDYW	IGH	2	None	
3	1189	CAAENPGGCSGGSCYSNYYYGMDVW	IGH	3	None	
3	1190	CATGFLEGYYKYYFDYW	IGH	2	None	
3	1191	CATMLSFWGGSYGAFDIW	IGH	4	None	

3	1192	CARVRGFFDGLFTGDWLDPW	IGH	21	None	
3	1193	CAREPLDSGSSMYYFDYW	IGH	8	None	
3	1194	CAKDGSNYDWFDPW	IGH	21	None	
3	1195	CAREIYGSGSYYNLYYFDYW	IGH	2	None	
3	1196	CARDPWGGADTAMVLGFRYKYYYYGMDVW	IGH	3	None	
3	1197	CAKDPQFGELLLTADWYFDLW	IGH	2	None	
3	1198	CAKARSTLLLLRSALDVW	IGH	4	None	
3	1199	CARAQDRHYFAMDVW	IGH	2	None	
3	1200	CAKSWGGSYFAFFDYW	IGH	2	None	
3	1201	CQAWDRDTALYVF	IGL	14	None	
3	1202	CARPHSGSYRTASDYW	IGH	2	None	
3	1203	CVRRFYYGLGRSPDPVGGTSLIPSNAFDIW	IGH	5	None	
3	1204	CQHLNSFPPMYAF	IGK	2	C1	7BZ5
3	1205	CALPICLRGMYQYESAGYRGWLDPW	IGH	3	None	
3	1207	CARAAYDSSYFDYW	IGH	8	None	
3	1209	CAHRRPGFRSPAFDIW	IGH	2	None	
3	1210	CAKGFDGYGVPSFDSW	IGH	22	None	
3	1211	CARDARGGQQLVRNWYFDLW	IGH	2	None	
3	1212	CARDLNPDIVVVLAAADVW	IGH	2	None	
3	1213	CASMGATRFDPW	IGH	8	None	
3	1214	CAREFWGGFYRGGYHLDYW	IGH	8	None	
3	1215	CASDYGDYTNYYYYYGMDVW	IGH	4	None	
3	1216	CAKAQEQWLVPKAGFDYW	IGH	2	None	
3	1217	CVLYMGSGIWVF	IGL	69	None	
3	1218	CVRDYDSAGHYVYFQHW	IGH	4	None	
3	1219	CARSYYGSGSSYNYKYYYYGMDVW	IGH	2	None	
3	1220	CARDLGASYDILTGYYRVGAFDIW	IGH	2	None	
3	1221	CARDLSDNWNDYWFDYW	IGH	2	None	
3	1223	CAKDLVPAPMGGLDYW	IGH	3	None	
3	1224	CASHRTHIAAAGAFDYW	IGH	13	None	
3	1225	CACRQLSGAFDIW	IGH	5	None	
3	1226	CARGFPYCSDVTGCHSELFYYNAMDVW	IGH	2	None	
3	1227	CAKDKIGGDGYWVFDYW	IGH	9	None	
3	1228	CAKDYMITFGGVIEGWFDPW	IGH	3	None	
3	1229	CAHVDYGDNGELFDFW	IGH	5	None	
3	1230	CAREVLMVYANYYYYGMDVW	IGH	7	None	
3	1231	CAKADEYSSFRGTFDYW	IGH	2	None	
3	1232	CAKGGHITMIVVAILQGYYFDYW	IGH	2	None	
3	1233	CTKIAAAGTNSDYW	IGH	10	None	
3	1234	CGADHGSGSDFVRVF	IGL	95	None	
3	1235	CTRDTIFDGIPDYW	IGH	6	None	
3	1236	CAHNTLYCSGGSCYPDAFDIW	IGH	2	None	
3	1237	CAALYDSSAIGDSDGMDVW	IGH	2	None	
3	1238	CARTQYYYDSSGYYPLDFDYW	IGH	20	None	
3	1239	CARALGSSDGWDPRFDYW	IGH	4	None	
3	1240	CARDRGYNWNYTYYYGMDVW	IGH	6	None	
3	1241	CASHMGKDFIVAGGCDYW	IGH	2	None	
3	1243	CARDLAPIVGAPHYYYGMDVW	IGH	6	None	
3	1244	CQSYDSRNEKWVF	IGL	9	C4	7JVC
3	1245	CVRPRDIPTDRGVRVTPGLWWWFESW	IGH	11	None	
3	1246	CAKDMDSGYDGYRWFDPW	IGH	2	None	
3	1247	CARTSIAAAGTALDYW	IGH	22	None	
3	1248	CVRESGYVNLFDFW	IGH	2	None	
3	1249	CAKAKITMIVVAPDYW	IGH	2	None	
3	1250	CARQRLSDITIFGVVIHNWFDPW	IGH	4	None	
3	1251	CAKGRSSIFDAFNFW	IGH	3	None	
3	1252	CARRRGPSVDDAFDIW	IGH	3	None	
3	1253	CAKIGTYYYDSSGSRGDYW	IGH	2	None	
3	1254	CATSRYLYGTPDWFDPW	IGH	4	None	
3	1255	CAIWFGESSEDDYW	IGH	5	None	
3	1256	CAKDLELMVYAFDYW	IGH	2	None	
3	1257	CARDQGPPGLFVAYYYGMDVW	IGH	6	None	
3	1258	CARLAKSGTTIRGGWFDPW	IGH	2	None	
3	1259	CATDPLWIRVFDPW	IGH	2	None	
3	1260	CCVRASGVGADDAFDIW	IGH	3	None	
3	1261	CAKDIIAITMVRGVTYFDYW	IGH	4	None	

3	1262	CARDPDDSSFGTGYDALDFW	IGH	3	None	
3	1263	CASSGSPLGGWLQNRRGYYFDYW	IGH	5	None	
3	1264	CAKEGGWPNNGLDYW	IGH	18	None	
3	1265	CARDSRIQLWQDAFDIW	IGH	2	None	
3	1266	CATRPSGRVDWSTSSTAFDIW	IGH	5	None	
3	1267	CASLLQWDLPDYW	IGH	2	None	
3	1268	CATECTTSCPGPNRGFDYW	IGH	2	None	
3	1269	CGAWDDTLNGLYVF	IGL	7	None	
3	1270	CVTYYDVFTGYYYHFDYW	IGH	2	None	
3	1271	CAKDMRATTPGPFDYW	IGH	2	None	
3	1272	CARMWAIYAGAFDIW	IGH	8	None	
3	1273	CARANRRHFSDGYFDYW	IGH	10	None	
3	1274	CARVSSGSYLGWFDPW	IGH	12	None	
3	1275	CARDDRGSHLDYW	IGH	7	None	
3	1276	CAREPPEKYYFDYW	IGH	2	None	
3	1277	CAKDGYSSSSEGWFDPW	IGH	3	None	
3	1278	CARGNLPAAGGAFDIW	IGH	2	None	
3	1279	CAREGEIILFLDYW	IGH	5	None	
3	1280	CARALGAKDYYYGMDVW	IGH	2	None	
3	1281	CANGGVASYYYGMDVW	IGH	12	None	
3	1282	CSSYAGSNNLGVLF	IGL	132	C2	7BWJ
3	1283	CAKVTVDSSGYYYGQRPSVENYYFDYW	IGH	15	None	
3	1285	CANLAVHYYDSSGYYDDAFDIW	IGH	2	None	
3	1286	CARISGDLRGGYYRYYGMDVW	IGH	6	None	
3	1287	CARDWGPQWLQSYDAMDVW	IGH	2	None	
3	1288	CLSADSSRTAVIF	IGL	2	None	
3	1289	CARIGQYCSGGSCYFDHW	IGH	3	None	
3	1290	CARDRSDSWSLDYYYDGMDVW	IGH	2	None	
3	1291	CQKYNSAPPLTF	IGK	567	C3	7K8R
3	1292	CARDDFGVHDTFDLW	IGH	2	None	
3	1293	CLRGMYQYESAGYRGWLDPW	IGH	168	None	
3	1294	CARDGRYFDWANYMTETTNSYYYYYYGMDVW	IGH	5	None	
3	1295	CMRQMGMGRWYFDYW	IGH	3	None	
3	1296	CARSSPIILDPASVVNVFDIW	IGH	2	None	
3	1297	CATWSKSWYYYYYGLDVW	IGH	4	None	
3	1298	CARLREFCDGATCYAAVNYFDFW	IGH	2	None	
3	1300	CATLWFGEHMPVDYYYGMDVW	IGH	4	None	
3	1301	CARGPFGDLLGYYFDLW	IGH	2	None	
3	1302	CAKQGVEQLVEFYFDYW	IGH	2	None	
3	1303	CARDKYDNSGSPLDYW	IGH	3131	None	
3	1304	CAKAGYSDYDYEWFDPW	IGH	3	None	
3	1305	CARGCSGGNCNADYW	IGH	2	None	
3	1306	CTTAVGPDNNIVTGYYKTFDNW	IGH	10	None	
3	1307	CTRDRGGSYSGGYNWFDPW	IGH	3	None	
3	1308	CARGCSSTSCYYNWFDPW	IGH	3	None	
3	1309	CAKAHEVAVYYYYYGMDVW	IGH	2	None	
3	1310	CARLPGIAVAGYVFDYW	IGH	2	None	
3	1313	CVKGSNSGWYYVALDYW	IGH	2	None	
3	1314	CARAPIWFGETETGSFDIW	IGH	6	None	
3	1315	CATGTLYEVTTDYYYGMDVW	IGH	16	None	
3	1316	CARAGYGSEEGWFDPW	IGH	2	None	
3	1317	CMQALQTPPVICTF	IGK	4	None	
3	1318	CARALNGGSYYEGFDYW	IGH	2	None	
3	1319	CARGWITLVRGVVNWFDPW	IGH	2	None	
3	1320	CAKDEATRVYGDYYFDYW	IGH	2	None	
3	1321	CAHRLWGSGYSRYGFDYW	IGH	4	None	
3	1323	CARGFRGVYATDYW	IGH	2	None	
3	1324	CAKDYEDIVVVPAAPGYYFDYW	IGH	3	None	
3	1325	CVGGYFDDYW	IGH	22	None	
3	1326	CAGYGSGSGLGFDPW	IGH	4	None	
3	1327	CAKTNYDSSGYRVGYFDYW	IGH	2	None	
3	1328	CARSRLPSLYGDSASASFYFQYW	IGH	5	None	
3	1330	CARVHPKYEWLALYYYYGMDVW	IGH	5	None	
3	1332	CARGLDYRHYYYYGMDVW	IGH	16	None	
3	1333	CAKDFGYDSSGYYFDYW	IGH	24	None	
3	1334	CARESNGLGTYYDILTGYFLHKAFDYW	IGH	6	None	

3	1335	CNSRDSSGNHLGYVF	IGL	40	None	
3	1336	CVRDAYCTNVNCFPSRSLENVFDLW	IGH	2	None	
3	1338	CAKEGYYGSGSYYKIYYYYGMDVW	IGH	10	None	
3	1339	CAHRRRYCTSVTCFFGFDIW	IGH	5	None	
3	1340	CAHRRAATGIDYW	IGH	7	None	
3	1341	CARERGSGLGDHFDYW	IGH	3	None	
3	1342	CATVGYSSSWYDDAFDIW	IGH	8	None	
3	1343	CATLLGYCSSTSCSPFDYW	IGH	7	None	
3	1344	CVKYDGAAVAGTSHFDSW	IGH	70	None	
3	1345	CARTPPTPDSRFYYYYYGMDVW	IGH	3	None	
3	1346	CAKEHHSSGWSYYYYGMDVW	IGH	2	None	
3	1347	CTRSNYYALDVW	IGH	4	None	
3	1348	CAKGYTSQVLSTADYW	IGH	2	None	
3	1350	CAALDYGDSHTGYNWFDPW	IGH	2	None	
3	1351	CASVAAADPETYYYYGMDVW	IGH	3	None	
3	1352	CARPESTGSRDGYRHSFDYW	IGH	2	None	
3	1353	CAVAVAGSGVSFDIW	IGH	3	None	
3	1354	CVRGSLWFGELQGFDPW	IGH	4	None	
3	1355	CARDKIRDCSGGSCHTIDYYYYGMDVW	IGH	2	None	
3	1356	CVRDRGVTRSMDWFDPW	IGH	12	None	
3	1358	CAKESNDNSPFRHFDSW	IGH	18	None	
3	1359	CARDGPVLTGSCPDYW	IGH	9	None	
3	1360	CASPWGCSSTSCYDPFYYYYGMDVW	IGH	2	None	
3	1361	CARRGDWWYFDLW	IGH	18	None	
3	1362	CARTYSYGPHFYYDSSGYYYNDAFDIW	IGH	4	None	
3	1363	CLVHQYYYGMDVW	IGH	2	None	
3	1364	CARADYDILTGSKVEYYYGMDVW	IGH	7	None	
3	1365	CARATDRFNFDSSGYSLPFDVW	IGH	2	None	
3	1366	CVRDDVGRCSGGTCFDPW	IGH	2	None	
3	1367	CAKDIYPSLYYFDYW	IGH	11	None	
3	1368	CARDHPFYYDSSGYYYAPNYYYYGMDVW	IGH	2	None	
3	1369	CAREGYYDSRAFDIW	IGH	2	None	
3	1370	CATGPPYCSSTSCPNWFDPW	IGH	4	None	
3	1371	CAKDAGRNGAELFDFW	IGH	6	None	
3	1372	CATAPVVTTSYWFDPW	IGH	9	None	
3	1373	CARDPLGYCSSTSCPWYYYGMDVW	IGH	18	None	
3	1375	CAKLPRWDSNYDYW	IGH	2	None	
3	1376	CARDGFTGTTQSGYRYYGMDVW	IGH	3	None	
3	1377	CARGGCSSTSCYVVGDWFDPW	IGH	2	None	
3	1378	CANQEGTTLFYYYYGMDVW	IGH	2	None	
3	1379	CARSPGYSTNWYLPNFDSW	IGH	7	None	
3	1380	CAREGGCSGGSCYGKFDPW	IGH	6	None	
3	1381	CATSSSWPHYYYGMDVW	IGH	3	None	
3	1382	CAITLSPGGYSHYYGFDVW	IGH	5	None	
3	1384	CARGSIAVAGTGDTEYYYYYYGMDVW	IGH	6	None	
3	1385	CAKHPYYGAVLRFLEWRNWFDPW	IGH	2	None	
3	1386	CATAPVLRDILTGYSQSYYYGMDVW	IGH	2	None	
3	1387	CARDLDDSETLYYGFYDYW	IGH	4	None	
3	1388	CSLKASDSAIYYCAKYARPTYYYNYAVDVW	IGH	2	None	
3	1389	CARHPVYCSSDSCYFDYW	IGH	8	None	
3	1390	CAKEGVGSGSYSDAFDIW	IGH	2	None	
3	1391	CARPGVSAATQYWYFDLW	IGH	2	None	
3	1392	CATYFDWLTFFDSW	IGH	2	None	
3	1393	CATSWAYCGGDCYPFDYW	IGH	14	None	
3	1394	CATEYPYDFWSGPLFDYW	IGH	4	None	
3	1395	CQSYDSDLRGDVLF	IGL	7	C4	7JVC
3	1396	CARIRYCSSTSCLVGYFDYW	IGH	2	None	
3	1397	CARYFGSGVSDWFDPW	IGH	3	None	
3	1398	CAKDHWYQDIVVGAAFDIW	IGH	2	None	
3	1399	CAKDHYDYVWGSYRIPFDYW	IGH	3	None	
3	1400	CARVLRQEYFWSGPTGWFDPW	IGH	2	None	
3	1401	CATANPMVRANWFDPW	IGH	27	None	
3	1402	CAKISYDGYNSDYW	IGH	2	None	
3	1403	CARDRNSVVVVPADYYYYALDVW	IGH	2	None	
3	1404	CATASVKDHHYYDTGSPSYYYYGMDVW	IGH	2	None	
3	1406	CARAPIDDILTGTGWNWFDPW	IGH	3	None	

3	1407	CARGFYDILTGYYYGMDVW	IGH	49	None	
3	1408	CARDSGFWTGYYDYW	IGH	4	None	
3	1409	CAGGYDSSGYYYANYYYGMDVW	IGH	2	None	
3	1410	CAKATVTLENRYFDYW	IGH	2	None	
3	1411	CARYSSGWPALDYW	IGH	2	None	
3	1413	CASRTWIQLWYSYYYYGMDVW	IGH	2	None	
3	1414	CQVWESSSEHYVF	IGL	6	None	
3	1415	CARDFTIVVVPAAIEDYYYGMDVW	IGH	3	None	
3	1416	CTRFIYCSGGSCYFDYW	IGH	4	None	
3	1417	CVKGTWTWFDPW	IGH	2	None	
3	1418	CAKGAQGYSGYANDAFDIW	IGH	5	None	
3	1419	CAKEKGYNYGYYYYGMDVW	IGH	26	None	
3	1420	CAKDPGGYYDSSGYYGWYFDLW	IGH	2	None	
3	1421	CARDPSLGYCSGGSCPPLDYW	IGH	2	None	
3	1422	CARLPPWGGRWYFDLW	IGH	4	None	
3	1423	CAKENRDGYSSSWPGWFDPW	IGH	2	None	
3	1424	CARGGRYYYDSSGYYFDYYYGMDVW	IGH	17	None	
3	1425	CAKQRHFMIVGPTTIDWFDPW	IGH	6	None	
3	1426	CAREEVGAGPNNDFDFW	IGH	5	None	
3	1427	CVKDLYDSSGFHYFDSW	IGH	2	None	
3	1428	CARDPTTVTTGPLCAFDIW	IGH	2	None	
3	1429	CAKDTYRQWLVLSWYFDLW	IGH	2	None	
3	1430	CARASVLDWLFRSEIGGWFDPW	IGH	2	None	
3	1433	CAKDIPYYDSSGYPFAGAFDIW	IGH	2	None	
3	1434	CARVPTLTTDLYFDYW	IGH	2	None	
3	1435	CAKASGILWFGEGRGAFEIW	IGH	6	None	
3	1436	CARGSTSYGDFKYW	IGH	2	None	
3	1437	CARHPPATEGYGFYYYNGMDVW	IGH	3	None	
3	1438	CAREGIVGATTGPDFDYW	IGH	2	None	
3	1439	CARDFVRGYGGNAFDIW	IGH	4	None	
3	1440	CQQTNNFPPYIF	IGK	4	C1	7BZ5
3	1441	CAKEDVDKGFYFDSW	IGH	2	None	
3	1442	CASRQYSSGWHAFDIW	IGH	2	None	
3	1443	CASELWFGELWDYW	IGH	2	None	
3	1444	CARVTRWWSDAFDIW	IGH	4	None	
3	1445	CATAPVLLWSSSKYYYYYGMDVW	IGH	2	None	
3	1446	CAKDNWAAFGKYFDYW	IGH	12	None	
3	1447	CARPSPTYSDSSSYFFDSW	IGH	2	None	
3	1448	CAKDRRSYSNACDYW	IGH	6	None	
3	1449	CARDLDGDPFYYYYGMDVW	IGH	8	None	
3	1450	CAKDWGARMVGDWFDPW	IGH	2	None	
3	1451	CAKLSSYDSSGYYYRGWFDPW	IGH	11	None	
3	1452	CAGQLSPYYYGMDVW	IGH	286	None	
3	1453	CARDKGPSSIASNPRLLTYYYYGMDVW	IGH	2	None	
3	1454	CAKLSGSYYEMSDYW	IGH	2	None	
3	1455	CAKDRGTGDIVIQGSYFDYW	IGH	4	None	
3	1456	CAKGTAATGLTPRLDYW	IGH	2	None	
3	1457	CAKGLDYYDSSGYYSIGYYYYGMDVW	IGH	2	None	
3	1458	CAKDPIRDGYNYGAPDYW	IGH	3	None	
3	1460	CAKDGEATTLDFFDHW	IGH	2	None	
3	1461	CTTDPELYYDILTGLRLRNWFDPW	IGH	4	None	
3	1462	CAATSSSWYNWYFDLW	IGH	18	None	
3	1463	CATDSAGVYFDSW	IGH	2	None	
3	1464	CASGSNFDWLSADYW	IGH	3	None	
3	1465	CAATSGYSSSWYCDFW	IGH	2	None	
3	1467	CARSLAAAVLWFDPW	IGH	3	None	
3	1468	CAKPAHLVYNYFDPW	IGH	4	None	
3	1469	CVRGKYQVLGNDVFEIW	IGH	2	None	
3	1470	CAKAGGYSSPSDYW	IGH	6	None	
3	1472	CVKDVGQTTVTRYFDLW	IGH	5	None	
3	1473	CAKLPYYYDSSGWGYYYGMDVW	IGH	2	None	
3	1475	CARLTYCSGGSCGWFDPW	IGH	2	None	
3	1476	CVKGGGRGLEWLLNFDSW	IGH	3	None	
3	1477	CASALVWVGMIDYW	IGH	6	None	
3	1478	CARLMVYAPSYYFDYW	IGH	2	None	
3	1479	CARGGGYRGPFDLW	IGH	9	None	

3	1480	CARSKYDSSTSAGDYW	IGH	15	None	
3	1481	CARGAPGIRTIFGVVIRYFDYW	IGH	2	None	
3	1482	CARVLTDTDRYLKYW	IGH	4	None	
3	1483	CAKDIGDSSGSNFDYW	IGH	3	None	
3	1484	CARSTSERYFGSYGWFDPW	IGH	2	None	
3	1485	CAKARVDVIVTPPGYYYGMDVW	IGH	2	None	
3	1486	CATDIVGDHSYGFDYW	IGH	4	None	
3	1487	CAKDLWGVVVMGVGFDYW	IGH	4	None	
3	1488	CAREPVGGYVVFDYW	IGH	4	None	
3	1489	CVTWELSDYW	IGH	11	None	
3	1490	CQSSDRSGIDVLF	IGL	8	None	
3	1491	CAHTHMVRGVGYYYGMDVW	IGH	6	None	
3	1492	CVRGRHSSSPYRYFLYW	IGH	10	None	
3	1493	CAAWDDSSDGLCAF	IGL	10	None	
3	1494	CARDGGWYLQHDNEFDYW	IGH	17	None	
3	1495	CAKDIFDYGDYGLADYW	IGH	13	None	
3	1496	CARTYTTVSFDYW	IGH	5	None	
3	1497	CAREDCSGDGCRRGAFDIW	IGH	2	None	
3	1498	CQQYDNLLSLTF	IGK	320	C3	6XDG
3	1499	CARDCSSTSCYPNSDAFDIW	IGH	2	None	
3	1500	CASGGFDSGYDRNWFDPW	IGH	5	None	
3	1501	CATVGISGWYSGDDYW	IGH	16	None	
3	1502	CAKLIDWNDVYFDYW	IGH	5	None	
3	1503	CATSSAAAGTGWFDPW	IGH	14	None	
3	1504	CARDGDGEQWFQYYFDYW	IGH	3	None	
3	1505	CVRLDDYVWGSVDKGQNWFDPW	IGH	3	None	
3	1506	CARGARVGYFDWGNWFDPW	IGH	2	None	
3	1507	CARMNSYFEDSSGFYYDHW	IGH	2	None	
3	1508	CARDSSIWHWYFDLW	IGH	6	None	
3	1509	CVKDVGWAVANGFDHW	IGH	2	None	
3	1510	CAKDASPFSLAIMTVTESEYFLSW	IGH	3	None	
3	1511	CAKDYYDSSDSPYYYGMDVW	IGH	3	None	
3	1512	CQQYNSYSSFGTF	IGK	4	C3	7K8R
3	1513	CAKDLNVLLWFGDPPRGFDYW	IGH	15	None	
3	1514	CASLTHYDILTGYPYYYYYGMDVW	IGH	6	None	
3	1515	CARHCTYYYDSSGYYYVRFFDYW	IGH	4	None	
3	1516	CARDSDYGDYGGTDYYYYYGMDVW	IGH	14	None	
3	1518	CARTHDLSGNTWFDPW	IGH	2	None	
3	1520	CAKDPSEFRWLRRDYYYGMDVW	IGH	2	None	
3	1521	CAKAASITFVRGVILSDAFDIW	IGH	2	None	
3	1522	CAKDRRTAYSTYAFDIW	IGH	5	None	
3	1523	CARHIRTEYSSSWYTPYYYYYGMDVW	IGH	4	None	
3	1524	CVKGGGGYDAPGNWLDSW	IGH	6	None	
3	1525	CARLSWGDSSSPQNGPLIPRLGSDVYYYYGMDVW	IGH	6	None	
3	1526	CARVLVVRGVIVYYYYGMDVW	IGH	10	None	
3	1527	CAREKGTSYGDYGPYYYGMDVW	IGH	5	None	
3	1528	CATVATPYPFDIW	IGH	2	None	
3	1529	CARDRAPYYYGSGSLYYYGMDVW	IGH	15	None	
3	1530	CAKDRHLYSGSLAGAMDVW	IGH	4	None	
3	1531	CAKDKDCSSTSCYGVGGDAFDIW	IGH	6	None	
3	1532	CARYYYDSSGYWGPVENYFDYW	IGH	10	None	
3	1533	CARDNWNYNPFVFDYW	IGH	6	None	
3	1534	CARDTLYGDLGRFDFW	IGH	6	None	
3	1535	CARDTLHAVLTGYYTSDYW	IGH	2	None	
3	1536	CARDRVGGDGDALDVW	IGH	4	None	
3	1537	CAWENYYDSGGVFHYW	IGH	2	None	
3	1538	CAKDAAQWFGELYGYYYGMDVW	IGH	2	None	
3	1539	CAKLAAATRRDSYYYYYGMDVW	IGH	2	None	
3	1540	CARERTSGSYYNSDHFYYYGMDVW	IGH	2	None	
3	1541	CAKDTHLYSSELDYW	IGH	43	None	
3	1542	CAKAKSITMIVVVIQGPFDYW	IGH	2	None	
3	1543	CAKLCHGDYCFDYW	IGH	4	None	
3	1544	CARVSAGMTRDSYYYYAMDVW	IGH	2	None	
3	1545	CASTQAVGATNLHYYYGMDVW	IGH	3	None	
3	1546	CSSTSCPNWFDPW	IGH	2	None	
3	1547	CATSTPFGVVTHWFDPW	IGH	5	None	

3	1548	CARTWGIAAAGTEYPNYYYGMDVW	IGH	2	None	
3	1549	CATTQATSSYYYYGMDVW	IGH	10	None	
3	1550	CQSYDSRLSAHVLF	IGL	15	C4	7JVC
3	1551	CARVTYSGYALMWDWFDPW	IGH	3	None	
3	1552	CARDPSAGLYGSGSWFDYW	IGH	4	None	
3	1553	CARDTPPTYYYDSSGYPDYW	IGH	12	None	
3	1554	CARDGHGYSDYDPPGPFDYW	IGH	2	None	
3	1555	CARLGGTGTTLFDYW	IGH	17	None	
3	1556	CAHSDWNDVGSFDYW	IGH	2	None	
3	1557	CAKHETGAPYYLDSW	IGH	2	None	
3	1558	CAREAVLIENLSASPWSHNNWFDPW	IGH	3	None	
3	1559	CARTPSSIYWTDYDILTGGQISYYYGMDVW	IGH	2	None	
3	1560	CARVNWWVGARGYFDNW	IGH	6	None	
3	1561	CATDPAVLTGSYLWYW	IGH	5	None	
3	1562	CAKDGIVGAMFDYW	IGH	12	None	
3	1563	CARMSSWDFLTGPPYFDYW	IGH	2	None	
3	1564	CARYYWAITIFGVLTQAFDYW	IGH	2	None	
3	1565	CTRHEDHRFDPW	IGH	8	None	
3	1566	CARHTTTSGSYYYGMDVW	IGH	2	None	
3	1567	CARYGLGPYAPPYDYW	IGH	3	None	
3	1568	CARDAYSTAWPYYLDYW	IGH	4	None	
3	1569	CTRALSKRGYYYYGMDVW	IGH	10	None	
3	1570	CARDLRYFDWDGMDVW	IGH	9	None	
3	1571	CARETTGSYSDWFAPW	IGH	7	None	
3	1572	CAKDSSSWYLKYAFDIW	IGH	2	None	
3	1573	CASYPPHSTYSSAWYVDYW	IGH	5	None	
3	1574	CATQFGPYYYDSSGYILDYW	IGH	3	None	
3	1577	CQVWDTSSDPPVIF	IGL	75	None	
3	1578	CAKMPEDPNPFDYW	IGH	3	None	
3	1579	CAREHRRITMIVVVIYAFDIW	IGH	2	None	
3	1580	CAKEIFPHWRKRGWFDPW	IGH	2	None	
3	1581	CAKDRATIFGVVNPDYW	IGH	11	None	
3	1582	CAKDRGDGYNFDAFDIW	IGH	4	None	
3	1583	CAHRRRGIYYFDYW	IGH	103	None	
3	1584	CARGTSVSGYDFGVLRYYDSSGYFGPHGFDYW	IGH	2	None	
3	1585	CARDWGSYGDSYYYYGMDVW	IGH	27	None	
3	1586	CTTGLEAVAGTPNYYYAMDVW	IGH	6	None	
3	1587	CARPLAVAGGPYYYYGMDVW	IGH	2	None	
3	1588	CTTDGLVTTQDYYYYYGMDVW	IGH	3	None	
3	1589	CASGIVVVPAANPYYYGMDVW	IGH	5	None	
3	1590	CETRRDAFPSDYW	IGH	2	None	
3	1591	CAKARENYYDSSGYYPADYW	IGH	2	None	
3	1592	CARPFSSSWTTSIVYYYYMDVW	IGH	2	None	
3	1593	CATTRIYLVEPVFYYFDNW	IGH	2	None	
3	1594	CTTLITYYDILTGYYPGFDYW	IGH	4	None	
3	1595	CARVGVLPDVLLWFGELFYFDYW	IGH	2	None	
3	1596	CAAAAVTNPPYYYYGMDVW	IGH	3	None	
3	1597	CAKDRLGLVLVPSGDFDYW	IGH	2	None	
3	1598	CARIRLDITYYFDYW	IGH	6	None	
3	1599	CAREFYSDNTGWYFDLW	IGH	24	None	
3	1600	CVTHYDSSEYYFDYW	IGH	3	None	
3	1601	CARSIAVAGLGVDYW	IGH	7	None	
3	1602	CARGRYLEISFGGYYFDYW	IGH	4	None	
3	1603	CATLPTPPISSGYYSGYYYYGMDVW	IGH	3	None	
3	1604	CAREGSWYHFDYW	IGH	12	None	
3	1605	CARDLANGGERGFDYW	IGH	16	None	
3	1606	CARDDKYYDILTGYLGAIYYYYYGMDVW	IGH	2	None	
3	1607	CARDRGGSYRDVDYW	IGH	5	None	
3	1608	CARHTRLGGLLWFGELFPDYW	IGH	13	None	
3	1609	CARIKYYYDSSGFWFYFDYW	IGH	3	None	
3	1610	CAKDRRGSVGWWYFDSW	IGH	2	None	
3	1611	CTTDPLYGSGRAGEPWDYW	IGH	2	None	
3	1612	CARPPRGRRDGYNYYFDQW	IGH	2	None	
3	1613	CAKDPGDAFDIW	IGH	58	None	
3	1614	CAREADIAVAGTGLDYW	IGH	2	None	
3	1615	CARDFRFGESARGFDPW	IGH	2	None	

3	1616	CAGNPGGSYGDYW	IGH	4	None	
3	1617	CARDRVSSSWWTYYFDYW	IGH	6	None	
3	1618	CARLGLLYDNRSGATQFDSW	IGH	4	None	
3	1619	CARRQFRSWGYFDYW	IGH	2	None	
3	1621	CAREGVAVITIIDYW	IGH	2	None	
3	1622	CAREVSPTDFDIGYFDYW	IGH	2	None	
3	1623	CAREGYGVGATYFDYW	IGH	2	None	
3	1624	CARSVISERVRGVAFDFW	IGH	5	None	
3	1625	CATLPRYYILTGYLDGMDVW	IGH	4	None	
3	1626	CARRLSTSYYFYGMDVW	IGH	5	None	
3	1627	CARAFDSRGLGTDYW	IGH	4	None	
3	1628	CQQRSKWPPECSF	IGK	4	C1	7BZ5
3	1630	CASSYYYDSSGQPDYW	IGH	5	None	
3	1631	CAKDTRGSSWYQYNWFDPW	IGH	2	None	
3	1632	CARDDGSFYDTFNGPFQWLDPW	IGH	4	None	
3	1633	CARERMWDDILTRLGDYYYYGMDVW	IGH	2	None	
3	1634	CARAHRITIFGVVRYFDYW	IGH	3	None	
3	1635	CARDFCTGGRCYVDYW	IGH	2	None	
3	1636	CARMGIVGAKEAFDIW	IGH	2	None	
3	1637	CASVPHYYGSGRGWYFDLW	IGH	4	None	
3	1638	CTSQSMSFFDYW	IGH	6	None	
3	1639	CANVGSSWYPLDYW	IGH	2	None	
3	1640	CAKDNSGYCSSTSCYARGKYFEDW	IGH	2	None	
3	1641	CARRFSVVTIFGITGGWFDPW	IGH	4	None	
3	1642	CARDGADYGDEGGYYYYGMDVW	IGH	2	None	
3	1643	CTTFPLRINMKVVDYW	IGH	2	None	
3	1644	CARVTVTPILFYFYDGLDVW	IGH	6	None	
3	1645	CARDDGSFYDVVTGPYNWLDPW	IGH	6	None	
3	1646	CARELDHYDSSGYPPFGYW	IGH	2	None	
3	1647	CARSYSMIVDDAFDIW	IGH	2	None	
3	1649	CVGENSSSWYQSWFDPW	IGH	7	None	
3	1650	CQQYDTVPRIF	IGK	51	C3	6WPS
3	1651	CARVIQIWVFDHW	IGH	3	None	
3	1652	CARDLIMITFGGFDYW	IGH	3	None	
3	1653	CARQEFFSLVREVTTSFDYW	IGH	2	None	
3	1654	CGRAGLGKFQLQPPDYW	IGH	11	None	
3	1656	CARAGRYGGYYMDVW	IGH	2	None	
3	1657	CAKEWYYDSSGYGGFDYW	IGH	8	None	
3	1658	CATLFGASGSLLPYYYYGMDVW	IGH	4	None	
3	1659	CARVAQWLAEGVFDMW	IGH	2	None	
3	1660	CATLANKGLFTGSLYYYAMDIW	IGH	4	None	
3	1661	CAAFFGGARSSEADAFDIW	IGH	551	None	
3	1662	CARTPSSWRGYYFDYW	IGH	11	None	
3	1663	CARRKNVFCGGDCYSTLSFGYW	IGH	2	None	
3	1664	CAKVFDSGYVLYYFDYW	IGH	131	None	
3	1665	CAHRQKTITVTSSFDYW	IGH	4	None	
3	1666	CAKGGSGYFGEYFQHW	IGH	3	None	
3	1667	CANLCECGFDIW	IGH	2	None	
3	1668	CAGYSSQSLFDYW	IGH	4	None	
3	1669	CAARRVVKATTCFDYW	IGH	2	None	
3	1670	CARILMTDFHWFDPW	IGH	4	None	
3	1671	CARDHVLRYSFDYW	IGH	2	None	
3	1673	CAKDRMGYYDFWSGYPYYFDYW	IGH	11	None	
3	1674	CARYRGSLESGYAYYFDYW	IGH	2	None	
3	1675	CARHSSPDLDNSDDSGFSSPWVDGMDVW	IGH	5	None	
3	1676	CQRYGSSAYMYTF	IGK	23	C1	7K8N
3	1677	CARALDGYSYWYFDLW	IGH	8	None	
3	1678	CAAVYCSSTSCYDAFDIW	IGH	5	None	
3	1679	CATYLLFGELYADFW	IGH	3	None	
3	1680	CAGHNSGWYYFDYW	IGH	4	None	
3	1681	CAHRRFGLYDTSDFFGFFDSW	IGH	4	None	
3	1682	CAREGGWGAAAGLDYW	IGH	17	None	
3	1683	CASRNYYNSRGYFWYYFDYW	IGH	4	None	
3	1684	CARGGLWFAAAYYYYGMDVW	IGH	3	None	
3	1685	CTRDRGATSTARDGYDRDYW	IGH	15	None	
3	1686	CAKLGLLGYCSGDPCYDWFDPW	IGH	12	None	

3	1688	CAKDLGIAAAGLLSYYYYYGMDVW	IGH	6	None	
3	1689	CARVTALRFLDSYYYYYGMDVW	IGH	2	None	
3	1690	CAKVPYPYYSGSTAFYIPFFEYW	IGH	8	None	
3	1691	CARRRSGVGFPGYYYYYGMDVW	IGH	3	None	
3	1692	CVKIPGSHLGYCTGNTCSDRGVWGLHFDYW	IGH	2	None	
3	1693	CAKDRAVGERTYYYYYGMDVW	IGH	2	None	
3	1694	CAKSNVLFWFGQFKRDAFDIW	IGH	42	None	
3	1695	CARAFGVVVIAYFDYW	IGH	4	None	
3	1696	CARDLGPYDYVWGSYRTDAFDIW	IGH	2	None	
3	1697	CARGEYEQLVSYYYGMDVW	IGH	2	None	
3	1698	CARDDDSSGPYDAFDIW	IGH	3	None	
3	1699	CARVSIAVVPAANYYYGMDVW	IGH	2	None	
3	1700	CARGFYYYGSDGMDVW	IGH	2	None	
3	1701	CARTPRGWMDQYRFDYW	IGH	2	None	
3	1702	CARPWAYSGSSYFDHW	IGH	2	None	
3	1703	CAKDRGVGWHGIDYW	IGH	2	None	
3	1705	CAIKQLVRRNWFDPW	IGH	2	None	
3	1706	CARDKYYNQNWFDPW	IGH	12	None	
3	1707	CATSPGYYYGSGSPNWFDPW	IGH	3	None	
3	1708	CARVGSYGYPYYYGLDVW	IGH	81	None	
3	1709	CAKAGSMSMIIVVDFDNW	IGH	3	None	
3	1710	CGKDMYGSGASRGLIDYW	IGH	3	None	
3	1712	CAIRQTTTSEFYYYYGMDVW	IGH	2	None	
3	1713	CARDPTLVGANDYYYGMDVW	IGH	28	None	
3	1714	CAKIVSRPGHWYFDLW	IGH	2	None	
3	1715	CAKDIWYYDILTGYWPGRVNYYYYGMDVW	IGH	6	None	
3	1716	CARDLKTPYCTNGVCYYYYGMDVW	IGH	3	None	
3	1717	CAILPQGQHVTLDYW	IGH	2	None	
3	1719	CARAVVVTALGYFDPW	IGH	45	None	
3	1720	CASGYYYDSSGYGIFDYW	IGH	13	None	
3	1721	CAKAEEDSSGYYWYFDYW	IGH	15	None	
3	1722	CAKDINTSGSGYYYYGMDVW	IGH	8	None	
3	1723	CARVSSHDFWSGYPTCFDYW	IGH	4	None	
3	1724	CASQGYSSFWYW	IGH	3	None	
3	1725	CAAWDDSQSALYVF	IGL	3	None	
3	1726	CARSSFPSGSIYFQHW	IGH	2	None	
3	1727	CARRRYYNNRGYYFYYFDDW	IGH	3	None	
3	1728	CAREHSSGWRHCDYW	IGH	3	None	
3	1729	CAKDQSYDYGENWFDPW	IGH	21	None	
3	1730	CAKDPFSIVATSYFDYW	IGH	3	None	
3	1731	CAKTAIFGVVPKYYAMDVW	IGH	4	None	
3	1732	CQSYDSSTPHVIF	IGL	10	C4	7JVC
3	1733	CAKGPWGGSPYHHDTSGYSIDAFDIW	IGH	2	None	
3	1734	CATPVHSSGYSTTSDYW	IGH	4	None	
3	1735	CARDHGVGAAADYFFDDW	IGH	3	None	
3	1736	CASESPTYYYDSSGYYTDYYFDYW	IGH	6	None	
3	1737	CARDPPEWEIRVFDHW	IGH	2	None	
3	1738	CAKVGATVVSPVIDFDYW	IGH	2	None	
3	1739	CARDGPAAGTSFGLMFDPW	IGH	11	None	
3	1740	CAKDNPGYDSSGYYSVGGYFDYW	IGH	2	None	
3	1741	CARVFPSMVVYANNWFDPW	IGH	3	None	
3	1742	CATPQEYCSGGRCSFDYW	IGH	2	None	
3	1744	CATLDYGDAWEAFDIW	IGH	5	None	
3	1745	CASGPHYYYGSGRGAFDIW	IGH	5	None	
3	1746	CAKNNGVATTHFDFW	IGH	5	None	
3	1747	CARHPPQYGTSEGDGMDVW	IGH	4	None	
3	1750	CARLFIGSGSWYVYNWFDPW	IGH	2	None	
3	1751	CAIRPVNYDILTGYRYYGMDVW	IGH	5	None	
3	1753	CARDFRACSGGSCTYYFDYW	IGH	2	None	
3	1754	CTRESGEGWEQEYYYYYGIDVW	IGH	6	None	
3	1755	CAKWPIAVASFDYW	IGH	2	None	
3	1757	CAKLVGATTGDAFDIW	IGH	10	None	
3	1758	CQSPDSSATIRIF	IGL	4	None	
3	1759	CARTYLSEAGGYVAYW	IGH	3	None	
3	1760	CASSNYYGSGSYYYYYGMDVW	IGH	2	None	
3	1761	CARDSFTNFDRLFFDNSGYFDYW	IGH	9	None	

3	1762	CAKADCSGGSCSQFDSW	IGH	3	None	
3	1763	CARDQITMVRGLLKPSFDYW	IGH	19	None	
3	1764	CATLPYYYGSGSYAYYGMDVW	IGH	2	None	
3	1765	CTGGVVATFDYW	IGH	2	None	
3	1766	CARVYSSSSGRAFDIW	IGH	2	None	
3	1767	CARPRPTVKGLFDSW	IGH	2	None	
3	1768	CAKGLGFYGSGSYWFDPW	IGH	2	None	
3	1769	CAKEGNGDYPVPQPLEFDYW	IGH	8	None	
3	1770	CTPTGRTIPNAFDFW	IGH	7	None	
3	1771	CARGYSSGQIDYW	IGH	20	None	
3	1772	CAREGTMVRGVIIKASFDYW	IGH	4	None	
3	1773	CARSLSGYGYAFDLW	IGH	647	None	
3	1774	CARDGLDYGRGFDIW	IGH	4	None	
3	1775	CTRLVGYYDGRSGYIFDYW	IGH	4	None	
3	1776	CARDSVYGGYPDDYW	IGH	3	None	
3	1777	CASPLRYFDWYAFDIW	IGH	2	None	
3	1778	CAKVGILTGYPVRLDAFDIW	IGH	3	None	
3	1779	CARINNLYYYDSSGYYYDDYYGMDVW	IGH	3	None	
3	1781	CARPLKGYYDSSGYYYDAFDIW	IGH	2	None	
3	1782	CARDMSLGSTLYRWVDPW	IGH	7	None	
3	1784	CARDSDTAMGWGQWFDPW	IGH	2	None	
3	1785	CARVRYDYYYAMDVW	IGH	17	None	
3	1786	CSTDNVYCSGSSCPGNFDHW	IGH	4	None	
3	1787	CAKDQNQYGSGIEDYW	IGH	6	None	
3	1788	CARHGWENVGRGYSYGTFDYW	IGH	2	None	
3	1789	CLRSEDTALYYCARDSSRAFGYW	IGH	2	None	
3	1791	CAIAPERYSGYQHLGDYW	IGH	8	None	
3	1792	CATSLPRLPAAMGGGYYYYGMDVW	IGH	4	None	
3	1793	CARGGTYYYDNNGPHYFDYW	IGH	3	None	
3	1794	CSRGNAVSGDYW	IGH	3	None	
3	1795	CAKDNSGSYPESWFDPW	IGH	5	None	
3	1796	CAKANCSSTSCFNWFDPW	IGH	15	None	
3	1798	CARVYSSNWLWWFDPW	IGH	19	None	
3	1799	CAAQVNYYDSSGYYRDYW	IGH	2	None	
3	1800	CARGKGDYVWGSYRYTWFDPW	IGH	2	None	
3	1801	CARIFPGDFDYFQGYKYYGMDVW	IGH	2	None	
3	1802	CARDARYCSSTSCYIPNWFDPW	IGH	4	None	
3	1803	CAKLSSGYYYGSGSSDYW	IGH	4	None	
3	1804	CARVMWVPEVIVVVPAALDYW	IGH	2	None	
3	1805	CATGGVVTGYYYYGMDVW	IGH	6	None	
3	1806	CASHTSGWPNLFDLW	IGH	2	None	
3	1807	CARGPNTYFDWFPNGGYYFDYW	IGH	9	None	
3	1808	CARDGETPDGSYSDYYYYGMDVW	IGH	3	None	
3	1809	CARAGYRVARPLYYYYGMDVW	IGH	2	None	
3	1810	CAKDQGLRYFDWLLIRYYYYYGMDVW	IGH	5	None	
3	1811	CVRLIKGGAFDFW	IGH	7	None	
3	1812	CAVWDDSMSGPLF	IGL	2	None	
3	1813	CAKVKSGAVVVVAAPFDYW	IGH	2	None	
3	1814	CQQYGALPGITF	IGK	2	C2	7K8U
3	1815	CAREGTRVVITDAFDIW	IGH	2	None	
3	1817	CAKDYGGSLPDAFDVW	IGH	6	None	
3	1818	CARVTVAGTPKELNWFDPW	IGH	3	None	
3	1819	CAREGRDGYNWRVLEFDPW	IGH	3	None	
3	1820	CARLSGSYYSPLDYW	IGH	5	None	
3	1821	CARITLLYIRGDTGWFDPW	IGH	7	None	
3	1822	CARDAPVGVTSTYFDSW	IGH	4	None	
3	1823	CAREDEDYGHSQGFDVW	IGH	6	None	
3	1825	CAKDPHFDWPSVYYYYGMDVW	IGH	2	None	
3	1827	CAKDKESWGYNWFDPW	IGH	12	None	
3	1828	CSRITNYYENFGDFPPTGYFDYW	IGH	2	None	
3	1829	CARGGGSRSGGVDYW	IGH	4	None	
3	1830	CATAPAVAGPIYYYYGMDVW	IGH	4	None	
3	1831	CAKDIVGYCSSTSCLGAFDIW	IGH	2	None	
3	1832	CARAPRGDYDSWLYYYGMDVW	IGH	5	None	
3	1833	CQVWDSSSDSGSWVF	IGL	12	None	
3	1834	CVKDLSPAAIRPLFDDW	IGH	43	None	

3	1835	CTTEEQWAGYYYDSSGYYFFDYW	IGH	3	None	
3	1836	CATNVGTSSLVLVWYFDLW	IGH	6	None	
3	1837	CARDQALGTADHWAVVVTAILDYW	IGH	2	None	
3	1838	CARPSLAYCDGDCSGFDLW	IGH	9	None	
3	1839	CATFPEYSYGRSYYYYYGMDVW	IGH	2	None	
3	1840	CAHTAFHYYGSGRTFDPW	IGH	2	None	
3	1841	CAKDWGPFLYCSGGSCYPFDYW	IGH	3	None	
3	1842	CAKEVLVAGTLPWFDPW	IGH	29	None	
3	1843	CARDDFERALAGTFYDYYYAVDVW	IGH	2	None	
3	1844	CARGPIITMVRGVIPWFDPW	IGH	2	None	
3	1845	CAKCGSGSYYNVGVDYW	IGH	4	None	
3	1846	CAKDRNSHPPGNYFDYW	IGH	3	None	
3	1847	CAKEGKWELGPYYYGMDVW	IGH	2	None	
3	1848	CARHNSGKFRANWFDPW	IGH	3	None	
3	1849	CYSADSSVKVF	IGL	29	None	
3	1850	CARVGNDYVWGSYRLTDAFDIW	IGH	3	None	
3	1851	CQNYNSAPQSTFTF	IGK	6	C3	7K8R
3	1852	CVKEMGLGFAEFPPNYFDSW	IGH	2	None	
3	1853	CAREYDILTGYYTGDYYYYYGMDVW	IGH	10	None	
3	1856	CARDRSGFGGVIVNYYYGMDVW	IGH	2	None	
3	1857	CAHRHSNSYYTYW	IGH	4	None	
3	1858	CACLQLGYSSSWYAYFDLW	IGH	3	None	
3	1859	CARSNYDILTGYLSSFDYW	IGH	37	None	
3	1860	CAKDAGGDCSSTSCSPSDYW	IGH	2	None	
3	1861	CAHSLYFPNPFDYW	IGH	21	None	
3	1863	CAHTYDFWSGLDYW	IGH	4	None	
3	1864	CARDADQLLEVVPVTAPFDPW	IGH	2	None	
3	1865	CAKDPSISAAGYYFDSW	IGH	2	None	
3	1866	CARTYIVATTNYYYYYGMDVW	IGH	2	None	
3	1867	CARVRYFDWLLQTGYYYGMDVW	IGH	2	None	
3	1868	CARDLLGITGPDYW	IGH	4	None	
3	1869	CARERGKQHLPALDYW	IGH	2	None	
3	1870	CARTRTVASARHFFDYW	IGH	23	None	
3	1871	CAKFAQLWPGTAFDIW	IGH	4	None	
3	1872	CAKAPDFYDGGSSVFDDW	IGH	2	None	
3	1873	CAKVGYSYGDDFYDYW	IGH	2	None	
3	1874	CARVFVNSAFDLW	IGH	2	None	
3	1875	CAKRGPYYDFWTLDYW	IGH	2	None	
3	1876	CAKDRPLHDYGDFDYW	IGH	7	None	
3	1877	CASGYSGYDFLDYFDYW	IGH	13	None	
3	1878	CARGYSSSWEDGMDVW	IGH	8	None	
3	1879	CAKPWQWLEPGDYW	IGH	2	None	
3	1880	CARAAGPIAVAGTGDWFDPW	IGH	3	None	
3	1881	CARGKGYDILNGLDYW	IGH	4	None	
3	1882	CAKGSSDGSGSWAFDIW	IGH	2	None	
3	1883	CTRDLRFCAVGSCWPSWYDPW	IGH	2	None	
3	1884	CAKAVREETAEWLDPW	IGH	4	None	
3	1885	CARGPVVGAIPYYFDYW	IGH	5	None	
3	1886	CARRHTRYDSSGYDTFDIW	IGH	4	None	
3	1887	CASMIYGGYCSGGSCHSADYW	IGH	2	None	
3	1888	CAREDVVGTSLTFDPW	IGH	2	None	
3	1889	CAREGHDSSGYYDLYAFDIW	IGH	4	None	
3	1890	CARGLDCSGGSCYMGFDPW	IGH	2	None	
3	1891	CTRVSSSWYLDYFDYW	IGH	6	None	
3	1892	CARSYIARGEMDSDYW	IGH	3	None	
3	1893	CARARGPYCHGDCPFDYW	IGH	5	None	
3	1894	CARAGYADTHFDYW	IGH	6	None	
3	1895	CARAPHCVGDSCYYYYYYGMDVW	IGH	27	None	
3	1896	CARRLYDSSAWFDPW	IGH	16	None	
3	1897	CARILYSSGLGFDPW	IGH	3	None	
3	1898	CTKHPAWDDPW	IGH	2	None	
3	1899	CAHSSDYIWGKFRYYDAFDIW	IGH	15	None	
3	1900	CAKVPSGDSPFGYW	IGH	9	None	
3	1902	CAKGQYSSSWSHFDYW	IGH	8	None	
3	1903	CVRGNYYDSSGYYSGRYYFDSW	IGH	4	None	
3	1904	CATAPIDYVRWFDPW	IGH	7	None	

3	1905	CAKDFDSSGHWDYW	IGH	13	None	
3	1906	CARLDWFGGIWDPNFWYFDPW	IGH	2	None	
3	1907	CARIYGTPWGSYSIDYW	IGH	8	None	
3	1908	CARSQNIAAAPIYYYYYGMDVW	IGH	6	None	
3	1909	CAKEGRPSGGNSGRDYW	IGH	2	None	
3	1910	CAKALYSYGYLFDYW	IGH	8	None	
3	1911	CAADPTAAAGTGFDYW	IGH	2	None	
3	1912	CQSNDSNNHKIF	IGL	10	C4	7JVC
3	1913	CARARGLGAARWFDPW	IGH	9	None	
3	1914	CVREGFSSKWNGYFHHW	IGH	23	None	
3	1915	CASIYDILTGSYYYYGMDVW	IGH	5	None	
3	1916	CASPYPTRDFDIW	IGH	5	None	
3	1917	CAKNPGLLIRGTDAFDIW	IGH	24	None	
3	1918	CARHNTRGQIGFAIDYW	IGH	2	None	
3	1919	CASGAGSYYDYFDYW	IGH	6	None	
3	1920	CVRGPSYYILTGYLNSDAFDIW	IGH	42	None	
3	1921	CARSSLRGSGSRYNWFDPW	IGH	16	None	
3	1922	CARQGGSGNYYRPRWQFNWFGPW	IGH	2	None	
3	1923	CAKPSGDASGYYSSYYYAMDVW	IGH	5	None	
3	1924	CARDMMFGELSDYW	IGH	22	None	
3	1925	CARRAAVVAGGTYSFDIW	IGH	2	None	
3	1926	CASNYDILTGYYLPRDYYYGMDVW	IGH	6	None	
3	1927	CARSYSGLRFPGVYYYYGMDVW	IGH	3	None	
3	1928	CKSEWLPVDYW	IGH	12	None	
3	1929	CARHLPFNTAPRFDPW	IGH	2	None	
3	1930	CVRDRPAVKFFDYW	IGH	2	None	
3	1932	CARHVGYSYGIDYW	IGH	6	None	
3	1933	CAGAVADPTAHFDYW	IGH	4	None	
3	1934	CAHSPRQGPTSGTTFDYW	IGH	2	None	
3	1935	CARGNTDYYDSSGYLDYW	IGH	52	None	
3	1937	CASPMSGHSDYW	IGH	7	None	
3	1938	CGRAMTGYYKDAFDIW	IGH	6	None	
3	1939	CARDRGEEFGEFLWNFDLW	IGH	2	None	
3	1940	CAPFHYYYYYGMDVW	IGH	47	None	
3	1941	CARDNLGHCLSTSCYDVYW	IGH	2	None	
3	1942	CAKESSTQQQDDYW	IGH	2	None	
3	1943	CARVLGGGETHELDYW	IGH	12	None	
3	1944	CARGNSYFDSYYNYAMDVW	IGH	9	None	
3	1945	CTRFPRSPTYFFGSGENPLDYW	IGH	2	None	
3	1946	CARVPLRDYDILTGYYRGPFDPW	IGH	2	None	
3	1947	CAKSPVPMVVTLAFDIW	IGH	2	None	
3	1949	CAKVHIAAVYFDYW	IGH	4	None	
3	1950	CARMIGPQYTTSWWFDPW	IGH	8	None	
3	1951	CARGVLDTAMVKSYRFDPW	IGH	4	None	
3	1952	CAKDMLPIAAAGHGLDYW	IGH	2	None	
3	1953	CARMSYSSSFRRGWFDPW	IGH	3	None	
3	1954	CARRGYCNRITCPVVDYW	IGH	4	None	
3	1956	CGRLDVLGGYYEDYW	IGH	27	None	
3	1957	CAKSGYSSTPDACDIW	IGH	8	None	
3	1959	CARGLYYYGSGSVRGWFDSW	IGH	2	None	
3	1960	CMQRIEFPLYTF	IGK	45	None	
3	1961	CAKDMWPAAGTIFDYW	IGH	4	None	
3	1962	CAKDRSGEYCSGGSCYSLYW	IGH	2	None	
3	1963	CAKDLRDYGGQWGYLDSW	IGH	2	None	
3	1964	CLTDDLQVSTSWYGLDAFNLW	IGH	2	None	
3	1965	CARHKGNGITIFGVVTLNNWFDPW	IGH	8	None	
3	1966	CVRSPPAGDHTFDFW	IGH	4	None	
3	1967	CARDGDYYDSSGYYYRGPPYWYFDLW	IGH	2	None	
3	1968	CAKDVRPQTVSDAFEIW	IGH	2	None	
3	1969	CAKDSSGWANNWFDPW	IGH	11	None	
3	1970	CTTNLGGQVAGQGYWFDPW	IGH	3	None	
3	1971	CAKLVGATRGGFDYW	IGH	54	None	
3	1972	CAIMPPHGIAVAGIMGGDYYYYGMDVW	IGH	2	None	
3	1973	CAHSGQKELWPFDFW	IGH	8	None	
3	1974	CARGIFEGEFDYW	IGH	2	None	
3	1975	CAGAHSGSYLFGFDYW	IGH	2	None	

3	1976	CAKDIYGDYDGHVDYW	IGH	4	None	
3	1977	CARAPGLYDFWSGYPHDYYYGMDVW	IGH	2	None	
3	1978	CQSADNDGTTFPVVF	IGL	3	None	
3	1979	CARETVTTVIYYYYGMYVW	IGH	3	None	
3	1980	CARGGYDFWSGYNNWFDPW	IGH	3	None	
3	1981	CQHYPSSPLAF	IGK	33	C1	7K8N
3	1982	CAKPTVSDLQLWFSYYYGMDVW	IGH	5	None	
3	1983	CAKDIRGWEGEAFDIW	IGH	4	None	
3	1984	CARDRVGSYYNPLYYYYGMDVW	IGH	6	None	
3	1985	CAKADSDSYFPPSYFDSW	IGH	20	None	
3	1986	CASTHLRLGELSLYYYYGMDVW	IGH	3	None	
3	1987	CAREQTTVTTYWHFDLW	IGH	2	None	
3	1988	CAKSPPLSYDRDDAFDIW	IGH	6	None	
3	1989	CARGYFDWLGPGWFDPW	IGH	8	None	
3	1991	CAKDVGLAYVVYAYYYYGMDVW	IGH	7	None	
3	1992	CQQYITYSTMLTF	IGK	9	None	
3	1993	CARELRNSYGELLWFGEVYYYYGMDVW	IGH	6	None	
3	1994	CASNVIVLRYFDWLLEATKDDYW	IGH	2	None	
3	1995	CASERSTYYYDSSGLDYW	IGH	3	None	
3	1996	CAKETDSRQSHDTDYW	IGH	4	None	
3	1997	CAKTVLVRGVISADYFDYW	IGH	2	None	
3	1998	CARDRKELGYCSGGSCDYWYFDLW	IGH	4	None	
3	1999	CARSAGWSRDYWYFDLW	IGH	2	None	
3	2000	CQTWGTGIRVF	IGL	198	None	
3	2001	CARELGGDGYCSSTSCPAYYYGMDVW	IGH	3	None	
3	2002	CQVWDNSIDHWEF	IGL	5	None	
3	2003	CHQSSALPYTF	IGK	172	None	
3	2004	CAKFASGWYSDYYYGMDVW	IGH	2	None	
3	2005	CARDPLRYFDWFEVLVSTQDYYYGMDVW	IGH	4	None	
3	2006	CARSPICSGAGCYLGRVFDIW	IGH	8	None	
3	2007	CVRDWYCSGGRCYWTGYYYYGMDVW	IGH	2	None	
3	2008	CAKDSHGYGSGVYLFHYW	IGH	4	None	
3	2009	CAAWLVVAFDIW	IGH	2	None	
3	2010	CAKDKRVTSSWSHDAFDIW	IGH	2	None	
3	2011	CARDRGRYDFWSGYGDYYGMDVW	IGH	2	None	
3	2012	CARDGGGDCYSWDLCWFDPW	IGH	4	None	
3	2013	CARGRYCSSPSCYKANWFDPW	IGH	2	None	
3	2014	CAREMYYDILTGYRGPLDYW	IGH	2	None	
3	2015	CAKDWHILTGYYFFDYW	IGH	2	None	
3	2016	CTKLVGVSVLDYW	IGH	4	None	
3	2017	CARDEAYCSGGSCYSYFDYW	IGH	14	None	
3	2019	CARARALGMLVVIDAFDIW	IGH	18	None	
3	2020	CAGEPPGIVGAGWDYW	IGH	10	None	
3	2021	CMHAAQFPPFIYTF	IGK	2	None	
3	2022	CARTFLNTLYDNSNYFDFW	IGH	5	None	
3	2023	CAKGEAVAGTFTYW	IGH	3	None	
3	2024	CATSYGGNSRGWFDPW	IGH	9	None	
3	2025	CAKDSVKHIVVVTAIRYYYYGMDVW	IGH	2	None	
3	2026	CAIGAPSYCGGDCYYYFDYW	IGH	2	None	
3	2027	CAGDQKDIAVAGIHYYYGMDVW	IGH	19	None	
3	2028	CAKDPISYGSGSYPNWFDPW	IGH	6	None	
3	2029	CARYCSGGNCYPKDDSFDIW	IGH	2	None	
3	2030	CTTDLHDFGDYDYW	IGH	40	None	
3	2031	CARALPLGVMITFGGPFDYW	IGH	2	None	
3	2032	CAKDIVGATAYYFDYW	IGH	14	None	
3	2034	CAREYDLLTAYYNPPGWFDPW	IGH	2	None	
3	2035	CATAFAVVVTPRFDYW	IGH	5	None	
3	2036	CARGFDYW	IGH	200	None	
3	2037	CATFYGSGTTYYYGMDVW	IGH	3	None	
3	2038	CAKRAMAYCGGDCYSYYFDYW	IGH	3	None	
3	2039	CAREGDSITMVRGVIPDYYYYGMDVW	IGH	20	None	
3	2040	CASHRILYYDSSVFDYW	IGH	2	None	
3	2041	CAREPLYYDILTGYYNSYYYGMDVW	IGH	3	None	
3	2042	CARGLGTPFDYW	IGH	18	None	
3	2043	CATGPAVKLTGYYSSYYYGMDVW	IGH	4	None	
3	2044	CAKEFYCDSTKCYRAFDYW	IGH	4	None	

3	2045	CARTINWAWLDPW	IGH	2	None	
3	2046	CATYCSSMSCYNDYW	IGH	6	None	
3	2047	CARLRVLLWFGDLFAFDIW	IGH	8	None	
3	2048	CARVGTKYLLGVVVSKHFDYW	IGH	2	None	
3	2049	CATNHYGDNGFDPW	IGH	4	None	
3	2050	CARDFWPPYYGSGSYLPLDYW	IGH	8	None	
3	2051	CAKVRKYEFYTGFDSW	IGH	14	None	
3	2052	CARGDLAGDQGGVDYW	IGH	3	None	
3	2053	CARYQRDFWSGRHRGWYFDLW	IGH	2	None	
3	2054	CAKLGYSGYDLNNFDYW	IGH	14	None	
3	2055	CAREDYDYIWGNYRHDYYYGMDVW	IGH	5	None	
3	2056	CAQDLGYTYGVVEDGNWFDPW	IGH	6	None	
3	2057	CISYCGGDCPRDYW	IGH	4	None	
3	2058	CARRGLITSAFHVW	IGH	4	None	
3	2059	CARGAGYCSSTSCLLPHWYFDLW	IGH	2	None	
3	2060	CARVNIVSYAMDVW	IGH	4	None	
3	2061	CARDHYYYENSGYHVILSYLDNW	IGH	7	None	
3	2062	CANLEPDYYDSSGYYSAFDIW	IGH	4	None	
3	2063	CAREQQASFSGSYDYW	IGH	3	None	
3	2064	CAKLPSDGDGYYYGMDVW	IGH	2	None	
3	2065	CVRSVVCYVTSYCYFDYW	IGH	16	None	
3	2066	CAKGSTSWERGLFDYW	IGH	5	None	
3	2067	CAHCYYDILTGYSQDFDYW	IGH	4	None	
3	2068	CARDSPYYDFFTGSIFDLW	IGH	2	None	
3	2069	CARPTVSGNWFDPW	IGH	2	None	
3	2070	CSSFTSSDTFAF	IGL	9	C2	7K90
3	2071	CARDPYSYAESPFDYW	IGH	2	None	
3	2073	CAKDKGVGRFLEWLSNYYYGMDVW	IGH	2	None	
3	2074	CVRGGSSGWYAPFDYW	IGH	2	None	
3	2075	CAKAIRVYSSPRGDAFDIW	IGH	2	None	
3	2076	CARGDYGDYPRDYYYYYGMDVW	IGH	3	None	
3	2077	CARASVEGWYYFDYW	IGH	6	None	
3	2078	CTTDQGLHTTTYYYDSSGFDAFDIW	IGH	2	None	
3	2079	CARDYSSGSYYAVLWFDYW	IGH	2	None	
3	2080	CARDGITMVRGVIRWIFDYW	IGH	31	None	
3	2081	CARDGVDTAMADFDYW	IGH	5	None	
3	2082	CAKDQRYCSGGSCSVFEYYYYGMDVW	IGH	2	None	
3	2083	CGAGILLLVDYW	IGH	4	None	
3	2084	CARVFLARDVLLWFGELTVGYFDYW	IGH	2	None	
3	2085	CASDRRYGDYPPDYYYGMDVW	IGH	2	None	
3	2086	CARDKDVYDILTGYYTYYYYGMDVW	IGH	8	None	
3	2087	CAAPGHSYDSSGYYHYW	IGH	3	None	
3	2088	CARTYYDFWSGYYNFPFDYW	IGH	2	None	
3	2089	CARRPPLWFGEADFDYW	IGH	4	None	
3	2090	CAREHGHNDWYFDLW	IGH	2	None	
3	2091	CATRVTYCSSTNCRYFDFW	IGH	7	None	
3	2092	CARDLMDGYCSGGSCYGDAFDIW	IGH	3	None	
3	2093	CARFVGDYGGVNGFDPW	IGH	2	None	
3	2094	CSIDDPYNYNGAGDYW	IGH	3	None	
3	2095	CAKGDNPSTYSSSWYDYW	IGH	6	None	
3	2096	CVRHCASSSCYGYFDIW	IGH	2	None	
3	2097	CVKEVGVSLDGGFDIW	IGH	4	None	
3	2098	CARSAYSSSNRIDYW	IGH	2	None	
3	2099	CARLDFWSGYYQDYW	IGH	2	None	
3	2100	CANEMGTSVFDYW	IGH	9	None	
3	2101	CARDSQGPVVLIYGGWFDPW	IGH	2	None	
3	2102	CARGGFEELLYPFDYW	IGH	10	None	
3	2103	CARWGTDSGGYYLFDYW	IGH	2	None	
3	2104	CARDLILLNTFMYYYGMDVW	IGH	2	None	
3	2105	CAKLIEMATITDDYFDYW	IGH	3	None	
3	2106	CARGLYSGYEYYFDYW	IGH	30	None	
3	2107	CAKDLSAIFTSWFDPW	IGH	7	None	
3	2108	CARDQPAHDYGDYPDYW	IGH	6	None	
3	2109	CTREISSSWLDPW	IGH	2	None	
3	2110	CARPHSGSYDPHYFDYW	IGH	3	None	
3	2111	CARTYYDDHWVSYRQNWFDSW	IGH	2	None	

3	2112	CAKVESGSYWGYLDYW	IGH	5	None	
3	2113	CTRKEYFNTIYYFDYW	IGH	2	None	
3	2114	CARDSDAYSSSWYTFNYYYYGMDVW	IGH	3	None	
3	2115	CTRHVDDILTGYYSYYYYGMDVW	IGH	2	None	
3	2116	CAAGLLEPIDYW	IGH	2	None	
3	2117	CASARASRLSRSPFDYW	IGH	2	None	
3	2118	CAKHASLRWVPRDAFDVW	IGH	8	None	
3	2119	CASYKPPLFDCW	IGH	2	None	
3	2120	CARDPLTYCDGDCYPPSYFDYW	IGH	4	None	
3	2121	CAKDADSGSYSGGIDYW	IGH	2	None	
3	2122	CARILRQQPYYYYGMDVW	IGH	3	None	
3	2123	CARENEGYDILTGYLDYYYYGMDVW	IGH	3	None	
3	2124	CARAPDPFYRGWFDPW	IGH	2	None	
3	2125	CGRSGYFGSGSYPDYW	IGH	2	None	
3	2126	CTRDAYLASESNPWYFDYW	IGH	6	None	
3	2127	CTVKWYNYETSGYHLDYW	IGH	2	None	
3	2128	CAHVPAAMWDDAFDIW	IGH	5	None	
3	2129	CAKDITYYDILTGYLMPMNYYYYGMDVW	IGH	3	None	
3	2130	CTTESRFFASATGTYSYYFDYW	IGH	2	None	
3	2131	CARDLEGESDYGSGSYLDYW	IGH	2	None	
3	2132	CAKDIYSGFADSGYGHYYYGMDVW	IGH	6	None	
3	2133	CAKDGSRGYSGYARYYYYGMDVW	IGH	2	None	
3	2134	CARLNLGSGYPNFDLW	IGH	2	None	
3	2135	CVRGCSSVTCTGYFDPW	IGH	2	None	
3	2136	CTSYTSSSTFVVF	IGL	4620	C2	7K90
3	2137	CTTDMGGRIVVVTAINGGFDYW	IGH	2	None	
3	2138	CEKVGTLYYYDSSGYYQGWYFFDYW	IGH	2	None	
3	2139	CARDVSPSGSYPTAFDYW	IGH	9	None	
3	2140	CARDLLYYDFWSGYYFGSVPPGGASYYYGMDVW	IGH	2	None	
3	2141	CAKHSSDSSPYNNFDHW	IGH	4	None	
3	2143	CARRVGANWGDNWFDPW	IGH	3	None	
3	2144	CARGGCSSTSCYDYYYYGMDVW	IGH	6	None	
3	2145	CARHHLWGGNPYDAFDIW	IGH	2	None	
3	2146	CARDYCSSPSCYDWAMDVW	IGH	6	None	
3	2147	CAKDRGHIVVVVAATPEFDYW	IGH	6	None	
3	2148	CAKDITDSGYNSFFSLPSNYFDYW	IGH	2	None	
3	2149	CAKVPEAFYSGFLNWFDPW	IGH	3	None	
3	2151	CAKVLGAWFGGAWSPFDNW	IGH	2	None	
3	2152	CAHKLDYRHYFDYW	IGH	16	None	
3	2153	CARVGVMTTVTGSPSYYYGMDVW	IGH	2	None	
3	2154	CARHEPSSTTGYSSNWGWFDPW	IGH	2	None	
3	2156	CLLYYSGAVIF	IGL	491	None	
3	2157	CATGHDTAMVIKDGMDVW	IGH	2	None	
3	2159	CAKSYQLLTIVQLEYYFDYW	IGH	3	None	
3	2160	CARVVLDPQDIVVVPAAPSYYYYYGMDVW	IGH	2	None	
3	2161	CAKLVVSSLNYYYYGMDVW	IGH	13	None	
3	2162	CAKDRDDIFWSGYAMDVW	IGH	13	None	
3	2166	CAGTTAMVENWFDPW	IGH	12	None	
3	2167	CARDGDDILTGYSADYYYGMDVW	IGH	19	None	
3	2169	CARVPTVTSSYYYYGMDVW	IGH	41	None	
3	2170	CTSRDYDLDYW	IGH	4	None	
3	2171	CAHGDVSYTQEGYFDYW	IGH	2	None	
3	2172	CARDYVRLDYDILTGYYYPGHFDYW	IGH	4	None	
3	2173	CQSVDISGTSLIF	IGL	1084	None	
3	2174	CARDLNSIWVAAIESYFDYW	IGH	7	None	
3	2175	CTTAALWFGESLDYW	IGH	3	None	
3	2176	CARGQYYYDSSGYQNAWFDPW	IGH	2	None	
3	2177	CARGPWRETYYYDSSGYSYYYYYGMDVW	IGH	5	None	
3	2178	CARDQRPHDYDILTGYFPYYFDYW	IGH	2	None	
3	2179	CARDGDYYDSSGYAAHAFDIW	IGH	2	None	
3	2181	CARGSGWPPYYYYYGMDVW	IGH	2	None	
3	2182	CTTTPFYYDYW	IGH	2	None	
3	2183	CATSFAITGTPRHYYYYYGMDVW	IGH	17	None	
3	2184	CAHTLVGSYYDSSGYSDYW	IGH	7	None	
3	2185	CAKAQSSLWYSENWFDPW	IGH	7	None	
3	2186	CAKDIDSSGWYHWFDPW	IGH	14	None	

3	2187	CASSVLLWFGDFDYW	IGH	98	None	
3	2188	CAKDQGVSRGSGSYYYPPRWDYYYGMDVW	IGH	2	None	
3	2189	CAKGDQSTPYSSSWYQNWFDPW	IGH	3	None	
3	2190	CTTDPLEGTIFGVVIIYPGYW	IGH	3	None	
3	2191	CASQTSIRDAFDIW	IGH	2	None	
3	2192	CARGGVVAGISGWIDPW	IGH	3	None	
3	2193	CALYHDFLSGRRFDPW	IGH	2	None	
3	2194	CARQDVWFGELLYFDYW	IGH	2	None	
3	2195	CATTEGWEGFDKW	IGH	2	None	
3	2196	CATEWAPEGATSDYYYYGMDVW	IGH	4	None	
3	2197	CVKPRETDYDYIWGSSRRGFDHW	IGH	2	None	
3	2198	CVREQYYHRSGNTYNEENWFDPW	IGH	2	None	
3	2199	CAHSYYDILTGIAIYFDYW	IGH	16	None	
3	2200	CARANIVVVVDEVWFDRW	IGH	3	None	
3	2201	CARVQGHDSSAHPWCFDYW	IGH	3	None	
3	2202	CAREESDGEYFRHW	IGH	2	None	
3	2203	CAGEGELGVDTAMVNTNYYYYYGMDVW	IGH	2	None	
3	2205	CARDVLFCSASRCWASYFDYW	IGH	2	None	
3	2206	CAHIWGQGDSWGQLYGDYPGWAFDIW	IGH	2	None	
3	2207	CARDVPGYSSSWYGRGHYYYGMDVW	IGH	4	None	
3	2208	CAREGTAPVPGAFDYW	IGH	2	None	
3	2209	CASLVQLEELRSWFDPW	IGH	4	None	
3	2210	CARHSMGSTMIVGVITTPAWVDPW	IGH	2	None	
3	2211	CAREYLSSWYGWFDPW	IGH	10	None	
3	2212	CAREDPTCSGGTCFDHW	IGH	8	None	
3	2213	CAKDSGDYGDYGVYW	IGH	16	None	
3	2214	CARDMGYYYDSSGYQYYYYYGMDVW	IGH	3	None	
3	2215	CARVFYYDSTAEPFEHW	IGH	6	None	
3	2216	CARSLDEQYLAAYYFDYW	IGH	2	None	
3	2217	CARASRNYSILTAYNVFFDYW	IGH	4	None	
3	2218	CALVTMVRGDYW	IGH	2	None	
3	2219	CTTDSPPRFLEWLLRRGHQYYYYYGMDVW	IGH	2	None	
3	2220	CTREPDNKDKFDFW	IGH	4	None	
3	2221	CARVPTYPFCSSVSCYGFDPW	IGH	6	None	
3	2223	CARDKYPGLAVAGNWFDPW	IGH	14	None	
3	2224	CARGYYDTDNWFDPW	IGH	2	None	
3	2225	CATVRDMFHLGESSPKFDSW	IGH	678	None	
3	2226	CARDGDFTYYYDSSGYQPPRLHYYYYGMDVW	IGH	3	None	
3	2227	CAKWGGEAKKDFWSGPFDFW	IGH	2	None	
3	2228	CARDTPNWTYFDSW	IGH	2	None	
3	2229	CAKDLSSVTSRRHFDYW	IGH	8	None	
3	2230	CARVHQYYDILTGLGYDYYYGMDVW	IGH	2	None	
3	2231	CARDGEGLRLYEAYYFDYW	IGH	2	None	
3	2232	CAKLAGTGESVDYW	IGH	8	None	
3	2233	CQSAESGGTYVWVF	IGL	4	None	
3	2234	CARIPVGATGGGVDYW	IGH	3	None	
3	2235	CAGDPIYCNSTRCPQWFDPW	IGH	2	None	
3	2236	CATGLGYCPSTRCYDDGLTDYW	IGH	2	None	
3	2237	CARDVDSSHVFVGDALDIW	IGH	3	None	
3	2238	CARPHCSFTRCFDAMGVW	IGH	2	None	
3	2240	CARDQSPDDYVWGTIAAWFDPW	IGH	5	None	
3	2241	CAGSDNWSPKGRFDPW	IGH	6	None	
3	2242	CVKDKPDASAFDAFDFW	IGH	6	None	
3	2243	CSSYTSSSTLPSHVVF	IGL	4	C2	7K90
3	2244	CARHAYGYTRGSYYYGMDVW	IGH	9	None	
3	2245	CARVLGAYSTFYITYYNYGMDVW	IGH	23	None	
3	2246	CVAGTHLGFYYHYGMDVW	IGH	3	None	
3	2247	CARTRGNGDIVVVPARWFDPW	IGH	3	None	
3	2248	CARRYCDDLTCYLFFDSW	IGH	2	None	
3	2249	CAKASNYYDSSGYYWYFDLW	IGH	4	None	
3	2251	CARGKYEFLIPLYYYYAMDVW	IGH	9	None	
3	2252	CAKATGYYDSSGYPNWFDPW	IGH	2	None	
3	2253	CARFVPDYGDYPQGDYYYYGMDVW	IGH	2	None	
3	2254	CSSYTTSSTTSVSF	IGL	8	None	
3	2255	CARGRGYSYGLPSFDIW	IGH	2	None	
3	2257	CNRDRHYPIAGALGLGIYNYYGMDVW	IGH	2	None	

3	2258	CARAKYDILTGYSLGYFFDYW	IGH	2	None	
3	2259	CAKDAGFGDSSGYAPITRVWGVYYYYGMDVW	IGH	2	None	
3	2260	CARGGRPTIFGVVISGYYYYGMDVW	IGH	4	None	
3	2262	CARRSPYSSYQYYFDSW	IGH	2	None	
3	2263	CAKEDLNGYSSGWTYYYYGMDVW	IGH	4	None	
3	2265	CAKGGAFNEYSGGWPRGLFDSW	IGH	2	None	
3	2266	CARDGGYYAPFPGYYYGMDVW	IGH	2	None	
3	2268	CKVHTGLRTF	IGK	5	None	
3	2269	CTRGGEMTGYRVTAFDCW	IGH	3	None	
3	2270	CARGHPSGSYQLEFDYW	IGH	2	None	
3	2271	CARGVLTSSRWFDPW	IGH	3	None	
3	2272	CARDSLTTVVYYYGMDVW	IGH	12	None	
3	2273	CAKVENPNHSFQTDYW	IGH	2	None	
3	2274	CATDGRGYSGYDDEWGYYYYGMDVW	IGH	2	None	
3	2275	CAKEDCSGGSCYSDYW	IGH	8	None	
3	2276	CAKIPTGSYESRAFDIW	IGH	10	None	
3	2277	CAHLGAYYYGSGSWGYNWFDPW	IGH	5	None	
3	2278	CAKDFTRYSGYETNFDYW	IGH	2	None	
3	2279	CAKAEEETTSTQGNYYYYYGMDVW	IGH	4	None	
3	2280	CASPRDYGDYGGAFDYW	IGH	15	None	
3	2281	CARDVRSISMIVVVVTTFPSYAMDVW	IGH	6	None	
3	2282	CARTKRYCTNGICFQEWFDSW	IGH	5	None	
3	2283	CARGEFFARGYPEHWFDPW	IGH	5	None	
3	2284	CARGRGWYGNYFDYW	IGH	9	None	
3	2285	CAKLRSTVTTLWDAMDVW	IGH	2	None	
3	2286	CAKDRAIAAAGQVDYW	IGH	2	None	
3	2287	CAIDIVVVTAIPLLDYW	IGH	5	None	
3	2288	CARDVNQRGYSYGYYW	IGH	3	None	
3	2289	CQQYYSYSPTYTF	IGK	34	None	
3	2290	CAKIDYGDYQGSPDYW	IGH	9	None	
3	2291	CARIVATINSFDYW	IGH	8	None	
3	2292	CTKAYIIGTPSGFDYW	IGH	2	None	
3	2293	CAGGDDILTGYFYW	IGH	6	None	
3	2294	CAKDRWSPDSSGYIDYW	IGH	6	None	
3	2295	CAREEQYYDSGTYYNYKMDYW	IGH	3	None	
3	2296	CQQHKNWPLMYTF	IGK	14	C3	6XDG
3	2297	CANWVLFGSTKVFDVW	IGH	2	None	
3	2298	CARDSRGHRRYYYYYGMDVW	IGH	4	None	
3	2299	CARMGEYCSRSSCYFDFW	IGH	244	None	
3	2300	CARDSHYDTTGYVAYW	IGH	3	None	
3	2301	CARTFQQWRNFGYFDYW	IGH	7	None	
3	2302	CARMWTDYSRRPYYYGMDVW	IGH	2	None	
3	2303	CAKDIGSPYGSGSGRDYYYYGMDVW	IGH	3	None	
3	2304	CARLIAPYNWFDPW	IGH	5	None	
3	2305	CATPRYYGSGGFDYW	IGH	6	None	
3	2306	CQAWDSSTVVF	IGL	3151	C4	7JVC
3	2307	CARVNRLDTNPTTPLFFDFW	IGH	2	None	
3	2308	CARTDYDSKGCRFDSW	IGH	3	None	
3	2309	CAHRRTSYDSGGNFDSW	IGH	27	None	
3	2310	CARYINLEVIFFSDYW	IGH	2	None	
3	2311	CASLFINWGLPDLYYYYGMDVW	IGH	2	None	
3	2312	CARGHGSWFDPW	IGH	50	None	
3	2313	CAKGWAGLLWFGEFYDYW	IGH	4	None	
3	2314	CARDDDYYDSSGYILPFDYW	IGH	2	None	
3	2315	CTHRLHRRDSSGPGYFHHW	IGH	13	None	
3	2316	CARAAGITMIVVVIAAFDIW	IGH	19	None	
3	2317	CASLPYGSGVIRFDPW	IGH	8	None	
3	2318	CARIPPPYYFNNSDYYSRRSGPAAFDIW	IGH	2	None	
3	2319	CASSYSGSYYSWFDPW	IGH	28	None	
3	2320	CATAPGIAVAGGGGNWFDPW	IGH	20	None	
3	2321	CAREIRLVGADYYYYGMDVW	IGH	8	None	
3	2322	CAKDLAPSIAAAGGDYW	IGH	4	None	
3	2323	CARVPDDIDTTVVDYW	IGH	3	None	
3	2324	CARFGFDIVATPHGAFDIW	IGH	2	None	
3	2325	CAREHRIGRTIDYW	IGH	2	None	
3	2326	CAREDYSGDPPFDHW	IGH	3	None	

3	2327	CNSRDSSSNVLGVIF	IGL	4	None	
3	2328	CARGQGSITMVRGFDPW	IGH	2	None	
3	2329	CAKDIRAERGYSYGLYYYYGMDVW	IGH	2	None	
3	2330	CVKAPDYYDILTGYPYYFDAW	IGH	2	None	
3	2331	CARHTGVTVTTEVLDYW	IGH	2	None	
3	2332	CAKDSRLGIDEVIFDYW	IGH	2	None	
3	2333	CARAPSGGTIFGVVIIAYYYGMDVW	IGH	3	None	
3	2334	CATDRGPYSSSLGFDPW	IGH	21	None	
3	2335	CARADDILTGYSVYFDYW	IGH	8	None	
3	2336	CARDPIVLMVYPPVGWFDPW	IGH	3	None	
3	2337	CARMYYDILTGYSNWFDPW	IGH	16	None	
3	2338	CAAWDDSLNGWVF	IGL	3093	None	
3	2339	CATGGYEMATILRGGVLRGFDPW	IGH	9	None	
3	2341	CARDITPGYNIGWHFGFDYW	IGH	4	None	
3	2342	CAKDQDYGDYDDKPRGFDYW	IGH	22	None	
3	2343	CARDWILGELSGPLDYW	IGH	7	None	
3	2344	CASYVVVTAITNWFDPW	IGH	2	None	
3	2346	CATHYGDYASYYYYGMDVW	IGH	8	None	
3	2347	CTTEAVYCSGSSCPGYFDFW	IGH	21	None	
3	2348	CARDLNPLVEMATFLFDYW	IGH	2	None	
3	2349	CARVPVGRGSPPGYYYYGMDVW	IGH	4	None	
3	2350	CARPGHGYNDFHYW	IGH	4	None	
3	2351	CARVDPPRSNYDILTGLLGANYYYYGMDVW	IGH	2	None	
3	2352	CARSNSYSGVPRDFDYW	IGH	6	None	
3	2353	CAKDLWDLALWFGELFPIPDYW	IGH	2	None	
3	2354	CARDYYDSLTDFYDYYFDQW	IGH	2	None	
3	2355	CARDVSTMVRGVIITFDYYYGMDVW	IGH	3	None	
3	2356	CARLSDCDEDVCYRGDYW	IGH	2	None	
3	2357	CARDHYYSSWAYYKFDYW	IGH	2	None	
3	2358	CARRVWRVAASRDRKSGPNYFDLW	IGH	2	None	
3	2359	CAKDLLQTSGGYYYYYYGMDVW	IGH	4	None	
3	2360	CQSYDNSLSGRLWLF	IGL	3	C4	7JVC
3	2361	CGRRQKYFESGFYQYAFDMW	IGH	3	None	
3	2362	CAKDVYRSGYDPQIDYW	IGH	2	None	
3	2363	CAKDLQLSDWTPDYW	IGH	2	None	
3	2364	CARDSFDSSGYLGWYFDYW	IGH	9	None	
3	2365	CARDTTVVAATPEVEGGYYYYGMDVW	IGH	4	None	
3	2367	CQQYHSWPRGYTF	IGK	14	C4	6W41
3	2368	CAKIATNWPGPSFDYW	IGH	5	None	
3	2369	CGRIAAAGTGPIDYW	IGH	2	None	
3	2371	CAKDNDFWSGGDWFDPW	IGH	10	None	
3	2372	CARDIVVVPAAQGDYYYYYGMDVW	IGH	15	None	
3	2373	CARIRRTTGTTSVGVPFDYW	IGH	5	None	
3	2374	CASHRITMIVVVILDYW	IGH	25	None	
3	2375	CASGEYDILTGYYSEGYYYGMDVW	IGH	2	None	
3	2376	CATGLYYDRSGYYVGIDYW	IGH	2	None	
3	2377	CATGSGTVTTGWFDPW	IGH	4	None	
3	2378	CQHLNSHPLPF	IGK	4	C1	7BZ5
3	2379	CARDGGITGTTPALPNNNYYYGMDVW	IGH	4	None	
3	2381	CATTFYVVVVAAPFDYW	IGH	3	None	
3	2382	CARGSEDITMIVVVTNNDAFDIW	IGH	2	None	
3	2384	CTHRRGYGSTARFDSW	IGH	2	None	
3	2385	CAKDKVVVVPAAMGGYYYYYGMDVW	IGH	4	None	
3	2386	CARVSKASQWSSIPFAYFDSW	IGH	3	None	
3	2387	CAKIERLWAGIDYW	IGH	2	None	
3	2388	CAKDLGEWPPDYW	IGH	11	None	
3	2389	CAKDKGLWFGEQYYFDYW	IGH	2	None	
3	2390	CARNSEQPRYNWARGWFDPW	IGH	2	None	
3	2391	CTTLGGATDYW	IGH	15	None	
3	2392	CARHLITMVRGVIIAGYYYYGMDVW	IGH	2	None	
3	2393	CARAGVGLSYYGSGSYLPRWDWFDPW	IGH	2	None	
3	2395	CVREGRHLGVAAYGDCW	IGH	2	None	
3	2396	CSRGSANSGDVVVLLGAEIHFDYW	IGH	5	None	
3	2397	CAKPPHVVRGDDYYSGFDVW	IGH	2	None	
3	2398	CARRVEDYYGSGSPLSEYYFDYW	IGH	8	None	
3	2399	CARAVEYYYDSSGYYLEYFQHW	IGH	6	None	

3	2400	CARDPTQTVSSSSFYYYYGMDVW	IGH	38	None	
3	2401	CARGSWKYFDISTGYYNPGSFDYW	IGH	3	None	
3	2402	CAKDLDSGYNLGETHDSFDMW	IGH	23	None	
3	2403	CAGPHDLHALLMLREVDVYYYGMDVW	IGH	10	None	
3	2404	CARARHSSSWYEGGNWFDPW	IGH	3	None	
3	2405	CARVPDTWYDYW	IGH	47	None	
3	2406	CAREPGYCSRGSCYTSFDPW	IGH	3	None	
3	2407	CARDRGKVVPLGAATDAFDLW	IGH	7	None	
3	2408	CQHRNNWPLSF	IGK	14	None	
3	2409	CANLGGFGESAFDIW	IGH	3	None	
3	2410	CATAIVVVPAATNAVQPQNAQNYYYGMDVW	IGH	2	None	
3	2411	CARGASSPNSSGWYGYYYYGMDVW	IGH	2	None	
3	2412	CSGGSCYSRAPPETRYYYYGMDVW	IGH	2	None	
3	2413	CARMAFKWLPGEDAFDIW	IGH	2	None	
3	2415	CAKLLEWELQGDAFDIW	IGH	4	None	
3	2416	CARGGLWEAHSNGYQESDYW	IGH	3	None	
3	2417	CAIWYSNTYVF	IGL	168	None	
3	2418	CAKEGPTVTTKYYYYYYGMDVW	IGH	4	None	
3	2419	CARGPDCSGGSCFVAPFDPW	IGH	2	None	
3	2420	CASSSYIYGTFGTRYYAIDVW	IGH	5	None	
3	2421	CQQSGYSPVSFTF	IGK	2	None	
3	2422	CQSVDTSGTSLVF	IGL	11430	None	
3	2424	CHSATDNNVPYVF	IGL	2	None	
3	2425	CAKAPSSGYYADAFDIW	IGH	5	None	
3	2426	CARHLGCSGGSCYWGYDYW	IGH	6	None	
3	2427	CAKAHDNNGYYGSDFDSW	IGH	2	None	
3	2428	CAKIAAADTDAFDIW	IGH	12	None	
3	2429	CAKVAEYYYGSGDYW	IGH	2	None	
3	2430	CARDGAARYDSSGYYYPPYYYYGMDVW	IGH	5	None	
3	2431	CARALSGYSYGLDYW	IGH	2	None	
3	2432	CAKDLIVVVPKGYFDSW	IGH	2	None	
3	2434	CTKGPVSSDRGWYFDLW	IGH	15	None	
3	2435	CAKLTDGYNHYFDYW	IGH	29	None	
3	2436	CATAFIITSQNTQAYYYYYGMDVW	IGH	2	None	
3	2437	CAKADDYAQQRGAFDIW	IGH	7	None	
3	2438	CAKDGGGITMIVVVISPTFDYW	IGH	12	None	
3	2439	CARGLRYFDWLVRSNYYYGMDVW	IGH	4	None	
3	2440	CARTYTDDGDYGGGALDIW	IGH	3	None	
3	2441	CAKGYNSAWYGGVDYW	IGH	6	None	
3	2442	CAKDVFLDDYDIFEPQAFDPW	IGH	2	None	
3	2443	CARDQDYDILTGYYQWGYGGPVNWFDPW	IGH	4	None	
3	2445	CARGRITIFGVVIGWFDPW	IGH	5	None	
3	2446	CAKIEAGTGTTDYW	IGH	2	None	
3	2447	CATYFLDGMDVW	IGH	35	None	
3	2448	CTTGSSTTIFGVVIILGAFDIW	IGH	4	None	
3	2449	CARGITCSGGSCYSELDYW	IGH	3	None	
3	2450	CVKDGDDHPAFDIW	IGH	4	None	
3	2451	CALMALSPTDTYFPSW	IGH	4	None	
3	2452	CARVGAGVETTIDYW	IGH	16	None	
3	2453	CTIRVDTAMAPIFDYW	IGH	4	None	
3	2454	CTRDRGYGYPELVFDIW	IGH	3	None	
3	2455	CAKVAYYDSSGYLGYYFDYW	IGH	4	None	
3	2457	CARVDGDRTYNWFDPW	IGH	7	None	
3	2458	CAKDLKYYDILTGYFAPPNYYYYYGMDVW	IGH	9	None	
3	2459	CAADVRYYDWFFSGSDAYEVW	IGH	3	None	
3	2460	CIAEDPPYYYYGMDVW	IGH	3	None	
3	2461	CYCSADNNRVF	IGL	348	None	
3	2462	CGRMTGDFWRGSPQPNSMYAMDVW	IGH	4	None	
3	2463	CARHVWGHGDSGLDYW	IGH	2	None	
3	2466	CARVPRHYDETSGGLGYFDLW	IGH	2	None	
3	2468	CARERWYPIAAAVYYYYGMDVW	IGH	2	None	
3	2470	CARNPVGIAAPLGWFDPW	IGH	5	None	
3	2471	CASIAAAEGATYYYYGMDVW	IGH	69	None	
3	2472	CAKGTSLGDDAFDIW	IGH	4	None	
3	2473	CATLYDSSGYPHSSTVDYW	IGH	4	None	
3	2474	CATTQTHSSSWYRGDPFDVW	IGH	3	None	

3	2475	CQSSDYSGDNRWVF	IGL	59	None	
3	2476	CAKDLAYYDILTGYFPAGSSSTYYYYGMDVW	IGH	4	None	
3	2479	CAKSFPSGWYHFDYW	IGH	4	None	
3	2480	CAKGGIGSYFTYFDYW	IGH	7	None	
3	2481	CAKAPDSSGWYGAFSYW	IGH	2	None	
3	2482	CAREFCSSTSCYFDYW	IGH	5	None	
3	2483	CARRPYCSSTTCFLHFDFW	IGH	2	None	
3	2484	CARSFLHYSGSSGYYSGSFDYW	IGH	4012	None	
3	2485	CAKVGYYDSSGYLYPGWFDPW	IGH	2	None	
3	2486	CAKRRVRPDPWIQLWLPSYYYGMDVW	IGH	7	None	
3	2487	CQSRSDWPVAF	IGK	8	None	
3	2488	CARRGRYGGPDYW	IGH	21	None	
3	2489	CAREAVAVWLSTDYW	IGH	4	None	
3	2490	CASLIDVEDYYYGMDVW	IGH	2	None	
3	2491	CATGPQRWLQLDYW	IGH	4	None	
3	2492	CAKVGGIAAAGSFYYYGMDVW	IGH	4	None	
3	2493	CASEGVINRDAIIDYW	IGH	2	None	
3	2494	CASHYVWGSYRQYGGGFDYW	IGH	3	None	
3	2496	CQTWDTVIPGLF	IGL	2	None	
3	2497	CQHYNNGPPVMYTF	IGK	2	C3	6XDG
3	2498	CATAPSVRYDILTGSYYYYYGMDVW	IGH	3	None	
3	2499	CARDMVGTTVRTVHNWFDPW	IGH	3	None	
3	2500	CARAEIQVDDFWSGYFHMGAQYYYGMDVW	IGH	3	None	
3	2501	CATRRKFDWLLLDYW	IGH	3	None	
3	2502	CAKAYLSGSQTTNYYYYGMDVW	IGH	8	None	
3	2503	CARARRGITMIVVVITTTFDYW	IGH	2	None	
3	2504	CARDISSGWYVDYYGMDVW	IGH	2	None	
3	2505	CATSNLNRAMDVW	IGH	3	None	
3	2506	CTPTYDFWSGNYFDYW	IGH	2	None	
3	2507	CAHMFYDILTGYYINWFDPW	IGH	2	None	
3	2508	CARASHGVLAAGTNYYYGMDVW	IGH	5	None	
3	2509	CARQRDTMIVVVEYYFDYW	IGH	26	None	
3	2510	CARDQIPVTGDRYYYYGMDVW	IGH	2	None	
3	2512	CASGHATYYDILTGYYIGQDYFDYW	IGH	4	None	
3	2513	CARGHAVAGTYDYW	IGH	31	None	
3	2514	CVKNTEYDFWSGYAPFYFDSW	IGH	6	None	
3	2515	CARPSYGDPSYYYYYYGMDVW	IGH	2	None	
3	2516	CAKDIERFWYHYYYGMDVW	IGH	7	None	
3	2517	CAKPHGGYYYGYFDYW	IGH	2	None	
3	2518	CAKPNFDWLWDAFDVW	IGH	2	None	
3	2519	CAAWDDNLSGPIWVF	IGL	6	None	
3	2520	CSSTDSSGTYRMF	IGL	9	None	
3	2521	CARPDSSASYWFFDFW	IGH	2	None	
3	2522	CAKDSVWFGEFFVFDYW	IGH	3	None	
3	2523	CAKLPYCSSTSCYVGDYW	IGH	2	None	
3	2524	CARFGYGDYVSLTYYHYGMDVW	IGH	6	None	
3	2525	CAKDGIPANGRWDSFDLW	IGH	2	None	
3	2526	CAKDSTAAGLWDYW	IGH	4	None	
3	2527	CASYAGSNNVIF	IGL	50	C2	7BWJ
3	2528	CAKDKGEGSGWSPPYFDYW	IGH	2	None	
3	2529	CTTVLLWFGEPLGDDYW	IGH	4	None	
3	2530	CGESHTIGGQVGWVF	IGL	4	None	
3	2531	CARDSVNGHDFWSGHVDYW	IGH	4	None	
3	2532	CARTPYSEQWLVRWGYYYGMDVW	IGH	2	None	
3	2533	CARAEEGGFDWLFNSDYYYYGMDVW	IGH	3	None	
3	2534	CARDGGVLTGSRNYFDYW	IGH	2	None	
3	2535	CARGIRLNYDILTGYWWEYYYYGMDVW	IGH	5	None	
3	2536	CAKAPGCSGGSCPRIWFDPW	IGH	2	None	
3	2537	CARAPNPTYSSGHYFDYW	IGH	2	None	
3	2538	CARLRIAVAGSGVYYYYGMDVW	IGH	2	None	
3	2539	CARDPVKVGATGFDPW	IGH	4	None	
3	2540	CATQRVGADHFDYW	IGH	2	None	
3	2541	CAHRGGIAADGLRGVFDYW	IGH	6	None	
3	2542	CTPSWLHELFPNTGSFSYW	IGH	4	None	
3	2543	CGRSVFDSTLYYFEYW	IGH	6	None	
3	2544	CARVDWFGDISNPFFWYFDPW	IGH	96	None	

3	2545	CARISAAAGSAIDYW	IGH	8	None	
3	2546	CARAGTGYSSSWYLYWYFDLW	IGH	2	None	
3	2547	CARDGPGTPFGYW	IGH	3	None	
3	2548	CARSDCSSSVCPWGWDYW	IGH	2	None	
3	2549	CAKDAGDGYYYDSSGYPRYFDLW	IGH	4	None	
3	2550	CVKQYQAIAAAGFFDYW	IGH	2	None	
3	2551	CAKMYYSDNSGYFYGPPHAFDTW	IGH	2	None	
3	2552	CARGRRGPTTLLGPFGGVIVDKEFDYW	IGH	2	None	
3	2553	CAFPLSIVRGGASDYW	IGH	4	None	
3	2555	CARVYCTNGVCVDYW	IGH	2	None	
3	2556	CARDPTKVWGSYPTYYFDYW	IGH	6	None	
3	2557	CARSGDTVVVPAGEARSNWLDPW	IGH	2	None	
3	2558	CARHVGYSSGYPTPPYYFDYW	IGH	3	None	
3	2559	CQSADTGGPYVVF	IGL	11	None	
3	2560	CAREGSYGDLLTVKGYAFDIW	IGH	3	None	
3	2561	CQQYSSYSPSMYIF	IGK	3	C3	7K8R
3	2562	CARTYIAAGSLGFDPW	IGH	5	None	
3	2563	CARVRDARYCSAGRCSGWFDPW	IGH	3	None	
3	2564	CARVWNYYHSSGDYLYYIDYW	IGH	17	None	
3	2565	CARLVDTAMGYYYYGMDVW	IGH	3	None	
3	2566	CARHGGTMMVVVITVFDYW	IGH	6	None	
3	2567	CAKHDYGDYSGTFDIW	IGH	4	None	
3	2568	CATSRGIVNEYDFWSGPIRGESASAVDVW	IGH	4	None	
3	2569	CARGVTFGGPRGLYPRGNYALDVW	IGH	2	None	
3	2570	CARDLGYFGTAKWAWLDPW	IGH	2	None	
3	2571	CARVGLGIFGVVTLGFDPW	IGH	2	None	
3	2572	CATATVLVWRTTGYYYYYGMDVW	IGH	17	None	
3	2573	CAGKLRGRESRWFDPW	IGH	4	None	
3	2575	CANLLGYYYGSGSNTFDYW	IGH	4	None	
3	2576	CARGKAVVGIMGMWFDPW	IGH	3	None	
3	2577	CAGGLINDYSNYYYYTFDVW	IGH	2	None	
3	2579	CAKVSSSFDPSDYW	IGH	2	None	
3	2580	CASPRNDYDILTGSFDYW	IGH	3	None	
3	2581	CATFPRYWFGEFWYW	IGH	11	None	
3	2583	CAKDQWYGSGSYYNPNYYYYGMDVW	IGH	3	None	
3	2584	CQSYDTSLTDNYVF	IGL	27	C4	7JVC
3	2585	CARGYAGSAHYYKSPFDYW	IGH	5	None	
3	2586	CTKGDLSAMTSLWGYFQHW	IGH	4	None	
3	2587	CCSYAGSYLF	IGL	22	C2	7K8X
3	2588	CAKNVIWGKWSPLYFDSW	IGH	2	None	
3	2589	CARDGVWNEAFDIW	IGH	4	None	
3	2590	CARDRVDGSGWSYYFDYW	IGH	6	None	
3	2591	CARGGYDYTPLFDYW	IGH	7	None	
3	2592	CAKIPGGQWLVSDAFDIW	IGH	6	None	
3	2593	CARVGIEVVPNFDYW	IGH	3	None	
3	2594	CARSNAGPAVLDYW	IGH	12	None	
3	2595	CAHLVYGDSLGFDYW	IGH	10	None	
3	2596	CAKLNNRYSYGDYW	IGH	6	None	
3	2597	CASHPRGYDILTGYDYW	IGH	2	None	
3	2598	CARNDDSRTYYDILTGYYRNYGMDVW	IGH	6	None	
3	2599	CARLTTLHDYVWGSYRFNWFDPW	IGH	2	None	
3	2600	CARAPREAYDSTGYPDYW	IGH	2	None	
3	2601	CTRGVEYATSGYSVYW	IGH	3	None	
3	2602	CARGVVSPGYYILDQFSWFDPW	IGH	2	None	
3	2603	CAKAAGDYYDSSGYSDYW	IGH	22	None	
3	2604	CARVGYCSDGRCHNWFDPW	IGH	2	None	
3	2605	CAHRRFISRADYFDYW	IGH	2	None	
3	2606	CVKDLDYYDGSAYYFDHPFDSW	IGH	4	None	
3	2607	CARADNGELLRNNWFDSW	IGH	2	None	
3	2608	CAKAEIKITFGGLIVPSYYYGMDVW	IGH	2	None	
3	2609	CARVKAGIGSWTLYYYYGMDVW	IGH	5	None	
3	2611	CTAWSRGEEWFDPW	IGH	4	None	
3	2613	CAREDDGVIDYW	IGH	121	None	
3	2614	CQSADDSGGYDYVLF	IGL	10	None	
3	2615	CVRGQFYGLGGTFDKW	IGH	3	None	
3	2616	CARDFVVVPPAMYYYYGMDVW	IGH	6	None	

3	2617	CAREISYGDSTYFDYW	IGH	2	None	
3	2618	CARHATMIVVVITPWFDPW	IGH	3	None	
3	2619	CAPLSDLLDYW	IGH	4	None	
3	2620	CARDVRVPAGGKSYYYDGMDVW	IGH	5	None	
3	2621	CAKDISPIIAAAGTGYFDYW	IGH	3	None	
3	2622	CARAERVAVAGSYWYFDLW	IGH	5	None	
3	2623	CAKATLLLTGYLYYFDYW	IGH	6	None	
3	2624	CARANWVSMTVENWFDPW	IGH	2	None	
3	2625	CARNGRTIGEGPLDYW	IGH	2	None	
3	2626	CAANILVVESFDLW	IGH	8	None	
3	2627	CATAPTVVTPEDYYYYYGMDVW	IGH	2	None	
3	2628	CARDLEPPPAAVDAFDLW	IGH	8	None	
3	2630	CARTINYYGSGSPDYW	IGH	2	None	
3	2631	CARGGTLAWYFDLW	IGH	5	None	
3	2632	CATGVLLWFGGDNWFDPW	IGH	4	None	
3	2633	CAKGIEYGRTDENYFDYW	IGH	2	None	
3	2635	CTKIRRARNYYDYAMDVW	IGH	14	None	
3	2636	CARLEKGQWHGYYFDYW	IGH	2	None	
3	2637	CAHRLPSSWYGEWFDPW	IGH	7	None	
3	2638	CARVGPFYETLTGFWTQMNWFDPW	IGH	2	None	
3	2639	CARGPLGGSPDWCFDLW	IGH	2	None	
3	2640	CAKDLRYYDILTGYYPSHQYYYYYGMDVW	IGH	6	None	
3	2641	CTTDLPKDSSVYYQDDAFDIW	IGH	2	None	
3	2643	CAKLSSGWYEGFDFDYW	IGH	8	None	
3	2644	CAKGSFGIAAASVDYW	IGH	2	None	
3	2645	CAKKPGIAAAGTSGWFDPW	IGH	2	None	
3	2646	CARDVGDCPDYW	IGH	16	None	
3	2647	CLLSYSGLGGGYVVF	IGL	4	None	
3	2648	CARVGRTLLWFGEFSYFDYW	IGH	4	None	
3	2649	CARAGYCSGGSCYYYYGMDVW	IGH	14	None	
3	2650	CAALMITFGGIMPVEDGLDVW	IGH	9	None	
3	2652	CAKDGRDYYGSGSYGASFDYW	IGH	3	None	
3	2653	CARMYYDILTGYLGGYYYYGMDVW	IGH	15	None	
3	2654	CARARRWQQMVEAFDYW	IGH	2	None	
3	2655	CATGALLRGSWFDPW	IGH	4	None	
3	2656	CAHRIYDSGGYYDYYFNYW	IGH	5	None	
3	2657	CAKFNRLLWFGELSDDAFDIW	IGH	4	None	
3	2658	CARGLEDPLDVW	IGH	2	None	
3	2659	CTTTLRYFPGYYYYGMDVW	IGH	2	None	
3	2660	CARVIPGIAAEYFDYW	IGH	2	None	
3	2661	CARDLGSYDSSGWAFDIW	IGH	2	None	
3	2662	CARGPYSGTYYGHFDYW	IGH	6	None	
3	2663	CAKMDHHETYYYGSGTYDYW	IGH	3	None	
3	2664	CATIHYYDFWSGFYYGDYW	IGH	2	None	
3	2665	CVKDHEQWLLEYFHRW	IGH	2	None	
3	2666	CARTFRGVIRDDAFDIW	IGH	5	None	
3	2667	CASITYYYDSTPGYFDYW	IGH	4	None	
3	2669	CARDLLSSPEWFGELLSPFSYYYYGMDVW	IGH	10	None	
3	2670	CARGEVVLTSDLCDYW	IGH	2	None	
3	2671	CARDGDDYYGSGYSNWFDPW	IGH	2	None	
3	2672	CAKALGETYYGSGSRDYW	IGH	2	None	
3	2673	CARAPSGTLNPFNFDYW	IGH	15	None	
3	2674	CAREEVGATWVNWYFDLW	IGH	5	None	
3	2675	CAKEGGLNIAAAGYGVYFDYW	IGH	4	None	
3	2676	CAKDHICNSGCTAWFDSW	IGH	6	None	
3	2677	CMQALQTPLTF	IGK	4562	None	
3	2678	CARQNGGPGPFDIW	IGH	6	None	
3	2679	CARGSPVEMATITMDYW	IGH	2	None	
3	2680	CARQSRLGDTRNAFDIW	IGH	20	None	
3	2681	CARWKAVPAMADYGDYPGYFDYW	IGH	10	None	
3	2682	CARHILGPYGSGSYHFDYW	IGH	4	None	
3	2683	CARGITMIVDDGMDVW	IGH	2	None	
3	2684	CAGEGVSKGYYTGLPGNDALDAW	IGH	2	None	
3	2685	CARDNGEWFPHDYW	IGH	3	None	
3	2686	CAKVGEAGTPFDYW	IGH	4	None	
3	2687	CAKAPDIVVVVAASHFDYW	IGH	2	None	

3	2688	CARSPITMVRGFYWTGLIVSDYFDYW	IGH	2	None	
3	2689	CAHDDDGRHYYYVSWFDNW	IGH	6	None	
3	2690	CARNRRIEVGGTTVDAFDSW	IGH	3	None	
3	2691	CASPRGYSYGPFDYW	IGH	19	None	
3	2692	CARVGAPITMVRGVIRYWFDPW	IGH	3	None	
3	2693	CAKFEGGLNPGPYYYDSTGDYW	IGH	6	None	
3	2694	CARDYSSGWYGGNYFDYW	IGH	12	None	
3	2695	CAKAKIDPDYYDSSDFFDALDVW	IGH	2	None	
3	2696	CARRSSSWYGTPGDWFDPW	IGH	12	None	
3	2697	CARELVTQAAAGVYYYYGMDVW	IGH	24	None	
3	2698	CAKDRGYGDYDWLGNYYYYGMDVW	IGH	5	None	
3	2699	CARELLMGSSFFDSW	IGH	3	None	
3	2700	CARFGYSYSGDADAFDIW	IGH	3	None	
3	2701	CAGGPSFYIYYYGMDVW	IGH	2	None	
3	2702	CAREGALTPGFDLW	IGH	8	None	
3	2704	CARDRDYYGSGSYSLYYYYGMDVW	IGH	5	None	
3	2705	CAKDSLLGYYDSSMGWFDPW	IGH	3	None	
3	2706	CAREDDDFWSGYYTRYFDYW	IGH	3	None	
3	2707	CAAHLSVRAVFDYW	IGH	6	None	
3	2708	CAKDKGDGYNPRYYYFDYW	IGH	4	None	
3	2709	CARVLRGTASIMRFDAW	IGH	3	None	
3	2710	CQQYHNWPLAF	IGK	56	C3	6XDG
3	2712	CATFMVAATRGQQTYYYGMDVW	IGH	2	None	
3	2713	CTRVWALGAAGTAWFDPW	IGH	2	None	
3	2714	CARGARRVRATITPPAADYW	IGH	4	None	
3	2715	CARRVSDILVRRPFDYW	IGH	4	None	
3	2717	CAKHDCSSTSCPPDYW	IGH	9	None	
3	2718	CQSADSSGTYRGNWVF	IGL	8	None	
3	2719	CARGVARHCSSTSCYVDYYYYYGMDVW	IGH	2	None	
3	2720	CATTENSDFDWLRGRYYYGMDVW	IGH	4	None	
3	2721	CARATIGYCSGGSCLPGFDYW	IGH	4	None	
3	2722	CARIVGGVVGGWPSVVATDDALEIW	IGH	3	None	
3	2723	CATELPRVRSSGWYLGYW	IGH	9	None	
3	2724	CARMTPTDYDILTGYFADAFDIW	IGH	2	None	
3	2725	CAKDRTYSYGNYFDYW	IGH	4	None	
3	2726	CARDTPGFYYYFDNW	IGH	2	None	
3	2727	CAGQLVESLDYW	IGH	15	None	
3	2729	CASNRRSGYYYYYYDMDVW	IGH	4	None	
3	2730	CAKVSLDLQVPADGFKYYFDYW	IGH	7	None	
3	2731	CVKDHVEGGAGWFDSW	IGH	3	None	
3	2732	CVKGFWSGYHPFDYW	IGH	2	None	
3	2733	CVKGRSYSYYNFDYW	IGH	2	None	
3	2734	CARFSTFSLVRAWFDPW	IGH	49	None	
3	2735	CANLPTVTPGSDGAFDIW	IGH	2	None	
3	2736	CAKDITEFYEVVGDDGFDIW	IGH	2	None	
3	2737	CARSHMAAPGPNAERGPWLDPW	IGH	4	None	
3	2738	CQQLHSYKITF	IGK	35	C1	7BZ5
3	2739	CAKGSGSGYYSSTSCEGDYFDYW	IGH	3	None	
3	2740	CAKDMWDGYSYGYGGLKYYYGMDVW	IGH	3	None	
3	2741	CARAAVLRYFDWLLPAVNWFDPW	IGH	2	None	
3	2742	CAKDNHLSDWNYGIDYW	IGH	2	None	
3	2743	CARDQMATIMGYNWFDPW	IGH	3	None	
3	2744	CARDAGYPGSISYKFDQW	IGH	3	None	
3	2745	CAREEPGIAAAENYFDSW	IGH	2	None	
3	2746	CAKIPTPYCSSTSCRDYW	IGH	5	None	
3	2747	CARQPIPLLLKAPGWFDPW	IGH	6	None	
3	2748	CTRSQWQQLEFDSW	IGH	2	None	
3	2749	CAKVTSEGDFDFDYW	IGH	15	None	
3	2750	CVGEEDGGTHAFDIW	IGH	3	None	
3	2751	CARLSGQWLASGWFDPW	IGH	4	None	
3	2752	CARDEPGYYGDWGPFDYW	IGH	2	None	
3	2753	CQHYGSSPPLTF	IGK	214	C1	7K8N
3	2754	CARDWAGATYAFDIW	IGH	4	None	
3	2755	CAKIAIVGAPGDYW	IGH	4	None	
3	2756	CATPRQQLVPIWYFDYW	IGH	10	None	
3	2757	CARAASAVVVMVYAGDAFDLW	IGH	3	None	

3	2758	CAKDGGLPMPFYYFDYW	IGH	4	None	
3	2760	CAGGYSYGYVDYW	IGH	3	None	
3	2761	CARESGFDDTGYLHYLDLW	IGH	2	None	
3	2762	CAKEGYDSSGYYYHYW	IGH	3	None	
3	2763	CAKQLYDILTGYYIPPFDYW	IGH	8	None	
3	2764	CVKGVPGALDGVDIW	IGH	4	None	
3	2765	CTNLDILTGHHDYW	IGH	2	None	
3	2766	CAKDRGPIPASMDYW	IGH	2	None	
3	2767	CAASNYYDSSGYAFDIW	IGH	2	None	
3	2768	CARDQFAFWYFDLW	IGH	4	None	
3	2769	CARDMVEQWLGYFDYW	IGH	5	None	
3	2770	CARSYDYGEYIGGPEPCFDYW	IGH	10	None	
3	2771	CARDKGGRWPFDYW	IGH	9	None	
3	2772	CARDGDIAMVTDALDVW	IGH	2	None	
3	2773	CARGQRDRSNTWYGFDYW	IGH	5	None	
3	2774	CAKDGVMAPGAFDIW	IGH	2	None	
3	2775	CARGGSGWDSLGFFDYW	IGH	13	None	
3	2776	CAKDGDSSSWYDYYYYYGMDVW	IGH	2	None	
3	2777	CARSGPLGGVDYW	IGH	11	None	
3	2778	CARDGENYYDSSSGYYYYGMDVW	IGH	3	None	
3	2779	CHSVDSSGAFRVF	IGL	8	None	
3	2780	CARSPQTRVQRDLYFFDYW	IGH	2	None	
3	2781	CAKDIGTYYYDSSGYADAFDIW	IGH	4	None	
3	2782	CTPIDSSDWWEWFDPW	IGH	4	None	
3	2784	CAHRLPGTGGWFDPW	IGH	2	None	
3	2785	CAILGWDTVVVPVDEDYFDSW	IGH	3	None	
3	2786	CGRAQSGYTYIDHW	IGH	9	None	
3	2787	CARNKNYYHTSGYFDSW	IGH	14	None	
3	2788	CARGGFDALAGFGEIYYFAMDVW	IGH	2	None	
3	2789	CATAHVDCWNTNCYSYYHFYAMDVW	IGH	2	None	
3	2791	CASGLPGYTSRPHFDYW	IGH	7	None	
3	2792	CAKGGLPVAGFVDYW	IGH	2	None	
3	2793	CARSTTSADFDNW	IGH	47	None	
3	2795	CARDRSSDLVGWFDPW	IGH	2	None	
3	2796	CASLEFFGVVMTKYFVYW	IGH	2	None	
3	2797	CAKMGNEYGDYDDYW	IGH	2	None	
3	2798	CARLPFGDPYFDYW	IGH	4	None	
3	2799	CAREGVVGRVWGSYRYYGMDVW	IGH	2	None	
3	2800	CVHSLADYDAFDLW	IGH	2	None	
3	2801	CASHHTPWAVAGSFDYW	IGH	3	None	
3	2802	CTREVLGGFYYW	IGH	2	None	
3	2803	CARGGAYYDFWSGYSPYYYYGMDVW	IGH	2	None	
3	2804	CARDPAYYDILTGYLSPAYYYYYGMDVW	IGH	2	None	
3	2805	CARYSYSNYEFGYYYGMDVW	IGH	2	None	
3	2806	CAREEIAAAGTNWFDPW	IGH	6	None	
3	2807	CARDPLSGELANALDIW	IGH	8	None	
3	2808	CARSHLTLGPSSQLDGMDVW	IGH	44	None	
3	2809	CARTNYDYVWGRSGYFDYW	IGH	2	None	
3	2810	CARDNTQRSGYTYWFDPW	IGH	17	None	
3	2811	CTRHDPLAYYDSSGRLDDYYYGMDVW	IGH	2	None	
3	2812	CARDSGGYASRLLDYW	IGH	4	None	
3	2813	CAKAKPRGYSGYEGFDYW	IGH	2	None	
3	2814	CARDPRVRYSGYVVEPGYFDYW	IGH	2	None	
3	2815	CAKCGLSGGNCYSSAMDVW	IGH	2	None	
3	2816	CARVSSSYYRYWHFDLW	IGH	4	None	
3	2817	CAKPLRRQEPALTLVNDWGLTDYW	IGH	2	None	
3	2818	CAREEGVIGGYDYW	IGH	2	None	
3	2819	CARDFPLYAFWSGFYPNSFDPW	IGH	2	None	
3	2820	CARDLKWLRSGGDYYYAMDVW	IGH	45	None	
3	2821	CAKDMRPTVTGFDYW	IGH	11	None	
3	2822	CAKDHLYLYYGSGSYYGIDYW	IGH	2	None	
3	2823	CTTGLGRTYYYDSWGWRHFRAFDYW	IGH	5	None	
3	2824	CAKVRIAAAGPFDYW	IGH	30	None	
3	2825	CAREGDIVVVTDCMDVW	IGH	4	None	
3	2827	CARDSPPLSGWSDRDYW	IGH	5	None	
3	2828	CAKDGGGYYYDSSGYYSPRYYYYGMDVW	IGH	4	None	

3	2829	CSRYWELFSHDAFDIW	IGH	2	None	
3	2830	CAHRPAPYNSNWYSWVDPW	IGH	2	None	
3	2831	CAKDKYPSGGTDYYYGMDVW	IGH	3	None	
3	2832	CARTDHPEENYYESSGYYYYFDYW	IGH	16	None	
3	2833	CARSGLAVAAMPLDYW	IGH	2	None	
3	2834	CLSAHSSGSYWVF	IGL	13	None	
3	2835	CARHPYYYDSSGRNGHFDYW	IGH	6	None	
3	2836	CVRDFGNGTPYYFDFW	IGH	11	None	
3	2837	CAKDMGPYLGWFDPW	IGH	9	None	
3	2838	CTKGYYYDQSRFDSW	IGH	4	None	
3	2839	CARDVRYYDSSGYYSPYYYYGMDVW	IGH	28	None	
3	2840	CAKGGGSGSYSLYYYYGMDVW	IGH	5	None	
3	2841	CARDSQWELLDYW	IGH	13	None	
3	2842	CASRPAYSFETGHYSFDSW	IGH	2	None	
3	2843	CAKQGSIGLYFYYGMDVW	IGH	65	None	
3	2844	CAKSGGYDYYW	IGH	11	None	
3	2845	CVKVSSYYNILIGYYSSFDHW	IGH	4	None	
3	2846	CAKDMGDGYSSGQGHFDYW	IGH	2	None	
3	2847	CARVRLIQDKYYFDYW	IGH	2	None	
3	2848	CAHNYILTDYWDAFDLW	IGH	2	None	
3	2849	CARARVGATTFLSDYW	IGH	4	None	
3	2850	CARQTGAIFGVVIMYYYYGMDVW	IGH	3	None	
3	2851	CARGGAGPAAIYDYW	IGH	12	None	
3	2852	CAHSLSAYYDILTGYPTDAFDIW	IGH	2	None	
3	2853	CAHAYYYDSSGYYYYGYW	IGH	2	None	
3	2854	CQSIDDSGMVF	IGL	4	None	
3	2855	CAREEWLRLGYSYGYGAPGLDYW	IGH	6	None	
3	2856	CARRMSRYSSSWCYFDYW	IGH	2	None	
3	2857	CQSYDSGPHPLFVF	IGL	7	C4	7JVC
3	2858	CARLGPYDILTGWDSDYYYGMDVW	IGH	2	None	
3	2859	CARDSAIFLGLGLFPLDHSSGYYKNHDAFDIW	IGH	2	None	
3	2860	CARLLRNMMTGDPRYWYFDLW	IGH	2	None	
3	2861	CAKERDYDYVWGSYPGPFDYW	IGH	2	None	
3	2862	CARDPQGYCSSTSCYYYYNMDVW	IGH	2	None	
3	2863	CARSRGLLWFGELRTEFDYW	IGH	4	None	
3	2864	CAKVSRYEPSEFSFYFDDW	IGH	3	None	
3	2865	CARAAARSALYNWFAPW	IGH	2	None	
3	2866	CSTAPVRGVRGTIDYW	IGH	4	None	
3	2867	CAKDRRDGYNWGGDYYYGMDVW	IGH	4	None	
3	2868	CARDLGASYTPLDYW	IGH	2	None	
3	2869	CARDSIVVVVAATAKYYYYGMDVW	IGH	2	None	
3	2870	CASDYDILTGYYKGLGLFDYW	IGH	2	None	
3	2871	CAREAYYYDSSGYPPLYYYYGMDVW	IGH	2	None	
3	2872	CARETVWFVFAYW	IGH	12	None	
3	2873	CARDAPGIEDFDSW	IGH	2	None	
3	2874	CAGRRNGDSPFEYW	IGH	4	None	
3	2875	CARVVSYDSNGFHGFPDYW	IGH	2	None	
3	2876	CAKPGGLWYFQDW	IGH	2	None	
3	2877	CARATSGRNVGHIDYW	IGH	2	None	
3	2878	CAKDIGYGANSPDYW	IGH	15	None	
3	2879	CTTVVTPSHYYYGMDVW	IGH	6	None	
3	2880	CAKLSKLVWFGEKLGDYW	IGH	2	None	
3	2881	CARLIGSLLRSFDWSGGQFDPW	IGH	5	None	
3	2882	CAKMNLGYYYDSSGYYPDDYW	IGH	3	None	
3	2883	CAKSAYSYGQGYYYGMDVW	IGH	5	None	
3	2884	CAYRDSYCNDDNCYSGFDSW	IGH	2	None	
3	2885	CSRDSRRCSGRKICLDYW	IGH	2	None	
3	2886	CAKDRQYYDFWSGYLTPHPQYYYYYYGMDVW	IGH	2	None	
3	2887	CARDGLGYSNNWYSWDYW	IGH	2	None	
3	2888	CARVYSSGWRDDYYYGMDVW	IGH	7	None	
3	2889	CARSPAYFDWLFQSDNLYYYGMDVW	IGH	3	None	
3	2890	CARRPLGWAFDIW	IGH	33	None	
3	2891	CARVNYMIRGPFDPW	IGH	4	None	
3	2892	CARIRVDSSSWYHYYYYGMDVW	IGH	2	None	
3	2893	CAKLSYDILTGYLDAFDIW	IGH	5	None	
3	2894	CARANWGSYYFDYW	IGH	68	None	

3	2895	CARGIGTGLVFGELSPYYYGMDVW	IGH	3	None	
3	2896	CARFRLEDTSGYLDRW	IGH	4	None	
3	2897	CARAMAGKNFFDYW	IGH	24	None	
3	2898	CARLSPRITMVRGVIITEYYFDYW	IGH	2	None	
3	2899	CAKMGGTTEGDYW	IGH	2	None	
3	2900	CAKDNWYDSSGSTFDPW	IGH	4	None	
3	2901	CARKMPYASNLGWFDPW	IGH	2	None	
3	2902	CAKLPSHYDILTGHESSYYFDYW	IGH	7	None	
3	2903	CARASDHGYFESSGYYYNPLDYW	IGH	4	None	
3	2904	CARVYFDWLLWVYYFDYW	IGH	4	None	
3	2905	CARVTTSWAYYVSSDYWLRPFKYW	IGH	3	None	
3	2906	CARHGVYDSGYPRYFDHW	IGH	3	None	
3	2907	CAKNMAVAGLSQYYYAMDVW	IGH	2	None	
3	2908	CASGYSSSWPPAPHYYYGMDVW	IGH	3	None	
3	2910	CAKAGMYCSGGSCYSNDFDYW	IGH	2	None	
3	2911	CARDGPAVTNYFDYW	IGH	3	None	
3	2912	CARAFRRPRLLLLYYFDYW	IGH	3	None	
3	2913	CAKDTVAGTRDYW	IGH	29	None	
3	2914	CAKDQTLVTAIPTYYFDYW	IGH	4	None	
3	2915	CARQYFDNSGYFHFDYW	IGH	4	None	
3	2916	CARADLYDTSAPGWNALDIW	IGH	2	None	
3	2917	CARDASNSPEYNYYGMDVW	IGH	2	None	
3	2918	CARDSCGGNSCYRVGWFDPW	IGH	2	None	
3	2919	CATIGGAVADMDRNPTRYYYYYGMDVW	IGH	2	None	
3	2920	CAKSLEYSRTWNPLDYW	IGH	3	None	
3	2921	CQQSYSTGAF	IGK	200	C2	7K8O
3	2922	CLYLQMYSLRPEDTALYYCVKADSDSYFPPSYFDSW	IGH	2	None	
3	2923	CATAGAMIVVALDYW	IGH	2	None	
3	2924	CVKETFSMVRQGGSFDKW	IGH	20	None	
3	2926	CARAYCDGDCYYSDPLDIW	IGH	2	None	
3	2927	CARSGEVVTAIHYFDYW	IGH	5	None	
3	2928	CAKDEYFDWIPSSDGMDVW	IGH	8	None	
3	2929	CASPLDCSSTSCPSVGYYYGMDVW	IGH	2	None	
3	2930	CTTDSEEGAYDYVWGSYRYYW	IGH	3	None	
3	2931	CAKEGGTGSPSYYGFDVW	IGH	2	None	
3	2932	CAKEKITMVRGVILYYYGMDVW	IGH	11	None	
3	2933	CATDRVTMVRALDYYYYGMDVW	IGH	2	None	
3	2934	CAKMGSIVVVVAATENWFDPW	IGH	2	None	
3	2935	CARTGESGYWFDFW	IGH	2	None	
3	2936	CMESLQTPYAF	IGK	2	None	
3	2937	CTTVRAWYYFDYW	IGH	38	None	
3	2938	CAKSQISSSSWYTGDGYFQHW	IGH	6	None	
3	2939	CARPSADPYDVTSYDYDYYGMDVW	IGH	4	None	
3	2940	CAKAPITIFGVVIHRLYYFDYW	IGH	2	None	
3	2941	CARDSTGYYGSGSPLDYW	IGH	2	None	
3	2943	CAKGKACSGGGCPFDYW	IGH	3	None	
3	2944	CATTKGSWHPIDFDYW	IGH	4	None	
3	2945	CTTDRRPYHYDTSGYFSAW	IGH	5	None	
3	2946	CAKDFNGDGYPLRAFDIW	IGH	4	None	
3	2947	CAKIDSYGQEIDYW	IGH	6	None	
3	2948	CFSTDSNGNHRGFF	IGL	19	None	
3	2949	CASTSYYYDSSGYLLPYYFDYW	IGH	2	None	
3	2950	CARTYVVVTAAAFDIW	IGH	9	None	
3	2951	CARDPSGSYGQGCWFDPW	IGH	2	None	
3	2952	CARAGGWEYSSSADFDYW	IGH	2	None	
3	2953	CAKYGLTYYYHYGMDVW	IGH	2	None	
3	2954	CARAYGGNYFAAFDIW	IGH	2	None	
3	2955	CANFGDEWSPRDYW	IGH	4	None	
3	2956	CASMSTILSSLDYW	IGH	4	None	
3	2957	CAKTHPSGAADFCWFDPW	IGH	2	None	
3	2958	CARVHVWLQLPDYW	IGH	2	None	
3	2959	CARGLARDGSFPWFDPW	IGH	2	None	
3	2960	CATDDLGTGTLGHYYYYGMDVW	IGH	2	None	
3	2961	CARDQGRYNWNDVMAFDIW	IGH	10	None	
3	2962	CARDQEFPLTSFDYW	IGH	39	None	
3	2963	CAKDLGSSWGGDYW	IGH	6	None	

3	2964	CAKLPLGSGLDAFDIW	IGH	2	None	
3	2965	CAHLERQSGGWLKSFDYW	IGH	3	None	
3	2966	CARAGITIFGVVNPIFDYW	IGH	5	None	
3	2967	CARGQHFSDGSGYYFDFW	IGH	5	None	
3	2968	CAKDKGTQGRYFDYW	IGH	16	None	
3	2969	CAKGISPLYYSEGYFDNW	IGH	2	None	
3	2970	CQSADSNVSGSFGVF	IGL	4	None	
3	2971	CAGETYYYDSSALNWFDPW	IGH	2	None	
3	2972	CARLTYDNSGYYYIDYW	IGH	4	None	
3	2973	CARDSRVSWSGEVSYYFDFW	IGH	2	None	
3	2974	CAKDPTPGYIYLDYW	IGH	2	None	
3	2975	CSRDLVYGSGSLDYW	IGH	3	None	
3	2976	CAKSDILTGSPPDYW	IGH	7	None	
3	2977	CASEFSSWANYYYYGMDVW	IGH	10	None	
3	2978	CARGLSQRITIFGVVRYYFDYW	IGH	6	None	
3	2979	CITAAYYDILTGYSPLDYW	IGH	5	None	
3	2980	CARESANDYDFWSGRPNWFDPW	IGH	3	None	
3	2981	CAKESAKYSGYHWSDYW	IGH	3	None	
3	2982	CARSDYDILTGYYEIDYW	IGH	2	None	
3	2983	CSVDLYCIVGRCEFFDHW	IGH	7	None	
3	2984	CSSYAGSNIPYDF	IGL	2	C2	7BWJ
3	2985	CARLDGDVVAARRGWFDPW	IGH	9	None	
3	2986	CAHRLGILSGFDIW	IGH	2	None	
3	2987	CARTYYDILTGYYQSREGSWFDPW	IGH	4	None	
3	2988	CQVWDRSTASLF	IGL	8	None	
3	2989	CAKEGDGKLGARFDYW	IGH	4	None	
3	2990	CAREGLVGGADDAADIW	IGH	18	None	
3	2991	CARLGSDTYYDILTGLPLNYYYYGMDVW	IGH	4	None	
3	2992	CAREGSTYYDILTGYHRSYYFDYW	IGH	4	None	
3	2994	CAKSSAAWLRHFDNW	IGH	3	None	
3	2995	CARDTYYYDSSGFPHDAFDIW	IGH	2	None	
3	2996	CARHFEEYYDSSGFFDYW	IGH	2	None	
3	2997	CARDKNPAAVANIDYW	IGH	2	None	
3	2998	CARDRVATIGIVFALGNTYYYYGMDVW	IGH	2	None	
3	2999	CTTFVCSSTSCYAAWGPGFDYW	IGH	3	None	
3	3000	CQVWDSTNDHMIF	IGL	10	C4	7JVC
3	3001	CARVSYFSETTGYYSLFDNW	IGH	4	None	
3	3002	CARDVVSGGTYYYDSSGYPAYW	IGH	2	None	
3	3003	CARDIFAAAAGHYYYYGMDVW	IGH	5	None	
3	3004	CATLGTRPDYYYYYGMDVW	IGH	10	None	
3	3005	CAKDMGDGYNPKISAFDIW	IGH	2	None	
3	3006	CARGYAIFAPLSGYYYGMDVW	IGH	3	None	
3	3007	CLQTTHWPYSF	IGK	17	None	
3	3008	CAKGAGADSSSWYGHSLWYYFDYW	IGH	3	None	
3	3009	CAKDDRPGCTNGVCYTPSGYSYAPDYW	IGH	2	None	
3	3010	CARGDGYKSPCDYW	IGH	10	None	
3	3011	CAREAANFDWLFRTDYYYYYGMDVW	IGH	3	None	
3	3012	CARHHLYDSSGSYLRSSFAYW	IGH	4	None	
3	3013	CASPGAQQGASWYTETWFDYW	IGH	2	None	
3	3014	CARQWVRDVIVLRTHFDYW	IGH	2	None	
3	3015	CARPYSDSGGHHLATFDYW	IGH	3	None	
3	3016	CAKHKKGIAVAGQFDYW	IGH	2	None	
3	3017	CAKEKSGNNYGDFQYW	IGH	9	None	
3	3018	CAKFPQWLDDAFDIW	IGH	2	None	
3	3019	CAKDHGADVVAYFDHW	IGH	11	None	
3	3020	CATFSTMGRVPGYYYYGMDVW	IGH	2	None	
3	3021	CARDGIIYDYVWGSPDEWYYFDYW	IGH	2	None	
3	3022	CAKQEVTMIVVVTYFDYW	IGH	11	None	
3	3023	CARHTDFRSGYGYVDYW	IGH	14	None	
3	3024	CATDRTYYDFWSGSSALDYW	IGH	2	None	
3	3025	CAKEELDSASPLPAVGSEAPALNWYFDLW	IGH	6	None	
3	3026	CAKDAPDDILTGGPFDYW	IGH	4	None	
3	3027	CGRHMNVAVRPFDYW	IGH	2	None	
3	3028	CARTPYYDFWSGYYLGYYYYGMDVW	IGH	2	None	
3	3029	CQVWESSRDHRGNVVF	IGL	2	None	
3	3030	CAKGIFGVVTIDGMDVW	IGH	8	None	

3	3031	CAKSGVRNFDLW	IGH	2	None	
3	3032	CAKAQADYGDYGAFDIW	IGH	9	None	
3	3033	CARDIPHYDVRGYQDFHFDLW	IGH	2	None	
3	3034	CTTESVYYYGMDVW	IGH	2	None	
3	3035	CARDMYYFDSSGYFSDYYAMDVW	IGH	4	None	
3	3036	CAANYYDSSGYYDYYGMDVW	IGH	13	None	
3	3037	CAVQLHRSDYYYYGMDVW	IGH	4	None	
3	3038	CAHSRSSPRHYYFDYW	IGH	2	None	
3	3039	CAKDIGYSDGSGTPPDYW	IGH	2	None	
3	3040	CARDATDPTSPGYYDYW	IGH	7	None	
3	3041	CARRVPDYYDSSGYYYRGQNWYFDLW	IGH	7	None	
3	3042	CARTYYDILGAVQYYFDYW	IGH	3	None	
3	3043	CARAGGYGDYDEYFDYW	IGH	2	None	
3	3044	CARMYYDILTGYYAAVFDYW	IGH	2	None	
3	3045	CATWVTTDAFDIW	IGH	2	None	
3	3046	CTTVWGFGELSLDYW	IGH	2	None	
3	3047	CARLEGVYYDILTGYYLDYW	IGH	15	None	
3	3048	CARGKFGGFEGWFEPW	IGH	5	None	
3	3049	CARIPYGSGSPMGFDPW	IGH	3	None	
3	3050	CARDLAGYYDFWSGHGADYYYGMDVW	IGH	2	None	
3	3051	CQESYNTPQVTF	IGK	3	C2	7K8O
3	3052	CARDSDDYGDYDFMDVW	IGH	3	None	
3	3053	CARVIRAAGPIYYYYGMDVW	IGH	7	None	
3	3054	CVRDGYGVVAAARSGFDVW	IGH	23	None	
3	3055	CAIGTTKITSVFVYW	IGH	2	None	
3	3057	CATLQASGIITGYFHFW	IGH	2	None	
3	3058	CAKASFGDYDYYGMDVW	IGH	6	None	
3	3059	CARELRGYGSGFSDYW	IGH	4	None	
3	3060	CAREFLCTGGVCYSGYFDYW	IGH	2	None	
3	3061	CARGGIYYYDTGYYFDHW	IGH	2	None	
3	3062	CAKDQEAHDYGDYEGYFDYW	IGH	22	None	
3	3063	CQGTGTKDGDYFDYW	IGH	4	None	
3	3064	CAKESHPGGMATTDYW	IGH	4	None	
3	3065	CARSQYFNSPGDYYYYGMDVW	IGH	3	None	
3	3066	CARSLGTYDRFDYW	IGH	6	None	
3	3067	CTTEDYYDSSGYNLGSTHFDYW	IGH	3	None	
3	3068	CATSFPIVPPDYYYYGMDVW	IGH	4	None	
3	3069	CTTDERLLRYFDWLFRDAFDIW	IGH	4	None	
3	3070	CAKLPTGENDYGDSGDYW	IGH	9	None	
3	3071	CVRDGGFGELGFDYW	IGH	11	None	
3	3072	CLSYDISLSGFWVF	IGL	42	None	
3	3073	CAKGEISYYRTDAFDIW	IGH	2	None	
3	3074	CARFIMVRGVTPAADYW	IGH	2	None	
3	3075	CAKDQSPVYYGSGSYFKQW	IGH	3	None	
3	3076	CAKGGVLLWFGESSPLDYW	IGH	2	None	
3	3077	CARAAYECSGSDCYAGPWFDRW	IGH	6	None	
3	3078	CARYPGYCSGGSCWFDSW	IGH	5	None	
3	3080	CAKVGGSGYYAYYVDSW	IGH	181	None	
3	3081	CARPMYYYDSSGYYSFATYAFDIW	IGH	2	None	
3	3082	CAKDTFGGSYSTYWYFDLW	IGH	8	None	
3	3083	CARDSSYYYDSSGYSNWFDPW	IGH	8	None	
3	3084	CARRADYYDLFSGSPKPFDYW	IGH	3	None	
3	3085	CATDQPSYSSDYYYGMDVW	IGH	3	None	
3	3086	CAKDRLQVLRYFDWSPTFDYW	IGH	2	None	
3	3087	CARDLWVSFEPGASWNRGDYW	IGH	6	None	
3	3089	CARDLWPVTTVTTSYYYYGMDVW	IGH	3	None	
3	3090	CTRNYYGSGSFYYYGMDVW	IGH	6	None	
3	3091	CQQRTTWPFALTF	IGK	6	C3	7K8P
3	3092	CARTRWNLTGYLHLSAWFDPW	IGH	11	None	
3	3093	CATLGGSWYDIDYYYYYGLDIW	IGH	3	None	
3	3094	CARGNYGSNSGDSFDIW	IGH	3	None	
3	3095	CARDLEMSGGYDYW	IGH	6	None	
3	3096	CAARYCSGHTCFFSFDYW	IGH	4	None	
3	3097	CARAPGSIIVGAENFWFDPW	IGH	3	None	
3	3098	CAKDDGSGWPRGWYFDLW	IGH	4	None	
3	3099	CAKDEGAWELLIDYW	IGH	12	None	

3	3100	CARGRADSTTWYAREHFGNWLDPW	IGH	2	None	
3	3101	CARDSGGSGSYSHYYGMDVW	IGH	4	None	
3	3102	CPTTKNLATPGSDYW	IGH	2	None	
3	3103	CAGLKDYGDYEPWYYYGMDVW	IGH	8	None	
3	3104	CARDRGLITMIVWPNWFDPW	IGH	11	None	
3	3105	CAKGDDRRIMITFGGVIAPFDYW	IGH	2	None	
3	3106	CAKEDGDSSGYKGLDYW	IGH	2	None	
3	3107	CARGDYSGYDSLCFDYW	IGH	2	None	
3	3108	CARGDCGGDCYYHYYYYGMDVW	IGH	3	None	
3	3109	CVKDVSGPGKYYYGMDVW	IGH	25	None	
3	3110	CAKIEDDYGDSTDYW	IGH	2	None	
3	3111	CAKGTYYDILTGYFQNRYYYGMDVW	IGH	5	None	
3	3112	CARDLEPGPSLRLGELSSYYYYGMDVW	IGH	11	None	
3	3113	CARDHSGNDEDFDYW	IGH	11	None	
3	3114	CARLKGSYDYIDSW	IGH	2	None	
3	3115	CALVSTVTTTFYFASW	IGH	2	None	
3	3116	CARIKAMVTLGGYYYYGMDVW	IGH	7	None	
3	3117	CAKSTRPNTVTTLYFDYW	IGH	3	None	
3	3118	CTKGFYGFGYNYFDYW	IGH	2	None	
3	3119	CARDPDFAEDNGDYSFDYW	IGH	2	None	
3	3120	CATPRHENIAAAGSFDYW	IGH	7	None	
3	3121	CAKDLGPSIEAHGSWFDPW	IGH	35	None	
3	3122	CAGISGHLDYW	IGH	36	None	
3	3123	CARGEGSSGPQQGWFDPW	IGH	3	None	
3	3124	CARAPLKYDILTGYYTYYGMDVW	IGH	2	None	
3	3125	CARVSGDYVWGSYRPYFDYW	IGH	3	None	
3	3126	CARPSRGQLWWFDPW	IGH	3	None	
3	3128	CARRQSDYSGHFDYW	IGH	3	None	
3	3129	CARHFRQWLDLGPYYFDYW	IGH	8	None	
3	3130	CARQSLRPYCSGGSCYEGWFDPW	IGH	2	None	
3	3131	CVKDLRDYSYGYYGFDVW	IGH	2	None	
3	3132	CARVKETYYDSKGAMDVW	IGH	2	None	
3	3133	CARVEGAKRLDYW	IGH	5	None	
3	3134	CARDQYYDGNPYSHFDYW	IGH	2	None	
3	3135	CATLGGGNSGAYYYYGMDVW	IGH	2	None	
3	3136	CAKDQVTGNRGLGVFDVW	IGH	5	None	
3	3137	CARGPYESSGAAAARGWFDPW	IGH	12	None	
3	3138	CAKDRLYYDILTGYYRQINYYYYYGMDVW	IGH	2	None	
3	3139	CAREIRAPRGSFDPW	IGH	7366	None	
3	3140	CAREIPDQTSSGGYFDYW	IGH	4	None	
3	3142	CARVGQDESTGSDALDIW	IGH	6	None	
3	3143	CARTHAPTYYDFWSGYPSLFGQNYYYGMDVW	IGH	2	None	
3	3144	CAREGGSGWTGEVDYW	IGH	2	None	
3	3145	CARTLTMTRGYGVFNAFDTW	IGH	5	None	
3	3146	CVHKLGSWTIVGTGSFDYW	IGH	2	None	
3	3147	CARDSGVFDYGDNFDYW	IGH	6	None	
3	3148	CSRDRGFCSAGTCYSGWFDPW	IGH	4	None	
3	3149	CARDMYYDILTGYYPLDYW	IGH	10	None	
3	3150	CTRRGDCSGGSCYSGGYYYYNGMDVW	IGH	2	None	
3	3151	CMHALQSPFSF	IGK	3	None	
3	3152	CARDHRIGVSDNWFDPW	IGH	2	None	
3	3153	CAREEVWFGELFGPGGDDYW	IGH	3	None	
3	3154	CAKEGHSSGYSIARDAFDIW	IGH	7	None	
3	3155	CVAGGVYYYGMDVW	IGH	10	None	
3	3156	CVSYSDDYGDADYW	IGH	9	None	
3	3157	CVPIVDTAMAFFDYW	IGH	5	None	
3	3158	CARNRITASQFDYW	IGH	2	None	
3	3159	CAKDGIWQADYFDYW	IGH	15	None	
3	3160	CTLLHCSSNVCPPYFHFHAMDVW	IGH	4	None	
3	3161	CATLPSLAAGYDTYYYGMDVW	IGH	2	None	
3	3162	CATGSVVPSRYFDWERESVARYWFDPW	IGH	4	None	
3	3163	CARVSRAMVFDFW	IGH	15	None	
3	3164	CARVGPNDSSGYTAFDIW	IGH	6	None	
3	3165	CATATVVMGATNYYYYGMDVW	IGH	10	None	
3	3166	CARVDLAADGTYPEWFDPW	IGH	3	None	
3	3167	CATPAAYDYVWGSYRYDYFDYW	IGH	2	None	

3	3168	CTTGGGITIFGVVITFDAFDIW	IGH	2	None	
3	3170	CARHVAGYFDGSDYYEVYFDYW	IGH	3	None	
3	3171	CAKDPPPSMIVVVITYFDYW	IGH	6	None	
3	3172	CARSRWATMTGPDYW	IGH	2	None	
3	3173	CARGFHYGDSHFDTW	IGH	2	None	
3	3174	CAKTFHDYGDYAGDYW	IGH	6	None	
3	3175	CARGPILRFLEWLSPHYYYGMDVW	IGH	2	None	
3	3176	CAKILSIAVAGTGDYW	IGH	2	None	
3	3177	CAADSSGYRGRFDPW	IGH	10	None	
3	3178	CFSPDSSGPAF	IGL	2	None	
3	3179	CASMPAAMWMPFDYW	IGH	4	None	
3	3180	CTTGPSGFWRGYDYW	IGH	2	None	
3	3181	CARHFSSGWYERGWFDPW	IGH	2	None	
3	3182	CARDPLSLYGDVYYFDYW	IGH	2	None	
3	3184	CATERVALAGPYYFDYW	IGH	4	None	
3	3185	CARSGGWYRNWFDPW	IGH	4	None	
3	3186	CAHSTLWFGEERPPNWFDPW	IGH	3	None	
3	3187	CARPDHPLGYCSGGSCYAWYYFDYW	IGH	5	None	
3	3188	CVKAGSKNYDSSAYFPYNWFDSW	IGH	2	None	
3	3190	CASHAPAYGSAYYYNDSPFDPW	IGH	3	None	
3	3191	CARQQLVMNYYQYAMDVW	IGH	2	None	
3	3192	CARDTHDSGWFGYW	IGH	150	None	
3	3193	CARDKGCGTKCRNWFDPW	IGH	3	None	
3	3194	CAKDIYPIYGDYLLDYW	IGH	2	None	
3	3195	CTKCPTARSAWFDSW	IGH	8	None	
3	3196	CAKLPLVRAPFDYW	IGH	6	None	
3	3197	CAKDSQSLWFGKVYYYYGMDVW	IGH	9	None	
3	3198	CAKDHGVISYYFDYW	IGH	4	None	
3	3199	CARDFPYCSSTSCPDAEYFQHW	IGH	2	None	
3	3200	CAKAYYDFWSGYSLTFGGVIVTAAFDIW	IGH	2	None	
3	3201	CAKDPAPYYGEGGDYW	IGH	5	None	
3	3202	CARISVGGYDWAFDYW	IGH	3	None	
3	3203	CATGPASYYDSSGELYYYYYGMDVW	IGH	2	None	
3	3204	CAHPARYSSRIDYW	IGH	2	None	
3	3205	CAKDISPIGVWPAFDYW	IGH	2	None	
3	3206	CQQYDSSSRVVTF	IGK	3	C3	6XDG
3	3207	CVRDIEQQLIYSGGFDFW	IGH	2	None	
3	3208	CAKVSSDYTDYW	IGH	6	None	
3	3209	CARAAGLLWFGELLYPPGWFDPW	IGH	6	None	
3	3210	CARGLSGRGFSSGNDYW	IGH	2	None	
3	3211	CTAAPNYYYFNYW	IGH	3	None	
3	3212	CARASNDLGVVVPGGRTRDRFDPW	IGH	4	None	
3	3213	CARQGYDTTGSYPSVNWFDPW	IGH	5	None	
3	3214	CTGELAYCTNGVCSYPFDNW	IGH	9	None	
3	3215	CARHYISGWNDYW	IGH	2	None	
3	3216	CARHVRVIGLLNFDYW	IGH	6	None	
3	3219	CARGRGSFSLDYW	IGH	15	None	
3	3221	CARAPLDNPGGNDYW	IGH	4	None	
3	3222	CAKDLYDYVWGSYRYTGSSFDYW	IGH	2	None	
3	3223	CVRDEGYYYGSGNWGSAWFAPW	IGH	4	None	
3	3224	CARMNLMYYDILTGYYTGPFDMW	IGH	2	None	
3	3225	CARAASGYYYLGAFDIW	IGH	4	None	
3	3226	CANGNYYLSGYYYGMDVW	IGH	22	None	
3	3229	CAKDMWAAAAYYFDSW	IGH	12	None	
3	3230	CAKLPAPFYGDYAFFDAFDIW	IGH	5	None	
3	3231	CAKLFYDRSDAFDIW	IGH	2	None	
3	3232	CAKDVWYYDSSALVGAFDIW	IGH	2	None	
3	3233	CAKGVYGYSTSDFDYW	IGH	3	None	
3	3234	CAVVPAAPLGYYYYYGMDVW	IGH	5	None	
3	3235	CARQFHESVAGNDYW	IGH	12	None	
3	3236	CLQHNSYPYTF	IGK	617	C3	7K8P
3	3237	CARRPLGYCSGGSCSQSWFDPW	IGH	8	None	
3	3238	CAKPNYYGRGASNFWAFDHW	IGH	2	None	
3	3239	CARTPNYYDSSGYYAALFDYW	IGH	5	None	
3	3240	CQSLDSSLSQTVVF	IGL	3	None	
3	3241	CARDQEGLLLWFGELSVFLDYW	IGH	4	None	

3	3242	CQVWDSSPDQRGFF	IGL	2	None	
3	3243	CARIRDWGSYGWFDPW	IGH	16	None	
3	3244	CAKLKGDMVRGVSDGMDVW	IGH	6	None	
3	3245	CARVWRTGSDYPYFDLW	IGH	4	None	
3	3246	CARGHTPMSNNWLDPW	IGH	4	None	
3	3247	CARHSPPSSSWPRGWFDPW	IGH	2	None	
3	3248	CARVRGDIVAKPNQNWFDPW	IGH	2	None	
3	3249	CAKDPDDGYSSSWSFDYW	IGH	4	None	
4	1	CTNNWRDSTGGYVPAAFEVW	IGH	3	None	
4	2	CALPIYKTGHYSWGFDFW	IGH	2	None	
4	3	CARVFLGNTGPRGDYMDVW	IGH	4	None	
4	4	CARYYYDSYGYFPERSFDLW	IGH	7	None	
4	5	CTREPNNGYDVGEIDYW	IGH	6	None	
4	6	CARRSRRYGGQGFYWYFDLW	IGH	3	None	
4	8	CARGMYGGAASYYGYYFDYW	IGH	4	None	
4	9	CARVIIAFGGYYYYMDVW	IGH	21	None	
4	10	CARELGTTFRNYYFDLW	IGH	3	None	
4	11	CARTVLWFGSGYFDSW	IGH	3	None	
4	12	CGRQGRDFDGNGYSRQRFYIDVW	IGH	2	None	
4	15	CARGRTRYSSGDDAFDIW	IGH	4	None	
4	16	CARLRIFGVVRDAFDIW	IGH	13	None	
4	17	CAREVEYSSTWYSGYHYLDVW	IGH	2	None	
4	18	CTRGSHFDDRRTYTNWLDPW	IGH	2	None	
4	19	CARHKYYYGYVADIDYW	IGH	13	None	
4	20	CATAPRAIPSAHWWGDYW	IGH	2	None	
4	21	CARGEGDFWSGYPLSFEYW	IGH	2	None	
4	22	CARSQIPITMIYDYW	IGH	9	None	
4	23	CARASGYSYGLTHYYYYYMDVW	IGH	2	None	
4	24	CAREELLGRYYFDSW	IGH	4	None	
4	25	CAKGPSGYSYGVDYW	IGH	3	None	
4	26	CARDYGSFRMRSFFDNW	IGH	4	None	
4	27	CQQRAKWPPTTPITF	IGK	5	None	
4	28	CARAPSTAMVAQCLDYW	IGH	6	None	
4	29	CARKGSNGWWDYYMDVW	IGH	7	None	
4	30	CARQFCGSSDCSWDYYYYMDVW	IGH	7	None	
4	31	CARGRGTRRDSSNYLYYFDYW	IGH	4	None	
4	32	CARGAGESLEWFDPW	IGH	4	None	
4	33	CAKDTGRYYYDSNSYPFDIW	IGH	4	None	
4	34	CARDLGGHGEAFEIW	IGH	6	None	
4	35	CASRRPGPDGSGTYLDLW	IGH	2	None	
4	36	CAREGLNQWVHYYDSSTYSNYFDSW	IGH	3	None	
4	38	CVRGYSSFDHW	IGH	3	None	
4	39	CARILMYDSGDYVYAFNIW	IGH	2	None	
4	40	CARGRRISITMGNWFDPW	IGH	3	None	
4	41	CARGNYYDSSGYYTRRGPTLDYW	IGH	3	None	
4	42	CTTRGGKHGVDYW	IGH	8	None	
4	43	CAKEKGYLSGCPDYW	IGH	3	None	
4	44	CARGGESQSSVGAFDIW	IGH	4	None	
4	45	CARAPIYGDHPKTFDYW	IGH	2	None	
4	46	CARDLGYCSGGSCYSGDTADYW	IGH	2	None	
4	47	CATNPRQGSTWYDSFYYYMDFW	IGH	4	None	
4	48	CARGIGGSYYSDYW	IGH	8	None	
4	49	CAKDRHTSAPYYFDFW	IGH	6	None	
4	50	CGKDRFGSSPLDAFDIW	IGH	17	None	
4	51	CAKDLGVASPFSFDLW	IGH	2	None	
4	52	CVKIRGFSDHDSIQTVSYYFDYW	IGH	2	None	
4	53	CAAHWASNGYFTYW	IGH	4	None	
4	54	CARDYYYGSDKYPPHFDNW	IGH	5	None	
4	55	CARHPAYESSGYPFDNW	IGH	4	None	
4	56	CARSEFPEDRGRYRLDFDSW	IGH	2	None	
4	57	CARETLETYDFWSGYYYYYYMDVW	IGH	4	None	
4	59	CARRGYVAARPDGWYFDLW	IGH	4	None	
4	60	CARHVAGGGEFDYW	IGH	5	None	
4	61	CARHAGRLMVVEFDFW	IGH	2	None	
4	62	CARGIAYCSSTSCYGGYYYMDVW	IGH	2	None	
4	63	CARDGRFLEWLLSHYYYYMDVW	IGH	2	None	

4	64	CAKRVEYGSSSGVFSFDSW	IGH	3	None	
4	65	CARDYDQQWLIDYW	IGH	2	None	
4	66	CARRVVPAAKSYWYFDLW	IGH	4	None	
4	67	CARLGYCTNGVCYYIDYW	IGH	6	None	
4	68	CATFPLNYLYYFESW	IGH	8	None	
4	69	CARYKRDWYDASGNSFYFDSW	IGH	2	None	
4	71	CAREAGYSSTWYEYLDYW	IGH	2	None	
4	72	CARQHVLDWLWGGRSWFDPW	IGH	3	None	
4	73	CARGPDYDFMTGQAFFAAFDIW	IGH	5	None	
4	74	CARGSGRTVTNDDFDYW	IGH	2	None	
4	75	CAREWFEDSSGYYWKRSDGQHFDYW	IGH	2	None	
4	76	CARGSSVTTTYWYYDIW	IGH	2	None	
4	78	CARPTGDAVPISGGYHYYYYMDVW	IGH	4	None	
4	79	CAKGDGSGNYYITFDSW	IGH	10	None	
4	80	CARHLRRGSYYHGFDVW	IGH	2	None	
4	81	CAKGSTHGFPPNWYFDLW	IGH	29	None	
4	82	CARLIRALSGGGSPPLYYFDYW	IGH	5	None	
4	83	CARDINQYDNSGYYHGWFDTW	IGH	4	None	
4	84	CARDGVGATIYFDYW	IGH	8	None	
4	85	CARPDNLGYCSGGSCYSVDYW	IGH	3	None	
4	86	CARDNGDYEDNW	IGH	2	None	
4	87	CASLLDPDFAFDMW	IGH	18	None	
4	88	CVHRPVAGFFDYW	IGH	11	None	
4	89	CARIPSLVVQPWGDGFDFW	IGH	3	None	
4	90	CMQGTHWPWTF	IGK	4658	None	
4	91	CAKDRASHRLYYFHSW	IGH	3	None	
4	92	CASGPYYYESIGYRTFDIW	IGH	2	None	
4	93	CARHPYASSPSNYLDLW	IGH	2	None	
4	94	CALSHYGDYEGNWFDPW	IGH	3	None	
4	95	CVRDDPGIGLDYW	IGH	110	None	
4	96	CSAWDGRLSAWVF	IGL	2	None	
4	97	CARGGPGNSRYRYMDVW	IGH	10	None	
4	99	CATPGLGYTSSWQDYYYYYKDVW	IGH	2	None	
4	100	CARLNSFMVEYAHYFQYW	IGH	2	None	
4	101	CQVWDRSTDQRNSVHWVF	IGL	28	None	
4	102	CARSPGAARRYYYMDVW	IGH	14	None	
4	103	CARNPALWFGESRGVFDIW	IGH	7	None	
4	104	CARARAGSNGWYSPLDYW	IGH	5	None	
4	105	CAGQDSTSWPRRFDFW	IGH	4	None	
4	106	CARDQLNRESILTLALDYW	IGH	2	None	
4	107	CARVVIASYYYMDVW	IGH	5	None	
4	108	CAHSDISAAGAFDYW	IGH	2	None	
4	109	CARRNYFDSSGSLWKYFDNW	IGH	2	None	
4	110	CMIWHSSAVVF	IGL	175	None	
4	111	CAREGQGDLRHSFDYW	IGH	4	None	
4	112	CAKGGASSGGHPPLDYW	IGH	2	None	
4	113	CARRIGSSWFDPW	IGH	96	None	
4	114	CASQGGEDYFDFW	IGH	3	None	
4	115	CARGIYKFTSGSPQDWFDTW	IGH	3	None	
4	116	CAKDLEYYDSSPYYPVDAFDVW	IGH	20	None	
4	117	CARHDYVWGSYPPGSRYYYMDVW	IGH	3	None	
4	118	CAKANAVTTYFFDYW	IGH	4	None	
4	119	CAKDLYRLPQGGYYFDYW	IGH	3	None	
4	120	CARMVLGGNSDYW	IGH	2	None	
4	121	CARGPSTGAARPFDYW	IGH	8	None	
4	122	CAKTPYVWGSYRHVTVSCFDYW	IGH	6	None	
4	123	CAKLSGDTSDWYGSPDVFDVW	IGH	21	None	
4	124	CARDSPRVPLKGEIKTHQRSGSEIGLFDYW	IGH	2	None	
4	125	CARDRKWLDYYYYMDVW	IGH	4	None	
4	126	CSNLVDEGPKDYW	IGH	6	None	
4	127	CARMIVAGYWYFDIW	IGH	18	None	
4	128	CARDLWSGYSPFDYW	IGH	8	None	
4	129	CASYDSRGYQYFQHW	IGH	14	None	
4	130	CARSPHLDSRGYYERPYYFDYW	IGH	7	None	
4	131	CARPHREGSSTYFDYW	IGH	29	None	
4	132	CAREGVAARRGRYDYYYYMDVW	IGH	58	None	

4	133	CVRGVGSFPPPYYYYMDVW	IGH	15	None	
4	134	CARAPPLRYYDILTGSAYYFVYW	IGH	2	None	
4	135	CASPHETPYSSSPHGLFDYW	IGH	3	None	
4	136	CAKRGRVFMLYENSHYMDVW	IGH	206	None	
4	137	CTLYLQMNSLGVEDTAVYYCARDFRAGRRYDVFEIW	IGH	2	None	
4	138	CATLPQLRKPLDYW	IGH	4	None	
4	139	CARAKGRISLRHFDWLFHAFDLW	IGH	13	None	
4	140	CATQKPNCNSTMCYYDGFDMW	IGH	2	None	
4	141	CAENWNFNWFDPW	IGH	3	None	
4	142	CARGRGQWLLVDYW	IGH	3	None	
4	143	CARDHLEYNISPADYW	IGH	3	None	
4	144	CAREGGLAASNWFDPW	IGH	11	None	
4	145	CARHGRGGYSGSYYSAFDIW	IGH	7	None	
4	146	CARGQGSWHLRRGYYYYMDVW	IGH	2	None	
4	147	CAREHQLLYRCLDYW	IGH	2	None	
4	148	CAREHYYDYIWQTSRSYYFDFW	IGH	3	None	
4	150	CARGRRPHHFWTYSYYYMDVW	IGH	7	None	
4	151	CAHRPGCSGGNCYSGAFNIW	IGH	3	None	
4	152	CAITKYDYSNYSPLDAFDIW	IGH	6	None	
4	153	CARVSPLADYVKPFFDYW	IGH	9	None	
4	154	CCSYAGSYTFDVVF	IGL	26	C2	7K8X
4	155	CARVSLGTGYSYSVGWFDPW	IGH	2	None	
4	156	CARDPLEYSSSLDWYFDLW	IGH	3	None	
4	157	CARTMMTKLYPDAFDLW	IGH	2	None	
4	158	CKVGALDNDYW	IGH	3	None	
4	159	CARLKYSDVWSRYPQFYYMDVW	IGH	148	None	
4	160	CQSTDGSGNPVF	IGL	34	None	
4	161	CARVETGAPDYW	IGH	29	None	
4	162	CVKALGSGTGHLFDIW	IGH	4	None	
4	163	CAKIGIVGGTTYYYYYMDVW	IGH	2	None	
4	164	CARKYSSSREGPFDYW	IGH	8	None	
4	165	CARRYTRAYSSYWYYW	IGH	8	None	
4	166	CARHVRITIFGVVISPGYFDYW	IGH	6	None	
4	167	CAKPYVWGSYRQESFDFW	IGH	2	None	
4	168	CAKLTSDTIFGATAGYFDHW	IGH	3	None	
4	169	CAKAYSSSWYFWYFDLW	IGH	2	None	
4	170	CAKAQYSYGSGSYYDRPLDYW	IGH	6	None	
4	171	CARDWFLYGGYGVVFAENAFDIW	IGH	9	None	
4	173	CAKDGSSGYVYYYMDVW	IGH	9	None	
4	174	CATLPSGDEENWFDPW	IGH	10	None	
4	175	CARYYYETTGYHLIDYW	IGH	4	None	
4	176	CARAERFLDKIYYYYMDVW	IGH	10	None	
4	177	CARGRCTSATCAFEYW	IGH	3	None	
4	179	CATDRAVRTNGDAFDIW	IGH	2	None	
4	180	CARGNSGWPSYSDNW	IGH	8	None	
4	181	CAKGQGYIYGDSFDYW	IGH	8	None	
4	182	CARLKTTIAAQNFDYW	IGH	3	None	
4	183	CARRHIVGGTRNFDLW	IGH	2	None	
4	184	CARDLSSSWYEYYFDYW	IGH	16	None	
4	185	CARGPGLEISKRYLDFW	IGH	12	None	
4	186	CARHAYFYDSIGYYDAFDIW	IGH	7	None	
4	187	CARSWSAADFDYW	IGH	17	None	
4	188	CARVVTARADYW	IGH	11	None	
4	189	CARYENQLVRKAWFDFW	IGH	2	None	
4	190	CARDGGDFWGGYYAFDCW	IGH	3	None	
4	191	CARASAHLRFAFDIW	IGH	42	None	
4	192	CARDFSTYYFESYGYFNGFFDCW	IGH	2	None	
4	193	CARDSNSITMIVVVPTEIDYW	IGH	14	None	
4	194	CVRGTSRGSYDLDFDFW	IGH	7	None	
4	195	CARDFLKSSRDGYNYYFDYW	IGH	4	None	
4	196	CARHAALSPSIDSSWYIDHW	IGH	2	None	
4	197	CARGSSSSYRDNWFDPW	IGH	4	None	
4	198	CARHHDFWSSPSDYW	IGH	9	None	
4	199	CVKEALERSSSWDDSW	IGH	4	None	
4	200	CARHFRTSEYQLLLWFDPW	IGH	2	None	
4	201	CARDSGRPGAYWYFGLW	IGH	3	None	

4	202	CARDRSASYNDYFDYW	IGH	12	None	
4	203	CAKHRHVGILTGYYSPFDYW	IGH	4	None	
4	204	CVKDEVLRTYMDVW	IGH	3	None	
4	205	CAKAPSRWLYYYYYMDAW	IGH	2	None	
4	206	CTGGSCSSDYSYYMDVW	IGH	2	None	
4	207	CVRLDTSGWYRPYYDHW	IGH	2	None	
4	208	CTRRLDYYDSSGYHDYW	IGH	4	None	
4	209	CASGRDVVFDYW	IGH	6	None	
4	210	CQTADTSGNDWVF	IGL	68	None	
4	211	CVRTEAWATGNDYW	IGH	8	None	
4	212	CTRAGPVGYYMDVW	IGH	8	None	
4	213	CARGLRGWRRYFYYLDLW	IGH	4	None	
4	214	CAKAQYSSDTLDYW	IGH	2	None	
4	215	CSSFTTSSTLDVVF	IGL	6	C2	7K90
4	216	CARYGLGDSAAFDLW	IGH	5	None	
4	217	CLSYDGDNVRVF	IGL	2	None	
4	218	CVRAHSMTGPMRMAFDVW	IGH	3	None	
4	219	CARGYSGSDGKLGNFYFDYW	IGH	11	None	
4	220	CARNMIVMVGGAFDLW	IGH	2	None	
4	221	CASGRPGSVYLDVW	IGH	9	None	
4	222	CATYFERASESFFDAVDIW	IGH	10	None	
4	223	CASRMKRTVTGSTRPFDYW	IGH	2	None	
4	224	CARPNSSNWGISYYMDVW	IGH	105	None	
4	225	CANALGQFSPFDYW	IGH	2	None	
4	226	CAKAHNPSIAAAAAEGHYYYYMDVW	IGH	2	None	
4	227	CYSTDSSANRRGVF	IGL	7	None	
4	228	CVKEIGSGSYRLLIDYW	IGH	2	None	
4	229	CAKDALRARFVYNWFDPW	IGH	2	None	
4	230	CARQDRGTYLRGPLDYW	IGH	6	None	
4	231	CARTENYYGSGRPDFDYW	IGH	4	None	
4	232	CARFSLSDTSLVTDYYYMDVW	IGH	8	None	
4	233	CAKDILVGAFDLW	IGH	4	None	
4	235	CVRLPHVVGVPVGSYYFDYW	IGH	5	None	
4	236	CVKDTHYQGSGTYFYYFDSW	IGH	3	None	
4	237	CARDLGGADYVATGEYYFDYW	IGH	2	None	
4	238	CARVSINNNWFDPW	IGH	3	None	
4	239	CARHWATVISGGWFDPW	IGH	3	None	
4	240	CAKFSGSYYYPLDYW	IGH	3	None	
4	241	CRSITWYGAVDYW	IGH	3	None	
4	242	CARDRLDDNGYYGGSFDLW	IGH	2	None	
4	243	CAKDSEYSGYDLALGYFDYW	IGH	21	None	
4	244	CARGGRCYDFWSGYYTCAFDIW	IGH	3	None	
4	245	CARVPYSGWGHFFDYW	IGH	4	None	
4	246	CAREIPYGTYALDIW	IGH	4	None	
4	247	CAREYRDMTGTQRAVDIW	IGH	4	None	
4	248	CATAPGIAVAGNWFDPW	IGH	15	None	
4	249	CARVSSDFWNGYYRRYFDCW	IGH	2	None	
4	250	CARINRSPYDYDAFDIW	IGH	6	None	
4	252	CAAIYETSTFDLW	IGH	2	None	
4	253	CASGTYYDLVTHQLAFHYW	IGH	2	None	
4	254	CARGRGSIAAGGTGFDYW	IGH	2	None	
4	255	CARDPFSGDRNNFDYW	IGH	2	None	
4	256	CARLGYCSSRDCSPFYFDYW	IGH	4	None	
4	257	CARGVVALTYFDNW	IGH	5	None	
4	258	CARGGRFLEWLLLDYW	IGH	6	None	
4	259	CAMPLGRGKNYGSRVYDYYYMDVW	IGH	2	None	
4	260	CAKHPYTTSSAYYVDYW	IGH	4	None	
4	261	CARVVFLAISAYYMDIW	IGH	5	None	
4	262	CARRIDPGYSYGYAGHWFDPW	IGH	2	None	
4	263	CARGWGDATTNWFDPW	IGH	3	None	
4	264	CAKDRQGLGYSAFDFW	IGH	2	None	
4	265	CTKDLLGAAGPWYFGFW	IGH	2	None	
4	266	CATLTNLYGSGSYDGFDVW	IGH	3	None	
4	267	CASCNFDSAGYRYDVDYW	IGH	2	None	
4	268	CVCMLSFPLSYYYMDVW	IGH	3	None	
4	269	CANWVGELVDFDQW	IGH	4	None	

4	270	CARGGRIRDWSGHFIFDYW	IGH	7	None	
4	271	CARLSGYCSGYSCYPHWYFDLW	IGH	4	None	
4	272	CARRAYCSSTSCFYYLDHW	IGH	2	None	
4	273	CARQTGLHVHYYYYYYMDVW	IGH	3	None	
4	274	CAHSQTGGGYDPYFDYW	IGH	3	None	
4	275	CARRLNWSYEQYFDTW	IGH	4	None	
4	276	CVRHREGDFWSGYSPYYVDFW	IGH	6	None	
4	277	CARVVLGVGATPAVRTYWYFDLW	IGH	10	None	
4	278	CARGSTVTFPVYYYYMDVW	IGH	3	None	
4	279	CARVAYSYGPVSTYYYMDVW	IGH	18	None	
4	280	CAHSALFPIAVAPPSCAFDIW	IGH	7	None	
4	281	CAKDIASAVPSNGFHYW	IGH	2	None	
4	282	CATTKVRGYSYNYPFDYW	IGH	14	None	
4	283	CAKGLQNYRSRPEYFDYW	IGH	2	None	
4	284	CVRAPTAVRDISGYYYASFDSW	IGH	2	None	
4	285	CAMDLTPEDHQLVHPDYW	IGH	2	None	
4	286	CTTASRVTMIVVVITTGGFDYW	IGH	3	None	
4	287	CQSADNSGVYGVLF	IGL	355	None	
4	288	CARHGHDWYPDYW	IGH	4	None	
4	290	CARVGETNWNYEDHW	IGH	8	None	
4	291	CARGGDYYGLGPLDYW	IGH	2	None	
4	292	CARVFGIAAARQYFDYW	IGH	2	None	
4	293	CARASGAARGWHHSFDFW	IGH	14	None	
4	294	CATAHGSETFYFDHW	IGH	17	None	
4	295	CVREDVAKGWNTFDIW	IGH	31	None	
4	296	CARGQYSSSFRFDPW	IGH	13	None	
4	297	CARGGYYDSSGYYYMDVDYW	IGH	40	None	
4	298	CARVHSDSSGWYNDFDYW	IGH	2	None	
4	299	CARIPSPFWSGYYSLFDYW	IGH	2	None	
4	300	CATHLTGYGNDAFDIW	IGH	2	None	
4	301	CAREGLAKKRIWIFDSW	IGH	2	None	
4	302	CARVGGDSSGIHYYYYYYMDVW	IGH	3	None	
4	303	CQQYRGSPAMYAF	IGK	4	C1	7K8N
4	304	CALQGAYVGYLDVW	IGH	2	None	
4	305	CARVIRVATILYYFDYW	IGH	5	None	
4	306	CARGGNYPVYMDVW	IGH	4	None	
4	307	CAKAGGKYQLLYGGDRFDSW	IGH	2	None	
4	308	CARSYTNRDGLDVW	IGH	46	None	
4	310	CARDLGYAGKYERVDYW	IGH	2	None	
4	311	CTGGHSYAFFYW	IGH	7	None	
4	313	CARILAGTTGADYW	IGH	3	None	
4	314	CARGPVEYTSASSFDYW	IGH	2	None	
4	315	CARHPIRGHSYGFDFW	IGH	2	None	
4	316	CARDPKWGTAADGTDYW	IGH	2	None	
4	318	CVYHYLWGPAWPNAFDFW	IGH	4	None	
4	319	CARRWSDSSGYPKNRYYYMDVW	IGH	2	None	
4	320	CARGRRVLLWFGELFDSYYMDVW	IGH	3	None	
4	321	CARSGGDITGTTRSFDYW	IGH	2	None	
4	322	CAAGAPNLSFDSW	IGH	5	None	
4	323	CARGRYCTEGSCFFDYW	IGH	2	None	
4	324	CARAPKGSSYHYIDSW	IGH	4	None	
4	325	CQQFNVAPYSF	IGK	7	C3	7K8P
4	326	CARDRAEFWSGRAFDVW	IGH	2	None	
4	328	CARQSVDVFRDGNWFDPW	IGH	3	None	
4	329	CAHTRTLTGSFDYW	IGH	2	None	
4	330	CAKDRGATMSVLDYW	IGH	2	None	
4	331	CATVRSNYDRDGYFIHFDNW	IGH	17	None	
4	332	CARGGATGGGDCTRCAFDYW	IGH	8	None	
4	333	CARDIYSSGWYGPKYYFDYW	IGH	2	None	
4	334	CARVSQGYYDSSGYHYFDYW	IGH	14	None	
4	335	CARATTGYANSPTDYW	IGH	3	None	
4	336	CARDGLTITTRGWFDSW	IGH	5	None	
4	337	CAKVDSFDWSYSYYYMDVW	IGH	6	None	
4	338	CVKDDYYASGNFASNFDCW	IGH	40	None	
4	339	CARRVPRWGYYYMDVW	IGH	23	None	
4	340	CARPFPPGYFSGWSGFDYW	IGH	2	None	

4	341	CTRGAYFDLW	IGH	4	None	
4	342	CNSWDSSGNRNVLF	IGL	2	None	
4	343	CVKEEVGAAYYLDFW	IGH	3	None	
4	344	CAKRSWEYGTGYYMDVW	IGH	4	None	
4	345	CARVADSSSTAAFDYW	IGH	6	None	
4	346	CARGGLEQWAERNYFNYW	IGH	2	None	
4	347	CVHTRYFYDAGAFDIW	IGH	2	None	
4	348	CARGTVTTSVINNYYYDSW	IGH	4	None	
4	349	CARSFMTGGPNDVFDIW	IGH	6	None	
4	350	CARGAYWYGSGMDENPNAFDIW	IGH	18	None	
4	351	CARRNALKYEFWSGYSKKGPYDYW	IGH	6	None	
4	352	CSSLETTFDVW	IGH	17	None	
4	353	CATRLDYGAPGPFDPW	IGH	10	None	
4	354	CVAADDYIWGNYPYTGVDYW	IGH	2	None	
4	355	CARENCAGTCYWGDYMDVW	IGH	30	None	
4	356	CARVRRRLHASGWYNYFDLW	IGH	18	None	
4	357	CARHLAARRLDYYYMDVW	IGH	2	None	
4	358	CARMNYYDSPFDPW	IGH	6	None	
4	359	CQTYDSSLSGHTYVF	IGL	17	C4	7JVC
4	360	CAKDFSDIWTAYPLDYW	IGH	4	None	
4	361	CASLDRAPYYMDVW	IGH	7	None	
4	362	CQSYDSSLSDSDVVFGDSDVVF	IGL	12	None	
4	363	CAKDFRAGRRYDVFDIW	IGH	113	None	
4	364	CARTGPGGSDQLGLFDPW	IGH	5	None	
4	365	CAKDLDRRGYCSGGSCFPKFDSW	IGH	4	None	
4	366	CARAWDSIDNWFDFW	IGH	7	None	
4	367	CARHRGSGWFYYYDSW	IGH	7	None	
4	368	CARIAHRFGRAFDIW	IGH	10	None	
4	369	CARHWGWLQLSYFDYW	IGH	4	None	
4	370	CARDIDDLTTSGAVVNWFDPW	IGH	4	None	
4	371	CAAWDGSLSVAMF	IGL	2	None	
4	372	CATLYYDFWSGDFTGTWFDPW	IGH	2	None	
4	373	CARVKDVVADWYFDLW	IGH	32	None	
4	374	CAHRPLSYRGSSHYFDFW	IGH	11	None	
4	375	CAKVLTMIVVDPFDYW	IGH	2	None	
4	376	CAKDREASSYDSSGSDYW	IGH	2	None	
4	377	CALRRYRDAFDIW	IGH	16	None	
4	378	CASFLDLGYCSGGSCFDYW	IGH	2	None	
4	379	CASHEGWTDDWFDPW	IGH	7	None	
4	380	CARFLGFADYRNNWYDPW	IGH	2	None	
4	381	CARDGISQHAFDIW	IGH	17	None	
4	382	CAKDKRYFRYSDYENWFDPW	IGH	2	None	
4	384	CARGPHIVVVIAAYWYFDLW	IGH	2	None	
4	385	CVNLIKVQQRPYDAFDIW	IGH	3	None	
4	386	CARHRRAGYSSGWHEAFDIW	IGH	2	None	
4	387	CVRAPFSYYRRLNYFDPW	IGH	2	None	
4	388	CTRSRGPIYAYFFDYW	IGH	2	None	
4	389	CARSIAVFGVVPGDAFDMW	IGH	2	None	
4	391	CARGRKAGTMAGTFDYW	IGH	2	None	
4	392	CARHLHIPKFDIW	IGH	2	None	
4	393	CATDGQYSDNSDQDALNIW	IGH	3	None	
4	394	CAGSMVRGVISAFHIW	IGH	2	None	
4	395	CAKIFPRGWTTLGAFDFW	IGH	5	None	
4	396	CARVFVPPSTFYYYYYMDVW	IGH	2	None	
4	397	CARAFRSSGRPWDYFNYW	IGH	2	None	
4	398	CARSSSSWDFDAFDFW	IGH	3	None	
4	399	CAHRRSGAGVRFDFW	IGH	2	None	
4	400	CARSYNSGFNWFDPW	IGH	5	None	
4	401	CAAIESMVRGVIRKGWFDSW	IGH	2	None	
4	402	CTRDLFESSGYYVPWYFESW	IGH	8	None	
4	403	CANQYSNGWFESW	IGH	13	None	
4	404	CVKEPHYDLWRSYYMDVW	IGH	2	None	
4	405	CARHRLDYGALYFFDYW	IGH	4	None	
4	406	CARSPTYTTSGSDYHYYMDVW	IGH	3	None	
4	407	CASMVWGFDITGFDYW	IGH	4	None	
4	408	CQHYGSSRYTF	IGK	1236	C1	7K8N

4	409	CARGGEVTGTGAFDIW	IGH	5	None	
4	410	CARDPGPESGSLPSHLDYW	IGH	6	None	
4	411	CTRGGYGLYTDFDYW	IGH	4	None	
4	412	CAREHDGEHDGLGSGHWFYYMDVW	IGH	5	None	
4	413	CAAHGSGSHRNFDHW	IGH	7	None	
4	414	CLQHDDFLWTF	IGK	6	None	
4	415	CARVPMVRGVTDYYFYYMDVW	IGH	2	None	
4	416	CAARPGYSYRDMW	IGH	4	None	
4	417	CARLYNNSPYYFDSW	IGH	2	None	
4	418	CVHRTRGWGADWFDPW	IGH	2	None	
4	420	CVKGDPQAVNAPDAFDLW	IGH	3	None	
4	421	CAAAFHSSGYYSIYYYYMDVW	IGH	4	None	
4	422	CARDGQWMALPYFDDW	IGH	4	None	
4	423	CATESYLWGSYRNFAYW	IGH	2	None	
4	424	CAREDIIAARLWDYW	IGH	6	None	
4	425	CARKRGGHGDNGLFDVW	IGH	6	None	
4	426	CVKDDKAGYWDGFDIW	IGH	2	None	
4	427	CARREYYSDSSGKISDAFDIW	IGH	3	None	
4	428	CARDDSAVAGVEIDYW	IGH	2	None	
4	429	CAKGLAAAGKIYYYYKDVW	IGH	6	None	
4	430	CARDDLGATTVDYW	IGH	4	None	
4	431	CTTEGSGTQLSYGHSYYMDVW	IGH	4	None	
4	432	CAKDYEMVIAIGVFDYW	IGH	2	None	
4	433	CAREYSSGWDYNWFDPW	IGH	4	None	
4	434	CARRVVADWFDPW	IGH	76	None	
4	435	CAREKDIVVVVSTSRGSAFNIW	IGH	4	None	
4	436	CVHRRLSSTSADYFLHW	IGH	29	None	
4	437	CASVGFCSGGTCYDDYW	IGH	3	None	
4	438	CVRDMGCGGDCYESGSPDYW	IGH	2	None	
4	439	CARHVGERRRQLDFFDYW	IGH	13	None	
4	440	CARGAFYGSGTFYNDFDYW	IGH	2	None	
4	441	CARDQRGNTYGSDYW	IGH	12	None	
4	442	CARVTTTSRIGAFDIW	IGH	16	None	
4	443	CARTPPLYSSSSQGAFDIW	IGH	3	None	
4	444	CARQGRYASSSFDYW	IGH	2	None	
4	445	CARASSWKYFHDSSDHHVSYYYIDVW	IGH	19	None	
4	446	CSRLPIVDEPVDYW	IGH	54	None	
4	447	CARREDTSMNFDYW	IGH	13	None	
4	449	CASTYTYYFDGSGYGIDYW	IGH	4	None	
4	450	CAHSDMDLLVVWHAFDFW	IGH	14	None	
4	451	CTTLSLWQLRMDGGFDTW	IGH	9	None	
4	453	CATDRGGPNSSGFPTDYW	IGH	2	None	
4	454	CAKDHGSLEWLLYLDVW	IGH	3	None	
4	455	CARGKEVAWFGDLDYW	IGH	2	None	
4	456	CARGALRDKHMPFDPW	IGH	4	None	
4	457	CAKDGGWELLGKDYFDYW	IGH	8	None	
4	458	CARAVPVSAANYFYMDVW	IGH	2	None	
4	459	CAKDITKWMVTPPGFDYW	IGH	2	None	
4	460	CARGGSRGLMPFDVW	IGH	6	None	
4	461	CARDQFCYGDCYSQGYFDFW	IGH	15	None	
4	462	CAGHTLVFAINGISSVFEYW	IGH	2	None	
4	463	CARDWSSYYQYFFDFW	IGH	5	None	
4	464	CARHGPNFDLWSGYAPRWFDPW	IGH	2	None	
4	465	CALRMMAGSNFDYW	IGH	2	None	
4	466	CAKVPRPYSYGTDDAFDIW	IGH	3	None	
4	467	CARDRGYSSYFDLW	IGH	11	None	
4	468	CAAGDDSMNGPMF	IGL	3	None	
4	469	CARDNRYYDSSGYSDAFDIW	IGH	732	None	
4	470	CARDFPNYGSGVDAFDIW	IGH	3	None	
4	471	CARFRYSSSWTPPFDYW	IGH	12	None	
4	472	CTKDVYHGSGSYYRGLYFDYW	IGH	2	None	
4	473	CAHRDQDDFDNW	IGH	2	None	
4	474	CASPPPKADSLGYYVRAYFEYW	IGH	2	None	
4	475	CARDNSSEYSSSSAFDYW	IGH	2	None	
4	476	CARGSLSYYYESSGYPPHDYW	IGH	3	None	
4	477	CARSGRLQRIYWDYW	IGH	4	None	

4	478	CARESIAATYYYYFDSW	IGH	20	None	
4	479	CARSRSYGSGSQTLIDYW	IGH	2	None	
4	480	CVRQKFGDHLDYW	IGH	5	None	
4	481	CARDWLDDYGDYWGLDLW	IGH	4	None	
4	482	CAKGGAQERRIISNMADSW	IGH	2	None	
4	483	CAGLYSTLSHFDYW	IGH	2	None	
4	484	CAKDPKYRGSGSHYNPDYW	IGH	3	None	
4	485	CARLGYNLNDVDYW	IGH	2	None	
4	486	CARDFRASSYCTNGVCGAFDIW	IGH	2	None	
4	487	CARYDTIRHASDYW	IGH	17	None	
4	488	CARETYWYENGDYYYTFDYW	IGH	2	None	
4	489	CTTDLLRFSHYMDVW	IGH	3	None	
4	490	CAKDFRFLEWSHSGYFDSW	IGH	5	None	
4	491	CAPPGSQEDTYFGYW	IGH	2	None	
4	492	CAREDMSEPSFDLW	IGH	7	None	
4	493	CAKIQCVRNFCSLYYFDHW	IGH	2	None	
4	494	CASTPHHSSDYYFHFDHW	IGH	6	None	
4	495	CARARRQTYLDFWSGYFESW	IGH	2	None	
4	496	CTTIRVTIFGNYFDYW	IGH	2	None	
4	497	CTRTYGGHADFDYW	IGH	9	None	
4	498	CLQYNIWPPWTF	IGK	1865	C1	7BZ5
4	499	CVRGGRAYSFDFW	IGH	4	None	
4	500	CARELGYCSSQSCYGSGFDYW	IGH	2	None	
4	501	CVRDVGHWSSFDVW	IGH	2	None	
4	503	CAKCPFDWPTLCPFDYW	IGH	2	None	
4	504	CQQYATWPPGPF	IGK	4	C2	7K8U
4	505	CARRVWGKYRLDYW	IGH	99	None	
4	506	CARSLRGYSGYGYFDYW	IGH	7	None	
4	507	CVRGDYDENKYRWFDPW	IGH	4	None	
4	508	CARVTSYYYDSDDYW	IGH	2	None	
4	509	CARPILRWPREFAFEIW	IGH	2	None	
4	510	CARLSALRSAFDYW	IGH	53	None	
4	511	CAKDPGRDPHESYFDYW	IGH	2	None	
4	512	CARGGFDTNGFTFAYW	IGH	11	None	
4	513	CARHLTLTLDAALPFDMW	IGH	2	None	
4	514	CASDLSSSWGWGQFDYW	IGH	2	None	
4	515	CAAHVVIVPAATFDYW	IGH	2	None	
4	516	CVKWGLSAVARPSDWFDPW	IGH	2	None	
4	517	CAKPKYSSSWSDAFDIW	IGH	2	None	
4	518	CASHVWQWLVPAQARGWFDPW	IGH	5	None	
4	519	CVRGAPSWGSNYFDYW	IGH	8	None	
4	520	CARESAGKLDAFDIW	IGH	12	None	
4	521	CAREGWDSYTSSWYYFDYW	IGH	2	None	
4	522	CARGYTSSLKTLYYMDVW	IGH	2	None	
4	523	CAKDLSSRSDYWRGYWGADYFDYW	IGH	2	None	
4	524	CASGITVTTFDYW	IGH	12	None	
4	525	CVRNAYITTWYHFDHW	IGH	2	None	
4	526	CGRVSGDSSVWTIDYW	IGH	2	None	
4	527	CARVRRMAFLIGAFDIW	IGH	12	None	
4	528	CTTVGCSSVGCYYFDFW	IGH	4	None	
4	530	CARHGWDLTHFDFW	IGH	2	None	
4	531	CARDHVLGDYYDSSGSHWFDPW	IGH	3	None	
4	532	CVHSPYYFDSSGYKEYFHHW	IGH	2	None	
4	533	CVRDYYDSSGLTYNAFDIW	IGH	8	None	
4	534	CARQSLYSDSTSYPPDYW	IGH	6	None	
4	535	CATYNWRDGSRRPDALDIW	IGH	2	None	
4	536	CATDHSMNSGSYFPPWFDPW	IGH	8	None	
4	537	CAKAPPGSSWYLGWFDPW	IGH	4	None	
4	538	CARARSAADDLWSSKTYFDYW	IGH	3	None	
4	539	CAHSRKSNSGNLAKYLGYHYYMDVW	IGH	2	None	
4	540	CARDQSYRVNWNDEDYW	IGH	7	None	
4	541	CARVWGAMEWQLWDYW	IGH	5	None	
4	542	CARDREPKDYDSSGYYYALGFDYW	IGH	2	None	
4	543	CVGGSSGWYKFDYW	IGH	5	None	
4	544	CATRPSGSTWYPYFDYW	IGH	4	None	
4	545	CARGDSSGYFHGDYW	IGH	2	None	

4	547	CARHPHDYSTYADDYW	IGH	60	None	
4	548	CARDRFMLFTPAAGNFDEW	IGH	3	None	
4	549	CARSPIAAGIDYW	IGH	9	None	
4	550	CARVGARNYADYPLDYW	IGH	2	None	
4	551	CGHRPIVGAAHFDHW	IGH	2	None	
4	553	CARSATWRVKNACDIW	IGH	22	None	
4	554	CVRDGTDGIVPWAVDYW	IGH	5	None	
4	555	CARLRVVYSHYFYYMDDW	IGH	7	None	
4	556	CAKDVAPTPFGRFDPW	IGH	8	None	
4	557	CATYQPPYFDDW	IGH	12	None	
4	558	CARGPLRCNKVTCHPGSFDPW	IGH	2	None	
4	559	CARQVDFSNPYYYYLDVW	IGH	3	None	
4	560	CQQFNSYPPAITF	IGK	157	C1	7BZ5
4	561	CAKLEEQELYFDNW	IGH	4	None	
4	562	CAADRGQPNSFDPW	IGH	2	None	
4	565	CARPRTYGALFVDYW	IGH	4	None	
4	566	CARLLISYDSSGSVGGYFDYW	IGH	7	None	
4	567	CASLDPFRGGFDYW	IGH	2	None	
4	568	CAREYYSSTFRSFDLW	IGH	2	None	
4	569	CASGPTTVRLSYMDIW	IGH	2	None	
4	570	CARGSPRGNWNYVDYFDYW	IGH	2	None	
4	571	CARHWDDSSGYSWDSIDYW	IGH	9	None	
4	573	CARDLGSASHRYFDSW	IGH	21	None	
4	574	CATRPPDTSMVYFFDYW	IGH	2	None	
4	575	CQSADTSRTYHVIF	IGL	11	None	
4	576	CTTRFASGSYYYSDYTDVW	IGH	2	None	
4	577	CQTWGTGFWDTGFWVF	IGL	6	None	
4	578	CARQATYYYDTDNYYYYMDVW	IGH	3	None	
4	579	CARRGSGFRLYHFDYW	IGH	2	None	
4	581	CARDSVTIIRGIIMYYFDYW	IGH	3	None	
4	582	CARDLEFVVVTAGFDYW	IGH	6	None	
4	583	CARPHQYSSFDYW	IGH	12	None	
4	584	CAREGVGASNALDIW	IGH	5	None	
4	587	CARGKTTVPHYYYMDVW	IGH	23	None	
4	588	CAKETGKYYFGSGSPTPMDYW	IGH	3	None	
4	589	CAKDHASHTSGLLDWYFDLW	IGH	3	None	
4	590	CATEPQGFRLDYMDVW	IGH	4	None	
4	591	CETWDSRLSVVAF	IGL	2	None	
4	592	CLRVEDTALYFCARGGVSGDSYWFFDYW	IGH	2	None	
4	593	CTREYYGGADSNRGFDYW	IGH	2	None	
4	594	CARGVVVAATRLFDPW	IGH	2	None	
4	595	CARETYAAAHAFDDW	IGH	2	None	
4	596	CARHKSGFSSGLDYW	IGH	5	None	
4	597	CVTTSNDILTWDYW	IGH	6	None	
4	598	CARGIFGVVILPYYKYYMDVW	IGH	2	None	
4	599	CARGRGYSPYEGYYFDYW	IGH	29	None	
4	600	CAKDVVWGYSSETEDYFDFW	IGH	3	None	
4	601	CARRTLGGFGDLPPFDFW	IGH	5	None	
4	602	CASLGSYGDYARDYW	IGH	18	None	
4	603	CAITYYYASGTYYDEEESDYW	IGH	3	None	
4	604	CARRTAVAGTWEDYYYYYMDVW	IGH	2	None	
4	605	CARFPTVDTTMGTANHDYW	IGH	4	None	
4	606	CARAYGSGTYFHFDYW	IGH	7	None	
4	607	CSSYAGTDNFHYVF	IGL	2	C2	7BWJ
4	608	CARRVQYYYGSGSYSHYFDFW	IGH	2	None	
4	609	CATGARRGYDFYYMDVW	IGH	26	None	
4	610	CAPDRQGFDPW	IGH	10	None	
4	611	CARMECLGGICSMYYFDNW	IGH	13	None	
4	612	CAMRPIYNDFWSGQRSWFDPW	IGH	3	None	
4	613	CARKSHDDIGAFYNWFDPW	IGH	2	None	
4	615	CVRDRSNYDPNYFDYW	IGH	2	None	
4	616	CTSDGPRTGYGFWSGYFDHW	IGH	3	None	
4	617	CTKDINSRFYNFYYFDVW	IGH	7	None	
4	618	CARESGESNNWPRRSYWYFDFW	IGH	2	None	
4	619	CARGNKVKRADYW	IGH	6	None	
4	620	CARGDYHDSGGYPLSFDYW	IGH	2	None	

4	621	CAKETVERIQLTQFDYW	IGH	4	None	
4	622	CARDPFRAYGSGWFTPFSFDYW	IGH	3	None	
4	623	CAGGWGPRASSGAFDIW	IGH	3	None	
4	624	CASPPYAGDWYYFDYW	IGH	2	None	
4	626	CARGRGDRFLRAFDFW	IGH	2	None	
4	627	CTRDLVYDYVWRYDYW	IGH	3	None	
4	628	CAKERGPMVRGGLAPIDYW	IGH	4	None	
4	629	CARWGHYGSGTYYDDW	IGH	7	None	
4	630	CARVELGIPRATFDSW	IGH	2	None	
4	631	CARQVNYDRSGYTQSTSAFDCW	IGH	4	None	
4	632	CAKPLAVRDYGGIRSAAFNIW	IGH	2	None	
4	633	CARRSLEPGMGGFDYW	IGH	2	None	
4	634	CAKDTGYSSGWHLVPGAVDYW	IGH	10	None	
4	635	CARERFAQIEKGYMDVW	IGH	7	None	
4	636	CARDLGSTCSSTSCYWRDWYVDYW	IGH	4	None	
4	637	CARADITDDVFDIW	IGH	6	None	
4	638	CAHRSPIGTSWYQDVW	IGH	3	None	
4	639	CARESPGDITEVVTFDVW	IGH	5	None	
4	640	CQQSFSTQCTF	IGK	42	C4	6ZDH
4	641	CVRGRGYSYGHGDYW	IGH	4	None	
4	643	CARGPYSSGYYAPLTFDYW	IGH	2	None	
4	644	CARTEYYDFRSGYATKFDYYYMDVW	IGH	2	None	
4	645	CQQTYSNPLVIF	IGK	2	C4	6W41
4	646	CTNTIQWRRFPFDFW	IGH	9	None	
4	647	CAKSMGWQWLTQGYFDSW	IGH	2	None	
4	649	CARHLDYGSGTYWGFDSW	IGH	7	None	
4	650	CARDGGSYDPDHYFDYW	IGH	3	None	
4	651	CARGRVGYKYWYFDLW	IGH	4	None	
4	652	CARGFWNGEKYYDYW	IGH	11	None	
4	653	CARPLGVVTAYDAFDIW	IGH	7	None	
4	654	CAKDIFVTGSGKHIHDYW	IGH	3	None	
4	655	CARENWESYYFDLW	IGH	2	None	
4	656	CASIFGLWAFDLW	IGH	9	None	
4	657	CTRDFLMVIPNYFDPW	IGH	6	None	
4	658	CARHSYGDSRGGFDYW	IGH	4	None	
4	659	CAHTYYDFWSGYSWFDPW	IGH	6	None	
4	660	CAKAPEDYGDLYFDYW	IGH	4	None	
4	661	CASNDLYDSSDYYVESVNWFKFW	IGH	8	None	
4	662	CARDLVTELSGYYPFDYW	IGH	8	None	
4	663	CARGFWMTTVTTGWSGGYYYYMDVW	IGH	4	None	
4	664	CAKHIEGLEKYYFEYW	IGH	2	None	
4	665	CAKDLHDYVWQSYRPPLDYW	IGH	2	None	
4	666	CAKSSSSSSDDPFDVW	IGH	2	None	
4	667	CAHRLGGESGSGWDQGAFDFW	IGH	5	None	
4	668	CVRSHFWSAYHLGWFDPW	IGH	138	None	
4	669	CARGKSGSFPYFFDSW	IGH	4	None	
4	670	CAKDKTSWYGESTPYFDHW	IGH	12	None	
4	671	CARDPQEWELLLDYW	IGH	4	None	
4	672	CARVSSYDDLDYLGHNYGDDAFDIW	IGH	2	None	
4	674	CMQDAQDPPAF	IGK	3	None	
4	675	CARSSGGNGFFDNW	IGH	4	None	
4	676	CAKARIQFGVLIYDFDFW	IGH	2	None	
4	678	CAKEGGGPVPWYFEQW	IGH	4	None	
4	679	CARDLWNYGGEPNFDYW	IGH	11	None	
4	680	CARDPRNYYDSSGYYYASWFDPW	IGH	4	None	
4	681	CAREVLYYDFWSGPQYFQHW	IGH	2	None	
4	682	CVRDWDSSLDYW	IGH	2	None	
4	683	CSSYAGSYTDTTYVF	IGL	2	C2	7BWJ
4	684	CARDQFPADWSVADPGGFDYW	IGH	2	None	
4	685	CARQVGNYNWNSAVRGMLYFFDYW	IGH	2	None	
4	686	CARVLTRTDLGVYHFDHW	IGH	3	None	
4	687	CAKAFTSGYYPEDVFDVW	IGH	3	None	
4	688	CARGCGGDCSDVFDIW	IGH	35	None	
4	689	CARHWGSVAADRILDYW	IGH	2	None	
4	690	CAKLGYSSGWYGYYFDSW	IGH	2	None	
4	692	CAHAGGPYSSSWYSFDYW	IGH	7	None	

4	693	CARDEGVWDTAMDYMDVW	IGH	2	None	
4	694	CARGPATFTDDSFDIW	IGH	9	None	
4	695	CAKYEGSGTYPQKTFDYW	IGH	4	None	
4	696	CARDTRPQVLFGESFSSFDAFDIW	IGH	5	None	
4	697	CTRDREYCSHTSCYRGWFDPW	IGH	8	None	
4	698	CASGGFFDGFDSW	IGH	6	None	
4	699	CARHGGAQLQRRPYFEYW	IGH	4	None	
4	700	CAREHRPRPATWGQSASDAFDVW	IGH	7	None	
4	701	CAADIIRFGGVIVPNGWFDPW	IGH	5	None	
4	702	CAREYYLDSSGRLDYW	IGH	2	None	
4	703	CARIGYYYDSSGYYHVDWFDPW	IGH	2	None	
4	704	CARRRGSSWGYNFDYW	IGH	4	None	
4	705	CARAPSYTDDPYGGDAFDIW	IGH	10	None	
4	706	CARVGIVMVAAAEFDYW	IGH	8	None	
4	707	CARDKGRFLEWQPFDMW	IGH	6	None	
4	708	CARRYYDNRGYYFFDYW	IGH	2	None	
4	709	CAHRHWGTFDYW	IGH	4	None	
4	710	CARDYSFEQLGRDYW	IGH	2	None	
4	711	CARRHGVAATLPPNDAFDIW	IGH	6	None	
4	712	CAPEVITPHDVFDIW	IGH	7	None	
4	713	CARRRVYGGSYFFDYW	IGH	5	None	
4	714	CARVGKDYIYRLYFDYW	IGH	2	None	
4	715	CARWDFDYGRNDDGGAFDVW	IGH	4	None	
4	716	CARGLRSLERFNPGDNFYYW	IGH	3	None	
4	717	CATEYGEDLAYFATW	IGH	6	None	
4	718	CAHKPSASTHFDYW	IGH	2	None	
4	719	CARLPGDFWNSYYPGRHWFDPW	IGH	3	None	
4	720	CARHRSYREPLDYW	IGH	3	None	
4	721	CARVKGSTEIDDAFDIW	IGH	6	None	
4	722	CARGMYSGSFPTVKLDYW	IGH	4	None	
4	723	CVRPKDYW	IGH	120	None	
4	724	CQSADRSGKMVIF	IGL	158	None	
4	725	CAHSLLYGSGRQRPLPFDYW	IGH	3	None	
4	726	CARDEALSVAGTSYYYYYMDVW	IGH	6	None	
4	727	CAKVEGNGWFNFEYW	IGH	5	None	
4	728	CAKESQIAGTYKPYLDYW	IGH	2	None	
4	729	CVRNPVRTGTFDPW	IGH	3	None	
4	730	CAKESGWYGDLLYYFDYW	IGH	2	None	
4	731	CARLRLSSWEYYYVDVW	IGH	2	None	
4	732	CAKFPGLQRLGYYYYYMEVW	IGH	6	None	
4	733	CVRPKAEGTGFFHYYYMDVW	IGH	3	None	
4	734	CARPQAEWDLLLPFDMW	IGH	13	None	
4	735	CARSAVRSIFGVVSAFDIW	IGH	2	None	
4	736	CARAHGSGNYYNYYYHYMDVW	IGH	2	None	
4	738	CARGPLYLGYSDDAFDIW	IGH	3	None	
4	740	CARSVLMVDVVTGWFDSW	IGH	2	None	
4	741	CARDPPGNYGSVRIHFDSW	IGH	2	None	
4	742	CARQDVWFGELLYFDYW	IGH	2	None	
4	743	CASGQDTSIWYGYPFHYW	IGH	6	None	
4	744	CARVHSGSYQGAFDYW	IGH	20	None	
4	747	CVRFLESEEELGGFDIW	IGH	3	None	
4	748	CAKHYDFWSGYPNAFDYW	IGH	6	None	
4	749	CARGTTLLVVTEPDGFDIW	IGH	5	None	
4	750	CAINFFDSRGSGLTFDHW	IGH	2	None	
4	751	CARSQGTWLQDIKYFDFW	IGH	10	None	
4	752	CAPQYSSGWGGFDPW	IGH	4	None	
4	753	CATHLSYYDSSGYPNYYFDSW	IGH	4	None	
4	755	CVRDCVDGVCYLFDPW	IGH	3	None	
4	756	CAKGGDYGGPRGPFDSW	IGH	2	None	
4	757	CAKVGNTYGYLSWFDLW	IGH	13	None	
4	758	CARVPPDSGVLTAYSYYFDYW	IGH	2	None	
4	759	CLLYYGVTQIWVF	IGL	12	None	
4	760	CAKYLAMIVDKGYFDYW	IGH	2	None	
4	761	CQSAASSETYMLF	IGL	3	None	
4	762	CVRHLRYGDSSDYW	IGH	14	None	
4	764	CVREAWEQWLTNWYFDLW	IGH	4	None	

4	765	CARHAPPGISFDPW	IGH	4	None	
4	766	CARQYSGYDPYFDCW	IGH	2	None	
4	767	CARDKKGSNYYYFDYW	IGH	2	None	
4	769	CARGSGTAYDYYLDVW	IGH	2	None	
4	770	CARGWTKQWLVTWYIDHW	IGH	3	None	
4	771	CARARTGWELNALDIW	IGH	2	None	
4	772	CATLSAGYTYGEYYFDYW	IGH	6	None	
4	773	CVLDPLWFGELVVVGRDYW	IGH	2	None	
4	774	CATGTGSGDGYNPPFEYW	IGH	2	None	
4	775	CARDVVVSAPKYFQHW	IGH	2	None	
4	776	CQRYRSYSPYMYTF	IGK	8	C1	7BZ5
4	777	CAKAATGYHYDSSGYRFDYW	IGH	3	None	
4	778	CARNRYYYDSGGYIGPDYW	IGH	3	None	
4	779	CARGANYDFWSGSYFDHW	IGH	2	None	
4	780	CARLKRYGAEPKGIFDHW	IGH	2	None	
4	782	CAHRRCSSCQFDYW	IGH	2	None	
4	783	CARDSGYYSPYYFDYW	IGH	49	None	
4	784	CARAAGGSYYAHFDYW	IGH	4	None	
4	785	CARDSPITPLGLVRTPDYYMDVW	IGH	4	None	
4	786	CARHWALGFRVVVPAAIVGAFDIW	IGH	2	None	
4	787	CAGRSRGIAATSQNWFDPW	IGH	6	None	
4	788	CARDEPFITGSHYYFRYW	IGH	5	None	
4	789	CARDFHNWNYARFYYW	IGH	2	None	
4	790	CTKDAPGDGSGDHIDYW	IGH	2	None	
4	791	CARYGPKLRVTNGFEYW	IGH	4	None	
4	792	CVRGGTDTLTKMGDYW	IGH	2	None	
4	793	CAREDCSGGSCYWNYFDYW	IGH	4	None	
4	794	CAKAHEELGKWDVYFDYW	IGH	5	None	
4	795	CARDPSIGWVTAVACDYW	IGH	2	None	
4	797	CARDRYESGNYPNWFDSW	IGH	3	None	
4	798	CVKGDAIGYYYYMDVW	IGH	6	None	
4	799	CARTECSDINCYAGDIDYW	IGH	5	None	
4	800	CQQYNNIPPGGGITF	IGK	4	C3	7K8R
4	801	CARERGDRFLDSLPNYYDHW	IGH	2	None	
4	802	CAKDFRKMNYYDRSGYPLDYW	IGH	2	None	
4	803	CATDGHGYGLDYW	IGH	13	None	
4	804	CARERRRGSSGLDASDIW	IGH	6	None	
4	806	CARARGDVYNPSHLDYW	IGH	4	None	
4	807	CARGWDWNNIRGSWFDPW	IGH	17	None	
4	808	CARAFSGTAPADYW	IGH	5	None	
4	809	CAHSDLHIWSHLDYW	IGH	3	None	
4	810	CARDRAHYSSSFDHW	IGH	2	None	
4	811	CAKDDAHSTSYSDYW	IGH	2	None	
4	812	CAKAYYYESDGYYYFAFDLW	IGH	2	None	
4	813	CARDRLGYTYTYYFDYW	IGH	5	None	
4	814	CANSRSSGYYIRGTFDYW	IGH	2	None	
4	815	CARDHKQWLVDALDTW	IGH	2	None	
4	816	CARAPWDGYKEIHYYYYYMDVW	IGH	3	None	
4	817	CARRRIRDREPYFDSW	IGH	3	None	
4	818	CASAFDYGDNNWFDSW	IGH	4	None	
4	819	CARGRGTMVRGAMGYSYMDVW	IGH	5	None	
4	820	CARGSRTAAYASDIW	IGH	59	None	
4	821	CAKDRHGVAAAGWGFDDW	IGH	2	None	
4	822	CATNRGTTFGIVLTDAADVW	IGH	3	None	
4	823	CARDHPTFTMIYAFDIW	IGH	2	None	
4	824	CAMSPSSLRQYYFQIW	IGH	2	None	
4	825	CMQSIQLPIAF	IGK	17	None	
4	826	CARRGTYDSIGWAEFDPW	IGH	2	None	
4	827	CAKEGRGYTSDEYLDSW	IGH	2	None	
4	828	CAKGAIAAAAYNWFDPW	IGH	7	None	
4	830	CGTWDNSLSTDHWVF	IGL	3	None	
4	831	CARTDIVVVVAATYDYW	IGH	2	None	
4	832	CQQAYNLPFNF	IGK	36	None	
4	834	CARHINGGNFVPFGYW	IGH	7	None	
4	835	CAKDWDGITFGGPTPRFDPW	IGH	2	None	
4	836	CATSPGHGGNAKFDHW	IGH	7	None	

4	838	CARGLSGSNNRYFQHW	IGH	4	None	
4	839	CANSAQDLLWFGHFDYW	IGH	4	None	
4	840	CSGDRTVVTAILDDYW	IGH	2	None	
4	842	CARQGRKWNYGSYDLDAFDVW	IGH	4	None	
4	843	CVKDVDAHGDYEAFHYW	IGH	3	None	
4	844	CARDVGYYSGLSKWLDPW	IGH	3	None	
4	846	CAKGRGYSGGSFDHW	IGH	7	None	
4	847	CARAIIAITFYFDYW	IGH	2	None	
4	848	CVRDLMTSENFDYW	IGH	3	None	
4	849	CARSRDAYSHFDYW	IGH	117	None	
4	850	CARGDTSSGWETFFDYW	IGH	2	None	
4	851	CATHARIEAAGTPFDYW	IGH	4	None	
4	852	CARDSVTYSSSWYYFDYW	IGH	5	None	
4	853	CARDRSDFSAFDLW	IGH	8	None	
4	854	CARGLSDEWHSGYRAPRLDYW	IGH	5	None	
4	855	CTRGSSSWYPLDYW	IGH	2	None	
4	857	CARDRFGGFGESLPLGDYW	IGH	3	None	
4	858	CAKHRKSFVLRGAHFDDW	IGH	2	None	
4	859	CAKEQVSKNRYYFFYYDYW	IGH	4	None	
4	860	CAKDLAAHNDYRYYMDVW	IGH	3	None	
4	861	CARGGDGGYSDSNGYYYFDYW	IGH	6	None	
4	862	CARRLSPAAFSYYFDFW	IGH	2	None	
4	863	CARIELVTTRKGFDYW	IGH	2	None	
4	864	CGRSSSSSYFHLYYFDYW	IGH	2	None	
4	865	CARDRGSSSGRGVDYW	IGH	4	None	
4	866	CQQYNNLLALTF	IGK	36	C3	6XDG
4	867	CARDPDSSGWPYFFDLW	IGH	21	None	
4	869	CARDLGGYDRSGHRSGFFDDW	IGH	7	None	
4	870	CIASGYDPDNFDYW	IGH	24	None	
4	871	CGNSPYYYDSSASNAFDIW	IGH	7	None	
4	872	CAREGPVTGDFVDYW	IGH	6	None	
4	874	CHQYNNWPYVF	IGK	49	C3	7K8P
4	875	CARVKRFNRGFDQW	IGH	2	None	
4	876	CARDQIRAGYGSGSSDYW	IGH	6	None	
4	877	CAKDRGMSIAALSEAFDIW	IGH	2	None	
4	878	CVSGFYDFWSGYPFAYW	IGH	2	None	
4	879	CIREVRNRRGGSGLWIDPW	IGH	5	None	
4	880	CARVAHNDYLWFFDLW	IGH	2	None	
4	881	CARTTFWSGYRYFDFW	IGH	4	None	
4	882	CARVRVPNTAIDYW	IGH	6	None	
4	883	CAKAGITIFGVVPWFDPW	IGH	24	None	
4	884	CVRDRGEVARQYYFDHW	IGH	2	None	
4	885	CAREMAGPFGVVRQYNFDSW	IGH	5	None	
4	886	CAKIGGGESVGWYFDYW	IGH	5	None	
4	887	CASSIIEAGTIDYW	IGH	2	None	
4	888	CARLHGSGSYYTRNLHYYMDVW	IGH	2	None	
4	889	CAYSSVGGTLLGAFEIW	IGH	4	None	
4	890	CARDGTQFTTWGLYYLDVW	IGH	13	None	
4	891	CARHLPAAALSYGPFDYW	IGH	6	None	
4	892	CEQFDSYPPSF	IGK	22	C1	7BZ5
4	893	CVRGLYSGNPIFDYW	IGH	2	None	
4	894	CARGSSASGDYGDNW	IGH	3	None	
4	895	CARGTHFDFWRGSYAYIDLW	IGH	3	None	
4	896	CARSRYDWNDRYFDFW	IGH	18	None	
4	897	CARDLMAGIIAGCFDPW	IGH	4	None	
4	898	CARHSPYYYGSGSPSYFDYW	IGH	15	None	
4	899	CVRGLAGRMNDAFEIW	IGH	2	None	
4	900	CARAKPDFWIDSW	IGH	16	None	
4	901	CARDGTVPAATTSEYFQHW	IGH	2	None	
4	902	CARHGTYHDFLSVYQDPHWIDSW	IGH	84	None	
4	903	CVKHEINVVAHFFDSW	IGH	2	None	
4	904	CARGDQKPAFSLDYW	IGH	11	None	
4	905	CVRDKSELRDVDYYYYYMDVW	IGH	4	None	
4	906	CARHESSAWYRVAAFDVW	IGH	87	None	
4	907	CARDTWNFEKFYYYYMDVW	IGH	3	None	
4	908	CARIWRSSIAARPWYFDLW	IGH	2	None	

4	909	CARDPRMSGFCASINPDYFADW	IGH	4	None	
4	910	CAKSMHGPFDYW	IGH	45	None	
4	911	CVRRGISSWSSTGSWYFDRW	IGH	3	None	
4	912	CARHETPKDRNAYYDFWSGFYYMDVW	IGH	3	None	
4	913	CTKEYDFWSGPDYW	IGH	21	None	
4	914	CARATSITIFGVVSGAWFDPW	IGH	4	None	
4	915	CARDSSHLWFFDYW	IGH	4	None	
4	916	CALPICTVYMELSSLSSEDTGIYYCARDTSRGFGYW	IGH	4	None	
4	917	CARGLGSSGWHIDYW	IGH	2	None	
4	918	CARPRRWSYFENW	IGH	2	None	
4	919	CATVPLRYLVSGFDPW	IGH	5	None	
4	920	CTRQRSATGEHYFDSW	IGH	2	None	
4	921	CSTDDIWGRDYW	IGH	2	None	
4	922	CARGAPRAYYEMAEYFQHW	IGH	2	None	
4	923	CARAGEQFRSSLTLFDSW	IGH	2	None	
4	924	CATWDDTLSGPKFVF	IGL	2	None	
4	925	CAKDRAVVVFPILDYW	IGH	4	None	
4	926	CAREGHSSGYAGTFDVW	IGH	2	None	
4	927	CVRADGPYSSSFAFDHW	IGH	2	None	
4	928	CARAPQMLDCSGGSCHISYYYYSMDVW	IGH	2	None	
4	929	CARDTYYDSSGYYRGAGSYYYYYMDVW	IGH	2	None	
4	930	CARVVARAAAGTFNYYYYYYMDVW	IGH	2	None	
4	931	CAKKTDNYYYRSGYSSDAFDIW	IGH	41	None	
4	932	CAMVDGGYYYYYYMDVW	IGH	2	None	
4	933	CATGPDYYEGSGYYYGKYYFDYW	IGH	2	None	
4	934	CARDWPPDGATSGNFAYW	IGH	6	None	
4	935	CARDHPDNDYGDYAGYW	IGH	18	None	
4	936	CALPISESSDSSAYFLFW	IGH	3	None	
4	937	CQVWDYSGDHRNYVF	IGL	2	None	
4	938	CARHLGYCSSTSCFLLNNWFDPW	IGH	2	None	
4	939	CAREGPTRDFDSW	IGH	6	None	
4	940	CARADGVGAQFDYW	IGH	4	None	
4	941	CARAHYTVMVQHFDSW	IGH	6	None	
4	942	CARGQPLLYPLDYW	IGH	14	None	
4	943	CARCNYDYVWGSYRPSGYFDYW	IGH	2	None	
4	944	CARRGRGTHALWYFDHW	IGH	4	None	
4	945	CARKRGHSYGSWAYFDNW	IGH	4	None	
4	946	CQQTYTTPRALSF	IGK	19	C2	7K8U
4	947	CTTDARGYPDYW	IGH	3	None	
4	948	CGAWDSSLSGGHYVF	IGL	2	None	
4	949	CARDNLVYDSWSGRSFDYW	IGH	3	None	
4	950	CAREALAAAGYYFDYW	IGH	12	None	
4	951	CAREGTLEAAGLFDSW	IGH	4	None	
4	952	CARRLFLPAAHPYDAFDIW	IGH	10	None	
4	953	CAREGSGSNYLNDGFDIW	IGH	3	None	
4	954	CARTPLQWLVLDYW	IGH	36	None	
4	955	CVRSPGGEPLHPFDYW	IGH	15	None	
4	956	CARDGHTVGWYKADYYNYMDVW	IGH	9	None	
4	957	CARDREYSSSLHYFDYW	IGH	8	None	
4	959	CAREYVAVASYYMDVW	IGH	14	None	
4	960	CARGSADDGDYDLDYW	IGH	4	None	
4	961	CASQRRDYGGNWYFDLW	IGH	13	None	
4	962	CARPRRVNSWSDFDYW	IGH	2	None	
4	963	CARPIGGYVGGFDVW	IGH	4	None	
4	964	CLLYF	IGL	2	None	
4	965	CACRLAVAGTKGAFDLW	IGH	2	None	
4	966	CARASHSILRTLDYW	IGH	2	None	
4	967	CARRGWNYDAFDFW	IGH	7	None	
4	968	CSKTPFHFYDSSAYPAGDAFDIW	IGH	2	None	
4	969	CAKHTLTRRFLEWTIEGHFDYW	IGH	2	None	
4	970	CARGSARWLFSSVFDYW	IGH	2	None	
4	971	CARHLSSGAGRPFDYW	IGH	2	None	
4	972	CAGAPGGVRGFDVW	IGH	2	None	
4	973	CARAWYYYDSSGYSLGWFDPW	IGH	2	None	
4	974	CTPTYDISGYYPYYFDYW	IGH	4	None	
4	975	CARDSPVTTPLAAFDLW	IGH	2	None	

4	976	CARIPDSYSAGGVFDYW	IGH	2	None	
4	977	CARHWFGAIAGPDYW	IGH	5	None	
4	978	CARDWGTQERFDYW	IGH	2	None	
4	979	CQHRSNYPLITF	IGK	21	None	
4	980	CASFYFNADYW	IGH	3	None	
4	981	CSHRVHGEEFNYW	IGH	7	None	
4	984	CARIRSDYGVVISRGGWFDPW	IGH	2	None	
4	985	CARFYGSGSHFFDYW	IGH	9	None	
4	987	CARRPDIAAAGTYAFDIW	IGH	2	None	
4	988	CARHPLGRGGAVDYW	IGH	14	None	
4	989	CVVCAFWSLDYNFDYW	IGH	5	None	
4	990	CTKEAPLGGTTARPGGFAFDGW	IGH	85	None	
4	991	CASMITSFFYYMDVW	IGH	3	None	
4	992	CVREACSGGSCFFDPW	IGH	7	None	
4	993	CARDAKNLALLDYW	IGH	2	None	
4	994	CARADSGYGPIYYDSW	IGH	2	None	
4	995	CVRDRRGCSTTSCYSYFDQW	IGH	2	None	
4	996	CARATFDYLDVW	IGH	23	None	
4	998	CASYRITIFGPNWFDPW	IGH	2	None	
4	999	CAKAFYFDFWSGYYGPSDYW	IGH	62	None	
4	1000	CARDEAGSYYMNWFDPW	IGH	4	None	
4	1003	CARARIPARWYWFDPW	IGH	6	None	
4	1004	CARDRRGYCSGGSCYSGFFDYW	IGH	4	None	
4	1005	CARVASYFGSGSKDDYW	IGH	2	None	
4	1006	CAKDASWYSSSWYFDYW	IGH	14	None	
4	1007	CARLLYPVYSSSSNWLDPW	IGH	2	None	
4	1008	CAKAYYGSGSYYKEFFDYW	IGH	25	None	
4	1009	CARDRGTYYYENYYMDVW	IGH	14	None	
4	1010	CASRAGDDYNSFYYYYMDVW	IGH	4	None	
4	1011	CAHRRHRGGNPFDFW	IGH	2	None	
4	1012	CASPRSVLGVTTSWFDPW	IGH	2	None	
4	1013	CSSYTGSSTGSSSPVLF	IGL	73	None	
4	1014	CASTGDNWNYYYYYYMDVW	IGH	2	None	
4	1015	CAKKGGNYYYDSSGSYFDHW	IGH	2	None	
4	1016	CARDTEVGSGWSTDYFDSW	IGH	2	None	
4	1017	CARDQNSHSRSPFDYW	IGH	4	None	
4	1018	CARTILGGSGSGADYW	IGH	2	None	
4	1019	CARVGFIAGFFYYYMDVW	IGH	3	None	
4	1020	CASYTSSRTFKF	IGL	35	C2	7K90
4	1021	CAREDYDYVWGTYRTFDYW	IGH	5	None	
4	1022	CARVTEETASITQFDYW	IGH	4	None	
4	1023	CARRARGAEWQPFDNW	IGH	2	None	
4	1024	CARGEIERFTGWENFDFW	IGH	2	None	
4	1026	CAKDIKTAKGWYFDLW	IGH	2	None	
4	1027	CAKDPKFWSGSYYYYMDVW	IGH	2	None	
4	1028	CARHFEGAGFCYGGGCYYLDSW	IGH	3	None	
4	1029	CARDLLASCGGDCPKWYFDLW	IGH	6	None	
4	1030	CARIHLGYGDNFNALDVW	IGH	2	None	
4	1031	CARSPWAILDRYYYYYMDVW	IGH	2	None	
4	1032	CVRGRPLYYMDVW	IGH	2	None	
4	1034	CAREKPYDTRTYFYYYMDVW	IGH	70	None	
4	1035	CARLDFGSGSYIYYYFMDVW	IGH	2	None	
4	1036	CARHTYGYKDYW	IGH	13	None	
4	1037	CARTLMITFEGVIAHDSFDIW	IGH	4	None	
4	1038	CTRDRSGDFDYW	IGH	6	None	
4	1039	CATGQWDCTNNVCSSAEYFHDW	IGH	2	None	
4	1040	CARSRGYYDGSGYYYFFDYW	IGH	2	None	
4	1041	CARDHGGELPPTDYW	IGH	37	None	
4	1042	CARHAWHGGNHLDYW	IGH	3	None	
4	1043	CARCPVGAAGSCYYYYYMDVW	IGH	2	None	
4	1044	CARLGRSSTSWRVYYMDVW	IGH	2	None	
4	1045	CARAGSYSSSWYDWFDPW	IGH	2	None	
4	1046	CARADYYDTDGNYLRVFDLW	IGH	9	None	
4	1047	CAAWDDNYSGKWLF	IGL	26	None	
4	1048	CTRDPSGNNPLPFDAW	IGH	2	None	
4	1049	CARVEEITIFGVVIIPGYMDVW	IGH	5	None	

4	1050	CAREVPSGLGGYFDPW	IGH	5	None	
4	1051	CVGGDSYGYSHYW	IGH	3	None	
4	1052	CARRLEDCSSTSCYASYWFDPW	IGH	5	None	
4	1053	CARGGSWSGDSNAFDIW	IGH	4	None	
4	1054	CAKGTDYGDYYYYMDVW	IGH	6	None	
4	1055	CARQAGVYKWNDPLYYYYYMDVW	IGH	4	None	
4	1056	CARVLGYYDSSGYYLGWFDSW	IGH	2	None	
4	1058	CAKAPVHSSSWSLDYW	IGH	6	None	
4	1059	CARGRDFYCSGGSCYSSDFDYW	IGH	5	None	
4	1060	CAIPPRGAGTMEYYMDVW	IGH	2	None	
4	1061	CQSAASIGIWLF	IGL	9	None	
4	1062	CARENEEYCGADCQWAYYYYMDVW	IGH	3	None	
4	1063	CARVQTYCSGGSCSSWFDPW	IGH	2	None	
4	1064	CAREGGDYEGWYFDFW	IGH	4	None	
4	1065	CARGSLSGWVFGEKWFDPW	IGH	2	None	
4	1066	CASQLTIAVTGHFDSW	IGH	7	None	
4	1067	CARDAVAGIGYWYFEHW	IGH	3	None	
4	1068	CARETTEMAAVGRGWFDPW	IGH	2	None	
4	1069	CARHVITIFGVVIISGGWFDPW	IGH	4	None	
4	1070	CARRHGLWKHPFDSW	IGH	158	None	
4	1071	CARDLYRPGDASDIW	IGH	4	None	
4	1072	CARGHDIIAVPGGPLYNWVDPW	IGH	2	None	
4	1073	CARGYDTTGYHSFDYW	IGH	2	None	
4	1074	CARTQYSTSWYDYW	IGH	5	None	
4	1075	CCSYAGSYSFVF	IGL	839	C2	7K8X
4	1076	CARVGWELLGHFDYW	IGH	2	None	
4	1077	CARRSNGLGGGNAFDLW	IGH	3	None	
4	1078	CAAVGSWGEFRYW	IGH	3	None	
4	1079	CANSHARRWYSYDAFDIW	IGH	6	None	
4	1080	CARDGGDYPPAWYFDLW	IGH	7	None	
4	1081	CARSIDGGDTLYYFDYW	IGH	2	None	
4	1082	CSSYSSSNPVKRVF	IGL	2	C4	7JVC
4	1083	CARSDIAVAGSYYYYYYMDVW	IGH	2	None	
4	1084	CASGLKQLVLGSANYYYMDVW	IGH	5	None	
4	1085	CAREAFGLSKPFDIW	IGH	2	None	
4	1086	CAREVLFCTNGICSFYYFDHW	IGH	21	None	
4	1087	CARDWNGGRSYWYFDLW	IGH	2	None	
4	1088	CARERSNSNYVWLRPFDYW	IGH	3	None	
4	1090	CTKDSANGLYRDGHIFDFW	IGH	2	None	
4	1091	CVRAPLGLQQQLDYW	IGH	5	None	
4	1092	CAKGRTNSPLWYFDSW	IGH	4	None	
4	1093	CARHSGGLRYFDWLLQIGFFDYW	IGH	3	None	
4	1094	CQVWDGSRDHLFWVF	IGL	2	None	
4	1095	CARHDTRSGVWTGPFDCW	IGH	5	None	
4	1096	CNQYKSYPYSF	IGK	10	C3	7K8P
4	1097	CARATKDKGDRRWFDPW	IGH	2	None	
4	1098	CGRQYLSTERDLGDVFDSW	IGH	7	None	
4	1099	CVRSSPGYFNNWAYYYMDVW	IGH	5	None	
4	1100	CVRDAGYTTVGGYFDYW	IGH	3	None	
4	1101	CARHAAMVNSPFDYW	IGH	3	None	
4	1102	CARGRISGSSWYLDYW	IGH	5	None	
4	1104	CARTHGSGLMLFEYW	IGH	10	None	
4	1105	CARGTGYYDTVGGWYFDYW	IGH	3	None	
4	1106	CARDNAVLLWFGESFTGAFDIW	IGH	2	None	
4	1107	CARDPGGFCIGGTCSYWYFSLW	IGH	3	None	
4	1108	CARLLRRGAVAGNYFDYW	IGH	13	None	
4	1109	CARVVNLSSGWYYYYYMDVW	IGH	2	None	
4	1111	CARGGGTYSRHNWFDPW	IGH	4	None	
4	1112	CARSQTSVHGSATRSVATDYFDLW	IGH	2	None	
4	1113	CATFSGFIAARPGFDYW	IGH	4	None	
4	1114	CARGPWGYWYFDLW	IGH	43	None	
4	1116	CARLNFDFWSSYYNGRNFDHW	IGH	2	None	
4	1118	CAKDGGFRFLERLFDPW	IGH	2	None	
4	1119	CVKDITHTWGLLNDGFDVW	IGH	4	None	
4	1120	CARGPMVVADQNWFDPW	IGH	9	None	
4	1121	CARDPGRGVGAKIGSSWYFDLW	IGH	24	None	

4	1122	CVRDGGGSYHGHYYYMDVW	IGH	4	None	
4	1123	CARRMAVVGAFGANGASDIW	IGH	2	None	
4	1124	CAKGVGAARSRLGFLDYW	IGH	4	None	
4	1125	CTKERTYQGLNLVYYFDPW	IGH	3	None	
4	1126	CARMYYYDSSGSRPDFDYW	IGH	9	None	
4	1127	CAKSIQGDDYAFDYW	IGH	2	None	
4	1128	CARETGDYSSSYFDYW	IGH	4	None	
4	1129	CARDGEQYQLLSLSYYYYYMDVW	IGH	10	None	
4	1130	CARDTEFRSAPKFDYW	IGH	2	None	
4	1131	CATHRRDCSGVSCFPIFYAYYMDVW	IGH	2	None	
4	1133	CVKDKSTGFALMVGFDYW	IGH	10	None	
4	1134	CLKTAHFFDGSGFHYPFDYW	IGH	2	None	
4	1135	CAALYGDHYSYYLDVW	IGH	2	None	
4	1136	CARRDYSSGWLHDAFDIW	IGH	6	None	
4	1137	CGKRSSGWPLDDYW	IGH	3	None	
4	1138	CAREPIPLLGIHFDYW	IGH	2	None	
4	1139	CAKLLQFIVAHTDYFDYW	IGH	2	None	
4	1141	CAKNANFPPEAPPYFDSW	IGH	2	None	
4	1142	CVRAPGVVIPGVEYSYYMDVW	IGH	3	None	
4	1143	CLKDGLPSRPFDSW	IGH	2	None	
4	1144	CAKTGSRSPTGWNYYLDLW	IGH	2	None	
4	1145	CQSYDGDLSGSKVF	IGL	811	None	
4	1146	CARDPGYSSGWVTFDPW	IGH	4	None	
4	1147	CARGDSSSSSVHGHALDIW	IGH	4	None	
4	1148	CARFNMDYYASGAGPRDYW	IGH	5	None	
4	1149	CARTEGAYGDQSYYHFDYW	IGH	2	None	
4	1150	CARLTGSWFNFDYW	IGH	5	None	
4	1151	CARGAPERSFEWLIAYYYMDVW	IGH	2	None	
4	1153	CARHDFWSGYYHHFDYW	IGH	8	None	
4	1154	CARGEGHNWNVPIFFDLW	IGH	2	None	
4	1155	CARIRGRGYMDVW	IGH	8	None	
4	1156	CAREFLGGYNYAGPQGWFDPW	IGH	5	None	
4	1157	CARVDRYYDSRGHYEETYLDFW	IGH	2	None	
4	1158	CASRPFSFGPFDHW	IGH	4	None	
4	1159	CARDRIAARPKGPYYFDNW	IGH	2	None	
4	1160	CAKDSLDFNILTGYSYMDVW	IGH	10	None	
4	1161	CARTPNTNPADYW	IGH	2	None	
4	1162	CAKDFWSHNGIYDAFDVW	IGH	2	None	
4	1163	CARGYYYGSGSYARYYYMDVW	IGH	2	None	
4	1165	CARGRDDSGTYSTPFDFW	IGH	3	None	
4	1166	CARAHRWIGYDAVTNWFDPW	IGH	2	None	
4	1167	CTTYSSGYYYGDYW	IGH	6	None	
4	1168	CARVLGRGSYWYGDYW	IGH	3	None	
4	1169	CARLTMTMDVQPIDYW	IGH	6	None	
4	1170	CATATQYYTSYEYYFEYW	IGH	5	None	
4	1171	CARHAEERDDYNFYYYYMDVW	IGH	13	None	
4	1172	CAKNYCSGGSCFRKYYFYYYMDVW	IGH	3	None	
4	1173	CVKGGRFTAPDYW	IGH	9	None	
4	1174	CAKGRWSGDYVPNFDFW	IGH	6	None	
4	1175	CARRDFWGSYCDYW	IGH	14	None	
4	1176	CARQGHILTGYYYYYMDVW	IGH	7	None	
4	1177	CATARYSSSSEFYFHYW	IGH	2	None	
4	1178	CATKPPGGGTYYDYW	IGH	5	None	
4	1179	CARKEDDSSASDYW	IGH	4	None	
4	1180	CATVRGVVVAGIRGGWFDPW	IGH	11	None	
4	1181	CAREGNTMVDGAMDVW	IGH	4	None	
4	1182	CARGGDSYGAYW	IGH	3	None	
4	1183	CARGATYSRGWYIRDYW	IGH	4	None	
4	1184	CVRYGSGTYTDSW	IGH	4	None	
4	1185	CQRYDNVPSFTF	IGK	668	C3	6XDG
4	1186	CASYWDYYDSRGYYHHDDFDIW	IGH	2	None	
4	1187	CARDRNWRSGYNYNWFEPW	IGH	3	None	
4	1188	CARPATFISVFDYW	IGH	2	None	
4	1190	CAKDPHLYCSSTSCFNYYFDFW	IGH	9	None	
4	1191	CARLDAARRTFDYW	IGH	9	None	
4	1193	CAADLWRDGIWHVFDIW	IGH	8	None	

4	1194	CCAKDRPYGDYGAPPFDAFDMW	IGH	2	None	
4	1195	CAKHYYYDSSGYSSFDYW	IGH	3	None	
4	1196	CARDYCGTMACDIEGAFDVW	IGH	9	None	
4	1197	CARGPPKRELRGGLNYYYYMDVW	IGH	2	None	
4	1198	CARESNGYTYYFDFW	IGH	3	None	
4	1199	CAHRLWIGQWNYPFDHW	IGH	2	None	
4	1200	CARRGGNHYDSSGFYVKWFDPW	IGH	2	None	
4	1201	CARSPGPWLVNHYFDYW	IGH	9	None	
4	1202	CARRRARGEYAFDFW	IGH	5	None	
4	1203	CARHIKRWLQLGGGLWYFDYW	IGH	3	None	
4	1204	CVRLWGLGLAWYFDVW	IGH	3	None	
4	1205	CARPGGSYEGYFEYW	IGH	2	None	
4	1206	CARDSWAGQDLFGFHFFDYW	IGH	2	None	
4	1207	CAKDTTYYYDSSGYYYGGRDVDYYLDSW	IGH	18	None	
4	1208	CARNRGTSSGVVISDASDVW	IGH	7	None	
4	1209	CARENRGFCSGGICHSYLDYW	IGH	4	None	
4	1210	CAREGTSYRWYFDLW	IGH	14	None	
4	1211	CAKGHWDYGGNSDIADYW	IGH	2	None	
4	1212	CARHKGYLSSSTASDYW	IGH	2	None	
4	1213	CGRSGGTYWYVDLW	IGH	3	None	
4	1214	CAREGTFYYGSGSLNWYFDLW	IGH	2	None	
4	1215	CARSRGGWYRHFDLW	IGH	2	None	
4	1216	CAKAGGHISIFPKWFDPW	IGH	2	None	
4	1217	CTTARGVLRFLEWLRSSFDYW	IGH	10	None	
4	1218	CVKGHISMNGSPFDYW	IGH	6	None	
4	1219	CARAKRVVTTPDFWYFDLW	IGH	2	None	
4	1220	CARQLGGRVVAALDYW	IGH	6	None	
4	1221	CARGTTYDTLTGGLWTWFDSW	IGH	6	None	
4	1222	CARDQGDSGGWNFDIW	IGH	4	None	
4	1223	CAKDMGGHGDMLGYFDYW	IGH	6	None	
4	1224	CARHSAILTGDFDLW	IGH	2	None	
4	1225	CARGQNYYGSGSYYWFDPW	IGH	5	None	
4	1226	CARHAPVWQWSYGGFYFDYW	IGH	9	None	
4	1227	CARMYSIYYMDVW	IGH	9	None	
4	1229	CARHVGSVGALILDAFDVW	IGH	4	None	
4	1230	CARLRREGETTIFGVVNPKYYFDYW	IGH	2	None	
4	1231	CARGNYYDNTGYYAFDIW	IGH	3	None	
4	1232	CAKDLFRFPYACDIW	IGH	5	None	
4	1233	CARVMTTVTKYYFDYW	IGH	5	None	
4	1234	CARLNSGYDWGPGYYYYYMDVW	IGH	4	None	
4	1235	CARVAGWQWNDVFDIW	IGH	8	None	
4	1236	CARLTGSSRWYAFEYW	IGH	2	None	
4	1237	CARGLPSLRFLEWLLIGDYW	IGH	7	None	
4	1238	CARGPRGYTSGIDYW	IGH	6	None	
4	1239	CARPNTRGYSDYDHWYFDLW	IGH	2	None	
4	1240	CQQLNTYVYPF	IGK	12	C1	7BZ5
4	1241	CAREYAEVTIFGVVRGNWFDPW	IGH	2	None	
4	1242	CAKEAWFGPRNYMDVW	IGH	2	None	
4	1243	CARGAGGSYSNFDSW	IGH	2	None	
4	1244	CAKGPRSGYYGQDTWFDPW	IGH	4	None	
4	1245	CAREPGIAESNRYFDLW	IGH	2	None	
4	1246	CAKYGSSWYLFDYW	IGH	9	None	
4	1247	CARDGRIWFGELLYLFYW	IGH	4	None	
4	1248	CAHTRRSHFNWLSGNWFDPW	IGH	4	None	
4	1249	CARHDYGWGYFDPW	IGH	6	None	
4	1250	CATGGTYGSRWSYFDYW	IGH	2	None	
4	1251	CAKYIIGSMIDYW	IGH	5	None	
4	1252	CARGTQRTDAFDIW	IGH	32	None	
4	1254	CAKDRNIVPTSNDYW	IGH	5	None	
4	1255	CAKEDMILIPAAVDYW	IGH	2	None	
4	1256	CARSGSYHQIPDYW	IGH	2	None	
4	1259	CARGEYSDTNAFDMW	IGH	7	None	
4	1260	CQETYSTPLLTF	IGK	219	C4	6W41
4	1261	CARVWGKWLNRHNWFDPW	IGH	3	None	
4	1262	CAKDRFYYDSTGPFDSW	IGH	2	None	
4	1263	CARHTPSYGFWSGYRNWFDPW	IGH	49	None	

4	1264	CARLRSGYNYESYYFYYYMDVW	IGH	3	None	
4	1265	CARPSNSWYGALDYW	IGH	2	None	
4	1266	CQVLDSSSDQYVF	IGL	58	None	
4	1267	CAKDLRPTTSRVVAPDAFDLW	IGH	28	None	
4	1268	CVKGGYSIFGVAIRDYW	IGH	4	None	
4	1270	CARSPDGYGGQDYFFDYW	IGH	2	None	
4	1271	CARSVVLLFLDWSGSKFHYYMDVW	IGH	4	None	
4	1272	CAKDRRFRVPSVFDYW	IGH	7	None	
4	1273	CARSPRTLRFTMVRGVRDAFDVW	IGH	2	None	
4	1274	CARDPGSYYDSSGYPQGAFDFW	IGH	4	None	
4	1275	CARDRMDGGNTGWYFDLW	IGH	10	None	
4	1276	CAKFLPYCEGDCYPSNYFDLW	IGH	13	None	
4	1277	CARPADYSSGTFDAFDIW	IGH	5	None	
4	1278	CARLKRGSSWYKTFDIW	IGH	3	None	
4	1281	CTKNSGASHGTALALAHDYW	IGH	4	None	
4	1283	CARYSSNWFEAFDIW	IGH	3	None	
4	1284	CARDSLQAFDVW	IGH	26	None	
4	1285	CARSTVMTGFDYW	IGH	8	None	
4	1286	CAKWEKTTVPMTYWHLDLW	IGH	2	None	
4	1287	CARGFQTYYSADYW	IGH	9	None	
4	1288	CAGWDDSLTGPNWLF	IGL	2	None	
4	1290	CARTPSHWGTHSYYYYMDVW	IGH	5	None	
4	1291	CARLSTPAAGVLEMLSPWYFDLW	IGH	127	None	
4	1292	CARGKSVAGLTRDFDFW	IGH	84	None	
4	1293	CQSYDISLSVDLVDVIF	IGL	8	C4	7JVC
4	1294	CVRHPGTTIVDGFQIW	IGH	2	None	
4	1295	CARGLGAATGEALDIW	IGH	3	None	
4	1297	CARRLGRLTSSTAFFGATYDYW	IGH	2	None	
4	1298	CARHIWIFGVISYPYYFDYW	IGH	14	None	
4	1299	CASRNCQSTTCYEKIGHFYFYYMDVW	IGH	4	None	
4	1300	CVREIITRSLSFDYW	IGH	27	None	
4	1301	CARPIRMRVFDAFDIW	IGH	6	None	
4	1302	CTRDCSGGSCYLNDAFDIW	IGH	5	None	
4	1303	CAKGRSARAVDWFDPW	IGH	2	None	
4	1304	CARPGSGGYTYGWGDAFDIW	IGH	3	None	
4	1306	CAVGDCTTTSCYYYYHYYMDVW	IGH	2	None	
4	1307	CARVPPVTTVTLDYW	IGH	2	None	
4	1308	CAKVMYSGWSLGATDFDYW	IGH	30	None	
4	1309	CAKLYDYFYMDAW	IGH	12	None	
4	1310	CARESKSRSLSFDLW	IGH	6	None	
4	1311	CAHRLTGTRIWNYGTFDIW	IGH	6	None	
4	1312	CARVRRDSSSGRYMDVW	IGH	2	None	
4	1313	CARLPYSGSDMDVFDLW	IGH	3	None	
4	1314	CARETESALGWYFDLW	IGH	2	None	
4	1315	CARDQAFGDVMIHSFDYW	IGH	10	None	
4	1316	CARDEYGSGNYSGLPDYW	IGH	3	None	
4	1317	CARRWAIFGPGAPFDPW	IGH	2	None	
4	1318	CQQSYSTHSF	IGK	297	C2	7K8O
4	1319	CVRVTEEMTTIGGSFDTW	IGH	2	None	
4	1321	CARERDTSGYWDHW	IGH	4	None	
4	1322	CARLYLTEYYQSNCFDSW	IGH	5	None	
4	1323	CAKGTNVDTPLAAPSDYYYYLDVW	IGH	4	None	
4	1324	CARDRYYDSSGYYTAGIDYW	IGH	2	None	
4	1325	CASWYYYDSWIDYW	IGH	3	None	
4	1326	CARGKYDFQYRVFDYW	IGH	4	None	
4	1327	CARAVVTTTGGRFDIW	IGH	9	None	
4	1328	CAKDPDNECRPPGGFDPW	IGH	2	None	
4	1329	CARGRWGYYGSDNDSFYYMDVW	IGH	3	None	
4	1330	CARGKAAYYDSSGYLYNWFDPW	IGH	2	None	
4	1331	CARWKGDTAMAPDYW	IGH	4	None	
4	1332	CVKEVGYTSSSEPFDIW	IGH	2	None	
4	1333	CATKDGLYYYLDVW	IGH	2	None	
4	1334	CARVRLDSHTTWRDAFDIW	IGH	6	None	
4	1335	CARRRGACSGGSCYSDDGFDIW	IGH	4	None	
4	1336	CTRAPKFSDFKDNAFDIW	IGH	2	None	
4	1337	CAREMFSGSSPSFDYW	IGH	2	None	

4	1338	CARLKQGWLQSTGYFDLW	IGH	2	None	
4	1339	CARGGADSPYYYYYMDVW	IGH	11	None	
4	1340	CARPPYSSSAWYYYYMDVW	IGH	15	None	
4	1341	CAKGPIYYSDNSGYLRDYFDYW	IGH	5	None	
4	1342	CARSLFMGAFPDSFFYW	IGH	2	None	
4	1343	CARDGGSCTGGSCYTNLDYW	IGH	4	None	
4	1344	CARALPGGDAYFDFW	IGH	2	None	
4	1345	CARPKPLDYSNYGRFLYYFDQW	IGH	2	None	
4	1346	CARGFKVRGVRYYFDYW	IGH	3	None	
4	1347	CARRHRFFGDDDFYYYMDVW	IGH	2	None	
4	1348	CAREGGTLIYAGNDWLSEYYFDSW	IGH	3	None	
4	1349	CIRDHTGPVDYW	IGH	2	None	
4	1350	CAKEPRTDYQLFFDSW	IGH	2	None	
4	1351	CARAPGDYTPYTTDAFDIW	IGH	6	None	
4	1352	CARGGRWLRRDDAFDIW	IGH	2	None	
4	1353	CARGAHGSSWYTNVDYW	IGH	54	None	
4	1354	CARDLFCGSDCSNLDYW	IGH	2	None	
4	1355	CAKESPYDSSGYYPDAFDIW	IGH	2	None	
4	1356	CARAPILTGYNAEVDYW	IGH	5	None	
4	1357	CARGRGSSLYRGAFDIW	IGH	3	None	
4	1358	CARRLAASGTFWYFDLW	IGH	2	None	
4	1359	CVRLVAPPNWNFGYFDPW	IGH	2	None	
4	1360	CARGRRVFSSTPGYYCDSW	IGH	2	None	
4	1361	CVYRSLKYYDRGDRWFHPW	IGH	10	None	
4	1362	CARLTSWVTTSTNWFDPW	IGH	4	None	
4	1363	CARSSFFLTYHERNYYYYYMDVW	IGH	3	None	
4	1366	CARDGQWFGEFPLDYW	IGH	7	None	
4	1367	CAHRRGLSAYGRFDPW	IGH	4	None	
4	1368	CTRGRTDFGDYVAFYYFDYW	IGH	2	None	
4	1369	CQSADDSDTHFF	IGL	2	None	
4	1370	CDNAYYYFDSW	IGH	3	None	
4	1372	CARDEGLTGTNWFDPW	IGH	3	None	
4	1373	CGGSDGPFYGFYAYMDVW	IGH	2	None	
4	1374	CARDGEGYGESWVAYHFDAW	IGH	2	None	
4	1376	CAKLALPLQISTNYYMDVW	IGH	2	None	
4	1377	CTTACLSGFCSGTGLSYFDYW	IGH	2	None	
4	1378	CAKGGGCHPYSSASCSLRGLDASDIW	IGH	4	None	
4	1379	CAKAETTVTTVDYW	IGH	5	None	
4	1380	CARESGYRATSYRHYEDVW	IGH	3	None	
4	1381	CARVVPHMGSLQWEQLGAGAFDIW	IGH	10	None	
4	1383	CARPRGSDVYDFWEFDYW	IGH	3	None	
4	1384	CAREIYHHRSDFLHTQYFQHW	IGH	5	None	
4	1385	CARGGYCGGECYYWYFDLW	IGH	3	None	
4	1386	CQAWKSSTAPYVF	IGL	9	None	
4	1387	CARDLSDSSNDALDTW	IGH	2	None	
4	1388	CARLGAARGHEAFDYW	IGH	2	None	
4	1389	CTRNPVGPPYFYYYMDVW	IGH	3	None	
4	1390	CARDTFPWWELLSYFDYW	IGH	4	None	
4	1391	CQQYNGHSGLTF	IGK	2	C3	7K8R
4	1392	CARVSTYYNFWSGAPDYW	IGH	2	None	
4	1393	CARSYCRGGNCYSNEAWDYW	IGH	2	None	
4	1395	CAVGVDLWSWVLDYW	IGH	82	None	
4	1396	CAKFMSASYITISGVFDIW	IGH	2	None	
4	1397	CTRDRGTTQHFLDYW	IGH	3	None	
4	1398	CARAPPEGGWNTIFGVVSYSDYMDVW	IGH	2	None	
4	1399	CARETQTPYDSHGYCPYFDFW	IGH	3	None	
4	1400	CAKDSGMGVHDAFDIW	IGH	6	None	
4	1401	CARSSSGDYGFDIW	IGH	35	None	
4	1402	CARVEYYYGSGSTQYYMDVW	IGH	13	None	
4	1403	CARDISSGYYSQFSYFDYW	IGH	12	None	
4	1405	CARGRTTIFGVDTYNWFDPW	IGH	3	None	
4	1406	CAREKTFYDFWSGSAYYFDYW	IGH	3	None	
4	1407	CTRDQGGAWQDYW	IGH	2	None	
4	1408	CTKAIFVANFDHW	IGH	2	None	
4	1409	CAKEDVDEYYFQYW	IGH	6	None	
4	1410	CARQYCSITTCYKFFDYW	IGH	6	None	

4	1411	CSRGGDFDSSGYPYYFDYW	IGH	2	None	
4	1412	CARDPEGGPSVDYW	IGH	3	None	
4	1413	CARETDQQGYYYMDVW	IGH	8	None	
4	1414	CAREVTMVRGVRKPDYW	IGH	3	None	
4	1415	CARLRCSSISCSGDLWFDPW	IGH	63	None	
4	1416	CARWVYYDSRYFDYW	IGH	6	None	
4	1417	CARSRTYGDWGPFFDYW	IGH	2	None	
4	1418	CARAGSWHYFDYW	IGH	17	None	
4	1419	CARDRGTGTSSSWLDPW	IGH	124	None	
4	1420	CARGARDSSSWYTGYYFYYMDVW	IGH	5	None	
4	1421	CARRLRVALDYRNYYFDYW	IGH	7	None	
4	1422	CARQALILKQQLLNFDYW	IGH	4	None	
4	1423	CARDLGYCSSTSCPGGVDYW	IGH	6	None	
4	1424	CARDPFLDYYESSGYYDYW	IGH	2	None	
4	1425	CARVFAASLRRIYDDYYMDVW	IGH	4	None	
4	1426	CARSEGIRYFDWSPFFHWFDPW	IGH	2	None	
4	1427	CVKDGAALWFNGFDVW	IGH	10	None	
4	1428	CAAAVGGTSHNWFDPW	IGH	3	None	
4	1429	CARHGENYDLLTAYYPDYW	IGH	4	None	
4	1430	CARSSDWHEPFDYW	IGH	16	None	
4	1431	CARDQNTVFRGDLDYW	IGH	32	None	
4	1432	CAKDGGTSSWYFDSW	IGH	21	None	
4	1433	CYSVADDNPQWLF	IGL	2	None	
4	1434	CTRADWGTLKYFDFW	IGH	2	None	
4	1435	CAKGALWPETLFDYW	IGH	3	None	
4	1436	CAREVWFGEGEDYLDVW	IGH	3	None	
4	1437	CTSGLYYDTSAFFDFW	IGH	4	None	
4	1438	CAKGDNWNPPYGLFDFW	IGH	8	None	
4	1439	CASSQGNDFWRHYRYMDVW	IGH	2	None	
4	1440	CASEVFDYGDYEGYYMDVW	IGH	2	None	
4	1441	CARGITELAVAGQGYYYYYYMDVW	IGH	3	None	
4	1442	CARALESFMIFGDFDFW	IGH	4	None	
4	1443	CARMTVDSTMVHYPYYYYMDVW	IGH	2	None	
4	1444	CAKEKDRKYYPLNDASDIW	IGH	3	None	
4	1445	CARSGPYSGTYWQAFDIW	IGH	6	None	
4	1446	CARGTHFGSGTYYCFDYW	IGH	2	None	
4	1447	CARVANRPYYFDFW	IGH	2	None	
4	1448	CVRDDYGTSSHFDYW	IGH	10	None	
4	1449	CAKEFKRPIDYW	IGH	2	None	
4	1450	CATDGHSNSWFGNWFDPW	IGH	3	None	
4	1451	CAREVLYCSGGSCYGGYFDLW	IGH	2	None	
4	1452	CARSNSRTSRAFDIW	IGH	14	None	
4	1453	CARVDFHDFWLGRNWFDTW	IGH	5	None	
4	1454	CARVVKPNFGIGVSQIYYQYYYMDIW	IGH	2	None	
4	1455	CARGGSSAYDHVWGNYPVRPFDMW	IGH	3	None	
4	1456	CVKGGGGLLHYYMDVW	IGH	4	None	
4	1457	CVKGSPNWERRWVFDYW	IGH	2	None	
4	1458	CARVVGSWPRHPGAFDIW	IGH	2	None	
4	1461	CAKDYSLTGLTIDYW	IGH	6	None	
4	1462	CARDLDTAMIHRGPFDTW	IGH	2	None	
4	1463	CQSSDSSGSSLIF	IGL	26	None	
4	1464	CARLRGGVIMGTIRGYIDTW	IGH	7	None	
4	1465	CAKEIRGSWHAFDFW	IGH	7	None	
4	1466	CFCAADNNQGGF	IGL	5	None	
4	1467	CAHRRDLGSTWSYGDFDYW	IGH	5	None	
4	1468	CARQELVGARGFDIW	IGH	2	None	
4	1469	CARSPSGDFWSGSSSHYFDYW	IGH	3	None	
4	1470	CARHLLDHYDFWSGYYSDQSLPPNWFDPW	IGH	5	None	
4	1471	CARVLFDSNGYFVDYW	IGH	7	None	
4	1472	CAKTRSAAMGGDAFDIW	IGH	7	None	
4	1474	CARARAGHNIGWYYFDHW	IGH	4	None	
4	1475	CARSAILTGLFLWFDAW	IGH	3	None	
4	1476	CARGYCRGAGCLRFFDYW	IGH	2	None	
4	1477	CVRRDGSTWVHDYW	IGH	2	None	
4	1478	CARNQGEFYFNHYMDVW	IGH	5	None	
4	1479	CAAGSSFSQFDYW	IGH	2	None	

4	1480	CARDSLTPTGRKWYFDLW	IGH	4	None	
4	1481	CVSDARPVGMPTIGYYFDYW	IGH	3	None	
4	1482	CAKDPTYYYDSSGYYPGWFDPW	IGH	7	None	
4	1483	CTTAGWNYYFDAFDIW	IGH	2	None	
4	1484	CAREKYFDSSAHHGIDYW	IGH	2	None	
4	1485	CAKGPRDTVVVVAVNGRWFDPW	IGH	4	None	
4	1486	CARERGEGYQQGYFDCW	IGH	16	None	
4	1487	CARKAAVTANTLFDYW	IGH	4	None	
4	1488	CATLPPAEFFFNYW	IGH	5	None	
4	1490	CAKGGRSNAYSYVDVW	IGH	7	None	
4	1491	CARPSYSSSWLPGYMDVW	IGH	8	None	
4	1492	CARHRYCSSTSCRRLAFDYW	IGH	3	None	
4	1493	CARVAFAAAAALYFDLW	IGH	3	None	
4	1494	CARSNCARSSCYEDYW	IGH	4	None	
4	1495	CARVFRYCSSANCLNYYMDVW	IGH	2	None	
4	1496	CARVLFPRRSFDIW	IGH	2	None	
4	1497	CVLYMGSGIWVF	IGL	292	None	
4	1498	CAKGASGSYSPTDYW	IGH	2	None	
4	1499	CAKDKGLRYLELPSGYFDLW	IGH	63	None	
4	1500	CASGEANNFFDTW	IGH	2	None	
4	1501	CARVRDRYYDSSGYLDYW	IGH	30	None	
4	1502	CARHSSSWPFDYW	IGH	85	None	
4	1504	CARDLGHTNNWWAFDYW	IGH	3	None	
4	1505	CTKRYCSTDSCSYFDLW	IGH	8	None	
4	1506	CARVPRRLATIGGDAFHIW	IGH	3	None	
4	1507	CQQYNEWQDF	IGK	7	None	
4	1508	CARRGYTASGQPWYFDLW	IGH	39	None	
4	1509	CVKVAAPLRLGEERNYLEYW	IGH	2	None	
4	1510	CAHRPGIAAAGQPTPYFDYW	IGH	4	None	
4	1511	CARGGYCSGTTCYLLNAFDIW	IGH	4	None	
4	1512	CVRRGSTWYGHYFDSW	IGH	4	None	
4	1513	CIYSSWYSYYFYYMDVW	IGH	7	None	
4	1515	CARKVYAIVHDAFDIW	IGH	2	None	
4	1516	CARDYGRVGLEPDAFDVW	IGH	2	None	
4	1517	CQSYSGSNHWMF	IGL	8	C4	7JVC
4	1518	CARHGSVSRLFDSW	IGH	2	None	
4	1519	CAREIPIFGVAPGAFDIW	IGH	3	None	
4	1520	CARGRLVLRVVRAYFDSW	IGH	8	None	
4	1521	CARGETYYWDSSGYFYFDYW	IGH	3	None	
4	1522	CAKYYYSSGGYYFFDLW	IGH	2	None	
4	1524	CAKVNRAFRYGLDYYYMDVW	IGH	4	None	
4	1525	CVRGVRWIQRAEWYFEVW	IGH	2	None	
4	1526	CTTDHRETIAARPATGWFDPW	IGH	2	None	
4	1527	CAREVAIAGHGDWFDPW	IGH	2	None	
4	1528	CVRGKRITVASIDYYYMDVW	IGH	4	None	
4	1530	CVRAPVGAPSGWFDPW	IGH	11	None	
4	1531	CAKTRRWLPRDAGFDIW	IGH	2	None	
4	1532	CARGDILTGYYNYRYSYWFDPW	IGH	31	None	
4	1534	CARGFSYSSSPPSFDYW	IGH	9	None	
4	1535	CARSLNYYDTSGDIFGYYFDKW	IGH	4	None	
4	1536	CAKRARFSEWSSSLASLDHLDVW	IGH	3	None	
4	1537	CARWWVSGISAIDYW	IGH	3	None	
4	1538	CAREATYSYDSRGYLVARYFYYYMDVW	IGH	2	None	
4	1539	CARGYWYFNLW	IGH	41	None	
4	1540	CQQFDHYPRLIF	IGK	4	C3	7K8R
4	1541	CAKGHRIFGVVITTAPLDYW	IGH	10	None	
4	1542	CARRHSSWFSESSYYFDYW	IGH	2	None	
4	1543	CARERVPGGGRYFDAFDIW	IGH	2	None	
4	1544	CVRDGSRGQYPDYYFDNW	IGH	5	None	
4	1545	CLLSYSGAARSWVF	IGL	669	None	
4	1546	CAKAGAVDPIWTAHYSVPYYFDNW	IGH	8	None	
4	1547	CARRGYISSSSNYYHYYMDVW	IGH	2	None	
4	1548	CARQSGSNTRGYFDYW	IGH	9	None	
4	1549	CVRDGEFASGTTWVFDYW	IGH	4	None	
4	1550	CAGAAMPVPARVYYYYYYMDVW	IGH	3	None	
4	1551	CAIRSGAHSYYMDVW	IGH	3	None	

4	1552	CARHIVALAPHITNYNYLDVW	IGH	2	None	
4	1553	CMQGTHWPPRDLLTF	IGK	3	None	
4	1554	CARERALHDYSTYYFDYW	IGH	8	None	
4	1555	CAKGTTYDFWSGYSYDYW	IGH	2	None	
4	1556	CARETGYTYGHPYFDFW	IGH	8	None	
4	1557	CAKDWSRVRGIIMGDFDYW	IGH	2	None	
4	1558	CARATARLNYAFDIW	IGH	3	None	
4	1559	CAKRGIAAAGVYSYFDYW	IGH	6	None	
4	1560	CARQAHDSHSTGFVDYW	IGH	5	None	
4	1561	CARGNASFFFGLFDYW	IGH	68	None	
4	1562	CARVGFNCVGGGSCYSPPQFGLDYW	IGH	2	None	
4	1563	CARMDSFYDRLTGYYSNSNAFDIW	IGH	2	None	
4	1564	CARSSIWLFPFDQW	IGH	4	None	
4	1565	CAKDPLLDFWSTNYRELNYMDVW	IGH	7	None	
4	1566	CARVTQSGMSPRVFDYW	IGH	3	None	
4	1567	CATDSGYSVLHYYYYMDVW	IGH	6	None	
4	1568	CAGGIRQLLYGSAPFDYW	IGH	2	None	
4	1569	CAREMYNNGWHPAFDYW	IGH	3	None	
4	1570	CAKGGDSSRVPYYYYYMDVW	IGH	24	None	
4	1571	CAKFQFWEGYNGYDVAFDVW	IGH	2	None	
4	1572	CARVALSSAWYFDSW	IGH	4	None	
4	1573	CAKGDDNSGYYLEGFDYW	IGH	23	None	
4	1574	CAAWDDSLNGPMF	IGL	6192	None	
4	1575	CARDGSSWFGGLSQPFDNW	IGH	5	None	
4	1576	CARQRDVFNRAYFEYW	IGH	5	None	
4	1577	CARGHLYYYDSSGLDYW	IGH	2	None	
4	1578	CATLRGWSSSSTSYNWFDPW	IGH	3	None	
4	1579	CARVQYRGFDYSEAFDIW	IGH	11	None	
4	1580	CAKDLFYETSGALDVW	IGH	9	None	
4	1582	CQSYDGSLRGPLF	IGL	39	C4	7JVC
4	1583	CARVYCGGDCSRPSDFYYYMDVW	IGH	3	None	
4	1584	CARDFDPSECSTAICSNLVDYW	IGH	5	None	
4	1585	CARIIRYYDSGGYYYYYMDVW	IGH	5	None	
4	1586	CARVGQSCNSGWCYFDYW	IGH	2	None	
4	1587	CARHATTYTSNWYFDYW	IGH	2	None	
4	1588	CARGTEIQPPYFDYW	IGH	6	None	
4	1589	CASHRGWPPHLGFDYW	IGH	2	None	
4	1590	CVRHPSGVVTPRWIDSW	IGH	3	None	
4	1591	CARGGMSYYSYYYMDVW	IGH	8	None	
4	1592	CQAWDSTTAWGLF	IGL	6	None	
4	1593	CARDMASGWYGGHDAFEIW	IGH	4	None	
4	1594	CQQYGNVPMTFF	IGK	2	C2	7K8U
4	1595	CTIRGTNRRITMLRGVPLFGYW	IGH	2	None	
4	1596	CTRDSSSSWYRVVDDYYMDVW	IGH	4	None	
4	1597	CARAGLGGTGTYYPPFFEYW	IGH	3	None	
4	1598	CVRDGHETQWYYYMDVW	IGH	2	None	
4	1599	CAKGLQWYGSGSYWSDSW	IGH	23	None	
4	1601	CVRDTEGAYYFDNW	IGH	2	None	
4	1602	CARMVWLSGTRSFDSW	IGH	4	None	
4	1603	CARRRAISSWEALDYW	IGH	2	None	
4	1604	CARRMTGQFAPVGYYYYMDVW	IGH	2	None	
4	1605	CARHPGHTMIVVVTPYYFDYW	IGH	11	None	
4	1606	CASRVREATPVYYHYMDVW	IGH	5	None	
4	1607	CATYSNYLSDYYMDVW	IGH	2	None	
4	1608	CAHRRTIRGFDPW	IGH	3	None	
4	1609	CARLNTRHGGNLDAFDMW	IGH	6	None	
4	1610	CAKEGAYRSGWFDHIDVW	IGH	16	None	
4	1611	CVRVSVVAGTDYW	IGH	10	None	
4	1612	CAGAIVATTRGHYYYYYMDVW	IGH	4	None	
4	1613	CARGETLGATTVGAFHIW	IGH	4	None	
4	1614	CSNPRDYYDSSRDAFDIW	IGH	2	None	
4	1615	CAKDSRITSFRVVRPDAFDIW	IGH	6	None	
4	1616	CARGNCVVDCFSPRRPYHSFYMDVW	IGH	3	None	
4	1617	CVRRTLWMTTSPKPMFYFDSW	IGH	3	None	
4	1618	CTRDRGGLKSAFDIW	IGH	5	None	
4	1619	CVKSGARRGAAEYFDNW	IGH	2	None	

4	1620	CARESSNTVLHPRIDYW	IGH	4	None	
4	1621	CVRDPPELDAFDLW	IGH	2	None	
4	1622	CARRGGSPKYDAFDIW	IGH	2	None	
4	1623	CAREGEGYCYSTSCKKYFDSW	IGH	8	None	
4	1624	CARSFAAAGTGFDYW	IGH	6	None	
4	1625	CARHWMVSYYIDSW	IGH	5	None	
4	1626	CARGLSLYYGSESYYKARWFDPW	IGH	2	None	
4	1627	CARAVAVWFGELSGWFDPW	IGH	6	None	
4	1628	CARDGILATSYFDSW	IGH	8	None	
4	1629	CAKESLGFLLWFGEPWVYFDYW	IGH	2	None	
4	1630	CARPDSFNYGGDGFDLW	IGH	4	None	
4	1632	CARGGRDDDESWYFDLW	IGH	4	None	
4	1633	CAKENANWGRQFDSW	IGH	3	None	
4	1634	CARGTVVTPDFYYYMDVW	IGH	3	None	
4	1635	CARYPLSDWTYGRRAGHFDHW	IGH	2	None	
4	1636	CAKLGGATCSASCYHDYW	IGH	6	None	
4	1637	CARLGPVWGTYRSHSPFDYW	IGH	2	None	
4	1638	CARRGYDYDSGRDLHGFDLW	IGH	2	None	
4	1639	CARQTWETLKYHYMDVW	IGH	9	None	
4	1640	CARDVFGSSTYLDHW	IGH	18	None	
4	1642	CARHARLSATIKGPFDLW	IGH	3	None	
4	1643	CARDGGYCSTPRCLQFDNW	IGH	3	None	
4	1644	CAREETYCSGGGCYNGAFDFW	IGH	7	None	
4	1645	CARQRSQYSSSSFDWFDPW	IGH	2	None	
4	1646	CARLSYGYSTGWYYFDYW	IGH	19	None	
4	1647	CTRALRDIVATRGFDSW	IGH	6	None	
4	1648	CTKANSSSPPEFDPW	IGH	3	None	
4	1650	CGRRRRNCVGPTCTNLFDYW	IGH	3	None	
4	1651	CVKGLRYYGSGSFDYFDYW	IGH	6	None	
4	1652	CARTYDYGATPYYFDYW	IGH	5	None	
4	1653	CARGAEDSTYRLNWFDPW	IGH	6	None	
4	1654	CARAPTYQWPFAGPFEYW	IGH	10	None	
4	1655	CAETYCGPDCYFVDAFDIW	IGH	2	None	
4	1656	CAKEGLRLVYSGDAFDMW	IGH	3	None	
4	1657	CARLVRGAQYGDFWYFDPW	IGH	6	None	
4	1658	CGRDGYQVDAIDVW	IGH	3	None	
4	1659	CTRIIYPGDWFDLW	IGH	4	None	
4	1660	CAKEGGYYGSGSYLDSW	IGH	2	None	
4	1661	CATAFGFKSGWFDTW	IGH	2	None	
4	1662	CTRLMGDFWSGYSDYW	IGH	15	None	
4	1663	CARKGPYYTGSEKKKGAFDVW	IGH	2	None	
4	1664	CARAKMGSNWNPDFDYW	IGH	8	None	
4	1665	CSAWDDTLNGHVVF	IGL	6	None	
4	1667	CATTYFYDSSGHFDNW	IGH	3	None	
4	1668	CARTEYFQAFDFW	IGH	10	None	
4	1669	CSSYGGSSTFELF	IGL	4	C2	7K8X
4	1670	CARGWFGKLLSDTFDMW	IGH	25	None	
4	1671	CARLWGGTYYGSGFDIW	IGH	2	None	
4	1672	CARPYYYDDIGQPGGFDSW	IGH	8	None	
4	1673	CARRGSHYEGLDYW	IGH	2	None	
4	1674	CAKGRRITMIRAPFDYW	IGH	2	None	
4	1675	CARDGTEFGTSIYYFDHW	IGH	2	None	
4	1676	CARGAIRDAYSPDAFEIW	IGH	5	None	
4	1677	CARDSLTNSGDNFDYW	IGH	2	None	
4	1678	CARGYYYGSARLNWFNPW	IGH	2	None	
4	1679	CARDAYGSGSYWGYYFDYW	IGH	26	None	
4	1680	CVRSGPPAAIANWFDSW	IGH	4	None	
4	1681	CARGANLCSSTSCRRYYFDYW	IGH	2	None	
4	1682	CAKSSRSCSTTSCYTHFDYW	IGH	2	None	
4	1683	CTSDYGDYYFDYW	IGH	10	None	
4	1684	CDDAAVYYCARGNASFFFGLFDYW	IGH	2	None	
4	1685	CTGDLNSGWGWLDPW	IGH	11	None	
4	1686	CAKQGDYDFWSGSIYNWFDPW	IGH	2	None	
4	1687	CAGYCSSTSCYAAKAFDIW	IGH	4	None	
4	1689	CARDDYGDYFIPYFDYW	IGH	2	None	
4	1691	CQSVDSSGAYGSYVLF	IGL	4	None	

4	1692	CARAAWVFCTSTRCYRHFDYW	IGH	2	None	
4	1693	CARERDELIVPSAVPYYYYYVDVW	IGH	5	None	
4	1694	CAGPSHKGQLWYFDYW	IGH	2	None	
4	1695	CVRENSGHPYYYYMDVW	IGH	3	None	
4	1696	CAREGADSISWDRNDAFDIW	IGH	4	None	
4	1697	CQVWDISNDHSGVF	IGL	2	None	
4	1698	CARHGGKRSGSSVFDYW	IGH	5	None	
4	1699	CARVCGQHYGSGSYYNADYW	IGH	2	None	
4	1700	CAREEYDTFLGGGGYFYYYYLDVW	IGH	4	None	
4	1701	CLQYNDWPPSRPF	IGK	15	C3	7K8P
4	1702	CARIPDYGGNSMAFDIW	IGH	2	None	
4	1703	CARRRAGSTGFDVW	IGH	2	None	
4	1704	CARYSSSWFRRFFDYW	IGH	2	None	
4	1705	CAKPLTGTSSNYLDYW	IGH	2	None	
4	1706	CAKDSGYDNSGYYAWQNAFDVW	IGH	12	None	
4	1707	CARFSYNSQFDSW	IGH	3	None	
4	1708	CARAYLDYGDNYGLEYMDVW	IGH	3	None	
4	1709	CARDNPYDDVPFDYW	IGH	3	None	
4	1710	CARGEWERLSYYHRYYMDVW	IGH	7	None	
4	1711	CARHVTWNFDLW	IGH	2	None	
4	1712	CAKAAYTYDSSGPTHYYYMDVW	IGH	2	None	
4	1713	CARARDYLGYYLDYW	IGH	6	None	
4	1714	CASLPFGYSDRWYSYYYYMDVW	IGH	12	None	
4	1715	CAREVSILSALHYYYMDVW	IGH	5	None	
4	1716	CARDWIYYDGSSYSVHFYYMDVW	IGH	3	None	
4	1718	CARMGAYCSGGDCFGFDFW	IGH	2	None	
4	1719	CATDREWEQGGPFDYW	IGH	10	None	
4	1721	CARVGKEFWRGYQREMAYYYYMDVW	IGH	2	None	
4	1722	CARVVITLGHDASDMW	IGH	4	None	
4	1723	CARDSPNVKRWFGELAVHAWFDPW	IGH	4	None	
4	1724	CARRAYYSGYDPSYSYYYMDVW	IGH	3	None	
4	1725	CARDRNNYYDIGYFEFW	IGH	2	None	
4	1727	CARQEGVVFMVYAPYDYW	IGH	3	None	
4	1728	CARRGAEYQLLFSHYYYTDVW	IGH	4	None	
4	1729	CAKSGGSYGWFDPW	IGH	29	None	
4	1730	CARGAYCSGYSCYSDYFDYW	IGH	2	None	
4	1731	CARKNPGWIQIWTRHWFDPW	IGH	2	None	
4	1732	CAHSSLYSSNWYGGGFDPW	IGH	11	None	
4	1733	CATAQKHYYDSIGALGFDTW	IGH	2	None	
4	1734	CARDGIASWAIFDSW	IGH	4	None	
4	1735	CARKGNWNYLGDGFDIW	IGH	4	None	
4	1736	CVKDNLRFSEWLPAAFDMW	IGH	4	None	
4	1737	CARPRGWRCYSSSCSYYMDVW	IGH	2	None	
4	1738	CARDFVGATHRAFDPW	IGH	2	None	
4	1739	CARSSLAYSYAPYYFYLDVW	IGH	3	None	
4	1740	CARGGLWWELDRYFFDSW	IGH	2	None	
4	1741	CVRPYCSGGSCFYYMDVW	IGH	8	None	
4	1742	CARVRTYYYDSSGDRDAFDIW	IGH	6	None	
4	1743	CARGSDCTVGVCDTWGNAFDVW	IGH	5	None	
4	1744	CARLGILVVTGIPLQDFYMDVW	IGH	11	None	
4	1745	CARDGPIMYCSGSSCFEYLFDSW	IGH	2	None	
4	1746	CARAPSYFGAFYFDNW	IGH	2	None	
4	1747	CAREVGSPRYNWFDPW	IGH	7	None	
4	1748	CATAGDYDFWNGYKNNHYMDVW	IGH	26	None	
4	1750	CVRGFSGAAREDYW	IGH	70	None	
4	1751	CALPICSYAPFHYYTDVW	IGH	3	None	
4	1752	CARGDYDILTHYHRFFDYW	IGH	2	None	
4	1753	CARERAVASETSRPAFDIW	IGH	4	None	
4	1754	CARAAYCGGDCYYYMDVW	IGH	4	None	
4	1756	CESSDSSGSLEVF	IGL	9	None	
4	1758	CARGHLGGYSYGHTLLWFDPW	IGH	6	None	
4	1759	CARGTWVYFDNW	IGH	44	None	
4	1760	CAPGWNIAVRRWFDPW	IGH	2	None	
4	1761	CAREVYGNSVSRYMDVW	IGH	2	None	
4	1762	CARGDGYNSDWHVDYW	IGH	2	None	
4	1763	CARDSGSGRGWFDPW	IGH	21	None	

4	1764	CARQWAVVPTATNYFDYW	IGH	2	None	
4	1765	CARDGSSTDNWIDPW	IGH	2	None	
4	1766	CASGGNSALDAFDIW	IGH	3	None	
4	1767	CAIYSYGDSLFDFW	IGH	10	None	
4	1768	CARDPPLRSSYYSYYMDVW	IGH	6	None	
4	1769	CARAIQTVAGTHANLYYYYMDVW	IGH	5	None	
4	1770	CARDYWGSWREHRFDSW	IGH	2	None	
4	1771	CARVGEDSRGYYFGAFDFW	IGH	4	None	
4	1772	CAKQGDRSSRFDPW	IGH	2	None	
4	1774	CARSPNVEYGISGDYFDYW	IGH	79	None	
4	1775	CVTERDPFGAYWRAYFPYW	IGH	2	None	
4	1776	CQQLNGYPRLSF	IGK	44	C1	7BZ5
4	1777	CARTDYYRSGTPYMDVW	IGH	3	None	
4	1778	CTTDWEETTVVRAAFDIW	IGH	2	None	
4	1779	CTTVGSLAGDYW	IGH	3	None	
4	1780	CAHLRSVYANNWYFFDYW	IGH	11	None	
4	1781	CAKDFRVKWDPDGYFNYW	IGH	2	None	
4	1782	CAREIDNWIDPW	IGH	27	None	
4	1783	CTGRAATRPVFFDYW	IGH	5	None	
4	1784	CARGEGRYQLFSSWYFDLW	IGH	2	None	
4	1785	CARHWTSATGSNFDFW	IGH	2	None	
4	1786	CAKRLVPLRATYAIDYW	IGH	3	None	
4	1787	CAKDYYNFGYW	IGH	2	None	
4	1788	CGSRNYDFLTGTTDYYMDVW	IGH	9	None	
4	1789	CVMDTSMYFDHW	IGH	3	None	
4	1790	CAHHPPHCTNGVCYHNWFDPW	IGH	4	None	
4	1791	CARRRSLYDENTAYTFLHFDFW	IGH	2	None	
4	1792	CARISTNNNYDSSGYYSYW	IGH	3	None	
4	1793	CAKGLLLYYYDSSANIRLTDYW	IGH	2	None	
4	1794	CARTGDGYSGMILDFDNW	IGH	8	None	
4	1795	CTTVRYGDHDYW	IGH	3	None	
4	1796	CVKDAYCGGNCYWDYYYMDVW	IGH	2	None	
4	1797	CARGGLRFSEYYFDLW	IGH	2	None	
4	1798	CAAGNDSPLFDNW	IGH	2	None	
4	1799	CATGASGTYYSSSFVYW	IGH	5	None	
4	1800	CARGYLYCQQTQCPRYYMDVW	IGH	2	None	
4	1801	CARGRALMFTFGGVIGKFFDYW	IGH	3	None	
4	1802	CARGPGSGTHLSAFDIW	IGH	4	None	
4	1803	CASYKKINDDFYATFPYW	IGH	2	None	
4	1804	CARDHSIGESWDYW	IGH	3	None	
4	1805	CARASGDRWLDPW	IGH	2	None	
4	1807	CAKPPREIQFWIDSFDVW	IGH	8	None	
4	1808	CARTAGKNCAVGFCYMGGLDYW	IGH	38	None	
4	1809	CARDQYSSSWGCFDYW	IGH	7	None	
4	1810	CQSFDASLSGVIF	IGL	10	C4	7JVC
4	1811	CARVLHGSETYAPDYW	IGH	2	None	
4	1812	CTTLAATASESFDYW	IGH	3	None	
4	1813	CATDFSSSWRNNWFDPW	IGH	2	None	
4	1814	CARDLFYNPGYSSGGIDYW	IGH	3	None	
4	1815	CARDQDGVPRPYFFDYW	IGH	2	None	
4	1816	CARVKIPAAGTPDVFDIW	IGH	3	None	
4	1818	CARHASLYSTFDYW	IGH	7	None	
4	1819	CARSPIGDGYTFQYYMDVW	IGH	4	None	
4	1820	CAHNPSTAYDYVWGSYQLDYW	IGH	2	None	
4	1821	CARVGPAYYDSSGYPYYCYYMDVW	IGH	3	None	
4	1822	CERDPRGTPPTDYW	IGH	2	None	
4	1823	CQQRNSWHTF	IGK	50	C1	7BZ5
4	1824	CTAPQPSIALPGIFDYW	IGH	4	None	
4	1825	CAKDYFDNSDNYNWSDPW	IGH	5	None	
4	1826	CVRDHLSFEWFDPW	IGH	4	None	
4	1827	CARFPGENWNPTPWFDPW	IGH	4	None	
4	1828	CNSRDTTGNHLLF	IGL	10	None	
4	1829	CARTPHHSTVITPAFDFW	IGH	9	None	
4	1830	CNSRDNSGHHVQVIF	IGL	3	None	
4	1831	CVRDRDVVVQDYMDVW	IGH	4	None	
4	1832	CARVEYSSSWSYFYYFYYMDVW	IGH	2	None	

4	1833	CAREGGSGSSHYDYW	IGH	2	None	
4	1834	CAKGFHDDTNTYDNYW	IGH	2	None	
4	1835	CARLQGLGATGFSWFDPW	IGH	3	None	
4	1836	CAREIPDYGDQSGSYYMDVW	IGH	4	None	
4	1837	CARPAYTNLYFYYYMDVW	IGH	2	None	
4	1838	CARGYAYYFLYMDVW	IGH	4	None	
4	1839	CTRDAIFGGYDAFDIW	IGH	4	None	
4	1840	CGKGYRVCSSDSCYGGTDYW	IGH	2	None	
4	1841	CARDLGVRRWLQSEYYFDYW	IGH	11	None	
4	1842	CARDLSPSGNYRPDYFDYW	IGH	2	None	
4	1843	CARQRRDGGGIDAFDFW	IGH	2	None	
4	1845	CARGPYYDFWSGYYTTDYW	IGH	6	None	
4	1846	CAREGLFGDYGVGAFHIW	IGH	2	None	
4	1848	CAKDFGIAENYYDFWSGYIDYW	IGH	6	None	
4	1849	CASVAGNQYWYFDRW	IGH	18	None	
4	1850	CTTAFGGRDDAFDFW	IGH	5	None	
4	1851	CLLYYGGGQGWLF	IGL	23	None	
4	1852	CASPYYYDSSGHYYHYMDVW	IGH	4	None	
4	1853	CARDCLTLSARLCGETFDSW	IGH	7	None	
4	1854	CARRPLGSTSYNAFDIW	IGH	4	None	
4	1855	CARLDCGSTSCFGRYLEYW	IGH	32	None	
4	1856	CASAPIATAWRYFDFW	IGH	2	None	
4	1857	CARDGKALVVPAASFDYW	IGH	2	None	
4	1858	CAKDMGRWGYYDSSGYYYKDYW	IGH	4	None	
4	1859	CARSELQKTVIIPVVGVSYYMDVW	IGH	2	None	
4	1860	CARDPHWGIFGEGGFDYW	IGH	3	None	
4	1861	CARYKYGGHPNIDYYYYMDVW	IGH	2	None	
4	1862	CVRGFGSGNGFTWYLDVW	IGH	2	None	
4	1863	CAKDFGNTWGFYYYMDVW	IGH	2	None	
4	1864	CARLYLSSSGYYKGGFDPW	IGH	11	None	
4	1865	CASANYHFWSDYFSSYYIDVW	IGH	2	None	
4	1866	CARLRPGTLSFFDFW	IGH	2	None	
4	1867	CAGDPALSRNVFDLW	IGH	6	None	
4	1868	CTRDYPLSMAARPGFDPW	IGH	2	None	
4	1869	CATDPRHRGSEFLDAFDFW	IGH	25	None	
4	1870	CATTPDSSGQDVFDIW	IGH	3	None	
4	1871	CASGLRGVTKGFDYW	IGH	5	None	
4	1872	CARMPEYSTSSVGFDFW	IGH	2	None	
4	1873	CARVATYTSGFPFDSW	IGH	2	None	
4	1874	CARLKGDFWSGYYNYFDYW	IGH	3	None	
4	1875	CARSGTPKYSGHDEFKYW	IGH	7	None	
4	1876	CAREPHLGVAGPYYYMDVW	IGH	2	None	
4	1877	CARTTGYSNGWAEDCWFDPW	IGH	3	None	
4	1878	CARDPQILPNNWFDPW	IGH	8	None	
4	1879	CTTDKLHYGPSSFDYW	IGH	3	None	
4	1880	CARDRTAEAHTYLGSYSYYMDVW	IGH	2	None	
4	1881	CAKDGIGFYDNSGYYYDYW	IGH	2	None	
4	1882	CAKDISIGSGWPRGFDKW	IGH	6	None	
4	1883	CTRHSSQLLWLGGSGGFDVW	IGH	3	None	
4	1884	CARSLGDLWSGYYKVPGFFDSW	IGH	7	None	
4	1885	CVRDTAMVTGSSDGFDIW	IGH	3	None	
4	1886	CARHLSGTYGRRDWYFDLW	IGH	2	None	
4	1887	CARAAGYYDSSGPSPFDYW	IGH	14	None	
4	1888	CAREQGSGSYYWAPFDMW	IGH	3	None	
4	1889	CVRQTIYFGSGFFDLW	IGH	2	None	
4	1890	CAKDRGGYSGYGPRFDYW	IGH	2	None	
4	1891	CAKDPALYEQWLTRGGFDFW	IGH	4	None	
4	1892	CARVFCWGGTCYDDRGSFDIW	IGH	4	None	
4	1893	CARLGYCNSNGCPGYYYMDVW	IGH	2	None	
4	1894	CAKNYDNSGYYSQFYFDYW	IGH	3	None	
4	1895	CARGESSGWYRAPTYFDFW	IGH	2	None	
4	1896	CAREIAVAAFDSYYFDYW	IGH	6	None	
4	1897	CARAPLGYCSSTSCPGNWFDPW	IGH	3	None	
4	1898	CAREPYSSSSVDYW	IGH	29	None	
4	1899	CANLGGYRSYYYDSSGYFVDYW	IGH	2	None	
4	1900	CARVGPLAGYYPNAYFTYW	IGH	5	None	

4	1901	CARDLSRVDYGVLSGYYQGGILDYW	IGH	24	None	
4	1902	CAKHLTNRPYYYYMDVW	IGH	6	None	
4	1903	CARAPKYITMVRGTYYYYYYMDVW	IGH	55	None	
4	1904	CTKGGLVTPARSLGFDPW	IGH	4	None	
4	1905	CAKDYHPLLRYFDWLSPWGPFDIW	IGH	2	None	
4	1906	CARAWLEAELLIDYW	IGH	2	None	
4	1909	CHQYGSAPPVFAF	IGK	2	C1	7K8N
4	1910	CAKGTGTTQGYFDYW	IGH	5	None	
4	1911	CARAGPDSGWYPAFDIW	IGH	2	None	
4	1912	CARHPWISFYDNSYIDVW	IGH	40	None	
4	1913	CAKDFQVLGSHYYYMDVW	IGH	2	None	
4	1914	CARAEVTVVKFDYW	IGH	7	None	
4	1915	CVKIYYIYWSNYSSFDYW	IGH	2	None	
4	1916	CARPIYRYGSGSFDLW	IGH	3	None	
4	1917	CQVWDDRSDHVIF	IGL	22	None	
4	1918	CATSSVVYRYYFDFW	IGH	2	None	
4	1919	CVKGYCGIISCSRSIPFDSW	IGH	2	None	
4	1920	CARENPRWEPYRAFDIW	IGH	3	None	
4	1921	CSLYSGSFDYW	IGH	3	None	
4	1922	CARSYYYERSAPEDYW	IGH	5	None	
4	1923	CAREAGLYDFFDFW	IGH	2	None	
4	1924	CARGRGYNWNYHYYYMDVW	IGH	12	None	
4	1925	CARDANIVVVVGHAFDIW	IGH	2	None	
4	1926	CARAVTPSHYYMDVW	IGH	2	None	
4	1927	CARDYYDSSGYYYPGAFDIW	IGH	11	None	
4	1928	CARGSRQPIIFYYYMDVW	IGH	3	None	
4	1929	CQSADNSGTSRWMF	IGL	26	None	
4	1930	CAKTRIAYCGGDSCSGLDYW	IGH	4	None	
4	1931	CATLRRGSELGRGYYFDYW	IGH	2	None	
4	1932	CARGIVGATRVIYFDFW	IGH	5	None	
4	1933	CARGRYSSSSGNVNTHDYW	IGH	2	None	
4	1934	CARDWARRDYYDHSNFDAVDIW	IGH	3	None	
4	1935	CASPSTVTMLSFDTW	IGH	5	None	
4	1936	CARLFDSTSFYYVQDYYYMDVW	IGH	2	None	
4	1937	CARSYNFWREGYFDYW	IGH	4	None	
4	1938	CARDTITMTVVDRGLVGYFDLW	IGH	2	None	
4	1939	CARGRSTYYDSKNYYNYFDFW	IGH	5	None	
4	1940	CARDSSGWGGRPDYW	IGH	4	None	
4	1941	CARMRRTIFGVATTFDAFDIW	IGH	2	None	
4	1942	CARGDGYNRRVFDYW	IGH	26	None	
4	1943	CARRGDPVVDHDYW	IGH	2	None	
4	1944	CARGPVPTYYGSGSYADYW	IGH	2	None	
4	1945	CARDQHPNIVVVPTYYMDVW	IGH	4	None	
4	1946	CARGLTASAYCPNGLCYRYFDYW	IGH	17	None	
4	1947	CARMPGGRGYTYGFFDSW	IGH	5	None	
4	1948	CAKDFYCSRSGCYGFDYW	IGH	5	None	
4	1949	CARLLGDGYIARAFDFW	IGH	2	None	
4	1950	CARHKWPAAIDYYMDVW	IGH	4	None	
4	1951	CARSDYDSSGYGPFEYW	IGH	2	None	
4	1952	CARLAWEVEDDAFDIW	IGH	2	None	
4	1953	CAGGYSGYGPSDYW	IGH	2	None	
4	1954	CATTGIAVSGGDYW	IGH	4	None	
4	1955	CARGPTYCTNGVCYNPNYFDYW	IGH	3	None	
4	1956	CARDVQGYYGSGSEFDPW	IGH	2	None	
4	1957	CAHRLGGYNWNDCYFDYW	IGH	6	None	
4	1958	CARDPGKYGGYYMDVW	IGH	2	None	
4	1959	CATSHGVAAAGPDYW	IGH	4	None	
4	1960	CARDWLGKRYYVSGSYQDYW	IGH	2	None	
4	1961	CARVTAGSDTGWCLDYW	IGH	2	None	
4	1962	CAKDIGLINPLYYFDYW	IGH	3	None	
4	1963	CARGDPAPEFVYW	IGH	3	None	
4	1964	CARRGSGYSSGWPYYFDYW	IGH	7	None	
4	1965	CARTLVKDYFYYMDVW	IGH	4	None	
4	1966	CTRAREDSGYPFYDYW	IGH	5	None	
4	1967	CARVRERLAQDAFDIW	IGH	6	None	
4	1969	CGRPGRPHYYYFYMDVW	IGH	10	None	

4	1970	CARAKPFASTGLYYFDYW	IGH	93	None	
4	1972	CARGKAYSFGTPPRPYDYW	IGH	2	None	
4	1973	CAKSRGAIRVLYYFDHW	IGH	3	None	
4	1974	CARRSYDSRGYGGGYYFDSW	IGH	2	None	
4	1975	CAHRQGPNSSGYFDYW	IGH	2	None	
4	1976	CASTVVAYFDYW	IGH	68	None	
4	1977	CARDMSLFWFGEGRRGFDPW	IGH	2	None	
4	1978	CVKPASYGTAAANQDYYYYYYMDVW	IGH	2	None	
4	1979	CARSRDAHYAILTSYSLAPPSLFDSW	IGH	4	None	
4	1980	CARVGGSRSTTYNPPGGGRRPVQYSFRYYIDVW	IGH	2	None	
4	1981	CARDVDGQQLDWHFDLW	IGH	2	None	
4	1982	CANSVGPIATRPFDYW	IGH	4	None	
4	1983	CARVHHWGQGWFGPW	IGH	4	None	
4	1984	CARLSVAGTSWEFDYW	IGH	11	None	
4	1985	CARHTAFWYSSSYGWFDPW	IGH	15	None	
4	1986	CATGYYHDSTSFDYW	IGH	4	None	
4	1987	CARHSGIGWYGGEDYW	IGH	3	None	
4	1988	CARDTYDNTGYYYKDFDYW	IGH	4	None	
4	1989	CARARQLRQYFDCW	IGH	35	None	
4	1990	CARGAPGLVVPFDYW	IGH	12	None	
4	1991	CARHRWELPFDSW	IGH	2	None	
4	1992	CAYSKSYPPYYFDYW	IGH	6	None	
4	1993	CARNFGVQNFDSW	IGH	10	None	
4	1994	CARRRMAIPGRNFFYMDVW	IGH	3	None	
4	1995	CASHSYDFWSGYYGFEYW	IGH	2	None	
4	1996	CARSTSSWAQEGYWFDFW	IGH	16	None	
4	1997	CAGFRDGLQGTNYFDYW	IGH	2	None	
4	1999	CARDSLSRSSGWSYPFSGWFDPW	IGH	5	None	
4	2000	CARDHNWNDAGVDYW	IGH	2	None	
4	2001	CARVDFTTWSSRDYFDSW	IGH	2	None	
4	2002	CTSRPIVKRVGYFDYW	IGH	4	None	
4	2003	CAKDVVANYYDSSSYYYVPPRYNFDYW	IGH	2	None	
4	2004	CAKQTSSSWYGGAFDIW	IGH	7	None	
4	2005	CARHCGRDCVFDTLDYW	IGH	2	None	
4	2006	CARGLVYFDSTAYYGYW	IGH	17	None	
4	2007	CASSRHSSSPPFDSW	IGH	7	None	
4	2008	CATKWLGIVARLGYMDVW	IGH	3	None	
4	2009	CATRTWGPVTGSLTLYYMDVW	IGH	2	None	
4	2012	CAQGMAVGPLDYW	IGH	6	None	
4	2013	CAKGSRWDNWNYFDFW	IGH	2	None	
4	2014	CARFSVAASSWAFDIW	IGH	2	None	
4	2015	CARDHNSGWSKEEYFQHW	IGH	4	None	
4	2016	CARTEGATTGDAFDVW	IGH	12	None	
4	2017	CARGRVDVTMTIVVVTAGSYYLDFW	IGH	4	None	
4	2018	CAKDPGYDSSGYIDYW	IGH	3	None	
4	2019	CARDREPLVGAAGQSAFDIW	IGH	4	None	
4	2020	CVKDNNEFAFDYW	IGH	3	None	
4	2021	CAHSPYYSDTITYSPHFDYW	IGH	10	None	
4	2022	CVRDPSGERDYHFDLW	IGH	4	None	
4	2023	CARGPRKQQLAPYYFDYW	IGH	2	None	
4	2024	CARGRAEVGLYKRPYYFDSW	IGH	4	None	
4	2025	CQHYNGYSGTF	IGK	1070	None	
4	2026	CAKEMGENDSSFDSW	IGH	3	None	
4	2027	CAHSDESIAVVWHAFHIW	IGH	40	None	
4	2028	CARDGRSSRWSVDWFDPW	IGH	12	None	
4	2029	CARLPFLRLSTYSYYQYMDVW	IGH	5	None	
4	2030	CAKESVLVVIPPAWFDQW	IGH	3	None	
4	2031	CARVVSGWGDYFDSW	IGH	4	None	
4	2032	CARMYSYYFGAGSSRPFDYW	IGH	3	None	
4	2033	CARDRGRYFDWPRGDDAFDIW	IGH	4	None	
4	2034	CARVDGWYSTAWNWYFDLW	IGH	4	None	
4	2035	CARDPPSVNDYYFDYW	IGH	4	None	
4	2036	CARDHDDFGDYGGSNRFDPW	IGH	2	None	
4	2037	CAGQRGNTKYFQHW	IGH	2	None	
4	2038	CAREDMNHYYMDVW	IGH	2	None	
4	2039	CKQALQPPLVF	IGK	5	None	

4	2040	CAKALGVATTRLSWFDPW	IGH	2	None	
4	2041	CASNIAGRLRAPYYYYMDVW	IGH	2	None	
4	2043	CARDRIAAAELYDYW	IGH	5	None	
4	2044	CARAEHPEYNSSPRDPCDVW	IGH	2	None	
4	2045	CARQANSGDYGNHDAFDIW	IGH	14	None	
4	2046	CARGRLGSGSYFDYYMDVW	IGH	4	None	
4	2047	CAKRVIPAANIYFDYW	IGH	2	None	
4	2048	CANSYSRGPLYYFDYW	IGH	3	None	
4	2049	CARADYGANSITAFHIW	IGH	2	None	
4	2050	CAKGTVGATRGQSDYW	IGH	10	None	
4	2052	CASQMFQPHAFDLW	IGH	23	None	
4	2053	CATAHYDYVWGTYRPPDYW	IGH	4	None	
4	2054	CARMTCWGGDCYGYYFDSW	IGH	3	None	
4	2055	CARVAGRWLHIDYW	IGH	3	None	
4	2056	CARATYYYDTSGPIGGYFDFW	IGH	3	None	
4	2057	CGATGTHFFDYW	IGH	6	None	
4	2058	CAKAVRGLSSGYYYRQGGFDPW	IGH	4	None	
4	2059	CVKTSQWLAANVYSFDHW	IGH	2	None	
4	2060	CAREVRQWLVLSYYYMDVW	IGH	8	None	
4	2061	CAHRHIGQAAAFFDSW	IGH	8	None	
4	2062	CTRLGPEDAFDIW	IGH	5	None	
4	2063	CTRDPTGGSWPYFDLW	IGH	2	None	
4	2064	CARTENIVLDAAAGAFDLW	IGH	5	None	
4	2065	CARGNPHYSGLGDYW	IGH	4	None	
4	2066	CARRPGGLWSGSYNWFDPW	IGH	5	None	
4	2067	CHQSSSLPWTF	IGK	419	C3	7K8R
4	2068	CAKGEKLGFGAGSHSDFGYW	IGH	2	None	
4	2069	CARVALLGYFATSGHYAPDYFDPW	IGH	4	None	
4	2070	CARGGHCSGTRCNPDNYYFYGMDVW	IGH	2	None	
4	2071	CLSADSTGAWVF	IGL	16	None	
4	2072	CARAHLVDEKEWDLDAFDIW	IGH	7	None	
4	2073	CARGGIVLITNDPFDIW	IGH	7	None	
4	2074	CAIFRTKWDNEDWFDPW	IGH	2	None	
4	2075	CARGGPWGRGWSAEPFYYYYMDVW	IGH	18	None	
4	2076	CARHFYYSGWPYYFDLW	IGH	5	None	
4	2077	CARDPPPYYYDSSGYRFDYW	IGH	4	None	
4	2078	CVRTQITMVRDWRPDPFDIW	IGH	6	None	
4	2079	CARLPMQTLDYYHFYMDVW	IGH	2	None	
4	2080	CAKAGTSQYYHYYYTDVW	IGH	6	None	
4	2081	CARGGAYSSSSSPLPFHFDYW	IGH	6	None	
4	2082	CATTSGIAVAGNTYFEYW	IGH	9	None	
4	2083	CARAAFGRNPNWLDSW	IGH	3	None	
4	2084	CAIDLLSRVSAAPDYW	IGH	5	None	
4	2085	CAKSRPSNWGVFGPFYYYYYMDVW	IGH	4	None	
4	2086	CARRPLTFGAYIADGFDSW	IGH	21	None	
4	2087	CARDSYRGNPNDAFDIW	IGH	20	None	
4	2088	CARRGWTGGSSTSSNWFDPW	IGH	6	None	
4	2089	CARGIYTTDYGDYVPTNDQSYYYIDVW	IGH	4	None	
4	2090	CARDADTAMPTPFDYW	IGH	2	None	
4	2091	CARGEGEWLLFDHW	IGH	21	None	
4	2092	CARDRGIAAGGDDPFDIW	IGH	6	None	
4	2093	CARDHYDSSGYSHSFDVW	IGH	2	None	
4	2094	CARAQRIGAALSSQSFDSW	IGH	5	None	
4	2095	CARIIRGIGIDYYYYYMDVW	IGH	10	None	
4	2096	CARDQRWYGEPRGFDVW	IGH	4	None	
4	2097	CARLPNRVVYRPNTVTTFYDGFDIW	IGH	2	None	
4	2098	CAKRRAVFMLYSGNEAFDIW	IGH	76	None	
4	2099	CARGSWGGSTGFYPYSFDYW	IGH	2	None	
4	2100	CARLGVPASMPISRYMDVW	IGH	5	None	
4	2101	CARRQNVGADIAFDIW	IGH	2	None	
4	2102	CARARDVVVAPGGSSRNNHAEYFQHW	IGH	2	None	
4	2103	CARLGRKGYYGSGADYW	IGH	2	None	
4	2104	CARDFKVRAAAGEVYFDYW	IGH	4	None	
4	2105	CAKDSRLFLMGADYHFDYW	IGH	42	None	
4	2106	CARTYSIGYTEKDAFDVW	IGH	4	None	
4	2107	CARVGPQAFDYW	IGH	31	None	

| | | | | | | |
[truncated: 467,243 more chars]
